# The microRNA toolkit of insects

**DOI:** 10.1038/srep37736

**Published:** 2016-11-24

**Authors:** Guillem Ylla, Bastian Fromm, Maria-Dolors Piulachs, Xavier Belles

**Affiliations:** 1 Institute of Evolutionary Biology (CSIC-Universitat Pompeu Fabra), Passeig Marítim 37, 08003 Barcelona, Spain; 2 Department of Tumor Biology, Institute for Cancer Research, The Norwegian Radium Hospital, Oslo University Hospital, Nydalen, N-0424, Oslo, Norway

## Abstract

Is there a correlation between miRNA diversity and levels of organismic complexity? Exhibiting extraordinary levels of morphological and developmental complexity, insects are the most diverse animal class on earth. Their evolutionary success was in particular shaped by the innovation of holometabolan metamorphosis in endopterygotes. Previously, miRNA evolution had been linked to morphological complexity, but astonishing variation in the currently available miRNA complements of insects made this link unclear. To address this issue, we sequenced the miRNA complement of the hemimetabolan *Blattella germanica* and reannotated that of two other hemimetabolan species, *Locusta migratoria* and *Acyrthosiphon pisum*, and of four holometabolan species, *Apis mellifera*, *Tribolium castaneum*, *Bombyx mori* and *Drosophila melanogaster*. Our analyses show that the variation of insect miRNAs is an artefact mainly resulting from poor sampling and inaccurate miRNA annotation, and that insects share a conserved microRNA toolkit of 65 families exhibiting very low variation. For example, the evolutionary shift toward a complete metamorphosis was accompanied only by the acquisition of three and the loss of one miRNA families.

Although discovered more than 20 years ago, microRNAs (miRNAs) have appeared as important players in the post-transcriptional regulation of gene expression^1^. During these years, thousands of new miRNAs have been discovered, the pathways of miRNA biogenesis have been unveiled, and the mechanisms governing target regulation have been basically understood^2^. Today, the contribution of miRNAs to the regulation of almost all cellular processes is considered crucial^3^. These processes rely on the coordinated expression of genes, which is implemented through gene-regulatory networks, and the participation of miRNAs in them increase functionality precision and reduce intrinsic noise^4^. Moreover, it is plausible that miRNAs have played a role in the emergence of evolutionary innovations and increase of organismic complexity in animals, as apparition of new clades is accompanied by the emergence of new miRNAs, which became highly conserved^2^,^5^,^6^.

Insects, with about 1 million species described, represent approximately 90% of recorded metazoan species living on Earth and exhibit an extraordinary level of morphological and developmental diversity. Moreover, they show growing levels of organismal complexity within the class, from the most “primitive” Palaeoptera, to Polyneoptera and Paraneoptera (including Condylognatha), until to the more modified and megadiverse Endopterygota (=Holometabola)^7^. An important innovation that explains most of the insect evolutionary success has been the holometabolan metamorphosis, by which the juvenile stages can adopt a body plan very different from that of the adult, thus allowing the exploitation of new resources^8^. Recent reviews emphasize the importance that miRNAs play in insects^9^,^10^, also in metamorphosis^11^.

Moreover, a substantial number of insect miRNAs has been published and the correlated number of deposited miRNA sequences in miRBase is significant (26 insect species and 3,119 miRNAs)^12^. However, the representation of insect-groups is highly biased, as we found that out of the 26 insect species represented, there are two hemimetabolan species (1polyneopteran and 1 paraneopteran) and 24 holometabolan (endopterygotes). Moreover, the information is also unequal, as the number of miRNAs ranges from 7 in the locust, *Locusta migratoria*, to 487 in the silkworm, *Bombyx mori*, while a recent paper described 833 miRNA genes from the locust^13^. Therefore, this underrepresentation of hemimetabolan species and the dramatic inequality regarding the completeness of the miRNA complement of each species precludes any serious analysis on whether miRNAs played any role in insect evolution. To address this problem, we have studied in detail the miRNAs of the cockroach *Blattella germanica* (a hemimetabolan species), and we have revised the data of the hemimetabolan and holometabolan species that have the most robust information available on miRNAs.

Concerning *B. germanica*, we used an initial miRNA catalogue^14^, together with the information of a number of new small RNA-seq datasets (including Dicer-knockdown libraries, to validate miRNA candidates) and the newly available genome [ https://www.hgsc.bcm.edu/arthropods/german-cockroach-genome-project]. The criteria used to establish the miRNA complement of this cockroach were those reported by Fromm *et al*.^15^. Concerning insect species having a good level of miRNA knowledge, we have chosen *L. migratoria* (paleopteran, hemimetabolan), with 833 described miRNA genes^13^, the pea aphid *Acyrthosiphon pisum* (paraneopteran, hemimetabolan), for which 176 miRNAs have been reported^16^, and the endopterygotes and holometabolan *Apis mellifera*, the honeybee, with 254 miRNAs recorded in miRBase, *Tribolium castaneum*, the flour beetle, with 220 miRNAs recorded in miRBase and 123 recently reported^17^, *B. mori*, with 487 miRNAs recorded in miRBase, and *Drosophila melanogaster*, the fruit fly, with 165 miRNAs described by Fromm *et al*.^15^.

Our goal was to study and identify the miRNA complement in insects in a robust manner, which can be useful in different fronts. For example, the study can allow to test if the great disparity of miRNA numbers in insects is real or only apparent. Data obtained would contribute to understand miRNA evolution in insects, with special attention to the transition from hemimetaboly to holometaboly, and facilitate comparisons with the equivalent miRNA complement in other clades in order to derive evolutionary conclusions. Finally, establishing the essential miRNA toolkit in insects can allow to address general evolutionary questions, like testing whether miRNA number correlate with genome size in insects, or contributing to the debate on the parallelism between the evolution of complex organisms and the evolution of gene regulation mechanisms.

## Results

### *B. germanica* miRNA complement

To identify the miRNAs of *B. germanica*, we started using two prediction tools, applied to all the small RNA-seq reads available and the *B. germanica* genome assembly. The mirDeep* tool produced an output file containing the coordinates for 1,724 miRNA candidates. The other tool, a modified version of mirDeep2 (https://www.mdc-berlin.de/8551903/en)^18^, predicted 1,104 putative miRNAs. Both predictions were merged into a single file containing 2,761 miRNA candidates.

Comparisons of our predictions with miRBase data led to the identification of previously described, conserved miRNAs, which were then grouped into families according to similarity, especially in the seed region. When families contained miRNAs in different loci, we compared their sequences and flanking regions to discard eventual assembly artefacts. With this approach, we discarded three miRNA loci that would correspond to MIR-137, MIR-29 and MIR-276 families, as they resulted from misassembling artefacts. Reciprocal antisense miRNAs, like Mir-iab-8 and Mir-iab-4, were considered as single miRNA genes. Following these premises, we ended with 59 miRNA families containing 86 miRNA genes under the category of conserved in *B. germanica* (Supplementary Table S1). Two miRNA families expected to be present were not found: MIR-36, which emerged with protostomes, and MIR-309, which emerged with pancrustaceans^6^). To confirm their apparent absence, we assessed that there were no reads corresponding to these miRNAs in the small RNAseq data. Additionally, using the miRNA precursor sequence of other insect species where it was available, we carried out homology searches on the *B. germanica* genome assembly and we assessed that there were no significant hits. Conversely, MIR-2001, MIR-932, MIR-3770 and MIR-6012 families that were considered exclusive of dipterans^6^, were now found in *B. germanica*.

**Figure 1:**
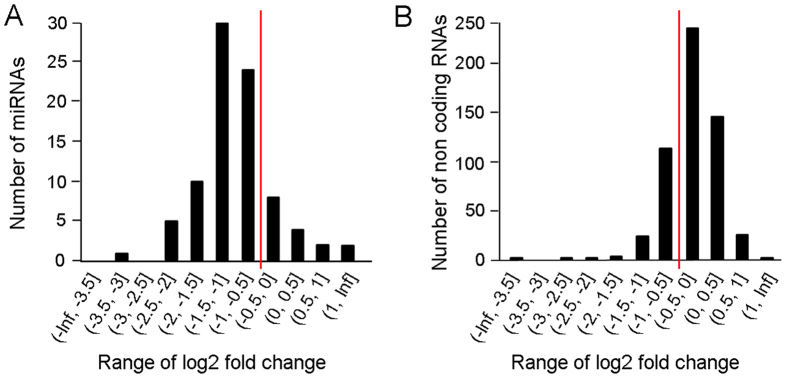
Expression changes due to Dicer-1 depletion in *Blattella germanica* expressed as ranges of fold change in log2 scale. (**A**) For conserved miRNAs. (**B**) For non-coding RNAs from regions of *B. germanica* genome that not contain miRNAs, used as negative control. The expression was computed as the number of reads per million reads of each feature in each library. The red line indicates the log2 fold change = 0.

In addition, our predictions gave 2,675 miRNA candidates with no significant similarity with any previously known miRNA. To discard false positives, we depleted Dicer-1 by RNAi, which resulted in significant reduction of miRNA levels, in general, as previously reported^19^. Subsequently, we prepared and sequenced two libraries from *B. germanica* treated with a dsRNA targeting Dicer-1, and two libraries from control specimens (treated with an unspecific dsRNA). The reads obtained from sequencing them are shown in Table 1. Then, the read counts for each arm of each miRNA candidate were recorded (Supplementary Table S2). To set the limits for discarding false positives, we considered the effects of Dicer-1 depletion on conserved miRNAs. Thus, we recorded the read counts for conserved miRNAs (Supplementary Table S3). The expression of 90% of the conserved miRNAs was reduced in some degree, whereas that of 81% of them was reduced with a log2(FC) lower than −0.5 (Fig. 1A). As a negative control, we selected 558 non-coding RNA regions and we compared the expression change of them in Dicer-1 libraries and in control libraries, which resulted in a median fold change of −0.18 (Fig. 1B). These results suggest that miRNA expression differences between control and dsDicer-1libraries are due to Dicer-1 processing of miRNA precursors and not to unspecific effects.

Subsequently, we excluded the miRNA candidates that were not significantly reduced after Dicer-1 depletion by setting a threshold of log2(FC) = −0.5. Moreover, we also excluded the candidates that did not have at least 1 read-pair from the two control libraries in each arm. This rendered 213 miRNA candidates. Finally, we further filtered these candidates by using the criteria detailed in the methods section (secondary structure of the precursor forming a hairpin and with free energy InitialdG <−22 kcal/mol; occurrence of more than 16 nucleotides paired between the mature and star sequence; and occurrence of two bases overhang from each arm). After that filter we obtained 13 *bona fide* novel miRNA loci belonging to 9 miRNA families (Table 2). One of these families was MIR-bg5, which had been previously described in *B. germanica* as “candidate 1”^14^. It contains two miRNA genes and, as this MIR-bg5 family was also found in *L. migratoria* and *A. pisum* (see below), we will later consider it as conserved in insects. Therefore, we ended with 11 miRNAs novel genes belonging to the 8 novel miRNA families specific of *B. germanica*.

Using the mirPLOT bioinformatic tool available at GitHub (https://github.com/labP64/mirPLOT) that was developed ad hoc, we generated a graphical report for each conserved (Supplementary Data S1) and specific (Supplementary Data S2) *B. germanica* miRNAs, which contains all structural and genomic information on every miRNA.

### Properties of the *B. germanica* miRNA complement

**Figure 2:**
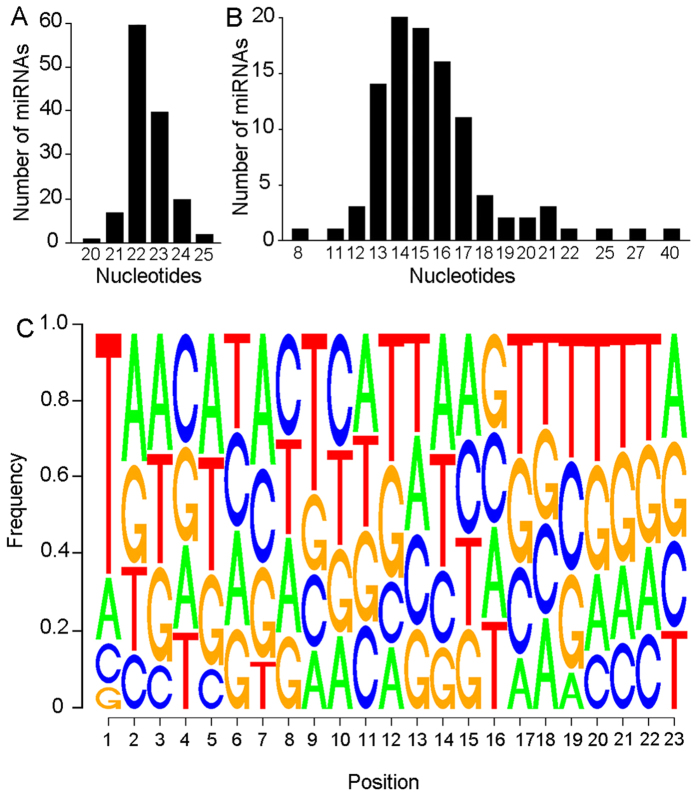
Properties of the miRNAs identified in *Blattella germanica*. (**A**) Length range of the mature miRNA sequence. (**B**) Length range of the loop of the miRNA precursor. (**C**) Sequence logo showing the proportion of each nucleotide in each position on the mature mRNA. In the mature miRNA, the most frequent nucleotide in the first position is Uracil (represented as T), whereas the other positions show similar frequencies of each one of the 4 possible bases.

The frequencies of occurrence of the mature miRNA in the 5p or 3p arm of the hairpin precursor are consistently similar in the 86 conserved miRNAs: 49% are placed in the 3p and 44% in 5p. When both are similarly expressed and the ratio between the most abundant divided by the least abundant is <2, we considered the miRNAs of both arms as co-mature, which represent a 7% of the miRNAs. The sample of novel miRNAs is smaller, and the frequencies of occurrence of the mature miRNA in the 5p or 3p arm are not so similar (3p = 64%, 5p = 27%, co-mature = 9%, n = 11) (Supplementary Table S1). The length of mature miRNAs ranges between 20 and 25 nucleotides, the most common being 22 nucleotides (Fig. 2A). Moreover, the loop length of the precursor ranges between 8 and 40 nucleotides, the most common being 14–15 nucleotides (Fig. 2B). In 66% of the cases the first nucleotide of the mature miRNA is U, as occurs in miRNAs, in general^20^, followed by A (17%), C (11%) and G (6%) (Fig. 2C).

**Figure 3:**
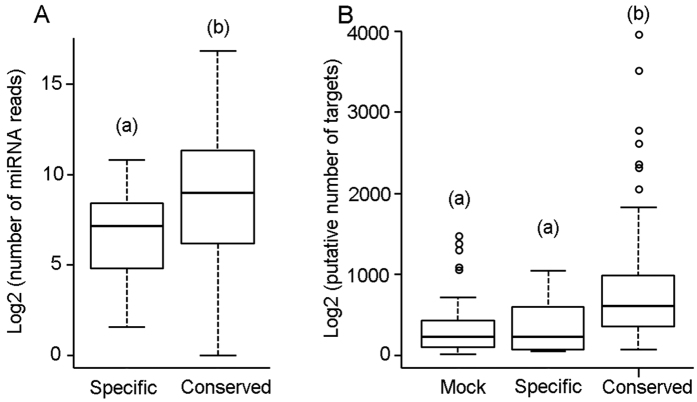
Expression of miRNAs of *Blattella germanica* and potential miRNA targets depicted as boxplots in log2 scale. (**A**) Expression of specific and conserved miRNAs. (**B**) Number of predicted targets for Mock miRNAs (randomly generated sequences of 22 nucleotides different to any known miRNA), for specific miRNAs and for conserved miRNAs. Different letters in parenthesis at the top of each boxplot indicate significant differences (t-test, p-value < 0.05).

In terms of number of reads, specific miRNAs are significantly less expressed than conserved miRNAs (Fig. 3A). We then wondered whether specific miRNAs might have more or less potential targets than conserved miRNAs. Thus, we estimated the number of potential targets in both cases, considering the protein coding genes in the *B. germanica* genome using the algorithms RNAhybrid, miRanda and RNA22. For conserved miRNAs, RNAhybrid predicted 2,362,584 unique miRNA-mRNA pairs, miRanda 371,604 and RNA22 a total of 426,515. Then, we selected the 75,549 miRNA-mRNA pairs predicted by the three algorithms, and ended with an average of 803.96 mean targets predicted per conserved miRNA. The same approach gave 363.36 mean targets predicted per specific miRNA, which is significantly lower value (t-test p-value < 0.05). As a baseline reference, we predicted potential targets in the *B. germanica* genome for randomly generated 100 different sequences of 20 nucleotides each. The number of targets predicted for these 100 mock miRNAs was 31,270, which gives an average of 312.70 targets per mock miRNA. This value is significantly lower than the 803.96 putative targets per conserved miRNA, and a slightly lower than the 363.36 targets per specific miRNA (Fig. 3B).

### The miRNA complement of *B. germanica* and the insect context

Current data on insect miRNAs recorded in miRBase (Supplementary Table S4) show that the number of miRNAs per insect species ranges between 100 and 200, although *B. mori* is an exception with 487 reported miRNAs. However, miRBase information on different species is unequal, as shown, for example, by the seven miRNAs recorded for *L. migratoria*, whereas a recent work^13^ reports 833 miRNAs for this species. This inequality suggests that comparisons should be performed with species that have the miRNA complement accurately established, especially on the basis of robust sequencing data. In addition to *B. germanica*, examples of species that accomplish this criterion are *L. migratoria*, *A. pisum*, *T. castaneum*, *A. mellifera* and *D. melanogaster*^12^,^13^,^15^,^16^ (Supplementary Table S5).

**Figure 4:**
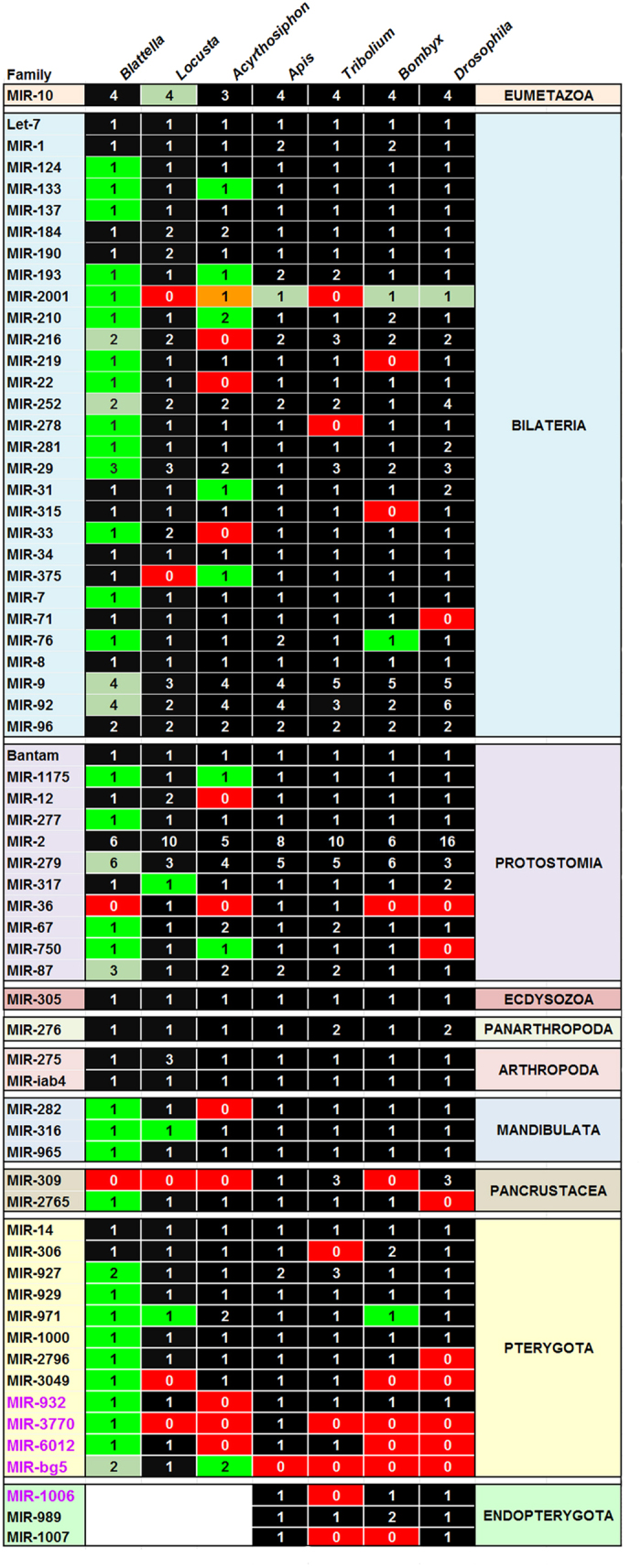
miRNA families and miRNA genes found in the seven species of insects studied. *Blattella germanica*, *Locusta migratoria*, *Acyrtosiphon pisum*, *Apis mellifera*, *Tribolium castaneum*, *Bombyx mori* and *Drosophila melanogaster*. The lineages where the miRNA families originated is indicated. miRNAs highlighted in bright green are those identified in the present study for the first time. Those in light green are miRNAs added to a previously known family. Orange colour highlights a miRNA gene found in the genome but without expression data. The origin of the miRNA families indicated in violet was updated.

**Figure 5:**
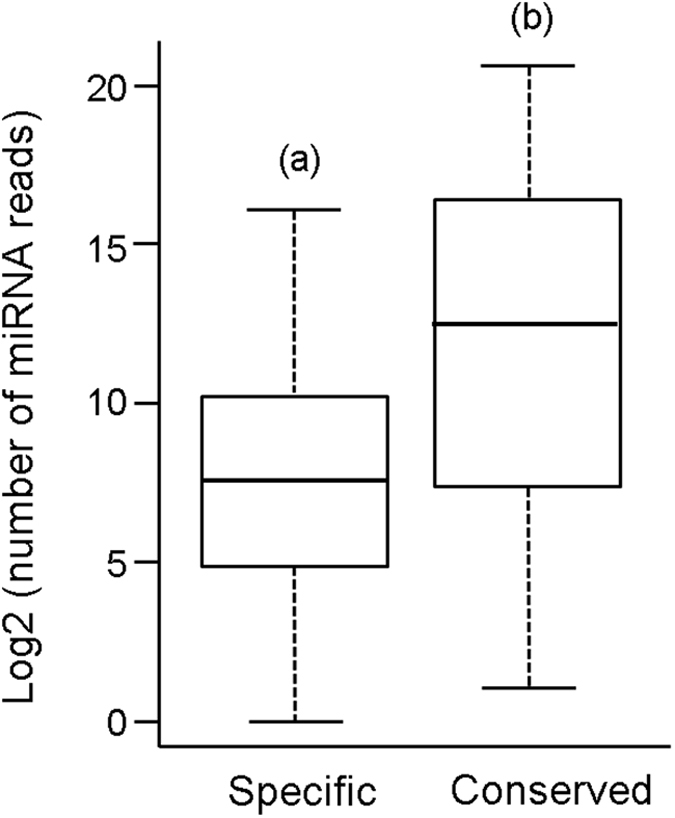
Expression of specific and conserved miRNAs of *Locusta migratoria* depicted as boxplots in log2 scale. Different letters in parenthesis at the top of each boxplot indicate significant differences (t-test, p-value < 0.05).

From an evolutionary point of view, *L. migratoria* is the closest relative to *B. germanica*, for which 833 miRNAs have been reported^13^. In order to compare this species with *B. germanica*, we submitted the miRNAs described by Wang *et al*.^13^ to the filtering procedure described herein. Results showed that 532 of the originally described *L. migratoria* miRNAs fulfilled the filtering criteria. From these, we discarded seven candidates that were identified as miRNA duplicated loci resulting from genome assembly artefacts. We also noticed that seven miRNA families present in *B. germanica* (MIR-2001, MIR-375, MIR-3049, MIR-3770, MIR-317, MIR-316 and MIR-971) were not recorded in *L. migratoria*. Blast approaches on the genome assembly and on small RNA-seq reads led to find three of them: MIR-317, MIR-316 and MIR-971, as well as a new Mir-10 gene. In summary, 85 conserved miRNA belonging to 57 families were identified in *L. migratoria*, including a MIR-bg5 gene (Fig. 4). The 444 remaining candidate genes are *L. migratoria* specific, which gave 365 different mature miRNAs (as some genes expressed identical mature miRNAs) that were grouped into 312 families. Supplementary Data S3 shows the complete reports of these *L. migratoria* miRNAs, and Supplementary Table S6 shows the correspondence between these miRNAs, the family assigned by us and the miRNA identifiers of Wang *et al*.^13^. *L. migratoria* specific miRNAs are clearly less expressed than conserved miRNAs, as occurs in *B. germanica* (Fig. 5).

As an example of paraneopteran, we used the 176 miRNAs reported in *A. pisum*^16^, as well as small RNA-seq data used for the predictions (Supplementary Table S5). Among them we found 65 conserved miRNAs belonging to 44 families (Fig. 4). In an attempt to find possible missing conserved miRNAs, we examined the *A. pisum* genome and the available expression data, finding the additional conserved miRNAs: Mir-133, Mir-193, two miRNAs of the MIR-210 family, Mir-750, Mir-375 and two miRNAs of the MIR-bg5 family. The case of Mir-133 is of note, as the precursor has a loop of about 200 nucleotides (Supplementary Fig. S1). However, it shows a good star-mature complementarity, both are supported by small RNA-seq reads and present the typical two overhanging nucleotides resulting from Dicer-1 and Drosha cleavage. Importantly, both arms show clear similarity with Mir-133 of other insect species. We included the ancestral Mir-2001 in the *A. pisum* complement since we found a genome locus highly similar to that of *B. germanica* Mir-2001, despite expression data for this miRNA was not available in *A. pisum*. Moreover, using a newer version of *A. pisum* genome (ABLF00000000.2), we identified Mir-31 and Mir-1175. We ended with 76 conserved miRNAs belonging to 52 families, including MIR-bg5 (Fig. 4, Supplementary Data S4). As members of the MIR-bg5 family were found in *A. pisum*, *B. germanica* and *L. migratoria*, we considered it as a miRNA conserved in insects. Concerning specific miRNAs, 34 fitted our filtering criteria and were considered *bona fide* specific miRNAs of *A. pisum* (Supplementary Data S4).

With respect to endopterygotes, we used the 254 miRNAs of *A. mellifera* and the 487 of *B. mori* available in miRBase. We also examined the 220 miRNAs of *T. castaneum* deposited in miRBase, and 123 specific miRNAs recently described^17^. In the case of *D. melanogaster* we directly used the miRNA data reported by Fromm *et al*.^15^, who followed the filtering criteria used herein. In the case of *B. mori*, we additionally identified Mir-971 and Mir-76. Concerning Mir-1007 of *D. melanogaster* and Mir-6037 of *A. mellifera*, we considered that they were homologues (using the older name Mir-1007 for both), because conservation extends not only to the seed sequence and most of the mature miRNA, but also because there is significant conservation in the hairpin loop (Supplementary Fig. S2). Finally, we recorded 92 conserved miRNAs belonging to 64 families in *A. mellifera* (Fig. 4, Supplementary Data S5), 94 conserved miRNAs belonging to 58 families in *T. castaneum* (Fig. 4, Supplementary Data S6) and 81 conserved miRNAs belonging to 56 families in *B. mori* (Fig. 4, Supplementary Data S7). With respect to *bona fide* specific miRNAs, we found 22 in *A. mellifera* (Supplementary Data S5), 127 in *T. castaneum* (Supplementary Data S6) and 14 in *B. mori* (Supplementary Data S7). According to Fromm *et al*.^15^, *D. melanogaster* have 98 conserved miRNAs belonging to 56 families (Fig. 5) and 65 specific miRNAs.

### miRNA gains and losses in the context of insect evolution

**Figure 6:**
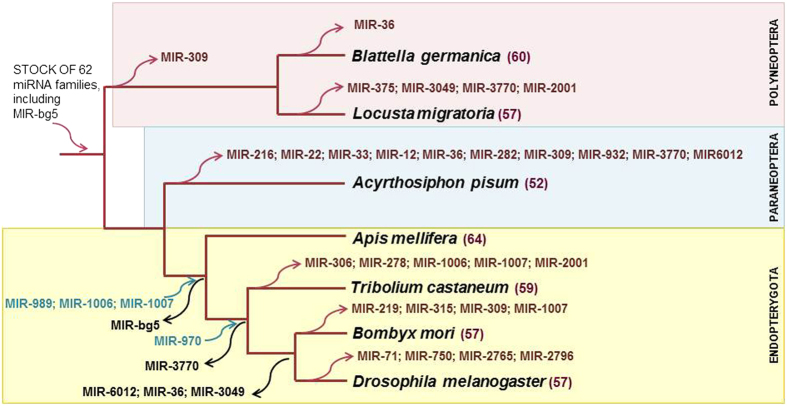
Gains and losses of miRNA families during cladogenesis of the seven species studied. The phylogenetic tree is based on Misof *et al*.^21^. The number of conserved miRNA families is shown besides the name of each species.

Most of the conserved miRNAs come from an ancestral complement of 62 miRNA families accumulated from the emergence of eumetazoans until the hexapodan last common ancestor (Fig. 4). We considered MIR-bg5 family in this complement, as it is present in the polyneopterans *B. germanica* and *L. migratoria*, and the paraneopteran *A. pisum*, although it is absent in the endopterygotes *A. mellifera*, *T. castaneum*, *B. mori* and *D. melanogaster*. Thus, according the more robust phylogenetic reconstruction available^21^, it is more parsimonious to hypothesize a loss of MIR-bg5 in the endopterygotes than two independent gains in polyneopterans and paraneopterans. From these 62 miRNA families, it is apparent the loss of MIR-309 in the branch leading to the paraneopteran *A. pisum*. Then, one miRNA family appears lost in *B. germanica* and four in *L. migratoria* (Fig. 6). No apparent changes are observed in the node clustering paraneopterans and endopterygotes, but 10 miRNA family losses can be recorded in the branch leading to *A. pisum*.

Examination of the miRNAs only found in the four endopterygotes (Fig. 6) indicates that MIR-989 family is present in all them, MIR-1006 in *A. mellifera*, *B. mori* and *D. melanogaster*, and MIR-1007 in *A. mellifera* and *D. melanogaster*. This suggests that these three miRNA families originated with the endopterygotes, whereas MIR-bg5 was lost. Then, MIR-970 family appears to have originated in the last common ancestor of coleopterans + panorpids, whereas MIR-3770 family was apparently lost. Finally, MIR-6012, MIR-3049 and MIR-36 families were lost in the panorpid branch. Specific gains in the branch leading to the hymenopteran *A. mellifera* (5 gains), and specific gains and losses in the respective branches leading to the coleopteran *T. castaneum* (2 gains, 5 losses), the lepidopteran *B. mori* (7 gains, 4 losses) and the dipteran *D. melanogaster* (3 gains, 4 losses), complete the picture (Fig. 6).

### Conservation in number and genomic organization of the conserved miRNAs

Despite discrete gains and losses in particular branches of insect evolution, a look to the Fig. 4 readily suggests that the common and ancestral miRNA complement in the seven studied species is remarkably conserved. The conservation not only refers to miRNA families but it is also well apparent at the level of the number of miRNA genes of each family.

**Figure 7:**
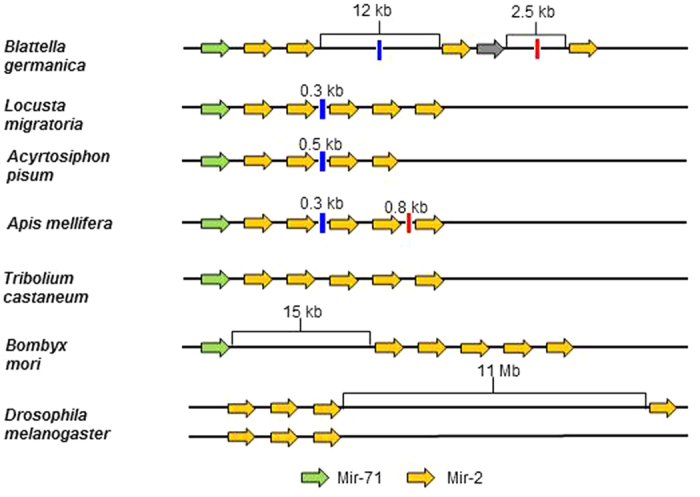
The structure of the miR-2 cluster in the seven species of insects studied. The lines symbolize the genome assembly locus that contains the miRNAs. Segments between miRNA genes are not at size scale, but especially long sequence stretches have been indicated with longer segments and the real length has been indicated. In the cluster of *Blattella germanica* it is remarkable the distance between the second and the third copy of miR-2, where it is inserted a TIGD4 sequence. Blue and red bars indicate homologous regions.

**Figure 8:**
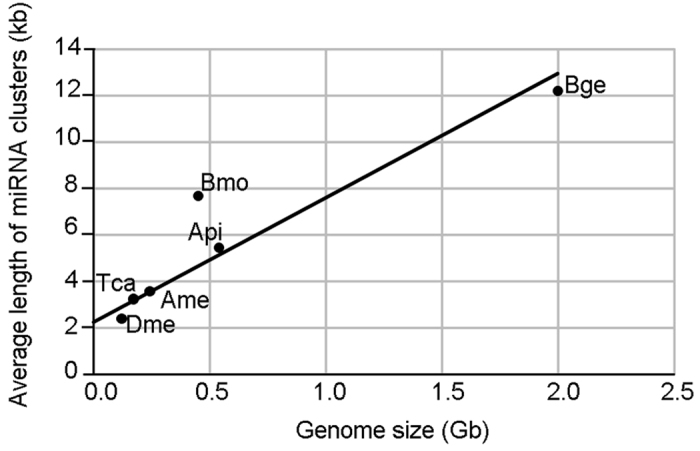
Correlation between genome size and average length of miRNA clusters. Considering the species *Blattella germanica* (Bge), *Acyrtosiphon pisum* (Api), *Apis mellifera* (Ame), *Tribolium castaneum* (Tca), *Bombyx mori* (Bmo) and *Drosophila melanogaster* (Dme), the correlation coefficient is notably high (R^2^ = 0.874). *Locusta migratoria* data was excluded from the analysis because the genome assembly of this species (with a total size of 5.8 Gbs) is composed by small scaffolds (N50 = 9.587 bp), which artificially split large clusters.

Moreover, we identified 13 miRNA clusters in the genome of *B. germanica*, whose organization is conserved across most of the species studied (Supplementary Table S7). *A. pisum* lost the entire cluster Mir-12/Mir-216, which contains two Mir-216 and one Mir-12 precursors in the other six species studied. Notably, this cluster is located in the X chromosome in *D. melanogaster*^22^. Other clusters lost are Mir-306/Mir-9 in *T. castaneum* and Mir-750 in *D. melanogaster*. At specific level, cluster length can be highly variable, like in *B. germanica*, where it ranges from 0.18 to 51.60 kb. Moreover, cluster length average in different species can vary between 2.39 ± 3.20 kb (n = 9) in *D. melanogaster* to 12.19 ± 15.82 kb (n = 13) in *B. germanica* (Supplementary Table S7). A representative example is the Mir-71/Mir-2 cluster, which is usually composed by one Mir-71 precursor followed by a number of Mir-2 precursors^23^. In *B. germanica* genome this cluster is formed by Mir-71 followed by five Mir-2 (=Mir-13) precursors^11^. However, a major difference with respect to other species is the distance between the second and third Mir-2 precursors, which in *B. germanica* exceeds 12 kb, whereas in other species this distance is much shorter (Fig. 7). Within the 12 kb cluster of *B. germanica* we have found a sequence homologous to “Tigger transposable element derived protein 4” (TIGD4) for which there are expression data, as it appears in tergal gland transcriptomes^24^. It is worth noting that many copies of TIGD4 are spread in the genome of *B. germanica*. Interestingly, cluster length appears to correlate with the genome size of the species. If we exclude the case of *L. migratoria*, where clusters length is probably underestimated due to the small scaffold size (N50 = 9,587, while in *B. germanica* N50 = 1,056,071), which provokes the splitting of large clusters, then there is a strong correlation (r^2^ = 0.874) between cluster length and genome size in the six other studied species (Fig. 8).

## Discussion

### Lessons from the *B. germanica* miRNA complement

Using highly stringent criteria^15^ to identify the miRNAs of *B. germanica*, we recovered the miRNAs conserved in other insects, and 11 novel miRNAs belonging to 8 novel miRNA families, out of 2,675 miRNA putative candidates resulting from prediction algorithms. Especially efficient was Dicer-1 depletion for candidate discrimination, as this approach allowed discarding 2,117 out of the 2,675 candidates in a first filter, results that are in concordance with those of other authors^25^,^26^,^27^. It is worth noting, however, that Dicer-1 depletion did not significantly affect 10% of the conserved miRNA, which might be Dicer-1 independent miRNAs^28^.

The mirPLOT bioinformatic tool developed to generate a graphical report for each miRNA has been a useful device. The report shows the precursor folding, the mature/star arms overlap, and the number of bases matched between both arms and the expression levels of each nucleotide in the precursor. On the basis of the graphical information initially generated by mirPLOT, each miRNA can be readily adjusted to show the right mature and star arms, and the correct 5′ or 3′ annotation.

Concerning the conserved miRNAs (89 miRNA genes belonging to 60 miRNA families), the only microRNA family that was expected to be found in hemimetabolan species^6^ but was not found is MIR-36 (which was neither found in *A. pisum*, see below). On the other hand, three families considered to have appeared in holometabolan insects^6^ were identified in *B. germanica* (MIR-932, MIR-3770 and MIR-6012). Additionally, we confirmed the new MIR-bg5 family in *B. germanica*, which we also subsequently found in *L. migratoria* and *A. pisum*.

With respect to the *B. germanica* novel, specific miRNAs, we found a total of 11 *bona fide* new miRNA loci belonging to 8 new miRNA families. These miRNAs are less expressed than the conserved ones, which is in agreement with other studies^29^,^30^,^31^, and have less potential targets than the conserved ones, but more potential targets than mock 22-nucleotide sequences. It has been suggested that a newly emerged miRNA is initially likely to target many genes simply by chance, because only short sequences are required for target recognition^2^,^31^. Our predictions with mock miRNAs afford quantitative data to this suggestion.

Intriguingly, the number of *B. germanica* specific miRNAs appears very low, in comparison with the 365 specific miRNAs (belonging to 312 families) of *L. migratoria*. This difference can be hardly explained by differences in genome size between *L. migratoria* and *B. germanica* (5.8 and 2.0 Gbp, respectively). Rather, the low number of specific miRNAs found *in B. germanica* must be mainly due to less extensive representation of tissues and ages in the libraries used in this species. Novel miRNAs are expressed generally at lower levels than conserved miRNAs and in specific tissues and/or stages^2^,^29^,^30^,^31^; and *B. germanica* libraries did not cover specific tissues and stages as important as nervous system and the embryonic development, for example.

### The miRNA toolkit of insects

Our analyses indicate that the complement of 65 conserved miRNA families is quite constant in the species studied, which are representative of the three more diverse insect suborders: Polyneoptera, Paraneoptera and Endopterygota, which contain 98% of insects species^32^, spanning about 390 my of insect evolution^21^. This complement varies from 52 miRNA families in *A. pisum* to 64 in *A. mellifera* (average 58.00 ± 3.65, n = 7) (Fig. 4). Equally, the number of miRNA genes in each miRNA family is notably stable. Most families have an average of 1 miRNA gene (37 families out of 65: 57%) or between 1 and 2 (18 families out of 65: 28%). Then, 5 families (8%) have between 2 and 3 genes, 4 families (6%) have between 3 and 5, and only one family (MIR-2) that have between 5 and 16 genes (Supplementary Table S8).

There is no correlation between the number of miRNA genes in a given family and the time of emergence of the family (MIR-2 family, which is the one that have more genes, appeared with the protostomes, whereas there are many monogenic miRNA families that emerged with the bilaterians) (Fig. 4). Thus, the quite constant and generally low number of genes in a family suggest that this number is evolutionarily constrained. Of note, this set of 65 miRNA families is independent of genome size, which varies from 0.12 Gbp in *D. melanogaster* (with 57 miRNA families) to 5.80 Gbp in *L. migratoria* (also with 57 miRNA families).

The last common ancestor of the species studied emerged some 390 million years ago^21^ and in this temporal frame, a total of four families have been acquired, three at the origin of endopterygotes, and one at the origin of coleopterans + panorpids) (Fig. 6). At species level, the losses of miRNA families range from 1 (considering the loss of MIR-bg5 in endopterygotes) in *A. mellifera* to 10 in *A. pisum* (Fig. 6). It is surprising that the emergence of the endopterygotes, the most successful group of metazoans on Earth in terms of diversity^33^, entailed the appearance of only three microRNAs: Mir-989, Mir-1006 and Mir-1007. One of the more derived features of this subclass and possibly the most influential for diversification, is the holometabolan mode of metamorphosis^34^, which implies key developmental divergences in the embryo stage^8^. Thus, further efforts to identify miRNAs in the embryo of holometabolan species should possibly increase the number of specific endopterygote miRNAs.

Concerning losses, data suggest that miRNAs that appeared later in evolution are more prone to disappear. For example, 7 out of 12 miRNA families of apterygotes (58%) (Fig. 4) were lost in one or other species and often in more than one species, whereas only 8 out of 29 miRNA families of bilaterians (28%) were lost in one or other species, but rarely in more than one species. The pea aphid *A. pisum* is notable for the large number of miRNA family losses (9 out of the 62 miRNAs families). Aphids display an XX/X0 sex-determination system and combine an unusual (autosome-like) inheritance of the X chromosome with the alternation of sexual and asexual reproduction. This has led to an accelerated evolution of sex chromosomes^35^ and to a masculinization of the X chromosome^36^. In *D. melanogaster*, three of the miRNA families lost in *A. pisum* (MIR-216, MIR-22 and MIR-12) are located on the X chromosome^22^. If they were also located in the X chromosome of *A. pisum*, then it would be plausible that these three families were lost in the context of the unusual evolution of the X chromosome. Finally, it is important to take into account that the absence of a given miRNA family in the complement of a given species can be also due to an incomplete genome and/or small RNA sequencing. Thus, it is always possible that some of the reported losses (Fig. 6) might be attributable to incomplete data.

Our analyses have also revealed that genomic miRNA clusters are conserved in the species studied, and that losses of some miRNA genes are associated to the loss of the entire cluster. Intriguingly, the size of the cluster is notably variable and correlate with genome size, which suggests that cluster size is not constrained by natural selection. Transposable elements could be one of the sources of the miRNA cluster expansions as we observed on *B. germanica* Mir-71/Mir-2 cluster.

Concerning specific miRNAs, it is premature to draw general trends, as the data in the different species is unequal. In principle, the number of specific miRNAs might be correlated with the more or less derived features of the species, but this does not fit with the data available, as the list is headed by *L. migratoria* with 365 miRNAs, followed by *T. castaneum* (127 miRNAs), *D. melanogaster* (65 miRNAs), *A. pisum* (34 miRNAs), *A. mellifera* (22 miRNAs), *B. mori* (14 miRNAs) and *B. germanica* (11 miRNAs). Again, sampling effort, in terms of tissues and stages, are the factors that by the moment determine the number of specific miRNA in each species. Possibly, genome size have also some influence, especially on the number of miRNAs rather than on the number of families, but data on specific miRNA is still too imperfect to establish correlations.

From a practical point of view, the accurate establishment of the miRNA toolkit in a given clade has predictive power, thus facilitating the empirical identification of the conserved miRNA complement in a given species of the clade. From an evolutionary point of view, the miRNA toolkit can enable comparisons between different clades of the tree of life using comparable data, and derive possible correlations between changes in the toolkit and changes in the organization and complexity during evolution.

## Methods

### Insect Colony

*B. germanica* specimens used were from a colony reared in the dark at 29 ± 1 °C and 60–70% r.h. Dissections and tissue sampling were carried out on carbon dioxide-anesthetized specimens. Tissues were frozen on liquid nitrogen and stored at −80 °C until use.

### miRNA prediction

We used the 7 small RNA-seq libraries previously produced in our laboratory^14^, available at Gene Expression Omnibus repository^37^ with accession number GSE22892. These libraries include the small RNAs from whole body and ovaries of different developmental stages from *B. germanica* females (Supplementary Table S9). The reads were mapped to *B. germanica* genome assembly (https://www.hgsc.bcm.edu/arthropods/german-cockroach-genome-project) with Bowtie2^38^. The sam files were converted to the binary format (bam) with SAMtools^39^. The bam files together with the genome assembly were used as input for mirDeep*^40^ which predicted miRNA candidates. Additionally, we ran another miRNA prediction tool, a modified version of mirDeep2 (https://www.mdc-berlin.de/8551903/en)^18^ using a file with unique reads between 16–26 nucleotides from the 7 small RNAseq libraries. The results of both predictions were merged in a single file of miRNA candidates.

### Identification of conserved miRNAs on *B. germanica*

The miRNAs predicted from *B. germanica* were blasted against miRBase mature insect sequences. Those predictions showing an alignment longer than 18 nucleotides with a maximum of a single mismatch were considered conserved miRNAs. For each conserved miRNA we retrieved the corresponding precursor from the genome assembly and the complementary arm (star). To avoid misidentifications due to assembly artefacts, we examined all cases where two miRNAs from different loci had the same precursor sequence. In these cases, we retrieved the precursor plus 50 flanking nucleotides, aligned them and, if a perfect identity of the extended precursors was found, we considered that there was an assembly artefact. We grouped the conserved miRNA genes in miRNA families on the basis of sequence similarity, especially in the seed region. When a family that should be present in a species according to phylogenetic criteria^6^ was not found, we carried out specific analyses to confirm the apparent absence. First, we assessed that there were no reads corresponding to the missing miRNA in the available small RNAseq data. Additionally, we used the miRNA precursor sequence of other insect species where it was available, to carry out homology searches on the *B. germanica* genome assembly in order to assess that there were no significant hits.

### miRNA libraries from Dicer-1-depleted specimens

We had demonstrated that a decrease of Dicer-1 by RNAi results in the decrease of mature miRNA levels^19^. This was useful to validate the first canonical mature miRNAs and a few mature novel miRNA in *B. germanica*^14^. Following this approach, 5-day-old fifth instar female nymphs were treated with a 3 μg of dsBgDicer-1 twice, the first dose applied just after the emergency and the second 3 days later. RNA extraction in these animals was carried out 2 days after molting to the last (sixth) nymphal instar^11^. As control dsRNA (dsMock), was used a fragment (300 bp) of the sequence of *Autographa californica nucleopolyhedrovirus* (GenBank: K01149)^41^, at the same dose and conditions. qRT-PCR measurements indicated that Dicer-1 depletion *in vivo* results in reduced levels of mature miRNAs^11^, as occurred in previous studies^19^. Then, the whole body of dsBgDicer-1-treated and dsMock-treated nymphs was processed to construct the respective small RNA libraries. Total small RNAs were isolated using miRNeasy Mini Kit (Qiagen), and the libraries were prepared with 500 ng of total small RNAs using the NEBNext^®^ Small RNA Library Prep Set for Illumina^®^ (Multiplex Compatible) (New England BioLabs). The samples were multiplexed in a single flow cell and sequenced in 30-cycles paired-end run of Illumina Mi-Seq. Low quality reads and adapters were removed with Trimmomatic^42^. Then, the reads were mapped against the *B. germanica* genome assembly using the Bowtie2^38^. And the number of reads for each miRNA candidate was retrieved with the R version of FeatureCounts package^43^. Count number was transformed into reads per million reads (RPM), and the base 2 logarithm of the fold change (log2(FC)) between control libraries and Dicer-1-depleted libraries was computed. To ensure that the decrease of miRNAs expression on the RNA-seq libraries was due to Dicer-1 depletion and not to a normalization effect, we computed the log2(FC) of other non-coding RNAs that should not be processed by Dicer-1. Since tRNA or rRNAs, which were used in similar studies^25^, are not annotated in *B. germanica* genome, we used genomic sequences shorter than 500 nucleotides that were completely covered by at least 10 reads in each control library, which did contain neither miRNAs nor coding regions.

### Identification of novel, specific miRNAs

To be considered a *bona fide* novel miRNA, candidates had to meet the following 7 criteria^6^,^15^. 1) Expression data of both arms (mature and star). 2) Expression reduction in Dicer-1 depleted libraries (log2(FC) <−0.5). 3) Homogeneity in the 5′ extreme of the miRNA reads. 4) Secondary structure of the precursor (predicted with mfold^44^) showing the typical miRNA hairpin. 5) Secondary structure with free energy InitialdG <−22 Kcal/mol (the minimum free energy we observed in the conserved miRNAs). 6) More than 16 nucleotides paired between the mature and star sequence. 7) Two bases overhang from each arm (resulting from to Dicer-1/Drosha cleavage). Then, the novel miRNAs were grouped into families on the basis of their seed sequence. The nomenclature used for novel miRNAs is the usually followed for previously described miRNAs but using, as number designator, the code “bg” (from *Blattella germanica*) followed by the number assigned to the novel miRNA. Thus, the most abundantly expressed *bona fide* novel miRNA has been named “Bge-Mir-bg1”, and the corresponding family “MIR-bg1”. The comparison of the expression between specific miRNAs and conserved miRNAs was carried out in the control libraries, considering the total number of reads mapped to the mature miRNAs; statistical differences between the two groups were evaluated with the Welch’s T-test. Computation of the mature sequences length, loop length and nucleotide frequencies in the mature miRNA sequences was performed with the Shortread package^45^.

### Development of mirPLOT software

To generate a graphical support showing the precursor folding, the mature/star arms overlapping, the number of bases matched between both arms and the expression levels of each nucleotide in the precursor, we developed a mirPLOT software. The paired-end reads were assembled using the PEAR software^46^. Then, RNA fragments between 18–26 nucleotides were selected and aligned to the miRNA precursor sequences extended with 30 flanking nucleotides. Bowtie^47^ was used to map the RNA fragments on the extended precursors, forcing perfect matches in the seed region. The miRPLOT software generated the report, enriched with the mature arm alignments against other insect’s miRNAs and the number of reads from controls and Dicer-1-depleted libraries.

### Examination of other insect miRNAs

Other hemimetabolan insects for which the miRNA complement had been studied combining small RNA-seq and genomic data are the hemipteran *A. pisum* and the orthopteran *L. migratoria*. To assess their respective miRNA complements, we followed the same approach as in the case of *B. germanica*. In *A. pisum* we analysed the 176 miRNA precursors reported by Legeai *et al*.^16^ and the small RNA-seq data made public (SRX016814) by the same authors. For *L. migratoria,* we analysed the 833 miRNA candidates previously reported by Wang *et al*.^13^. For these analyses we used the precursor sequences provided by Wang *et al*. and the small RNA-seq data made public (SRP062155) by the same authors. As holometabolan species, we analysed the miRNAs available in miRBase of *A. mellifera* (254 miRNAs), *B. mori* (488 miRNAs) and *T. castaneum* (227 miRNAs), to which 123 miRNAs were recently added^17^. For each species we used available small RNA-seq data publicly available (Supplementary Table S5). In the case of *D. melanogaster,* we used the miRNAs reported by Fromm *et al*.^15^ which were filtered with the criteria used in the present work.

### Prediction of miRNAs targets

We identified potential miRNA targets on the protein coding genes in the *B. germanica* genome using RNAhybrid^48^, miRanda^49^, and RNA22^50^ algorithms. As potential targets, we accepted those predicted by the three algorithms, using a free energy threshold of −12 Kcal/mol in all cases. Although combination of miRNA target predictions is not always recommended^51^, the general scope of the analysis and the large number of potential targets expected led us to choose these high levels of astringency.

## Additional Information

**How to cite this article**: Ylla, G. *et al*. The microRNA toolkit of insects. *Sci. Rep.* **6**, 37736; doi: 10.1038/srep37736 (2016).

**Publisher’s note:** Springer Nature remains neutral with regard to jurisdictional claims in published maps and institutional affiliations.

## Supplementary Material

Supplementary Information

Supplementary Table 1

Supplementary Table 2

Supplementary Table 3

Supplementary Table 5

Supplementary Table 6

Supplementary Table 7

Supplementary Table 8

## Figures and Tables

**Table 1:** Number of reads obtained in the four small RNA-seq libraries from the Dicer-1 depletion experiment.

Library	Raw read pairs	Readpairs after Trimmomatic filtering	Mapped read pairs
Control 1	5,319,915	5,266,309	4,005,167
Control 2	4,324,534	4,237,459	3,448,926
Dicer-depleted 1	5,689,294	5,652,183	4,368,767
Dicer-depleted 2	4,520,535	4,483,662	3,403,451

Fifth-day-old fifth instar female nymphs were treated with a 3 μg of dsBgDicer-1 twice, first just after the emergency and then 3 days later. RNA was extracted 2 days after molting to the last (sixth) nymphal instar. As control dsRNA, we used a fragment (300 bp) of the sequence of *Autographa californica nucleopolyhedrovirus* (GenBank: K01149), using the same dose and conditions.

**Table 2:** The 13 novel miRNA genes identified in *Blattella germanica*.

miRNA	Family	Mature sequence
Bge-Mir-bg1	MIR-bg1	TGACTCCAGACCTTGTTGCTGA
Bge-Mir-bg2	MIR-bg2	TCGGACGAAGTGCACTTATTTACGT
Bge-Mir-bg3a	MIR-bg3	A**TGAAAT**GGACGATTGGCTGTG
Bge-Mir-bg3b	MIR-bg3	T**TGAAAT**GGACGATTGTCTGTG
Bge-Mir-bg4a	MIR-bg4	T**ACATAA**CCGCAATCACCGATT
Bge-Mir-bg4b	MIR-bg4	T**ACATAA**CCGCAACCACCGACT
Bge-Mir-bg4c	MIR-bg4	T**ACATAA**CCGCAACCACCGACT
Bge-Mir-bg5a	MIR-bg5	T**GTGATG**TGCATGTGGGCTTTCC
Bge-Mir-bg5b	MIR-bg5	T**GTGATG**TGCATGTGGGCTTTCC
Bge-Mir-bg6	MIR-bg6	TCACAACTTTCTGTCCAGAAC
Bge-Mir-bg7	MIR-bg7	CTACGAACCAGAATGACATCGCG
Bge-Mir-bg8	MIR-bg8	CATAGGCGCTATTTCCTCTGCC
Bge-Mir-bg9	MIR-bg9	CATTCTTCCTAGAATGGTCCGT

They are grouped into families according to the seed sequence (indicated in bold in multimember families). The MIR-bg5 was initially discovered in *B. germanica*, but we have identified it also in *Acyrthosiphon pisum* and *Locusta migratoria*.

## References

[b1] Bartel D. P. MicroRNAs: genomics, biogenesis, mechanism, and function. Cell 116, 281–297 (2004). 14744438 10.1016/s0092-8674(04)00045-5

[b2] Berezikov E. Evolution of microRNA diversity and regulation in animals. Nature reviews. Genetics 12, 846–860 (2011). 10.1038/nrg3079 22094948

[b3] Bushati N. & Cohen S. M. microRNA functions. Annual review of cell and developmental biology 23, 175–205 (2007). 10.1146/annurev.cellbio.23.090506.123406 17506695

[b4] Herranz H. & Cohen S. M. MicroRNAs and gene regulatory networks: managing the impact of noise in biological systems. Genes and Development 24, 1339–1344 (2010). 20595229 10.1101/gad.1937010 PMC2895193

[b5] Peterson K. J., Dietrich M. R. & McPeek M. A. MicroRNAs and metazoan macroevolution: insights into canalization, complexity, and the Cambrian explosion. BioEssays: news and reviews in molecular, cellular and developmental biology 31, 736–747 (2009). 10.1002/bies.200900033 19472371

[b6] Tarver J. E. . miRNAs: small genes with big potential in metazoan phylogenetics. Molecular biology and evolution 30, 2369–2382 (2013). 23913097 10.1093/molbev/mst133

[b7] Grimaldi D. & Engel M. S. Evolution of the Insects. (Cambridge University Press, 2005).

[b8] Belles X. Origin and Evolution of Insect Metamorphosis. In Encyclopedia of Life Sciences (ELS) 1–11 (John Wiley & Sons, 2011).

[b9] Belles X., Cristino A. S., Tanaka E. D., Rubio M. & Piulachs M.-D. In Insect Molecular Biology and Biochemistry (ed. Gilbert L. I.) 30–56 (Elsevier-Academic Press, 2011).

[b10] Lucas K. L., Zhao B., Liu L. & Raikhel A. S. Regulation of physiological processes by microRNAs in insects. Current Opinion in Insect Science 11, 1–7 (2015). 26251827 10.1016/j.cois.2015.06.004 PMC4522942

[b11] Lozano J., Montanez R. & Belles X. MiR-2 family regulates insect metamorphosis by controlling the juvenile hormone signaling pathway. Proceedings of the National Academy of Sciences of the United States of America 112, 3740–3745 (2015). 25775510 10.1073/pnas.1418522112 PMC4378413

[b12] Kozomara A. & Griffiths-Jones S. miRBase: integrating microRNA annotation and deep-sequencing data. Nucleic Acids Research 39, D152–157 (2011). 21037258 10.1093/nar/gkq1027 PMC3013655

[b13] Wang Y. . Evidence for the expression of abundant microRNAs in the locust genome. Scientific Reports 5, 13608 (2015). 26329925 10.1038/srep13608 PMC4556993

[b14] Cristino A. S., Tanaka E. D., Rubio M., Piulachs M. D. & Belles X. Deep sequencing of organ- and stage-specific microRNAs in the evolutionarily basal insect *Blattella germanica* (L.) (Dictyoptera, Blattellidae). PLoS One 6, e19350 (2011). 21552535 10.1371/journal.pone.0019350 PMC3084283

[b15] Fromm B. . A Uniform System for the Annotation of Vertebrate microRNA Genes and the Evolution of the Human microRNAome. Annual Review of Genetics 49, 213–242 (2015). 10.1146/annurev-genet-120213-092023 PMC4743252 26473382

[b16] Legeai F. . Bioinformatic prediction, deep sequencing of microRNAs and expression analysis during phenotypic plasticity in the pea aphid, *Acyrthosiphon pisum*. BMC Genomics 11, 281 (2010). 20444247 10.1186/1471-2164-11-281 PMC2880305

[b17] Ninova M., Ronshaugen M. & Griffiths-Jones S. MicroRNA evolution, expression, and function during short germband development in *Tribolium castaneum*. Genome research 26, 85–96 (2016). 26518483 10.1101/gr.193367.115 PMC4691753

[b18] Fromm B., Worren M. M., Hahn C., Hovig E. & Bachmann L. Substantial loss of conserved and gain of novel MicroRNA families in flatworms. Molecular biology and evolution 30, 2619–2628 (2013). 24025793 10.1093/molbev/mst155 PMC3840308

[b19] Gomez-Orte E. & Belles X. MicroRNA-dependent metamorphosis in hemimetabolan insects. Proceedings of the National Academy of Sciences of the United States of America 106, 21678–21682 (2009). 19966227 10.1073/pnas.0907391106 PMC2799836

[b20] Lau N. C., Lim L. P., Weinstein E. G. & Bartel D. P. An abundant class of tiny RNAs with probable regulatory roles in *Caenorhabditis elegans*. Science 294, 858–862 (2001). 11679671 10.1126/science.1065062

[b21] Misof B. . Phylogenomics resolves the timing and pattern of insect evolution. Science 346, 763–767 (2014). 25378627 10.1126/science.1257570

[b22] Christodoulou F. . Ancient animal microRNAs and the evolution of tissue identity. Nature 463, 1084–1088 (2010). 20118916 10.1038/nature08744 PMC2981144

[b23] Marco A., Hooks K. & Griffiths-Jones S. Evolution and function of the extended miR-2 microRNA family. RNA Biology 9, 242–248 (2012). 22336713 10.4161/rna.19160 PMC3384581

[b24] Ylla G. & Belles X. Towards understanding the molecular basis of cockroach tergal gland morphogenesis. A transcriptomic approach. Insect Biochemistry and Molecular Biology 63, 104–112 (2015). 26086932 10.1016/j.ibmb.2015.06.008

[b25] Friedlander M. R., Mackowiak S. D., Li N., Chen W. & Rajewsky N. miRDeep2 accurately identifies known and hundreds of novel microRNA genes in seven animal clades. Nucleic Acids Research 40, 37–52 (2012). 21911355 10.1093/nar/gkr688 PMC3245920

[b26] Li N. . Global profiling of miRNAs and the hairpin precursors: insights into miRNA processing and novel miRNA discovery. Nucleic Acids Research 41 (2013). 10.1093/nar/gkt072 PMC3616697 23396444

[b27] Langenberger D., Cakir M. V., Hoffmann S. & Stadler P. F. Dicer-processed small RNAs: rules and exceptions. Journal of Experimental Zoology Part B Molecular and Developmental Evolution 320, 35–46 (2013). 10.1002/jez.b.22481 23165937

[b28] Yang J. S. & Lai E. C. Alternative miRNA biogenesis pathways and the interpretation of core miRNA pathway mutants. Molecular Cell 43, 892–903 (2011). 21925378 10.1016/j.molcel.2011.07.024 PMC3176435

[b29] Liang H. & Li W. H. Lowly expressed human microRNA genes evolve rapidly. Molecular biology and evolution 26, 1195–1198 (2009). 19299536 10.1093/molbev/msp053 PMC2727378

[b30] Franca G. S., Vibranovski M. D. & Galante P. A. Host gene constraints and genomic context impact the expression and evolution of human microRNAs. Nature communications 7, 11438 (2016). 10.1038/ncomms11438 PMC4848552 27109497

[b31] Chen K. & Rajewsky N. The evolution of gene regulation by transcription factors and microRNAs. Nature reviews. Genetics 8, 93–103 (2007). 10.1038/nrg1990 17230196

[b32] Mayhew P. J. Why are there so many insect species? Perspectives from fossils and phylogenies. Biological reviews of the Cambridge Philosophical Society 82, 425–454 (2007). 17624962 10.1111/j.1469-185X.2007.00018.x

[b33] Kristensen N. P. Phylogeny of endopterygote insects, the most successful lineage of living organisms. European Journal of Entomology 96, 237–254 (1999).

[b34] Rainford J. L., Hofreiter M., Nicholson D. B. & Mayhew P. J. Phylogenetic distribution of extant richness suggests metamorphosis is a key innovation driving diversification in insects. PLoS One 9, e109085 (2014). 25275450 10.1371/journal.pone.0109085 PMC4183542

[b35] Jaquiery J. . Accelerated evolution of sex chromosomes in aphids, an x0 system. Molecular biology and evolution 29, 837–847 (2012). 21998277 10.1093/molbev/msr252

[b36] Jaquiery J. . Masculinization of the x chromosome in the pea aphid. PLoS Genetics 9, e1003690 (2013). 23950732 10.1371/journal.pgen.1003690 PMC3738461

[b37] Edgar R., Domrachev M. & Lash A. E. Gene Expression Omnibus: NCBI gene expression and hybridization array data repository. Nucleic Acids Research 30, 207–210 (2002). 11752295 10.1093/nar/30.1.207 PMC99122

[b38] Langmead B. & Salzberg S. L. Fast gapped-read alignment with Bowtie 2. Nature Methods 9, 357–359 (2012). 22388286 10.1038/nmeth.1923 PMC3322381

[b39] Li H. . The Sequence Alignment/Map format and SAMtools. Bioinformatics 25, 2078–2079 (2009). 19505943 10.1093/bioinformatics/btp352 PMC2723002

[b40] Anders S. . Count-based differential expression analysis of RNA sequencing data using R and Bioconductor. Nature Protocols 8, 1765–1786 (2013). 23975260 10.1038/nprot.2013.099

[b41] Lozano J. & Belles X. Conserved repressive function of Kruppel homolog 1 on insect metamorphosis in hemimetabolous and holometabolous species. Scientific Reports 1, 163 (2011). 22355678 10.1038/srep00163 PMC3240953

[b42] Bolger A. M., Lohse M. & Usadel B. Trimmomatic: a flexible trimmer for Illumina sequence data. Bioinformatics 30, 2114–2120 (2014). 24695404 10.1093/bioinformatics/btu170 PMC4103590

[b43] Liao Y., Smyth G. K. & Shi W. featureCounts: an efficient general purpose program for assigning sequence reads to genomic features. Bioinformatics 30, 923–930 (2014). 24227677 10.1093/bioinformatics/btt656

[b44] Zuker M. Mfold web server for nucleic acid folding and hybridization prediction. Nucleic Acids Research 31, 3406–3415 (2003). 12824337 10.1093/nar/gkg595 PMC169194

[b45] Morgan M. . ShortRead: a bioconductor package for input, quality assessment and exploration of high-throughput sequence data. Bioinformatics 25, 2607–2608 (2009). 19654119 10.1093/bioinformatics/btp450 PMC2752612

[b46] Zhang J., Kobert K., Flouri T. & Stamatakis A. PEAR: a fast and accurate Illumina Paired-End reAd mergeR. Bioinformatics 30, 614–620 (2014). 24142950 10.1093/bioinformatics/btt593 PMC3933873

[b47] Langmead B., Trapnell C., Pop M. & Salzberg S. L. Ultrafast and memory-efficient alignment of short DNA sequences to the human genome. Genome Biology 10, R25 (2009). 19261174 10.1186/gb-2009-10-3-r25 PMC2690996

[b48] Kruger J. & Rehmsmeier M. RNAhybrid: microRNA target prediction easy, fast and flexible. Nucleic Acids Research 34, W451–454 (2006). 16845047 10.1093/nar/gkl243 PMC1538877

[b49] Enright A. J. . MicroRNA targets in *Drosophila*. Genome Biology 5, R1 (2003). 14709173 10.1186/gb-2003-5-1-r1 PMC395733

[b50] Loher P. & Rigoutsos I. Interactive exploration of RNA22 microRNA target predictions. Bioinformatics 28, 3322–3323 (2012). 23074262 10.1093/bioinformatics/bts615

[b51] Alexiou P., Maragkakis M., Papadopoulos G. L., Reczko M. & Hatzigeorgiou A. G. Lost in translation: an assessment and perspective for computational microRNA target identification. Bioinformatics 25, 3049–3055 (2009). 19789267 10.1093/bioinformatics/btp565

