# Supplementary material for: The microRNA toolkit of insects
Source: Sci Rep. 2016 Nov 24;6:37736. doi: 10.1038/srep37736 (PMC5121899; doi:10.1038/srep37736)
Supplement: Supplementary Information [file srep37736-s1.pdf]

The microRNA toolkit of insects

Guillem Ylla, Bastian Fromm, Maria-Dolors Piulachs, Xavier Belles

SUPPLEMENTARY PDF FILES

Supplementary Data S1. Report generated with MirPlot of the 88 conserved miRNAs of *Blattella germanica*.

Supplementary Data S2. Report generated with MirPlot of the 11 specific miRNAs of *Blattella germanica*.

Supplementary Data S3. Report generated with MirPlot for the 833 *Locusta migratoria* miRNA candidates from Wang et al. (2015).

Supplementary Data S4. Report generated with MirPlot of the 176 *Acyrtosiphon pisum* miRNA candidates from Legeai et al. (2010) plus the 10 newly found miRNAs.

Supplementary Data S5. Report generated with MirPlot of the 254 miRNAs of *Apis mellifera* available in miRBase.

Supplementary Data S6. Report generated with MirPlot for the 342 miRNAs of *Tribolium castaneum* from miRBase and Ninova et al. (2016).

Supplementary Data S7. Report generated with MirPlot for the 554 miRNAs of *Bombyx mori* available in miRBase.

Supplementary Table S4. Currently available on insect miRNAs in miRBase 21.

Supplementary Table S9. Description of the *Blattella germanica* small RNA-seq libraries used for the miRNA predictions. The accession number of each library, the number of raw reads and the number of mapped reds is shown.

Supplementary Fig. S1. Structure of the Mir-133 precursor in *Acyrtosyphon pisum*. The mature miRNA is indicated in red and the star miRNA in blue. Note the hairpin loop comprising about 200 nucleotides.

Supplementary Fig. S2. Alignment of Mir-1007 precursor of *Drosophila melanogaster* (Dme-Mir-1007) and Mir-6037 of *Apis mellifera* (Ame-Mir-6037).

Supplementary Data S1. Report generated with MirPlot of the 88 conserved miRNAs of *Blattella germanica* . For each miRNA the following information is shown:

- The miRNA precursor plus 11 flanking nucleotides, with the star arm colored blue and mature arm colored red.
- The 5p and 3p arms, colored red the mature and in blue color and an asterisk on the name for the star.
- The precursor folding in Vienna format.
- The number of complementary bases between the mature and star arms.
- The number of reads of both arms in control and dsDicer-1 treated libraries.
- The free energy of the precursor folding.
- The folding of the miRNA precursor with colored mature and star arms.
- The alignment of the mature with homologous miRNA from other species.
- The genomic coordinates of the precursor and both arms for the *B.germancia* genome assembly.
- A plot showing all the expressed nucleotides of the precursor (grey), the reads per million in control libraries (green) and in dsDicer-1 libraries (purple).

>Bge-Mir-29b_pre+_11
TGCTTGAGGAC**TGGTTTCAAGTTGGTGGTAAG**ATTCAGCTTCATTCAAAACTC**TAGCACCATTTGAAATCAGT**GCTTCAGTAGG

>Bge-Mir-29b_3p
TAGCACCATTTGAAATCAGT

>Bge-Mir-29b_5p*
TGGTTTCAAGTTGGTGGTAAG

```
UGCUUGAGGACUGGUUUCAAGUUGGUGGUAAGAUUCAGCUUCAUUCAAAACUCUAGCACCAUUUGAAAUCAGUGCUUCAGUAGG
((((.(((.(((((((((((((.((((.((.((..................)))).))))))))))))))))).))).)))).. (-29.20)
```

Matched bases: 17

Mature reads in Control: 3   Mature reads in dsDicer: 0
Star reads in Control: 0   Star reads in dsDicer: 0

Initial dG =   -29.20

```
          10        20        30        40
--|   U   G             U    G  A  UUCAGCUU
  UGCU GAG ACUGGUUUCAAGU GGUG UA GA        C
  AUGA UUC UGACUAAAGUUUA CCAC AU CU        A
GG^   C   G             -    G  -  CAAAACUU
   80        70         60         50
```

```
Bge-Mir-29b      UAGCACCAUUUGAAAUCAGU
ame-mir-29b      UAGCACCAUUUGAAAUCAGU
nvi-mir-29b      UAGCACCAUUUGAAAUCAGU
                 ********************
```

Genomic coordinates:
Bge-Mir-29b_3p: Scaffold1018:201471-201490:-
Bge-Mir-29b_pre: Scaffold1018:201471-201532:-
Bge-Mir-29b_5p*: Scaffold1018:201512-201532:-

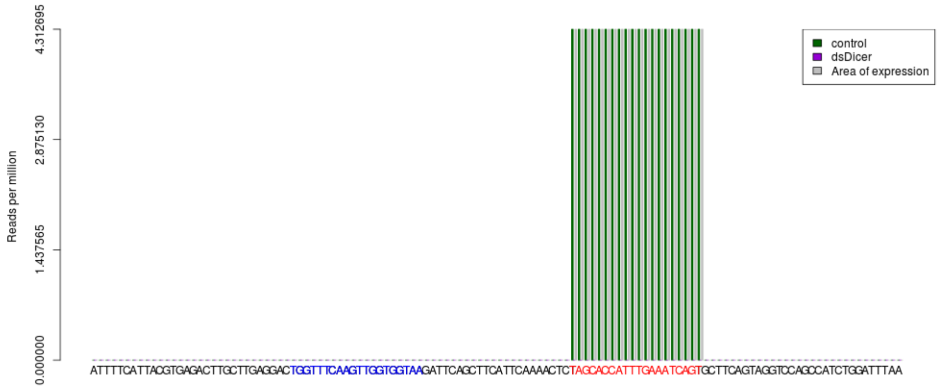

>Bge-Mir-932_pre+_11
GTCCTTCTACT**TCAATTCCGTAGTGCATTGCAGT**GTTTGATGTAGATC**TGCAAGCAGTGCGGAAGTGAGG**TGGAAGAGGCT

>Bge-Mir-932_3p
TGCAAGCAGTGCGGAAGTGAGG

>Bge-Mir-932_5p*
TCAATTCCGTAGTGCATTGCAGT

```
GUCCUUCUACUUCAAUUCCGUAGUGCAUUGCAGUGUUUGAUGUAGAUCUGCAAGCAGUGCGGAAGUGAGGUGGAAGAGGCU
(((((((((((((.(((((((.(((.((((((.(((((...)))))))))))))))).))))))).))))))))))).))). (-44.00)
```

Matched bases: 18

Mature reads in Control: 4
Star reads in Control: 2

Mature reads in dsDicer: 2
Star reads in dsDicer: 2

Initial dG = -44.00

```
         10        20        30        40
-   -|         A       G   A      U     A
 GUC CUUCUACUUCA UUCCGUA UGC UUGCAG GUUUG \
 CGG GAAGGUGGAGU AAGGCGU ACG AACGUC UAGAU U
U   A^         G       G   -      -     G
80       70        60         50
```

```
Bge-Mir-932         UGCAAGCAGUGCGGAAGUGAGG
tca-mir-932-3p      UGCAAGCAGUGCGGAAGUGAG-
dme-mir-932-3p      UGCAAGCGCUGCGGAUUUGGCA
                    *******  ******  **
```

Genomic coordinates:
Bge-Mir-932_5p*: Scaffold104:302165-302187:+
Bge-Mir-932_pre: Scaffold104:302165-302223:+
Bge-Mir-932_3p: Scaffold104:302202-302223:+

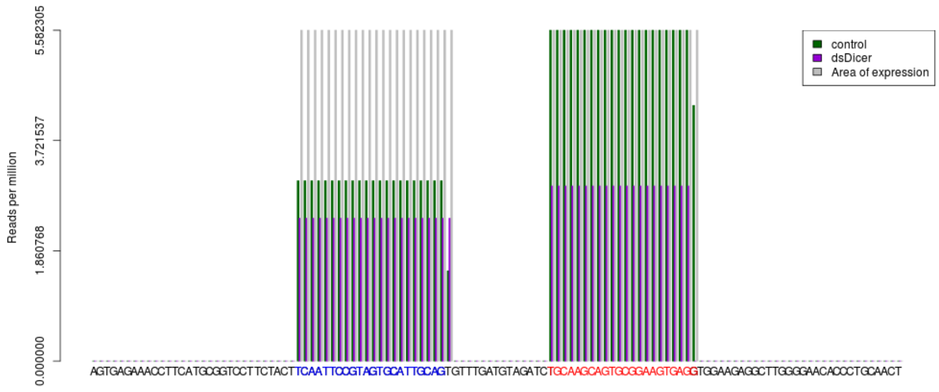

>Bge-Mir-281_pre+_11
TCCGCACTATC**AAGAGAGCTATCCGTCGACAGT**ATTGTTTATAAAC**TGTCATGGAGTTGCTCTCTTTA**TGGACGGGCTG

>Bge-Mir-281_3p
TGTCATGGAGTTGCTCTCTTTA

>Bge-Mir-281_5p*
AAGAGAGCTATCCGTCGACAGT

```
UCCGCACUAUCAAGAGAGCUAUCCGUCGACAGUAUUGUUUAUAAACUGUCAUGGAGUUGCUCUCUUUAUGGACGGGCUG
((((..((((.((((((((..(((((.((((((...........)))))))))))...)))))))).)))).))))... (-33.50)
```

Matched bases: 17

Mature reads in Control: 913   Mature reads in dsDicer: 281
Star reads in Control: 862   Star reads in dsDicer: 184

Initial dG =   -33.50

```
           10        20          30
---|   CA    C        UA-      C       AUUGU
   UCCG  CUAU AAGAGAGC   UCCGU GACAGU      \
   GGGC  GGUA UUCUCUCG   AGGUA CUGUCA      U
GUC^   A-    U        UUG      -       AAUAU
         70        60          50        40
```

```
Bge-Mir-281         --UGUCAUGGAGUUGCUCUCUUUA-
tca-mir-281-3p      --UGUCAUGGAGUUGCUCUCUUU--
nvi-mir-281         --UGUCAUGGACUUGCUCUCUUUGU
dme-mir-281-3p      --UGUCAUGGAAUUGCUCUCUUUGU
ame-mir-281         --UGUCAUGGAGUUGCUCUCUUUGU
api-mir-281         --UGUCAUGGAGUUGCUCUCUUU--
bmo-mir-281-3p      ACUGUCAUGGAGUUGCUCUCUU---
mse-mir-281         -CUGUCAUGGAGUUGCUCUCUUU--
                      ********* **********
```

Genomic coordinates:
Bge-Mir-281_5p: Scaffold1129:274505-274526:+
Bge-Mir-281_pre: Scaffold1129:274505-274561:+
Bge-Mir-281_3p*: Scaffold1129:274540-274561:+

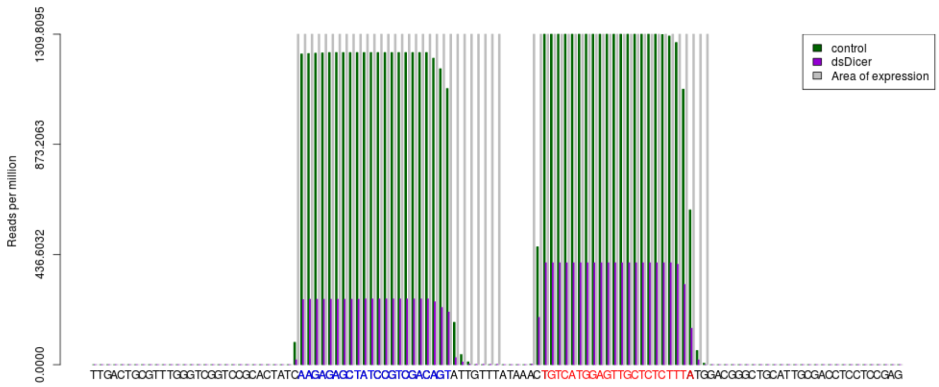

>Bge-Mir-279_pre+_11
CTACCTCAGGT**GACGGATGTTGCTCTGGTGCACG**TTATATGAATAGTCG**TGACTAGAGTGACACTCGTCGA**CTGAGGAAGTC

>Bge-Mir-279_3p
TGACTAGAGTGACACTCGTCGA

>Bge-Mir-279_5p*
GACGGATGTTGCTCTGGTGCACG

```
CUACCUCAGGUGACGGAUGUUGCUCUGGUGCACGUUAUAUGAAUAGUCGUGACUAGAGUGACACUCGUCGACUGAGGAAGUC
((.((((((.(((((..(((..(((((((.((((((((....)))).)))))))))))..)))..))))).)))))).)).. (-43.80)
```

Matched bases: 16

Mature reads in Control: 547   Mature reads in dsDicer: 296
Star reads in Control: 127   Star reads in dsDicer: 26

Initial dG =   -43.80

```
          10        20        30         40
--| A      G      GA    UG        G     -     A
  CU CCUCAG UGACG  UGU  CUCUGGU CACG UUAU U
  GA GGAGUC GCUGC  ACA  GAGAUCA GUGC GAUA G
CU^ A      A      UC    GU        -     U     A
 80        70        60          50
```

```
nvi-mir-279         UGACUAGAUCCACACUCAUUAA
dme-mir-279-3p      UGACUAGAUCCACACUCAUUAA
Bge-Mir-279         UGACUAGAGUGACACUCGUCGA
                    ********   ****** *  *
```

Genomic coordinates:
Bge-Mir-279_5p*: Scaffold1178:243923-243945:+
Bge-Mir-279_pre: Scaffold1178:243923-243982:+
Bge-Mir-279_3p: Scaffold1178:243961-243982:+

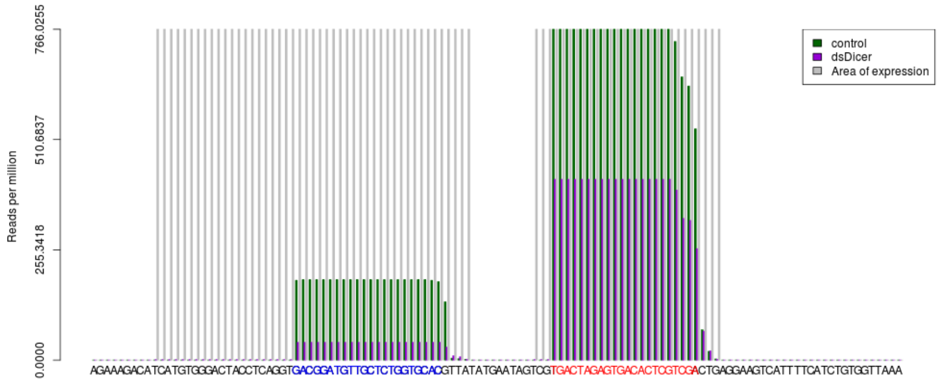

>Bge-Mir-252a_pre+_11
CTCTTCCGTTC**CTAAGTACTAGTGCCGCAGGAG**TGTGCTTGAGTAATTTCT**CCTGCTGCTCAAGTGCTTATCA**ATGGGAAGGGA

>Bge-Mir-252a_5p
CTAAGTACTAGTGCCGCAGGAG

>Bge-Mir-252a_3p*
CCTGCTGCTCAAGTGCTTATCA

```
CUCUUCCGUUCCUAAGUACUAGUGCCGCAGGAGUGUGCUUGAGUAAUUUCUCCUGCUGCUCAAGUGCUUAUCACUGAGGAAGUC
((((((((((..((((((((...((.(((((((..(((....)))....))))))).))...))))))))..))))).))))). (-37.00)
```

Matched bases: 15

Mature reads in Control: 472   Mature reads in dsDicer: 345
Star reads in Control: 5   Star reads in dsDicer: 1

Initial dG =   -37.00

```
          10        20        30           40
-     -     CC        AGU   C       UG--|  U
 CUCUU CCGUU  UAAGUACU   GC GCAGGAG     UGC U
 GGGAA GGUAA  AUUCGUGA   CG CGUCCUC     AUG G
A     G     CU        ACU   U       UUUA^  A
   80        70        60        50
```

```
Bge-Mir-252a         CUAAGUACUAGUGCCGCAGGAG
tca-mir-252a-5p      CUAAGUACUAGUGCCGCAGG--
api-mir-252a         CUAAGUACUAGUGCCGCGGGAG
ame-mir-252a         AUAAGUACUAGUGCCGCAGGAG
                      **************** **
```

Genomic coordinates:
Bge-Mir-252a_3p*: Scaffold1231:310874-310895:-
Bge-Mir-252a_pre: Scaffold1231:310874-310935:-
Bge-Mir-252a_5p: Scaffold1231:310914-310935:-

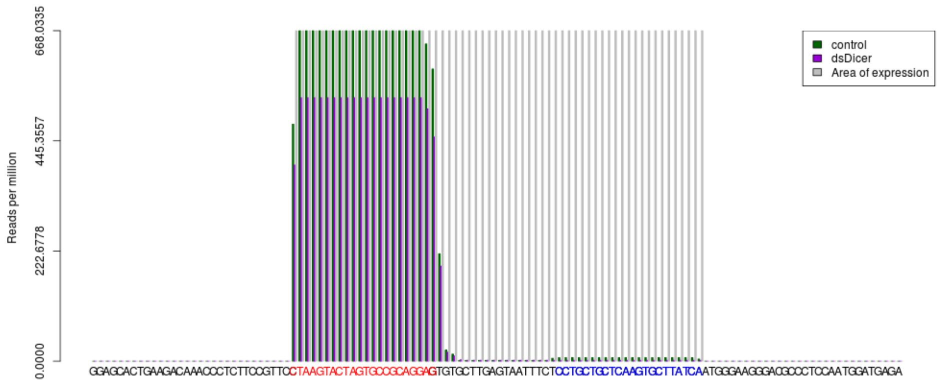

>Bge-Mir-252b_pre+_11
TTCTTCGTGGT**GTAAGTAGTTCTGCCGAAGACAA**TTGACATTAGCTACATT**GTCTGCGCAATTCTACTTAAAC**CACTGAGACAG

>Bge-Mir-252b_3p
GTCTGCGCAATTCTACTTAAAC

>Bge-Mir-252b_5p*
GTAAGTAGTTCTGCCGAAGACAA

```
UUCUUCGUGGUGUAAGUAGUUCUGCCGAAGACAAUUGACAUUAGCUACAUUGUCUGCGCAAUUCUACUUAAACCACUGAGACAG
.((((.(((((.(((((((...(((...(((((((.............)))))))..)))...))))))).))))).))))... (-28.60)
```

Matched bases: 14

Mature reads in Control: 626   Mature reads in dsDicer: 299
Star reads in Control: 9   Star reads in dsDicer: 2

Initial dG =   -28.60

```
          10        20        30        40
U--|   C     G       UUC   CGA       UGACAU
   UCUU GUGGU UAAGUAG   UGC   AGACAAU      \
   AGAG CACCA AUUCAUC   ACG   UCUGUUA      U
GAC^   U     A       UUA   CG-       CAUCGA
   80        70        60         50
```

```
tca-miR-252a-3p      CCUGCUGCUCAAGUG--CUU-AUC
Bge-Mir-252b         -GU-CUGCGCAAUUCUACUUAAAC
                       * **** **   *    *** * *
```

Genomic coordinates:
Bge-Mir-252b_3p*: Scaffold1231:351498-351519:-
Bge-Mir-252b_pre: Scaffold1231:351498-351559:-
Bge-Mir-252b_5p: Scaffold1231:351537-351559:-

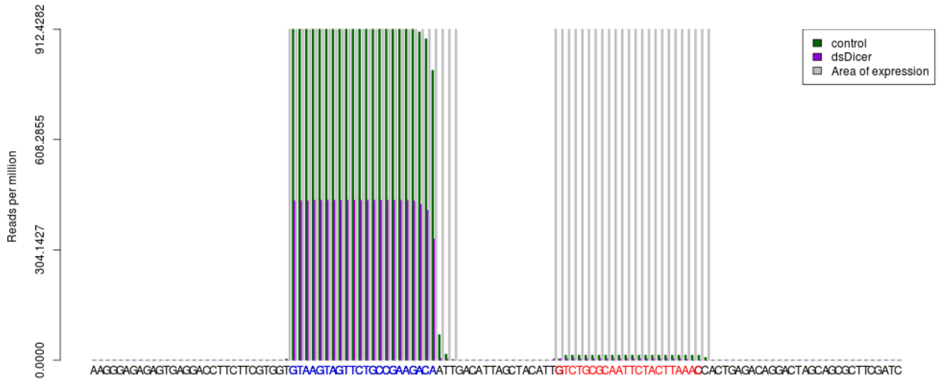

>Bge-Mir-31_pre+_11
AGGGGTAGAGA**AGGCAAGATGTCGGCATAGCTGA**TTATACTTGAAATTC**AGCTGTGTCACATCAAGCCAAC**TCTCACTTCTG

>Bge-Mir-31_5p
AGGCAAGATGTCGGCATAGCTGA

>Bge-Mir-31_3p*
AGCTGTGTCACATCAAGCCAAC

```
AGGGGUAGAGAAGGCAAGAUGUCGGCAUAGCUGAUUAUACUUGAAAUUCAGCUGUGUCACAUCAAGCCAACCACUGAGACAG
((((((((((..(((..(((((.(((((((((((............))))))))))))))))..))).))))).))))))). (-38.60)
```

Matched bases: 17

Mature reads in Control: 6003　　Mature reads in dsDicer: 1313
Star reads in Control: 0　　Star reads in dsDicer: 0

Initial dG = -38.60

```
          10        20        30        40
-      -|   AA   AA      C           UUAUAC
 AGGGGU AGAG  GGC  GAUGU GGCAUAGCUGA      \
 UCUUCA UCUC  CCG  CUACA CUGUGUCGACU      U
G      C^   AA   AA      -           UAAAGU
 80        70        60         50
```

```
Bge-Mir-31         --AGGCAAGAUGUCGGCAUAGCUGA
tca-mir-31-5p      --AGGCAAGAUGUCGGCAUAGCU--
bmo-mir-31-5p      ---GGCAAGAAGUCGGCAUAGCUG-
                      ******* **********
```

Genomic coordinates:
Bge-Mir-31_3p*: Scaffold1307:353359-353380:-
Bge-Mir-31_pre: Scaffold1307:353359-353418:-
Bge-Mir-31_5p: Scaffold1307:353396-353418:-

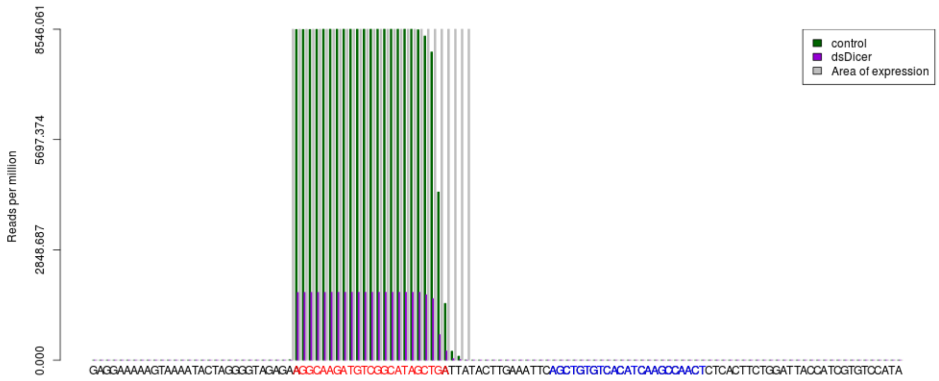

>Bge-Mir-278_pre+_11
TGGTGGCACAT**ACGGATGATCGTCTCCGCCAACC**GTGAATATAATAAGG**TCGGTGGGACTTTCGTCCGTTT**GGGACACCTTG

>Bge-Mir-278_3p
TCGGTGGGACTTTCGTCCGTTT

>Bge-Mir-278_5p*
ACGGATGATCGTCTCCGCCAACC

```
UGGUGGCACAUACGGAUGAUCGUCUCCGCCAACCGUGAAUAUAAUAAGGUCGGUGGGACUUUCGUCCGUUUGGGACACCUUG
.((((...((.((((((((..(((.(((((.(((.............))).))))))))..)))))))).))...)))).... (-38.50)
```

Matched bases: 17

Mature reads in Control: 2199   Mature reads in dsDicer: 1537
Star reads in Control: 11   Star reads in dsDicer: 4

Initial dG =   -38.50

```
         10        20        30        40
U--|   GCA  U        UC    U     A    GUGAAU
   GGUG   CA ACGGAUGA  GUC CCGCC ACC      \
   CCAC   GU UGCCUGCU  CAG GGUGG UGG      A
GUU^   AGG  U        UU    -     C    AAUAAU
 80        70        60         50
```

```
dme-mir-278-3p      UCGGUGGGACUUUCGUCCGUUU
ame-mir-278         UCGGUGGGACUUUCGUCCGUUU
Bge-Mir-278         UCGGUGGGACUUUCGUCCGUUU
api-mir-278         UCGGUGGGACUUUCGUUCGUUU
bmo-mir-278-3p      UCGGUGGGAUCUUCGUCCGUUU
mse-mir-278         UCGGUGGGAUCUUCGUCCGUUU
                    *********  ***** *****
```

Genomic coordinates:
Bge-Mir-278_5p*: Scaffold132:158579-158601:+
Bge-Mir-278_pre: Scaffold132:158579-158638:+
Bge-Mir-278_3p: Scaffold132:158617-158638:+

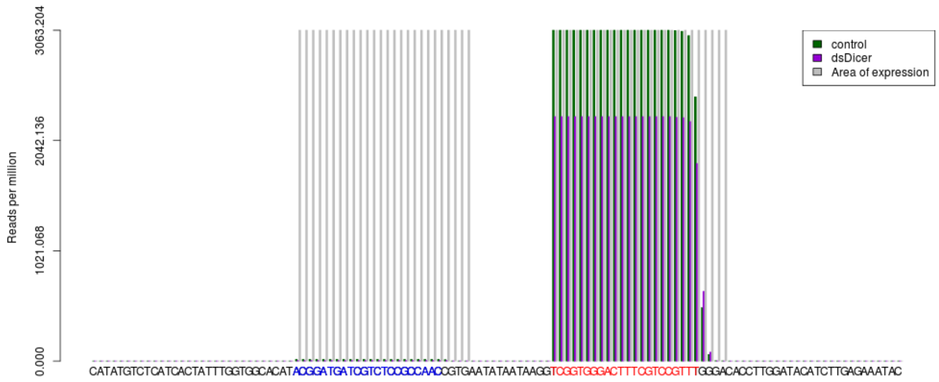

>Bge-Mir-1175_pre+_11
GTTCTTGGACT**AAGTGGAGAAGTGGTCTCTTC**ACTTTGATTTTAAG**TGAGATTCAACTCCTCCAACTTAT**TTCAGCCACGG

>Bge-Mir-1175_3p
TGAGATTCAACTCCTCCAACTTAT

>Bge-Mir-1175_5p*
AAGTGGAGAAGTGGTCTCTTC

```
GUUCUUGGACUAAGUGGAGAAGUGGUCUCUUCACUUUGAUUUUAAGUGAGAUUCAACUCCUCCAACUUAUUUCAGCCACGG
((..(((((.(((((((((.(((....(((.(((((.......)))))))).....))).))))).)))).)))))..)).. (-23.70)
```

Matched bases: 15

Mature reads in Control: 5 Mature reads in dsDicer: 2
Star reads in Control: 1 Star reads in dsDicer: 3

Initial dG = -23.70

```
          10         20        30
--   UC      C     -|     A    GGUC    U      UGA
  GU   UUGGA UAAG UGGAG AGU     UCU CACUU    \
  CA   GACUU AUUC ACCUC UCA     AGA GUGAA    U
GG   CC      U     A^     C    ACUU    -      UUU
80         70         60        50
```

```
bmo-mir-1175-3p      UGAGAUUCAACUCCUCCAACUUAA
ame-mir-1175         UGAGAUUCA-CUCCUCCAACUUAC
Bge-Mir-1175         UGAGAUUCAACUCCUCCAACUUAU
tca-mir-1175-3p      UGAGAUUCAACUCCUCCAUCUC--
                     ********* ******** **
```

Genomic coordinates:
Bge-Mir-1175_3p: Scaffold1358:321652-321675:-
Bge-Mir-1175_pre: Scaffold1358:321652-321710:-
Bge-Mir-1175_5p*: Scaffold1358:321690-321710:-

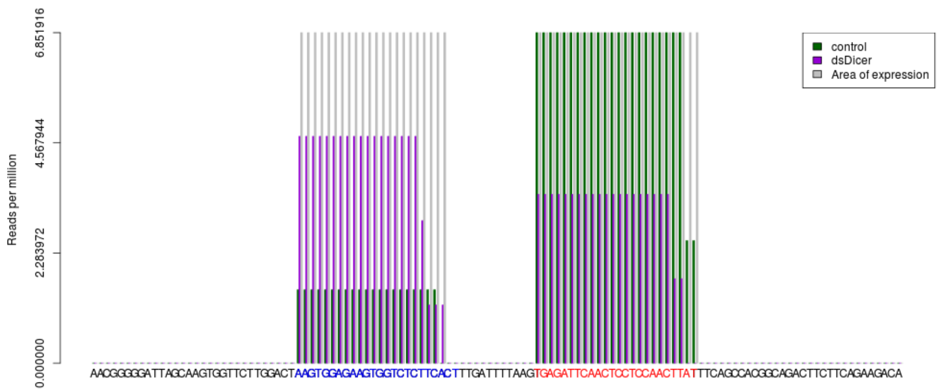

>Bge-Mir-750_pre+_11
GGGTGTCTGTT**AGTTGGAAGTGGGGATCTAGGCA**CAGAATTGCGTAATTTTAAAACTGCAACTTCCAGATAGTG**CCAGATCTAACTCTTC
CAGCTCA**TGGACATTCGG

>Bge-Mir-750_3p
CCAGATCTAACTCTTCCAGCTCA

>Bge-Mir-750_5p*
AGTTGGAAGTGGGGATCTAGGCA

```
GGGUGUCUGUUAGUUGGAAGUGGGGAUCUAGGCACAGAAUUGCGUAAUUUUAAAACUGCAACUUCCAGAUAGUGCCAGAUCUAACUCUUCCAGCUCAUGGACAUUCGG
((((((((((.(((((((((...((((((.(((((.((((((((...........))))).))).......)))))))))))....))))))))).))))))))))).. (-44.40)
```

Matched bases: 17

Mature reads in Control: 222   Mature reads in dsDicer: 43
Star reads in Control: 0   Star reads in dsDicer: 0

Initial dG =   -44.40

```
         10        20         30              40        50
--          U         UGG-       A     A-----    -|     UAAUU
  GGGUGUCUGU AGUUGGAAG    GGAUCU GGCAC      GAA UUGCG      U
  CUUACAGGUA UCGACCUUC    UCUAGA CCGUG      CUU AACGU      U
GG          C         UCAA       -     AUAGAC    C^     CAAAA
      100        90        80         70        60
```

```
bmo-mir-750-3p      CCAGAUCUAUCU-UUCCAGCU----
mse-mir-750         CCAGAUCUAUCU-UUCCAGCUCA--
Bge-Mir-750         CCAGAUCUAACUCUUCCAGCUCA--
ame-mir-750         CCAGAUCUAACUCUUCCAGCUC---
tca-mir-750-3p      CCAGAUCUAACUCUUCCAUAUGACG
                    ********* ** *****  *
```

Genomic coordinates:
Bge-Mir-750_3p: Scaffold1358:325731-325753:-
Bge-Mir-750_pre: Scaffold1358:325731-325816:-
Bge-Mir-750_5p*: Scaffold1358:325794-325816:-

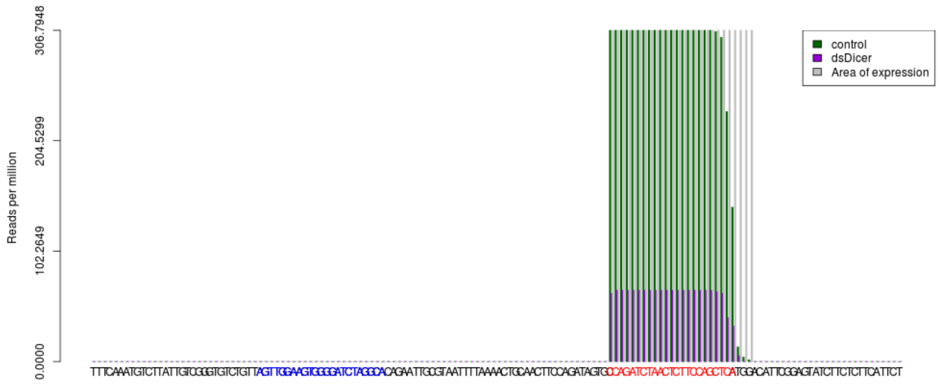

>Bge-Mir-219_pre+_11
GTTTCAGGCTG**TGATTGTCCAAACGCAATTCTTG**TGGTTAGTCTAGTCA**AGGACTGTGTGTGGACATCAAT**GCTTGGAACGT

>Bge-Mir-219_5p
TGATTGTCCAAACGCAATTCTTG

>Bge-Mir-219_3p*
AGGACTGTGTGTGGACATCAAT

```
GUUUCAGGCUGUGAUUGUCCAAACGCAAUUCUUGUGGUUAGUCUAGUCAAGGACUGUGUGUGGACAUCAAUUGGACAUUCGG
((((((((..(((.((((((.(((((.(((((((((.....)))..)))))).))))).)))))))))..))))))))).. (-36.30)
```

Matched bases: 18

Mature reads in Control: 1 Mature reads in dsDicer: 5
Star reads in Control: 0 Star reads in dsDicer: 0

Initial dG = -36.30

```
         10        20        30
--         UG   U      A     A      --|  UU
  GUUUCAGGC  UGA UGUCCA ACGCA UUCUUG  UGG  \
  CAAGGUUCG  ACU ACAGGU UGUGU AGGAAC  AUC  A
UG         UA   -      G     C      UG^  UG
 80        70         60        50
```

```
Bge-Mir-219         UGAUUGUCCAAACGCAAUUCUUG
tca-mir-219-5p      UGAUUGUCCAAACGCAAUUCU--
nvi-mir-219         UGAUUGUCCAAACGCAAUUCUUG
dme-mir-219-5p      UGAUUGUCCAAACGCAAUUCU--
api-mir-219         UGAUUGUCCAAACGCAAUUCUUG
ame-mir-219         UGAUUGUCCAAACGCAAUUCUUG
                    *********************
```

Genomic coordinates:
Bge-Mir-219_3p: Scaffold1386:268868-268889:-
Bge-Mir-219_pre: Scaffold1386:268868-268927:-
Bge-Mir-219_5p*: Scaffold1386:268905-268927:-

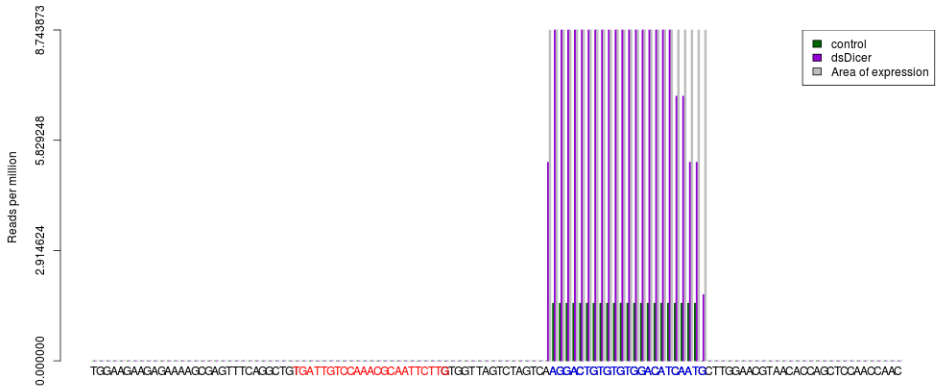

>Bge-Mir-190_pre+_11
TATATCCTGTGAGATATGTTTGATATTCTTGGTTGTTGAATTGAATCACCCAGGAATCAAACATATTATTACAGTGTATAGT

>Bge-Mir-190_5p
AGATATGTTTGATATTCTTGGTTG

>Bge-Mir-190_3p*
CCCAGGAATCAAACATATTATTA

```
UAUAUCCUGUGAGAUAUGUUUGAUAUUCUUGGUUGUUGAAUUGAAUCACCCAGGAAUCAAACAUAUUAUUAUGGACAUUCGG
((((..(((((((((((((((((..((((((.((..........)).))))))))))))))))))).)))))).)))).. (-29.10)
```

Matched bases: 19

Mature reads in Control: 442   Mature reads in dsDicer: 166
Star reads in Control: 8   Star reads in dsDicer: 0

Initial dG =   -29.10

```
          10         20         30         40
--    UC      -|           AU       U   UUGAA
  UAUA  CUGUGA GAUAUGUUUGAU  UCUUGG UG      \
  AUAU  GACAUU UUAUACAAACUA  AGGACC AC      U
UG    GU      A^           --       C   UAAGU
 80        70        60          50
```

```
ame-mir-190         AGAUAUGUUUGAUAUUCUUGGUUGUU
mse-mir-190         AGAUAUGUUUGAUAUUCUUGGUUG--
api-mir-190         AGAUAUGUUUGAUAUUCUUGGUUG--
dme-mir-190-5p      AGAUAUGUUUGAUAUUCUUGGUUG--
bmo-mir-190-5p      AGAUAUGUUUGAUAUUCUUGGUU---
tca-mir-190-5p      AGAUAUGUUUGAUAUUCUUGG-----
Bge-Mir-190         AGAUAUGUUUGAUAUUCUUGGUUG--
nvi-mir-190         AGAUAUGUUUGAUAUUCUUGGUUGUA
                    *********************
```

Genomic coordinates:
Bge-Mir-190_5p: Scaffold142:425546-425569:+
Bge-Mir-190_pre: Scaffold142:425546-425605:+
Bge-Mir-190_3p*: Scaffold142:425583-425605:+

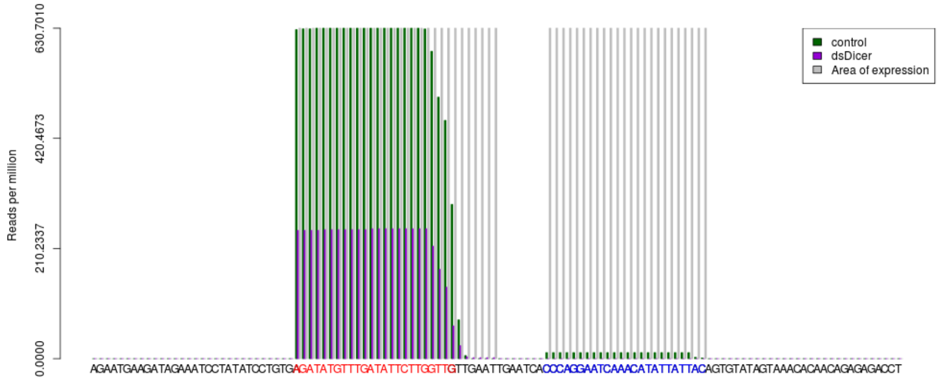

>Bge-Mir-927b_pre+_11
GCTCGCTTCGT**TTTAGAATTTGTACGCTTTGT**TGTAATTTTTTTAAAAATCAC**AAAGCATATGGATTCTTGAGC**GGATTGTGCAA

>Bge-Mir-927b_5p
TTTAGAATTTGTACGCTTTGT

>Bge-Mir-927b_3p*
AAAGCATATGGATTCTTGAGC

```
GCUCGCUUCGUUUUAGAAUUUGUACGCUUUGUUGUAAUUUUUUUAAAAAUCACAAAGCAUAUGGAUUCUUGAGCUGGACAUUCGG
((.((.(((((((.((((((..((.(((((((....(((((....))))).))))))).))..)))))).))))))).)).)).. (-28.90)
```

Matched bases: 15

Mature reads in Control: 10 Mature reads in dsDicer: 12
Star reads in Control: 1 Star reads in dsDicer: 0

Initial dG = -28.90

```
          10        20        30        40
--| U  C       U      UG  C       UGUA     U
  GC CG UUCGUUU AGAAUU  UA GCUUUGU    AUUUU U
  CG GU AGGCGAG UCUUAG  AU CGAAACA    UAAAA U
AA^ U  U       U      GU  A       C---     A
    80        70        60            50
```

```
Bge-Mir-927b         -UUUAGAAUUUGUACGCUUUGU
ame-mir-927b         UUUUAGAAUUUGUACGCUCUGU
tca-mir-927b-5p      -UUUAGAAUCUGUACGCUUUGU
                      ******** ******** ***
```

Genomic coordinates:
Bge-Mir-927b_5p: Scaffold1421:425040-425060:+
Bge-Mir-927b_pre: Scaffold1421:425040-425102:+
Bge-Mir-927b_3p*: Scaffold1421:425082-425102:+

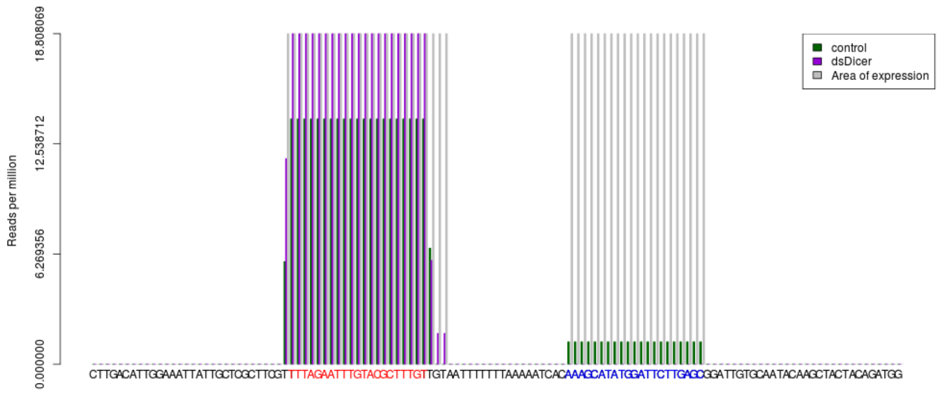

>Bge-Mir-279c_pre+_11
TGGTTTCTGTA**GGCGGGTCTGGGTCTGGATTACG**CAATTTTAAGACG**TGACTAGATCCATACTCGTCTA**TAATAATCAGG

>Bge-Mir-279c_3p
TGACTAGATCCATACTCGTCTA

>Bge-Mir-279c_5p*
GGCGGGTCTGGGTCTGGATTACG

```
UGGUUUCUGUAGGCGGGUCUGGGUCUGGAUUACGCAAUUUUAAGACGUGACUAGAUCCAUACUCGUCUAUAAUAAUCAGG
(((((..(((((((((((.(((((((((.(((((...........))))))))))))))).)))))))))))..))))).. (-36.40)
```

Matched bases: 19

Mature reads in Control: 284   Mature reads in dsDicer: 246
Star reads in Control: 5   Star reads in dsDicer: 2

```
 Initial dG =    -36.40

         10        20        30
--|    UC          C         A     CAAUU
  UGGUU  UGUAGGCGGGU UGGGUCUGG UUACG     \
  ACUAA  AUAUCUGCUCA ACCUAGAUC AGUGC     U
GG^    UA          U         -     AGAAU
.        70        60         50
```

```
Bge-Mir-279c         UGACUAGAUCCA-UACUCGUCUA
bmo-mir-279c-3p      UGACUAGAUCCA-UACUCGUCUG
ame-mir-279c         UGACUAGAGUCA-CACUCGUCCA
mse-mir-279c         UGACUAGAUUUU-CACUUAUCCU
tca-mir-279c-3p      UGACUAGAUCGAACAUUCGCUCG
                     ********      * *
```

Genomic coordinates:
Bge-Mir-279c_3p: Scaffold147:2087677-2087698:-
Bge-Mir-279c_pre: Scaffold147:2087677-2087734:-
Bge-Mir-279c_5p*: Scaffold147:2087712-2087734:-

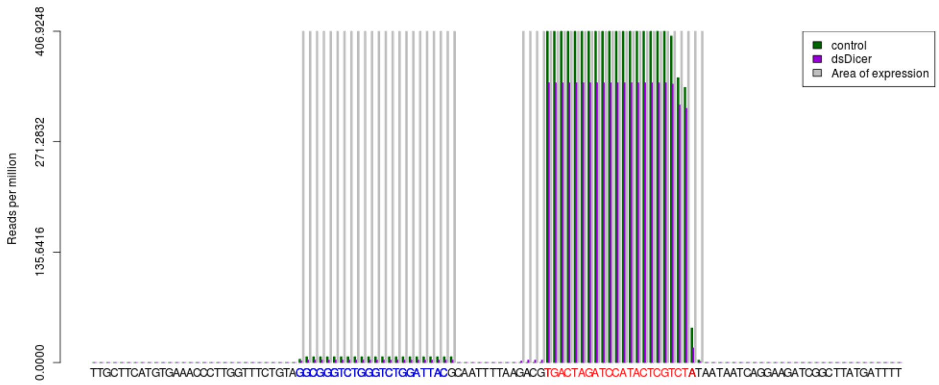

>Bge-Mir-279d_pre+_11
TTACAGCCTGT**GATGAGTGTAGGTCTGGTGCACA**TGTGTTAAAAAGCTG**TGACTAGATCCACACTCATCAC**TGGAGGTGAGA

>Bge-Mir-279d_3p
TGACTAGATCCACACTCATCAC

>Bge-Mir-279d_5p*
GATGAGTGTAGGTCTGGTGCACA

```
UUACAGCCUGUGAUGAGUGUAGGUCUGGUGCACAUGUGUUAAAAAGCUGUGACUAGAUCCACACUCAUCACUGGAGGUGAGA
((((..((.(((((((((((.((((((((.((((.((........)))))))))))))).)))))))))).))..)))).. (-41.90)
```

Matched bases: 19

Mature reads in Control: 3373   Mature reads in dsDicer: 2546
Star reads in Control: 98   Star reads in dsDicer: 62

Initial dG = -41.90

```
          10        20        30        40
--|   AG  U           A        G     U  GUU
  UUAC  CC GUGAUGAGUGU GGUCUGGU CACA GU   A
  AGUG  GG CACUACUCACA CUAGAUCA GUGU CG   A
AG^   GA  U           C        -     -  AAA
 80        70        60         50
```

```
bmo-mir-279d-3p      UGACUAGAUUUUCACUUAUCCU---
ame-mir-279d         UGACUAGAUCCACACUCAUCCA---
tca-mir-279d-3p      UGACUAGAUCCAUACUCGUCUAUAG
mse-mir-279d         UGACUAGAUCCAUACUCGUCUGC--
Bge-Mir-279d         UGACUAGAUCCACACUCAUCAC---
                     *********    ***  **
```

Genomic coordinates:
Bge-Mir-279d_3p: Scaffold147:2089234-2089255:-
Bge-Mir-279d_pre: Scaffold147:2089234-2089293:-
Bge-Mir-279d_5p*: Scaffold147:2089271-2089293:-

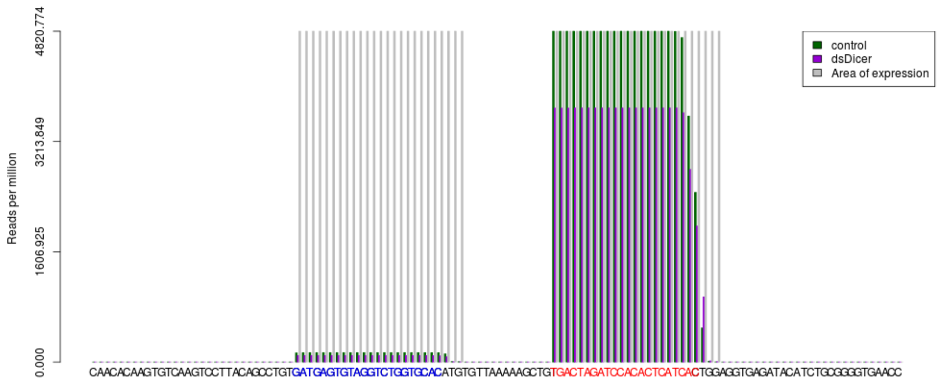

>Bge-Mir-2765_pre+_11
GTAGTGAGGTG**TGGTAACTCCACCACCGTTGGC**TGATTGAATGAAGTTGT**CAACGGTAGAGGAGTTCCTTAT**CCTCACTGCAT

>Bge-Mir-2765_5p
TGGTAACTCCACCACCGTTGGC

>Bge-Mir-2765_3p*
CAACGGTAGAGGAGTTCCTTAT

```
GUAGUGAGGUGUGGUAACUCCACCACCGUUGGCUGAUUGAAUGAAGUUGUCAACGGUAGAGGAGUUCCUUAUUGGAGGUGAGA
((((((((...((.((((((...(((((((((.((((......)))))))))))))...))))))))....))))))))).. (-42.20)
```

Matched bases: 15

Mature reads in Control: 1139   Mature reads in dsDicer: 135
Star reads in Control: 69   Star reads in dsDicer: 18

Initial dG =   -42.20

```
          10         20        30        40
--         UGU-| U      ACC         U    GA
  GUAGUGAGG   GG AACUCC   ACCGUUGGC GAUU  A
  CGUCACUCC   CC UUGAGG   UGGCAACUG UUGA  U
UA         UAUU^ -      AGA         -    AG
  80        70         60        50
```

```
Bge-Mir-2765         UGGUAACUCCA--CCACCGUUGGC
tca-mir-2765-5p      UGGUAACUCCA--CCACCGUUGGC
bmo-mir-2765         UGGUAACUCCA--CCACCGUUGGC
ame-mir-2765-5p      UGGUAACUCCA--CCACCGUUGGC
mse-mir-2765         UGGUAACUCCA--CCACCGUUGGC
api-mir-2765         UGGUAACUCCAAACCAUUGCCGG-
                     ***********  ***  *  **
```

Genomic coordinates:
Bge-Mir-2765_5p: Scaffold1486:250952-250973:+
Bge-Mir-2765_pre: Scaffold1486:250952-251012:+
Bge-Mir-2765_3p*: Scaffold1486:250991-251012:+

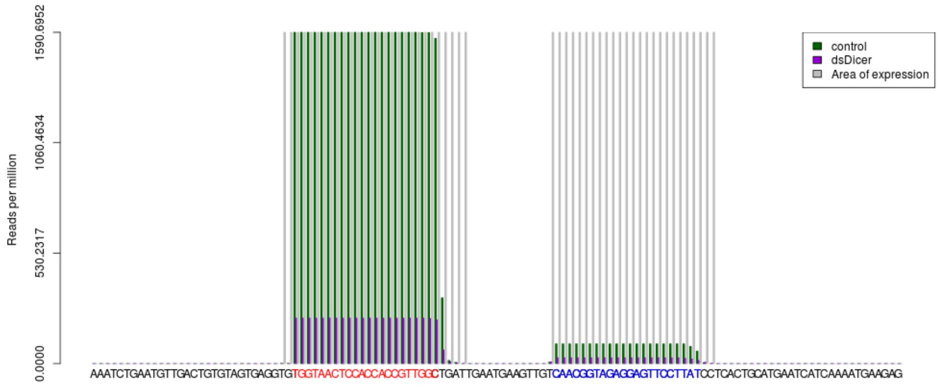

>Bge-Mir-1_pre+_11
TGGCGAAAGTT**CCATACTTCCTTACTTCTCATA**TTGCTTTTAAAACTTA**TGGAATGTAAAGAAGTATGGAG**CGAACGCGGGC

>Bge-Mir-1_3p
TGGAATGTAAAGAAGTATGGAG

>Bge-Mir-1_5p*
CCATACTTCCTTACTTCTCATA

```
UGGCGAAAGUUCCAUACUUCCUUACUUCUCAUAUUGCUUUUAAAACUUAUGGAAUGUAAAGAAGUAUGGAGCGAACGCGGGC
..(((...((((((((((((.(((((((.((((.............))))))).)))).)))))))))))))...))).... (-30.60)
```

Matched bases: 18

Mature reads in Control: 38847 Mature reads in dsDicer: 22080
Star reads in Control: 23 Star reads in dsDicer: 14

```
 Initial dG =    -30.60

         10        20         30        40
UG--   AAA            C    -|  U    UUGCUU
    GCG   GUUCCAUACUUC UUAC UUC CAUA      U
    CGC   CGAGGUAUGAAG AAUG AAG GUAU      U
CGGG   AAG            A    U^  -    UCAAAA
 80        70        60         50
```

```
Bge-Mir-1              UGGAAUGUAAAGAAGUAUGGAG
tca-mir-1-3p           UGGAAUGUAAAGAAGUAUGGAG
ame-mir-1              UGGAAUGUAAAGAAGUAUGGAG
api-mir-1              UGGAAUGUAAAGAAGUAUGGAG
dme-mir-1-3p           UGGAAUGUAAAGAAGUAUGGAG
nvi-mir-1              UGGAAUGUAAAGAAGUAUGGAG
                       **********************
```

Genomic coordinates:
Bge-Mir-1_5p*: Scaffold15:2616714-2616735:+
Bge-Mir-1_pre: Scaffold15:2616714-2616773:+
Bge-Mir-1_3p: Scaffold15:2616752-2616773:+

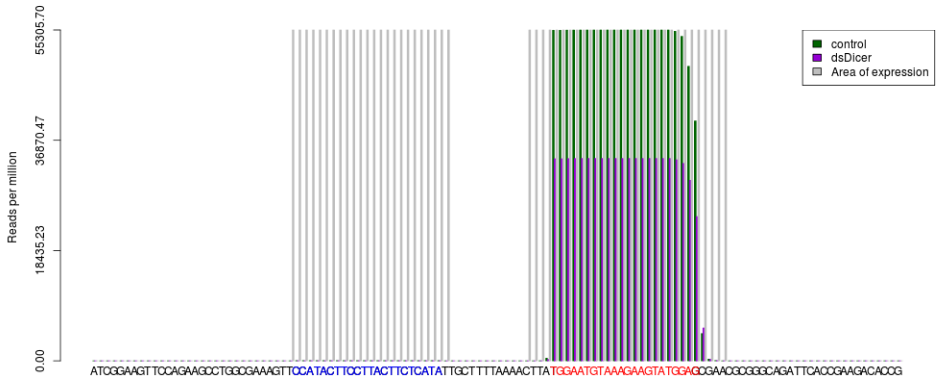

>Bge-Mir-133_pre+_11

TGCTACGCTTT**AGCTGGTTGAATCCGGGCCAAAT**TGTACGAGAGTTGAAGACAT**TTGGTCCCCTTCAACCAGCTGT**AGTTGGCATTC

>Bge-Mir-133_3p

TTGGTCCCCTTCAACCAGCTGT

>Bge-Mir-133_5p*

AGCTGGTTGAATCCGGGCCAAAT

```
UGCUACGCUUUAGCUGGUUGAAUCCGGGCCAAAUUGUACGAGAGUUGAAGACAUUUGGUCCCCUUCAACCAGCUGUAGUUGGCAUUC
(((((.(((.((((((((((((...((((((((.(((((....)).....)))))))))))..)))))))))))).)))))))).... (-39.80)
```

Matched bases: 18

```
Mature reads in Control: 993          Mature reads in dsDicer: 604
Star reads in Control: 3              Star reads in dsDicer: 2

 Initial dG =    -39.80

           10        20        30             40
---     C   U            UCC        U   -----| G
   UGCUA GCU UAGCUGGUUGAA   GGGCCAAA UGU     AC A
   ACGGU UGA GUCGACCAACUU   CCUGGUUU ACA     UG G
CUU     -   U            CC-        -   GAAGU^ A
      80         70         60         50


Bge-Mir-133         UUGGUCCCCUUCAACCAGCUGU
tca-mir-133-3p      UUGGUCCCCUUCAACCAGCUGU
bmo-mir-133         UUGGUCCCCUUCAACCAGCUGU
nvi-mir-133         UUGGUCCCCUUCAACCAGCUGU
dme-mir-133-3p      UUGGUCCCCUUCAACCAGCUGU
mse-mir-133         UUGGUCCCCUUCAACCAGCUGU
ame-mir-133         UUGGUCCCCUUCAACCAGCUGU
                    **********************
```

Genomic coordinates:

Bge-Mir-133_5p*: Scaffold15:2988224-2988246:+

Bge-Mir-133_pre: Scaffold15:2988224-2988288:+

Bge-Mir-133_3p: Scaffold15:2988267-2988288:+

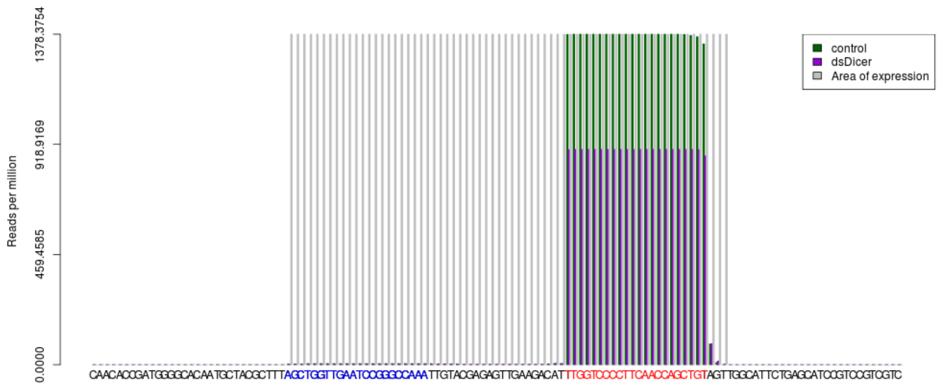

>Bge-Mir-8_pre+_11
GTTCGTGTATA**CATCTTACCGGGCAGCATTAGA**ATGTGTTAGAGCTC**TAATACTGTCAGGTAAAGATGTC**CTCCGAACTCC

>Bge-Mir-8_3p
TAATACTGTCAGGTAAAGATGTC

>Bge-Mir-8_5p*
CATCTTACCGGGCAGCATTAGA

```
GUUCGUGUAUACAUCUUACCGGGCAGCAUUAGAAUGUGUUAGAGCUCUAAUACUGUCAGGUAAAGAUGUCCUCCGAACUCC
(((((.....((((((((((.(((((.((((((..((......)))))))).))))).))).)))))))....)))))... (-28.40)
```

Matched bases: 18

Mature reads in Control: 57479 Mature reads in dsDicer: 35729
Star reads in Control: 3885 Star reads in dsDicer: 1532

Initial dG = -28.40

```
           10         20        30        40
---|    UGUAU       -   G      C      AU  GU
   GUUCG     ACAUCUU ACC GGCAG AUUAGA  GU  U
   CAAGC     UGUAGAA UGG CUGUC UAAUCU  CG  A
CCU^    CUCC-       A   A      A      --  AG
80        70         60        50
```

```
Bge-Mir-8             UAAUACUGUCAGGUAAAGAUGUC
tca-mir-8-3p          UAAUACUGUCAGGUAAAGAUGUC
ame-mir-8             UAAUACUGUCAGGUAAAGAUGUC
mse-mir-8             UAAUACUGUCAGGUAAAGAUGUC
dme-mir-8-3p          UAAUACUGUCAGGUAAAGAUGUC
nvi-mir-8             UAAUACUGUCAGGUAAAGAUGUC
bmo-mir-8-3p          UAAUACUGUCAGGUAAAGAUGUC
                      ***********************
```

Genomic coordinates:
Bge-Mir-8_5p*: Scaffold1545:268925-268946:+
Bge-Mir-8_pre: Scaffold1545:268925-268983:+
Bge-Mir-8_3p: Scaffold1545:268961-268983:+

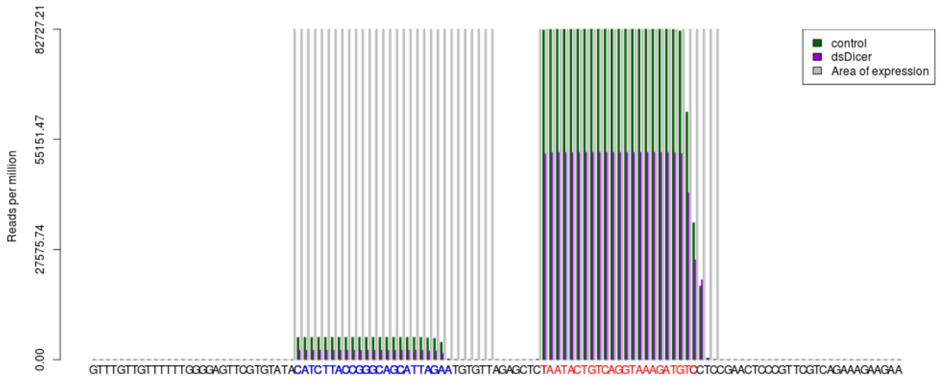

>Bge-Mir-316_pre+_11
GTCTGTGGATG**TGTCTTTTTCCGCTTTGCTGCCG**CTGTTGTTTAGAGATTCG**ACAGCAAAGTGGAAGAGGCCGAT**TCATGGGGCAA

>Bge-Mir-316_5p
TGTCTTTTTCCGCTTTGCTGCCG

>Bge-Mir-316_3p*
ACAGCAAAGTGGAAGAGGCCGAT

```
GUCUGUGGAUGUGUCUUUUUCCGCUUUGCUGCCGCUGUUGUUUAGAGAUUCGACAGCAAAGUGGAAGAGGCCGAUCUCCGAACUCC
.((((((((.((.((((((((((((((((.((..(((.......))).)).))))))))))))))))).))))))))))).... (-35.90)
```

Matched bases: 19

Mature reads in Control: 407 Mature reads in dsDicer: 234
Star reads in Control: 22 Star reads in dsDicer: 16

Initial dG = -35.90

```
           10        20        30        40
G---|        G  U                 C  CU   GUU
    UCUGUGGAU UG CUUUUUCCGCUUUGCUG CG  GUU   \
    GGGUACUUA GC GGAGAAGGUGAAACGAC GC  UAG   U
AACG^        -  C                 A  U-   AGA
     80         70        60        50
```

```
bmo-mir-316-5p      UGUCUUUUUCCGCUUUGCUGCUG
mse-mir-316         UGUCUUUUUCCGCUUUGCUGCU-
tca-mir-316-5p      UGUCUUUUUCCGCUUUGCUGC--
ame-mir-316         UGUCUUUUUCCGCUUUGCUGCCG
api-mir-316         UGUCUUUUUCCGCUUUGCUGCCG
Bge-Mir-316         UGUCUUUUUCCGCUUUGCUGCCG
dme-mir-316-5p      UGUCUUUUUCCGCUU-ACUGGCG
                    ***************  ***
```

Genomic coordinates:
Bge-Mir-316_3p*: Scaffold16:2921376-2921398:-
Bge-Mir-316_pre: Scaffold16:2921376-2921439:-
Bge-Mir-316_5p: Scaffold16:2921417-2921439:-

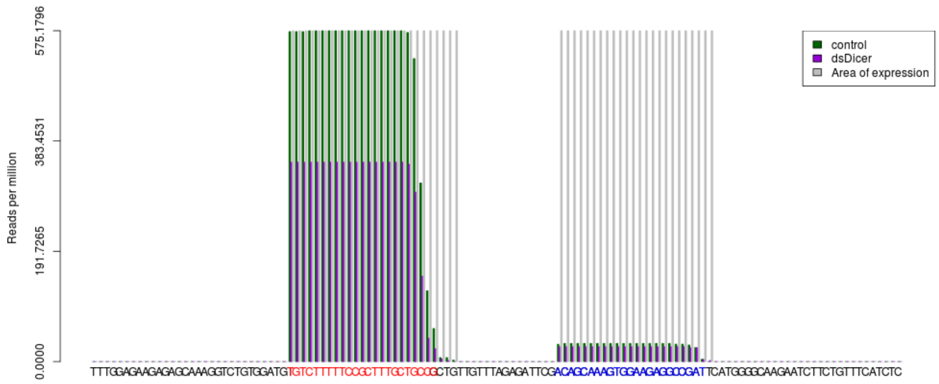

>Bge-Mir-279b_pre+_11
CGTTTGCTTCG**GATGGCTGTGTTTCTGGTGCATG**TGCATTTGAAGATCTCA**TGACTAGATCCACACTCATCCA**TAGCAGACGGT

>Bge-Mir-279b_3p
TGACTAGATCCACACTCATCCA

>Bge-Mir-279b_5p*
GATGGCTGTGTTTCTGGTGCATG

```
CGUUUGCUUCGGAUGGCUGUGUUUCUGGUGCAUGUGCAUUUGAAGAUCUCAUGACUAGAUCCACACUCAUCCAUAGCAGACGGU
((((((((..(((((..((((..((((((.((((...(((....)))..))))))))))..)))).)))))..)))))))).. (-38.10)
```

Matched bases: 16

Mature reads in Control: 4047 Mature reads in dsDicer: 2240
Star reads in Control: 134 Star reads in dsDicer: 51

Initial dG = -38.10

```
          10        20        30        40
--|       UC      GC     UU       G     UGC    U
  CGUUUGCU  GGAUG  UGUG  UCUGGU CAUG   AUU G
  GCAGACGA  CCUAC  ACAC  AGAUCA GUAC   UAG A
UG^       UA      UC     CU       -     UC-    A
   80        70        60          50
```

```
tca-mir-279b-3p      UGACUAGAUCCA--CACUCAUCCA
ame-mir-279b         UGACUAGAUCGAAAUACUCGUCCC
Bge-Mir-279b         UGACUAGAUCCA--CACUCAUCCA
bmo-mir-279b-3p      UGACUAGAUCUA--CACUCAUUGA
mse-mir-279b         UGACUAGAUCUA--CACUCAUUGA
api-mir-279b         UGACUAGAUCUA--CACUCAUCA-
                     ********** *   **** *
```

Genomic coordinates:
Bge-Mir-279b_3p: Scaffold164:1557127-1557148:-
Bge-Mir-279b_pre: Scaffold164:1557127-1557188:-
Bge-Mir-279b_5p*: Scaffold164:1557166-1557188:-

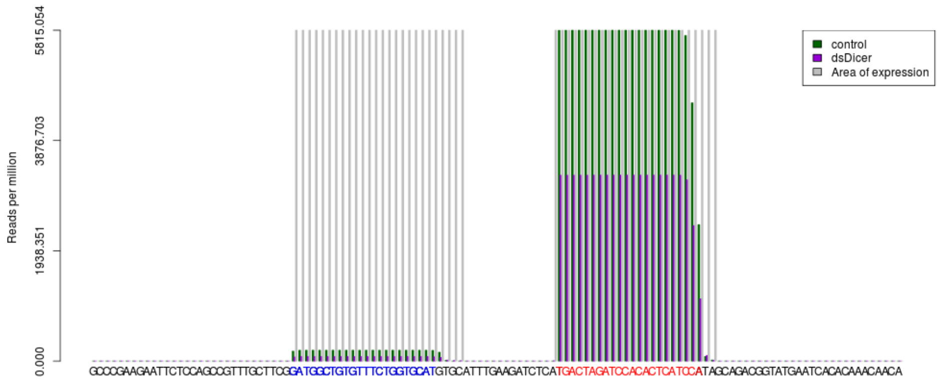

>Bge-Mir-315_pre+_11
GTTCTTTATGT**TTTTGATTGTTGCTCAGAAAGCC**GTGACGTTGAAGCAGG**CTTTCGAGCAATAATCACAATC**AGAAAGGAGCA

>Bge-Mir-315_5p
TTTTGATTGTTGCTCAGAAAGCC

>Bge-Mir-315_3p*
CTTTCGAGCAATAATCACAATC

```
GUUCUUUAUGUUUUUGAUUGUUGCUCAGAAAGCCGUGACGUUGAAGCAGGCUUUCGAGCAAUAAUCACAAUCUAGCAGACGGU
((((((.((.((.((((((((((((.(((((((((.........)).))))))))))))))))))).)).))..))))))). (-35.50)
```

Matched bases: 19

Mature reads in Control: 972   Mature reads in dsDicer: 310
Star reads in Control: 0   Star reads in dsDicer: 0

```
 Initial dG =    -35.50

          10        20        30         40
-       A-| U  U            A       -  GACG
 GUUCUUU  UG UU UGAUUGUUGCUC GAAAGCC GU    \
 CGAGGAA  AC AA ACUAAUAACGAG CUUUCGG CG    U
A       AG^ U  C            -       A  AAGU
  80        70        60         50
```

```
Bge-Mir-315         UUUUGAUUGUUGCUCAGAAAGCC
tca-mir-315         UUUUGAUUGUUGCUCAGAAAGC-
nvi-mir-315         UUUUGAUUGUUGCUCAGAAAGC-
dme-mir-315-5p      UUUUGAUUGUUGCUCAGAAAGC-
api-mir-315         UUUUGAUUGUUGCUCAGAAAGCC
ame-mir-315         UUUUGAUUGUUGCUCAGAAAGC-
                    **********************
```

Genomic coordinates:
Bge-Mir-315_5p: Scaffold164:2198035-2198057:+
Bge-Mir-315_pre: Scaffold164:2198035-2198095:+
Bge-Mir-315_3p*: Scaffold164:2198074-2198095:+

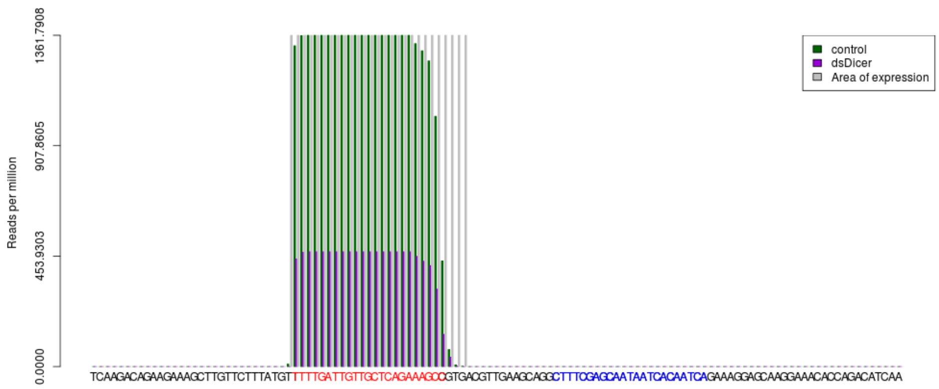

>Bge-Mir-279a_pre+_11

GTTTGCTTCGC**ATGGCTGCGTTTCTGGTGCATG**TGCATTTGAAGATCTCA**TGACTAGATCCACACTCATCC**ATAGCAGACGA

>Bge-Mir-279a_3p

TGACTAGATCCACACTCATCC

>Bge-Mir-279a_5p*

ATGGCTGCGTTTCTGGTGCATG

```
GUUUGCUUCGCAUGGCUGCGUUUCUGGUGCAUGUGCAUUUGAAGAUCUCAUGACUAGAUCCACACUCAUCCAUAGCAGACGA
((((((.....(((..((....((((((.((((...(((....)))..))))))))))....))..)))....))))))).. (-22.10)
```

Matched bases: 13

Mature reads in Control: 4038   Mature reads in dsDicer: 2253
Star reads in Control: 0   Star reads in dsDicer: 0

```
 Initial dG =    -22.40

         10        20          30
--      UCGC    C  C  .-U| UG    A
  GUUUGCU    AUGG UG GU   UC  GUGC U
  CAGACGA    UACC AC CA   AG  UACG G
AG      ----    U  U  \ -^ UU    U
 80           70         40
                              50
                         A--     CAU
                            GAUCU   \
                            CUAGA   G
                         CAC     UCA
                           60
```

```
api-mir-279a         UGACUAGAUCCACACUCAUCC-
mse-mir-279a         UGACUAGAUCCACACUCAUCCA
Bge-Mir-279a         UGACUAGAUCCACACUCAUCC-
bmo-mir-279a         UGACUAGAUCCACACUCAU---
tca-mir-279a-3p      UGACUAGAUCCACACUCAUUAA
ame-mir-279a         UGACUAGAUCCACACUCAUUAA
                     *******************
```

Genomic coordinates:

Bge-Mir-279a_3p: Scaffold17444:3002-3022:-
Bge-Mir-279a_pre: Scaffold17444:3002-3061:-
Bge-Mir-279a_5p*: Scaffold17444:3040-3061:-

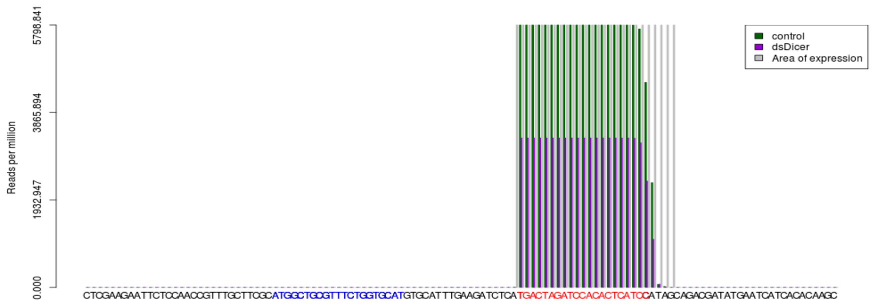

>Bge-Mir-210_pre+_11
TAACCCACGGC**AGCTGCTGGACACTGCACAAGAT**TAGCGATTAAAACT**CTTGTGCGTGTGACAGCGGCTAT**TGTAGTTGCTC

>Bge-Mir-210_3p
CTTGTGCGTGTGACAGCGGCTAT

>Bge-Mir-210_5p*
AGCTGCTGGACACTGCACAAGAT

```
UAACCCACGGCAGCUGCUGGACACUGCACAAGAUUAGCGAUUAAAACUCUUGUGCGUGUGACAGCGGCUAUUGUAGUUGCUC
((((..((((.((((((((.((((.((((((((.............)))))))))))).)))))))).)))).)))).... (-32.70)
```

Matched bases: 19

Mature reads in Control: 11   Mature reads in dsDicer: 3
Star reads in Control: 7   Star reads in dsDicer: 2

```
 Initial dG =    -32.70

           10        20         30        40
---|   CC    C         G-     U         UUAGCG
   UAAC  ACGG AGCUGCUG  ACAC GCACAAGA      A
   GUUG  UGUU UCGGCGAC  UGUG CGUGUUCU      U
CUC^   A-     A         AG     -        CAAAAU
 80         70        60         50
```

```
Bge-Mir-210         CUUGUGCGUGUGACAGCGGCUAU
bmo-mir-210         -UUGUGCGUGUGACAGCGGCU--
tca-mir-210-3p      -UUGUGCGUGUGACAGCGGCUA-
nvi-mir-210         -UUGUGCGUGUGACAGCGGCUA-
dme-mir-210-3p      CUUGUGCGUGUGACAGCGGCUAU
ame-mir-210         -UUGUGCGUGUGACAGCGGCUA-
mse-mir-210         CUUGUGCGUGUGACAGCGGCUAU
api-mir-210         -UUGUGCGUGUGACAUCGGCUA-
                     ************** *****
```

Genomic coordinates:
Bge-Mir-210_5p*: Scaffold1748:131360-131382:+
Bge-Mir-210_pre: Scaffold1748:131360-131419:+
Bge-Mir-210_3p: Scaffold1748:131397-131419:+

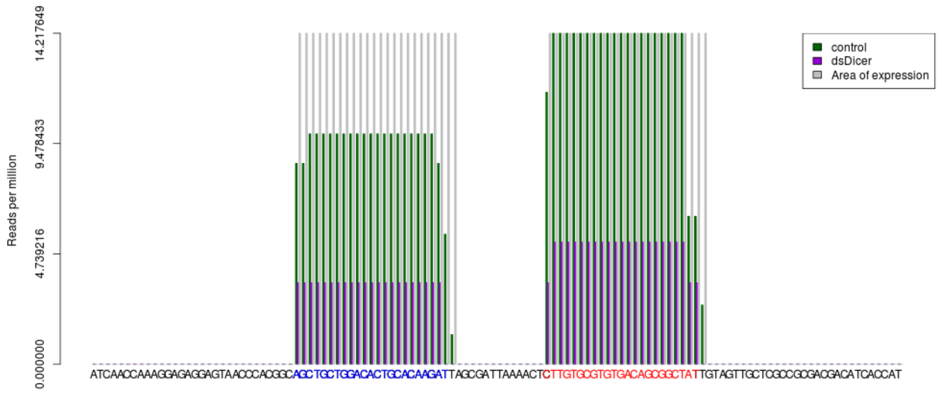

>Bge-Mir-7_pre+_11
CTATTGTTGTGTGGAAGACTAGTGATTTTGTTGTTCTTTAATTGAGTAACAAGGAATCACTAATCATCCTACAAAGATGGCT

>Bge-Mir-7_5p
TGGAAGACTAGTGATTTTGTTGTT

>Bge-Mir-7-3_3p*
CAAGGAATCACTAATCATCCTA

```
CUAUUGUUGUGUGGAAGACUAGUGAUUUUGUUGUUCUUUAAUUGAGUAACAAGGAAUCACUAAUCAUCCUAUGUAGUUGCUC
(((((.(((((.(((.((.((((((((((.((((((((.....))).))))))))))))))).)).)))))))).))))).. (-28.00)
```

Matched bases: 18

Mature reads in Control: 2748 Mature reads in dsDicer: 1427
Star reads in Control: 0 Star reads in dsDicer: 1

```
 Initial dG =    -28.00

         10        20        30         40
--|    G     U   A  C         G     -   UA
  CUAUU UUGUG GGA GA UAGUGAUUUU UUGUU CUU  \
  GGUAG AACAU CCU CU AUCACUAAGG AACAA GAG  A
UC^    A     -   A  A         -     U   UU
 80        70         60         50
```

```
api-mir-7         UGGAAGACUAGUGAUUUUGUUGUU-
mse-mir-7         UGGAAGACUAGUGAUUUUGUUGUU-
ame-mir-7         UGGAAGACUAGUGAUUUUGUUGU--
dme-mir-7-5p      UGGAAGACUAGUGAUUUUGUUGU--
nvi-mir-7         UGGAAGACUAGUGAUUUUGUUGU--
bmo-mir-7-5p      UGGAAGACUAGUGAUUUUGUUGU--
tca-mir-7-5p      UGGAAGACUAGUGAUUUUGUUGUUC
Bge-Mir-7         UGGAAGACUAGUGAUUUUGUUGUU-
                  ***********************
```

Genomic coordinates:
Bge-Mir-7_pre: Scaffold1783:352146-352205:-
Bge-Mir-7_5p: Scaffold1783:352182-352205:-

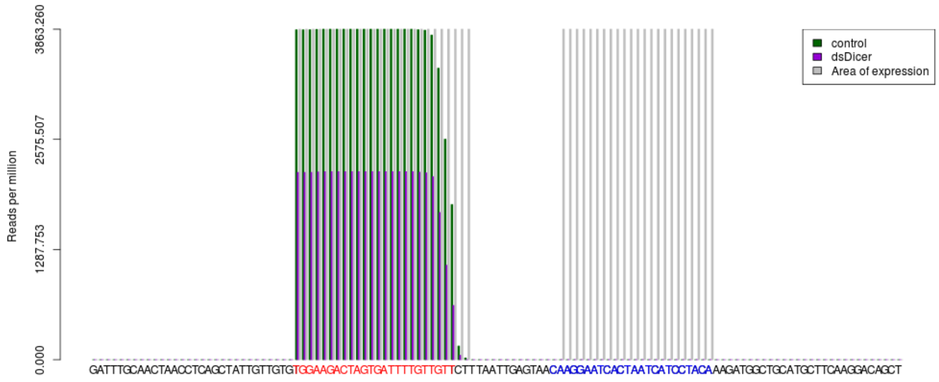

>Bge-Mir-100_pre+_11
TTAGGGACCTA**AACCCGTAGATCCGAACTTGTG**CTAGTTCGATTCA**CAAGCTCGATTCTATGGGTCTA**GGTAGCTAATG

>Bge-Mir-100_5p
AACCCGTAGATCCGAACTTGTG

>Bge-Mir-100_3p*
CAAGCTCGATTCTATGGGTCTA

```
UUAGGGACCUAAACCCGUAGAUCCGAACUUGUGCUAGUUCGAUUCACAAGCUCGAUUCUAUGGGUCUAUGUAGUUGCUC
((((..(((((.(((((((((..(((.((((((...........)))))).)))..))))))))).))))))..)))).. (-34.60)
```

Matched bases: 16

Mature reads in Control: 23200   Mature reads in dsDicer: 10210
Star reads in Control: 1   Star reads in dsDicer: 2

Initial dG =   -34.60

```
          10        20        30
--|   GG     A         UC   A      CUAGU
  UUAG  ACCUA ACCCGUAGA  CGA CUUGUG     \
  AAUC  UGGAU UGGGUAUCU  GCU GAACAC     U
GU^   GA     C         UA   C      UUAGC
        70        60        50        40
```

```
Bge-Mir-100         AACCCGUAGAUCCGAACUUGUG--
tca-mir-100-5p      AACCCGUAGAUCCGAACUUGUGGG
bmo-mir-100         AACCCGUAGAUCCGAACUUGUG--
nvi-mir-100         AACCCGUAGAUCCGAACUUGUG--
api-mir-100         GACCCGUAGAUCCGAACUUGUG--
mse-mir-100         AACCCGUAGAUCCGAACUUGUG--
ame-mir-100         AACCCGUAGAUCCGAACUUGUG--
dme-mir-100-5p      AACCCGUAAAUCCGAACUUGUG--
                     ******* *************
```

Genomic coordinates:
Bge-Mir-100_5p: Scaffold202:1024563-1024584:+
Bge-Mir-100_pre: Scaffold202:1024563-1024619:+
Bge-Mir-100_3p*: Scaffold202:1024598-1024619:+

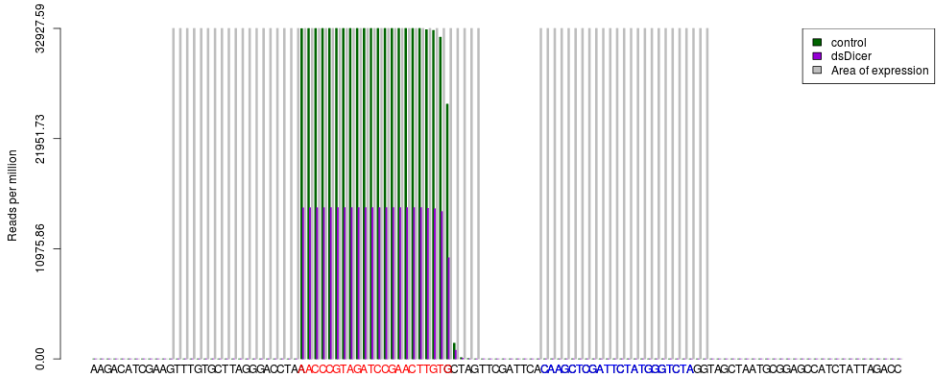

>Bge-let-7_pre+_11
TGTGGCCAGGG**TGAGGTAGTAGGTTGTATAGTT**ATGAACTACATCATTTTTGGGAGAA**CTGTACAACTTGCTAACTTTCC**TTGTTCACGC
T

>Bge-let-7_5p
TGAGGTAGTAGGTTGTATAGTT

>Bge-let-7_3p*
CTGTACAACTTGCTAACTTTCC

```
UGUGGCCAGGGUGAGGUAGUAGGUUGUAUAGUUAUGAACUACAUCAUUUUUGGGAGAACUGUACAACUUGCUAACUUUCCUGUAGUUGCUC
.(((..(((((.(((.((((((((((((((((.....(((.........)))...)))))))))))))))).))).))))).)))... (-37.80)
```

Matched bases: 18

Mature reads in Control: 761   Mature reads in dsDicer: 224
Star reads in Control: 96   Star reads in dsDicer: 7

```
 Initial dG =    -37.80

          10        20        30        40
U--|  GC     U   G                AUGAA   CAUC
   GUG  CAGGG GAG UAGUAGGUUGUAUAGUU     CUA    \
   CAC  GUUCC UUC AUCGUUCAACAUGUCAA     GGU    A
UCG^  UU     U   A                GAG--   UUUU
90        80        70        60         50
```

```
Bge-let-7         UGAGGUAGUAGGUUGUAUAGUU
bmo-let-7-5p      UGAGGUAGUAGGUUGUAUAGU-
nvi-let-7         UGAGGUAGUAGGUUGUAUAGU-
dme-let-7-5p      UGAGGUAGUAGGUUGUAUAGU-
ame-let-7         UGAGGUAGUAGGUUGUAUAGU-
tca-let-7-5p      UGAGGUAGUAGGUUGUAUAG--
api-let-7         UGAGGUAGUUGGUUGUAUAGU-
                  ********* **********
```

Genomic coordinates:
Bge-let-7_5p: Scaffold202:1027262-1027283:+
Bge-let-7_pre: Scaffold202:1027262-1027330:+
Bge-let-7_3p*: Scaffold202:1027309-1027330:+

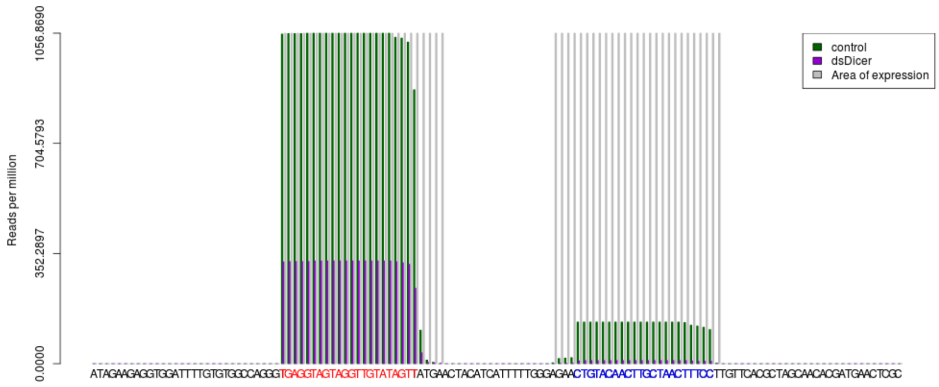

>Bge-Mir-125_pre+_11
CAGCAGCCCATTCCCTGAGACCCTAACTTGTGACGTATTATGTAAAATCTCACAGGCTAGAATCTCAGGTATGGGAGGTTCGG

>Bge-Mir-125_5p
TCCCTGAGACCCTAACTTGTGA

>Bge-Mir-125_3p*
ACAGGCTAGAATCTCAGGTAT

```
CAGCAGCCCAUUCCCUGAGACCCUAACUUGUGACGUAUUAUGUAAAAUCUCACAGGCUAGAAUCUCAGGUAUUGUAGUUGCUC
.(((..(((((..(((((((..(((.(((((((.((.((....)).)).))))))).)))..))))))).))))).)..)))... (-31.40)
```

Matched bases: 15

Mature reads in Control: 1189   Mature reads in dsDicer: 590
Star reads in Control: 48   Star reads in dsDicer: 16

Initial dG =   -31.40

```
          10        20        30        40
C--|  AG     UC       CC    A       C   A  A
   AGC  CCCAU  CCUGAGA  CUA CUUGUGA GU UU U
   UUG  GGGUA  GGACUCU  GAU GGACACU UA AA G
GGC^  GA     U-       AA    C       C   A  U
  80        70         60        50
```

```
Bge-Mir-125         UCCCUGAGACCCUAACUUGUGA
tca-mir-125-5p      UCCCUGAGACCCUAACUUGUGA
nvi-mir-125         UCCCUGAGACCCUAACUUGUGA
dme-mir-125-5p      UCCCUGAGACCCUAACUUGUGA
ame-mir-125         CCCCUGAGACCCUAACUUGUGA
                     *********************
```

Genomic coordinates:
Bge-Mir-125_5p: Scaffold202:1087715-1087736:+
Bge-Mir-125_pre: Scaffold202:1087715-1087775:+
Bge-Mir-125_3p*: Scaffold202:1087755-1087775:+

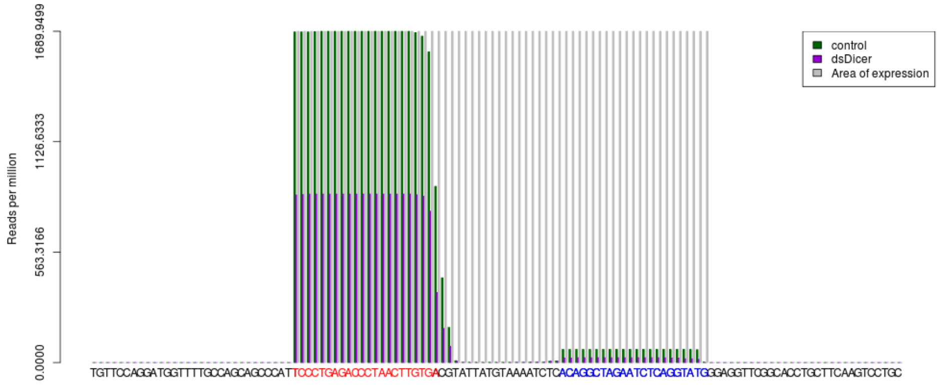

>Bge-Mir-275_pre+_11
GGTCACTCGCA**CGTGCTGCATCAGGTGCTAGTGACT**GTGAATAAATTGTCAG**TCAGGTACCTGAAGTAGCGCGCG**GAGAGACTCAG

>Bge-Mir-275_3p
TCAGGTACCTGAAGTAGCGCGCG

>Bge-Mir-275_5p
CGTGCTGCATCAGGTGCTAGTGACT

```
GGUCACUCGCACGUGCUGCAUCAGGUGCUAGUGACUGUGAAUAAAUUGUCAGUCAGGUACCUGAAGUAGCGCGCGGAGAGACUCAG
((((.(((...((((((((.(((((((((..((((((...........))))))))))))))).))))))))..))).))))... (-41.90)
```

Matched bases: 20

Mature reads in Control: 5312 Mature reads in dsDicer: 2210
Star reads in Control: 157 Star reads in dsDicer: 50

```
 Initial dG =    -41.90

           10        20        30        40
---|   A   GCA       A         AG       UGAAU
   GGUC CUC   CGUGCUGC UCAGGUGCU  UGACUG     A
   UCAG GAG   GCGCGAUG AGUCCAUGG  ACUGAC     A
GAC^   A   GC-       A         --       UGUUA
     80         70        60          50
```

```
Bge-Mir-275         UCAGGUACCUGAAGUAGCGCGCG
tca-mir-275-3p      UCAGGUACCUGAAGUAGCGCGCG
bmo-mir-275-3p      UCAGGUACCUGAAGUAGCGCGCG
nvi-mir-275         UCAGGUACCUGAAGUAGCGCGCG
dme-mir-275-3p      UCAGGUACCUGAAGUAGCGCGCG
ame-mir-275         UCAGGUACCUGAAGUAGCGCGCG
mse-mir-275         UCAGGUACCUGAAGUAGCGCGCG
api-mir-275         UCAGGUACCUGAAGUAGCGCG--
                    *********************
```

Genomic coordinates:
Bge-Mir-275_5p: Scaffold207:2106422-2106446:+
Bge-Mir-275_pre: Scaffold207:2106422-2106485:+
Bge-Mir-275_3p: Scaffold207:2106463-2106485:+

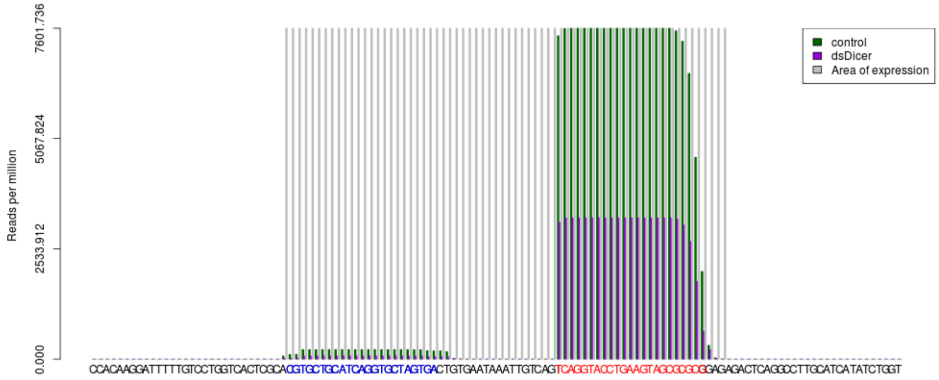

>Bge-Mir-305_pre+_11
GTCCCGTGTTC**ATTGTACTTCATCAGGTGCTCTGG**TAGGTTCAGTATC**CCGGCATCTGTTGGAGTGCAACTGA**TAGGATGGCGC

>Bge-Mir-305_5p
ATTGTACTTCATCAGGTGCTCTGG

>Bge-Mir-305_3p*
CCGGCATCTGTTGGAGTGCAACTGA

```
GUCCCGUGUUCAUUGUACUUCAUCAGGUGCUCUGGUAGGUUCAGUAUCCCGGCAUCUGUUGGAGUGCAACUGAGAGAGACUCAG
((((.....((((((((((((.((((((((..((((.......))))..)))))))).))))))))))).))).)).)).))).. (-31.90)
```

Matched bases: 19

Mature reads in Control: 3017   Mature reads in dsDicer: 904
Star reads in Control: 692   Star reads in dsDicer: 62

Initial dG =   -31.90

```
            10         20         30        40
--   --| GUGU   -          U        CU     GGU
  GUC  CC     UCA UUGUACUUCA CAGGUGCU  GGUA   \
  CGG  GG     AGU AACGUGAGGU GUCUACGG  CUAU   U
CG   UA^ AU--   C          U        CC     GAC
   80          70         60         50
```

```
Bge-Mir-305         AUUGUACUUCAUCAGGUGCUCUGG
tca-mir-305-5p      AUUGUACUUCAUCAGGUGCUC---
bmo-mir-305-5p      AUUGUACUUCAUCAGGUGCUCUG-
nvi-mir-305         AUUGUACUUCAUCAGGUGCUCUG-
ame-mir-305         AUUGUACUUCAUCAGGUGCUCUG-
dme-mir-305-5p      AUUGUACUUCAUCAGGUGCUCUG-
                    *********************
```

Genomic coordinates:
Bge-Mir-305_5p: Scaffold207:2106558-2106581:+
Bge-Mir-305_pre: Scaffold207:2106558-2106619:+
Bge-Mir-305_3p*: Scaffold207:2106595-2106619:+

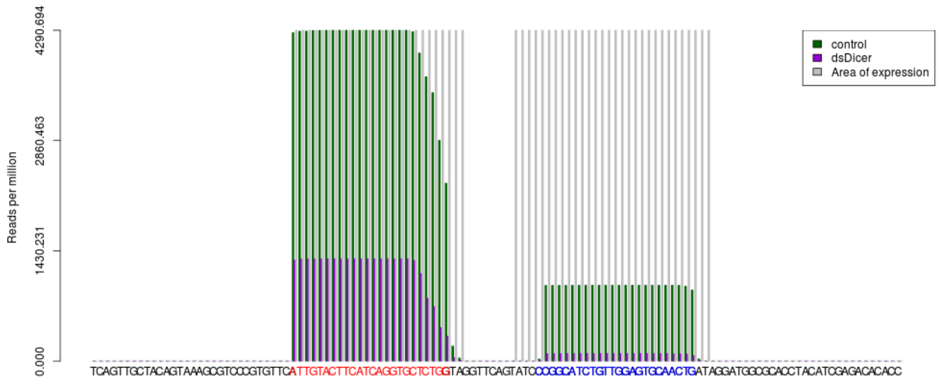

>Bge-Mir-1000_pre+_11
GGTGGACTGTG**ATATTGTCCTGTCACAGCAGT**ACTGATCACAAGAGCC**TACTGCTGTTTCGGGACATA**GCCACAGACCC

>Bge-Mir-1000_3p
ATATTGTCCTGTCACAGCAGT

>Bge-Mir-1000_5p*
TACTGCTGTTTCGGGACATA

GGUGGACUGUG**AUAUUGUCCUGUCACAGCAGU**ACUGAUCACAAGAGCC**UACUGCUGUUUCGGGACAUA**GAGAGACUCAG
...((.((((....(((((((..(((((((((....((....))...)))))))))..))))))).....))))).)). (-34.70)

Matched bases: 15

Mature reads in Control: 5 Mature reads in dsDicer: 6
Star reads in Control: 1 Star reads in dsDicer: 1

Initial dG = -34.70

```
        10        20        30        40
GGU| A     AUAU       UC         CUGA  A
   GG CUGUG    UGUCCUG  ACAGCAGUA    UC C
   CC GACAC    ACAGGGC  UGUCGUCAU    AG A
C--^ A     CGAU       UU         CCG-  A
          70        60        50
```

```
Bge-Mir-1000         AUAUUGUCCUGUCACAGCAGU-
tca-mir-1000-5p      AUAUUGUCCUGUCACAGC----
bmo-mir-1000         AUAUUGUCCUGUCACAGCAGU-
dme-mir-1000-5p      AUAUUGUCCUGUCACAGCAGU-
api-mir-1000         AUAUUGUCCUGUCACAGCAGUA
mse-mir-1000         AUAUUGUCCUGUCACAGCAGUA
ame-mir-1000         AUAUUGUCUUGUCACAGCAGU-
                     ******** *********
```

Genomic coordinates:
Bge-Mir-1000_5p: Scaffold226:2796-2816:+
Bge-Mir-1000_pre: Scaffold226:2796-2852:+
Bge-Mir-1000_3p*: Scaffold226:2833-2852:+

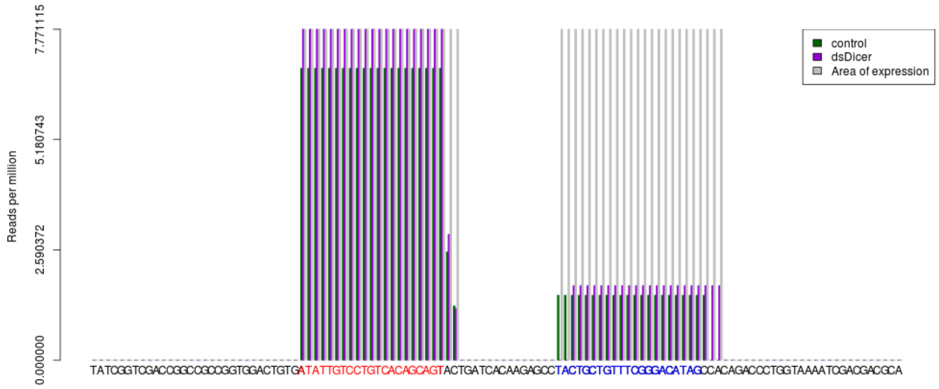

>Bge-Mir-276_pre+_11
AACTTGCCAGT**AGCGAGGTATAGAGTTCCTACG**TGGTATTTGTAATAG**TAGGAACTTCATACCGTGCTCT**TGGAAGTGCCA

>Bge-Mir-276_3p
TAGGAACTTCATACCGTGCTCT

>Bge-Mir-276_5p*
AGCGAGGTATAGAGTTCCTACG

```
AACUUGCCAGUAGCGAGGUAUAGAGUUCCUACGUGGUAUUUGUAAUAGUAGGAACUUCAUACCGUGCUCUUGGAAGUGCCA
.((((.((((.((((.(((((..(((((((((..............)))))))))..))))).)))).)))))))).... (-34.10)
```

Matched bases: 17

Mature reads in Control: 117004 Mature reads in dsDicer: 54929
Star reads in Control: 22 Star reads in dsDicer: 9

Initial dG = -34.10

```
           10        20        30
A---|   G    U    A     AG         GUGGUAU
    ACUU CCAG AGCG GGUAU  AGUUCCUAC       \
    UGAA GGUU UCGU CCAUA  UCAAGGAUG       U
ACCG^   -    C    G     CU         AUAAUGU
80         70        60        50
```

```
Bge-Mir-276         UAGGAACUUCAUACCGUGCUCU
tca-mir-276-3p      UAGGAACUUCAUACCGUGCUCU
bmo-mir-276-3p      UAGGAACUUCAUACCGUGCUCU
nvi-mir-276         UAGGAACUUCAUACCGUGCUCU
ame-mir-276         UAGGAACUUCAUACCGUGCUCU
mse-mir-276         UAGGAACUUCAUACCGUGCUCU
api-mir-276         UAGGAACUUCAUACCGUGCUCU
                    **********************
```

Genomic coordinates:
Bge-Mir-276_3p: Scaffold22706:2023-2044:-
Bge-Mir-276_pre: Scaffold22706:2023-2081:-
Bge-Mir-276_5p*: Scaffold22706:2060-2081:-

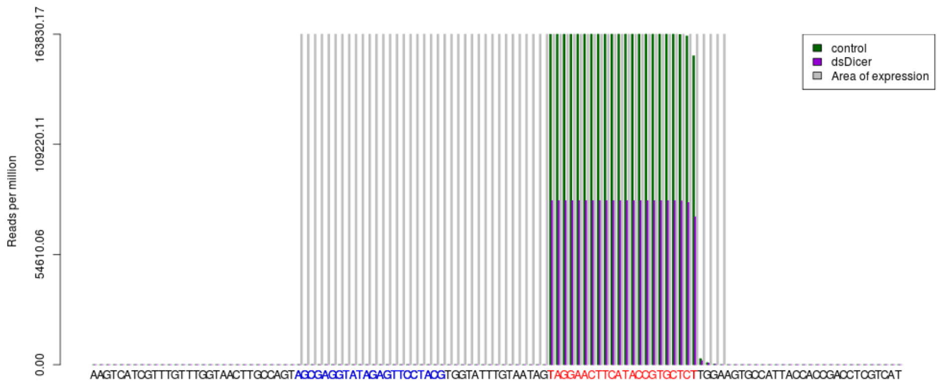

>Bge-Mir-9d_pre+_11
CGCTGATTTTTTCTTTGGTTATCTAGCTGTATGAGTGAAATGAGCGTCATAAAGCTAGGTTACCGAAGTTAAGAACAGCACC

>Bge-Mir-9d_5p
TCTTTGGTTATCTAGCTGTATGA

>Bge-Mir-9d_3p*
ATAAAGCTAGGTTACCGAAGTTA

```
CGCUGAUUUUUUCUUUGGUUAUCUAGCUGUAUGAGUGAAAUGAGCGUCAUAAAGCUAGGUUACCGAAGUUAUGGAAGUGCCA
.((((.((((..(((((((.((((((((.(((((((.......)).))))).)))))))).)))))))..)))).)))).... (-35.20)
```

Matched bases: 18

Mature reads in Control: 28965 Mature reads in dsDicer: 14046
Star reads in Control: 42 Star reads in dsDicer: 93

Initial dG = -35.20

```
          10        20        30
C--    A    UU        U         G     -| GAA
   GCUG UUUU  CUUUGGU AUCUAGCU UAUGA GU   \
   CGAC AGAA  GAAGCCA UGGAUCGA AUACU CG   A
CCA    A    UU        U         A     G^ AGU
 80        70        60        50
```

```
Bge-Mir-9d         UCUUUGGUUAUCUAGCUGUAUGA
tca-mir-9d-5p      -CUUUGGUGAUCUAACU-UAUGA
bmo-mir-9d         -AUAAAGCUAAUUCACUGAGUGU
                     *   *  *  *  **   **
```

Genomic coordinates:
Bge-Mir-9d_5p: Scaffold240:1038521-1038543:+
Bge-Mir-9d_pre: Scaffold240:1038521-1038580:+
Bge-Mir-9d_3p*: Scaffold240:1038558-1038580:+

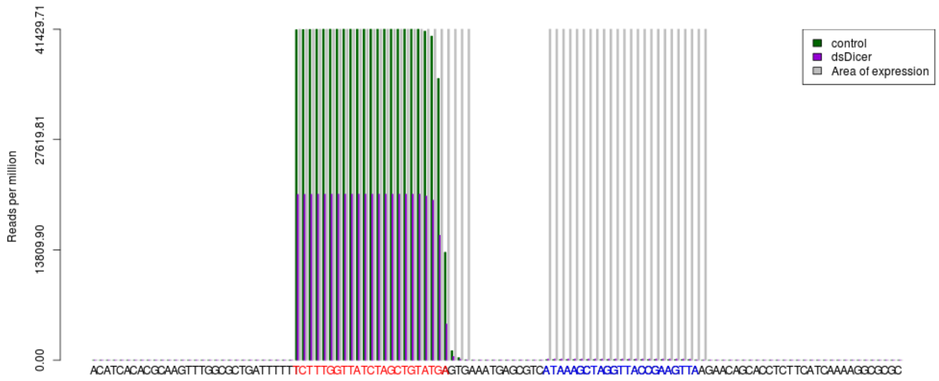

>Bge-Mir-22_pre+_11
CTACCTCGGTG**CGGCTCATCACCGGGCATCTTGC**GTGACGTTTGACTCTGC**AAGCTGCCTAGTGAAGGGCAACA**TTGGGGTGGAG

>Bge-Mir-22_3p
AAGCTGCCTAGTGAAGGGCAACA

>Bge-Mir-22_5p*
CGGCTCATCACCGGGCATCTTGC

```
CUACCUCGGUGCGGCUCAUCACCGGGCAUCUUGCGUGACGUUUGACUCUGCAAGCUGCCUAGUGAAGGGCAACAUUGGGGUGGAG
((((((((((..((((.((((..((((.((((((.((........)))))))).)))).)))).)))).)))))))))))).. (-46.40)
```

Matched bases: 15

Mature reads in Control: 33 Mature reads in dsDicer: 8
Star reads in Control: 11 Star reads in dsDicer: 7

Initial dG = -46.40

```
          10        20        30        40
--|          CG    A    CG    U      U  CGU
  CUACCUCGGUG  GCUC UCAC  GGCA CUUGCG GA   U
  GGUGGGGUUAC  CGGG AGUG  CCGU GAACGU CU   U
GA^          AA    A    AU    C      -  CAG
    80        70        60        50
```

Genomic coordinates:
Bge-Mir-22_5p*: Scaffold2523:49274-49296:+
Bge-Mir-22_pre: Scaffold2523:49274-49336:+
Bge-Mir-22_3p: Scaffold2523:49314-49336:+

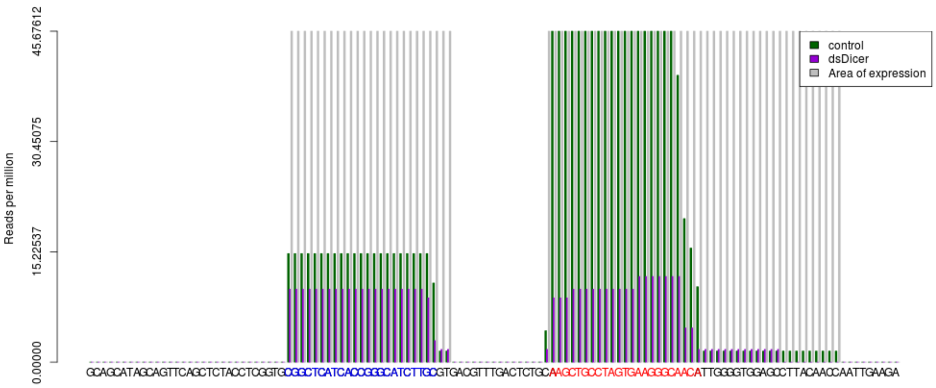

>Bge-Mir-137_pre+_11
TTGGTTGGCCA**CGCGTATTCTTGGGGAATTAACA**CACATGTAGCGATG**TTATTGCTTGAGAATACACGTA**GCTGACAAGTT

>Bge-Mir-137_3p
TTATTGCTTGAGAATACACGTA

>Bge-Mir-137_5p*
CGCGTATTCTTGGGGAATTAACA

```
UUGGUUGGCCACGCGUAUUCUUGGGGAAUUAACACACAUGUAGCGAUGUUAUUGCUUGAGAAUACACGUAGCUGACAAGUU
...((..((.(((.(((((((..((.(((.((((...........))))))).))..))))))).))).))..)).....  (-28.80)
```

Matched bases: 16

Mature reads in Control: 24   Mature reads in dsDicer: 30
Star reads in Control: 0   Star reads in dsDicer: 1

Initial dG =   -28.80

```
          10        20         30        40
UUG--| UG  C   C       UG  G   U    CACAU
     GU  GC ACG GUAUUCU  GG AAU AACA     G
     CA  CG UGC CAUAAGA  UC UUA UUGU     U
UUGAA^ GU  A   A       GU  G   -    AGCGA
80        70        60         50
```

```
Bge-Mir-137         UUAUUGCUUGAGAAUACACGUA-
tca-mir-137-3p      UUAUUGCUUGAGAAUACACGUA-
bmo-mir-137         UUAUUGCUUGAGAAUACACGUA-
nvi-mir-137         UUAUUGCUUGAGAAUACACGUA-
ame-mir-137         UUAUUGCUUGAGAAUACACGUA-
dme-mir-137-3p      -UAUUGCUUGAGAAUACACGUAG
api-mir-137         -UAUUGCUUGAGAAUACACGUAG
mse-mir-137         -UAUUGCUUGAGAAUACACGUAG
                     *********************
```

Genomic coordinates:
Bge-Mir-137_3p: Scaffold29:2455180-2455201:-
Bge-Mir-137_pre: Scaffold29:2455180-2455238:-
Bge-Mir-137_5p*: Scaffold29:2455216-2455238:-

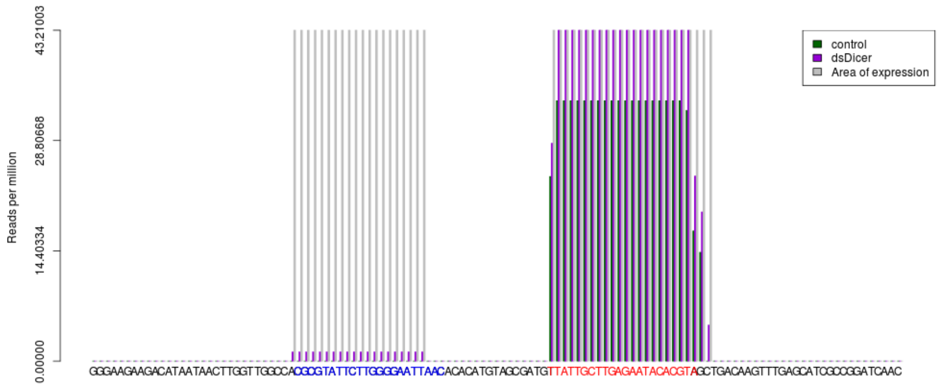

>Bge-Mir-279e_pre+_11
TTCTTGTGGTG**GATGGGTGTATCTCTAGTGCATG**TTTTTTTATATCTCA**TGACTAGATTTTCACTCGTTCA**TCAAGAGATTT

>Bge-Mir-279e_3p
TGACTAGATTTTCACTCGTTCA

>Bge-Mir-279e_5p*
GATGGGTGTATCTCTAGTGCATG

```
UUCUUGUGGUGGAUGGGUGUAUCUCUAGUGCAUGUUUUUUUAUAUCUCAUGACUAGAUUUUCACUCGUUCAUCAAGAGAUUU
.((((.(((((((((((((....((((((.((((.............))))))))))....))))))))))))).))))... (-31.40)
```

Matched bases: 16

```
Mature reads in Control: 909          Mature reads in dsDicer: 506
Star reads in Control: 55             Star reads in dsDicer: 28

 Initial dG =    -31.40

          10        20        30        40
U--|   G            UAUC      G     UUUUUU
   UCUU UGGUGGAUGGGUG    UCUAGU CAUG      \
   AGAG ACUACUUGCUCAC    AGAUCA GUAC      U
UUU^   A            UUUU      -     UCUAUA
 80        70        60          50


tca-mir-279e-3p      UGACUAG-AUCGAACACUCGCUUGC
bmo-mir-279e         UGACUAGCGAUACACUCCGGCU---
Bge-Mir-279e         UGACUAG--AUUUUCACUCGUUCA-
                     *******       * *   * *
```

Genomic coordinates:
Bge-Mir-279e_3p: Scaffold309:303135-303156:-
Bge-Mir-279e_pre: Scaffold309:303135-303194:-
Bge-Mir-279e_5p*: Scaffold309:303172-303194:-

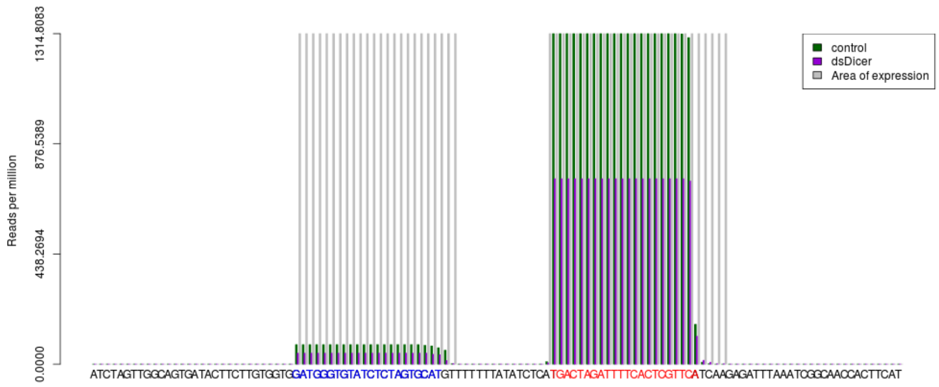

>Bge-Mir-34_pre+_11
TCAGAGCACGA**TGGCAGTGTGGTTAGCTGGTTGT**GTAGTTGTTAATCA**CGACCGCTTCTGCACTGCCCTC**GTGAGTTGATT

>Bge-Mir-34_5p
TGGCAGTGTGGTTAGCTGGTTGT

>Bge-Mir-34_3p*
CGACCGCTTCTGCACTGCCCTC

UCAGAGCACGA**UGGCAGUGUGGUUAGCUGGUUGU**GUAGUUGUUAAUCA**CGACCGCUUCUGCACUGCCCUC**UCAAGAGAUUU
((((..(((((.(((((((..(..(((.(((((((..........)))))))))).)..))))))).))))).)..)))).. (-42.80)

Matched bases: 16

Mature reads in Control: 1159 Mature reads in dsDicer: 327
Star reads in Control: 9 Star reads in dsDicer: 4

Initial dG = -42.80

```
          10        20        30        40
--|   AG      U        UG UU    U        UAGUU
  UCAG  CACGA GGCAGUG  G  AGC GGUUGUG      \
  AGUU  GUGCU CCGUCAC  C  UCG CCAGCAC      G
UU^   GA      C        GU U-    -        UAAUU
80        70        60          50
```

```
Bge-Mir-34          UGGCAGUGUGGUUAGCUGGUUGU-
tca-mir-34-5p       UGGCAGUGUGGUUAGCUGGU----
nvi-mir-34          UGGCAGUGUGGUUAGCUGGUUG--
bmo-mir-34-5p       UGGCAGUGUGGUUAGCUGGUUG--
ame-mir-34          UGGCAGUGUUGUUAGCUGGUUG--
mse-mir-34          UGGCAGUGUGGUUAGCUGGUUGU-
dme-mir-34-5p       UGGCAGUGUGGUUAGCUGGUUGUG
api-mir-34          UGGCAGUGUGAUUAGCUGGUU---
                    *********  *********
```

Genomic coordinates:
Bge-Mir-34_3p*: Scaffold338:858241-858262:-
Bge-Mir-34_pre: Scaffold338:858241-858299:-
Bge-Mir-34_5p: Scaffold338:858277-858299:-

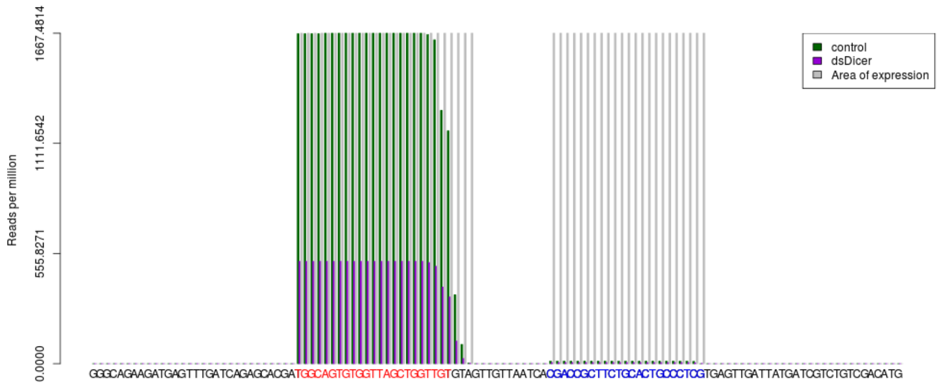

>Bge-Mir-277_pre+_11
GTTCTGGAGTG**CGTGTCAGAGGCGCGTTTACA**AAGCTGCTGTTAGAAATTCTG**TAAATGCACTATCTGGTACGACA**TTCCAGCACTT

>Bge-Mir-277_3p
TAAATGCACTATCTGGTACGACA

>Bge-Mir-277_5p
CGTGTCAGAGGCGCGTTTACA

```
GUUCUGGAGUGCGUGUCAGAGGCGCGUUUACAAAGCUGCUGUUAGAAAUUCUGUAAAUGCACUAUCUGGUACGACAUUCCAGCACUU
((.((((((((((..((((((.(((((((((...(((....)))......))))))))).)).)))))..))).)))))))).)).. (-36.40)
```

Matched bases: 16

Mature reads in Control: 2880 Mature reads in dsDicer: 1379
Star reads in Control: 0 Star reads in dsDicer: 2

```
 Initial dG =    -36.40

          10         20         30           40
--  U        -   GU     -  C         AAG---|  C
  GU CUGGAGUG CGU  CAGA GG GCGUUUACA      CUG U
  CA GACCUUAC GCA  GUCU UC CGUAAAUGU      GAU G
UU  C        A   UG     A  A         CUUAAA^  U
      80        70        60        50
```

```
Bge-Mir-277         UAAAUGCACUAUCUGGUACGACA
tca-mir-277-3p      UAAAUGCACUAUCUGGUACGACA
bmo-mir-277-3p      UAAAUGCACUAUCUGGUACGACA
nvi-mir-277         UAAAUGCACUAUCUGGUACGACA
dme-mir-277-3p      UAAAUGCACUAUCUGGUACGACA
ame-mir-277         UAAAUGCACUAUCUGGUACGACA
mse-mir-277         UAAAUGCACUAUCUGGUACGACA
api-mir-277         UAAAUGCACUAUCUGGUACGACA
                    ***********************
```

Genomic coordinates:
Bge-Mir-277_3p: Scaffold338:873459-873481:-
Bge-Mir-277_pre: Scaffold338:873459-873523:-
Bge-Mir-277_5p: Scaffold338:873503-873523:-

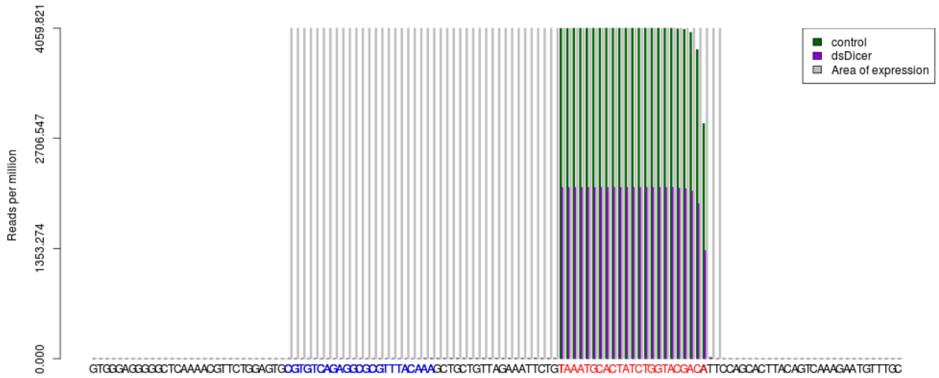

>Bge-Mir-317_pre+_11
TGGTGTGGGAC**GGGGAGCCACACTGAGTTCACT**CGAATATGAGAAACGAG**TGAACACAGCTGGTGGTATCTCAGT**TTCATGCCAGG

>Bge-Mir-317_3p
TGAACACAGCTGGTGGTATCTCAGT

>Bge-Mir-317_5p*
GGGGAGCCACACTGAGTTCACT

```
UGGUGUGGGACGGGGAGCCACACUGAGUUCACUCGAAUAUGAGAAACGAGUGAACACAGCUGGUGGUAUCUCAGUUUCAUGCCAGG
((((((..((.(((((((((.(((.(((((((((..........))))))))).)))...))))).)))).))..)))))).. (-41.90)
```

Matched bases: 17

Mature reads in Control: 916   Mature reads in dsDicer: 423
Star reads in Control: 0   Star reads in dsDicer: 1

```
 Initial dG =    -41.90

         10          20           30         40
--       GG  G    -     A--|  A          AAUAU
  UGGUGUG  AC GGGA GCCAC   CUG GUUCACUCG     \
  ACCGUAC  UG CUCU UGGUG   GAC CAAGUGAGC     G
GG       UU  A    A     GUC^  A          AAAGA
     80        70         60         50
```

```
Bge-Mir-317         --UGAACACAGCUGGUGGUAUCUCAGU
dme-mir-317-3p      --UGAACACAGCUGGUGGUAUC-CAGU
tca-mir-317-3p      --UGAACACAGCUGGUGGUAUCUCAGU
nvi-mir-317         --UGAACACAGCUGGUGGUAUCUCAGU
ame-mir-317         --UGAACACAGCUGGUGGUAUCUCAGU
mse-mir-317         --UGAACACAGCUGGUGGUAUCUCA--
api-mir-317         --UGAACACAGCUGGUGGUAUCU----
bmo-mir-317-3p      AGUGAACACAGCUGGUGGUAUC-----
                      ********************
```

Genomic coordinates:
Bge-Mir-317_3p: Scaffold338:892361-892385:-
Bge-Mir-317_pre: Scaffold338:892361-892424:-
Bge-Mir-317_5p*: Scaffold338:892403-892424:-

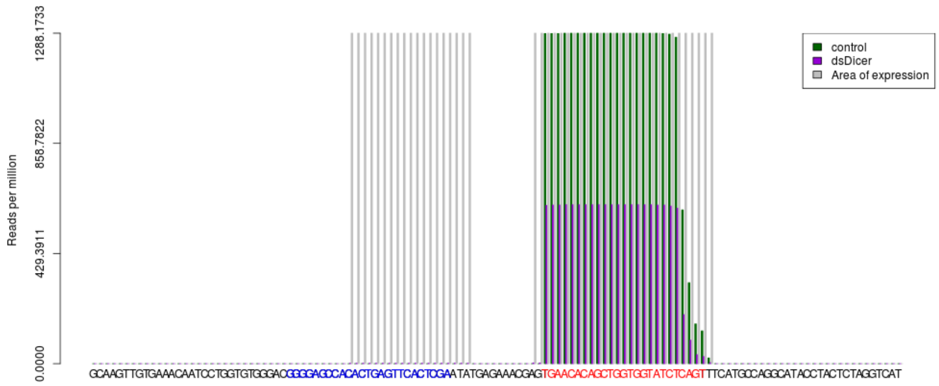

>Bge-Mir-995b_pre+_11
GCTCTTGGCCG**AGTTGAATTTTGTGGCTGCTTCA**CATAAATTAATTG**TAGCACCATGGGATTCAGCTTA**GTCCATAGCCA

>Bge-Mir-995b_3p
TAGCACCATGGGATTCAGCTTA

>Bge-Mir-995b_5p*
AGTTGAATTTTGTGGCTGCTTCA

GCUCUUGGCCG**AGUUGAAUUUUGUGGCUGCUUCA**CAUAAAUUAAUUG**UAGCACCAUGGGAUUCAGCUUA**GUCCAUAGCCA
(((..((((..((((((((((..(((.((((..(((.........))))))))))..))))))))))..).))).))).. (-32.00)

Matched bases: 18

Mature reads in Control: 71 Mature reads in dsDicer: 49
Star reads in Control: 3 Star reads in dsDicer: 0

Initial dG = -32.00

```
           10        20        30        40
--|  CU   - CG          UG   C    UC   UAAA
  GCU  UGG C  AGUUGAAUUU  UGG UGCU  ACA    \
  CGA  ACC G  UCGACUUAGG  ACC ACGA  UGU    U
AC^  U-   U AU          GU   -    --   UAAU
.         70        60         50
```

Genomic coordinates:
Bge-Mir-995b_3p: Scaffold339:440856-440877:-
Bge-Mir-995b_pre: Scaffold339:440856-440913:-
Bge-Mir-995b_5p*: Scaffold339:440891-440913:-

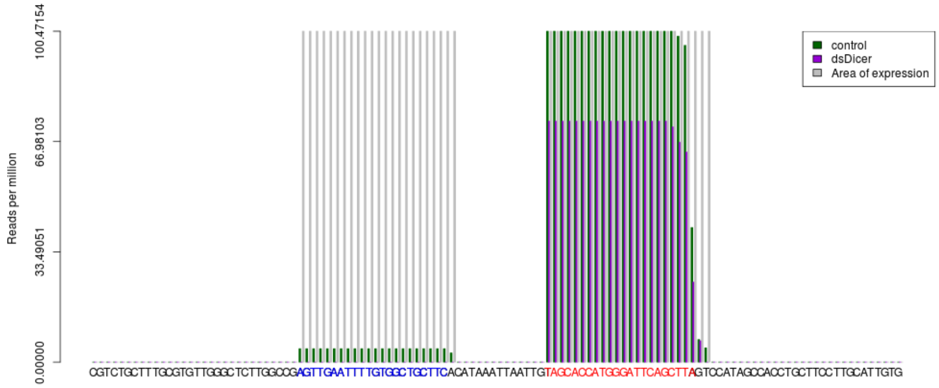

>Bge-Mir-11_pre+_11
TGCTTTGGTGC**CAGAACTCTGGCTGTGACATTGTG**CAGTATATAGGTCA**CATCACAGACAGAGTTCTAGTT**ACCCGTGCATC

>Bge-Mir-11_3p
CATCACAGACAGAGTTCTAGTT

>Bge-Mir-11_5p*
CAGAACTCTGGCTGTGACATTGTG

```
UGCUUUGGUGCCAGAACUCUGGCUGUGACAUUGUGCAGUAUAUAGGUCACAUCACAGACAGAGUUCUAGUUACCCGUGCAUC
(((...(((((.(((((((((.((((((...(((((.........).)))))))))).))))))))).)).)))...))).. (-32.00)
```

Matched bases: 17

Mature reads in Control: 501   Mature reads in dsDicer: 414
Star reads in Control: 71   Star reads in dsDicer: 21

```
 Initial dG =    -32.00

           10        20        30         40
--   UUU   -| C         G      CAU     - AGUA
  UGC   GGU GC AGAACUCUG CUGUGA   UGUG C    \
  ACG   CCA UG UCUUGAGAC GACACU   ACAC G    U
CU   UGC   U^ A         A      ---    U GAUA
 80        70        60           50
```

```
dme-mir-11-3p      CAUCACAGUCUGAGUUCUUGC-
mse-mir-11         CAUCACAGUCAGAGUUCUAGCU
bmo-mir-11-3p      CAUCACAGUCAGAGUUCUAGCU
Bge-Mir-11         CAUCACAGACAGAGUUCUAGUU
ame-mir-11         CAUCACAGGCAGAGUUCUAGUU
tca-mir-11-3p      CAUCACAGGCAGAGUUCUAGCU
                   ******** * ******* *
```

Genomic coordinates:
Bge-Mir-11_3p: Scaffold339:447257-447278:-
Bge-Mir-11_pre: Scaffold339:447257-447316:-
Bge-Mir-11_5p*: Scaffold339:447293-447316:-

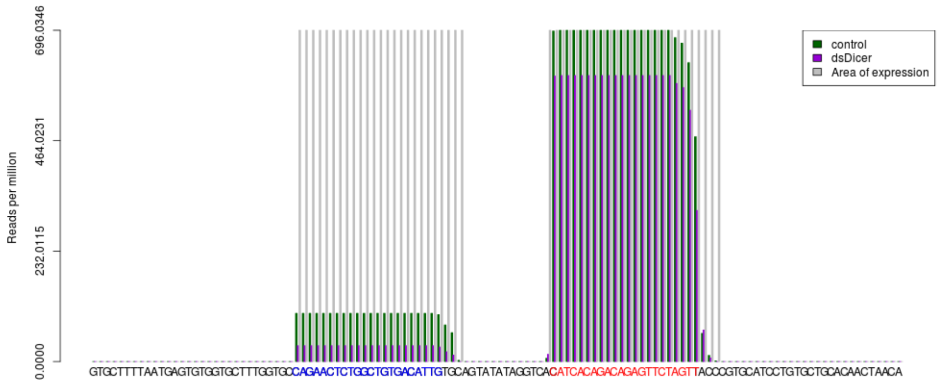

>Bge-Mir-3049_pre+_11
CCTTCGCTGAG**TCGGGAAGGCAGTTGCGGCGGATT**GTGACGTCAGACGAG**TCCGTCCAACTCCTTTCCGTCT**CGGCGTCGGGC

>Bge-Mir-3049_5p
TCGGGAAGGCAGTTGCGGCGGATT

>Bge-Mir-3049_3*p
TCCGTCCAACTCCTTTCCGTCT

```
CCUUCGCUGAGUCGGGAAGGCAGUUGCGGCGGAUUGUGACGUCAGACGAGUCCGUCCAACUCCUUUCCGUCUACCCGUGCAUC
((..(((((((.((((((((.(((((.((((((((((........)).))))))))))))))))))))).))))))).)).. (-48.60)
```

Matched bases: 19

Mature reads in Control: 7   Mature reads in dsDicer: 3
Star reads in Control: 0   Star reads in dsDicer: 2

```
 Initial dG =    -48.60

          10        20        30         40
--| UU       U        C     C         -   GAC
  CC   CGCUGAG CGGGAAGG AGUUG GGCGGAUU GU   G
  GG   GCGGCUC GCCUUUCC UCAAC CUGCCUGA CA   U
CG^ CU       U        -     -         G   GAC
  80        70         60        50
```

```
tca-mir-3049-5p      UCGGGAAGACAGUUGCGGCGGA--
api-mir-3049         UCGGGAAGGCAGCUGCGGCGGACU
Bge-Mir-3049         UCGGGAAGGCAGUUGCGGCGGAUU
ame-mir-3049-5p      UCGGGAAGGUAGUUGCGGCGGAU-
                     ********  ** *********
```

Genomic coordinates:
Bge-Mir-3049_5p: Scaffold36:2815142-2815165:+
Bge-Mir-3049_pre: Scaffold36:2815142-2815202:+
Bge-Mir-3049_3*p: Scaffold36:2815181-2815202:+

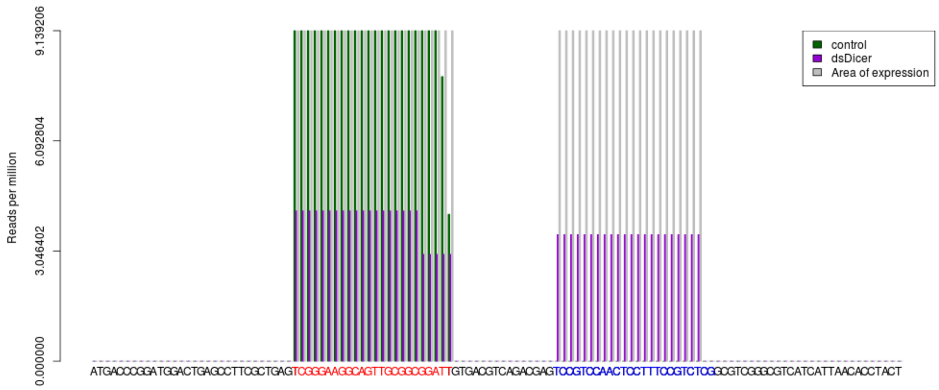

>Bge-Mir-76_pre+_11
CTCACATGTTA**CGGGTTTCGCGACGATTGAGCC**GGATAAAGTTGAACAGG**TTCGTTGTCGACGAAACCTGCA**CAATGTGGGGA

>Bge-Mir-76_3p
TTCGTTGTCGACGAAACCTGCA

>Bge-Mir-76_5p*
CGGGTTTCGCGACGATTGAGCC

```
CUCACAUGUUACGGGUUUCGCGACGAUUGAGCCGGAUAAAGUUGAACAGGUUCGUUGUCGACGAAACCUGCACAAUGUGGGGA
((((((....(((((((((((((((.(((((...............)))))))))))).))))))))....))))))).. (-38.50)
```

Matched bases: 19

Mature reads in Control: 141   Mature reads in dsDicer: 29
Star reads in Control: 1   Star reads in dsDicer: 0

Initial dG =   -38.50

```
          10        20         30        40
--       GUUA          -|       U      GGAUAAA
  CUCACAU    CGGGUUUCG CGACGAU GAGCC        \
  GGGUGUA    GUCCAAAGC GCUGUUG CUUGG        G
AG       ACAC          A^      -      ACAAGUU
  80        70        60         50
```

Genomic coordinates:
Bge-Mir-76_5p*: Scaffold367:763980-764001:+
Bge-Mir-76_pre: Scaffold367:763980-764040:+
Bge-Mir-76_3p: Scaffold367:764019-764040:+

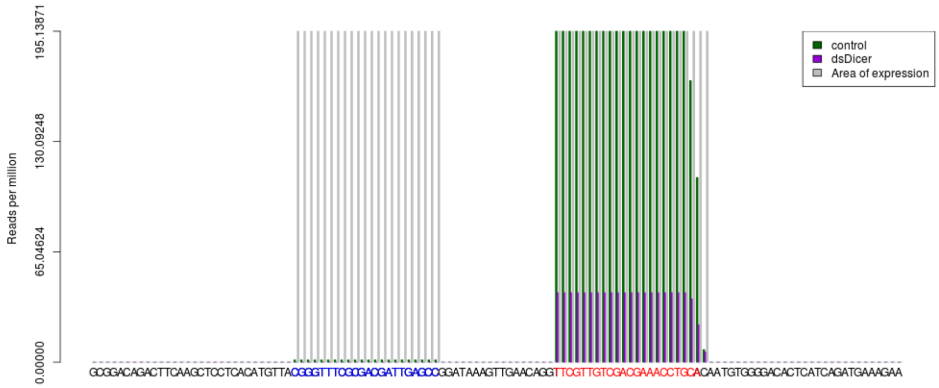

>Bge-Mir-12_pre+_11
GCGTCGAGCCG**TGAGTATTACATCAGGTACTGGT**GAGACATTAGAACAACC**CAGTACTTGTGTTATACTTTCG**ATTCGGCTGCT

>Bge-Mir-12_5p
TGAGTATTACATCAGGTACTGGT

>Bge-Mir-12_3p
CAGTACTTGTGTTATACTTTCG

```
GCGUCGAGCCGUGAGUAUUACAUCAGGUACUGGUGAGACAUUAGAACAACCCAGUACUUGUGUUAUACUUUCGCAAUGUGGGGA
((((((..((.((((((.(((.((((((((((................))))))))))))).)))))).))..))))).)). (-33.70)
```

Matched bases: 18

Mature reads in Control: 405   Mature reads in dsDicer: 116
Star reads in Control: 5   Star reads in dsDicer: 1

```
 Initial dG =    -33.70

          10        20        30        40
-  -|    GC  U      U   U          UGAGACAU
 GC GUCGA  CG GAGUAU ACA CAGGUACUGG        \
 CG CGGCU  GC UUCAUA UGU GUUCAUGACC        U
U  U^    UA  U      U   -          CAACAAGA
   80        70         60        50
```

```
Bge-Mir-12         UGAGUAUUACAUCAGGUACUGGU
tca-mir-12-5p      UGAGUAUUACAUCAGGUACUG--
nvi-mir-12         UGAGUAUUACAUCAGGUACUGGU
dme-mir-12-5p      UGAGUAUUACAUCAGGUACUGGU
ame-mir-12         UGAGUAUUACAUCAGGUACUGGU
bmo-mir-12         UGAGUAUUACUUCAGGUACUGGU
mse-mir-12         UGAGUAUUACUUCAGGUACUGGU
                   ********** **********
```

Genomic coordinates:
Bge-Mir-12_3p: Scaffold383:709800-709821:-
Bge-Mir-12_pre: Scaffold383:709800-709861:-
Bge-Mir-12_5p: Scaffold383:709839-709861:-

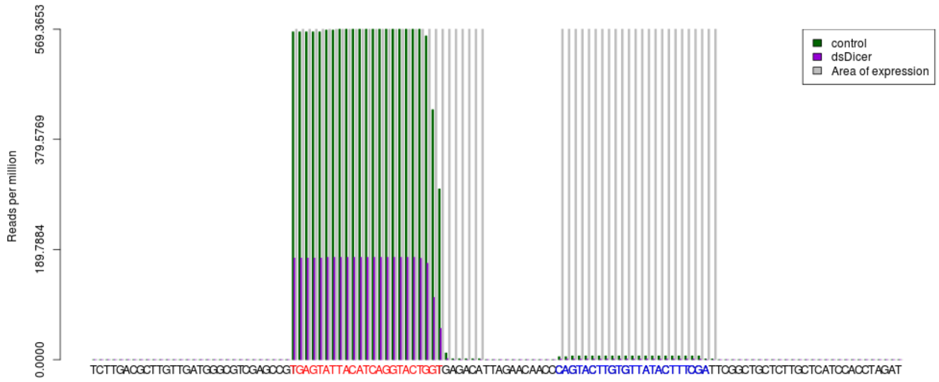

>Bge-Mir-216a_pre+_11
GTGTATAGGTG**TAATCTCATGTGGTAACTGTGA**GTGATTTGCGCAACCT**CAGATTGCCACGTGGGGTTTCA**CGTGTGCACTC

>Bge-Mir-216a_5p
TAATCTCATGTGGTAACTGTGA

>Bge-Mir-216a_3p*
CAGATTGCCACGTGGGGTTTCA

```
GUGUAUAGGUGUAAUCUCAUGUGGUAACUGUGAGUGAUUUGCGCAACCUCAGAUUGCCACGUGGGGUUUCACAAUGUGGGGA
((((((.(((.((((((((((((((((((...(((.....))).....))).))))))))))))))).))).))))))).. (-39.50)
```

Matched bases: 18

Mature reads in Control: 6028   Mature reads in dsDicer: 2562
Star reads in Control: 3   Star reads in dsDicer: 1

Initial dG =   -39.50

```
          10        20         30
--       G   U               -   UGA--|  AU
  GUGUAUA GUG AAUCUCAUGUGGUAA CUG     GUG  \
  CACGUGU CAC UUGGGGUGCACCGUU GAC     CGC  U
CU       G   U               A   UCCAA^  GU
 80        70        60        50        40
```

Genomic coordinates:
Bge-Mir-216a_3p*: Scaffold383:716662-716683:-
Bge-Mir-216a_pre: Scaffold383:716662-716721:-
Bge-Mir-216a_5p: Scaffold383:716700-716721:-

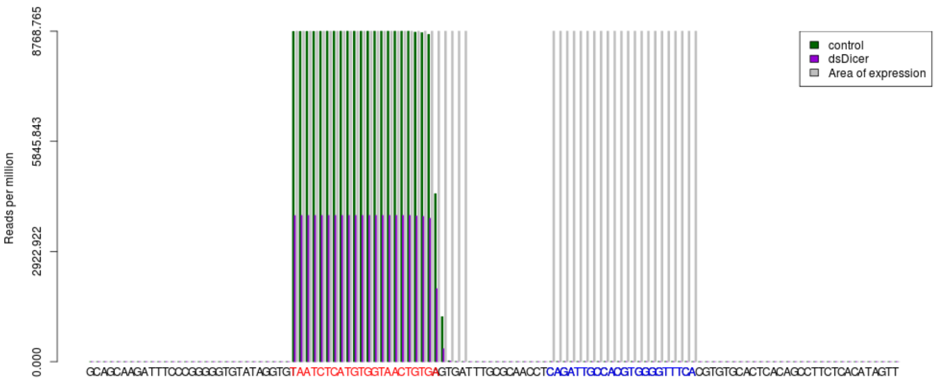

>Bge-Mir-216b_pre+_11
AGATCTCGGCC**AAATATCAGCTGGTAATTCTGGG**AAACATGAAATACC**CAGATTGCTCGCTGGTATCTGG**CTGCGACTGGC

>Bge-Mir-216b_5p
AAATATCAGCTGGTAATTCTGGG

>Bge-Mir-216b_3p*
CAGATTGCTCGCTGGTATCTGG

```
AGAUCUCGGCCAAAUAUCAGCUGGUAAUUCUGGGAAACAUGAAAUACCCAGAUUGCUCGCUGGUAUCUGGCAAUGUGGGGA
((.((.((((((.((((((((.((((((.(((((...........)))))))))))).)))))))).)))))).)))).... (-36.90)
```

Matched bases: 18

Mature reads in Control: 8491   Mature reads in dsDicer: 3381
Star reads in Control: 9   Star reads in dsDicer: 3

```
 Initial dG =    -36.90

           10        20        30        40
---| A  U      A        U      U     AAACA
   AG UC CGGCCA AUAUCAGC GGUAAU CUGGG     U
   UC AG GUCGGU UAUGGUCG UCGUUA GACCC     G
CGG^ -  C      C        C      -     AUAAA
80         70        60         50
```

Genomic coordinates:
Bge-Mir-216b_3p*: Scaffold383:717880-717901:-
Bge-Mir-216b_pre: Scaffold383:717880-717938:-
Bge-Mir-216b_5p: Scaffold383:717916-717938:-

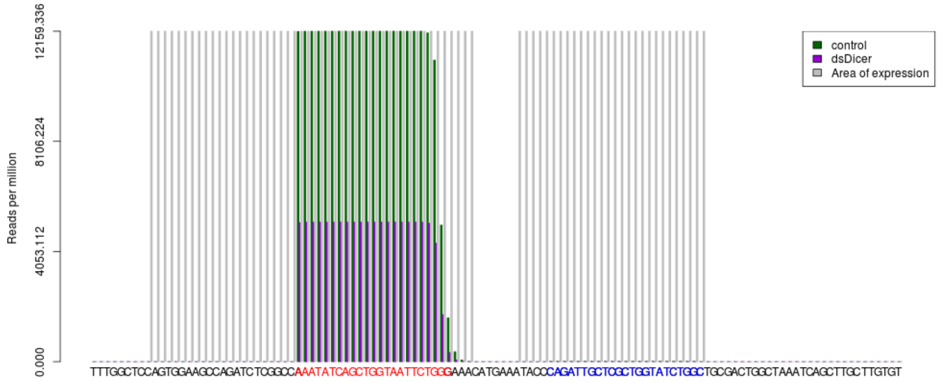

>Bge-Mir-193_pre+_11
CAGTGTGGCTT**GGGCCTTAGCGGTTCAGTGGG**TGTCGGTGTTACCGATTATCAAGTGTC**TACTGGCCTGCTAAGTCCCAA**GTTGCTCTGA
G

>Bge-Mir-193_3p
TACTGGCCTGCTAAGTCCCAA

>Bge-Mir-193_5p*
GGGCCTTAGCGGTTCAGTGGG

```
CAGUGUGGCUUGGGCCUUAGCGGUUCAGUGGGUGUCGGUGUUACCGAUUAUCAAGUGUCUACUGGCCUGCUAAGUCCCAAGUUGCUCUGAG
(((.(..(((((((.((((((((..((((((..(((((.....))))........)..))))))..)))))))).))))))).).).))).. (-48.50)
```

Matched bases: 16

Mature reads in Control: 286   Mature reads in dsDicer: 156
Star reads in Control: 0   Star reads in dsDicer: 0

Initial dG =   -48.50

```
          10        20        30                  40
--   U UG       C        UU      GU --------|   UG
  CAG G  GCUUGGG CUUAGCGG  CAGUGG  G        UCGG  \
  GUC C  UGAACCC GAAUCGUC  GUCAUC  U        AGCC  U
GA   U GU       U        CG      UG GAACUAUU^   AU
90        80        70        60        50
```

```
dme-mir-193-3p      UACUGGCCUACUAAGUCCCAAC-
ame-mir-193         UACUGGCCUGCUAAGUCCCAA--
tca-mir-193-3p      UACUGGCCUGUUAAGUCCCAAGU
mse-mir-193         UACUGGCCUGCUAAGUCCCAAG-
Bge-Mir-193         UACUGGCCUGCUAAGUCCCAA--
                    *********  **********
```

Genomic coordinates:
Bge-Mir-193_3p: Scaffold409:46059-46079:-
Bge-Mir-193_pre: Scaffold409:46059-46127:-
Bge-Mir-193_5p*: Scaffold409:46107-46127:-

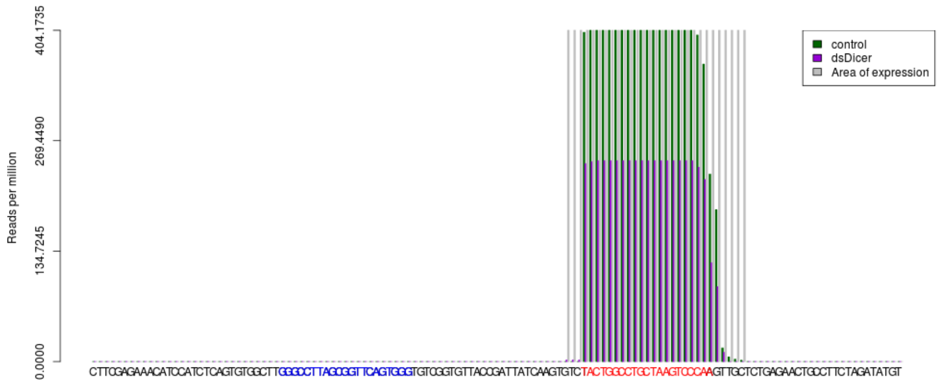

>Bge-Mir-282_pre+_11
AGACTCTGATT**TAGCCTCTCCTAGGCTTTGTCTGT**TCAGAGAATTACATCAC**TAGACATAGCCTAAAAGAGGTTAG**TTCAAGTTATC

>Bge-Mir-282_5p
TAGCCTCTCCTAGGCTTTGTCTGT

>Bge-Mir-282_3p*
TAGACATAGCCTAAAAGAGGTTAG

```
AGACUCUGAUUUAGCCUCUCCUAGGCUUUGUCUGUUCAGAGAAUUACAUCACUAGACAUAGCCUAAAAGAGGUUAGGUUGCUCUGAG
.((((.(((.(((((((((..((((((.((((((....((........))..)))))).))))))..))))))))).))))))).... (-33.40)
```

Matched bases: 20

Mature reads in Control: 12 Mature reads in dsDicer: 8
Star reads in Control: 3 Star reads in dsDicer: 1

Initial dG = -33.40

```
         10        20        30        40
A--|   C   U         CC       U      UUCA  GAA
   GACU UGA UUAGCCUCU  UAGGCU UGUCUG    GA   U
   UUGA ACU GAUUGGAGA  AUCCGA ACAGAU    CU   U
CUA^   -   U         AA       U      CA--  ACA
      80        70        60          50
```

```
nvi-mir-282          GAUUUAGCCUCUCCUAGGCUUUGUCUGU
ame-mir-282          GAUUUAGCCUCUCCUAGGCUUUGUCUGU
dme-mir-282-5p       ----UAGCCUCUACUAGGCUUUGUCUGU
tca-mir-282-5p       ----UAGCCUCUCCUAGGCUUUGUCU--
Bge-Mir-282          ----UAGCCUCUCCUAGGCUUUGUCUGU
bmo-mir-282-5p       -ACCUAGCCUCUCCUUGGCUUUGUCUGU
mse-mir-282          ----UAGCCUCUCCUUGGCUUUGUCU--
                         ******** ** **********
```

Genomic coordinates:
Bge-Mir-282_5p: Scaffold413:167896-167919:+
Bge-Mir-282_pre: Scaffold413:167896-167960:+
Bge-Mir-282_3p*: Scaffold413:167937-167960:+

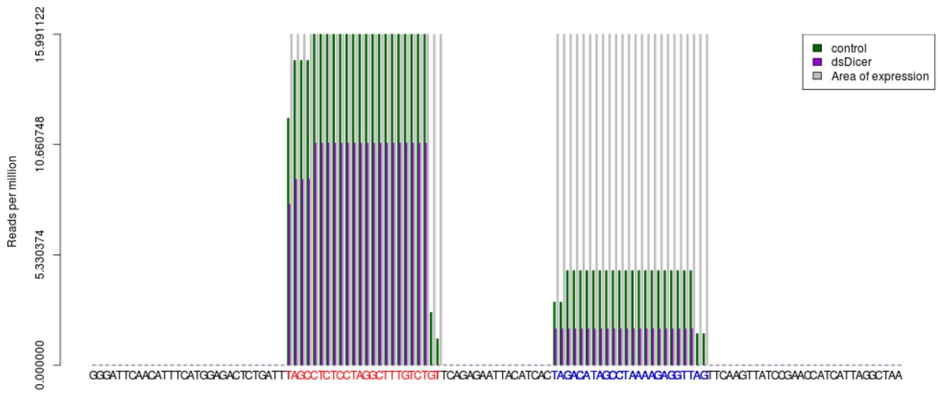

>Bge-Mir-993_pre+_11
CATTCGTAATC**TACCCTGTAGATCCGGGCTTTTG**TAGAACCCCTGTAGATATCA**GAAGCTCGTCTCTACAGGTATCT**TACGGATGGGA

>Bge-Mir-993_5p
TACCCTGTAGATCCGGGCTTTTG

>Bge-Mir-993_3p*
GAAGCTCGTCTCTACAGGTATCT

CAUUCGUAAUC**UACCCUGUAGAUCCGGGCUUUUG**UAGAACCCCUGUAGAUAUCA**GAAGCUCGUCUCUACAGGUAUCU**GUUGCUCUGAG
((((((((..((.((((((((..((((((((((((..((....))...)).))))))))))..))))))))))..)))))))))... (-37.40)

Matched bases: 18

Mature reads in Control: 24 Mature reads in dsDicer: 35
Star reads in Control: 50 Star reads in dsDicer: 91

Initial dG = -37.40

```
           10        20        30          40
---         UC  C        UC          -  GA-| C
   CAUUCGUAA  UA CCUGUAGA  CGGGCUUUUG UA   AC C
   GUAGGCAUU  AU GGACAUCU  GCUCGAAGAC AU   UG C
AGG         CU  -        CU          U  AGA^ U
       80         70        60        50
```

```
Bge-Mir-993         -UACCCUGUAGAUCCGGGCUUUUG
mse-mir-993         -UACCCUGUAGAUCCGGGCUUUUG
tca-mir-993-5p      -UACCCUGUAGAUCCGGGCUU---
dme-mir-993-5p      -UACCCUGUAGUUCCGGGCUUUUG
nvi-mir-993         GAAGCUCGUCUCUACAGGUAUCU-
ame-mir-993         GAAGCUCGUCUCUACAGGUAUCU-
api-mir-993         GAAGCUCGUCUCUACAGGUAUCU-
                      * *   **    * * **   *
```

Genomic coordinates:
Bge-Mir-993_5p: Scaffold415:148381-148403:+
Bge-Mir-993_pre: Scaffold415:148381-148446:+
Bge-Mir-993_3p*: Scaffold415:148424-148446:+

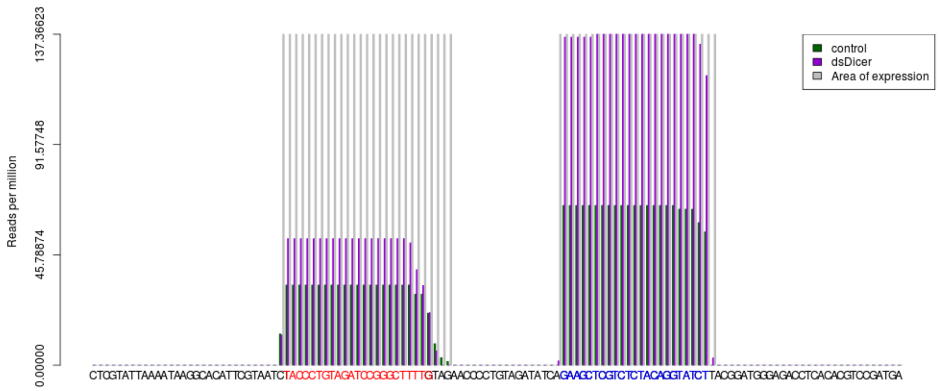

>Bge-Mir-10_pre+_11
TGCTCTACATC**TACCCTGTAGATCCGAATTTGT**TTGACATTGGGCGAC**AAATTCGGTTCTAGAGAGGTTTG**TGTGGTGCATA

>Bge-Mir-10_5p
TACCCTGTAGATCCGAATTTGT

>Bge-Mir-10_3p*
AAATTCGGTTCTAGAGAGGTTTG

```
UGCUCUACAUCUACCCUGUAGAUCCGAAUUUGUUUGACAUUGGGCGACAAAUUCGGUUCUAGAGAGGUUUGGUUGCUCUGAG
(((.((((((..(((((.((((.(((((((((((..........))))))))))).)))).)).))).)..)))))).))).. (-36.40)
```

Matched bases: 17

Mature reads in Control: 10584   Mature reads in dsDicer: 6274
Star reads in Control: 924   Star reads in dsDicer: 487

Initial dG =   -36.40

```
          10         20        30
--   U      CU   -| G    U           UGACA
  UGC CUACAU  ACC CU UAGA CCGAAUUUGUU     \
  ACG GGUGUG  UGG GA AUCU GGCUUAAACAG     U
AU   U      UU   A^ G    U           CGGGU
 80        70        60        50
```

```
dme-mir-10-5p      -ACCCUGUAGAUCCGAAUUUGUU
api-mir-10         -ACCCUGUAGAUCCGAAUUUGU-
ame-mir-10         -ACCCUGUAGAUCCGAAUUUGU-
nvi-mir-10         -ACCCUGUAGAUCCGAAUUUGU-
bmo-mir-10-5p      -ACCCUGUAGAUCCGAAUUUGU-
Bge-Mir-10         UACCCUGUAGAUCCGAAUUUGU-
tca-mir-10-5p      UACCCUGUAGAUCCGAAUUUGU-
                    *********************
```

Genomic coordinates:
Bge-Mir-10_3p*: Scaffold415:411318-411340:-
Bge-Mir-10_pre: Scaffold415:411318-411377:-
Bge-Mir-10_5p: Scaffold415:411356-411377:-

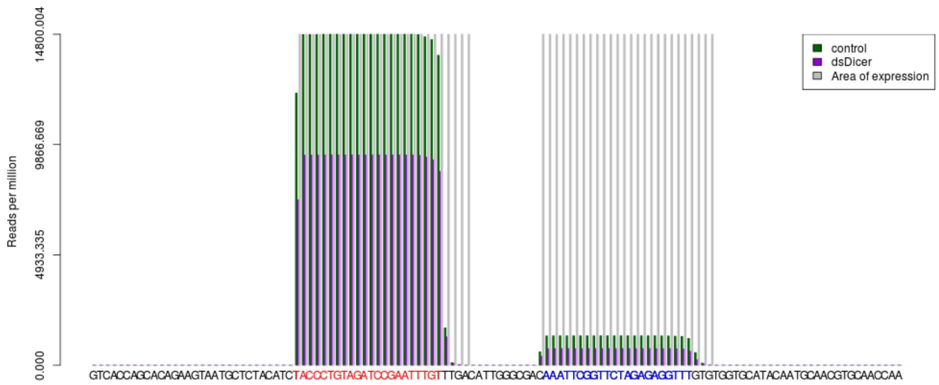

>Bge-Mir-184_pre+_11
TTCCTACGTTC**CCTTGTCATTCTCGGGTCCGGT**GTTGCCATCTGACGAC**TGGACGGAGAACTGATAAGGGC**TTGTGGGAAGT

>Bge-Mir-184_3p
TGGACGGAGAACTGATAAGGGC

>Bge-Mir-184_5p*
CCTTGTCATTCTCGGGTCCGGT

```
UUCCUACGUUCCCUUGUCAUUCUCGGGUCCGGUGUUGCCAUCUGACGACUGGACGGAGAACUGAUAAGGGCUUGUGGGAAGU
((((((((..((((((((((((((..((((((((((.......))).))))))).))))).))))))))))..)))))))).. (-42.40)
```

Matched bases: 18

Mature reads in Control: 5120 Mature reads in dsDicer: 1931
Star reads in Control: 4 Star reads in dsDicer: 1

Initial dG = -42.40

```
          10         20        30
--        UU         -|     GG        -    GCC
  UUCCUACG  CCCUUGUCA UUCUC  GUCCGGU GUU    \
  AAGGGUGU  GGGAAUAGU AAGAG  CAGGUCA CAG    A
UG        UC         C^     G-        G    UCU
 80        70        60         50
```

```
Bge-Mir-184         --UGGACGGAGAACUGAUAAGGGC
tca-mir-184-3p      --UGGACGGAGAACUGAUAAGGGC
bmo-mir-184-3p      ACUGGACGGAGAACUGAUAAGGGC
nvi-mir-184         --UGGACGGAGAACUGAUAAGGGC
dme-mir-184-3p      --UGGACGGAGAACUGAUAAGGGC
mse-mir-184         --UGGACGGAGAACUGAUAAGGGC
ame-mir-184         --UGGACGGAGAACUGAUAAGGGC
                      **********************
```

Genomic coordinates:
Bge-Mir-184_5p*: Scaffold42:1295078-1295099:+
Bge-Mir-184_pre: Scaffold42:1295078-1295137:+
Bge-Mir-184_3p: Scaffold42:1295116-1295137:+

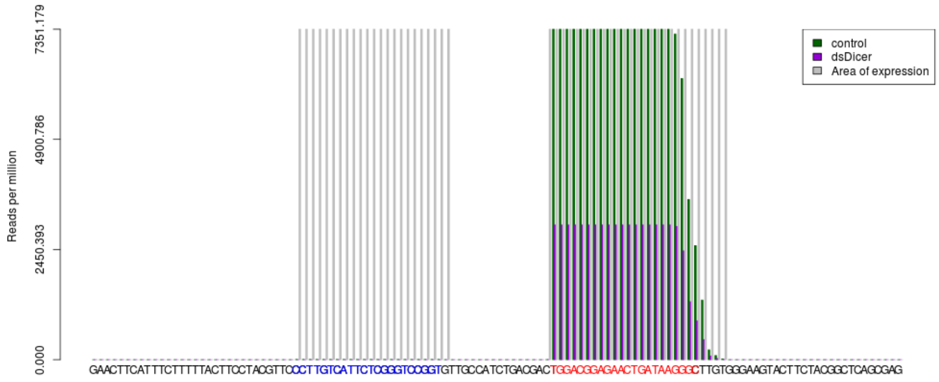

>Bge-Mir-14_pre+_11
GGGGTGAGCAT**GGGAGGGGGGAACGGGCTTCGACT**CTCGTCTGCAGCAG**TCAGTCTTTTTCTCTCTCCTAT**GCTCGCACCGA

>Bge-Mir-14_3p
TCAGTCTTTTTCTCTCTCCTAT

>Bge-Mir-14_5*p
GGGAGGGGGGAACGGGCTTCGACT

```
GGGGUGAGCAUGGGAGGGGGGAACGGGCUUCGACUCUCGUCUGCAGCAGUCAGUCUUUUUCUCUCUCCUAUGCUCGCACCGA
((.((((((((((((((((((((.(((((..((((((.......)).))))))))).))))))))))))))))))))).)).. (-46.20)
```

Matched bases: 19

Mature reads in Control: 6347 Mature reads in dsDicer: 4498
Star reads in Control: 15 Star reads in dsDicer: 3

Initial dG = -46.20

```
          10        20        30         40
--| G                   C     UC    -   CGU
  GG GUGAGCAUGGGAGGGGGGAA GGGCU  GACU CU   \
  CC CGCUCGUAUCCUCUCUCUUU UCUGA  CUGA GA   C
AG^ A                   U     --    C   CGU
 80        70        60          50
```

```
Bge-Mir-14          UCAGUCUUUUUCUCUCUCCUAU
tca-mir-14-3p       UCAGUCUUUUUCUCUCUCCUAU
bmo-mir-14-3p       UCAGUCUUUUUCUCUCUCCUA-
nvi-mir-14          UCAGUCUUUUUCUCUCUCCUA-
dme-mir-14-3p       UCAGUCUUUUUCUCUCUCCUAU
ame-mir-14          UCAGUCUUUUUCUCUCUCCUA-
mse-mir-14          UCAGUCUUUUUCUCUCUCCUAU
api-mir-14          UCAGUCUUUUUCUCUCUCCUAU
                    *********************
```

Genomic coordinates:
Bge-Mir-14_3p: Scaffold467:268887-268908:-
Bge-Mir-14_pre: Scaffold467:268887-268946:-
Bge-Mir-14_5*p: Scaffold467:268923-268946:-

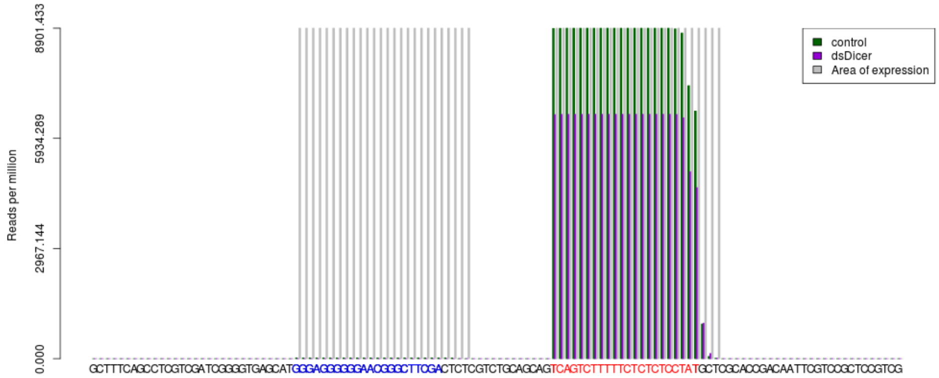

>Bge-Mir-995a_pre+_11
GCTGTGAGCAA**AGCTGACTCGTGCGGTTGCCACG**TTGTGTCTGAGATGCG**TAGCACCACATGATTCAGCTTA**GTTCGCAGTAG

>Bge-Mir-995a_3p
TAGCACCACATGATTCAGCTTA

>Bge-Mir-995a_5p*
AGCTGACTCGTGCGGTTGCCACG

```
GCUGUGAGCAAAGCUGACUCGUGCGGUUGCCACGUUGUGUCUGAGAUGCGUAGCACCACAUGAUUCAGCUUAGUUCGCAGUAG
((((((((.(((((((.(((((.(((.((.((((...........)))).))))).))))).))))))).))))))))).. (-41.50)
```

Matched bases: 17

Mature reads in Control: 29 Mature reads in dsDicer: 22
Star reads in Control: 4 Star reads in dsDicer: 3

Initial dG = -41.50

```
          10        20        30        40
--|        A       C      C   U  C    UGUGU
  GCUGUGAGC AAGCUGA UCGUG GGU GC ACGU     C
  UGACGCUUG UUCGACU AGUAC CCA CG UGCG     U
GA^        A       U      A   -  A    UAGAG
  80        70        60         50
```

Genomic coordinates:
Bge-Mir-995a_3p: Scaffold5:240447-240468:-
Bge-Mir-995a_pre: Scaffold5:240447-240507:-
Bge-Mir-995a_5p*: Scaffold5:240485-240507:-

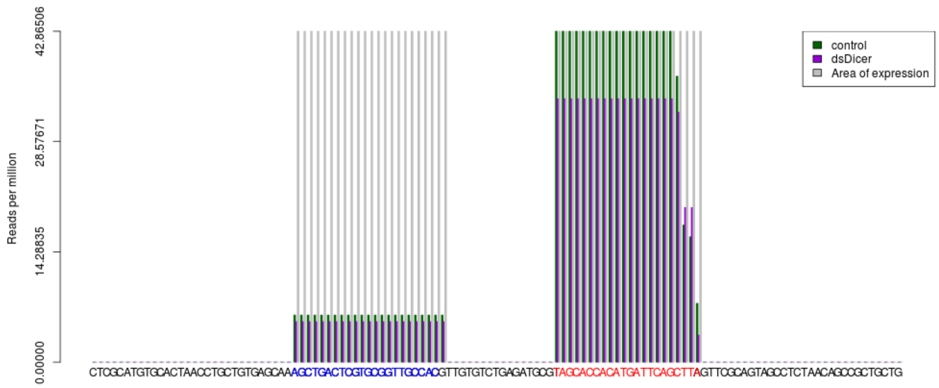

>Bge-bantam_pre+_11
ATGAGACGAAG**CCGGTTTTCACAGTGATTTGCCAGA**TATGTTAAATTC**TGAGATCATTGTGAAAGCTGATT**TTGTTGAGTAG

>Bge-bantam_3p
TGAGATCATTGTGAAAGCTGATT

>Bge-bantam_5p*
CCGGTTTTCACAGTGATTTGCCAGA

```
AUGAGACGAAGCCGGUUUUCACAGUGAUUUGCCAGAUAUGUUAAAUUCUGAGAUCAUUGUGAAAGCUGAUUUUGUUGAGUAG
((..((((((..((((((((((((((((((..((((.........))))))))))))))))))))))..))))))..)).. (-32.20)
```

Matched bases: 20

Mature reads in Control: 36330 Mature reads in dsDicer: 22971
Star reads in Control: 16 Star reads in dsDicer: 4

Initial dG = -32.20

```
          10        20        30        40
--| GA      GC                  GC     UAUG
  AU  GACGAA  CGGUUUUCACAGUGAUUU  CAGA     U
  UG  UUGUUU  GUCGAAAGUGUUACUAGA  GUCU     U
GA^ AG      UA                  --     UAAA
 80        70        60          50
```

```
dme-bantam-3p      UGAGAUCAUUUUGAAAGCUGAUU
ame-bantam         UGAGAUCAUUGUGAAAGCUGAUU
nvi-bantam         UGAGAUCAUUGUGAAAGCUGAUU
tca-bantam-3p      UGAGAUCAUUGUGAAAGCUGAUU
Bge-bantam         UGAGAUCAUUGUGAAAGCUGAUU
bmo-bantam-3p      UGAGAUCAUUGUGAAAGCUAAUU
mse-bantam         UGAGAUCAUUGUGAAAGCUAAU-
api-bantam         UGAGAUCAUUGUGAAAGCUAAUU
                   ********** ******** **
```

Genomic coordinates:
Bge-bantam_5p*: Scaffold510:618998-619022:+
Bge-bantam_pre: Scaffold510:618998-619057:+
Bge-bantam_3p: Scaffold510:619035-619057:+

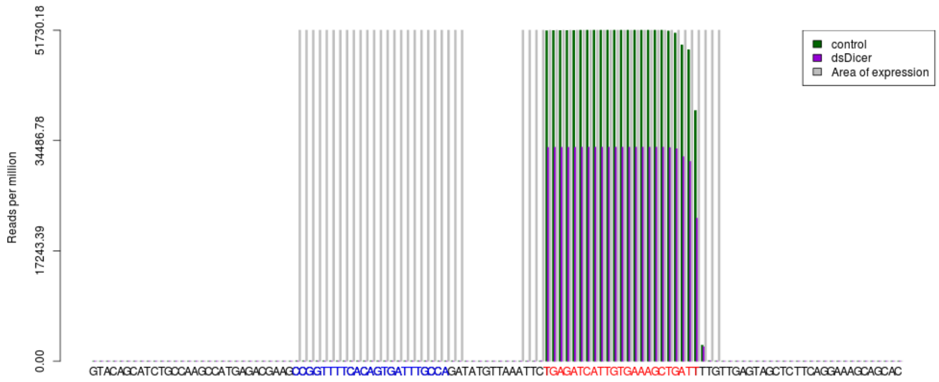

>Bge-Mir-9a_pre+_11
CTGTTGTCCTC**TCTTTGGTGATCTAGCTGTATGA**GTGTAAAACGACATC**ATAAAGCTAGATTACCGAAGTCA**GGAAAACAGTA

>Bge-Mir-9a_5p
TCTTTGGTGATCTAGCTGTATGA

>Bge-Mir-9a_3p*
ATAAAGCTAGATTACCGAAGTCA

```
CUGUUGUCCUCUCUUUGGUGAUCUAGCUGUAUGAGUGUAAAACGACAUCAUAAAGCUAGAUUACCGAAGUCAUUGUUGAGUAG
(((((.((((..((((((((((((((((.(((((.(((......)))))))).))))))))))))))))..)))).))))).. (-42.90)
```

Matched bases: 19

```
Mature reads in Control: 3703         Mature reads in dsDicer: 2056
Star reads in Control: 332            Star reads in dsDicer: 220

 Initial dG =    -42.90

          10        20        30        40
--|    G    CU                G     G    AA
  CUGUU UCCU  CUUUGGUGAUCUAGCU UAUGA UGU   A
  GACAA AGGA  GAAGCCAUUAGAUCGA AUACU ACA   A
AU^    A    CU                A     -    GC
  80        70        60        50


ame-mir-9a          UCUUUGGUUAUCUAGCUGUAUGA-
api-mir-9a          UCUUUGGUUAUCUAGCUGUAUGA-
dme-mir-9a-5p       UCUUUGGUUAUCUAGCUGUAUGA-
nvi-mir-9a          UCUUUGGUUAUCUAGCUGUAUGA-
bmo-mir-9a-5p       UCUUUGGUUAUCUAGCUGUAUGA-
tca-mir-9a-5p       UCUUUGGUUAUCUAGCUGUAU---
Bge-Mir-9a          UCUUUGGUGAUCUAGCUGUAUGA-
                    ******** ************
```

Genomic coordinates:
Bge-Mir-9a_3p*: Scaffold5210:3836-3858:-
Bge-Mir-9a_pre: Scaffold5210:3836-3896:-
Bge-Mir-9a_5p: Scaffold5210:3874-3896:-

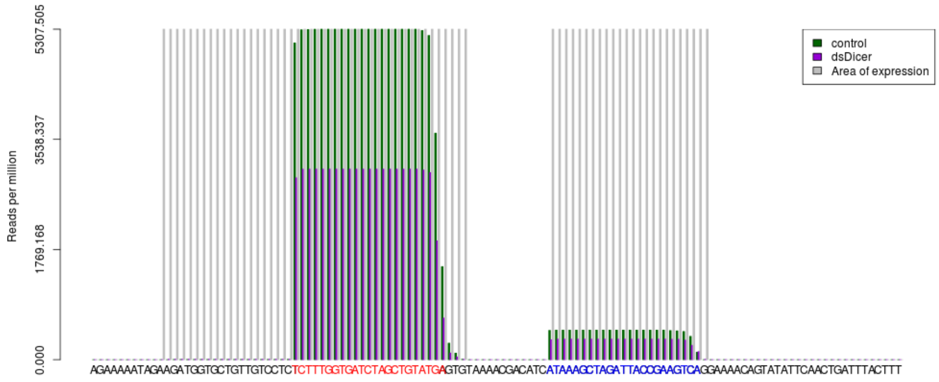

>Bge-Mir-9b_pre+_11
CATTTGTCTCT**TCTTTGGTGATGTAGCTGTATGA**TTGCTGTAGAAGGTC**ATAAAGCTAGATTACCAAAGCA**GAGACATTGGC

>Bge-Mir-9b_3p
ATAAAGCTAGATTACCAAAGCA

>Bge-Mir-9b_5p*
TCTTTGGTGATGTAGCTGTATGA

```
CAUUUGUCUCUUCUUUGGUGAUGUAGCUGUAUGAUUGCUGUAGAAGGUCAUAAAGCUAGAUUACCAAAGCAGAGACAUUGGC
((..(((((((.((((((((((.(((((.(((((((.........))))))).))))).)))))))))).))))))).)).. (-38.60)
```

Matched bases: 18

Mature reads in Control: 1069 Mature reads in dsDicer: 441
Star reads in Control: 70 Star reads in dsDicer: 63

Initial dG = -38.60

```
          10        20        30        40
--| UU       U          G     G       GCUG
  CA  UGUCUCU CUUUGGUGAU UAGCU UAUGAUU    \
  GU  ACAGAGA GAAACCAUUA AUCGA AUACUGG    U
CG^ U-       C          G     A       AAGA
 80        70        60        50
```

```
Bge-Mir-9b         AUAAAGCUAGAUUACCAAAGCA------
tca-mir-9b-3p      AUAAAGCUAGAUUACCAAAGCA------
mse-mir-9b         AUAAAGCUAGGUUACCGGAGUUA-----
bmo-mir-9b-3p      ACGGAGCUAAAUCGCCAAAGCG------
dme-mir-9b-3p      -UAGAGCUUUAUUACCAAAAACC-----
ame-mir-9b         -----GCUUUGGUAAUCUAGCUUUAUGA
api-mir-9b         -----UCUUUGGUACUUUAGCUGUAG--
                        **          *
```

Genomic coordinates:
Bge-Mir-9b_3p*: Scaffold5210:5672-5693:-
Bge-Mir-9b_pre: Scaffold5210:5672-5731:-
Bge-Mir-9b_5p: Scaffold5210:5709-5731:-

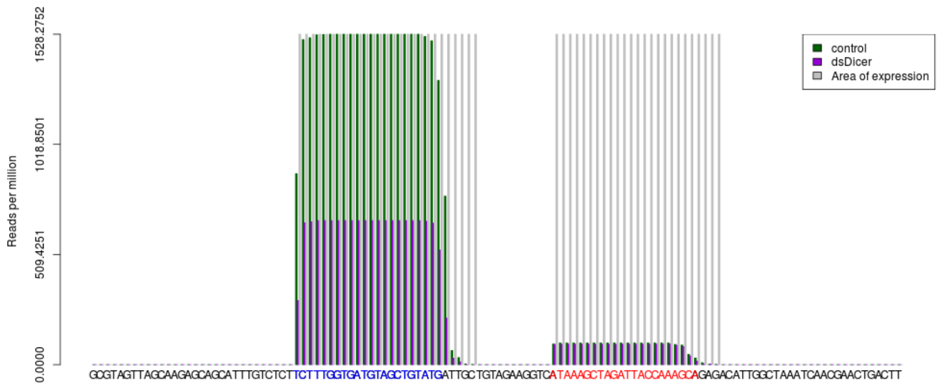

>Bge-Mir-306_pre+_11
TGTTTCCTCTG**TCAGGTACTGAGTGACTCTGAG**TAATTTAATTCT**CAGAGGCACTTGGTTTCTGGCC**GAGTGAACAGT

>Bge-Mir-306_5p
TCAGGTACTGAGTGACTCTGAG

>Bge-Mir-306_3p*
CAGAGGCACTTGGTTTCTGGCC

```
UGUUUCCUCUGUCAGGUACUGAGUGACUCUGAGUAAUUUAAUUCUCAGAGGCACUUGGUUUCUGGCCGAGACAUUGGC
((((..(((.((((((.((..((((.(((((((..........))))))).))))..)).)))))).)))..)))).. (-38.40)
```

Matched bases: 16

Mature reads in Control: 2858 Mature reads in dsDicer: 1800
Star reads in Control: 4 Star reads in dsDicer: 4

Initial dG = -38.40

```
          10        20        30
--|   UC   U      U  UG     A        UAAU
  UGUU  CUC GUCAGG AC  AGUG CUCUGAG    U
  ACAA  GAG CGGUCU UG  UCAC GAGACUC    U
UG^   GU   C      U  GU     G        UUAA
       70        60        50        40
```

```
Bge-Mir-306         UCAGGUACUGAGUGACUCUGAG
ame-mir-306         UCAGGUACUGAGUGACUCUGAG
dme-mir-306-5p      UCAGGUACUUAGUGACUCUCAA
mse-mir-306         UCAGGUACUAGGUGACUCUGA-
api-mir-306         UCAGGUACCAAGUGAUUUCUGA
                    ********   **** *
```

Genomic coordinates:
Bge-Mir-306_3p*: Scaffold5210:5780-5801:-
Bge-Mir-306_pre: Scaffold5210:5780-5835:-
Bge-Mir-306_5p: Scaffold5210:5814-5835:-

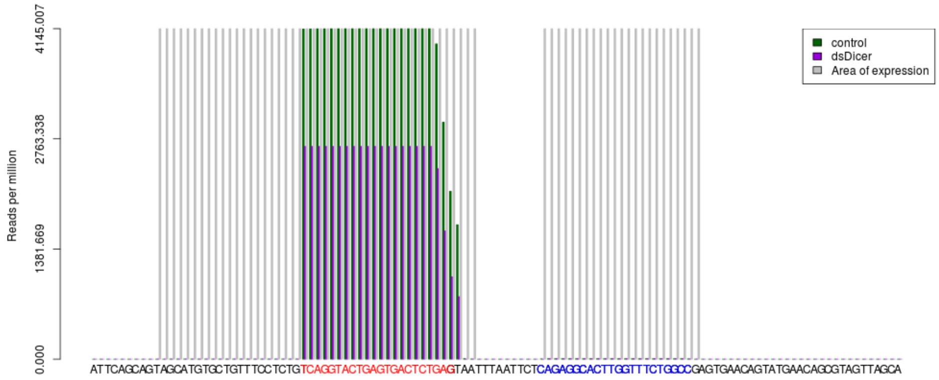

>Bge-Mir-9c_pre+_11
CTGTGGTCCTG**TCTTTGGTATCCTAGCTGTAGG**TTGTATTAGCTGCTCC**TAAAGCTATTGTACCAAAGTAA**GGAACAGTGTT

>Bge-Mir-9c_5p
TCTTTGGTATCCTAGCTGTAGG

>Bge-Mir-9c_3p*
TAAAGCTATTGTACCAAAGTAA

```
CUGUGGUCCUGUCUUUGGUAUCCUAGCUGUAGGUUGUAUUAGCUGCUCCUAAAGCUAUUGUACCAAAGUAAGAGACAUUGGC
((((..((((..(((((((((..(((((.((((..(((.....))).)))).)))))..)))))))))..))))))))).... (-32.00)
```

Matched bases: 16

Mature reads in Control: 11780 Mature reads in dsDicer: 3041
Star reads in Control: 11 Star reads in dsDicer: 9

Initial dG = -32.00

```
            10        20        30        40
----|   GG    GU         CC      G     UU    UU
    CUGU  UCCU  CUUUGGUAU  UAGCU UAGG  GUA  \
    GACA  AGGA  GAAACCAUG  AUCGA AUCC  CGU  A
UUGU^   --    AU         UU      A     U-    CG
 80          70        60        50
```

```
Bge-Mir-9c         UCUUUGGUAUCCUAGCUGUAGG
ame-mir-9a         UCUUUGGUUAUCUAGCUGUAUGA-
api-mir-9a         UCUUUGGUUAUCUAGCUGUAUGA-
dme-mir-9a-5p      UCUUUGGUUAUCUAGCUGUAUGA-
nvi-mir-9a         UCUUUGGUUAUCUAGCUGUAUGA-
bmo-mir-9a-5p      UCUUUGGUUAUCUAGCUGUAUGA-
tca-mir-9a-5p      UCUUUGGUUAUCUAGCUGUAU---
                   ********   **********
```

Genomic coordinates:
Bge-Mir-9c_3p*: Scaffold5210:9882-9903:-
Bge-Mir-9c_pre: Scaffold5210:9882-9941:-
Bge-Mir-9c_5p: Scaffold5210:9920-9941:-

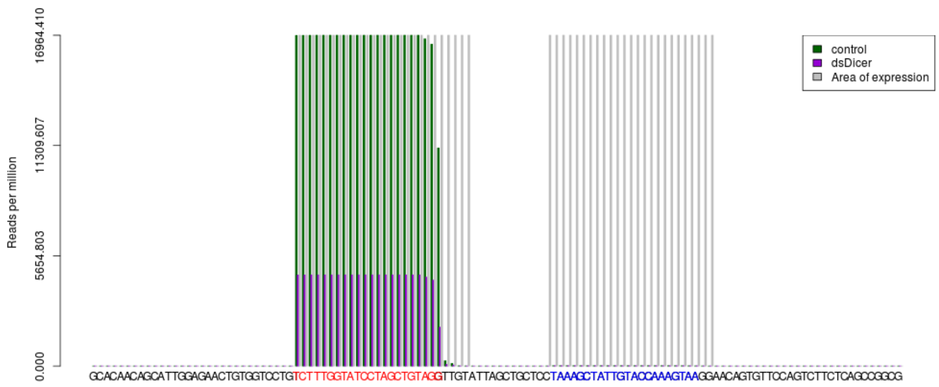

>Bge-Mir-375_pre+_11
GCGTCCTTCTG**ACTTGCGCTTTGCGGACAAACT**GTTGCATTCCAAGGCG**TTTGTTCGTTCGGCTCGAGTTA**AAAAGGGCTGC

>Bge-Mir-375_3p
TTTGTTCGTTCGGCTCGAGTTA

>Bge-Mir-375_5p*
ACTTGCGCTTTGCGGACAAACT

```
GCGUCCUUCUGACUUGCGCUUUGCGGACAAACUGUUGCAUUCCAAGGCGUUUGUUCGUUCGGCUCGAGUUAAAAAGGGCUGC
(((((((.(((((((.(((..((((((((((.(((.........)))))))))))))..))).))))))).)))))).)) (-33.40)
```

Matched bases: 17

Mature reads in Control: 85 | Mature reads in dsDicer: 44
Star reads in Control: 1 | Star reads in dsDicer: 0

Initial dG = -33.40

```
         10        20        30        40
  -      C-|      C    UU          U    GCAU
GC GUCCUU  UGACUUG GCU  GCGGACAAAC GUU    \
CG CGGGAA  AUUGAGC CGG  UGCUUGUUUG CGG    U
  U      AA^      U    CU          -    AACC
 80        70        60        50
```

```
tca-mir-375-3p      UUUGUUCGUGU-GGCUCGAGUUA
dme-mir-375-3p      UUUGUUCGUUU-GGCUUAAGUUA
Bge-Mir-375         UUUGUUCGUUC-GGCUCGAGUUA
nvi-mir-375         UUUGUUCGUUC-GGCUCGAGUUA
ame-mir-375         UUUGUUCGUUC-GGCUCGAGUUA
bmo-mir-375-3p      UUUGUUCGCCCCGGCUCGUGUCG
                    ********    ****   **
```

Genomic coordinates:
Bge-Mir-375_3p: Scaffold551:487185-487206:-
Bge-Mir-375_pre: Scaffold551:487185-487244:-
Bge-Mir-375_5p*: Scaffold551:487223-487244:-

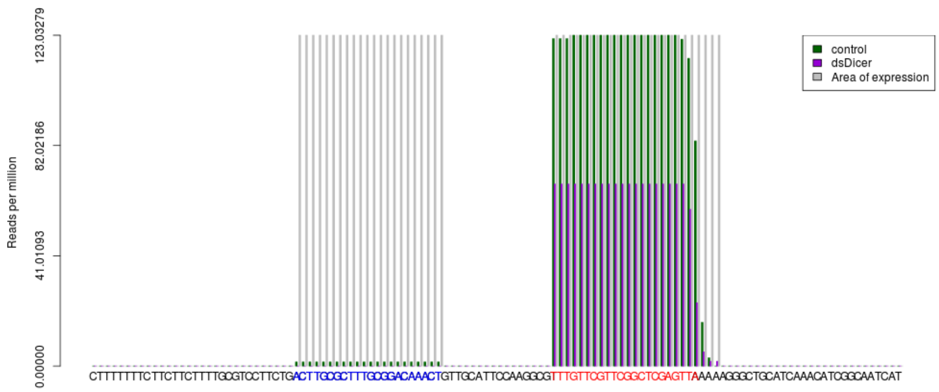

>Bge-Mir-67_pre+_11
CTAGCTTGGTT**ACTCACTCAACCTGGGTGTGATG**TGTTTATGGAGCCCA**TCACAACCTCCTTGAGTGAGCGA**CTAGGCGCGCC

>Bge-Mir-67_3p
TCACAACCTCCTTGAGTGAGCGA

>Bge-Mir-67_5p*
ACTCACTCAACCTGGGTGTGATG

```
CUAGCUUGGUUACUCACUCAACCUGGGUGUGAUGUGUUUAUGGAGCCCAUCACAACCUCCUUGAGUGAGCGACUAGGCGCGCC
...(((((((..(((((((((...((.(((((((.((((...)))).))))))).))...)))))))))..))))))).....  (-40.40)
```

Matched bases: 16

Mature reads in Control: 143   Mature reads in dsDicer: 77
Star reads in Control: 1   Star reads in dsDicer: 0

Initial dG =   -40.40

```
          10        20        30        40
CUA--|      UA         CCU  G       U    A
     GCUUGGU  CUCACUCAA   GG UGUGAUG GUUU \
     CGGAUCA  GAGUGAGUU   CC ACACUAC CGAG U
CCGCG^      GC         CCU  A       C    G
  80        70        60        50
```

Genomic coordinates:
Bge-Mir-67_3p: Scaffold552:140449-140471:-
Bge-Mir-67_pre: Scaffold552:140449-140509:-
Bge-Mir-67_5p*: Scaffold552:140487-140509:-

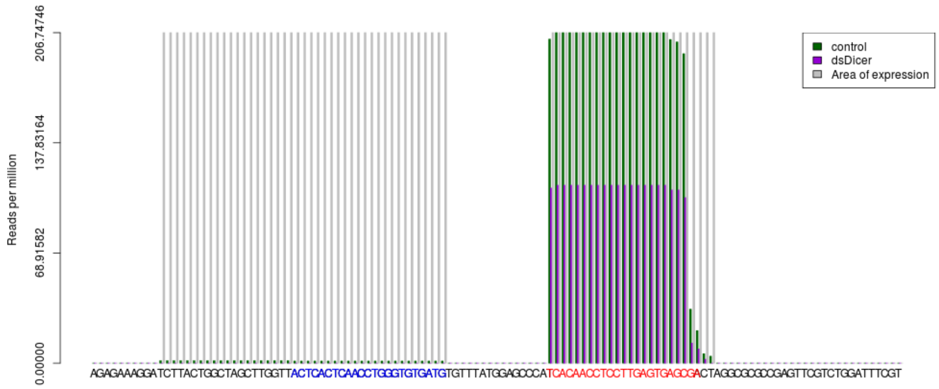

>Bge-Mir-3770_pre+_11
ACAGTGGGTGA**AATCCTGCATCAAGTGCGTTGCT**TGGATTTCAAACAG**CAACGCTTTGACAGCAGGAATTT**ATCCATGGTGT

>Bge-Mir-3770_5p
AATCCTGCATCAAGTGCGTTGCT

>Bge-Mir-3770_3p*
CAACGCTTTGACAGCAGGAATTT

```
ACAGUGGGUGAAAUCCUGCAUCAAGUGCGUUGCUUGGAUUUCAAACAGCAACGCUUUGACAGCAGGAAUUUCUAGGCGCGCC
((.(((((((((.((((((.(((((.((((((((.(.........))))))))))))))..)))))).))))))))).)).. (-38.00)
```

Matched bases: 18

Mature reads in Control: 503　　　Mature reads in dsDicer: 199
Star reads in Control: 5　　　Star reads in dsDicer: 2

Initial dG =   -38.00

```
          10        20         30        40
--   A          A      A-|    U         U GAUU
  AC GUGGGUGAA UCCUGC  UCAAG GCGUUGCU G    \
  UG UACCUAUUU AGGACG  AGUUU CGCAACGA C    U
UG   G          A      AC^    -         - AAAC
 80        70        60         50
```

```
Bge-Mir-3770      AAUCCUGCAUCAAGUGCGUUGCU
ame-mir-3770      AAUCCUGCAUCAAGUGCGUUGU-
                  ********************* 
```

Genomic coordinates:
Bge-Mir-3770_3p*: Scaffold57:2529895-2529917:-
Bge-Mir-3770_pre: Scaffold57:2529895-2529954:-
Bge-Mir-3770_5p: Scaffold57:2529932-2529954:-

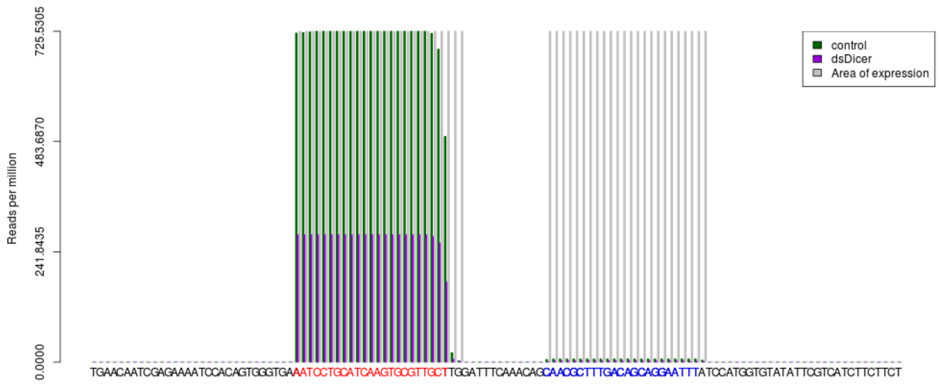

>Bge-Mir-927a_pre+_11
GTGTGGTTTGC**TTTAGAATTCCTACGCTTTACC**GTTTCACTTGAAAGG**CAAAGCGTTTGAATTCTGAAAC**GAACTGCCCTT

>Bge-Mir-927a_5p
TTTAGAATTCCTACGCTTTACC

>Bge-Mir-927a_3p*
CAAAGCGTTTGAATTCTGAAAC

```
GUGUGGUUUGCUUUAGAAUUCCUACGCUUUACCGUUUCACUUGAAAGGCAAAGCGUUUGAAUUCUGAAACCUAGGCGCGCC
..(..((((.((((((((((..(((((((.((.((((....)))))).))))))).))))))))))).))))).)).... (-34.50)
```

Matched bases: 17

Mature reads in Control: 125   Mature reads in dsDicer: 134
Star reads in Control: 8   Star reads in dsDicer: 12

Initial dG =   -34.50

```
          10        20        30        40
GU--|UG     C          CU        A   G     A
    G  GUUUG UUUAGAAUUC  ACGCUUU CC UUUC C
    C  CAAGC AAGUCUUAAG  UGCGAAA GG AAAG U
UUCC^GU     A          UU        C   -     U
80        70        60        50
```

```
Bge-Mir-927a         -UUUAGAAUUCCUACGCUUUACC
ame-mir-927a         UUUUAGAAUUCCUACGCUUUACC
tca-mir-927a-5p      -UUUAGAAUUCCUACGCUUUA--
                      ********************
```

Genomic coordinates:
Bge-Mir-927a_5p: Scaffold63:1177093-1177114:+
Bge-Mir-927a_pre: Scaffold63:1177093-1177151:+
Bge-Mir-927a_3p*: Scaffold63:1177130-1177151:+

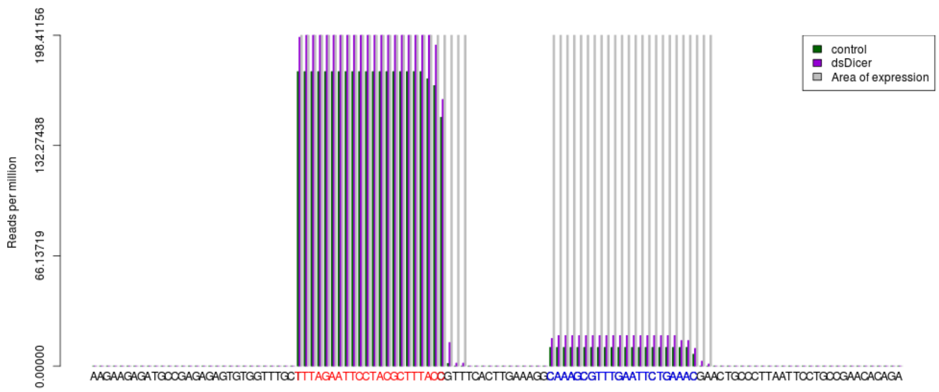

>Bge-Mir-87-1_pre+_11
GTTCCCCTCAT**CGCCTGATACTTGCTTCAACCT**GGGTATTTGACAAG**GTGAGCAAAGTTTCAGGTGTGT**GGGTGGAACAC

>Bge-Mir-87-1_3p
GTGAGCAAAGTTTCAGGTGTGT

>Bge-Mir-87-1_5p*
CGCCTGATACTTGCTTCAACCT

```
GUUCCCCUCAUCGCCUGAUACUUGCUUCAACCUGGGUAUUUGACAAGGUGAGCAAAGUUUCAGGUGUGUGGGUGGAACAC
(((((.((((((((((((.((((((((..((((..((.....)).)))))))))).)).))))))).))))).))))).. (-37.20)
```

Matched bases: 17

Mature reads in Control: 1645   Mature reads in dsDicer: 676
Star reads in Control: 2   Star reads in dsDicer: 0

```
 Initial dG =    -37.20

          10         20         30
--     C     -       U  -|     CA    GG  AU
  GUUCC CUCAU CGCCUGA AC UUGCUU  ACCU  GU  \
  CAAGG GGGUG GUGGACU UG AACGAG  UGGA  CA  U
CA     U     U       U  A^     --    A-  GU
.        70        60        50
```

```
Bge-Mir-87-1       GUGAGCAAAGUUUCAGGUGUGU
ame-mir-87         GUGAGCAAAGUUUCAGGUGU--
bmo-mir-87         -UGAGCAAACUUUCAGGUGUGU
mse-mir-87         GUGAGCAAACUUUCAGGUGUGU
dme-mir-87-3p      UUGAGCAAAAUUUCAGGUGUG-
                    ******** **********
```

Genomic coordinates:
Bge-Mir-87-1_3p: Scaffold70:22446-22467:-
Bge-Mir-87-1_pre: Scaffold70:22446-22503:-
Bge-Mir-87-1_5p*: Scaffold70:22482-22503:-

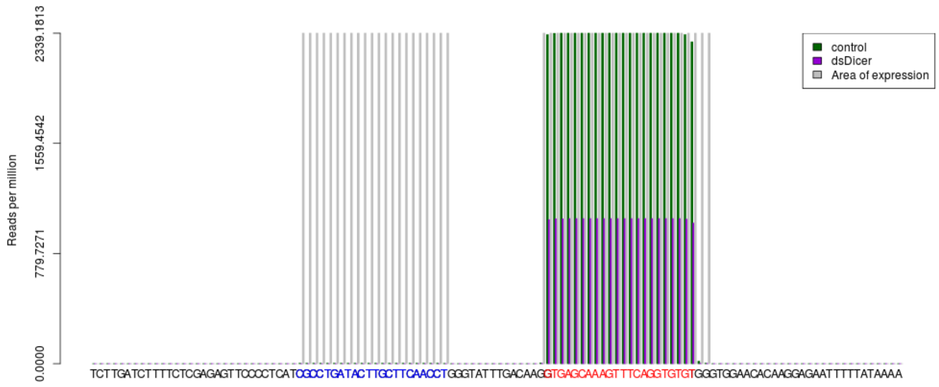

>Bge-Mir-87-2_pre+_11
TTACTTCTGAC**GGGCCTGAATCCTTGCTCAGCCT**GATGTAAAAAG**GTGAGCAAAGTTTCAGGTGTGT**TGGTGGTAAGG

>Bge-Mir-87-2_3p
GTGAGCAAAGTTTCAGGTGTGT

>Bge-Mir-87-2_5p*
GGGCCTGAATCCTTGCTCAGCCT

```
UUACUUCUGACGGGCCUGAAUCCUUGCUCAGCCUGAUGUAAAAAGGUGAGCAAAGUUUCAGGUGUGUUGGUGGUAAGG
(((((.(..(((.(((((((...(((((((.(((.........))))))))))...))))))).))).).))))).. (-35.60)
```

Matched bases: 16

Mature reads in Control: 1743 Mature reads in dsDicer: 725
Star reads in Control: 0 Star reads in dsDicer: 0

Initial dG = -35.60

```
          10        20        30
--|    U UG   G       UCC       G   GAUG
  UUACU C  ACG GCCUGAA   UUGCUCA CCU    \
  AAUGG G  UGU UGGACUU   AACGAGU GGA    U
GG^    U GU   G       UGA       -   AAAA
       70        60        50         40
```

```
Bge-Mir-87-2       GUGAGCAAAGUUUCAGGUGUGU
ame-mir-87         GUGAGCAAAGUUUCAGGUGU--
bmo-mir-87         -UGAGCAAACUUUCAGGUGUGU
mse-mir-87         GUGAGCAAACUUUCAGGUGUGU
dme-mir-87-3p      UUGAGCAAAAUUUCAGGUGUG-
                    ******** **********
```

Genomic coordinates:
Bge-Mir-87-2_3p: Scaffold70:23160-23181:-
Bge-Mir-87-2_pre: Scaffold70:23160-23215:-
Bge-Mir-87-2_5p*: Scaffold70:23193-23215:-

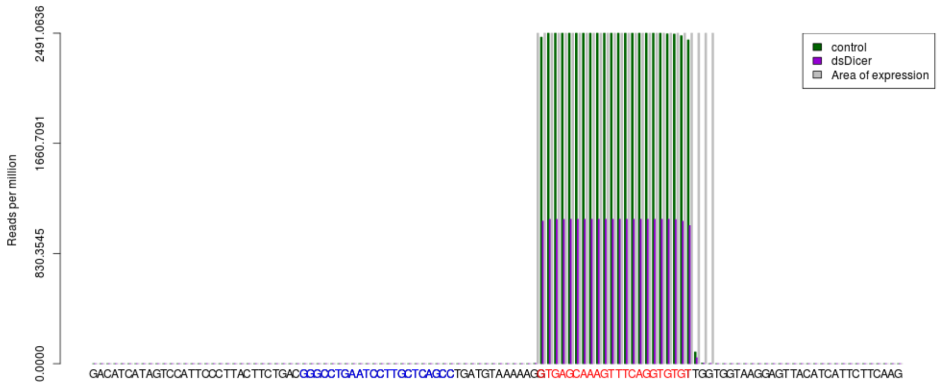

>Bge-Mir-87-3_pre+_11
GTTCCCCTCAT**CGCCTGATACTTGCTTTAACCT**GAGTATTTGACAAG**GTGAGCAAAGTTTCAGGTGTGT**GGGTGGAGCGG

>Bge-Mir-87-3_3p
GTGAGCAAAGTTTCAGGTGTGT

>Bge-Mir-87-3_5p*
CGCCTGATACTTGCTTTAACCT

```
GUUCCCCUCAUCGCCUGAUACUUGCUUUAACCUGAGUAUUUGACAAGGUGAGCAAAGUUUCAGGUGUGUGGGUGGAGCGG
(((((.((((((((((((.((((((((..((((..((.....)).)))))))))).)).))))))).))))).))))).. (-36.60)
```

Matched bases: 17

Mature reads in Control: 1699 Mature reads in dsDicer: 684
Star reads in Control: 0 Star reads in dsDicer: 0

Initial dG = -36.60

```
          10         20         30
--     C     -       U  -|     UA    GA  AU
  GUUCC CUCAU CGCCUGA AC UUGCUU  ACCU  GU  \
  CGAGG GGGUG GUGGACU UG AACGAG  UGGA  CA  U
GG     U     U       U  A^     --    A-  GU
.        70        60        50
```

```
Bge-Mir-87-3       GUGAGCAAAGUUUCAGGUGUGU
ame-mir-87         GUGAGCAAAGUUUCAGGUGU--
bmo-mir-87         -UGAGCAAACUUUCAGGUGUGU
mse-mir-87         GUGAGCAAACUUUCAGGUGUGU
dme-mir-87-3p      UUGAGCAAAAUUUCAGGUGUG-
                    ******** **********
```

Genomic coordinates:
Bge-Mir-87-3_3p: Scaffold70:1851925-1851946:-
Bge-Mir-87-3_pre: Scaffold70:1851925-1851982:-
Bge-Mir-87-3_5p*: Scaffold70:1851961-1851982:-

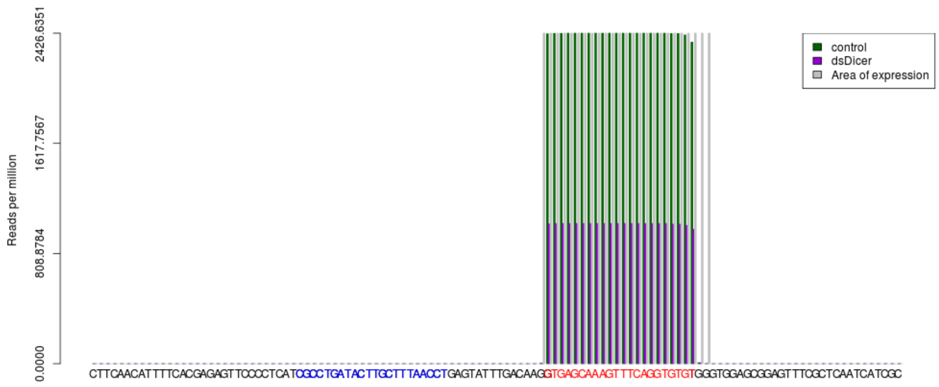

>Bge-Mir-92a_pre+_11
CAGTCGTGGTC**AGTCCGTGACTTGTGGCAATATT**ATCATTGTATACAAA**TATTGCACTTGTCCCGGCCTAT**CCAAGGCAGTA

>Bge-Mir-92a_3p
TATTGCACTTGTCCCGGCCTAT

>Bge-Mir-92a_5p
AGTCCGTGACTTGTGGCAATATT

```
CAGUCGUGGUCAGUCCGUGACUUGUGGCAAUAUUAUCAUUGUAUACAAAUAUUGCACUUGUCCCGGCCUAUCCAAGGCAGUA
..(((.(((..((.(((.(((..(((.(((((((.............))))))))))..))).))).))..))).))).... (-26.00)
```

Matched bases: 16

Mature reads in Control: 75 Mature reads in dsDicer: 38
Star reads in Control: 112 Star reads in dsDicer: 79

Initial dG = -26.00

```
          10        20        30        40
CA--|  G   UC  U   U   UU   G       AUCAUU
    GUC UGG  AG CCG GAC  GUG CAAUAUU      \
    CGG ACC  UC GGC CUG  CAC GUUAUAA      G
AUGA^  A   UA  C   C   UU   -       ACAUAU
 80        70        60         50
```

```
nvi-mir-92a         -AUUGCACUUGUCCCGGCCUAU
dme-mir-92a-3p      CAUUGCACUUGUCCCGGCCUAU
ame-mir-92a         -AUUGCACUUGUCCCGGCCUAU
Bge-Mir-92a         UAUUGCACUUGUCCCGGCCUAU
bmo-mir-92a         UAUUGCACCAGUCCCGGCCUAU
mse-mir-92a         UAUUGCACCAGUCCCGGCCUAU
tca-mir-92a-3p      -AUUGCACUAGUCCCGGCCUA-
api-mir-92a         UAUUGCACAUGUCCCGGCCAAU
                     *******  ********* *
```

Genomic coordinates:
Bge-Mir-92a_5p: Scaffold702:1049047-1049069:+
Bge-Mir-92a_pre: Scaffold702:1049047-1049106:+
Bge-Mir-92a_3p: Scaffold702:1049085-1049106:+

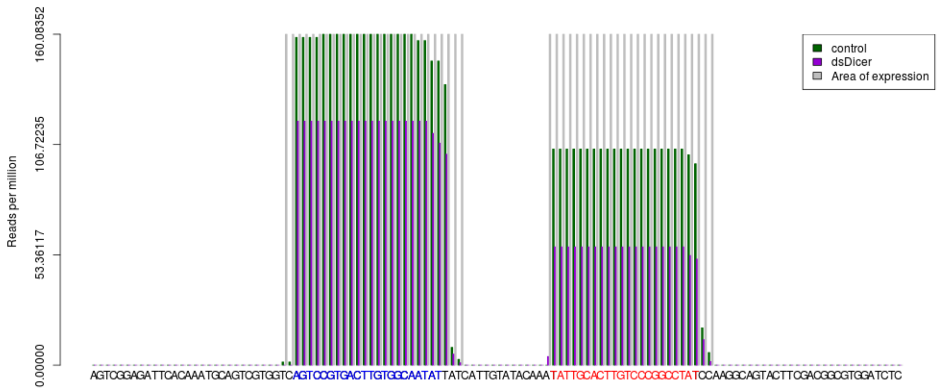

>Bge-Mir-92b_pre+_11
GTGAGCAGCAC**AGGCCGTGTCAGGTGCACTTTG**TAATATTCGCTCA**AATTGCACTTGTCCCGGCCTGC**CTTGCCCACGC

>Bge-Mir-92b_3p
AATTGCACTTGTCCCGGCCTGC

>Bge-Mir-92b_5p*
AGGCCGTGTCAGGTGCACTTTG

```
GUGAGCAGCACAGGCCGUGUCAGGUGCACUUUGUAAUAUUCGCUCAAAUUGCACUUGUCCCGGCCUGCCUUGCCCACGC
(((.((((..(((((((...((((((((.((((...........)))).))))))))...))))))).)..)))).))).. (-37.20)
```

Matched bases: 16

Mature reads in Control: 169   Mature reads in dsDicer: 123
Star reads in Control: 30   Star reads in dsDicer: 16

Initial dG =   -37.20

```
         10        20        30
--|  A    CA       UGU        C    UAAUA
  GUG GCAG  CAGGCCG   CAGGUGCA UUUG     \
  CAC CGUU  GUCCGGC   GUUCACGU AAAC     U
CG^  C    CC       CCU        U    UCGCU
       70        60        50        40
```

```
tca-mir-92b-3p      -AUUGCACUUGUCCCGGCCUG-
api-mir-92b         UAUUGCACUUGACCCAGCCUGC
Bge-Mir-92b         AAUUGCACUUGUCCCGGCCUGC
bmo-mir-92b         AAUUGCACCAAUCCCGGCCUGC
mse-mir-92b         AAUUGCACCAAUCCCGGCCUGC
ame-mir-92b         AAUUGCACCCGUCCCGGCCUGA
dme-mir-92b-3p      AAUUGCACUAGUCCCGGCCUGC
                     *******     *** *****
```

Genomic coordinates:
Bge-Mir-92b_5p*: Scaffold702:1049172-1049193:+
Bge-Mir-92b_pre: Scaffold702:1049172-1049228:+
Bge-Mir-92b_3p: Scaffold702:1049207-1049228:+

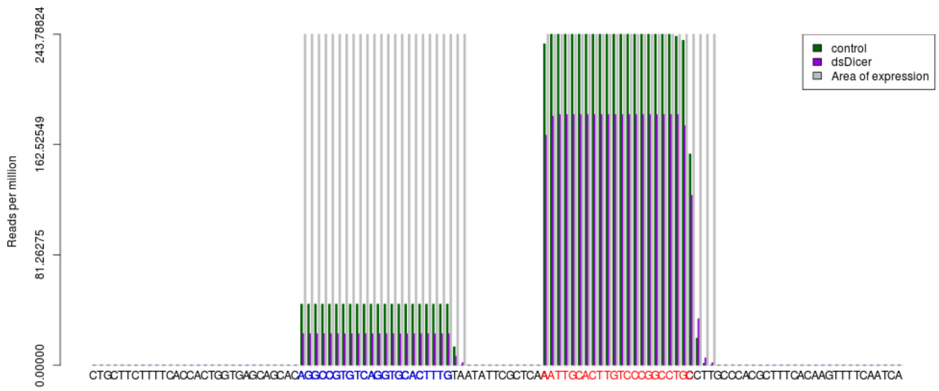

>Bge-Mir-2796_pre+_11
GGCTCAGGGGC**AGGGGTTTCTTTCGGCCTCCAG**CCGACATGGAATGTT**GTAGGCCGGCGGAAACTACTTGC**CTCTGAGCCAC

>Bge-Mir-2796_3p
GTAGGCCGGCGGAAACTACTTGC

>Bge-Mir-2796_5p*
AGGGGTTTCTTTCGGCCTCCAG

```
GGCUCAGGGGCAGGGGUUUCUUUCGGCCUCCAGCCGACAUGGAAUGUUGUAGGCCGGCGGAAACUACUUGCCUCUGAGCCAC
(((((((((((((((((((.(((((((.((((..........)))).))))))).))))))).)))))))))))))).. (-53.10)
```

Matched bases: 18

```
Mature reads in Control: 278          Mature reads in dsDicer: 146
Star reads in Control: 0              Star reads in dsDicer: 0

 Initial dG =    -53.10

         10         20        30
--              -|       U       C     CGACA
  GGCUCAGGGGCAGG GGUUUCU UCGGCCU CAGC       \
  CCGAGUCUCCGUUC UCAAAGG GGCCGGA GUUG       U
CA              A^       C       U     UAAGG
 80        70         60        50


Bge-Mir-2796         GUAGGCCGGCGGAAACUACUUGC
nvi-mir-2796         --AGGCCGGCGGAAACUACUUGC
tca-mir-2796-3p      GUAGGCCGGCGGAAACUACUUGC
bmo-mir-2796-3p      GUAGGCCGGCGGAAACUACUUGC
ame-mir-2796         GUAGGCCGGCGGAAACUACUUGC
mse-mir-2796         GUAGGCCGGCGGAAACUACUUGC
api-mir-2796         GUAGGCCGGCGGAAACUACUAG-
                       ****************** *
```

Genomic coordinates:
Bge-Mir-2796_3p: Scaffold708:496196-496218:-
Bge-Mir-2796_pre: Scaffold708:496196-496255:-
Bge-Mir-2796_5p*: Scaffold708:496234-496255:-

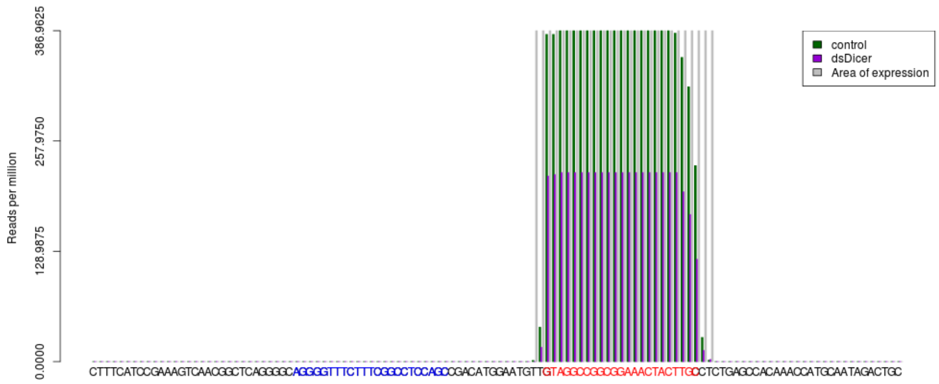

>Bge-Mir-33_pre+_11
TGATTTCTGGG**GTGCATTGTAGTTGCATTGCA**TGTTGTCATGAAACTATG**CAATACTTCTATAGTGCAACACC**AGCGATCAACA

>Bge-Mir-33_5p
GTGCATTGTAGTTGCATTGCA

>Bge-Mir-33_3p*
CAATACTTCTATAGTGCAACACC

```
UGAUUUCUGGGGUGCAUUGUAGUUGCAUUGCAUGUUGUCAUGAAACUAUGCAAUACUUCUAUAGUGCAACACCCUCUGAGCCAC
(((((.((((..((((((((((....((((((((((.......))).)))))))....))))))))))...)))).)))))... (-33.50)
```

Matched bases: 14

```
Mature reads in Control: 13          Mature reads in dsDicer: 3
Star reads in Control: 4             Star reads in dsDicer: 2

 Initial dG =    -33.50

           10         20        30
---     U    GG-          UUGC       -|  GUC
   UGAUU CUGG   UGCAUUGUAG    AUUGCAU GUU   \
   ACUAG GACC   ACGUGAUAUC    UAACGUA CAA   A
ACA     C    ACA          UUCA       U^  AGU
   80        70         60        50


Bge-Mir-33          GUGCAUUGUAGUUGCAUUGCA--
tca-mir-33-5p       GUGCAUUGUAGUUGCAUUGCAUG
bmo-mir-33-5p       GUGCAUUGUAGUUGCAUUGCA--
mse-mir-33          GUGCAUUGUAGUUGCAUUGCA--
ame-mir-33          GUGCAUUGUAGUUGCAUUG----
nvi-mir-33          GUGCAUUGUAGUUGCAUUG----
dme-mir-33-5p       GUGCAUUGUAGUCGCAUUGUC--
                    ************ ******
```

Genomic coordinates:
Bge-Mir-33_3p*: Scaffold711:287928-287950:-
Bge-Mir-33_pre: Scaffold711:287928-287989:-
Bge-Mir-33_5p: Scaffold711:287969-287989:-

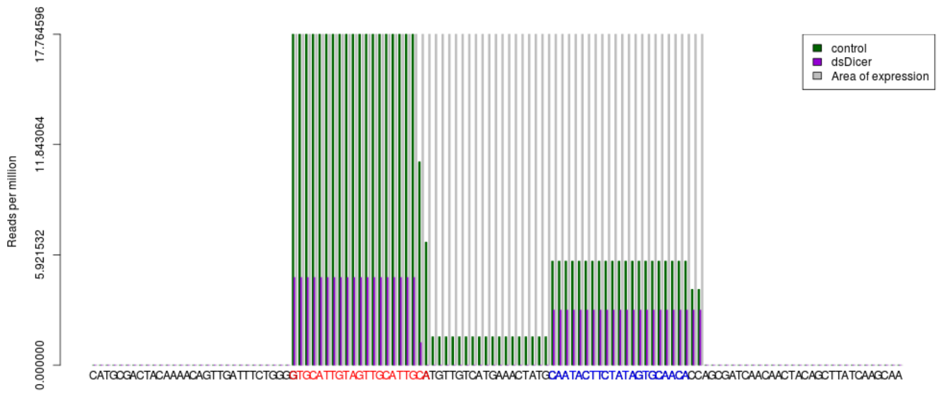

>Bge-Mir-965_pre+_11
TTTGGCTTGAA**CGGGAGAAGCTGTAACGATTATG**TTTTATTACCATCA**TAAGCGTATAGCTTTTCCCCTT**CAAACTATATC

>Bge-Mir-965_5p
CGGGAGAAGCTGTAACGATTATG

>Bge-Mir-965_3p*
TAAGCGTATAGCTTTTCCCCTT

```
UUUGGCUUGAACGGGAGAAGCUGUAACGAUUAUGUUUUAUUACCAUCAUAAGCGUAUAGCUUUUCCCCUUCUCUGAGCCAC
..(((.(((((.((((((((((((((.((.(((((...........))))).))))))))))))))).))))).))).... (-30.50)
```

Matched bases: 18

Mature reads in Control: 222   Mature reads in dsDicer: 128
Star reads in Control: 14   Star reads in dsDicer: 14

Initial dG =   -30.50

```
          10        20        30        40
UU--|  C     C             A  A     UUUUA
    UGG UUGAA GGGAGAAGCUGUA CG UUAUG     U
    AUC AACUU CCCUUUUCGAUAU GC AAUAC     U
CUAU^  A     C             -  G     UACCA
80        70        60         50
```

```
api-mir-965         UAAGCGUAUAGCUUUU-CCCCUU---
mse-mir-965         UAAGCGUAUAGCUUUU-CCCCUU---
tca-mir-965-5p      --GGUGGAAAGUUGUA-CCGCU----
dme-mir-965-5p      --GGGGUAAAACUGUA-CGUUAUAUG
Bge-Mir-965         --CGGGAGAAGCUGUAACGAU-UAUG
bmo-mir-965-5p      --CGGGAGAAGCUAUAGCGCUAUAUG
ame-mir-965-5p      --GGGGAAAGGUUAUAGCGAU-UAUG
                       * *       * *   *
```

Genomic coordinates:
Bge-Mir-965_3p*: Scaffold718:609142-609163:-
Bge-Mir-965_pre: Scaffold718:609142-609200:-
Bge-Mir-965_5p: Scaffold718:609178-609200:-

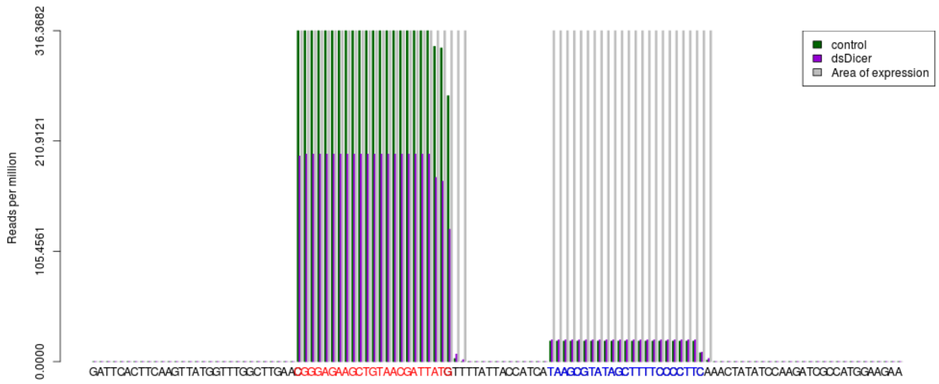

>Bge-Mir-92c_pre+_11
TAGCTCCTTTC**AGGCTGTGACTTGTGCAAGTTTT**GTGATATATGATCA**AATTGCACTTGTCCCGGCCTGA**AGGTACTAGTT

>Bge-Mir-92c_3p
AATTGCACTTGTCCCGGCCTGA

>Bge-Mir-92c_5p*
AGGCTGTGACTTGTGCAAGTTTT

```
UAGCUCCUUUCAGGCUGUGACUUGUGCAAGUUUUGUGAUAUAUGAUCAAAUUGCACUUGUCCCGGCCUGAAGGUACUAGUU
(((..((((.(((((((.(((..((((((......((((.....))))..))))))..))).)))))))))))..)))... (-35.10)
```

Matched bases: 15

Mature reads in Control: 248   Mature reads in dsDicer: 109
Star reads in Control: 125   Star reads in dsDicer: 82

Initial dG =   -35.10

```
           10        20        30        40
---|  CU    U       U   UU      GUUUUG    AU
   UAG  CCUU CAGGCUG GAC  GUGCAA      UGAU  \
   AUC  GGAA GUCCGGC CUG  CACGUU      ACUA  A
UUG^  AU    -       C   UU      AA----    GU
80        70         60        50
```

```
Bge-Mir-92c         AAUUGCACUUGUCCCGGCCUGA----
tca-mir-92c-3p      UAUUGCACCAGUCCCGGCCUGAC---
                     *******  ************
```

Genomic coordinates:
Bge-Mir-92c_3p: Scaffold721:145973-145994:-
Bge-Mir-92c_pre: Scaffold721:145973-146031:-
Bge-Mir-92c_5p*: Scaffold721:146009-146031:-

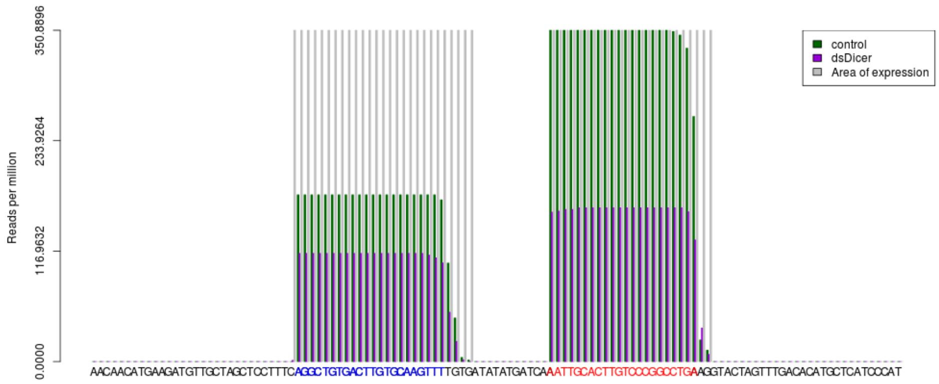

>Bge-Mir-92d_pre+_11
CTGGTTCCTTT**CAGGATATGACATTTGCAAATTT**TGTGATTTAAAA**AATTGCACTTGTTATATTCTTGA**AAGCACCAGAG

>Bge-Mir-92d_3p
AATTGCACTTGTTATATTCTTGA

>Bge-Mir-92d_5p*
CAGGATATGACATTTGCAAATTT

```
CUGGUUCCUUUCAGGAUAUGACAUUUGCAAAUUUUGUGAUUUAAAAAAUUGCACUUGUUAUAUUCUUGAAAGCACCAGAG
(((((..((((((((((((((((..(((((.((((........)))).)))))..))))))).))))))))).))))).. (-31.80)
```

Matched bases: 18

Mature reads in Control: 2 Mature reads in dsDicer: 2
Star reads in Control: 1 Star reads in dsDicer: 0

Initial dG = -31.80

```
          10         20        30
--|    UC          -       UU      A    GUG
  CUGGU  CUUUCAGGA UAUGACA  UGCAA UUUU   A
  GACCA  GAAAGUUCU AUAUUGU  ACGUU AAAA   U
GA^    C-          U       UC      A    AUU
.         70        60        50        40
```

Genomic coordinates:
Bge-Mir-92d_5p*: Scaffold721:346194-346216:+
Bge-Mir-92d_pre: Scaffold721:346194-346251:+
Bge-Mir-92d_3p: Scaffold721:346229-346251:+

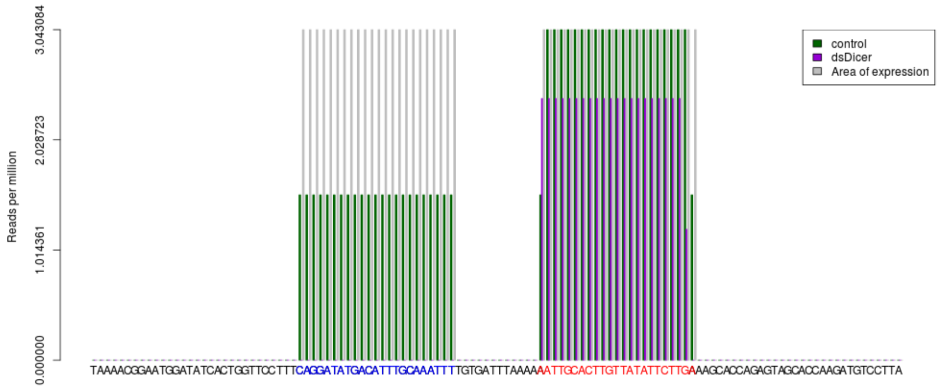

>Bge-Mir-2a_pre+_11
ATCTCATTGCC**CTCGTCAGAGTGGTTATGATGTG**TCTATATTAATTTCA**TATCACAGCCAGCTTTGATGAGCG**CAAAGGGATTC

>Bge-Mir-2a_3p
TATCACAGCCAGCTTTGATGAGCG

>Bge-Mir-2a_5p
CTCGTCAGAGTGGTTATGATGTG

AUCUCAUUGCC**CUCGUCAGAGUGGUUAUGAUGUG**UCUAUAUUAAUUUCA**UAUCACAGCCAGCUUUGAUGAGCG**CAAAGGGAUUC
(((((.((((.(((((((((((((((.(((((((.............))))))).))))).)))))))))).)))).))))).. (-37.30)

Matched bases: 20

Mature reads in Control: 438   Mature reads in dsDicer: 191
Star reads in Control: 8   Star reads in dsDicer: 5

```
 Initial dG =    -37.30

         10        20         30        40
--      A    C          -|     A       UCUAUA
  AUCUC UUGC CUCGUCAGAG UGGUU UGAUGUG       \
  UAGGG AACG GAGUAGUUUC ACCGA ACUAUAC       U
CU      A    C          G^     C       UUUAAU
   80        70        60        50
```

```
Bge-Mir-2a         UAUCACAGCCAGCUUUGAUGAGCG
nvi-mir-2a         UAUCACAGCCAGCUUUGUUGAGC-
tca-mir-2a-3p      UAUCACAGCCAGCUUUGAUGAGC-
bmo-mir-2a-3p      UAUCACAGCCAGCUUUGAUGAGC-
dme-mir-2a-3p      UAUCACAGCCAGCUUUGAUGAGC-
api-mir-2a         UAUCACAGCCAGCUUUGAUGAGCG
mse-mir-2a         --UCACAGCCAGCUUUGAUGAGCA
                     *************** *****
```

Genomic coordinates:
Bge-Mir-2a_3p: Scaffold804:804858-804881:-
Bge-Mir-2a_pre: Scaffold804:804858-804919:-
Bge-Mir-2a_5p: Scaffold804:804897-804919:-

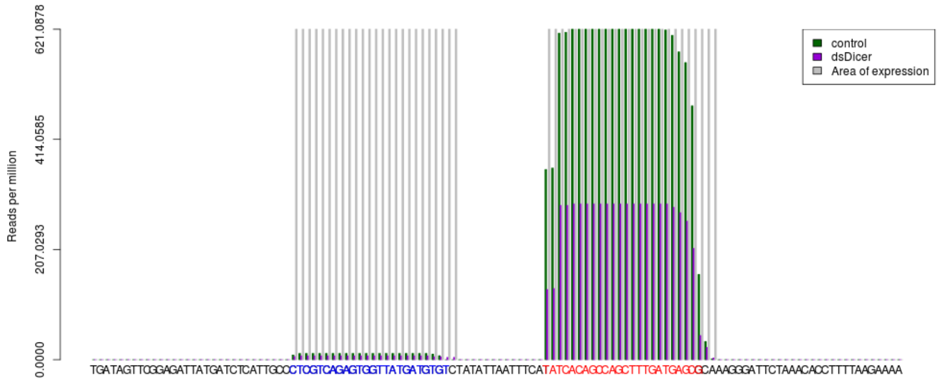

>Bge-Mir-2b_pre+_11
TGGTGTGCTGC**TCACAAAGCTGTTGTGATGTG**TCGATTTGAGTTCA**TATCACAGCCAGCTTTGATGAGC**GGCACACTAAG

>Bge-Mir-2b_3p
TATCACAGCCAGCTTTGATGAGC

>Bge-Mir-2b_5p*
TCACAAAGCTGTTGTGATGTG

```
UGGUGUGCUGCUCACAAAGCUGUUGUGAUGUGUCGAUUUGAGUUCAUAUCACAGCCAGCUUUGAUGAGCGGCACACUAAG
((((((((((((((((((((((((((((((((...........))))))))))).))))))).)))))))))))))).. (-49.40)
```

Matched bases: 19

Mature reads in Control: 436   Mature reads in dsDicer: 191
Star reads in Control: 0   Star reads in dsDicer: 0

```
 Initial dG =    -49.40

          10         20         30
--              -       -|          UCGAU
  UGGUGUGCUGCUCA CAAAGCU GUUGUGAUGUG     U
  AUCACACGGCGAGU GUUUCGA CGACACUAUAC     U
GA              A       C^          UUGAG
.        70        60        50        40
```

```
Bge-Mir-2b         -UAUCACAGCCAGCUUUG--AUGAGC-
mse-mir-2b         ---UCACAGCCAGCUUUG--AUGAGC-
tca-mir-2b-3p      -UAUCACAGCCAGCUUUG--AUGAGC-
dme-mir-2b-3p      -UAUCACAGCCAGCUUUG--AGGAGC-
bmo-mir-2b-3p      -UAUCACAGCCAGCUUUG--UUGAGU-
api-mir-2b         -UAUCACAGCCAGCUUUGU-AUGAGCA
nvi-mir-2b         -UAUCACAGCCAGCUUUG--AUGUGC-
ame-mir-2b         UCAUCAAAGCUGGCUGUGAUAUGA---
                      *** ***   *** **     *
```

Genomic coordinates:
Bge-Mir-2b_3p: Scaffold804:807622-807644:-
Bge-Mir-2b_pre: Scaffold804:807622-807679:-
Bge-Mir-2b_5p*: Scaffold804:807659-807679:-

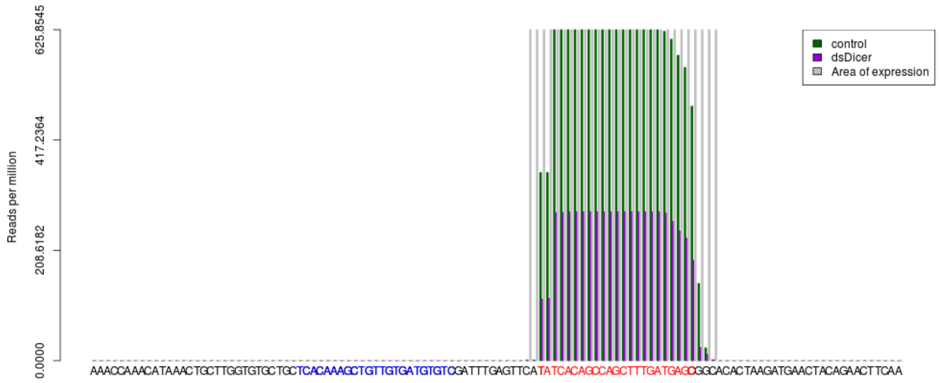

>Bge-Mir-13a_pre+_11
CTATGTCCCAACAGTCAAAATGACTGTGATGTGTAGATTTAAGTCATATCACAGCCATTTTTGACGAGTTGGGCTGTAGGA

>Bge-Mir-13a_3p
TATCACAGCCATTTTTGACGAGTT

>Bge-Mir-13a_5p*
CAGTCAAAATGACTGTGATGTG

```
CUAUGUCCCAACAGUCAAAAUGACUGUGAUGUGUAGAUUUAAGUCAUAUCACAGCCAUUUUUGACGAGUUGGGCUGUAGGA
(((((.((((((.(((((((((.((((((((((...........)))))))))).)))))).))))..)))))).))))).. (-35.30)
```

Matched bases: 18

Mature reads in Control: 809   Mature reads in dsDicer: 348
Star reads in Control: 707   Star reads in dsDicer: 101

Initial dG =   -35.30

```
          10          20         30
--      U      A-    -|      A          UAGAU
  CUAUG CCCAAC  GUC AAAAUG CUGUGAUGUG      \
  GAUGU GGGUUG  CAG UUUUAC GACACUAUAC      U
AG      C      AG    U^      C          UGAAU
80        70        60        50        40
```

```
Bge-Mir-13a         UAUCACAGCCAUUUUUGACGAGUU
dme-mir-13a-3p      UAUCACAGCCAUUUU-GAUGAGU-
nvi-mir-13a         UAUCACAGCCAUUUU-GAUGAG--
ame-mir-13a         UAUCACAGCCAUUUU-GAUGAG--
tca-mir-13a-3p      UAUCACAGCCACUUU-GAUGAGC-
bmo-mir-13a-3p      UAUCACAGCCACUUU-GAUGUG--
api-mir-13a         UAUCACAGCCACUUU-GAUGAGC-
                    *********** *** ** * *
```

Genomic coordinates:
Bge-Mir-13a_3p: Scaffold804:807734-807757:-
Bge-Mir-13a_pre: Scaffold804:807734-807792:-
Bge-Mir-13a_5p*: Scaffold804:807771-807792:-

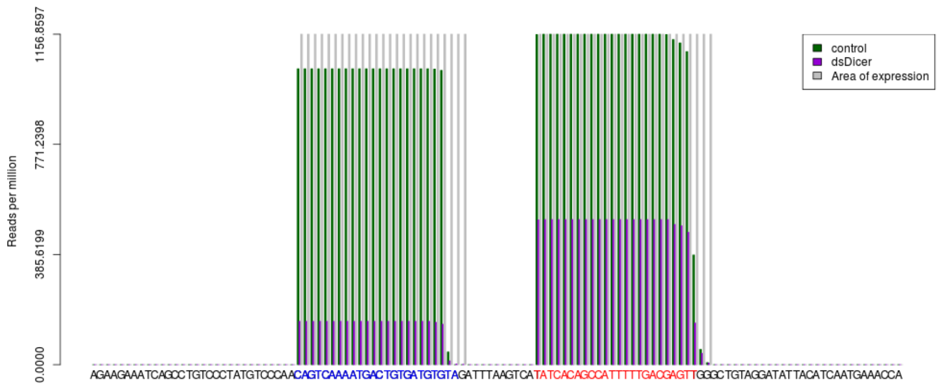

>Bge-Mir-13b_pre+_11
CGTTGCACTGG**TCGTCAATTTGGTTGTGAAATG**TTGTATTAAAAATTCA**TATCACAGCCACTTTGATGAGC**TAGTTTTTCGG

>Bge-Mir-13b_3p
TATCACAGCCACTTTGATGAGC

>Bge-Mir-13b_5p*
TCGTCAATTTGGTTGTGAAATG

CGUUGCACUGG**UCGUCAAUUUGGUUGUGAAAUG**UUGUAUUAAAAAUUCA**UAUCACAGCCACUUUGAUGAGC**UAGUUUUUCGG
((....((((((((((((..(((((((((.(((..............))).)))))))))..))))))).))))))....)). (-29.80)

Matched bases: 17

Mature reads in Control: 503   Mature reads in dsDicer: 365
Star reads in Control: 3   Star reads in dsDicer: 2

Initial dG =   -29.80

```
         10         20         30         40
-  UUGC      -|       UU          A    UUGUAU
 CG     ACUGG UCGUCAA  UGGUUGUGA AUG       U
 GC     UGAUC AGUAGUU  ACCGACACU UAC       A
G  UUUU      G^       UC          A    UUAAAA
 80        70         60         50
```

```
bmo-mir-13b-3p      UAUCACAGCCAUUUUUGACGAGU
ame-mir-13b         UAUCACAGCCAUUUUUGACGAUU
nvi-mir-13b         UAUCACAGCCAUUUUUGACGAGU
dme-mir-13b-3p      UAUCACAGCCAUUUU-GACGAGU
tca-mir-13b-3p      UAUCACAGCCAUUUU-GACGAGU
Bge-Mir-13b         UAUCACAGCCACUUU-GAUGAGC
                    *********** *** ** **
```

Genomic coordinates:
Bge-Mir-13b_3p: Scaffold804:820526-820547:-
Bge-Mir-13b_pre: Scaffold804:820526-820585:-
Bge-Mir-13b_5p*: Scaffold804:820564-820585:-

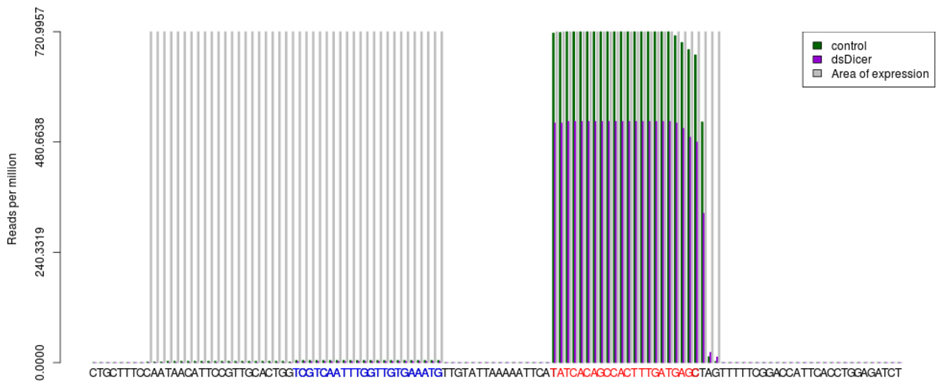

>Bge-Mir-2c_pre+_11
GCAGTGTTCTG**GCATCAAATCTGGTTTGTCATA**GATTGCTTGAGTCCAC**TATCACAGCCAGCTTTGATGAGC**AGAAAGTTGTC

>Bge-Mir-2c_3p
TATCACAGCCAGCTTTGATGAGC

>Bge-Mir-2c_5p*
GCATCAAATCTGGTTTGTCATA

```
GCAGUGUUCUGGCAUCAAAUCUGGUUUGUCAUAGAUUGCUUGAGUCCACUAUCACAGCCAGCUUUGAUGAGCAGAAAGUUGUC
((((..(((((.(((((((.((((((.((.((((...((....))...)))).)))))))).))))))).)))))..)))). (-28.80)
```

Matched bases: 18

Mature reads in Control: 649 Mature reads in dsDicer: 303
Star reads in Control: 3 Star reads in dsDicer: 0

Initial dG = -28.80

```
         10         20        30        40
-    UG      G-|       U       U  C     AUU  U
 GCAG  UUCUG  CAUCAAA CUGGUU GU AUAG   GC U
 UGUU  AAGAC  GUAGUUU GACCGA CA UAUC   UG G
C    GA      GA^       C       -  C     ACC  A
  80         70        60         50
```

```
Bge-Mir-2c          -UAUCACAGCCAGCUUUGAUGAGC
tca-mir-2c-3p       -UAUCACAGCCAGCUUUGAUGAGC
nvi-mir-2c          -UAUCACAGCCAGCUUUGAUGAGC
dme-mir-2c-3p       -UAUCACAGCCAGCUUUGAUGGGC
api-mir-2c          CCGUCAAAGCGGGCUGUCGUA---
                       *** ***   *** *   *
```

Genomic coordinates:
Bge-Mir-2c_3p: Scaffold804:820664-820686:-
Bge-Mir-2c_pre: Scaffold804:820664-820724:-
Bge-Mir-2c_5p*: Scaffold804:820703-820724:-

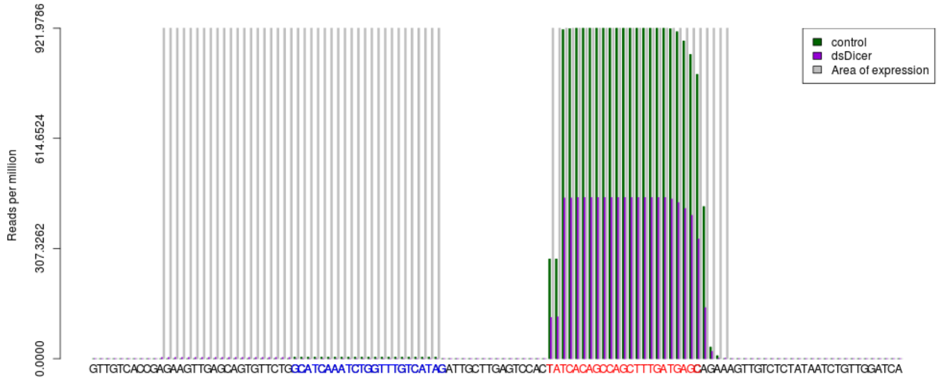

>Bge-Mir-71_pre+_11
TTGGGACTGTG**TGAAAGACATGGGTAGTGAGATG**TTGGTTGTATCTTGCA**TCTCACTACCTTGTCTTTCATG**TGGTCCTAACG

>Bge-Mir-71_5p
TGAAAGACATGGGTAGTGAGATG

>Bge-Mir-71_3p*
TCTCACTACCTTGTCTTTCATG

```
UUGGGACUGUGUGAAAGACAUGGGUAGUGAGAUGUUGGUUGUAUCUUGCAUCUCACUACCUUGUCUUUCAUGAGAAAGUUGUC
((((((..((((((((((.((((((((((((((.((......)).))))))))))))))))))))))))))..))))))).. (-52.40)
```

Matched bases: 20

Mature reads in Control: 974   Mature reads in dsDicer: 478
Star reads in Control: 254   Star reads in dsDicer: 166

Initial dG =   -52.40

```
         10        20        30        40
--|      UG          U              U  UU
  UUGGGAC  UGUGAAAGACA GGGUAGUGAGAUGU GG  G
  AAUCCUG  GUACUUUCUGU UCCAUCACUCUACG UC  U
GC^      GU          -              U  UA
  80        70         60        50
```

```
Bge-Mir-71         UGAAAGACAUGGGUAGUGAGAUG
tca-mir-71-5p      UGAAAGACAUGGGUAGUGAGAU-
bmo-mir-71-5p      UGAAAGACAUGGGUAGUGA----
nvi-mir-71         UGAAAGACAUGGGUAGUGA----
ame-mir-71         UGAAAGACAUGGGUAGUGA----
mse-mir-71         UGAAAGACAUGGGUAGUGAGAUG
api-mir-71         UGAAAGACAUGGGUAGUGAGAUG
                   *******************
```

Genomic coordinates:
Bge-Mir-71_3p*: Scaffold804:820775-820796:-
Bge-Mir-71_pre: Scaffold804:820775-820835:-
Bge-Mir-71_5p: Scaffold804:820813-820835:-

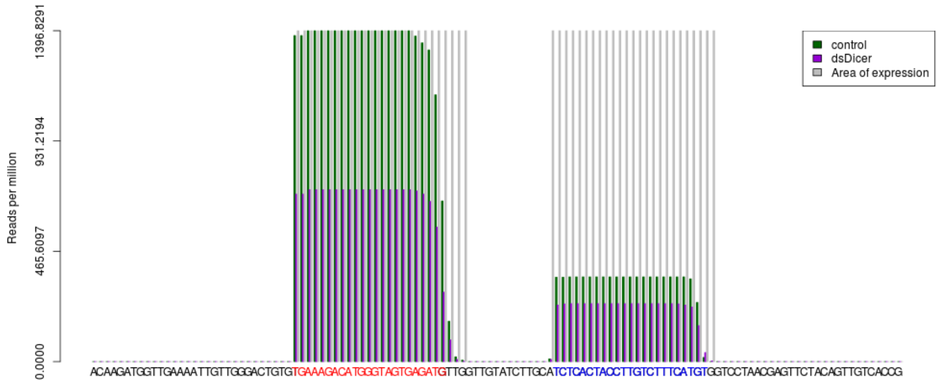

>Bge-Mir-971_pre+_11
TGAGGTCCGTC**CACTCTAAGCTCGAACACCAAGC**CTGGAACCTGTACCGT**TTGGTGTTCTACCTTACAGTGA**GCGGACTTCAG

>Bge-Mir-971_5p
CACTCTAAGCTCGAACACCAAGC

>Bge-Mir-971_3p*
TTGGTGTTCTACCTTACAGTGA

```
UGAGGUCCGUCCACUCUAAGCUCGAACACCAAGCCUGGAACCUGUACCGUUUGGUGUUCUACCUUACAGUGAAGAAAGUUGUC
((((((((..((((.((((...(((((((((((..((........)))))))))))))...)))).)))).)))))))))). (-42.00)
```

Matched bases: 17

Mature reads in Control: 7 Mature reads in dsDicer: 12
Star reads in Control: 0 Star reads in dsDicer: 3

Initial dG = -42.00

```
         10        20        30        40
-|        UC    C    CUC           CU  AAC
 UGAGGUCCG  CACU UAAG   GAACACCAAGC  GG   C
 ACUUCAGGC  GUGA AUUC   CUUGUGGUUUG  CC   U
G^        GA    C    CAU           --  AUG
  80        70        60        50
```

```
dme-mir-971-5p      G-CUGUAAAUUGUAAUCAUCAAAGC
Bge-Mir-971         CACUCUAAGCUCGAA-CACCAAGC-
                      ** ***  *  ** ** ***
```

Genomic coordinates:
Bge-Mir-971_5p: Scaffold824:229881-229903:+
Bge-Mir-971_pre: Scaffold824:229881-229941:+
Bge-Mir-971_3p*: Scaffold824:229920-229941:+

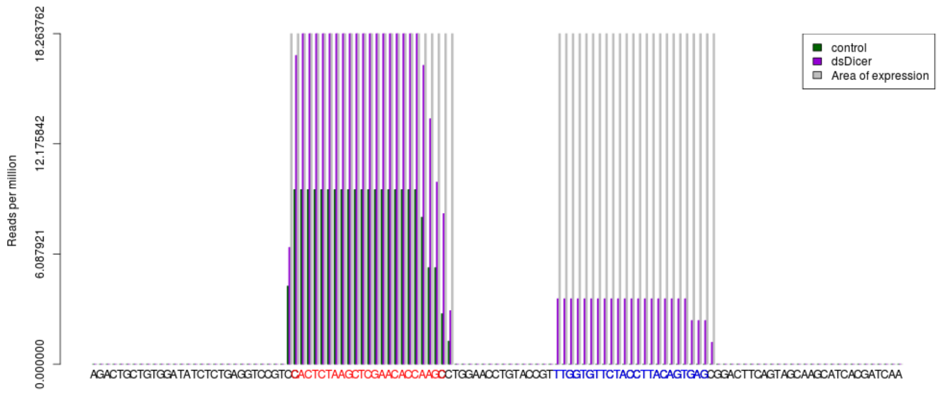

>Bge-Mir-929_pre+_11
CAACCGGGGTC**AAATTGACTCTAGTAGGGAGTC**CGTATTATAAACATAGTGA**CTCCCTAACGGAGTCAGATTGA**CCTCTTTGAAG

>Bge-Mir-929_5p
AAATTGACTCTAGTAGGGAGTC

>Bge-Mir-929_3p
CTCCCTAACGGAGTCAGATTGA

```
CAACCGGGGUCAAAUUGACUCUAGUAGGGAGUCCGUAUUAUAAACAUAGUGACUCCCUAACGGAGUCAGAUUGAAGAAAGUUGUC
.....((((((((.((((((((..(((((((((...(((((....))))))))))))))..)))))))).))))))))....... (-40.90)
```

Matched bases: 17

Mature reads in Control: 40 Mature reads in dsDicer: 27
Star reads in Control: 12 Star reads in dsDicer: 3

Initial dG = -41.20

```
          10        20        30        40
CAACC--|       A         AG          CGU      A
       GGGGUCAA UUGACUCU  UAGGGAGUC   AUUAU A
       CUCCAGUU GACUGAGG  AUCCCUCAG   UGAUA A
GAAGUUU^       A         CA          ---      C
    80        70        60           50
```

```
nvi-mir-929         --AUUGACUCUAGUAGGGAGUCC
ame-mir-929         --AUUGACUCUAGUAGGGAGUCC
bmo-mir-929-5p      --AUUGACUCUAGUAGGGAGUCC
Bge-Mir-929         AAAUUGACUCUAGUAGGGAGUC-
api-mir-929         AAAUUGACUCUAGUAGGGAG---
dme-mir-929-5p      AAAUUGACUCUAGUAGGGAGUC-
tca-mir-929-5p      -AAUUGACUCUAGUAGGGAGUC-
                      *****************
```

Genomic coordinates:
Bge-Mir-929_5p: Scaffold860:479316-479337:+
Bge-Mir-929_pre: Scaffold860:479316-479378:+
Bge-Mir-929_3p: Scaffold860:479357-479378:+

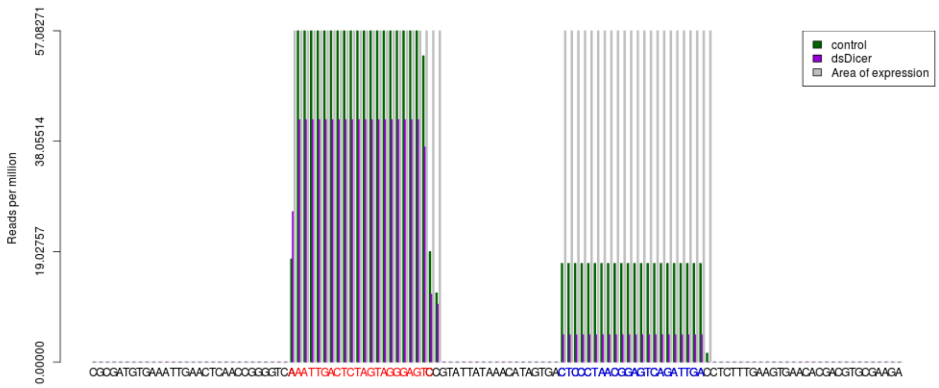

>Bge-Mir-iab-4_pre+_11
CAACCCCCTGT**ACGTATACTGAATGTATCCTGA**GTGTATTCTTTTC**CGGTATACCTTCAGTATACGTAACA**GGAGGCCACAC

>Bge-Mir-iab-4_5p
ACGTATACTGAATGTATCCTGA

>Bge-Mir-iab-4_3p*
CGGTATACCTTCAGTATACGTAACA

```
CAACCCCCUGUACGUAUACUGAAUGUAUCCUGAGUGUAUUCUUUUCCGGUAUACCUUCAGUAUACGUAACAAGAAAGUUGUC
...((.((((((((((((((((.((((((.(((.........))).)).)))).)))))))))))).)))).))...... (-34.00)
```

Matched bases: 18

Mature reads in Control: 77 Mature reads in dsDicer: 60
Star reads in Control: 1 Star reads in dsDicer: 0

Initial dG = -34.00

```
           10         20         30
CAA---   C    -              U    -| U   UGUA
      CC CCUG UACGUAUACUGAA GUAU CC GAG    \
      GG GGAC AUGCAUAUGACUU CAUA GG CUU    U
CACACC   A    A              C    U^ C   UUCU
 80        70        60        50        40
```

```
Bge-Mir-iab-4         ACGUAUACUGAAUGUAUCCUGA
tca-mir-iab-4-5p      ACGUAUACUGAAUGUAUCCUGA
ame-mir-iab-4         ACGUAUACUGAAUGUAUCCUGA
mse-mir-iab-4-5p      ACGUAUACUGAAUGUAUCCUGA
dme-mir-iab-4-5p      ACGUAUACUGAAUGUAUCCUGA
bmo-mir-iab-4-5p      ACGUAUACUGAAUGUAUCCUGA
api-mir-iab-4         ACGUAUACUAAAUGUAUCCUGA
                      ********* ************
```

Genomic coordinates:
Bge-Mir-iab-4_pre: Scaffold951:1153211-1153270:+
Bge-Mir-iab-4_3p*: Scaffold951:1153246-1153270:+
Bge-Mir-iab-4_5p: Scaffold951:1153211-1153232:+

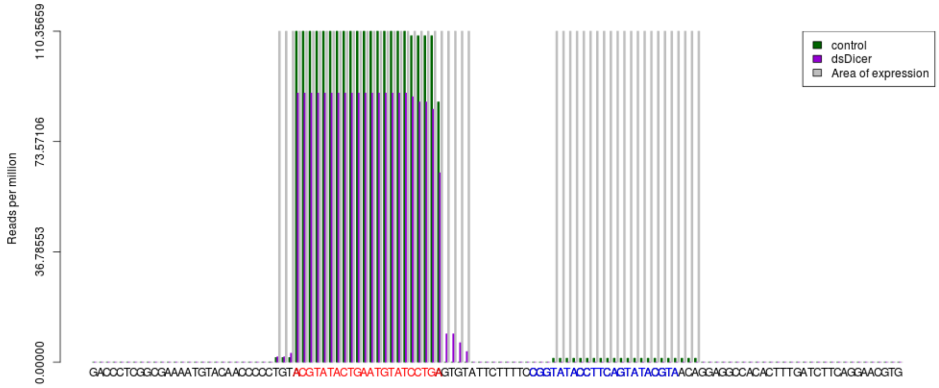

>Bge-Mir-124_pre+_11
GTCTTGCTCCT**CGTGTTCACTGTTGGCCTTGATG**TGTCGTATTGTGGATCA**TAAGGCACGCGGTGAATGCCAA**GAGCGGACTTG

>Bge-Mir-124_3p
TAAGGCACGCGGTGAATGCCAA

>Bge-Mir-124_5p*
CGTGTTCACTGTTGGCCTTGATG

```
GUCUUGCUCCUCGUGUUCACUGUUGGCCUUGAUGUGUCGUAUUGUGGAUCAUAAGGCACGCGGUGAAUGCCAAGAGCGGACUUG
((((.((((...(((((((((((..((((((.((.(((........)))))))))))..)))))))))))...)))))))).... (-34.50)
```

Matched bases: 17

Mature reads in Control: 13   Mature reads in dsDicer: 13
Star reads in Control: 12   Star reads in dsDicer: 15

```
 Initial dG =    -34.50

           10        20        30        40
---|   U    CUC          UG      A  U   GUA
   GUCU GCUC   GUGUUCACUGU  GCCUUG UG GUC   U
   CAGG CGAG   CGUAAGUGGCG  CGGAAU AC UAG   U
GUU^   -    AAC          CA      -  -   GUG
   80         70        60         50
```

```
Bge-Mir-124         UAAGGCACGCGGUGAAUGCCAA-
tca-mir-124-3p      UAAGGCACGCGGUGAAUGCCAAG
bmo-mir-124         UAAGGCACGCGGUGAAUGCCAAG
nvi-mir-124         UAAGGCACGCGGUGAAUGCCAAG
dme-mir-124-3p      UAAGGCACGCGGUGAAUGCCAAG
ame-mir-124         UAAGGCACGCGGUGAAUGCCAAG
mse-mir-124         UAAGGCACGCGGUGAAUGCCA--
api-mir-124         UAAGGCACGCGGUGAAUGCCA--
                    *********************
```

Genomic coordinates:
Bge-Mir-124_3p: Scaffold989:613885-613906:-
Bge-Mir-124_pre: Scaffold989:613885-613946:-
Bge-Mir-124_5p*: Scaffold989:613924-613946:-

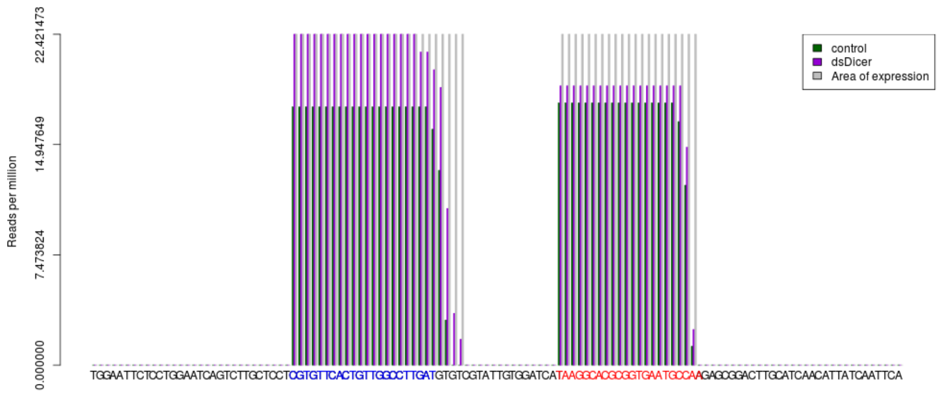

>Bge-Mir-6012_pre+_11
GCGGGGTTTAC**AGGCTGACCTCTTCACCTGTGGAAAT**GTGAATTGAGATTAGACAT**TTCGGCGATGAGATCAGCCAGT**AGAACCCAGTC

>Bge-Mir-6012_3p
TTCGGCGATGAGATCAGCCAGT

>Bge-Mir-6012_5p*
AGGCTGACCTCTTCACCTGTGGAAAT

```
GCGGGGUUUACAGGCUGACCUCUUCACCUGUGGAAAUGUGAAUUGAGAUUAGACAUUUCGGCGAUGAGAUCAGCCAGUAGAACCCAGUC
..(((.(((((.((((((.(((.((.((....(((((((.(((....)))..))))))))).)).))).)))))).))))).)))).... (-34.10)
```

Matched bases: 16

Mature reads in Control: 2
Star reads in Control: 0

Mature reads in dsDicer: 3
Star reads in dsDicer: 1

Initial dG =   -34.10

```
          10        20        30        40
GC--|  G     A      C   U  A  UGUG      G-   U
    GGG UUUAC GGCUGA CUC UC CC    GAAAUGU  AAU G
    CCC AGAUG CCGACU GAG AG GG    CUUUACA  UUA A
CUGA^  A     A      A   U  C  ----      GA   G
        80        70        60            50
```

```
tca-mir-6012-3p      UUCGGCGAUGAGAUCAGCCUGUC
ame-mir-6012         UUCGGCGAUGAGAUCAGCCUGU-
Bge-Mir-6012         UUCGGCGAUGAGAUCAGCCAGU-
                     ******************* **
```

Genomic coordinates:
Bge-Mir-6012_3p: Scaffold991:499982-500003:-
Bge-Mir-6012_pre: Scaffold991:499982-500048:-
Bge-Mir-6012_5p*: Scaffold991:500023-500048:-

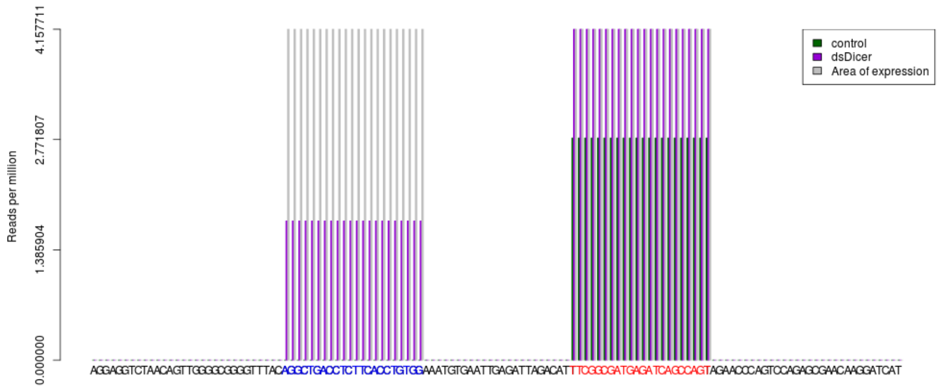

>Bge-Mir-96b_pre+_11
AGCCTGTGCGC**CTTGGCACTGGAAGAATTCACAGA**TATTTTTGAAATC**GTGGATCTCCCTGTGCCAAAGC**ATACGGCAACG

>Bge-Mir-96b_5p
CTTGGCACTGGAAGAATTCACAGA

>Bge-Mir-96b_3p*
GTGGATCTCCCTGTGCCAAAGC

```
AGCCUGUGCGCCUUGGCACUGGAAGAAUUCACAGAUAUUUUUGAAAUCGUGGAUCUCCCUGUGCCAAAGCAGAACCCAGUC
.(((.(((.((.(((((((.((.(((.(((((.(((.........))))))))))).)).))))))).)).)))))).... (-32.50)
```

Matched bases: 17

Mature reads in Control: 1199 Mature reads in dsDicer: 395
Star reads in Control: 25 Star reads in dsDicer: 3

Initial dG = -32.50

```
           10        20        30        40
A---|  U   C  C       U  A   A     A   AUUU
    GCC GUG GC UUGGCAC GG AGA UUCAC GAU    \
    CGG CAU CG AACCGUG CC UCU AGGUG CUA    U
GCAA^  -   A  A       U  C   -     -   AAGU
80         70        60         50
```

Genomic coordinates:
Bge-Mir-96b_3p*: Scaffold999:164595-164616:-
Bge-Mir-96b_pre: Scaffold999:164595-164653:-
Bge-Mir-96b_5p: Scaffold999:164630-164653:-

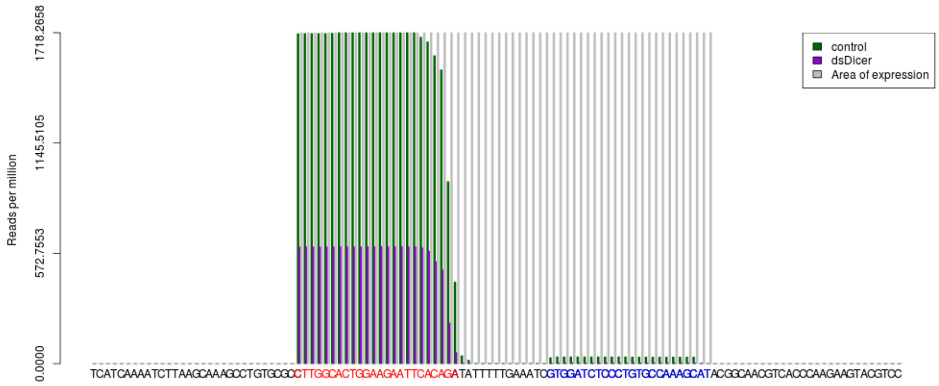

>Bge-Mir-96a_pre+_11
CGACTTCTGGT**AATGGCACTGGAAGAATTCACGGG**GAATAAAAACGCC**CGTGATTCCCCGGTGCCATCCC**CAGCGGTCGGG

>Bge-Mir-96a_5p
AATGGCACTGGAAGAATTCACGGG

>Bge-Mir-96a_3p*
CGTGATTCCCCGGTGCCATCCC

```
CGACUUCUGGUAAUGGCACUGGAAGAAUUCACGGGGAAUAAAAACGCCCGUGAUUCCCCGGUGCCAUCCCAGAACCCAGUC
(((((.((((..((((((((((..((((.((((((..........)))))))))).))))))))))..)))).))))).. (-46.20)
```

Matched bases: 18

Mature reads in Control: 2419 Mature reads in dsDicer: 975
Star reads in Control: 2 Star reads in dsDicer: 0

Initial dG = -46.20

```
          10        20        30        40
--|    U    UA          AA    U      GAAUA
  CGACU CUGG  AUGGCACUGG  GAAU CACGGG     \
  GCUGG GACC  UACCGUGGCC  CUUA GUGCCC     A
GG^    C    CC          C-    -      GCAAA
80        70        60          50
```

Genomic coordinates:
Bge-Mir-96a_3p*: Scaffold999:192259-192280:-
Bge-Mir-96a_pre: Scaffold999:192259-192317:-
Bge-Mir-96a_5p: Scaffold999:192294-192317:-

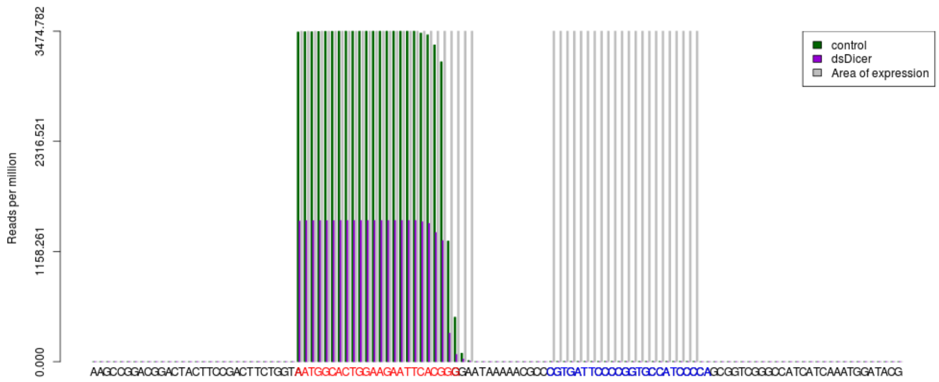

>Bge-2001_pre+_11
AGGGATTTTGG**TTTGTGACCGCCATAATGGGCA**TTTCTTACAATTCACTACAATG**CTCGTTATCACGTTCGCAATT**CCGACGCCCTT

>Bge-Mir-2001_5p
TTTGTGACCGCCATAATGGGCA

>Bge-Mir-2001_3p*
CTCGTTATCACGTTCGCAATT

```
AGGGAUUUUGGUUUGUGACCGCCAUAAUGGGCAUUUCUUACAAUUCACUACAAUGCUCGUUAUCACGUUCGCAAUUCCGACGCCCUU
((((...((((.((((((.((..((((((((((((...............))))))))))))..)).))))))..)))))..)))). (-29.40)
```

Matched bases: 16

	Control	dsDicer
**Mature reads**	21	7
**Star reads**	1	0

```
 Initial dG =    -29.40

        10         20        30        40
-|   AUU     U-      C   CC            UCUUACA
 AGGG   UUGG  UUGUGA CG  AUAAUGGGCAUU        A
 UCCC   AGCC  AACGCU GC  UAUUGCUCGUAA        U
U^   GC-     UU      U   AC            CAUCACU
      80         70        60        50
cte-mir-2001         -UUGUGACCGUUAUAAUGGGCAUG
aisc-mir-2001        -UUGUGACCGUUACAAUGGGCAUG
Bge-Mir-2001_5p      UUUGUGACCGCCAUAAUGGGC---
                      *********  * *******
```

Genomic coordinates:
Bge-Mir-**2001**_3p: Scaffold1231:362451-362471:-
Bge-Mir-**2001**_pre: Scaffold1231:362451-362515:-
Bge-Mir-**2001**_5p*: Scaffold1231:362494-362515:-

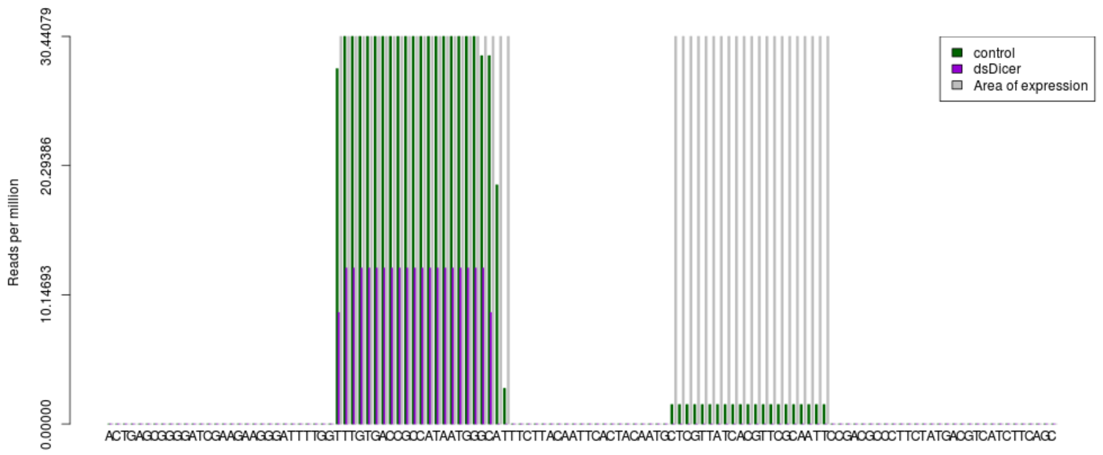

>Bge-Mir-bg5a_pre+_11
GCTTTGGGCCA**TGTGATGTGCATGTGGGCTTTCC**GAAATTTAACAGG**TGGCCCACAAGTACATCATTAGC**GACCAAGGTTG

>Bge-Mir-bg5a_5p
TGTGATGTGCATGTGGGCTTTCC

>Bge-Mir-bg5a_3p*
TGGCCCACAAGTACATCATTAGC

GCUUUGGGCCA**UGUGAUGUGCAUGUGGGCUUUCC**GAAAUUUAACAGG**UGGCCCACAAGUACAUCAUUAGC**GACCAAGGUUG
(((((((((...(((((((((.((((((((..((...........)).)))))))).)))))))))..))..))))))).. (-38.50)

Matched bases: 17

	Control	dsDicer
**Mature reads**	217	11
**Star reads**	3	0

```
 Initial dG =    -38.50

            10        20        30
--       --| CAU         A        UU   GAAAU
  GCUUUGG   GC   GUGAUGUGC UGUGGGCU  CC     \
  UGGAACC  CG   UACUACAUG ACACCCGG  GG     U
GU       AG^ AU-         A        U-   ACAAU
80        70         60        50
```

Genomic coordinates:
Bge-Mir-bg5a_3p*: Scaffold1379:119400-119422:-
Bge-Mir-bg5a_pre: Scaffold1379:119400-119458:-
Bge-Mir-bg5a_5p: Scaffold1379:119436-119458:-

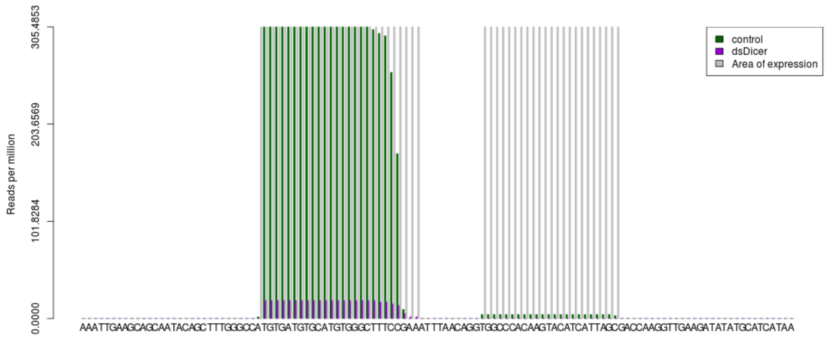

>Bge-Mir-bg5b_pre+_11
GTTATGGGCCA**TGTGATGTGCATGTGGGCTTTCC**GATATATGACAGG**TGGCCCACACTTACATCACTTGC**GACCGTAACTG

>Bge-Mir-bg5b_5p
TGTGATGTGCATGTGGGCTTTCC

>Bge-Mir-bg5b_3p*
TGGCCCACACTTACATCACTTGC

```
GUUAUGGGCCAUGUGAUGUGCAUGUGGGCUUUCCGAUAUAUGACAGGUGGCCCACACUUACAUCACUUGCGACCGUAACUG
((((((..((.((((((((..((((((((..((...........)).))))))))..)))))))).))...))))))).. (-37.80)
```

Matched bases: 16

	Control	dsDicer
**Mature reads**	211	6
**Star reads**	4	0

```
 Initial dG =    -37.80

           10        20        30
--       GC-| U        CA        UU  GAUAU
  GUUAUGG   CA GUGAUGUG  UGUGGGCU  CC     \
  CAAUGCC   GU CACUACAU  ACACCCGG  GG     A
GU       AGC^ U        UC        U-  ACAGU
80        70        60        50
```

Genomic coordinates:
Bge-Mir-bg5b_3p*: Scaffold1379:123394-123416:-
Bge-Mir-bg5b_pre: Scaffold1379:123394-123452:-
Bge-Mir-bg5b_5p: Scaffold1379:123430-123452:-

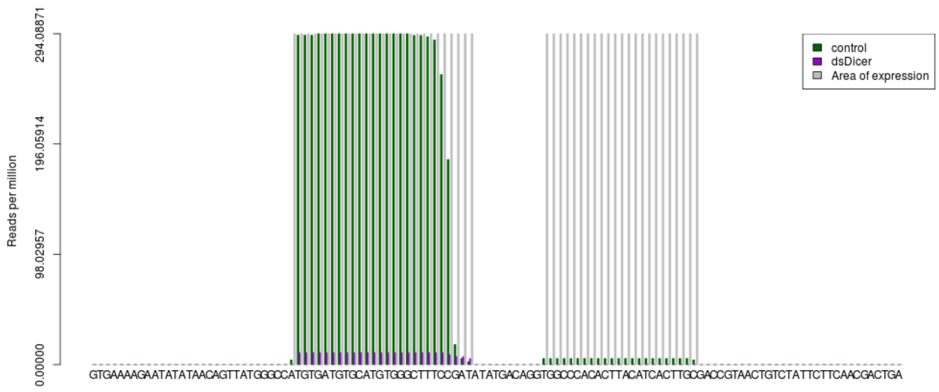

Supplemental Data S2. Report generated with MirPlot of the 11 specific miRNAs of *Blattella germanica* . For each miRNA the following information is shown:

- The miRNA precursor plus 11 flanking nucleotides, with the star arm colored blue and mature arm colored red.
- The 5p and 3p arms, colored red the mature and in blue color and an asterisk on the name for the star.
- The precursor folding in Vienna format.
- The number of complementary bases between the mature and star arms.
- The number of reads of both arms in control and dsDicer-1 treated libraries.
- The free energy of the precursor folding.
- The folding of the miRNA precursor with colored mature and star arms.
- The genomic coordinates of the precursor and both arms for the *B.germancia* genome assembly.
- A plot showing all the expressed nucleotides of the precursor (grey), the reads per million in control libraries (green) and in dsDicer-1 libraries (purple).

>Bge-Mir-bg1_pre+_11
CGGGCATCAAC**AGCGGCAAGGTCTGGAGTGAAG**TTTTCAAAGTAATCT**TGACTCCAGACCTTGTTGCTGA**TGACGTCTGTC

>Bge-Mir-bg1_3p
TGACTCCAGACCTTGTTGCTGA

>Bge-Mir-bg1_5p*
AGCGGCAAGGTCTGGAGTGAAG

```
CGGGCAUCAAC AGCGGCAAGGUCUGGAGUGAAGUUUUCAAAGUAAUCUUGACUCCAGACCUUGUUGCUGAUGACGUCUGUC
(((((.(((.((((((((((((((((((.(((.((.......)).))).)))))))))))))))))).))).))))).. (-47.80)
```

Mated bases: 19

	Control	dsDicer
**Mature reads**	1788	344
**Star reads**	19	6

```
 Initial dG =    -47.80

         10        20        30
--|    A   A                   G    U   UCA
  CGGGC UCA CAGCGGCAAGGUCUGGAGU AAG UU     \
  GUCUG AGU GUCGUUGUUCCAGACCUCA UUC AA     A
CU^    C   A                   G    U   UGA
80       70        60        50
```

Genomic coordinates:
Bge-Mir-bg1_5p*: Scaffold105:401215-401236:+
Bge-Mir-bg1_pre: Scaffold105:401215-401273:+
Bge-Mir-bg1_3p: Scaffold105:401252-401273:+

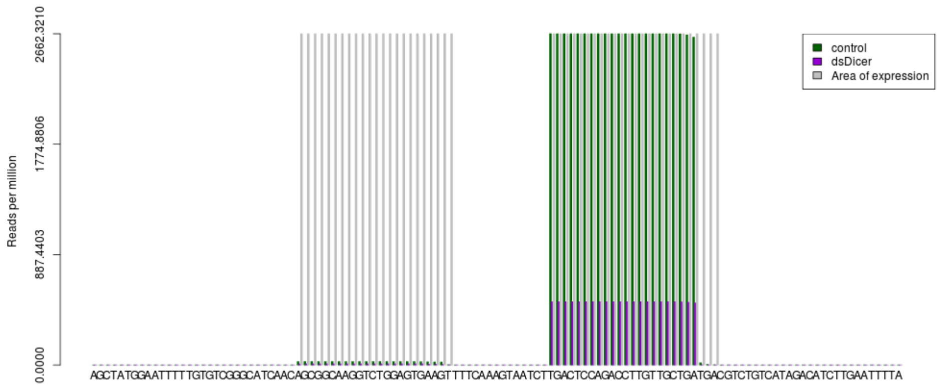

>Bge-Mir-bg2_pre+_11
AGCCTTTTCCCTCGGACGAAGTGCACTTATTTACGTAGTTATGAATTTCCGTTAAGGGCACATCGTTCGAGCATAAGGGCTTT

>Bge-Mir-bg2_5p
TCGGACGAAGTGCACTTATTTACGT

>Bge-Mir-bg2_3p*
GTTAAGGGCACATCGTTCGAGC

```
AGCCUUUUCCCUCGGACGAAGUGCACUUAUUUACGUAGUUAUGAAUUUCCGUUAAGGGCACAUCGUUCGAGCAUAAGGGCUUU
((((((...(((((((((.((((.((((...(((.((........)).))))))).)))).)))))))))...))))))).. (-32.60)
```

Matched bases: 18

	Control	dsDicer
**Mature reads**	636	41
**Star reads**	476	93

```
 Initial dG =    -32.60

         10        20        30        40
--|      UCC          A    A    UUU   U  UUA
  AGCCUUU   CUCGGACGA GUGC CUUA   ACG AG   U
  UCGGGAA   GAGCUUGCU CACG GAAU   UGC UU   G
UU^      UAC          A    G    ---   C  UAA
  80        70         60            50
```

Genomic coordinates:
Bge-Mir-bg2_5p: Scaffold23:3661643-3661667:+
Bge-Mir-bg2_pre: Scaffold23:3661643-3661703:+
Bge-Mir-bg2_3p*: Scaffold23:3661682-3661703:+

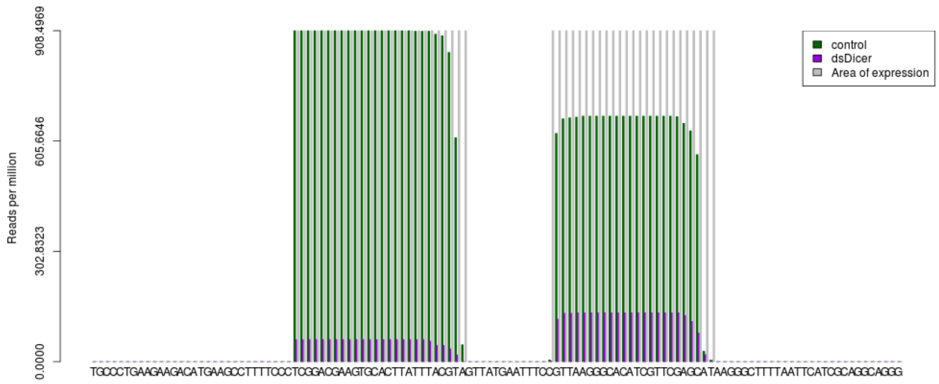

>Bge-Mir-bg3a_pre+_11
CTTTGCTTTCC**CAGCTAATCGTCCATTTCAATTG**TGTTGTTTGAAATCA**ATGAAATGGACGATTGGCTGTG**TGAGTACAAGC

>Bge-Mir-bg3a_3p
ATGAAATGGACGATTGGCTGTG

>Bge-Mir-bg3a_5p*
CAGCTAATCGTCCATTTCAATTG

```
CUUUGCUUUCCCAGCUAAUCGUCCAUUUCAAUUGUGUUGUUUGAAAUCAAUGAAAUGGACGAUUGGCUGUGUGAGUACAAGC
((((((((...(((((((((((((((((((.(((.(((......)))))))))))))))))))))))))))...))))).))). (-36.60)
```

Mated bases: 20

	Control	dsDicer
**Mature reads**	500	307
**Star reads**	33	23

```
 Initial dG =    -36.60

         10        20        30        40
-   -|    UCC                   A   U   GU
 CUU UGCUU   CAGCUAAUCGUCCAUUUCA UUG GUU  U
 GAA AUGAG   GUCGGUUAGCAGGUAAAGU AAC UAA  U
C   C^    UGU                   -   -   AG
 80        70        60          50
```

Genomic coordinates:
Bge-Mir-bg3a_3p: Scaffold90:1658230-1658251:-
Bge-Mir-bg3a_pre: Scaffold90:1658230-1658289:-
Bge-Mir-bg3a_5p*: Scaffold90:1658267-1658289:-

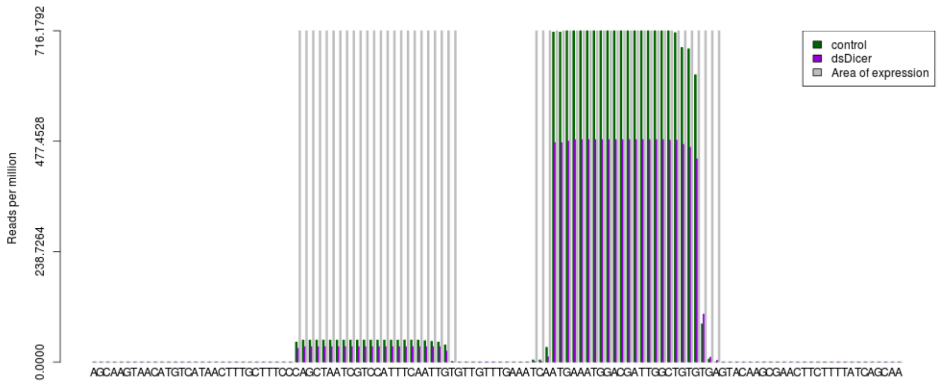

>Bge-Mir-bg3b_pre+_11
CTCTGCTCACG**CAGGTAATTGTCCATATCAGATG**TAATATTTGTAATCA**TTGAAATGGACGATTGTCTGTG**TGAGTAGAAGA

>Bge-Mir-bg3b_3p
TTGAAATGGACGATTGTCTGTG

>Bge-Mir-bg3b_5p*
CAGGTAATTGTCCATATCAGATG

```
CUCUGCUCACGCAGGUAAUUGUCCAUAUCAGAUGUAAUAUUUGUAAUCAUUGAAAUGGACGAUUGUCUGUGUGAGUAGAAGA
.((((((((((((..((((((((((.((((.((.............)))))).))))))))))..))))))))))))).... (-41.10)
```

Mated bases: 17

	Control	dsDicer
**Mature reads**	14	3
**Star reads**	1	0

Initial dG =   -41.10

```
          10         20         30         40
C--|             GU           A     A  UAAUAU
   UCUGCUCACGCAG  AAUUGUCCAU UCAG UG       \
   AGAUGAGUGUGUC  UUAGCAGGUA AGUU AC       U
AGA^             UG           A     -  UAAUGU
 80         70         60         50
```

Genomic coordinates:
Bge-Mir-bg3b_5p*: Scaffold255:1397077-1397099:+
Bge-Mir-bg3b_pre: Scaffold255:1397077-1397136:+
Bge-Mir-bg3b_3p: Scaffold255:1397115-1397136:+

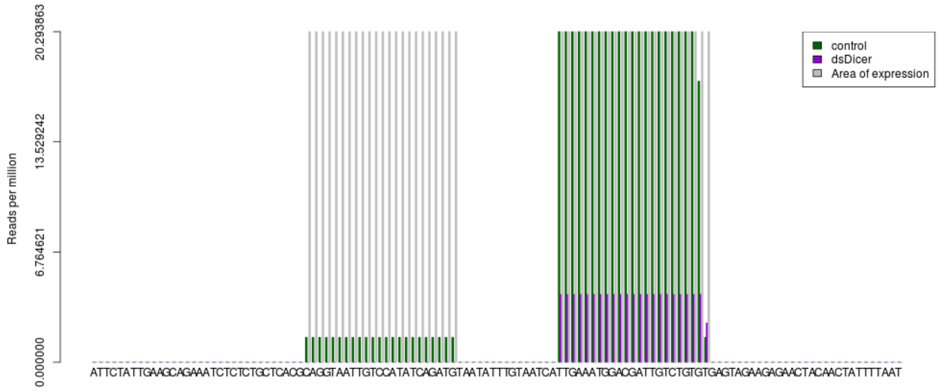

>Bge-Mir-bg4a_pre+_11
GGCATGGTGTC**TACATAACCGCAATCACCGATT**GTAATGTTATAGGTAA**TCGGTGATTGCGGTTGTGTGGA**TATCGAGCTGC

>Bge-Mir-bg4a_5p
TACATAACCGCAATCACCGATT

>Bge-Mir-bg4a_3p*
TCGGTGATTGCGGTTGTGTGGA

```
GGCAUGGUGUCUACAUAACCGCAAUCACCGAUUGUAAUGUUAUAGGUAAUCGGUGAUUGCGGUUGUGUGGAUAUCGAGCUGC
(((.((((((((((((((((((((((((((((((............)))))))))))))))))))))))))))))).))).. (-51.30)
```

Matched bases: 20

	Control	dsDicer
**Mature reads**	242	114
**Star reads**	34	12

```
 Initial dG =    -51.30

          10        20        30        40
--|  A                              UAAUG
  GGC UGGUGUCUACAUAACCGCAAUCACCGAUUG     U
  UCG GCUAUAGGUGUGUUGGCGUUAGUGGCUAAU     U
CG^  A                              GGAUA
 80        70        60        50
```

Genomic coordinates:
Bge-Mir-bg4a_3p*: Scaffold683:127413-127434:-
Bge-Mir-bg4a_pre: Scaffold683:127413-127472:-
Bge-Mir-bg4a_5p: Scaffold683:127451-127472:-

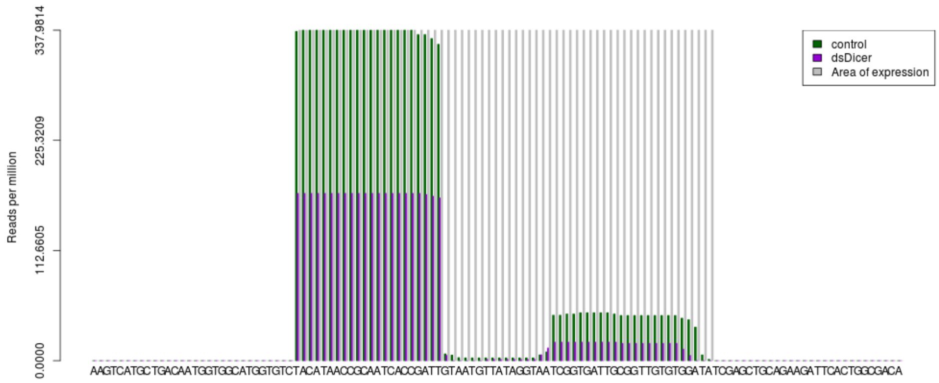

>Bge-Mir-bg4b_pre+_11
ACTCCTGTATT**TACATAACCGCAACCACCGACT**GTGATGTTAAGTGTAG**TCGGTGGTTGCGGTTGTGTGGA**CATAGGCTTGC

>Bge-Mir-bg4b_5p
TACATAACCGCAACCACCGACT

>Bge-Mir-bg4b_3p*
TCGGTGGTTGCGGTTGTGTGGA

ACUCCUGUAUU**UACAUAACCGCAACCACCGACU**GUGAUGUUAAGUGUAG**UCGGUGGUUGCGGUUGUGUGGA**CAUAGGCUUGC
```
...(((((.(((((((((((((((((((((((..(........)..))))))))))))))))))))))).))))).....  (-48.50)
```

Matched bases: 20

	Control	dsDicer
**Mature reads**	178	82
**Star reads**	3	3

```
 Initial dG =    -48.50

          10        20        30        40
ACU--|    A                        GU  AUG
     CCUGU UUUACAUAACCGCAACCACCGACU  G   U
     GGAUA AGGUGUGUUGGCGUUGGUGGCUGA  U   U
CGUUC^    C                        UG  GAA
 80        70        60        50
```

Genomic coordinates:
Bge-Mir-bg4b_3p*: Scaffold608:474852-474873:-
Bge-Mir-bg4b_pre: Scaffold608:474852-474911:-
Bge-Mir-bg4b_5p: Scaffold608:474890-474911:-

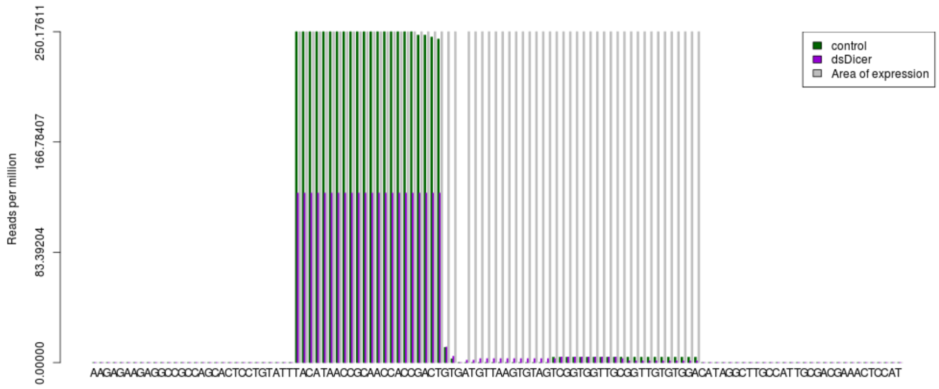

>Bge-Mir-bg4c_pre+_11
GCTCTTGTATT**TACATAACCGCAACCACCGACT**GTGATGTTAAGTGTAG**TCGGTGGTTGCGGTTGTGTGGA**TGCCGGGTCGC

>Bge-Mir-bg4c_5p
TACATAACCGCAACCACCGACT

>Bge-Mir-bg4c_3p*
TCGGTGGTTGCGGTTGTGTGGA

```
GCUCUUGUAUUUACAUAACCGCAACCACCGACUGUGAUGUUAAGUGUAGUCGGUGGUUGCGGUUGUGUGGAUGCCGGGUCGC
((((..(((((((((((((((((((((((((((..(........)..))))))))))))))))))))))))))).)))).... (-50.00)
```

Matched bases: 20

	Control	dsDicer
**Mature reads**	141	77
**Star reads**	2	1

```
 Initial dG =    -50.00

           10        20        30        40
---|   UU                           GU  AUG
   GCUC  GUAUUUACAUAACCGCAACCACCGACU  G   U
   UGGG  CGUAGGUGUGUUGGCGUUGGUGGCUGA  U   U
CGC^   C-                           UG GAA
 80         70        60        50
```

Genomic coordinates:
Bge-Mir-bg4c_3p*: Scaffold608:338133-338154:-
Bge-Mir-bg4c_pre: Scaffold608:338133-338192:-
Bge-Mir-bg4c_5p: Scaffold608:338171-338192:-

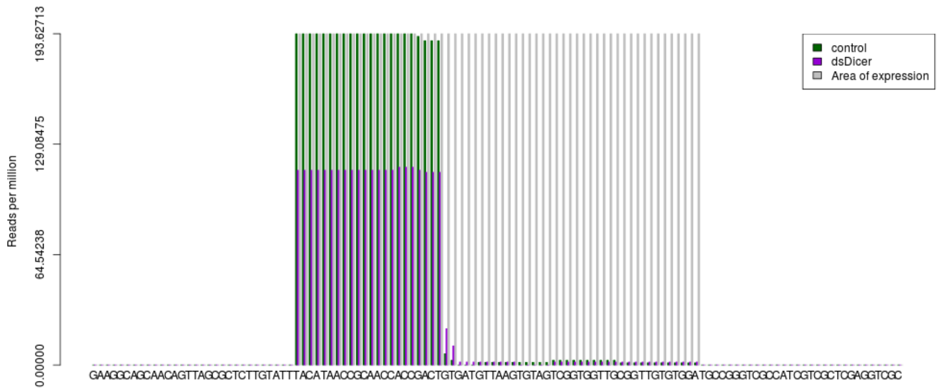

>Bge-Mir-bg6_pre+_11
CAGGATTCGAA**GCTGGGCAGCGGTGGTGTGGGT**GTTATAAC**TCACAACTTTCTGTCCAGAAC**GATTCAGGGGC

>Bge-Mir-bg6_3p
TCACAACTTTCTGTCCAGAAC

>Bge-Mir-bg6_5p*
GCTGGGCAGCGGTGGTGTGGGT

```
CAGGAUUCGAAGCUGGGCAGCGGUGGUGUGGGUGUUAUAACUCACAACUUUCUGUCCAGAACGAUUCAGGGGC
...((.(((...((((((((.(((..(((((((......))))))))))..))))))))..))).))...... (-27.90)
```

Mated bases: 16

	Control	dsDicer
**Mature reads**	54	25
**Star reads**	1	0

```
 Initial dG =    -27.90

           10        20         30
CAG---| U   AAG        C-    GG        GU
      GA UCG   CUGGGCAG  GGU  UGUGGGU  U
      CU AGC   GACCUGUC  UCA  ACACUCA  A
CGGGGA^ U   AA-        UU    --        AU
  70         60         50          40
```

Genomic coordinates:
Bge-Mir-bg6_3p: Scaffold16:2921158-2921178:-
Bge-Mir-bg6_pre: Scaffold16:2921158-2921208:-
Bge-Mir-bg6_5p*: Scaffold16:2921187-2921208:-

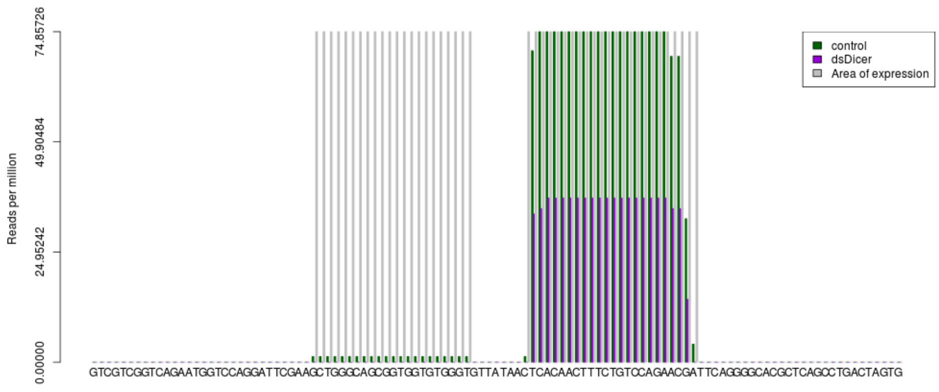

>Bge-Mir-bg7_pre+_11
TTCCCACGTATCGATGTCATTTTGGTTCGTAATGTTGTAATTGTCTCACTACGAACCAGAATGACATCGCGTTGTTCGAAGA

>Bge-Mir-bg7_3p
CTACGAACCAGAATGACATCGCG

>Bge-Mir-bg7_5p*
CGATGTCATTTTGGTTCGTAATG

```
UUCCCACGUAUCGAUGUCAUUUUGGUUCGUAAUGUUGUAAUUGUCUCACUACGAACCAGAAUGACAUCGCGUUGUUCGAAGA
(((..(((...((((((((((((((((((((.((...........)).))))))))))))))))))))...)))..))).. (-33.60)
```

Mated bases: 20

	Control	dsDicer
**Mature reads**	63	35
**Star reads**	3	1

```
 Initial dG =    -33.60

          10        20        30        40
--|  CC   UAU                   A  UUGUA
  UUC  ACG   CGAUGUCAUUUUGGUUCGUA UG     A
  AAG  UGU   GCUACAGUAAGACCAAGCAU AC     U
AG^  CU   UGC                   C  UCUGU
 80        70        60        50
```

Genomic coordinates:
Bge-Mir-bg7_5p*: Scaffold45:2099417-2099439:+
Bge-Mir-bg7_pre: Scaffold45:2099417-2099476:+
Bge-Mir-bg7_3p: Scaffold45:2099454-2099476:+

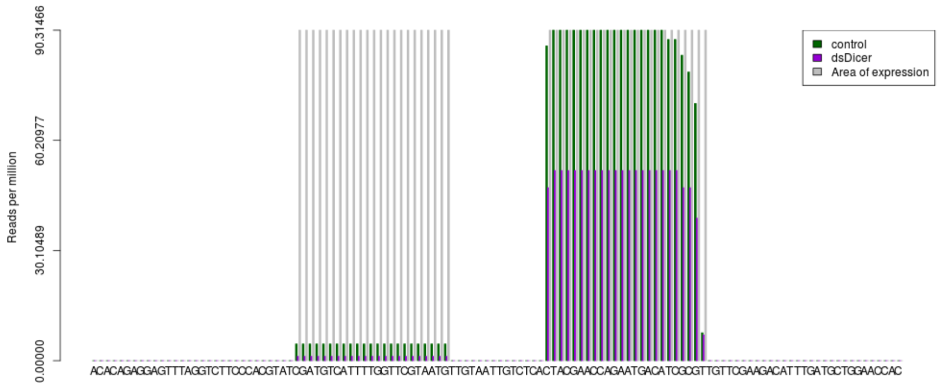

>Bge-Mir-bg8_pre+_11
CTTTTATCTGG**AAGGGGAAGTGGTGCGTTAGA**TTTGCTTTAAATTATGTC**CATAGGCGCTATTTCCTCTGCC**AGAAGGAAGAG

>Bge-Mir-bg8_3p
CATAGGCGCTATTTCCTCTGCC

>Bge-Mir-bg8_5p*
AAGGGGAAGTGGTGCGTTAGA

```
CUUUUAUCUGGAAGGGGAAGUGGUGCGUUAGAUUUGCUUUAAAUUAUGUCAUAGGCGCUAUUUCCUCUGCCAGAAGGAAGAG
((((..(((((.((((((((((((((.((((((..............))).)))))))))))))))).)))))..)))).. (-34.10)
```

Mated bases: 18

	Control	dsDicer
**Mature reads**	5	2
**Star reads**	1	0

```
 Initial dG =    -34.10

         10        20        30         40
--|   UA     A              G   -   UUGCUU
  CUUU  UCUGG AGGGGAAGUGGUGC UUA GAU      U
  GAAG  AGACC UCUCCUUUAUCGCG GAU CUG      A
GA^   GA     G              -   A   UAUUAA
 80        70        60         50
```

Genomic coordinates:
Bge-Mir-bg8_5p*: Scaffold3298:27925-27945:+
Bge-Mir-bg8_pre: Scaffold3298:27925-27984:+
Bge-Mir-bg8_3p: Scaffold3298:27963-27984:+

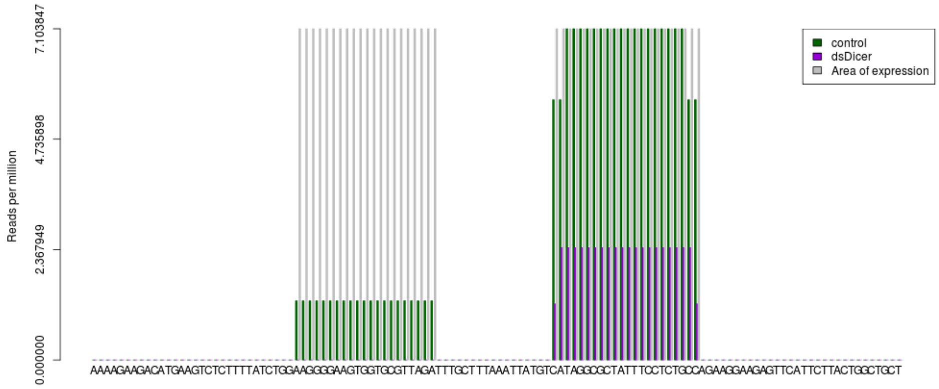

>Bge-Mir-bg9_pre+_11
GAAGGATGGAT**GGATCGTTCCAGTAGGACTGTG**TAGAATTTTATCA**CATTCTTCCTAGAATGGTCCGT**TTATTCTTCTT

>Bge-Mir-bg9_3p
CATTCTTCCTAGAATGGTCCGT

>Bge-Mir-bg9_5p*
GGATCGTTCCAGTAGGACTGTG

GAAGGAUGGAU**GGAUCGUUCCAGUAGGACUGUG**UAGAAUUUUAUCA**CAUUCUUCCUAGAAUGGUCCGU**UUAUUCUUCUU
((((((((((((((((((.((.((((.(((((((....))).)))).)))).)).)))))))))))))))))))).. (-35.50)

Mated bases: 17

	Control	dsDicer
**Mature reads**	3	0
**Star reads**	1	0

```
 Initial dG =    -35.50

         10        20        30
--                    C  U    C    -|  A
  GAAGGAUGGAUGGAUCGUUC AG AGGA UGUG UAG A
  CUUCUUAUUUGCCUGGUAAG UC UUCU ACAC AUU U
UU                    A  C    U    U^  U
       70        60        50        40
```

Genomic coordinates:
Bge-Mir-bg9_5p*: Scaffold9:1984709-1984730:+
Bge-Mir-bg9_pre: Scaffold9:1984709-1984765:+
Bge-Mir-bg9_3p: Scaffold9:1984744-1984765:+

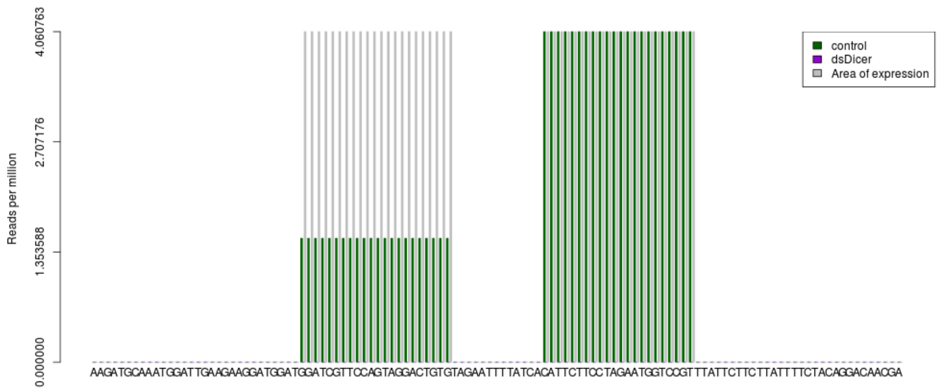

Supplementary Data S3. Report generated with MirPlot for the 833 *Locusta migratoria* miRNA candidates from Wang et al. (2015). For each candidate the following information is shown:

- The miRNA precursor mature arm colored red.
- The 5p and 3p arms. The mature arm colored red and the predicted star arm colored in blue.
- The precursor folding in Vienna format.
- The number of complementary bases between the mature and predicted star arm.
- The free energy of the precursor folding.
- The number of reads of both the precursor each small RNA-seq libraries.
- The folding of the miRNA precursor with colored mature arm.
- A plot showing all the expressed nucleotides of the precursor (grey), the reads per million in each library.

>ID1001
AGTAATACATGGATGGAGGG**TGATATGCGTTTGATGGAAACAG**TTTATAAAGGTTCTGTTCCATCACTCTCATATCACCCACATTACATG
ACAAGCAGCAACGGGTGGCC

>ID1001-5P
TGATATGCGTTTGATGGAAACAG

>miRNA precursor
AGUAAUACAUGGAUGGAGGG**UGAUAUGCGUUUGAUGGAAACAG**UUUAUAAAGGUUCUGUUCCAUCACUCUCAUAUCACCCACAUUACAUGACAAGCAGCAACGGGUGGCC
.......((((((((..((((((((((....((((((((.(((............))))))))))).....)))))))))).)))).)))).....((.((.....)).)).

>Predicted STAR seq:
GTTCCATCACTCTCATATCACC

Complementarity: 16

Initial dG =   -37.50

```
          10         20        30        40
.-AGUAAUA    -|   GA          CGUU        A   UUUAU
         CAUG GAUG  GGGUGAUAUG    UGAUGGAA CAG     A
         GUAC UUAC  CCCACUAUAC    ACUACCUU GUC     A
\ -------    A^   A-          UCUC        -   UUGGA
        90         80        70        60         50



         100
  ACAA  A  AA
      GC GC  \
      CG UG  C
  C---  G  GG
110
```

Reads in: lib-42, lib-45, lib-47, lib-48, lib-49, lib-50, lib-72
Number of reads="ID1001",1,6,0,0,1,0,0

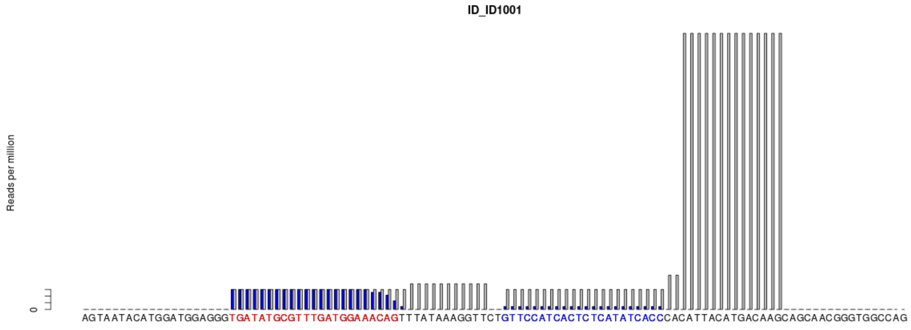

>ID1002
AGTAATTCATATATGGTGGG**TGATGGTTGTGTGGCATTAACA**CGTTATAAAAAATGTTGTTGAAGTTGCACAACTATCACTCACTGTGCA
TGACAGGCATCAGATGTTCT

>ID1002-5P
TGATGGTTGTGTGGCATTAACA

>miRNA precursor
AGUAAUUCAUAUAUGGUGGG**UGAUGGUUGUGUGGCAUUAACA**CGUUAUAAAAAAUGUUGUUGAAGUUGCACAACUAUCACUCACUGUGCAUGACAGGCAUCAGAUGUUCU
```
.((...((((.((((((((((((((((((((..((.((((((((((......))))).))))).))..)))))))))))))))))))).))))...))...........
```

>Predicted STAR seq:
GTTGAAGTTGCACAACTATCACT

Complementarity: 17

Initial dG =   -48.50

```
                   10        20        30        40
A-----------  AAU    A                   UG  A     -|    AU
            GU   UCAU UAUGGUGGGUGAUGGUUGUG  GC UUAAC ACGUU  A
            CG   AGUA GUGUCACUCACUAUCAACAC  UG AGUUG UGUAA  A
UCUUGUAGACUA  GAC    C                   GU  A     U^    AA
.       100        90        80        70        60        50
```

Reads in: lib-42, lib-45, lib-47, lib-48, lib-49, lib-50, lib-72
Number of reads="ID1002",0,3,0,0,0,0,0

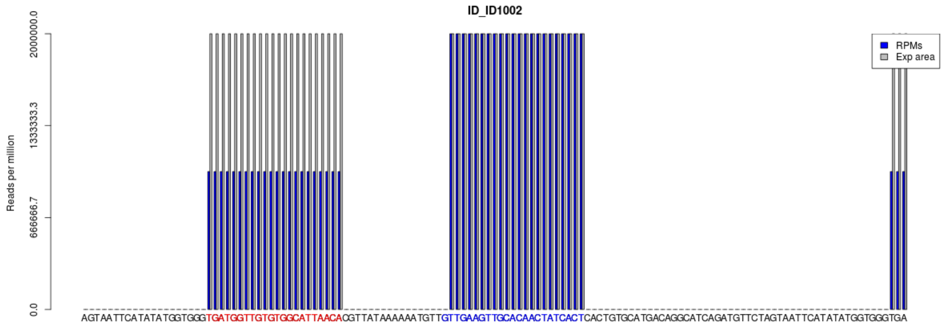

>ID1003
AGTAATACATGGATGGAGGG**TGATGTGCGTTTGATGGAACTAG**GTTATAAAGGTTCTGTTCCATCACTCTCATATCACCCACTTTACATG
ACAAGCAGCAACGGGTGG

>ID1003-5P
TGATGTGCGTTTGATGGAACTAG

>miRNA precursor
AGUAAUACAUGGAUGGAGGG**UGAUGUGCGUUUGAUGGAACUAG**GUUAUAAAGGUUCUGUUCCAUCACUCUCAUAUCACCCACUUUACAUGACAAGCAGCAACGGGUGG
```
.......((((...((.((((((((((....(((((((((.(((..........))))))))))))....)))))))))).))...)))).................
```

>Predicted STAR seq:
GTTCCATCACTCTCATATCACC

Complementarity: 16

Initial dG = -36.60

```
                   10        20        30        40
AGUAAUA-----------|   GAU   A          CGUU         U   UUAU
                  CAUG   GG GGGUGAUGUG    UGAUGGAAC AGG    A
                  GUAC   UC CCCACUAUAC    ACUACCUUG UCU    A
GGUGGGCAACGACGAACA^   AUU   A          UCUC         -   UGGA
      100        90        80        70        60         50
```

Reads in: lib-42, lib-45, lib-47, lib-48, lib-49, lib-50, lib-72
Number of reads="ID1003",18,28,1,0,0,1,0

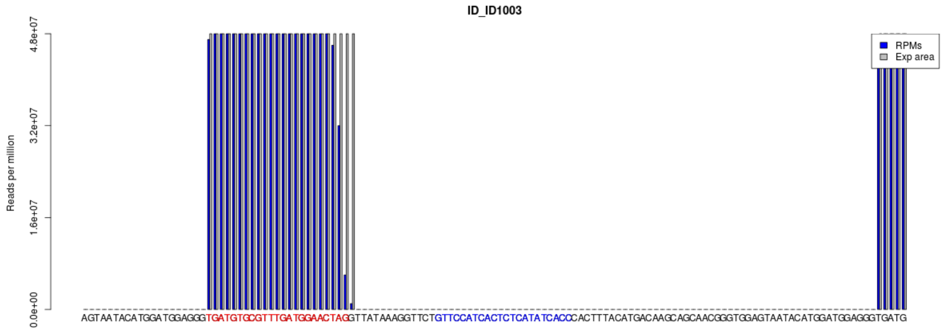

>ID1004

TGTTTCAGTGACAGTAATTCATGCATGGAGGGTGATGTCTGTGTGGGTTCAACATGTTATAAAATTACTG**TTGAACATACACGGCCATCACT**CACTGTGCATGGTAAC

>ID1004-3P

TTGAACATACACGGCCATCACT

>miRNA precursor

UGUUUCAGUGACAGUAAUUCAUGCAUGGAGGGUGAUGUCUGUGUGGGUUCAACAUGUUAUAAAAUUACUG**UUGAACAUACACGGCCAUCACU**CACUGUGCAUGGUAAC

```
((((.....)))).....((((((((((.((((((((.(((((((.((((((((.((.........)))))))))).))))))).)))))))).))))))))))....
```

>Predicted STAR seq:

GTGATGTCTGTGTGGGTTCAA

Complementarity: 18

ERROR 4: Strange structure

Initial dG = -47.20

```
  |   UC
--UGUU  \
  ACAG  A
\ ^   UG
    10
```

```
       20        30        40        50        60
  GUAAU          A        U       G        U  UAUA
       UCAUGCAUGG GGGUGAUG CUGUGUG GUUCAACA GU    \
       GGUACGUGUC CUCACUAC GGCACAU CAAGUUGU CA    A
  CAAU-          A        C       A        -  UUAA
         100        90        80        70
```

Reads in: lib-42, lib-45, lib-47, lib-48, lib-49, lib-50, lib-72

Number of reads="ID1004",3,9,0,0,0,0,0

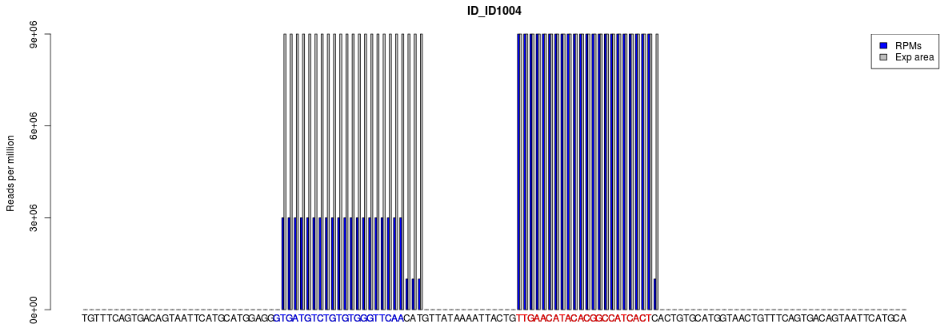

>ID1005
TACCATGATAATAGTAATTCGTGCATGGAGGGTGGTGACTATGTGGCCCCCACATGTTATTAAATTAATG**TTGAGGCCATGTAGTCATCA**
CTCACTGTGCATAACTGG

>ID1005-3P
TTGAGGCCATGTAGTCATCACT

>miRNA precursor
UACCAUGAUAAUAGUAAUUCGUGCAUGGAGGGUGGUGACUAUGUGGCCCCCACAUGUUAUUAAAUUAAUG**UUGAGGCCAUGUAGUCAUCACU**CACUGUGCAUAACUGG
...........((((.....((((((((.(((((((((((((((((((..((.((((((......)))))))).)))))))))))))))))))).)))))))).)))).

>Predicted STAR seq:
GTGGTGACTATGTGGCCCCCAC

Complementarity: 19

Initial dG =   -45.70

```
        10        20        30        40        50        60
UACCAUGAUAA|   AAUUC        A                   CC   C      UU
           UAGU     GUGCAUGG GGGUGGUGACUAUGUGGCC  CA AUGUUA  A
           GUCA     UACGUGUC CUCACUACUGAUGUACCGG  GU UGUAAU  A
G----------^   A----        A                   A-   -      UA
                    100        90        80          70
```

Reads in: lib-42, lib-45, lib-47, lib-48, lib-49, lib-50, lib-72
Number of reads="ID1005",23,73,0,0,1,0,0

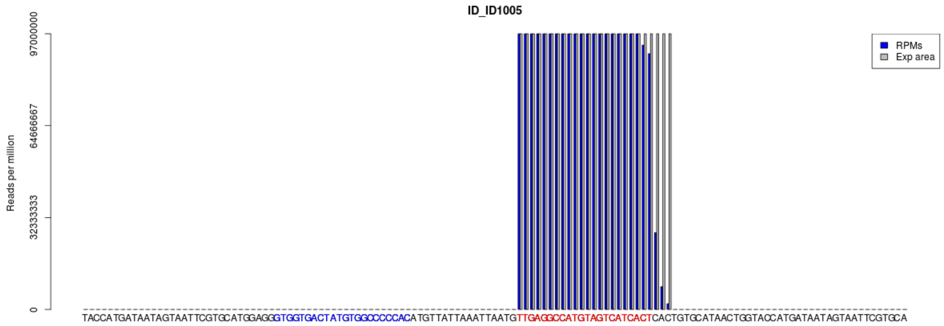

>ID1006
AGTAATCCATGCGTGGAGGG**TGATGTGCGTTTGATGGAAACAG**GCTATAAAGGTTCTGTTCCATGACTCTCACATCACCCACTTTACATG
ACAAGCAGCAACGGGTGG

>ID1006-5P
TGATGTGCGTTTGATGGAAACAG

>miRNA precursor
AGUAAUCCAUGCGUGGAGGG**UGAUGUGCGUUUGAUGGAAACAG**GCUAUAAAGGUUCUGUUCCAUGACUCUCACAUCACCCACUUUACAUGACAAGCAGCAACGGGUGG
......((((.(((...((((((((((.(((..((((((.((((((.....)).)))))))))))))...)))))))))).(((........))).....))).))))

>Predicted STAR seq:
GTTCCATGACTCTCACATCACC

Complementarity: 17

Initial dG =   -36.10

```
        10          20          30         40
AGUAAU    G   .-GGA          C--|  UG       A    -  AU
      CCAU CGU     GGGUGAUGUG   GUU  AUGGAA CAGG CU  \
      GGUG GCA     CCCACUACAC   CAG  UACCUU GUCU GG  A
------    G   \ ---          UCU^  --       -    U  AA
                  80        70          60         50



                        90
                ACUUUACA  AC
                        UG  \
                        AC  A
                ACG-----  GA
              100
```

Reads in: lib-42, lib-45, lib-47, lib-48, lib-49, lib-50, lib-72
Number of reads="ID1006",2,2,0,0,0,0,0

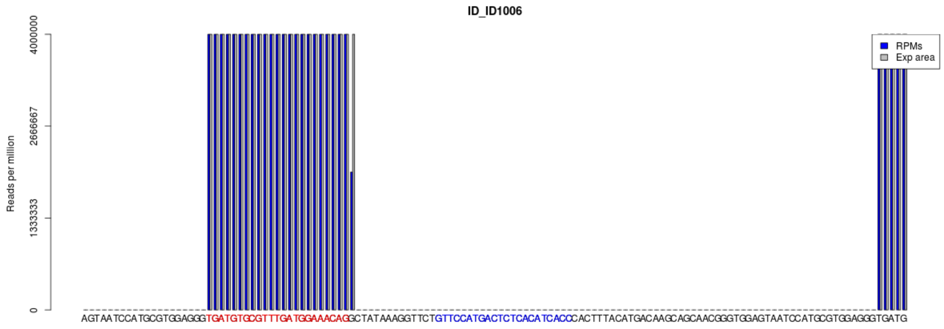

>ID1007
TGTAATCCATGTATAGGAAT**TGGCGATTGCATGACTAATACA**GGTTATATTTTACTGTTTTAGTCACCCAATCGTCAGATCCTGTTGCAT
GGCATGCCACAGAGTTGTATCT

>ID1007-5P
TGGCGATTGCATGACTAATACA

>miRNA precursor
UGUAAUCCAUGUAUAGGAAU**UGGCGAUUGCAUGACUAAUACA**GGUUAUAUUUUACUGUUUUAGUCACCCAAUCGUCAGAUCCUGUUGCAUGGCAUGCCACAGAGUUGUAUCU
......(((((((((((.((((((((((..(((((((.((((...........)))).))))))).)))))))))).))))).))))))).((((.((...)).))))..

>Predicted STAR seq:
TTTTAGTCACCCAATCGTCAGA

Complementarity: 17

Initial dG =   -44.30

```
                      10         20        30        40
U--------------   AU      -|     A          CA        U    GUUAU
               GUA  CCAUGU AUAGGA UUGGCGAUUG  UGACUAA ACAG     \
               CGU  GGUACG UGUCCU GACUGCUAAC  ACUGAUU UGUC     A
UCUAUGUUGAGACAC   AC      U^     A          CC        U    AUUUU
110       100        90        80        70        60        50
```

Reads in: lib-42, lib-45, lib-47, lib-48, lib-49, lib-50, lib-72
Number of reads="ID1007",3,9,0,0,0,0,0

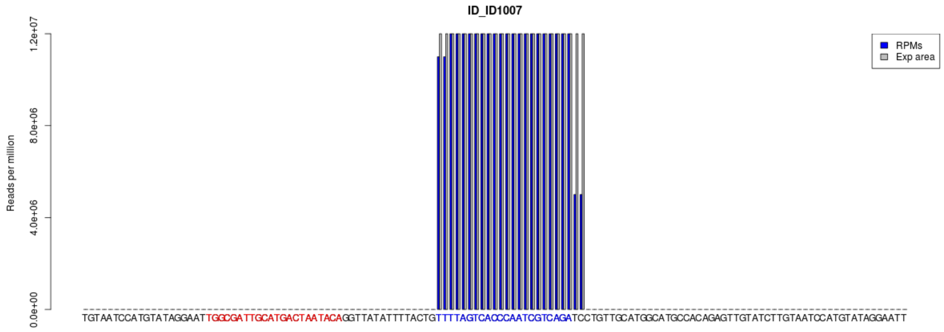

>ID1009
TCTAATTCGTGTATAGGGTT**CGGCGACTGCATGAACACCACT**TGTTATTTACTACTGTGGCAGTCACACAGTCGTCAGATCCTGTGCATG
GTATGCGACAAAATTGTGTCTG

>ID1009-5P
CGGCGACTGCATGAACACCACT

>miRNA precursor
UCUAAUUCGUGUAUAGGGUU**CGGCGACUGCAUGAACACCACU**UGUUAUUUACUACUGUGGCAGUCACACAGUCGUCAGAUCCUGUGCAUGGUAUGCGACAAAAUUGUGUCUG
```
......(((((((((((((..((((((((..(((...((((..((........)).))))...)))..))))))))..))))))))))))).....((((......)))).. 
```

>Predicted STAR seq:
GGCAGTCACACAGTCGTCAGAT

Complementarity: 13

Initial dG =   -44.70

```
          10        20        30        40
.-UCUAAU|            UC        CA   ACA    UU  UAU
        UCGUGUAUAGGGU  GGCGACUG  UGA   CCAC  GU   U
        GGUACGUGUCCUA  CUGCUGAC  ACU   GGUG  CA   U
\ ------^            GA        AC   GAC    U-  UCA
        90        80        70        60         50



        100
  UAUGC    AA
       GACA  A
       CUGU  U
  GU---    GU
     110
```

Reads in: lib-42, lib-45, lib-47, lib-48, lib-49, lib-50, lib-72
Number of reads="ID1009",4,1,1,0,0,1,0

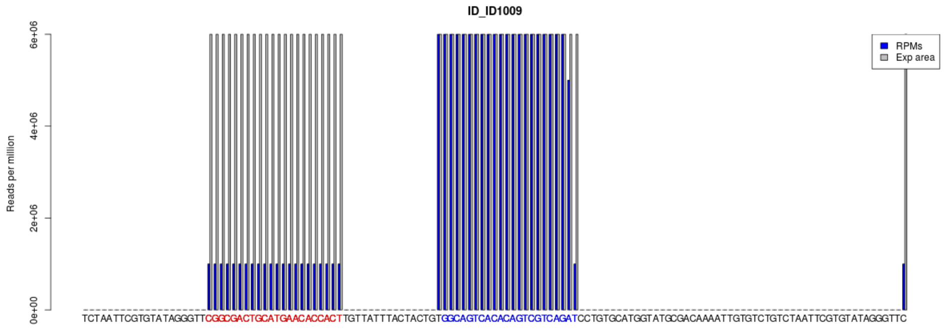

>ID1010
AATAGTACATGCATGGAGGG**TTGTGGCTGTGTATGACAACA**CGTTATGAAATCAGTGTTGTACATACACAGACACCACCCACTGTGCATG
GGAATCATCAGCCGTTGGTC

>ID1010-5P
TTGTGGCTGTGTATGACAACA

>miRNA precursor
AAUAGUACAUGCAUGGAGGG**UUGUGGCUGUGUAUGACAACA**CGUUAUGAAAUCAGUGUUGUACAUACACAGACACCACCCACUGUGCAUGGGAAUCAUCAGCCGUUGGUC
.......(((((((((.((((.(((.((((((((((((((((...........))))))).))))))))).))).)))).)))))))))...((((........)))).

>Predicted STAR seq:
TTGTACATACACAGACACCACC

Complementarity: 17

```
 Initial dG =    -48.40

          10        20        30         40
.-AAUAGUA         A    U   G         -|      GUUAU
         CAUGCAUGG GGGU GUG CUGUGUAUG ACAACAC     G
         GUACGUGUC CCCA CAC GACACAUAC UGUUGUG     A
\ -------         A    C   A         A^      ACUAA
        90        80        70        60        50



         100
  GGA    UCA
     AUCA   G
     UGGU   C
  C--    UGC
110
```

Reads in: lib-42, lib-45, lib-47, lib-48, lib-49, lib-50, lib-72
Number of reads="ID1010",5,10,0,1,0,0,0

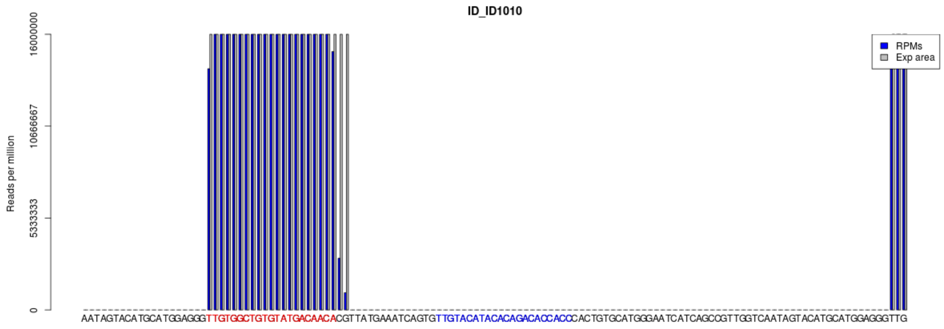

>ID1011
TGTTGCAGTGTCAGTAATTCATGCATGGAGGG**TGATGTCTGTGTGGGTCCAAC**ACATTATGAGATTACTGTTAAGCACACACGGCCATCA
CTCACTGTGCATGATAACCA

>ID1011-5P
TGATGTCTGTGTGGGTCCAAC

>miRNA precursor
UGUUGCAGUGUCAGUAAUUCAUGCAUGGAGGG**UGAUGUCUGUGUGGGUCCAAC**ACAUUAUGAGAUUACUGUUAAGCACACACGGCCAUCACUCACUGUGCAUGAUAACCA
.(((((.......)))))((((((((((.((((((((.(((((((.((..((((.............))))..)).))))))).)))))))).))))))))))......

>Predicted STAR seq:
TAAGCACACACGGCCATCACT

Complementarity: 15

Initial dG =   -40.70

ERROR 3: Strange structure
Initial dG =   -40.70

```
.-U|    AGU
   GUUGC   \
   UAAUG   G
\ -^    ACU
```

```
        20        30         40        50        60
  ------          A         U       G   CC     CAUUAU
        UCAUGCAUGG GGGUGAUG CUGUGUG GU  AACA      G
        AGUACGUGUC CUCACUAC GGCACAC CG  UUGU      A
  ACCAAU          A         C       A   AA     CAUUAG
110       100        90        80        70
```

Reads in: lib-42, lib-45, lib-47, lib-48, lib-49, lib-50, lib-72
Number of reads="ID1011",0,4,0,0,0,0,0

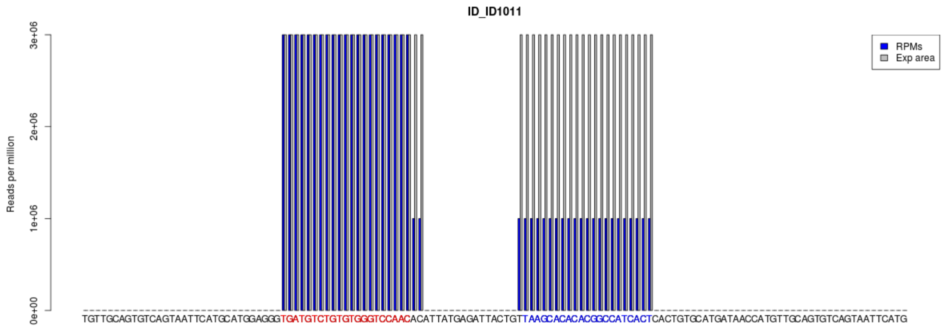

>ID1012
CTGCAGTTACAGTGCTAATTACTGCATAGTGGTTGACGGTTGTGTGGCTGTCACGTGTTATTTACTACCG**TCACAGACGCCCAACCGTTA**
CCTGCTGTGAATGGTATACCA

>ID1012-3P
TCACAGACGCCCAACCGTTACCT

>miRNA precursor
CUGCAGUUACAGUGCUAAUUACUGCAUAGUGGUUGACGGUUGUGUGGCUGUCACGUGUUAUUUACUACCG**UCACAGACGCCCAACCGUUACCU**GCUGUGAAUGGUAUACCA
.........(((((.....)))))(((((..(.(((((((((.(((.((((.(((.((........))))).)))).))).))))))))).)..)))))..(((....)))

>Predicted STAR seq:
TAATTACTGCATAGTGGTTGACGGTTGTGTG

Complementarity: 16
ERROR 4: Strange structure
Initial dG = -39.60

```
          10
.-CUGCAGUUA|    CU
           CAGUG  \
           GUCAU  A
\ ---------^    UA
               20



        30        40        50        60
         UG U         U   G    C   U  UAU
  --CAUAG  G UGACGGUUG GUG CUGU ACG GU   U
    GUGUC  C AUUGCCAAC CGC GACA UGC CA   U
  \      GU C         C   A    C   -  UCA
            90        80        70
```

Reads in: lib-42, lib-45, lib-47, lib-48, lib-49, lib-50, lib-72
Number of reads="ID1012",2,14,0,0,1,0,0

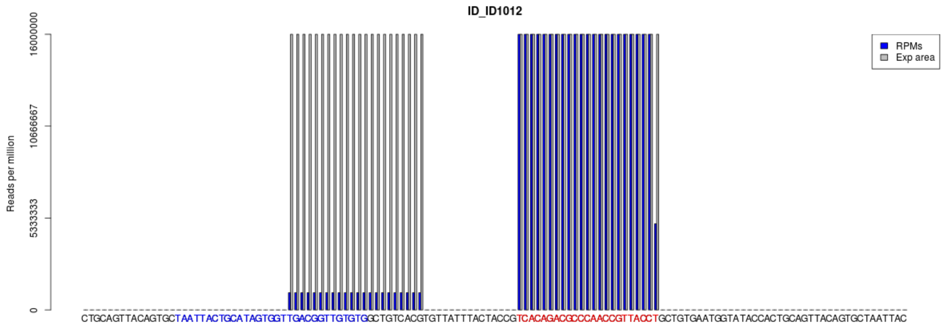

>ID1013
TCGCTGTGGCATTAATTCATGCATGGAGGGTGATGTGTGTGTATGGTGCCAACACATTATACAATTACTG**TTGCACTTCCACACACATCA**
CTCACCGTGCATGACAAGCA

>ID1013-3P
TTGCACTTCCACACACATCACT

>miRNA precursor
UCGCUGUGGCAUUAAUUCAUGCAUGGAGGGUGAUGUGUGUGUAUGGUGCCAACACAUUAUACAAUUACUG**UUGCACUUCCACACACAUCACU**CACCGUGCAUGACAAGCA
..((....))......((((((((((.((((((((((((((...(((((.((((.............)))))))))..))))))))))))))).))))))))))......

>Predicted STAR seq:
GTGATGTGTGTGTATGGTGCCAA

Complementarity: 18
ERROR 4: Strange structure
Initial dG = -51.40

```
.-UC| U
    GC G
    CG U
\ --^ G
   10



          20        30        40        50        60
  AUUAAU          A              UAU      C     CAUUAU
        UCAUGCAUGG GGGUGAUGUGUGUG   GGUGC AACA      A
        AGUACGUGCC CUCACUACACACAC   UCACG UUGU      C
  ACGAAC          A              CU-      -     CAUUAA
110       100        90        80          70
```

Reads in: lib-42, lib-45, lib-47, lib-48, lib-49, lib-50, lib-72
Number of reads="ID1013",1,2,0,0,0,0,0

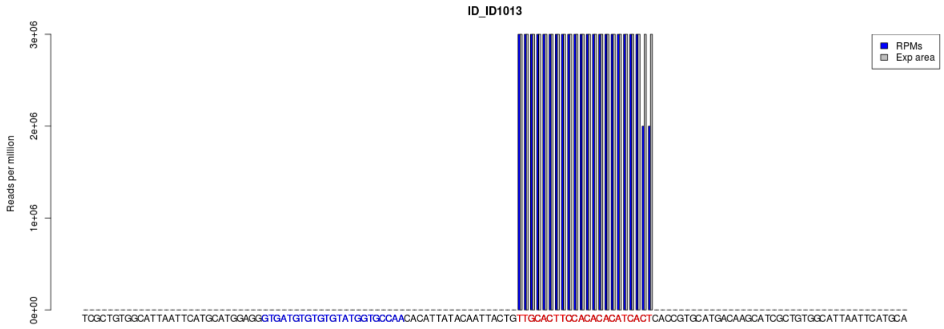

>ID1014
TTCTGATGACCAATAAGTTTCAGTGAATCCCGAG**TGACGGAAGCGAGGTCTGTACAAG**TTTTTAAATTACTTGAGATCTCCCTTCCGTTACTTGGTGTTCTGGATAAGCGTC

>ID1014-5P
TGACGGAAGCGAGGTCTGTACAAG

>miRNA precursor
UUCUGAUGACCAAUAAGUUUCAGUGAAUCCCGAG**UGACGGAAGCGAGGUCUGUACAAG**UUUUUAAAUUACUUGAGAUCUCCCUUCCGUUACUUGGUGUUCUGGAUAAGCGUC
..((((.(((......))))))).(((..(((((((((((((.(((((((...(((((.........)))))))))))).)))))))))))))..)))............

>Predicted STAR seq:
TGAGATCTCCCTTCCGTTACT

Complementarity: 18

Initial dG = -43.50

```
          10
ERROR 3: Strange structure
 Initial dG =    -43.50

          10
.-UU|   U   CA
    CUGA GAC  A
    GACU UUG  U
\ --^   -   AA
      20


                  30        40        50        60
  U-----------   UC              C       GUA      UUUU
              GAA  CCGAGUGACGGAAG GAGGUCU   CAAGU    \
              CUU  GGUUCAUUGCCUUC CUCUAGA   GUUCA    A
  CUGCGAAUAGGU   GU              C       ---      UUAA
  110       100        90        80            70
```

Reads in: lib-42, lib-45, lib-47, lib-48, lib-49, lib-50, lib-72
Number of reads="ID1014",23,51,2,1,0,0,0

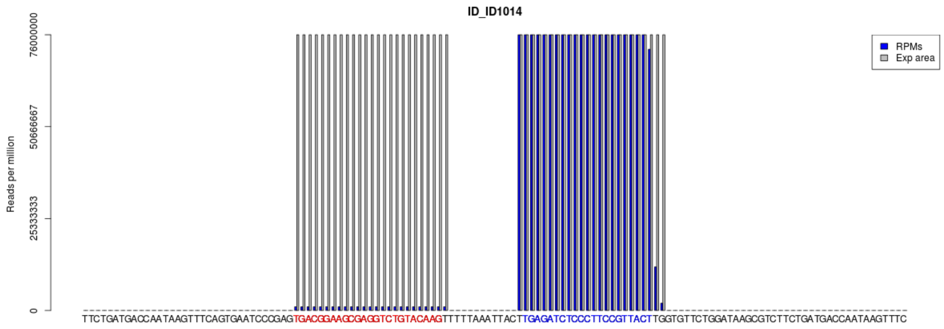

>ID1015
ACGTTGAAGTGAAAGTAATTCATGCATGGAGGG**TGATGGCTGTGTCTACCAGCA**CGTTATAAAGTCACTGTTGTATAGACCCAGCCATCA
CTCACTGTGCATGGCAACCATC

>ID1015-5P
TGATGGCTGTGTCTACCAGCA

>miRNA precursor
ACGUUGAAGUGAAAGUAAUUCAUGCAUGGAGGG**UGAUGGCUGUGUCUACCAGCA**CGUUAUAAAGUCACUGUUGUAUAGACCCAGCCAUCACUCACUGUGCAUGGCAACCAUC
..((((.............(((((((((.((((((((((((.(((((.(((((.((.........)))))))..))))).)))))))))))).))))))))))))))....

>Predicted STAR seq:
TTGTATAGACCCAGCCATCACT

Complementarity: 17

Initial dG =   -45.80

```
          10        20        30        40         50        60
AC--|   AAGUGAAAGUAAU          A            U     C-     C  UAUA
    GUUG             UCAUGCAUGG GGGUGAUGGCUG GUCUA  CAGCA GU    \
    CAAC             GGUACGUGUC CUCACUACCGAC CAGAU  GUUGU CA    A
CUAC^   -------------          A            C     AU     -  CUGA
110                   100        90        80        70
```

Reads in: lib-42, lib-45, lib-47, lib-48, lib-49, lib-50, lib-72
Number of reads="ID1015",4,2,0,0,0,0,0

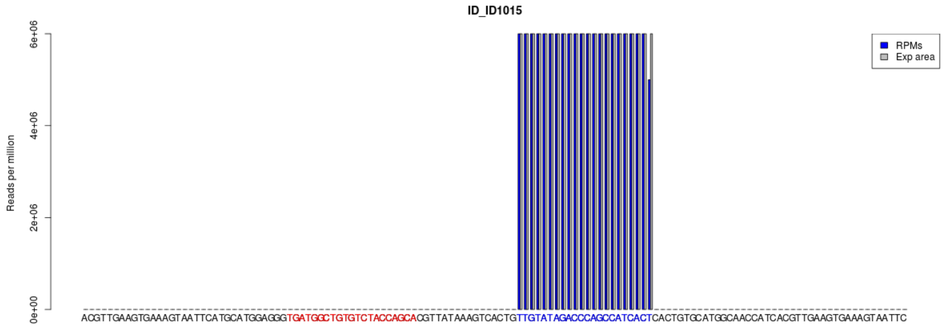

>ID1016
TGTTGCAGTGTCAGTAATTCATGCATGGAGGG**TGATGTCTGTGTGGGTCCAAC**ACGTTTTAAAATTACTGTTGAAGTTACACAGCCATCA
CTCACTGTGCATGGTAACCATC

>ID1016-5P
TGATGTCTGTGTGGGTCCAAC

>miRNA precursor
UGUUGCAGUGUCAGUAAUUCAUGCAUGGAGGG**UGAUGUCUGUGUGGGUCCAAC**ACGUUUUAAAAUUACUGUUGAAGUUACACAGCCAUCACUCACUGUGCAUGGUAACCAUC
.(((((.......)))))((((((((((.((((((((.((((((((...(((((.((.........)))))))...)))))))).)))))))).))))))))))........

>Predicted STAR seq:
TGAAGTTACACAGCCATCACT

Complementarity: 15

Initial dG =   -41.10

ERROR 3: Strange structure
Initial dG =   -41.10

```
.-U|    AGU
   GUUGC   \
   UAAUG   G
\ -^    ACU
```

```
          20        30        40        50        60
  --------          A         U        GUC       C  UUUA
          UCAUGCAUGG GGGUGAUG CUGUGUGG   CAACA GU    \
          GGUACGUGUC CUCACUAC GACACAUU   GUUGU CA    A
  CUACCAAU          A         C        GAA       -  UUAA
  110       100        90        80        70
```

Reads in: lib-42, lib-45, lib-47, lib-48, lib-49, lib-50, lib-72
Number of reads="ID1016",0,0,0,0,0,0,0

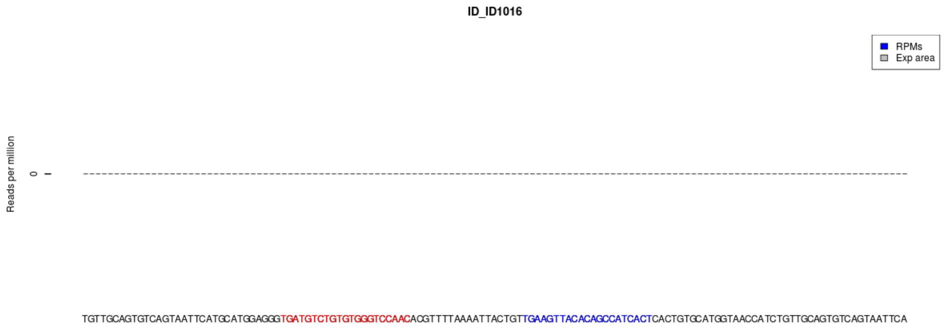

>ID1017
TGTTGCACTGTCAGTAATTCATGCATGGAGGGTGATGTCTGTGTGGGTCCAACACATTATGAGATTACTG**TTGAACACACACGGCTATCA**
CTCACTGTGCATGATAACCAT

>ID1017-3P
TTGAACACACACGGCTATCACT

>miRNA precursor
UGUUGCACUGUCAGUAAUUCAUGCAUGGAGGGUGAUGUCUGUGUGGGUCCAACACAUUAUGAGAUUACUG**UUGAACACACACGGCUAUCACU**CACUGUGCAUGAUAACCAU
.(((((.......)))))((((((((((.((((((((.(((((((.((.(((((.............))))).)).))))))).)))))))).))))))))))).......

>Predicted STAR seq:
GTGATGTCTGTGTGGGTCCAA

Complementarity: 17
ERROR 4: Strange structure
Initial dG =   -41.90

```
.-U|    ACU
   GUUGC   \
   UAAUG   G
\ -^    ACU
```

```
         20        30        40        50        60
  -------          A         U       G  C      CAUUAU
         UCAUGCAUGG GGGUGAUG CUGUGUG GU CAACA      G
         AGUACGUGUC CUCACUAU GGCACAC CA GUUGU      A
  UACCAAU          A         C       A  A      CAUUAG
 110       100        90        80        70
```

Reads in: lib-42, lib-45, lib-47, lib-48, lib-49, lib-50, lib-72
Number of reads="ID1017",4,5,0,0,0,0,0

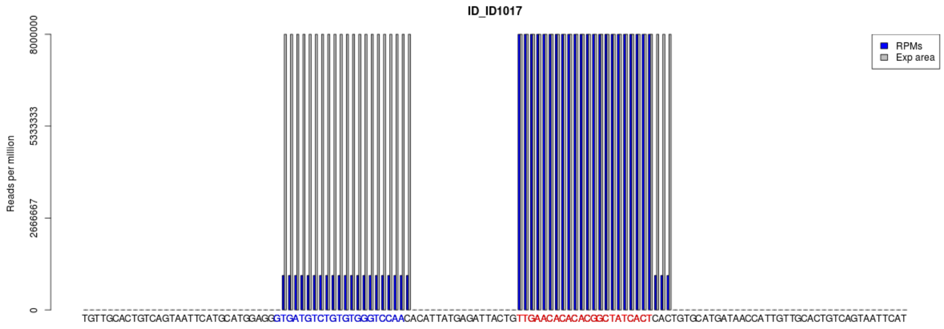

>ID1019
TACTACTTCAGGTGTTACTGAATGCATAGAGGCCGGCGATTTCGTGGTTGTTACATGTTATTTAGCACTG**TCACAATCACCCAGTCGTCA**
GCTCCTGTGCATGGAATACCAC

>ID1019-3P
TCACAATCACCCAGTCGTCAGCT

>miRNA precursor
UACUACUUCAGGUGUUACUGAAUGCAUAGAGGCCGGCGAUUUCGUGGUUGUUACAUGUUAUUUAGCACUG**UCACAAUCACCCAGUCGUCAGCU**CCUGUGCAUGGAAUACCAC
..........((((((.....((((((((..((.(((((((..((((((((.(((((((....)))).))).))))))))..))))))).))..))))))))..))))))..

>Predicted STAR seq:
GGCCGGCGAUUUCGUGGUUGUU

Complementarity: 16

Initial dG =   -46.60

```
        10        20        30        40        50         60
UACUACUUCA|     ACUGA        AG  C       UC        U   -    A
          GGUGUU     AUGCAUAG  GC GGCGAUU  GUGGUUGU ACA UGUU U
          CCAUAA     UACGUGUC  CG CUGCUGA  CACUAACA UGU ACGA U
CA--------^     GG---        CU  A       CC        C   C    U
        110          100        90        80        70
```

Reads in: lib-42, lib-45, lib-47, lib-48, lib-49, lib-50, lib-72
Number of reads="ID1019",14,2,0,0,0,0,0

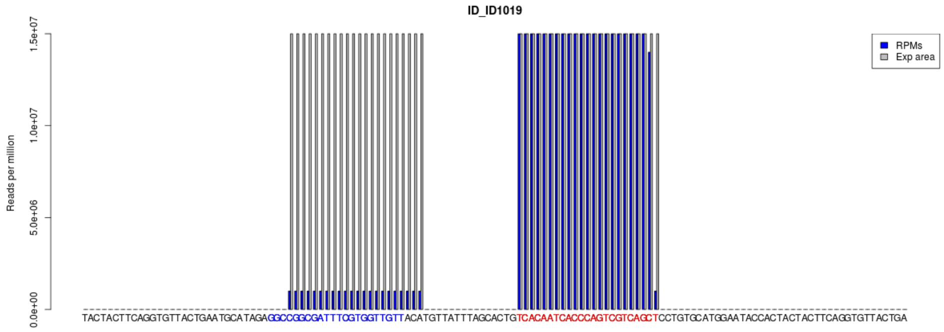

>ID102

ACTACAGAAGCATTCTGGAG**TTTCGAATGTATTCCTATCACG**TATTTTACTAATCGTTATAGGAATTCATTCGTTTCTCTATGGTGCACA
TTGTGCTTCAAACATTTATCCA

>ID102-5P

TTTCGAATGTATTCCTATCACG

>miRNA precursor

ACUACAGAAGCAUUCUGGAG**UUUCGAAUGUAUUCCUAUCACG**UAUUUUACUAAUCGUUAUAGGAAUUCAUUCGUUUCUCUAUGGUGCACAUUGUGCUUCAAACAUUUAUCCA

..(((((..((((..(((((...((((((.((((((((.(((.(((.....)))))).)))))))).))))))...)))))..))))...))))).................

>Predicted STAR seq:

TTATAGGAATTCATTCGTTTCT

Complementarity: 15

Initial dG = -34.90

```
                        10        20        30        40
AC---------------     AA-|   UC     UUU      U        C   U   UU
                 UACAG   GCAU  UGGAG   CGAAUG AUUCCUAU ACG AUU  \
                 GUGUU   CGUG  AUCUC   GCUUAC UAAGGAUA UGC UAA  A
ACCUAUUUACAAACUUC     ACA^   GU     UUU      U        U   -   UC
110       100        90        80        70        60         50
```

Reads in: lib-42, lib-45, lib-47, lib-48, lib-49, lib-50, lib-72

Number of reads="ID102",0,1,0,0,0,0,0

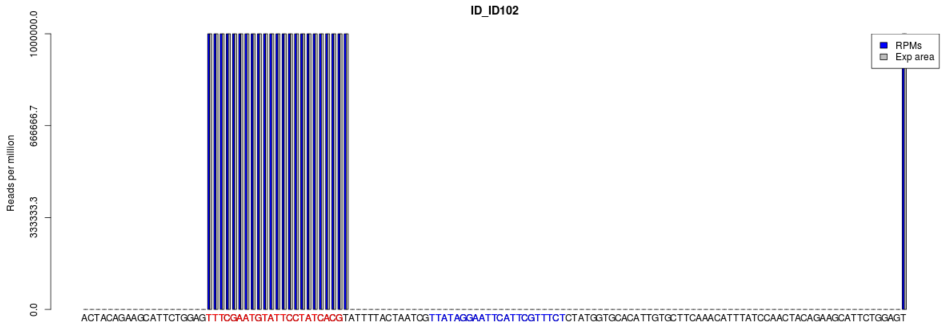

>ID1020
TTAGTTCAGTAAAGATTGTAATTCATGTATAGGGGC**CGGCGATTGCATGACTACCACA**CATTTTTTACTGTTGTAGTCACCCAGTCGTCA
GTCCCTGTGTATGATATACTAC

>ID1020-5P
CGGCGATTGCATGACTACCACA

>miRNA precursor
UUAGUUCAGUAAAGAUUGUAAUUCAUGUAUAGGGGC**CGGCGAUUGCAUGACUACCACA**CAUUUUUUACUGUUGUAGUCACCCAGUCGUCAGUCCCUGUGUAUGAUAUACUAC
................(((..((((((((((((((.((((((((..(((((((.(((..........))).)))))))..)))))))).))))))))))))))..)))...

>Predicted STAR seq:
TTGTAGTCACCCAGTCGTCAGT

Complementarity: 16

Initial dG = -43.50

```
        10        20        30        40        50        60
UUAGUUCAGUAAAGAUU|  AU              C        CA       C   CAUU
                 GUA  UCAUGUAUAGGGGC GGCGAUUG  UGACUAC ACA    U
                 CAU  AGUAUGUGUCCCUG CUGCUGAC  ACUGAUG UGU    U
CAU--------------^  AU              A        CC       U   CAUU
110                    100        90        80        70
```

Reads in: lib-42, lib-45, lib-47, lib-48, lib-49, lib-50, lib-72
Number of reads="ID1020",1,0,0,0,0,0,0

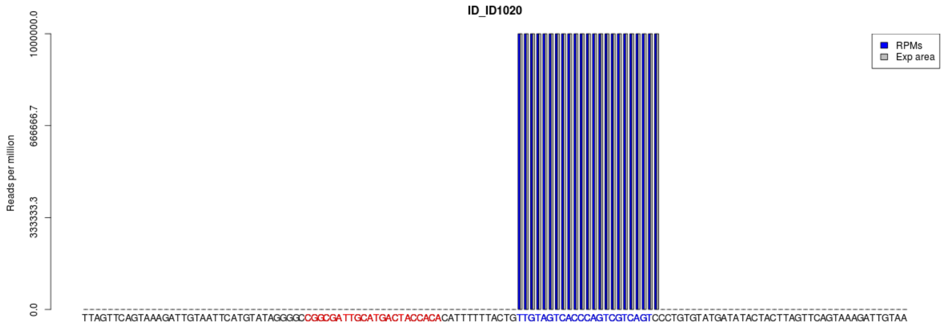

>ID1021
ATGTTGGAGTGAAAGTAATTCATGCATGGAGGG**TGATGGCTGTGTCTACCAGCA**CGTTATAAAGTCACTGTTGTATAGACCCAGCGATTA
CTCACTGTGTATGGCAACCATC

>ID1021-5P
TGATGGCTGTGTCTACCAGCA

>miRNA precursor
AUGUUGGAGUGAAAGUAAUUCAUGCAUGGAGGG**UGAUGGCUGUGUCUACCAGCA**CGUUAUAAAGUCACUGUUGUAUAGACCCAGCGAUUACUCACUGUGUAUGGCAACCAUC
..((((.(((.......)))(((((((((.(((((((.((((.(((((.(((((.((.........)))))))..))))).)))).))))))).))))))))).)))).....

>Predicted STAR seq:
TTGTATAGACCCAGCGATTACT

Complementarity: 16

```
 Initial dG =    -37.00

          10
ERROR 3: Strange structure
 Initial dG =    -37.00

          10
AUGU   .-AG|G
    UGG    U A
    ACC    G A
CU--   \ --^A
  110


            20        30        40         50        60
         UAAU          A       G    U     C-     C  UAUA
             UCAUGCAUGG GGGUGAU GCUG GUCUA  CAGCA GU    \
             GGUAUGUGUC CUCAUUA CGAC CAGAU  GUUGU CA    A
         AAC-          A       G    C     AU     -  CUGA
               100        90        80        70
```

Reads in: lib-42, lib-45, lib-47, lib-48, lib-49, lib-50, lib-72
Number of reads="ID1021",6,5,0,0,0,0,0

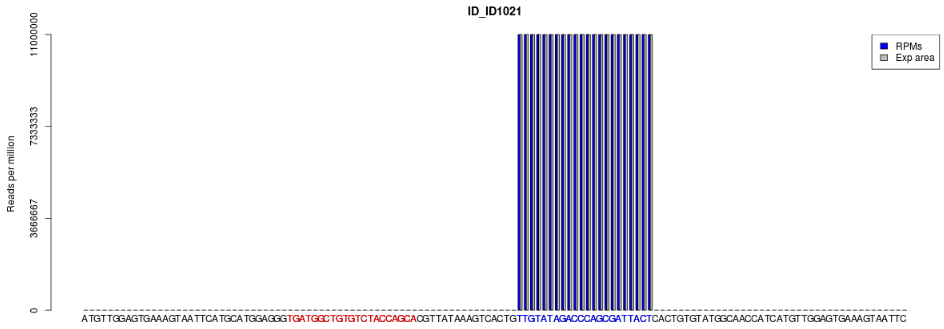

>ID1022
ATGTTGCAGTGAGAGTAATTCATATATGGAGAGTGATAGCTGTGTCTGCCAACACGTTATATAATCACTG**TTGTATAGGCCCAGCTATCA**
TTCACTGTGCATGATAAGCATC

>ID1022-3P
TTGTATAGGCCCAGCTATCATT

>miRNA precursor
AUGUUGCAGUGAGAGUAAUUCAUAUAUGGAGAGUGAUAGCUGUGUCUGCCAACACGUUAUAUAAUCACUG**UUGUAUAGGCCCAGCUAUCAUU**CACUGUGCAUGAUAAGCAUC
```
..............((...((((.(((((.((((((((((((.(((((.(((((.((.........)))))))..))))).))))))))))))).))))).))))...))...
```

>Predicted STAR seq:
GTGATAGCTGTGTCTGCCAA

Complementarity: 17

Initial dG =   -35.20

```
        10        20        30        40         50        60
AUGUUGCAGUGAGA  AAU    A     A            U     C-|    C  UAUA
              GU   UCAU UAUGG GAGUGAUAGCUG GUCUG  CAACA GU    \
              CG   AGUA GUGUC CUUACUAUCGAC CGGAU  GUUGU CA    U
CUA-----------  AAU    C     A            C     AU^    -  CUAA
110                 100        90        80        70
```

Reads in: lib-42, lib-45, lib-47, lib-48, lib-49, lib-50, lib-72
Number of reads="ID1022",0,0,0,0,0,0,0

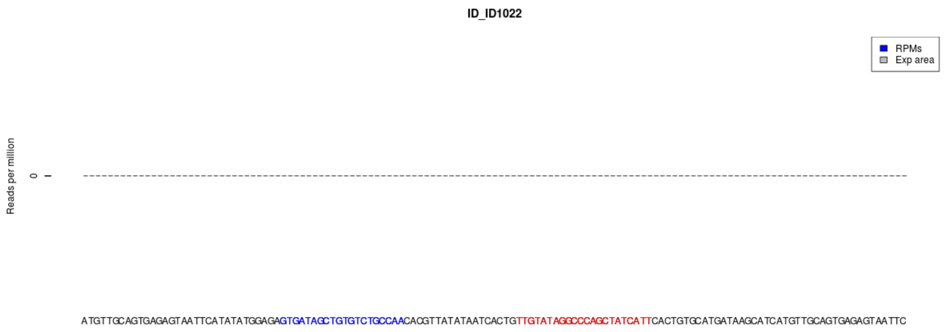

>ID1023
TGTTTCAGTGACAGTAATTCATGCATGGAGGGTGATGTCTGTGTGTGTCCAACATGTTATAAAATTACTG**TTGAGCATACACTGTCATCA**
CTCTCTGTGCATGGTAACCA

>ID1023-3P
TTGAGCATACACTGTCATCACT

>miRNA precursor
UGUUUCAGUGACAGUAAUUCAUGCAUGGAGGGUGAUGUCUGUGUGUGUCCAACAUGUUAUAAAAUUACUG**UUGAGCAUACACUGUCAUCACU**CUCUGUGCAUGGUAACCA
((((.....)))).....(((((((((((((((((((...(((((((..(((((.((.........)))))))..)))))))...)))))))))))))))))))......

>Predicted STAR seq:
GTGATGTCTGTGTGTGTCCAA

Complementarity: 15
ERROR 4: Strange structure
Initial dG =   -49.50

```
  |   UC
--UGUU  \
  ACAG  A
\ ^   UG
    10
```

```
        20        30        40        50        60
  GUAAU-                   UCU       UC     U  UAUA
        UCAUGCAUGGAGGGUGAUG   GUGUGUG  CAACA GU    \
        GGUACGUGUCUCUCACUAC   CACAUAC  GUUGU CA    A
  ACCAAU                   UGU       GA     -  UUAA
110       100        90        80        70
```

Reads in: lib-42, lib-45, lib-47, lib-48, lib-49, lib-50, lib-72
Number of reads="ID1023",1,11,1,0,0,0,0

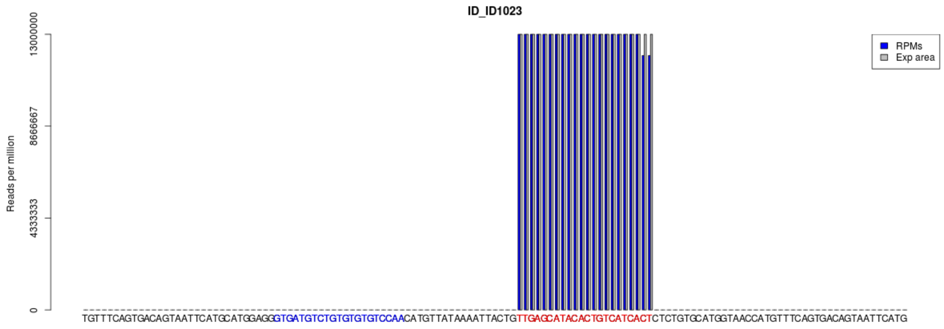

>ID1024
ACTTCAAAGAAGATTCTAATCATGTATAGGGGTTGGCGATTGCATGGCTGTCAAACATAGATTATTACTG**TTTCAGTCACCCAATCGTCA**
GCTCCTGTACATGGGATACCA

>ID1024-3P
TTTCAGTCACCCAATCGTCAGC

>miRNA precursor
ACUUCAAAGAAGAUUCUAAUCAUGUAUAGGGGUUGGCGAUUGCAUGGCUGUCAAACAUAGAUUAUUACUG**UUUCAGUCACCCAAUCGUCAGC**UCCUGUACAUGGGAUACCA
.((((...)))).......((((((((((((((((((((((..((((((..(((((...........)))))))))))..))))))))))))))))))))))).......

>Predicted STAR seq:
TTGGCGATTGCATGGCTGTCAAA

Complementarity: 18
ERROR 4: Strange structure
Initial dG =   -46.90

```
.-A|   A
   CUUC \
   GAAG A
\ -^   A
    10



        20        30        40        50        60
  AUUCUAA                       CA       UC      UAGAU
         UCAUGUAUAGGGGUUGGCGAUUG  UGGCUG  AAACA      \
         GGUACAUGUCCUCGACUGCUAAC  ACUGAC  UUUGU      U
  ACCAUAG                       CC       --      CAUUA
 110       100        90        80          70
```

Reads in: lib-42, lib-45, lib-47, lib-48, lib-49, lib-50, lib-72
Number of reads="ID1024",16,16,0,0,0,2,0

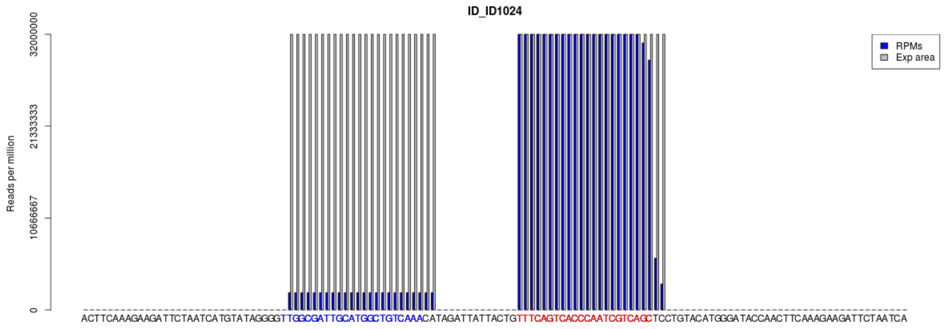

>ID1025
AAACCTATTGATTCTAATACATGCCATGGAGGTTGGCGGAAGTGTGCCTGTTACACGTTATTTTGTACTG**TAACAGACACCCTGACGTCA**
GCTGCCGTGCATGGTACACT

>ID1025-3P
TAACAGACACCCTGACGTCAGC

>miRNA precursor
AAACCUAUUGAUUCUAAUACAUGCCAUGGAGGUUGGCGGAAGUGUGCCUGUUACACGUUAUUUUGUACUG**UAACAGACACCCUGACGUCAGC**UGCCGUGCAUGGUACACU
..................(((((.((((.((((((((..((.(((.((((((((.((........))))))))))).))).))..)))))))).)))))))).......

>Predicted STAR seq:
TTGGCGGAAGTGTGCCTGTTA

Complementarity: 16

Initial dG = -42.50

```
        10        20        30        40        50        60
AAACCUAUUGAUUCUAAUA|    C    A        GA  U   C        C  UAU
                   CAUGC AUGG GGUUGGCG  AG GUG CUGUUACA GU   U
                   GUACG UGCC UCGACUGC  UC CAC GACAAUGU CA   U
UCACAUG------------^    -    G        AG  C   A        -  UGU
.                   100        90        80        70
```

Reads in: lib-42, lib-45, lib-47, lib-48, lib-49, lib-50, lib-72
Number of reads="ID1025",26,33,0,1,1,0,0

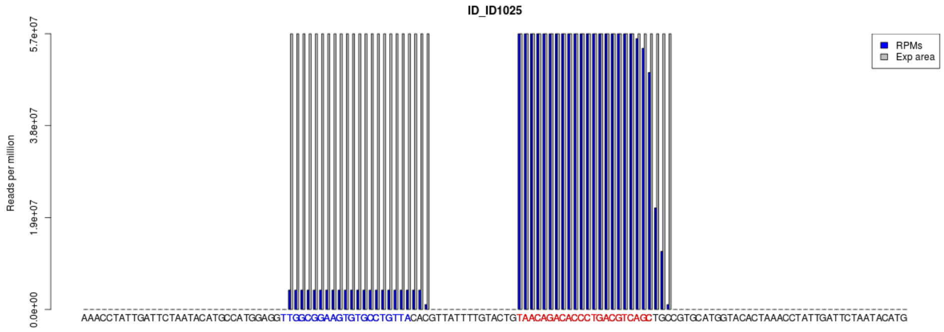

>ID1026

CTGTAATAACGATGCTACTGAGTGCATAGAGGCCGGCGATTCCGTGGTTGTCACAAGTTATTTACCACTG**TCACAACCACCCAATCGTCA
GCT**CCTGTGCATGGTATACCAC

>ID1026-3P

TCACAACCACCCAATCGTCAGCT

>miRNA precursor

CUGUAAUAACGAUGCUACUGAGUGCAUAGAGGCCGGCGAUUCCGUGGUUGUCACAAGUUAUUUACCACUG**UCACAACCACCCAAUCGUCAGCU**CCUGUGCAUGGUAUACCAC

```
...........(((((.....((((((((..((.(((((((..((((((((.(((.((........))))).))))))))..))))))).))..)))))))))))))).....
```

>Predicted STAR seq:

GGCCGGCGAUUCCGUGGUUGUC

Complementarity: 16

Initial dG =   -41.60

```
        10        20        30        40        50        60
CUGUAAUAACG|    ACUGA        AG  C        CC        C   A  UAU
           AUGCU     GUGCAUAG  GC GGCGAUU  GUGGUUGU ACA GU   U
           UAUGG     UACGUGUC  CG CUGCUAA  CACCAACA UGU CA   U
CACCA------^    -----        CU  A        CC        C   -  CCA
110                 100        90        80        70
```

Reads in: lib-42, lib-45, lib-47, lib-48, lib-49, lib-50, lib-72

Number of reads="ID1026",16,23,0,0,0,0,0

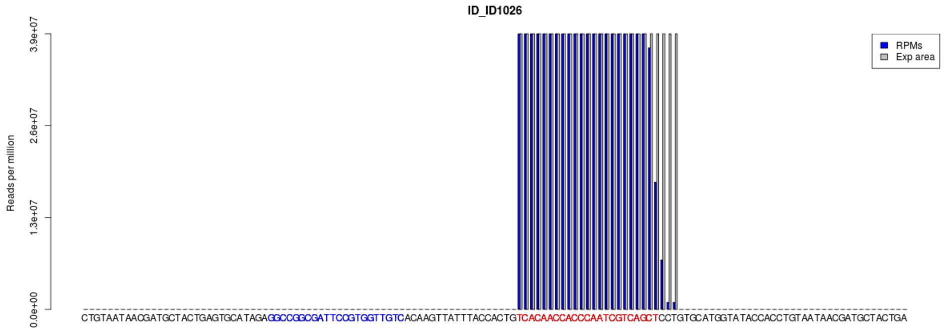

>ID1027
CGTCATCGCACGATGTGGTCTGGTGGACCGCAGCCGCTGCTGCTGCCGAGCACAGTGTTTTAAGGTG**TTCGCCAGCAACAGTAGCGG**
GCGCGGTGCTCTCCATGGA

>ID1027-3P
TTCGCCAGCAACAGTAGCGGGC

>miRNA precursor
CGUCAUCGCACGAUGUGGUCUGGUGGACCGCAGCCGCUGCUGCUGCUGCCGAGCACAGUGUUUUAAGGUG**UUCGCCAGCAACAGUAGCGGGC**GCGGUGCUCUCCAUGGA
.(.((((((....(((((((.....))))))).(((((((((.(((((.(((((((..((...))..))))))).))))).)))))))))..)))))))..........

>Predicted STAR seq:
CGATGTGGTCTGGTGGACCGCAGCCGCTGCTGCTG

Complementarity: 16

Initial dG =   -55.60

```
                   10        20
C--------- U      .-ACGA|      UG
          G CAUCGC      UGUGGUC  \
          C GUGGCG      ACGCCAG  G
AGGUACCUCU -      \ ----^      GU
       100               30
```

Reads in: lib-42, lib-45, lib-47, lib-48, lib-49, lib-50, lib-72
Number of reads="ID1027",8,19,0,0,0,0,0

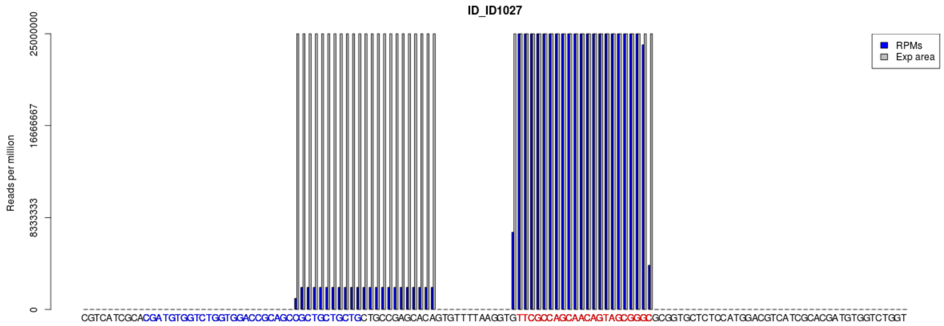

>ID1028
TGTTGCAGTGTCAGTAATTCATGCATGGAGGGTGATGTCTGTGTGGGTCCAACACATTATGAGATTACTG**TTGAACAGACACGGCCATCACT**CACTGTGCATGATATT

>ID1028-3P
TTGAACAGACACGGCCATCACT

>miRNA precursor
UGUUGCAGUGUCAGUAAUUCAUGCAUGGAGGGUGAUGUCUGUGUGGGUCCAACACAUUAUGAGAUUACUG**UUGAACAGACACGGCCAUCACU**CACUGUGCAUGAUAUU
.(((((.......)))))((((((((((.((((((((.((((((..((.(((((..............))))).))..)))))).)))))))).))))))))))....

>Predicted STAR seq:
GTGATGTCTGTGTGGGTCCAA

Complementarity: 16
ERROR 4: Strange structure
Initial dG = -41.60

```
.-U|    AGU
   GUUGC   \
   UAAUG   G
\ -^    ACU
```

```
      20        30        40        50        60
  ----          A         U      GG   C      CAUUAU
      UCAUGCAUGG GGGUGAUG CUGUGU  GU CAACA      G
      AGUACGUGUC CUCACUAC GGCACA  CA GUUGU      A
  UUAU          A         C      GA   A      CAUUAG
        100        90        80        70
```

Reads in: lib-42, lib-45, lib-47, lib-48, lib-49, lib-50, lib-72
Number of reads="ID1028",6,5,0,0,0,0,0

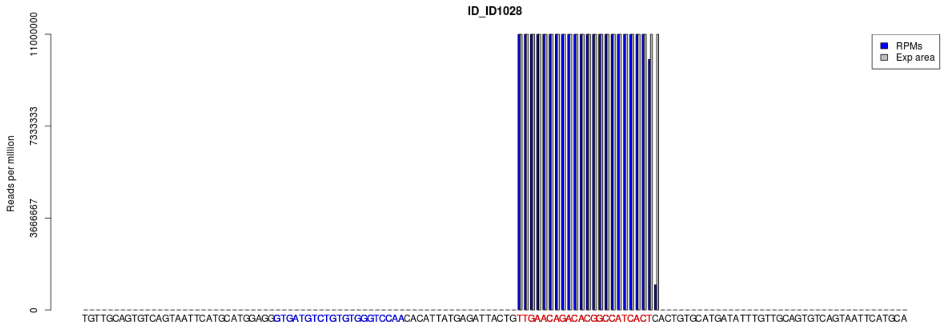

>ID1029
ATGTTGCAGTGAGAGTAATTTATGCATGGAGGG**TGATAGCTGTGTCTACCAACA**CGTTATATAATCACTGTTGTATAGGCCCAGCTATCA
TTCACTGTGCATGATAACCATC

>ID1029-5P
TGATAGCTGTGTCTACCAACA

>miRNA precursor
AUGUUGCAGUGAGAGUAAUUUAUGCAUGGAGGG**UGAUAGCUGUGUCUACCAACA**CGUUAUAUAAUCACUGUUGUAUAGGCCCAGCUAUCAUUCACUGUGCAUGAUAACCAUC
..(((((.......)))))(((((((((.((((((((((((.(((((.(((((.((.........)))))))..))))).))))))))))))).))))))))))........

>Predicted STAR seq:
TTGTATAGGCCCAGCTATCATT

Complementarity: 17

```
 Initial dG =    -40.10

          10
ERROR 3: Strange structure
 Initial dG =    -40.10

          10
.-AU|    AGU
    GUUGC   \
    UAAUG   G
\ --^    AGA



         20        30        40         50        60
  --------         A            U     C-     C  UAUA
          UUAUGCAUGG GGGUGAUAGCUG GUCUA  CAACA GU    \
          AGUACGUGUC CUUACUAUCGAC CGGAU  GUUGU CA    U
  CUACCAAU         A            C     AU     -  CUAA
  110       100        90        80        70
```

Reads in: lib-42, lib-45, lib-47, lib-48, lib-49, lib-50, lib-72
Number of reads="ID1029",0,0,0,0,0,0,0

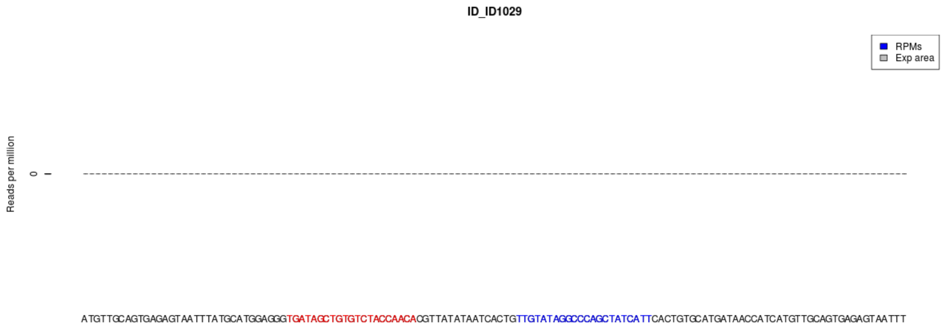

>ID103
TATTATTCTCACACGAAGGGTGACGCATGTGTCGCGGATACACGTTATTTAATTTGTG**TCTCTGAGACCCATACGTCACT**CATTGTGCGA
GTTTCACATCAAATTCCATCA

>ID103-3P
TCTCTGAGACCCATACGTCACT

>miRNA precursor
UAUUAUUCUCACACGAAGGGUGACGCAUGUGUCGCGGAUACACGUUAUUUAAUUUGUG**UCUCUGAGACCCAUACGUCACU**CAUUGUGCGAGUUUCACAUCAAAUUCCAUCA
.......(((.(((((.((((((((.(((.(((.((((.(((((..........))))).)))).))).))).)))))))).))))).)))...................

>Predicted STAR seq:
GTGACGCATGTGTCGCGGAT

Complementarity: 16

Initial dG = -39.40

```
                     10        20        30        40
UAUUAUU-------------|  A     A        C   U   G    U     UUAU
                    CUC CACGA GGGUGACG AUG GUC CGGA ACACG    U
                    GAG GUGUU CUCACUGC UAC CAG GUCU UGUGU    U
ACUACCUUAAACUACACUUU^  C     A        A   C   A    C     UUAA
 .       100        90        80        70        60        50
```

Reads in: lib-42, lib-45, lib-47, lib-48, lib-49, lib-50, lib-72
Number of reads="ID103",1,9,0,0,1,0,0

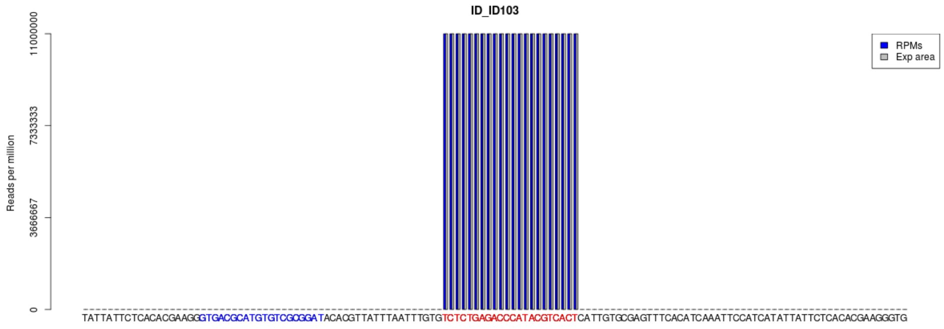

>ID1030
CGTGAACGCACGATGTTGTGTGCTGGTCAGCAGT**GGGGGCTGCTGCAGGCGAGCA**TAGTGTTTTGAGGTGCGCGCCTGCAGCAGCCAGCC
CGCTGCTGGCCATCGAACAAACG

>ID1030-5P
GGGGGCTGCTGCAGGCGAGCA

>miRNA precursor
CGUGAACGCACGAUGUUGUGUGCUGGUCAGCAGU**GGGGGCUGCUGCAGGCGAGCA**UAGUGUUUUGAGGUGCGCGCCUGCAGCAGCCAGCCCGCUGCUGGCCAUCGAACAAACG
((((....))))...((((.((.(((((((((((((((((((((((((((.((((..(.....)..)))).)))))))))))))))..))))))))))))))).)).))))...

>Predicted STAR seq:
CGCGCCTGCAGCAGCCAGCCCGC

Complementarity: 18

Initial dG =   -73.50

ERROR 3: Strange structure
Initial dG =   -73.50

```
  |   A
--CGUG A
  GCAC C
\ ^   G
   10


       20        30          40        50        60
  AUG    G  C               --              A    AG GU
     UUGU UG UGGUCAGCAGUGGG  GGCUGCUGCAGGCG GCAU  U  \
     AACA GC ACCGGUCGUCGCCC  CCGACGACGUCCGC CGUG  G  U
  GCA    A  U               GA              G    GA UU
   110       100        90        80        70
```

Reads in: lib-42, lib-45, lib-47, lib-48, lib-49, lib-50, lib-72
Number of reads="ID1030",4,3,0,0,0,0,0

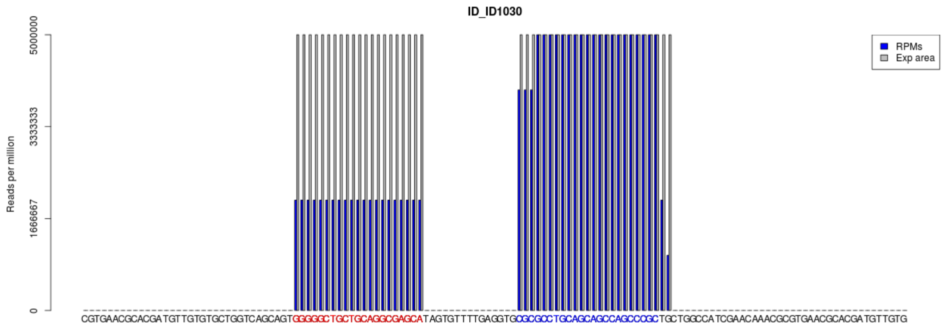

>ID1031
GATAATTACTGCATAATGGT**TGGCGATTGTGTGACTGTTACG**TCTTATTTACTACCGTCACAGCTGCGCAATCGTCAGCTGCTGTGCACA
GTATACCACATTATTGTCAAC

>ID1031-5P
TGGCGATTGTGTGACTGTTACG

>miRNA precursor
GAUAAUUACUGCAUAAUGGU**UGGCGAUUGUGUGACUGUUACG**UCUUAUUUACUACCGUCACAGCUGCGCAAUCGUCAGCUGCUGUGCACAGUAUACCACAUUAUUGUCAAC
((((((((((((((..(((((((((((((((..((((.(((.............))).))))..)))))))))))))))..))))))..))).........))))))...

>Predicted STAR seq:
TCACAGCTGCGCAATCGTCAGC

Complementarity: 17

Initial dG =   -41.60

```
                      10        20        30        40
---      ---------   --|     AU               GA    U   UCUUAU
   GAUAAU         UAC  UGCAUA  GGUUGGCGAUUGUGU  CUGU ACG      \
   CUGUUA         AUG  ACGUGU  UCGACUGCUAACGCG  GACA UGC      U
CAA      UUACACCAU   AC^     CG               UC    C   CAUCAU
 .       100        90        80        70        60        50
```

Reads in: lib-42, lib-45, lib-47, lib-48, lib-49, lib-50, lib-72
Number of reads="ID1031",1,2,0,0,0,0,0

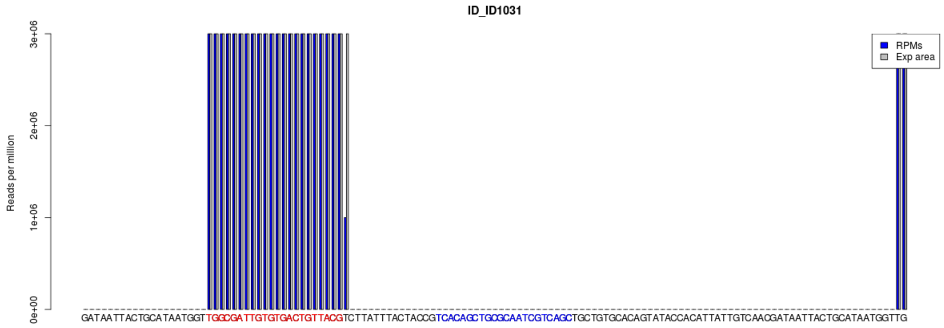

>ID1032
CTTCAAGGAAGATTCTAATTCATGTATAGGGTTCAGCGACTGCGTGAACGCCACATGTTATTTACTACTG**TGGCAGTGACGCAGTCGTCA GA**TCCTGTGCATGGTATACCAC

>ID1032-3P
TGGCAGTGACGCAGTCGTCAGA

>miRNA precursor
CUUCAAGGAAGAUUCUAAUUCAUGUAUAGGGUUCAGCGACUGCGUGAACGCCACAUGUUAUUUACUACUG**UGGCAGUGACGCAGUCGUCAGA**UCCUGUGCAUGGUAUACCAC
((((...))))........((((((((((((((..((((((((((..((((((((.((........)))))))).)).))))))))))..))))))))))))))........

>Predicted STAR seq:
TCAGCGACTGCGTGAACGCCA

Complementarity: 16
ERROR 4: Strange structure
Initial dG =   -50.50

```
  |   A
--CUUC \
  GAAG A
\ ^   G
  10


        20        30        40         50        60
  AUUCUAAU              CA           GA   -      U  UAU
          UCAUGUAUAGGGUU  GCGACUGCGU  AC GCCACA GU   U
          GGUACGUGUCCUAG  UGCUGACGCA  UG CGGUGU CA   U
  CACCAUAU              AC           G-   A      -  UCA
  110       100         90         80         70
```

Reads in: lib-42, lib-45, lib-47, lib-48, lib-49, lib-50, lib-72
Number of reads="ID1032",6,0,0,2,0,0,0

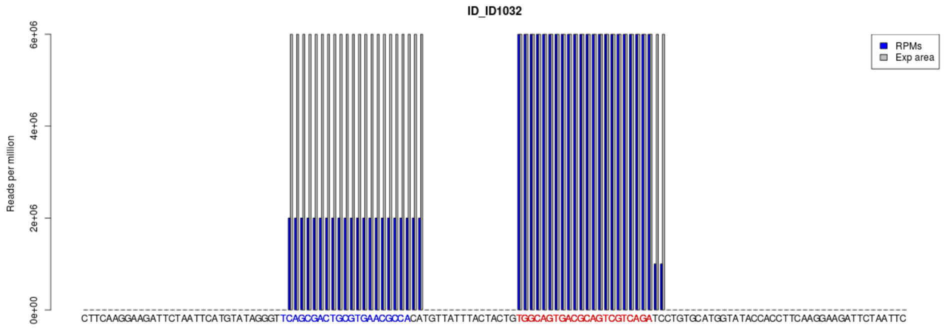

>ID1033
AGTAATTCATGCACGGAGGG**TGATATCTGTGTGGGCCCAAC**ACGATATAAAATTACTGTTGAGCATACACGGCTATCACCCACTGTGCAT
GGTAATCATCAGTTTTGGAG

>ID1033-5P
TGATATCTGTGTGGGCCCAAC

>miRNA precursor
AGUAAUUCAUGCACGGAGGG**UGAUAUCUGUGUGGGCCCAAC**ACGAUAUAAAAUUACUGUUGAGCAUACACGGCUAUCACCCACUGUGCAUGGUAAUCAUCAGUUUUGGAG
......((((((((((.((((((((.(((((((.((.(((((.(((.....)))..))))).)).))))))).)))))))).))))))))))).................

>Predicted STAR seq:
TGAGCATACACGGCTATCACC

Complementarity: 16

Initial dG =   -46.40

```
                    10        20        30        40
AGUAAU------------          A        U       G  C     C-|  AU
                  UCAUGCACGG GGGUGAUA CUGUGUG GC CAACA  GAU  \
                  GGUACGUGUC CCCACUAU GGCACAU CG GUUGU  UUA  A
GAGGUUUUGACUACUAAU          A        C       A  A     CA^  AA
.       100        90        80        70        60        50
```

Reads in: lib-42, lib-45, lib-47, lib-48, lib-49, lib-50, lib-72
Number of reads="ID1033",0,5,0,0,0,0,0

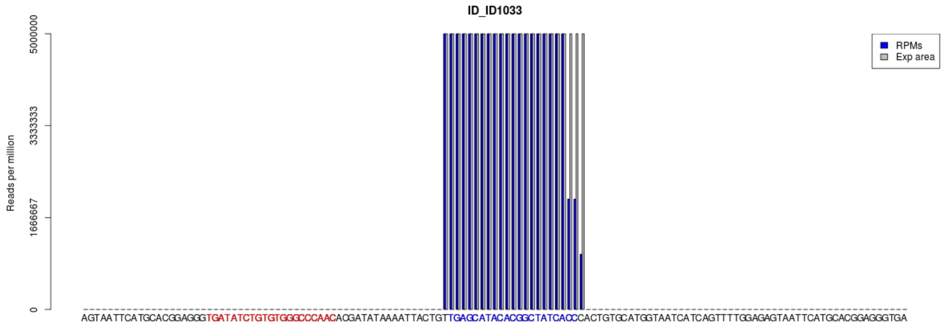

>ID1034

GGTAATTCCTGCATGGAGGG**TTATGGTAGAGTGGTGACAACA**AGTCATTGAATTTCTGTTGCCCACACCCTACCATTACCCACTGTGTAT
GGGAAAGCAAATAAGACA

>ID1034-5P

TTATGGTAGAGTGGTGACAACA

>miRNA precursor

GGUAAUUCCUGCAUGGAGGG**UUAUGGUAGAGUGGUGACAACA**AGUCAUUGAAUUUCUGUUGCCCACACCCUACCAUUACCCACUGUGUAUGGGAAAGCAAAUAAGACA
.((..(((((((((((.((((.(((((((.((((..(((..((((......)))).)))..))...)).))))))).)))).))))))).))))..))..........

>Predicted STAR seq:

TGTTGCCCACACCCTACCATTACC

Complementarity: 14

Initial dG = -37.00

```
                  10        20        30           40
G---------  AA    -       A    U       A  ---| UG   AC    CA
          GU  UUCC UGCAUGG GGGU AUGGUAG GU   GG  ACA  AAGU  U
          CG  AGGG AUGUGUC CCCA UACCAUC CA   CC  UGU  UUUA  U
ACAGAAUAAA  AA    U       A    U       C  CAC^ GU   C-    AG
      100        90        80        70        60         50
```

Reads in: lib-42, lib-45, lib-47, lib-48, lib-49, lib-50, lib-72

Number of reads="ID1034",0,2,0,0,0,0,0

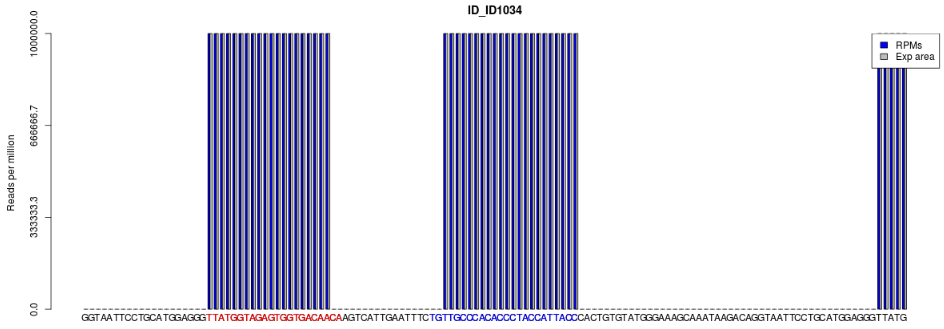

>ID1035
TGGGTTTCGTGCATCCCGAG**TAACGGAAGCCGAGGCTACACAAG**TTTATTTAAAGTACTTGAGCCTCAGCTCCCGTTTCCCGGTGTGTAC
GAAAAGCGACAAGTATGGAC

>ID1035-5P
TAACGGAAGCCGAGGCTACACAAG

>miRNA precursor
UGGGUUUCGUGCAUCCCGAG**UAACGGAAGCCGAGGCUACACAAG**UUUAUUUAAAGUACUUGAGCCUCAGCUCCCGUUUCCCGGUGUGUACGAAAAGCGACAAGUAUGGAC
((..(((((((((..(((...(((((.(((.((((((...(((((...........))))))))))).))).)))))...)))..)))))))))..))...........

>Predicted STAR seq:
TGAGCCTCAGCTCCCGTTTCCCG

Complementarity: 16

Initial dG =   -43.70

```
                    10        20        30        40        50
------------| GG         UC   AGU     A   C      ACA     UUAUU
            UG  UUUCGUGCA  CCG   AACGG AGC GAGGCU   CAAGU     \
            GC  AAAGCAUGU  GGC   UUGCC UCG CUCCGA   GUUCA     U
CAGGUAUGAACA^ GA         GU   CCU     C   A      ---     UGAAA
.       100        90        80        70           60
```

Reads in: lib-42, lib-45, lib-47, lib-48, lib-49, lib-50, lib-72
Number of reads="ID1035",1,3,0,0,0,0,0

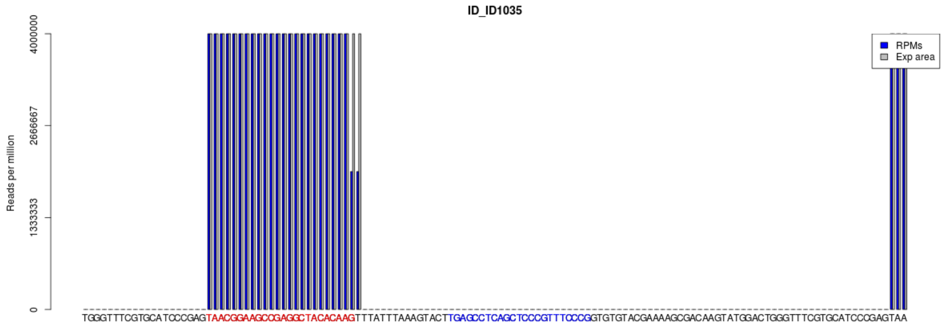

>ID1036
ATGTTGGAGTGAAAGTAATTCATGCATGGAGGGTGATGGCTGTGTCTGCCAACACGTTATAAAATCACTG**TTGTTAAGACCCAGCCATCA**
CTCACTGTGCATGGCAACC

>ID1036-3P
TTGTTAAGACCCAGCCATCACT

>miRNA precursor
AUGUUGGAGUGAAAGUAAUUCAUGCAUGGAGGGUGAUGGCUGUGUCUGCCAACACGUUAUAAAAUCACUG**UUGUUAAGACCCAGCCAUCACU**CACUGUGCAUGGCAACC
..((((.............((((((((((.((((((((((((.((((..(((((.((.........)))))))...)))).))))))))))))).)))))))))))))).

>Predicted STAR seq:
GTGATGGCTGTGTCTGCCAA

Complementarity: 16

Initial dG =   -45.60

```
        10        20        30        40         50        60
AU|   GAGUGAAAGUAAU          A            U    GC-     C  UAUA
  GUUG             UCAUGCAUGG GGGUGAUGGCUG GUCU   CAACA GU    \
  CAAC             GGUACGUGUC CUCACUACCGAC CAGA   GUUGU CA    A
C-^   -------------          A            C    AUU     -  CUAA
                    100        90        80        70
```

Reads in: lib-42, lib-45, lib-47, lib-48, lib-49, lib-50, lib-72
Number of reads="ID1036",8,12,0,0,0,0,0

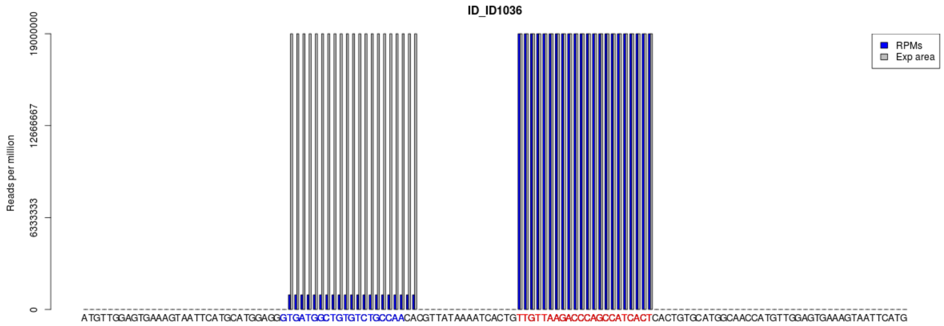

>ID1037
TACTATGATAATAGTAATTCGTGCATGGAGGGTGGTGACTATGTGGCCCCCACATGTTATTAAATGAATG**TTGAGGCCATGTAGTCATCA**
CTCACTGTGCATAGCTGG

>ID1037-3P
TTGAGGCCATGTAGTCATCACT

>miRNA precursor
UACUAUGAUAAUAGUAAUUCGUGCAUGGAGGGUGGUGACUAUGUGGCCCCCACAUGUUAUUAAAUGAAUG**UUGAGGCCAUGUAGUCAUCACU**CACUGUGCAUAGCUGG
```
((((((....))))))....((((((((.(((((((((((((((((((...((((............))))...)))))))))))))))))))).))))))))......
```

>Predicted STAR seq:
GTGGTGACTATGTGGCCC

Complementarity: 17
ERROR 4: Strange structure
Initial dG =   -49.30

```
  |     G
--UACUAU A
  AUGAUA U
\ ^     A
       10



    20          30        40        50        60
  AUUC--        A                   CCC     GUUAU
        GUGCAUGG GGGUGGUGACUAUGUGGCC   ACAU      U
        UACGUGUC CUCACUACUGAUGUACCGG   UGUA      A
  GGUCGA        A                   AGU     AGUAA
        100        90        80        70
```

Reads in: lib-42, lib-45, lib-47, lib-48, lib-49, lib-50, lib-72
Number of reads="ID1037",20,61,0,1,1,0,0

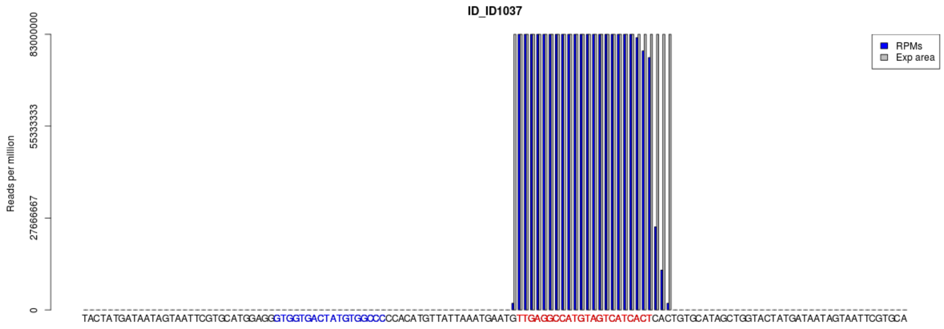

>ID1038
CTAATACATGTCATGGAGAT**TGACGGATGTCTGACTGGAACAT**GTTATTTCACATTGTACCAGTCACCCATTCGTTAGCCGCCCTGTATG
ATAGACGACAAAGATGCT

>ID1038-5P
TGACGGATGTCTGACTGGAACAT

>miRNA precursor
CUAAUACAUGUCAUGGAGAU**UGACGGAUGUCUGACUGGAACAU**GUUAUUUCACAUUGUACCAGUCACCCAUUCGUUAGCCGCCCUGUAUGAUAGACGACAAAGAUGCU
........(((((((.((.((((((((((..(((((((.((((((......)))).)).)))))))..))))))))))).....)).))))))).............

>Predicted STAR seq:
TGTACCAGTCACCCATTCGTTAGC

Complementarity: 18

Initial dG = -34.00

```
               10            20        30        40
CUAAUACA-------       G  A----          UC        A  -|   UA
               UGUCAUG AG     UUGACGGAUG  UGACUGG AC AUGU  U
               AUAGUAU UC     GAUUGCUUAC  ACUGACC UG UACA  U
UCGUAGAAACAGCAG       G  CCGCC          CC        A  U^   CU
      100        90        80        70        60        50
```

Reads in: lib-42, lib-45, lib-47, lib-48, lib-49, lib-50, lib-72
Number of reads="ID1038",3,3,0,0,0,0,0

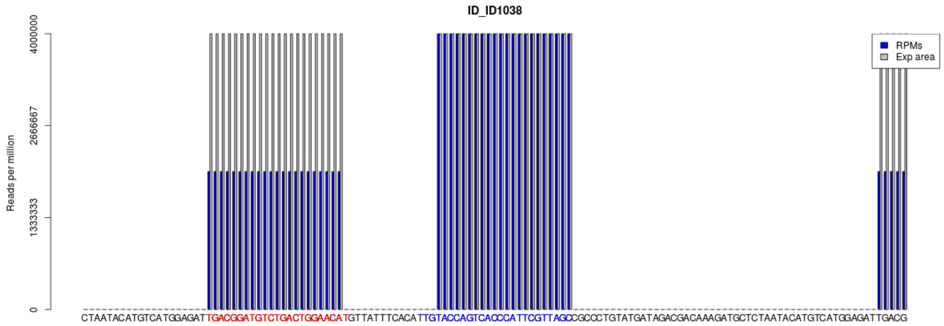

>ID1039
ACTTCGCTGAACATCCTTATCCATTTCAGGGGCTGGCCATTGTACGACTGTCACACATAATTTATTGGTG**TTTCAGTCATGCAATCGTCA**
GCTCCTGTGCATGGTATACC

>ID1039-3P
TTTCAGTCATGCAATCGTCAGC

>miRNA precursor
ACUUCGCUGAACAUCCUUAUCCAUUUCAGGGGCUGGCCAUUGUACGACUGUCACACAUAAUUUAUUGGUG**UUUCAGUCAUGCAAUCGUCAGC**UCCUGUGCAUGGUAUACC
................((((((..(((((((((((.((((((.(((((..((((.(((....)))))))..))))).)))))).)))))))))))...)))).)))..

>Predicted STAR seq:
CTGGCCATTGTACGACTGT

Complementarity: 16

Initial dG = -42.60

```
        10        20          30        40        50        60
ACUUCGCUGAACAUCCU   -    UU-|          C      C     UC    A   U
                 UAU CCAU   CAGGGGCUGGC AUUGUA GACUG  ACAC UAA U
                 AUA GGUA   GUCCUCGACUG UAACGU CUGAC  UGUG GUU U
CC---------------   U    CGU^          C      A     UU    -   A
.                      100        90        80        70
```

Reads in: lib-42, lib-45, lib-47, lib-48, lib-49, lib-50, lib-72
Number of reads="ID1039",24,13,0,0,1,0,0

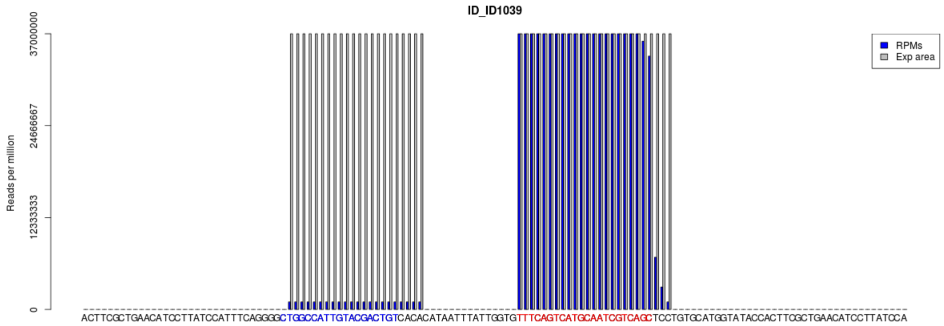

>ID1040
CTGCAGATAAGTTGATAATTACTGCATAATGGTTGGCGATTGTGTGGCTGCCACGTCTTATTTACTACCG**TCACAGCCGCGCAATCGTCA**
GCTGCTGTGCACAGTATACCA

>ID1040-3P
TCACAGCCGCGCAATCGTCAGC

>miRNA precursor
CUGCAGAUAAGUUGAUAAUUACUGCAUAAUGGUUGGCGAUUGUGUGGCUGCCACGUCUUAUUUACUACCG**UCACAGCCGCGCAAUCGUCAGC**UGCUGUGCACAGUAUACCA
```
..................((((((((..((((((((((((((((((((..(((.............)))..))))))))))))))))))))..))))))..))).....
```

>Predicted STAR seq:
TTGGCGATTGTGTGGCTGC

Complementarity: 18

Initial dG =   -45.20

```
        10        20          30        40        50        60
CUGCAGAUAAGUUGAUAAU   --|     AU                    CC   UCUUAU
                   UAC  UGCAUA  GGUUGGCGAUUGUGUGGCUG  ACG      \
                   AUG  ACGUGU  UCGACUGCUAACGCGCCGAC  UGC      U
ACCAU--------------   AC^     CG                    AC   CAUCAU
 .                      100        90        80        70
```

Reads in: lib-42, lib-45, lib-47, lib-48, lib-49, lib-50, lib-72
Number of reads="ID1040",22,36,0,1,0,1,0

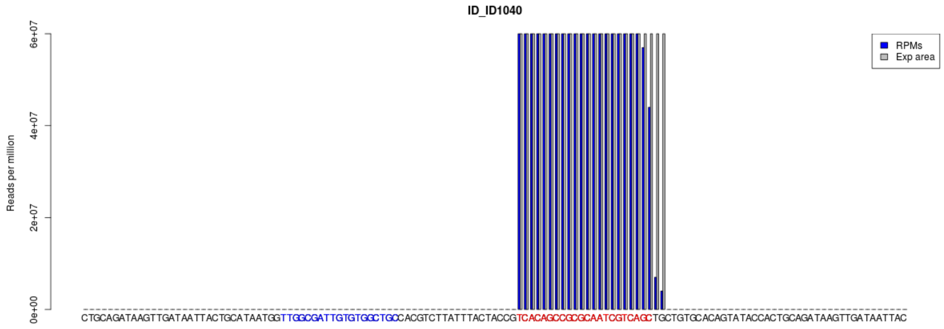

>ID1041

CGTTGCAGTGTCAGTAATTCATGCATGGAGGGTGATGGCTGTGTGGGTCCAACACATTATAAGATTACTG**TTGAACAGACACGGCCATCACT**CACTGTGCATGATATT

>ID1041-3P

TTGAACAGACACGGCCATCACT

>miRNA precursor

CGUUGCAGUGUCAGUAAUUCAUGCAUGGAGGGUGAUGGCUGUGUGGGUCCAACACAUUAUAAGAUUACUG**UUGAACAGACACGGCCAUCACU**CACUGUGCAUGAUAUU

```
.(((((.......)))))((((((((((.((((((((((((((((..((.(((((.............))))).))..)))))))))))))))).))))))))))....
```

>Predicted STAR seq:

GTGATGGCTGTGTGGGTCCAA

Complementarity: 17

ERROR 4: Strange structure

Initial dG =   -48.80

```
.-C|    AGU
   GUUGC   \
   UAAUG   G
\ -^    ACU
```

```
      20        30        40        50        60
  ----          A               GG   C      CAUUAU
      UCAUGCAUGG GGGUGAUGGCUGUGU  GU CAACA       A
      AGUACGUGUC CUCACUACCGGCACA  CA GUUGU       A
  UUAU          A               GA   A      CAUUAG
        100        90        80        70
```

Reads in: lib-42, lib-45, lib-47, lib-48, lib-49, lib-50, lib-72

Number of reads="ID1041",5,13,0,0,0,0,0

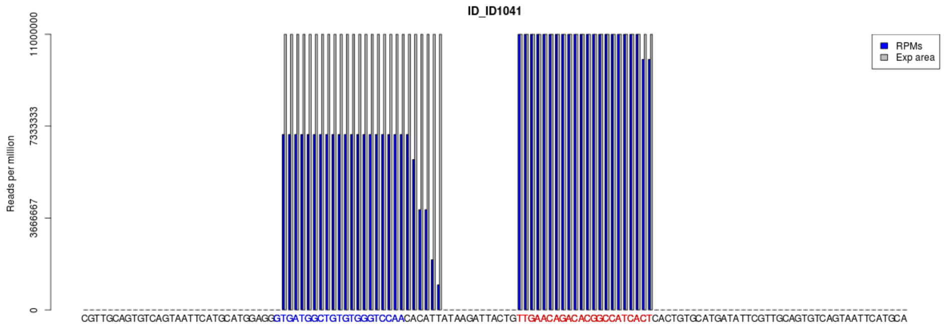

>ID1043
TGTAATTCATGTATAGGGTT**CTCCGACTGCATGAACGCCACA**TGTCATTTACTACTGTGGCAGTCACGCAGTCGTCAGATCCTGTGCATG
GTATACCACAGAATTGTGCCT

>ID1043-5P
CTCCGACTGCATGAACGCCACA

>miRNA precursor
UGUAAUUCAUGUAUAGGGUU**CUCCGACUGCAUGAACGCCACA**UGUCAUUUACUACUGUGGCAGUCACGCAGUCGUCAGAUCCUGUGCAUGGUAUACCACAGAAUUGUGCCU
.(((..((((((((((((((...(((((((.(((..((((((.((........)))))))))..))).)))))))...))))))))))))))..)))(((......)))...

>Predicted STAR seq:
TGGCAGTCACGCAGTCGTCAGA

Complementarity: 14

Initial dG =   -46.40

```
          10        20        30        40
.-U|  AU              CUC       A   AC      U  CAU
   GUA  UCAUGUAUAGGGUU   CGACUGC UGA  GCCACA GU   U
   CAU  GGUACGUGUCCUAG   GCUGACG ACU  CGGUGU CA   U
\ -^  AU              ACU       C   GA      -  UCA
        90        80        70        60         50



      100
  ---   AG
     CAC  A
     GUG  A
  UCC   UU
 110
```

Reads in: lib-42, lib-45, lib-47, lib-48, lib-49, lib-50, lib-72
Number of reads="ID1043",14,10,0,0,0,0,0

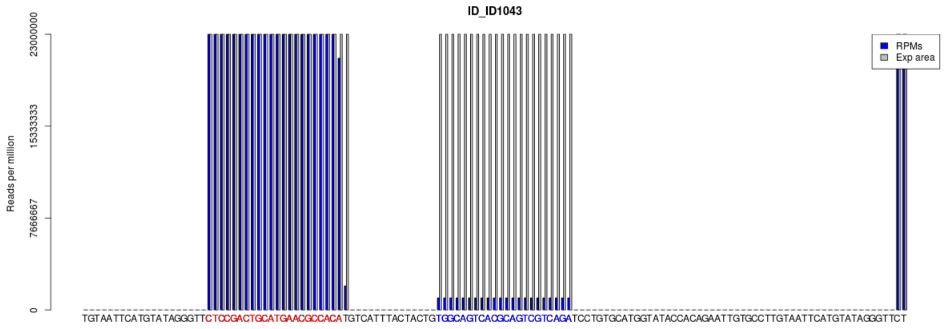

>ID1044
CTAATACATGTCATGGAGGT**TGACGGATGTCTGACTGGAACAT**GTTATTTTATATTGTTCAAGTCACCCATTCGTTAGCCGCCCTGCATG
GTAGACGACGAAGATGCT

>ID1044-5P
TGACGGATGTCTGACTGGAACAT

>miRNA precursor
CUAAUACAUGUCAUGGAGGU**UGACGGAUGUCUGACUGGAACAU**GUUAUUUUAUAUUGUUCAAGUCACCCAUUCGUUAGCCGCCCUGCAUGGUAGACGACGAAGAUGCU
......((((.((.((.((((((((((((..(((((.((((((((......))).))))).))))).))))))))))))).)).))))))................

>Predicted STAR seq:
TGTTCAAGTCACCCATTCGTTAGC

Complementarity: 18

Initial dG =   -40.20

```
                    10        20        30        40
CUAAUA------------|   U  U  A            UC      G     -   UA
                  CAUG CA GG GGUUGACGGAUG  UGACU GAACA UGU  U
                  GUAC GU CC CCGAUUGCUUAC  ACUGA CUUGU AUA  U
UCGUAGAAGCAGCAGAUG^   -  C  G            CC      A     U   UU
      100        90         80        70        60        50
```

Reads in: lib-42, lib-45, lib-47, lib-48, lib-49, lib-50, lib-72
Number of reads="ID1044",4,2,0,0,0,0,0

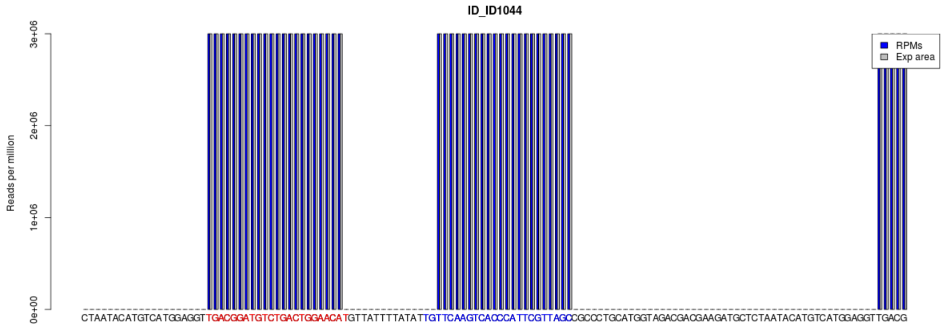

>ID1046
AATAGTTCGTACATGGAGGG**TTGTGGCTGTGTATGACAACA**CGTTATGAAATCAGTGTTGTACCTACACAGCCATCACCCACTGTGTATG
GTAAGCATCAGCCGTTGGTC

>ID1046-5P
TTGTGGCTGTGTATGACAACA

>miRNA precursor
AAUAGUUCGUACAUGGAGGG**UUGUGGCUGUGUAUGACAACA**CGUUAUGAAAUCAGUGUUGUACCUACACAGCCAUCACCCACUGUGUAUGGUAAGCAUCAGCCGUUGGUC
......((((((((((.((((.(((((((((((..(((((((...........)))))))...))))))))))).)))).))))))))))...((....))........

>Predicted STAR seq:
TTGTACCTACACAGCCATCACC

Complementarity: 16

Initial dG =   -47.20

```
          10        20        30         40
.-AAUAGU          A    U           UG-|      GUUAU
        UCGUACAUGG GGGU GUGGCUGUGUA   ACAACAC     G
        GGUAUGUGUC CCCA UACCGACACAU   UGUUGUG     A
\ ------          A    C           CCA^      ACUAA
        90        80        70        60        50
```

```
  UAA-----  A
         GC U
         CG C
  CUGGUUGC  A
110       100
```

Reads in: lib-42, lib-45, lib-47, lib-48, lib-49, lib-50, lib-72
Number of reads="ID1046",3,14,0,0,0,0,0

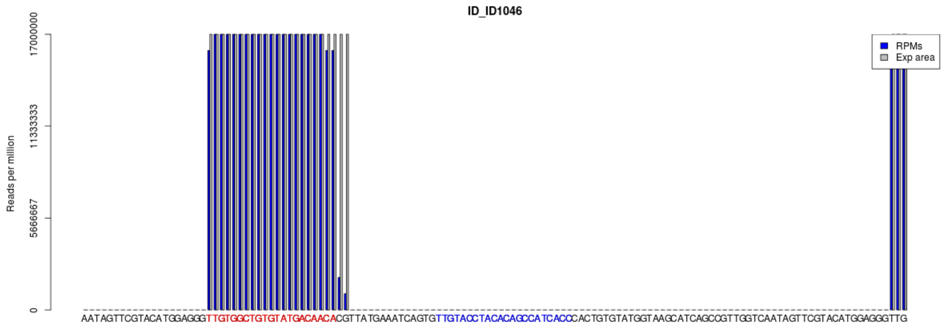

>ID1047
ATTCTGAAGACCAATATGTTTCGTGAATCCCGAGCGACGGAAGCGAGATCTGTACAAGTTTTTAAATTAC**TTGAGATCTCGATTCCGTTA**
CTCGGTGTTCTCGATATTCG

>ID1047-3P
TTGAGATCTCGATTCCGTTACT

>miRNA precursor
AUUCUGAAGACCAAUAUGUUUCGUGAAUCCCGAGCGACGGAAGCGAGAUCUGUACAAGUUUUUAAAUUAC**UUGAGAUCUCGAUUCCGUUACU**CGGUGUUCUCGAUAUUCG
...................(((.(((..(((((..((((((.((((((((...(((((.........))))))))))))).))))))..)))))..))).)))......

>Predicted STAR seq:
GCGACGGAAGCGAGAUCUGUACAA

Complementarity: 17

Initial dG =   -43.30

```
        10        20        30        40        50        60
AUUCUGAAGACCAAUAUGUU|  U   UC      CG      G        GUA     UUUU
                    UCG GAA  CCGAG  ACGGAA CGAGAUCU   CAAGU    \
                    AGC CUU  GGCUC  UGCCUU GCUCUAGA   GUUCA    A
GCUUAU--------------^  U   GU      AU      A        ---     UUAA
.                    100        90        80           70
```

Reads in: lib-42, lib-45, lib-47, lib-48, lib-49, lib-50, lib-72
Number of reads="ID1047",14,15,7,0,0,0,0

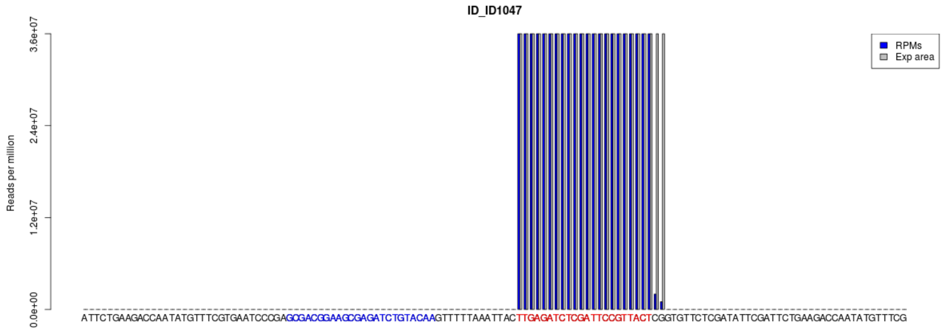

>ID1048
TGGTTGAGTGGTGGTAAGACCTACATCGAAGGTGGTGGTAGAGTAGTGCCAACACATTATTTAATTGCTG**TGTGGCCTGCCCTACCATCA**
CTCTCTGTGTATGGGCTAGGGA

>ID1048-3P
TGTGGCCTGCCCTACCATCACT

>miRNA precursor
UGGUUGAGUGGUGGUAAGACCUACAUCGAAGGUGGUGGUAGAGUAGUGCCAACACAUUAUUUAAUUGCUG**UGUGGCCUGCCCUACCAUCACU**CUCUGUGUAUGGGCUAGGGA
................((.(((((((.((..((((((((((.((((.((((.((((.((......)).))))))))))))).))))))))))..)).))))).)).)).....

>Predicted STAR seq:
GGTGGTGGTAGAGTAGTGCCAACA

Complementarity: 18

Initial dG =   -44.50

```
        10        20         30        40        50        60
UGGUUGAGUGGUGGUA  A  -|    C  AG          A    U    A    U  UU
                AG CC UACAU GA  GUGGUGGUAG GUAG GCCA CACA UA  U
                UC GG AUGUG CU  CACUACCAUC CGUC CGGU GUGU GU  A
AGGGA-----------  G  U^    U  CU          C    -    -    C  UA
110                 100        90        80          70
```

Reads in: lib-42, lib-45, lib-47, lib-48, lib-49, lib-50, lib-72
Number of reads="ID1048",1,0,0,0,0,0,0

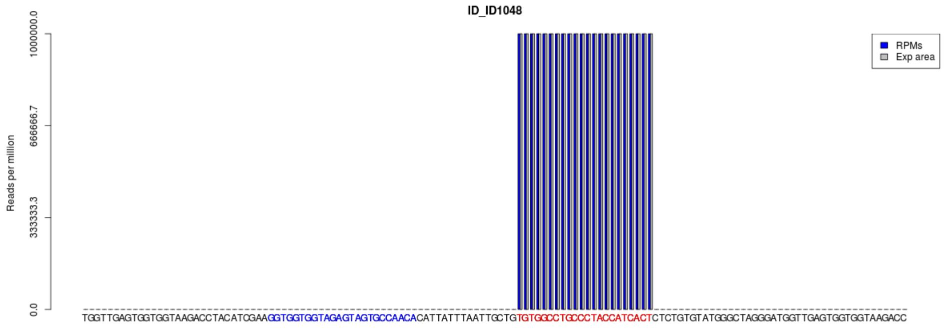

>ID1049
TGTTGTGTGCTGGTCAGCAG**TGGGGGCTGCTGCAGACGAGCAT**AGTGTTTTGAGGTGCGCGCCTGCAGCAGCCAGCCCGCTGCTGACCAT
CGAACAACCGTCCACTGCTGTC

>ID1049-5P
TGGGGGCTGCTGCAGACGAGCAT

>miRNA precursor
UGUUGUGUGCUGGUCAGCAG**UGGGGGCUGCUGCAGACGAGCAU**AGUGUUUUGAGGUGCGCGCCUGCAGCAGCCAGCCCGCUGCUGACCAUCGAACAACCGUCCACUGCUGUC
.(((((.((.((((((((((((((((((((((((((.((.((((..(.....)..)))).)).))))))))))))..)))))))))))))))).)).))))).............

>Predicted STAR seq:
GCGCGCCTGCAGCAGCCAGCCCGCT

Complementarity: 19

Initial dG = -66.00

```
                     10        20          30        40
U-------------     G  C              --|          A  A    AG GU
              GUUGU UG UGGUCAGCAGUGGG  GGCUGCUGCAG CG GCAU  U  \
              CAACA GC ACCAGUCGUCGCCC  CCGACGACGUC GC CGUG  G  U
CUGUCGUCACCUGC     A  U              GA^          C  G    GA UU
110       100        90        80        70        60        50
```

Reads in: lib-42, lib-45, lib-47, lib-48, lib-49, lib-50, lib-72
Number of reads="ID1049",2,3,0,1,0,0,0

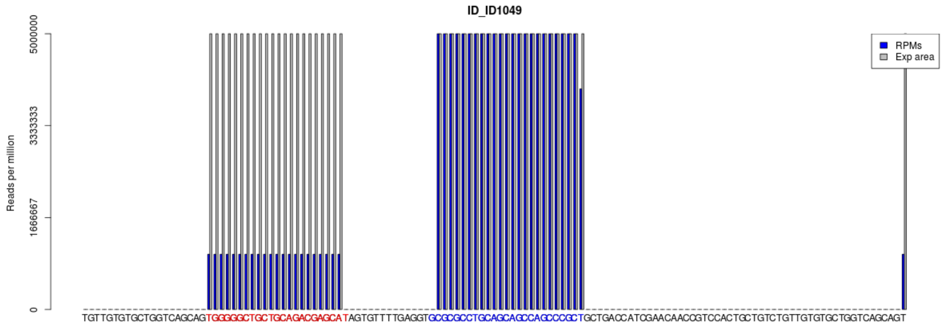

>ID1050
ATGTTGGAGTGAAAGTAAATCATGCATGGAGGG**TGATGGCTATGTCTACCAACA**CGTTATAAAATCACTGTTGAATAGACCCAGCCATCA
CTCACTGTGCATGGCAAGCATC

>ID1050-5P
TGATGGCTATGTCTACCAACA

>miRNA precursor
AUGUUGGAGUGAAAGUAAAUCAUGCAUGGAGGG**UGAUGGCUAUGUCUACCAACA**CGUUAUAAAAUCACUGUUGAAUAGACCCAGCCAUCACUCACUGUGCAUGGCAAGCAUC
..............((...((((((((((.(((((((((((..(((((.(((((.((.........)))))))..)))))..))))))))))).))))))))))...))...

>Predicted STAR seq:
TTGAATAGACCCAGCCATCACT

Complementarity: 16

Initial dG = -42.70

```
        10        20        30        40         50        60
AUGUUGGAGUGAAA  AAA          A           AU      C-|    C  UAUA
              GU   UCAUGCAUGG GGGUGAUGGCU  GUCUA  CAACA GU    \
              CG   GGUACGUGUC CUCACUACCGA  CAGAU  GUUGU CA    A
CUA-----------  AAC          A           CC      AA^    -  CUAA
110                  100        90        80        70
```

Reads in: lib-42, lib-45, lib-47, lib-48, lib-49, lib-50, lib-72
Number of reads="ID1050",2,5,0,0,0,0,0

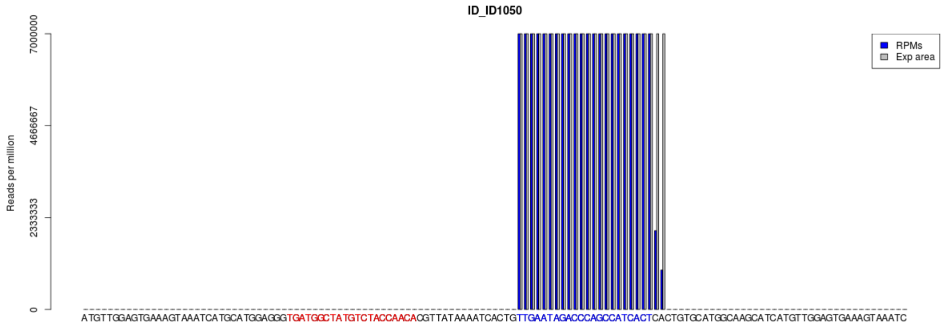

>ID1052
ACTAATTCATGTATAGGGTT**TGGCGACCGCATGAACACCACT**TATTATTTACTACAGTGGCGGTCACGCGGTCGTCAGATCCTGTGCATT
GTATACCACAGAATTGTGTCTG

>ID1052-5P
TGGCGACCGCATGAACACCACT

>miRNA precursor
ACUAAUUCAUGUAUAGGGUU**UGGCGACCGCAUGAACACCACU**UAUUAUUUACUACAGUGGCGGUCACGCGGUCGUCAGAUCCUGUGCAUUGUAUACCACAGAAUUGUGUCUG
........(((((((((((((((((((((.(((...(((((.............)))))...))).))))))))))))))))))))))).........((((......))))

>Predicted STAR seq:
TGGCGGTCACGCGGTCGTCAGA

Complementarity: 16

```
 Initial dG =    -48.20

          10        20        30        40
.-ACUAAUUC|                     A   ACA      UAUUAU
          AUGUAUAGGGUUUGGCGACCGC UGA   CCACU       \
          UACGUGUCCUAGACUGCUGGCG ACU   GGUGA       U
\ --------^                     C   GGC      CAUCAU
                  80        70        60        50



 90       100
  UGUAUACCA    AU
           CAGA  U
           GUCU  G
  ---------    GU
           110
```

Reads in: lib-42, lib-45, lib-47, lib-48, lib-49, lib-50, lib-72
Number of reads="ID1052",7,36,0,0,1,0,0

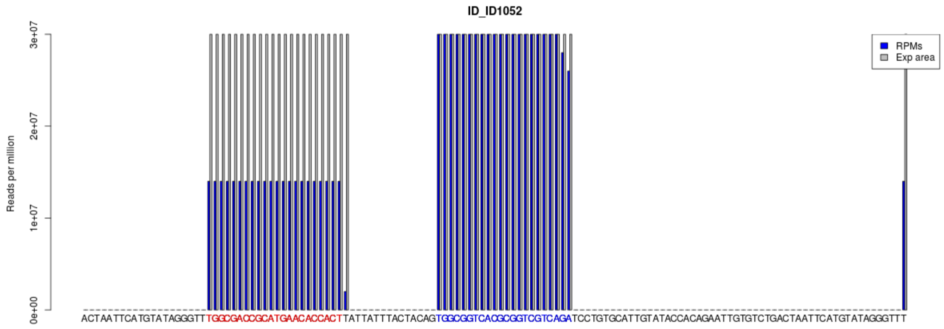

>ID1053
ATTCTGAAGACCAATATGTTTCGTGAATCTCGAGCGACGGAAGCGAGGTCTGTACAAGTTTTTAAATTAC**TTGAGATCTCGATTCCGTTA CT**CGGTGTTCTCGATATTCG

>ID1053-3P
TTGAGATCTCGATTCCGTTACT

>miRNA precursor
AUUCUGAAGACCAAUAUGUUUCGUGAAUCUCGAGCGACGGAAGCGAGGUCUGUACAAGUUUUUAAAUUAC**UUGAGAUCUCGAUUCCGUUACU**CGGUGUUCUCGAUAUUCG
...................(((.((((..((((..((((((.((((((((...(((((.........))))))))))))).))))))..)))).)))).))).......

>Predicted STAR seq:
GCGACGGAAGCGAGGTCTGTACAA

Complementarity: 17

Initial dG = -40.20

```
        10        20        30        40        50        60
AUUCUGAAGACCAAUAUGUU|  U    CU    CG      G        GUA     UUUU
                    UCG GAAU  CGAG  ACGGAA CGAGGUCU   CAAGU    \
                    AGC CUUG  GCUC  UGCCUU GCUCUAGA   GUUCA    A
GCUUAU--------------^  U    UG    AU      A        ---     UUAA
.                     100        90        80            70
```

Reads in: lib-42, lib-45, lib-47, lib-48, lib-49, lib-50, lib-72
Number of reads="ID1053",18,20,13,0,1,1,0

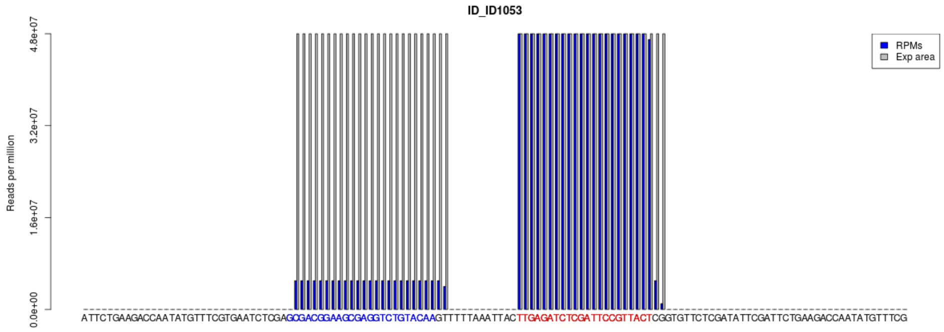

>ID1054

AATAGTTCATGCATGGAGGG**TTGTGGCTGTGTATGACAACA**CGTTATGAAATCAGTGTTGTACATACACAGCCATCACCCACTGTGCATG
GTAATCATCACCAGTTGGTCT

>ID1054-5P

TTGTGGCTGTGTATGACAACA

>miRNA precursor

AAUAGUUCAUGCAUGGAGGG**UUGUGGCUGUGUAUGACAACA**CGUUAUGAAAUCAGUGUUGUACAUACACAGCCAUCACCCACUGUGCAUGGUAAUCAUCACCAGUUGGUCU

```
......((((((((((.((((.(((((((((((((((((((((...........))))))).)))))))))))))).)))).))))))))))........(((....)))..
```

>Predicted STAR seq:

TTGTACATACACAGCCATCACC

Complementarity: 18

Initial dG =   -55.30

```
          10        20        30          40
.-AAUAGU          A     U              -|       GUUAU
        UCAUGCAUGG GGGU GUGGCUGUGUAUG ACAACAC      G
        GGUACGUGUC CCCA UACCGACACAUAC UGUUGUG      A
\ ------          A     C              A^       ACUAA
        90        80        70         60        50



        100
  UAAUCAUC   A
          ACC G
          UGG U
  UC------   U
 110
```

Reads in: lib-42, lib-45, lib-47, lib-48, lib-49, lib-50, lib-72

Number of reads="ID1054",1,17,0,0,0,0,0

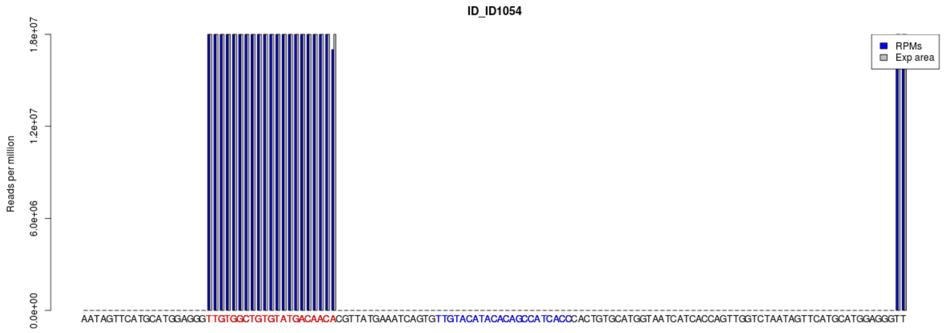

>ID1055

TGTTGCAGTGTCAGTAATTCATGCATGGAGGGTGATGTCTGTGTGGGTCCAACACATTATGAGATTACTG**TTGAACACACACGGCTATCA**
CTCACTGTGCCTGATAACCAT

>ID1055-3P

TTGAACACACACGGCTATCACT

>miRNA precursor

UGUUGCAGUGUCAGUAAUUCAUGCAUGGAGGGUGAUGUCUGUGUGGGUCCAACACAUUAUGAGAUUACUG**UUGAACACACACGGCUAUCACU**CACUGUGCCUGAUAACCAU
...((...((((((........((((((.((((((((.(((((((.((.(((((.............))))).)).))))))).)))))))).))))))))))))..)).

>Predicted STAR seq:

GTGATGTCTGTGTGGGTCCAA

Complementarity: 17

Initial dG =   -37.80

```
        10        20        30        40        50        60
UGU| CAG      UAAUUCAU      A        U       G  C     CAUUAU
   UG   UGUCAG        GCAUGG GGGUGAUG CUGUGUG GU CAACA      G
   AC   AUAGUC        CGUGUC CUCACUAU GGCACAC CA GUUGU      A
U--^ CA-      --------      A        C       A  A     CAUUAG
 110               100        90        80        70
```

Reads in: lib-42, lib-45, lib-47, lib-48, lib-49, lib-50, lib-72

Number of reads="ID1055",8,7,0,0,0,0,0

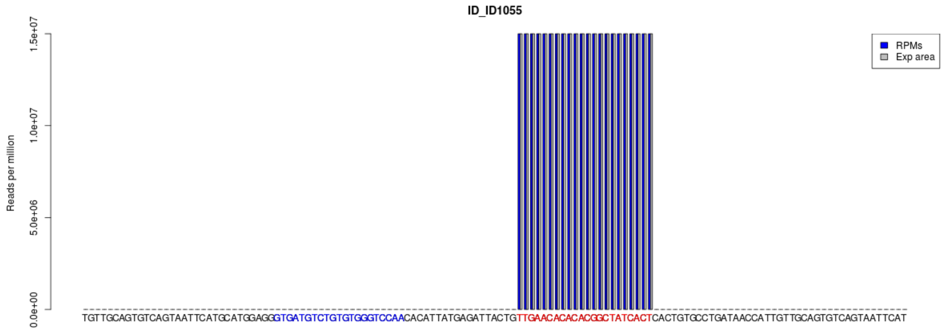

>ID1057
TCTAATTCGTGTATAGGGTT**CGGCGACTGCATGAACACCACT**TGTTATTTACTACAGTGGTAGTCACGCAGTCATCACATCCTGTGCATG
GTATCCCACGCAATTGTATCTG

>ID1057-5P
CGGCGACTGCATGAACACCACT

>miRNA precursor
UCUAAUUCGUGUAUAGGGUU**CGGCGACUGCAUGAACACCACU**UGUUAUUUACUACAGUGGUAGUCACGCAGUCAUCACAUCCUGUGCAUGGUAUCCCACGCAAUUGUAUCUG
......(((((((((((((..(..((((((.(((..((((((.((........)))))))).)))).))))))..)..)))))))))))))....................

>Predicted STAR seq:
TGGTAGTCACGCAGTCATCACAT

Complementarity: 14

Initial dG =   -40.80

```
                       10        20        30        40
UCUAAU---------------|            UC GC       A   AC       U  UAU
                     UCGUGUAUAGGGU  G  GACUGC UGA  ACCACU GU   U
                     GGUACGUGUCCUA  C  CUGACG ACU  UGGUGA CA   U
GUCUAUGUUAACGCACCCUAU^            CA UA       C   GA       -  UCA
110       100        90        80        70        60         50
```

Reads in: lib-42, lib-45, lib-47, lib-48, lib-49, lib-50, lib-72
Number of reads="ID1057",0,0,0,0,0,1,0

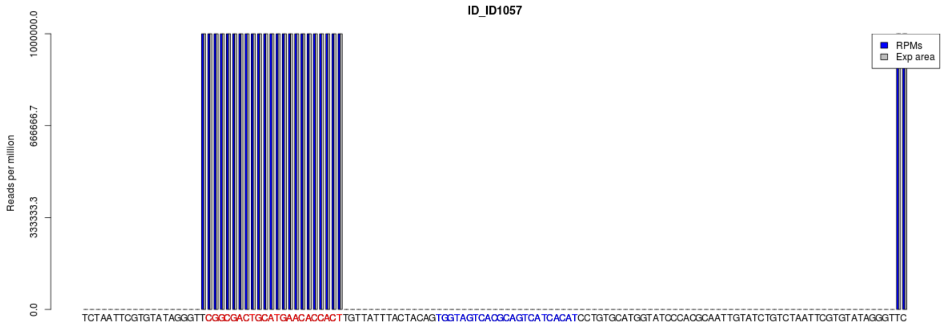

>ID1059

TGTTTCAGTGACAATAATTCATGCATGGAGGGTGCTGTGTGTGTGATGCCAACACGTTATAAAAATTCTG**TTGCATTTGCACACACATCA**
CCCACTTTGCATGACAAGCA

>ID1059-3P

TTGCATTTGCACACACATCACC

>miRNA precursor

UGUUUCAGUGACAAUAAUUCAUGCAUGGAGGGUGCUGUGUGUGUGAUGCCAACACGUUAUAAAAAUUCUG**UUGCAUUUGCACACACAUCACC**CACUUUGCAUGACAAGCA
((((.....)))).....(((((((.((.(((((.((((((((..((((.((((.(((.....)))..))))))).)..)))))))).))))).)).)))))))......

>Predicted STAR seq:

GTGCTGTGTGTGTGATGCCAA

Complementarity: 16
ERROR 4: Strange structure
Initial dG =   -43.30

```
  |   UC
--UGUU  \
  ACAG  A
\ ^   UG
    10



        20        30        40         50         60
  AUAAU-       U  A     C        UG -    C    C-   AU
        UCAUGCA GG GGGUG UGUGUGUG  A UGC AACA  GUU  \
        AGUACGU UC CCCAC ACACACAC  U ACG UUGU  UAA  A
  ACGAAC       U  A     U        GU U    -    CU   AA
110      100        90        80         70
```

Reads in: lib-42, lib-45, lib-47, lib-48, lib-49, lib-50, lib-72

Number of reads="ID1059",0,8,0,0,1,0,0

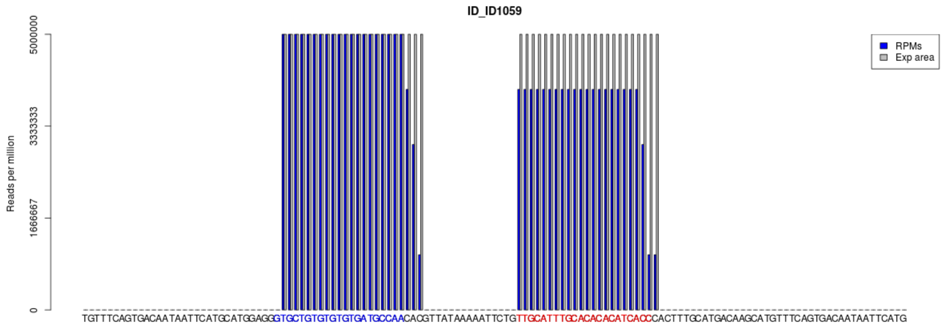

>ID106
CAGTGAAAATTCTCCCAAGGGACTTGCACAGGGACTGGTTTCAAGTGTGTGGTAAGATCATTTGAAATTC**TAGCACCATTTGAAATCAGTGC**TTTGGTAGGTCTTATCTT

>ID106-3P
TAGCACCATTTGAAATCAGTGC

>miRNA precursor
CAGUGAAAAUUCUCCCAAGGGACUUGCACAGGGACUGGUUUCAAGUGUGUGGUAAGAUCAUUUGAAAUUC**UAGCACCAUUUGAAAUCAGUGC**UUUGGUAGGUCUUAUCUU
```
..................(((((((((.(((((((((((((((((((.(((.((..((........))..)).))))))))))))))))))))).))))))))).....
```

>Predicted STAR seq:
ACTGGTTTCAAGTGTGTGGTA

Complementarity: 18

Initial dG = -39.90

```
        10        20        30         40        50        60
CAGUGAAAAUUCUCCCAA|        A     -              U   G  AG   CAU
                  GGGACUUGC CAGGG ACUGGUUUCAAGUG GUG UA  AU    U
                  UUCUGGAUG GUUUC UGACUAAAGUUUAC CAC AU  UA    U
UUCUA-------------^        -     G              -   G  CU   AAG
.                    100         90        80         70
```

Reads in: lib-42, lib-45, lib-47, lib-48, lib-49, lib-50, lib-72
Number of reads="ID106",338,655,74,2239,341,60,0

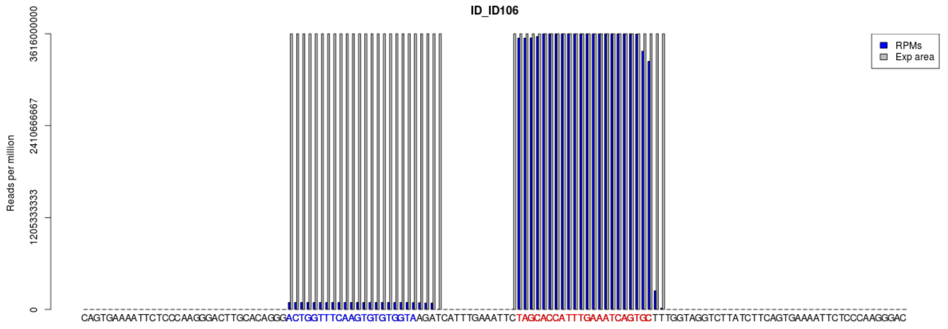

>ID1060
AGTAATACATGGATAGAGGG**TGATGTGCGTTTGATGGAAACAG**GTTATAAAGGTTCTGTTCCATCACTCTCACATCATCCACTTTACATG
ACAAGCAGCAGTCGCTGG

>ID1060-5P
TGATGTGCGTTTGATGGAAACAG

>miRNA precursor
AGUAAUACAUGGAUAGAGGG**UGAUGUGCGUUUGAUGGAAACAG**GUUAUAAAGGUUCUGUUCCAUCACUCUCACAUCAUCCACUUUACAUGACAAGCAGCAGUCGCUGG

```
.......((((...((.((((((((((....((((((((.((((..........))))))))))))....)))))))))).))...)))).....((((....)))).
```

>Predicted STAR seq:
GTTCCATCACTCTCACATCATC

Complementarity: 16

Initial dG =   -40.90

```
          10        20        30        40
.-AGUAAUA|   GAU  A          CGUU        A    UUAU
         CAUG   AG GGGUGAUGUG    UGAUGGAA CAGG    A
         GUAC   UC CCUACUACAC    ACUACCUU GUCU    A
\ -------^   AUU  A          UCUC        -    UGGA
        90        80        70        60         50


         100
  ACAAG    A
       CAGC G
       GUCG U
  G----    C
```

Reads in: lib-42, lib-45, lib-47, lib-48, lib-49, lib-50, lib-72
Number of reads="ID1060",1,1,0,0,0,0,0

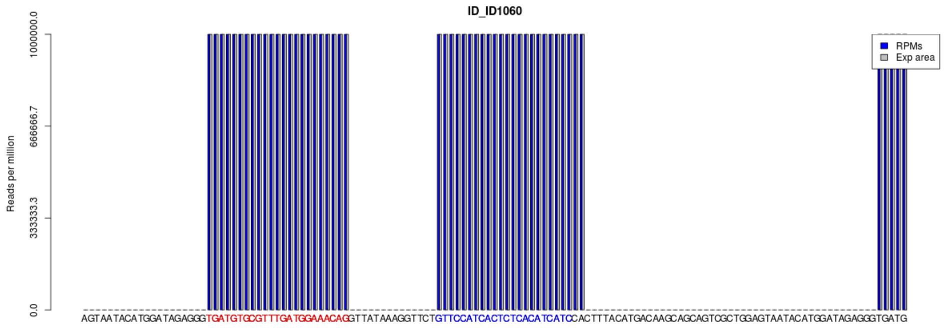

>ID1068
TTCATTACCACATCGCTTTTCGTTAGATGTCACCCTTAACCATTCAGCGTCCCGGAGTGGAGGGTGCACG**AGATGCCGAGTGGTTAAGGC**
GACATCTGCGTTCCAGTC

>ID1068-3P
AGATGCCGAGTGGTTAAGGCGA

>miRNA precursor
UUCAUUACCACAUCGCUUUUCGUUAGAUGUCACCCUUAACCAUUCAGCGUCCCGGAGUGGAGGGUGCACG**AGAUGCCGAGUGGUUAAGGCGA**CAUCUGCGUUCCAGUC
..............(((...(((.(((((((..(((((((((((((.(((((.((..((.......)).)).))))).))))))))))))).))))))))))....))).

>Predicted STAR seq:
CACCCTTAACCATTCAGCGTC

Complementarity: 17

Initial dG =   -41.30

```
        10         20          30         40         50         60
UUCAUUACCACAUC   UUU-|  U       AC            A      C  GA   GGA
              GCU    CGU AGAUGUC  CCUUAACCAUUC GCGUC CG  GU   \
              UGA    GCG UCUACAG  GGAAUUGGUGAG CGUAG GC  CG   G
C-------------   CCUU^  -       C-            C      A  A-   UGG
                     100         90          80         70
```

Reads in: lib-42, lib-45, lib-47, lib-48, lib-49, lib-50, lib-72
Number of reads="ID1068",63,204,123,1001,201,64,0

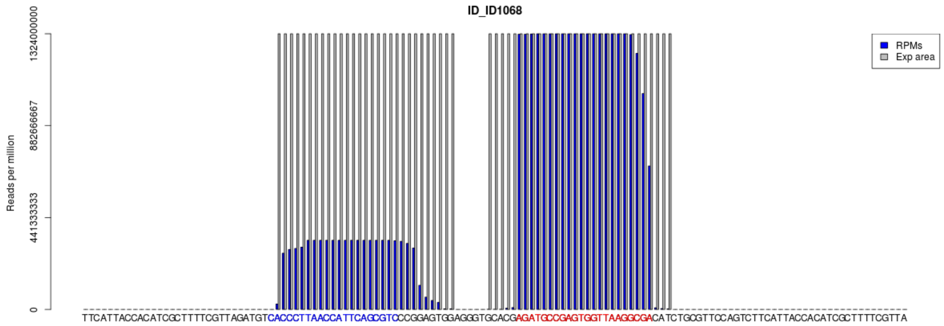

>ID1069
GTTACCGGCGTGTCCCGTTC**CTAAGTACTAGTGCCGCAGGAG**TGTGACCTCTAGGCCTCCTGCTGCTCAAGTGCTTATCAACGGGAGGGC
CCACACACGCGACCAGTGC

>ID1069-5P
CTAAGTACTAGTGCCGCAGGAG

>miRNA precursor
GUUACCGGCGUGUCCCGUUC**CUAAGUACUAGUGCCGCAGGAG**UGUGACCUCUAGGCCUCCUGCUGCUCAAGUGCUUAUCAACGGGAGGGCCCACACACGCGACCAGUGC
..(((.(((((((((((((..((((((((...((.(((((((.((.........))))))))).))...))))))))..))))))).........)))).)).))).

>Predicted STAR seq:
CCTGCTGCTCAAGTGCTTATCAA

Complementarity: 15

Initial dG =   -43.10

```
         10                    20        30        40
GU   C   -    -----------|      CC        AGU  C       U  GACC
  UAC GG CGUG           UCCCGUU  UAAGUACU   GC GCAGGAG GU    \
  GUG CC GCGC           AGGGCAA  AUUCGUGA   CG CGUCCUC CG    U
C-   A  A    ACACACCCGGG^      CU        ACU  U       -  GAUC
        100        90        80        70        60
```

Reads in: lib-42, lib-45, lib-47, lib-48, lib-49, lib-50, lib-72
Number of reads="ID1069",1121,3379,1616,29015,3289,787,0

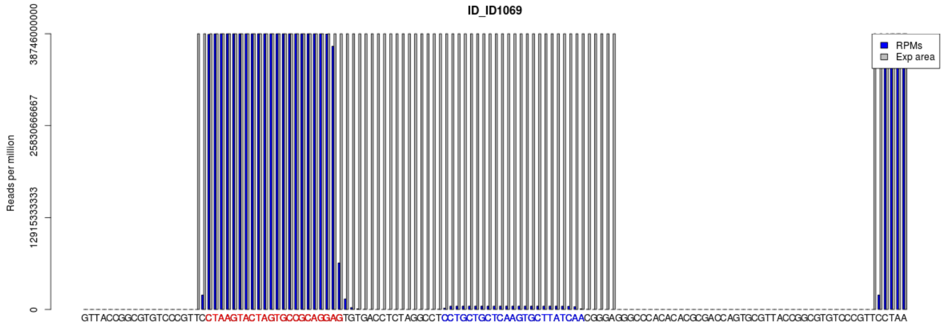

>ID107
GCTGATTGGGAGCTGACAGG**TAGTCTCAGGCTGGAGCAACGGT**GACAGACAAAAACAGTTGCTGAGCCCGAGGTCTACCTTGTCAGCTTC
ATCACGAACATCGCCTGCATG

>ID107-5P
TAGTCTCAGGCTGGAGCAACGGT

>miRNA precursor
GCUGAUUGGGAGCUGACAGG**UAGUCUCAGGCUGGAGCAACGGU**GACAGACAAAAACAGUUGCUGAGCCCGAGGUCUACCUUGUCAGCUUCAUCACGAACAUCGCCUGCAUG
((((.((((((((((((((((((((((.((((..((((((.((...........)).)))))).)))).)))).))))).))))))))))....))).))..)).......

>Predicted STAR seq:
AGTTGCTGAGCCCGAGGTCTACC

Complementarity: 17

Initial dG =   -46.30

```
             10         20         30        40
---  .-UGAUUG         -       -|   A    GG       G  GACAG
   GC        GGAGCUGAC AGGUAG UCUC GGCU  AGCAAC GU     \
   CG        CUUCGACUG UCCAUC GGAG CCGA  UCGUUG CA     A
GUA  \ ------         U      U^   C    G-       A  AAAAC
 .          90        80        70         60        50



       AUCA  AA
           CG  \
           GC  C
       UCC-  UA
            100
```

Reads in: lib-42, lib-45, lib-47, lib-48, lib-49, lib-50, lib-72
Number of reads="ID107",0,11,0,318,6,1,0

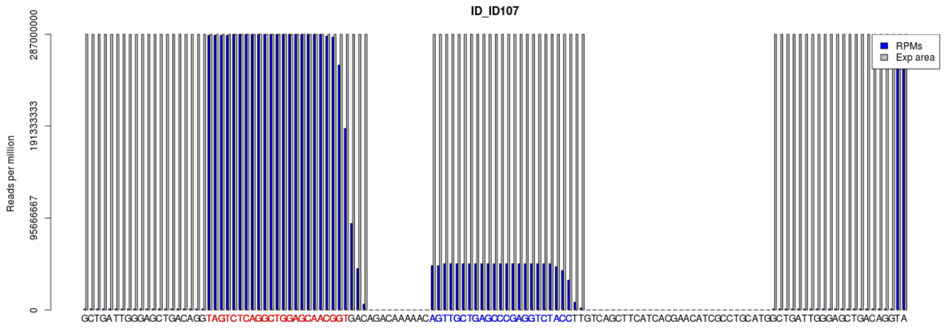

>ID1074
AACTATGTTGCACGCTTCGTTTTAGAATTTGTACGCTTTGTTGTAAAATATTTACAATCACAAAGCATATGGATTCTTGAGCGAATCGTT
CAATACTTTCATCTCCAACGC

>ID1074-5P
TTTTAGAATTTGTACGCTTTGT

>miRNA precursor
AACUAUGUUGCACGCUUCGUUUUAGAAUUUGUACGCUUUGUUGUAAAAUAUUUACAAUCACAAAGCAUAUGGAUUCUUGAGCGAAUCGUUCAAUACUUUCAUCUCCAACGC
.....((((.(((.(((((((.((((((..((.(((((((((((((...))))))...))))))).))..)))))).))))))).))).)))))...............

>Predicted STAR seq:
AAAGCATATGGATTCTTGAGCG

Complementarity: 16

Initial dG =   -35.60

```
                   10        20        30        40
AACUA-----------     C   C       U      UG  C       ---|     A
                UGUUG ACG UUCGUUU AGAAUU  UA GCUUUGU   UGUAAA \
                AUAAC UGC AAGCGAG UCUUAG  AU CGAAACA   ACAUUU U
CGCAACCUCUACUUUC     U   U       U      GU  A       CUA^     A
 .       100       90        80        70        60        50
```

Reads in: lib-42, lib-45, lib-47, lib-48, lib-49, lib-50, lib-72
Number of reads="ID1074",35,164,59,1759,207,11,0

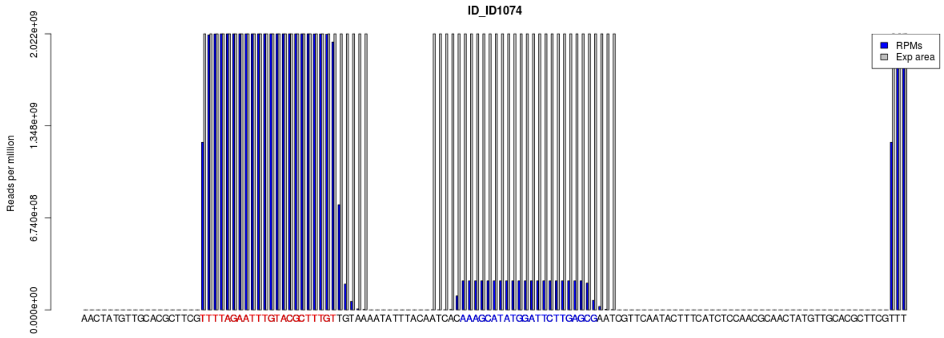

>ID1082
CAGAGTGCTGCCTCCCTCCGCCG**CACGGCCCACTCGCTGGAGCGC**TCTCAGTTTACTCGTTGACGGCCGCCCTCCACGAATTGGGCCGCG
CGGTGCAGGAGGGCCCCCTGCC

>ID1082-5P
CACGGCCCACTCGCTGGAGCGC

>miRNA precursor
CAGAGUGCUGCCUCCCUCCGCCG**CACGGCCCACUCGCUGGAGCGC**UCUCAGUUUACUCGUUGACGGCCGCCCUCCACGAAUUGGGCCGCGCGGUGCAGGAGGGCCCCCUGCC
.........(((..(((.((((((.(((((((.(((.(((((.(((.((((........)))).))).....))))))))..))))))).)))))).)))..)))........

>Predicted STAR seq:
CGCCCTCCACGAATTGGGCCGCGCG

Complementarity: 16

Initial dG = -54.40

```
        10         20         30          40            50
CAGAGUGCU   UC    C       A       C-    C      C---|  C    UUU
         GCC  CCU CGCCGC CGGCCCA  UCG UGGAG     GCU UCAG   A
         CGG  GGA GUGGCG GCCGGGU  AGC ACCUC     CGG AGUU   C
CCGUCCCC-   GA    C       C       UA    -      CCGC^  C    GCU
110        100        90         80          70         60
```

Reads in: lib-42, lib-45, lib-47, lib-48, lib-49, lib-50, lib-72
Number of reads="ID1082",3,7,0,43,21,1,0

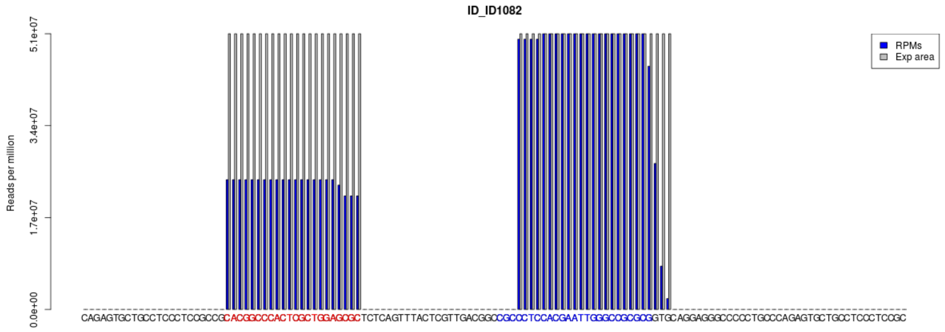

>ID1086
TACTAGTGAGCACTTACTAT**GCAGAACTCAGACATTTACTAG**GCAGCTACGAAGACAGAAATAGTGACTGCCTGAGTTCTAACTAGTAAT
TGCCCTAACTCCGAACGCG

>ID1086-5P
GCAGAACTCAGACATTTACTAG

>miRNA precursor
UACUAGUGAGCACUUACUAU**GCAGAACUCAGACAUUUACUAG**GCAGCUACGAAGACAGAAAUAGUGACUGCCUGAGUUCUAACUAGUAAUUGCCCUAACUCCGAACGCG
```
....(((..(((.((((((...(((((((((.((.((((((.....((........))...)))))).)).)))))))))...)))))).))).....))).........
```

>Predicted STAR seq:
GTGACTGCCTGAGTTCTAACTA

Complementarity: 15

Initial dG =   -28.90

```
               10        20        30        40        50
UACU-----   GA--|  C      UGC        A  U      GGCAG  ACG
         AGU    GCA UUACUA   AGAACUCAG CA UUACUA     CU   A
         UCA    CGU AAUGAU   UCUUGAGUC GU AGUGAU     GA   A
GCGCAAGCC   AUCC^  U      CAA        C  C      AAA--  CAG
       100        90        80        70        60
```

Reads in: lib-42, lib-45, lib-47, lib-48, lib-49, lib-50, lib-72
Number of reads="ID1086",25,39,16,925,18,10,0

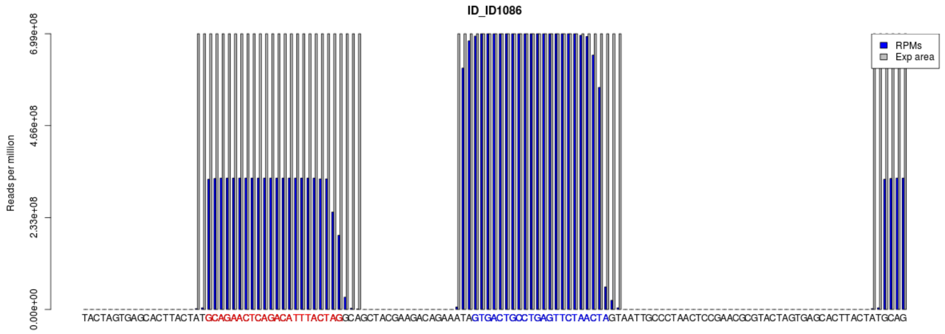

>ID1088
CTAAGGTTTCTGTCAGTATAAATGCATCACTGTTGTTGGAGGTGTCTCTTGCACATGTTTGATAGTAATG**TTTAAGAGGCACTTCTGACG**
ACTGTTGTGCATTATTGCAT

>ID1088-3P
TTTAAGAGGCACTTCTGACGACT

>miRNA precursor
CUAAGGUUUCUGUCAGUAUAAAUGCAUCACUGUUGUUGGAGGUGUCUCUUGCACAUGUUUGAUAGUAAUG**UUUAAGAGGCACUUCUGACGACU**GUUGUGCAUUAUUGCAU
...............(((..(((((((.((.(((((..(((((((((((((.((((...........)))).)))))))))))))..))))).)).)))))))..)))..

>Predicted STAR seq:
GTTGTTGGAGGTGTCTCTTGC

Complementarity: 17

Initial dG =   -44.80

```
        10        20        30        40        50        60
CUAAGGUUUCUGUCA|  UA       C  U     UG            C    GUUUG
               GUA  AAUGCAU AC GUUGU  GAGGUGUCUCUUG ACAU     \
               CGU  UUACGUG UG CAGCA  CUUCACGGAGAAU UGUA     A
UA-------------^  UA       U  U     GU            U    AUGAU
.                    100        90        80        70
```

Reads in: lib-42, lib-45, lib-47, lib-48, lib-49, lib-50, lib-72
Number of reads="ID1088",1,19,0,1,0,0,0

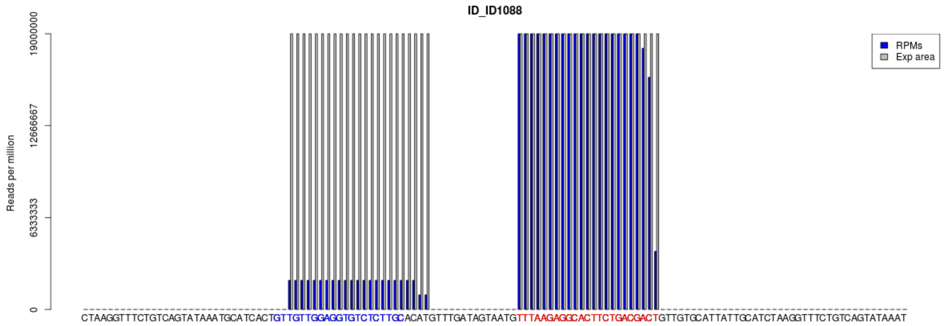

>ID1089
TCAAGTTTTCAGTCTGTACAAATGCATCGCTGTTGTTGGAAGTGTCTTTTTCACACGTTTCAAGGCAGTG**TTGATGAGGCACTTCTAACG**
ATTGTTGTGCATTATTTCA

>ID1089-3P
TTGATGAGGCACTTCTAACGAT

>miRNA precursor
UCAAGUUUUCAGUCUGUACAAAUGCAUCGCUGUUGUUGGAAGUGUCUUUUUCACACGUUUCAAGGCAGUG**UUGAUGAGGCACUUCUAACGAU**UGUUGUGCAUUAUUUCA
...................((((((.((.(((((((((((((((((..(((((((((....))).)))).)).)))))))))))))))))).)).)))))))......

>Predicted STAR seq:
GTTGTTGGAAGTGTCTTTTTCA

Complementarity: 18

Initial dG = -39.70

```
        10        20        30        40        50          60
UCAAGUUUUCAGUCUGUACA       C  U                 UU  -    -|  U
                    AAUGCAU GC GUUGUUGGAAGUGUCUU  UC ACAC GUU C
                    UUACGUG UG UAGCAAUCUUCACGGAG  AG UGUG CGG A
ACUUUA--------------       U  U                 U-  U    A^  A
                     100        90        80         70
```

Reads in: lib-42, lib-45, lib-47, lib-48, lib-49, lib-50, lib-72
Number of reads="ID1089",1,4,0,0,0,0,0

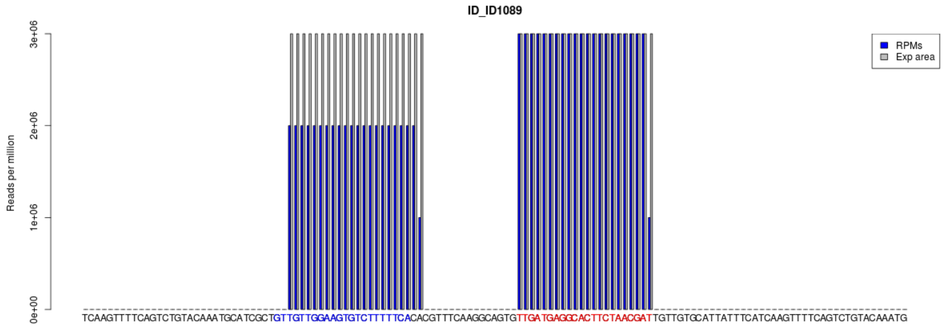

>ID1093
CATTTTGGGCGCCGTGGCCGGCGACAGGGGAGGCTGTC**ACTCGCACAGTGCGTGCGGG**GCCGGCAGGTCCTCGCACACACTGTGCGAGCG
GAAAAGCCTCTGGGGGCATCC

>ID1093-5P
ACTCGCACAGTGCGTGCGGG

>miRNA precursor
CAUUUUGGGCGCCGUGGCCGGCGACAGGGGAGGCUGUC**ACUCGCACAGUGCGUGCGGG**GCCGGCAGGUCCUCGCACACACUGUGCGAGCGGAAAAGCCUCUGGGGGCAUCC
...((((..(((((....))))).)))).((((((.((.(((((((((((.((((((((((....))))).))))).))))))))))).))...)))))).(((....)))

>Predicted STAR seq:
TCGCACACACTGTGCGAGCGG

Complementarity: 16

```
 Initial dG =    -65.10

          10
ERROR 3: Strange structure
 Initial dG =    -65.10

          10
.-CAU|   GG     U
     UUUG  CGCCG G
     GGAC  GCGGC G
\ ---^   A-     C
             20


    30          40        50         60
  .-G      G--  A           C     -     G
     GAGGCU   UC CUCGCACAGUG GUGCG GGGCC G
     CUCCGA   GG GAGCGUGUCAC CACGC CCUGG C
  \ -      AAA  C           A     U     A
   100        90        80        70
```

Reads in: lib-42, lib-45, lib-47, lib-48, lib-49, lib-50, lib-72
Number of reads="ID1093",1,3,1,51,19,0,0

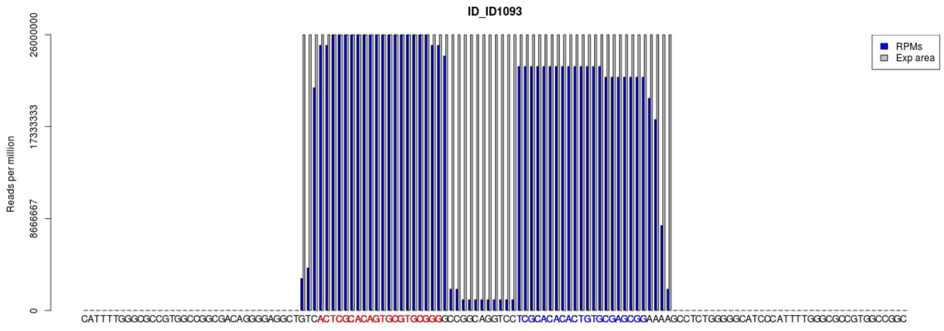

>ID1095

TGCTGATGGACTTTACCTGA**CGGGTGAAGCTATAGCGATTATG**ATAATTACGAAACCATAAGCGTATAGCTTTTCCCCTTGGGTTCAGTT
CTTTATACATCATTGCTGTCAGG

>ID1095-5P

CGGGTGAAGCTATAGCGATTATG

>miRNA precursor

UGCUGAUGGACUUUACCUGA**CGGGUGAAGCUAUAGCGAUUAUG**AUAAUUACGAAACCAUAAGCGUAUAGCUUUUCCCCUUGGGUUCAGUUCUUUAUACAUCAUUGCUGUCAGG
..((((((((((..(((..(.(((.(((((((((.((.(((((............))))).))))))))))).))).)..)))..)))))......((....))..))))).

>Predicted STAR seq:

TAAGCGTATAGCTTTTCCCCTT

Complementarity: 17

Initial dG =   -36.20

```
          10        20        30         40
UG       |    UU   UG C   U         G  A     AUAAUU
  CUGAU--GGACU  ACC  A GGG GAAGCUAUA CG UUAUG      \
  GACUG  CUUGA  UGG  U CCC UUUCGAUAU GC AAUAC      A
G-     \ ^    CU   GU C   U         -  G     CAAAGC
  110    90        80        70         60



               100
         UUUAUA  U
               CA C
               GU A
         UC----  U
```

Reads in: lib-42, lib-45, lib-47, lib-48, lib-49, lib-50, lib-72

Number of reads="ID1095",296,337,267,489,756,257,0

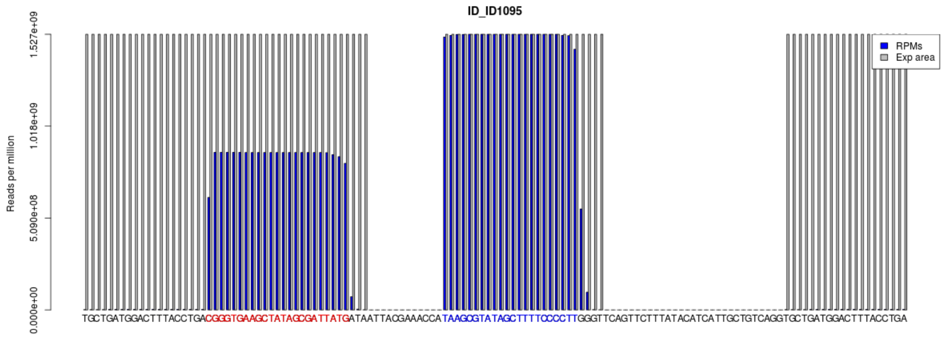

>ID1110
AGAATCCCAGTGCCTCCACACTGCTTCGCAAGGTGTGCTTTCTGTGCACGCTAGTCTATATCTATATCAG**CGTACACAGAAAGTATCACC**
ATCTGCGACAGTGACTGCCAA

>ID1110-3P
CGTACACAGAAAGTATCACCATC

>miRNA precursor
AGAAUCCCAGUGCCUCCACACUGCUUCGCAAGGUGUGCUUUCUGUGCACGCUAGUCUAUAUCUAUAUCAG**CGUACACAGAAAGUAUCACCAUC**UGCGACAGUGACUGCCAA
.......((((.......(((((..(((((.((((((((((((((.(((((.((.((....)).)).))))).))))))))))).)))).)))...)))))))))))))....

>Predicted STAR seq:
GGTGTGCTTTCTGTGCACG

Complementarity: 16

Initial dG =   -41.40

```
        10        20        30          40        50        60
AGAAUCC|   GCCUCCA     CU     A--    -          C     A  C  U
       CAGU       CACUG  UCGCA   GGUG UGCUUUCUGUG ACGCU GU UA A
       GUCA       GUGAC  AGCGU   CCAC AUGAAAGACAC UGCGA UA AU U
AACC---^   -------     --     CUA    U          A     C  U  C
 .                 100          90        80        70
```

Reads in: lib-42, lib-45, lib-47, lib-48, lib-49, lib-50, lib-72
Number of reads="ID1110",30,22,3,992,125,2,0

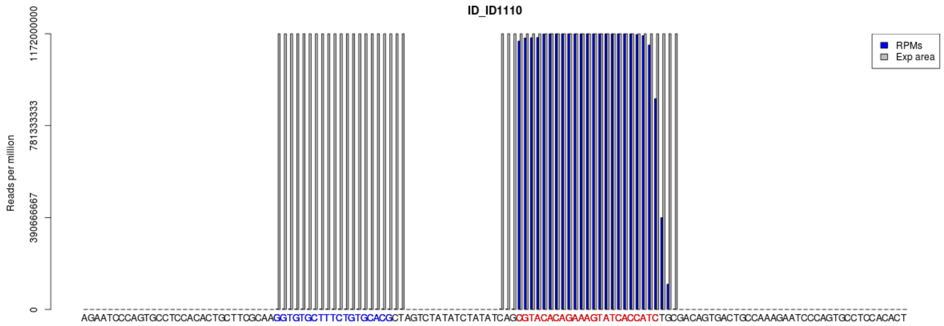

>ID1111
AGAATCCCAGAGCCTCTACACTGCTTCGGATGGTGTACTTCACATGCACGCTAGTCCATATCTATATCAG**CGTACCTGTAAAGTAACACC**
ATCTGCGACAGTGACTGCCA

>ID1111-3P
CGTACCTGTAAAGTAACACCATC

>miRNA precursor
AGAAUCCCAGAGCCUCUACACUGCUUCGGAUGGUGUACUUCACAUGCACGCUAGUCCAUAUCUAUAUCAG**CGUACCUGUAAAGUAACACCAUC**UGCGACAGUGACUGCCA
```
.................(((((...((((((((((((((.(((...(((((................)))))...))).))))).)))))))))...))))).......
```

>Predicted STAR seq:
ATGGTGTACTTCACATGCACG

Complementarity: 16

Initial dG =   -32.30

```
        10        20        30         40        50        60
AGAAUCCCAGAGCCUCUA     CUU         -|     C   UGC      AGUCCAU
                  CACUG   CGGAUGGUG UACUU ACA   ACGCU       A
                  GUGAC   GUCUACCAC AUGAA UGU   UGCGA       U
ACCGUCA-----------     AGC         A^     A   CCA      CUAUAUC
.                  100       90        80        70
```

Reads in: lib-42, lib-45, lib-47, lib-48, lib-49, lib-50, lib-72
Number of reads="ID1111",131,61,2,3960,101,3,0

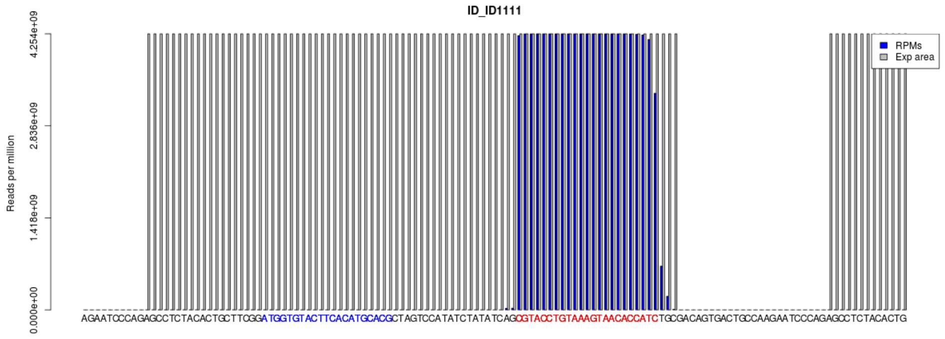

>ID1112

AGAATCCCAGGGCCTCTACACTGCTTCGGATGGTGTACTTTACGTGCTCGCTAGTACATATCTATATCAG**CATACCTGTAAAGTATCACC**
ATCTGAGGCAGTGGCTGCCAAC

>ID1112-3P

CATACCTGTAAAGTATCACCATCT

>miRNA precursor

```
AGAAUCCCAGGGCCUCUACACUGCUUCGGAUGGUGUACUUUACGUGCUCGCUAGUACAUAUCUAUAUCAGCAUACCUGUAAAGUAUCACCAUCUGAGGCAGUGGCUGCCAAC
..........(((.....((((((((((((((((((((((((((((((...(((.......)))....)))))....)))))))).)))))))))))))))))...)))...
```

>Predicted STAR seq:

CTGCTTCGGATGGTGTAC

Complementarity: 17

Initial dG = -49.00

```
        10        20        30         40            50
AGAAUCCCAG   CUCUA                 -        ----     CGC-|  UAC
          GGC     CACUGCUUCGGAUGGUG UACUUUAC    GUGCU    UAG   \
          CCG     GUGACGGAGUCUACCAC AUGAAAUG    UACGA    AUC   A
CAA-------   UCG--                 U        UCCA     CUAU^  UAU
110              100        90        80        70        60
```

Reads in: lib-42, lib-45, lib-47, lib-48, lib-49, lib-50, lib-72

Number of reads="ID1112",9,0,0,126,10,0,0

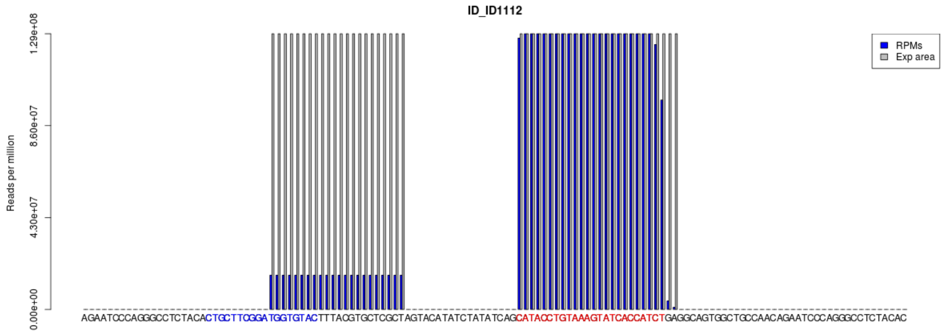

>ID1114
AGAATCCCAGTGCCTCTACACTGCTTCGGATGGTGTACTTCACATGCACGCTAGTCCATATCTATATCAG**CGTACCTGTAAAGTAACACC**
ATCTGCGACAGTGACTGCCA

>ID1114-3P
CGTACCTGTAAAGTAACACCATC

>miRNA precursor
AGAAUCCCAGUGCCUCUACACUGCUUCGGAUGGUGUACUUCACAUGCACGCUAGUCCAUAUCUAUAUCAG**CGUACCUGUAAAGUAACACCAUC**UGCGACAGUGACUGCCA
```
.......((((.......(((((...(((((((((((((.(((...(((((................)))))...))).)))).))))))))))...)))))))))...
```

>Predicted STAR seq:
ATGGTGTACTTCACATGCACG

Complementarity: 16

Initial dG =   -32.70

```
        10        20        30         40        50        60
AGAAUCC|   GCCUCUA     CUU          -    C   UGC     AGUCCAU
       CAGU       CACUG   CGGAUGGUGU ACUU ACA   ACGCU       A
       GUCA       GUGAC   GUCUACCACA UGAA UGU   UGCGA       U
ACC----^   -------     AGC          A    A   CCA     CUAUAUC
.                  100       90        80        70
```

Reads in: lib-42, lib-45, lib-47, lib-48, lib-49, lib-50, lib-72
Number of reads="ID1114",98,55,2,4015,86,2,0

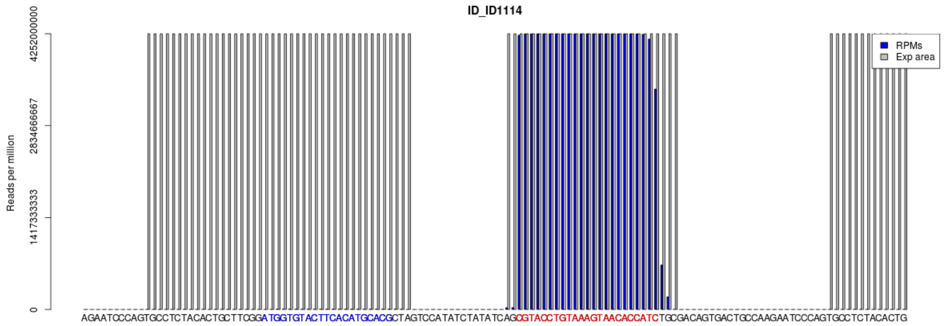

>ID1115
AGAATCCCAGGGCCTCTACACTGCTTCGGATGGTGTACTTTACGTGCTCGCTAGTACATATCTATATCAG**CATACCTGTAAAGTATCACC**
ATCTGAGGCAGTGGCTGCCAAC

>ID1115-3P
CATACCTGTAAAGTATCACCATCT

>miRNA precursor
AGAAUCCCAGGGCCUCUACACUGCUUCGGAUGGUGUACUUUACGUGCUCGCUAGUACAUAUCUAUAUCAG**CAUACCUGUAAAGUAUCACCAUCU**GAGGCAGUGGCUGCCAAC
..........(((.....((((((((((((((((((((((((((((((...(((.......)))....)))))....)))))))).))))))))))))))))))...)))...

>Predicted STAR seq:
CTGCTTCGGATGGTGTAC

Complementarity: 17

Initial dG =   -49.00

```
        10        20        30         40            50
AGAAUCCCAG   CUCUA                 -        ----     CGC-|  UAC
          GGC     CACUGCUUCGGAUGGUG UACUUUAC    GUGCU    UAG   \
          CCG     GUGACGGAGUCUACCAC AUGAAAUG    UACGA    AUC   A
CAA-------   UCG--                 U        UCCA     CUAU^  UAU
110                100        90        80        70        60
```

Reads in: lib-42, lib-45, lib-47, lib-48, lib-49, lib-50, lib-72
Number of reads="ID1115",4,2,0,114,10,0,0

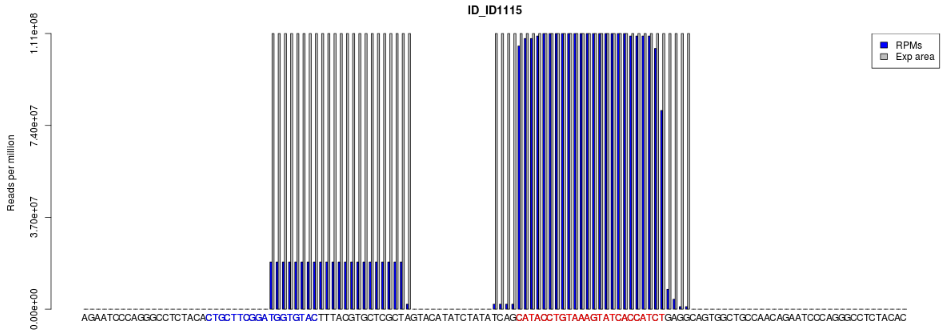

>ID1117
AGAATCCCAGAGCCTCTACACTGCTTCGGATGGTGTACTTCACATGCACGCTAGTCCATATCTATATCAG**CGTACCTGTAAAGTAACACC**
ATCTGCGACAGTGACTGCCA

>ID1117-3P
CGTACCTGTAAAGTAACACCATC

>miRNA precursor

```
AGAAUCCCAGAGCCUCUACACUGCUUCGGAUGGUGUACUUCACAUGCACGCUAGUCCAUAUCUAUAUCAGCGUACCUGUAAAGUAACACCAUCUGCGACAGUGACUGCCA
.................(((((...((((((((((((((.(((...(((((................)))))...))).))))).)))))))))...))))).......
```

>Predicted STAR seq:
ATGGTGTACTTCACATGCACG

Complementarity: 16

Initial dG =   -32.30

```
        10        20        30         40        50        60
AGAAUCCCAGAGCCUCUA     CUU         -|    C   UGC     AGUCCAU
                  CACUG   CGGAUGGUG UACUU ACA   ACGCU       A
                  GUGAC   GUCUACCAC AUGAA UGU   UGCGA       U
ACCGUCA-----------     AGC         A^    A   CCA     CUAUAUC
.                  100        90        80        70
```

Reads in: lib-42, lib-45, lib-47, lib-48, lib-49, lib-50, lib-72
Number of reads="ID1117",100,62,6,4044,92,0,0

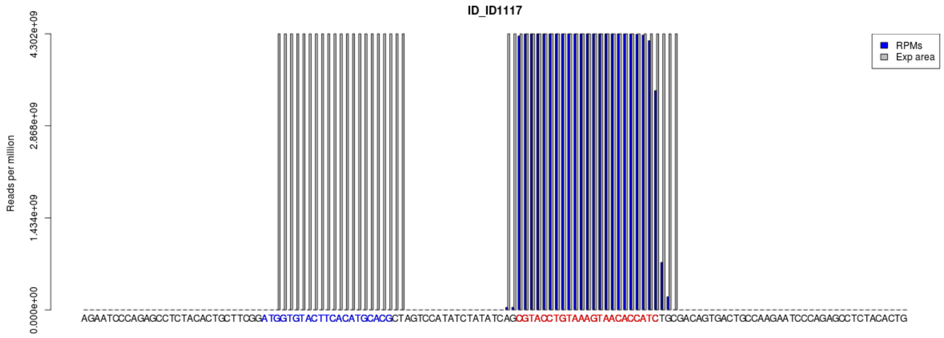

>ID1118
AGAATCCCAGTGCCTCCACACTGCTTCGGAAGGTGTGCTTTCTGTGCACGCTAGTCTATATCTATATCAG**CGTACACAGAAAGTATCACC**
ATCTGCGACAGTGACTGCCAA

>ID1118-3P
CGTACACAGAAAGTATCACCATC

>miRNA precursor
AGAAUCCCAGUGCCUCCACACUGCUUCGGAAGGUGUGCUUUCUGUGCACGCUAGUCUAUAUCUAUAUCAG**CGUACACAGAAAGUAUCACCAUC**UGCGACAGUGACUGCCAA
.......((((.......(((((...((((.(((((((((((((((.(((((.((.((....)).)).))))).))))))))))).)))).))))...)))))))))....

>Predicted STAR seq:
AAGGTGTGCTTTCTGTGCACG

Complementarity: 18

Initial dG =   -40.60

```
        10        20        30         40        50        60
AGAAUCC|   GCCUCCA     CUU    A    -           C     A  C  U
       CAGU       CACUG   CGGA GGUG UGCUUUCUGUG ACGCU GU UA A
       GUCA       GUGAC   GUCU CCAC AUGAAAGACAC UGCGA UA AU U
AACC---^   -------     AGC    A    U           A     C  U  C
 .                 100        90        80        70
```

Reads in: lib-42, lib-45, lib-47, lib-48, lib-49, lib-50, lib-72
Number of reads="ID1118",27,22,1,961,123,1,0

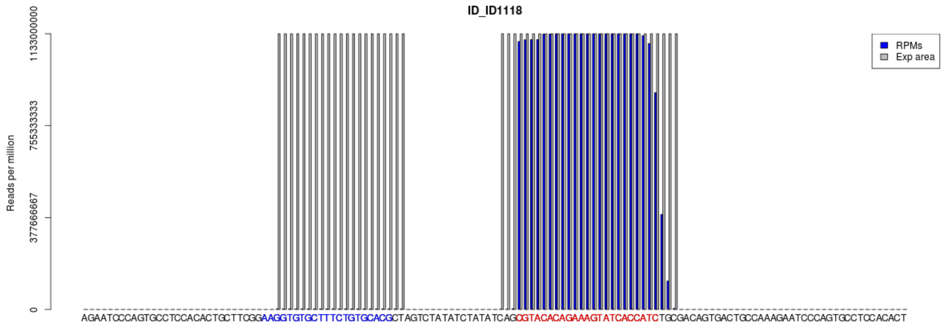

>ID1119
CTGGACATTGGTGCGTCCCTCACATGAGCCGGGTTTCGCGATGAGCGAGCCGTACACGTTGTTATCGTGG**TTCGTTGTCGACGAAACCTG**
CACGATGTGAGGGCGTTCG

>ID1119-3P
TTCGTTGTCGACGAAACCTGC

>miRNA precursor
CUGGACAUUGGUGCGUCCCUCACAUGAGCCGGGUUUCGCGAUGAGCGAGCCGUACACGUUGUUAUCGUGG**UUCGUUGUCGACGAAACCUGC**ACGAUGUGAGGGCGUUCG
.........((.(((.((((((((((.((.((((((((((((.((((((((....(((.......))))))))))))))).))))))))))).).)))))))))))))).

>Predicted STAR seq:
GCCGGGTTTCGCGATGAGCGAG

Complementarity: 18

Initial dG = -49.50

```
        10        20         30         40        50        60
CUGGACAUU| U   U          - A  C         -    G         GUAC   UUG
         GG GCG CCCUCACAU G GC GGGUUUCG CGAU AGCGAGCC    ACG   \
         CU UGC GGGAGUGUA C CG UCCAAAGC GCUG UUGCUUGG    UGC   U
G--------^ -   -          G A  -         A    -         ----   UAU
                 100        90         80         70
```

Reads in: lib-42, lib-45, lib-47, lib-48, lib-49, lib-50, lib-72
Number of reads="ID1119",70,78,132,3193,2143,71,0

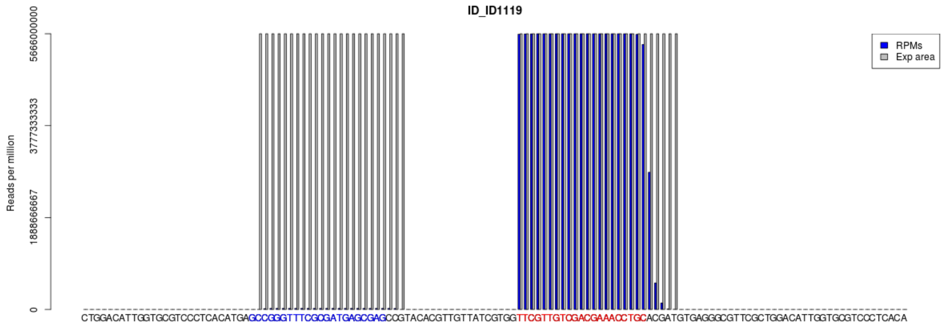

>ID113
TTTTGGAACACATATTATTCTCACACGAAGGGTGACGCATGTGTCGCGGGTACACGTTATTTAATTTGTG**TCCCTGAGACCCATACGTTACT**CATTGTGCGAGTTGCACATC

>ID113-3P
TCCCTGAGACCCATACGTTACT

>miRNA precursor
UUUUGGAACACAUAUUAUUCUCACACGAAGGGUGACGCAUGUGUCGCGGGUACACGUUAUUUAAUUUGUG**UCCCUGAGACCCAUACGUUACU**CAUUGUGCGAGUUGCACAUC
..................(((.(((((.((((((((.(((.(((.((((.(((((..........))))).)))).))).))).)))))))).))))).)))..........

>Predicted STAR seq:
GTGACGCATGTGTCGCGGGT

Complementarity: 16

Initial dG = -38.70

```
        10         20         30         40         50         60
UUUUGGAACACAUAUUAUU|  A     A        C   U   G    U     UUAU
                   CUC CACGA GGGUGACG AUG GUC CGGG ACACG    U
                   GAG GUGUU CUCAUUGC UAC CAG GUCC UGUGU    U
CUACACGUU----------^  C     A        A   C   A    C     UUAA
110                 100        90         80         70
```

Reads in: lib-42, lib-45, lib-47, lib-48, lib-49, lib-50, lib-72
Number of reads="ID113",1,17,0,0,0,0,0

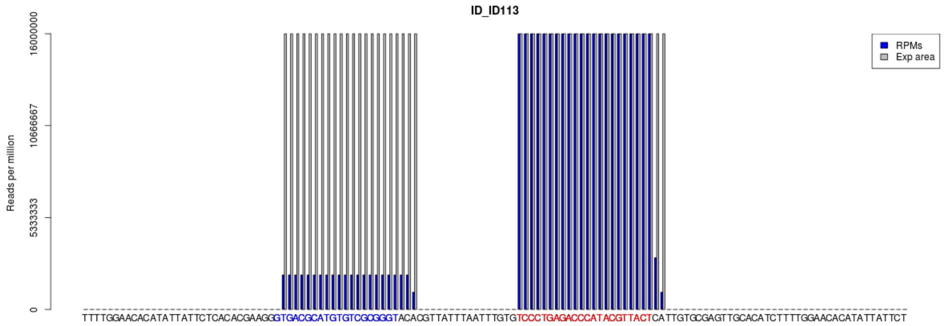

>ID1131
GGAGTGTGTCCGGCGTGGGGTCGGGCGATGCGTCCGGACGATCGTCCTCGCCGGCTATGTGTTGGATGGG**TCGGTGGGACTTTCGTCCGT**
TTGCATCGCCTAAGTGGTTC

>ID1131-3P
TCGGTGGGACTTTCGTCCGTTT

>miRNA precursor
GGAGUGUGUCCGGCGUGGGGUCGGGCGAUGCGUCCGGACGAUCGUCCUCGCCGGCUAUGUGUUGGAUGGG**UCGGUGGGACUUUCGUCCGUUU**GCAUCGCCUAAGUGGUUC
```
..........((((.....))))(((((((((..(((((((..((((.((((((((............))))))))))))..))))))).)))))))))..........
```

>Predicted STAR seq:
TCCGGACGAUCGUCCUCGCCGG

Complementarity: 16
ERROR 4: Strange structure
Initial dG =   -55.40

```
  |  GU
--GGA  \
  CCU  G
\ ^  GU
  10


         20        30        40        50        60
  GGCGUGGGGUC          UC        UC     U         AUGUG
             GGGCGAUGCG  CGGACGA  GUCC CGCCGGCU     U
             UCCGCUACGU  GCCUGCU  CAGG GUGGCUGG     U
  CUUGGUGAA--          UU        UU     -         GUAGG
110         100        90        80          70
```

Reads in: lib-42, lib-45, lib-47, lib-48, lib-49, lib-50, lib-72
Number of reads="ID1131",2073,10817,31902,8586,21636,11228,0

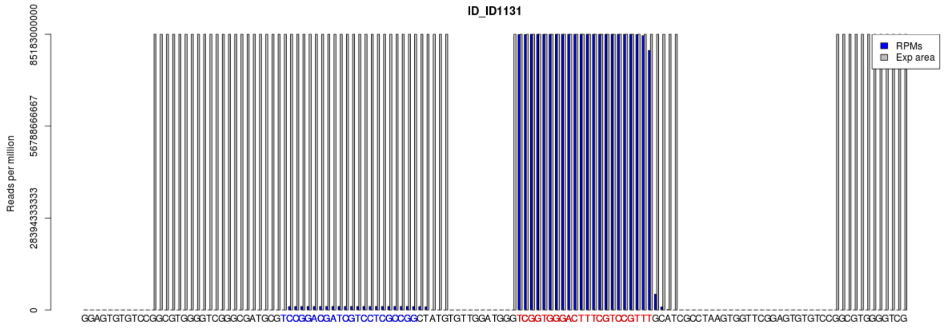

>ID1132
AATGACCGTTATATTTTGGCTTACAACCACTCCGCAATCATCAGGTGGTAATAATGAAATTCTATCCGTA**TCACCTGACGATGGCGGAAC**
GATTATTCGCCGAAACCTCGTG

>ID1132-3P
TCACCTGACGATGGCGGAACGA

>miRNA precursor
AAUGACCGUUAUAUUUUGGCUUACAACCACUCCGCAAUCAUCAGGUGGUAAUAAUGAAAUUCUAUCCGUA**UCACCUGACGAUGGCGGAACGA**UUAUUCGCCGAAACCUCGUG
............((((((..........(((((.(((.((((((((((...(((......)))...)))))))))).))).)))))..........)))))).......

>Predicted STAR seq:
TACAACCACTCCGCAATCATCAGGTGG

Complementarity: 14

Initial dG =   -33.60

```
        10        20        30        40        50
AAUGACCGUUAUA|      UUACAACCAC     A   A          AUA   AA
             UUUUGGC          UCCGC AUC UCAGGUGGUA   AUG  A
             AAAGCCG          AGGCG UAG AGUCCACUAU   UAU  U
GUGCUCC------^      CUUAUUAGCA     G   C          GCC   CU
110             100        90        80        70        60
```

Reads in: lib-42, lib-45, lib-47, lib-48, lib-49, lib-50, lib-72
Number of reads="ID1132",3,7,0,1,1,3,0

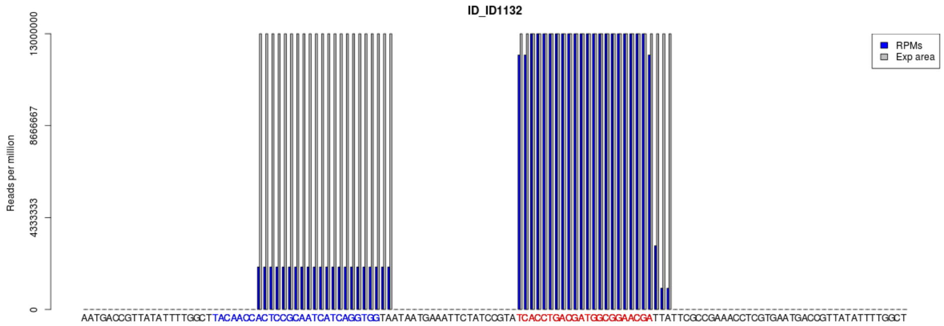

>ID1134

GCAAACCAGACACTTGCAGACGAATTCGCTAGGAGGGAGGTGACCGGGTGCAGCGTGGAAATAGCAACCT**GTACCCGGTCACCTCCTTCC**
TGACGTATTCTGACCTCGCAAC

>ID1134-3P

GTACCCGGTCACCTCCTTCCTGA

>miRNA precursor

```
GCAAACCAGACACUUGCAGACGAAUUCGCUAGGAGGGAGGUGACCGGGUGCAGCGUGGAAAUAGCAACCUGUACCCGGUCACCUCCUUCCUGACGUAUUCUGACCUCGCAAC
.............((((((..((((.((.(((((((((((((((((((((((((.((..........))))))))))))))))))))))))))))).)).)))))....)).)))).
```

>Predicted STAR seq:

TAGGAGGGAGGTGACCGGGTGC

Complementarity: 20

Initial dG =   -58.80

```
        10         20          30         40         50         60
GCAAACCAGACAC     -  AC--|   U  C                       C   GGAA
             UUGC AG    GAAU CG UAGGAGGGAGGUGACCGGGUGCAG GU    A
             AACG UC    CUUA GC GUCCUUCCUCCACUGGCCCAUGUC CA    U
C------------    C  CAGU^   U  A                        -  ACGA
            110       100        90         80         70
```

Reads in: lib-42, lib-45, lib-47, lib-48, lib-49, lib-50, lib-72

Number of reads="ID1134",222,840,1008,411,1261,455,0

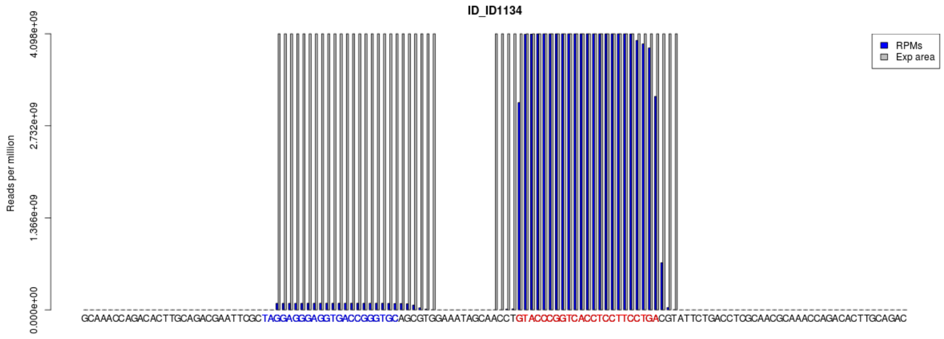

>ID114

TTGTGAAACTAATGGTATTCTCGCACGAAGGGTGACGTATGTGTCGACAGTACACGTTCCTTAATTTGTG**TACTTGAGGCACATTCGTTA**
CTCGTCGTGAGAGGTTAACATC

>ID114-3P

TACTTGAGGCACATTCGTTACT

>miRNA precursor

UUGUGAAACUAAUGGUAUUCUCGCACGAAGGGUGACGUAUGUGUCGACAGUACACGUUCCUUAAUUUGUG**UACUUGAGGCACAUUCGUUACU**CGUCGUGAGAGGUUAACAUC
...........(((....((((.(((((.((((((((.(((((((...((((((((..........))))))))...))))))).)))))))).))))).))))....))).

>Predicted STAR seq:

GTGACGTATGTGTCGACAGTA

Complementarity: 16

Initial dG = -45.60

```
        10        20        30        40        50        60
UUGUGAAACUA|  GUAU    G     A        U       GAC        UUCC
           AUG    UCUC CACGA GGGUGACG AUGUGUC   AGUACACG    U
           UAC    GGAG GUGCU CUCAUUGC UACACGG   UCAUGUGU    U
C----------^  AAUU    A     G        U       AGU        UUAA
          110       100        90        80        70
```

Reads in: lib-42, lib-45, lib-47, lib-48, lib-49, lib-50, lib-72

Number of reads="ID114",0,9,0,2,0,0,0

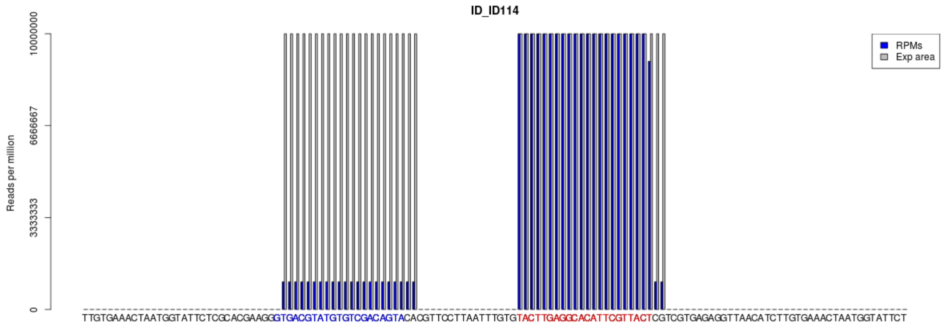

>ID1141
ACTTACCGTCGGCCAGTGCG**TTAAGTAGTAGCGCCAGAGTGG**CGACTGGGCGCTACTGTGGCGTTGCTGCTTATCTCATTGGTCGACAAA
CTCATCGCGGTGGACTTC

>ID1141-5P
TTAAGTAGTAGCGCCAGAGTGG

>miRNA precursor
ACUUACCGUCGGCCAGUGCG**UUAAGUAGUAGCGCCAGAGUGG**CGACUGGGCGCUACUGUGGCGUUGCUGCUUAUCUCAUUGGUCGACAAACUCAUCGCGGUGGACUUC
```
.......((((((((((...(((((((((((((((.(((((((......))))))).)))))))))))))))...)))))))))))....((((....)))).....
```

>Predicted STAR seq:
ACTGTGGCGTTGCTGCTTATCTCA

Complementarity: 18

Initial dG =   -59.20

```
          10        20        30        40
.-ACUUACC|          CGU               G       AC
         GUCGGCCAGUG   UUAAGUAGUAGCGCCA AGUGGCG  U
         CAGCUGGUUAC   AUUCGUCGUUGCGGU UCAUCGC  G
\ -------^          UCU               G       GG
               80        70        60        50



   90
  AAAC-    C
      UCAU G
      GGUG C
  CUUCA    G
       100
```

Reads in: lib-42, lib-45, lib-47, lib-48, lib-49, lib-50, lib-72
Number of reads="ID1141",9195,37,10,30,3,1,0

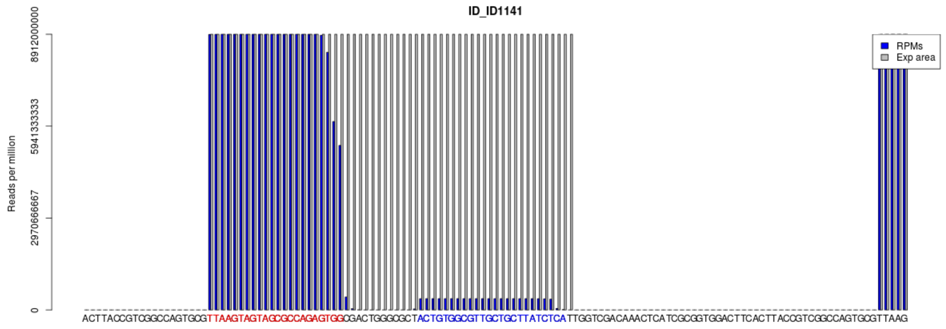

>ID1142
ACAACGGCAGAGAAGACGTATTCAGGTCAAGCCAGTGTCACTGTCGGGACAGTCACCATTTTTCTGAATG**ACTATTCTGTTAGTGACACT**
GATTCCACCTGCAATAACTCCTC

>ID1142-3P
ACTATTCTGTTAGTGACACTGAT

>miRNA precursor
ACAACGGCAGAGAAGACGUAUUCAGGUCAAGCCAGUGUCACUGUCGGGACAGUCACCAUUUUUCUGAAUG**ACUAUUCUGUUAGUGACACUGAU**UCCACCUGCAAUAACUCCUC
.........(((.((...(((((((((..(..(((((((((((.(((((.(((((.((......))..))))).))))).)))))))))))..)..))))).)))).)).)))

>Predicted STAR seq:
CCAGTGTCACTGTCGGGACAGT

Complementarity: 17

Initial dG =   -37.90

```
        10        20         30        40        50         60
ACAACGGCA|  A  ACG    -     CA GC           U     C     C-  UU
         GAG AG   UAUU CAGGU  A  CAGUGUCACUG CGGGA AGUCA  CA  U
         CUC UC   AUAA GUCCA  U  GUCACAGUGAU GUCUU UCAGU  GU  U
---------^  C  A--    C     CC UA           U     A     AA  CU
          110         100        90        80        70
```

Reads in: lib-42, lib-45, lib-47, lib-48, lib-49, lib-50, lib-72
Number of reads="ID1142",3504,4077,16755,1034,42563,9787,0

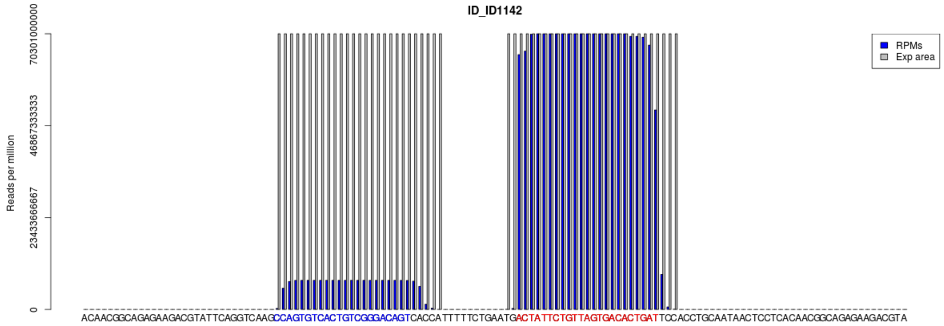

>ID1149

TTGCTTTGAATTATATTTTT**TGGAACACGAGGTAGATTTGTC**TGAGTTATACTGAATGAGACAGCTCTACTTCGTGCTCCAGAAAATATG
AGAAACCATCATCACATATGGA

>ID1149-5P

TGGAACACGAGGTAGATTTGTC

>miRNA precursor

UUGCUUUGAAUUAUAUUUUU**UGGAACACGAGGUAGAUUUGUC**UGAGUUAUACUGAAUGAGACAGCUCUACUUCGUGCUCCAGAAAAUAUGAGAAACCAUCAUCACAUAUGGA
..........((((((((((((((.(((((((((((.((((((..(((......))).)))))).))))))))))).))))))))))))))....((((........)))).

>Predicted STAR seq:

CAGCTCTACTTCGTGCTCCAGA

Complementarity: 18

Initial dG = -42.90

```
          10        20        30        40        50
.-UUGCUUUGAA|             A           U      GA   AU
            UUAUAUUUUUUGGA CACGAGGUAGA UUGUCU  GUU  A
            AGUAUAAAAGACCU GUGCUUCAUCU GACAGA  UAA  C
\ ----------^             C           C      G-   GU
            90        80        70        60



        100
  GAAA    CAU
      CCAU   C
      GGUA   A
  A---    UAC
     110
```

Reads in: lib-42, lib-45, lib-47, lib-48, lib-49, lib-50, lib-72

Number of reads="ID1149",3,21,8,5,8,7,0

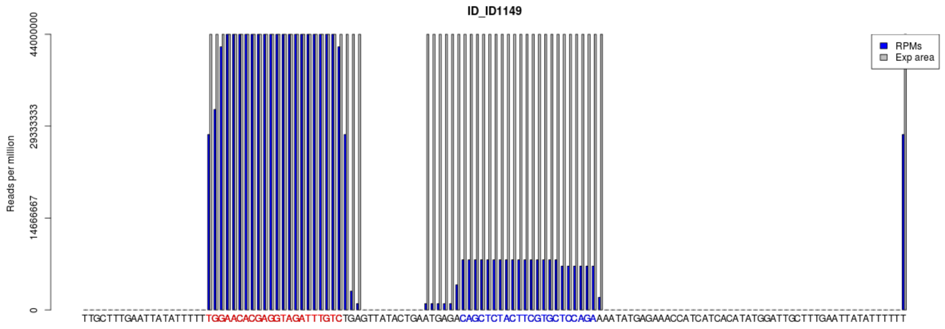

>ID115
ATGTGGGACAAATGTCATGTTCGCATCACGGCTGATGTATGTGTCAGGGGTACATGTTTTTTCAATTGTG**TTACTCTGGTACATACGTTA**
CTCGTTGTGCGTGTAAAAAGA

>ID115-3P
TTACTCTGGTACATACGTTACT

>miRNA precursor
AUGUGGGACAAAUGUCAUGUUCGCAUCACGGCUGAUGUAUGUGUCAGGGGUACAUGUUUUUUCAAUUGUG**UUACUCUGGUACAUACGUUACU**CGUUGUGCGUGUAAAAAGA
```
......(((....))).((..(((((.(((..((((((((((..(((((..(((((..........)))))..)))))..))))))))))..))).)))))..))......
```

>Predicted STAR seq:
CTGATGTATGTGTCAGGGG

Complementarity: 14
ERROR 4: Strange structure
Initial dG =   -38.30

```
          10
.-AUGUGG|  A
        GAC A
        CUG A
\ ------^  U




         20        30        40        50        60
  A-----  UU      C   GC          GU      GU      UUUU
        UG  CGCAU ACG  UGAUGUAUGU  CAGGG  ACAUG    U
        AU  GCGUG UGC  AUUGCAUACA  GUCUC  UGUGU    U
  AGAAAA  GU      U   UC          UG      AU      UAAC
 110       100        90         80        70
```

Reads in: lib-42, lib-45, lib-47, lib-48, lib-49, lib-50, lib-72
Number of reads="ID115",2,14,1,0,0,1,0

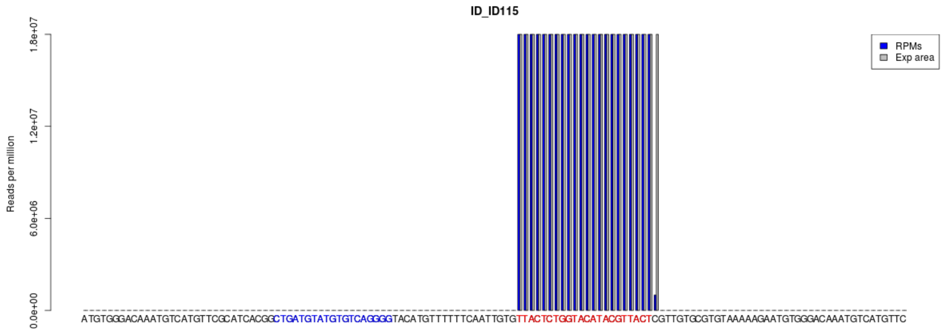

>ID1153
ACAGAAGATCAAGGCTAATGGTCAGGGGTAGGGAGAGCTGGGTACAGAAAAATGTTTTATTCTAGTCATT**TTACGTGCTCAGCTTTCCTT**
GCTTCTTTCCATAGATTGTG

>ID1153-3P
TTACGTGCTCAGCTTTCCTTG

>miRNA precursor
ACAGAAGAUCAAGGCUAAUGGUCAGGGGUAGGGAGAGCUGGGUACAGAAAAAUGUUUUAUUCUAGUCAUU**UUACGUGCUCAGCUUUCCUUG**CUUCUUUCCAUAGAUUGUG
.........(((..(((.(((..(((((((((((((((((((((...((((((.((......)).))))))..))))))))))))))))))))))..)))))).)))..

>Predicted STAR seq:
AGGGAGAGCTGGGTACAGAAA

Complementarity: 17

Initial dG =   -44.30

```
        10         20         30         40         50         60
ACAGAAGAU|  GG   A   UC                      AGA      U  UA
         CAA  CUA UGG  AGGGGUAGGGAGAGCUGGGUAC   AAAAUG UU  U
         GUU  GAU ACC  UCUUCGUUCCUUUCGACUCGUG   UUUUAC GA  U
GU-------^  A-   -   UU                      CA-      U  UC
.                100        90         80         70
```

Reads in: lib-42, lib-45, lib-47, lib-48, lib-49, lib-50, lib-72
Number of reads="ID1153",0,0,0,0,0,0,0

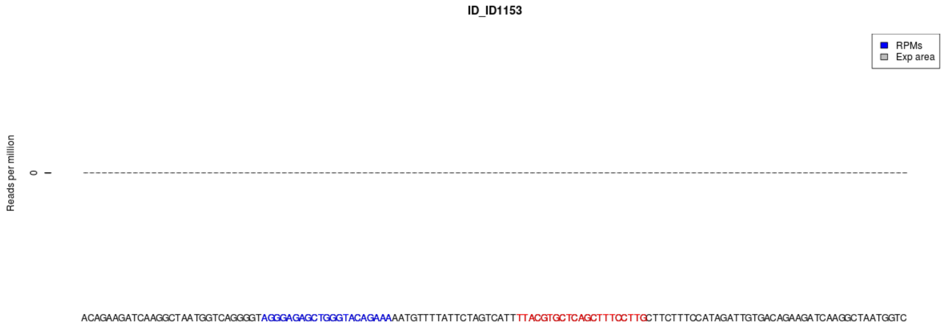

>ID1154
CATCAAGATAGACAAGGTAATGGTTAGTGGCAGGGGTGCAAAGATTCATGAAGTGTTATATTCTAGCGAC**TTCGTGAGTTTTTGCCTCTC**
TAGCTGCTTTCCATATTGTCAGC

>ID1154-3P
TTCGTGAGTTTTTGCCTCTCTAG

>miRNA precursor
CAUCAAGAUAGACAAGGUAAUGGUUAGUGGCAGGGGUGCAAAGAUUCAUGAAGUGUUAUAUUCUAGCGAC**UUCGUGAGUUUUUGCCUCUCUAG**CUGCUUUCCAUAUUGUCAGC
..........(((((....((((..((..(((((((.((((((((((((((((((((((.....)))).))))))))))))))))).))))).))..))..)))).)))))...

>Predicted STAR seq:
AGGGGTGCAAAGATTCATGAA

Complementarity: 19

Initial dG =   -48.80

```
        10        20        30         40        50         60
CAUCAAGAUA|    GGUA    UU  UG  -     U                 -    UA
          GACAA    AUGG  AG  GC AGGGG GCAAAGAUUCAUGAAGU GUUA  \
          CUGUU    UACC  UC  CG UCUCU CGUUUUUGAGUGCUUCA CGAU  U
CGA-------^    A---    UU  GU  A     C                 G    CU
        110          100        90        80        70
```

Reads in: lib-42, lib-45, lib-47, lib-48, lib-49, lib-50, lib-72
Number of reads="ID1154",0,4,0,0,0,0,0

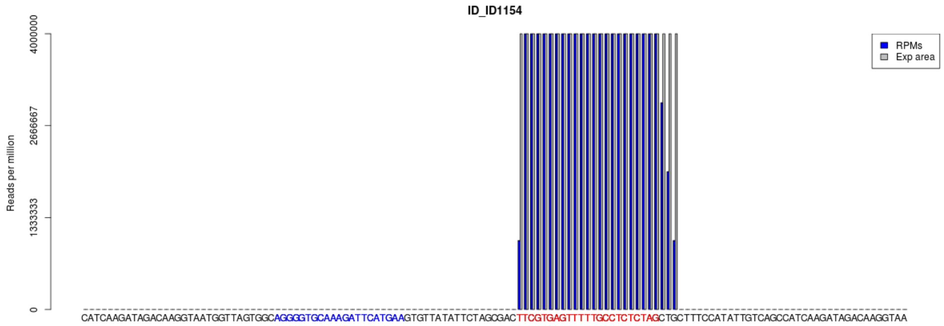

>ID1156
AGAAGATCAAATTATAATGGTCAGAGGTAGGGGAAGCTAGGAGACACATAAGTGTTTTATTCTAGGCATT**TAATGTCCTTAGCTTCTCTTGC**TTCTTTCCATTGACTGTCC

>ID1156-3P
TAATGTCCTTAGCTTCTCTTGC

>miRNA precursor
AGAAGAUCAAAUUAUAAUGGUCAGAGGUAGGGGAAGCUAGGAGACACAUAAGUGUUUUAUUCUAGGCAUU**UAAUGUCCUUAGCUUCUCUUGC**UUCUUUCCAUUGACUGUCC
```
.............((((((..(((((((((((((((((((.((((..(((((((((......))))))))).)))))))))))))))))))))))..))))))).......
```

>Predicted STAR seq:
TAGGGGAAGCTAGGAGACACATA

Complementarity: 19

Initial dG =   -47.70

```
        10        20        30        40        50        60
AGAAGAUCAAAUUA|     UC                  A    CA         UA
              UAAUGG  AGAGGUAGGGGAAGCUAGG GACA  UAAGUGUUU  U
              GUUACC  UCUUCGUUCUCUUCGAUUC CUGU  AUUUACGGA  U
CCUGUCA-------^     UU                  -    A-         UC
 .              100        90        80          70
```

Reads in: lib-42, lib-45, lib-47, lib-48, lib-49, lib-50, lib-72
Number of reads="ID1156",0,3,0,1,0,0,0

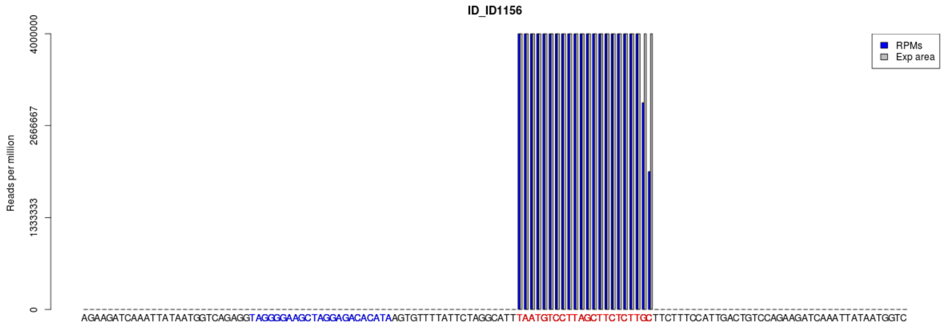

>ID116
ACGTGGAACAAATGGTATGTTGGCACCACGATTGATGTATGTGTTGGGGGAACATATTGTTTCATTTGTG**TTCCTGTGGTACATACGTTA**
CTCGTTGTGCGAGTATAAAG

>ID116-3P
TTCCTGTGGTACATACGTTACT

>miRNA precursor
ACGUGGAACAAAUGGUAUGUUGGCACCACGAUUGAUGUAUGUGUUGGGGGAACAUAUUGUUUCAUUUGUG**UUCCUGUGGUACAUACGUUACU**CGUUGUGCGAGUAUAAAG
.(((.......))).((((((.((((.((((.(((((((((((..(.(((((((((..........))))))))).)..))))))))))).)))).)))).))))))...

>Predicted STAR seq:
TTGATGTATGTGTTGGGGGAA

Complementarity: 16

Initial dG =   -42.70

```
        10        20        30        40        50        60
ACGUGGAACAAAUGG|     G    C    U           UU G         UUGU
               UAUGUU GCAC ACGA UGAUGUAUGUG  G GGGAACAUA    U
               AUAUGA CGUG UGCU AUUGCAUACAU  U UCCUUGUGU    U
GAA------------^     G    U    C           GG G         UUAC
.                   100        90        80        70
```

Reads in: lib-42, lib-45, lib-47, lib-48, lib-49, lib-50, lib-72
Number of reads="ID116",1,4,0,0,0,0,0

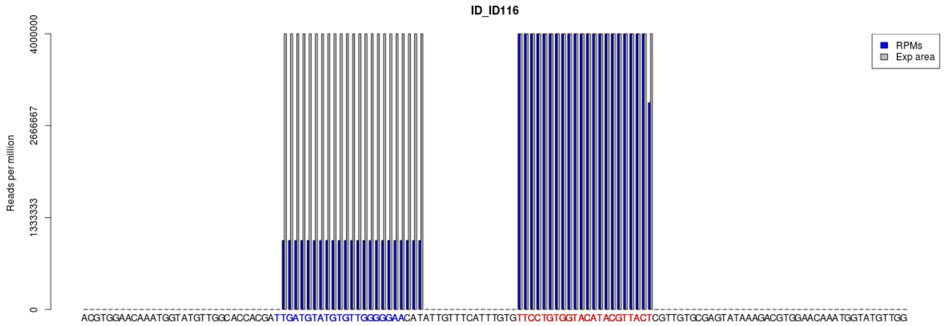

>ID1160
ACAGAAGATCAAGGCTAATGGTCAGGGGT**AGGGAGAGCTGGGTACATAGAAG**TGTTTTATTGTAGTCACTTCATGTACTCTGCATTCCTT
GCTTCTTTCCATATAGTGCC

>ID1160-5P
AGGGAGAGCTGGGTACATAGAAG

>miRNA precursor
ACAGAAGAUCAAGGCUAAUGGUCAGGGGU**AGGGAGAGCUGGGUACAUAGAAG**UGUUUUAUUGUAGUCACUUCAUGUACUCUGCAUUCCUUGCUUCUUUCCAUAUAGUGCC
```
.............((((((((..((((((((((((.((.((((((((.((((((.((......)).))))))))))))))).)).))))))))))))..)))).))))...
```

>Predicted STAR seq:
TCATGTACTCTGCATTCCTTGC

Complementarity: 18

Initial dG =   -45.00

```
        10         20         30         40         50         60
ACAGAAGAUCAAG    -|   UC            A  U        A      U  UA
             GCUA AUGG  AGGGGUAGGGAG GC GGGUACAU GAAGUG UU  U
             UGAU UACC  UCUUCGUUCCUU CG CUCAUGUA CUUCAC GA  U
CCG----------    A^   UU            A  U        -      U  UG
.                 100        90         80         70
```

Reads in: lib-42, lib-45, lib-47, lib-48, lib-49, lib-50, lib-72
Number of reads="ID1160",3,18,0,0,1,0,0

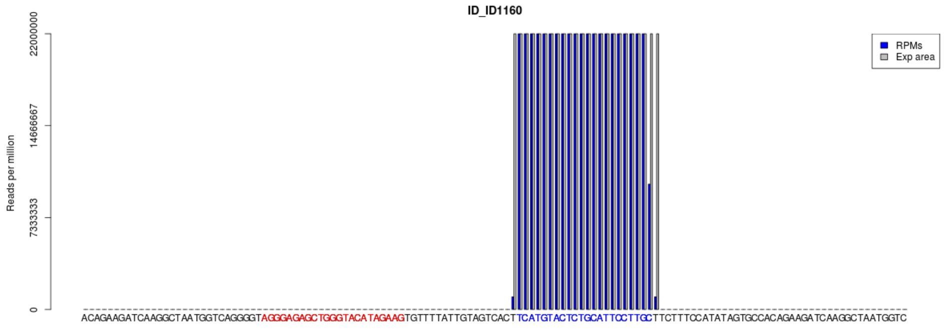

>ID1163

AGAAGATAAAGATCTAATAGTCAGAGG**TAGGGGAAGCTAGGGTACTTAGA**AGTGTTTTATTCTAGTCACTTCAAGTCCTTATCTTCTCTT
GCTTCTTTCCATTGAGTG

>ID1163-5P

TAGGGGAAGCTAGGGTACTTAGA

>miRNA precursor

AGAAGAUAAAGAUCUAAUAGUCAGAGG**UAGGGGAAGCUAGGGUACUUAGA**AGUGUUUUAUUCUAGUCACUUCAAGUCCUUAUCUUCUCUUGCUUCUUUCCAUUGAGUG
...((((....)))).......((((((((((((((.(((((.((((.((((((.((......)).)))))))))))))))).))))))))))))))...........

>Predicted STAR seq:

AAGTCCTTATCTTCTCTTGCT

Complementarity: 18

Initial dG =   -36.30

ERROR 3: Strange structure
Initial dG =   -36.30

```
.-AGA|   A
     AGAU A
     UCUA A
\ ---^   G
         10


     20             30        40        50        60
  AAUAGUC-----              C     U    A      U  UA
              AGAGGUAGGGGAAG UAGGG ACUU GAAGUG UU  U
              UCUUCGUUCUCUUC AUUCC UGAA CUUCAC GA  U
  GUGAGUUACCUU              U     -    -      U  UC
        100        90        80          70
```

Reads in: lib-42, lib-45, lib-47, lib-48, lib-49, lib-50, lib-72

Number of reads="ID1163",1,1,0,0,0,0,0

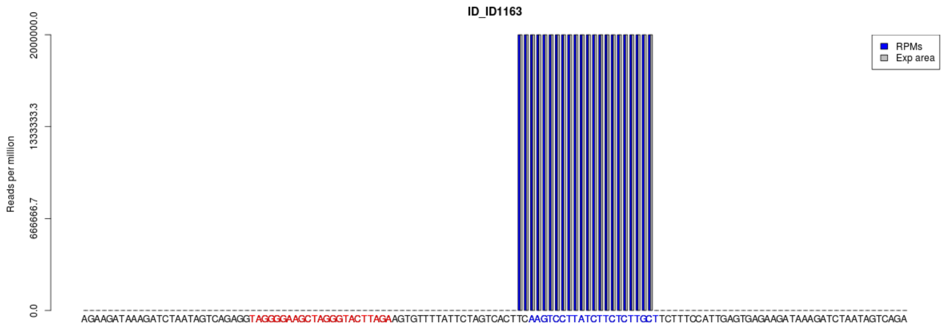

>ID1165
CGAGGATCAAGATCTAATGGTCAGAGGTAGGGGAAGCTAGGGTACTCAGAAGTGTTTTATTCTAGTCATT**TCAAGTCCTTAGTTTCTCTT**
GCATCTTTCCATTGATTGTCCG

>ID1165-3P
TCAAGTCCTTAGTTTCTCTTGC

>miRNA precursor
CGAGGAUCAAGAUCUAAUGGUCAGAGGUAGGGGAAGCUAGGGUACUCAGAAGUGUUUUAUUCUAGUCAUU**UCAAGUCCUUAGUUUCUCUUGC**AUCUUUCCAUUGAUUGUCCG
...((((...((((.(((((..(((.((((((((((((((((.(((..((((((.((......)).)))))).))))))))))))))))))).)))..)))))))))))))).

>Predicted STAR seq:
GTAGGGGAAGCTAGGGTACTCAGA

Complementarity: 19

Initial dG =   -45.50

```
        10        20        30        40        50        60
CGA|   CAA    U     UC    G                U    CA      U   UA
   GGAU   GAUC AAUGG  AGA GUAGGGGAAGCUAGGG ACU  GAAGUG UU  U
   CCUG   UUAG UUACC  UCU CGUUCUCUUUGAUUCC UGA  CUUUAC GA  U
G--^   ---    -     UU    A                -   A-      U   UC
  110           100        90        80          70
```

Reads in: lib-42, lib-45, lib-47, lib-48, lib-49, lib-50, lib-72
Number of reads="ID1165",1,10,0,0,0,0,0

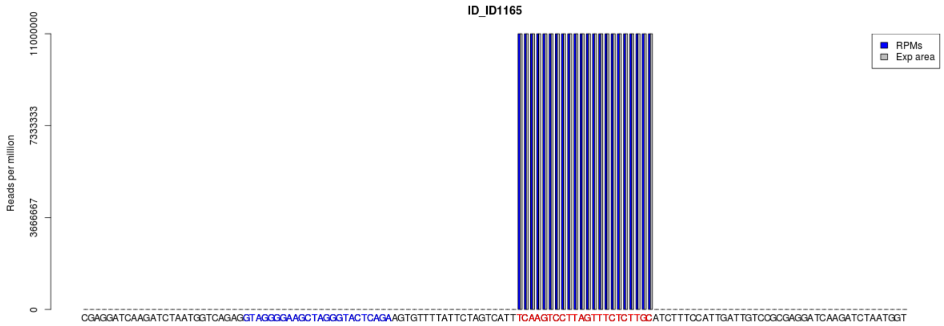

>ID1167
CAAAATTAATGGTCTGAGAT**AGGGGAAGCTAGGGTACTCAGA**CGTGTTTTATTCTACTCACTTCAAGTCCTTATCTTCTCTTGCTTCATT
CCATTGAGTGTCCGCATCAACG

>ID1167-5P
AGGGGAAGCTAGGGTACTCAGA

>miRNA precursor
CAAAAUUAAUGGUCUGAGAU**AGGGGAAGCUAGGGUACUCAGA**CGUGUUUUAUUCUACUCACUUCAAGUCCUUAUCUUCUCUUGCUUCAUUCCAUUGAGUGUCCGCAUCAACG
.....(((((((..((((.(((((((((.(((((.(((..((.(((...........))).)).)))))))).))))))))).)))).)))))))..............

>Predicted STAR seq:
AAGTCCTTATCTTCTCTTGC

Complementarity: 16

Initial dG =   -32.20

```
                  10        20        30        40        50
CAAAA----------|      UC    A         C     U   CA  C   UUUUA
               UUAAUGG  UGAG UAGGGGAAG UAGGG ACU  GA GUG     U
               AGUUACC  ACUU GUUCUCUUC AUUCC UGA  CU CAC     U
GCAACUACGCCUGUG^      UU    C         U     -   A-  U   UCAUC
110       100        90        80        70         60
```

Reads in: lib-42, lib-45, lib-47, lib-48, lib-49, lib-50, lib-72
Number of reads="ID1167",0,0,0,0,0,0,0

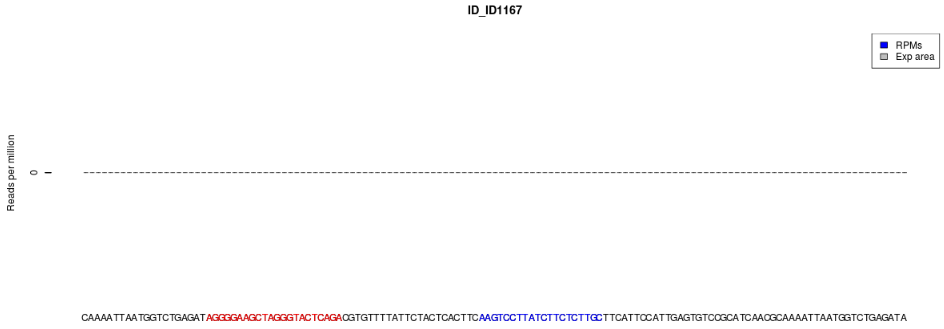

>ID117
ATGTGGAACAAATGTCAACCTCGTACTACGGCTGACATATGTGGTAGGTGTGCACGTTATTTCGTTAGTG**TGCCACAACCACATACGTCA**
CTCGTTGTTCGTGTTTAAAGA

>ID117-3P
TGCCACAACCACATACGTCACT

>miRNA precursor
AUGUGGAACAAAUGUCAACCUCGUACUACGGCUGACAUAUGUGGUAGGUGUGCACGUUAUUUCGUUAGUG**UGCCACAACCACAUACGUCACU**CGUUGUUCGUGUUUAAAGA
```
................(((..((.((.(((..((((.((((((((..(((.((((((((......))))))))))).))))))))).)))).)))).)).)).)))......
```

>Predicted STAR seq:
CTGACATATGTGGTAGGTGTGCA

Complementarity: 16

Initial dG =   -35.40

```
       10        20        30        40        50        60
AUGUGGAACAAAUGUC|  CU  U  U   GC    A        AG   U        UU
                AAC  CG AC ACG  UGAC UAUGUGGU  GUG GCACGUUA  U
                UUG  GC UG UGC  ACUG AUACACCA  CAC CGUGUGAU  C
AGAAAU----------^  U-  U  U   UC    C        A-   -        UG
 .                  100        90        80          70
```

Reads in: lib-42, lib-45, lib-47, lib-48, lib-49, lib-50, lib-72
Number of reads="ID117",1,2,0,0,0,0,0

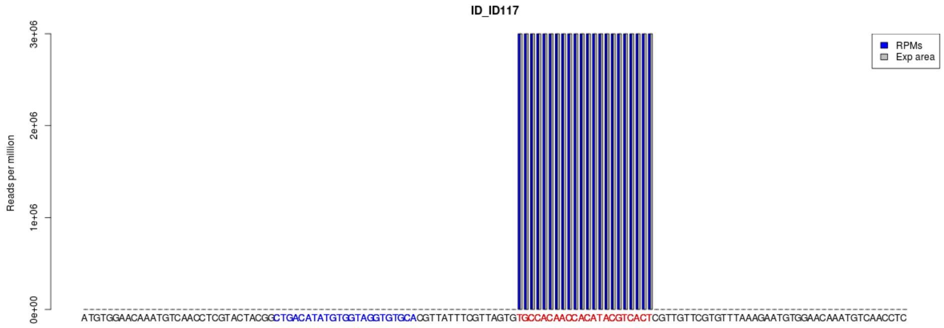

>ID1176
ACGTAGTCACAGTGGTTTAGTTAGAAGAGTGCTTGTTTCCTTAAGGACATAGTTTCCCAACAAGCAAACT**AGCCAACCGCTCGTGACTAC**
ACATACTATTTTTAAGAAAGAAA

>ID1176-3P
AGCCAACCGCTCGTGACTACACA

>miRNA precursor
ACGUAGUCACAGUGGUUUAGUUAGAAGAGUGCUUGUUUCCUUAAGGACAUAGUUUCCCAACAAGCAAACU**AGCCAACCGCUCGUGACUACACA**UACUAUUUUUAAGAAAGAAA
```
..((((((((((((((..(((((.....(((((((((((....)))...........))))))))..))))).))))))).)))))))).......................
```
Error predicting STAR on 5p
Initial dG =   -36.60

```
                            10         20         30                    40
AC---------------------         -       UA      AAGAG        -----------|  U
                       GUAGUCAC AGUGGUU  GUUAG     UGCUUGUU           UCC U
                       CAUCAGUG UCGCCAA  CGAUC     ACGAACAA           AGG A
AAAGAAAGAAUUUUUAUCAUACA         C       C-      AA---        CCCUUUGAUAC^  A
 110       100        90         80         70           60         50
```

Reads in: lib-42, lib-45, lib-47, lib-48, lib-49, lib-50, lib-72
Number of reads="ID1176",0,41,0,0,0,0,0

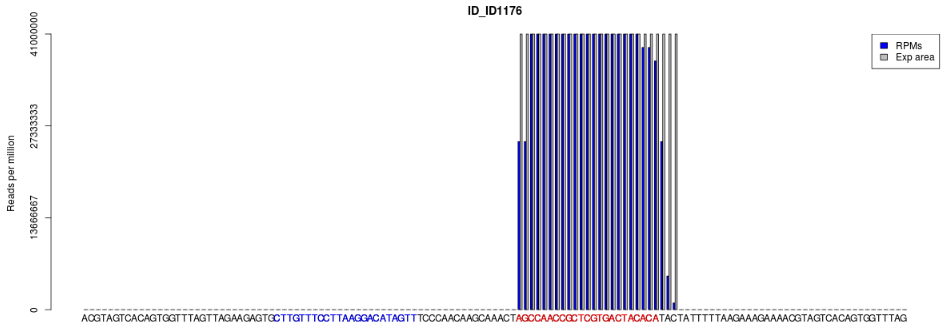

>ID1177
GGAGACTCTTGTTCCTGACA**CCACTGCAGAACCCTCAAACTCT**TCAGAGCCATCCTCGGAAACTGATAAGCTGAAGAGACATGTGAGCCA
GCTGATGGTGAAAAATGAACAAT

>ID1177-5P
CCACTGCAGAACCCTCAAACTCT

>miRNA precursor
GGAGACUCUUGUUCCUGACA**CCACUGCAGAACCCUCAAACUCU**UCAGAGCCAUCCUCGGAAACUGAUAAGCUGAAGAGACAUGUGAGCCAGCUGAUGGUGAAAAAUGAACAAU
........((((((....(((((...(((....((((..((((((((........((((...))))....))))))))).....)))).....))).)))))......)))))).

>Predicted STAR seq:
AGACATGTGAGCCAGCTGATGGTG

Complementarity: 12

Initial dG =   -27.80

```
        10          20        30           40        50        60
GGAGACUC      CUGA--     CUG   AACC    AA---|       AGCCAUCC    A
        UUGUUC      CACCA   CAG    CUCA     CUCUUCAG        UCGG \
        AACAAG      GUGGU   GUC    GAGU     GAGAAGUC        AGUC A
U-------      UAAAAA     A--   GACC    GUACA^       GAAU----    A
        110       100         90        80        70
```

Reads in: lib-42, lib-45, lib-47, lib-48, lib-49, lib-50, lib-72
Number of reads="ID1177",138,96,68,243,317,122,0

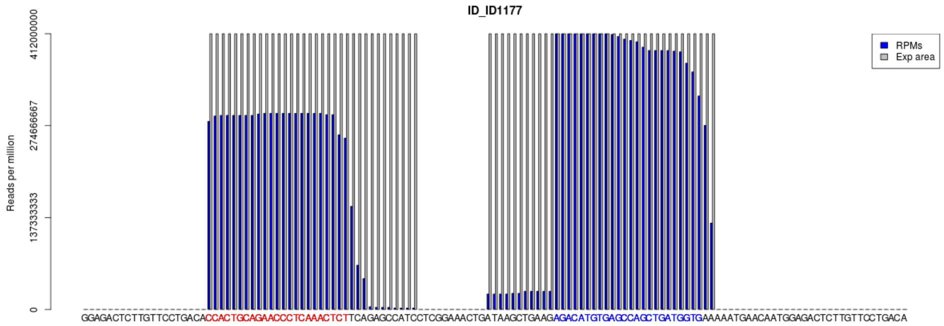

>ID1179
CATTGCGGGCGCCGTGGCCGGCAGCAGCACAGGCTGTCACTCGCACAGTGCGTGCGGGGCCGGCAGGTCG**CCGCACGCACTGTGCGAGCG**
GAATGGCCTCTGCAGGCGCGC

>ID1179-3P
CCGCACGCACTGTGCGAGCGGA

>miRNA precursor
CAUUGCGGGCGCCGUGGCCGGCAGCAGCACAGGCUGUCACUCGCACAGUGCGUGCGGGGCCGGCAGGUCG**CCGCACGCACUGUGCGAGCGGA**AUGGCCUCUGCAGGCGCGC
```
..(((((((.(((((..(((...(((((....)))))..((((((((((((((((((((((....)))).))))))))))))))))))))).))))).)))))))......
```

>Predicted STAR seq:
GCCGGCAGCAGCACAGGCTGTCACTCGCACAGTGCG

Complementarity: 19

Initial dG =   -73.30

```
            10        20           30
CA----       C     GG    .-GCA|     A
      UUGCGGG GCCGU  CCG      GCAGC C
      GACGUCU CGGUA  GGC      UGUCG A
CGCGCG       C     A-    \ ---^     G
 .       100         90
```

Reads in: lib-42, lib-45, lib-47, lib-48, lib-49, lib-50, lib-72
Number of reads="ID1179",34,201,173,389,1838,56,0

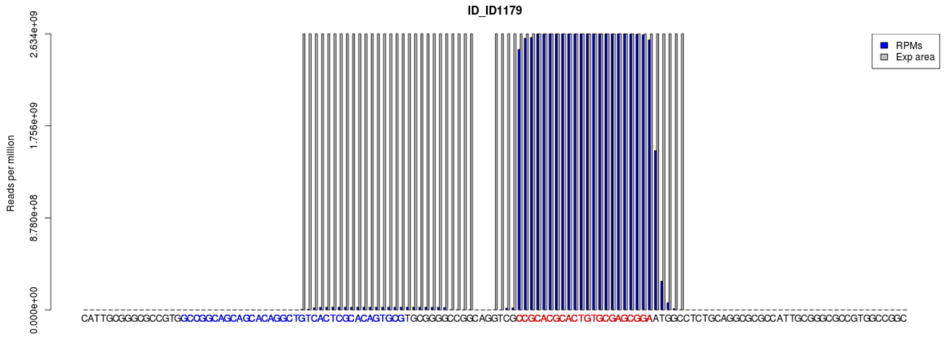

>ID118
CGGCATCCTCGCATCACGGG**TGACGTATGTGGTGGGGGAACAC**GTTGATTCATTTGTGTCTCCCGCGCACATACGTTACACGTTGTGCGA
GGCAAACGATTACCGGACCA

>ID118-5P
TGACGTATGTGGTGGGGGAACAC

>miRNA precursor
CGGCAUCCUCGCAUCACGGG**UGACGUAUGUGGUGGGGGAACAC**GUUGAUUCAUUUGUGUCUCCCGCGCACAUACGUUACACGUUGUGCGAGGCAAACGAUUACCGGACCA
((....((((((((.(((.((((((((((((((((((((.((((..........))))))))))))).))))))))))))).))).)))))))).....))...........

>Predicted STAR seq:
GTCTCCCGCGCACATACGTTACA

Complementarity: 20

Initial dG =   -56.70

```
                    10        20        30         40
------------  GCAU        C   G            -|       A    UUGA
            CG    CCUCGCAU ACG GUGACGUAUGUG GUGGGGGA CACG    U
            GC    GGAGCGUG UGC CAUUGCAUACAC CGCCCUCU GUGU    U
ACCAGGCCAUUA    AAAC      U   A            G^       -    UUAC
.       100        90        80        70        60         50
```

Reads in: lib-42, lib-45, lib-47, lib-48, lib-49, lib-50, lib-72
Number of reads="ID118",0,0,0,0,0,0,0

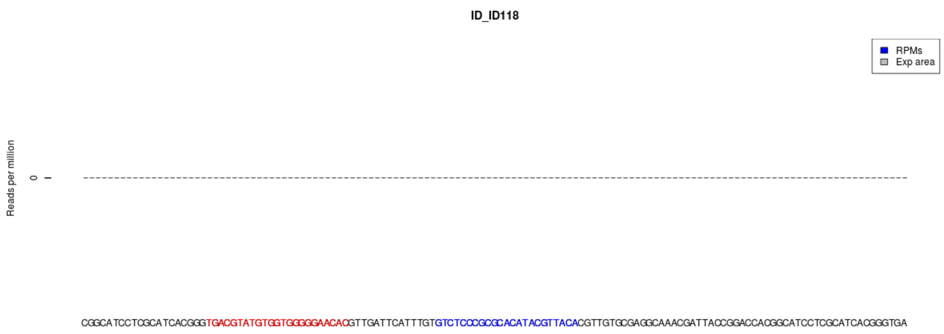

>ID1181
TTCAGCTGGGTC**TGATGCTGCAGGAGTTGTTGTGA**TCCTCCAGTGATGCCAACAGCTCCGGCAGTGTCGGATCCAGCAGAACGCTATCGC
CGGTGTCGTCGTGTG

>ID1181-5P
TGATGCTGCAGGAGTTGTTGTGA

>miRNA precursor
UUCAGCUGGGUC**UGAUGCUGCAGGAGUUGUUGUGA**UCCUCCAGUGAUGCCAACAGCUCCGGCAGUGUCGGAUCCAGCAGAACGCUAUCGCCGGUGUCGUCGUGUG
```
....((((((((((((((((.((((((((((..(((.......)))..)))))))))).))))))))))))))))).((.((((......)))))).......
```

>Predicted STAR seq:
CCAACAGCUCCGGCAGUGUCGGA

Complementarity: 18

Initial dG =   -54.00

```
          10        20        30        40
.-UUCA|                A          UG   CUC
      GCUGGGUCUGAUGCUGC GGAGUUGUUG  AUC   \
      CGACCUAGGCUGUGACG CCUCGACAAC  UAG   C
\ ----^                G          CG   UGA
            70        60        50



          80
  A-------  A    AU
          GA CGCU  C
          CU GUGG  G
  GUGUGCUG  -    CC
     100         90
```

Reads in: lib-42, lib-45, lib-47, lib-48, lib-49, lib-50, lib-72
Number of reads="ID1181",6,30,100,32,24,15,0

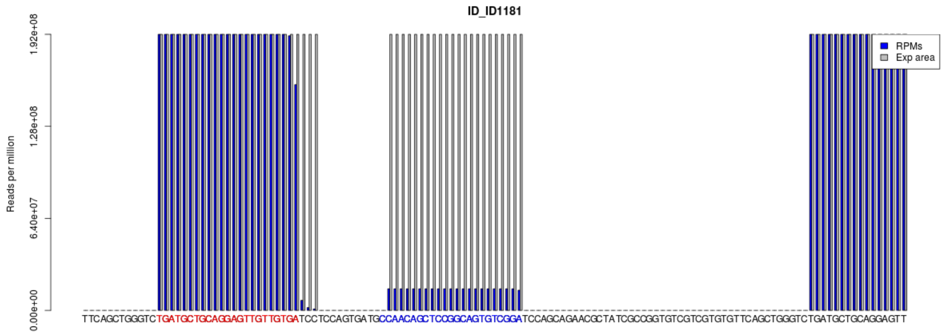

>ID1183
CACTTAAGGTGTTGGATTT**GCTTTCTTCTTGTGGTTTGACTGT**TGTTCATTTATTTAAAAACATTACGAACAATTGGTTGAATCAAAGGG
ACAGTGACAAAACATTTAAATTTA

>ID1183-5P
GCTTTCTTCTTGTGGTTTGACTGT

>miRNA precursor
CACUUAAGGUGUUGGAUUU**GCUUUCUUCUUGUGGUUUGACUGU**UGUUCAUUUAUUUAAAAACAUUACGAACAAUUGGUUGAAUCAAAGGGACAGUGACAAAACAUUUAAAUUUA
......((((((....(..((.(((.(((.(((((..((((((((((...................)))))).)))))..))))))))))).))..)...)))))))......

>Predicted STAR seq:
TGGTTGAATCAAAGGGACAGTGAC

Complementarity: 16

Initial dG =   -25.00

```
         10        20        30        40         50
CACUUA-|      GGAU UG  U   U   G     UG    -      AUUUAUUUA
       AGGUGUU    U  CU UCU CUU UGGUU  ACUG UUGUUC         \
       UUUACAA    A  GA AGG GAA ACUAA  UGGU AACAAG         A
AUUUAAA^      AAC- GU  C   -   -     GU    U      CAUUACAAA
  110       100        90          80        70        60
```

Reads in: lib-42, lib-45, lib-47, lib-48, lib-49, lib-50, lib-72
Number of reads="ID1183",72,31,16,51,48,40,0

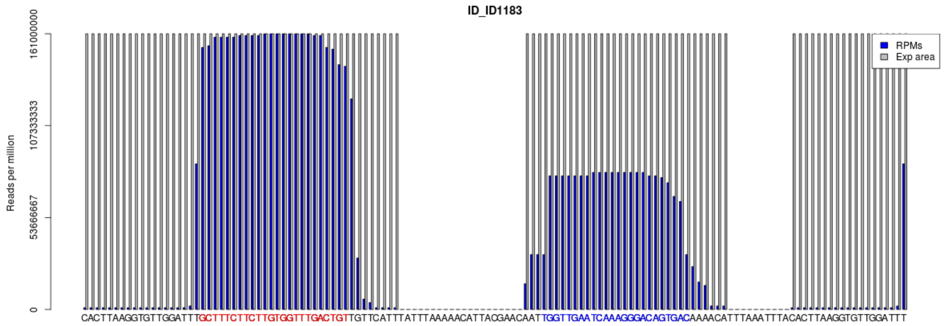

>ID1188
ATGTGCTGATGGCTGGCCTCGGCACTTGTCTACATTGCCGACCATCTCTTCCCTACGAAAAAATGAAACA**CAGGGAAGACATGTTCAGTG**
ATGTAGACGAGCTGCACTGTCTG

>ID1188-3P
CAGGGAAGACATGTTCAGTGATG

>miRNA precursor
AUGUGCUGAUGGCUGGCCUCGGCACUUGUCUACAUUGCCGACCAUCUCUUCCCUACGAAAAAAUGAAACA**CAGGGAAGACAUGUUCAGUGAUG**UAGACGAGCUGCACUGUCUG
```
..(((((((.((....)))))))))((((((((((..(.((.(((.((((((((................)))))))).))).)).)..))))))))))............
```

>Predicted STAR seq:
GGCTGGCCTCGGCACTTGTCTACATTGCCGACCA

Complementarity: 15
ERROR 4: Strange structure
Initial dG =   -44.40

```
          10
.-AU|      U  C
    GUGCUGA GG U
    CACGGCU CC G
\ --^      -  G
        20



                 30        40        50        60
  -------------          UG C  C   C        ACGAAAAA
             UUGUCUACAU  C GA CAU UCUUCCCU        \
             AGCAGAUGUA  G CU GUA AGAAGGGA        A
  GUCUGUCACGUCG          GU A  U   C        CACAAAGU
   110       100        90        80        70
```

Reads in: lib-42, lib-45, lib-47, lib-48, lib-49, lib-50, lib-72
Number of reads="ID1188",26,38,22,70,374,33,0

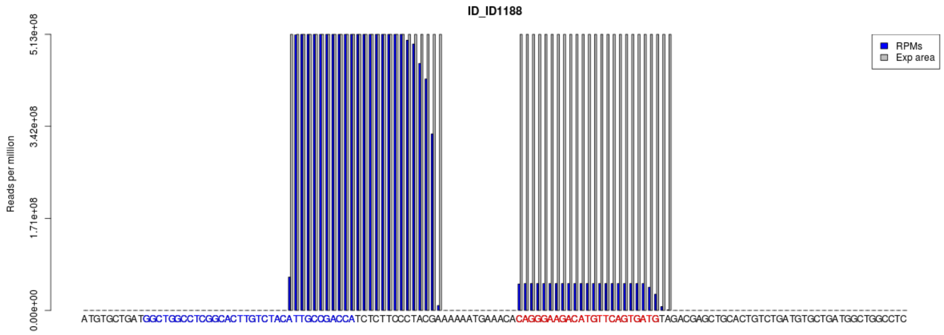

>ID119
TTGTGGGACTAATTGTATTCTCGCACGAAGGGTGACGTATGTGTCGCAGGTACATTTTCCTTGATTTGTG**TATCTGAGGCCCATTCGTTA**
CTTGTCGTGTGAGTTTAACAAT

>ID119-3P
TATCTGAGGCCCATTCGTTACT

>miRNA precursor
UUGUGGGACUAAUUGUAUUCUCGCACGAAGGGUGACGUAUGUGUCGCAGGUACAUUUUCCUUGAUUUGUG**UAUCUGAGGCCCAUUCGUUACU**UGUCGUGUGAGUUUAACAAU
```
...........(((((..(((((((((..(((((((.(((.(((.(((((((((..((...))...))))))))).))).))).)))))))..)))))))))..)).))))
```

>Predicted STAR seq:
GGGTGACGTATGTGTCGCAGGTA

Complementarity: 17

Initial dG =   -46.00

```
        10         20        30        40        50         60
UUGUGGGACUA    -  UU         AG       U   U   G         UU-| C
           AUUG UA  CUCGCACGA  GGUGACG AUG GUC CAGGUACAU   UC \
           UAAC AU  GAGUGUGCU  UCAUUGC UAC CGG GUCUAUGUG   AG U
-----------    A  UU         GU       U   C   A         UUU^ U
           110       100        90        80        70
```

Reads in: lib-42, lib-45, lib-47, lib-48, lib-49, lib-50, lib-72
Number of reads="ID119",2,2,0,0,0,0,0

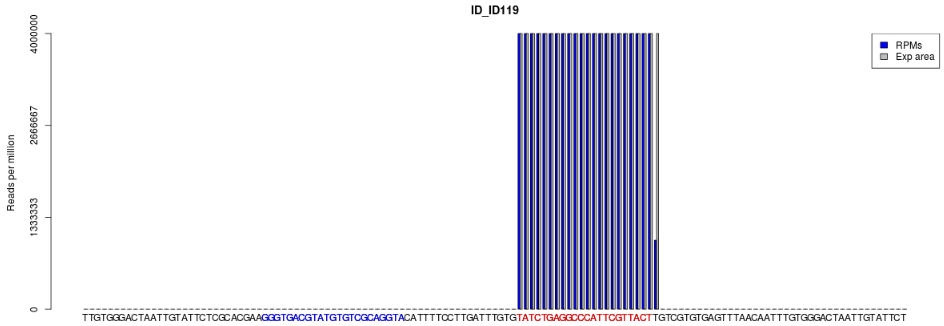

>ID1193
AAGAACATGCTGTTGTCCTC**TCTTTGGTGATCTAGCTGTATGA**GCATAGGGATGGCATCATAAAGCTAGATTACCGAAGTCAGGAAAACA
GTGCAGACTTCGAGTTCATCT

>ID1193-5P
TCTTTGGTGATCTAGCTGTATGA

>miRNA precursor
AAGAACAUGCUGUUGUCCUC**UCUUUGGUGAUCUAGCUGUAUGA**GCAUAGGGAUGGCAUCAUAAAGCUAGAUUACCGAAGUCAGGAAAACAGUGCAGACUUCGAGUUCAUCU
..((((..((((((.((((..((((((((((((((((.(((((((.........)).))))).))))))))))))))))..)))).))))))..........))))....

>Predicted STAR seq:
ATAAAGCTAGATTACCGAAGTCAG

Complementarity: 19

Initial dG = -45.60

```
                   10        20        30        40
AA--    AU---------      G    CU                G     -| AUAG
    GAAC           GCUGUU UCCU  CUUUGGUGAUCUAGCU UAUGA GC    \
    CUUG           UGACAA AGGA  GAAGCCAUUAGAUCGA AUACU CG    G
UCUA    AGCUUCAGACG      A    CU                A     A^ GUAG
 .       100       90        80        70        60
```

Reads in: lib-42, lib-45, lib-47, lib-48, lib-49, lib-50, lib-72
Number of reads="ID1193",20770,29948,214749,26890,64134,17424,0

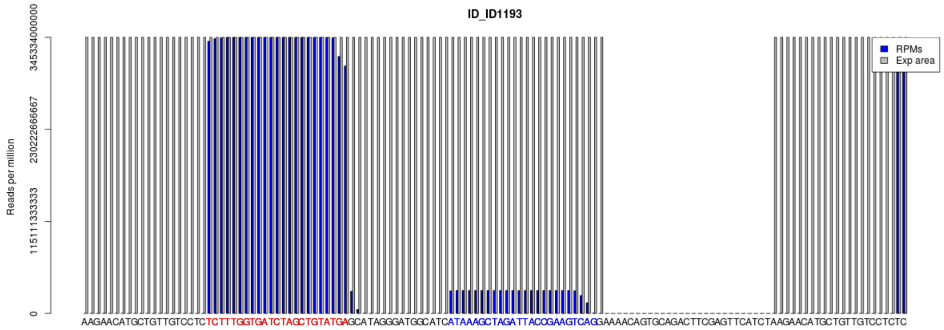

>ID1194
TAACCTTGCTCTGGGCCTTG**CTTTGGTGATGTAGCTGTATGA**TTGATTACTATTTCATAAAGCTAGATTACCAAAGCATGGCTATGAGCT
TATGCTACATGTGTGGACAGAA

>ID1194-5P
CTTTGGTGATGTAGCTGTATGA

>miRNA precursor
UAACCUUGCUCUGGGCCUUG**CUUUGGUGAUGUAGCUGUAUGA**UUGAUUACUAUUUCAUAAAGCUAGAUUACCAAAGCAUGGCUAUGAGCUUAUGCUACAUGUGUGGACAGAA
.......((((..((((.((((((((((((.(((((.(((((............))))).))))).)))))))))))).))))..))))...((((((....)))).))...

>Predicted STAR seq:
ATAAAGCTAGATTACCAAAGCA

Complementarity: 18

Initial dG =   -48.00

```
          10        20        30        40
.-UAACCUU|   UG    U            G     G     UUGAU
         GCUC  GGCC UGCUUUGGUGAU UAGCU UAUGA     U
         CGAG  UCGG ACGAAACCAUUA AUCGA AUACU     A
\ -------^   UA    U            G     A     UUAUC
                 80        70        60        50



 90        100
  UUA  -    A
     UG CUAC U
     AC GGUG G
  AAG  A    U
  110
```

Reads in: lib-42, lib-45, lib-47, lib-48, lib-49, lib-50, lib-72
Number of reads="ID1194",4927,5308,5729,5567,10136,2710,0

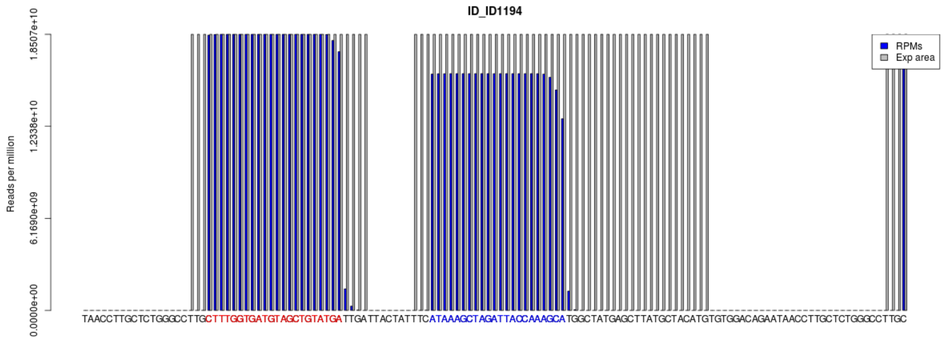

>ID1195
TTCGAATGCATGTGGTCTTG**TCTTTGGTATCCTAGCTGTAGG**TGATTTGTTCAGTGAACCCTAAAGCTAGCGTACCATAGTCAAGAGCAT
GTGACTAAACTTCTGAGCAGA

>ID1195-5P
TCTTTGGTATCCTAGCTGTAGG

>miRNA precursor
UUCGAAUGCAUGUGGUCUUG**UCUUUGGUAUCCUAGCUGUAGG**UGAUUUGUUCAGUGAACCCUAAAGCUAGCGUACCAUAGUCAAGAGCAUGUGACUAAACUUCUGAGCAGA
........((((((.(((((.((.((((((.((((((.((((......((((...)))))))).)))))).)))))).)).))))).)))))).................

>Predicted STAR seq:
TAAAGCTAGCGTACCATAGTCA

Complementarity: 16

Initial dG =   -38.40

```
                  10        20        30        40        50
UUCGAAUG----------|     G     U  U      C      G    UGAUUU    A
                  CAUGUG UCUUG CU UGGUAU CUAGCU UAGG      GUUC \
                  GUGUAC AGAAC GA ACCAUG GAUCGA AUCC      CAAG G
AGACGAGUCUUCAAAUCA^     G     U  U      C      A    ------    U
 .       100        90        80        70        60
```

Reads in: lib-42, lib-45, lib-47, lib-48, lib-49, lib-50, lib-72
Number of reads="ID1195",22349,31040,49608,30879,65682,21718,0

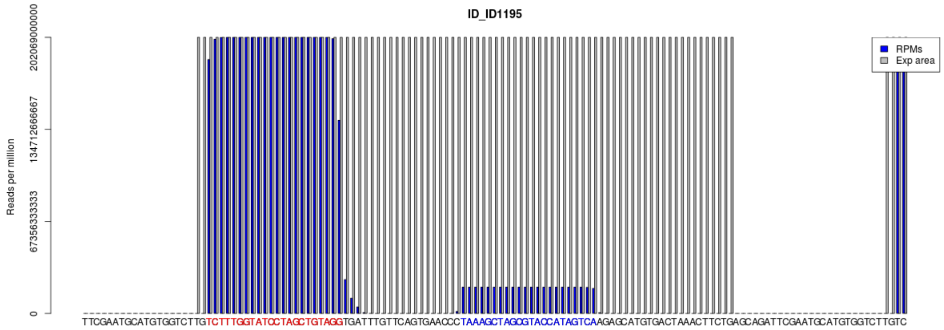

>ID1196
CGGTAAGCTGTGCTCTGTGG**TCAGGTACTGAGTGACTCTGAG**ATGTTAACTCAGGGGCACTTGGTACCTAATCAGGAAGGACAGCAACGC
ATCACAGTACTCTTGTATC

>ID1196-5P
TCAGGTACTGAGTGACTCTGAG

>miRNA precursor
CGGUAAGCUGUGCUCUGUGG**UCAGGUACUGAGUGACUCUGAG**AUGUUAACUCAGGGGCACUUGGUACCUAAUCAGGAAGGACAGCAACGCAUCACAGUACUCUUGUAUC
......(((((.((((.((((.((((((..((((.(((((((.......))))))).))))..)))))).))))))).).)))))..........((((....)))).

>Predicted STAR seq:
CAGGGGCACTTGGTACCTAATC

Complementarity: 16

Initial dG =   -44.90

```
          10          20         30         40
.-CGGUAA     G -|  G     C       UG     A        AUG
        GCUGU C UCU UGGU AGGUAC  AGUG CUCUGAG   \
        CGACA G AGG ACUA UCCAUG  UCAC GGGACUC   U
\ ------     G A^  -     A       GU     G        AAU
            80          70         60         50



     90        100
  AACGCAUCACA    U
             GUAC C
             UAUG U
  C----------    U
```

Reads in: lib-42, lib-45, lib-47, lib-48, lib-49, lib-50, lib-72
Number of reads="ID1196",16596,29748,71094,27237,66137,19688,0

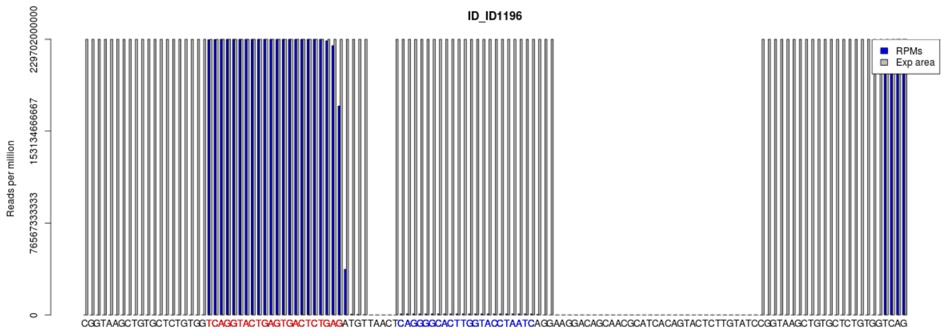

>ID1203
GACTGTCACTGAGTTGGCCCTCTCAGTTGTTGTAGGGACGGATAAACTCCTATGCATCTCTACAGTAACTTGAGCGGGTCGGGTCAGTAC
TCAACGAAATTAACTAGTGTGC

>ID1203-5P
TCTCAGTTGTTGTAGGGACGGA

>miRNA precursor
GACUGUCACUGAGUUGGCCCUCUCAGUUGUUGUAGGGACGGAUAAACUCCUAUGCAUCUCUACAGUAACUUGAGCGGGUCGGGUCAGUACUCAACGAAAUUAACUAGUGUGC
....((.(((((.(((((((.((((((((((((((((.(((.....)))......)))))))))))))).))).))))))).))))))).....................

>Predicted STAR seq:
CTATGCATCTCTACAGTAACTTGAGCGG

Complementarity: 18

Initial dG = -39.60

```
                          10        20         30             40
GACU------------------  C     G       U   -              C-----|  UA
                      GU ACUGA UUGGCCC CUC AGUUGUUGUAGGGA      GGA  \
                      CA UGACU GGCUGGG GAG UCAAUGACAUCUCU      CCU  A
CGUGUGAUCAAUUAAAGCAACU  -     G       C   U              ACGUAU^  CA
110       100        90         80        70         60        50
```

Reads in: lib-42, lib-45, lib-47, lib-48, lib-49, lib-50, lib-72
Number of reads="ID1203",143,60,80,1912,1101,17,0

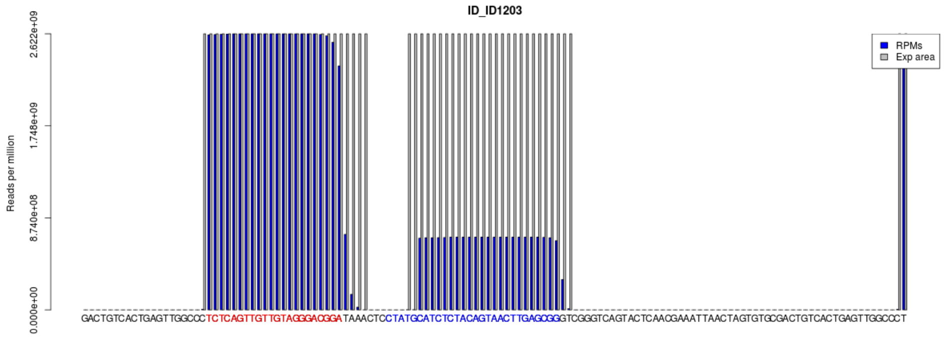

>ID1205
TCGCTGCTGAGAGCAGATTGTCACTGAGCTGCCCCGCTTCAGTTGCTGTAGGAGTAGTTATGTGCATCT**CTACAGCAACTGAAGCGGGTT**
GGGTCGGTGGGCAACGAAACTG

>ID1205-3P
CTACAGCAACTGAAGCGGGTTG

>miRNA precursor
UCGCUGCUGAGAGCAGAUUGUCACUGAGCUGCCCCGCUUCAGUUGCUGUAGGAGUAGUUAUGUGCAUCU**CUACAGCAACUGAAGCGGGUUG**GGUCGGUGGGCAACGAAACUG
...((((.....)))).((((((((((.((..((((((((((((((((((((((((......))).)).)))))))))))))))))))))..)).))))).))))).........

>Predicted STAR seq:
GCCCCGCTTCAGTTGCTGTAG

Complementarity: 18
ERROR 4: Strange structure
Initial dG = -60.00

```
.-UCG|   UG
     CUGC  \
     GACG  A
\ ---^   AG



           20         30         40         50
  A-------      -      G   GC                   -  -   GU
          UUGUC ACUGA CU  CCCGCUUCAGUUGCUGUAG GA GUA  U
          AACGG UGGCU GG  GGGCGAAGUCAACGACAUC CU CGU  A
  GUCAAAGC      G      G  UU                  U  A   GU
  110       100        90         80         70         60
```

Reads in: lib-42, lib-45, lib-47, lib-48, lib-49, lib-50, lib-72
Number of reads="ID1205",44,20,23,861,245,7,0

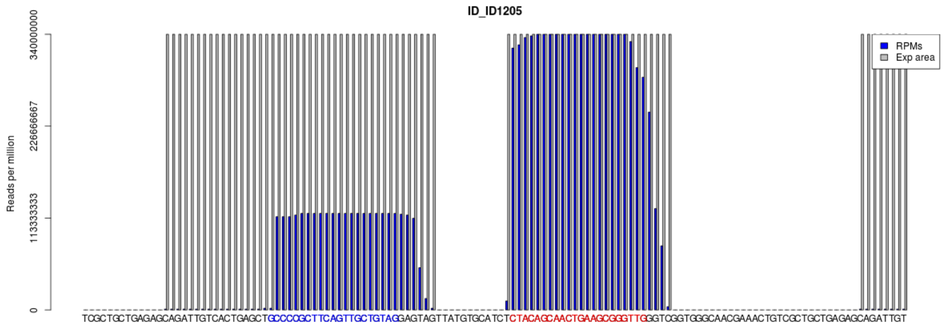

>ID1206

CACTTTGCTGCTGAGGGCAGACTGGCACTGAGCCGGCCCGCTCAGTTGCTGTAGGGACAGATGTGCATC**TCTACAGCAACTGAACTGGGC**
TGGGTCGGTGGTCAACAAAACTG

>ID1206-3P

TCTACAGCAACTGAACTGGGCTGG

>miRNA precursor

CACUUUGCUGCUGAGGGCAGACUGGCACUGAGCCGGCCCGCUCAGUUGCUGUAGGGACAGAUGUGCAUC**UCUACAGCAACUGAACUGGGCUGG**GUCGGUGGUCAACAAAACUG

```
.......((((.....))))..((((((((.((((((((.((((((((((((((((..........)))))))))))))))).)))))))).))))).)))).........
```

>Predicted STAR seq:

CCGGCCCGCTCAGTTGCTGTAGG

Complementarity: 20
ERROR 4: Strange structure
Initial dG =   -58.10

```
          10
.-CACUUUG|   UG
         CUGC  \
         GACG  A
\ -------^   GG
        20


                 30        40          50         60
  AC-------    -     G        C-                CAGA
           UGGC ACUGA CCGGCCCG  UCAGUUGCUGUAGGGA    U
           ACUG UGGCU GGUCGGGU  AGUCAACGACAUCUCU    G
  GUCAAAACA    G     G        CA                ACGU
   110       100        90         80         70
```

Reads in: lib-42, lib-45, lib-47, lib-48, lib-49, lib-50, lib-72
Number of reads="ID1206",39,27,3,501,117,5,0

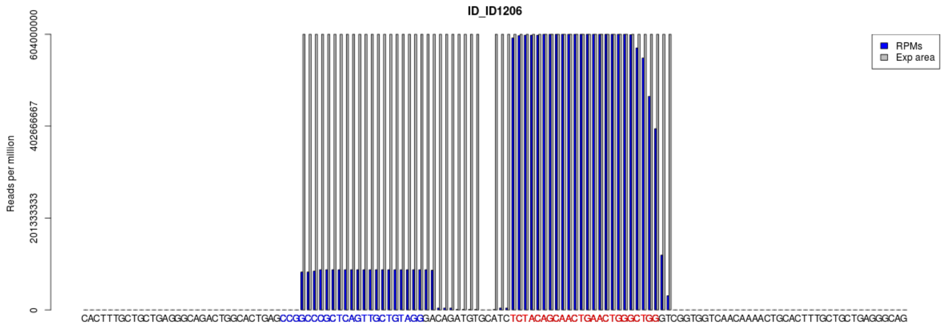

>ID1207
TCGCTGCTGAGGGCAGACTGGCACTGAGCCGGCCCGCTTCAGTTGCTGTAGGAGTAGTTATGTGCATCTCTACACCAACTGAAGCGGGTC
GGGTCGGTGTTCAACGGAACCG

>ID1207-3P
CTACACCAACTGAAGCGGGTCG

>miRNA precursor
UCGCUGCUGAGGGCAGACUGGCACUGAGCCGGCCCGCUUCAGUUGCUGUAGGAGUAGUUAUGUGCAUCUCUACACCAACUGAAGCGGGUCGGGUCGGUGUUCAACGGAACCG
```
...((((.....)))).(((((((((.((((((((((((((((.((((((((((......))).)).))))).))))))))))))))))).))))))))....))).....
```

>Predicted STAR seq:
GGCCCGCTTCAGTTGCTGTAG

Complementarity: 19
ERROR 4: Strange structure
Initial dG =   -61.30

```
.-UCG|   UG
     CUGC  \
     GACG  A
\ ---^   GG
```

```
      20        30        40        50
  .-ACU        G                 C     -  -   GU
       GGCACUGA CCGGCCCGCUUCAGUUG UGUAG GA GUA  U
       UUGUGGCU GGCUGGGCGAAGUCAAC ACAUC CU CGU  A
  \ ---        G                 C     U  A   GU
      100        90        80        70        60
```

Reads in: lib-42, lib-45, lib-47, lib-48, lib-49, lib-50, lib-72
Number of reads="ID1207",225,223,75,8742,2462,48,0

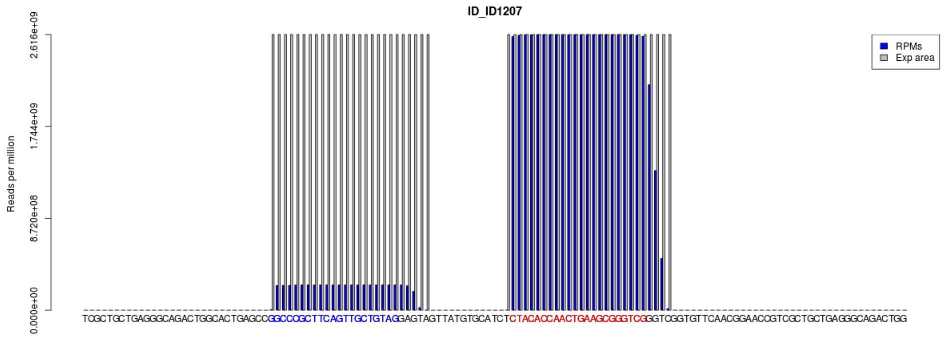

>ID1208
CACTTTGCTGCTGAGGGCAGACTGGCACTGAGCCGGCTCGCTCAGTTGCTGTAGGGACAGATGTGCATC**TCTACAGCAACTGAACTGGGC TGG**GTCGGTGGTCAACAAAACTA

>ID1208-3P
TCTACAGCAACTGAACTGGGCTGG

>miRNA precursor
CACUUUGCUGCUGAGGGCAGACUGGCACUGAGCCGGCUCGCUCAGUUGCUGUAGGGACAGAUGUGCAUC**UCUACAGCAACUGAACUGGGCUGG**GUCGGUGGUCAACAAAACUA
```
.......((((.....))))..((((((((.((((((((.((((((((((((((((..........))))))))))))))))..)))))))).))))).)))).........
```

>Predicted STAR seq:
CCGGCTCGCTCAGTTGCTGTAGG

Complementarity: 20
ERROR 4: Strange structure
Initial dG = -55.10

```
          10
.-CACUUUG|   UG
         CUGC  \
         GACG  A
\ -------^   GG
        20


                30        40         50        60
  AC-------    -     G        C-                CAGA
          UGGC ACUGA CCGGCUCG  UCAGUUGCUGUAGGGA    U
          ACUG UGGCU GGUCGGGU  AGUCAACGACAUCUCU    G
  AUCAAAACA    G     G        CA                ACGU
   110       100        90        80        70
```

Reads in: lib-42, lib-45, lib-47, lib-48, lib-49, lib-50, lib-72
Number of reads="ID1208",50,33,4,529,137,2,0

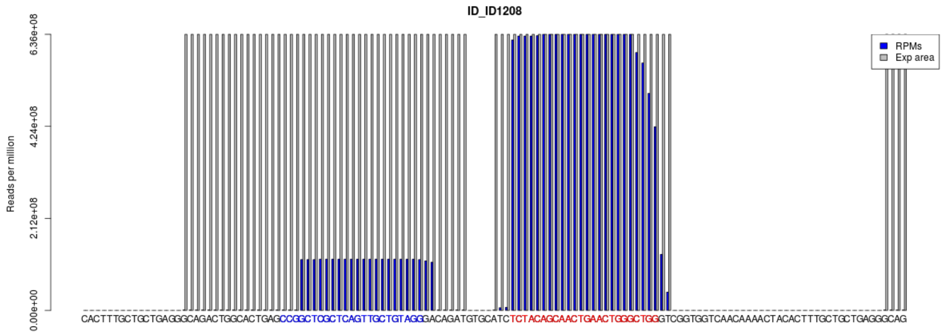

>ID1210

TCGCTGCTGAGGGCAGATTGTCACTGAGCTGGCCCGCTTCAGTTGCTGTAGGAGTAGTTATGTGCATCT**CTACACCAACTGAAGCGGGTC**
GGGTCGGTGGGCAACGAAACTG

>ID1210-3P

CTACACCAACTGAAGCGGGTCG

>miRNA precursor

UCGCUGCUGAGGGCAGAUUGUCACUGAGCUGGCCCGCUUCAGUUGCUGUAGGAGUAGUUAUGUGCAUCU**CUACACCAACUGAAGCGGGUCG**GGUCGGUGGGCAACGAAACUG

```
...((((.....)))).(((((((((.((((((((((((((((.((((((((((......))).)).))))).))))))))))))))))).))))).)))))........
```

>Predicted STAR seq:

GGCCCGCTTCAGTTGCTGTAG

Complementarity: 19

ERROR 4: Strange structure

Initial dG =   -59.30

```
.-UCG|   UG
     CUGC  \
     GACG  A
\ ---^   GG
```

```
           20         30        40        50
  A-------     -     G                 C     -  -   GU
          UUGUC ACUGA CUGGCCCGCUUCAGUUG UGUAG GA GUA  U
          AACGG UGGCU GGCUGGGCGAAGUCAAC ACAUC CU CGU  A
  GUCAAAGC     G     G                 C     U  A   GU
  110       100        90        80        70        60
```

Reads in: lib-42, lib-45, lib-47, lib-48, lib-49, lib-50, lib-72

Number of reads="ID1210",123,66,51,3040,584,14,0

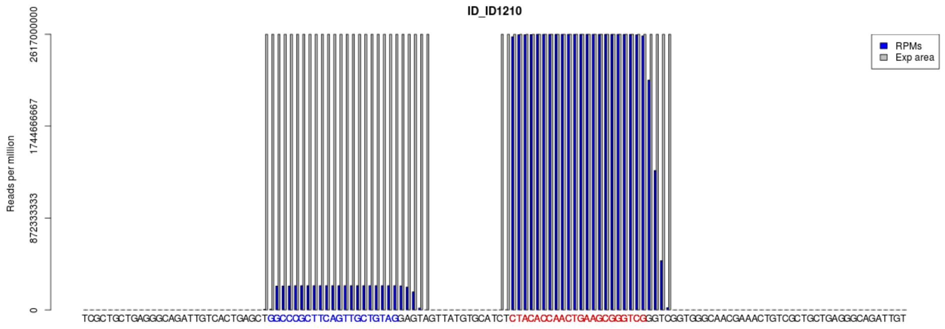

>ID1211
CACTTTGCTTCTGAGGGCAGACTGGCACTGAGCCGGCCCGCTCAGTTGCTGTAGGGACAGATGTGCATC**TCTACAGCAACTGAACTGGGC**
TGGGTCGGTGGTCAACAAAACTA

>ID1211-3P
TCTACAGCAACTGAACTGGGCTGG

>miRNA precursor
CACUUUGCUUCUGAGGGCAGACUGGCACUGAGCCGGCCCGCUCAGUUGCUGUAGGGACAGAUGUGCAUC**UCUACAGCAACUGAACUGGGCUGG**GUCGGUGGUCAACAAAACUA
((.((((((......)))))).)).(((((.((((((((.((((((((((((((((.........))))))))))))))))..)))))))).))))))............

>Predicted STAR seq:
TTCTGAGGGCAGACTGGCACTGA

Complementarity: 20
ERROR 4: Strange structure
Initial dG = -58.00

```
          10
  | C      UC
--CA UUUGCU  U
  GU AGACGG  G
\ ^ C      GA
     20


                  30        40         50        60
  G------------      G        C-                CAGA
             CACUGA CCGGCCCG  UCAGUUGCUGUAGGGA    U
             GUGGCU GGUCGGGU  AGUCAACGACAUCUCU    G
  AUCAAAACAACUG      G        CA                ACGU
   110       100        90         80        70
```

Reads in: lib-42, lib-45, lib-47, lib-48, lib-49, lib-50, lib-72
Number of reads="ID1211",39,35,6,499,81,3,0

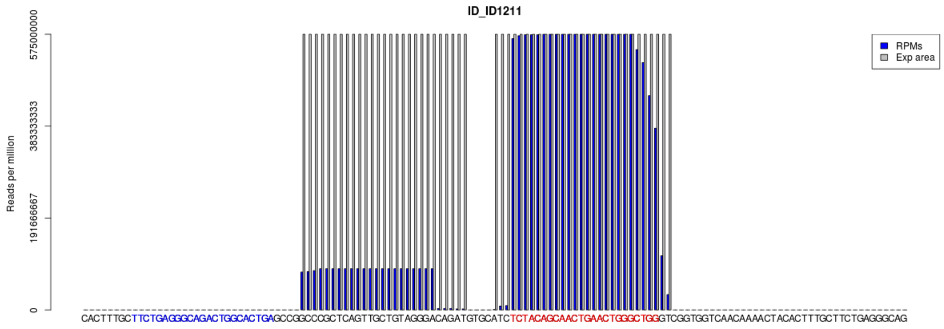

>ID1212
TCGCTGCTGAGGGCAGACTGTCACTGAGCCGGCCCACTTTAGTTGCTGTAGGAGGAGATATGTGCATCT**CTATAGCAACTGAATCGGGCG**
GGGTCGGTGTCAACGAAACTGC

>ID1212-3P
CTATAGCAACTGAATCGGGCGGG

>miRNA precursor
UCGCUGCUGAGGGCAGACUGUCACUGAGCCGGCCCACUUUAGUUGCUGUAGGAGGAGAUAUGUGCAUCU**CUAUAGCAACUGAAUCGGGCGGG**GUCGGUGUCAACGAAACUGC
((((((.....))))..((.((((((.((.((((..((((((((((((....((((((......))))))))))))))))))..)))).)).)))))).)).)))......

>Predicted STAR seq:
CCGGCCCACTTTAGTTGCTGTAGGAGG

Complementarity: 18
ERROR 4: Strange structure
Initial dG =   -51.20

```
------     |   UG
      UCG--CUGC  \
      AGC  GACG  A
CGUCAA   \ ^   GG
110
```

```
             20        30        40        50        60
           AC  U      G  G    AC            AGGA      AU
             UG CACUGA CC GCCC  UUUAGUUGCUGU    GGAGAU  G
             AC GUGGCU GG CGGG  AAGUCAACGAUA    UCUCUA  U
           A-  U      G  G    CU            ----      CG
             100        90        80            70
```

Reads in: lib-42, lib-45, lib-47, lib-48, lib-49, lib-50, lib-72
Number of reads="ID1212",326,150,25,6552,1966,31,0

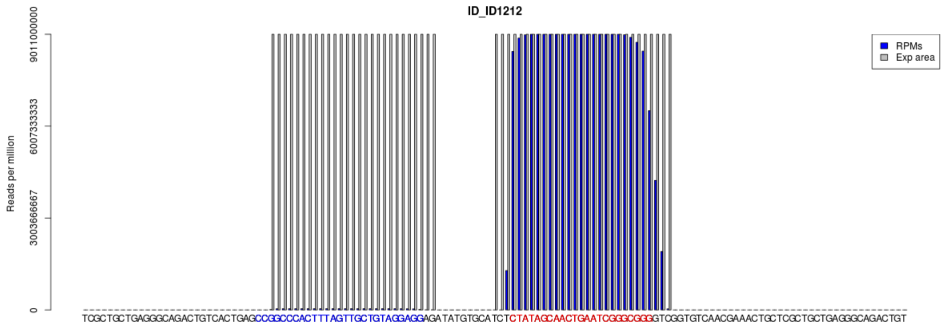

>ID1213
TCGCTGCTGAGAGCAGATTGTCACTGAGCTGCCCCGCTTCAGTTGCTGTAGGAGTAGTTATGTGCATCT**CTACAGCAACTGAAGCGGGTT**
GGGTCGGTGGGCAACGAAACTG

>ID1213-3P
CTACAGCAACTGAAGCGGGTTG

>miRNA precursor
UCGCUGCUGAGAGCAGAUUGUCACUGAGCUGCCCCGCUUCAGUUGCUGUAGGAGUAGUUAUGUGCAUCU**CUACAGCAACUGAAGCGGGUUG**GGUCGGUGGGCAACGAAACUG
...((((.....)))).(((((((((.((..(((((((((((((((((((((((((......))).)).)))))))))))))))))))..)).))))).)))))........

>Predicted STAR seq:
GCCCCGCTTCAGTTGCTGTAG

Complementarity: 18
ERROR 4: Strange structure
Initial dG = -60.00

```
.-UCG|   UG
     CUGC  \
     GACG  A
\ ---^   AG
```

```
           20         30        40        50
  A-------     -     G  GC                  -  -   GU
          UUGUC ACUGA CU  CCCGCUUCAGUUGCUGUAG GA GUA  U
          AACGG UGGCU GG  GGGCGAAGUCAACGACAUC CU CGU  A
  GUCAAAGC     G     G  UU                  U  A   GU
  110       100        90        80        70        60
```

Reads in: lib-42, lib-45, lib-47, lib-48, lib-49, lib-50, lib-72
Number of reads="ID1213",34,26,19,867,194,10,0

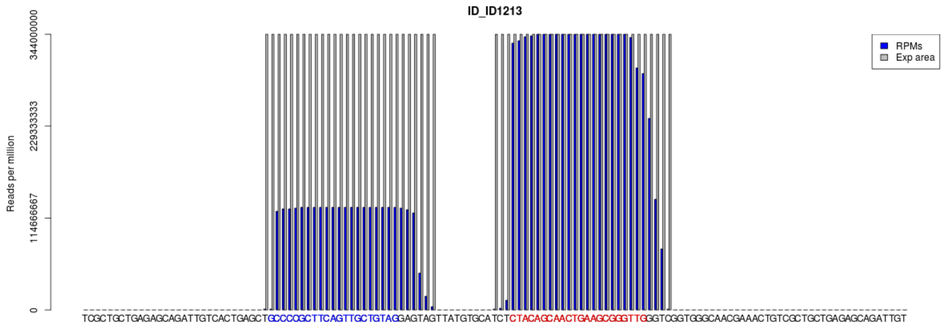

>ID1214
CAGCTGCTGAGAGCTGATTGCCACTGAGCCGAGCCGCCTCAGTTGACTGTTTAGGAGACATGTGCATCCC**TAAAGCAACTGAGGCGAGTG**
GGTTCGGTGGTCGACGAATCT

>ID1214-3P
TAAAGCAACTGAGGCGAGTGG

>miRNA precursor
CAGCUGCUGAGAGCUGAUUGCCACUGAGCCGAGCCGCCUCAGUUGACUGUUUAGGAGACAUGUGCAUCCC**UAAAGCAACUGAGGCGAGUGG**GUUCGGUGGUCGACGAAUCU
(((((......)))))...((((((((((..((((((((((((.((..(((((.((........)))))))))))))))))))).)).)))))))))))..........

>Predicted STAR seq:
GAGCCGCCTCAGTTGACTGTTTA

Complementarity: 17
ERROR 4: Strange structure
Initial dG = -53.60

```
  |    GC
--CAGCU  U
  GUCGA  G
\ ^    GA
       10


           20        30         40        50        60
  AUU-------           GA   -           A   GU      A   CAU
            GCCACUGAGCC  GC CGCCUCAGUUG CU  UUAGG GA   G
            UGGUGGCUUGG  UG GCGGAGUCAAC GA  AAUCC CU   U
  UCUAAGCAGC           G-   A           -   --      -   ACG
 110       100        90         80            70
```

Reads in: lib-42, lib-45, lib-47, lib-48, lib-49, lib-50, lib-72
Number of reads="ID1214",59,10,14,963,384,4,0

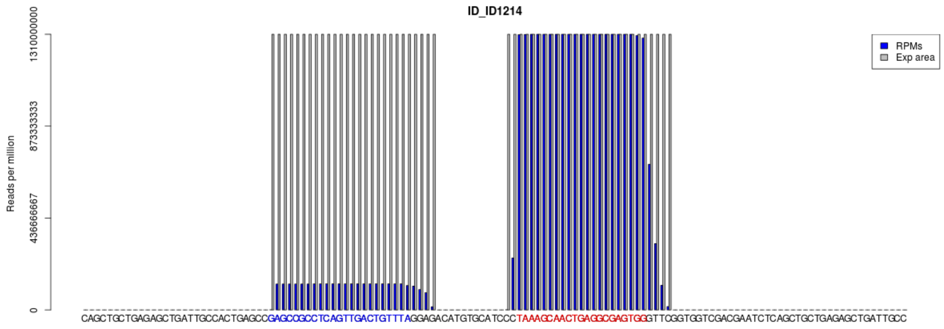

>ID1215
TCGCTGCTTAGCTCTGATTGTCACTGAGCCGGCCCGCTTCAGTTACTGTTGGAGGCGTTTTGTGCATCT**CTAGAGCAACTGAAGTGGACC**
GGGTCGGTGTTCAACGAAACT

>ID1215-3P
CTAGAGCAACTGAAGTGGACCG

>miRNA precursor
UCGCUGCUUAGCUCUGAUUGUCACUGAGCCGGCCCGCUUCAGUUACUGUUGGAGGCGUUUUGUGCAUCU**CUAGAGCAACUGAAGUGGACCG**GGUCGGUGUUCAACGAAACU
```
..(((....))).....(((.((((((.((((.(((((((((((.((.((((((..((.....))..)))))))).))))))))))).)))).))))))..))).......
```

>Predicted STAR seq:
GGCCCGCTTCAGTTACTGTTGG

Complementarity: 18
ERROR 4: Strange structure
Initial dG = -48.40

```
.-UC|  G
    GCU C
    CGA U
\ --^  U
     10


          20         30        40        50        60
  UCUGA--   U-      G    C          A  G      GC  UU
         UUG  CACUGA CCGG CCGCUUCAGUU CU UUGGAG  GU  \
         AAC  GUGGCU GGCC GGUGAAGUCAA GA GAUCUC  CG  U
  UCAAAGC   UU      G    A          C  -      UA  UG
 110       100        90        80         70
```

Reads in: lib-42, lib-45, lib-47, lib-48, lib-49, lib-50, lib-72
Number of reads="ID1215",114,78,19,3377,1095,11,0

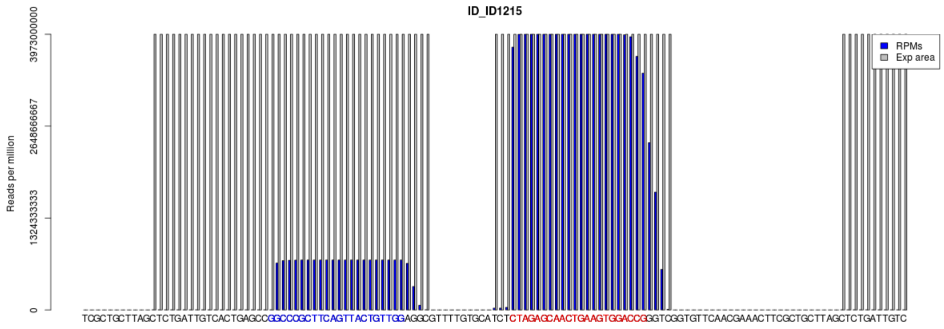

>ID1218

AACTGTCACTGAGTTGGCCCTCTCAGTTGTTGTAGGGACGGATAAACTCCTGTGCATCTCTATAGCAACTGAAGTGGGCCAGCTCGGTAT
TCAACAAAACTGCCTAGTGCAC

>ID1218-5P

TCTCAGTTGTTGTAGGGACGGA

>miRNA precursor

AACUGUCACUGAGUUGGCCCUCUCAGUUGUUGUAGGGACGGAUAAACUCCUGUGCAUCUCUAUAGCAACUGAAGUGGGCCAGCUCGGUAUUCAACAAAACUGCCUAGUGCAC
.......((((((((((((.((((((((((((((((((((........)))....))))))))))))))))).).))))))))))))))...........(((.....))).

>Predicted STAR seq:

GTGCATCTCTATAGCAACTGAAGTGG

Complementarity: 18

Initial dG = -50.60

```
          10        20         30            40
.-AACUGUC             U -                ----|  AUA
         ACUGAGUUGGCCC C UCAGUUGUUGUAGGGA     CGG   A
         UGGCUCGACCGGG G AGUCAACGAUAUCUCU     GUC   A
\ -------             U A               ACGU^  CUC
               80         70        60        50
```

```
  90       100
  AUUCAACAAAAC   CU
              UGC  \
              ACG  A
  C-----------   UG
             110
```

Reads in: lib-42, lib-45, lib-47, lib-48, lib-49, lib-50, lib-72

Number of reads="ID1218",131,65,80,1596,1070,16,0

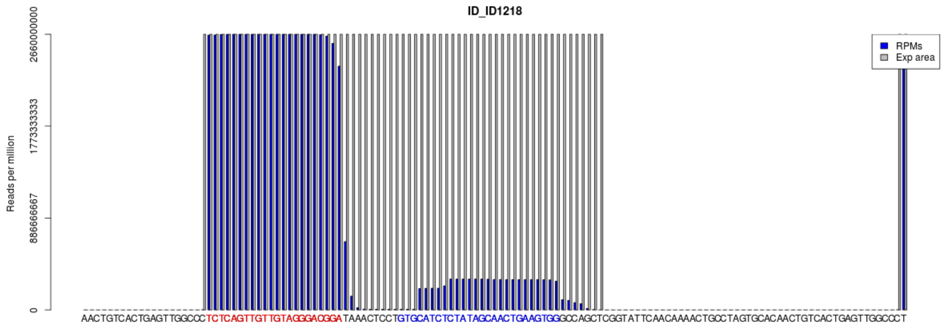

>ID1225
GTCCATGCAGCACCGCCTGA**CGGACAGACTTGAGCTGCGCCG**CGCCACGTCACGAAATCACGGCAGAGCGCAAGTCTCTCTTCAGGCGGT
GTTGACCCACGAGGACAGAG

>ID1225-5P
CGGACAGACTTGAGCTGCGCCG

>miRNA precursor
GUCCAUGCAGCACCGCCUGA**CGGACAGACUUGAGCUGCGCCG**CGCCACGUCACGAAAUCACGGCAGAGCGCAAGUCUCUCUUCAGGCGGUGUUGACCCACGAGGACAGAG
((((.(((((((((((((((.(((.(((((((.(((..((((.................))))..))).))))))).)))))))))))))))).....)).))))....

>Predicted STAR seq:
GCAGAGCGCAAGTCTCTCTTC

Complementarity: 15

Initial dG =   -52.80

```
                 10        20        30        40        50
----    A  -----|            C   C       A   GC     CGCCACGU
    GUCC UG     CAGCACCGCCUGA GGA AGACUUG GCU  GCCG        C
    CAGG GC     GUUGUGGCGGACU UCU UCUGAAC CGA  CGGC        A
GAGA    A  ACCCA^            -   C       G   GA     ACUAAAGC
.       100        90         80        70        60
```

Reads in: lib-42, lib-45, lib-47, lib-48, lib-49, lib-50, lib-72
Number of reads="ID1225",59,9,4,619,93,4,0

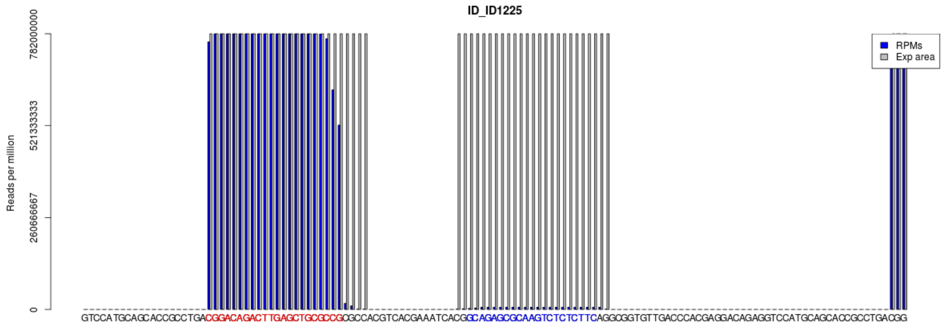

>ID1226

TCTGCAGCAGCACCAGGAACACCTTCAGACTGTGCAGTAGCAGCTGTTTAAGCAGCAGCAAGAACACCTG**TACGGCCTGAAGATGTTCCT**
AGTACTGCAGCAGAAGAACA

>ID1226-3P

TACGGCCTGAAGATGTTCCTAGT

>miRNA precursor

UCUGCAGCAGCACCAGGAACACCUUCAGACUGUGCAGUAGCAGCUGUUUAAGCAGCAGCAAGAACACCUG**UACGGCCUGAAGAUGUUCCUAGU**ACUGCAGCAGAAGAACA
(((((.((((.((.(((((((.((((((.((((((((..((.(((((....))))).))........)))))))).))))))).))))))).)).)))).))))).....

>Predicted STAR seq:

ACCAGGA

Complementarity: 18

Initial dG =   -53.20

```
              10        20        30              40
------     A    C  C       C      A        UA------| A     U
      UCUGC GCAG AC AGGAACA CUUCAG CUGUGCAG        GC GCUGU U
      AGACG CGUC UG UCCUUGU GAAGUC GGCAUGUC        CG CGACG A
ACAAGA     A    A  A       A      C        CACAAGAA^ A     A
.       100        90        80        70        60        50
```

Reads in: lib-42, lib-45, lib-47, lib-48, lib-49, lib-50, lib-72

Number of reads="ID1226",384,24,16,9,7,1,0

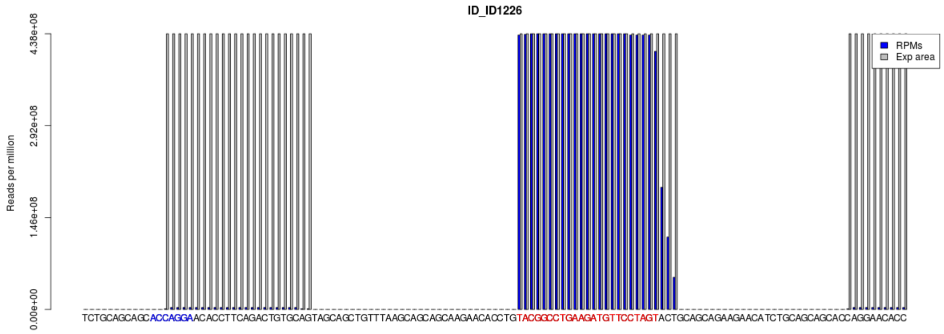

>ID1228
TTTTTGACTTACCCAAAGCGTGGGCAGACATTTGTAGAAAAAGACATTTGTAGAAATGGCTTTTCTACAAATGTCTGCCTATGTGTGTTT
GCGTGTGGATATATGTGTGT

>ID1228-5P
GTGGGCAGACATTTGTAGAAA

>miRNA precursor
UUUUUGACUUACCCAAAGCGUGGGCAGACAUUUGUAGAAAAAGACAUUUGUAGAAAUGGCUUUUCUACAAAUGUCUGCCUAUGUGUGUUUGCGUGUGGAUAUAUGUGUGU
................((((((((((((((((((((((((...(((((....)))))..)))))))))))))))))))))))))......((((((....)))))).....

>Predicted STAR seq:
TCTACAAATGTCTGCCTATGT

Complementarity: 19

Initial dG = -43.10

```
          10         20         30         40         50
.-UUUUUGACUUACCCAAA|                       AGA      G
                   GCGUGGGCAGACAUUUGUAGAAAA   CAUUU U
                   UGUAUCCGUCUGUAAACAUCUUUU   GUAAA A
\ -----------------^                       CG-      G
                     80         70         60



      90
  GUGUUU      G
        GCGUGU G
        UGUAUA A
  UGUG--      U
110         100
```

Reads in: lib-42, lib-45, lib-47, lib-48, lib-49, lib-50, lib-72
Number of reads="ID1228",2,5,1,6,2,9,0

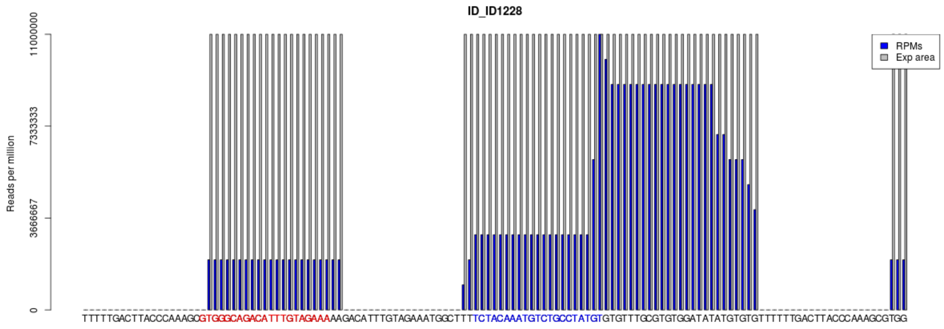

>ID1233

GATGACAGCAGAAAGAGGCGGAGCCACTCTCTTCTGAGTGTTGGACAGAGGGCTGGTACTGGGGAGACC**CTCTGTCTATCATTTGGAGGA**
CAGTTGCCTGGCCGTAGCTTCG

>ID1233-3P

CTCTGTCTATCATTTGGAGGACA

>miRNA precursor

GAUGACAGCAGAAAGAGGCGGAGCCACUCUCUUCUGAGUGUUGGACAGAGGGCUGGUACUGGGGAGACC**CUCUGUCUAUCAUUUGGAGGACA**GUUGCCUGGCCGUAGCUUCG

```
...((.(((.......(((((.((.(((.(((((..((((.(((((((((((((..........)).)))))))))))).)))).)))))..))).)))).))).)))...))))).
```

>Predicted STAR seq:

CTCTTCTGAGTGTTGGACAGAG

Complementarity: 17

Initial dG =   -47.10

```
        10         20        30        40        50
GAU| C   AGAAAGA   -  A  C   C     UG    U           -  GGUA
   GA AGC       GGC GG GC ACU UCUUC  AGUG UGGACAGAGGG CU    C
   CU UCG       CCG UC CG UGA AGGAG  UUAC AUCUGUCUCCC GA    U
G--^ -   AUG----   G  -  U   C     GU    U           A  GGGG
  110            100        90        80        70        60
```

Reads in: lib-42, lib-45, lib-47, lib-48, lib-49, lib-50, lib-72

Number of reads="ID1233",32,79,25,3339,445,7,0

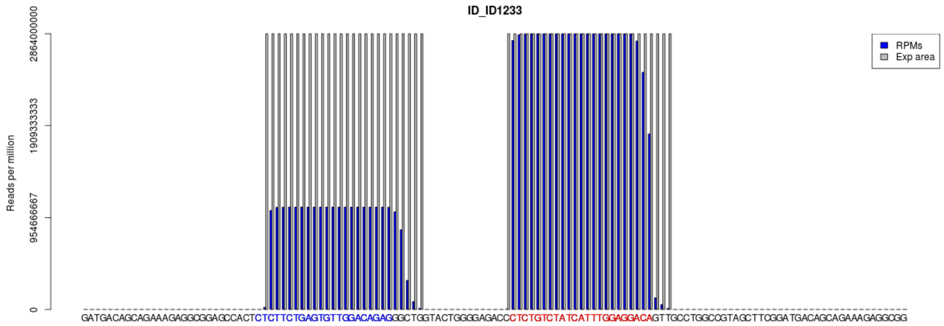

>ID1234
CAGCTCCAGATCTCTGTGCTGTGCCACGGCTTGGGCCTTAGCGGCTCAGTGGCCGACCATGAGCACCGCC**TACTGGCCTGCTAAGTCCCA**
AGCTGGCGCCAGCAGTAC

>ID1234-3P
TACTGGCCTGCTAAGTCCCAAG

>miRNA precursor
CAGCUCCAGAUCUCUGUGCUGUGCCACGGCUUGGGCCUUAGCGGCUCAGUGGCCGACCAUGAGCACCGCC**UACUGGCCUGCUAAGUCCCAAG**CUGGCGCCAGCAGUAC
......(((....)))(((((.((..(((((((((.(((((((((.((((((.((...........)).))))))))).)))))).)))))))))..)))))))....

>Predicted STAR seq:
TTGGGCCTTAGCGGCTCAGTG

Complementarity: 18
ERROR 4: Strange structure
Initial dG =   -52.40

```
          10
.-CAGCUC|  A
        CAG U
        GUC C
\ ------^  U
```

```
        20         30         40           50
  ----     U  CA         C      .-G      G   C
      UGCUG GC  CGGCUUGGG CUUAGC   GCUCA UGG C
      ACGAC CG  GUCGAACCC GAAUCG   CGAGU ACC G
  CAUG     -  CG         U      \ -      -   A
        100         90         80      60
```

Reads in: lib-42, lib-45, lib-47, lib-48, lib-49, lib-50, lib-72
Number of reads="ID1234",98,416,2947,179,3165,1622,0

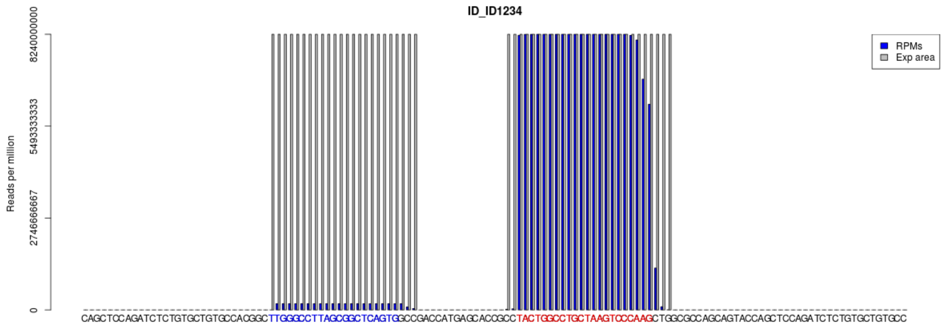

>ID1235
CGGCGGCATCGGCGCGTTGCAGCAGTAATGGGGTTTCTAAGCGGCATTTGCCTTTCATGGCTCCGTCATG**CAATGCCCTTGGAAATCCCA**
AACGTGCTCAACAACATC

>ID1235-3P
CAATGCCCTTGGAAATCCCAAA

>miRNA precursor
CGGCGGCAUCGGCGCGUUGCAGCAGUAAUGGGGUUUCUAAGCGGCAUUUGCCUUUCAUGGCUCCGUCAUG**CAAUGCCCUUGGAAAUCCCAAA**CGUGCUCAACAACAUC
((.((....)).)).((((.((((((..(((((((((((((.((((((.((.....(((((...))))))))))))))))))))))))))).)).)))))))).......

>Predicted STAR seq:
AATGGGGTTTCTAAGCGGCATTT

Complementarity: 19
ERROR 4: Strange structure
Initial dG =   -43.50

```
  | G  G
--CG CG C
  GC GC A
\ ^ G  U
     10
```

```
           20         30        40        50        60
  C-----    C    -  AA             C      U  CUUUC     U
        GUUG AGCA GU  UGGGGUUUCUAAG GGCAUU GC     AUGGC \
        CAAC UCGU CA  ACCCUAAAGGUUC CCGUAA CG     UACUG C
  CUACAA    -    G  A-             -      -  -----     C
        100          90        80          70
```

Reads in: lib-42, lib-45, lib-47, lib-48, lib-49, lib-50, lib-72
Number of reads="ID1235",22,20,412,20,305,134,0

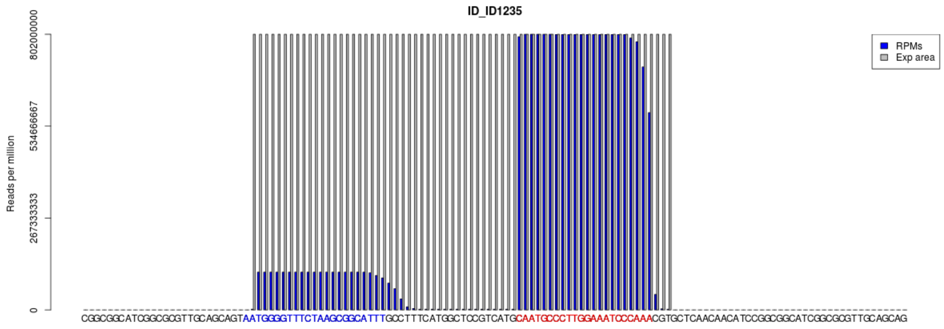

>ID1236
AGTTAAACCTGGTGTCTGGGAGAGTGTCAGGAATGTGACTCGTGGCTTATTTGGAAATCGAAGCACATTTCTGACAATGTCATGACCGCC
AGGTGATTCTTCATGGCATC

>ID1236-5P
AGAGTGTCAGGAATGTGACTCG

>miRNA precursor
AGUUAAACCUGGUGUCUGGGAGAGUGUCAGGAAUGUGACUCGUGGCUUAUUUGGAAAUCGAAGCACAUUUCUGACAAUGUCAUGACCGCCAGGUGAUUCUUCAUGGCAUC
```
......((((((((((...((...(((((((((((((..(((...((.....))....)))..))))))))))))))...))..)).))))))))(((.((....)).)))
```

>Predicted STAR seq:
AAGCACATTTCTGACAATGTCA

Complementarity: 15

Initial dG =   -40.70

```
            10        20        30        40
A--    .-A       -|  UGG  GAG             AC   UGG-  UA
   GUUA   ACCUGGU GUC   GA   UGUCAGGAAUGUG  UCG    CU  \
   CGGU   UGGACCG CAG   CU   ACAGUCUUUACAC  AGC    GG  U
CUA    \ -       C^  UA-  GUA             GA   UAAA  UU
.            90        80        70        60



         -  U
          GA U
          CU C
         A  U
          100
```

Reads in: lib-42, lib-45, lib-47, lib-48, lib-49, lib-50, lib-72
Number of reads="ID1236",4,111,49,9,12,8,0

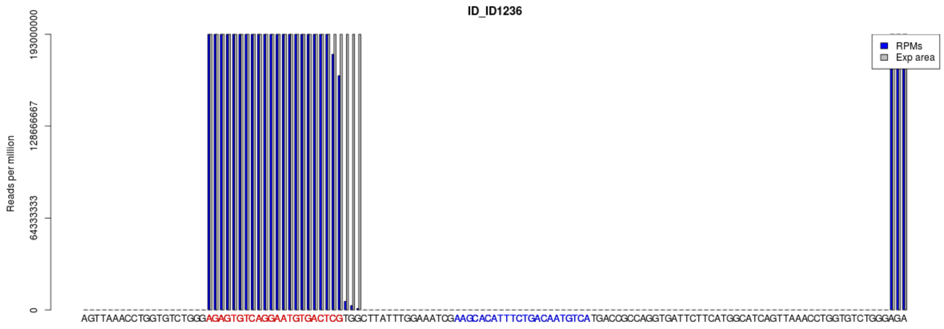

>ID1238
TTGCTTTGAATTATATTTTT**TGGAACACGAGGTAGATTTGTC**TGAGTTATACTGAATGAGACAGCTCTACTTCGTGCTCCAGAAAATATG
AGAAACCATCATCACATATG

>ID1238-5P
TGGAACACGAGGTAGATTTGTC

>miRNA precursor
UUGCUUUGAAUUAUAUUUUU**UGGAACACGAGGUAGAUUUGUC**UGAGUUAUACUGAAUGAGACAGCUCUACUUCGUGCUCCAGAAAAUAUGAGAAACCAUCAUCACAUAUG
..........((((((((((((((.(((((((((((.((((((..(((......))).)))))).))))))))))).))))))))))))))..................

>Predicted STAR seq:
CAGCTCTACTTCGTGCTCCAGA

Complementarity: 18

Initial dG = -40.40

```
        10                  20        30        40        50
UUGCUUUGAA---------|             A           U       GA    AU
                   UUAUAUUUUUUGGA CACGAGGUAGA UUGUCU  GUU   A
                   AGUAUAAAAGACCU GUGCUUCAUCU GACAGA  UAA   C
GUAUACACUACUACCAAAG^             C           C       G-    GU
.       100        90         80        70        60
```

Reads in: lib-42, lib-45, lib-47, lib-48, lib-49, lib-50, lib-72
Number of reads="ID1238",6,23,14,4,10,7,0

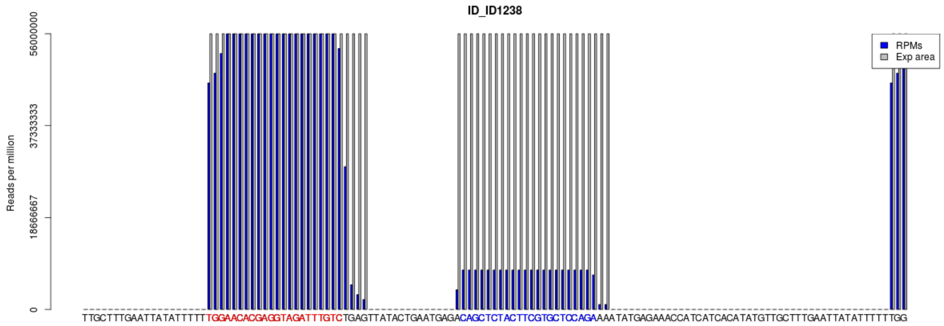

>ID1242
TTGGACGCGTCGACAGGCTTTCCGTCTGCGTTCCAGCACGCTTTCTGGCGTTCTGGCGGTCTGATTACG**TGGATGGTAAGCCTATCGACG**
TGTCCATTGGACGACGTCTCCC

>ID1242-3P
TGGATGGTAAGCCTATCGACGTG

>miRNA precursor
UUGGACGCGUCGACAGGCUUUCCGUCUGCGUUCCAGCACGCUUUCUGGCGUUCUGGCGGUCUGAUUACG**UGGAUGGUAAGCCUAUCGACGUG**UCCAUUGGACGACGUCUCCC
.((((((((((((.((((((.(((((..(((.((((.(((((....))))).)))).........)))..))))).)))))).)))))))))))))..(((.......))).

>Predicted STAR seq:
GACGCGTCG

Complementarity: 17

Initial dG =   -58.70

```
          10        20        30                  40
.-U            C      U     UG   U---------|   C     U
   UGGACGCGUCGA AGGCUU CCGUC  CGU          CCAG ACGCU U
   ACCUGUGCAGCU UCCGAA GGUAG  GCA          GGUC UGCGG C
\ -            A      U     GU   UUAGUCUGGC^   U     U
        90        80        70        60        50
```

Reads in: lib-42, lib-45, lib-47, lib-48, lib-49, lib-50, lib-72
Number of reads="ID1242",2,11,1,216,2,0,0

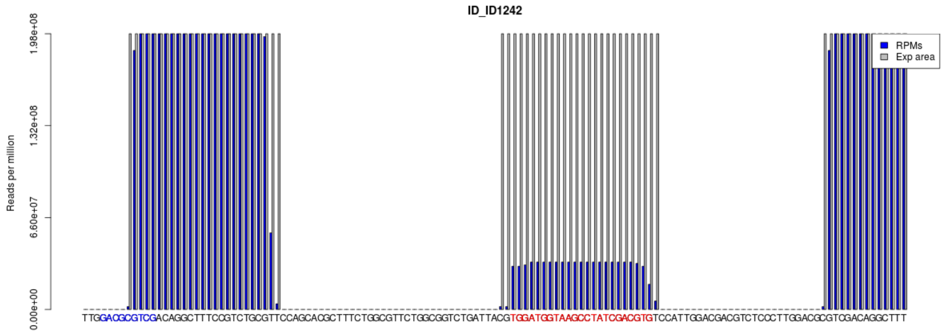

>ID1244
GTATGATGAAGTGGAAGTATGATATGTACATATGCACTGCTATGGAGCTGTCATGTTAATACACTCTACA**TCACAGCTCCATAACGGTGC**
ATATGAACATATACATGATTC

>ID1244-3P
TCACAGCTCCATAACGGTGCAT

>miRNA precursor
GUAUGAUGAAGUGGAAGUAUGAUAUGUACAUAUGCACUGCUAUGGAGCUGUCAUGUUAAUACACUCUACA**UCACAGCUCCAUAACGGUGCAU**AUGAACAUAUACAUGAUUC
```
.............(((.((((((((((.(((((((((((.(((((((((((.((((...........)))).))))))))))).))))))))))).)))))).)))).)))
```

>Predicted STAR seq:
GATATGTACATATGCACTGC

Complementarity: 18

Initial dG =   -48.90

```
        10        20         30        40        50        60
GUAUGAUGAAGUG   G    -|     A           C           C    UAAUA
             GAA UAUG AUAUGU CAUAUGCACUG UAUGGAGCUGU AUGU     \
             CUU GUAC UAUACA GUAUACGUGGC AUACCUCGACA UACA     C
-------------   A    A^     A           A           C    UCUCA
            110       100        90        80        70
```

Reads in: lib-42, lib-45, lib-47, lib-48, lib-49, lib-50, lib-72
Number of reads="ID1244",5,11,26,20,43,6,0

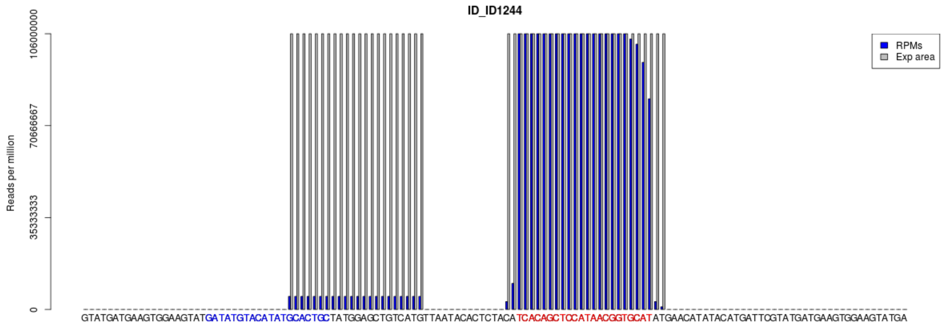

>ID1254
GTTAAGAAATCACTGAAATGTTCATATGGCCCATGATGGGTGTAGGTCTTGTGTACATATATTGGAATTG**TGACTAGATCCACACTCATC**
AAGGGATGTATGACTGCC

>ID1254-3
TGACTAGATCCACACTCATCA

>miRNA precursor
GUUAAGAAAUCACUGAAAUGUUCAUAUGGCCCAUGAUGGGUGUAGGUCUUGUGUACAUAUAUUGGAAUUG**UGACUAGAUCCACACUCAUCA**AGGGAUGUAUGACUGCC
....................((((((..(((.((((((((((.(((((.((.((((...........)))))).))))).)))))))))).))).))..)))))).....

>Predicted STAR seq:
TGATGGGTGTAGGTCTTGTGTA

Complementarity: 17

Initial dG =   -35.50

```
        10        20        30        40        50        60
GUUAAGAAAUCACUGAAAUGU|     GG   A          A     U  G    UAUAU
                     UCAUAU  CCC UGAUGGGUGU GGUCU GU UACA     \
                     AGUAUG  GGG ACUACUCACA CUAGA CA GUGU     U
CCGUC----------------^     UA   A          C     U  -    UAAGG
                      100        90        80         70
```

Reads in: lib-42, lib-45, lib-47, lib-48, lib-49, lib-50, lib-72
Number of reads="ID1254",19060,19928,51081,10786,64470,16079,0

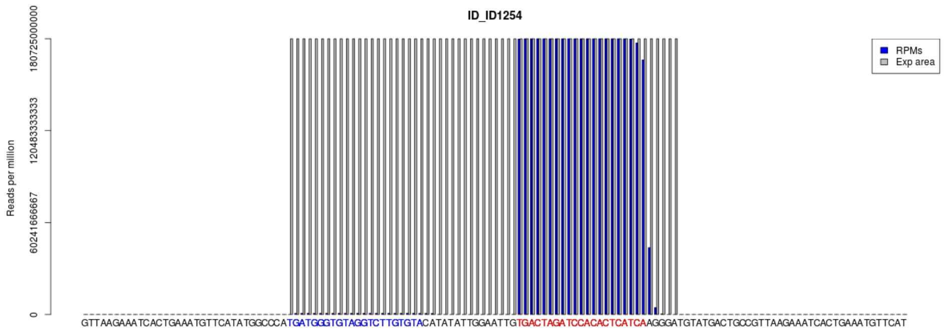

>ID1262

AATGTGTGCAGCAAATAACTGGCCTGTTCCGTGCCCTTGTCATTCTCCTGTCCAGTGTGTATGTGACTAC**TGGACGGAGATATGATAAGG**
TCTTGGGGCAGGCAGAGT

>ID1262-3P

TGGACGGAGATATGATAAGGTC

>miRNA precursor

AAUGUGUGCAGCAAAUAACUGGCCUGUUCCGUGCCCUUGUCAUUCUCCUGUCCAGUGUGUAUGUGACUAC**UGGACGGAGAUAUGAUAAGGUC**UUGGGGCAGGCAGAGU
```
..(((.....)))....(((.((((((((((...((((((((((((((.((((((((.((.....))))))))))))))))).)))))))))...))))))))))..)))
```

>Predicted STAR seq:

GCCCTTGTCATTCTCCTGTCCA

Complementarity: 17
ERROR 4: Strange structure
Initial dG =   -52.00

```
.-AA|  GU
    UGU  \
    ACG  G
\ --^  AC
      10



       20         30        40         50        60
  AAUA   G-          UGC         -      U        U  AU
      ACU  GCCUGUUCCG   CCUUGUCAU UCUCC GUCCAGUG GU  \
      UGA  CGGACGGGGU   GGAAUAGUA AGAGG CAGGUCAU CA  G
  ----   GA          UCU         U      -        -  GU
            100        90        80         70
```

Reads in: lib-42, lib-45, lib-47, lib-48, lib-49, lib-50, lib-72

Number of reads="ID1262",12811,36150,20092,73383,11184,10166,0

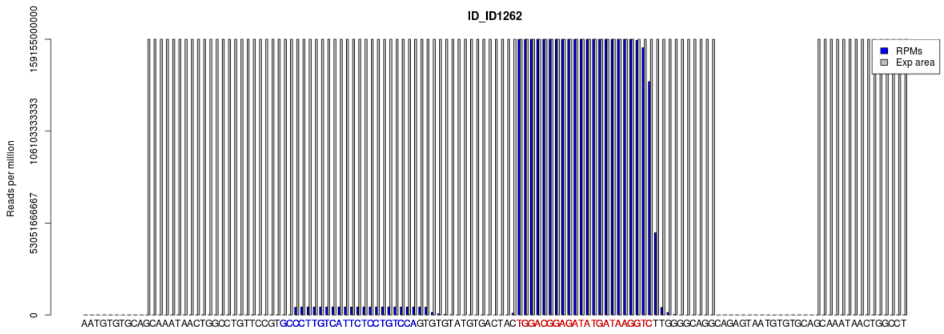

>ID1264
CAGAGGTTGCTCCTCGAAATGCTCCTTGAAGAAGTTCGCGACACCCAGGTGGCACTGTATTGGCATCCAC**TTTGTCACCGGCCGTCGTGA**
ACTTTTACATGAAGCACCTT

>ID1264-3P
TTTGTCACCGGCCGTCGTGAACT

>miRNA precursor
CAGAGGUUGCUCCUCGAAAUGCUCCUUGAAGAAGUUCGCGACACCCAGGUGGCACUGUAUUGGCAUCCAC**UUUGUCACCGGCCGUCGUGAACU**UUUACAUGAAGCACCUU
..((((.....))))....((((...((.(((((((((((((..((.(((((((..((...((...))))..)))))))))..))))))))))))).))...)))).....

>Predicted STAR seq:
GTTCGCGACACCCAGGTGGCA

Complementarity: 17
ERROR 4: Strange structure
Initial dG =   -42.40

```
.-CA|   UU
    GAGG  \
    CUCC  G
\ --^   UC
        10
```

```
     20        30        40        50        60
  GAAA    CCU  A             AC  A       CU  AUU  C
      UGCU   UG AGAAGUUCGCGAC  CC GGUGGCA  GU   GG \
      ACGA   AC UUUUCAAGUGCUG  GG CCACUGU  CA   CC A
  UUCC    AGU  A             CC  -       UU  ---  U
110       100        90        80         70
```

Reads in: lib-42, lib-45, lib-47, lib-48, lib-49, lib-50, lib-72
Number of reads="ID1264",0,374,19,0,0,5,0

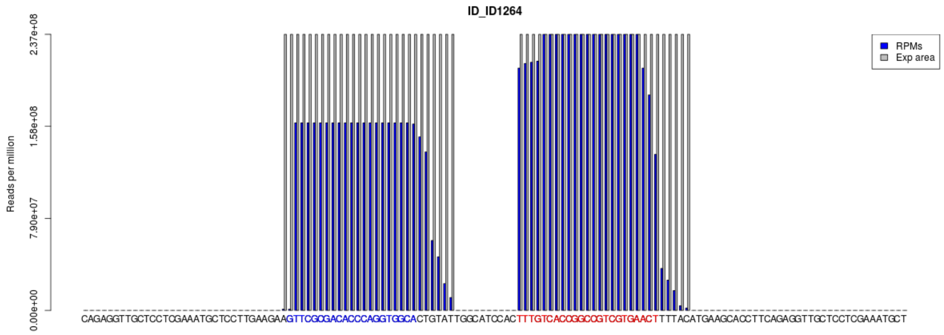

>ID1269
TTGATATGTGTAGTGAGGAA**TGGTAACTCCACCACCGTTGGC**GTGCTACTATAAACTTTTGCCAACGGTTGCGGAGTTCCTTAACCTCAT
TGCATTGGATCAACAGGGCCCA

>ID1269-5P
TGGTAACTCCACCACCGTTGGC

>miRNA precursor
UUGAUAUGUGUAGUGAGGAA**UGGUAACUCCACCACCGUUGGC**GUGCUACUAUAAACUUUUGCCAACGGUUGCGGAGUUCCUUAACCUCAUUGCAUUGGAUCAACAGGGCCCA
(((((..(((((((((((...((.((((((...((((((((((...............))))))))))...))))))))....)))))))))))...))))).........

>Predicted STAR seq:
CAACGGTTGCGGAGTTCCTTAACC

Complementarity: 15

Initial dG =   -46.90

```
                  10        20         30        40        50
---------     AU-           AAU-| U      ACC          UGCUACU
         UUGAU   GUGUAGUGAGG    GG AACUCC   ACCGUUGGCG       A
         AACUA   UACGUUACUCC    CC UUGAGG   UGGCAACCGU       U
ACCCGGGAC     GGU           AAUU^ -      CGU          UUUCAAA
110       100        90        80         70        60
```

Reads in: lib-42, lib-45, lib-47, lib-48, lib-49, lib-50, lib-72
Number of reads="ID1269",16490,9969,38933,3937,75016,5649,0

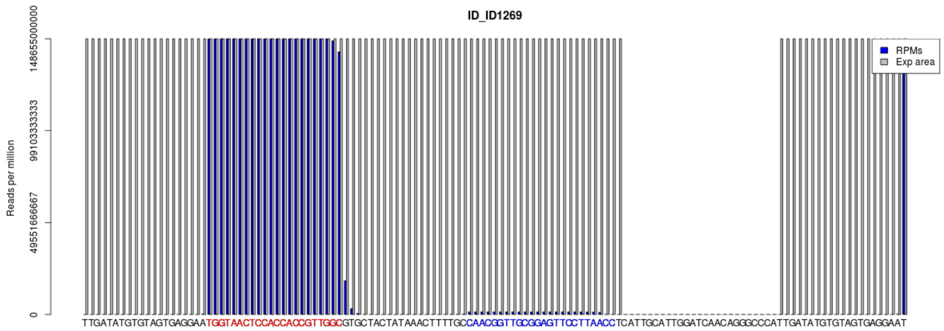

>ID1271
AACGGCCGGCGGTTCTTCCTGTGGGTGTGTCTTTTTCCGCTTTGCTGCCGCGTGTGATATCTCCAGGACG**CCAGCAAAGTGGAAGAGGCC**
GATTCACGGGCAAGTGTCTCTG

>ID1271-3P
CCAGCAAAGTGGAAGAGGCCGA

>miRNA precursor
AACGGCCGGCGGUUCUUCCUGUGGGUGUGUCUUUUUCCGCUUUGCUGCCGCGUGUGAUAUCUCCAGGACG**CCAGCAAAGUGGAAGAGGCCGA**UUCACGGGCAAGUGUCUCUG
......(((.((..(((((((((((.((.((((((((((((((((..((((.((.......))..)))))))))))))))))))))).))))))))))).)))..)).)))

>Predicted STAR seq:
GTGTCTTTTTCCGCTTTGCTGC

Complementarity: 19

Initial dG =   -47.30

```
        10          20         30         40         50
AACGGC   C  UU   -|        G  U                 CC    G-  AUA
      CGG GG  CUU CCUGUGGGU UG CUUUUUCCGCUUUGCUG  GCGU  UG   \
      GUC CU  GAA GGGCACUUA GC GGAGAAGGUGAAACGAC  CGCA  AC   U
------   U  GU   C^        -  C                 --    GG  CUC
      110       100         90         80          70
```

Reads in: lib-42, lib-45, lib-47, lib-48, lib-49, lib-50, lib-72
Number of reads="ID1271",324,3925,2951,484,636,2238,0

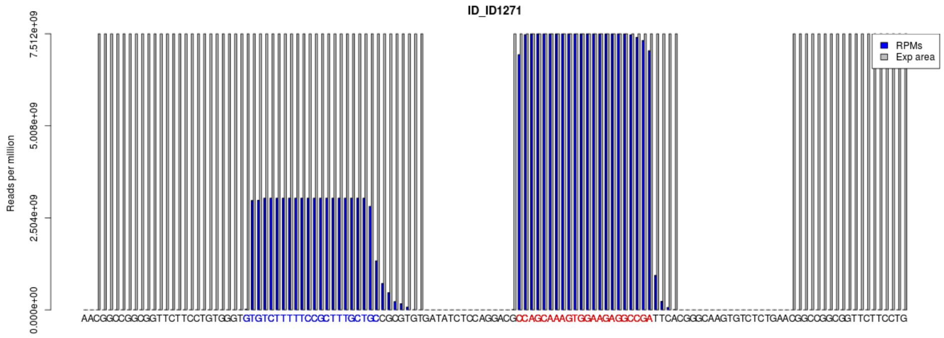

>ID1272
GTTTGCGCACAATGAGTCGGGGCTCGGGGCGCTGCTGGTGCGGAGGCCGTGTGAGTTGGATGTTCAGA**CCTCCGCATCACCAGCCTTCCG**
AGCCCCGCATCACTGGCGCCGA

>ID1272-3P
CCTCCGCATCACCAGCCTTCCGA

>miRNA precursor
GUUUGCGCACAAUGAGUCGGGGCUCGGGGCGCUGCUGGUGCGGAGGCCGUGUGAGUUGGAUGUUCAGA**CCUCCGCAUCACCAGCCUUCCGA**GCCCCGCAUCACUGGCGCCGA
....((((.((.(((..((((((((((((.((((.(((((((((((...(.((((.......)))).))))))))))))).)))).))))))))))))..))).))))))...

>Predicted STAR seq:
CGGGGCGCTGCTGGTGCGGAGG

Complementarity: 19

Initial dG =   -66.30

```
        10        20        30        40        50
GUUU|   A  A   GU           C    C           CCG G    UUG
    GCGC CA UGA  CGGGGCUCGGGG GCUG UGGUGCGGAGG   U UGAG   \
    CGCG GU ACU  GCCCCGAGCCUU CGAC ACUACGCCUCC   A ACUU   G
AGC-^   -  C   AC           C    C           --- G    GUA
110         100        90        80        70           60
```

Reads in: lib-42, lib-45, lib-47, lib-48, lib-49, lib-50, lib-72
Number of reads="ID1272",16,26,1,456,215,3,0

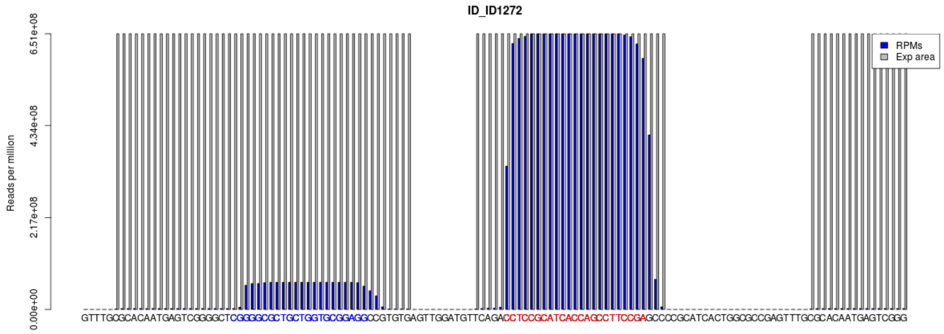

>ID1275
GTCAGCAGCAGGAGAGAGACGTTGGTGCTGGGGAGTCACCTGGTATCACAGGCCACTGGAGAGAGTCAGG**TGGACTGTGCTAGCAGCGGA**
CTCTCCGGCGTCTCCAGGCAAC

>ID1275-3P
TGGACTGTGCTAGCAGCGGACT

>miRNA precursor
GUCAGCAGCAGGAGAGAGACGUUGGUGCUGGGGAGUCACCUGGUAUCACAGGCCACUGGAGAGAGUCAGG**UGGACUGUGCUAGCAGCGGACU**CUCCGGCGUCUCCAGGCAAC
```
.......((.(((((...((....))((((((((((((..(((.((.(((((.(((((.((.....)).))))).))))).)).)))..))))))))))))).)))))..))...
```

>Predicted STAR seq:
GTCACCTGGTATCACAGGCCA

Complementarity: 15
ERROR 4: Strange structure
Initial dG = -52.00

```
        10            20
GUCAGCA  A-     .-GAG| G
       GC  GGAGA     AC U
       CG  CCUCU     UG U
CAA----  GA     \ ---^ G
110
```

```
                     30        40        50        60
                  -           AC   G  U     G     G  GA
                   GCUGGGGAGUC  CUG UA CACAG CCACU GA  \
                   CGGCCUCUCAG  GAC AU GUGUC GGUGG CU  G
                  G           GC   G  C     A     A  GA
                100        90        80        70
```

Reads in: lib-42, lib-45, lib-47, lib-48, lib-49, lib-50, lib-72
Number of reads="ID1275",7,7,1,3,2,0,0

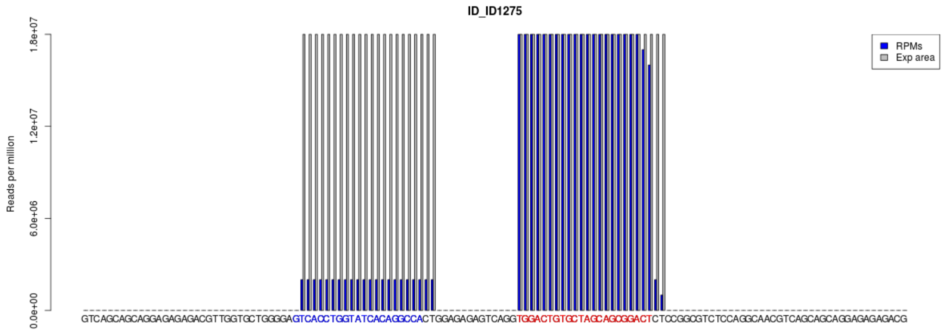

>ID1277
AACCTGCGCATCCCATATGTGGTTCGCTGCATGGCCCCGGTTGTGTGTCTCCGTGGCTATTTGATGTCGG**ATATACATAATCGGTGCTTT**
GTTGCTATCCACGATACTCTTC

>ID1277-3P
ATATACATAATCGGTGCTTTGT

>miRNA precursor
AACCUGCGCAUCCCAUAUGUGGUUCGCUGCAUGGCCCCGGUUGUGUGUCUCCGUGGCUAUUUGAUGUCGG**AUAUACAUAAUCGGUGCUUUGU**UGCUAUCCACGAUACUCUUC
................(((((...((.(((.(((.((((((((((((.((((..((........)))))).))))))))))))).))).))).))...))))).........

>Predicted STAR seq:
GCATGGCCCCGGTTGTGTGTC

Complementarity: 17

Initial dG =   -39.10

```
        10        20        30        40        50        60
AACCUGCGCAUCCCAUA|    UUC  U   U   C           C    UG  UAU
                 UGUGG   GC GCA GGC CCGGUUGUGUGU UCCG  GC   U
                 GCACC   CG UGU UCG GGCUAAUACAUA AGGC  UG   U
CUUCUCAUA--------^    UAU  U   U   U           U    --  UAG
110               100        90        80        70
```

Reads in: lib-42, lib-45, lib-47, lib-48, lib-49, lib-50, lib-72
Number of reads="ID1277",0,0,0,0,0,0,0

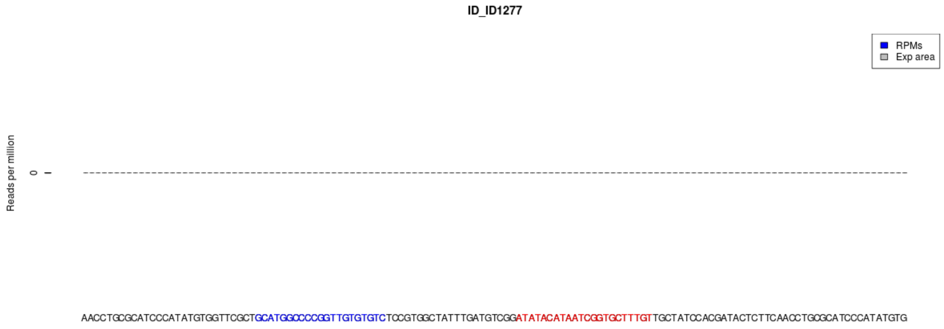

>ID128
AGCTTTCGTCGCAGTCAAGGCGGTCCTTCTGCTTCAATTCCGTAGTGCATTGCAGTGTTTGTTGTAGGTC**TGCAAGCAGTGCGGAAGTGA**
GGTGGAAGAGGCCTCAGAGAC

>ID128-3P
TGCAAGCAGTGCGGAAGTGAG

>miRNA precursor
AGCUUUCGUCGCAGUCAAGGCGGUCCUUCUGCUUCAAUUCCGUAGUGCAUUGCAGUGUUUGUUGUAGGUC**UGCAAGCAGUGCGGAAGUGAG**GUGGAAGAGGCCUCAGAGAC
.((.......)).(((..((.((((((((..(((((.(((((((.(((.((((((..............))))))))).))))))).))))).)))).))))))...)))

>Predicted STAR seq:
TCAATTCCGTAGTGCATTGCA

Complementarity: 17
ERROR 4: Strange structure
Initial dG =   -46.90

```
.-A| UUU
   GC   \
   CG   C
\ -^ CUG
    10
```

```
         20         30        40        50        60
  A   AA-  C    -    UG     A       G   A      UGUUUG
   GUC   GG GGUC CUUC  CUUCA UUCCGUA UGC UUGCAG      U
   CAG   CU CCGG GAAG  GGAGU AAGGCGU ACG AACGUC      U
  -   AGA  -    A    GU     G       G   -      UGGAUG
  110       100        90        80         70
```

Reads in: lib-42, lib-45, lib-47, lib-48, lib-49, lib-50, lib-72
Number of reads="ID128",10,14,1,643,6,1,0

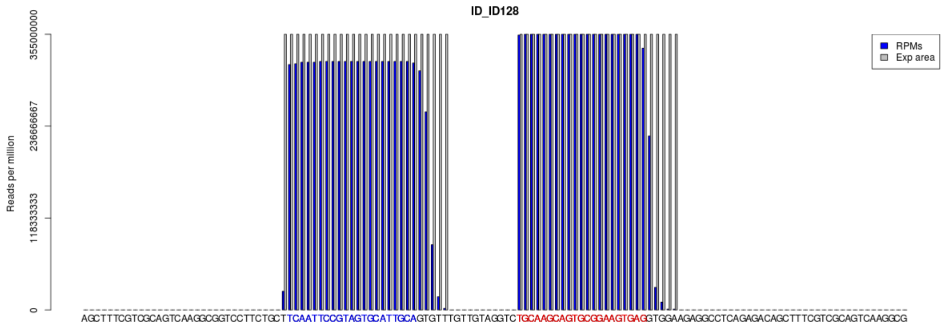

>ID1281
CTTTCCAATGCTACCATCGACATGGTGGGAGATTGTCACATTTAGTGGCCATACGTAATTAATGACAGTA**TCGCTATTATCTGTGACGAT**
ATTCCACCATGTCAATCGCACT

>ID1281-3P
TCGCTATTATCTGTGACGATAT

>miRNA precursor
CUUUCCAAUGCUACCAUCGACAUGGUGGGAGAUUGUCACAUUUAGUGGCCAUACGUAAUUAAUGACAGUA**UCGCUAUUAUCUGUGACGAUAU**UCCACCAUGUCAAUCGCACU
.................((((((((((((.(((((((((..(((((((.((((((........)).)))).))))))).)).))))))))).))))))))))))).........

>Predicted STAR seq:
GATTGTCACATTTAGTGGCC

Complementarity: 16

Initial dG =   -46.40

```
        10        20        30        40        50         60
CUUUCCAAUGCUACCAUC            G         UU       C    -| AAU
                  GACAUGGUGGGA AUUGUCACA  UAGUGGC AUAC GU   U
                  CUGUACCACCUU UAGCAGUGU  AUUAUCG UAUG CA   A
UCACGCUAA---------            A         CU       C    A^ GUA
110                100        90        80        70
```

Reads in: lib-42, lib-45, lib-47, lib-48, lib-49, lib-50, lib-72
Number of reads="ID1281",792,1190,1712,1305,1769,727,0

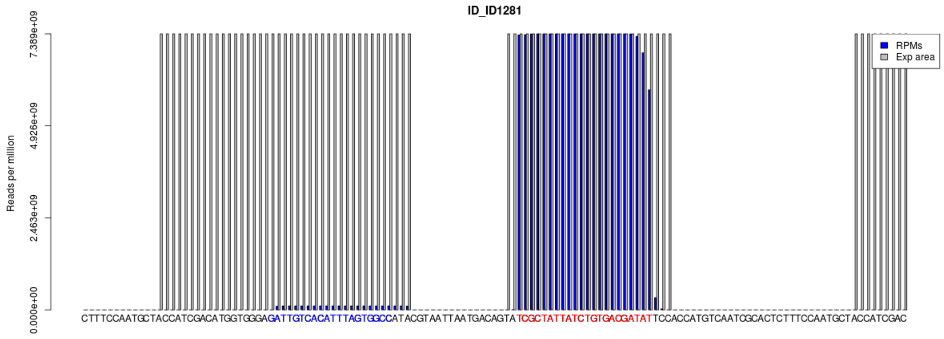

>ID1283
CTTTCTGATGATACCATTGACAAGGTGGGAGCCTGTCACGCCTGCTGGCAGTACTTAGTGAAAGATAGTA**TTGCCAGTAGGTGTGACAGA**
GATCCACCATGTCAACTGCATT

>ID1283-3P
TTGCCAGTAGGTGTGACAGAGA

>miRNA precursor
CUUUCUGAUGAUACCAUUGACAAGGUGGGAGCCUGUCACGCCUGCUGGCAGUACUUAGUGAAAGAUAGUA**UUGCCAGUAGGUGUGACAGAGA**UCCACCAUGUCAACUGCAUU
......(((......((((((.((((((...(((((((((((((((((((((((...........)))))))))))))))))))))))...)))))).))))))...))))

>Predicted STAR seq:
GCCTGTCACGCCTGCTGGCAG

Complementarity: 17

Initial dG =   -59.70

```
        10        20        30        40        50        60
CUUUCU|   AUACCA      A      AGC                       UAGUG
      GAUG      UUGACA GGUGGG   CUGUCACGCCUGCUGGCAGUACU     \
      UUAC      AACUGU CCACCU   GACAGUGUGGAUGACCGUUAUGA     A
------^   GUC---      A      AGA                       UAGAA
      110          100        90        80        70
```

Reads in: lib-42, lib-45, lib-47, lib-48, lib-49, lib-50, lib-72
Number of reads="ID1283",1610,1014,2007,2552,3255,753,0

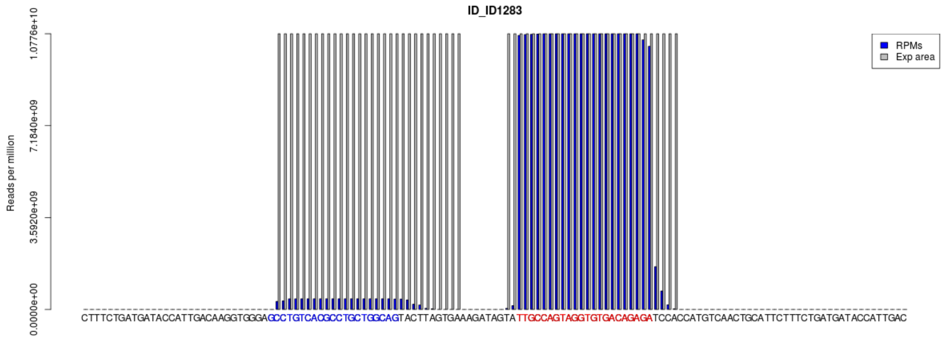

>ID1284
AATCAGTTTGGACGAACCTTGCCTGTTTACGTGTTGTCATTATTCTGCCTCCTCTGTGGTTGAGGAGTCA**GGGGAGTCGGAACAATGGCA**
GCATGTGGCAGGAGCCTACNNN

>ID1284-3P
GGGGAGTCGGAACAATGGCAGC

>miRNA precursor
AAUCAGUUUGGACGAACCUUGCCUGUUUACGUGUUGUCAUUAUUCUGCCUCCUCUGUGGUUGAGGAGUCA**GGGGAGUCGGAACAAUGGCAGC**AUGUGGCAGGAGCCUACNNN
.....((((....))))....((((((.(((((((((((((.(((((.((((((((...........)))))))).))))).)))))))))))))))))))..........

>Predicted STAR seq:
TTGTCATTATTCTGCCTCCTC

Complementarity: 18
ERROR 4: Strange structure
Initial dG = -45.50

```
          10
.-AAUCA|   G
       GUUU G
       CAAG A
\ -----^   C



   20              30        40        50        60
  CUUG------      U             A      C        UGGUU
            CCUGUU ACGUGUUGUCAUU UUCUG CUCCUCUG     G
            GGACGG UGUACGACGGUAA AAGGC GAGGGGAC     A
  NNNCAUCCGA      -             C      U        UGAGG
  110       100         90        80        70
```

Reads in: lib-42, lib-45, lib-47, lib-48, lib-49, lib-50, lib-72
Number of reads="ID1284",29,5,1,68,53,0,0

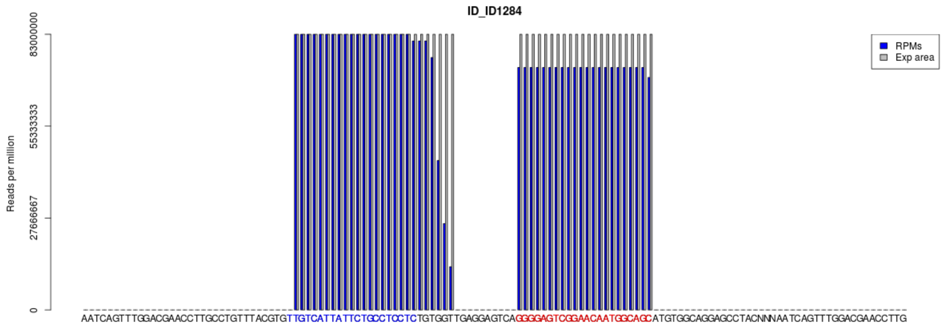

>ID1290
AACCAGTTTGGACGAAGCTTGCCTGCTGACGTGTTGTCATTATTCTGCCTCCTCTGTGGTTGAGGAGTCA**GAGGAGTCGGAACAATGGCA**
GCACGTGGCAGGAGCCTACA

>ID1290-3P
GAGGAGTCGGAACAATGGCAGC

>miRNA precursor
AACCAGUUUGGACGAAGCUUGCCUGCUGACGUGUUGUCAUUAUUCUGCCUCCUCUGUGGUUGAGGAGUCA**GAGGAGUCGGAACAAUGGCAGC**ACGUGGCAGGAGCCUACA
```
..((.....)).....(((..((((((.((((((((((((.(((((.((((((((...........)))))))).))))).)))))))))))))))))))))).....
```

>Predicted STAR seq:
TTGTCATTATTCTGCCTCCTC

Complementarity: 18
ERROR 4: Strange structure
Initial dG =   -52.70

```
.-AA| AG
    CC  \
    GG  U
\ --^ UU
    10
```

```
         20        30        40        50        60
  ACGAA   UG      G            A     C        UGGUU
       GCU  CCUGCU ACGUGUUGUCAUU UUCUG CUCCUCUG     G
       CGA  GGACGG UGCACGACGGUAA AAGGC GAGGAGAC     A
  ACAUC   --      -            C     U        UGAGG
110        100        90        80        70
```

Reads in: lib-42, lib-45, lib-47, lib-48, lib-49, lib-50, lib-72
Number of reads="ID1290",37,5,2,66,70,0,0

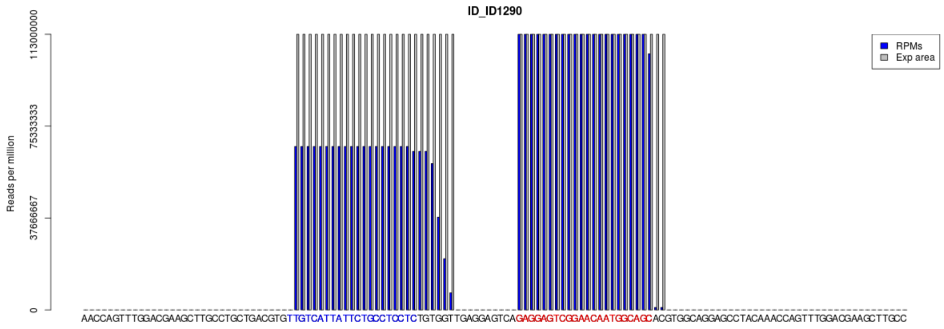

>ID1292
AATGGCCTTTATTTGTGTGA**CTTGGCACTGGAAGAATTCACAGA**TAGTTTGGTAAATCGTGGATCTCCTGGTGCCAAAGCATACATATAA
AAACTGCAACAATGCCGAGTGGTA

>ID1292-5P
CTTGGCACTGGAAGAATTCACAGA

>miRNA precursor
AAUGGCCUUUAUUUGUGUGA**CUUGGCACUGGAAGAAUUCACAGA**UAGUUUGGUAAAUCGUGGAUCUCCUGGUGCCAAAGCAUACAUAUAAAAACUGCAACAAUGCCGAGUGGUA
....((.(((((.((((((..(((((((..(.(((.(((((.(((..........))))))))))).)..)))))))..)))))).)))))....)).....((((....))))

>Predicted STAR seq:
GTGGATCTCCTGGTGCCAAAGCA

Complementarity: 16

Initial dG =   -36.40

```
          10        20        30        40        50
AAUG   .-U|   U      AC        UG A   A     A   AGUU
    GCC   UUAU UGUGUG  UUGGCAC  G AGA UUCAC GAU    U
    UGG   AAUA ACAUAC  AACCGUG  C UCU AGGUG CUA    G
A---   \ -^   U      GA        GU C   -     -   AAUG
         90        80        70         60



                100
         AAACU   A
              GCA C
              CGU A
         UGAGC   A
       110
```

Reads in: lib-42, lib-45, lib-47, lib-48, lib-49, lib-50, lib-72
Number of reads="ID1292",4248,3687,8016,1975,77335,3040,0

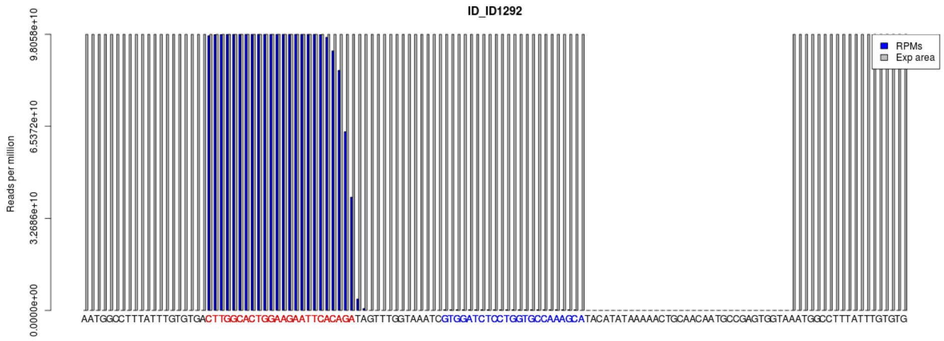

>ID1293
GCGACGGCCGCCGCCCTAGC**AATGGCACTGGAAGAATTCACGG**GGTCTCTTCCTGCCGTGGATTCTCGGTGCCGTCGCTAGTGCCGCGGC
CACTCGACGCTCCCTCGACGTCG

>ID1293-5P
AATGGCACTGGAAGAATTCACGG

>miRNA precursor
GCGACGGCCGCCGCCCUAGC**AAUGGCACUGGAAGAAUUCACGG**GGUCUCUUCCUGCCGUGGAUUCUCGGUGCCGUCGCUAGUGCCGCGGCCACUCGACGCUCCCUCGACGUCG
.....((((((.((.(((((.(((((((((..((((((((((((((......))..)))))))))))))))))))))).))))).)).))))))...(((((.((....)))))))

>Predicted STAR seq:
GTGGATTCTCGGTGCCGTCGC

Complementarity: 18

Initial dG =   -63.60

```
          10        20        30        40
.-GCGAC|     C  C     A         GA          --  UC
       GGCCGC GC CUAGC AUGGCACUG  AGAAUUCACGG  GG  U
       CCGGCG CG GAUCG UGCCGUGGC  UCUUAGGUGCC  CC  C
\ -----^     C  U     C         --          GU  UU
       90        80        70         60        50



        100
  ACU     C  C
     CGACG UC C
     GCUGC AG U
  ---     -  C
      110
```

Reads in: lib-42, lib-45, lib-47, lib-48, lib-49, lib-50, lib-72
Number of reads="ID1293",4446,4942,12733,1681,107832,4777,0

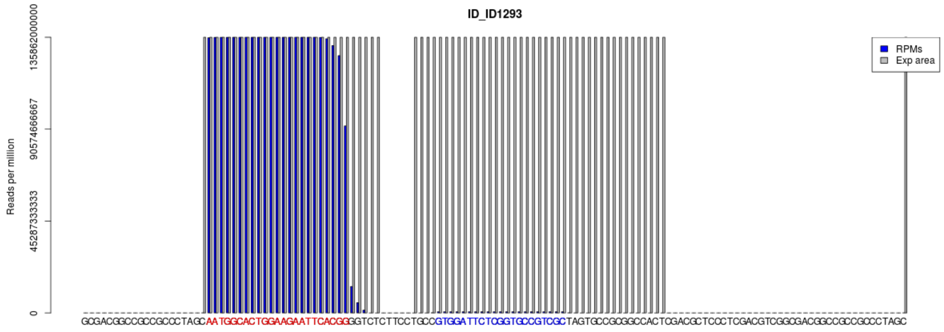

>ID1298
GTGTGACATAGTGTCCAACAGTTTTGATTCTCTGCCTTTTACAAACATGGGGTTTTAGTGTGAATAGAGA**AACCTTATTTTTGTAAATGG**
CAAAGCGTTGAAACAATTCTTC

>ID1298-3P
AACCTTATTTTTGTAAATGGCA

>miRNA precursor
```
GUGUGACAUAGUGUCCAACAGUUUUGAUUCUCUGCCUUUUACAAACAUGGGGUUUUAGUGUGAAUAGAGAAACCUUAUUUUUGUAAAUGGCAAAGCGUUGAAACAAUUCUUC
.((.(((.....)))))...((((..((.((.((((.((((((((.((((((((((...........))))))))))).)))))))).)))).)).))..))))........
```

>Predicted STAR seq:
TGCCTTTTACAAACATGGGGTT

Complementarity: 18
ERROR 4: Strange structure
Initial dG =   -34.70

```
.-G| U   AU
   UG GAC  \
   AC CUG  A
\ -^ -   UG
```

```
   20             30        40        50        60
  ACA-----    UG  U  C    U        C          AGUGU
          GUUU  AU CU UGCC UUUACAAA AUGGGGUUUU     G
          CAAA  UG GA ACGG AAAUGUUU UAUUCCAAAG     A
  CUUCUUAA    GU  C  A    U        U          AGAUA
  110       100        90        80        70
```

Reads in: lib-42, lib-45, lib-47, lib-48, lib-49, lib-50, lib-72
Number of reads="ID1298",251,675,668,807,539,250,0

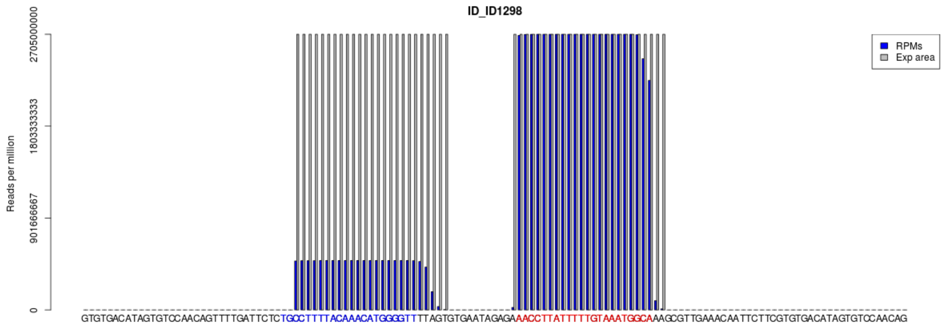

>ID130
TTAAATGTATTGTAGCTGTAAGTGTAAGTATTCAGCTATTCGTATTG**AGCCCATATGGAGTCAGCTGGCA**AATTCCTTTGAATGTGTTGG
CGTTACACCTATGGGAGATC

>ID130-5P
AGCCCATATGGAGTCAGCTGGCA

>miRNA precursor
UUAAAUGUAUUGUAGCUGUAAGUGUAAGUAUUCAGCUAUUCGUAUUG**AGCCCAUAUGGAGUCAGCUGGCA**AAUUCCUUUGAAUGUGUUGGCGUUACACCUAUGGGAGAUC

```
......((((.(((((((..............)))))))..))))....((((((.((.((..((..(((.((((....)))).)))..))...)).)))))))).....
Error predicting STAR on 5pERROR 4: Strange structure
 Initial dG =    -31.00

          10         20
.-UUAAAU    U-|      UAAGUG
        GUAU  GUAGCUG      U
        UAUG  UAUCGAC      A
\ ------    CU^      UUAUGA
            40        30



      50        60         70
  UGAG-      U  A  CA-  UG   A    C
       CCCAUA GG GU   GC  GCA AUUC U
       GGGUAU CC CA   CG  UGU UAAG U
  CUAGA      -  A  UUG  GU   G    U
110       100        90        80
```

Reads in: lib-42, lib-45, lib-47, lib-48, lib-49, lib-50, lib-72
Number of reads="ID130",196,124,832,79,25,100,0

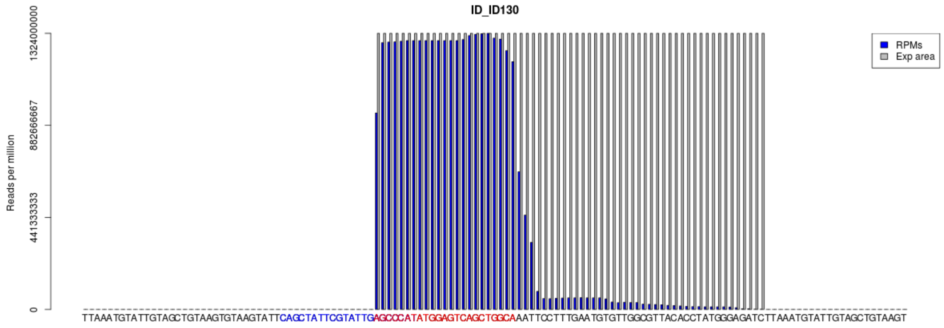

>ID1300
CTCGCAGACTGGACGACTGCTGCGCAGCGCGGACACCCGTTTTGTTCGCTGCGCTGCTATTAATCTCTCG**CGCGTGAACAGAACGGGAGG**
CCGCGAGGCGCGAGGGCGTGCC

>ID1300-3P
CGCGTGAACAGAACGGGAGGCC

>miRNA precursor
CUCGCAGACUGGACGACUGCUGCGCAGCGCGGACACCCGUUUUGUUCGCUGCGCUGCUAUUAAUCUCUCG**CGCGUGAACAGAACGGGAGGCC**GCGAGGCGCGAGGGCGUGCC
```
..........(((((.((..(((((..(((((...((((((((((((((.((((...............))))))))))))))))))...))))).)))))..))))).))
```

>Predicted STAR seq:
GACACCCGTTTTGTTCGCTGCG

Complementarity: 17

Initial dG =   -55.90

```
        10         20        30        40        50        60
CUCGCAGACU  -|  A  GC     AG     ACA              U    UGCUAUU
          GG ACG CU  UGCGC  CGCGG   CCCGUUUUGUUCGC GCGC       \
          CC UGC GG  GCGCG  GCGCC   GGGCAAGACAAGUG CGCG       A
----------  G^  -  GA     GA     GGA              -    CUCUCUA
          110        100        90        80         70
```

Reads in: lib-42, lib-45, lib-47, lib-48, lib-49, lib-50, lib-72
Number of reads="ID1300",4,1,7,2,11,0,0

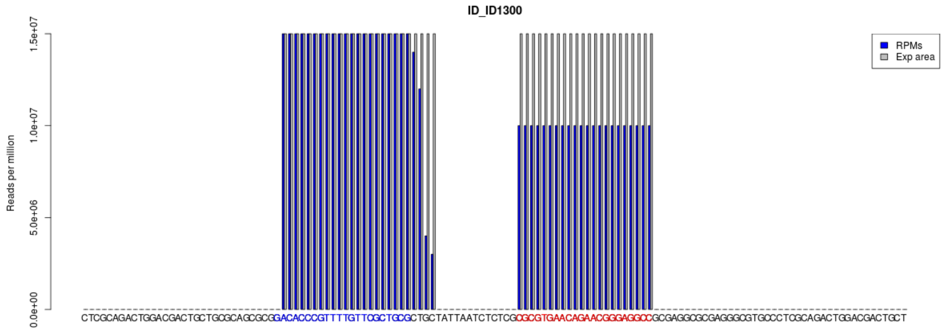

>ID1310
AAGTTGGAAGTGTGAAGTTG**TAAGCTCGTCTTTCTGAGCAGT**AGTGAATATTATCTACTGCCCAAAGGACGAATTTACAAGTTTATACTG
CAAGGTACATCTAGTATGCTAC

>ID1310-5P
TAAGCTCGTCTTTCTGAGCAGT

>miRNA precursor
AAGUUGGAAGUGUGAAGUUG**UAAGCUCGUCUUUCUGAGCAGU**AGUGAAUAUUAUCUACUGCCCAAAGGACGAAUUUACAAGUUUAUACUGCAAGGUACAUCUAGUAUGCUAC
...(((..((((((((.((((((.((((((((.((.(((((((..........))))))).)))))))))).))))))).)))))))).))).((((.....)))).....

>Predicted STAR seq:
TGCCCAAAGGACGAATTTACA

Complementarity: 17

Initial dG =   -39.60

```
          10         20         30         40
.-AAG|  GA         G        C         C  A        UGAA
     UUG  AGUGUGAA UUGUAAG UCGUCUUU UG GCAGUAG    U
     AAC  UCAUAUUU AACAUUU AGCAGGAA AC CGUCAUC    A
\ ---^  G-         G       A        -  C        UAUU
       90         80         70         60         50



          100
  G----    AU
       GUAC  \
       UAUG  C
  CAUCG    AU
  110
```

Reads in: lib-42, lib-45, lib-47, lib-48, lib-49, lib-50, lib-72
Number of reads="ID1310",650,1170,1694,1932,1535,719,0

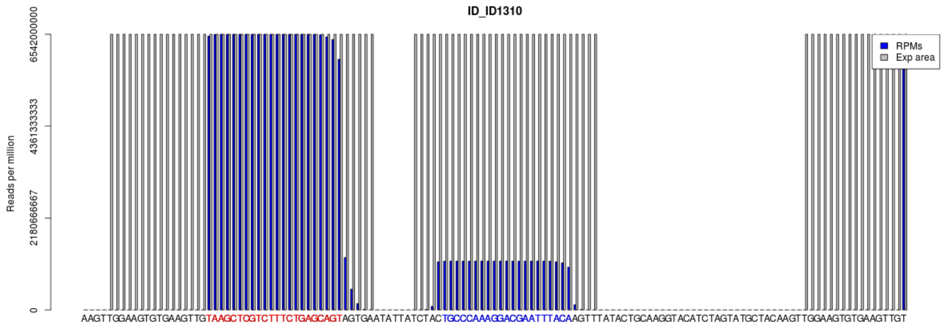

>ID1312
TTTATTATATAATTATAAGGTGACCGGTTTCAATCTATGCAGCATTTCGAAATTTGTCTATGCAGCATTT**AGGTCGAAACCGGTCACCTT**
ATTACAAAATAAATTGTAAAAAC

>ID1312-3P
AGGTCGAAACCGGTCACCTTAT

>miRNA precursor
UUUAUUAUAUAAUUAUAAGGUGACCGGUUUCAAUCUAUGCAGCAUUUCGAAAUUUGUCUAUGCAGCAUUU**AGGUCGAAACCGGUCACCUUAU**UACAAAAUAAAUUGUAAAAAC
..............(((((((((((((((.((((((((.((((..(((...)))...)))).)))..))))).)))))))))))))))(((((......))))).....

```
Error predicting STAR on 5p
 Initial dG =    -41.70

          10         20         30           40          50
.-UUUAUUAUAUAAUU                 A      --    A     UU-|  A
                AUAAGGUGACCGGUUUC AUCUA  UGC GCAU    CGA \
                UAUUCCACUGGCCAAAG UGGAU  ACG CGUA    GUU A
\ --------------                 C     UU    A     UCU^  U
                 90         80         70         60
```

Reads in: lib-42, lib-45, lib-47, lib-48, lib-49, lib-50, lib-72
Number of reads="ID1312",1361,988,349,3321,1079,769,0

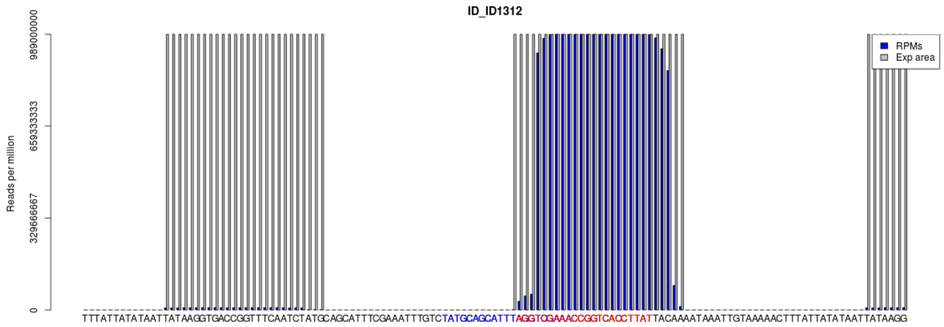

>ID1313
TTTTTTTTGTCTATGCACGA**AAGGATGTGACTGTAGCAGATG**TAGTTGCAGAAAAGCGTTGTTACACTGCTTTGTTACATGCCTGAACTG
CAAGGGACACTGCAACAACGGATT

>ID1313-5P
AAGGATGTGACTGTAGCAGATG

>miRNA precursor
UUUUUUUUGUCUAUGCACGA**AAGGAUGUGACUGUAGCAGAUG**UAGUUGCAGAAAAGCGUUGUUACACUGCUUUGUUACAUGCCUGAACUGCAAGGGACACUGCAACAACGGAUU
.......(((((.((((....((((((((((...(((((.(((((..((........))..))))))))))..))))))).)))....))))..)))))(((......)))...

>Predicted STAR seq:
CTGCTTTGTTACATGCCTGAACTG

Complementarity: 15

Initial dG =   -34.60

```
                10         20         30        40        50
UUUUUUU--------     A-    CGAA   -|      UGU     A     UU  AGA
               UGUCU  UGCA    AGG AUGUGAC   AGCAG UGUAG  GC   A
               ACAGG  ACGU    UCC UACAUUG   UCGUC ACAUU  UG   A
UUAGGCAACAACGUC     GA    CAAG   G^      UU-     -     GU  CGA
  110       100        90        80         70         60
```

Reads in: lib-42, lib-45, lib-47, lib-48, lib-49, lib-50, lib-72
Number of reads="ID1313",7,6,1,5,18,0,0

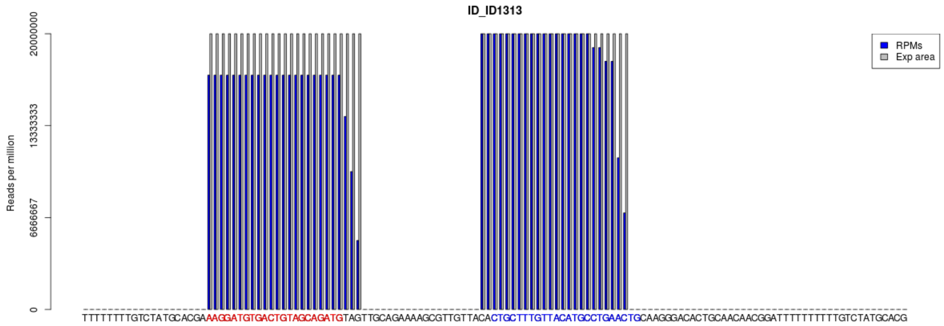

>ID1314
CGCCAGACCACGAGAAGTGTTCGCTAGAGGCCCGGGTACTGCTCACTCTTTCTAGTGTGTGAGAAGCATA**GCAGGAGTAGGCAGTATCAC**
GGGGCTCTTGTGAAGAGCT

>ID1314-3P
GCAGGAGTAGGCAGTATCACG

>miRNA precursor
CGCCAGACCACGAGAAGUGUUCGCUAGAGGCCCGGGUACUGCUCACUCUUUCUAGUGUGUGAGAAGCAUA**GCAGGAGUAGGCAGUAUCACG**GGGCUCUUGUGAAGAGCU
```
..............(((.(((((.((((.((((((((((((..((((((.(((.(((.......)))))).))))))..)))))))).)))).)))).)))))..)))
```

>Predicted STAR seq:
GGGTACTGCTCACTCTTT

Complementarity: 15
ERROR 4: Strange structure
Initial dG = -49.30

```
          10
C  .-CAGAC|  GA
 GC       CAC  \
 CG       GUG  G
U  \ -----^  AA




      20        30         40        50        60
     --      U    G    -        UC      U   G   GUG
       UUCGC AGAG CCCG GGUACUGC  ACUCUU CUA UGU   \
       AAGUG UCUC GGGC CUAUGACG  UGAGGA GAU ACG   A
     AG      U    G    A        GA      C   -   AAG
          100        90        80        70
```

Reads in: lib-42, lib-45, lib-47, lib-48, lib-49, lib-50, lib-72
Number of reads="ID1314",258,436,590,1258,1254,146,0

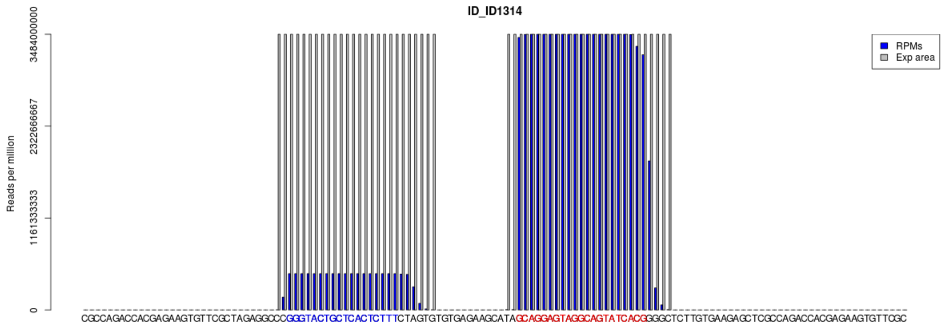

>ID1318
GGACTGTTAAAATACAAAAATGCCAGTGGCTTTTGGCGGGTCTAGGTCTAGGTGACGTTGTGTTTGAACG**TGACTAGATCCATACTCGTC**
TATAACTACTGGTACATAC

>ID1318-3P
TGACTAGATCCATACTCGTCT

>miRNA precursor
GGACUGUUAAAAUACAAAAAUGCCAGUGGCUUUUGGCGGGUCUAGGUCUAGGUGACGUUGUGUUUGAACG**UGACUAGAUCCAUACUCGUCU**AUAACUACUGGUACAUAC
....(((......)))....(((((((((.....(((((((...(((((((.(.(((((.......))))).))))))))...))))))).....))))))))).....

>Predicted STAR seq:
TTTTGGCGGGTCTAGGTCTAGGTG

Complementarity: 14
ERROR 4: Strange structure
Initial dG = -36.50

```
          10
.-GGAC|  UA
      UGU  A
      ACA  A
\ ----^  UA



    20         30         40         50         60
  AAAA-         CUUUU       CUA       G G      GUG
       UGCCAGUGG     GGCGGGU   GGUCUAG U ACGUU    \
       AUGGUCAUC     CUGCUCA   CUAGAUC A UGCAA    U
  CAUAC         AAUAU       UAC       - G      GUU
         100        90         80         70
```

Reads in: lib-42, lib-45, lib-47, lib-48, lib-49, lib-50, lib-72
Number of reads="ID1318",33879,27041,52696,17437,63728,17603,0

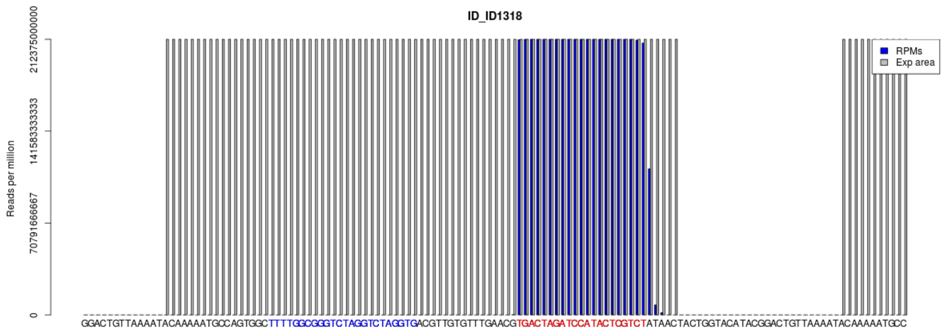

>ID1321
TAACACTGGCTATTGTTGTG**TGGAAGACTAGTGATTTTGTTGT**TCTTTACATGAGTAACAAGGAATCACTAATCATCCTACAAAAGTAGCTGCATGCTTCAGTTAAGTCAACGG

>ID1321-5P
TGGAAGACTAGTGATTTTGTTGT

>miRNA precursor
UAACACUGGCUAUUGUUGUG**UGGAAGACUAGUGAUUUUGUUGU**UCUUUACAUGAGUAACAAGGAAUCACUAAUCAUCCUACAAAAGUAGCUGCAUGCUUCAGUUAAGUCAACGG
.......(((((((.(((((.(((.((.((((((((((.((((((((.....))).)))))))))))))))).)).)))))))).))))))).......................

>Predicted STAR seq:
AAGGAATCACTAATCATCCTA

Complementarity: 17

Initial dG = -33.40

```
                        10        20        30        40
UAACACU----------------|      G     U   A  C          G     -   UA
                       GGCUAUU UUGUG GGA GA UAGUGAUUUU UUGUU CUU  \
                       UCGAUGA AACAU CCU CU AUCACUAAGG AACAA GAG  C
GGCAACUGAAUUGACUUCGUACG^      A     -   A  A          -     U   UA
  110       100       90        80         70         60
```

Reads in: lib-42, lib-45, lib-47, lib-48, lib-49, lib-50, lib-72
Number of reads="ID1321",5339,8047,7406,46144,286427,1830,0

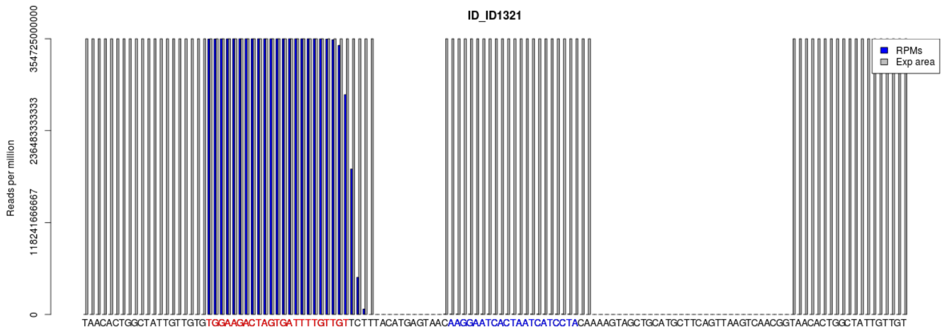

>ID1323
AGAAGCATCATCTCTTCGAGTGGTGGGTTGCGGTCGATGGAGTAATGACAGTCCACGTGGTAATAAGCCG**TGACTTTCGCTACTCCATCT**
AGCTGCTGCCCACTCCACAGCA

>ID1323-3P
TGACTTTCGCTACTCCATCTAG

>miRNA precursor
AGAAGCAUCAUCUCUUCGAGUGGUGGGUUGCGGUCGAUGGAGUAAUGACAGUCCACGUGGUAAUAAGCCG**UGACUUUCGCUACUCCAUCUAG**CUGCUGCCCACUCCACAGCA
.((((........))))..(((((((((.(((((.(((((((((.(((.((((.((..(((.....))))))))).))).)))))))))..))))).))))).)))....

>Predicted STAR seq:
CGATGGAGTAATGACAGTCC

Complementarity: 16
ERROR 4: Strange structure
Initial dG =   -47.00

```
.-A|   CAU
   GAAG   C
   CUUC   A
\ -^   UCU
         10


     20          30         40        50        60
  GA--    --     U     C-         A   C    C  GU   AA
      GUGG  UGGGU GCGGU  GAUGGAGUA UGA AGUC AC  GGU  \
      CACC  ACCCG CGUCG  CUACCUCAU GCU UCAG UG  CCG  U
  ACGA    UC     U     AU         C   U    -  --   AA
  110       100        90        80         70
```

Reads in: lib-42, lib-45, lib-47, lib-48, lib-49, lib-50, lib-72
Number of reads="ID1323",0,0,0,0,0,0,0

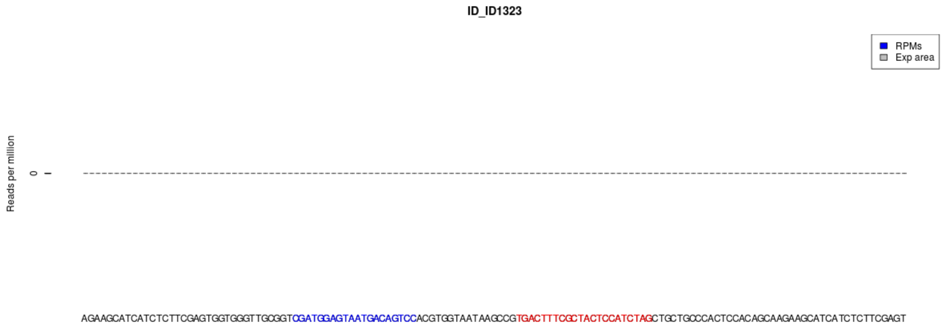

>ID1326
TTGAAGATTGAACTGAGTCA**TGCTTGATAGGACAGACACTGG**TGTATTTTTAAAAATCCCGGTGTTTCTCATATCAAGTGTGGAACTGTT
CAATCCAGCAGCAGTTCAGCATT

>ID1326-5P
TGCTTGATAGGACAGACACTGG

>miRNA precursor
UUGAAGAUUGAACUGAGUCA**UGCUUGAUAGGACAGACACUGG**UGUAUUUUUAAAAAUCCCGGUGUUUCUCAUAUCAAGUGUGGAACUGUUCAAUCCAGCAGCAGUUCAGCAUU
.....((((((((....((((((((((((.((.(((((((((..(..........)..))))))))).)).))))))))))))....)))))))).....((......))...

>Predicted STAR seq:
GGTGTTTCTCATATCAAGTGTG

Complementarity: 18

Initial dG = -39.60

```
          10         20         30         40         50
.-UUGAA|       UGAG            G  C         UG AUUU
       GAUUGAAC    UCAUGCUUGAUA GA AGACACUGG  U    U
       CUAACUUG    GGUGUGAACUAU CU UUUGUGGCC  A    U
\ -----^       UCAA            A  C         CU AAAA
           90         80         70         60



    100
  CAGCA  AG
       GC  U
       CG  U
  UUA--  AC
     110
```

Reads in: lib-42, lib-45, lib-47, lib-48, lib-49, lib-50, lib-72
Number of reads="ID1326",593,546,3193,5026,2365,268,0

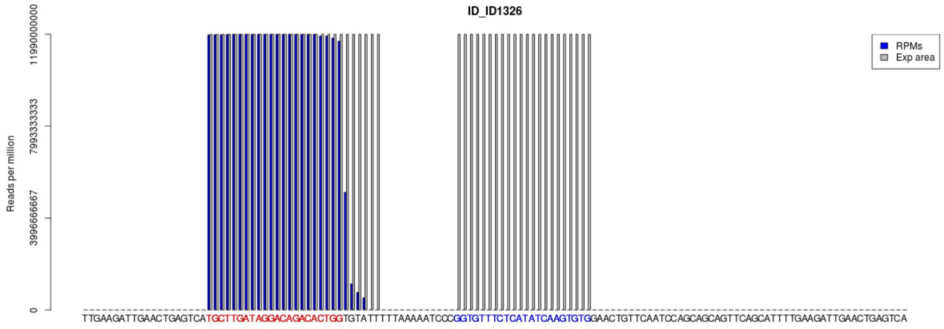

>ID1327
TATATCTATGGATGGTCACA**TGCTTGATAGGACAGACACTGG**TGTAATGTTAAAGATCCCGGTGTCTCTCATATCAGGTGTGGAACTGTT
CATTGCAACAACAGTGAAGAATT

>ID1327-5P
TGCTTGATAGGACAGACACTGG

>miRNA precursor
UAUAUCUAUGGAUGGUCACA**UGCUUGAUAGGACAGACACUGG**UGUAAUGUUAAAGAUCCCGGUGUCUCUCAUAUCAGGUGUGGAACUGUUCAUUGCAACAACAGUGAAGAAUU
.......((((((((..(((((((((((.((.(((((((((..(..........)..))))))))).)).)))))))))))..)))))))))...................

>Predicted STAR seq:
GGTGTCTCTCATATCAGGTGTG

Complementarity: 18

Initial dG =   -38.80

```
                     10        20        30        40        50
UAUAUCU-------------|         CA            G  C         UG AAUG
                    AUGGAUGGU  CAUGCUUGAUA GA AGACACUGG  U    U
                    UACUUGUCA  GUGUGGACUAU CU UCUGUGGCC  A    U
UUAAGAAGUGACAACAACGU^         AG            A  C         CU GAAA
 110       100        90        80        70        60
```

Reads in: lib-42, lib-45, lib-47, lib-48, lib-49, lib-50, lib-72
Number of reads="ID1327",577,603,3207,5055,2258,245,0

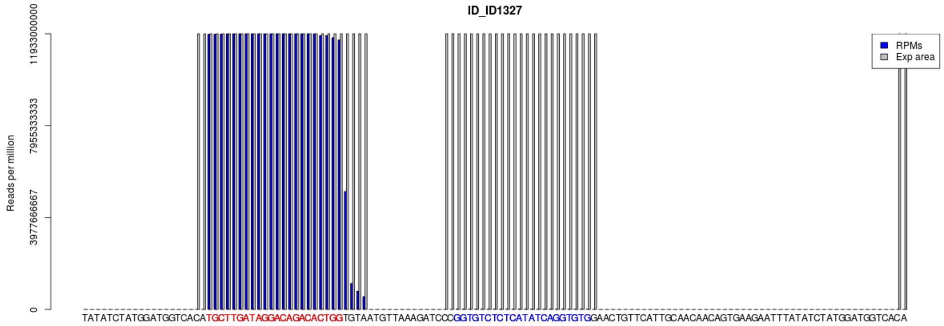

>ID1328
TAAATCTATGGACAGTCACA**TGCTTGATAGGACAGACACTGG**TGTAATGTTAAAGATCCCGGTGTCTCTCATATCAGGTGTGGAACTGTT
CATTGCAACAGCAGTGAAGAATT

>ID1328-5P
TGCTTGATAGGACAGACACTGG

>miRNA precursor
UAAAUCUAUGGACAGUCACA**UGCUUGAUAGGACAGACACUGG**UGUAAUGUUAAAGAUCCCGGUGUCUCUCAUAUCAGGUGUGGAACUGUUCAUUGCAACAGCAGUGAAGAAUU
....(((...((((((..(((((((((((.((.(((((((((..(..........)..))))))))).)).)))))))))))..))))))(((((....))))).)))...

>Predicted STAR seq:
GGTGTCTCTCATATCAGGTGTG

Complementarity: 18

Initial dG = -44.70

```
          10        20        30        40        50
UAAA   .-AUG|     CA           G  C         UG AAUG
    UCU     GACAGU  CAUGCUUGAUA GA AGACACUGG  U    U
    AGA     UUGUCA  GUGUGGACUAU CU UCUGUGGCC  A    U
UUA-   \ ---^     AG           A  C         CU GAAA
  110      90        80        70        60



         -      A
          CAUUGC A
          GUGACG C
         A      A
              100
```

Reads in: lib-42, lib-45, lib-47, lib-48, lib-49, lib-50, lib-72
Number of reads="ID1328",556,569,3176,5109,2278,244,0

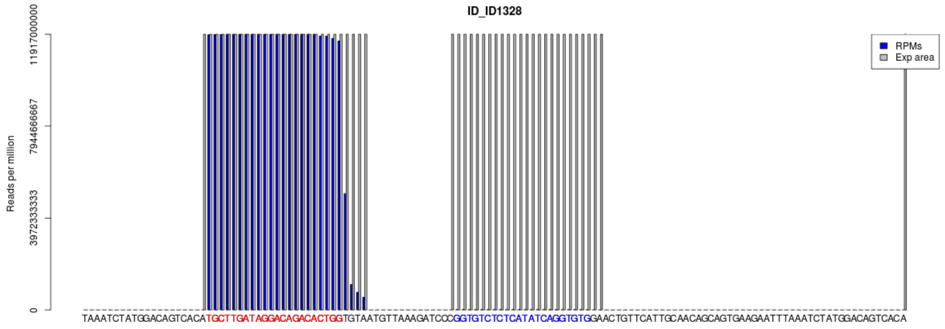

>ID1329
CAATAAATTGAATTGATTCG**TGCTTCATATGACAGACACAGG**TGTATTGTTAAAAATCCCTGTGTCTCTCATATTGGGCACGGAACTGTT
CAGTCTAACTGCAGTTTAGCAT

>ID1329-5P
TGCTTCATATGACAGACACAGG

>miRNA precursor
CAAUAAAUUGAAUUGAUUCG**UGCUUCAUAUGACAGACACAGG**UGUAUUGUUAAAAAUCCCUGUGUCUCUCAUAUUGGGCACGGAACUGUUCAGUCUAACUGCAGUUUAGCAU
...((.(((((((...((((((((..((((((.(((((((((..(.((....)).)..))))))))).))))))..))))))))...))))))).))..(((......))).

>Predicted STAR seq:
TGTGTCTCTCATATTGGGCACG

Complementarity: 17

Initial dG =   -42.40

```
          10        20        30        40        50
.-CAA| A       UGA       UC      C         UG A  G
     UA AUUGAAU   UUCGUGCU  AUAUGA AGACACAGG  U UU U
     AU UGACUUG   AGGCACGG  UAUACU UCUGUGUCC  A AA U
\ ---^ C       UCA       GU      C         CU A  A
           90        80        70        60



  100
  AC   AG
    UGC  U
    ACG  U
  U-   AU
   110
```

Reads in: lib-42, lib-45, lib-47, lib-48, lib-49, lib-50, lib-72
Number of reads="ID1329",11,3,1,53,7,1,0

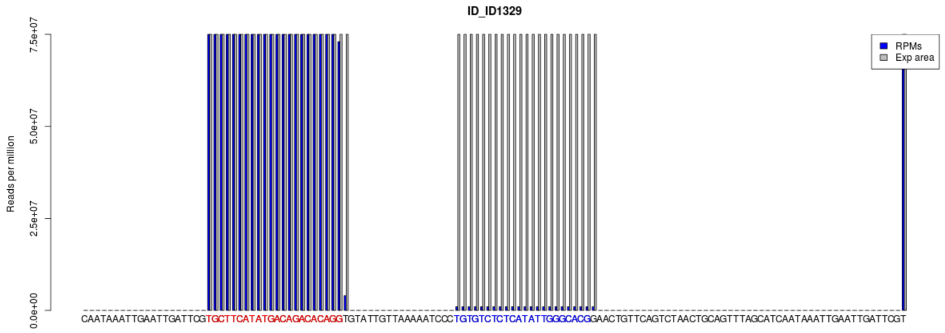

>ID133
CGTGTCCGCGCGGGTCCGGT**TTGGTATTTCGGGTTCCTGCCTG**CAGGATATTCAGGGCTCTCAGGCGGCGATCCGAAATACCGGCTGGAT
CCGCTGCAAATGACGCCTACTC

>ID133-5P
TTGGTATTTCGGGTTCCTGCCTG

>miRNA precursor
CGUGUCCGCGCGGGUCCGGU**UUGGUAUUUCGGGUUCCUGCCUG**CAGGAUAUUCAGGGCUCUCAGGCGGCGAUCCGAAAUACCGGCUGGAUCCGCUGCAAAUGACGCCUACUC
.(((((.(((((((((((((.((((((((((((.(((((((.(((...........)))))))))).))))))))))))))))))))))))).)).....))))).....

>Predicted STAR seq:
GGCGGCGATCCGAAATACCGGC

Complementarity: 19

Initial dG = -61.50

```
                 10        20        30        40        50
C-----     C---  -|           U             C       C   AUAUU
      GUGUC    GC GCGGGUCCGGUU GGUAUUUCGGGUU CUGCCUG AGG     \
      CGCAG    CG CGCCUAGGUCGG CCAUAAAGCCUAG GGCGGAC UCU     C
CUCAUC     UAAA  U^           -             C       -   CGGGA
110       100        90         80        70         60
```

Reads in: lib-42, lib-45, lib-47, lib-48, lib-49, lib-50, lib-72
Number of reads="ID133",0,0,0,17,1,0,0

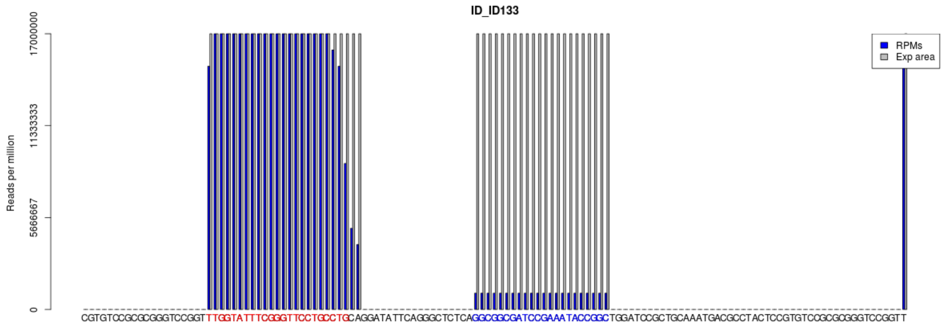

>ID1330
TATATATATGGACTGTCCCA**TGCTTGATAGGACAGACACTGG**TGTAATGTTAAAGATCCCGGTGTCTCTCATATCAGGTGTGGAACTGTT
CATTGCAACAGCAGTTAAGAATT

>ID1330-5P
TGCTTGATAGGACAGACACTGG

>miRNA precursor
UAUAUAUAUGGACUGUCCCA**UGCUUGAUAGGACAGACACUGG**UGUAAUGUUAAAGAUCCCGGUGUCUCUCAUAUCAGGUGUGGAACUGUUCAUUGCAACAGCAGUUAAGAAUU
```
.......((((((.((.((((((((((((.((.(((((((((..(..........)..))))))))).)).))))))))))))).)).))))))(((....)))..........
```

>Predicted STAR seq:
GGTGTCTCTCATATCAGGTGTG

Complementarity: 18

Initial dG =   -40.90

```
          10        20        30        40        50
.-UAUAUAU|     U  C            G  C         UG AAUG
         AUGGAC GU CCAUGCUUGAUA GA AGACACUGG  U    U
         UACUUG CA GGUGUGGACUAU CU UCUGUGGCC  A    U
\ -------^     U  A            A  C         CU GAAA
           90        80        70        60



  ----------   A
            UGC A
            ACG C
  UUAAGAAUUG   A
   110       100
```

Reads in: lib-42, lib-45, lib-47, lib-48, lib-49, lib-50, lib-72
Number of reads="ID1330",548,572,3367,5063,2290,250,0

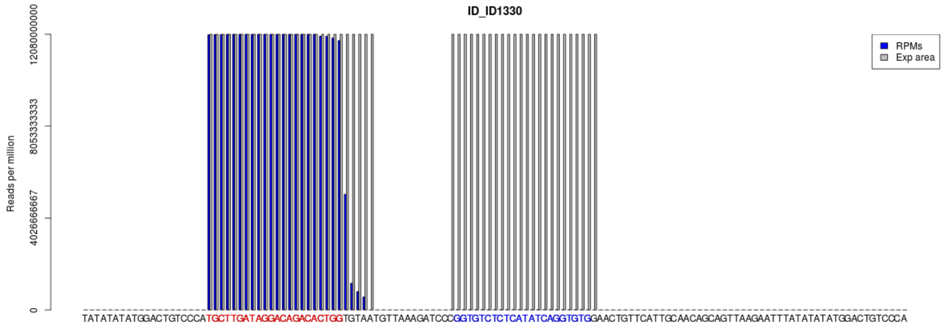

>ID1331
TAAATCTATGGACTGTCTCA**TTCTTTATATGACAGACACTGG**TGTAATGTTAAAGTTCCCGGTGTCTCTTATATCAAGTGTGGAACTGCT
CATTGCATCAGCAGTGAAGAAT

>ID1331-5P
TTCTTTATATGACAGACACTGG

>miRNA precursor
UAAAUCUAUGGACUGUCUCA**UUCUUUAUAUGACAGACACUGG**UGUAAUGUUAAAGUUCCCGGUGUCUCUUAUAUCAAGUGUGGAACUGCUCAUUGCAUCAGCAGUGAAGAAU
..............((..(((.(((.((((((.(((((((((...(((......))).))))))))).)))))).))).)))..))...(((((((....))))))).....

>Predicted STAR seq:
GGTGTCTCTTATATCAAGTGTG

Complementarity: 17

Initial dG =   -32.70

```
          10        20        30        40        50
.-UAAAUCUAUGGACU| CU   U   U      C         UGU   GU
                GU  CAU CUU AUAUGA AGACACUGG   AAU  U
                CA  GUG GAA UAUAUU UCUGUGGCC   UUG  A
\ --------------^ AG   U   C      C         C--   AA
                     80        70        60



      90
  UGC--      A
       UCAUUGC U
       AGUGACG C
  UAAGA      A
  110       100
```

Reads in: lib-42, lib-45, lib-47, lib-48, lib-49, lib-50, lib-72
Number of reads="ID1331",8,0,0,62,3,0,0

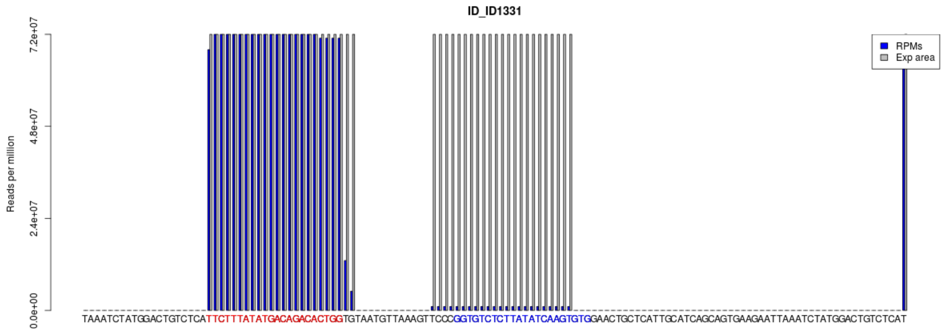

>ID1332
TATATATATGGACAGTCACA**TGCTTGATAGGACAGACACTGG**TGTAATGTTAAAGATCCCGGTGTCTCTCATATCAGGTGTGGAACTGTT
CATTGCAACAGCAGTGAAGAATT

>ID1332-5P
TGCTTGATAGGACAGACACTGG

>miRNA precursor
UAUAUAUAUGGACAGUCACA**UGCUUGAUAGGACAGACACUGG**UGUAAUGUUAAAGAUCCCGGUGUCUCUCAUAUCAGGUGUGGAACUGUUCAUUGCAACAGCAGUGAAGAAUU
...........(((((..(((((((((((.((.(((((((((..(..........)..))))))))).)).)))))))))))..)))))(((((((....)))))))......

>Predicted STAR seq:
GGTGTCTCTCATATCAGGTGTG

Complementarity: 18

Initial dG = -44.30

```
          10        20        30        40        50
.-UAUAUAUAUGG|    CA           G  C         UG AAUG
             ACAGU  CAUGCUUGAUA GA AGACACUGG  U    U
             UGUCA  GUGUGGACUAU CU UCUGUGGCC  A    U
\ -----------^    AG           A  C         CU GAAA
                     80        70        60
```

```
       90
  ------       A
        UCAUUGC A
        AGUGACG C
  UUAAGA       A
   110       100
```

Reads in: lib-42, lib-45, lib-47, lib-48, lib-49, lib-50, lib-72
Number of reads="ID1332",577,580,3342,5066,2322,269,0

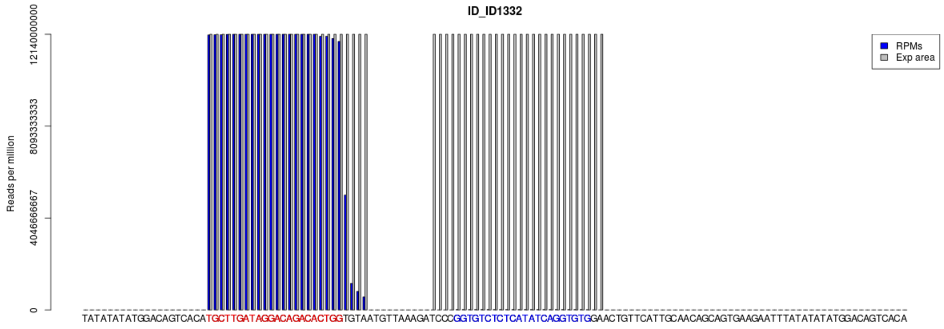

>ID1333
TAAATTTATGGACAGAGCCG**TGCTTGATAGGACAGACACTGG**CGTAATGTTAAAAATCCCGGTGTCTCTCTTATCAGGTGCGGAGCTGTT
CAGCCCAACAGCAGTGATGTATT

>ID1333-5P
TGCTTGATAGGACAGACACTGG

>miRNA precursor
UAAAUUUAUGGACAGAGCCG**UGCUUGAUAGGACAGACACUGG**CGUAAUGUUAAAAAUCCCGGUGUCUCUCUUAUCAGGUGCGGAGCUGUUCAGCCCAACAGCAGUGAUGUAUU
........(((((((..(((..((((((((((.(((((((((.((..........)).))))))))).))))))))))..)))..))))))).........(((....)))...

>Predicted STAR seq:
GGTGTCTCTCTTATCAGGTGCG

Complementarity: 17

Initial dG =   -51.00

```
          10         20         30         40         50
.-UAAAUUUA|      AG   UG          C         C  AAUG
          UGGACAG  CCG  CUUGAUAGGA AGACACUGG GU    U
          ACUUGUC  GGC  GGACUAUUCU UCUGUGGCC UA    U
\ --------^      GA   GU          C         C  AAAA
           90         80         70         60



       100
  GCCCAACA   G
          GCA U
          UGU G
  UUA-----   A
        110
```

Reads in: lib-42, lib-45, lib-47, lib-48, lib-49, lib-50, lib-72
Number of reads="ID1333",511,525,2840,4205,2038,210,0

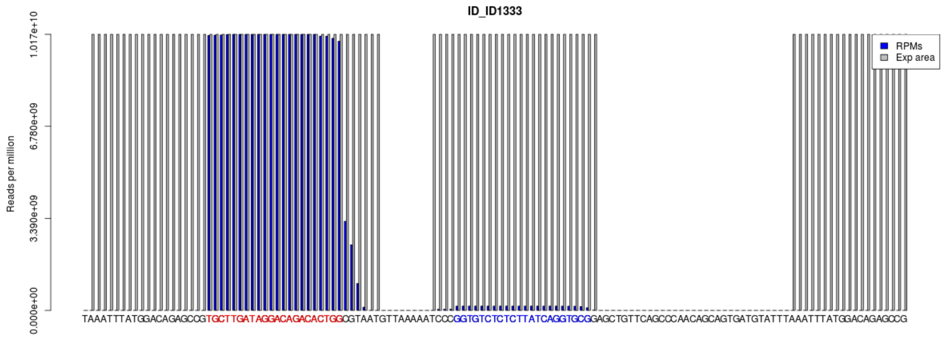

>ID1334
TAGATCTATGGCCGGTCACA**TGCTTGATAGGACAGACACAGG**TGTAATGTTAAAGATCCCGGTGTCTCTCATATCAGGTGTGGAACTGTT
CATTGCAACAGCAGTGAAGA

>ID1334-5P
TGCTTGATAGGACAGACACAGG

>miRNA precursor
UAGAUCUAUGGCCGGUCACA**UGCUUGAUAGGACAGACACAGG**UGUAAUGUUAAAGAUCCCGGUGUCUCUCAUAUCAGGUGUGGAACUGUUCAUUGCAACAGCAGUGAAGA
............((((..(((((((((((.((.((((((.((..(..........)..)).)))))).)).)))))))))))..))))((((((((....))))))))..

>Predicted STAR seq:
GGTGTCTCTCATATCAGGTGTG

Complementarity: 17

Initial dG =   -38.80

```
          10        20        30        40        50
.-UAGAUCUAUGGC|   CA          G  C      A  UG AAUG 
              CGGU  CAUGCUUGAUA GA AGACAC GG  U    U
              GUCA  GUGUGGACUAU CU UCUGUG CC  A    U
\ ------------^   AG          A  C      G  CU GAAA 
                     80        70        60


    90
  --       A
    UUCAUUGC A
    AAGUGACG C
  AG       A
110       100
```

Reads in: lib-42, lib-45, lib-47, lib-48, lib-49, lib-50, lib-72
Number of reads="ID1334",286,242,2906,5685,295,40,0

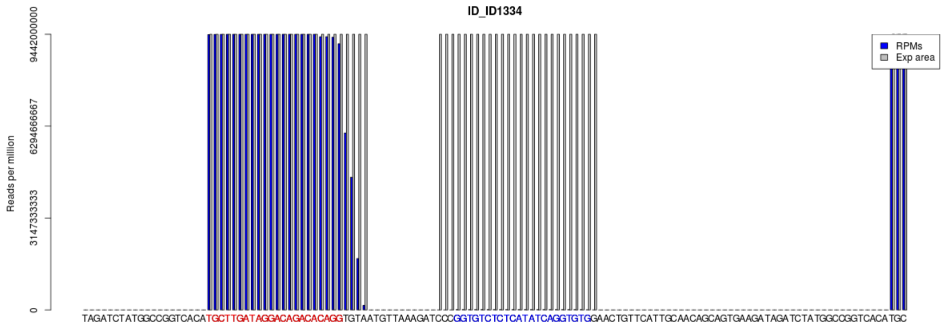

>ID1340
CTGCAGCAGCACCAGGAACACCTTCAGACAGTGCAGTAGCAGCTGCTTAAGCAGCAGCAAGAACACCTG**TACCGCCTGAAGATGTTCCTA**
GTACTGTGGCAGAAGAACATC

>ID1340-3P
TACCGCCTGAAGATGTTCCTAG

>miRNA precursor
CUGCAGCAGCACCAGGAACACCUUCAGACAGUGCAGUAGCAGCUGCUUAAGCAGCAGCAAGAACACCUG**UACCGCCUGAAGAUGUUCCUAG**UACUGUGGCAGAAGAACAUC
((((.((((.((.(((((((.((((((...((((((..((.(((((....))))).))........))))))...)))))).))))))).)).)))).)))).........

>Predicted STAR seq:
CAGGAACACCTTCAGACAGTG

Complementarity: 15

Initial dG = -47.60

```
                 10        20        30               40
---------    A    C  C       C      ACA      UA------| A     U
         CUGC GCAG AC AGGAACA CUUCAG   GUGCAG        GC GCUGC U
         GACG UGUC UG UCCUUGU GAAGUC   CAUGUC        CG CGACG A
CUACAAGAA    G    A  A       A      CGC      CACAAGAA^ A     A
 .       100        90        80        70        60        50
```

Reads in: lib-42, lib-45, lib-47, lib-48, lib-49, lib-50, lib-72
Number of reads="ID1340",11,1,0,0,0,0,0

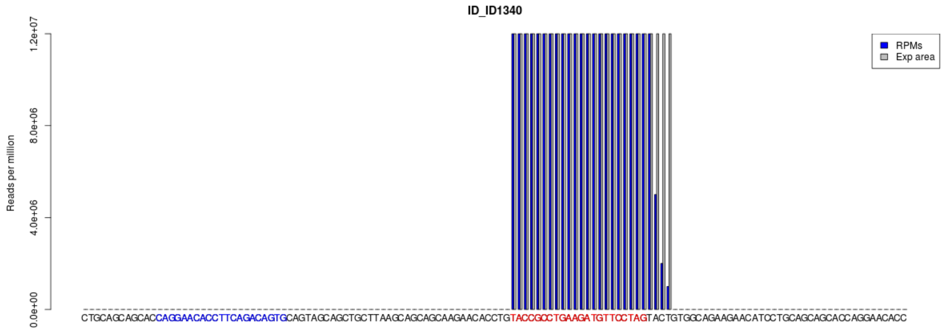

>ID1341
CCTGCAGCAGCACCAGGAACACCTTCAGACAGTGCAGTAGCAGCTGCTTAAGCAGCAGCAAGAACACCTG**TACGGCCTGAAGATGTTCCT**
AGTACTGCAGCAGAAGAACATCA

>ID1341-3P
TACGGCCTGAAGATGTTCCTAGT

>miRNA precursor
CCUGCAGCAGCACCAGGAACACCUUCAGACAGUGCAGUAGCAGCUGCUUAAGCAGCAGCAAGAACACCUG**UACGGCCUGAAGAUGUUCCUAGU**ACUGCAGCAGAAGAACAUCA
.((((.((((.((.(((((((.((((((...((((((..((.(((((....))))).))........))))))...))))))).))))))).)).)))).)))).........

>Predicted STAR seq:
ACCAGGAACACCTTCAGACAGTG

Complementarity: 16

Initial dG =   -49.90

```
                 10        20        30              40
C---------    A    C  C        C      ACA      UA------| A     U
          CUGC GCAG AC AGGAACA CUUCAG   GUGCAG        GC GCUGC U
          GACG CGUC UG UCCUUGU GAAGUC   CAUGUC        CG CGACG A
ACUACAAGAA    A    A  A        A      CGG      CACAAGAA^ A     A
 110       100        90        80        70        60        50
```

Reads in: lib-42, lib-45, lib-47, lib-48, lib-49, lib-50, lib-72
Number of reads="ID1341",368,17,15,7,2,1,0

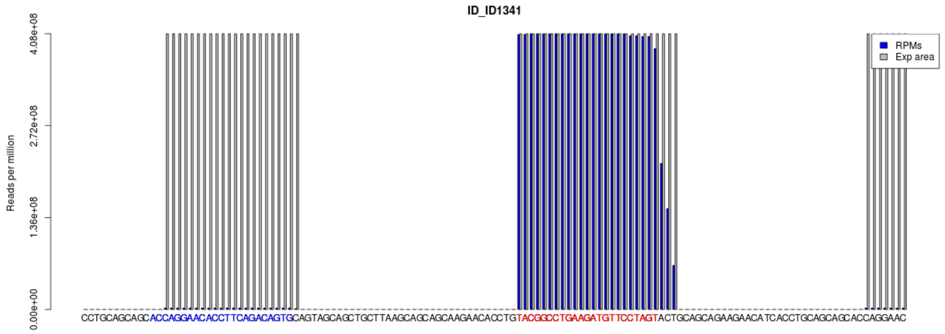

>ID1345

TTTTATTGGTTATGCATGTG**TAATCTCATGTGGTAACTGTGAG**TGGAGTAAGAAAACTCAGATTGCCATGTGGGGTTTCATATGCTTATG
ACCATATCACCAACATCAAAA

>ID1345-5P

TAATCTCATGTGGTAACTGTGAG

>miRNA precursor

```
UUUUAUUGGUUAUGCAUGUGUAAUCUCAUGUGGUAACUGUGAGUGGAGUAAGAAAACUCAGAUUGCCAUGUGGGGUUUCAUAUGCUUAUGACCAUAUCACCAACAUCAAAA
......((((((((((((((.((((((((((((((((...(((((...........)))))..)))))))))))))))).))))))))..))))))).................
```

>Predicted STAR seq:

CAGATTGCCATGTGGGGTTTCA

Complementarity: 17

Initial dG =   -39.70

```
                   10          20        30        40
UUUUAU-----------       --|      U               CUG      GGAGU
                 UGGUUAU  GCAUGUG AAUCUCAUGUGGUAA   UGAGU      \
                 ACCAGUA  CGUAUAC UUGGGGUGUACCGUU   ACUCA      A
AAAACUACAACCACUAU       UU^      U               AG-      AAAGA
 .       100        90        80        70         60
```

Reads in: lib-42, lib-45, lib-47, lib-48, lib-49, lib-50, lib-72

Number of reads="ID1345",3903,27674,12483,5545,11620,3388,0

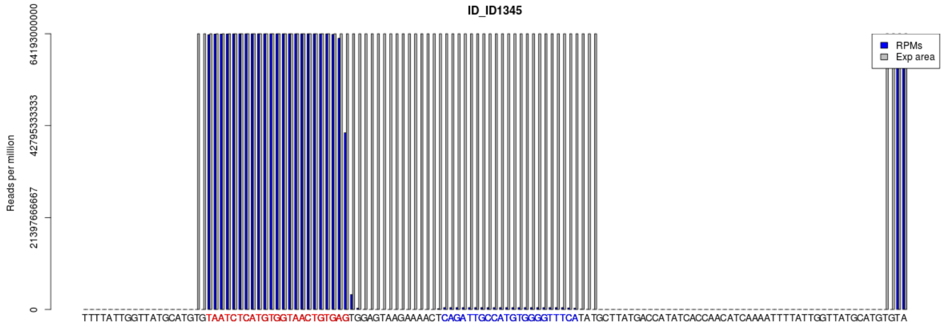

>ID1346
GATGCTGGCGGTCCAAGGCG**TGAGTATTACATCAGGTACTGA**CGGGCTGTGCAACGCCAGTACTTGTGTCATACTTTCACTTTGCCGTCC
TATTCAGCTGGGTGTATCCACT

>ID1346-5P
TGAGTATTACATCAGGTACTGA

>miRNA precursor
GAUGCUGGCGGUCCAAGGCG**UGAGUAUUACAUCAGGUACUGA**CGGGCUGUGCAACGCCAGUACUUGUGUCAUACUUUCACUUUGCCGUCCUAUUCAGCUGGGUGUAUCCACU
...((((((((..(((((...((((((.(((.(((((((((.((.((...))..)).))))))))))))).))))))...)))))))))......)))).((((....)))...

>Predicted STAR seq:
GTACTTGTGTCATACTTTCACT

Complementarity: 16

Initial dG =   -38.10

```
                10        20        30        40
.-GAU    ------|   UC      CGU       U   U          A  G-  U
     GCUG      GCGG  CAAGG   GAGUAU ACA CAGGUACUG CG  GC \
     CGAC      UGCC  GUUUC   UUCAUA UGU GUUCAUGAC GC  CG G
\ ---    UUAUCC^   --      ACU       C   -          C  AA  U
            90          80        70         60        50



   100
  U--   U
     GGG G
     CCU U
  UCA   A
  110
```

Reads in: lib-42, lib-45, lib-47, lib-48, lib-49, lib-50, lib-72
Number of reads="ID1346",1636,12446,4988,1734,2821,885,0

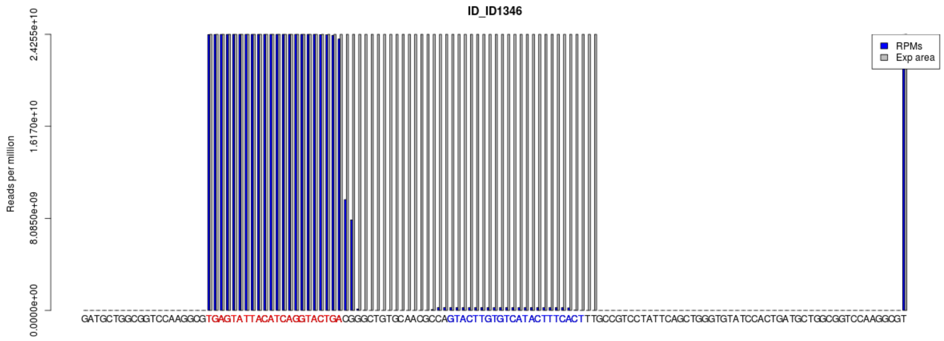

>ID1347
TGAAACTGTGGCTCAAGGCC**AAATATCAGTTGGTAATTCTGG**GGTGAAATGATGTGAAAACCCAGATTGCTTTCTGATATGAGGCCTTTA
GCCACGATTTTCTTCTTT

>ID1347-5P
AAATATCAGTTGGTAATTCTGG

>miRNA precursor
UGAAACUGUGGCUCAAGGCC**AAAUAUCAGUUGGUAAUUCUGG**GGUGAAAUGAUGUGAAAACCCAGAUUGCUUUCUGAUAUGAGGCCUUUAGCCACGAUUUUCUUCUUU
.((((.(((((((.((((((..(((((((..((((((.(((((.................)))))))))))..)))))))..)))))).)))))))..)))).....

>Predicted STAR seq:
AGATTGCTTTCTGATATGAGG

Complementarity: 15

Initial dG =   -48.60

```
              10        20        30        40        50
U-----    C-|      C      AA        UU      U     GUGAAAUG
      GAAA  UGUGGCU AAGGCC  AUAUCAG  GGUAAU CUGGG        \
      CUUU  GCACCGA UUCCGG  UAUAGUC  UCGUUA GACCC        A
UUUCUU    UA^      U      AG        UU      -     AAAAGUGU
      100        90        80        70         60
```

Reads in: lib-42, lib-45, lib-47, lib-48, lib-49, lib-50, lib-72
Number of reads="ID1347",5909,36482,16423,7918,13080,4244,0

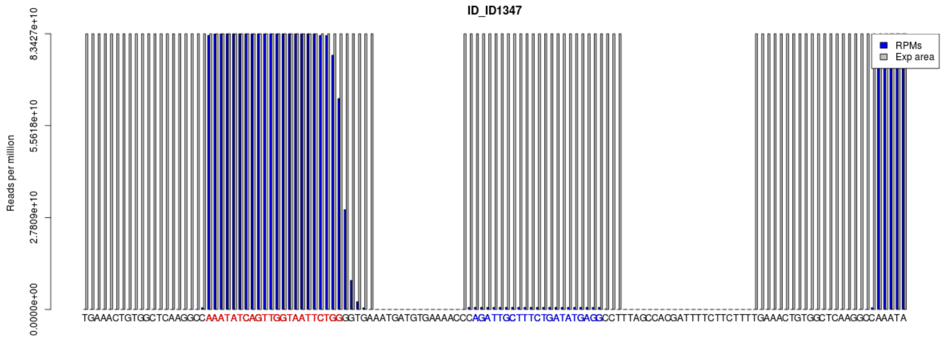

>ID1352
TCTTGCACTGACAGTAATACATGGATGGAGGGTGATGTGCGTGTGATAACAACACGTGATAAAGGTTCTG**TTTTATCACCCTCACATCAC**
CCACTTTACATGACAAACAGC

>ID1352-3P
TTTTATCACCCTCACATCACC

>miRNA precursor
UCUUGCACUGACAGUAAUACAUGGAUGGAGGGUGAUGUGCGUGUGAUAACAACACGUGAUAAAGGUUCUG**UUUUAUCACCCUCACAUCACC**CACUUUACAUGACAAACAGC
..((((.......))))..((((...((.((((((((((...(((((((.((((...((.......)))))))))))))...)))))))))).))...)))).........

>Predicted STAR seq:
GTGATGTGCGTGTGATAACAA

Complementarity: 16
ERROR 4: Strange structure
Initial dG = -33.80

```
.-UC|   ACU
    UUGC   \
    AAUG   G
\ --^   ACA
```

```
          20        30          40
  UA-------    GAU  A          .-C    GAU
         CAUG   GG GGGUGAUGUG   GUGU   \
         GUAC   UC CCCACUACAC   CACA   A
  CGACAAACA    AUU  A          \ -    ACA
 110       100        90              50
```

Reads in: lib-42, lib-45, lib-47, lib-48, lib-49, lib-50, lib-72
Number of reads="ID1352",1,0,0,0,0,0,0

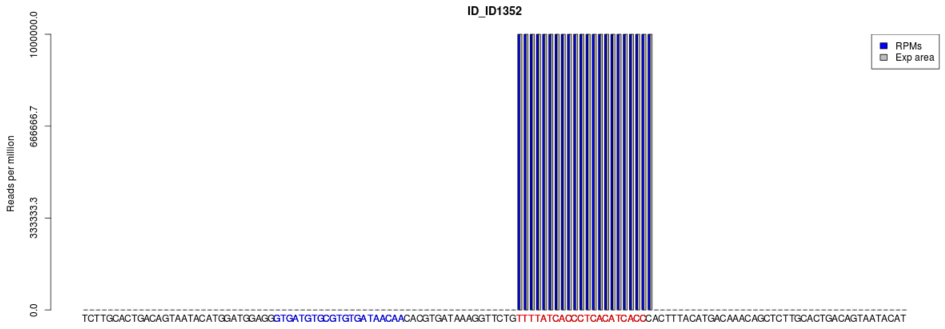

>ID1353
GCCAGTTGTTACTATTTCTAGTTATAATCAGATCTAGGTCAACTGACAATTGGCA**ACCTAGATCTGATTATAACTAG**GGCAAGTAACAAG
CATATATTTGAAAACTCCAA

>ID1353-3P
ACCTAGATCTGATTATAACTAG

>miRNA precursor
GCCAGUUGUUACUAUUUCUAGUUAUAAUCAGAUCUAGGUCAACUGACAAUUGGCA**ACCUAGAUCUGAUUAUAACUAG**GGCAAGUAACAAGCAUAUAUUUGAAAACUCCAA
((...((((((((..((((((((((((((((((((((((...(..(...)..)..))))))))))))))))))))))))..))))))))))...................

>Predicted STAR seq:
CTAGTTATAATCAGATCTAGG

Complementarity: 20

Initial dG =   -44.10

```
                           10        20        30        40
-------------------| CAG        AU                       CAA UG C
                   GC   UUGUUACU  UUCUAGUUAUAAUCAGAUCUAGGU   C  A \
                   CG   AACAAUGA  GGGAUCAAUAUUAGUCUAGAUCCA   G  U A
AACCUCAAAAGUUUAUAUA^ ---        AC                       AC- GU A
.       100        90           80        70        60         50
```

Reads in: lib-42, lib-45, lib-47, lib-48, lib-49, lib-50, lib-72
Number of reads="ID1353",40,234,144,496,219,94,0

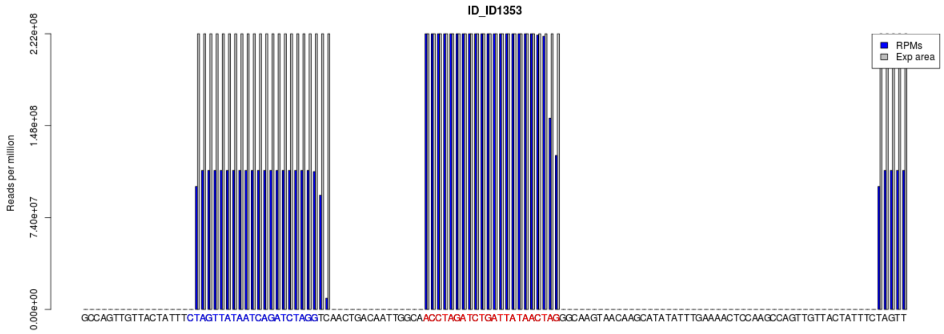

>ID1354

TATATGCTTGTTACTTGCCC**TAGTTATAATCAGATCTAGGTT**GCCAATTGTCAGTTGACCTAGATCTGATTATAACTAGAAATAGTAACA
ACTGGCAACGGGAAGCTCAAGA

>ID1354-5P

TAGTTATAATCAGATCTAGGTT

>miRNA precursor

UAUAUGCUUGUUACUUGCCC**UAGUUAUAAUCAGAUCUAGGUU**GCCAAUUGUCAGUUGACCUAGAUCUGAUUAUAACUAGAAAUAGUAACAACUGGCAACGGGAAGCUCAAGA

```
.....((((((((((....((((((((((((((((((((((...((((.....))))))))))))))))))))))))))....))))))..(((....))).)))).......
```

>Predicted STAR seq:

CTAGATCTGATTATAACTAGA

Complementarity: 19

Initial dG =   -48.70

```
           10        20        30        40        50
UAUAU-      |     UGCC                      UGC    UG
      GCUU--GUUACU    CUAGUUAUAAUCAGAUCUAGGU   CAAU  \
      CGAA  CAAUGA    GAUCAAUAUUAGUCUAGAUCCA   GUUG  U
AGAACU    \ ^     UAAA                      ---    AC
110                 80        70        60



           90
            AA   G
              CUG C
              GGC A
            G-   A
             100
```

Reads in: lib-42, lib-45, lib-47, lib-48, lib-49, lib-50, lib-72

Number of reads="ID1354",648,1543,1904,1325,1960,772,0

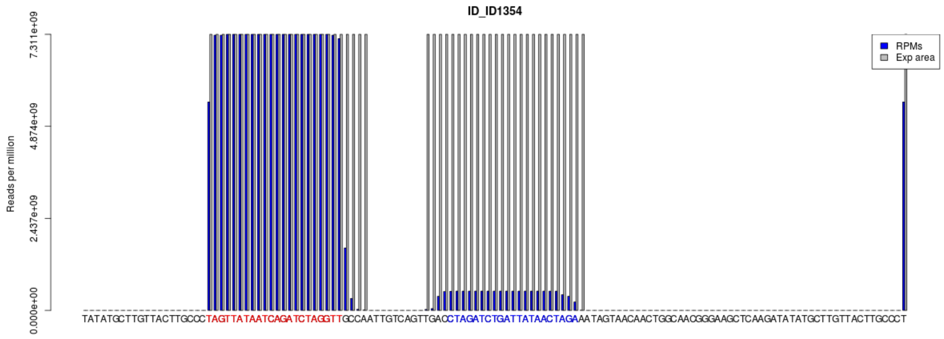

>ID136

CCGCCATCTCCGGATCTGATGCTGTTGCTGAGTCTCACCGAACTAATGCCAACTTGTTATCTCTGGCTT**GGCATCTGTTCGGTGAAGCTG**
AGCAAGAGCTTCTACAGCTTCA

>ID136-3P

GGCATCTGTTCGGTGAAGCTGAG

>miRNA precursor

```
CCGCCAUCUCCGGAUCUGAUGCUGUUGCUGAGUCUCACCGAACUAAUGCCAACUUGUUAUCUCUGGCUUGGCAUCUGUUCGGUGAAGCUGAGCAAGAGCUUCUACAGCUUCA
...........(((.(((..(((.(((((.((..(((((((((..(((((((...((((....)))))))))))..)))))))))..)).))))).))).....))).))).
```

>Predicted STAR seq:

TGAGTCTCACCGAACTAATGCC

Complementarity: 16

Initial dG =   -48.10

```
        10        20            30        40        50        60
CCGCCAUCUCC   U   AU---|  G      G  UC          UA        CUU    U
           GGA CUG     GCU UUGCU AG  UCACCGAAC  AUGCCAA   GUUA C
           CUU GAC     CGA AACGA UC  AGUGGCUUG  UACGGUU   CGGU U
A----------   C   AUCUU^  G      G  GA          UC        ---    C
          110       100         90        80        70
```

Reads in: lib-42, lib-45, lib-47, lib-48, lib-49, lib-50, lib-72

Number of reads="ID136",10,61,17,734,81,17,0

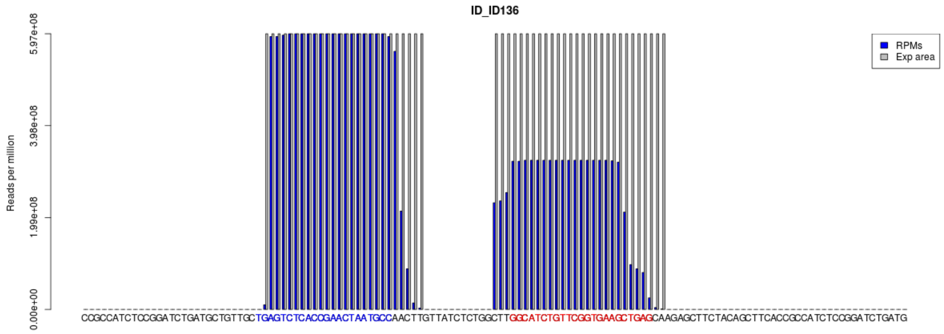

>ID1361
CCAGATAACTGTGTGCGAACCTCAATGTCGCCTGTAATGCCTTTTATTTTGCCAGGGCAGTACTGCCAACATTGGGGATTGCGCACATGT
TATAACATCGTGGTTGTCACAA

>ID1361-5P
CTCAATGTCGCCTGTAATGCCT

>miRNA precursor
CCAGAUAACUGUGUGCGAACCUCAAUGUCGCCUGUAAUGCCUUUUAUUUUGCCAGGGCAGUACUGCCAACAUUGGGGAUUGCGCACAUGUUAUAACAUCGUGGUUGUCACAA
....((((((((((((((.(((((((((.((..(((.((((((..........)))))).))).))..))))))))).))))))))).))))).......(((.....)))..

>Predicted STAR seq:
GCAGTACTGCCAACATTGGGGA

Complementarity: 16

Initial dG = -42.90

```
           10        20          30        40
.-CCAG     -         A          C-| CU   A       UUAU
      AUAAC UGUGUGCGA CCUCAAUGU  GC  GUA UGCCUU    U
      UAUUG ACACGCGUU GGGGUUACA  CG  CAU ACGGGA    U
\ ----     U         A          AC^ U-   G       CCGU
        90        80        70          60        50



      100
  AACAUC   GU
        GUG  \
        CAC  U
  AA----   UG
      110
```

Reads in: lib-42, lib-45, lib-47, lib-48, lib-49, lib-50, lib-72
Number of reads="ID1361",32,12,3,870,12,1,0

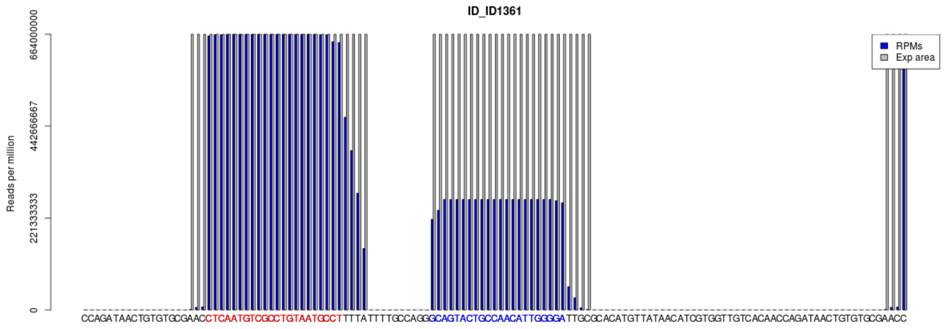

>ID1362
CATACTTACTTATTGTGGCTTCTCCTGGTTTGCTTTCGATATATGAACTACTCGTAAAATATTACGAAAA**AAGGAGGTGAAATGTCGAGC**
GGTAAATACTGCAATGAGTGAGTA

>ID1362-3P
AAGGAGGTGAAATGTCGAGCGGTA

>miRNA precursor
CAUACUUACUUAUUGUGGCUUCUCCUGGUUUGCUUUCGAUAUAUGAACUACUCGUAAAAUAUUACGAAAA**AAGGAGGUGAAAUGUCGAGCGGUA**AAUACUGCAAUGAGUGAGUA
..((((((((((((..(.........((((((((((((((....(((.(((((((....))))))......).)))...)))))))).))))))).)..)))))))))))))

>Predicted STAR seq:
GCTTTCGATATATGAACTA

Complementarity: 14

Initial dG =   -32.60

```
        10        20        30         40        50
CA|            UG CUUCUCCUG       -        AUGA   A ------      A
  UACUUACUUAUUG  G         GUUUGCU UUCGAUAU    ACU C      UCGUAA A
  AUGAGUGAGUAAC  C         UAAAUGG GAGCUGUA    UGG G      AGCAUU U
--^            GU A--------       C        AAG-   A GAAAAA      A
    110       100                90        80         70        60
```

Reads in: lib-42, lib-45, lib-47, lib-48, lib-49, lib-50, lib-72
Number of reads="ID1362",0,0,0,0,0,1,0

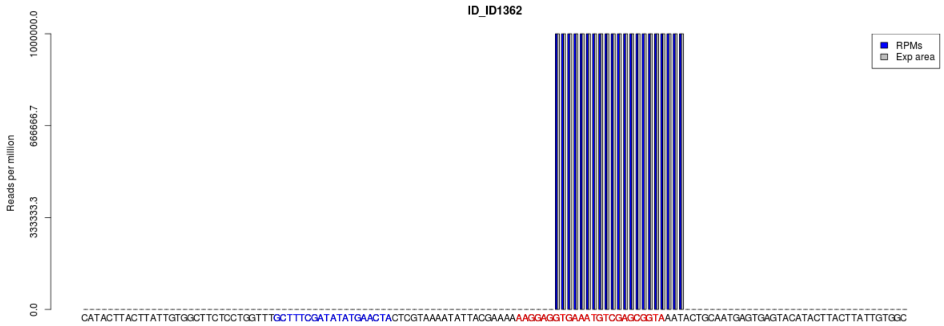

>ID1365
ACAGCAGAAGCAGGAGCAGCAGAAGCAGGAACATGAGTACTTGCAGCATCAGTTGTAAGAGCACCAGTTC**ATGCTTCTGTTTCTCCTGCT**
GCTGCTGTAGCAGCTATAAC

>ID1365-3P
ATGCTTCTGTTTCTCCTGCTGCT

>miRNA precursor
ACAGCAGAAGCAGGAGCAGCAGAAGCAGGAACAUGAGUACUUGCAGCAUCAGUUGUAAGAGCACCAGUUC**AUGCUUCUGUUUCUCCUGCUGCU**GCUGUAGCAGCUAUAAC
((((((.((((((((.((((((((((.((((.((.((.((((((((....)))))))).))..)))))).)))))))))).)))))))).))))))).. ....

>Predicted STAR seq:
GAAG

Complementarity: 18

Initial dG =   -59.30

```
                    10        20        30         40
------------|      A        C          G    A  A-  A        A
            ACAGCAG AGCAGGAG AGCAGAAGCA GAAC UG  GU CUUGCAGC U
            UGUCGUC UCGUCCUC UUGUCUUCGU CUUG AC  CG GAAUGUUG C
CAAUAUCGACGA^      G        U          A    -  CA  A        A
.       100        90        80         70         60        50
```

Reads in: lib-42, lib-45, lib-47, lib-48, lib-49, lib-50, lib-72
Number of reads="ID1365",15,5,0,1,1,0,0

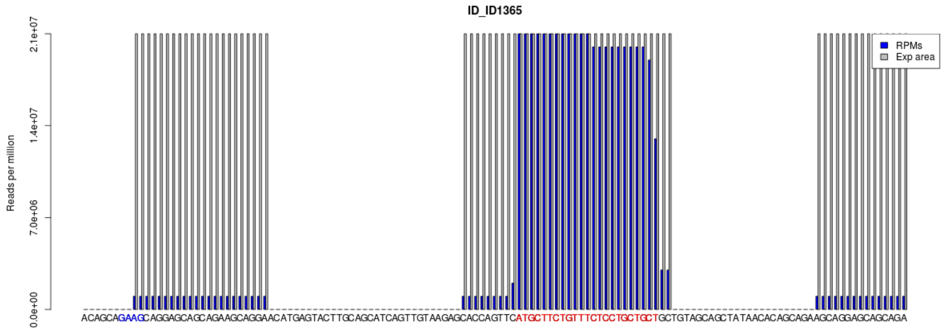

>ID1367
CCTACATGGACATATTTCTGGGACGGGCAGATTCTATTTGGGGTCGGTCGAGTGTTGGATCTCATATTCA**TTCGACGGCCTCAAGAATCT**
CCCCGTTCTACACGAATCGACC

>ID1367-3P
TTCGACGGCCTCAAGAATCTCC

>miRNA precursor
CCUACAUGGACAUAUUUCUGGGACGGGCAGAUUCUAUUUGGGGUCGGUCGAGUGUUGGAUCUCAUAUUCA**UUCGACGGCCUCAAGAAUCUCC**CCGUUCUACACGAAUCGACC
```
((.....)).........((((((((.(((((((...((((((((.(((((((..............)))))))))))))))))))))).))))))))...........
```

>Predicted STAR seq:
CAGATTCTATTTGGGGTCGGTCGAG

Complementarity: 19
ERROR 4: Strange structure
Initial dG =   -43.40

```
  | UA
--CC  \
  GG  C
\ ^ UA
```

```
 10            20        30        40        50        60
  ACAUAUUUC---          C       AUU        G       UUGGAU
              UGGGACGGG AGAUUCU   UGGGGUCG UCGAGUG      C
              AUCUUGCCC UCUAAGA   ACUCCGGC AGCUUAC      U
  CCAGCUAAGCAC          C       ---        -       UUAUAC
  110       100        90           80         70
```

Reads in: lib-42, lib-45, lib-47, lib-48, lib-49, lib-50, lib-72
Number of reads="ID1367",7,35,13,2332,244,7,0

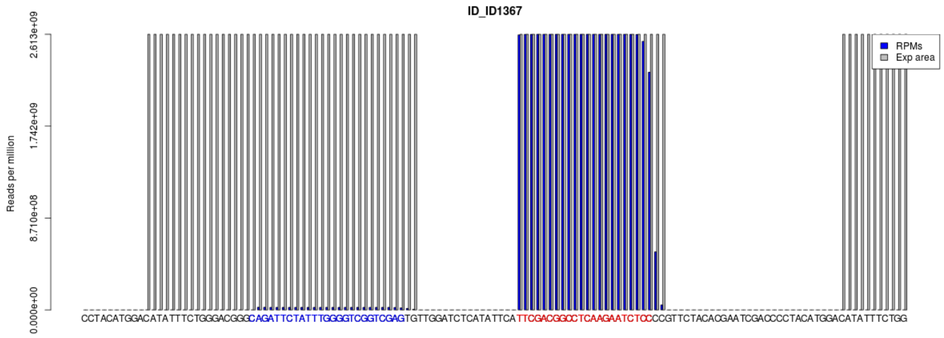

>ID1368
GGTTGTGTTTGCATCCTCC**CGAACTATCAATTCTATTGCTGA**CGTATTCAAACGCAGCGGGGGAGTTGATGTTTCGGAAGGGTTCAGGCA
AATGCTTCGTGAAGATGGAAAA

>ID1368-5P
CGAACTATCAATTCTATTGCTGA

>miRNA precursor
GGUUGUGUUUGCAUCCUCC**CGAACUAUCAAUUCUAUUGCUGA**CGUAUUCAAACGCAGCGGGGGAGUUGAUGUUUCGGAAGGGUUCAGGCAAAUGCUUCGUGAAGAUGGAAAA
(((..((((((.(((((.(((((.((((((((((.((((((.(((......))))))))).)))))))))).))))).))))).))))))...)))...............

>Predicted STAR seq:
GCGGGGGAGTTGATGTTTCGGA

Complementarity: 18

Initial dG =   -44.60

```
                         10        20        30        40
----------------   UG-|     C     C     C          A      A   AU
                GGU   UGUUUG AUCCU CCGAA UAUCAAUUCU UUGCUG CGU  U
                UCG   ACGGAC UGGGA GGCUU GUAGUUGAGG GGCGAC GCA  C
AAAAGGUAGAAGUGCU   UAA^     U     A     U          G      -   AA
110       100        90        80        70        60         50
```

Reads in: lib-42, lib-45, lib-47, lib-48, lib-49, lib-50, lib-72
Number of reads="ID1368",1,5,0,443,24,1,0

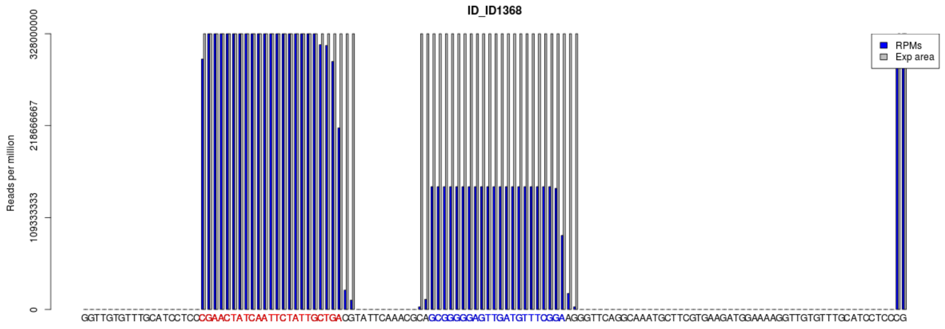

>ID1371
AGAGCAGCGCCCGCCCCCC**TCCGTTCACCTGCCTTTCCGCGGG**CAGGTTGCACTCCGTGGCACTGCGGGTGACGGGGGGGTGCGGCCGCA
CCACCAGCTCCAGGGCGCTCCAG

>ID1371-5P
TCCGTTCACCTGCCTTTCCGCGGG

>miRNA precursor
AGAGCAGCGCCCGCCCCCC**UCCGUUCACCUGCCUUUCCGCGGG**CAGGUUGCACUCCGUGGCACUGCGGGUGACGGGGGGGUGCGGCCGCACCACCAGCUCCAGGGCGCUCCAG
.((((.(((.((((.(((((((((.(((((((....(((((((..........)))))))....)))))))))))))))).)))).))).......(((....)))))))...

>Predicted STAR seq:
CGTGGCACTGCGGGTGACGGGGG

Complementarity: 17

Initial dG = -66.00

```
            10        20        30        40
A--    .-A|  C    C         U       CUUU       CAGG
   GAGC   GCG CCGC CCCCUCCGU CACCUGC    CCGCGGG    U
   CUCG   CGC GGCG GGGGGGGCA GUGGGCG    GGUGCCU    U
GAC    \ -^  C    U         -       UCAC       CACG
 110             80         70        60        50


        90       100
         ACCACCA   C
                GCU C
                CGG A
         -------   G
```

Reads in: lib-42, lib-45, lib-47, lib-48, lib-49, lib-50, lib-72
Number of reads="ID1371",11,8,4,994,95,1,0

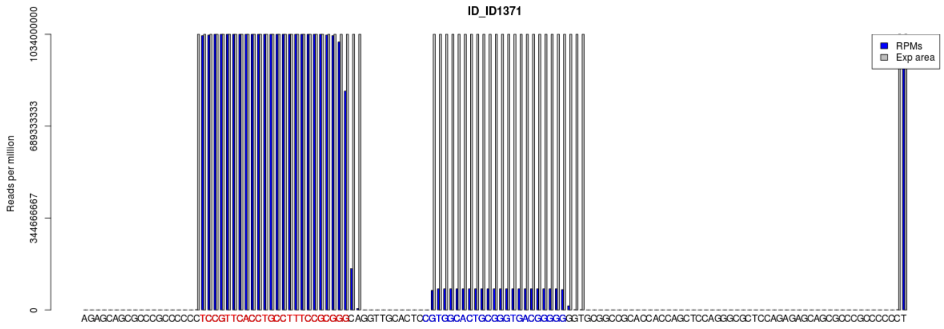

>ID1378
CACCATTGCTTCTTCATATCTAAATAGTTCCATTTGAATATCATTGCACATTGTTGCGTGCTGGAAAAAAATGAGCAATGTTATTCAAATGGAGTTGTTTAGAATCTCAGT

>ID1378-3P
ATGAGCAATGTTATTCAAATGG

>miRNA precursor
CACCAUUGCUUCUUCAUAUCUAAAUAGUUCCAUUUGAAUAUCAUUGCACAUUGUUGCGUGCUGGAAAAAAUGAGCAAUGUUAUUCAAAUGGAGUUGUUUAGAAUCUCAGU
.................((((((((..((((((((((((.((((((.((((................)))).)))))).))))))))))))..))))))))........

>Predicted STAR seq:
CATTTGAATATCATTGCACAT

Complementarity: 18

Initial dG = -35.80

```
        10        20        30        40        50        60
CACCAUUGCUUCUUCAUA|       GU            U      A    GUUGCGUG
                  UCUAAAUA  UCCAUUUGAAUA CAUUGC CAUU        \
                  AGAUUUGU  AGGUAAACUUAU GUAACG GUAA        C
UGACUCUA----------^       UG            U      A    AAAAAGGU
 .                 100        90        80        70
```

Reads in: lib-42, lib-45, lib-47, lib-48, lib-49, lib-50, lib-72
Number of reads="ID1378",11024,24955,19181,70307,42453,6729,0

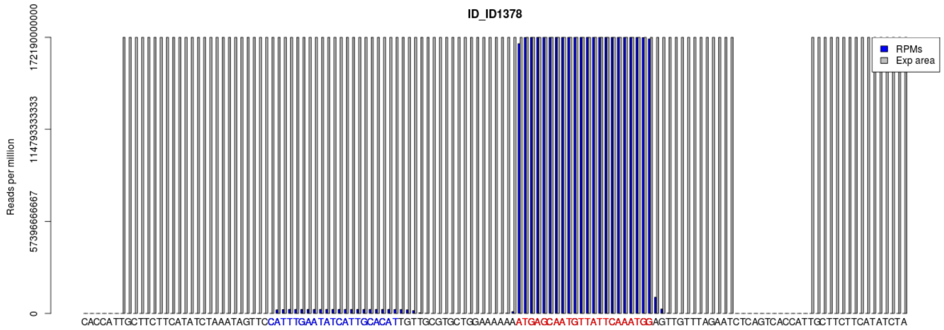

>ID1381
AGAAGCAGCAATACGTCGTGTGATGGGTAGCGGTAGATGGAATTGTGCCAGTTAACGTGAAAATAATTCG**TCACTGGCACGATTCCATCA**
TCTGCTTCCCAATCAACAGCTCA

>ID1381-3P
TCACTGGCACGATTCCATCAT

>miRNA precursor
AGAAGCAGCAAUACGUCGUGUGAUGGGUAGCGGUAGAUGGAAUUGUGCCAGUUAACGUGAAAAUAAUUCG**UCACUGGCACGAUUCCAUCAU**CUGCUUCCCAAUCAACAGCUCA
.(.(((.((.....)).((.(((((((.(((((..(((((((((((((((((..(((...........))).)))))))))))))))))..))))).))).)))))).)))).

>Predicted STAR seq:
AGATGGAATTGTGCCAGTT

Complementarity: 16
ERROR 4: Strange structure
Initial dG = -48.10

```
          10
A A   .-A| AA
 G AGC   GC  \
 C UCG   UG  U
A -   \ -^ CA
  110



          20         30         40         50         60
        C  G    -   U     UA                 UA   UGAAA
         GU UGAU GGG AGCGG  GAUGGAAUUGUGCCAGU  ACG     \
         CA ACUA CCC UCGUC  CUACCUUAGCACGGUCA  UGC     A
        A  -    A   U     UA                 C-   UUAAU
               100        90         80         70
```

Reads in: lib-42, lib-45, lib-47, lib-48, lib-49, lib-50, lib-72
Number of reads="ID1381",0,0,0,0,1,0,0

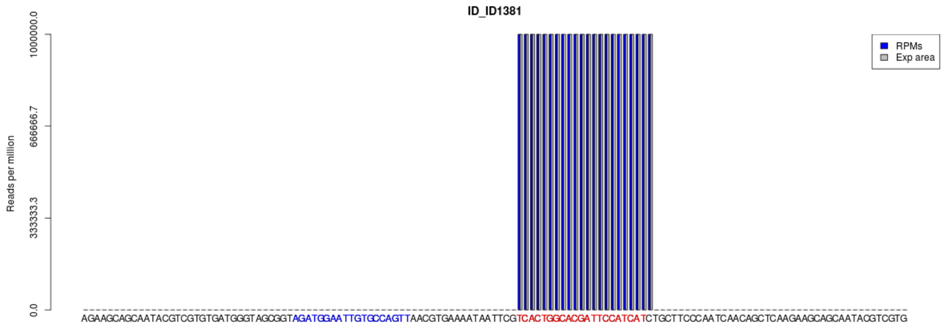

>ID1386
ACCTACACCAGAGGTAAAGA**AGGCAAGATGTCGGCATAGCTGA**AGTGTGTTTTAGTAACCGGCTATGTCACATCAAGCCAGCTTTCACTT
CTGGAATTTCAACAGTCAC

>ID1386-5P
AGGCAAGATGTCGGCATAGCTGA

>miRNA precursor
ACCUACACCAGAGGUAAAGA**AGGCAAGAUGUCGGCAUAGCUGA**AGUGUGUUUUAGUAACCGGCUAUGUCACAUCAAGCCAGCUUUCACUUCUGGAAUUUCAACAGUCAC
.......((((((((((((..(((..(((((.((((((((((.................)))))))))))))))..)))..)))).))))))))...............

>Predicted STAR seq:
GCTATGTCACATCAAGCCAGCT

Complementarity: 16

Initial dG =   -40.80

```
                10         20        30        40        50
ACCUACA--------        -|   AA   AA     C          AAGUGUGU
               CCAGAGGU AAAG  GGC  GAUGU GGCAUAGCUG        \
               GGUCUUCA UUUC  CCG  CUACA CUGUAUCGGC        U
CACUGACAACUUUAA        C^   GA   AA     -          CAAUGAUU
       100        90        80        70         60
```

Reads in: lib-42, lib-45, lib-47, lib-48, lib-49, lib-50, lib-72
Number of reads="ID1386",3597,5684,11250,6466,9584,7336,0

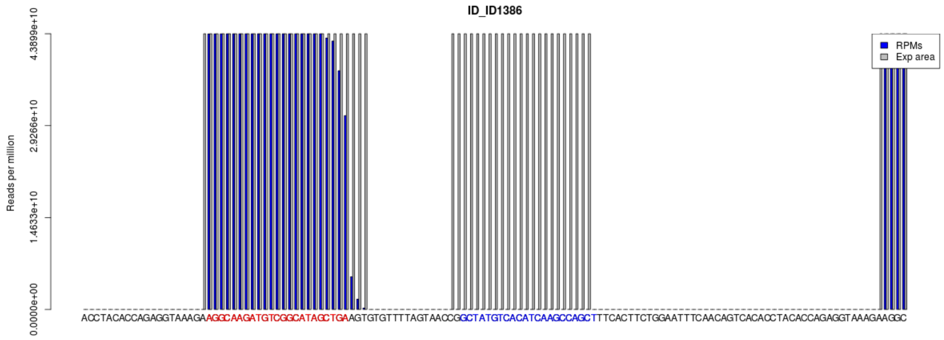

>ID1387
AGCCCTCTGTTACTTTGTTA**CGGAATTTGTCTGTCCTGTCTGGT**ACAACTGAAGTAATCCAGACAGGTTGACAAAGCCCTAAACAAGGTT
TTCAGAATCATCATTGGCTGACT

>ID1387-5P
CGGAATTTGTCTGTCCTGTCTGGT

>miRNA precursor
AGCCCUCUGUUACUUUGUUA**CGGAAUUUGUCUGUCCUGUCUGGU**ACAACUGAAGUAAUCCAGACAGGUUGACAAAGCCCUAAACAAGGUUUUCAGAAUCAUCAUUGGCUGACU
((((.((((..((((((((..((..((((((...((((((((((((.......)))..)))))))))..))))))..))..))))))))...)))).........))))....

>Predicted STAR seq:
TCCAGACAGGTTGACAAAGCCCTAAA

Complementarity: 16

Initial dG =   -41.80

```
                    10         20         30         40
----    C--------    UU-         AC  AA       UGU         --|  AAC
    AGCC         UCUG   ACUUUGUU  GG  UUUGUC   CCUGUCUGG  UAC   \
    UCGG         AGAC   UGGAACAA  CC  AAACAG   GGACAGACC  AUG   U
UCAG    UUACUACUA    UUU         AU  CG       UU-         UA^  AAG
 110       100        90         80         70         60
```

Reads in: lib-42, lib-45, lib-47, lib-48, lib-49, lib-50, lib-72
Number of reads="ID1387",3290,5697,3408,4582,4603,1177,0

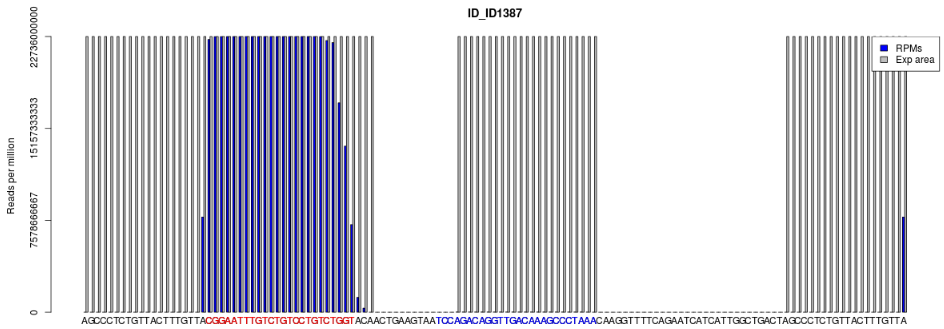

>ID1388
CGGCGGGGTGCGAGGTCGGGGTTTACCGGCTGACCTTGTCTTCCAGAGAAATGTGAGTGCTGTCAGTCAT**TTCGGCGATGAGATCAGCCA**
GTCGAACCCCGACTCGCAGCGA

>ID1388-3P
TTCGGCGATGAGATCAGCCAGT

>miRNA precursor
CGGCGGGGUGCGAGGUCGGGGUUUACCGGCUGACCUUGUCUUCCAGAGAAAUGUGAGUGCUGUCAGUCAU**UUCGGCGAUGAGAUCAGCCAGU**CGAACCCCGACUCGCAGCGA
..((....((((((.(((((((((((.((((((.((..((..((.(((....((((.((....)).))))))))).))..)).)))))).)).)))))))))))))))))..

>Predicted STAR seq:
CCGGCTGACCTTGTCTTCCAGAG

Complementarity: 15

Initial dG =   -50.50

```
        10        20         30        40        50        60
CG| GGGG      G          -  C      C  UG  UU  A   AAAU    G  C
  GC    UGCGAG UCGGGGUUU AC GGCUGA CU  UC  CC GAG    GUGA UG U
  CG    ACGCUC AGCCCCAAG UG CCGACU GA  AG  GG CUU    UACU AC G
AG^ ----      -          C  A      A  GU  C-  -   ----    G  U
110            100        90        80              70
```

Reads in: lib-42, lib-45, lib-47, lib-48, lib-49, lib-50, lib-72
Number of reads="ID1388",5,37,10,696,15,3,0

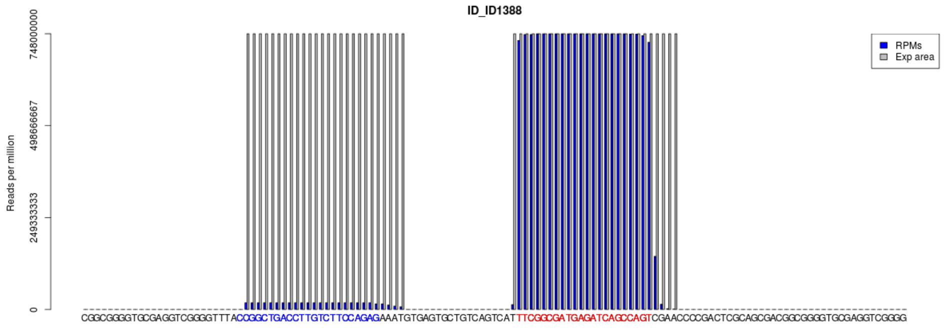

>ID1389

TGAAATTTCCGGTCTGTGTAAATGCATCACGGTCGTCGGAGGTGTATTATGCACACGTTTCATAGCAATG**TCCATTATACACTTCTGACGAC**TGTTGTGCATTATTGCATCA

>ID1389-3P

TCCATTATACACTTCTGACGAC

>miRNA precursor

UGAAAUUUCCGGUCUGUGUAAAUGCAUCACGGUCGUCGGAGGUGUAUUAUGCACACGUUUCAUAGCAAUG**UCCAUUAUACACUUCUGACGAC**UGUUGUGCAUUAUUGCAUCA

```
..............(((((((((((.((((((((((((((((((((.(((.(((.(((....)))..))).))).))))))))))))))))))))).))))))))..))))).. 
```

>Predicted STAR seq:

TCGTCGGAGGTGTATTATGC

Complementarity: 18

Initial dG = -46.90

```
        10        20          30        40        50         60
UGAAAUUUCCGGUCU     --        C                   U   C   C-|  U
               GUGUA  AAUGCAU ACGGUCGUCGGAGGUGUAU AUG ACA  GUU C
               UACGU  UUACGUG UGUCAGCAGUCUUCACAUA UAC UGU  CGA A
AC-------------     UA        U                   U   C   AA^  U
             110       100        90        80        70
```

Reads in: lib-42, lib-45, lib-47, lib-48, lib-49, lib-50, lib-72

Number of reads="ID1389",5,18,0,1,1,0,0

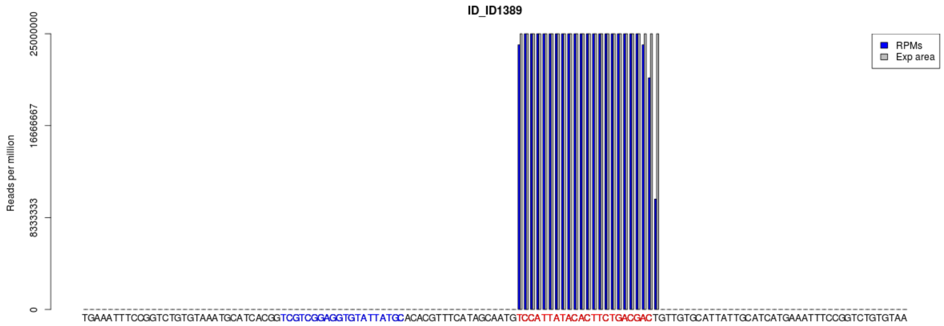

>ID1393
TAGTGTTCTCCTCAACCAGCTCATGCTGTAGGGCAACATACCTTGTGTATGTAACTGATTGTGAAATGGA**CATACTCAATATATGTTGCT**
CTGCATCACGAGTTGCTACACA

>ID1393-3P
CATACTCAATATATGTTGCTCT

>miRNA precursor
UAGUGUUCUCCUCAACCAGCUCAUGCUGUAGGGCAACAUACCUUGUGUAUGUAACUGAUUGUGAAAUGGA**CAUACUCAAUAUAUGUUGCUCU**GCAUCACGAGUUGCUACACA
..((((..........((((((.((.((((((((((((((..(((.((((((..((.(((....))))))))))).)))..)))))))))))))).)).))))))..)))).

>Predicted STAR seq:
GGGCAACATACCTTGTGTATG

Complementarity: 17

Initial dG =   -40.50

```
        10        20        30        40        50        60
UA|   UCUCCUCAAC      A  C              CC   U      AA  G   G
  GUGU          CAGCUC UG UGUAGGGCAACAUA  UUG GUAUGU  CU AUU U
  CACA          GUUGAG AC ACGUCUCGUUGUAU  AAC CAUACA  GG UAA G
A-^   UC--------      C  U              AU   U      --  -   A
 110               100       90        80        70
```

Reads in: lib-42, lib-45, lib-47, lib-48, lib-49, lib-50, lib-72
Number of reads="ID1393",37,20,9,1039,79,1,0

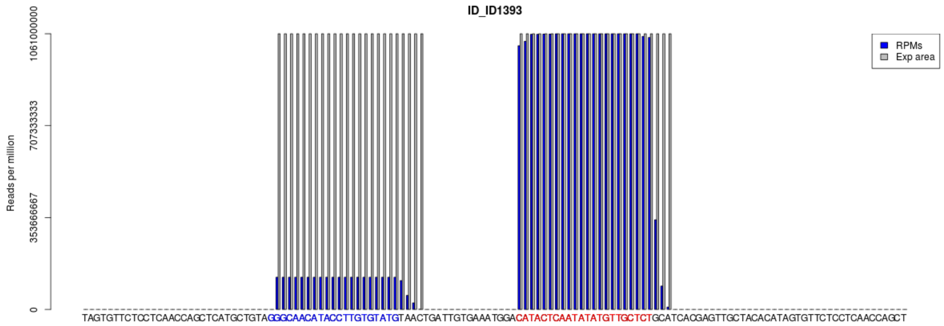

>ID1402
AACAGTAATCCATTTCACAAAATGGTCCGCAGGTGTCATAATTACCGGTCTACATTGTATACAAAACTTG**TAGGACGGTCCTTATGACAC**
CTTGCTGGTCATTAAATAA

>ID1402-3P
TAGGACGGTCCTTATGACACCT

>miRNA precursor
AACAGUAAUCCAUUUCACAAAAUGGUCCGCAGGUGUCAUAAUUACCGGUCUACAUUGUAUACAAAACUUG**UAGGACGGUCCUUAUGACACCU**UGCUGGUCAUUAAAUAA
...................((((..(.(((((((((((((..((((..(((((..((.......)).)))))..))))..)))))))))))).)).)..))))......

>Predicted STAR seq:
GGTGTCATAATTACCGGTCTA

Complementarity: 16

Initial dG =   -39.40

```
        10        20        30         40        50        60
AACAGUAAUCCAUUUCACAA    GU C  -|          UU    GU     UU  AUA
                    AAUG  C GC AGGUGUCAUAA  ACCG  CUACA  GU   \
                    UUAC  G CG UCCACAGUAUU  UGGC  GAUGU  CA   C
AAUAAA--------------    UG U  U^          CC    AG     U-  AAA
                     100        90        80        70
```

Reads in: lib-42, lib-45, lib-47, lib-48, lib-49, lib-50, lib-72
Number of reads="ID1402",14,20,0,0,0,0,0

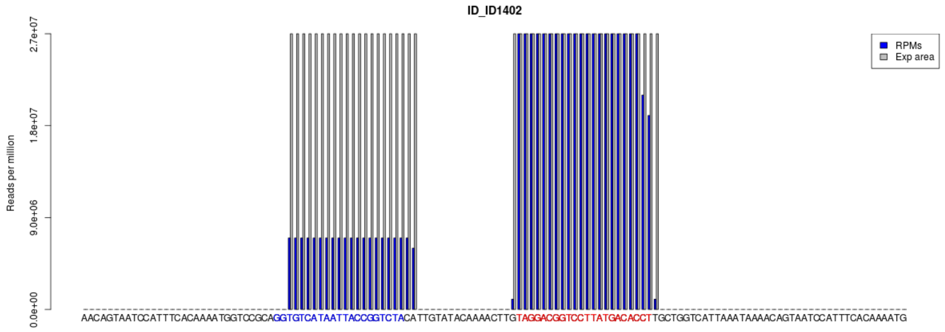

>ID1403
TAACCTAGGTAACTAGTGGG**AGGGTGTGGCTAGTAATCGACAT**ATTATTAAGAATATGTCATTGCTAACTACAGCCATCCTATAGGTTGC
CAGCAACGTCTGTAGCTG

>ID1403-5P
AGGGTGTGGCTAGTAATCGACAT

>miRNA precursor
UAACCUAGGUAACUAGUGGG**AGGGUGUGGCUAGUAAUCGACAU**AUUAUUAAGAAUAUGUCAUUGCUAACUACAGCCAUCCUAUAGGUUGCCAGCAACGUCUGUAGCUG
.......((((((.((((((((.(((((.(((((((.((((((((......))))))))))))))).))))).)).)))))).))))))).((.((....)).))..

>Predicted STAR seq:
GTCATTGCTAACTACAGCCATCC

Complementarity: 18

Initial dG =   -46.00

```
          10        20          30        40
.-UAACCUA       A      -| G      C       C        AU
         GGUAACU GUGGGA GG UGUGG UAGUAAU GACAUAUU  U
         CCGUUGG UAUCCU CC ACAUC AUCGUUA CUGUAUAA  A
\ -------       A      A^ G      A       -        GA
         90        80        70          60        50
```

```
  A-  A  G
    GC AC U
    CG UG C
  GU  A  U
        100
```

Reads in: lib-42, lib-45, lib-47, lib-48, lib-49, lib-50, lib-72
Number of reads="ID1403",14,2,0,0,0,0,0

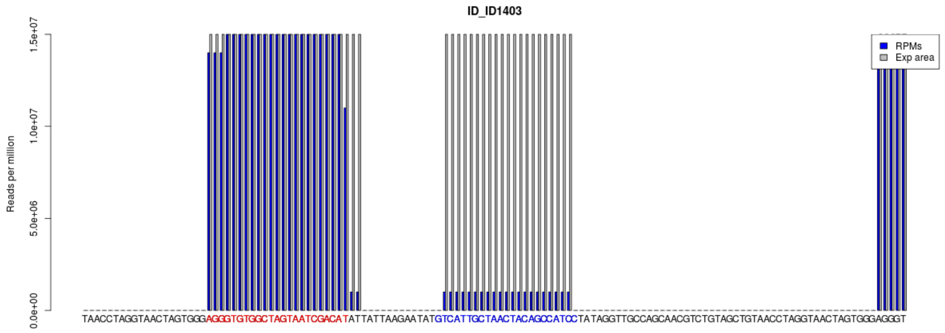

>ID1404
TTAGAGATGGTTTTCCCAGC**AGAAGAACATCAGCTGGCTTACA**AGTAATAGATATGTAGTCAGCTAGTGCTCTTTTACTGGTGGCCATTA
CGATCCACAACAGTTTCAACC

>ID1404-5P
AGAAGAACATCAGCTGGCTTACA

>miRNA precursor
UUAGAGAUGGUUUUCCCAGC**AGAAGAACAUCAGCUGGCUUACA**AGUAAUAGAUAUGUAGUCAGCUAGUGCUCUUUUACUGGUGGCCAUUACGAUCCACAACAGUUUCAACC
.....(((((((...((((.((((((.(((.((((((((.(((.((.....)).))))))))))).))).)))))).)))).))))))).....................

>Predicted STAR seq:
TAGTCAGCTAGTGCTCTTTTACT

Complementarity: 18

Initial dG =   -34.60

```
                         10        20        30        40
UUAGA----------------|      UUC    C      A   C        U   A  AA
                      GAUGGUU   CCAG AGAAGA CAU AGCUGGCU ACA GU  \
                      UUACCGG   GGUC UUUUCU GUG UCGACUGA UGU UA  U
CCAACUUUGACAACACCUAGCA^      U--    A      C   A        -   A  GA
 .       100        90          80        70        60          50
```

Reads in: lib-42, lib-45, lib-47, lib-48, lib-49, lib-50, lib-72
Number of reads="ID1404",1,2,0,0,0,0,0

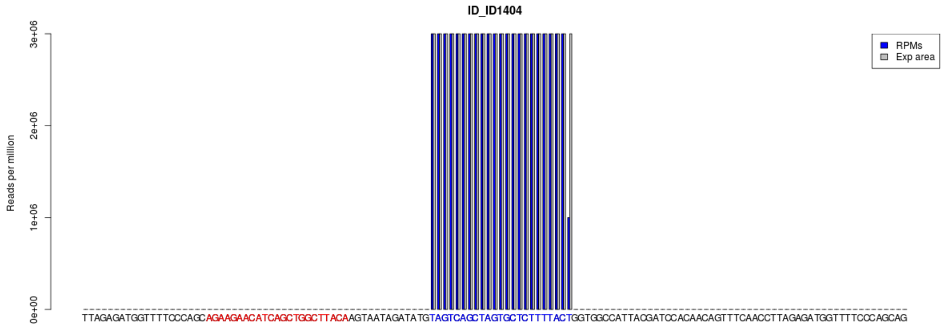

>ID1405

TTTGGTGTGTCCACCAATAAAGATGGTCTGCAGG**CGTCATAAGGGCAGTGAGCA**CTGTTTGGAGAAAGTGCACATTGCCCCTATGACCGC
CTTGCCGGTCATCATCAACAAC

>ID1405-5P

CGTCATAAGGGCAGTGAGCA

>miRNA precursor

```
UUUGGUGUGUCCACCAAUAAAGAUGGUCUGCAGGCGUCAUAAGGGCAGUGAGCACUGUUUGGAGAAAGUGCACAUUGCCCCUAUGACCGCCUUGCCGGUCAUCAUCAACAAC
.((((((....))))))....((((..(.(((((((((((.((((((((.(((((.........))))).)))))))).))))).)))).))).)..)))).........
```

>Predicted STAR seq:

CACATTGCCCCTATGACCGCC

Complementarity: 16

Initial dG =   -55.10

ERROR 3: Strange structure

Initial dG =   -55.10

```
.-U|      U
   UUGGUG G
   AACCAC U
\ -^      C
          10


   20             30          40        50        60
  UAAA-----     GU U    -     -     A         A      GUUU
           GAUG  C GCA GGCG UCAUA GGGCAGUG GCACU    G
           CUAC  G CGU CCGC AGUAU CCCGUUAC CGUGA    G
  CAACAACUA     UG C    U     C     C         A      AAGA
  110       100        90         80         70
```

Reads in: lib-42, lib-45, lib-47, lib-48, lib-49, lib-50, lib-72

Number of reads="ID1405",7,8,0,0,0,0,0

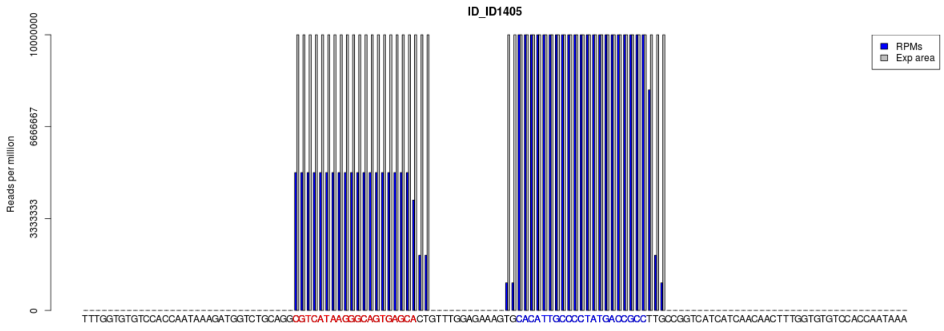

>ID1406
GCCGAGACATTGCTTCCAAC**GGGGGAAATTGACTGGTGAG**CATGGCTTGTAAATAATGCTACCAGCCAAGTTCCTCCGCCTGGCGGCAAT
GACAGCCTTCACTGGTTTCA

>ID1406-5P
GGGGGAAATTGACTGGTGAG

>miRNA precursor
GCCGAGACAUUGCUUCCAAC**GGGGGAAAUUGACUGGUGAG**CAUGGCUUGUAAAUAAUGCUACCAGCCAAGUUCCUCCGCCUGGCGGCAAUGACAGCCUUCACUGGUUUCA
```
...((..(((((((.(((.((((((((.(((.(((((.(((((...........)))))))))).))).))))))))..))).)))))))..((((......)))))).
```

>Predicted STAR seq:
ACCAGCCAAGTTCCTCCGC

Complementarity: 15

Initial dG =   -45.40

```
          10         20        30        40
GCC  .-GA       U   A-|       A   A     G     GGCUU
   GA     CAUUGCU CCA  CGGGGGAA UUG CUGGU AG CAU     G
   CU     GUAACGG GGU  GCCUCCUU AAC GACCA UCGUA     U
A--  \ --       C   CC^       G   C     -     AUAAA
.        90        80        70         60        50


             100
       AC     UU
         AGCC  C
         UUGG  A
       --     UC
```

Reads in: lib-42, lib-45, lib-47, lib-48, lib-49, lib-50, lib-72
Number of reads="ID1406",96,9,0,0,0,1,0

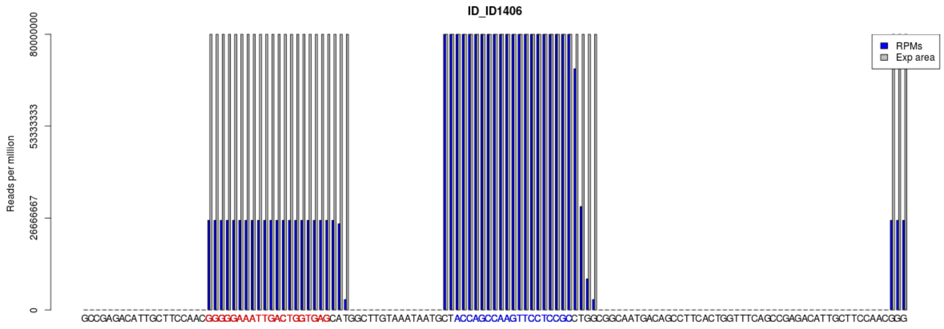

>ID1407
AGGAGGAGCACCAGCACACGTAATGGTGGGCAGGTGTCGTAAGAGGCCAAACACATTATTATAGAACATG**TCTTTGGTCTCTTACGACAC**
CTTGCCGTCCATGAAATAGCGC

>ID1407-3P
TCTTTGGTCTCTTACGACACCT

>miRNA precursor
AGGAGGAGCACCAGCACACGUAAUGGUGGGCAGGUGUCGUAAGAGGCCAAACACAUUAUUAUAGAACAUG**UCUUUGGUCUCUUACGACACCU**UGCCGUCCAUGAAAUAGCGC
....((....)).((.......((((..(((((((((((((((((((((.((((...........)))).)))))))))))))))))))).)))))..)))).......))..

>Predicted STAR seq:
CAGGTGTCGTAAGAGGCCA

Complementarity: 18

Initial dG =   -50.60

```
                     10
A-----------   .-GGAG     --| A
            GGA      CACCA  GC C
            CCU      GUGGU  UG A
CGCGAUAAAGUA   \ ----     AA^ C
110       100              20
```

Reads in: lib-42, lib-45, lib-47, lib-48, lib-49, lib-50, lib-72
Number of reads="ID1407",16,26,0,0,0,0,0

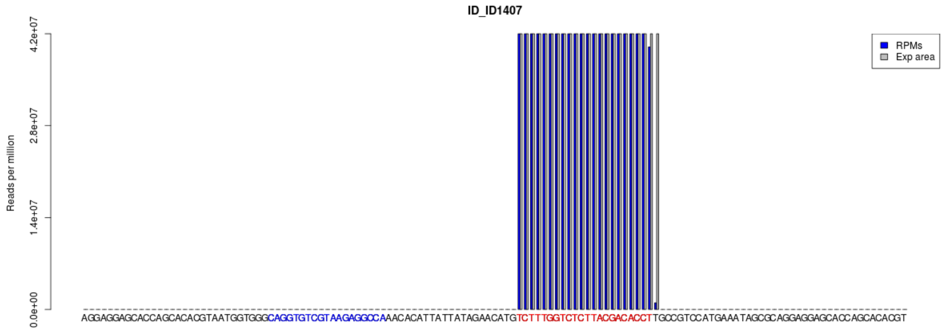

>ID1408
TTCACTTTGCAGCAGAAGAATAGTGGTCACCGGTGAGCAACAATGGTTTTACAAACTTGCTTTTTAAGAG**CTTGTAATTCCATTGTTGTTCG**CTGTGGATCATACGGCT

>ID1408-3P
CTTGTAATTCCATTGTTGTTCG

>miRNA precursor
UUCACUUUGCAGCAGAAGAAUAGUGGUCACCGGUGAGCAACAAUGGUUUUACAAACUUGCUUUUUAAGAG**CUUGUAAUUCCAUUGUUGUUCG**CUGUGGAUCAUACGGCU
....((((......))))....((((((..(((((((((((((((..((((((....((((.....))))))))))..)))))))))))))))..))))))......

>Predicted STAR seq:
GAGCAACAATGGTTTTACAAA

Complementarity: 18
ERROR 4: Strange structure
 Initial dG =   -43.10

```
          10
.-UUCA|   GC
      CUUU  A
      GAAG  G
\ ----^   AC




  20          30         40         50         60
  AAUA--      AC                UU       ACUU     UU
        GUGGUC  CGGUGAGCAACAAUGG  UUACAA     GCUU  \
        UACUAG  GUCGCUUGUUGUUACC  AAUGUU     CGAG  U
  UCGGCA      GU                UU       ----     AA
         100         90         80             70
```

Reads in: lib-42, lib-45, lib-47, lib-48, lib-49, lib-50, lib-72
Number of reads="ID1408",387,36,8,1,0,1,0

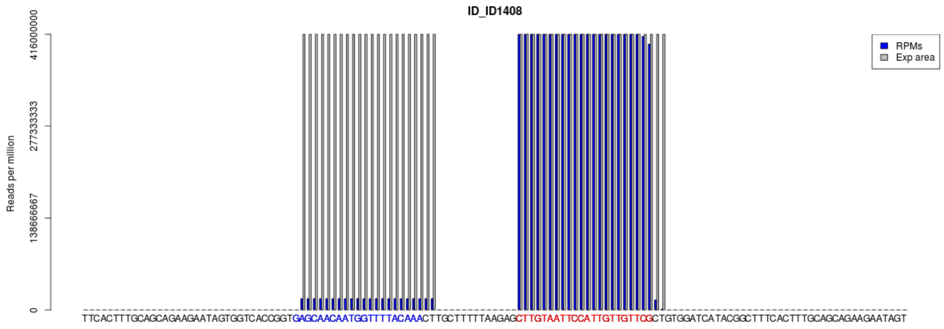

>ID1409

ATAAGGAAGTGGTATGCACC**TGACAACAGTTGGTGTAGAAAC**TTGCATTTTAGAAGTTACTACCTCAATTGTTGTTTAGATGCAGATCAC
ACAGCAAACACACGTCATAATA

>ID1409-5P

TGACAACAGTTGGTGTAGAAAC

>miRNA precursor

AUAAGGAAGUGGUAUGCACC**UGACAACAGUUGGUGUAGAAAC**UUGCAUUUUAGAAGUUACUACCUCAAUUGUUGUUUAGAUGCAGAUCACACAGCAAACACACGUCAUAAUA

```
........(((((.((((.((((((((((((((.((((.(((((.........))))).)))).))))))))))))).)).)))).))))).....................
```

>Predicted STAR seq:

TACTACCTCAATTGTTGTTTAGA

Complementarity: 18

Initial dG =   -34.30

```
                      10        20         30        40
AUAAGGAA--------------     A    C  -|          U    A     GCAU
                      GUGGU UGCA CU GACAACAGUUGG GUAG AACUU    \
                      CACUA ACGU GA UUGUUGUUAACU CAUC UUGAA    U
AUAAUACUGCACACAAACGACA     G    A  U^          C    A     GAUU
110       100       90        80        70        60        50
```

Reads in: lib-42, lib-45, lib-47, lib-48, lib-49, lib-50, lib-72

Number of reads="ID1409",426,15,2,2,0,4,0

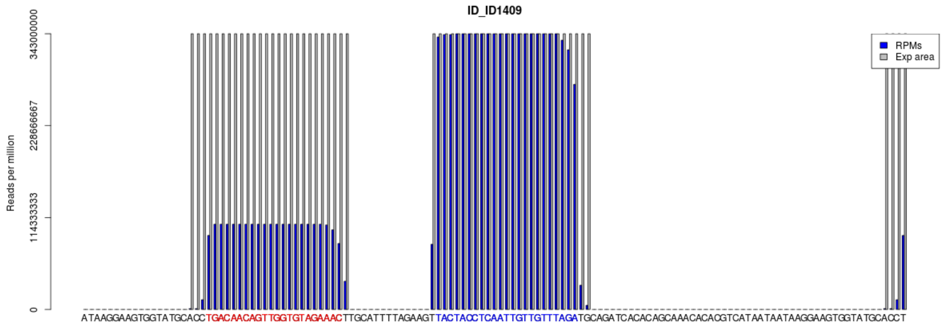

>ID1410
CAAGAAGAGCACACTTAACATAATGGTCAGCAGGTGTCATAAGTTACCAACGGCAGTGAGAAAAGAATTT**GACGCTGGTTACTTGTGGCA**
GCTCGTCGGCCATTTCCTCT

>ID1410-3P
GACGCTGGTTACTTGTGGCAG

>miRNA precursor
```
CAAGAAGAGCACACUUAACAUAAUGGUCAGCAGGUGUCAUAAGUUACCAACGGCAGUGAGAAAAGAAUUUGACGCUGGUUACUUGUGGCAGCUCGUCGGCCAUUUCCUCU
.....((((...........(((((((.((((.((((((((((.((((.((.(((...........))).)).)))).)))))))))).)).)).)))))))..))))
```

>Predicted STAR seq:
TGTCATAAGTTACCAACGG

Complementarity: 15

Initial dG =   -34.30

```
        10        20        30          40        50        60
CAAGA|   CACACUUAACAU       A  -  G          U    A  G   UGAGA
     AGAG            AAUGGUC GC AG UGUCAUAAGU ACCA CG CAG     A
     UCUC            UUACCGG UG UC ACGGUGUUCA UGGU GC GUU     A
-----^   CU----------       C  C  G          U    C  A   UAAGA
   110                100        90        80        70
```

Reads in: lib-42, lib-45, lib-47, lib-48, lib-49, lib-50, lib-72
Number of reads="ID1410",12,4,1,0,0,0,0

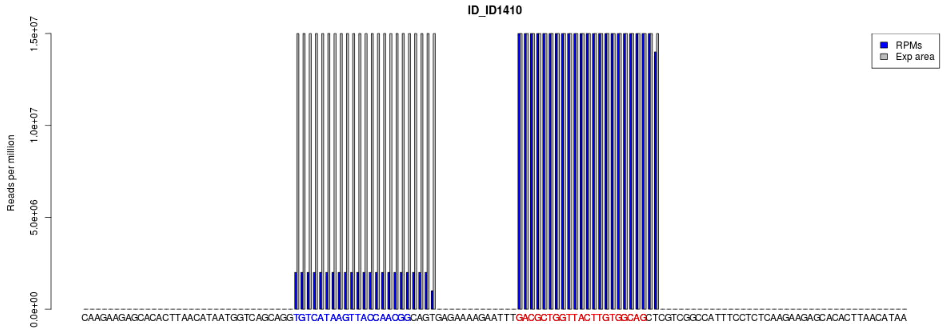

>ID1411
TTTGGTGTGTCCACCAATAAAGATGGTCTGCAGGCGTCATAAGGGCAGTGAGCACTGTTTGGAGAAAGTG**CACTCTGCCCCTATGACGCC**
TTGCCGGTCATCATTAACG

>ID1411-3P
CACTCTGCCCCTATGACGCCT

>miRNA precursor
UUUGGUGUGUCCACCAAUAAAGAUGGUCUGCAGGCGUCAUAAGGGCAGUGAGCACUGUUUGGAGAAAGUG**CACUCUGCCCCUAUGACGCCU**UGCCGGUCAUCAUUAACG
.((((((....))))))....((((..(.((((((((((((.((((((...(((((..........)))))...)))))).))))))))).))).)..)))).......

>Predicted STAR seq:
GCGTCATAAGGGCAGT

Complementarity: 14
ERROR 4: Strange structure
 Initial dG =   -56.00

```
.-U|     U
   UUGGUG G
   AACCAC U
\ -^     C
         10



   20           30         40        50        60
  UAAA---    GU U    -         A      UGA      GUUU
         GAUG  C GCA GGCGUCAUA GGGCAG   GCACU    G
         CUAC  G CGU CCGCAGUAU CCCGUC   CGUGA    G
  GCAAUUA    UG C    U         C      UCA      AAGA
         100        90         80        70
```

Reads in: lib-42, lib-45, lib-47, lib-48, lib-49, lib-50, lib-72
Number of reads="ID1411",24,36,0,0,0,0,0

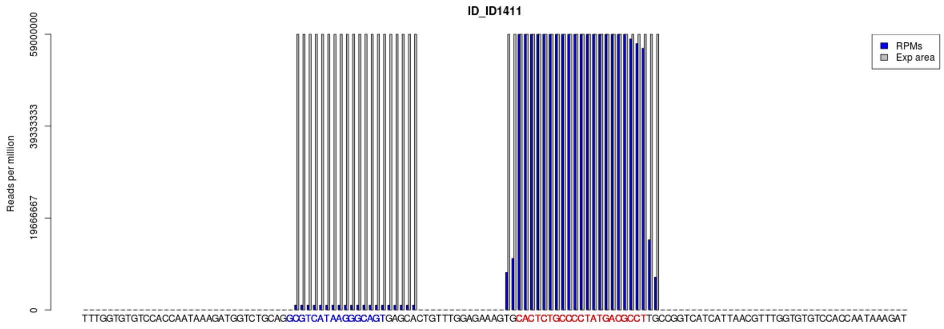

>ID1412
CATCACCACAACCAAGTAAGATGGTCAGCAGCAAATAGGACCAGACGGAACACACAGTGATTAGAATATGTGTTCTGCTGACCCTGTTAC
GCTCCTCTCCATTTCTTTCTGG

>ID1412-5P
AAATAGGACCAGACGGAACACA

>miRNA precursor
CAUCACCACAACCAAGUAAGAUGGUCAGCAGCAAAUAGGACCAGACGGAACACACAGUGAUUAGAAUAUGUGUUCUGCUGACCCUGUUACGCUCCUCUCCAUUUCUUUCUGG
...........(((((..((((((..((.(((.((((((..(((.((((((((((..((.......)).))))))))))))..))))))..))).))..))))))..)).)))

>Predicted STAR seq:
TGTTCTGCTGACCCTGTTACGC

Complementarity: 16

Initial dG =   -33.50

```
        10         20         30         40         50         60
CAUCACCACAA   -   UA       UC   C    A-|      AC    A          CA   GAU
           CCA AG   AGAUGG   AG AGC  AAUAGG  CAG CGGAACACA  GU    \
           GGU UU   UUUACC   UC UCG  UUGUCC  GUC GUCUUGUGU  UA    U
-----------   C  UC       UC   C    CA^      CA    -          A-   AGA
           110       100        90         80         70
```

Reads in: lib-42, lib-45, lib-47, lib-48, lib-49, lib-50, lib-72
Number of reads="ID1412",73,95,1,2,0,0,0

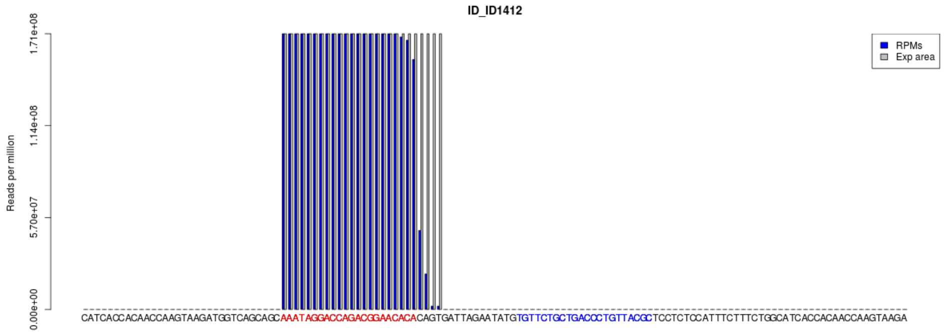

>ID1413
TTGGTGTGTGCACAAATAAAGATGGTCTGCAGGCGTCATAAGAGCCAATGAGCACTGTTTGTAGAAAGTG**CCCATTGACCTTGTGACGCC**
TTGCCGGTTATCATCAACA

>ID1413-3P
CCCATTGACCTTGTGACGCCT

>miRNA precursor
UUGGUGUGUGCACAAAUAAAGAUGGUCUGCAGGCGUCAUAAGAGCCAAUGAGCACUGUUUGUAGAAAGUG**CCCAUUGACCUUGUGACGCCU**UGCCGGUUAUCAUCAACA
...(((....)))........((((..(.((((((((((((((...(((((.(((((..........))))).)))))..)))))))))))).))).)..)))).......

>Predicted STAR seq:
GCGTCATAAGAGCCAATGA

Complementarity: 15
ERROR 4: Strange structure
Initial dG =   -44.00

```
.-UUG|  U
     GUG G
     CAC U
\ ---^  G
       10


       20        30         40        50        60
  AAAUAAA    GU U   -          AGC     A     GUUU
         GAUG  C GCA GGCGUCAUAAG   CAAUG GCACU    G
         CUAU  G CGU CCGCAGUGUUC   GUUAC CGUGA    U
  ACAACUA    UG C   U          CA-     C     AAGA
         100        90        80         70
```

Reads in: lib-42, lib-45, lib-47, lib-48, lib-49, lib-50, lib-72
Number of reads="ID1413",39,69,0,0,0,0,0

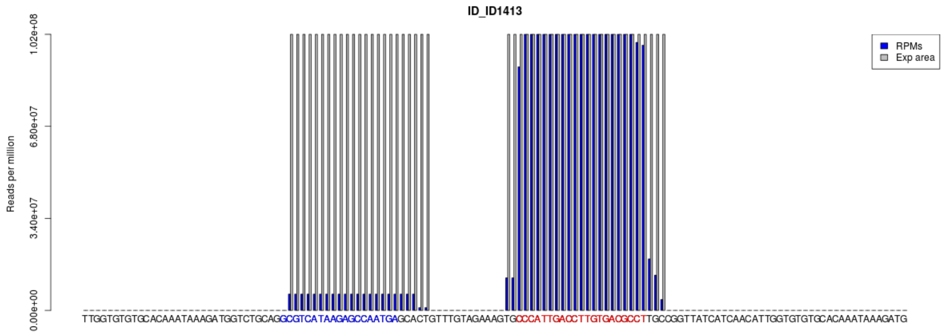

>ID1414
TTTGGTGTGTGCACAAATAAAGATGGTCTGCAGGCGTCATAAGGGCAGTGAGCACTGTTTGTAGAAAGTG**CACTCTGCCCCTATGACGCC**
TTGCCGGTCATCATCAACG

>ID1414-3P
CACTCTGCCCCTATGACGCCT

>miRNA precursor
UUUGGUGUGUGCACAAAUAAAGAUGGUCUGCAGGCGUCAUAAGGGCAGUGAGCACUGUUUGUAGAAAGUG**CACUCUGCCCCUAUGACGCCU**UGCCGGUCAUCAUCAACG
....(((....)))).......((((..(.((((((((((((.((((((...(((((..........)))))...)))))).))))))))).))).)..)))).......

>Predicted STAR seq:
GCGTCATAAGGGCAGT

Complementarity: 14
ERROR 4: Strange structure
Initial dG = -52.30

```
.-UUUG|  U
      GUG G
      CAC U
\ ----^  G
         10


      20        30         40        50        60
  AAAUAAA    GU U   -          A       UGA      GUUU
         GAUG  C GCA GGCGUCAUA GGGCAG   GCACU    G
         CUAC  G CGU CCGCAGUAU CCCGUC   CGUGA    U
  GCAACUA    UG C   U          C       UCA      AAGA
         100        90         80        70
```

Reads in: lib-42, lib-45, lib-47, lib-48, lib-49, lib-50, lib-72
Number of reads="ID1414",31,49,0,2,0,0,0

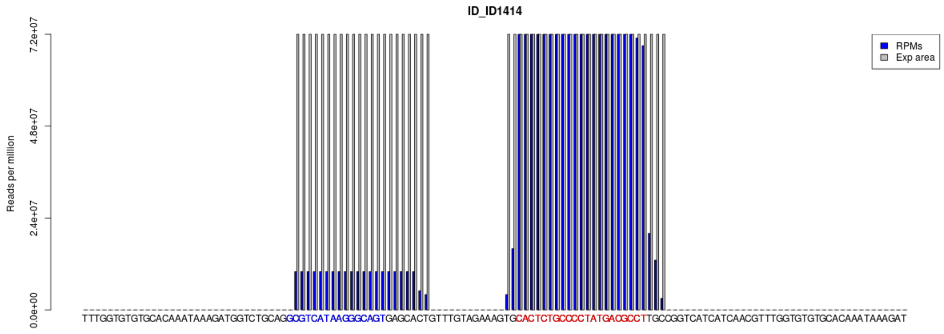

>ID1415

TTTGGTGTGTGTACCAATAAAGATGGTCTGCAGGCGTCATAAGGGCAGTGAGCATTGTTTGTAGAAAGTG**CATTCTGCCCCTATGACGCC**
TTGCCGGTCATCATCAACA

>ID1415-3P

CATTCTGCCCCTATGACGCCT

>miRNA precursor

UUUGGUGUGUGUACCAAUAAAGAUGGUCUGCAGGCGUCAUAAGGGCAGUGAGCAUUGUUUGUAGAAAGUG**CAUUCUGCCCCUAUGACGCCU**UGCCGGUCAUCAUCAACA
.((((((....))))))....((((..(.(((((((((((.((((((...(((((..........)))))...)))))).))))))))).))).)..)))).......

>Predicted STAR seq:

GCGTCATAAGGGCAGT

Complementarity: 14
ERROR 4: Strange structure
 Initial dG =   -52.00

```
.-U|     U
   UUGGUG G
   AACCAU U
\ -^     G
         10



   20           30         40        50        60
  UAAA---    GU U   -         A       UGA      GUUU
         GAUG  C GCA GGCGUCAUA GGGCAG   GCAUU    G
         CUAC  G CGU CCGCAGUAU CCCGUC   CGUGA    U
  ACAACUA    UG C   U         C       UUA      AAGA
         100        90         80        70
```

Reads in: lib-42, lib-45, lib-47, lib-48, lib-49, lib-50, lib-72

Number of reads="ID1415",12,6,0,0,0,0,0

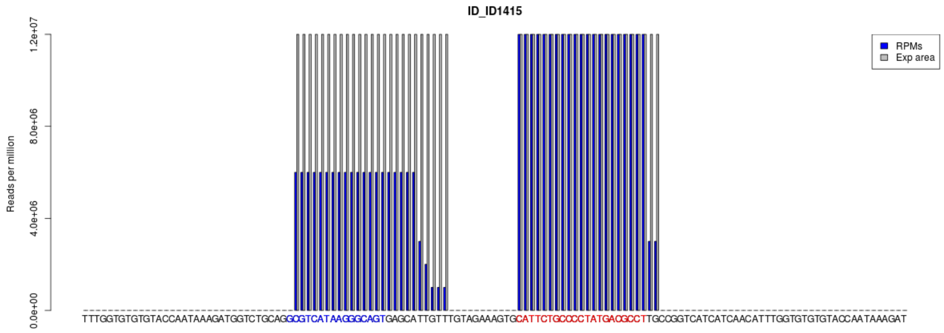

>ID1416
TTTGGTGTGTGCACCAATAAAGATGGTCTGCAGGCGTCATAAGGGCAGTGAGCACTGTTTGTAGAAAGTG**CACTCTGCTCCTATGACGCC**
TTGCCGGTCATCATCAAC

>ID1416-3P
CACTCTGCTCCTATGACGCCT

>miRNA precursor
UUUGGUGUGUGCACCAAUAAAGAUGGUCUGCAGGCGUCAUAAGGGCAGUGAGCACUGUUUGUAGAAAGUG**CACUCUGCUCCUAUGACGCCU**UGCCGGUCAUCAUCAAC
.((((((....))))))....((((..(.(((((((((((.((((((...(((((..........)))))...)))))).))))))))).))).)..))))......

>Predicted STAR seq:
GCGTCATAAGGGCAGT

Complementarity: 14
ERROR 4: Strange structure
Initial dG =   -54.20

```
.-U|     U
   UUGGUG G
   AACCAC U
\ -^     G
         10



   20          30         40        50        60
  UAAA--    GU U   -         A      UGA     GUUU
        GAUG  C GCA GGCGUCAUA GGGCAG   GCACU    G
        CUAC  G CGU CCGCAGUAU CUCGUC   CGUGA    U
  CAACUA    UG C   U         C      UCA     AAGA
        100       90        80        70
```

Reads in: lib-42, lib-45, lib-47, lib-48, lib-49, lib-50, lib-72
Number of reads="ID1416",9,19,0,0,0,0,0

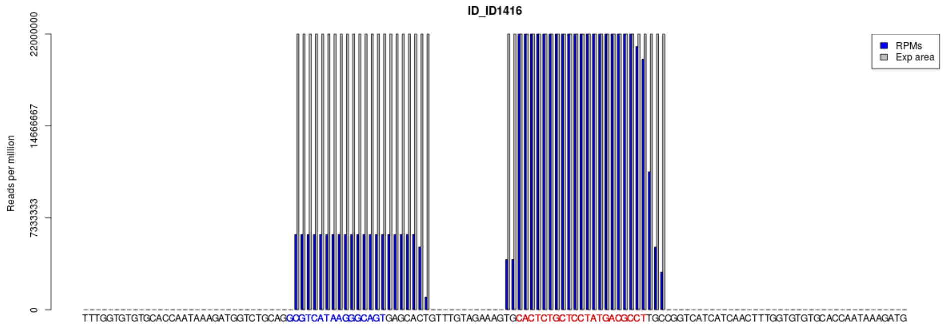

>ID1418
TTAGCATTGCAAAACGATAGAAGTGGTCTGCAACGACCGACAGTTGCAGCAAACAACTGCCATTTAAAAG**TTGGTGCTGCCACTGTTGGC**
GGTTGCAAATCATACAGCATCC

>ID1418-3P
TTGGTGCTGCCACTGTTGGCGG

>miRNA precursor
UUAGCAUUGCAAAACGAUAGAAGUGGUCUGCAACGACCGACAGUUGCAGCAAACAACUGCCAUUUAAAAG**UUGGUGCUGCCACUGUUGGCGG**UUGCAAAUCAUACAGCAUCC
```
...............(((....(((((.((((((..((((((((.((((((..(((((..........))))).)))))).))))))))..)))))).))))).....))).
```

>Predicted STAR seq:
GACCGACAGUUGCAGCAAACAA

Complementarity: 16
ERROR 4: Strange structure
Initial dG =   -42.20

```
           10
UUA-  .-A|  CA
    GC   UUG  \
    CG   AGC  A
CCUA  \ -^  AA
110



         20        30        40        50        60
        UAGAA     C      GA        U      AA      GCCA
             GUGGU UGCAAC  CCGACAGU GCAGCA  CAACU    U
             UACUA ACGUUG  GGUUGUCA CGUCGU  GUUGA    U
        ACA--     A      GC        C      G-      AAAU
              100        90        80         70
```

Reads in: lib-42, lib-45, lib-47, lib-48, lib-49, lib-50, lib-72
Number of reads="ID1418",42,0,0,0,0,0,0

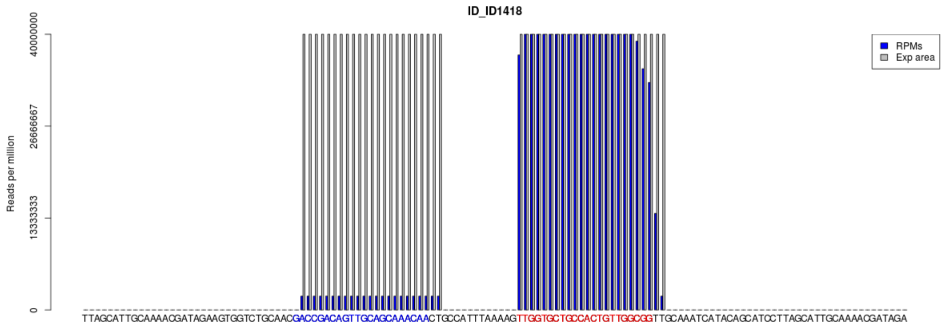

>ID1419
CAATAAAGATGGTCTGCAGGTGTCACAAGGACAGTGAGCACTGTTTGGAGAAAG**TGGACACTGGCCTTATGACGCCT**TGCCGCTCATCAC
CAACGACGTTTCTGTTTCCT

>ID1419-3P
TGGACACTGGCCTTATGACGCCT

>miRNA precursor
CAAUAAAGAUGGUCUGCAGGUGUCACAAGGACAGUGAGCACUGUUUGGAGAAAG**UGGACACUGGCCUUAUGACGCCU**UGCCGCUCAUCACCAACGACGUUUCUGUUUCCU
```
.......((((((..((((((((((.((((.(((((..((((..........))))..))))).)))).))))))).))).)).)))).....................
```

>Predicted STAR seq:
GTGTCACAAGGACAGTGAGCA

Complementarity: 16

Initial dG =   -37.10

```
                       10          20        30        40
CAAUAAA---------------    -| CU   -       C    A     AG    GUUU
                      GAUG GU  GCA GGUGUCA AAGG CAGUG  CACU    G
                      CUAC CG  CGU CCGCAGU UUCC GUCAC  GUGA    G
UCCUUUGUCUUUGCAGCAACCA    U^ C-   U       A    G     AG    AAGA
.       100        90         80        70        60        50
```

Reads in: lib-42, lib-45, lib-47, lib-48, lib-49, lib-50, lib-72
Number of reads="ID1419",5,1,0,0,0,0,0

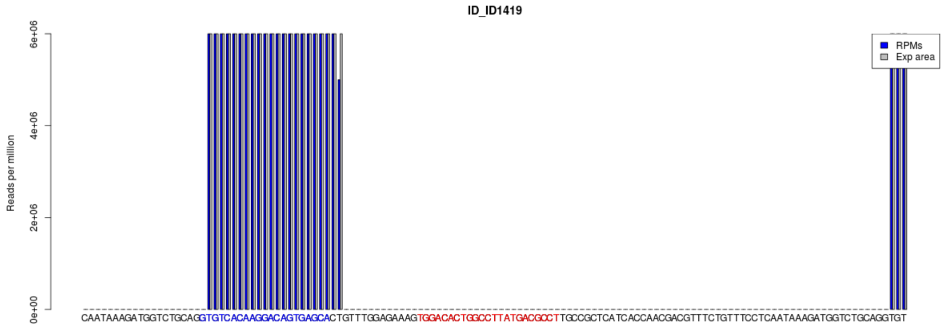

>ID1420
TGTAATATTGCCGCAAAGGACAGTGTTCTGCGGCGAGCAGCAGAGGTTGTACAGACTTACATGTAAGAAA**GTTGTACTTCCTTTGATGCT**
CGCTGCGGTTCACACAGCCAC

>ID1420-3P
GTTGTACTTCCTTTGATGCTCG

>miRNA precursor
UGUAAUAUUGCCGCAAAGGACAGUGUUCUGCGGCGAGCAGCAGAGGUUGUACAGACUUACAUGUAAGAAA**GUUGUACUUCCUUUGAUGCUCG**CUGCGGUUCACACAGCCAC
```
...((((((((.....)).)))))))((((((((((((.((((((..((((((.((((....))))....))))))..)))))).)))))))))))))............
```

>Predicted STAR seq:
CCGCAAAGGACAGTGTTCTGCGGCGAGCAGCAG

Complementarity: 16

Initial dG =   -46.90

```
        10        20         30        40        50           60
UGUAAUAUUGCCGCAAA  ACA-    UU            G      UU      A---|   C
                 GG    GUG  CUGCGGCGAGCA CAGAGG  GUACAG     CUUA A
                 CC    CAC  GGCGUCGCUCGU GUUUCC  CAUGUU     GAAU U
CA---------------  GACA    UU            A      UU      GAAA^   G
 .                      100        90        80        70
```

Reads in: lib-42, lib-45, lib-47, lib-48, lib-49, lib-50, lib-72
Number of reads="ID1420",50,6,1,0,0,3,0

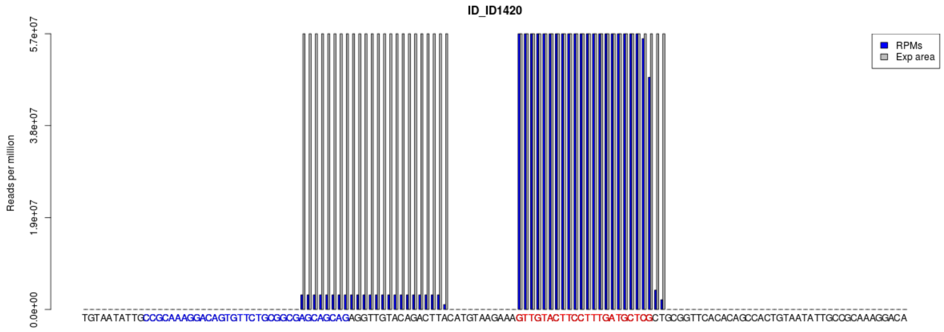

>ID1421
AGGATTGTCTGTCTGCGAACAGCGGTCTGCAGGAGTCGTAAGTGCCTAGACACAATGTAATTGGAATGTG**TGTCTCGTCGCTTATGACGCCT**TGCCGTCCGCATACCACAA

>ID1421-3P
TGTCTCGTCGCTTATGACGCCT

>miRNA precursor
AGGAUUGUCUGUCUGCGAACAGCGGUCUGCAGGAGUCGUAAGUGCCUAGACACAAUGUAAUUGGAAUGUG**UGUCUCGUCGCUUAUGACGCCU**UGCCGUCCGCAUACCACAA
.((((.....)))).......((((...(((((.((((((((((...(((((((.............)))))))...))))))))))..)))))...)))).........

>Predicted STAR seq:
GAGTCGTAAGTGCCTAGACA

Complementarity: 15
ERROR 4: Strange structure
Initial dG = -39.00

```
             10
A---  .-A|   CU
    GG   UUGU  G
    CC   AGCG  U
AACA  \ -^   UC
 .


        20        30         40        50        60
        ACA    UCU     A-          CCU       AUGUAA
           GCGG   GCAGG  GUCGUAAGUG   AGACACA      U
           CGCC   CGUUC  CAGUAUUCGC   UCUGUGU      U
        AUA    UGC     CG          UGC       GUAAGG
           100        90        80        70
```

Reads in: lib-42, lib-45, lib-47, lib-48, lib-49, lib-50, lib-72
Number of reads="ID1421",10,10,0,0,0,0,0

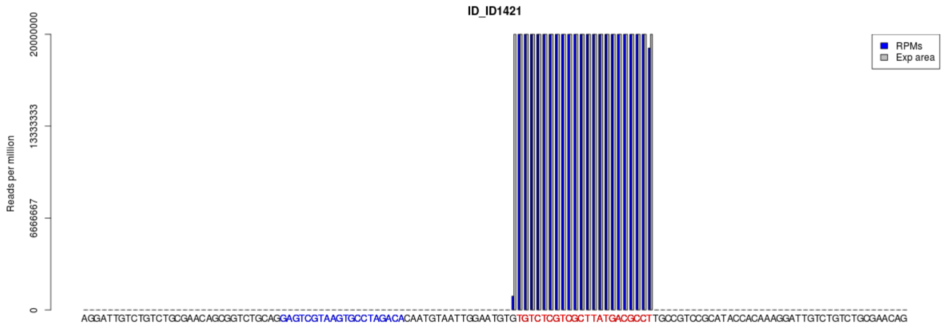

>ID1422

ACGGACAGTGGACTACGGTG**AGTGGCAATGGTTTTACACACT**TGCATTTTAGAAGCTTGTAATTCCATTGGCGTTTGCTGTAGTTCATAC
GACTCCCACATGGCACAGAACT

>ID1422-5P

AGTGGCAATGGTTTTACACACT

>miRNA precursor

ACGGACAGUGGACUACGGUG**AGUGGCAAUGGUUUUACACACU**UGCAUUUUAGAAGCUUGUAAUUCCAUUGGCGUUUGCUGUAGUUCAUACGACUCCCACAUGGCACAGAACU
..(((..(((((((((((..((((.((((((..(((((..(((.........)))..)))))..)))))).)))))..)))))))))))).....)))................

>Predicted STAR seq:

CTTGTAATTCCATTGGCGTTTGCT

Complementarity: 15

Initial dG =   -37.70

```
                          10        20        30        40
AC--------------   CA---|          UG    G       UU      CA   GCAU
                GGA     GUGGACUACGG  AGUG CAAUGG  UUACA  CUU    \
                CCU     UACUUGAUGUC  UUGC GUUACC  AAUGU  GAA    U
UCAAGACACGGUACAC   CAGCA^          GU    G       UU      UC   GAUU
110       100       90        80        70        60        50
```

Reads in: lib-42, lib-45, lib-47, lib-48, lib-49, lib-50, lib-72

Number of reads="ID1422",129,4,1,1,0,1,0

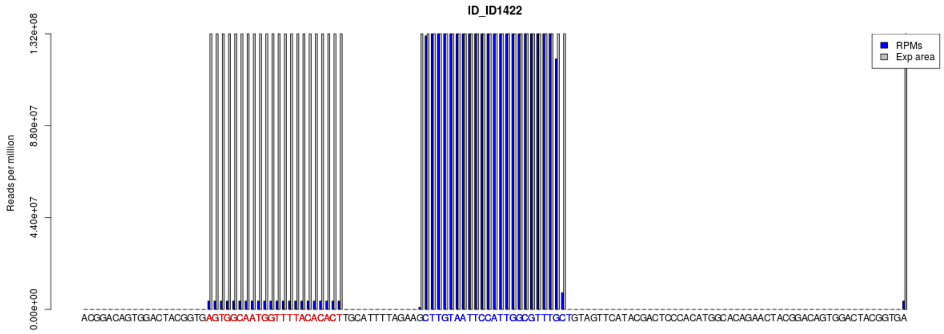

>ID1423
AAAGGAAGTTCATTTTGCAGAATGGTCCGCAGGAGTCACAAGGACTGAGCTACATTGCATAAAAAATATG**TAGCTCTGGACTTATGACGCCT**TGCTGATCGTTAAATA

>ID1423-3P
TAGCTCTGGACTTATGACGCCT

>miRNA precursor
AAAGGAAGUUCAUUUUGCAGAAUGGUCCGCAGGAGUCACAAGGACUGAGCUACAUUGCAUAAAAAAUAUG**UAGCUCUGGACUUAUGACGCCU**UGCUGAUCGUUAAAUA
...................(((((((.(((((.((((.(((....(((((((((...........)))))))))....))).)))).)))))).))))))).....

>Predicted STAR seq:
GAGTCACAAGGACTGAGCTA

Complementarity: 13

Initial dG =   -35.10

```
        10        20        30         40        50        60
AAAGGAAGUUCAUUUUGCAG       C     A-|   C   GACU         UGCAU
                    AAUGGUC GCAGG  GUCA AAG    GAGCUACAU     A
                    UUGCUAG CGUUC  CAGU UUC    CUCGAUGUA     A
AUAAA---------------       U     CG^   A   AGGU         UAAAA
                     100       90        80        70
```

Reads in: lib-42, lib-45, lib-47, lib-48, lib-49, lib-50, lib-72
Number of reads="ID1423",16,17,0,1,0,1,0

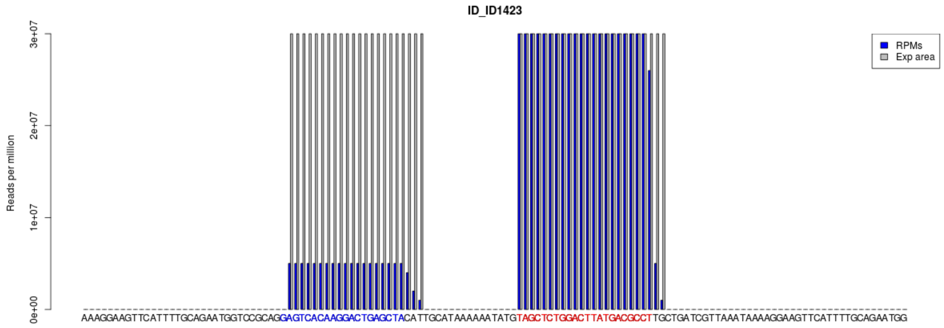

>ID1424
GTTTGGTATGTGCAACAATAAAGATGGCCTGCAGGCGTTATAAGGGCCAGTGAGCACTGTTTGCAGAAAG**TGCGCATTGACCTTATGACA**
CCTTGCCGGTCATTATCAAC

>ID1424-3P
TGCGCATTGACCTTATGACACCT

>miRNA precursor
GUUUGGUAUGUGCAACAAUAAAGAUGGCCUGCAGGCGUUAUAAGGGCCAGUGAGCACUGUUUGCAGAAAG**UGCGCAUUGACCUUAUGACACCU**UGCCGGUCAUUAUCAAC
```
.....................(((((((.(((((.(((((((((..(((((.(((((..........))))).))))).))))))))).))).)).)))))))......
```

>Predicted STAR seq:
GGCGTTATAAGGGCCAGTGAGCA

Complementarity: 18

Initial dG =   -43.00

```
        10        20        30          40        50        60
GUUUGGUAUGUGCAACAAUAAA       U  -|  C         GC      A      GUUU
                      GAUGGCC GC AGG GUUAUAAGG  CAGUG GCACU    G
                      UUACUGG CG UCC CAGUAUUCC  GUUAC CGUGA    C
CAACUA----------------       C  U^  A         A-      G      AAGA
.                       100        90        80          70
```

Reads in: lib-42, lib-45, lib-47, lib-48, lib-49, lib-50, lib-72
Number of reads="ID1424",7,5,0,0,0,0,0

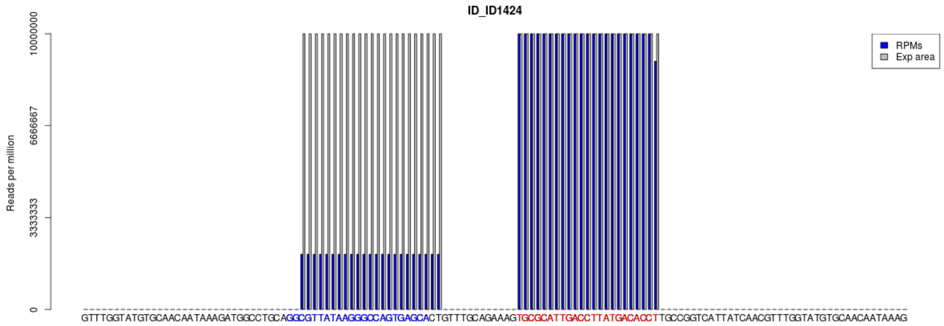

>ID1425
CTGAAAGCATGTCTCCCTGC**AAAAGAAAATGGCTGTTTAACA**TTGCTTATAAACGATGTTAAGAGCTAATTTTCTTTTGCTGGTGGACAT
GACGGCCTTCATCAATTTCAGT

>ID1425-5P
AAAAGAAAATGGCTGTTTAACA

>miRNA precursor
CUGAAAGCAUGUCUCCCUGC**AAAAGAAAAUGGCUGUUUAACA**UUGCUUAUAAACGAUGUUAAGAGCUAAUUUUCUUUUGCUGGUGGACAUGACGGCCUUCAUCAAUUUCAGU
((((((.((((((..((.(((((((((((((((.((((((((((........))))))))))))))).))))))))))).))..)))))).((......))..)))))).

>Predicted STAR seq:
TTAAGAGCTAATTTTCTTTTGC

Complementarity: 19

Initial dG = -44.80

```
           10        20          30         40
-      .-G      UC  U           -|    G          CUU
 CUGAAA   CAUGUC  CC GCAAAAGAAAA UGGCU UUUAACAUUG   A
 GACUUU   GUACAG  GG CGUUUUCUUUU AUCGA GAAUUGUAGC   U
U      \ -      GU  U           A^    -          AAA
110       90        80        70         60        50




         AC  CC
           GG  U
           CU  U
         AA  AC
            100
```

Reads in: lib-42, lib-45, lib-47, lib-48, lib-49, lib-50, lib-72
Number of reads="ID1425",12,1,0,0,0,0,0

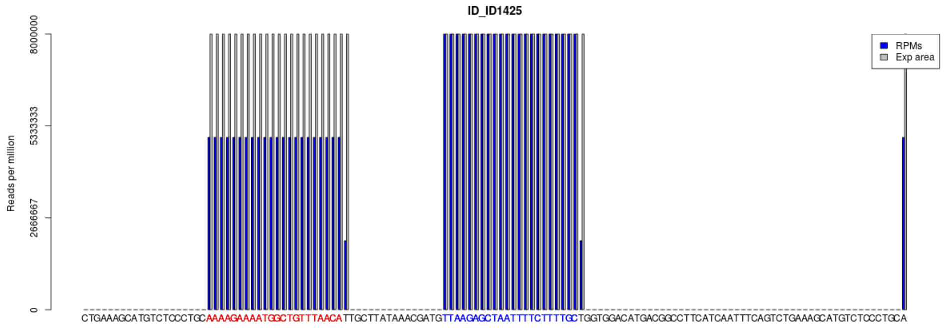

>ID1426
TTTGGTGTGTGCACCAATAAAGATGGTCTGCAGGCGTCATAAGGGCAGTGAGCACTGTTTGTAGAAAGTG**CACTCTGCTCCTATGACGCC**
TTGCCGGTCATCATCAAC

>ID1426-3P
CACTCTGCTCCTATGACGCCT

>miRNA precursor
UUUGGUGUGUGCACCAAUAAAGAUGGUCUGCAGGCGUCAUAAGGGCAGUGAGCACUGUUUGUAGAAAGUG**CACUCUGCUCCUAUGACGCCU**UGCCGGUCAUCAUCAAC
.((((((....))))))....((((..(.(((((((((((.((((((...(((((..........)))))...)))))).))))))))).))).)..))))......

>Predicted STAR seq:
GCGTCATAAGGGCAGT

Complementarity: 14
ERROR 4: Strange structure
Initial dG = -54.20

```
.-U|     U
   UUGGUG G
   AACCAC U
\ -^     G
         10



   20          30         40        50        60
  UAAA--    GU U    -         A       UGA      GUUU
        GAUG  C GCA GGCGUCAUA GGGCAG   GCACU    G
        CUAC  G CGU CCGCAGUAU CUCGUC   CGUGA    U
  CAACUA    UG C    U         C       UCA      AAGA
        100        90         80        70
```

Reads in: lib-42, lib-45, lib-47, lib-48, lib-49, lib-50, lib-72
Number of reads="ID1426",9,12,0,0,0,1,0

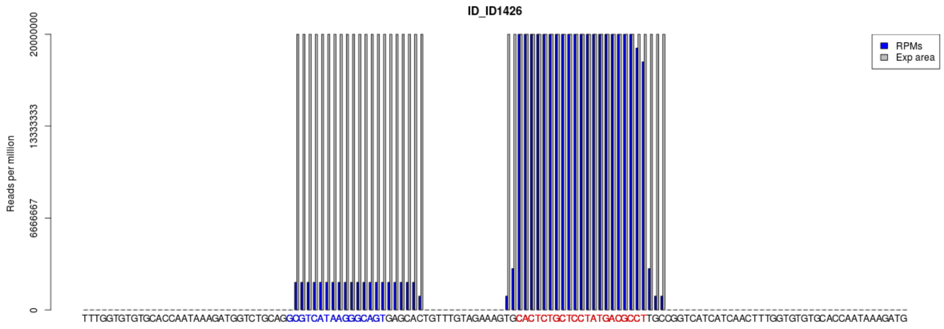

>ID1427
TTTGGTGTGTGCACCAATAAAGATGGTCTGCAGGCGTCATAAGGGCAGTGAGCACTGTTTGTAGAAAGTG**CATTCTGCCCCTATGACGCC**
TTGCCGGTCATCATCAACA

>ID1427-3P
CATTCTGCCCCTATGACGCCT

>miRNA precursor
UUUGGUGUGUGCACCAAUAAAGAUGGUCUGCAGGCGUCAUAAGGGCAGUGAGCACUGUUUGUAGAAAGUG**CAUUCUGCCCCUAUGACGCCU**UGCCGGUCAUCAUCAACA
.((((((....))))))....((((..(.(((((((((((.((((((...(((((..........)))))...)))))).))))))))).))).)..)))).......

>Predicted STAR seq:
GCGTCATAAGGGCAGT

Complementarity: 14
ERROR 4: Strange structure
Initial dG = -57.20

```
.-U|     U
   UUGGUG G
   AACCAC U
\ -^     G
         10



   20           30         40        50        60
  UAAA---    GU U    -         A       UGA      GUUU
         GAUG  C GCA GGCGUCAUA GGGCAG   GCACU    G
         CUAC  G CGU CCGCAGUAU CCCGUC   CGUGA    U
  ACAACUA    UG C    U         C       UUA      AAGA
         100        90         80        70
```

Reads in: lib-42, lib-45, lib-47, lib-48, lib-49, lib-50, lib-72
Number of reads="ID1427",10,5,0,0,0,0,0

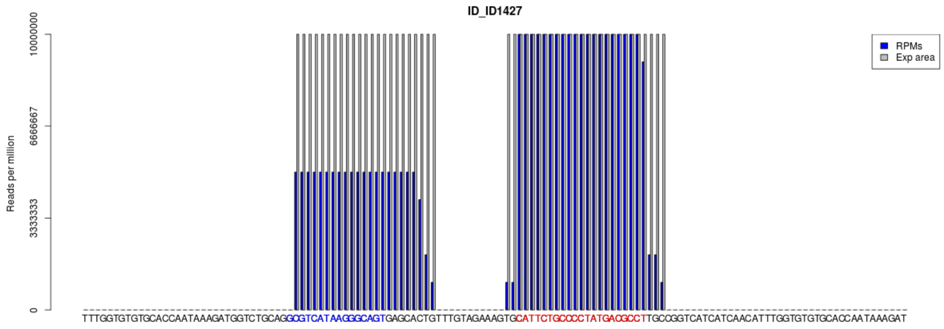

>ID1428
TTTGGACCATATTGACCAGC**AGGAGAGACGCAGCAGTGGAACAT**GTCTTGATAAACGTTGTTCCCTGCTGCTTCTCTCCTGCTGGTCGAC
ATGGCAACATCCTTCGTTTTCTG

>ID1428-5P
AGGAGAGACGCAGCAGTGGAACAT

>miRNA precursor
UUUGGACCAUAUUGACCAGC**AGGAGAGACGCAGCAGUGGAACAU**GUCUUGAUAAACGUUGUUCCCUGCUGCUUCUCUCCUGCUGGUCGACAUGGCAACAUCCUUCGUUUUCUG
...((((((.((((((((((((((((.(((((((.(((((((((........))).)))))))))))))).))))))))))))))))).)))).....)))...........

>Predicted STAR seq:
TGTTCCCTGCTGCTTCTCTCCTGC

Complementarity: 20

Initial dG =   -64.60

```
                     10        20        30        40         50
UUU--------   -----|   A                 C       U       -   CUU
           GGA     CCAU UUGACCAGCAGGAGAGA GCAGCAG GGAACA UGU   G
           CCU     GGUA AGCUGGUCGUCCUCUCU CGUCGUC CCUUGU GCA   A
GUCUUUUGCUU   ACAAC^   C                 U       -       U   AAU
 110       100        90        80        70         60
```

Reads in: lib-42, lib-45, lib-47, lib-48, lib-49, lib-50, lib-72
Number of reads="ID1428",28,3,0,0,0,0,0

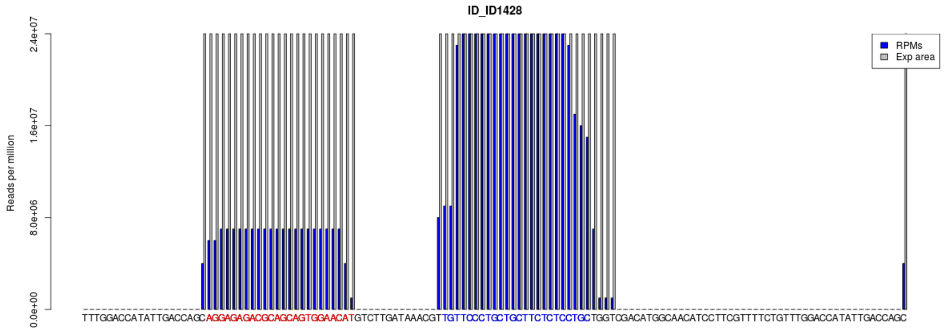

>ID1430
CTAAAAGCGTAGCTACCTGG**AGAAGTGCAGTAGTTGACGAACA**TGGCATATAGAAGGTGTTCGTTGCTATTGCACTTTCCATGGTGGCTA
TGACGATCTTCAACCATTTC

>ID1430-5P
AGAAGTGCAGTAGTTGACGAACA

>miRNA precursor
CUAAAAGCGUAGCUACCUGG**AGAAGUGCAGUAGUUGACGAACA**UGGCAUAUAGAAGGUGUUCGUUGCUAUUGCACUUUCCAUGGUGGCUAUGACGAUCUUCAACCAUUUC
.......((((((((((((((((.((((((((((.(((((((((............))))))))))))))))))))))))))).)))))))))).................

>Predicted STAR seq:
TTCGTTGCTATTGCACTTTCC

Complementarity: 19

Initial dG =   -47.90

```
                   10         20        30        40        50
CUAAAAG-----------          -|     A          U          GGCAU
                  CGUAGCUACC UGGAGA GUGCAGUAGU GACGAACAU     A
                  GUAUCGGUGG ACCUUU CACGUUAUCG UUGCUUGUG     U
CUUUACCAACUUCUAGCA          U^     -          -          GAAGA
.       100        90        80        70        60
```

Reads in: lib-42, lib-45, lib-47, lib-48, lib-49, lib-50, lib-72
Number of reads="ID1430",1,69,0,0,1,0,0

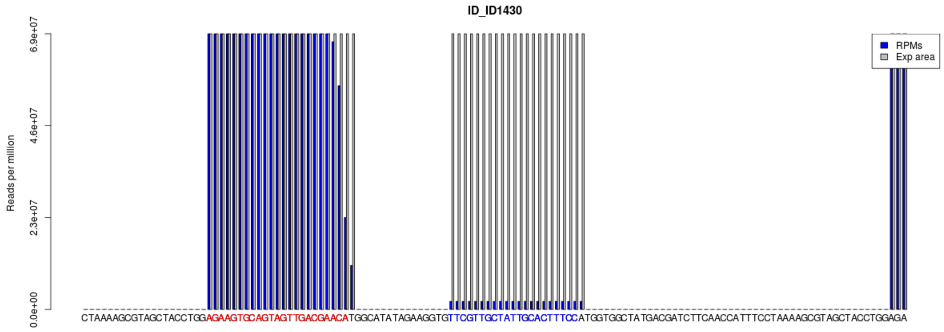

>ID1431

TTGGGTAGTGGATTGCAGACGTCATAAGTGCGTAACCACATTGTATTATGAAATTG**TGGTTGCATGCTTATGACGTCA**TGCAGTCCATAA
CGTT

>ID1431-3P

TGGTTGCATGCTTATGACGTCA

>miRNA precursor

UUGGGUAGUGGAUUGCAGACGUCAUAAGUGCGUAACCACAUUGUAUUAUGAAAUUG**UGGUUGCAUGCUUAUGACGUCA**UGCAGUCCAUAACGUU
....((.((((((((((((((((((((((..(((((((((..............)))))))))..)))))))))))).)))))))))).))...

>Predicted STAR seq:

ACGTCATAAGTGCGTAACCA

Complementarity: 17

Initial dG = -50.60

```
        10         20        30        40
UUGG  A          -|           GC          UUGUAU
    GU GUGGAUUGCA GACGUCAUAAGU  GUAACCACA      U
    CA UACCUGACGU CUGCAGUAUUCG  CGUUGGUGU      A
UUG-  A          A^           UA          UAAAGU
    90        80        70        60        50
```

Reads in: lib-42, lib-45, lib-47, lib-48, lib-49, lib-50, lib-72

Number of reads="ID1431",19,35,0,0,1,0,0

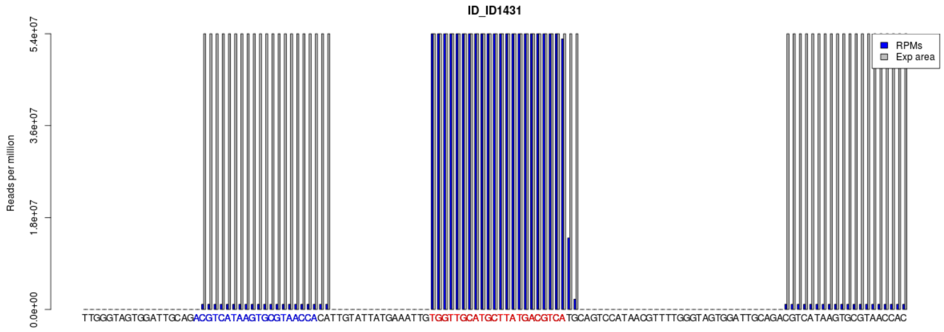

>ID1432

GTGGTGTGTGCACCAATAACGGTGGTCTGCAGGTGTCATAAGTGCCAATGAACACTGTTTGGAGAATTTG**TTCATTGGCCTTGTGACACC**
TTGCCGGTCATCATCAACG

>ID1432-3P

TTCATTGGCCTTGTGACACCT

>miRNA precursor

GUGGUGUGUGCACCAAUAACGGUGGUCUGCAGGUGUCAUAAGUGCCAAUGAACACUGUUUGGAGAAUUUG**UUCAUUGGCCUUGUGACACCU**UGCCGGUCAUCAUCAACG
.(((((....))))).....((((..(.((((((((((((((.(((((((((((..((((...)))).)))))))))))))))))))))))).))).)..)))).......

>Predicted STAR seq:

GTGTCATAAGTGCCAATGAA

Complementarity: 18
ERROR 4: Strange structure
Initial dG =   -55.60

```
.-G|    U
   UGGUG G
   ACCAC U
\ -^    G
       10
```

```
     20          30         40        50        60
  AUAAC--    GU U   -           U           CU    G
         GGUG  C GCA GGUGUCAUAAG GCCAAUGAACA  GUUU \
         CUAC  G CGU CCACAGUGUUC CGGUUACUUGU  UAAG G
  GCAACUA    UG C   U           -           U-    A
         100        90        80         70
```

Reads in: lib-42, lib-45, lib-47, lib-48, lib-49, lib-50, lib-72
Number of reads="ID1432",13,25,1,1,1,2,0

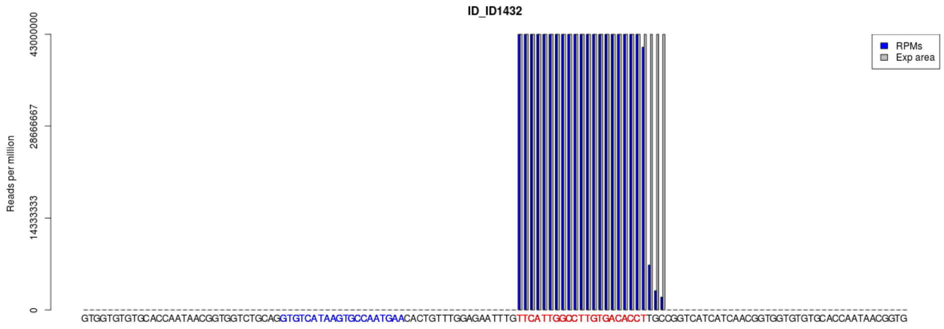

>ID1435
AGACACAGCAGCAGCCTGCCAAGTGGTCTGCAGGTGTCTTAAGAGGTCAAACACATTATTATTGAAGATG**TCTTTGGCCTCTTATGACTCCT**TGCCGTCCATGAAATATCG

>ID1435-3P
TCTTTGGCCTCTTATGACTCCT

>miRNA precursor
AGACACAGCAGCAGCCUGCCAAGUGGUCUGCAGGUGUCUUAAGAGGUCAAACACAUUAUUAUUGAAGAUG**UCUUUGGCCUCUUAUGACUCCU**UGCCGUCCAUGAAAUAUCG
.(((...((.((((((........)).)))).((.(((.((((((((((((.(((((.........))))).)))))))))))).))).))..)).)))...........

>Predicted STAR seq:
GCAGCCTGCCAAGTGGTCTGCAGGTGTCTTAAGAGG

Complementarity: 16
ERROR 4: Strange structure
Initial dG = -39.70

```
          10
A  .-CACA|   C
 GA      GCAG A
 CU      CGUC G
G  \ ----^   C
 .


    20           30         40        50        60
     CAA---    UCU   -  U   U            C     AUUA
           GUGG   GCA GG GUC UAAGAGGUCAAA ACAUU    \
           UACC   CGU CC CAG AUUCUCCGGUUU UGUAG    U
     AUAAAG    UGC   U  U   U            C     AAGU
           100        90        80        70
```

Reads in: lib-42, lib-45, lib-47, lib-48, lib-49, lib-50, lib-72
Number of reads="ID1435",31,65,0,0,2,0,0

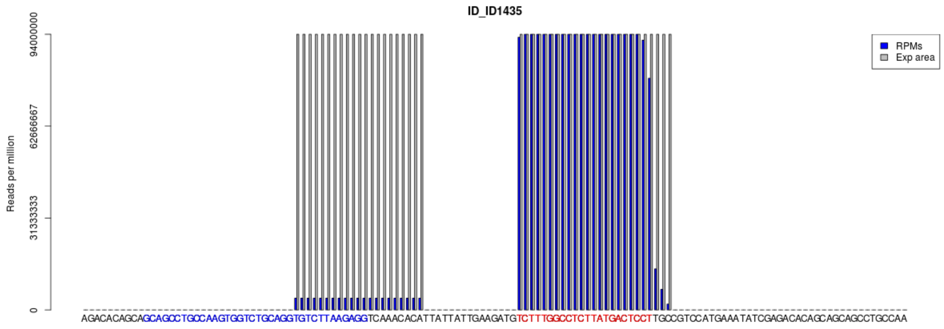

>ID1440
GAAAACTGGTGGAAGGCGTCGAATCAGGACAGTTACACTTTGCACCTGTGCAGGGTTCCCAAAAATAAAA**TGGGAACCTTACACAGGTGTCT**AGTGTTCCTGTCACTTCATC

>ID1440-3P
TGGGAACCTTACACAGGTGTCT

>miRNA precursor
GAAAACUGGUGGAAGGCGUCGAAUCAGGACAGUUACACUUUGCACCUGUGCAGGGUUCCCAAAAAUAAAA**UGGGAACCUUACACAGGUGUCU**AGUGUUCCUGUCACUUCAUC
```
(((..(((((.((.....))..)))))(((((..(((((..(((((((((.((((((((((.........)))))))))).)))))))))..)))))..)))))..)))...
```

>Predicted STAR seq:
TGCACCTGTGCAGGGTTCCCA

Complementarity: 17

Initial dG =   -53.00

```
             10
---   .-AA     G-| AG
   GAA    CUGGU  GA  \
   CUU    GACUA  CU  G
CUA   \ --     AG^ GC
110             20
```

Reads in: lib-42, lib-45, lib-47, lib-48, lib-49, lib-50, lib-72
Number of reads="ID1440",1239,2332,3553,2125,3891,1502,0

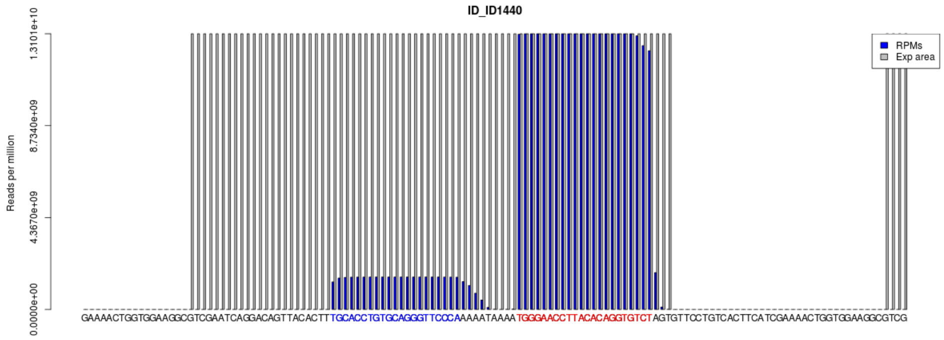

>ID1443
CACAGAGTCCGTGAATTTTC**CGTTAATTTCATGTGCAAGCA**AGTGTAATTAGTTCTTTGCTTTCACGTGAAACTAGCGGAGAAAGTAATG
GACGCTTCTACGAGAACGTCC

>ID1443-5P
CGTTAATTTCATGTGCAAGCA

>miRNA precursor
CACAGAGUCCGUGAAUUUUC**CGUUAAUUUCAUGUGCAAGCA**AGUGUAAUUAGUUCUUUGCUUUCACGUGAAACUAGCGGAGAAAGUAAUGGACGCUUCUACGAGAACGUCC
```
...((.((((((...((((((((((.(((((((((.(((((((...........))))))).))))))))).))))))))))....)))))).)).............
```

>Predicted STAR seq:
CTTTCACGTGAAACTAGCGGA

Complementarity: 17

Initial dG =   -39.30

```
                    10          20         30         40
CAC------------  A      GAA-|         A          C        UGUAA
               AG GUCCGU    UUUUCCGUUA UUUCAUGUG AAGCAAG     U
               UC CAGGUA    AAGAGGCGAU AAAGUGCAC UUCGUUU     U
CCUGCAAGAGCAUCU  G      AUGA^         C          U        CUUGA
 .       100        90        80         70         60         50
```

Reads in: lib-42, lib-45, lib-47, lib-48, lib-49, lib-50, lib-72
Number of reads="ID1443",202,461,613,4154,817,246,0

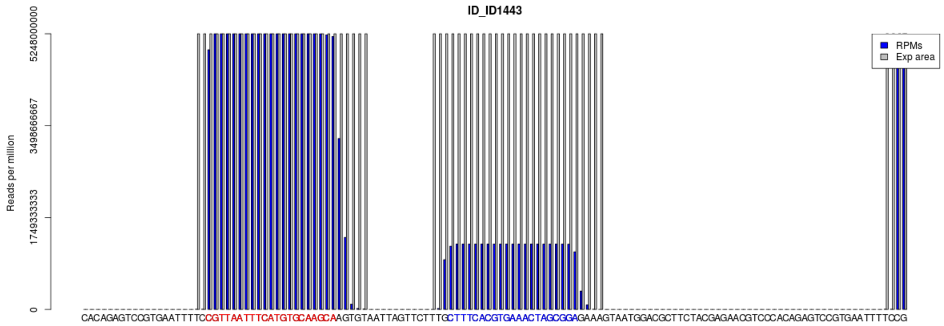

>ID1447
CCACAACTCTTCAAAATGCCTGCGAGAACTGCATGTCAGCAAATCCAGGAGCGCTGCAGTTTTGCCCACG**GCGCGCCGGGGTTTGCTGCG**
TGCAGCTCTCGCACCTCACAAA

>ID1447-3P
GCGCGCCGGGGTTTGCTGCGTG

>miRNA precursor
CCACAACUCUUCAAAAUGCCUGCGAGAACUGCAUGUCAGCAAAUCCAGGAGCGCUGCAGUUUUGCCCACG**GCGCGCCGGGGUUUGCUGCGUG**CAGCUCUCGCACCUCACAAA
....................(((((((.(((((((.((((((((((.((.((((((............)))))).)).))))))))))))))))).))))))).........

>Predicted STAR seq:
ATGTCAGCAAATCCAGGAGCGC

Complementarity: 18

Initial dG = -53.70

```
        10        20        30        40        50        60
CCACAACUCUUCAAAAUGCC|      A       U          A  A      CAGUU
                    UGCGAGA CUGCAUG CAGCAAAUCC GG GCGCUG     U
                    ACGCUCU GACGUGC GUCGUUUGGG CC CGCGGC     U
AAACACUCC-----------^      C       -          G  G      ACCCG
110                  100        90         80        70
```

Reads in: lib-42, lib-45, lib-47, lib-48, lib-49, lib-50, lib-72
Number of reads="ID1447",6,1,1,63,28,0,0

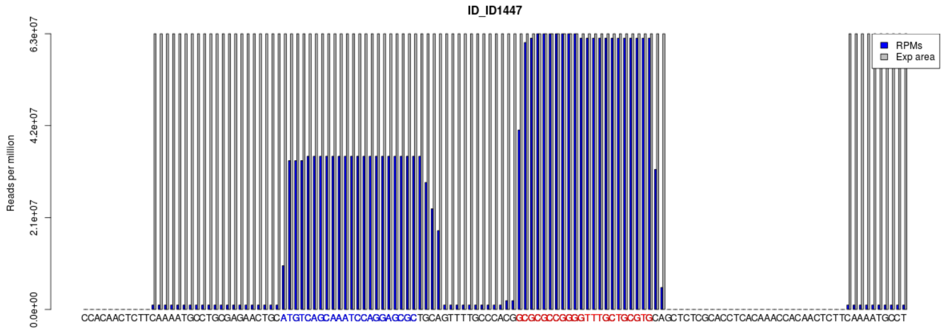

>ID1454
GGCGGTATAAACAAGGCAAGGTTGCGGCAGTTGTCTCTGCTGTGGTCTTCACGACTTTGTGCAGCATCTG**AAGATCTAGTAGAGACGGCT**
GCCGAGGCGTTGCGTCAT

>ID1454-3P
AAGATCTAGTAGAGACGGCTGC

>miRNA precursor
GGCGGUAUAAACAAGGCAAGGUUGCGGCAGUUGUCUCUGCUGUGGUCUUCACGACUUUGUGCAGCAUCUG**AAGAUCUAGUAGAGACGGCUGC**CGAGGCGUUGCGUCAU
```
..............((((.((..(((((((((((((((((.((((((((.((...((.....))))))))))))))))))))))))))))))))..)).)))).....
```

>Predicted STAR seq:
CAGTTGTCTCTGCTGTGGTCTT

Complementarity: 20

Initial dG =   -53.70

```
        10        20        30        40        50        60
GGCGGUAUAAACAAG|   G  UG                  U        C  CUU  UG
               GCAA GU  CGGCAGUUGUCUCUGCUG GGUCUUCA GA   UG  \
               CGUU CG  GCCGUCGGCAGAGAUGAU CUAGAAGU CU   AC  C
UACUG----------^   G  GA                  -        -  ---  GA
                100        90        80        70
```

Reads in: lib-42, lib-45, lib-47, lib-48, lib-49, lib-50, lib-72
Number of reads="ID1454",240,700,409,5092,1596,117,0

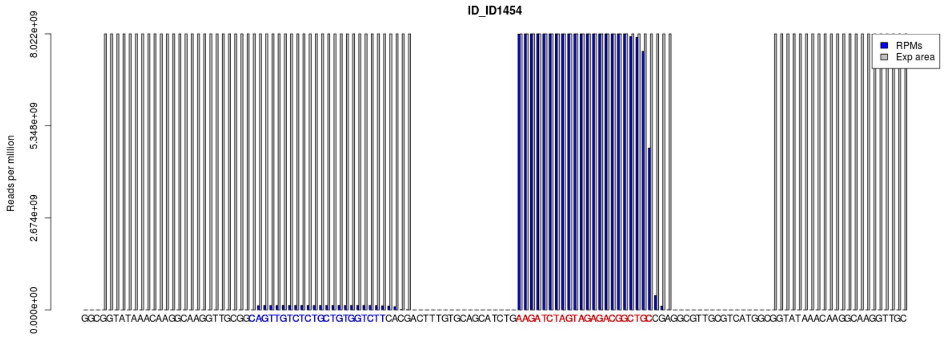

>ID1455
CATCAATAACTTTCTTAATA**TTGATTATGGGCCGAAGCATT**GTATTAAAATATGTACCAATGTGGCGGCTTATAATCATTATCATAGATA
AAGTTAACTCATCACGTCTT

>ID1455-5P
TTGATTATGGGCCGAAGCATT

>miRNA precursor
CAUCAAUAACUUUCUUAAUA**UUGAUUAUGGGCCGAAGCAUU**GUAUUAAAAUAUGUACCAAUGUGGCGGCUUAUAAUCAUUAUCAUAGAUAAAGUUAACUCAUCACGUCUU
......((((((((..(((.(((((((((((((..((((((((........)))).)))))..))))))))))))).)))...)))..)))))).............

>Predicted STAR seq:
TGTGGCGGCTTATAATCATTA

Complementarity: 16

Initial dG =   -33.80

```
                10          20        30        40
CAUCAA--------      --   UA-   U             AA      -|   UAA
              UAACUU  UCU   AUA UGAUUAUGGGCCG  GCAUU GUAU   A
              AUUGAA  AGA   UAU ACUAAUAUUCGGC  UGUAA CAUG   A
UUCUGCACUACUCA      AU   UAC   U             GG      C^   UAU
.       100        90        80        70        60        50
```

Reads in: lib-42, lib-45, lib-47, lib-48, lib-49, lib-50, lib-72
Number of reads="ID1455",1,13,0,53,25,4,0

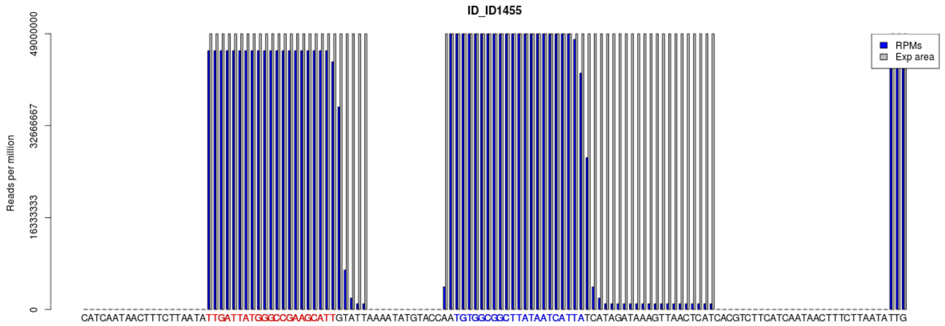

>ID1458
GTCGTTTCGTGGGTAGCGGT**AGATGGAGTTGAGCCGGAATACG**TGAAATTAAGTCGTATCTGGCCCGATTCCATTTCCTGCTGCCTACTG
CACAGCATCAGGAACTGAGCCA

>ID1458-5P
AGATGGAGTTGAGCCGGAATACG

>miRNA precursor
GUCGUUUCGUGGGUAGCGGU**AGAUGGAGUUGAGCCGGAAUACG**UGAAAUUAAGUCGUAUCUGGCCCGAUUCCAUUUCCUGCUGCCUACUGCACAGCAUCAGGAACUGAGCCA
```
........((((((((((.(((((((((((.((((((.((((..........)))))))))).))))))))))).)))))))))))......((.((((...))))))..
```

>Predicted STAR seq:
TATCTGGCCCGATTCCATTTCCT

Complementarity: 19

Initial dG =   -51.00

```
          10        20        30        40
.-GUCGUUUC|          U           A      A    UGAAA
          GUGGGUAGCGG AGAUGGAGUUG GCCGGA UACG     \
          CAUCCGUCGUC UUUACCUUAGC CGGUCU AUGC     U
\ --------^          C           C      -    UGAAU
                 80        70        60         50



  90       100
  UGCACA  A    G
        GC UCAG \
        CG AGUC A
  AC----  -    A
      110
```

Reads in: lib-42, lib-45, lib-47, lib-48, lib-49, lib-50, lib-72
Number of reads="ID1458",0,0,0,0,0,0,0

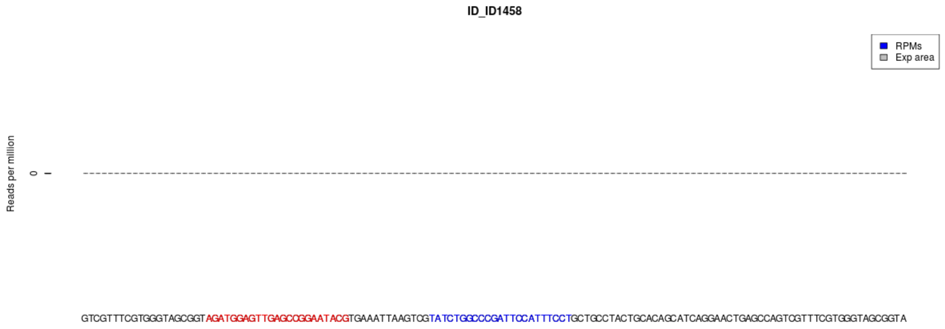

>ID1460
GTCGTTTCGTGGGTAGCGGT**AGATGGAGTTGAGCCGGAATACG**TGAAATTAAGTCGTATCTGGCCCGATTCCATTTCCTGCTGCCTACTG
CACAGCATCAGGAACTGAGCCA

>ID1460-5P
AGATGGAGTTGAGCCGGAATACG

>miRNA precursor
GUCGUUUCGUGGGUAGCGGU**AGAUGGAGUUGAGCCGGAAUACG**UGAAAUUAAGUCGUAUCUGGCCCGAUUCCAUUUCCUGCUGCCUACUGCACAGCAUCAGGAACUGAGCCA
........((((((((((.(((((((((((.((((((.((((...........)))))))))).))))))))))).)))))))))))......((.((((...))))))..

>Predicted STAR seq:
TATCTGGCCCGATTCCATTTCCT

Complementarity: 19

Initial dG = -51.00

```
          10        20        30        40
.-GUCGUUUC|          U           A      A    UGAAA
          GUGGGUAGCGG AGAUGGAGUUG GCCGGA UACG     \
          CAUCCGUCGUC UUUACCUUAGC CGGUCU AUGC     U
\ --------^          C           C      -    UGAAU
                 80        70        60         50



  90       100
  UGCACA  A    G
        GC UCAG \
        CG AGUC A
  AC----  -    A
      110
```

Reads in: lib-42, lib-45, lib-47, lib-48, lib-49, lib-50, lib-72
Number of reads="ID1460",0,0,0,0,0,0,0

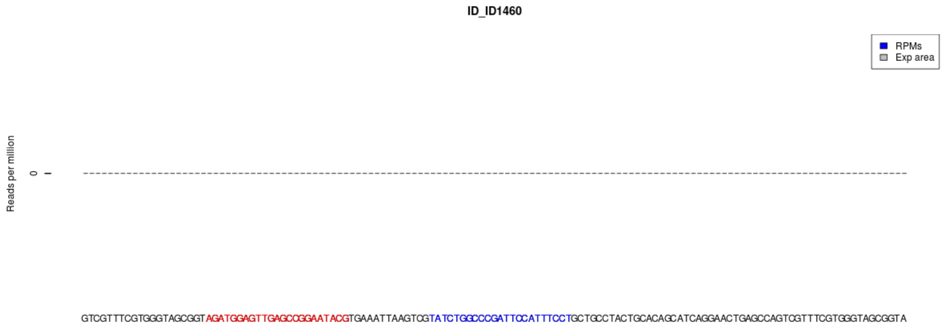

>ID1465

GGGGGACTATTTTACATCTGGAATATTTTACCCAAGAGGATGCCATCATCATCTGTTCATTCAGTAGAGC**GGCATGTCCTTGGGAAAAGT**
AACGGATGTAGTTTTCCCTTGTT

>ID1465-3P

GGCATGTCCTTGGGAAAAGTAACG

>miRNA precursor

GGGGGACUAUUUUACAUCUGGAAUAUUUUACCCAAGAGGAUGCCAUCAUCAUCUGUUCAUUCAGUAGAGC**GGCAUGUCCUUGGGAAAAGUAACG**GAUGUAGUUUUCCCUUGUU
((((((.....(((((((((...((((((.((((((.(((((((....((..(((......)))..))..))))).)))))))).)))))).))))))))))...))))))...

>Predicted STAR seq:

GGAATATTTTACCCAAGAGGATGCC

Complementarity: 19

Initial dG =   -40.30

```
           10        20        30         40        50
---|     CUAUU         GAA      A      A   -      AUCA  AU   UU
   GGGGGA     UUACAUCUG   UAUUUU CCCAAG GG AUGCC    UC  CUG  C
   UUCCCU     GAUGUAGGC   AUGAAA GGGUUC CU UACGG    AG  GAC  A
UUG^     UUU--         A--      A      -  G      CG--  AU   UU
 110        100          90        80         70          60
```

Reads in: lib-42, lib-45, lib-47, lib-48, lib-49, lib-50, lib-72

Number of reads="ID1465",18,31,20,27,129,48,0

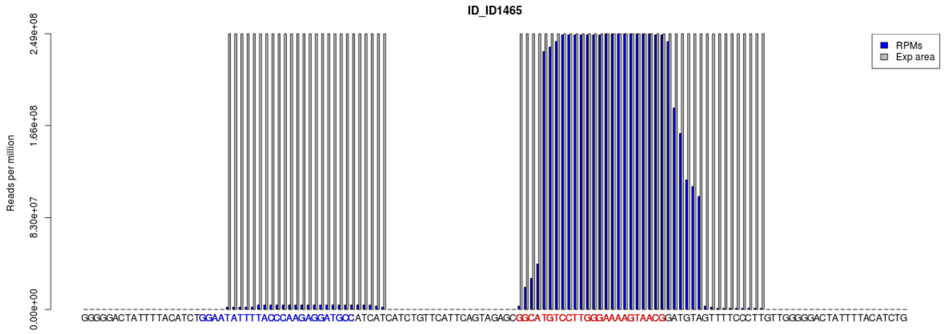

>ID1468
TTACTTTGGAATACGCCTCG**AACGATGGTACTGTACAGTATGTG**CTGCTTAACTCCAAATGCACATACACAATACGATCGTTGGCGGTGT
GTTTATCGAGCCAGCGCTTTGCAGC

>ID1468-5P
AACGATGGTACTGTACAGTATGTG

>miRNA precursor
UUACUUUGGAAUACGCCUCG**AACGAUGGUACUGUACAGUAUGUG**CUGCUUAACUCCAAAUGCACAUACACAAUACGAUCGUUGGCGGUGUGUUUAUCGAGCCAGCGCUUUGCAGC
```
........((((((((((.((((((.(((.(((...((((((((...............)))))))))).))).)))))))).)))))))))...((((....))))......
```

>Predicted STAR seq:
CATACACAATACGATCGTTGG

Complementarity: 17

Initial dG =   -36.60

```
          10          20         30         40         50
.-UUACUUUG         -| G       G   C   ACA        UGCUUAA
          GAAUACGCC UC AACGAU GUA UGU   GUAUGUGC       \
          UUUGUGUGG GG UUGCUA CAU ACA   CAUACACG       C
\ --------         C^ -      G   A   ---        UAAACCU
             90         80         70           60



        100
  AUC---    C
        GAGC A
        UUCG G
  CGACGU    C
     110
```

Reads in: lib-42, lib-45, lib-47, lib-48, lib-49, lib-50, lib-72
Number of reads="ID1468",60,58,67,1317,138,12,0

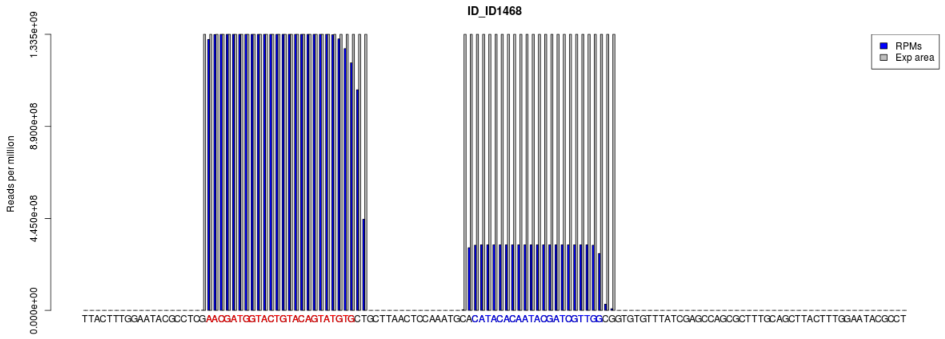

>ID1478
AATAACAAAATAACTAACAATCCTGTTCCATTCTTCAGTTGAGCTGTATGCTATGTCTATCAAAGGCAAA**CATATATAGATCAACTGAAA**
GATGGGATACGAGAACTACCAG

>ID1478-3P
CATATATAGATCAACTGAAAGA

>miRNA precursor
AAUAACAAAAUAACUAACAAUCCUGUUCCAUUCUUCAGUUGAGCUGUAUGCUAUGUCUAUCAAAGGCAAA**CAUAUAUAGAUCAACUGAAAGA**UGGGAUACGAGAACUACCAG
....................((.((((((((..(((((((((.(((((((...(((((.....)))))....))))))).)))))))))..)))))))).))..........

>Predicted STAR seq:
TCTTCAGTTGAGCTGTATG

Complementarity: 15

Initial dG =   -34.70

```
        10        20        30        40        50         60
AAUAACAAAAUAACUAACAA  C        UC         G       CUA-|     AU
                    UC UGUUCCAU  UUCAGUUGA CUGUAUG    UGUCU  \
                    AG AUAGGGUA  AAGUCAACU GAUAUAU    ACGGA  C
GACCAUCAAG----------  C        GA         A       ACAA^     AA
110                 100        90        80        70
```

Reads in: lib-42, lib-45, lib-47, lib-48, lib-49, lib-50, lib-72
Number of reads="ID1478",1,3,0,5,3,1,0

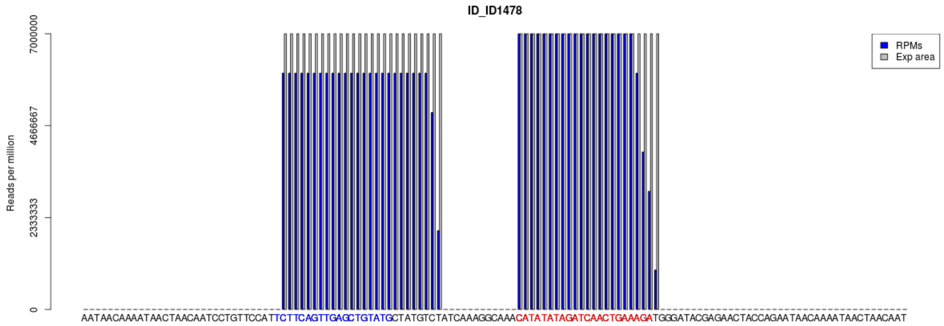

>ID1479
ACATTAGAAGTCTGCCGTTGACATACCTGACTTCTATACACTAGGTCAAGTTGACAAATGTGGTTGAGTG**TATTTGGCAGAGTGTATGGA**
AATCAGTTGTGGCTCTAGCAACG

>ID1479-3P
TATTTGGCAGAGTGTATGGAAAT

>miRNA precursor
ACAUUAGAAGUCUGCCGUUGACAUACCUGACUUCUAUACACUAGGUCAAGUUGACAAAUGUGGUUGAGUG**UAUUUGGCAGAGUGUAUGGAAAU**CAGUUGUGGCUCUAGCAACG
.........((.(((......((((.((((.(((((((((((..(((((((..((............))..)))))))..))))))))))).)))).))))......))))).

>Predicted STAR seq:
CTTCTATACACTAGGTCAAGT

Complementarity: 17

Initial dG =   -34.50

```
        10        20        30        40        50        60
ACAUUAGAA| C   CGUUGA    C    C           AG        UG  AAAUG
         GU UGC      CAUA CUGA UUCUAUACACU  GUCAAGU  AC     U
         CA ACG      GUGU GACU AAGGUAUGUGA  CGGUUUA  UG     G
G--------^ -   AUCUCG    U    A           GA        UG  AGUUG
          110       100        90        80        70
```

Reads in: lib-42, lib-45, lib-47, lib-48, lib-49, lib-50, lib-72
Number of reads="ID1479",271,204,313,334,181,98,0

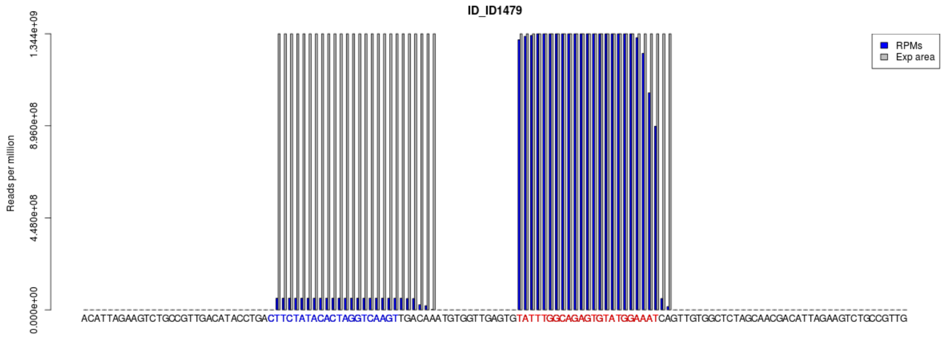

>ID1488
GTGCCATTTTCAGGCAGATG**TGAGATTGCACGGGAATTGGCAGC**AGAAAGTAAAGCAGCCAACCCTTTCAATCTCATTCCTGCCCGAAAA
CTTTCATCACGTCGCTGT

>ID1488-5P
TGAGATTGCACGGGAATTGGCAGC

>miRNA precursor
GUGCCAUUUUCAGGCAGAUG**UGAGAUUGCACGGGAAUUGGCAGC**AGAAAGUAAAGCAGCCAACCCUUUCAAUCUCAUUCCUGCCCGAAAACUUUCAUCACGUCGCUGU
(((...(((((.(((((..(((((((((...(((..(((((.((..........)).))))))))...)))))))))..))))).))))).......)))........

>Predicted STAR seq:
AGCCAACCCTTTCAATCTCATT

Complementarity: 16

Initial dG =   -38.20

```
                    10        20        30        40
--------   CCA----|    A     AU         CAC   AA     A  AGAA
        GUG       UUUUC GGCAG  GUGAGAUUG   GGG  UUGGC GC    A
        CAC       AAAAG CCGUC  UACUCUAAC   CCC  AACCG CG    G
UGUCGCUG   UACUUUC^    C     CU         UUU   --     A  AAAU
      100       90        80        70         60        50
```

Reads in: lib-42, lib-45, lib-47, lib-48, lib-49, lib-50, lib-72
Number of reads="ID1488",255,3,10,1,1,3,0

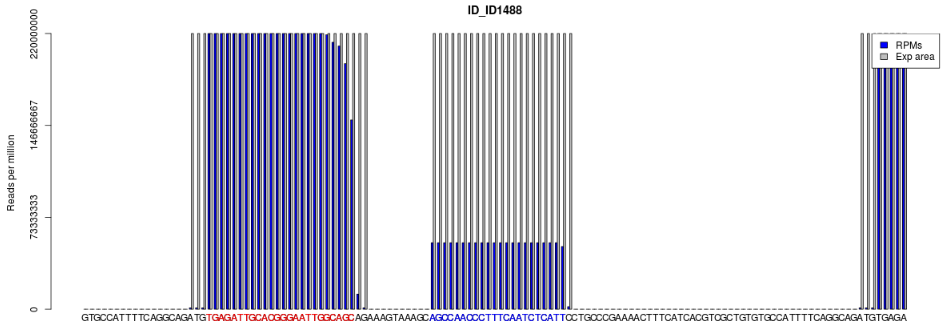

>ID1489
CGGAAAACACTGGGAGGCAT**TGGACACGTGGCTGGCCTTGCA**TTTTATGAGCTTGTGAGATCAGCCACGAGGCTGTATGCCTTCCAGGTG
GAATATAGCTGTCATCATGGGA

>ID1489-5P
TGGACACGTGGCTGGCCTTGCA

>miRNA precursor
CGGAAAACACUGGGAGGCAU**UGGACACGUGGCUGGCCUUGCA**UUUUAUGAGCUUGUGAGAUCAGCCACGAGGCUGUAUGCCUUCCAGGUGGAAUAUAGCUGUCAUCAUGGGA
```
.......((((((((((((((((...((((((((..((..((...........))..))..))))))))...))).))))))))))).)).....................
```

>Predicted STAR seq:
TGAGATCAGCCACGAGGCTGTAT

Complementarity: 13

Initial dG =   -44.80

```
                        10        20         30        40
CGGAAAA---------------  -          -|  ACA        GC  UG  UUUUA
                      CA CUGGGAGGCAU UGG   CGUGGCUG  CU  CA     \
                      GU GACCUUCCGUA GUC   GCACCGAC  GA  GU     U
AGGGUACUACUGUCGAUAUAAG  G          U^  GGA        UA  GU  UCGAG
110       100       90        80        70        60        50
```

Reads in: lib-42, lib-45, lib-47, lib-48, lib-49, lib-50, lib-72
Number of reads="ID1489",78,0,1,1,0,1,0

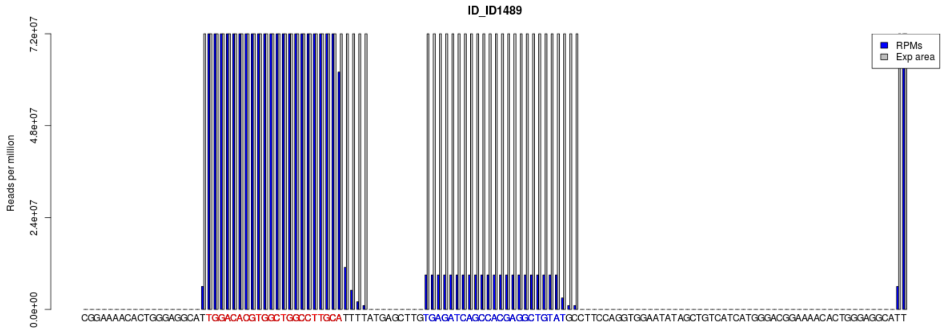

>ID1490
TAAACTGCACTGGGAGGCAT**CAGGCGCGAGGTAGATCTAACA**TTTTATGAGCATATGTGAGATCTCCCCCGAAGCCGTATGCCTTCCAGG
TGCCAACATCGCAACTGAATGC

>ID1490-5P
CAGGCGCGAGGTAGATCTAACA

>miRNA precursor
UAAACUGCACUGGGAGGCAU**CAGGCGCGAGGUAGAUCUAACA**UUUUAUGAGCAUAUGUGAGAUCUCCCCCGAAGCCGUAUGCCUUCCAGGUGCCAACAUCGCAACUGAAUGC
```
......((((((((((((((..(((.((.((.((((((.((((..........)))).)))))).)).)).)))..))))))))))).))).......(((......)))
```

>Predicted STAR seq:
TGAGATCTCCCCCGAAGCCGTAT

Complementarity: 14

Initial dG = -43.30

```
           10        20         30        40
.-UAAACU   -           CA   G-| A  U      A    UUUAU
        GCA CUGGGAGGCAU  GGC  CG GG AGAUCU ACAU     \
        CGU GACCUUCCGUA  CCG  GC CC UCUAGA UGUA     G
\ ------   G           UG   AA^ C  C      G    UACGA
          90        80        70        60        50



      100
  CAACAUC   AC
         GCA  U
         CGU  G
  -------   AA
         110
```

Reads in: lib-42, lib-45, lib-47, lib-48, lib-49, lib-50, lib-72
Number of reads="ID1490",363,4,4,1,0,6,0

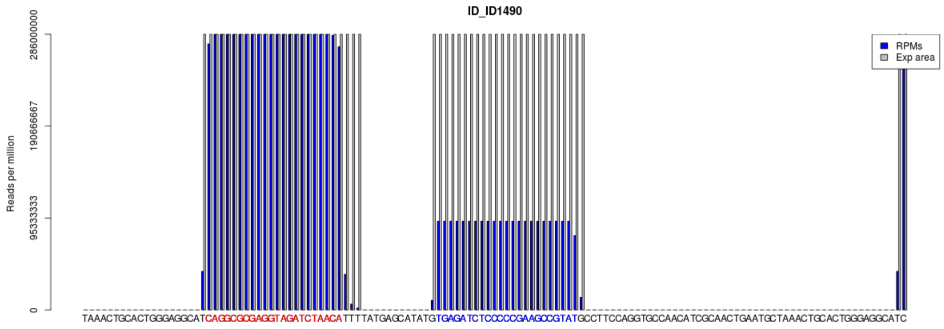

>ID1494
GTGTTGACTGCAACTAAAAGATTTATTTTACACCAAAAGTAATCGCTGACAGCACGAAGGATCTCAA**GTGCTGTCAGCGATTACT**TTTGG
TGTAAAATAAATCTCTCAAT

>ID1494-3P
GTGCTGTCAGCGATTACT

>miRNA precursor
GUGUUGACUGCAACUAAAAGAUUUAUUUUACACCAAAAGUAAUCGCUGACAGCACGAAGGAUCUCAA**GUGCUGUCAGCGAUUACU**UUUGGUGUAAAAUAAAUCUCUCAAU
```
..((((....))))....((((((((((((((((((((((((((((((((((((((((...........)))))))))))))))))))))))))))))))))))))))))......
```

>Predicted STAR seq:
ATTTATTTTACACCAAA

Complementarity: 16
ERROR 4: Strange structure
Initial dG = -62.40

```
.-GU|   A
    GUUG C
    CAAC U
\ --^   G
       10


        20        30        40        50        60
  UAAA--                                      GAAGG
        AGAUUUAUUUUACACCAAAAGUAAUCGCUGACAGCAC     A
        UCUAAAUAAAAUGUGGUUUUCAUUAGCGACUGUCGUG     U
  UAACUC                                      AACUC
110       100        90        80        70
```

Reads in: lib-42, lib-45, lib-47, lib-48, lib-49, lib-50, lib-72
Number of reads="ID1494",23884,14067,14488,1303,2340,4715,0

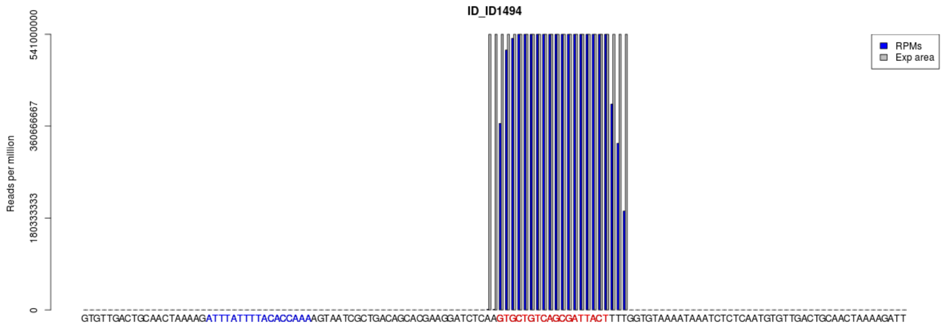

>ID1495

AGACTTCTGTGGAAAGTATTTTGTTGATGATTTTGGTGAGTGGCAGTAAAATTTCATTCTTTACATTTTT**TATTACAAAATTTGAGAGTCCAT**AGAAGTCTTCTGCTCTAGAAT

>ID1495-3P

TATTACAAAATTTGAGAGTCCAT

>miRNA precursor

AGACUUCUGUGGAAAGUAUUUUGUUGAUGAUUUUGGUGAGUGGCAGUAAAAUUUCAUUCUUUACAUUUUU**UAUUACAAAAUUUGAGAGUCCAU**AGAAGUCUUCUGCUCUAGAAU
((((((((((((((....((((((.((((((...((..((((((..........))))))....))....))))))))))))).......)))))))))))))............

>Predicted STAR seq:

TGGAAAGTATTTTGTTGATG

Complementarity: 14

Initial dG = -30.20

```
                     10           20        30           40
-------------             AAGU---      U      UUU-  GU--|     CAGU
             AGACUUCUGUGGA       AUUUUG UGAUGA    UG    GAGUGG    A
             UCUGAAGAUACCU       UAAAAC AUUAUU    AC    CUUACU    A
UAAGAUCUCGUCU             GAGAGUU      -      UUUU  AUUU^     UUAA
  110       100        90        80         70        60        50
```

Reads in: lib-42, lib-45, lib-47, lib-48, lib-49, lib-50, lib-72

Number of reads="ID1495",0,0,0,0,0,0,0

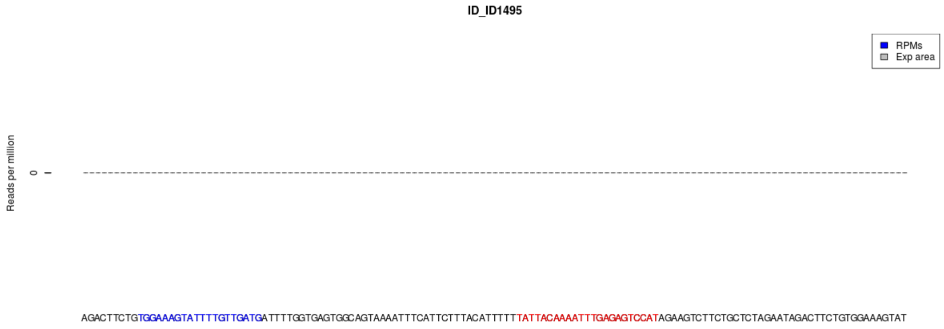

>ID15
CTATGGCAACTTATCAAAAAGTGGGTCACTTT**AGGTGGAGGAATGCCTTTGTACG**TGGTAAAATATACGTGAAAGACGTTCTTCCATCTA
CAGTTATCCATATACCACCTG

>ID15-5P
AGGTGGAGGAATGCCTTTGTACG

>miRNA precursor
```
CUAUGGCAACUUAUCAAAAAGUGGGUCACUUUAGGUGGAGGAAUGCCUUUGUACGUGGUAAAAUAUACGUGAAAGACGUUCUUCCAUCUACAGUUAUCCAUAUACCACCUG
...................((((((.(((.((((((((((((((.((((.((((((........)))))))))).))))))))))))))).))).)))))).........
```

>Predicted STAR seq:
TGAAAGACGTTCTTCCATCTAC

Complementarity: 19

Initial dG =   -39.10

```
        10        20        30        40        50        60
CUAUGGCAACUUAUCAAAAA|     C   U              C    G       GUA
                    GUGGGU ACU UAGGUGGAGGAAUG CUUU UACGUG   A
                    UACCUA UGA AUCUACCUUCUUGC GAAA GUGCAU   A
GUCCACCAUA----------^     U   C              A    -       AUA
 .                 100        90        80         70
```

Reads in: lib-42, lib-45, lib-47, lib-48, lib-49, lib-50, lib-72
Number of reads="ID15",1,1,0,0,0,0,0

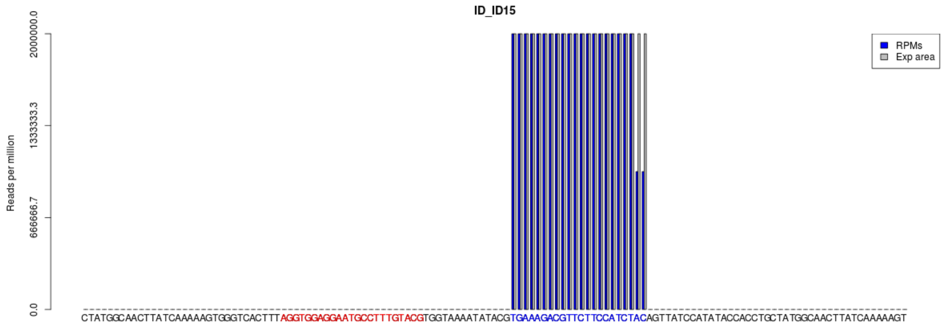

>ID1505
GCGAACCACAGGCACTTGCAGAAGAATTCACTAGGAGGGAGGTGACCGGGTGCAGAGTGGTAATATCGAT**CTGTACCTGGTCACCTTCTT**
CCTGGCGTATTCTGACCTCGCAA

>ID1505-3P
CTGTACCTGGTCACCTTCTTCCT

>miRNA precursor
GCGAACCACAGGCACUUGCAGAAGAAUUCACUAGGAGGGAGGUGACCGGGUGCAGAGUGGUAAUAUCGAU**CUGUACCUGGUCACCUUCUUCCU**GGCGUAUUCUGACCUCGCAA
((((.......((....))...(((((...(((((((((((((((((((((((((.((((...)))).)))))))))))))))))))))))))...))))).....))))..

>Predicted STAR seq:
AGGAGGGAGGTGACCGGGTGCA

Complementarity: 21
ERROR 4: Strange structure
 Initial dG =   -61.50

```
            10
--    .-ACCACAG| A
  GCGA         GC C
  CGCU         CG U
AA    \ -------^ U
 110


       20         30        40        50        60
        AGA-     UCA                          G    A
            AGAAU   CUAGGAGGGAGGUGACCGGGUGCAGA UGGU \
            UCUUA   GGUCCUUCUUCCACUGGUCCAUGUCU GCUA A
        CCAG     UGC                          A    U
             100        90        80        70
```

Reads in: lib-42, lib-45, lib-47, lib-48, lib-49, lib-50, lib-72
Number of reads="ID1505",136,125,98,132,201,34,0

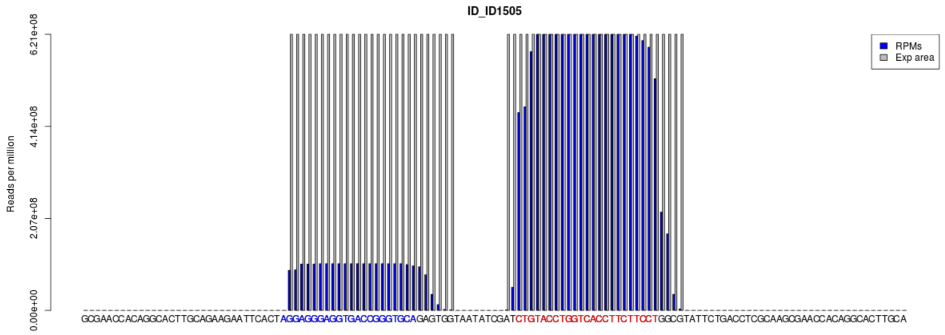

>ID1507
CGGCGTCGACGCAGTCCCTCGTGCCGCTGGCGGTGGACGGGTGCACTCTGGGTCACGCGTATTCGAGTCG**TGACTAGAGTGACACTCGTC**
AACCGACTGCGGATTTTTCTGC

>ID1507-3P
TGACTAGAGTGACACTCGTCAA

>miRNA precursor
CGGCGUCGACGCAGUCCCUCGUGCCGCUGGCGGUGGACGGGUGCACUCUGGGUCACGCGUAUUCGAGUCG**UGACUAGAGUGACACUCGUCAA**CCGACUGCGGAUUUUUCUGC
```
(((((.(((.........))))))))..((((((.((((((((((((((((.((((((........).))))))))))))))).)))))))).)))).))(((((....)))))
```

>Predicted STAR seq:
CGACGCAGTCCCTCGTGCCGCTGG

Complementarity: 18
ERROR 4: Strange structure
 Initial dG =   -53.40

```
          10
  |    U   CGCA
--CGGCG CGA    \
  GCCGU GCU    G
\ ^    -   CCCU
        20



      30         40         50         60
  .-CU  -    G        -        G     - GUA
      GG CGGU GACGGGUG CACUCUGG UCACG C   U
      UC GCCA CUGCUCAC GUGAGAUC AGUGC G   U
  \ --  A    A        A        -     U AGC
             90        80         70
```

Reads in: lib-42, lib-45, lib-47, lib-48, lib-49, lib-50, lib-72
Number of reads="ID1507",6669,7384,18970,4645,17880,9501,0

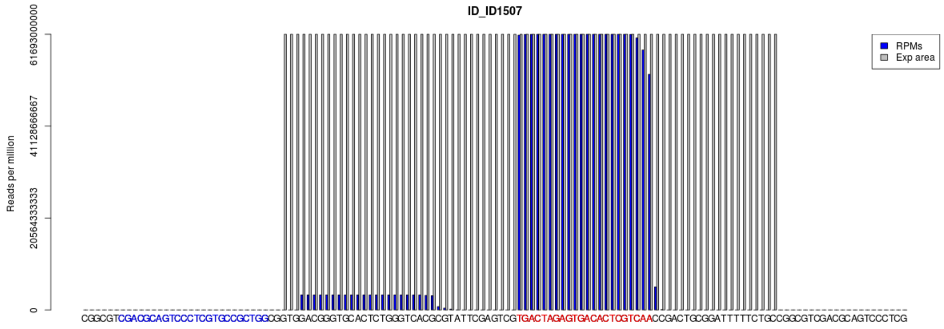

>ID1509
GTTCTGGAACAAACAGCATTCTTGCATAATGAG**TGATGGGCGTGCGATTTCACAT**ATTGATATATGATTATGTGTATCGTATGCTGATCT
CTCACTGTGCACGACTTC

>ID1509-5P
TGATGGGCGTGCGATTTCACAT

>miRNA precursor
GUUCUGGAACAAACAGCAUUCUUGCAUAAUGAG**UGAUGGGCGUGCGAUUUCACAU**AUUGAUAUAUGAUUAUGUGUAUCGUAUGCUGAUCUCUCACUGUGCACGACUUC
...(((.......)))...((.((((((.((((.(((.((((((((((..((((((...........)))))).)))))))))).))).)))).)))))).))....

>Predicted STAR seq:
GTGTATCGTATGCTGATCTCT

Complementarity: 16

Initial dG =   -40.70

ERROR 3: Strange structure
Initial dG =   -40.70

```
.-GUU|  GAA
     CUG   \
     GAC   C
\ ---^  AAA
```

```
     20        30        40        50        60
  CAU-  U      A    U   G          UU       UUGAU
      UC UGCAUA UGAG GAU GGCGUGCGAU  CACAUA      A
      AG ACGUGU ACUC CUA UCGUAUGCUA  GUGUAU      U
  CUUC  C      C    U   G          U-       UAGUA
        100        90        80         70
```

Reads in: lib-42, lib-45, lib-47, lib-48, lib-49, lib-50, lib-72
Number of reads="ID1509",0,0,0,0,4,0,0

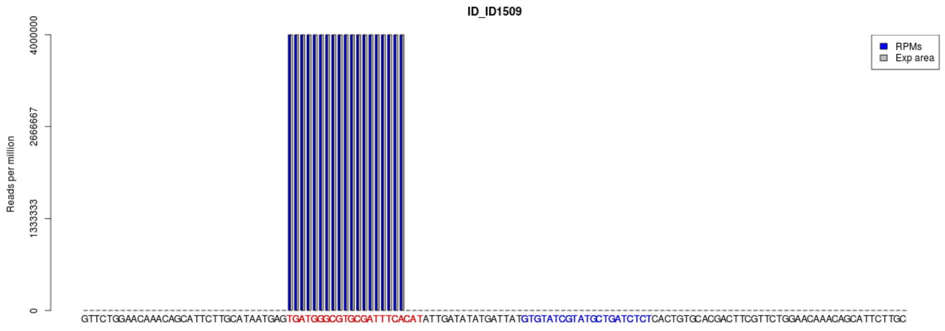

>ID1510
GAGAATGGTGGCCTGTGGTA**CGGTGGAAACCTACACGAACACG**GTATTTGAGCAGTGTCGTGTGCGTTTCTACCTTACTACGCGCTGCCT
CCTACATCGTCTACGAATCTC

>ID1510-5P
CGGTGGAAACCTACACGAACACG

>miRNA precursor
GAGAAUGGUGGCCUGUGGUA**CGGUGGAAACCUACACGAACACG**GUAUUUGAGCAGUGUCGUGUGCGUUUCUACCUUACUACGCGCUGCCUCCUACAUCGUCUACGAAUCUC
((((..((..((.(((((((.(((((((((.((((((((.(((.((......)).))))))))))).))))))))).))))))).))..))........(((....))).))))

>Predicted STAR seq:
GTCGTGTGCGTTTCTACCTTA

Complementarity: 17

Initial dG = -50.70

```
          10        20        30        40
    .-AU| UG  C       C         C       A   G  AU
GAGA    GG  GC UGUGGUA GGUGGAAAC UACACGA CAC GU  U
CUCU    CC  CG GCAUCAU CCAUCUUUG GUGUGCU GUG CG  U
    \ --^ GU  C       U         C       -   A  AG
 .              80        70        60         50


     90       100
      UCCUACA   U
             UCG C
             AGC U
      A------   A
```

Reads in: lib-42, lib-45, lib-47, lib-48, lib-49, lib-50, lib-72
Number of reads="ID1510",1,4,0,0,0,0,0

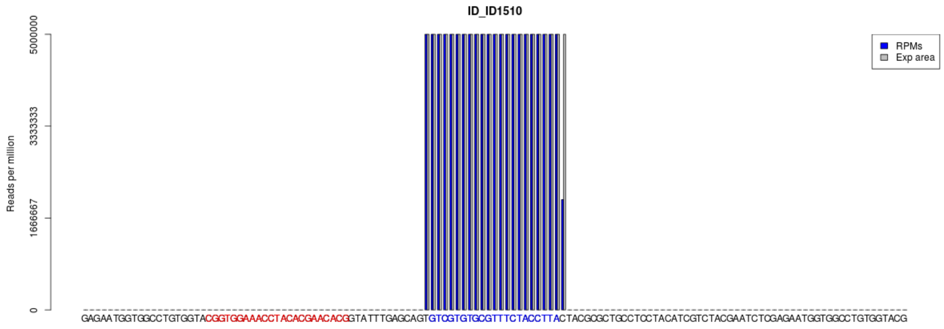

>ID1511
GAGAACGGTGGTCTGTGGTA**CGGAGGAAACCTACACGAACACG**GTATTTAAGCAGTGTTGTGTGCGTTTCAACCTAACTACACGCTGCCT
CCTACATCGTCTACGAGTC

>ID1511-5P
CGGAGGAAACCTACACGAACACG

>miRNA precursor
GAGAACGGUGGUCUGUGGUA**CGGAGGAAACCUACACGAACACG**GUAUUUAAGCAGUGUUGUGUGCGUUUCAACCUAACUACACGCUGCCUCCUACAUCGUCUACGAGUC
......((..((.((((((..((..(((((.(((((.(((((.((......)).))))))))))).))))).))..)))))).))..)).......(((....)))...

>Predicted STAR seq:
GTTGTGTGCGTTTCAACCTAAC

Complementarity: 15

Initial dG =   -37.60

```
          10        20        30        40
.-GAGAAC| UG  C      AC  AG     C     G     G  AU
        GG  GU UGUGGU  GG  GAAAC UACAC AACAC GU  U
        CC  CG ACAUCA  CC  CUUUG GUGUG UUGUG CG  U
\ ------^ GU  C      AU  AA     C     -     A  AA
                80        70        60         50



 90       100
  UCCUACA   U
         UCG C
         AGC U
  CUG----   A
```

Reads in: lib-42, lib-45, lib-47, lib-48, lib-49, lib-50, lib-72
Number of reads="ID1511",1,0,0,0,0,0,0

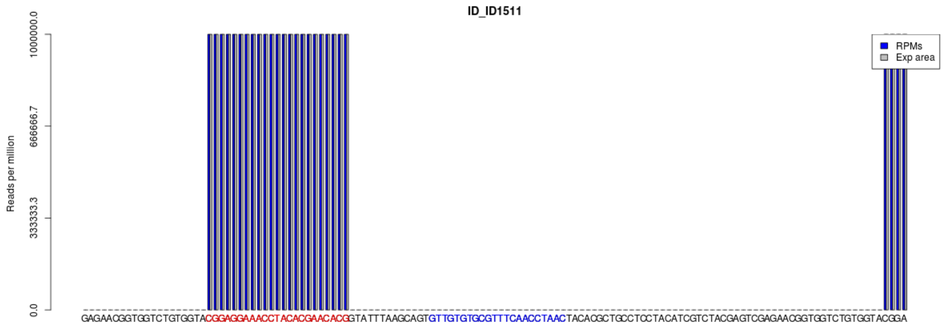

>ID1513
ACGGATTGCATGTTCCTTGG**TCACAGAAGCGTTAGTTGCACA**TGTTATTAATTTACTGTGTGACTTTCCCTTCCGTGTCTCAAGGAGCAT
GACATACGCCAGCACCATCGT

>ID1513-5P
TCACAGAAGCGTTAGTTGCACA

>miRNA precursor
ACGGAUUGCAUGUUCCUUGG**UCACAGAAGCGUUAGUUGCACA**UGUUAUUAAUUUACUGUGUGACUUUCCCUUCCGUGUCUCAAGGAGCAUGACAUACGCCAGCACCAUCGU
```
..((..((((((((((((((.(((.((((....(((..((((.((.........))))))..)))....)))).))).).))))))))))).))....))...........
```

>Predicted STAR seq:
TGTGACTTTCCCTTCCGTGTCT

Complementarity: 12

Initial dG =   -41.00

```
                    10         20        30        40
AC---------  AU--  -           -|U   A    CGUU   UG     U  UAUU
           GG    UG CAUGUUCCUUG G CAC GAAG    AGU  CACA GU    \
           CC    AC GUACGAGGAAC C GUG CUUC    UCA  GUGU CA    A
UGCUACCACGA  GCAU  A           U^U   C    CCUU   GU     -  UUUA
 .       100        90        80        70        60
```

Reads in: lib-42, lib-45, lib-47, lib-48, lib-49, lib-50, lib-72
Number of reads="ID1513",4,11,0,0,1,0,0

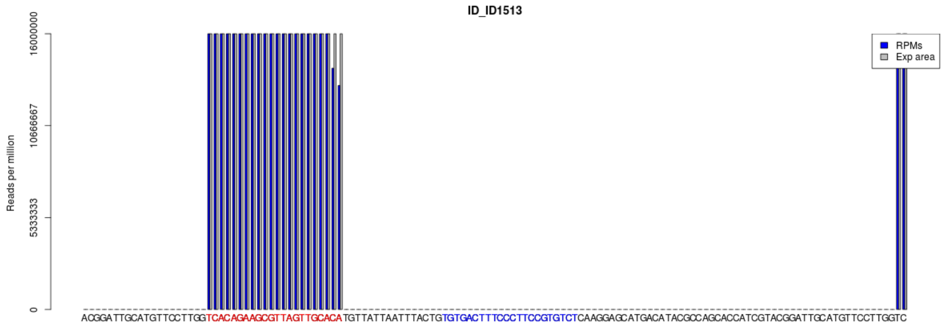

>ID1516
TAGCTGCAGTAGCGCC**AGCCACTACTACAGATGGAACA**GCTGCTGCTGCAACAGTAGCAAGAGAACCTGTTCCGACTGTAGTAGTTCCTG
ATGCTGCCGCAGCAAGTTCAC

>ID1516-5P
AGCCACTACTACAGATGGAACA

>miRNA precursor
UAGCUGCAGUAGCGCC**AGCCACUACUACAGAUGGAACA**GCUGCUGCUGCAACAGUAGCAAGAGAACCUGUUCCGACUGUAGUAGUUCCUGAUGCUGCCGCAGCAAGUUCAC
..(((((.((((((.(((..((((((((((.(((((((((((((((((...))))))))).).....)))))))).))))))))))..))).)))))).))))).........

>Predicted STAR seq:
TTCCGACTGTAGTAGTTCCTGA

Complementarity: 17

Initial dG = -58.90

```
              10        20        30             40
UA------     A      C   CC          A        ----- -|       C
        GCUGC GUAGCG CAG  ACUACUACAG UGGAACAG     C UGCUGCUG \
        CGACG CGUCGU GUC  UGAUGAUGUC GCCUUGUC     G ACGAUGAC A
CACUUGAA     C      A   CU          A        CAAGA A^       A
 .       100        90        80        70        60
```

Reads in: lib-42, lib-45, lib-47, lib-48, lib-49, lib-50, lib-72
Number of reads="ID1516",5,0,0,0,0,0,0

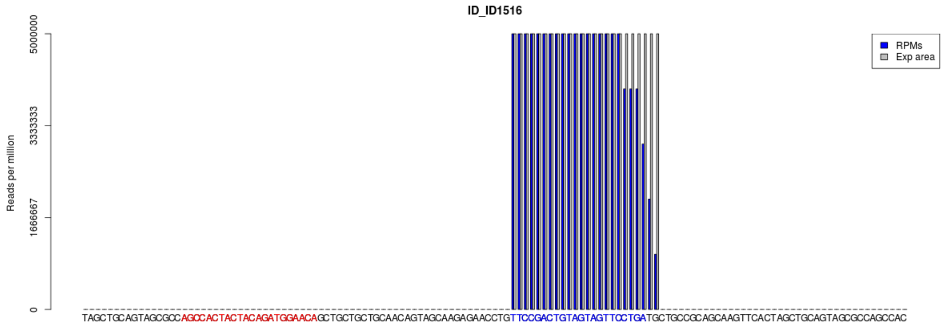

>ID1517
TGCAGTAGCGCCAGCGACTACTTCAGATAGAACAACAGCCCGGCTGCTGCAACAGTAGCTAGAGAACCTG**TTCCGACTGTAGTAGTTCCT**
GGTGCTGCCACAGCAAGTTCAC

>ID1517-3P
TTCCGACTGTAGTAGTTCCTGG

>miRNA precursor
UGCAGUAGCGCCAGCGACUACUUCAGAUAGAACAACAGCCCGGCUGCUGCAACAGUAGCUAGAGAACCUG**UUCCGACUGUAGUAGUUCCUGG**UGCUGCCACAGCAAGUUCAC
(((.((((((((((.(((((((.(((...((((((.......((((((((...))))))))........)))))...))).))))))).))))))))))....))).......

>Predicted STAR seq:
CA

Complementarity: 15

Initial dG =   -46.40

```
                  10        20        30        40         50
-------   A---          C       U   AUA     ACAGCCC-|       C
       UGC    GUAGCGCCAG GACUACU CAG   GAACA        GGCUGCUG \
       ACG    CGUCGUGGUC UUGAUGA GUC   CUUGU        UCGAUGAC A
CACUUGA   ACAC          C       U   AGC     CCAAGAGA^       A
110       100        90        80        70        60
```

Reads in: lib-42, lib-45, lib-47, lib-48, lib-49, lib-50, lib-72
Number of reads="ID1517",8,1,0,0,0,0,0

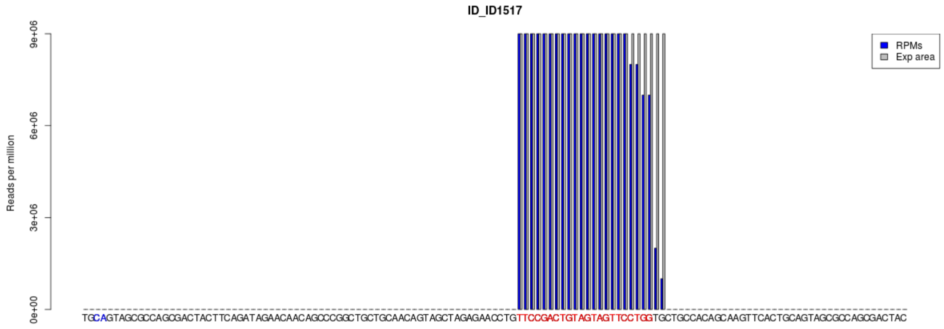

>ID1520
ACAGCAGAAGCAGGAGCAACAGCAGCAGGAACATGAGTACTTGCAGCACCAGTTGTAAGAGCACCAGTTC**ATGCTTCTGTTTCTCCTGCT**
GCTGCTGTAGCAGCTAAAACACC

>ID1520-3P
ATGCTTCTGTTTCTCCTGCTGCT

>miRNA precursor
ACAGCAGAAGCAGGAGCAACAGCAGCAGGAACAUGAGUACUUGCAGCACCAGUUGUAAGAGCACCAGUUC**AUGCUUCUGUUUCUCCUGCUGCU**GCUGUAGCAGCUAAAACACC
((((((.((((((((.(((((.((((.((((.((.((.((((((((....)))))))).)).))..)))))).)))).))))).)))))))).)))))))..............

>Predicted STAR seq:
GAAG

Complementarity: 17

Initial dG =   -54.90

```
                       10        20        30         40
---------------|      A        C     C    G    A  A-  A        A
               ACAGCAG AGCAGGAG AACAG AGCA GAAC UG  GU CUUGCAGC C
               UGUCGUC UCGUCCUC UUGUC UCGU CUUG AC  CG GAAUGUUG C
CCACAAAAUCGACGA^      G        U     U    A    -  CA  A        A
 110       100       90        80        70         60        50
```

Reads in: lib-42, lib-45, lib-47, lib-48, lib-49, lib-50, lib-72
Number of reads="ID1520",20,1,0,2,0,0,0

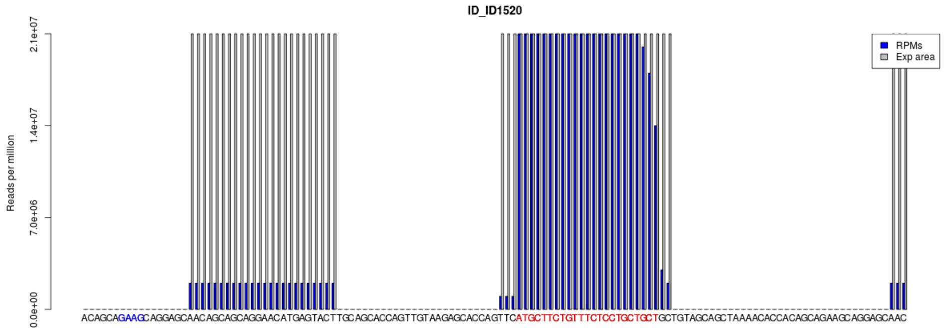

>ID1523
AATGGGGCACCAGTATCTGCAGTAGCACCAGCCGCAACTACAGATGGGACAGCAGTTGCAAGAACACCTG**TCCTAACTGTAGTTGCTGCT**
GGTGCTGAGGCAGAAACAACAC

>ID1523-3P
TCCTAACTGTAGTTGCTGCTGG

>miRNA precursor
AAUGGGGCACCAGUAUCUGCAGUAGCACCAGCCGCAACUACAGAUGGGACAGCAGUUGCAAGAACACCUG**UCCUAACUGUAGUUGCUGCUGG**UGCUGAGGCAGAAACAACAC
..(((....)))((.(((((..((((((((((.((((((((((.((((((((..(((.....)))..)))))))).)))))))))).))))))))))..))))).)).....

>Predicted STAR seq:
CAGCCGCAACTACAGATGGGA

Complementarity: 18
ERROR 4: Strange structure
Initial dG = -64.70

```
.-AA|  G
    UGG G
    ACC C
\ --^  A
     10


             20        30        40        50
  -----  A     AG          C          A        CA   GC
       GU UCUGC  UAGCACCAGC GCAACUACAG UGGGACAG  GUU  \
       CA AGACG  GUCGUGGUCG CGUUGAUGUC AUCCUGUC  CAA  A
  CACAA  A     GA          U          A        CA   GA
  110       100        90        80        70
```

Reads in: lib-42, lib-45, lib-47, lib-48, lib-49, lib-50, lib-72
Number of reads="ID1523",191,6,3,11,3,2,0

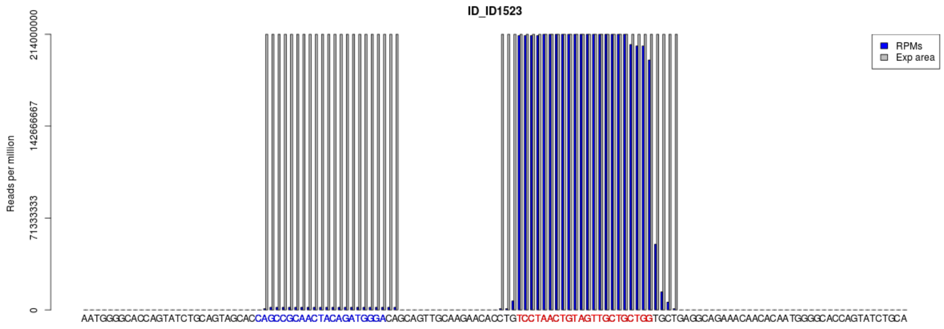

>ID1524
ACAATGGGGACACCAGTATCTGCAGTAGCACTAGCCGCAACTACAGATGGGACAGCACTCAGAACACCTG**TCCTAACTGTAGTTGCTGCT**
GGTGCTGAGGCAGAAACAACAC

>ID1524-3P
TCCTAACTGTAGTTGCTGCTGG

>miRNA precursor
ACAAUGGGGACACCAGUAUCUGCAGUAGCACUAGCCGCAACUACAGAUGGGACAGCACUCAGAACACCUG**UCCUAACUGUAGUUGCUGCUGG**UGCUGAGGCAGAAACAACAC
....((....))...((.(((((..(((((((((.(((((((((.((((((((...........)))))))).))))))))).)))))))))..))))).)).....

>Predicted STAR seq:
TAGCCGCAACTACAGATGGGA

Complementarity: 18
ERROR 4: Strange structure
 Initial dG =   -60.30

```
.-ACAA| G
      UG G
      AC G
\ ----^ A
       10


          20         30         40         50         60
  CCA--   A      AG           C           A         CACUC
       GU UCUGC  UAGCACUAGC GCAACUACAG UGGGACAG      A
       CA AGACG  GUCGUGGUCG CGUUGAUGUC AUCCUGUC      G
  CACAA   A      GA           U           A         CACAA
  110       100         90         80         70
```

Reads in: lib-42, lib-45, lib-47, lib-48, lib-49, lib-50, lib-72
Number of reads="ID1524",228,14,3,6,3,2,0

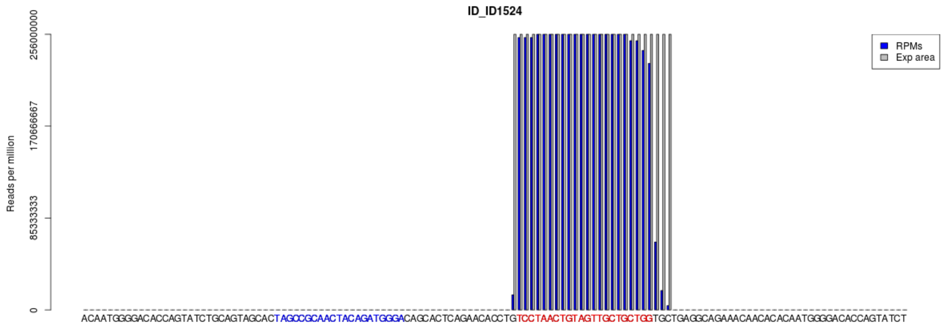

>ID1529
AGCTTTCGTCGCAGTCAAGGCGGTCCTTCTGCTTCAATTCCGTAGTGCATTGCAGTGTTTGTTGTAGGTC**TGCAAGCAGTGCGGAAGTGA**
GGTGGAAGAGGCCTCAGAG

>ID1529-3P
TGCAAGCAGTGCGGAAGTGAG

>miRNA precursor
AGCUUUCGUCGCAGUCAAGGCGGUCCUUCUGCUUCAAUUCCGUAGUGCAUUGCAGUGUUUGUUGUAGGUC**UGCAAGCAGUGCGGAAGUGAG**GUGGAAGAGGCCUCAGAG
```
.......(((........)))((((((((..(((((.(((((((.(((.((((((.............))))))))).))))))).))))))..)))).))))......
```

>Predicted STAR seq:
TCAATTCCGTAGTGCATTGCA

Complementarity: 17
ERROR 4: Strange structure
Initial dG =   -46.50

```
.-A| UUU
   GC   \
   CG   C
\ -^ CUG
    10


        20         30         40         50         60
  AGUCAA  C    -    UG      A       G    A       UGUUUG
        GG GGUC CUUC  CUUCA UUCCGUA UGC UUGCAG       U
        CU CCGG GAAG  GGAGU AAGGCGU ACG AACGUC       U
  GAGA--  -    A    GU      G       G    -       UGGAUG
            100        90         80         70
```

Reads in: lib-42, lib-45, lib-47, lib-48, lib-49, lib-50, lib-72
Number of reads="ID1529",6,12,1,626,8,0,0

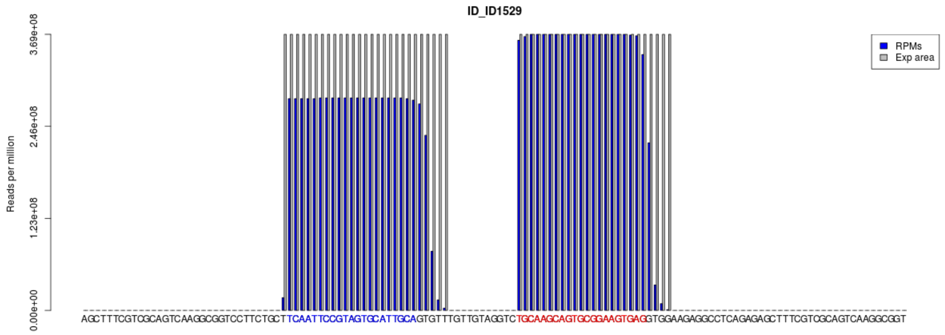

>ID153
TCCGGCGGCAGCGGCGGGAGTCACTGTTGCCATAGTTCCGGGTGTTGCGAGGCCCGGAACTGTGGTTCCAGGGACTCCCTACGCTGCCCA
CAGCGCCTCACCGACGCCGCTG

>ID153-5P
TCACTGTTGCCATAGTTCCGGG

>miRNA precursor
UCCGGCGGCAGCGGCGGGAGUCACUGUUGCCAUAGUUCCGGGUGUUGCGAGGCCCGGAACUGUGGUUCCAGGGACUCCCUACGCUGCCCACAGCGCCUCACCGACGCCGCUG
```
......(((((((..(((((((.(((..(((((((((((((((........)))))))))))))))..))).)))))))..)))))))..((((((.((...)).).)))))
```

>Predicted STAR seq:
CGGAACTGTGGTTCCAGGGACT

Complementarity: 17

Initial dG =   -72.30

```
        10          20         30         40
UC       AG   .-C|      A   UU               GUU
  CGGCGGC  CGG   GGGAGUC CUG  GCCAUAGUUCCGGGU   G
  GUCGCCG  GCC   CCCUCAG GAC  UGGUGUCAAGGCCCG   C
--       CA   \ -^      G   CU               GAG
  110                   70         60         50



               80
                UA---     C
                     CGCUG C
                     GCGAC C
                ACUCC     A
              100        90
```

Reads in: lib-42, lib-45, lib-47, lib-48, lib-49, lib-50, lib-72
Number of reads="ID153",11,4,0,1,0,0,0

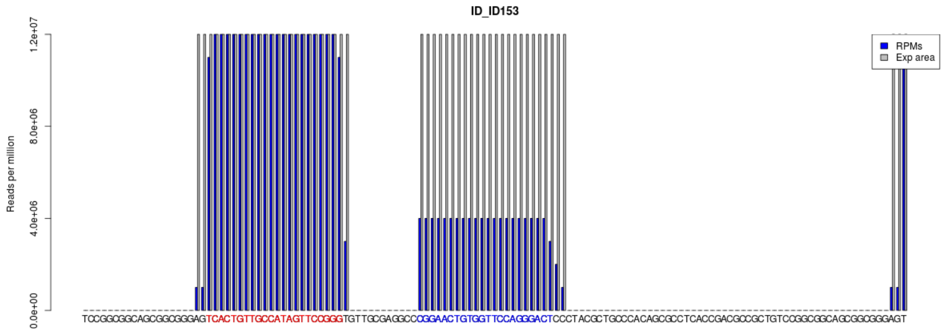

>ID1534
ACAAAAGATACCTCGTGGAAT**TCGGGTGAACTGCCGGCGATCC**GTGTCTTCTAAATACGTTCATCGGCAGTACGCCCGATTCCACGAGATG
TCAACGAATCGCCTGGAAAG

>ID1534-5P
TCGGGTGAACTGCCGGCGATCC

>miRNA precursor
ACAAAAGAUACCUCGUGGAA**UCGGGUGAACUGCCGGCGAUCC**GUGUCUUCUAAAUACGUUCAUCGGCAGUACGCCCGAUUCCACGAGAUGUCAACGAAUCGCCUGGAAAG
```
......((((.((((((((((((((((.(((((((..((..(((((.......))))).))..))))))).)))))))))))))))).)))).................
```

>Predicted STAR seq:
TCATCGGCAGTACGCCCGATT

Complementarity: 15

Initial dG = -56.00

```
                    10        20        30        40
ACAAAA------------|   C                A       GC  UC      CUU
                  GAUA CUCGUGGAAUCGGGUG ACUGCCG  GA  CGUGU   \
                  CUGU GAGCACCUUAGCCCGC UGACGGC  CU  GCAUA   C
GAAAGGUCCGCUAAGCAA^   A                A       UA  U-      AAU
.       100        90        80        70        60
```

Reads in: lib-42, lib-45, lib-47, lib-48, lib-49, lib-50, lib-72
Number of reads="ID1534",1,19,29,4,62,25,0

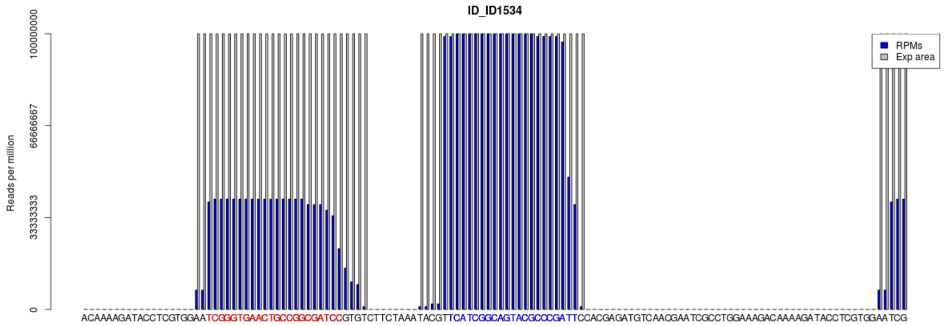

>ID1535
GTGGCAGTGCCGCTCT**CATCTGAGTGCTGGACAGAGGGC**TAGCATTGCGGAGATCCTCTGTCCAGCACTCAGAGGGGAACTGCTTGGTGG
CAGCTTCGATACCATGACTCCA

>ID1535-5P
CATCTGAGTGCTGGACAGAGGGC

>miRNA precursor
GUGGCAGUGCCGCUCU**CAUCUGAGUGCUGGACAGAGGGC**UAGCAUUGCGGAGAUCCUCUGUCCAGCACUCAGAGGGGAACUGCUUGGUGGCAGCUUCGAUACCAUGACUCCA
(((((...))))).(((.((((((((((((((((((((((.((...))..)).)))))))))))))))))))))))((((((((....))))).)))..............

>Predicted STAR seq:
TCCTCTGTCCAGCACTCAGAGGG

Complementarity: 20

Initial dG =   -58.00

ERROR 3: Strange structure
Initial dG =   -58.00

```
  |    A
--GUGGC \
  CGCCG G
\ ^    U
    10
```

```
         20        30         40
  .-U   A                   -  A-  A
     CUC UCUGAGUGCUGGACAGAGGG CU  GC \
     GGG AGACUCACGACCUGUCUCCU GA  CG U
  \ -   -                   A  GG  U
           70        60        50
```

Reads in: lib-42, lib-45, lib-47, lib-48, lib-49, lib-50, lib-72
Number of reads="ID1535",0,1,0,47,8,0,0

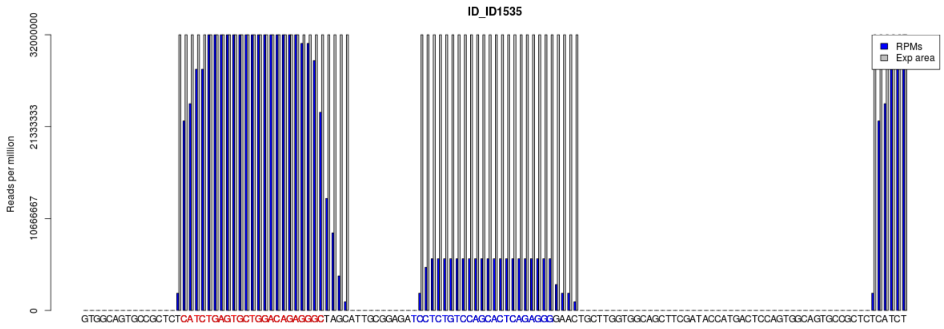

>ID1538
CACGGTTCGGAGCTTTGCAG**TGAAGGTGGGAGTCTCTTGTGC**CGCCACTGTTCTCCATGGCACTGGGATTTCTGTGGTCACTGCTGCGAA
CCTGGCCTCTACCCGAGCTG

>ID1538-5P
TGAAGGTGGGAGTCTCTTGTGC

>miRNA precursor
CACGGUUCGGAGCUUUGCAG**UGAAGGUGGGAGUCUCUUGUGC**CGCCACUGUUCUCCAUGGCACUGGGAUUUCUGUGGUCACUGCUGCGAACCUGGCCUCUACCCGAGCUG
...((((((.(((.....(((((..(..((((((((..((((((.............)))))).)))))))))..)..)))))))).)))))).((.(((.....))))).

>Predicted STAR seq:
ACTGGGATTTCTGTGGTCACT

Complementarity: 14

Initial dG =   -44.90

```
        10          20         30         40         50
CA        .-AGCUUU|       AG UG        UU       CCACUG
  CGGUUCGG        GCAGUGA  G  GGAGUCUC  GUGCCG      \
  GUCGAGCC        CGUCACU  U  CUUUAGGG  CACGGU      U
--        \ ------^       GG GU        U-       ACCUCU
110                  80         70         60



                     90
            U-----  GAA
                  GC   \
                  CG   C
            CAUCUC  GUC
            100
```

Reads in: lib-42, lib-45, lib-47, lib-48, lib-49, lib-50, lib-72
Number of reads="ID1538",1,27,34,20,67,20,0

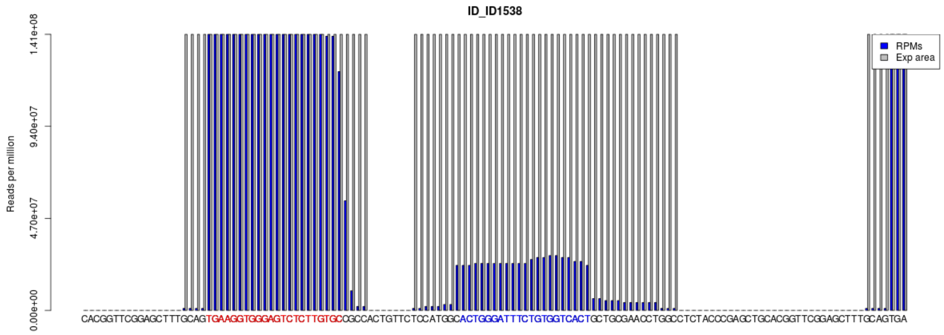

>ID1539
CGTCGATGTCTACAAATTCGGCACGTGCGCATGGGAGATAGAAACAGGCTATGACTCTGTGATAAAGTAG**TCAGTCTTTTTCTCTCTCCT**
ATCCAACGGCCGGACAGT

>ID1539-3P
TCAGTCTTTTTCTCTCTCCTAT

>miRNA precursor
CGUCGAUGUCUACAAAUUCGGCACGUGCGCAUGGGAGAUAGAAACAGGCUAUGACUCUGUGAUAAAGUAG**UCAGUCUUUUUCUCUCUCCUAU**CCAACGGCCGGACAGU
```
................((((((.(((....((((((((.(((((.(((((..((((((.......)).)))))))))).))))).))))))))...))))))))).....
```

>Predicted STAR seq:
CGCATGGGAGATAGAAACAGGCTATGA

Complementarity: 18

Initial dG =   -38.40

```
        10        20        30        40        50         60
CGUCGAUGUCUACAAA|     A   GCGC         U     C     AU    -  GUG
                UUCGGC CGU    AUGGGAGA AGAAA AGGCU  GACU CU   \
                AGGCCG GCA    UAUCCUCU UCUUU UCUGA  CUGA GA   A
UGAC------------^     -   ACC-         C     U     --    U  AAU
                  100          90        80          70
```

Reads in: lib-42, lib-45, lib-47, lib-48, lib-49, lib-50, lib-72
Number of reads="ID1539",989,67867,187281,43000,252264,95041,0

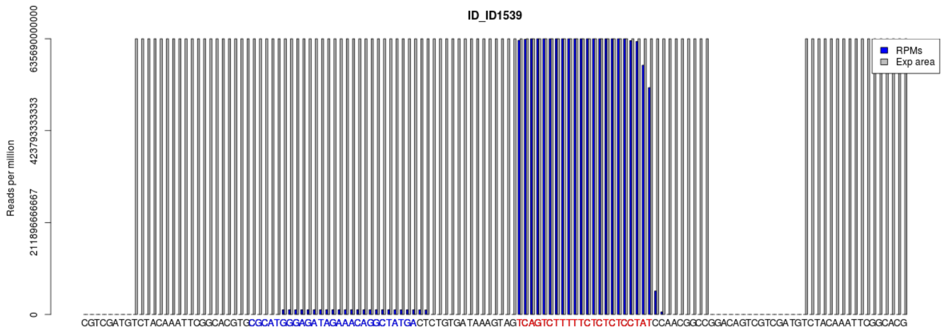

>ID1540
GTGAAATGACAAGAGTTCAG**TGATGTCTGTCTGTATTGTTGA**TAAAGCTTTTTGGCAACAGCTTTACATTCAGACATTAGTGAAATTCTT
GTTTTAAGCAGTAGTACCAATG

>ID1540-5P
TGATGTCTGTCTGTATTGTTGA

>miRNA precursor
GUGAAAUGACAAGAGUUCAG**UGAUGUCUGUCUGUAUUGUUGA**UAAAGCUUUUUGGCAACAGCUUUACAUUCAGACAUUAGUGAAAUUCUUGUUUUAAGCAGUAGUACCAAUG
((.....((((((((((((.(((((((((..((((..((((.....(((....)))..))))..))))..))))))))).)).))))))))))....))............

>Predicted STAR seq:
GCTTTACATTCAGACATTAGTG

Complementarity: 15

Initial dG =   -37.90

```
                     10          20         30         40         50
-------------| GAAAU           -  G         UC     UU     AUAAA   U
             GU     GACAAGAGUU CA UGAUGUCUG  UGUA  GUUG     GCU U
             CG     UUGUUCUUAA GU AUUACAGAC  ACAU  CGAC     CGG U
GUAACCAUGAUGA^ AAUU-          A  G         UU     UU     AA---   U
110       100         90         80         70         60
```

Reads in: lib-42, lib-45, lib-47, lib-48, lib-49, lib-50, lib-72
Number of reads="ID1540",4,17,29,5,30,2,0

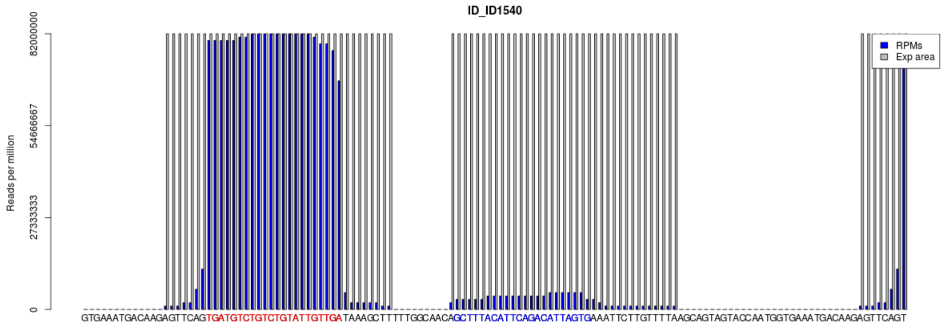

>ID1544
AAGAAGTACATAGTCTTTGATAAAAAGGAAACAGCGATCAATCGCGAGGAATTTGAGTTGTTTAGTAAA**TTTCTCGCGGATAATTACTGT**
TTTCTTTTATCTTCCATTTCAA

>ID1544-3P
TTTCTCGCGGATAATTACTGTTT

>miRNA precursor
AAGAAGUACAUAGUCUUUGAUAAAAAGGAAACAGCGAUCAAUCGCGAGGAAUUUGAGUUGUUUAGUAAA**UUUCUCGCGGAUAAUUACUGUUU**UCUUUUAUCUUCCAUUUCAA
..((((........))))(((((((.((((((((..((...((((((((((((((..........))))))))))))))...))..))))))))))))))).........

>Predicted STAR seq:
AACAGCGAUCAAUCGCGAGGAA

Complementarity: 16
ERROR 4: Strange structure
Initial dG = -32.20

```
          10
.-AA|   UAC
    GAAG   A
    UUUC   U
\ --^   UGA



             20        30        40        50        60
  -----------       A        CG  CAA            AGUU
             GAUAAAA GGAAACAG  AU   UCGCGAGGAAUUUG    G
             CUAUUUU CUUUUGUC  UA   GGCGCUCUUUAAAU    U
  AACUUUACCUU       -        AU  AUA            GAUU
  110       100         90        80        70
```

Reads in: lib-42, lib-45, lib-47, lib-48, lib-49, lib-50, lib-72
Number of reads="ID1544",3,0,0,28,1,0,0

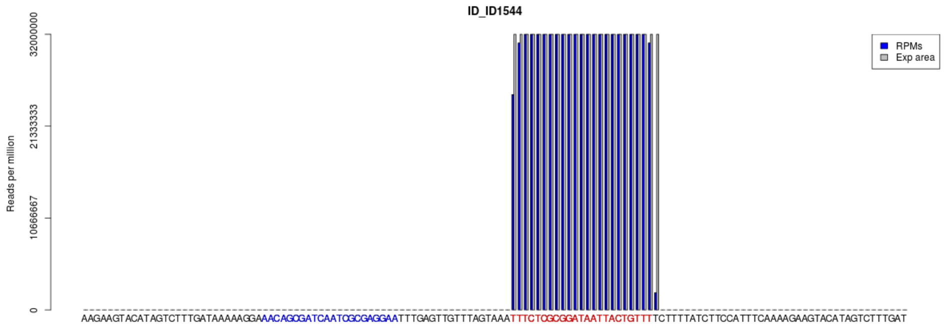

>ID155
TCACCACGGTACTTGCTACGCTGGAGCCGGGGTATCACATACAGCCCCTCCTTCCGTGACGTCAGCAGCG**GAGGGGGGCTGTGTGTGGTG**
CCTCGTCTCTGGCTGGTCGA

>ID155-3P
GAGGGGGGCTGTGTGTGGTGCC

>miRNA precursor
UCACCACGGUACUUGCUACGCUGGAGCCGGGGUAUCACAUACAGCCCCUCCUUCCGUGACGUCAGCAGCG**GAGGGGGGCUGUGUGUGGUGCC**UCGUCUCUGGCUGGUCGA
..............((((.((..(((.((((((((((((((((((((.(((.((((..........))))))))))))))))))))))))))))).)))..))))))...

>Predicted STAR seq:
GTATCACATACAGCCCCTCCTT

Complementarity: 20
ERROR 4: Strange structure
Initial dG =   -66.20

```
           10
  -   .-AC|  A
UC ACC    GGU C
AG UGG    UCG U
  C   \ --^  U
.


         20        30        40        50        60
        AC  UG   C                    U   U    GACG
          GC  GAG CGGGGUAUCACAUACAGCCCC CCU CCGU    U
          CG  CUC GCUCCGUGGUGUGUGUCGGGG GGA GGCG    C
        U-  GU   U                    -   -    ACGA
           100        90        80          70
```

Reads in: lib-42, lib-45, lib-47, lib-48, lib-49, lib-50, lib-72
Number of reads="ID155",18,17,1,2,0,1,0

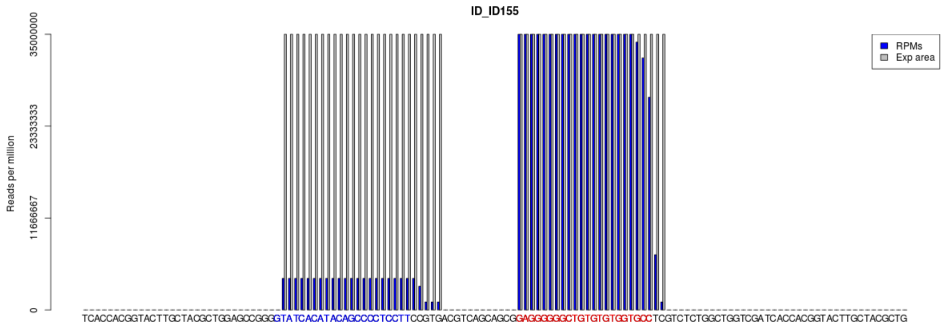

>ID1550
CGTCCACCCAGCGGATTGGCTGTTGGCGCAGCTGAGCGGCGAGCGTTCTGCAACAGTGTGCAAAGTAATG**CATAACGCTCTTTGGTCAGCTGT**GCCAATGTAGCATGACAGCA

>ID1550-3P
CATAACGCTCTTTGGTCAGCTGT

>miRNA precursor
CGUCCACCCAGCGGAUUGGCUGUUGGCGCAGCUGAGCGGCGAGCGUUCUGCAACAGUGUGCAAAGUAAUG**CAUAACGCUCUUUGGUCAGCUGU**GCCAAUGUAGCAUGACAGCA
..........((......((((((((((((((((.(((.(((((((.((((((..........))..)))).))))))).))).)))))))))))))))..)))......)).

>Predicted STAR seq:
CAGCTGAGCGGCGAGCGTTCTG

Complementarity: 18

Initial dG =   -50.20

```
        10        20           30        40        50
CGUCCACCCA  GGAUUG   --              G   C        C    --| AGUG
          GC      GCU  GUUGGCGCAGCUGA CGG GAGCGUU UGCA  AC    U
          CG      CGA  UAACCGUGUCGACU GUU CUCGCAA ACGU  UG    G
A---------  ACAGUA   UG              G   U        U    AA^ AAAC
          110       100        90        80        70        60
```

Reads in: lib-42, lib-45, lib-47, lib-48, lib-49, lib-50, lib-72
Number of reads="ID1550",29,18,8,349,64,3,0

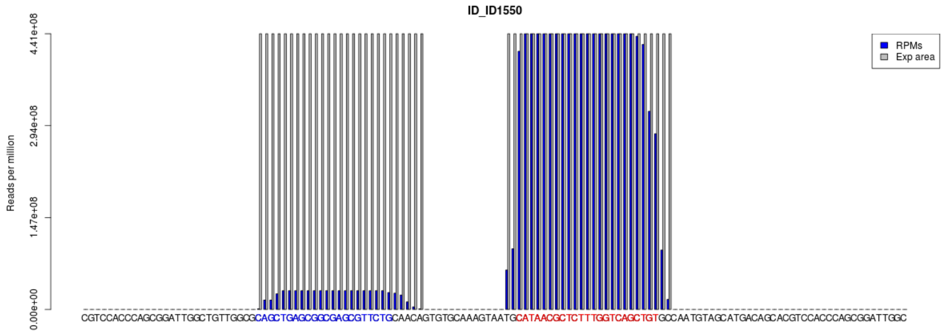

>ID156

CGCCAGTGAAGCCACCAGACGCTGGCGCCAGAGTCTCTACCTGCTGCCTTGTCGCTGCTGTTTCGTTTGC**GAAGGCTTCAGGTGGAGAAC**
GCTGGAGTCAGCTGTGTC

>ID156-3P

GAAGGCTTCAGGTGGAGAACGC

>miRNA precursor

CGCCAGUGAAGCCACCAGACGCUGGCGCCAGAGUCUCUACCUGCUGCCUUGUCGCUGCUGUUUCGUUUGC**GAAGGCUUCAGGUGGAGAACGC**UGGAGUCAGCUGUGUC

```
(((((((..........))))))))((((..((((((((((..((((..((((.((......))..))))))))..))))))))))...)))).............
Error predicting STAR on 5pERROR 4: Strange structure
 Initial dG =    -50.90

            10
--   .-CA|  A
  CGC    GUG A
  GUG    CAC G
CU   \ --^  C



          20        30         40        50         60
       CAGAC      G    AG-          CU    UG    U-  UG
            GCUGGC CCAG   UCUCUACCUG  GCCU  UCGC  GC  U
            CGACUG GGUC   AGAGGUGGAC  CGGA  AGCG  UG  U
       U----      A    GCA          UU    --    UU  CU
            100        90        80          70
```

Reads in: lib-42, lib-45, lib-47, lib-48, lib-49, lib-50, lib-72

Number of reads="ID156",18,19,0,1,1,1,0

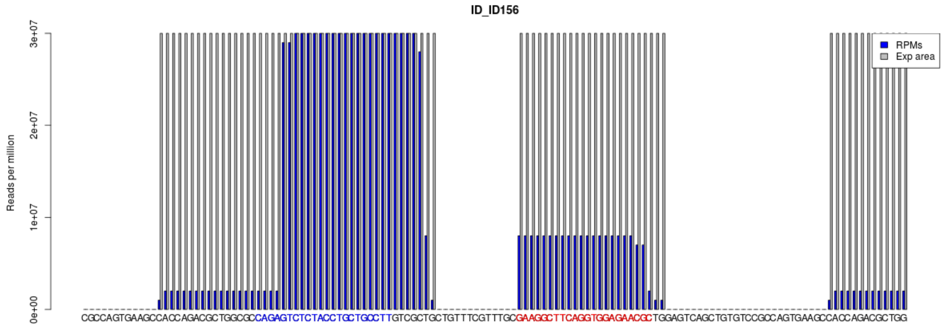

>ID1561
CACCAAGCTGAGGCACTCAC**AGAGACAGATTTTGTTAAGTTA**CTGTTTAAAGTCCTTAACTTCACAATTTCTGTGTCTGAGTGCTTCAGT
TGCATTCGACGAGAATTCTGTT

>ID1561-5P
AGAGACAGATTTTGTTAAGTTA

>miRNA precursor
CACCAAGCUGAGGCACUCAC**AGAGACAGAUUUUGUUAAGUUA**CUGUUUAAAGUCCUUAACUUCACAAUUUCUGUGUCUGAGUGCUUCAGUUGCAUUCGACGAGAAUUCUGUU
.....((((((((((((..(((.(((((..((((.((((((((......)))....))))).)))).)))))).))))))))))))))))..((((.....)))).......

>Predicted STAR seq:
CTTAACTTCACAATTTCTGTGTCTGA

Complementarity: 16

Initial dG =   -41.10

```
          10        20        30        40
.-CACCA             AC   G     UU    U     ----|  GU
       AGCUGAGGCACUC  AGA ACAGA  UUGU AAGUU    ACU  U
       UUGACUUCGUGAG  UCU UGUCU  AACA UUCAA    UGA  U
\ -----             --   G     UU    C     UUCC^  AA
       90        80          70        60        50
```

```
  GC----    GA
        AUUC  \
        UAAG  C
  UUGUCU    AG
  110
```

Reads in: lib-42, lib-45, lib-47, lib-48, lib-49, lib-50, lib-72
Number of reads="ID1561",6,6,4,151,43,2,0

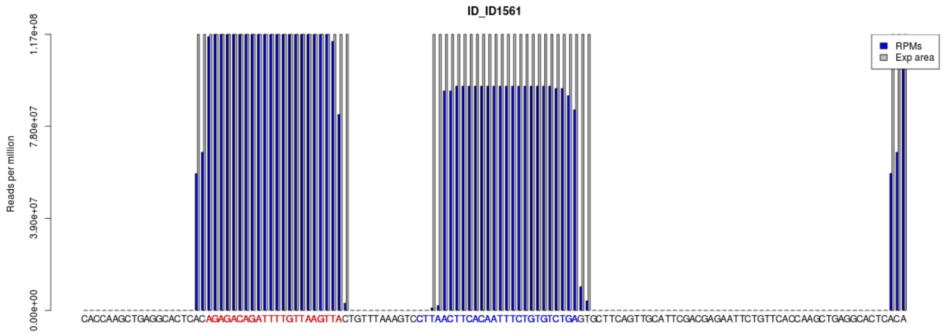

>ID1562

GACACCAAACTGAGGCACTC**AGACACAGGAGTTGTTAAGTTA**CTGTTTAAAGTCCATAACTTTACAATTTCTGTTCTGAGTGCTTCAGTT
GCATTCGACGAGAATTGTATTA

>ID1562-5P

AGACACAGGAGTTGTTAAGTTA

>miRNA precursor

```
GACACCAAACUGAGGCACUCAGACACAGGAGUUGUUAAGUUACUGUUUAAAGUCCAUAACUUUACAAUUUCUGUUCUGAGUGCUUCAGUUGCAUUCGACGAGAAUUGUAUUA
.......((((((((((((((((.(((((((((((.((((((((......)))....))))).)))))))))))))))))))))))))))(((((.....))).))).....
```

>Predicted STAR seq:

CATAACTTTACAATTTCTGTTCTGA

Complementarity: 18

Initial dG =   -46.50

```
          10        20        30        40
.-GACACCA                C           U     ----|  GU
         AACUGAGGCACUCAGA ACAGGAGUUGU AAGUU    ACU  U
         UUGACUUCGUGAGUCU UGUCUUUAACA UUCAA    UGA  U
\ -------                -           U     UACC^  AA
        90        80         70        60        50
```

```
  ----   -   GA
      GCA UUC  \
      UGU AAG  C
  AUUA   U   AG
  110       100
```

Reads in: lib-42, lib-45, lib-47, lib-48, lib-49, lib-50, lib-72

Number of reads="ID1562",7,10,4,125,15,3,0

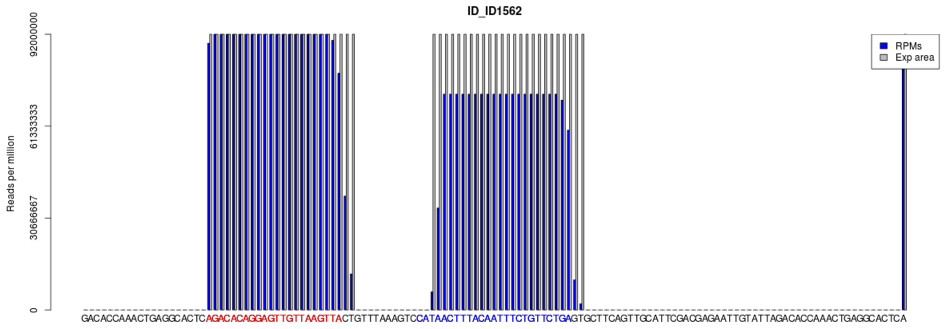

>ID1563
GACACCAAACTGAGGCACTC**CCAGAGACAGATTTTGTTAAGTT**ACTGTTTAAAGTCCTTAACTTCACATTTTCTGTGTCTGAGTGCTTCA
GTTGCATTCCACGAGAATTGAAT

>ID1563-5P
CCAGAGACAGATTTTGTTAAGTT

>miRNA precursor
GACACCAAACUGAGGCACUC**CCAGAGACAGAUUUUGUUAAGUU**ACUGUUUAAAGUCCUUAACUUCACAUUUUCUGUGUCUGAGUGCUUCAGUUGCAUUCCACGAGAAUUGAAU
.......(((((((((((((..(((.(((((...(((.((((((((......)))....))))).)))...))))).)))))))))))))))))...................

>Predicted STAR seq:
CTTCACATTTTCTGTGTCTGA

Complementarity: 14

Initial dG =   -37.90

```
                    10        20        30        40
GACACCA-------------             CC   G     UUU   U     ----|  GU
                    AACUGAGGCACUC  AGA ACAGA   UGU AAGUU    ACU  U
                    UUGACUUCGUGAG  UCU UGUCU   ACA UUCAA    UGA  U
UAAGUUAAGAGCACCUUACG             --   G     UUU   C     UUCC^  AA
 110       100        90         80        70        60        50
```

Reads in: lib-42, lib-45, lib-47, lib-48, lib-49, lib-50, lib-72
Number of reads="ID1563",11,8,14,179,36,6,0

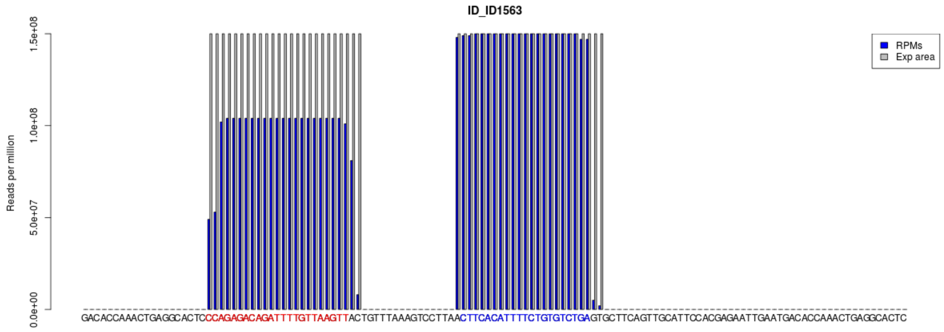

>ID1564
CACCAAACTGAGGCACTC**AGAAACAGAAGTTGTTAAGTTA**CTGTTTAAAGTCCTTAACTTAACTATTTCTGCTTCTAAGTGTTTCAGTTG
CATTCGACGAGATTTGTATT

>ID1564-5P
AGAAACAGAAGTTGTTAAGTTA

>miRNA precursor
CACCAAACUGAGGCACUC**AGAAACAGAAGUUGUUAAGUUA**CUGUUUAAAGUCCUUAACUUAACUAUUUCUGCUUCUAAGUGUUUCAGUUGCAUUCGACGAGAUUUGUAUU
.....((((((((((((.((((.(((((((.((((((((((......)))....)))))))).))))))).)))).)))))))))))))(((.((.....))..)))...

>Predicted STAR seq:
CTTAACTTAACTATTTCTGCTTCTAAG

Complementarity: 18

Initial dG =   -37.80

```
          10        20        30            40
.-CACCA            C    A       U        ----|  GU
       AACUGAGGCACU AGAA CAGAAGU GUUAAGUU    ACU  U
       UUGACUUUGUGA UCUU GUCUUUA CAAUUCAA    UGA  U
\ -----            A    C       U        UUCC^  AA
               80        70        60        50



    90
  ---   U-   GA
     GCA  UC  \
     UGU  AG  C
  UUA   UU   AG
110       100
```

Reads in: lib-42, lib-45, lib-47, lib-48, lib-49, lib-50, lib-72
Number of reads="ID1564",1,3,0,53,14,2,0

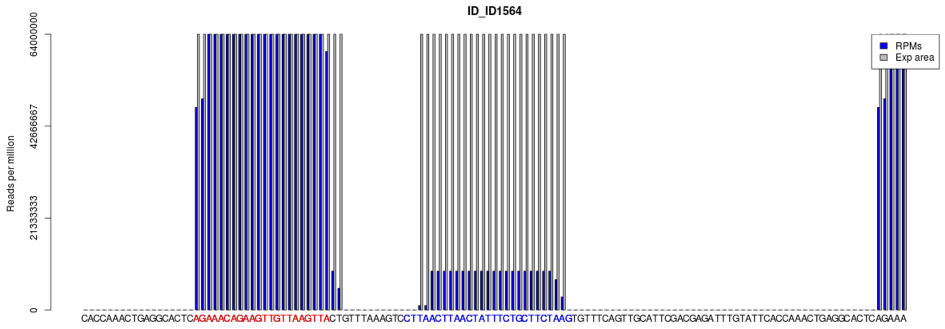

>ID1565
GACACCAAACTGAGGCACTC**AGACACAGGAGTTGTTAAGTTA**CTGTTTAAAGTCCATAACTTTACAATTTCTGTTCTGAGTGCTTCAGTT
GCATTCGACGAGAATAGTATTA

>ID1565-5P
AGACACAGGAGTTGTTAAGTTA

>miRNA precursor
GACACCAAACUGAGGCACUC**AGACACAGGAGUUGUUAAGUUA**CUGUUUAAAGUCCAUAACUUUACAAUUUCUGUUCUGAGUGCUUCAGUUGCAUUCGACGAGAAUAGUAUUA
.......((((((((((((((((.(((((((((((.((((((((......)))....))))).))))))))))))))))))))))))))))..((((.....)))).......

>Predicted STAR seq:
CATAACTTTACAATTTCTGTTCTGA

Complementarity: 18

Initial dG =   -46.70

```
         10        20        30        40
.-GACACCA                C           U     ----|  GU
         AACUGAGGCACUCAGA ACAGGAGUUGU AAGUU    ACU  U
         UUGACUUCGUGAGUCU UGUCUUUAACA UUCAA    UGA  U
\ -------                -           U     UACC^  AA
       90        80         70        60        50




  GC-----    GA
         AUUC  \
         UAAG  C
  AUUAUGA    AG
  110       100
```

Reads in: lib-42, lib-45, lib-47, lib-48, lib-49, lib-50, lib-72
Number of reads="ID1565",2,14,9,122,25,3,0

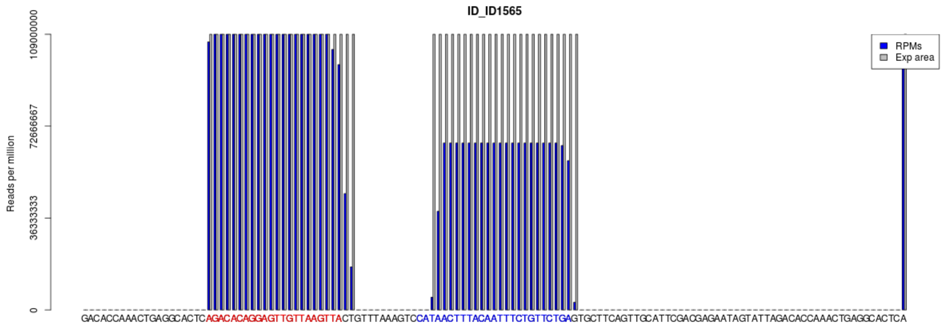

>ID1566
GACACCAAACTGAGGCACTC**AGACATAGGAGTTGTTAAGTTA**CTGTTTAAAGTCCATAACTTTACAATTTCTGTTCTGAGTGCTTCAGTT
GCATTCGACGAGAATAGTATTA

>ID1566-5P
AGACATAGGAGTTGTTAAGTTA

>miRNA precursor
GACACCAAACUGAGGCACUC**AGACAUAGGAGUUGUUAAGUUA**CUGUUUAAAGUCCAUAACUUUACAAUUUCUGUUCUGAGUGCUUCAGUUGCAUUCGACGAGAAUAGUAUUA
```
.......((((((((((((((((.(((((((((((.((((((((......)))....))))).)))))))))))))))))))))))))))..((((.....)))).......
```

>Predicted STAR seq:
CATAACTTTACAATTTCTGTTCTGA

Complementarity: 18

Initial dG =   -44.80

```
         10        20        30        40
.-GACACCA                C           U     ----|  GU
         AACUGAGGCACUCAGA AUAGGAGUUGU AAGUU    ACU  U
         UUGACUUCGUGAGUCU UGUCUUUAACA UUCAA    UGA  U
\ -------                -           U     UACC^  AA
       90        80         70        60        50



  GC-----    GA
         AUUC  \
         UAAG  C
  AUUAUGA    AG
  110       100
```

Reads in: lib-42, lib-45, lib-47, lib-48, lib-49, lib-50, lib-72
Number of reads="ID1566",5,8,4,144,28,6,0

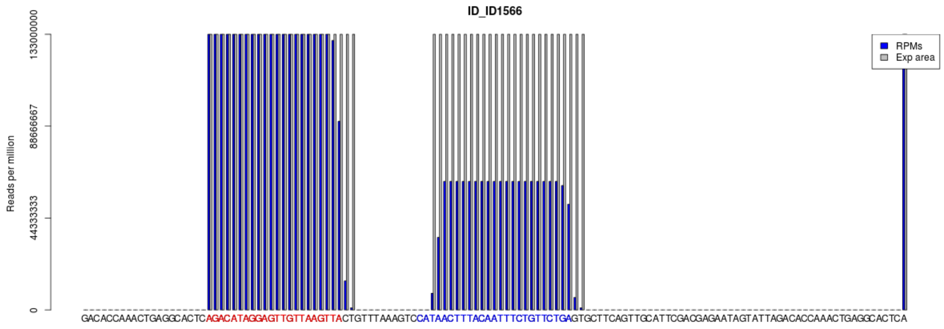

>ID157

GTCAGCAGCAGGAGAGAGACGTTGGTGCTGGGGAGTCACCTGGTATCACAGGCCACTGGAGAGAGTCAGG**TGGACTGTGCTAGCAGCGGA**
CTCTCCGGCGTCTCCAGG

>ID157-3P

TGGACTGTGCTAGCAGCGGACT

>miRNA precursor

GUCAGCAGCAGGAGAGAGACGUUGGUGCUGGGGAGUCACCUGGUAUCACAGGCCACUGGAGAGAGUCAGG**UGGACUGUGCUAGCAGCGGACU**CUCCGGCGUCUCCAGG
..........(((((...((....))((((((((((((..(((.((.(((((.(((((.((.....)).))))).))))).)).)))..))))))))))))).)))))...

>Predicted STAR seq:

GTCACCTGGTATCACAGGCCA

Complementarity: 15

Initial dG =   -50.20

```
        10          20
GUCAGCAGCA     .-GAG| G
          GGAGA     AC U
          CCUCU     UG U
GGA-------     \ ---^ G
```

Reads in: lib-42, lib-45, lib-47, lib-48, lib-49, lib-50, lib-72

Number of reads="ID157",4,12,0,0,1,0,0

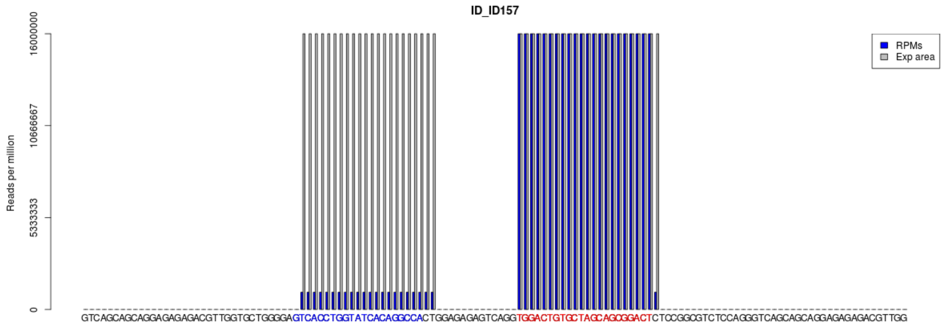

>ID1576
GAAAGGGAAG**GATATGGGTTTTAAGGAAGTGG**AAGGGACTAGAATGACTCCTTTTCCACTTCCTTAAAACCCGTATCCTTCTCTTTCTCC
TCTTTCTTCCTCTTTTCCT

>ID1576-5P
GATATGGGTTTTAAGGAAGTGG

>miRNA precursor
GAAAGGGAAG**GAUAUGGGUUUUAAGGAAGUGG**AAGGGACUAGAAUGACUCCUUUUCCACUUCCUUAAAACCCGUAUCCUUCUCUUUCUCCUCUUUCUUCCUCUUUUCCU
(((((((((((((((((((((((((((((((((((((...........))))..))))))))))))))))))))))))))))))))).....................

>Predicted STAR seq:
ACTTCCTTAAAACCCGTATCCT

Complementarity: 20

Initial dG =   -61.10

```
                              10        20        30         40
----------------------                                  --|   ACUAG
                      GAAAGGGAAGGAUAUGGGUUUUAAGGAAGUGGA  AGGG     \
                      CUUUCUCUUCCUAUGCCCAAAAUUCCUUCACCU  UCCU     A
UCCUUUUCUCCUUCUUUCUCCU                                  UU^   CAGUA
       100       90        80        70        60        50
```

Reads in: lib-42, lib-45, lib-47, lib-48, lib-49, lib-50, lib-72
Number of reads="ID1576",51,66,88,74,161,250,0

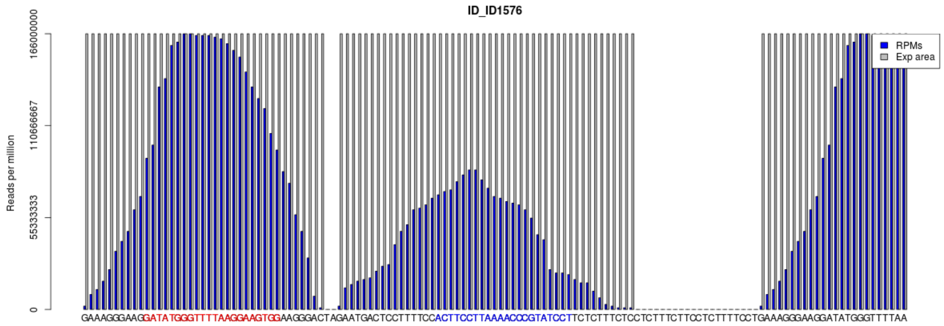

>ID1578
GCTGATACGTGCAATATCTT**TGGACTTTGATCGGGATGGGCTAG**GAAGGAAGAATTAGAAACTTCTGCCTCCGTTAATAGACTGATTAAT
ATTCGTGATATTGGAATTCGAACT

>ID1578-5P
TGGACTTTGATCGGGATGGGCTAG

>miRNA precursor
GCUGAUACGUGCAAUAUCUU**UGGACUUUGAUCGGGAUGGGCUAG**GAAGGAAGAAUUAGAAACUUCUGCCUCCGUUAAUAGACUGAUUAAUAUUCGUGAUAUUGGAAUUCGAACU
...........((((((..((((..((((((((..((.....(((.((.((((........)))).))))).....))..))))))))..)))).)))))))...........

>Predicted STAR seq:
TTAATAGACTGATTAATATTCGTGA

Complementarity: 13

Initial dG =   -28.80

```
        10        20         30       40         50
GCUGAUACGUG|      UU    CU        GA  GGCUA   A  A    UUA
           CAAUAUC  UGGA  UUGAUCGG  UG     GGA GG AGAA   G
           GUUAUAG  GCUU  AAUUAGUC  AU     CCU CC UCUU   A
UCAAGCUUAAG^      U-    AU        AG  AAUUG   -  G    CAA
  110       100         90        80        70         60
```

Reads in: lib-42, lib-45, lib-47, lib-48, lib-49, lib-50, lib-72
Number of reads="ID1578",0,0,1,0,0,1,0

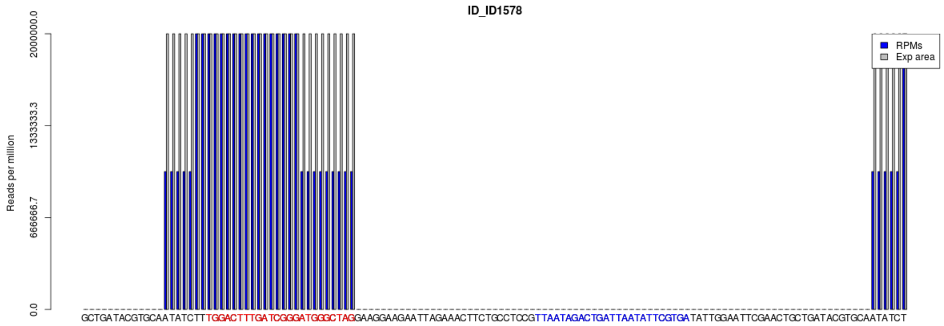

>ID158
TGCAGTGCCAGTGTGACCTCGAGGCGTCGGCGGCGGGCAGCGCAGCTGTAGTCCCCGCTGTTGGAGTCGG**ATATAGCTGTCCTGTCCGCG**
TCCGACGCCACGGCTCGC

>ID158-3P
ATATAGCTGTCCTGTCCGCGTC

>miRNA precursor
UGCAGUGCCAGUGUGACCUCGAGGCGUCGGCGGCGGGCAGCGCAGCUGUAGUCCCCGCUGUUGGAGUCGG**AUAUAGCUGUCCUGUCCGCGUC**CGACGCCACGGCUCGC
............((((.(.((.((((((((..((((((((.(((((((((.(((..(((.....))).))))))))))))).))))))))..)))))))).))).))))

>Predicted STAR seq:
CGGCGGGCAGCGCAGCTGTAG

Complementarity: 17

Initial dG =   -56.10

```
        10        20        30        40        50        60
UGCAGUGCCAGU|   C U  A        CG        C         G   CC   GU
            GUGA C CG GGCGUCGG  GCGGGCAG GCAGCUGUA UCC  GCU  \
            CGCU G GC CCGCAGCC  CGCCUGUC UGUCGAUAU AGG  UGA  U
------------^   C -  A        UG        C         -   C-   GG
                   100        90        80         70
```

Reads in: lib-42, lib-45, lib-47, lib-48, lib-49, lib-50, lib-72
Number of reads="ID158",36,50,3,3,6,1,0

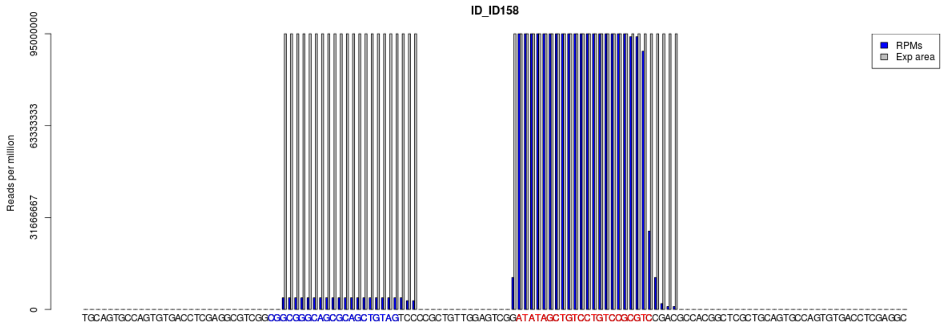

>ID1580
ATAATTAAATCACATGCAGTATATGTTGGTGTAGCTCAGGTGGGGATGGGAGTGGTGGCTTACTC**CTGTAATCCAGCTACTTGGA**GGCCG
CGTGTGGAGGATGGTTTGA

>ID1580-3P
CTGTAATCCAGCTACTTGGA

>miRNA precursor
AUAAUUAAAUCACAUGCAGUAUAUGUUGGUGUAGCUCAGGUGGGGAUGGGAGUGGUGGCUUACUC**CUGUAAUCCAGCUACUUGGA**GGCCGCGUGUGGAGGAUGGUUUGA
.........((((((((.((.....(..(.((((((..((.....((((((((((....))))))))))...))))))))).)..)).))))))))...........

>Predicted STAR seq:
TATGTTGGTGTAGCTCAGGTGGGGATGG

Complementarity: 12

Initial dG =   -33.40

```
           10        20        30        40        50
AUAAUUAAA---|       A  AUAUG UG U      CA  UGGGG         U
            UCACAUGC GU     U  G GUAGCU  GG     AUGGGAGUGG G
            GGUGUGCG CG     G  U CAUCGA  CC     UGUCCUCAUU G
AGUUUGGUAGGA^       C  GA--- GU -      --  UAA--         C
       100       90            80         70          60
```

Reads in: lib-42, lib-45, lib-47, lib-48, lib-49, lib-50, lib-72
Number of reads="ID1580",516,374,987,89,82,347,0

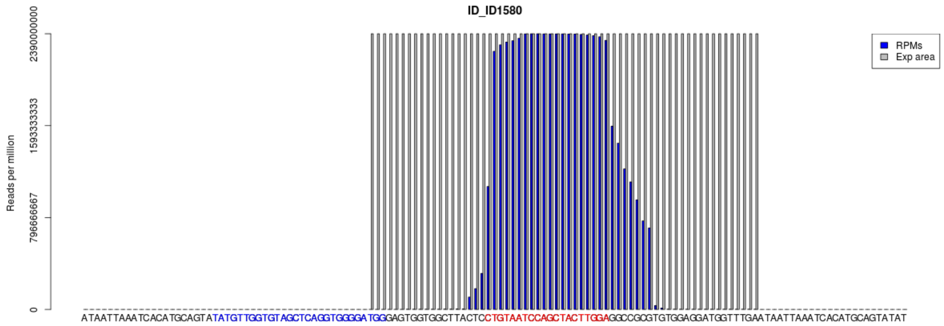

>ID1582
CTGGAACGTGCTCTTTGTGT**TTTTGATTGTTGCTCAGAAAGC**CGTGAATTTGAACAGGCTTTCTAGCTACAATCATAATCAGAAAAGAGC
AAACTATCATCTTGTACATCTG

>ID1582-5P
TTTTGATTGTTGCTCAGAAAGC

>miRNA precursor
CUGGAACGUGCUCUUUGUGU**UUUUGAUUGUUGCUCAGAAAGC**CGUGAAUUUGAACAGGCUUUCUAGCUACAAUCAUAAUCAGAAAAGAGCAAACUAUCAUCUUGUACAUCUG
.(((....((((((((.((.((.(((((((.(((.((((((((((........)).))))))))))).))))))).)).)).))..))))))))..))).................

>Predicted STAR seq:
TTTCTAGCTACAATCATAATCA

Complementarity: 17

Initial dG =   -37.20

```
                       10          20         30         40
C---------------|  AACG        G-  U  U       U   C        -  GAA
                UGG    UGCUCUUU  UG UU UGAUUGU GCU AGAAAGCC GU   U
                AUC    ACGAGAAA  AC AA ACUAACA CGA UCUUUCGG CA   U
GUCUACAUGUUCUACU^  AA--        AG  U  U       U   -        A  AGU
110       100          90        80        70         60        50
```

Reads in: lib-42, lib-45, lib-47, lib-48, lib-49, lib-50, lib-72
Number of reads="ID1582",2435,3307,842,275,451,477,0

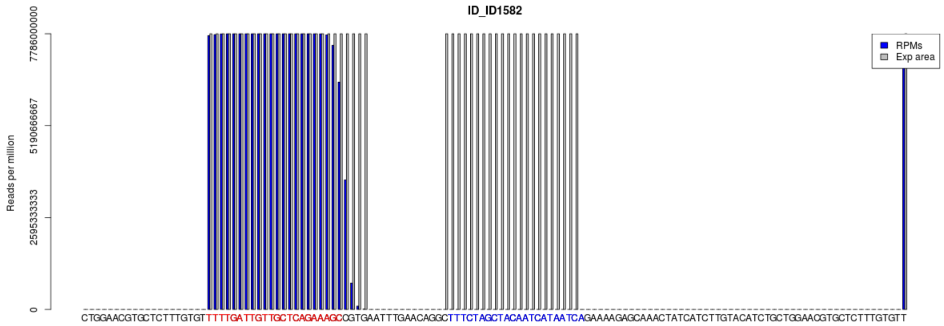

>ID1584
AGTAAGGTTGTGATTATTGAGAGTGTACACTTTGCTAGGCGC**GCGGATGAGATTGCAGAAGCGTG**GGGACCGTGGTTGTGAGCAGTTGTCGTTTCGGCGGGAATGCCG

>ID1584-5P
GCGGATGAGATTGCAGAAGCGTG

>miRNA precursor
AGUAAGGUUGUGAUUAUUGAGAGUGUACACUUUGCUAGGCGC**GCGGAUGAGAUUGCAGAAGCGUG**GGGACCGUGGUUGUGAGCAGUUGUCGUUUCGGCGGGAAUGCCG

```
((((((((.((((((......))).))))))))))).(((((((...((((((.((((..((.....(((....)))....))..)))).))))))))))....)))).
Error predicting STAR on 5pERROR 4: Strange structure
 Initial dG =    -27.80

          10
  |       U   -   AU
--AGUAAGGU GUG AUU  U
  UCGUUUCA CAU UGA  G
\ ^       -   G   GA
       30         20



    40
  A   .-GC    AU
   GGC    GCGG  G
   CCG    CGUU  A
  G   \ --    AG
               50
```

Reads in: lib-42, lib-45, lib-47, lib-48, lib-49, lib-50, lib-72
Number of reads="ID1584",0,0,1,0,0,0,0

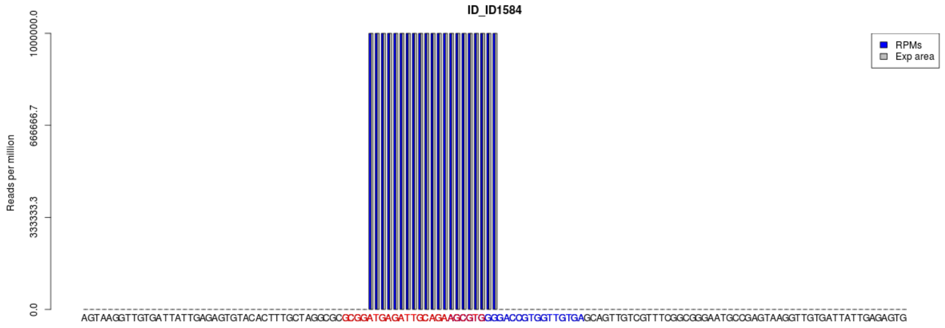

>ID1586
CCACGTGGAGTCGCCGTGTTCCCCCAGTCCGAGCCCTTGTCATTCTCATGTCCGGTGTGTATATGGCTAC**TGGACGGAGAACTGATAAGG**
GCCCGTGCTGGGGCAGGCAGCG

>ID1586-3P
TGGACGGAGAACTGATAAGGGC

>miRNA precursor
CCACGUGGAGUCGCCGUGUUCCCCCAGUCCGAGCCCUUGUCAUUCUCAUGUCCGGUGUGUAUAUGGCUAC**UGGACGGAGAACUGAUAAGGGC**CCGUGCUGGGGCAGGCAGCG
((....)).((.(((.((...((((((..((.(((((((((((((((.(((((((((.((.....)))))))))))))))).)))))))))).))..))))))))))).)).

>Predicted STAR seq:
GCCCTTGTCATTCTCATGTCCG

Complementarity: 19
ERROR 4: Strange structure
 Initial dG =   -60.80

```
  | A
--CC C
  GG G
\ ^ U
```

```
  10        20        30        40         50        60
  A  C   G  UUC      UC  A          -     A         U  AU
   GU GCC UG   CCCCAG  CG GCCCUUGUCA UUCUC UGUCCGGUG GU  \
   CG CGG AC   GGGGUC  GC CGGGAAUAGU AAGAG GCAGGUCAU CG  A
  G  A   -  ---      GU  C          C     -         -  GU
  110          100       90        80         70
```

Reads in: lib-42, lib-45, lib-47, lib-48, lib-49, lib-50, lib-72
Number of reads="ID1586",17469,39210,24495,82306,8404,14163,0

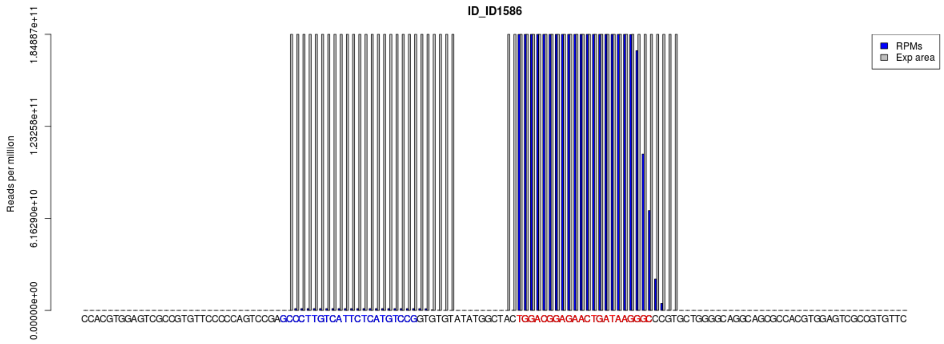

>ID1589
TGGTAAACTGAAGTACTTCG**ACTGTTCATGTGGAGAGCTCCT**TGGTACTATAAGAAACAGGAGGTCCTGCATGGGTAGCTGAAGTGATTT
GTTTGCGACAGTGTTAGTCTCA

>ID1589-5P
ACTGTTCATGTGGAGAGCTCCT

>miRNA precursor
UGGUAAACUGAAGUACUUCG**ACUGUUCAUGUGGAGAGCUCCU**UGGUACUAUAAGAAACAGGAGGUCCUGCAUGGGUAGCUGAAGUGAUUUGUUUGCGACAGUGUUAGUCUCA
..((((((.....(((((((.(((..((((..(.((.(((((.(.............)))))).))).)))).)))).))))))).....))))))(((.......)))...

>Predicted STAR seq:
GAGGTCCTGCATGGGTAGCTG

Complementarity: 13

Initial dG =   -38.90

```
                      10        20        30        40        50
UG--------------|     UGAAG       A    UU     UG A   G      U GUACUA
                GUAAAC     UACUUCG CUG  CAUG  G GA CUCCU G       \
                CGUUUG     GUGAAGU GAU  GUAC  C CU GAGGA C       U
ACUCUGAUUGUGACAG^     UUUA-       C    GG     GU -   G      - AAAGAA
110       100       90         80        70         60
```

Reads in: lib-42, lib-45, lib-47, lib-48, lib-49, lib-50, lib-72
Number of reads="ID1589",35,17,7,20,31,6,0

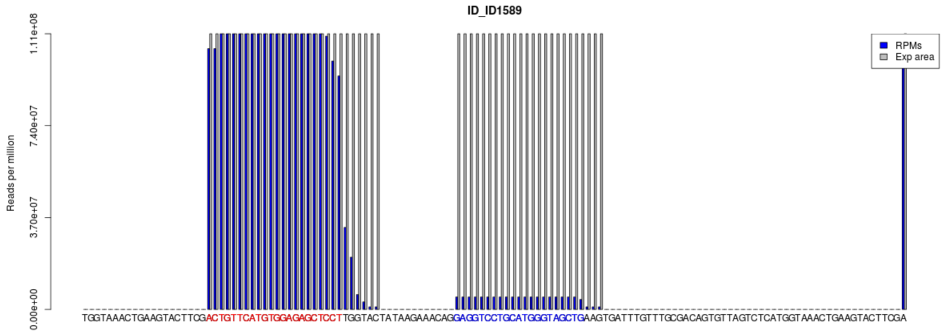

>ID1591
AAACCTGCGCATCCCATATGTGGTTCGCTACATAGCCCCGGTTGTGTGTCTCCGTGGCTATCTGATGTCG**GATCTACATAATCGGTGCTG**
TGTTGCTATCCACGATACTCTTC

>ID1591-3P
GATCTACATAATCGGTGCTGTGT

>miRNA precursor
AAACCUGCGCAUCCCAUAUGUGGUUCGCUACAUAGCCCCGGUUGUGUGUCUCCGUGGCUAUCUGAUGUCG**GAUCUACAUAAUCGGUGCUGUGU**UGCUAUCCACGAUACUCUUC
.................(((((...((.(((((((.((((((((((((..((((..((........))))))..)))))))))))).))))))).))...))))).........

>Predicted STAR seq:
ACATAGCCCCGGTTGTGTGTCTC

Complementarity: 18

Initial dG =   -41.00

```
        10        20        30        40        50        60
AAACCUGCGCAUCCCAUA|    UUC  U       C           UC    UG  UAU
                  UGUGG   GC ACAUAGC CCGGUUGUGUG  UCCG  GC   C
                  GCACC   CG UGUGUCG GGCUAAUACAU  AGGC  UG   U
CUUCUCAUA---------^    UAU  U       U           CU    --  UAG
 110               100        90        80        70
```

Reads in: lib-42, lib-45, lib-47, lib-48, lib-49, lib-50, lib-72
Number of reads="ID1591",0,1,0,2,1,0,0

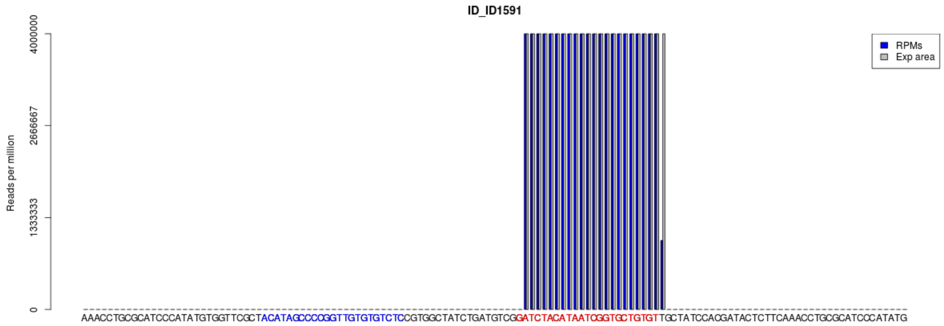

>ID1592
AAACCTGCGCATCCCATATGTGGTTCGCTACATAGCCCCGGTTGTGTGTCTCCGTGGCTATCTGATGTCG**GATCTACATAATCGGTGCTG**
TGTTGCTATCCACGATACTCTTC

>ID1592-3P
GATCTACATAATCGGTGCTGTGT

>miRNA precursor
AAACCUGCGCAUCCCAUAUGUGGUUCGCUACAUAGCCCCGGUUGUGUGUCUCCGUGGCUAUCUGAUGUCG**GAUCUACAUAAUCGGUGCUGUGU**UGCUAUCCACGAUACUCUUC
.................(((((...((.(((((((.(((((((((((..((((..((........))))))..))))))))))).))))))).))...))))).........

>Predicted STAR seq:
ACATAGCCCCGGTTGTGTGTCTC

Complementarity: 18

Initial dG =   -41.00

```
        10        20        30        40        50        60
AAACCUGCGCAUCCCAUA|    UUC  U       C           UC    UG  UAU
                  UGUGG   GC ACAUAGC CCGGUUGUGUG  UCCG  GC   C
                  GCACC   CG UGUGUCG GGCUAAUACAU  AGGC  UG   U
CUUCUCAUA---------^    UAU  U       U           CU    --  UAG
 110                100        90        80        70
```

Reads in: lib-42, lib-45, lib-47, lib-48, lib-49, lib-50, lib-72
Number of reads="ID1592",0,0,0,5,0,0,0

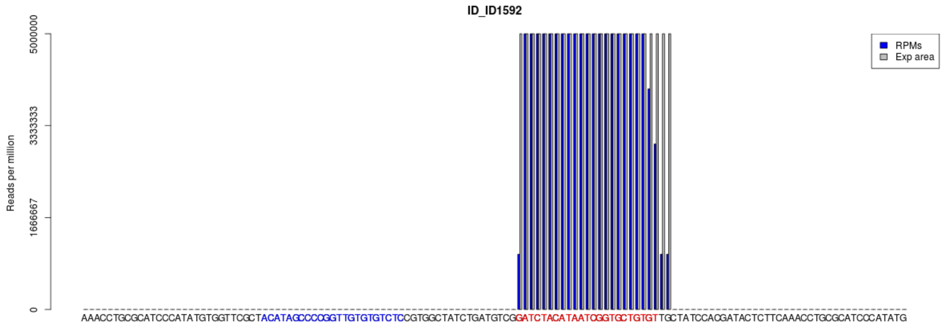

>ID1594
CGGATTGCTTCCTTGGGAGA**TGAGACAGACAAAGGGAGAAAG**ATTTTGTCTTGACACCTCCTTTCTCTCTTTATCTTTCCTGTCTCCCTG
GAAGAGAACCTGTGATATGGAA

>ID1594-5P
TGAGACAGACAAAGGGAGAAAG

>miRNA precursor
CGGAUUGCUUCCUUGGGAGA**UGAGACAGACAAAGGGAGAAAG**AUUUUGUCUUGACACCUCCUUUCUCUCUUUAUCUUUCCUGUCUCCCUGGAAGAGAACCUGUGAUAUGGAA
.((.(..(((((..(((((((..((.(((.((((((((((((....(((....)))....)))))))))))).))).))..))))))).))))).).)).)............

>Predicted STAR seq:
TTCTCTCTTTATCTTTCCTGTC

Complementarity: 16

Initial dG =   -44.70

```
                   10         20         30         40         50
C-----------| A UG     UU        GA  C   C            AUUU   C
            GG U  CUUCC  GGGAGAU  GA AGA AAAGGGAGAAAG    UGU U
            CC A  GAAGG  CCCUCUG  CU UCU UUUCUCUCUUUC    ACA U
AAGGUAUAGUGU^ A GA     U-        UC  U   A            CUCC   G
110       100        90         80         70         60
```

Reads in: lib-42, lib-45, lib-47, lib-48, lib-49, lib-50, lib-72
Number of reads="ID1594",46,197,107,1090,274,44,0

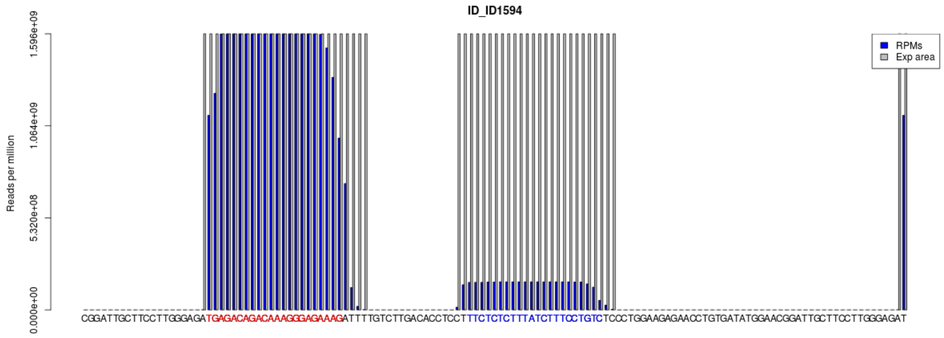

>ID1595
TTTAAGTATATATAGTATCTCCAAATTCCGTCGCTTCAAAATTGTGTGCCTGATAAGGATGTGAGAAATC**CTGTCAGACACACAATTTTATT**GAAATAATACTTAGTAT

>ID1595-3P
CTGTCAGACACACAATTTTATT

>miRNA precursor
UUUAAGUAUAUAUAGUAUCUCCAAAUUCCGUCGCUUCAAAAUUGUGUGCCUGAUAAGGAUGUGAGAAAUC**CUGUCAGACACACAAUUUUAUU**GAAAUAAUACUUAGUAU
```
.((((((((....(((................)))..(((((((((((.((((((.((((.......)))))))))).)))))))))))).........))))))))...
```

>Predicted STAR seq:
TATAGTATCTCCAAATTCCGTCGCTTCA

Complementarity: 16

Initial dG =   -28.50

```
           10        20
U--        .-AUAU|  AUCUCCA
   UUAAGUAU      AGU       A
   GAUUCAUA      UCG       A
UAU        \ ----^  CUGCCUU
       100           30
```

Reads in: lib-42, lib-45, lib-47, lib-48, lib-49, lib-50, lib-72
Number of reads="ID1595",87,44,30,26,19,29,0

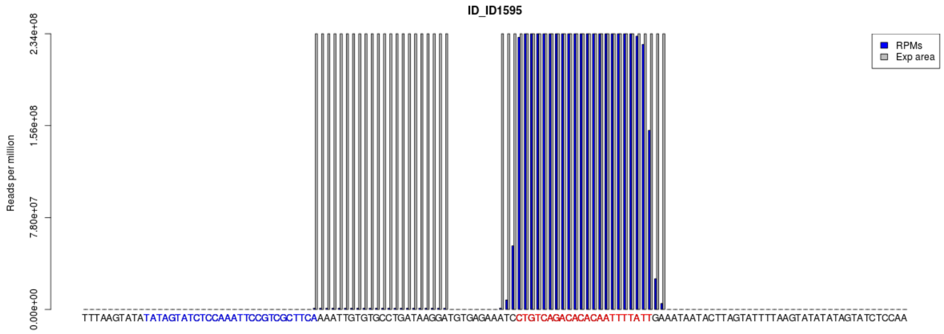

>ID1603
GCCAACGCAAGAAGAGGCGCGGGGGCGGCGCAGGGGCTGTCAGTTCCTGAGGTGGCGTCTCTGCTCAAG**CACCAGAGGAACTCACAGCTC**
CTGAGCCGCTCCACAACAGCT

>ID1603-3P
CACCAGAGGAACTCACAGCTCC

>miRNA precursor
GCCAACGCAAGAAGAGGCGCGGGGGCGGCGCAGGGGCUGUCAGUUCCUGAGGUGGCGUCUCUGCUCAAG**CACCAGAGGAACUCACAGCUCC**UGAGCCGCUCCACAACAGCU
(((...........)))...((((((((.((((((((((.(((((((..(((((((....))).....))))..))))))).)))))))))).)))))))).........

>Predicted STAR seq:
GGGCTGTCAGTTCCTGAGGTG

Complementarity: 17
ERROR 4: Strange structure
Initial dG =   -58.60

```
           10
-  .-CAA|  AAG
 GC     CGC   A
 CG     GCG   A
U  \ ---^  GAG
 .


    20            30        40        50
     CG----        G          C       GA    -----   U
           GGGGCGGC CAGGGGCUGU AGUUCCU  GGUG     GCG C
           CCUCGCCG GUCCUCGACA UCAAGGA  CCAC     CGU U
     ACAACA        A          C       GA    GAACU   C
           100        90        80        70        60
```

Reads in: lib-42, lib-45, lib-47, lib-48, lib-49, lib-50, lib-72
Number of reads="ID1603",11,11,0,614,8,0,0

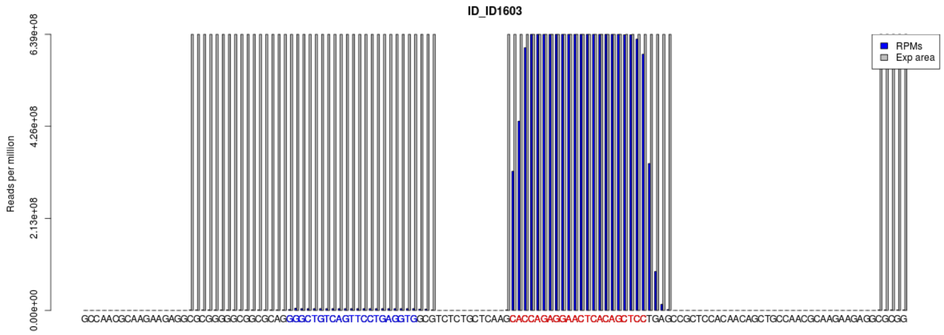

>ID1614
ACCAGCTGACACCGACTGGTACTTTGTTTTTGACGTTCGACAGAGTTGAAC**TCCCGTCGAAGGTCAAAGCAGGGT**ACAAGGTTATCTCCG
TCCGCCCTTATATACCAAACCCA

>ID1614-3P
TCCCGTCGAAGGTCAAAGCAGGGT

>miRNA precursor
ACCAGCUGACACCGACUGGUACUUUGUUUUUGACGUUCGACAGAGUUGAAC**UCCCGUCGAAGGUCAAAGCAGGGU**ACAAGGUUAUCUCCGUCCGCCCUUAUAUACCAAACCCA
....((.((((((.....((((((((((((.(((.((((((.(((.....)))..)))))).)))))))))))))))..))).......))).)).................

>Predicted STAR seq:
CTTTGTTTTTGACGTTCGACAGA

Complementarity: 19

Initial dG =   -36.70

```
                      10                20        30        40
ACCA--------------  U   -------|  GACUG            U   G      A-   UU
                  GC GAC       ACC     GUACUUUGUUUU GAC UUCGAC  GAG  \
                  CG CUG       UGG     CAUGGGACGAAA CUG AAGCUG  CUC  G
ACCCAAACCAUAUAUUCC  C   CCUCUAU^  AA---            -   G      CC   AA
 110       100        90        80           70         60        50
```

Reads in: lib-42, lib-45, lib-47, lib-48, lib-49, lib-50, lib-72
Number of reads="ID1614",17,16,60,25,13,36,0

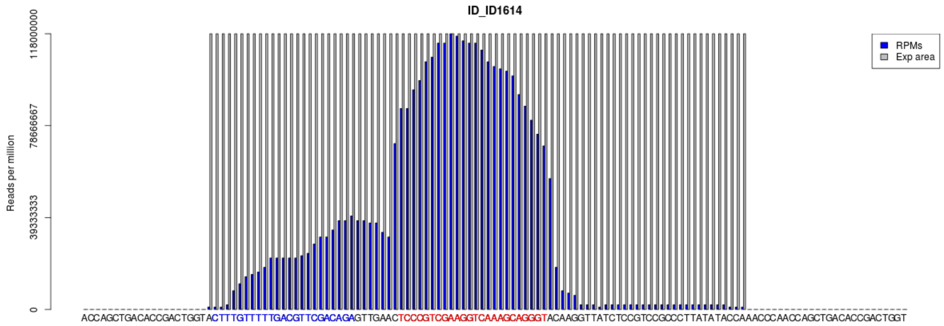

>ID1617
AGAGGAGAAACGCCGACTTGCGCCCGTTGTGGCGGTCCTCACGTTGCCAATTGGCTTTCTCAATCCGAAG**CCAGAGGGCTGTGTGGTGAC**
CGCTGCTCCGACGCCTCAAC

>ID1617-3P
CCAGAGGGCTGTGTGGTGACCG

>miRNA precursor
AGAGGAGAAACGCCGACUUGCGCCCGUUGUGGCGGUCCUCACGUUGCCAAUUGGCUUUCUCAAUCCGAAG**CCAGAGGGCUGUGUGGUGACCG**CUGCUCCGACGCCUCAAC
```
.((((.....(((......)))..((..(..((((((.(((((..(((..((((((((........))))))))..)))..))))).))))))..)..))...))))...
```

>Predicted STAR seq:
GGTCCTCACGTTGCCAATTGG

Complementarity: 15
ERROR 4: Strange structure
Initial dG = -49.70

```
            10
A--    .-AGAAA|  CG
   GAGG       CGC  A
   CUCC       GCG  C
CAA    \ -----^  UU
.              20


                30        40        50        60
         CC-  UU UG      C     UU   AA        CUC
            CG  G  GCGGUC UCACG  GCC  UUGGCUUU   A
            GC  C  CGCCAG GGUGU  CGG  GACCGAAG   A
         GCA  CU GU      U     GU   GA        CCU
          100        90        80        70
```

Reads in: lib-42, lib-45, lib-47, lib-48, lib-49, lib-50, lib-72
Number of reads="ID1617",4,1,2,35,4,0,0

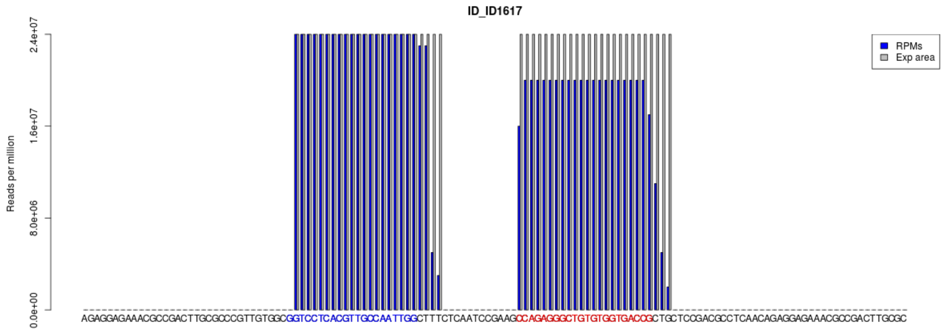

>ID1619
CATGCATTACAGCTGAAGGCAGAAAGGGCAAGCCACTCTCTTCTGAGTGCTGGCCAGAGGACTTGTACCC**TCTGTCAGCACTCAGTGGAC**
AGTTGCTTGGCTGCAGCTT

>ID1619-3P
TCTGTCAGCACTCAGTGGACA

>miRNA precursor
CAUGCAUUACAGCUGAAGGCAGAAAGGGCAAGCCACUCUCUUCUGAGUGCUGGCCAGAGGACUUGUACCC**UCUGUCAGCACUCAGUGGACA**GUUGCUUGGCUGCAGCUU
```
...((......))...((((.....((.(((((.(((.(((.((((((((((((.(((((........))))))))))))))))).))).))).))))).))...))))
```

>Predicted STAR seq:
CTCTTCTGAGTGCTGGCCAGA

Complementarity: 17
ERROR 4: Strange structure
 Initial dG =   -50.80

```
            10
CA--   .-AUUACA| UG
    UGC        GC  \
    ACG        CG  A
UUCG   \ ------^ GA
              20



                 30        40        50        60
         AGAAA  G     C   C   U            C     ACU
              GG CAAGC ACU UCU CUGAGUGCUGGC AGAGG   U
              UC GUUCG UGA AGG GACUCACGACUG UCUCC   G
         -----  G     U   C   U            -     CAU
              100        90        80         70
```

Reads in: lib-42, lib-45, lib-47, lib-48, lib-49, lib-50, lib-72
Number of reads="ID1619",2,8,4,345,104,2,0

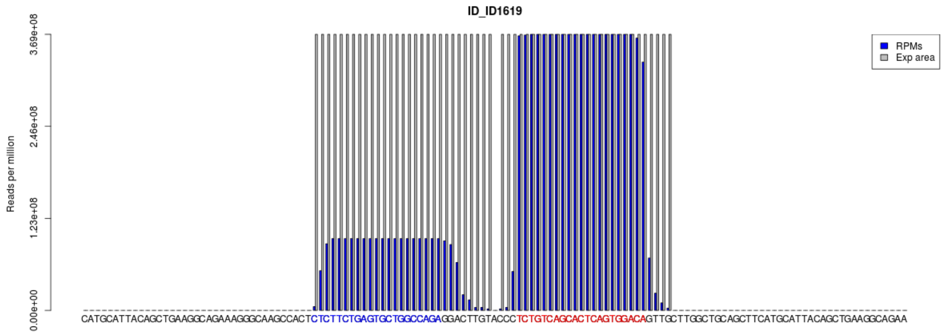

>ID162
GCTCTCCGGCCGGGGCTGGG**ATATTGTCCTGTCACAGCAGTA**CTAAGAAAAAAGCTTACTGCTGTTTCGGGACATAGCCTCAGACCCGGC
CGCCCGCAGCTTCAGTGTTT

>ID162-5P
ATATTGTCCTGTCACAGCAGTA

>miRNA precursor
GCUCUCCGGCCGGGGCUGGG**AUAUUGUCCUGUCACAGCAGUA**CUAAGAAAAAAGCUUACUGCUGUUUCGGGACAUAGCCUCAGACCCGGCCGCCCGCAGCUUCAGUGUUU
(((...((((((((.(((((....(((((((..(((((((((((........))..)))))))))..))))))).....))))).))))))))).....)))..........

>Predicted STAR seq:
CTGCTGTTTCGGGACATAGCCT

Complementarity: 14

Initial dG =   -52.80

```
                    10        20        30        40
----------   CUC--        G      AUAU       UC          --| AAG
          GCU     CGGCCGGG CUGGG    UGUCCUG  ACAGCAGUA  CU   A
          CGA     GCCGGCCC GACUC    ACAGGGC  UGUCGUCAU  GA   A
UUUGUGACUU   CGCCC        A      CGAU       UU          UC^ AAA
.       100        90        80        70        60        50
```

Reads in: lib-42, lib-45, lib-47, lib-48, lib-49, lib-50, lib-72
Number of reads="ID162",1236,2774,534,16930,5103,135,0

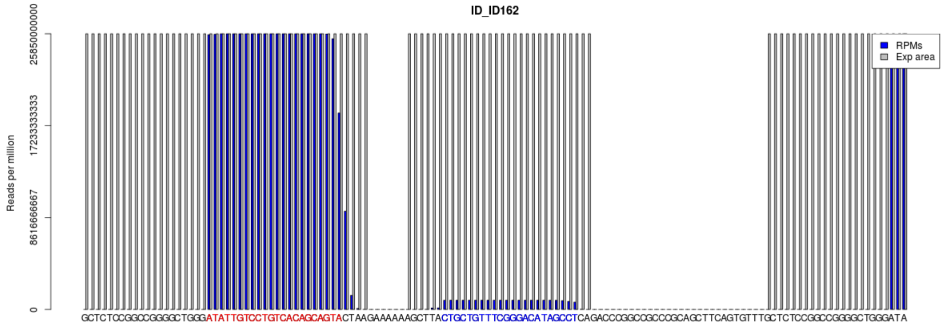

>ID1621
TTGGACACTTGTTCCTGACACCACTGCAGAACCCTCAAACTCTTCAGAGCCATCCTCGGAAACTGATAAGCTGAAGAGACATGTGAGCCA
GCTGATGGTGAAAAATGAACAAT

>ID1621-5P
CCACTGCAGAACCCTCAAACTCT

>miRNA precursor

```
UUGGACACUUGUUCCUGACACCACUGCAGAACCCUCAAACUCUUCAGAGCCAUCCUCGGAAACUGAUAAGCUGAAGAGACAUGUGAGCCAGCUGAUGGUGAAAAAUGAACAAU
........((((((....(((((...(((....((((..((((((((........((((...))))....))))))))).....))))....))).)))))......)))))).
```

>Predicted STAR seq:
AGACATGTGAGCCAGCTGATGGTG

Complementarity: 12

Initial dG =   -27.80

```
        10          20        30           40         50         60
UUGGACAC      CUGA--     CUG   AACC    AA---|       AGCCAUCC    A
        UUGUUC      CACCA   CAG    CUCA     CUCUUCAG        UCGG \
        AACAAG      GUGGU   GUC    GAGU     GAGAAGUC        AGUC A
U-------      UAAAAA     A--   GACC    GUACA^       GAAU----    A
        110       100         90        80         70
```

Reads in: lib-42, lib-45, lib-47, lib-48, lib-49, lib-50, lib-72
Number of reads="ID1621",115,97,73,237,312,113,0

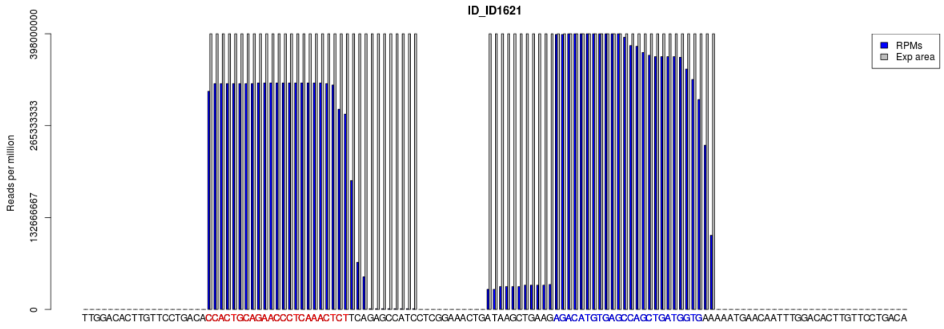

>ID1623
CGGCGAGGATTCCTTTCGTA**AACTCTGTGGTGCTAGTGACAG**ATGTTATGTTAGAAGCTCTGTCGCAAGCACCACATAGTTAACGACGGG
CGGTCCCAGCGCAACGCCAC

>ID1623-5P
AACTCTGTGGTGCTAGTGACAG

>miRNA precursor
CGGCGAGGAUUCCUUUCGUA**AACUCUGUGGUGCUAGUGACAG**AUGUUAUGUUAGAAGCUCUGUCGCAAGCACCACAUAGUUAACGACGGGCGGUCCCAGCGCAACGCCAC
.((((.((((((((.((((.((((.(((((((((.((((((((.(((........))))))))))).))))))))).)))).)))).))).))))).......))))..

>Predicted STAR seq:
GTCGCAAGCACCACATAGTTAAC

Complementarity: 18

Initial dG =   -53.30

```
                10         20        30        40        50
C-    A-------     -|  U    A    C         A        U   AUG
  GGCG        GGAUU CCU UCGU AACU UGUGGUGCU GUGACAGA GUU   U
  CCGC        CCUGG GGG AGCA UUGA ACACCACGA CGCUGUCU CGA   U
CA    AACGCGAC     C^  C    A    U         A        -   AGA
.       100        90        80        70        60
```

Reads in: lib-42, lib-45, lib-47, lib-48, lib-49, lib-50, lib-72
Number of reads="ID1623",634,803,969,786,1191,341,0

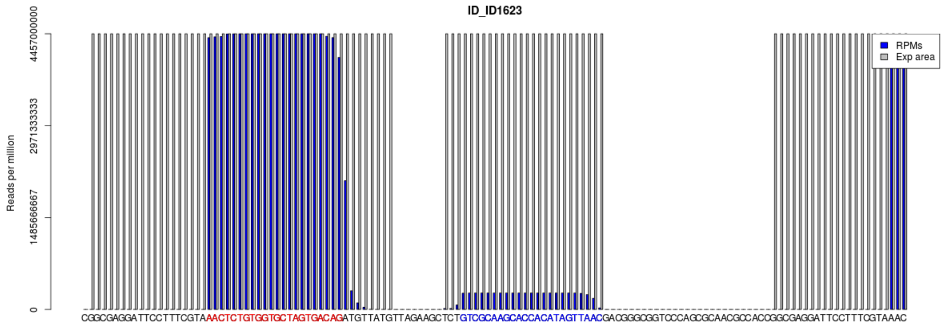

>ID1624
GCCACTGGCTGCGCGGCTTG**AACCCGTAGATCCGAACTTGTG**TCTGAGCGGATCGCAAGCTCGATTCTATGGGTCTAAGTCGCCAGCGGC
AGTCCCCGCCGGACTACGAGAC

>ID1624-5P
AACCCGTAGATCCGAACTTGTG

>miRNA precursor
GCCACUGGCUGCGCGGCUUG**AACCCGUAGAUCCGAACUUGUG**UCUGAGCGGAUCGCAAGCUCGAUUCUAUGGGUCUAAGUCGCCAGCGGCAGUCCCCGCCGGACUACGAGAC
(((....((((.((((((((.(((((((((..(((.(((((((((....))).))))))).))).))..))))))))).))))))))))))))).((((.....)))).......

>Predicted STAR seq:
CAAGCTCGATTCTATGGGTCTA

Complementarity: 16

Initial dG =   -56.30

```
          10        20        30        40
  |  ACUG    C         A         UC   A       -   G
--GCC    GCUG GCGGCUUG ACCCGUAGA  CGA CUUGUG UCU A
  CGG    CGAC CGCUGAAU UGGGUAUCU  GCU GAACGC AGG G
\ ^  ----    -         C         UA   C      U   C
 90            80        70        60        50



  A-------    CC
          GUCC  \
          CAGG  G
  CAGAGCAU    CC
  110       100
```

Reads in: lib-42, lib-45, lib-47, lib-48, lib-49, lib-50, lib-72
Number of reads="ID1624",9831,49515,70022,39111,105096,35261,0

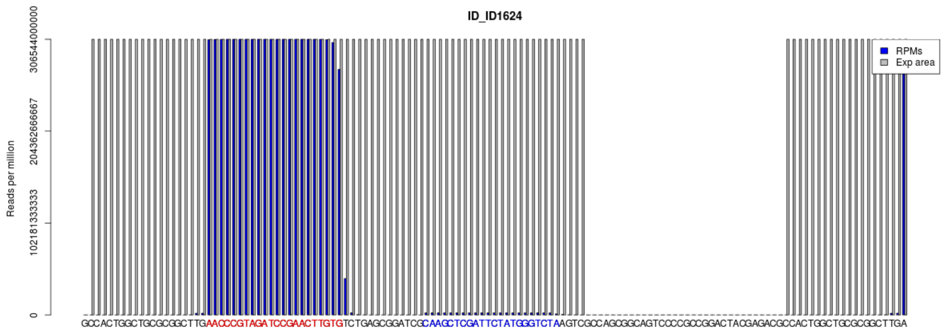

>ID1625
CCGAGTCGAGGGCGCCGGGC**TGAGGTAGTAGGTTGTATAGTT**ATGAACTACATCTTTATTGGGAGAACTGTACAACTTGCTAACTTTCCC
CGTCGCTTCCTCAAATCGCCAC

>ID1625-5P
TGAGGTAGTAGGTTGTATAGTT

>miRNA precursor
CCGAGUCGAGGGCGCCGGGC**UGAGGUAGUAGGUUGUAUAGUU**AUGAACUACAUCUUUAUUGGGAGAACUGUACAACUUGCUAACUUUCCCCGUCGCUUCCUCAAAUCGCCAC
.(((...(((((((.((((..(((.((((((((((((((((.....(((.........)))...)))))))))))))))).)))..)))).)))..))))...)))....

>Predicted STAR seq:
CTGTACAACTTGCTAACTTTCCC

Complementarity: 18

Initial dG =   -45.60

```
           10          20         30         40         50
C---   GUC    --|  C    CU   G                 AUGAA   CAUC
    CGA   GAGG  GCG CGGG  GAG UAGUAGGUUGUAUAGUU     CUA    \
    GCU   CUCC  CGC GCCC  UUC AUCGUUCAACAUGUCAA     GGU    U
CACC   AAA    UU^  U    CU   A                 GAG--   UAUU
110       100        90         80         70         60
```

Reads in: lib-42, lib-45, lib-47, lib-48, lib-49, lib-50, lib-72
Number of reads="ID1625",1051,31434,50811,41243,47629,23240,0

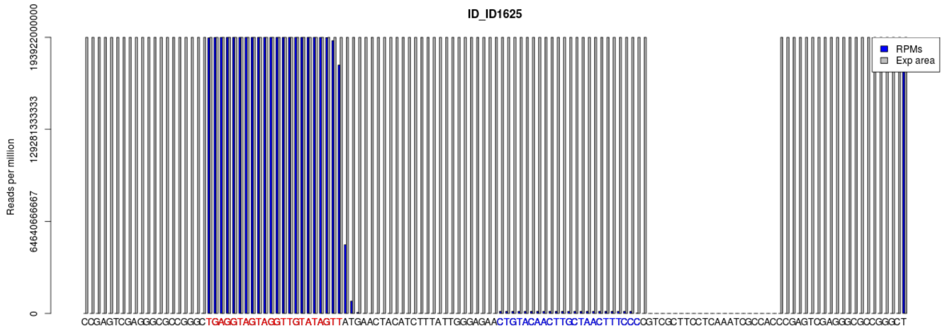

>ID1626
CATATACTGCTGTGGGTGGA**ATCCTGCATCATGTGCATTGCT**TTATGTAAGGATAGCAATGCTCTGGAAGCAGGATATCATCCAGGGCAC
AAACAAATCTAGTGAGGAATCT

>ID1626-5P
ATCCTGCATCATGTGCATTGCT

>miRNA precursor
CAUAUACUGCUGUGGGUGGA**AUCCUGCAUCAUGUGCAUUGCU**UUAUGUAAGGAUAGCAAUGCUCUGGAAGCAGGAUAUCAUCCAGGGCACAAACAAAUCUAGUGAGGAAUCU
.......((((.(((((((.(((((((.((....((((((((............))))))))....)).))))))).))))))).))))......................

>Predicted STAR seq:
CAATGCTCTGGAAGCAGGATATC

Complementarity: 15

Initial dG = -42.70

```
                        10        20        30        40
CAUAUAC----------------|   G       A       A  AUGU        UUAUG
                       UGCU UGGGUGG AUCCUGC UC    GCAUUGCU     U
                       ACGG ACCUACU UAGGACG AG    CGUAACGA     A
UCUAAGGAGUGAUCUAAACAAAC^   G       A       A  GUCU        UAGGA
110       100       90        80        70        60        50
```

Reads in: lib-42, lib-45, lib-47, lib-48, lib-49, lib-50, lib-72
Number of reads="ID1626",35,5,7,11,1,0,0

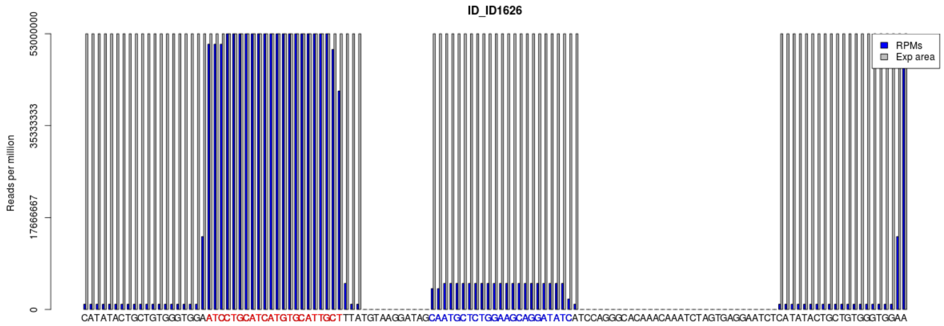

>ID1628
ACATTTGCACGGGTGCGCTTGGGCGGACATACTGTAGAACTGTCAAGTTCTGCAGTATGTCTGCCTAAGTGAGCTTGCGTCTGTGTAAAT
GTGTGGGTGTGTGTGTTTAC

>ID1628-5P
TTGGGCGGACATACTGTAGAAC

>miRNA precursor
ACAUUUGCACGGGUGCGCUUGGGCGGACAUACUGUAGAACUGUCAAGUUCUGCAGUAUGUCUGCCUAAGUGAGCUUGCGUCUGUGUAAAUGUGUGGGUGUGUGUGUUUAC
((((((((((((..((((((((((((((((((((((((((((....))))))))))))))))))))))))))))......))..))))))))))))(((((........)))))

>Predicted STAR seq:
TCTGCAGTATGTCTGCCTAAGT

Complementarity: 20

Initial dG =   -63.40

```
          10               20        30        40
              GU  -----|                        G
--ACAUUUGCACGG  GC      GCUUGGGCGGACAUACUGUAGAACU U
  UGUAAAUGUGUC  CG      UGAAUCCGUCUGUAUGACGUCUUGA C
\             UG  UUCGAG^                        A
   90        80        70        60        50



       100
       UGU
  GUGGG   G
  CAUUU   U
       GUG
110
```

Reads in: lib-42, lib-45, lib-47, lib-48, lib-49, lib-50, lib-72
Number of reads="ID1628",23,60,28,41,50,105,0

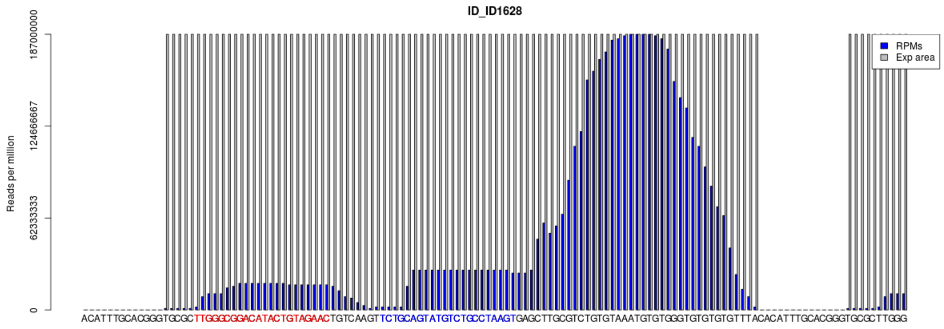

>ID1630
CCTAAAACCTCAGAGTTCTCATTTAAGAAGACGGTCGCCATAAACCCTAATTATAGTGTCTTATTTATTA**TAATTATGGTAAATGGAGGC**
TGTTTTCTTAAATACCTACCAGC

>ID1630-3P
TAATTATGGTAAATGGAGGCTGT

>miRNA precursor
```
CCUAAAACCUCAGAGUUCUCAUUUAAGAAGACGGUCGCCAUAAACCCUAAUUAUAGUGUCUUAUUUAUUAUAAUUAUGGUAAAUGGAGGCUGUUUUCUUAAAUACCUACCAGC
...................((((((((((((((((.((((..(((.(((((((((((.......))))))))))).)))..)))).))))))))))))))))..........
```

>Predicted STAR seq:
CGGTCGCCATAAACCCTAATTA

Complementarity: 17

Initial dG =   -36.90

```
        10        20        30        40        50        60
CCUAAAACCUCAGAGUUCUC|               G    AA   C           UCU
                    AUUUAAGAAGACGGUC CCAU  ACC UAAUUAUAGUG   \
                    UAAAUUCUUUUGUCGG GGUA  UGG AUUAAUAUUAU   U
CGACCAUCCA----------^               A    AA   U           UUA
 110                 100        90        80        70
```

Reads in: lib-42, lib-45, lib-47, lib-48, lib-49, lib-50, lib-72
Number of reads="ID1630",9,28,0,1032,170,2,0

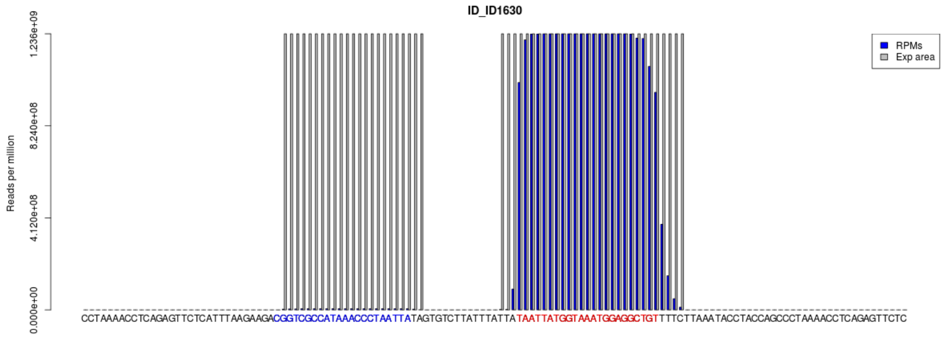

>ID1634

CTACGGGTGATCCAACGGTTCGAAGATGGGTCGGACTGTTCAATCAAGGACGTAATCAAGTGCAGCAGTC**CTCTTTTGAATAGTCTGACC**
CATCTTCTAATCACCAACTGTA

>ID1634-3P

CTCTTTTGAATAGTCTGACCCA

>miRNA precursor

```
CUACGGGUGAUCCAACGGUUCGAAGAUGGGUCGGACUGUUCAAUCAAGGACGUAAUCAAGUGCAGCAGUCCUCUUUUGAAUAGUCUGACCCAUCUUCUAAUCACCAACUGUA
....(((...)))...((((.((((((((((((((((((((((...(((((................)))))...)))))))))))))))))))))).)))).........
```

>Predicted STAR seq:

GGGTCGGACTGTTCAATCAAG

Complementarity: 17
ERROR 4: Strange structure
Initial dG = -51.30

```
          10
CUACG--        | A
       GGUGAU--CC \
       CCACUA  GG A
AUGUCAA      \ ^ C
110       100


               20        30        40        50
               UUC                      UCA      GUAAUCA
                  GAAGAUGGGUCGGACUGUUCAA   AGGAC        A
                  CUUCUACCCAGUCUGAUAAGUU   UCCUG        G
               AU-                      UUC      ACGACGU
                        90        80        70        60
```

Reads in: lib-42, lib-45, lib-47, lib-48, lib-49, lib-50, lib-72

Number of reads="ID1634",53,20,11,1485,279,2,0

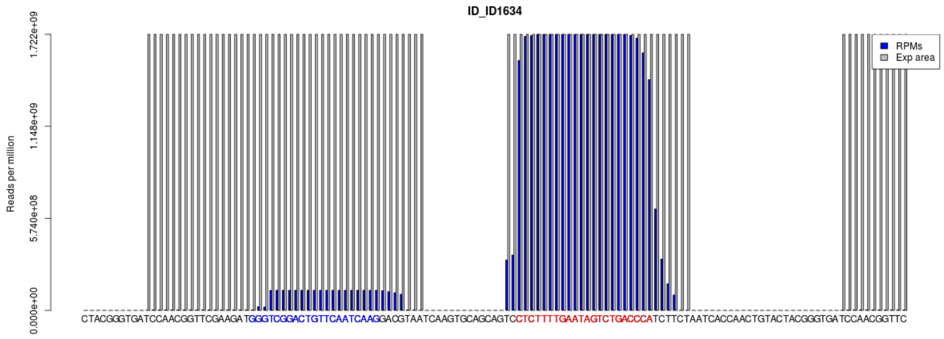

>ID1643
AAATTATAGTACCCTAGTAT**TACCCTGTGTTGTTTGAGAGAAA**CTCTTCAGTAAGAGTTTACTCATAGGTCACAGGGAGAATAATTGCAA
AGGTTACCACAGTTTGACTGTCT

>ID1643-5P
TACCCTGTGTTGTTTGAGAGAAA

>miRNA precursor
AAAUUAUAGUACCCUAGUAU**UACCCUGUGUUGUUUGAGAGAAA**CUCUUCAGUAAGAGUUUACUCAUAGGUCACAGGGAGAAUAAUUGCAAAGGUUACCACAGUUUGACUGUCU
```
........(((.(((..((((.(((((((.....((((..((((((((....)))))))).)))).....))))))).)))).......))).))).((((.....)))).. 
```

>Predicted STAR seq:
TACTCATAGGTCACAGGGAGAAT

Complementarity: 13

Initial dG =   -30.20

```
          10             20          30         40         50
.-AAAUUAUA   C   AG-----    A-|       UUGUU    AG         C
          GUA CCU       UAUU  CCCUGUG     UGAG  AAACUCUU A
          CAU GGA       AUAA  GGGACAC     ACUC  UUUGAGAA G
\ --------   U   AACGUUA    GA^       UGGAU    A-         U
                90         80         70          60



   100
  C-    UU
    ACAG  \
    UGUC  U
  UC    AG
   110
```

Reads in: lib-42, lib-45, lib-47, lib-48, lib-49, lib-50, lib-72
Number of reads="ID1643",32,51,283,15,15,31,0

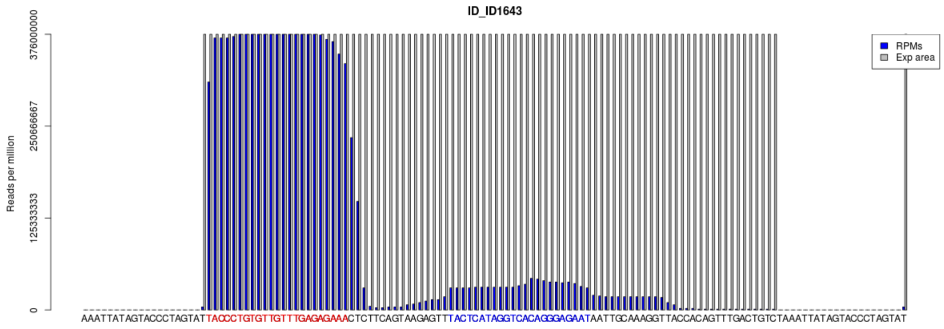

>ID1646
GACAGCATTTCGCATCCTCACTGCTGGTACCAGC**GGGGTGACATGTACCAGCAGTG**AGGCATCTCTCACACTGCGCATACATGTCGCCCT
CCTGGCGCTAGCATGTGTCGTCT

>ID1646-5P
GGGGTGACATGTACCAGCAGTG

>miRNA precursor
GACAGCAUUUCGCAUCCUCACUGCUGGUACCAGC**GGGGUGACAUGUACCAGCAGUG**AGGCAUCUCUCACACUGCGCAUACAUGUCGCCCUCCUGGCGCUAGCAUGUGUCGUCU
(((.((.....)).....((((((((..((((.(((((((((((((...((((((.((......)).))))))...))))))))))))).)))))..))))))).)))..))).

>Predicted STAR seq:
CTGCGCATACATGTCGCCCTCCT

Complementarity: 17

Initial dG =   -58.70

ERROR 3: Strange structure
Initial dG =   -58.70

```
-   .-A| AU
 GAC   GC  \
 CUG   CG  U
U   \ -^ CU
 110     10


           20         30         40         50         60
      AUCCU    -      UA     C              CCA       A   CA
           CAC UGCUGG   CCAG GGGGUGACAUGUA   GCAGUG GG   U
           GUG ACGAUC   GGUC UCCCGCUGUACAU   CGUCAC CU   C
      CU---    U      GC     C              ACG       A   CU
                100        90         80         70
```

Reads in: lib-42, lib-45, lib-47, lib-48, lib-49, lib-50, lib-72
Number of reads="ID1646",1,1,8,10,1,0,0

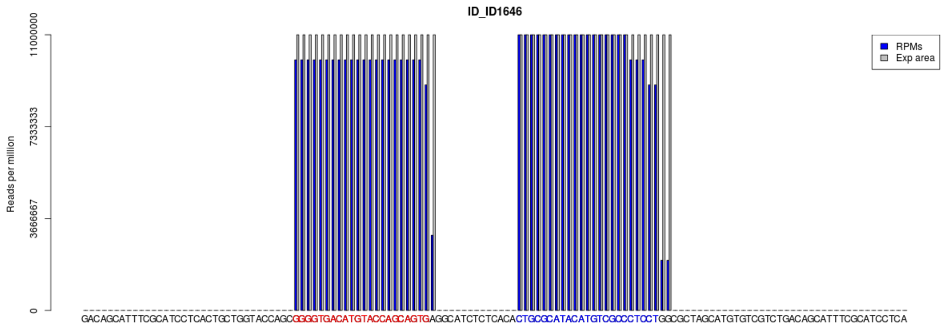

>ID1647
GGACGCGGCCCCGGGTCCCC**TCGAGATTCCTGGAATGCCACG**CGTGCTGTAGTCTGCCTGGCGTTACTGGAACGGCGGGAGGAACCGGGG
CCAGCGCGACGCACAGCGAGAA

>ID1647-5P
TCGAGATTCCTGGAATGCCACG

>miRNA precursor
GGACGCGGCCCCGGGUCCCC**UCGAGAUUCCUGGAAUGCCACG**CGUGCUGUAGUCUGCCUGGCGUUACUGGAACGGCGGGAGGAACCGGGGCCAGCGCGACGCACAGCGAGAA
...((((((((((.(((.((((...((((...(((((((.(((.((....)).))).)))))))...))))...)))).))).)))))))).)))...(((...)))....

>Predicted STAR seq:
TGGCGTTACTGGAACGGCGGGA

Complementarity: 13

Initial dG = -56.80

```
           10        20        30        40
.-GGA   -|       G   C    AGA    UGG       C   U  U
     CGC GGCCCCGG UCC CUCG   UUCC   AAUGCCA GCG GC G
     GCG CCGGGGCC AGG GGGC   AAGG   UUGCGGU CGU UG U
\ ---   A^       A   A    GGC    UCA       C   C  A
          90        80        70        60        50



    100
  CGA-   A
      CGC \
      GCG C
  AAGA   A
  110
```

Reads in: lib-42, lib-45, lib-47, lib-48, lib-49, lib-50, lib-72
Number of reads="ID1647",31,10,1,1080,82,2,0

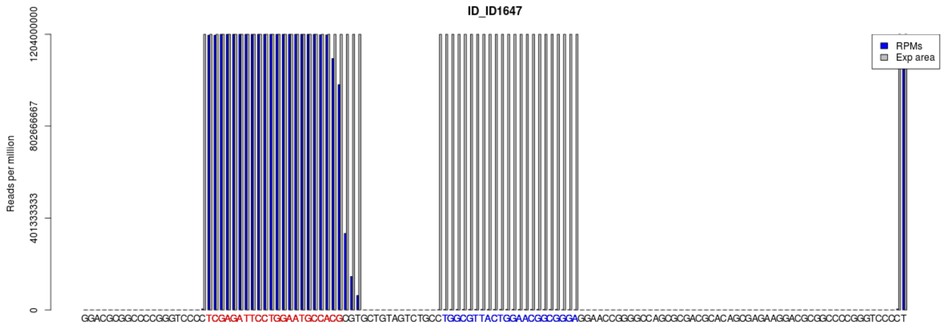

>ID1648
CTGAGACCTGGTGCATCTTT**TACAGGACTCTGCAACTTACCG**ATTCTCGCCGGCGGTCAGTTACTGAGGCCTGTGAAAGACGCAGCAGGG
TAAAGACGGTGCTGAAACTCGT

>ID1648-5P
TACAGGACTCTGCAACTTACCG

>miRNA precursor
CUGAGACCUGGUGCAUCUUU**UACAGGACUCUGCAACUUACCG**AUUCUCGCCGGCGGUCAGUUACUGAGGCCUGUGAAAGACGCAGCAGGGUAAAGACGGUGCUGAAACUCGU
```
......((((.(((.(((((((((((.(((...((((.((((...........)))).))))...))).)))))))))))).))).))))......(((((......)).)))
```

>Predicted STAR seq:
GTCAGTTACTGAGGCCTGTGAA

Complementarity: 15

Initial dG =   -43.40

```
          10        20        30        40
.-CUGAGA|   G   A           A   UGC    U    AUUCU
        CCUG UGC UCUUUUACAGG CUC   AACU ACCG     \
        GGAC ACG AGAAAGUGUCC GAG   UUGA UGGC     C
\ ------^   G   C           G   UCA    C    GGCCG
                80        70        60        50



 90        100
  GUAAAG   -  GC
        ACG GU  U
        UGC CA  G
  ------   U  AA
        110
```

Reads in: lib-42, lib-45, lib-47, lib-48, lib-49, lib-50, lib-72
Number of reads="ID1648",20,8,0,471,83,0,0

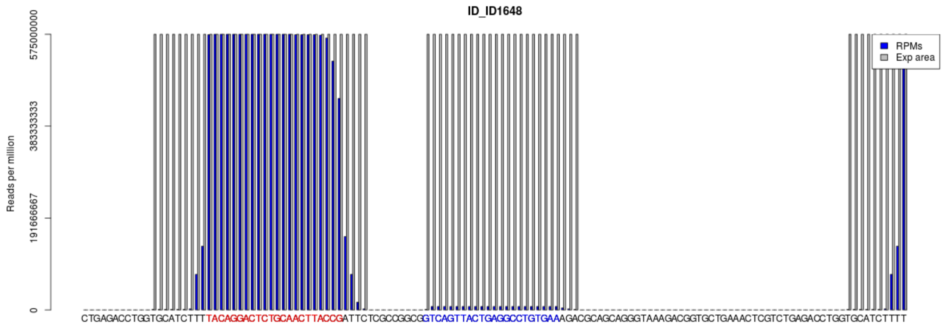

>ID1650
CTGAAGTCCCCGACAGCATAGCGCCTCTGGCCATCTGGTATGTGGGTCACTTGCAGGGGTTTAGGAGCTG**CACGTGACTCACCTACCGGA**
TAGTCAGTGAGACGCCAGGAAC

>ID1650-3P
CACGTGACTCACCTACCGGATAG

>miRNA precursor
CUGAAGUCCCCGACAGCAUAGCGCCUCUGGCCAUCUGGUAUGUGGGUCACUUGCAGGGGUUUAGGAGCUG**CACGUGACUCACCUACCGGAUAG**UCAGUGAGACGCCAGGAAC
(((..((.....))......(((.(((((((.((((((((.(((((((((.(((((...........))))).))))))))).)))))))).)))).)))..))).)))))).....

>Predicted STAR seq:
CATCTGGTATGTGGGTCACTTG

Complementarity: 18

Initial dG =   -46.80

```
              10
----   AA   .-CCCGACA| AU
    CUG  GUC         GC  \
    GAC  CAG         CG  A
CAAG   CG   \ -------^ CG
110
```

Reads in: lib-42, lib-45, lib-47, lib-48, lib-49, lib-50, lib-72
Number of reads="ID1650",2,178,18,24,41,32,0

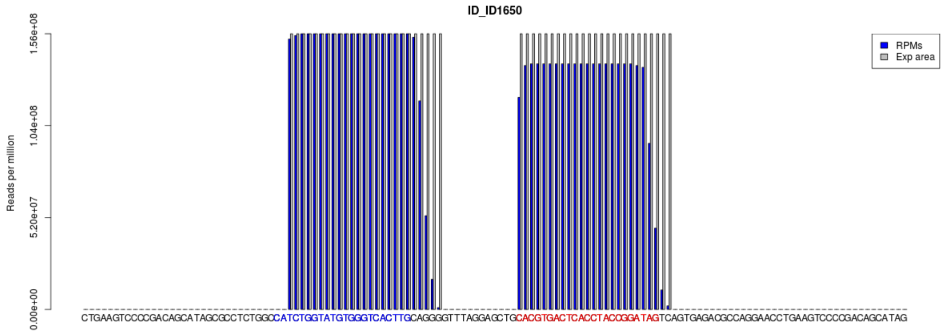

>ID1651

GGTGCTTAGCTGGTCTCTGA**TCAGGTACCAAGAATGTGATTC**GTGTATTATTTCAGTCGAATAACATTCTAGGTATCTCCTCGGATGCCA
TCTAAATTGTAACATTCACA

>ID1651-5P

TCAGGTACCAAGAATGTGATTC

>miRNA precursor

GGUGCUUAGCUGGUCUCUGA**UCAGGUACCAAGAAUGUGAUUC**GUGUAUUAUUUCAGUCGAAUAACAUUCUAGGUAUCUCCUCGGAUGCCAUCUAAAUUGUAACAUUCACA
.....((((.((((.(((((..(((((((.(((((((.(((((((........))..))))).))))))).)))))))..))))).)))).))))...............

>Predicted STAR seq:

ATAACATTCTAGGTATCTCCTC

Complementarity: 16

Initial dG = -36.90

```
                  10        20        30        40
GGUGC----------    C    C     UC       A       G     --| UAU
               UUAG UGGU UCUGA  AGGUACC AGAAUGU AUUCG  UG   U
               AAUC ACCG AGGCU  UCUAUGG UCUUACA UAAGC  AC   A
ACACUUACAAUGUUA    U    U     CC       A       A     UG^ UUU
.       100        90        80        70        60        50
```

Reads in: lib-42, lib-45, lib-47, lib-48, lib-49, lib-50, lib-72

Number of reads="ID1651",0,2,8,1,2,5,0

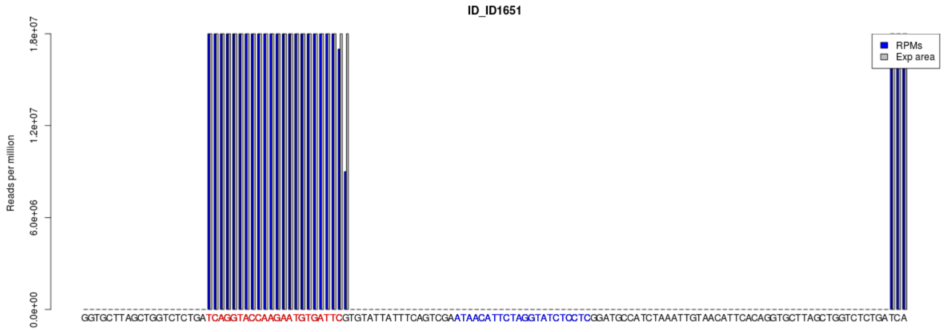

>ID1652
AACGACGAGTCGCTGCAGTGCTCTGGTCCCTGAGAAAGTGCCAAGTGCGACATTCGCGTTTTTCGAGGCG**AATCTCTCTCCTGACACTGTCT**CAGTAGCCAGGGAGGACAGA

>ID1652-3P
AATCTCTCTCCTGACACTGTCT

>miRNA precursor
AACGACGAGUCGCUGCAGUGCUCUGGUCCCUGAGAAAGUGCCAAGUGCGACAUUCGCGUUUUUCGAGGCG**AAUCUCUCUCCUGACACUGUCU**CAGUAGCCAGGGAGGACAGA
```
..(((....)))(((.....(((((((..((((((.((((.((.....((.((((((.((....)).)))))).)).....)).)))).))))))..))))))).....))).
```

>Predicted STAR seq:
GAAAGTGCCAAGTGCGACATT

Complementarity: 12
ERROR 4: Strange structure
Initial dG = -39.90

```
.-AA|  C
    CGA G
    GCU A
\ --^  G
     10


        20        30        40        50        60
  -   CAGUG       CC       A    C  AGUGC  C      G  U
   CUG     CUCUGGU  CUGAGA AGUG CA     GA AUUCGC UU U
   GAC     GGGACCG  GACUCU UCAC GU     CU UAAGCG AG U
  A   AGGA-       AU       G    A  CCUCU  C      G  C
  110       100       90        80        70
```

Reads in: lib-42, lib-45, lib-47, lib-48, lib-49, lib-50, lib-72
Number of reads="ID1652",0,4,8,0,1,2,0

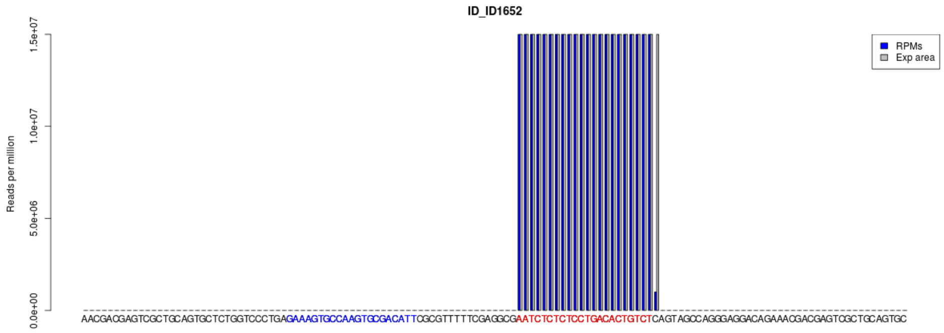

>ID1653
TGAGGTCAATAAGTGCTCTTGGTGTCTGGGAGAGTGTCAGGAATGTGACTCGTGGCTTATTTGGAAATCG**AAGCACATTTCTGACACTGT**
CACGGCTGCCAGGTGATTCTT

>ID1653-3P
AAGCACATTTCTGACACTGTCA

>miRNA precursor
UGAGGUCAAUAAGUGCUCUUGGUGUCUGGGAGAGUGUCAGGAAUGUGACUCGUGGCUUAUUUGGAAAUCG**AAGCACAUUUCUGACACUGUCA**CGGCUGCCAGGUGAUUCUU
.(((((((.........((((((.(((.((.(((((((((((((((..(((...((.....))....)))..))))))))))))))).)).))).))))))))))).)))

>Predicted STAR seq:
GAGAGTGTCAGGAATGTGA

Complementarity: 16

Initial dG = -43.50

```
         10        20        30        40        50
U   -|   AUAAGUGCU       U   G  G               AC   UGG-  UA
 GAG GUCA         CUUGGUG CUG GA AGUGUCAGGAAUGUG  UCG    CU  \
 UUC UAGU         GGACCGU GGC CU UCACAGUCUUUACAC  AGC    GG  U
-   U^   ---------       C   A  G               GA   UAAA  UU
110             100        90        80        70
```

Reads in: lib-42, lib-45, lib-47, lib-48, lib-49, lib-50, lib-72
Number of reads="ID1653",9,323,152,42,67,46,0

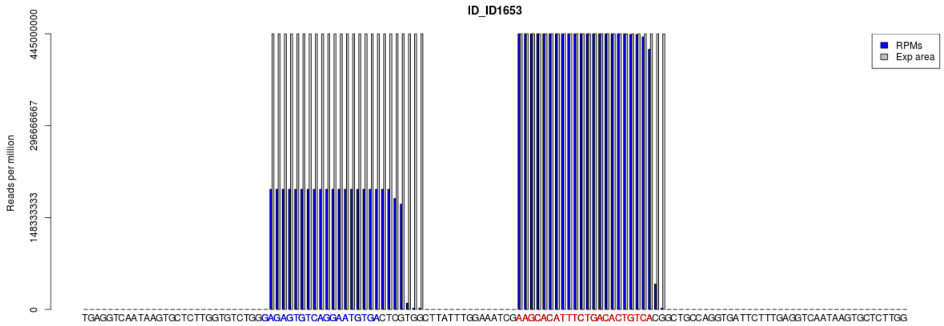

>ID1654
GGAATTCGGTTGGTTGGAAC**TGAAGCTCCTCATATCTGACCT**GTTTTGACGTCACTTGACCAGGTGAGATGTGATGAGCTTCACGTTCCC
ACCTGCAATTTCCAACTTCTTC

>ID1654-5P
TGAAGCTCCTCATATCTGACCT

>miRNA precursor
GGAAUUCGGUUGGUUGGAAC**UGAAGCUCCUCAUAUCUGACCU**GUUUUGACGUCACUUGACCAGGUGAGAUGUGAUGAGCUUCACGUUCCCACCUGCAAUUUCCAACUUCUUC
((((....((.(((.(((((((((((((.((((((((.(((((.......(((....)))))))).)))))))).)))))))).))))).))).))...))))).........

>Predicted STAR seq:
GTGAGATGTGATGAGCTTCACGT

Complementarity: 18

Initial dG =   -52.70

```
                 10        20         30        40        50
---------|   UUCG  U   U     -        C        G     UUUUGAC   A
         GGAA    GU GGU GGAAC UGAAGCUC UCAUAUCU ACCUG       GUC C
         CCUU    CG CCA CCUUG ACUUCGAG AGUGUAGA UGGAC       CAG U
CUUCUUCAA^   UAA-  U   C     C        U        G     -------   U
110       100        90        80        70              60
```

Reads in: lib-42, lib-45, lib-47, lib-48, lib-49, lib-50, lib-72
Number of reads="ID1654",2,25,8,2,8,0,0

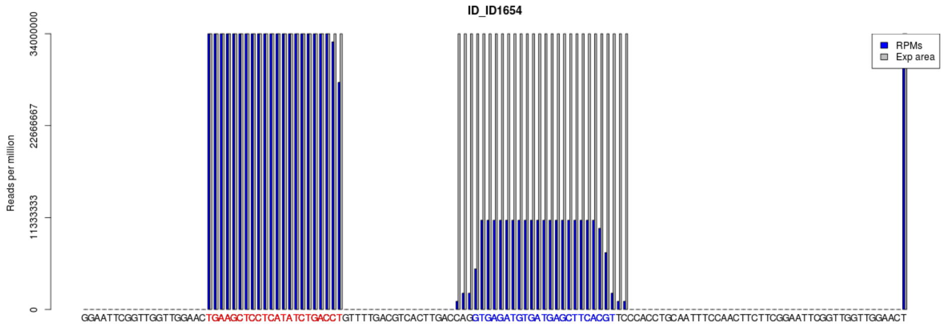

>ID1655
GATCCCAGAACGGCGCAGGGCACCCCCCGCCAACCAGCACGTGGGACTGAGGACACTGCCATCAGAGCTG**TCCTGTTGCACCTGTTGGTTGAC**GGTGGGCAGCCACTACA

>ID1655-3P
TCCTGTTGCACCTGTTGGTTGAC

>miRNA precursor
GAUCCCAGAACGGCGCAGGGCACCCCCCGCCAACCAGCACGUGGGACUGAGGACACUGCCAUCAGAGCUG**UCCUGUUGCACCUGUUGGUUGAC**GGUGGGCAGCCACUACA
................(((((..((((((.(((((((((.(((.(((..((((((((........)).))))))))).))).))))))))).))).)))..))).))...

>Predicted STAR seq:
CCAACCAGCACGTGGGACTGAGGA

Complementarity: 18

Initial dG =   -46.40

```
        10         20         30        40        50         60
GAUCCCAGAACGGCGC  -   AC   -|  C         C   G   UG      -  GCC
                AG GGC  CCC CCG CAACCAGCA GUG GAC  AGGACA CU   A
                UC CCG  GGG GGC GUUGGUUGU CAC UUG  UCCUGU GA   U
ACA-------------  A   AC   U^  A         C   G   --      C  GAC
.                    100        90        80          70
```

Reads in: lib-42, lib-45, lib-47, lib-48, lib-49, lib-50, lib-72
Number of reads="ID1655",6,213,168,16,47,21,0

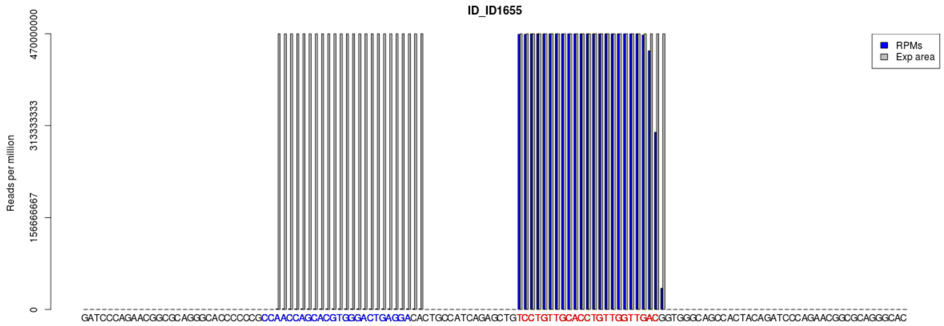

>ID1656
AAACGGATTTTGGCGGTTGCCATATTCCGCCACTGAGCCTTGACGCCAGAACGCTGGGACTCTAGAGACG**TTCTGGCTCAATGCTCAGTC**
GCGGCAGACGGCACAATC

>ID1656-3P
TTCTGGCTCAATGCTCAGTCG

>miRNA precursor
AAACGGAUUUUGGCGGUUGCCAUAUUCCGCCACUGAGCCUUGACGCCAGAACGCUGGGACUCUAGAGACG**UUCUGGCUCAAUGCUCAGUCG**CGGCAGACGGCACAAUC
.....((((.((.((.(((((.......((.(((((((.((((.(((((((((((((....))))...))))))))))))).))))))).))))))).)).)).))))

>Predicted STAR seq:
CACTGAGCCTTGACGCCAGAA

Complementarity: 17

Initial dG = -48.60

```
        10         20         30         40         50
AAACG|   U   G   G      AUAUUCC   C         C    C          ---    G
     GAUU UG CG UUGCC          GC ACUGAGC UUGA GCCAGAACG   CUGG A
     CUAA AC GC GACGG          CG UGACUCG AACU CGGUCUUGC   GAUC C
-----^   C   G   A      -------   C         U    -          AGA    U
           100                90         80          70         60
```

Reads in: lib-42, lib-45, lib-47, lib-48, lib-49, lib-50, lib-72
Number of reads="ID1656",1,5,6,1,5,2,0

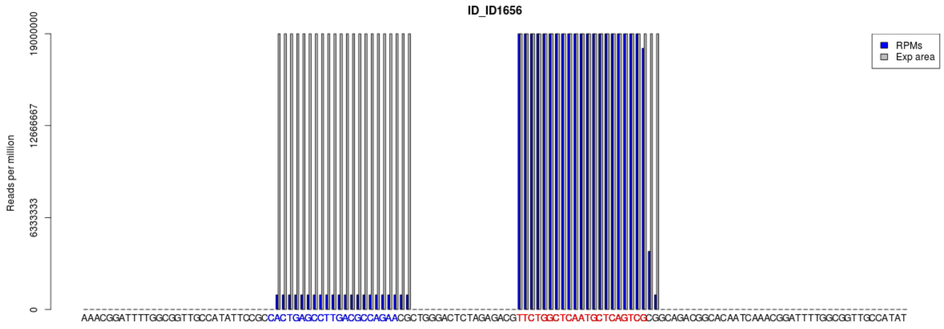

>ID1657
CTGACGTCCATTACAGGCAGTGCGACTCTGGCAATCTGGTAGGTGGGTCAGTCTCAGGGGTTTAGAACTG**GAACTGACTCAACTACCGGA**
CAGTCAGTGAGACGCCAGA

>ID1657-3P
GAACTGACTCAACTACCGGACAG

>miRNA precursor
CUGACGUCCAUUACAGGCAGUGCGACUCUGGCAAUCUGGUAGGUGGGUCAGUCUCAGGGGUUUAGAACUG**GAACUGACUCAACUACCGGACAG**UCAGUGAGACGCCAGA
(((.((((..((((.(((..(((.......))).((((((((.(((((((((..(((..........)))..))))))))).))))))))..))).)))))))).))).

>Predicted STAR seq:
AGTGCGACTCTGGCAATCTGGTAGGTGGGTC

Complementarity: 16

Initial dG =   -46.60

```
            10
-   A    - .-AUUACAG| A
 CUG CGUC C         GC \
 GAC GCAG G         CG G
A   C    A \ -------^ U
       100
```

Reads in: lib-42, lib-45, lib-47, lib-48, lib-49, lib-50, lib-72
Number of reads="ID1657",3,29,3,5,4,10,0

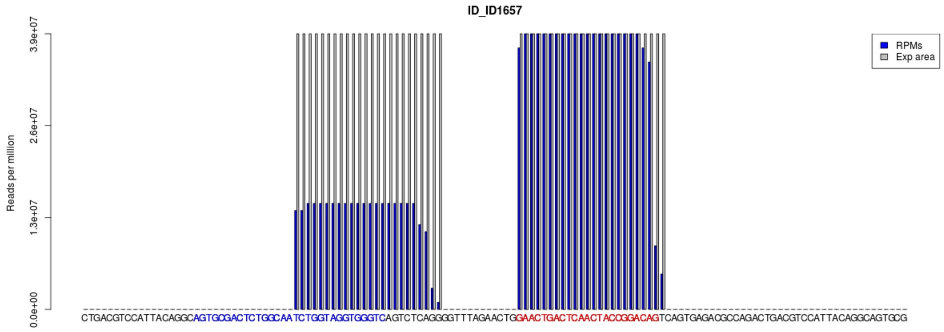

>ID1659
GTAGCCGGGTGGCTGGAACTGAAGCTCATCAGATGTGACCCGCTCTCTCACAAGACCGGGTCACATCTGGTTAGCCTCAGTCCCTCACCA
GCAACTGCCATCTCTCTGT

>ID1659-5P
GAAGCTCATCAGATGTGACCCGC

>miRNA precursor
GUAGCCGGGUGGCUGGAACUGAAGCUCAUCAGAUGUGACCCGCUCUCUCACAAGACCGGGUCACAUCUGGUUAGCCUCAGUCCCUCACCAGCAACUGCCAUCUCUCUGU
((((...(((((..((.(((((.(((.((((((((((((((.(((......))).))))))))))))))).))).))))).))))))).....))))...........

>Predicted STAR seq:
GGTCACATCTGGTTAGCCTCAG

Complementarity: 18

Initial dG =   -52.50

```
                     10        20        30        40
-----------    CCG--|    CU  A     A   C               C   CU
           GUAG     GGUGG  GG ACUGA GCU AUCAGAUGUGACCCG UCU  C
           CGUC     CCACU  CC UGACU CGA UGGUCUACACUGGGC AGA  A
UGUCUCUCUAC    AACGA^    --  C     C   U               C   AC
       100       90          80        70        60        50
```

Reads in: lib-42, lib-45, lib-47, lib-48, lib-49, lib-50, lib-72
Number of reads="ID1659",0,4,1,1,0,1,0

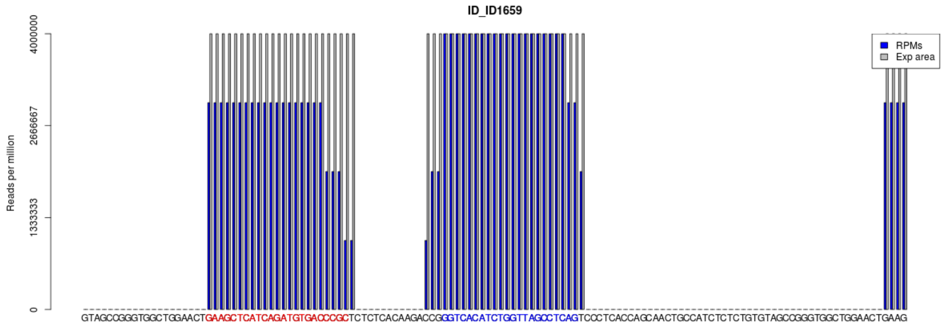

>ID1660

GCGTTATGCGGCGCTGGGGG**TGATGTGAAGCTCGTGCTGCTGA**CTGTGGGACCAGCAGTGGGGGCTACACATCGCCCCCAGTGGCGTCCA
GTCAGCAGGGACAGCGGC

>ID1660-5P

TGATGTGAAGCTCGTGCTGCTGA

>miRNA precursor

GCGUUAUGCGGCGCUGGGGG**UGAUGUGAAGCUCGUGCUGCUGA**CUGUGGGACCAGCAGUGGGGGCUACACAUCGCCCCCAGUGGCGUCCAGUCAGCAGGGACAGCGGC
.......(((.(((((((((((((((.(((((.((((((((..........)))))))).))))).))))))))))))))).)))...(((.((.......)))))

>Predicted STAR seq:

GCAGTGGGGGCTACACATCGCC

Complementarity: 18

Initial dG =   -64.60

```
             10        20        30        40
---  .-GUUAU|  G               A     G        ACUG
   GC       GCG CGCUGGGGGUGAUGUG AGCUC UGCUGCUG    U
   CG       UGC GUGACCCCCGCUACAC UCGGG GUGACGAC    G
CGG  \ -----^  G               A     G        CAGG
                  80        70        60        50



        90
       CCA   AG
          GUC  C
          CAG  A
       A--   GG
          100
```

Reads in: lib-42, lib-45, lib-47, lib-48, lib-49, lib-50, lib-72

Number of reads="ID1660",4,74,13,7,29,16,0

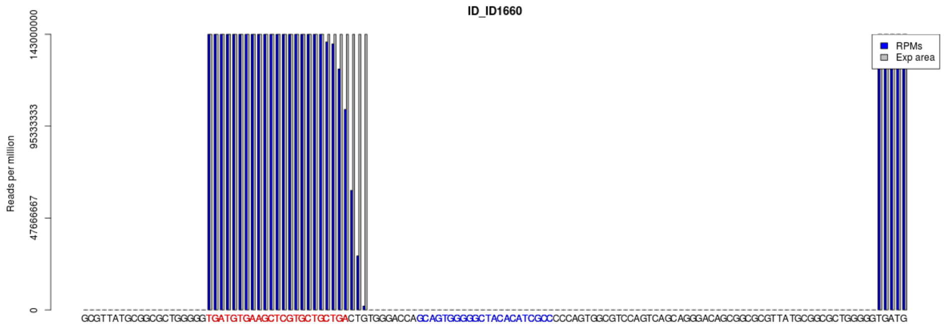

>ID1661
AACTAACATTCAGCTGGGTC**TGATGCTGCAGGAGTTGTTGTGA**TCCTCCAGTGATGCCAACAGCTCCGGCAGTGTCGGATCCAGCAGAAC
GCTATCGCCGGTGTCGTCG

>ID1661-5P
TGATGCTGCAGGAGTTGTTGTGA

>miRNA precursor
AACUAACAUUCAGCUGGGUC**UGAUGCUGCAGGAGUUGUUGUGA**UCCUCCAGUGAUGCCAACAGCUCCGGCAGUGUCGGAUCCAGCAGAACGCUAUCGCCGGUGUCGUCG
............((((((((((((((((.((((((((((..(((.......)))..)))))))))).))))))))))))))))).((((((......)))))..)).

>Predicted STAR seq:
CCAACAGCTCCGGCAGTGTCGGA

Complementarity: 18

Initial dG =   -54.00

```
          10        20        30        40
.-AACUAACAUUCA|                A          UG    CUC
              GCUGGGUCUGAUGCUGC GGAGUUGUUG  AUC   \
              CGACCUAGGCUGUGACG CCUCGACAAC  UAG   C
\ ------------^                G          CG    UGA
                  80        70        60        50



       90
  A  --     AU
   GA  ACGCU  C
   CU  UGUGG  G
  G  GC     CC
         100
```

Reads in: lib-42, lib-45, lib-47, lib-48, lib-49, lib-50, lib-72
Number of reads="ID1661",8,45,85,21,30,19,0

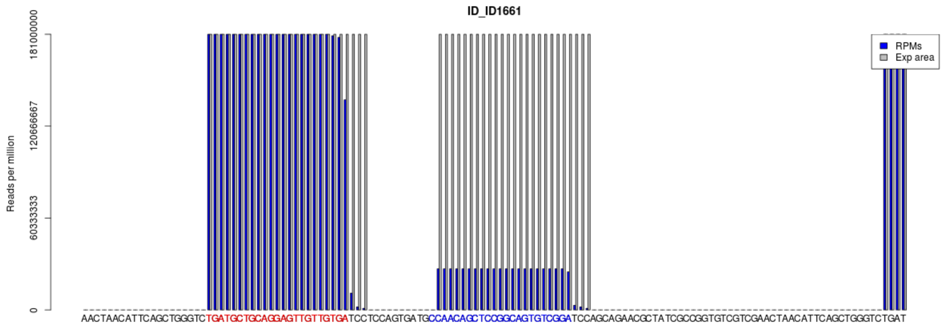

>ID1662
GATCCCAGAACGACGCAGGGCACCCCCCGCCAACCAGCACGTGGGACTGAGGACACTGCCATCAGAGCTG**TCCTGTTGCACCTGTTGGTT**
GACGGTGGGCAGCCCCTACA

>ID1662-3P
TCCTGTTGCACCTGTTGGTTGAC

>miRNA precursor
GAUCCCAGAACGACGCAGGGCACCCCCCGCCAACCAGCACGUGGGACUGAGGACACUGCCAUCAGAGCUG**UCCUGUUGCACCUGUUGGUUGAC**GGUGGGCAGCCCCUACA
................((((..((((((.(((((((((.(((.(((..((((((((........)).))))))))).))).))))))))).))).)))..)))).....

>Predicted STAR seq:
CCAACCAGCACGTGGGACTGAGGA

Complementarity: 18

Initial dG =   -48.60

```
        10        20         30        40        50         60
GAUCCCAGAACGACGCA    AC   -|  C         C   G   UG      -  GCC
                 GGGC  CCC CCG CAACCAGCA GUG GAC  AGGACA CU   A
                 CCCG  GGG GGC GUUGGUUGU CAC UUG  UCCUGU GA   U
ACAUC------------    AC   U^  A         C   G   --      C  GAC
.                   100        90        80          70
```

Reads in: lib-42, lib-45, lib-47, lib-48, lib-49, lib-50, lib-72
Number of reads="ID1662",5,208,145,19,39,24,0

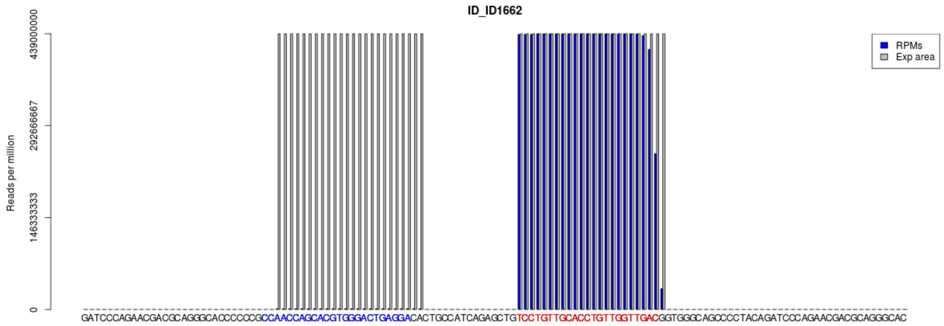

>ID1663
GGTCACTGCTGACGTCTGAG**TTAGCAGAAGGTAAGTGACTCGT**GCCATGAGTGCAAATCGAATCACTTTCCTTCTGCTGACTCAGCTGTC
AGATGCTACATACAGGCA

>ID1663-5P
TTAGCAGAAGGTAAGTGACTCGT

>miRNA precursor
GGUCACUGCUGACGUCUGAG**UUAGCAGAAGGUAAGUGACUCGU**GCCAUGAGUGCAAAUCGAAUCACUUUCCUUCUGCUGACUCAGCUGUCAGAUGCUACAUACAGGCA
........((((((.((((((((((((((((.((((((.((((((.......)))...))).)))))).))))))))))))))))).))))))..(((.......))).

>Predicted STAR seq:
ATCGAATCACTTTCCTTCTGCTGACT

Complementarity: 19

Initial dG =   -52.20

```
          10        20        30        40
.-GGUCACUG      U                U      C   ---|  CAU
          CUGACG CUGAGUUAGCAGAAGG AAGUGA UCG    UGC   \
          GACUGU GACUCAGUCGUCUUCC UUCACU AGC    ACG   G
\ --------      C                U      A   UAA^  UGA
           90        80        70        60        50



       100
  AU   ACA
    GCU   \
    CGG   U
  A-   ACA
```

Reads in: lib-42, lib-45, lib-47, lib-48, lib-49, lib-50, lib-72
Number of reads="ID1663",4,157,24,16,60,31,0

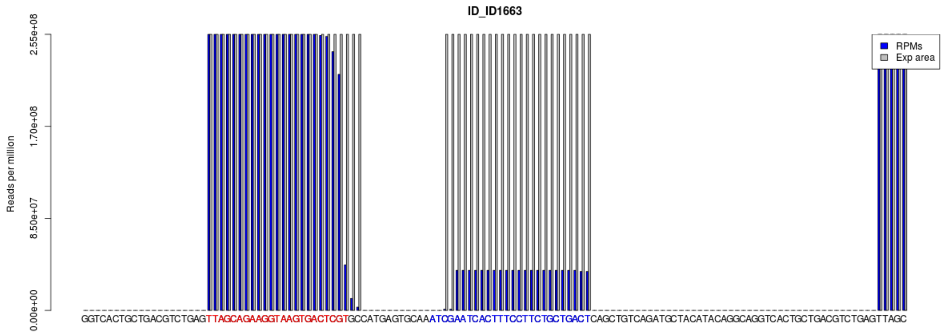

>ID1664
TCAAGGACATACAGAGAGGCAGTCCCCATCAACTGGGTTTTGGGCACAAGAACGTTGCCAGATCTTGCCG**CCCTGGTCCAACACCCAGTC**
GATGGGGTTGTCAGTTCTTTCT

>ID1664-3P
CCCTGGTCCAACACCCAGTCGA

>miRNA precursor
UCAAGGACAUACAGAGAGGCAGUCCCCAUCAACUGGGUUUUGGGCACAAGAACGUUGCCAGAUCUUGCCG**CCCUGGUCCAACACCCAGUCGA**UGGGGUUGUCAGUUCUUUCU
............((((.(((((.(((((((.(((((((.((((((.((.(..((..((........))))..))))))))).))))))).))))))))))))..))))....

>Predicted STAR seq:
CAACTGGGTTTTGGGCACAA

Complementarity: 16

Initial dG =   -43.40

```
        10         20        30        40        50        60
UCAAGGACAUAC    A-|    U       A       U      A  A AA  UU  CAG
            AGAG  GGCAG CCCCAUC ACUGGGU UUGGGC CA G  CG  GC   A
            UCUU  CUGUU GGGGUAG UGACCCA AACCUG GU C  GC  CG   U
UCUU--------    GA^    -       C       C      -  - CC  --  UUC
110               100        90        80          70
```

Reads in: lib-42, lib-45, lib-47, lib-48, lib-49, lib-50, lib-72
Number of reads="ID1664",2,2,0,2,0,0,0

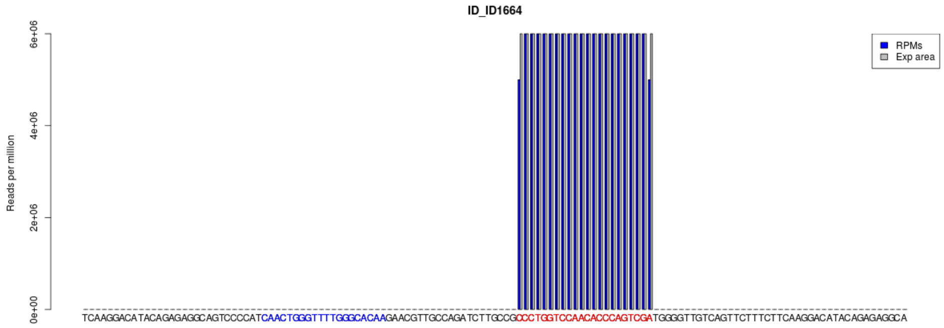

>ID1665
GAGATCTGACGTAGCCAGTTGGCTGGAACTCAAGCTTATCAAATATGTCTTGCTGTAGCGTCACGTGACC**AGGACACATCTGATAAGCTT**
CGCTTCCCCAACTGAAACTTC

>ID1665-3P
AGGACACATCTGATAAGCTTCG

>miRNA precursor
GAGAUCUGACGUAGCCAGUUGGCUGGAACUCAAGCUUAUCAAAUAUGUCUUGCUGUAGCGUCACGUGACC**AGGACACAUCUGAUAAGCUUCG**CUUCCCCAACUGAAACUUC
```
...............(((((((..((((...((((((((((.((.(((((((..((.(((...))).))))))))).)).))))))))))...)))))))))))).......
```

>Predicted STAR seq:
TCAAGCTTATCAAATATGTCTT

Complementarity: 16

Initial dG = -36.90

```
        10        20        30        40        50        60
GAGAUCUGACGUAGC|      CU    CUC          A  A       CU  A   U
               CAGUUGG  GGAA   AAGCUUAUCA AU UGUCUUG  GU GCG \
               GUCAACC  CCUU   UUCGAAUAGU UA ACAGGAC  CA UGC C
CUUCAAA--------^      --    CGC          C  C       --  G   A
 .               100         90        80        70
```

Reads in: lib-42, lib-45, lib-47, lib-48, lib-49, lib-50, lib-72
Number of reads="ID1665",0,1,0,2,2,2,0

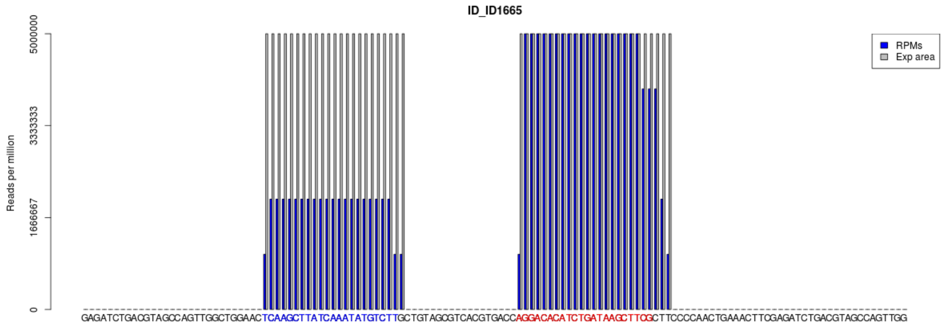

>ID1666
GGTCTCTGCCAGCGTCTGTG**ATAGTAACGGCGATGCGTCTCG**TGTCCTGGTTGCAAATCGAAACACATTACCGTTACTTTCACAGCCGCT AGGCACTTTCCAGTTAATCA

>ID1666-5P
ATAGTAACGGCGATGCGTCTCG

>miRNA precursor
GGUCUCUGCCAGCGUCUGUG**AUAGUAACGGCGAUGCGUCUCG**UGUCCUGGUUGCAAAUCGAAACACAUUACCGUUACUUUCACAGCCGCUAGGCACUUUCCAGUUAAUCA
((....((((((((.((((((.((((((((..(((.((.((((((.......)))...))).)).))).)))..)))))))).)))))).)))).)))).....)).........

>Predicted STAR seq:
AAACACATTACCGTTACTTTCA

Complementarity: 15

Initial dG = -45.90

```
                 10         20        30        40
---------  UCUC    -    U      U        CG   C  C   ---|  CCU
         GG    UGCC AGCG CUGUGA AGUAACGG  AUG GU UCG   UGU   \
         CC    ACGG UCGC GACACU UCAUUGCC  UAC CA AGC   ACG   G
ACUAAUUGA  UUUC    A    C      U        AU   A  A   UAA^  UUG
.       100        90        80        70        60        50
```

Reads in: lib-42, lib-45, lib-47, lib-48, lib-49, lib-50, lib-72
Number of reads="ID1666",3,69,22,7,16,14,0

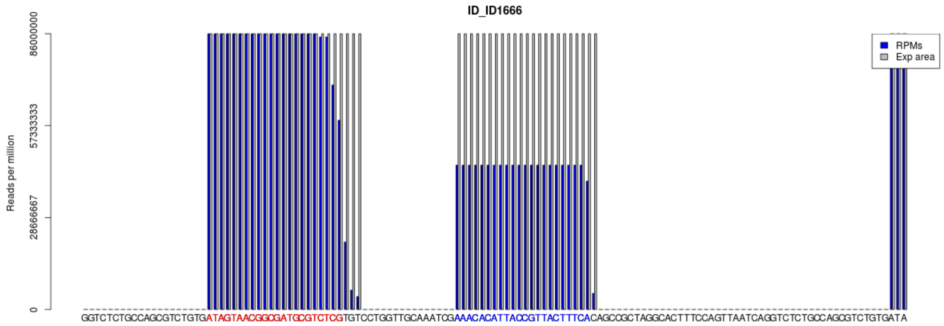

>ID1667
TCGGGCAGCTGGTCTGCGTG**TCCTGAGCCACGGTACAGGACG**TGGTTAGCGCCGTTCTGTCCCGCCGCTCAGACACGCAGCCCAGCACCG
CCGTCGGTCGTCGGAGCGACCAG

>ID1667-5P
TCCTGAGCCACGGTACAGGACG

>miRNA precursor
UCGGGCAGCUGGUCUGCGUG**UCCUGAGCCACGGUACAGGACG**UGGUUAGCGCCGUUCUGUCCCGCCGCUCAGACACGCAGCCCAGCACCGCCGUCGGUCGUCGGAGCGACCAG
...(((.(((((.(((((((((.(((((..(((.((((((((((.....)).)))))))).)))..)))))))))))))).)))))...)))...((((((....))))))..

>Predicted STAR seq:
TTCTGTCCCGCCGCTCAGACA

Complementarity: 16

Initial dG = -65.80

```
            10        20        30        40
.-UCG   A--|    U         C     CA   U         -  GU
     GGC   GCUGG CUGCGUGUC UGAGC  CGG ACAGGACG UG  \
     CCG   CGACC GACGCACAG ACUCG  GCC UGUCUUGC GC  U
\ ---   CCA^    C         -     CC   C         C  GA
      90        80         70        60        50



       100
  GUC      C
     GGUCGU G
     CCAGCG G
  GA-      A
    110
```

Reads in: lib-42, lib-45, lib-47, lib-48, lib-49, lib-50, lib-72
Number of reads="ID1667",3,48,4,11,6,5,0

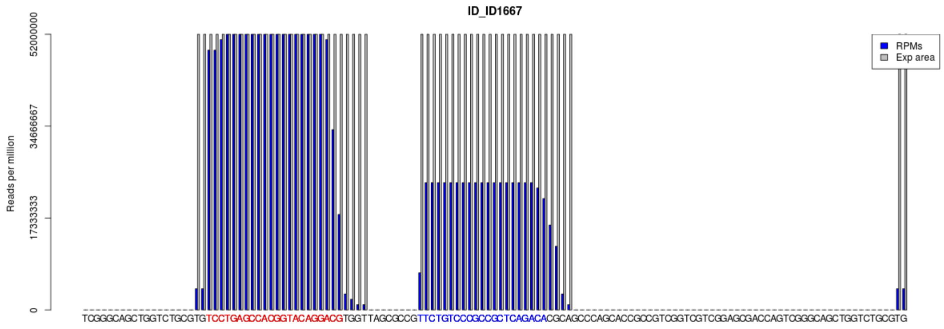

>ID1668
GCGAGACCCCCATTGTATTGCTCGCTTCCAGGGGCTACGTGCAGTTCTTGCTGCTGACTGGGAAAAATGC**TGCGAGAGCTGTACATGACC**
CCTGGGGGCGCGCAGTCAGC

>ID1668-3P
TGCGAGAGCTGTACATGACCC

>miRNA precursor
GCGAGACCCCCAUUGUAUUGCUCGCUUCCAGGGGCUACGUGCAGUUCUUGCUGCUGACUGGGAAAAAUGC**UGCGAGAGCUGUACAUGACCC**CUGGGGGCGCGCAGUCAGC
```
............(((.(((((.((((((((((((.((.(((((((((((((.((..............)).)))))))))))))).)).))))))))))))).)))))))).
```

>Predicted STAR seq:
GGCTACGTGCAGTTCTTGC

Complementarity: 16

Initial dG =   -56.90

```
        10        20        30        40        50        60
GCGAGACCCCCA|  U     U            C  C             U  UGACUG
            UUG AUUGC CGCUUCCAGGGG UA GUGCAGUUCUUGC GC      G
            GAC UGACG GCGGGGGUCCCC GU CAUGUCGAGAGCG CG      G
C-----------^  -     C            A  A             U  UAAAAA
.                   100        90        80        70
```

Reads in: lib-42, lib-45, lib-47, lib-48, lib-49, lib-50, lib-72
Number of reads="ID1668",0,3,18,1,1,0,0

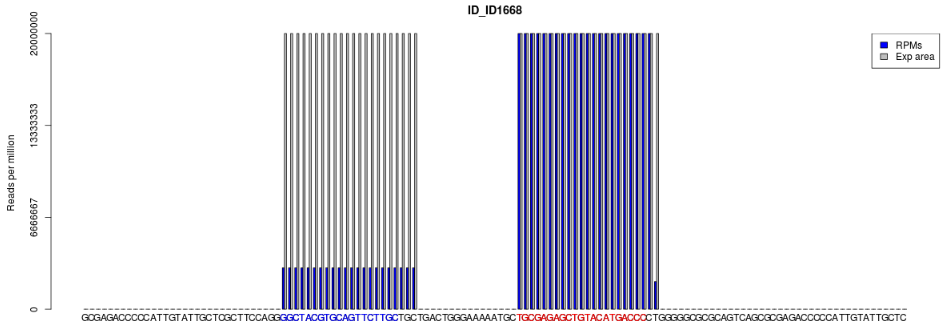

>ID167
GATGGAAGAAGATTTCTGAT**GTGAGAAGATAGCACTCTAGTT**GTGGTAATTTTGTTCAGATAGAGTGAAGTTTTCTCACAGCAGCATTCT
TCATGTTTGGTTGGTGAAGCAA

>ID167-5P
GTGAGAAGATAGCACTCTAGTT

>miRNA precursor
GAUGGAAGAAGAUUUCUGAU**GUGAGAAGAUAGCACUCUAGUU**GUGGUAAUUUUGUUCAGAUAGAGUGAAGUUUUCUCACAGCAGCAUUCUUCAUGUUUGGUUGGUGAAGCAA
```
.......(((((...(((.(((((((((((..(((((((.(((.............))).)))))))..)))))))))))).))).....))))).(((((........))))).
```

>Predicted STAR seq:
ATAGAGTGAAGTTTTCTCACAG

Complementarity: 17

Initial dG =   -37.00

```
          10        20        30        40
.-GAUGGAA|    UUU   A           AG      G   UGGUAA
         GAAGA   CUG UGUGAGAAGAU  CACUCUA UUG      \
         CUUCU   GAC ACACUCUUUUG  GUGAGAU GAC      U
\ -------^    UAC   G           AA      A   UUGUUU
          90        80        70        60



       100
  A     GGU
   UGUUU   U
   ACGAA   G
  A     GUG
  110
```

Reads in: lib-42, lib-45, lib-47, lib-48, lib-49, lib-50, lib-72
Number of reads="ID167",262,135,57,280,203,41,0

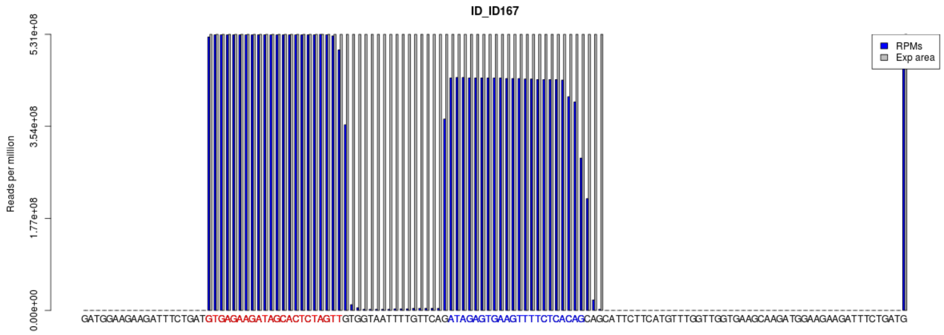

>ID1670
GTAACCTAGGAGCCATCCGA**TGAGGTATCCAGGATGAGATT**CGTTTAATAGAAAGAATACCATTCTCGATGCCTCTTCGGATGGCATCTA
CATTCGACGTTCGCAGCT

>ID1670-5P
TGAGGTATCCAGGATGAGATT

>miRNA precursor
GUAACCUAGGAGCCAUCCGA**UGAGGUAUCCAGGAUGAGAUU**CGUUUAAUAGAAAGAAUACCAUUCUCGAUGCCUCUUCGGAUGGCAUCUACAUUCGACGUUCGCAGCU
......(((..(((((((((.((((((((.((((((..((((.(((....))).))))..)))))).)))))))).)))))))))..)))..................

>Predicted STAR seq:
TACCATTCTCGATGCCTCTTC

Complementarity: 15

Initial dG =   -44.70

```
                    10        20        30        40
GUAACC------------|  GA         U        C      AG    G   A
                  UAG  GCCAUCCGA GAGGUAUC AGGAUG  AUUC UUU A
                  AUC  CGGUAGGCU CUCCGUAG UCUUAC  UAAG AAG U
UCGACGCUUGCAGCUUAC^  UA         U        C      CA    A   A
      100       90        80        70        60        50
```

Reads in: lib-42, lib-45, lib-47, lib-48, lib-49, lib-50, lib-72
Number of reads="ID1670",2,35,2,2,7,4,0

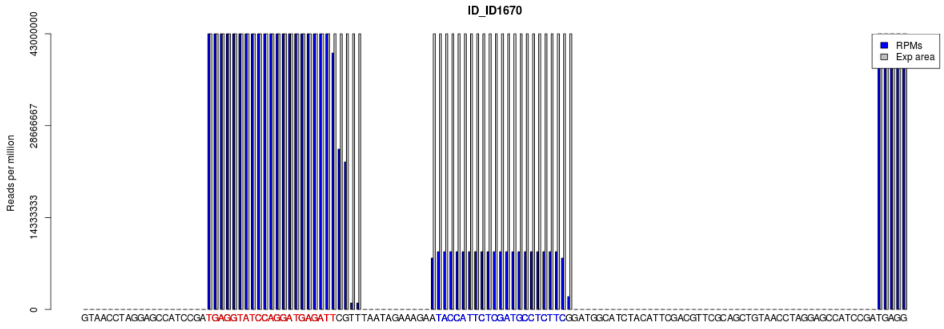

>ID1671
CGGAGACCGGAGTCGACAACTACAGCCTGTGTCTGCAGTCAGGAAAATGGTCCGGTCAGGGGGCGAGTGACC**CGACTGCAGACGCAGACT**
GCAGTGGTGGGCTGCGGCAGT

>ID1671-3P
CGACTGCAGACGCAGACTGCAG

>miRNA precursor
CGGAGACCGGAGUCGACAACUACAGCCUGUGUCUGCAGUCAGGAAAAUGGUCCGGUCAGGGGGCGAGUGACC**CGACUGCAGACGCAGACUGCAG**UGGUGGGCUGCGGCAGU
```
......(((.((((.((.(((.(((.((((((((((((((.((...((.((((.......))))..))..)).))))))))))))))).))).))).)).)))).)))....
```

>Predicted STAR seq:
TACAGCCTGTGTCTGCAGTC

Complementarity: 17

Initial dG =   -55.70

```
        10        20        30        40         50
CGGAGA|  G    G  A   A   C              A  AAA  G-    GGU
      CCG AGUC AC ACU CAG CUGUGUCUGCAGUC GG   AU  GUCC   \
      GGC UCGG UG UGA GUC GACGCAGACGUCAG CC   UG  CGGG   C
UGAC--^  G    G  G   C   A              C  AG-  AG    GGA
 .         100        90        80        70         60
```

Reads in: lib-42, lib-45, lib-47, lib-48, lib-49, lib-50, lib-72
Number of reads="ID1671",0,10,0,0,0,0,0

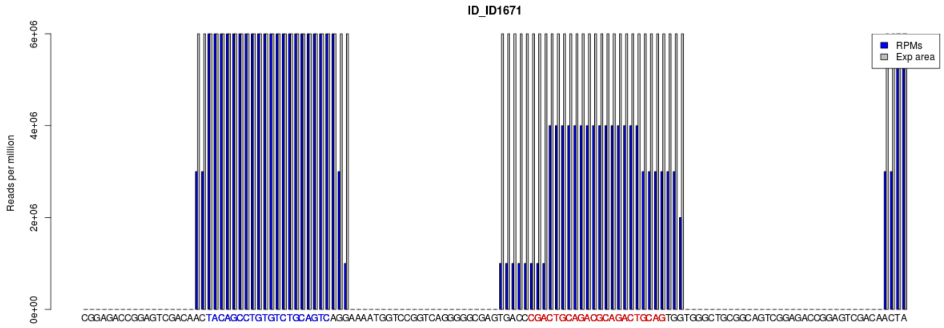

>ID1672
CATCATTATCAGGTAGTGCGCTTTGCTGGCGAAGGAATAGGACAGTCAACCTGCTCAGTTTATGATTTGC**ACTGACTGCCCTATTTCTTT GC**CAGCTAGACGCCAGAAACT

>ID1672-3P
ACTGACTGCCCTATTTCTTTGC

>miRNA precursor
CAUCAUUAUCAGGUAGUGCGCUUUGCUGGCGAAGGAAUAGGACAGUCAACCUGCUCAGUUUAUGAUUUGC**ACUGACUGCCCUAUUUCUUUGC**CAGCUAGACGCCAGAAACU
................(((((..((((((((((((((((.((((((...((((((.....)))...))).)))))).))))))))))))))))).)).)))........

>Predicted STAR seq:
CGAAGGAATAGGACAGTCAA

Complementarity: 18

Initial dG =   -46.00

```
        10        20         30        40        50
CAUCAUUAUCAGGUAGU   -| UU                A      ACC   ---   GU
                 GCG CU  GCUGGCGAAGGAAUAGG CAGUCA   UGC   UCA  \
                 CGC GA  CGACCGUUUCUUUAUCC GUCAGU   ACG   AGU  U
UCAAAGAC---------   A^ U-                C      C--   UUU   AU
 .                100        90        80          70
```

Reads in: lib-42, lib-45, lib-47, lib-48, lib-49, lib-50, lib-72
Number of reads="ID1672",6,87,76,14,28,20,0

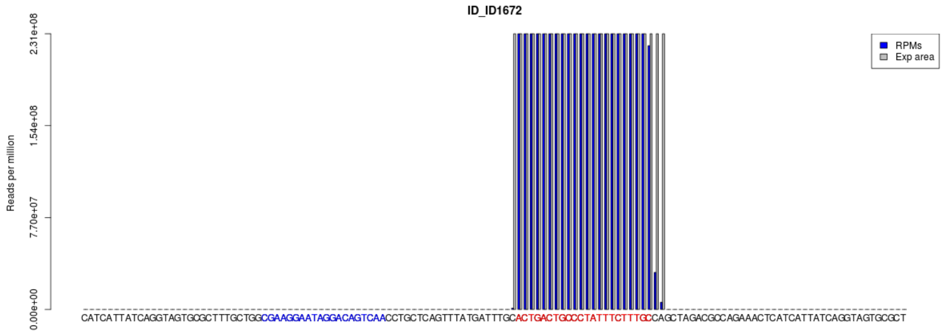

>ID1673
AAACGGCGCATATTGCTGTG**ATCGGAACAAGATGGTCTAATGC**TGATTATGAATTGCATAAACCACCCTGTTTCGACCACAGAGGTATGT
CAATGAGTGTTCCAGAAGACGT

>ID1673-5P
ATCGGAACAAGATGGTCTAATGC

>miRNA precursor
AAACGGCGCAUAUUGCUGUG**AUCGGAACAAGAUGGUCUAAUGC**UGAUUAUGAAUUGCAUAAACCACCCUGUUUCGACCACAGAGGUAUGUCAAUGAGUGUUCCAGAAGACGU
...((..((((((..(((((.((((((((...((((...((((...........))))..))))...)))))))).)))))..))))))...)).((.((....)).))..

>Predicted STAR seq:
ATAAACCACCCTGTTTCGACCA

Complementarity: 14

Initial dG =   -32.50

```
           10        20        30        40
.-AAA  GC-|     UG      A         AGA    CUA     UGAUU
     CG   GCAUAU  CUGUG UCGGAACA   UGGU   AUGC      A
     GU   UGUAUG  GACAC AGCUUUGU   ACCA   UACG      U
\ ---  AAC^     GA      C         CCC    AA-     UUAAG
         90        80        70        60          50


     100
  A-   G  C
    GU UU C
    CA AA A
  UG   G  G
  110
```

Reads in: lib-42, lib-45, lib-47, lib-48, lib-49, lib-50, lib-72
Number of reads="ID1673",8,209,37,21,50,17,0

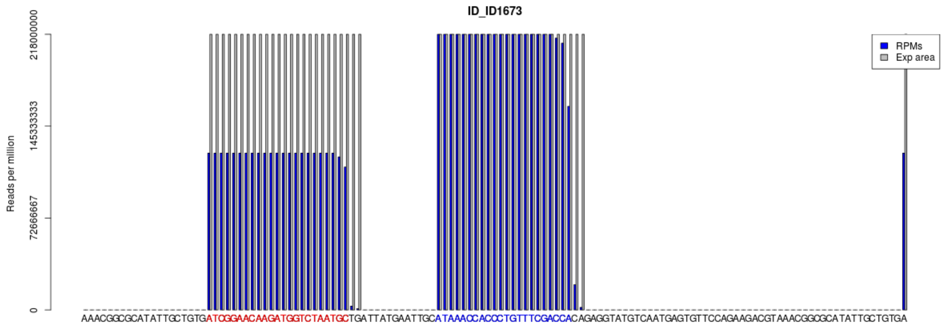

>ID1674
AGGTCCTCAGAGGGGACTCCTGGTGTCTGTGAGAGTCCCAGGCGAGCAATTCTTGGATTTGTCAGAAAAG**AATTGCAGCCCAGGTACTCTCA**CAGCCGCTTGGGAATTCAT

>ID1674-3P
AATTGCAGCCCAGGTACTCTCA

>miRNA precursor
```
AGGUCCUCAGAGGGGACUCCUGGUGUCUGUGAGAGUCCCAGGCGAGCAAUUCUUGGAUUUGUCAGAAAAGAAUUGCAGCCCAGGUACUCUCACAGCCGCUUGGGAAUUCAU
.((((((.....))))))((.((((.((((((((((((..(((..(((((((((............))))))))).)))..)).))))))))))).)))).))........
```

>Predicted STAR seq:
GAGAGTCCCAGGCGAGCAATT

Complementarity: 16
ERROR 4: Strange structure
Initial dG = -51.70

```
.-A|     CA
   GGUCCU  \
   UCAGGG  G
\ -^     GA
```

```
          20        30         40        50        60
  --------  U    U          -  CA   GA         GGAUUU
          CC GGUG CUGUGAGAGU CC  GGC  GCAAUUCUU      \
          GG UCGC GACACUCUCA GG  CCG  CGUUAAGAA      G
  UACUUAAG  U    C          U  AC   A-         AAGACU
 110       100        90        80         70
```

Reads in: lib-42, lib-45, lib-47, lib-48, lib-49, lib-50, lib-72
Number of reads="ID1674",0,13,3,1,3,3,0

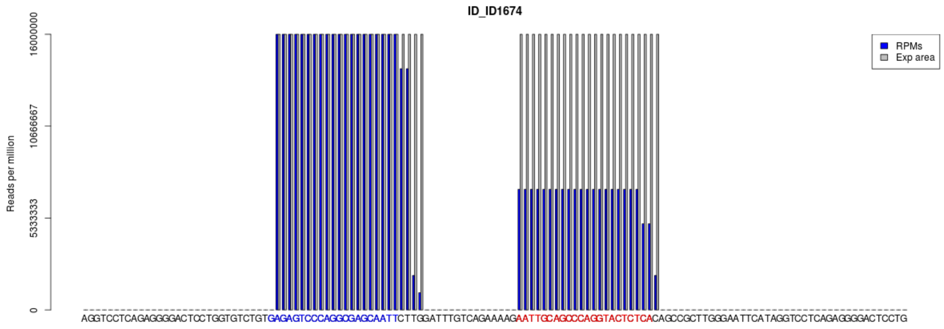

>ID1675
TTCGTGAATGGTGACTCCTGGTGCCTGTG**TCTGTACCAGGAATATGAATC**GTACCTTGTTAGTAAAATCGAAGCATATTCCTGATACAGA
CACAGCTGCCAGGCATTCTT

>ID1675-5P
TCTGTACCAGGAATATGAATC

>miRNA precursor
UUCGUGAAUGGUGACUCCUGGUGCCUGUG**UCUGUACCAGGAAUAUGAAUC**GUACCUUGUUAGUAAAAUCGAAGCAUAUUCCUGAUACAGACACAGCUGCCAGGCAUUCUU
.....((((......((((((..(((((((((((.((((((((((..((((((.......)))....)))..)))))))))).)))))))))))..)))))))))))..

>Predicted STAR seq:
AGCATATTCCTGATACAGACA

Complementarity: 16

Initial dG = -50.00

```
        10        20        30        40        50
UUCGU|    GUGACU      GC          C          AA   ----   CUU
     GAAUG      CCUGGU  CUGUGUCUGUA CAGGAAUAUG  UCG    UAC   \
     CUUAC      GGACCG  GACACAGACAU GUCCUUAUAC  AGC    AUG   G
UU---^    ------      UC          A          GA   UAAA   AUU
.                100       90        80        70        60
```

Reads in: lib-42, lib-45, lib-47, lib-48, lib-49, lib-50, lib-72
Number of reads="ID1675",3,45,8,11,4,6,0

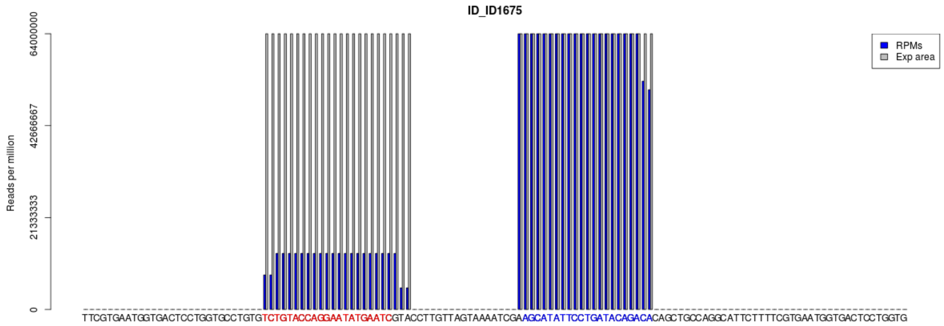

>ID1676
GGAGTCCTCCAGGGGTCTGT**GTGAGTACGCGGGTTGTGATTC**TTGACATTCTCATAAGATGAATCACAGCTCCCGTACTCCCGCAGAAAT
TCGGAGCTTCAACGTGCAATC

>ID1676-5P
GTGAGTACGCGGGTTGTGATTC

>miRNA precursor
GGAGUCCUCCAGGGGUCUGU**GUGAGUACGCGGGUUGUGAUUC**UUGACAUUCUCAUAAGAUGAAUCACAGCUCCCGUACUCCCGCAGAAAUUCGGAGCUUCAACGUGCAAUC
((((..((((..(..((((((.(((((((.((((((((((((.................)))))))))))).))))))).))))))..)..))))))))...........

>Predicted STAR seq:
ATCACAGCTCCCGTACTCCCGC

Complementarity: 18

Initial dG = -48.60

```
                   10        20        30        40        50
-----------|   UC    AG GG      U       C            UUGACAUU
           GGAG  CUCC  G  UCUGUG GAGUACG GGGUUGUGAUUC        C
           CUUC  GAGG  U  AGACGC CUCAUGC CUCGACACUAAG        U
CUAACGUGCAA^   --    CU AA      C       C            UAGAAUAC
 .       100         90        80        70        60
```

Reads in: lib-42, lib-45, lib-47, lib-48, lib-49, lib-50, lib-72
Number of reads="ID1676",1,22,3,7,2,1,0

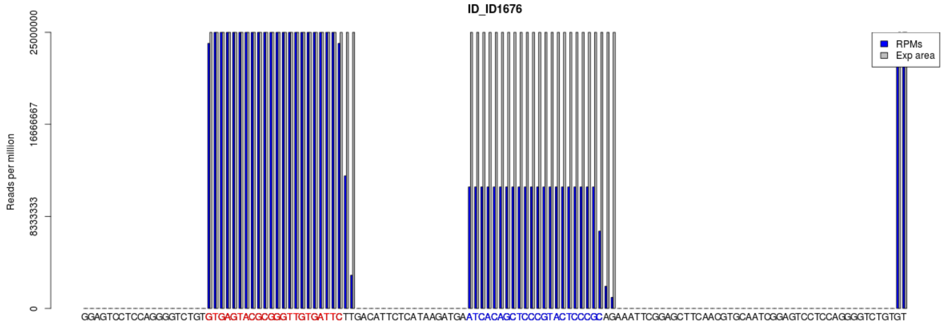

>ID1677
GATGCCAGAACGACGCAGGGCGCCCCCCACCAACCAGGTCTTGGGCCTGAGGGCACTGCCATCAGAGCTG**TCCTGGTCCACCACCTGGTT GA**CGGTGGGCAGCCACTTCAC

>ID1677-3P
TCCTGGTCCACCACCTGGTTGA

>miRNA precursor
GAUGCCAGAACGACGCAGGGCGCCCCCCACCAACCAGGUCUUGGGCCUGAGGGCACUGCCAUCAGAGCUG**UCCUGGUCCACCACCUGGUUGA**CGGUGGGCAGCCACUUCAC
..(((.........))).(((((((.((..(((((((((..((((((.((.(((.(((....))).))).)).))))))..)))))))))..)).)))).))).......

>Predicted STAR seq:
CCAACCAGGTCTTGGGCCTGA

Complementarity: 15
ERROR 4: Strange structure
Initial dG = -53.40

```
.-GA|  CAGA
    UGC    \
    ACG    A
\ --^  CAGC
```

```
         20         30        40         50        60
  G------   -    C  AC         CU      U-  G   A   C
         GGC GCCC CC  CAACCAGGU  UGGGCC  GA GGC CUG C
         CCG CGGG GG  GUUGGUCCA  ACCUGG  CU UCG GAC A
  CACUUCA   A    U  CA         CC      UC  G   A   U
 110       100        90        80        70
```

Reads in: lib-42, lib-45, lib-47, lib-48, lib-49, lib-50, lib-72
Number of reads="ID1677",1,43,15,3,15,13,0

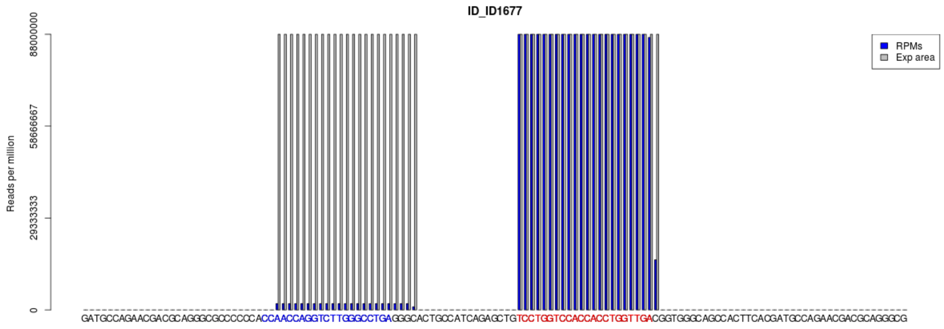

>ID1678
AGCGAGAAGACGCTGCGGTGGTCTGGAGCTTGGGACGGTGTCAGGGGGGAGATTCGCAGTTTCTGGGGCG**TCTCTCACTCCTGACGCTGCC**CCAATTGCCAGGCAGGA

>ID1678-3P
TCTCTCACTCCTGACGCTGCC

>miRNA precursor
AGCGAGAAGACGCUGCGGUGGUCUGGAGCUUGGGACGGUGUCAGGGGGGAGAUUCGCAGUUUCUGGGGCG**UCUCUCACUCCUGACGCUGCC**CCAAUUGCCAGGCAGGA
((((......))))......((((((...(((((.((((((((((((.((((..(((..........)))..)))).))))))))))))).)))))...))))))....

>Predicted STAR seq:
ACGGTGTCAGGGGGGAGA

Complementarity: 15
ERROR 4: Strange structure
Initial dG =   -54.10

```
  |   AG
--AGCG  A
  UCGC  A
\ ^   AG
     10
```

```
      20        30        40        50        60
  GCGGUG      AGC     A           G    UU   AGUU
        GUCUGG   UUGGG CGGUGUCAGGGG GAGA  CGC    U
        CGGACC   AACCC GUCGCAGUCCUC CUCU  GCG    C
  AGGA--      GUU     C           A    CU   GGGU
          100        90        80        70
```

Reads in: lib-42, lib-45, lib-47, lib-48, lib-49, lib-50, lib-72
Number of reads="ID1678",1,84,43,7,34,32,0

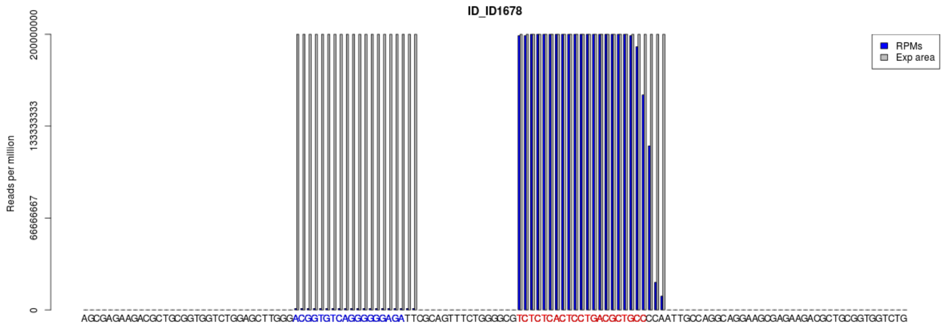

>ID1679
GGATTAGGACAGCGAAAGACAGCTCCCATCATCTGGGTTTTGGGCACCAGAACGTTGCCAGATCTTGCCG**CCCTGGTCCAACACCCAGTC**
GAAGGTGGTTGTCAGTCAACAC

>ID1679-3P
CCCTGGTCCAACACCCAGTCGA

>miRNA precursor
GGAUUAGGACAGCGAAAGACAGCUCCCAUCAUCUGGGUUUUGGGCACCAGAACGUUGCCAGAUCUUGCCG**CCCUGGUCCAACACCCAGUCGA**AGGUGGUUGUCAGUCAACAC
.............((..((((((..((.((..((((((.((((..(((((..((..((........))))..))))))))).))))))..)).))..))))))..)).....

>Predicted STAR seq:
TCATCTGGGTTTTGGGCACCAG

Complementarity: 15

Initial dG =   -40.90

```
        10        20        30        40        50        60
GGAUUAGGACAGC| AA      UC  A  AU      U    GC     AA  UU  CAG
             GA  GACAGC  CC UC  CUGGGU UUGG  ACCAG  CG  GC   A
             CU  CUGUUG  GG AG  GACCCA AACC  UGGUC  GC  CG   U
CACAA--------^ GA      GU  A  CU      C    --     CC  --  UUC
110               100       90        80          70
```

Reads in: lib-42, lib-45, lib-47, lib-48, lib-49, lib-50, lib-72
Number of reads="ID1679",1,2,0,1,0,0,0

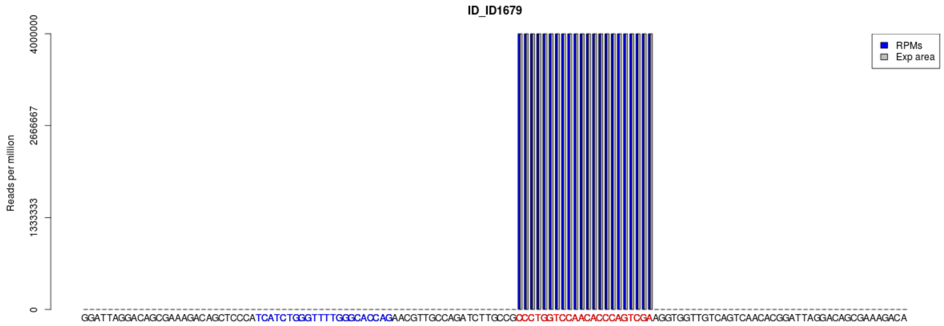

>ID168
GAAGCATTGGCGAATGGAAGAAGTTTTCTGTTGTGAGAAAGCATCACTCTAGTTGTGTTAATTTTGTTCA**TGTAGAGCGAAGTTTTCTCA**
CGGCAGCATTCTTCATGTTT

>ID168-3P
TGTAGAGCGAAGTTTTCTCACGG

>miRNA precursor
GAAGCAUUGGCGAAUGGAAGAAGUUUUCUGUUGUGAGAAAGCAUCACUCUAGUUGUGUUAAUUUUGUUCA**UGUAGAGCGAAGUUUUCUCACGG**CAGCAUUCUUCAUGUUU
...((....)).....((((((.....(((((((((((((((.((.(((((..((.............))..))))).)).)))))))))))))))..))))))......

>Predicted STAR seq:
TGTGAGAAAGCAUCACUCTA

Complementarity: 17
ERROR 4: Strange structure
Initial dG = -37.30

```
.-GAA| A
     GC U
     CG U
\ ---^ G
     10



          20        30        40        50        60
  GAAUG-      GUUUU               A  A     GU  UGUUAA
        GAAGAA     CUGUUGUGAGAAAGC UC CUCUA  UG      \
        CUUCUU     GACGGCACUCUUUUG AG GAGAU  AC      U
  UUUGUA      AC---               A  C     GU  UUGUUU
110       100           90        80        70
```

Reads in: lib-42, lib-45, lib-47, lib-48, lib-49, lib-50, lib-72
Number of reads="ID168",1024,886,556,2525,992,139,0

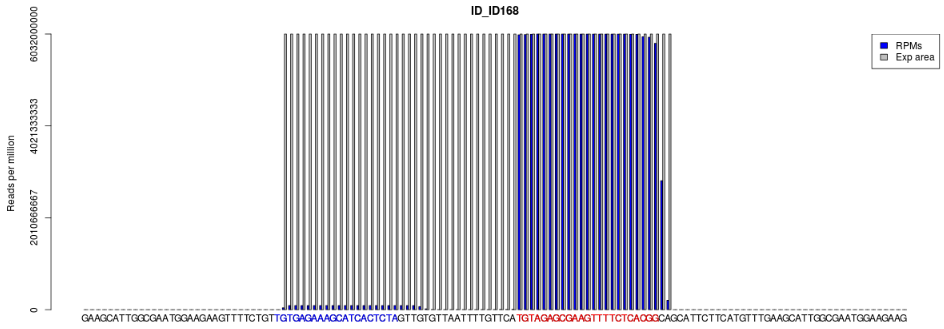

>ID1680
ACATCAATGATAGGCAGTGCGTTTCGCGGGT**GAAGAGATAGAGGAGTCAACT**GCTGGATTTTTGAATTGCACTGACTTCTCCATCTCTTT
GCCAGCGACACGCCAGAAAC

>ID1680-5P
GAAGAGATAGAGGAGTCAACT

>miRNA precursor
ACAUCAAUGAUAGGCAGUGCGUUUCGCGGGU**GAAGAGAUAGAGGAGUCAACU**GCUGGAUUUUUGAAUUGCACUGACUUCUCCAUCUCUUUGCCAGCGACACGCCAGAAAC
```
............(((.(((....((((.((..(((((((.(((((((((..(((..............))).))))))))).)))))))..)).))))))))))......
```

>Predicted STAR seq:
TGACTTCTCCATCTCTTTGCC

Complementarity: 15

Initial dG =   -45.30

```
        10        20        30        40        50        60
ACAUCAAUGAUA|  A   CGUU    G  UG       A         AC   UGGAUU
            GGC GUG    UCGC GG  AAGAGAU GAGGAGUCA  UGC      U
            CCG CAC    AGCG CC  UUCUCUA CUCUUCAGU  ACG      U
CAAAGA------^  -   ----    A  GU       C         C-   UUAAGU
.              100           90         80         70
```

Reads in: lib-42, lib-45, lib-47, lib-48, lib-49, lib-50, lib-72
Number of reads="ID1680",3,86,11,4,24,17,0

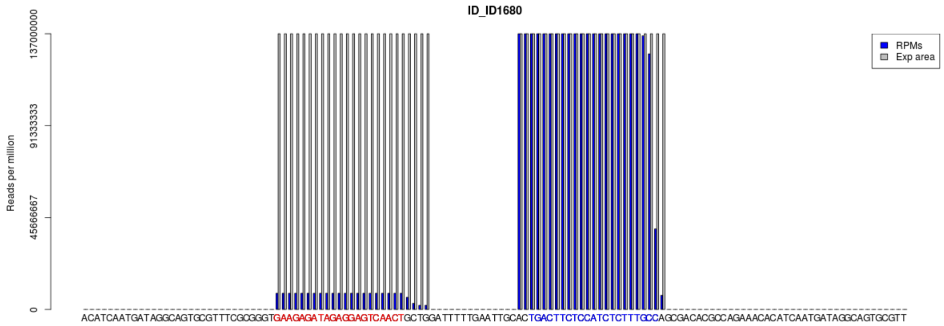

>ID1681
CATACCCAATGCCGAATCGACTGATTATATGAGCGGTCGGCACATATGCATCTACATGTACATATACATG**CAGGTACAAATGCACCGACC**
GCTGGTGTAATCAACTTAC

>ID1681-3P
CAGGTACAAATGCACCGACCGC

>miRNA precursor
CAUACCCAAUGCCGAAUCGACUGAUUAUAUGAGCGGUCGGCACAUAUGCAUCUACAUGUACAUAUACAUG**CAGGUACAAAUGCACCGACCGC**UGGUGUAAUCAACUUAC
....................(((((((((.(((((((((..(((.((.((((.((((((....)))))).)))).)).)))..))))))))).))))))))).......

>Predicted STAR seq:
CGGTCGGCACATATGCATCT

Complementarity: 15

Initial dG = -42.60

```
        10        20        30        40        50        60
CAUACCCAAUGCCGAAUCGAC|        G         CA   A  C    A      C
                     UGAUUAUAU AGCGGUCGG  CAU UG AUCU CAUGUA A
                     ACUAAUGUG UCGCCAGCC  GUA AC UGGA GUACAU U
CAUUCA---------------^        G         AC   A  A    C      A
                      100        90        80        70
```

Reads in: lib-42, lib-45, lib-47, lib-48, lib-49, lib-50, lib-72
Number of reads="ID1681",0,4,0,0,0,0,0

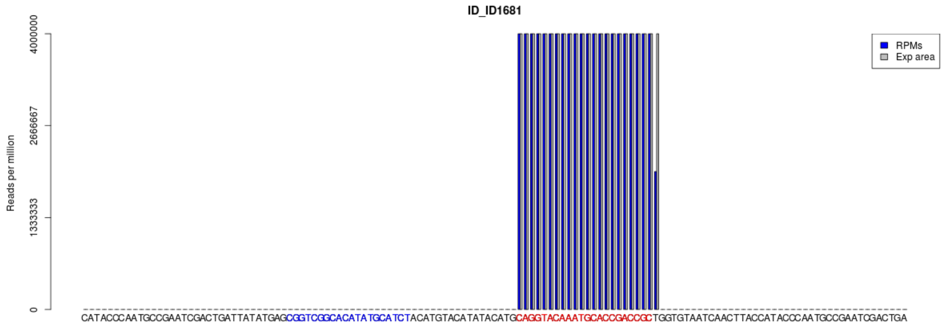

>ID1682
CCCCGGTCGCCCTGGTGGGG**AGGAAGATACGACGGGTCGTTTG**CCTGCTGCCCTGCGCAACGACACTCATATCTTCCACCCCAACGGGGC
GCGAGCGACTGTTTGGAAGACT

>ID1682-5P
AGGAAGATACGACGGGTCGTTTG

>miRNA precursor
CCCCGGUCGCCCUGGUGGGG**AGGAAGAUACGACGGGUCGUUUG**CCUGCUGCCCUGCGCAACGACACUCAUAUCUUCCACCCCAACGGGGCGCGAGCGACUGUUUGGAAGACU
((.((((((((((.(((((.((((((((.((...((((((.((..((......))))))))))..)).)))))))).))))).))))))......)))))...))......

>Predicted STAR seq:
ACGACACTCATATCTTCCACC

Complementarity: 15

Initial dG =   -52.30

```
          10        20        30        40        50
CCCC      |     G     A        C  CGG      U  CU  UG
    GGUC--GCCCUG UGGGG GGAAGAUA GA   GUCGUU GC  GC  C
    UCAG  CGGGGC ACCCC CCUUCUAU CU   CAGCAA CG  CG  C
----    \ ^     A     A        A  CA-      -  --  UC
    110  90        80        70         60
```

```
          G--     G
             CGAGC A
             GUUUG C
          AAG     U
                100
```

Reads in: lib-42, lib-45, lib-47, lib-48, lib-49, lib-50, lib-72
Number of reads="ID1682",6,100,37,17,59,23,0

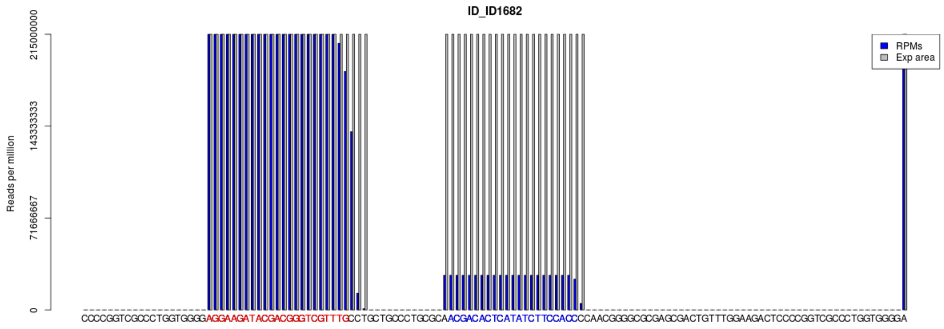

>ID1684
AGCTACTCCTGGTGTCTGCG**ATGGTGTCAGGAATATGAGTCG**TGCCTTGTTTGTAAACCGACACATATTCCTGATACTGACACAGCTGCC
AGGACTCTTCAAGGCCTAAAC

>ID1684-5P
ATGGTGTCAGGAATATGAGTCG

>miRNA precursor
AGCUACUCCUGGUGUCUGCG**AUGGUGUCAGGAAUAUGAGUCG**UGCCUUGUUUGUAAACCGACACAUAUUCCUGAUACUGACACAGCUGCCAGGACUCUUCAAGGCCUAAAC
......((((((((.(((...((((((((((((((((.(((((((.......)))...)))).)))))))))))))))))...))).))))))))................

>Predicted STAR seq:
ACACATATTCCTGATACTGACACA

Complementarity: 18

Initial dG =   -42.40

```
                  10        20        30        40
AGCUAC-----------        U   CGA                A    ---|  CUU
                 UCCUGGUG CUG   UGGUGUCAGGAAUAUG GUCG   UGC   \
                 AGGACCGU GAC   GUCAUAGUCCUUAUAC CAGC   AUG   G
CAAAUCCGGAACUUCUC        C   ACA                A    CAA^  UUU
 .       100       90        80        70        60        50
```

Reads in: lib-42, lib-45, lib-47, lib-48, lib-49, lib-50, lib-72
Number of reads="ID1684",1,40,33,5,10,4,0

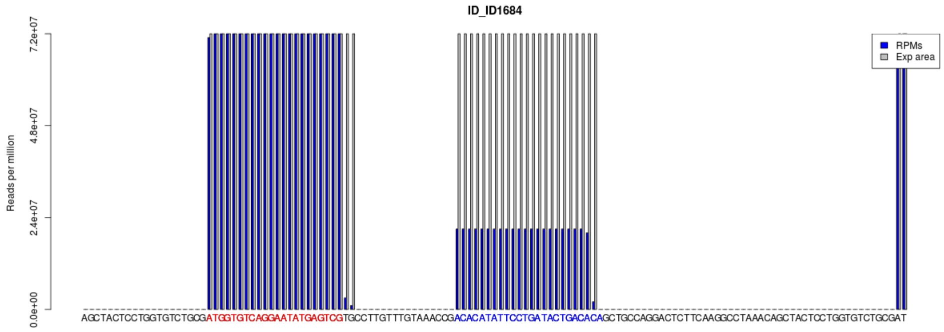

>ID1685
GACGCAGGGCGCACCCCGCC**AACCAGGCGTTGGTCTTCAGGGCA**CTGCCATCAGAGCTGTCCTGGTCCACCACCTGGTTGACAGTGAGCA
GCCACTCCTCTGCTCGCCGT

>ID1685-5P
AACCAGGCGTTGGTCTTCAGGGCA

>miRNA precursor
GACGCAGGGCGCACCCCGCC**AACCAGGCGUUGGUCUUCAGGGCA**CUGCCAUCAGAGCUGUCCUGGUCCACCACCUGGUUGACAGUGAGCAGCCACUCCUCUGCUCGCCGU
...((.(((....))).))((((((((...((((..((((((((((........)).))))))))...))))))))))))...((((((((........))))))))...

>Predicted STAR seq:
TCCTGGTCCACCACCTGGTTGA

Complementarity: 17

```
 Initial dG =    -46.00

          10
ERROR 3: Strange structure
 Initial dG =    -46.00

          10
.-GAC| A   C
     GC GGG G
     CG CCC C
\ ---^ C   A



   20        30         40         50
           CGU    CU-         -  GCC
  --CAACCAGG   UGGU   UCAGGGCA CU   A
    GUUGGUCC   ACCA   GGUCCUGU GA   U
  \         ---    CCU         C  GAC
   80            70         60
```

Reads in: lib-42, lib-45, lib-47, lib-48, lib-49, lib-50, lib-72
Number of reads="ID1685",2,153,37,20,19,11,0

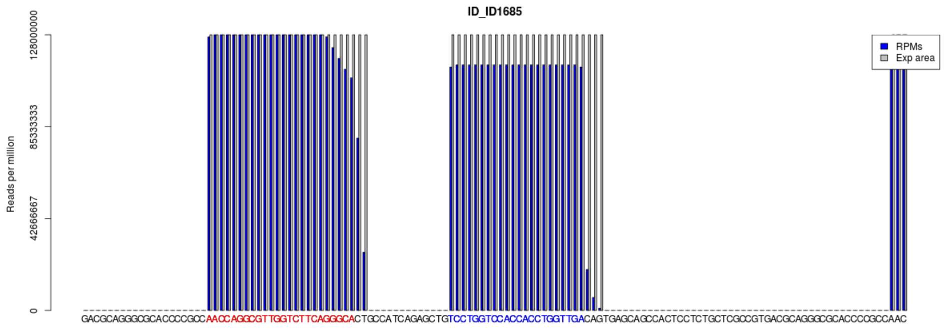

>ID1686
GGTACTTAGCTGGTCTCTGA**TCAGGTACCCAGTTTGTGATTCG**TGTACTGTTTCAGTCGAATAACATTCTAGGTACCTCCTCGGATGCCA
TCTAAATTAAAACATTCGC

>ID1686-5P
TCAGGTACCCAGTTTGTGATTCG

>miRNA precursor
GGUACUUAGCUGGUCUCUGA**UCAGGUACCCAGUUUGUGAUUCG**UGUACUGUUUCAGUCGAAUAACAUUCUAGGUACCUCCUCGGAUGCCAUCUAAAUUAAAACAUUCGC
.....((((.((((.(((((..(((((((.((..(((.(((((...((((...))))))))).)))..)).)))))))..))))).)))).)))).............

>Predicted STAR seq:
AATAACATTCTAGGTACCTCCTC

Complementarity: 15

Initial dG =   -37.40

```
                 10        20        30        40        50
GGUAC---------|   C    C      UC        C  UU   G     UGU    U
              UUAG UGGU UCUGA  AGGUACC AG  UGU AUUCG   ACUG \
              AAUC ACCG AGGCU  UCCAUGG UC  ACA UAAGC   UGAC U
CGCUUACAAAAUUA^   U    U     CC        A  UU   A     ---    U
       100        90        80        70        60
```

Reads in: lib-42, lib-45, lib-47, lib-48, lib-49, lib-50, lib-72
Number of reads="ID1686",0,44,3,4,17,4,0

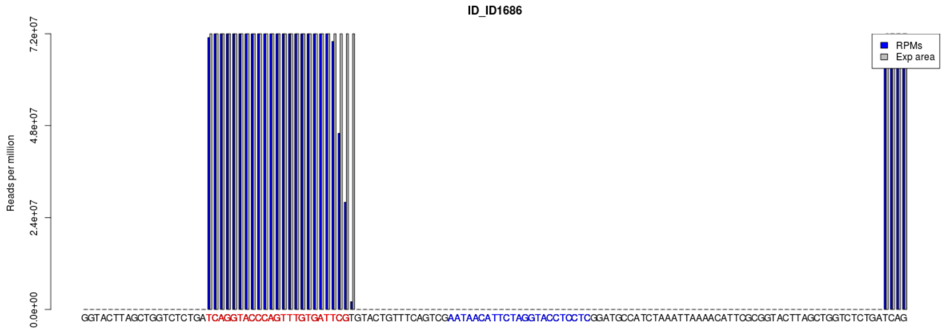

>ID1687
AGCAAAAGACAGCCCCCATC**AACTGGGTTTTGGGCACCAGAACG**TTTCCAGCTCTTACCGCTCTGGTCCAACACCCAGTCGATGGTGGTT
GTCAGATCCGTACTCACCACT

>ID1687-5P
AACTGGGTTTTGGGCACCAGAACG

>miRNA precursor
AGCAAAAGACAGCCCCCAUC**AACUGGGUUUUGGGCACCAGAACG**UUUCCAGCUCUUACCGCUCUGGUCCAACACCCAGUCGAUGGUGGUUGUCAGAUCCGUACUCACCACU
.......(((((((.(((((.(((((((.((((..((((((.((..............)).)))))))))).))))))).))))).)))))))..................

>Predicted STAR seq:
CTCTGGTCCAACACCCAGTCGA

Complementarity: 17

Initial dG =   -46.30

```
                  10        20        30        40        50
AGCAAAA-----------|      C     A       U    GC       A  UUUCCA
                  GACAGCC CCAUC ACUGGGU UUGG  ACCAGA CG      G
                  CUGUUGG GGUAG UGACCCA AACC  UGGUCU GC      C
UCACCACUCAUGCCUAGA^      U     C       C    --       C  CAUUCU
 .       100        90        80        70          60
```

Reads in: lib-42, lib-45, lib-47, lib-48, lib-49, lib-50, lib-72
Number of reads="ID1687",3,25,5,8,4,0,0

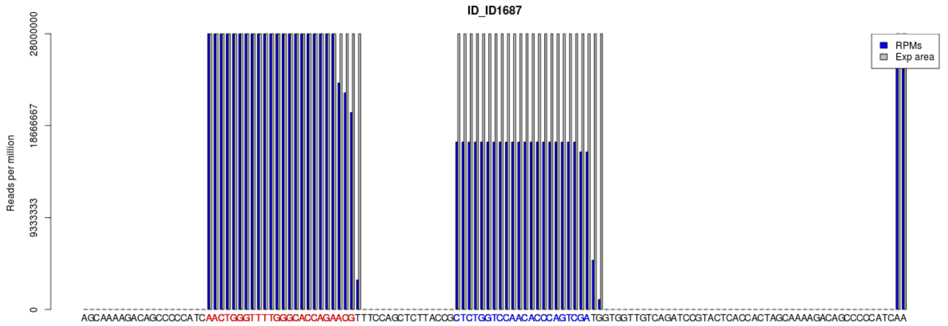

>ID1688

AGTAGAGGTTATAACAGGCAGTGCGTTTCTGGTGACGAGTTAGCTGGGTCAGGATCAGGGGCTCTGAGCT**GAAGTGACCCCCCTAACTCG**
TTGTCAGTGAGACGCCTGAAAC

>ID1688-3P

GAAGTGACCCCCCTAACTCGTT

>miRNA precursor

AGUAGAGGUUAUAACAGGCAGUGCGUUUCUGGUGACGAGUUAGCUGGGUCAGGAUCAGGGGCUCUGAGCU**GAAGUGACCCCCCUAACUCGUU**GUCAGUGAGACGCCUGAAAC
.................(((.((((((((((..(((((((((..((((((((.((((.....)))).))....))))))..)))))))))..)))..))))))))))....

>Predicted STAR seq:

GACGAGTTAGCTGGGTCA

Complementarity: 13

Initial dG =   -46.40

```
        10        20          30        40        50            60
AGUAGAGGUUAUAACAGG   U        --   UG         CU       ----| A    GG
                  CAG GCGUUUC  UGG  ACGAGUUAG  GGGUCA    GG UCAG  \
                  GUC CGCAGAG  ACU  UGCUCAAUC  CCCAGU    UC AGUC  G
CAAA--------------   -        UG   GU         CC       GAAG^ G    UC
110                     100        90         80        70
```

Reads in: lib-42, lib-45, lib-47, lib-48, lib-49, lib-50, lib-72

Number of reads="ID1688",0,0,0,1,0,0,0

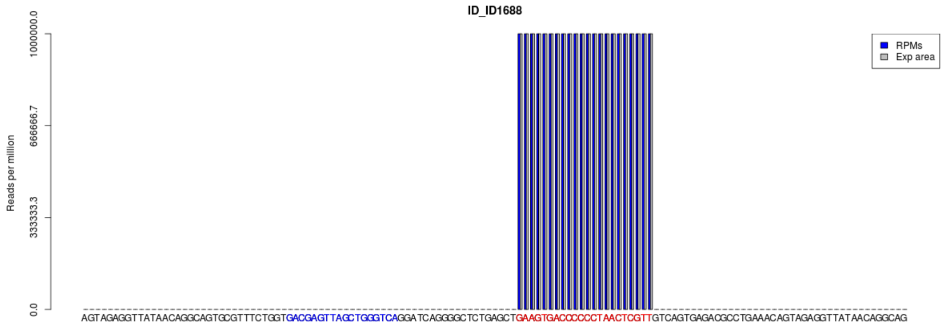

>ID1689
GGCTACTGTGCCCCACGGGG**TGTGACTCAGCGAAACCACAGC**TGATGTCCGGACCAGCTGAGGTGGCACTAAGCCACACCCCGTGGTGTC
TCATTCCATCACATCTTGACG

>ID1689-5P
TGTGACTCAGCGAAACCACAGC

>miRNA precursor
GGCUACUGUGCCCCACGGGG**UGUGACUCAGCGAAACCACAGC**UGAUGUCCGGACCAGCUGAGGUGGCACUAAGCCACACCCCGUGGUGUCUCAUUCCAUCACAUCUUGACG
(((......)))((((((((((((.((.((....(((.((((((..........)))))).)))....)).)).)))))))))))).(((................))).

>Predicted STAR seq:
TGAGGTGGCACTAAGCCACACC

Complementarity: 13

Initial dG = -45.70

ERROR 3: Strange structure
Initial dG = -45.70

```
  |  UA
--GGC  C
  CCG  U
\ ^  UG
   10
```

```
          20        30        40
                A  C  CGAA   A      AUGU
  --CCACGGGGUGUG CU AG    ACC CAGCUG    C
    GGUGCCCCACAC GA UC    UGG GUCGAC    C
  \             C  A  ACGG   A      CAGG
         80        70        60        50
```

Reads in: lib-42, lib-45, lib-47, lib-48, lib-49, lib-50, lib-72
Number of reads="ID1689",0,1,0,0,0,1,0

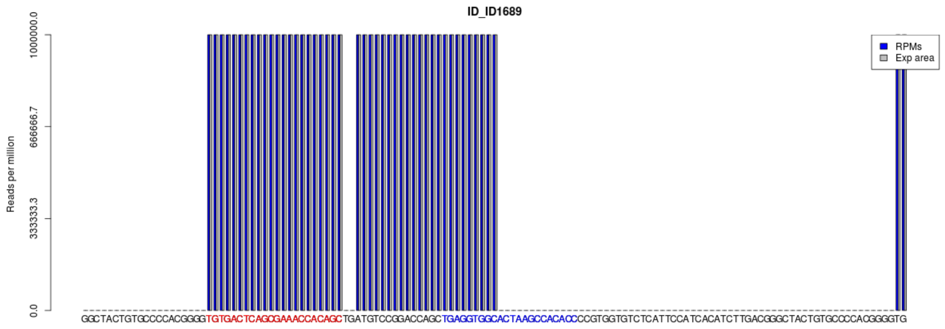

>ID169
TGGCAATGTGGATGGAAAACAAGATTTCTGAT**GTGAGAAAGCGGCATTCTAGTTG**TGTTAATTTTGTTCATCTGGAGTGAAGTTTTCTCA
CAGCAGCATTCTTCATGG

>ID169-5P
GTGAGAAAGCGGCATTCTAGTTG

>miRNA precursor
UGGCAAUGUGGAUGGAAAACAAGAUUUCUGAU**GUGAGAAAGCGGCAUUCUAGUUG**UGUUAAUUUUGUUCAUCUGGAGUGAAGUUUUCUCACAGCAGCAUUCUUCAUGG
```
...................((((...(((.(((((((((((..((((((((.((.............)).))))))))..))))))))))).)))...)))).....
```

>Predicted STAR seq:
TCTGGAGTGAAGTTTTCTCACAG

Complementarity: 18

Initial dG =   -32.60

```
         10        20        30        40        50        60
UGGCA   U-|    GAAAACAAGAUUU   A           GG         U  UGUUAA
     AUG  GGAUG             CUG UGUGAGAAAGC  CAUUCUAG UG      \
     UAC  CUUAC             GAC ACACUCUUUUG  GUGAGGUC AC      U
GG---   UU^    -------------   G           AA         U  UUGUUU
         100                     90        80        70
```

Reads in: lib-42, lib-45, lib-47, lib-48, lib-49, lib-50, lib-72
Number of reads="ID169",493,287,94,817,481,82,0

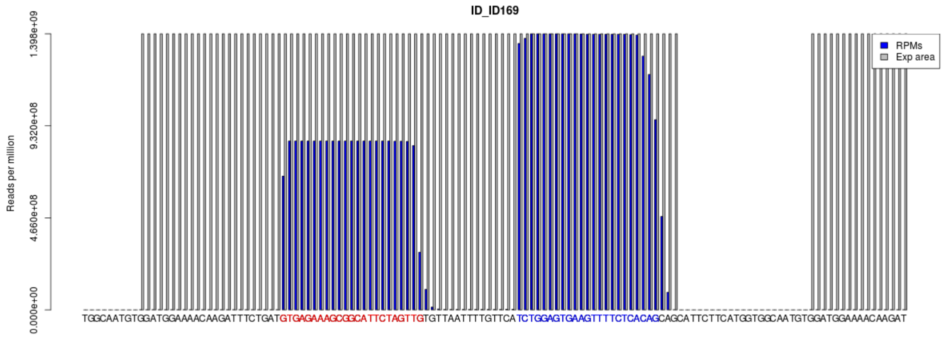

>ID1690
GACGCAGGGCGCCCCCCGCG**AACCAGGCGTTGGTCTTCAGGACA**CTGCCATCAGAGCTGTCCTGGTCCACCGCCTGGTTGACGGTGGGCA
GCCACTTCACTGCTCGTCGT

>ID1690-5P
AACCAGGCGTTGGTCTTCAGGACA

>miRNA precursor
GACGCAGGGCGCCCCCCGCG**AACCAGGCGUUGGUCUUCAGGACA**CUGCCAUCAGAGCUGUCCUGGUCCACCGCCUGGUUGACGGUGGGCAGCCACUUCACUGCUCGUCGU
((((((((((((((.(((..(((((((((.(((...((((((((((........)).)))))))).))).)))))))))..))).)))).))).......)))).)))..

>Predicted STAR seq:
TCCTGGTCCACCGCCTGGTTGACG

Complementarity: 18

Initial dG =   -57.90

```
                  10         20        30         40         50
--   --    ------   -|   C   CG         U   UCU         -  GCC
  GAC  GCAG      GGC GCCC CCG  AACCAGGCG UGG   UCAGGACA CU   A
  CUG  CGUC      CCG CGGG GGC  UUGGUCCGC ACC   GGUCCUGU GA   U
UG   CU    ACUUCA   A^   U   AG         C   U--         C  GAC
.       100        90         80        70          60
```

Reads in: lib-42, lib-45, lib-47, lib-48, lib-49, lib-50, lib-72
Number of reads="ID1690",4,129,23,8,21,8,0

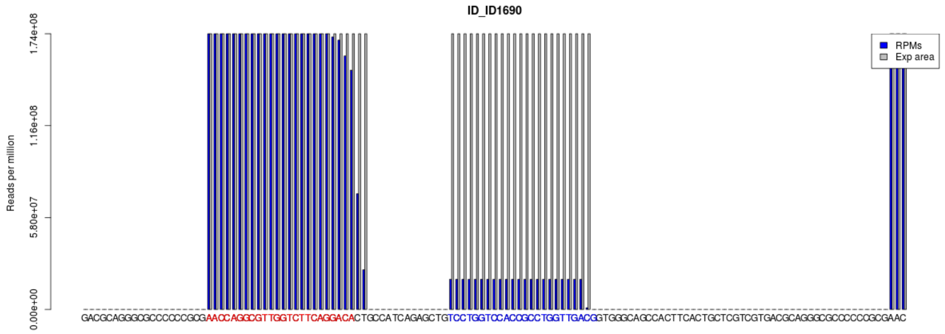

>ID1691
GCTCCTTAGAGAGTAATCCTGGGTGTCTGTGAGAGTACCAGGTAAGTAATTCTTGCAATTGTCAGAAAAG**AATTACAGCCTGAGTACTCT**
CACAGCCGCCTGGGACTTCA

>ID1691-3P
AATTACAGCCTGAGTACTCTCA

>miRNA precursor
GCUCCUUAGAGAGUAAUCCUGGGUGUCUGUGAGAGUACCAGGUAAGUAAUUCUUGCAAUUGUCAGAAAAG**AAUUACAGCCUGAGUACUCUCA**CAGCCGCCUGGGACUUCA
((((......))))..(((..((((.((((((((((((((((..(((((((((............))))))))).))))).))))))))))))).)))).)))).....

>Predicted STAR seq:
GAGAGTACCAGGTAAGTAATT

Complementarity: 18
ERROR 4: Strange structure
 Initial dG =   -55.70

```
  |   CU
--GCUC  U
  UGAG  A
\ ^   AG
     10


         20        30          40        50        60
  AA---   UG    U            -     AA         GCAAUU
       UCC  GGUG CUGUGAGAGUAC CAGGU  GUAAUUCUU      \
       AGG  CCGC GACACUCUCAUG GUCCG  CAUUAAGAA      G
  ACUUC   GU    C            A     A-         AAGACU
110       100        90        80         70
```

Reads in: lib-42, lib-45, lib-47, lib-48, lib-49, lib-50, lib-72
Number of reads="ID1691",8,167,54,13,30,23,0

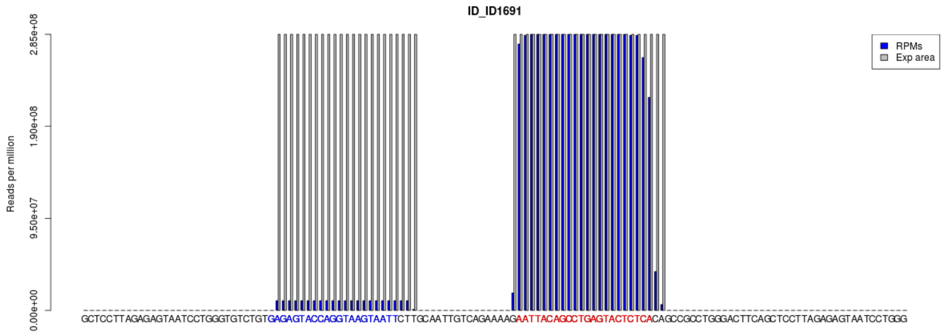

>ID1692
ACATCGTTCACGGGCAGTGCGTTTCGCTGGCAAAGAGATGGGAGAGTCAATTGCTGGATTTCAGAGTTGC**AAGAACTCTGCCATTTCTTT**
GCTAGTGTGACGCAAGAAACT

>ID1692-3P
AAGAACTCTGCCATTTCTTTGC

>miRNA precursor
ACAUCGUUCACGGGCAGUGCGUUUCGCUGGCAAAGAGAUGGGAGAGUCAAUUGCUGGAUUUCAGAGUUGC**AAGAACUCUGCCAUUUCUUUGC**UAGUGUGACGCAAGAAACU
.....(((....)))..((((((.((((((((((((((((.((((((((((.(((.....)))))))))).....))))).))))))))))))))))).)))))).......

>Predicted STAR seq:
CAAAGAGATGGGAGAGT

Complementarity: 15
ERROR 4: Strange structure
 Initial dG =   -49.90

```
          10
.-ACAUC|  C
       GUU A
       CGG C
\ -----^  G




          20        30        40             50
  AG-----      U                 G     -----     G   GA
         UGCGUU CGCUGGCAAAGAGAUGG AGAGU     CAAUU CUG  \
         ACGCAG GUGAUCGUUUCUUUACC UCUCA     GUUGA GAC  U
  UCAAAGA      U                 G     AGAAC     -   UU
 110       100        90        80        70         60
```

Reads in: lib-42, lib-45, lib-47, lib-48, lib-49, lib-50, lib-72
Number of reads="ID1692",10,432,197,37,99,64,0

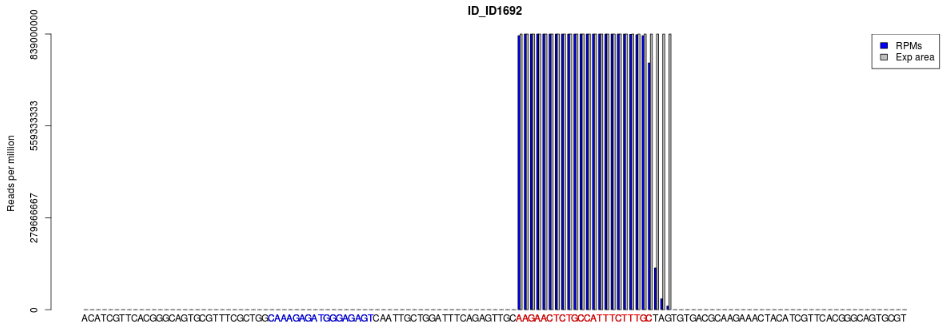

>ID1693
CCGGCGCCTCAGAGAGTATTCCCGGCGGCTGTGTGAGTACCAGGTATGTGATTCCTGTGTCGTAAAACGA**ATCACAGCCTCCGTACTCAC**
ACAGACGTCTGGGGCTTCG

>ID1693-3P
ATCACAGCCTCCGTACTCACA

>miRNA precursor
CCGGCGCCUCAGAGAGUAUUCCCGGCGGCUGUGUGAGUACCAGGUAUGUGAUUCCUGUGUCGUAAAACGA**AUCACAGCCUCCGUACUCACA**CAGACGUCUGGGGCUUCG
```
.(((.(((((((...........(((.((((((((((((.((((.((((((((..((........))))))))))))))..)))))))))))).)))))))))).)))
```

>Predicted STAR seq:
GTGAGTACCAGGTATGTGAT

Complementarity: 17

Initial dG =   -49.30

```
        10        20        30        40         50        60
C|  C       AGAGUAUUCCCG   G            C-     A        CU   GUC
 CGG GCCUCAG            GCG CUGUGUGAGUAC  AGGU UGUGAUUC  GU   G
 GCU CGGGGUC            UGC GACACACUCAUG  UCCG ACACUAAG  CA   U
-^  U       ------------   A            CC     -        --  AAA
        100                   90        80         70
```

Reads in: lib-42, lib-45, lib-47, lib-48, lib-49, lib-50, lib-72
Number of reads="ID1693",0,51,1,1,9,5,0

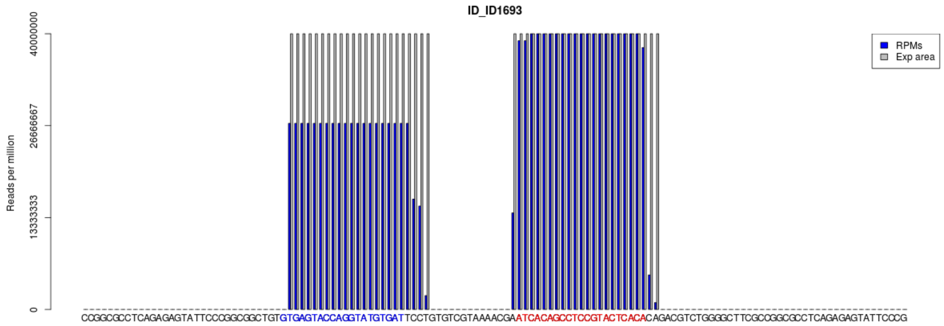

>ID1694
AGCTACTCCCGGTGTCTGTG**AGAGTGCCAGGCGAGCAATTCT**TGCTTTTGTCGCGGAAAGAATTGCAGCCCGGGCACTCGCACAGCCGCC
TGGGACTTCATCGTGCAAGCCA

>ID1694-5P
AGAGTGCCAGGCGAGCAATTCT

>miRNA precursor
AGCUACUCCCGGUGUCUGUG**AGAGUGCCAGGCGAGCAAUUCU**UGCUUUUGUCGCGGAAAGAAUUGCAGCCCGGGCACUCGCACAGCCGCCUGGGACUUCAUCGUGCAAGCCA
.((.((((((((((.(((((.(((((((.(((..(((((((((.((........)).))))))))).)))..))))))).))))).)))).)))).......)))).....

>Predicted STAR seq:
AATTGCAGCCCGGGCACTCGCA

Complementarity: 16

Initial dG = -52.60

```
                    10         20         30        40        50
A-----  U  -------    -    U     A       A-|  GA        G  UUU
      GC AC       UCCC GGUG CUGUG GAGUGCC  GGC  GCAAUUCUU CU   G
      CG UG       AGGG CCGC GACAC CUCACGG  CCG  CGUUAAGAA GG   U
ACCGAA  -  CUACUUC    U    C     G       GC^  A-        A  CGC
110       100        90        80        70         60
```

Reads in: lib-42, lib-45, lib-47, lib-48, lib-49, lib-50, lib-72
Number of reads="ID1694",0,17,4,3,2,1,0

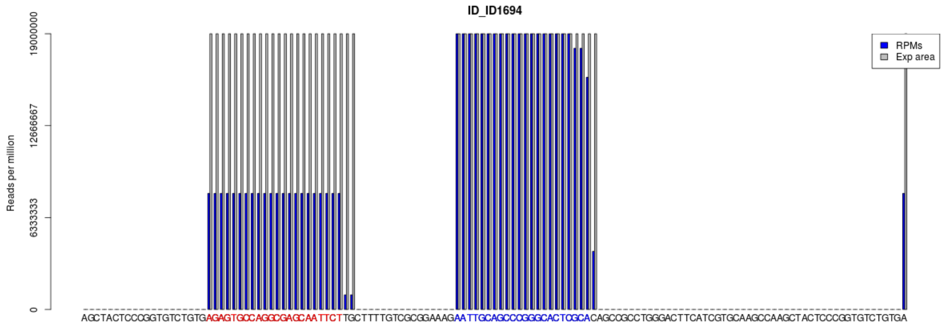

>ID1695

GATCCCAGAACGACGCAGGGCGCCCCCCGCCAACCAGCACGTGGAACTGAGGACACTGCCATCAGAGCTG**TCCTGTTGCACCTGTTGGTT**
GACGGTGGGCAGCCACTACA

>ID1695-3P

TCCTGTTGCACCTGTTGGTTGAC

>miRNA precursor

GAUCCCAGAACGACGCAGGGCGCCCCCCGCCAACCAGCACGUGGAACUGAGGACACUGCCAUCAGAGCUG**UCCUGUUGCACCUGUUGGUUGAC**GGUGGGCAGCCACUACA
................((((((((.(((.(((((((((.(((.(((..((((((((........)).))))))))).))).))))))))).))).)))).))).))...

>Predicted STAR seq:

CCAACCAGCACGTGGAACTGAGGA

Complementarity: 18

Initial dG =   -49.70

```
        10         20         30        40        50         60
GAUCCCAGAACGACGC  -    -|   C   C         C   G   UG      -  GCC
                AG GGC GCCC CCG CAACCAGCA GUG AAC  AGGACA CU   A
                UC CCG CGGG GGC GUUGGUUGU CAC UUG  UCCUGU GA   U
ACA-------------  A   A^   U   A         C   G   --      C  GAC
.                   100        90        80          70
```

Reads in: lib-42, lib-45, lib-47, lib-48, lib-49, lib-50, lib-72

Number of reads="ID1695",6,238,125,17,51,30,0

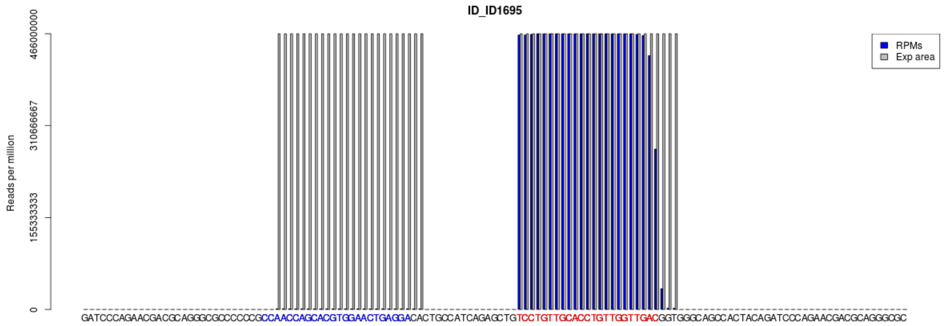

>ID1696
CAACGAGAAGTCGCTGTGGTACTCTGGTCCCCGGGAAGTGTCAAGGGCGAAATTCGCAGTTTTCGAGACG**ATTTTCGCTCCTGACACTTA**
CCCGGTGGCCAGGGAGAACGT

>ID1696-3P
ATTTTCGCTCCTGACACTTACC

>miRNA precursor
CAACGAGAAGUCGCUGUGGUACUCUGGUCCCCGGGAAGUGUCAAGGGCGAAAUUCGCAGUUUUCGAGACG**AUUUUCGCUCCUGACACUUACC**CGGUGGCCAGGGAGAACGU
```
((.((....))).)).....((((((((.((((((((((((.((((((((.(((...........))).)))))))).)))))))).))))).))))))).......
```

>Predicted STAR seq:
GAAGTGTCAAGGGCGAAAT

Complementarity: 17
ERROR 4: Strange structure
Initial dG =   -52.60

```
  | A   G
--CA CGA A
  GU GCU A
\ ^ C   G
       10
```

```
    20          30         40        50        60
  UGGUA--        C      -        A        U   CAGUU
         CUCUGGUC CCGGG AAGUGUCA GGGCGAAA UCG     U
         GGGACCGG GGCCC UUCACAGU CUCGCUUU AGC     U
  UGCAAGA        U      A        C        U   AGAGC
 110       100        90        80        70
```

Reads in: lib-42, lib-45, lib-47, lib-48, lib-49, lib-50, lib-72
Number of reads="ID1696",6,127,43,11,27,18,0

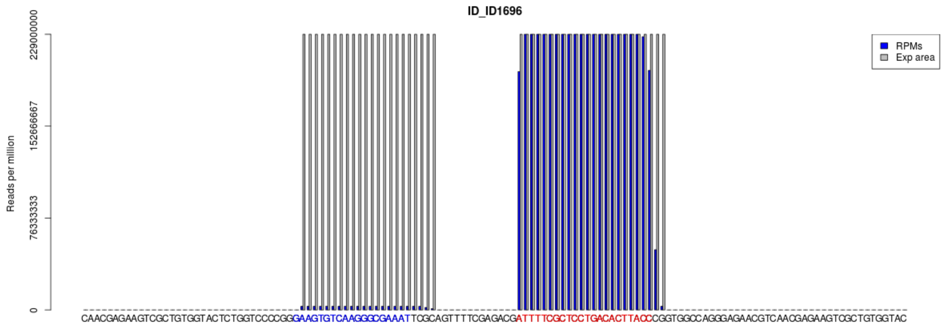

>ID1697
GTGGCGACCTGGTGCCTGAG**AGAGTGTCAAGAGCGCGACTC**GTTGTTTTGAGGGCGAATCTCACTCATGACACTGTCCCAATTGCCAGGC
AGAGCAGAATCACAGCCATC

>ID1697-5P
AGAGTGTCAAGAGCGCGACTC

>miRNA precursor
GUGGCGACCUGGUGCCUGAG**AGAGUGUCAAGAGCGCGACUC**GUUGUUUUGAGGGCGAAUCUCACUCAUGACACUGUCCCAAUUGCCAGGCAGAGCAGAAUCACAGCCAUC
(((((..(((((((..((.((.(((((((.((((((..((((......)))).)))).......))).))))))).)).))..))))))))..((......))...))))).

>Predicted STAR seq:
GGGCGAATCTCACTCATGACACTGTCC

Complementarity: 15

Initial dG = -41.80

```
           10        20        30                40
-     .-GA       CC   A  G        A   -------|  GA    UU
 GUGGC    CCUGGUG  UG GA AGUGUCA GAG       CGC  CUCG  G
 UACCG    GGACCGU  AC CU UCACAGU CUC       GCG  GAGU  U
C     \ --       UA   C  G        A   ACUCUAA^  G-    UU
.                  80        70        60         50



       90
        CA-  GC
           GA  A
           CU  G
        ACA  AA
          100
```

Reads in: lib-42, lib-45, lib-47, lib-48, lib-49, lib-50, lib-72
Number of reads="ID1697",5,24,5,8,19,4,0

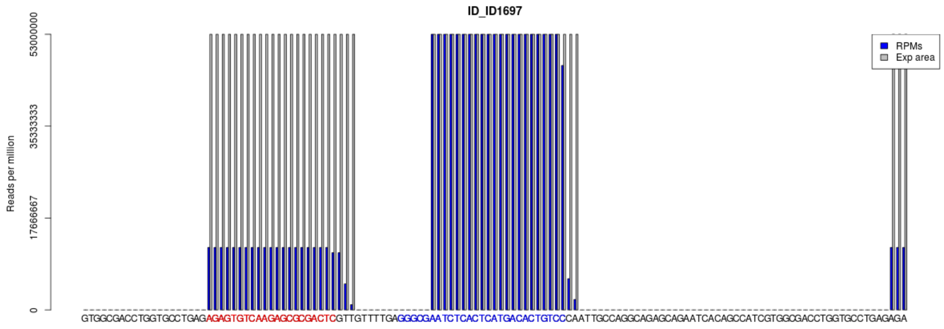

>ID1698

CCTCTGCTCTGTCATTTCTGACGTCTGAGTTATGCGGAAGGAATGTGACTCGTGCCATGAGTGCAAATCG**AATCACTTTCCTTCTGCTGA**
CTCAGCTGTCAGATGCTGCATT

>ID1698-3P

AATCACTTTCCTTCTGCTGACT

>miRNA precursor

CCUCUGCUCUGUCAUUUCUGACGUCUGAGUUAUGCGGAAGGAAUGUGACUCGUGCCAUGAGUGCAAAUCG**AAUCACUUUCCUUCUGCUGACU**CAGCUGUCAGAUGCUGCAUU
.........(((....(((((((.((((((((.((((((((((.((((.((((((.......)))...))).)))).)))))))))))))))))).))))))).....)))..

>Predicted STAR seq:

GTTATGCGGAAGGAATGTGAC

Complementarity: 18

Initial dG =   -49.10

```
        10         20         30         40         50
CCUCUGCUC   CAUU       U         U          U     C   ---|  CAU
         UGU    UCUGACG CUGAGUUA GCGGAAGGAA GUGA UCG   UGC   \
         ACG    AGACUGU GACUCAGU CGUCUUCCUU CACU AGC   ACG   G
UU-------   UCGU       C         -          U     A   UAA^  UGA
       110       100        90         80        70        60
```

Reads in: lib-42, lib-45, lib-47, lib-48, lib-49, lib-50, lib-72

Number of reads="ID1698",0,41,9,8,19,8,0

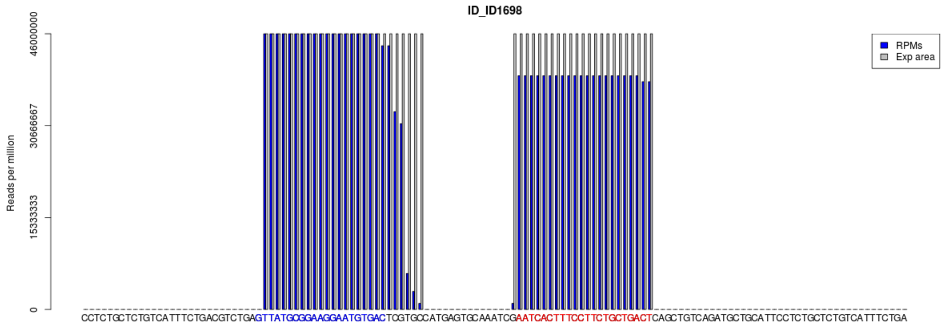

>ID1699
TTCCTCTGGTGTCGCTGGGG**AGGTTGTCATGGGACGACTGCC**GCTGTGAGTGCCGGCAGTGGTCCACTGACTTCCTCCTCGTCGTCACCC
CACCAGCTACAGTGCTCGACG

>ID1699-5P
AGGTTGTCATGGGACGACTGCC

>miRNA precursor
UUCCUCUGGUGUCGCUGGGG**AGGUUGUCAUGGGACGACUGCC**GCUGUGAGUGCCGGCAGUGGUCCACUGACUUCCUCCUCGUCGUCACCCCACCAGCUACAGUGCUCGACG
.......((((.((.((((((((..((((..((((.((((((((.......).))))))).)))))..)))))..)))))))).)).)))).(((........))).......

>Predicted STAR seq:
CAGTGGTCCACTGACTTCCTCC

Complementarity: 15

Initial dG =   -53.00

```
          10        20        30        40
.-UUCCUCU    U  C        UU    UG    G      -| UGU
         GGUG CG UGGGGAGG  GUCA  GGAC ACUGCC GC   \
         CCAC GC GCUCCUCC  CAGU  CCUG UGACGG CG   G
\ -------    U  U        UU    CA    G      C^ UGA
                 80        70        60        50



 90       100
  CCACC   UA
       AGC  C
       UCG  A
  GCAGC   UG
 110
```

Reads in: lib-42, lib-45, lib-47, lib-48, lib-49, lib-50, lib-72
Number of reads="ID1699",0,10,6,6,9,2,0

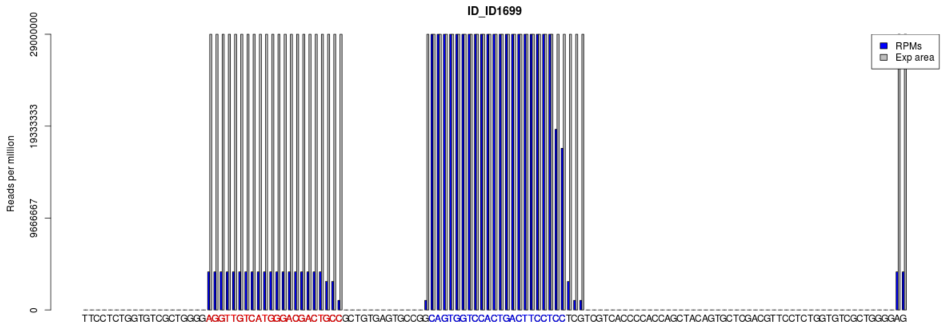

>ID170
GGAAGAACAAGATTTCTGAT**GTGAAAAAGTAGCACTCTAGTT**GTGTTAAAAATTTTGTTCAACTAGAGTGAAGCTTTCTCACATCAGCAT
TCTTCAGAAACAGCTGCTGAAA

>ID170-5P
GTGAAAAAGTAGCACTCTAGTT

>miRNA precursor
GGAAGAACAAGAUUUCUGAU**GUGAAAAAGUAGCACUCUAGUU**GUGUUAAAAAUUUUGUUCAACUAGAGUGAAGCUUUCUCACAUCAGCAUUCUUCAGAAACAGCUGCUGAAA
```
.((((((........(((((((((.(((((..(((((((((((...((((...))))..)))))))))))..))))).)))))))))..)))))).....(((...)))...
```

>Predicted STAR seq:
CTAGAGTGAAGCTTTCTCACAT

Complementarity: 17

Initial dG =   -39.30

```
          10        20        30        40        50
.-G|     CAAGAUUU         A     AG           UGU    A
   GAAGAA        CUGAUGUGA AAAGU  CACUCUAGUUG   UAAA \
   CUUCUU        GACUACACU UUUCG  GUGAGAUCAAC   GUUU A
\ -^     AC------         C     AA           UU-    U
       90              80        70        60


    100
  AGAAA   C
       CAG \
       GUC U
  AAA--   G
  110
```

Reads in: lib-42, lib-45, lib-47, lib-48, lib-49, lib-50, lib-72
Number of reads="ID170",1121,1368,1267,2522,3070,781,0

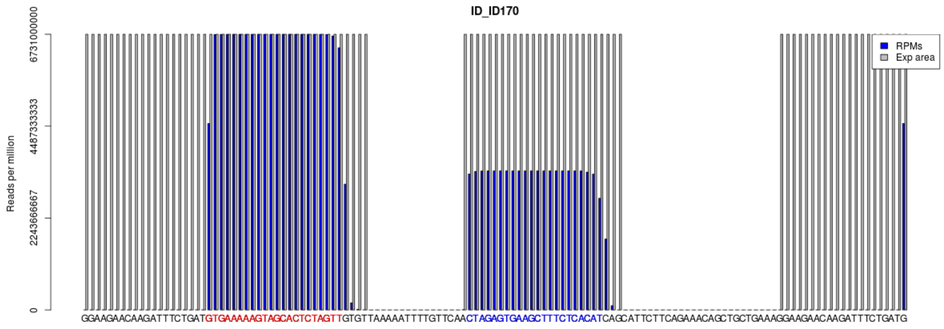

>ID1700
GACTCCAGCTGGTGTCTGGG**CGCGTACCTGGGAACTGGGCCG**CGTATATTTCTGCAGACCGAACCAGTCCCCCGGTACGTCCTCGGCCGC
CAGCTACTTCATCCTGGCCTCA

>ID1700-5P
CGCGTACCTGGGAACTGGGCCG

>miRNA precursor
GACUCCAGCUGGUGUCUGGG**CGCGUACCUGGGAACUGGGCCG**CGUAUAUUUCUGCAGACCGAACCAGUCCCCCGGUACGUCCUCGGCCGCCAGCUACUUCAUCCUGGCCUCA
......((((((((.(((((.(((((((.(((.(((((..((.((...........))))..))))).))).))))))).))))).))))))))................

>Predicted STAR seq:
AACCAGTCCCCCGGTACGTCCT

Complementarity: 15

Initial dG = -50.50

```
                 10        20        30        40        50
GACUCC-----------|       U     C       U   A     GC  C  AUAUU
                 AGCUGGUG CUGGG GCGUACC GGG ACUGG  CG GU     U
                 UCGACCGC GGCUC UGCAUGG CCC UGACC  GC CA     C
ACUCCGGUCCUACUUCA^       C     C       C   C     AA  -  GACGU
110       100       90        80        70        60
```

Reads in: lib-42, lib-45, lib-47, lib-48, lib-49, lib-50, lib-72
Number of reads="ID1700",8,141,13,27,47,13,0

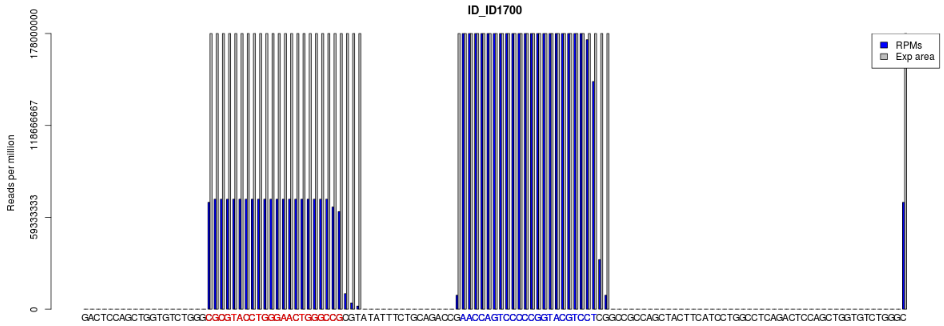

>ID1701
TGTGGTTGCGCCGTTGGGCG**TGACTGGGTGATGCAGCAG**TTGGTGTTGAGGCAACTGCGGTATCGCCCAGTCACACCTCACGGTGCCTCG
CGACCGGCACTTCTGGAC

>ID1701-5P
TGACTGGGTGATGCAGCAG

>miRNA precursor
UGUGGUUGCGCCGUUGGGCG**UGACUGGGUGAUGCAGCAG**UUGGUGUUGAGGCAACUGCGGUAUCGCCCAGUCACACCUCACGGUGCCUCGCGACCGGCACUUCUGGAC
.((((..(((((((.(((.((((((((((((((.(((((((.........))))))).))))))))))))))).))).))))))).))))..((((.....))))..

>Predicted STAR seq:
GCGGTATCGCCCAGTCACA

Complementarity: 16

Initial dG = -64.70

```
          10        20        30        40
.-U|   UU       U   C               A       GUGU
   GUGG  GCGCCGU GGG GUGACUGGGUGAUGC GCAGUUG    \
   CGCU  CGUGGCA UCC CACUGACCCGCUAUG CGUCAAC    U
\ -^   C-       C   A               G       GGAG
   90         80        70        60        50
```

```
  GA    CA
    CCGG  \
    GGUC  C
  CA    UU
```

Reads in: lib-42, lib-45, lib-47, lib-48, lib-49, lib-50, lib-72
Number of reads="ID1701",0,4,5,1,3,0,0

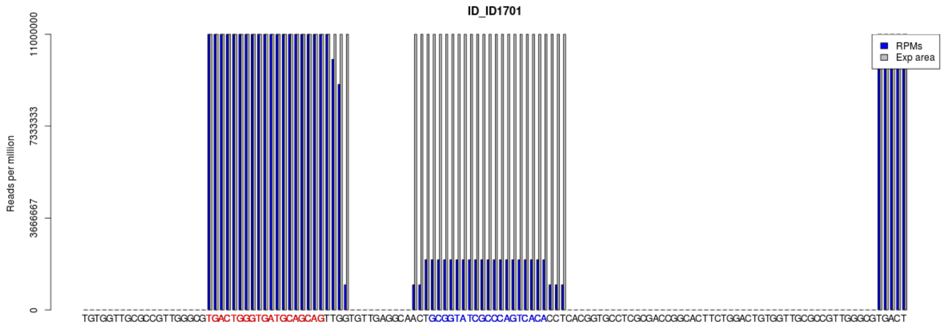

>ID1702
TCCACATCCACTCCTGAGGG**AGGCTCCAGTGATGCGACGGTG**AAGTTGAGGACATCGTCGCGTCATTGGACCTCCTTCCGGAGCGCGACA
GCAGCTGAAGGTGCGGGG

>ID1702-5P
AGGCTCCAGTGATGCGACGGTG

>miRNA precursor
UCCACAUCCACUCCUGAGGG**AGGCUCCAGUGAUGCGACGGUG**AAGUUGAGGACAUCGUCGCGUCAUUGGACCUCCUUCCGGAGCGCGACAGCAGCUGAAGGUGCGGGG
(((.((((..((((.((((((((.((((((((((((((((((.........)))))))))))))))))))))))))))).)))).((....)).......)))).))).

>Predicted STAR seq:
TCGTCGCGTCATTGGACCTCC

Complementarity: 19

Initial dG = -61.00

```
           10        20        30        40
-   A    .-CA|   U         C                  AAGU
 UCC CAUC    CUCC GAGGGAGG UCCAGUGAUGCGACGGUG    U
 GGG GUGG    GAGG CUUCCUCC AGGUUACUGCGCUGCUAC    G
G   C    \ --^   C         -                  AGGA
      100      80         70        60        50




           C------  G
                  GC A
                  CG C
           AAGUCGA  A
                   90
```

Reads in: lib-42, lib-45, lib-47, lib-48, lib-49, lib-50, lib-72
Number of reads="ID1702",1,62,11,13,15,7,0

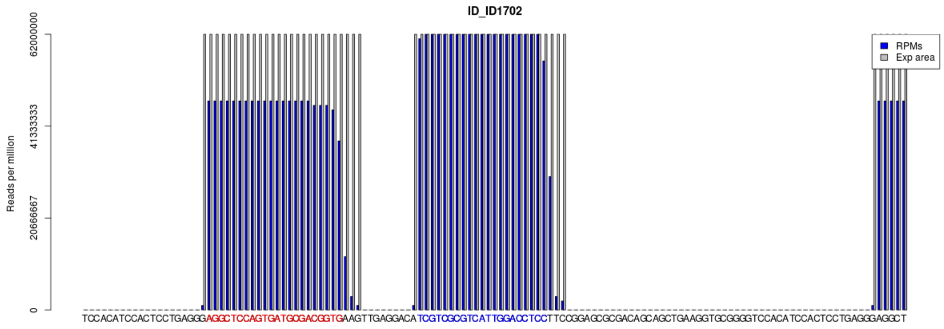

>ID1703
AGTTAAACCTGGTGTCTGGG**AGAGTGTCAGGAATGTGACTCG**TGGCTTATTTGGAAATCGAAGCACATTTCTGACAATGTCATGACCGCC
AGGTGATTCTTCATGGCATC

>ID1703-5P
AGAGTGTCAGGAATGTGACTCG

>miRNA precursor
AGUUAAACCUGGUGUCUGGG**AGAGUGUCAGGAAUGUGACUCG**UGGCUUAUUUGGAAAUCGAAGCACAUUUCUGACAAUGUCAUGACCGCCAGGUGAUUCUUCAUGGCAUC
......((((((((((...((...(((((((((((((..(((...((.....))....)))..)))))))))))))...))..)).))))))))(((.((....)).)))

>Predicted STAR seq:
AAGCACATTTCTGACAATGTCA

Complementarity: 15

Initial dG = -40.70

```
            10         20         30         40
A--    .-A       -|  UGG  GAG             AC   UGG-  UA
   GUUA   ACCUGGU GUC   GA   UGUCAGGAAUGUG  UCG    CU  \
   CGGU   UGGACCG CAG   CU   ACAGUCUUUACAC  AGC    GG  U
CUA    \ -       C^  UA-  GUA             GA   UAAA  UU
.            90         80         70         60



         -  U
          GA U
          CU C
         A  U
          100
```

Reads in: lib-42, lib-45, lib-47, lib-48, lib-49, lib-50, lib-72
Number of reads="ID1703",2,91,47,6,17,11,0

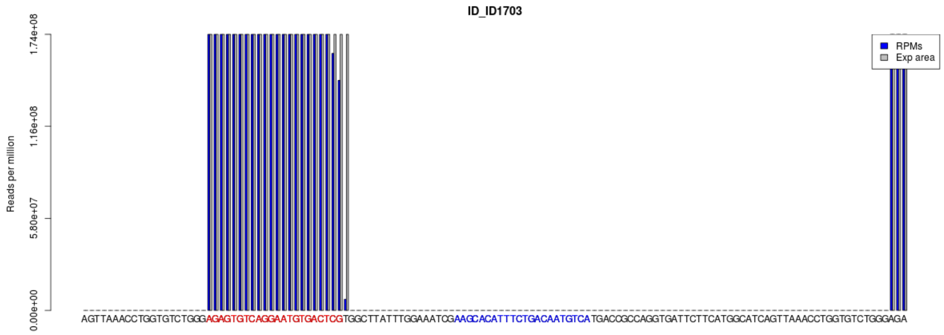

>ID1704
AGCAAAAGACAGCCCCCATCAACTGAGTTTTGGGCACCAGAGCGTTGCCAAATCTTGCCG**CCCTGGACCAACACCCAGTCGA**TGGTGGTT
GTCAGTTCCCTATTACCAGTA

>ID1704-3P
CCCTGGACCAACACCCAGTCGA

>miRNA precursor
AGCAAAAGACAGCCCCCAUCAACUGAGUUUUGGGCACCAGAGCGUUGCCAAAUCUUGCCG**CCCUGGACCAACACCCAGUCGA**UGGUGGUUGUCAGUUCCCUAUUACCAGUA
```
.......(((((((.(((((.((((.((.((((...((((.(((..((........))))).)))).)))).)).)))).))))).))))))).................
```

>Predicted STAR seq:
CAACTGAGTTTTGGGCACCAGA

Complementarity: 15

Initial dG =   -41.30

```
                  10        20        30        40        50
AGCAAAA-----------|      C     A     A  U    GCA     A   UU  CAA
                  GACAGCC CCAUC ACUG GU UUGG   CCAG GCG  GC   A
                  CUGUUGG GGUAG UGAC CA AACC   GGUC CGC  CG   U
AUGACCAUUAUCCCUUGA^      U     C     C  C    A--     C   --  UUC
 .       100        90        80        70          60
```

Reads in: lib-42, lib-45, lib-47, lib-48, lib-49, lib-50, lib-72
Number of reads="ID1704",2,28,1,10,7,2,0

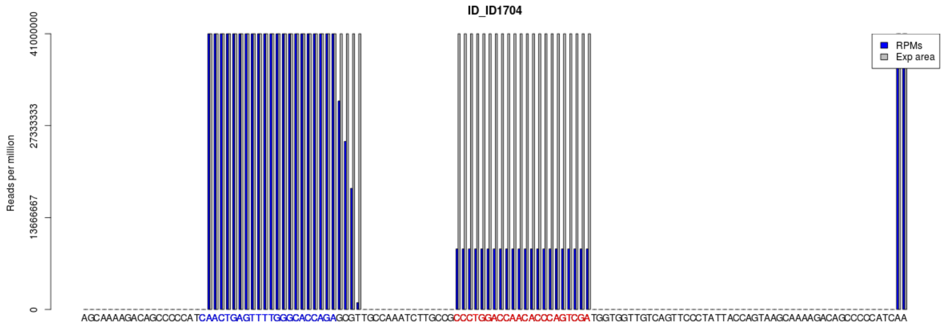

>ID1705
CTGACGTCCATTACAGGCAGTGCGACTCTGGCAATCTGGTAGGTGGGTCAGTCTCAGGGGTTTAGAACTG**GAACTGACTCAACTACCGGA**
CAGTCAGTGAGACGCCAGA

>ID1705-3P
GAACTGACTCAACTACCGGACAG

>miRNA precursor
CUGACGUCCAUUACAGGCAGUGCGACUCUGGCAAUCUGGUAGGUGGGUCAGUCUCAGGGGUUUAGAACUG**GAACUGACUCAACUACCGGACAG**UCAGUGAGACGCCAGA
(((.((((..((((.(((..(((.......))).((((((((.(((((((((..(((..........)))..))))))))).))))))))..))).))))))))).))).

>Predicted STAR seq:
AGTGCGACTCTGGCAATCTGGTAGGTGGGTC

Complementarity: 16

Initial dG =   -46.60

```
            10
-   A    - .-AUUACAG| A
 CUG CGUC C         GC \
 GAC GCAG G         CG G
A   C    A \ -------^ U
       100
```

Reads in: lib-42, lib-45, lib-47, lib-48, lib-49, lib-50, lib-72
Number of reads="ID1705",1,24,6,3,6,2,0

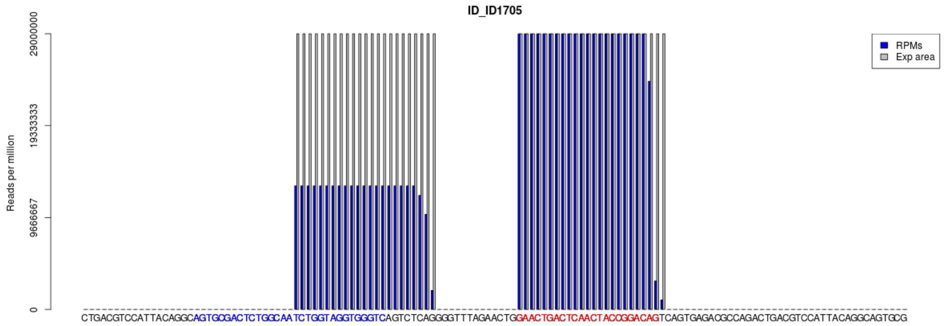

>ID1706

GGGCAGTGCGTTTTGCTGGC**GAAGAGATAGGACAGTCAATC**TGCTGGTATTTTGATTGCACTGACTGCCCTATTTCTTTGCCAGCTAGAC
GCCAGAAACTACAGTGCTTCA

>ID1706-5P

GAAGAGATAGGACAGTCAATC

>miRNA precursor

GGGCAGUGCGUUUUGCUGGC**GAAGAGAUAGGACAGUCAAUC**UGCUGGUAUUUUGAUUGCACUGACUGCCCUAUUUCUUUGCCAGCUAGACGCCAGAAACUACAGUGCUUCA
```
(((((.(((((((.((((((((((((((((((.((((((...(((.............))).)))))).)))))))))))))))))).))))))..........).))))).. 
```

>Predicted STAR seq:

ACTGCCCTATTTCTTTGC

Complementarity: 15

Initial dG =   -50.70

```
                    10        20        30        40        50
--     G ----------|     U                 A      AUC   UGGUAU
  GGGCA U          GCGUUU GCUGGCGAAGAGAUAGG CAGUCA   UGC      \
  UUCGU A          CGCAGA CGACCGUUUCUUUAUCC GUCAGU   ACG      U
AC     G CAUCAAAGAC^     U                 C      C--   UUAGUU
 .       100       90        80        70          60
```

Reads in: lib-42, lib-45, lib-47, lib-48, lib-49, lib-50, lib-72

Number of reads="ID1706",2,99,74,17,46,14,0

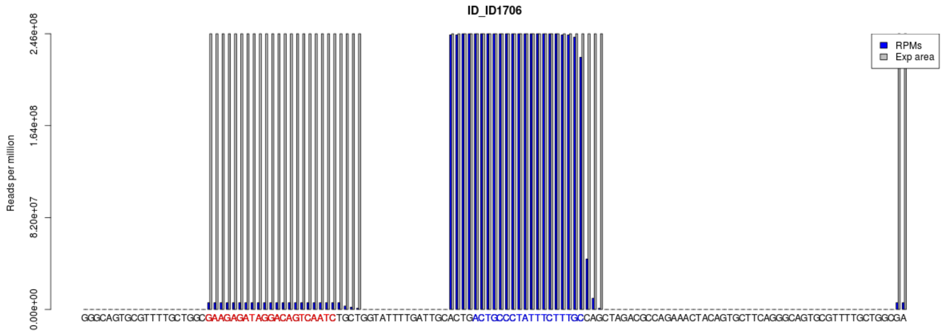

>ID1707
GATGCCAGAACGACCCAGGGCGCCCCCCACCAACCAGCTCGTGGGACTGAGGACACTGCCATCAGAGCTG**TTCTCTTCCACCATCTGGTT**
GACGGTGGGCAGCCACTTCACT

>ID1707-3P
TTCTCTTCCACCATCTGGTTGA

>miRNA precursor
GAUGCCAGAACGACCCAGGGCGCCCCCCACCAACCAGCUCGUGGGACUGAGGACACUGCCAUCAGAGCUG**UUCUCUUCCACCAUCUGGUUGA**CGGUGGGCAGCCACUUCACU
...........((...((((((((.((..(((((((...((((((..(((((((((........)).))))))))))))))...))))))))..)).)))).))).)))).)))...

>Predicted STAR seq:
CCAACCAGCTCGTGGGACTGAGGA

Complementarity: 16

Initial dG = -43.80

```
        10         20         30         40         50         60
GAUGCCAGAAC|  CCC  -   -    C  AC        CUC       CU        -  GCC
           GA   AG GGC GCCC CC  CAACCAG   GUGGGA  GAGGACA CU   A
           CU   UC CCG CGGG GG  GUUGGUC   CACCUU  CUCUUGU GA   U
UCA--------^ ---  A   A    U  CA        UAC       --        C  GAC
110                 100        90         80           70
```

Reads in: lib-42, lib-45, lib-47, lib-48, lib-49, lib-50, lib-72
Number of reads="ID1707",2,71,43,8,15,8,0

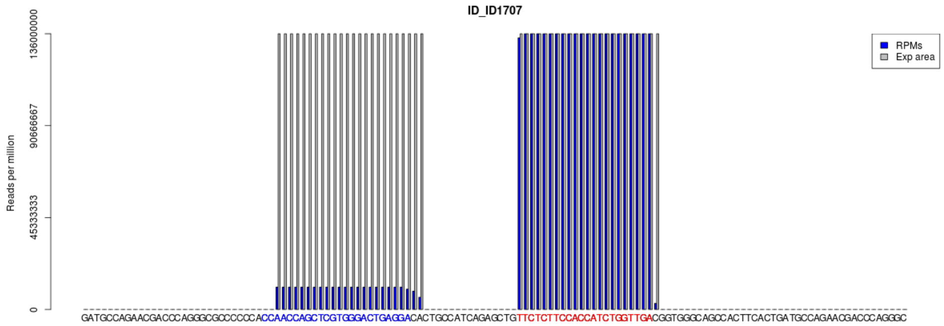

>ID1708
GCCACAGCGGTCGCCGGGGG**TAAGTCTCATCGGATATGACCA**CATGTGACTCCGGTCATATTGGATAAGGCTGACCCCCTGTCACCTAAC
ACCGACGGTTTATGTATG

>ID1708-5P
TAAGTCTCATCGGATATGACCA

>miRNA precursor
GCCACAGCGGUCGCCGGGGG**UAAGUCUCAUCGGAUAUGACCA**CAUGUGACUCCGGUCAUAUUGGAUAAGGCUGACCCCCUGUCACCUAACACCGACGGUUUAUGUAUG
........(((.((.((((((.(((((.(((.(((((((((............))))))))).))).))))).)))))).)).)))........(((.....)))...

>Predicted STAR seq:
TCATATTGGATAAGGCTGACC

Complementarity: 16

Initial dG =   -42.20

```
          10        20        30        40
GCC   .-GC|  C  C      A     C   G         ACAUG
   ACA    GGU GC GGGGGU AGUCU AUC GAUAUGACC     U
   UGU    CCA UG CCCCCA UCGGA UAG UUAUACUGG     G
GUA   \ --^  C  U      G     A   G         CCUCA
               80        70        60        50



          90
        UAAC   G
            ACC \
            UGG A
        AUU-   C
        100
```

Reads in: lib-42, lib-45, lib-47, lib-48, lib-49, lib-50, lib-72
Number of reads="ID1708",0,18,6,1,2,4,0

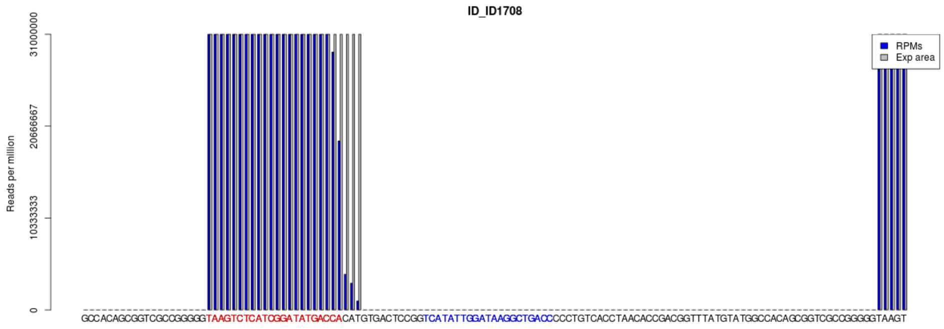

>ID1709
AGCGACAGACAGCCCCCATC**AACTGGGTTTTGGGCACCAGAACG**CTTCCAGATTTTGCCGCCCTGGTCCAACACCCAGTCGAAGGTGCTT
GTCAGTACCCTACTTAGCACT

>ID1709-5P
AACTGGGTTTTGGGCACCAGAACG

>miRNA precursor
AGCGACAGACAGCCCCCAUC**AACUGGGUUUUGGGCACCAGAACG**CUUCCAGAUUUUGCCGCCCUGGUCCAACACCCAGUCGAAGGUGCUUGUCAGUACCCUACUUAGCACU
.((....((((((..((.((.(((((((.((((..(((((..(((...........).))..))))))))).))))))).)).)).))).))).((.....))...))...

>Predicted STAR seq:
CCCTGGTCCAACACCCAGTCGA

Complementarity: 16

Initial dG =   -35.20

```
            10         20        30        40         50
A--  .-GACA   -|  CC  A  A       U    GC     AA  - UUCCA
   GC      GAC AGC  CC UC ACUGGGU UUGG  ACCAG  CG C     \
   CG      CUG UCG  GG AG UGACCCA AACC  UGGUC  GC G     G
UCA  \ ----   U^  U-  A  C       C    --     CC  C UUUUA
 .           90        80        70          60



       A--  AC
          GU  \
          CA  C
       AUU  UC
           100
```

Reads in: lib-42, lib-45, lib-47, lib-48, lib-49, lib-50, lib-72
Number of reads="ID1709",0,12,3,7,3,3,0

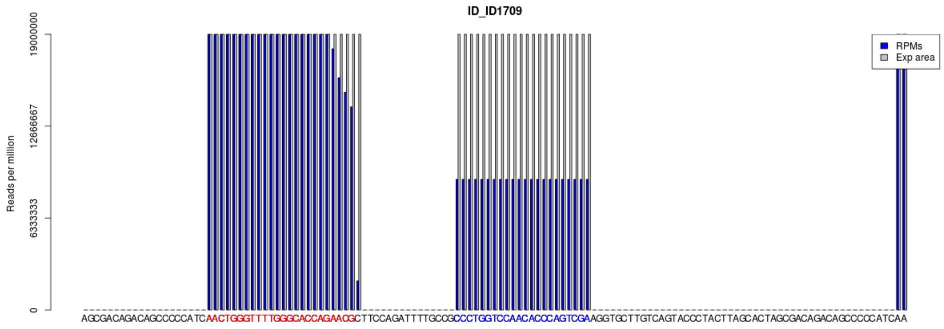

>ID171

TGGTGTGAATGGATGAACAAGATTTCTGATGTGAAAAAGTAGCACACTAGTTGTGTTAAAAATTGGTCAA**CTAGTGTGAAGCTTTCTCACATT**GGCATTCTTCAAAAAC

>ID171-3P

CTAGTGTGAAGCTTTCTCACATT

>miRNA precursor

UGGUGUGAAUGGAUGAACAAGAUUUCUGAUGUGAAAAAGUAGCACACUAGUUGUGUUAAAAAUUGGUCAA**CUAGUGUGAAGCUUUCUCACAUU**GGCAUUCUUCAAAAAC

.....((((.(((((..........(..((((((.(((((..(((((((((((..............)))))))))))..))))).)))))).)))))))))).....

>Predicted STAR seq:

GATGTGAAAAAGTAGCACACTAG

Complementarity: 17

Initial dG =   -36.60

```
        10        20        30        40        50        60
UGGUG|   U     AACAAGAUUU UG      A     AG          UGUUAA
     UGAA GGAUG          C  AUGUGA AAAGU  CACACUAGUUG      A
     ACUU CUUAC          G  UACACU UUUCG  GUGUGAUCAAC      A
CAAAA^   -     ---------- GU      C     AA          UGGUUA
        100                 90        80        70
```

Reads in: lib-42, lib-45, lib-47, lib-48, lib-49, lib-50, lib-72

Number of reads="ID171",772,975,1484,961,895,336,0

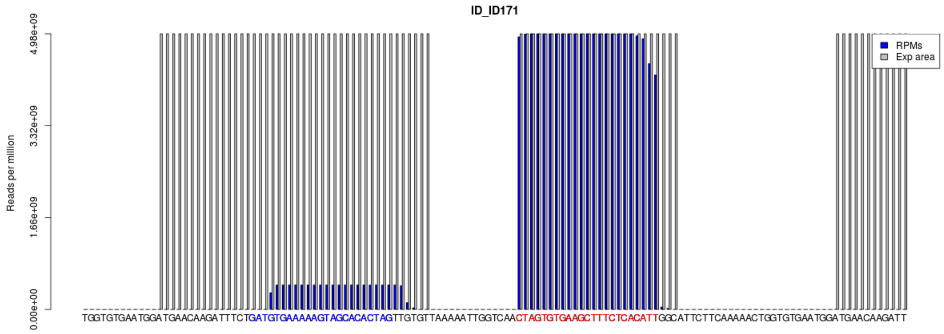

>ID1710
AGTAGAGGTTATAACAGGCAGTGCGTTTCTGGTGACGAGTTAGCTGGGTCAGGATCAGGGGCTCTGAGCT**GAAGTGACCCCCCTAACTCG**
TTGTCAGTGAGACGCCTGAAAC

>ID1710-3P
GAAGTGACCCCCCTAACTCGTT

>miRNA precursor
AGUAGAGGUUAUAACAGGCAGUGCGUUUCUGGUGACGAGUUAGCUGGGUCAGGAUCAGGGGCUCUGAGCU**GAAGUGACCCCCCUAACUCGUU**GUCAGUGAGACGCCUGAAAC
.................(((.((((((((((..(((((((((..((((((((.((((.....)))).))....))))))..)))))))))..)))..))))))))))....

>Predicted STAR seq:
GACGAGTTAGCTGGGTCA

Complementarity: 13

Initial dG =   -46.40

```
        10        20          30        40        50            60
AGUAGAGGUUAUAACAGG   U       --   UG         CU       ----| A    GG
                  CAG GCGUUUC  UGG  ACGAGUUAG  GGGUCA    GG UCAG  \
                  GUC CGCAGAG  ACU  UGCUCAAUC  CCCAGU    UC AGUC  G
CAAA--------------   -       UG   GU         CC       GAAG^ G    UC
110                    100        90        80        70
```

Reads in: lib-42, lib-45, lib-47, lib-48, lib-49, lib-50, lib-72
Number of reads="ID1710",0,1,0,1,1,1,0

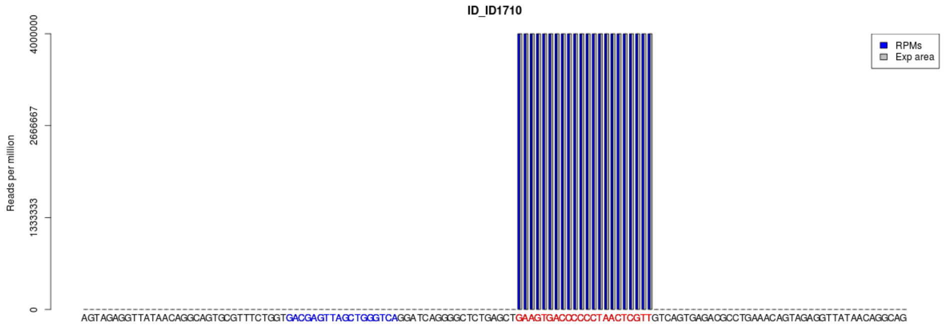

>ID1711
ATCAGATGCTGGCGTCTGGG**AAGTAGAAGGAACGTGAATCG**TGTCGTGTTCAAAAGTCGATTCATGGTCCTTCTACTGACACAGCCGTCA
GGAGATTCTTCAGTGCAT

>ID1711-5P
AAGTAGAAGGAACGTGAATCG

>miRNA precursor
AUCAGAUGCUGGCGUCUGGG**AAGUAGAAGGAACGUGAAUCG**UGUCGUGUUCAAAAGUCGAUUCAUGGUCCUUCUACUGACACAGCCGUCAGGAGAUUCUUCAGUGCAU
```
........((((((.(((...((((((((((.(((((((((................))))))))).))))))))))....))).))))))................
```

>Predicted STAR seq:
ATTCATGGTCCTTCTACTGACACA

Complementarity: 17

Initial dG = -41.00

```
                 10        20         30        40
AUCAGAUG---------      U   GGA-|         A         UGUCGUG
                 CUGGCG CUG    AGUAGAAGGA CGUGAAUCG       U
                 GACUGC GAC    UCAUCUUCCU GUACUUAGC       U
UACGUGACUUCUUAGAG      C   ACAG^         G         UGAAAAC
      100        90        80        70        60        50
```

Reads in: lib-42, lib-45, lib-47, lib-48, lib-49, lib-50, lib-72
Number of reads="ID1711",1,201,53,19,44,25,0

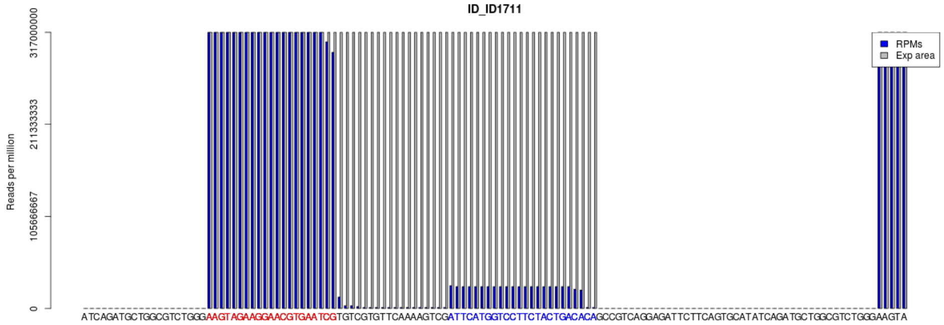

>ID1712
GATGAATTCTGGCGTCTGAG**TTATGTAGTAGGAAAGTGAATC**GTGCTATGACTGGAAATCGATTCACTTTCCTGCTACTGACTCAGCTGT
CAGATACTACATCTGTGCA

>ID1712-5P
TTATGTAGTAGGAAAGTGAATC

>miRNA precursor
GAUGAAUUCUGGCGUCUGAG**UUAUGUAGUAGGAAAGUGAAUC**GUGCUAUGACUGGAAAUCGAUUCACUUUCCUGCUACUGACUCAGCUGUCAGAUACUACAUCUGUGCA
((((...(((((((.((((((((.(((((((((((((((((((..(((....)))....)))))))))))))))))))))))))))).))))))).....))))......

>Predicted STAR seq:
TTCACTTTCCTGCTACTGACT

Complementarity: 19

Initial dG =   -56.80

```
                10        20        30        40          50
------    AAU--       U         U                   UG--|  U
      GAUG     UCUGGCG CUGAGUUA GUAGUAGGAAAGUGAAUCG    CUA G
      CUAC     AGACUGU GACUCAGU CAUCGUCCUUUCACUUAGC    GGU A
ACGUGU    AUCAU       C         -                   UAAA^  C
       100       90        80          70        60
```

Reads in: lib-42, lib-45, lib-47, lib-48, lib-49, lib-50, lib-72
Number of reads="ID1712",1,16,35,6,13,6,0

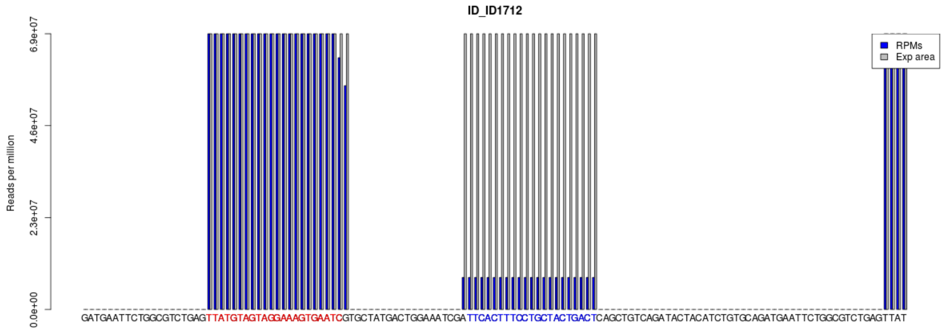

>ID1713
AGCGAAAGACAGCCCTCGTC**AACTGGGTTTTGGGCACCAGAACG**TTGCTAGATCTTACCGCCCTGGTCCAAAACCCAGTCGATGGTGTTG
TCTGCTCTCTTCTCGCCATTA

>ID1713-5P
AACTGGGTTTTGGGCACCAGAACG

>miRNA precursor
AGCGAAAGACAGCCCUCGUC**AACUGGGUUUUGGGCACCAGAACG**UUGCUAGAUCUUACCGCCCUGGUCCAAAACCCAGUCGAUGGUGUUGUCUGCUCUCUUCUCGCCAUUA
.((((.(((((((.(.((((.((((((((((((..(((((..((.((........)).))..))))))))))))))))).))))).)))))))).........)))).....

>Predicted STAR seq:
CCCTGGTCCAAAACCCAGTCGA

Complementarity: 17

Initial dG =   -43.00

```
                    10        20        30        40        50
A----    A--------|      C U    A            GC      AA  U  CUA
     GCGA         AGACAGC C CGUC ACUGGGUUUUGG  ACCAG  CG UG   G
     CGCU         UCUGUUG G GUAG UGACCCAAAACC  UGGUC  GC AU   A
AUUAC    CUUCUCUCG^      U -    C            --      CC  C  UCU
 .       100       90         80        70         60
```

Reads in: lib-42, lib-45, lib-47, lib-48, lib-49, lib-50, lib-72
Number of reads="ID1713",0,18,6,6,3,1,0

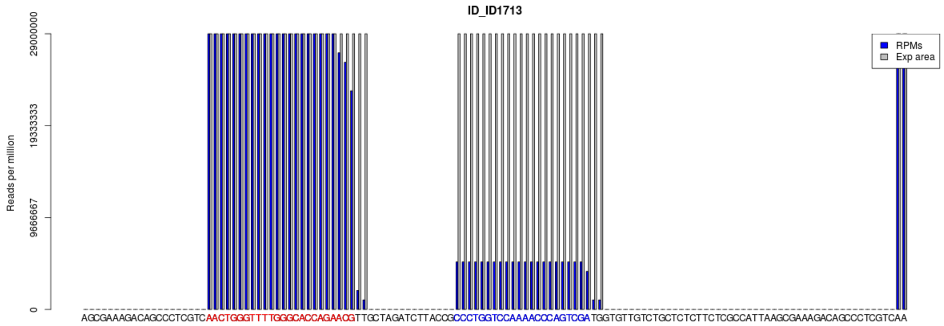

>ID1714
GGCTACTCCTGGTGTCTACA**ATGGTGTCAGGAATATGAGTCG**TGCCTTCTTCGTAAACCGAGACATATTCCTGATACTAACACAGCTGCC
AGGATTCTTCATGGCCTAAACA

>ID1714-5P
ATGGTGTCAGGAATATGAGTCG

>miRNA precursor
GGCUACUCCUGGUGUCUACA**AUGGUGUCAGGAAUAUGAGUCG**UGCCUUCUUCGUAAACCGAGACAUAUUCCUGAUACUAACACAGCUGCCAGGAUUCUUCAUGGCCUAAACA
```
(((((.((((((((.((....((((((((((((((((..((((((.......)))...)))..)))))))))))))))))....)).))))))))).......))))).......
```

>Predicted STAR seq:
AGACATATTCCTGATACTAACACAG

Complementarity: 17

Initial dG =   -41.70

```
                    10        20        30        40
------     C------        U  ACAA                AG    ---|  CUU
      GGCUA       UCCUGGUG CU    UGGUGUCAGGAAUAUG  UCG   UGC   \
      CCGGU       AGGACCGU GA    AUCAUAGUCCUUAUAC  AGC   AUG   C
ACAAAU     ACUUCUU        C  CACA                AG   CAA^  CUU
110       100        90        80        70        60        50
```

Reads in: lib-42, lib-45, lib-47, lib-48, lib-49, lib-50, lib-72
Number of reads="ID1714",0,67,31,10,8,6,0

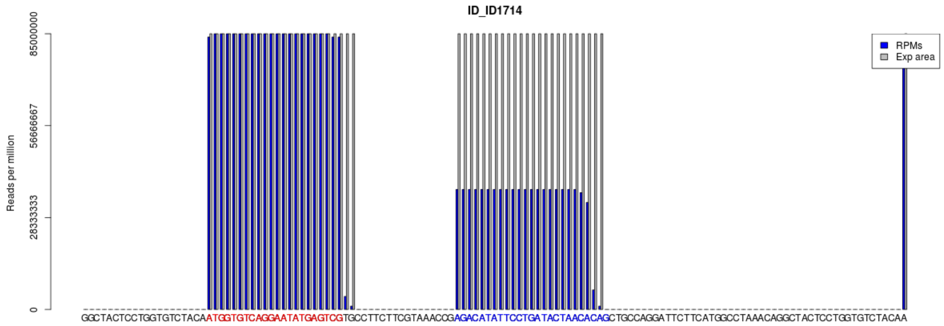

>ID1725
GAAGTGAGGGTGTGTCCTTG**TGAAAGACATGGGTAGTGAGAT**GATACTTGAAGTTTCCTTGCATCTCACTACCTTGTCTTTCATGAGGCT
CCAGCCTACAGACCTACTAG

>ID1725-5P
TGAAAGACATGGGTAGTGAGAT

>miRNA precursor
GAAGUGAGGGUGUGUCCUUG**UGAAAGACAUGGGUAGUGAGAU**GAUACUUGAAGUUUCCUUGCAUCUCACUACCUUGUCUUUCAUGAGGCUCCAGCCUACAGACCUACUAG
...((.(((.((...(((..(((((((((.((((((((((((......((....))....)))))))))))))))))))))..)))...)).)))))..........

>Predicted STAR seq:
CTCACTACCTTGTCTTTCATGAG

Complementarity: 19

Initial dG =   -48.00

```
                10        20        30        40        50
GAA--------| G   G  UGU   UG        U             AUACUU  A
           GU AGG UG   CCU  UGAAAGACA GGGUAGUGAGAUG      GA G
           CA UCC AC   GGA  ACUUUCUGU UCCAUCACUCUAC      CU U
GAUCAUCCAGA^ -   G  CUC   GU        -             GUUC--  U
.       100         90        80         70        60
```

Reads in: lib-42, lib-45, lib-47, lib-48, lib-49, lib-50, lib-72
Number of reads="ID1725",20297,15138,24606,15992,48906,12479,0

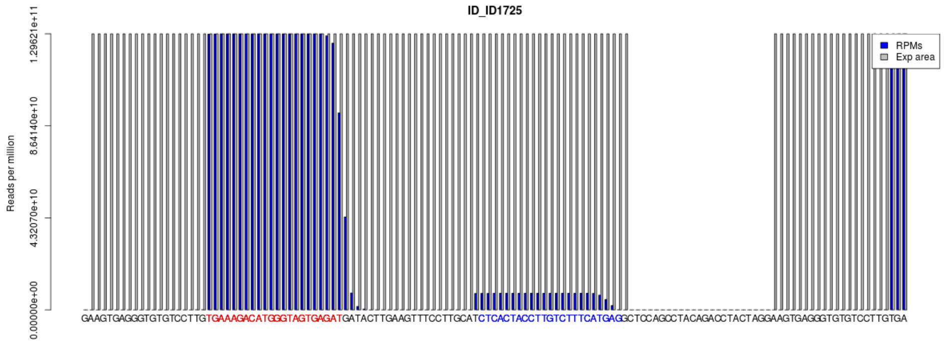

>ID1726
GCAACTGATGAAGCTGTGGGTCAGATTGTCGCATCAAAGCTGGTTTGTCATAGAGTTGTCCGAGAGGCGC**TATCACAGCCAGCTTTGATG**
AGCACAAACTGGCCTCTACTC

>ID1726-3P
TATCACAGCCAGCTTTGATGAGC

>miRNA precursor
GCAACUGAUGAAGCUGUGGGUCAGAUUGUCGCAUCAAAGCUGGUUUGUCAUAGAGUUGUCCGAGAGGCGC**UAUCACAGCCAGCUUUGAUGAGC**ACAAACUGGCCUCUACUC
(((.((.....)).))).((((((.((((..((((((((((((((.((.((((...((.((....)))))))).))))))))))))))))...)))).))))))).......

>Predicted STAR seq:
GCATCAAAGCTGGTTTGTCATA

Complementarity: 17
ERROR 4: Strange structure
Initial dG =   -44.60

```
  |  A  GA
--GCA CU  \
  UGU GA  U
\ ^  C  AG
        10


         20        30         40        50        60
  G------      A    CG-             U  C    AGU  U  G
         GGUCAG UUGU   CAUCAAAGCUGGUU GU AUAG   UG CC A
         CCGGUC AACA   GUAGUUUCGACCGA CA UAUC   GC GG G
  CUCAUCU      A    CGA             -  C    ---  -  A
 110       100        90        80         70
```

Reads in: lib-42, lib-45, lib-47, lib-48, lib-49, lib-50, lib-72
Number of reads="ID1726",4985,8377,15156,4964,9604,3672,0

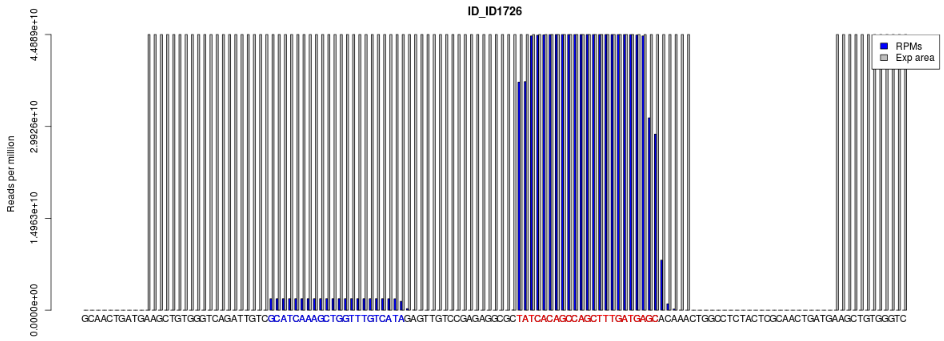

>ID1727
AACTGTACATGGTGAGATATCCCTGTGGCCTACCAGTCAAATTGGTTGTGATGTGTTGGAATTTAGATCA**TATCACAGCCATTTTTGACG**
AGTTGGGCCATTGGTTATTCC

>ID1727-3P
TATCACAGCCATTTTTGACGAGTT

>miRNA precursor
AACUGUACAUGGUGAGAUAUCCCUGUGGCCUACCAGUCAAAUUGGUUGUGAUGUGUUGGAAUUUAGAUCA**UAUCACAGCCAUUUUUGACGAGUU**GGGCCAUUGGUUAUUCC
.(((......)))..((((.((..(((((((((..((((((.((((((((((((((((........)).))))))))))))))..)))))).)..).)))))))).)).))))..

>Predicted STAR seq:
CCAGTCAAATTGGTTGTGATG

Complementarity: 17
ERROR 4: Strange structure
Initial dG = -42.30

```
.-A|  GU
   ACU  A
   UGG  C
\ -^  UA
     10


       20        30         40         50         60
  GA    U  CU        - CA      U-              -  GGA
    GAUA CC  GUGGCCUA C  GUCAAA  UGGUUGUGAUGUG UU   A
    UUAU GG  UACCGGGU G  CAGUUU  ACCGACACUAUAC AG   U
  CC    U  U-        U AG      UU              U  AUU
 110        100        90        80        70
```

Reads in: lib-42, lib-45, lib-47, lib-48, lib-49, lib-50, lib-72
Number of reads="ID1727",22780,15315,30390,24287,38431,11940,0

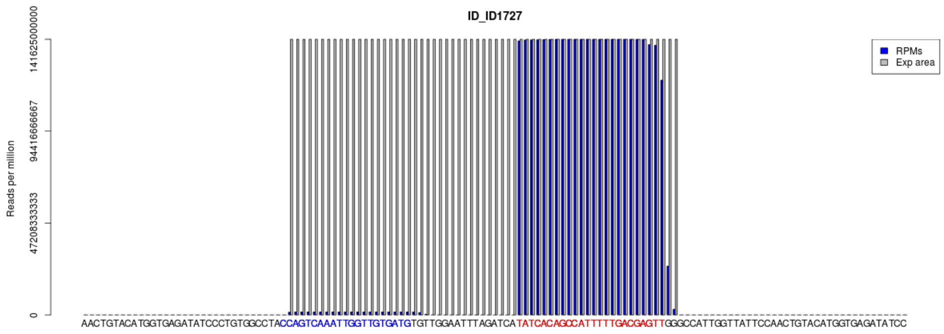

>ID1728
CCGACTTCAGCAGATTGGAAATAGCTGCACTGTGTTCGTCAAAGTGGCTGTGATGTGTGTGTTGTTGTCA**TATCACAGCCAGCTTTGATG**
AGCATATTGTGGCATGAGCAA

>ID1728-3P
TATCACAGCCAGCTTTGATGAGC

>miRNA precursor
CCGACUUCAGCAGAUUGGAAAUAGCUGCACUGUGUUCGUCAAAGUGGCUGUGAUGUGUGUGUUGUUGUCA**UAUCACAGCCAGCUUUGAUGAGC**AUAUUGUGGCAUGAGCAA
((((((.....)).)))).....((..((.(((((((((((((((((((((((((...........))))))))))))).))))))))))))).))..))........

>Predicted STAR seq:
TGTTCGTCAAAGTGGCTGTGA

Complementarity: 20
ERROR 4: Strange structure
Initial dG =   -52.00

```
      -| UC
--CCGA CU  \
  GGUU GA   A
\     A^ CG
         10
```

```
  20           30        40          50        60
  AAAUA---  UG  C               -             UGUGU
          GC  CA UGUGUUCGUCAAAG UGGCUGUGAUGUG     \
          CG  GU AUACGAGUAGUUUC ACCGACACUAUAC     U
  AACGAGUA  GU  U               G             UGUUG
 110       100        90         80         70
```

Reads in: lib-42, lib-45, lib-47, lib-48, lib-49, lib-50, lib-72
Number of reads="ID1728",5869,9073,15706,6017,12148,4136,0

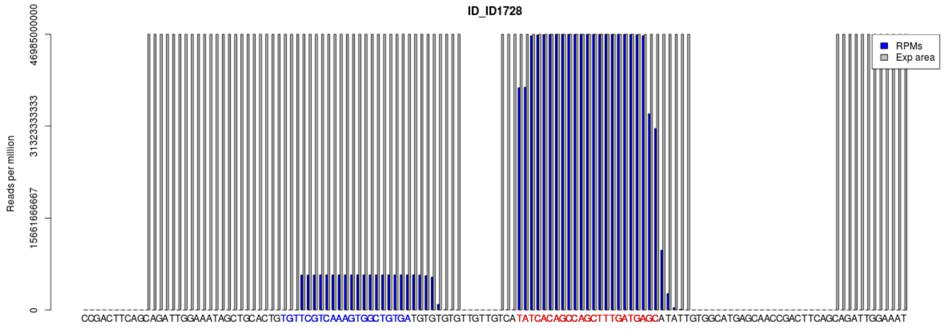

>ID1729
GCCATAGCTGCAGTTAGCAGTAACCAGTTGCTCTGCTCACAAAGCTGTTGTGATGTGTCGTTGTTTATCA**TATCACAGCCAGCTTTGATG**
AGCGGTGCGGCAGGTGTCACT

>ID1729-3P
TATCACAGCCAGCTTTGATGA

>miRNA precursor
GCCAUAGCUGCAGUUAGCAGUAACCAGUUGCUCUGCUCACAAAGCUGUUGUGAUGUGUCGUUGUUUAUCA**UAUCACAGCCAGCUUUGAUGA**GCGGUGCGGCAGGUGUCACU
(((...(((((.....))))).....(((((.(((((((((((((((((((((((((...........))))))))))).)))))))).))))))).))))).)))......

>Predicted STAR seq:
GCTCACAAAGCTGTTGTG

Complementarity: 17
ERROR 4: Strange structure
Initial dG =   -53.70

```
                10
------   .-AUA|    AG
      GCC     GCUGC  \
      UGG     UGACG  U
UCACUG   \ ---^    AU
 .            20
```

```
                  30         40         50        60
           AACCA     U       -        -          UCGUU
                GUUGC CUGCUCA CAAAGCUG UUGUGAUGUG     \
                CGGCG GGCGAGU GUUUCGAC GACACUAUAC     G
           A----     U       A        C          UAUUU
               100        90        80        70
```

Reads in: lib-42, lib-45, lib-47, lib-48, lib-49, lib-50, lib-72
Number of reads="ID1729",4623,7944,18520,4719,9670,3532,0

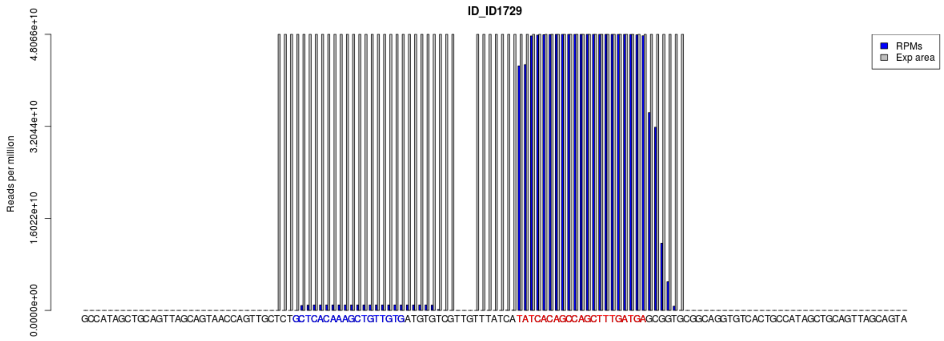

>ID1730
AACTTCGAGCTGACTGCCTATTTGCCAAGGCCGGCCCGTCAACCTGGCTGTGCCATGTCCCGTCTGTGCA**TATCACAGCCACTTTGATGA**
GCTCGGACTTGGCCCAAGAT

>ID1730-3P
TATCACAGCCACTTTGATGAGCT

>miRNA precursor
AACUUCGAGCUGACUGCCUAUUUGCCAAGGCCGGCCCGUCAACCUGGCUGUGCCAUGUCCCGUCUGUGCA**UAUCACAGCCACUUUGAUGAGCU**CGGACUUGGCCCAAGAU
....((..((.....))......((((((.(((((.((((((..((((((((..((((.........))))..))))))))..)))))).)).))).))))))....)).

>Predicted STAR seq:
CCCGTCAACCTGGCTGTGC

Complementarity: 15
ERROR 4: Strange structure
Initial dG = -42.00

```
          10
AACU  .-GA| UG
    UC    GC  \
    AG    CG  A
U---  \ --^ UC
.



         20        30         40        50        60
        CUAUUU      G    -   C      CC        CC     CCCG
              GCCAAG CCG GC CGUCAA  UGGCUGUG  AUGU    \
              CGGUUC GGC CG GUAGUU  ACCGACAC  UACG    U
        AACC--      A    U   A      UC        UA     UGUC
               100        90        80        70
```

Reads in: lib-42, lib-45, lib-47, lib-48, lib-49, lib-50, lib-72
Number of reads="ID1730",23664,16990,23271,18542,22188,8151,0

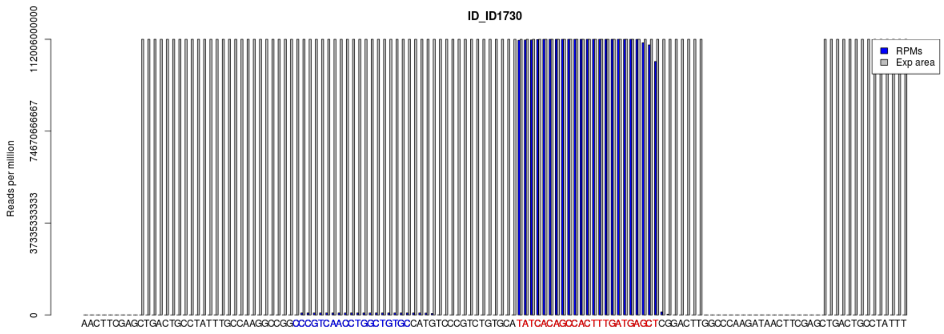

>ID1735
ACTATCTGCCTGCGGAGCTCCATCACGCGCCTGACATTGGAGTTTCCAGTAACTAATAATTCCTACGTTA**CTGGGTACTCCAATGTCAGG**
GGTGTGATGGAGCGCACTCCCC

>ID1735-3P
CTGGGTACTCCAATGTCAGGGG

>miRNA precursor
ACUAUCUGCCUGCGGAGCUCCAUCACGCGCCUGACAUUGGAGUUUCCAGUAACUAAUAAUUCCUACGUUA**CUGGGUACUCCAAUGUCAGGGG**UGUGAUGGAGCGCACUCCCC
....((((....))))((((((((((((.((((((((((((((.(((((((((............))))))))).))))))))))))))).)))))))))))).........

>Predicted STAR seq:
GCCTGACATTGGAGTTTCCAG

Complementarity: 18
ERROR 4: Strange structure
Initial dG = -67.70

```
          10
.-ACUA|   C
      UCUG C
      AGGC U
\ ----^   G
```

```
             20        30        40        50
  ---------            G              U         UAAUAA
           GCUCCAUCACGC CCUGACAUUGGAGU UCCAGUAAC      \
           CGAGGUAGUGUG GGACUGUAACCUCA GGGUCAUUG      U
  CCCCUCACG            G              U         CAUCCU
  110       100        90        80        70
```

Reads in: lib-42, lib-45, lib-47, lib-48, lib-49, lib-50, lib-72
Number of reads="ID1735",288,387,563,3164,620,265,0

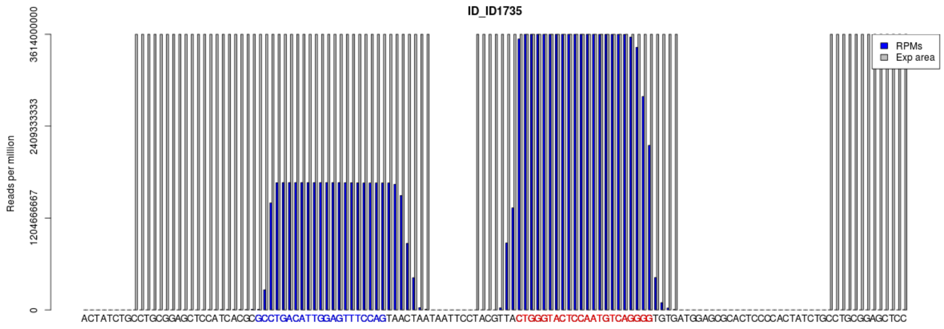

>ID174
TGACTGTCTGTCAACGGAGGAGCCTTCGACAGTCTGGAGCGAACTGCCTGGGGGGCAGCAGCGCGACGTG**CCGACCAGCCAGTTCGTTCC**
AGACTGCCAGGAGGTGCGCGC

>ID174-3P
CCGACCAGCCAGTTCGTTCCAGA

>miRNA precursor
UGACUGUCUGUCAACGGAGGAGCCUUCGACAGUCUGGAGCGAACUGCCUGGGGGGCAGCAGCGCGACGUG**CCGACCAGCCAGUUCGUUCCAGA**CUGCCAGGAGGUGCGCGC
((((.....))))........((((((..(((((((((((((((((.((((..((((.(........)))))..)))).)))))))))))))))))...))))))......

>Predicted STAR seq:
CTGGAGCGAACTGCCTGGGGGG

Complementarity: 18
ERROR 4: Strange structure
Initial dG =   -58.40

```
  |   UG
--UGAC  \
  ACUG  U
\ ^   UC
    10
```

```
       20         30         40         50         60
  ACGGAGGA      GA-                 C     GG     G AGC
          GCCUUC   CAGUCUGGAGCGAACUG CUGG  GGCA C   G
          UGGAGG   GUCAGACCUUGCUUGAC GACC  CCGU G   C
  CGCGCG--      ACC                 C     AG     - CAG
 110        100        90         80         70
```

Reads in: lib-42, lib-45, lib-47, lib-48, lib-49, lib-50, lib-72
Number of reads="ID174",680,931,1259,1766,4176,501,0

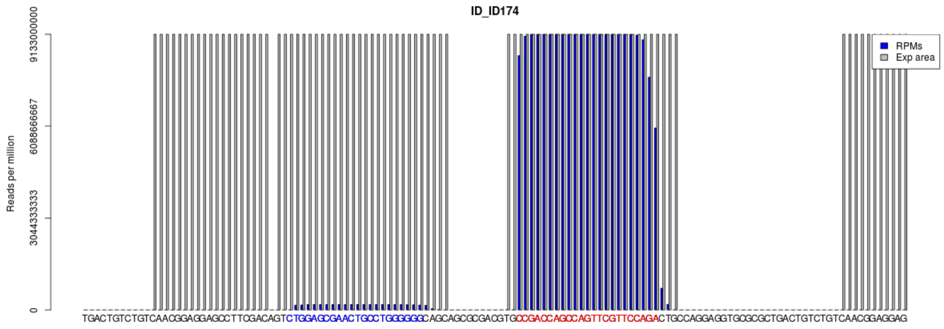

>ID1742
CAGTACTTCGTGGGTAGCAGTAGATGCAGTTCTGCCAGTTGACGTGGGAATAAGTCG**TCAGTGGTTGAACTACAACTATT**TGCTGTCCAC
GCCAGTCAACGAAGAGAACAGG

>ID1742-3P
TCAGTGGTTGAACTACAACTATT

>miRNA precursor
CAGUACUUCGUGGGUAGCAGUAGAUGCAGUUCUGCCAGUUGACGUGGGAAUAAGUCG**UCAGUGGUUGAACUACAACUAUU**UGCUGUCCACGCCAGUCAACGAAGAGAACAGG
........(((((..((((((((.((.(((((.((((.((((((...........)))))))))).))))).)).))).)))))..)))))((..((........))...))

>Predicted STAR seq:
TAGATGCAGTTCTGCCAGTTGA

Complementarity: 17

Initial dG =   -39.50

```
                     10         20        30        40
CA----------  --   U-     GU   -|    A  C     U    G      UGGGA
            GU  ACU  CGUGG  AGC AGUAG UG AGUUC GCCA UUGACG     \
            CA  UGA  GCACC  UCG UUAUC AC UCAAG UGGU GACUGC     A
GGACAAGAGAAG  AC   CC     UG   U^    A  A     U    -      UGAAU
110       100        90        80        70         60
```

Reads in: lib-42, lib-45, lib-47, lib-48, lib-49, lib-50, lib-72
Number of reads="ID1742",0,0,0,1,1,0,0

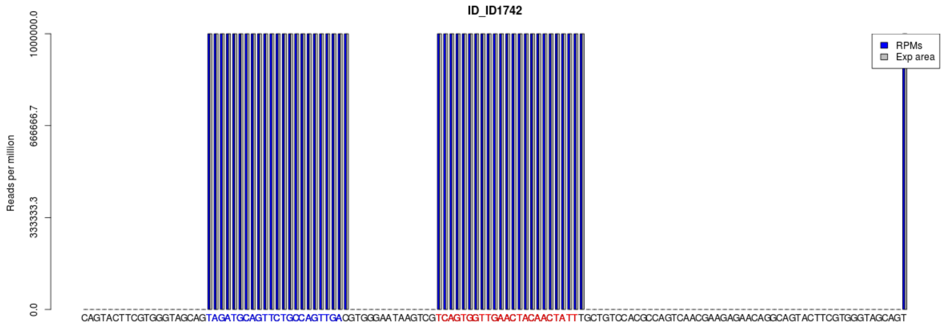

>ID1743
GCCCCCACGAGCACAATGCTACGCTTTAGCTGGTTGAATCCGGGCCAAATTGTACCACAGTTGAAGACAT**TTGGTCCCCTTCAACCAGCT**
GTAGTTGGCATTACGAGC

>ID1743-3P
TTGGTCCCCTTCAACCAGCTGT

>miRNA precursor
GCCCCCACGAGCACAAUGCUACGCUUUAGCUGGUUGAAUCCGGGCCAAAUUGUACCACAGUUGAAGACAU**UUGGUCCCCUUCAACCAGCUGU**AGUUGGCAUUACGAGC
((........))..(((((((.(((.((((((((((((...(((((((((.((..((....))...)))))))))))..)))))))))))).))))))))))......

>Predicted STAR seq:
TAGCTGGTTGAATCCGGGCCAA

Complementarity: 17
ERROR 4: Strange structure
Initial dG = -41.70

```
  | CCC
--GC   C
  CG   A
\ ^ AGC
   10


            20        30        40        50
  AC----       C   U            UCC         U  AC-  C
        AAUGCUA GCU UAGCUGGUUGAA   GGGCCAAAU GU   CA A
        UUACGGU UGA GUCGACCAACUU   CCUGGUUUA CA   GU G
  CGAGCA       -   U            CC-         -  GAA  U
        100        90        80         70         60
```

Reads in: lib-42, lib-45, lib-47, lib-48, lib-49, lib-50, lib-72
Number of reads="ID1743",112,3709,2565,2123,476,1409,0

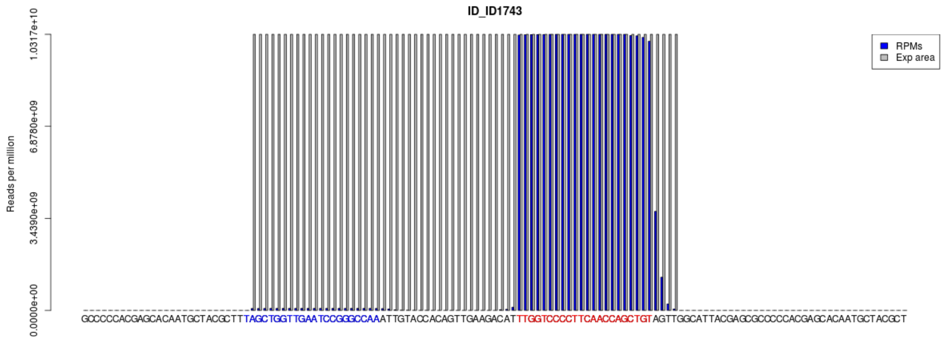

>ID1745
NNNNNNNGGCGGCGACGGCCTGGCGCCAGTTCCGTACTTCCTTACTTCTCATATTGATGTTTGAAATTTA**TGGAATGTAAAGAAGTATGG**
AGCGATCGCGGGCAGCTT

>ID1745-3P
TGGAATGTAAAGAAGTATGGAG

>miRNA precursor
NNNNNNNGGCGGCGACGGCCUGGCGCCAGUUCCGUACUUCCUUACUUCUCAUAUUGAUGUUUGAAAUUUA**UGGAAUGUAAAGAAGUAUGGAG**CGAUCGCGGGCAGCUU
.........((....))(((((.((...(((((((((((((.(((((((.((((...............))))))).)))).)))))))))))))...))))))).....

>Predicted STAR seq:
TCCGTACTTCCTTACTTCTCA

Complementarity: 18
ERROR 4: Strange structure
Initial dG =   -37.50

```
          10
.-NNNNNNNGG| G
           CG C
           GC G
\ ---------^ A




        20        30        40         50        60
  -----     G  CCA            C    -    U    UUGAUGU
       GCCUG CG   GUUCCGUACUUC UUAC UUC CAUA       \
       CGGGC GC   CGAGGUAUGAAG AAUG AAG GUAU       U
  UUCGA     -  UAG            A    U    -    UUAAAGU
        100        90        80         70
```

Reads in: lib-42, lib-45, lib-47, lib-48, lib-49, lib-50, lib-72
Number of reads="ID1745",12779,375524,55465,27305,18476,101262,0

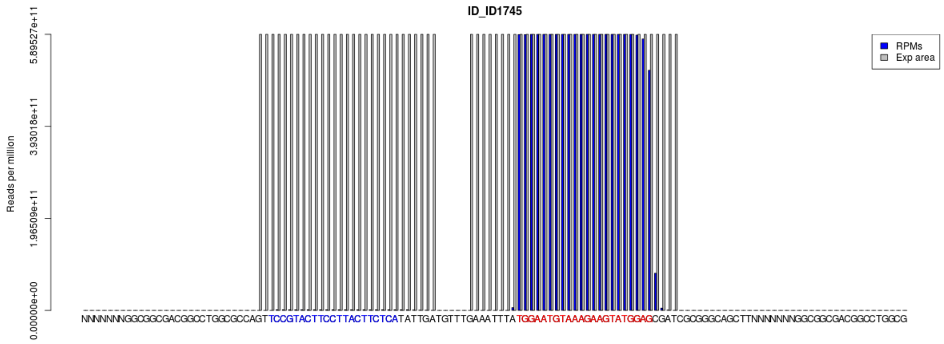

>ID1747
CTGTAAAACACCAGTTGCC**CGGCTAACAGTAGTGCGCCCCGGC**GAGACAGAGGATGCCCGGGGCCGGCTACTGTTAGGCGGGTGACGGGT
GAGTCACGCATCGATGGAGGAGG

>ID1747-5P
CGGCTAACAGTAGTGCGCCCCGGC

>miRNA precursor
CUGUAAAACACCAGUUGCC**CGGCUAACAGUAGUGCGCCCCGGC**GAGACAGAGGAUGCCCGGGGCCGGCUACUGUUAGGCGGGUGACGGGUGAGUCACGCAUCGAUGGAGGAGG
```
........((((.((..((((.(((((((((((..(((((((((..........)).)))))))..))))))))))).)))).)).)))).(((.......)))........
```

>Predicted STAR seq:
CCGGGGCCGGCTACTGTTAGGCGGG

Complementarity: 19

Initial dG = -55.90

```
                      10        20        30        40
CUGUAAAA--------------    A  UG    G           GC     -|   AGAC
                      CACC GU  CCCG CUAACAGUAGU  GCCCC GGCG    A
                      GUGG CA  GGGC GAUUGUCAUCG  CGGGG CCGU    G
GGAGGAGGUAGCUACGCACUGA    G  GU    G           GC     C^   AGGA
 110       100        90        80        70        60        50
```

Reads in: lib-42, lib-45, lib-47, lib-48, lib-49, lib-50, lib-72
Number of reads="ID1747",144,138,107,382,261,26,0

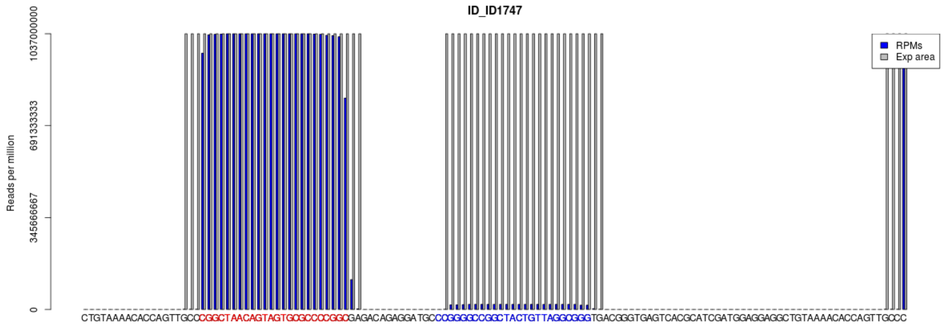

>ID1748
GGCGCCGTGCACACTCAGGTACGTGTGTCCTGTCGTGCTGAACAGTCGAGTATTCCGCCGGTTGTTCCAG**TACCAGCCGGATATTCGACT**
GTTCATCACGACAACGCCTCGC

>ID1748-3P
TACCAGCCGGATATTCGACTGT

>miRNA precursor
GGCGCCGUGCACACUCAGGUACGUGUGUCCUGUCGUGCUGAACAGUCGAGUAUUCCGCCGGUUGUUCCAG**UACCAGCCGGAUAUUCGACUGU**UCAUCACGACAACGCCUCGC
```
(((((((((........))))).))))).(((((((.(((((((((((((((((.((.(((.........))).)).))))))))))))))))).))))))).........
```

>Predicted STAR seq:
CACTCAGGTACGTGTGTCCTGTCGTGCTG

Complementarity: 17

Initial dG =   -56.40

```
              10
----    .-CCGU|     UCA
    GGCG      GCACAC   G
    CCGC      UGUGUG   G
CGCU    \ ----^     CAU
110                  20
```

Reads in: lib-42, lib-45, lib-47, lib-48, lib-49, lib-50, lib-72
Number of reads="ID1748",3,227,41,163,9,12,0

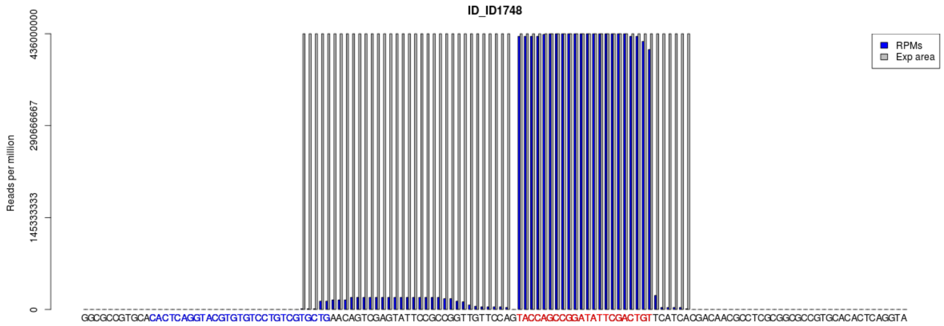

>ID1752
CTCCTAAGATTACCCCGGGACGGCAGACCATGATCTCGACGGCCCAGACTGAGAAGAGGCAACTCTTCAT**AAAGTCGAAAGCTGAAGAAT**
CTGGTGCGGATCTCAAGAAGAGAT

>ID1752-3P
AAAGTCGAAAGCTGAAGAATCTGG

>miRNA precursor
CUCCUAAGAUUACCCCGGGACGGCAGACCAUGAUCUCGACGGCCCAGACUGAGAAGAGGCAACUCUUCAU**AAAGUCGAAAGCUGAAGAAUCUGG**UGCGGAUCUCAAGAAGAGAU
.((((..........))))..((..((((.(((.((..((((...((((..((((((....))))))....))))....))))..))))))))))).(((((.....)))))

>Predicted STAR seq:
AAGATTACCCCGGGACGG

Complementarity: 14
ERROR 4: Strange structure
 Initial dG =   -34.90

```
          10
.-C|   AAGAU
   UCCU     \
   AGGG     U
\ -^   CCCCA
  20



            30        40         50          60
  .-CG  AG    U   C  GA    CCA-    GA--       G
      GC  ACCA GAU UC  CGGC    GACU    GAAGAG C
      CG  UGGU CUA AG  GUCG    CUGA    CUUCUC A
  \ --  --    -   -  AA    AAAG    AAUA       A
               90         80        70
```

Reads in: lib-42, lib-45, lib-47, lib-48, lib-49, lib-50, lib-72
Number of reads="ID1752",50,28,21,36,83,43,0

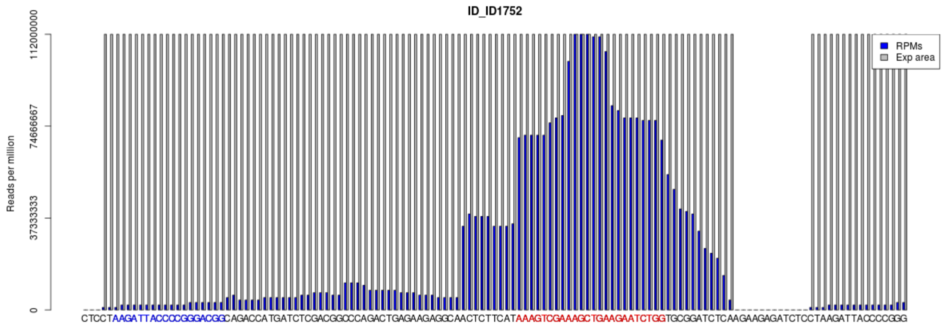

>ID1754
GTGTCTCTCCGTATGTGTTTGCGTGTCTGTCATCGCCTCAGTCTTTGCCCCATATTCATCATCAACATTA**TCGGCACAGAGTGAGGTGAT**
GGTAGACACGCAGCGGTCGACG

>ID1754-3P
TCGGCACAGAGTGAGGTGATGG

>miRNA precursor
GUGUCUCUCCGUAUGUGUUUGCGUGUCUGUCAUCGCCUCAGUCUUUGCCCCAUAUUCAUCAUCAACAUUA**UCGGCACAGAGUGAGGUGAUGG**UAGACACGCAGCGGUCGACG
```
..(((...((((.......(((((((((..((((((((((.(((.((((......................)))).))).))))))))))..)))))))))))))..))).
```

>Predicted STAR seq:
TCATCGCCTCAGTCTTTGCC

Complementarity: 15

Initial dG =   -53.30

```
        10        20        30        40        50        60
GU|  UCU    AUGUGUU         GU           G   U    CCAUAUUCAUC
  GUC   CCGU       UGCGUGUCU  CAUCGCCUCA UCU UGCC           \
  CAG   GGCG       ACGCACAGA  GUAGUGGAGU AGA ACGG           A
G-^  CU-    -------         UG           G   C    CUAUUACAACU
 110              100        90        80        70
```

Reads in: lib-42, lib-45, lib-47, lib-48, lib-49, lib-50, lib-72
Number of reads="ID1754",3,10,0,591,3,0,0

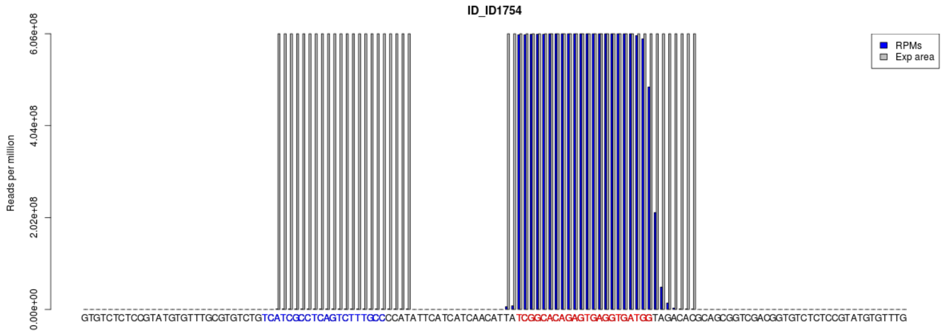

>ID1764
TAAGGTGACCGGTTTCGATC**TACGAAAAATTCATCATCCGCC**CGAAAATTCGTCGCAACATTGGCGCCGCACCCTCATCTCGTCGGGTGA
TGAATTCTGCGTAAATCGAAAC

>ID1764-5P
TACGAAAAATTCATCATCCGCC

>miRNA precursor
UAAGGUGACCGGUUUCGAUC**UACGAAAAAUUCAUCAUCCGCC**CGAAAAUUCGUCGCAACAUUGGCGCCGCACCCUCAUCUCGUCGGGUGAUGAAUUCUGCGUAAAUCGAAAC
...........((((((((.((((...(((((((((((((..(((.....((.(((.......))).)).........))).))))))))))))))...)))).))))))))

>Predicted STAR seq:
GGTGATGAATTCTGCGTAAAT

Complementarity: 15

Initial dG =   -36.20

```
        10        20        30        40        50
UAAGGUGACCG        C    AAA             CC   AAAUU-----| U   AAC
           GUUUCGAU UACG   AAUUCAUCAUCCG  CGA          CG CGC   \
           CAAAGCUA AUGC   UUAAGUAGUGGGC  GCU          GC GCG   A
-----------        A    GUC             U-   CUACUCCCAC^ C   GUU
           110       100        90         80        70
```

Reads in: lib-42, lib-45, lib-47, lib-48, lib-49, lib-50, lib-72
Number of reads="ID1764",363,128,354,514,296,232,0

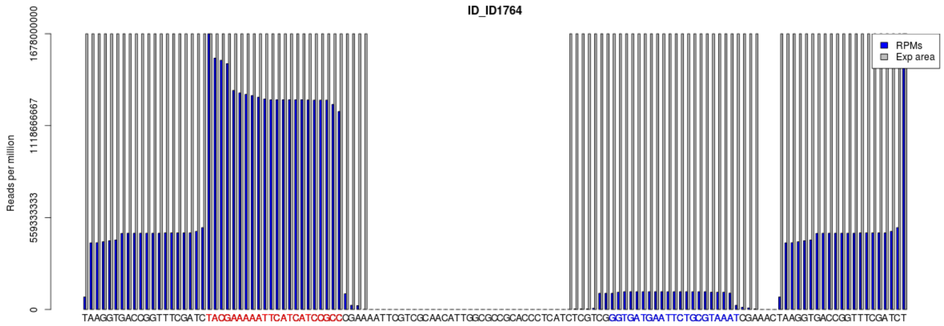

>ID1766
TAATTCATTTGTGATATAAACTGTGGTCTGTTC**TGCTGGACACTGAATACAGCAG**TTTGGTGTGTTATCTGCTGTAGGCAGTGTCCAGTG
TGCCGACTACAGACAGTCATC

>ID1766-5P
TGCTGGACACTGAATACAGCAG

>miRNA precursor
UAAUUCAUUUGUGAUAUAAACUGUGGUCUGUUC**UGCUGGACACUGAAUACAGCAG**UUUGGUGUGUUAUCUGCUGUAGGCAGUGUCCAGUGUGCCGACUACAGACAGUCAUC
..........(((((.....((((((((.((...(((((((((((..((((((((.............))))))))..)))))))))))..)).))))))))...))))).

>Predicted STAR seq:
GCTGTAGGCAGTGTCCAGTGTGC

Complementarity: 17

Initial dG =   -49.90

```
        10        20        30        40        50        60
UAAUUCAUUU|    AUAAA        U  UCU           AA         UUUGGU
          GUGAU     CUGUGGUC GU   GCUGGACACUG  UACAGCAG       \
          UACUG     GACAUCAG CG   UGACCUGUGAC  AUGUCGUC       G
C---------^    ACA--        C  UG-           GG         UAUUGU
        110        100        90         80        70
```

Reads in: lib-42, lib-45, lib-47, lib-48, lib-49, lib-50, lib-72
Number of reads="ID1766",6,65,214,528,21,14,0

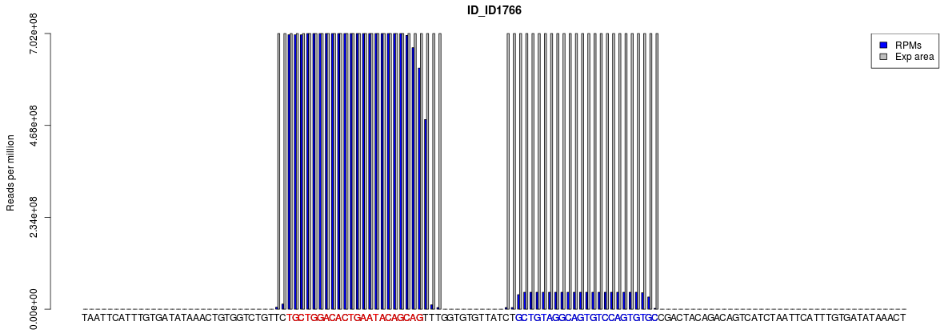

>ID1767
CCATAATCTGTGGTCTGTCC**TGCTGGACACTGAATACAGCGG**TTTAGTGTGTTATCTGCTGTAGACAGTGTCCAGTGTGCAGACTACAGA
CAATCATCGGTCTTCGGCGAGT

>ID1767-5P
TGCTGGACACTGAATACAGCGG

>miRNA precursor
CCAUAAUCUGUGGUCUGUCC**UGCUGGACACUGAAUACAGCGG**UUUAGUGUGUUAUCUGCUGUAGACAGUGUCCAGUGUGCAGACUACAGACAAUCAUCGGUCUUCGGCGAGU
......((((((((((((...(((((((((((..((((((((.............))))))))..)))))))))))..)))))))))))))...((..((.....))..))..

>Predicted STAR seq:
GCTGTAGACAGTGTCCAGTGTGC

Complementarity: 17

Initial dG = -52.90

```
          10        20        30        40
.-CCAUAA|           CCU           AA        UUUAGU
        UCUGUGGUCUGU   GCUGGACACUG  UACAGCGG      \
        AGACAUCAGACG   UGACCUGUGAC  AUGUCGUC      G
\ ------^           UG-           AG        UAUUGU
       90        80         70        60        50



         100
  CAA  AU  GU
     UC  CG  \
     AG  GC  C
  UG-  CG  UU
   110
```

Reads in: lib-42, lib-45, lib-47, lib-48, lib-49, lib-50, lib-72
Number of reads="ID1767",12,61,172,1275,3,10,0

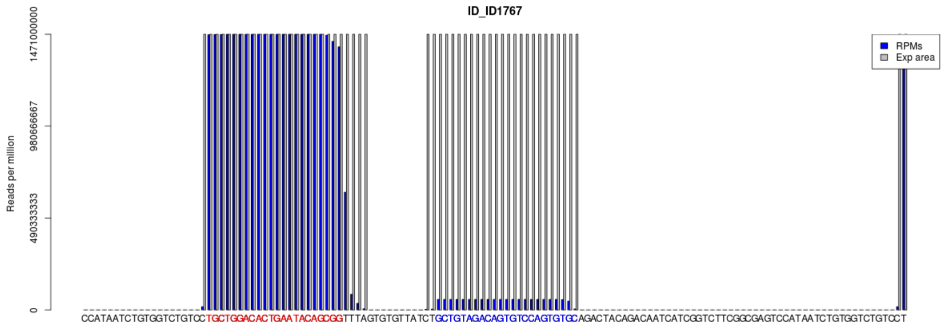

>ID1768
ATGACTGTCTGTAGTCGGCA**CACTGGACACTGCCTACAGCAG**ATAACACACCAAACTGCTGTATTCAGTGTCCAGCAGAACAGACCACAG
TTTATATCACAAATGAATTATA

>ID1768-5P
CACTGGACACTGCCTACAGCAG

>miRNA precursor
AUGACUGUCUGUAGUCGGCA**CACUGGACACUGCCUACAGCAG**AUAACACACCAAACUGCUGUAUUCAGUGUCCAGCAGAACAGACCACAGUUUAUAUCACAAAUGAAUUAUA
```
.(((....((((.(((......((((((((((..((((((((.............))))))))..))))))))))).......))).)))).....))).............
```

>Predicted STAR seq:
GCTGTATTCAGTGTCCAGCAGAACAGA

Complementarity: 16

Initial dG =   -37.60

```
                      10        20         30        40
A------------   CUGU--    A   GGCACA-|         CC         AUAACA
             UGA      CUGU GUC       CUGGACACUG  UACAGCAG      \
             ACU      GACA CAG       GACCUGUGAC  AUGUCGUC      C
AUAUUAAGUAAAC   AUAUUU    C   ACAAGAC^         UU         AAACCA
110       100        90        80        70        60        50
```

Reads in: lib-42, lib-45, lib-47, lib-48, lib-49, lib-50, lib-72
Number of reads="ID1768",14,294,139,1156,83,52,0

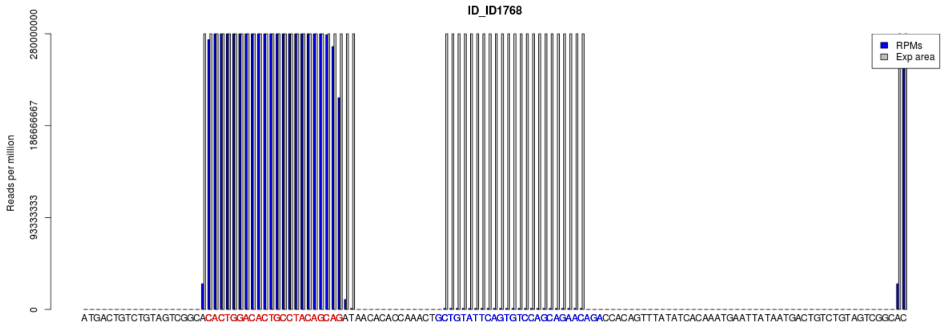

>ID1769
GATACAGCAATCCATGTATGATATAATCTGTGGTCTGTTC**TGCTGGACACTGAATACAGCAGT**TTAGTGTGTTATCTGCTGTAGACAGTG
TCCAGTGTGCAGACTACAGA

>ID1769-5P
TGCTGGACACTGAATACAGCAGT

>miRNA precursor
GAUACAGCAAUCCAUGUAUGAUAUAAUCUGUGGUCUGUUC**UGCUGGACACUGAAUACAGCAGU**UUAGUGUGUUAUCUGCUGUAGACAGUGUCCAGUGUGCAGACUACAGA
.(((((........))))).......((((((((((((...(((((((((((..((((((((............))))))))..)))))))))))..))))))))))))
Error predicting STAR on 5pERROR 4: Strange structure
Initial dG = -55.00

```
          10
.-G|    GCA
   AUACA   A
   UAUGU   U
\ -^    ACC
```

```
 20        30        40        50        60
  GAUAUAA            UCU           AA        UUUAGU
         UCUGUGGUCUGU   GCUGGACACUG  UACAGCAG      \
         AGACAUCAGACG   UGACCUGUGAC  AUGUCGUC      G
  -------            UG-           AG        UAUUGU
      110       100        90        80        70
```

Reads in: lib-42, lib-45, lib-47, lib-48, lib-49, lib-50, lib-72
Number of reads="ID1769",11,32,173,529,7,15,0

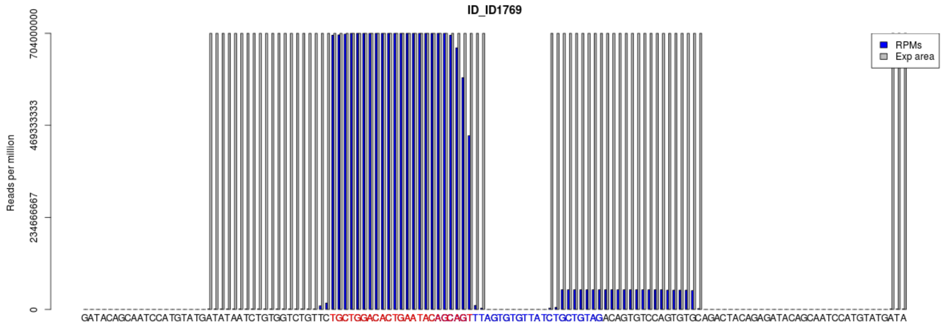

>ID177
AACCTGCGCATCCCATATGTGGTTAGCTACATGGCCCCGGTTGTGTGTCTCCGTGGCTATTTGATGTTGG**ATATACATAATCGGTGCTGT**
ATTGCTATCCACGATACTCTCC

>ID177-3P
ATATACATAATCGGTGCTGTAT

>miRNA precursor
AACCUGCGCAUCCCAUAUGUGGUUAGCUACAUGGCCCCGGUUGUGUGUCUCCGUGGCUAUUUGAUGUUGG**AUAUACAUAAUCGGUGCUGUAU**UGCUAUCCACGAUACUCUCC
```
................(((((.((((...(((((.((((((((((((.((((..((........)))))).))))))))))))).))))).....)))).))))).........
```

>Predicted STAR seq:
ACATGGCCCCGGTTGTGTGTC

Complementarity: 16

Initial dG =   -38.60

```
        10        20        30        40        50        60
AACCUGCGCAUCCCAUA|    U    UAC     C            C    UG   UAU
                 UGUGG UAGC   AUGGC CCGGUUGUGUGU UCCG  GC   U
                 GCACC AUCG   UGUCG GGCUAAUACAUA AGGU  UG   U
CCUCUCAUA--------^    U    UUA     U            U    --   UAG
110              100       90        80        70
```

Reads in: lib-42, lib-45, lib-47, lib-48, lib-49, lib-50, lib-72
Number of reads="ID177",0,0,0,14,1,0,0

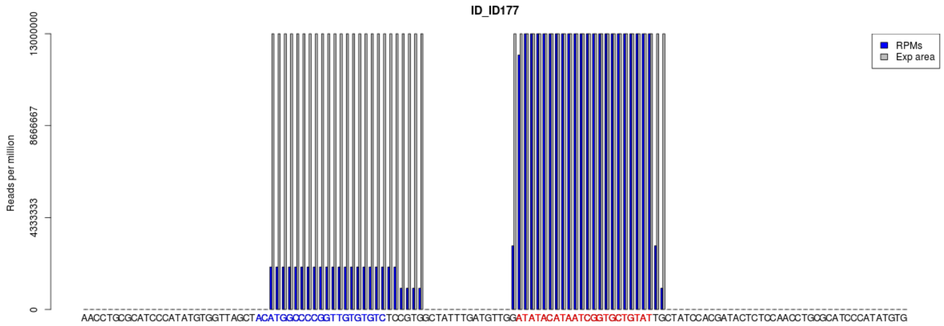

>ID1770

GATGCAGCAATCCATGTGTAATATAATCTGTGGTCTGTTC**TGCTGGACACTGAATACAGCAGT**TTAGTGTGTCATCTGCTGTAGACAGTG
TCCAGTGTGCAGACTACAGA

>ID1770-5P

TGCTGGACACTGAATACAGCAGT

>miRNA precursor

```
GAUGCAGCAAUCCAUGUGUAAUAUAAUCUGUGGUCUGUUCUGCUGGACACUGAAUACAGCAGUUUAGUGUGUCAUCUGCUGUAGACAGUGUCCAGUGUGCAGACUACAGA
.(((((........)))))).......((((((((((((...(((((((((((..((((((((............))))))))..))))))))))))..))))))))))))
Error predicting STAR on 5pERROR 4: Strange structure
 Initial dG =    -54.00

          10
.-G|    GCA
   AUGCA   A
   UGUGU   U
\ -^    ACC



 20        30        40        50        60
  AAUAUAA            UCU           AA        UUUAGU
         UCUGUGGUCUGU   GCUGGACACUG  UACAGCAG      \
         AGACAUCAGACG   UGACCUGUGAC  AUGUCGUC      G
  -------            UG-           AG        UACUGU
      110       100         90        80        70
```

Reads in: lib-42, lib-45, lib-47, lib-48, lib-49, lib-50, lib-72

Number of reads="ID1770",4,41,186,488,4,13,0

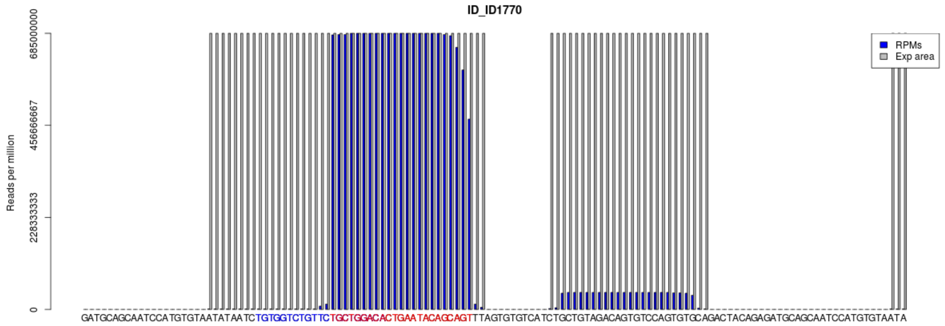

>ID1771
CCATAATCTGTGGTCTGTCC**TGCTGGACACTGAATACAGCGG**TTTAGTGTGTTATCTGCTGTAGACAGTGTCCAGTGTGCAGACTACAGA
CAATCATCAGTCTTCGGCGAGT

>ID1771-5P
TGCTGGACACTGAATACAGCGG

>miRNA precursor
CCAUAAUCUGUGGUCUGUCC**UGCUGGACACUGAAUACAGCGG**UUUAGUGUGUUAUCUGCUGUAGACAGUGUCCAGUGUGCAGACUACAGACAAUCAUCAGUCUUCGGCGAGU
......((((((((((((...(((((((((((..((((((((.............))))))))..)))))))))))..)))))))))))))).........((.....))....

>Predicted STAR seq:
GCTGTAGACAGTGTCCAGTGTGC

Complementarity: 17

Initial dG = -51.80

```
          10        20        30        40
.-CCAUAA|           CCU           AA        UUUAGU
        UCUGUGGUCUGU   GCUGGACACUG  UACAGCGG      \
        AGACAUCAGACG   UGACCUGUGAC  AUGUCGUC      G
\ ------^           UG-           AG        UAUUGU
       90        80         70        60        50



         100
  CAAUCAUCA  CU
           GU  \
           CG  U
  UGAG-----  GC
  110
```

Reads in: lib-42, lib-45, lib-47, lib-48, lib-49, lib-50, lib-72
Number of reads="ID1771",10,68,166,1312,2,13,0

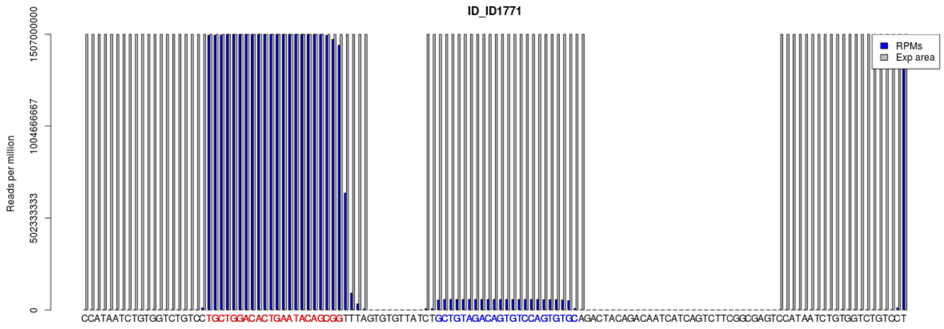

>ID1772
GATGCAGCAATCCATGTGTGATATAATCTGTGGTCTGTTC**TGCTGGACACTGAATACAGCAGT**TTAGGGTGTTATCTGCTGTAGACAGTG
TCCAGTGTGCAGACTACAGA

>ID1772-5P
TGCTGGACACTGAATACAGCAGT

>miRNA precursor
GAUGCAGCAAUCCAUGUGUGAUAUAAUCUGUGGUCUGUUC**UGCUGGACACUGAAUACAGCAGU**UUAGGGUGUUAUCUGCUGUAGACAGUGUCCAGUGUGCAGACUACAGA
.(((((........))))).......((((((((((((...(((((((((((..((((((((............))))))))..)))))))))))..))))))))))))
Error predicting STAR on 5pERROR 4: Strange structure
 Initial dG =   -54.00

```
          10
.-G|    GCA
   AUGCA   A
   UGUGU   U
\ -^    ACC
```

```
 20        30        40        50        60
  GAUAUAA            UCU            AA         UUUAGG
         UCUGUGGUCUGU   GCUGGACACUG  UACAGCAG      \
         AGACAUCAGACG   UGACCUGUGAC  AUGUCGUC      G
  -------            UG-            AG         UAUUGU
      110       100         90        80        70
```

Reads in: lib-42, lib-45, lib-47, lib-48, lib-49, lib-50, lib-72
Number of reads="ID1772",5,47,215,484,2,11,0

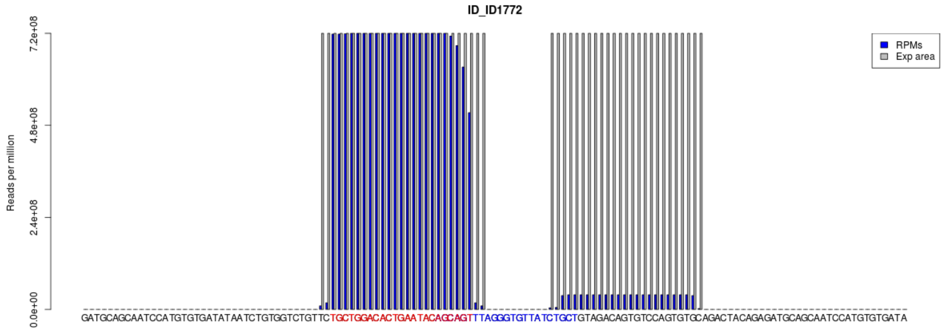

>ID1773

ATATAATCTGTGGTCTGTTCTGCTGGACACTGAATACAGCGGTTTAGTGTGTTATCTGCTGTAGACAGTGTCCAGTGTGCAGACTAGAGA
CAATCATCAGTCTTCGGCAAGT

>ID1773-5P

TGCTGGACACTGAATACAGCGG

>miRNA precursor

AUAUAAUCUGUGGUCUGUUCUGCUGGACACUGAAUACAGCGGUUUAGUGUGUUAUCUGCUGUAGACAGUGUCCAGUGUGCAGACUAGAGACAAUCAUCAGUCUUCGGCAAGU
......(((.((((((((...(((((((((((..((((((((.............))))))))..)))))))))))..)))))))).))).........((.....))....

>Predicted STAR seq:

GCTGTAGACAGTGTCCAGTGTGC

Complementarity: 17

Initial dG =   -48.60

```
          10        20        30        40
.-AUAUAA|  G        UCU           AA        UUUAGU
        UCU UGGUCUGU   GCUGGACACUG  UACAGCGG      \
        AGA AUCAGACG   UGACCUGUGAC  AUGUCGUC      G
\ ------^  G        UG-           AG        UAUUGU
       90        80         70        60        50



         100
  CAAUCAUCA  CU
           GU  \
           CG  U
  UGAA-----  GC
  110
```

Reads in: lib-42, lib-45, lib-47, lib-48, lib-49, lib-50, lib-72

Number of reads="ID1773",12,67,162,1226,4,16,0

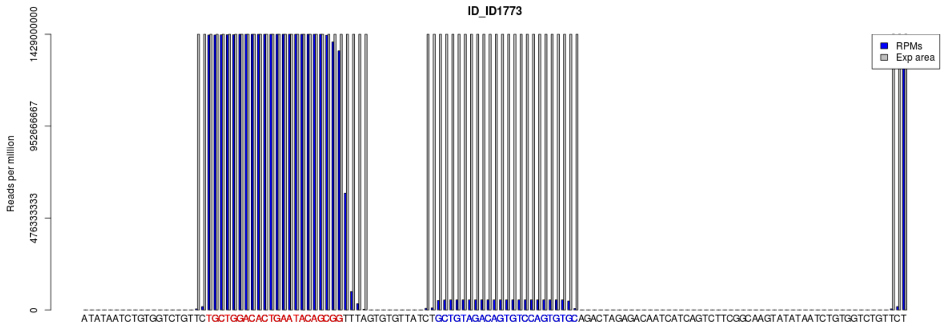

>ID1774
CCATAATCTGTGGTCTGTCC**TGCTGGACACTGAATACAGCGG**TTTAGTGTGTTATCTGCTGTAGACAGTGTCCAGTGTGCAGACTACAGA
CAATCATCGGTCTTCGGCGAGT

>ID1774-5P
TGCTGGACACTGAATACAGCGG

>miRNA precursor
CCAUAAUCUGUGGUCUGUCC**UGCUGGACACUGAAUACAGCGG**UUUAGUGUGUUAUCUGCUGUAGACAGUGUCCAGUGUGCAGACUACAGACAAUCAUCGGUCUUCGGCGAGU
......((((((((((((...(((((((((((..((((((((.............))))))))..)))))))))))..)))))))))))))...((..((.....))..))..

>Predicted STAR seq:
GCTGTAGACAGTGTCCAGTGTGC

Complementarity: 17

Initial dG =   -52.90

```
          10        20        30        40
.-CCAUAA|           CCU           AA        UUUAGU
        UCUGUGGUCUGU   GCUGGACACUG  UACAGCGG      \
        AGACAUCAGACG   UGACCUGUGAC  AUGUCGUC      G
\ ------^           UG-           AG        UAUUGU
       90        80         70        60        50



         100
  CAA  AU  GU
     UC  CG  \
     AG  GC  C
  UG-  CG  UU
   110
```

Reads in: lib-42, lib-45, lib-47, lib-48, lib-49, lib-50, lib-72
Number of reads="ID1774",6,74,177,1227,2,10,0

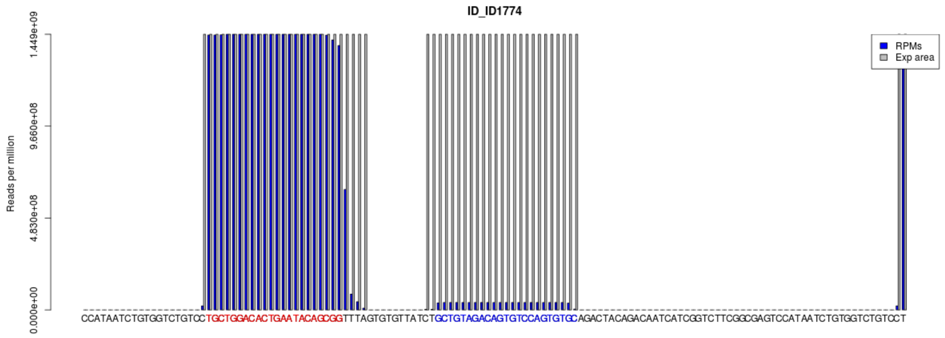

>ID1775
ATATAATCTGTGGTCTGATC**TGCTGGACACTGAATACAGCGG**TTTAATCTATCATCTGCTGTAGACAGTTTCCAGTGTACAGACTATAGA
CAGTCAGCAGTCTCAGCGAATT

>ID1775-5P
TGCTGGACACTGAATACAGCGG

>miRNA precursor
AUAUAAUCUGUGGUCUGAUC**UGCUGGACACUGAAUACAGCGG**UUUAAUCUAUCAUCUGCUGUAGACAGUUUCCAGUGUACAGACUAUAGACAGUCAGCAGUCUCAGCGAAUU
```
......((((((((((...(((((((.((((..((((((((.............))))))))..)))).)))))))..)))))))))))......((.......)).....
```

>Predicted STAR seq:
GCTGTAGACAGTTTCCAGTGTACA

Complementarity: 17

Initial dG =   -42.20

```
          10        20        30        40
.-AUAUAA|          AUC       C     AA        UUUAAU
        UCUGUGGUCUG   UGCUGGA ACUG  UACAGCGG      \
        AGAUAUCAGAC   GUGACCU UGAC  AUGUCGUC      C
\ ------^          AU-       U     AG        UACUAU
       90        80         70        60        50
```

```
         100
  CAGUCA  AGU
        GC   \
        CG   C
  UUAAG-  ACU
  110
```

Reads in: lib-42, lib-45, lib-47, lib-48, lib-49, lib-50, lib-72
Number of reads="ID1775",9,71,200,1199,4,8,0

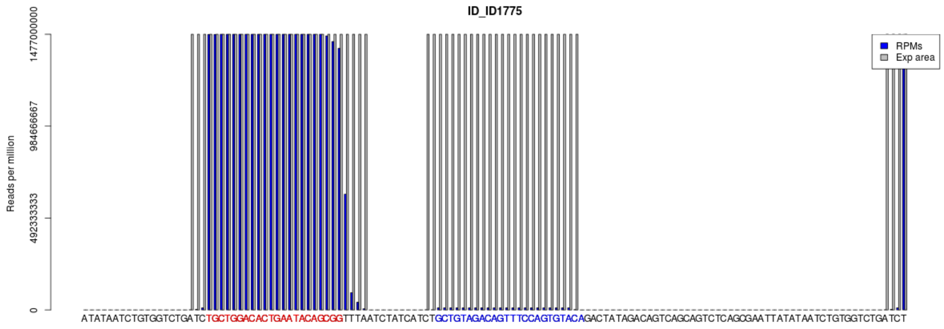

>ID1790
TAATTGCGACTTCCCGCTGC**AGGAGGCGACAATAGCTGCCAA**TTGTATCTGGTATTTGGTACCTACTGTCGGCTCCGGCAGCTGTTAGTA
GCCACCAAACTGTAACTGCTGA

>ID1790-5P
AGGAGGCGACAATAGCTGCCAA

>miRNA precursor
UAAUUGCGACUUCCCGCUGC**AGGAGGCGACAAUAGCUGCCAA**UUGUAUCUGGUAUUUGGUACCUACUGUCGGCUCCGGCAGCUGUUAGUAGCCACCAAACUGUAACUGCUGA
```
..............(((((.((((.(((((.(((.((((((............)))))).))).))))).)))).)))))..(((((((..((......))..)))))))
```

>Predicted STAR seq:
GGTACCTACTGTCGGCTCCGGC

Complementarity: 16

Initial dG =   -41.20

```
          10        20        30        40
.-UAAUUGCGACUUCCC|    A    G     A   C      UUGUAU
                 GCUGC GGAG CGACA UAG UGCCAA      \
                 CGACG CCUC GCUGU AUC AUGGUU      C
\ ---------------^    G    G     C   C      UAUGGU
                  80        70        60        50



        90
  UG       CC  CA
    UUAGUAG  AC  A
    AGUCGUC  UG  A
  --       AA  UC
    110       100
```

Reads in: lib-42, lib-45, lib-47, lib-48, lib-49, lib-50, lib-72
Number of reads="ID1790",0,0,0,10,3,1,0

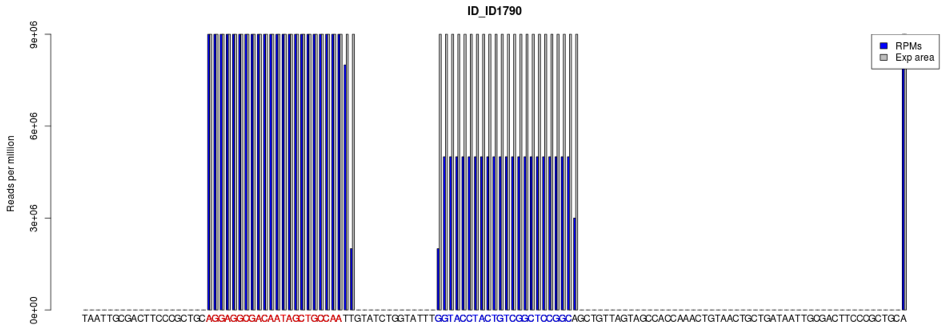

>ID1791
CAGCAGCGACCACCGAGTCTCAGAGGTGGCAGGATCAGCGGAGGTGGACGGTAGGTTATGTTGGCATCAC**TGTCTACCTCCACTGATCCCGCT**GCCTCTGCGGCATGACGACT

>ID1791-3P
TGTCTACCTCCACTGATCCCGCT

>miRNA precursor
CAGCAGCGACCACCGAGUCUCAGAGGUGGCAGGAUCAGCGGAGGUGGACGGUAGGUUAUGUUGGCAUCAC**UGUCUACCUCCACUGAUCCCGCU**GCCUCUGCGGCAUGACGACU
```
...............(((.((((((..((.(((((((.(((((((((((((..(((.....)))...))))))))))))).))))))).))..)))))).)))........
```

>Predicted STAR seq:
GGCAGGATCAGCGGAGGTGGACG

Complementarity: 18

Initial dG =   -61.80

```
        10        20        30        40        50
CAGCAGCGACCACCGA   U       UG  A       C             AG-|  AU
                GUC CAGAGG  GC GGAUCAG GGAGGUGGACGGU   GUU  \
                CGG GUCUCC  CG CCUAGUC CCUCCAUCUGUCA   CGG  G
UCAGCAGUA-------   C       GU  C       A             CUA^  UU
 110              100        90        80        70
```

Reads in: lib-42, lib-45, lib-47, lib-48, lib-49, lib-50, lib-72
Number of reads="ID1791",825,1909,4750,12558,4269,646,0

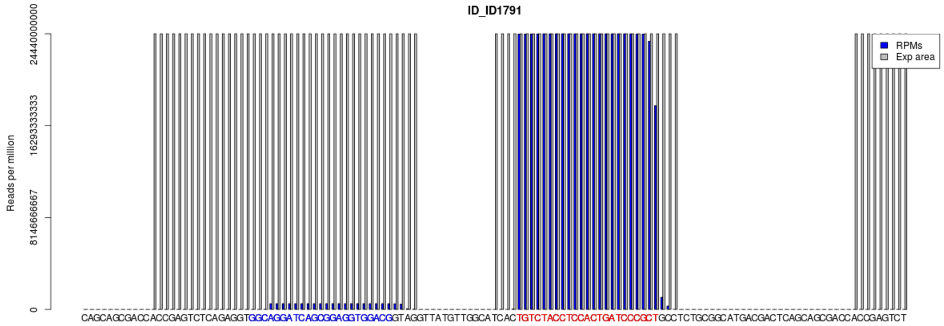

>ID1793
CTCATTTTCTGGTCGACTGTGATGATACAGCTCCCTGCTACCTGCTTCACCGCTTCTGTTTTTGAGCTAA**GCGGTGAGGTATGTAGCAAA**
GAGGGGTATCGTCAAAGCTACA

>ID1793-3P
GCGGTGAGGTATGTAGCAAAGA

>miRNA precursor
CUCAUUUUCUGGUCGACUGUGAUGAUACAGCUCCCUGCUACCUGCUUCACCGCUUCUGUUUUUGAGCUAA**GCGGUGAGGUAUGUAGCAAAGA**GGGGUAUCGUCAAAGCUACA
```
...........((...((.(((((((((..(((..((((((.(((((((((((((..(((....))).))))))))))))).))))))..)))..))))))))).))..)).
```

>Predicted STAR seq:
CCCTGCTACCTGCTTCACCGC

Complementarity: 17

Initial dG =   -49.20

```
        10        20        30        40        50        60
CUCAUUUUCUG| CGA  G         AG   CC      C             CU   U
           GU   CU UGAUGAUAC  CUC  UGCUAC UGCUUCACCGCUU  GUU U
           CA   GA ACUGCUAUG  GAG  ACGAUG AUGGAGUGGCGAA  CGA U
A----------^ UC-  A         GG   AA      U             U-   G
          110        100       90        80        70
```

Reads in: lib-42, lib-45, lib-47, lib-48, lib-49, lib-50, lib-72
Number of reads="ID1793",0,4,0,0,0,0,0

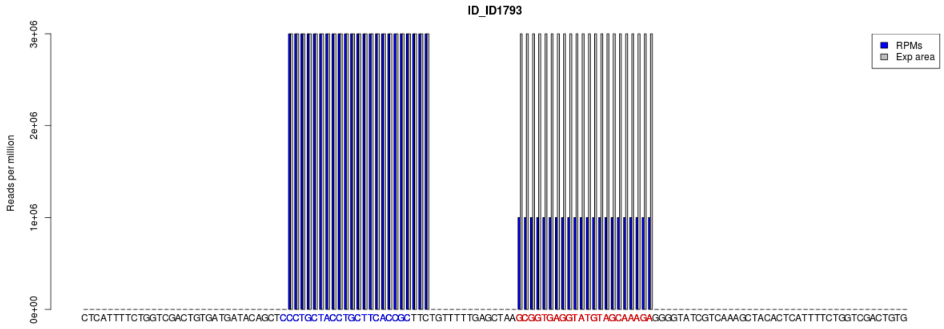

>ID1794
AAGAGAAAGAGGACTCCATT**GTGTCCTGTCAGCGTCGATCTTCG**TATATACGAATCGAAGATCCGTTGATAGTACAAGATGGTGTACTCA
TGACATCTCGCAACAAAAGTGATAA

>ID1794-5P
GTGTCCTGTCAGCGTCGATCTTCG

>miRNA precursor
AAGAGAAAGAGGACUCCAUU**GUGUCCUGUCAGCGUCGAUCUUCG**UAUAUACGAAUCGAAGAUCCGUUGAUAGUACAAGAUGGUGUACUCAUGACAUCUCGCAACAAAAGUGAUAA
........(((.((.(((((.(((.(((((((((..((((((((...........))))))))))))))))).))).))))).)).)))........((((.......))))...

>Predicted STAR seq:
AAGATCCGTTGATAGTACAAGA

Complementarity: 18

Initial dG =   -39.50

```
          10        20        30        40
.-AAGAGAAA|  G  U     G   C         UC        UAUAU
          GAG AC CCAUU UGU CUGUCAGCG  GAUCUUCG     \
          CUC UG GGUAG ACA GAUAGUUGC  CUAGAAGC     A
\ --------^  A  U     A   U         --        UAAGC
                  80        70          60



 90       100
  AUGACAUC    AAC
          UCGC   \
          AGUG   A
  AAU-----    AAA
          110
```

Reads in: lib-42, lib-45, lib-47, lib-48, lib-49, lib-50, lib-72
Number of reads="ID1794",1,16,1,0,4,2,0

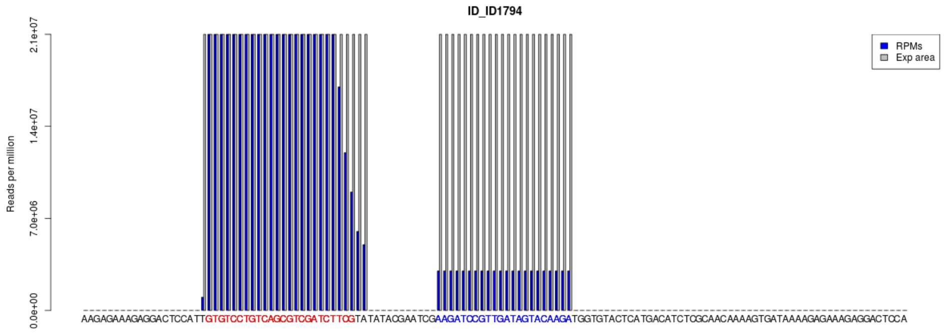

>ID1795
CGTGACGGTGGGTGATCCTG**TGTTGCTCTCATCGTCATCATC**TTGTCAAATGATGATGACATGGTGAGCCACACAGGAACGCTTCACACG
AGCTGCTATCATCTGACGCATC

>ID1795-5P
TGTTGCTCTCATCGTCATCATC

>miRNA precursor
CGUGACGGUGGGUGAUCCUG**UGUUGCUCUCAUCGUCAUCAUC**UUGUCAAAUGAUGAUGACAUGGUGAGCCACACAGGAACGCUUCACACGAGCUGCUAUCAUCUGACGCAUC
.....(((((((((.((((((((.((((((((.(((((((((.........))))))))))))).)))).)))))))).))).)))).))...(((..((....)).)))..

>Predicted STAR seq:
TGATGACATGGTGAGCCACACA

Complementarity: 18

Initial dG =   -48.70

```
        10          20         30        40
C   A  GU                U    -|   C         UUGU
 GUG CG  GGGUGA--UCCUGUGU GCUC UCAU GUCAUCAUC    \
 UAC GC  UCUACU  AGGACACA CGAG GGUA CAGUAGUAG    C
C   -  AG      \         C    U^   -         UAAA
110       100          70         60        50



                 80
                 AC---    CA
                      GCUU  \
                      CGAG  C
                 AUCGU    CA
                        90
```

Reads in: lib-42, lib-45, lib-47, lib-48, lib-49, lib-50, lib-72
Number of reads="ID1795",12,27,2,3,3,0,0

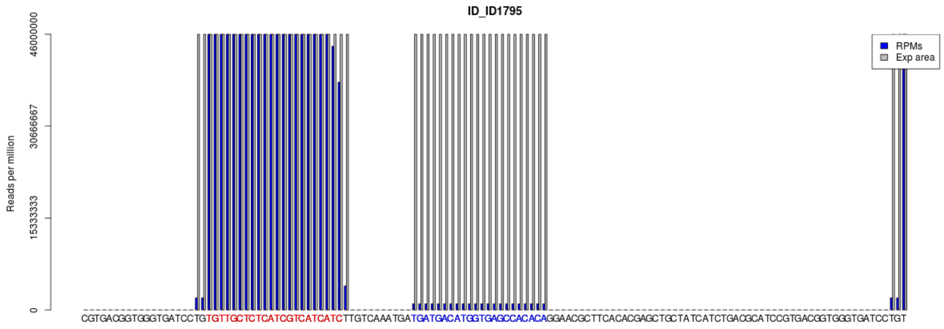

>ID1797

ATAATGCGATTATCTCATGAAGTTTCCTGCTGTCACCGAATGTGTTCGATCACTGCACGAACGAGTAAAC**TTTCGTGCACCGATCGAACA**
CGTTCTGCGTCAGCGTTACCA

>ID1797-3P

TTTCGTGCACCGATCGAACAC

>miRNA precursor

AUAAUGCGAUUAUCUCAUGAAGUUUCCUGCUGUCACCGAAUGUGUUCGAUCACUGCACGAACGAGUAAAC**UUUCGUGCACCGAUCGAACAC**GUUCUGCGUCAGCGUUACCA
...(((.(.....).)))..........((((.....((((((((((((((..(((((((..(((....)))))))))).))))))))))))))).....)))).......

>Predicted STAR seq:

TGTTCGATCACTGCACGAACGA

Complementarity: 17

Initial dG =   -42.20

```
           10          20
A--       -   .-UAUCUCA   -|  UU
   UAAUGC GAU         UGA AGU  \
   AUUGCG CUG         ACU UCG  C
ACC      A   \ -------   G^  UC
 .      100              30
```

Reads in: lib-42, lib-45, lib-47, lib-48, lib-49, lib-50, lib-72

Number of reads="ID1797",3,19,1,0,6,2,0

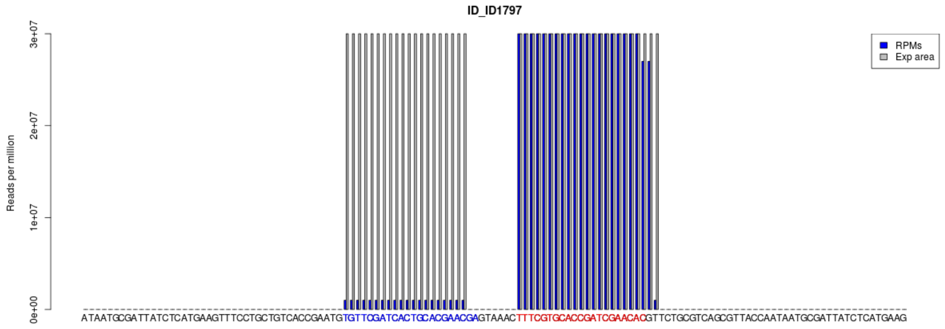

>ID1798

AGAGAAAGAGAATACCATTG**TGTAATACCAGCGCCTGTCTTC**GTATGTACGAATCGAAGATTGTCGTTGATAATACACGAAGGTGTACTC
CTGACATCTCGCAAGAAAACTG

>ID1798-5P

TGTAATACCAGCGCCTGTCTTC

>miRNA precursor

AGAGAAAGAGAAUACCAUUG**UGUAAUACCAGCGCCUGUCUUC**GUAUGUACGAAUCGAAGAUUGUCGUUGAUAAUACACGAAGGUGUACUCCUGACAUCUCGCAAGAAAACUG
.((((..(((.(((((.(((((((.((.(((((...(((((((.((......)))))))))...))))).)).))))))).))))).)))......))))...........

>Predicted STAR seq:

AGATTGTCGTTGATAATACACG

Complementarity: 15

Initial dG =   -35.90

```
                       10        20        30        40
A-----------    AA----|  A     A       A  C      CCU       U  GU
            GAGA      GAG AUACC UUGUGUA UA CAGCG   GUCUUCG AU  A
            CUCU      CUC UGUGG AGCACAU AU GUUGC   UAGAAGC UA  C
GUCAAAAGAACG    ACAGUC^  A     A       A  A      UGU       -  AG
110       100        90        80        70        60         50
```

Reads in: lib-42, lib-45, lib-47, lib-48, lib-49, lib-50, lib-72

Number of reads="ID1798",2,1,1,1,1,0,0

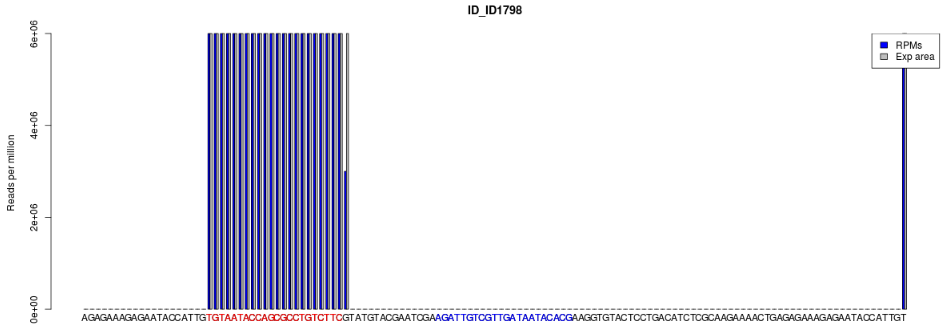

>ID1799
CTTGGTACAGTTCAGCGACAGCACGGCTTCAGAGTAGCTCTCGTCGTTTCTGCTTGTATGAGGCTTCAAG**TAGGAGAAACGAGAGCTACT**
CTGCAACTGGAGTCTGCAGG

>ID1799-3P
TAGGAGAAACGAGAGCTACT

>miRNA precursor
CUUGGUACAGUUCAGCGACAGCACGGCUUCAGAGUAGCUCUCGUCGUUUCUGCUUGUAUGAGGCUUCAAG**UAGGAGAAACGAGAGCUACU**CUGCAACUGGAGUCUGCAGG
.((((......))))).....(((.((((((((((((((((((((..((((((((((..........))))))))))..))))))))))))))))).......))))))))...

>Predicted STAR seq:
GTAGCTCTCGTCGTTTCTG

Complementarity: 16
ERROR 4: Strange structure
Initial dG = -47.50

```
.-C|   UA
   UUGG  C
   GACU  A
\ -^   UG
       10


     20              30        40        50        60
  CGACA   C     ------              CG          UAUG
       GCA GGCUU      CAGAGUAGCUCUCGU  UUUCUGCUUG    A
       CGU CUGAG      GUCUCAUCGAGAGCA  GAGGAUGAAC    G
  GGA--   -     GUCAAC              AA          UUCG
110         100        90        80        70
```

Reads in: lib-42, lib-45, lib-47, lib-48, lib-49, lib-50, lib-72
Number of reads="ID1799",4,16,3,0,1,0,0

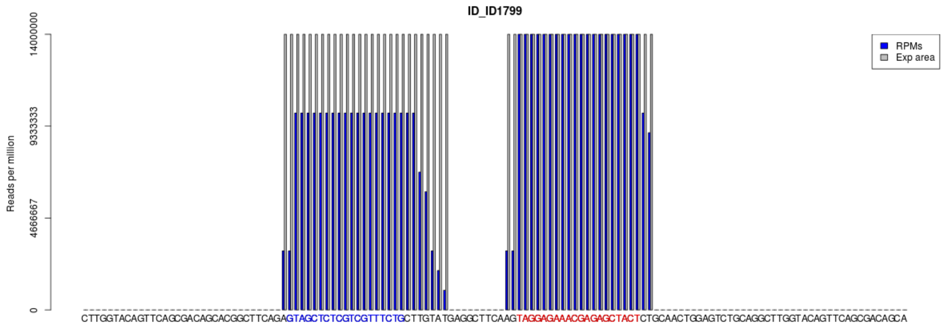

>ID18
GAAGAGGGTGGGCATGCAGT**CGATGGAATTGTGAACCCTGGC**GTGGTAATCATTTGGCAGGGTGCACAATTCTGTCTGCCGCTACCCATA
AAGACATCTTCCAGCCGTCGAC

>ID18-5P
CGATGGAATTGTGAACCCTGGC

>miRNA precursor
GAAGAGGGUGGGCAUGCAGU**CGAUGGAAUUGUGAACCCUGGC**GUGGUAAUCAUUUGGCAGGGUGCACAAUUCUGUCUGCCGCUACCCAUAAAGACAUCUUCCAGCCGUCGAC
(((((..(((((...((.((.((((((((((((.((((((.((...........)).)))))).))))))))))))).)).))..)))))).......))))).........

>Predicted STAR seq:
CAGGGTGCACAATTCTGTCTGC

Complementarity: 17

Initial dG =   -44.60

```
                        10        20        30        40
-----------     GG-----|    CAU  A  C            A      G  UGGUA
           GAAGA       GUGGG   GC GU GAUGGAAUUGUG ACCCUG CG     \
           CUUCU       UACCC   CG CG CUGUCUUAACAC UGGGAC GU     A
CAGCUGCCGAC     ACAGAAA^    AU-  C  U            G      G  UUACU
110       100        90         80        70        60        50
```

Reads in: lib-42, lib-45, lib-47, lib-48, lib-49, lib-50, lib-72
Number of reads="ID18",4,7,0,0,1,0,0

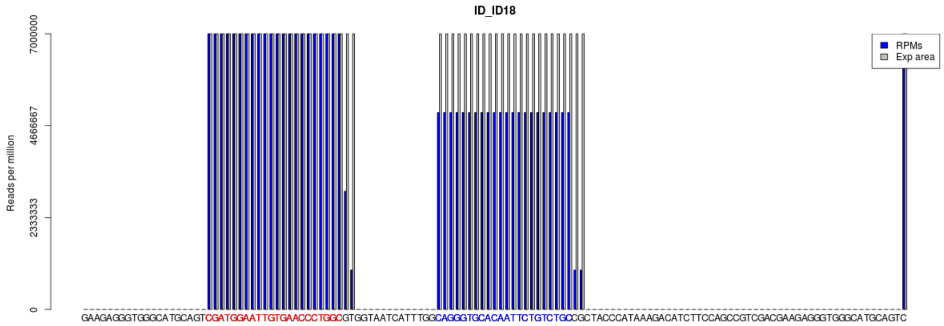

>ID1800
CGCTCGCGTCTTGCAAGAAGGAGACAGTGGCCTGGAAATGTTCTCCCAGTTAACACTGTGTAACAGTAAG**TGGTAACTGGCAGAACTTTT**
TCAGGAGGCTGTTCGTCATCCG

>ID1800-3P
TGGTAACTGGCAGAACTTTTTC

>miRNA precursor
CGCUCGCGUCUUGCAAGAAGGAGACAGUGGCCUGGAAAUGUUCUCCCAGUUAACACUGUGUAACAGUAAG**UGGUAACUGGCAGAACUUUUUC**AGGAGGCUGUUCGUCAUCCG
..((.((.....)).))..((((((((..(((((((.(((((.(((((((.((((...........)))).))))))).))))).))))))))..))))))).....))).

>Predicted STAR seq:
GAAATGTTCTCCCAGTTAACA

Complementarity: 17
ERROR 4: Strange structure
Initial dG =   -43.40

```
.-CG| C  GU
    CU GC  \
    GA CG  C
\ --^ A  UU
```

```
   20             30        40        50        60
  AA   -----      GG        U     C       A     GUGUA
    GGA     GACAGU  CCUGGAAA GUUCU CCAGUUA CACU     \
    CCU     UUGUCG  GGACUUUU CAAGA GGUCAAU GUGA     A
  G-   ACUGC      GA        U     C       G     AUGAC
   110       100        90        80        70
```

Reads in: lib-42, lib-45, lib-47, lib-48, lib-49, lib-50, lib-72
Number of reads="ID1800",15,49,0,1,18,4,0

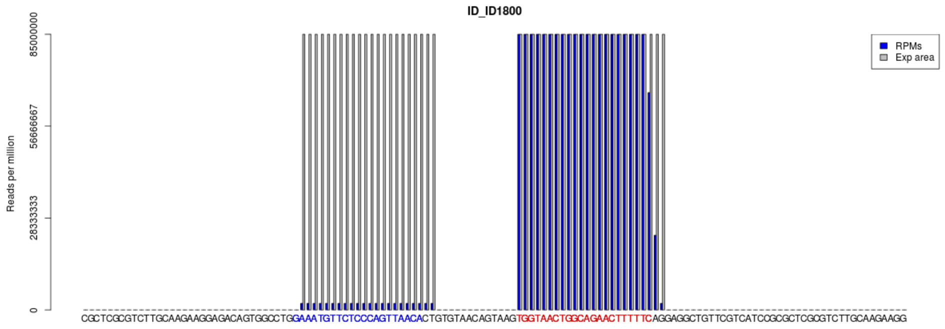

>ID1801
AGATGCTGCTTACTTCCATCGGCGCTGGCCGCTTTGCTCTTTGTCCATCTACCAGCTAGCTGTCTGTTGT**TGGTGGATGCCTCAAAGTGC**
AAAGTGTATGGTGCCTAATCAC

>ID1801-3P
TGGTGGATGCCTCAAAGTGCAA

>miRNA precursor
AGAUGCUGCUUACUUCCAUCGGCGCUGGCCGCUUUGCUCUUUGUCCAUCUACCAGCUAGCUGUCUGUUGU**UGGUGGAUGCCUCAAAGUGCAA**AGUGUAUGGUGCCUAAUCAC
.((((..........))))(((((((..((((((((.(((((..(((((((((((.(((.....)))))))))))))))...))))).))))))))..))))))).......

>Predicted STAR seq:
TGCTCTTTGTCCATCTACCA

Complementarity: 16

Initial dG = -48.40

```
        10        20        30        40         50        60
AGAUGCUGCUUACUUCCAUC       GC        U     UC-|          U   UG
                    GGCGCUG  CGCUUUGC CUUUG   CAUCUACCAGC AGC  \
                    CCGUGGU  GUGAAACG GAAAC   GUAGGUGGUUG UUG  U
CACUAAU-------------       AU        U     UCC^          -   UC
110                   100        90        80        70
```

Reads in: lib-42, lib-45, lib-47, lib-48, lib-49, lib-50, lib-72
Number of reads="ID1801",0,2,0,0,0,0,0

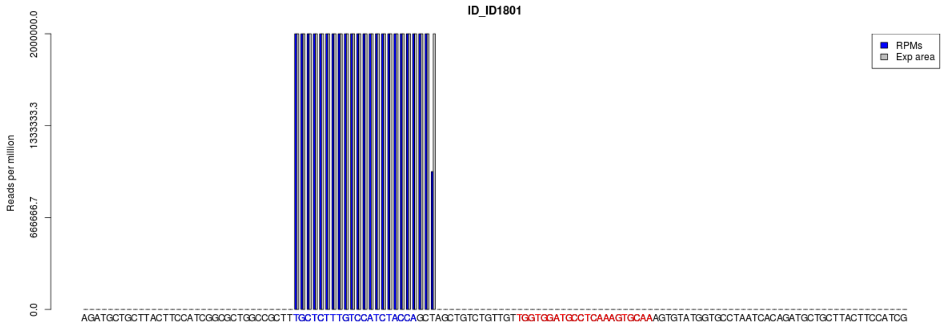

>ID1802

GAGAAAGAGAACACCATTG**TGTCCTATCAGTGTCGGTCTTC**GTATGTACGAATCGAAGATCGTCGTTGATAATACACGAGGGTGTACTCT
TGACATCACGCAACAAAACTG

>ID1802-5P

TGTCCTATCAGTGTCGGTCTTC

>miRNA precursor

GAGAAAGAGAACACCAUUG**UGUCCUAUCAGUGUCGGUCUUC**GUAUGUACGAAUCGAAGAUCGUCGUUGAUAAUACACGAGGGUGUACUCUUGACAUCACGCAACAAAACUG
((..(((((.(((((.((((((..((((((((.(((((((((.((......))))))))))).))))))))..)))))).))))).)))))....))..............

>Predicted STAR seq:

AGATCGTCGTTGATAATACACG

Complementarity: 17

Initial dG = -43.40

```
                        10        20        30        40
--------------  GA--|    A     A      CC        U         U  GU
              GA    AAGAG ACACC UUGUGU  UAUCAGUG CGGUCUUCG AU  A
              CU    UUCUC UGUGG AGCACA  AUAGUUGC GCUAGAAGC UA  C
GUCAAAACAACGCA  ACAG^    A     G      UA        U         -  AG
 .       100       90        80        70        60         50
```

Reads in: lib-42, lib-45, lib-47, lib-48, lib-49, lib-50, lib-72

Number of reads="ID1802",10,22,0,1,2,1,0

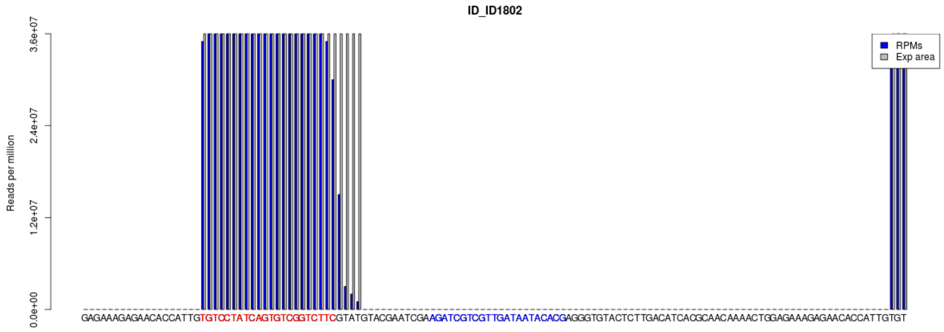

>ID1803
AAAAAAAAGAGAATACCAT**TGTGTATTGTCAGCGTCGGTCTTC**GTATATACGAAACGAAGATCCGTTGATAATACACGATGGTGTACTCC
AGACATCTCGCAACAAAGGTGATA

>ID1803-5P
TGTGTATTGTCAGCGTCGGTCTTC

>miRNA precursor
AAAAAAAAGAGAAUACCAU**UGUGUAUUGUCAGCGUCGGUCUUC**GUAUAUACGAAACGAAGAUCCGUUGAUAAUACACGAUGGUGUACUCCAGACAUCUCGCAACAAAGGUGAUA
```
........(((.((((((((((((((((((((((..(((((((((.........))))))))))))))))))))))))))))))))).))).....(((((........)))))...
```

>Predicted STAR seq:
AGATCCGTTGATAATACACGAT

Complementarity: 20

```
 Initial dG =    -50.30

          10        20        30        40
.-AAAAAAAA|  A                      UC         AUAU
          GAG AUACCAUUGUGUAUUGUCAGCG  GGUCUUCGU    \
          CUC UGUGGUAGCACAUAAUAGUUGC  CUAGAAGCA    A
\ --------^  A                      --         AAGC
                  80        70          60



 90       100
  CAGA      CGC
      CAUCU   A
      GUGGA   A
  AUA-      AAC
     110
```

Reads in: lib-42, lib-45, lib-47, lib-48, lib-49, lib-50, lib-72
Number of reads="ID1803",18,44,1,0,4,1,0

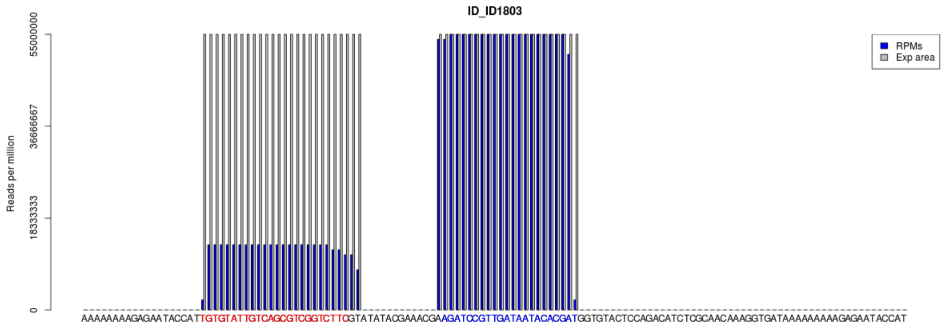

>ID1804
CAGAGAGAGAACACCGTTG**TGCCTTATCAGCCTCGGTCTTCG**TATGTTCGAAAAGAAGATCGTCGTTGATAAAACACGAGGATGTACTCT
TGACCTCTCGTGACATGTAAGA

>ID1804-5P
TGCCTTATCAGCCTCGGTCTTCG

>miRNA precursor
CAGAGAGAGAACACCGUUG**UGCCUUAUCAGCCUCGGUCUUCG**UAUGUUCGAAAAGAAGAUCGUCGUUGAUAAAACACGAGGAUGUACUCUUGACCUCUCGUGACAUGUAAGA
.((((((((.(((((.(((((..((((((((..((((((((.............))))))))..)))))))))..))))))).))).)))).....)))).............

>Predicted STAR seq:
AGATCGTCGTTGATAAAACACG

Complementarity: 16

Initial dG =   -37.00

```
                         10         20        30        40
C-------------    ----    A   -| G     CC        CU        GUAUGU
              AGAG    AGAG ACA CC UUGUG  UUAUCAGC  CGGUCUUC      \
              UCUC    UCUC UGU GG AGCAC  AAUAGUUG  GCUAGAAG      U
AGAAUGUACAGUGC    CAGU    A   A^ -     AA        CU        AAAAGC
110       100        90        80         70        60        50
```

Reads in: lib-42, lib-45, lib-47, lib-48, lib-49, lib-50, lib-72
Number of reads="ID1804",1,2,0,0,0,0,0

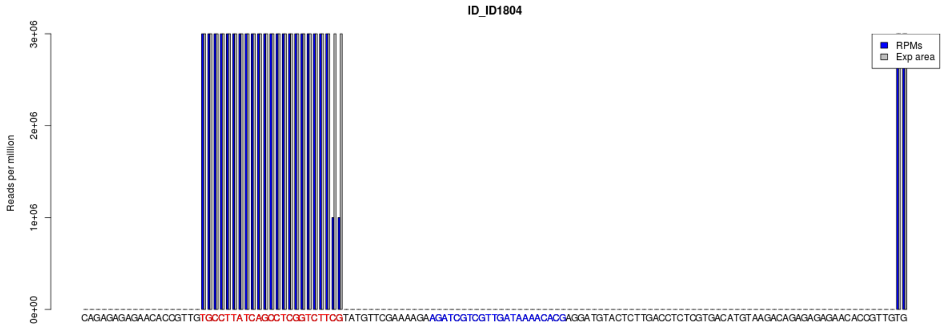

>ID1805
GAGAGAGAGAATGCCATTG**TGTCCTATCAGCGTCGGTCTTCG**TATGTACGAATCGAAGATCGTCGTTGATAAAACACGAAGGTGTACTCC
CGACATCTGGCAACAAAACTGA

>ID1805-5P
TGTCCTATCAGCGTCGGTCTTCG

>miRNA precursor
GAGAGAGAGAAUGCCAUUG**UGUCCUAUCAGCGUCGGUCUUCG**UAUGUACGAAUCGAAGAUCGUCGUUGAUAAAACACGAAGGUGUACUCCCGACAUCUGGCAACAAAACUGA
```
......(((.(((((.((((((..((((((((.(((((((((.((......))))))))))).))))))))..)))))).))))).)))........((....)).......
```

>Predicted STAR seq:
AAGATCGTCGTTGATAAAACACG

Complementarity: 18

Initial dG =   -43.30

```
         10        20        30        40
.-GAGAGA|  A     A      CC        U         U  GU
        GAG AUGCC UUGUGU  UAUCAGCG CGGUCUUCG AU  A
        CUC UGUGG AGCACA  AUAGUUGC GCUAGAAGC UA  C
\ ------^  A     A      AA        U         -  AG
               80        70        60         50



 90       100
  CCGACAUC  G
          UG C
          AC A
  AGUCAAA-  A
  110
```

Reads in: lib-42, lib-45, lib-47, lib-48, lib-49, lib-50, lib-72
Number of reads="ID1805",9,16,7,4,5,1,0

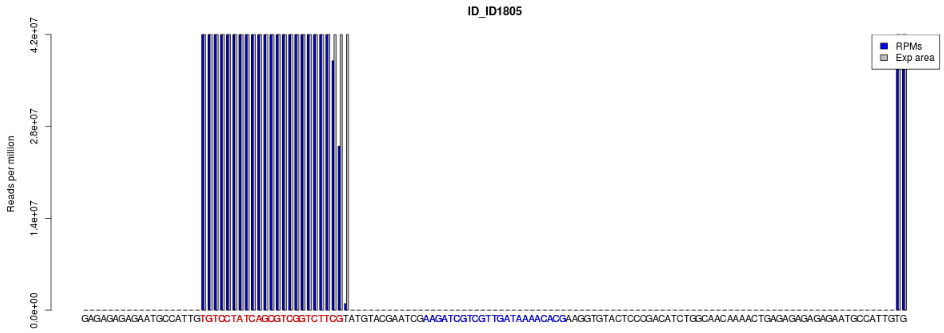

>ID1806
AGCAGAGGAATAGGCTCCAG**TGTTGCTCCGTGATTCTATAGT**TGTTTGTCAGCATCAATTATAGAGTCAAAGAGCATCGCTGGAACTGAT
TCCAGAATATTCGCCAAGTTTT

>ID1806-5P
TGTTGCTCCGTGATTCTATAGT

>miRNA precursor
AGCAGAGGAAUAGGCUCCAG**UGUUGCUCCGUGAUUCUAUAGU**UGUUUGUCAGCAUCAAUUAUAGAGUCAAAGAGCAUCGCUGGAACUGAUUCCAGAAUAUUCGCCAAGUUUU
......(((((.((.(((((((.(((((..((((((((((((((..((....)).))))))))))))))..))))).))))))).)).))))).................

>Predicted STAR seq:
TATAGAGTCAAAGAGCATCGCT

Complementarity: 17

Initial dG =   -47.40

```
                     10        20        30        40        50
AGCAGA-------------|    A  C        U     CG              UU  U
                   GGAAU GG UCCAGUG UGCUC  UGAUUCUAUAGUUG  UG C
                   CCUUA UC AGGUCGC ACGAG  ACUGAGAUAUUAAC  AC A
UUUUGAACCGCUUAUAAGA^    G  A        U     AA              U-  G
110       100       90        80        70        60
```

Reads in: lib-42, lib-45, lib-47, lib-48, lib-49, lib-50, lib-72
Number of reads="ID1806",8,30,25,2,9,2,0

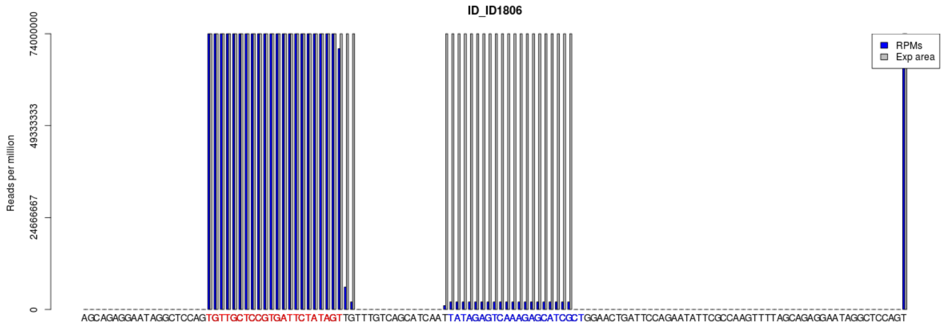

>ID182
CCACTTGGTGCTTTTTCGTGGGTTGACAGGCCTCTATGTGTTAGAGAGTAATTAATATTCTGGTGTACGC**TTTGACACTCAGAGGCCTGACA**GTCCACGAGAAAAGTCCTAC

>ID182-3P
TTTGACACTCAGAGGCCTGACA

>miRNA precursor
CCACUUGGUGCUUUUUCGUGGGUUGACAGGCCUCUAUGUGUUAGAGAGUAAUUAAUAUUCUGGUGUACGC**UUUGACACUCAGAGGCCUGACA**GUCCACGAGAAAAGUCCUAC
.....(((..((((((((((..((.(((((((((..(((((((((.(((((((......)))).))).)))))))))..))))))))).))..)))))))))).)..))).

>Predicted STAR seq:
TTGACAGGCCUCTATGTGTTAGA

Complementarity: 17

Initial dG =   -54.10

```
        10         20        30        40        50
CCACU   UG -          GU  A         AU         A   -|   AU
     UGG  C UUUUUCGUGG  UG CAGGCCUCU  GUGUUAGAG GUA AUUA  A
     AUC  G AAAGAGCACC  AC GUCCGGAGA  CACAGUUUC CAU UGGU  U
C----   CU A          UG  A         CU         G   G^   CU
    110       100        90        80        70        60
```

Reads in: lib-42, lib-45, lib-47, lib-48, lib-49, lib-50, lib-72
Number of reads="ID182",22,13,1,494,503,2,0

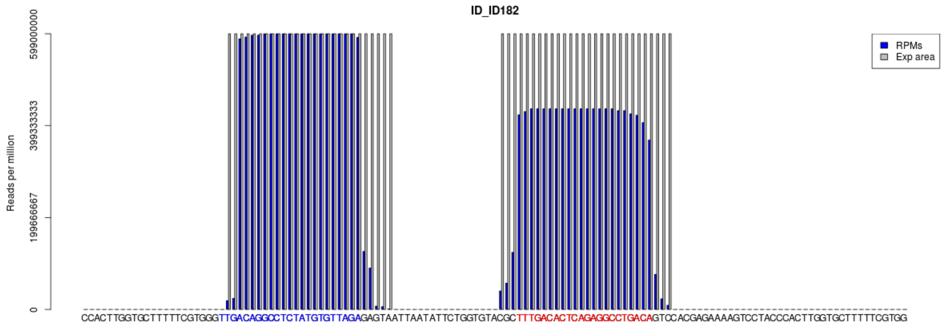

>ID184
AGCACCGCAGCGTAGCGCGGGTGGCCTCGGTGGAGCTTGCTGCACGCTCGACGTGTTCTCATTTCGCTC**TCGAGTGCAGTAACTTCCCCG**
AGGACTCCTGCCCAGCGTCTG

>ID184-3P
TCGAGTGCAGTAACTTCCCCGA

>miRNA precursor
AGCACCGCAGCGUAGCGCGGGUGGCCUCGGUGGAGCUUGCUGCACGCUCGACGUGUUCUCAUUUCGCUC**UCGAGUGCAGUAACUUCCCCGA**GGACUCCUGCCCAGCGUCUG
.......((((((...(((((...((((((.((((.(((((((..((((((.(((.........)))..))))))))))))))).))))))))))...)))))...))).)))

>Predicted STAR seq:
CGGTGGAGCTTGCTGCACGCTCGA

Complementarity: 19

Initial dG =   -50.60

```
        10         20         30         40         50
AGCACCG   -|  AGC     UGG       U    C       AC       C-    UUCU
       CAG CGU   GCGGG   CCUCGG GGAG UUGCUGC  GCUCGA  GUG     \
       GUC GCG   CGUCC   GGAGCC CCUU AAUGACG  UGAGCU  CGC     C
-------   U^  ACC     UCA      -    C       --      CU    UUUA
      110       100        90         80          70
```

Reads in: lib-42, lib-45, lib-47, lib-48, lib-49, lib-50, lib-72
Number of reads="ID184",15,15,3,375,131,1,0

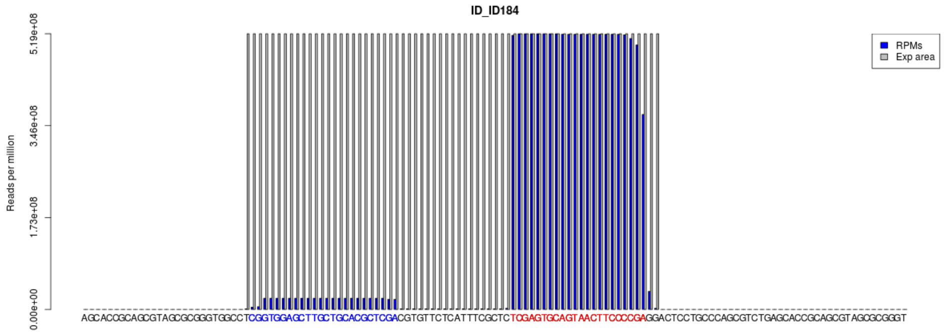

>ID186
GTAATAATTCTTCGTAGTCTCTTCTGGTTTGCTGCCAGATCGTAAAATCGACGA**TCCGGCAGCAAACCCGAAGAGA**CTACGAAGAACATT
CCACACCGCCAACGCCTCGCGTG

>ID186-3P
TCCGGCAGCAAACCCGAAGAGA

>miRNA precursor
GUAAUAAUUCUUCGUAGUCUCUUCUGGUUUGCUGCCAGAUCGUAAAAUCGACGA**UCCGGCAGCAAACCCGAAGAGA**CUACGAAGAACAUUCCACACCGCCAACGCCUCGCGUG
.......((((((((((((((((.(((((((((((.((((((.......)))))).))))))))))).)))))))))))))))))..............((((...)))).

>Predicted STAR seq:
CTCTTCTGGTTTGCTGCCAGA

Complementarity: 18

Initial dG = -62.40

```
          10        20        30        40
.-GUAAUAA|                U           A      AAA
         UUCUUCGUAGUCUCUUC GGUUUGCUGCC GAUCGU   \
         AAGAAGCAUCAGAGAAG CCAAACGACGG CUAGCA   A
\ -------^                C           C      GCU
              80        70        60        50
```

Reads in: lib-42, lib-45, lib-47, lib-48, lib-49, lib-50, lib-72
Number of reads="ID186",65,59,5,381,52,2,0

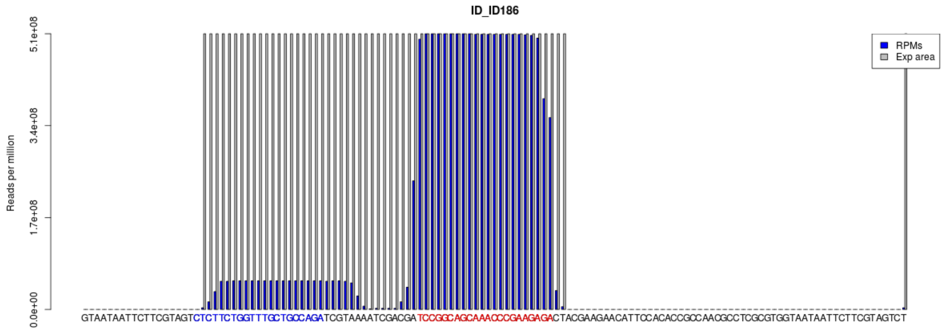

>ID189
TGGCCAAGTAGTCCACAGCAACTTGATTGGCCACGCGTATTCTTGGGGAATTAACACACATTGAGAGCTG**TTATTGCTTGAGAATACACG**
TAGCTGACAAGTTAAAGCGA

>ID189-3P
TTATTGCTTGAGAATACACGT

>miRNA precursor
UGGCCAAGUAGUCCACAGCAACUUGAUUGGCCACGCGUAUUCUUGGGGAAUUAACACACAUUGAGAGCUG**UUAUUGCUUGAGAAUACACGU**AGCUGACAAGUUAAAGCGA
((((......).)))..((((((((.(..((.(((.(((((((..((.(((.((((...........))))))).))..))))))).))).)).))..)))))))...))..

>Predicted STAR seq:
ACGCGTATTCTTGGGGAATTAA

Complementarity: 15
ERROR 4: Strange structure
 Initial dG =   -33.80

```
     -|CA
--UGG C  A
  ACC G  G
\    U^AU
      10
```

```
        20        30        40        50        60
  CA  ---      A UG  C   C       UG  G   U    CACAU
    GC   AACUUG U  GC ACG GUAUUCU  GG AAU AACA     U
    CG   UUGAAC A  CG UGC CAUAAGA  UC UUA UUGU     G
  AG  AAA      - GU  A   A       GU  G   -    CGAGA
110       100        90        80         70
```

Reads in: lib-42, lib-45, lib-47, lib-48, lib-49, lib-50, lib-72
Number of reads="ID189",7,7,6,61,4,2,0

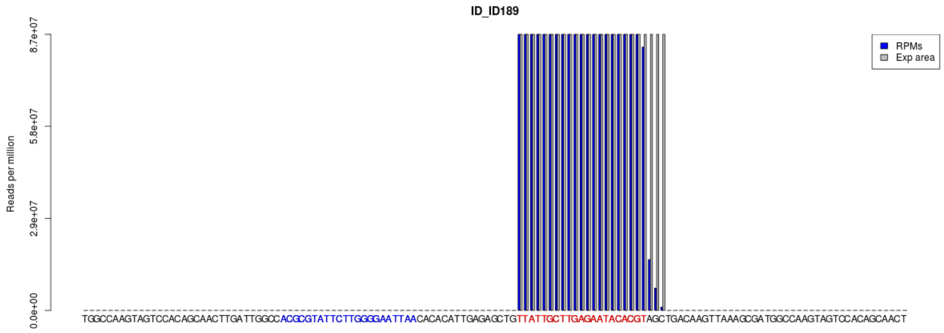

>ID19
TGTTTCTCGGAAAGTGTTTCGCAATTCTCTCCGACACACGTCCCCTGTGTGTGTGTTTGTCACTTTAACA**TACAGAAGACGTGTGTCAGA**
GAGAATTAAGAAACACCAGC

>ID19-3P
TACAGAAGACGTGTGTCAGAGA

>miRNA precursor
UGUUUCUCGGAAAGUGUUUCGCAAUUCUCUCCGACACACGUCCCCUGUGUGUGUGUUUGUCACUUUAACA**UACAGAAGACGUGUGUCAGAGA**GAAUUAAGAAACACCAGC
```
.............((((((..(((((((((.((((((((((..(((((((((((.....)))....))))))))..)))))))))).))))))))).)))))))....
```

>Predicted STAR seq:
CTCCGACACACGTCCCCTGTG

Complementarity: 17

Initial dG = -53.00

```
        10        20        30        40        50
UGUUUCUCGGAAA       GC          C           CC        ----|  UU
             GUGUUUC  AAUUCUCUC GACACACGUC  CUGUGUGU    GUG  \
             CACAAAG  UUAAGAGAG CUGUGUGCAG  GACAUACA    CAC  U
CGAC---------       AA          A           AA        AUUU^  UG
.                100        90        80        70        60
```

Reads in: lib-42, lib-45, lib-47, lib-48, lib-49, lib-50, lib-72
Number of reads="ID19",86,67,33,966,38,15,0

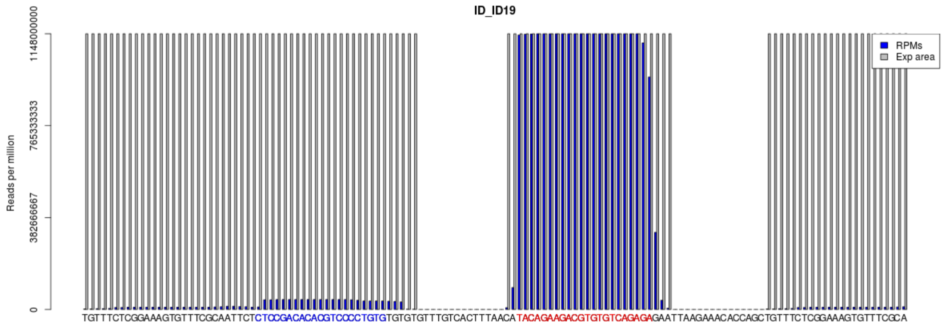

>ID190

TGGCCAAGTAGTCCACAGCAACTTGATTGGCCACGCGTATTCTTGGGGAATTAACACACATTGAGAGCTG**TTATTGCTTGAGAATACACG**
TAGCTGACAAGTTAAAGCGA

>ID190-3P

TTATTGCTTGAGAATACACGT

>miRNA precursor

UGGCCAAGUAGUCCACAGCAACUUGAUUGGCCACGCGUAUUCUUGGGGAAUUAACACACAUUGAGAGCUG**UUAUUGCUUGAGAAUACACGU**AGCUGACAAGUUAAAGCGA
((((......).)))..((((((((.(..((.(((.(((((((..((.(((.((((...........))))))).))..))))))).))).))..)))))))...))..

>Predicted STAR seq:

ACGCGTATTCTTGGGGAATTAA

Complementarity: 15
ERROR 4: Strange structure
 Initial dG =   -33.80

```
     -|CA
--UGG C  A
  ACC G  G
\    U^AU
      10



        20        30        40        50        60
  CA  ---      A UG  C   C       UG  G   U    CACAU
    GC   AACUUG U  GC ACG GUAUUCU  GG AAU AACA     U
    CG   UUGAAC A  CG UGC CAUAAGA  UC UUA UUGU     G
  AG  AAA      - GU  A   A       GU  G   -    CGAGA
110       100        90        80         70
```

Reads in: lib-42, lib-45, lib-47, lib-48, lib-49, lib-50, lib-72
Number of reads="ID190",11,9,8,69,7,2,0

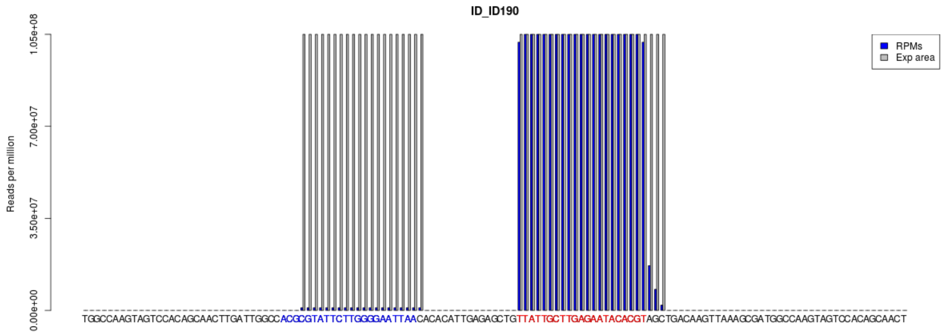

>ID193
GTCGCTCTGGCCGAGCGGTG**TGCGGTTCGCGCCTGGCGGTACGA**GTGTGTGCAGTCTGCCGCCGGGGCGAGCCGGGTACTGTTTCGGCTA
ACCGCCTCCCGTGGATCTCGAACC

>ID193-5P
TGCGGTTCGCGCCTGGCGGTACGA

>miRNA precursor
GUCGCUCUGGCCGAGCGGUG**UGCGGUUCGCGCCUGGCGGUACGA**GUGUGUGCAGUCUGCCGCCGGGGCGAGCCGGGUACUGUUUCGGCUAACCGCCUCCCGUGGAUCUCGAACC
.(((...(((((((((((((.((((((((.((((((((((.((.((....)).)))))))))))))))))))).)))))))).)))))).((((.....))))....)))...

>Predicted STAR seq:
TGCCGCCGGGGCGAGCCGGGTA

Complementarity: 19

Initial dG =   -64.50

```
            10         20        30        40
G--   .-CUC      -|       G        G          C  G  U
   UCG     UGGCCG AGCGGUGU CGGUUCGC CCUGGCGGUA GA UG G
   AGC     AUCGGC UUGUCAUG GCCGAGCG GGGCCGCCGU CU AC U
CCA   \ ---      U^       G        -          -  G  G
  110     90        80        70         60         50




        A---    CU
            CCGC  \
            GGUG  C
        UCUA    CC
              100
```

Reads in: lib-42, lib-45, lib-47, lib-48, lib-49, lib-50, lib-72
Number of reads="ID193",253,105,106,75,277,14,0

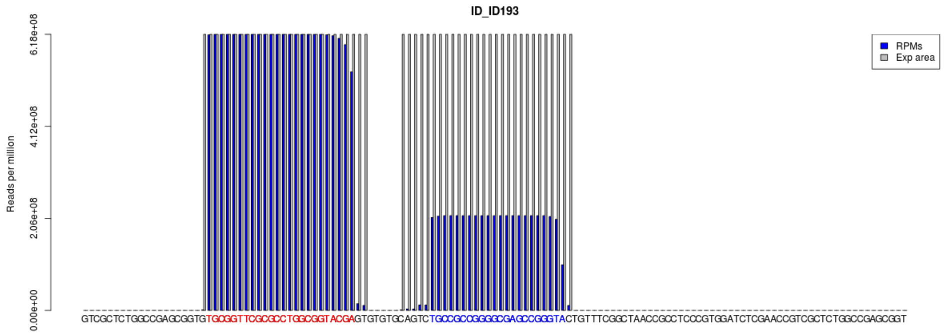

>ID196
GACACTTTGTCTGCAGCCA**CGACACATTCGTGGGACTCTGTGC**GGTATTGCAACACTTACAGCTTCACATGAATATGTCCAAGCTGCAGA
CAAACAAGCGTCAATTTTCTGTA

>ID196-5P
CGACACATTCGTGGGACTCTGTGC

>miRNA precursor
GACACUUUGUCUGCAGCCA**CGACACAUUCGUGGGACUCUGUGC**GGUAUUGCAACACUUACAGCUUCACAUGAAUAUGUCCAAGCUGCAGACAAACAAGCGUCAAUUUUCUGUA
(((.((((((((((((...((((.(((((((.((..(((((.(((........))))))))..)).))))))).))))...)))))))))))...)).)))..........

>Predicted STAR seq:
CAGCTTCACATGAATATGTCCAAGC

Complementarity: 16

Initial dG =   -43.20

```
                      10        20        30        40        50
-----------   A  ---|          CAC    C       G  CU     C   AUU
           GAC CU   UUGUCUGCAGC   GACA AUUCGUG GA  CUGUG GGU   G
           CUG GA   AACAGACGUCG   CUGU UAAGUAC CU  GACAU UCA   C
AUGUCUUUUAA   C  ACA^          AAC    A       A  UC     -   CAA
 110       100        90        80        70        60
```

Reads in: lib-42, lib-45, lib-47, lib-48, lib-49, lib-50, lib-72
Number of reads="ID196",81,63,12,3703,85,12,0

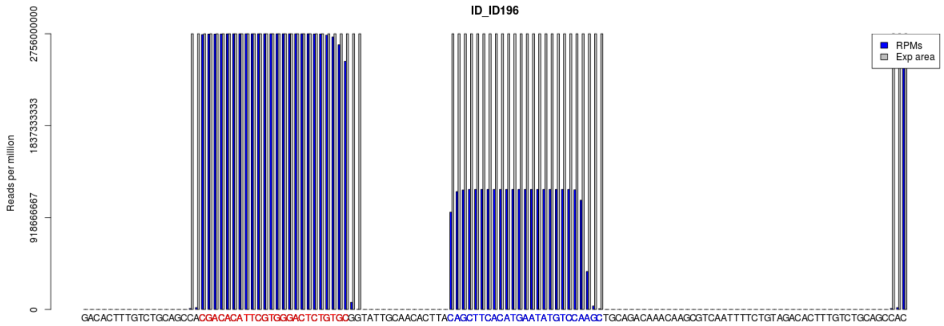

>ID197

TATTCAGCCATCTGCTGTC**TGACGTTTGCATCAAACAGCTGC**TGTGCTGGAGTCAGCTATCTGATGCAAACGGCAGATGGCAACTGGCAA
GTGTCAACTATCAACTGCCTGT

>ID197-5P

TGACGTTTGCATCAAACAGCTGC

>miRNA precursor

UAUUCAGCCAUCUGCUGUC**UGACGUUUGCAUCAAACAGCUGC**UGUGCUGGAGUCAGCUAUCUGAUGCAAACGGCAGAUGGCAACUGGCAAGUGUCAACUAUCAACUGCCUGU
......((((((((.......(((((((((((...(((((((.......)).)))))...)))))))))))))))))))))).((.((((..((........))..)))).))

>Predicted STAR seq:

CAGCTATCTGATGCAAACGGC

Complementarity: 15

Initial dG =   -46.30

```
          10        20        30        40
.-UAUUCA|        UGUCUGA           AAC     -  GUG
        GCCAUCUGC       CGUUUGCAUCA   AGCUG CU   \
        CGGUAGACG       GCAAACGUAGU   UCGAC GA   C
\ ------^        -------           CUA     U  GGU
        80               70        60        50



         90
  A  U    AG  UCA
   AC GGCA  UG   A
   UG CCGU  AC   C
  -  U    CA  UAU
   110       100
```

Reads in: lib-42, lib-45, lib-47, lib-48, lib-49, lib-50, lib-72

Number of reads="ID197",42,35,6,1098,22,2,0

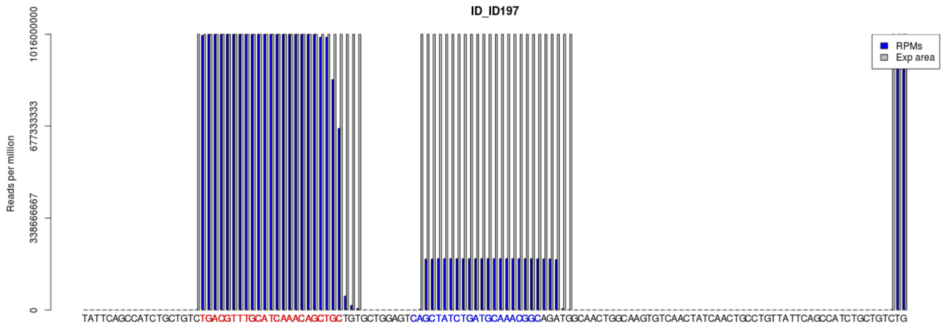

>ID20
GACGGCATTTCGCATCCTCACTGCTGGTACCAGC**GGGGTGACATGTACCAGCAGTG**AGGCATCTCTCACACTGCGCATACATGTCGCCCT
CCTGGCGCTAGCATGTGTCGTCT

>ID20-5P
GGGGTGACATGTACCAGCAGTG

>miRNA precursor
GACGGCAUUUCGCAUCCUCACUGCUGGUACCAGC**GGGGUGACAUGUACCAGCAGUG**AGGCAUCUCUCACACUGCGCAUACAUGUCGCCCUCCUGGCGCUAGCAUGUGUCGUCU
```
((((((((.............((((((..((((.((((((((((((((...((((((.((......)).))))))...)))))))))))))).)))))..)))))).)))))))).
```

>Predicted STAR seq:
CTGCGCATACATGTCGCCCTCCT

Complementarity: 17

Initial dG =   -59.60

```
         10        20        30        40        50        60
-|       UUCGCAUCCUCAC      UA     C             CCA       A   CA
 GACGGCAU             UGCUGG  CCAG GGGGUGACAUGUA   GCAGUG GG  U
 CUGCUGUG             ACGAUC  GGUC UCCCGCUGUACAU   CGUCAC CU  C
U^       U------------      GC     C             ACG       A   CU
 110                   100        90        80        70
```

Reads in: lib-42, lib-45, lib-47, lib-48, lib-49, lib-50, lib-72
Number of reads="ID20",0,0,3,9,0,0,0

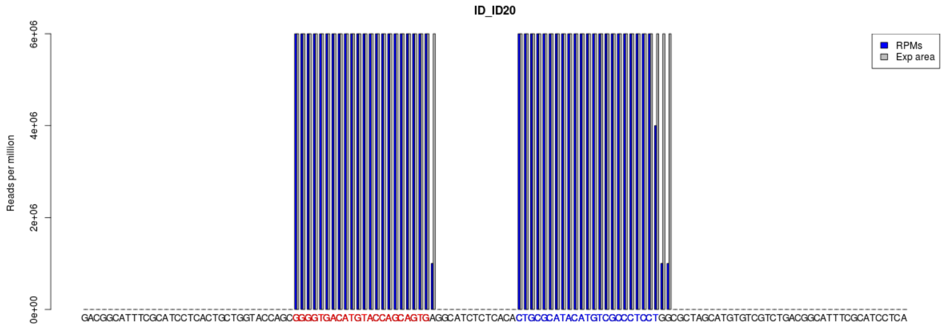

>ID200
AAAGTGACGGGGCCGCTCTCGTCTGAGTGCTGGACAGAGGGCTGGTACTGGGGAGATCCTCTGTCCAGCACTCAGAGGGGAATCGCTTGG
CTGCAGCTTCGATACCATGGCT

>ID200-5P
CGTCTGAGTGCTGGACAGAGGGC

>miRNA precursor
AAAGUGACGGGGCCGCUCUCGUCUGAGUGCUGGACAGAGGGCUGGUACUGGGGAGAUCCUCUGUCCAGCACUCAGAGGGGAAUCGCUUGGCUGCAGCUUCGAUACCAUGGCU
...(((.((((((...((((.((((((((((((((((((((...............))))))))))))))))))))).))))...((......)).)))))).))).......

>Predicted STAR seq:
CTCTGTCCAGCACTCAGAGGGG

Complementarity: 19

Initial dG =   -58.40

```
            10          20        30        40
AAA----   A      .-CGC|   G                    CUGGUAC
       GUG CGGGGC     UCUC UCUGAGUGCUGGACAGAGGG       \
       CAU GCUUCG     AGGG AGACUCACGACCUGUCUCCU       U
UCGGUAC   A      \ ---^   G                    AGAGGGG
110       100         80        70        60        50




                   AUC  UU
                      GC  G
                      CG  G
                   A--  UC
                         90
```

Reads in: lib-42, lib-45, lib-47, lib-48, lib-49, lib-50, lib-72
Number of reads="ID200",2,5,1,193,38,1,0

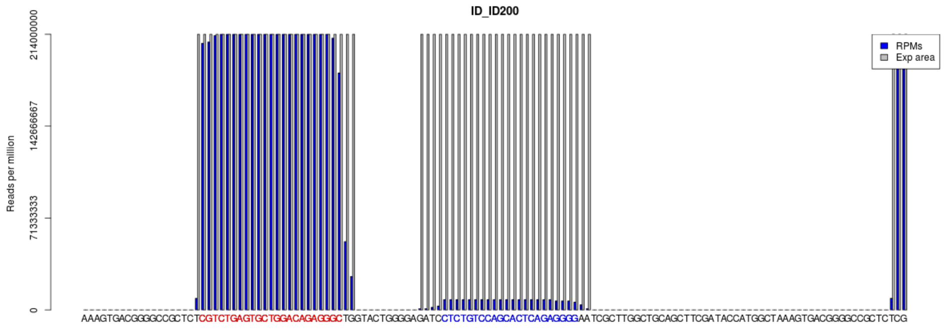

>ID210
GATAAAAGTAAATGTCGCTCGAACTGTCCACGGGTGTACTACTGATCCATAGTGCTCGTTGGATAATA**TGAACCAGCAGCACACCCGTGG**
ACTGTTCCGGCAACAAATACGCC

>ID210-3P
TGAACCAGCAGCACACCCGTGG

>miRNA precursor
GAUAAAAGUAAAUGUCGCUCGAACUGUCCACGGGUGUACUACUGAUCCAUAGUGCUCGUUGGAUAAUA**UGAACCAGCAGCACACCCGUGG**ACUGUUCCGGCAACAAAUACGCC
```
.......(((..(((.(((.((((.((((((((((((.((.((((((((..........))))).........))).)).))))))))))))).)))).))).)))..)))...
```

>Predicted STAR seq:
CACGGGTGTACTACTG

Complementarity: 13

Initial dG =   -45.30

```
        10        20        30        40                  50
GAUAAAA   AA   C   C    U            A  A   ---------|    UAGU
       GUA  UGU GCU GAAC GUCCACGGGUGU CU CUG         AUCCA    G
       CAU  ACA CGG CUUG CAGGUGCCCACA GA GAC         UAGGU    C
CCG----   AA   A   C    U            C  C   CAAGUAUAA^    UGCU
     110       100        90        80        70        60
```

Reads in: lib-42, lib-45, lib-47, lib-48, lib-49, lib-50, lib-72
Number of reads="ID210",1,0,0,4,0,0,0

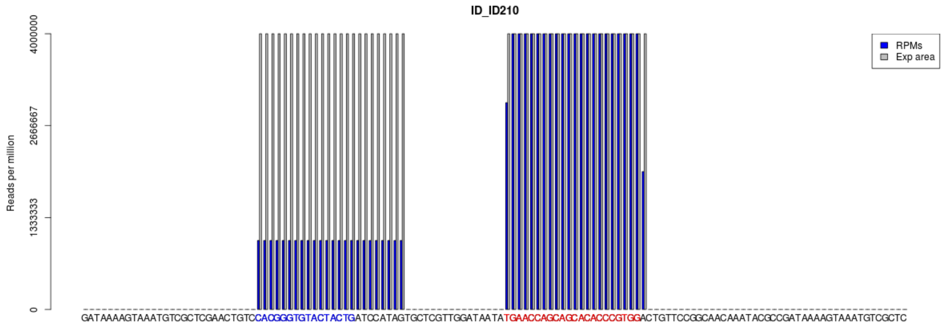

>ID212
ACAGATAACTGTGTGCGAAC**CTCAATGTCGCCTGTAATGCCT**TTCTTTTCCCAGGGCAGTACTGCCAACATTGGGGATTGCGCACAAGTT
AGAACATCGTGGTTGTTGCAAC

>ID212-5P
CTCAATGTCGCCTGTAATGCCT

>miRNA precursor
ACAGAUAACUGUGUGCGAAC**CUCAAUGUCGCCUGUAAUGCCU**UUCUUUUCCCAGGGCAGUACUGCCAACAUUGGGGAUUGCGCACAAGUUAGAACAUCGUGGUUGUUGCAAC
.....(((((((((((((.(((((((((.((..(((.((((((.........)))))).))).))..))))))))).))))))))).)))).((((.......)))).....

>Predicted STAR seq:
GCAGTACTGCCAACATTGGGGA

Complementarity: 16

Initial dG =   -40.60

```
           10        20          30        40
.-ACAGA    -         A         C-| CU   A      UCUU
       UAAC UGUGUGCGA CCUCAAUGU  GC  GUA UGCCUU    \
       AUUG ACACGCGUU GGGGUUACA  CG  CAU ACGGGA    U
\ -----    A         A         AC^ U-   G      CCCU
       90        80        70          60        50



  G----    UCG
       AACA   \
       UUGU   U
  CAACG    UGG
  110
```

Reads in: lib-42, lib-45, lib-47, lib-48, lib-49, lib-50, lib-72
Number of reads="ID212",34,7,1,688,7,0,0

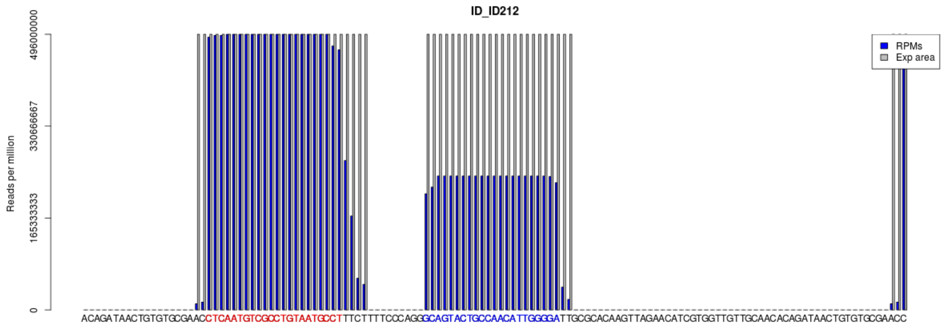

>ID22
GTTATAGAAAAATTGCTGGT**ATAGAGCAGAGTTGACTTCATTG**TAAAAATTAATGACCAGTGATCCAACACTGTTCTGCTGCCAGCGTTT
TTCGATCATTTGGCTCAAAAGAC

>ID22-5P
ATAGAGCAGAGTTGACTTCATTG

>miRNA precursor
GUUAUAGAAAAAUUGCUGGU**AUAGAGCAGAGUUGACUUCAUUG**UAAAAAUUAAUGACCAGUGAUCCAACACUGUUCUGCUGCCAGCGUUUUUCGAUCAUUUGGCUCAAAAGAC
```
......((((((.((((((((((((((.((((...((((((..............))))))..)))).)))))))).)))))))))))))...................
```

>Predicted STAR seq:
GTGATCCAACACTGTTCTGCTGC

Complementarity: 17

Initial dG = -36.90

```
                      10        20         30        40        50
GUUAUA--------------|      U       -        A    ACU       UAAAAA
                    GAAAAAU GCUGGUA UAGAGCAG GUUG   UCAUUG       U
                    CUUUUUG CGACCGU GUCUUGUC CAAC   AGUGAC       U
CAGAAAACUCGGUUUACUAG^      -       C        A    CU-       CAGUAA
 110       100        90         80        70         60
```

Reads in: lib-42, lib-45, lib-47, lib-48, lib-49, lib-50, lib-72
Number of reads="ID22",19,13,10,28,27,4,0

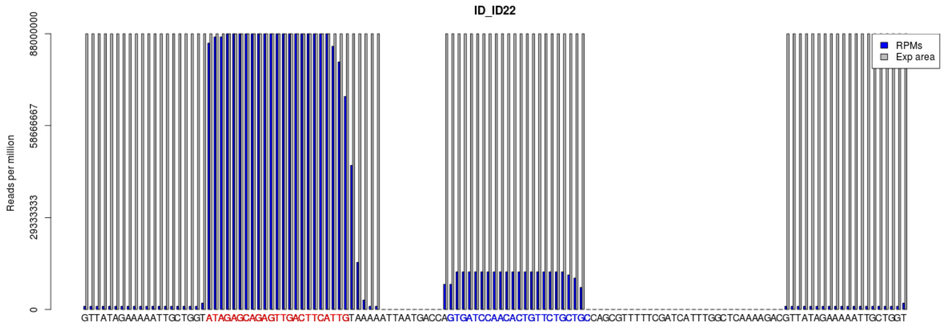

>ID220
AGAGAAAGAGAATACCATTG**TGTAATACCAGCGCCTGTCTTC**GTATGTACGAATCGAAGATTGTCGTTGATAATACACGAAGGTGTACTC
CTGACATCTTCCTAAGTGGAC

>ID220-5P
TGTAATACCAGCGCCTGTCTTC

>miRNA precursor
AGAGAAAGAGAAUACCAUUG**UGUAAUACCAGCGCCUGUCUUC**GUAUGUACGAAUCGAAGAUUGUCGUUGAUAAUACACGAAGGUGUACUCCUGACAUCUUCCUAAGUGGAC
(((....(((.(((((.(((((((.((.(((((...(((((((.((......)))))))))...))))).)).))))))).))))).)))......)))(((.....))).

>Predicted STAR seq:
AGATTGTCGTTGATAATACACG

Complementarity: 15

Initial dG =   -35.40

```
          10        20        30        40
.-AGAGAAA|  A     A       A  C     CCU       U  GU
         GAG AUACC UUGUGUA UA CAGCG   GUCUUCG AU  A
         CUC UGUGG AGCACAU AU GUUGC   UAGAAGC UA  C
\ -------^  A     A       A  A     UGU       -  AG
        90        80        70        60         50



         100
  CUGACAUCU   UA
           UCC  \
           AGG  A
  C--------   UG
         110
```

Reads in: lib-42, lib-45, lib-47, lib-48, lib-49, lib-50, lib-72
Number of reads="ID220",0,2,0,0,0,0,0

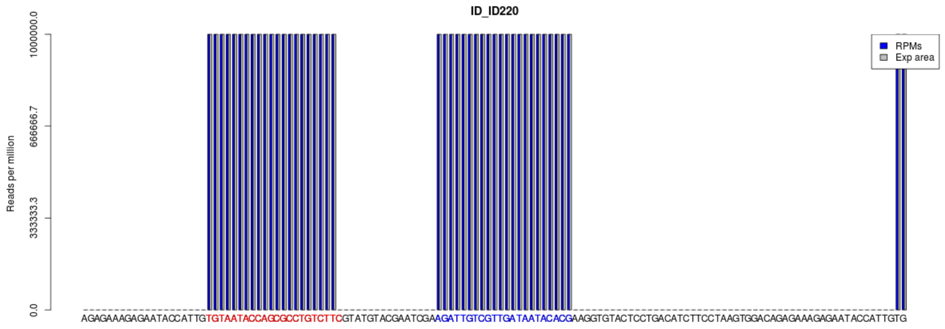

>ID23
CGCCATGTGGACAGGGGTCCCTAGCACAAGTTGAATTCTGTGGTGTCTGTGCTGCTACGTTTACGAGTCG**TAGCACCACATGATTCAGCT**
TAGCTCGGGACCGCACAGTCGG

>ID23-3P
TAGCACCACATGATTCAGCTTA

>miRNA precursor
CGCCAUGUGGACAGGGGUCCCUAGCACAAGUUGAAUUCUGUGGUGUCUGUGCUGCUACGUUUACGAGUCG**UAGCACCACAUGAUUCAGCUUA**GCUCGGGACCGCACAGUCGG
((...((((......((((((.(((..((((((((((.((((((((...(((.(((.((....))))).)))))))))))).)))))))))).))).))))))).)))))..)).

>Predicted STAR seq:
CAAGTTGAATTCTGTGGTGTCTGTG

Complementarity: 18

Initial dG =   -48.20

```
         10        20        30        40        50        60
-| CCA    GACAGG      U   AC          C        CUG   U   A  U
 CG   UGUG      GGUCCC AGC  AAGUUGAAUU UGUGGUGU   UGC GCU CG U
 GC   ACAC      CCAGGG UCG  UUCGACUUAG ACACCACG   AUG UGA GC U
G^ UG-    G-----      C   A-          U        ---   C   -  A
110            100         90        80           70
```

Reads in: lib-42, lib-45, lib-47, lib-48, lib-49, lib-50, lib-72
Number of reads="ID23",2690,1005,1118,3054,5670,240,0

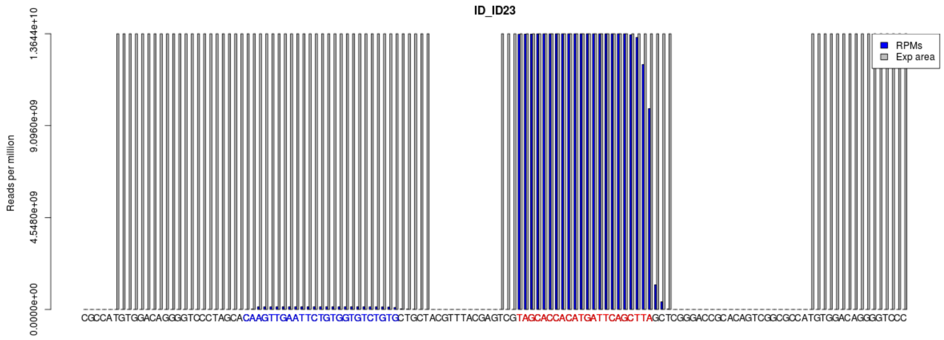

>ID231
AATGCCTGCGAGGGCTGCA**TGTTAGCAAACCCAGCCGCGCTGC**AGTTTTGCCCACGGCGCACCGGGGTTTGCTGCGTGCAGCTCTCGCAC
CTCACACCAGTGATGTCTAACAG

>ID231-5P
TGTTAGCAAACCCAGCCGCGCTGC

>miRNA precursor
AAUGCCUGCGAGGGCUGCA**UGUUAGCAAACCCAGCCGCGCUGC**AGUUUUGCCCACGGCGCACCGGGGUUUGCUGCGUGCAGCUCUCGCACCUCACACCAGUGAUGUCUAACAG
```
......((((((((((((((((.(((((((((....((((((............))))))....))))))))))))))))))))))))))..((((....)))).........
```

>Predicted STAR seq:
GCGCACCGGGGTTTGCTGCGTG

Complementarity: 16

```
 Initial dG =    -58.60

          10        20        30        40
.-AAUGCC|              U         AGCC      CAGUU
        UGCGAGGGCUGCAUGU AGCAAACCC    GCGCUG     U
        ACGCUCUCGACGUGCG UCGUUUGGG    CGCGGC     U
\ ------^              -         GCCA      ACCCG
                80         70        60        50



 90
  CC--------   A
          UCAC C
          AGUG C
  GACAAUCUGU   A
   110       100
```

Reads in: lib-42, lib-45, lib-47, lib-48, lib-49, lib-50, lib-72
Number of reads="ID231",55,127,18,2425,584,27,0

\---

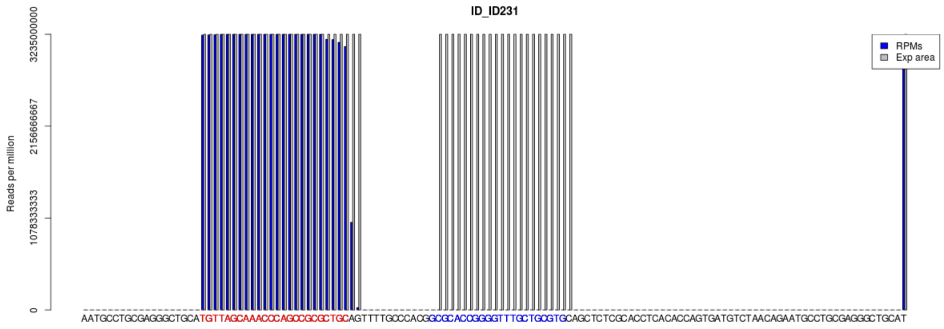

>ID241

GCAGTGGTCTCCTCGACTGATGGAGTTTGACGGGCTGGGTCGGCTGCCGGGTGCTGTTATACTCTTGTAC**AGCAACTCGGCAGCCAACCT**
GGCCCACCATCCCCGTAACTTTG

>ID241-3P

AGCAACTCGGCAGCCAACCTGGC

>miRNA precursor

GCAGUGGUCUCCUCGACUGAUGGAGUUUGACGGGCUGGGUCGGCUGCCGGGUGCUGUUAUACUCUUGUAC**AGCAACUCGGCAGCCAACCUGGC**CCACCAUCCCCGUAACUUUG
.....((((.....)))).((((....((..((((..(((.((((((((((((((((.(((....)))))))).)))))))))))).)))..)))).)).))...)))).......

>Predicted STAR seq:

CTGGGTCGGCTGCCGGGTGCT

Complementarity: 17

Initial dG =   -56.30

```
                    10
GCA----------    .-U    -| G
             GUGG   CUCC UC A
             UACC   GAGG AG C
GUUUCAAUGCCCC    \ -    U^ U
 110       100          20
```

Reads in: lib-42, lib-45, lib-47, lib-48, lib-49, lib-50, lib-72

Number of reads="ID241",4,5,0,161,4,1,0

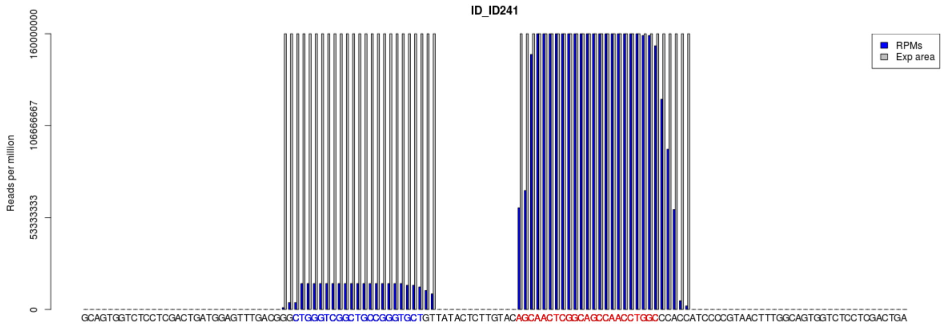

>ID242
AGTGTTCTTCGAGACTGAACGGAGTTTGACAGGGTGGGTCGGCTGCCGGGTGCTGTTATATTCTTGTAC**AGCAACTTGGCAGTCGGCCCA TC**CTATCAGCGCCATAATTTCGA

>ID242-3P
AGCAACTTGGCAGTCGGCCCATC

>miRNA precursor
AGUGUUCUUCGAGACUGAACGGAGUUUGACAGGGUGGGUCGGCUGCCGGGUGCUGUUAUAUUCUUGUAC**AGCAACUUGGCAGUCGGCCCAUC**CUAUCAGCGCCAUAAUUUCGA

```
........((((((.((...((.(((.((.((((((((((((((((((((((((((.(((....)))))))).))))))))))))))))))))).))))).)).)).))))))
```

>Predicted STAR seq:
GGTGGGTCGGCTGCCGGGTGC

Complementarity: 20

Initial dG =   -58.20

```
        10        20        30        40        50         60
AGUGUUCU|     C  AAC  A   U  C                    -     U   U
        UCGAGA UG   GG GUU GA AGGGUGGGUCGGCUGCCGGGU GCUGU AUA U
        AGCUUU AU   CC CGA CU UCCUACCCGGCUGACGGUUCA CGACA UGU C
--------^     A  A--  G   -  A                    A     -   U
         110         100        90        80        70
```

Reads in: lib-42, lib-45, lib-47, lib-48, lib-49, lib-50, lib-72
Number of reads="ID242",20,2,0,234,5,1,0

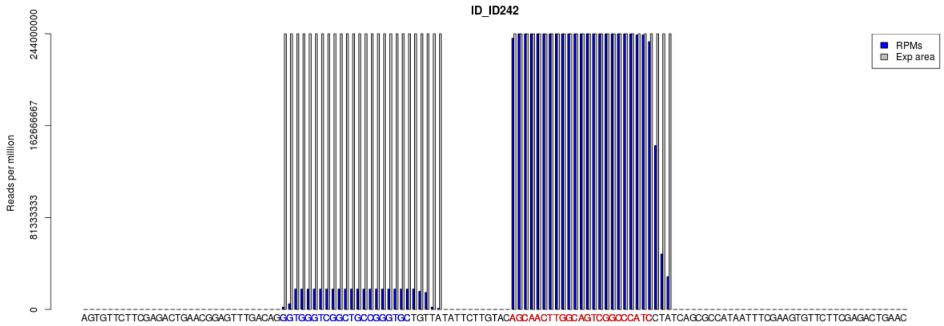

>ID243

GGTCTTCTCGATTGGGCGGAGTTTGATGGGGTGTGCAGGGCGGCGTCTGCTGTCCCACTTTTGTACGAG**CAGACGTCTCGATGTACTCCC**
CACCTGACCCGCAAATCGTCAC

>ID243-3P

CAGACGTCTCGATGTACTCCCCA

>miRNA precursor

GGUCUUCUCGAUUGGGCGGAGUUUGAUGGGGUGUGCAGGGCGGCGUCUGCUGUCCCACUUUUGUACGAG**CAGACGUCUCGAUGUACUCCCCA**CCUGACCCGCAAAUCGUCAC
```
........(((((..((((.(((...(((((.(((((.((.((((((((((((...((....)))).))))))))))))..))))).))))).....)))))).)))))....
```

>Predicted STAR seq:

GATGGGGTGTGCAGGGCGGCGTCTG

Complementarity: 18

Initial dG =   -49.60

```
        10        20        30         40        50         60
GGUCUUCU|    GG    A   UGA     U     G-  C          -  CCC  U
        CGAUU  GCGG GUU   UGGGG GUGCA  GG GGCGUCUGCU GU   AC U
        GCUAA  CGCC CAG   ACCCC CAUGU  CU CUGCAGACGA CA   UG U
CACU----^    A-    -   UCC     U     AG  -          G  ---  U
110            100        90        80         70
```

Reads in: lib-42, lib-45, lib-47, lib-48, lib-49, lib-50, lib-72

Number of reads="ID243",27,29,1,640,7,5,0

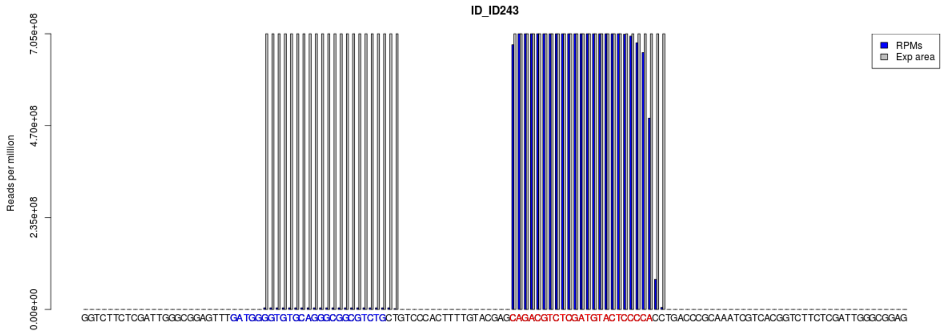

>ID245
TTGTCTCTTCGATCGCGCGGGAGTTGACGGGTGGAGTAGGCTGCCAGCTGCTGTTCCACTTTTGTACGAG**CATCTGGCAGGGGCGCCACC**
CACCAGCTCCTGCAAATCTCCA

>ID245-3P
CATCTGGCAGGGGCGCCACCCA

>miRNA precursor
UUGUCUCUUCGAUCGCGCGGGAGUUGACGGGUGGAGUAGGCUGCCAGCUGCUGUUCCACUUUUGUACGAG**CAUCUGGCAGGGGCGCCACCCA**CCAGCUCCUGCAAAUCUCCA
```
..........(((...((((((((((..((((((.((...(((((((.((((((...((....)))).)))).)))))))..)).))))))..))))))))))..)))....
```

>Predicted STAR seq:
CGGGTGGAGTAGGCTGCCAGCTG

Complementarity: 15

Initial dG =   -52.20

```
        10         20         30         40         50          60
UUGUCUCUUC|  CGC          AC       A   AGG        C     -   UCC   U
          GAU   GCGGGAGUUG  GGGUGG GU   CUGCCAG UGCU GU   AC  U
          CUA   CGUCCUCGAC  CCCACC CG   GACGGUC ACGA CA   UG  U
ACCU------^  AA-          CA       G   GG-        U     G   ---   U
110              100        90         80         70
```

Reads in: lib-42, lib-45, lib-47, lib-48, lib-49, lib-50, lib-72
Number of reads="ID245",4,6,1,20,0,0,0

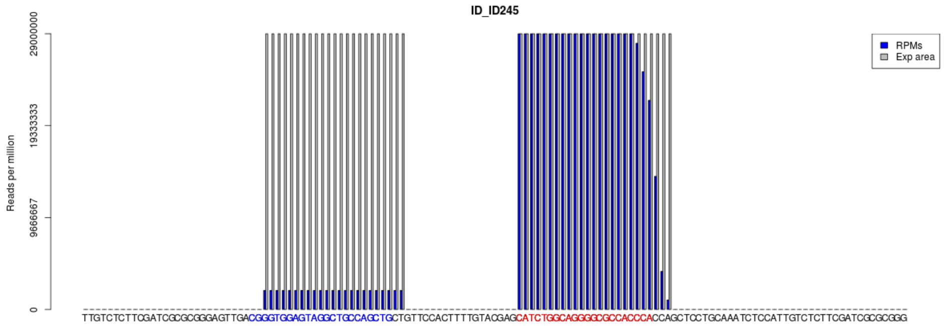

>ID246

GGTCTTCTCGATTGAGCGGAGTTTGATGGGGGGTACAGCGTGGCACCTGCTGTCCCACTTCTGCACGAG**CAGGTGCCTCGCTATGCTCCA**
CCATCTGCCATCGCAAATCGCC

>ID246-3P

CAGGTGCCTCGCTATGCTCCACC

>miRNA precursor

GGUCUUCUCGAUUGAGCGGAGUUUGAUGGGGGGUACAGCGUGGCACCUGCUGUCCCACUUCUGCACGAG**CAGGUGCCUCGCUAUGCUCCACC**AUCUGCCAUCGCAAAUCGCC
........((((..((((.((..(((((((((((.((((.((((((((((((...........)).)))))))))).)))).)))))).))))).))..)))).)))))..

>Predicted STAR seq:

GGGGGTACAGCGTGGCACCTG

Complementarity: 18

Initial dG =   -53.70

```
        10        20          30        40        50
GGUCUUCU     GA    A-   UU     -      C    U          -| CCCAC
        CGAUU  GCGG  GU  GAUGG GGGGUA AGCG GGCACCUGCU GU     \
        GCUAA  CGCU  CG  CUACC CCUCGU UCGC CCGUGGACGA CA     U
CC------     A-    AC  U-     A      A    U          G^ CGUCU
      110       100         90        80        70        60
```

Reads in: lib-42, lib-45, lib-47, lib-48, lib-49, lib-50, lib-72

Number of reads="ID246",5,16,3,86,7,6,0

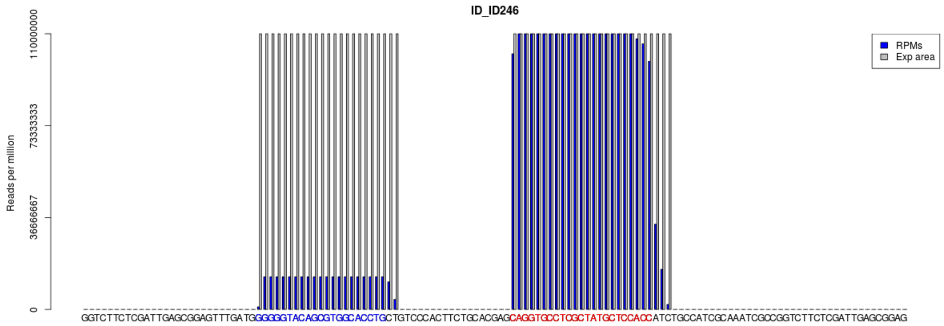

>ID247
GATTGAGCGGAGTTTGATGG**GGGGAGCTGGCTGCCGATTGCT**GTTCCACTCTTGTACGAGCAACTCAGCAGCCAGCCCCCCTATAAGCCC
CGAATATTCGCACCCAGGATTG

>ID247-5P
GGGGAGCTGGCTGCCGATTGCT

>miRNA precursor
GAUUGAGCGGAGUUUGAUGG**GGGGAGCUGGCUGCCGAUUGCU**GUUCCACUCUUGUACGAGCAACUCAGCAGCCAGCCCCCCUAUAAGCCCCGAAUAUUCGCACCCAGGAUUG
((.((..(((.(((((.(((((((.(((((((((.(((((((((...((....)))).))))).)).))))))))))))))))))))).)))..)).)).............

>Predicted STAR seq:
CAACTCAGCAGCCAGCCCCCCT

Complementarity: 18

Initial dG =   -51.60

```
                     10        20        30         40         50
-------------| U  AG   A     A       A         C  -     -  UCC  U
             GA UG  CGG GUUUG UGGGGGG GCUGGCUGC GA UUGCU GU   AC C
             CU AU  GCC CGAAU AUCCCCC CGACCGACG CU AACGA CA   UG U
GUUAGGACCCACG^ U  AA   C     -       -         A  C     G  ---  U
110       100       90         80         70        60
```

Reads in: lib-42, lib-45, lib-47, lib-48, lib-49, lib-50, lib-72
Number of reads="ID247",1,3,0,32,1,2,0

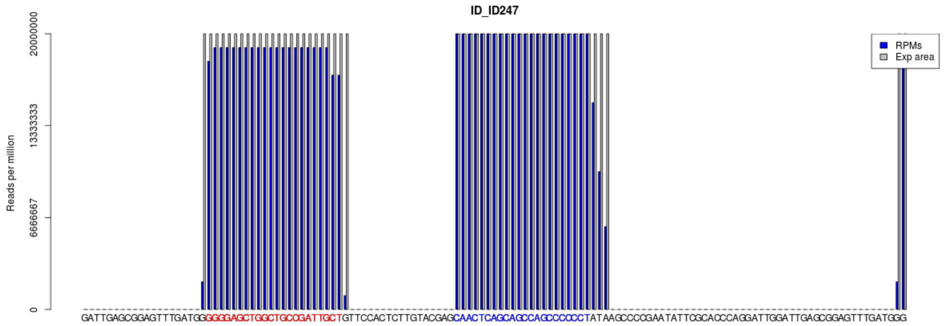

>ID248
AGTGGTCTCCTTGGCTGAACGGTGTTTGACGGGT**TGGGGTGGCTGCCGGGTGCTGT**TATATTCTTGTACAGCAACTCGGCAGTCGGCCCA
TCCTATCAGTTCCAAAAGTTCGC

>ID248-5P
TGGGGTGGCTGCCGGGTGCTGT

>miRNA precursor
AGUGGUCUCCUUGGCUGAACGGUGUUUGACGGGU**UGGGGUGGCUGCCGGGUGCUGU**UAUAUUCUUGUACAGCAACUCGGCAGUCGGCCCAUCCUAUCAGUUCCAAAAGUUCGC
...((((.....))))((((((...((((..((.((((.(((((((((((((((((.(((....)))))))).)))))))))))).)))).))..)))).))....))))..

>Predicted STAR seq:
AGCAACTCGGCAGTCGGCCCATCC

Complementarity: 19

Initial dG = -53.50

ERROR 3: Strange structure
Initial dG = -53.50

```
.-AGU|   UC
     GGUC  \
     UCGG  C
\ ---^   UU
```

```
      20            30        40        50         60
  --    ----  UGU    CG  U    G            -     U   U
    GAAC    GG   UUGA  GG UGGG UGGCUGCCGGGU GCUGU AUA U
    CUUG    CC   GACU  CC ACCC GCUGACGGCUCA CGACA UGU C
  CG    AAAA  UU-    AU  U    G            A     -   U
   110       100         90        80        70
```

Reads in: lib-42, lib-45, lib-47, lib-48, lib-49, lib-50, lib-72
Number of reads="ID248",7,7,1,81,7,2,0

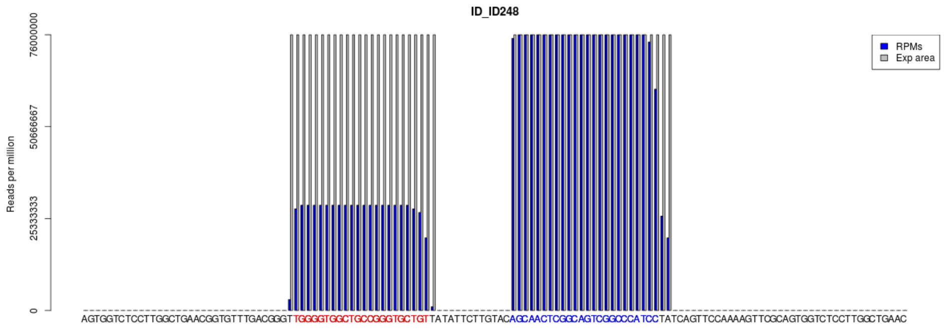

>ID250

AGTGGTCTCCTCGACTGAACGGAGTTTGACGGGGTGGGGCGGCTGCCGGGTGCTGTTATATTCTTGCAC**AGCAACTCAGCAGTCGGCCCA TCC**TATCAGCCCCATAATTTCGC

>ID250-3P

AGCAACTCAGCAGTCGGCCCATCC

>miRNA precursor

AGUGGUCUCCUCGACUGAACGGAGUUUGACGGGGUGGGGCGGCUGCCGGGUGCUGUUAUAUUCUUGCAC**AGCAACUCAGCAGUCGGCCCAUCC**UAUCAGCCCCAUAAUUUCGC

...((((.....))))....((.(((.((..(((((((.(((((((.(((((((((((......)).))))).)))).))))))).)))))))..))))).))..........

>Predicted STAR seq:

GGGGTGGGGCGGCTGCCGGGTGCT

Complementarity: 19

Initial dG =   -51.60

```
                 10
A-------    .-U|   UCGA
        GUGG   CUCC    C
        UACC   GAGG    U
CGCUUUAA    \ -^   CAAG
 110              20
```

Reads in: lib-42, lib-45, lib-47, lib-48, lib-49, lib-50, lib-72

Number of reads="ID250",16,22,2,524,10,3,0

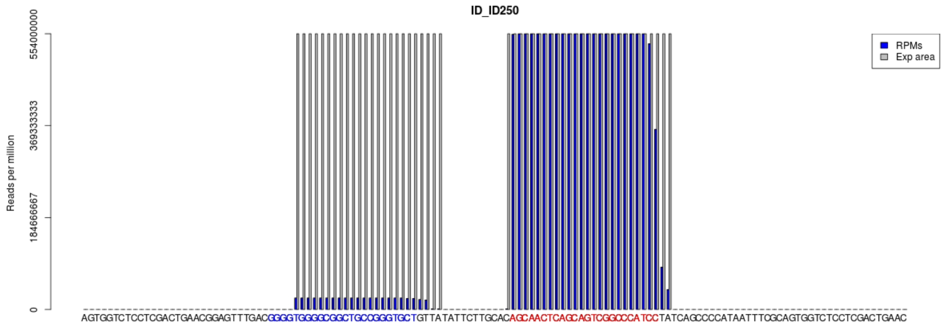

>ID252
CTGAACGGAGTTTTACGGGG**TGGGTCGGCTGCCGGGTGCTGT**TATATTCTTGTACAGCAACTCGGCAGCTGACCCATCCTACCAACCCCA
AAAGTTTGCACACAGGACTGCC

>ID252-5P
TGGGTCGGCTGCCGGGTGCTGT

>miRNA precursor
```
CUGAACGGAGUUUUACGGGGUGGGUCGGCUGCCGGGUGCUGUUAUAUUCUUGUACAGCAACUCGGCAGCUGACCCAUCCUACCAACCCCAAAAGUUUGCACACAGGACUGCC
......((.(((....((((((((((((((((((((((((((.(((....)))))))).)))))))))))))))))))))...))).))...(((((.......)))))...
```

>Predicted STAR seq:
AGCAACTCGGCAGCTGACCCATC

Complementarity: 20

Initial dG =   -56.60

```
          10        20        30         40
.-CUGAAC| A   UUAC                    -     U   U
        GG GUU    GGGGUGGGUCGGCUGCCGGGU GCUGU AUA U
        CC CAA    UCCUACCCAGUCGACGGCUCA CGACA UGU C
\ ------^ C   CCA-                    A     -   U
                 80        70        60         50



 90       100
  AAA     GCA
     AGUUU   \
     UCAGG   C
  CCG     ACA
  110
```

Reads in: lib-42, lib-45, lib-47, lib-48, lib-49, lib-50, lib-72
Number of reads="ID252",4,6,0,75,0,0,0

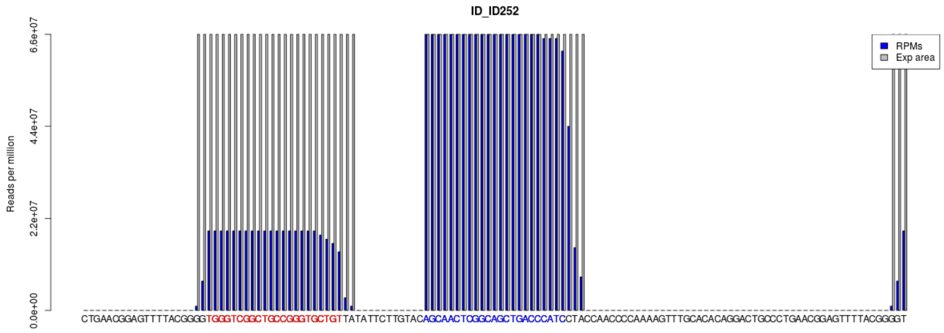

>ID254
GTGATCTTCAAACTGAGAGGAGTTTGATGGGGGGGCCGGCTGCCAATTGCTGTTCCACTTTTGTACGA**ACTATTTGACAGCCAGTCCCCC**
TATCAGCTCCTGCAAATCGG

>ID254-3P
ACTATTTGACAGCCAGTCCCCC

>miRNA precursor
GUGAUCUUCAAACUGAGAGGAGUUUGAUGGGGGGGCCGGCUGCCAAUUGCUGUUCCACUUUUGUACGA**ACUAUUUGACAGCCAGUCCCCC**UAUCAGCUCCUGCAAAUCGG
```
............((((.(((((((.(((((((((((.(((((.(((.....((((.((....))..))))...))).))))).))))))))))))))))))).....))))
```

>Predicted STAR seq:
GGGGGCCGGCTGCCAATTGCTGT

Complementarity: 15

Initial dG =   -47.70

```
        10            20         30         40         50          60
GUGAUCUUCAAA    G----|      U           C      C   UUGCU    C-   U
            CUGA     AGGAGUU GAUGGGGGGGC GGCUG CAA     GUUC  AC U
            GGCU     UCCUCGA CUAUCCCCCUG CCGAC GUU     CAAG  UG U
------------    AAACG^      -           A      A   UAU--    CA   U
          110       100         90         80          70
```

Reads in: lib-42, lib-45, lib-47, lib-48, lib-49, lib-50, lib-72
Number of reads="ID254",5,21,3,68,10,2,0

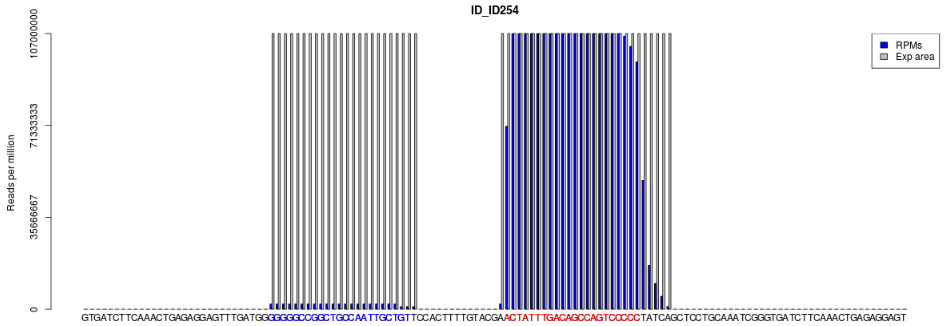

>ID256
GACTCAGCGGAGTTTGATGG**GGAGAGTCGGCTGCCAGTTGCT**GTCCACTCTTGTACCAGCAACTCGGCTGCCGGTTCCCTGTCAGCCCCG
CAAATTCGCACCCAGTCCAGTC

>ID256-5P
GGAGAGTCGGCTGCCAGTTGCT

>miRNA precursor
GACUCAGCGGAGUUUGAUGG**GGAGAGUCGGCUGCCAGUUGCU**GUCCACUCUUGUACCAGCAACUCGGCUGCCGGUUCCCUGUCAGCCCCGCAAAUUCGCACCCAGUCCAGUC
((((..((((.(((.(((((((((..(((((.(((((((((((...((....))..)))))))).))).))))))))))))))))).)))).............)))).....

>Predicted STAR seq:
CAACTCGGCTGCCGGTTCCCT

Complementarity: 17

Initial dG =   -49.50

```
          10        20        30        40        50
    .-CA|   A   U         AG      U   -        UCC  U
GACU    GCGG GUU GAUGGGGAG  UCGGC GCC AGUUGCUG   AC C
CUGA    CGCC CGA CUGUCCCUU  GGCCG CGG UCAACGAC   UG U
    \ --^   C   -         --      U   C        CA-  U
110     90         80          70        60


            100
      AAAUUC  AC
            GC  \
            UG  C
      CC----  AC
```

Reads in: lib-42, lib-45, lib-47, lib-48, lib-49, lib-50, lib-72
Number of reads="ID256",4,8,0,47,4,0,0

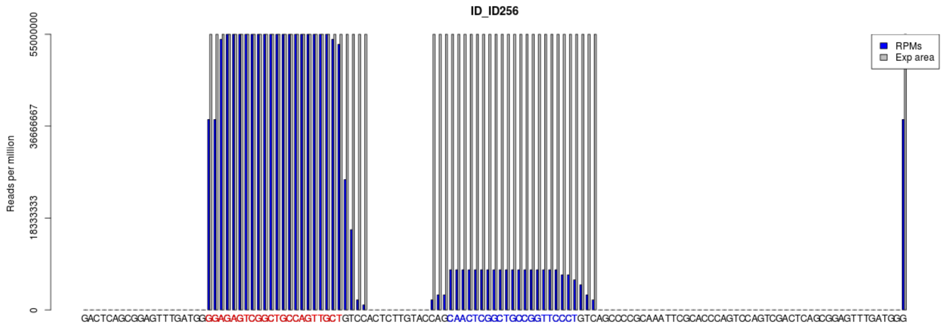

>ID257
CTAAACGGAGTTTGATGGGG**TGGGTCGGCTGCCGGGTGCTGT**TATATTCTTGTACAGCAACTCGGCAGCTGACACATCCTATCAACCCCA
AAAATTCGCCCCCAGGACTGCT

>ID257-5P
TGGGTCGGCTGCCGGGTGCTGT

>miRNA precursor
CUAAACGGAGUUUGAUGGGG**UGGGUCGGCUGCCGGGUGCUGU**UAUAUUCUUGUACAGCAACUCGGCAGCUGACACAUCCUAUCAACCCCAAAAAUUCGCCCCCAGGACUGCU
......((.(((.(((((((((.((((((((((((((((((.(((....)))))))).)))))))))))))))).))))))))))))).)).......................

>Predicted STAR seq:
AGCAACTCGGCAGCTGACACATC

Complementarity: 19

Initial dG =   -55.00

```
                         10        20        30         40
CUAAAC-----------------| A   U         G               -     U   U
                       GG GUU GAUGGGGUG GUCGGCUGCCGGGU GCUGU AUA U
                       CC CAA CUAUCCUAC CAGUCGACGGCUCA CGACA UGU C
UCGUCAGGACCCCCGCUUAAAAA^ C   -         A              A     -   U
110       100        90         80        70        60         50
```

Reads in: lib-42, lib-45, lib-47, lib-48, lib-49, lib-50, lib-72
Number of reads="ID257",3,6,0,108,2,1,0

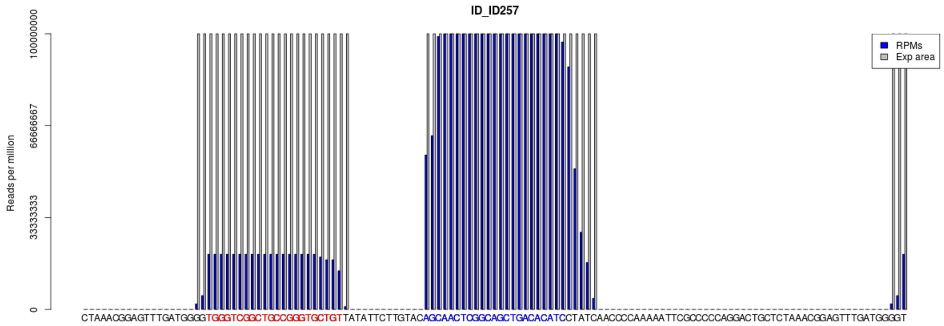

>ID258
GTGACCTTCAAGCTGAGCGGAGTTTGATGGGGGGGGCCGGCTGCCAATTGCTGTTCCAATTTTGTACGA**ACTATTTGACAGCCAGTCCCCC**
TATCAGCACCTGCAAATCGG

>ID258-3P
ACTATTTGACAGCCAGTCCCCC

>miRNA precursor
GUGACCUUCAAGCUGAGCGGAGUUUGAUGGGGGGGGCCGGCUGCCAAUUGCUGUUCCAAUUUUGUACGA**ACUAUUUGACAGCCAGUCCCCC**UAUCAGCACCUGCAAAUCGG
```
............((((((((.((((((((((((((((.(((((.(((.....((((...........))))...))).))))).))))))))))))).))))))...))))
```

>Predicted STAR seq:
GGGGGCCGGCTGCCAATTGCTGT

Complementarity: 15

Initial dG =   -45.30

```
        10           20          30         40         50         60
GUGACCUUCAAG    ---|   A  -             C     C   UUGCU    CAAUU
            CUGA   GCGG GU UUGAUGGGGGGGC GGCUG CAA     GUUC     \
            GGCU   CGUC CA GACUAUCCCCCUG CCGAC GUU     CAAG     U
------------    AAA^   -  C             A     A   UAU--    CAUGU
          110       100         90         80          70
```

Reads in: lib-42, lib-45, lib-47, lib-48, lib-49, lib-50, lib-72
Number of reads="ID258",9,13,1,58,8,3,0

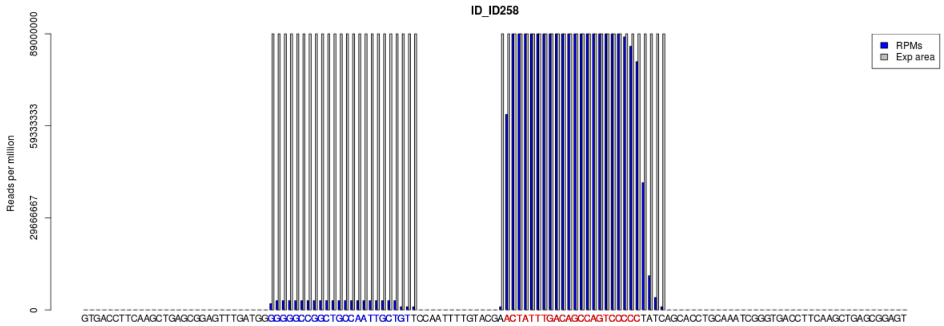

>ID262
CCAGCACGAGCAGTACGCCA**CGCAGAATCCGACGGTGACGTCC**GTGGCGACGCCGGCGTCGGATGCTGAGTGGAGTGCTGCGTCGACACC
GACGTCACCGTCTACGTCATCA

>ID262-5P
CGCAGAATCCGACGGTGACGTCC

>miRNA precursor
CCAGCACGAGCAGUACGCCA**CGCAGAAUCCGACGGUGACGUCC**GUGGCGACGCCGGCGUCGGAUGCUGAGUGGAGUGCUGCGUCGACACCGACGUCACCGUCUACGUCAUCA
......((((((((((.((((.(((.((((((((.((.((((......)))).)).)))))))).))).)))).))))))).)))).....(((((........)))))....

>Predicted STAR seq:
CGCCGGCGTCGGATGCTGAGTG

Complementarity: 16

Initial dG = -55.20

```
           10        20        30        40
.-CCAGCA   -|      G    G   A        G  A    CG
        CGA GCAGUAC CCAC CAG AUCCGACG UG CGUC  U
        GCU CGUCGUG GGUG GUC UAGGCUGC GC GCAG  G
\ ------   G^     A    A   G        G  C    CG
            80        70        60        50



     90
  ACACC     CAC
       GACGU   C
       CUGCA   G
  ACUA-     UCU
  110        100
```

Reads in: lib-42, lib-45, lib-47, lib-48, lib-49, lib-50, lib-72
Number of reads="ID262",0,5,1,43,0,0,0

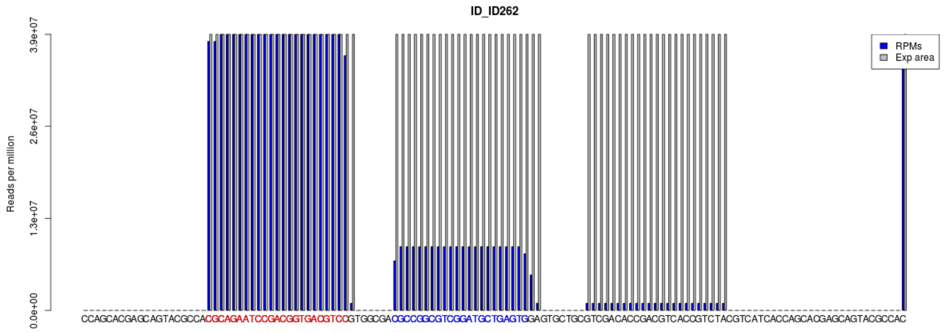

>ID266

TAAAAAAATAAAA**TTAGTATAGTATAGGTGGGAGTT**GTGACAATTTATTGTCATAACGTCAATGAAATACCACTATAATTATTATTTTTT
TAATTAAATGATAAATATAG

>ID266-5P

TTAGTATAGTATAGGTGGGAGTT

>miRNA precursor

UAAAAAAAUAAAA**UUAGUAUAGUAUAGGUGGGAGUU**GUGACAAUUUAUUGUCAUAACGUCAAUGAAAUACCACUAUAAUUAUUAUUUUUUAAUUAAAUGAUAAAUAUAG
((((((((((......((((((...(((((..((((((((((....))))))))))..).......))))))))))....))))))))))).................

>Predicted STAR seq:

CGTCAATGAAATACCACTATAATTATT

Complementarity: 12

Initial dG =   -23.40

```
                          10        20        30               40
------------------           AAUUAG      AUA    -------|GA          U
                  UAAAAAAAUAA      UAUAGU   GGUG       G  GUUGUGACAA U
                  AUUUUUUUAUU      AUAUCA   CCAU       C  CAAUACUGUU U
GAUAUAAAUAGUAAAUUA           AUUA--      ---    AAAGUAA^UG          A
.       100        90        80             70        60        50
```

Reads in: lib-42, lib-45, lib-47, lib-48, lib-49, lib-50, lib-72

Number of reads="ID266",2,497,5,1,14,6,0

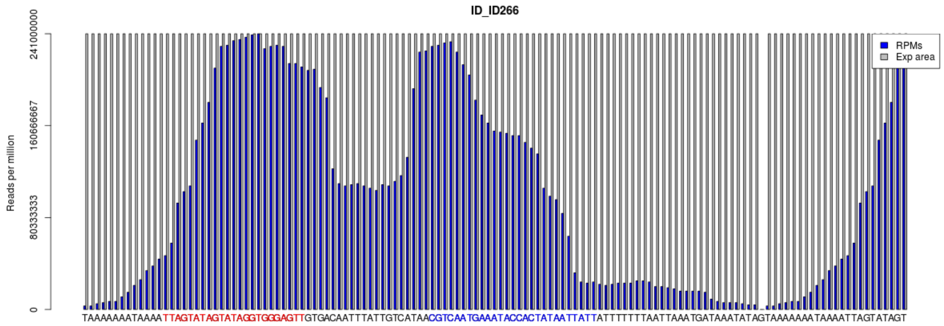

>ID272
GCGGCGGCAACTGTAAGTGGCACGTGTACACCCTCTCTCGAGATGGCTACCCTCTAGGGGGCCGTGTTGT**CCTCTTGAGACAGGTTGTCT**
GCGTGAGGCTTGCACGCACACC

>ID272-3P
CCTCTTGAGACAGGTTGTCTGC

>miRNA precursor
GCGGCGGCAACUGUAAGUGGCACGUGUACACCCUCUCUCGAGAUGGCUACCCUCUAGGGGGCCGUGUUGU**CCUCUUGAGACAGGUUGUCUGC**GUGAGGCUUGCACGCACACC
...(((.....(((((((..(((((..(((.(((.(((((((((((((.(((....))))))))........))))))))).))).))).)).))))).)))))))))).....

>Predicted STAR seq:
TGTACACCCTCTCTCGAGA

Complementarity: 14

Initial dG = -47.20

```
          10        20        30        40                 50
GCG--   GCAAC       GG      GU   C   C        --------|    A   U
     GCG     UGUAAGU  CACGU  ACA CCU UCUCGAGA        UGGCU CCC C
     CGC     ACGUUCG  GUGCG  UGU GGA AGAGUUCU        GCCGG GGG U
CCACA   -----       GA      UC   U   C        CCUGUUGU^    -   A
110            100        90        80        70        60
```

Reads in: lib-42, lib-45, lib-47, lib-48, lib-49, lib-50, lib-72
Number of reads="ID272",518,650,575,1190,1146,218,0

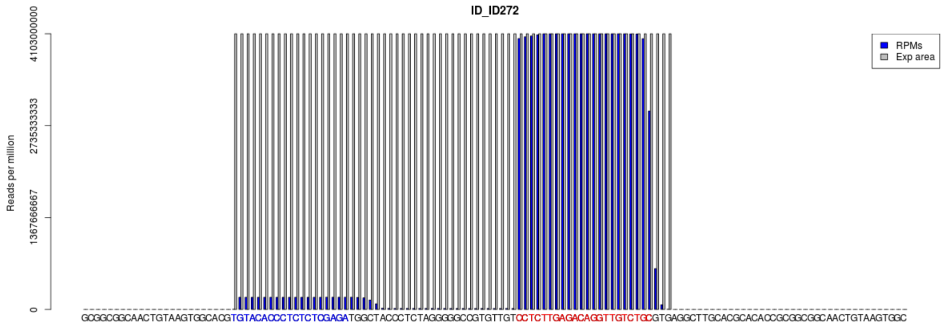

>ID277

CAAAAAGATCTGAGGCACTC**AGAATCAGTAGTTGCTGAGTCA**AGGCTTTTAAAATGTGACTTGACAATTTCTGTTCCTAAGTGCTTCAAA TATTTCCTACTGCTCCGCCGTT

>ID277-5P

AGAATCAGTAGTTGCTGAGTCA

>miRNA precursor

CAAAAAGAUCUGAGGCACUC**AGAAUCAGUAGUUGCUGAGUCA**AGGCUUUUAAAAUGUGACUUGACAAUUUCUGUUCCUAAGUGCUUCAAAUAUUUCCUACUGCUCCGCCGUU

```
..........(((((((((.((...(((.(((((..((((((..............))))))..))))).)))...)).)))))))))........................
```

>Predicted STAR seq:

ACTTGACAATTTCTGTTCCTAAG

Complementarity: 14

Initial dG = -27.80

```
        10                      20        30        40
CAAAAAGAUC--------------|        C  AAU   U     CU      AGGCUU
                        UGAGGCACU AG   CAG AGUUG  GAGUCA      U
                        ACUUCGUGA UC   GUC UUAAC  UUCAGU      U
UUGCCGCCUCGUCAUCCUUUAUAA^        A  CUU   U     AG      GUAAAA
110       100        90        80        70        60        50
```

Reads in: lib-42, lib-45, lib-47, lib-48, lib-49, lib-50, lib-72

Number of reads="ID277",0,1,0,12,0,0,0

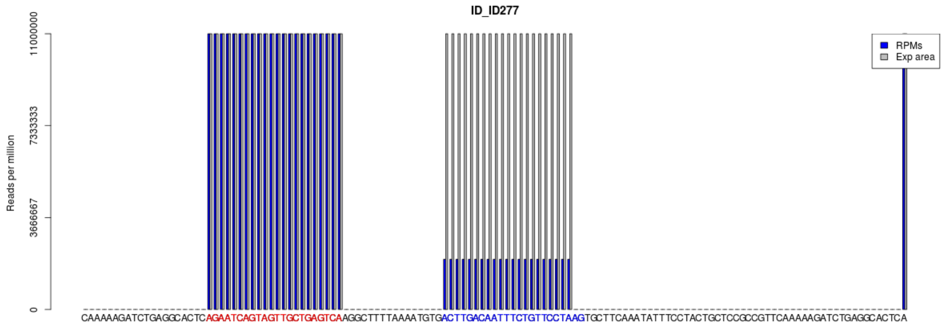

>ID278
CAAAAAGATCTGAGGCACTC**AGAATCAGTAGTTGCTGAGTCA**AGGCTTTTAAAATGTGACTTGACAATTTCTGTTCCTAAGTGCTTCAAA
TATTTCCTACTGCTCCGCCGAA

>ID278-5P
AGAATCAGTAGTTGCTGAGTCA

>miRNA precursor
CAAAAAGAUCUGAGGCACUC**AGAAUCAGUAGUUGCUGAGUCA**AGGCUUUUAAAAUGUGACUUGACAAUUUCUGUUCCUAAGUGCUUCAAAUAUUUCCUACUGCUCCGCCGAA
```
..........(((((((((.((...(((.(((((..((((((..............))))))..))))).)))...)).)))))))))........................
```

>Predicted STAR seq:
ACTTGACAATTTCTGTTCCTAAG

Complementarity: 14

Initial dG =   -27.80

```
        10                       20        30        40
CAAAAAGAUC--------------|        C  AAU   U     CU       AGGCUU
                        UGAGGCACU AG   CAG AGUUG  GAGUCA      U
                        ACUUCGUGA UC   GUC UUAAC  UUCAGU      U
AAGCCGCCUCGUCAUCCUUUAUAA^        A  CUU   U     AG       GUAAAA
110       100        90        80        70        60        50
```

Reads in: lib-42, lib-45, lib-47, lib-48, lib-49, lib-50, lib-72
Number of reads="ID278",0,0,0,15,0,0,0

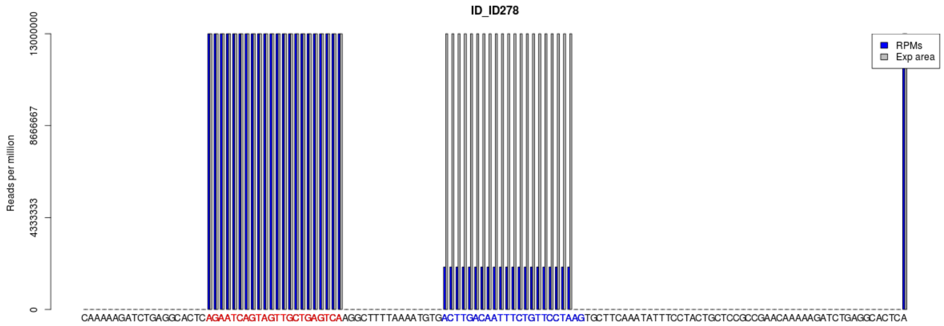

>ID286

GGATTAGAAGTAGTAGTTCCAAGGAACTATTGATGTGATATGTTAGTAGAATCC**TATCACATTCTTAGTTCTTTCT**GAACCTACTTCCAA
AGTCACCGAAGGGATTGTCAGG

>ID286-3P

TATCACATTCTTAGTTCTTTCT

>miRNA precursor

GGAUUAGAAGUAGUAGUUCCAAGGAACUAUUGAUGUGAUAUGUUAGUAGAAUCC**UAUCACAUUCUUAGUUCUUUCU**GAACCUACUUCCAAAGUCACCGAAGGGAUUGUCAGG
```
.((((.(((((((..((((.(((((((((..(((((((((.(((.....)))..)))))))))..)))))))))..)))))))))))...)))).((....)).........
```

>Predicted STAR seq:

AAGGAACTATTGATGTGATA

Complementarity: 16

Initial dG =   -34.60

```
            10        20         30        40
.-G    A--|      UA    C-         UU         U-   AG
   GAUU   GAAGUAG  GUUC  AAGGAACUA  GAUGUGAUA  GUU  \
   CUGA   CUUCAUC  CAAG  UUUCUUGAU  UUACACUAU  UAA  U
\ -    AAC^      --    UC         UC         CC   GA
      90          80        70        60        50
```

Reads in: lib-42, lib-45, lib-47, lib-48, lib-49, lib-50, lib-72

Number of reads="ID286",4376,28,2,6,12,4,0

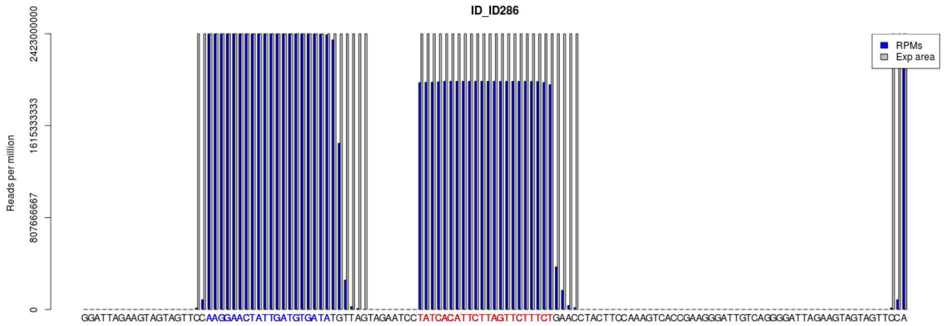

>ID287
AAATATGTTGAGATAAAATTTTTCGGCAGACTGTAAGCGTTGAACTACGCCATGTGTGTTGGAATAGAAT**TCACCGGGTGCAGTTCAATA
CT**TTCAGCATGTCATAATCAAC

>ID287-3P
TCACCGGGTGCAGTTCAATACT

>miRNA precursor
AAAUAUGUUGAGAUAAAAUUUUUCGGCAGACUGUAAGCGUUGAACUACGCCAUGUGUGUUGGAAUAGAAU**UCACCGGGUGCAGUUCAAUACU**UUCAGCAUGUCAUAAUCAAC
.....((((((((.......))))))))(((((((((..(((((((.((((..(((.(((.......))).)))..)))).)))))))..)))...))).)))........

>Predicted STAR seq:
TAAAATTTTTCGGCAGACTGTAAGCGTTGAAC

Complementarity: 14
ERROR 4: Strange structure
 Initial dG =   -30.00

```
          10
.-AAAUA|       UAA
       UGUUGAGA   \
       ACGGCUUU   A
\ -----^       UUA
              20



           30            40        50        60
  ---------   -   ---   CG       A     AU    U    GGA
           GAC UGU   AAG  UUGAACU CGCC  GUG GUU    \
           CUG ACG   UUC  AACUUGA GUGG  CAC UAA    A
  CAACUAAUA   U   ACU   AU       C     GC    U    GAU
  110       100        90        80        70
```

Reads in: lib-42, lib-45, lib-47, lib-48, lib-49, lib-50, lib-72
Number of reads="ID287",1242,11,1,1,5,0,0

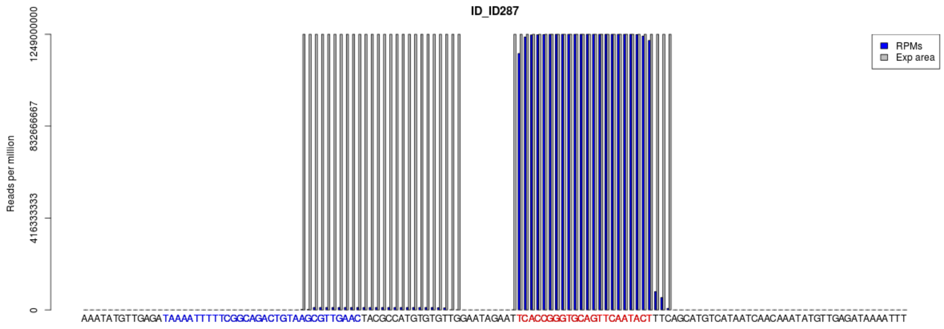

>ID29
GGCTCTTGCGTTCATTTGTAAGTGGTATTTGGGCTAAGTGGAGAAGTGGTCTCTTCACTTTGTTTACAAG**TGAGATTCAACTCCTCCAAC TTAT**TTCTGCCACTTCAGTCATC

>ID29-3P
TGAGATTCAACTCCTCCAACTTAT

>miRNA precursor
GGCUCUUGCGUUCAUUUGUAAGUGGUAUUUGGGCUAAGUGGAGAAGUGGUCUCUUCACUUUGUUUACAAG**UGAGAUUCAACUCCUCCAACUUAU**UUCUGCCACUUCAGUCAUC
.((....))......(((.((((((((...(((.(((((((((.(((....(((.(((((.(....)))))))))....))).)))).))))).)))))))))))))).....

>Predicted STAR seq:
TAAGTGGAGAAGTGGTCTCTTCA

Complementarity: 15
ERROR 4: Strange structure
Initial dG = -32.00

```
.-G| U
   GC C
   CG U
\ -^ U
```

```
 10        20        30          40        50        60
  GUUCAU    U        UUU    C      -     A    GGUC    U      U U
        UUG AAGUGGUA   GGG UAAGU GGAG AGU    UCU CACUU G U
        GAC UUCACCGU   CUU AUUCA CCUC UCA    AGA GUGAA C U
  CUACU-    -        ---    U      A     C    ACUU    -      - A
   110         100            90        80          70
```

Reads in: lib-42, lib-45, lib-47, lib-48, lib-49, lib-50, lib-72
Number of reads="ID29",77,4954,385,30,5,57,0

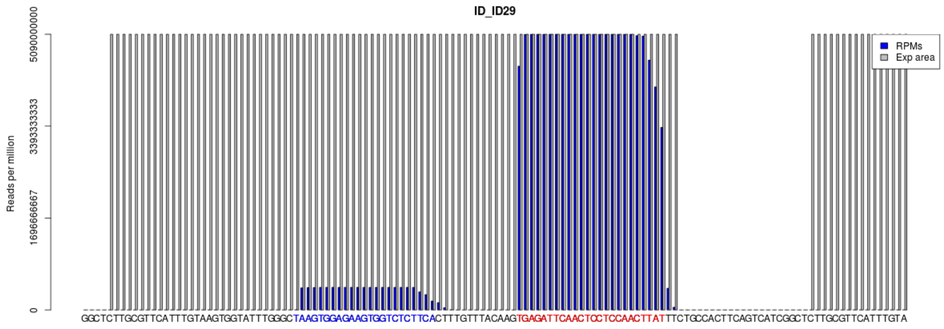

>ID294

ACAGCAGAAGCAGGAGCGGCAGAAGCAGGAACATGAGTACTTGCAGCACTATTTGTAAGAGCACCAGTTC**ATGCTGCTGTCTCTCCTGCT GCT**GCTGTAGCAGCCAAAACAC

>ID294-3P

ATGCTGCTGTCTCTCCTGCTGCT

>miRNA precursor

ACAGCAGAAGCAGGAGCGGCAGAAGCAGGAACAUGAGUACUUGCAGCACUAUUUGUAAGAGCACCAGUUC**AUGCUGCUGUCUCUCCUGCUGCU**GCUGUAGCAGCCAAAACAC

```
((((((.((((((((.(((((.((((.((((.((.((.(((((((......))))))).))..)))))).)))).))))).)))))))).))))))).............
```

>Predicted STAR seq:

GAAG

Complementarity: 17

Initial dG =   -53.30

```
                      10        20        30         40
--------------|      A         C     A    G    A  A-  A       CA
              ACAGCAG AGCAGGAG GGCAG AGCA GAAC UG  GU CUUGCAG  C
              UGUCGUC UCGUCCUC CUGUC UCGU CUUG AC  CG GAAUGUU  U
CACAAAACCGACGA^      G         U     G    A    -  CA  A       UA
110       100       90        80        70         60        50
```

Reads in: lib-42, lib-45, lib-47, lib-48, lib-49, lib-50, lib-72

Number of reads="ID294",9,0,0,0,0,0,0

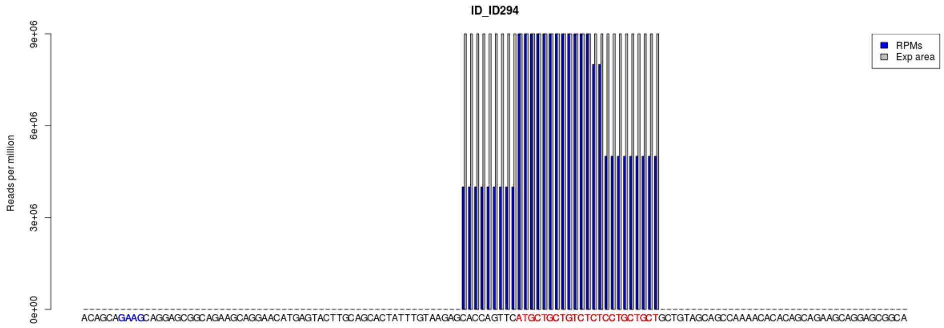

>ID295
CGACGTGGAGGCTGTGGTT**TAGCCTCTCCTAGGCTTTGTCT**GTGAAATATTAGTGGTTGAGACATAGCCTAAAAGAGGTTAGGTCCAAGC
CTCCAGTCTCTGCACAGCTGA

>ID295-5P
TAGCCTCTCCTAGGCTTTGTCT

>miRNA precursor

```
CGACGUGGAGGCUGUGGUUUAGCCUCUCCUAGGCUUUGUCUGUGAAAUAUUAGUGGUUGAGACAUAGCCUAAAAGAGGUUAGGUCCAAGCCUCCAGUCUCUGCACAGCUGA
.(((.((((((((.(((((((((((((..((((((.(((((.................))))).))))))..)))))))))).))))))))))))))............
```

>Predicted STAR seq:
ACATAGCCTAAAAGAGGTTAGG

Complementarity: 17

Initial dG =   -50.60

```
                    10         20        30        40        50
C------------|  G        G   -          CC       U      GUGAAAUA
             GAC UGGAGGCU UGG UUUAGCCUCU  UAGGCU UGUCU        U
             CUG ACCUCCGA ACC GGAUUGGAGA  AUCCGA ACAGA        U
AGUCGACACGUCU^  -        -   U          AA       U      GUUGGUGA
 .       100        90        80        70        60
```

Reads in: lib-42, lib-45, lib-47, lib-48, lib-49, lib-50, lib-72
Number of reads="ID295",60,181,541,172,334,238,0

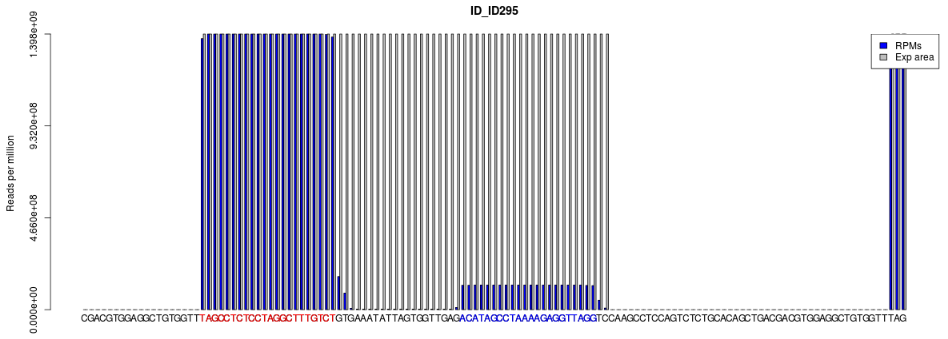

>ID298

ATGTGGCGTTGGCGCTTGTGTCTTGCTCCTCGTGTTCACTGTTGGCCTTGATGTGTCGTTTTGAGGATCA**TAAGGCACGCGGTGAATGCC**
AAGAGCAGACTGAAAGTC

>ID298-3P

TAAGGCACGCGGTGAATGCCA

>miRNA precursor

AUGUGGCGUUGGCGCUUGUGUCUUGCUCCUCGUGUUCACUGUUGGCCUUGAUGUGUCGUUUUGAGGAUCA**UAAGGCACGCGGUGAAUGCCA**AGAGCAGACUGAAAGUC

```
....((((....))))...((((.((((...(((((((((((..((((((.((.(((........)))))))))))..)))))))))))...))))))))........
```

>Predicted STAR seq:

TCGTGTTCACTGTTGGCCTTG

Complementarity: 15
ERROR 4: Strange structure
Initial dG =   -39.50

```
          10
.-AUGU|   U
      GGCG U
      UCGC G
\ ----^   G



         20        30        40        50        60
  UGU-----    U    CUC          UG       A  U   GUU
          GUCU GCUC   GUGUUCACUGU  GCCUUG UG GUC   U
          CAGA CGAG   CGUAAGUGGCG  CGGAAU AC UAG   U
  CUGAAAGU    -    AAC          CA       -  -   GAG
        100        90        80         70
```

Reads in: lib-42, lib-45, lib-47, lib-48, lib-49, lib-50, lib-72
Number of reads="ID298",453,128,43,8888,277,15,0

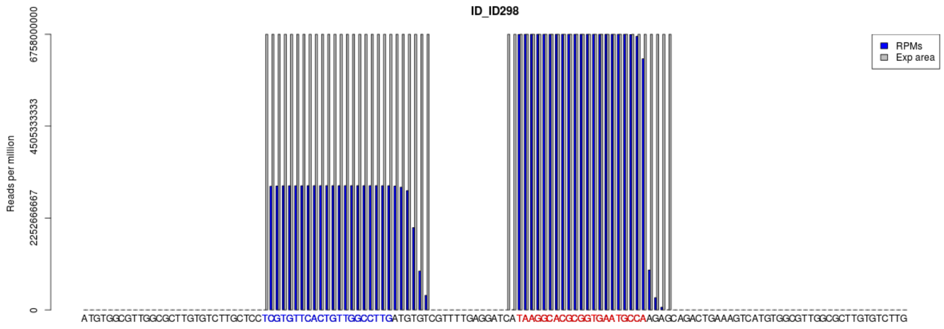

>ID3
GTTGACAACAGAAAGTGGCGGGGTCGCTCTC**CGTCTGAGTGCTGGACAGAGGGC**TAGCACTGGGGAGACCCTTTGTCCAGCACTCAGAGG
GGGGCTGNNNNNNNNNNNN

>ID3-5P
CGTCTGAGTGCTGGACAGAGGGC

>miRNA precursor
GUUGACAACAGAAAGUGGCGGGGUCGCUCUC**CGUCUGAGUGCUGGACAGAGGGC**UAGCACUGGGGAGACCCUUUGUCCAGCACUCAGAGGGGGGCUGNNNNNNNNNNNN
```
...(((..((.....)).....)))((((((.((((((((((((((((((((((.........)).))))))))))))))))))))))))))).............
```

>Predicted STAR seq:
CCCTTTGTCCAGCACTCAGAGGG

Complementarity: 20

```
 Initial dG =    -53.90

            10          20
ERROR 3: Strange structure
 Initial dG =    -53.90

            10          20
GUUGACAA----   .-AA|     G
            CAG    AGUGGC \
            GUC    UCGCUG G
NNNNNNNNNNNN   \ --^     G
       100


                  30        40        50         60
                 -    G                    -  AGCA
                  CUCC UCUGAGUGCUGGACAGAGGG CU    C
                  GGGG AGACUCACGACCUGUUUCCC GA    U
                 G    G                    A  GGGG
                    90        80        70
```

Reads in: lib-42, lib-45, lib-47, lib-48, lib-49, lib-50, lib-72
Number of reads="ID3",1,19,3,857,128,6,0

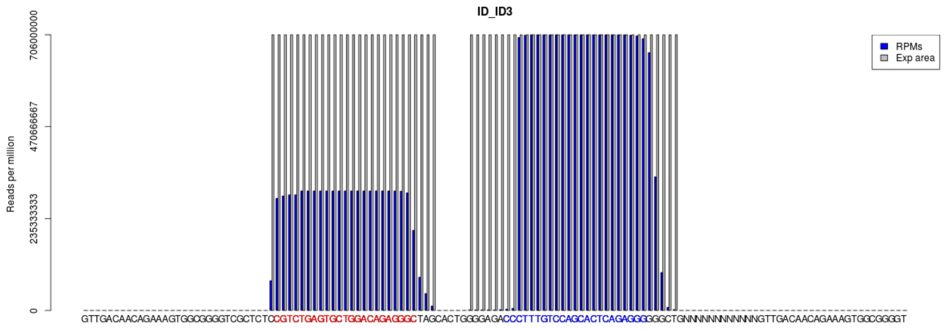

>ID30

CGGGCAGCGGGCGACCGGGCGGCCGTAAGTTGGAAGTGGGGATCTCGGCACGTGTTTCTCGAGTCCGCTG**CCAGATCTAACTCTTCCAGCTCA**CGGACGGCCCGATATT

>ID30-3P

CCAGATCTAACTCTTCCAGCTCA

>miRNA precursor

CGGGCAGCGGGCGACCGGGCGGCCGUAAGUUGGAAGUGGGGAUCUCGGCACGUGUUUCUCGAGUCCGCUG**CCAGAUCUAACUCUUCCAGCUCA**CGGACGGCCCGAUAUU

```
(((((..(((....)))..((.((((.(((((((((...((((((.((((((..(((...)))..)).))))))))))....))))))))).)))).))))))).....
```

>Predicted STAR seq:

AAGTTGGAAGTGGGGATCTCGG

Complementarity: 16

Initial dG =   -53.10

```
               10
-----     .-AG|  G
     CGGGC    CGG C
     GCCCG    GCC G
UUAUA     \ --^  A
       100
```

Reads in: lib-42, lib-45, lib-47, lib-48, lib-49, lib-50, lib-72

Number of reads="ID30",2317,98775,7292,562,108,1940,0

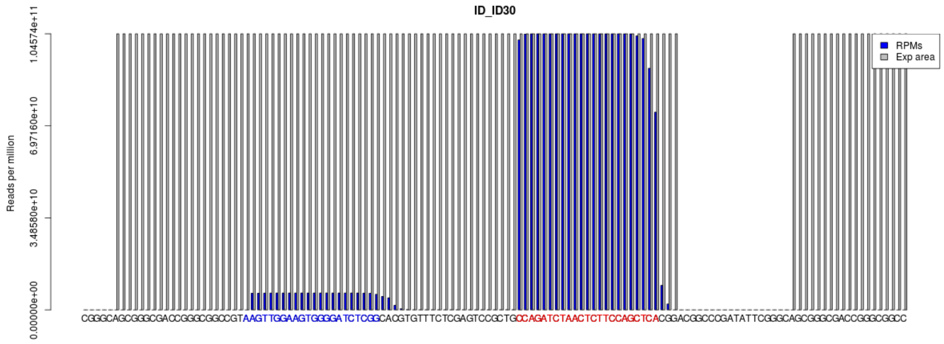

>ID303
TATGAGGACTAAATGTGAAAAATTTGAAGCACTCACAAGCAGAAGTTGTTAAGTCATTTTCTTAAAGCTT**TGACTTAACAATTTCTGCAC**
CTGAGTGTTTCATTCATCTTCA

>ID303-3P
TGACTTAACAATTTCTGCACCT

>miRNA precursor
UAUGAGGACUAAAUGUGAAAAAUUUGAAGCACUCACAAGCAGAAGUUGUUAAGUCAUUUUCUUAAAGCUU**UGACUUAACAAUUUCUGCACCU**GAGUGUUUCAUUCAUCUUCA
```
..((((((.......((((.....((((((((((((...((((((((((((((((((.............))))))))))))))))))...)))))))))))))))))))))
```

>Predicted STAR seq:
CAAGCAGAAGTTGTTAAGTCA

Complementarity: 17

Initial dG =   -45.10

```
        10        20        30        40        50        60
UA|     CUAAAUG    AAAUU          CAA                 UUUUCU
  UGAGGA       UGAA     UGAAGCACUCA   GCAGAAGUUGUUAAGUCA      U
  ACUUCU       ACUU     ACUUUGUGAGU   CGUCUUUAACAAUUCAGU      A
--^     -------    -----          CCA                 UUCGAA
  110                 100        90        80        70
```

Reads in: lib-42, lib-45, lib-47, lib-48, lib-49, lib-50, lib-72
Number of reads="ID303",6,2,0,10,8,2,0

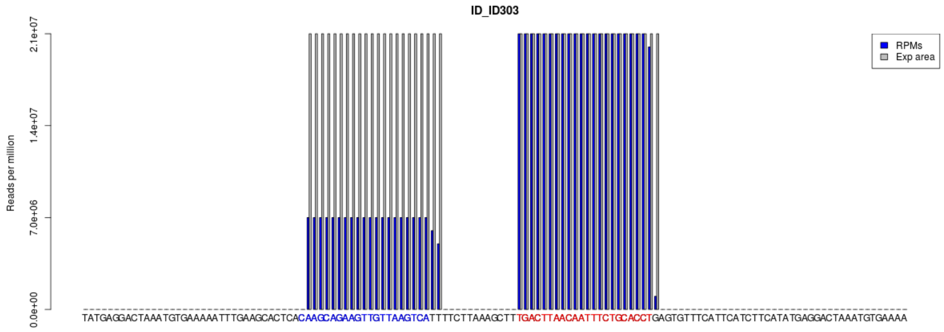

>ID306
GTTCTCGGGCGGCTGCGGACAGTCCGTGACTTGTGGCAGTATTGCTGATGGCCGAA**TATTGCACTTGTCCCGGCCTAT**CCGCGCCGCCCC
GCTCGCCGCAGCAGACGTCGCCG

>ID306-3P
TATTGCACTTGTCCCGGCCTAT

>miRNA precursor
GUUCUCGGGCGGCUGCGGACAGUCCGUGACUUGUGGCAGUAUUGCUGAUGGCCGAA**UAUUGCACUUGUCCCGGCCUAU**CCGCGCCGCCCCGCUCGCCGCAGCAGACGUCGCCG
......(((((((.(((((.((.(((.(((..(((.(((((((((.....))..))))))))))..))).))).)).))))))))))))((((.((....)))).))......

>Predicted STAR seq:
CAGTCCGTGACTTGTGGCAGTA

Complementarity: 15

Initial dG = -50.20

```
                 10        20        30        40
-------   .-CUC|      U     C  U   U   UU   G       --  UG
       GUU     GGGCGGC GCGGA AG CCG GAC  GUG CAGUAUU  GC  \
       CAG     CCCGCCG CGCCU UC GGC CUG  CAC GUUAUAA  CG  A
GCCGCUG   \ ---^      -     A  C   C   UU   -       GC  GU
 110                   80        70         60        50
```

Reads in: lib-42, lib-45, lib-47, lib-48, lib-49, lib-50, lib-72
Number of reads="ID306",609,200,629,1208,1825,269,0

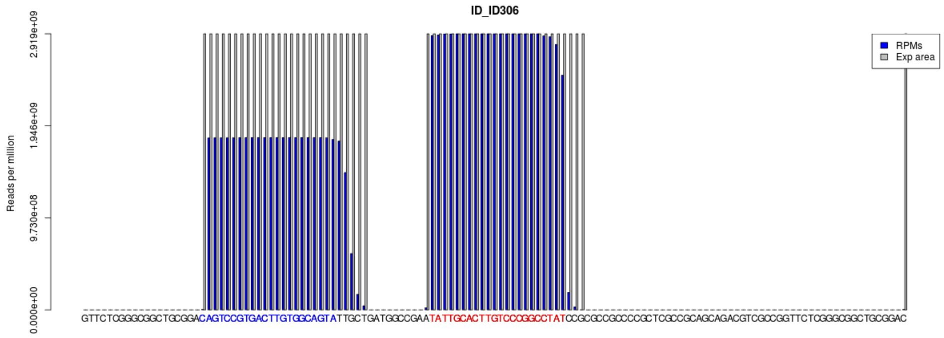

>ID31
TGATGGTGTTTGCGAAGAGCTCTGGAAGTTTCAGGACATGATTGGTGAGTACTTTTTGCTGTTGCAGAAA**TGCTTGTAGAAGTTCTTAGA**
CGGTGGCAGGCATGATTGAACAT

>ID31-3P
TGCTTGTAGAAGTTCTTAGACGGT

>miRNA precursor
UGAUGGUGUUUGCGAAGAGCUCUGGAAGUUUCAGGACAUGAUUGGUGAGUACUUUUUGCUGUUGCAGAAA**UGCUUGUAGAAGUUCUUAGACGGU**GGCAGGCAUGAUUGAACAU
.(((.((((((((..((....))....((((.(((((.......(..((((.(((((((....)))))))))))..)....))))).))))....)))))))).)))......

>Predicted STAR seq:
AAGAGCTCTGGAAGTTTCAGGACATGATTGGTGAG

Complementarity: 12

Initial dG =   -30.70

```
             10
U-----   G        .-GA| A
      GAU GUGUUUGC    AG G
      UUA UACGGACG    UC C
UACAAG   G        \ --^ U
 110       100          20
```

Reads in: lib-42, lib-45, lib-47, lib-48, lib-49, lib-50, lib-72
Number of reads="ID31",1,2,0,4,1,0,0

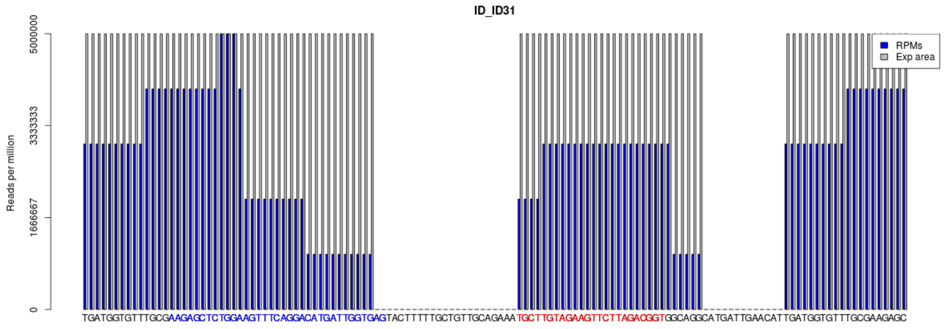

>ID314
ATTTGTTTGTCGATGCACGA**AAGGATGTAACAGTGGCAGATGGG**GTTGCAGAAAAGCATTGTTACACTGCTCTGTTACATGCCTGAACTG
CAAGGACAACTGCAACAACGG

>ID314-5P
AAGGATGTAACAGTGGCAGATGGG

>miRNA precursor
AUUUGUUUGUCGAUGCACGA**AAGGAUGUAACAGUGGCAGAUGGG**GUUGCAGAAAAGCAUUGUUACACUGCUCUGUUACAUGCCUGAACUGCAAGGACAACUGCAACAACGG
..((((((((..((((....((((((((((((.(((((.((....(((......)))......))))))))))))))))).))).....)))).)))))..)))).....

>Predicted STAR seq:
CTGCTCTGTTACATGCCTGAACTG

Complementarity: 17

Initial dG = -37.80

```
              10        20         30        40          50
AU----    --     GA    CGAA   -|        U     A  GGGU--   AG
      UUGU  UUGUC  UGCA    AGG AUGUAACAG GGCAG UG      UGC  A
      AACG  AACAG  ACGU    UCC UACAUUGUC UCGUC AC      ACG  A
GGCAAC    UC     GA    CAAG   G^        -     -  AUUGUU   AA
 .       100        90        80         70         60
```

Reads in: lib-42, lib-45, lib-47, lib-48, lib-49, lib-50, lib-72
Number of reads="ID314",5,5,2,2,6,2,0

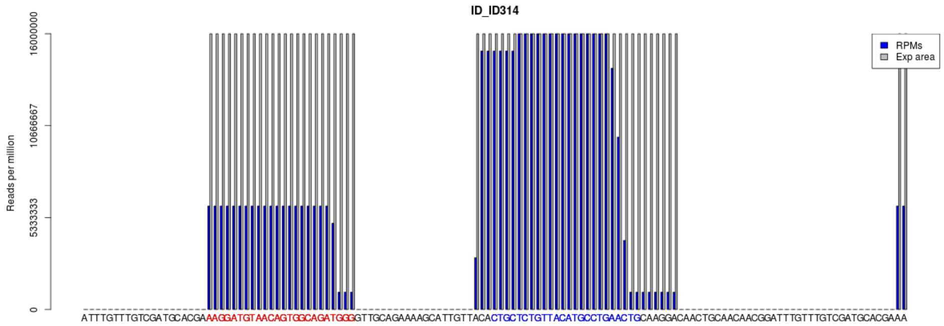

>ID319

GTGGTGCTGCATTCTTCAAGGGTGCATTTTCAAATGTCCTCAGAGGCACTGGTGGTGCTCTTGTACTTGTGTTGTATGATGAAATCAAGG
CACTCATTTAAAGTGTGCAGCG

>ID319-3P

GTTGTATGATGAAATCAAGGCAC

>miRNA precursor

GUGGUGCUGCAUUCUUCAAGGGUGCAUUUUCAAAUGUCCUCAGAGGCACUGGUGGUGCUCUUGUACUUGUGUUGUAUGAUGAAAUCAAGGCACUCAUUUAAAGUGUGCAGCG
.....(((((((.(((...((((((..(((((.......(((..(((((....(((((....))))).))))).....))))))))).....)))))).....))).))))))).

>Predicted STAR seq:

TGCATTTTCAAATGTCCTCAGAGGC

Complementarity: 13

Initial dG =   -34.00

```
        10          20           30         40          50         60
GUGGU       U   CAA--      AU---|    AAUGUCC   GA-     UGGU     U
     GCUGCAU CUU     GGGUGC     UUUCA       UCA   GGCAC    GGUGC C
     CGACGUG GAA     CUCACG     AAAGU       AGU   UUGUG    UCAUG U
G----       U   AUUUA      GAACU^    -------   AUG     U---     U
    110       100        90        80               70
```

Reads in: lib-42, lib-45, lib-47, lib-48, lib-49, lib-50, lib-72

Number of reads="ID319",13,26,20,6,6,15,0

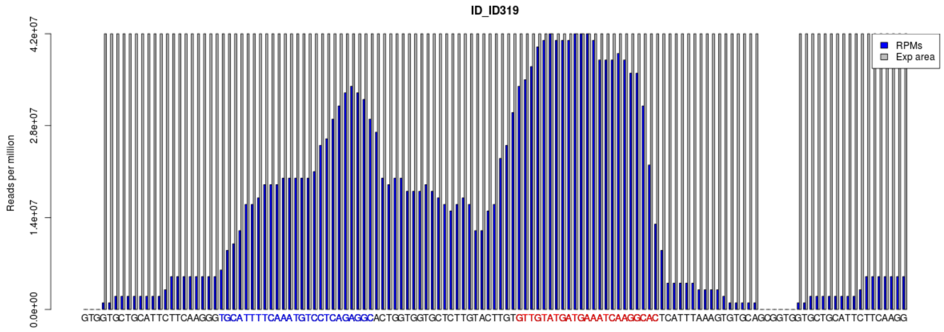

>ID322
GGCGGTGATGTGAGCTGCCC**AGAAGGGCATCGACGGCAGCTTG**CGGGCGTCAGCAACGAGCGGCCGCTGGTGCCCTGCTGANAGTGGCAG
CAGCTGAAGGCAGCGACAGGAA

>ID322-5P
AGAAGGGCATCGACGGCAGCTTG

>miRNA precursor
GGCGGUGAUGUGAGCUGCCC**AGAAGGGCAUCGACGGCAGCUUG**CGGGCGUCAGCAACGAGCGGCCGCUGGUGCCCUGCUGANAGUGGCAGCAGCUGAAGGCAGCGACAGGAA
((((((.......))))))(((.(((((((((.((((.(((((...((....))..))))).)))).))))))))).)))...((.((.((........)).)).)).....

>Predicted STAR seq:
AGCGGCCGCUGGUGCCCUGCUGA

Complementarity: 18

Initial dG =   -53.90

```
        10               20        30        40        50
GGCGGUGA   .-GA      -----|  A         A    A     CGG  G
        UGU    GCUGCC     CAG AGGGCAUCG CGGC GCUUG   GC U
        ACA    CGACGG     GUC UCCCGUGGU GCCG CGAGC   CG C
AAGG----   \ --      UGANA^  G         C    G     AA-  A
110            90        80        70        60




             A    A
              GCUG A
              CGAC G
             G    G
                100
```

Reads in: lib-42, lib-45, lib-47, lib-48, lib-49, lib-50, lib-72
Number of reads="ID322",6,5,1,66,26,1,0

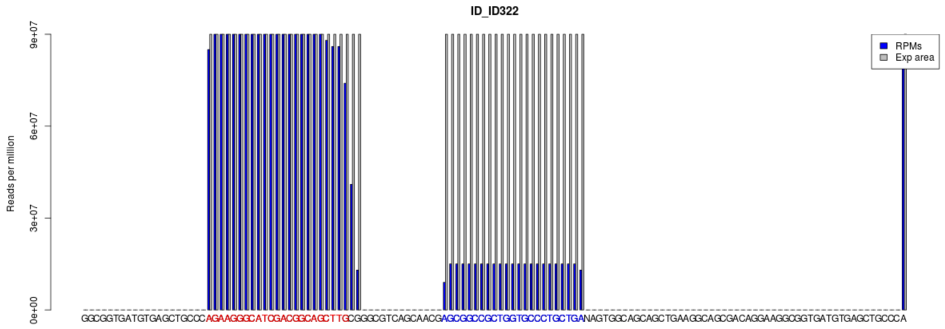

>ID323
CGGAGCGACACAGCGGCGAGTTGCGAAGAGGGAAGTTTCACCGGGGGATCCTGCCTGAATGTGAATCTGG**ATCCCCTGGTGAAACTTTCC**
GCTTCACGACCCGAAGGTCT

>ID323-3P
ATCCCCTGGTGAAACTTTCCGC

>miRNA precursor

```
CGGAGCGACACAGCGGCGAGUUGCGAAGAGGGAAGUUUCACCGGGGGAUCCUGCCUGAAUGUGAAUCUGGAUCCCCUGGUGAAACUUUCCGCUUCACGACCCGAAGGUCU
......(((....(((...((((.((((.((((((((((((((((((((((.................)))))))))))))))))))))).)))).)))))))...))).
```

>Predicted STAR seq:
AGGGAAGTTTCACCGGGGGAT

Complementarity: 19

Initial dG = -59.50

```
        10        20        30        40        50
CGGAGC|  ACAG   CGA    C    A                      UGCCUGAA
      GAC    CGG   GUUG GAAG GGGAAGUUUCACCGGGGGAUCC        \
      CUG    GCC   CAGC CUUC CCUUUCAAAGUGGUCCCCUAGG        U
U-----^  GAA-   ---    A    G                      UCUAAGUG
.                100       90        80        70
```

Reads in: lib-42, lib-45, lib-47, lib-48, lib-49, lib-50, lib-72
Number of reads="ID323",179,454,523,985,788,141,0

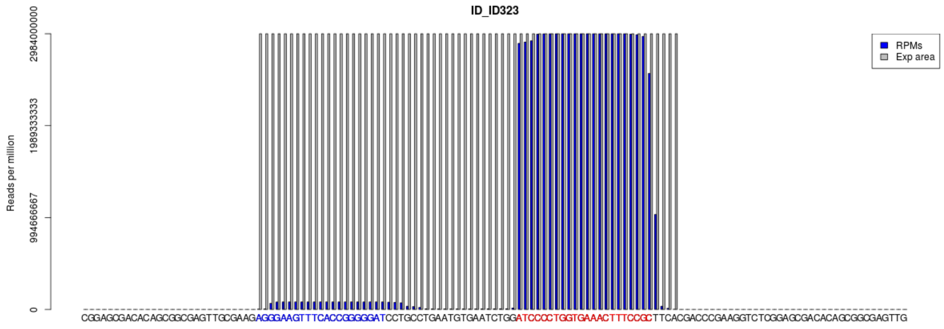

>ID326
TCACGTTGGGAACCGTCTGT**ACGTATACTGAATGTATCC**TGAGTGCTAGATATCCCTTCCGGTATACCTTCAGTATACGTAACAGGGGGT
GGTGTCCCGTTTCTGCGAA

>ID326-5P
ACGTATACTGAATGTATCC

>miRNA precursor
UCACGUUGGGAACCGUCUGU**ACGUAUACUGAAUGUAUCC**UGAGUGCUAGAUAUCCCUUCCGGUAUACCUUCAGUAUACGUAACAGGGGGUGGUGUCCCGUUUCUGCGAA
...((((((((((.((((((((((((((((.((((((.(((.............))).)).)))).))))))))))))).)))).))).....))))).....)))..

>Predicted STAR seq:
TATACCTTCAGTATACGTAA

Complementarity: 16

Initial dG =   -40.90

```
             10             20        30         40
UCA   -----     ----   G    -             U    -| U   UGCUAG
   CGU     UGGGA    ACC UCUG UACGUAUACUGAA GUAU CC GAG      \
   GCG     GCCCU    UGG GGAC AUGCAUAUGACUU CAUA GG CUU      A
AA-   UCUUU     GUGG   G    A             C    U^ C   CCCUAU
        100        90         80        70        60
```

Reads in: lib-42, lib-45, lib-47, lib-48, lib-49, lib-50, lib-72
Number of reads="ID326",884,6,0,2,3,1,0

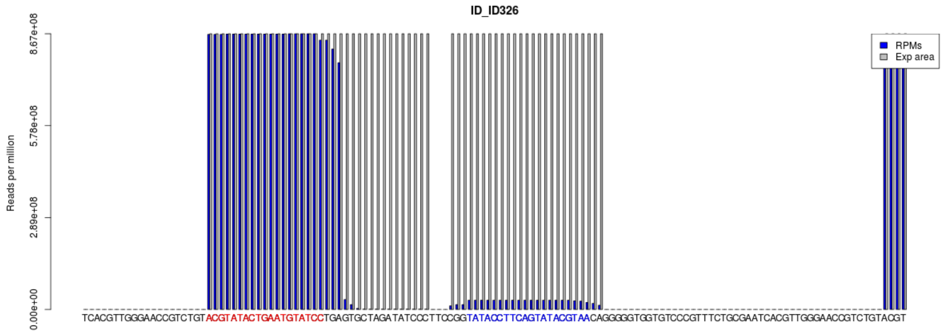

>ID335

CGAAACACTAACCAACTTGCTCGAACAGTCCACAGGTGTAATGCCGGTTCATAGTAACTGTTGGACACTA**TGAACTGGCAGTGCACCTGT GG**ACTGTTCGAGCAACAAATAC

>ID335-3P

TGAACTGGCAGTGCACCTGTGG

>miRNA precursor

```
CGAAACACUAACCAACUUGCUCGAACAGUCCACAGGUGUAAUGCCGGUUCAUAGUAACUGUUGGACACUAUGAACUGGCAGUGCACCUGUGGACUGUUCGAGCAACAAAUAC
................((((((((((((((((((((((.((((((((((((((..(.....)..)))))))))))))).))))))))))))))))))))))).......
```

>Predicted STAR seq:

CACAGGTGTAATGCCGGTTCA

Complementarity: 19

Initial dG = -69.60

```
        10        20        30        40        50        60
CGAAACACUAACCAAC|                      A              AA UG
                UUGCUCGAACAGUCCACAGGUGUA UGCCGGUUCAUAGU  C  \
                AACGAGCUUGUCAGGUGUCCACGU ACGGUCAAGUAUCA  G  U
CAUAAAC---------^                      G              CA GU
110              100        90        80        70
```

Reads in: lib-42, lib-45, lib-47, lib-48, lib-49, lib-50, lib-72

Number of reads="ID335",10,8,3,2,13,1,0

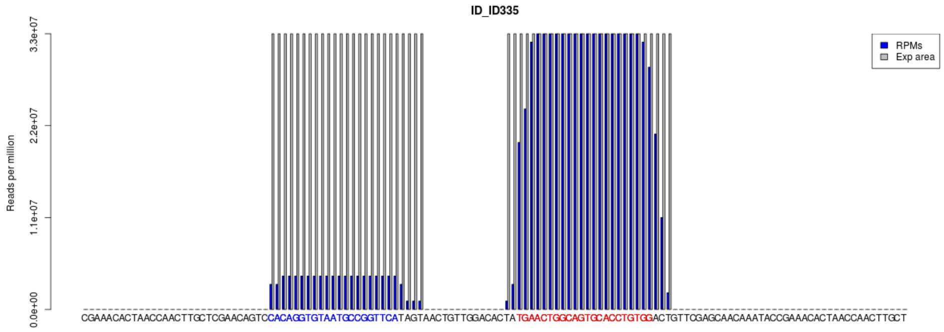

>ID342
TTTTCATTCAGTATTACAAT**AGACGAATGCATCTGTGGAGGCT**ACGTAGAGAACGAACGTCTCCAGCTTGCATTCGTCATTGTCGTACTG
ACCCAGCACCTGACTGAAGCACA

>ID342-5P
AGACGAATGCATCTGTGGAGGCT

>miRNA precursor
UUUUCAUUCAGUAUUACAAU**AGACGAAUGCAUCUGUGGAGGCU**ACGUAGAGAACGAACGUCUCCAGCUUGCAUUCGUCAUUGUCGUACUGACCCAGCACCUGACUGAAGCACA
.(((((.(((((((.(((((.((((((((((....(((((((..(((.....)))...)))))))...))))))))))))))).)))))))..(((...)))..)))))....

>Predicted STAR seq:
CTCCAGCTTGCATTCGTCAT

Complementarity: 15

Initial dG =   -42.80

```
             10        20        30         40
U---     .-U|      U     A          UCUG       UA-   AG
    UUUCA   UCAGUAU ACAAU GACGAAUGCA    UGGAGGC   CGU  \
    GAAGU   AGUCAUG UGUUA CUGCUUACGU    ACCUCUG   GCA  A
ACAC     \ -^      C     -          UCG-       CAA   AG
 110        90        80         70         60




           CC   C
             CAG \
             GUC A
           CA   C
             100
```

Reads in: lib-42, lib-45, lib-47, lib-48, lib-49, lib-50, lib-72
Number of reads="ID342",117,17,2,282,256,5,0

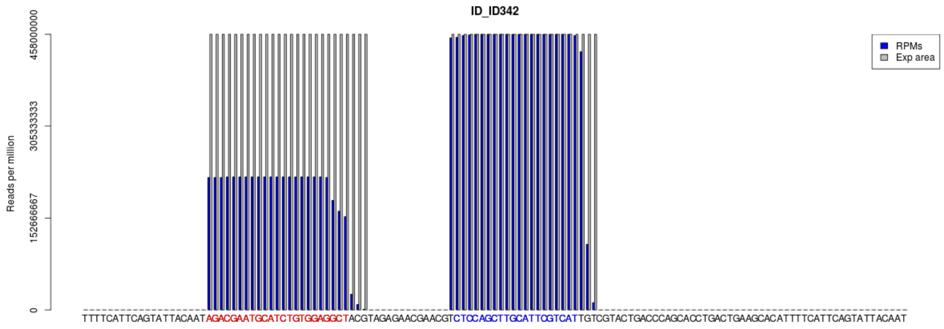

>ID343
ATTGACCAGACGTTTTCAGTTGCTGAGAGATATGGAGAATGTGCTGGCCAGAGCAACAGTCGAACATTTTCCGTATCCAGAAAGGCCCGT
ACAGGACCTGGAACATGCGGTCATG

>ID343-5P
TGAGAGATATGGAGAATGTGCTGG

>miRNA precursor
AUUGACCAGACGUUUUCAGUUGCUGAGAGAUAUGGAGAAUGUGCUGGCCAGAGCAACAGUCGAACAUUUUCCGUAUCCAGAAAGGCCCGUACAGGACCUGGAACAUGCGGUCAUG
```
..(((((...(((.(((.....(((...((((((((((((((((((((....))..))))...))))))))))))))))))..(((.((.....)).))).))).))).))))).. 
```

>Predicted STAR seq:
ACAGTCGAACATTTTCCGTATCCAGA

Complementarity: 19

Initial dG =   -36.80

```
        10          20         30         40              50
AU      AGA   U   .-AGUUG   AGA              ---     --| C
  UGACC   CGU UUC       CUG   GAUAUGGAGAAUGU   GCUG   GC A
  ACUGG   GUA AAG       GAC   CUAUGCCUUUUACA   UGAC   CG G
GU      C--   C   \ -----   ---              AGC     AA^ A
   110               80           70         60



                           90
                  AA    C   GU
                    AGG CC   \
                    UCC GG   A
                  G-    A   AC
                100
```

Reads in: lib-42, lib-45, lib-47, lib-48, lib-49, lib-50, lib-72
Number of reads="ID343",36,34,35,37,29,43,0

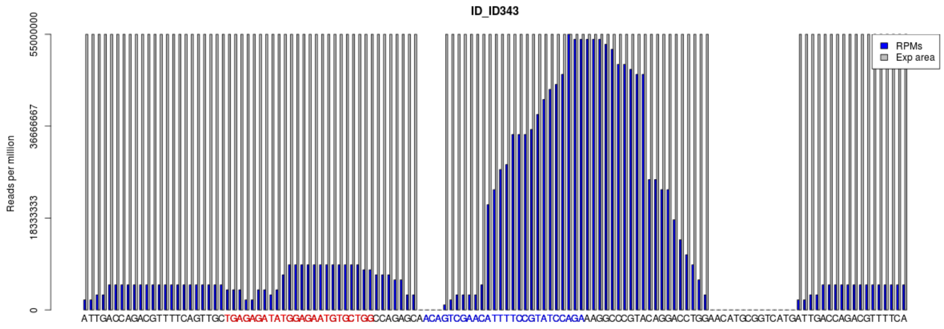

>ID351
AATGCGGAAACTGACTGGGCCGCTGTCATCTGAGTGCTGGACTGAGGGCTGGATCTGGGATGATTGTC**TAGCTCATAGTCCCACACTCGG**
AGGGGAGCTGCTTAACCACAGCA

>ID351-3P
TAGCTCATAGTCCCACACTCGG

>miRNA precursor
AAUGCGGAAACUGACUGGGCCGCUGUCAUCUGAGUGCUGGACUGAGGGCUGGAUCUGGGAUGAUUGUC**UAGCUCAUAGUCCCACACUCGG**AGGGGAGCUGCUUAACCACAGCA
..(((......((..(((((.(((.((.((((((((..((((((.(((((((((..........))))))))).))))))..)))))))).)).))).)))))..))..)))

>Predicted STAR seq:
TGAGTGCTGGACTGAGGGCTG

Complementarity: 17

Initial dG =   -47.50

```
        10        20        30        40        50
AA|  GGAAAC  AC     C   G  A        CU      A         CUGGG
  UGC      UG  UGGGC GCU UC UCUGAGUG  GGACUG GGGCUGGAU     \
  ACG      AC  AUUCG CGA GG AGGCUCAC  CCUGAU CUCGAUCUG     A
--^  AC----  CA     U   G  G        AC      A         UUAGU
   110           100        90        80        70
```

Reads in: lib-42, lib-45, lib-47, lib-48, lib-49, lib-50, lib-72
Number of reads="ID351",2,12,10,270,191,9,0

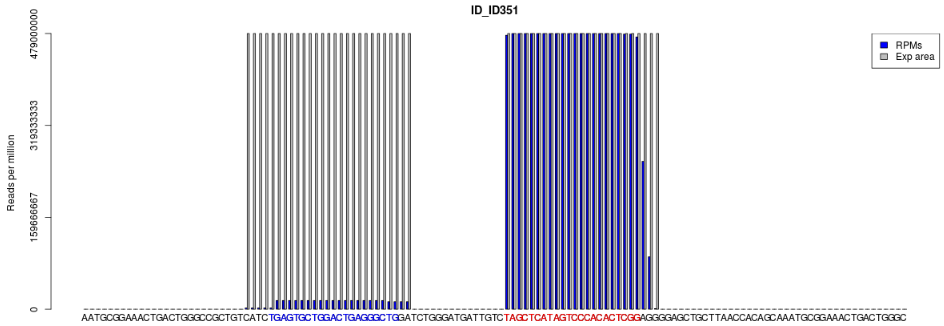

>ID352
GAATGCGGAAACTGGCTGGGCTGCTGTCATCTGAGTGCTGGACTGAGGGCCGGATCTGGGATGATTGTC**CAGCTCGTAGTCCCACACTCGGA**GGGGAGCTGCTTAACCACAG

>ID352-3P
CAGCTCGTAGTCCCACACTCGGA

>miRNA precursor
GAAUGCGGAAACUGGCUGGGCUGCUGUCAUCUGAGUGCUGGACUGAGGGCCGGAUCUGGGAUGAUUGUC**CAGCUCGUAGUCCCACACUCGGA**GGGGAGCUGCUUAACCACAG
............(((.(((((.(((.((.((((((((..((((((.((((.(((((......))...))).)))).))))))..)))))))).)).))).))))).)))...

>Predicted STAR seq:
TCTGAGTGCTGGACTGAGGGCC

Complementarity: 17

Initial dG =   -47.80

```
        10        20        30        40        50
GAAUGCGGAAAC   C     U   G  A        CU       A    C   ---| UG
            UGG UGGGC GCU UC UCUGAGUG  GGACUG GGGC GGA   UC  G
            ACC AUUCG CGA GG AGGCUCAC  CCUGAU CUCG CCU   AG  G
GAC---------   A     U   G  G        AC       G    A   GUU^ UA
110               100        90        80        70        60
```

Reads in: lib-42, lib-45, lib-47, lib-48, lib-49, lib-50, lib-72
Number of reads="ID352",2,5,12,163,67,0,0

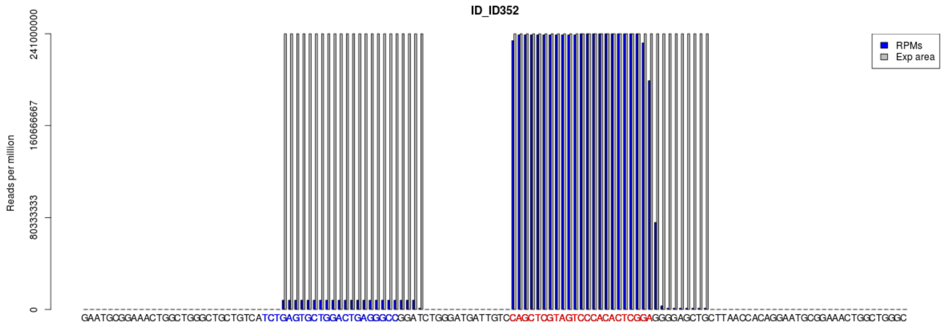

>ID353
AATGCGGAAACTGACTGGGCCGCTGTCATCTGAGTGCTGGACTGAGGGCTGGATCTGGGATGATTGTC**TAGCTCATAGTCCCACACTCGG**
AGGGGAGCTGCTTAACCACAGCA

>ID353-3P
TAGCTCATAGTCCCACACTCGG

>miRNA precursor
AAUGCGGAAACUGACUGGGCCGCUGUCAUCUGAGUGCUGGACUGAGGGCUGGAUCUGGGAUGAUUGUC**UAGCUCAUAGUCCCACACUCGG**AGGGGAGCUGCUUAACCACAGCA
..(((......((..(((((.(((.((.((((((((..((((((.(((((((((..........))))))))).))))))..)))))))).)).))).)))))..))..)))

>Predicted STAR seq:
TGAGTGCTGGACTGAGGGCTG

Complementarity: 17

Initial dG =   -47.50

```
        10        20        30        40        50
AA|  GGAAAC  AC      C   G  A        CU      A         CUGGG
  UGC      UG  UGGGC GCU UC UCUGAGUG  GGACUG GGGCUGGAU     \
  ACG      AC  AUUCG CGA GG AGGCUCAC  CCUGAU CUCGAUCUG     A
--^  AC----  CA      U   G  G        AC      A         UUAGU
   110           100        90        80        70
```

Reads in: lib-42, lib-45, lib-47, lib-48, lib-49, lib-50, lib-72
Number of reads="ID353",2,10,8,284,174,7,0

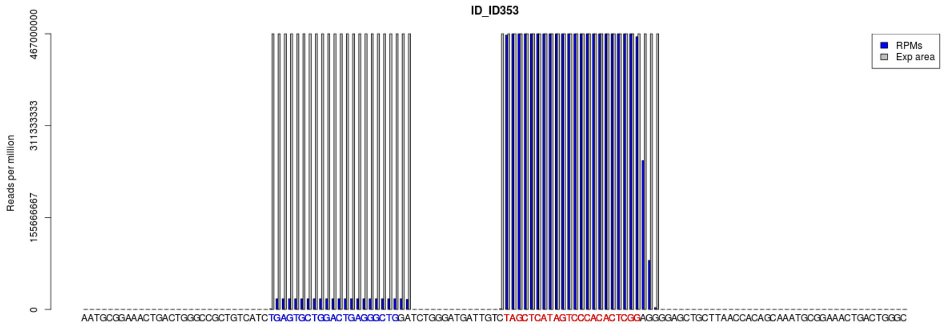

>ID354
GAATGCGGAAACTGGCTGGGCTGCTGTCATCTGAGTGCTGGACTGAGGGCCGGATCTGGGATGATTGTC**CAGCTCGTAGTCCCACACTCGGA**GGGGAGCTGCTTGACCACAG

>ID354-3P
CAGCTCGTAGTCCCACACTCGGA

>miRNA precursor
GAAUGCGGAAACUGGCUGGGCUGCUGUCAUCUGAGUGCUGGACUGAGGGCCGGAUCUGGGAUGAUUGUC**CAGCUCGUAGUCCCACACUCGGA**GGGGAGCUGCUUGACCACAG
```
............(((..((((.(((.((.((((((((..((((((.((((.(((((......))...))).)))).))))))..)))))))).)).))).))))..)))...
```

>Predicted STAR seq:
TCTGAGTGCTGGACTGAGGGCC

Complementarity: 17

Initial dG = -47.60

```
        10        20        30        40        50
GAAUGCGGAAAC   CU    U   G  A        CU      A    C   ---| UG
            UGG  GGGC GCU UC UCUGAGUG  GGACUG GGGC GGA   UC  G
            ACC  UUCG CGA GG AGGCUCAC  CCUGAU CUCG CCU   AG  G
GAC---------   AG    U   G  G        AC      G    A   GUU^ UA
110              100        90        80        70        60
```

Reads in: lib-42, lib-45, lib-47, lib-48, lib-49, lib-50, lib-72
Number of reads="ID354",1,5,14,136,68,1,0

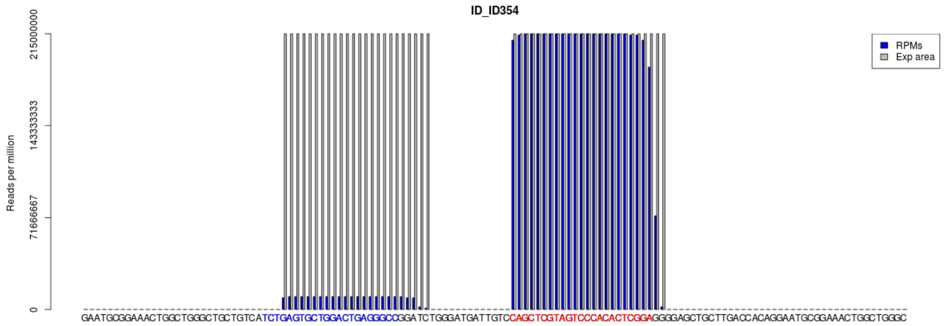

>ID355
AGTGGCGGGGCCGCTCTCATCAAGTGTGCTGGACAGAGGACTGGCATTGGGGAGGTCCTCTGTCCAGCACTCTGAGATGAGCTGCTTGGC
TTCAGCTTCGGCATCATGCTC

>ID355-5P
TCAAGTGTGCTGGACAGAGGAC

>miRNA precursor
AGUGGCGGGGCCGCUCUCAUCAAGUGUGCUGGACAGAGGACUGGCAUUGGGGAGGUCCUCUGUCCAGCACUCUGAGAUGAGCUGCUUGGCUUCAGCUUCGGCAUCAUGCUC
...(((.((((.((((...(((.(.((((((((((((((((..........))))))))))))))))).))))...)))).)))).)))...((....)).........

>Predicted STAR seq:
CCTCTGTCCAGCACTCTGAGATGA

Complementarity: 18

Initial dG = -60.40

```
             10           20          30         40
A---   .-GC      --  U-      A-| U                 GGCAU
    GUG    GGGGCC  GC  CUCAUC  AG GUGCUGGACAGAGGACU     \
    UAC    CUUCGG  CG  GAGUAG  UC CACGACCUGUCUCCUGG     U
CUCG   \ --      UU  UC      AG^ U                 AGGGG
 .           90         80         70         60         50
```

```
         A-  U
           GC U
           CG C
         UA  G
           100
```

Reads in: lib-42, lib-45, lib-47, lib-48, lib-49, lib-50, lib-72
Number of reads="ID355",21,27,27,808,255,4,0

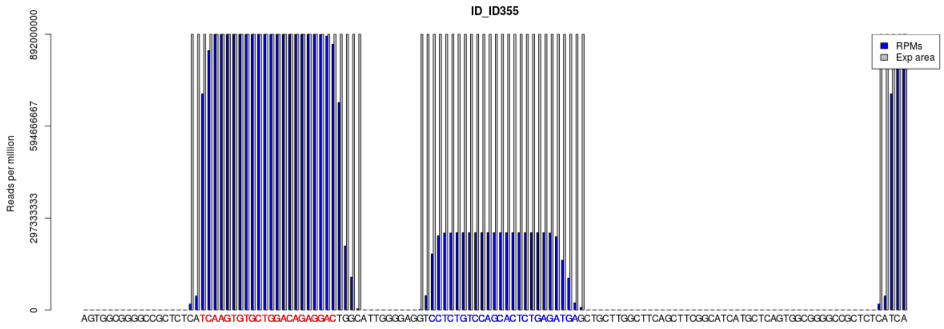

>ID356
GAATGCGGAAACTGGCTGGGCTGCTGTCATCTGAGTGCTGGACTGAGGGCCGGATCTGGGATGATTGTC**CAGCTCGTAGTCCCACACTCGGA**GGGGAGCTGCTTAACCACAG

>ID356-3P
CAGCTCGTAGTCCCACACTCGGA

>miRNA precursor
GAAUGCGGAAACUGGCUGGGCUGCUGUCAUCUGAGUGCUGGACUGAGGGCCGGAUCUGGGAUGAUUGUC**CAGCUCGUAGUCCCACACUCGGA**GGGGAGCUGCUUAACCACAG

```
............(((.(((((.(((.((.((((((((..((((((.((((.(((((......))...))).)))).))))))..)))))))).)).))).))))).)))...
```

>Predicted STAR seq:
TCTGAGTGCTGGACTGAGGGCC

Complementarity: 17

Initial dG = -47.80

```
        10        20        30        40        50
GAAUGCGGAAAC   C     U   G  A        CU       A    C   ---| UG
            UGG UGGGC GCU UC UCUGAGUG  GGACUG GGGC GGA   UC  G
            ACC AUUCG CGA GG AGGCUCAC  CCUGAU CUCG CCU   AG  G
GAC---------   A     U   G  G        AC       G    A   GUU^ UA
110               100       90        80        70        60
```

Reads in: lib-42, lib-45, lib-47, lib-48, lib-49, lib-50, lib-72
Number of reads="ID356",1,4,10,139,60,1,0

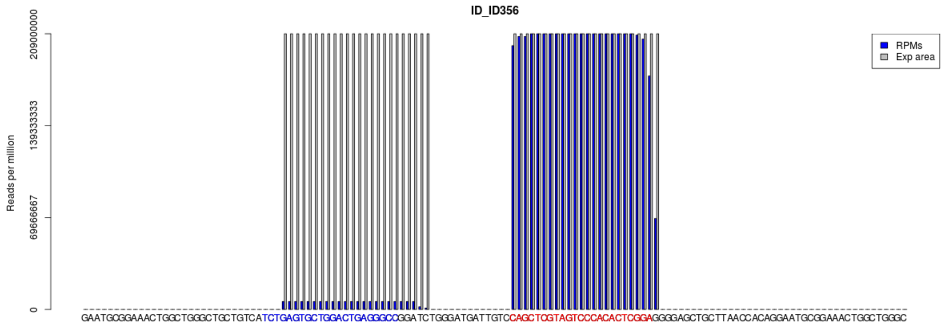

>ID357
TGAATGCGGAAACTGGCTGGGCTGCTGTCATCT**GAGTGCTGGACTGAGGGCCGGA**TCTGGGATGATTGTCCAGCTCGTAGTCCCACACTC
GGAGGGGAGCTGCTTGACCGCA

>ID357-5P
GAGTGCTGGACTGAGGGCCGGA

>miRNA precursor
UGAAUGCGGAAACUGGCUGGGCUGCUGUCAUCU**GAGUGCUGGACUGAGGGCCGGA**UCUGGGAUGAUUGUCCAGCUCGUAGUCCCACACUCGGAGGGGAGCUGCUUGACCGCA
....(((((.........((((.(((.((.((((((((..((((((.((((.((((...........)))).)))).))))))..)))))))).)).))).)))).)))))

>Predicted STAR seq:
CAGCTCGTAGTCCCACACTCGG

Complementarity: 16

Initial dG =   -47.10

```
        10        20        30        40        50        60
UGAA|    AAACUGGCU    U   G  A         CU       A    C    CUGGG
    UGCGG         GGGC GCU UC UCUGAGUG  GGACUG GGGC GGAU     \
    ACGCC         UUCG CGA GG AGGCUCAC  CCUGAU CUCG CCUG     A
----^    AG-------    U   G  G         AC       G    A    UUAGU
    110              100       90        80        70
```

Reads in: lib-42, lib-45, lib-47, lib-48, lib-49, lib-50, lib-72
Number of reads="ID357",1,4,6,137,76,3,0

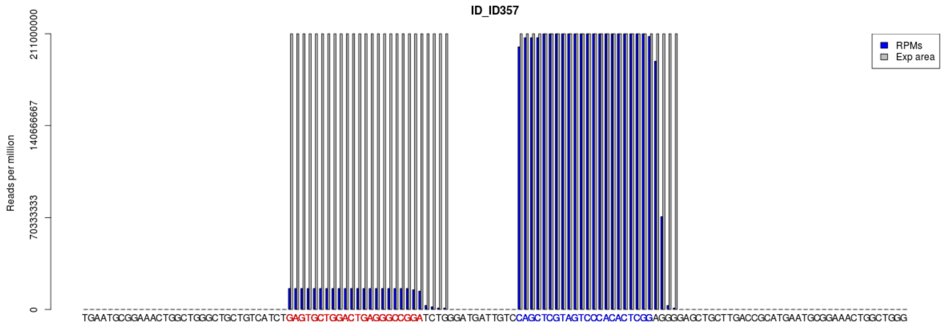

>ID358

TGAATGCGGAAACTGGCTGGGCTGCTGTCATCT**GAGTGCTGGACTGAGGGCCGGA**TCTGGGATGATTGTCCAGCTCGTAGTCCCACACTC
GGAGGGGAGCTGCTTGACCGCA

>ID358-5P

GAGTGCTGGACTGAGGGCCGGA

>miRNA precursor

UGAAUGCGGAAACUGGCUGGGCUGCUGUCAUCU**GAGUGCUGGACUGAGGGCCGGA**UCUGGGAUGAUUGUCCAGCUCGUAGUCCCACACUCGGAGGGGAGCUGCUUGACCGCA
....(((((.........((((.(((.((.((((((((..((((((.((((.((((...........)))).)))).))))))..)))))))).)).))).)))).)))))

>Predicted STAR seq:

CAGCTCGTAGTCCCACACTCGG

Complementarity: 16

Initial dG =   -47.10

```
        10        20        30        40        50        60
UGAA|    AAACUGGCU    U   G  A         CU       A    C    CUGGG
    UGCGG         GGGC GCU UC UCUGAGUG  GGACUG GGGC GGAU     \
    ACGCC         UUCG CGA GG AGGCUCAC  CCUGAU CUCG CCUG     A
----^    AG-------    U   G  G         AC       G    A    UUAGU
    110              100        90        80        70
```

Reads in: lib-42, lib-45, lib-47, lib-48, lib-49, lib-50, lib-72

Number of reads="ID358",0,9,13,125,61,0,0

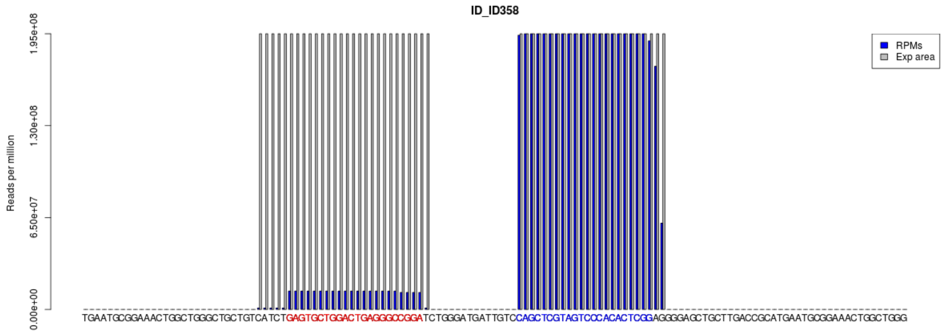

>ID36
CTAGTTGATCGAACAGTCC**ACTGACGTACTGCCGGTTCATAG**TATCCAACGGCCACTATGAACCGGCAGTACGCCCGCGGACTGCTTGAT
GACGAGATACACCGGGAGGAAC

>ID36-5P
ACTGACGTACTGCCGGTTCATAG

>miRNA precursor
CUAGUUGAUCGAACAGUCC**ACUGACGUACUGCCGGUUCAUAG**UAUCCAACGGCCACUAUGAACCGGCAGUACGCCCGCGGACUGCUUGAUGACGAGAUACACCGGGAGGAAC
((.(((.(((((.((((((.....((((((((((((((((((..((...))..))))))))))))))))))))).....)))))).)))))))).)).....((....))...

>Predicted STAR seq:
ATGAACCGGCAGTACGCCCGCGG

Complementarity: 16

Initial dG =   -55.20

```
          10        20        30        40
  | A   G     A      ACUGA                   AU  A
--CU GUU AUCGA CAGUCC     CGUACUGCCGGUUCAUAGU  CC \
  GA CAG UAGUU GUCAGG     GCAUGACGGCCAAGUAUCA  GG A
\ ^ G   -     C      CGCCC                   CC  C
        90        80        70        60        50



   100
  AUACA  G
       CC G
       GG G
  CAA--  A
  110
```

Reads in: lib-42, lib-45, lib-47, lib-48, lib-49, lib-50, lib-72
Number of reads="ID36",1,7,1,1,5,2,0

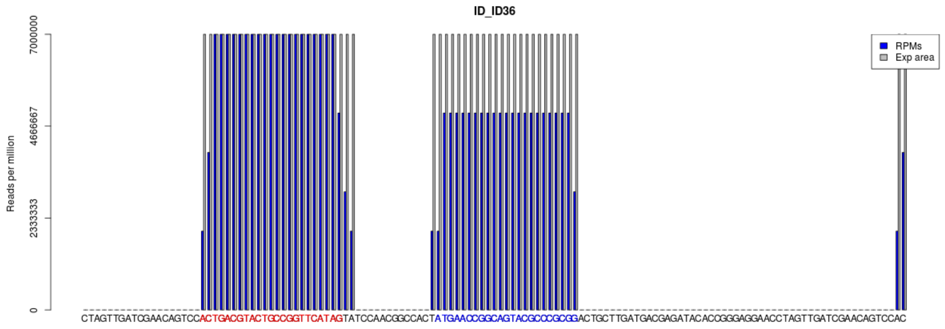

>ID360
AAACCAAAAAAATAGTA**AATTAACGTTTACTTCTGAT**AAGGTAAATTATAAACGTAATCCCTCAAATATCACCAGTAATATGATTTTCAC
TATTCATTATATTTTAAATA

>ID360-5P
AATTAACGTTTACTTCTGAT

>miRNA precursor
AAACCAAAAAAAUAGUA**AAUUAACGUUUACUUCUGAU**AAGGUAAAUUAUAAACGUAAUCCCUCAAAUAUCACCAGUAAUAUGAUUUUCACUAUUCAUUAUAUUUUAAAUA
..........((((((((((((....(((((..((((((((...(((((....))))).)))....)))))..)))))..)))))))..)))))).................

>Predicted STAR seq:
CACCAGTAATATGATTTT

Complementarity: 12

Initial dG =   -12.90

```
        10                 20        30             40        50
AAACCAAAAA------      --      ACGU      UC      ----|  UAA      A
                AAUAGU  AAAUUA    UUACU  UGAUA    AGG   AUUAU A
                UUAUCA  UUUAGU    AAUGA  ACUAU    UCC   UAAUG A
AUAAAUUUUAUAUUAC      CU      AU--      CC      AAAC^  C--      C
.       100        90        80          70        60
```

Reads in: lib-42, lib-45, lib-47, lib-48, lib-49, lib-50, lib-72
Number of reads="ID360",779,4230,2406,197,129,1153,0

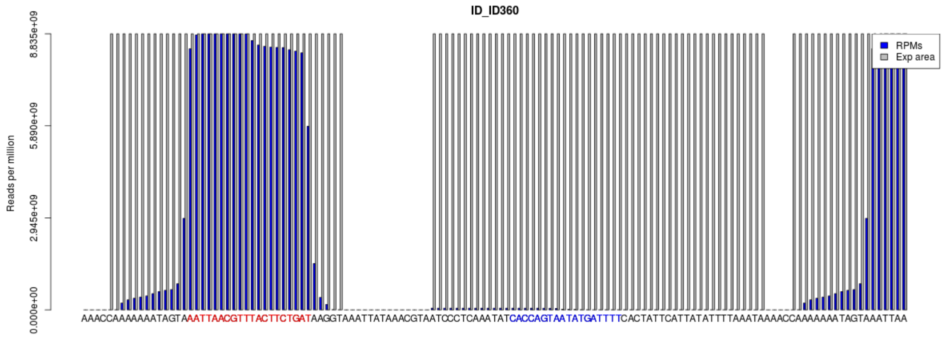

>ID366

CAGCTATGACGGAAGTTGTG**ACGTGCATCCCCAATGGAGTCG**TAGCCCTACTCTCCCGACTCCATTGTGAATGCACGTTACGACTTTCGC
CAACGGTACACGACACTATCGC

>ID366-5P

ACGTGCATCCCCAATGGAGTCG

>miRNA precursor

CAGCUAUGACGGAAGUUGUG**ACGUGCAUCCCCAAUGGAGUCG**UAGCCCUACUCUCCCGACUCCAUUGUGAAUGCACGUUACGACUUUCGCCAACGGUACACGACACUAUCGC
......((.((((((((((((((((((...((((((((((((.............)))))))))))...)))))))))))))))))))).)).(((((........))))).

>Predicted STAR seq:

ACTCCATTGTGAATGCACGTTA

Complementarity: 17

Initial dG =   -49.40

```
          10        20        30        40
.-CAGCUA| A                   CCC          UAGCCC
        UG CGGAAGUUGUGACGUGCAU   CAAUGGAGUCG      U
        AC GCUUUCAGCAUUGCACGUA   GUUACCUCAGC      A
\ ------^ C                   AGU          CCUCUC
         90        80        70        60        50



       100
  A     CAC
   CGGUA   G
   GCUAU   A
  C     CAC
  110
```

Reads in: lib-42, lib-45, lib-47, lib-48, lib-49, lib-50, lib-72

Number of reads="ID366",3,13,0,628,0,0,0

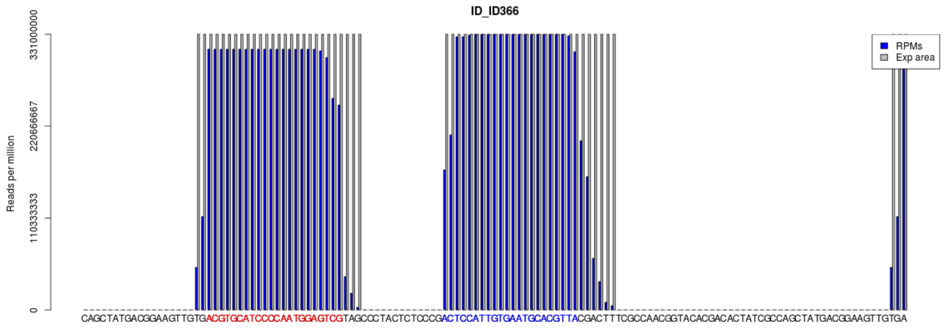

>ID369
TGCACGGACACGGGTGCAC**CTGGGCGGACATACTGTAGAAA**AGCCAGTTGAAAATATCTAAGTAAAATATCTTTTCTACAGTATGTCTGC
CTAGGTGTACTTGTGTCTGT

>ID369-5P
CTGGGCGGACATACTGTAGAAA

>miRNA precursor
UGCACGGACACGGGUGCAC**CUGGGCGGACAUACUGUAGAAA**AGCCAGUUGAAAAUAUCUAAGUAAAAUAUCUUUUCUACAGUAUGUCUGCCUAGGUGUACUUGUGUCUGU
...((...(((................)))..)))

>Predicted STAR seq:
TCTACAGTATGTCTGCCTAGGT

Complementarity: 20

Initial dG = -69.10

```
        10        20        30        40        50
UGC|                                       CCA    GAAAAUA
   ACGGACACGGGUGCACCUGGGCGGACAUACUGUAGAAAAG   GUU       U
   UGUCUGUGUUCAUGUGGAUCCGUCUGUAUGACAUCUUUUC   UAA       C
---^                                       UA-    AAUGAAU
 110       100        90        80        70         60
```

Reads in: lib-42, lib-45, lib-47, lib-48, lib-49, lib-50, lib-72
Number of reads="ID369",32,67,33,48,50,121,0

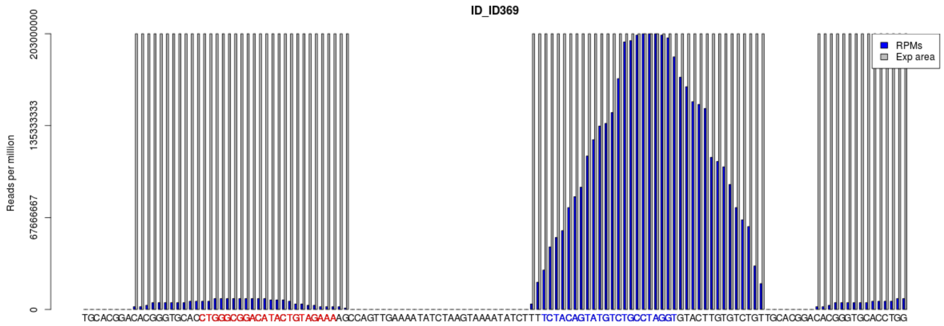

>ID373
TCTTGCACTGACAGTAATACATGGATGGAGGGTGATGTGCGTGTGATAACAACACGTGATAAAGGTTCTG**TTTTATCACCCTCACATCAC**
CCACTTTACATGACAAACAGC

>ID373-3P
TTTTATCACCCTCACATCACC

>miRNA precursor
UCUUGCACUGACAGUAAUACAUGGAUGGAGGGUGAUGUGCGUGUGAUAACAACACGUGAUAAAGGUUCUG**UUUUAUCACCCUCACAUCACC**CACUUUACAUGACAAACAGC
..((((.......))))..((((...((.((((((((((...(((((((.((((...((.......)))))))))))))...)))))))))).))...)))).........

>Predicted STAR seq:
GTGATGTGCGTGTGATAACAA

Complementarity: 16
ERROR 4: Strange structure
Initial dG =   -33.80

```
.-UC|   ACU
    UUGC   \
    AAUG   G
\ --^   ACA
```

```
          20        30          40
  UA-------    GAU  A          .-C    GAU
           CAUG   GG GGGUGAUGUG   GUGU   \
           GUAC   UC CCCACUACAC   CACA   A
  CGACAAACA    AUU  A          \ -    ACA
 110       100        90              50
```

Reads in: lib-42, lib-45, lib-47, lib-48, lib-49, lib-50, lib-72
Number of reads="ID373",0,0,0,0,0,0,0

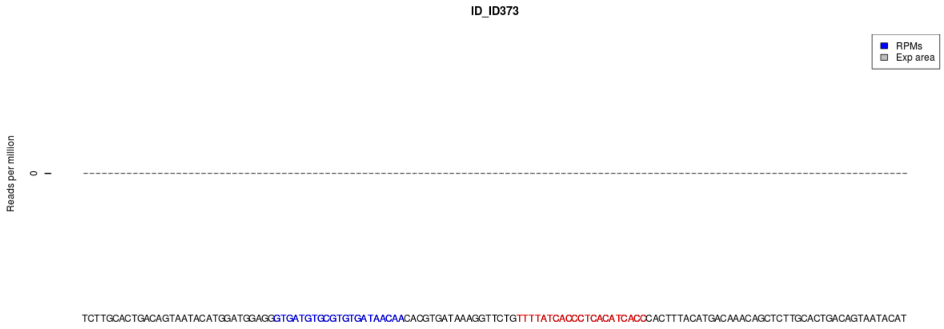

>ID374
GGACCTAGTGGCCCATTTGG**ACCTTCTGGTGGTCGACCAGGA**GGACCTGGTCAGCCAGGATTCCCAGGGACAGGAAGGCCAGGTACACCT
GGTTCACCAGGTGGTCCAGGAG

>ID374-5P
ACCTTCTGGTGGTCGACCAGGA

>miRNA precursor
GGACCUAGUGGCCCAUUUGG**ACCUUCUGGUGGUCGACCAGGA**GGACCUGGUCAGCCAGGAUUCCCAGGGACAGGAAGGCCAGGUACACCUGGUUCACCAGGUGGUCCAGGAG
```
((.((....)).)).((((((((.(((((((...((((((...((((((((..((....(((....)))..))..))))))))...))))))))))))))).)))))))).. 
```

>Predicted STAR seq:
TGGTTCACCAGGTGGTCC

Complementarity: 15

Initial dG =   -56.50

ERROR 3: Strange structure
Initial dG =   -56.50

```
   | A  U
--GG CC A
  CC GG G
\ ^ C  U
     10


       20        30        40        50        60
  A-        U       GUC       AGG        AG  AGGA   C
    UUUGGACC UCUGGUG   GACCAGG   ACCUGGUC  CC    UUC C
    GGACCUGG GGACCAC   UUGGUCC   UGGACCGG  GG    AGG A
  GA        U       ---       ACA        AA  AC--   G
  110       100            90        80          70
```

Reads in: lib-42, lib-45, lib-47, lib-48, lib-49, lib-50, lib-72
Number of reads="ID374",0,0,4,0,25,1,0

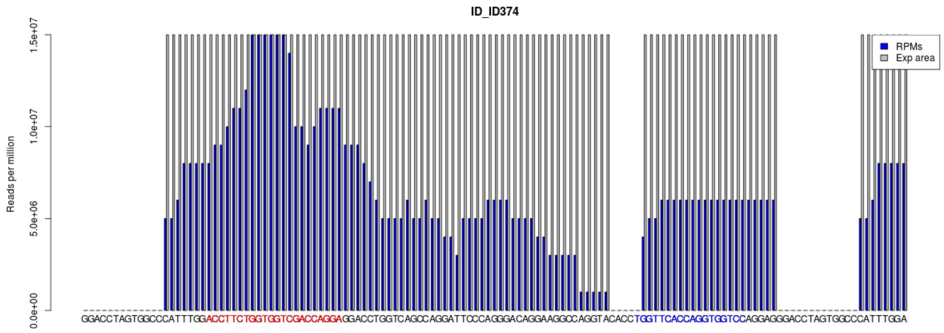

>ID384
TGCATGCGTCGCTCTGGCTTGGCAGCAGGAGCCCCGTCTTCGTCGCCATGTTGTGCTTCCTATGTTCCG**CAACATAGCGGCGAACACGGG**
TTTCCTGACTGCCATCACGTG

>ID384-3P
CAACATAGCGGCGAACACGGGT

>miRNA precursor
UGCAUGCGUCGCUCUGGCUUGGCAGCAGGAGCCCCGUCUUCGUCGCCAUGUUGUGCUUCCUAUGUUCCG**CAACAUAGCGGCGAACACGGGU**UUCCUGACUGCCAUCACGUG
.((..((...))....)).((((((((((((.(((((.((((((((.((((((((...........)))))))).)))))))).))))).)))))).)))))).......

>Predicted STAR seq:
CCCGTCTTCGTCGCCATGTTG

Complementarity: 17
ERROR 4: Strange structure
 Initial dG =   -56.10

```
          10
UGCAU    .-C| U
     GCGU   GC C
     UGCA   CG U
G----    \ -^ G
   110



            20         30        40        50        60
           U-      -      C      C         C        CUUCC
             UGGCAG CAGGAG CCCGU UUCGUCGC AUGUUGUG     U
             ACCGUC GUCCUU GGGCA AAGCGGCG UACAACGC     A
           CU      A      U      C         A        CUUGU
               100        90        80        70
```

Reads in: lib-42, lib-45, lib-47, lib-48, lib-49, lib-50, lib-72
Number of reads="ID384",6024,8568,12117,11623,17681,5650,0

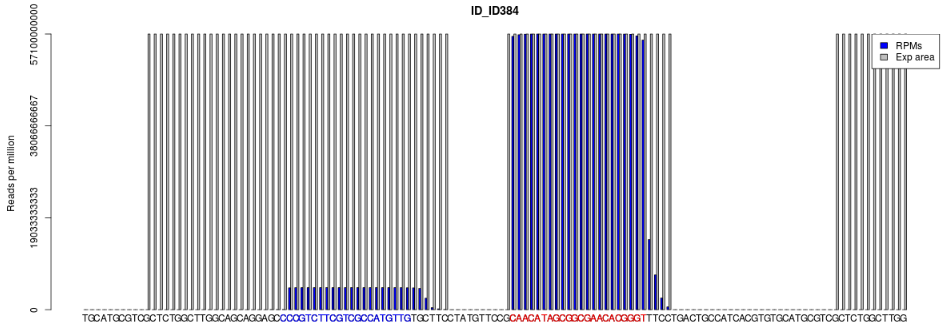

>ID387
ATTTTAAAGATAGAAGTCTC**TCAGGAAATCAATCGTGTAAGT**GGTGAATTTTACTATGCCTTACACAGCTGGTTTCCTGGGATACTTTTA
TCAGGGTCAACATGGAGGACA

>ID387-5P
TCAGGAAATCAATCGTGTAAGT

>miRNA precursor
AUUUUAAAGAUAGAAGUCUC**UCAGGAAAUCAAUCGUGUAAGU**GGUGAAUUUUACUAUGCCUUACACAGCUGGUUUCCUGGGAUACUUUUAUCAGGGUCAACAUGGAGGACA
........((((((((.(((((((((((((...(((((((((((((...))))))...)))))))...)))))))))))))).)))))))))...(((.........))).

>Predicted STAR seq:
GCCTTACACAGCTGGTTTCCTGGGA

Complementarity: 17

Initial dG =   -41.90

```
          10        20        30        40
.-AUUUUAAA         C             AUC       ---|     A
          GAUAGAAGU UCUCAGGAAAUCA   GUGUAAG    UGGUGA \
          CUAUUUUCA AGGGUCCUUUGGU   CACAUUC    AUCAUU U
\ --------         U             CGA       CGU^     U
           90        80        70        60        50



       100
  AGG   AACA
     GUC    \
     CAG    U
  A--   GAGG
   110
```

Reads in: lib-42, lib-45, lib-47, lib-48, lib-49, lib-50, lib-72
Number of reads="ID387",2885,3461,2793,10089,5705,1399,0

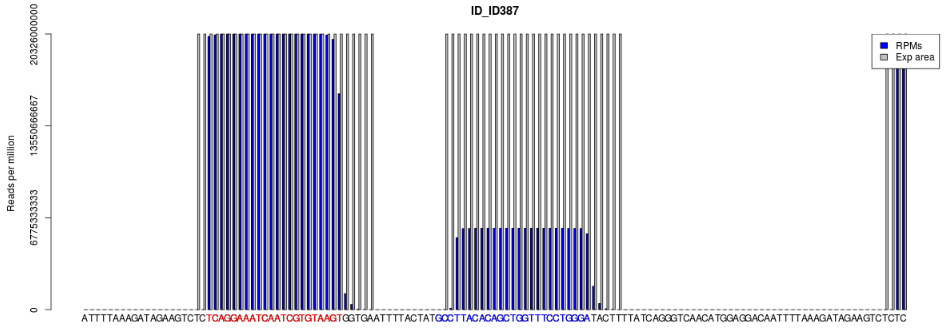

>ID388
GCCTGAAGAGGACAGCATTTACTTAAGACGAACCCGGTTTTCACAGTGATTATCCAGATGTATTTGATTC**TGAGATCATTGTGAAAGCTG**
ATTTTGTTGTTGCGTCGC

>ID388-3P
TGAGATCATTGTGAAAGCTGATT

>miRNA precursor
GCCUGAAGAGGACAGCAUUUACUUAAGACGAACCCGGUUUUCACAGUGAUUAUCCAGAUGUAUUUGAUUC**UGAGAUCAUUGUGAAAGCUGAUU**UUGUUGUUGCGUCGC
..........(((.(((.........((((((..(((((((((((((((((...((((..........)))).)))))))))))))))))..)))))))..)))))))..

>Predicted STAR seq:
CCCGGTTTTCACAGTGATTATCCA

Complementarity: 18

Initial dG =   -34.70

```
        10        20        30        40        50        60
GCCUGAAGAG|  A   UUUACUUAA      CC                  AUC    UGUA
          GAC GCA         GACGAA  CGGUUUUCACAGUGAUU   CAGA    U
          CUG CGU         UUGUUU  GUCGAAAGUGUUACUAG   GUCU    U
CG--------^  -   UG-------      UA                  A--    UAGU
               100              90        80           70
```

Reads in: lib-42, lib-45, lib-47, lib-48, lib-49, lib-50, lib-72
Number of reads="ID388",36043,90982,138843,60840,194807,75115,0

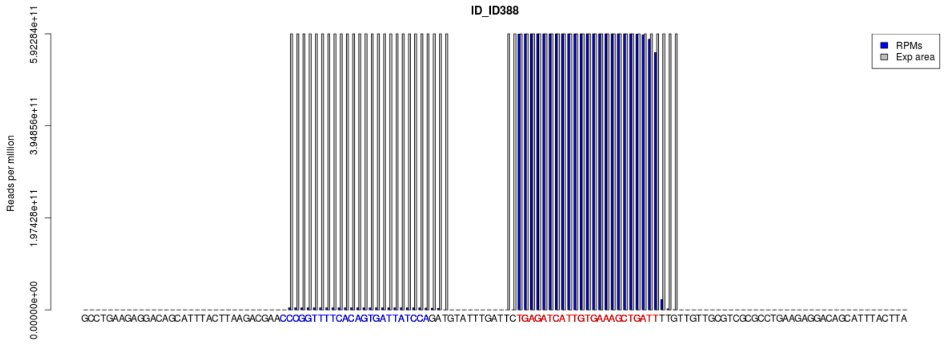

>ID390
ACGGCTTCGGGGGGCCGGCCATTGCTTAACTGGGTACGTGGGACAGGCATGGCGGCTG**TTACGTATCAGTTAAGTAATAG**CCGTCCCCCC
TCCTGGCGCGAGACGCGGGCCCCGG

>ID390-3P
TTACGTATCAGTTAAGTAATAG

>miRNA precursor
ACGGCUUCGGGGGGCCGGCCAUUGCUUAACUGGGUACGUGGGACAGGCAUGGCGGCUG**UUACGUAUCAGUUAAGUAAUAG**CCGUCCCCCCUCCUGGCGCGAGACGCGGGCCCCGG
.(((....((((((.((((.(((((((((((((.(((((..(((((.(......))))))))))))))))))))))))))).)))).))))))....(((.((.....)).)))))).

>Predicted STAR seq:
CATTGCTTAACTGGGTACGTGGGA

Complementarity: 19

Initial dG =   -59.20

```
          10        20        30        40        50
A   .-CUUC|     C    C             G     GG      G AU
 CGG      GGGGGG CGGC AUUGCUUAACUGG UACGU  GACAG C  G
 GCC      CCCCCC GCCG UAAUGAAUUGACU AUGCA  UUGUC G  G
G   \ ----^     U    A             -     --      - GC
         90        80        70           60
```

Reads in: lib-42, lib-45, lib-47, lib-48, lib-49, lib-50, lib-72
Number of reads="ID390",76,3,0,0,0,0,0

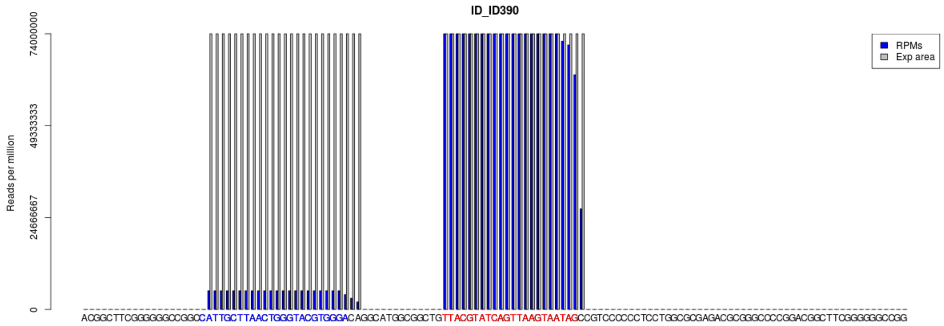

>ID395

AAGAGAAGCATGTTGTTCGT**TTTAGAATTCCTACGCTTTACC**GTGGAGACATCAGGCAAAGCGTTTGAATTCTGAAACGAAAGGCATGCA
CAGCAGCTTCGTCGCCTTCAAC

>ID395-5P

TTTAGAATTCCTACGCTTTACC

>miRNA precursor

AAGAGAAGCAUGUUGUUCGU**UUUAGAAUUCCUACGCUUUACC**GUGGAGACAUCAGGCAAAGCGUUUGAAUUCUGAAACGAAAGGCAUGCACAGCAGCUUCGUCGCCUUCAAC
..((((((.(((((((((((((((((((..(((((((.(((((....)))..)).)))))))..)))))))))))))))).........))))))))))).)).........

>Predicted STAR seq:

CAAAGCGTTTGAATTCTGAAAC

Complementarity: 17

Initial dG =   -48.30

```
           10        20        30        40
AA-   .-AA      UG              CU       A  --|  G
   GAG    GCAUGU  UUCGUUUUAGAAUUC  ACGCUUU CC  GUG A
   CUU    CGUACG  AAGCAAAGUCUUAAG  UGCGAAA GG  UAC G
CAA   \ --      GA              UU       C  AC^  A
110              80        70        60        50


       90       100
        ACAGCA  UU
              GC  C
              CG  G
        C-----  CU
```

Reads in: lib-42, lib-45, lib-47, lib-48, lib-49, lib-50, lib-72

Number of reads="ID395",218,65,82,3593,1523,29,0

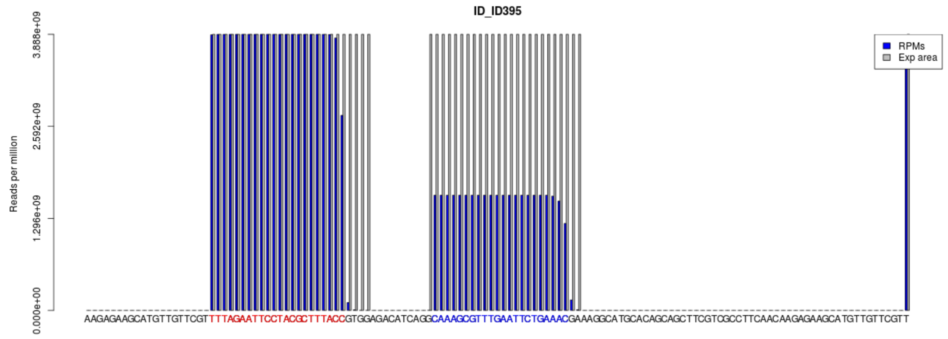

>ID396
TTACTTTGGAATACGCCTCG**AACGATGGTACTGTACAGTATGTG**CTGCTTAACTCCAAATGCACATACACAATACGATCGTTGGCGGTGT
GTTTATCGAGCCAGCGCTTTGC

>ID396-5P
AACGATGGTACTGTACAGTATGTG

>miRNA precursor
UUACUUUGGAAUACGCCUCG**AACGAUGGUACUGUACAGUAUGUG**CUGCUUAACUCCAAAUGCACAUACACAAUACGAUCGUUGGCGGUGUGUUUAUCGAGCCAGCGCUUUGC
........((((((((((.((((((.(((.(((...((((((((...............)))))))))).))).)))))))).)))))))))...((((....))))...

>Predicted STAR seq:
CATACACAATACGATCGTTGG

Complementarity: 17

Initial dG =   -36.60

```
          10         20        30        40        50
.-UUACUUUG         -| G       G   C   ACA        UGCUUAA
          GAAUACGCC UC AACGAU GUA UGU   GUAUGUGC       \
          UUUGUGUGG GG UUGCUA CAU ACA   CAUACACG       C
\ --------         C^ -       G   A   ---        UAAACCU
             90         80        70           60



     100
  AUC    C
     GAGC A
     UUCG G
  CGU    C
  110
```

Reads in: lib-42, lib-45, lib-47, lib-48, lib-49, lib-50, lib-72
Number of reads="ID396",47,60,75,1302,133,10,0

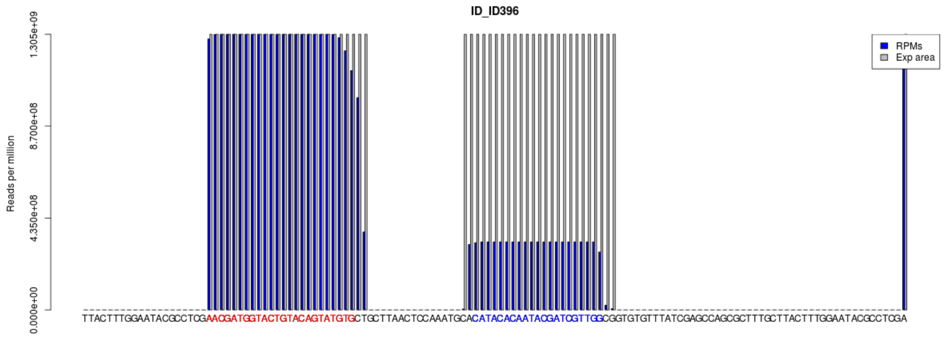

>ID4
AGTTGATGGTAGAAAGTGGCGGGGCCGCTCCCCTCTGAGTGCTGGACAGAAGGCTGGTACTGGGGAGATC**CTCTGTCCAGCACTCAGAGC**
GGGGCTGCTTGGCCGCAGCTTC

>ID4-3P
CTCTGTCCAGCACTCAGAGCGGG

>miRNA precursor
AGUUGAUGGUAGAAAGUGGCGGGGCCGCUCCCCUCUGAGUGCUGGACAGAAGGCUGGUACUGGGGAGAUC**CUCUGUCCAGCACUCAGAGCGGG**GCUGCUUGGCCGCAGCUUC
..............(((((.((((.(((((.((((((((((((((((((...........(((....)))))))))))))))))))))).))))).)))).))))).......

>Predicted STAR seq:
CCCCTCTGAGTGCTGGACAGAA

Complementarity: 20

Initial dG = -65.30

```
        10        20        30        40        50        60
AGUUGAUGGUAGAAA|    G    C     C                    AGGCUGGUACU   G
               GUGGC GGGC GCUCC CUCUGAGUGCUGGACAGA           GGG A
               CGCCG UUCG CGGGG GAGACUCACGACCUGUCU           CCU G
CUUCGA---------^    G    U     C                    -----------   A
110                100        90        80                  70
```

Reads in: lib-42, lib-45, lib-47, lib-48, lib-49, lib-50, lib-72
Number of reads="ID4",4,11,5,276,105,3,0

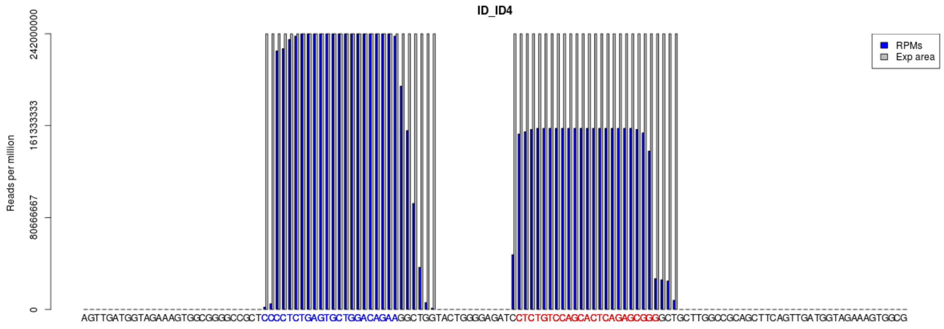

>ID41

TTTCGGGCGCGGTGGCCGTCGGCAGCGGAGGCTGTCACTCGCACAGTGCGTGCGAGGCCGGTAGGTCC**TCGCACGCACTGTGCGAGCGGA**
AATGCCTCTGTGGGCGCACAC

>ID41-3P

TCGCACGCACTGTGCGAGCGGAA

>miRNA precursor

UUUCGGGCGCGGUGGCCGUCGGCAGCGGAGGCUGUCACUCGCACAGUGCGUGCGAGGCCGGUAGGUCC**UCGCACGCACUGUGCGAGCGGAA**AUGCCUCUGUGGGCGCACAC
```
......((((....(((...))).((((((((..((.((((((((((((((((((((((....)).))))))))))))))))))))).))....))))))))..)))).....
```

>Predicted STAR seq:

GTCACTCGCACAGTGCGTGCGA

Complementarity: 18
ERROR 4: Strange structure
Initial dG = -74.40

```
        10
UUUCGG    .-GGUG|  G
      GCGC      GCC \
      CGCG      CGG U
CACA--    \ ----^  C
 .                20



                  30          40         50          60
            A-        UG--  A                    -  G
              GCGGAGGC    UC CUCGCACAGUGCGUGCGAGG CC G
              UGUCUCCG    GG GAGCGUGUCACGCACGCUCC GG U
            GG        UAAA  C                    U  A
             100        90        80         70
```

Reads in: lib-42, lib-45, lib-47, lib-48, lib-49, lib-50, lib-72

Number of reads="ID41",49,367,252,10892,814,16,0

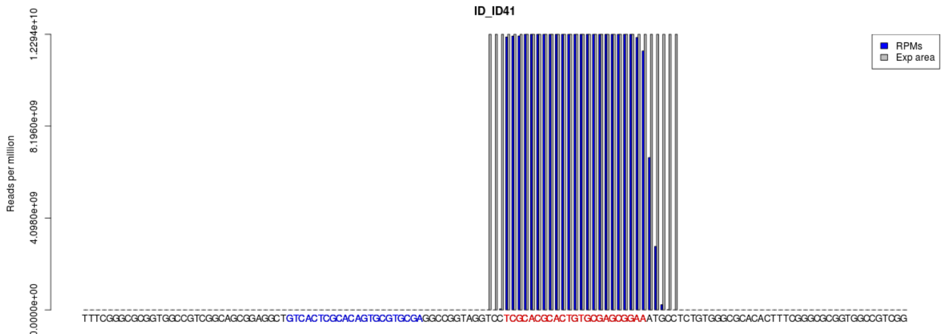

>ID417
CTGTGAAAATTGATTATAAGA**GGTACTCCGTCTTATGCTGACT**ATAATTGCAGTCAGCATAAGACGGATTACCTCTTATAATCAACCACA
CACTTTCACTAACATCCCATT

>ID417-5P
GGTACTCCGTCTTATGCTGACT

>miRNA precursor
CUGUGAAAAUUGAUUAUAAGA**GGUACUCCGUCUUAUGCUGACU**AUAAUUGCAGUCAGCAUAAGACGGAUUACCUCUUAUAAUCAACCACACACUUUCACUAACAUCCCAUU
.((((....((((((((((((((((.(((((((((((((((((........))))))))))))))))).)))))))))))))))))...)))).................

>Predicted STAR seq:
TCAGCATAAGACGGATTACCTC

Complementarity: 19

Initial dG = -56.00

```
                          10        20        30        40
C------------------|   AAAA                C                  AUA
                   UGUG    UUGAUUAUAAGAGGUA UCCGUCUUAUGCUGACU   A
                   ACAC    AACUAAUAUUCUCCAU AGGCAGAAUACGACUGA   U
UUACCCUACAAUCACUUUC^   ACC-                U                  CGU
 .       100       90         80        70        60        50
```

Reads in: lib-42, lib-45, lib-47, lib-48, lib-49, lib-50, lib-72
Number of reads="ID417",2,4,2,199,32,1,0

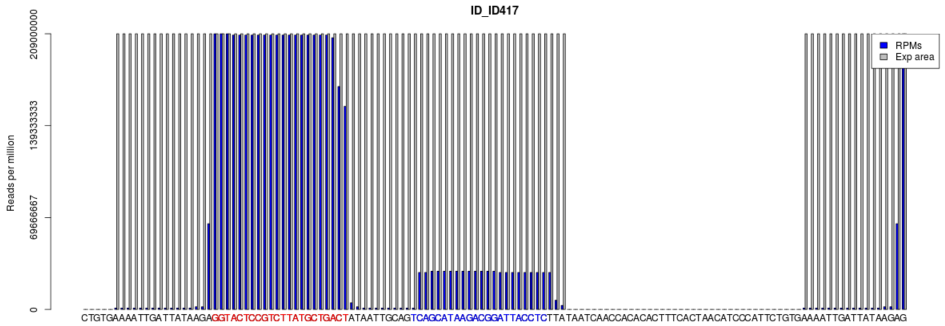

>ID424
ATGAATTGGTACCATGTCTGTGTCAGTGGAATAGAAAGTGTTCTTGGTTTTGGTAGCTGTGTGTATGGTT**GCCTGAATAGGGGAACAAAC**
ATTTTTAATTCCAGTATGAACATA

>ID424-3P
GCCTGAATAGGGGAACAAACATTT

>miRNA precursor
AUGAAUUGGUACCAUGUCUGUGUCAGUGGAAUAGAAAGUGUUCUUGGUUUUGGUAGCUGUGUGUAUGGUU**GCCUGAAUAGGGGAACAAACAUUU**UUAAUUCCAGUAUGAACAUA
..((..((....))..)).((((((.((((((.(((((((((((.((((.(((((((((....))))))))).))))..))))....)))))))).))))))...)).)))).

>Predicted STAR seq:
AGTGTTCTTGGTTTTGGT

```
Complementarity: 15
ERROR 4: Strange structure
 Initial dG =    -29.10

          10
.-AU| AU  G
    GA  UG U
    CU  AC A
\ --^ GU  C




  20           30        40             50        60
  U    -  G--      A        ----    G-    U         G
   GUGU CA   UGGAAU GAAAGUGU    UCUU  GUUU GGUAGCUGU U
   UACA GU   ACCUUA UUUUUACA    AGGG  UAAG CCGUUGGUA G
  A    A  AUG      A        AACA    GA    U         U
    110       100        90        80        70
```

Reads in: lib-42, lib-45, lib-47, lib-48, lib-49, lib-50, lib-72
Number of reads="ID424",47,28,115,18,12,41,0

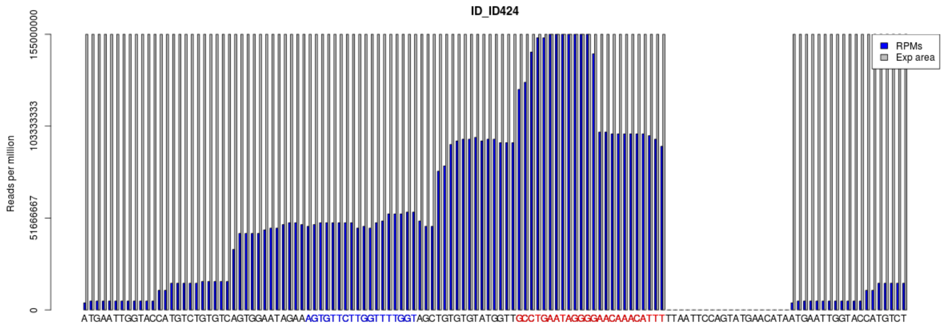

>ID425

CTACAGGTCTTTAAAACCTTCCATTATAATGAGCTCTGTTTGAATCTGTTATAGCTTGTAAGAGCTATTT**TGGATAAGGAAAACATGAGC**
ATCTTAGTGGAAGGAGAGAAACTG

>ID425-3P

TGGATAAGGAAAACATGAGCATCT

>miRNA precursor

CUACAGGUCUUUAAAACCUUCCAUUAUAAUGAGCUCUGUUUGAAUCUGUUAUAGCUUGUAAGAGCUAUUU**UGGAUAAGGAAAACAUGAGCAUCU**UAGUGGAAGGAGAGAAACUG
...(((.(((((....(((((((((....((((((((((..(((((..(((((((....)))))))..)))))).....))))).)))).)).)))))))))))))))..)))

>Predicted STAR seq:

AATGAGCTCTGTTTGAATCTG

Complementarity: 14

Initial dG =   -37.90

```
         10        20        30          40            50
CUA   G-|    AAAA          UAAU   -     -      GA---     UU       G
   CAG  UCUUU    CCUUCCAUUA    GA GCUC UGUUU      AUCUG  AUAGCUU U
   GUC  AGAGA    GGAAGGUGAU    CU CGAG ACAAA      UAGGU  UAUCGAG A
---   AA^    ----          U---   A     U      AGGAA     UU       A
     110           100           90        80        70         60
```

Reads in: lib-42, lib-45, lib-47, lib-48, lib-49, lib-50, lib-72

Number of reads="ID425",26,25,37,5,3,14,0

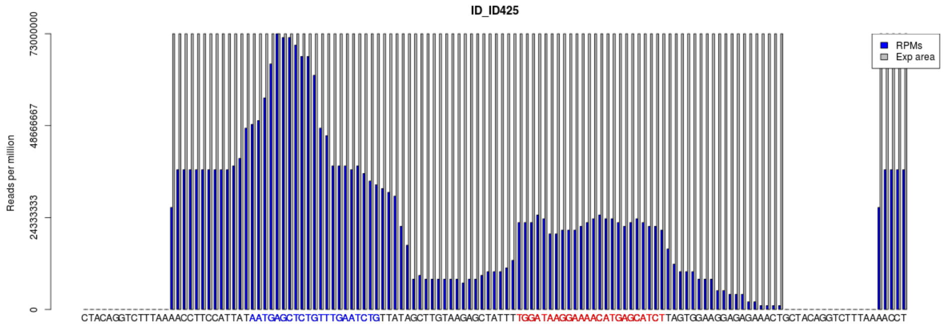

>ID427
GTCACGTCACACTGATAGGCCGCTGTCGTTAGTAGACTTGCTGGCTGATTAGAGGCAATAGGCTGTACC**CAGGTCACCAACAAGTCTGCT**
AGCGATCGCGGCCTGCTCTTCG

>ID427-3P
CAGGTCACCAACAAGTCTGCTAG

>miRNA precursor
GUCACGUCACACUGAUAGGCCGCUGUCGUUAGUAGACUUGCUGGCUGAUUAGAGGCAAUAGGCUGUACC**CAGGUCACCAACAAGUCUGCUAG**CGAUCGCGGCCUGCUCUUCG
.............((((((((((.((((((((((((((((.(((.(((((...(((.....))).......)))))))).)))))))))))))))).)))))))).)).....

>Predicted STAR seq:
TAGTAGACTTGCTGGCTGATT

Complementarity: 18

Initial dG = -52.60

```
        10         20        30        40        50
GUCACGUCACACU  -         U                C   C      AGA----|  AA
             GA UAGGCCGC GUCGUUAGUAGACUUG UGG UGAUU       GGC  \
             CU GUCCGGCG UAGCGAUCGUCUGAAC ACC ACUGG       UCG  U
GCUU---------  C         C                A   -      ACCCAUG^  GA
110               100        90        80         70        60
```

Reads in: lib-42, lib-45, lib-47, lib-48, lib-49, lib-50, lib-72
Number of reads="ID427",1,3,0,205,9,2,0

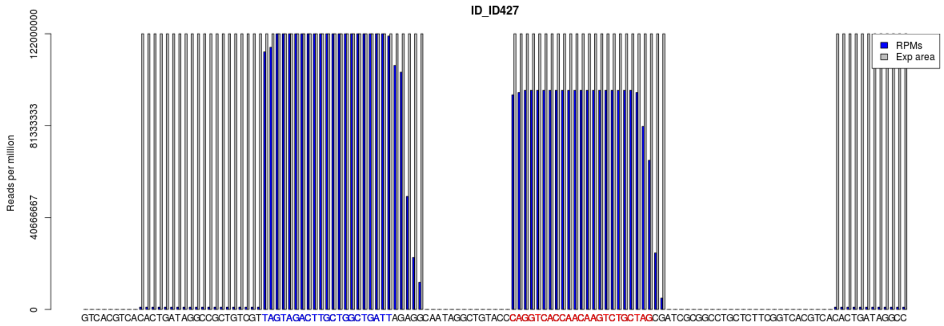

>ID428
CTGATAGGCTGCGATCGTT**AGCTGACTTGTTGGCTGATTCGAA**GCAATAGGCTGCACACCGGTCACTAACAAGTCAGCCAGCGATCGCGG
CCTGGACTTCGTCCAGGATGATG

>ID428-5P
AGCTGACTTGTTGGCTGATTCGAA

>miRNA precursor
CUGAUAGGCUGCGAUCGUU**AGCUGACUUGUUGGCUGAUUCGAA**GCAAUAGGCUGCACACCGGUCACUAACAAGUCAGCCAGCGAUCGCGGCCUGGACUUCGUCCAGGAUGAUG
....((((((((((((((.(((((((((((((.((((.((..(((......)))....))))))))))))))))))).)))))))))))))))....(((((...)))))..

>Predicted STAR seq:
GTCACTAACAAGTCAGCCAG

Complementarity: 17

Initial dG =   -57.70

```
          10        20        30        40
.-CUGA|              A             C    U  AA--   AU
      UAGGCUGCGAUCGUU GCUGACUUGUUGG UGAU CG     GCA  A
      GUCCGGCGCUAGCGA CGACUGAACAAUC ACUG GC     CGU  G
\ ----^              C             -    -  CACA   CG
         90        80        70         60        50



     100
  GACU     C
      UCGUC \
      AGUAG A
  GU--     G
     110
```

Reads in: lib-42, lib-45, lib-47, lib-48, lib-49, lib-50, lib-72
Number of reads="ID428",2,4,0,87,0,0,0

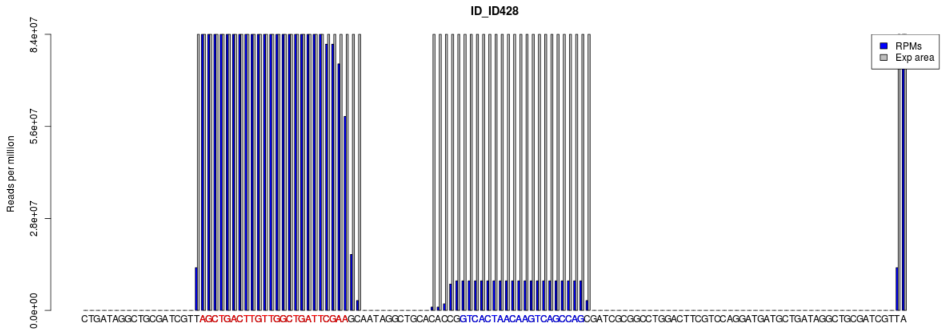

>ID429
ACTGATAGGCTGCGATCGTT**AGCTGACTTGTTGGCTGATTCGAG**GCAATAGGCTGCACCCCGGTCACCAACAAGTCAGCCAGCGATCGCA
GCCTGGACGTCGTCCAGGATGATGA

>ID429-5P
AGCTGACTTGTTGGCTGATTCGAG

>miRNA precursor
ACUGAUAGGCUGCGAUCGUU**AGCUGACUUGUUGGCUGAUUCGAG**GCAAUAGGCUGCACCCCGGUCACCAACAAGUCAGCCAGCGAUCGCAGCCUGGACGUCGUCCAGGAUGAUGA
.....((((((((((((((.(((((((((((((.((((.((..(((......)))....))))))))))))))))))).)))))))))))))))..(((((((...))))))).

>Predicted STAR seq:
GTCACCAACAAGTCAGCCAG

Complementarity: 17

Initial dG = -65.90

```
          10        20        30        40          50
.-ACUGA|              A             C    U  AG--   AU
       UAGGCUGCGAUCGUU GCUGACUUGUUGG UGAU CG    GCA  A
       GUCCGACGCUAGCGA CGACUGAACAACC ACUG GC    CGU  G
\ -----^              C             -    -  CCCA   CG
           90        80        70          60



    100
  GA       C
    CGUCGUC \
    GUAGUAG A
  A-       G
      110
```

Reads in: lib-42, lib-45, lib-47, lib-48, lib-49, lib-50, lib-72
Number of reads="ID429",8,2,1,282,6,1,0

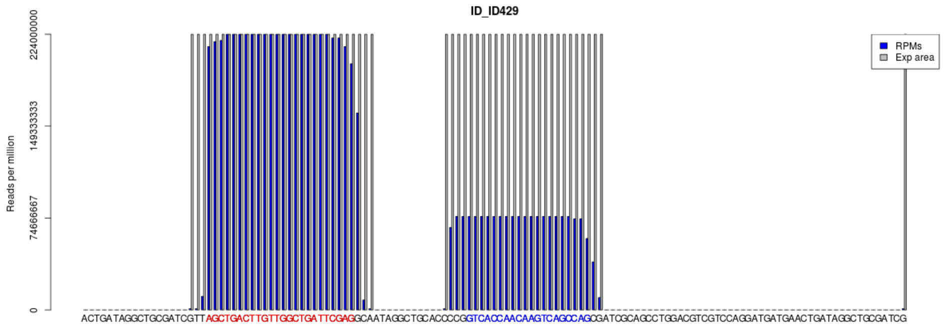

>ID43
AGTTGCGGCCAGGCAACCCCG**CTCTGAGTGCTGGACAGAGGAT**CTCTCCAGTGCCAGCCCTCTGTCCAGCACTCAGAGGGGAGTGGCCCC
GCCATTTTCTGCCGTCAACTG

>ID43-5P
CTCTGAGTGCTGGACAGAGGAT

>miRNA precursor
AGUUGCGGCCAGGCAACCCCG**CUCUGAGUGCUGGACAGAGGAU**CUCUCCAGUGCCAGCCCUCUGUCCAGCACUCAGAGGGGAGUGGCCCCGCCAUUUUCUGCCGUCAACUG
((((((((..(((.(((((.((((((((((((((((((((................))))))))))))))))))))))).)).)))...........)))).))))).

>Predicted STAR seq:
TCTGTCCAGCACTCAGAGGG

Complementarity: 18

Initial dG =   -63.80

```
          10          20        30        40
-     -   C    .-CAAC|  G                    AUCUCUCC
 AGUUG CGG CAGG      CCC CUCUGAGUGCUGGACAGAGG        \
 UCAAC GCC GUCU      GGG GAGACUCACGACCUGUCUCC        A
G     U   -    \ ----^  -                    CGACCGUG
 .        100        80         70        60
```

```
                 A-    CC
                   GUGG  \
                   UACC  C
                 UU    GC
                       90
```

Reads in: lib-42, lib-45, lib-47, lib-48, lib-49, lib-50, lib-72
Number of reads="ID43",7,55,10,1348,176,4,0

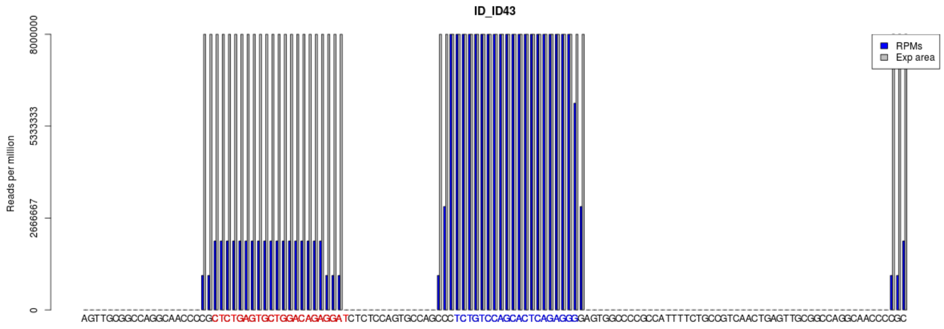

>ID430
GTCACGTCACACTGATAGGCCGCTGTCGTTAGTAGACTTGCTGGCTGATTCGAGGCAATAGGCTGCTCC**CAGGTCACCAACAAGTCTGCC**
AGCGATCGCGGCCTGCTCTTCG

>ID430-3P
CAGGTCACCAACAAGTCTGCCAG

>miRNA precursor
GUCACGUCACACUGAUAGGCCGCUGUCGUUAGUAGACUUGCUGGCUGAUUCGAGGCAAUAGGCUGCUCC**CAGGUCACCAACAAGUCUGCCAG**CGAUCGCGGCCUGCUCUUCG
.............(((((((((.((((((.(((((((((.(((.(((((...((((......))))....)))))))).))))))))).)))))).)))))))).))....

>Predicted STAR seq:
TAGTAGACTTGCTGGCTGATT

Complementarity: 17

Initial dG =   -50.20

```
        10         20        30        40        50         60
GUCACGUCACACU  -|       U      A         C   C     CGA-    AU
             GA UAGGCCGC GUCGUU GUAGACUUG UGG UGAUU    GGCA  A
             CU GUCCGGCG UAGCGA CGUCUGAAC ACC ACUGG    UCGU  G
GCUU---------  C^       C      C         A   -     ACCC    CG
110               100        90        80         70
```

Reads in: lib-42, lib-45, lib-47, lib-48, lib-49, lib-50, lib-72
Number of reads="ID430",16,15,2,497,21,3,0

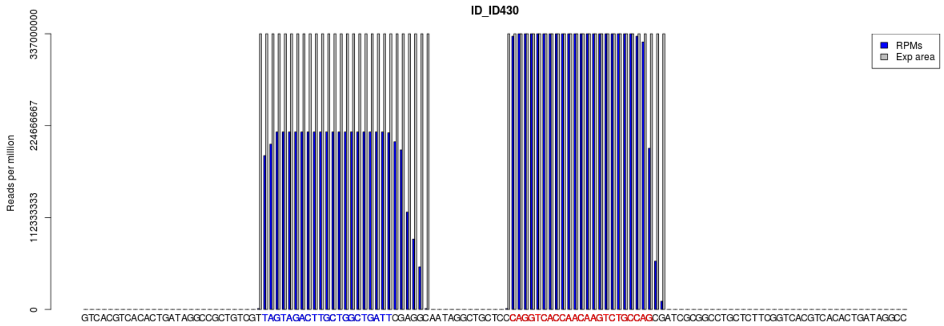

>ID431
GTCACGTCACACTGATAGGCCGCTGTCGTTAGTAGACTTGCTGGCTGATTAGAGGCAATAGGCTGTACC**CAGGTCACCAACAAGTCTGCTAG**CGATCGCGGCCTGCTCTTCG

>ID431-3P
CAGGTCACCAACAAGTCTGCTAG

>miRNA precursor
GUCACGUCACACUGAUAGGCCGCUGUCGUUAGUAGACUUGCUGGCUGAUUAGAGGCAAUAGGCUGUACC**CAGGUCACCAACAAGUCUGCUAG**CGAUCGCGGCCUGCUCUUCG
.............((((((((((.((((((((((((((((.(((.(((((...(((.....))).......)))))))).)))))))))))))))).)))))))).))....

>Predicted STAR seq:
TAGTAGACTTGCTGGCTGATT

Complementarity: 18

Initial dG = -52.60

```
        10         20        30        40        50
GUCACGUCACACU  -        U                C   C     AGA----|  AA
             GA UAGGCCGC GUCGUUAGUAGACUUG UGG UGAUU       GGC  \
             CU GUCCGGCG UAGCGAUCGUCUGAAC ACC ACUGG       UCG  U
GCUU---------  C        C                A   -     ACCCAUG^  GA
110                100       90        80         70        60
```

Reads in: lib-42, lib-45, lib-47, lib-48, lib-49, lib-50, lib-72
Number of reads="ID431",1,3,1,168,6,0,0

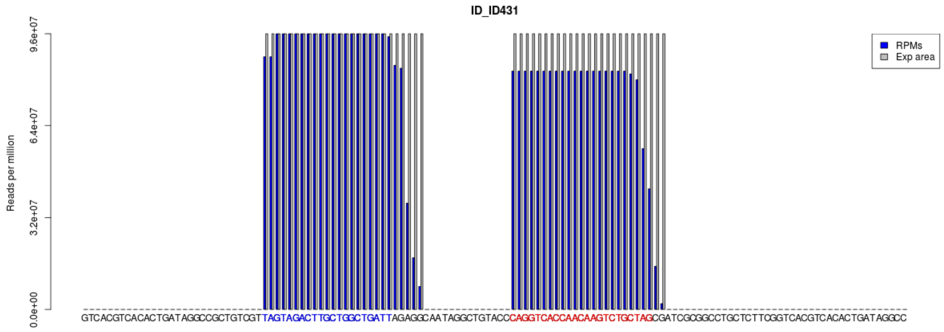

>ID439
ACTTTAGCCATTCGGGAATGGCCGCCTGAGTTTCGAGTCCTCCTCGGGCATAGGTGTATTTGCTGTCC**TATGCCCGAGGGAGGGCTCGAG**
GCTCCGACGGCAAGCAACCGCC

>ID439-3P
TATGCCCGAGGGAGGGCTCGAGG

>miRNA precursor
ACUUUAGCCAUUCGGGAAUGGCCGCCUGAGUUUCGAGUCCUCCUCGGGCAUAGGUGUAUUUGCUGUCC**UAUGCCCGAGGGAGGGCUCGAGG**CUCCGACGGCAAGCAACCGCC
......((((((....)))))).(((.(((((((((((((((((((((((...........))))))))))).))))))))))))....)))..((....)).

>Predicted STAR seq:
GGGAATGGCCGCCTGAGTTTCGAGTCCTCCTCG

Complementarity: 20
ERROR 4: Strange structure
Initial dG =   -63.10

```
          10
.-ACUUUA|     C
        GCCAUU G
        CGGUAA G
\ ------^     G
         20



             30        40         50        60
  .-C   U---                -           UGUAU
     GCC    GAGUUUCGAGUCCUCC UCGGGCAUAGG     U
     CGG    CUCGGAGCUCGGGAGG AGCCCGUAUCC     U
  \ -   CAGC                G           UGUCG
    100        90        80        70
```

Reads in: lib-42, lib-45, lib-47, lib-48, lib-49, lib-50, lib-72
Number of reads="ID439",58,826,160,5922,235,67,0

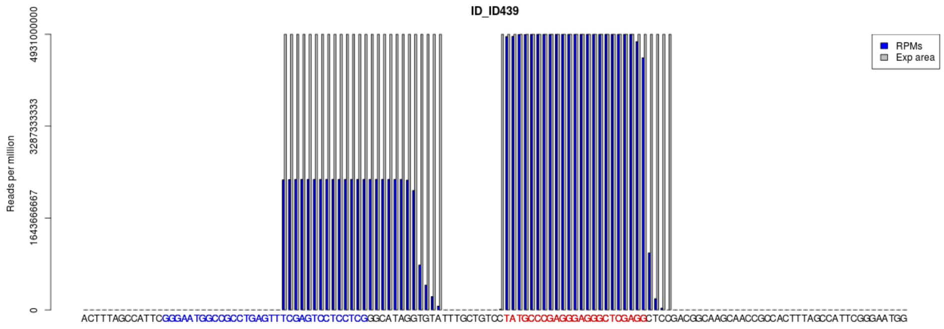

>ID44
GAAAGTGGCGGGGTTGCTCT**CATCTGAATGCTGGACAGAGGA**CCAGATCTGGGGAGCTCCTCTGTCTAGCACTCAGAGGGGAGCTGCTTG
GCTATAGCGTCGACACCGTGCC

>ID44-5P
CATCTGAATGCTGGACAGAGGA

>miRNA precursor
GAAAGUGGCGGGGUUGCUCU**CAUCUGAAUGCUGGACAGAGGA**CCAGAUCUGGGGAGCUCCUCUGUCUAGCACUCAGAGGGGAGCUGCUUGGCUAUAGCGUCGACACCGUGCC
....(((((.((((.((((((.(((((.(((((((((((((((((....))).....))))))))))))))).))))).)))))).)))).))))).(((.((....))))).

>Predicted STAR seq:
CTCTGTCTAGCACTCAGAGGGG

Complementarity: 18

Initial dG =   -59.90

```
          10        20        30        40
.-GAAA     G    U      A     A              -----|  G
      GUGGC GGGU GCUCUC UCUGA UGCUGGACAGAGGA     CCA A
      UAUCG UUCG CGAGGG AGACU ACGAUCUGUCUCCU     GGU U
\ ----     G    U      G     C              CGAGG^  C
          90        80        70        60        50
```

```
    100
  A   U  A
   GCG CG C
   CGU GC A
  C   -  C
  110
```

Reads in: lib-42, lib-45, lib-47, lib-48, lib-49, lib-50, lib-72
Number of reads="ID44",1,6,4,93,23,0,0

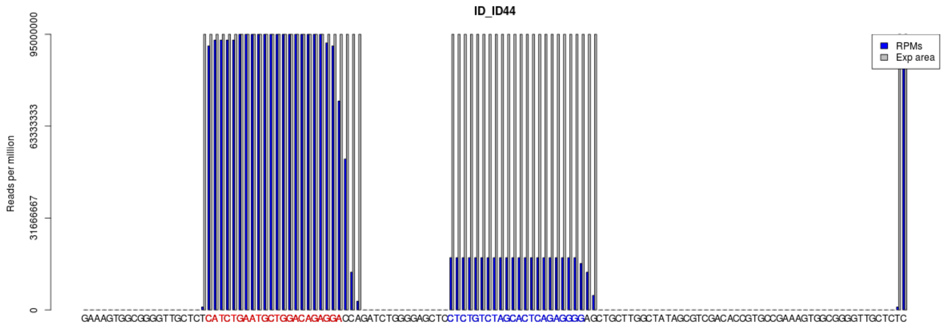

>ID441
TTGATTGTGGAGGAACTGGGCGTTATAAAGGACGCCATGCATTGTGTCCACCGCGGTGATTTGGATAAGC**GGTGGATGTGGTGCACGGCG**
TCCTTCGTAATGCCTCTACGCA

>ID441-3P
GGTGGATGTGGTGCACGGCGTC

>miRNA precursor
UUGAUUGUGGAGGAACUGGGCGUUAUAAAGGACGCCAUGCAUUGUGUCCACCGCGGUGAUUUGGAUAAGC**GGUGGAUGUGGUGCACGGCGUC**CUUCGUAAUGCCUCUACGCA
......(((((.......(((((((..(((((((((.(((((..((((((((((.............))))))))))..))))).)))))))))..))))))))))))...

>Predicted STAR seq:
ACGCCATGCATTGTGTCCACC

Complementarity: 17

Initial dG =   -62.00

```
        10         20         30         40         50         60
UUGAUU|    GGAACUG       UA          A      UG           GGUGAU
      GUGGA       GGCGUUA  AAGGACGCC UGCAU  UGUCCACCGC      U
      CAUCU       CCGUAAU  UUCCUGCGG ACGUG  GUAGGUGGCG      U
ACG---^    -------       GC          C      GU           AAUAGG
110                 100        90         80         70
```

Reads in: lib-42, lib-45, lib-47, lib-48, lib-49, lib-50, lib-72
Number of reads="ID441",75,16,17,592,467,14,0

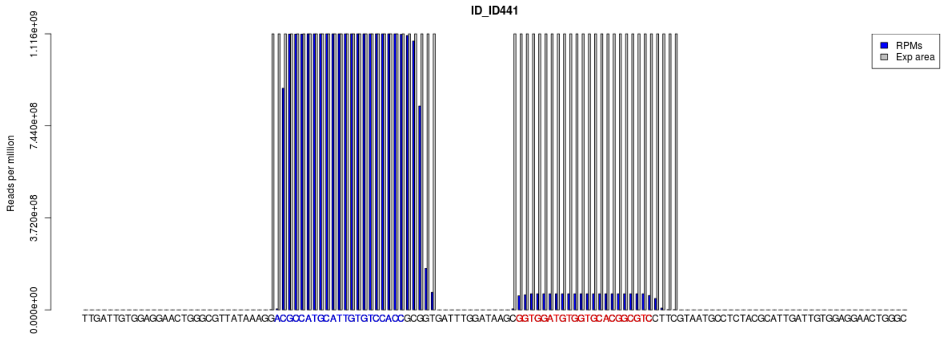

>ID443

CACAAGGAGGCATGTCGTAC**TGGGAATCGAACTTGTGTCCTTT**GGCTTTCGTATCAAGGTCCCAAGTTCGAGTACCGGTTCGACGTGCAG
TATCAATCGGCCAGGGAAG

>ID443-5P

TGGGAATCGAACTTGTGTCCTTT

>miRNA precursor

CACAAGGAGGCAUGUCGUAC**UGGGAAUCGAACUUGUGUCCUUU**GGCUUUCGUAUCAAGGUCCCAAGUUCGAGUACCGGUUCGACGUGCAGUAUCAAUCGGCCAGGGAAG

```
.........((((((((.(((((...(((((((((...((((..((....))...))))...)))))))))...))))).))))))))....................
```

>Predicted STAR seq:

GGTCCCAAGTTCGAGTACCGGT

Complementarity: 14

Initial dG =   -40.50

```
                    10        20        30        40
CACAAGGAG------------        U     GAA        UGU    UG-| U
                     GCAUGUCG ACUGG   UCGAACUUG   CCUU   GC U
                     CGUGCAGC UGGCC   AGCUUGAAC   GGAA   UG U
GAAGGGACCGGCUAACUAUGA        U     AUG        CCU    CUA^ C
       100       90        80        70        60        50
```

Reads in: lib-42, lib-45, lib-47, lib-48, lib-49, lib-50, lib-72

Number of reads="ID443",25,13,18,751,23,3,0

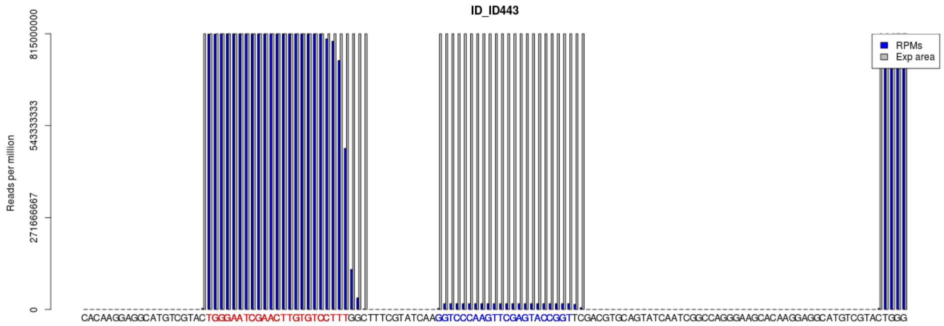

>ID445
CGAGACAGTTAAAAGCACATTCGTAATCTACCCTGTAGATCCGGGCTTTTGTAGAGTCTGCTTTACGTCA**GAAGCTCGACTCTACAGGTA**
TCTTACGGATGTGAGACTTCGCT

>ID445-3P
GAAGCTCGACTCTACAGGTATCT

>miRNA precursor
CGAGACAGUUAAAAGCACAUUCGUAAUCUACCCUGUAGAUCCGGGCUUUUGUAGAGUCUGCUUUACGUCA**GAAGCUCGACUCUACAGGUAUCU**UACGGAUGUGAGACUUCGCU
...((.((((.....((((((((((..((((.((((((..(((((((((((((((....))))))))...))))))))..))))))))))..))))))))))).))))))...

>Predicted STAR seq:
TCTACCCTGTAGATCCGGGCTTT

Complementarity: 17

Initial dG =   -45.90

```
        10        20        30        40           50
CGA| C    AAAAG          UC    C      UC        ---       U
   GA AGUU     CACAUUCGUAA  UACC UGUAGA  CGGGCUUU   UGUAGAG C
   CU UCAG     GUGUAGGCAUU  AUGG ACAUCU  GCUCGAAG   GCAUUUC U
UCG^ -    A----          CU    -      CA        ACU       G
 110            100       90         80        70        60
```

Reads in: lib-42, lib-45, lib-47, lib-48, lib-49, lib-50, lib-72
Number of reads="ID445",517,63,0,306,1,3,0

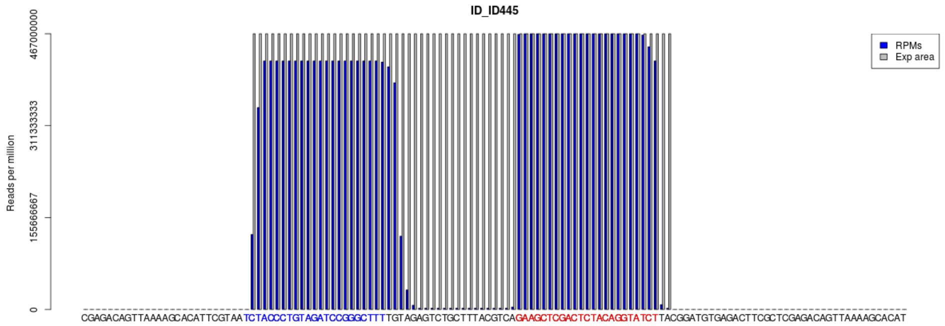

>ID45

AAGTGGCGGGGTCGCCCTC**CGTCTGAGTGCTGGACAGAGGGA**TAGCACTGGGGAGACCCTTTGTCCAGCACTCAGAGGGGGGCTGCTTTG
TGGCAGCTTCGACTCCGTGGCT

>ID45-5P

CGTCTGAGTGCTGGACAGAGGGA

>miRNA precursor

AAGUGGCGGGGUCGCCCUC**CGUCUGAGUGCUGGACAGAGGGA**UAGCACUGGGGAGACCCUUUGUCCAGCACUCAGAGGGGGGCUGCUUUGUGGCAGCUUCGACUCCGUGGCU
.(((.((((((((.(((((.((((((((((((((((((((..............)))))))))))))))))))))))))(((((....))))))..))))))))).)))

>Predicted STAR seq:

CTTTGTCCAGCACTCAGAGGG

Complementarity: 19

Initial dG = -75.30

```
        10          20         30         40
A   G         .-C|    G                   AUAGCAC
 AGU GCGGGGUCG   CCUCC UCUGAGUGCUGGACAGAGGG       \
 UCG UGCCUCAGC   GGGGG AGACUCACGACCUGUUUCCC       U
-   G         \ -^    -                   AGAGGGG
 110       100   80         70         60         50




                --      U
                  GCUGCU U
                  CGACGG G
                UU      U
                        90
```

Reads in: lib-42, lib-45, lib-47, lib-48, lib-49, lib-50, lib-72

Number of reads="ID45",1,14,1,868,123,5,0

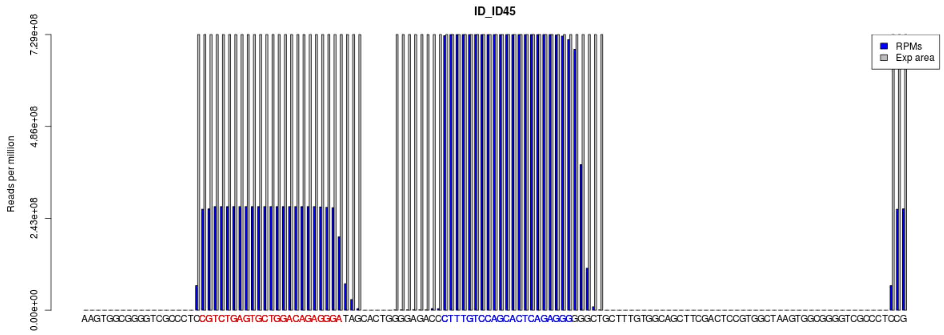

>ID456

GCTACATGGCCCCGGTTGTGTGTCTCCGTGGCTATTTGATGTTGG**ATATACATAATCGGTGCTGTAT**TGCTATCCACGATACTCTCC

>ID456-5?P

ATATACATAATCGGTGCTGTAT

>miRNA precursor

```
GCUACAUGGCCCCGGUUGUGUGUCUCCGUGGCUAUUUGAUGUUGGAUAUACAUAAUCGGUGCUGUAUUGCUAUCCACGAUACUCUCC
((...(((((.((((((((((((.((((..((........)))))).))))))))))))).)))))...))................
Error predicting STAR on 5p
 Initial dG =    -27.80

                         10        20        30
-----------------| UAC     C            C    UG   UAU
                 GC   AUGGC CCGGUUGUGUGU UCCG  GC   U
                 CG   UGUCG GGCUAAUACAUA AGGU  UG   U
CCUCUCAUAGCACCUAU^ UUA     U            U    --   UAG
      80        70        60        50          40
```

Reads in: lib-42, lib-45, lib-47, lib-48, lib-49, lib-50, lib-72

Number of reads="ID456",0,0,0,23,2,0,0

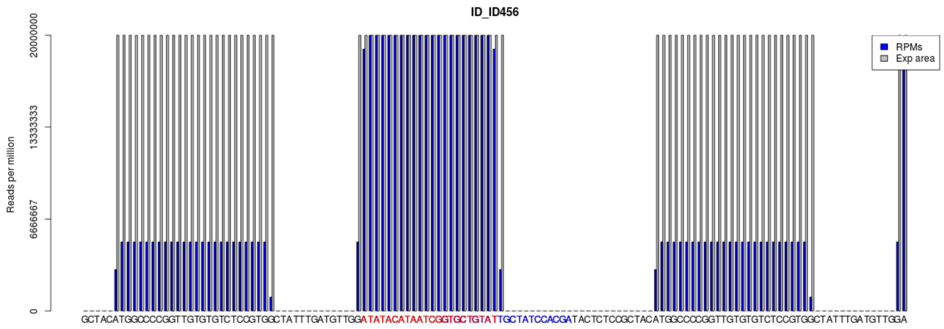

>ID458

AACCTGCGCATCCCATATGTGGTTCGCTACATGGCCCCGGTTGTGTGTCTCCGTGGCTATTTGATGTCG**GATCTACATAATCGGTGCTGT**
GTTGCTATCCACGATACTCTTC

>ID458-3P

GATCTACATAATCGGTGCTGTGT

>miRNA precursor

AACCUGCGCAUCCCAUAUGUGGUUCGCUACAUGGCCCCGGUUGUGUGUCUCCGUGGCUAUUUGAUGUCG**GAUCUACAUAAUCGGUGCUGUGU**UGCUAUCCACGAUACUCUUC
................(((((...((.(((((((.(((((((((((..((((..((........))))))..))))))))))).))))))).))...))))).........

>Predicted STAR seq:

ACATGGCCCCGGTTGTGTGTCTC

Complementarity: 18

Initial dG =   -39.70

```
        10        20        30        40        50        60
AACCUGCGCAUCCCAUA|    UUC  U       C           UC    UG  UAU
                 UGUGG   GC ACAUGGC CCGGUUGUGUG  UCCG  GC   U
                 GCACC   CG UGUGUCG GGCUAAUACAU  AGGC  UG   U
CUUCUCAUA--------^    UAU  U       U           CU    --  UAG
110              100       90        80        70
```

Reads in: lib-42, lib-45, lib-47, lib-48, lib-49, lib-50, lib-72

Number of reads="ID458",1,0,0,7,0,0,0

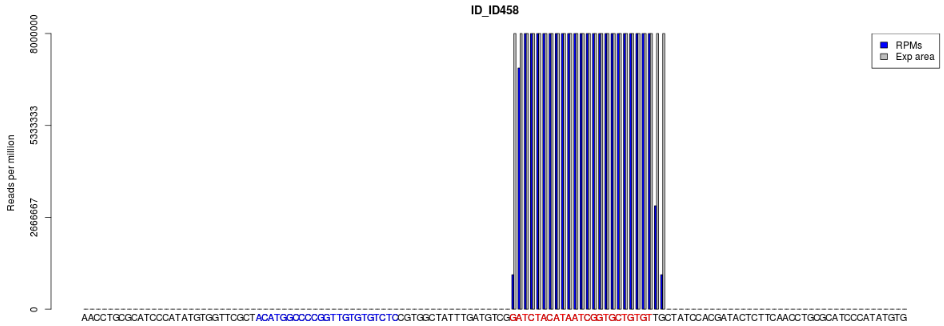

>ID46
TGACGGCAGAAAATGGCGGGGCCACTCCCCTCTGAGTGCTGGACAGAGGGCTGGCACTGGAGAGATC**CTCTGTCCAGCACTCAGAGCGGG**
GTTGCCTGGCCGCAACTTAGA

>ID46-3P
CTCTGTCCAGCACTCAGAGCGGG

>miRNA precursor
UGACGGCAGAAAAUGGCGGGGCCACUCCCCUCUGAGUGCUGGACAGAGGGCUGGCACUGGAGAGAUC**CUCUGUCCAGCACUCAGAGCGGG**GUUGCCUGGCCGCAACUUAGA
............((((.((((.(((((.((((((((((((((((((((...............))))))))))))))))))))).))))).)))).)))).........

>Predicted STAR seq:
CCCCTCTGAGTGCTGGACAGAG

Complementarity: 20

Initial dG =   -67.60

```
        10        20        30        40        50
UGACGGCAGAAAA|   G    C     C                     CUGGCAC
             UGGC GGGC ACUCC CUCUGAGUGCUGGACAGAGGG       \
             GCCG UCCG UGGGG GAGACUCACGACCUGUCUCCU       U
AGAUUCAAC----^   G    U     C                     AGAGAGG
 .           100        90        80        70        60
```

Reads in: lib-42, lib-45, lib-47, lib-48, lib-49, lib-50, lib-72
Number of reads="ID46",4,6,2,202,60,2,0

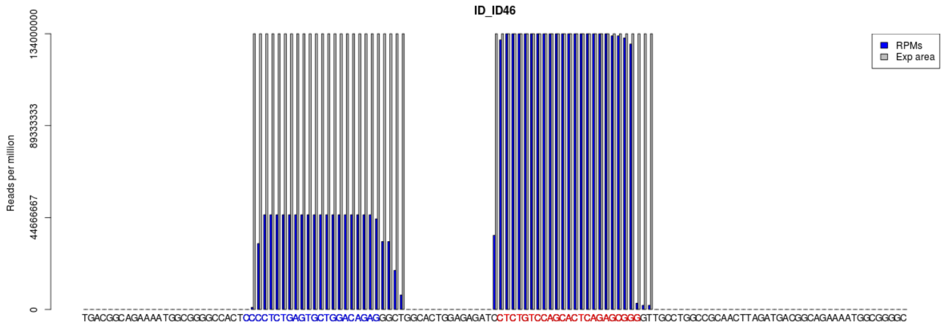

>ID461
CTGACAGCGGCATCTGGTCTGCGCAGTTTCATAGTTTACAGCGACCAGTATCCTGCTACTAGATTTGGTA**CAGGTCGGTGTAAATTGTGT**
GACTGTGTGGACACAAAA

>ID461-3P
CAGGTCGGTGTAAATTGTGTGA

>miRNA precursor
```
CUGACAGCGGCAUCUGGUCUGCGCAGUUUCAUAGUUUACAGCGACCAGUAUCCUGCUACUAGAUUUGGUACAGGUCGGUGUAAAUUGUGUGACUGUGUGGACACAAAA
....(((......)))(((..(((((((.(((((((((((.(((((.(((((...(.....)....))))).))))).)))))))))))).)))))))..)))......
```

>Predicted STAR seq:
TTCATAGTTTACAGCGACCA

Complementarity: 17

Initial dG =   -46.50

```
        10         20        30        40        50
CUGACAGCGGCAUC  -   UG       U           G      A      CUG-|UA
              UG GUC  CGCAGUU CAUAGUUUACA CGACC GUAUC    C  \
              AC CAG  GUGUCAG GUGUUAAAUGU GCUGG CAUGG    G  C
AAA-----------  A   GU       U           G      A      UUUA^AU
                 100       90        80        70        60
```

Reads in: lib-42, lib-45, lib-47, lib-48, lib-49, lib-50, lib-72
Number of reads="ID461",94,48,5,3247,34,1,0

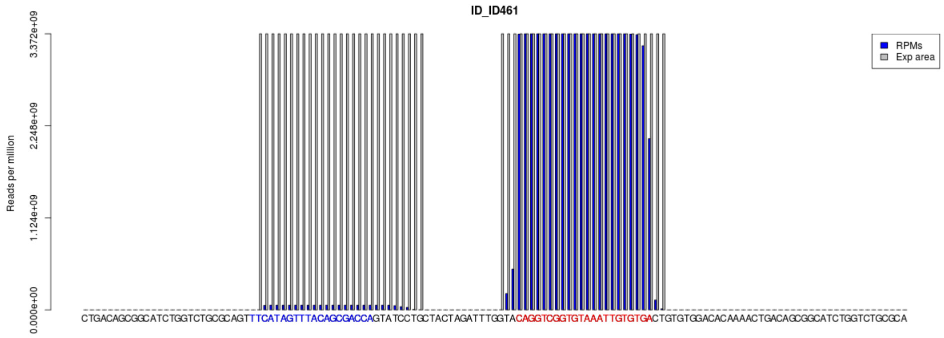

>ID465
AAAATTGGCAGGGCCATTCTCA**TCTGAGTGCTGGACAGAGGGC**TGGCACTCGGGAGATCCTCTGTCCAGCACTCAGAGGGGAGTTGCTTA
GCTGCCGCTTCAACACCACAG

>ID465-5P
TCTGAGTGCTGGACAGAGGGC

>miRNA precursor
AAAAUUGGCAGGGCCAUUCUCA**UCUGAGUGCUGGACAGAGGGC**UGGCACUCGGGAGAUCCUCUGUCCAGCACUCAGAGGGGAGUUGCUUAGCUGCCGCUUCAACACCACAG
......((((((((.((((((.((((((((((((((((((((...............)))))))))))))))))))).)))))).)))...))))).............. .

>Predicted STAR seq:
CTCTGTCCAGCACTCAGAGGG

Complementarity: 18

Initial dG =   -60.00

```
                 10           20        30        40
AAAAUU---------     ---|  C      A                    CUGGCAC
               GGCAG   GGC AUUCUC UCUGAGUGCUGGACAGAGGG       \
               CCGUC   UCG UGAGGG AGACUCACGACCUGUCUCCU       U
GACACCACAACUUCG     GAU^  U      G                    AGAGGGC
 .       100        90        80        70        60
```

Reads in: lib-42, lib-45, lib-47, lib-48, lib-49, lib-50, lib-72
Number of reads="ID465",0,2,0,47,11,0,0

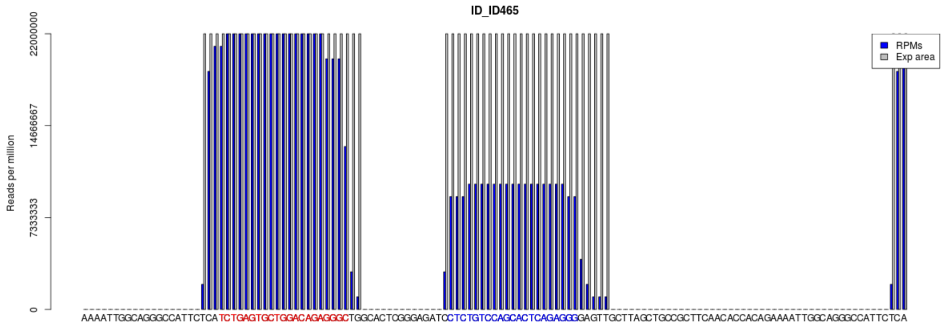

>ID466
TCTTTGCGCGGTGGGGGCTA**TTATTTGGCACTCATGGAATCG**ATGTTCATCTTACATCAATTCTTTGCGTGTCAGGGTCGTCCTCCCCGT
CATCCGACTTAGTAAGCGACTT

>ID466-5P
TTATTTGGCACTCATGGAATCG

>miRNA precursor
UCUUUGCGCGGUGGGGGCUA**UUAUUUGGCACUCAUGGAAUCG**AUGUUCAUCUUACAUCAAUUCUUUGCGUGUCAGGGUCGUCCUCCCCGUCAUCCGACUUAGUAAGCGACUU
....((.((((.((((((.....((((((((.((.(((((.(((((.......))))).))))).)).))))))))...)))))).))))))..((.(((...)))))....

>Predicted STAR seq:
ATTCTTTGCGTGTCAGGGTCGT

Complementarity: 14

```
 Initial dG =    -37.20

          10        20        30        40
UCU    .-GC| U      UAUUA        U  U     C     UCA
   UUGC    GG GGGGGC     UUUGGCAC CA GGAAU GAUGU   \
   AGCG    CC CUCCUG     GGACUGUG GU UCUUA CUACA   U
UUC    \ --^ -      CUG--        C  U     A     UUC
110               80          70        60



                90
         C------   A
                GUC U
                CAG C
         AAUGAUU   C
            100
```

Reads in: lib-42, lib-45, lib-47, lib-48, lib-49, lib-50, lib-72
Number of reads="ID466",0,1,0,49,13,1,0

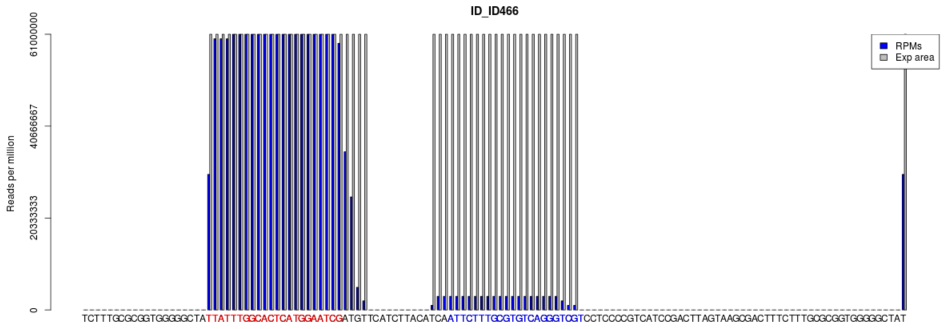

>ID467
AGAAATGGAGAGCCACTCTC**GTCTGGGTGCTGGACAGACCGC**TAGCACTGGAGAGGTGCTCTGTCCAGCACCCAGCAGGGAGTTGCTTGG
GTCCATCTTCAACACCGTGGGA

>ID467-5P
GTCTGGGTGCTGGACAGACCGC

>miRNA precursor
AGAAAUGGAGAGCCACUCUC**GUCUGGGUGCUGGACAGACCGC**UAGCACUGGAGAGGUGCUCUGUCCAGCACCCAGCAGGGAGUUGCUUGGGUCCAUCUUCAACACCGUGGGA
.(((((((((((.((((((..((((((((((((((((......(((((.....))))))))))))))))))))))..))))))).))))...))))).)))...........

>Predicted STAR seq:
TGTCCAGCACCCAGCAGG

Complementarity: 14

Initial dG =   -59.10

```
                       10        20        30        40        50
A-----------   -     ---|   C      GU                CCGCUA     GG
            GAA AUGGA   GAGC ACUCUC  CUGGGUGCUGGACAGA      GCACU  \
            CUU UACCU   UUCG UGAGGG  GACCCACGACCUGUCU      CGUGG  A
AGGGUGCCACAA   C     GGG^   U      AC                ------     AG
110       100       90        80        70        60
```

Reads in: lib-42, lib-45, lib-47, lib-48, lib-49, lib-50, lib-72
Number of reads="ID467",3,3,0,173,25,1,0

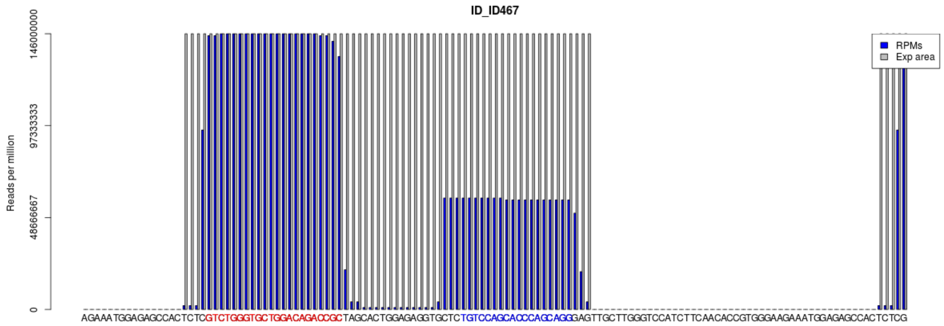

>ID468
AGAAATGGCGAGCCACTTCG**TCCGCGTGTTGGACAGAGCGC**TAGCACTGTGGATGTGCTCTGTCCAGCACTCAGTAGAAAGTGACTTGTG
CCAATCTTCGACACCGTGGG

>ID468-5P
TCCGCGTGTTGGACAGAGCGC

>miRNA precursor
AGAAAUGGCGAGCCACUUCG**UCCGCGUGUUGGACAGAGCGC**UAGCACUGUGGAUGUGCUCUGUCCAGCACUCAGUAGAAAGUGACUUGUGCCAAUCUUCGACACCGUGGG
.(((.(((((((.(((((..((..((((((((((((((((............))))))))))))))))...)..))))))).))).)))))...)))...........

>Predicted STAR seq:
GCTCTGTCCAGCACTCAGTAGAAA

Complementarity: 17

Initial dG =   -43.00

```
            10        20            30        40
.-AGAAA    --   C      CG  CG ---|               UAGCAC
       UGGC  GAG CACUU  UC  C   GUGUUGGACAGAGCGC      \
       ACCG  UUC GUGAA  AG  G   CACGACCUGUCUCGUG      U
\ -----    UG   A      --  AU ACU^               UAGGUG
         90        80           70        60        50
```

```
      100
  AU  U  A
    CU CG C
    GG GC A
  G-  U  C
110
```

Reads in: lib-42, lib-45, lib-47, lib-48, lib-49, lib-50, lib-72
Number of reads="ID468",4,14,13,501,100,0,0

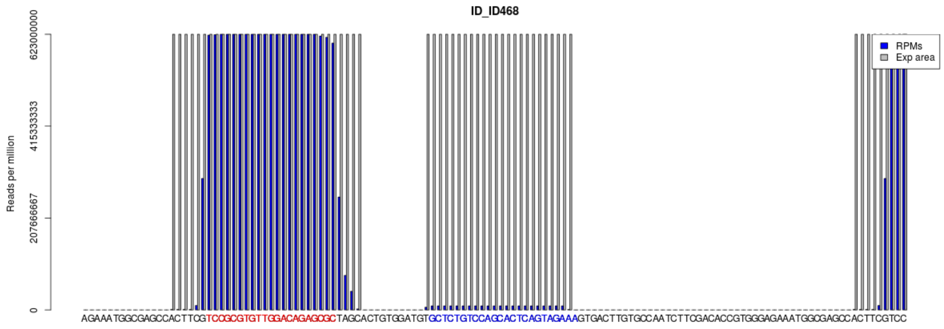

>ID469
CAGAAATTGCAAGCCGCTCT**CGTCTGTGTGCTGGGCAGAGC**GCTAGCACTGGAGAGGTGCTCTGTCCAGCACCCAGTTGGGAGTTGCTTG
GGCGCATCTTCGACTACACGG

>ID469-5P
CGTCTGTGTGCTGGGCAGAGC

>miRNA precursor
CAGAAAUUGCAAGCCGCUCU**CGUCUGUGUGCUGGGCAGAGC**GCUAGCACUGGAGAGGUGCUCUGUCCAGCACCCAGUUGGGAGUUGCUUGGGCGCAUCUUCGACUACACGG
..(((..(((((((.(((((((.(((.(((((((((((((((((..........))))))))))))))))).))).))))))).)))))....)))..))).........

>Predicted STAR seq:
TCTGTCCAGCACCCAGTTGGG

Complementarity: 17

Initial dG =   -56.20

```
                10            20         30         40
CA--------   AU   ----|   C        U   U                 AGCAC
          GAA  UGC    AAGC GCUCUCG CUG GUGCUGGGCAGAGCGCU      \
          CUU  ACG    UUCG UGAGGGU GAC CACGACCUGUCUCGUGG      U
GGCACAUCAG   CU   CGGG^   U        U   C                 AGAGG
 .       100        90         80         70         60
```

Reads in: lib-42, lib-45, lib-47, lib-48, lib-49, lib-50, lib-72
Number of reads="ID469",5,15,3,138,65,5,0

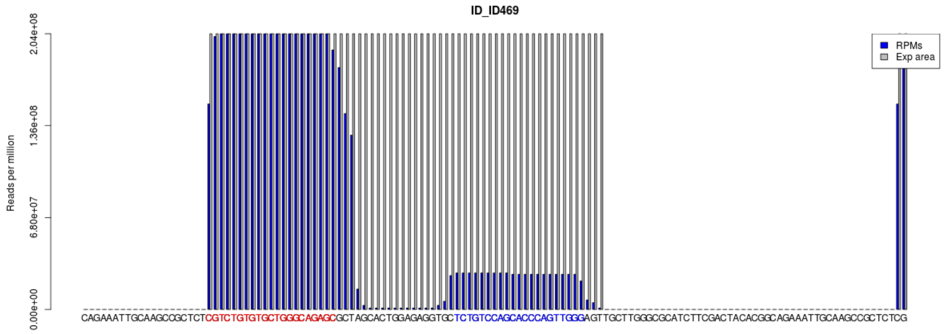

>ID47

ACACTGGTGGGGCCGCTCT**CGATCTGAGTGCTGGACAGAGCAC**TAGCACTGGGGAGATCCTCTGTGCAGCACTCAGAGGGGAGCTGCTTG
GCCGCAGCTCCGACACCTTGGAT

>ID47-5P

CGATCTGAGTGCTGGACAGAGCAC

>miRNA precursor

ACACUGGUGGGGCCGCUCU**CGAUCUGAGUGCUGGACAGAGCAC**UAGCACUGGGGAGAUCCUCUGUGCAGCACUCAGAGGGGAGCUGCUUGGCCGCAGCUCCGACACCUUGGAU

```
.....(((.((((.((((((..(((((((((((.((((((..(((....)))..................)))))).))))))))))).)))))).)))).)))).....((((....))))).
```

>Predicted STAR seq:

GAGATCCTCTGTGCAGCACTCAGAGGGG

Complementarity: 17

Initial dG =   -58.00

```
          10         20         30          40
.-ACACU   G    C      GA            G     .-A| A
       GGU GGGC GCUCUC  UCUGAGUGCUG ACAG    GC C
       CCG UUCG CGAGGG  AGACUCACGAC UGUC    CG U
\ -----   G    U      G-            G     \ -^ A
         90         80          70



                                           50
                                          ACU   G
                                             GGG A
                                             CCU G
                                          U--   A
                                            60
```

Reads in: lib-42, lib-45, lib-47, lib-48, lib-49, lib-50, lib-72

Number of reads="ID47",0,2,0,33,4,0,0

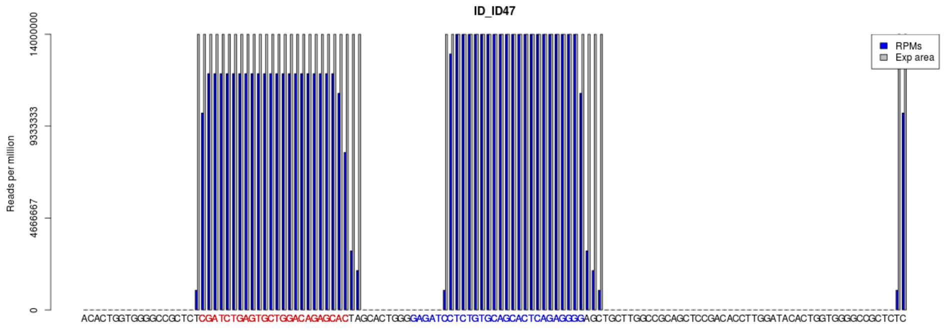

>ID470
TACATGCACCACGGATGTGGCGGGGCCGCTCCCCTCTGAGTGCTGGACAGAGGGCTGGTATTGGGAGATC**CCTGCTCCAGCACTCAGAGG**
GGAGTTGCTTGGCCAAAGCTTC

>ID470-3P
CCTGCTCCAGCACTCAGAGGGG

>miRNA precursor
UACAUGCACCACGGAUGUGGCGGGGCCGCUCCCCUCUGAGUGCUGGACAGAGGGCUGGUAUUGGGAGAUC**CCUGCUCCAGCACUCAGAGGGG**AGUUGCUUGGCCAAAGCUUC
```
............((...((((.((((.(((((((((((((((((((((((.(((..............)))))).))))))))))))))))))))).)))).)))).)))...))..
```

>Predicted STAR seq:
TCCCCTCTGAGTGCTGGACA

Complementarity: 19

Initial dG =   -66.20

```
        10        20        30        40         50        60
UACAUGCACCAC  AUG    G    C                    -|  A   CUGGUA
            GG   UGGC GGGC GCUCCCCUCUGAGUGCUGGA CAG GGG      U
            UC   ACCG UUCG UGAGGGGAGACUCACGACCU GUC CCU      U
CU----------  GAA    G    U                    C^  -   AGAGGG
          110       100        90        80         70
```

Reads in: lib-42, lib-45, lib-47, lib-48, lib-49, lib-50, lib-72
Number of reads="ID470",9,39,27,1306,302,14,0

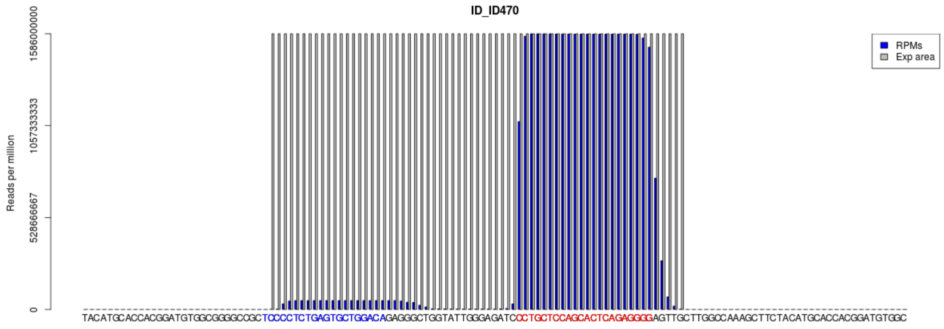

>ID471
AAAGTGGCGGGACCACTCTC**GCTTTGAGTGCTGGACAGAGGGC**TGGTACTTGGAGGTCCTCTGTCCAGCACTCAGAGCGGAGTTGCTTGG
CCAAAGCTTCGACACCGAGGGAC

>ID471-5P
GCTTTGAGTGCTGGACAGAGGGC

>miRNA precursor
AAAGUGGCGGGACCACUCUC**GCUUUGAGUGCUGGACAGAGGGC**UGGUACUUGGAGGUCCUCUGUCCAGCACUCAGAGCGGAGUUGCUUGGCCAAAGCUUCGACACCGAGGGAC
....((((((...((((.((((((((((((((((((((((((((..........)))))))))))))))))))))))))))))..)))).)))...(((((....)))))...

>Predicted STAR seq:
CCTCTGTCCAGCACTCAGAGCGG

Complementarity: 21

Initial dG =   -67.30

```
           10        20        30        40
.-AAAG   -|   ACC    U                         GGUA
      UGG CGGG   ACUC CGCUUUGAGUGCUGGACAGAGGGCU    C
      ACC GUUC   UGAG GCGAGACUCACGACCUGUCUCCUGG    U
\ ----   G^   GU-    -                         AGGU
        90         80         70         60         50
```

```
      100
  AAG     A
     CUUCG C
     GGAGC A
  CAG     C
   110
```

Reads in: lib-42, lib-45, lib-47, lib-48, lib-49, lib-50, lib-72
Number of reads="ID471",7,32,34,884,373,11,0

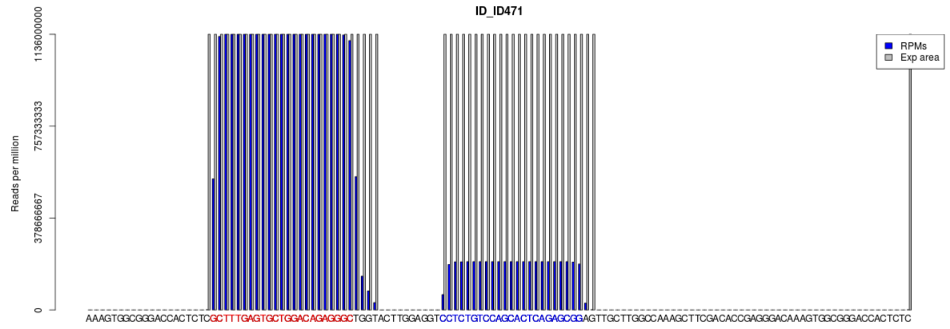

>ID472
AGAAATAGCAAGTCACTCTC**ATCAGGGTGCTGGACAGAGCGC**TAGCACTGGAGAGGTGCTTTGTCTAACACTCAGAGGGGAGTTGCTTGG
GCACTTCTTCGACACCGTGGGA

>ID472-5P
ATCAGGGTGCTGGACAGAGCGC

>miRNA precursor
AGAAAUAGCAAGUCACUCUC**AUCAGGGUGCUGGACAGAGCGC**UAGCACUGGAGAGGUGCUUUGUCUAACACUCAGAGGGGAGUUGCUUGGGCACUUCUUCGACACCGUGGGA
........(((((.((((((.((.(((((.(((((((((((((...........))))))))))))).))))).)).)))))).))))).....((((.((....)).))))

>Predicted STAR seq:
GCTTTGTCTAACACTCAGAGGG

Complementarity: 17

Initial dG =   -43.50

```
          10        20        30        40
.-AGAAAUAG|    C      A  A     C            AGCAC
          CAAGU ACUCUC UC GGGUG UGGACAGAGCGCU     \
          GUUCG UGAGGG AG CUCAC AUCUGUUUCGUGG     U
\ --------^    U      G  A     A            AGAGG
                  80        70        60        50
```

```
 90       100
  GGCAC    U  A
       UUCU CG C
       AGGG GC A
  -----    U  C
       110
```

Reads in: lib-42, lib-45, lib-47, lib-48, lib-49, lib-50, lib-72
Number of reads="ID472",6,20,8,543,175,6,0

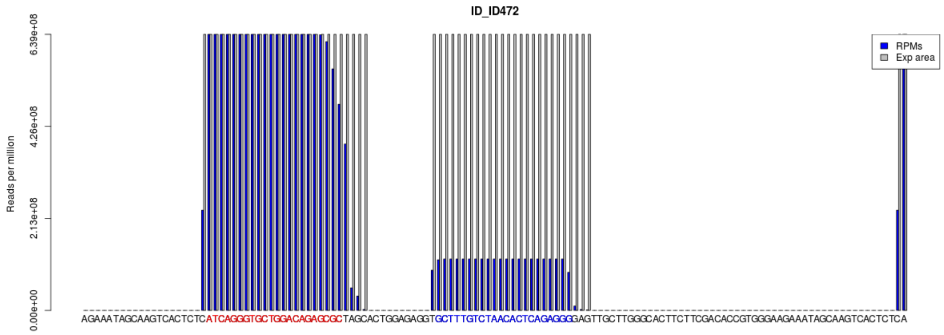

>ID474
AGATGACGGCAGACACTGGCGGGGCCGCTCTCGTCGGAGTGCTGGACAGAGCACTAGCACTGGAGAGATC**CTCTGTTCAGCACTCTGAGG**
GGAGCTGCTTGGCCAGAGCTT

>ID474-3P
CTCTGTTCAGCACTCTGAGGGG

>miRNA precursor
AGAUGACGGCAGACACUGGCGGGGCCGCUCUCGUCGGAGUGCUGGACAGAGCACUAGCACUGGAGAGAUC**CUCUGUUCAGCACUCUGAGGGG**AGCUGCUUGGCCAGAGCUU
```
...............(((((.((((.((((((.((((((((((((((((((..(((....)))........)))))))))))))))))).)))))).)))).))))).....
```

>Predicted STAR seq:
TCGTCGGAGTGCTGGACAGAG

Complementarity: 19

Initial dG = -63.00

```
        10        20        30        40          50
AGAUGACGGCAGACA     G    C      G                .-A| A
               CUGGC GGGC GCUCUC UCGGAGUGCUGGACAG   GC C
               GACCG UUCG CGAGGG AGUCUCACGACUUGUC   CG U
UUCGA----------     G    U      G                \ -^ A
 .                 100        90        80
```

Reads in: lib-42, lib-45, lib-47, lib-48, lib-49, lib-50, lib-72
Number of reads="ID474",65,52,44,2379,500,35,0

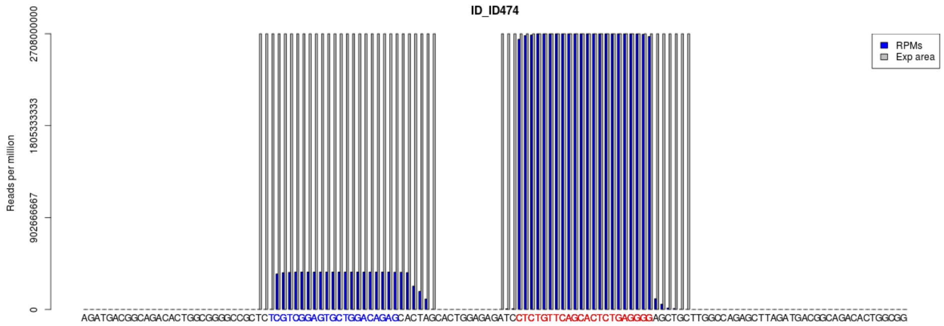

>ID475
GAAAGTGACGGGGCCGCTCT**CGTCTGAGTGCTGGACAGAGGGC**TGGTACTGGGGAGATCCTCTGTCCAGCACTCAGAGGGGAATCGCTTG
GCTGCAGCTTCGACACCATGG

>ID475-5P
CGTCTGAGTGCTGGACAGAGGGC

>miRNA precursor
GAAAGUGACGGGGCCGCUCU**CGUCUGAGUGCUGGACAGAGGGC**UGGUACUGGGGAGAUCCUCUGUCCAGCACUCAGAGGGGAAUCGCUUGGCUGCAGCUUCGACACCAUGG
....(((.((((((...((((.((((((((((((((((((((...............)))))))))))))))))))).))))...((......)).)))))).))).....

>Predicted STAR seq:
CTCTGTCCAGCACTCAGAGGGG

Complementarity: 19

Initial dG = -59.80

```
         10          20         30         40
GAAA-   A      .-CGC|   G                   CUGGUAC
     GUG CGGGGC     UCUC UCUGAGUGCUGGACAGAGGG       \
     CAC GCUUCG     AGGG AGACUCACGACCUGUCUCCU       U
GGUAC   A      \ ---^   G                   AGAGGGG
 .       100         80         70         60


                        90
                 AUC   UU
                    GC   G
                    CG   G
                 A--   UC
```

Reads in: lib-42, lib-45, lib-47, lib-48, lib-49, lib-50, lib-72
Number of reads="ID475",0,9,0,161,33,0,0

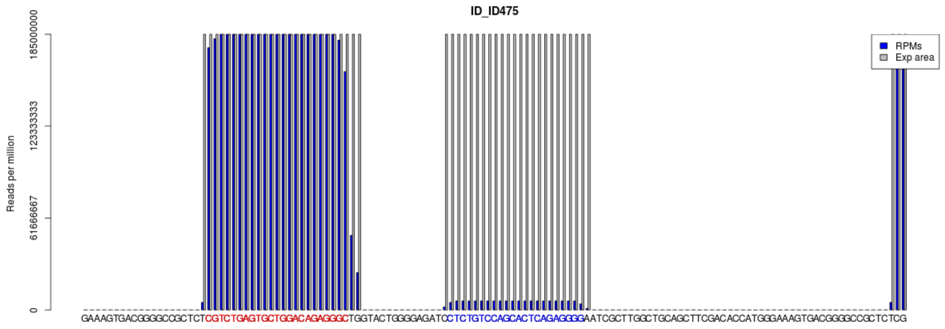

>ID476

AATAAATGCGGAGTCACTCTCATCTGGGTACTGGACAGATCAGTAGCCCAGAGAT**CCTCTGTCCAGCACTCAGAGGGG**AGTTGCTTGGGC
ACATCATCGACATCATGGGACAA

>ID476-3P

CCTCTGTCCAGCACTCAGAGGGG

>miRNA precursor

AAUAAAUGCGGAGUCACUCUCAUCUGGGUACUGGACAGAUCAGUAGCCCAGAGAU**CCUCUGUCCAGCACUCAGAGGGG**AGUUGCUUGGGCACAUCAUCGACAUCAUGGGACAA

```
......(((.((((.((((((.(((((((.(((((((((.................))))))))).))))))).)))))).))))..))).....................
```

>Predicted STAR seq:

TCATCTGGGTACTGGACAGA

Complementarity: 17

Initial dG =   -39.40

```
                        10         20        30        40
AAUAAA----------------   G-|   C      A       A         UCAGUAGC
                      UGC  GAGU ACUCUC UCUGGGU CUGGACAGA        C
                      ACG  UUCG UGAGGG AGACUCA GACCUGUCU        C
AACAGGGUACUACAGCUACUAC   GG^   U      G       C         CCUAGAGA
 110       100        90        80        70        60        50
```

Reads in: lib-42, lib-45, lib-47, lib-48, lib-49, lib-50, lib-72

Number of reads="ID476",0,0,0,26,3,0,0

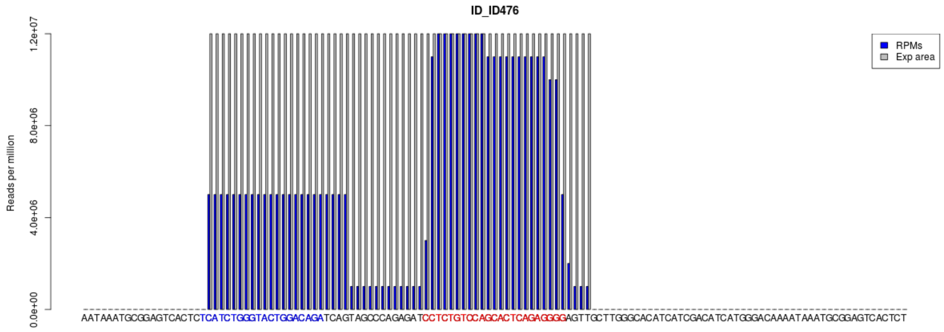

>ID477
AATAAATGCGGAGTCACTCT**CGTCTGGGTGCTGGACAGATCAG**TAGCACAGAGAGATCCTCTGTCCAGCACTCAGATGGGAGTTGCTTGG
GCACATCTTCAACATCGTGGGA

>ID477-5P
CGTCTGGGTGCTGGACAGATCAG

>miRNA precursor
AAUAAAUGCGGAGUCACUCU**CGUCUGGGUGCUGGACAGAUCAG**UAGCACAGAGAGAUCCUCUGUCCAGCACUCAGAUGGGAGUUGCUUGGGCACAUCUUCAACAUCGUGGGA
......(((.((((.((((((((((((((((((((((((....................)))))))))))))))))))))))).))))..)))...................

>Predicted STAR seq:
TGTCCAGCACTCAGATGGG

Complementarity: 17

Initial dG =   -49.80

```
                     10         20        30        40
AAUAAA-------------   G-|   C                        UCAGUAGCA
                   UGC  GAGU ACUCUCGUCUGGGUGCUGGACAGA         C
                   ACG  UUCG UGAGGGUAGACUCACGACCUGUCU         A
AGGGUGCUACAACUUCUAC   GG^   U                        CCUAGAGAG
110       100       90         80        70        60        50
```

Reads in: lib-42, lib-45, lib-47, lib-48, lib-49, lib-50, lib-72
Number of reads="ID477",1,5,2,205,40,1,0

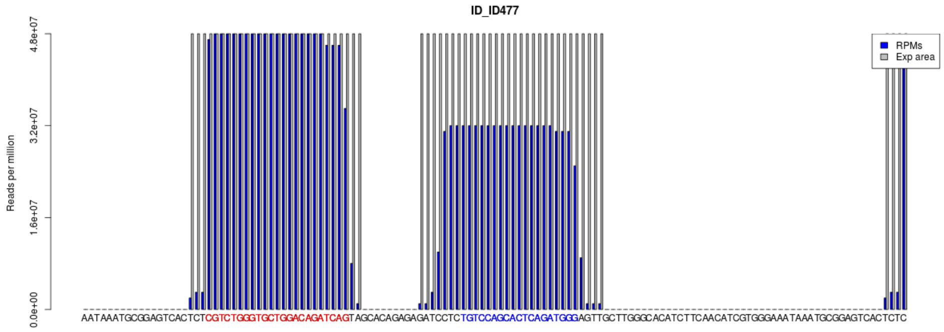

>ID478
TTCGATGGCAGAAAATGGCTGGGACGCTCTCGGACTGCGTGCTGGACAGCTGACTGGCAGTTGGGAGATC**AGCTGTCGGGCACGCAGAGG**
GAAGTGGCCCGGCCGCAGCT

>ID478-3P
AGCTGTCGGGCACGCAGAGGGA

>miRNA precursor
UUCGAUGGCAGAAAAUGGCUGGGACGCUCUCGGACUGCGUGCUGGACAGCUGACUGGCAGUUGGGAGAUC**AGCUGUCGGGCACGCAGAGGGA**AGUGGCCCGGCCGCAGCU
```
..............((((((((.(((((((...(((((((((.(((((((((((..........)).))))))))).))))))))).)))).))).))))))))).....
```

>Predicted STAR seq:
CTCGGACTGCGTGCTGGACAGCT

Complementarity: 18

Initial dG =   -57.90

```
        10         20          30         40         50          60
UUCGAUGGCAGAAAA        A    -|   GGA          G          -   GGCA
               UGGCUGGG CGC UCUC   CUGCGUGCU GACAGCUGA CU    G
               GCCGGCCC GUG AGGG   GACGCACGG CUGUCGACU GA    U
UCGAC----------        G   A^   A--          G          A   GGGU
.                 100        90          80         70
```

Reads in: lib-42, lib-45, lib-47, lib-48, lib-49, lib-50, lib-72
Number of reads="ID478",0,0,0,74,5,0,0

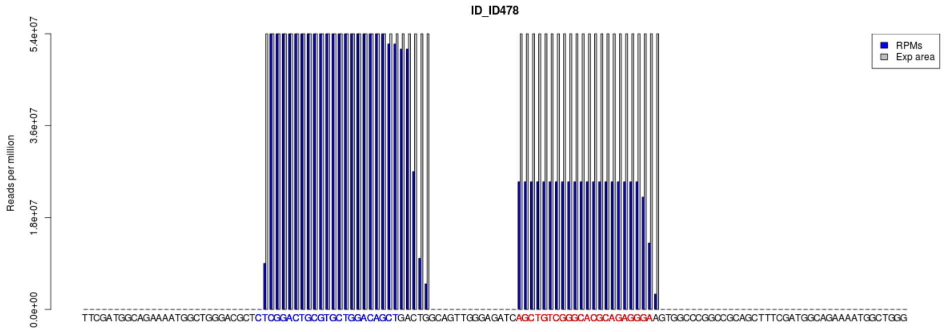

>ID498

GGATGAGATGGTCATCTTCG**CCTTCATCTCCCTAGCAAGACT**GGTTAAAATTGTAAGCCTTGCTTGTGGGATGAAGGTGGAGCTCACCGT
CTGGACATCGTCGACGGGACTG

>ID498-5P

CCTTCATCTCCCTAGCAAGACT

>miRNA precursor

GGAUGAGAUGGUCAUCUUCG**CCUUCAUCUCCCUAGCAAGACU**GGUUAAAAUUGUAAGCCUUGCUUGUGGGAUGAAGGUGGAGCUCACCGUCUGGACAUCGUCGACGGGACUG
.((((((((((...(((((((((((((((...((((((.((.............)).))))))...)))))))))))))))...))))))))...))))(((.....)))..

>Predicted STAR seq:

CCTTGCTTGTGGGATGAAGGTG

Complementarity: 16

Initial dG =   -45.40

```
             10        20        30        40
.-G    ---|      CAU               CCU      A  GGUUAA
   GAUG   AGAUGGU   CUUCGCCUUCAUCUC   AGCAAG CU      \
   CUAC   UCUGCCA   GAGGUGGAAGUAGGG   UCGUUC GA      A
\ -    AGG^      CUC               UGU      C  AUGUUA
           90        80        70        60        50



  100
  --   GA
    GUC  \
    CAG  C
  GU   GG
  110
```

Reads in: lib-42, lib-45, lib-47, lib-48, lib-49, lib-50, lib-72

Number of reads="ID498",81,14,1,1485,23,1,0

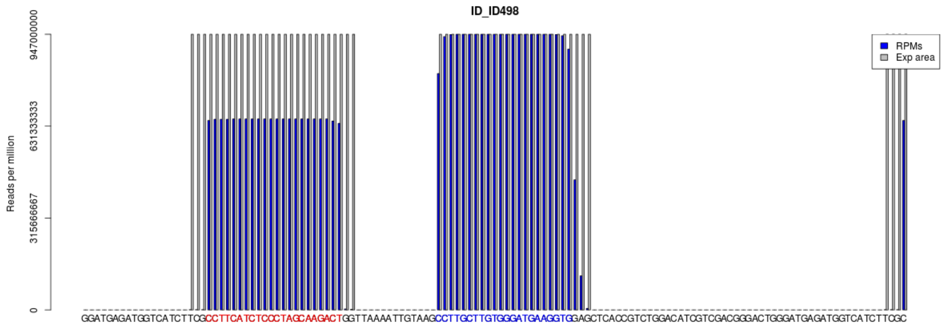

>ID5
GAAAGTGGCGGGGTTGCTCT**CATCTGAATGCTGGACAGAGGA**CCAGATCTGGGGAGCTCCTCTGTCTAGCACTCAGAGGGGAGCTGCTTG
GCTATAGCGTCGACACCGTGG

>ID5-5P
CATCTGAATGCTGGACAGAGGA

>miRNA precursor
GAAAGUGGCGGGGUUGCUCU**CAUCUGAAUGCUGGACAGAGGA**CCAGAUCUGGGGAGCUCCUCUGUCUAGCACUCAGAGGGGAGCUGCUUGGCUAUAGCGUCGACACCGUGG
....(((((.((((.((((((.(((((.((((((((((((((((((....))).....))))))))))))))).))))).)))))).)))).))))).(((.......)))..

>Predicted STAR seq:
CTCTGTCTAGCACTCAGAGGGG

Complementarity: 18

Initial dG = -57.60

```
                    10        20        30        40
----------  AA--     G    U       A     A              -----|  G
          GA    GUGGC GGGU GCUCUC UCUGA UGCUGGACAGAGGA     CCA A
          CU    UAUCG UUCG CGAGGG AGACU ACGAUCUGUCUCCU     GGU U
GGUGCCACAG  GCGA     G    U       G     C              CGAGG^  C
 .       100       90        80        70        60        50
```

Reads in: lib-42, lib-45, lib-47, lib-48, lib-49, lib-50, lib-72
Number of reads="ID5",0,3,7,95,27,0,0

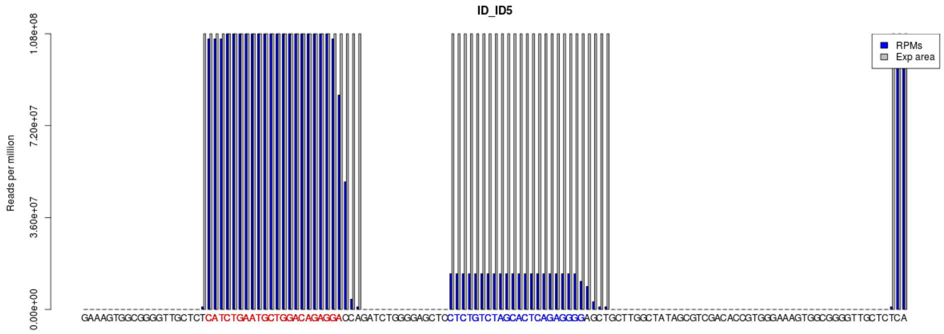

>ID50
GCGCGTAGCGCCTCATGTTC**ATTGTACTTCATCAGGTGCTCTGG**TGTGACGGAACCCGGCACCTGTTGGAGTGCAATTGATAGGAAGGCG
CCCATCCACGCGGCCGGCACTGCT

>ID50-5P
ATTGTACTTCATCAGGTGCTCTGG

>miRNA precursor
GCGCGUAGCGCCUCAUGUUC**AUUGUACUUCAUCAGGUGCUCUGG**UGUGACGGAACCCGGCACCUGUUGGAGUGCAAUUGAUAGGAAGGCGCCCAUCCACGCGGCCGGCACUGCU
.(((((.(((((((.((((.(((((((((((.((((((((..(((........))).)))))))).))))))))))).)))).)).)))))......))))).........,..

>Predicted STAR seq:
CGGCACCTGTTGGAGTGCAATTGA

Complementarity: 19

Initial dG = -52.00

```
                        10          20         30         40
G-----------     A-----     -| A     C           U         CU    GUG
            CGCGU      GCGCC UC UGUU AUUGUACUUCA CAGGUGCU  GGU    A
            GCGCA      CGCGG AG AUAG UAACGUGAGGU GUCCACGG  CCA    C
UCGUCACGGCCG     CCUACC     A^ G     U           U         C-    AGG
  110       100        90         80         70         60          50
```

Reads in: lib-42, lib-45, lib-47, lib-48, lib-49, lib-50, lib-72
Number of reads="ID50",10507,11726,39971,7739,26477,9749,0

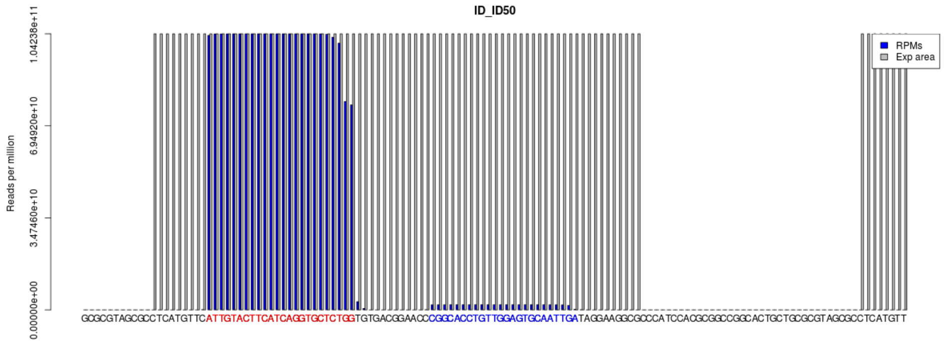

>ID501
ACTTTATATAGTCGGTATGGTTTCTGGAGCGTGAAGAAGCATCACCAGGGGGGTCGTGTGTTGGTCTG**ACCACTGGTGATGCGTCTCAAC**
GTTCCTCAGACCTCTTCATCCA

>ID501-3P
ACCACTGGTGATGCGTCTCAACG

>miRNA precursor
ACUUUAUAUAGUCGGUAUGGUUUCUGGAGCGUGAAGAAGCAUCACCAGGGGGGUCGUGUGUUGGUCUG**ACCACUGGUGAUGCGUCUCAACG**UUCCUCAGACCUCUUCAUCCA
(((......))).((...(((((..(((((((..(((.((((((((((((((..((.....))..))..)).))))))))))).)))..))))))).)))))......)).

>Predicted STAR seq:
GTGAAGAAGCATCACCAGGG

Complementarity: 16

Initial dG =   -47.30

```
        10        20        30        40         50
ACUUUAUAUAGUC  UAU     CU       GA   A          -  --| GU  UG
             GG   GGUUU  GGAGCGU  AGA GCAUCACCAG GG  GG  CG  \
             CU   CCAGA  CCUUGCA  UCU CGUAGUGGUC CC  UC  GU  U
ACCUA--------  UCU     CU       AC   G          A  AG^ UG  UG
110               100        90        80       70         60
```

Reads in: lib-42, lib-45, lib-47, lib-48, lib-49, lib-50, lib-72
Number of reads="ID501",29,5,0,0,0,0,0

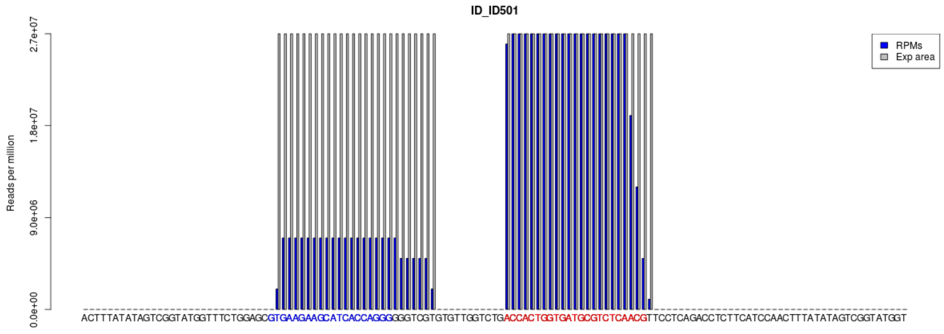

>ID511

CACACCCGCTGCTTCGGGGTCCGGTGGCCATGACGAATTGATGCCAGTATTGGAATCATC**AAAACGTCGTAGCTGCTTTGGCAC**TCCGGG
ACAGCAAGCACTCAACGTCTAG

>ID511-3P

AAAACGTCGTAGCTGCTTTGGCAC

>miRNA precursor

CACACCCGCUGCUUCGGGGUCCGGUGGCCAUGACGAAUUGAUGCCAGUAUUGGAAUCAUC**AAAACGUCGUAGCUGCUUUGGCAC**UCCGGGACAGCAAGCACUCAACGUCUAG

```
.......((((..(((((((((((..((.((((((..((((((((......))...))))))..)))))).))..))..)).)))))))..)))).................
```

>Predicted STAR seq:

TCCGGTGGCCATGACGAATT

Complementarity: 14

Initial dG =   -42.40

```
                  10        20           30        40
CACACCC----------    CU       -  --  UG  C       AA      ---| AG
                 GCUG  UCGGGGU CC  GG  GC AUGACG  UUGAUG   CC  U
                 CGAC  GGCCUCA GG  UC  CG UGCUGC  AACUAC   GG  A
GAUCUGCAACUCACGAA    AG       C  UU  GU  A       AA      UAA^ UU
110       100        90        80        70        60        50
```

Reads in: lib-42, lib-45, lib-47, lib-48, lib-49, lib-50, lib-72

Number of reads="ID511",6,3,0,254,10,0,0

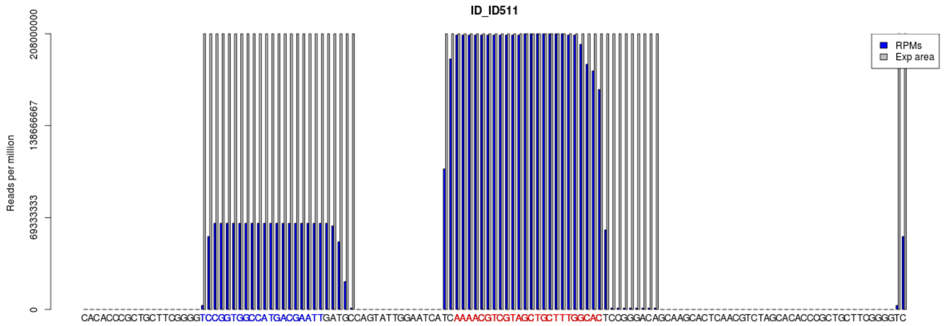

>ID513
GAGGTGATACTGCCCTACAGCTGGCCTGGGGTCCAGCAGACATGGCGGCTTGTTGCCAGTATTGGAATCA**TCAAACGTCATAGCTGCTTT**
GGTACTCCAGGTCAGCAAGCACTCA

>ID513-3P
TCAAACGTCATAGCTGCTTTGGTA

>miRNA precursor
GAGGUGAUACUGCCCUACAGCUGGCCUGGGGUCCAGCAGACAUGGCGGCUUGUUGCCAGUAUUGGAAUCA**UCAAACGUCAUAGCUGCUUUGGUA**CUCCAGGUCAGCAAGCACUCA
```
..(((......))).....((((((((((((((((((..((((((...((...((......))...))).....))))))..)))).))).)))))))))))))).........
```

>Predicted STAR seq:
CCAGCAGACAUGGCG

Complementarity: 12
ERROR 4: Strange structure
Initial dG = -48.80

```
.-GA|  GA
    GGU  U
    CCG  A
\ --^  UC
       10
```

```
          20         30            40         50           60
  CUACA----              -    --     AC       GCU--  UUG   AG
           GCUGGCCUGGGGU CCA  GCAG  AUGGCG      UG   CC  U
           CGACUGGACCUCA GGU  CGUC  UACUGC      AC   GG  A
  ACUCACGAA             U    UU     GA       AAACU  UAA   UU
     110        100        90         80         70
```

Reads in: lib-42, lib-45, lib-47, lib-48, lib-49, lib-50, lib-72
Number of reads="ID513",18,6,2,488,74,0,0

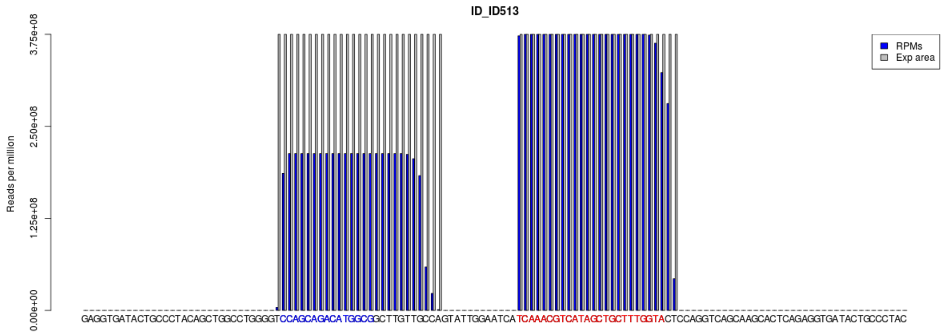

>ID514
GAGGTGATACTACCCAACAGCTGCCCCGGGGTCCAACAGCTATGACGGATTGTTGCCGGTATGGGAATCA**TCAATACGTGTAGCTGCTTT**
GGAATTCCGGGTCAGCAAACACTCA

>ID514-3P
TCAATACGTGTAGCTGCTTTGGAA

>miRNA precursor
GAGGUGAUACUACCCAACAGCUGCCCCGGGGUCCAACAGCUAUGACGGAUUGUUGCCGGUAUGGGAAUCA**UCAAUACGUGUAGCUGCUUUGGAA**UUCCGGGUCAGCAAACACUCA
```
..((((....)))).....((((.((((((((((((((((((.(((.((((.((((.((((......))...)).)))).)))))))))))..)))).)))))))).)))).........
```

>Predicted STAR seq:
CCAACAGCTATGACGGATTG

Complementarity: 17
ERROR 4: Strange structure
Initial dG =   -43.40

```
.-GA|   A
    GGUG U
    CCAU A
\ --^   C
       10


          20        30            40        50           60
  CAACA----    C        -    --       G   G    U  ---  GG
           GCUG CCCGGGGU CCAA  CAGCUAU ACG AUUG UG   CC  U
           CGAC GGGCCUUA GGUU  GUCGAUG UGC UAAC AC   GG  A
  ACUCACAAA    U        A    UC       -   A    U  UAA  GU
     110       100        90        80         70
```

Reads in: lib-42, lib-45, lib-47, lib-48, lib-49, lib-50, lib-72
Number of reads="ID514",78,51,18,3967,410,3,0

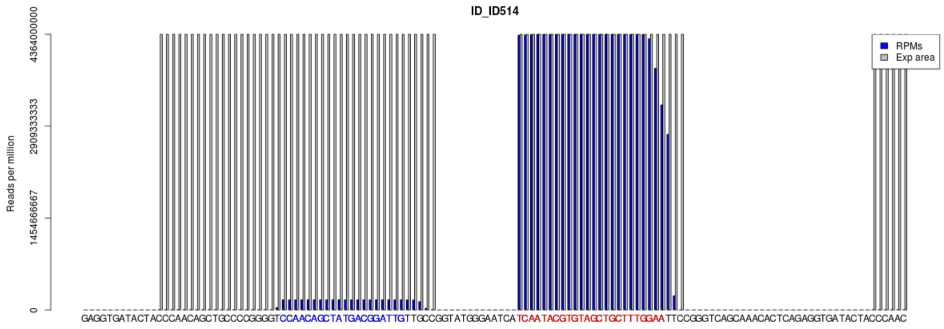

>ID515

TTATCACACGCTGTAGTGCA**AAAAGCACCGACGAATTTACCT**GTGGATACGAAATGGTAAAACCGACTGTGCTTTTGATGCTGCAGCGAA
GCTCTGACCCCTTTAACCCTCA

>ID515-5P

AAAAGCACCGACGAATTTACCT

>miRNA precursor

UUAUCACACGCUGUAGUGCA**AAAAGCACCGACGAAUUUACCU**GUGGAUACGAAAUGGUAAAACCGACUGUGCUUUUGAUGCUGCAGCGAAGCUCUGACCCCUUUAACCCUCA

```
........((((((((((..((((((((...((..((((((..((....))....))))))..))...))))))))..))))))))))........................
```

>Predicted STAR seq:

TAAAACCGACTGTGCTTTTGATG

Complementarity: 14

Initial dG =   -33.90

```
                        10        20        30        40
UUAUCACA----------------          CA        CGA  AA       UG--| G
                        CGCUGUAGUG  AAAAGCAC   CG  UUUACC    UG A
                        GCGACGUCGU  UUUUCGUG   GC  AAAUGG    GC U
ACUCCCAAUUUCCCCAGUCUCGAA          AG        UCA  CA       UAAA^ A
110       100       90        80        70        60        50
```

Reads in: lib-42, lib-45, lib-47, lib-48, lib-49, lib-50, lib-72

Number of reads="ID515",7,16,0,994,29,0,0

\---

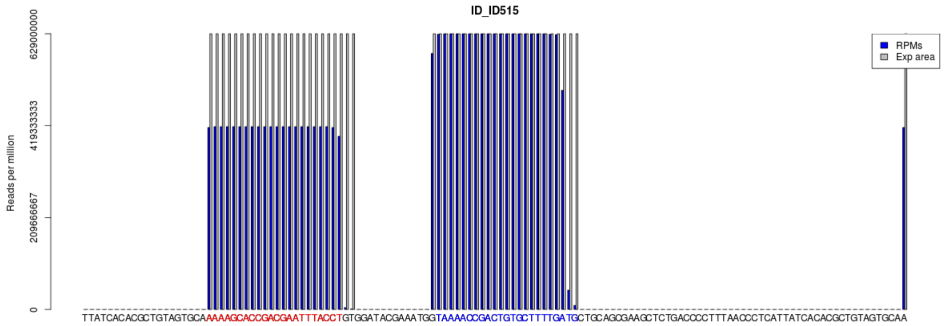

>ID516
ACTTCTGTATTGATTATTAGAGGTACTCCGTCTTATGCTGACTGTCAGTTAATCAGC**CAGCATAAGACAGATTACTTCT**TATAATCAATA
TCAACAGCTGCTGCGGAAGAT

>ID516-3P
CAGCATAAGACAGATTACTTCT

>miRNA precursor
ACUUCUGUAUUGAUUAUUAGAGGUACUCCGUCUUAUGCUGACUGUCAGUUAAUCAGC**CAGCAUAAGACAGAUUACUUCU**UAUAAUCAAUAUCAACAGCUGCUGCGGAAGAU
.((((.(((((((((((.(((((((.((.(((((((((((.(((.........))).))))))))))).)).))))))).)))))))))))).....((....)).))))..

>Predicted STAR seq:
AGAGGTACTCCGTCTTATGCTG

Complementarity: 18

Initial dG =   -46.30

```
           10        20        30        40
A-     .-U|         U       C  C           A   UCAG
  CUUC   GUAUUGAUUAU AGAGGUA UC GUCUUAUGCUG CUG    \
  GAAG   UAUAACUAAUA UCUUCAU AG CAGAAUACGAC GAC    U
UA     \ -^         U       U  A           C   UAAU
 .       90        80        70        60        50
```

Reads in: lib-42, lib-45, lib-47, lib-48, lib-49, lib-50, lib-72
Number of reads="ID516",2,10,1,387,43,0,0

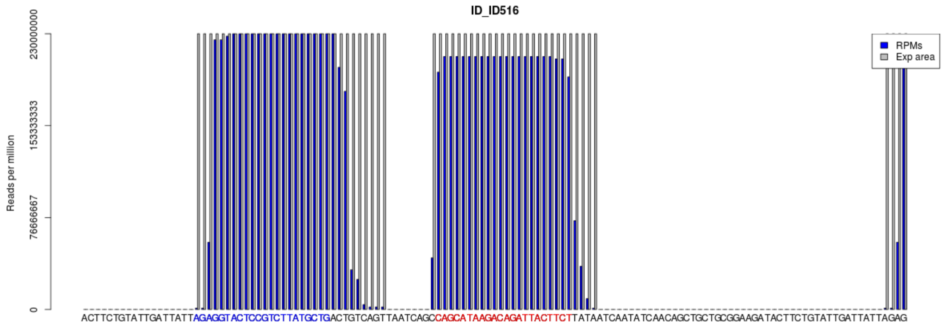

>ID517
AAGTTGATACTGCCCTACAGCTGGCCTGGGGTCCAGCAGACATGGCGGCTTGTTGCCCGTATTGGAATCA**TCAAACGTCATAGCTGCTTTGGTA**CTCCAGGTCAGCATGAACTCA

>ID517-3P
TCAAACGTCATAGCTGCTTTGGTA

>miRNA precursor
AAGUUGAUACUGCCCUACAGCUGGCCUGGGGUCCAGCAGACAUGGCGGCUUGUUGCCCGUAUUGGAAUCA**UCAAACGUCAUAGCUGCUUUGGUA**CUCCAGGUCAGCAUGAACUCA
.(((....)))......((((((((((((((((((((((..((((((..(((.((.((.....))...)).))).))))))..))))..))).))))))))))))).))......

>Predicted STAR seq:
CCAGCAGACATGGCGGCTTG

Complementarity: 15
ERROR 4: Strange structure
Initial dG = -46.20

```
.-A|  U
   AGU G
   UCA A
\ -^  U
   10


          20        30           40        50          60
  GCCCUA  -             -   --    AC       GC    U  C--  GU
        CA GCUGGCCUGGGGU CCA  GCAG  AUGGCG  UUG UG   CC  \
        GU CGACUGGACCUCA GGU  CGUC  UACUGC  AAC AC   GG  A
  ACUCAA  A             U   UU    GA       A-    U  UAA  UU
     110       100        90        80         70
```

Reads in: lib-42, lib-45, lib-47, lib-48, lib-49, lib-50, lib-72
Number of reads="ID517",15,4,0,521,66,1,0

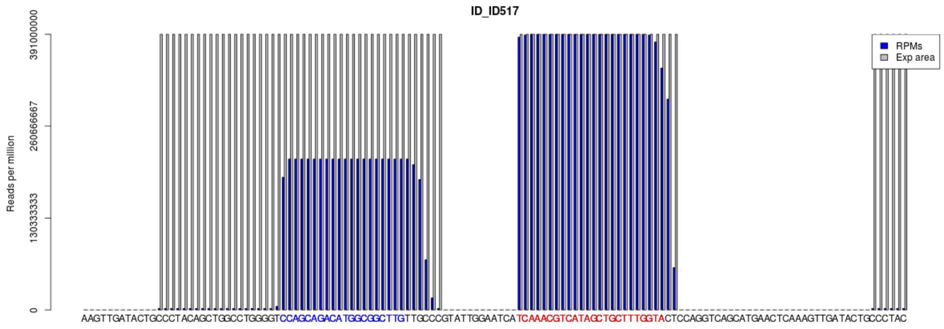

>ID518

GTTGATACTGCCCAATAGCTGACCTGGGGTCCAGCAGCCATGACGGATTTTTGCCAGTATGGGAATCATC**AAAACGTCGTAGCTGCTTTG**
GAATTCCGGGTCAGCAAACGCTCA

>ID518-3P

AAAACGTCGTAGCTGCTTTGGA

>miRNA precursor

GUUGAUACUGCCCAAUAGCUGACCUGGGGUCCAGCAGCCAUGACGGAUUUUUGCCAGUAUGGGAAUCAUC**AAAACGUCGUAGCUGCUUUGGA**AUUCCGGGUCAGCAAACGCUCA
((((........)))).((((((((((((((((((((.(((((((((..((.((.....)).)).)..))).....)))))).)))))..))).)))))))))))))).........

>Predicted STAR seq:

CAGCAGCCATGACG

Complementarity: 12
ERROR 4: Strange structure
 Initial dG =   -47.90

```
  |   AUA
--GUUG   C
  UAAC   U
\ ^   CCG
       10
```

```
            20        30            40             50
  A--------             -    --      C       ----   UU   G   AG
           GCUGACCUGGGGU CCA  GCAGC AUGACG    GAU  UU CC  \
           CGACUGGGCCUUA GGU  CGUCG UGCUGC    CUA  AA GG  U
  ACUCGCAAA             A    UU      A      AAAA   CU   G   UA
    110       100        90         80        70         60
```

Reads in: lib-42, lib-45, lib-47, lib-48, lib-49, lib-50, lib-72
Number of reads="ID518",2,2,0,174,19,0,0

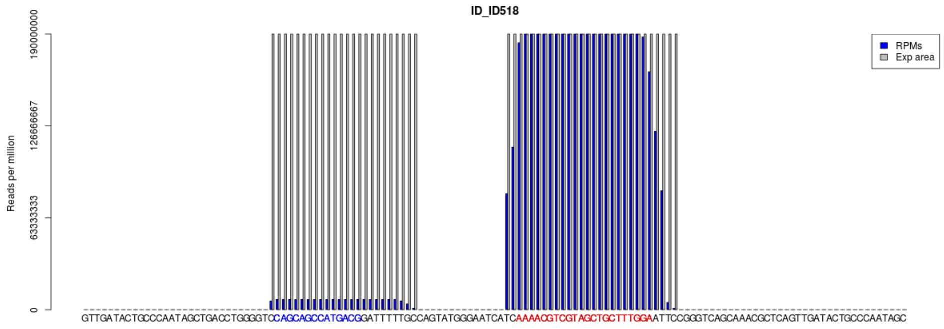

>ID520
AGTTGGAAACTAGCATAAA**TTCTCGAGTTTCCATCCGAGTG**ACGAAACTTCACTCGGATGGAAGCTCGAGAACTTTGTATTAGTTTCGCT
CGCCGAGAAAGCCGAGATCAA

>ID520-5P
TTCTCGAGTTTCCATCCGAGTG

>miRNA precursor
AGUUGGAAACUAGCAUAAA**UUCUCGAGUUUCCAUCCGAGUG**ACGAAACUUCACUCGGAUGGAAGCUCGAGAACUUUGUAUUAGUUUCGCUCGCCGAGAAAGCCGAGAUCAA
.....((((((((.((((((((((((((((((((((((((((((.......)))))))))))))))))))))))))).))))).)))))))).(((((.......).)))).....

>Predicted STAR seq:
CTCGGATGGAAGCTCGAGAACTT

Complementarity: 20

Initial dG =   -55.80

```
          10          20         30         40
.-AGUUG        C      -|                      CGA
       GAAACUAG AUAAA UUCUCGAGUUUCCAUCCGAGUGA    \
       CUUUGAUU UGUUU AAGAGCUCGAAGGUAGGCUCACU    A
\ -----        A      C^                      UCA
             80         70         60         50



       90
  G----    - CGA
       CUCG C   \
       GAGC G   G
  AACUA    C AAA
 110       100
```

Reads in: lib-42, lib-45, lib-47, lib-48, lib-49, lib-50, lib-72
Number of reads="ID520",0,3,1,45,3,0,0

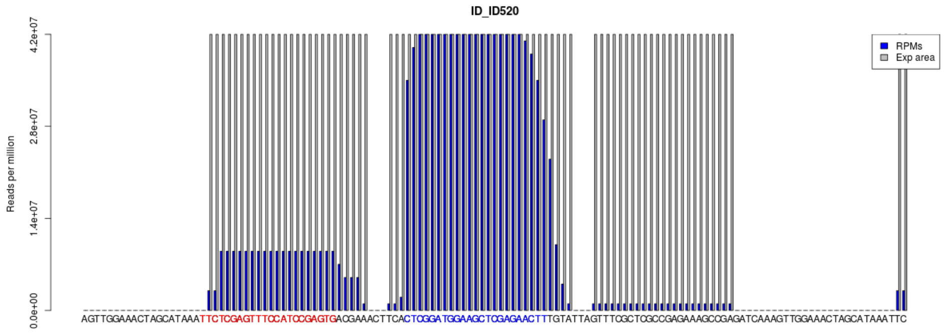

>ID521
AAAAGTACCGATGTCGTAG**TCTGGACCAGCCATTGCTCGTCA**ATATTCAGAAGCGAGCTGTGGCTGGTCTAGGCCATAACATTGGTCCTG
CAGCGCCCGAAGACATCAAACA

>ID521-5P
TCTGGACCAGCCATTGCTCGTCA

>miRNA precursor
AAAAGUACCGAUGUCGUAG**UCUGGACCAGCCAUUGCUCGUCA**AUAUUCAGAAGCGAGCUGUGGCUGGUCUAGGCCAUAACAUUGGUCCUGCAGCGCCCGAAGACAUCAAACA
```
...((.((((((((..(.((((((((((((((((.((((((...........)))))).))))))))))))))))).)..)))))))).)).......................
```

>Predicted STAR seq:
GAGCTGTGGCTGGTCTAGGCC

Complementarity: 18

Initial dG =   -50.80

```
                            10        20        30        40
AAA--------------------| U        CG A               U      CAAUA
                       AG ACCGAUGU  U GUCUGGACCAGCCAU GCUCGU     U
                       UC UGGUUACA  A CGGAUCUGGUCGGUG CGAGCG     U
ACAAACUACAGAAGCCCGCGACG^ C        AU C               U      AAGAC
110       100       90        80        70        60        50
```

Reads in: lib-42, lib-45, lib-47, lib-48, lib-49, lib-50, lib-72
Number of reads="ID521",56,63,12,4032,519,5,0

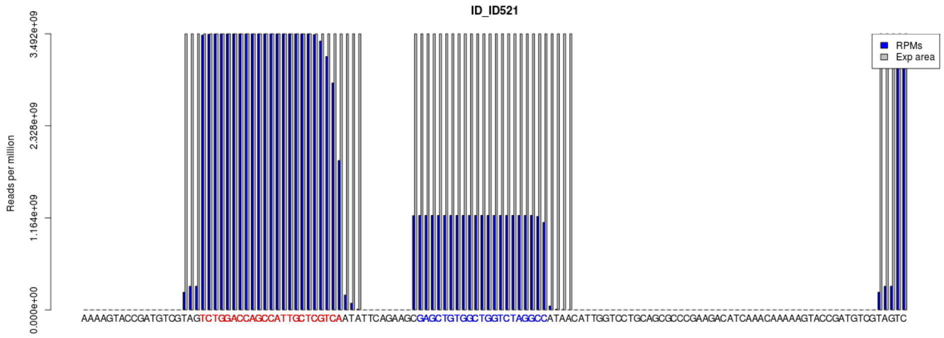

>ID522
GAAGAGACGATGCCCAACAGCTGCTCCGGGGCCCGGGAGCCATGACGAATTGTTGCCAGAATGGGAATCA**TCAAATCGTCGTAGCTGCTT
TGGC**CCCGGTGCAGCACGTGCTCA

>ID522-3P
TCAAATCGTCGTAGCTGCTTTGGC

>miRNA precursor
GAAGAGACGAUGCCCAACAGCUGCUCCGGGGCCCGGGAGCCAUGACGAAUUGUUGCCAGAAUGGGAAUCA**UCAAAUCGUCGUAGCUGCUUUGGC**CCCGGUGCAGCACGUGCUCA
```
........((.((......((((.((((((((..(((((.(((((((.(((((.((.....)).))....))).))))))).))).))..)))))))).)))))....)))).
```

>Predicted STAR seq:
CCCGGGAGCCATGACGAATTG

Complementarity: 16
ERROR 4: Strange structure
Initial dG =   -54.20

```
           10
GAA   -   .-AU|CC
   GAG ACG    G  \
   CUC UGC    C  C
A--   G   \ --^AA
    110
```

```
            20        30         40        50
            A     U        CG  -   C       A   ----  G  AG
             GCUGC CCGGGGCC  GG AGC AUGACGA UUG    UU CC  \
             CGACG GGCCCCGG  UC UCG UGCUGCU AAC    AA GG  A
            A     U        UU  G   A       A   UACU  G  UA
                100        90         80        70
```

Reads in: lib-42, lib-45, lib-47, lib-48, lib-49, lib-50, lib-72
Number of reads="ID522",88,55,75,5280,418,8,0

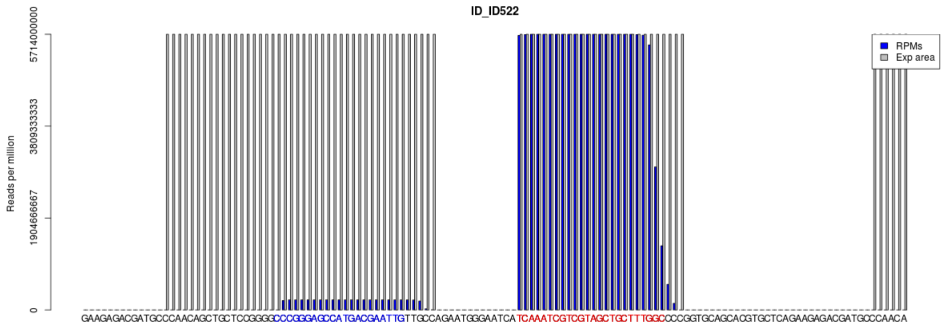

>ID523
GGATATACTGTACAACAGCTGCCCTGGGGTCCGGTGGCCATGACGGATTGATGCCAGTATTGGAATCATC**AAAACGTCGTAGCTGCTTTG**
GAACTCCGGGACAGCAACAACTCAA

>ID523-3P
AAAACGTCGTAGCTGCTTTGGAAC

>miRNA precursor
GGAUAUACUGUACAACAGCUGCCCUGGGGUCCGGUGGCCAUGACGGAUUGAUGCCAGUAUUGGAAUCAUC**AAAACGUCGUAGCUGCUUUGGAAC**UCCGGGACAGCAACAACUCAA
................((((.((((((((((((..((.((((((..((((((((......))...)))))))..)))))).))..))..))).))))))).)))).........

>Predicted STAR seq:
TCCGGTGGCCATGACGGATT

Complementarity: 14

Initial dG = -47.20

```
        10        20         30          40        50
GGAUAUACUGUACAACA    C       -   --  UG  C      GA      ---| AG
                 GCUG CCUGGGG UCC  GG  GC AUGACG  UUGAUG   CC  U
                 CGAC GGGCCUC AGG  UC  CG UGCUGC  AACUAC   GG  A
AACUCAACAA-------    A       A   UU  GU  A      AA      UAA^ UU
   110              100        90         80        70        60
```

Reads in: lib-42, lib-45, lib-47, lib-48, lib-49, lib-50, lib-72
Number of reads="ID523",11,6,1,323,23,0,0

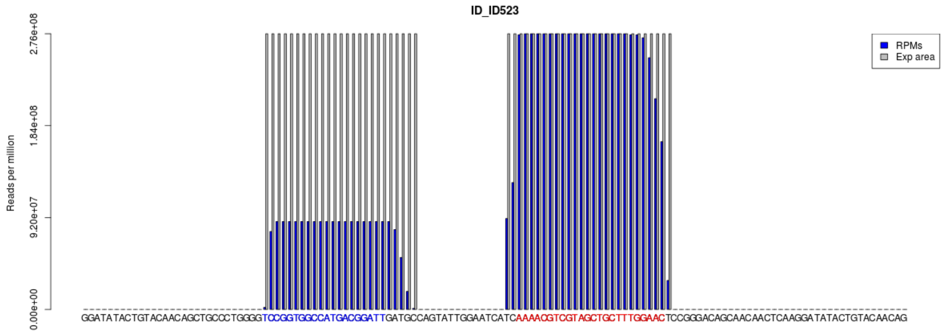

>ID524
GCACAACACTGCCTCGGGG**TCCGGTGGCAATGACGGATTGA**TGCTAGTATTGGAATCATCAAAACGTCGTAGCTGCTGTGGGACTCCGGG
ACAGCAACAACTCAACGGCCA

>ID524-5P
TCCGGTGGCAATGACGGATTGA

>miRNA precursor
GCACAACACUGCCUCGGGG**UCCGGUGGCAAUGACGGAUUGA**UGCUAGUAUUGGAAUCAUCAAAACGUCGUAGCUGCUGUGGGACUCCGGGACAGCAACAACUCAACGGCCA
........(((.((((((((((.(..(((((((((..(((((((((....)))...))))))..))))))...)))..).))))))))).))).................

>Predicted STAR seq:
AAAACGTCGTAGCTGCTGTGGGACT

Complementarity: 15

Initial dG = -43.30

```
                 10        20            30        40
GCACAACA---------   C          G UG   ---      GA      ---|  G
                 CUG CUCGGGGUCC G  GCA   AUGACG  UUGAUG   CUA U
                 GAC GGGCCUCAGG U  CGU   UGCUGC  AACUAC   GGU A
ACCGGCAACUCAACAAC   A          G GU   CGA      AA      UAA^  U
 .       100        90        80        70        60        50
```

Reads in: lib-42, lib-45, lib-47, lib-48, lib-49, lib-50, lib-72
Number of reads="ID524",3,2,0,189,15,1,0

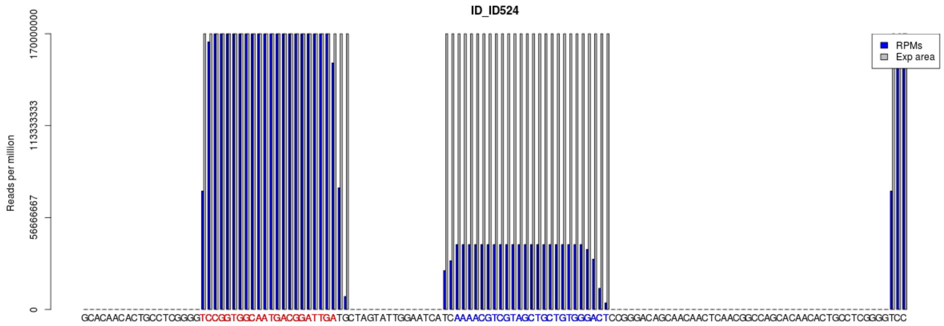

>ID525
GAGGTGGTACTACCCAACAGCTGATCCGGTGTCCAGCAGCTATGACGGATTGTTGCCAGAATGGGAATCA**TCAATACACGTAGCTGCTTTGGA**ATTCCGGGTCAGCAAACACT

>ID525-3P
TCAATACACGTAGCTGCTTTGGA

>miRNA precursor
GAGGUGGUACUACCCAACAGCUGAUCCGGUGUCCAGCAGCUAUGACGGAUUGUUGCCAGAAUGGGAAUCA**UCAAUACACGUAGCUGCUUUGGA**AUUCCGGGUCAGCAAACACU
..((((....))))).....((((((((((..((((((((((((((...(((.(((.((.....)).))).))).......)))))))))..)))))...))))))))))).......

>Predicted STAR seq:
GTCCAGCAGCTATGACGGA

Complementarity: 13
ERROR 4: Strange structure
Initial dG = -49.10

```
.-GA|   G
    GGUG U
    CCAU A
\ --^   C
       10


        20        30           40           50
  CAACA--          UG-    --         ACG---   U   G  AG
         GCUGAUCCGG   UCCA  GCAGCUAUG      GAU GUU CC  \
         CGACUGGGCC   AGGU  CGUCGAUGC      CUA UAA GG  A
  UCACAAA          UUA    UU         ACAUAA   C   G  UA
   110       100        90         80         70
```

Reads in: lib-42, lib-45, lib-47, lib-48, lib-49, lib-50, lib-72
Number of reads="ID525",8,13,1,1304,41,1,0

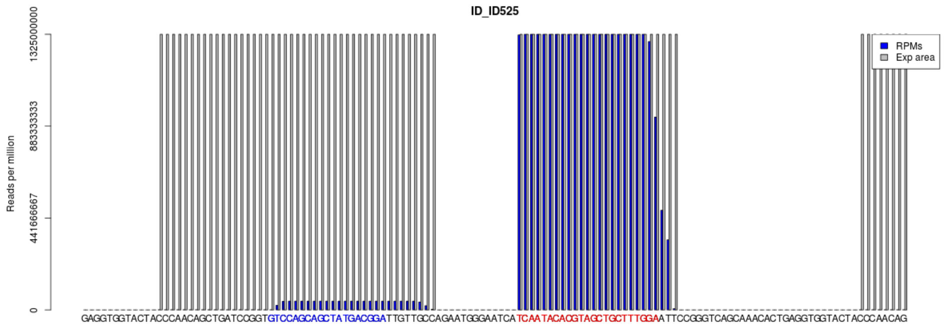

>ID526

CAGTTGGAAACTAGCATAAA**TTCTCGAGTTTCCATCCGAGTG**ACGAAACTTCACTCGGATGGAAGCTCGAGAACTTTGTATTAGTTTCGCTCGCCGAGAAAGCCGAGATCAA

>ID526-5P

TTCTCGAGTTTCCATCCGAGTG

>miRNA precursor

CAGUUGGAAACUAGCAUAAA**UUCUCGAGUUUCCAUCCGAGUG**ACGAAACUUCACUCGGAUGGAAGCUCGAGAACUUUGUAUUAGUUUCGCUCGCCGAGAAAGCCGAGAUCAA

```
......((((((((.(((((((((((((((((((((((((((((.......))))))))))))))))))))))))).))))).)))))))).(((((.......).)))).....
```

>Predicted STAR seq:

CTCGGATGGAAGCTCGAGAACTT

Complementarity: 20

Initial dG = -55.80

```
         10        20         30        40
.-CAGUUG        C     -|                      CGA
        GAAACUAG AUAAA UUCUCGAGUUUCCAUCCGAGUGA   \
        CUUUGAUU UGUUU AAGAGCUCGAAGGUAGGCUCACU   A
\ ------        A     C^                      UCA
               80        70        60        50



      90
  G----    - CGA
       CUCG C   \
       GAGC G   G
  AACUA    C AAA
  110       100
```

Reads in: lib-42, lib-45, lib-47, lib-48, lib-49, lib-50, lib-72

Number of reads="ID526",1,2,0,47,1,0,0

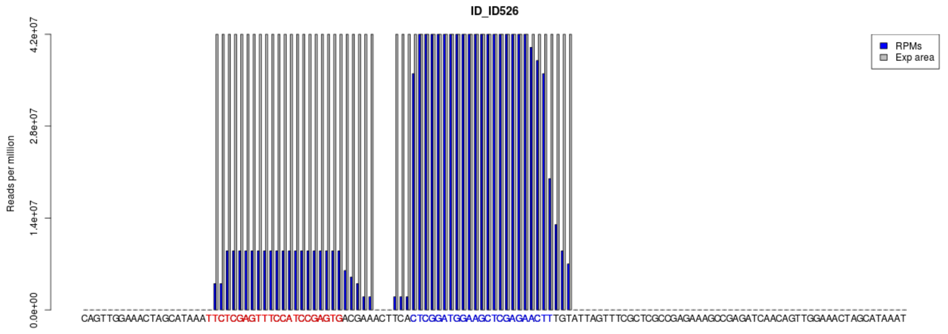

>ID527
GGTGATACTGCACAATAGCTGCCTCAGGG**TCCGGTGGCAATGACGGATTGA**TGTCAGTATTGGAATCATCAAAACGTTGTAGCTGCTTTA
GGACTCTGGGACAGCAAGCACTCAA

>ID527-5P
TCCGGTGGCAATGACGGATTGA

>miRNA precursor
GGUGAUACUGCACAAUAGCUGCCUCAGGG**UCCGGUGGCAAUGACGGAUUGA**UGUCAGUAUUGGAAUCAUCAAAACGUUGUAGCUGCUUUAGGACUCUGGGACAGCAAGCACUCAA
.(((......)))....((((.((((((((((((..((.(..(((..((((((............))))))..)))..).))..))...)))))))))).)))).........

>Predicted STAR seq:
AAAACGTTGTAGCTGCTTTAGGACT

Complementarity: 13

Initial dG =   -45.70

ERROR 3: Strange structure
Initial dG =   -45.70

```
.-G|  AU
   GUG  A
   CAC  C
\ -^  GU
     10


             20        30           40        50
  AAUA------    C          ---  UG  A UG   GA      UCAGUA
            GCUG CUCAGGGUCC   GG  GC A  ACG  UUGAUG      \
            CGAC GGGUCUCAGG   UC  CG U  UGC  AACUAC      U
  AACUCACGAA    A          AUU  GU  A GU   AA      UAAGGU
     110       100        90        80        70
```

Reads in: lib-42, lib-45, lib-47, lib-48, lib-49, lib-50, lib-72
Number of reads="ID527",6,0,0,208,7,0,0

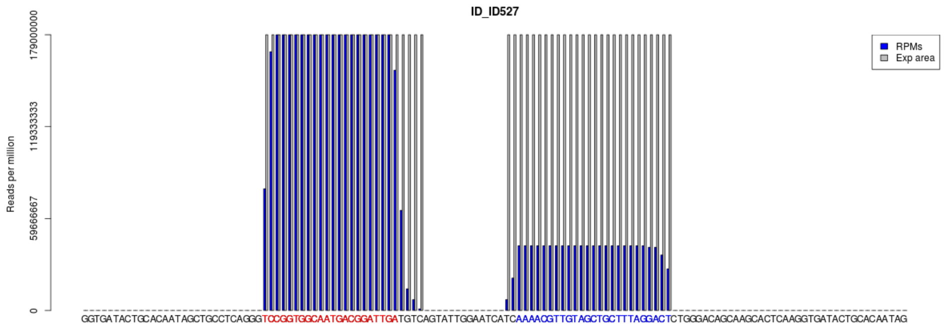

>ID528
TGATCACATGATGCTCCTT**TCTTCTGATGAGTGTGCTGTGGGT**GTTGAGGAATTCCCGCAGCACACTCATCAGGAGAGATGAGTAACATG
AATGAACGCATAATATTGTGTGA

>ID528-5P
TCTTCTGATGAGTGTGCTGTGGGT

>miRNA precursor
UGAUCACAUGAUGCUCCUU**UCUUCUGAUGAGUGUGCUGUGGGU**GUUGAGGAAUUCCCGCAGCACACUCAUCAGGAGAGAUGAGUAACAUGAAUGAACGCAUAAUAUUGUGUGA
...(((((((.(((((.((((((((((((((((((((((((((.(((....))).)))))))))))))))))))))))))).))))).)))).)))..))).((((((....)))))).

>Predicted STAR seq:
CGCAGCACACTCATCAGGAGAGA

Complementarity: 21

Initial dG =   -63.90

```
          10        20        30        40
.-UGAUCA|   A     C                        U   G
        CAUG UGCUC UUUCUUCUGAUGAGUGUGCUGUGGG GUU A
        GUAC AUGAG AGAGAGGACUACUCACACGACGCCC UAA G
\ ------^   A     U                        U   G
       90        80        70        60        50



         100
  AAUGAA      A
        CGCAUA U
        GUGUGU A
  A-----      U
        110
```

Reads in: lib-42, lib-45, lib-47, lib-48, lib-49, lib-50, lib-72
Number of reads="ID528",120,282,87,20221,1757,41,0

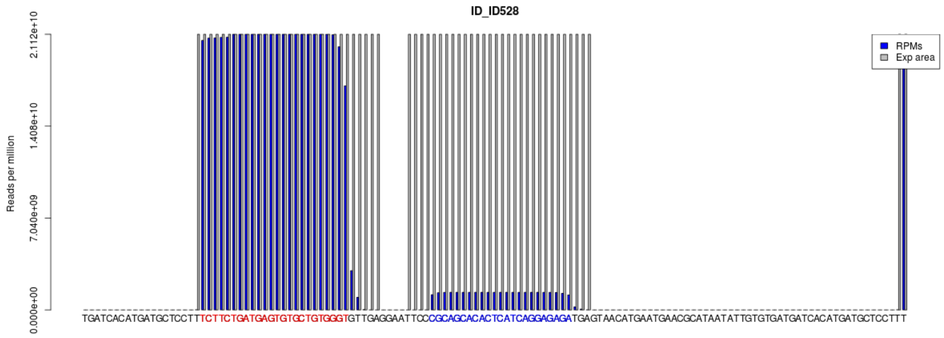

>ID529
CACAACAGCTGCCTCGGGGTCCGGTGGCAATGACGGATTGATGCCAGTATTGGAATCATCAAAACGTCGTAGCTGCTTTGGGACTTCGGG
GAGCAACCACTCAACGGCCAA

>ID529-5P
TCCGGTGGCAATGACGGATTGA

>miRNA precursor
CACAACAGCUGCCUCGGGGUCCGGUGGCAAUGACGGAUUGAUGCCAGUAUUGGAAUCAUCAAAACGUCGUAGCUGCUUUGGGACUUCGGGGAGCAACCACUCAACGGCCAA
.......(((.(((((((((((((..((.((((((..((((((((......))...))))))..))))))).))..))...)))))))))))))).................

>Predicted STAR seq:
AAAACGTCGTAGCTGCTTTGGGACT

Complementarity: 15

Initial dG = -47.20

```
                  10        20           30        40
CACAACA----------   G           ---  UG  A      GA      ---| AG
                 GCU CCUCGGGGUCC   GG  GC AUGACG  UUGAUG   CC  U
                 CGA GGGGCUUCAGG   UC  CG UGCUGC  AACUAC   GG  A
AACCGGCAACUCACCAA   -           GUU  GU  A      AA      UAA^ UU
 .       100         90        80        70        60        50
```

Reads in: lib-42, lib-45, lib-47, lib-48, lib-49, lib-50, lib-72
Number of reads="ID529",13,1,0,247,16,1,0

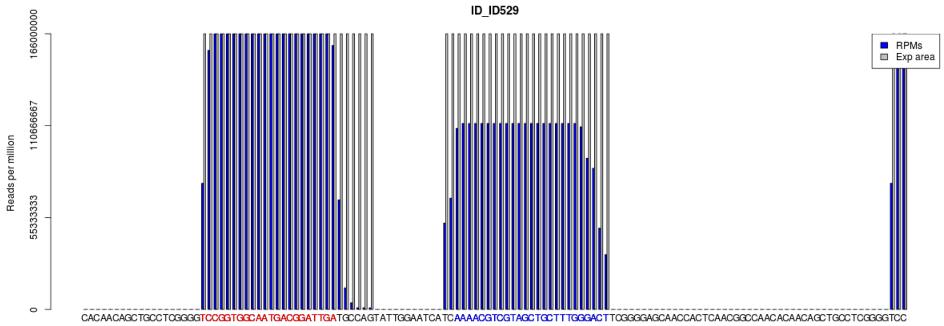

>ID530

ACAACAGCTGCCTCCGAGTC**CAGTGGCAATGACGGATTGATG**CCAGTATTGGAATCATCAAACGTCGTAGCTGCTTTGGGACTTTGGGAC
AGCAAGCACTCAACGGCCAGCA

>ID530-5P

CAGTGGCAATGACGGATTGATG

>miRNA precursor

ACAACAGCUGCCUCCGAGUC**CAGUGGCAAUGACGGAUUGAUG**CCAGUAUUGGAAUCAUCAAACGUCGUAGCUGCUUUGGGACUUUGGGACAGCAAGCACUCAACGGCCAGCA
......((((.(((.((((((((..((.((((((..((((((((......))...)))))).)))))).))..))...)))))).))).))))..((........)).....

>Predicted STAR seq:

TCAAACGTCGTAGCTGCTTTGGGA

Complementarity: 15

Initial dG =   -41.30

```
          10        20           30        40
.-ACAACA    C   C      ---  UG  A      GA       ---| AG
        GCUG CUC GAGUCC   AG  GC AUGACG  UUGAUG   CC  U
        CGAC GGG UUCAGG   UC  CG UGCUGC  AACUAC   GG  A
\ ------    A   U      GUU  GU  A      A-       UAA^ UU
          90        80        70          60        50



         100
  AA---  ACU
       GC   C
       CG   A
  ACGAC  GCA
  110
```

Reads in: lib-42, lib-45, lib-47, lib-48, lib-49, lib-50, lib-72

Number of reads="ID530",10,4,0,447,31,0,0

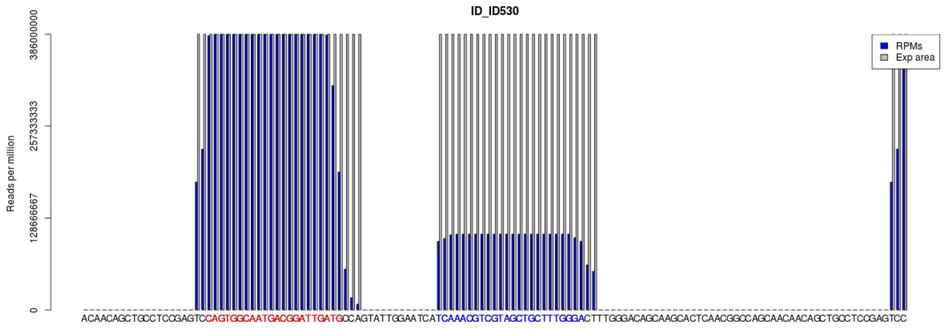

>ID531

GAGGTAGCGTTGCCCAACAGCTGTTCCGGGGCCCGGGAGCCATGACGAATTGTTGCCATTTTGGGAATCA**TCAAATCGTCGTAGCTGCTT**
TGGCCCCTGGGCAGCCAACGCTCC

>ID531-3P

TCAAATCGTCGTAGCTGCTTTGGC

>miRNA precursor

GAGGUAGCGUUGCCCAACAGCUGUUCCGGGGCCCGGGAGCCAUGACGAAUUGUUGCCAUUUUGGGAAUCA**UCAAAUCGUCGUAGCUGCUUUGGC**CCCUGGGCAGCCAACGCUCC

```
..((.(((((((.......(((((((.((((((..(((((.(((((((.(((((.((.....)).))....))).))))))).))).))..)))))).))))))))))))))))
```

>Predicted STAR seq:

CCCGGGAGCCAUGACGAAUUG

Complementarity: 16

Initial dG =   -54.50

```
        10        20        30         40        50
GA| U       CCCAACA       C      CG  -   C       A   ----  G  AU
  GG AGCGUUG       GCUGUUC GGGGCC  GG AGC AUGACGA UUG    UU CC  \
  CC UCGCAAC       CGACGGG CCCCGG  UC UCG UGCUGCU AAC    AA GG  U
--^ -       -------       U      UU  G   A       A   UACU  G  UU
     110              100        90        80        70
```

Reads in: lib-42, lib-45, lib-47, lib-48, lib-49, lib-50, lib-72

Number of reads="ID531",73,73,54,5255,463,7,0

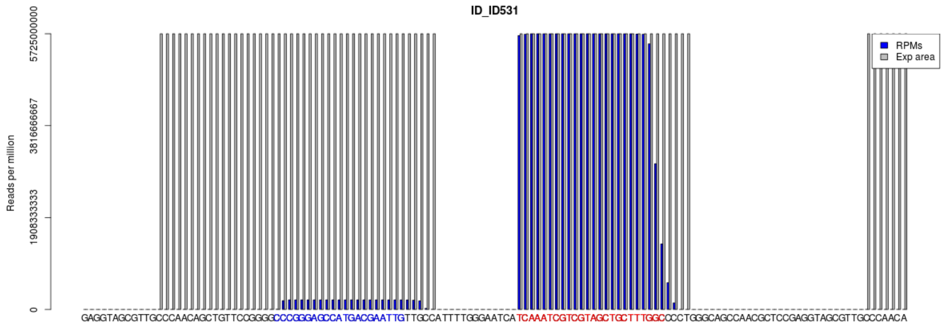

>ID533
CACAACAGCTGTCTCGGGGT**CCGGTGGCAATGACGGATTGAT**GCTAGTATTGGAATCATCAAAACGCCGTAGCTGCTTTGGGACTCCGGG
ACAGCAAGCACTCAACGTCCAC

>ID533-5P
CCGGTGGCAATGACGGATTGAT

>miRNA precursor
CACAACAGCUGUCUCGGGGU**CCGGUGGCAAUGACGGAUUGAU**GCUAGUAUUGGAAUCAUCAAAACGCCGUAGCUGCUUUGGGACUCCGGGACAGCAAGCACUCAACGUCCAC
```
.......((((((((((((((((..((.(((.((..(((((((((....)))...))))))..)).))).))..))...)))))))))))))))................
```

>Predicted STAR seq:
CAAAACGCCGTAGCTGCTTTGGGAC

Complementarity: 14

Initial dG =   -47.40

```
                  10        20          30        40
CACAACA----------               ---  UG  A   A  GA      ---|  G
                 GCUGUCUCGGGGUCC   GG  GC AUG CG  UUGAUG   CUA U
                 CGACAGGGCCUCAGG   UC  CG UGC GC  AACUAC   GGU A
CACCUGCAACUCACGAA               GUU  GU  A   C  AA      UAA^  U
110       100       90        80        70        60        50
```

Reads in: lib-42, lib-45, lib-47, lib-48, lib-49, lib-50, lib-72
Number of reads="ID533",3,2,0,212,9,0,0

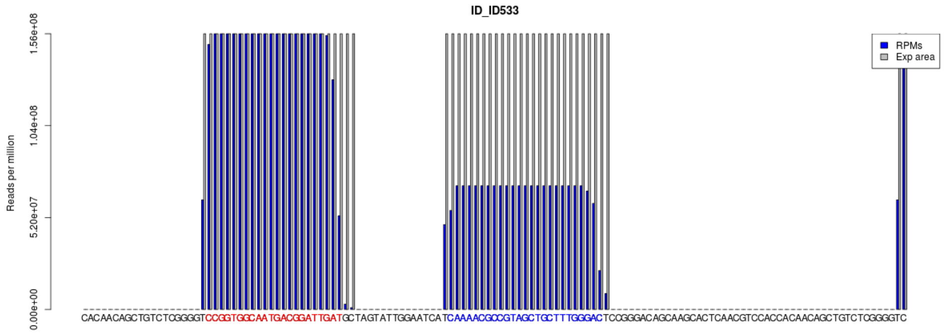

>ID534
ACAATAACTGCCTCGGAGTC**CAGTGGCAATGACGGATTGATG**CCAGTATTGGAATCATCAAACGTCGTAGCTGCTTTGGGACTTCGGGAC
AGCAAGCAGTCAACGGTCAGCA

>ID534-5P
CAGTGGCAATGACGGATTGATG

>miRNA precursor
ACAAUAACUGCCUCGGAGUC**CAGUGGCAAUGACGGAUUGAUG**CCAGUAUUGGAAUCAUCAAACGUCGUAGCUGCUUUGGGACUUCGGGACAGCAAGCAGUCAACGGUCAGCA
......((((((((((((((((..((.((((((..((((((((......))...)))))).)))))).))..))...)))))))))).......)))))...........

>Predicted STAR seq:
TCAAACGTCGTAGCTGCTTTGGGA

Complementarity: 15

Initial dG =   -42.10

```
              10                20            30         40
ACAAUA------     -------          ---  UG  A       GA       ---| AG
            ACUGC       CUCGGAGUCC   AG  GC AUGACG  UUGAUG   CC  U
            UGACG       GGGCUUCAGG   UC  CG UGCUGC  AACUAC   GG  A
ACGACUGGCAAC     AACGACA          GUU  GU  A       A-       UAA^ UU
110       100        90         80        70          60        50
```

Reads in: lib-42, lib-45, lib-47, lib-48, lib-49, lib-50, lib-72
Number of reads="ID534",9,4,0,461,30,0,0

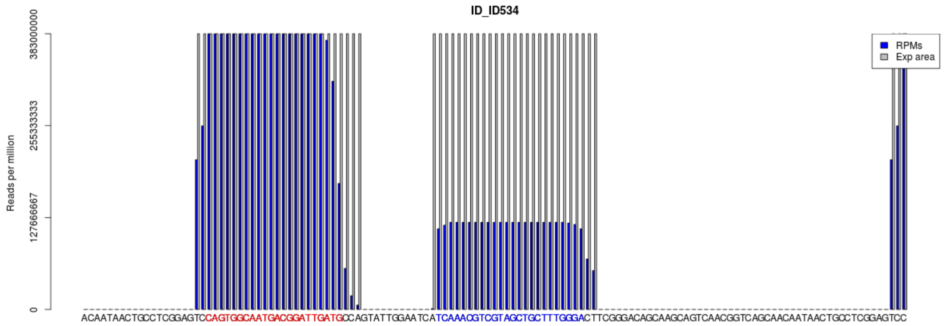

>ID537
AGAAAAAATGGCTCAGCTGTGTGTCTGTGCTTTCTTGTCATCTATCTTGCATCACTGTGAATTAAATAG**TGAAGCAGTAGAAGCCAAGAC**
AGCCTTGCATGCTTTACAACT

>ID537-3P
TGAAGCAGTAGAAGCCAAGACA

>miRNA precursor
AGAAAAAAUGGCUCAGCUGUGUGUCUGUGCUUUCUUGUCAUCUAUCUUGCAUCACUGUGAAUUAAAUAG**UGAAGCAGUAGAAGCCAAGACA**GCCUUGCAUGCUUUACAACU
.................(((((....(((.(((((...(((((..(((.(((((((.......))))))).))))))))...))))).)))...)))))).........

>Predicted STAR seq:
TTCTTGTCATCTATCTTGCATCA

Complementarity: 15

Initial dG = -29.10

```
        10        20        30        40        50        60
AGAAAAAAUGGCUCAGCU|     CUGU   U     UCA     CU   A       GAA
                  GUGUGU    GCU UCUUG   UCUAU  UGC UCACUGU   \
                  CGUACG    CGA AGAAC   AGAUG  ACG AGUGAUA   U
UCAACAUUU---------^     UUC-   C     CGA     --   A       AAU
 .                100         90        80          70
```

Reads in: lib-42, lib-45, lib-47, lib-48, lib-49, lib-50, lib-72
Number of reads="ID537",22,6,0,0,1,0,0

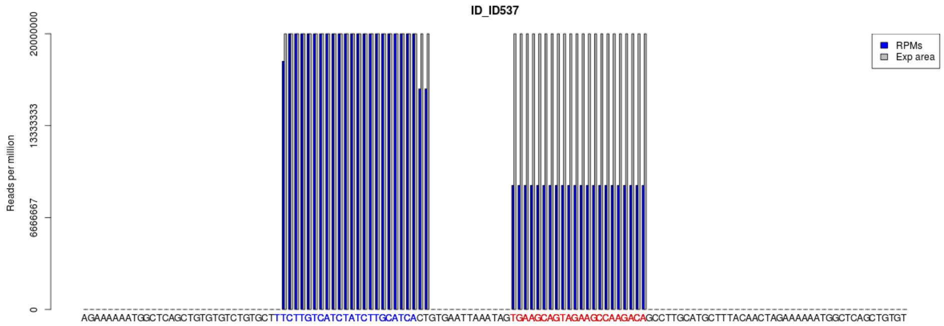

>ID539

CTGTTCTCACAAGGAAACATTTTTACTTGGAGCAAATATTCAGACTCGAAATTGGGTTCATTCGGCGAAT**ATTTGGTCCAGGCAAAAATG**
CTCACTTGTTAGGAGAAATGTC

>ID539-3P

ATTTGGTCCAGGCAAAAATGCT

>miRNA precursor

CUGUUCUCACAAGGAAACAUUUUUACUUGGAGCAAAUAUUCAGACUCGAAAUUGGGUUCAUUCGGCGAAU**AUUUGGUCCAGGCAAAAAUGCU**CACUUGUUAGGAGAAAUGUC
...((((((((((((..(((((((.((((((.(((((((((....(((((..((....))))))).))))))))).)))))).))))))).)).)))))...))))).....

>Predicted STAR seq:

AACATTTTTACTTGGAGCAAAT

Complementarity: 17

Initial dG =   -35.90

```
             10         20         30         40          50
CUG--     ---      -   AA        A       G          .-A|      A
     UUCUC   ACAAG GA  CAUUUUU CUUGGA CAAAUAUUC   GACUCG A
     AAGAG   UGUUC CU  GUAAAAA GGACCU GUUUAUAAG   UUGGGU A
CUGUA     GAU      A  C-        C       G          \ -^      U
110       100          90         80         70
```

Reads in: lib-42, lib-45, lib-47, lib-48, lib-49, lib-50, lib-72

Number of reads="ID539",46,5,0,0,2,1,0

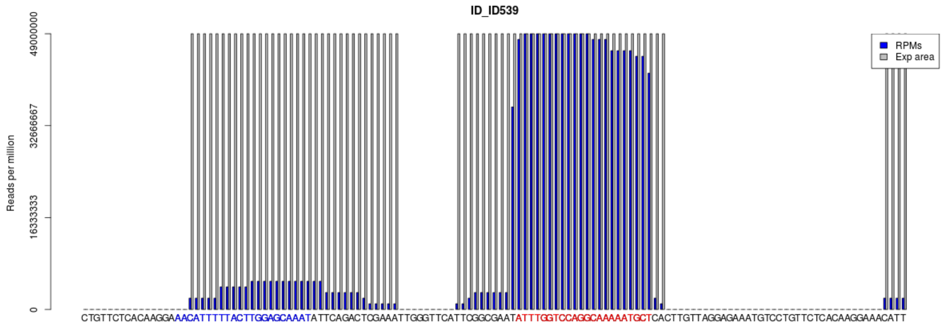

>ID541
TGGTGACACAGTAAGAACATTGATCTCTAATCTCAAGAATGTTGCCTGCACTTAATATCATAACAAAATA**TCAAGTGAGGTAACATTTTA**
GAGATGGGGGATTACTATCCAC

>ID541-3P
TCAAGTGAGGTAACATTTTAGA

>miRNA precursor
UGGUGACACAGUAAGAACAUUGAUCUCUAAUCUCAAGAAUGUUGCCUGCACUUAAUAUCAUAACAAAAUA**UCAAGUGAGGUAACAUUUUAGA**GAUGGGGGAUUACUAUCCAC
((....))...........((((((((.(((((.((((((((((((.(((((.((((.........)))).))))))))))))))))).))))).))))))))........

>Predicted STAR seq:
CAAGAATGTTGCCTGCACTTA

Complementarity: 18
ERROR 4: Strange structure
Initial dG = -38.30

```
  | G
--UG U
  AC G
\ ^ A



  10        20        30        40        50        60
  CAGUAAGAACAU        A     A            G     A    CAUA
              UGAUCUCU AUCUC AGAAUGUUGCCU CACUU AUAU    \
              AUUAGGGG UAGAG UUUUACAAUGGA GUGAA UAUA    A
  CACCUAUC----        G     A            -     C    AAAC
  110          100        90        80         70
```

Reads in: lib-42, lib-45, lib-47, lib-48, lib-49, lib-50, lib-72
Number of reads="ID541",48,13,0,1,0,0,0

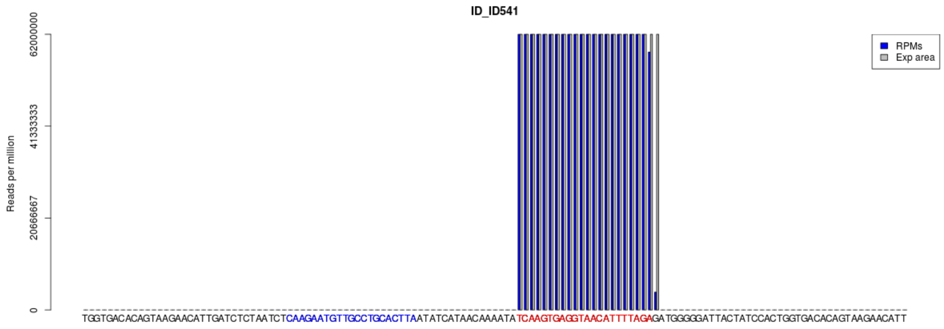

>ID542
TTCACCAGGAGTAAGGATAGTGCTGTACGCAGACCCACTTCTGGACCGTCCCTGTGCTAATTAATCAAG**AGACGTGCCGGAAGTGCGGGT CAG**CGTGCAGCACACTGACAACA

>ID542-3P
AGACGTGCCGGAAGTGCGGGTCAG

>miRNA precursor
UUCACCAGGAGUAAGGAUAGUGCUGUACGCAGACCCACUUCUGGACCGUCCCUGUGCUAAUUAAUCAAG**AGACGUGCCGGAAGUGCGGGUCAG**CGUGCAGCACACUGACAACA
```
....((........))...((((((((((.((((((((((((((..((((.((..............)).))))..)))))))))..))))).))))))))))).........
```

>Predicted STAR seq:
AGACCCACTTCTGGACCGTC

Complementarity: 16
ERROR 4: Strange structure
Initial dG = -50.70

```
          10
.-UUCA| AGG
      CC   A
      GG   G
\ ----^ AAU



           20        30          40        50        60
  AUA-------           A     --        AC    C  GUGCUA
            GUGCUGUACGC GACCC  ACUUCUGG  CGUC CU      A
            CACGACGUGCG CUGGG  UGAAGGCC  GCAG GA      U
  ACAACAGUCA           A     CG        GU    A  ACUAAU
   110       100        90        80        70
```

Reads in: lib-42, lib-45, lib-47, lib-48, lib-49, lib-50, lib-72
Number of reads="ID542",16,2,0,0,0,0,0

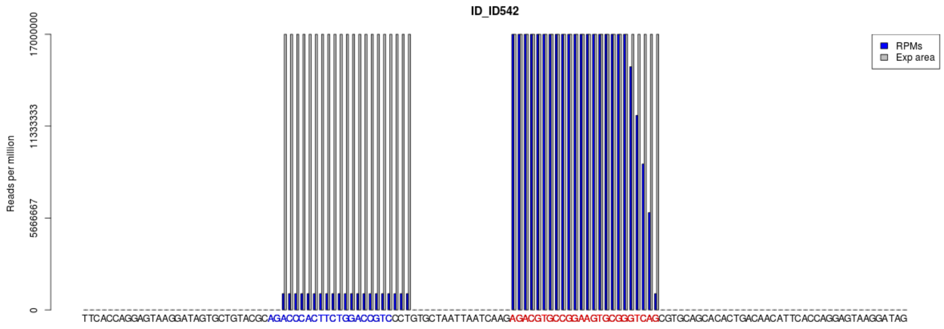

>ID543
TCGACGCCGCGTAGGCACTG**TAGAGGTCAGTTGTTAAGATT**AGTCAATTTTTGTTGCTAATCGTAACAACCGACCTTCACAGTGCCTAGG
CGGCCAATCAACGGCAGCGAC

>ID543-5P
TAGAGGTCAGTTGTTAAGATT

>miRNA precursor
UCGACGCCGCGUAGGCACUG**UAGAGGUCAGUUGUUAAGAUU**AGUCAAUUUUUGUUGCUAAUCGUAACAACCGACCUUCACAGUGCCUAGGCGGCCAAUCAACGGCAGCGAC
.....((((.((((((((((..(((((.(((((((.((((((.((((....)))))))))).))))))).))))).)).)))))))))).))))).................

>Predicted STAR seq:
TCGTAACAACCGACCTTCACA

Complementarity: 15

Initial dG =   -60.50

```
                    10        20        30        40        50
UCGAC------------|    G          AG      A       A      U    U
                 GCCGC UAGGCACUGU  AGGUC GUUGUUA GAUUAG CAAU U
                 CGGCG AUCCGUGACA  UCCAG CAACAAU CUAAUC GUUG U
CAGCGACGGCAACUAAC^    G          CU      C       G      -    U
 .       100       90        80        70        60
```

Reads in: lib-42, lib-45, lib-47, lib-48, lib-49, lib-50, lib-72
Number of reads="ID543",15,8,11,280,347,7,0

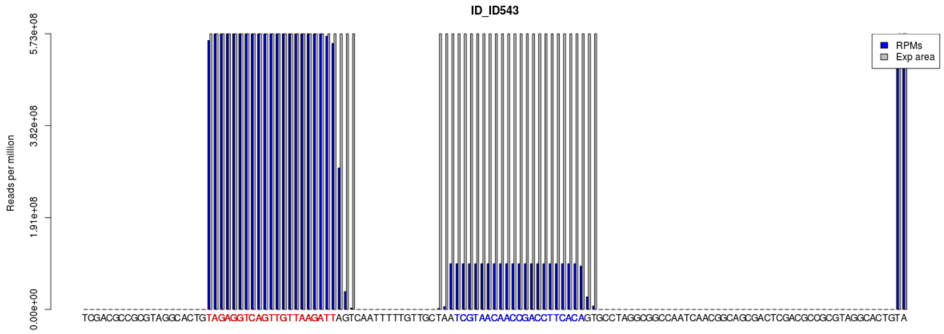

>ID544
GATCGCTGTATAATTGGATCAAAGCCAGTCTTTCCGGCCG**CAGTGGTCGAGTGGTCTGAGGC**GCGCCAGCCACAGTCTGCCCGACCGCTA
CGGTCGGGGGTACGTATCCTCC

>ID544-5P
CAGTGGTCGAGTGGTCTGAGGC

>miRNA precursor
GAUCGCUGUAUAAUUGGAUCAAAGCCAGUCUUUCCGGCCG**CAGUGGUCGAGUGGUCUGAGGC**GCGCCAGCCACAGUCUGCCCGACCGCUACGGUCGGGGGUACGUAUCCUCC
```
(((.((.((((.(((((.......)))))...((((((((.((((((((.(..(.(((.(((......))).))).)..).)))))))).)))))))).)))))))))....
Error predicting STAR on 5pERROR 4: Strange structure
 Initial dG =    -51.10

          10
  |   GCUGU
--GAUC     A
  CUAG     U
\ ^   GUUAA
 20



            30        40        50        60
  AAAGCCA--  C UU       C        A UG U   A   GC
         GU U  CCGGCCG AGUGGUCG G  G CUG GGC  G
         CA G  GGCUGGC UCGCCAGC C  C GAC CCG  C
  CCUCCUAUG  U GG       A        C GU U   A   AC
  110       100        90        80        70
```

Reads in: lib-42, lib-45, lib-47, lib-48, lib-49, lib-50, lib-72
Number of reads="ID544",27,8,0,384,493,0,0

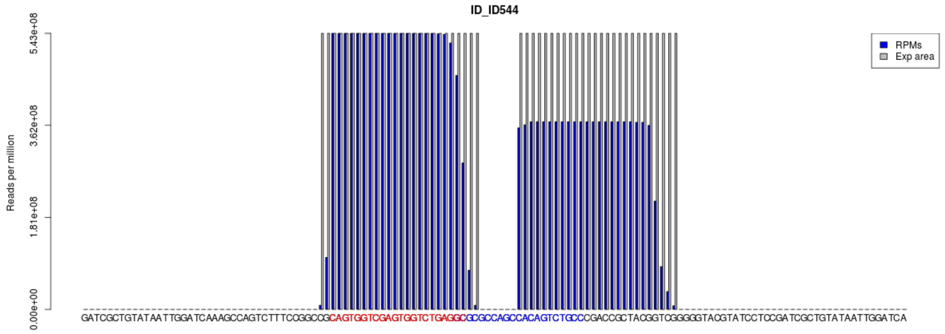

>ID546
GCGAAAGCGAACATTATCTT**TGGACTTTGATCGGGATAGGA**CGGAAGAATTAGAAACTTCTGCCTTCCGTAATAAACTGACGAACATTCC
AGAGAGATTAATACAATCAAACTTT

>ID546-5P
TGGACTTTGATCGGGATAGGA

>miRNA precursor
GCGAAAGCGAACAUUAUCUU**UGGACUUUGAUCGGGAUAGGA**CGGAAGAAUUAGAAACUUCUGCCUUCCGUAAUAAACUGACGAACAUUCCAGAGAGAUUAAUACAAUCAAACUUU
((....))........((((((((.((((.((((..((..((((((((...((((....)))).))))))))..))..))))))))...))))))))((((.....)))).......

>Predicted STAR seq:
ATAAACTGACGAACATTCCAGA

Complementarity: 13

Initial dG =   -34.10

ERROR 3: Strange structure
Initial dG =   -34.10

```
  | G
--GC A
  CG A
\ ^ A
```

```
    10        20          30        40        50
  .-GAACAUUA        C--     A     GA   GG        AAU     A
            UCUUUGGA   UUUG UCGG  UA   ACGGAAG    UAGA A
            AGAGACCU   AAGC AGUC  AU   UGCCUUC    GUCU C
  \ --------        UAC     -     AA   AA        C--     U
                90          80        70          60
```

Reads in: lib-42, lib-45, lib-47, lib-48, lib-49, lib-50, lib-72
Number of reads="ID546",0,2,4,0,0,1,0

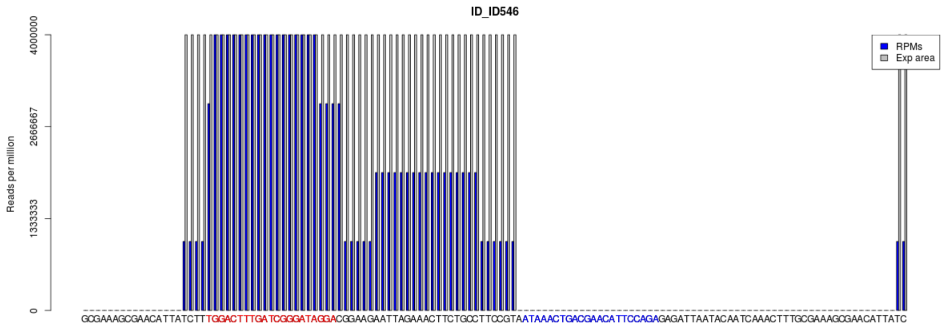

>ID551
ACGGGAGGCGCGCAGCCCGC**TGGGGTATCTCGGCAAGTGTCG**TCGTGTCGTGCGCGCGTTGCAGGGCCGCCAACTGCCGAGATAGCTCAG
CGGGACGCGCGCCTGGACTTCA

>ID551-5P
TGGGGTATCTCGGCAAGTGTCG

>miRNA precursor
ACGGGAGGCGCGCAGCCCGC**UGGGGUAUCUCGGCAAGUGUCG**UCGUGUCGUGCGCGCGUUGCAGGGCCGCCAACUGCCGAGAUAGCUCAGCGGGACGCGCGCCUGGACUUCA
.....((((((((..(((((((((.((((((((((.((...(.((.(((.((((.....)))).))))))..))))))))))))).)))))))))..))))))))........

>Predicted STAR seq:
AACTGCCGAGATAGCTCAGC

Complementarity: 15

Initial dG =   -68.80

```
           10        20        30        40        50
ACGGG---|       AG         G          A  GUC U  U   G    CG
        AGGCGCGC  CCCGCUGGG UAUCUCGGCA GU   G CG GUC UGCG  \
        UCCGCGCG  GGGCGACUC AUAGAGCCGU CA   C GC CGG ACGU  C
ACUUCAGG^       CA         G          -  AC- -  -   G    UG
110       100        90        80          70          60
```

Reads in: lib-42, lib-45, lib-47, lib-48, lib-49, lib-50, lib-72
Number of reads="ID551",37,14,1,1517,21,6,0

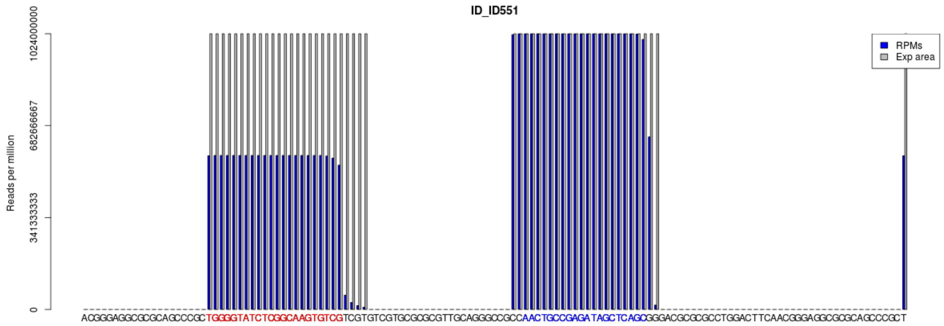

>ID553
AAGCAGCAGTAAACCTAAAGCCACAACCACACCAGTTGGACCGCACTGTGCCAAACTATGATAAGATGTT**TGTTAAAGTGCAGTTCAGCC**
GGCGTGGACGGCCACCAACGC

>ID553-3P
TGTTAAAGTGCAGTTCAGCCGG

>miRNA precursor
AAGCAGCAGUAAACCUAAAGCCACAACCACACCAGUUGGACCGCACUGUGCCAAACUAUGAUAAGAUGUU**UGUUAAAGUGCAGUUCAGCCGG**CGUGGACGGCCACCAACGC
```
..................(((....((((.((.(((((((.(((((.((.(((((...........))))).)).))))).))))))).)).))))..)))........
```

>Predicted STAR seq:
CCAGTTGGACCGCACTGTGCCA

Complementarity: 16

Initial dG =   -34.90

```
        10        20         30         40         50         60
AAGCAGCAGUAAACCUAAA|  ACAA    A  A       C      G  C     UAUGA
                   GCC    CCAC CC GUUGGAC GCACU UG CAAAC     \
                   CGG    GGUG GG CGACUUG CGUGA AU GUUUG     U
CGCAACCAC----------^  CA--    C  C       A      A  U     UAGAA
 .                 100          90         80         70
```

Reads in: lib-42, lib-45, lib-47, lib-48, lib-49, lib-50, lib-72
Number of reads="ID553",5,7,2,397,54,2,0

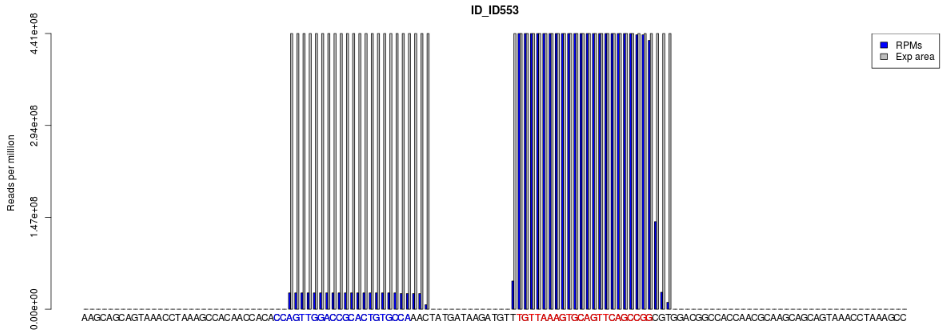

>ID559
ATATGATTGGAATGGTTGTTCACAGCACAAGGCACAGGCCATCACGTATAAGAAATTG**GTCTGTGCCTCCTGCTATGAGA**AAAACGTTCC
AATATCGCAAAGAAAAGAAGAA

>ID559-3P
GTCTGTGCCTCCTGCTATGAGA

>miRNA precursor
AUAUGAUUGGAAUGGUUGUUCACAGCACAAGGCACAGGCCAUCACGUAUAAGAAAUUG**GUCUGUGCCUCCUGCUAUGAGA**AAAACGUUCCAAUAUCGCAAAGAAAAGAAGAA
.....((((((((((....((((.((((..((((((((((((...............))))))))))))..)))).)))).....)))))))))..................

>Predicted STAR seq:
TTGTTCACAGCACAAGGCACAGGC

Complementarity: 16

Initial dG = -42.60

```
                      10          20         30         40
AUAUG--------------         GUUG-|   C     CA            UCACGUA
                   AUUGGAAUG     UUCA AGCA  AGGCACAGGCCA       \
                   UAACCUUGC     GAGU UCGU  UCCGUGUCUGGU       U
AAGAAGAAAAGAAACGCUA         AAAAA^   A     CC            UAAAGAA
110       100        90         80         70         60         50
```

Reads in: lib-42, lib-45, lib-47, lib-48, lib-49, lib-50, lib-72
Number of reads="ID559",4,4,0,230,11,0,0

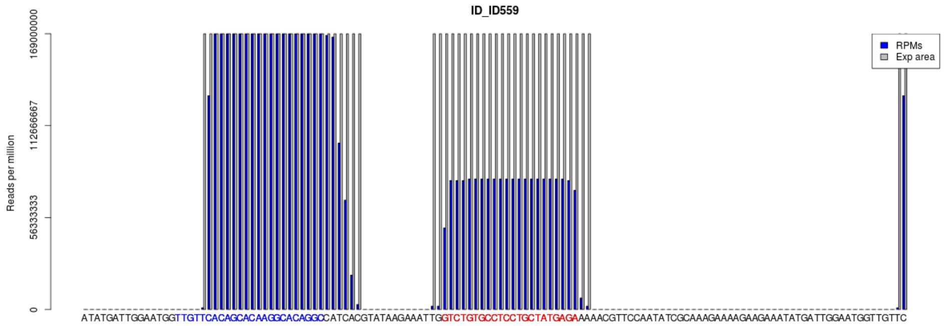

>ID562
TTTTGGAACACATATTATTCTCACACGAAGGGTGACGAATGTGTCGCGGATACACGTTATTTAATTTGTG**TCTCTGAGACCCATACGTCA**
CTCATTGTGCGAGTTTCACATC

>ID562-3P
TCTCTGAGACCCATACGTCACT

>miRNA precursor
UUUUGGAACACAUAUUAUUCUCACACGAAGGGUGACGAAUGUGUCGCGGAUACACGUUAUUUAAUUUGUG**UCUCUGAGACCCAUACGUCACU**CAUUGUGCGAGUUUCACAUC
..................(((.(((((.((((((((.(((.(((.((((.(((((.........))))).)))).))).))).)))))))).))))).)))........

>Predicted STAR seq:
GTGACGAATGTGTCGCGGAT

Complementarity: 16

Initial dG =   -39.40

```
        10        20        30        40        50        60
UUUUGGAACACAUAUUAUU|  A     A        A   U   G    U     UUAU
                   CUC CACGA GGGUGACG AUG GUC CGGA ACACG    U
                   GAG GUGUU CUCACUGC UAC CAG GUCU UGUGU    U
CUACACUUU----------^  C     A        A   C   A    C     UUAA
110                 100        90        80        70
```

Reads in: lib-42, lib-45, lib-47, lib-48, lib-49, lib-50, lib-72
Number of reads="ID562",1,4,0,2,3,1,0

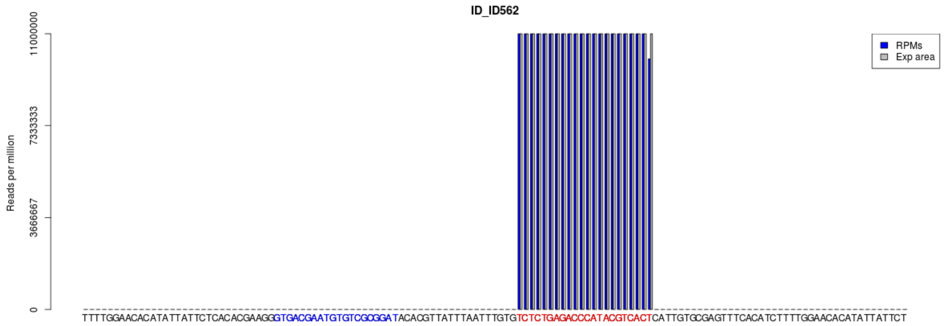

>ID564

ATTTGGATCTCATAGTGTTCTCGCATGAAGGGTGACGTATGTGTCGCGGGTACACGTTGTTTAATTTGTG**TTGCTGAGGCCCATACGTTA**
CTCATTATGCGAGTTTCACATC

>ID564-3P

TTGCTGAGGCCCATACGTTACT

>miRNA precursor

AUUUGGAUCUCAUAGUGUUCUCGCAUGAAGGGUGACGUAUGUGUCGCGGGUACACGUUGUUUAAUUUGUG**UUGCUGAGGCCCAUACGUUACU**CAUUAUGCGAGUUUCACAUC

```
..............(((..(((((((((.((((((((((((.(((.(((..(((((..........)))))..))).))).)))))))))))).)))))))))...)))...
```

>Predicted STAR seq:

GTGACGTATGTGTCGCGGG

Complementarity: 16

Initial dG =   -47.10

```
        10         20        30        40        50        60
AUUUGGAUCUCAUA   UU-|        A            U   G   GU     UUGU
              GUG   CUCGCAUGA GGGUGACGUAUG GUC CGG  ACACG    U
              CAC   GAGCGUAUU CUCAUUGCAUAC CGG GUC  UGUGU    U
CUA-----------   UUU^        A            C   A   GU     UUAA
110                 100        90        80        70
```

Reads in: lib-42, lib-45, lib-47, lib-48, lib-49, lib-50, lib-72

Number of reads="ID564",0,14,0,0,0,0,0

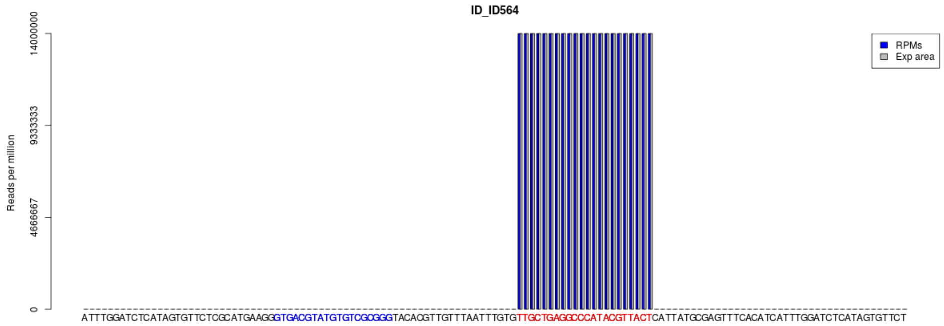

>ID565
TTGTGGAACTAATGGTATTCTTGCACGAAGGGTGACGTATGTGTCGCAGGTACACTTTCCTTGATTTGTG**TATCTGAGGCCCATTCGTTA**
CTTGTCGTGTGAGTTTAAAAAC

>ID565-3P
TATCTGAGGCCCATTCGTTACT

>miRNA precursor
UUGUGGAACUAAUGGUAUUCUUGCACGAAGGGUGACGUAUGUGUCGCAGGUACACUUUCCUUGAUUUGUG**UAUCUGAGGCCCAUUCGUUACU**UGUCGUGUGAGUUUAAAAAC
...............((..((..(((((..(((((((.(((.(((.(((((((((...........))))))))).))).))).))))))).)..)))))..))..)).....

>Predicted STAR seq:
GGGTGACGTATGTGTCGCAGGTA

Complementarity: 17

Initial dG =   -43.50

```
        10        20        30        40        50        60
UUGUGGAACUAAUGG| UU  UG     AG       U   U   G         UUUCC
               UA  CU  CACGA  GGUGACG AUG GUC CAGGUACAC     U
               AU  GA  GUGCU  UCAUUGC UAC CGG GUCUAUGUG     U
CAAAA----------^ UU  GU     GU       U   C   A         UUUAG
110               100        90        80        70
```

Reads in: lib-42, lib-45, lib-47, lib-48, lib-49, lib-50, lib-72
Number of reads="ID565",2,3,0,0,0,0,0

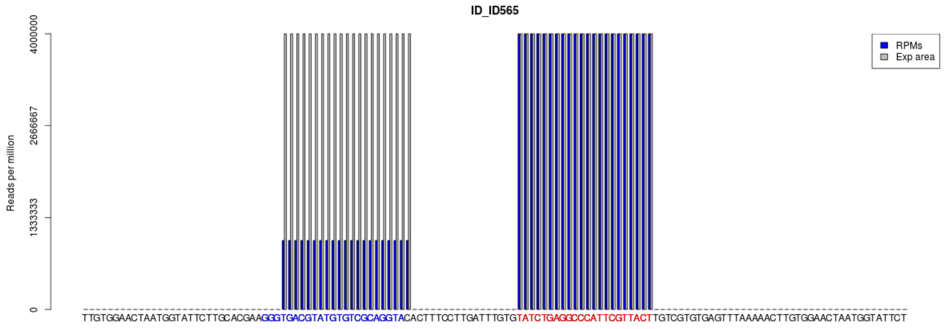

>ID568
TACGCTAACTAAGCATCACT**AAAGTTCTGGTAGAAGAGAG**GGTTAAATCGCAAAGTGGCTCTCCCTTTATTTAGAACTTTCGGTGATGCT
ACACTGAACATCAAATTCA

>ID568-5P
AAAGTTCTGGTAGAAGAGAG

>miRNA precursor
UACGCUAACUAAGCAUCACU**AAAGUUCUGGUAGAAGAGAG**GGUUAAAUCGCAAAGUGGCUCUCCCUUUAUUUAGAACUUUCGGUGAUGCUACACUGAACAUCAAAUUCA
```
...........(((((((((((((((((((..((((.((((((((..........)))))))).))))..))))))))))).))))))))).......................
```

>Predicted STAR seq:
CCCTTTATTTAGAACTTTCGG

Complementarity: 15

Initial dG =   -35.40

```
        10                 20         30         40         50
UACGCUAACUA--------          -|          UA    A         AAUC
                   AGCAUCACU AAAGUUCUGG  GAAG GAGGGUUA    G
                   UCGUAGUGG UUUCAAGAUU  UUUC CUCUCGGU    C
ACUUAAACUACAAGUCACA          C^          UA    C         GAAA
       100        90         80         70         60
```

Reads in: lib-42, lib-45, lib-47, lib-48, lib-49, lib-50, lib-72
Number of reads="ID568",40,33,28,46,83,22,0

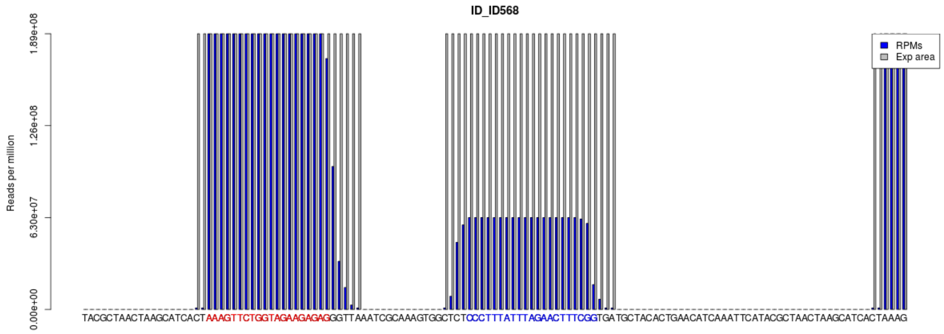

>ID569
AAAGCAATAACGTGTA**AATTGCTACGTAGGTAATCAGA**AACGCGCACGATGCCTACGCTCTGATTAATACAACGTGTTGCGCAGAACCGT
CAGCGCAGCGCTGCATTCCCC

>ID569-5P
AATTGCTACGTAGGTAATCAGA

>miRNA precursor
AAAGCAAUAACGUGUA**AAUUGCUACGUAGGUAAUCAGA**AACGCGCACGAUGCCUACGCUCUGAUUAAUACAACGUGUUGCGCAGAACCGUCAGCGCAGCGCUGCAUUCCCC
..((((((.........))))))..(((..((((((((...(((...........)))))))))))).)))...(((((((((.((....)).)))))))))..........

>Predicted STAR seq:
TACGTAGGTAATCAGAAACGCGCACGATGCCTACGCTCTGATT

Complementarity: 9

Initial dG =   -32.70

```
              10        20        30        40
AAA----  .-AAUAAC|   A     CUA        A  AGAAA
       GC        GUGU AAUUG   CGUAGGUA UC     C
       CG        CAUA UUAGU   GCAUCCGU AG     G
CCCCUUA  \ ------^   A     CUC        -  CACGC
 .              70        60        50



                   80
           AAC         A  A
              GUGUUGCGC GA C
              CGCGACGCG CU C
           U--         A  G
             100        90
```

Reads in: lib-42, lib-45, lib-47, lib-48, lib-49, lib-50, lib-72
Number of reads="ID569",21,25,8,313,30,13,0

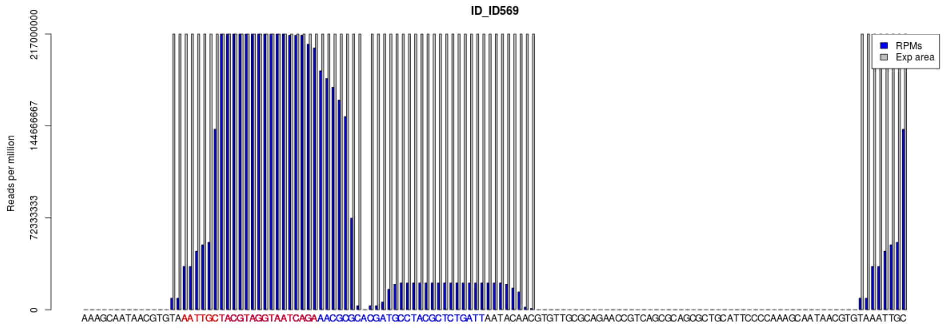

>ID576

TGAGGAGTACAGAACAGAATACAGAGCAGCCTTGGTTTCACACTATGACTGCATATTTGGTAAGTGCATG**TGTAGCCATAGTGCTAAGCCGA**GGCCGCTCTGAAGTAGCCAG

>ID576-3P

TGTAGCCATAGTGCTAAGCCGA

>miRNA precursor

UGAGGAGUACAGAACAGAAUACAGAGCAGCCUUGGUUUCACACUAUGACUGCAUAUUUGGUAAGUGCAUG**UGUAGCCAUAGUGCUAAGCCGA**GGCCGCUCUGAAGUAGCCAG

```
...((..(((...........((((((.((((((((((..(((((((.((((((((...((....)))))))))).)))))))..)))))))))).))))))..))).))..
```

>Predicted STAR seq:

TGGTTTCACACTATGACTGCA

Complementarity: 17
ERROR 4: Strange structure
 Initial dG =   -46.60

```
          10
UGA  .-A| AC
   GG   GU  \
   CC   CA  A
GA-  \ -^ AG
 110



          20        30        40        50        60
       AGAAUA      A          CA        A        UUG  A
             CAGAGC GCCUUGGUUU  CACUAUG CUGCAUAU   GU A
             GUCUCG CGGAGCCGAA  GUGAUAC GAUGUGUA   CG G
       GAUGAA      C          UC        C        ---  U
             100        90        80        70
```

Reads in: lib-42, lib-45, lib-47, lib-48, lib-49, lib-50, lib-72

Number of reads="ID576",4,76,30,564,139,9,0

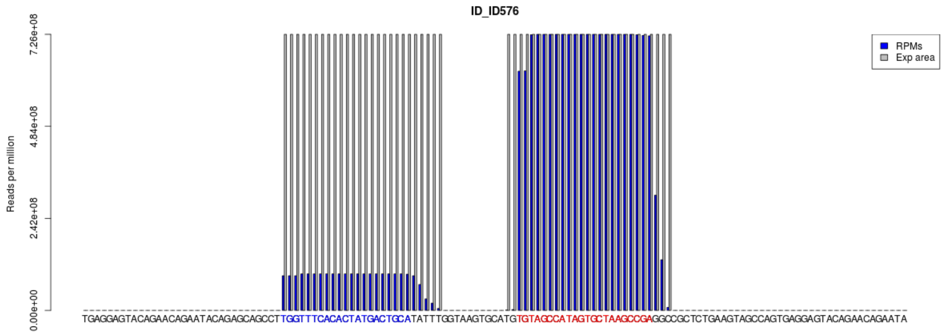

>ID583

TTAGGTGTTCTTTAATAACATCATTTTTGTTTTGGCTTTTCGCCATTTTTGAGCCATCTGCGATATATTG**CAGGTGACTTGAAAATGGCG**
AAAAGCCGAAATGAGATCGTC

>ID583-3P

CAGGTGACTTGAAAATGGCGA

>miRNA precursor

UUAGGUGUUCUUUAAUAACAUCAUUUUUGUUUUGGCUUUUCGCCAUUUUUGAGCCAUCUGCGAUAUAUUG**CAGGUGACUUGAAAAUGGCGA**AAAGCCGAAAUGAGAUCGUC
...((((((.......))))))..(((..((((((((((((((((((((..((.(((((((((....))))))))).))..)))))))))))))))))))))..))).....

>Predicted STAR seq:

CGCCATTTTTGAGCCATCTG

```
Complementarity: 16
ERROR 4: Strange structure
 Initial dG =    -56.80

          10
.-UUA|     CUU
     GGUGUU   \
     CUACAA   U
\ ---^     UAA
      20



           30        40        50        60
  AU---   UG                    UG  C         U
       UUU  UUUUGGCUUUUCGCCAUUUU  AG CAUCUGCGA A
       AGA  AAAGCCGAAAAGCGGUAAAA  UC GUGGACGUU U
  CUGCU   GU                    GU  A         A
 110       100        90        80        70
```

Reads in: lib-42, lib-45, lib-47, lib-48, lib-49, lib-50, lib-72

Number of reads="ID583",5,12,3,5,22,2,0

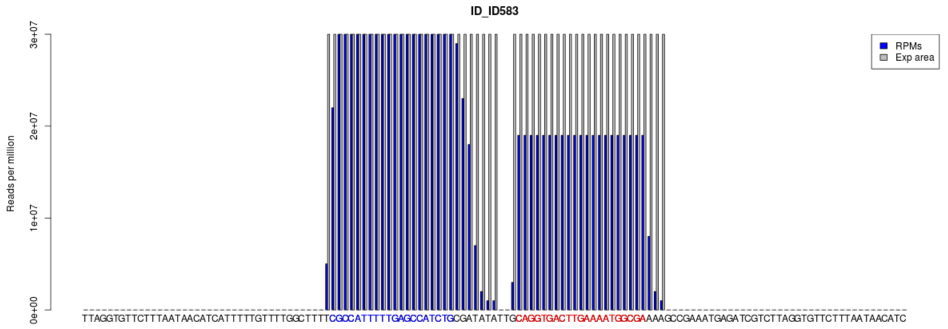

>ID589

AAACCAGACACTTGCAGACGAATTCGCTAGGAGGGAGGTGACCGGGTGCAGCGTGGAAATAGCAACCT**GTACCCGGTCACCTCCTTCCTG**
ACGTATTCTGACCTCGCAACT

>ID589-3P

GTACCCGGTCACCTCCTTCCTGA

>miRNA precursor

```
AAACCAGACACUUGCAGACGAAUUCGCUAGGAGGGAGGUGACCGGGUGCAGCGUGGAAAUAGCAACCUGUACCCGGUCACCUCCUUCCUGACGUAUUCUGACCUCGCAACU
..........((((((..((((.((.((((((((((((((((((((((((.((..........))))))))))))))))))))))))))).)).)))).....)).))))..
```

>Predicted STAR seq:

TAGGAGGGAGGTGACCGGGTGC

Complementarity: 20

Initial dG =   -58.80

```
        10           20         30         40         50
AAACCAGACAC    -  AC--|   U  C                       C  GGAA
           UUGC AG    GAAU CG UAGGAGGGAGGUGACCGGGUGCAG GU    A
           AACG UC    CUUA GC GUCCUUCCUCCACUGGCCCAUGUC CA    U
UC---------    C  CAGU^   U  A                       -  ACGA
 .                 100        90         80         70         60
```

Reads in: lib-42, lib-45, lib-47, lib-48, lib-49, lib-50, lib-72

Number of reads="ID589",250,826,970,346,1243,481,0

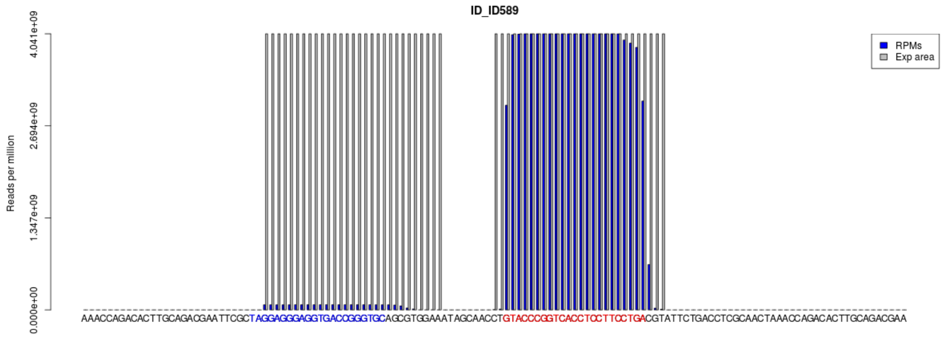

>ID59
CAAATGGCATTACCTGCAGCAGCAGCATCGCTTTCATCACCAGGGAGAACATCAATACTTGCATCAGTTC**TCCAAGGTGGAGAAATCGGT**
TCTGCTGCAGCAGCCAAGACAT

>ID59-3P
TCCAAGGTGGAGAAATCGGTTC

>miRNA precursor
CAAAUGGCAUUACCUGCAGCAGCAGCAUCGCUUUCAUCACCAGGGAGAACAUCAAUACUUGCAUCAGUUC**UCCAAGGUGGAGAAAUCGGUUC**UGCUGCAGCAGCCAAGACAU

```
....((((......(((.(((((((.((((.((((..((((..(((((((...............)))))))..))))..)))).)))).))))))).)))))))......
```

>Predicted STAR seq:
CATCGCTTTCATCACCAGGGA

Complementarity: 14

Initial dG =   -47.00

```
          10        20        30        40        50
CAAA--|   AUUACC   A       C    C    AU    AG        AUCAAUA
      UGGC      UGC GCAGCAG AUCG UUUC  CACC  GGAGAAC       C
      ACCG      ACG CGUCGUC UGGC AAAG  GUGG  CCUCUUG       U
UACAGA^   ------   A       U    U    AG    AA        ACUACGU
110             100       90        80        70        60
```

Reads in: lib-42, lib-45, lib-47, lib-48, lib-49, lib-50, lib-72
Number of reads="ID59",26,0,0,0,0,0,0

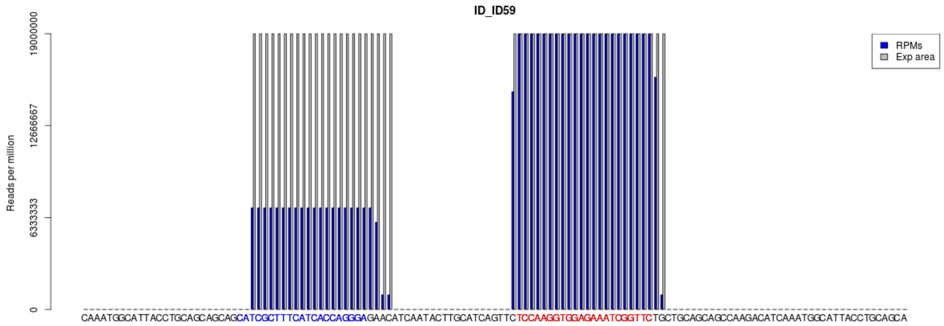

>ID592
GGCGGTATAAACAAGGCAAGGTTGCGGCAGTTGTCTCTGCTGTGGTCTTCACGACTTTGTGCAGCATCTG**AAGATCTAGTAGAGACGGCT**
GCCGAGGCGTTGCGTCAT

>ID592-3P
AAGATCTAGTAGAGACGGCTGC

>miRNA precursor
GGCGGUAUAAACAAGGCAAGGUUGCGGCAGUUGUCUCUGCUGUGGUCUUCACGACUUUGUGCAGCAUCUG**AAGAUCUAGUAGAGACGGCUGC**CGAGGCGUUGCGUCAU
```
..............((((.((..((((((((((((((((((.((((((((.((...((.....)))))))))))))))))))))))))))))))))..)).)))).....
```

>Predicted STAR seq:
CAGTTGTCTCTGCTGTGGTCTT

Complementarity: 20

Initial dG = -53.70

```
        10        20        30        40        50        60
GGCGGUAUAAACAAG|   G  UG                 U        C  CUU  UG
               GCAA GU  CGGCAGUUGUCUCUGCUG GGUCUUCA GA   UG  \
               CGUU CG  GCCGUCGGCAGAGAUGAU CUAGAAGU CU   AC  C
UACUG----------^   G  GA                 -        -  ---  GA
                100        90        80         70
```

Reads in: lib-42, lib-45, lib-47, lib-48, lib-49, lib-50, lib-72
Number of reads="ID592",256,703,427,5061,1609,92,0

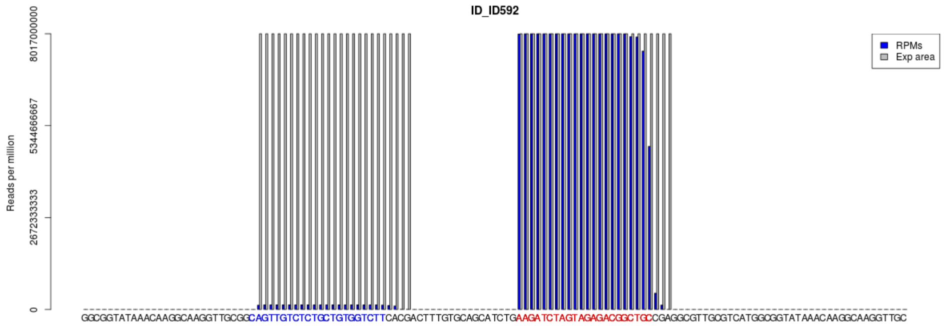

>ID593
CATCAATAACTTTCTTAATA**TTGATTATGGGCCGAAGCATT**GTATTAAAATATGTACCAATGTGGCGGCTTATAATCATTATCATAGATA
AAGTTAACTCATCACGTCTT

>ID593-5P
TTGATTATGGGCCGAAGCATT

>miRNA precursor
CAUCAAUAACUUUCUUAAUA**UUGAUUAUGGGCCGAAGCAUU**GUAUUAAAAUAUGUACCAAUGUGGCGGCUUAUAAUCAUUAUCAUAGAUAAAGUUAACUCAUCACGUCUU
......((((((((..(((.(((((((((((((..(((((((((........)))).)))))..)))))))))))))).)))...)))..)))))).............

>Predicted STAR seq:
TGTGGCGGCTTATAATCATTA

Complementarity: 16

Initial dG =   -33.80

```
                10          20        30        40
CAUCAA--------      --   UA-   U             AA     -|   UAA
              UAACUU  UCU   AUA UGAUUAUGGGCCG  GCAUU GUAU   A
              AUUGAA  AGA   UAU ACUAAUAUUCGGC  UGUAA CAUG   A
UUCUGCACUACUCA      AU   UAC   U             GG     C^   UAU
.       100        90        80        70        60        50
```

Reads in: lib-42, lib-45, lib-47, lib-48, lib-49, lib-50, lib-72
Number of reads="ID593",5,7,1,45,24,0,0

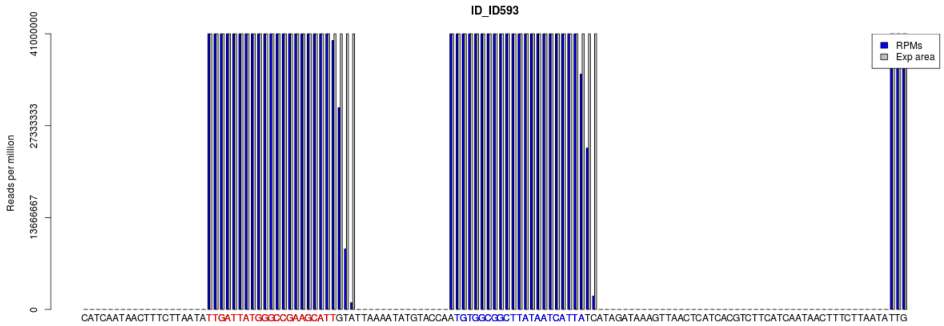

>ID594
TTACAGAACATACTCTGGGAAGGTCCGATACTGCTAGTTCCGGCTTAAATGAGTGCCGTATTGAGAGCCG**CACGCAGTGACAATGACCTT**
CCCTTTTTTATTATTCCTTTTTATT

>ID594-3P
CACGCAGTGACAATGACCTTCCCT

>miRNA precursor
UUACAGAACAUACUCUGGGAAGGUCCGAUACUGCUAGUUCCGGCUUAAAUGAGUGCCGUAUUGAGAGCCG**CACGCAGUGACAAUGACCUUCCCU**UUUUUAUUAUUCCUUUUUAUU
................(((((((((...((((((..((..((((((.((((.......))))..)))))).)))))))).....)))))))))......................
Error predicting STAR on 5p
Initial dG =   -32.00

```
        10              20          30        40         50
UUACAGAACAUACUCU------         CGA--|     UA  UC      A-    AGU
                      GGGAAGGUC     UACUGC  GU  CGGCUU  AAUG   \
                      CCCUUCCAG     GUGACG  CA  GCCGAG  UUAU   G
UUAUUUUUCCUUAUUAUUUUUU         UAACA^     --  C-      AG    GCC
   110       100        90        80           70        60
```

Reads in: lib-42, lib-45, lib-47, lib-48, lib-49, lib-50, lib-72
Number of reads="ID594",164,16,21,11,13,35,0

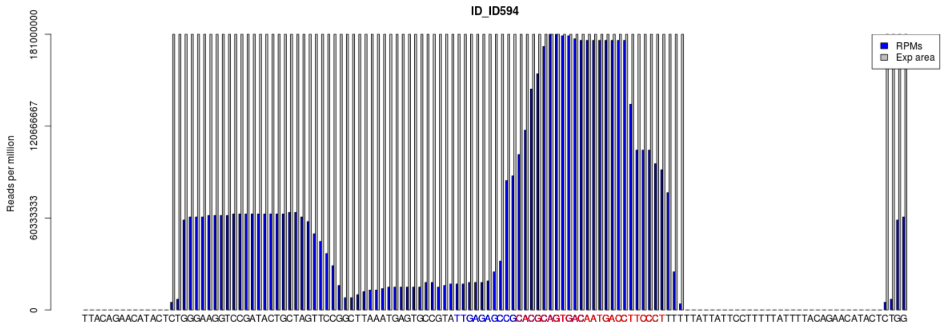

>ID595
GTTCGTGAGGGCGGAATGTG**TGGCAGGAAGTTTCGTTTTCTAGA**TGCTTTCAGGACTCTAGATTCGGCTCCTCCTCCACGTGTTTCGCGC
CGCACGGTCTGCTGTTCTGCTCGC

>ID595-5P
TGGCAGGAAGTTTCGTTTTCTAGA

>miRNA precursor
GUUCGUGAGGGCGGAAUGUG**UGGCAGGAAGUUUCGUUUUCUAGA**UGCUUUCAGGACUCUAGAUUCGGCUCCUCCUCCACGUGUUUCGCGCCGCACGGUCUGCUGUUCUGCUCGC
..(((((.((((((((..(((((.((((....(((...((((((..((....))..))))))..)))....)))))))))..)))))).)).))))).. .((......))....

>Predicted STAR seq:
TAGATTCGGCTCCTCCTCCACG

Complementarity: 14

Initial dG = -44.20

```
          10         20         30         40         50
.-GU      A  -|      UG      C     AGUU   UUU       UG  U
    UCGUG GG GCGGAA  UGUGG AGGA    UCG   UCUAGA  CU U
    GGCAC CC CGCUUU  GCACC UCCU    GGC   AGAUCU  GG C
\ --      G  G^      GU      -     CCUC   UU-       CA  A
          90        80         70          60



  100
  UCU-  UG
      GC  U
      CG  U
  CGCU  UC
    110
```

Reads in: lib-42, lib-45, lib-47, lib-48, lib-49, lib-50, lib-72
Number of reads="ID595",3,2,0,78,18,1,0

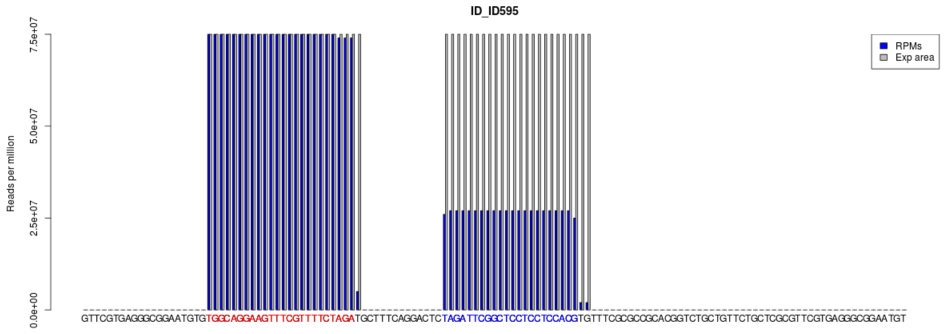

>ID599
CACACGTCACCGAGAAGTAGGCTTCCCTCCTGGCCATCTAGGCAGAGTCCCGAGCGATCATGAACAGTC**TCGGGTCTGCCTCGGTGGCTA**
GGATGCCAGCCAATAGCTG

>ID599-3P
TCGGGTCTGCCTCGGTGGCTAG

>miRNA precursor
CACACGUCACCGAGAAGUAGGCUUCCCUCCUGGCCAUCUAGGCAGAGUCCCGAGCGAUCAUGAACAGUC**UCGGGUCUGCCUCGGUGGCUAG**GAUGCCAGCCAAUAGCUG
............((..((.((((....(((((((((((.(((((((..(((((((...........).)))))))))))))).)))))))))))....)))).))..)).

>Predicted STAR seq:
TGGCCATCTAGGCAGAGTCCCGA

Complementarity: 19

Initial dG = -49.70

```
        10        20        30        40        50         60
CACACGUCACCG| AA  A    UCCC           U       GU      - GAUCA
            AG  GU GGCU    UCCUGGCCAUC AGGCAGA  CCCGAG C     \
            UC  UA CCGA    AGGAUCGGUGG UCCGUCU  GGGCUC G     U
G-----------^ GA  A    CCGU           C       --      U ACAAG
                  100        90        80          70
```

Reads in: lib-42, lib-45, lib-47, lib-48, lib-49, lib-50, lib-72
Number of reads="ID599",60,15,3,1117,47,2,0

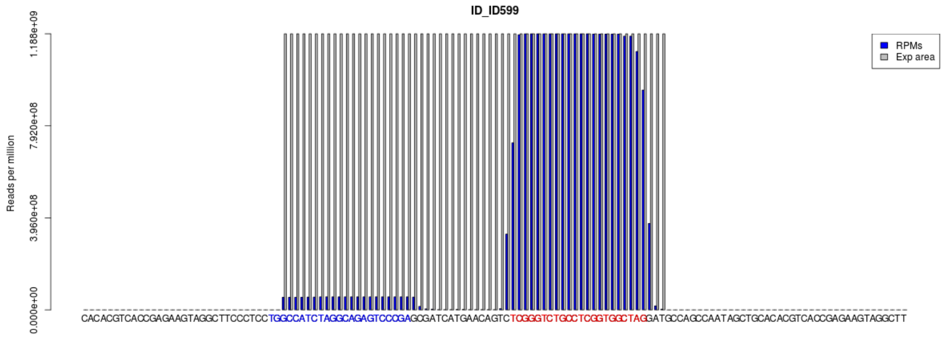

>ID60
GTACCTGCAGTAGCGCCAGCCACTACTACAGATGGAACAGCTGCTGCTGCAGCAGTAGCAAGAGAACCTG**TTCCGACTGTAGTAGTTCCTGG**TGCTGCCGCAGCAAGTACAC

>ID60-3P
TTCCGACTGTAGTAGTTCCTGG

>miRNA precursor
GUACCUGCAGUAGCGCCAGCCACUACUACAGAUGGAACAGCUGCUGCUGCAGCAGUAGCAAGAGAACCUG**UUCCGACUGUAGUAGUUCCUGG**UGCUGCCGCAGCAAGUACAC
((((((((.((((((((((..((((((((((.((((((((((((((((....))))))))).........))))))).)))))))))))..))))))))))).)))))...)))))..

>Predicted STAR seq:
CAGCCACTA

Complementarity: 17

Initial dG =   -66.10

```
             10        20        30                  40
--    ---    A          CC          A       ---------|       U
  GUAC   CUGC GUAGCGCCAG  ACUACUACAG UGGAACA         GCUGCUGC G
  CAUG   GACG CGUCGUGGUC  UGAUGAUGUC GCCUUGU         CGAUGACG C
CA    AAC    C          CU          A       CCAAGAGAA^       A
110       100        90        80        70        60        50
```

Reads in: lib-42, lib-45, lib-47, lib-48, lib-49, lib-50, lib-72
Number of reads="ID60",8,0,0,0,0,0,0

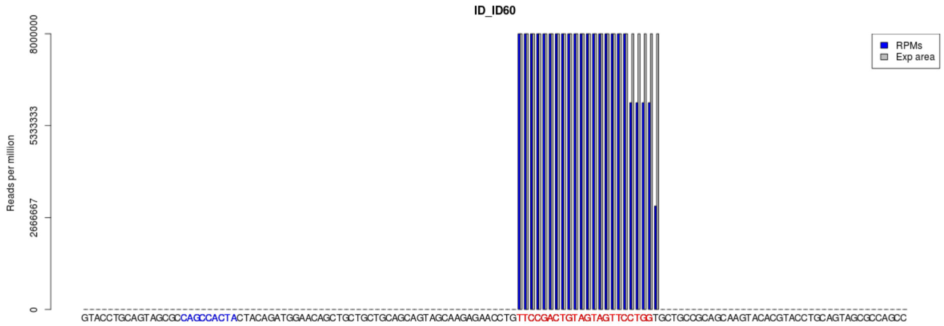

>ID603
ATATATGAAAGTGAAACAAAGCATTAGCGTGTGGTCGGGCGTCTGTTCCTTGTTGTCAGAAATAGAATCA**CCAGGTAACAGATCCTCGACCG**TGTGCTGATGCTCATTCTAC

>ID603-3P
CCAGGTAACAGATCCTCGACCG

>miRNA precursor
AUAUAUGAAAGUGAAACAAAGCAUUAGCGUGUGGUCGGGCGUCUGUUCCUUGUUGUCAGAAAUAGAAUCA**CCAGGUAACAGAUCCUCGACCG**UGUGCUGAUGCUCAUUCUAC
```
...(((....)))......((((((((((..((((((((.((((((((((.((..((.......))...)).))).))))))).))))))))..))))))))))........
```

>Predicted STAR seq:
GTGGTCGGGCGTCTGTTCCTT

Complementarity: 17
ERROR 4: Strange structure
Initial dG = -45.90

```
.-AUA|  G
     UAU A
     GUG A
\ ---^  A
       10


         20        30        40         50         60
  AAACAA--          UG        C        -   U  UG-   AGA
          AGCAUUAGCG  UGGUCGGG GUCUGUU CCU GU    UC   \
          UCGUAGUCGU  GCCAGCUC UAGACAA GGA CA    AG   A
  CAUCUUAC          GU        C       U   C  CUA   AUA
  110       100        90        80        70
```

Reads in: lib-42, lib-45, lib-47, lib-48, lib-49, lib-50, lib-72
Number of reads="ID603",2223,3762,5117,9921,6290,1077,0

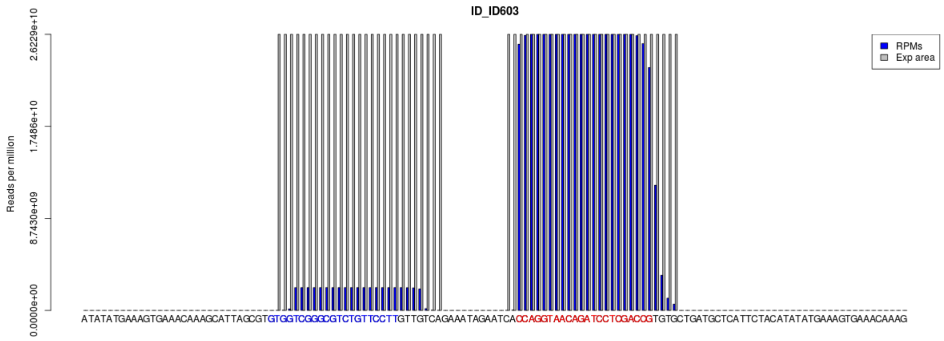

>ID604

GTATGAAAGTGAAATATGAGGCATTAGCGTATAGTCGGGTATCTGTGCCTCTTAGTGAAAATTGGATCAC**TAGGTAACAGAACCCCGAGT**
GTATGCTGGTGCTCATTCTACG

>ID604-3P

TAGGTAACAGAACCCCGAGTGT

>miRNA precursor

GUAUGAAAGUGAAAUAUGAGGCAUUAGCGUAUAGUCGGGUAUCUGUGCCUCUUAGUGAAAAUUGGAUCAC**UAGGUAACAGAACCCCGAGUGU**AUGCUGGUGCUCAUUCUACG
........(((....(((((.((((((((((((.(((((..(((((....((((((((........))))))))..))))).))))).)))))))))))))))))..))).

>Predicted STAR seq:

TAGTCGGGTATCTGTGCCTCTTA

Complementarity: 15

Initial dG =   -50.40

```
        10         20         30         40         50         60
GUAUGAAA|  AAAU     G            G     UA      GCCU         AAA
        GUG    AUGAG CAUUAGCGUAUA UCGGG  UCUGU    CUUAGUGA   U
        CAU    UACUC GUGGUCGUAUGU AGCCC  AGACA    GGAUCACU   U
G-------^  CU--     -            G     CA      AU--         AGG
       110          100        90         80          70
```

Reads in: lib-42, lib-45, lib-47, lib-48, lib-49, lib-50, lib-72

Number of reads="ID604",675,980,3254,1603,1898,449,0

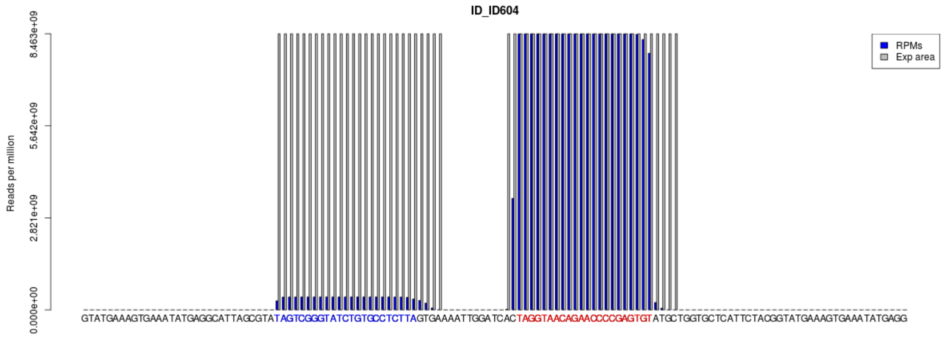

>ID605
TATATAAAAGTGAAATTAAAGCATTAGTGTGTGGTCGGGCGTCTGTTCCTTGTTGTCAGAAATAGAATCA**CCAGGTAACAGATCCTCGAC**
CGTGTGCTGATGCTCTTTCTAC

>ID605-3P
CCAGGTAACAGATCCTCGACCG

>miRNA precursor
UAUAUAAAAGUGAAAUUAAAGCAUUAGUGUGUGGUCGGGCGUCUGUUCCUUGUUGUCAGAAAUAGAAUCA**CCAGGUAACAGAUCCUCGACCG**UGUGCUGAUGCUCUUUCUAC
...........((((....((((((((((..((((((((.((((((((((.((..((.......))...)).))).))))))).))))))))..)))))))))).))))...

>Predicted STAR seq:
GTGGTCGGGCGTCTGTTCCTT

Complementarity: 17

Initial dG =   -43.90

```
        10        20        30        40         50         60
UAUAUAAAAGU|   UUAA         UG        C        -   U  UG-  AGA
           GAAA    AGCAUUAGUG  UGGUCGGG GUCUGUU CCU GU   UC   \
           CUUU    UCGUAGUCGU  GCCAGCUC UAGACAA GGA CA   AG   A
CAU--------^   C---         GU        C        U   C  CUA  AUA
110                  100        90        80        70
```

Reads in: lib-42, lib-45, lib-47, lib-48, lib-49, lib-50, lib-72
Number of reads="ID605",2304,3846,5145,9917,6163,1008,0

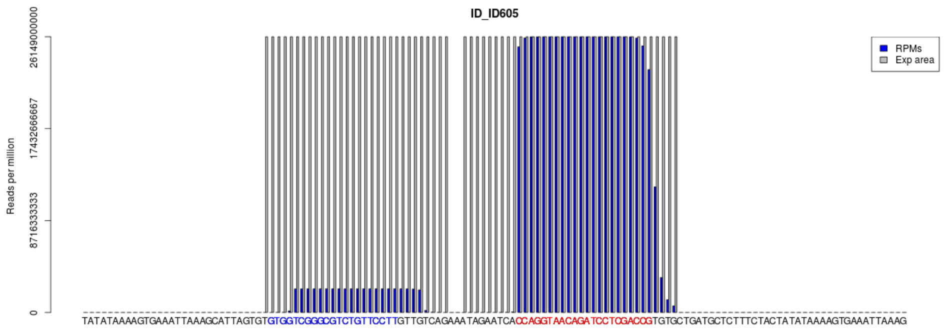

>ID607

GTATGGAAGTGAAATATGAAGTATTAGCATATGGTCGGGTATCTGTGCCTTGTAGTGAAAATTGGATCAC**TAGGTAACAGAACCCCGAGTGT**ATGCTGGTGCTCATTCTACA

>ID607-3P

TAGGTAACAGAACCCCGAGTGT

>miRNA precursor

GUAUGGAAGUGAAAUAUGAAGUAUUAGCAUAUGGUCGGGUAUCUGUGCCUUGUAGUGAAAAUUGGAUCAC**UAGGUAACAGAACCCCGAGUGU**AUGCUGGUGCUCAUUCUACA

```
........(((....((((.((((((((((((((.(((((..(((((((((...(((((........))))))))).)))))..))))).))))))))))))))))))..))).
```

>Predicted STAR seq:

TGGTCGGGTATCTGTGCCTT

Complementarity: 16

Initial dG = -46.30

```
        10         20         30         40          50         60
GUAUGGAA|  AAAU    A             G      UA      -     UGU      AAA
        GUG    AUGA GUAUUAGCAUAUG UCGGG  UCUGU GCCU   AGUGA   U
        CAU    UACU CGUGGUCGUAUGU AGCCC  AGACA UGGA   UCACU   U
A-------^  CU--    -             G      CA      A     ---      AGG
       110          100        90         80           70
```

Reads in: lib-42, lib-45, lib-47, lib-48, lib-49, lib-50, lib-72

Number of reads="ID607",779,1072,3382,2305,2142,469,0

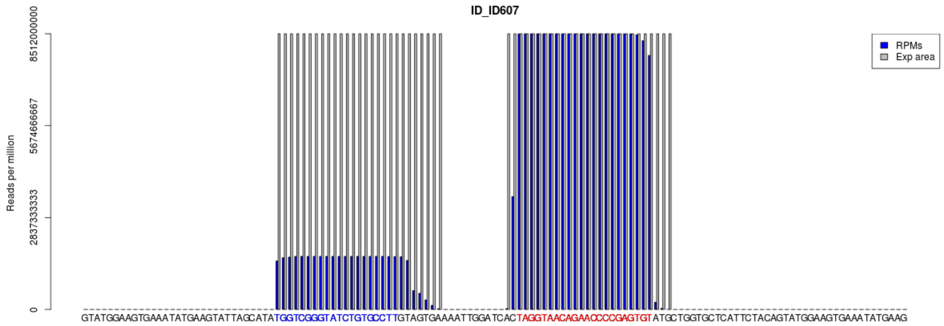

>ID608

GTACAGAAGTGAAATATGAAGCATTAGCGTATAGTCGGGCATCTGTGCCTTTTAGTGAGAATTGGATCAC**TAGGTAACAGAACCCCGAGT**
GTATGCTGGTGCTCATTCTACG

>ID608-3P

TAGGTAACAGAACCCCGAGTGT

>miRNA precursor

GUACAGAAGUGAAAUAUGAAGCAUUAGCGUAUAGUCGGGCAUCUGUGCCUUUUAGUGAGAAUUGGAUCAC**UAGGUAACAGAACCCCGAGUGU**AUGCUGGUGCUCAUUCUACG
........(((....((((.((((((((((((((.(((((..(((((((((...(((((........))))))))).)))))..))))).)))))))))))))))))..))).

>Predicted STAR seq:

TAGTCGGGCATCTGTGCCTT

Complementarity: 16

Initial dG =   -49.00

```
        10         20         30         40          50         60
GUACAGAA|  AAAU    A             G      CA      -    UUU      GAA
        GUG    AUGA GCAUUAGCGUAUA UCGGG  UCUGU GCCU   AGUGA   U
        CAU    UACU CGUGGUCGUAUGU AGCCC  AGACA UGGA   UCACU   U
G-------^  CU--    -             G      CA      A    ---      AGG
       110          100        90        80            70
```

Reads in: lib-42, lib-45, lib-47, lib-48, lib-49, lib-50, lib-72

Number of reads="ID608",697,1024,3291,1604,1895,387,0

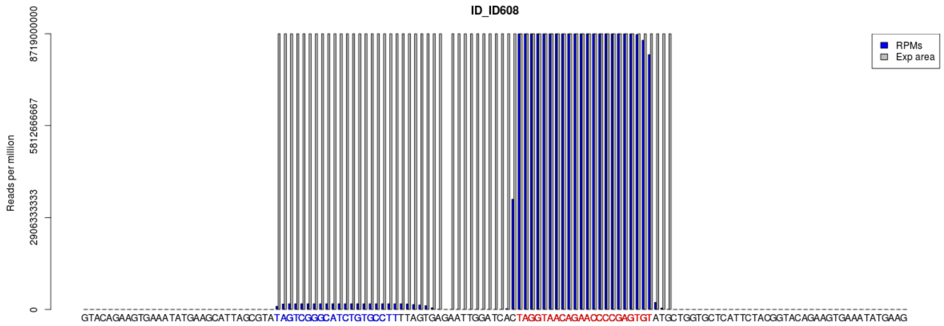

>ID609
TGAAATGAGGCATTAGCGTG**TGGTCGGGTATCTGTGCCTTGT**AGTGAAAATAGGATCACTAGGTAACAGAACCCCGAGTATATGCTGGTG
CTCATCCTACAAAACCACATCG

>ID609-5P
TGGTCGGGTATCTGTGCCTTGT

>miRNA precursor
UGAAAUGAGGCAUUAGCGUG**UGGUCGGGUAUCUGUGCCUUGU**AGUGAAAAUAGGAUCACUAGGUAACAGAACCCCGAGUAUAUGCUGGUGCUCAUCCUACAAAACCACAUCG
....(((((.((((((((((((.(((((..(((((((((...(((((........))))))))).))))).))))).))))))))))))))))).................

>Predicted STAR seq:
GTAACAGAACCCCGAGTATA

Complementarity: 14

Initial dG =   -47.40

```
                     10        20        30         40        50
UGAA-------------|    G            G     UA     -    UGU     AAA
                 AUGAG CAUUAGCGUGUG UCGGG  UCUGU GCCU   AGUGA   U
                 UACUC GUGGUCGUAUAU AGCCC  AGACA UGGA   UCACU   A
GCUACACCAAAACAUCC^    -            G     CA     A    ---     AGG
110       100        90        80        70           60
```

Reads in: lib-42, lib-45, lib-47, lib-48, lib-49, lib-50, lib-72
Number of reads="ID609",470,627,311,1655,1227,222,0

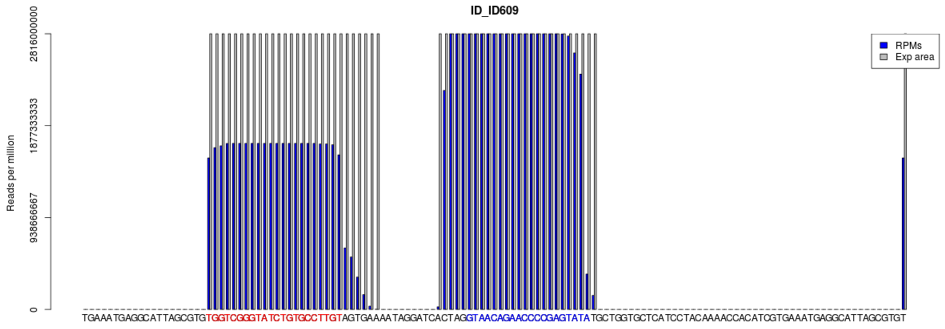

>ID61
CAATTACCTGCAGCAGCCCCAGGAACACCTTCAGACTGTGCAGTAGCAGTTGCTTAAGGAGCAGCAAGAACACCTG**TACGGCCTGAAGAT**
GTTCCTAGGACTGCAGCAGAAG

>ID61-3P
TACGGCCTGAAGATGTTCCTAGG

>miRNA precursor
CAAUUACCUGCAGCAGCCCCAGGAACACCUUCAGACUGUGCAGUAGCAGUUGCUUAAGGAGCAGCAAGAACACCUG**UACGGCCUGAAGAUGUUCCUAGG**ACUGCAGCAGAAG
.......((((.((((.((.(((((((.((((((.((((((((.....(((((((...))))))).........)))))))).)))))).))))))).)).)))).))))...

>Predicted STAR seq:
CCCAGGAACACCTTCAGACTGTG

Complementarity: 18

Initial dG =   -53.30

```
        10        20        30        40           50
CAAUUAC    A    C  C       C      A        UAGCA---|      A
       CUGC GCAG CC AGGAACA CUUCAG CUGUGCAG        GUUGCUU \
       GACG CGUC GG UCCUUGU GAAGUC GGCAUGUC        CGACGAG A
GAA----    A    A  A       A      C        CACAAGAA^      G
110           100        90        80        70        60
```

Reads in: lib-42, lib-45, lib-47, lib-48, lib-49, lib-50, lib-72
Number of reads="ID61",130,4,3,3,1,1,0

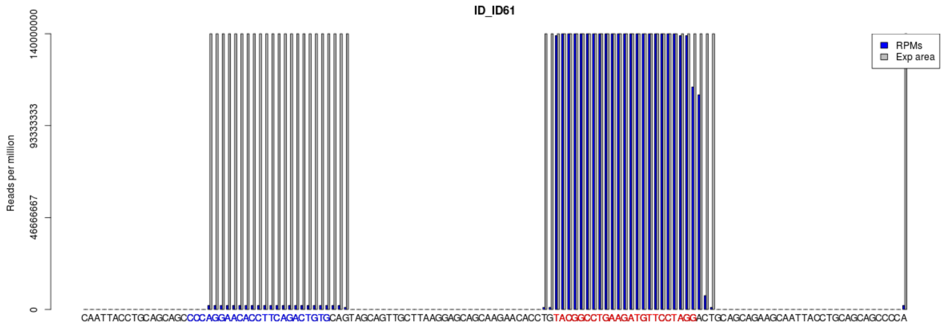

>ID610
GTACGAAAGTGAAATACGAAGCATTAGCGTATAGTCGGGTATCTGTGCCTCTTAGTGAGAATTGGATCAC**TAGGTAACAGAACCCCGAGTGT**ATGCTGGTGTTCATTCTACG

>ID610-3P
TAGGTAACAGAACCCCGAGTGT

>miRNA precursor
GUACGAAAGUGAAAUACGAAGCAUUAGCGUAUAGUCGGGUAUCUGUGCCUCUUAGUGAGAAUUGGAUCAC**UAGGUAACAGAACCCCGAGUGU**AUGCUGGUGUUCAUUCUACG
.(((....)))......(((.((((((((((((.(((((..(((((....((((((((........))))))))..))))).)))))).)))))))))))))))........

>Predicted STAR seq:
TAGTCGGGTATCTGTGCCTCTTA

Complementarity: 15
ERROR 4: Strange structure
Initial dG =   -49.50

```
.-G|  G
   UAC A
   GUG A
\ -^  A
   10


           20        30        40        50        60
  AAAUAC--   G            G      UA      GCCU        GAA
          GAA CAUUAGCGUAUA UCGGG  UCUGU    CUUAGUGA   U
          CUU GUGGUCGUAUGU AGCCC  AGACA    GGAUCACU   U
  GCAUCUUA   -            G      CA      AU--        AGG
  110        100        90        80          70
```

Reads in: lib-42, lib-45, lib-47, lib-48, lib-49, lib-50, lib-72
Number of reads="ID610",709,996,3211,1644,1983,429,0

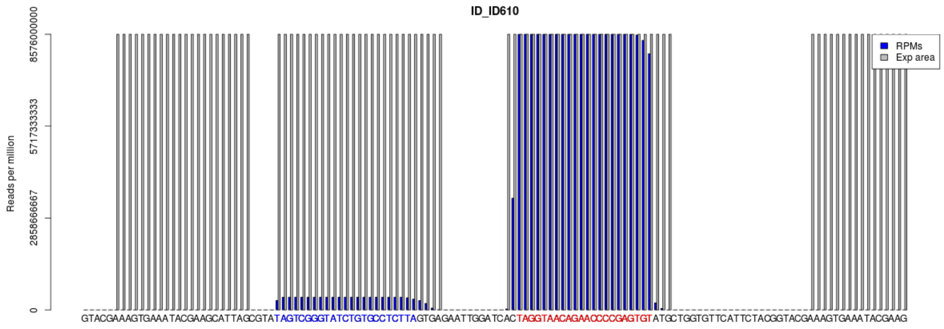

>ID611
TAATATTACTAGTTATGGTA**TGTTATTCTGATTGACAGCTGT**GTGAATATGTTTCACAGCTGCAGATTACAATAACATGCTGAAATCAGT
AATGTGAATATTTTGATTCTTA

>ID611-5P
TGTTATTCTGATTGACAGCTGT

>miRNA precursor
UAAUAUUACUAGUUAUGGUA**UGUUAUUCUGAUUGACAGCUGU**GUGAAUAUGUUUCACAGCUGCAGAUUACAAUAACAUGCUGAAAUCAGUAAUGUGAAUAUUUUGAUUCUUA
..((((((((.(((.((((((((((((.(((((..((((((((..........))))))))..))))).))))))))))))).))).))))))))((((......))))...

>Predicted STAR seq:
AGCTGCAGATTACAATAACATG

Complementarity: 17

Initial dG =   -37.60

```
          10        20        30        40
.-UA|       A   A           C     GA        UGAAU
    AUAUUACU GUU UGGUAUGUUAUU UGAUU  CAGCUGUG     \
    UGUAAUGA UAA GUCGUACAAUAA AUUAG  GUCGACAC     A
\ --^       C   A           C     AC        UUUGU
        90        80        70        60        50



       100
  ---    AU
     GAAU  U
     CUUA  U
  AUU    GU
  110
```

Reads in: lib-42, lib-45, lib-47, lib-48, lib-49, lib-50, lib-72
Number of reads="ID611",67,75,35,151,94,23,0

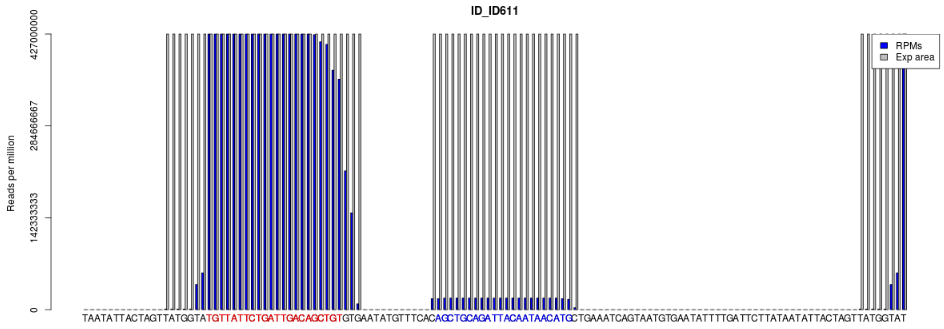

>ID631
CCGGCTCGGACACCACTGGTTGTTTCGTCTGCACCTCGCCCTGTAGGCTGTACCGCAAGCTGGGCCACCT**TGCAGTACAGTCTACGGGGC**
GAGGTACAGTGGACTCCGTCAA

>ID631-3P
TGCAGTACAGTCTACGGGGCGA

>miRNA precursor
CCGGCUCGGACACCACUGGUUGUUUCGUCUGCACCUCGCCCUGUAGGCUGUACCGCAAGCUGGGCCACCU**UGCAGUACAGUCUACGGGGCGA**GGUACAGUGGACUCCGUCAA
..(((..(((..((((((..(((.......)))((((((((((((((((((((.(((((.((...)).))))).))))))))))))))))))))..))))))..))))))..

>Predicted STAR seq:
TTCGTCTGCACCTCGCCCTGTAGGCTGTAC

Complementarity: 19

Initial dG =   -61.90

```
        10          20
CC   UC   CA       .-GU|  UUC
  GGC  GGA  CCACUG    UGU   \
  CUG  CCU  GGUGAC    ACG   G
AA   --   CA       \ --^  UCU
110         100         30
```

Reads in: lib-42, lib-45, lib-47, lib-48, lib-49, lib-50, lib-72
Number of reads="ID631",3,7,0,393,63,0,0

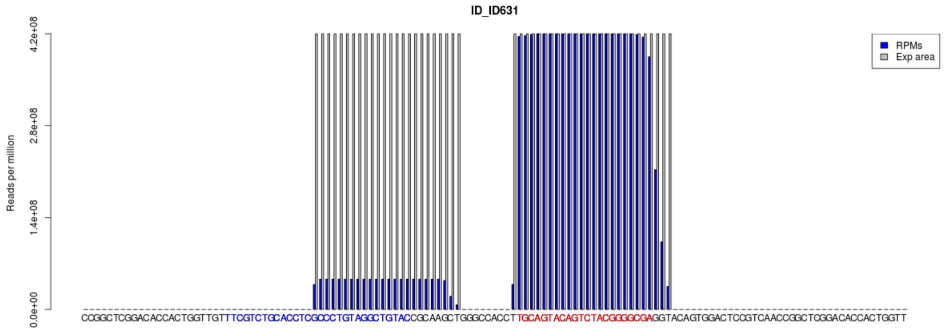

>ID639
TACATGCACCACGGATGTGGCGGGGCCGCTCCCCTCTGAGTGCTGGACAGAGGGCTGGTATTGGGAGATC**CCTGCTCCAGCACTCAGAGG**
GGAGTTGCTTGGCCAAAGCTTC

>ID639-3P
CCTGCTCCAGCACTCAGAGGGG

>miRNA precursor
UACAUGCACCACGGAUGUGGCGGGGCCGCUCCCCUCUGAGUGCUGGACAGAGGGCUGGUAUUGGGAGAUC**CCUGCUCCAGCACUCAGAGGGG**AGUUGCUUGGCCAAAGCUUC
............((...((((.((((.(((((((((((((((((((((((.(((..............)))))).)))))))))))))))))))).)))).))))...))..

>Predicted STAR seq:
TCCCCTCTGAGTGCTGGACA

Complementarity: 19

Initial dG =   -66.20

```
        10        20        30        40         50        60
UACAUGCACCAC  AUG    G    C                    -|  A   CUGGUA
            GG   UGGC GGGC GCUCCCCUCUGAGUGCUGGA CAG GGG      U
            UC   ACCG UUCG UGAGGGGAGACUCACGACCU GUC CCU      U
CU----------  GAA    G    U                    C^  -   AGAGGG
          110       100        90        80         70
```

Reads in: lib-42, lib-45, lib-47, lib-48, lib-49, lib-50, lib-72
Number of reads="ID639",18,26,26,1288,270,12,0

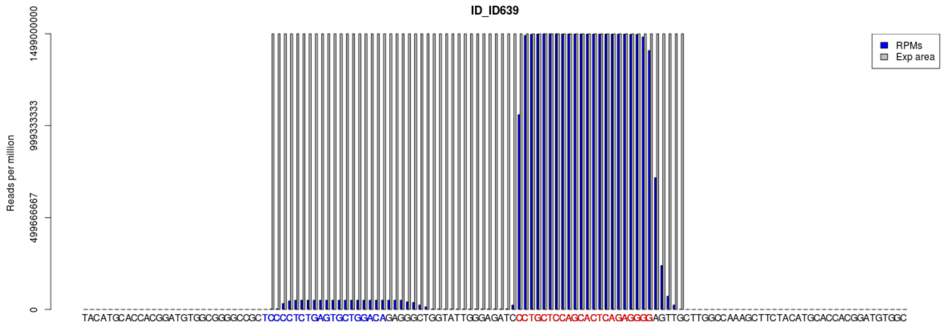

>ID64
GTACCTGCAGTAGCACCAGCCACTACTACAGATGGAACAGCTGCTGCTGCAGCAGTAGCAAGAGAACCTG**TTCCGACTGTAGTAGTTCCT**
GGTGCTGCCGCGGCAAGTACAC

>ID64-3P
TTCCGACTGTAGTAGTTCCTGG

>miRNA precursor
GUACCUGCAGUAGCACCAGCCACUACUACAGAUGGAACAGCUGCUGCUGCAGCAGUAGCAAGAGAACCUG**UUCCGACUGUAGUAGUUCCUGG**UGCUGCCGCGGCAAGUACAC
```
(((((((.((((((((((..((((((((((.((((((((((((((((....)))))))).........))))))).))))))))))..)))))))))).)))).....)))))..
```

>Predicted STAR seq:
CAGCCACTA

Complementarity: 17

Initial dG =   -65.80

```
             10        20        30                  40
--    ---    A          CC          A       ---------|       U
  GUAC   CUGC GUAGCACCAG  ACUACUACAG UGGAACA         GCUGCUGC G
  CAUG   GGCG CGUCGUGGUC  UGAUGAUGUC GCCUUGU         CGAUGACG C
CA    AAC    C          CU          A       CCAAGAGAA^       A
110       100        90        80        70        60        50
```

Reads in: lib-42, lib-45, lib-47, lib-48, lib-49, lib-50, lib-72
Number of reads="ID64",8,0,0,0,0,0,0

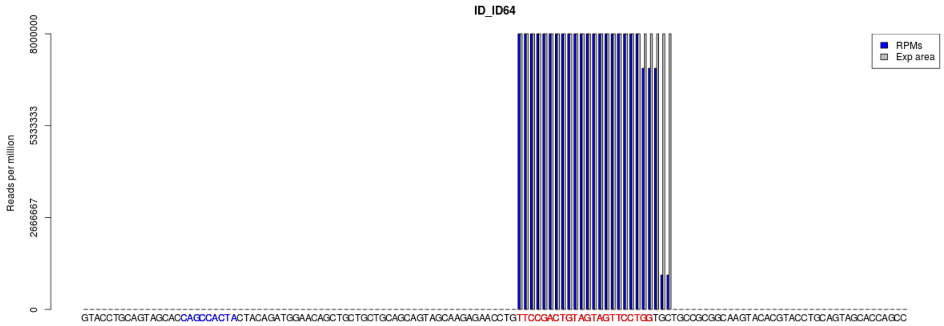

>ID641

AAGATCGTCCAACCCAGGTT**CGTGTCGGCATGTTGAGACGCG**AAGTATCTGGTCTAAAAATGCGGCTCAGTATTCCGAGACGAGTCTGGA
TTGGACCAACAACAGGAGCGAC

>ID641-5P

CGTGTCGGCATGTTGAGACGCG

>miRNA precursor

AAGAUCGUCCAACCCAGGUU**CGUGUCGGCAUGUUGAGACGCG**AAGUAUCUGGUCUAAAAAUGCGGCUCAGUAUUCCGAGACGAGUCUGGAUUGGACCAACAACAGGAGCGAC
......((((((.((((..((((.((((.((((((((.((((...((.......))....)))).)))))))).)))).)))).)).)))).)))))).................

>Predicted STAR seq:

CGGCTCAGTATTCCGAGACGAG

Complementarity: 17

Initial dG =   -42.40

```
                  10        20        30        40          50
AAGAUC----------      C    GU    G    C        A    AAG-| UCU
                GUCCAA CCAG  UCGU UCGG AUGUUGAG CGCG    UA   \
                CAGGUU GGUC  AGCA AGCC UAUGACUC GCGU    AU   G
CAGCGAGGACAACAAC      A    UG    G    U        G    AAAA^ CUG
110       100        90        80        70        60
```

Reads in: lib-42, lib-45, lib-47, lib-48, lib-49, lib-50, lib-72

Number of reads="ID641",57,47,12,2642,256,7,0

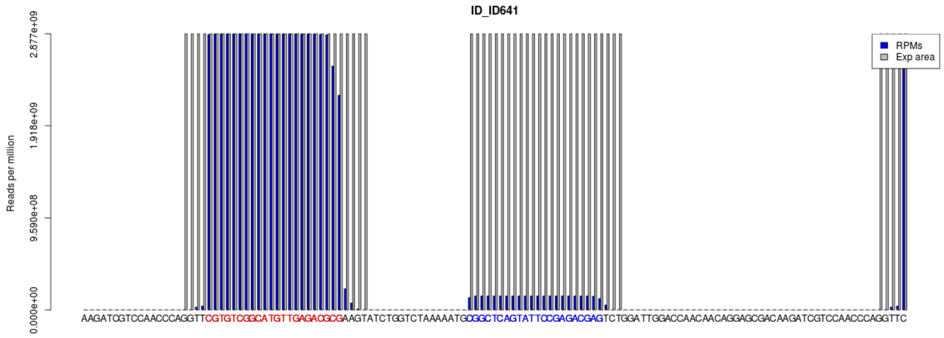

>ID642
CCATGAGCCCCACCGCTGTG**AGATATGTTTGATATTCTTGGTT**GTAATATCAGATTATCACCCAGGAATCAAACATATTATTACAGAGGG
GGGTCCAGCAGCATCTTCA

>ID642-5P
AGATATGTTTGATATTCTTGGTT

>miRNA precursor
CCAUGAGCCCCACCGCUGUG**AGAUAUGUUUGAUAUUCUUGGUU**GUAAUAUCAGAUUAUCACCCAGGAAUCAAACAUAUUAUUACAGAGGGGGGUCCAGCAGCAUCUUCA
```
...((..((((.((.((((((((((((((((((.((((.(((.(((((.....))))).))).)))))))))))))))))).)))))).))))))..))...........
```

>Predicted STAR seq:
CCAGGAATCAAACATATTATTA

Complementarity: 18

Initial dG =   -43.90

```
                 10        20         30        40        50
CCA---------| AG    A  G      -           A    U   U     AU
            UG  CCCC CC CUGUGA GAUAUGUUUGAU UUCU GGU GUAAU  \
            AC  GGGG GG GACAUU UUAUACAAACUA AGGA CCA UAUUA  C
ACUUCUACGACG^ CU    -  A      A           -    C   C     GA
       100       90         80        70         60
```

Reads in: lib-42, lib-45, lib-47, lib-48, lib-49, lib-50, lib-72
Number of reads="ID642",324,494,525,1160,326,128,0

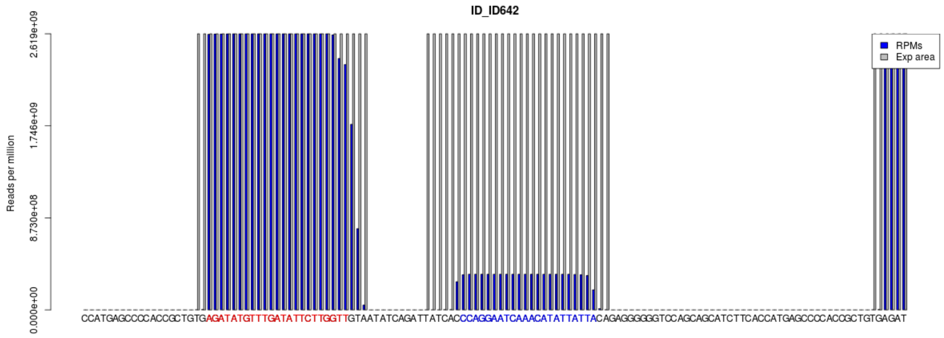

>ID643
GAGTGCCAGACTACAAATCG**GAAGGTCGCGGGTTCGTTCCC**AGCCAGGTCCTGGGATTTTTATCTGTCACTTTCTACTTCTTTCACCTCT
GGCAGTATTGCTGATGTGAAA

>ID643-5P
GAAGGTCGCGGGTTCGTTCCC

>miRNA precursor
GAGUGCCAGACUACAAAUCG**GAAGGUCGCGGGUUCGUUCCC**AGCCAGGUCCUGGGAUUUUUAUCUGUCACUUUCUACUUCUUUCACCUCUGGCAGUAUUGCUGAUGUGAAA
...(((((((.........(((((((.((((((....((((((.......))))))......)))))).))))))).............)))))))...(..(....)..)..

>Predicted STAR seq:
GATTTTTATCTGTCACTTTCTA

Complementarity: 14

Initial dG =   -33.20

```
          10            20         30          40
.-GAG       CUACAAAUC---        C       UCGU-|     CCA
     UGCCAGA            GGAAGGU GCGGGU      UCCCAG   \
     ACGGUCU            UCUUUCA UGUCUA      AGGGUC   G
\ ---       CCACUUUCUUCA        C       UUUUU^     CUG
        90        80         70         60        50
```

```
     100
  GUA UG U
     U  C G
     A  G A
  AA- GU U
 110
```

Reads in: lib-42, lib-45, lib-47, lib-48, lib-49, lib-50, lib-72
Number of reads="ID643",41,47,219,48,29,91,0

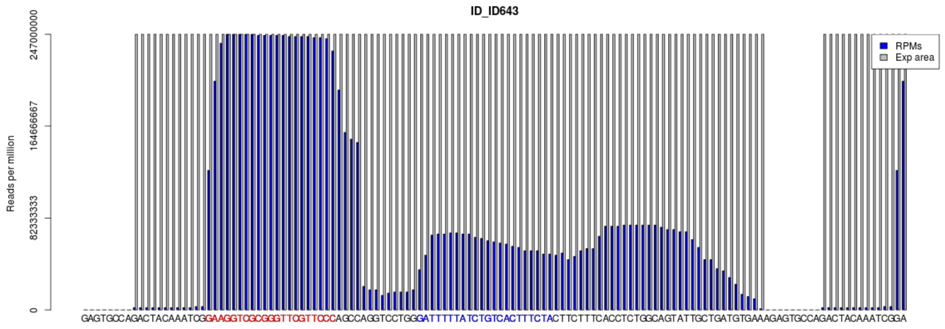

>ID645

GTGCGCTCTATTTCCCATCTCTTCACAGGGCAGCATAAAAATTCAACGGTTCAATAATATATTAAATCTA**TGCTGCCCTGTGCAGAGGTT**
GATTACAAAATTTTAAACATA

>ID645-3P

TGCTGCCCTGTGCAGAGGTTGA

>miRNA precursor

```
GUGCGCUCUAUUUCCCAUCUCUUCACAGGGCAGCAUAAAAAUUCAACGGUUCAAUAAUAUAUUAAAUCUAUGCUGCCCUGUGCAGAGGUUGAUUACAAAAUUUUAAACAUA
...............((((((.((((((((((((((..........((((.((((...)))).)))))))))))))))))))).))))))......................
Error predicting STAR on 5p
 Initial dG =    -32.70

        10               20        30        40        50
GUGCGCUCUAUUUCCC------|     U             AAAAUUCAAC    C    A
                      AUCUCU CACAGGGCAGCAUA          GGUU AAUA \
                      UGGAGA GUGUCCCGUCGUAU          CUAA UUAU U
AUACAAAUUUUAAAACAUUAGU^     C             ----------    A    A
 .       100        90        80        70                  60
```

Reads in: lib-42, lib-45, lib-47, lib-48, lib-49, lib-50, lib-72

Number of reads="ID645",0,8,1,0,0,1,0

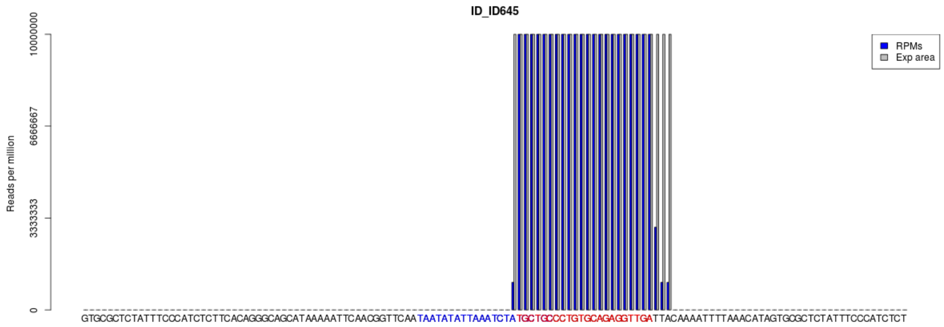

>ID646

AGAAGAAGCAGTGCGTCATGAGGTGGGTAGCGGTAAATTGAGTTGTGCCGGTAAACGTGAAAATAAGTCG**TTATCGGATCAACTCAATTT**
AACTGCTGCTCACTCTACAACA

>ID646-3P

TTATCGGATCAACTCAATTTAAC

>miRNA precursor

AGAAGAAGCAGUGCGUCAUGAGGUGGGUAGCGGUAAAUUGAGUUGUGCCGGUAAACGUGAAAAUAAGUCG**UUAUCGGAUCAACUCAAUUUAAC**UGCUGCUCACUCUACAACA

```
.............((..((..(((((((((((((((((((((((..(((((((.((...........)))))))))..))))))))))))).))))))))))..))..)).
```

>Predicted STAR seq:

TAAATTGAGTTGTGCCGGTAA

Complementarity: 18

Initial dG = -45.20

```
        10        20        30         40        50        60
AGAAGAAGCAGUGC  CA  AG           -|           UG       A  UGAAA
              GU  UG  GUGGGUAGCGG UAAAUUGAGUUG  CCGGUAA CG     \
              CA  AU  CACUCGUCGUC AUUUAACUCAAC  GGCUAUU GC     A
A-------------  AC  CU          A^           UA       -  UGAAU
             110       100        90        80         70
```

Reads in: lib-42, lib-45, lib-47, lib-48, lib-49, lib-50, lib-72

Number of reads="ID646",4,0,1,6,0,0,0

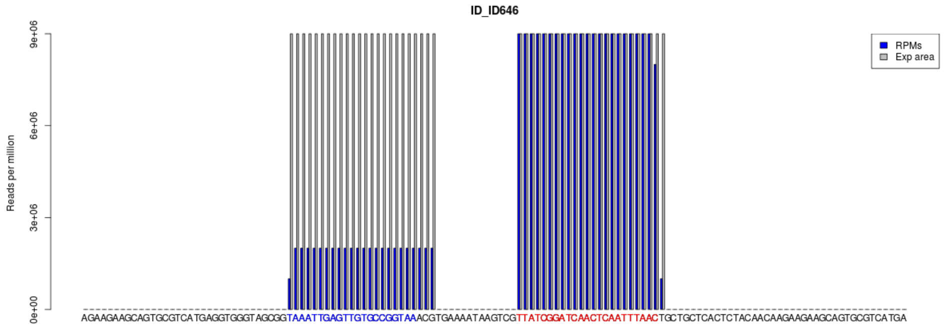

>ID649
TTAATTGATAAGCCAAATTTCTTTAGCAATGAA**AGACAGTCGTGGTTGCAACTTT**TTTAATAATGAATAAAGTTGCAACAATGACTGTCT
TTCATAGCTAAACAATTTTGTT

>ID649-5P
AGACAGTCGTGGTTGCAACTTT

>miRNA precursor
UUAAUUGAUAAGCCAAAUUUCUUUAGCAAUGAA**AGACAGUCGUGGUUGCAACUUU**UUUAAUAAUGAAUAAAGUUGCAACAAUGACUGUCUUUCAUAGCUAAACAAUUUUGUU
.............((((.((.((((((.(((((((((((((((.(((((((((((((((....)))).))))))))))).))))))))))))))).)))))).)).))))..

>Predicted STAR seq:
AGTTGCAACAATGACTGTCTTT

Complementarity: 19

Initial dG = -47.60

```
        10        20        30        40        50         60
UUAAUUGAUAAGC    U  C      A               G           -|   A
             CAAA UU UUUAGC AUGAAAGACAGUCGU GUUGCAACUUU UUUA U
             GUUU AA AAAUCG UACUUUCUGUCAGUA CAACGUUGAAA AAGU A
UU-----------    U  C      A               A           U^   A
           110       100        90        80        70
```

Reads in: lib-42, lib-45, lib-47, lib-48, lib-49, lib-50, lib-72
Number of reads="ID649",15,36,25,8,56,14,0

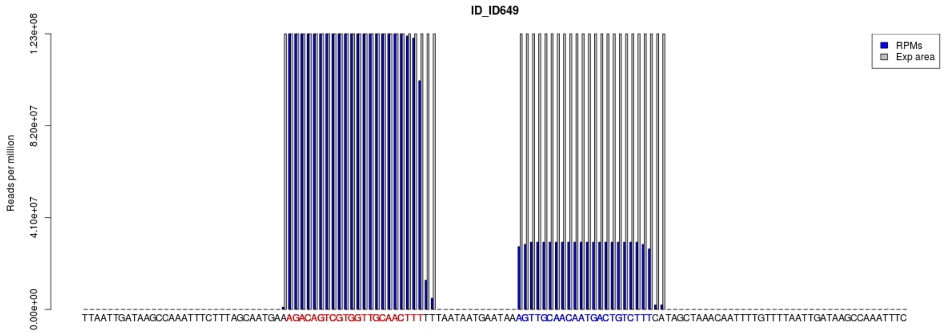

>ID65

ATGAAGCCACCAGTATCTGCAGTAGCACTAGCCGTAACTACAGATGGGGCAGCAGTTGCAAGAACACCTG**TCCTAACTGTAGTTGCTGCT**
GGTGCTGAGGCAGAAACA

>ID65-3P

TCCTAACTGTAGTTGCTGCTGG

>miRNA precursor

AUGAAGCCACCAGUAUCUGCAGUAGCACUAGCCGUAACUACAGAUGGGGCAGCAGUUGCAAGAACACCUG**UCCUAACUGUAGUUGCUGCUGG**UGCUGAGGCAGAAACA
............((.(((((..((((((((((.((((((((((.((((((((..(((.....)))..)))))))).)))))))))).)))))))))).)))))).)).

>Predicted STAR seq:

TAGCCGTAACTACAGATGGGG

Complementarity: 18

Initial dG = -58.00

```
        10        20        30        40        50
AUGAAGCCACCA| A     AG          C          A        CA   GC
            GU UCUGC  UAGCACUAGC GUAACUACAG UGGGGCAG  GUU  \
            CA AGACG  GUCGUGGUCG CGUUGAUGUC AUCCUGUC  CAA  A
A-----------^ A     GA          U          A        CA   GA
                 100        90        80        70
```

Reads in: lib-42, lib-45, lib-47, lib-48, lib-49, lib-50, lib-72

Number of reads="ID65",210,10,4,8,3,3,0

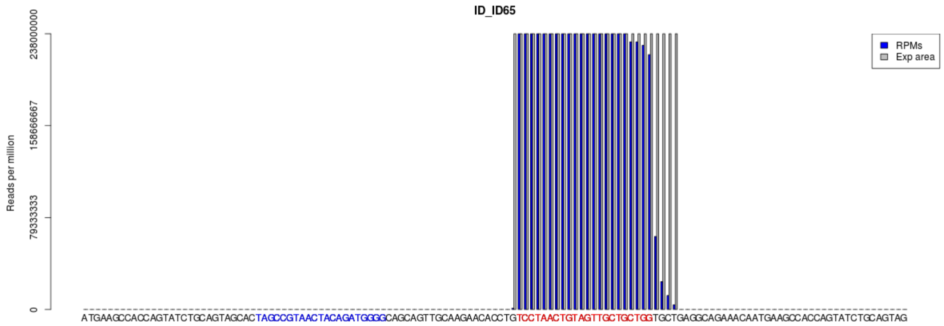

>ID656
GGTGTTCGCGCCACAGTCTC**TGGTCGACTTTCTTCTGAGGTGC**AATTATGATCGAGTCTCCACAGAATGGAAATCGGTCGAGACTGTGGT
GCCGGTGCTACGATTGACCCTT

>ID656-5P
TGGTCGACTTTCTTCTGAGGTGC

>miRNA precursor
GGUGUUCGCGCCACAGUCUC**UGGUCGACUUUCUUCUGAGGUGC**AAUUAUGAUCGAGUCUCCACAGAAUGGAAAUCGGUCGAGACUGUGGUGCCGGUGCUACGAUUGACCCUU
((..(..((((((((((((((.(..(((.(((((((((.((.((............))..)).))))).)))).)))..)))))))))))))))..)..))............

>Predicted STAR seq:
CTCCACAGAATGGAAATCGGTCGA

Complementarity: 15

Initial dG =   -47.00

```
                     10        20        30         40
-------------  UG UC            U GU   C    -     A  U-| AAUUA
             GG  U  GCGCCACAGUCUC G  CGA UUUC UUCUG GG  GC     U
             UC  G  CGUGGUGUCAGAG C  GCU AAGG AAGAC CC  UG     G
UUCCCAGUUAGCA  GU GC            - UG   A    U     A  UC^ AGCUA
110       100       90        80         70        60        50
```

Reads in: lib-42, lib-45, lib-47, lib-48, lib-49, lib-50, lib-72
Number of reads="ID656",39,6,0,70,10,0,0

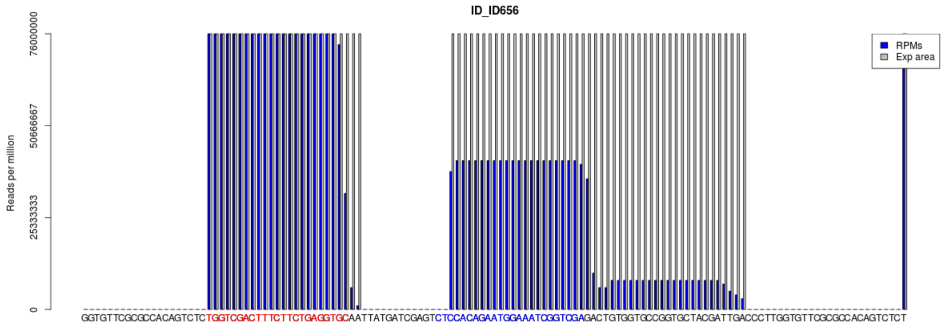

>ID659
CCTGCTGCCCTGGGCCGCGGTGGCACTGGGGTCTCTCAGGCGACTGCTTCGCTGCCTCTGGAACATTTAG**CATCGCCTGGGATCCCTGAT**
GCCAACGGGCCTGCCGGTC

>ID659-3P
CATCGCCTGGGATCCCTGATG

>miRNA precursor
CCUGCUGCCCUGGGCCGCGGUGGCACUGGGGUCUCUCAGGCGACUGCUUCGCUGCCUCUGGAACAUUUAG**CAUCGCCUGGGAUCCCUGAUG**CCAACGGGCCUGCCGGUC
```
......(((..(((((.((.(((((..((((..((((((((((.((((......((...)).......)))))))))))))).)))))..))))).)))))))...))).
```

>Predicted STAR seq:
ACTGGGGTCTCTCAGGCGACTG

Complementarity: 16

Initial dG =   -53.60

```
        10         20         30         40         50
CCUGCU   CU-|    G  G      CU    UC          C    UCGCUG-  U
      GCC   GGGCC CG UGGCA  GGGG  UCUCAGGCGA UGCU       CC \
      UGG   UCCGG GC ACCGU  UCCC  AGGGUCCGCU ACGA       GG C
C-----   CCG^    -  A     AG    U-          -    UUUACAA  U
            100         90         80         70        60
```

Reads in: lib-42, lib-45, lib-47, lib-48, lib-49, lib-50, lib-72
Number of reads="ID659",50,45,157,774,127,40,0

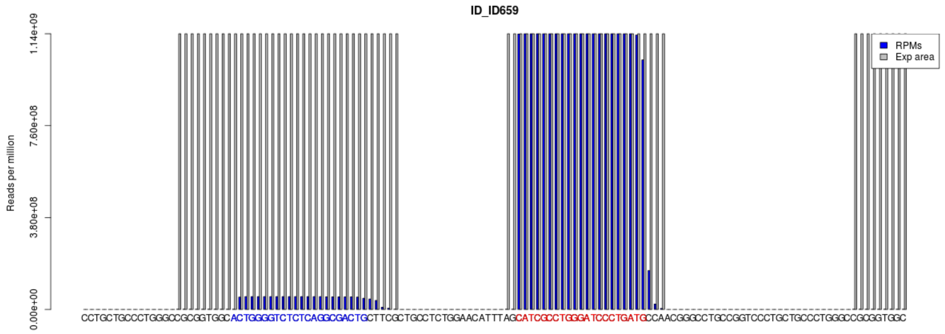

>ID66
GTATTACCTGCAGCAGCACC**AGGAACACCTTCAGACTGTGCA**GTAGCAGCTGCTTAAGCAGCAGCAAGAACACCTGTACGGCCTGAAGAT
GTTCCTTAGTACTGCACAGAAG

>ID66-5P
AGGAACACCTTCAGACTGTGCA

>miRNA precursor
GUAUUACCUGCAGCAGCACC**AGGAACACCUUCAGACUGUGCA**GUAGCAGCUGCUUAAGCAGCAGCAAGAACACCUGUACGGCCUGAAGAUGUUCCUUAGUACUGCACAGAAG
.......(((..((((.((.(((((((.((((((.((((((((..((.(((((....))))).))........)))))))).)))))).))))))).)).)))).)))...

>Predicted STAR seq:
TACGGCCTGAAGATGTTCCTTAGT

Complementarity: 18

Initial dG =   -47.30

```
        10        20         30        40               50
GUAUUAC   CA    C  C-        C      A        UA------| A     U
       CUG  GCAG AC  AGGAACA CUUCAG CUGUGCAG        GC GCUGC U
       GAC  CGUC UG  UCCUUGU GAAGUC GGCAUGUC        CG CGACG A
GAA----   A-    A  AU       A      C        CACAAGAA^ A     A
110            100        90        80        70        60
```

Reads in: lib-42, lib-45, lib-47, lib-48, lib-49, lib-50, lib-72
Number of reads="ID66",18,3,0,0,0,1,0

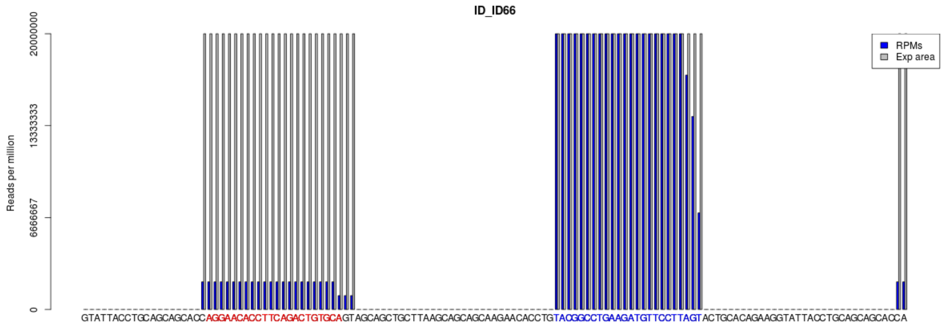

>ID660
GTGATACAGGATGAATCCACACCACCT**AGAAGACAAGGCCTGACAACTTG**TCATGGCTACGTCACACCAAGTTGTCAGGCCATGTCTTCT
AGGTGGTGTTGAATGAATCCAA

>ID660-5P
AGAAGACAAGGCCTGACAACTTG

>miRNA precursor
GUGAUACAGGAUGAAUCCACACCACCU**AGAAGACAAGGCCUGACAACUUG**UCAUGGCUACGUCACACCAAGUUGUCAGGCCAUGUCUUCUAGGUGGUGUUGAAUGAAUCCAA
........((((...((.((((((((((((((((.((((((((((((((...((((...))))...)))))))))))))).))))))))))))))))).)).....))))..

>Predicted STAR seq:
AGTTGTCAGGCCATGTCTTCTAG

Complementarity: 20

Initial dG =   -67.70

```
        10         20        30        40        50
GUGAUACA    GAA-| C                  A              UCA    U
        GGAU    UC ACACCACCUAGAAGACA GGCCUGACAACUUG   UGGC \
        CCUA    AG UGUGGUGGAUCUUCUGU CCGGACUGUUGAAC   ACUG A
AA------    AGUA^ U                  A              CAC    C
      110       100        90        80        70        60
```

Reads in: lib-42, lib-45, lib-47, lib-48, lib-49, lib-50, lib-72
Number of reads="ID660",15,71,35,29,72,25,0

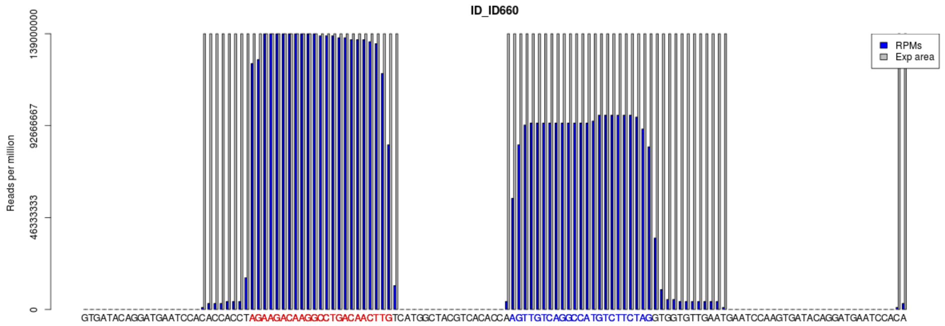

>ID661
CCATACTACTTTATAGCTGC**TGATAGATCTTCCAGGTTGTAAG**TTTTATGGACCACAGCCTTATAACCTGGAAGATCTACCAGCAGCTGT
AACGTTTGTCATGAAAGGCTT

>ID661-5P
TGATAGATCTTCCAGGTTGTAAG

>miRNA precursor
CCAUACUACUUUAUAGCUGC**UGAUAGAUCUUCCAGGUUGUAAG**UUUUAUGGACCACAGCCUUAUAACCUGGAAGAUCUACCAGCAGCUGUAACGUUUGUCAUGAAAGGCUU
```
.(((((.((.((((((((((((.((((((((((((((((((((.....((.....))..))))))))))))))))))))).))))))))))))).))..)).))).........
```

>Predicted STAR seq:
TATAACCTGGAAGATCTACCAGC

Complementarity: 20

Initial dG =   -55.10

```
                 10        20        30        40        50
C-------   -  U-| U            A                    UUUUA  GA
        CAU AC  AC UUAUAGCUGCUG UAGAUCUUCCAGGUUGUAAG     UG  \
        GUA UG  UG AAUGUCGACGAC AUCUAGAAGGUCCAAUAUUC     AC  C
UUCGGAAA   C  UU^ C            C                    CG---  AC
 .       100        90        80        70        60
```

Reads in: lib-42, lib-45, lib-47, lib-48, lib-49, lib-50, lib-72
Number of reads="ID661",19,39,6,16,23,5,0

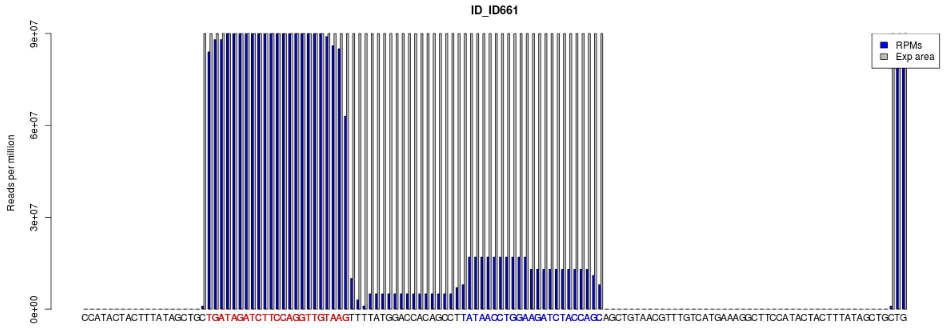

>ID666
CGGCGGCAGCGGAGCCCGTCACTCGCACAGTGCCTGCGGGGCCGGCGTGACGCCGCATGCACTGTGCAAGCGGAATGGCCTCTGCAGGCG
CGACACCTCGCGCCACTGCT

>ID666-5P
ACTCGCACAGTGCCTGCGGG

>miRNA precursor
CGGCGGCAGCGGAGCCCGUCACUCGCACAGUGCCUGCGGGGCCGGCGUGACGCCGCAUGCACUGUGCAAGCGGAAUGGCCUCUGCAGGCGCGACACCUCGCGCCACUGCU
```
.(((((..((((((((..((.((.(((((((((.(((((...((......))))))).))))))))).)).))...))).))))).(((((((....))))))).)))))
```

>Predicted STAR seq:
GCATGCACTGTGCAAGCGG

Complementarity: 14

Initial dG =   -61.30

```
          10          20         30         40
C     .-CA     -   CG-| A  C         C     GGC  GC
 GGCGG    GCGGA GCC   UC CU GCACAGUGC UGCGG   CG  G
 UCGUC    CGUCU CGG   GG GA CGUGUCACG ACGCC   GC  U
-     \ --     C   UAA^ C  A         U     ---  AG
 .           80        70        60           50



           90
        A       C
         GGCGCGA A
         CCGCGCU C
        A       C
           100
```

Reads in: lib-42, lib-45, lib-47, lib-48, lib-49, lib-50, lib-72
Number of reads="ID666",4,42,32,118,42,11,0

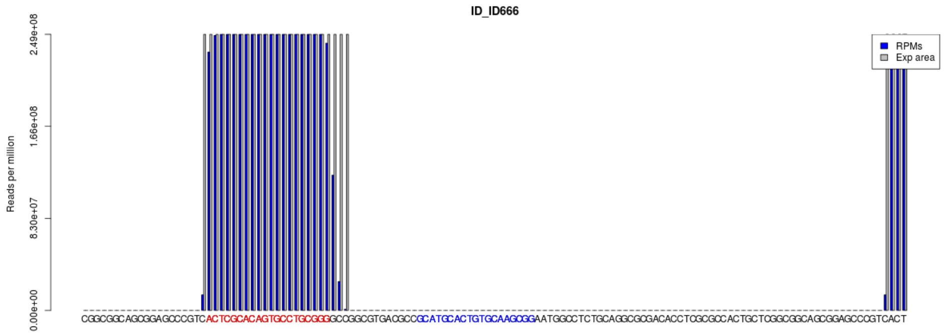

>ID67
GTACCTGCAGTAGCACCAGCCACTACTACAGATGGAACAGCTGCTGCAGCAGCAGTAGCAAGAGAACCTG**TTCCGACTGTAGTAGTTCCT**
GGTGCTGCCACAGCAAGTACAC

>ID67-3P
TTCCGACTGTAGTAGTTCCTGG

>miRNA precursor
GUACCUGCAGUAGCACCAGCCACUACUACAGAUGGAACAGCUGCUGCAGCAGCAGUAGCAAGAGAACCUG**UUCCGACUGUAGUAGUUCCUGG**UGCUGCCACAGCAAGUACAC
((((.(((.((((((((((..((((((((((.((((((((((((((((....)))))))).........))))))).)))))))))).)))))))))))....))).)))))..

>Predicted STAR seq:
CAGCCACTACTACAGATGGAA

Complementarity: 17

Initial dG =   -63.40

```
             10        20        30                  40
--    C   A---          CC          A       ---------|       A
  GUAC UGC    GUAGCACCAG  ACUACUACAG UGGAACA         GCUGCUGC G
  CAUG ACG    CGUCGUGGUC  UGAUGAUGUC GCCUUGU         CGAUGACG C
CA    A   ACAC          CU          A       CCAAGAGAA^       A
110       100       90        80        70        60        50
```

Reads in: lib-42, lib-45, lib-47, lib-48, lib-49, lib-50, lib-72
Number of reads="ID67",7,1,0,1,0,0,0

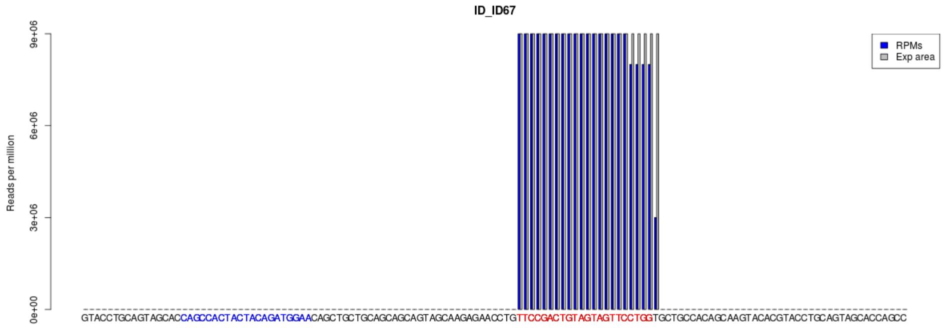

>ID670
AAGCAGAATGAAGAGACTTGAGGTGAATTCTATGGCTGTTCGTCAAATTCAGTTAATTAAAGAAGCTCTG**AATTTTATGAGCAACCGTGG**
GGTTCCCCTCAAGAAGCAGCCA

>ID670-3P
AATTTTATGAGCAACCGTGGGG

>miRNA precursor
AAGCAGAAUGAAGAGACUUGAGGUGAAUUCUAUGGCUGUUCGUCAAAUUCAGUUAAUUAAAGAAGCUCUG**AAUUUUAUGAGCAACCGUGGGG**UUCCCCUCAAGAAGCAGCCA
```
................(((((((.(((((((((((.(((((((.((((((((...............)))))))).))))))).)))))))))))).))))))).........
```

>Predicted STAR seq:
TCTATGGCTGTTCGTCAAATT

Complementarity: 18

Initial dG = -42.50

```
        10        20        30        40        50
AAGCAGAAUGAAGAGA|      U           C       C        UUAAUUA
                CUUGAGG GAAUUCUAUGG UGUUCGU AAAUUCAG       \
                GAACUCC CUUGGGGUGCC ACGAGUA UUUAAGUC       A
ACCGACGAA-------^      C           A       U        UCGAAGA
110              100       90        80        70
```

Reads in: lib-42, lib-45, lib-47, lib-48, lib-49, lib-50, lib-72
Number of reads="ID670",55,34,16,1634,355,12,0

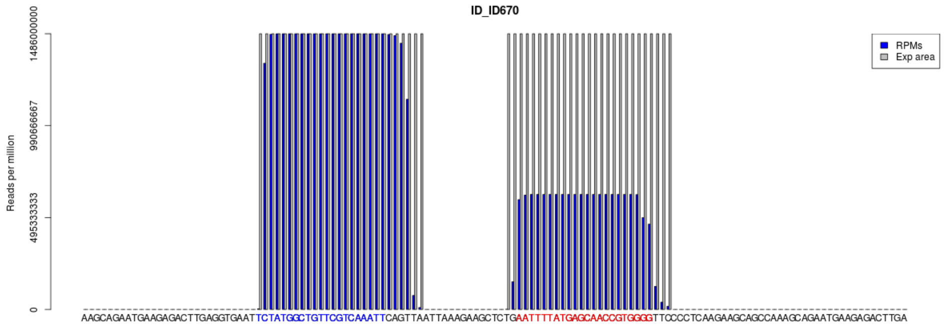

>ID671
GCACGTACCGTTATGAGACTTGAGGTGAATTCTAAGGTTGTTCGTCATATTCAGACAGTTTAAGTAACTG**GATGTTATGAGGGACCTTAG
AGT**GCACCTCAGGAAGCAGCCAA

>ID671-3P
GATGTTATGAGGGACCTTAGAGT

>miRNA precursor
GCACGUACCGUUAUGAGACUUGAGGUGAAUUCUAAGGUUGUUCGUCAUAUUCAGACAGUUUAAGUAACUG**GAUGUUAUGAGGGACCUUAGAGU**GCACCUCAGGAAGCAGCCAA
((.((((....))))...((((((((.(((((((((((.(((((.((((((((((.......))..)))))))).))))).))))))))))).)))))))))..))......

>Predicted STAR seq:
ATTCTAAGGTTGTTCGTCATATT

Complementarity: 19
ERROR 4: Strange structure
 Initial dG =   -46.70

```
------  .-A|   C
      GC   CGUA C
      CG   GUAU G
AACCGA  \ -^   U
 110           10
```

```
             20        30        40        50
          AGA         A           G      C         --  AGU
             CUUGAGGUG AUUCUAAGGUU UUCGU AUAUUCAG  AC   \
             GGACUCCAC UGAGAUUCCAG GAGUA UGUAGGUC  UG   U
          AA-         G           G      U         AA  AAU
              100        90        80        70
```

Reads in: lib-42, lib-45, lib-47, lib-48, lib-49, lib-50, lib-72
Number of reads="ID671",14,1,0,247,34,2,0

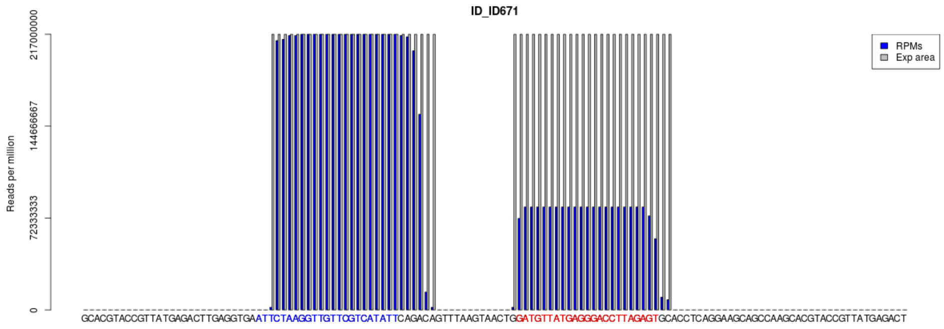

>ID673
TCATCGGGAACGGACAACTCAACTGGGGTCAAATTGACTCTAGTAGGGAGTCCGTGATTATGAGATGCGA**CTCCCTAACGGAGTCAGGTT**
GACCTCGTTGTAGTGAACACA

>ID673-3P
CTCCCTAACGGAGTCAGGTTG

>miRNA precursor
UCAUCGGGAACGGACAACUCAACUGGGGUCAAAUUGACUCUAGUAGGGAGUCCGUGAUUAUGAGAUGCGA**CUCCCUAACGGAGUCAGGUUG**ACCUCGUUGUAGUGAACACA
```
...(((....)))...(((((((.((((((((.((((((((..((((((((((((.........))).)))))))))..)))))))).))))))))))))).))).......
```

>Predicted STAR seq:
AAATTGACTCTAGTAGGGAG

Complementarity: 16
ERROR 4: Strange structure
Initial dG =   -50.90

```
.-UCA|  G
     UCG G
     GGC A
\ ---^  A
       10



            20        30        40        50
  ACA----   -    U        A        AG         -   GAUU
         ACU CAAC GGGGUCAA UUGACUCU  UAGGGAGUC CGU    \
         UGA GUUG CUCCAGUU GACUGAGG  AUCCCUCAG GUA    A
  ACACAAG   U    -        G        CA         C   GAGU
 110       100         90        80        70
```

Reads in: lib-42, lib-45, lib-47, lib-48, lib-49, lib-50, lib-72
Number of reads="ID673",4,65,2,1325,388,7,0

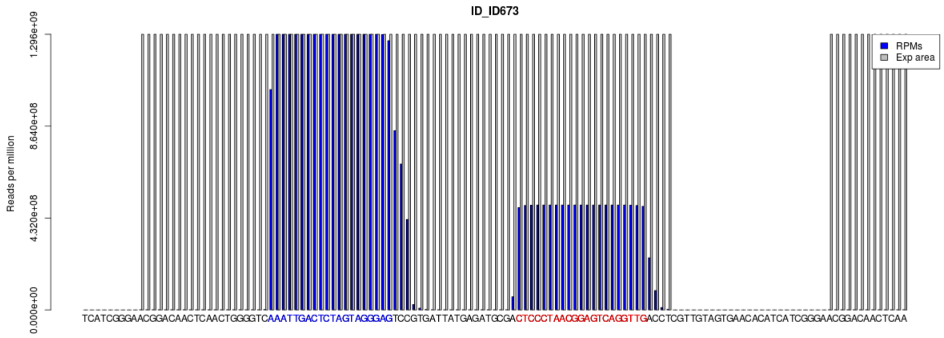

>ID675

CTCTACTGTATGAATATATGATGATGGCATCCTCTTGGGTAAAATATTCCGGGTGTAAAATAGTCCCCA**TTCGGATCTCTTACTCAGGA**
AGATGTCATCATCAGAAACGA

>ID675-3P

TTCGGATCTCTTACTCAGGAAG

>miRNA precursor

CUCUACUGUAUGAAUAUAUGAUGAUGGCAUCCUCUUGGGUAAAAUAUUCCGGGUGUAAAAUAGUCCCCA**UUCGGAUCUCUUACUCAGGAAG**AUGUCAUCAUCAGAAACGA
.................((((((((((((.((((((((((.....((((((((.............))))))))....)))))))))).)))))))))))).......

>Predicted STAR seq:

CTCTTGGGTAAAATATTCCGGG

Complementarity: 15

Initial dG =   -41.80

```
        10        20        30        40        50        60
CUCUACUGUAUGAAUAUA|            C          AAUAU        UAAAAU
                  UGAUGAUGGCAUC UCUUGGGUAA     UCCGGGUG      \
                  ACUACUACUGUAG AGGACUCAUU     AGGCUUAC      A
AGCAAAG-----------^            A          CUCU-        CCCCUG
 .                  100        90        80         70
```

Reads in: lib-42, lib-45, lib-47, lib-48, lib-49, lib-50, lib-72

Number of reads="ID675",113,51,45,75,88,41,0

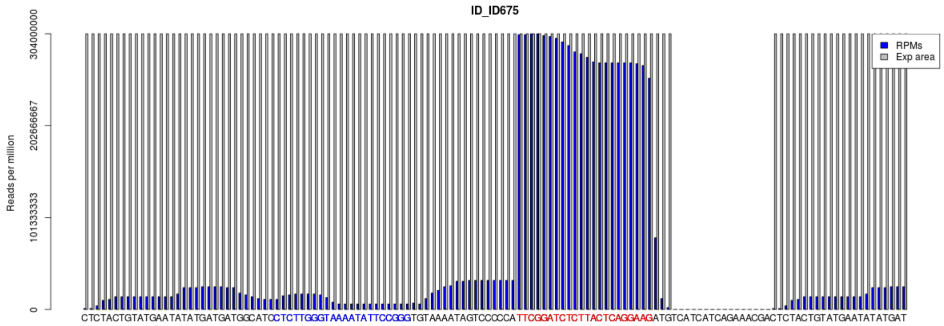

>ID677
GTGACAA**GGGATTGAACACCTGGCGGTTC**CGATTTGCTTTATCTATGTTATCAGTGAAAAGCTGATTCTAATCGCCAAGTGTTCCAACTC
TTGACAATTCCGCAAGCACAGC

>ID677-5P
GGGATTGAACACCTGGCGGTTC

>miRNA precursor
GUGACAA**GGGAUUGAACACCUGGCGGUUC**CGAUUUGCUUUAUCUAUGUUAUCAGUGAAAAGCUGAUUCUAAUCGCCAAGUGUUCCAACUCUUGACAAUUCCGCAAGCACAGC
.((.((((((...((((((.((((((((..((((.(((((.((..((....))..))))))).))))..)))))))).))))))...)))))).))...............

>Predicted STAR seq:
TCGCCAAGTGTTCCAACTCTT

Complementarity: 15

Initial dG =   -35.30

```
                       10        20        30        40
G-------------| A      AUU      C        CC    U     A  UA  U
               UG CAAGGG   GAACAC UGGCGGUU  GAUU GCUUU UC  UG U
               AC GUUCUC   CUUGUG ACCGCUAA  UUAG CGAAA AG  AC A
CGACACGAACGCCUUA^ A      AAC      A        UC    U     -  UG  U
110       100       90        80        70        60         50
```

Reads in: lib-42, lib-45, lib-47, lib-48, lib-49, lib-50, lib-72
Number of reads="ID677",48,25,3,243,79,2,0

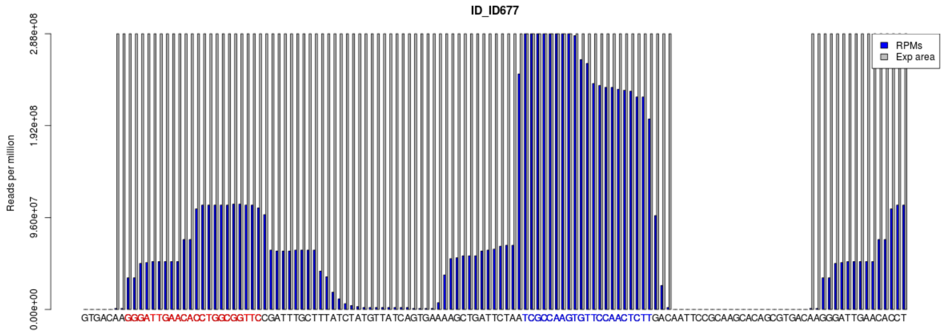

>ID679
TTTTTTCTAAAGTCCTGTAT**TTTGTTGATATTTTCGATGCACT**GTGGCAACTTGTTGTATAGAATTTTATCAGCGTTTATTGGATCTTTG
TCACTTAATTGTAACCGTTTACT

>ID679-5P
TTTGTTGATATTTTCGATGCACT

>miRNA precursor
UUUUUUCUAAAGUCCUGUAU**UUUGUUGAUAUUUUCGAUGCACU**GUGGCAACUUGUUGUAUAGAAUUUUAUCAGCGUUUAUUGGAUCUUUGUCACUUAAUUGUAACCGUUUACU
.......((((((((.(((...((((((((..(((.((((((.((....))..)).)))).)))...))))))))..))).))).)))))..........((((....)))).

>Predicted STAR seq:
TGTATAGAATTTTATCAGCGTTTA

Complementarity: 15

```
 Initial dG =    -21.90

          10          20         30          40
.-UUUUUUC     -   U   UUU        UU-   G     -  U-| G
         UAAAG UCC GUA   UGUUGAUA   UUC AUGC AC  GU G
         GUUUC AGG UAU   GCGACUAU   AAG UAUG UG  CA C
\ -------     U   U   UU-        UUU   A     U  UU^ A
        90        80         70         60        50



         100
  UCACUUAAUU   C
            GUAA C
            CAUU G
  U---------   U
            110
```

Reads in: lib-42, lib-45, lib-47, lib-48, lib-49, lib-50, lib-72
Number of reads="ID679",4,3,1,2,12,4,0

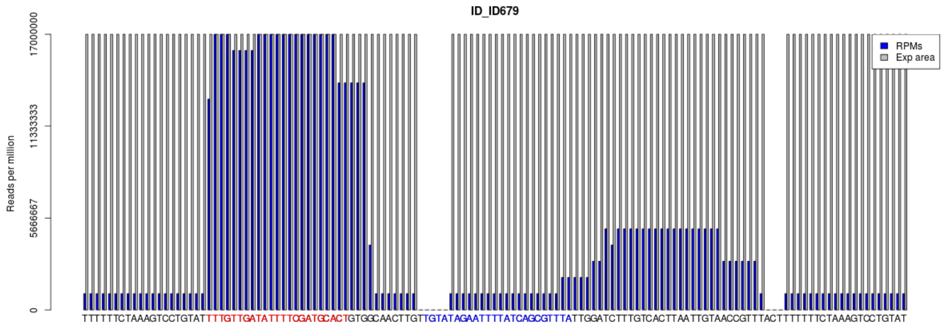

>ID68
CAAATGGTATTACCTGCAGCAGCAGCATCGCTTTCATCACCAGGGAGAACATCAATACTTGCATCAGTTC**TCCAAGGTGGAGAAATCGGT**
TCTGCTGCAGCAGCCAAGACAT

>ID68-3P
TCCAAGGTGGAGAAATCGGTTC

>miRNA precursor
CAAAUGGUAUUACCUGCAGCAGCAGCAUCGCUUUCAUCACCAGGGAGAACAUCAAUACUUGCAUCAGUUC**UCCAAGGUGGAGAAAUCGGUUC**UGCUGCAGCAGCCAAGACAU
```
......((.....((((.(((((((.((((.((((.((((..((((..(((((((................)))))))..))))..)))).)))).))))))).)))).....))..
```

>Predicted STAR seq:
CATCGCTTTCATCACCAGGGA

Complementarity: 14

Initial dG = -46.40

```
        10        20        30        40        50
CAAAUG| AUUAC    A       C    C    AU    AG        AUCAAUA
      GU     CUGC GCAGCAG AUCG UUUC  CACC  GGAGAAC       C
      CA     GACG CGUCGUC UGGC AAAG  GUGG  CCUCUUG       U
UA----^ GAACC    A       U    U    AG    AA        ACUACGU
    110       100        90        80        70        60
```

Reads in: lib-42, lib-45, lib-47, lib-48, lib-49, lib-50, lib-72
Number of reads="ID68",22,0,0,1,0,0,0

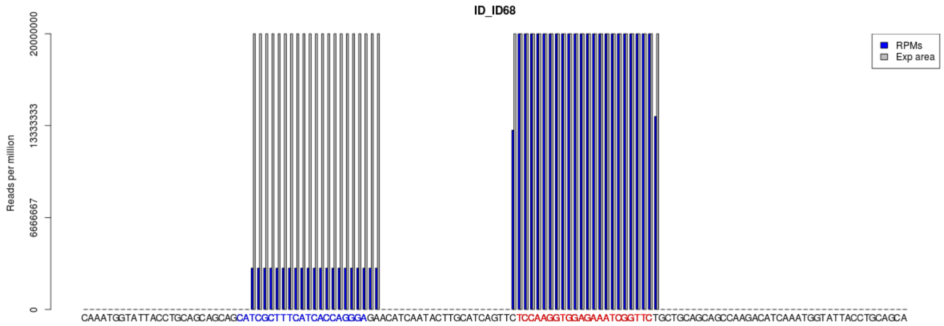

>ID680
TTGGCACAATTTATTATATT**TCTTGCAAGGGATATTGCTAGGGG**GGGATTTGTCCCAATTGGGAATATTCCGTGGGCATGAGGACAGCTC
TGGTATTCACCTTACAGCCAACAG

>ID680-5P
TCTTGCAAGGGATATTGCTAGGGG

>miRNA precursor
UUGGCACAAUUUAUUAUAUU**UCUUGCAAGGGAUAUUGCUAGGGG**GGGAUUUGUCCCAAUUGGGAAUAUUCCGUGGGCAUGAGGACAGCUCUGGUAUUCACCUUACAGCCAACAG
```
(((((............((((.(((..((((((((.((((..((((.....))))..)))).)))))))))....))).))))........(((....))).....)))))...
```

>Predicted STAR seq:
ATTGGGAATATTCCGTGGGCATGAGG

Complementarity: 16

Initial dG =   -36.50

```
             10        20          30        40
---     .-ACAAUUUAUUAUA    U   AA--|       G    GG    AU
   UUGGC               UUUC UGC    GGGAUAUU CUAG  GGGG  \
   AACCG               GGAG ACG    CCUUAUAA GGUU  CCCU  U
GAC     \ -------------    U   GGUG^       G    AA    GU
  110                    80        70        60



                 90
          ACAGCUCU   A
                  GGU U
                  CCA U
          ACAUU---   C
                   100
```

Reads in: lib-42, lib-45, lib-47, lib-48, lib-49, lib-50, lib-72
Number of reads="ID680",57,5,3,15,37,11,0

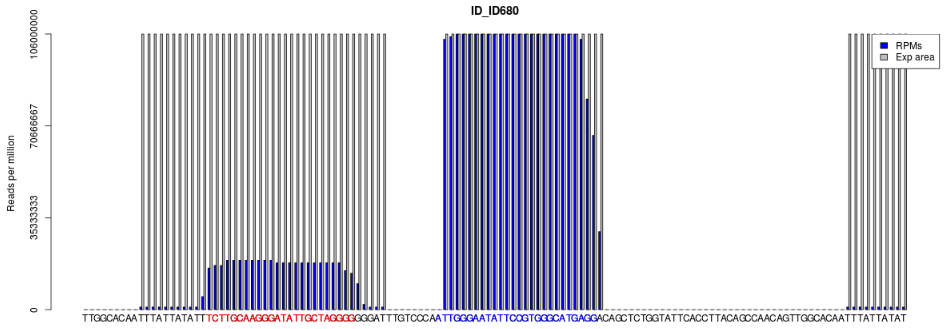

>ID684
TTTATATTTAGTTTCAGGCA**CTCGCGCTTGAGCCATATACGTT**GTGTGATACTTTAGACGTCTTGGTTCAATTACGAGTGCTTAAAACTA
AGCATAACGCCGCTGAAGCCAAG

>ID684-5P
CTCGCGCTTGAGCCATATACGTT

>miRNA precursor
UUUAUAUUUAGUUUCAGGCA**CUCGCGCUUGAGCCAUAUACGUU**GUGUGAUACUUUAGACGUCUUGGUUCAAUUACGAGUGCUUAAAACUAAGCAUAACGCCGCUGAAGCCAAG
.((((.((((((((.(((((((((...((((((((...(((((..(.....)....)))))..))))))))...))))))))).)))))))).)))).....((....))....

>Predicted STAR seq:
CGTCTTGGTTCAATTACGAGTG

Complementarity: 15

Initial dG =   -37.60

```
          10        20        30        40
.-U    A        C         CGC        UAU     GU--|UG
   UUAU UUUAGUUU AGGCACUCG   UUGAGCCA   ACGUU    G  \
   AAUA GAAUCAAA UUCGUGAGC   AACUUGGU   UGCAG    C  A
\ -    C        A         AUU        UC-     AUUU^AU
         90        80        70        60        50



  100
  CGCC  U
      GC G
      CG A
  GAAC  A
   110
```

Reads in: lib-42, lib-45, lib-47, lib-48, lib-49, lib-50, lib-72
Number of reads="ID684",1,0,0,54,70,0,0

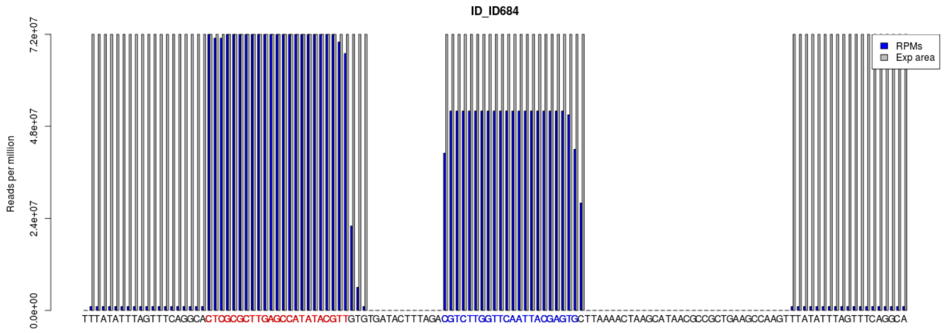

>ID685
TGGCCAAGTAGTCCACAGCAACTTGATTGGCCACGCGTATTCTTGGGGAATTAACACACATTGAGAGCTG**TTATTGCTTGAGAATACACG**
TAGCTGACAAGTTAAAGCGAC

>ID685-3P
TTATTGCTTGAGAATACACGT

>miRNA precursor
UGGCCAAGUAGUCCACAGCAACUUGAUUGGCCACGCGUAUUCUUGGGGAAUUAACACACAUUGAGAGCUG**UUAUUGCUUGAGAAUACACGU**AGCUGACAAGUUAAAGCGAC
((((......).)))..((((((((.(..((.(((.(((((((..((.(((.((((...........))))))).))..))))))).))).))..)))))))...))...

>Predicted STAR seq:
ACGCGTATTCTTGGGGAATTAA

Complementarity: 15
ERROR 4: Strange structure
Initial dG =   -33.80

```
     -|CA
--UGG C  A
  ACC G  G
\    U^AU
      10


         20        30        40        50        60
  CA-  ---      A UG   C   C       UG   G   U    CACAU
     GC   AACUUG U  GC ACG GUAUUCU  GG AAU AACA     U
     CG   UUGAAC A  CG UGC CAUAAGA  UC UUA UUGU     G
  CAG  AAA      - GU   A   A       GU   G   -    CGAGA
 110       100         90        80         70
```

Reads in: lib-42, lib-45, lib-47, lib-48, lib-49, lib-50, lib-72
Number of reads="ID685",10,7,6,71,10,4,0

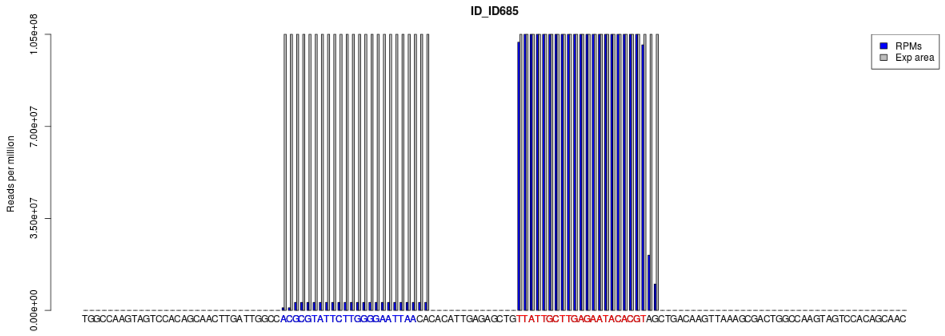

>ID698
AGGAGATGGTGATGATGTTG**AGGACAATGACGATGACGATGA**TGACAACTATCGCAATCTCATGGTGATCCTCATAGTCCTCCTCATTAT
CATTAAGGCATCGCGTCATCAT

>ID698-5P
AGGACAATGACGATGACGATGA

>miRNA precursor
AGGAGAUGGUGAUGAUGUUG**AGGACAAUGACGAUGACGAUGA**UGACAACUAUCGCAAUCUCAUGGUGAUCCUCAUAGUCCUCCUCAUUAUCAUUAAGGCAUCGCGUCAUCAU
......((((((((((..((((((.((((.(((.((.((((.((............)))))).)).))).)))).))))))..))))))))))).(((.....))).....

>Predicted STAR seq:
ATGGTGATCCTCATAGTCCTCC

Complementarity: 16

Initial dG =   -38.60

```
          10        20        30        40        50
.-AGGAGA|          UU       A    C   G  G    U  CAACU
        UGGUGAUGAUG  GAGGAC AUGA GAU AC AUGA GA      A
        AUUACUAUUAC  CUCCUG UACU CUA UG UACU CU      U
\ ------^          UC       A    C   G  G    -  AACGC
            90        80        70        60



        100
  A----   AU
       GGC  \
       CUG  C
  UACUA   CG
  110
```

Reads in: lib-42, lib-45, lib-47, lib-48, lib-49, lib-50, lib-72
Number of reads="ID698",15,27,1,1926,48,1,0

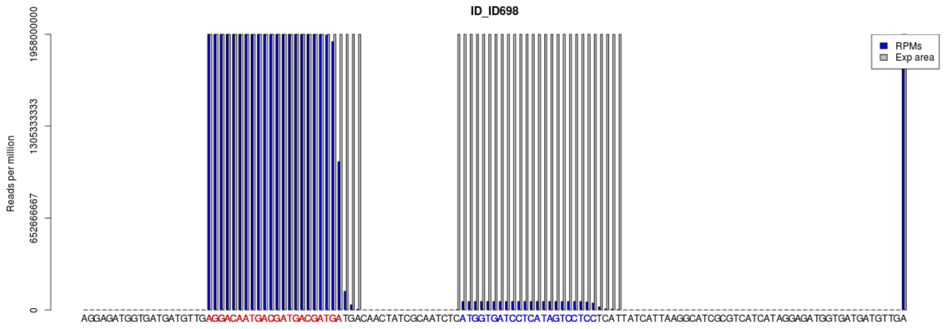

>ID699
ATTCTGAAGACCAATATGTTTCGTGAATCCCGAGCGACGGAAGTGAGATCTGTACAAGTTTTTAAATTAC**TTGAGATCTCGATTCCGTTA**
CTCGGTGTTCTCGATATTCG

>ID699-3P
TTGAGATCTCGATTCCGTTACT

>miRNA precursor
AUUCUGAAGACCAAUAUGUUUCGUGAAUCCCGAGCGACGGAAGUGAGAUCUGUACAAGUUUUUAAAUUAC**UUGAGAUCUCGAUUCCGUUACU**CGGUGUUCUCGAUAUUCG
...................(((.(((..(((((..((((((.((((((((...(((((.........)))))))))))))).))))))..))))))..))).)))......

>Predicted STAR seq:
GCGACGGAAGTGAGATCTGTACAA

Complementarity: 17

Initial dG =   -41.70

```
        10        20        30        40        50        60
AUUCUGAAGACCAAUAUGUU|  U   UC     CG      G        GUA     UUUU
                    UCG GAA  CCGAG  ACGGAA UGAGAUCU   CAAGU    \
                    AGC CUU  GGCUC  UGCCUU GCUCUAGA   GUUCA    A
GCUUAU--------------^  U   GU     AU      A        ---     UUAA
.                     100        90        80           70
```

Reads in: lib-42, lib-45, lib-47, lib-48, lib-49, lib-50, lib-72
Number of reads="ID699",11,26,9,0,0,1,0

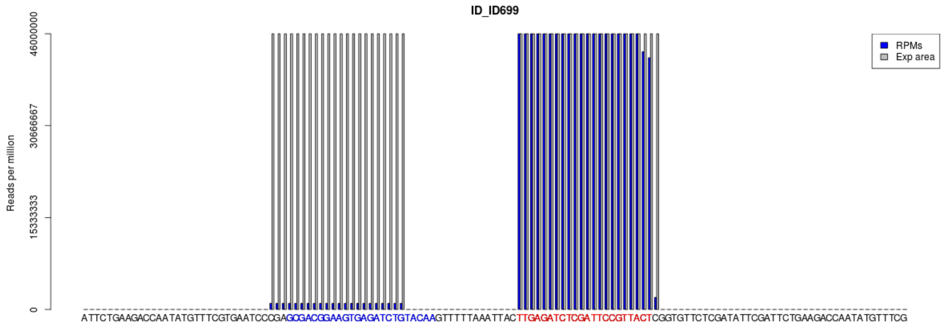

>ID7
AGAAGCATCATCTCTTCGACTGGTGGGTAGCGGTAGATGGAGTAATGACAGTCCACGTGATAATAAACCG**TGACTTTCGCTACTCCATCT**
AGCTGCTACCCACTCCACAGCA

>ID7-3P
TGACTTTCGCTACTCCATCTAG

>miRNA precursor
AGAAGCAUCAUCUCUUCGACUGGUGGGUAGCGGUAGAUGGAGUAAUGACAGUCCACGUGAUAAUAAACCG**UGACUUUCGCUACUCCAUCUAG**CUGCUACCCACUCCACAGCA
.((((........))))....(((((((((((((((((((((((.(((.((((.(((...........))))))).))).)))))))))).))))))))))))).......

>Predicted STAR seq:
AGATGGAGTAATGACAGTCC

Complementarity: 17
ERROR 4: Strange structure
Initial dG =   -50.60

```
.-A|   CAU
   GAAG   C
   CUUC   A
\ -^   UCU
         10
```

```
   20            30         40        50        60
  GACU----             -          A    C    C   UGAUA
          GGUGGGUAGCGGU AGAUGGAGUA UGA AGUC ACG      \
          UCACCCAUCGUCG UCUACCUCAU GCU UCAG UGC      A
  ACGACACC             A          C    U    -   CAAAU
  110       100        90         80         70
```

Reads in: lib-42, lib-45, lib-47, lib-48, lib-49, lib-50, lib-72
Number of reads="ID7",0,0,0,0,0,0,0

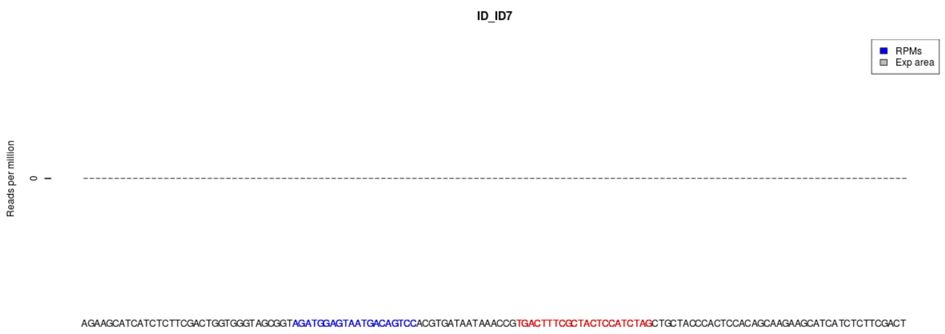

>ID700
TTCTGAAGACCAATATGTTTCAGTGAATCCCGAG**TGACGGAAGCGAGGTCTGTACAAG**TTTTAAAATTACTTGAGATCTCCCTTCTGTCA
CACGGTGTTCTCGATAAGCGTC

>ID700-5P
TGACGGAAGCGAGGTCTGTACAAG

>miRNA precursor
UUCUGAAGACCAAUAUGUUUCAGUGAAUCCCGAG**UGACGGAAGCGAGGUCUGUACAAG**UUUUAAAAUUACUUGAGAUCUCCCUUCUGUCACACGGUGUUCUCGAUAAGCGUC
..((((.(((......))))))).(((..(((.((((((((((.(((((((...(((((.........)))))))))))).)))))))))).))))..))).........

>Predicted STAR seq:
TGAGATCTCCCTTCTGTCACA

Complementarity: 18

```
 Initial dG =    -40.50

          10
ERROR 3: Strange structure
 Initial dG =    -40.50

          10
.-UU|   A   CA
    CUGA GAC  A
    GACU UUG  U
\ --^   -   UA
      20


                  30        40        50        60
  U-----------   UC   A          C       GUA      UUUA
            GAA  CCG GUGACGGAAG GAGGUCU   CAAGU    \
            CUU  GGC CACUGUCUUC CUCUAGA   GUUCA    A
  CUGCGAAUAGCU   GU   A          C       ---      UUAA
  110       100        90        80           70
```

Reads in: lib-42, lib-45, lib-47, lib-48, lib-49, lib-50, lib-72
Number of reads="ID700",1,0,0,0,0,0,0

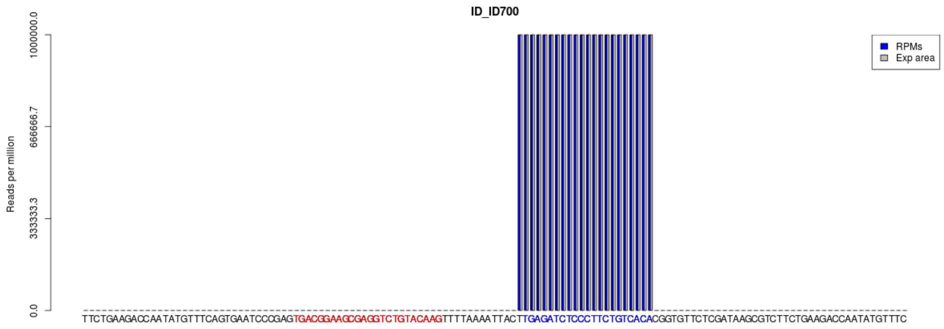

>ID702
TTCTGAAGACCAATATGTTTCAGTGAATCCCGAG**TGACGGAAGCGAGGTCTGTACAAG**TTTTAAAATTACTTGAGATCTCCCTTCTGTCA
CACGGTGTTCTCGATAAGCGTC

>ID702-5P
TGACGGAAGCGAGGTCTGTACAAG

>miRNA precursor
UUCUGAAGACCAAUAUGUUUCAGUGAAUCCCGAG**UGACGGAAGCGAGGUCUGUACAAG**UUUUAAAAUUACUUGAGAUCUCCCUUCUGUCACACGGUGUUCUCGAUAAGCGUC
..((((.(((......))))))).(((..(((.((((((((((.(((((((...(((((.........))))))))))))).)))))))))).))).).))).........

>Predicted STAR seq:
TGAGATCTCCCTTCTGTCACA

Complementarity: 18

```
 Initial dG =    -40.50

          10
ERROR 3: Strange structure
 Initial dG =    -40.50

          10
.-UU|   A   CA
    CUGA GAC  A
    GACU UUG  U
\ --^   -   UA
      20


                  30        40        50        60
  U-----------   UC   A          C       GUA      UUUA
            GAA  CCG GUGACGGAAG GAGGUCU   CAAGU    \
            CUU  GGC CACUGUCUUC CUCUAGA   GUUCA    A
  CUGCGAAUAGCU   GU   A          C       ---      UUAA
  110       100        90        80            70
```

Reads in: lib-42, lib-45, lib-47, lib-48, lib-49, lib-50, lib-72
Number of reads="ID702",1,0,0,0,0,0,0

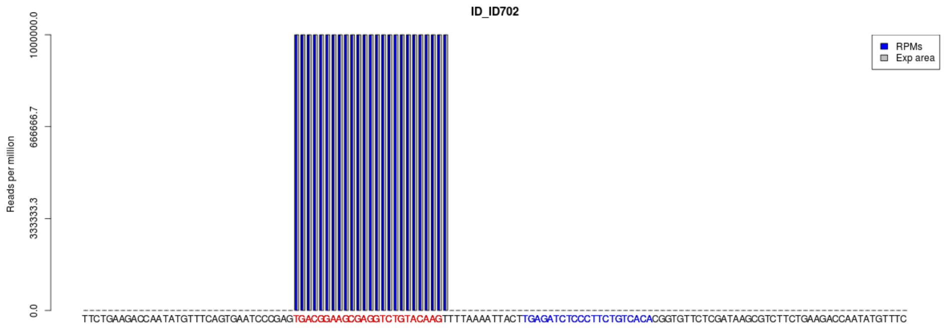

>ID703
ATTCTGAAGACCAATATGTTTCGTGAATCTCGAGCGACGGAAGCGAGATCTGTATAAGTTTTTAAATTAC**TTGAGATCTCGATTCCGTTA**
CTCAGTGTTCTCGATATTCG

>ID703-3P
TTGAGATCTCGATTCCGTTACT

>miRNA precursor
AUUCUGAAGACCAAUAUGUUUCGUGAAUCUCGAGCGACGGAAGCGAGAUCUGUAUAAGUUUUUAAAUUAC**UUGAGAUCUCGAUUCCGUUACU**CAGUGUUCUCGAUAUUCG
```
...................(((.((((((.(((..((((((.((((((((...(((((.........))))))))))))).))))))..))))).)))).))).......
```

>Predicted STAR seq:
GCGACGGAAGCGAGAUCUGUAUAA

Complementarity: 17

Initial dG = -35.00

```
        10        20         30        40        50        60
AUUCUGAAGACCAAUAUGUU   U    -| C   CG      G        GUA     UUUU
                    UCG GAAU CU GAG  ACGGAA CGAGAUCU   UAAGU    \
                    AGC CUUG GA CUC  UGCCUU GCUCUAGA   GUUCA    A
GCUUAU--------------   U    U^ -   AU      A        ---     UUAA
.                     100         90        80           70
```

Reads in: lib-42, lib-45, lib-47, lib-48, lib-49, lib-50, lib-72
Number of reads="ID703",17,16,10,0,0,0,0

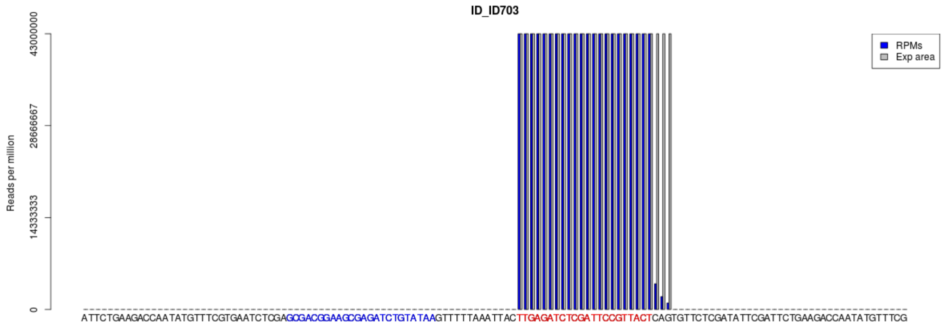

>ID71
TGCTGCTGGCTGGCAAGCACAGTGCCGGGACCCGTGCCAGAGGCGCGTTTACACGGCCTTCAGAATTCTG**TAAATGCACTATCTGGTACG ACA**CCCCGGCACTACCCACCACC

>ID71-3P
TAAATGCACTATCTGGTACGACA

>miRNA precursor
UGCUGCUGGCUGGCAAGCACAGUGCCGGGACCCGUGCCAGAGGCGCGUUUACACGGCCUUCAGAAUUCUG**UAAAUGCACUAUCUGGUACGACA**CCCCGGCACUACCCACCACC
((((((....)))).))).(((((((((...((((((((((.(((((((((.((..........)))))))))))).)).))))))))).....))))))))).........

>Predicted STAR seq:
CCCGTGCCAGAGGCGCGTTTA

Complementarity: 16
ERROR 4: Strange structure
Initial dG =   -50.50

```
     -|   G
--UGC UGCU G
  ACG ACGG C
\    A^   U
          10



 20                30         40         50        60
  C---------         ACC-         -  C         C  CCUU
            AGUGCCGGG    CGUGCCAGA GG GCGUUUACA GG    C
            UCACGGCCC    GCAUGGUCU UC CGUAAAUGU CU    A
  CCACCACCCA         CACA         A  A         -  UAAG
   110       100        90         80        70
```

Reads in: lib-42, lib-45, lib-47, lib-48, lib-49, lib-50, lib-72
Number of reads="ID71",293,75190,11672,2330,2203,5311,0

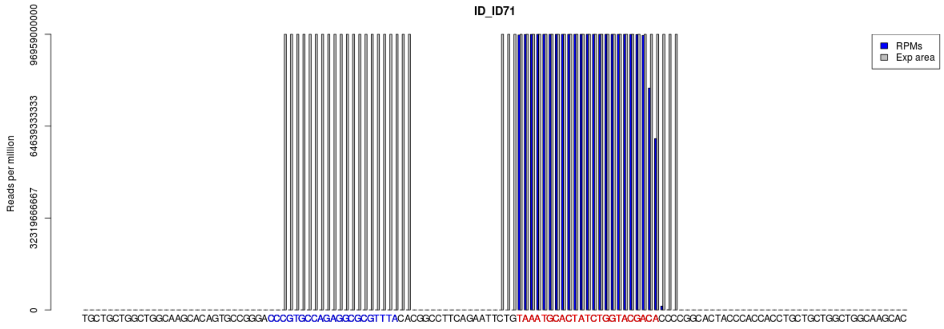

>ID710
ATCCAGCGACCACCTAGCGTCAGAGGTGAC**AGGATCAGCGGAAGTGGACGGT**TGGTTATGTTGGCAGCACTGTCTACTTCCACTGATCCA
GGCATGGCGACCGGCGGCGTG

>ID710-5P
AGGATCAGCGGAAGTGGACGGT

>miRNA precursor
AUCCAGCGACCACCUAGCGUCAGAGGUGAC**AGGAUCAGCGGAAGUGGACGGU**UGGUUAUGUUGGCAGCACUGUCUACUUCCACUGAUCCAGGCAUGGCGACCGGCGGCGUG
.....(((.((.((...(((((....((...(((((((.(((((((((((((..(((.....)))...))))))))))))).)))))))...)))))))...)).))))).

>Predicted STAR seq:
TGTCTACTTCCACTGATCCAGGCA

Complementarity: 18

Initial dG = -50.30

```
        10        20        30        40        50
AUCCA|  A  A  UAG     GAGG  ACA       C             UG-   AU
     GCG CC CC   CGUCA    UG   GGAUCAG GGAAGUGGACGGU   GUU  \
     UGC GG GG   GCGGU    AC   CCUAGUC CCUUCAUCUGUCA   CGG  G
G----^  -  C  CCA     ----  GGA       A             CGA   UU
   110       100           90        80        70
```

Reads in: lib-42, lib-45, lib-47, lib-48, lib-49, lib-50, lib-72
Number of reads="ID710",59,62,103,375,145,9,0

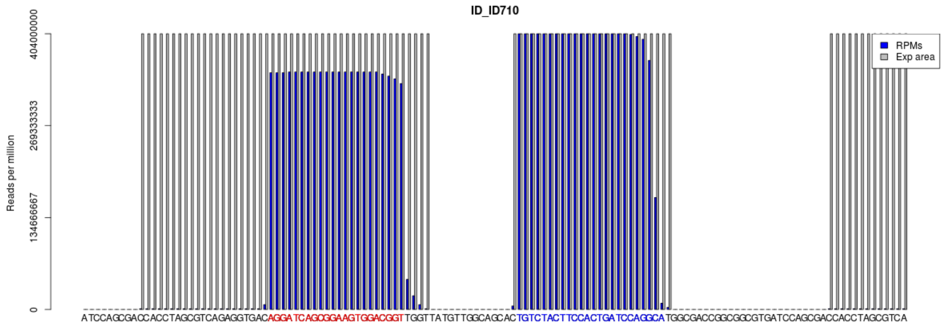

>ID711

CACCAGCGACCACCTAGCGTCAGAGGTGACAGGATCAGCGGAAGTGGACGGTTGGTTATGTTGGCAGCAC**TGTCTACTTCCACTGATCCC**
GCCGCACCTGGGGCATGGCGA

>ID711-3P

TGTCTACTTCCACTGATCCCGCC

>miRNA precursor

CACCAGCGACCACCUAGCGUCAGAGGUGACAGGAUCAGCGGAAGUGGACGGUUGGUUAUGUUGGCAGCAC**UGUCUACUUCCACUGAUCCCGCC**GCACCUGGGGCAUGGCGA
......((.(((....((.((((..(((...(((((((.(((((((((((((..(((.....)))...))))))))))))).)))))))...)))..)))).)).))))).

>Predicted STAR seq:

ACAGGATCAGCGGAAGTGGACG

Complementarity: 17

Initial dG =   -51.80

```
        10         20         30         40         50
CACCAG| A   CCUA  G    AG   ACA        C             UG-   AU
      CG CCA    GC UCAG  GUG   GGAUCAG GGAAGUGGACGGU   GUU  \
      GC GGU    CG GGUC  CGC   CCUAGUC CCUUCAUCUGUCA   CGG  G
A-----^ -   A---  G    CA   CGC       A             CGA   UU
    110           100        90        80        70
```

Reads in: lib-42, lib-45, lib-47, lib-48, lib-49, lib-50, lib-72

Number of reads="ID711",757,1865,3047,12980,4477,560,0

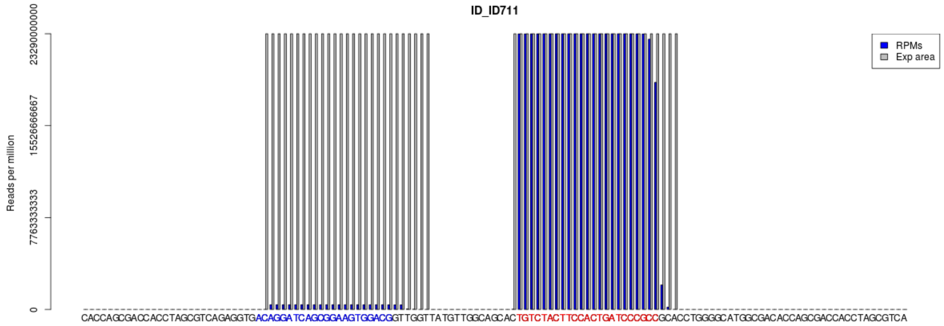

>ID712
CACCAGCGACCACCTAGCGTCAGAGGTGAC**AGGATCAGCGGAAGTGGACGGT**TGGTTATGTTGGCAGCACTGTCTACTTCCACTGATCCC
CCCGCCGCTGGGGCATGGCGAC

>ID712-5P
AGGATCAGCGGAAGTGGACGGT

>miRNA precursor
CACCAGCGACCACCUAGCGUCAGAGGUGAC**AGGAUCAGCGGAAGUGGACGGU**UGGUUAUGUUGGCAGCACUGUCUACUUCCACUGAUCCCCCCGCCGCUGGGGCAUGGCGAC
```
......((.(((....((.((((.((((...(((((((.(((((((((((((..(((.....)))...)))))))))))))).))))))))...)))).)))).)).))))).. 
```

>Predicted STAR seq:
TGTCTACTTCCACTGATCCCCCCG

Complementarity: 18

Initial dG =   -54.20

```
        10        20        30        40        50
CACCAG| A   CCUA  G    A    ACA       C             UG-   AU
      CG CCA    GC UCAG GGUG   GGAUCAG GGAAGUGGACGGU   GUU  \
      GC GGU    CG GGUC CCGC   CCUAGUC CCUUCAUCUGUCA   CGG  G
CA----^ -   A---  G    G    CCC       A             CGA   UU
    110           100        90        80        70
```

Reads in: lib-42, lib-45, lib-47, lib-48, lib-49, lib-50, lib-72
Number of reads="ID712",186,717,653,5409,1450,169,0

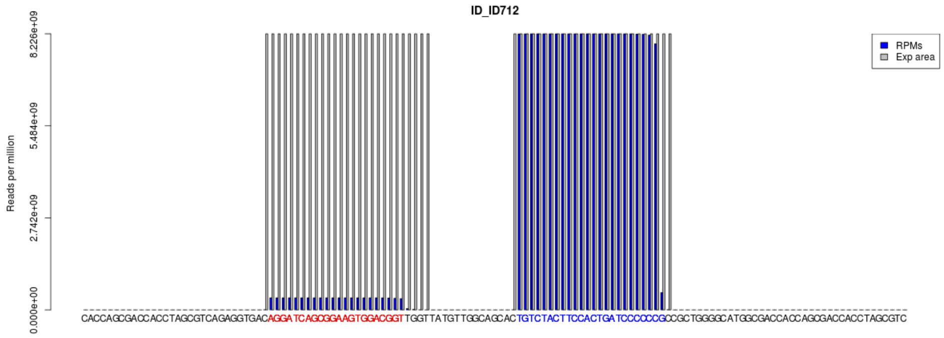

>ID714
CACCAGAGACCACCTAGCGTCAGAGGTGACAGGATCAGCGGAAGTGGACGGTTGGTTATGTTGGCAGCAC**TGTCTACTTCCACTGATCCC**
GCCGCCCCTGGGGCATGGCGA

>ID714-3P
TGTCTACTTCCACTGATCCCGCC

>miRNA precursor
CACCAGAGACCACCUAGCGUCAGAGGUGACAGGAUCAGCGGAAGUGGACGGUUGGUUAUGUUGGCAGCAC**UGUCUACUUCCACUGAUCCCGCC**GCCCCUGGGGCAUGGCGA
((((...(((........)))...))))...(((((((.(((((((((((((..(((.....)))...))))))))))))).)))))))((((((((...)))).)).)))).

>Predicted STAR seq:
GAGA

Complementarity: 16
ERROR 4: Strange structure
Initial dG = -52.60

```
          10
  |   AGA   CAC
--CACC   GAC   C
  GUGG   CUG   U
\ ^   AGA   CGA
         20



    30        40        50
  .-ACA       C             UG-   AU
       GGAUCAG GGAAGUGGACGGU   GUU  \
       CCUAGUC CCUUCAUCUGUCA   CGG  G
  \ ---       A             CGA   UU
               80        70
```

Reads in: lib-42, lib-45, lib-47, lib-48, lib-49, lib-50, lib-72
Number of reads="ID714",745,1788,3003,12820,4419,555,0

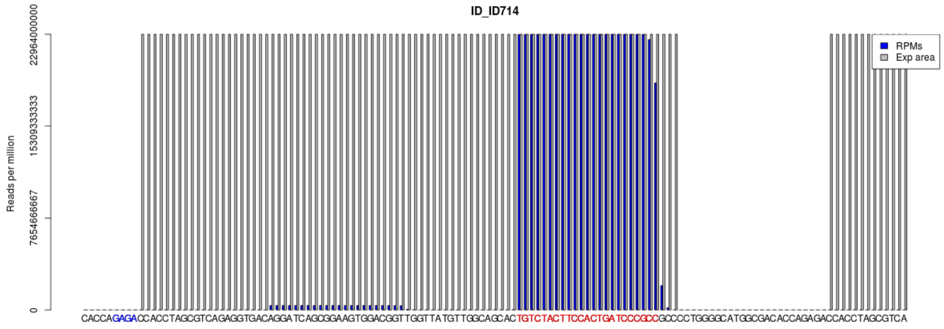

>ID715
CACCAGCGACCACCTAGCGTCAGAGGTGAC**AGGATCAGCGGAAGTGGACGGT**TGGTTATGTTGGCAGCACTGTCTACTTCCACTGATCCCCCCGCCCCCGCGACCGGCGGCG

>ID715-5P
AGGATCAGCGGAAGTGGACGGT

>miRNA precursor
CACCAGCGACCACCUAGCGUCAGAGGUGAC**AGGAUCAGCGGAAGUGGACGGU**UGGUUAUGUUGGCAGCACUGUCUACUUCCACUGAUCCCCCCGCCCCCGCGACCGGCGGCG
((((...(((........)))...))))...(((((((.(((((((((((((..(((.....)))...))))))))))))).))))))))...((((.(((....))).))))

>Predicted STAR seq:
TGTCTACTTCCACTGATCCCCCCGCCCCCGCGACCG

Complementarity: 18

```
 Initial dG =    -51.40

          10
ERROR 3: Strange structure
 Initial dG =    -51.40

          10
  |   AGC   CAC
--CACC   GAC   C
  GUGG   CUG   U
\ ^   AGA   CGA
         20



    30        40        50
  .-ACA       C            UG-   AU
       GGAUCAG GGAAGUGGACGGU   GUU  \
       CCUAGUC CCUUCAUCUGUCA   CGG  G
  \ ---       A            CGA   UU
               80        70
```

Reads in: lib-42, lib-45, lib-47, lib-48, lib-49, lib-50, lib-72
Number of reads="ID715",185,734,582,5481,1457,190,0

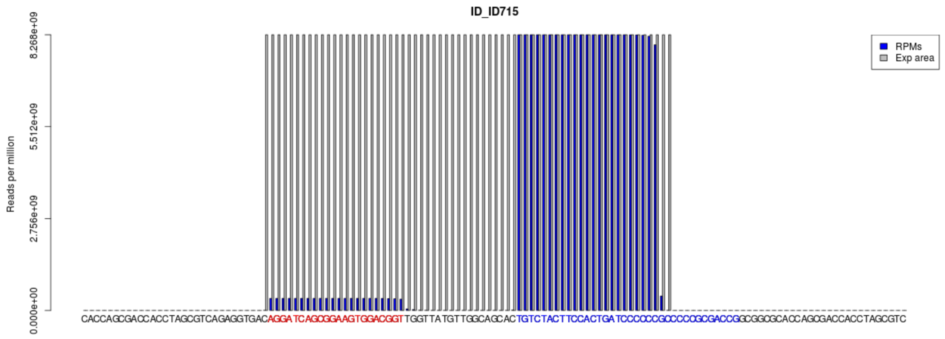

>ID716
CACCAGCGACCCCCTAGCGTCAGAGGTGACA**GGATCAGCGGAAGTGGACGGT**TGGTTATGTTGGCAGCACTGTCTACTTCCACTGATCCC
GCCGCCGCTGGGGCATGGCGAC

>ID716-5P
GGATCAGCGGAAGTGGACGGT

>miRNA precursor
CACCAGCGACCCCCUAGCGUCAGAGGUGACA**GGAUCAGCGGAAGUGGACGGU**UGGUUAUGUUGGCAGCACUGUCUACUUCCACUGAUCCCGCCGCCGCUGGGGCAUGGCGAC
```
......((.((((((((((.....((((...(((((((.((((((((((((..(((.....)))...)))))))))))))).)))))))))))..))))))))...)))).. 
```

>Predicted STAR seq:
TGTCTACTTCCACTGATCCCG

Complementarity: 18

Initial dG =   -56.50

```
        10            20         30         40         50
CACCAG   A   ---|       UCAGA     ACA        C             UG-    AU
      CG CC   CCCUAGCG       GGUG    GGAUCAG GGAAGUGGACGGU   GUU   \
      GC GG   GGGGUCGC       CCGC    CCUAGUC CCUUCAUCUGUCA   CGG   G
CA----   -  UAC^       CG---     ---        A             CGA    UU
    110        100           90            80         70
```

Reads in: lib-42, lib-45, lib-47, lib-48, lib-49, lib-50, lib-72
Number of reads="ID716",712,1884,2938,12995,4341,525,0

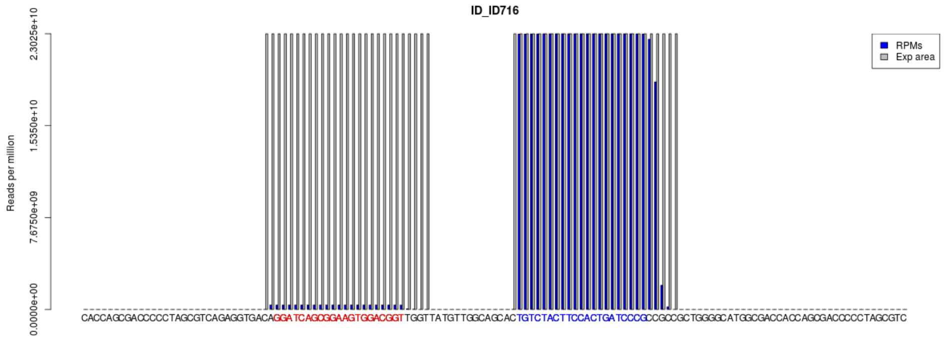

>ID717
CTGCAGCGGCCACCGAGTCTTAGAGGCGACAGGGTCAGTGGAGGTGGACGGTCGGTTATGTTGGCAGCAC**TGTCTACCTCCACTGATCCC**
GCTGCCTCTGCGACATGAC

>ID717-3P
TGTCTACCTCCACTGATCCCGCT

>miRNA precursor
CUGCAGCGGCCACCGAGUCUUAGAGGCGACAGGGUCAGUGGAGGUGGACGGUCGGUUAUGUUGGCAGCAC**UGUCUACCUCCACUGAUCCCGCU**GCCUCUGCGACAUGAC
```
......(((...))).(((.((((((((...((((((((((((((((((((..(((.....)))...)))))))))))))))))))))...)))))))).))).....
```

>Predicted STAR seq:
ACAGGGTCAGTGGAGGTGGACG

Complementarity: 18
ERROR 4: Strange structure
Initial dG = -59.30

```
          10
.-CUGCAG|  C
        CGG \
        GCC C
\ ------^  A




         20        30        40        50
  A----   U        ACA                    CG-   AU
     GUC UAGAGGCG   GGGUCAGUGGAGGUGGACGGU   GUU  \
     CAG GUCUCCGU   CCUAGUCACCUCCAUCUGUCA   CGG  G
  CAGUA   C        CGC                    CGA   UU
         100        90        80        70
```

Reads in: lib-42, lib-45, lib-47, lib-48, lib-49, lib-50, lib-72
Number of reads="ID717",826,1904,4599,12559,4247,614,0

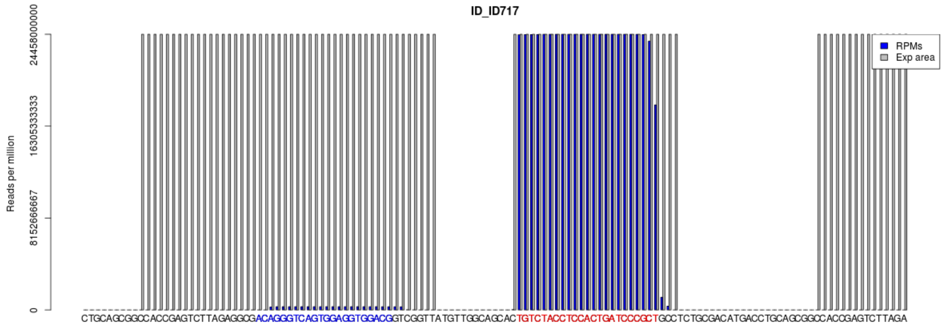

>ID718
CACCAGCGACCACCTAGCGTCAGAGGTGACAGGGTCAGCGGAAGTGGACGGTTGGTTATGTTGGCAGCAC**TGTCTACTTCCACTGATCCC**
GCCGCCCCTGGGGAATGGCGA

>ID718-3P
TGTCTACTTCCACTGATCCCGCC

>miRNA precursor
CACCAGCGACCACCUAGCGUCAGAGGUGACAGGGUCAGCGGAAGUGGACGGUUGGUUAUGUUGGCAGCAC**UGUCUACUUCCACUGAUCCCGCC**GCCCCUGGGGAAUGGCGA
((((...(((........)))...))))...(((((((.(((((((((((((..(((.....)))...))))))))))))).)))))))(((((((...))).)))))).

>Predicted STAR seq:
GCGA

Complementarity: 16
ERROR 4: Strange structure
Initial dG =   -49.50

```
          10
  |   AGC   CAC
--CACC   GAC   C
  GUGG   CUG   U
\ ^   AGA   CGA
         20



    30        40        50
  .-ACA       C             UG-   AU
       GGGUCAG GGAAGUGGACGGU   GUU  \
       CCUAGUC CCUUCAUCUGUCA   CGG  G
  \ ---       A             CGA   UU
               80        70
```

Reads in: lib-42, lib-45, lib-47, lib-48, lib-49, lib-50, lib-72
Number of reads="ID718",853,1879,2984,12972,4421,553,0

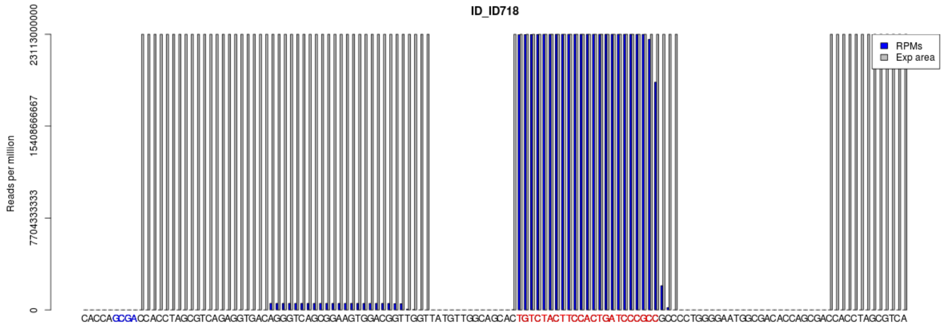

>ID719
CACGGGTGCGGATCCTTTAGGGGGATGCTTGTCCACTGTTGGTCGAGCCGATCTCCTGATCTCTACGA**GTTTACGATAGTAACTCGGGCG**
TCCGATCCTAGATTACAAGCACCC

>ID719-3P
GTTTACGATAGTAACTCGGGCGTC

>miRNA precursor
CACGGGUGCGGAUCCUUUAGGGGGAUGCUUGUCCACUGUUGGUCGAGCCGAUCUCCUGAUCUCUACGA**GUUUACGAUAGUAACUCGGGCGUC**CGAUCCUAGAUUACAAGCACCC
...((((((......((((((((((((((((...(((((((.(((((..((((....)))).)).))).....)))))))....)))))))))..))))))).......))))))

>Predicted STAR seq:
ATGCTTGTCCACTGTTG

Complementarity: 13

Initial dG =   -42.60

```
        10        20          30         40             50
CAC      GGAUCC       --          UCC-       G----   -| CC    U
   GGGUGC      UUUAGGG  GGAUGCUUG    ACUGUUG      UCG AG  GAUC C
   CCCACG      AGAUCCU  CCUGCGGGC    UGAUAGC      AGC UC  CUAG C
---      AACAUU       AG          UCAA       AUUUG   A^ U-    U
     110       100        90        80        70         60
```

Reads in: lib-42, lib-45, lib-47, lib-48, lib-49, lib-50, lib-72
Number of reads="ID719",1,1,5,4,2,3,0

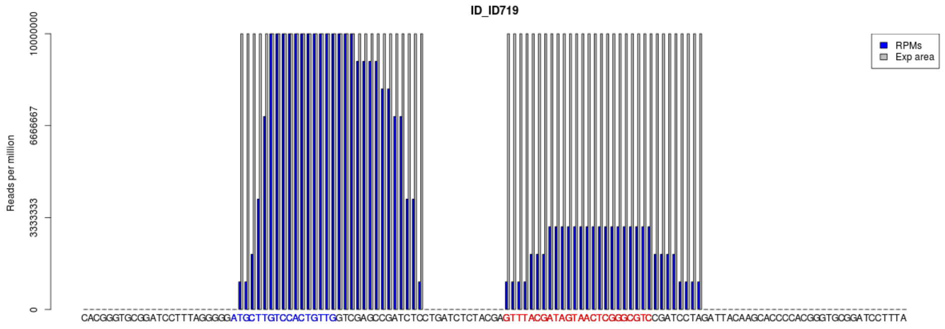

>ID72
CGGGAAGCGCGCGGCCGCAG**TGGCAGTGTGGTTAGCTGGTTGTG**TGGAATTTGCTCACTGCCACTATCCGCACTGCCTTGCGACCCGCCA
TCTGTCGACGCGTCGAGCGCCCAA

>ID72-5P
TGGCAGTGTGGTTAGCTGGTTGTG

>miRNA precursor
CGGGAAGCGCGCGGCCGCAG**UGGCAGUGUGGUUAGCUGGUUGUG**UGGAAUUUGCUCACUGCCACUAUCCGCACUGCCUUGCGACCCGCCAUCUGUCGACGCGUCGAGCGCCCAA
.(((..((((((((((((((.((((((((((.(((.((((.(((.((......))))).))))))).))))))))))))))))...........)))).))))).......)))..

>Predicted STAR seq:
CTGCCACTATCCGCACTGCCTT

Complementarity: 18

Initial dG = -55.00

```
           10        20        30        40        50
C-   .-AAGC|  C  C     U          U   C    U   U  AA
  GGG      GCG GG CGCAG GGCAGUGUGG UAG UGGU GUG GG  U
  CCC      CGC CC GCGUU CCGUCACGCC AUC ACCG CAC UC  U
AA   \ ----^  -  A     -          U   -    U   -  GU
  110              80        70         60


       90       100
       CAUCUG    C
             UCGA G
             AGCU C
       GCG---    G
```

Reads in: lib-42, lib-45, lib-47, lib-48, lib-49, lib-50, lib-72
Number of reads="ID72",1049,9612,5239,7896,8756,2041,0

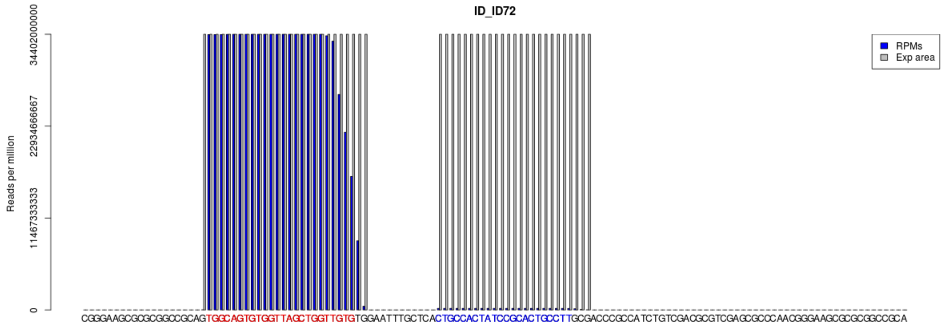

>ID720
CTGTGGGGGCGCCTGGCCGGGAGACGGGGGGGGCTGTCACTCGCACAGTGCGTGCGGGGCCGGCAGGTCC**TCGCACGCACTGTGCGAGCG**
GAAAAGCCTGTGGGGGCGCCC

>ID720-3P
TCGCACGCACTGTGCGAGCGGAA

>miRNA precursor
CUGUGGGGGCGCCUGGCCGGGAGACGGGGGGGGCUGUCACUCGCACAGUGCGUGCGGGGCCGGCAGGUCC**UCGCACGCACUGUGCGAGCGGAA**AAGCCUGUGGGGGCGCCC
......((((((((..(((.....)))...(((((.((.((((((((((((((((((((((....))))).))))))))))))))))).))...)))))....))))))))

>Predicted STAR seq:
TCACTCGCACAGTGCGTGCG

Complementarity: 17
ERROR 4: Strange structure
 Initial dG =   -76.20

```
        10          20
CUGUGG        .-GG|  GG
      GGGCGCCU    CCG  \
      CCCGCGGG    GGC  A
------        \ --^  AG
     110


                 30           40         50         60
                GGG-     G--  A                 -     G
                    GGGCU   UC CUCGCACAGUGCGUGCG GGGCC G
                    UCCGA   GG GAGCGUGUCACGCACGC CCUGG C
                GGUG     AAA  C                 U     A
                 100        90        80         70
```

Reads in: lib-42, lib-45, lib-47, lib-48, lib-49, lib-50, lib-72
Number of reads="ID720",55,359,282,8548,804,24,0

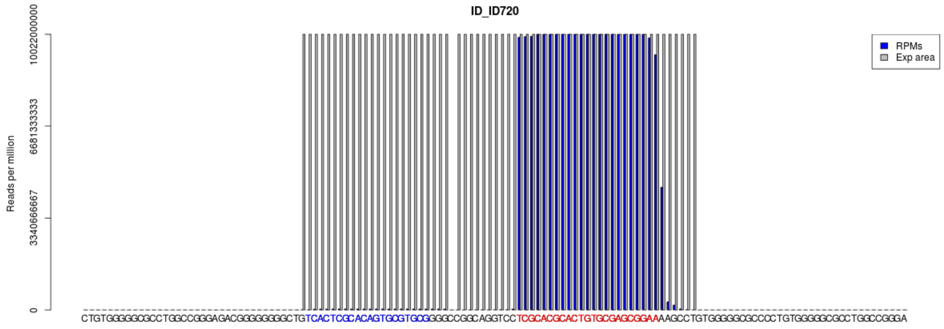

>ID724
TTCCTGTTAGCAACCACATG**CATTGTGATGATAATGCTTTGGTC**CGTGTTGCTGACTAGCACTGATAATAAAACAAGGCACTGATTGATG
CTTGTGGCTGCTAAATGTAG

>ID724-5P
CATTGTGATGATAATGCTTTGGTC

>miRNA precursor
UUCCUGUUAGCAACCACAUG**CAUUGUGAUGAUAAUGCUUUGGUC**CGUGUUGCUGACUAGCACUGAUAAUAAAACAAGGCACUGAUUGAUGCUUGUGGCUGCUAAAUGUAG
......((((((.(((((.(((((.....(((..((((((((((.((((((.....)))))).))).......)))))))...)))))))).))))).))))))......

>Predicted STAR seq:
TAATAAAACAAGGCACTGATTGATGCT

Complementarity: 15

Initial dG =   -33.00

```
        10        20        30         40                50
UUCCUG      A     U     GUGAU   AA-        ------|  C      CU
      UUAGCA CCACA GCAUU     GAU   UGCUUUG       GUC GUGUUG  \
      AAUCGU GGUGU CGUAG     UUA   ACGGAAC       UAG CACGAU  G
GAUGUA      C     U     -----   GUC       AAAAUAA^  U      CA
.       100        90              80         70         60
```

Reads in: lib-42, lib-45, lib-47, lib-48, lib-49, lib-50, lib-72
Number of reads="ID724",150,95,330,41,81,59,0

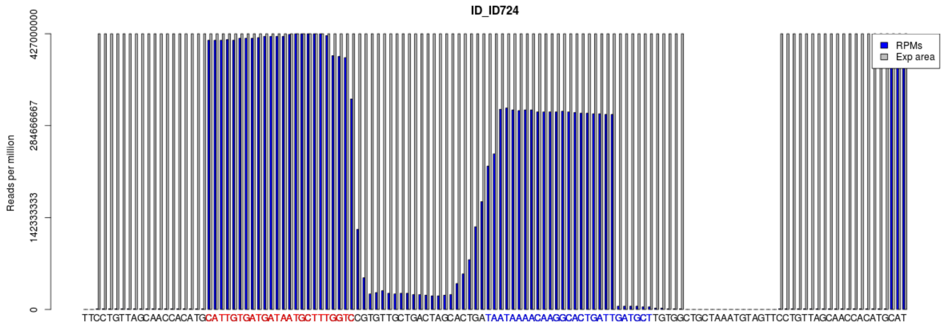

>ID729
TATACAGTGGTTTGGAACTTGGGTGTTGGCGAGGGGCAATATAAATGTTGATGTAGTGGTACTATAATACATCACATTTGTATTGTCTCA
CATCCACATGCATCATTCATCA

>ID729-3P
CATCACATTTGTATTGTCTCACA

>miRNA precursor
UAUACAGUGGUUUGGAACUUGGGUGUUGGCGAGGGGCAAUAUAAAUGUUGAUGUAGUGGUACUAUAAUACAUCACAUUUGUAUUGUCUCACAUCCACAUGCAUCAUUCAUCA
............((((...((.((((.((...((((((((((((((((.((((((.((......)).)))))))))))))))))))))))....)))))).))...))))...

>Predicted STAR seq:
GAGGGGCAATATAAATGTTGATG

Complementarity: 18

Initial dG =   -35.00

```
        10        20        30         40        50        60
UAUACAGUGGUU|   ACU  G    U  CGA-                U      G  GU
            UGGA   UG GUGU GG    GGGGCAAUAUAAAUGU GAUGUA UG  A
            ACUU   AC UACA CC    CUCUGUUAUGUUUACA CUACAU AU  C
ACU---------^   ACU  G    -  UACA                -      A  AU
110               100         90        80         70
```

Reads in: lib-42, lib-45, lib-47, lib-48, lib-49, lib-50, lib-72
Number of reads="ID729",180,80,25,598,86,17,0

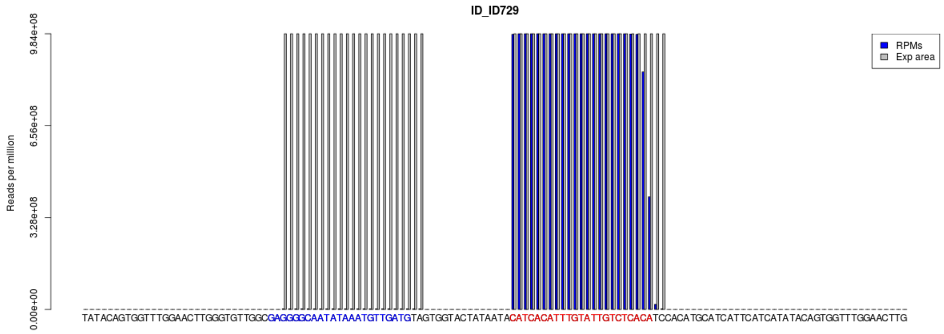

>ID730
AACGAACGGACTTGCTGTTGACATACGAGTGTGCACCTGTACACCGTATGATGTGATACAGGTAGCGTA**TACATCATAGGTGTGCGGGTG**
TTGTCTGGTATGTAATCGCCC

>ID730-3P
TACATCATAGGTGTGCGGGTGT

>miRNA precursor
AACGAACGGACUUGCUGUUGACAUACGAGUGUGCACCUGUACACCGUAUGAUGUGAUACAGGUAGCGUA**UACAUCAUAGGUGUGCGGGUGU**UGUCUGGUAUGUAAUCGCCC
....(((((.....))))).((((((.((...(((((((((((((.(((((((((.(((.......)))))))))))))))))))))))))...)).))))))........

>Predicted STAR seq:
GTGCACCTGTACACCGTATGATGTG

Complementarity: 20
ERROR 4: Strange structure
Initial dG =   -49.00

```
          10
.-AACG|    AC
      AACGG  \
      UUGUC  U
\ ----^    GU



 20               30        40        50        60
  G-------      G  UGU             G         A   AGG
          ACAUAC AG   GCACCUGUACACC UAUGAUGUG UAC   \
          UGUAUG UC   UGUGGGCGUGUGG AUACUACAU AUG   U
  CCCGCUAA      G  UGU             -         -   CGA
 110       100        90        80         70
```

Reads in: lib-42, lib-45, lib-47, lib-48, lib-49, lib-50, lib-72
Number of reads="ID730",163,100,77,4212,794,29,0

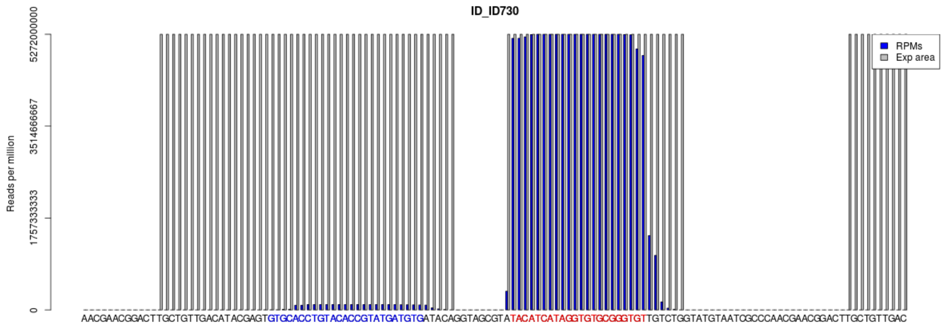

>ID731
TCTTCAATGGCTGGAGACTTGTATGTTGGTGTGGGGCCATAGAAATGTTGATGTAGTGGAACTGTAATA**CATCACATTTGTATGGTCTCA**
TATCCACATGCACCATCACTGA

>ID731-3P
CATCACATTTGTATGGTCTCATA

>miRNA precursor
UCUUCAAUGGCUGGAGACUUGUAUGUUGGUGUGGGGCCAUAGAAAUGUUGAUGUAGUGGAACUGUAAUA**CAUCACAUUUGUAUGGUCUCAUA**UCCACAUGCACCAUCACUGA
...(((.(((.(((.....(((((((.((((((((((((((.((((((.((((((...........))))))))))))).)))))))))))))).))))))))))))).)))

>Predicted STAR seq:
GTGGGGCCATAGAAATGTTGATG

Complementarity: 20

Initial dG =   -46.70

```
        10        20        30        40        50        60
UCU|  A   C   AGACU       U              G      U      GUGGA
   UCA UGG UGG     UGUAUGU GGUGUGGGGCCAUA AAAUGU GAUGUA     A
   AGU ACU ACC     ACGUACA CUAUACUCUGGUAU UUUACA CUACAU     C
---^  C   -   -----       C              G      -      AAUGU
   110             100        90        80         70
```

Reads in: lib-42, lib-45, lib-47, lib-48, lib-49, lib-50, lib-72
Number of reads="ID731",489,519,467,6295,490,143,0

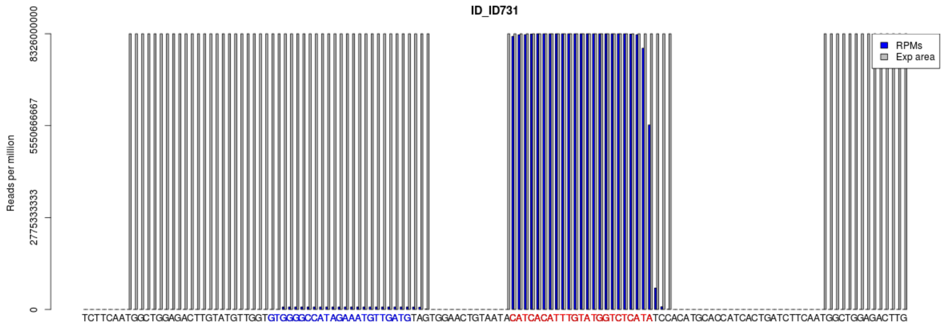

>ID732
ATGAAGAAATGTTTGTTATAAGTCCCAGGCTCTGATTGTCCAAACGCAATTCTTGTAGTTATGCAAATCA**AGGACTGTGTGTGGACATCA**
ACGCTTGGGGCAGTACCACGT

>ID732-3P
AGGACTGTGTGTGGACATCAAC

>miRNA precursor
AUGAAGAAAUGUUUGUUAUAAGUCCCAGGCUCUGAUUGUCCAAACGCAAUUCUUGUAGUUAUGCAAAUCA**AGGACUGUGUGUGGACAUCAAC**GCUUGGGGCAGUACCACGU
....................(((((((((..((((.(((((.(((((.((((((............)))))).))))).))))).)))))))))..))))))))).........

>Predicted STAR seq:
CTGATTGTCCAAACGCAATTCT

Complementarity: 16

Initial dG = -40.10

```
        10        20        30        40        50        60
AUGAAGAAAUGUUUGUUAUAA|        UC    U     A     A      UAGUUA
                     GUCCCAGGC  UGAU GUCCA ACGCA UUCUUG      \
                     CGGGGUUCG  ACUA CAGGU UGUGU AGGAAC      U
UGCACCAUGA-----------^        CA    -     G     C      UAAACG
 .                  100        90         80        70
```

Reads in: lib-42, lib-45, lib-47, lib-48, lib-49, lib-50, lib-72
Number of reads="ID732",12,6,1,236,1,0,0

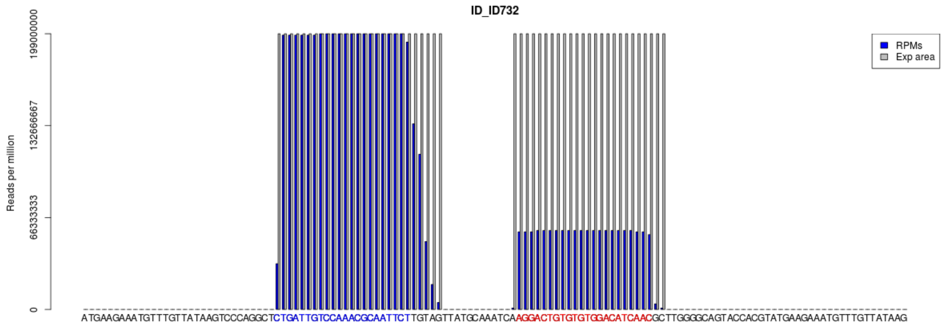

>ID735
GTTCGTTCCTGAAGGTGGTG**TCTGGCTCAGGATTGCCTGCCAT**TCAGTGAGCGTAGATGGCAGCCAACCCTGAGCCAGATACTACCTCCA
GAAATGCGGCGAAAAAATGCTC

>ID735-5P
TCTGGCTCAGGATTGCCTGCCAT

>miRNA precursor
GUUCGUUCCUGAAGGUGGUG**UCUGGCUCAGGAUUGCCUGCCAU**UCAGUGAGCGUAGAUGGCAGCCAACCCUGAGCCAGAUACUACCUCCAGAAAUGCGGCGAAAAAAUGCUC
...((((.(((.((((((((((((((((((.(((.((((((((...........)))))))).))).)))))))))))))))))))).))).)))).((((......)))).

>Predicted STAR seq:
GGCAGCCAACCCTGAGCCAGATA

Complementarity: 19

Initial dG =   -59.20

```
                     10        20        30        40
------------   -|   C   A                   A   C        CAGUG
            GUU CGUU CUG AGGUGGUGUCUGGCUCAGG UUG CUGCCAUU     \
            CGG GUAA GAC UCCAUCAUAGACCGAGUCC AAC GACGGUAG     A
CUCGUAAAAAAG   C^   A   C                   C   C        AUGCG
110       100       90        80        70        60
```

Reads in: lib-42, lib-45, lib-47, lib-48, lib-49, lib-50, lib-72
Number of reads="ID735",50,129,151,83,118,51,0

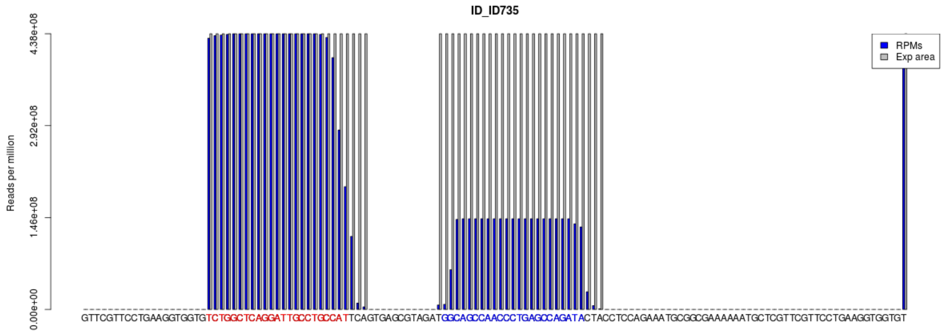

>ID737
GTGATGTCACGTCGCAAAGAGTTAGCCGGGTACGCGTAGCGATATGGCTGCCCGTTATGGGATACATCGG**TCAGCCTGATTGCTAGGCGA**
ACTCGGCCGACTCCGTGGTC

>ID737-3P
TCAGCCTGATTGCTAGGCGA

>miRNA precursor
GUGAUGUCACGUCGCAAAGAGUUAGCCGGGUACGCGUAGCGAUAUGGCUGCCCGUUAUGGGAUACAUCGG**UCAGCCUGAUUGCUAGGCGA**ACUCGGCCGACUCCGUGGUC
((((((...))))))...((((..(((((((.(((.(((((((..(((((.(((..(((.....)))))).))))).)..))))))).))).))))))).)..)))).......

>Predicted STAR seq:
CGCGTAGCGATATGGCTG

Complementarity: 13
ERROR 4: Strange structure
Initial dG =   -55.90

```
  |     U
--GUGAUG \
  CGCUGC C
\ ^     A
      10



       20        30        40        50        60
  AAA----    UA       A   G       AU      C   UU   GG
         GAGU  GCCGGGU CGC UAGCGAU  GGCUG CCG  AUG  \
         CUCA  CGGCUCA GCG AUCGUUA  CCGAC GGC  UAC  A
  CUGGUGC    GC       A   G       GU      U   --   AU
110       100        90        80        70
```

Reads in: lib-42, lib-45, lib-47, lib-48, lib-49, lib-50, lib-72
Number of reads="ID737",5,7,2,234,79,0,0

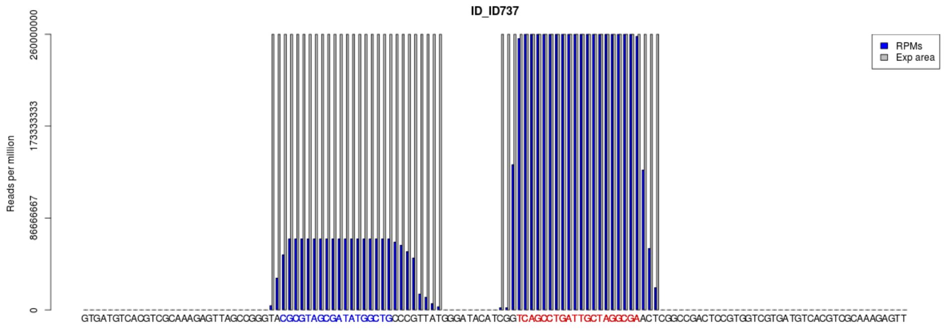

>ID745
CCGGTACCGGAAAAAAATTTTAAGTGACTAATTTTCTGTAATAAATACGGATAAATACAGAAAATTAGTACTTTTTAACAAGAAATCTAAA
AACTGTTGGTAACAGAAGAG

>ID745-5P
AAGTGACTAATTTTCTGTAATAA

>miRNA precursor
CCGGUACCGGAAAAAAAUUUUAAGUGACUAAUUUUCUGUAAUAAAUACGGAUAAAUACAGAAAAUUAGUACUUUUUAACAAGAAAUCUAAAAACUGUUGGUAACAGAAGAG
(((....)))..........(((((.((((((((((((((...............)))))))))))))))))))....................(((((...))))).....

>Predicted STAR seq:
CAGAAAATTAGTACTTTTTAACAAGAAATCTAAAAACTGTTGGTAA

Complementarity: 16

Initial dG = -23.10

ERROR 3: Strange structure
Initial dG = -23.10

```
  |  G
--CCG U
  GGC A
\ ^  C
 10
```

```
           20        30        40
  .-AAAAAAAUUUU     A             AUAAAUA
              AAGUG CUAAUUUUCUGUA       \
              UUCAU GAUUAAAAGACAU       C
  \ ----------     -             AAAUAGG
               70         60        50
```

Reads in: lib-42, lib-45, lib-47, lib-48, lib-49, lib-50, lib-72
Number of reads="ID745",66,342,210,326,230,104,0

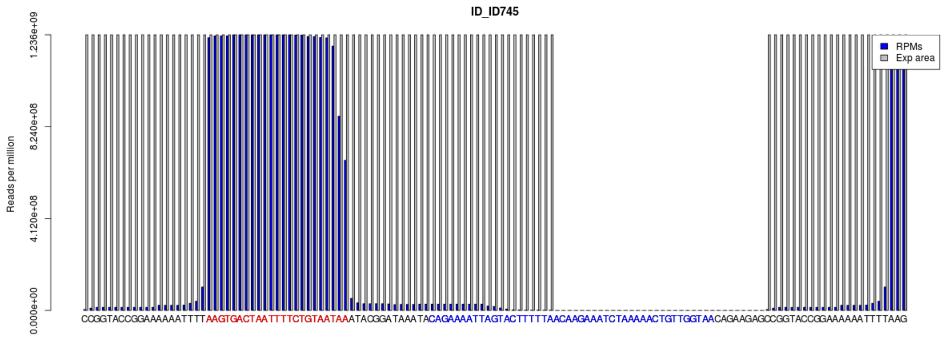

>ID747
TTCACCAGGAGTAAGGATAGTGCTGTACGCAGACCCACTTCTGGACCGTCCCTGTGCTAATTAATCAAG**AGACGTGCCGGAAGTGCGGGT CAG**CGTGCAGCACACTGACAACA

>ID747-3P
AGACGTGCCGGAAGTGCGGGTCAG

>miRNA precursor
```
UUCACCAGGAGUAAGGAUAGUGCUGUACGCAGACCCACUUCUGGACCGUCCCUGUGCUAAUUAAUCAAGAGACGUGCCGGAAGUGCGGGUCAGCGUGCAGCACACUGACAACA
....((........))...(((((((((((.(((((((((((((..((((.((..............)).))))..)))))))))..))))).)))))))))))..........
```

>Predicted STAR seq:
AGACCCACTTCTGGACCGTC

Complementarity: 16
ERROR 4: Strange structure
Initial dG = -50.70

```
          10
.-UUCA| AGG
      CC   A
      GG   G
\ ----^ AAU



           20        30          40        50        60
  AUA-------           A      --        AC     C   GUGCUA
            GUGCUGUACGC GACCC  ACUUCUGG  CGUC CU       A
            CACGACGUGCG CUGGG  UGAAGGCC  GCAG GA       U
  ACAACAGUCA           A      CG        GU     A   ACUAAU
   110       100        90        80        70
```

Reads in: lib-42, lib-45, lib-47, lib-48, lib-49, lib-50, lib-72
Number of reads="ID747",5,1,0,0,0,0,0

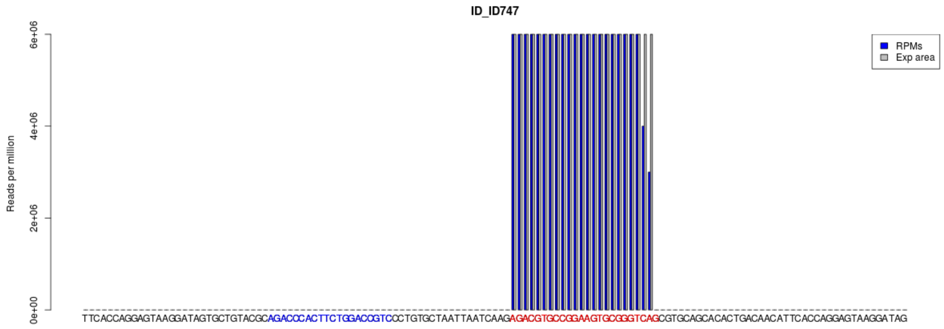

>ID748
TGGTGACACAGTAAGAACATTGATCTCTAATCTCAAGAATGTTGCCTGCACTTAATATCATAACAAAATA**TCAAGTGAGGTAACATTTTA**
GAGATGGGGGATTACTATCCAC

>ID748-3P
TCAAGTGAGGTAACATTTTAGA

>miRNA precursor
UGGUGACACAGUAAGAACAUUGAUCUCUAAUCUCAAGAAUGUUGCCUGCACUUAAUAUCAUAACAAAAUA**UCAAGUGAGGUAACAUUUUAGA**GAUGGGGGAUUACUAUCCAC
((....))...........((((((((.(((((.((((((((((((.(((((.((((.........)))).))))))))))))))))).))))).))))))))........

>Predicted STAR seq:
CAAGAAUGUUGCCUGCACUUA

```
Complementarity: 18
ERROR 4: Strange structure
 Initial dG =    -38.30


  | G
--UG U
  AC G
\ ^ A



  10        20        30        40        50        60
  CAGUAAGAACAU        A     A           G     A    CAUA
              UGAUCUCU AUCUC AGAAUGUUGCCU CACUU AUAU    \
              AUUAGGGG UAGAG UUUUACAAUGGA GUGAA UAUA    A
  CACCUAUC----        G     A           -     C    AAAC
  110          100        90        80         70
```

Reads in: lib-42, lib-45, lib-47, lib-48, lib-49, lib-50, lib-72
Number of reads="ID748",56,17,0,0,0,0,0

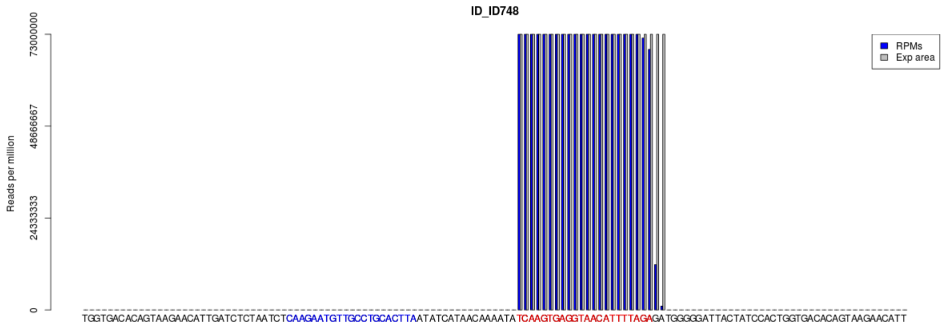

>ID75
GAGAACGGTGGTCTGTGGTA**CGGAGGAAACCTACACGAACACG**GTATTTAAGCAGTGTTGTGTGCGTTTCAACCTAACTACACGCTGCCT
CCTACATCGTCTACGAGTC

>ID75-5P
CGGAGGAAACCTACACGAACACG

>miRNA precursor
GAGAACGGUGGUCUGUGGUA**CGGAGGAAACCUACACGAACACG**GUAUUUAAGCAGUGUUGUGUGCGUUUCAACCUAACUACACGCUGCCUCCUACAUCGUCUACGAGUC
......((..((.((((((..((..(((((.(((((.(((((.((......)).)))))))))).)))))..))..)))))).))..)).......(((....)))...

>Predicted STAR seq:
GTTGTGTGCGTTTCAACCTAAC

Complementarity: 15

Initial dG = -37.60

```
          10        20        30        40
.-GAGAAC| UG  C      AC  AG     C     G     G  AU
        GG  GU UGUGGU  GG  GAAAC UACAC AACAC GU  U
        CC  CG ACAUCA  CC  CUUUG GUGUG UUGUG CG  U
\ ------^ GU  C      AU  AA     C     -     A  AA
                80        70        60         50



 90       100
  UCCUACA   U
         UCG C
         AGC U
  CUG----   A
```

Reads in: lib-42, lib-45, lib-47, lib-48, lib-49, lib-50, lib-72
Number of reads="ID75",2,2,0,0,0,0,0

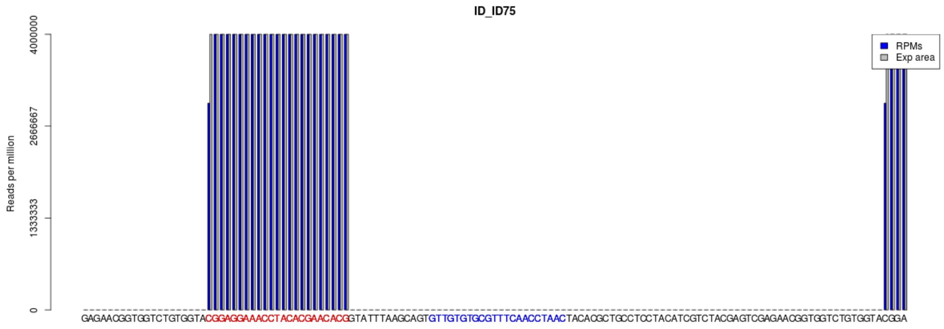

>ID753

CTGTTCTCACAAGGAAACATTTTTACTTGGAGCAAATATTCAGACTCGAAATTGGGTTCATTCGGCGAAT**ATTTGGTCCAGGCAAAAATG**
CTCACTTGTTAGGAGAAATGTC

>ID753-3P

ATTTGGTCCAGGCAAAAATGCT

>miRNA precursor

CUGUUCUCACAAGGAAACAUUUUUACUUGGAGCAAAUAUUCAGACUCGAAAUUGGGUUCAUUCGGCGAAU**AUUUGGUCCAGGCAAAAAUGCU**CACUUGUUAGGAGAAAUGUC
...((((((((((((..(((((((.((((((.(((((((((....(((((..((....))))))).))))))))).)))))).))))))).)).)))))...))))).....

>Predicted STAR seq:

AACATTTTTACTTGGAGCAAAT

Complementarity: 17

Initial dG =   -35.90

```
             10         20        30        40          50
CUG--     ---      -   AA       A       G         .-A|     A
     UUCUC   ACAAG GA  CAUUUUU CUUGGA CAAAUAUUC   GACUCG A
     AAGAG   UGUUC CU  GUAAAAA GGACCU GUUUAUAAG   UUGGGU A
CUGUA     GAU      A  C-       C      G         \ -^     U
110       100         90        80        70
```

Reads in: lib-42, lib-45, lib-47, lib-48, lib-49, lib-50, lib-72

Number of reads="ID753",30,9,0,0,1,0,0

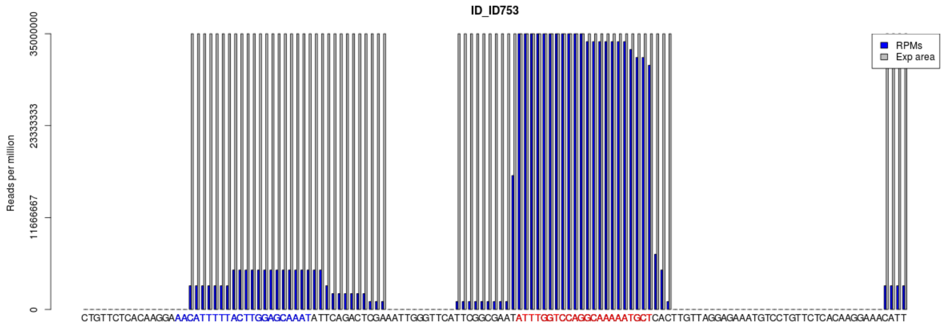

>ID754

AGAAAAAATGGCTCAGCTGTGTGTCTGTGCTTTCTTGTCATCTATCTTGCATCACTGTGAATTAAATAG**TGAAGCAGTAGAAGCCAAGAC**
AGCCTTGCATGCTTTACAACT

>ID754-3P

TGAAGCAGTAGAAGCCAAGACA

>miRNA precursor

AGAAAAAAUGGCUCAGCUGUGUGUCUGUGCUUUCUUGUCAUCUAUCUUGCAUCACUGUGAAUUAAAUAG**UGAAGCAGUAGAAGCCAAGACA**GCCUUGCAUGCUUUACAACU
.................(((((....(((.(((((...(((((..(((.(((((((.......))))))).))))))))...))))).)))...))))))........

>Predicted STAR seq:

TTCTTGTCATCTATCTTGCATCA

Complementarity: 15

Initial dG =   -29.10

```
        10        20        30        40        50        60
AGAAAAAAUGGCUCAGCU|     CUGU   U     UCA     CU   A       GAA
                  GUGUGU    GCU UCUUG   UCUAU  UGC UCACUGU   \
                  CGUACG    CGA AGAAC   AGAUG  ACG AGUGAUA   U
UCAACAUUU---------^     UUC-   C     CGA     --   A       AAU
 .                100         90        80          70
```

Reads in: lib-42, lib-45, lib-47, lib-48, lib-49, lib-50, lib-72

Number of reads="ID754",42,3,0,1,0,0,0

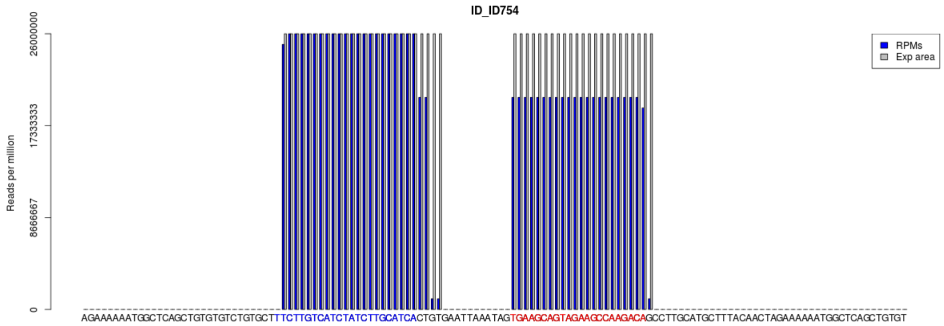

>ID76
GAGAATGGTTGTCTGTGGTA**CGGTGGAAACCTACACGAACACG**GTATTTGTGCAGTGGCGTGTGCGTTTCTACCTTACTACGTGCTGCCT
CCTACATCGTCTACGAGTCTCAT

>ID76-5P
CGGTGGAAACCTACACGAACACG

>miRNA precursor
GAGAAUGGUUGUCUGUGGUA**CGGUGGAAACCUACACGAACACG**GUAUUUGUGCAGUGGCGUGUGCGUUUCUACCUUACUACGUGCUGCCUCCUACAUCGUCUACGAGUCUCAU
((((..(((.((.(((((((.(((((((((.((((((..(((.(((....))).))).)))))).))))))))).))))))).)).)))).......((((....))).))))..

>Predicted STAR seq:
GGCGTGTGCGTTTCTACCTTA

Complementarity: 16

Initial dG =   -46.30

```
            10        20        30        40
--    .-AU|  U  C       C         C      AA   G   U
  GAGA    GGU GU UGUGGUA GGUGGAAAC UACACG  CAC GUA U
  CUCU    CCG CG GCAUCAU CCAUCUUUG GUGUGC  GUG CGU U
UA    \ --^  U  U       U         C      G-   A   G
 110              80        70        60         50



       90       100
        UCCUACA   U
               UCG C
               AGC U
        G------   A
```

Reads in: lib-42, lib-45, lib-47, lib-48, lib-49, lib-50, lib-72
Number of reads="ID76",0,3,0,0,0,0,0

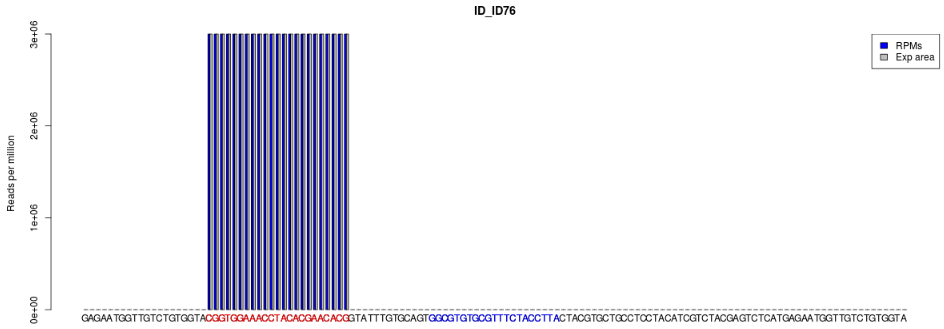

>ID766
GAGGTAGAGTCTCAACGAGC**TTGGAAAGAGACGACTGCATGAAA**CGTAGCGAATCAAGGAAGCGCAGCGTAATTTTTGGTGTCATTTGTT
TAGAGA

>ID766-5P
TTGGAAAGAGACGACTGCATGAAA

>miRNA precursor
GAGGUAGAGUCUCAACGAGC**UUGGAAAGAGACGACUGCAUGAAA**CGUAGCGAAUCAAGGAAGCGCAGCGUAAUUUUUGGUGUCAUUUGUUUAGAGA
((((.....))))......(((..(((.(((.(((..((.(((((((.(((..((...))..))).))))..))).))..))).))).))).))).

>Predicted STAR seq:
ATTTTTGGTGTCATTTGTTTAGAGA

Complementarity: 15

Initial dG =   -18.30

ERROR 3: Strange structure
Initial dG =   -18.30

```
  |   UA
--GAGG  \
  CUCU  G
\ ^   GA
    10
```

```
       20        30        40          50
  AACGAG   GG   G   C   UG  U   --    A   AA  A
        CUU  AAA AGA GAC  CA GAA  ACGU GCG  UC \
        GAG  UUU UUU CUG  GU UUU  UGCG CGC  AG A
  A-----   A-   G   A   UG  U   AA    A   GA  G
             90        80        70        60
```

Reads in: lib-42, lib-45, lib-47, lib-48, lib-49, lib-50, lib-72
Number of reads="ID766",361,57,26,241,166,33,0

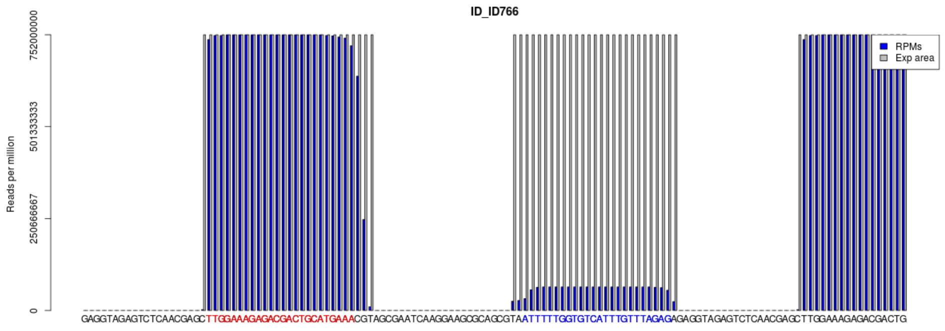

>ID772
AGAAGAACAACAGCCATGCCGCGGGCCGCACACTCTAAGCTTGAACACCAAGCCTGGTTGCTGACAACGT**TTGGTGTTCTACCTTACAGT**
GAGCGGACCGCGGCAAGCACCA

>ID772-3P
TTGGTGTTCTACCTTACAGTGA

>miRNA precursor
AGAAGAACAACAGCCAUGCCGCGGGCCGCACACUCUAAGCUUGAACACCAAGCCUGGUUGCUGACAACGU**UUGGUGUUCUACCUUACAGUGA**GCGGACCGCGGCAAGCACCA
```
............((..((((((((.((((.((((.((((...(((((((((((...((((....))))))))))))))))...)))).)))).)))).)))))))).))....
```

>Predicted STAR seq:
CACTCTAAGCTTGAACACCAA

Complementarity: 15

Initial dG = -55.40

```
        10        20        30        40        50        60
AGAAGAACAACA| CA        G    A    C    CUU           CUG    C
            GC  UGCCGCGG CCGC CACU UAAG   GAACACCAAGC   GUUG U
            CG  ACGGCGCC GGCG GUGA AUUC   CUUGUGGUUUG   CAAC G
ACCA--------^ A-        A    A    C    CAU           ---    A
110                100        90        80        70
```

Reads in: lib-42, lib-45, lib-47, lib-48, lib-49, lib-50, lib-72
Number of reads="ID772",4,31,27,2254,141,7,0

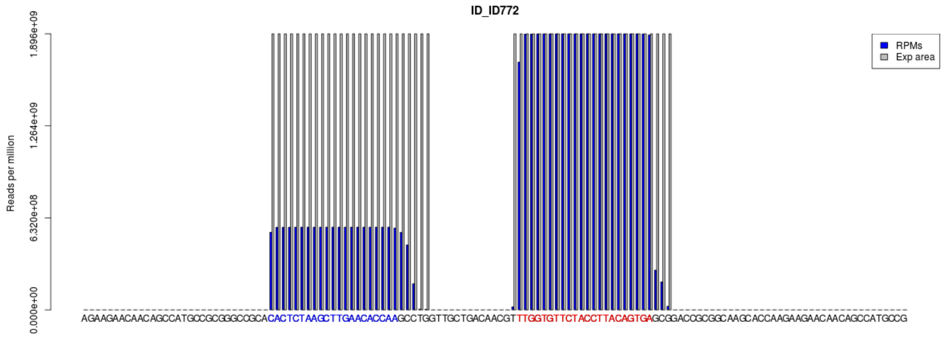

>ID78
GAGAATGGTGGTCTGTGGTA**CGGTGGAAACCTACACGAACACG**GTATTTGAGCAGTGTCGTGAGCGTTTCCACCTTACTACGTGCTGCCT
CCTACATCGTCTACGAGTC

>ID78-5P
CGGTGGAAACCTACACGAACACG

>miRNA precursor
GAGAAUGGUGGUCUGUGGUA**CGGUGGAAACCUACACGAACACG**GUAUUUGAGCAGUGUCGUGAGCGUUUCCACCUUACUACGUGCUGCCUCCUACAUCGUCUACGAGUC
```
......((..((.(((((((.(((((((((((.(((((.(((.((......)).)))))))))).))))))))).))))))).))..)).......(((....)))...
```

>Predicted STAR seq:
GTCGTGAGCGTTTCCACCTTA

Complementarity: 17

Initial dG =   -47.00

```
          10        20        30         40
.-GAGAAU  UG  C        C         -| A     A   G  AU
        GG  GU UGUGGUA GGUGGAAAC CU CACGA CAC GU  U
        CC  CG GCAUCAU CCACCUUUG GA GUGCU GUG CG  U
\ ------  GU  U        U         C^ -     -   A  AG
                80        70         60         50
```

```
 90       100
  UCCUACA   U
         UCG C
         AGC U
  CUG----   A
```

Reads in: lib-42, lib-45, lib-47, lib-48, lib-49, lib-50, lib-72
Number of reads="ID78",0,0,0,0,0,0,0

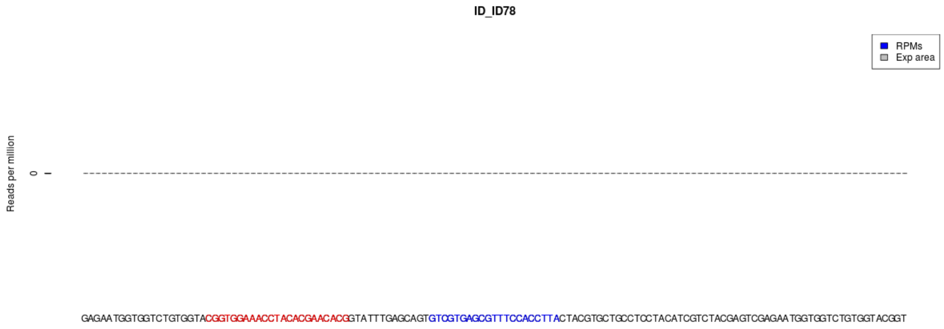

>ID782
TGGTGAGTTGTCAGTCGGTG**TCGCGCAGCTGGAGACCTTCGG**AGGAAATACCAGAATCCCGAAGGTCCCGGTTGCGGGGCGCCGGCAGGC
ACAAAAGACAGCGCAACAGCAG

>ID782-5P
TCGCGCAGCTGGAGACCTTCGG

>miRNA precursor
UGGUGAGUUGUCAGUCGGUG**UCGCGCAGCUGGAGACCUUCGG**AGGAAAUACCAGAAUCCCGAAGGUCCCGGUUGCGGGGCGCCGGCAGGCACAAAAGACAGCGCAACAGCAG
((.((.((((((.(((((((((.(((((((((.(((((((((................)))))))))))))))))).))))))))).........)))))).)).))....

>Predicted STAR seq:
GAAGGTCCCGGTTGCGGGGCG

Complementarity: 18

Initial dG =   -57.80

```
            10                  20         30         40         50
----  G  A      A---------|         G          A          AGGAAAU
    UG UG GUUGUC          GUCGGUGUC CGCAGCUGG GACCUUCGG       A
    AC AC CGACAG          CGGCCGCGG GCGUUGGCC CUGGAAGCC       C
GACG  A  G      AAAACACGGA^         G          -          CUAAGAC
110       100        90         80         70          60
```

Reads in: lib-42, lib-45, lib-47, lib-48, lib-49, lib-50, lib-72
Number of reads="ID782",5,16,1,2,3,1,0

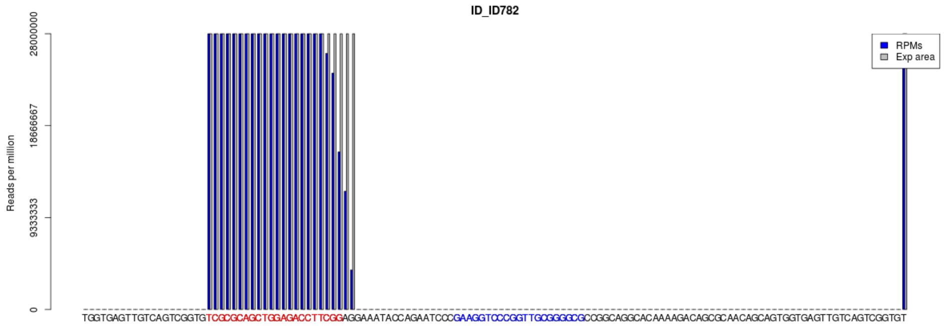

>ID783
ACCCAGAGCCCTCGCCGGCG**ACCCCTGTCGGCTGCCTCTTCGCT**GACTGAGAGATCGCGAAAGGGGCCGACGGTGGACGCTGGCCGAGGG
CACAGCAGCGACTGGCCACCTGCT

>ID783-5P
ACCCCTGTCGGCTGCCTCTTCGCT

>miRNA precursor
ACCCAGAGCCCUCGCCGGCG**ACCCCUGUCGGCUGCCUCUUCGCU**GACUGAGAGAUCGCGAAAGGGGCCGACGGUGGACGCUGGCCGAGGGCACAGCAGCGACUGGCCACCUGCU
.......((((((((((((.((.(((((((((.(((.(((((.((........))))))))))))))))))).)).))))))).))))))..(((((..........)))))

>Predicted STAR seq:
GAAAGGGGCCGACGGTGGACG

Complementarity: 17

Initial dG =   -60.70

```
          10         20        30        40        50
.-ACCCAGA      -|      A  C          G   C     U  CUG
         GCCCUC GCCGGCG CC CUGUCGGCU CCU UUCGC GA   A
         CGGGAG CGGUCGC GG GGCAGCCGG GGA AAGCG CU   G
\ -------      C^      A  U          -   -     -  AGA
         90        80        70          60



        100
  AC      CGACU
    AGCAG     \
    UCGUC     G
  --     CACCG
      110
```

Reads in: lib-42, lib-45, lib-47, lib-48, lib-49, lib-50, lib-72
Number of reads="ID783",21,10,1,1,2,1,0

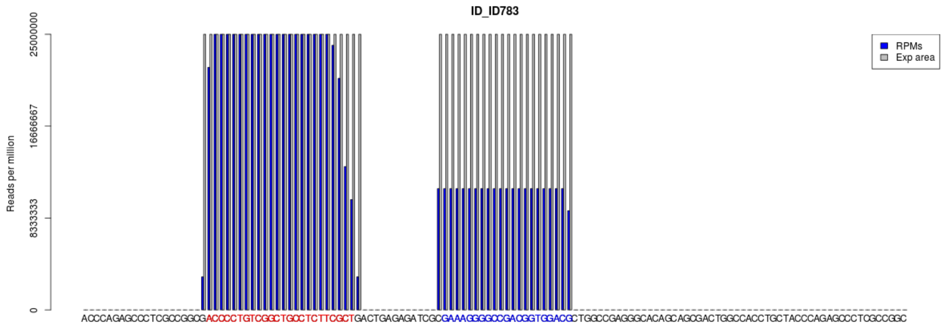

>ID784
CGGCCACCAGTCACCCAGAGCCCTCGCCAGCGACCCCTGTCGGCTGCCTCTTCGCTGACCTTGAGACAG**CGAAAGGGGCCGACAGTGGAC**
GCTGGCCGAGGGCACAGCAGCG

>ID784-3P
CGAAAGGGGCCGACAGTGGACGC

>miRNA precursor
CGGCCACCAGUCACCCAGAGCCCUCGCCAGCGACCCCUGUCGGCUGCCUCUUCGCUGACCUUGAGACAG**CGAAAGGGGCCGACAGUGGACGC**UGGCCGAGGGCACAGCAGCG
```
.(((.....))).......((((((((((((.((.(((((((((.(((.(((((((.........))))))))))))))))))).)).)))))).))))))).........
```

>Predicted STAR seq:
CGACCCCTGTCGGCTGCCTCTTCG

Complementarity: 19
ERROR 4: Strange structure
Initial dG =   -63.30

```
.-C|  CA
   GGC  \
   CUG  C
\ -^  AC
    10



          20         30        40        50        60
  ACCCAGA--       -      A  C         G   C       ACCU
           GCCCUCG CCAGCG CC CUGUCGGCU CCU UUCGCUG    \
           CGGGAGC GGUCGC GG GACAGCCGG GGA AAGCGAC    U
  GCGACGACA       C      A  U         -   -       AGAG
  110       100        90        80          70
```

Reads in: lib-42, lib-45, lib-47, lib-48, lib-49, lib-50, lib-72
Number of reads="ID784",9,12,0,2,2,0,0

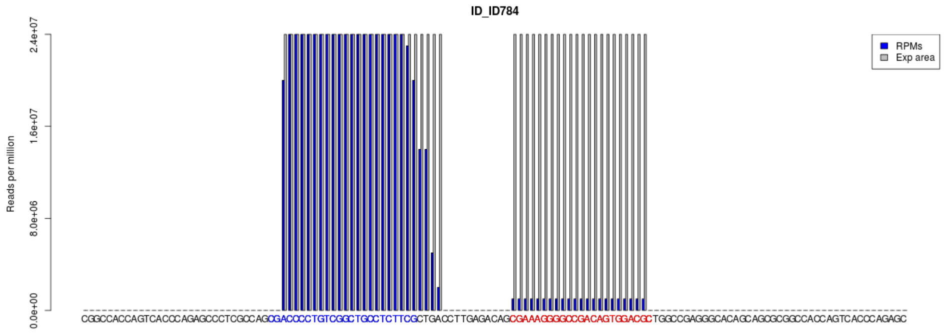

>ID785
CGCCAGTGAAGCCACCAGATGCTGGCGCCAGAGTCTCTACCTGCTGCCTTGTCGCTGCTGTTTCGTTTGC**GAAGGCTTCAGGTGGAGAAC**
GCTGGAGTCGGCTACTTCTACC

>ID785-3P
GAAGGCTTCAGGTGGAGAACGC

>miRNA precursor
CGCCAGUGAAGCCACCAGAUGCUGGCGCCAGAGUCUCUACCUGCUGCCUUGUCGCUGCUGUUUCGUUUGC**GAAGGCUUCAGGUGGAGAACGC**UGGAGUCGGCUACUUCUACC
.....(((....))).....((((((.((((..((((((((((..((((..((((.((......))..))))))))..))))))))))...)))).))))))..........

>Predicted STAR seq:
AGTCTCTACCTGCTGCCTTGTC

Complementarity: 15
ERROR 4: Strange structure
Initial dG =   -49.20

```
          10
.-CGCCA|  A
       GUG A
       CAC G
\ -----^  C



     20             30         40        50         60
  CAGAU-----      G    AG-          CU     UG     U-  UG
            GCUGGC CCAG   UCUCUACCUG  GCCU  UCGC  GC  U
            CGGCUG GGUC   AGAGGUGGAC  CGGA  AGCG  UG  U
  CCAUCUUCAU      A    GCA          UU     --     UU  CU
  110       100        90        80          70
```

Reads in: lib-42, lib-45, lib-47, lib-48, lib-49, lib-50, lib-72
Number of reads="ID785",9,17,2,0,2,1,0

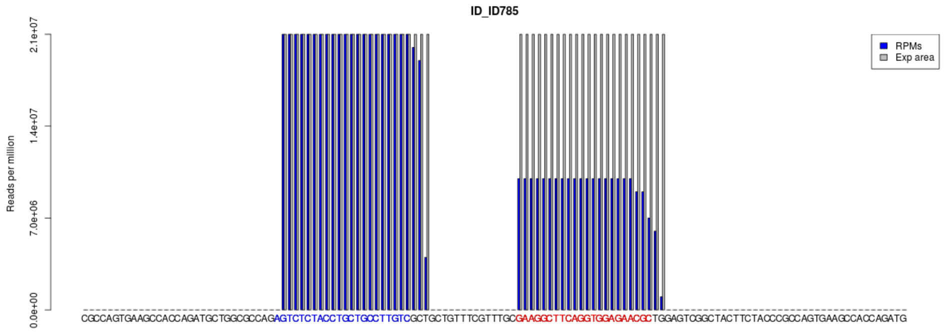

>ID787
TGGTGAGTTGTCAGTCGGCG**TCTCACAGCTGGAAATCTTCGGA**GGAAACAACAGAGTCCCGAAGGTCCCGGTTGCGGGATGCTGGCAGGC
GCCAGAGACAGCATGACAATAGG

>ID787-5P
TCTCACAGCTGGAAATCTTCGGA

>miRNA precursor
UGGUGAGUUGUCAGUCGGCG**UCUCACAGCUGGAAAUCUUCGGA**GGAAACAACAGAGUCCCGAAGGUCCCGGUUGCGGGAUGCUGGCAGGCGCCAGAGACAGCAUGACAAUAGG
......((((((.(((((((((((.(((((((..((((((((.((...........)))))))))).))))))).))))))))))))....((........))..))))))...

>Predicted STAR seq:
GAAGGTCCCGGTTGCGGGATG

Complementarity: 17

Initial dG =   -46.80

```
        10          20        30        40        50
UGGUGA      .-A|          A       AA        A  AAACA
      GUUGUC   GUCGGCGUCUC CAGCUGG  AUCUUCGG GG     \
      UAACAG   CGGUCGUAGGG GUUGGCC  UGGAAGCC CU     A
GGA---      \ -^          C       C-        -  GAGAC
    110             80        70         60



                90
              AGGC  CAG
                  GC   A
                  CG   G
              UA--  ACA
                  100
```

Reads in: lib-42, lib-45, lib-47, lib-48, lib-49, lib-50, lib-72
Number of reads="ID787",5,3,0,0,0,0,0

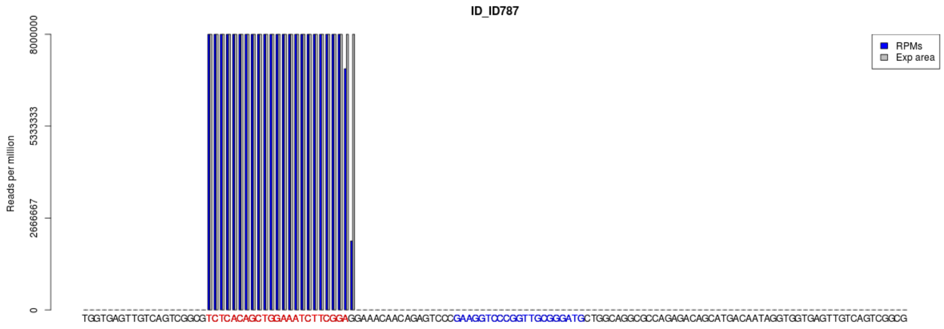

>ID788

CAGGAGTTGTCAGTCGGCG**TCTCACAGCTGGAGATCTTTGGAG**GAAATACCAGAGTCCCGAAGGTCCCAGTTGCGGGGCACCGGCAGGCA
CCAGAGACAGCGCGACAGCAGGT

>ID788-5P

TCTCACAGCTGGAGATCTTTGGAG

>miRNA precursor

CAGGAGUUGUCAGUCGGCG**UCUCACAGCUGGAGAUCUUUGGAG**GAAAUACCAGAGUCCCGAAGGUCCCAGUUGCGGGGCACCGGCAGGCACCAGAGACAGCGCGACAGCAGGU
..((...((((.(((((.(((((.(((((((.(((((((((.((...........)))))))))))))))))).))))).))))).)))))).......((......))....

>Predicted STAR seq:

CGAAGGTCCCAGTTGCGGGGCA

Complementarity: 19

Initial dG =   -50.20

```
        10          20         30         40
CAGGA      .-A|    C      A       A         A  AAAUA
     GUUGUC   GUCGG GUCUC CAGCUGG GAUCUUUGG GG     \
     CGACAG   CGGCC CGGGG GUUGACC CUGGAAGCC CU     C
UGGA-      \ -^    A      C       -         -  GAGAC
 110             80         70         60



                90
             AG  ACCA
               GC    G
               CG    A
             CG  ACAG
              100
```

Reads in: lib-42, lib-45, lib-47, lib-48, lib-49, lib-50, lib-72

Number of reads="ID788",14,23,0,2,5,0,0

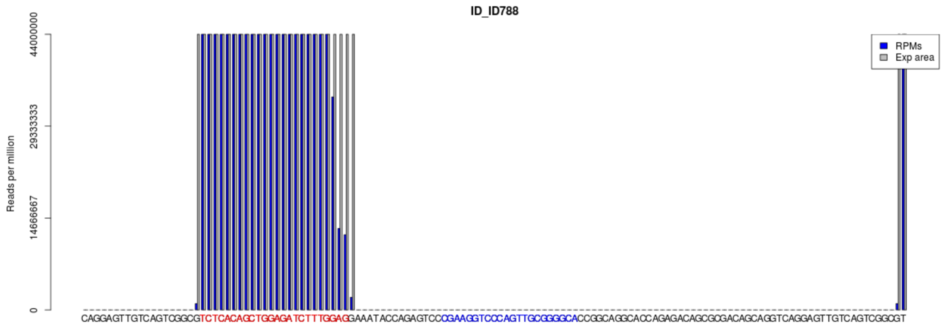

>ID789

TGAGGAGTTGTCAGTCGGTG**TCGCGCAGCTGGAGACATTCGG**AGGATATACAATAATTCCGAAGGTCCCGGTTGCGTCACGCCGGCAGGC
ACCAAAGACAGCGCGACAGCAG

>ID789-5P

TCGCGCAGCTGGAGACATTCGG

>miRNA precursor

UGAGGAGUUGUCAGUCGGUG**UCGCGCAGCUGGAGACAUUCGG**AGGAUAUACAAUAAUUCCGAAGGUCCCGGUUGCGUCACGCCGGCAGGCACCAAAGACAGCGCGACAGCAG
...((...((((.((((((((.((((((((((.(((.(((((((...........))))))).))))))))))))))).)))))))).)))))).......((......))..

>Predicted STAR seq:

GAAGGTCCCGGTTGCGTCACG

Complementarity: 17

Initial dG =   -54.50

```
        10          20        30        40        50
UGAGGA      .-A|       C          A   A       GAUAU
      GUUGUC   GUCGGUGU GCGCAGCUGG GAC UUCGGAG     A
      CGACAG   CGGCCGCA UGCGUUGGCC CUG AAGCCUU     C
GA----      \ -^       C          -   G       AAUAA
    110             80        70         60



                90
              AG  ACCA
                GC    A
                CG    A
              CG  ACAG
                100
```

Reads in: lib-42, lib-45, lib-47, lib-48, lib-49, lib-50, lib-72

Number of reads="ID789",3,9,0,2,1,0,0

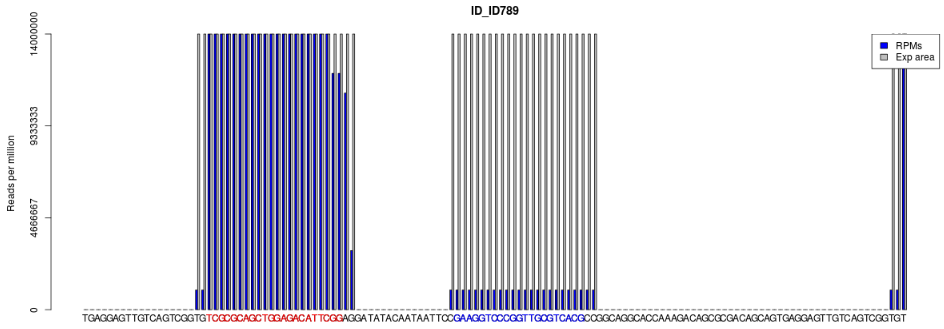

>ID791

TGAGGGGTTGTCAGTCGGCG**TCGCGCAGCTGGAGACATTCGG**AGGATATACAAGAATTCTGAAGGCCCCAGTTGCGTGACGCCGGCAGTC
ACCAAAGACAGCGCGACAGCAG

>ID791-5P

TCGCGCAGCTGGAGACATTCGG

>miRNA precursor

UGAGGGGUUGUCAGUCGGCG**UCGCGCAGCUGGAGACAUUCGG**AGGAUAUACAAGAAUUCUGAAGGCCCCAGUUGCGUGACGCCGGCAGUCACCAAAGACAGCGCGACAGCAG
......((((((.((((((((((((((((((((.....(((((((............)))))))....))))))))))))))))))))).(((......)))......))))))..

>Predicted STAR seq:

GAAGGCCCCAGTTGCGTGACG

Complementarity: 15

Initial dG =   -57.00

```
        10          20        30        40        50
UGAGGG      .-A|                  AGACA       GAUAU
      GUUGUC   GUCGGCGUCGCGCAGCUGG     UUCGGAG     A
      CGACAG   CGGCCGCAGUGCGUUGACC     AAGUCUU     C
GA----      \ -^                  CCGG-       AAGAA
    110             80        70         60
```

```
                    90
              A----   AC
                   GUC  C
                   CAG  A
              CGCGA   AA
                100
```

Reads in: lib-42, lib-45, lib-47, lib-48, lib-49, lib-50, lib-72

Number of reads="ID791",3,6,0,0,1,0,0

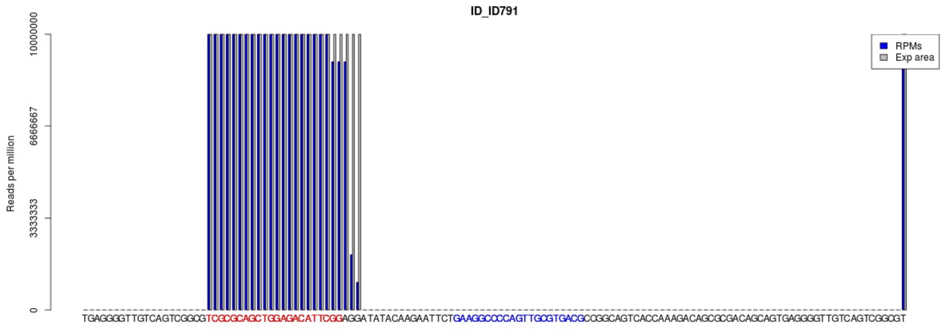

>ID792
CAGCCACCAGTCACCCAGAGCCCTCGCCGGCGACCCCTGTCGGCTGCCTCTTCGCTGACCTTGAGACAG**CGAAAGGGGCCGACAGTGGAC**
GCTGGCAGAGAGCACACCAGCG

>ID792-3P
CGAAAGGGGCCGACAGTGGACGC

>miRNA precursor
CAGCCACCAGUCACCCAGAGCCCUCGCCGGCGACCCCUGUCGGCUGCCUCUUCGCUGACCUUGAGACAG**CGAAAGGGGCCGACAGUGGACGC**UGGCAGAGAGCACACCAGCG
..................((.((((((((((.((.(((((((((.(((.(((((((.........))))))))))))))))))).)).))))))).))).)).........

>Predicted STAR seq:
CGACCCCTGTCGGCTGCCTCTTCG

Complementarity: 19

Initial dG =   -54.30

```
        10        20         30        40        50        60
CAGCCACCAGUCACCCAGA  C   -|      A  C         G   C       ACCU
                   GC CUC GCCGGCG CC CUGUCGGCU CCU UUCGCUG    \
                   CG GAG CGGUCGC GG GACAGCCGG GGA AAGCGAC    U
GCGACCACA----------  A   A^      A  U         -   -       AGAG
110                100        90        80          70
```

Reads in: lib-42, lib-45, lib-47, lib-48, lib-49, lib-50, lib-72
Number of reads="ID792",14,18,0,2,1,1,0

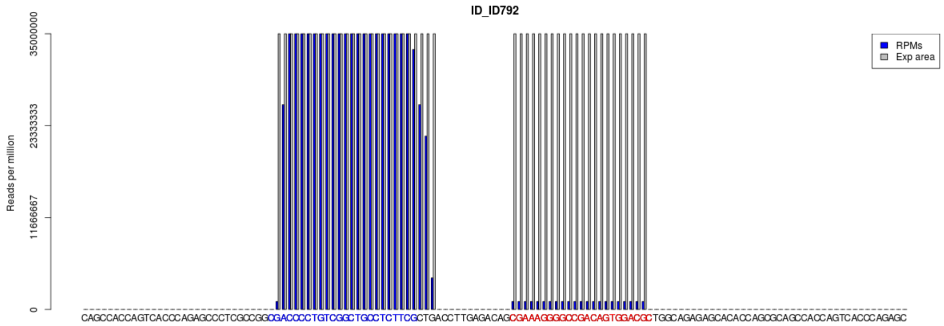

>ID793
TGGTGAGGAGTCGTCAGTTGTCAGTTGGCG**TCCCGCAACCGGCGACCCTCGGA**GAAAATACCAGAGTCCCGAAGGTCCCGGTTGCGGGGC
GCTGGCAGGCGCCGGAGACAGC

>ID793-5P
TCCCGCAACCGGCGACCCTCGGA

>miRNA precursor
UGGUGAGGAGUCGUCAGUUGUCAGUUGGCG**UCCCGCAACCGGCGACCCUCGGA**GAAAAUACCAGAGUCCCGAAGGUCCCGGUUGCGGGGCGCUGGCAGGCGCCGGAGACAGC
.........(((..(.((.(((.((..((((((((((((((.((((.((((.((...........)))))).)))))))))))))))))))..)).))))).)..)))...

>Predicted STAR seq:
GAAGGTCCCGGTTGCGGGGCG

Complementarity: 18

Initial dG =   -62.80

```
        10        20        30        40        50        60
UGGUGAGGA|  GU A  U   A  UG               C    C    A  AAAUA
         GUC  C GU GUC GU  GCGUCCCGCAACCGG GACC UCGG GA     \
         CAG  G CG CGG CG  CGCGGGGCGUUGGCC CUGG AGCC CU     C
CGA------^  AG C  -   A  GU               -    A    -  GAGAC
110             100       90        80         70
```

Reads in: lib-42, lib-45, lib-47, lib-48, lib-49, lib-50, lib-72
Number of reads="ID793",1,1,0,1,0,0,0

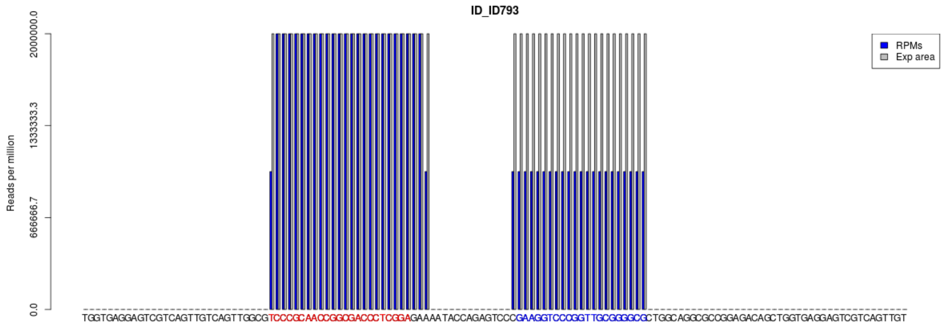

>ID799
CGGCGGCATCGGCGCGTTGCAGCAGTAATGGGGTTTCTAAGCGGCATTTGCCTTTCATGGCTCCGTCATG**CAATGCCCTTGGAAATCCCA**
AACGTGCTCAACAACATC

>ID799-3P
CAATGCCCTTGGAAATCCCAAA

>miRNA precursor
CGGCGGCAUCGGCGCGUUGCAGCAGUAAUGGGGUUUCUAAGCGGCAUUUGCCUUUCAUGGCUCCGUCAUG**CAAUGCCCUUGGAAAUCCCAAA**CGUGCUCAACAACAUC
((.((....)).)).((((.((((((..((((((((((((.((((((.((.....(((((...))))))))))))))))))))))))))))).)).))))))))......

>Predicted STAR seq:
AATGGGGTTTCTAAGCGGCATTT

Complementarity: 19
ERROR 4: Strange structure
Initial dG = -43.50

```
  | G  G
--CG CG C
  GC GC A
\ ^ G  U
     10
```

```
           20         30        40        50        60
  C-----    C    -  AA            C      U  CUUUC     U
        GUUG AGCA GU  UGGGGUUUCUAAG GGCAUU GC     AUGGC \
        CAAC UCGU CA  ACCCUAAAGGUUC CCGUAA CG     UACUG C
  CUACAA    -    G  A-            -      -  -----     C
        100          90        80          70
```

Reads in: lib-42, lib-45, lib-47, lib-48, lib-49, lib-50, lib-72
Number of reads="ID799",20,24,395,21,329,120,0

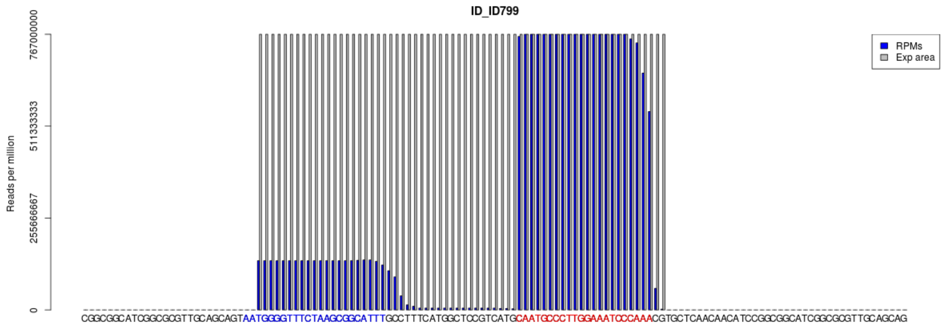

>ID8
AGACTTCTGTGGAAAGTATTTTGTTGATGATTTTGGTTAGTGGCAGTAAAATTTCATTCTTTACATTTTT**TATTACAAAATTTGAGAGTC**
CATAGAAGTCTTCTGCTTTAGAAT

>ID8-3P
TATTACAAAATTTGAGAGTCCAT

>miRNA precursor
AGACUUCUGUGGAAAGUAUUUUGUUGAUGAUUUUGGUUAGUGGCAGUAAAAUUUCAUUCUUUACAUUUUU**UAUUACAAAAUUUGAGAGUCCAU**AGAAGUCUUCUGCUUUAGAAU
(((((((((((((....((((((.((((((...((...(((((..........))))).....))....)))))))))))).......)))))))))))))............

>Predicted STAR seq:
TGGAAAGTATTTTGTTGATG

Complementarity: 14

Initial dG =   -26.30

```
                     10           20        30
-------------             AAGU---      U      .-UUUUG| UA
             AGACUUCUGUGGA       AUUUUG UGAUGA       GU  \
             UCUGAAGAUACCU       UAAAAC AUUAUU       CG  G
UAAGAUUUCGUCU             GAGAGUU      -      \ -----^ GU
  110       100        90        80         70
```

Reads in: lib-42, lib-45, lib-47, lib-48, lib-49, lib-50, lib-72
Number of reads="ID8",0,0,0,0,0,0,0

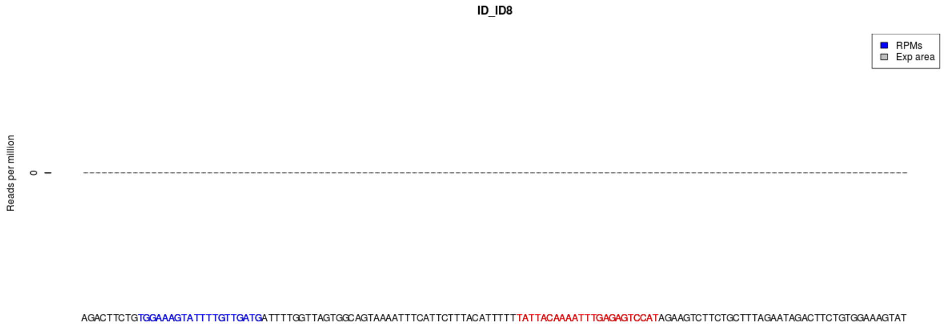

>ID802
CGGAGGAGGGGGCTGGGCGG**TGTGATGTGCATGTGGGCTTCCC**GTGCGCTCCCCGGTGGCCCACACTCACATTACTAACGCCCGGCCCT
CCACACCACCGCAATGCCA

>ID802-5P
TGTGATGTGCATGTGGGCTTCCC

>miRNA precursor
CGGAGGAGGGGGCUGGGCGG**UGUGAUGUGCAUGUGGGCUUCCC**GUGCGCUCCCCGGUGGCCCACACUCACAUUACUAACGCCCGGCCCUCCACACCACCGCAAUGCCA
(((.((.((((((((((((..((((((((..((((((((..(((..........))).))))))))..))))))))...))))))))))))....)).)))........

>Predicted STAR seq:
TGGCCCACACTCACATTACTAACG

Complementarity: 16

Initial dG =   -61.20

```
                   10        20         30        40
--------   A  A---            GU-|       CA        UC   UGCG
        CGG GG    GGGGGCUGGGCG   GUGAUGUG  UGUGGGCU  CCG    C
        GCC CC    CUCCCGGCCCGC   CAUUACAC  ACACCCGG  GGC    U
ACCGUAAC   A  ACAC            AAU^       UC        U-   CCCC
       100        90        80        70        60         50
```

Reads in: lib-42, lib-45, lib-47, lib-48, lib-49, lib-50, lib-72
Number of reads="ID802",4,623,1,0,1,0,0

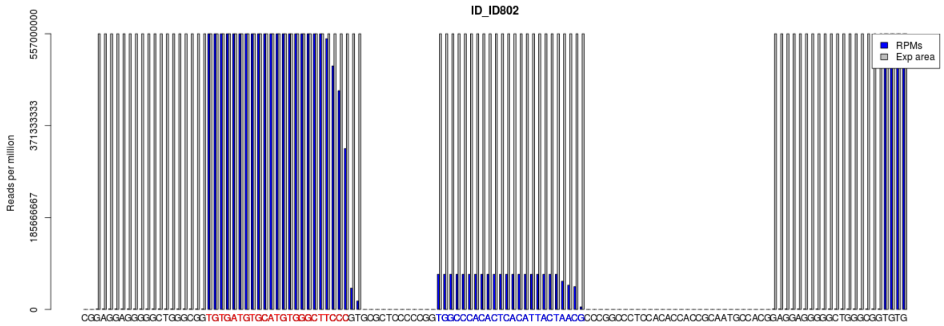

>ID803
GCGCGTAGCGCCTCATGTTC**ATTGTACTTCATCAGGTGCTCTGG**TGTGACGGAACCCGGCACCTGTTGGAGTGCAATTGATAGGAAGGCG
CCCATCCACGCGGCCGGCACTGCT

>ID803-5P
ATTGTACTTCATCAGGTGCTCTGG

>miRNA precursor
GCGCGUAGCGCCUCAUGUUC**AUUGUACUUCAUCAGGUGCUCUGG**UGUGACGGAACCCGGCACCUGUUGGAGUGCAAUUGAUAGGAAGGCGCCCAUCCACGCGGCCGGCACUGCU
.(((((.(((((((.((((.(((((((((((.((((((((..(((........))).)))))))).))))))))))).)))).)).))))).....)))))...........

>Predicted STAR seq:
CGGCACCTGTTGGAGTGCAATTGA

Complementarity: 19

Initial dG =   -52.00

```
                        10         20        30        40
G-----------     A-----     -| A     C           U         CU    GUG
            CGCGU      GCGCC UC UGUU AUUGUACUUCA CAGGUGCU  GGU    A
            GCGCA      CGCGG AG AUAG UAACGUGAGGU GUCCACGG  CCA    C
UCGUCACGGCCG     CCUACC     A^ G     U           U         C-    AGG
  110       100        90        80        70        60         50
```

Reads in: lib-42, lib-45, lib-47, lib-48, lib-49, lib-50, lib-72
Number of reads="ID803",10629,11744,40374,7702,26537,10085,0

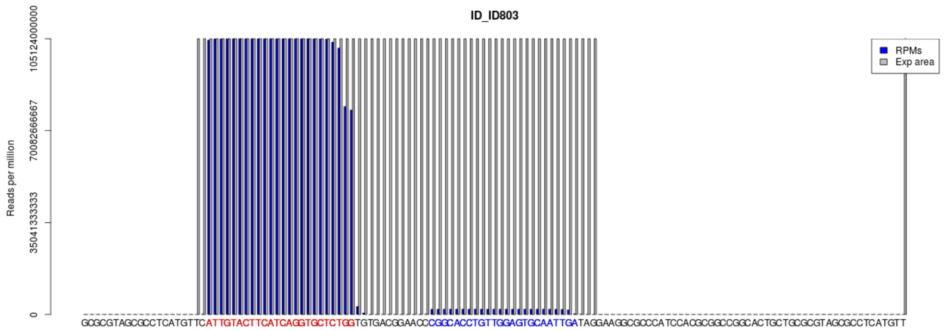

>ID804

GAACAATGTTGTCAACATTTGTCTGGAACAAGTTGTGAACTATGTCGCGAACAAAAACATGTTCCGAACT**TCAGTGTAAGGACTTGTACC**
ATGGCAGAATTATTATACTTAAGTG

>ID804-3P

TCAGTGTAAGGACTTGTACCATGG

>miRNA precursor

GAACAAUGUUGUCAACAUUUGUCUGGAACAAGUUGUGAACUAUGUCGCGAACAAAAACAUGUUCCGAACU**UCAGUGUAAGGACUUGUACCAUGG**CAGAAUUAUUAUACUUAAGUG
................((((((((.(((((((.((.(((...(((.(((((......))))))))).....))).)).)).))))))).))).))))))................

>Predicted STAR seq:

CTGGAACAAGTTGTGAACT

Complementarity: 15

Initial dG =   -26.50

```
        10        20         30         40          50
GAACAAUGUUGUCAACA      -   A       G-  A   AUG--|  C     AA
                 UUUGUC UGG ACAAGUU  UG ACU     UCG GAACA  A
                 AGACGG ACC UGUUCAG  AU UGA     AGC CUUGU  A
GUGAAUUCAUAUUAUUA      U   A       GA  G   CUUCA^  -     AC
   110       100        90        80        70         60
```

Reads in: lib-42, lib-45, lib-47, lib-48, lib-49, lib-50, lib-72

Number of reads="ID804",1,0,0,0,0,0,0

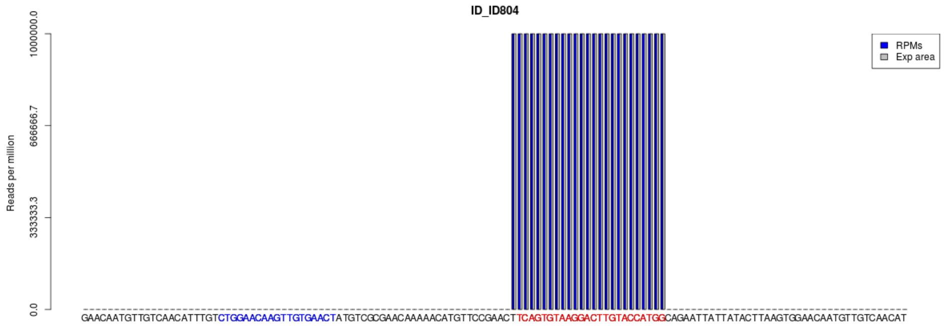

>ID805
CGCGTGTTGAGTTGTCCTCGTCATTCGCACGTGCTGCATCAGGTGCTAGTGACTGTGATGTAATGGTCAG**TCAGGTACCTGAAGTAGCGC**
GCGGAGAGGCGAGGGATAGCGG

>ID805-3P
TCAGGTACCTGAAGTAGCGCGC

>miRNA precursor
CGCGUGUUGAGUUGUCCUCGUCAUUCGCACGUGCUGCAUCAGGUGCUAGUGACUGUGAUGUAAUGGUCAG**UCAGGUACCUGAAGUAGCGCGC**GGAGAGGCGAGGGAUAGCGG
..........(((((((((((.(((((..(((((((.(((((((((..((((((...........))))))))))))))).))))))))))))..))))))).)))))..

>Predicted STAR seq:
CACGTGCTGCATCAGGTGCTAGTGA

Complementarity: 19

Initial dG = -55.60

```
        10         20         30        40        50        60
CGCGUGUUGA     -       A-|    AC        A         AG       UGAUG
          GUUGU CCUCGUC  UUCGC  GUGCUGC UCAGGUGCU  UGACUG      U
          CGAUA GGAGCGG  AGGCG  CGCGAUG AGUCCAUGG  ACUGAC      A
GG--------     G       AG^    --        A         --       UGGUA
        110       100         90        80          70
```

Reads in: lib-42, lib-45, lib-47, lib-48, lib-49, lib-50, lib-72
Number of reads="ID805",57532,49646,78624,42174,126874,41211,0

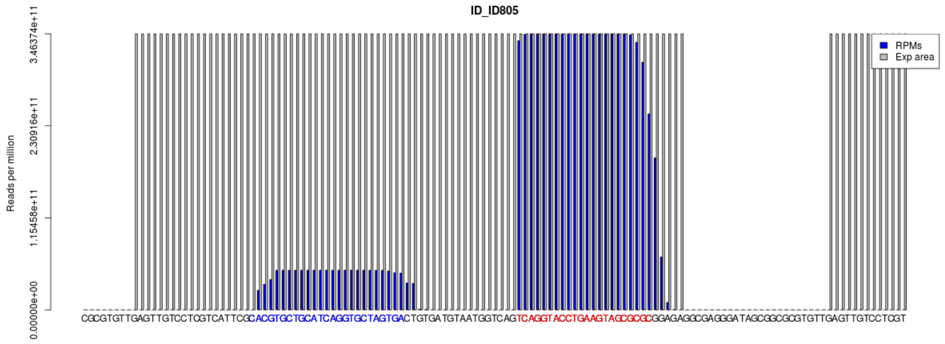

>ID808

CAGTTGATGGCAGAAAGTGGCTGGGCCACTCTCGTCGGAGTGCTGGACAGACTATAGCACTCTGGAGATA**CTCTGTCAAGCACTCAGAGG**
GGAGTTGCTCACCCGAATCTTC

>ID808-3P

CTCTGTCAAGCACTCAGAGGGG

>miRNA precursor

CAGUUGAUGGCAGAAAGUGGCUGGGCCACUCUCGUCGGAGUGCUGGACAGACUAUAGCACUCUGGAGAUA**CUCUGUCAAGCACUCAGAGGGG**AGUUGCUCACCCGAAUCUUC
...........(((...(((.(((((.((((((.((.(((((((.((((((.(((..(.....)...))).)))))).))))))).)).)))))).))))).)))..)))..

>Predicted STAR seq:

TCGTCGGAGTGCTGGACAGA

Complementarity: 16

Initial dG =   -46.10

```
        10        20        30        40        50         60
CAGUUGAUGGC|  AAG   C     C      G  G       G      C   AG- AC
           AGA   UGG UGGGC ACUCUC UC GAGUGCU GACAGA UAU   C  \
           UCU   GCC ACUCG UGAGGG AG CUCACGA CUGUCU AUA   G  U
CU---------^  AA-   C     U      G  A       A      C   GAG UC
         110        100        90        80        70
```

Reads in: lib-42, lib-45, lib-47, lib-48, lib-49, lib-50, lib-72

Number of reads="ID808",9,18,9,458,113,0,0

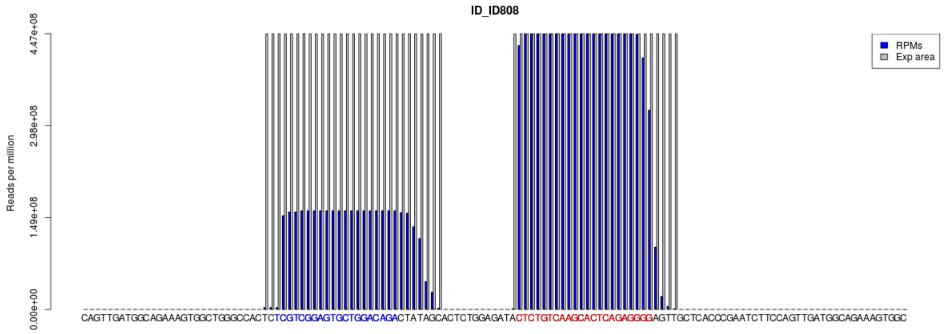

>ID809
CAGTTAACGGCAGAATATGGCAGGCCAGTCCCCTCTGAGTGCTGGACAGAGGGCTGGCATTGGGAGATC**CTTTATCCAGCACTCAGAGGG**
GAGCTGCTTGGCCGCAGCTTC

>ID809-3P
CTTTATCCAGCACTCAGAGGGG

>miRNA precursor
CAGUUAACGGCAGAAUAUGGCAGGCCAGUCCCCUCUGAGUGCUGGACAGAGGGCUGGCAUUGGGAGAUC**CUUUAUCCAGCACUCAGAGGGG**AGCUGCUUGGCCGCAGCUUC
.((((..((((.........(((((.(((((((((((((((((((.((((((..............)))))).))))))))))))))))))).))))))))))).))))..

>Predicted STAR seq:
CCCTCTGAGTGCTGGACAGAG

Complementarity: 19

Initial dG =   -58.90

```
         10        20         30        40        50        60
C-|   AA    AGAAUAUGG     C   -                  C      CUGGCA
  AGUU  CGGC         CAGGC AG UCCCCUCUGAGUGCUGGA AGAGGG      U
  UCGA  GCCG         GUUCG UC AGGGGAGACUCACGACCU UUUCCU      U
CU^   C-    ---------     -  G                  A      AGAGGG
 .                100        90        80        70
```

Reads in: lib-42, lib-45, lib-47, lib-48, lib-49, lib-50, lib-72
Number of reads="ID809",4,6,5,510,59,4,0

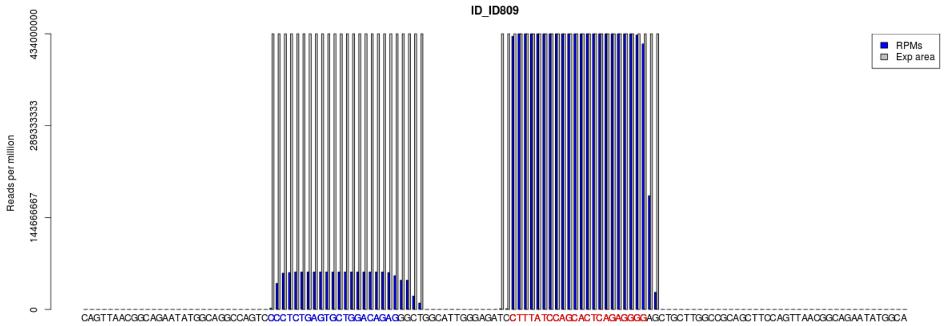

>ID81

ATGTGGAACAAATGTCAACCTCGTACTACGGCTGACATATGTGGTAGGTGTGCACGTTATTTCGTTAGTG**TGCCACAACCACATACGTCACT**CGTTGTTCGTGTTTAAAGAC

>ID81-3P

TGCCACAACCACATACGTCACT

>miRNA precursor

AUGUGGAACAAAUGUCAACCUCGUACUACGGCUGACAUAUGUGGUAGGUGUGCACGUUAUUUCGUUAGUG**UGCCACAACCACAUACGUCACU**CGUUGUUCGUGUUUAAAGAC

```
.............((((((..((.((.(((..((((.((((((((..(((.((((((((......))))))))))).)))))))).))))..))).)).)).)))....)))
```

>Predicted STAR seq:

CTGACATATGTGGTAGGTGTGCA

Complementarity: 16

Initial dG =   -36.00

```
        10            20         30         40         50         60
AUGUGGAACAAAU   ----|  CU  U  U   GC     A         AG   U         UU
             GUC    AAC  CG AC ACG  UGAC UAUGUGGU  GUG GCACGUUA  U
             CAG    UUG  GC UG UGC  ACUG AUACACCA  CAC CGUGUGAU  C
-------------   AAAU^  U-  U  U   UC     C         A-   -         UG
             110        100        90         80          70
```

Reads in: lib-42, lib-45, lib-47, lib-48, lib-49, lib-50, lib-72

Number of reads="ID81",1,5,0,0,0,0,0

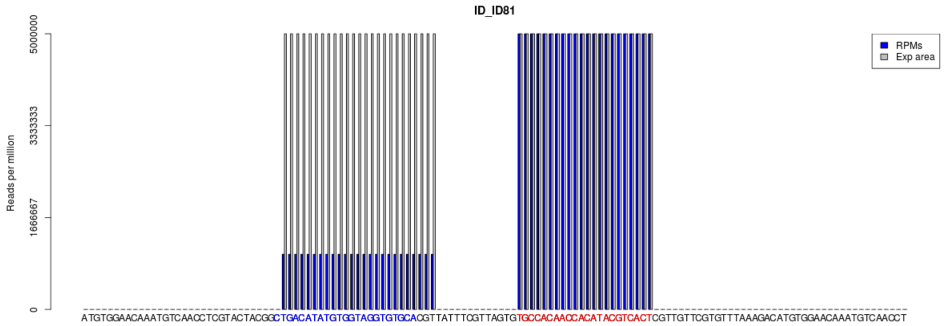

>ID810
GAAACTGGTAGGGTCACTCT**CGCTGAATGCTGGACAGAGGACT**GGTTCTGGGGGTCCTCTGTCCAGCACTCAGCGGGAGTTGCCTGGCCG
AAGCGTCGACGCCGCAGCTCCGT

>ID810-5P
CGCTGAATGCTGGACAGAGGACT

>miRNA precursor
```
GAAACUGGUAGGGUCACUCUCGCUGAAUGCUGGACAGAGGACUGGUUCUGGGGGUCCUCUGUCCAGCACUCAGCGGGAGUUGCCUGGCCGAAGCGUCGACGCCGCAGCUCCGU
.....((((.((((.(((((((((((.((((((((((((((((.........)))))))))))))))).))))))))))).)))).)))).((((....)))...........
```

>Predicted STAR seq:
TCCTCTGTCCAGCACTCAGCGGG

Complementarity: 20

Initial dG =   -66.40

```
          10        20        30        40
.-GAAAC|   A    C          A              GGUU
       UGGU GGGU ACUCUCGCUGA UGCUGGACAGAGGACU    \
       GCCG UCCG UGAGGGCGACU ACGACCUGUCUCCUGG    C
\ -----^   G    U          C              GGGU
      90        80        70        60        50




  AA---------   U
             GCG C
             CGC G
  UGCCUCGACGC   A
   110       100
```

Reads in: lib-42, lib-45, lib-47, lib-48, lib-49, lib-50, lib-72
Number of reads="ID810",80,495,231,15927,3647,114,0

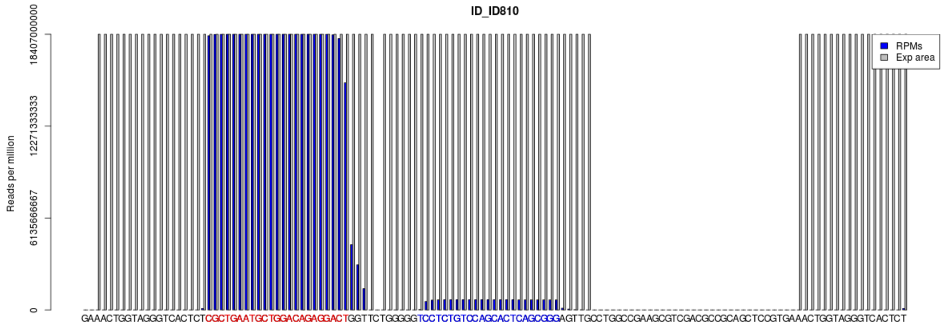

>ID813
AGCAAGTCCAACGTGAACT**TATCCCGGACAAGCCCCCA**AATGCGAGGGTGAAGGTCGGGGTGAGAGTTTAAAATAGAGAAAGTTTAATTT
TTTTTTAAACTCACAACC

>ID813-5P
TATCCCGGACAAGCCCCCA

>miRNA precursor
AGCAAGUCCAACGUGAACU**UAUCCCGGACAAGCCCCCA**AAUGCGAGGGUGAAGGUCGGGGUGAGAGUUUAAAAUAGAGAAAGUUUAAUUUUUUUUUAAACUCACAACC
............(((..((((((((((....((((..........))))).....)))))))))).((((((((.(((((........))))))))))))).))).....

>Predicted STAR seq:
GTGAAGGTCGGGGTGAG

Complementarity: 10

Initial dG =   -28.70

```
        10          20         30         40
AGCAAGUCCAAC    .-A          ACAA-|    CCAA
            GUGA   CUUAUCCCGG     GCCC    A
            CACU   GAGUGGGGCU     UGGG    U
CCAA--------    \ -          GGAAG^    AGCG
                      60         50



                      70         80
                  AGUUUAAAA         UU
                           UAGAGAAAG  U
                           AUUUUUUUU  A
                  CAA------         UA
                100              90
```

Reads in: lib-42, lib-45, lib-47, lib-48, lib-49, lib-50, lib-72
Number of reads="ID813",1,4,6,3,10,2,0

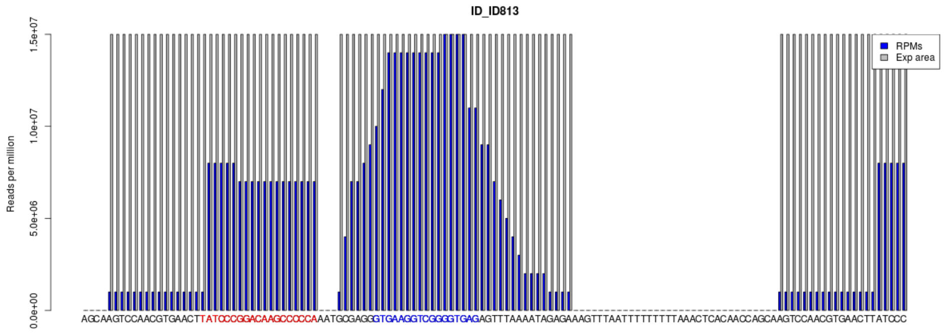

>ID814

GGAGAGACGTGGCGTGCTCT**TCTCGGACGAGGGCAGGTTCAG**TGTGAGTTGTTGTTCTGCAGCTGCCCTCATCTGACGAGAGCTCGTGGC
ACGTAATGCAACGGAGAATT

>ID814-5P

TCTCGGACGAGGGCAGGTTCAG

>miRNA precursor

GGAGAGACGUGGCGUGCUCU**UCUCGGACGAGGGCAGGUUCAG**UGUGAGUUGUUGUUCUGCAGCUGCCCUCAUCUGACGAGAGCUCGUGGCACGUAAUGCAACGGAGAAUU

........((.(((.((((((.(((((.((((((((...(((.(..(....)..).)))...)))))))).))))).)))))).))).)).(((......))).......

>Predicted STAR seq:

GCAGCTGCCCTCATCTGACGAG

Complementarity: 15

Initial dG =   -45.10

```
                  10          20         30         40
GGAGAG----------     --|  U      C     C        GUU   U UG G
                ACGUG  GCG GCUCUU UCGGA GAGGGCAG   CAG G  A U
                UGCAC  UGC CGAGAG AGUCU CUCCCGUC   GUC U  U U
UUAAGAGGCAACGUAA     GG^  U      C     A        GAC   U GU G
.       100        90         80         70         60         50
```

Reads in: lib-42, lib-45, lib-47, lib-48, lib-49, lib-50, lib-72

Number of reads="ID814",25,81,11,2569,472,19,0

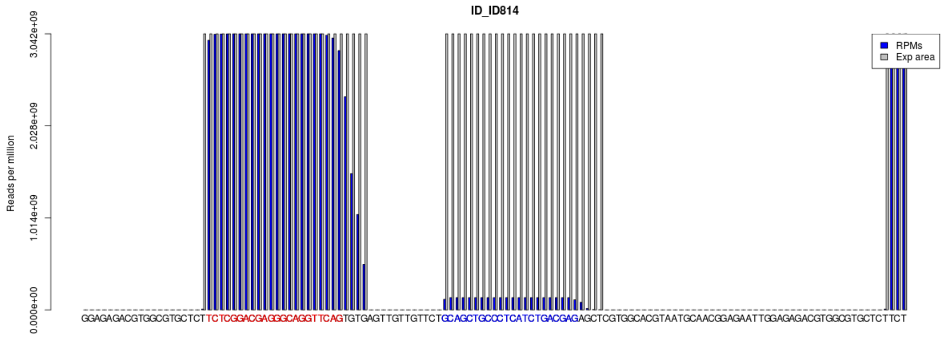

>ID816
TTGATGGCAGAAAGTGGCGGGGCCGCTCCCCTCTGAGTGCTGGACAGAGGGCTGGTACTGGAGAGATCCTCTGTCCAGCACTCAGAGTGG
GGTTGCCTGGCCACAACTTCGA

>ID816-5P
CCTCTGAGTGCTGGACAGAGGGC

>miRNA precursor
UUGAUGGCAGAAAGUGGCGGGGCCGCUCCCCUCUGAGUGCUGGACAGAGGGCUGGUACUGGAGAGAUCCUCUGUCCAGCACUCAGAGUGGGGUUGCCUGGCCACAACUUCGA
............(((((.((((.(((((.((((((((((((((((((((((...............))))))))))))))))))))))).))))).)))).))))).......

>Predicted STAR seq:
CTCTGTCCAGCACTCAGAGTGG

Complementarity: 19

Initial dG = -72.40

```
        10        20        30        40        50
UUGAUGGCAGAAA|    G    C     C                    CUGGUAC
             GUGGC GGGC GCUCC CUCUGAGUGCUGGACAGAGGG       \
             CACCG UCCG UGGGG GAGACUCACGACCUGUCUCCU       U
AGCUUCAA-----^    G    U     U                    AGAGAGG
110            100        90        80        70        60
```

Reads in: lib-42, lib-45, lib-47, lib-48, lib-49, lib-50, lib-72
Number of reads="ID816",0,9,1,126,21,2,0

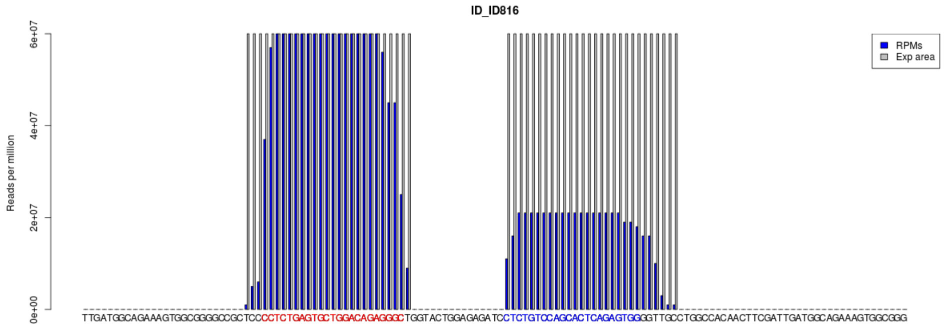

>ID817
TGGCAGGTGGCGGGCAGCTC**TGGCTGAGTGCTGGCCAGAGGG**CTAGCACTGGGAGATCCTCTGTCCAGCACTCAGCCGGGAGCTTCTTGG
GCACAACTTCGACACTGTGACT

>ID817-5P
TGGCTGAGTGCTGGCCAGAGGG

>miRNA precursor
UGGCAGGUGGCGGGCAGCUC**UGGCUGAGUGCUGGCCAGAGGG**CUAGCACUGGGAGAUCCUCUGUCCAGCACUCAGCCGGGAGCUUCUUGGGCACAACUUCGACACUGUGACU
..(((((((.((((.((((((((((((((((((((.(((((((.............))))))).))))))))))))).))))))).)))).)))..)))..........)))))....

>Predicted STAR seq:
CTCTGTCCAGCACTCAGCCGGGA

Complementarity: 19

Initial dG =   -58.20

```
        10           20        30        40
UGGCA   G    .-GC    -|              C       CUAGCA
     GGU GCGG    AGCU CUGGCUGAGUGCUGG CAGAGGG      C
     UCA UGUC    UCGA GGCCGACUCACGACC GUCUCCU      U
-----   G    \ --    G^              U       AGAGGG
     110            80        70        60        50



                   90
               UC     CA
                 UUGGG  C
                 AGCUU  A
               AC     CA
                 100
```

Reads in: lib-42, lib-45, lib-47, lib-48, lib-49, lib-50, lib-72
Number of reads="ID817",9,20,12,631,155,1,0

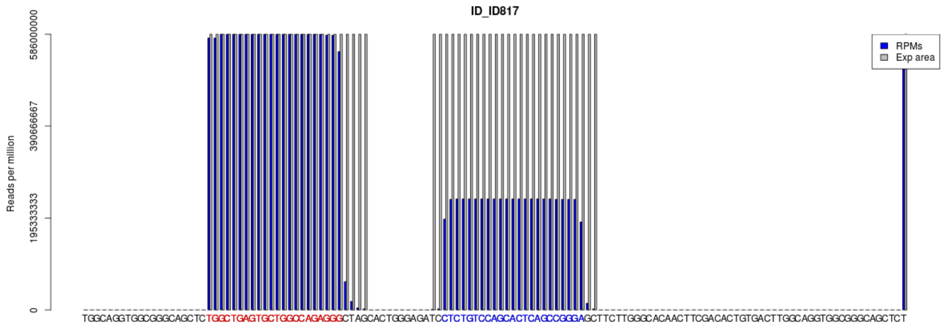

>ID82
ACGGATTGCATGTTCCTTGG**TCACAGAAGCGTTAGTTGCACA**TGTTATTAATTTACTGTGTGACTTTCCCTTCCGTGTCTCAAGGAGCAT
GGCATGCGCCAGTGCCATCGTC

>ID82-5P
TCACAGAAGCGTTAGTTGCACA

>miRNA precursor
ACGGAUUGCAUGUUCCUUGG**UCACAGAAGCGUUAGUUGCACA**UGUUAUUAAUUUACUGUGUGACUUUCCCUUCCGUGUCUCAAGGAGCAUGGCAUGCGCCAGUGCCAUCGUC
((((.....(((((((((((.(((.((((....(((..((((.((.........))))))..)))....)))).))).).))))))))))(((((......))))).)))).

>Predicted STAR seq:
TGTGACTTTCCCTTCCGTGTCT

Complementarity: 12

Initial dG =   -42.50

```
        10           20         30         40
ACG   U              -|U   A    CGUU   UG     U  UAUU
   GAU GCAU--GUUCCUUG G CAC GAAG    AGU  CACA GU    \
   CUA CGUG  CGAGGAAC C GUG CUUC    UCA  GUGU CA    A
CUG   C    \         U^U   C    CCUU   GU     -  UUUA
110                 80         70         60


             90
             AU   A
               GGC U
               CCG G
             A-   C
             100
```

Reads in: lib-42, lib-45, lib-47, lib-48, lib-49, lib-50, lib-72
Number of reads="ID82",0,9,0,0,1,0,0

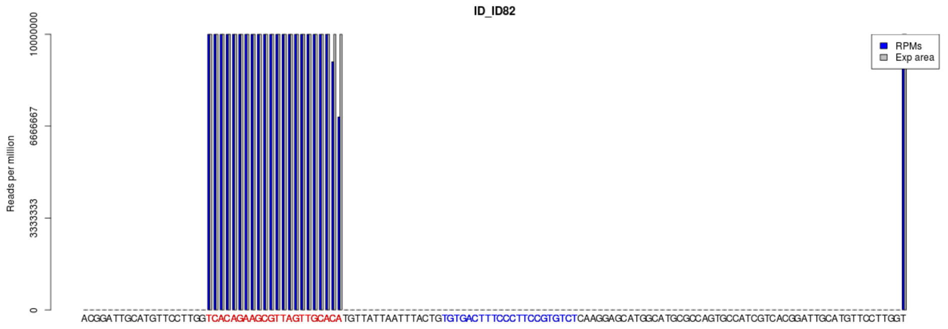

>ID822
CTGGTTTCGGAGGGTAGGCT**CCCATCATCAGGTGCAGTCGCCT**GGTTTTTATTATTGCGACTGCACCTGATGGTGGAGGCTAGCCCTCTG
TCTGCGACGCACAGTCACCACTA

>ID822-5P
CCCATCATCAGGTGCAGTCGCCT

>miRNA precursor
CUGGUUUCGGAGGGUAGGCU**CCCAUCAUCAGGUGCAGUCGCCU**GGUUUUUAUUAUUGCGACUGCACCUGAUGGUGGAGGCUAGCCCUCUGUCUGCGACGCACAGUCACCACUA
.(((...((((((((.(((..((((((((((((((((((((.((((....))))..))))))))))))))))))))..))).)))))))).....(((.....))).)))...

>Predicted STAR seq:
TGCGACTGCACCTGATGGTGGAGGC

Complementarity: 19

Initial dG =   -66.70

```
            10        20        30        40
C--   .-UUU       A   UC                    C-|   U
   UGG     CGGAGGGU GGC  CCAUCAUCAGGUGCAGUCGC  UGGU U
   ACC     GUCUCCCG UCG  GGUGGUAGUCCACGUCAGCG  AUUA U
AUC   \ ---       A   GA                    UU^   U
 110      90        80        70        60        50



               100
        UCUGC   GC
             GAC  \
             CUG  A
        A----   AC
```

Reads in: lib-42, lib-45, lib-47, lib-48, lib-49, lib-50, lib-72
Number of reads="ID822",916,54,62,1531,90,27,0

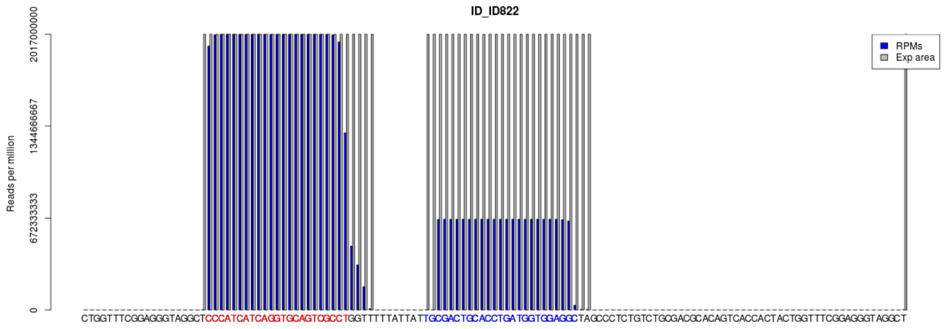

>ID823
CTAGTTTCGGAAGGCAGGCT**CCCATCATCAGGTGCAGTCGCCT**AGATATTTTTAACGCGACTGCACCTGATGGCGGAAGCTAGCCCTCCG
TCTGCGACGGACCGTCACCATTC

>ID823-5P
CCCATCATCAGGTGCAGTCGCCT

>miRNA precursor
CUAGUUUCGGAAGGCAGGCU**CCCAUCAUCAGGUGCAGUCGCCU**AGAUAUUUUUAACGCGACUGCACCUGAUGGCGGAAGCUAGCCCUCCGUCUGCGACGGACCGUCACCAUUC
...((..((((.(((.((((.((..(((((((((((((((.((((....))))..)))))))))))))))..)).)))).))).))))...))((((...)))).......

>Predicted STAR seq:
CGCGACTGCACCTGATGGCGGAAG

Complementarity: 17

Initial dG =   -58.90

```
          10          20        30        40
CUA--  UU   .-AA   A    C  AU                C-|   U
     GU  CGG    GGC GGCU CC  CAUCAGGUGCAGUCGC  UAGA A
     CA  GCC    CCG UCGA GG  GUAGUCCACGUCAGCG  AUUU U
CUUAC  CU   \ --   A    A  CG                CA^   U
 110               80        70        60        50



                 90
              C      U
               UCCGUC \
               AGGCAG G
              -      C
              100
```

Reads in: lib-42, lib-45, lib-47, lib-48, lib-49, lib-50, lib-72
Number of reads="ID823",794,37,60,1115,74,25,0

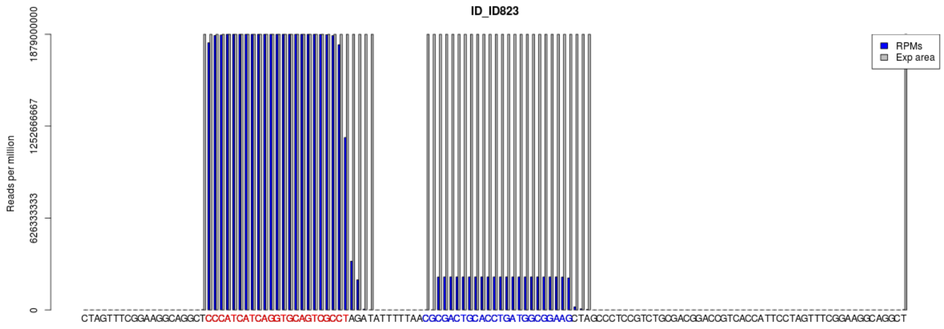

>ID824
TGGATTGGGAATGCTTGCT**CCCATCATCAGGTGCAGTCGTTC**AGTTATCACTACGACTGCACCTGATGGTGGAAGCTAGCCGTCCCACTG
CGACGAACCATCACCATCCCGG

>ID824-5P
CCCATCATCAGGTGCAGTCGTTC

>miRNA precursor
UGGAUUGGGAAUGCUUGCU**CCCAUCAUCAGGUGCAGUCGUUC**AGUUAUCACUACGACUGCACCUGAUGGUGGAAGCUAGCCGUCCCACUGCGACGAACCAUCACCAUCCCGG
(((..(((((..(((.(((.((((((((((((((((((((..(((....))))))))))))))))))))))))).))).)))((((.......)))).....)).))).)))).

>Predicted STAR seq:
GACTGCACCTGATGGTGGAAG

Complementarity: 18

Initial dG = -57.50

```
            10        20        30        40
U--      |    AU   U   C                    UC   U
   GGAU--UGGGA  GCU GCU CCAUCAUCAGGUGCAGUCGU  AGU A
   CCUA  ACCCU  CGA CGA GGUGGUAGUCCACGUCAGCA  UCA U
GGC    \ ^    GC   U   A                    --   C
110          80        70        60          50



          90
         CUGCGAC  A
                GA C
                CU C
         CCA----  A
                100
```

Reads in: lib-42, lib-45, lib-47, lib-48, lib-49, lib-50, lib-72
Number of reads="ID824",1133,82,84,1379,103,26,0

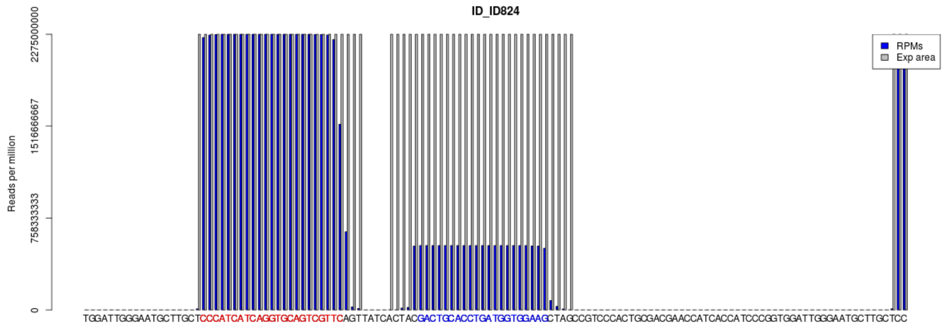

>ID826
CTACTATGGGAAGGCAGGCT**TCCATCACCAGGTGCAGCCGTCT**AGCTATTTAATGCGACTGCACCTGGTGGAGGAAGCTAGCCTTCTTTC
TGCGACGGATCAACAGCATGACA

>ID826-5P
TCCATCACCAGGTGCAGCCGTCT

>miRNA precursor
CUACUAUGGGAAGGCAGGCU**UCCAUCACCAGGUGCAGCCGUCU**AGCUAUUUAAUGCGACUGCACCUGGUGGAGGAAGCUAGCCUUCUUUCUGCGACGGAUCAACAGCAUGACA
```
.......((((((((.(((((((..((((((((((((.(((.............))).))))))))))))..))))))).))))))))(((((...........))).))..
```

>Predicted STAR seq:
GACTGCACCTGGTGGAGGAAG

Complementarity: 16

Initial dG =   -54.40

```
          10        20        30        40
.-CUACUAU|       A       AU            C   CUAGCU
         GGGAAGGC GGCUUCC  CACCAGGUGCAG CGU      \
         UUCUUCCG UCGAAGG  GUGGUCCACGUC GCG      A
\ -------^       A       AG            A   UAAUUU
                80        70        60        50



    90
  --  -   GACGG
    UC UGC     A
    AG ACG     U
  AC  U   ACAAC
   110       100
```

Reads in: lib-42, lib-45, lib-47, lib-48, lib-49, lib-50, lib-72
Number of reads="ID826",403,35,30,1072,112,17,0

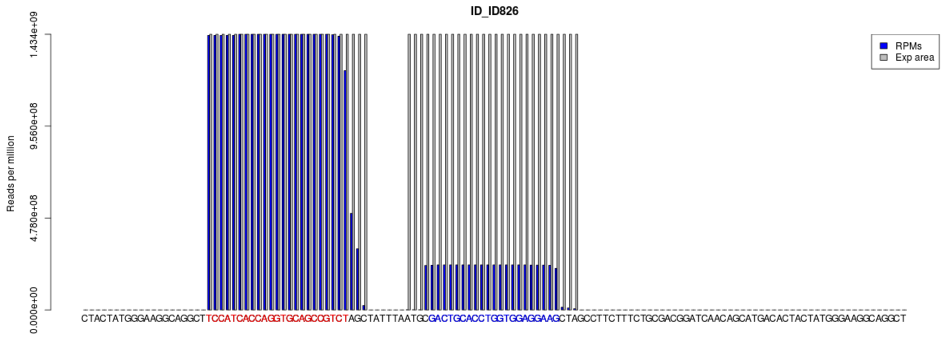

>ID827
TACCAACGTCCTCCTCGTTTCGGAAGGCAGGCTCCCATCATCAGGAGCAGTCGCCTAAATGTTATTAATG**CGACTGCACCTGACGGCGGA**
GGCCAGCTCTCTGTCTGCGACGC

>ID827-3P
CGACTGCACCTGACGGCGGAGGC

>miRNA precursor
UACCAACGUCCUCCUCGUUUCGGAAGGCAGGCUCCCAUCAUCAGGAGCAGUCGCCUAAAUGUUAUUAAUG**CGACUGCACCUGACGGCGGAGGC**CAGCUCUCUGUCUGCGACGC
..............((((..((((..((.(((..((..(.(((((.((((((((.(((......)))..)))))))).))))).)..))..))).))..))))...))))...

>Predicted STAR seq:
CTCCCATCATCAGGAGCAGTCG

Complementarity: 15

Initial dG = -47.50

```
        10        20         30        40        50         60
UACCAACGUCCUCC    UU-    AG  A   UC  AU A     A        C-|  AU
              UCGU   CGGA  GC GGC  CC  C UCAGG GCAGUCGC  UAA  G
              AGCG   GUCU  CG CCG  GG  G AGUCC CGUCAGCG  AUU  U
CGC-----------    UCU    CU  A   GA  CG C     A        UA^  AU
            110       100        90       80        70
```

Reads in: lib-42, lib-45, lib-47, lib-48, lib-49, lib-50, lib-72
Number of reads="ID827",369,42,5,757,41,13,0

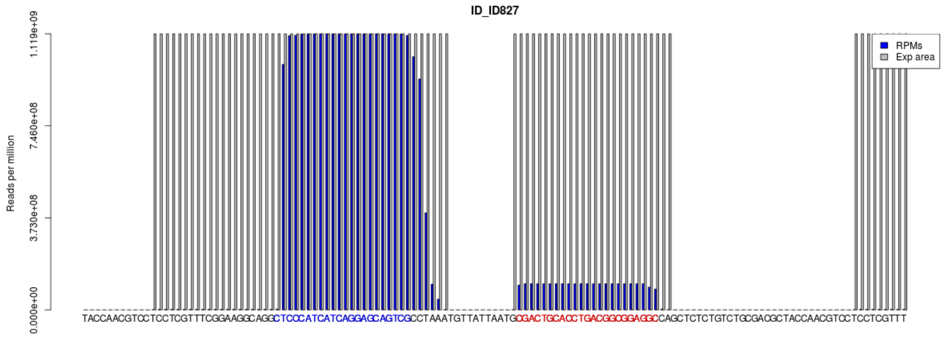

>ID830

ATGTAATCTGCGGTCTGTTC**TGCTGGATACTGAATACAGCGG**TTTATACTGTCATCCGCTGTAGGCAGTATCCAGTCTACAGACTGCAGA
CAGTCGTCAATTTTCGCCA

>ID830-5P

TGCTGGATACTGAATACAGCGG

>miRNA precursor

AUGUAAUCUGCGGUCUGUUC**UGCUGGAUACUGAAUACAGCGG**UUUAUACUGUCAUCCGCUGUAGGCAGUAUCCAGUCUACAGACUGCAGACAGUCGUCAAUUUUCGCCA

```
......((((((((((((...(((((((((((..((((((((.............))))))))..)))))))))))..)))))))))))))..................
```

>Predicted STAR seq:

GCTGTAGGCAGTATCCAGTCTAC

Complementarity: 17

Initial dG =   -55.00

```
                     10        20        30        40
AUGUAA------------|           UCU          AA         UUUAUA
                   UCUGCGGUCUGU   GCUGGAUACUG  UACAGCGG      \
                   AGACGUCAGACA   UGACCUAUGAC  AUGUCGCC      C
ACCGCUUUUAACUGCUGAC^           UC-          GG         UACUGU
       100       90        80         70        60        50
```

Reads in: lib-42, lib-45, lib-47, lib-48, lib-49, lib-50, lib-72

Number of reads="ID830",3,10,0,275,5,1,0

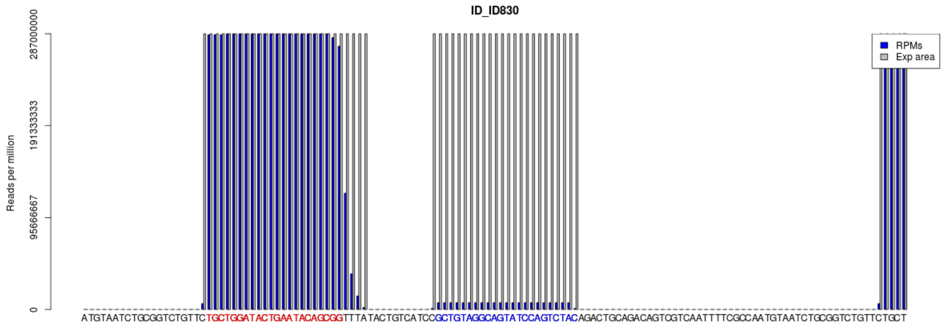

>ID836

GTCGCACTGAAGATTGGGAGACAAGTTTTCATTGGATGGGTGTGGGTCTGGTGCATGTGATGATAATTCA**TGACTAGATTCACACTCATCCT**ATGAGAATTGTCAATAT

>ID836-3P

TGACTAGATTCACACTCATCCT

>miRNA precursor

GUCGCACUGAAGAUUGGGAGACAAGUUUUCAUUGGAUGGGUGUGGGUCUGGUGCAUGUGAUGAUAAUUCA**UGACUAGAUUCACACUCAUCCU**AUGAGAAUUGUCAAUAU
.((.((........)).))(((((.(((((((.((((((((((((((((((.((((.(((....)))))))))))))))))))))))))).)))))))))))).....

>Predicted STAR seq:

GGATGGGTGTGGGTCTGGTGCA

Complementarity: 19
ERROR 4: Strange structure
Initial dG =   -48.50

```
          10
.-G| G  CUG
   UC CA   A
   AG GU   A
\ -^ G  UAG
```

```
      20        30        40        50        60
  -----     G       U                   G    U   G
       GACAA UUUUCAU GGAUGGGUGUGGGUCUGGU CAUG GAU A
       CUGUU AAGAGUA CCUACUCACACUUAGAUCA GUAC UUA U
  UAUAA     -       U                   -    -   A
         100         90        80         70
```

Reads in: lib-42, lib-45, lib-47, lib-48, lib-49, lib-50, lib-72
Number of reads="ID836",6512,5202,19947,4864,16629,6818,0

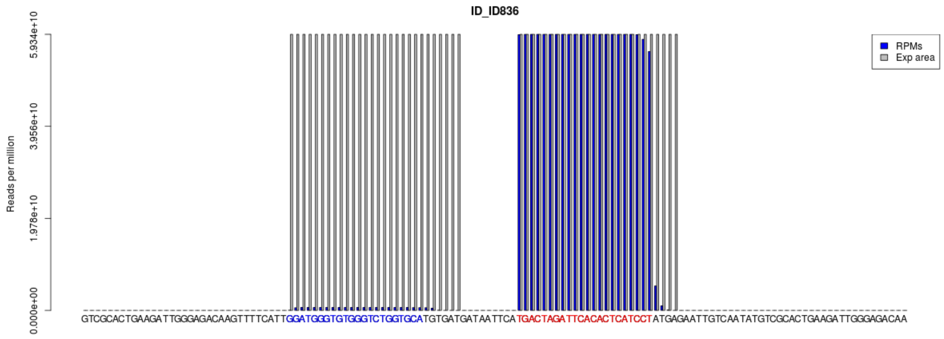

>ID84

GAGAATGGTGGCCTGTGGTA**CGGTGGAAACCTACACGAACACG**GTATTTGAGGAGTGTCGTGTGCGTTTCAACCTAACTACCCGCTGCCT
CCTACATCGTCTACGAGTCTCAT

>ID84-5P

CGGTGGAAACCTACACGAACACG

>miRNA precursor

GAGAAUGGUGGCCUGUGGUA**CGGUGGAAACCUACACGAACACG**GUAUUUGAGGAGUGUCGUGUGCGUUUCAACCUAACUACCCGCUGCCUCCUACAUCGUCUACGAGUCUCAU
((((..((..((..(((((..(((.(((((.(((((((.(((...........))))))))))).))))).))).))..)))))..))..))......(((....))).))))..

>Predicted STAR seq:

GTCGTGTGCGTTTCAACCTAAC

Complementarity: 16

Initial dG =   -41.80

```
            10        20        30        40
--    .-AU| UG  CU     AC   G     C       A   GGUAU
  GAGA    GG  GC  GUGGU  GGU GAAAC UACACGA CAC     U
  CUCU    CC  CG  CAUCA  CCA CUUUG GUGUGCU GUG     U
UA    \ --^ GU  CC     AU   A     C       -   AGGAG
 110             80        70        60         50



       90       100
        UCCUACA   U
               UCG C
               AGC U
        G------   A
```

Reads in: lib-42, lib-45, lib-47, lib-48, lib-49, lib-50, lib-72

Number of reads="ID84",0,2,0,0,0,0,0

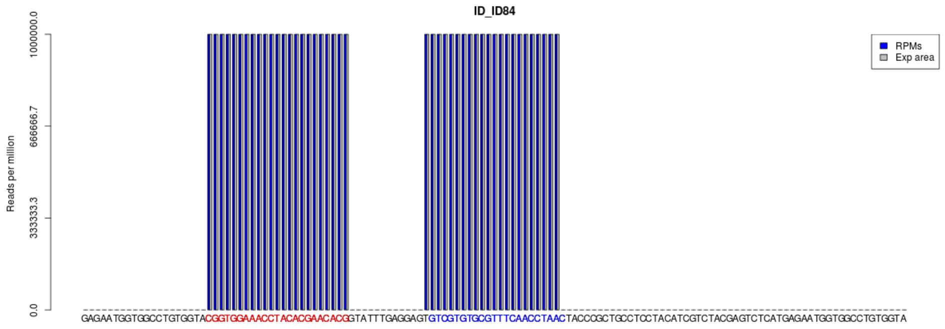

>ID843

AGTAGCATCAACTCTTCGAGTGGTGGGTAGCGGTAGATGGAGTAATGACAGTCCACGTGATAATAAGCCG**TGACTATCACTATTCTGTCT AGC**TGCTGCCCACTCTACAGCA

>ID843-3P

TGACTATCACTATTCTGTCTAGC

>miRNA precursor

AGUAGCAUCAACUCUUCGAGUGGUGGGUAGCGGUAGAUGGAGUAAUGACAGUCCACGUGAUAAUAAGCCG**UGACUAUCACUAUUCUGUCUAGC**UGCUGCCCACUCUACAGCA

```
.((((.....((((...)))).((((((((((((((((((((.(((.((((.(((...........))))))).))).)))))))))).))))))))))))).)))).... 
```

>Predicted STAR seq:

TAGATGGAGTAATGACAGTCC

Complementarity: 18

ERROR 4: Strange structure

Initial dG =   -47.60

```
             10
A---    .-CAUCA|   U
    GUAG       ACUC \
    CAUC       UGAG U
ACGA    \ -----^   C
110            20
```

```
                 30         40        50        60
          G            -          A    C    C   UGAUA
           GUGGGUAGCGGU AGAUGGAGUA UGA AGUC ACG      \
           CACCCGUCGUCG UCUGUCUUAU ACU UCAG UGC      A
          U            A          C    A    -   CGAAU
            100        90        80         70
```

Reads in: lib-42, lib-45, lib-47, lib-48, lib-49, lib-50, lib-72

Number of reads="ID843",2,4,0,92,28,0,0

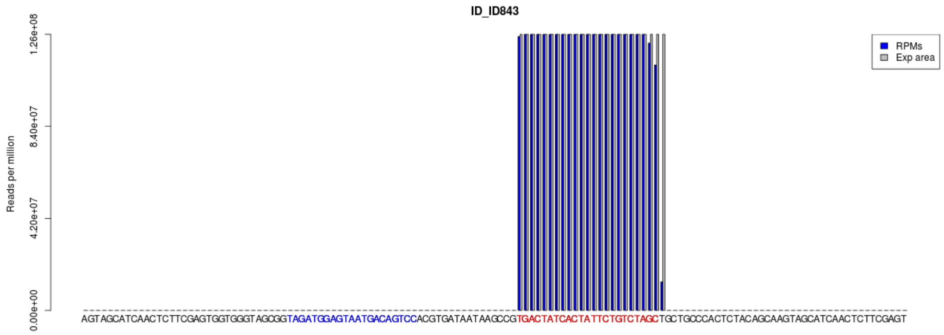

>ID85
TTGTGAAACTAATGGTATTCTCGCACGAAGGATGACGTATGTGTCGACAGTACACGTTCCTTAATTTGTG**TACTTGAGGCACATTCGTTA**
CTCGTCGTGCGAGGTTAACATC

>ID85-3P
TACTTGAGGCACATTCGTTACT

>miRNA precursor
UUGUGAAACUAAUGGUAUUCUCGCACGAAGGAUGACGUAUGUGUCGACAGUACACGUUCCUUAAUUUGUG**UACUUGAGGCACAUUCGUUACU**CGUCGUGCGAGGUUAACAUC
...........(((....(((((((((.(..(((((.(((((((...((((((((..........))))))))...))))))).)))))..).))))))))))....))).

>Predicted STAR seq:
ATGACGTATGTGTCGACAGTA

Complementarity: 14

Initial dG =   -46.40

```
        10        20        30        40        50        60
UUGUGAAACUA|  GUAU          A GA      U       GAC        UUCC
           AUG    UCUCGCACGA G  UGACG AUGUGUC   AGUACACG    U
           UAC    GGAGCGUGCU C  AUUGC UACACGG   UCAUGUGU    U
C----------^  AAUU          G UC      U       AGU        UUAA
          110       100        90        80        70
```

Reads in: lib-42, lib-45, lib-47, lib-48, lib-49, lib-50, lib-72
Number of reads="ID85",0,10,0,0,0,0,0

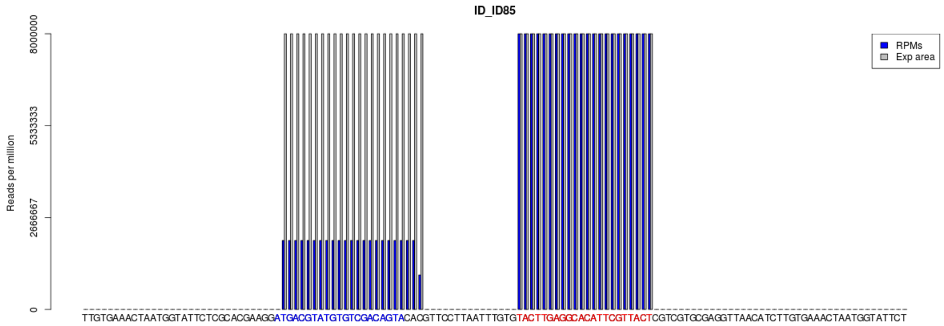

>ID851

CAACAGCTGGGCGTCGCGGCCCGTTCCGCAAGCTCTCGTAAACGCAGCGCACATTGCAGTGAATAAGGTG**CGGCGTTTGCCATAGCTTGC**
GGAAAGGGTCGCGTGTCGACG

>ID851-3P

CGGCGTTTGCCATAGCTTGCG

>miRNA precursor

CAACAGCUGGGCGUCGCGGCCCGUUCCGCAAGCUCUCGUAAACGCAGCGCACAUUGCAGUGAAUAAGGUG**CGGCGUUUGCCAUAGCUUGCG**GAAAGGGUCGCGUGUCGACG
.........((((.((((((((.(((((((((((...((((((((.((((..(((......)))...)))).))))))))...))))))))))).)))))))))))))....

>Predicted STAR seq:

GCAAGCTCTCGTAAACGCA

Complementarity: 15

Initial dG =   -59.40

```
        10        20        30        40        50
CAACAGCUG|   U        G          CUC        A    AC-   GC
         GGCG CGCGGCCC UUCCGCAAGCU   GUAAACGC GCGC   AUU  A
         CUGU GCGCUGGG AAGGCGUUCGA   CGUUUGCG CGUG   UAA  G
GCAG-----^   -        A          UAC        G    GAA   GU
 .             100       90        80        70        60
```

Reads in: lib-42, lib-45, lib-47, lib-48, lib-49, lib-50, lib-72

Number of reads="ID851",5,8,3,635,11,1,0

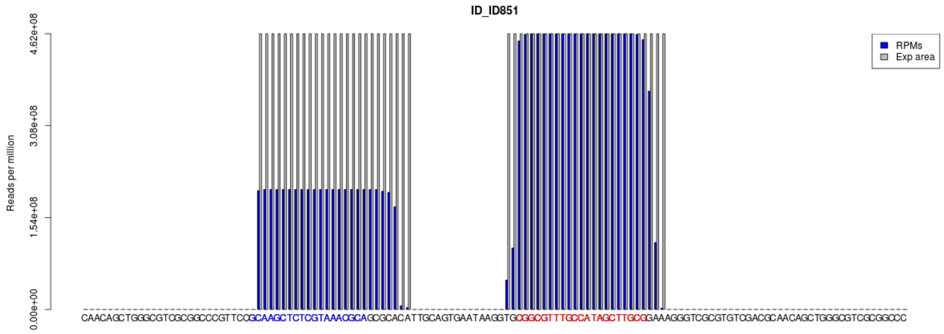

>ID856
AGTGTCAGATCTCAAATGGTGGGCTTGTAAGGGGGATTTGGTGAAGGCAGATTGTATTTAAAATGTTGTC**TACCTTCACCAAATCTTCCT**
CACAATTCTGCTGTCAAACATA

>ID856-3P
TACCTTCACCAAATCTTCCTCA

>miRNA precursor
AGUGUCAGAUCUCAAAUGGUGGGCUUGUAAGGGGGAUUUGGUGAAGGCAGAUUGUAUUUAAAAUGUUGUC**UACCUUCACCAAAUCUUCCUCA**CAAUUCUGCUGUCAAACAUA
.((((..(((.......((..(..((((.((((((((((((((((((.((((..((((...))))..)))).))))))))))))))))))).)))).).)..)))))..)))).

>Predicted STAR seq:
AAGGGGGATTTGGTGAAGGC

Complementarity: 18

Initial dG =   -46.20

```
        10        20        30        40        50
A|   CA   CUCAAAU  UG GC    A                  C    UG    U
 GUGU  GAU       GG  G  UUGU AGGGGGAUUUGGUGAAGG AGAU  UAUU \
 UACA  CUG       UC  C  AACA UCCUUCUAAACCACUUCC UCUG  GUAA A
A^   AA   -------  GU UU    C                  A    UU    A
110              100       90        80        70
```

Reads in: lib-42, lib-45, lib-47, lib-48, lib-49, lib-50, lib-72
Number of reads="ID856",0,0,0,0,33,1,0

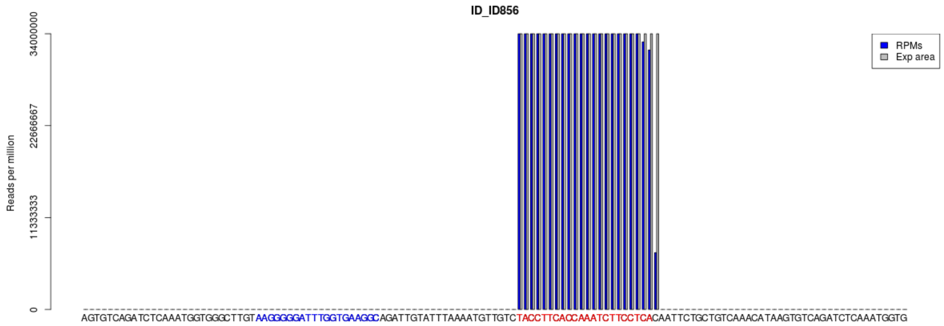

>ID86
GTGTGGAACTAATGGTATTCTCGCACGAAGGGTGACGTATGTGTCGCGGGTACGCGTTGTTTCGTCAGTG**TTCACGGGACCCATACGTTA**
CTCTTACTGCGAGTTCAGCAAC

>ID86-3P
TTCACGGGACCCATACGTTACT

>miRNA precursor
GUGUGGAACUAAUGGUAUUCUCGCACGAAGGGUGACGUAUGUGUCGCGGGUACGCGUUGUUUCGUCAGUG**UUCACGGGACCCAUACGUUACU**CUUACUGCGAGUUCAGCAAC
.(((.((((...........(((((..((((((((((((((.(((.((...((((............))))...)).))).))))))))))))))..))))))))).)))..

>Predicted STAR seq:
GTGACGTATGTGTCGCGG

Complementarity: 15

Initial dG =   -43.20

```
         10        20        30        40        50        60
G-|  G    UAAUGGUAUUC     CG              U   G  GGU    GUUGU
  UGU GAAC           UCGCA  AAGGGUGACGUAUG GUC CG   ACGC     U
  ACG CUUG           AGCGU  UUCUCAUUGCAUAC CAG GC   UGUG     U
CA^  A    -----------     CA              C   G  ACU    ACUGC
110                100        90        80        70
```

Reads in: lib-42, lib-45, lib-47, lib-48, lib-49, lib-50, lib-72
Number of reads="ID86",10,29,1,0,5,0,0

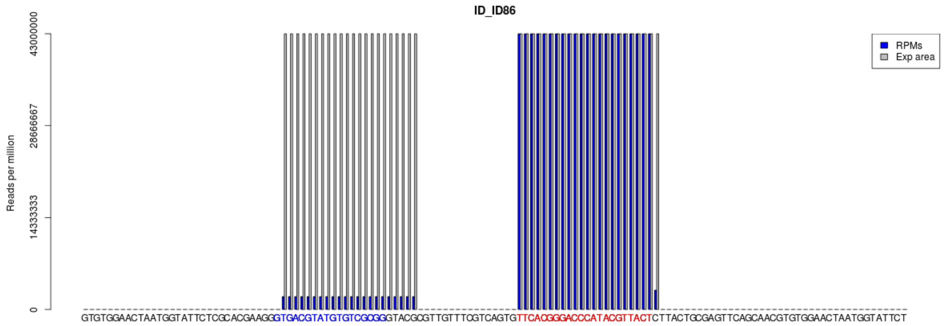

>ID862
TTCTACAGCATACCTTAACA**TCCAAATGATTTCCCACAGGCTGC**AAATATGAGTACAGTCTTGGGAGGTCCTTTGGGTATGAGTGTGTGC
ACCATTGACATTTTTAACTG

>ID862-5P
TCCAAATGATTTCCCACAGGCTGC

>miRNA precursor
UUCUACAGCAUACCUUAACA**UCCAAAUGAUUUCCCACAGGCUGC**AAAUAUGAGUACAGUCUUGGGAGGUCCUUUGGGUAUGAGUGUGUGCACCAUUGACAUUUUUAACUG
.......((((((((((..(((((((.(((((((((.((((((............))))))))))))))).))))))).)))).))))))...................

>Predicted STAR seq:
GTCTTGGGAGGTCCTTTGGGTA

Complementarity: 19

Initial dG =   -39.00

```
                     10         20        30        40
UUCUACA-------------      -|   AC       U         C      CAAAU
                    GCAUAC CUUA  AUCCAAA GAUUUCCCA AGGCUG     A
                    CGUGUG GAGU  UGGGUUU CUGGAGGGU UCUGAC     U
GUCAAUUUUUACAGUUACCA      U^   A-       C         -      AUGAG
.       100        90         80         70         60         50
```

Reads in: lib-42, lib-45, lib-47, lib-48, lib-49, lib-50, lib-72
Number of reads="ID862",149,148,39,2139,660,51,0

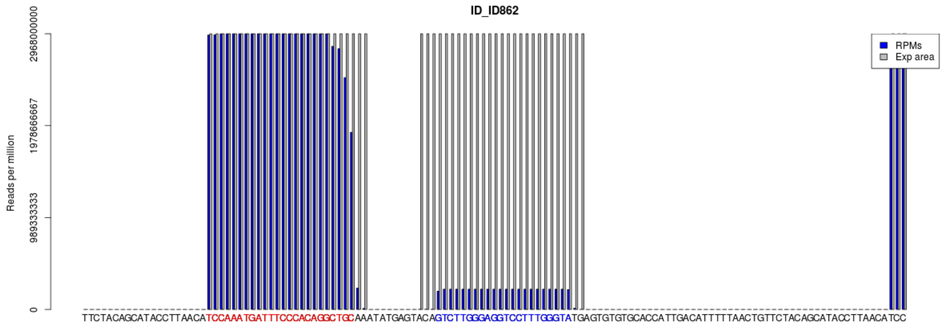

>ID863
TGCTATAGCTTTTTCTGACG**TCTGAAGGATTTGTTACAGACTG**TAATTAATCAATCAGTTTTGGCAAATCCTTCGGGTGTTTGGAGGAGC
CCAGACTACTTAGCAAAGAT

>ID863-5P
TCTGAAGGATTTGTTACAGACTG

>miRNA precursor
UGCUAUAGCUUUUUCUGACG**UCUGAAGGAUUUGUUACAGACUG**UAAUUAAUCAAUCAGUUUUGGCAAAUCCUUCGGGUGUUUGGAGGAGCCCAGACUACUUAGCAAAGAU
(((((..((((((((.(((..((((((((((((((((.((((((............))))))))))))))))))))))..))).)))))))).........))))).....

>Predicted STAR seq:
GTTTTGGCAAATCCTTCGGGTGT

Complementarity: 19

Initial dG = -42.80

```
                     10        20        30        40
-----     UA--------|       U   GU               C      UAAUU
     UGCUA          GCUUUUUC GAC  CUGAAGGAUUUGUUA AGACUG     A
     ACGAU          CGAGGAGG UUG  GGCUUCCUAAACGGU UUUGAC     A
UAGAA     UCAUCAGACC^       U   UG               -      UAACU
.       100        90        80        70         60        50
```

Reads in: lib-42, lib-45, lib-47, lib-48, lib-49, lib-50, lib-72
Number of reads="ID863",207,288,1199,2982,565,85,0

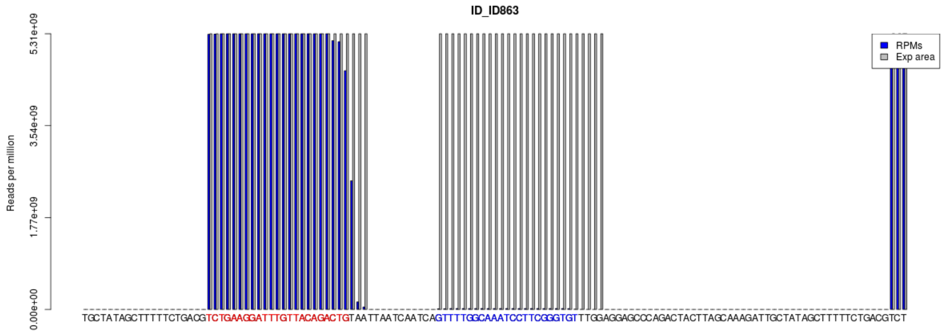

>ID864
TGCTATAGCTTCTTCTGTCA**TCTAAAGGATTTGTTACAGACTG**TAATTAATCAATCAGTTTTGGCAAATCCTTTGGGTGTTGGGAGGAGC
GCAGACTACTTTGCAAAGC

>ID864-5P
TCTAAAGGATTTGTTACAGACTG

>miRNA precursor
UGCUAUAGCUUCUUCUGUCA**UCUAAAGGAUUUGUUACAGACUG**UAAUUAAUCAAUCAGUUUUGGCAAAUCCUUUGGGUGUUGGGAGGAGCGCAGACUACUUUGCAAAGC
.(((...((((((((((.(((((((((((((((((((.((((((...........)))))))))))))))))))))))))).))))))))))(((((....)))))..)))

>Predicted STAR seq:
GTTTTGGCAAATCCTTTGGGTG

Complementarity: 20

Initial dG =   -46.10

```
          10        20        30        40
U   .-AUA|         U                  C       UAAUU
 GCU     GCUUCUUCUG CAUCUAAAGGAUUUGUUA AGACUG     A
 CGA     CGAGGAGGGU GUGGGUUUCCUAAACGGU UUUGAC     A
-   \ ---^         U                  -       UAACU
        90        80        70         60        50
```

```
      --     C
        GCAGA U
        CGUUU A
      AA     C
          100
```

Reads in: lib-42, lib-45, lib-47, lib-48, lib-49, lib-50, lib-72
Number of reads="ID864",439,584,1423,5334,1509,243,0

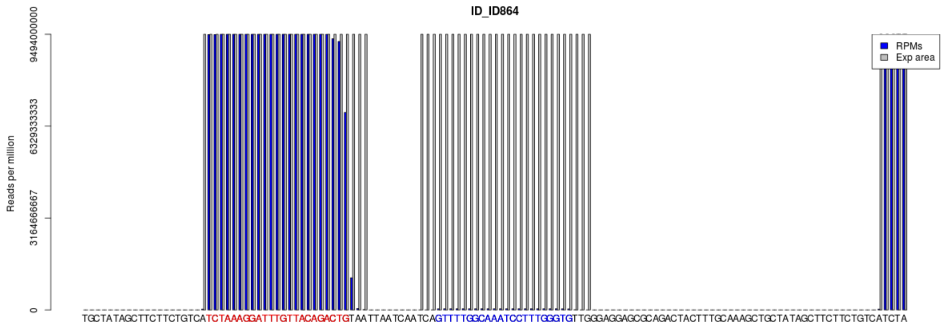

>ID865

TGCTATAGCTTCTTCTGTCG**TCTAAAGTATTTGTTACAGACT**GTAATTAATCAATCAGTTTTGACAAATCCTTTGGGTGTTGGGAGGAGC
GGAGACTACTTTGCAAAGCT

>ID865-5P

TCTAAAGTATTTGTTACAGACT

>miRNA precursor

UGCUAUAGCUUCUUCUGUCG**UCUAAAGUAUUUGUUACAGACU**GUAAUUAAUCAAUCAGUUUUGACAAAUCCUUUGGGUGUUGGGAGGAGCGGAGACUACUUUGCAAAGCU
.(((...((((((((((.(..((((((.((((((((.((((((...........)))))))))))))).))))))..).)))))))))).(((....))).....))).

>Predicted STAR seq:

TTTTGACAAATCCTTTGGGTGT

Complementarity: 17

Initial dG =   -34.90

```
          10        20        30        40
U   .-AUA|         U GU       U         C       UAAUU
 GCU      GCUUCUUCUG C  CUAAAG AUUUGUUA AGACUG      A
 CGA      CGAGGAGGGU G  GGUUUC UAAACAGU UUUGAC      A
U   \ ---^         U UG       C         -       UAACU
.       90        80        70         60        50




      G----   A
           GAG C
           UUC U
      AACGU   A
          100
```

Reads in: lib-42, lib-45, lib-47, lib-48, lib-49, lib-50, lib-72

Number of reads="ID865",230,256,895,2600,471,118,0

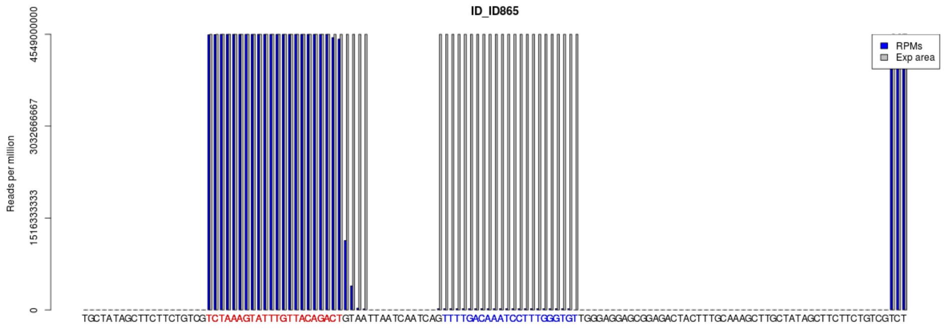

>ID866

TGCTATAGCTTTTTCTGACG**TCTGAAGGATTTGTTACAGACTG**TAATTAATCAATCAGTTTTGGCAAATCCTTCGGGTGTTTGGAGGAGC
CCAGACTACTTAGCAAAGAT

>ID866-5P

TCTGAAGGATTTGTTACAGACTG

>miRNA precursor

```
UGCUAUAGCUUUUUCUGACGUCUGAAGGAUUUGUUACAGACUGUAAUUAAUCAAUCAGUUUUGGCAAAUCCUUCGGGUGUUUGGAGGAGCCCAGACUACUUAGCAAAGAU
(((((..((((((((.(((..((((((((((((((.((((((............)))))))))))))))))))))..))).)))))))).........))))).....
```

>Predicted STAR seq:

GTTTTGGCAAATCCTTCGGGTGT

Complementarity: 19

Initial dG =   -42.80

```
                     10        20        30        40
-----     UA--------|       U   GU               C      UAAUU
     UGCUA          GCUUUUUC GAC  CUGAAGGAUUUGUUA AGACUG     A
     ACGAU          CGAGGAGG UUG  GGCUUCCUAAACGGU UUUGAC     A
UAGAA     UCAUCAGACC^       U   UG               -      UAACU
.       100        90        80        70         60        50
```

Reads in: lib-42, lib-45, lib-47, lib-48, lib-49, lib-50, lib-72

Number of reads="ID866",225,269,1200,2941,584,82,0

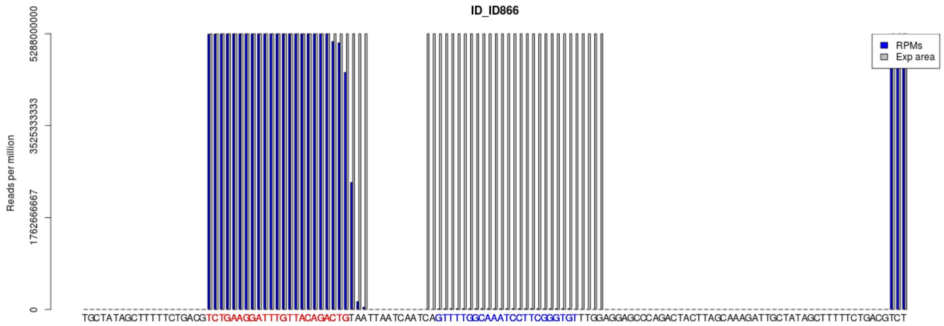

>ID867
TTTTACAGCTTCTTCTGTCA**TGTAAAGGACTTGCCACAAGCTG**TAATAAATGGATCAGTTTGGGCAGATCCTTTACGTGTTGGGAGGTGC
CAAAGTTGGATTTATATGAA

>ID867-5P
TGTAAAGGACTTGCCACAAGCTG

>miRNA precursor
UUUUACAGCUUCUUCUGUCA**UGUAAAGGACUUGCCACAAGCUG**UAAUAAAUGGAUCAGUUUGGGCAGAUCCUUUACGUGUUGGGAGGUGCCAAAGUUGGAUUUAUAUGAA
```
.......(((((((....(((((((((((.(((((.(((((((............))))))))))))).))))))))))))..))))))).(((....)))...........
```

>Predicted STAR seq:
GTTTGGGCAGATCCTTTACGTG

Complementarity: 19

Initial dG = -41.10

```
               10               20        30        40
UUUUA-------     ------|       U           C     A       UAAUA
            CAGCU      UCUUCUG CAUGUAAAGGA UUGCC CAAGCUG     A
            GUUGA      GGAGGGU GUGCAUUUCCU GACGG GUUUGAC     A
AAGUAUAUUUAG     AACCGU^       U           A     -       UAGGU
.       100        90        80        70         60        50
```

Reads in: lib-42, lib-45, lib-47, lib-48, lib-49, lib-50, lib-72
Number of reads="ID867",167,109,66,471,271,27,0

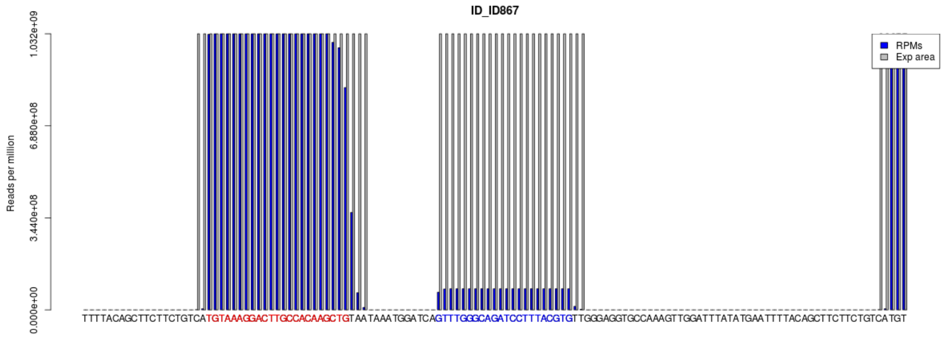

>ID868
TGCTATAGCTTCTTCTGACG**TCTGAAGGATTTGTTACAGACTG**TAATTAATCAATCAGTTTTGGCAAATCCTTTGGGTGTTGGGAGGAGC
CCAGTCTACTTTGCTAAGCC

>ID868-5P
TCTGAAGGATTTGTTACAGACTG

>miRNA precursor
UGCUAUAGCUUCUUCUGACG**UCUGAAGGAUUUGUUACAGACUG**UAAUUAAUCAAUCAGUUUUGGCAAAUCCUUUGGGUGUUGGGAGGAGCCCAGUCUACUUUGCUAAGCC
.(((...((((((((..((..(..((((((((((((.((((((...........)))))))))))))))))))..)..))..))))))))..)))..............

>Predicted STAR seq:
GTTTTGGCAAATCCTTTGGGTGT

Complementarity: 17

Initial dG =   -44.80

```
          10        20        30        40
U   .-AUA|       UG  GU UG            C      UAAUU
 GCU     GCUUCUUC  AC  C  AAGGAUUUGUUA AGACUG     A
 CGA     CGAGGAGG  UG  G  UUCCUAAACGGU UUUGAC     A
C   \ ---^       GU  UG GU            -      UAACU
.       90        80        70         60        50
```

```
      CC   CUA
        AGU   \
        UCG   C
      A-   UUU
           100
```

Reads in: lib-42, lib-45, lib-47, lib-48, lib-49, lib-50, lib-72
Number of reads="ID868",210,279,1155,2909,583,92,0

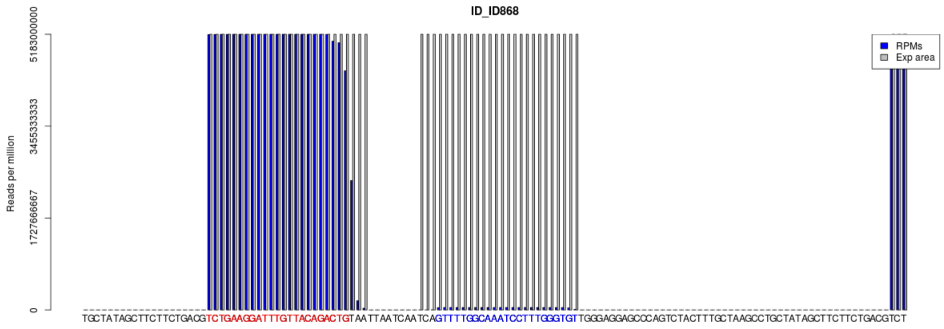

>ID869
TGCTATAGCTTCTTCTGTCG**TCTAAAGGATTTGTTACAGACTG**TAATTAATCAATCAGTTTTGGCAAATCCTTTGGGTGTTGGGAGGAGC
GCAGACTACTTTGCAAAGC

>ID869-5P
TCTAAAGGATTTGTTACAGACTG

>miRNA precursor
UGCUAUAGCUUCUUCUGUCG**UCUAAAGGAUUUGUUACAGACUG**UAAUUAAUCAAUCAGUUUUGGCAAAUCCUUUGGGUGUUGGGAGGAGCGCAGACUACUUUGCAAAGC
.(((...((((((((((.(..((((((((((((((((.((((((...........))))))))))))))))))))))..).))))))))))(((((....)))))..)))

>Predicted STAR seq:
GTTTTGGCAAATCCTTTGGGTGT

Complementarity: 19

Initial dG =   -43.50

```
          10        20        30        40
U   .-AUA|         U GU               C       UAAUU
 GCU     GCUUCUUCUG C  CUAAAGGAUUUGUUA AGACUG      A
 CGA     CGAGGAGGGU G  GGUUUCCUAAACGGU UUUGAC      A
-   \ ---^         U UG               -       UAACU
        90        80        70         60        50




      --     C
        GCAGA U
        CGUUU A
      AA     C
          100
```

Reads in: lib-42, lib-45, lib-47, lib-48, lib-49, lib-50, lib-72
Number of reads="ID869",395,608,1463,5372,1533,270,0

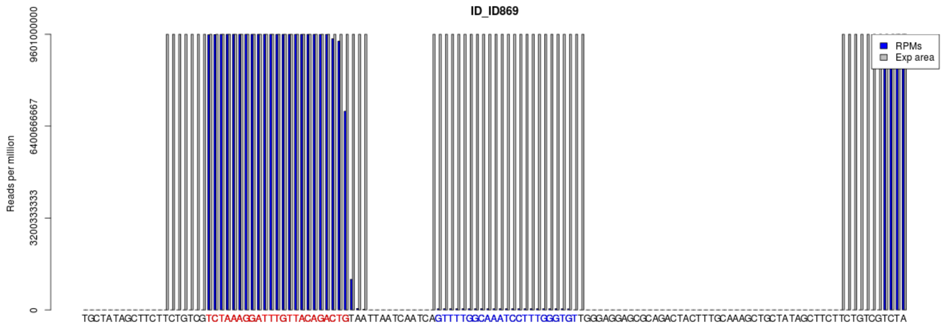

>ID870
TCTTGCGGCATCTCCTGGCA**TGTGAAAGATTTGCCACAAACTGT**AAGCAATGTCTCAGTTTTGGCAAATCCTTCACTTGCTGAGAAATGC
TTAAGTAGGTCTCATATCAGAT

>ID870-5P
TGTGAAAGATTTGCCACAAACTGT

>miRNA precursor
UCUUGCGGCAUCUCCUGGCA**UGUGAAAGAUUUGCCACAAACUGU**AAGCAAUGUCUCAGUUUUGGCAAAUCCUUCACUUGCUGAGAAAUGCUUAAGUAGGUCUCAUAUCAGAU
.((((((((((.((..((((.(((((.(((((((((.((((((............))))))))))))))).))))).)))).)).))))).)))...))))).............

>Predicted STAR seq:
GTTTTGGCAAATCCTTCACTTG

Complementarity: 18

Initial dG =   -41.50

```
                       10        20        30        40
U------------     ---|    C  CU    U     A         C      UAAGC
             CUUGC   GGCAU UC  GGCA GUGAA GAUUUGCCA AAACUG     A
             GGAUG   UCGUA AG  UCGU CACUU CUAAACGGU UUUGAC     A
UAGACUAUACUCU     AAU^    A  AG    U     C         -      UCUGU
110       100        90        80        70         60        50
```

Reads in: lib-42, lib-45, lib-47, lib-48, lib-49, lib-50, lib-72
Number of reads="ID870",32,67,38,514,106,19,0

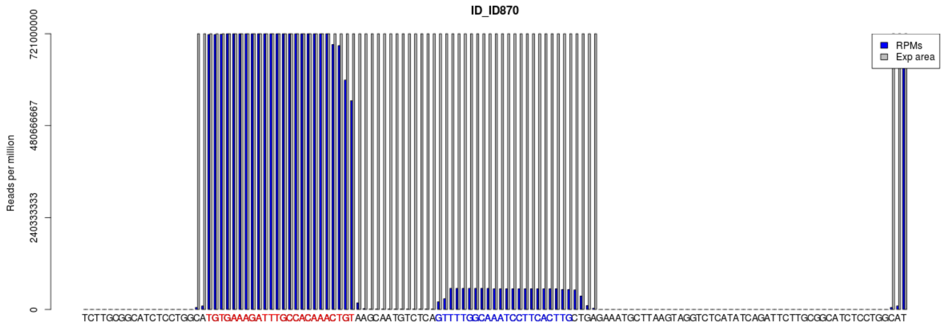

>ID873
CACAGAGTCCGTGAATTTTC**CGTTAATTTCATGTGCAAGCA**AGTGTAATTAGTTCTTTGCTTTCACGTGAAACTAGCGGAGAAAGTAATG
GACGCTTCTACGAGAACTGCC

>ID873-5P
CGTTAATTTCATGTGCAAGCA

>miRNA precursor
CACAGAGUCCGUGAAUUUUC**CGUUAAUUUCAUGUGCAAGCA**AGUGUAAUUAGUUCUUUGCUUUCACGUGAAACUAGCGGAGAAAGUAAUGGACGCUUCUACGAGAACUGCC
..(((.((((((...((((((((((.(((((((((.(((((((...........))))))).))))))))).))))))))))....)))))).(((....)))..)))..

>Predicted STAR seq:
CTTTCACGTGAAACTAGCGGA

Complementarity: 17

Initial dG = -40.00

```
          10          20         30         40
CA    .-A      GAA-|         A          C       UGUAA
  CAG    GUCCGU    UUUUCCGUUA UUUCAUGUG AAGCAAG      U
  GUC    CAGGUA    AAGAGGCGAU AAAGUGCAC UUCGUUU      U
CC    \ -      AUGA^         C          U       CUUGA
 .        90         80        70         60         50
```

```
       G-   C
         CUU U
         GAG A
       AA   C
           100
```

Reads in: lib-42, lib-45, lib-47, lib-48, lib-49, lib-50, lib-72
Number of reads="ID873",208,443,611,4173,813,288,0

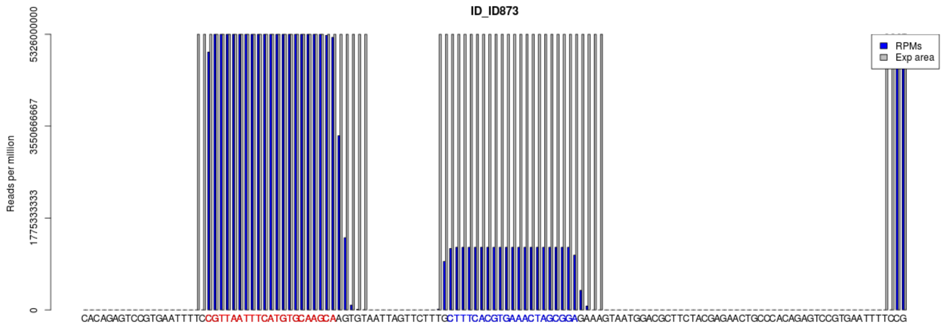

>ID876

CACCACCTTCAGGAGAAACTGCTGCAGCTGACACACGCTGACGAGTGATGCCTAGGCGGCTGTTTATGCC**TTGAGCATCATACGTCTGTGTGA**TTCAGCTTGTAGTGCCACAT

>ID876-3P

TTGAGCATCATACGTCTGTGTGA

>miRNA precursor

CACCACCUUCAGGAGAAACUGCUGCAGCUGACACACGCUGACGAGUGAUGCCUAGGCGGCUGUUUAUGCC**UUGAGCAUCAUACGUCUGUGUGA**UUCAGCUUGUAGUGCCACAU

```
.........(((......)))((((((((((..(((((.((((.(((((((..(((((........)))))...))))))).)))).)))))..))))).)))))........
```

>Predicted STAR seq:

ACACGCTGACGAGTGATGCC

Complementarity: 15

ERROR 4: Strange structure

Initial dG = -46.90

```
.-CACCA| U
       CC U
       GG C
\ -----^ A
         10


       20         30        40        50         60
  AGAAACU      -     CA     U    A       CU-     GCU
         GCUGCA GCUGA  CACGC GACG GUGAUGC   AGGCG   G
         UGAUGU CGACU  GUGUG CUGC UACUACG   UCCGU   U
  UACACCG      U     UA     U    A       AGU     AUU
   110       100        90        80        70
```

Reads in: lib-42, lib-45, lib-47, lib-48, lib-49, lib-50, lib-72

Number of reads="ID876",1,10,7,540,125,1,0

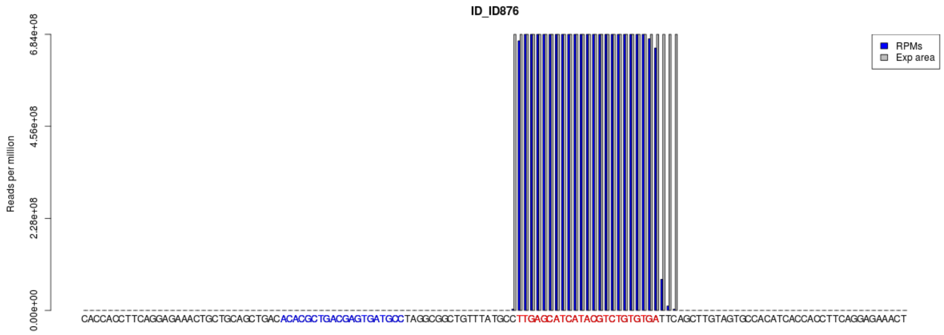

>ID877
CGGAGCGACACAGCGGCGAGTTGCGAAGAGGGAAGTTTCACCGGGGGATCCTGCCTGAATGTGAATCTGG**ATCCCCTGGTGAAACTTTCC**
GCTTCACGACCCGAAGGTCT

>ID877-3P
ATCCCCTGGTGAAACTTTCCGC

>miRNA precursor
CGGAGCGACACAGCGGCGAGUUGCGAAGAGGGAAGUUUCACCGGGGGAUCCUGCCUGAAUGUGAAUCUGG**AUCCCCUGGUGAAACUUUCCGC**UUCACGACCCGAAGGUCU
......(((....(((...((((.((((.((((((((((((((((((((((.................)))))))))))))))))))))).)))).))))))))...))).

>Predicted STAR seq:
AGGGAAGTTTCACCGGGGGAT

Complementarity: 19

Initial dG =   -59.50

```
        10        20        30        40        50
CGGAGC|  ACAG   CGA    C    A                      UGCCUGAA
      GAC    CGG   GUUG GAAG GGGAAGUUUCACCGGGGGAUCC        \
      CUG    GCC   CAGC CUUC CCUUUCAAAGUGGUCCCCUAGG        U
U-----^  GAA-   ---    A    G                      UCUAAGUG
.                100       90        80        70
```

Reads in: lib-42, lib-45, lib-47, lib-48, lib-49, lib-50, lib-72
Number of reads="ID877",174,420,553,1020,755,165,0

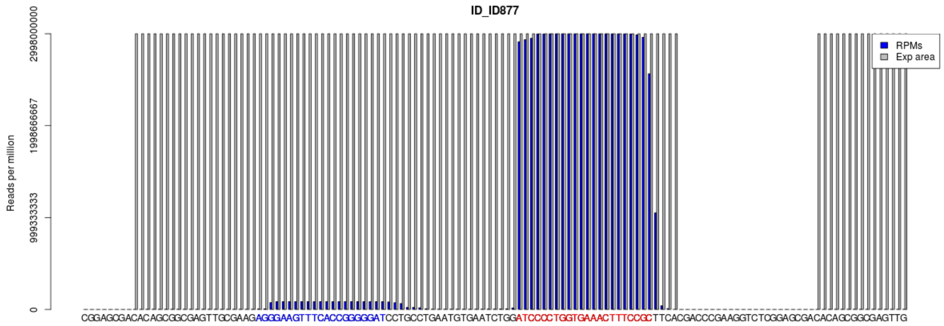

>ID878

TTTGAGTCATGATCCCTGGT**GTGCATTGTAGTTGCATTGCA**TGTGTCATATAAAAATGCAGTACTTCTACAATGCAACCCCAGTGTTCAG
AAGCATCAGCAAAGTGCA

>ID878-5P

GTGCATTGTAGTTGCATTGCA

>miRNA precursor

UUUGAGUCAUGAUCCCUGGU**GUGCAUUGUAGUUGCAUUGCA**UGUGUCAUAUAAAAAUGCAGUACUUCUACAAUGCAACCCCAGUGUUCAGAAGCAUCAGCAAAGUGCA
..((..((.(((...((((..((((((((((....((((((.............)))))))....))))))))))...))))...)))))..))...((.....)).

>Predicted STAR seq:

CAGTACTTCTACAATGCAACCCC

Complementarity: 14

Initial dG =   -33.50

```
          10        20         30        40
.-UU|  AG   A   UCC    UG-          UUGC       GUGUCA
    UG  UC UGA   CUGG   UGCAUUGUAG    AUUGCAU      \
    AC  AG ACU   GACC   ACGUAACAUC    UGACGUA      U
\ --^  GA   -   UGU    CCA          UUCA       AAAAUA
        90         80        70        60        50


    100
  UCA  AA
     GC  \
     CG  A
  A--  UG
```

Reads in: lib-42, lib-45, lib-47, lib-48, lib-49, lib-50, lib-72

Number of reads="ID878",226,513,419,352,482,118,0

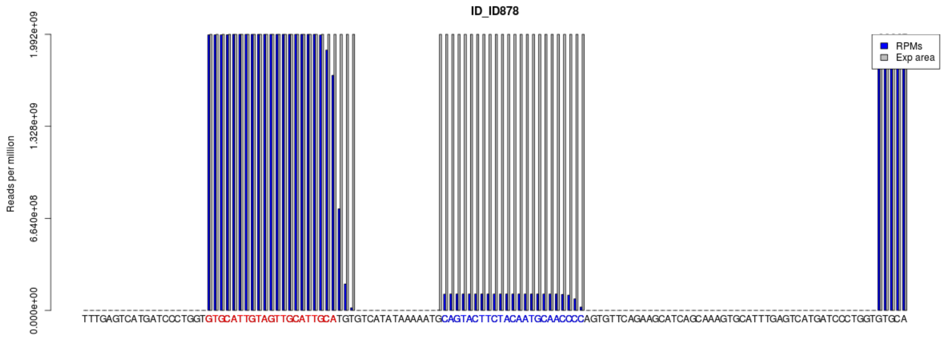

>ID88
ACGTGGAACAAATGGTATGTTCGCAGCACGATTGATGTATGTGTTGGGGGAACATACTGTTTCATTTGTG**TTCCTGTGGTACATACGTTA**
CTCGTTGTGCGTGTATAAAGAC

>ID88-3P
TTCCTGTGGTACATACGTTACT

>miRNA precursor
ACGUGGAACAAAUGGUAUGUUCGCAGCACGAUUGAUGUAUGUGUUGGGGGAACAUACUGUUUCAUUUGUG**UUCCUGUGGUACAUACGUUACU**CGUUGUGCGUGUAUAAAGAC
.(((.......))).((((..((((..((((.(((((((((((..(.(((((((((..........))))))))).)..))))))))))).)))).)))))..)))).....

>Predicted STAR seq:
TTGATGTATGTGTTGGGGGAA

Complementarity: 16

Initial dG = -39.60

```
        10        20        30        40        50        60
ACGUGGAACAAAUGG|   UU    GC    U           UU G         CUGU
               UAUG  CGCA  ACGA UGAUGUAUGUG  G GGGAACAUA    U
               AUAU  GCGU  UGCU AUUGCAUACAU  U UCCUUGUGU    U
CAGAA----------^   GU    GU    C           GG G         UUAC
110              100        90        80        70
```

Reads in: lib-42, lib-45, lib-47, lib-48, lib-49, lib-50, lib-72
Number of reads="ID88",1,2,0,0,0,0,0

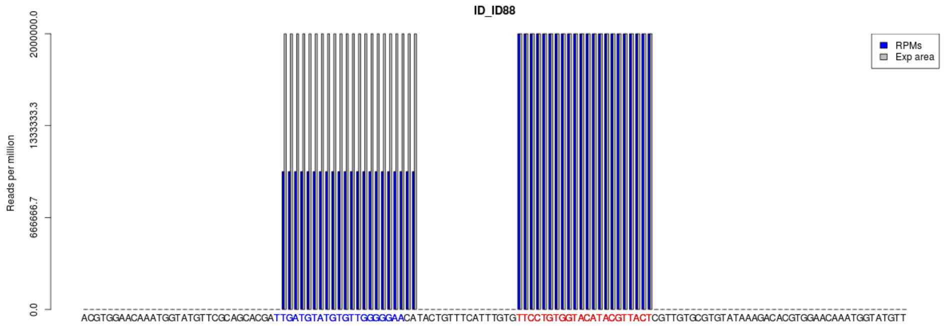

>ID885
TGTTACAAACATGACTCTCCGTCGGTGCCGAACTTCGATGGTACACACTGTCTGGTGCGACATAAATAGC**ACAGAAATTGTGTATTGTCGA**AGATCGGTGCTGAGAAC

>ID885-3P
ACAGAAATTGTGTATTGTCGA

>miRNA precursor
UGUUACAAACAUGACUCUCCGUCGGUGCCGAACUUCGAUGGUACACACUGUCUGGUGCGACAUAAAUAGC**ACAGAAAUUGUGUAUUGUCGA**AGAUCGGUGCUGAGAAC
.((((......))))......((((..((((.((((((..(((((((...((((.(((..........)))))))...))))))))..)))))).)))))..)))).....

>Predicted STAR seq:
CGATGGTACACACTGTCTGG

Complementarity: 14
ERROR 4: Strange structure
Initial dG = -46.60

```
.-U|   CA
   GUUA  A
   CAGU  A
\ -^   AC
       10


     20        30        40        50        60
  UCUCCG    UG    A      UG       CUG    G   GACA
        UCGG  CCGA CUUCGA  GUACACA   UCUG UGC    U
        AGUC  GGCU GAAGCU  UAUGUGU   AGAC ACG    A
  CAAG--    GU    A      GU       UAA    -   AUAA
          100        90        80         70
```

Reads in: lib-42, lib-45, lib-47, lib-48, lib-49, lib-50, lib-72
Number of reads="ID885",27,69,3,2912,9,1,0

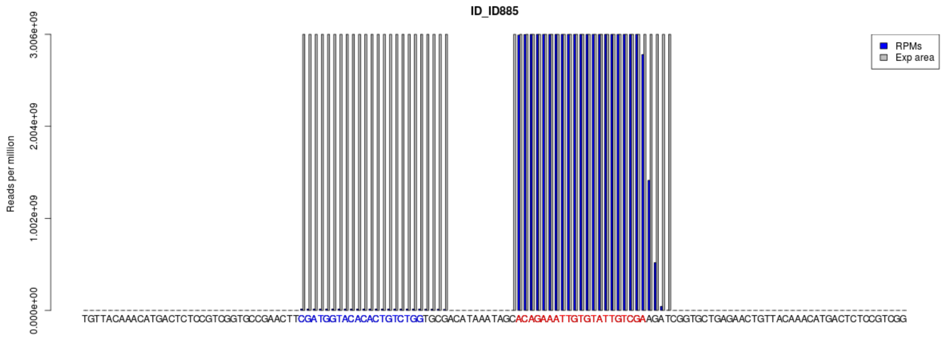

>ID89
ATGCTTGCACTTTTCCTGTACAAATTCACGGTTGTTGGAGGTGTCTTTTTCACACGTTTGAAGGCAGTG**TTGATCAGGCACTTCTAACGA**
TTGTTGTGCATTATTTCAT

>ID89-3P
TTGATCAGGCACTTCTAACGAT

>miRNA precursor
AUGCUUGCACUUUUCCUGUACAAAUUCACGGUUGUUGGAGGUGUCUUUUUCACACGUUUGAAGGCAGUG**UUGAUCAGGCACUUCUAACGAU**UGUUGUGCAUUAUUUCAU
................((((((.....(((((((((((((((((((..((.(((((((....))).)))).))..)))))))))))))))))))))))))).........

>Predicted STAR seq:
TTGTTGGAGGTGTCTTTTTC

Complementarity: 17

Initial dG =   -36.00

```
        10        20        30        40        50         60
AUGCUUGCACUUUUCC|     AAUUC                   UU  C    -   U
                UGUACA     ACGGUUGUUGGAGGUGUCU  UU ACAC GUU G
                ACGUGU     UGUUAGCAAUCUUCACGGA  AG UGUG CGG A
UACUUUAUU-------^     -----                   CU  U    A   A
              100             90        80        70
```

Reads in: lib-42, lib-45, lib-47, lib-48, lib-49, lib-50, lib-72
Number of reads="ID89",2,6,0,1,1,0,0

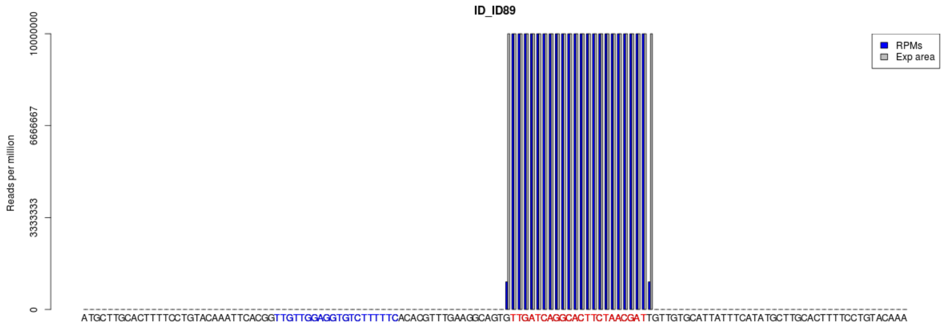

>ID890
TAAGGAAGCAGCTTTCAGCACGGGCACAATGGCTAAGTGGTCAGCGCCACAGATACACCAGGACTGGTTC**CTTGTACTGCGACGCTAACA**
ACTTACATTTGTGCCCGTGCT

>ID890-3P
CTTGTACTGCGACGCTAACAA

>miRNA precursor
UAAGGAAGCAGCUUUCAGCACGGGCACAAUGGCUAAGUGGUCAGCGCCACAGAUACACCAGGACUGGUUC**CUUGUACUGCGACGCUAACAA**CUUACAUUUGUGCCCGUGCU
...(((((...))))).((((((((((((....(((((.((.((((...(((.((((..((((.....)))))))))))...)))).)).)))))...)))))))))))).

>Predicted STAR seq:
GGTCAGCGCCACAGATACACCAG

Complementarity: 14
ERROR 4: Strange structure
Initial dG =   -45.80

```
.-UAA|    C
     GGAAG \
     CUUUC A
\ ---^    G
```

```
    20        30        40        50        60
  A            UGGC      G  C     CCA    A     CC     CU
   GCACGGGCACAA    UAAGU GU AGCG   CAG UACA  AGGA  \
   CGUGCCCGUGUU    AUUCA CA UCGC   GUC AUGU  UCCU  G
  U            UAC-      A  A     AGC    -     --     UG
 110       100          90        80            70
```

Reads in: lib-42, lib-45, lib-47, lib-48, lib-49, lib-50, lib-72
Number of reads="ID890",2,14,0,0,1,0,0

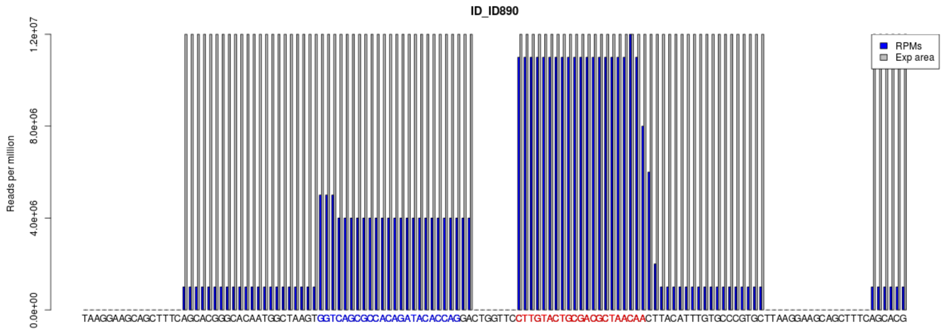

>ID891

AATTCAAGTTGATTCTATAGTAACTCGTGCAACTGAACATGGTCGAGAGAGGTATTTACATTTTTAGCGC**TTCTCCTGCTTTGTTCAGTT**
GTATAAGTTGCTGCGCAGCG

>ID891-3P

TTCTCCTGCTTTGTTCAGTTGT

>miRNA precursor

AAUUCAAGUUGAUUCUAUAGUAACUCGUGCAACUGAACAUGGUCGAGAGAGGUAUUUACAUUUUUAGCGC**UUCUCCUGCUUUGUUCAGUUGU**AUAAGUUGCUGCGCAGCG
.......((((......((((((((..((((((((((((.((.((.((((((..((.........))..)))))).)))).))))))))))))..))))))))..)))).

>Predicted STAR seq:

CAACTGAACATGGTCGAGAGAG

Complementarity: 18

Initial dG =   -41.80

```
        10        20        30        40        50        60
AAUUCAA|   AUUCUA        CG           U  U  A      UA  UACA
       GUUG      UAGUAACU  UGCAACUGAACA GG CG GAGAGG  UU    \
       CGAC      GUCGUUGA  AUGUUGACUUGU UC GU CUCUUC  GA    U
G------^   GC----        AU           U  -  C      GC  UUUU
.                 100        90        80         70
```

Reads in: lib-42, lib-45, lib-47, lib-48, lib-49, lib-50, lib-72

Number of reads="ID891",10,7,6,16,27,1,0

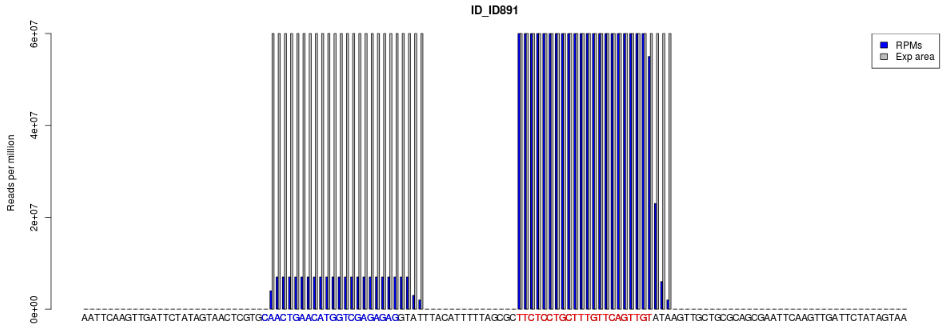

>ID893
TGGTGCCGTTTGCTTTGCTATGTGCCCTGAGCTTAGTTGGAGCCTGTGTATGCTACGTATATTATTACCG**TAGCACCATGGGATTCAGCTGA**GTTCGGGATTGCTTAG

>ID893-3P
TAGCACCATGGGATTCAGCTGA

>miRNA precursor
UGGUGCCGUUUGCUUUGCUAUGUGCCCUGAGCUUAGUUGGAGCCUGUGUAUGCUACGUAUAUUAUUACCG**UAGCACCAUGGGAUUCAGCUGA**GUUCGGGAUUGCUUAG
```
....(((((..((...)).))).))(((((((((((((((.((((((..(((((((((......)).))))))).)))))).))))))))))))))).........
```

>Predicted STAR seq:
TTGCTATGTGCCCTGAGCTTAGTTGGAGCCTG

Complementarity: 18
ERROR 4: Strange structure
Initial dG = -42.40

```
           10
.-UGGU  -|  UU  U
      GC CGU  GC \
      CG GUA  CG U
\ ----  U^  U-  U
          20



              30        40        50         60
  ---------                G      UA       -  UA
           CCUGAGCUUAGUUGGA CCUGUG  UGCUACG UA  U
           GGGCUUGAGUCGACUU GGGUAC  ACGAUGC AU  U
  GAUUCGUUA                A      C-       C  UA
        100        90         80         70
```

Reads in: lib-42, lib-45, lib-47, lib-48, lib-49, lib-50, lib-72
Number of reads="ID893",9336,1169,3159,3561,15801,958,0

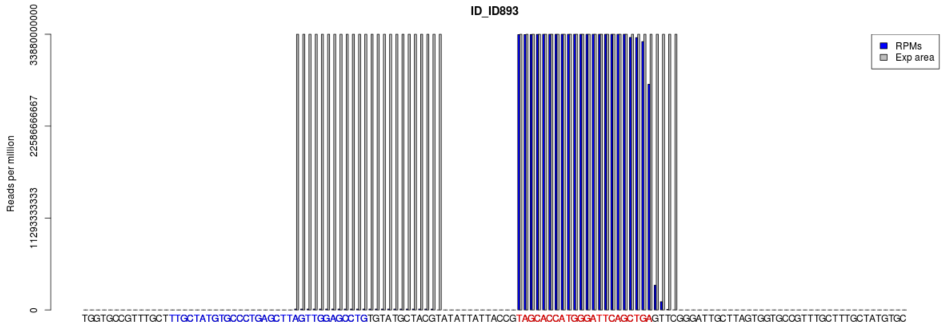

>ID895
TGAGAGACAATTGTACTGGTGTGGTAGTGCCAGATCTCTGCCTGTGACTTTGTGCTGTCCTACATAACCA**CATCACAGACAGAGTTCTAG**
TTACTACCAGATCAAAACCT

>ID895-3P
CATCACAGACAGAGTTCTAGTT

>miRNA precursor
UGAGAGACAAUUGUACUGGUGUGGUAGUGCCAGAUCUCUGCCUGUGACUUUGUGCUGUCCUACAUAACCA**CAUCACAGACAGAGUUCUAGUU**ACUACCAGAUCAAAACCU
```
................((((.(((((((((.(((.(((((.((((((...((((.((.....))....)))))))))).))))).))).)).))))))).))))......
```

>Predicted STAR seq:
CCAGATCTCTGCCTGTGACTTTG

Complementarity: 16

Initial dG =   -36.90

```
        10        20         30        40        50
UGAGAGACAAUUGUAC    G        -| C   U     C      CUU    C---  UC
                UGGU UGGUAGU GC AGA CUCUG CUGUGA   UGUG    UG  \
                ACUA ACCAUCA UG UCU GAGAC GACACU   ACAC    AC  C
UCCAAA----------    G        U^ A   U     A      ---    CAAU  AU
.                  100        90        80           70
```

Reads in: lib-42, lib-45, lib-47, lib-48, lib-49, lib-50, lib-72
Number of reads="ID895",2844,717,1373,1210,3633,419,0

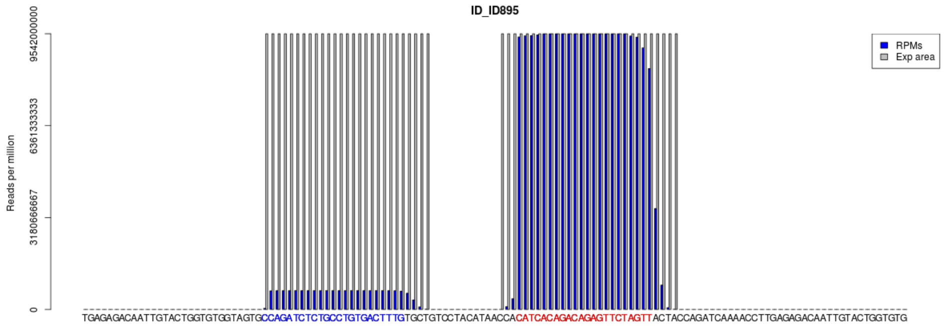

>ID903
TGGGAAATAGCTGCACTGTGTTCGTCAAAGTGGCTGTGATGTGTGTGTTGTTGTCA**TATCACAGCCAGCTTTGATGAGC**ATATTGTGGCA
TGAGCAACA

>ID903-3P
TATCACAGCCAGCTTTGATGAGC

>miRNA precursor
UGGGAAAUAGCUGCACUGUGUUCGUCAAAGUGGCUGUGAUGUGUGUUGUUGUCA**UAUCACAGCCAGCUUUGAUGAGC**AUAUUGUGGCAUGAGCAACA
.........((..((.(((((((((((((((((((((((((((...........))))))))))))).)))))))))))))).))..))..........

>Predicted STAR seq:
TGTTCGTCAAAGTGGCTGTGA

Complementarity: 20

Initial dG =   -49.90

```
         10        20        30         40
UGGGAAAUA-  UG  C              -|           UGUGU
          GC  CA UGUGUUCGUCAAAG UGGCUGUGAUGUG     \
          CG  GU AUACGAGUAGUUUC ACCGACACUAUAC     U
ACAACGAGUA  GU  U              G^           UGUUG
        90        80        70        60        50
```

Reads in: lib-42, lib-45, lib-47, lib-48, lib-49, lib-50, lib-72
Number of reads="ID903",5756,9025,15749,5842,12000,4136,0

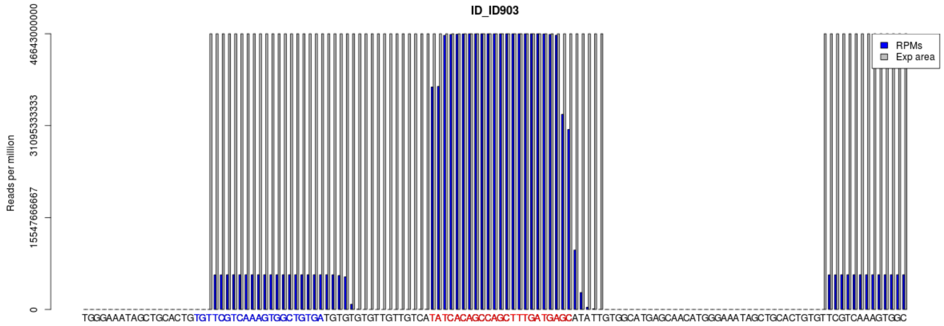

>ID904

AAAAGAATGAAGCTTTAGTCTCAGCTGCAGTGCGGCTCATCTCTGGGCACCTTGTATAATGATTAATCAC**TAGCTGCCTAGAGAAGGGCA**
TCACTGACTGAGACTTTAGTCAA

>ID904-3P

TAGCTGCCTAGAGAAGGGCATCA

>miRNA precursor

AAAAGAAUGAAGCUUUAGUCUCAGCUGCAGUGCGGCUCAUCUCUGGGCACCUUGUAUAAUGAUUAAUCAC**UAGCUGCCUAGAGAAGGGCAUCA**CUGACUGAGACUUUAGUCAA
...........(((..((((((((...(((((..((((.((((((((((.((.((.............)).)).)))))))))).)))))..))))).))))))))..)))...

>Predicted STAR seq:

GCGGCTCATCTCTGGGCACCT

Complementarity: 16

Initial dG =   -45.80

```
        10         20         30         40         50         60
AAAAGAAUGAA|  UU         CUG      CG     A           C  U  AUAAUG
           GCU  AGUCUCAG   CAGUG  GCUC UCUCUGGGCA CU GU      \
           UGA  UCAGAGUC   GUCAC  CGGG AGAGAUCCGU GA CA      A
AAC--------^  UU         A--      UA     A           C  U  CUAAUU
         110        100          90         80         70
```

Reads in: lib-42, lib-45, lib-47, lib-48, lib-49, lib-50, lib-72

Number of reads="ID904",258,3390,2380,1549,615,341,0

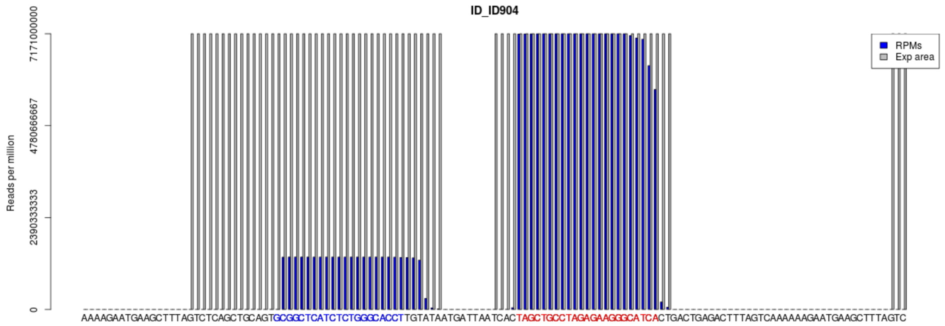

>ID91
GAGAACGGTGGTCTGTGGTA**CGGAGGAAACCTACACGAACACG**GTATTTAAGCAGTGTTGTGTGCGTTTCAACCTAACTACACGCTGCCT
CCTACATCGTCTACGAGTCGCAT

>ID91-5P
CGGAGGAAACCTACACGAACACG

>miRNA precursor
GAGAACGGUGGUCUGUGGUA**CGGAGGAAACCUACACGAACACG**GUAUUUAAGCAGUGUUGUGUGCGUUUCAACCUAACUACACGCUGCCUCCUACAUCGUCUACGAGUCGCAU
..((..((..((.((((((..((..(((((.(((((.(((((.((......)).))))))))))).)))))..))..))))))).))..)).......(((....))).))....

>Predicted STAR seq:
GTTGTGTGCGTTTCAACCTAAC

Complementarity: 15

Initial dG = -37.60

```
            10        20        30        40
GA--  .-AC| UG  C      AC  AG      C     G     G  AU
    GA    GG  GU UGUGGU  GG  GAAAC UACAC AACAC GU  U
    CU    CC  CG ACAUCA  CC  CUUUG GUGUG UUGUG CG  U
UACG  \ --^ GU  C      AU  AA      C     -     A  AA
 110              80        70        60         50


       90       100
        UCCUACA   U
               UCG C
               AGC U
        G------   A
```

Reads in: lib-42, lib-45, lib-47, lib-48, lib-49, lib-50, lib-72
Number of reads="ID91",0,1,0,0,0,0,0

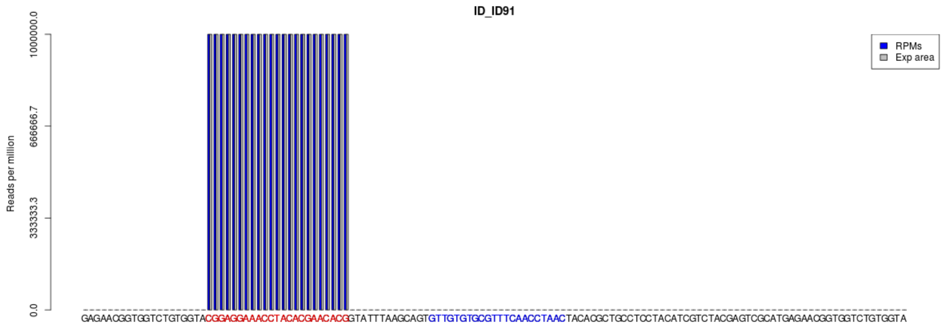

>ID914
GTTCGTTCCTGGAGGTGGTG**TCTTCGTCGCGGTTTGCTGCCAT**ACAATGGGCGCAGATGGCTGCCAATCGCGACGGATGTACTACCTCCA
GAAATGCGGCGAAAAAAATGCTC

>ID914-5P
TCTTCGTCGCGGTTTGCTGCCAT

>miRNA precursor
GUUCGUUCCUGGAGGUGGUG**UCUUCGUCGCGGUUUGCUGCCAU**ACAAUGGGCGCAGAUGGCUGCCAAUCGCGACGGAUGUACUACCUCCAGAAAUGCGGCGAAAAAAAUGCUC
...((((.((((((((((((..((((((((((((.((.(((((............))))).)).))))))))))))..))))))))))))).)))).((((......)))).

>Predicted STAR seq:
GGCTGCCAATCGCGACGGATGTA

Complementarity: 17

Initial dG = -55.20

```
                     10        20        30        40
------------   -|   C             UC             U  U     ACAAUG
            GUU CGUU CUGGAGGUGGUG  UUCGUCGCGGUU GC GCCAU      \
            CGG GUAA GACCUCCAUCAU  AGGCAGCGCUAA CG CGGUA      G
CUCGUAAAAAAG   C^   A             GU             C  U     GACGCG
110       100       90        80        70        60
```

Reads in: lib-42, lib-45, lib-47, lib-48, lib-49, lib-50, lib-72
Number of reads="ID914",2137,6136,4498,5043,8956,2073,0

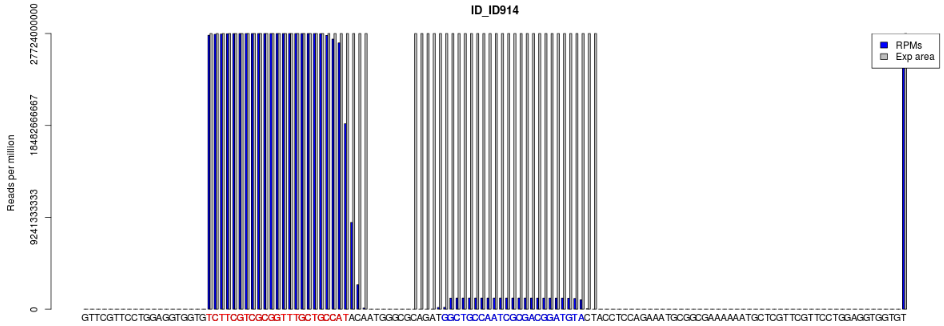

>ID917
TACAACCATTCAAAGTTTC**GGGGGCGTAGCTCATTGGC**CGAGTGGCTATCCCCTGGGGCACGGCCCACTGTGTCATGACCACCGGTCAGT
CAGCTACGCTGTCGTAAC

>ID917-5P
GGGGGCGTAGCTCATTGGC

>miRNA precursor
UACAACCAUUCAAAGUUUC**GGGGGCGUAGCUCAUUGGC**CGAGUGGCUAUCCCCUGGGGCACGGCCCACUGUGUCAUGACCACCGGUCAGUCAGCUACGCUGUCGUAAC
..............(((.(((.(((((((((.((((((((.((((.(((.......(((((((....)))))))))).))))))))))))).))))))))).))).)))

>Predicted STAR seq:
CAGTCAGCTACGCTGTCGT

Complementarity: 15

Initial dG =   -47.90

```
        10        20        30        40        50        60
UACAACCAUUCAAA|  U   G         C        A    C   CCCCUGG       C
              GUU CGG GGCGUAGCU AUUGGCCG GUGG UAU       GGCACGG C
              CAA GCU UCGCAUCGA UGACUGGC CACC GUA       CUGUGUC C
--------------^  U   G         C        -    A   -------       A
                    100        90         80               70
```

Reads in: lib-42, lib-45, lib-47, lib-48, lib-49, lib-50, lib-72
Number of reads="ID917",4,5,9,3,4,8,0

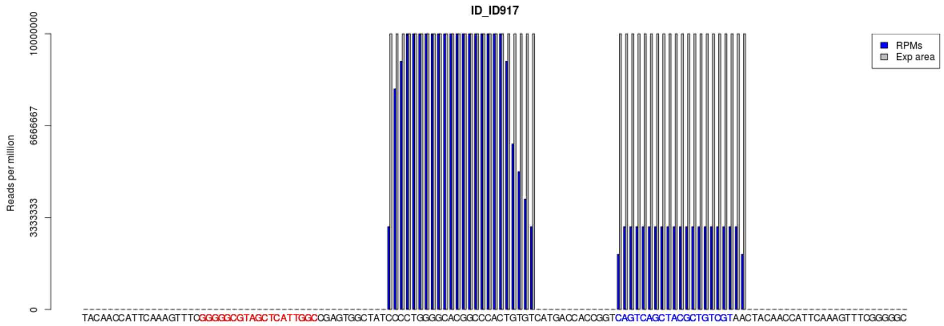

>ID92
TGAAATTTCCGGTCTGTGTAAATGCATCACGGTCGTCGGAGGTGTATTATGCACACGTTTCATAGCAATG**TCCATTATACACTTCTGACG**
ACTGTTGTGCATTATTGCATCA

>ID92-3P
TCCATTATACACTTCTGACGAC

>miRNA precursor
UGAAAUUUCCGGUCUGUGUAAAUGCAUCACGGUCGUCGGAGGUGUAUUAUGCACACGUUUCAUAGCAAUG**UCCAUUAUACACUUCUGACGAC**UGUUGUGCAUUAUUGCAUCA
..............((((((((((((.((((((((((((((((((((.(((.(((.(((....)))..))).))).)))))))))))))))))))).))))))..)))))..

>Predicted STAR seq:
TCGTCGGAGGTGTATTATGC

Complementarity: 18

Initial dG =   -46.90

```
        10        20          30        40        50         60
UGAAAUUUCCGGUCU     --       C                   U   C   C-|  U
               GUGUA  AAUGCAU ACGGUCGUCGGAGGUGUAU AUG ACA  GUU C
               UACGU  UUACGUG UGUCAGCAGUCUUCACAUA UAC UGU  CGA A
AC-------------     UA       U                   U   C   AA^  U
             110       100        90        80        70
```

Reads in: lib-42, lib-45, lib-47, lib-48, lib-49, lib-50, lib-72
Number of reads="ID92",2,23,0,1,0,0,0

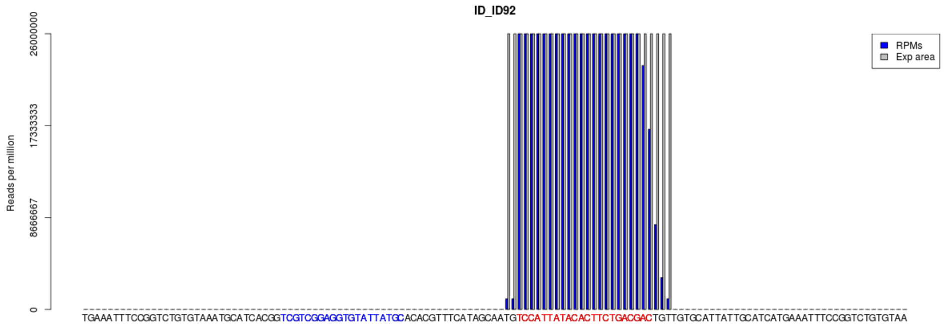

>ID925
GGTGGCGGGATATCTTGATGTACGCAGTTGCAAAACTACTTCTGCATGACGCGACAGTCGTTGGGAAGGA**CCCGTCATGCACAAGTAGCT**
TCGCGGCTGTCTGCACATGTC

>ID925-3P
CCCGTCATGCACAAGTAGCTTCG

>miRNA precursor
GGUGGCGGGAUAUCUUGAUGUACGCAGUUGCAAAACUACUUCUGCAUGACGCGACAGUCGUUGGGAAGGA**CCCGUCAUGCACAAGUAGCUUCG**CGGCUGUCUGCACAUGUC
((((......)))).((.((((.((((((((.((.((((((.(((((((((.....(((.((...)).))).))))))))).)))))).)).)))))))).))))))....

>Predicted STAR seq:
AAAACTACTTCTGCATGACGC

Complementarity: 17

Initial dG =   -40.50

```
             10
G--   -    .-G|   C
   GUG GCGG   AUAU U
   UAC CGUC   UGUA U
CUG   A    \ -^   G
 .            20
```

Reads in: lib-42, lib-45, lib-47, lib-48, lib-49, lib-50, lib-72
Number of reads="ID925",95,6127,1385,417,212,1109,0

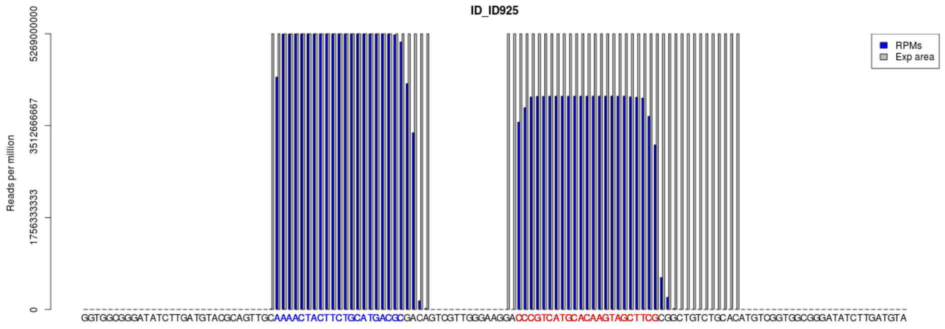

>ID926
ATGTAATCTGCGGTCTGTTC**TGCTGGATACTGAATACAGCGG**TTTATACTGTCATCCGCTGTAGGCAGTATCCAGTCTACAGACTGCAGACAGTCGTCAATTTTCGCCAACT

>ID926-5P
TGCTGGATACTGAATACAGCGG

>miRNA precursor
AUGUAAUCUGCGGUCUGUUC**UGCUGGAUACUGAAUACAGCGG**UUUAUACUGUCAUCCGCUGUAGGCAGUAUCCAGUCUACAGACUGCAGACAGUCGUCAAUUUUCGCCAACU
```
......((((((((((((...(((((((((((..((((((((.............))))))))..)))))))))))..))))))))))))....................
```

>Predicted STAR seq:
GCTGTAGGCAGTATCCAGTCTAC

Complementarity: 17

Initial dG = -55.00

```
                        10        20        30        40
AUGUAA----------------|           UCU            AA         UUUAUA
                      UCUGCGGUCUGU   GCUGGAUACUG  UACAGCGG      \
                      AGACGUCAGACA   UGACCUAUGAC  AUGUCGCC      C
UCAACCGCUUUUAACUGCUGAC^           UC-            GG         UACUGU
110       100       90        80         70        60        50
```

Reads in: lib-42, lib-45, lib-47, lib-48, lib-49, lib-50, lib-72
Number of reads="ID926",3,12,1,293,8,0,0

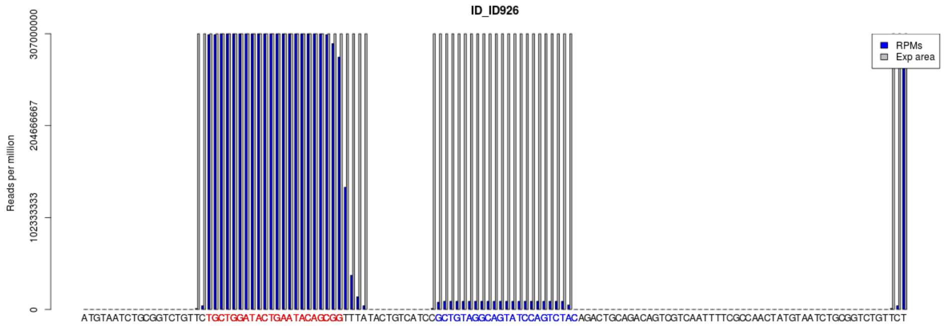

>ID927
ATATAACCTGTAGTCTGTTC**TGCTGGAGACTGAATACAGCGGT**TTAGACTGTAGTCCGCTGTAGGCAGTCTTCAGCCTACAGACTGCAGA
CAGTCGTCAGTCTTCGCCAAGTC

>ID927-5P
TGCTGGAGACTGAATACAGCGGT

>miRNA precursor
AUAUAACCUGUAGUCUGUUC**UGCUGGAGACUGAAUACAGCGGU**UUAGACUGUAGUCCGCUGUAGGCAGUCUUCAGCCUACAGACUGCAGACAGUCGUCAGUCUUCGCCAAGUC
.......(((((((((((...(((((((((((..((((((((.(((.....))).))))))))..)))))))))))..)))))))))))........................

>Predicted STAR seq:
GCTGTAGGCAGTCTTCAGCCTAC

Complementarity: 17

Initial dG = -53.90

```
                         10        20        30        40
AUAUAAC-----------------|          UCU            AA        U    GA
                        CUGUAGUCUGU   GCUGGAGACUG  UACAGCGG UUA   \
                        GACGUCAGACA   CGACUUCUGAC  AUGUCGCC GAU   C
CUGAACCGCUUCUGACUGCUGACA^          UC-            GG        U    GU
 110       100       90        80         70        60        50
```

Reads in: lib-42, lib-45, lib-47, lib-48, lib-49, lib-50, lib-72
Number of reads="ID927",21,85,141,3343,143,4,0

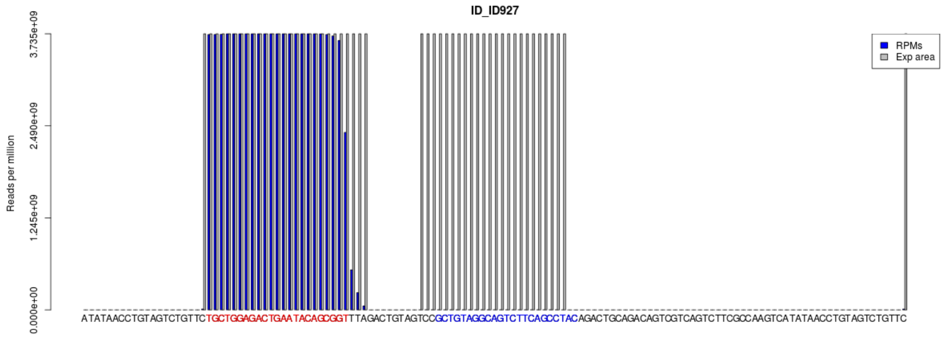

>ID928
GGTTGTGTTTGCATCCTCC**CGAACTATCAATTCTATTGCTGA**CGTATTCAAACGCAGCGGGGGAGTTGATGTTTCGGAAGGGTTCAGGCA
AATGCTTCGTGAAGATGGAAAA

>ID928-5P
CGAACTATCAATTCTATTGCTGA

>miRNA precursor
GGUUGUGUUUGCAUCCUCC**CGAACUAUCAAUUCUAUUGCUGA**CGUAUUCAAACGCAGCGGGGGAGUUGAUGUUUCGGAAGGGUUCAGGCAAAUGCUUCGUGAAGAUGGAAAA
(((..((((((.(((((.(((((.((((((((((.((((((.(((......))))))))).)))))))))).))))).))))).))))))...)))................

>Predicted STAR seq:
GCGGGGGAGTTGATGTTTCGGA

Complementarity: 18

Initial dG =   -44.60

```
                          10        20        30        40
----------------   UG-|     C     C     C          A      A   AU
                GGU   UGUUUG AUCCU CCGAA UAUCAAUUCU UUGCUG CGU  U
                UCG   ACGGAC UGGGA GGCUU GUAGUUGAGG GGCGAC GCA  C
AAAAGGUAGAAGUGCU   UAA^     U     A     U          G      -   AA
110       100       90        80        70        60         50
```

Reads in: lib-42, lib-45, lib-47, lib-48, lib-49, lib-50, lib-72
Number of reads="ID928",1,2,1,456,12,0,0

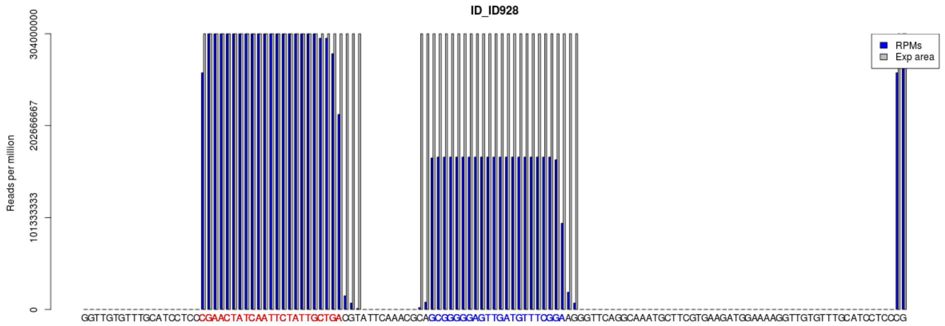

>ID929
ACTGGACACCTGTGGAGACGAGGGCTCCGGGGCAGGGGTTTCTTTCGGCCTCCAGCCGACATGGAATGTT**GTAGGCCGGCGGAAACTACT**
TGCCTCTGGGCCCCACACCGGCC

>ID929-3P
GTAGGCCGGCGGAAACTACTTGC

>miRNA precursor
ACUGGACACCUGUGGAGACGAGGGCUCCGGGGCAGGGGUUUCUUUCGGCCUCCAGCCGACAUGGAAUGUU**GUAGGCCGGCGGAAACUACUUGC**CUCUGGGCCCCACACCGGCC
```
.((((.....(((((.......(((((.(((((((((((((((.(((((((.((((..........)))).))))))).))))))).)))))))).))))))))))))))..
```

>Predicted STAR seq:
CAGGGGTTTCTTTCGGCCTC

Complementarity: 18
ERROR 4: Strange structure
Initial dG =   -56.40

```
           10
A-     .-ACACCUG| G
  CUGG         UG A
  GGCC         GC G
CC     \ -------^ A
 110          20


                   30         40         50        60
        A---       C         -        U       C     CGACA
            GGGCUC GGGGCAGG GGUUUCU UCGGCCU CAGC     \
            CCCGGG CUCCGUUC UCAAAGG GGCCGGA GUUG      U
        ACAC       U         A        C       U     UAAGG
             100        90         80         70
```

Reads in: lib-42, lib-45, lib-47, lib-48, lib-49, lib-50, lib-72
Number of reads="ID929",1190,3492,1648,193191,4287,666,0

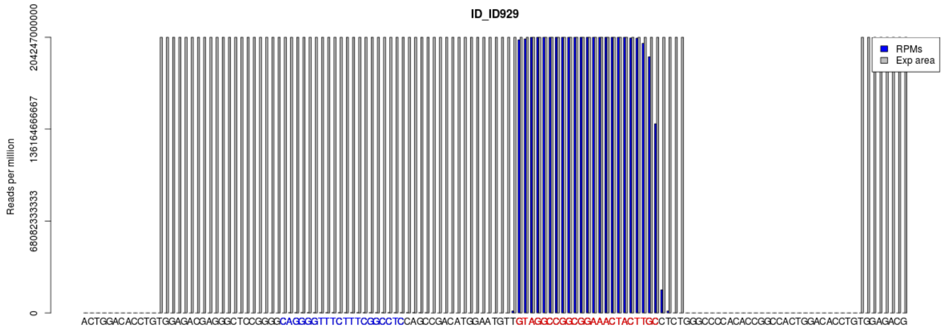

>ID930
GACAATACATGTCATGGAGG**TTGACGAGTGCCTGACTGGAACAT**ATTGTTTTATTGTTTCAGTCATCCATTCGTTAGCCGCCCTGCATGG
TACACTGCAATGATACTAAT

>ID930-5P
TTGACGAGTGCCTGACTGGAACAT

>miRNA precursor
GACAAUACAUGUCAUGGAGG**UUGACGAGUGCCUGACUGGAACAU**AUUGUUUUAUUGUUUCAGUCAUCCAUUCGUUAGCCGCCCUGCAUGGUACACUGCAAUGAUACUAAU
((((.....))))..((.((((((((((((..((((((((((((...........))))))))))))..))))))))))))).)).((((........))))...........

>Predicted STAR seq:
TTTCAGTCATCCATTCGTTAGCC

Complementarity: 19

Initial dG =   -42.70

```
                      10        20        30        40
GACAAUA--------------|   U  U  A            CC           UAUUG
                     CAUG CA GG GGUUGACGAGUG  UGACUGGAACA     \
                     GUAC GU CC CCGAUUGCUUAC  ACUGACUUUGU     U
UAAUCAUAGUAACGUCACAUG^   -  C  G            CU           UAUUU
.       100        90        80        70        60        50
```

Reads in: lib-42, lib-45, lib-47, lib-48, lib-49, lib-50, lib-72
Number of reads="ID930",6,31,0,0,0,1,0

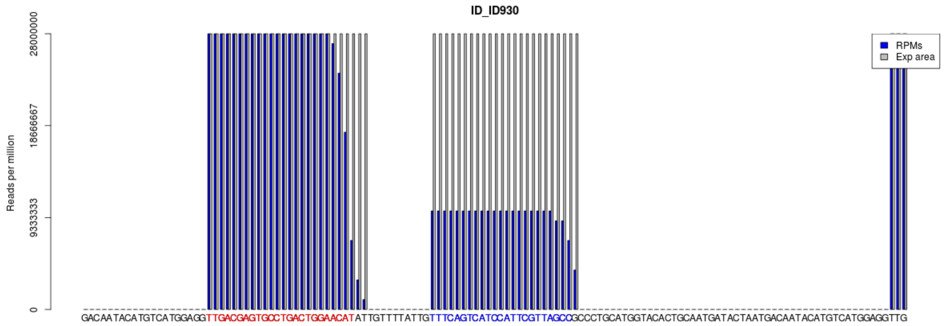

>ID931
AGTAATACATGGATAGAGGG**TGATGTGCGTTTGATGGAACTAG**ATTATAAAGGTTCTGTTCCATCACTCTCACATCACCCACTTTACATG
ACAAGCAGCAATGGGTGG

>ID931-5P
TGATGTGCGTTTGATGGAACTAG

>miRNA precursor
AGUAAUACAUGGAUAGAGGG**UGAUGUGCGUUUGAUGGAACUAG**AUUAUAAAGGUUCUGUUCCAUCACUCUCACAUCACCCACUUUACAUGACAAGCAGCAAUGGGUGG
```
.......((((...((.((((((((((....(((((((((.(((..........))))))))))))....)))))))))).))...)))).((..((....))..)).
```

>Predicted STAR seq:
GTTCCATCACTCTCACATCACC

Complementarity: 16

Initial dG =   -41.40

```
          10        20        30        40
.-AGUAAUA|   GAU  A          CGUU         U   UUAU
         CAUG   AG GGGUGAUGUG    UGAUGGAAC AGA    A
         GUAC   UC CCCACUACAC    ACUACCUUG UCU    A
\ -------^   AUU  A          UCUC         -   UGGA
        90        80        70        60         50
```

```
  A  AG  G
   CA  CA C
   GU  GU A
  G  GG  A
        100
```

Reads in: lib-42, lib-45, lib-47, lib-48, lib-49, lib-50, lib-72
Number of reads="ID931",23,24,0,0,2,0,0

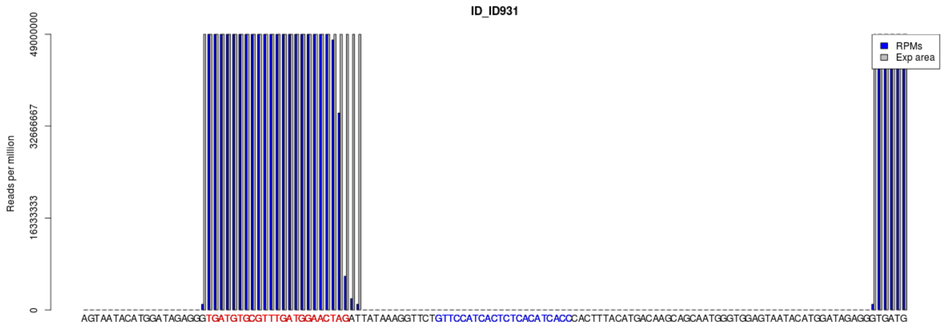

>ID932
AGTAATTCATGCATGGAGGGTTGTGACTGTGTATGACAACACGTTATGAAATCACTGTTGTACATACACAGCCACCACCCACTGTGCATG
GTAATCATCAGCCGTTGGTC

>ID932-5P
TTGTGACTGTGTATGACAACA

>miRNA precursor
AGUAAUUCAUGCAUGGAGGGUUGUGACUGUGUAUGACAACACGUUAUGAAAUCACUGUUGUACAUACACAGCCACCACCCACUGUGCAUGGUAAUCAUCAGCCGUUGGUC
......((((((((((.((((.(((.(((((((((((((((.((.........)))))))).))))))))).))).)))).))))))))))..((((........)))).

>Predicted STAR seq:
TTGTACATACACAGCCACCACC

Complementarity: 17

Initial dG = -44.90

```
          10        20        30         40
.-AGUAAU          A    U   A         -|     C  UAUG
        UCAUGCAUGG GGGU GUG CUGUGUAUG ACAACA GU    \
        GGUACGUGUC CCCA CAC GACACAUAC UGUUGU CA    A
\ ------          A    C   C         A^     -  CUAA
        90        80        70        60         50



        100
  UA    UCA
    AUCA   G
    UGGU   C
  C-    UGC
110
```

Reads in: lib-42, lib-45, lib-47, lib-48, lib-49, lib-50, lib-72
Number of reads="ID932",17,33,0,0,2,1,0

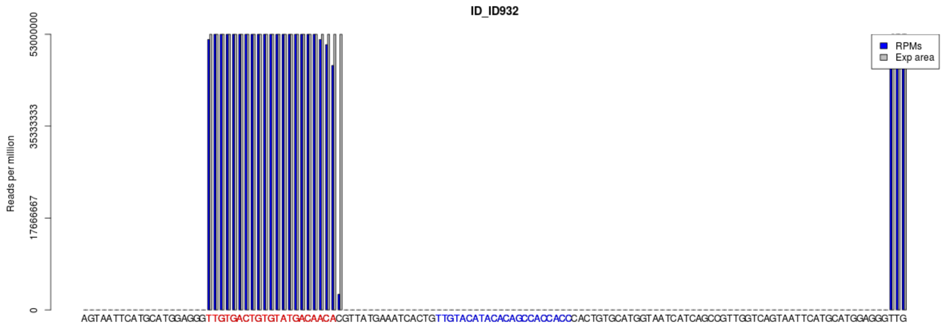

>ID933
TCTTGCACTGACAGTAATACATGGATGGAGGGTGATGTGCGTGTGATAACAACACGTGATAAAGGTTCTG**TTTTATCACCCTCACATCAC**
CCACTTTACATGACAAACAGC

>ID933-3P
TTTTATCACCCTCACATCACC

>miRNA precursor
UCUUGCACUGACAGUAAUACAUGGAUGGAGGGUGAUGUGCGUGUGAUAACAACACGUGAUAAAGGUUCUG**UUUUAUCACCCUCACAUCACC**CACUUUACAUGACAAACAGC
..((((.......))))..((((...((.((((((((((...(((((((.((((...((.......)))))))))))))...)))))))))).))...)))).........

>Predicted STAR seq:
GTGATGTGCGTGTGATAACAA

Complementarity: 16
ERROR 4: Strange structure
Initial dG =   -33.80

```
.-UC|   ACU
    UUGC   \
    AAUG   G
\ --^   ACA
```

```
          20        30          40
  UA-------    GAU  A          .-C    GAU
           CAUG   GG GGGUGAUGUG   GUGU   \
           GUAC   UC CCCACUACAC   CACA   A
  CGACAAACA    AUU  A          \ -    ACA
 110       100        90              50
```

Reads in: lib-42, lib-45, lib-47, lib-48, lib-49, lib-50, lib-72
Number of reads="ID933",0,0,0,0,0,0,0

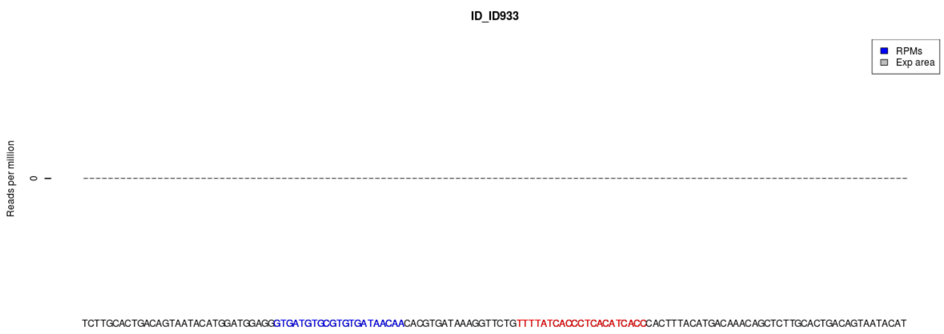

>ID934
GTCTGTTTTTATGCCAATACATGCCATGGAGGT**TGACGGATGTCTCACTGGAAA**ATGTTATTTTATATTGTACCAGTCACCCATTCGTTA
GCCGCCCTGCATTATACACGAC

>ID934-5P
TGACGGATGTCTCACTGGAAA

>miRNA precursor
GUCUGUUUUUAUGCCAAUACAUGCCAUGGAGGU**UGACGGAUGUCUCACUGGAAA**AUGUUAUUUUAUAUUGUACCAGUCACCCAUUCGUUAGCCGCCCUGCAUUAUACACGAC
```
((((((....((((.............((.((((((((((((....(((((..(((((......)))))...)))))....))))))))))))).))..)))))...))).)))
```

>Predicted STAR seq:
TACCAGTCACCCATTCGTTAGC

Complementarity: 13

Initial dG =   -32.40

```
         10        20        30        40        50         60
   -|  UUUU    CAAUACAUGCCAU  A            UCUC      AA-      UA
GUC UGU    AUGC             GG GGUUGACGGAUG    ACUGG   AAUGU  U
CAG ACA    UACG             CC CCGAUUGCUUAC    UGACC   UUAUA  U
   C^  UAU-    UC-----------  G            CCAC      AUG      UU
110        100                   90        80        70
```

Reads in: lib-42, lib-45, lib-47, lib-48, lib-49, lib-50, lib-72
Number of reads="ID934",1,0,0,0,0,0,0

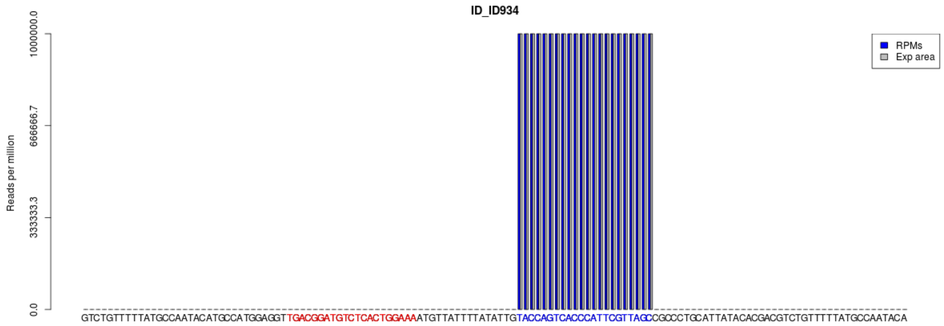

>ID936

AGTAATTCATGCATGGAGGG**TGATGTCTGTGTGGGCCCAACA**CGATATAAAATTACTGTTGAGCATACACGGCTATCACCCACTGTGCAT
GGTAATCATCAGTTTGGAGTCG

>ID936-5P

TGATGTCTGTGTGGGCCCAACA

>miRNA precursor

AGUAAUUCAUGCAUGGAGGG**UGAUGUCUGUGUGGGCCCAACA**CGAUAUAAAAUUACUGUUGAGCAUACACGGCUAUCACCCACUGUGCAUGGUAAUCAUCAGUUUGGAGUCG

```
......((((((((((.((((((((.(((((((.((.(((((.(((.....)))..))))).)).))))))).)))))))).))))))))))...................
```

>Predicted STAR seq:

TTGAGCATACACGGCTATCACC

Complementarity: 17

Initial dG =   -43.60

```
                      10        20        30        40
AGUAAU--------------          A        U       G  C     C-|  AU
                    UCAUGCAUGG GGGUGAUG CUGUGUG GC CAACA  GAU  \
                    GGUACGUGUC CCCACUAU GGCACAU CG GUUGU  UUA  A
GCUGAGGUUUGACUACUAAU          A        C       A  A     CA^  AA
110       100       90        80        70        60        50
```

Reads in: lib-42, lib-45, lib-47, lib-48, lib-49, lib-50, lib-72

Number of reads="ID936",1,3,0,0,0,0,0

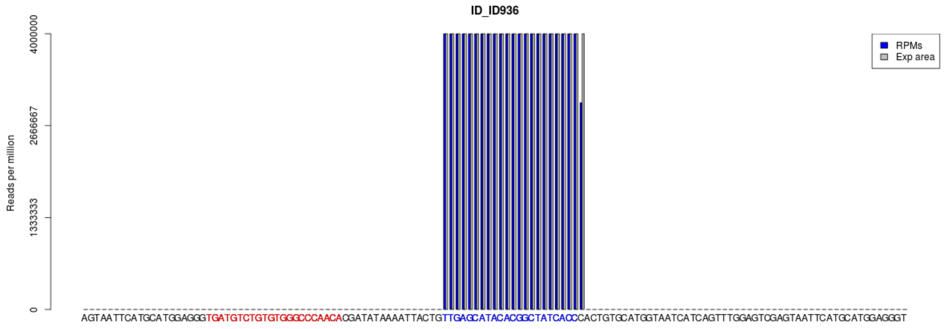

>ID937
GGTAATTCCTGCATGGAGGG**TTATGGTAGAGTGGTGACAACA**AATCATTGAATTTCTGTTGCCCACACCCTACTATTACCCACTGTGTAT
GGGAAAGCAAATAAGACA

>ID937-5P
TTATGGTAGAGTGGTGACAACA

>miRNA precursor
GGUAAUUCCUGCAUGGAGGG**UUAUGGUAGAGUGGUGACAACA**AAUCAUUGAAUUUCUGUUGCCCACACCCUACUAUUACCCACUGUGUAUGGGAAAGCAAAUAAGACA
.((..(((((((((((.((((.(((((((.((((..(((..((((......)))).)))..))...)).))))))).)))).))))))).))))..)).........

>Predicted STAR seq:
TGTTGCCCACACCCTACTATTACC

Complementarity: 14

Initial dG = -34.70

```
                  10        20        30           40
G---------  AA    -        A    U        A   ---| UG   AC    CA
          GU  UUCC UGCAUGG GGGU AUGGUAG GU    GG  ACA  AAAU  U
          CG  AGGG AUGUGUC CCCA UAUCAUC CA    CC  UGU  UUUA  U
ACAGAAUAAA  AA    U        A    U       C  CAC^ GU   C-    AG
      100        90        80        70        60         50
```

Reads in: lib-42, lib-45, lib-47, lib-48, lib-49, lib-50, lib-72
Number of reads="ID937",4,1,0,0,0,0,0

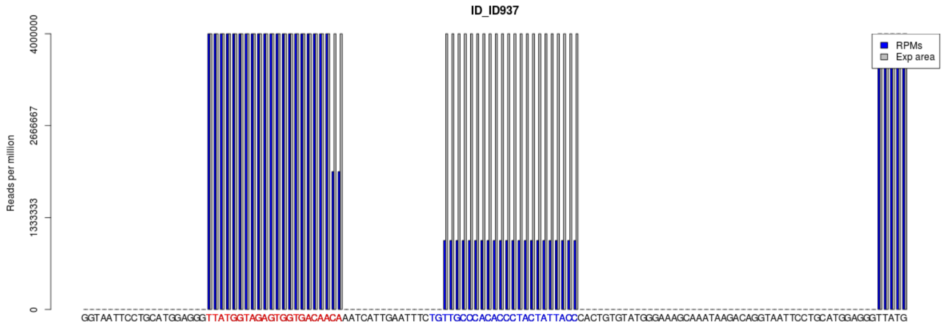

>ID938

TGTTGCAGTGTCAGTAATTCATGCATGGAGGGTGATGTCTGTGCTGGTCCAACACATTATAAGATTACTG**TTGAACAGACACGGCCATCA**
CTCAATGTGCATGATAAC

>ID938-3P

TTGAACAGACACGGCCATCACT

>miRNA precursor

UGUUGCAGUGUCAGUAAUUCAUGCAUGGAGGGUGAUGUCUGUGCUGGUCCAACACAUUAUAAGAUUACUG**UUGAACAGACACGGCCAUCACU**CAAUGUGCAUGAUAAC
```
.(((((.......)))))((((((((..((((((((.((((((((...(((((.............)))))..))).))))).))))))))..)))))))))....
```

>Predicted STAR seq:

GTGATGTCTGTGCTGGTCCAA

Complementarity: 16

ERROR 4: Strange structure

Initial dG =   -38.80

```
.-U|    AGU
   GUUGC   \
   UAAUG   G
\ -^    ACU
```

```
      20        30        40         50        60
  ----         GA        U      -   GUC      CAUUAU
      UCAUGCAUG  GGGUGAUG CUGUG CUG   CAACA      A
      AGUACGUGU  CUCACUAC GGCAC GAC   GUUGU      A
  CAAU         AA        C      A   AA-      CAUUAG
        100        90        80         70
```

Reads in: lib-42, lib-45, lib-47, lib-48, lib-49, lib-50, lib-72

Number of reads="ID938",5,5,0,1,0,0,0

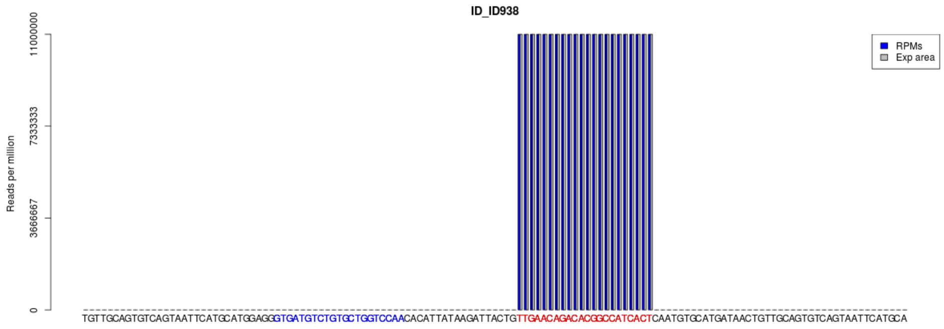

>ID939

TCTTGCACTGACAGTAATACATGGATGGAGGGTGATGTGCGTGTGATAACAACACGTGATAAAGGTTCTG**TTTTATCACCCTCACATCAC**
CCACTTTACATGACAAACAGC

>ID939-3P

TTTTATCACCCTCACATCACC

>miRNA precursor

UCUUGCACUGACAGUAAUACAUGGAUGGAGGGUGAUGUGCGUGUGAUAACAACACGUGAUAAAGGUUCUG**UUUUAUCACCCUCACAUCACC**CACUUUACAUGACAAACAGC
..((((.......))))..((((...((.((((((((((...(((((((.((((...((.......)))))))))))))...)))))))))).))...)))).........

>Predicted STAR seq:

GTGATGTGCGTGTGATAACAA

Complementarity: 16
ERROR 4: Strange structure
Initial dG =   -33.80

```
.-UC|   ACU
    UUGC   \
    AAUG   G
\ --^   ACA
```

```
          20        30          40
  UA-------    GAU  A          .-C    GAU
         CAUG   GG GGGUGAUGUG   GUGU   \
         GUAC   UC CCCACUACAC   CACA   A
  CGACAAACA    AUU  A          \ -    ACA
 110       100        90              50
```

Reads in: lib-42, lib-45, lib-47, lib-48, lib-49, lib-50, lib-72

Number of reads="ID939",0,0,0,0,0,0,0

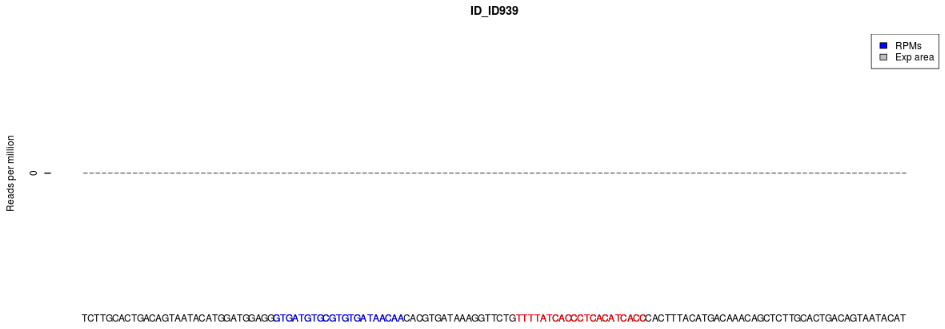

>ID94
ACTACAGAAGCATTCTGGAG**ATTCGGATGTATTCCCATCACG**TATTTTACTAATCGTTATGGTGATACATTCGTTTCTCTATGGTGCACA
TTGTGCTTCAAACATTTATCCA

>ID94-5P
ATTCGGATGTATTCCCATCACG

>miRNA precursor
ACUACAGAAGCAUUCUGGAG**AUUCGGAUGUAUUCCCAUCACG**UAUUUUACUAAUCGUUAUGGUGAUACAUUCGUUUCUCUAUGGUGCACAUUGUGCUUCAAACAUUUAUCCA
```
..(((((..((((..((((((..(((((((((..((((.(((.(((.....)))))).))))..)))))))))..))))))..))))...))))).................
```

>Predicted STAR seq:
TTATGGTGATACATTCGTTTCT

Complementarity: 15

Initial dG = -38.30

```
                        10        20        30        40
AC---------------     AA-|   UC      UU          UC    C   U   UU
                 UACAG   GCAU  UGGAGA  CGGAUGUAU  CCAU ACG AUU  \
                 GUGUU   CGUG  AUCUCU  GCUUACAUA  GGUA UGC UAA  A
ACCUAUUUACAAACUUC     ACA^   GU      UU          GU    U   -   UC
110       100        90        80        70        60         50
```

Reads in: lib-42, lib-45, lib-47, lib-48, lib-49, lib-50, lib-72
Number of reads="ID94",0,4,0,0,0,0,0

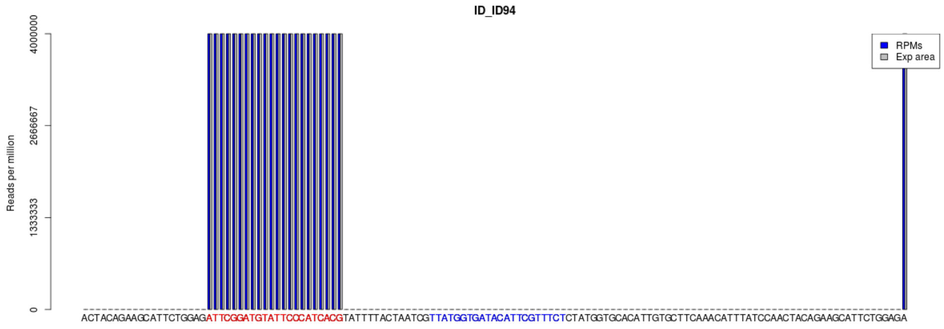

>ID940

ATATTGCAGTGACAGTAATTCATGCATGGAGGGCGATGACTGTGTCTGCCAACACTTTATTAAATCACTG**TTGTACAGACCCAGTTATCA**
CTCACTGTGTATGGTAAGCAT

>ID940-3P

TTGTACAGACCCAGTTATCACT

>miRNA precursor

AUAUUGCAGUGACAGUAAUUCAUGCAUGGAGGGCGAUGACUGUGUCUGCCAACACUUUAUUAAAUCACUG**UUGUACAGACCCAGUUAUCACU**CACUGUGUAUGGUAAGCAU
.(((((......)))))..((((((((((.(((.((((((((.(((((.(((((.............)))))..))))).)))))))).))).))))))))))).......

>Predicted STAR seq:

GCGATGACTGTGTCTGCCAA

Complementarity: 16
ERROR 4: Strange structure
Initial dG =   -37.70

```
.-A|    CA
   UAUUG  G
   AUGAC  U
\ -^    AG
         10



        20         30         40          50         60
  AU-----          A   C         U      C-      CUUUAU
         UCAUGCAUGG GGG GAUGACUG GUCUG  CAACA      U
         GGUAUGUGUC CUC CUAUUGAC CAGAC  GUUGU      A
  UACGAAU          A   A         C      AU      CACUAA
 110       100        90         80         70
```

Reads in: lib-42, lib-45, lib-47, lib-48, lib-49, lib-50, lib-72

Number of reads="ID940",10,9,0,0,2,1,0

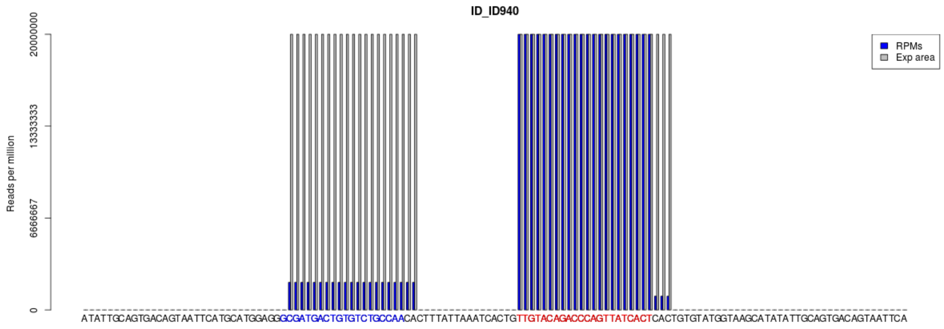

>ID941
TTGGTGTATTTCATAATAAAGGTGATCTGCAGGAGTCATAAGTGGCAATCAACACTGCATTAATGATGTG**TGGATTGCTCCTTCTGACGC**
CTTGCAGGTCATAGAAACGA

>ID941-3P
TGGATTGCTCCTTCTGACGCCT

>miRNA precursor
UUGGUGUAUUUCAUAAUAAAGGUGAUCUGCAGGAGUCAUAAGUGGCAAUCAACACUGCAUUAAUGAUGUG**UGGAUUGCUCCUUCUGACGCCU**UGCAGGUCAUAGAAACGA
...(((.....)))).......((((((((((((.((((.(((..((((((.((((..((....))..)))).)))))))..))).)))).)).))))))))))........

>Predicted STAR seq:
GAGTCATAAGTGGCAATCA

Complementarity: 14
ERROR 4: Strange structure
Initial dG = -41.50

```
.-UUG|  UA
     GUG  \
     UAC  U
\ ---^  UU
        10



      20         30         40        50        60
  AAUAAAG-            A-     U   UG       A     UG  U
          GUGAUCUGCAGG  GUCA AAG  GCAAUC ACAC  CA U
          UACUGGACGUUC  CAGU UUC  CGUUAG UGUG  GU A
  AGCAAAGA            CG     C   CU       G     UA  A
110       100        90        80        70
```

Reads in: lib-42, lib-45, lib-47, lib-48, lib-49, lib-50, lib-72
Number of reads="ID941",13,47,0,0,2,0,0

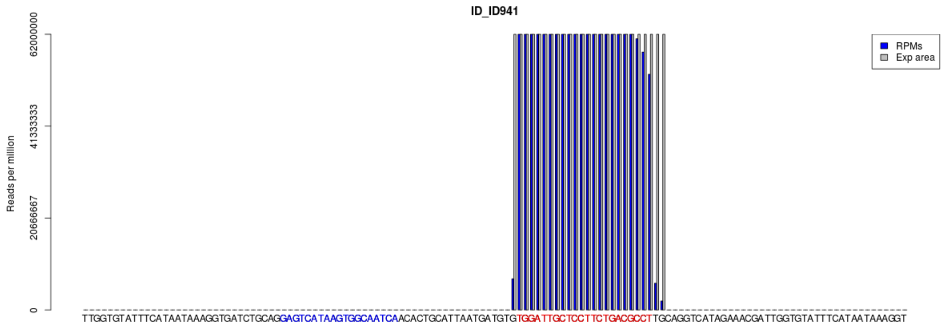

>ID942
ACTTCGCTGAAGATTCTAATCCATGTCAGGGGTTGGCCATTGTACGACTGACAAACAGAATTTATTGGTG**TTTCAGTCATGCAATCGTCA**
CCTCCTGTGTGGTATACCAC

>ID942-3P
TTTCAGTCATGCAATCGTCACC

>miRNA precursor
ACUUCGCUGAAGAUUCUAAUCCAUGUCAGGGGUUGGCCAUUGUACGACUGACAAACAGAAUUUAUUGGUG**UUUCAGUCAUGCAAUCGUCACC**UCCUGUGUGGUAUACCAC
.((((...))))........(((((.((((((.((((.((((((.((((((..((((...........)))))))))).)))))).)))).)))))))))))........

>Predicted STAR seq:
TTGGCCATTGTACGACTGACAAA

Complementarity: 17
ERROR 4: Strange structure
Initial dG = -34.90

```
.-A|   G
   CUUC \
   GAAG C
\ -^   U
    10
```

```
        20        30        40        50        60
  AUUCUAAU     U      U     C      C      CA     GAAUU
          CCAUG CAGGGG UGGC AUUGUA GACUGA  AACA      \
          GGUGU GUCCUC ACUG UAACGU CUGACU  UUGU      U
  CACCAUAU     -      C     C      A      --     GGUUA
110       100         90        80          70
```

Reads in: lib-42, lib-45, lib-47, lib-48, lib-49, lib-50, lib-72
Number of reads="ID942",2,13,0,0,0,0,0

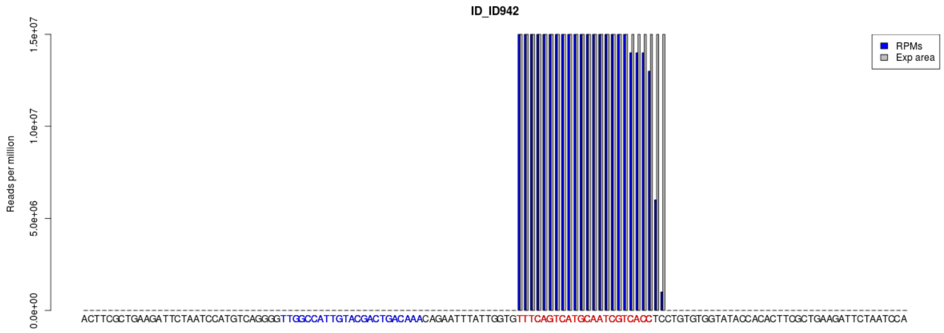

>ID943
GGTAAGACGTACATGGAGGG**TGATGGTAGAGTAGTGCCAACACA**TTATTTAATTGCTGTGTGGCCTGCCCTACCATCACTCACTGTGTAT
AGGACATGTCCGAAAGAACA

>ID943-5P
TGATGGTAGAGTAGTGCCAACACA

>miRNA precursor
GGUAAGACGUACAUGGAGGG**UGAUGGUAGAGUAGUGCCAACACA**UUAUUUAAUUGCUGUGUGGCCUGCCCUACCAUCACUCACUGUGUAUAGGACAUGUCCGAAAGAACA
........((((((((.((((((((((((.((((.((((.((((.((......)).))))))))))))).))))))))))))).)))))))).(((....))).........

>Predicted STAR seq:
TGTGGCCTGCCCTACCATCACT

Complementarity: 19

Initial dG =   -49.40

```
          10        20        30        40        50
.-GGUAAGAC|       A           A    U    A    U  UU
          GUACAUGG GGGUGAUGGUAG GUAG GCCA CACA UA  U
          UAUGUGUC CUCACUACCAUC CGUC CGGU GUGU GU  A
\ --------^       A           C    -    -    C  UA
         90        80        70          60



  A--------   C
           GGA A
           CCU U
  ACAAGAAAG   G
110       100
```

Reads in: lib-42, lib-45, lib-47, lib-48, lib-49, lib-50, lib-72
Number of reads="ID943",0,4,0,0,0,0,0

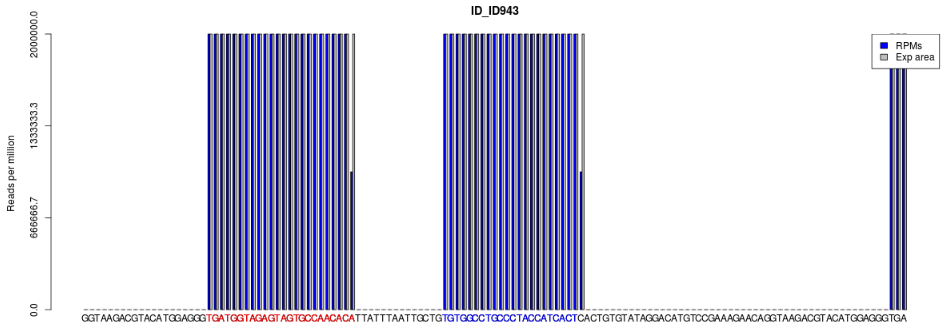

>ID944
GCCTAATGTGGTCCGCGGCT**AGCATGATCAGTGGGCACGAATT**GTGAATTGGTCAATTCGCTCCACCGCTCATGCAACACTGCGCTCCAC
ACAGTTCAACGTCGACGC

>ID944-5P
AGCATGATCAGTGGGCACGAATT

>miRNA precursor
GCCUAAUGUGGUCCGCGGCU**AGCAUGAUCAGUGGGCACGAAUU**GUGAAUUGGUCAAUUCGCUCCACCGCUCAUGCAACACUGCGCUCCACACAGUUCAACGUCGACGC
......(((((..(((((...((((((...(((((..(((((((.........))))))).)))))...))))))....)))))..))))).................

>Predicted STAR seq:
TTCGCTCCACCGCTCATGCAACACT

Complementarity: 15

Initial dG =   -35.80

```
                   10        20          30        40
GCCUAA-----------     UC     CUA-|     UCA     CA        UGAA
                 UGUGG  CGCGG    GCAUGA   GUGGG  CGAAUUG    \
                 ACACC  GCGUC    CGUACU   CACCU  GCUUAAC    U
CGCAGCUGCAACUUGAC     UC     ACAA^     CGC     C-        UGGU
      100        90        80        70         60        50
```

Reads in: lib-42, lib-45, lib-47, lib-48, lib-49, lib-50, lib-72
Number of reads="ID944",8,12,0,0,0,0,0

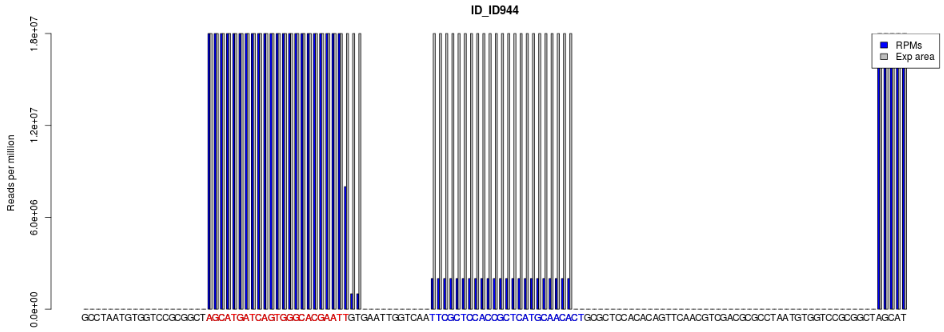

>ID945
CTTTGTGTATTTCTGAAGACCCTGTATACAGGTTGGCGATTGCTTGACTGCCACACTTGAATTATTACTG**TATCAGTCACCCAATCGTCA**
TTTCCTGTGCACAGTATACAA

>ID945-3P
TATCAGTCACCCAATCGTCATTT

>miRNA precursor
CUUUGUGUAUUUCUGAAGACCCUGUAUACAGGUUGGCGAUUGCUUGACUGCCACACUUGAAUUAUUACUG**UAUCAGUCACCCAAUCGUCAUUU**CCUGUGCACAGUAUACAA
..(((((((..((....))..((((.((((((.(((((((((..((((((..(((.............)))..))))))..)))))))))...)))))).)))))))))))

>Predicted STAR seq:
TGGCGATTGCTTGACTGC

Complementarity: 14
ERROR 4: Strange structure
Initial dG = -35.20

```
          10
CU       .-UU| U
  UUGUGUA    UC G
  AACAUAU    AG A
--       \ --^ A
 110
```

```
          20        30          40        50        60
           CC    A      U--         CU      CC   CUUGAA
             CUGU UACAGG   UGGCGAUUG  UGACUG  ACA      \
             GACA GUGUCC   ACUGCUAAC  ACUGAC  UGU      U
           --    C      UUU         CC      UA   CAUUAU
                 100        90        80        70
```

Reads in: lib-42, lib-45, lib-47, lib-48, lib-49, lib-50, lib-72
Number of reads="ID945",7,27,0,1,0,0,0

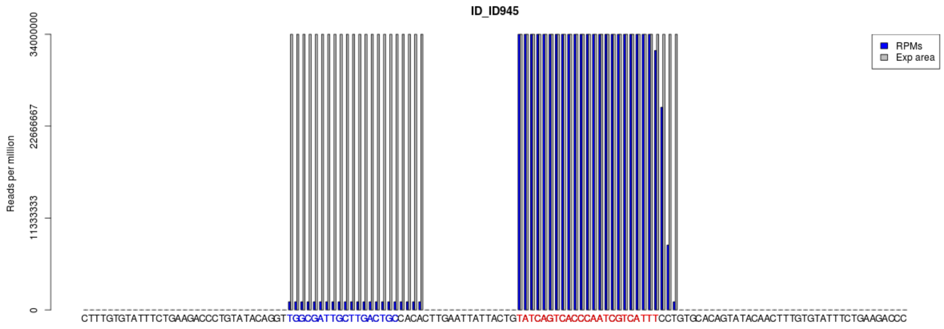

>ID946
TGTTGCAGTGTCAGTAATTCATGCATGGAGGGTGATGGCTGTGTGGGTCCAACACATTATGAGATTACTG**TTGAACAGACACGGCCATCACT**CACTGTGCATGATAAC

>ID946-3P
TTGAACAGACACGGCCATCACT

>miRNA precursor
UGUUGCAGUGUCAGUAAUUCAUGCAUGGAGGGUGAUGGCUGUGUGGGUCCAACACAUUAUGAGAUUACUG**UUGAACAGACACGGCCAUCACU**CACUGUGCAUGAUAAC
```
.(((((.......)))))((((((((((.(((((((((((((((..((.(((((.............))))).))..))))))))))))))).))))))))))....
```

>Predicted STAR seq:
GTGATGGCTGTGTGGGTCCAA

Complementarity: 17
ERROR 4: Strange structure
Initial dG = -48.70

```
.-U|    AGU
   GUUGC   \
   UAAUG   G
\ -^    ACU
```

```
      20        30        40        50        60
  ----          A               GG  C      CAUUAU
      UCAUGCAUGG GGGUGAUGGCUGUGU  GU CAACA      G
      AGUACGUGUC CUCACUACCGGCACA  CA GUUGU      A
  CAAU          A               GA  A      CAUUAG
        100        90        80        70
```

Reads in: lib-42, lib-45, lib-47, lib-48, lib-49, lib-50, lib-72
Number of reads="ID946",4,10,0,0,0,0,0

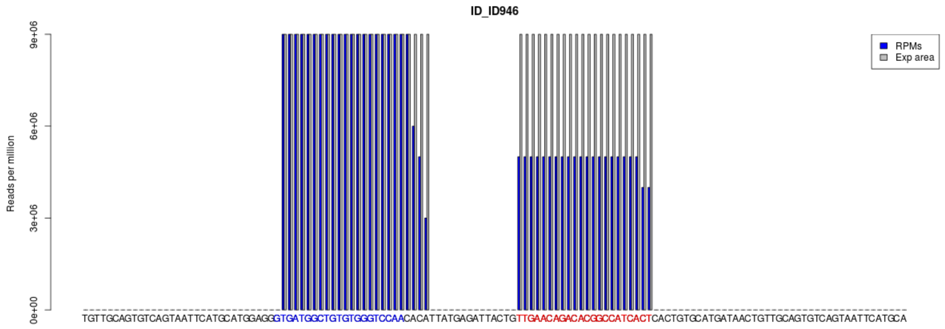

>ID947
TCAAAACATGTCAGGGAGGT**TGACGGATGTCTGACTGCAACAT**GTTATTTTATATTGTCTCAGTCACCCATTCGTTAGCCGCCTTGCATG
GTACACTGCAAAGATGCTAA

>ID947-5P
TGACGGATGTCTGACTGCAACAT

>miRNA precursor
UCAAAACAUGUCAGGGAGGU**UGACGGAUGUCUGACUGCAACAU**GUUAUUUUAUAUUGUCUCAGUCACCCAUUCGUUAGCCGCCUUGCAUGGUACACUGCAAAGAUGCUAA
......((((.(((((.((((((((((((..((((((..((((((......))).))).))))))..))))))))))))).)))))))))).......(((....)))...

>Predicted STAR seq:
TGTCTCAGTCACCCATTCGTTAGC

Complementarity: 17

Initial dG =   -43.80

```
          10        20        30        40
.-UCAAAA|   U     A           UC      CA   -   UA
        CAUG CAGGG GGUUGACGGAUG  UGACUG  ACA UGU  U
        GUAC GUUCC CCGAUUGCUUAC  ACUGAC  UGU AUA  U
\ ------^   -     G           CC      UC   U   UU
       90         80        70        60        50



         100
  GUACACU   A
         GCA A
         CGU G
  AAU----   A
110
```

Reads in: lib-42, lib-45, lib-47, lib-48, lib-49, lib-50, lib-72
Number of reads="ID947",10,11,1,2,0,0,0

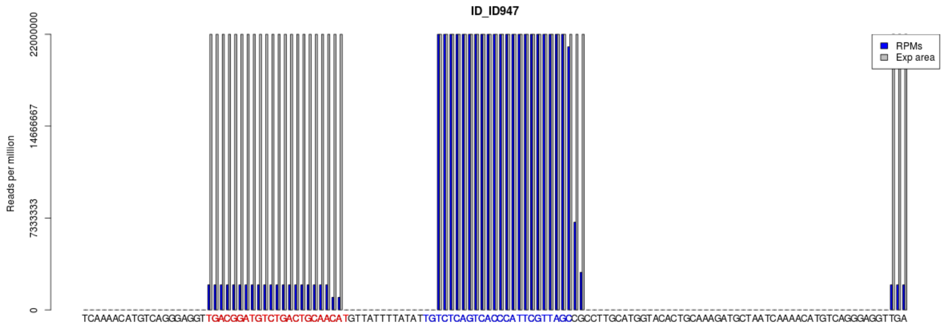

>ID948
AATAGTACATGCATGGAGGG**TTGTGGCTGTGTATGACAACA**CATTATGAAATCAGTGTTGTACATACACAGACACCACCCACTGTGCATG
GGAATCATCAGCCGTTGGTC

>ID948-5P
TTGTGGCTGTGTATGACAACA

>miRNA precursor
AAUAGUACAUGCAUGGAGGG**UUGUGGCUGUGUAUGACAACA**CAUUAUGAAAUCAGUGUUGUACAUACACAGACACCACCCACUGUGCAUGGGAAUCAUCAGCCGUUGGUC
.......(((((((((.((((.(((.((((((((((((((((...........))))))).))))))))).))).)))).)))))))))...((((........)))).

>Predicted STAR seq:
TTGTACATACACAGACACCACC

Complementarity: 17

Initial dG =   -47.70

```
          10        20        30         40
.-AAUAGUA         A    U   G         -|       AUUAU
         CAUGCAUGG GGGU GUG CUGUGUAUG ACAACAC     G
         GUACGUGUC CCCA CAC GACACAUAC UGUUGUG     A
\ -------         A    C   A         A^       ACUAA
        90        80        70        60        50



         100
  GGA    UCA
     AUCA   G
     UGGU   C
  C--    UGC
110
```

Reads in: lib-42, lib-45, lib-47, lib-48, lib-49, lib-50, lib-72
Number of reads="ID948",2,14,0,0,0,0,0

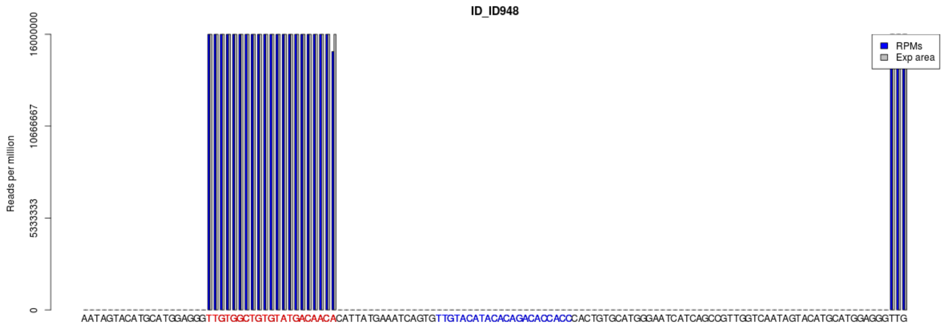

>ID949
TGTGAATGAGGAAGACATTTCATGCATAGGGGT**TGACGACTGTGTGGCTGCTGCA**TGTTATTTATTACTGTATCAGGCACTCAGTCGTTA
GCTCCTGTTCATCATATACC

>ID949-5P
TGACGACTGTGTGGCTGCTGCA

>miRNA precursor
UGUGAAUGAGGAAGACAUUUCAUGCAUAGGGGU**UGACGACUGUGUGGCUGCUGCA**UGUUAUUUAUUACUGUAUCAGGCACUCAGUCGUUAGCUCCUGUUCAUCAUAUACC
...(((((.......))))).(((.((((((((((((((((.(((.(((.((((.((........)))))).))).))).))))))))))))))))).))).......

>Predicted STAR seq:
TATCAGGCACTCAGTCGTTAGC

Complementarity: 17

```
 Initial dG =    -45.70

              10
ERROR 3: Strange structure
 Initial dG =    -45.70

              10
----    A      |   G
    UGUG AUGA--GGAA A
    AUAC UACU  CUUU C
CCAU    -    \ ^   A
.        100   20


                      30        40        50        60
               AUGC                 U   G   C    U  UAU
                   AUAGGGGUUGACGACUG GUG CUG UGCA GU   U
                   UGUCCUCGAUUGCUGAC CAC GAC AUGU CA   U
               ----                 U   G   U    -  UUA
                          90        80        70
```

Reads in: lib-42, lib-45, lib-47, lib-48, lib-49, lib-50, lib-72
Number of reads="ID949",12,23,0,1,1,2,0

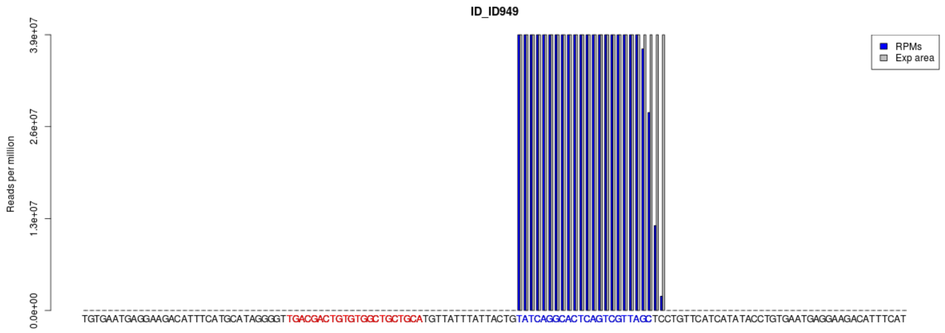

>ID950

GGTAATACCTGCATGGAGGG**TTATGGTAGAGTGGTGACAACA**AGTCACTGAATTTCTGTTGCCCACACCCTACCATTACCCACTGTGTAT
GGGAAAGCAAAACAGACA

>ID950-5P

TTATGGTAGAGTGGTGACAACA

>miRNA precursor

GGUAAUACCUGCAUGGAGGG**UUAUGGUAGAGUGGUGACAACA**AGUCACUGAAUUUCUGUUGCCCACACCCUACCAUUACCCACUGUGUAUGGGAAAGCAAAACAGACA

```
.((....((((((((((.((((.(((((((.((((..(((..((((......)))).)))..))...)).))))))).)))).))))))).))....))..........
```

>Predicted STAR seq:

TGTTGCCCACACCCTACCATTACC

Complementarity: 14

Initial dG =   -35.40

```
                  10        20        30          40
G---------  AAUA  -       A     U        A      |     AA
          GU    CC UGCAUGG GGGU AUGGUAG GUG--GUGAC  \
          CG    GG AUGUGUC CCCA UACCAUC CAC  CACUG  C
ACAGACAAAA  AAAG  U       A     U        C   \ ^     AA
      100        90        80        70
```

```
                                             50
                                             UG---   U
                                                  AAU U
                                                  UUG C
                                             ACCCG   U
                                                 60
```

Reads in: lib-42, lib-45, lib-47, lib-48, lib-49, lib-50, lib-72

Number of reads="ID950",1,2,0,0,0,0,0

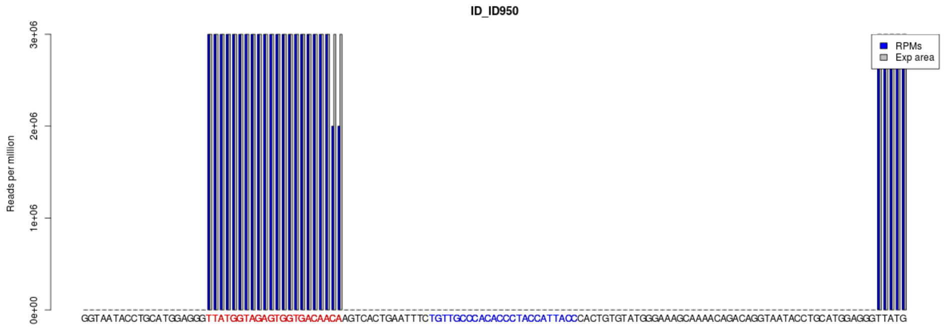

>ID951
CTGCAGATACAGTGCTAATTACCGCATAGTGGTTGACGGTTGTGTGGCTGTCACGTGTTATTTATTACCG**TCACAGACGCCCAACCGTTACCT**GCTGTGAATGGTATACCA

>ID951-3P
TCACAGACGCCCAACCGTTACCT

>miRNA precursor
CUGCAGAUACAGUGCUAAUUACCGCAUAGUGGUUGACGGUUGUGUGGCUGUCACGUGUUAUUUAUUACCG**UCACAGACGCCCAACCGUUACCU**GCUGUGAAUGGUAUACCA
..(((.......)))....((((((((((..(.(((((((((.(((.((((.(((.((........))))).)))).))).))))))))).)..))))))..))))).....

>Predicted STAR seq:
GGTTGACGGTTGTGTGGCTGTC

Complementarity: 16
ERROR 4: Strange structure
Initial dG =   -40.70

```
.-CU|  GAU
    GCA   \
    CGU   A
\ --^  GAC
        10
```

```
      20          30        40        50        60
  UAAU-     --     UG U         U   G    C   U  UAU
       UACCG  CAUAG  G UGACGGUUG GUG CUGU ACG GU   U
       AUGGU  GUGUC  C AUUGCCAAC CGC GACA UGC CA   U
  ACCAU     AA     GU C         C   A    C   -  UUA
 110       100        90        80        70
```

Reads in: lib-42, lib-45, lib-47, lib-48, lib-49, lib-50, lib-72
Number of reads="ID951",3,12,0,0,0,0,0

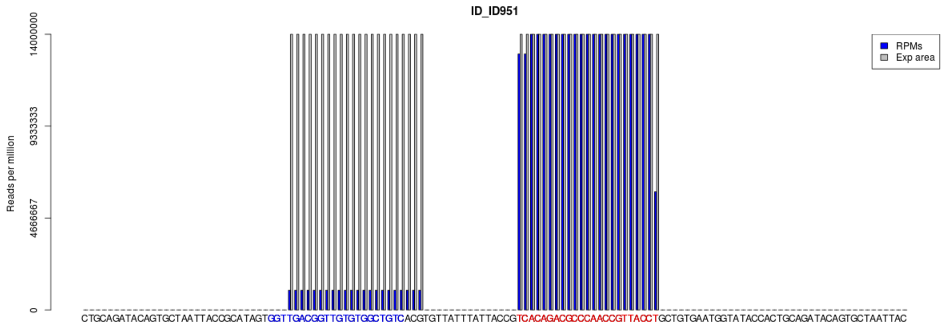

>ID952

CGTTTCAGTGGCAGTAATTCATGCATGGAGGGTGATGTGTGTGTGATGCCAACACGTTATAATAATTCTG**TTGCATTTGCACACACATCA**
CCCACTTTGCATGACAAGCA

>ID952-3P

TTGCATTTGCACACACATCACC

>miRNA precursor

CGUUUCAGUGGCAGUAAUUCAUGCAUGGAGGGUGAUGUGUGUGUGAUGCCAACACGUUAUAAUAAUUCUG**UUGCAUUUGCACACACAUCACC**CACUUUGCAUGACAAGCA
.(((.....))).((...(((((((.((.((((((((((((((..((((.((((.((((...))))..))))))).)..))))))))))))))).)).)))))))...)).

>Predicted STAR seq:

GTGATGTGTGTGTGATGCCAA

Complementarity: 17
ERROR 4: Strange structure
Initial dG =   -47.80

```
.-C|  UC
   GUU  \
   CGG  A
\ -^  UG
    10


        20        30        40         50         60
  A  AAU       U  A              UG -    C    C-    U
   GU   UCAUGCA GG GGGUGAUGUGUGUG  A UGC AACA  GUUA \
   CG   AGUACGU UC CCCACUACACACAC  U ACG UUGU  UAAU A
  A  AAC       U  A              GU U    -    CU    A
110       100        90        80         70
```

Reads in: lib-42, lib-45, lib-47, lib-48, lib-49, lib-50, lib-72

Number of reads="ID952",1,5,0,0,1,0,0

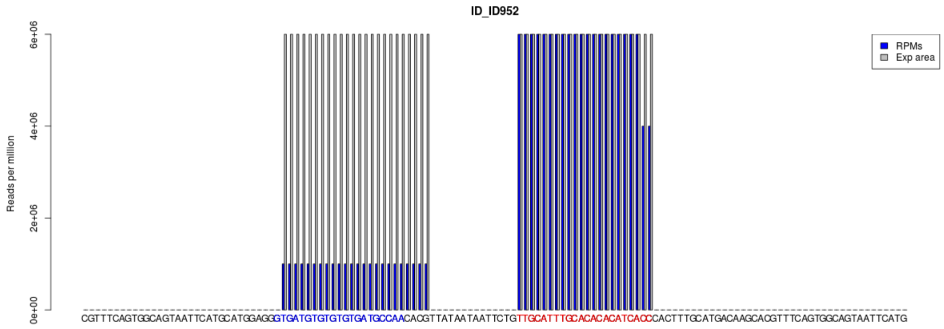

>ID954

TGTTGTGTGCTGGTCAGCAG**TGGGGGCTGCTGCAGACGAGCAT**AGTGTTTTGAGGTGCGCGCCTGCAGCAGCCATCCCGCTGCTGGCCAC
CGAACAGCCGTCCGCTGCTG

>ID954-5P

TGGGGGCTGCTGCAGACGAGCAT

>miRNA precursor

UGUUGUGUGCUGGUCAGCAG**UGGGGGCUGCUGCAGACGAGCAU**AGUGUUUUGAGGUGCGCGCCUGCAGCAGCCAUCCCGCUGCUGGCCACCGAACAGCCGUCCGCUGCUG
.(((((.((.((((((((((((((((((((((((((.((.((((..(.....)..)))).)).))))))))))))..)))))))))))))))).)).))))) ....

>Predicted STAR seq:

GCGCGCCTGCAGCAGCCATCCCGCT

Complementarity: 19

Initial dG =   -65.40

```
                   10        20          30        40
U-----------     G  C              --|           A  A    AG GU
            GUUGU UG UGGUCAGCAGUGGG  GGCUGCUGCAG CG GCAU  U  \
            CGACA GC ACCGGUCGUCGCCC  CCGACGACGUC GC CGUG  G  U
GUCGUCGCCUGC     A  C              UA^           C  G    GA UU
.       100        90        80        70        60        50
```

Reads in: lib-42, lib-45, lib-47, lib-48, lib-49, lib-50, lib-72

Number of reads="ID954",4,6,0,0,0,0,0

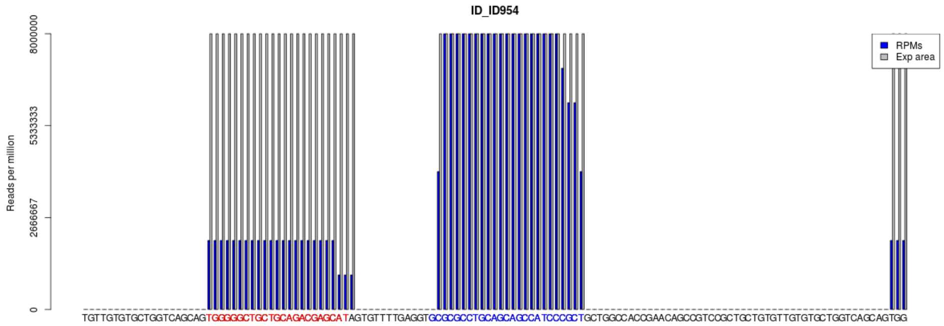

>ID956
ATTCTGTGGACCAATATCTTTCTTGCATCCCGAGCGACGGAAGGGAAGGCTCTAGGAGTTTTTAAATTAC**TCAGGACCGTCCCTTCCGTT**
TCTTGGTGTTCAAGATAAACGTC

>ID956-3P
TCAGGACCGTCCCTTCCGTTTCT

>miRNA precursor
AUUCUGUGGACCAAUAUCUUUCUUGCAUCCCGAGCGACGGAAGGGAAGGCUCUAGGAGUUUUUAAAUUAC**UCAGGACCGUCCCUUCCGUUUCU**UGGUGUUCAAGAUAAACGUC
........(((...((((((....(((..(((((.(((((((((((.((.(((..((((.........)))).))))).))))))))))).))))))))..))))))...)))

>Predicted STAR seq:
GCGACGGAAGGGAAGGCTCTAGGA

Complementarity: 18

Initial dG =   -41.70

```
        10        20        30        40        50        60
AUUCUGUG|  CAA      UCUU   UC      C           A  C   AG    UUUU
        GAC   UAUCUU    GCA  CCGAG GACGGAAGGGA GG UCU  GAGU    \
        CUG   AUAGAA    UGU  GGUUC UUGCCUUCCCU CC AGG  CUCA    A
--------^  CAA      CU--   --      U           G  -   A-    UUAA
         110       100            90        80          70
```

Reads in: lib-42, lib-45, lib-47, lib-48, lib-49, lib-50, lib-72
Number of reads="ID956",8,12,0,1,0,3,0

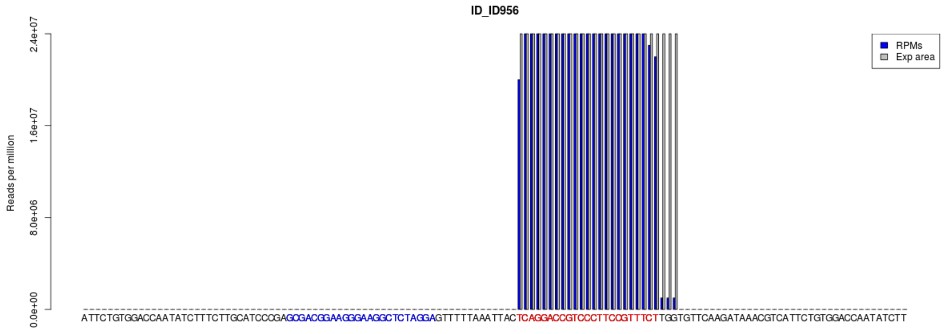

>ID957
CTGCAATGAAGAAACTGATGTATGCTTAGGGGGTGGCGATTACGTGAGTGAAACATGTTATTTATTATTG**TATCACTCTCCTAATCGTCA**
CCTCCTGTGCATAGCATACTG

>ID957-3P
TATCACTCTCCTAATCGTCACC

>miRNA precursor
CUGCAAUGAAGAAACUGAUGUAUGCUUAGGGGGUGGCGAUUACGUGAGUGAAACAUGUUAUUUAUUAUUG**UAUCACUCUCCUAAUCGUCACC**UCCUGUGCAUAGCAUACUG
```
................((((((((.((((((((((((((((...((((((.(((.............))).))))))...)))))))))))))))).))))).))).....
```

>Predicted STAR seq:
GTGGCGATTACGTGAGTGAA

Complementarity: 16

Initial dG =   -44.80

```
        10        20         30        40        50        60
CUGCAAUGAAGAAACUG   -|    U                CGU      A   UGUUAU
                 AUG UAUGC UAGGGGGUGGCGAUUA   GAGUGA ACA      \
                 UAC AUACG GUCCUCCACUGCUAAU   CUCACU UGU      U
GUCA-------------   G^    U                CCU      A   UAUUAU
 .                    100        90        80        70
```

Reads in: lib-42, lib-45, lib-47, lib-48, lib-49, lib-50, lib-72
Number of reads="ID957",6,35,0,0,1,0,0

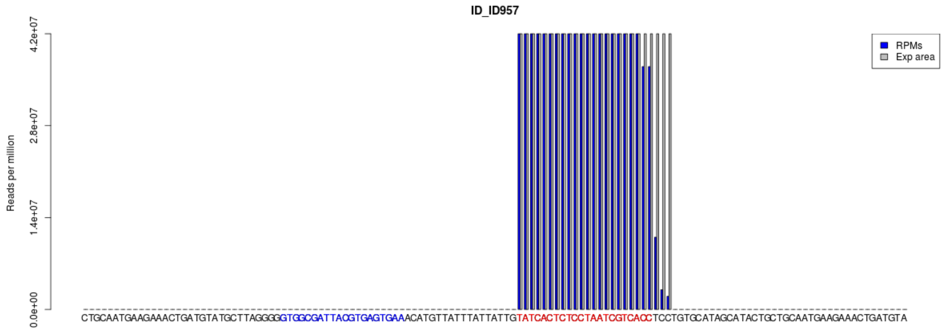

>ID958
TGTTTCAATGACAGTATTTCATGCCTGGAGGG**TGATGTCTGTGTGGGTCCAAC**ACGTTATAAAACTACTGTTGAACATACACGGCCATCT
CCCATTGTGCATGATAAC

>ID958-5P
TGATGTCTGTGTGGGTCCAAC

>miRNA precursor
UGUUUCAAUGACAGUAUUUCAUGCCUGGAGGG**UGAUGUCUGUGUGGGUCCAAC**ACGUUAUAAAACUACUGUUGAACAUACACGGCCAUCUCCCAUUGUGCAUGAUAAC
((((.....)))).....((((((.(((.(((.((((.(((((((.((.(((((.((.........))))))).)).))))))).)))).))).))).))))))....

>Predicted STAR seq:
TGAACATACACGGCCATCTCC

Complementarity: 15

Initial dG = -35.80

ERROR 3: Strange structure
Initial dG = -35.80

```
  |   UC
--UGUU  \
  ACAG  A
\ ^   UA
    10


       20        30        40        50        60
  GUAUU      C   A   U    U       G  C     C  UAUA
       UCAUGC UGG GGG GAUG CUGUGUG GU CAACA GU    \
       AGUACG GUU CCC CUAC GGCACAU CA GUUGU CA    A
  CAAU-      U   A   U    C       A  A     -  UCAA
         100        90        80        70
```

Reads in: lib-42, lib-45, lib-47, lib-48, lib-49, lib-50, lib-72
Number of reads="ID958",0,0,0,0,0,0,0

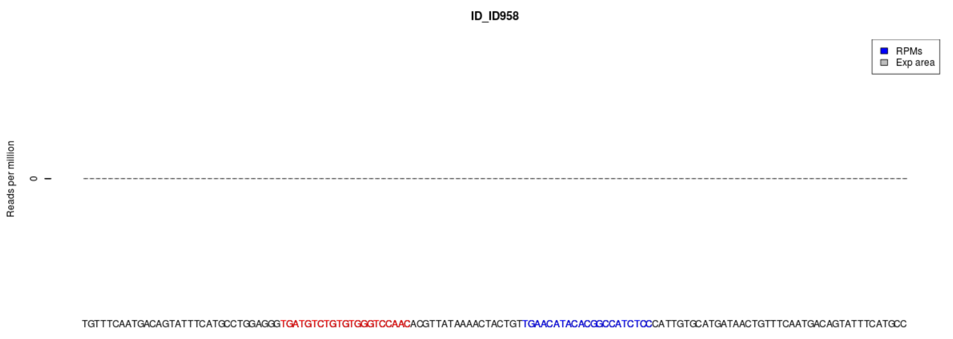

>ID959
ATTCTGAAGACCAATATGTTTCGTGAATCCCGAGCGACGGAAGCGAGGCCTGTACAAGTTTTTAAATTAC**TTGAGTACTCCCTTCCGTCA**
CTCGGTGTTCTCGATAAGCGTC

>ID959-3P
TTGAGTACTCCCTTCCGTCACT

>miRNA precursor
AUUCUGAAGACCAAUAUGUUUCGUGAAUCCCGAGCGACGGAAGCGAGGCCUGUACAAGUUUUUAAAUUAC**UUGAGUACUCCCUUCCGUCACU**CGGUGUUCUCGAUAAGCGUC
```
..............((((((((.(((..(((((.((((((((.(((..((...(((((.........)))))))..))).)))))))).)))))..))).))).)).))))).
```

>Predicted STAR seq:
GCGACGGAAGCGAGGCCTGTACAA

Complementarity: 16

Initial dG =   -42.00

```
        10        20          30        40        50        60
AUUCUGAAGACCAAU     --|  U   UC      C        C   GC  GUA     UUUU
               AUGUU  UCG GAA  CCGAG GACGGAAG GAG  CU   CAAGU    \
               UGCGA  AGC CUU  GGCUC CUGCCUUC CUC  GA   GUUCA    A
C--------------     AU^  U   GU      A        C   AU  ---     UUAA
              110       100        90        80           70
```

Reads in: lib-42, lib-45, lib-47, lib-48, lib-49, lib-50, lib-72
Number of reads="ID959",8,1,0,1,0,0,0

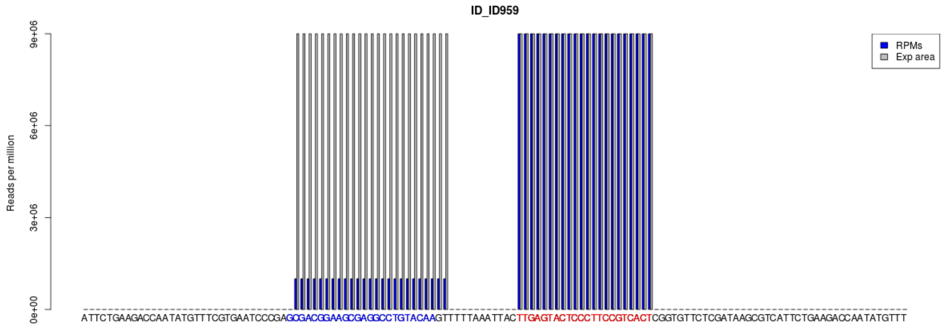

>ID96
GTAAACAAATGGGTCTACAAGTGCATGACCGTCATCAGAGTTGTATCTGGCACATGTCTAACAAAACTG**TGGCAGGTACATCTTTGATGG**
CTGTTGTGCGTTCCTGCATC

>ID96-3P
TGGCAGGTACATCTTTGATGG

>miRNA precursor
GUAAACAAAUGGGUCUACAAGUGCAUGACCGUCAUCAGAGUUGUAUCUGGCACAUGUCUAACAAAACUG**UGGCAGGUACAUCUUUGAUGG**CUGUUGUGCGUUCCUGCAUC
(((.((......)).)))....(((..((.((((((((((.((((((((.((((.((........)))))).)))))))).)))))))))).))..)))...........

>Predicted STAR seq:
CATCAGAGTTGTATCTGGCA

Complementarity: 17
ERROR 4: Strange structure
Initial dG =   -44.20

```
  |  A  AA
--GUA AC  A
  CAU UG  U
\ ^  C  GG
         10



  20               30        40        50        60
  AAGU-------   UG  C          U        G     U  CUA
           GCA  AC GUCAUCAGAG UGUAUCUG CACA GU   A
           CGU  UG CGGUAGUUUC ACAUGGAC GUGU CA   C
  CUACGUCCUUG   GU  U          U        G     -  AAA
110       100        90        80        70
```

Reads in: lib-42, lib-45, lib-47, lib-48, lib-49, lib-50, lib-72
Number of reads="ID96",1,1,0,0,0,0,0

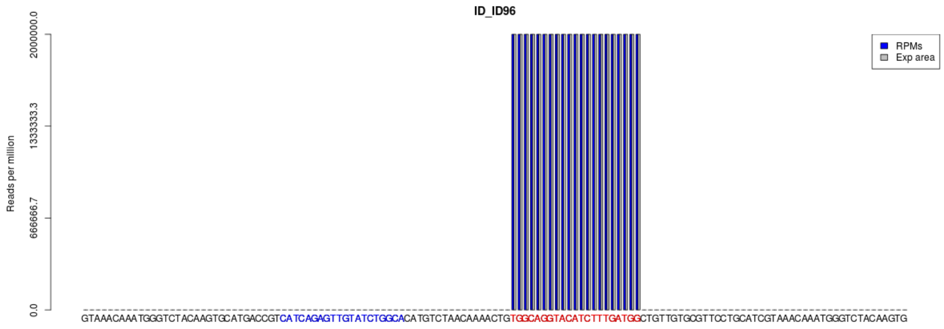

>ID960
CTGCTAATAAATCGCTAATGCATGTATAGGAGCTGGCGATTATGTGTGTGTTGCATGTTATTTAGTACTG**TCACATACGCCCAATCGTCA**
GCTGCTGTGCATGGTGTCCCA

>ID960-3P
TCACATACGCCCAATCGTCAGCT

>miRNA precursor
CUGCUAAUAAAUCGCUAAUGCAUGUAUAGGAGCUGGCGAUUAUGUGUGUGUUGCAUGUUAUUUAGUACUG**UCACAUACGCCCAAUCGUCAGCU**GCUGUGCAUGGUGUCCCA
```
...................((((((((.((((((((((((..((((((((.(((.((........))))).))))))))..)))))))))))).)))))))))........
```

>Predicted STAR seq:
AGCTGGCGATTATGTGTGTGTT

Complementarity: 18

Initial dG = -45.60

```
        10        20        30        40        50        60
CUGCUAAUAAAUCGCUAAUG|        G           AU         U   U  UAU
                    CAUGUAUAG AGCUGGCGAUU  GUGUGUGU GCA GU   U
                    GUACGUGUC UCGACUGCUAA  CGCAUACA UGU CA   U
ACCCUGUG------------^        G           CC         C   -  UGA
 .                   100        90        80        70
```

Reads in: lib-42, lib-45, lib-47, lib-48, lib-49, lib-50, lib-72
Number of reads="ID960",25,19,0,2,0,1,0

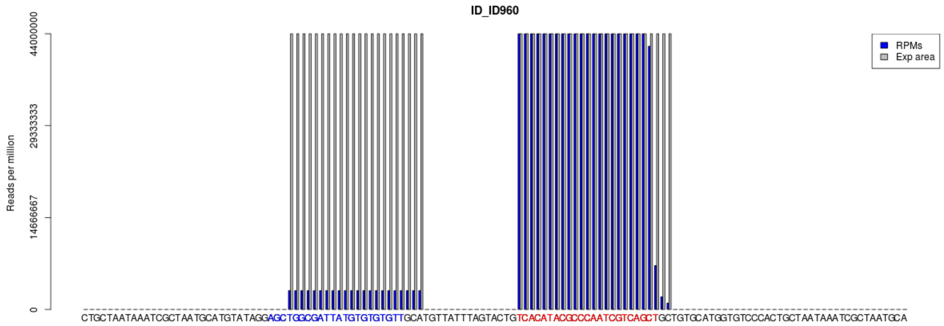

>ID961
GGTAATACCTGCATGGAGGG**TGATGGTAGAGTGGTGCCAACA**TGTCACTGAATTTCTGTTGCCCCCACCCTACTATCACCCACTGTGTAT
GGGACGAAAATAATACACTA

>ID961-5P
TGATGGTAGAGTGGTGCCAACA

>miRNA precursor
GGUAAUACCUGCAUGGAGGG**UGAUGGUAGAGUGGUGCCAACA**UGUCACUGAAUUUCUGUUGCCCCCACCCUACUAUCACCCACUGUGUAUGGGACGAAAAUAAUACACUA
.......((((((((.((((((((((((.((((...(((((..............)))))...)))).))))))))))))).))))))).)).................

>Predicted STAR seq:
TTGCCCCCACCCTACTATCACC

Complementarity: 16

Initial dG =   -40.90

```
                    10        20        30        40
GGUAAUA-----------  -|      A            A    UGC      UGUCAC
                  CC UGCAUGG GGGUGAUGGUAG GUGG   CAACA      U
                  GG AUGUGUC CCCACUAUCAUC CACC   GUUGU      G
AUCACAUAAUAAAAGCAG  U^      A            C    CCC      CUUUAA
.       100        90        80        70        60        50
```

Reads in: lib-42, lib-45, lib-47, lib-48, lib-49, lib-50, lib-72
Number of reads="ID961",3,1,0,0,0,0,0

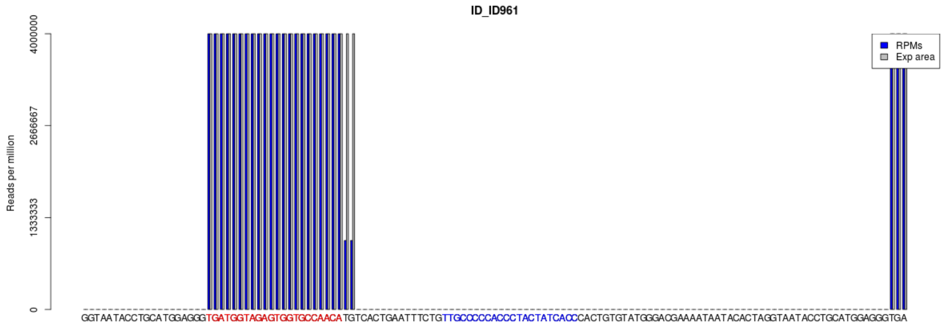

>ID962
TATGCTTCGTGAATCCCGAGCGACGAAAGCGAGGTCTGTACAAGTTTTTAAATTACTTGAGATCTCCCTTCCGTCACACGGTGTTCTCGA
TAAGCGTCACGTATGCACGACTCAA

>ID962-5P
CGACGAAAGCGAGGTCTGTACAAG

>miRNA precursor
UAUGCUUCGUGAAUCCCGAG**CGACGAAAGCGAGGUCUGUACAAG**UUUUUAAAUUACUUGAGAUCUCCCUUCCGUCACACGGUGUUCUCGAUAAGCGUCACGUAUGCACGACUCAA
.((((((((.(((..(((...((((.(((.(((((((...(((((.........))))))))))))).))).)))))...)))..))).))).)))..)))))..((((....)))......

>Predicted STAR seq:
TGAGATCTCCCTTCCGTCACACG

Complementarity: 16

Initial dG =   -36.10

```
            10        20        30        40
.-U     --|  U   UC   AGC    A   C       GUA     UUUU
   AUGCU  UCG GAA  CCG   GACG AAG GAGGUCU   CAAGU    \
   UGCGA  AGC CUU  GGC   CUGC UUC CUCUAGA   GUUCA    A
\ -     AU^  U   GU   ACA    C   C       ---     UUAA
         90        80        70        60



      100
  CA----   A
        CGU U
        GCA G
  AACUCA   C
     110
```

Reads in: lib-42, lib-45, lib-47, lib-48, lib-49, lib-50, lib-72
Number of reads="ID962",11,31,0,0,1,0,0

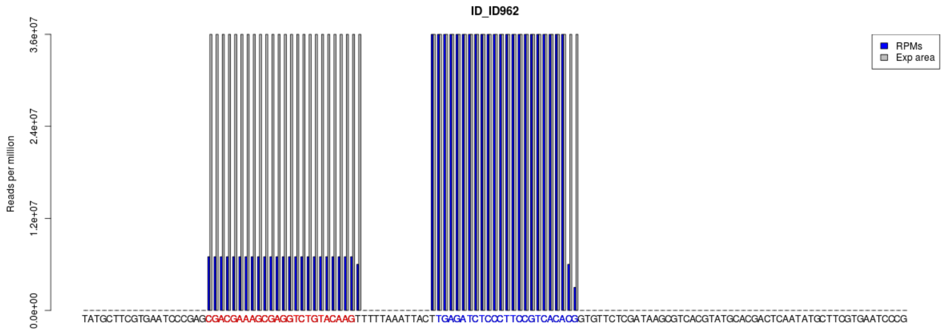

>ID963
ATATTGGAGTGATAGCAATTCATACATGGAGGGTGATGACTGTCTCTGCCAACACGTTATACAATCACTG**TTGTACAGACCCAGTTATCA**
CTCACTGTGTATGGTAAGCAT

>ID963-3P
TTGTACAGACCCAGTTATCACT

>miRNA precursor
AUAUUGGAGUGAUAGCAAUUCAUACAUGGAGGGUGAUGACUGUCUCUGCCAACACGUUAUACAAUCACUG**UUGUACAGACCCAGUUAUCACU**CACUGUGUAUGGUAAGCAU
```
..............((...((((((((((.((((((((((((..((((.(((((.((.........))))))).)))).))))))))))))).))))))))))...))..
```

>Predicted STAR seq:
GTGATGACTGTCTCTGCCAA

Complementarity: 16

Initial dG =   -41.50

```
        10        20        30        40         50        60
AUAUUGGAGUGAUA  AAU          A            UC     C-|    C  UAUA
              GC   UCAUACAUGG GGGUGAUGACUG  UCUG  CAACA GU    \
              CG   GGUAUGUGUC CUCACUAUUGAC  AGAC  GUUGU CA    C
UA------------  AAU          A            CC     AU^    -  CUAA
 .                    100        90        80        70
```

Reads in: lib-42, lib-45, lib-47, lib-48, lib-49, lib-50, lib-72
Number of reads="ID963",16,13,0,0,0,1,0

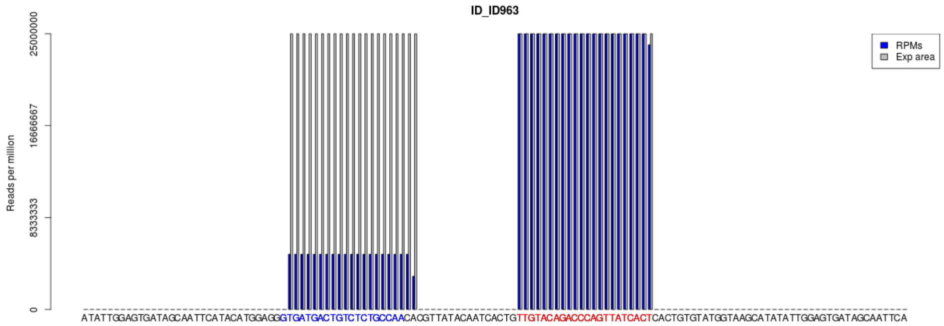

>ID964
GCCTAATGTGGTCCGCGGTT**AGCATGATCAGTGGGCACGA**ATTGTGAATTGGTCATTTCGCTCCACTGATCATGCAACACTGCGCTCCACCCAGTTCAACGTCGACGC

>ID964-5P
AGCATGATCAGTGGGCACGA

>miRNA precursor
GCCUAAUGUGGUCCGCGGUU**AGCAUGAUCAGUGGGCACGA**AUUGUGAAUUGGUCAUUUCGCUCCACUGAUCAUGCAACACUGCGCUCCACCCAGUUCAACGUCGACGC
.......((((..((((((..((((((((((((((..((((..((((.....)))))))).))))))))))))))...))))))..)))).................

>Predicted STAR seq:
GCTCCACTGATCATGCAACAC

Complementarity: 15

Initial dG =   -44.10

```
                   10         20          30         40
GCCUAAU-----------    UC       UA-|              CA     UU    AU
                  GUGG  CGCGGU    GCAUGAUCAGUGGG  CGAA  GUGA  \
                  CACC  GCGUCA    CGUACUAGUCACCU  GCUU  UACU  U
CGCAGCUGCAACUUGACC    UC       CAA^              C-     --    GG
      100        90         80         70         60
```

Reads in: lib-42, lib-45, lib-47, lib-48, lib-49, lib-50, lib-72
Number of reads="ID964",9,35,0,0,0,1,0

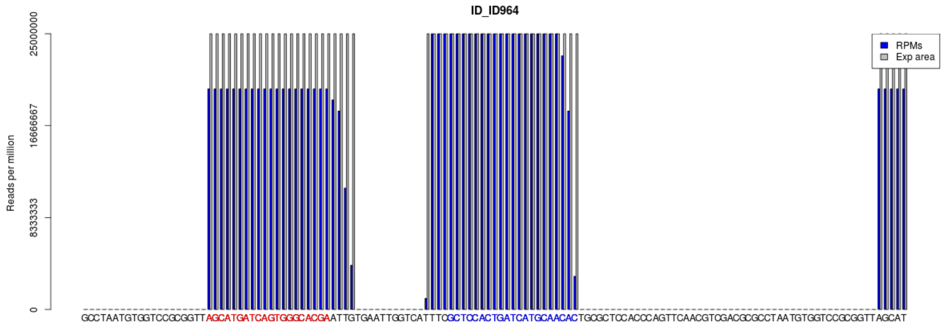

>ID965
TGTTGCACTGTCAGTAATTCATGCATGGAGGGTGATGTCTGTGTGGGTCCAACACATTATGAGATTACTG**TTGAACACACACGGCTATCA**
CTCACTGTGCATGATAACCAT

>ID965-3P
TTGAACACACACGGCTATCACT

>miRNA precursor
UGUUGCACUGUCAGUAAUUCAUGCAUGGAGGGUGAUGUCUGUGUGGGUCCAACACAUUAUGAGAUUACUG**UUGAACACACACGGCUAUCACU**CACUGUGCAUGAUAACCAU
```
.(((((.......)))))(((((((((.((((((((.(((((((.((.(((((.............))))).)).))))))).)))))))).))))))))).......
```

>Predicted STAR seq:
GTGATGTCTGTGTGGGTCCAA

Complementarity: 17
ERROR 4: Strange structure
Initial dG = -41.90

```
.-U|    ACU
   GUUGC   \
   UAAUG   G
\ -^    ACU
```

```
         20        30        40        50        60
  -------          A        U       G  C      CAUUAU
         UCAUGCAUGG GGGUGAUG CUGUGUG GU CAACA      G
         AGUACGUGUC CUCACUAU GGCACAC CA GUUGU      A
  UACCAAU          A        C       A  A      CAUUAG
 110       100        90        80        70
```

Reads in: lib-42, lib-45, lib-47, lib-48, lib-49, lib-50, lib-72
Number of reads="ID965",3,6,0,0,3,0,0

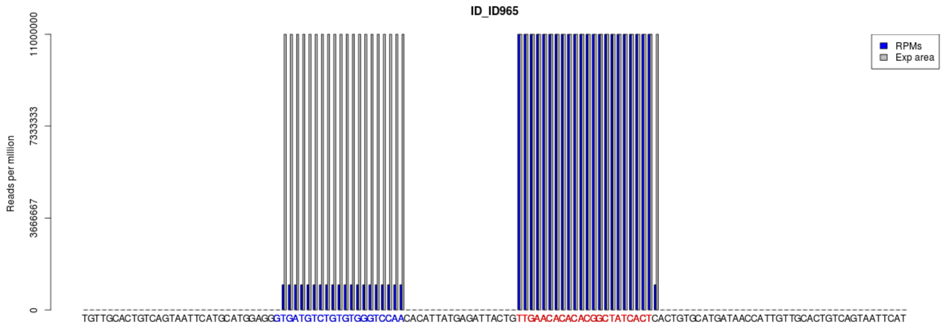

>ID966
CTAATACATGTCATGGAGGT**TGACGGATGTCTGACTGGAACAT**GTTATTTTATATTGTTCAAGTCACCCATTCGTTAGCCGCCCTGCATG
GTAGACGACGAAGATGCT

>ID966-5P
TGACGGATGTCTGACTGGAACAT

>miRNA precursor
CUAAUACAUGUCAUGGAGGU**UGACGGAUGUCUGACUGGAACAU**GUUAUUUUAUAUUGUUCAAGUCACCCAUUCGUUAGCCGCCCUGCAUGGUAGACGACGAAGAUGCU
......((((.((.((.((((((((((((..(((((.((((((((......))).))))).))))).)).))))))))))))).)).))))))..................

>Predicted STAR seq:
TGTTCAAGTCACCCATTCGTTAGC

Complementarity: 18

Initial dG =   -40.20

```
                    10        20        30        40
CUAAUA------------|   U  U  A            UC      G     -   UA
                  CAUG CA GG GGUUGACGGAUG  UGACU GAACA UGU  U
                  GUAC GU CC CCGAUUGCUUAC  ACUGA CUUGU AUA  U
UCGUAGAAGCAGCAGAUG^   -  C  G            CC      A     U   UU
      100        90         80        70        60        50
```

Reads in: lib-42, lib-45, lib-47, lib-48, lib-49, lib-50, lib-72
Number of reads="ID966",4,6,0,0,0,0,0

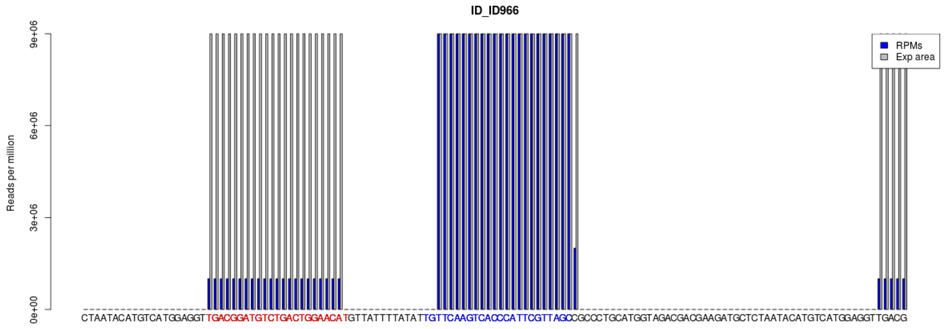

>ID967

ATGTTGGAGTGAAAGTAATTCATGCATGGAGGGTGATGGCTGTGTCTGCCAACACGTTATAAAATCACTG**TTGTTAAGACCCAGCCATCA**
CTCACTGTGCATGGCAACC

>ID967-3P

TTGTTAAGACCCAGCCATCACT

>miRNA precursor

AUGUUGGAGUGAAAGUAAUUCAUGCAUGGAGGGUGAUGGCUGUGUCUGCCAACACGUUAUAAAAUCACUG**UUGUUAAGACCCAGCCAUCACU**CACUGUGCAUGGCAACC
```
..((((.............((((((((((.((((((((((((.((((..(((((.((.........)))))))...)))).)))))))))))).)))))))))))))).
```

>Predicted STAR seq:

GTGATGGCTGTGTCTGCCAA

Complementarity: 16

Initial dG =   -45.60

```
        10        20        30        40         50         60
AU|   GAGUGAAAGUAAU          A            U    GC-     C  UAUA
  GUUG             UCAUGCAUGG GGGUGAUGGCUG GUCU   CAACA GU    \
  CAAC             GGUACGUGUC CUCACUACCGAC CAGA   GUUGU CA    A
C-^   -------------          A            C    AUU     -  CUAA
                     100        90        80        70
```

Reads in: lib-42, lib-45, lib-47, lib-48, lib-49, lib-50, lib-72

Number of reads="ID967",8,19,0,0,0,0,0

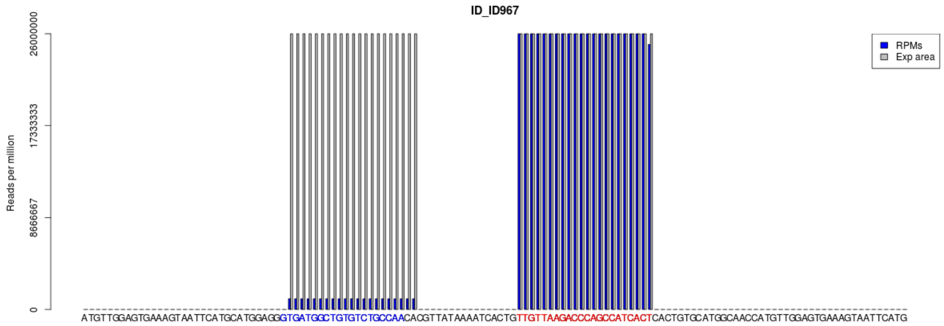

>ID968
TTACATTCATGCATAGAAAG**TAATGACTGCGTGACATTGACA**CATTATTAACTACTGTTGATGTTACGTAGTCATTACTTTCTGTGCATG
GCTTGCTGCATTCACTGGTTCT

>ID968-5P
TAATGACTGCGTGACATTGACA

>miRNA precursor
UUACAUUCAUGCAUAGAAAG**UAAUGACUGCGUGACAUUGACA**CAUUAUUAACUACUGUUGAUGUUACGUAGUCAUUACUUUCUGUGCAUGGCUUGCUGCAUUCACUGGUUCU
......((((((((((((((((((((((((((((((((..(((.............)))..)))))))))))))))))))))))))))))))))....................

>Predicted STAR seq:
TTGATGTTACGTAGTCATTACT

Complementarity: 18

Initial dG = -54.00

```
                       10        20        30        40
UUACAU---------------|                             UG   CAUUAU
                     UCAUGCAUAGAAAGUAAUGACUGCGUGACAU  ACA      \
                     GGUACGUGUCUUUCAUUACUGAUGCAUUGUA  UGU      U
UCUUGGUCACUUACGUCGUUC^                             GU   CAUCAA
110       100       90        80        70        60        50
```

Reads in: lib-42, lib-45, lib-47, lib-48, lib-49, lib-50, lib-72
Number of reads="ID968",28,77,1,0,2,0,0

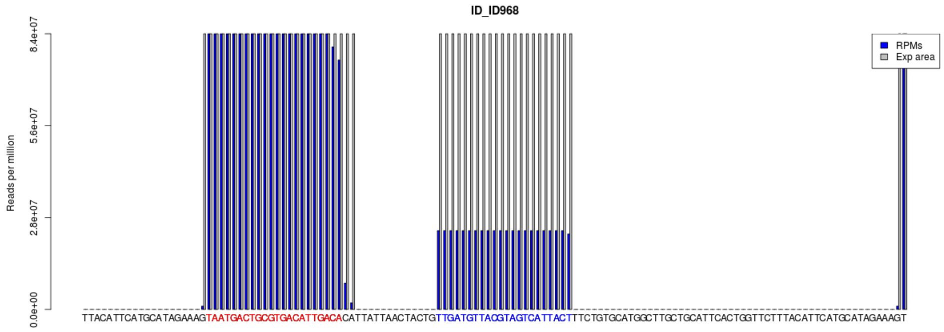

>ID97
GAGAATGGTTGTCTGTGGTA**CGGTGGAAACCTACACGAACACG**GTATTTGTGCAGTGGCGTGTGCGTTTCTACCTTACTACGTGCTGCCT
CCTACATCGTCTACGAGTCTCAT

>ID97-5P
CGGTGGAAACCTACACGAACACG

>miRNA precursor
GAGAAUGGUUGUCUGUGGUA**CGGUGGAAACCUACACGAACACG**GUAUUUGUGCAGUGGCGUGUGCGUUUCUACCUUACUACGUGCUGCCUCCUACAUCGUCUACGAGUCUCAU
((((..(((.((.(((((((.(((((((((.((((((..(((.(((....))).))).)))))).))))))))).))))))).)).)))........(((....))).))))..

>Predicted STAR seq:
GGCGTGTGCGTTTCTACCTTA

Complementarity: 16

Initial dG =   -46.30

```
            10         20         30         40
--    .-AU|  U  C       C         C      AA   G   U
  GAGA    GGU GU UGUGGUA GGUGGAAAC UACACG  CAC GUA U
  CUCU    CCG CG GCAUCAU CCAUCUUUG GUGUGC  GUG CGU U
UA    \ --^  U  U       U         C      G-   A   G
 110              80         70         60         50


       90       100
        UCCUACA   U
               UCG C
               AGC U
        G------   A
```

Reads in: lib-42, lib-45, lib-47, lib-48, lib-49, lib-50, lib-72
Number of reads="ID97",1,2,0,0,0,0,0

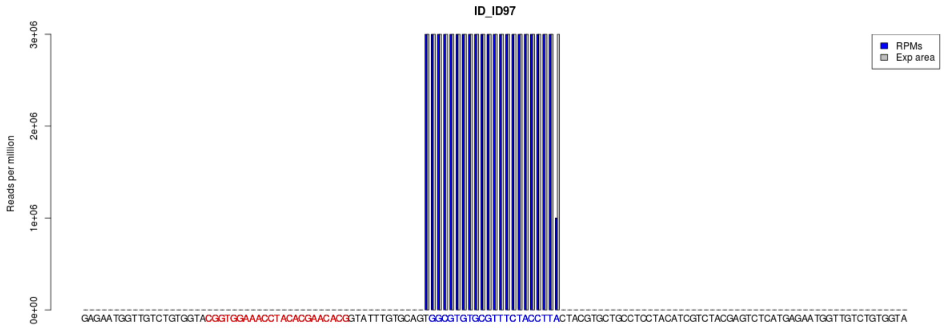

>ID970
TGGTTGAGTGGTGGTAAGACCTACATCGAAGGTGGTGGTAGAGTAGTGCCAACACATTATTTAATTGCTG**TGTGGCCTGCCCTACCATCA**
CTCTCTGTGTATGGGCTAGGGA

>ID970-3P
TGTGGCCTGCCCTACCATCACT

>miRNA precursor
UGGUUGAGUGGUGGUAAGACCUACAUCGAAGGUGGUGGUAGAGUAGUGCCAACACAUUAUUUAAUUGCUG**UGUGGCCUGCCCUACCAUCACU**CUCUGUGUAUGGGCUAGGGA
```
................((.(((((((.((..((((((((((.((((.((((.((((.((......)).))))))))))))).)))))))))))..)).))))).)).)).....
```

>Predicted STAR seq:
GGTGGTGGTAGAGTAGTGCCAACA

Complementarity: 18

Initial dG =   -44.50

```
        10        20         30        40        50        60
UGGUUGAGUGGUGGUA  A  -|    C  AG          A    U    A    U  UU
                AG CC UACAU GA  GUGGUGGUAG GUAG GCCA CACA UA  U
                UC GG AUGUG CU  CACUACCAUC CGUC CGGU GUGU GU  A
AGGGA-----------  G  U^    U  CU          C    -    -    C  UA
110                  100       90        80          70
```

Reads in: lib-42, lib-45, lib-47, lib-48, lib-49, lib-50, lib-72
Number of reads="ID970",0,1,0,0,0,0,0

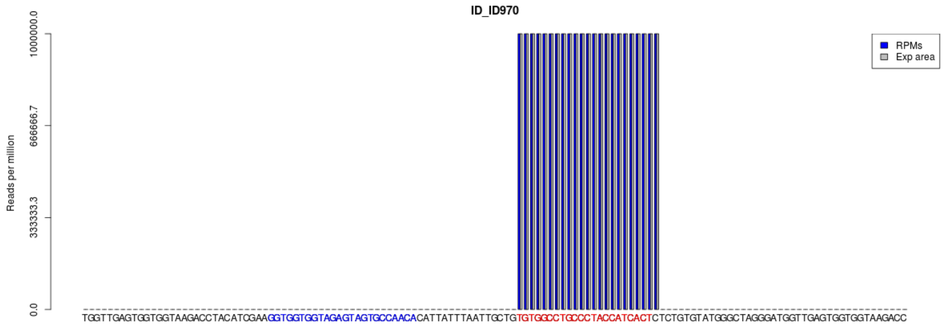

>ID971
GTCTATTACGATTCTAATACATGCCATGGAGGTTGGCGGAAGTTTGCCTGTTACACGTTATTTTGTACTG**TAACAGACACTCTGACGTCA**
GCTGCCGTGCATAGTACACTA

>ID971-3P
TAACAGACACTCTGACGTCAGC

>miRNA precursor
GUCUAUUACGAUUCUAAUACAUGCCAUGGAGGUUGGCGGAAGUUUGCCUGUUACACGUUAUUUUGUACUG**UAACAGACACUCUGACGUCAGC**UGCCGUGCAUAGUACACUA
.................(((((((.((((.((((((((..((..((.((((((((.((........)))))))))).))..))..)))))))).)))))))).))).....

>Predicted STAR seq:
TTGGCGGAAGTTTGCCTGTTA

Complementarity: 15

Initial dG =   -38.20

```
        10        20         30        40        50        60
GUCUAUUACGAUUCUAA   -|   C    A        GA  UU  C        C  UAU
                 UAC AUGC AUGG GGUUGGCG  AG  UG CUGUUACA GU   U
                 AUG UACG UGCC UCGACUGC  UC  AC GACAAUGU CA   U
AUCAC------------   A^   -    G        AG  UC  A        -  UGU
 .                    100         90        80        70
```

Reads in: lib-42, lib-45, lib-47, lib-48, lib-49, lib-50, lib-72
Number of reads="ID971",4,20,0,0,0,0,0

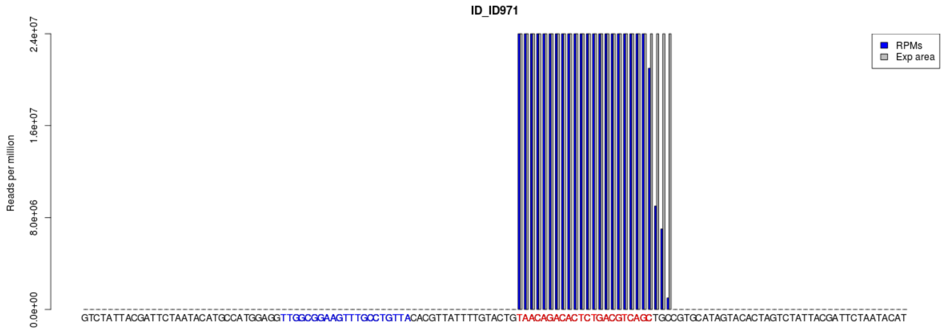

>ID973
ACTTCAAAGATAATTCTAATAATGTATAGGGACTGGCGATTGCATGGCTGTCAAACATAGATTATTACTG**TTTCAGTCACCCAATCGTCA**
GCTCCTGTGCATGGGATACCA

>ID973-3P
TTTCAGTCACCCAATCGTCAGC

>miRNA precursor
ACUUCAAAGAUAAUUCUAAUAAUGUAUAGGGACUGGCGAUUGCAUGGCUGUCAAACAUAGAUUAUUACUG**UUUCAGUCACCCAAUCGUCAGC**UCCUGUGCAUGGGAUACCA
```
.......(((....)))....(((((((((..((((((((((..((((((..(((((...........)))))))))))..)))))))))))..)))))))))((....)).
```

>Predicted STAR seq:
ACTGGCGATTGCATGGCTGTCAAA

Complementarity: 17
ERROR 4: Strange structure
Initial dG =   -40.40

```
          10
.-ACUUCAA|  U
         AGA A
         UCU A
\ -------^  U




     20        30        40        50        60
  .-AAUA         GA          CA       UC      UAGAU
        AUGUAUAGG  CUGGCGAUUG  UGGCUG  AAACA      \
        UACGUGUCC  GACUGCUAAC  ACUGAC  UUUGU      U
  \ ----         UC          CC       --      CAUUA
        100        90         80          70
```

Reads in: lib-42, lib-45, lib-47, lib-48, lib-49, lib-50, lib-72
Number of reads="ID973",14,10,1,1,0,0,0

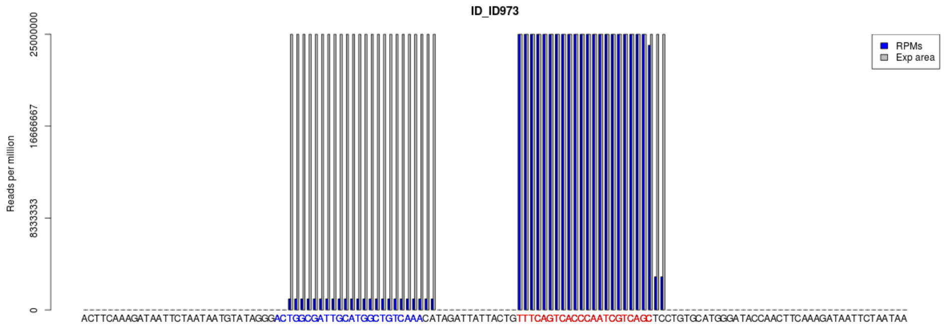

>ID975
CTGCAATGAAGAAACTGATGTATGCGTAGGGGGTGGCGATTGCGTGAGTGAAACAAGTTATTTATTATTG**TATCACCCTCCTAATCGTCA**
GCTCCTGTGCATAGCATA

>ID975-3P
TATCACCCTCCTAATCGTCAGC

>miRNA precursor
CUGCAAUGAAGAAACUGAUGUAUGCGUAGGGGGUGGCGAUUGCGUGAGUGAAACAAGUUAUUUAUUAUUG**UAUCACCCUCCUAAUCGUCAGC**UCCUGUGCAUAGCAUA
.................(((((((((((((((.(((((((((.....((((.((((...........)))).)))).....))))))))).))))))))))))).))).

>Predicted STAR seq:
GTGGCGATTGCGTGAGTGAA

Complementarity: 13

Initial dG = -36.60

```
        10        20         30        40        50        60
CUGCAAUGAAGAAACUG   -|           G         CGUGA    A    GUUAU
                 AUG UAUGCGUAGGGG UGGCGAUUG     GUGA ACAA     \
                 UAC AUACGUGUCCUC ACUGCUAAU     CACU UGUU     U
A----------------   G^           G         CCUCC    A    AUUAU
                      100        90        80        70
```

Reads in: lib-42, lib-45, lib-47, lib-48, lib-49, lib-50, lib-72
Number of reads="ID975",6,13,0,0,0,0,0

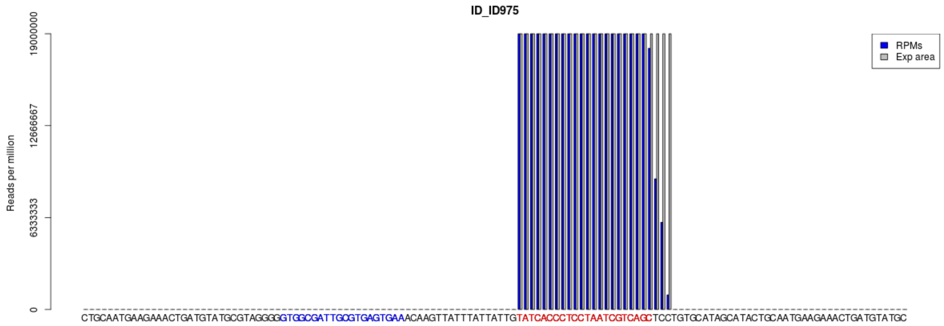

>ID977
ATTCTGAAGACCAATATGTTTCGTGAATCTCGAGCGACGGAAGCGAGATCTGTATAAGTTTTTAAATTAC**TTGAGATCTCGATTCCGTTA**
CTCAGTGTTCTCGATATTCG

>ID977-3P
TTGAGATCTCGATTCCGTTACT

>miRNA precursor
AUUCUGAAGACCAAUAUGUUUCGUGAAUCUCGAGCGACGGAAGCGAGAUCUGUAUAAGUUUUUAAAUUAC**UUGAGAUCUCGAUUCCGUUACU**CAGUGUUCUCGAUAUUCG
....................(((.((((((.(((..((((((.((((((((...(((((.........))))))))))))).))))))..))))).)))).)))......

>Predicted STAR seq:
GCGACGGAAGCGAGATCTGTATAA

Complementarity: 17

Initial dG =   -35.00

```
        10        20         30        40        50        60
AUUCUGAAGACCAAUAUGUU   U    -| C   CG      G        GUA     UUUU
                    UCG GAAU CU GAG  ACGGAA CGAGAUCU   UAAGU    \
                    AGC CUUG GA CUC  UGCCUU GCUCUAGA   GUUCA    A
GCUUAU--------------   U    U^ -   AU      A        ---     UUAA
.                     100         90        80           70
```

Reads in: lib-42, lib-45, lib-47, lib-48, lib-49, lib-50, lib-72
Number of reads="ID977",12,19,8,0,1,0,0

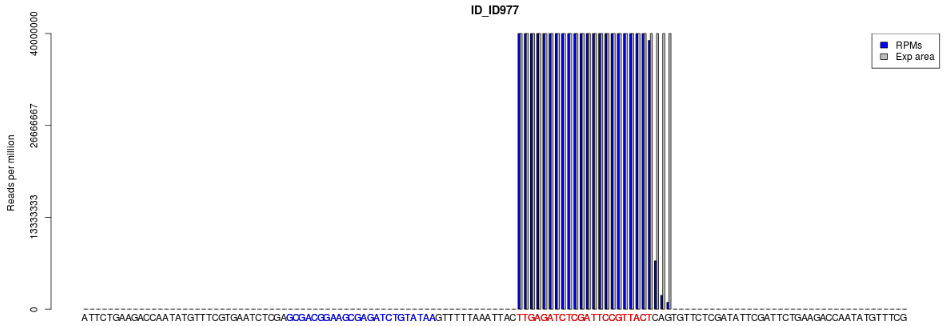

>ID979
AGTAATTCATGCATGGAGGG**TTGTGACTGTGTATGACAACA**CGTTATGAAATCAGTGTTGTACATACACAGCCATCACCCACTGTGCATG
GTAATCATCAGCCGTTGGTC

>ID979-5P
TTGTGACTGTGTATGACAACA

>miRNA precursor
AGUAAUUCAUGCAUGGAGGG**UUGUGACUGUGUAUGACAACA**CGUUAUGAAAUCAGUGUUGUACAUACACAGCCAUCACCCACUGUGCAUGGUAAUCAUCAGCCGUUGGUC
......((((((((((.((((.(((.((((((((((((((((...........))))))).))))))))).))).)))).))))))))))..((((........)))).

>Predicted STAR seq:
TTGTACATACACAGCCATCACC

Complementarity: 17

Initial dG =   -47.00

```
          10        20        30         40
.-AGUAAU          A    U   A         -|      GUUAU
        UCAUGCAUGG GGGU GUG CUGUGUAUG ACAACAC     G
        GGUACGUGUC CCCA UAC GACACAUAC UGUUGUG     A
\ ------          A    C   C         A^      ACUAA
        90        80        70        60        50



        100
  UA    UCA
    AUCA   G
    UGGU   C
  C-    UGC
110
```

Reads in: lib-42, lib-45, lib-47, lib-48, lib-49, lib-50, lib-72
Number of reads="ID979",23,40,0,0,2,0,0

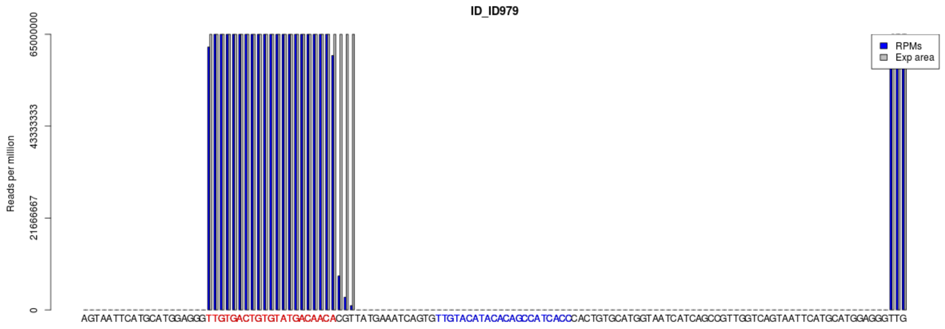

>ID98
GGCATCCTCGCATCACGGG**TGACGTATGTGGTGGGGGAACAC**TTTGATTCATTTGTGTTTCCCGCGCACATACGTCACACATTGTGCGAG
TCAGACGACTACCGGACCAGTT

>ID98-5P
TGACGTATGTGGTGGGGGAACAC

>miRNA precursor
GGCAUCCUCGCAUCACGGG**UGACGUAUGUGGUGGGGGAACAC**UUUGAUUCAUUUGUGUUUCCCGCGCACAUACGUCACACAUUGUGCGAGUCAGACGACUACCGGACCAGUU
```
......((((((.....(((((((((((((((((.(((((............))))))))))).))))))))))))).....)))))))......((((........))))
```

>Predicted STAR seq:
GTTTCCCGCGCACATACGTCACA

Complementarity: 20

Initial dG =   -48.30

```
              10        20        30         40
----  CA   .-U     CACGG            -|     G      UUUGA
    GG  UCC   CGCAU     GUGACGUAUGUG GUGGGG AACAC      U
    CC  AGG   GCGUG     CACUGCAUACAC CGCCCU UUGUG      U
UUGA  --   \ -     UUACA            G^     -      UUUAC
110                  80        70        60         50



                90
             A---   A
                 GUC G
                 CAG A
             CCAU   C
              100
```

Reads in: lib-42, lib-45, lib-47, lib-48, lib-49, lib-50, lib-72
Number of reads="ID98",2,6,0,0,0,1,0

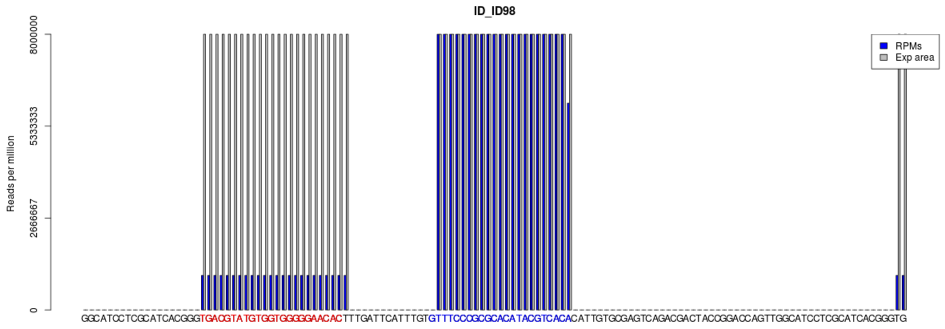

>ID980
TTGCTGTGGTAGTCATACATGCATGGAGGGTGATGTGTGTGTGTGGTGCCAACACATTATACAATTACTG**TTGCACTTCCACACACATCA**
CCCACCGTGCATGGCAAAC

>ID980-3P
TTGCACTTCCACACACATCACC

>miRNA precursor
UUGCUGUGGUAGUCAUACAUGCAUGGAGGGUGAUGUGUGUGUGUGGUGCCAACACAUUAUACAAUUACUG**UUGCACUUCCACACACAUCACC**CACCGUGCAUGGCAAAC
((((....)))).....((((((((.((((((((((((((...(((((.((((.............)))))))))..)))))))))))))).))))))))......

>Predicted STAR seq:
GTGATGTGTGTGTGTGGTGCCAA

Complementarity: 18
ERROR 4: Strange structure
 Initial dG =   -52.80

```
            10
--    .-U|  GU
  UUGC   GUG  \
  AACG   UAC  A
CA    \ -^  UG



          20        30        40        50        60
        A         A              UGU     C     CAUUAU
         CAUGCAUGG GGGUGAUGUGUGUG   GGUGC AACA      A
         GUACGUGCC CCCACUACACACAC   UCACG UUGU      C
        -         A              CU-     -     CAUUAA
          100        90        80          70
```

Reads in: lib-42, lib-45, lib-47, lib-48, lib-49, lib-50, lib-72
Number of reads="ID980",1,3,0,0,0,0,0

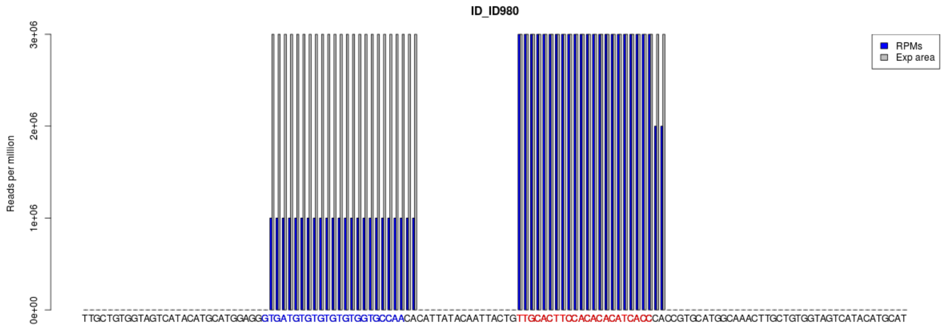

>ID983

CTGCAGATAAGTTGCTAATTACCGCATAATGGTTGACGAGTGTGTGGCTGTCACGTATTATTTACTACCG**TCACAGCCGCGCAATCGTCA**
GCTGTTGTGCAGCGTATAACA

>ID983-3P

TCACAGCCGCGCAATCGTCAGC

>miRNA precursor

CUGCAGAUAAGUUGCUAAUUACCGCAUAAUGGUUGACGAGUGUGUGGCUGUCACGUAUUAUUUACUACCG**UCACAGCCGCGCAAUCGUCAGC**UGUUGUGCAGCGUAUAACA
..((((.....)))).......((((((((((((((((.(((((((((((.(((((........)).))).))))))))))).))))))))))))))))..........

>Predicted STAR seq:

TTGACGAGTGTGTGGCTGTC

Complementarity: 18
ERROR 4: Strange structure
 Initial dG =   -49.20

```
.-CU|   AU
    GCAG  \
    CGUU  A
\ --^   GA
        10


     20           30         40         50          60
  UAAUUACC---                G           C    -   UUA
             GCAUAAUGGUUGACGA UGUGUGGCUGU ACG UA   U
             CGUGUUGUCGACUGCU ACGCGCCGACA UGC AU   U
  ACAAUAUGCGA                A           C    C   CAU
 110       100        90         80         70
```

Reads in: lib-42, lib-45, lib-47, lib-48, lib-49, lib-50, lib-72

Number of reads="ID983",23,45,1,0,2,2,0

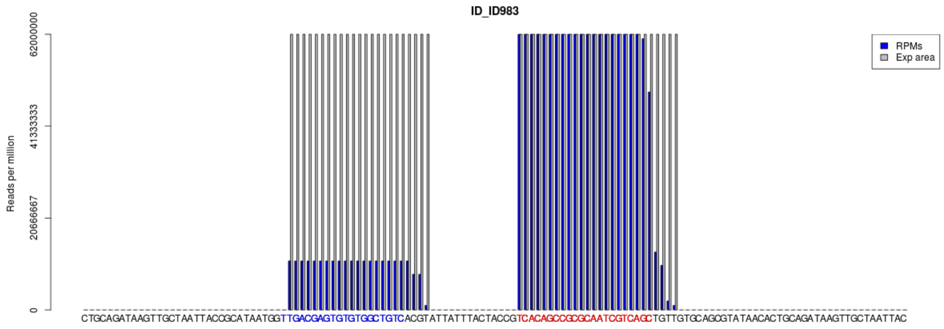

>ID984
GTCTATTATGATTATAATACATGCCATGGAGGTTGGCGGAAGTGTGCCTGTTACACGTTATTTTGTACTG**TAACAGACACCCTGACGTCA**
GCTGCCGTGCATGGTACACT

>ID984-3P
TAACAGACACCCTGACGTCAGC

>miRNA precursor
GUCUAUUAUGAUUAUAAUACAUGCCAUGGAGGUUGGCGGAAGUGUGCCUGUUACACGUUAUUUUGUACUG**UAACAGACACCCUGACGUCAGC**UGCCGUGCAUGGUACACU
...((((((....))))))((((.((((.((((((((..((.(((.((((((((.((........)))))))))).))).)).))..)))))))).)))))))).......

>Predicted STAR seq:
TTGGCGGAAGTGTGCCTGTTA

Complementarity: 16
ERROR 4: Strange structure
Initial dG =   -42.50

```
  |  UA
--GUC  U
  UAG  U
\ ^  UA
   10


        20        30        40        50        60
  UAUAAUA     C    A        GA  U   C        C  UAU
         CAUGC AUGG GGUUGGCG  AG GUG CUGUUACA GU   U
         GUACG UGCC UCGACUGC  UC CAC GACAAUGU CA   U
  UCACAUG     -    G        AG  C   A        -  UGU
110       100        90        80        70
```

Reads in: lib-42, lib-45, lib-47, lib-48, lib-49, lib-50, lib-72
Number of reads="ID984",29,33,0,0,1,1,0

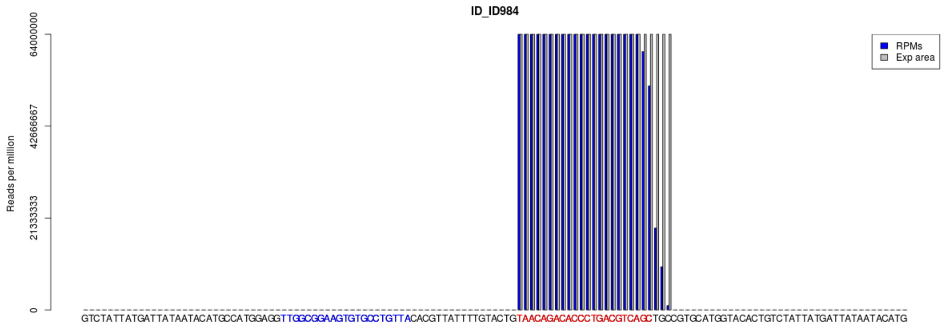

>ID985
AAACGTCAACACAAATGCAGTCATGGTCCGCAGCGGTATCTAATGCAAACGAACATTGTGTTTCAAGGTG**TTCGGTTGTACTAGATACCC**
GCTGCTGTTCATGGAATA

>ID985-3P
TTCGGTTGTACTAGATACCCGC

>miRNA precursor
AAACGUCAACACAAAUGCAGUCAUGGUCCGCAGCGGUAUCUAAUGCAAACGAACAUUGUGUUUCAAGGUG**UUCGGUUGUACUAGAUACCCGC**UGCUGUUCAUGGAAUA
```
...................((((((...(((((((((((((.(((((.(((((((..((...))..))))))).))))).))))))))).)))))...))))))....
```

>Predicted STAR seq:
CGGTATCTAATGCAAACGAA

Complementarity: 17

Initial dG =   -41.10

```
        10        20        30          40        50        60
AAACGUCAACACAAAUGCAG      UCC      -|       A      A       UG   U
                    UCAUGG   GCAGC GGUAUCUA UGCAA CGAACAU   UG \
                    GGUACU   CGUCG CCAUAGAU AUGUU GCUUGUG   AC U
AUAA----------------      UGU      C^       C      G       GA   U
                      100        90        80        70
```

Reads in: lib-42, lib-45, lib-47, lib-48, lib-49, lib-50, lib-72
Number of reads="ID985",2,14,0,0,0,0,0

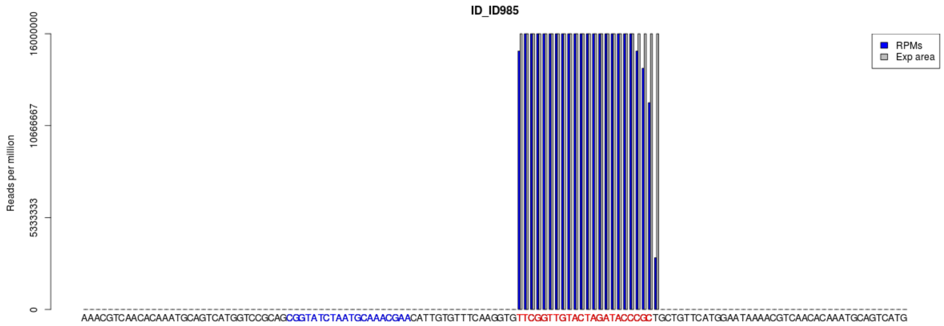

>ID986
ACTCTGAAGACCAATGTGTTTGGTGAATCCCGAGCGACGGAAGTGAGGTCTGTACAAGTTTCTTAATTAC**TTGAGATCTCGCTTCCGTTA**
CTCGGTGTTCTCGACAAGCG

>ID986-3P
TTGAGATCTCGCTTCCGTTACT

>miRNA precursor
ACUCUGAAGACCAAUGUGUUUGGUGAAUCCCGAGCGACGGAAGUGAGGUCUGUACAAGUUUCUUAAUUAC**UUGAGAUCUCGCUUCCGUUACU**CGGUGUUCUCGACAAGCG
................(((((..(((..(((((..(((((((((((((((...(((((.........))))))))))))))))))))..))))).)))....))))).

>Predicted STAR seq:
GCGACGGAAGTGAGGTCTGTACAA

Complementarity: 18

Initial dG = -48.60

```
        10        20          30        40        50        60
ACUCUGAAGACCAAUGU     GU--|  UC      CG               GUA     UUCU
                 GUUUG    GAA  CCGAG  ACGGAAGUGAGGUCU   CAAGU    \
                 CGAAC    CUU  GGCUC  UGCCUUCGCUCUAGA   GUUCA    U
G----------------     AGCU^  GU      AU               ---     UUAA
.                       100        90        80           70
```

Reads in: lib-42, lib-45, lib-47, lib-48, lib-49, lib-50, lib-72
Number of reads="ID986",30,56,16,0,5,0,0

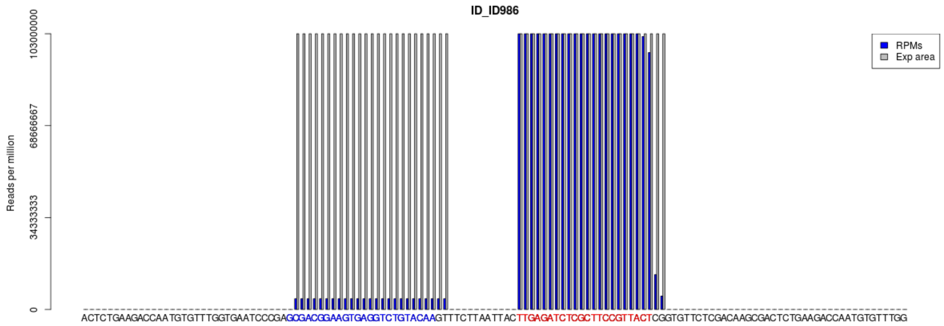

>ID987
TGTAATTCATGTATAGGGTT**CGGCGACTGCGTGGACGCCACA**TGTTATTTACTACTGTGGCAGTGACGCAGTCGTCAGATCCTGTGCATG
GTATGCCACAGAATTGTGTCTG

>ID987-5P
CGGCGACTGCGTGGACGCCACA

>miRNA precursor
UGUAAUUCAUGUAUAGGGUU**CGGCGACUGCGUGGACGCCACA**UGUUAUUUACUACUGUGGCAGUGACGCAGUCGUCAGAUCCUGUGCAUGGUAUGCCACAGAAUUGUGUCUG
.(((..(((((((((((((..(((((((((((..((((((((.((........)))))))).)).)))))))))))..)))))))))))))..)))(((......)))....

>Predicted STAR seq:
TGGCAGTGACGCAGTCGTCAGAT

Complementarity: 17

Initial dG =   -54.10

```
          10        20        30         40
.-U|  AU            UC          GG  -      U  UAU
   GUA  UCAUGUAUAGGGU  GGCGACUGCGU  AC GCCACA GU   U
   CGU  GGUACGUGUCCUA  CUGCUGACGCA  UG CGGUGU CA   U
\ -^  AU            GA          G-  A      -  UCA
        90        80        70         60         50



   100
  CA    AU
    CAGA  U
    GUCU  G
  --    GU
    110
```

Reads in: lib-42, lib-45, lib-47, lib-48, lib-49, lib-50, lib-72
Number of reads="ID987",2,0,0,0,0,0,0

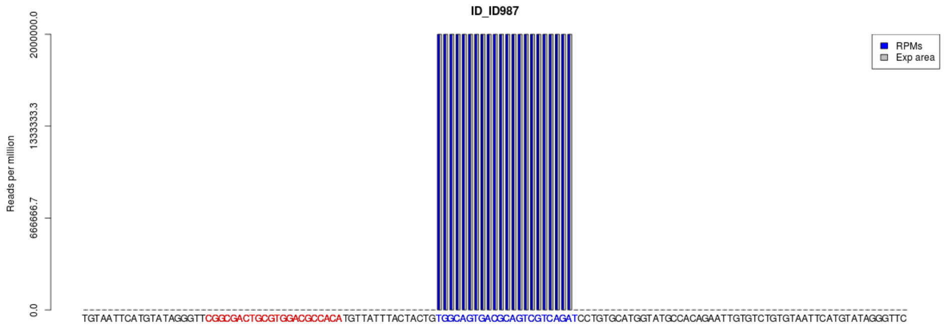

>ID989
ATTCTGAAGAACAATATGTTTCGTGAATCCCGAGCGACGGAAGAGAGGTCTGTACAAGTTTTTAAGTTAC**TTGGGATCTCCCTTCCGTTA**
CTCGGTGTTCTCGATAAGCGTC

>ID989-3P
TTGGGATCTCCCTTCCGTTACT

>miRNA precursor
AUUCUGAAGAACAAUAUGUUUCGUGAAUCCCGAGCGACGGAAGAGAGGUCUGUACAAGUUUUUAAGUUAC**UUGGGAUCUCCCUUCCGUUACU**CGGUGUUCUCGAUAAGCGUC
```
...............(((((((.(((..(((((..(((((((.(((((((...(((((.........)))))))))))).)))))))..)))))..))).)))..))))).
```

>Predicted STAR seq:
GCGACGGAAGAGAGGTCTGTACAA

Complementarity: 17

Initial dG = -44.80

```
        10        20          30        40        50        60
AUUCUGAAGAACAAU     --|  U   UC      CG       A        GUA     UUUU
               AUGUU  UCG GAA  CCGAG  ACGGAAG GAGGUCU   CAAGU    \
               UGCGA  AGC CUU  GGCUC  UGCCUUC CUCUAGG   GUUCA    A
C--------------     AU^  U   GU      AU       C        ---     UUGA
             110       100        90        80           70
```

Reads in: lib-42, lib-45, lib-47, lib-48, lib-49, lib-50, lib-72
Number of reads="ID989",5,5,0,0,0,0,0

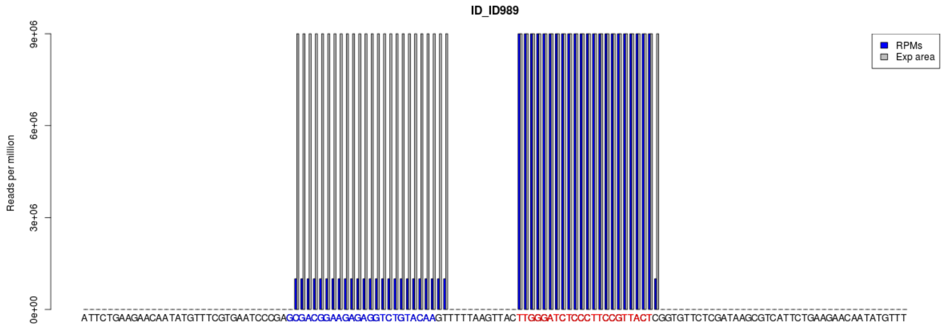

>ID99
CAGCATTCTTGCATAATGAG**TGATGGGCGTGCGATTACACAT**ATTGATATATGATTATGTGTATCGTATGCTGATCTCTCACTGTGCACG
ACTTCACTTATGGACTACTTTA

>ID99-5P
TGATGGGCGTGCGATTACACAT

>miRNA precursor
CAGCAUUCUUGCAUAAUGAG**UGAUGGGCGUGCGAUUACACAU**AUUGAUAUAUGAUUAUGUGUAUCGUAUGCUGAUCUCUCACUGUGCACGACUUCACUUAUGGACUACUUUA
......((.((((((.((((.(((.((((((((((.(((((((...........))))))))))))))))).))).)))).)))))).)).((((....))))........

>Predicted STAR seq:
GTGTATCGTATGCTGATCTCT

Complementarity: 17

Initial dG =   -41.20

```
                       10        20        30        40
CAGCAU---------------| U       A    U   G          U       UUGAU
                     UC UGCAUA UGAG GAU GGCGUGCGAU ACACAUA      A
                     AG ACGUGU ACUC CUA UCGUAUGCUA UGUGUAU      U
AUUUCAUCAGGUAUUCACUUC^ C       C    U   G          -       UAGUA
110      100       90        80        70         60        50
```

Reads in: lib-42, lib-45, lib-47, lib-48, lib-49, lib-50, lib-72
Number of reads="ID99",0,0,0,0,0,0,0

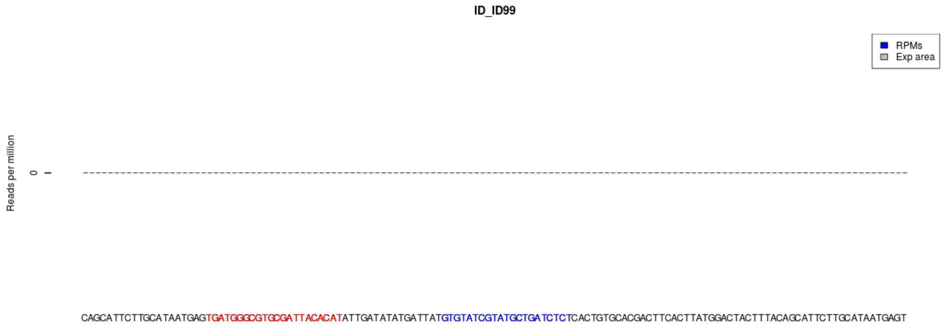

>ID990
CTTTTTGTACTTCTGAAGGCCATGTATACAGGTTGGCGATTGCTTGACTGCCACACGTCAATTATTACTG**TATCAGTCACCCAATCGTCA**
TTCCCTGTGCACGGAATACAA

>ID990-3P
TATCAGTCACCCAATCGTCATT

>miRNA precursor
CUUUUUGUACUUCUGAAGGCCAUGUAUACAGGUUGGCGAUUGCUUGACUGCCACACGUCAAUUAUUACUG**UAUCAGUCACCCAAUCGUCAUU**CCCUGUGCACGGAAUACAA
```
....(((((.(((((.......(((..(((((.(((((((((..((((((..(((.((........)))))..))))))..)))))))))...)))))))))))))))))))
```

>Predicted STAR seq:
TGGCGATTGCTTGACTGC

Complementarity: 14

Initial dG =   -33.90

```
        10        20        30          40        50        60
CUUU|    C     AAGGCCA   AU     U--         CU       CC   C  CAA
    UUGUA UUCUG       UGU  ACAGG   UGGCGAUUG  UGACUG  ACA GU   U
    AACAU AAGGC       ACG  UGUCC   ACUGCUAAC  ACUGAC  UGU CA   U
----^    -     -------   --     CUU         CC       UA   -  UUA
   110              100          90        80        70
```

Reads in: lib-42, lib-45, lib-47, lib-48, lib-49, lib-50, lib-72
Number of reads="ID990",3,7,0,0,0,0,0

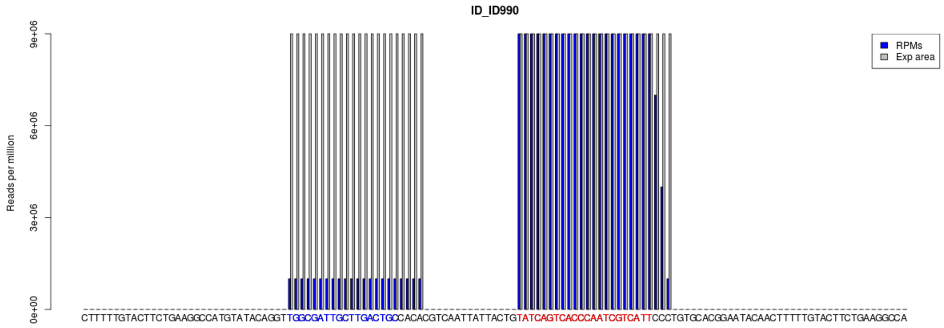

>ID991
TTTTCCTCCAGTTGCTAATTACCGCATAAAGGTTGACGATTGTGTGGCTGTCACGTCTTATCTACTACCG**TCACAGACGTGCAATCGTCA**
GCTGTTGTGCAGCGTATAACAT

>ID991-3P
TCACAGACGTGCAATCGTCAGC

>miRNA precursor
UUUUCCUCCAGUUGCUAAUUACCGCAUAAAGGUUGACGAUUGUGUGGCUGUCACGUCUUAUCUACUACCG**UCACAGACGUGCAAUCGUCAGC**UGUUGUGCAGCGUAUAACAU
............(((.......((((((.((((((((((((..((.((((.(((.............))).)))).))..)))))))))))).)))))))))........

>Predicted STAR seq:
TTGACGATTGTGTGGCTGTC

Complementarity: 16

Initial dG =   -38.00

```
        10        20        30        40        50        60
UUUUCCUCCAGU|   AAUUACC      A            UG  G    C   UCUUAU
            UGCU       GCAUAA GGUUGACGAUUG  UG CUGU ACG      \
            GCGA       CGUGUU UCGACUGCUAAC  GC GACA UGC      C
UACAAUAU----^   -------      G            GU  A    C   CAUCAU
110                  100        90        80        70
```

Reads in: lib-42, lib-45, lib-47, lib-48, lib-49, lib-50, lib-72
Number of reads="ID991",27,31,0,2,0,0,0

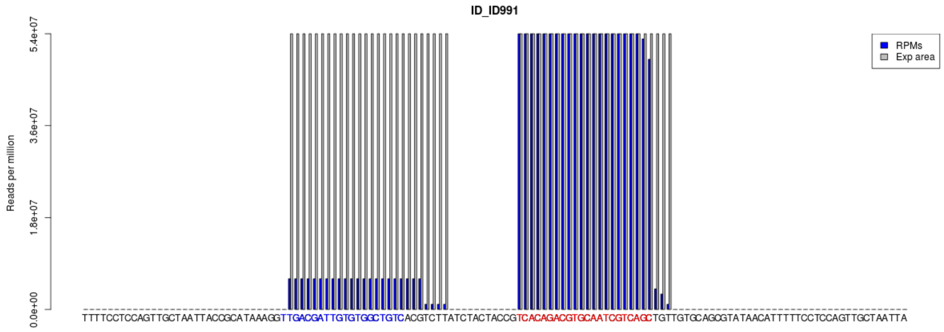

>ID993

GCTGTGGTCCAACGTATCTCTTGCATCCCGAGGAACAGAAGTGGAAGCCGTACATGTTTGTAACGATCTG**TAAGGCTCTCGTTTCGGTTC**
CTTGGTGTGCATTATAAGCGAC

>ID993-3P

TAAGGCTCTCGTTTCGGTTCCT

>miRNA precursor

GCUGUGGUCCAACGUAUCUCUUGCAUCCCGAGGAACAGAAGUGGAAGCCGUACAUGUUUGUAACGAUCUG**UAAGGCUCUCGUUUCGGUUCCU**UGGUGUGCAUUAUAAGCGAC

```
............(((......((((..(((((((((.((((((..((((.((((.(((......))).)))).)))).)))))).)))))))))..)))).......)))..
```

>Predicted STAR seq:

GGAACAGAAGUGGAAGCCGUA

Complementarity: 16

Initial dG =   -41.80

```
        10        20        30        40        50        60
GCUGUGGUCCAA|  AUCUCU    UC          A      GA    G    U   UG
            CGU      UGCA  CCGAGGAAC GAAGUG  AGCC UACA GUU  U
            GCG      ACGU  GGUUCCUUG CUUUGC  UCGG AUGU UAG  A
CA----------^  AAUAUU    GU          G      UC    A    C   CA
          110       100        90        80        70
```

Reads in: lib-42, lib-45, lib-47, lib-48, lib-49, lib-50, lib-72

Number of reads="ID993",11,1,0,0,0,0,0

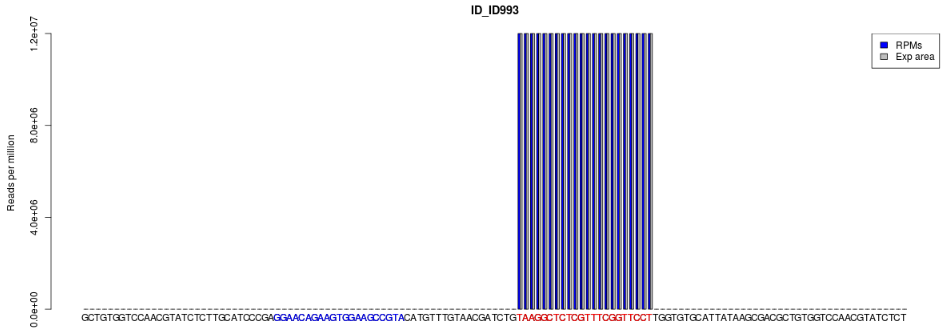

>ID994
CTGCAATGAAGAAACTGATGTATGCGTAGGGGGTGGCGATTGCGTGAGTGAAACAAGTTATTTATTATTG**TATCACCCTCCTAATCGTCA**
GCTCCTGTGCATAGCATA

>ID994-3P
TATCACCCTCCTAATCGTCAGC

>miRNA precursor
CUGCAAUGAAGAAACUGAUGUAUGCGUAGGGGGUGGCGAUUGCGUGAGUGAAACAAGUUAUUUAUUAUUG**UAUCACCCUCCUAAUCGUCAGC**UCCUGUGCAUAGCAUA
```
................((((((((((((((.(((((((((.....((((.((((...........)))).)))).....))))))))).))))))))))))).))).
```

>Predicted STAR seq:
GTGGCGATTGCGTGAGTGAA

Complementarity: 13

Initial dG =   -36.60

```
        10        20         30        40        50        60
CUGCAAUGAAGAAACUG   -|           G         CGUGA    A    GUUAU
                 AUG UAUGCGUAGGGG UGGCGAUUG     GUGA ACAA     \
                 UAC AUACGUGUCCUC ACUGCUAAU     CACU UGUU     U
A----------------   G^           G         CCUCC    A    AUUAU
                      100        90        80        70
```

Reads in: lib-42, lib-45, lib-47, lib-48, lib-49, lib-50, lib-72
Number of reads="ID994",5,16,0,0,0,0,0

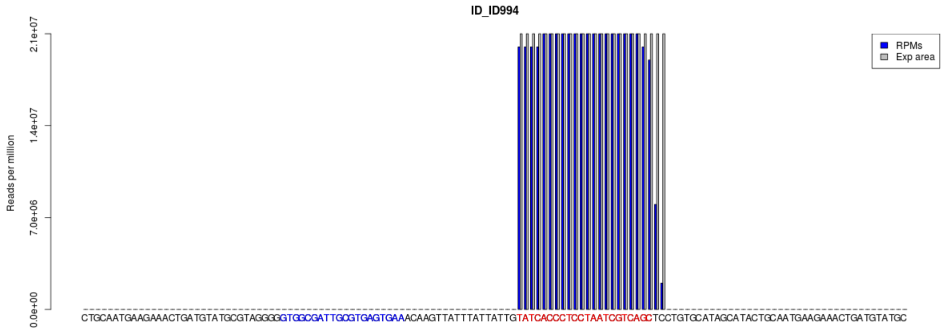

>ID995
GCCTACTGTGGTCCGCGGCT**AGCATGATCAGTGGCATGAATT**GTGAATTGGTCAATTCGTGTGACTGCTCATGCAACACTGCGCTCCGCT
CAGTTCAACGTCGACGTC

>ID995-5P
AGCATGATCAGTGGCATGAATT

>miRNA precursor
GCCUACUGUGGUCCGCGGCU**AGCAUGAUCAGUGGCAUGAAUU**GUGAAUUGGUCAAUUCGUGUGACUGCUCAUGCAACACUGCGCUCCGCUCAGUUCAACGUCGACGUC
.......((((..(((((...((((((.((((.((((((((((.........)))))))))).)))).))))))....))))).)))).......(((....))).

>Predicted STAR seq:
TTCGTGTGACTGCTCATGCAACACT

Complementarity: 17

Initial dG =   -42.30

```
          10        20         30        40
.-GCCUACU    UC      CUA-|      U    G           UGAA
         GUGG  CGCGG    GCAUGA CAGU GCAUGAAUUG    \
         CGCC  GCGUC    CGUACU GUCA UGUGCUUAAC    U
\ -------    UC      ACAA^      C    G           UGGU
                 80        70        60        50



 90       100
  UCAGUUCA   U
          ACG C
          UGC G
  C-------   A
```

Reads in: lib-42, lib-45, lib-47, lib-48, lib-49, lib-50, lib-72
Number of reads="ID995",15,23,2,0,0,0,0

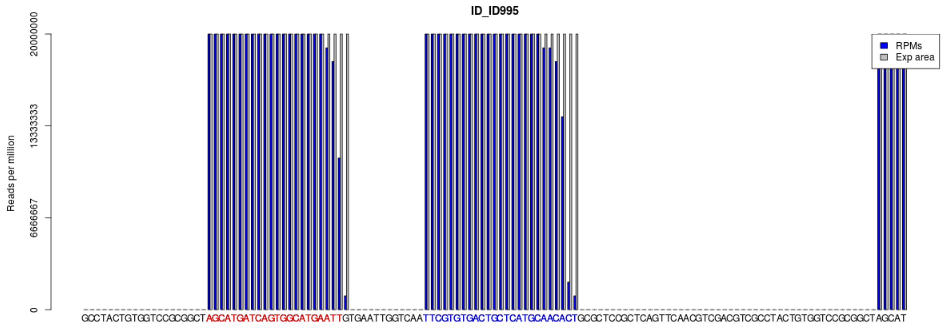

>ID996
TCTTGCACTGACAGTAATACATGGATGGAGGGTGATGTGCGTGTGATGACAACACGTGATAAAGGTTCTG**TTTTATCACCCTCACATCAC**
CCACTTTACATGACAAACAGC

>ID996-3P
TTTTATCACCCTCACATCACC

>miRNA precursor
UCUUGCACUGACAGUAAUACAUGGAUGGAGGGUGAUGUGCGUGUGAUGACAACACGUGAUAAAGGUUCUG**UUUUAUCACCCUCACAUCACC**CACUUUACAUGACAAACAGC
..((((.......))))..((((...((.((((((((((...(((((((.((((...((.......)))))))))))))...)))))))))).))...)))).........

>Predicted STAR seq:
GTGATGTGCGTGTGATGACAA

Complementarity: 16
ERROR 4: Strange structure
Initial dG = -33.80

```
.-UC|   ACU
    UUGC   \
    AAUG   G
\ --^   ACA
```

```
          20        30           40
  UA-------    GAU  A          .-C    GAU
           CAUG   GG GGGUGAUGUG   GUGU   \
           GUAC   UC CCCACUACAC   CACA   G
  CGACAAACA    AUU  A          \ -    ACA
 110       100        90              50
```

Reads in: lib-42, lib-45, lib-47, lib-48, lib-49, lib-50, lib-72
Number of reads="ID996",0,0,0,0,0,0,0

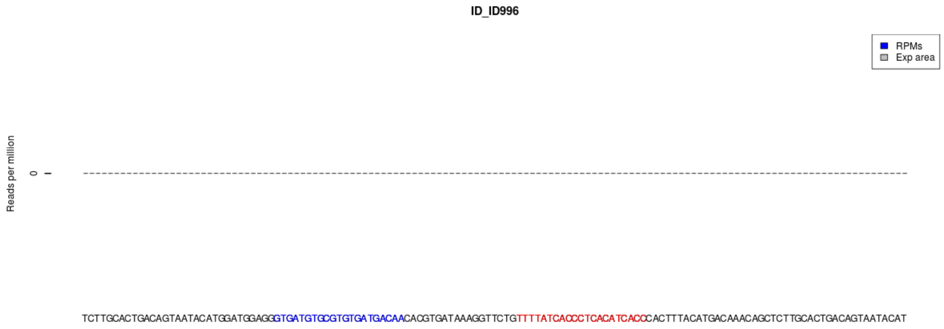

>ID997
CTGTAATAACGATGCTACTGAGTGCATAGAGGCCGGCGATTCCGTGGTTGTCACAAGTTATTTACCACTG**TCACAACCACCCAATCGTCA GCT**CCTGTGCATGGTATACCAC

>ID997-3P
TCACAACCACCCAATCGTCAGCT

>miRNA precursor
CUGUAAUAACGAUGCUACUGAGUGCAUAGAGGCCGGCGAUUCCGUGGUUGUCACAAGUUAUUUACCACUG**UCACAACCACCCAAUCGUCAGCU**CCUGUGCAUGGUAUACCAC
```
...........(((((.....((((((((..((.(((((((..((((((((.(((.((........))))).))))))))..))))))).))..))))))))))))).....
```

>Predicted STAR seq:
GGCCGGCGATTCCGTGGTTGTC

Complementarity: 16

Initial dG =   -41.60

```
        10         20         30         40         50         60
CUGUAAUAACG|    ACUGA        AG  C        CC         C   A  UAU
           AUGCU     GUGCAUAG  GC GGCGAUU  GUGGUUGU ACA GU   U
           UAUGG     UACGUGUC  CG CUGCUAA  CACCAACA UGU CA   U
CACCA------^    -----        CU  A        CC         C   -  CCA
110                  100        90         80         70
```

Reads in: lib-42, lib-45, lib-47, lib-48, lib-49, lib-50, lib-72
Number of reads="ID997",14,14,0,1,1,0,0

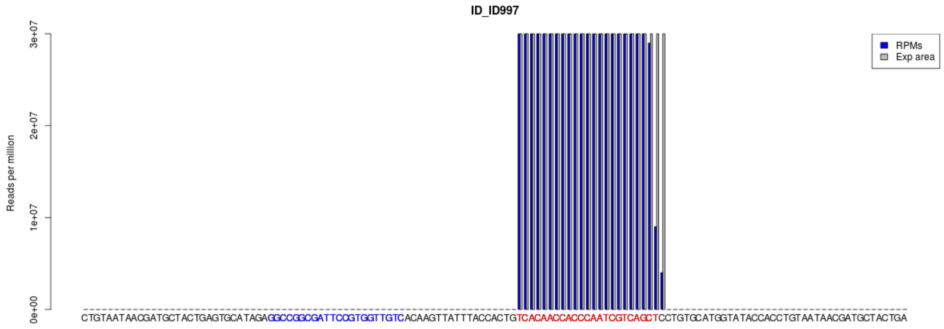

>ID998
ATTCTGAAAACCAATATGTTTCGCGAATCCCGGGCGACGGAAGCGAGGGCTGTACAAGTTTTTAAATTAC**TTGAGTACTCCCTTCCGTCA**
CTCGGTGTTCTCGCTAAGCGTC

>ID998-3P
TTGAGTACTCCCTTCCGTCACT

>miRNA precursor
AUUCUGAAAACCAAUAUGUUUCGCGAAUCCCGGGCGACGGAAGCGAGGGCUGUACAAGUUUUUAAAUUAC**UUGAGUACUCCCUUCCGUCACU**CGGUGUUCUCGCUAAGCGUC
...............((((((.(((((..(((((.((((((((.(((..((...(((((.........)))))))..))).)))))))).))))).)..)))).)))))).

>Predicted STAR seq:
GCGACGGAAGCGAGGGCTGTACAA

Complementarity: 16

Initial dG =   -41.80

```
        10        20          30        40        50        60
AUUCUGAAAACCAAU      C    --|UC     C        C   GG  GUA     UUUU
               AUGUUU GCGA  A  CCGGG GACGGAAG GAG  CU   CAAGU    \
               UGCGAA CGCU  U  GGCUC CUGCCUUC CUC  GA   GUUCA    A
C--------------      U    CU^GU     A        C   AU  ---     UUAA
             110       100        90        80           70
```

Reads in: lib-42, lib-45, lib-47, lib-48, lib-49, lib-50, lib-72
Number of reads="ID998",1,8,0,0,0,2,0

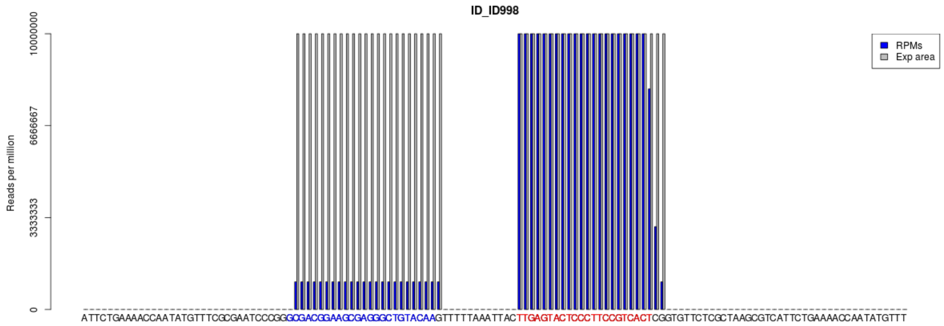

>ID999
TCTTGCACTGACAGTAATACATGGATGGAGGGTGATGTGCGTGTGATGACAACACGTGATAAAGGTTCTG**TTTTATCACCCTCACATCAC**
CCAGTTTACATGACAAACAGC

>ID999-3P
TTTTATCACCCTCACATCACC

>miRNA precursor
UCUUGCACUGACAGUAAUACAUGGAUGGAGGGUGAUGUGCGUGUGAUGACAACACGUGAUAAAGGUUCUG**UUUUAUCACCCUCACAUCACC**CAGUUUACAUGACAAACAGC
..((((.......))))..((((......((((((((((...(((((((.((((...((.......)))))))))))))...)))))))))).......)))).........

>Predicted STAR seq:
GTGATGTGCGTGTGATGACAA

Complementarity: 16
ERROR 4: Strange structure
Initial dG =   -31.40

```
.-UC|   ACU
    UUGC   \
    AAUG   G
\ --^   ACA
```

```
          20        30           40
  UA-------    GAUGGA          .-C    GAU
           CAUG      GGGUGAUGUG   GUGU   \
           GUAC      CCCACUACAC   CACA   G
  CGACAAACA    AUUUGA          \ -    ACA
 110       100        90              50
```

Reads in: lib-42, lib-45, lib-47, lib-48, lib-49, lib-50, lib-72
Number of reads="ID999",0,0,0,0,0,0,0

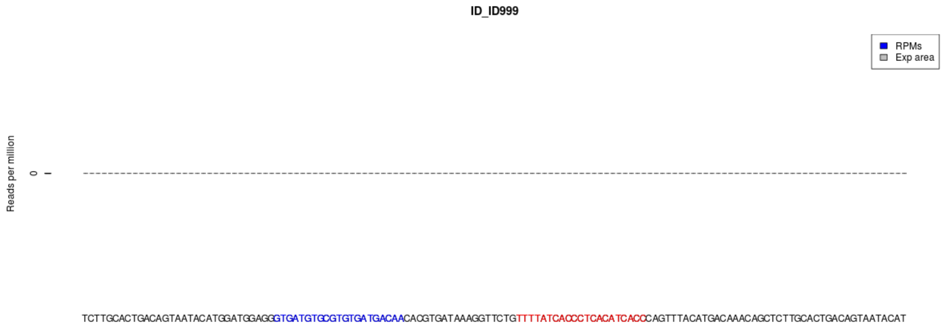

>IM1
CAGTTGCTGTCACTGCGTATGTCGTGTGAAGTCGTGTCGTGCCGCTGGCGACAGGCTGGGGCTGACTCAG**ACTCCTGCTGCAGCTGTTGG**
CGCATTTGACTGCTGTGTTCTCCACCGGCAGCTG

>IM1-5P
ACTCCTGCTGCAGCTGTTGGCG

>miRNA precursor
CAGUUGCUGUCACUGCGUAUGUCGUGUGAAGUCGUGUCGUGCCGCUGGCGACAGGCUGGGGCUGACUCAG**ACUCCUGCUGCAGCUGUUGGCG**CAUUUGACUGCUGUGUUCUCCACCGGCA
GCUG
((((((((((((.(((.....)))))))(((((.((.((((((..(((....(((.
((((((...))).)))).)))...)))..)))))))).))))).............)))))))))

>Predicted STAR seq:
GCCGCTGGCGACAGGCTGGGG

Complementarity: 13
ERROR 4: Strange structure
Initial dG =   -47.40

```
          10
           |   U   UA
CAGUUGCUG--UCAC GCG  \
GUCGACGGC  AGUG UGC  U
         \ ^   -   UG
  120



                        30        40        50        60
           --------------     U  C      CU    GACA   U    -   A
                         AGUCG GU GUGCCG  GGC    GGC GGGG CUG \
                         UCAGU UA CGCGGU  UCG    UCG CCUC GAC C
           CACCUCUUGUGUCG     U  -      UG    ACG-   U    A   U
              110       100         90        80         70
```

Reads in: lib-42, lib-45, lib-47, lib-48, lib-49, lib-50, lib-72
Number of reads="IM1",0,0,0,0,0,0,0

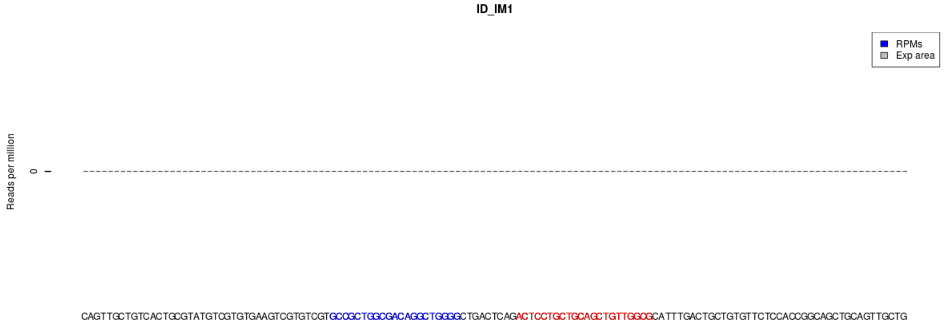

>IM10
CCTGAGACTGCCGGCAGAAGGGCTGAACATGGCTCTCACTGGCCTGCCAGTGTGGTGCCTAATGAGCTA**CCAGCAGTAGACGTGTGTG**AG
CGCGTGTGACGCAGCTCCCTGAGACCGGCAGCAGAAGG

>IM10-3?P
CCAGCAGTAGACGTGTGTG

>miRNA precursor
CCUGAGACUGCCGGCAGAAGGGCUGAACAUGGCUCUCACUGGCCUGCCAGUGUGGUGCCUAAUGAGCUA**CCAGCAGUAGACGUGUGUG**AGCGCGUGUGACGCAGCUCCCUGAGACCGGCA
GCAGAAGG
(((....((((((((((..((((((.(((((((((.(((((..((((..((.(((((.
((....)).))))))).)))).)).))).)))).)))))....)))))).)))...)))))))....)))

>Predicted STAR seq:
TCACTGGCCTGCCAGTGTGG

Complementarity: 13
ERROR 4: Strange structure
Initial dG = -52.70

```
        10        20          30         40
   GAGA       CAGA   .-GCUGAACAU   -| U      C
CCU    CUGCCGG    AGG           GGC UC CACUGG C
GGA    GACGGCC    UCC           CCG GG GUGACC U
   AGAC       AGAG   \ ---------   U^ U      G
      120       110                     50


                      60        70        80
                       UAAU     ACCA  AG  G      G
                           GAGCU    GC  UA ACGUGU U
                           CUCGA    CG  GU UGCGCG G
                       ----     ----  CA  G      A
                                    100        90
```

Reads in: lib-42, lib-45, lib-47, lib-48, lib-49, lib-50, lib-72
Number of reads="IM10",3,1,1,7,0,0,0

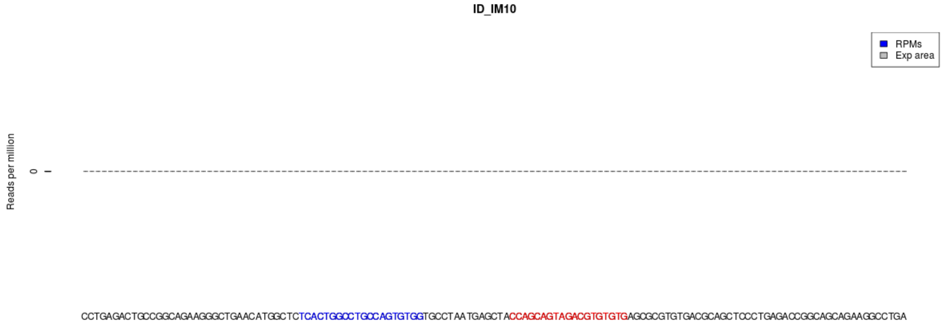

>IM11
GCTGGGCTGCCTCACCTGCAGAAGAAGAGGAAGT**AGGGCGCGGTGGTGAGCATG**ATGAAGACGGCGACCACGTCCTCGGCCTCGGTGTGG
CAGGACAGCCGCAGGAAGGGCATGCCCAGC

>IM11-5P
AGGGCGCGGTGGTGAGCATG

>miRNA precursor
GCUGGGCUGCCUCACCUGCAGAAGAAGAGGAAGU**AGGGCGCGGUGGUGAGCAUG**AUGAAGACGGCGACCACGUCCUCGGCCUCGGUGUGGCAGGACAGCCGCAGGAAGGGCAUGCCCAGC
((((((((((((..(((((.......((((....((((((.(((.((................)).))).))))))...)))).....(((......))))))))..))))).)))))))

>Predicted STAR seq:
GACCACGTCCTCGGCC

Complementarity: 9

Initial dG = -57.80

```
         10          20        30        40        50
       -     CA     .-AGAAGAA|   AAGU      C   G  GAGCAUG
GCUGGGC UGCCU  CCUGC         GAGG    AGGGCG GGU GU       A
CGACCCG ACGGG  GGACG         CUCC    UCCUGC CCA CG       U
       U     AA     \ -------^   GGC-      A   G  GCAGAAG
.       110                   80         70        60



                           90
                      GGUGU   AG
                           GGC  G
                           CCG  A
                      -----   AC
                         100
```

Reads in: lib-42, lib-45, lib-47, lib-48, lib-49, lib-50, lib-72
Number of reads="IM11",0,0,0,0,0,0,0

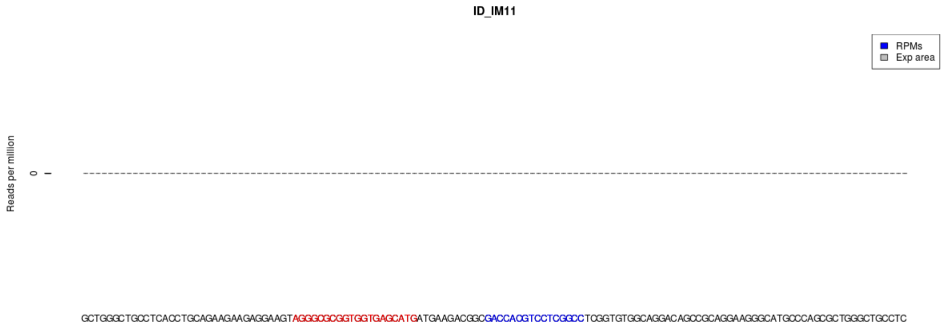

>IM12
GCACGCGGAAGTAATGCTTTACATCT**ACCCTGTAGATCCGAATTTGT**TTGACATAGGGCGACAAATTCGGTTCTAGAGAGGTTTGTGTGG
TGCATACAAGGCAACGTGC

>IM12-3P
ACCCTGTAGATCCGAATTTGT

>miRNA precursor
GCACGCGGAAGUAAUGCUUUACAUCU**ACCCUGUAGAUCCGAAUUUGU**UUGACAUAGGGCGACAAAUUCGGUUCUAGAGAGGUUUGUGUGGUGCAUACAAGGCAACGUGC
((((((....)..((((.((((((..(((((.((((.(((((((((((..........))))))))))).)))).)).))).)).))))))).)))).........)))))

>Predicted STAR seq:
AAATTCGGTTCTAGAGAGGTTTGT

Complementarity: 17

Initial dG = -41.10

ERROR 3: Strange structure
Initial dG = -41.10

```
       |G
GCACG--C G
CGUGC  G A
     \ ^A
       10



              20         30        40        50
       UA-------    U      CU    -  G     U           UGACA
                AUGC UUACAU  ACC CU UAGA CCGAAUUUGUU      \
                UACG GGUGUG  UGG GA AUCU GGCUUAAACAG      U
       AACGGAACA    U      UU    A  G     U           CGGGA
         100        90         80        70        60
```

Reads in: lib-42, lib-45, lib-47, lib-48, lib-49, lib-50, lib-72
Number of reads="IM12",11076,3771,19796,3699,2101,10752,0

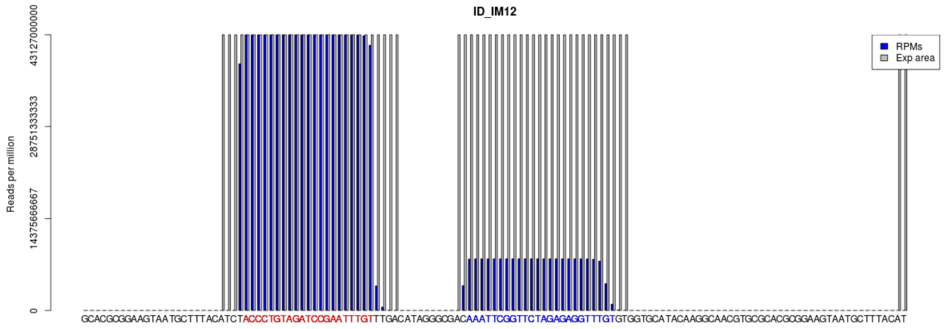

>IM13

GGCTGTTCACGGCACATACATGTCATTTACATTTTCTTTTTGGTTCCACACAACCAAAGACTTTTCACCA**CAAGGGAAAATATCTGTATG**
AGCAAAATATAAATGAAATGTATGTGGACGTGAGCAGCC

>IM13-5P

CAAGGGAAAATATCTGTATG

>miRNA precursor

GGCUGUUCACGGCACAUACAUGUCAUUUACAUUUUCUUUUUGGUUCCACACAACCAAAGACUUUUCACCA**CAAGGGAAAAUAUCUGUAUG**AGCAAAAUAUAAAUGAAAUGUAUGUGGACG
UGAGCAGCC

```
((((((((((((.(((((((((.(((((((.(((((((((((((((......)))))))...(((((.
((....)))))))).........))).))))).))))))).))))))))))..)))))))))))
```

>Predicted STAR seq:

CTGTTCA

Complementarity: 6

Initial dG =   -52.00

```
        10         20        30            40
           G-         G       C     -     |      CC
GGCUGUUCACG  CACAUACAU UCAUUUA AUUUU CUU--UUUGGUU  A
CCGACGAGUGC  GUGUAUGUA AGUAAAU UAAAA GAG  AAACCAA  C
           AG         A       A     C   \ ^      CA
       120       110       100        90         50
```

Reads in: lib-42, lib-45, lib-47, lib-48, lib-49, lib-50, lib-72

Number of reads="IM13",0,0,0,0,0,0

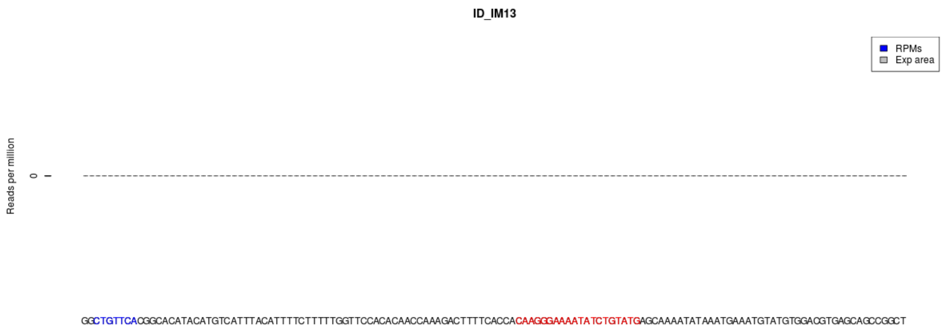

>IM14
TTTCACGTTATTTGCTTGTCTGGATCTTGAGTTATAAGGGAACAGATAGACAGACAACAAATGCGTCTTA**TAAGTATAGAGAGGATGTGG**
CAGGCATGTGGAA

>IM14-3P
TAAGTATAGAGAGGATGTGG

>miRNA precursor
UUUCACGUUAUUUGCUUGUCUGGAUCUUGAGUUAUAAGGGAACAGAUAGACAGACAACAAAUGCGUCUUA**UAAGUAUAGAGAGGAUGUGG**CAGGCAUGUGGAA
.(((((((..(((((.((((((..((((........))))..)))))).............(((((((............))))))).))))).))))))).

>Predicted STAR seq:
TTGTCTGG

Complementarity: 6

Initial dG =   -23.50

```
       10          20         30
U       UA     .-U|     GA    GAG
 UUCACGU  UUUGC   UGUCUG  UCUU   U
 AGGUGUA  GGACG   AUAGAC  GGGA   U
A       C-     \ -^     AA    AUA
 100         90          40
```

Reads in: lib-42, lib-45, lib-47, lib-48, lib-49, lib-50, lib-72
Number of reads="IM14",0,1,4,2,0,2,0

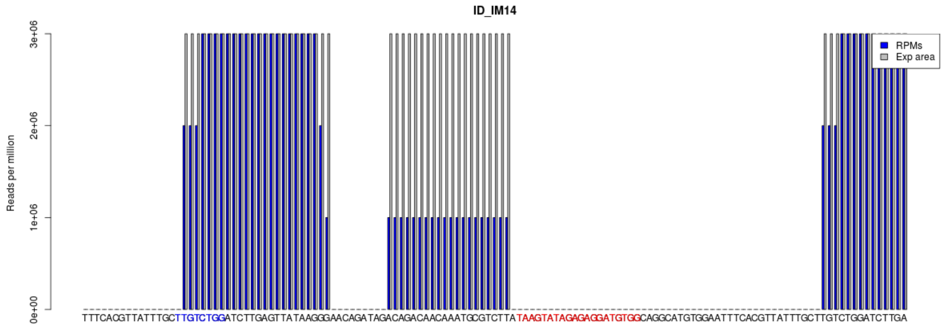

>IM15
TTAGTTGTAGCTAGTTCATAAGTAGTTTGTAAGTTTAGGGACTGATGAACCATAAGCTATACAAACATTT**GAACATCTGAGGAGCTAAA**T
AATAGGGAAAGTGTCATGCAGACATTTCAAAACTAA

>IM15-5P
GAACATCTGAGGAGCTAAA

>miRNA precursor
UUAGUUGUAGCUAGUUCAUAAGUAGUUUGUAAGUUUAGGGACUGAUGAACCAUAAGCUAUACAAACAUUU**GAACAUCUGAGGAGCUAAA**UAAUAGGGAAAGUGUCAUGCAGACAUUUCAA
AACUAA
.(((((.(((((..(((((((((.((((((((((((((((..........)).)))))).))))))))))))).......)))).))))).........((((.
((((.....))))))))..))))).

>Predicted STAR seq:
G

Complementarity: 8

Initial dG =   -29.10

```
          10              20        30          40
U     .-G     AG    ------     A       -       -| GACU
 UAGUU   UAGCU  UUCA      UAAGU GUUUGUA AGUUUA GG    G
 AUCAA   AUCGA  GAGU      GUUUA CAAACAU UCGAAU CC    A
A     \ -     G-    CUACAA     -       A      A^ AAGU
                80        70          60        50
```

Reads in: lib-42, lib-45, lib-47, lib-48, lib-49, lib-50, lib-72
Number of reads="IM15",806,1258,2066,1071,1135,2186,0

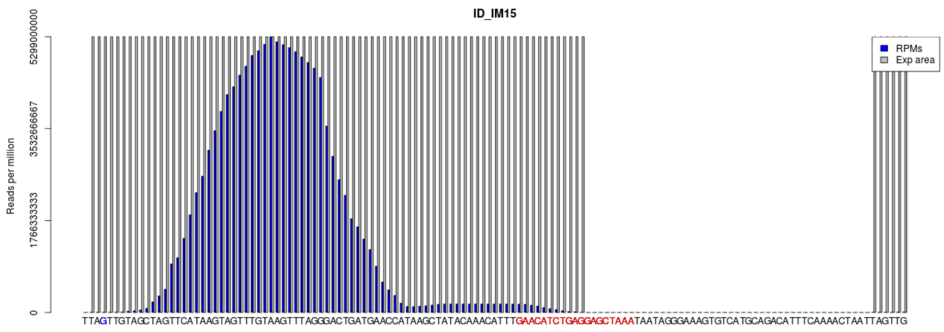

>IM16
GGAAATA**TAAAAGAGCCGCGGCGGA**ATAGCCGAGCGCATTGAGGCGCTATGCTCCGATGTGCGAGCCCGTTCCGTCGGGGGTTCGAACCC
TTCC

>IM16-3P
TAAAAGAGCCGCGGCGGA

>miRNA precursor
GGAAAUA**UAAAAGAGCCGCGGCGGA**AUAGCCGAGCGCAUUGAGGCGCUAUGCUCCGAUGUGCGAGCCCGUUCCGUCGGGGGUUCGAACCCUUCC
((((........(((((.(((((((((.((...((((((((..((.....))..))))))))..))..))))))))).).))))......))))

>Predicted STAR seq:
CGTCGGGGGTTCGAACCCTT

Complementarity: 10

Initial dG =   -40.00

```
        10          20          30         40
|   AUAUAAAA     - G         A-   CGA        AG   GC
GGAA        GAGC C CGGCGGAAU  GC   GCGCAUUG  GC   \
CCUU        CUUG G GCUGCCUUG  CG   CGUGUAGC  CG   U
^   CCCAAG--    G G          CC   AG-        CU   UA
   90          80         70          60         50
```

Reads in: lib-42, lib-45, lib-47, lib-48, lib-49, lib-50, lib-72
Number of reads="IM16",8,10,29,16,7,21,0

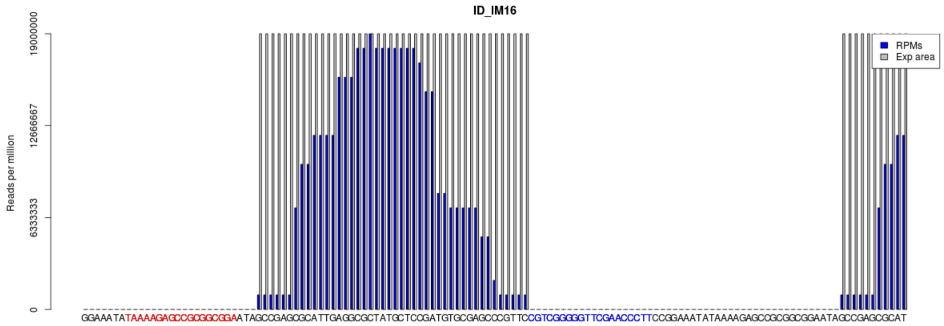

>IM17
GCTACGTTAGCATTTGATCATAACTTGCTCACTGAGTGCGTAACTACGTTTAAATTTGTTAAAAAATTAGTGAGGCAGTGCCATATCAAA
GCCCATTGGTAGC

>IM17-3P
ATCATAACTTGCTCACTGA

>miRNA precursor
GCUACGUUAGCAUUUGAUCAUAACUUGCUCACUGAGUGCGUAACUACGUUUAAAUUUGUUAAAAAAUUAGUGAGGCAGUGCCAUAUCAAAGCCCAUUGGUAGC
(((((....((.((((((....(((.(((((((((.....((((.............)))).....))))))).)))))....)))))))).......)))))

>Predicted STAR seq:
AGTGAGGCAGTGCCATATCA

Complementarity: 12

Initial dG =   -24.70

```
          10        20          30        40        50
     GUUA--| A      CAUA-   U  -       GUGCG    UACGUU
GCUAC      GC UUUGAU     ACU GC UCACUGA     UAAC      \
CGAUG      CG AAACUA     UGA CG AGUGAUU     AUUG      U
     GUUACC^ -      UACCG   -  G       AAAAA    UUUAAA
 100         90        80         70        60
```

Reads in: lib-42, lib-45, lib-47, lib-48, lib-49, lib-50, lib-72
Number of reads="IM17",0,0,0,0,0,0,0

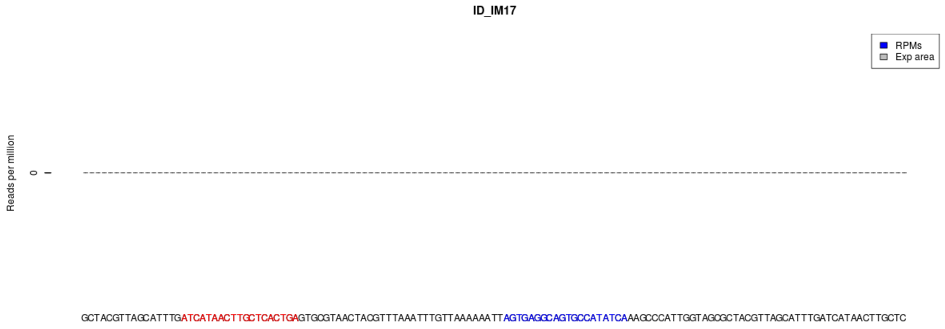

>IM18
TATTGTTGATATTTTTTCAAAAA**TCAGGTACTGAGTGACTCTGAG**AAAGCGTAATAGTGTCTCACGGTCTATAAGTGAAATGTGAGAGAA
TATTGTGAGTA

>IM18-3P
TCAGGTACTGAGTGACTCTGAG

>miRNA precursor
UAUUGUUGAUAUUUUUUCAAAAA**UCAGGUACUGAGUGACUCUGAG**AAAGCGUAAUAGUGUCUCACGGUCUAUAAGUGAAAUGUGAGAGAAUAUUGUGAGUA
((((..(((((((((((((.....((..((((....((((.(((((..((......)).))))).))))....))))...)))))))))))))))..))))

>Predicted STAR seq:
CACGGTCTATAAGTGAAATGTG

Complementarity: 12

Initial dG =   -24.80

```
        10        20         30        40        50
|   GU             AAAAU  GG-    GAGU    C     AA  GU
UAUU  UGAUAUUUUUUCA     CA   UACU    GACU UGAGA  GC  A
AUGA  GUUAUAAGAGAGU     GU   GUGA    CUGG ACUCU  UG  A
^   GU             -----  AAA    AUAU    C     G-  AU
 .        90             80        70        60
```

Reads in: lib-42, lib-45, lib-47, lib-48, lib-49, lib-50, lib-72
Number of reads="IM18",16354,29267,71217,26618,65723,19449,0

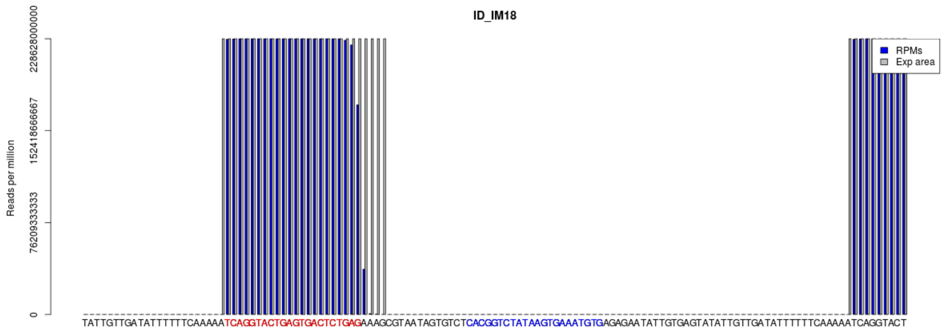

>IM19
TGACAGCGCGT**TGGGTTATTGTGCGCCGGC**CGCGGTGGCCGAGGTGACTTGTGCGTATGGTCGGCAGCGCCGGGCGCCGTGGCTCAGCGC
GCCCGGTCA

>IM19-5P
TGGGTTATTGTGCGCCGGC

>miRNA precursor
UGACAGCGCGU**UGGGUUAUUGUGCGCCGGC**CGCGGUGGCCGAGGUGACUUGUGCGUAUGGUCGGCAGCGCCGGGCGCCGUGGCUCAGCGCGCCCGGUCA
.(((.((((((((((((((.((((.((((((((.(((((((((....)))).)).))).).)))).....))))))).)))))))))))))))...))).

>Predicted STAR seq:
AGCGCCGGGCGCCGTGGCTCAGC

Complementarity: 15

Initial dG = -54.70

```
          10        20             30           40
U   A--               U    G    -----    - G   -   -|   G
 GAC   GCGCGUUGGGUUAU GUGC CCG     GCCG C GUG GC CGAG U
 CUG   CGCGCGACUCGGUG CGCG GGC     CGGC G UAU CG GUUC G
A   GCC               C    -    CGCGA    U G   G  U^   A
        90        80         70        60        50
```

Reads in: lib-42, lib-45, lib-47, lib-48, lib-49, lib-50, lib-72
Number of reads="IM19",126,377,194,208,138,147,0

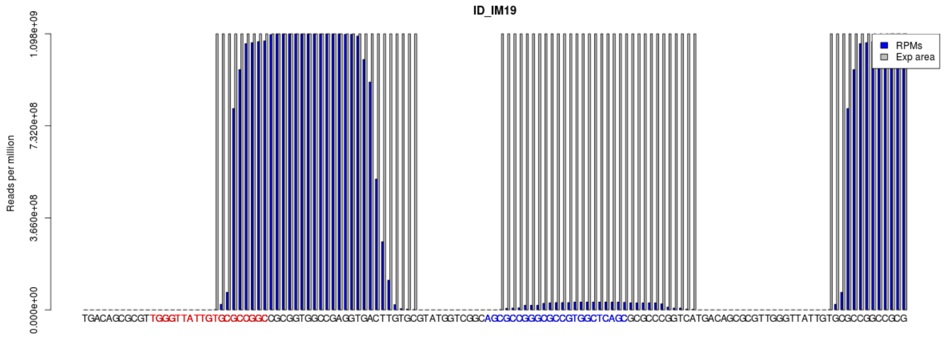

>IM2
CCGTCATTTCGAGCTGAGATGCAGCTCAGCTGTAGTTTTAGTAGCGGTAGCAAAAGCCAAAACGA**GCGGACGAACTAAAAGAAGAG**CTAC
CTGTCGCGAACTAAAATATACGCGATAGGTCGCGG

>IM2-3P
GCGGACGAACTAAAAGAAGAG

>miRNA precursor
CCGUCAUUUCGAGCUGAGAUGCAGCUCAGCUGUAGUUUUAGUAGCGGUAGCAAAAGCCAAAACGA**GCGGACGAACUAAAAGAAGAG**CUACCUGUCGCGAACUAAAAUAUACGCGAUAGGU
CGCGG
.((((..((((.(((((((((((((...))))))).)))))))...(((.......)))....))))..))))............((.
((((((((((............))))))))).))..

>Predicted STAR seq:
TCAT

Complementarity: 3
ERROR 4: Strange structure
Initial dG = -42.40

```
          10           20
.-C    AU    .-A        -|      U
   CGUC  UUCG   GCUGAGA UGCAGC \
   GCAG  GAGC   UGAUUUU AUGUCG C
\ -    GC    \ -        G^      A
    70           40        30



                      50
               AGC-   AGC
                   GGU   \
                   CCG   A
               AAAA   AAA
                60
```

Reads in: lib-42, lib-45, lib-47, lib-48, lib-49, lib-50, lib-72
Number of reads="IM2",7,4,20,13,11,19,0

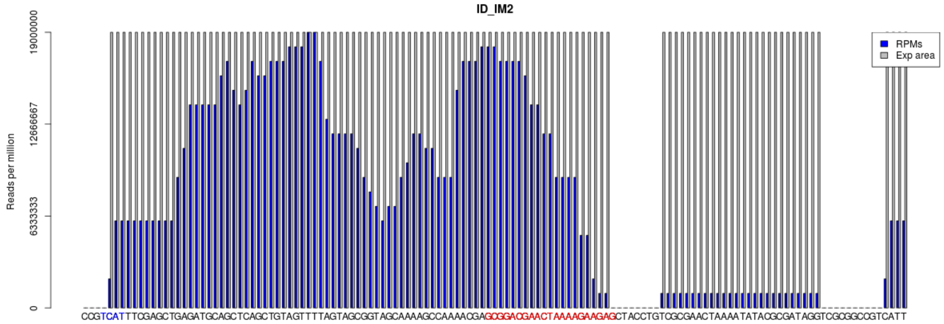

>IM20

ACGTAGATCGAATGCCCGGGGAAAAGACGAGACAGACGCCGATGCGAGAGACAACAGCAGAACG**AGTTGTTGACGTGGCTTTAGCG**GGTC
TGTACGGTTACGTCCCGAACATTTCTTTTAGT

>IM20-3P

AGTTGTTGACGTGGCTTTAGCG

>miRNA precursor

ACGUAGAUCGAAUGCCCGGGGAAAAGACGAGACAGACGCCGAUGCGAGAGACAACAGCAGAACG**AGUUGUUGACGUGGCUUUAGCG**GGUCUGUACGGUUACGUCCCGAACAUUUCUUUUA
GU

```
...((((..(((((..(((((....(((...((((((.(((....(((..((..
((((((......))))))..))..)))...)))))))))...)))...)))))..)))))..)))).. 
```

>Predicted STAR seq:

GATGCGAGAGACAACAGCAG

Complementarity: 11

Initial dG =   -37.50

```
       10        20        30        40        50        60
ACG|   UC     CC     AAAA   GAG      G   AUGC   AG  AA      AA
   UAGA  GAAUG  CGGGG    GAC   ACAGAC CCG    GAG  AC  CAGCAG  C
   AUUU  UUUAC  GCCCU    UUG   UGUCUG GGC    UUC  UG  GUUGUU  G
UG-^   UC     AA     GCA-   GCA      -   GAU-   GG  CA      GA
 120       110       100         90          80        70
```

Reads in: lib-42, lib-45, lib-47, lib-48, lib-49, lib-50, lib-72

Number of reads="IM20",0,0,0,0,0,0,0

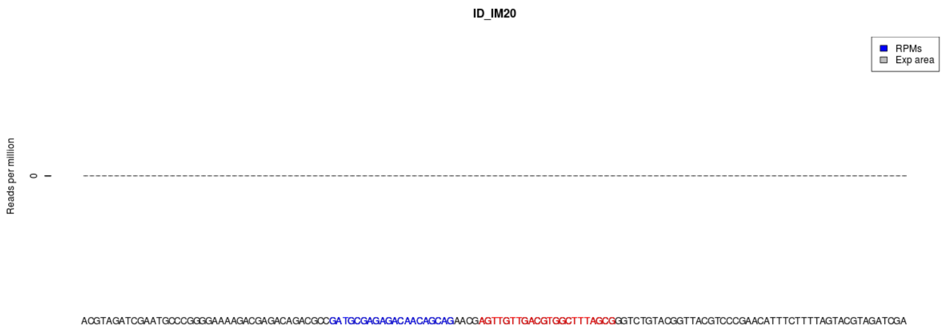

>IM21
CTCGAAC**CTGGGACCTTTGGCTTTCGGT**GGCAAGCGCACTACCGACTGAGCTATCGGTAGAGCGCTTGCCCGCGAAAGGCAAAGGTCCCA
GGTTCGAG

>IM21-5P
CTGGGACCTTTGGCTTTCGGT

>miRNA precursor
CUCGAAC**CUGGGACCUUUGGCUUUCGGU**GGCAAGCGCACUACCGACUGAGCUAUCGGUAGAGCGCUUGCCCGCGAAAGGCAAAGGUCCCAGGUUCGAG
((((((((((((((((((.((((((..(((((((((.(((((((........))))))).)))))))))..)))))).)))))))))))))))))))

>Predicted STAR seq:
AAAGGCAAAGGTCCCAGGT

Complementarity: 16

Initial dG =   -78.30

```
        10        20        30        40
|                  G      GU         A       CUG
CUCGAACCUGGGACCUUUG CUUUCG  GGCAAGCGC CUACCGA   A
GAGCUUGGACCCUGGAAAC GAAAGC  CCGUUCGCG GAUGGCU   G
^                  G      GC         A       AUC
       90        80        70        60        50
```

Reads in: lib-42, lib-45, lib-47, lib-48, lib-49, lib-50, lib-72
Number of reads="IM21",129,158,203,159,174,381,0

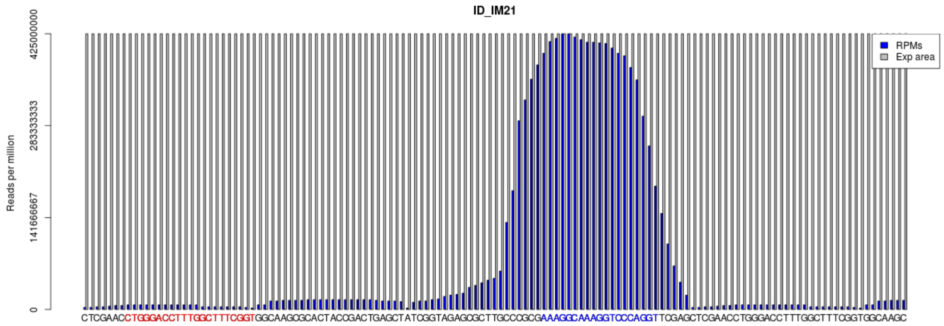

>IM22
AGTTATTATATTAAAATTCACTAGTTAAAAACATGACAGC**TTACAAAATATTCAGTATTTTG**TAAGCAGTCATGTTTTTAACTAGAGTAT
TTTAATATAGTGACT

>IM22-5P
TTACAAAATATTCAGTATTTTG

>miRNA precursor
AGUUAUUAUAUUAAAAUUCACUAGUUAAAAACAUGACAGC**UUACAAAAUAUUCAGUAUUUUG**UAAGCAGUCAUGUUUUUAACUAGAGUAUUUUAAUAUAGUGACU
(((((((((((((((((...(((((((((((((((((.(((((((((((((...))))))))))))).)))))))))))))))))...)))))))))))))))))

>Predicted STAR seq:
TTATTAT

Complementarity: 6

Initial dG =   -53.70

```
        10        20        30        40        50
|                UCA                A             U
AGUUAUUAUAUUAAAAU   CUAGUUAAAAACAUGAC GCUUACAAAAUAU \
UCAGUGAUAUAAUUUUA   GAUCAAUUUUUGUACUG CGAAUGUUUUAUG C
^                UGA                A             A
   100        90        80        70        60
```

Reads in: lib-42, lib-45, lib-47, lib-48, lib-49, lib-50, lib-72
Number of reads="IM22",0,0,3,0,2,2,0

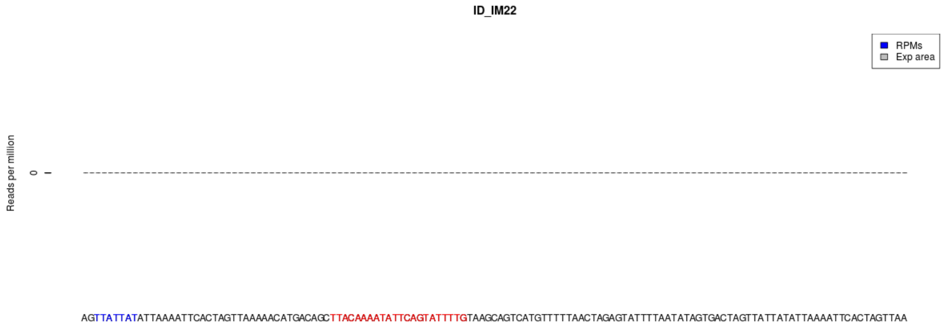

>IM23
GCAAGGCGGACTGTGAAGAGAGCTATCCGTCGACAGTATAGTGAGAAAAC**TGTCATGGAGTTGCTCTCTTTAT**GGACGACCTGC

>IM23-3P
TGTCATGGAGTTGCTCTCTTTAT

>miRNA precursor
GCAAGGCGGACUGUGAAGAGAGCUAUCCGUCGACAGUAUAGUGAGAAAAC**UGUCAUGGAGUUGCUCUCUUUAU**GGACGACCUGC
```
(((.((((..(((((((((((((..(((((.((((((...........)))))))))))...))))))))))))).)).)))))
```

>Predicted STAR seq:
TGAAGAGAGCTATCCGTCGACA

Complementarity: 18

Initial dG =   -39.50

```
         10        20         30        40
|  A  -  GA            UA-     C      AUAGU
GCA GG CG  CUGUGAAGAGAGC   UCCGU GACAGU     \
CGU CC GC  GGUAUUUCUCUCG   AGGUA CUGUCA     G
^  -  A  A-            UUG     -      AAAGA
    80        70        60         50
```

Reads in: lib-42, lib-45, lib-47, lib-48, lib-49, lib-50, lib-72
Number of reads="IM23",1378,16620,4112,170,4527,2070,0

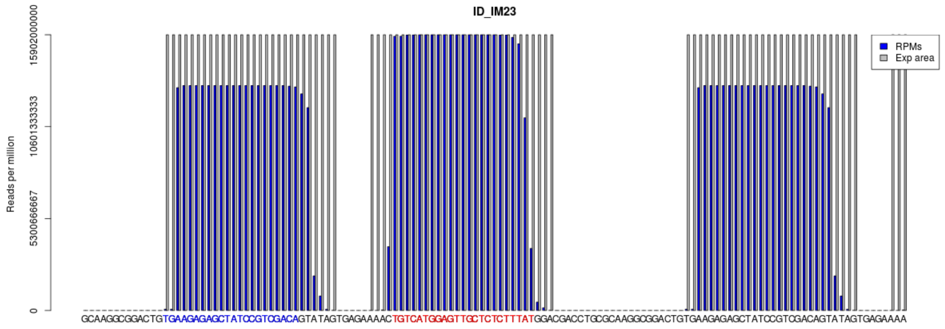

>IM24
ATTTAGTTGTGTCTGATTATAAAAATACGTATATTAAAACATCACAGCTTCTAAGTATCTTACAAG**CTTTGGTGTTTTAACTTATGA**ATT
TTTATATTTCCGCTAAGT

>IM24-5P
CTTTGGTGTTTTAACTTATGA

>miRNA precursor
AUUUAGUUGUGUCUGAUUAUAAAAAUACGUAUAUUAAAACAUCACAGCUUCUAAGUAUCUUACAAG**CUUUGGUGUUUUAACUUAUGA**AUUUUUAUAUUUCCGCUAAGU
((((((.......((.(((((((((.((((..(((((((((((.(((((.((((...)))).))))).)))))))))))..)))).))))))))).))..)))))))

>Predicted STAR seq:
CGTATATTAAAACATCACAG

Complementarity: 15

Initial dG =   -30.00

```
        10        20        30        40        50
|      UGUGUCU  U         A    UA           C     C    U
AUUUAGU       GA UAUAAAAAU CGUA  UUAAAACAUCA AGCUU UAAG \
UGAAUCG       UU AUAUUUUUA GUAU  AAUUUUGUGGU UCGAA AUUC A
^      CC-----  U         A    UC           U     C    U
      100             90        80        70        60
```

Reads in: lib-42, lib-45, lib-47, lib-48, lib-49, lib-50, lib-72
Number of reads="IM24",0,0,0,0,0,0,0

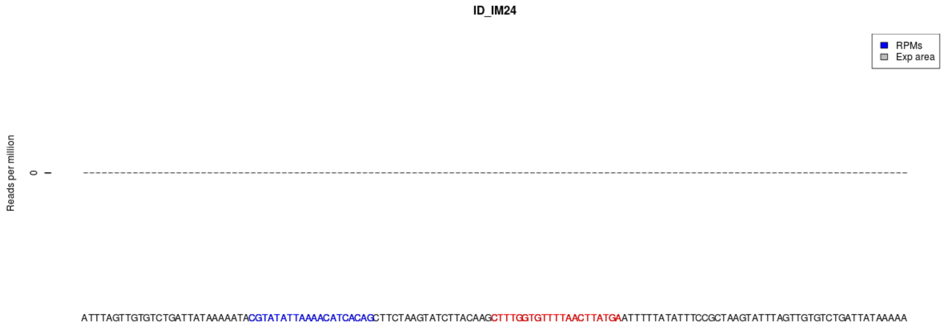

>IM25
AAACATGGTTTTAAACATCTAAAGCTTTTTGATCTTCATGCTGAAGATTGAACGTAATCTTCAATGAGAA**GATCGGAAAGTTTTATATGT**
TTTAAACCACTTGTTT

>IM25-5P
GATCGGAAAGTTTTATATGTT

>miRNA precursor
AAACAUGGUUUUAAACAUCUAAAGCUUUUUGAUCUUCAUGCUGAAGAUUGAACGUAAUCUUCAAUGAGAA**GAUCGGAAAGUUUUAUAUGUU**UUAAACCACUUGUUU
((((((((((.((((((.((((((((((((((((((.(..(((((((((....)))))))))..).)))))))))))))))))).)))))).))))))..)))))

>Predicted STAR seq:
ACATCTAAAGCTTT

Complementarity: 18

Initial dG =   -44.90

```
          10        20        30        40        50
     --|     U      C                  A GC         A
AAACA  UGGUUU AAACAU UAAAGCUUUUUGAUCUUC U  UGAAGAUUG A
UUUGU  ACCAAA UUUGUA AUUUUGAAAGGCUAGAAG G  ACUUCUAAU C
     UC^     U      U                  A UA         G
    100        90        80        70        60
```

Reads in: lib-42, lib-45, lib-47, lib-48, lib-49, lib-50, lib-72
Number of reads="IM25",0,0,0,0,0,0,0

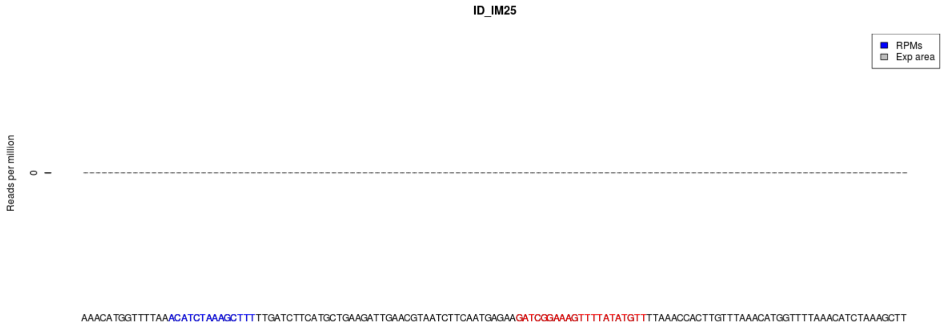

>IM26
GCCACGAGACGACAGAAGACAAAGCGCTGCTCGCGGTT**ACTCACTCAACCTGGGTGTGATGT**CCGTTGAGAGCCCGTCACAACCTCCTTG
AGTGAGCGACCGCGGCGGCCGACATCGGCACATCAGCCGCGGC

>IM26-5P
ACTCACTCAACCTGGGTGTGATGT

>miRNA precursor
GCCACGAGACGACAGAAGACAAAGCGCUGCUCGCGGUU**ACUCACUCAACCUGGGUGUGAUGU**CCGUUGAGAGCCCGUCACAACCUCCUUGAGUGAGCGACCGCGGCGGCCGACAUCGGCA
CAUCAGCCGCGGC
(((......................(((((.((((((..(((((((((...((.(((((((...((.....)).))))))).))...)))))))))..)))))))))))......
((((......)))).)))

>Predicted STAR seq:
TCACAACCTCCTTGAGTGAGCGAC

Complementarity: 16

Initial dG =   -57.90

```
          10        20        30        40        50        60
|  .-ACGAGACGACAGAAGACAAAGC     U      UA         CCU  G       UCC  UG
GCC                       GCUGC CGCGGU  CUCACUCAA   GG UGUGAUG   GU  \
CGG                       CGGCG GCGCCA  GAGUGAGUU   CC ACACUGC   CG  A
^  \ ----------------------     -      GC         CCU  A       C--  AG
                                 100        90        80         70



   110       120
     CGACAU    AC
           CGGC  A
           GCCG  U
     C-----    AC
   130
```

Reads in: lib-42, lib-45, lib-47, lib-48, lib-49, lib-50, lib-72
Number of reads="IM26",7829,11484,2861,23117,5284,1971,0

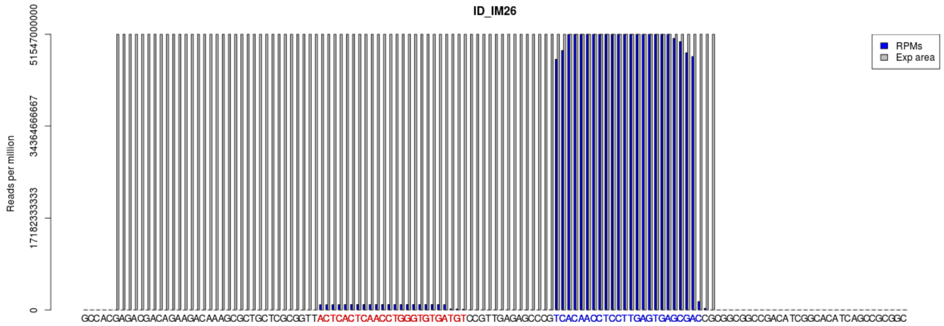

>IM27
GAAAAAAAAAAATCTCTTTATCCCGTACCTTAGGATACCGAATCTGTGTGCAGAGC**CGTTGATTTGGTGTCCTA**AGGAACGGGATAAGGCG
ATTTTTTTTTTT

>IM27-5P
CGTTGATTTGGTGTCCTA

>miRNA precursor
GAAAAAAAAAAAUCUCUUUAUCCCGUACCUUAGGAUACCGAAUCUGUGUGCAGAGC**CGUUGAUUUGGUGUCCUA**AGGAACGGGAUAAGGCGAUUUUUUUUUUU
((((((((((((.(((((((((((.((((((((((((((((((..((.((...)))).)))))))))))))))))))).))))))))))).)))))))))))))

>Predicted STAR seq:
AGGATACCGAATCTGTG

Complementarity: 14

Initial dG =   -59.30

```
        10        20        30        40        50
|            U           A                 UG  U  A
GAAAAAAAAAAAUC CUUUAUCCCGU CCUUAGGAUACCGAAUC  UG GC \
UUUUUUUUUUUAG GGAAUAGGGCA GGAAUCCUGUGGUUUAG  GC CG G
^            C           A                 UU  -  A
100        90        80        70        60
```

Reads in: lib-42, lib-45, lib-47, lib-48, lib-49, lib-50, lib-72
Number of reads="IM27",0,0,0,0,0,0,0

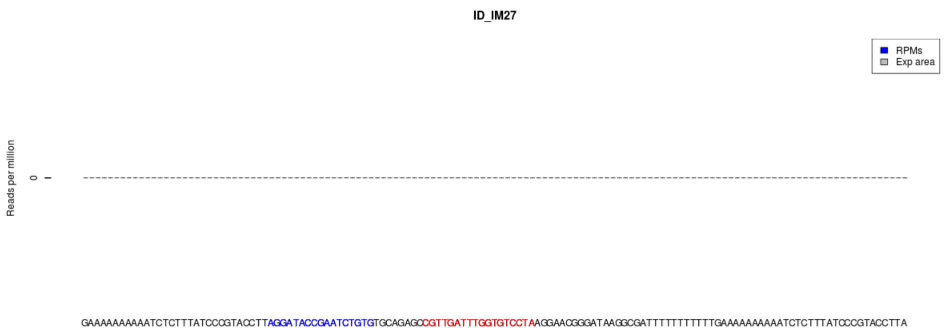

>IM28
CATCGTCGACAGGACCAACTGCAGTCCTCTGGTATAGAGCCGAGTGGAGGGTCTGATTCAGAGGCACAG**ACGGTTCTGTTACAGTGTAGA**
CTGCAGACTCCTTGACTTG

>IM28-5P
ACGGTTCTGTTACAGTGTAGA

>miRNA precursor
CAUCGUCGACAGGACCAACUGCAGUCCUCUGGUAUAGAGCCGAGUGGAGGGUCUGAUUCAGAGGCACAG**ACGGUUCUGUUACAGUGUAGA**CUGCAGACUCCUUGACUUG
```
....((((..((((....((((((((..(((..(((((((((..((.....((((...))))....))..)))))))))..)))....))))))))..))))))))...
```

>Predicted STAR seq:
CCTCTGGTATAGAGCCG

Complementarity: 12

Initial dG =   -39.80

```
        10        20          30        40        50
CAUC|   AC    CCAA        CU--   GU         AG  GAGGG    A
    GUCG  AGGA    CUGCAGUC    CUG  AUAGAGCCG  UG     UCUG \
    CAGU  UCCU    GACGUCAG    GAC  UGUCUUGGC  AC     AGAC U
GUU-^   --    CA--        AUGU   AU         AG  ACGG-    U
          100          90        80        70         60
```

Reads in: lib-42, lib-45, lib-47, lib-48, lib-49, lib-50, lib-72
Number of reads="IM28",1,0,2,2,0,5,0

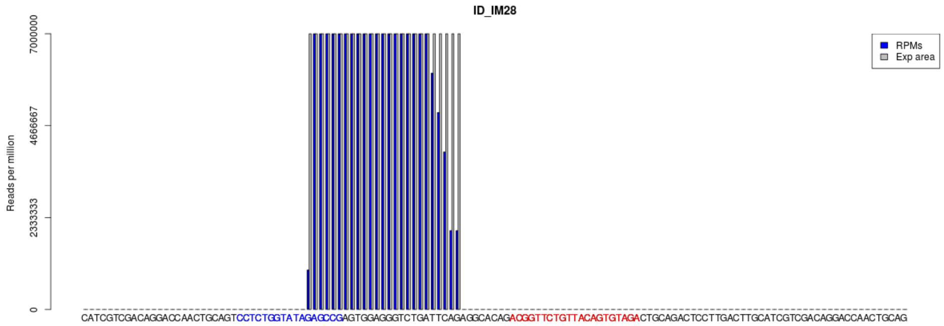

>IM29

AGACCATGATCA**TGATGGATGTTGGTGTAGAGG**AATGAGTAAGGTAAATGTCACTGTTGAAACACTTCCCTATACACCAACATCTATCAT
GCCCCCATGGTCT

>IM29-5P

TGATGGATGTTGGTGTAGAGG

>miRNA precursor

AGACCAUGAUCA**UGAUGGAUGUUGGUGUAGAGG**AAUGAGUAAGGUAAAUGUCACUGUUGAAACACUUCCCUAUACACCAACAUCUAUCAUGCCCCCAUGGUCU
((((((((..((((((((((((((((((((.(((...((((......(((......))).....)))).))).))))))))))))))))))))....))))))))

>Predicted STAR seq:

TATACACCAACATCTATCATG

Complementarity: 18

Initial dG =   -45.70

```
        10          20        30        40        50
        AU--|                 G   AAU    AAGGUA   GU
AGACCAUG    CAUGAUGGAUGUUGGUGUA AGG   GAGU      AAU  C
UCUGGUAC    GUACUAUCUACAACCACAU UCC   UUCA      UUG  A
        CCCC^                 A   C--    CAAAG-   UC
 100        90        80        70          60
```

Reads in: lib-42, lib-45, lib-47, lib-48, lib-49, lib-50, lib-72

Number of reads="IM29",0,0,1,1,1,3,0

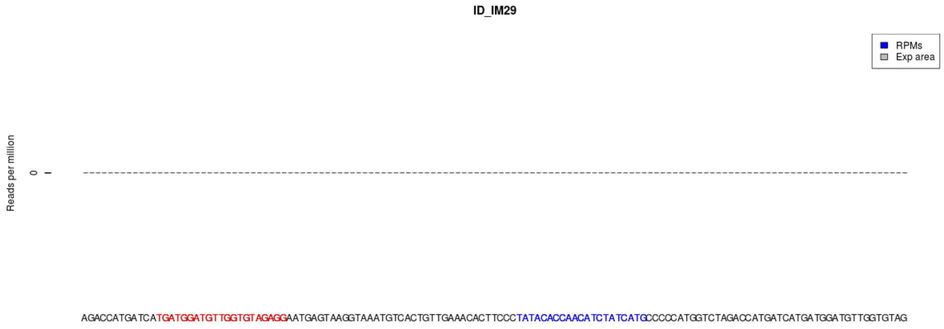

>IM3
GGTTGAGAGCAGCCTCCCTTGGTAACTTACCAGCAGCGAGGTATAGAGTTCCTACGTGTGTTGTTATAG**TAGGAACTTCATACCGTGCTC**
TTGGATTTGCCATCCTTATCGACGCTCTCACTTCC

>IM3-3P
TAGGAACTTCATACCGTGCTCT

>miRNA precursor
GGUUGAGAGCAGCCUCCCUUGGUAACUUACCAGCAGCGAGGUAUAGAGUUCCUACGUGUGUUGUUAUAG**UAGGAACUUCAUACCGUGCUCU**UGGAUUUGCCAUCCUUAUCGACGCUCUCA
CUUCC
...(((((((.........((((((....((((.((((.(((((..(((((((((.
((((....))))))))))))).))))).)))).)))).))))))...........))))))).....

>Predicted STAR seq:
CAGCGAGGTATAGAGTTCCTA

Complementarity: 16

Initial dG =   -48.20

```
         10         20        30        40        50        60
GGU--       AGCCUCCCU--|     CUUA    C    A     AG         G    U
     UGAGAGC           UGGUAA    CCAG AGCG GGUAU  AGUUCCUAC UGUG U
     ACUCUCG           ACCGUU    GGUU UCGU CCAUA  UCAAGGAUG AUAU G
CCUUC       CAGCUAUUCCU^     UA--    C    G     CU         -    U
   120       110       100          90        80        70
```

Reads in: lib-42, lib-45, lib-47, lib-48, lib-49, lib-50, lib-72
Number of reads="IM3",110771,212785,399102,244412,526637,179862,0

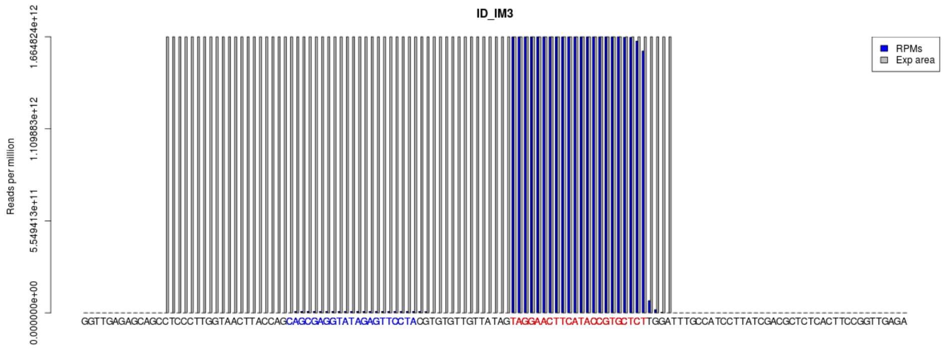

>IM30

GGTGGAGACATGGCCACAGCCTAAGGGATGTTTGCAGAAT**GAAACGTTCACTCTGCAGTTCT**CTGAAAGTTTCATTGTTTCCAGAATGAA
ACATCCCTTAGGCTGTGGCCATGTCTCCACC

>IM30-3P

GAAACGTTCACTCTGCAGTTCT

>miRNA precursor

GGUGGAGACAUGGCCACAGCCUAAGGGAUGUUUGCAGAAU**GAAACGUUCACUCUGCAGUUCU**CUGAAAGUUUCAUUGUUUCCAGAAUGAAACAUCCCUUAGGCUGUGGCCAUGUCUCCAC
C

```
((((((((((((((((((((((((((((((((((.((....((((((...(((...
(((....)))..))).....))))))).....)))))))))))))))))))))))))))))))))))
Error predicting STAR on 5pERROR 4: Strange structure
 Initial dG =    -79.80

        10        20        30         40
                                 |    A    A
GGUGGAGACAUGGCCACAGCCUAAGGGAUGUUU--GCAGA UGAA \
CCACCUCUGUACCGGUGUCGGAUUCCCUACAAA  CGUCU ACUU C
                                 \ ^    C    G
 .       110       100        90         50



                                   60        70
                                 AGUUC    A   UUU
                                      UCUG AAG   C
                                      AGAC UUU   A
                                 GUA--    C   GUU
                                          80
```

Reads in: lib-42, lib-45, lib-47, lib-48, lib-49, lib-50, lib-72

Number of reads="IM30",4,12,14,6,11,15,0

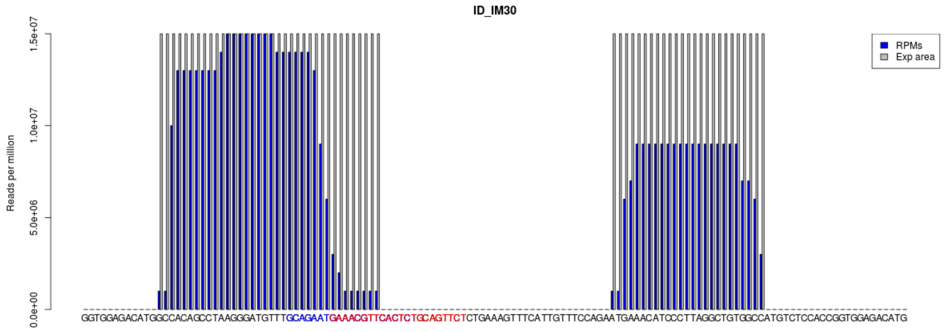

>IM31
ATGCGTAGCCAATTACTGTCCGAAGGGGTGAGACACAGTACAGATCGGGTTTTCGGGTGCGCTGTCCACT**TATCTGTATGTGCAGTCTTG**
CATGTTGGTTGGTTCTGGACGCGT

>IM31-3P
TATCTGTATGTGCAGTCTTGC

>miRNA precursor
AUGCGUAGCCAAUUACUGUCCGAAGGGGUGAGACACAGUACAGAUCGGGUUUUCGGGUGCGCUGUCCACU**UAUCUGUAUGUGCAGUCUUGC**AUGUUGGUUGGUUCUGGACGCGU
((((((..(((...((((.((((....(..((((...(((((...(((((....(((.......)))....)))))..))))).)))).)..)...)))).)))).))..)))))))))

>Predicted STAR seq:
GGGGTGAGACACAGTACAGATCGGGT

Complementarity: 13

Initial dG =   -37.30

```
            10          20
      ----         .-CUGU| G
AUGCGU    AGCCAAUUA      CC A
UGCGCA    UUGGUUGGU      GG A
      GGUC         \ ----^ G
  110       100
```

Reads in: lib-42, lib-45, lib-47, lib-48, lib-49, lib-50, lib-72
Number of reads="IM31",0,0,0,0,0,0

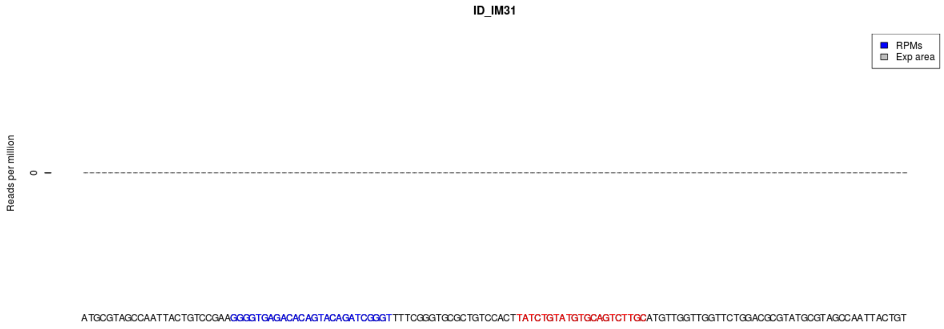

>IM32
GACAGAAGCCTCTCCCGCCCACCCTTCTTATGGGG**GGAAGGGGGAGCCGCGGTG**CAGTAGCCGAGTGGTCTAAAGCGACTCGCTGCGGAG
CGCGCGGTTGCTCGCCGTGGAGGTTCGAGTC

>IM32-5P
GGAAGGGGGAGCCGCGGTG

>miRNA precursor
GACAGAAGCCUCUCCCGCCCACCCUUCUUAUGGGG**GGAAGGGGGAGCCGCGGUG**CAGUAGCCGAGUGGUCUAAAGCGACUCGCUGCGGAGCGCGCGGUUGCUCGCCGUGGAGGUUCGAGU
C
(((.(((..(((((((.(((.(((.......))))))..)))))))((((((((.((((((((.(((.(((..
((((...))))..))).))).))))))))))))))))...)))..)))

>Predicted STAR seq:
CGGTTGCTC

Complementarity: 6

```
 Initial dG =    -65.80

           10         20
ERROR 3: Strange structure
 Initial dG =    -65.80

           10         20
   A-   .-GC       G-|  A   UUC
GAC  GAA    CUCUCCC  CCC CCC   \
CUG  CUU    GAGGGGG  GGG GGG   U
   AG   \ --       AA^  -   UAU
 .               40         30


               50        60        70
          ---       C        A   G   AA    A
             CCGCGGUG AGUAGCCG GUG UCU  AGCG \
             GGUGCCGC UCGUUGGC CGC AGG  UCGC C
          GGA       -        G   G   CG    U
           110       100        90        80
```

Reads in: lib-42, lib-45, lib-47, lib-48, lib-49, lib-50, lib-72
Number of reads="IM32",34,58,125,51,66,97,0

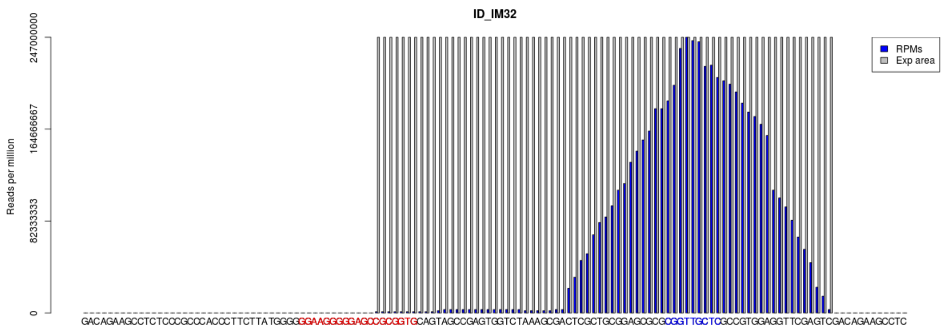

>IM33

GCAGGCCTGAGACAACGCGCTCGCTGGCTGACTGGCTGGCCGTCTTGGGACTGTTGGCTTGCCCCAGCGG**CCAGCTGCCAGCTGCGAGCG**
CGTTGTCTTAGGCCTGT

>IM33-5P

CCAGCTGCCAGCTGCGAGCGC

>miRNA precursor

GCAGGCCUGAGACAACGCGCUCGCUGGCUGACUGGCUGGCCGUCUUGGGACUGUUGGCUUGCCCCAGCGG**CCAGCUGCCAGCUGCGAGCGC**GUUGUCUUAGGCCUGU
((((((((((((((((((((((((.(((((...((((((((((..((((.............))))))))))))))..)))))))))))))))))))))))))))))

>Predicted STAR seq:

CGCTCGCTGGCTGACTGGCTGG

Complementarity: 17

Initial dG =   -82.90

```
        10        20        30        40        50
|                       U     ACU          CU    ACUGUU
GCAGGCCUGAGACAACGCGCUCGC GGCUG   GGCUGGCCGU  UGGG      \
UGUCCGGAUUCUGUUGCGCGAGCG UCGAC   UCGACCGGCG  ACCC      G
^                       -     CG-          --    CGUUCG
     100        90         80         70          60
```

Reads in: lib-42, lib-45, lib-47, lib-48, lib-49, lib-50, lib-72

Number of reads="IM33",0,0,0,0,0,0,0

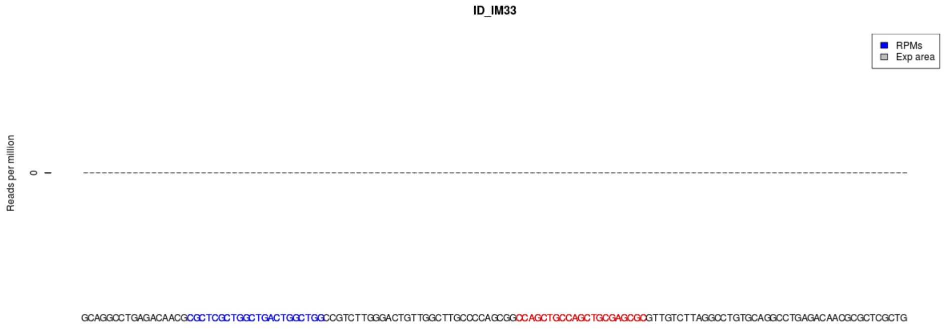

>IM34
AGT**TTACTGGACATTGTAAGAACGG**GCCTAAGGGAGATTCGCGACAGCTGATTTCCTTATAAGTTGTTCCTTAGAGCTCCTCGGGACT

>IM34-3P
TTACTGGACATTGTAAGAACGG

>miRNA precursor
AGU**UUACUGGACAUUGUAAGAACGG**GCCUAAGGGAGAUUCGCGACAGCUGAUUUCCUUAUAAGUUGUUCCUUAGAGCUCCUCGGGACU
(((((...(((..((.(((((((((.....(((((((((.((....)).))))))))).....))))).)))).)).)))...)))))

>Predicted STAR seq:
GTTCCTTAGAGCTCCTCGGGAC

Complementarity: 14

Initial dG = -23.30

```
       10        20         30        40
|    ACU   CA  G    -     GCCUA         C  G
AGUUU   GGA  UU UAAG AACGG     AGGGAGAUU GC A
UCAGG   CCU  GA AUUC UUGUU     UUCCUUUAG CG C
^    GCU   C-  G    C     GAAUA         U  A
      80         70        60        50
```

Reads in: lib-42, lib-45, lib-47, lib-48, lib-49, lib-50, lib-72
Number of reads="IM34",0,0,0,0,0,0,0

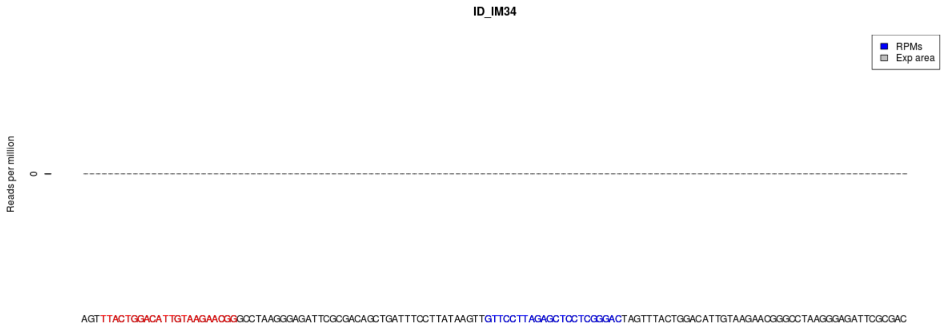

>IM35

GCGACTGTGTTGCCCCACTGCAGCTGCTGGACACTGCACAAGATTAGCGATTAAAAAACT**CTTGTGCGTGTGACAGCGGCTAT**TGTGGGT
GCTTCACCTGC

>IM35-3P

CTTGTGCGTGTGACAGCGGCTAT

>miRNA precursor

GCGACUGUGUUGCCCCACUGCAGCUGCUGGACACUGCACAAGAUUAGCGAUUAAAAAACU**CUUGUGCGUGUGACAGCGGCUAU**UGUGGGUGCUUCACCUGC
(((...(((..(((((((...((((((((.((((.((((((((................))))))))))))..))))))))...))))).))..))).)))

>Predicted STAR seq:

GCAGCTGCTGGACACTGCACAAG

Complementarity: 17

Initial dG =   -45.50

```
        10         20        30         40        50
|  ACU   UU  -     UGC        G-    U        UUAGCGA
GCG   GUG  GC CCCAC   AGCUGCUG  ACAC GCACAAGA       U
CGU   CAC  CG GGGUG   UCGGCGAC  UGUG CGUGUUCU       U
^  C--   UU  U     UUA        AG    -        CAAAAAA
 .          90        80        70         60
```

Reads in: lib-42, lib-45, lib-47, lib-48, lib-49, lib-50, lib-72

Number of reads="IM35",211,2838,214,4589,743,200,0

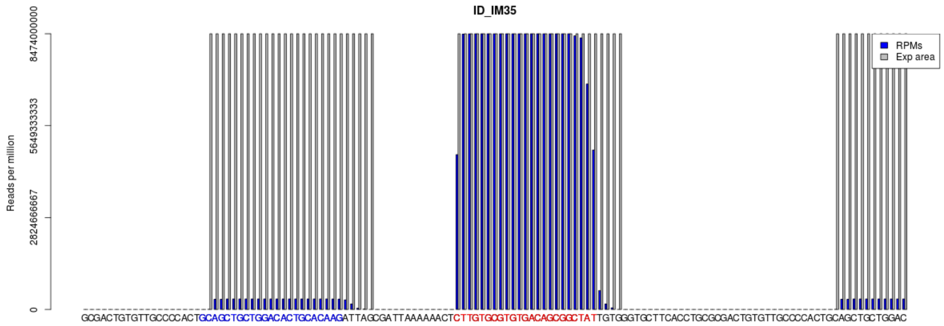

>IM36
CAGCCACTTGGGAGCGCCTGTCAACATCTTACCGGGCAGCATTAGATTGTGATTGTGATTTC**TAATACTGTCAGGTAACGATGTC**CTCCG
GGCTCCGTTCCGTCGAGACGCCGTCGCCGGCTG

>IM36-3P
TAATACTGTCAGGTAACGATGTC

>miRNA precursor
CAGCCACUUGGGAGCGCCUGUCAACAUCUUACCGGGCAGCAUUAGAUUGUGAUUGUGAUUUC**UAAUACUGUCAGGUAACGAUGUC**CUCCGGGCUCCGUUCCGUCGAGACGCCGUCGCCGG
CUG
(((((.((((((((((((((...((((((((((.(((((.((((((..............)))))).))))).))))).)))))....)))))...)))))..)))))..
((....)).)))))

>Predicted STAR seq:
CAACATCTTACCGGGCAGCATTA

Complementarity: 17

Initial dG =   -45.80

```
          10             20           30         40         50
     .-A    --     ---     UCA-     -|    G     C      UUGUGA
CAGCC   CUUG  GGAGC   GCCUG    ACAUC UUACC GGCAG AUUAGA      U
GUCGG   GAGC  CCUUG   CGGGC    UGUAG AAUGG CUGUC UAAUCU      U
     \ -    UG     CCU     CUCC     C^    A     A      UUAGUG
 120         100        90         80         70         60
```

Reads in: lib-42, lib-45, lib-47, lib-48, lib-49, lib-50, lib-72
Number of reads="IM36",168418,431229,308447,217136,131698,119848,0

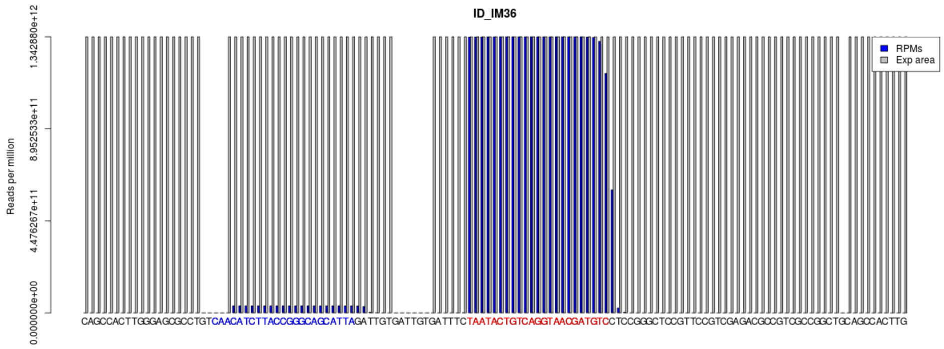

>IM37

A**CATCTTACCGGGCAGCATTAGA**TTGTGATTGTGATTTCTAATACTGTCAGGTAACGATGT

>IM37-3P

CATCTTACCGGGCAGCATTAGA

>miRNA precursor

A**CAUCUUACCGGGCAGCAUUAGA**UUGUGAUUGUGAUUUCUAAUACUGUCAGGUAACGAUGU

(((((((((.(((((.((((((..............)))))).))))).))))).)))))

>Predicted STAR seq:

TAATACTGTCAGGTAACGATGT

Complementarity: 18

Initial dG =   -22.00

```
        10        20        30
     -|    G     C      UUGUGA
ACAUC UUACC GGCAG AUUAGA      U
UGUAG AAUGG CUGUC UAAUCU      U
     C^    A     A      UUAGUG
60        50        40
```

Reads in: lib-42, lib-45, lib-47, lib-48, lib-49, lib-50, lib-72

Number of reads="IM37",21661,74944,20527,33557,35136,26540,0

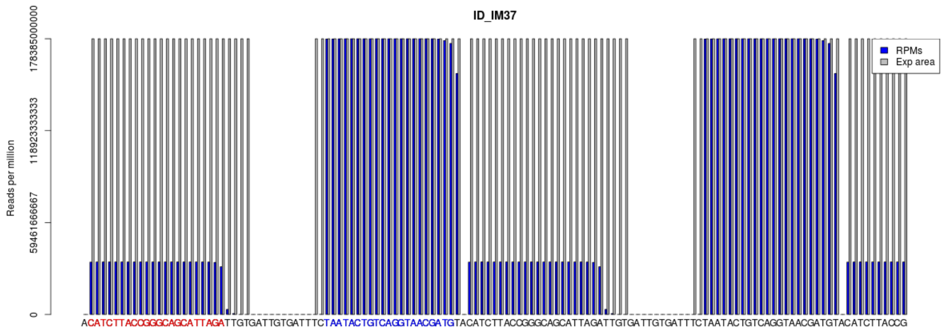

>IM38
AGTGTGCGTCACTAAATGAGGAACATCCATGTTCCCCCTCTGTCACAGTGATTGTCCTCTGCCTTAAT**TTTTGTGAAACTGGTGGCTGCT**
GAGCGAGCGGTCTAACGTAACGGTGTCACACT

>IM38-5P
TTTTGTGAAACTGGTGGCTGCT

>miRNA precursor
AGUGUGCGUCACUAAAUGAGGAACAUCCAUGUUCCCCCUCUGUCACAGUGAUUGUCCUCUGCCUUAAU**UUUUGUGAAACUGGUGGCUGCU**GAGCGAGCGGUCUAACGUAACGGUGUCACA
CU
((((((...((((..(((.((((((....)))))).((....((((((.(((((..........))))).))))))....)).
(((((((.....)))))))...)))...)))).))))))

>Predicted STAR seq:
TGATTGTCCTCTGCCTTAATTTTTGTGAAACTGGTGGC

Complementarity: 7
ERROR 4: Strange structure
Initial dG = -34.60

```
        10            20
      CGU    AA-    .-A|      U
AGUGUG   CACU   AUG   GGAACA C
UCACAC   GUGG   UGC   CCUUGU C
      U--    CAA   \ -^      A
120         110           30



                          40        50
                    .-C  UCUG      U     UCCU
                       CC    UCACAG GAUUG    C
                       GG    AGUGUU UUAAU    U
                    \ -  UCAA      U     UCCG
                         80        70        60
```

Reads in: lib-42, lib-45, lib-47, lib-48, lib-49, lib-50, lib-72
Number of reads="IM38",3,1,1,3,0,5,0

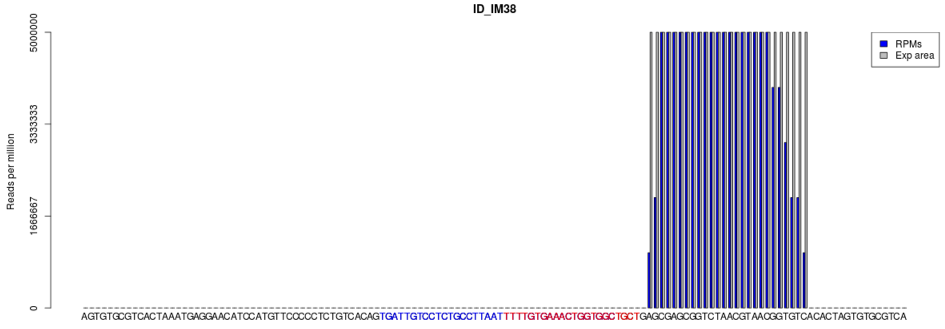

>IM39
GGGCAATATGCTAATGAGCGGCAGTACGCTGCGTACACA**TGTAGCTGCTTTGGGGCG**GACACAGCGAGTAGGTGTGTCCGTGCTGACGGA
CACGTAACACCACTCAAGTGTCGCTTGCCT

>IM39-5P
TGTAGCTGCTTTGGGGCG

>miRNA precursor
GGGCAAUAUGCUAAUGAGCGGCAGUACGCUGCGUACACA**UGUAGCUGCUUUGGGGCG**GACACAGCGAGUAGGUGUGUCCGUGCUGACGGACACGUAACACCACUCAAGUGUCGCUUGCCU
((((((...(((...(((((((.(((((...))))).......)))))))...))).(((((...((((.(((((((((((....))))))).....)))))))))..)))))..))))))

>Predicted STAR seq:

Complementarity: -2

 Initial dG =   -49.50

```
          10        20
ERROR 3: Strange structure
 Initial dG =    -49.50

          10        20
      .-UAU   AAU       A------|    C
GGGCAA     GCU   GAGCGGC       GUACG \
UCCGUU     CGG   UUCGUCG       CAUGC U
      \ ---   GGU       AUGUACA^    G
.              50        40


           60        70             80
        G-      AGC    A     -----      G
          GACAC   GAGU GGUGU     GUCCGU C
          CUGUG   CUCA CCACA     CAGGCA U
        CG      AA-    -     AUGCA      G
          110         100        90
```

Reads in: lib-42, lib-45, lib-47, lib-48, lib-49, lib-50, lib-72
Number of reads="IM39",0,0,0,0,0,0,0

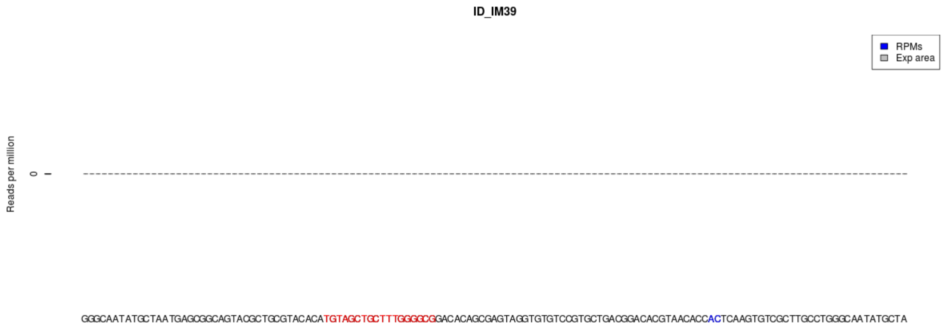

>IM4

CGCCACAAAGGCGTGCTGCACAGTTCACCTGCTCTGGATGCTTGGTACAGGGTGTTGCATTTTTGGGTGG**CCAGCAGTGTACGTTTGTG**G
TAGAGAGAGATAGAGACTGGCAGCTGCTTACGAAGGTG

>IM4-5P

CCAGCAGTGTACGTTTGTG

>miRNA precursor

CGCCACAAAGGCGUGCUGCACAGUUCACCUGCUCUGGAUGCUUGGUACAGGGUGUUGCAUUUUUGGGUGG**CCAGCAGUGUACGUUUGUG**GUAGAGAGAGAUAGAGACUGGCAGCUGCUUA
CGAAGGUG
((((....(((((.(((((.(((((...(((((((...((((..(((((..((((..
(........)..))..))..)))))......))))...)))).))).))))))))))))))).....))))

>Predicted STAR seq:

TGGTACAGGGTG

Complementarity: 7

Initial dG =   -40.50

```
         10        20        30         40            50          60
    ACAA-     U     A     CAC   -     GGA    UG----     GG  --| UG AUU
CGCC     AGGCG GCUGC CAGUU   CUG CUCU   UGCU      GUACA  GU  GU  C   U
GUGG     UUCGU CGACG GUCAG   GAU GAGA   AUGG      CAUGU  CG  CG  G   U
    AAGCA     -     -     A--   A     GAG    UGUUUG     GA  AC^ GU GGU
      120        110          100        90        80        70
```

Reads in: lib-42, lib-45, lib-47, lib-48, lib-49, lib-50, lib-72

Number of reads="IM4",0,0,0,0,0,0,0

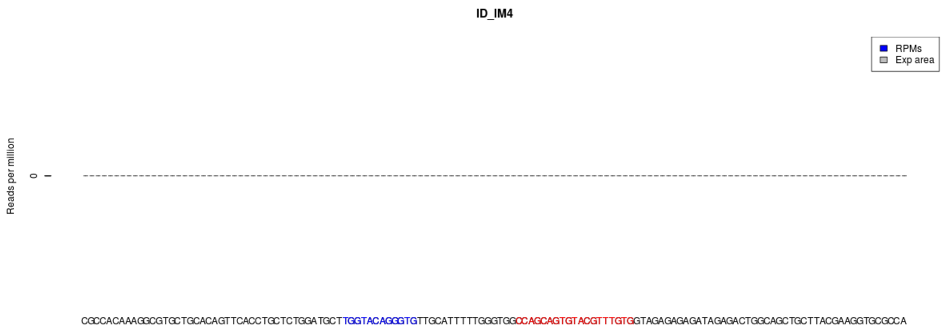

>IM40
TTGTTAAGTGCTGTGACAATGCTTCTGTATTTTGTTTCATCAGCAGTAACATGTGCTAACGCAAGCTGG**CACAGTTGCTGTTTCTCTTA**T
TGTGGCAGATCAATTATGCACACGAAGTATCCGACGA

>IM40-5P
CACAGTTGCTGTTTCTCTTA

>miRNA precursor
UUGUUAAGUGCUGUGACAAUGCUUCUGUAUUUUGUUUCAUCAGCAGUAACAUGUGCUAACGCAAGCUGG**CACAGUUGCUGUUUCUCUUA**UUGUGGCAGAUCAAUUAUGCACACGAAGUAU
CCGACGA
(((((..((((((((..(((...(((((.............((((((((.
(((((((........))))))))))))))))))...........)))))...)))...))))....)))))..)))))

>Predicted STAR seq:
TTTTGTTTCATCAGCAGTAACATGTG

Complementarity: 11
ERROR 4: Strange structure
Initial dG =   -35.10

```
        10            20
     AA    .-U U    --|   U
UUGUU  GUGC   G GACA  AUGC U
AGCAG  UAUG   C UUGU  UAUG C
     CC    \ - U    UU^   U
     120             30



              40        50        60
            .-AUC         A       ACG
                 AGCAGUAAC UGUGCUA   C
                 UUGUCGUUG ACACGGU   A
            \ ---         -       CGA
                  80         70
```

Reads in: lib-42, lib-45, lib-47, lib-48, lib-49, lib-50, lib-72
Number of reads="IM40",0,0,0,0,0,0,0

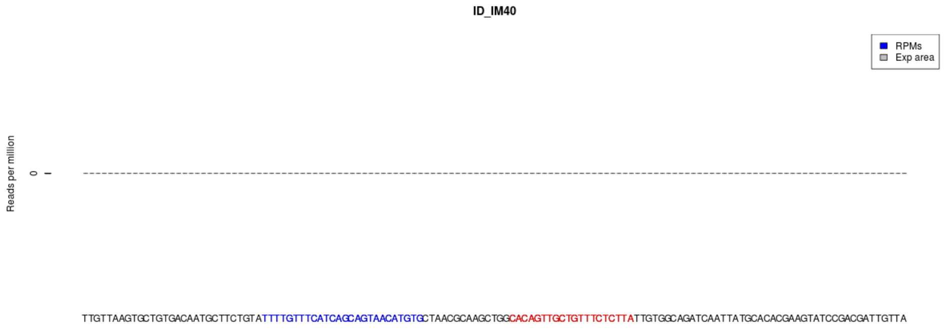

>IM41
GCCGTTTTTGGGACGTTATAGTCCCATCTTCAGGCGATAAATAAATTATAGCGTATGCGACCGG**GCAAGTCAATGACATTATTC**GTCATC
TTGAAGATGGGACAATAACGTGCAGAAACAGGC

>IM41-5P
GCAAGTCAATGACATTATTC

>miRNA precursor
GCCGUUUUUGGGACGUUAUAGUCCCAUCUUCAGGCGAUAAAUAAAUUAUAGCGUAUGCGACCGG**GCAAGUCAAUGACAUUAUUC**GUCAUCUUGAAGAUGGGACAAUAACGUGCAGAAACA
GGC
((((((((((..(((((((.((((((((((((((.((..(((((.((((.((...
(((......))).))..)))).)))))..))..)))))))))))))).))))))).))))))).)))

>Predicted STAR seq:
AAATAAATTATAGCGTATGC

Complementarity: 12

Initial dG =   -49.40

```
         10        20        30         40          50        60
   -|      GG       A             C-   UA      .-AUUAUAGCGUA    GA
GCC GUUUUUG  ACGUUAU GUCCCAUCUUCAGG  GA  AAUAA             UGC  C
CGG CAAAGAC  UGCAAUA CAGGGUAGAAGUUC  CU  UUAUU             ACG  C
   A^      G-       A             UA  GC     \ -----------    GG
 120        110       100        90        80
```

Reads in: lib-42, lib-45, lib-47, lib-48, lib-49, lib-50, lib-72
Number of reads="IM41",0,0,0,0,0,0,0

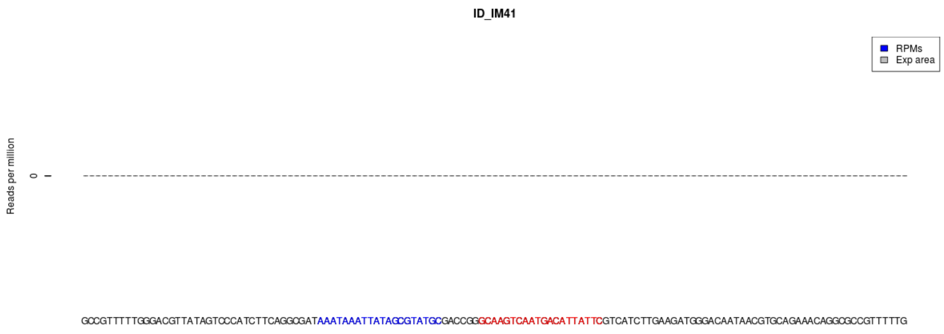

>IM42

GACACCGACGGCTGCCCTGGCGCGCCGGCTCAGGTCGTGACTAGTGCAATTTTGTGGAGCTCTGAGGCA**AATTGCACTTTTCCCGGCCTG**
CTTGTGCGCGCCGCATCGTCCAGCGCCGTGTC

>IM42-3?P

AATTGCACTTTTCCCGGCCTGC

>miRNA precursor

GACACCGACGGCUGCCCUGGCGCGCCGGCUCAGGUCGUGACUAGUGCAAUUUUGUGGAGCUCUGAGGCA**AAUUGCACUUUUCCCGGCCUGC**UUGUGCGCGCCGCAUCGUCCAGCGCCGUG
UC

```
(((((.(((((.(((...((((((((((..(((((((.((..(((((((((.
((((....))...))))))))))))..)).))))))).))).)))))))))))))))).......)))))
```

>Predicted STAR seq:

TCAGGTCGTGACTAGTGCAATT

Complementarity: 16

Initial dG =   -53.80

```
             10        20         30        40        50
     C------|    C   CCU       -   CU        U  CU          U  ---  A
GACAC       GACGG UGC   GGCGCGC CGG  CAGGUCG GA  AGUGCAAUUU GU   GG G
CUGUG       CUGCU ACG   CCGCGCG GUU  GUCCGGC CU  UCACGUUAAA CG   UC C
     CCGCGAC^    -   ---       U   C-        C  UU          -  GAG  U
120       110           100         90        80        70         60
```

Reads in: lib-42, lib-45, lib-47, lib-48, lib-49, lib-50, lib-72

Number of reads="IM42",1054,435,845,2144,2968,441,0

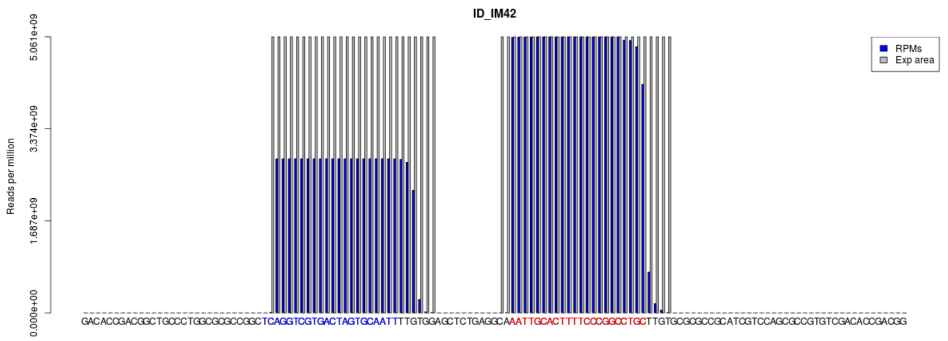

>IM43
GGTACTGAATCCCAATCCA**GTTGACCGAAACTGAGGAG**GTGCTCTTGGACACACAACAAGGAGGATGTCTTCAGTTTGGATCAGCTGGAA
TGAAAAGTACT

>IM43-5P
GTTGACCGAAACTGAGGAG

>miRNA precursor
GGUACUGAAUCCCAAUCCA**GUUGACCGAAACUGAGGAG**GUGCUCUUGGACACACAACAAGGAGGAUGUCUUCAGUUUGGAUCAGCUGGAAUGAAAAGUACU
((((((......((.((((((((((((.((((((((((.((.(((((...........))))).)).))))))))))))).))))))))).))...))))))

>Predicted STAR seq:
TTCAGTTTGGATCAGCTG

Complementarity: 15

Initial dG = -44.20

```
        10        20          30        40        50
|     GAAUCC  A          -  G          G  G     GGACA
GGUACU      CA UCCAGUUGA CC AAACUGAGGA GU CUCUU     C
UCAUGA      GU AGGUCGACU GG UUUGACUUCU UA GAGGA     A
^     AAA---  A          A  -          G  G     ACAAC
 .          90        80          70        60
```

Reads in: lib-42, lib-45, lib-47, lib-48, lib-49, lib-50, lib-72
Number of reads="IM43",0,0,0,0,0,0,0

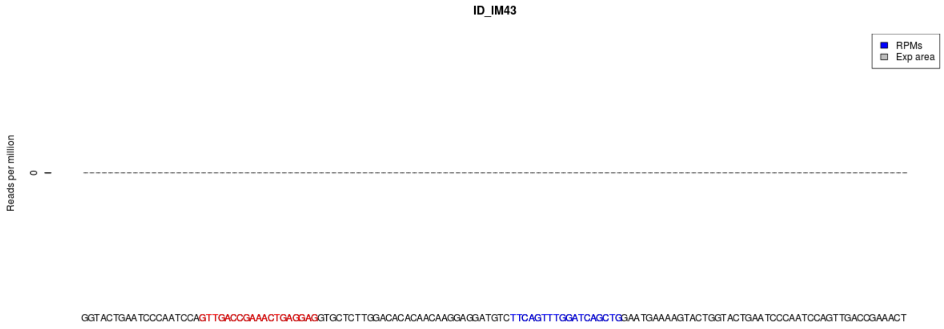

>IM44

GGTCTATTGCAGACCATCTTCAGAAGGATACCA**CCAGCAGTCAACGTCTGTG**CGGCCTGCTTTGTCACCGCCACCGCCTGCCATCATGGTGGTATCCACCTGAAGATGGTCTGCAAGAGACC

>IM44-3P

CCAGCAGTCAACGTCTGTG

>miRNA precursor

GGUCUAUUGCAGACCAUCUUCAGAAGGAUACCA**CCAGCAGUCAACGUCUGUG**CGGCCUGCUUUGUCACCGCCACCGCCUGCCAUCAUGGUGGUAUCCACCUGAAGAUGGUCUGCAAGAGACC

```
(((((.((((((((((((((((..((((((((((((((((....((..((.
((((............)))))).)).)))).....))))))))))..)))))))))))))))))).)))))
```

>Predicted STAR seq:

ACCGCCTGCCATCATGGTG

Complementarity: 10

Initial dG =   -68.80

```
       10        20        30             40        50        60
    A                 AA            -----|   UCAA  UC  U    CCUGC
GGUCU UUGCAGACCAUCUUCAG  GGAUACCACCA      GCAG    CG  UG GCGG     U
CCAGA AACGUCUGGUAGAAGUC  CCUAUGGUGGU      CGUC    GC  AC CGCC     U
    G                 CA            ACUAC^   C---  C-  -    ACUGU
120       110       100        90        80             70
```

Reads in: lib-42, lib-45, lib-47, lib-48, lib-49, lib-50, lib-72

Number of reads="IM44",0,1,2,0,1,0,0

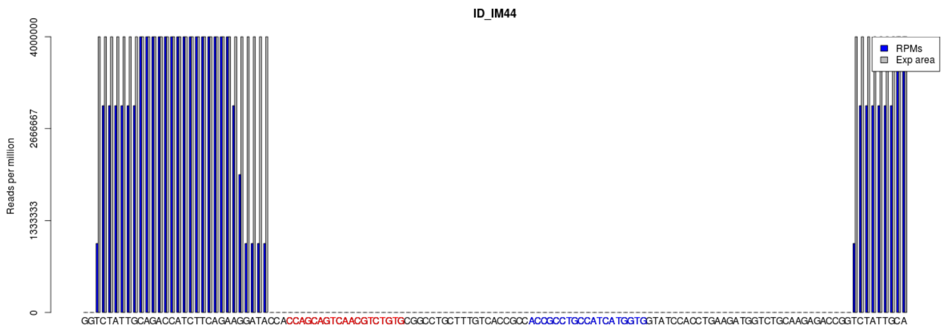

>IM45
CTCGTGCCCACCCCTGCAA**TAGACGGAGAACTGCTAAG**TGCGTCAGTGCGCGGCGCGTCGTTGCTGGGTGCGAGGCATCGCGGTTCTGCC
GCTAGAGGACGGCCGTG

>IM45-3P
TAGACGGAGAACTGCTAAG

>miRNA precursor
CUCGUGCCCACCCCUGCAA**UAGACGGAGAACUGCUAAG**UGCGUCAGUGCGCGGCGCGUCGUUGCUGGGUGCGAGGCAUCGCGGUUCUGCCGCUAGAGGACGGCCGUG
..((.(((....(((....(((.((((((((((...((((.((.(..(.(((((......))))).)..))).)))).)))))))).)))))).))).)).))))..

>Predicted STAR seq:
GCGGTTCTGCCGCTAGAG

Complementarity: 13

Initial dG =   -44.00

```
        10        20         30        40        50
CU| U   CACC   GCAA   A   -         UAA    G  A UG G     CG
  CG GCC    CCU    UAG CGG AGAACUGC   GUGC UC G  C CGGCG  U
  GC CGG    GGA    AUC GCC UCUUGGCG   UACG AG C  G GUCGU  C
GU^ -   CA--   G---   -   G        C--    G  - GU G     UG
      100              90        80            70        60
```

Reads in: lib-42, lib-45, lib-47, lib-48, lib-49, lib-50, lib-72
Number of reads="IM45",0,0,0,0,0,0,0

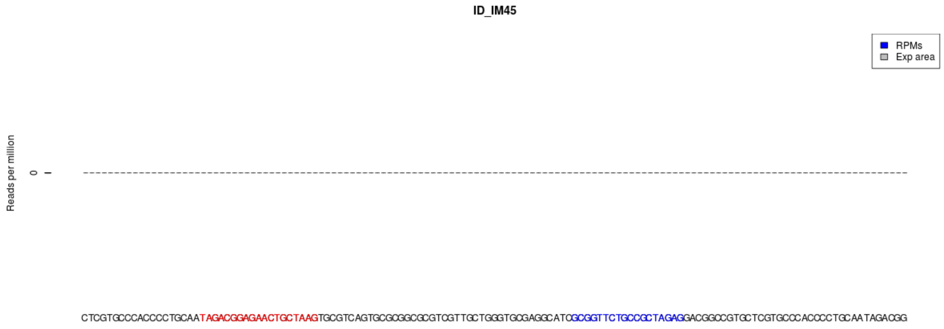

>IM46
CCATGTTTGTTGTGTTGCAGAGCTTCCCTGCGCAGCTGTACCGGAAGGAGGCGGCGGGCCGCACGAGCGC**CCTGCAGAGACATCTGTCGGACTC**TGAGTCAGACAGCGATTCGGACGACGAGCACCGG

>IM46-3P
CCTGCAGAGACATCTGTCGGACTC

>miRNA precursor
CCAUGUUUGUUGUGUUGCAGAGCUUCCCUGCGCAGCUGUACCGGAAGGAGGCGGCGGGCCGCACGAGCGC**CCUGCAGAGACAUCUGUCGGACUC**UGAGUCAGACAGCGAUUCGGACGACGAGCACCGG
((.((((((((((.((((((.(((((..(((((.(((((.((....))..)))))..)).))).))).)).)))))).((...(((((.(((.....))).)))))....))..))))))))))..))

>Predicted STAR seq:
CCTGCGC

Complementarity: 4

Initial dG =   -50.30

```
         10          20          30          40
  A-           .-G      A   -    CC    -   A-      A-| G
CC  UGUUUGUUGU   UUGCAG GC UUC  UGC GC  GCUGU  CC G
GG  ACGAGCAGCA   GACGUC CG GAG  ACG CG  CGGCG  GG A
  CC           \ -      C  C    C-    C  GG      GA^ A
      120               70         60         50
```

Reads in: lib-42, lib-45, lib-47, lib-48, lib-49, lib-50, lib-72
Number of reads="IM46",0,0,0,0,0,1,0

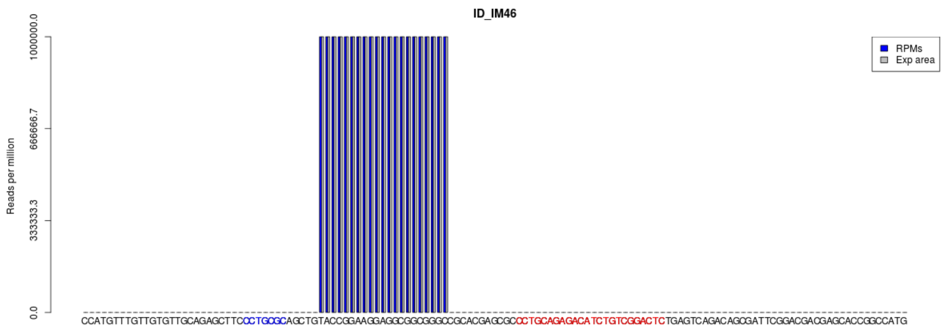

>IM47
CAGCAACCGGGTACTAATGGCGCTGATTTTTT**TCTTTGGTTATCTAGCTGTATGA**GTGCATGTGACTGTCATAAAGCTAGGTTACCGAAGT
TAAGAACAGCATCTGTTCATCGATGGTAGCTG

>IM47-5?P
TCTTTGGTTATCTAGCTGTATGA

>miRNA precursor
CAGCAACCGGGUACUAAUGGCGCUGAUUUUUU**UCUUUGGUUAUCUAGCUGUAUGA**GUGCAUGUGACUGUCAUAAAGCUAGGUUACCGAAGUUAAGAACAGCAUCUGUUCAUCGAUGGUAGC
UG
((((.((((......(((((.((((.((((..(((((((.((((((((.
(((((((.......))..))))).)))))))).)))))))..)))).)))).......))))).......)))).))))

>Predicted STAR seq:
ATAAAGCTAGGTTACCGAAGTTAA

Complementarity: 18

Initial dG =   -43.50

```
        10        20         30        40        50
    A    GGUACU     C-    A    UU        U        G     --| GCA
CAGC ACCG      AAUGG  GCUG UUUU  CUUUGGU AUCUAGCU UAUGA  GU   \
GUCG UGGU      UUGUC  CGAC AGAA  GAAGCCA UGGAUCGA AUACU  CA   U
    A    AGCUAC     UA    A    UU        U        A     GU^ GUG
120       110       100        90        80        70
```

Reads in: lib-42, lib-45, lib-47, lib-48, lib-49, lib-50, lib-72
Number of reads="IM47",18525,7578,113894,3228,63681,33280,0

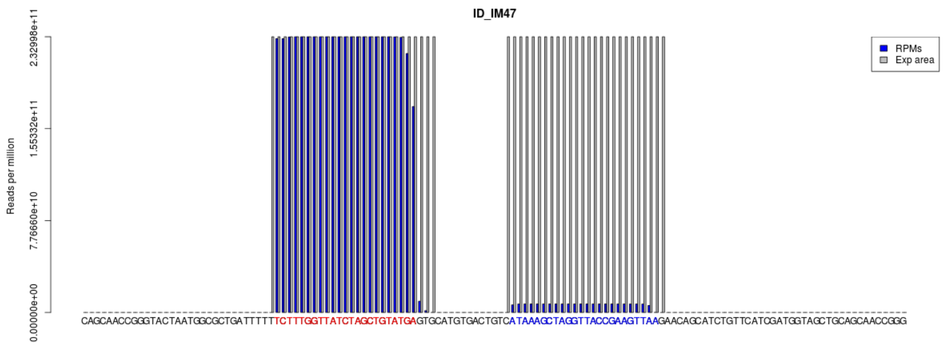

>IM48
CAGCAACCGGGTACTAATGGCGCTGATTTTTTCTTTGGTTATCTAGCTGTATGAGTGCATGTGACTGTC**ATAAAGCTAGGTTACCGAAGT**
TAAGAACAGCATCTGTTCATCGATGGTAGCTG

>IM48-5P
ATAAAGCTAGGTTACCGAAGTTA

>miRNA precursor
CAGCAACCGGGUACUAAUGGCGCUGAUUUUUUCUUUGGUUAUCUAGCUGUAUGAGUGCAUGUGACUGUC**AUAAAGCUAGGUUACCGAAGUUA**AGAACAGCAUCUGUUCAUCGAUGGUAGC
UG
((((.((((......(((((.((((.((((..(((((((.((((((((.
(((((((.......))..))))).))))))))).)))))))..)))).)))).)))))......)))).))))

>Predicted STAR seq:
TTCTTTGGTTATCTAGCTGTAT

Complementarity: 17

Initial dG =   -43.50

```
        10         20          30         40         50
    A     GGUACU      C-     A     UU        U         G      --| GCA
CAGC ACCG      AAUGG  GCUG UUUU  CUUUGGU AUCUAGCU UAUGA   GU    \
GUCG UGGU      UUGUC  CGAC AGAA  GAAGCCA UGGAUCGA AUACU   CA    U
    A     AGCUAC      UA     A     UU        U         A      GU^ GUG
120       110        100         90         80         70
```

Reads in: lib-42, lib-45, lib-47, lib-48, lib-49, lib-50, lib-72
Number of reads="IM48",18533,7608,113932,3266,63715,33252,0

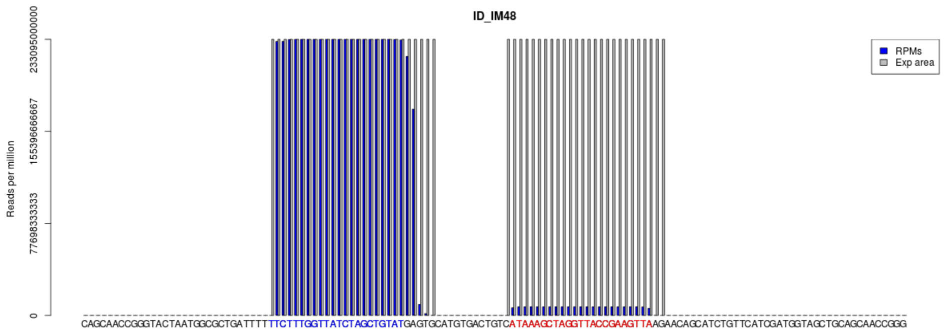

>IM49
TGTCTCTTAACTGGACATTGTAAAACAAAACTGCCGGTTAAGTTCCCGTAAAAGTTCCCGTAAACAGT**TTACTGGACATTGTAAAAACGG**
GCCTTACAGTTGCAAAAGTGGGTAACTTGAAAGGTGGCA

>IM49-3P
TTACTGGACATTGTAAAAACGG

>miRNA precursor
UGUCUCUUAACUGGACAUUGUAAAACAAAACUGCCGGUUAAGUUCCCGUAAAAGUUCCCGUAAACAGU**UUACUGGACAUUGUAAAAACGG**GCCUUACAGUUGCAAAAGUGGGUAACUUGA
AAGGUGGCA
.(((.(((((((((.(((((.....)))...))))))))))).. (((((...((((((.(((((...))))).))).))).......)))))((((.
((((((........))))).)).)))).))).

>Predicted STAR seq:
TAACTGGACATTGT

```
Complementarity: 12
ERROR 4: Strange structure
 Initial dG =    -32.70

          10          20
U   .-U         A  ---|  UA
 GUC   CUUAACUGG CA   UUG  \
 CGG   GAAUUGGCC GU   AAC  A
A   \ -         -  CAA^  AA
        40         30



              50            60
      .-UU     AAA---   -   C     C
         CCCGU      AGU UCC GUAAA \
         GGGCA      UUA AGG CAUUU A
      \ --     AAAAUG   C   U     G
         90        80        70
```

Reads in: lib-42, lib-45, lib-47, lib-48, lib-49, lib-50, lib-72
Number of reads="IM49",0,0,0,0,0,0,0

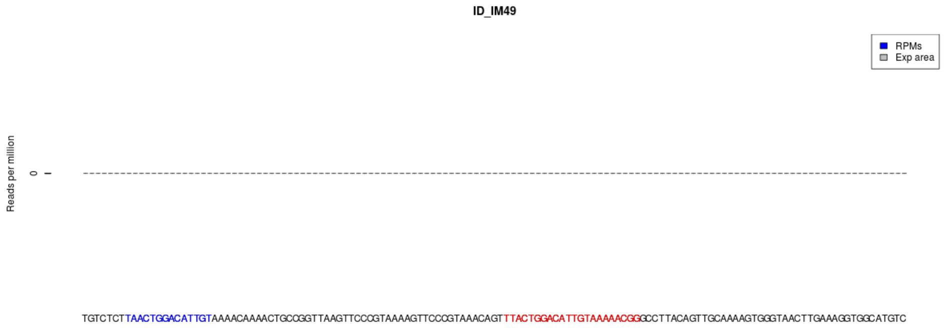

>IM5
TTTTTGTTATGGTCCTTTAGAAGTTTACAGAAAGAAAATTCAGGATAGTTTTACCTATGTAACTATTC**TGCATTTTCTTTCTGTAAAC**TT
TTTTTAGGACCATAACAAAAA

>IM5-5P
TGCATTTTCTTTCTGTAAAC

>miRNA precursor
UUUUUGUUAUGGUCCUUUAGAAGUUUACAGAAAGAAAAUUCAGGAUAGUUUUACCUAUGUAACUAUUC**UGCAUUUUCUUUCUGUAAAC**UUUUUUUAGGACCAUAACAAAAA
.(((((((((((((((..(((((((((((((((((((((.((((((((((..........)))))))))).)))))))))))))))))))))...))))))))))))))).

>Predicted STAR seq:
TTTACAGAAAGAAAATTCA

Complementarity: 17

Initial dG =   -60.80

```
        10         20         30         40         50
U               UU-|                    U          UUAC
 UUUUGUUAUGGUCCU   AGAAGUUUACAGAAAGAAAAU CAGGAUAGUU    C
 AAAACAAUACCAGGA   UUUUCAAAUGUCUUUCUUUUA GUCUUAUCAA    U
A               UUU^                    C          UGUA
 .       100        90         80         70         60
```

Reads in: lib-42, lib-45, lib-47, lib-48, lib-49, lib-50, lib-72
Number of reads="IM5",0,0,0,0,0,0,0

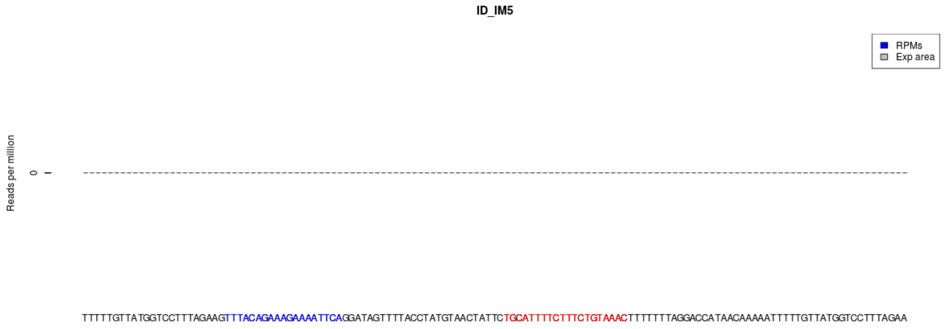

>IM50
AGAAGTATTCCGTCTTGTGCTGACTGCAATTGAGACATTTATTTTGTTTTCACGGAATGTATGACATT**AACTGCAAACGGGTAATGT**TGA
AAATGTGTACCCAGCCGGGATTCGAAAGCGGGTCT

>IM50-3P
AACTGCAAACGGGTAATGT

>miRNA precursor
AGAAGUAUUCCGUCUUGUGCUGACUGCAAUUGAGACAUUUAUUUUGUUUUCACGGAAUGUAUGACAUU**AACUGCAAACGGGUAAUGU**UGAAAAUGUGUACCCAGCCGGGAUUCGAAAGCG
GGUCU
....((.(((.((((((.((((..(((((((..((((((...(((((((.((...
((((.....))))...)).))))))).))))))...))).)))))..))))))))))..))).))......

>Predicted STAR seq:
CATTTATTTTGTTTTCA

Complementarity: 12

Initial dG =   -29.00

```
          10         20          30         40
AGAA--  A   C-       U  .-UGAC    A    GA-|  U        U
      GU UUC  GUCUUG GC      UGCA UUGA   CAU UAUUUUGU U
      CG AAG  UAGGGC CG      ACGU AAUU   GUA GUAAGGCA U
UCUGGG  A   CU       -  \ ----    C    ACA^  U        C
   120       110                  70        60        50
```

Reads in: lib-42, lib-45, lib-47, lib-48, lib-49, lib-50, lib-72
Number of reads="IM50",70,78,72,59,96,67,0

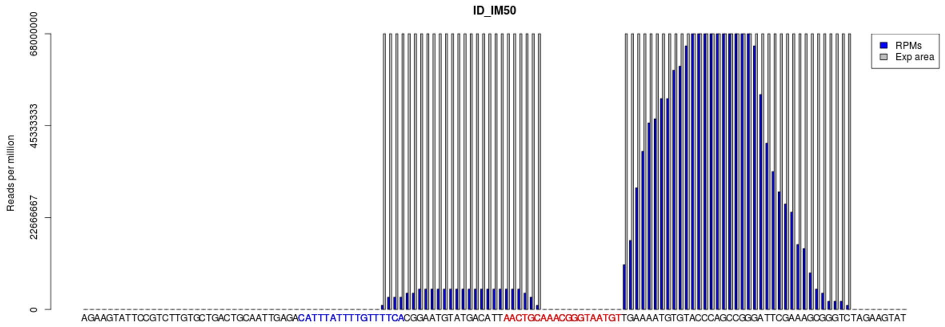

>IM51
ACCAAGACAAGTCGTCGAGAGAGTAGAAGACGGCTA**TAAAAGAGCCGCGGCGGA**TTAGCCGTGCGCACTGGGGCGCCGTGCAGTGGTGCG
CGCGGCCGTTCGCCTTCGTGTCCTTAGGT

>IM51-5P
TAAAAGAGCCGCGGCGGA

>miRNA precursor
ACCAAGACAAGUCGUCGAGAGAGUAGAAGACGGCUA**UAAAAGAGCCGCGGCGGA**UUAGCCGUGCGCACUGGGGCGCCGUGCAGUGGUGCGCGCGGCCGUUCGCCUUCGUGUCCUUAGGU
(((......(((((((...........)))))))....(((..(((.(((((((..((((((((((((((..((.....))..)))))))))))))).))))))))..)).)..))).)))

>Predicted STAR seq:
CGCCTTCGTGTCCTTAGG

Complementarity: 10

```
 Initial dG =    -53.30

          10        20
ERROR 3: Strange structure
 Initial dG =    -53.30

          10        20
   .-AAGACA|      GAGAG
ACC        AGUCGUC     A
UGG        UCGGCAG     G
   \ ------^      AAGAU
               30


       40          50        60        70
     AUAA   AG -  C-       UA            GG  GC
         AAG  C CG  GGCGGAU  GCCGUGCGCACUG  GC  \
         UUC  G GC  CCGCUUG  CGGCGCGCGUGGU  CG  C
     A---   CU U  UU       C-            GA  UG
            110       100         90        80
```

Reads in: lib-42, lib-45, lib-47, lib-48, lib-49, lib-50, lib-72
Number of reads="IM51",1,1,13,2,4,5,0

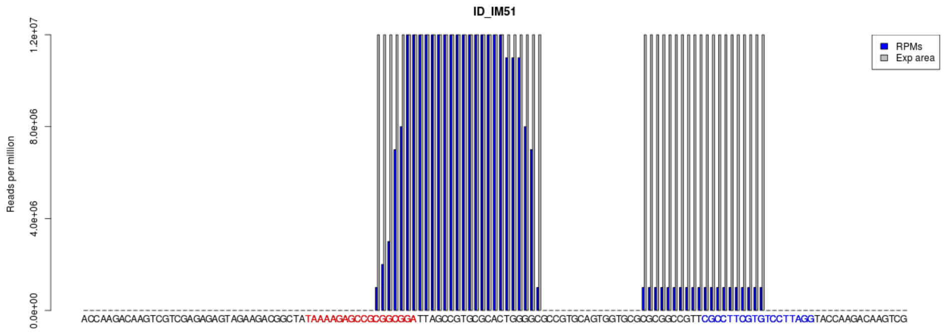

>IM52

CCTCTTAACTGGACATTATAAAATCAAATTGTCGGTAAAGTTCCCGTAAACAGT**TTACTGGACATTGTAAGAACGG**GCCTAAGTCTGTTA
AAATGCCTTACCCACAGTTCAAGAGG

>IM52-3P

TTACTGGACATTGTAAGAACGG

>miRNA precursor

CCUCUUAACUGGACAUUAUAAAAUCAAAUUGUCGGUAAAGUUCCCGUAAACAGU**UUACUGGACAUUGUAAGAACGG**GCCUAAGUCUGUUAAAAUGCCUUACCCACAGUUCAAGAGG
((((((((((.............(((((((((((........)))...)))))))...((.((((.....((((((......))))))..)))))).......))))).))))))

>Predicted STAR seq:

T

Complementarity: 0
ERROR 4: Strange structure
Initial dG =   -30.50

```
         10          20        30
      -     .-GACAUUAUAAAAUC       ---|  UAA
CCUCUU AACUG                AAAUUGU   CGG   A
GGAGAA UUGAC                UUUGACA   GCC   G
      C     \ --------------       AAU^  CUU
    110                          50        40


                    60        70        80
              ACU----  A    GUAAG      CC
                     GG CAUU     AACGGG  U
                     CC GUAA     UUGUCU  A
              ACCCAUU  -    AA---      GA
                100         90
```

Reads in: lib-42, lib-45, lib-47, lib-48, lib-49, lib-50, lib-72
Number of reads="IM52",0,0,0,0,0,0,0

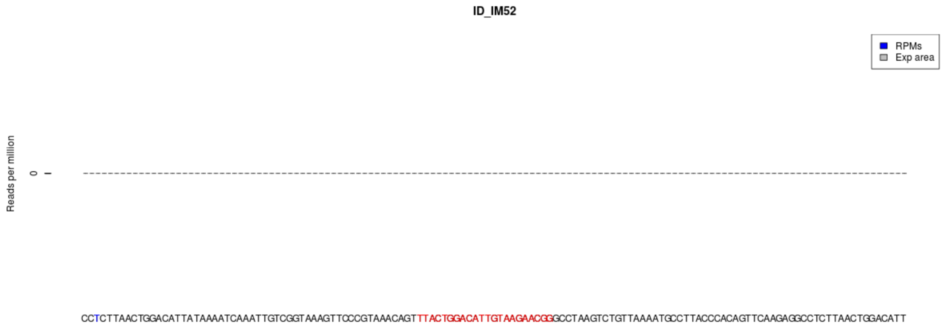

>IM53

ATCACGTGATGGGCGGTGGGCGAAGCATCCAGTACTTAAATCACAGCCGCACTGTTCCAA**CCAGCAGTTCACGTCTGTG**CAGCCTGCTGT
GTCACCGCCACCGCTTGCCATCATGGGT

>IM53-3P

CCAGCAGTTCACGTCTGTG

>miRNA precursor

AUCACGUGAUGGGCGGUGGGCGAAGCAUCCAGUACUUAAAUCACAGCCGCACUGUUCCAA**CCAGCAGUUCACGUCUGUG**CAGCCUGCUGUGUCACCGCCACCGCUUGCCAUCAUGGGU

```
(((.((((((((((((((.((..(((..(((((.......(((((.((.((((((......))))))...)).))))).....))))))))...)))))))))...)))))))))))
```

>Predicted STAR seq:

TTAAATCACAGCCGCACTGTT

Complementarity: 11

Initial dG =   -44.10

```
        10            20          30         40         50
   A         ---|       G   AA-    UC      CUUAAAU      C   C--       CC
AUC CGUGAUGG   GCGGUGG CG    GCA   CAGUA        CACAG CG    ACUGUU   A
UGG GUACUACC   CGCCACC GC    UGU   GUCGU        GUGUC GC    UGACGA   A
   -         GUU^       -   CAC    --      CCGAC--      U   ACU       CC
       110       100          90           80          70         60
```

Reads in: lib-42, lib-45, lib-47, lib-48, lib-49, lib-50, lib-72

Number of reads="IM53",0,0,0,1,0,0,0

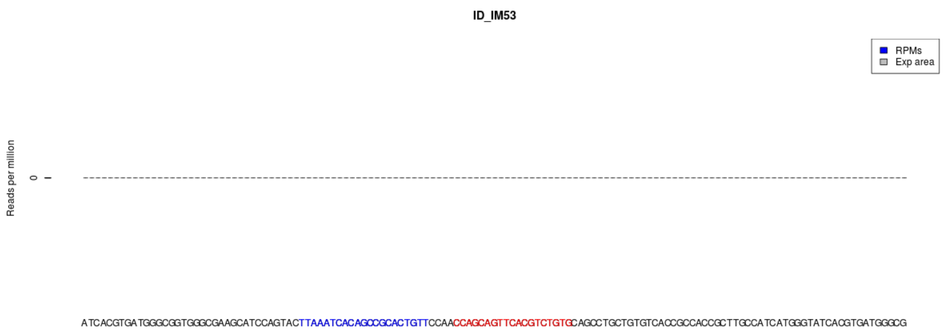

>IM54
GGAG**GAAAAGAGCCGCGGCGCA**GCGGCCGAGCGCGTTGAGGCGCTACGCTACGATGCGCGAGGCCGTTCCCCGCCGGGGGTTCGCATCCC
C

>IM54-5P
GAAAAGAGCCGCGGCGCA

>miRNA precursor
GGAG**GAAAAGAGCCGCGGCGCA**GCGGCCGAGCGCGUUGAGGCGCUACGCUACGAUGCGCGAGGCCGUUCCCCGCCGGGGGUUCGCAUCCCC
((.(((...(((((.(((((.(((((((..((((((((.((((...)))).))))))))..))))))))...))))).)).))).)))...)))))

>Predicted STAR seq:
CCCGCCGGGGGTTCGCATCCC

Complementarity: 11

Initial dG = -53.70

```
        10          20           30         40
  A   AAA   -  G     C--|      GA         A    C
GG GGA   GAG CC CGGCG   AGCGGCC  GCGCGUUG GGCG \
CC CCU   CUU GG GCCGC   UUGCCGG  CGCGUAGC UCGC U
  -   ACG   G  G     CCC^      AG         A    A
90         80         70         60         50
```

Reads in: lib-42, lib-45, lib-47, lib-48, lib-49, lib-50, lib-72
Number of reads="IM54",5,8,29,11,4,11,0

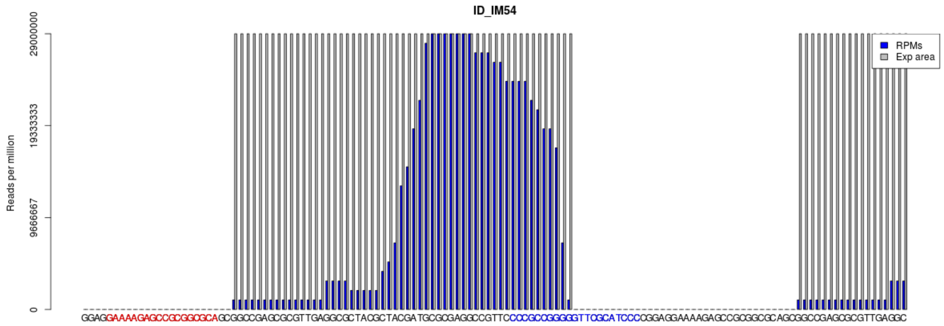

>IM55

TTTAGCCTAAGACCACCCTTTTCACTTTCGTTTTTTACCTTCAGCAACAGAGTAAAAACCGAAAGTGAAA**AGGCCGATCTTAGGCTAA**A

>IM55-5P

AGGCCGATCTTAGGCTAA

>miRNA precursor

```
UUUAGCCUAAGACCACCCUUUUCACUUUCGUUUUUUACCUUCAGCAACAGAGUAAAAACCGAAAGUGAAAAGGCCGAUCUUAGGCUAAA
.((((((((((....((((((((((((((.((((((((((........)).))))))).))))))))))))))....))))))))))).
Error predicting STAR on 5p
 Initial dG =    -42.90

        10        20        30         40
U           CCAC              U       -| UCA
 UUAGCCUAAGA    CCUUUUCACUUUCG UUUUUAC CU   G
 AAUCGGAUUCU    GGAAAAGUGAAAGC AAAAAUG GA   C
A           AGCC              C       A^ CAA
        80        70        60        50
```

Reads in: lib-42, lib-45, lib-47, lib-48, lib-49, lib-50, lib-72

Number of reads="IM55",0,0,0,0,0,0,0

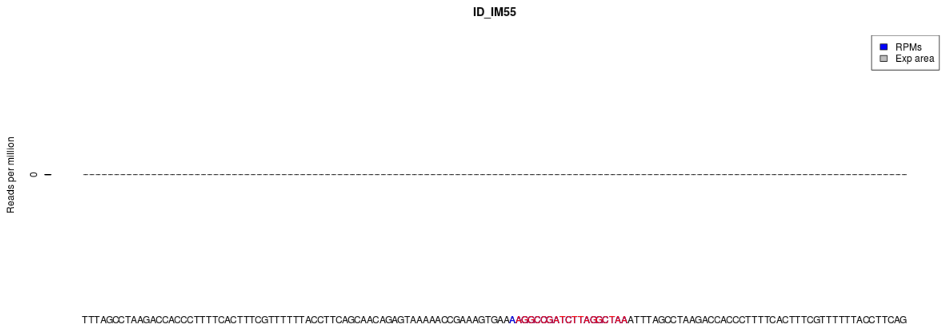

>IM56
GAGCTGTTTTGATGGCAAAGGAGCCTGTGCCCATGGGCGGAGTCATGGACGCAGTGAGGGTGCCTGCAC**TCACTGGGTGGAGTTTGGCT**G
GGCCTGGTTTAACTGATCATGTGATCACCAAAGCTC

>IM56-5P
TCACTGGGTGGAGTTTGGCT

>miRNA precursor
GAGCUGUUUUGAUGGCAAAGGAGCCUGUGCCCAUGGGCGGAGUCAUGGACGCAGUGAGGGUGCCUGCAC**UCACUGGGUGGAGUUUGGCU**GGGCCUGGUUUAACUGAUCAUGUGAUCACCA
AAGCUC
```
(((((....((((.(((...(((((...(((((..(.((((.((....((.(((((((.
((....)).))))))).)).)).)))).))))))..))))).........))).)))).....)))))
```

>Predicted STAR seq:
TGGGCGGAGTCATGGACGCAGTGA

Complementarity: 14

Initial dG =   -45.10

```
        10        20              30        40        50        60
     GUUU    G   AAG------|    UGU     UG G    G  AUGG  G       G  G
GAGCU    UGAU GCA         GAGCC   GCCCA  G CGGA UC    AC CAGUGAG GU C
CUCGA    ACUA UGU         UUUGG   CGGGU  C GUUU AG    UG GUCACUC CG C
     AACC    G   ACUAGUCAA^    UC-     -- G    G  G---  G       A  U
    120       110       100         90          80           70
```

Reads in: lib-42, lib-45, lib-47, lib-48, lib-49, lib-50, lib-72
Number of reads="IM56",0,0,0,0,0,0

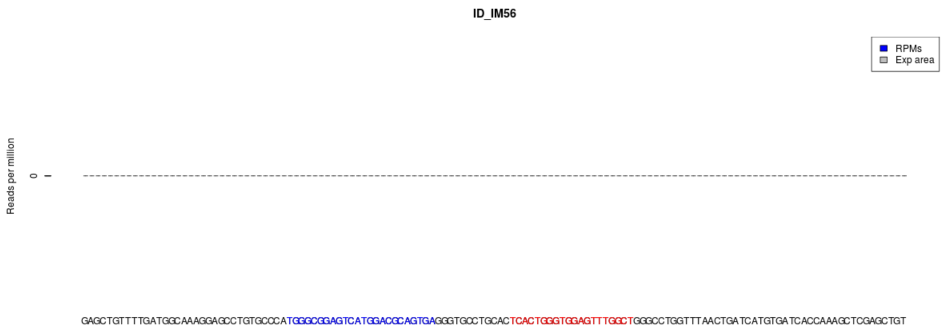

>IM57
CATTTCCTGGTGTCGTGTCGCCGGCCGCGGCGGGGGCGCGGCGCAACCGCGGACCGGCGGCGGTAGCGGC**AGCGGCGAGAGCACAGGAT**G
TG

>IM57-5P
AGCGGCGAGAGCACAGGAT

>miRNA precursor
CAUUUCCUGGUGUCGUGUCGCCGGCCGCGGCGGGGGCGCGGCGCAACCGCGGACCGGCGGCGGUAGCGGC**AGCGGCGAGAGCACAGGAU**GUG
(((.((((((((((((((((..(((((.((.((..(((((.....)))))..)).)).))))).)))))).)))).....)).))))).)))

>Predicted STAR seq:
TC

Complementarity: 10

Initial dG =   -52.90

```
         10              20         30         40
   U     -  ----    -|     CG     G  G  GG     CG
CAU UCCUG GU    GUCG UGUCGC  GCCGC GC GG  CGCGG  \
GUG AGGAC CG    CGGC ACGGCG  UGGCG CG CC  GCGCC  C
   U     A  AGAG    G^     A-     G  G  AG     AA
 90        80        70         60        50
```

Reads in: lib-42, lib-45, lib-47, lib-48, lib-49, lib-50, lib-72
Number of reads="IM57",0,0,0,0,0,0

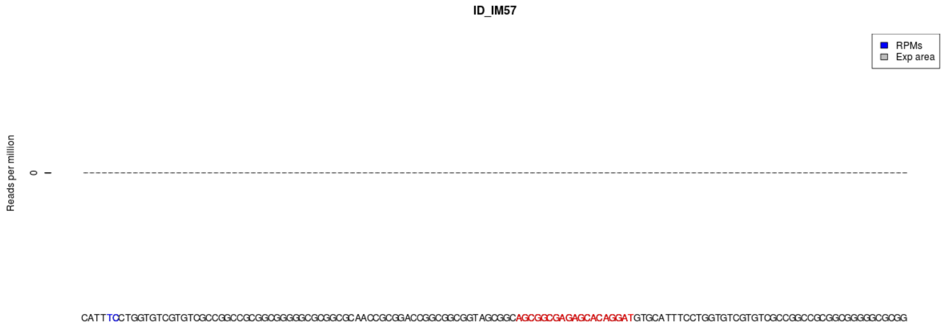

>IM58

TACATTCAACTGTGTGTGTGCCGTGGTGGAGT**ACCCGAGCGGTCTAAGGAAACT**CGCTGCGGAGCGCGCGGTTCATTGCTGTCAGAGATT
CGAGTCCTCCCTGGGGCATACGTGTGTGTGTGTA

>IM58-5P

ACCCGAGCGGTCTAAGGAAACT

>miRNA precursor

UACAUUCAACUGUGUGUGUGCCGUGGUGGAGU**ACCCGAGCGGUCUAAGGAAACU**CGCUGCGGAGCGCGCGGUUCAUUGCUGUCAGAGAUUCGAGUCCUCCCUGGGGCAUACGUGUGUGUG
UGUA

```
(((((...((.(..((((((((.(((.((((.((.((((((((...((....)).
(((((((.....))))))).....))))).......))).)).))))))).))))))))..)))..)))))
```

>Predicted STAR seq:

TCATTGCTGTCAGAGATTCGAGTCCT

Complementarity: 10

Initial dG =   -44.60

```
       10        20        30          40        50
     UCA  U UG        G   U    U  C      |    CUA  G
UACAU   AC G  UGUGUGCC UGG GGAG AC CGA--GCGGU   AG A
AUGUG   UG U  GCAUACGG GUC CCUC UG GCU  CGUCG   UC A
     UG-  - GU        G   -    C  A   \ ^    C--  A
  120        110       100       90 60



                                                 70
                                        GGAGCGC     UC
                                               GCGGU  \
                                               UGUCG  A
                                        UAGAGAC     UU
                                                 80
```

Reads in: lib-42, lib-45, lib-47, lib-48, lib-49, lib-50, lib-72

Number of reads="IM58",14,20,28,22,29,29,0

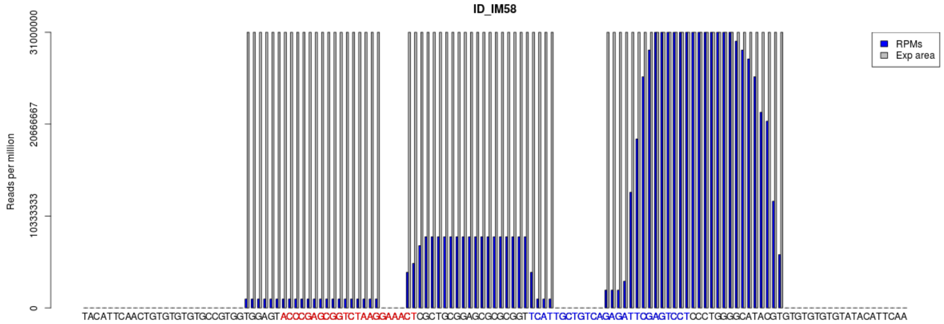

>IM59
CAGCAACCGGGTACTAATGGCGCTGATTTTTT**TCTTTGGTTATCTAGCTGTATGA**GTGCATGTGACTGTCATAAAGCTAGGTTACCGAAGT
TAAGAACAGCATCTGTTCATCGATGGTAGCTG

>IM59-5P
TCTTTGGTTATCTAGCTGTATGA

>miRNA precursor
CAGCAACCGGGUACUAAUGGCGCUGAUUUUUU**UCUUUGGUUAUCUAGCUGUAUGA**GUGCAUGUGACUGUCAUAAAGCUAGGUUACCGAAGUUAAGAACAGCAUCUGUUCAUCGAUGGUAGC
UG
((((.((((......(((((.((((.((((..(((((((.((((((((.
(((((((.......))..))))).))))))))).)))))))..)))).)))).)))))......)))).))))

>Predicted STAR seq:
ATAAAGCTAGGTTACCGAAGTTAA

Complementarity: 18

Initial dG =   -43.50

```
        10        20         30        40        50
    A    GGUACU     C-    A    UU       U        G     --| GCA
CAGC ACCG      AAUGG  GCUG UUUU  CUUUGGU AUCUAGCU UAUGA  GU   \
GUCG UGGU      UUGUC  CGAC AGAA  GAAGCCA UGGAUCGA AUACU  CA   U
    A    AGCUAC     UA    A    UU       U        A     GU^ GUG
120       110       100        90        80        70
```

Reads in: lib-42, lib-45, lib-47, lib-48, lib-49, lib-50, lib-72
Number of reads="IM59",18570,7716,113771,3164,63455,32943,0

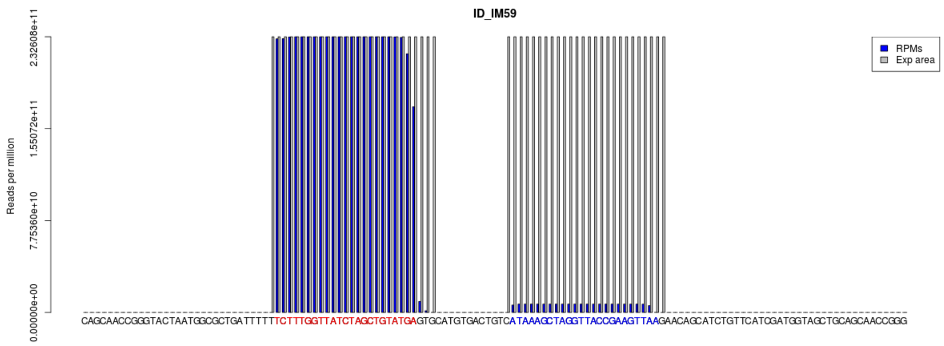

>IM6

CCGTTTCGACTAACGTATGGCAGCAAGTGAGGGCGATGGGGGACTGGGAGTGGGCACAAGGAGGATAGG**TGTTAACTGTCAGACTGTGAC**
TCTCCTATGATACATGCCATGCAACAGGCGTACCGG

>IM6-3P

TGTTAACTGTCAGACTGTGACT

>miRNA precursor

CCGUUUCGACUAACGUAUGGCAGCAAGUGAGGGCGAUGGGGGACUGGGAGUGGGCACAAGGAGGAUAGG**UGUUAACUGUCAGACUGUGACU**CUCCUAUGAUACAUGCCAUGCAACAGGCG
UACCGG
(((...((.((...((((((((((........)).((((((((((((.(((..
((((...........))))..))).)))).........))))))))......))))))))...)).))...)))

>Predicted STAR seq:

CTGGGAGTGGGCA

Complementarity: 9
ERROR 4: Strange structure
Initial dG = -37.20

```
        10        20
   UUU  A  AAC          | AAG
CCG   CG CU   GUAUGGCA--GC   U
GGC   GC GA   CGUACCGU  CG   G
   CAU  G  CAA        \ ^ GGA
    120       110          30
```

```
                                 40                50        60
                        G-----        --------    G   GG    AAGGA
                              AUGGGGGA        CUGG AGU  GCAC     \
                              UAUCCUCU        GACU UCA  UGUG     G
                        ACAUAG        CAGUGUCA    G   AU    GAUAG
                          100        90        80        70
```

Reads in: lib-42, lib-45, lib-47, lib-48, lib-49, lib-50, lib-72
Number of reads="IM6",0,0,0,0,0,1,0

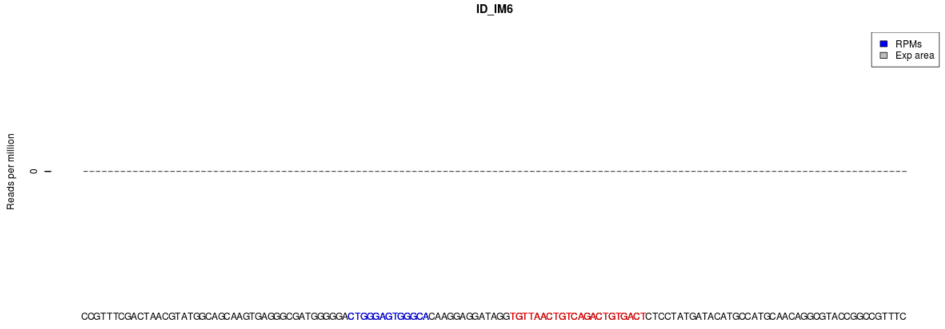

>IM60
CAGCAACCGGGTACTAATGGCGCTGATTTTTTCTTTGGTTATCTAGCTGTATGAGTGCATGTGACTGTC**ATAAAGCTAGGTTACCGAAGT**
TAAGAACAGCATCTGTTCATCGATGGTAGCTG

>IM60-3P
ATAAAGCTAGGTTACCGAAGTTA

>miRNA precursor
CAGCAACCGGGUACUAAUGGCGCUGAUUUUUUCUUUGGUUAUCUAGCUGUAUGAGUGCAUGUGACUGUC**AUAAAGCUAGGUUACCGAAGUUA**AGAACAGCAUCUGUUCAUCGAUGGUAGC
UG
((((.((((......(((((.((((.((((..(((((((.((((((((.
((((((((.......))..))))).)))))))).)))))))..)))).)))))..)))))......)))).))))

>Predicted STAR seq:
TTCTTTGGTTATCTAGCTGTAT

Complementarity: 17

Initial dG =   -43.50

```
        10        20         30        40        50
    A    GGUACU     C-    A    UU       U        G     --| GCA
CAGC ACCG      AAUGG  GCUG UUUU  CUUUGGU AUCUAGCU UAUGA  GU   \
GUCG UGGU      UUGUC  CGAC AGAA  GAAGCCA UGGAUCGA AUACU  CA   U
    A    AGCUAC     UA    A    UU       U        A     GU^ GUG
120       110       100        90        80        70
```

Reads in: lib-42, lib-45, lib-47, lib-48, lib-49, lib-50, lib-72
Number of reads="IM60",18737,7610,114128,3173,63849,33128,0

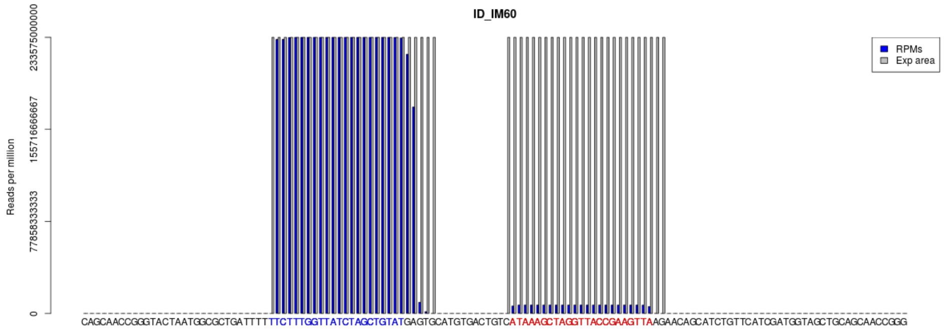

>IM61
GTGGATTTCGTCTCTTAGTTGGAAACAACAACAGCAAATGTGACACCAAACCACATCTAGTCCTTGGTGT**CACAGTTGCTGTTGTTTTTA**
CCAACTAAGAGACGAAATCCAC

>IM61-3P
CACAGTTGCTGTTGTTTTTA

>miRNA precursor
GUGGAUUUCGUCUCUUAGUUGGAAACAACAACAGCAAAUGUGACACCAAACCACAUCUAGUCCUUGGUGU**CACAGUUGCUGUUGUUUUUA**CCAACUAAGAGACGAAAUCCAC
```
((((((((((((((((((((((.((.(((((((((((.(((((((((((...((.....))..))))))))))).))))))))))).)).))))))))))))))))))))))
```

>Predicted STAR seq:
AACAACAACAGCAAATGTG

Complementarity: 15

Initial dG =   -73.00

```
        10        20        30        40        50
|                     A  C           A           ACC  AU
GUGGAUUUCGUCUCUUAGUUGG AA AACAACAGCAA UGUGACACCAA   AC  \
CACCUAAAGCAGAGAAUCAACC UU UUGUUGUCGUU ACACUGUGGUU   UG  C
^                     A  U           G           CC-  AU
110       100       90        80        70          60
```

Reads in: lib-42, lib-45, lib-47, lib-48, lib-49, lib-50, lib-72
Number of reads="IM61",2,1,1,0,0,4,0

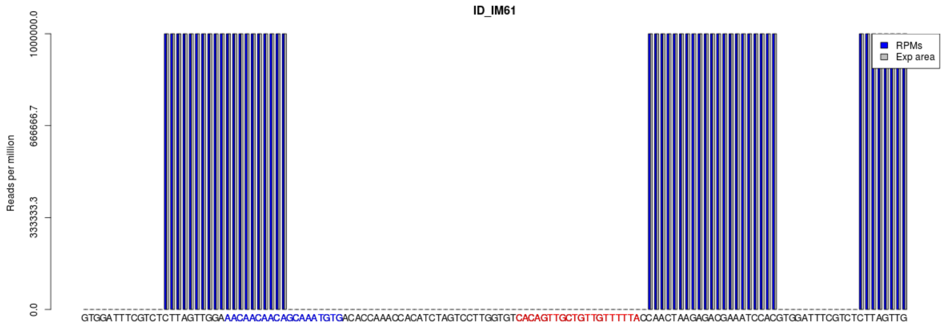

>IM62
ATCCCCCTTATCAGTATCACGGAGGAATACT**TCAGGTACCTGTAGTAGCGC**CGGCCGCAGTGGCCGAGGCGACGGTTGTTAGACAGTCAC
GGTCTGTGCGGCCACTACGGTCGGGGAT

>IM62-5P
TCAGGTACCTGTAGTAGCGC

>miRNA precursor
AUCCCCCUUAUCAGUAUCACGGAGGAAUACU**UCAGGUACCUGUAGUAGCGC**CGGCCGCAGUGGCCGAGGCGACGGUUGUUAGACAGUCACGGUCUGUGCGGCCACUACGGUCGGGGAU
.(((.((.............)).))).((((.(((....))).))))...(((((((.((((((((((((((.(((....))).)))...))))...)))))))).)))))))....

>Predicted STAR seq:
CGGTC

Complementarity: 2

```
 Initial dG =    -48.20

          10        20
ERROR 3: Strange structure
 Initial dG =    -48.20

          10        20
      .-CUUAUCA|    AC    GA
AUCCCC         GUAUC  GGAG  \
UAGGGG         CAUGG  CUUC  A
      \ -------^    A-    AU
                        30



         40        50        60        70
        --       AGCGCC       GU      AGGC   G   G
          CUGUAGU      GGCCGCA  GGCCG    GAC GUU U
          GGCAUCA      CCGGCGU  CUGGC    CUG CAG U
        CU       ------       GU      A---   A   A
        110             100        90           80
```

Reads in: lib-42, lib-45, lib-47, lib-48, lib-49, lib-50, lib-72
Number of reads="IM62",21,22,6543,12,47,19,0

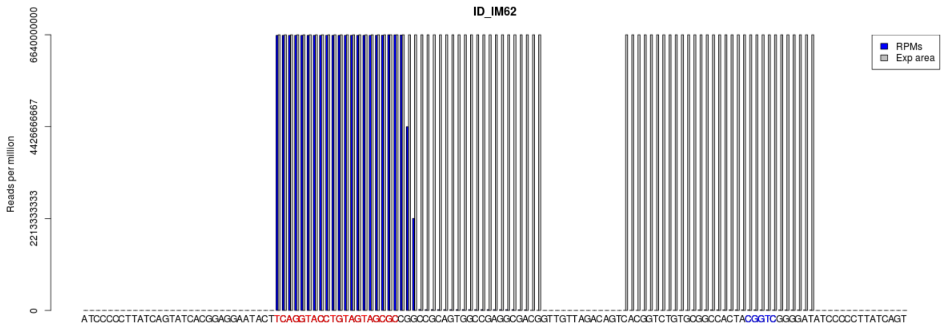

>IM63

GTCTTGTGGTCATCTACACACCTGACAGGTTTGGCGCGGTGCCAGGGGCGGCTCGATTGTAGTGCGAG**TTGTGCGTGTGACCTCGGCCA**G
TGCCTGCCACGTCACGTAGAGTATGTTGCGAGTGC

>IM63-3P

TTGTGCGTGTGACCTCGGCCA

>miRNA precursor

GUCUUGUGGUCAUCUACACACCUGACAGGUUUGGCGCGGUGCCAGGGGCGGCUCGAUUGUAGUGCGAG**UUGUGCGUGUGACCUCGGCCA**GUGCCUGCCACGUCACGUAGAGUAUGUUGCG
AGUGC
..((((..(.(((((....((.((((.(((..(((((...(((..
((((((((((((....)).))))))))........))..)))..))))).)))..)))).))..)).)))).))))...

>Predicted STAR seq:

GTGCCAGGGGCGG

Complementarity: 7

Initial dG =   -42.20

```
         10         20         30        40                 50
GU-    UG U   -  ACAC  C    A-   UU     GGU   AG  --------        -| U
   CUUG  G CAU CU    AC UGAC  GGU  GGCGC   GCC  GG        GCGGCUCG AU G
   GAGC  U GUA GA    UG ACUG  CCG  CCGUG   CGG  CC        UGUUGAGC UG U
CGU    GU -   U  GA--  C    CA   U-     AC-   CU  AGUGUGCG        G^ A
   120        110         100         90         80         70         60
```

Reads in: lib-42, lib-45, lib-47, lib-48, lib-49, lib-50, lib-72

Number of reads="IM63",0,0,0,0,0,0

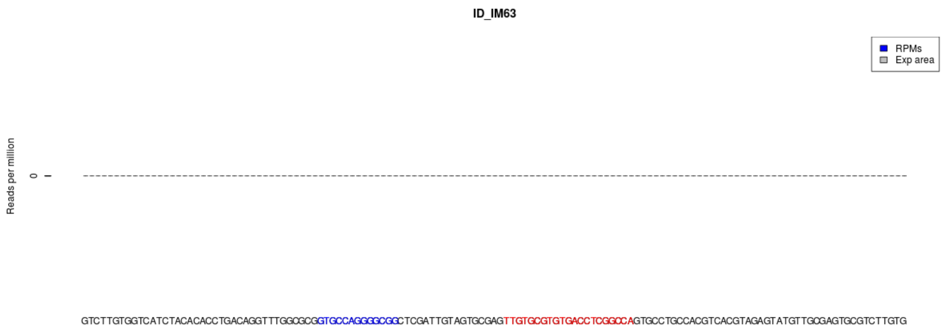

>IM64

TGTCGCAATTCCTCAGCACCTCAGCCTCCGTGATCCCTGGATTTAATTTGGTGATCATGGCGGTGTAGGT**GGGTGGTGGTGGAAGGGGGG**
GAAGGGATTTGGCGGCAGGAGGTGGGTGGAGAAAGAGA

>IM64-3P

GGGTGGTGGTGGAAGGGGGGG

>miRNA precursor

UGUCGCAAUUCCUCAGCACCUCAGCCUCCGUGAUCCCUGGAUUUAAUUUGGUGAUCAUGGCGGUGUAGGU**GGGUGGUGGUGGAAGGGGGGG**AAGGGAUUUGGCGGCAGGAGGUGGGUGGA
GAAAGAGA

```
((((((..(((((((.(((((..(((.((((((((.(..(((...)))..).)))))))).)))......))))).))).))))..
(((........))).)))))).....................
```

>Predicted STAR seq:

TCAGCACCTCAGCC

Complementarity: 10

Initial dG =   -42.70

```
                               10         20             30        40
---------------------      .-AA    -   G     CA----|  U        C UG   U
                     UGUCGC    UUCC UCA CACCU      GCC CCGUGAUC C  GAU \
                     ACGGCG    AAGG GGU GUGGG      UGG GGUACUAG G  UUA U
AGAGAAAGAGGUGGGUGGAGG      \ --    U   G     UGGAUG^  C        U GU   A
      120       110               80        70        60        50
```

Reads in: lib-42, lib-45, lib-47, lib-48, lib-49, lib-50, lib-72

Number of reads="IM64",0,1,0,0,1,0,0

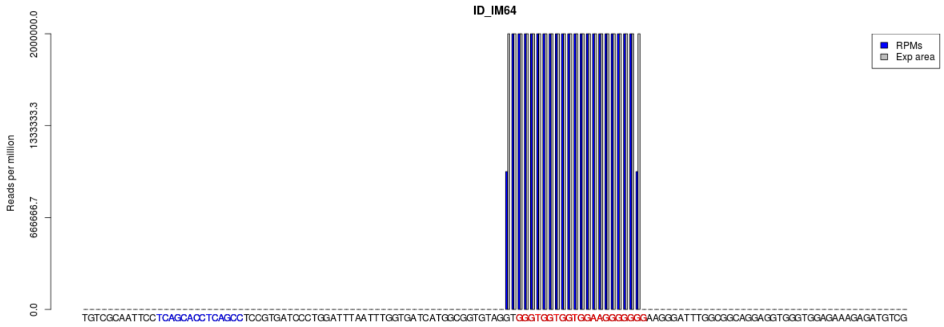

>IM65
CTAACCATTTATGAAATTTT**CATTTATATTTCATTCACGT**ATTTTAGTTAGTTCCGAAACTGACTAAAATTCGTGAATGAAATATAAAAG
AACACCTCATAAACGGTTAG

>IM65-3P
CATTTATATTTCATTCACGT

>miRNA precursor
CUAACCAUUUAUGAAAUUUU**CAUUUAUAUUUCAUUCACGU**AUUUUAGUUAGUUCCGAAACUGACUAAAAUUCGUGAAUGAAAUAUAAAAGAACACCUCAUAAACGGUUAG
((((((.(((((((....(((.((((((((((((((((((.(((((((((((((....))))))))))))).)))))))))))))))))).))).....))))))).))))))

>Predicted STAR seq:
TGAATGAAATATAAAAGAA

Complementarity: 16

Initial dG =   -50.30

```
        10        20        30        40        50
|     A       AAUU   A                 U             C
CUAACC UUUAUGA    UUC UUUAUAUUUCAUUCACG AUUUUAGUUAGUU C
GAUUGG AAAUACU    AAG AAAUAUAAAGUAAGUGC UAAAAUCAGUCAA G
^     C       CCAC   A                 U             A
.       100       90        80        70        60
```

Reads in: lib-42, lib-45, lib-47, lib-48, lib-49, lib-50, lib-72
Number of reads="IM65",0,0,0,0,0,0,0

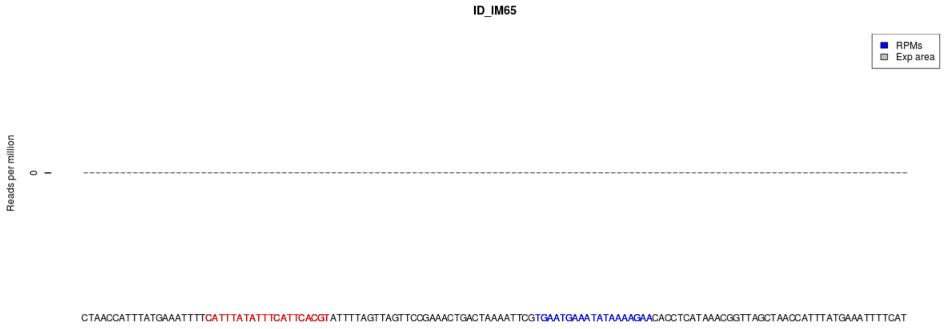

>IM66
CATCGTCGACAGGACCAACTGCAGTCCTCTGGTATAGAGCCGAGTGGAGGGTCTGATTCAGAGGCACAG**ACGGTTCTGTTACAGTGTAGA**
CTGCAGACTCCTTGACTTG

>IM66-5P
ACGGTTCTGTTACAGTGTAGA

>miRNA precursor
CAUCGUCGACAGGACCAACUGCAGUCCUCUGGUAUAGAGCCGAGUGGAGGGUCUGAUUCAGAGGCACAG**ACGGUUCUGUUACAGUGUAGA**CUGCAGACUCCUUGACUUG
....((((..((((....((((((((..(((..(((((((((..((.....((((...))))....))..)))))))))..))).....))))))))..))))))))...

>Predicted STAR seq:
CCTCTGGTATAGAGCCG

Complementarity: 12

Initial dG =   -39.80

```
        10        20          30        40        50
CAUC|   AC    CCAA        CU--   GU         AG  GAGGG    A
    GUCG  AGGA    CUGCAGUC    CUG  AUAGAGCCG  UG     UCUG \
    CAGU  UCCU    GACGUCAG    GAC  UGUCUUGGC  AC     AGAC U
GUU-^   --    CA--        AUGU   AU         AG  ACGG-    U
          100          90        80        70         60
```

Reads in: lib-42, lib-45, lib-47, lib-48, lib-49, lib-50, lib-72
Number of reads="IM66",0,1,2,1,2,1,0

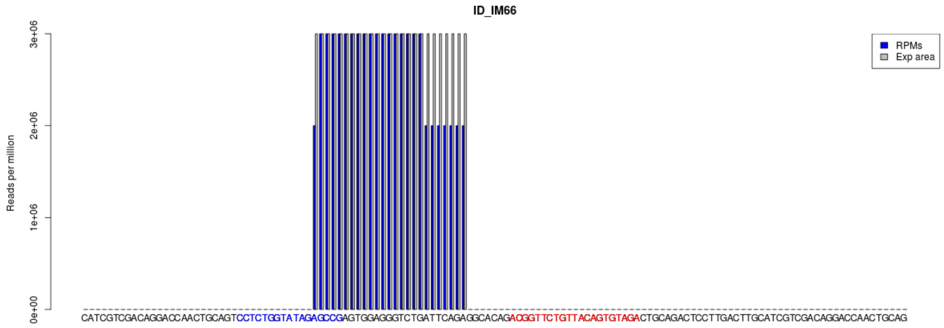

>IM67
TTGATGGTGGTATCCTCTCTGGTAAAATATTCCTGAGGTAAAATTCAGCCCCACCAACTGCTTTAAAT**TACTGAATTTTATGTGGAC**TTC
GACTACCACTGAGGGTGTCATCATCAG

>IM67-5P
TACTGAATTTTATGTGGAC

>miRNA precursor
UUGAUGGUGGUAUCCUCUCUGGUAAAAUAUUCCUGAGGUAAAAUUCAGCCCCACCAACUGCUUUAAAU**UACUGAAUUUUAUGUGGAC**UUCGACUACCACUGAGGGUGUCAUCAUCAG
.((((((((..((((((..(((((......(((....(((((((((((.....................)))))))))))..))).......)))))..))))))..)))))))).

>Predicted STAR seq:
AATATTCCTGAGGTAAAATTCAG

Complementarity: 12

Initial dG = -39.90

```
        10        20        30         40        50
U        GU      UC      AAAUAU-|  UGAG           CCCCACCAAC
 UGAUGGUG  AUCCUC  UGGUA       UCC    GUAAAAUUCAG          U
 ACUACUAC  UGGGAG  ACCAU       AGG    UAUUUUAAGUC          G
G        UG      UC      CAGCUUC^  UG--           AUUAAAUUUC
     110       100        90          80        70        60
```

Reads in: lib-42, lib-45, lib-47, lib-48, lib-49, lib-50, lib-72
Number of reads="IM67",0,0,0,0,0,0,0

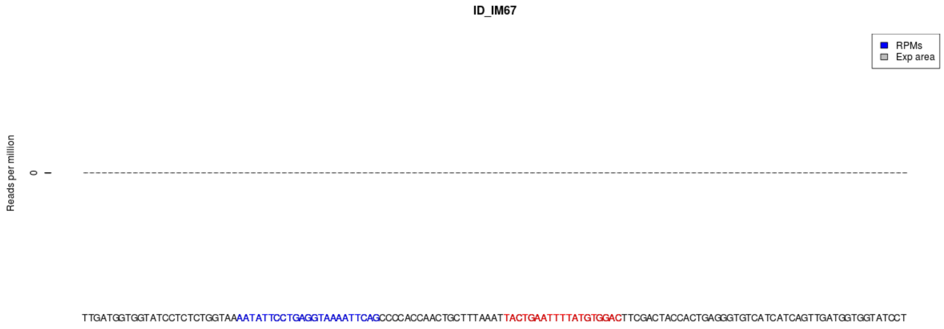

>IM68
CTTCGGGGCTAAGTTGCCATGGTCCGTCTGTGAAACTTTCTTCTCCCAACATTTCATTGCCTACTGC**AGGCAAGATCTTGAGCAG**TGAGT
GGACGACTGGCTGCTAGCCGCTGGAG

>IM68-3P
AGGCAAGATCTTGAGCAG

>miRNA precursor
CUUCGGGGCUAAGUUGCCAUGGUCCGUCUGUGAAACUUUCUUCUCCCAACAUUUCAUUGCCUACUGC**AGGCAAGAUCUUGAGCAG**UGAGUGGACGACUGGCUGCUAGCCGCUGGAG
((((((((((.((.((((..(((.((((.....((((.((.(((........((.((((((.....))))))))....))).)).))))))))))))))).))))))).))))))

>Predicted STAR seq:
CTTCTCCCAACATTTCATTGCCT

Complementarity: 11

Initial dG =   -40.60

```
         10        20        30        40        50        60
      -|    A  U    UG   C    GUGAA    U  U   CCAACAUU  A      AC
CUUCGG GGCUA GU GCCA  GUC GUCU     ACUU CU CUC        UC UUGCCU  \
GAGGUC CCGAU CG CGGU  CAG CAGG     UGAG GA GAG        AG AACGGA  U
      G^    -  U    --   -    -----    U  C   UUCU----  -      CG
    110        100               90        80             70
```

Reads in: lib-42, lib-45, lib-47, lib-48, lib-49, lib-50, lib-72
Number of reads="IM68",0,0,0,0,0,1,0

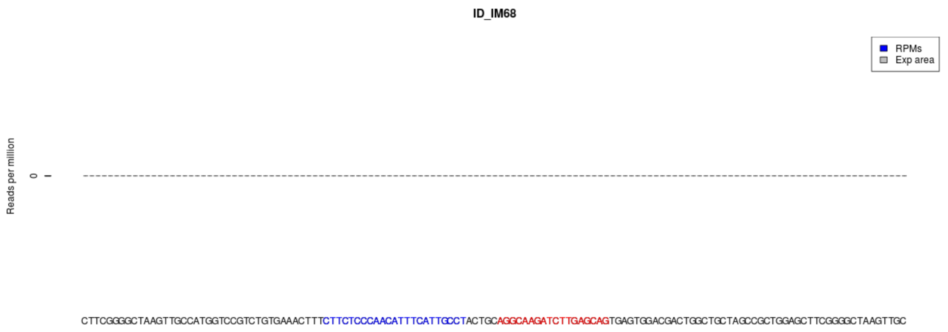

>IM69
CCTCCTACTTTCCAGACTTCATAGACGTGTAGTTTTGTCA**TGGGTTATAGTGTGCCGGC**CGCGGTGGCCCAGGGGACTGAGGCACTTCAG TCACGGTCTGCACGGCCACTACGGTCGGGGG

>IM69-3P
TGGGTTATAGTGTGCCGGC

>miRNA precursor
CCUCCUACUUUCCAGACUUCAUAGACGUGUAGUUUUGUCA**UGGGUUAUAGUGUGCCGGC**CGCGGUGGCCCAGGGGACUGAGGCACUUCAGUCACGGUCUGCACGGCCACUACGGUCGGGG G
....(((...(((((((.....((((.....)))).))).))))...)))....(((((((..((((((....((((((.
(((......))).))))))....))))))..)))))))...

>Predicted STAR seq:
AGACTTCATAG

Complementarity: 5
ERROR 4: Strange structure
Initial dG = -47.10

```
          10        20
.-CCUC   CUU    -|  UUCAU    GU
      CUA   UCCA GAC     AGAC  \
      GAU   GGGU CUG     UUUG  G
\ ----   AUU    A^  U----    AU
     50        40            30


          60        70        80
  UUGUG       CG       CAGG      A   AC
      CCGGCCG  GUGGCC    GGACUG GGC  U
      GGCUGGC  CACCGG    UCUGGC CUG  U
  GGG-        AU       CACG      A   AC
 120        110       100        90
```

Reads in: lib-42, lib-45, lib-47, lib-48, lib-49, lib-50, lib-72
Number of reads="IM69",234,524,628,456,536,905,0

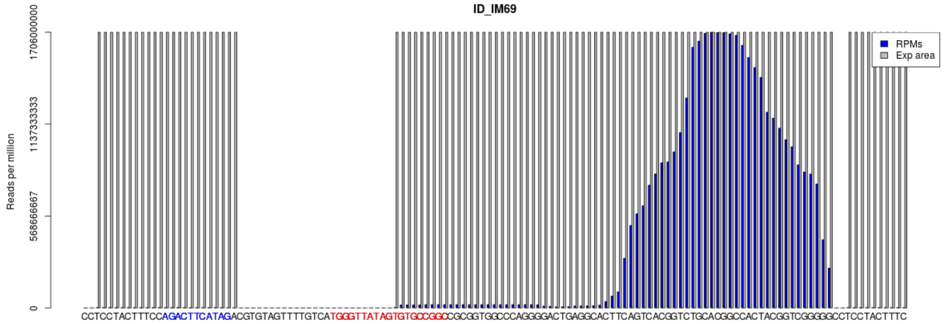

>IM7
CAGTTCGATGTAATGCTATTGCCAGTAAAGTGGGTTATAACTTGTGAAATGGAACTGTGTGCAACAAT**GTAAGGATTTAAATTAAATGTT**
TATGACTGGTCTGTATTGGCTATAGCATTGCACCGAACTG

>IM7-5P
GTAAGGATTTAAATTAAATGTT

>miRNA precursor
CAGUUCGAUGUAAUGCUAUUGCCAGUAAAGUGGGUUAUAACUUGUGAAAUGGAACUGUGUGCAACAAU**GUAAGGAUUUAAAUUAAAUGUU**UAUGACUGGUCUGUAUUGGCUAUAGCAUUG
CACCGAACUG
(((((((.(((((((((((.(((((((.(((.(((((((((...(((..((((.((...
((((....)))).)).))))..)))...))).)))))).).)).))))))).))))))))))).)))))))

>Predicted STAR seq:
ACTTGTGAAATGGAACTGTGTGC

Complementarity: 13

Initial dG =   -46.00

```
        10        20        30          40        50        60
       A           U       A  - G      -|  UUG   AA    A  GUG    A
CAGUUCG UGUAAUGCUAU GCCAGUA AG U GGUUAU AAC   UGA  UGGA CU   UGCA C
GUCAAGC ACGUUACGAUA CGGUUAU UC G UCAGUA UUG   AUU  AUUU GG   AUGU A
       C           U       G  U G      U^  UAA   AA    A  A--    A
.       120       110       100        90        80          70
```

Reads in: lib-42, lib-45, lib-47, lib-48, lib-49, lib-50, lib-72
Number of reads="IM7",0,1,0,0,0,1,0

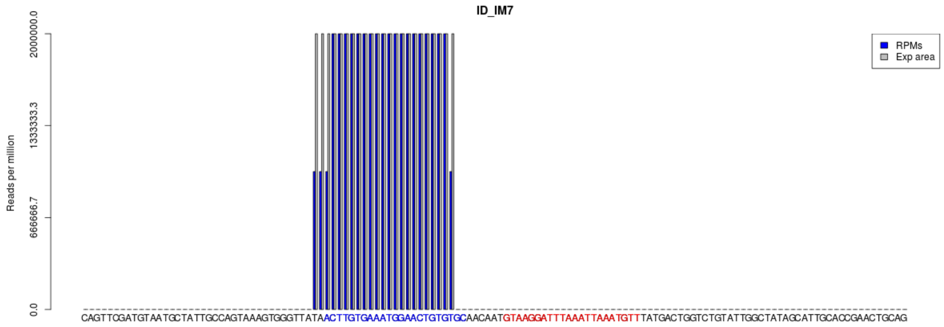

>IM70
CTGCCTGCAGCGTGAATGCAACAAGGCTGCCGGCGAGCAGCCAGCTGTTATAGGTGCTAGCAGCGAGCAG**CCAGCTGCCAGCTGCGAGCG**
GGCTGCATAAACGCATCCTAAGGTAG

>IM70-3P
CCAGCTGCCAGCTGCGAGCGG

>miRNA precursor
CUGCCUGCAGCGUGAAUGCAACAAGGCUGCCGGCGAGCAGCCAGCUGUUAUAGGUGCUAGCAGCGAGCAG**CCAGCUGCCAGCUGCGAGCGG**GCUGCAUAAACGCAUCCUAAGGUAG
((((((...((((..((((.....((((((......)))))).(((........((((.(((((..((((....))))..))))).))))))).)))))..))))......))))))

>Predicted STAR seq:
TGCCGGCGAGCAGCCAGCTGTTATAGGTGCTAGCA

Complementarity: 12

Initial dG =   -51.60

```
           10        20           30
      GCA---    GA      .-ACAA|     CG
CUGCCU      GCGU  AUGCA      GGCUGC  G
GAUGGA      CGCA  UACGU      CCGACG  C
      AUCCUA    AA      \ ----^     AG
    110       100             40
```

Reads in: lib-42, lib-45, lib-47, lib-48, lib-49, lib-50, lib-72
Number of reads="IM70",0,0,0,1,0,0,0

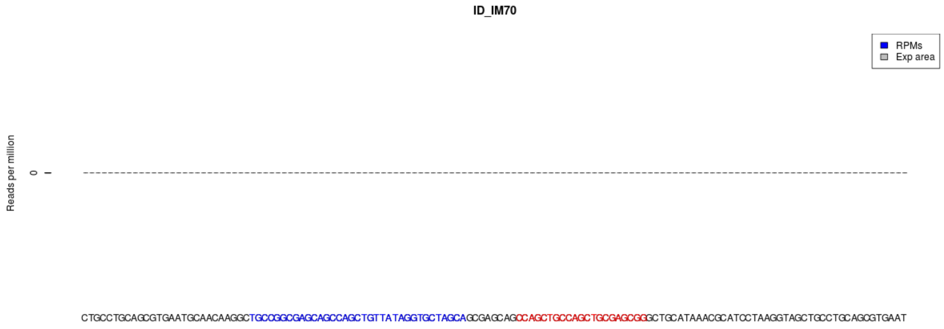

>IM71

ATTA**CCAATGTCCCACGCCTTGC**TTTGGCTTTAAATGTAATGTGCAGTGCCGGTCATATTTAAAGGCAAAGCAAGGCAGGCAACATTTGT
AAT

>IM71-5P

CCAATGTCCCACGCCTTGC

>miRNA precursor

AUUA**CCAAUGUCCCACGCCUUGC**UUUGGCUUUAAAUGUAAUGUGCAGUGCCGGUCAUAUUUAAAGGCAAAGCAAGGCAGGCAACAUUUGUAAU

```
(((((.(((((.((..((((((((((.((((((((((..((.((...)))).....)))))))))).))))))))))).))..))))).)))))
```

>Predicted STAR seq:

AAGGCAGGCAACATTTGTA

Complementarity: 13

Initial dG = -38.10

```
        10         20        30        40
     C     C-| AC           G          AA-  U  A
AUUAC AAUGU  CC  GCCUUGCUUUG CUUUAAAUGU   UG GC \
UAAUG UUACA  GG  CGGAACGAAAC GAAAUUUAUA   GC CG G
     U     AC^ A-           G          CUG  -  U
  90        80         70        60         50
```

Reads in: lib-42, lib-45, lib-47, lib-48, lib-49, lib-50, lib-72

Number of reads="IM71",0,0,0,0,0,0

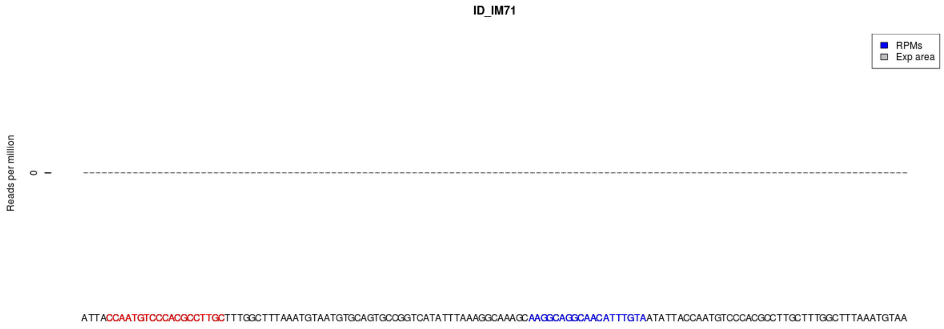

>IM72
TGGTGCCCAGAGCATTATCAGACCATGTTTCCACTGGTCGGTGGATTGGTAGAGGTAGCCCTTTTAACTGGCCTACTAGGTCACACGATT
TGAAGCTACTAGATTTCTTTCTGTGGGGGTATTA

>IM72-5P
ACTGGCCTACTAGGTCACA

>miRNA precursor
UGGUGCCCAGAGCAUUAUCAGACCAUGUUUCCACUGGUCGGUGGAUUGGUAGAGGUAGCCCUUUUAACUGGCCUACUAGGUCACACGAUUUGAAGCUACUAGAUUUCUUUCUGUGGGGGU
AUUA
((((((((...(((.......((((....((((((....)))))).))))...((((((.........
(((((.....)))))...........))))))...........)))..))))))))

>Predicted STAR seq:
GTGC

Complementarity: 3
ERROR 4: Strange structure
 Initial dG =   -36.00

```
          10        20          30
        .-AGAGCAUUAUCA    .-AUGUU|     G
UGGUGCCC              GACC       UCCACU G
AUUAUGGG              CUGG       AGGUGG U
        \ ------------    \ -----^     C
  120                  80           40



                               50        60
                            UUGGUAGA     C  UUU
                                    GGUAG CC   U
                                    UCAUC GG   A
                            A-------     C  UCA
                                          70
```

Reads in: lib-42, lib-45, lib-47, lib-48, lib-49, lib-50, lib-72
Number of reads="IM72",0,0,1,0,0,0,0

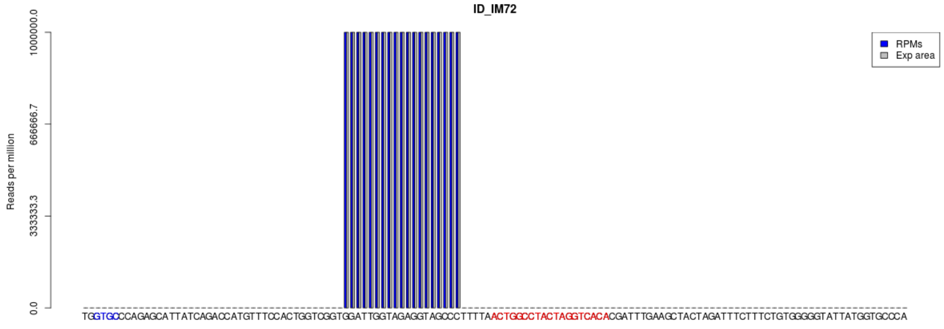

>IM73

CTGGCAGATTAAAATTGTGTGCCGGGACCAGGATCAGAAC**CTGGGACCTTTGCCTTACGGT**AGAGCGCTTGCCCGCGAAAGGCAAAGTTC
ACAGAATCCCGCTTCCGGCACACAATTTTAATCTGCCAG

>IM73-5P

CTGGGACCTTTGCCTTACGGT

>miRNA precursor

CUGGCAGAUUAAAAUUGUGUGCCGGGACCAGGAUCAGAAC**CUGGGACCUUUGCCUUACGGU**AGAGCGCUUGCCCGCGAAAGGCAAAGUUCACAGAAUCCCGCUUCCGGCACACAAUUUUA
AUCUGCCAG

```
((((((((((((((((((((((((((...((((......(((.((.(((((((((.(((..
((....))..)))...))))))))).)).))).)))).))))...))))))))))))))))))))))))))
```

Error predicting STAR on 5pERROR 4: Strange structure
 Initial dG =   -69.40

```
        10        20          30
                             |    AUC
CUGGCAGAUUAAAAUUGUGUGCCGGGA--CCAGG   \
GACCGUCUAAUUUUAACACACGGCCUU  GGUCC   A
                           \ ^    AAG
       120       110            40



                                        50          60
                             ------------  C         A--   UA  G
                                         GA CUUUGCCUU   CGG  GA C
                                         CU GAAACGGAA   GCC  UU G
                             CGCCCUAAGACA  U         AGC   CG  C
                             100        90        80        70
```

Reads in: lib-42, lib-45, lib-47, lib-48, lib-49, lib-50, lib-72

Number of reads="IM73",50,94,85,76,85,172,0

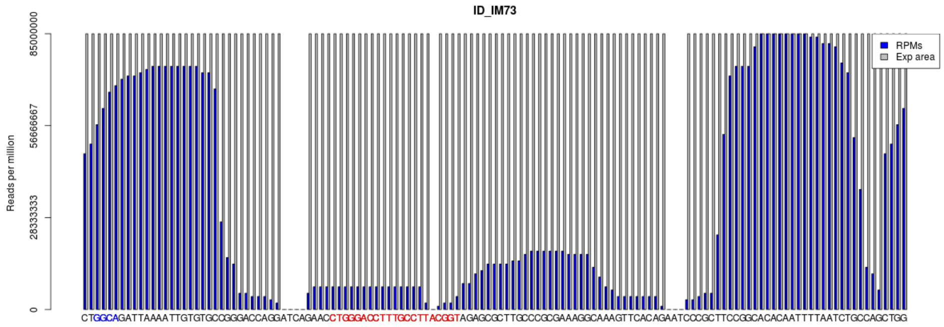

>IM8
CGGCGAGCAGTGCGGAGCCTGCAGCGTGAACGCAACGAGGCTGCCGGCGAGCAGCGAGCAGCGAGCAG**CCAGCTGCCAGCTGCGAGAGG**G
CAGCATAAACGCAGCCCTGCCTGTGCAGGCCG

>IM8-3P
CCAGCTGCCAGCTGCGAGAGG

>miRNA precursor
CGGCGAGCAGUGCGGAGCCUGCAGCGUGAACGCAACGAGGCUGCCGGCGAGCAGCGAGCAGCGAGCAG**CCAGCUGCCAGCUGCGAGAGG**GCAGCAUAAACGCAGCCCUGCCUGUGCAGGC
CG

```
((((..((((.((((.(.((((.(((....)))......((((((.....(((((..
(((((.........)))))..))))).....))))))......))))).))))))))....))))
```

>Predicted STAR seq:
GGCGAGCAGCGAGCAGC

Complementarity: 9
ERROR 4: Strange structure
 Initial dG =   -61.40

```
          10        20
    GA--    U    A C    .-A|  U
CGGC    GCAG GCGG G CUGC   GCG G
GCCG    UGUC CGUC C GACG   CGC A
    GACG    -    C -    \ -^  A
120        110               30


                              40        50        60
                          AACGAG      GGCGA     GA      GAGC
                                GCUGCC     GCAGC  GCAGC    \
                                CGACGG     CGUCG  CGUCG    A
                          CAAAUA      GAGAG     AC      ACCG
                        100        90        80        70
```

Reads in: lib-42, lib-45, lib-47, lib-48, lib-49, lib-50, lib-72
Number of reads="IM8",0,0,1,0,0,0,0

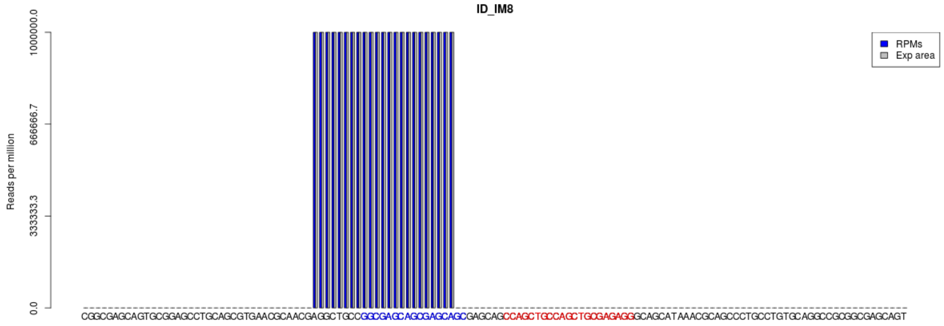

>IM9
AACTCTTCTGGCGGCTTATTGAAAAGAGACGAAGGACAATACTGCACACCTTTCTGTACAGGTGTTA**AGGAGGTGTGATCGGTTTG**TGCC
ATTAACTGAATTAAAGCTGCTGGTATGGTT

>IM9-5P
AGGAGGTGTGATCGGTTTG

>miRNA precursor
AACUCUUCUGGCGGCUUAUUGAAAAGAGACGAAGGACAAUACUGCACACCUUUCUGUACAGGUGUUA**AGGAGGUGUGAUCGGUUUG**UGCCAUUAACUGAAUUAAAGCUGCUGGUAUGGUU
((((...(..(((((((.(((........))).((((((.((((((((((((..((.((....)))).))))))))..))))))))).))............)))))))..)...))))

>Predicted STAR seq:
AAAGAGACGAAGGACAATACTGCACAC

Complementarity: 13
ERROR 4: Strange structure
Initial dG = -35.80

```
        10          20
    CUU UG       .-A|  AAA
AACU   C  GCGGCUU   UUG   A
UUGG   G  CGUCGAA   AGC   G
    UAU GU       \ -^  AGA
.       110          30
```

```
                      40          50         60
A------------  -    U    --        UC  U  A
             GG ACAA ACUG  CACACCUU  UG AC G
             CC UGUU UGGC  GUGUGGAG  AU UG G
AUUAAGUCAAUUA  G    -    UA        GA  -  U
 100        90         80        70
```

Reads in: lib-42, lib-45, lib-47, lib-48, lib-49, lib-50, lib-72
Number of reads="IM9",0,0,0,0,0,0,0

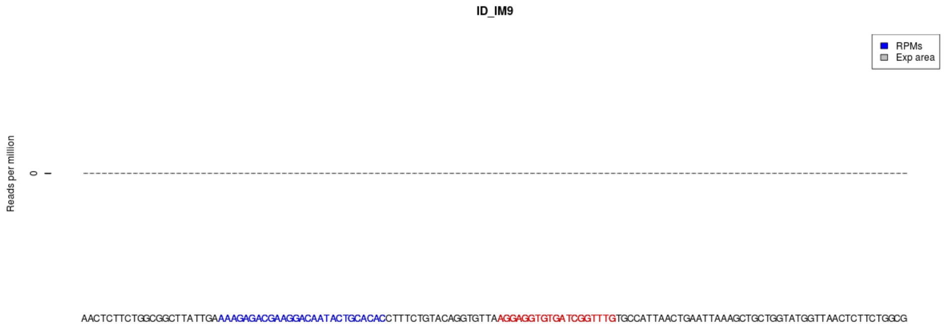

Supplementary Data S4. Report generated with MirPlot of the 176 *Acyrtosiphon pisum* miRNA candidates from Legeai et al. (2010) plus the 10 newly found miRNAs. For each candidate the following information is shown;

- The miRNA precursor with mature arm colored red.
- The mature arm colored red and indicating if it is 3' or 5'.
- The precursor folding in Vienna format.
- The free energy of the precursor folding.
- The folding of the miRNA precursor with colored mature arm.
- A plot showing all the expressed nucleotides of the precursor (grey), the reads per million in the available small RNA-seq libraries.

>Ap-bantam
TGATGCGAAGCTGGTTTTCACTTTGATCTGTCAGATTTATCATTGTTCATTC**TGAGATCATTGTGAAAGCTAAT**TTCGTAACTCA

>Ap-bantam-3p
TGAGATCATTGTGAAAGCTAAT

UGAUGCGAAGCUGGUUUUCACUUUGAUCUGUCAGAUUUAUCAUUGUUCAUUC**UGAGAUCAUUGUGAAAGCUAAU**UUCGUAACUCA
...(((((((.((((((((((..(((((..(((((...............))))))))))..)))))))))).))))))).....

Initial dG = -31.20

```
          10        20        30        40
UGA--|      C          UU      UG      UUUAUCA
     UGCGAAG UGGUUUUCAC  UGAUC  UCAGA       \
     AUGCUUU AUCGAAAGUG  ACUAG  AGUCU       U
ACUCA^      A          UU      --      UACUUGU
    80        70        60         50
```

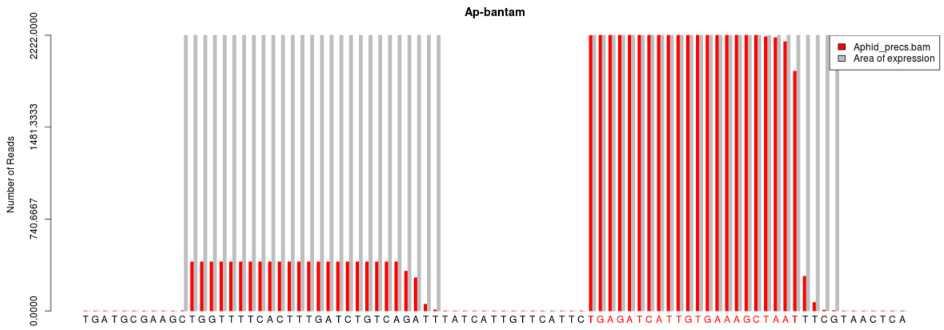

>Ap-let-7
GCTGACAAGAG**TGAGGTAGTTGGTTGTATAGT**AGTTTATTATTTGACTTTACTATACAATCGGCTAACTTTACTTCTGTCTGGCT

>Ap-let-7-5p
TGAGGTAGTTGGTTGTATAGT

GCUGACAAGAG**UGAGGUAGUUGGUUGUAUAGU**AGUUUAUUAUUUGACUUUACUAUACAAUCGGCUAACUUUACUUCUGUCUGGCU
(((((((..(((((((((((((((((((((((((((........))))..)))))))))))))))))).)))))))..)))).))).

```
 Initial dG =    -39.80

          10          20         30           40
-   -    AG        -                --|   UAU
 GCU GACA  AGUGAGG UAGUUGGUUGUAUAGU  AGUU   U
 CGG CUGU  UCAUUUC AUCGGCUAACAUAUCA  UCAG   A
U   U    CU        A                UU^   UUU
    80         70         60         50

-----------------------------------------------------
```

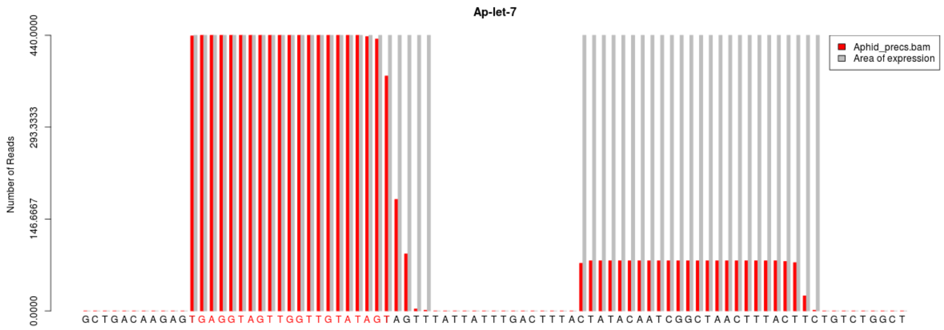

>Ap-mir-1

GCACCAGAGTTCCGTACTTCTTGTATGTTCGCATAGTGTGCGCGAGCCATTTGGCCGAATTTTACGACGTA**TGGAATGTAAAGAAGTATGGAG**CGGCGGGCCGG

>Ap-mir-1-3p

TGGAATGTAAAGAAGTATGGAG

```
GCACCAGAGUUCCGUACUUCUUGUAUGUUCGCAUAGUGUGCGCGAGCCAUUUGGCCGAAUUUUACGACGUAUGGAAUGUAAAGAAGUAUGGAGCGGCGGGCCGG
((.((...(((((((((((((.(((((((.((((((((..((.(((....))))).....))).)).)))))))))))))))))))))))...))))...

 Initial dG =    -41.30

           10        20        30          40           50
---  A  AGA              G       G    -  -   CG---| A   A
   GC CC   GUUCCGUACUUCUU UAUGUUC CAUA GU GUG     CG GCC U
   CG GG   CGAGGUAUGAAGAA AUGUAAG GUAU CA CAU     GC CGG U
GGC  -  CGG              -       -    G  G   UUUAA^ -   U
  100        90         80         70        60

-----------------------------------------------------
```

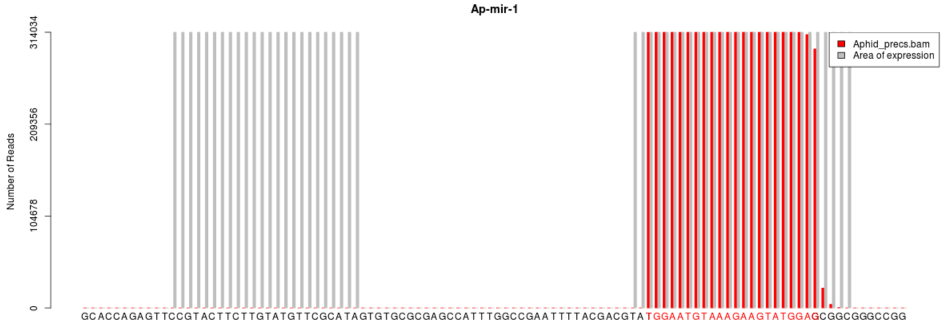

>Ap-mir-10
TCGGTGTAGTGATGCTCTACATC**TACCCTGTAGATCCGAATTTGT**TTGAAAAACGGCGACAAATTCGGTTCTAGAGAGGTTTGTGTGGTG
CATATTCCTTACGACCA

>Ap-mir-10-5p
TACCCTGTAGATCCGAATTTGT

UCGGUGUAGUGAUGCUCUACAUC**UACCCUGUAGAUCCGAAUUUGU**UUGAAAAACGGCGACAAAUUCGGUUCUAGAGAGGUUUGUGUGGUGCAUAUUCCUUACGACCA
..(((((((..((((.((((((..(((((.((((.(((((((((((...........))))))))))).)))).)).)))..)))))).)))).....)))).))).

Initial dG =   -43.30

```
         10            20          30         40         50
UC   -    UG---    U       CU   -| G    U           UGAAA
  GGU GUAG     AUGC CUACAU  ACC CU UAGA CCGAAUUUGUU      \
  CCA CAUU     UACG GGUGUG  UGG GA AUCU GGCUUAAACAG      A
A-   G    CCUUA    U       UU   A^ G    U           CGGCA
      100        90         80         70         60
```

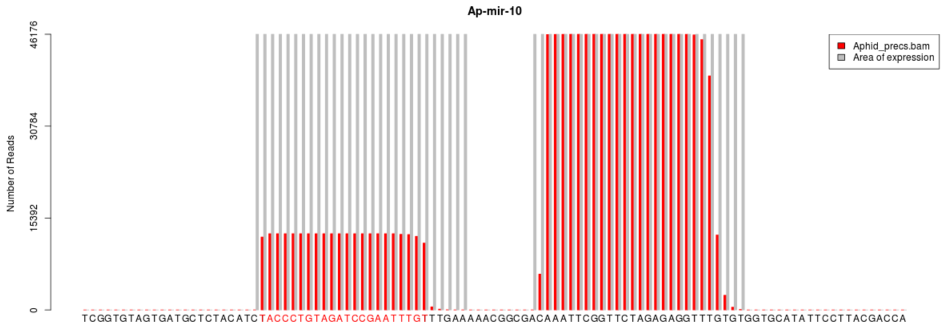

>Ap-mir-100
TGAGTTGCTAC**GACCCGTAGATCCGAACTTGTG**TCGCAAAGTTTAGTATCACAAGTTCGTTTCTAGAGGATACAGCCACTCAGT

>Ap-mir-100-5p
GACCCGTAGATCCGAACTTGTG

```
UGAGUUGCUACGACCCGUAGAUCCGAACUUGUGUCGCAAAGUUUAGUAUCACAAGUUCGUUUCUAGAGGAUACAGCCACUCAGU
(((((.(((....((..((((..((((((((((..((........))..))))))))))..))))..))....))).)))))..
```

```
 Initial dG =    -33.20

          10         20         30         40
--|    U   ACGA  CG    UC          UC   AAA
  UGAGU GCU    CC  UAGA  CGAACUUGUG  GC   G
  ACUCA CGA    GG  AUCU  GCUUGAACAC  UG   U
UG^    C   CAUA  AG    UU          UA   AUU
   80         70         60         50

-----------------------------------------------------
```

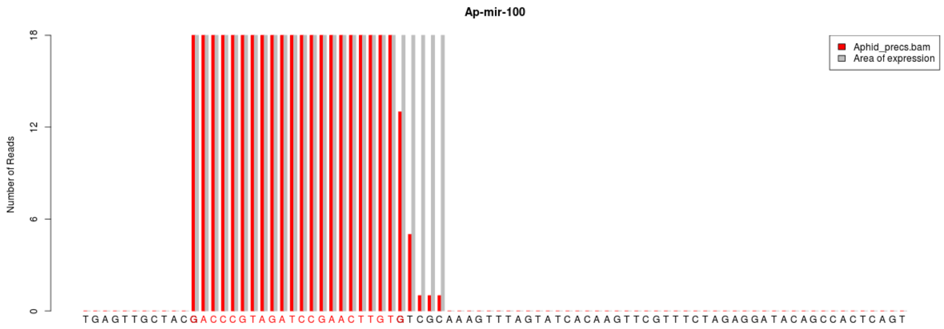

>Ap-mir-1000
ATAACACTCCG**ATATTGTCCTGTCACAGCAGTA**ATGTCACGTATCTGAATCGTACTGCTGTCGCGGGACATTTTCGGAGCTGTCGTC

>Ap-mir-1000-5p
ATATTGTCCTGTCACAGCAGTA

```
AUAACACUCCGAUAUUGUCCUGUCACAGCAGUAAUGUCACGUAUCUGAAUCGUACUGCUGUCGCGGGACAUUUUCGGAGCUGUCGUC
...(((((((((...((((((((.(((((((((...(((......)))....))))))))).))))))))...)))))).))).....

 Initial dG =    -42.40

          10        20        30         40
AUA-    -      UAU        C         AUG-|   CG
    ACA CUCCGA    UGUCCUGU ACAGCAGUA    UCA   U
    UGU GAGGCU    ACAGGGCG UGUCGUCAU    AGU   A
CUGC    C      UUU        C         GCUA^   CU
      80        70        60        50

-----------------------------------------------------
```

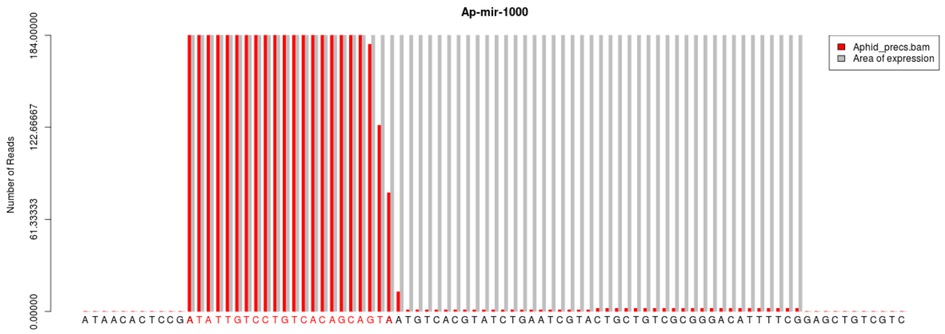

>Ap-mir-124
ATTCGCTCCTTGCGTTCACTGTTGAGCCTATATGTTGTTAATATGAATCA**TAAGGCACGCGGTGAATGCCA**AGATCGAATGA

>Ap-mir-124-3p
TAAGGCACGCGGTGAATGCCA

AUUCGCUCCUUGCGUUCACUGUUGAGCCUAUAUGUUGUUAAUAUGAAUCA**UAAGGCACGCGGUGAAUGCCA**AGAUCGAAUGA
(((((.((...(((((((((((.(.((((.((((...((.....))..)))))))).))))))))))))...)).)))))..

```
 Initial dG =    -28.70

          10        20        30        40
--|    C  CUU          U A    A    UUG  AA
  AUUCG UC   GCGUUCACUGU G GCCU UAUG   UU  \
  UAAGC AG   CGUAAGUGGCG C CGGA AUAC   AG  U
AG^    U  AAC          - A    -    UA-  UA
 80        70        60          50

-----------------------------------------------------
```

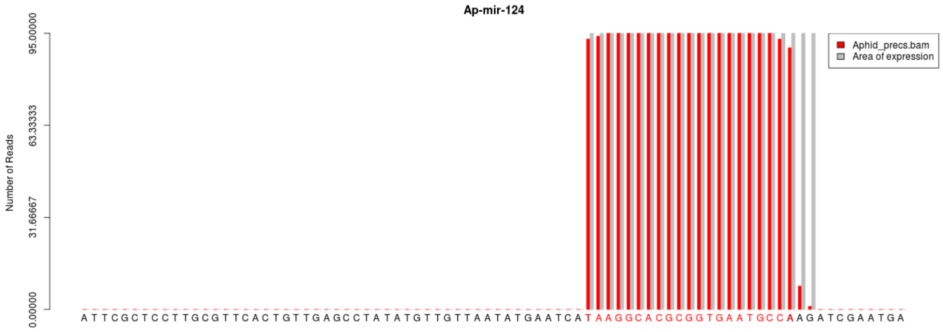

>Ap-mir-137

CGCATCTTCGTAGACTAACTTGGTTGGCCCCGCGTATTCTTGGGACATAACACATATTTAAGACTG**TTATTGCTTGAGAATACACGTA**GT
TGACAAGTTTAAACTTATTGTGCTGC

>Ap-mir-137-3p

TTATTGCTTGAGAATACACGTA

```
CGCAUCUUCGUAGACUAACUUGGUUGGCCCCGCGUAUUCUUGGGACAUAACACAUAUUUAAGACUGUUAUUGCUUGAGAAUACACGUAGUUGACAAGUUUAAACUUAUUGUGCUGC
.((((...........((((((.(..((..((.(((((((..((..((((((...........))))))..))..))))))).))..))..))))))).........))))...
```

```
 Initial dG =    -33.10

          10        20        30        40        50
C--|   CUUCGUAGACU      G UG  CC  C       UG  AC      CAUAU
   GCAU           AACUUG U  GC  CG GUAUUCU  GG  AUAACA     U
   CGUG           UUGAAC A  UG  GC CAUAAGA  UC  UAUUGU     U
CGU^   UUAUUCAAAU-      - GU  AU  A       GU  GU      CAGAA
    110       100         90        80        70        60
```

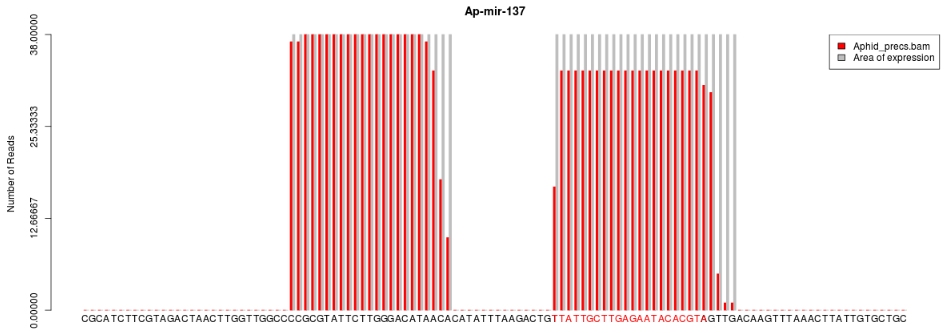

>Ap-mir-13a
CCAGACATAGGCCGTCAAAATGGATGTGAAGTGGTTTTTTTTACTATTCA**TATCACAGCCACTTTGATGAGC**CGATGTGTGTG

>Ap-mir-13a-3p
TATCACAGCCACTTTGATGAGC

```
CCAGACAUAGGCCGUCAAAAUGGAUGUGAAGUGGUUUUUUUUACUAUUCAUAUCACAGCCACUUUGAUGAGCCGAUGUGUGUG
.((.((((.((((((((((.(((.(((((((((((.......))))))....))))).))).))))))).))).)))).))..

 Initial dG =    -32.00

         10         20             30
C-  G    A   -       A   A     ----|     UUU
  CA ACAU GGC CGUCAAA UGG UGUGA    AGUGGU   \
  GU UGUA CCG GUAGUUU ACC ACACU    UUAUCA   U
GU  G    G   A       C   G     AUAC^     UUU
  80        70        60        50        40
```

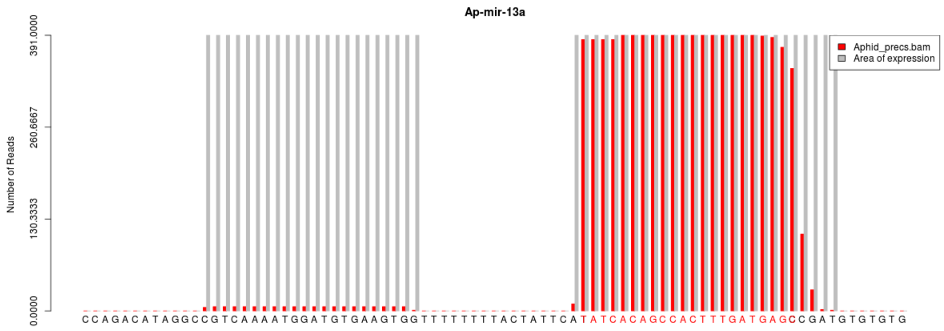

>Ap-mir-13b

AGGCGTCCAAATGGTCAAATCGGATGTGACGTGTTGATATTTATATTCA**TATCACAGCCGTTTTTGAC**AATTTTGGCGGCTAA

>Ap-mir-13b-3p

TATCACAGCCGTTTTTGAC

```
AGGCGUCCAAAUGGUCAAAUCGGAUGUGACGUGUUGAUAUUUAUAUUCAUAUCACAGCCGUUUUUGACAAUUUUGGCGGCUAA
((.((.(((((..((((((.(((.(((((..((..((((....))))))..))))).))).))))))...))))))).))..

 Initial dG =    -29.40

          10         20         30        40
--  G  U     UG-      U-|  A     CG  UU    U
  AG CG CCAAA   GUCAAA  CGG UGUGA  UG  GAUA U
  UC GC GGUUU   CAGUUU  GCC ACACU  AC  UUAU U
AA  G  -     UAA      UU^  G     AU  --    A
  80         70        60         50

-----------------------------------------------------
```

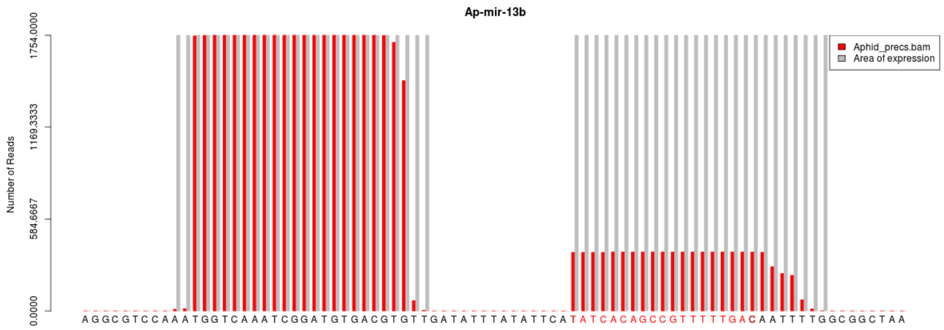

>Ap-mir-14
GATAAGATTATATAAATCGGAAGGGCATGCGTCGGAGAGAGATAACAGGCTTTGGCTTTTTATGTATTTTATTAG**TCAGTCTTTTTCTCT**
CTCCTATTTACCCACCGAACATTTCTACGT

>Ap-mir-14-3p
TCAGTCTTTTTCTCTCTCCTAT

```
GAUAAGAUUAUAUAAAUCGGAAGGGCAUGCGUCGGAGAGAGAUAACAGGCUUUGGCUUUUUAUGUAUUUUAUUAGUCAGUCUUUUUCUCUCUCCUAUUUACCCACCGAACAUUUCUACGU
................((((..(((..((.((.(((((((((....(((((..((((...((.......))..)))))))))...))))))))).)).))))).))))............
```

```
 Initial dG =    -30.30

        10        20        30        40        50        60
GAUAAGAUUAUAUAAA|   AA   CA  C  C         UAAC     UU    UUU  UGU
                UCGG  GGG  UG GU GGAGAGAGA    AGGCU  GGCU   UA   \
                AGCC  CCC  AU UA CCUCUCUCU    UCUGA  CUGA   AU   A
UGCAUCUUUACA----^   A-   --  U  U         UUU-     --    UU-  UUU
.       110              100        90         80           70

-----------------------------------------------------
```

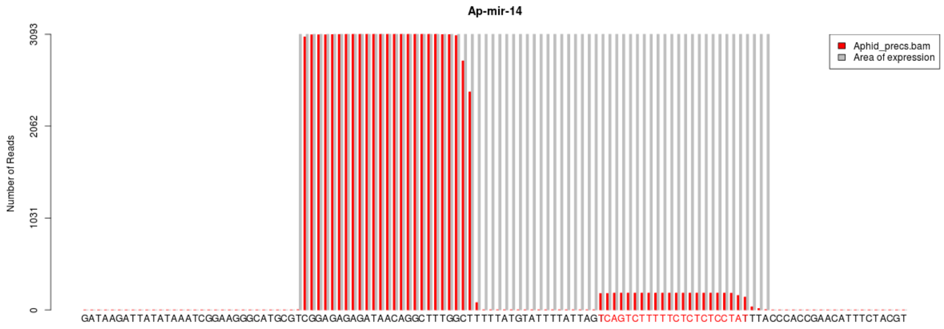

>Ap-mir-184a

CAATTACACTCCTTTATCATTCTCTGGTCTTGTGTGAATTTTATACTAC**TGGACGGAGAACTGATAAAGGA**AAGTGATGTGG

>Ap-mir-184a-3p

TGGACGGAGAACTGATAAAGGA

```
CAAUUACACUCCUUUAUCAUUCUCUGGUCUUGUGUGAAUUUUAUACUACUGGACGGAGAACUGAUAAAGGAAAGUGAUGUGG
..(((((..((((((((((((((((..(..((((((...))))))....)..))))))).)))))))))))..))))).....

 Initial dG =    -29.90

          10         20        30
CA--     AC          -        GU UU--|     A
    AUUAC  UCCUUUAUCA UUCUCUG  C    GUGUGA \
    UAGUG  AGGAAAUAGU AAGAGGC  G    CAUAUU U
GGUG     AA          C        AG UCAU^     U
 80        70        60        50        40

-----------------------------------------------------
```

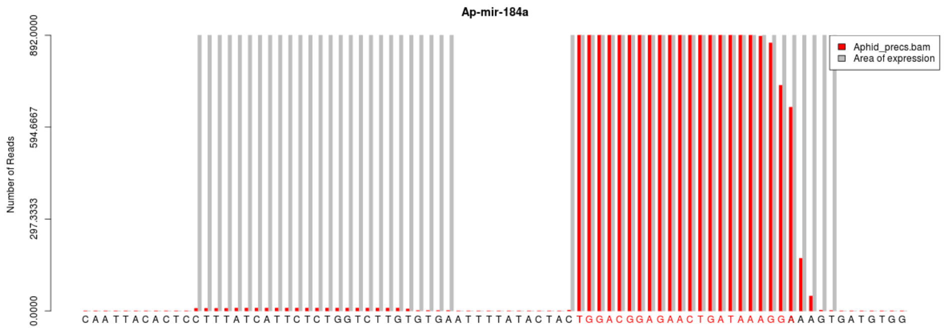

>Ap-mir-184b

TCGCATACCGGTCCTTATCATTCTTCGCCTTGTGTGCAATTTAAACAAC**TGGACGGAGAACTGATAAGGGCT**GGTATGTCGAT

>Ap-mir-184b-3p

TGGACGGAGAACTGATAAGGGCT

```
UCGCAUACCGGUCCUUAUCAUUCUUCGCCUUGUGUGCAAUUUAAACAACUGGACGGAGAACUGAUAAGGGCUGGUAUGUCGAU
((((((((((((((((((((((((((((...(((.........)))...)).))))))).)))))))))))))))))).))).

 Initial dG =    -44.40

          10        20          30
-   -                 -       -| UUG   GCAA
 UCG CAUACCGGUCCUUAUCA UUCUUCG CC   UGU    \
 AGC GUAUGGUCGGGAAUAGU AAGAGGC GG   ACA    U
U   U                 C       A^ UCA   AAUU
  80        70        60        50

-----------------------------------------------------
```

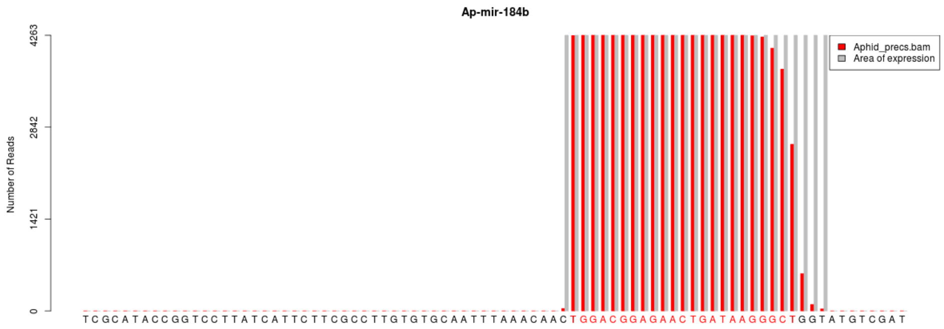

>Ap-mir-190
GTCGATTCGTG**AGATATGTTTGATATTCTTG**GTTGTTATATTTGAAACCGACCGAGAATCGGACATATTTTACGGTCACAGAA

>Ap-mir-190-5p
AGATATGTTTGATATTCTTG

```
GUCGAUUCGUGAGAUAUGUUUGAUAUUCUUGGUUGUUAUAUUUGAAACCGACCGAGAAUCGGACAUAUUUUACGGUCACAGAA
((.(((.(((((((((((((((((..(((((((((.............)))))))))))))))))))))))))))))))....

 Initial dG =    -32.80

            10        20        30        40
----| C   U                 AU          UUAUAU
    GU GAU CGUGAGAUAUGUUUGAU  UCUUGGUUG      \
    CA CUG GCAUUUUAUACAGGCUA  AGAGCCAGC      U
AAGA^ -   -                 --          CAAAGU
  80          70        60          50

-----------------------------------------------------
```

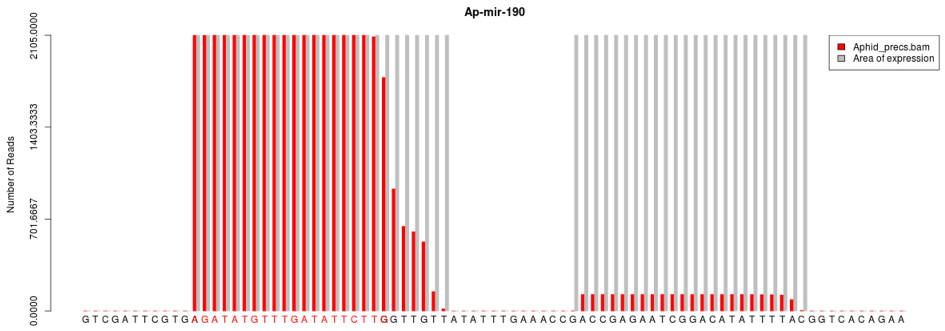

>Ap-mir-219
AGCGACAAGGTTCTCAGGCTG**TGATTGTCCAAACGCAATTCTTG**TTAAACGTATATATGCAATCAAGGATTGAGTAGGGACGTCAACGCT
TGAGACGTTTATTCGA

>Ap-mir-219-5p
TGATTGTCCAAACGCAATTCTTG

AGCGACAAGGUUCUCAGGCUG**UGAUUGUCCAAACGCAAUUCUUG**UUAAACGUAUAUAUGCAAUCAAGGAUUGAGUAGGGACGUCAACGCUUGAGACGUUUAUUCGA
..(((.((.((.(((((((..((((.((((..((.(((((((((.....(((....)))....))))))))).))..))))))))..))))))))).))...))).

Initial dG = -35.80

```
          10        20        30        40        50
AG   C--| G  U       UG    U    AA  G         UUAAA   A
  CGA   AA GU CUCAGGC  UGAU GUCC  AC CAAUUCUUG     CGU U
  GCU   UU CA GAGUUCG  ACUG CAGG  UG GUUAGGAAC     GUA A
A-   UAU^ G  -       CA    -    GA  A         UAAC-   U
     100        90         80        70        60
```

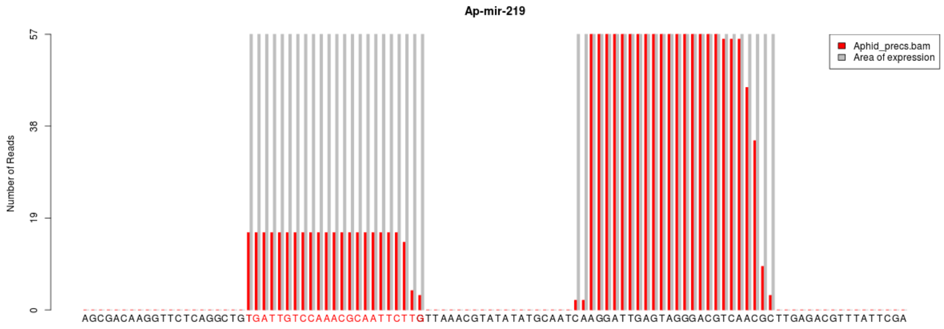

>Ap-mir-228
GTCTCACCGAA**AATGGCACTGAAAGAATTCACGGG**GTACATATTTACACATCACCCGTAGTTCTTCTGTGCCGTATTCGGTGGCCATT

>Ap-mir-228-5p
AATGGCACTGAAAGAATTCACGGG

GUCUCACCGAA**AAUGGCACUGAAAGAAUUCACGGG**GUACAUAUUUACACAUCACCCGUAGUUCUUCUGUGCCGUAUUCGGUGGCCAUU
...((((((((.(((((((.(((.(((((.(((((.................))))))))))))).))))))).)))))))).....

Initial dG =   -37.50

```
          10        20        30        40
GUC--|       A        U   A     C     GUACAUAU
     UCACCGAA AUGGCAC GAA GAAUU ACGGG        U
     GGUGGCUU UGCCGUG CUU CUUGA UGCCC        U
UUACC^       A        U   -     -     ACUACACA
       80        70         60        50
```

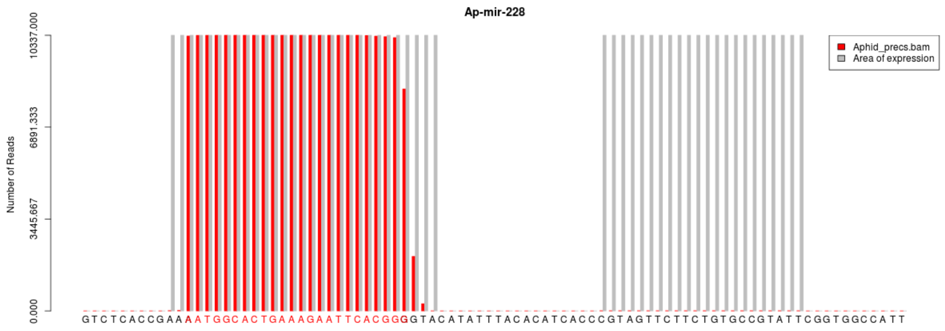

>Ap-mir-252
ATATTCTGTTC**CTAAGTACTAGTGCCGCGGGAG**TGATATCCGTTTTTTGTATCTCCTGCAGCTCAAGTGCTTATCAACGGGAAACAG

>Ap-mir-252-5p
CTAAGTACTAGTGCCGCGGGAG

AUAUUCUGUUC**CUAAGUACUAGUGCCGCGGGAG**UGAUAUCCGUUUUUUGUAUCUCCUGCAGCUCAAGUGCUUAUCAACGGGAAACAG
...(((((((..((((((((...((.(((((((..(((.........)))..))))))).))...))))))))..))))))).....

```
 Initial dG =    -30.50

          10        20        30        40
AUA--|      CC        AGU  C       UG   UCCG
     UUCUGUU  UAAGUACU   GC GCGGGAG  AUA    \
     AGGGCAA  AUUCGUGA   CG CGUCCUC  UGU    U
GACAA^      CU        ACU  A       UA   UUUU
      80        70        60        50
```

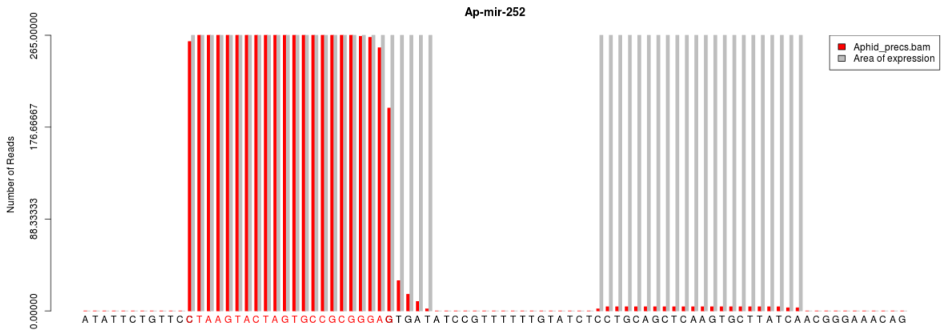

>Ap-mir-263b
GATCTGCGAAT**CTTGGCACTGGAAGAATTCACAGA**CTAATTATTGAAGATCGTGAGTCTCCTGGTGTCAACTATTGCGGATCGA

>Ap-mir-263b-5p
CTTGGCACTGGAAGAATTCACAGA

GAUCUGCGAAU**CUUGGCACUGGAAGAAUUCACAGA**CUAAUUAUUGAAGAUCGUGAGUCUCCUGGUGUCAACUAUUGCGGAUCGA
((((((((...(((((((..(.(((.(((((.((((.........)).)))))))))).)..)))))))...)))))))))..

```
 Initial dG =    -33.20

          10        20        30         40
--|        AUC       UG A    A     A  -  AAUU
  GAUCUGCGA   UUGGCAC  G AGA UUCAC GA CU    \
  CUAGGCGUU   AACUGUG  C UCU GAGUG CU GA    A
AG^        AUC       GU C    -     -  A  AGUU
   80        70        60         50

-----------------------------------------------------
```

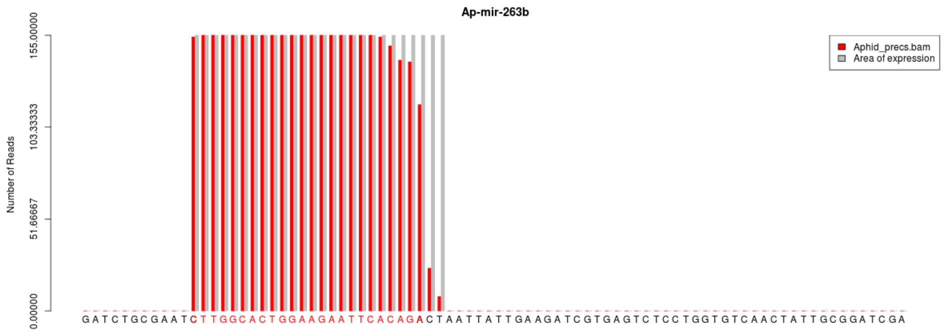

>Ap-mir-275
TTGTTTTCACGGGCTGCTACAGGCACTCTTAGACTCTTTAATGTTGACGAAG**TCAGGTACCTGAAGTAGCGCG**TGGAAATTTAA

>Ap-mir-275-3p
TCAGGTACCTGAAGTAGCGCG

UUGUUUUCACGGGCUGCUACAGGCACUCUUAGACUCUUUAAUGUUGACGAAG**UCAGGUACCUGAAGUAGCGCG**UGGAAAUUUAA
..(((((((((.((((((.((((.((.((..(((((.(((....))).).)))))))).)))).)))))).)))))))))....

Initial dG =   -32.30

```
          10        20        30         40
UU--|        G      A    C  U  UA    - U   A
    GUUUUCACG GCUGCU CAGG AC CU  GACU C UUA U
    UAAAGGUGC CGAUGA GUCC UG GA  CUGA G AGU G
AAUU^        G      A    A  -  --    A C   U
   80        70        60           50
```

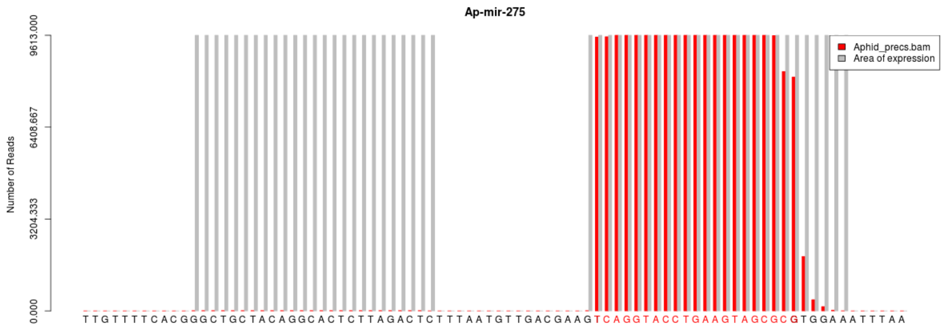

>Ap-mir-276
TAAAGCAATGACCACT**AGCGAGGTATAGAGTTCCTACG**TTCAAATTATTAATGTTACTCGTAGGAACTTCATACCGTGCTCTTGGACTTG
CGACAT

>Ap-mir-276-5p
AGCGAGGTATAGAGTTCCTACG

```
UAAAGCAAUGACCACUAGCGAGGUAUAGAGUUCCUACGUUCAAAUUAUUAAUGUUACUCGUAGGAACUUCAUACCGUGCUCUUGGACUUGCGACAU
....((((...(((..((((.(((((..((((((((((....(((.......)))...))))))))))..))))).))))..)))..)))).....

 Initial dG =    -35.60

         10        20        30        40
UAAA-|   UGA   CU    A     AG          UUCA   UAU
     GCAA   CCA  AGCG GGUAU  AGUUCCUACG    AAU   \
     CGUU   GGU  UCGU CCAUA  UCAAGGAUGC    UUG   U
UACAG^   CA-   UC    G     CU          UCA-   UAA
     90         80        70        60         50

-----------------------------------------------------
```

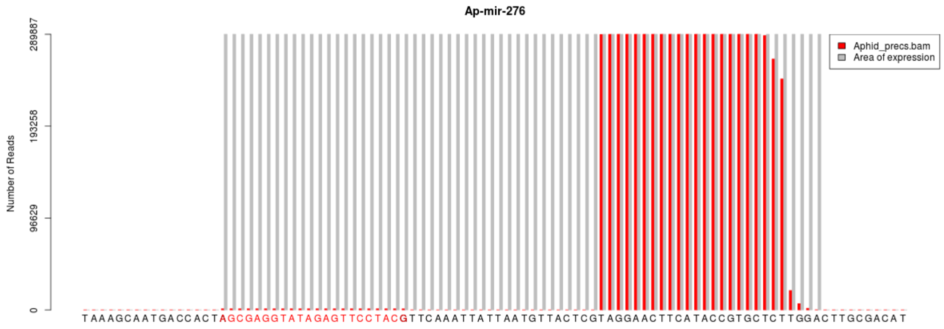

>Ap-mir-277
NNNNNNGAAGACCGTACCAGAAGTGTTTTTGACATGGACACACGGTTTTCGCCGTTCCCAAATCATG**TAAATGCACTATCTGGTACGACA**
CTTCGGCGCCC

>Ap-mir-277-3p
TAAATGCACTATCTGGTACGACA

```
NNNNNNGAAGACCGUACCAGAAGUGUUUUUGACAUGGACACACGGUUUUCGCCGUUCCCAAAUCAUGUAAAUGCACUAUCUGGUACGACACUUCGGCGCCC
......((((..((((((((((((((.(((.((((((....(((((....))))).......))))))))).))))).)))))))))...)))).......
```

```
 Initial dG =    -34.80

         10         20         30        40
NNNNNN-    AC-         -     U   G      ACAC---|    U
       GAAG   CGUACCAGA AGUGU UUU ACAUGG       ACGGU U
       CUUC   GCAUGGUCU UCACG AAA UGUACU       UGCCG U
CCCGCGG    ACA         A     U   -      AAACCCU^    C
 .        90        80        70         60        50

-----------------------------------------------------
```

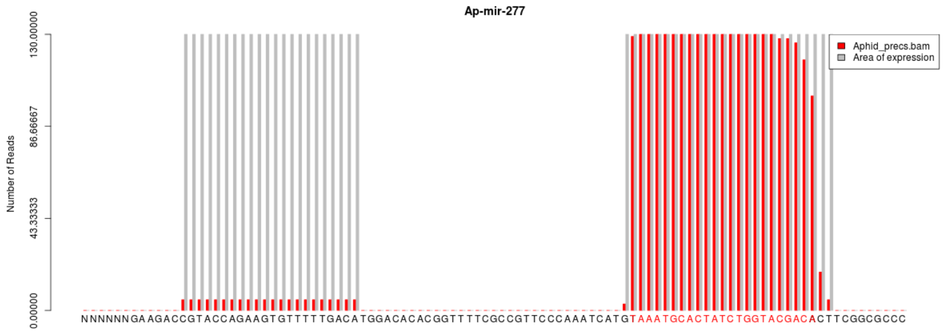

>Ap-mir-278
GTGCGCCACGGACGGACGAAGGTTTCATCAATCGTGTTACAGATAAACGTGTCGG**TCGGTGGGACTTTCGTTCGTTT**GGATCGCTCTC

>Ap-mir-278-3p
TCGGTGGGACTTTCGTTCGTTT

```
GUGCGCCACGGACGGACGAAGGUUUCAUCAAUCGUGUUACAGAUAAACGUGUCGGUCGGUGGGACUUUCGUUCGUUUGGAUCGCUCUC
..(((...((((((((((((((..((((.((((...(((........))).)))).)))).)))))))))))))))...)))....

 Initial dG =    -37.90

          10        20        30        40
GU--|  CCA               UU     A     UGU   AGA
    GCG   CGGACGGACGAAGGU  CAUC AUCG   UAC   U
    CGC   GUUUGCUUGCUUUCA  GUGG UGGC   GUG   A
CUCU^  UAG               GG     C     U--   CAA
       80        70        60          50

-----------------------------------------------------
```

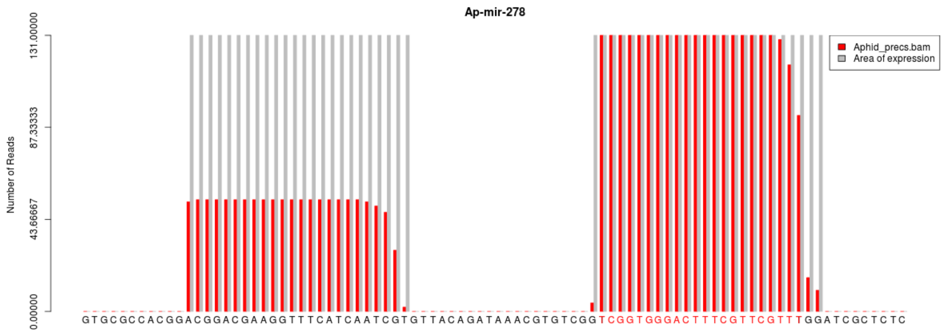

>Ap-mir-279a
CGGGATTGTTGATGGGTGTGTGTCTAGTGACATGCGTTTTATATTTGACAGACA**TGACTAGATCCACACTCATCC**ATGTTCCCGGG

>Ap-mir-279a-3p
TGACTAGATCCACACTCATCC

CGGGAUUGUUGAUGGGUGUGUGUCUAGUGACAUGCGUUUUAUAUUUGACAGACA**UGACUAGAUCCACACUCAUCC**AUGUUCCCGGG
```
(((((..((.((((((((((..((((((..((((.(((........)))...))))))))))..)))))))))).)).)))))..
```

```
 Initial dG =    -37.50

          10        20        30          40
--|    UU  U          UG       GA     C--    UUA
  CGGGA  GU GAUGGGUGUG  UCUAGU  CAUG   GUU   U
  GCCCU  UA CUACUCACAC  AGAUCA  GUAC   CAG   A
GG^    UG  C          CU       --     AGA    UUU
     80        70        60          50
```

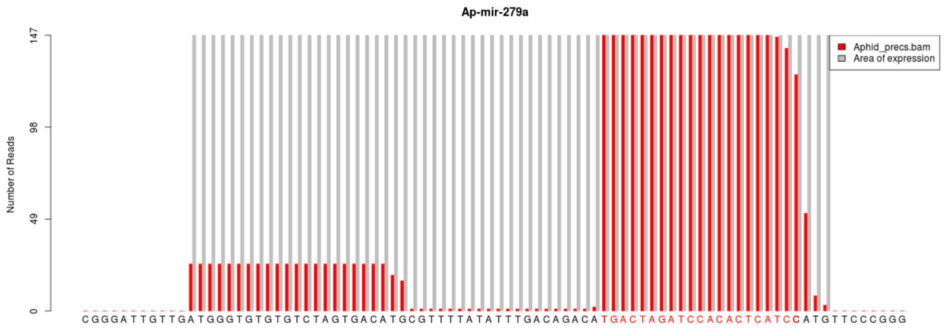

>Ap-mir-279b
TTAGACGGTCCGTGGGTGTTTTTCTAGTCGACACTGAATATTCAAAGCTG**TGACTAGATCTACACTCATCA**ACTGCTTACCT

>Ap-mir-279b-3p
TGACTAGATCTACACTCATCA

```
UUAGACGGUCCGUGGGUGUUUUUCUAGUCGACACUGAAUAUUCAAAGCUGUGACUAGAUCUACACUCAUCAACUGCUUACCU
..((.((((..((((((((...(((((((.(((((..........)).)))))))))))...))))))))..)))))).....

 Initial dG =    -27.00

           10         20         30          40
UU---| A    CC         UUU        G    -   GAAU
     AG CGGU  GUGGGUGU   UCUAGUC ACA CU     A
     UC GUCA  UACUCACA   AGAUCAG UGU GA     U
UCCAU^ -    AC         UCU        -    C   AACU
 80          70         60          50

-----------------------------------------------------
```

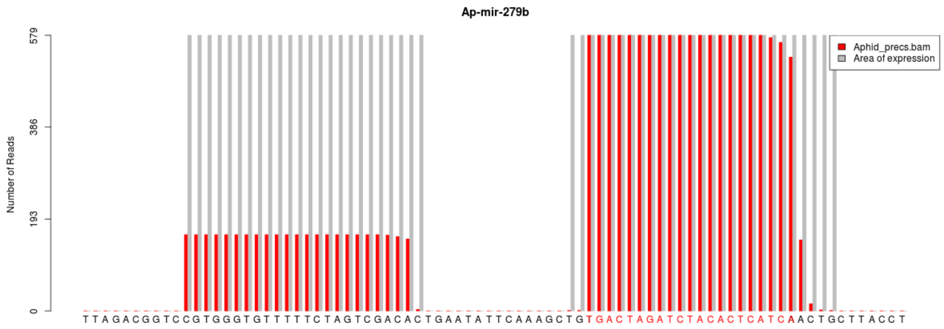

>Ap-mir-281

CTTGAACTATCAAGAGAGCTATCCGTCGACAGTATAATTTTTAACTACAC**TGTCATGGAGTTGCTCTCTTT**ATGGACAAGTCT

>Ap-mir-281-3p

TGTCATGGAGTTGCTCTCTTT

CUUGAACUAUCAAGAGAGCUAUCCGUCGACAGUAUAAUUUUUAACUACAC**UGUCAUGGAGUUGCUCUCUUU**AUGGACAAGUCU

((((..((((.((((((((..(((((.((((((.((.........)).)))))))))))...)))))))).)))).))))...

```
 Initial dG =    -31.50

           10         20          30         40
---|   AA     C        UA-      C       A   AUUU
   CUUG  CUAU AAGAGAGC   UCCGU GACAGU UA    \
   GAAC  GGUA UUCUCUCG   AGGUA CUGUCA AU    U
UCU^   A-     U        UUG      -       C   CAAU
  80          70         60          50

-----------------------------------------------------
```

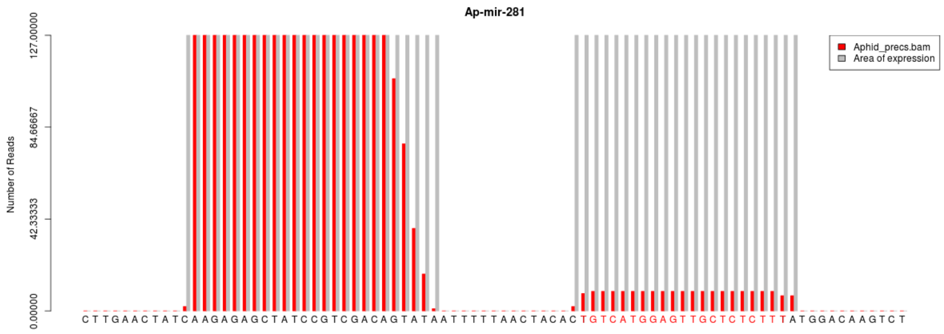

>Ap-mir-29b

TTGGTGAATACTGGTTTCGTATTGGTGCATAGAAGTATTAATTTTGTAACTTGTC**TAGCACCATTTGAAACCAGT**ACTTATCGACG

>Ap-mir-29b-3p

TAGCACCATTTGAAACCAGT

UUGGUGAAUACUGGUUUCGUAUUGGUGCAUAGAAGUAUUAAUUUUGUAACUUGUC**UAGCACCAUUUGAAACCAGU**ACUUAUCGACG

((((((.(((((((((((...((((((.((((..(((.......))).....))))))))))..))))))))))).)))))))..

```
 Initial dG =    -34.60

          10        20        30           40
--       A           UAU      A    AG---|  UAA
  UUGGUGA UACUGGUUUCG   UGGUGC UAGA     UAU   \
  AGCUAUU AUGACCAAAGU   ACCACG AUCU     AUG   U
GC       C           UU-      -    GUUCA^  UUU
     80        70         60         50
```

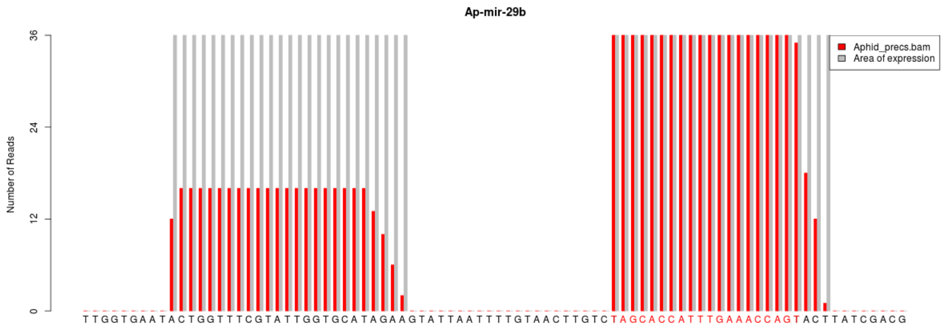

>Ap-mir-2a-1a
GGCTTGAGATGCTCACAAAGTAACTGTGAAATGTTGGTATATTGAGTCA**TATCACAGCCAGCTTTGATGAGCG**TTTCGAGTTGA

>Ap-mir-2a-1a-3p
TATCACAGCCAGCTTTGATGAGCG

GGCUUGAGAUGCUCACAAAGUAACUGUGAAAUGUUGGUAUAUUGAGUCA**UAUCACAGCCAGCUUUGAUGAGCG**UUUCGAGUUGA
((((((((((((((((((((..((((((.(((..............))).))))))...)))))).))))))))))))))))..

```
 Initial dG =    -36.00

          10         20         30        40
--                -      AA-|     A   UUGGUA
  GGCUUGAGAUGCUCA CAAAGU   CUGUGA AUG      U
  UUGAGCUUUGCGAGU GUUUCG   GACACU UAC      A
AG               A      ACC^     A   UGAGUU
   80        70         60        50
```

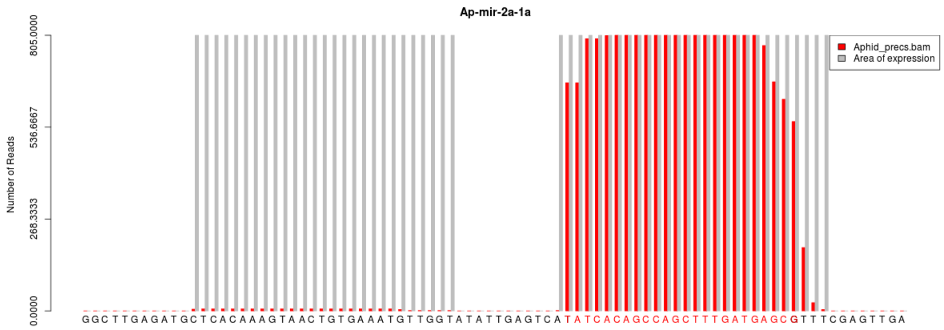

>Ap-mir-2a-1b
GAATTGTGGTTCTCGTCAAAGTGGTTGTGAAACGTTCTGGTTTTCATTTTAAATTCAATTAATATCG**TATCACAGCCAGCTTTGTATGAG**
CACTGCAATTTCT

>Ap-mir-2a-1b-3p
TATCACAGCCAGCTTTGTATGAGCA

GAAUUGUGGUUCUCGUCAAAGUGGUUGUGAAACGUUCUGGUUUUCAUUUUAAAUUCAAUUAAUAUCG**UAUCACAGCCAGCUUUGUAUGAGCA**CUGCAAUUUCU
```
((((((..((.((((((((((((((((((.(((...(((((.............)))))....))).))))))))).))))).))))).))..)))))).. 
```

```
 Initial dG =    -36.20

          10         20         30         40
--       UG   U      -      -         A    UUC-|     UUCAUU
  GAAUUG   GU CUCGU CAAAG UGGUUGUGA ACG     UGGUU      U
  UUUAAC   CA GAGUA GUUUC ACCGACACU UGC     AUUAA      U
UC       GU   C      U      G         A    UAUA^     CUUAAA
 100         90        80         70         60         50

-----------------------------------------------------
```

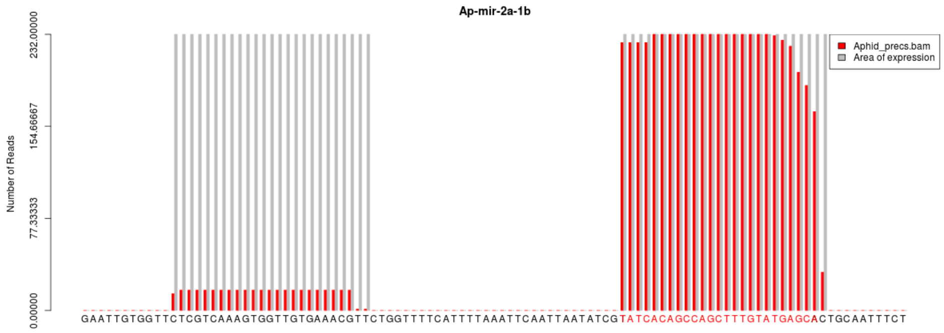

>Ap-mir-2b
TCGATCTGTTG**CCGTCAAAGCGGGCTGTCGTA**GAGTGTTCATTTTCTTTGACTATCACAGCCAGCTTTGATGAGCACCGGATCGATA

>Ap-mir-2b-5p
CCGTCAAAGCGGGCTGTCGTA

```
UCGAUCUGUUGCCGUCAAAGCGGGCUGUCGUAGAGUGUUCAUUUUCUUUGACUAUCACAGCCAGCUUUGAUGAGCACCGGAUCGAUA
((((((((.((((((((((((.((((((.((((.....(((.......))))))).)))))).))))))))).))).))))))))..

 Initial dG =    -43.80

          10         20         30         40
--         U    -|         G       C     AGUGU    UUU
  UCGAUCUG UGC CGUCAAAGC GGCUGU GUAG      UCA    \
  AGCUAGGC ACG GUAGUUUCG CCGACA UAUC      AGU    U
AU         C    A^         A       C     -----   UUC
      80        70        60              50

-----------------------------------------------------
```

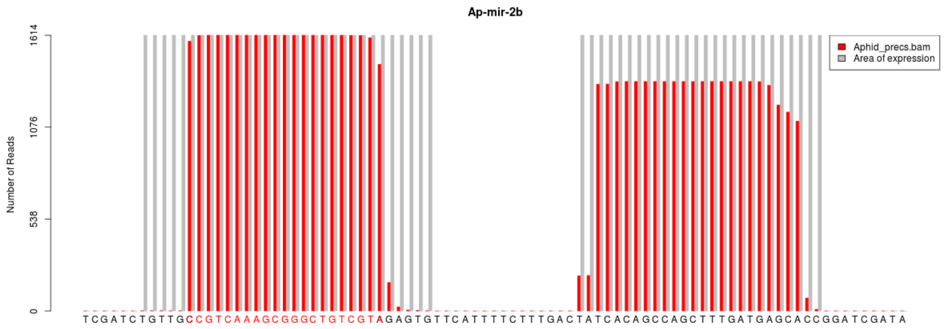

>Ap-mir-305_pre+11

TCAAACCACCTGTTG**ATTGTACTTCATCAGGTGCTCTG**GTAATGCTGAAAATCCCGGCACTTGTTGGAGTGCAATTCACTTGCTGGCATC

>Ap-mir-305-5p

ATTGTACTTCATCAGGTGCTCTG

```
UCAAACCACCUGUUGAUUGUACUUCAUCAGGUGCUCUGGUAAUGCUGAAAAUCCCGGCACUUGUUGGAGUGCAAUUCACUUGCUGGCAUC
.....(((...((.((((((((((((.((((((((..((.............)).)))))))).)))))))))))).))....)))....
```

```
 Initial dG =    -30.80

        10         20        30        40
UCAAA   CCU-| U            U        CU   UAAUGC
     CCA    GU GAUUGUACUUCA CAGGUGCU  GG      \
     GGU    CA UUAACGUGAGGU GUUCACGG  CC      U
CUAC-   CGUU^ C            U        C-   UAAAAG
.         80        70        60         50

-----------------------------------------------------
```

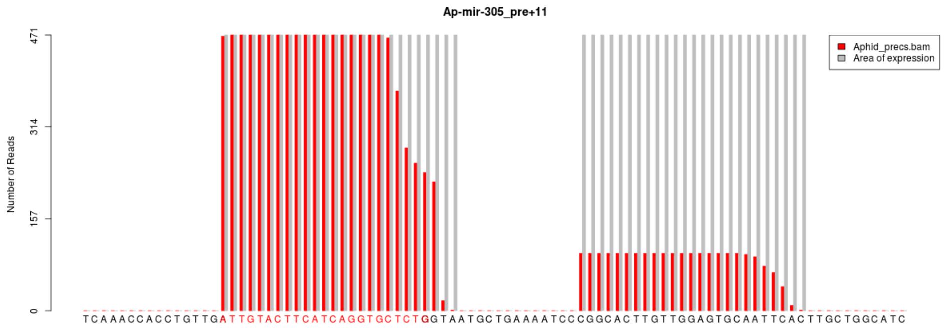

>Ap-mir-307-1
TATTCAGAGTTACTCACTCAATTTGGATGTGATGTTCAAATTGGCGCCA**CCACAACCTCCTTGAGTGAGCGA**CTCGGGGATCT

>Ap-mir-307-1-3p
CCACAACCTCCTTGAGTGAGCGA

```
UAUUCAGAGUUACUCACUCAAUUUGGAUGUGAUGUUCAAAUUGGCGCCACCACAACCUCCUUGAGUGAGCGACUCGGGGAUCU
.((((.((((..(((((((((...((.((((.((.((.....))...)).)))).))...)))))))))..)))).)))).)..
```

```
 Initial dG =    -29.10

          10        20        30
U-    A-    UA         UUU  A    A  U--| AA
  AUUC  GAGU  CUCACUCAA   GG UGUG UG   UC  \
  UAGG  CUCA  GAGUGAGUU   CC ACAC AC   GG  A
UC    GG    GC         CCU  A    C  CGC^ UU
  80        70        60        50
```

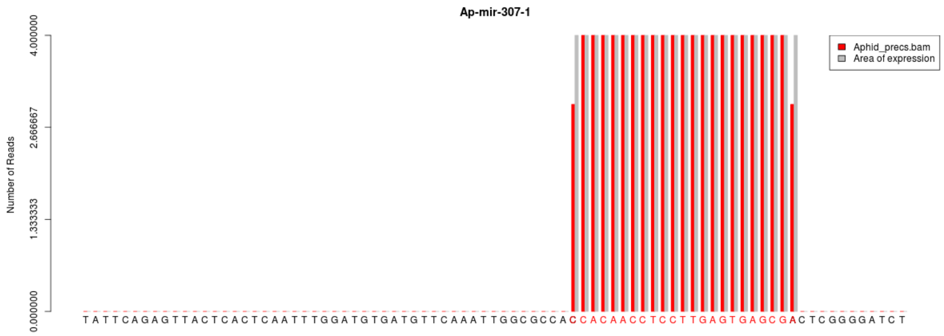

>Ap-mir-307-2
TATTCAGAGTTACTCACTCAATTTGGATGTGATGTTCAAATTGGCGCCA**CCACAACCTCCTTGAGTGAGCGA**CTCGGGGATCT

>Ap-mir-307-2-3p
CCACAACCTCCTTGAGTGAGCGA

```
UAUUCAGAGUUACUCACUCAAUUUGGAUGUGAUGUUCAAAUUGGCGCCACCACAACCUCCUUGAGUGAGCGACUCGGGGAUCU
.((((.((((..(((((((((...((.((((.((.((.....))...)).)))).))...)))))))))..)))).)))).. 

 Initial dG =    -29.10

          10        20        30
U-    A-    UA         UUU  A    A  U--| AA
  AUUC  GAGU  CUCACUCAA   GG UGUG UG   UC  \
  UAGG  CUCA  GAGUGAGUU   CC ACAC AC   GG  A
UC    GG    GC         CCU  A    C  CGC^ UU
  80        70        60        50

-----------------------------------------------------
```

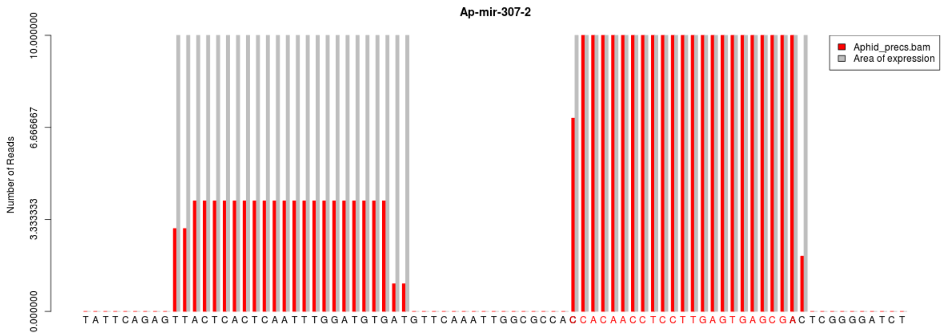

>Ap-mir-315
GCTCTAAATGA**TTTTGATTGTTGCTCAGAAAGCC**TTTCATATATTGTACGGTGGCTTTCTAGCAATAATCATGACCACAAAAGAGCA

>Ap-mir-315-5p
TTTTGATTGTTGCTCAGAAAGCC

GCUCUAAAUGA**UUUUGAUUGUUGCUCAGAAAGCC**UUUCAUAUAUUGUACGGUGGCUUUCUAGCAAUAAUCAUGACCACAAAAGAGCA
(((((...((.((.(((((((((((.((((((((...((.....))......)))))))))))))))))))).)).))....))))).

```
 Initial dG =    -33.30

          10        20        30           40
-     AAA-  A  U           C        UUU---| UA
 GCUCU    UG UU UGAUUGUUGCU AGAAAGCC      CA  \
 CGAGA    AC AG ACUAAUAACGA UCUUUCGG      GU  U
A     AAAC  C  U           -        UGGCAU^ UA
      80        70         60        50
```

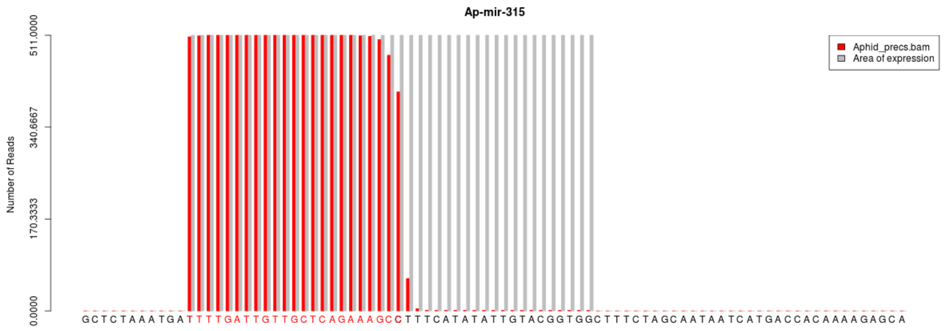

>Ap-mir-316
TCTTGTGGGTT**TGTCTTTTTCCGCTTTGCTGCCG**AAGTGTAAATACGATTTGACAGCAAAGTGAAAGAGACTGATTCATAGAGA

>Ap-mir-316-5p
TGTCTTTTTCCGCTTTGCTGCCG

```
UCUUGUGGGUUUGUCUUUUUCCGCUUUGCUGCCGAAGUGUAAAUACGAUUUGACAGCAAAGUGAAAGAGACUGAUUCAUAGAGA
(((((((((((.(((((((((.(((((((((.((((.(((....))).)))).)))))))))))))))))).)))))))).)))

 Initial dG =    -35.70

         10        20        30        40
   -|        U         C         C    G   A
UCU UGUGGGUU GUCUUUUUC GCUUUGCUG CGAA UGU A
AGA AUACUUAG CAGAGAAAG UGAAACGAC GUUU GCA A
   G^        U         -         A    A   U
   80        70         60        50

-----------------------------------------------------
```

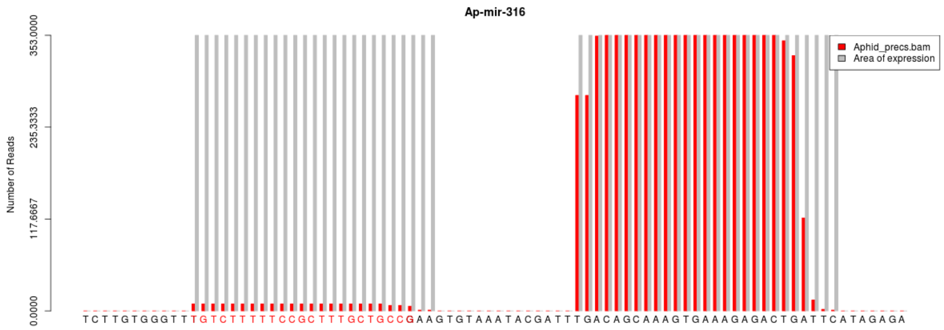

>Ap-mir-317
GTGGGACTGGGAACCTCGCTGTGTTAACTTGTCATGTTATTTCGATAATTATTCGATGAAATCAGAG**TGAACACAGCTGGTGGTATCT**CA
GTTCCATCG

>Ap-mir-317-3p
TGAACACAGCTGGTGGTATCT

```
GUGGGACUGGGAACCUCGCUGUGUUAACUUGUCAUGUUAUUUCGAUAAUUAUUCGAUGAAAUCAGAGUGAACACAGCUGGUGGUAUCUCAGUUCCAUCG
((((((((((((((((((((((((.((((......((...((((.......))))..))....)))).)))))))).)).))).))))))))))))..
```

Initial dG =   -40.20

```
          10           20        30        40
--            -   -  -|       A    GUCAUG  AUU    UAA
  GUGGGACUGGGA ACC UC GCUGUGUU ACUU      UU   UCGA   \
  UACCUUGACUCU UGG GG CGACACAA UGAG      AA   AGCU   U
GC            A   U  U^       G    ACUA--  GU-    UAU
        90        80        70          60         50

-----------------------------------------------------
```

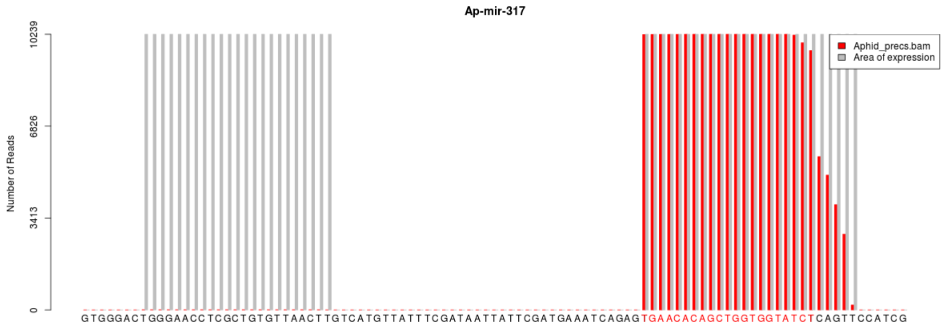

>Ap-mir-34
TTTTCGCACTG**TGGCAGTGTGATTAGCTGGTTG**TGTACATATTTGATATTATACTCACGGCCACTAAGCACATTCCACTGGGCGTCAGGC

>Ap-mir-34-5p
TGGCAGTGTGATTAGCTGGTTG

```
UUUUCGCACUGUGGCAGUGUGAUUAGCUGGUUGUGUACAUAUUUGAUAUUAUACUCACGGCCACUAAGCACAUUCCACUGGGCGUCAGGC
....(((...((((.((((((.((((.((((((((...(((........)))...)))))))))))).))))))))))...)))......

 Initial dG =    -32.50

          10         20         30         40
UUUU--|  ACU     C       A     C         UAC    UUU
      CGC   GUGG AGUGUG UUAG UGGUUGUG   AUA   G
      GCG   CACC UUACAC AAUC ACCGGCAC   UAU   A
CGGACU^  GGU     -       G     -         UCA    UAU
.        80         70         60         50

-----------------------------------------------------
```

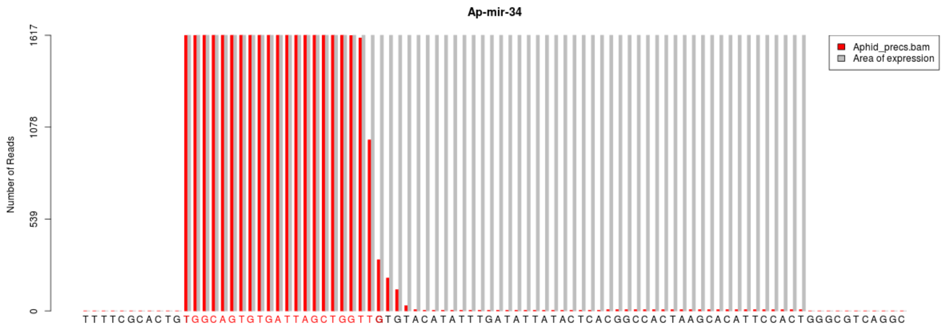

>Ap-mir-7
TAACTGTCGTA**TGGAAGACTAGTGATTTTGTTGTT**TTAAAAAAAAAGTATTTTCCTAACAAGAAGTCTCTAGTCTTCTTACGAAAGTATC
GA

>Ap-mir-7-5p
TGGAAGACTAGTGATTTTGTTGTT

```
UAACUGUCGUAUGGAAGACUAGUGAUUUUGUUGUUUUAAAAAAAAAGUAUUUUCCUAACAAGAAGUCUCUAGUCUUCUUACGAAAGUAUCGA
..(((.(((((.((((((((((.((((((.(((((..((((........))))...))))))))))).))))))))))))))).))).....

 Initial dG =    -30.00

           10         20         30          40
UA---|  G      U          U      G      UU-     AAA
     ACU UCGUA GGAAGACUAG GAUUUU UUGUU   AAAA   A
     UGA AGCAU UCUUCUGAUC CUGAAG AACAA   UUUU   A
AGCUA^  A      -          U      -      UCC     AUG
 90         80         70         60         50

-----------------------------------------------------
```

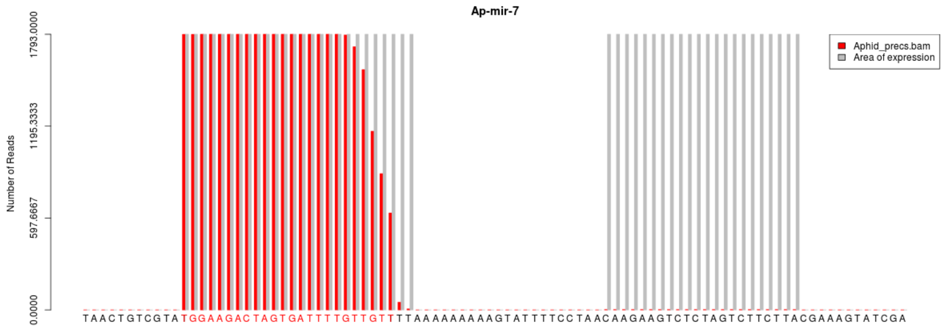

>Ap-mir-71
TCGTTGCCTAT**TGAAAGACATGGGTAGTGAGATG**CGGTATATTTAAGATTGTCTCACTATCTTGTCTTTCATGTTGCATCGATT

>Ap-mir-71-5p
TGAAAGACATGGGTAGTGAGATG

```
UCGUUGCCUAUUGAAAGACAUGGGUAGUGAGAUGCGGUAUAUUUAAGAUUGUCUCACUAUCUUGUCUUUCAUGUUGCAUCGAUU
(((.(((....((((((((.((((((((((((................))))))))))))))))))))....))).)))..

 Initial dG =    -33.60

          10        20        30        40
--|  U   CUAU         U            GCGGUAUA
  UCG UGC    UGAAAGACA GGGUAGUGAGAU        \
  AGC ACG    ACUUUCUGU UCUAUCACUCUG        U
UU^  U   UUGU         -            UUAGAAUU
   80        70         60        50

-----------------------------------------------------
```

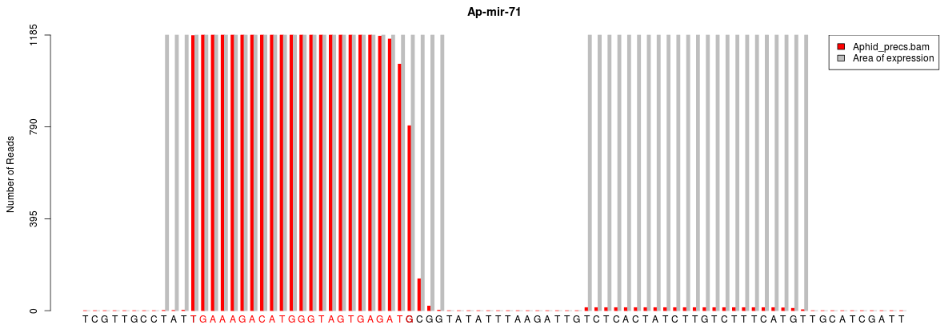

>Ap-mir-76
ATCAATTTATTTGGGTTTCTTGATGACTGGGACCTTGACATTATATTAATATGG**TTCGTTGTCGATGAAACCTTGA**TATTCTTGATA

>Ap-mir-76-3p
TTCGTTGTCGATGAAACCTTGA

AUCAAUUUAUUUGGGUUUCUUGAUGACUGGGACCUUGACAUUAUAUUAAUAUGG**UUCGUUGUCGAUGAAACCUUGA**UAUUCUUGAUA
(((((..((((.(((((((((((..((..(((((.................)))))))..)))).))))))).)))))...))))).

```
 Initial dG =    -26.00

          10          20         30         40
-     UU-    U        -|   UG  UG      UUGACAUU
 AUCAA   UAUU GGGUUUC UUGA  AC  GGACC         A
 UAGUU   AUAG UCCAAAG AGCU  UG  CUUGG         U
A     CUU    U        U^   GU  --      UAUAAUUA
      80        70         60           50

-----------------------------------------------------
```

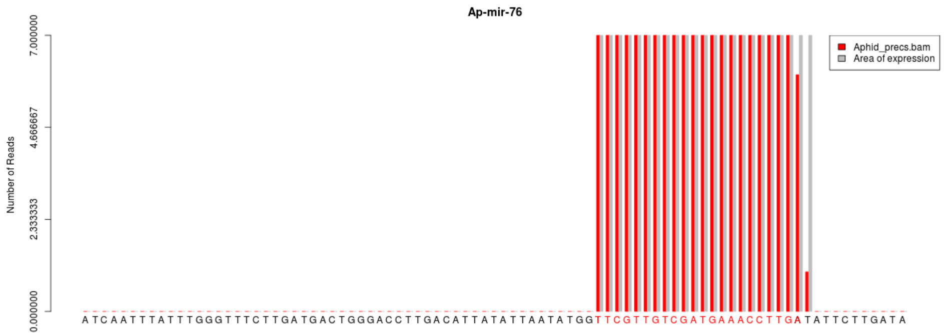

>Ap-mir-79
TCCATGCTTTGGCGCTTTAGCTGTATGATAGAATTTAATATTTCA**TAAAGCTAGATTACCAAAGCAT**GGA

>Ap-mir-79-3p
TAAAGCTAGATTACCAAAGCAT

```
UCCAUGCUUUGGCGCUUUAGCUGUAUGAUAGAAUUUAAUAUUUCAUAAAGCUAGAUUACCAAAGCAUGGA
((((((((((((...(((((((.(((((.............))))).)))))))...))))))))))))

 Initial dG =    -30.70

        10        20        30
|           CGC       G     UAGAAU
UCCAUGCUUUGG   UUUAGCU UAUGA      U
AGGUACGAAACC   AGAUCGA AUACU      U
^           AUU       A     UUAUAA
.        60        50        40

-----------------------------------------------------
```

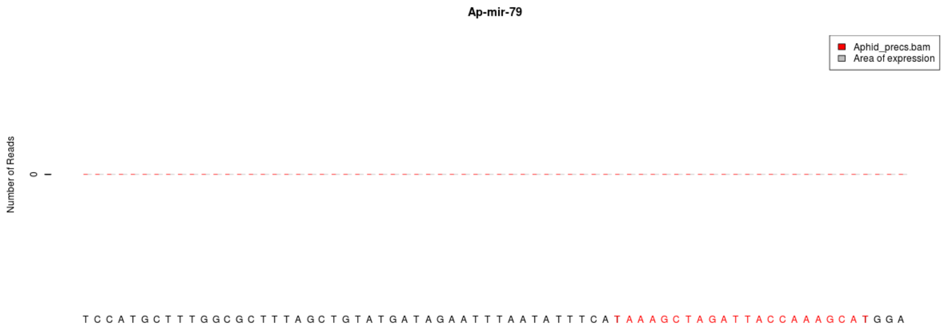

>Ap-mir-8

GGAACTGTTAACGTCTTACTTGGGCAGGATTAGAGTGACAATTGATATGTC**TAATACTGTCAGGTAATGATGTC**CTCAGTTACCC

>Ap-mir-8-3p

TAATACTGTCAGGTAATGATGTC

```
GGAACUGUUAACGUCUUACUUGGGCAGGAUUAGAGUGACAAUUGAUAUGUCUAAUACUGUCAGGUAAUGAUGUCCUCAGUUACCC
((((((...(((((((((((((.(((.(((((((((........))).)))))).))))))))))).)))))...))))).)).

 Initial dG =    -27.40

          10          20         30          40
-  -     UUA      -|        G   G       -    ACA
 GG AACUG   ACGUC UUACUUGG CAG AUUAGA GUG    A
 CC UUGAC   UGUAG AAUGGACU GUC UAAUCU UAU    U
C  A     UCC      U^        -   A       G    AGU
    80         70         60          50

-----------------------------------------------------
```

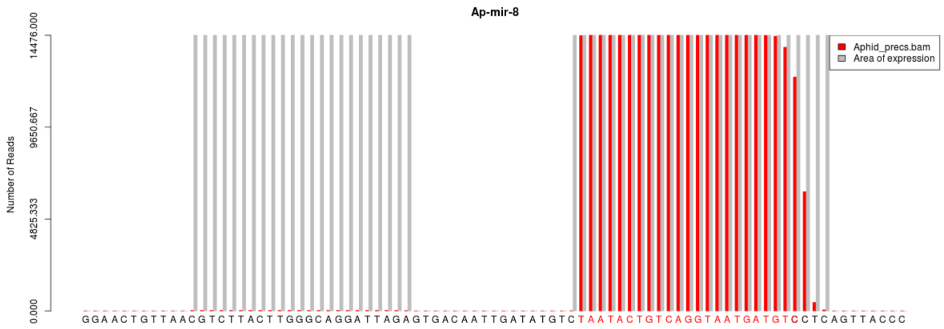

>Ap-mir-87a

CCATCCACATGTCGCCTGAAACTTGCTTTGACCTAAGTACATTTGCATCAAG**GTGAGCAAAGTTTCAGGCGTGT**AAGTGAGATCG

>Ap-mir-87a-3p

GTGAGCAAAGTTTCAGGCGTGT

CCAUCCACAUGUCGCCUGAAACUUGCUUUGACCUAAGUACAUUUGCAUCAAG**GUGAGCAAAGUUUCAGGCGUGU**AAGUGAGAUCG

..(((((....((((((((((((((((..((((..(((....)))....)))))))))))).))))))))))).....))).)))..

Initial dG =   -31.00

```
         10         20         30          40
CC   -   AUGU-          -|     UG     AA--   C
  AUC CAC     CGCCUGAAAC UUGCUU  ACCU    GUA A
  UAG GUG     GCGGACUUUG AACGAG  UGGA    CGU U
GC   A   AAUGU          A^     --     ACUA   U
    80        70        60          50
```

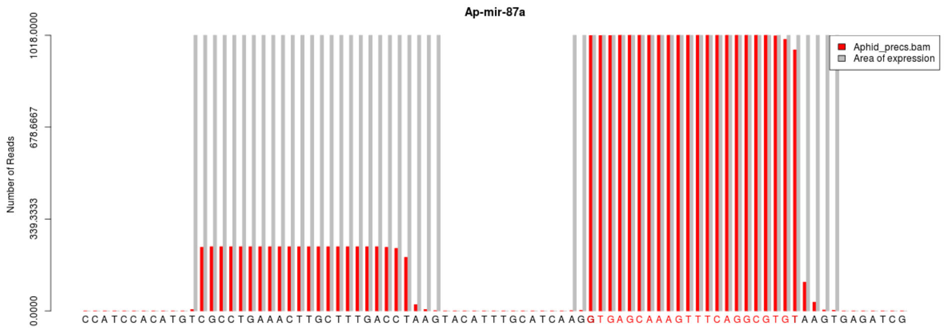

>Ap-mir-87b
ACAATGTTGGCGGGCCTGACTCATTGCTCAACCTGTGTTATTGATACGTTATTAG**GTGAGCAAAGTTTCAGGTGTGC**CAACGTTGCGA

>Ap-mir-87b-3p
GTGAGCAAAGTTTCAGGTGTGC

ACAAUGUUGGCGGGCCUGACUCAUUGCUCAACCUGUGUUAUUGAUACGUUAUUAG**GUGAGCAAAGUUUCAGGUGUGC**CAACGUUGCGA
.(((((((((((.((((((....(((((((.((((.................)))))))))))....)))))).)))))))))))...

```
 Initial dG =    -40.90

          10        20        30        40
A--|          G       CUCA       A     UGUUAUUG
   CAAUGUUGGCG GCCUGA    UUGCUCA CCUG        \
   GUUGCAACCGU UGGACU    AACGAGU GGAU        A
AGC^          G       UUGA       -     UAUUGCAU
       80        70        60         50
```

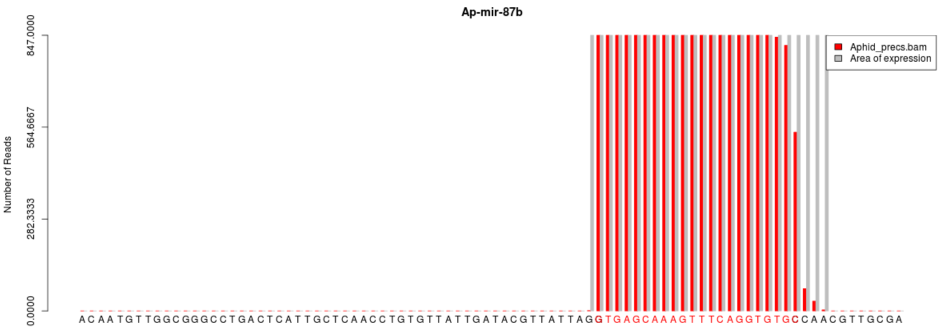

>Ap-mir-927
TGGTGTTTGGT**TTTAGAATTCCTACGCTTTACC**GTACAATTTTATCGGCAAAGCGTTTGAATTGTAAAACCGAACCCCAGT

>Ap-mir-927-5p
TTTAGAATTCCTACGCTTTACC

UGGUGUUUGGU**UUUAGAAUUCCUACGCUUUACC**GUACAAUUUUAUCGGCAAAGCGUUUGAAUUGUAAAACCGAACCCCAGU
(((.(((((((((((.(((((..(((((((.(((..........))).)))))))..))))).))))))))))).)))..

Initial dG = -36.40

```
          10        20        30
--|  U           G     CU        A    UACAA
  UGG GUUUGGUUUUA AAUUC  ACGCUUU CCG      \
  ACC CAAGCCAAAAU UUAAG  UGCGAAA GGC      U
UG^  C           G     UU        C    UAUUU
80        70        60        50
```

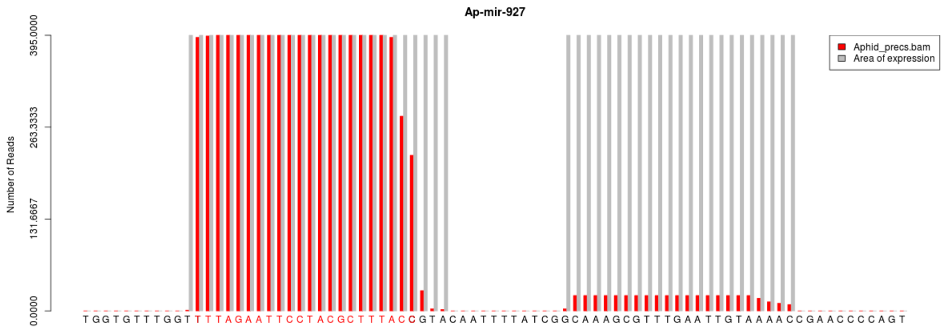

>Ap-mir-929
TTATGTGGTTC**AAATTGACTCTAGTAGGGAG**TCCTTATATTTAATGTGACTCCCTAATGGGGTCAGATTGAAACGCTGCAGC

>Ap-mir-929-5p
AAATTGACTCTAGTAGGGAG

```
UUAUGUGGUUCAAAUUGACUCUAGUAGGGAGUCCUUAUAUUUAAUGUGACUCCCUAAUGGGGUCAGAUUGAAACGCUGCAGC
...((..((((((.(((((((((.(((((((((..(((.....))).))))))))).))))))))).)))))...))..))..

 Initial dG =    -35.40

           10        20        30        40
UUA  UG  ---|   A         G         CU   AU
   UG  GU   UCAA UUGACUCUA UAGGGAGUC  UAU  \
   AC  CG   AGUU GACUGGGGU AUCCCUCAG  GUA  U
CG-  GU  CAA^   A         A         U-   AU
  80        70        60        50

-----------------------------------------------------
```

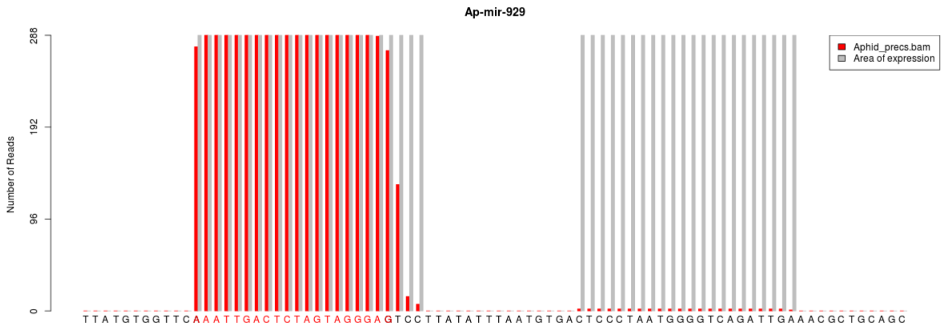

>Ap-mir-92a-1
GTTCTGCCAGTTGGTCGTCGACTTGTGCAACTATTGTACAATTTTAATCAGACTAA**TATTGCACATGTCCCGGCCAAT**TGGAGGATTTC

>Ap-mir-92a-1-3p
TATTGCACATGTCCCGGCCAAT

GUUCUGCCAGUUGGUCGUCGACUUGUGCAACUAUUGUACAAUUUUAAUCAGACUAA**UAUUGCACAUGUCCCGGCCAAU**UGGAGGAUUUC
(((((.((((((((((((..(((.(((((((.(((((................))))))))))))).))).))))))))))))))))...

Initial dG = -33.80

```
           10         20         30         40
---|    G           UC   U        C      UACAAUUU
   GUUCU CCAGUUGGUCG  GAC UGUGCAA UAUUG        \
   UAGGA GGUUAACCGGC  CUG ACACGUU AUAAU        U
CUU^    -           C-   U        -      CAGACUAA
         80         70          60         50
```

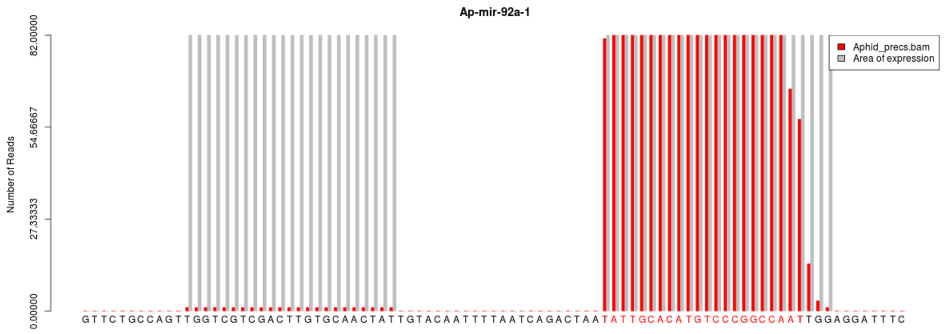

>Ap-mir-92a-2

GTTCTGCCAGTTGGTCGTCGACTTGTGCAACTATTGTACAATTTTAATCAGACTAA**TATTGCACATGTCCCGGCCAAT**TGGAGGATTTC

>Ap-mir-92a-2-3p

TATTGCACATGTCCCGGCCAAT

```
GUUCUGCCAGUUGGUCGUCGACUUGUGCAACUAUUGUACAAUUUUAAUCAGACUAAUAUUGCACAUGUCCCGGCCAAUUGGAGGAUUUC
(((((.(((((((((((..(((.(((((((.(((((................))))))))))))).))).))))))))))))))))...

 Initial dG =     -33.80

           10        20        30        40
---|    G           UC    U       C      UACAAUUU
   GUUCU CCAGUUGGUCG  GAC UGUGCAA UAUUG        \
   UAGGA GGUUAACCGGC  CUG ACACGUU AUAAU        U
CUU^    -           C-    U       -      CAGACUAA
         80        70         60         50

-----------------------------------------------------
```

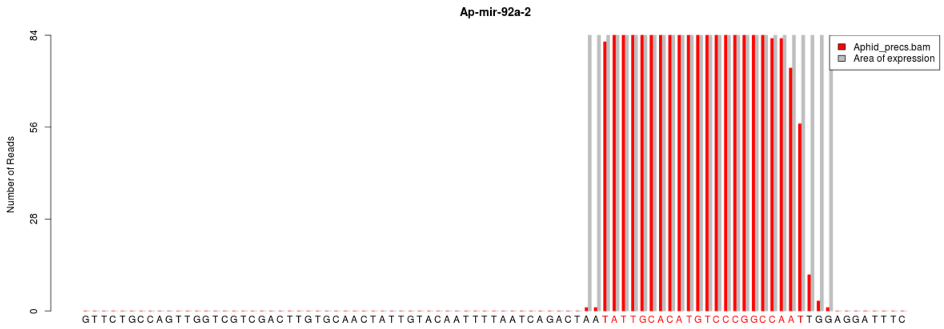

>Ap-mir-92b-1
TGTAGCAAATTAGGTTGTGTTGTTTGCAATATGTATTGTACATTACCGTGTGCGGTCCGTACACGATCTCA**TATTGCACTTGACCCAGCC**
TGCAATGCACATTT

>Ap-mir-92b-1-3p
TATTGCACTTGACCCAGCCTGC

UGUAGCAAAUUAGGUUGUGUUGUUUGCAAUAUGUAUUGUACAUUACCGUGUGCGGUCCGUACACGAUCUCA**UAUUGCACUUGACCCAGCCUGC**AAUGCACAUUU
(((.(((...(((((((.((((..(((((((((...(((((...((((....))))..)))))......)))))))))..)))).)))))))...))))))...

```
 Initial dG =    -37.20

           10        20        30           40        50
---   A   AAU       U    UU         UAU---|    AUU    U
   UGU GCA   UAGGUUG GUUG  UGCAAUAUG      UGUAC   ACCG G
   ACA CGU   GUCCGAC CAGU  ACGUUAUAC      ACAUG   UGGC U
UUU   -   AAC       C    UC         UCUAGC^    CC-    G
  100         90        80        70        60

-----------------------------------------------------
```

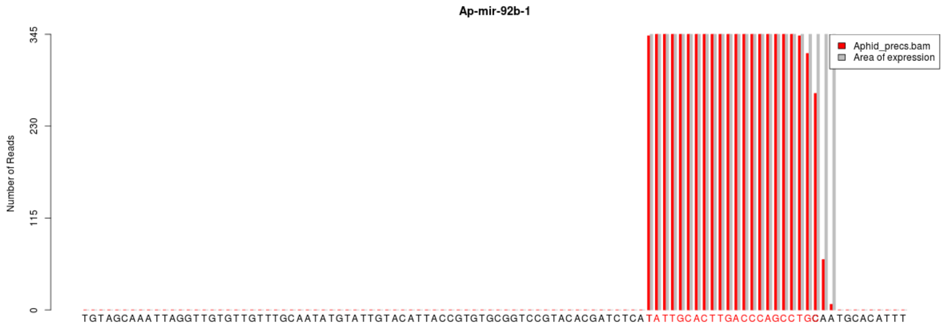

>Ap-mir-92b-2
TGTAGCAAATTAGGTTGTGTTGTTTGCAATATGTATTGTACATTACCGTGTGCGGTCCGTACACGATCTCA**TATTGCACTTGACCCAGCC**
TGCAATGCACATTT

>Ap-mir-92b-2-3p
TATTGCACTTGACCCAGCCTGC

UGUAGCAAAUUAGGUUGUGUUGUUUGCAAUAUGUAUUGUACAUUACCGUGUGCGGUCCGUACACGAUCUCA**UAUUGCACUUGACCCAGCCUGC**AAUGCACAUUU
(((.(((...(((((((.((((..(((((((((...(((((...((((....))))..))))).......)))))))))..)))).)))))))...))))))...

```
 Initial dG =    -37.20

           10        20        30           40        50
---   A   AAU       U    UU         UAU---|    AUU    U
   UGU GCA   UAGGUUG GUUG  UGCAAUAUG      UGUAC   ACCG G
   ACA CGU   GUCCGAC CAGU  ACGUUAUAC      ACAUG   UGGC U
UUU   -   AAC       C    UC         UCUAGC^    CC-    G
  100         90        80        70        60

-----------------------------------------------------
```

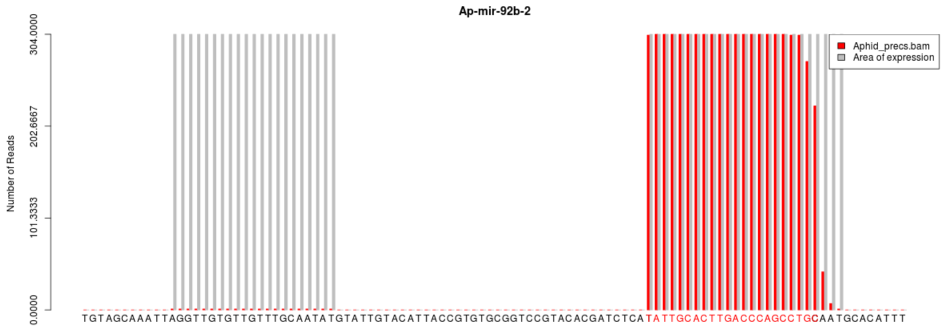

>Ap-mir-965
GTATGCTGAAAGAGGAAAAGTTATAACGGTTATGTTCATAATAACAGTCG**TAAGCGTATAGCTTTTCCCCTT**TTAGTGTATTC

>Ap-mir-965-3p
TAAGCGTATAGCTTTTCCCCTT

```
GUAUGCUGAAAGAGGAAAAGUUAUAACGGUUAUGUUCAUAAUAACAGUCGUAAGCGUAUAGCUUUUCCCCUUUUAGUGUAUUC
((((((((((.(((((((((((.((.(((((.............))))).))))))))))))))).))))))))))))..

 Initial dG =    -32.60

          10         20         30         40
--|           A             A   G      UUCAUA
  GUAUGCUGAAAG GGAAAAGUUAUA CG UUAUG      A
  UAUGUGAUUUUC CCUUUUCGAUAU GC AAUGC      U
CU^           C             -   G      UGACAA
  80         70         60          50

-----------------------------------------------------
```

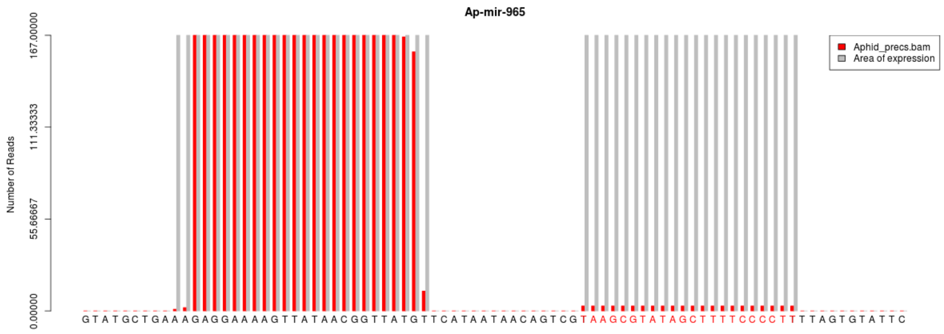

>Ap-mir-971-1
GTATGGCCGCCCACTTTAAGTTTGAGCACCAAGCCTCAGGCTTATTGT**TTGGTGTTCAACCTTACAGTGAG**CGGACGTACTC

>Ap-mir-971-1-3p
TTGGTGTTCAACCTTACAGTGAG

```
GUAUGGCCGCCCACUUUAAGUUUGAGCACCAAGCCUCAGGCUUAUUGUUUGGUGUUCAACCUUACAGUGAGCGGACGUACUC
(((((.((((.((((.((((.((((((((((((((...........)))))))))))))).)))).)))).)))).))))).)..

 Initial dG =    -40.10

          10         20         30         40
--|     G     C     U     U             CUCAG
  GUAUG CCGC CACU UAAG UUGAGCACCAAGC     G
  CAUGC GGCG GUGA AUUC AACUUGUGGUUUG     C
CU^     A     A     C     C             UUAUU
 80         70         60         50

-----------------------------------------------------
```

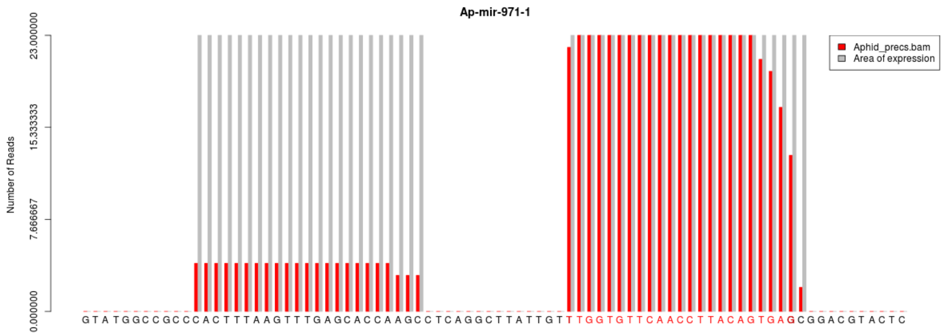

>Ap-mir-971-2
GTATGGCCGCCCACTTTAAGTTTGAGCACCAAGCCTCAGGCTTATTGT**TTGGTGTTCAACCTTACAGTGAG**CGGACGTACTC

>Ap-mir-971-2-3p
TTGGTGTTCAACCTTACAGTGAG

GUAUGGCCGCCCACUUUAAGUUUGAGCACCAAGCCUCAGGCUUAUUGU**UUGGUGUUCAACCUUACAGUGAG**CGGACGUACUC
```
(((((.((((.((((.((((.(((((((((((((...........))))))))))))).)))).)))).)))).))))).. 
```

```
 Initial dG =    -40.10

          10        20        30        40
--|    G    C    U    U             CUCAG
  GUAUG CCGC CACU UAAG UUGAGCACCAAGC     G
  CAUGC GGCG GUGA AUUC AACUUGUGGUUUG     C
CU^    A    A    C    C             UUAUU
 80        70        60        50
```

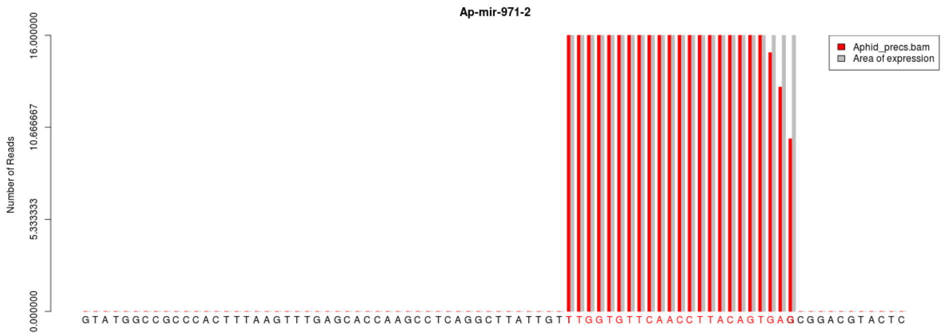

>Ap-mir-993
AATGTAACGCATCCGTGATCTACCCTGTAGATCCGGGCTTTTGTAGAATAATCGATATGTTCA**GAAGCTCGTCTCTACAGGTATCT**TACG
GATGGAAGTTTAC

>Ap-mir-993-3p
GAAGCTCGTCTCTACAGGTATCT

AAUGUAACGCAUCCGUGAUCUACCCUGUAGAUCCGGGCUUUUGUAGAAUAAUCGAUAUGUUCA**GAAGCUCGUCUCUACAGGUAUCU**UACGGAUGGAAGUUUAC

```
.........((((((((..((((.((((((..((((((((((....(((.....)))...))))))))))..))))))))))..))))))))).........
```

```
 Initial dG =    -41.60

        10        20        30        40        50
AAUGUAACG|         UC     C       UC           UAGA   AU
         CAUCCGUGA  UACC UGUAGA  CGGGCUUUUG    AUA  \
         GUAGGCAUU  AUGG ACAUCU  GCUCGAAGAC    UAU  C
CAUUUGAAG^         CU     -       CU           UUG-   AG
 100        90        80         70        60

-----------------------------------------------------
```

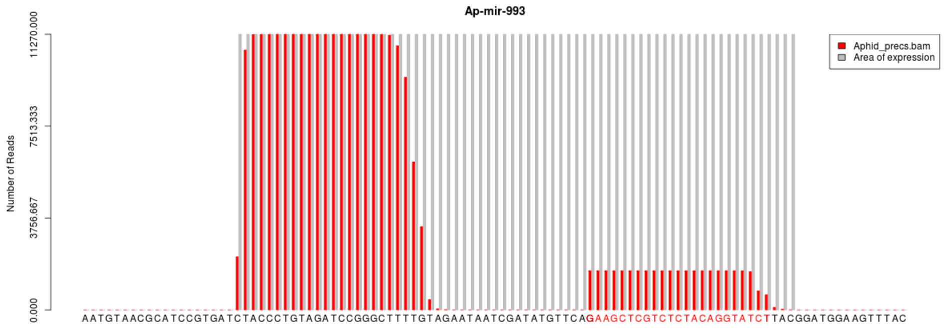

>Ap-mir-996-1
CATAGCATTTAACGGGTGTAGTCTTTAGTGCACGTTAATATTTATAGTCG**TGACTAGAGTTACACTCGTCA**AATTCTAAAGA

>Ap-mir-996-1-3p
TGACTAGAGTTACACTCGTCA

CAUAGCAUUUAACGGGUGUAGUCUUUAGUGCACGUUAAUAUUUAUAGUCG**UGACUAGAGUUACACUCGUCA**AAUUCUAAAGA
..(((.((((.((((((((((.(((((((.(((((((.......))).))))))))))))))))))))).)))).)))....

```
 Initial dG =    -29.30

          10        20        30         40
CA--|  C    A          U       G    -   AUA
    UAG AUUU ACGGGUGUAG CUUUAGU CACG UUA   \
    AUC UAAA UGCUCACAUU GAGAUCA GUGC GAU   U
AGAA^  U    C          -       -    U   AUU
 80        70        60          50
```

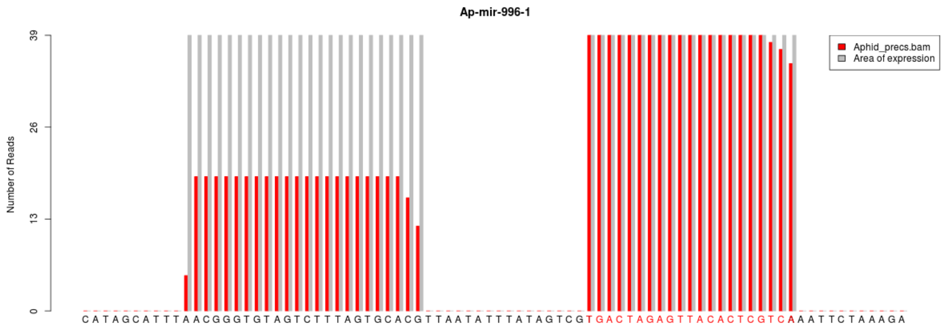

>Ap-mir-996-2
CATAGCATTTAACGGGTGTAGTCTTTAGTGCACGTTAATATTTATAGTCG**TGACTAGAGTTACACTCGTCA**AATTCTAAAGA

>Ap-mir-996-2-3p
TGACTAGAGTTACACTCGTCA

```
CAUAGCAUUUAACGGGUGUAGUCUUUAGUGCACGUUAAUAUUUAUAGUCGUGACUAGAGUUACACUCGUCAAAUUCUAAAGA
..(((.((((.((((((((((.(((((((.(((((((.......))).))))))))))))))))))))).)))).)))....

 Initial dG =    -29.30

          10        20        30         40
CA--|  C    A          U       G    -   AUA
    UAG AUUU ACGGGUGUAG CUUUAGU CACG UUA   \
    AUC UAAA UGCUCACAUU GAGAUCA GUGC GAU   U
AGAA^  U    C          -       -    U   AUU
 80        70        60          50

-----------------------------------------------------
```

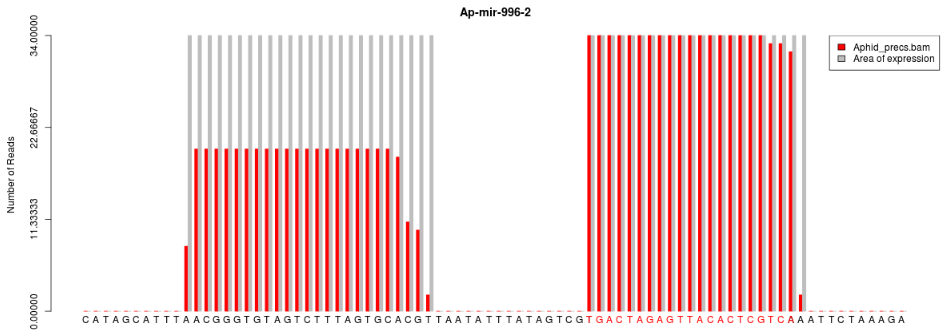

>Ap-mir-998
GTCGGTCGTCCGCTGAATTTTACGAGTGCCGCATGTAATGTATGTCTTG**TAGCACCATGGAATTCAGCTG**TCGCCTGGTGT

>Ap-mir-998-3p
TAGCACCATGGAATTCAGCTG

```
GUCGGUCGUCCGCUGAAUUUUACGAGUGCCGCAUGUAAUGUAUGUCUUGUAGCACCAUGGAAUUCAGCUGUCGCCUGGUGU
.((((.((...(((((((((((...((((..((..............))..))))..)))))))))))...)).))))...

 Initial dG =    -24.60

          10        20        30        40
G--|   U  UCC           CGA     CG  UGUAAU
   UCGG CG   GCUGAAUUUUA   GUGC  CA      G
   GGUC GC   CGACUUAAGGU   CACG  GU      U
UGU^   C  UGU           AC-     AU  UCUGUA
80        70        60         50

-----------------------------------------------------
```

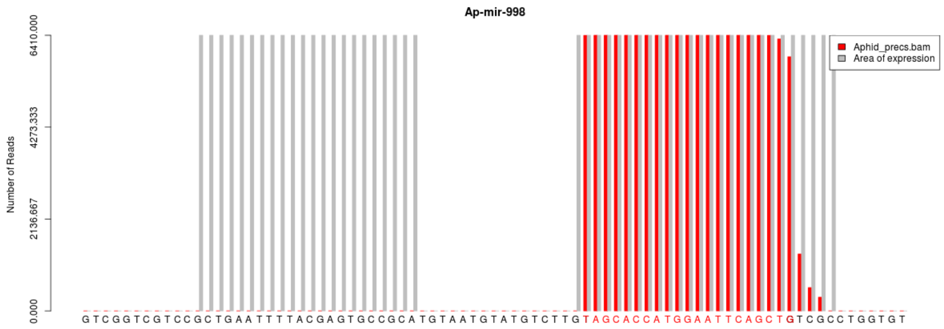

>Ap-mir-9a
GCTGGTTTTTC**TCTTTGGTTATCTAGCTGTATGA**GTGTTACGTATGAAGCTCCTCATAGGTCTAGGTGACCGAAGTTAAAAATCGCACC

>Ap-mir-9a-5p
TCTTTGGTTATCTAGCTGTATGA

```
GCUGGUUUUUCUCUUUGGUUAUCUAGCUGUAUGAGUGUUACGUAUGAAGCUCCUCAUAGGUCUAGGUGACCGAAGUUAAAAAUCGCACC
((.(((((((..((((((((((((((((.((((((.(((........)))..)))))))).)))))))))))))))..)))))))))...

 Initial dG =    -35.10

           10        20         30         40
---| U      CU             -  G      U-   ACG
   GC GGUUUUU  CUUUGGUUAUCUAG CU UAUGAG  GUU   U
   CG CUAAAAA  GAAGCCAGUGGAUC GG AUACUC  CGA   A
CCA^ -      UU             U  -      CU   AGU
         80        70        60         50

-----------------------------------------------------
```

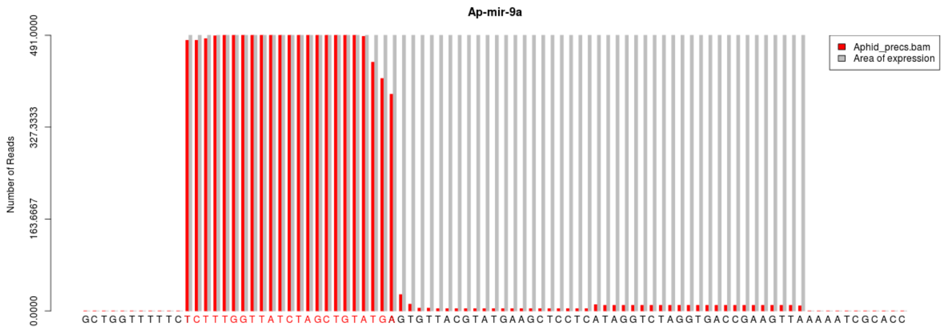

>Ap-mir-9c
ATGCAGTATCA**AATGCTAATAACTTTGGTACTAA**AGCTTTAGGATGTTTCTTAAAAATTTAACCTACAGCTAGAATACCAAAGAGATCAA
CTCTGACACATTAG

>Ap-mir-9c-3p
AATGCTAATAACTTTGGTACTAA

```
AUGCAGUAUCAAAUGCUAAUAACUUUGGUACUAAAGCUUUAGGAUGUUUCUUAAAAAUUUAACCUACAGCUAGAAUACCAAAGAGAUCAACUCUGACACAUUAG
..(((........)))......((((((((....((((.((((..((((.....))))....)))).))))....))))))))....................
ERROR 4: Strange structure
 Initial dG =    -20.30
```

```
.-AU|  GUA
    GCA   U
    CGU   C
\ --^  AAA
         10
```

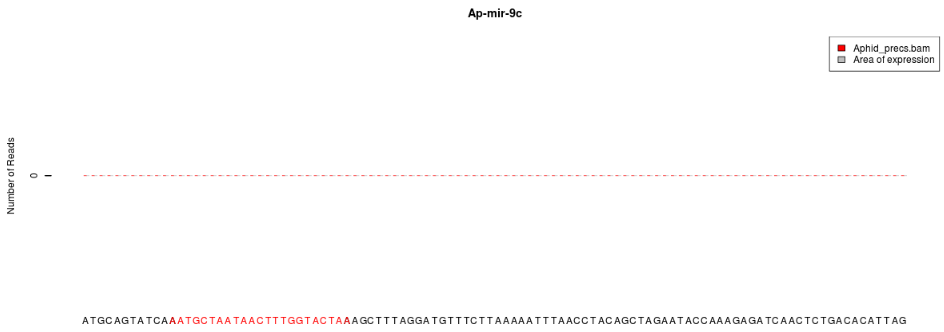

>Ap-mir-9c2

CAATGTTGATC**TCTTTGGTACTTTAGCTGTAG**GTATATTTTAAAGAGACGCCCTAAAGCTTCTGTACCAATGTTATTGGCAATTGA

>Ap-mir-9c2-5p

TCTTTGGTACTTTAGCTGTAG

```
CAAUGUUGAUCUCUUUGGUACUUUAGCUGUAGGUAUAUUUUAAAGAGACGCCCUAAAGCUUCUGUACCAAUGUUAUUGGCAAUUGA
((((((..((....(((((((...((((.((((..................)))).))))...))))))).....))..))).))).
```

```
 Initial dG =    -24.20

          10        20        30        40
-   -|  UG  CUCU       UUU    G     UAUAUUUU
 CAA UGU  AU    UUGGUAC   AGCU UAGG         A
 GUU ACG  UA    AACCAUG   UCGA AUCC         A
A   A^  GU  UUGU       UCU    A     CGCAGAGA
     80        70        60        50
```

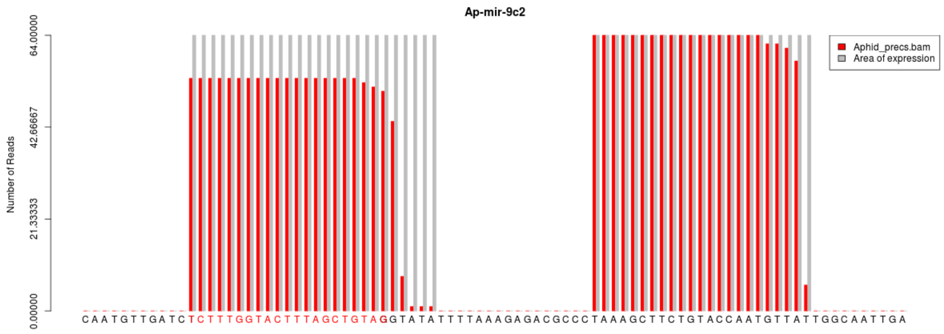

>Ap-mir-X100
TGCGTCCACAC**TGGAGAACGAATGCGAACGAGTGCGAA**CGAATGCAGATTCGGTCCAAAAATTCGTGGGTTCTATTTTTAGTGTGGACGT
ACT

>Ap-mir-X100-5p
TGGAGAACGAATGCGAACGAGTGCGAA

UGCGUCCACAC**UGGAGAACGAAUGCGAACGAGUGCGAA**CGAAUGCAGAUUCGGUCCAAAAAUUCGUGGGUUCUAUUUUUAGUGUGGACGUACU
(((((((((((((((((..(((((((((....((.((.(((((....))))).))))....)))))..))))..)))))))))))))))))..

```
 Initial dG =    -37.80

          10        20          30        40
--                 AC    --|    CGAG  C  A     G
  UGCGUCCACACUGGAGA  GAAU  GCGAA    UG GA CGAAU C
  AUGCAGGUGUGAUUUUU  CUUG  UGCUU    AC CU GCUUA A
UC                 AU    GG^    AAAA  -  G     G
  90        80        70        60         50

-----------------------------------------------------
```

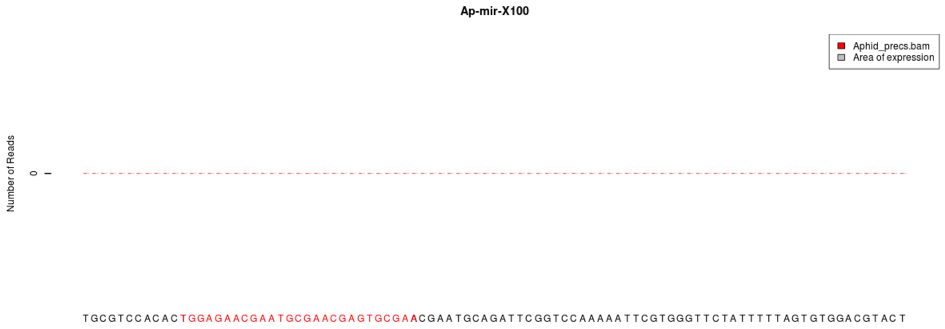

>Ap-mir-X101

CGGAGTTGTCA**AATCACTGTTACGCTAGAATGTT**GTAAAATATTAATCAACATTCTGCTTAACGTGATTATACAATTACGGT

>Ap-mir-X101-5p

AATCACTGTTACGCTAGAATGTT

CGGAGUUGUCA**AAUCACUGUUACGCUAGAAUGUU**GUAAAAUAUUAAUCAACAUUCUGCUUAACGUGAUUAUACAAUUACGGU

((.((((((..((((((.((((.((.(((((((((...........))))))))))).))))))))))..)))))).))..

```
 Initial dG =    -27.60

          10        20        30        40
--| G      CA      U    C  U         UAAAA
  CG AGUUGU  AAUCAC GUUA GC AGAAUGUUG     U
  GC UUAACA  UUAGUG CAAU CG UCUUACAAC     A
UG^ A      UA      -    U  -         UAAUU
 80        70         60         50

-----------------------------------------------------
```

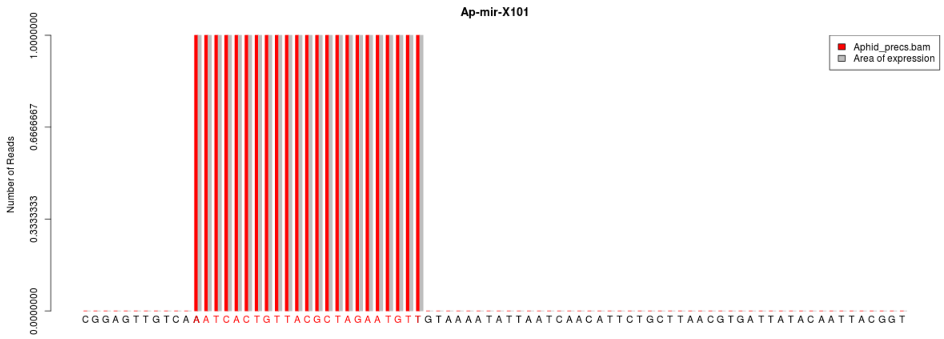

>Ap-mir-X102

TTGTATGAAGA**ATTAGGATATACCACGGACTAC**TACAGTAGTGTAGTAGTCCGTGGGATATACTAATGTGCATTATCATC

>Ap-mir-X102-5p

ATTAGGATATACCACGGACTAC

```
UUGUAUGAAGAAUUAGGAUAUACCACGGACUACUACAGUAGUGUAGUAGUCCGUGGGAUAUACUAAUGUGCAUUAUCAUC
.(((((.....(((((.((((.((((((((((((((((....)))))))))))))))).)))).))))))))))........

 Initial dG =    -34.80

               10        20        30
U-------|    GAAGA     G    A               G
        UGUAU     AUUAG AUAU CCACGGACUACUACA U
        ACGUG     UAAUC UAUA GGUGCCUGAUGAUGU A
CUACUAUU^    -----     A    G               G
.        70              60        50        40

-----------------------------------------------------
```

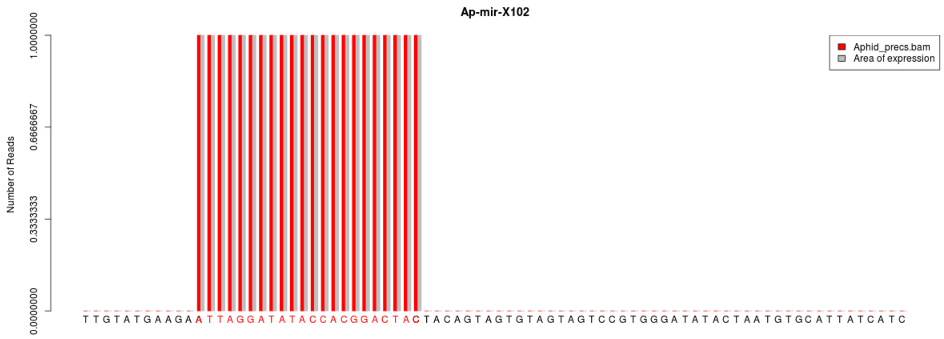

>Ap-mir-X103-1

TTGCATCGTTT**TGCGACGGAACAGCATGATCAGAAT**AGATAAATCGTCGGACTCGCTAGTCGTCGCACGACCGATGCAGC

>Ap-mir-X103-1-5p

TGCGACGGAACAGCATGATCAGAAT

```
UUGCAUCGUUUUGCGACGGAACAGCAUGAUCAGAAUAGAUAAAUCGUCGGACUCGCUAGUCGUCGCACGACCGAUGCAGC
(((((((((.((((((((...(((..((((......((....))....)).)))))..)))))))).))).))))))).

 Initial dG =    -27.70

         10        20          30
-       -   U        AAC   .-AUG|  AG
 UUGCAUC GUU UGCGACGG   AGC     AUC  A
 GACGUAG CAG ACGCUGCU   UCG     UAG  A
C       C   C        GA-   \ ---^  AU
.        70        60          40



                             AAA  G
                                UC U
                                AG C
                             CUC  G
                                50

-----------------------------------------------------
```

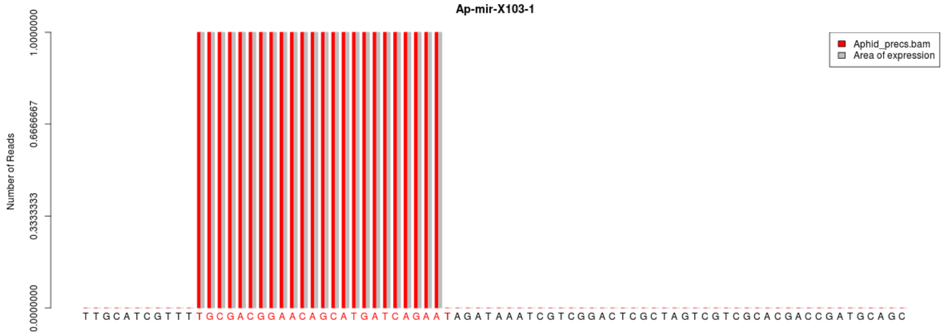

>Ap-mir-X103-2

TTGCATCGTTT**TGCGACGGAACAGCATGATCAGAAT**AGATAAATCGTCGGACTCGCTAGTCGTCGCACGACCGATGCAGC

>Ap-mir-X103-2-5p

TGCGACGGAACAGCATGATCAGAAT

```
UUGCAUCGUUUUGCGACGGAACAGCAUGAUCAGAAUAGAUAAAUCGUCGGACUCGCUAGUCGUCGCACGACCGAUGCAGC
(((((((((.((((((((...(((..((((......((....))....)).)))))..)))))))).))).))))))).

 Initial dG =    -27.70

         10        20          30
-       -   U        AAC   .-AUG|  AG
 UUGCAUC GUU UGCGACGG   AGC     AUC  A
 GACGUAG CAG ACGCUGCU   UCG     UAG  A
C       C   C        GA-   \ ---^  AU
.        70        60          40



                             AAA  G
                                UC U
                                AG C
                             CUC  G
                                50
```

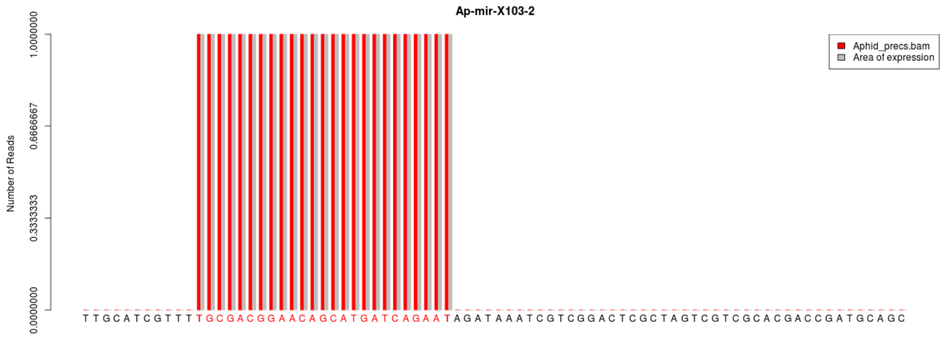

>Ap-mir-X104-1
GTTGTTGCAGTCGGTGCGTTTCTTTTTTGTTTCGCAACATATATTTTGCAACAGAATGTTGCG**TTGCGCAACAAAATGTAGCAGCCGGT**G
CGTTTCCAGC

>Ap-mir-X104-1-3p
TTGCGCAACAAAATGTAGCAGCCGGT

```
GUUGUUGCAGUCGGUGCGUUUCUUUUUUGUUUCGCAACAUAUAUUUUGCAACAGAAUGUUGCGUUGCGCAACAAAAUGUAGCAGCCGGUGCGUUUCCAGC
((((.((((.(((((..(((....(((((((.((((((..((((((((...))))))))...)))))).)))))))...))).)))))))))....))))

 Initial dG =    -32.60

           10        20        30        40
    U---|   G      GC    UCUU       U      AU-        C
GUUG    UGCA UCGGU  GUU    UUUUGUU CGCAAC   AUAUUUUG \
CGAC    GCGU GGCCG  CGA    AAAACAA GCGUUG   UGUAAGAC A
    CUUU^   -      A-   UGU-       C      CGU        A
.        90          80         70         60

-----------------------------------------------------
```

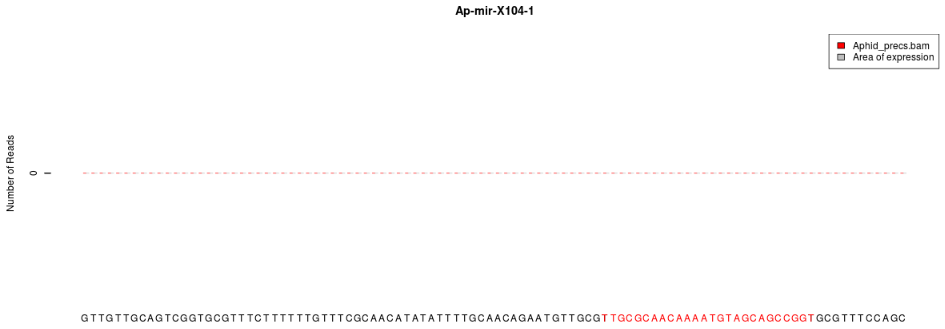

>Ap-mir-X104-2
GTTGTTGCAGTCGGTGCGTTTCTTTTTTGTTTCGCAACATATATTTTGCAACAGAATGTTGCG**TTGCGCAACAAAATGTAGCAGCCGGT**G
CGTTTCCAGC

>Ap-mir-X104-2-3p
TTGCGCAACAAAATGTAGCAGCCGGT

GUUGUUGCAGUCGGUGCGUUUCUUUUUUGUUUCGCAACAUAUAUUUUGCAACAGAAUGUUGCG**UUGCGCAACAAAAUGUAGCAGCCGGU**GCGUUUCCAGC
((((.((((.(((((..(((....(((((((.((((((..((((((((...))))))))...)))))).))))))).....))).))))))))).....))))

Initial dG = -32.60

```
           10        20        30        40
    U---|   G     GC   UCUU       U      AU-        C
GUUG    UGCA UCGGU  GUU    UUUUGUU CGCAAC   AUAUUUUG \
CGAC    GCGU GGCCG  CGA    AAAACAA GCGUUG   UGUAAGAC A
    CUUU^   -     A-   UGU-       C      CGU        A
.        90          80         70        60
```

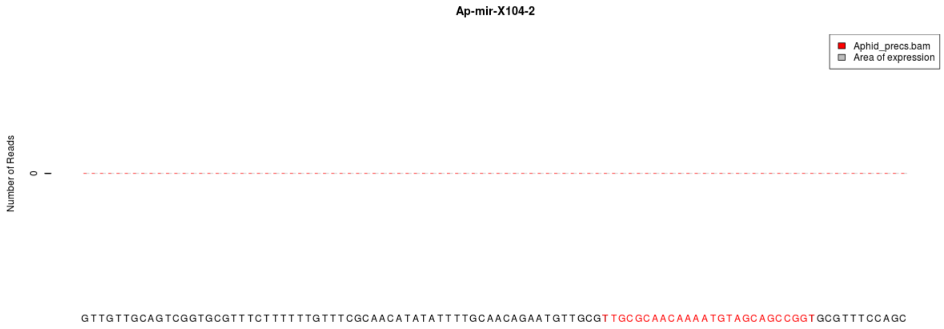

>Ap-mir-X105

TAAGTATAATGCCTGTATGCATAGTTAATTTATGTGACACA**CAAATTGACTATGCATATGGACA**TAAAACTTGTA

>Ap-mir-X105-3p

CAAATTGACTATGCATATGGACA

UAAGUAUAAUGCCUGUAUGCAUAGUUAAUUUAUGUGACACA**CAAAUUGACUAUGCAUAUGGACA**UAAAACUUGUA

```
(((((...(((.(((((((((((((((((((.((.....)).))))))))))))))))))).)))...)))))..

 Initial dG =    -28.40

          10        20        30
--|    AUA   C                   A  UG
  UAAGU   AUG CUGUAUGCAUAGUUAAUUU UG  \
  GUUCA   UAC GGUAUACGUAUCAGUUAAA AC  A
AU^    AAA   A                   C  AC
    70        60        50        40

-----------------------------------------------------
```

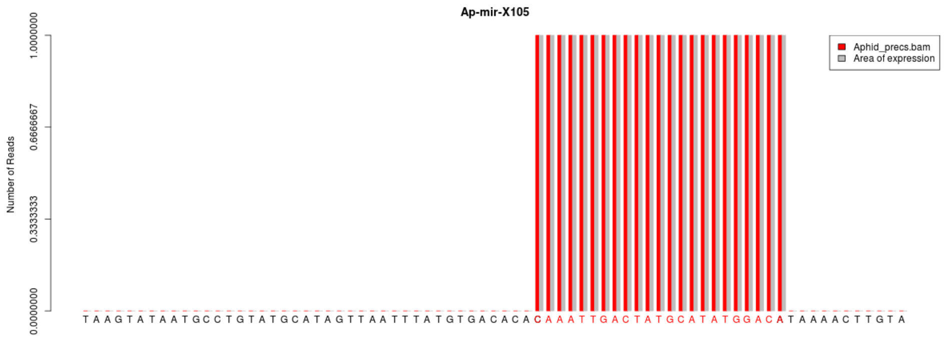

>Ap-mir-X106
CAGCAGTGTGC**AAGAACTTTCAACAGCTAGCA**AGTACTTAATTGCTCGTTGTTGCAAGTTCTTGCTCGATGTTGCA

>Ap-mir-X106-5p
AAGAACTTTCAACAGCTAGCA

CAGCAGUGUGCAAGAACUUUCAACAGCUAGCAAGUACUUAAUUGCUCGUUGUUGCAAGUUCUUGCUCGAUGUUGCA
(((((.((.((((((((((.(((((((.(((((........))))).))))))).))))))))))).)).)))))..

Initial dG = -36.90

```
          10        20        30
--|    G  U          U       U     GUA
  CAGCA UG GCAAGAACUU CAACAGC AGCAA   C
  GUUGU GC CGUUCUUGAA GUUGUUG UCGUU   U
AC^    A  U          C       C     AAU
     70        60        50        40
```

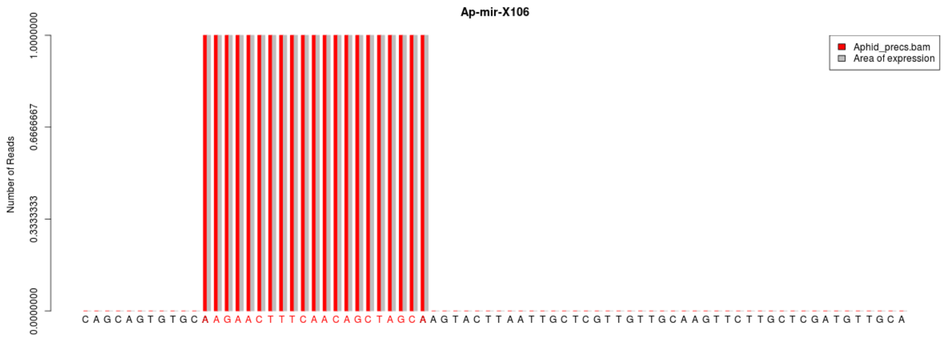

>Ap-mir-X107-1
CGCCTTCATCGTAGTTCTCCACTTCTTAATGGCTGTCAATCATATACATACATATACAACATAGAACAAAATCAAC**CAATAAGAAGTGGA GGACTACG**ACATGAGTCGG

>Ap-mir-X107-1-3p
CAATAAGAAGTGGAGGACTACG

```
CGCCUUCAUCGUAGUUCUCCACUUCUUAAUGGCUGUCAAUCAUAUACAUACAUAUACAACAUAGAACAAAAUCAACCAAUAAGAAGUGGAGGACUACGACAUGAGUCGG
((.(((..((((((((((((((((((((.(((.((.....................................)).))).))))))))))))))))))))...))).)).

 Initial dG =    -38.47

          10        20        30        40        50
-  C   CA-|                   A   C  UCAAUCAUAUACAUACAU
 CG CUU   UCGUAGUUCUCCACUUCUUA UGG UG                  \
 GC GAG   AGCAUCAGGAGGUGAAGAAU ACC AC                  A
G  U   UAC^                   A   A  UAAAACAAGAUACAACAU
       100        90        80        70        60

-----------------------------------------------------
```

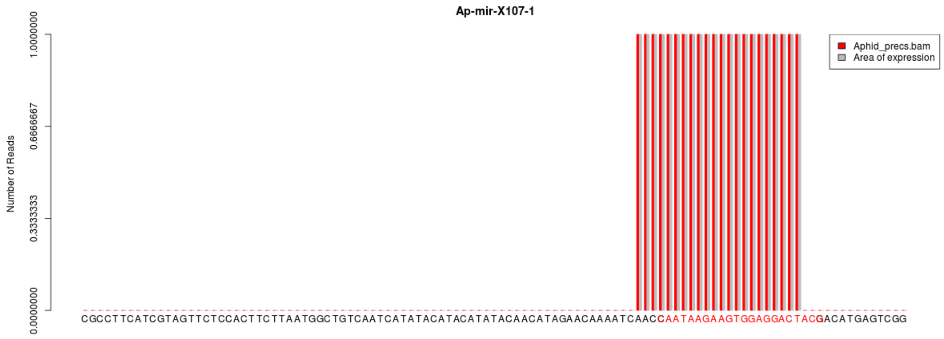

>Ap-mir-X107-2
CGCCTTCATCGTAGTTCTCCACTTCTTAATGGCTGTCAATCATATACATACATATACAACATAGAACAAAATCAAC**CAATAAGAAGTGGA GGACTACG**ACATGAGTCGG

>Ap-mir-X107-2-3p
CAATAAGAAGTGGAGGACTACG

```
CGCCUUCAUCGUAGUUCUCCACUUCUUAAUGGCUGUCAAUCAUAUACAUACAUAUACAACAUAGAACAAAAUCAACCAAUAAGAAGUGGAGGACUACGACAUGAGUCGG
((.(((..((((((((((((((((((((.(((.((...................................)).))).))))))))))))))))))))...))).)).

 Initial dG =    -38.47

          10        20        30        40        50
-  C   CA-|                   A   C  UCAAUCAUAUACAUACAU
 CG CUU   UCGUAGUUCUCCACUUCUUA UGG UG                  \
 GC GAG   AGCAUCAGGAGGUGAAGAAU ACC AC                  A
G  U   UAC^                   A   A  UAAAACAAGAUACAACAU
       100        90        80        70        60

-----------------------------------------------------
```

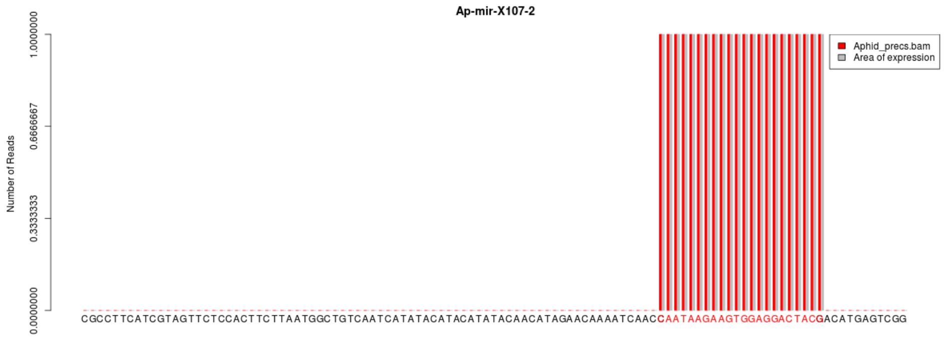

>Ap-mir-X108
GACATTTGATCAGTTGGTGTTTCGTTTAAGGGTTAGGCATATATAAATAACAA**CATTGAACGCAAGCATTAACGGA**TCAAATGGCTA

>Ap-mir-X108-3p
CATTGAACGCAAGCATTAACGGA

```
GACAUUUGAUCAGUUGGUGUUUCGUUUAAGGGUUAGGCAUAUAUAAAUAACAACAUUGAACGCAAGCAUUAACGGAUCAAAUGGCUA
..((((((((.(((((((((((((((((..((((...........))))....))))))).))))))))))).))))))))).....
```

```
 Initial dG =    -31.10

          10        20         30          40
GA--         A          -       GG--|   GGCAU
    CAUUUGAUC GUUGGUGUUU CGUUUAA    GUUA     A
    GUAAACUAG CAAUUACGAA GCAAGUU    CAAU     U
AUCG         G          C       ACAA^   AAAUA
      80        70        60        50
```

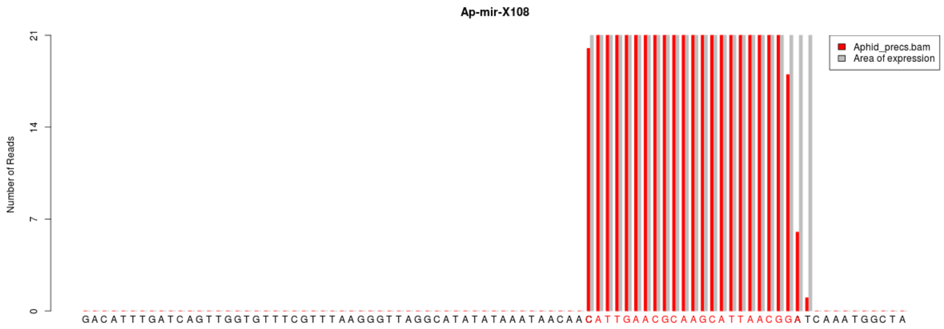

>Ap-mir-X109
AAAAGCGTTTTGGGTCTTCGTCAAGATGGAAGTGTGATTTCATTTCATTTCATTTTTCATTTCATTTCT**TCCATCTTGAAGAAGACCCAA**
AACGCTGAATT

>Ap-mir-X109-3p
TCCATCTTGAAGAAGACCCAA

AAAAGCGUUUUGGGUCUUCGUCAAGAUGGAAGUGUGAUUUCAUUUCAUUUCAUUUUUCAUUUCAUUUCU**UCCAUCUUGAAGAAGACCCAA**AACGCUGAAUU
```
...(((((((((((((((.((((((((((((.((((.......................))))..))))))))))))).)))))))))))))))).....
```

```
 Initial dG =    -50.30

          10        20        30         40
AAA--               G            U-|    UUUCAUUUCAU
     AGCGUUUUGGGUCUUC UCAAGAUGGAAG  GUGA           U
     UCGCAAAACCCAGAAG AGUUCUACCUUC  UACU           U
UUAAG               A            UU^    UUACUUUUUAC
 .        90        80        70        60        50

-----------------------------------------------------
```

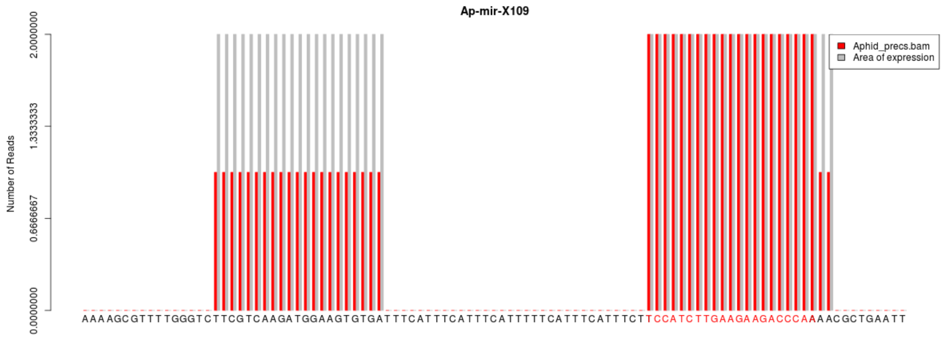

>Ap-mir-X11-1
TTTCAAAGGCTGGTTCTTACGACCTCCGGTTAAAGTTAACCGACACT**TTACTGGACATTGTAAGAACGG**CCCTTAATAAC

>Ap-mir-X11-1-3p
TTACTGGACATTGTAAGAACGG

```
UUUCAAAGGCUGGUUCUUACGACCUCCGGUUAAAGUUAACCGACACUUUACUGGACAUUGUAAGAACGGCCCUUAAUAAC
.......(((((.(((((((((..((((((.(((((........)))))))))))..))))))))))))))).........

 Initial dG =    -29.40

          10         20         30         40
UUUCAAA--|    G          CC       U      UAA
         GGCUG UUCUUACGA  UCCGGU AAAGU   C
         CCGGC AAGAAUGUU  AGGUCA UUUCA   C
CAAUAAUUC^    -          AC       -      CAG
.        70          60         50
```

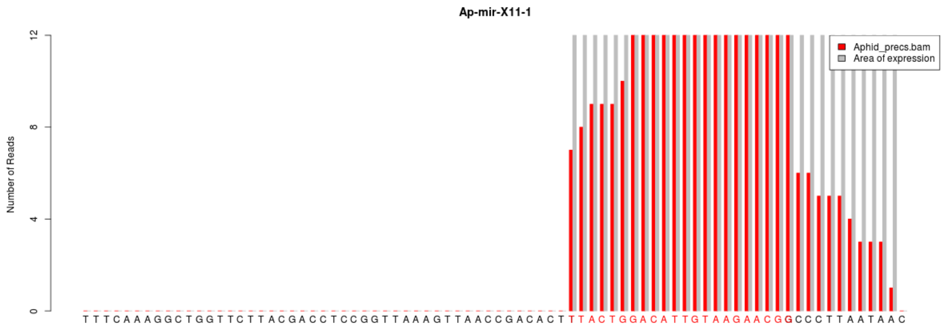

>Ap-mir-X11-2
CACTAAGGGCCTTTCTTACAATGTCAAAATTAACCGACACT**TTACTGGACATTGTAAGAACGG**CCCTAAGTTGT

>Ap-mir-X11-2-3p
TTACTGGACATTGTAAGAACGG

CACUAAGGGCCUUUCUUACAAUGUCAAAAUUAACCGACACU**UUACUGGACAUUGUAAGAACGG**CCCUAAGUUGU
.(((.((((((.(((((((((((((......................))))))))))))).)))))).)))...

Initial dG =   -31.50

```
          10        20        30
C--|  A      U             AAAAUUAACC
   ACU AGGGCC UUCUUACAAUGUC          G
   UGA UCCCGG AAGAAUGUUACAG          A
UGU^  A      C             GUCAUUUCAC
   70        60        50        40
```

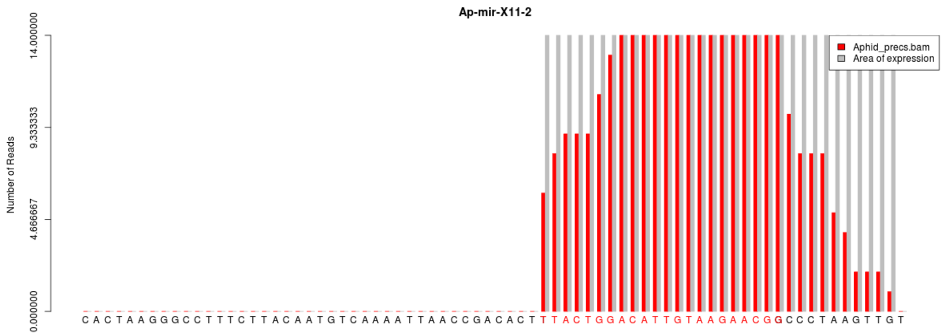

>Ap-mir-X110

AGAATTATCTG**ATACCAAAGAAAGGACCAAC**AATATACCTATACCTACCTGTTGGTTTTTTCTTTGGTATCAGATAATTCATA

>Ap-mir-X110-5p

ATACCAAAGAAAGGACCAAC

```
AGAAUUAUCUGAUACCAAAGAAAGGACCAACAAUAUACCUAUACCUACCUGUUGGUUUUUUCUUUGGUAUCAGAUAAUUCAUA
.(((((((((((((((((((((((((((((((.................)))))))))))))))))))))))))))))))...

 Initial dG =    -45.30

          10        20        30        40
A--|                              AUAUACCU
   GAAUUAUCUGAUACCAAAGAAAGGACCAACA        \
   CUUAAUAGACUAUGGUUUCUUUUUUGGUUGU        A
AUA^                              CCAUCCAU
  80        70        60        50

-----------------------------------------------------
```

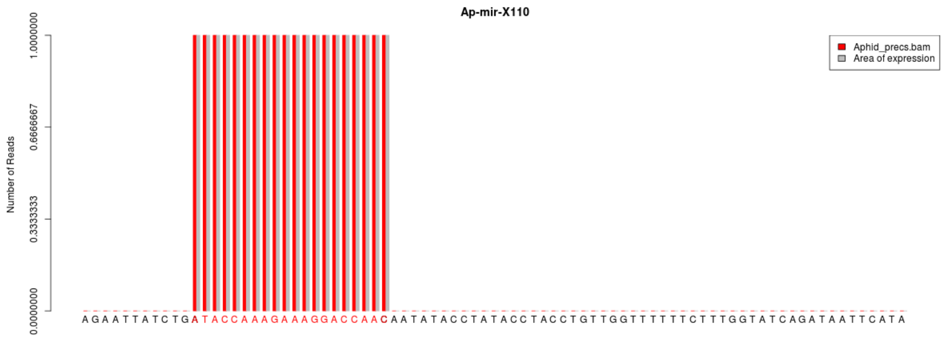

>Ap-mir-X111
GCTATCTGTAT**TTCGGATTGGTAGTCGCTTCGTCGGAA**TATCGATTGCGCGAGGCGGCGGTCGATAATGATTAACGGTAACGAG

>Ap-mir-X111-5p
TTCGGATTGGTAGTCGCTTCGTCGGAA

```
GCUAUCUGUAUUUCGGAUUGGUAGUCGCUUCGUCGGAAUAUCGAUUGCGCGAGGCGGCGGUCGAUAAUGAUUAACGGUAACGAG
.....((((...(((.(((((..(((((((((.((.(((....))).)))))))))))..)))))..)))...)))).......

 Initial dG =    -26.50

          10         20        30        40
GCUAU--    AUU   G-|    UA         U  G    A
       CUGU   UCG  AUUGG  GUCGCUUCG CG AAU U
       GGCA   AGU  UAGCU  CGGCGGAGC GC UUA C
GAGCAAU    AUU   AA^    GG         -  G    G
   80        70        60        50

-----------------------------------------------------
```

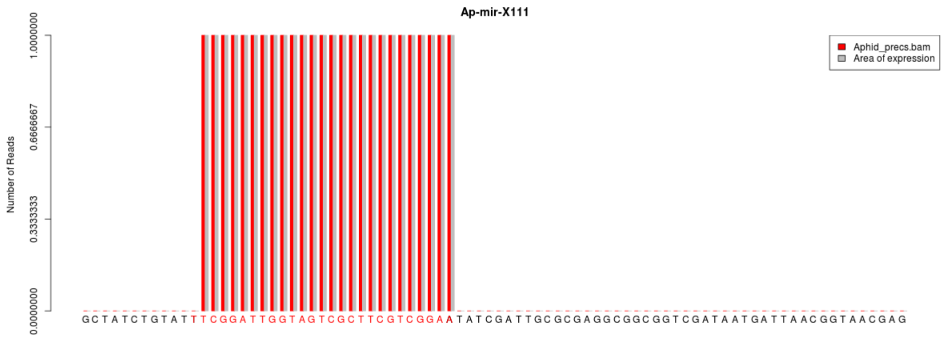

>Ap-mir-X12

GAAAGATTCCT**ATTGGTAACACATACGTCTTTAG**CCTTTTTGTGGAAATCTAAAGATGTTATTGTTACCTACGGAATTTCATAAG

>Ap-mir-X12-5p

ATTGGTAACACATACGTCTTTAG

GAAAGAUUCCU**AUUGGUAACACAUACGUCUUUAG**CCUUUUUGUGGAAAUCUAAAGAUGUUAUUGUUACCUACGGAAUUUCAUAAG

```
...(((((((....((((((...((((((((((((.......))....))))))))))...)))))))...)))))))......
```

```
 Initial dG =    -27.30

           10        20        30
GAA---       UAUU       CAU          ----| UUU
      AGAUUCC    GGUAACA   ACGUCUUUAG    CC   \
      UUUAAGG    CCAUUGU   UGUAGAAAUC    GG   U
GAAUAC       CAU-       UAU          UAAA^ UGU
    80        70         60        50

-----------------------------------------------------
```

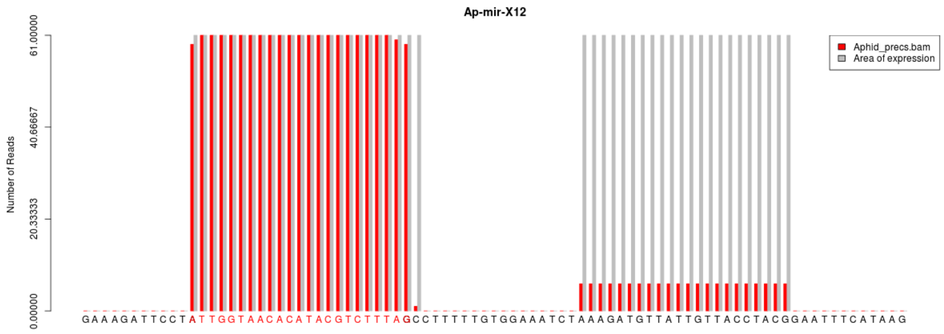

>Ap-mir-X14

CGGCGTCGGTGCCGTGTTCTATGATAGACTCGGCTGGCGCCTGC**CGGTCTGTCGCAGAACACGGCA**CCGCATACGGA

>Ap-mir-X14-3p

CGGTCTGTCGCAGAACACGGCA

```
CGGCGUCGGUGCCGUGUUCUAUGAUAGACUCGGCUGGCGCCUGCCGGUCUGUCGCAGAACACGGCACCGCAUACGGA
((....((((((((((((((.((((((((.((((........)))))))))))).))))))))))))))....))..

 Initial dG =    -49.70

          10         20         30
--| GCGU              A         U    UGG
  CG    CGGUGCCGUGUUCU UGAUAGAC CGGC   C
  GC    GCCACGGCACAAGA GCUGUCUG GCCG   G
AG^ AUAC              C         -    UCC
      70         60         50          40

-----------------------------------------------------
```

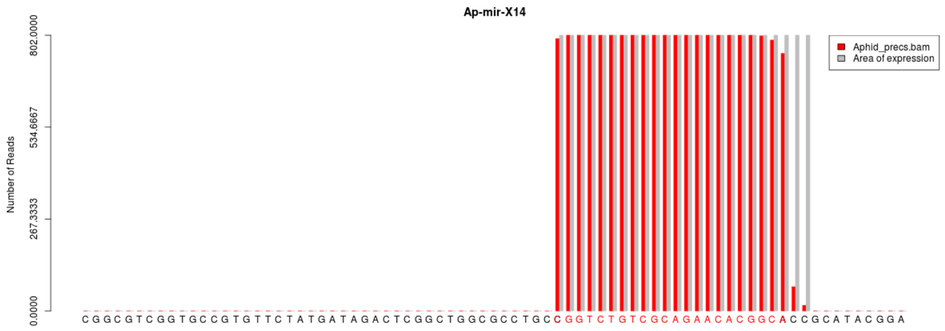

>Ap-mir-X15
TACAACTTAGGGCCGTTCTTACAATGTTCAGTTTAGTGTCAGTTAACTTTAA**CCGGACATTGTGAGAACGGCTC**TTAAAATGTAT

>Ap-mir-X15-3p
CCGGACATTGTGAGAACGGCTC

UACAACUUAGGGCCGUUCUUACAAUGUUCAGUUUAGUGUCAGUUAACUUUAA**CCGGACAUUGUGAGAACGGCUC**UUAAAAUGUAU
((((....((((((((((((((((((((.(((.(((........)))..))).))))))))))))))))))))).....)))).

```
 Initial dG =    -40.30

          10        20        30         40
-    ACUU-                    A   U-|  GUC
 UACA     AGGGCCGUUCUUACAAUGUUC GUU  AGU   A
 AUGU     UCUCGGCAAGAGUGUUACAGG CAA  UCA   G
U    AAAAU                    C   UU^  AUU
    80        70        60        50
```

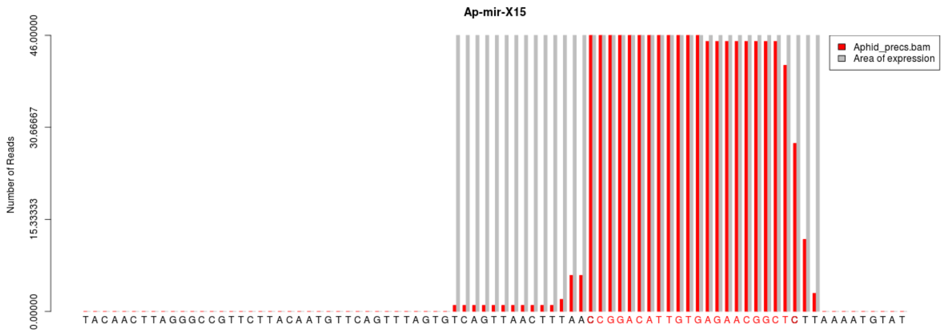

>Ap-mir-X18
GGTCTCTCTTACCTTCGATTTTCTATCCTTTTATAATTTTTGGAATA**AAAGAATAGAAAATCGAAGGTG**AGATGGACCGA

>Ap-mir-X18-3p
AAAGAATAGAAAATCGAAGGTG

```
GGUCUCUCUUACCUUCGAUUUUCUAUCCUUUUAUAAUUUUUGGAAUAAAAGAAUAGAAAAUCGAAGGUGAGAUGGACCGA
(((((.((((((((((((((((((((.(((((((..........))))))).)))))))))))))))))))).))))).. 

 Initial dG =    -43.30

          10        20        30
--|    C                    C       AAUU
  GGUCU UCUUACCUUCGAUUUUCUAU CUUUUAU    U
  CCAGG AGAGUGGAAGCUAAAAGAUA GAAAAUA    U
AG^    U                    A       AGGU
.        70        60        50        40

-----------------------------------------------------
```

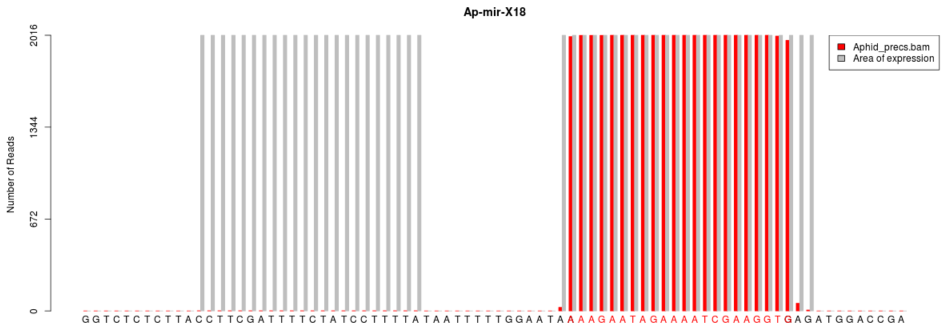

>Ap-mir-X19

TACAGCATGTAAATTAATTGGAGAGTCAGTAGCAGTATTATTATTATAGC**TACTGACTTTCTAATTGATTTA**CATACTGTATA

>Ap-mir-X19-3p

TACTGACTTTCTAATTGATTTA

```
UACAGCAUGUAAAUUAAUUGGAGAGUCAGUAGCAGUAUUAUUAUUAUAGCUACUGACUUUCUAAUUGAUUUACAUACUGUAUA
(((((.(((((((((((((((((((((((((((.(((.......))).))))))))))))))))))))))))))).))))).. 
```

```
 Initial dG =    -45.60

          10        20        30        40
--|    C                          A   UUA
  UACAG AUGUAAAUUAAUUGGAGAGUCAGUAGC GUA   \
  AUGUC UACAUUUAGUUAAUCUUUCAGUCAUCG UAU   U
AU^    A                          A   UAU
  80        70        60        50
```

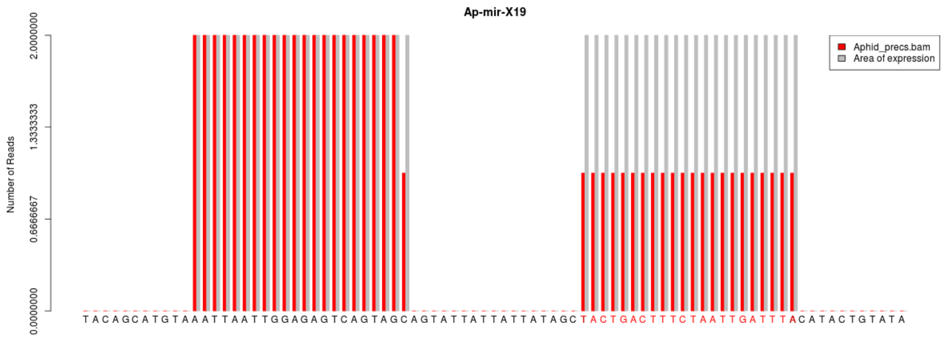

>Ap-mir-X2
ACCATACCAAG**GTAAGAATAATGATCCATTAGA**AGGTATACAAATAAATCATCTAATAGATCATTTTTCCGACAGGGTCTGGCATA

>Ap-mir-X2-5p
GTAAGAATAATGATCCATTAGA

ACCAUACCAAG**GUAAGAAUAAUGAUCCAUUAGA**AGGUAUACAAAUAAAUCAUCUAAUAGAUCAUUUUUCCGACAGGGUCUGGCAUA
.(((.(((...((..(((.(((((((.((((((.(((.((....)).))).)))))).))))))).)))..))..))).)))....

```
 Initial dG =    -25.70

           10        20        30        40
A---|  U   AAG  AA   U       C      A   A  C
    CCA ACC   GU  GAA AAUGAUC AUUAGA GGU UA A
    GGU UGG   CA  CUU UUACUAG UAAUCU CUA AU A
AUAC^  C   GA-  GC   U       A      A   A  A
     80         70        60        50
```

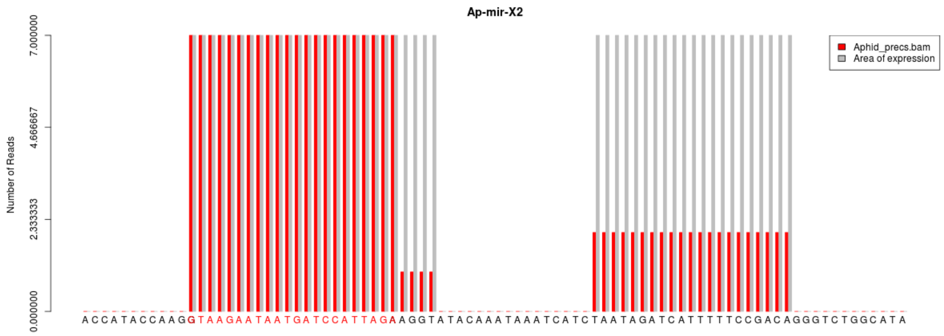

>Ap-mir-X20
ATTGGAGGAAA**TTGTCTTGTGTAGTCCATGATAGCATC**GAAACCATCCTCCATGGAAAATGGACAGCGCGGGTCATTCCTGGTTGCGGG

>Ap-mir-X20-5p
TTGTCTTGTGTAGTCCATGATAGCATC

```
AUUGGAGGAAAUUGUCUUGUGUAGUCCAUGAUAGCAUCGAAACCAUCCUCCAUGGAAAAUGGACAGCGCGGGUCAUUCCUGGUUGCGGG
.....(((((..((.(((((((.((((((.....(((.((........)).)))....)))))).))))))).)))))))).........
```

```
 Initial dG =    -25.60

            10        20          30
AUUGG----     AU  U       A        |  A
         AGGAA  UG CUUGUGU GUCCAU--GAU \
         UCCUU  AC GGGCGCG CAGGUA  CUA G
GGGCGUUGG     --  U       A      \ ^  C
        80          70        60


                                   40        50
                                   GAAACCA   UC
                                          UCC  \
                                          AGG  C
                                   AA-----   UA
```

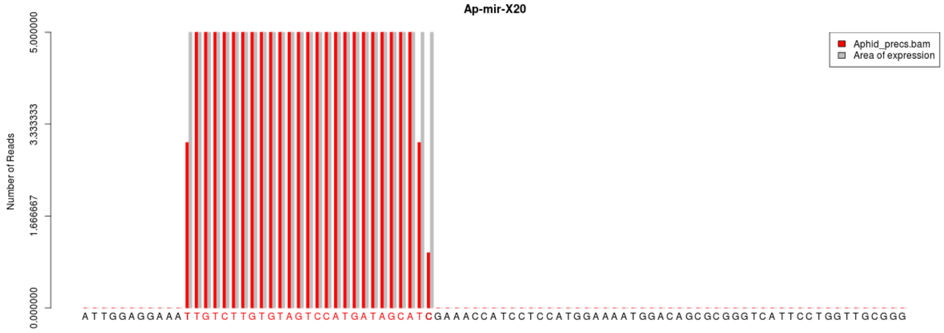

>Ap-mir-X22
CGATGATAACC**TCTCGTTATATCTGACGTCGGGGAA**TGGTATATATACCAGAACTCCAGCGTAGATACTCGATGCGCTATCAACCTC

>Ap-mir-X22-5p
TCTCGTTATATCTGACGTCGGGGAA

CGAUGAUAACC**UCUCGUUAUAUCUGACGUCGGGGAA**UGGUAUAUAUACCAGAACUCCAGCGUAGAUACUCGAUGCGCUAUCAACCUC
...(((((.....(((...((((((.(((.((((..(((((....)))))...)))).)))))))))..))).....))))).....

Initial dG = -25.10

```
          10        20        30         40
CGA--|    ACCUC   UUA      A   C    AA-     U
     UGAUA     UCG   UAUCUG CGU GGGG   UGGUA A
     ACUAU     AGC   AUAGAU GCG CCUC   ACCAU U
CUCCA^    CGCGU   UC-      -   A    AAG     A
      80        70          60        50
```

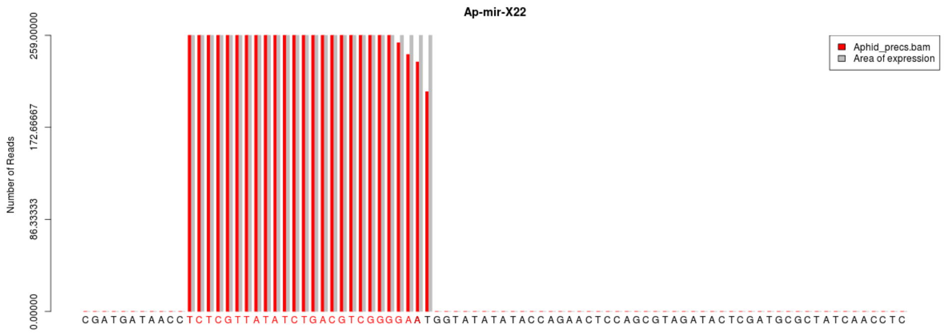

>Ap-mir-X24
ATACCATATTCGGCATTTCTGTTTTCAAGCCTTATTAAAATTTTAAAAT**AAGCTTGAATTCAGAAATGTCGA**ATGTTGAGTGT

>Ap-mir-X24-3p
AAGCTTGAATTCAGAAATGTCGA

AUACCAUAUUCGGCAUUUCUGUUUUCAAGCCUUAUUAAAAUUUUAAAAU**AAGCUUGAAUUCAGAAAUGUCGA**AUGUUGAGUGU
((((((((((((((((((((..(((((((.(((((..........))))))))))))..)))))))))))))))).).))))

```
 Initial dG =    -35.00

          10        20        30        40
    - -|               UU       C     AAAA
AUAC C AUAUUCGGCAUUUCUG  UUCAAGC UUAUU    U
UGUG G UGUAAGCUGUAAAGAC  AAGUUCG AAUAA    U
    A U^               UU       -     AAUU
  80        70        60         50

-----------------------------------------------------
```

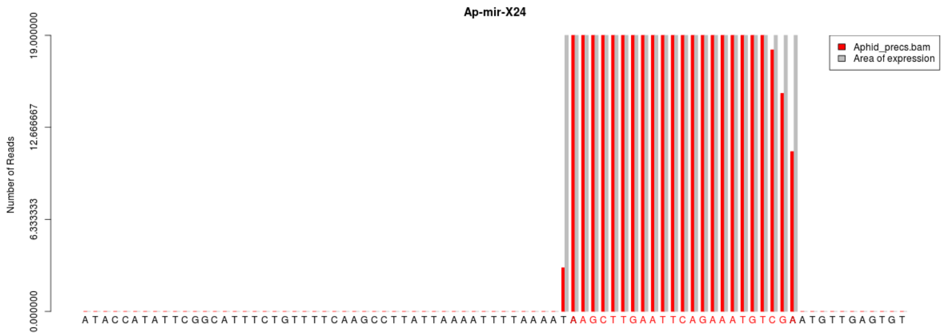

>Ap-mir-X25
TGGTTTAGTAT**TCTTTGGGATTTAATAGAGCCGGT**TGAAATTGTTTCGTATACCAGCTATCTTAAATTTCAAAATACTAGACTTTG

>Ap-mir-X25-5p
TCTTTGGGATTTAATAGAGCCGGT

```
UGGUUUAGUAUUCUUUGGGAUUUAAUAGAGCCGGUUGAAAUUGUUUCGUAUACCAGCUAUCUUAAAUUUCAAAAUACUAGACUUUG
.((((((((((..((((..((((((...(((.((((((((...)))))...))).))).)))...))))))..))))))))))))))...

 Initial dG =    -26.20

          10        20        30           40
U--          UC    GG      UAG   C    ---|    U
   GGUUUAGUAU  UUUG  AUUUAA   AGC GGU    UGAAA \
   UCAGAUCAUA  AAAC  UAAAUU   UCG CCA    GCUUU U
GUU          --    UU      CUA   A    UAU^    G
     80          70        60        50

-----------------------------------------------------
```

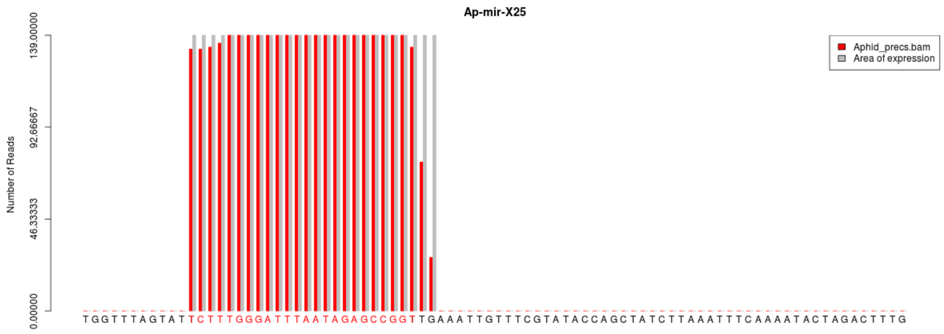

>Ap-mir-X26-1
TCTTCGAATTG**TGTGACTGTACTTTTCGTGGATGGGA**TCATTTAATATTTGTATTAAATGATCTAGTGAGCTGGGAAGAGGGGGTCGCCT
ACAACGTCGCTGTC

>Ap-mir-X26-1-5p
TGTGACTGTACTTTTCGTGGATGGGA

UCUUCGAAUUG**UGUGACUGUACUUUUCGUGGAUGGGA**UCAUUUAAUAUUUGUAUUAAAUGAUCUAGUGAGCUGGGAAGAGGGGGUCGCCUACAACGUCGCUGUC
....(((.((((((((((...((((((.(((...((((((((((((((....))))))))))))))......))).))))))..))))))..))))..))).....

Initial dG =   -33.10

```
          10          20         30            40
UCUU-   A-     --       GUA       G    AUG---|            U
     CGA  UUGU  GUGACU   CUUUUC UGG       GGAUCAUUUAAUA U
     GCU  AACA  CGCUGG   GAGAAG GUC       UCUAGUAAAUUAU U
CUGUC   GC     UC       GG-       G    GAGUGA^            G
  100        90         80         70         60         50
```

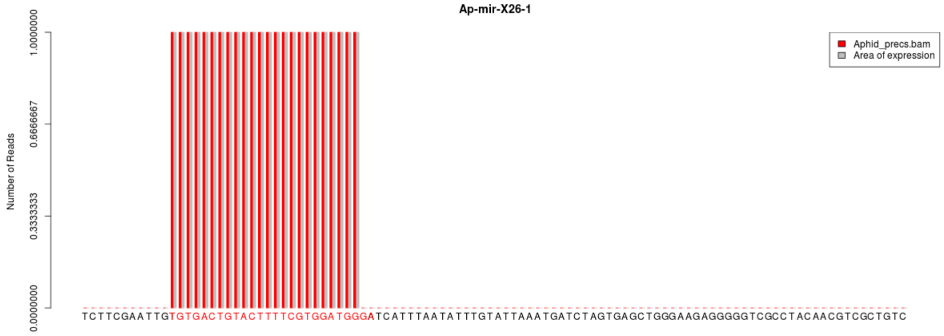

>Ap-mir-X26-2
TCTTCGAATTG**TGTGACTGTACTTTTCGTGGATGGGA**TCATTTAATATTTGTACTAAATGATCTGGTGAGCTGGGAAGGGTGTCAGGTTA
TGTGTTCGAATGAAT

>Ap-mir-X26-2-5p
TGTGACTGTACTTTTCGTGGATGGGA

UCUUCGAAUUG**UGUGACUGUACUUUUCGUGGAUGGGA**UCAUUUAAUAUUUGUACUAAAUGAUCUGGUGAGCUGGGAAGGGUGUCAGGUUAUGUGUUCGAAUGAAU
((((((((...((((((((((((((.(((((.((((((((((.((....)).)))))))))).))...))).)))))))).))).)))).)))))))).))..

```
 Initial dG =    -32.90

           10          20        30           40
--   -       UGU    -   -        G   ---| G          A  U
  UC UUCGAAU    GUGA CUG UACUUUUC UGG    AU GGAUCAUUUA UA U
  AG AAGCUUG    UAUU GAC GUGGGAAG GUC    UG UCUAGUAAAU AU U
UA   U       UG-    G   U        G   GAG^ G          C  G
   100         90        80        70        60        50

-----------------------------------------------------
```

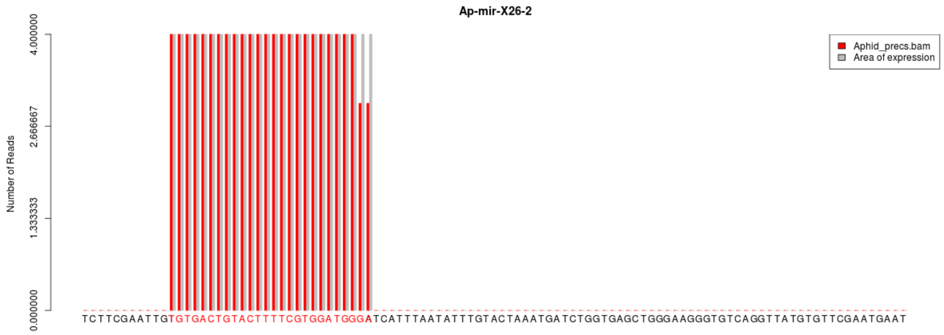

>Ap-mir-X27
TAAAGTATCCGTGGTCGTAGATGCCAAACCCAGGGTAAAACCTCACCC**TGGTCCCTACAGCAAGTGCCTCCTGACG**GGACGCAAACG

>Ap-mir-X27-3p
TGGTCCCTACAGCAAGTGCCTCCTGACG

```
UAAAGUAUCCGUGGUCGUAGAUGCCAAACCCAGGGUAAAACCUCACCCUGGUCCCUACAGCAAGUGCCUCCUGACGGGACGCAAACG
....((.(((((((..(((..(((.....(((((((........)))))))........)))..)))..))..))))))).......

 Initial dG =    -24.70

           10          20           30        40
UAAA---  A     --  UC   GA   CAAAC---|      AAA
       GU UCCGU  GG  GUA  UGC        CCAGGGU   A
       CA GGGCA  CC  CGU  ACG        GGUCCCA   C
GCAAACG  -     GU  UC   GA   ACAUCCCU^      CUC
      80         70        60        50

-----------------------------------------------------
```

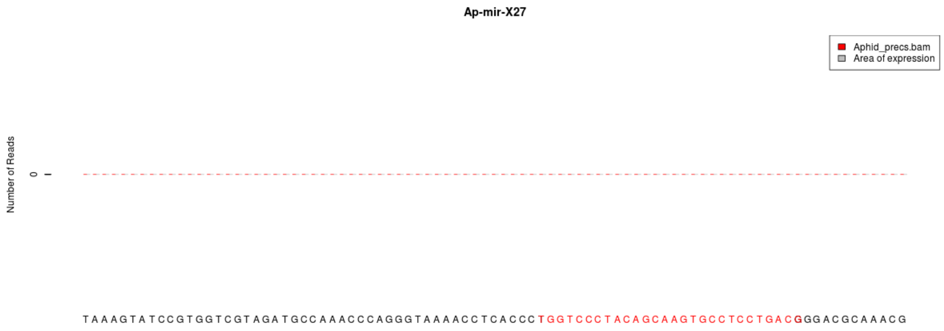

>Ap-mir-X29

GCTGGTCTTGG**AGTAGTAAGAGATCAGTTGGATA**TTGGAAGGGCTATATTTTGTCAAATTGATCTCTTATCACGCCAAGACCAGCAA

>Ap-mir-X29-5p

AGTAGTAAGAGATCAGTTGGATA

```
GCUGGUCUUGGAGUAGUAAGAGAUCAGUUGGAUAUUGGAAGGGCUAUAUUUUGUCAAAUUGAUCUCUUAUCACGCCAAGACCAGCAA
(((((((((((.((.((((((((((((((.((((.................)))).)))))))))))))).)).)))))))))))..
```

```
 Initial dG =    -45.00

          10        20        30        40
--|          A  A              G    UUGGAAGG
  GCUGGUCUUGG GU GUAAGAGAUCAGUU GAUA        \
  CGACCAGAACC CA UAUUCUCUAGUUAA CUGU        G
AA^          G  C              A    UUUAUAUC
      80        70        60        50
```

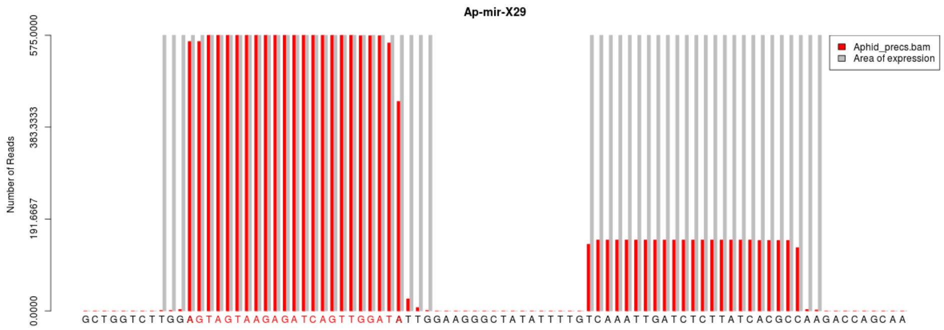

>Ap-mir-X30
GTCCGTCTCTT**TCGGCGTAACATGATGTGTGCG**CTGCATTTGCGAAGTCGGACACACGTGTTATGCCGCAAGAGATGGTCTA

>Ap-mir-X30-5p
TCGGCGTAACATGATGTGTGCG

```
GUCCGUCUCUUUCGGCGUAACAUGAUGUGUGCGCUGCAUUUGCGAAGUCGGACACACGUGUUAUGCCGCAAGAGAUGGUCUA
..((((((((.(((((((((((((.(((((.((((((....))..)).)).))))))))))))))))))).)))))))))....

 Initial dG =    -45.20

          10        20        30
GU--         U            A     G  -   --| A
    CCGUCUCUU CGGCGUAACAUG UGUGU CG CU  GC U
    GGUAGAGAA GCCGUAUUGUGC ACACA GC GA  CG U
AUCU         C            -     G  U  AG^ U
 80        70        60         50        40

-----------------------------------------------------
```

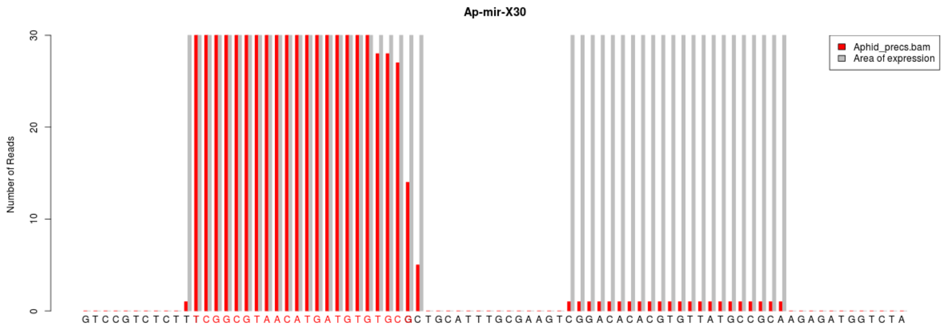

>Ap-mir-X31
TGTCGTCATGGGGGGATAATTGTAGGACTTACTGTGAATTTGTAATACAG**CCAGTCTTGCATTTATTCCACT**TTGACTGCAGA

>Ap-mir-X31-3p
CCAGTCTTGCATTTATTCCACT

UGUCGUCAUGGGGGGAUAAUUGUAGGACUUACUGUGAAUUUGUAAUACAG**CCAGUCUUGCAUUUAUUCCACU**UUGACUGCAGA
(((.((((.((.(((((((.(((((((((..(((((.........)))))..))))))))).))))))).)).)))).)))..

```
 Initial dG =    -32.60

          10        20        30        40
--|  C    U  G       U         UA      AAUU
  UGU GUCA GG GGGAUAA UGUAGGACU  CUGUG    \
  ACG CAGU UC CCUUAUU ACGUUCUGA  GACAU    U
AG^  U    U  A       U         CC      AAUG
  80        70        60        50
```

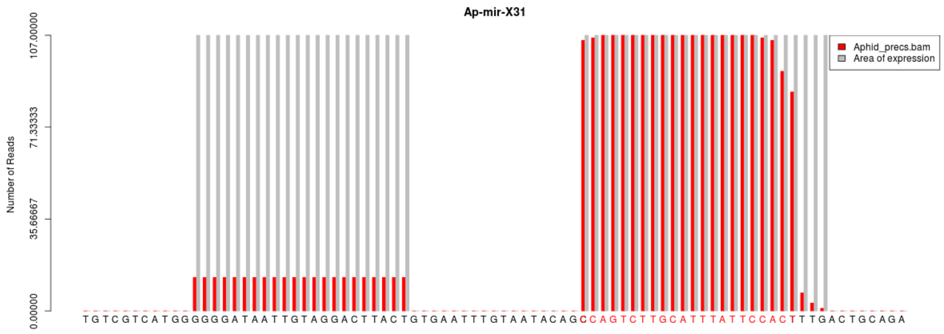

>Ap-mir-X32

AAACGTTGGACAATGAATAGTCTGCACTGTTCAATCTCTACCGAATTCGTG**TACGGTTTAGACTATTCATTTT**CCAGTGTTGCT

>Ap-mir-X32-3p

TACGGTTTAGACTATTCATTTT

```
AAACGUUGGACAAUGAAUAGUCUGCACUGUUCAAUCUCUACCGAAUUCGUGUACGGUUUAGACUAUUCAUUUUCCAGUGUUGCU
.(((((((((.(((((((((((((.(((((.((...((....)).....)).))))).))))))))))))).)))))))))...

 Initial dG =    -31.80

          10         20         30           40
A--         C             C      U  AUC--| U
   AACGUUGGA AAUGAAUAGUCUG ACUGU CA      UC A
   UUGUGACCU UUACUUAUCAGAU UGGCA GU      AG C
UCG         U             U      U  GCUUA^ C
   80         70         60         50

-----------------------------------------------------
```

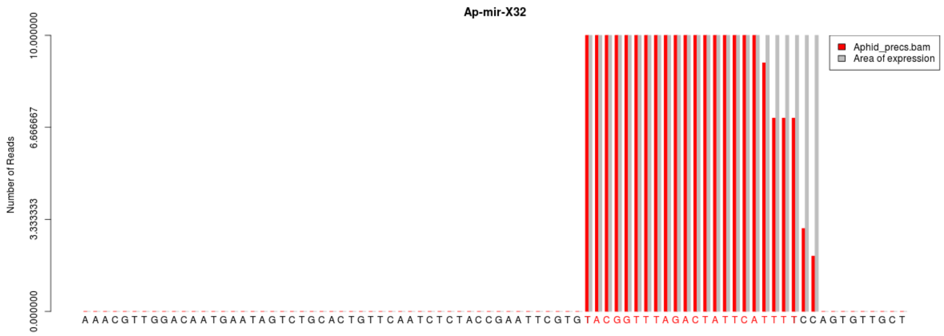

>Ap-mir-X33
GTGTACGCCGATTGGTAACATCAACCTCTTTAGCGAATATTTGACGCACTAAAGACGTAAATGTTACCTGCGGAGTTCCCATG

>Ap-mir-X33-3p
AAAGACGTAAATGTTACCTGCG

GUGUACGCCGAUUGGUAACAUCAACCUCUUUAGCGAAUAUUUGACGCACUAAAGACGUAAAUGUUACCUGCGGAGUUCCCAUG
(((.((.(((...((((((((..((.(((((((((.........)))..)))))).))..))))))))..))).))...))).

```
 Initial dG =    -24.50

           10        20        30
-   U--  G   AUU        CA  C       --|  AAUA
 GUG   AC CCG   GGUAACAU  AC UCUUUA  GCG    \
 UAC   UG GGC   CCAUUGUA  UG AGAAAU  CGC    U
G   CCU  A   GU-        AA  C       CA^  AGUU
  80        70         60        50
```

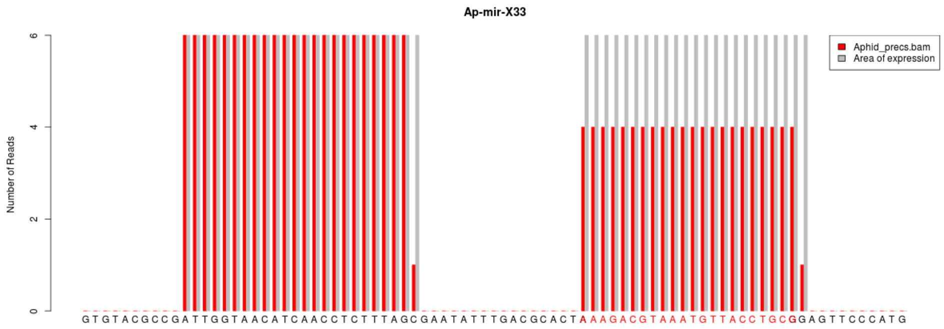

>Ap-mir-X34
TGAATACTATC**AGGTAACAGGTTTGTTTTGTGA**GTGATAAATTTAAAACTTCCCAGACGAACTCTGTTACCCCATAGTTTCAAGA

>Ap-mir-X34-5p
AGGTAACAGGTTTGTTTTGTGA

```
UGAAUACUAUCAGGUAACAGGUUUGUUUUGUGAGUGAUAAAUUUAAAACUUCCCAGACGAACUCUGUUACCCCAUAGUUUCAAGA
((((.(((((..((((((((((((((((...((((...........))))...)))))))).))))))))..)))))))))...

 Initial dG =    -28.00

           10        20         30        40
---|   U     CA        -         UGU    GAUAA
   UGAA ACUAU  GGUAACAG GUUUGUUU   GAGU      A
   ACUU UGAUA  CCAUUGUC CAAGCAGA   UUCA      U
AGA^   -     CC        U         CCC    AAAUU
    80         70        60        50

-----------------------------------------------------
```

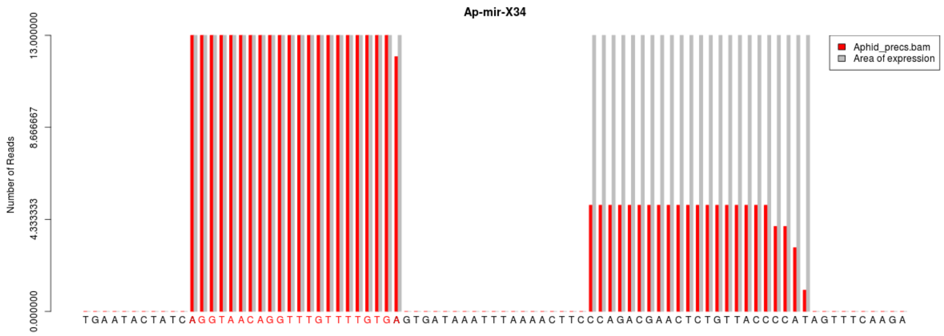

>Ap-mir-X35
TGTCTACAATAGGGAAATTTGTTAAGAAGCAAAGTGTTAAATATTCC**TTGCTTTTTAACAAGTTTCACTA**TGGTGGATGTA

>Ap-mir-X35-3p
TTGCTTTTTAACAAGTTTCACTA

UGUCUACAAUAGGGAAAUUUGUUAAGAAGCAAAGUGUUAAAUAUUCC**UUGCUUUUUAACAAGUUUCACUA**UGGUGGAUGUA
.((((((.((((.((((((((((((((((((((((((...)))))..))))))))))))))))))).)))).))))))...

```
 Initial dG =    -32.70

          10        20        30
U--      A    G                   --|    U
   GUCUAC AUAG GAAAUUUGUUAAGAAGCAA  AGUGU \
   UAGGUG UAUC CUUUGAACAAUUUUUCGUU  UUAUA A
AUG      G    A                   CC^    A
80        70        60        50        40
```

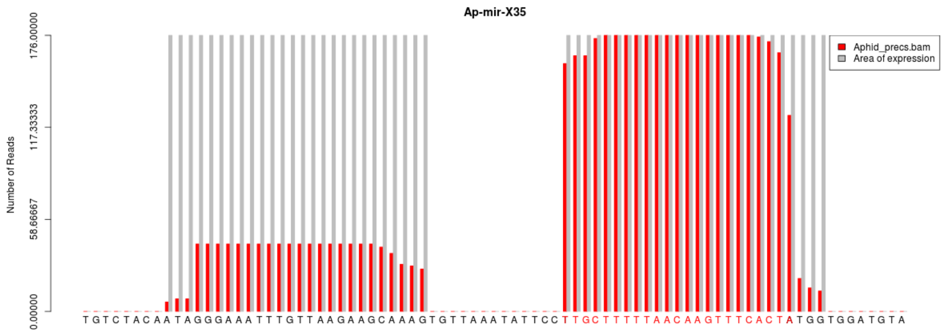

>Ap-mir-X36

TAACTATCAAC**TGTTAGTATAACTCTTAGTAACA**GTGAATTTTAAAATTGTGACTAGAGTTTATACTACCGATTGATGGTAACC

>Ap-mir-X36-5p

TGTTAGTATAACTCTTAGTAACA

```
UAACUAUCAACUGUUAGUAUAACUCUUAGUAACAGUGAAUUUUAAAAUUGUGACUAGAGUUUAUACUACCGAUUGAUGGUAACC
..((((((((.((.((((((((((((.(((.(((((..........))))).))))))))).)))))).)).))))))))....

 Initial dG =    -27.40

          10        20         30        40
UA--        C  U      -|      U   A     GAAU
    ACUAUCAA UG UAGUAU AACUCU AGU ACAGU    U
    UGGUAGUU GC AUCAUA UUGAGA UCA UGUUA    U
CCAA        A  C      U^      -   G     AAAU
   80        70        60         50

-----------------------------------------------------
```

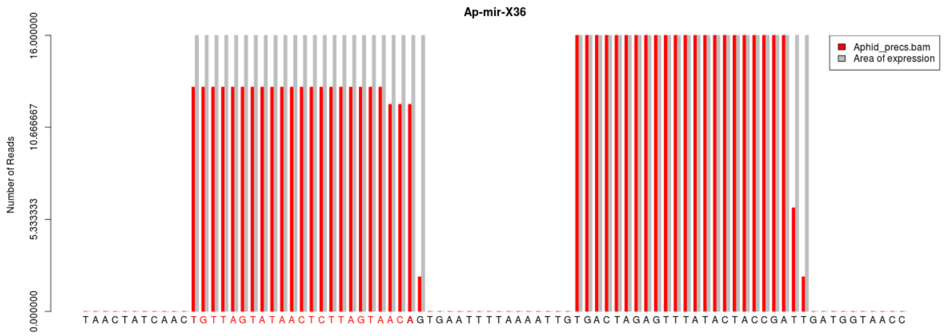

>Ap-mir-X37
CGATTACATACACAAGAATGGTTGCCCGTGAACGTGGTAATTTGATTCG**TCCACGGACATCCATTTTTGTGA**CGGTAAGCGAG

>Ap-mir-X37-3p
TCCACGGACATCCATTTTTGTGA

```
CGAUUACAUACACAAGAAUGGUUGCCCGUGAACGUGGUAAUUUGAUUCGUCCACGGACAUCCAUUUUUGUGACGGUAAGCGAG
((.((((...(((((((((((.((.(((((.(((.(((......)))))).))))).)).)))))))))))...)))).))..

 Initial dG =    -31.50

          10        20        30        40
--| A    AUA           U  C     A   U   AA
  CG UUAC   CACAAGAAUGG UG CCGUG ACG GGU  U
  GC AAUG   GUGUUUUUACC AC GGCAC UGC UUA  U
GA^ G    GCA           U  A     C   -   GU
  80        70        60        50

-----------------------------------------------------
```

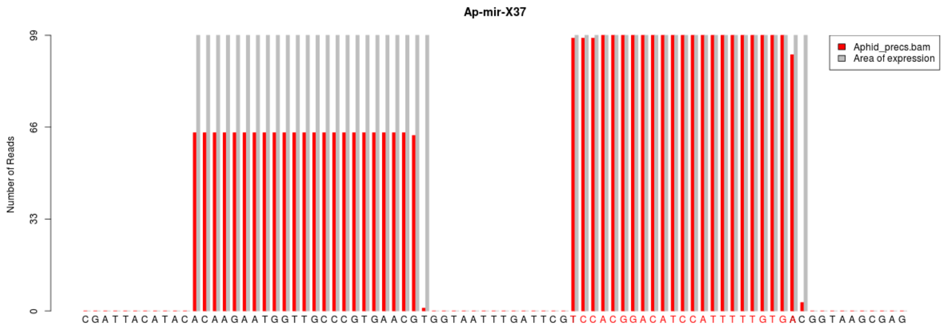

>Ap-mir-X38-1
TGACACCATAA**TGCAACTGTACAATGACAATAGTAGGA**TTATAGGGGTCTATAAACCTACTAATATAAGGTACAGCTGCATCTGGTGACA
ACT

>Ap-mir-X38-1-5p
TGCAACTGTACAATGACAATAGTAGGA

UGACACCAUAA**UGCAACUGUACAAUGACAAUAGUAGGA**UUAUAGGGGUCUAUAAACCUACUAAUAUAAGGUACAGCUGCAUCUGGUGACAACU

```
((.(((((..(((((.((((((........(((((((.((((((....)))))).))))))).......)))))).))))).))))).))...

 Initial dG =    -35.20

           10        20        30        40
---| A     UA     A      AAUGACAA       A      G
   UG CACCA  AUGCA CUGUAC        UAGUAGG UUAUAG G
   AC GUGGU  UACGU GACAUG        AUCAUCC AAUAUC G
UCA^ A     C-     C      GAAUAUA-       A      U
  90         80        70         60        50

-----------------------------------------------------
```

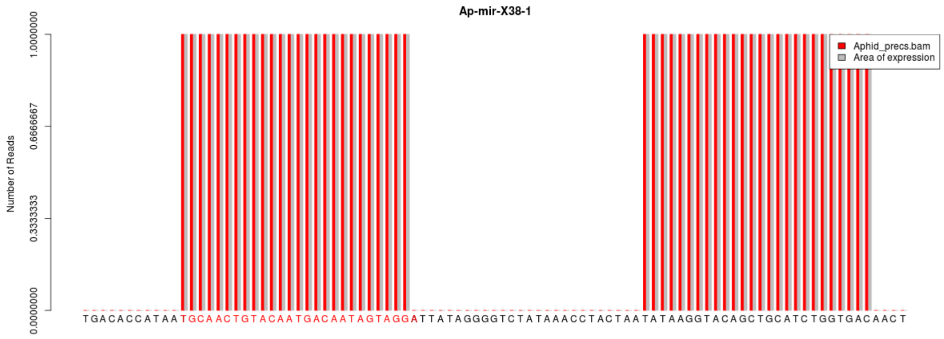

>Ap-mir-X38-2

TGACACCATAA**TGCAACTGTACAATGACAATAGTAGGA**TTATAGGGGTCTATAAACCTACTAATATAAGGTACAGCTGCATCTGGTGACA
ACT

>Ap-mir-X38-2-5p

TGCAACTGTACAATGACAATAGTAGGA

UGACACCAUAA**UGCAACUGUACAAUGACAAUAGUAGGA**UUAUAGGGGUCUAUAAACCUACUAAUAUAAGGUACAGCUGCAUCUGGUGACAACU
((.(((((..(((((.((((((........(((((((.((((((....)))))).)))))))........)))))).))))).))))).))...

```
 Initial dG =    -35.20

           10        20        30        40
---| A     UA     A      AAUGACAA       A      G
   UG CACCA  AUGCA CUGUAC        UAGUAGG UUAUAG G
   AC GUGGU  UACGU GACAUG        AUCAUCC AAUAUC G
UCA^ A     C-     C      GAAUAUA-       A      U
  90         80        70         60        50
```

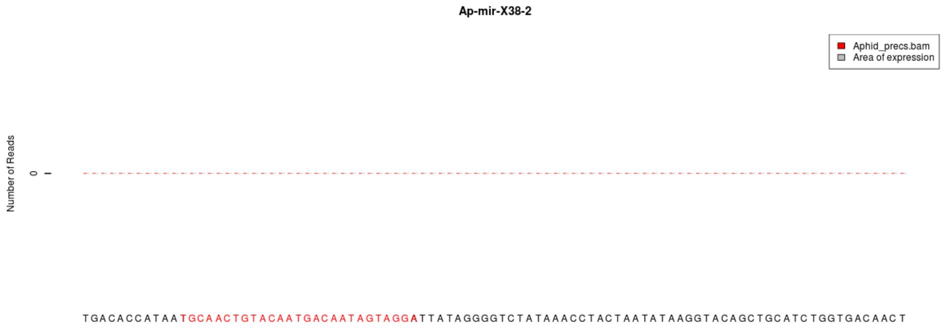

>Ap-mir-X39-1
AATGCGACCAAATTAAGACGATGACCAGATAACCGTCTAAAGCAGGTTTGGTCCCATG**TTACTGGTCATCGTCTTAACTT**GGTCGCCCTA
T

>Ap-mir-X39-1-3p
TTACTGGTCATCGTCTTAACTT

```
AAUGCGACCAAAUUAAGACGAUGACCAGAUAACCGUCUAAAGCAGGUUUGGUCCCAUGUUACUGGUCAUCGUCUUAACUUGGUCGCCCUAU
...((((((((.((((((((((((((((.((((...(((((.....)))))......))))))))))))))))))))).)))))))).....

 Initial dG =    -44.80

          10        20        30            40
AAU--        A                A    CGU---|     GC
     GCGACCAA UUAAGACGAUGACCAG UAAC       CUAAA  \
     CGCUGGUU AAUUCUGCUACUGGUC AUUG       GGUUU  A
UAUCC        C                -    UACCCU^     GG
90        80        70         60        50

-----------------------------------------------------
```

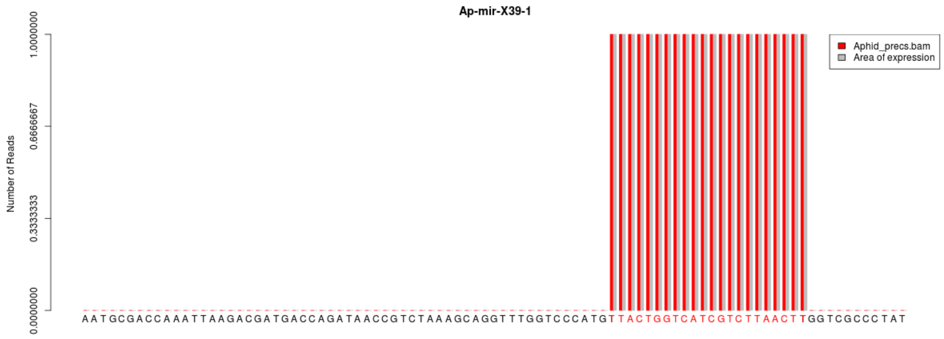

>Ap-mir-X39-2

AGTGCGACCAAATTAAGACGATGACCAGATAACCGTCTAAAGCAGGTTTGGTCCCATG**TTACTGGTCATCGTCTTAACTT**GGTCGCCCTA
T

>Ap-mir-X39-2-3p

TTACTGGTCATCGTCTTAACTT

AGUGCGACCAAAUUAAGACGAUGACCAGAUAACCGUCUAAAGCAGGUUUGGUCCCAUG**UUACUGGUCAUCGUCUUAACUU**GGUCGCCCUAU
((.((((((((.((((((((((((((((.((((...(((((.....)))))......)))))))))))))))))))).)))))))).))..

Initial dG =   -45.80

```
          10        20        30            40
--  U        A                A    CGU---|    GC
  AG GCGACCAA UUAAGACGAUGACCAG UAAC       CUAAA  \
  UC CGCUGGUU AAUUCUGCUACUGGUC AUUG       GGUUU  A
UA  C        C                -    UACCCU^    GG
90        80        70         60        50
```

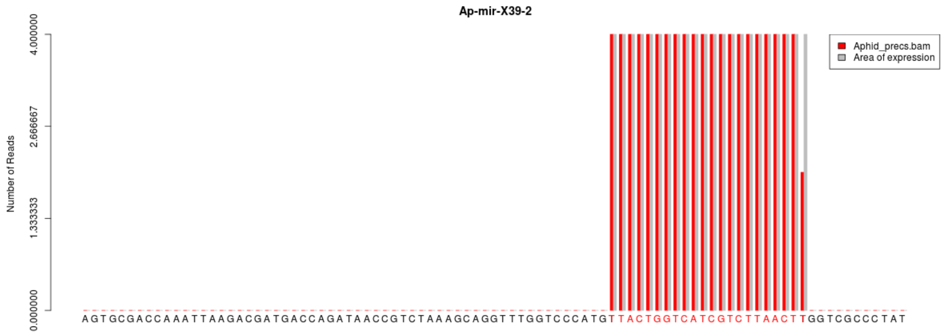

>Ap-mir-X4

AGACTAGAGTG**CTAAGTAGTAGCGCCAACGGTGA**ATTGATTTTAAATATGTCACCTGCGTACTACTGCTTACCATTCTGGCCTCC

>Ap-mir-X4-5p

CTAAGTAGTAGCGCCAACGGTGA

AGACUAGAGUG**CUAAGUAGUAGCGCCAACGGUGA**AUUGAUUUUAAAUAUGUCACCUGCGUACUACUGCUUACCAUUCUGGCCUCC
((.((((((((.((((((((((..(((..(((((....(((...)))...))))))).)..)))))))))).)))))))).))..

Initial dG = -31.50

```
          10        20          30        40
--   A         C          CG -| AC      AUUG    U
  AG CUAGAGUG UAAGUAGUAG  C CA  GGUGA     AUU \
  UC GGUCUUAC AUUCGUCAUC  G GU  CCACU     UAA U
CC   C         C          AU C^ --      GUA-    A
    80        70         60           50
```

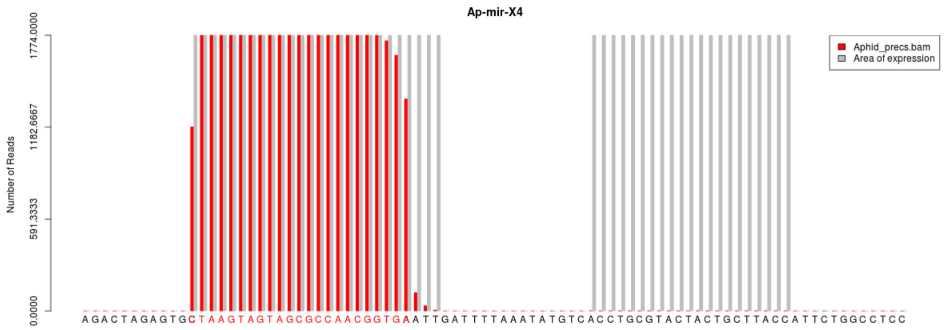

>Ap-mir-X40-1
ACATCGGTTTT**ATTGGTTTCTAGATCTCAGCA**TATAAATTGATTGTTGCTGGGACTTAGAAAGCAATATAACAGTTGTGC

>Ap-mir-X40-1-5p
ATTGGTTTCTAGATCTCAGCA

ACAUCGGUUUU**AUUGGUUUCUAGAUCUCAGCA**UAUAAAUUGAUUGUUGCUGGGACUUAGAAAGCAAUAUAACAGUUGUGC
```
(((...(((.(((((.(((((((.((((((((.((((.....)))))))))))).))))))).))))).)))...)))..
```

```
 Initial dG =    -28.90

          10        20        30
--|  UCG   U      G       A        U    AU
  ACA   GUU UAUUG UUUCUAG UCUCAGCA AUAA  \
  UGU   CAA AUAAC AAAGAUU AGGGUCGU UGUU  U
CG^  UGA   U      G       C        -    AG
.        70        60        50

-----------------------------------------------------
```

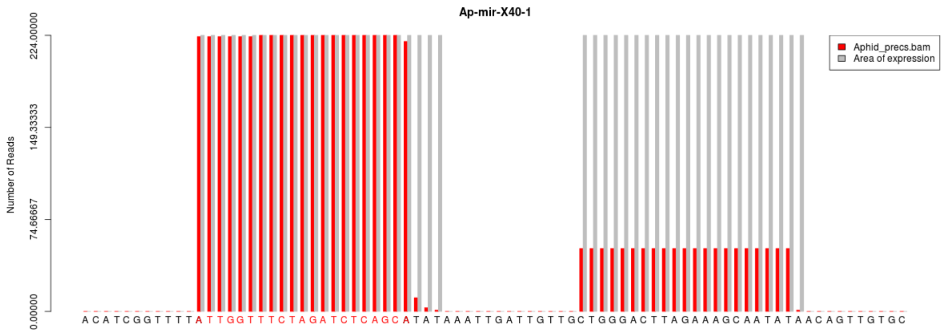

>Ap-mir-X40-2
ACATCGGTTTT**ATTGGTTTCTAGATCTCAGCA**TATAAATTGATTGTTGCTGGGACTTAGAAAGCAATATAACAGTTGTGC

>Ap-mir-X40-2-5p
ATTGGTTTCTAGATCTCAGCA

```
ACAUCGGUUUUAUUGGUUUCUAGAUCUCAGCAUAUAAAUUGAUUGUUGCUGGGACUUAGAAAGCAAUAUAACAGUUGUGC
(((...(((.(((((.(((((((.((((((((.((((.....)))))))))))).))))))).))))).)))...))).. 

 Initial dG =    -28.90

          10        20        30
--|  UCG   U     G       A        U    AU
  ACA   GUU UAUUG UUUCUAG UCUCAGCA AUAA  \
  UGU   CAA AUAAC AAAGAUU AGGGUCGU UGUU  U
CG^  UGA   U     G       C        -    AG
.        70        60        50

-----------------------------------------------------
```

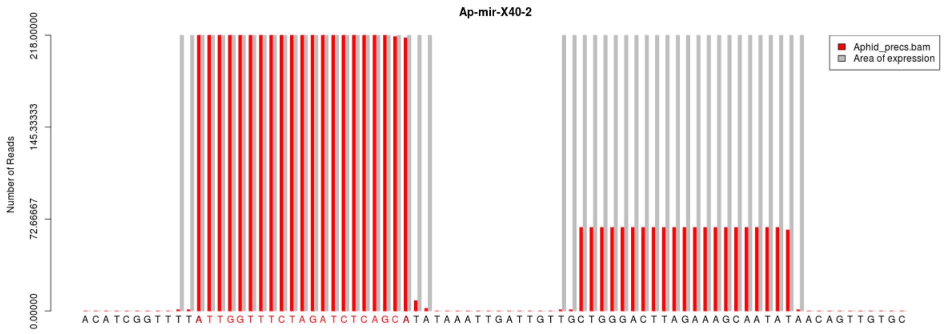

>Ap-mir-X40-3
ACATCGGTTTT**ATTGGTTTCTAGATCTCAGCA**TTTAAATTGATTGTTGCTGGGATTTAGAAACCAATATAACAGTTGTAC

>Ap-mir-X40-3-5p
ATTGGTTTCTAGATCTCAGCA

```
ACAUCGGUUUUAUUGGUUUCUAGAUCUCAGCAUUUAAAUUGAUUGUUGCUGGGAUUUAGAAACCAAUAUAACAGUUGUAC
(((...(((.((((((((((((((((((((((.............)))))))))))))))))))))).)))...)))..

 Initial dG =    -36.60

          10        20        30
--|  UCG   U                      UUUAAA
  ACA   GUU UAUUGGUUUCUAGAUCUCAGCA      U
  UGU   CAA AUAACCAAAGAUUUAGGGUCGU      U
CA^  UGA   U                      UGUUAG
.        70        60        50        40

-----------------------------------------------------
```

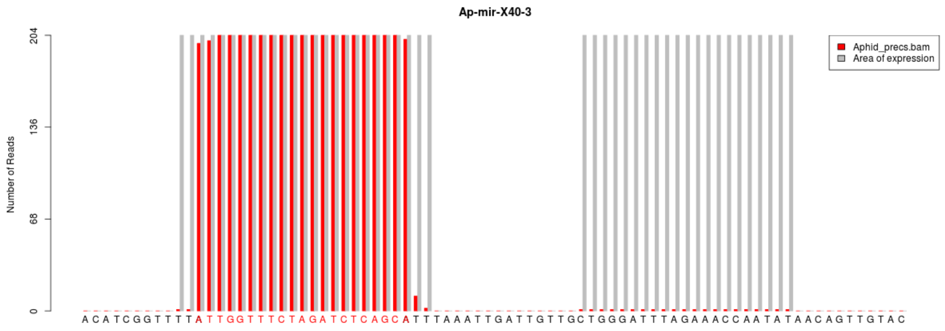

>Ap-mir-X40-4
ACATCTGTTTT**ATTGGTTTCTAGATCTCAGCA**TTTAAATTGATTGTTGCTGGGATTTAGAAAGCAATATAACAGTTGTGC

>Ap-mir-X40-4-5p
ATTGGTTTCTAGATCTCAGCA

```
ACAUCUGUUUUAUUGGUUUCUAGAUCUCAGCAUUUAAAUUGAUUGUUGCUGGGAUUUAGAAAGCAAUAUAACAGUUGUGC
(((.(((((.(((((.((((((((((((((((.............)))))))))))))))).))))).))))).)))..

 Initial dG =    -36.20

          10        20        30
--|  U     U     G                UUUAAA
  ACA CUGUU UAUUG UUUCUAGAUCUCAGCA      U
  UGU GACAA AUAAC AAAGAUUUAGGGUCGU      U
CG^  U     U     G                UGUUAG
.        70        60        50        40

-----------------------------------------------------
```

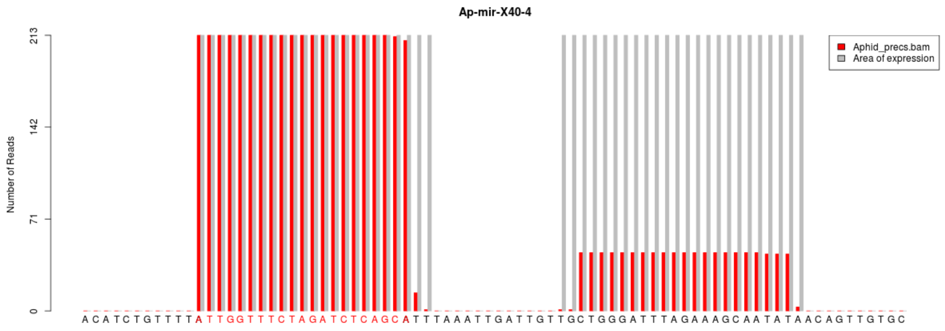

>Ap-mir-X40-5
CATCGGTTTT**ATTGGTTTCTAGATCTCAGCA**TTTAAATTGATTGTTGCTGGGATTTAGAAAGCAATATAACAGTTGTGC

>Ap-mir-X40-5-5p
ATTGGTTTCTAGATCTCAGCA

CAUCGGUUUU**AUUGGUUUCUAGAUCUCAGCA**UUUAAAUUGAUUGUUGCUGGGAUUUAGAAAGCAAUAUAACAGUUGUGC
((...(((.(((((.((((((((((((((((.............))))))))))))))))).))))).)))...))...

```
 Initial dG =    -30.30

           10        20        30
---| UCG   U     G                UUUAAA
   CA   GUU UAUUG UUUCUAGAUCUCAGCA      U
   GU   CAA AUAAC AAAGAUUUAGGGUCGU      U
CGU^ UGA   U     G                UGUUAG
        70        60        50        40
```

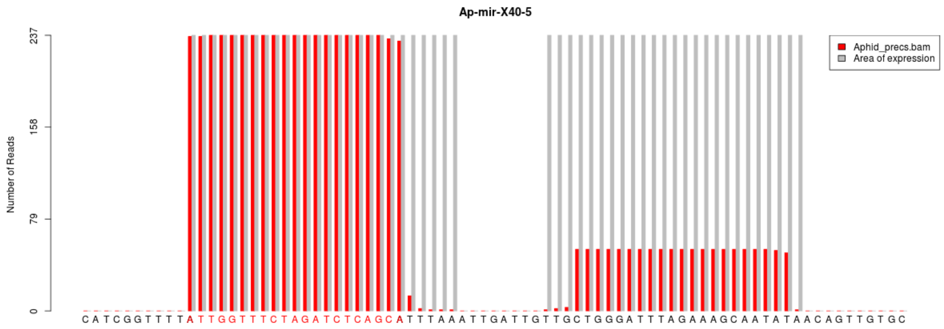

>Ap-mir-X41
TGAATAAATGGAAATTCTGAATGTTATTGTAGACATTCTATGATTTTAAATG**TTACAAAACATTCAGAATTTTG**TTTTATAGAGG

>Ap-mir-X41-3p
TTACAAAACATTCAGAATTTTG

UGAAUAAAUGGAAAUUCUGAAUGUUAUUGUAGACAUUCUAUGAUUUUAAAUG**UUACAAAACAUUCAGAAUUUUG**UUUUAUAGAGG
...(((((..(((((((((((((((.((((.((((((.((......)))))))))))))))))))))))))))..))))).....

```
 Initial dG =    -25.20

          10        20        30        40
UGA--|    UG              A    A      C   UG
     AUAAA  GAAAUUCUGAAUGUU UUGU GACAUU UA  A
     UAUUU  UUUUAAGACUUACAA AACA UUGUAA AU  U
GGAGA^    UG              -    -      -   UU
    80        70        60          50
```

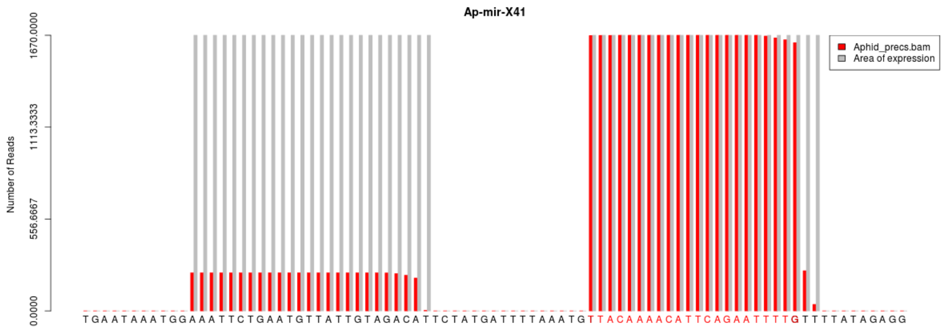

>Ap-mir-X43
TCCTCAAACCT**TGGGGTTTCAATAGGCATTTACC**CGACATTTAACGAGAGGCAATGCCCTTAGAAATCCCATGCGTAGAGGAGC

>Ap-mir-X43-5p
TGGGGTTTCAATAGGCATTTACC

```
UCCUCAAACCUUGGGGUUUCAAUAGGCAUUUACCCGACAUUUAACGAGAGGCAAUGCCCUUAGAAAUCCCAUGCGUAGAGGAGC
(((((......(((((((((....((((((...((..............)).))))))....)))))))))......)))))..
```

```
 Initial dG =    -27.30

          10        20        30        40
--|    AAACCU         AAUA      UAC   GACAUU
  UCCUC      UGGGGUUUC    GGCAUU   CC      U
  AGGAG      ACCCUAAAG    CCGUAA   GG      A
CG^    AUGCGU         AUUC      C--   AGAGCA
   80        70        60          50
```

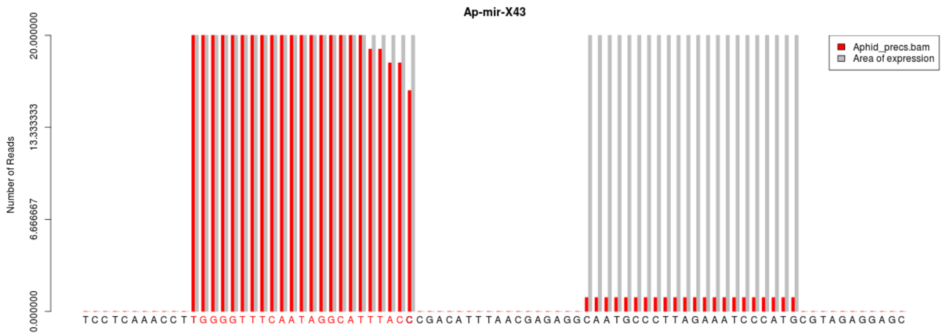

>Ap-mir-X44

CTAATACACAT**TCAGGTACCAAGTGATTTCTGA**GTAATTAAACAAACATCAGTAATCTCTGGGTATCTTAACTCTGTGTTAGA

>Ap-mir-X44-5p

TCAGGTACCAAGTGATTTCTGA

CUAAUACACAU**UCAGGUACCAAGUGAUUUCUGA**GUAAUUAAACAAACAUCAGUAAUCUCUGGGUAUCUUAACUCUGUGUUAGA

```
((((((((..((.(((((((.((.((((.((((((..........)).)))).)))).)).))))))).))...)))))))).
```

```
 Initial dG =    -25.10

         10          20         30          40
-        CA-  C       A  U    U    -| AAUU
 CUAAUACA   UU AGGUACC AG GAUU CUGA GU    A
 GAUUGUGU   AA UCUAUGG UC CUAA GACU CA    A
A        CUC  U       G  U    U    A^ AACA
  80        70         60         50
```

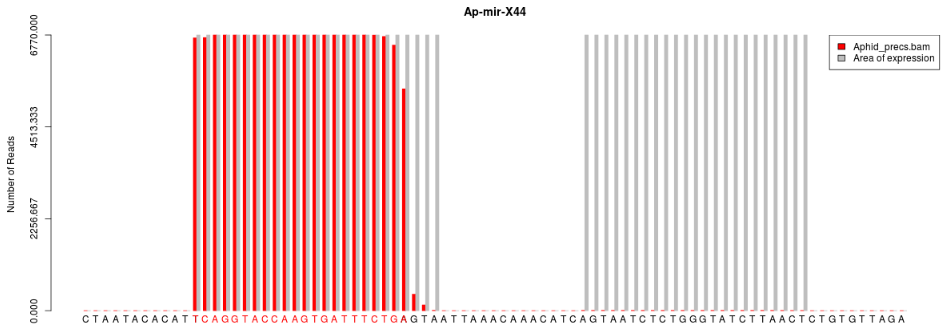

>Ap-mir-X45

CTGAGCGGGCCACCGTGTTTTCCCGGTTTTCTGTATCATCCATATCGTGG**AAAAACGTCAAAACACGGTGG**CCCACCCGTGG

>Ap-mir-X45-3p

AAAAACGTCAAAACACGGTGG

```
CUGAGCGGGCCACCGUGUUUUCCCGGUUUUCUGUAUCAUCCAUAUCGUGGAAAAACGUCAAAACACGGUGGCCCACCCGUGG
......(((((((((((((((..((.(((((..(............)..))))).))..)))))))))))))))........
```

```
 Initial dG =    -36.70

          10        20        30        40
CUGAGC--|              CC  G     UG AUCAU
        GGGCCACCGUGUUUU  CG UUUUC  U     C
        CCCGGUGGCACAAAA  GC AAAAG  G     C
GGUGCCCA^              CU  A     GU CUAUA
 80        70        60        50
```

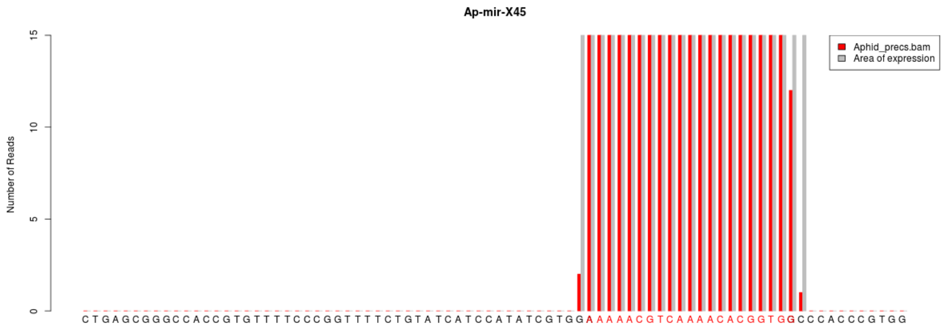

>Ap-mir-X47

GTAATGGGTTTTGGTAACTCCACCACCGATTGCGTTTTTAATTTTGTTCCG**CAGCCGGTGGTGACTGTTTCCACA**CGCTGTTACAA

>Ap-mir-X47-3p

CAGCCGGTGGTGACTGTTTCCACA

GUAAUGGGUUUUGGUAACUCCACCACCGAUUGCGUUUUUAAUUUUGUUCCG**CAGCCGGUGGUGACUGUUUCCACA**CGCUGUUACAA
((((((((..(((.(((..((((((((..((((...............))))..))))))))...))).))).)).)))))))..

```
 Initial dG =    -32.30

           10        20         30        40
--       -| UU   U   UC-        AU    UUUUUAA
  GUAAUGG GU  UGG AAC   CACCACCG  UGCG       \
  CAUUGUC CA  ACC UUG   GUGGUGGC  ACGC       U
AA       G^ C-   U   UCA        CG    CUUGUUU
     80         70        60        50
```

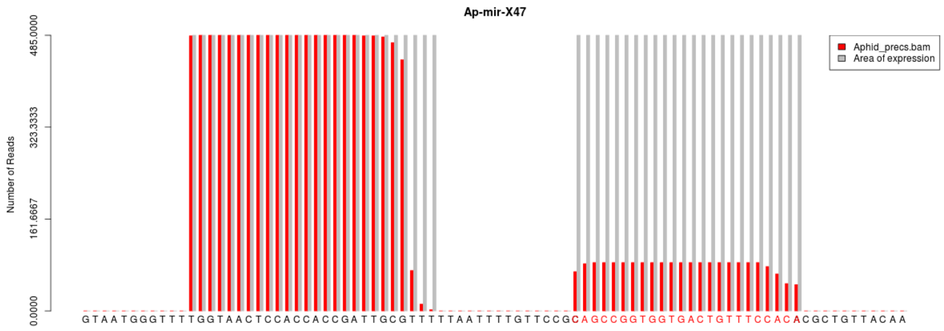

>Ap-mir-X48

GACGTACGTTTTCTTGTTCATCAAAGTTTTAGGTCGTACACGTCTATAGGAACAC**TTAAAGCTTTGATGACGGGATA**GTGTGCGCCGG

>Ap-mir-X48-3p

TTAAAGCTTTGATGACGGGATA

```
GACGUACGUUUUCUUGUUCAUCAAAGUUUUAGGUCGUACACGUCUAUAGGAACACUUAAAGCUUUGAUGACGGGAUAGUGUGCGCCGG
..((((((((.((((((.((((((((((((((((.((..............))))))))))))))))))))))))).)))))))))....

 Initial dG =    -32.20

          10        20        30        40
GA--|       U      U                 C   ACACGU
    CGUACGUU UCUUGU CAUCAAAGUUUUAGGU GU        C
    GCGUGUGA AGGGCA GUAGUUUCGAAAUUCA CA        U
GGCC^       U      -                 -   AGGAUA
       80        70         60         50

-----------------------------------------------------
```

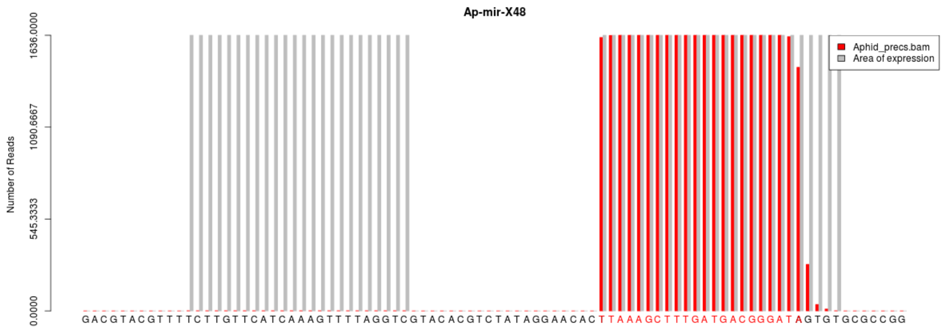

>Ap-mir-X49
TTGTCTGTTTG**GAGGGCAGATTATTTCTGATAC**GATTTTGACTTTTGACAGGTAACAGAGTAATTCTGTCCTTCTCACCGGCAATC

>Ap-mir-X49-5p
GAGGGCAGATTATTTCTGATAC

UUGUCUGUUUG**GAGGGCAGAUUAUUUCUGAUAC**GAUUUUGACUUUUGACAGGUAACAGAGUAAUUCUGUCCUUCUCACCGGCAAUC
(((((.((..((((((((((....(((((.(((..................))).)))))....))))))))))..)).)))))..

```
 Initial dG =    -28.30

          10        20        30        40
--|    U  UU          UUAU      A   GAUUUUGA
  UUGUC GU  GGAGGGCAGA    UUCUG UAC        C
  AACGG CA  CUUCCUGUCU    GAGAC AUG        U
CU^    C  CU          UAAU      A   GACAGUUU
     80        70        60        50
```

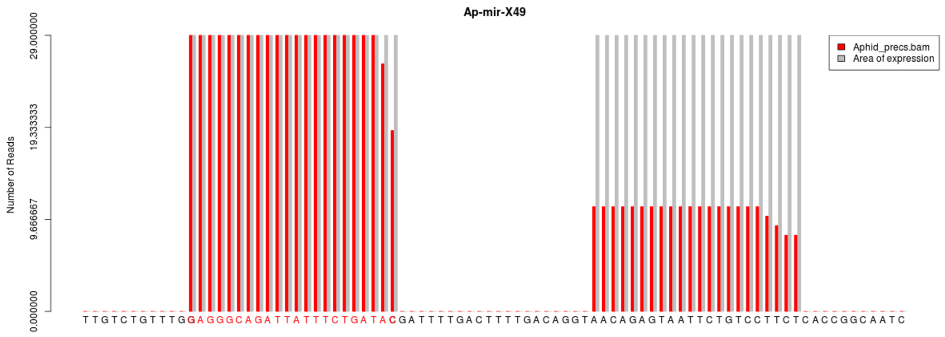

>Ap-mir-X5-1
TAGTTTTTTCTTCAGTATGTGTCCTTTTATGTGCTGTGCTATATTATCAAAA**CACATTAAGGACTCATATTGGAG**TAATGACCTAA

>Ap-mir-X5-1-3p
CACATTAAGGACTCATATTGGAG

```
UAGUUUUUUCUUCAGUAUGUGUCCUUUUAUGUGCUGUGCUAUAUUAUCAAAACACAUUAAGGACUCAUAUUGGAGUAAUGACCUAA
..(((....((((((((((.((((((..(((((...((.........))...))))).)))))).))))))))))....)))....

 Initial dG =    -27.00

          10        20        30        40
UA--|  UUUU          U      UU     CUG   CUAU
    GUU    CUUCAGUAUG GUCCUU  AUGUG   UG    \
    CAG    GAGGUUAUAC CAGGAA  UACAC   AC    A
AAUC^  UAAU          U      U-     AAA   UAUU
     80        70        60         50

-----------------------------------------------------
```

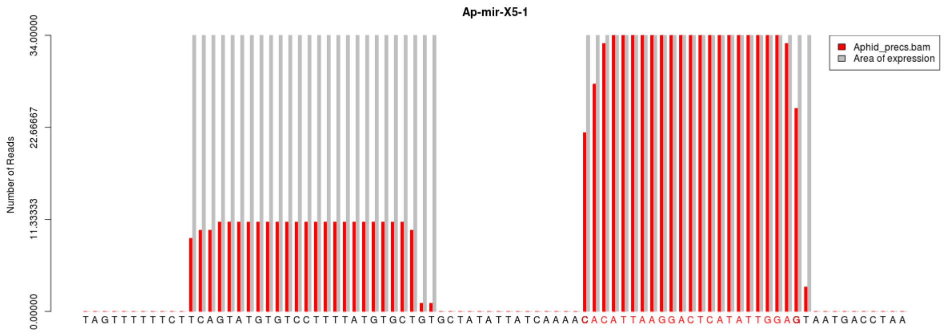

>Ap-mir-X5-2
TAGTTTTTTCTTCAGTATGTGTCCTTTTATGTGCTGTGCTATATTATCAAAA**CACATTAAGGACTCATATTGGAG**TAATGACCTTA

>Ap-mir-X5-2-3p
CACATTAAGGACTCATATTGGAG

```
UAGUUUUUUCUUCAGUAUGUGUCCUUUUAUGUGCUGUGCUAUAUUAUCAAAACACAUUAAGGACUCAUAUUGGAGUAAUGACCUUA
..(((....((((((((((.((((((..(((((...((.........))...))))).)))))).))))))))))....)))....

 Initial dG =    -27.00

          10        20        30        40
UA--|  UUUU          U      UU      CUG   CUAU
    GUU    CUUCAGUAUG GUCCUU  AUGUG   UG    \
    CAG    GAGGUUAUAC CAGGAA  UACAC   AC    A
AUUC^  UAAU          U      U-      AAA   UAUU
     80        70        60         50

-----------------------------------------------------
```

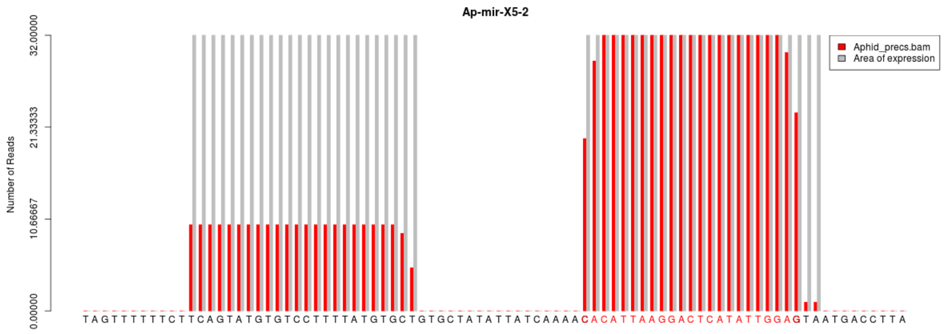

>Ap-mir-X5-3

AAGTTTTTTCTTCAGTATGTGTCCTTTTATGTGCTGTGCTATATTATCAAAA**CACATTAAGGACTCATATTGGAG**TAATGACCTTA

>Ap-mir-X5-3-3p

CACATTAAGGACTCATATTGGAG

AAGUUUUUUCUUCAGUAUGUGUCCUUUUAUGUGCUGUGCUAUAUUAUCAAAA**CACAUUAAGGACUCAUAUUGGAG**UAAUGACCUUA

```
..(((....((((((((((.((((((..(((((...((.........))...))))).)))))).))))))))))....)))....

 Initial dG =    -27.00

          10        20        30        40
AA--|  UUUU          U      UU      CUG   CUAU
    GUU    CUUCAGUAUG GUCCUU  AUGUG   UG    \
    CAG    GAGGUUAUAC CAGGAA  UACAC   AC    A
AUUC^  UAAU          U      U-      AAA   UAUU
     80        70        60         50

-----------------------------------------------------
```

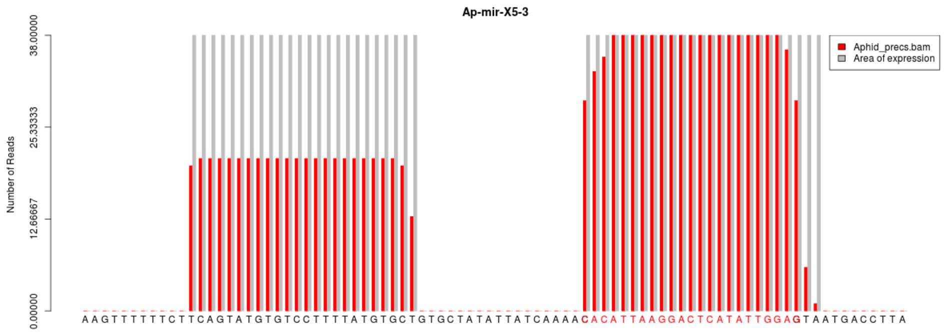

>Ap-mir-X51
TTTCTCCCGTA**TGAATTCTTATGTGAGCTTTGC**TAGGTTAACAAAACTCCTATTAGAATTTGTACAATTCATATGGGAGATAAA

>Ap-mir-X51-5p
TGAATTCTTATGTGAGCTTTGC

UUUCUCCCGUA**UGAAUUCUUAUGUGAGCUUUGC**UAGGUUAACAAAACUCCUAUUAGAAUUUGUACAAUUCAUAUGGGAGAUAAA
..(((((((((((((((....((..((.((((.((((...........)))).)))).))..)).)))))))))))))))....

```
 Initial dG =    -26.90

          10        20        30        40
UU--|               CUUA  UG   C    C    UUAAC
    UCUCCCGUAUGAAUU     UG  AG UUUG UAGG      \
    AGAGGGUAUACUUAA     AU  UU AGAU AUCC      A
AAAU^               C---  GU   A    U    UCAAA
   80        70           60        50
```

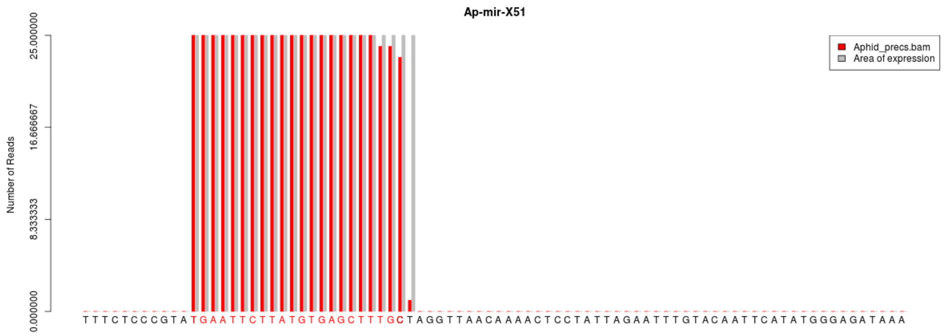

>Ap-mir-X52-1

CCGCGTCGGTGCCGTGTTCTGTGACTGACTCGGCTGGCAACGGACTGAGCCCTC**CGGTCTGTCACGGAACACGGGA**CCGGCCGCATC

>Ap-mir-X52-1-3p

CGGTCTGTCACGGAACACGGGA

```
CCGCGUCGGUGCCGUGUUCUGUGACUGACUCGGCUGGCAACGGACUGAGCCCUCCGGUCUGUCACGGAACACGGGACCGGCCGCAUC
..((((((((.((((((((((((((.(((.(((..(((..........)))..)))))).))))))))))))))).)))))).))...
```

```
 Initial dG =    -54.70

          10        20        30        40
CC-  -|     G              U   U   CU   AACG
   GC GUCGGU CCGUGUUCUGUGAC GAC CGG  GGC    G
   CG CGGCCA GGCACAAGGCACUG CUG GCC  CCG    A
CUA  C^     G              U   -   UC   AGUC
      80        70        60         50
```

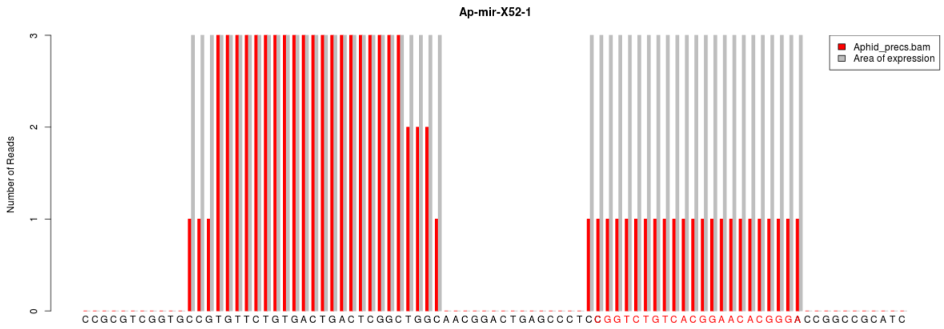

>Ap-mir-X52-2
CCGCGTCGGTGCCGTGTTCTGTGACTGACTCGGCTGGCAACGGACTGAGCCCTC**CGGTCTGTCACGGAACACGGGA**CCGGCCGCATC

>Ap-mir-X52-2-3p
CGGTCTGTCACGGAACACGGGA

```
CCGCGUCGGUGCCGUGUUCUGUGACUGACUCGGCUGGCAACGGACUGAGCCCUCCGGUCUGUCACGGAACACGGGACCGGCCGCAUC
..((((((((.((((((((((((((.(((.(((..(((..........)))..)))))).))))))))))))))).)))))).))...

 Initial dG =    -54.70

          10        20        30        40
CC-   -|     G              U   U   CU    AACG
   GC GUCGGU CCGUGUUCUGUGAC GAC CGG   GGC     G
   CG CGGCCA GGCACAAGGCACUG CUG GCC   CCG     A
CUA   C^     G              U   -   UC    AGUC
      80        70        60         50

-----------------------------------------------------
```

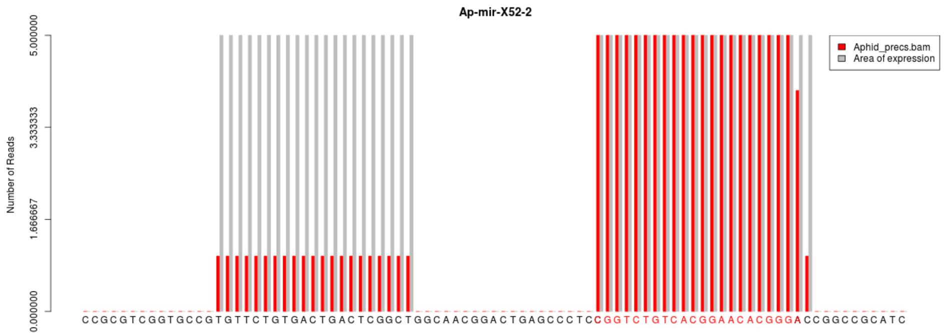

>Ap-mir-X52-3
ATTAGTCGGTGCCGTGTTCTGTGACTGACTCGGCTGGCGCCGGACTGGCGGGGGCCAGC**CGGTCTGTCACGGAACACGGGA**CCGGCCGCA
TC

>Ap-mir-X52-3-3p
CGGTCTGTCACGGAACACGGGA

AUUAGUCGGUGCCGUGUUCUGUGACUGACUCGGCUGGCGCCGGACUGGCGGGGGCCAGC**CGGUCUGUCACGGAACACGGGA**CCGGCCGCAUC
....((((((.((((((((((((((.(((.((((((((.((.........)).))))))))))).))))))))))))))).))))))......

```
 Initial dG =    -65.70

          10         20         30         40
AUUA--|      G              U   U         G  GGAC
      GUCGGU CCGUGUUCUGUGAC GAC CGGCUGGC CC    \
      CGGCCA GGCACAAGGCACUG CUG GCCGACCG GG    U
CUACGC^      G              U   -         G  GCGG
 90         80         70          60         50

-----------------------------------------------------
```

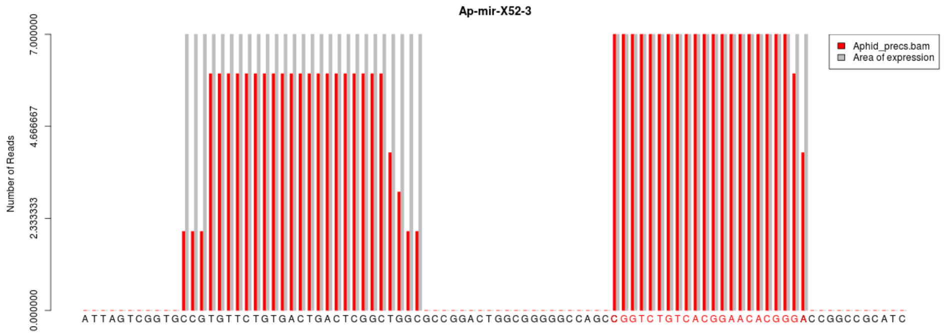

>Ap-mir-X54

TATAGCCTTTT**CAGAATAAGGGCACACTAGATTAACG**TACAAAACCAGTTTTGACCATATTCGCCTGGTGTGCTCTTATTCTGAATAGTC TATAGC

>Ap-mir-X54-5p

CAGAATAAGGGCACACTAGATTAACG

UAUAGCCUUUU**CAGAAUAAGGGCACACUAGAUUAACG**UACAAAACCAGUUUUGACCAUAUUCGCCUGGUGUGCUCUUAUUCUGAAUAGUCUAUAGC

```
(((((.((.(((((((((((((((((((((......((.(((((....))))))).........))))))))))))))))))))).)).)))))..
```

```
 Initial dG =    -40.30

          10        20        30           40
--     C  U                     AUUAAC---| A     C
  UAUAG CU UUCAGAAUAAGGGCACACUAG          GU CAAAA C
  AUAUC GA AAGUCUUAUUCUCGUGUGGUC          CA GUUUU A
CG     U  U                     CGCUUAUAC^ -     G
     90        80        70        60         50

-----------------------------------------------------
```

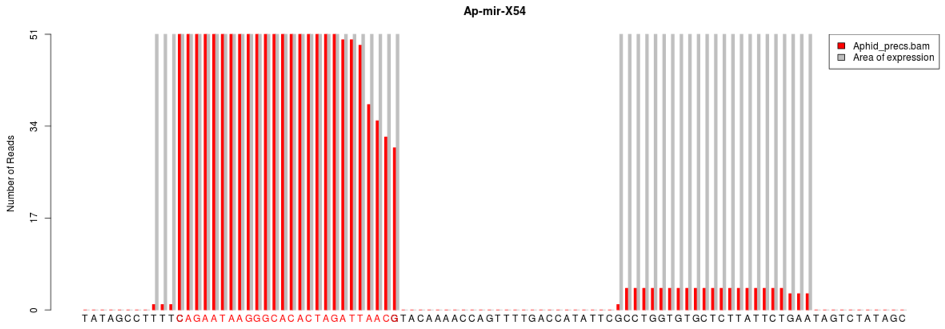

>Ap-mir-X55

ACATAGGTATGCGTTCTAACCACGGATTAAGATAATATAATATCTCGACGTA**TCTTAATTCGTGGTTATAGCAGT**ATACCATGTAT

>Ap-mir-X55-3p

TCTTAATTCGTGGTTATAGCAGT

```
ACAUAGGUAUGCGUUCUAACCACGGAUUAAGAUAAUAUAAUAUCUCGACGUAUCUUAAUUCGUGGUUAUAGCAGUAUACCAUGUAU
((((.((((((((((.((((((((((((((((((................)))))))))))))))))).))).))))))))))))..
```

```
 Initial dG =    -36.90

          10         20         30         40
--|   A       -   C                  AUAUAAU
  ACAU GGUAUGC GUU UAACCACGGAUUAAGAUA       A
  UGUA CCAUAUG CGA AUUGGUGCUUAAUUCUAU       U
UA^   -       A   U                  GCAGCUC
      80        70        60        50

-----------------------------------------------------
```

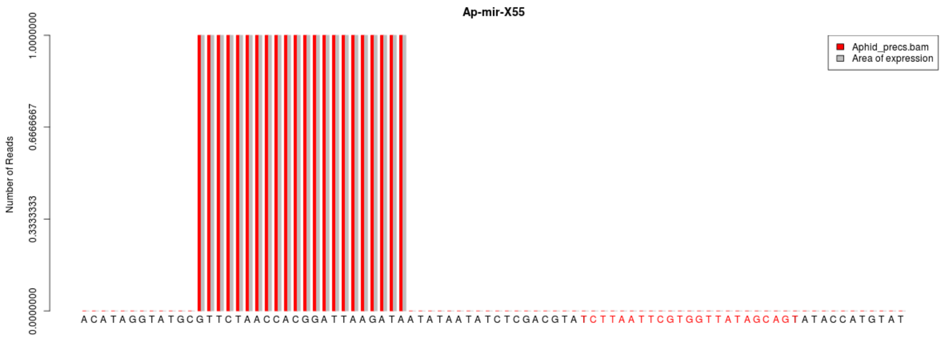

>Ap-mir-X56
TAATGTTGAAC**TTATAGATAATACAAGCATAT**AGTTTTTTTTTATTTTTTTTTCAAACGTATATGCTTGTACATATCTATATCTTCAATA
TTTTT

>Ap-mir-X56-5p
TTATAGATAATACAAGCATAT

UAAUGUUGAAC**UUAUAGAUAAUACAAGCAUAU**AGUUUUUUUUUAUUUUUUUUUCAAACGUAUAUGCUUGUACAUAUCUAUAUCUUCAAUAUUUUU
.((((((((..((((((((.((((((((((((((((.................)))).)))))))))))))..))))))))..)))))))))...

```
 Initial dG =    -30.30

          10        20         30         40
U--         CU        A-             -|    UUUUUUAU
   AAUGUUGAA  UAUAGAUA  UACAAGCAUAUA GUUU        \
   UUAUAACUU  AUAUCUAU  AUGUUCGUAUAU CAAA        U
UUU         CU        AC             G^    CUUUUUUU
    90        80        70        60        50

-----------------------------------------------------
```

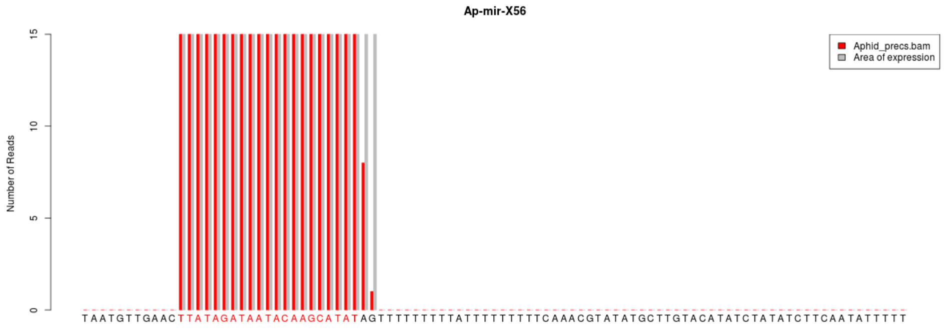

>Ap-mir-X57-1
CTATGGGTATTAGGGAGTTTTGAATGTTGTTGTAAAATATTTAAACAATA**CAAAACATTCAAAACTCCCTAC**TATCCTTAGGT

>Ap-mir-X57-1-3p
CAAAACATTCAAAACTCCCTAC

```
CUAUGGGUAUUAGGGAGUUUUGAAUGUUGUUGUAAAAUAUUUAAACAAUACAAAACAUUCAAAACUCCCUACUAUCCUUAGGU
(((.(((((.((((((((((((((((((.(((((..............))))))))))))))))))))))))).))))).)))..

 Initial dG =    -37.50

          10        20        30        40
--|  U     U                  G     AAAUAU
  CUA GGGUA UAGGGAGUUUUGAAUGUU UUGUA      U
  GAU CCUAU AUCCCUCAAAACUUACAA AACAU      U
UG^  U     C                  -     AACAAA
  80        70        60         50

-----------------------------------------------------
```

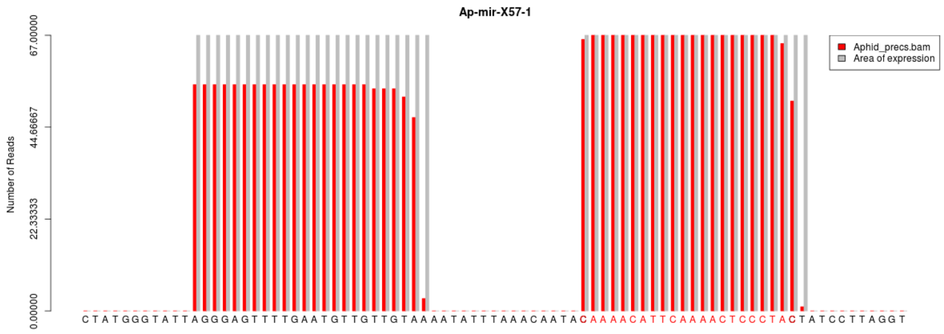

>Ap-mir-X57-2
CTATGGGTATTGGGGAGTTTTGAATGTTGTTGTATAATATTTAAACAATT**CAAAACATTCAAAACTCCCTAC**TATCCTTAGGT

>Ap-mir-X57-2-3p
CAAAACATTCAAAACTCCCTAC

```
CUAUGGGUAUUGGGGAGUUUUGAAUGUUGUUGUAUAAUAUUUAAACAAUUCAAAACAUUCAAAACUCCCUACUAUCCUUAGGU
(((.(((((.(((((((((((((((((((((((.(((...))).)))))....)))))))))))))))))).))))).)))..

 Initial dG =    -36.50

          10        20             30
--   U     U                  ----|    A   U
  CUA GGGUA UGGGGAGUUUUGAAUGUU    GUUGU UAA \
  GAU CCUAU AUCCCUCAAAACUUACAA    UAACA AUU A
UG   U     C                  AACU^    A   U
  80        70        60        50        40

-----------------------------------------------------
```

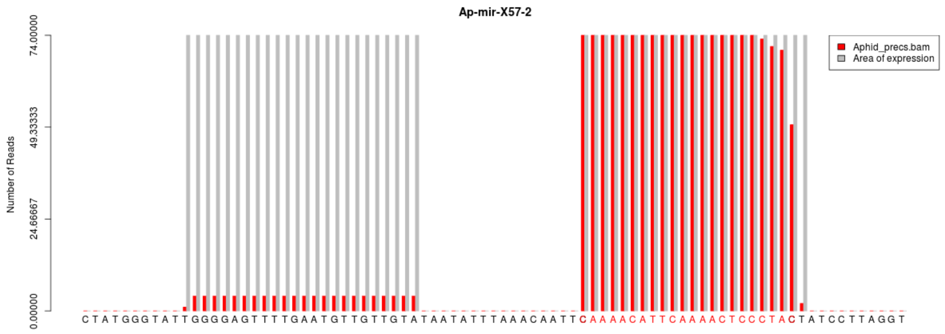

>Ap-mir-X58
GTATTGACTTA**TACAGTTGTACGCTAATAAATT**GTATTTATTTTATAGTAATTTATTAGCGTATAACTGTGTAAGTCAATACCA

>Ap-mir-X58-5p
TACAGTTGTACGCTAATAAATT

GUAUUGACUUA**UACAGUUGUACGCUAAUAAAUU**GUAUUUAUUUUAUAGUAAUUUAUUAGCGUAUAACUGUGUAAGUCAAUACCA
```
((((((((((((((((((((((((((((((((((((((.......))).)))))))))))))))))))))))))))))))))))))..
```

```
 Initial dG =    -47.30

          10         20         30          40
--                                   -|   UUA
  GUAUUGACUUAUACAGUUGUACGCUAAUAAAUUG UAU    \
  CAUAACUGAAUGUGUCAAUAUGCGAUUAUUUAAU AUA    U
AC                                   G^   UUU
   80         70         60         50
```

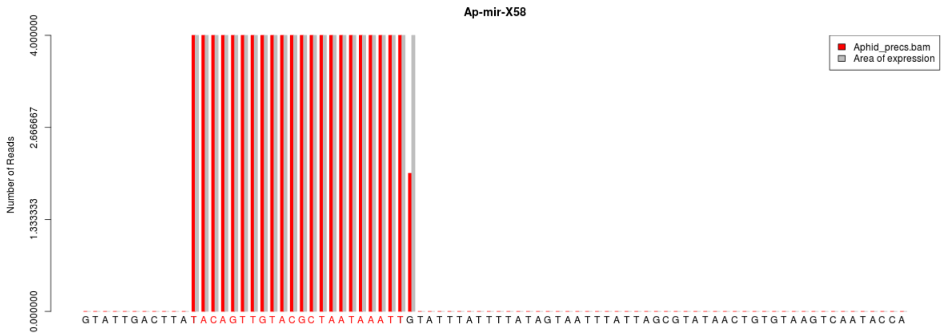

>Ap-mir-X59

GATTCAAGCTG**TGGTAACTCCAAACCATTGCCGG**CGTTTTATTTTGTATCCCGCAATGGTTGGAAGTTCCTCACTTTGGTCACG

>Ap-mir-X59-5p

TGGTAACTCCAAACCATTGCCGG

```
GAUUCAAGCUGUGGUAACUCCAAACCAUUGCCGGCGUUUUAUUUUGUAUCCCGCAAUGGUUGGAAGUUCCUCACUUUGGUCACG
(((..(((.((.((.((((((.(((((((((.((((........))...)).))))))))))))).))))).))))).)))...

 Initial dG =    -28.20

           10         20        30            40
---   UC   C  U  U   -   A         C  ---| UUU
   GAU  AAG UG GG AAC UCC AACCAUUGC GG   CG   U
   CUG  UUC AC CC UUG AGG UUGGUAACG CC   GU   A
GCA   GU   -  U  -   A   -         C  UAU^ UUU
   80         70          60        50

-----------------------------------------------------
```

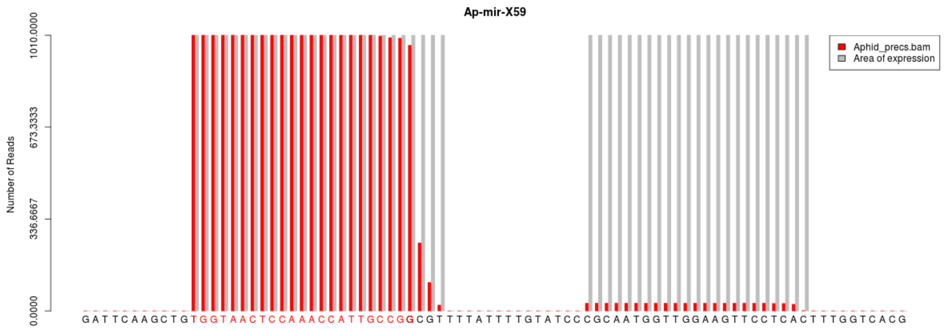

>Ap-mir-X60

CTGTACACATTGAGAGCATGACCAGTGTGCCATATAAAACCTTTTTCTCA**TGTGAACTGGTCATGCTCTTAA**TGTGCGATAGA

>Ap-mir-X60-3p

TGTGAACTGGTCATGCTCTTAA

```
CUGUACACAUUGAGAGCAUGACCAGUGUGCCAUAUAAAACCUUUUUCUCAUGUGAACUGGUCAUGCUCUUAAUGUGCGAUAGA
((((.((((((((((((((((((((....(((((.............))))).))))))))))))))))))))..)))).

 Initial dG =    -38.50

          10        20        30        40
-    A-|                    GUGC      AAAACC
 CUGU  CACAUUGAGAGCAUGACCAGU    CAUAU      U
 GAUA  GUGUAAUUCUCGUACUGGUCA    GUGUA      U
A    GC^                    A---      CUCUUU
  80        70        60          50

-----------------------------------------------------
```

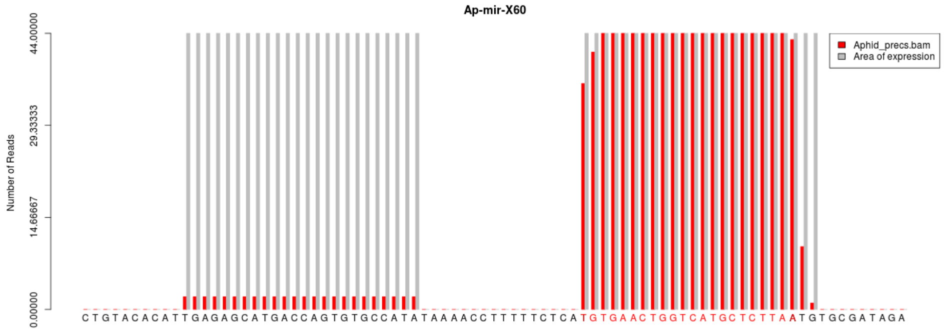

>Ap-mir-X61
GAGGAACGGAA**TCGGGAAGGCAGCTGCGGCGGACT**GTGCAGGTTCGTGCCCAACATTCCAGTCCGTCCAACTCCTTTCCGTTTTCGAACG
ACGC

>Ap-mir-X61-5p
TCGGGAAGGCAGCTGCGGCGGACT

GAGGAACGGAA**UCGGGAAGGCAGCUGCGGCGGACU**GUGCAGGUUCGUGCCCAACAUUCCAGUCCGUCCAACUCCUUUCCGUUUUCGAACGACGC
```
......(((((.((((((((.((.((.(((((((((((..(((....)))...))...))))))))))).))))))))))).))))).......
```

```
 Initial dG =    -38.30

          10        20        30           40
GAGGAA--     U        C  C  C         ---  CA-|  U
        CGGAA CGGGAAGG AG UG GGCGGACUG   UG   GGU C
        GCUUU GCCUUUCC UC AC CUGCCUGAC   AC   CCG G
CGCAGCAA     U        -  A  -         CUU  AAC^  U
   90        80         70         60        50
```

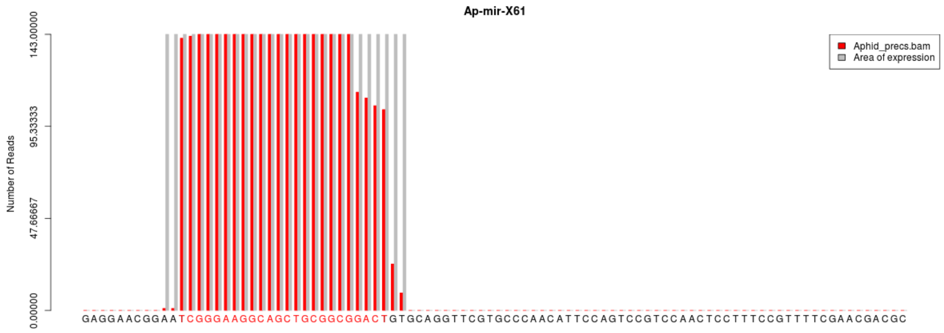

>Ap-mir-X62

TTGCGAGAGAC**TGATCGGTATTTGTTTTTGTCGTAGT**AGCGTATTTGTTGTACGGCATTAGTGATGATGGACGGTGTAATATAAGT

>Ap-mir-X62-5p

TGATCGGTATTTGTTTTTGTCGTAGT

UUGCGAGAGAC**UGAUCGGUAUUUGUUUUUGUCGUAGU**AGCGUAUUUGUUGUACGGCAUUAGUGAUGAUGGACGGUGUAAUAUAAGU

.........((((.((.((..(..((..(((((((.(((((....))))))))))))..))..)..)).))))))...........

```
 Initial dG =    -20.00

          10        20        30        40
UUGCGAGAG--|   A  G  AU UG  UU       G     U
           ACUG UC GU  U  UU  UGUCGUA UAGCG A
           UGGC AG UA  A  GA  ACGGCAU GUUGU U
UGAAUAUAAUG^   -  G  GU GU  UU       -     U
     80         70        60         50
```

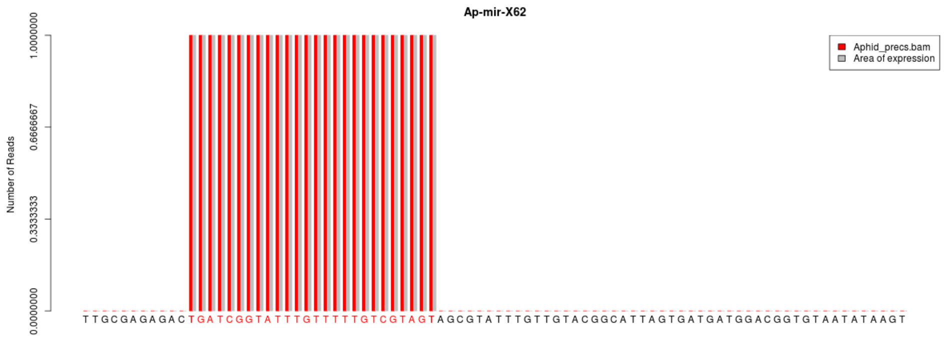

>Ap-mir-X63
TGATGTGGGCACCATGAATTGTTTCTAGTTGGTATTTGGTACTCATAATTAAACTCTA**TCACTGGTGACAATTCATGAAG**CATGCATTTT
A

>Ap-mir-X63-3p
TCACTGGTGACAATTCATGAAG

UGAUGUGGGCACCAUGAAUUGUUUCUAGUUGGUAUUUGGUACUCAUAAUUAAACUCUA**UCACUGGUGACAAUUCAUGAAG**CAUGCAUUUUA
.(((((..((..(((((((((((.((((((((..((((((.......))))))..)))..))))).)))))))))))..))..)))))...

Initial dG = -28.00

```
          10        20          30        40
U--      GG  AC           U     --|  UA      ACU
   GAUGU  GC  CAUGAAUUGUU CUAGU  UGG  UUUGGU   \
   UUACG  CG  GUACUUAACAG GGUCA  AUC  AAAUUA   C
AUU      UA  AA           U     CU^  UC      AUA
90        80        70        60        50
```

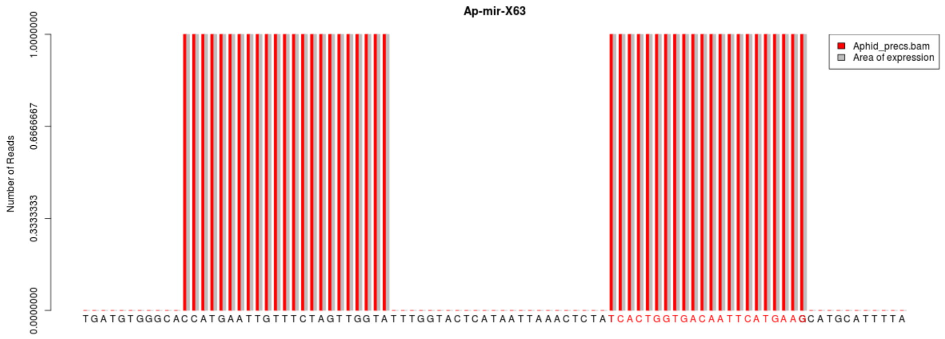

>Ap-mir-X64
GTCGGTAAAGCAGTTGTTTCAACCGGTCTTCAGCGACTATAAAACAATGTTT**GTAGGCCGGCGGAAACTACTAG**CTTCGCTGATC

>Ap-mir-X64-3p
GTAGGCCGGCGGAAACTACTAG

```
GUCGGUAAAGCAGUUGUUUCAACCGGUCUUCAGCGACUAUAAAACAAUGUUUGUAGGCCGGCGGAAACUACUAGCUUCGCUGAUC
(((((..(((((((.(((((..(((((((...((((.(((......))).)))))))))))..))))).))).)))))..))))).
```

```
 Initial dG =    -35.00

         10         20        30        40
-     UA    -|  U     AA       UCA     C    AA
 GUCGG  AAGC AGU GUUUC  CCGGUCU   GCGA UAU   A
 UAGUC  UUCG UCA CAAAG  GGCCGGA   UGUU GUA   A
C     GC    A^  U     GC       ---     U    AC
    80        70        60           50
```

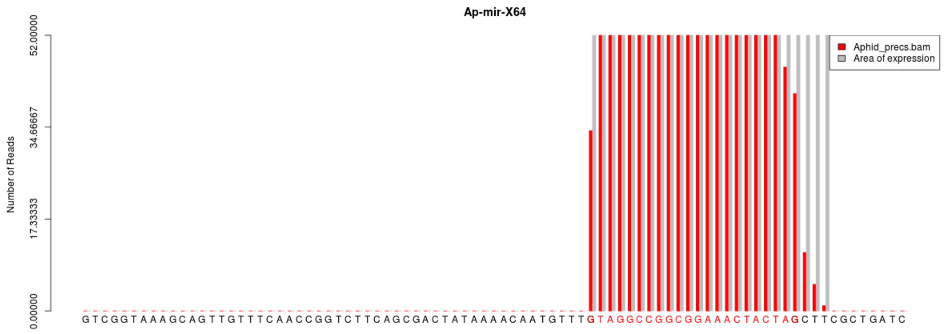

>Ap-mir-X66
TTGTTATTGAG**TGAGATCTTGATAAACTCGCCT**GTGAATTTTTAAAACTAGGCGAGGATATAAAGATCTTATTTGGTACTCATT

>Ap-mir-X66-5p
TGAGATCTTGATAAACTCGCCT

UUGUUAUUGAG**UGAGAUCUUGAUAAACUCGCCU**GUGAAUUUUUAAAACUAGGCGAGGAUAUAAAGAUCUUAUUUGGUACUCAUU
```
.((.(((..(((((((((((.(((..(((((((((...........)).)))))))..))).)))))))))))..)))..))..

 Initial dG =    -32.80

          10         20         30          40
U-  U-   UG          G   AA       -| GAAUU
  UG  UAU  AGUGAGAUCUU AUA  CUCGCCU GU     \
  AC  AUG  UUAUUCUAGAA UAU  GAGCGGA CA     U
UU  UC   GU          A   AG       U^ AAAUU
   80         70         60         50

-----------------------------------------------------
```

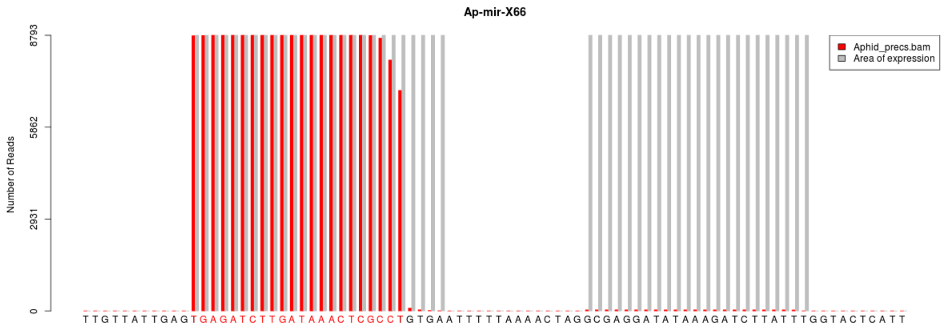

>Ap-mir-X67
TAGGCTCTCATTCGCTGTCTCGCTCAATCGCTCGTCGATCGCAATAACATTCTCGA**TGAGCGGATGAGTAGAGGCGTAG**AGCAGCAGAGC

>Ap-mir-X67-3p
TGAGCGGATGAGTAGAGGCGTAG

UAGGCUCUCAUUCGCUGUCUCGCUCAAUCGCUCGUCGAUCGCAAUAACAUUCUCGA**UGAGCGGAUGAGUAGAGGCGUAG**AGCAGCAGAGC
...((((((...((((.(((.(((((.(((((((((((.............))))))))))).)))))))))))))..))).))).....

Initial dG = -37.70

```
           10        20        30        40
UAG--   -|  AUU    G   C     A           UCGCAA
     GCU CUC   CGCU UCU GCUCA UCGCUCGUCGA      U
     CGA GAG   GCGG AGA UGAGU GGCGAGUAGCU      A
CGAGA   C^  AU-    -   -     A           CUUACA
.        80          70         60         50
```

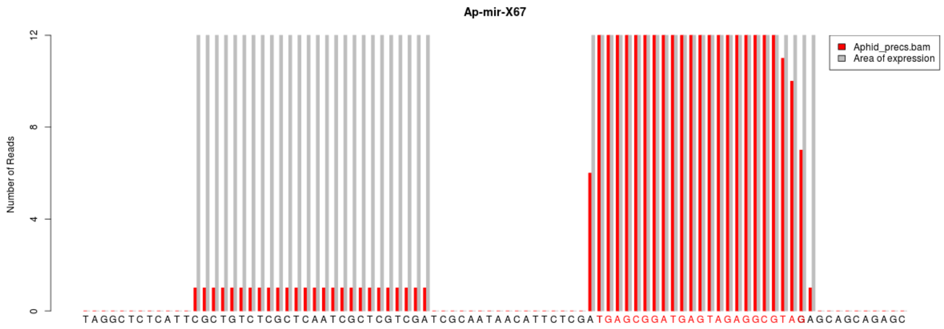

>Ap-mir-X68-1

TTTCATTTTTTAAGGAACGTTAAAAACCATTGTGTATATGTTTAAATCCTTAAATGTTAAAACAACAATGGTGTTTTTCGTTTCTATAAA
TGATGTTAA

>Ap-mir-X68-1-5p

AAGGAACGTTAAAAACCATTGT

```
UUUCAUUUUUUAAGGAACGUUAAAAACCAUUGUGUAUAUGUUUAAAUCCUUAAAUGUUAAAACAACAAUGGUGUUUUUCGUUUCUAUAAAUGAUGUUAA
..((((((....(((((((..(((.((((((((((..((((((((....))))))))....))).))))))).))).))))))))..))))))......

 Initial dG =    -26.30

            10        20        30            40
UU----      UUUA        UU    A        -   AU--|       A
      UCAUUU    AGGAACG  AAA ACCAUUG UGU    AUGUUUAA U
      AGUAAA    UCUUUGC  UUU UGGUAAC ACA    UGUAAAUU C
AAUUGU      UA--        UU    G        A   AAAU^       C
        90          80        70        60        50

-----------------------------------------------------
```

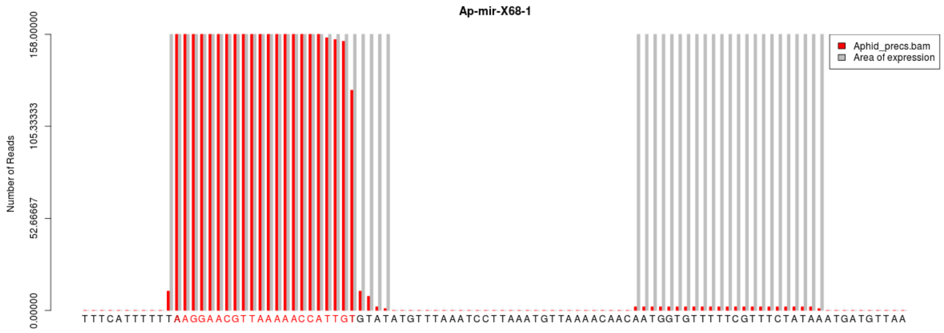

>Ap-mir-X68-2
TTTCATTTTTTAAGGAACGTTAAAAACCATTGTGTATATGTTTGAATCCTTAACTTTTACAACGACAATGGTGTTTTTCGTTTCTATAAA
TGATGTTAA

>Ap-mir-X68-2-5p
AAGGAACGTTAAAAACCATTGT

UUUCAUUUUUUAAGGAACGUUAAAAACCAUUGUGUAUAUGUUUGAAUCCUUAACUUUUACAACGACAAUGGUGUUUUUCGUUUCUAUAAAUGAUGUUAA
..((((((....(((((((..(((.(((((((((((...(((.........)))...)))....)))))))).))).)))))))..))))))......

```
 Initial dG =    -23.70

            10        20        30            40
UU----      UUUA       UU   A        ----|  UAU   UGAA
      UCAUUU    AGGAACG  AAA ACCAUUGU    GUA   GUU    \
      AGUAAA    UCUUUGC  UUU UGGUAACA    CAU   CAA    U
AAUUGU      UA--       UU   G        GCAA^  UUU   UUCC
        90          80        70        60        50
```

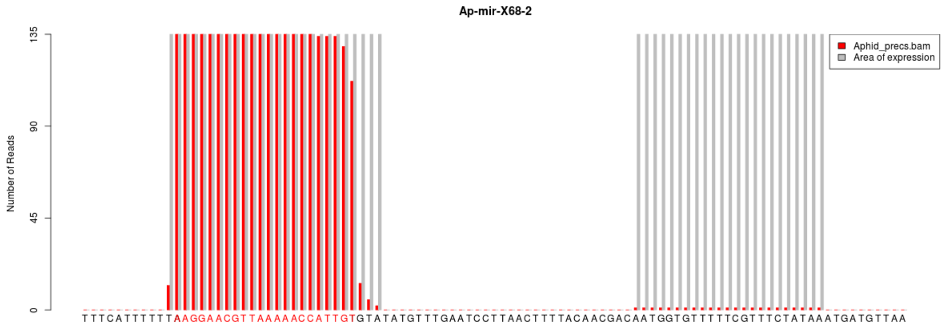

>Ap-mir-X69
CGCGTTCTCGGCACATCATGATTCTCGTGAAGAGGGAGTTTACTCTCTCCCA**TGCATTCTTTGAGCTTAGCATGATGGCT**GCTGTGTTGTC

>Ap-mir-X69-3p
TGCATTCTTTGAGCTTAGCATGATGGCT

```
CGCGUUCUCGGCACAUCAUGAUUCUCGUGAAGAGGGAGUUUACUCUCUCCCAUGCAUUCUUUGAGCUUAGCAUGAUGGCUGCUGUGUUGUC
((((....((((.(((((((...((((.((((((((((....)))))))).......)))..)))).....))))))))))).)))).....

 Initial dG =    -28.00

             10        20           30               40
-----    UUCU    A       AUU--    U-   -------|      U
     CGCG    CGGC CAUCAUG     CUCG  GAA       GAGGGAG U
     GUGU    GUCG GUAGUAC     GAGU  CUU       CUCUCUC U
CUGUU    C---    -       GAUUC    UU   ACGUACC^      A
90           80         70        60        50

-----------------------------------------------------
```

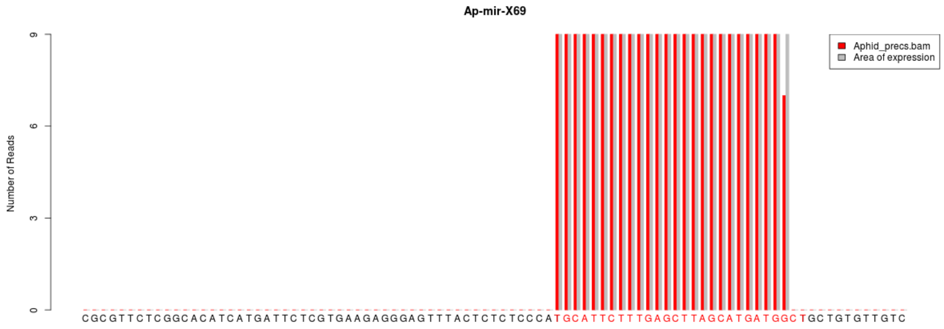

>Ap-mir-X70-1
CACTTAGAGCTGTTCTTACAATTGCCGGTTAAGTGTCGATTAACTTT**AACCAGATGTTGTAAGAACGA**CCCTTAATGTT

>Ap-mir-X70-1-3p
AACCAGATGTTGTAAGAACGA

CACUUAGAGCUGUUCUUACAAUUGCCGGUUAAGUGUCGAUUAACUUU**AACCAGAUGUUGUAAGAACGA**CCCUUAAUGUU
```
......(((.((((((((((((....((((((((........)))).))))....)))))))))))).....))).......
```

```
 Initial dG =    -20.90

        10          20         30
CACUUA   C--            UGCC    -|   GUC
      GAG   UGUUCUUACAAU    GGUU AAGU   G
      UUC   GCAAGAAUGUUG    CCAA UUCA   A
UUGUAA   CCA            UAGA    U^   AUU
        70         60         50         40
```

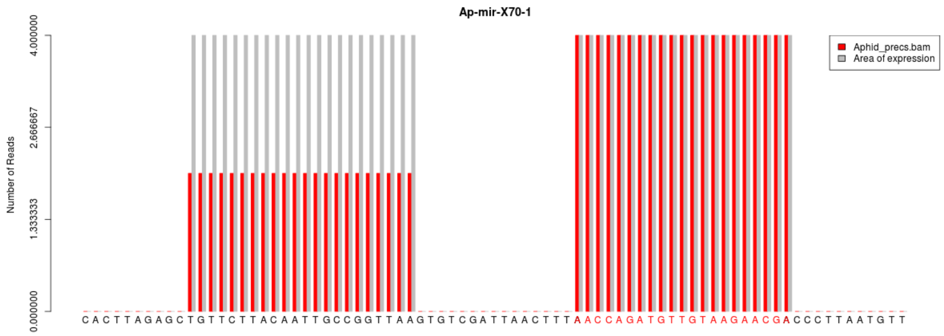

>Ap-mir-X70-2

CACTTAGAGCTGTTCTTACAATTGCCGGTTAAGTGTCGATTAACTTT**AACCAGATGTTGTAAGAACGA**CCCTTAATGTT

>Ap-mir-X70-2-3p

AACCAGATGTTGTAAGAACGA

```
CACUUAGAGCUGUUCUUACAAUUGCCGGUUAAGUGUCGAUUAACUUUAACCAGAUGUUGUAAGAACGACCCUUAAUGUU
......(((.((((((((((((....((((((((........)))).)))).....)))))))))))).....))).......
```

```
 Initial dG =    -20.90

        10          20         30
CACUUA   C--            UGCC    -|   GUC
      GAG   UGUUCUUACAAU    GGUU AAGU   G
      UUC   GCAAGAAUGUUG    CCAA UUCA   A
UUGUAA   CCA            UAGA    U^   AUU
        70         60         50         40

-----------------------------------------------------
```

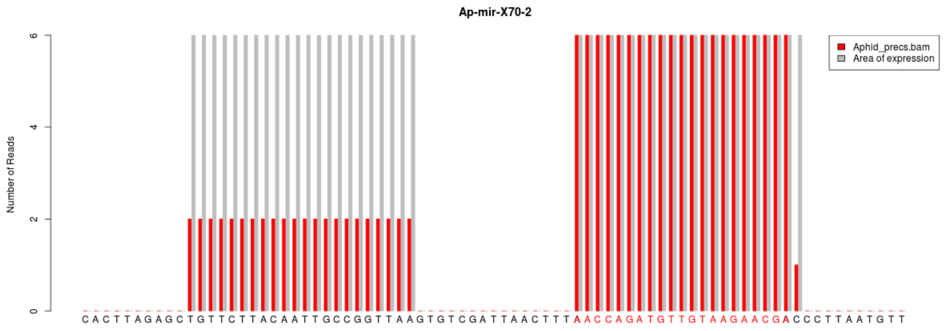

>Ap-mir-X71

GTCATACATCAAGGTAACTTATTCACTGTGCGAGTGTTGTGTTTGAAAATC**CCACGGTTGAATAAGGTACCATA**ATGTATGATGA

>Ap-mir-X71-3p

CCACGGTTGAATAAGGTACCATA

GUCAUACAUCAAGGUAACUUAUUCACUGUGCGAGUGUUGUGUUUGAAAAUC**CCACGGUUGAAUAAGGUACCAUA**AUGUAUGAUGA

```
((((((((...((((.(((((((((((((.((...((......))...)).)))))).))))))).)))))...)))))))))..

 Initial dG =    -31.20

          10          20          30         40
--         CAA    A       -|     C  GUG  GU
  GUCAUACAU   GGUA CUUAUUC ACUGUG GA   UU  G
  UAGUAUGUA   CCAU GAAUAAG UGGCAC CU   AG  U
AG         AUA    G       U^     C  AAA  UU
    80        70        60        50

-----------------------------------------------------
```

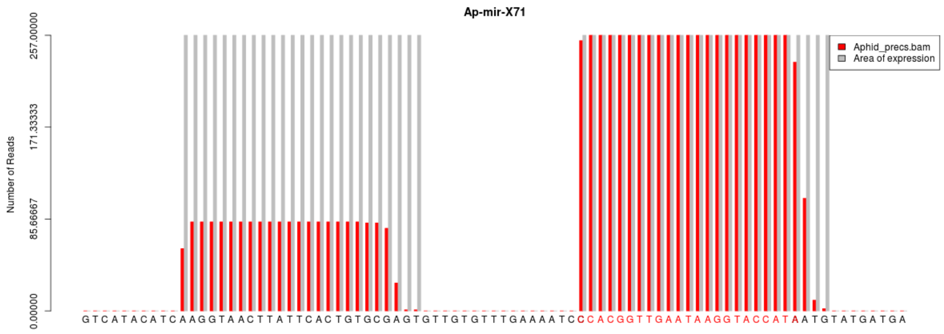

>Ap-mir-X72

GATGGACGGTT**TGGGGCATGTGTAGAGGAATGCAG**CTATAACTGCTGCGTCTTGCACATGTTCTCGACCATCTACCCT

>Ap-mir-X72-5p

TGGGGCATGTGTAGAGGAATGCAG

```
GAUGGACGGUUUGGGGCAUGUGUAGAGGAAUGCAGCUAUAACUGCUGCGUCUUGCACAUGUUCUCGACCAUCUACCCU
..((((.((((.((((((((((((..(((.((((((.......)))))))))))))))))))))).)))).))))....

 Initial dG =    -36.30

          10        20        30
GA--|   C    U            GA   A      UAU
    UGGA GGUU GGGGCAUGUGUA  GGA UGCAGC   \
    AUCU CCAG UCUUGUACACGU  UCU GCGUCG   A
UCCC^   A    C            --   -      UCA
       70        60          50
```

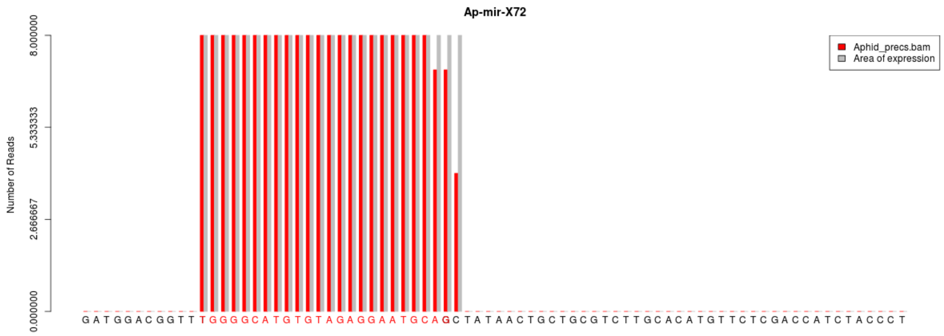

>Ap-mir-X73

CATTCATTTCGTCGTGGTAGTCTGCTGTGTTTTTTTTGGAC**TCAAAACACAGTAGACCACCCTCGACG**AAACCCTCTGC

>Ap-mir-X73-3p

TCAAAACACAGTAGACCACCCTCGACG

```
CAUUCAUUUCGUCGUGGUAGUCUGCUGUGUUUUUUUUGGACUCAAAACACAGUAGACCACCCUCGACGAAACCCUCUGC
......((((((((.(((.((((((((((((((..........)))))))))))))).)))..))))))))........
```

Initial dG = -34.30

```
          10          20         30
CAUUCA--        U-|  A              UUUU
        UUUCGUCG  GGU GUCUGCUGUGUUUU    G
        AAAGCAGC  CCA CAGAUGACACAAAA    G
CGUCUCCC        UC^  C              CUCA
        70         60         50         40
```

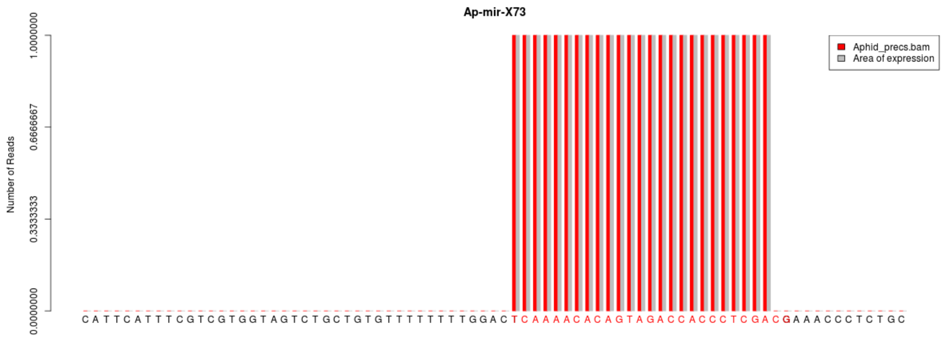

>Ap-mir-X74
TATACTCTATTCAGAATAAAAGCAGACCAGATTTATGTAGCAAATCCAGTTTTGACCGTGGCC**GCCTGGTGTGCTCTTATTCTGAA**TAAA
GTATATT

>Ap-mir-X74-3p
GCCTGGTGTGCTCTTATTCTGAA

UAUACUCUAUUCAGAAUAAAAGCAGACCAGAUUUAUGUAGCAAAUCCAGUUUUGACCGUGGCC**GCCUGGUGUGCUCUUAUUCUGAA**UAAAGUAUAUU
((((((.((((((((((((.((((.(((((..(((((...((((......))))..)))))....))))).)))).))))))))))))).))))))..

```
 Initial dG =    -37.90

          10        20        30          40
--      C            A    G     AU--|     UAG     UC
  UAUACU UAUUCAGAAUAA AGCA ACCAG     UUAUG    CAAA  C
  AUAUGA AUAAGUCUUAUU UCGU UGGUC     GGUGC    GUUU  A
UU      A            C    G     CGCC^     CA-     UG
      90        80        70        60          50
```

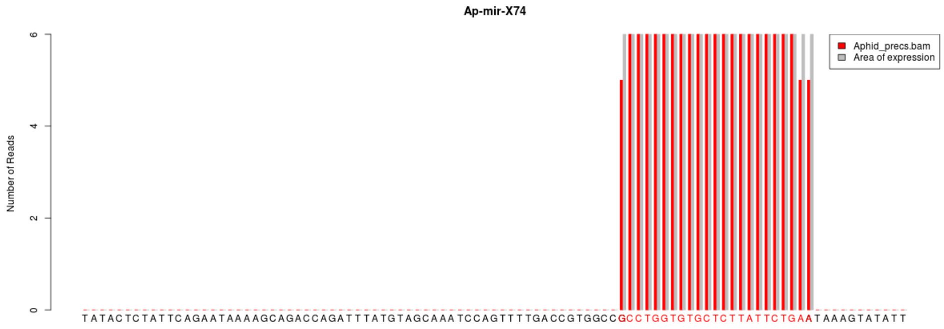

>Ap-mir-X76
CATTATTACTA**AAGTAGCTAATTGGGAAGCTGT**TGCAAATGATAAACACAGCTTCTTAATTAACTAATTTGGAAATAATGGA

>Ap-mir-X76-5p
AAGTAGCTAATTGGGAAGCTGT

```
CAUUAUUACUAAAGUAGCUAAUUGGGAAGCUGUUGCAAAUGAUAAACACAGCUUCUUAAUUAACUAAUUUGGAAAUAAUGGA
((((((.(((((.(((.((((((((((((((((.............)))))))))))))))).))).))))).)))))))..

 Initial dG =    -29.30

          10        20        30        40
--|      A     G   C               UGCAAA
  CAUUAUU CUAAA UAG UAAUUGGGAAGCUGU      U
  GUAAUAA GGUUU AUC AUUAAUUCUUCGACA      G
AG^      A     A   A               CAAAUA
 80        70        60        50

-----------------------------------------------------
```

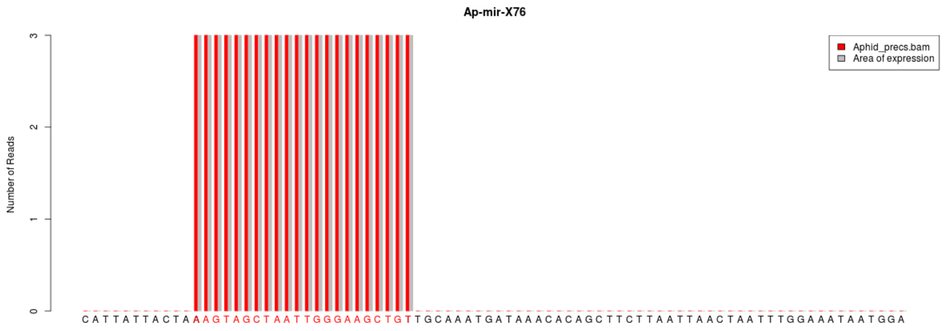

>Ap-mir-X77
TGTGTAAATAT**TACACATTGGGGTAAATTGACGA**TCGTCATCTTACCCGCGTCAATTTACCCCAACTGGTAATATTTTACATAA

>Ap-mir-X77-5p
TACACATTGGGGTAAATTGACGA

UGUGUAAAUAU**UACACAUUGGGGUAAAUUGACGA**UCGUCAUCUUACCCGCGUCAAUUUACCCCAACUGGUAAUAUUUUACAUAA
((((((((((((((.((((((((((((((((((...((......))...)))))))))))))))).))))))))))).))))).

Initial dG =   -38.30

```
          10          20         30         40
-     -|        A  -                AUC  CA
 UGUGU AAAUAUUAC CA UUGGGGUAAAUUGACG   GU  U
 AUACA UUUAUAAUG GU AACCCCAUUUAACUGC   CA  C
A     U^        -  C                GCC  UU
   80        70         60        50
```

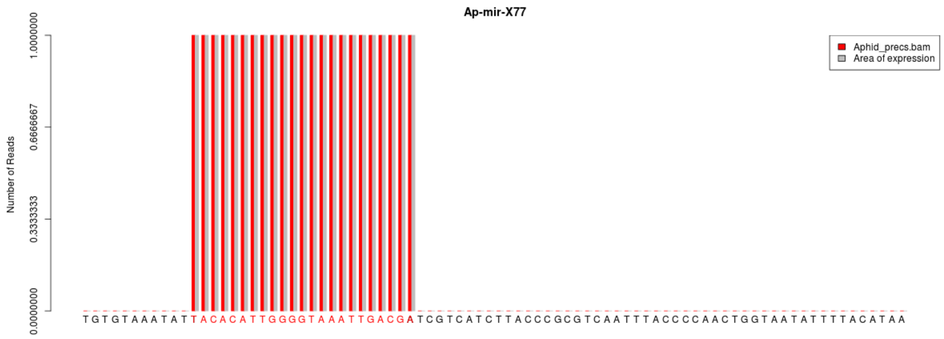

>Ap-mir-X78
CACAAATTGTGGTCGACCGACCCATAGATATTAAAGATATCTATAATATCTTTA**ATATCTATGGACCGACCGGCAA**AACACAGATAA

>Ap-mir-X78-3p
ATATCTATGGACCGACCGGCAA

CACAAAUUGUGGUCGACCGACCCAUAGAUAUUAAAGAUAUCUAUAAUAUCUUUA**AUAUCUAUGGACCGACCGGCAA**AACACAGAUAA
```
......((((((((..((..((((((((((((((((((((.....))))))))))))))))))))..))..)))))....))))).....
```

```
 Initial dG =    -33.40

        10             20         30         40
CACAAA     ----|   AC  AC                   CU
      UUGUG    GUCG  CG  CCAUAGAUAUUAAAGAUAU  \
      GACAC    CGGC  GC  GGUAUCUAUAAUUUCUAUA  A
AAUA--     AAAA^   CA  CA                   AU
        80         70         60         50

-----------------------------------------------------
```

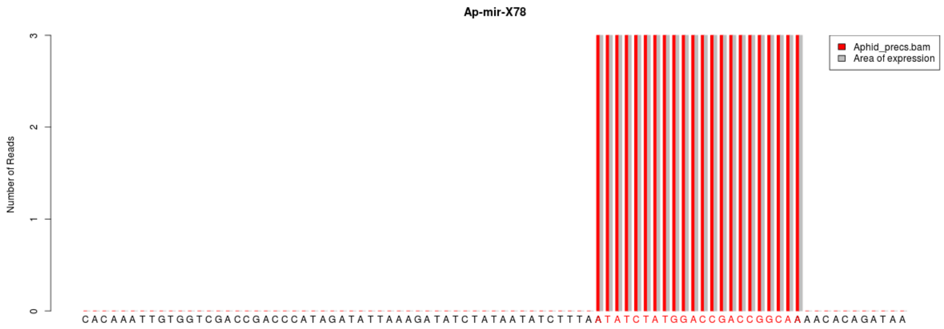

>Ap-mir-X79
AAATACTATAG**ATTGCACACTGGACGGTTTTTC**AGTAGCACAAAATATTTACTGCAACCGAAAAACCATTCAGTGAGCAATCTATAGTAT
TTAT

>Ap-mir-X79-5p
ATTGCACACTGGACGGTTTTTC

AAAUACUAUAG**AUUGCACACUGGACGGUUUUUC**AGUAGCACAAAAUAUUUACUGCAACCGAAAAACCAUUCAGUGAGCAAUCUAUAGUAUUUAU
```
((((((((((((((((.(((((((.((((((((.((.(((...........))).)).)))))))).))))))).)))))))))))))))))..
```

```
 Initial dG =    -45.40

          10        20        30        40
--|                 A       C        A  A   CAAAA
  AAAUACUAUAGAUUGC CACUGGA GGUUUUUC GU GCA     U
  UUUAUGAUAUCUAACG GUGACUU CCAAAAAG CA CGU     A
UA^                 A       A        C  A   CAUUU
   90        80        70        60        50

-----------------------------------------------------
```

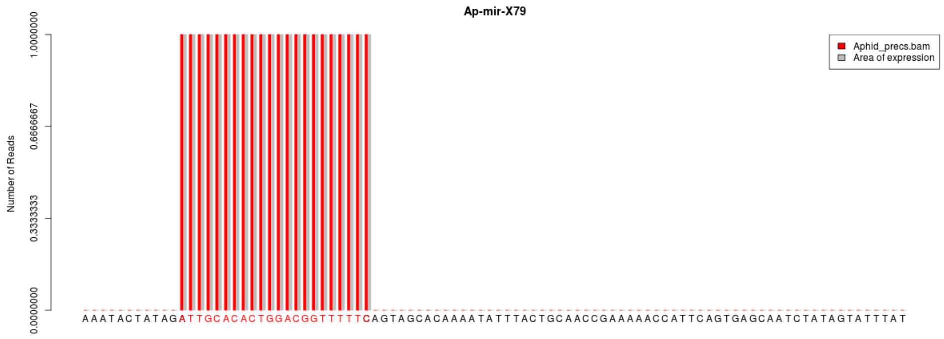

>Ap-mir-X8
GCTCTTGCAGC**TTAGTGGATTCGCCTGTTGACACCGGC**ACCCTGGTCATGACCACCATCTCACCAGGGTTAGGTTAGGCAGGTCCGCCAG
GGTAGCATAATTC

>Ap-mir-X8-5p
TTAGTGGATTCGCCTGTTGACACCGGC

```
GCUCUUGCAGCUUAGUGGAUUCGCCUGUUGACACCGGCACCCUGGUCAUGACCACCAUCUCACCAGGGUUAGGUUAGGCAGGUCCGCCAGGGUAGCAUAAUUC
.....(((.((((.((((((..(((((.....(((...((((((((.(((.....)))...))))))))..))))))))..))))))..)))).)))......
```

```
 Initial dG =    -40.20

         10          20         30         40           50
GCUCU-    A     A-|      UC      UUGAC    GGC          C--    AC
      UGC GCUU  GUGGAU  GCCUG      ACC    ACCCUGGU    AUG  \
      ACG UGGG  CGCCUG  CGGAU      UGG    UGGGACCA    UAC  C
CUUAAU    A     AC^      GA      -----    AU-          CUC    CA
 100        90         80              70          60
```

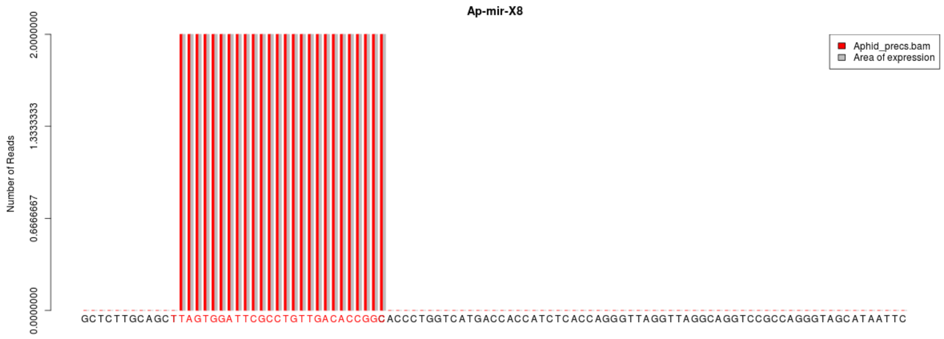

>Ap-mir-X80

TAACTGGGTCGATCGTTTATCGCGAACCGCTATGTGATTTAAAATTTA**GCAGTTCGCGATAAGCGGCCG**AATCAAGTTTT

>Ap-mir-X80-3p

GCAGTTCGCGATAAGCGGCCG

```
UAACUGGGUCGAUCGUUUAUCGCGAACCGCUAUGUGAUUUAAAAUUUAGCAGUUCGCGAUAAGCGGCCGAAUCAAGUUUU
.(((((..(((..((((((((((((((.((((.((........)).)))).))))))))))))))..)))..)).)))..

 Initial dG =    -36.60

          10        20        30
U-   -| GG   AU             C    U  GAU
  AAC UG  UCG  CGUUUAUCGCGAAC GCUA GU   U
  UUG AC  AGC  GCGAAUAGCGCUUG CGAU UA   U
UU   A^ UA   CG             A    U  AAA
.        70        60        50        40

-----------------------------------------------------
```

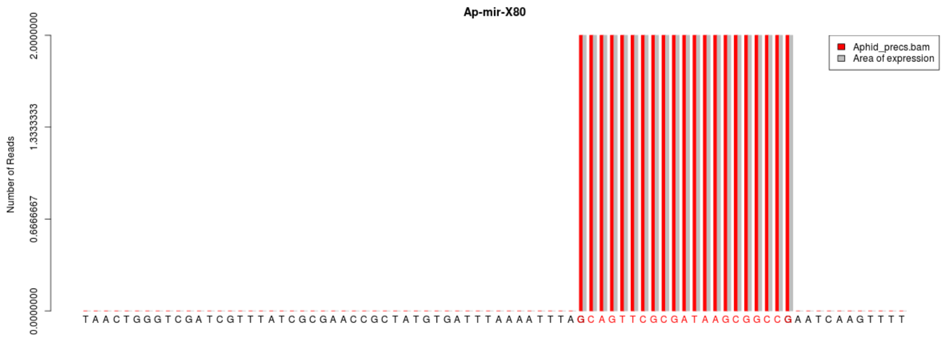

>Ap-mir-X81

CGACACTTTAC**CGGACATTGTAAGAACGGCCCA**TAGGCTTGTTATTATAATTTCCTGTTAAGTGTCGGT

>Ap-mir-X81-5p

CGGACATTGTAAGAACGGCCCA

```
CGACACUUUACCGGACAUUGUAAGAACGGCCCAUAGGCUUGUUAUUAUAAUUUCCUGUUAAGUGUCGGU
((((((((.((.(((.(((((((.(((((((....)))).))).))))))).))).)).))))))))..

 Initial dG =    -29.40

          10        20         30
--        U  C   C       G   -|   C
  CGACACUU AC GGA AUUGUAA AAC GGCC A
  GCUGUGAA UG CCU UAAUAUU UUG UCGG U
UG        U  U   U       A   U^   A
        60        50        40
```

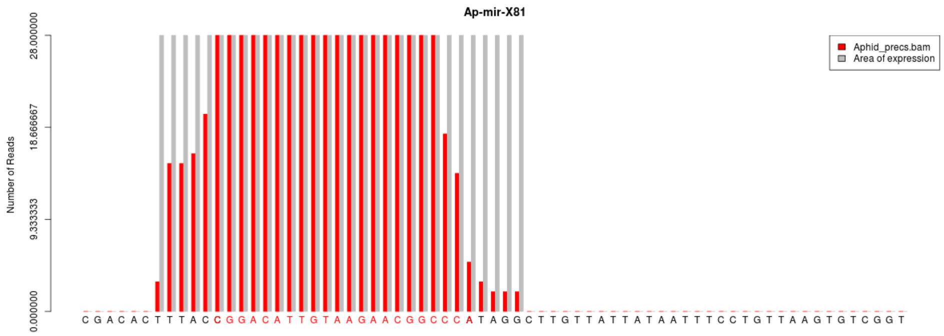

>Ap-mir-X82

CTGTCATTCCCCATTTGTTAGCCACCCGTCCAGTGGCGAAATTACGATCAAAC**ACTGGAGTTGGCTAACAGATGAA**AAATGACGGTG

>Ap-mir-X82-3p

ACTGGAGTTGGCTAACAGATGAA

```
CUGUCAUUCCCCAUUUGUUAGCCACCCGUCCAGUGGCGAAAUUACGAUCAAACACUGGAGUUGGCUAACAGAUGAAAAAUGACGGUG
((((((((...(((((((((((((....(((((((..((........))...)))))))..)))))))))))))...))))))))..
```

```
 Initial dG =    -38.30

          10        20        30         40
--|       CCC             CCCG       GC-   AAU
  CUGUCAUU   CAUUUGUUAGCCA    UCCAGUG   GA    U
  GGCAGUAA   GUAGACAAUCGGU    AGGUCAC   CU    A
GU^       AAA             UG--       AAA   AGC
      80        70        60          50

-----------------------------------------------------
```

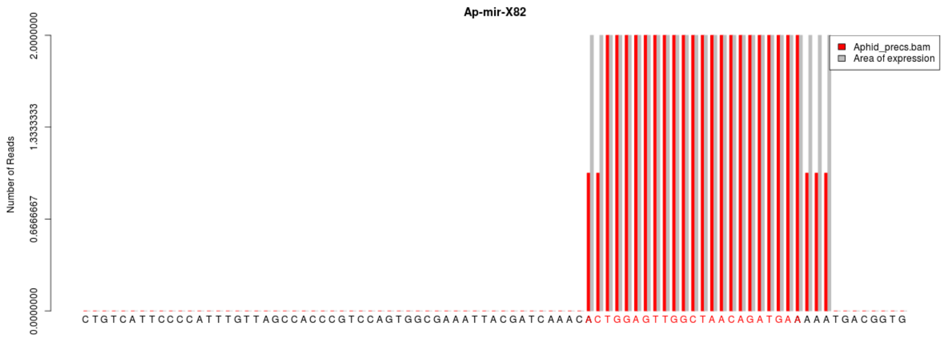

>Ap-mir-X83
GTAAGTATTTA**AAGTTTAGATTGGTCGAGCGGAGT**GGTACGGGGTTACCCCGCAAAATGTTTGTCCACTACGCTGCTTGTCTAAACTTTA
AATACTTATAA

>Ap-mir-X83-5p
AAGTTTAGATTGGTCGAGCGGAGT

GUAAGUAUUUA**AAGUUUAGAUUGGUCGAGCGGAGU**GGUACGGGGUUACCCCGCAAAAUGUUUGUCCACUACGCUGCUUGUCUAAACUUUAAAUACUUAUAA
((((((((((((((((((((.(((..((((.(((((..(((((...)))))((((...)))).))))).))))))).)))))))))))))))))))))))..

```
 Initial dG =    -45.70

          10        20        30          40
--                     U   CG    G     .-UA|    U
  GUAAGUAUUUAAAGUUUAGAU GGU  AGCG AGUGG    CGGGG \
  UAUUCAUAAAUUUCAAAUCUG UCG  UCGC UCACC    GCCCC U
AA                     U   --    A     \ --^    A
 .        90        80          70          50



                                         -    A
                                          CAAA \
                                          GUUU U
                                         U    G
                                            60

-----------------------------------------------------
```

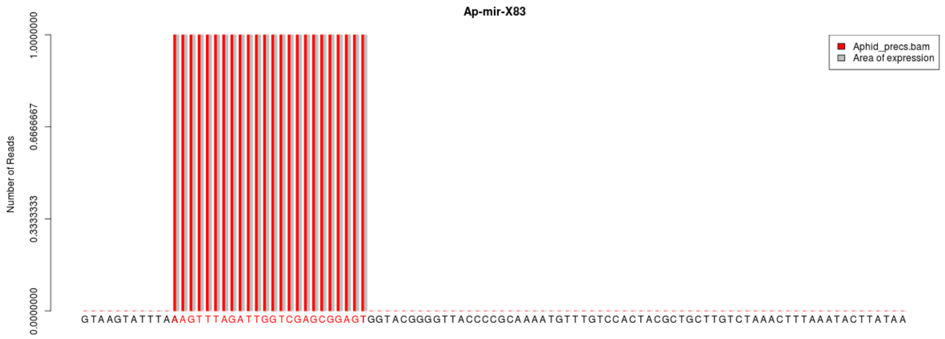

>Ap-mir-X84
GTAAGAACATT**ATCTGTTTTTGTTTGTCGGTTT**TTAAAAAACCAACATACGAACACAGATAATGCCTTTACCA

>Ap-mir-X84-5p
ATCTGTTTTTGTTTGTCGGTTT

GUAAGAACAUU**AUCUGUUUUUGUUUGUCGGUUU**UUAAAAAACCAACAUACGAACACAGAUAAUGCCUUUACCA
(((((..((((((((((.(((((.(((.((((((...)))))).))).))))).))))))))))..)))))..

Initial dG = -27.20

```
          10        20        30
--|    AA          U     U   C      U
  GUAAG  CAUUAUCUGU UUUGU UGU GGUUUU \
  CAUUU  GUAAUAGACA AAGCA ACA CCAAAA A
AC^    CC          C     U   A      A
  70        60        50        40
```

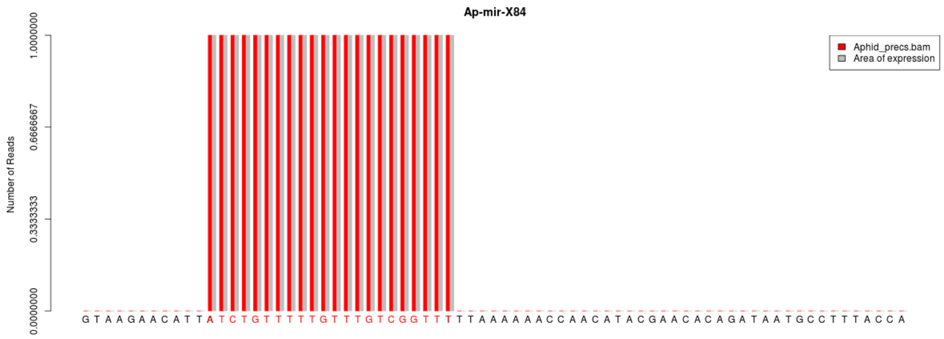

>Ap-mir-X85

GAGTGAAGTCT**TGTTAATTTAGTCGGTGGCGT**AAACCAGTGCTTTGATTAAACAGCACGGGCGGCGCTAGGGATTGAATTAGCAAGATAG
AAATAAA

>Ap-mir-X85-5p

TGTTAATTTAGTCGGTGGCGT

```
GAGUGAAGUCUUGUUAAUUUAGUCGGUGGCGUAAACCAGUGCUUUGAUUAAACAGCACGGGCGGCGCUAGGGAUUGAAUUAGCAAGAUAGAAAUAAA
.......((((((((((((((((..((((((...((.(((((((.....)).)))))))...))))))..)))))))))))))))).........
```

```
 Initial dG =    -38.70

          10        20        30        40
GAGUGAA--|                GG      AAA  A     -  GA
         GUCUUGUUAAUUUAGUC  UGGCGU   CC GUGCU UU  \
         UAGAACGAUUAAGUUAG  AUCGCG   GG CACGA AA  U
AAAUAAAGA^                GG      GCG  -     C  AU
      90        80        70        60         50
```

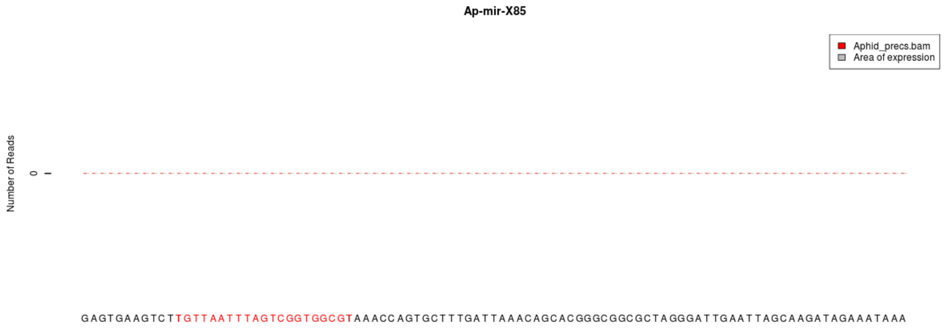

>Ap-mir-X86

NNTATAAGAGCCGTTCTTACAATATCTGGTTAAGTGTGGGTTAACTTT**AACCGGACATTGTAAGAATGGC**CTTAAGATTCA

>Ap-mir-X86-3p

AACCGGACATTGTAAGAATGGC

NNUAUAAGAGCCGUUCUUACAAUAUCUGGUUAAGUGUGGGUUAACUUU**AACCGGACAUUGUAAGAAUGGC**CUUAAGAUUCA

```
....((((.((((((((((((((.((((((((((.((......)))))))))))).)))))))))))))))))).......
```

```
 Initial dG =    -37.70

           10        20        30        40
NNUA---|   A              A          U  GG
       UAAG GCCGUUCUUACAAU UCUGGUUAAG GU  G
       AUUC CGGUAAGAAUGUUA AGGCCAAUUU CA  U
ACUUAGA^   -              C          -  AU
80         70        60        50

-----------------------------------------------------
```

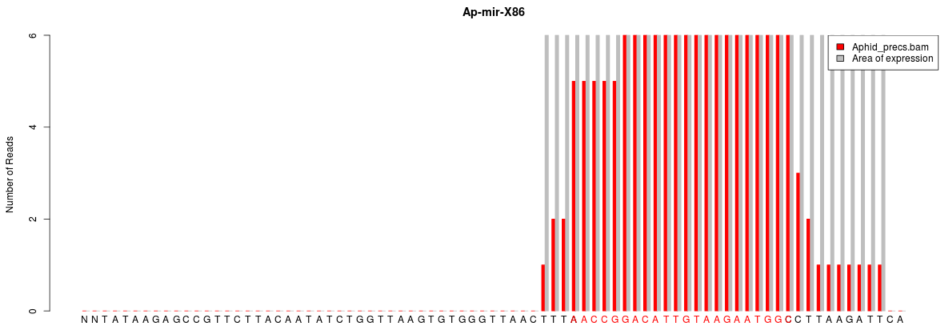

>Ap-mir-X87
CGGCTCGTACGAGTCCTTTATACATACATGCGCACAACAAATCTGCTCCAC**CTAGCTATGTATAAAGGACTCG**CACGAGCCGTC

>Ap-mir-X87-3p
CTAGCTATGTATAAAGGACTCG

CGGCUCGUACGAGUCCUUUAUACAUACAUGCGCACAACAAAUCUGCUCCAC**CUAGCUAUGUAUAAAGGACUCG**CACGAGCCGUC
((((((((.((((((((((((((((((...((......................))))))))))))))))))))).)))))))))..

Initial dG = -41.30

```
          10        20        30        40
--|       A                  CAU   GCACAACAAAU
  CGGCUCGU CGAGUCCUUUAUACAUA   GC           \
  GCCGAGCA GCUCAGGAAAUAUGUAU   CG           C
CU^       C                  ---   AUCCACCUCGU
   80        70        60          50
```

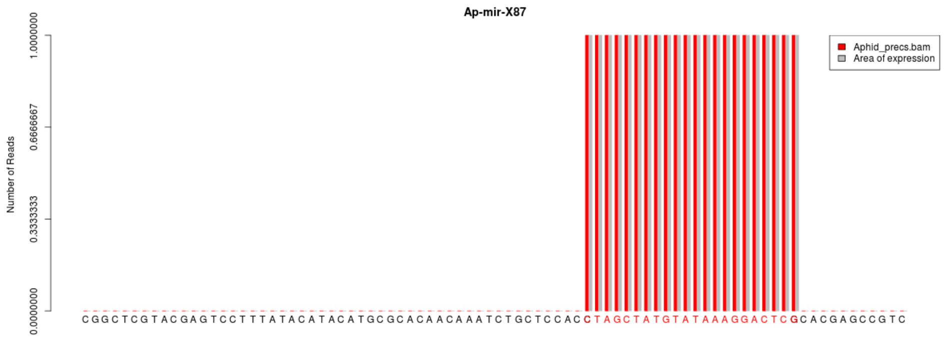

>Ap-mir-X88
TTACTGCGACTATCGTTTTTAACGTATTACTATGTGTATATATTAAAAC**ATAGAACCCGTTTAAGCGGTAG**TTGAGGTAAAG

>Ap-mir-X88-3p
ATAGAACCCGTTTAAGCGGTAG

```
UUACUGCGACUAUCGUUUUUAACGUAUUACUAUGUGUAUAUAUUAAAACAUAGAACCCGUUUAAGCGGUAGUUGAGGUAAAG
(((((.((((((((((((..((((..((.((((((...........))))))))..)))).))))))))))))).))))).. 

 Initial dG =    -28.50

          10        20        30        40
--|    G         UU     UA   A      GUAUA
  UUACU CGACUAUCGUUU  AACG  UU CUAUGU     U
  AAUGG GUUGAUGGCGAA  UUGC  AA GAUACA     A
GA^    A         U-     CC   -      AAAUU
 80        70         60         50
```

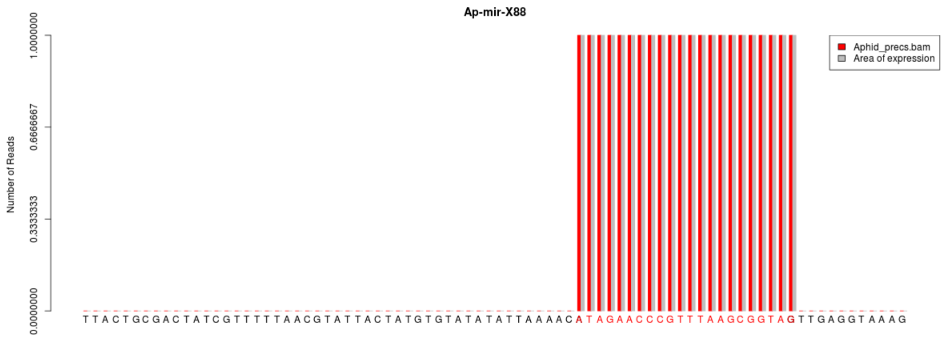

>Ap-mir-X89

GCTTCTGCTTT**TCATTTGATGGATTGAAGCGAT**TCTAAACTTTTTCATCGCTTCAATCCATCAAATGAAAGAAAAGTTTAG

>Ap-mir-X89-5p

TCATTTGATGGATTGAAGCGAT

```
GCUUCUGCUUUUCAUUUGAUGGAUUGAAGCGAUUCUAAACUUUUUCAUCGCUUCAAUCCAUCAAAUGAAAGAAAAGUUUAG
((((...((((.(((((((((((((((((((((.............)))))))))))))))))))))))))..))))....

 Initial dG =    -36.20

            10        20        30
----|   CUG    U                    UCUAAA
    GCUU   CUUU CAUUUGAUGGAUUGAAGCGAU      \
    UGAA   GAAA GUAAACUACCUAACUUCGCUA      C
GAUU^   AA-    -                    CUUUUU
80         70        60        50

-----------------------------------------------------
```

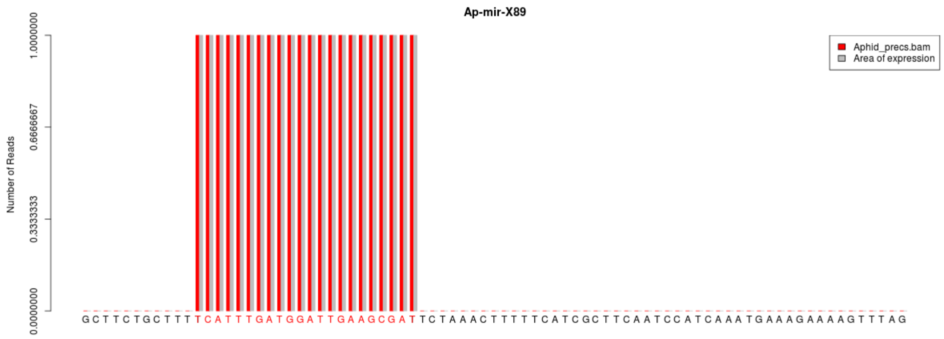

>Ap-mir-X9
ATTTTAAATCA**TAAGACATTATCTGAAGATTTC**ATTAGAGAATTTTCAGATAAAGTTTATTGTTGTAATATAT

>Ap-mir-X9-5p
TAAGACATTATCTGAAGATTTC

AUUUUAAAUCA**UAAGACAUUAUCUGAAGAUUUC**AUUAGAGAAUUUUCAGAUAAAGUUUAUUGUUGUAAUAUAU
```
...(((...((..((((.((((((((((((((.......))))))))))))))).))))..))...))).....
```

Initial dG = -16.70

```
          10        20        30
AUU--|  AAU  UA    A              CAU
     UUA   CA  AGAC UUAUCUGAAGAUUU   \
     AAU   GU  UUUG AAUAGACUUUUAAG   U
UAUAU^  GUU  UA    A              AGA
  70        60        50        40
```

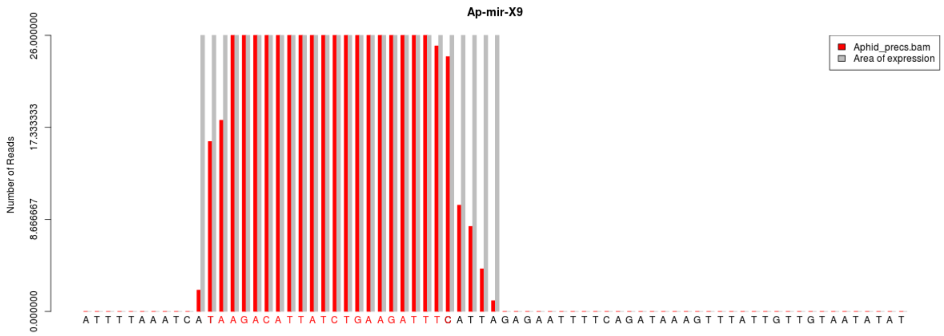

>Ap-mir-X90
TTAATACTAGGAGCTTCTTCCTGTTGTCATTGTTTGAAATAATTATTAAATTAC**AATGACAACAGTTGGCAGCCAC**AAGTGTTGATT

>Ap-mir-X90-3p
AATGACAACAGTTGGCAGCCAC

```
UUAAUACUAGGAGCUUCUUCCUGUUGUCAUUGUUUGAAAUAAUUAUUAAAUUACAAUGACAACAGUUGGCAGCCACAAGUGUUGAUU
((((((((....(((.((..((((((((((((((((........))))...))))))))))))))..)).)))....))))))))..

 Initial dG =    -29.60

          10        20        30           40
--        AGGA   U  UC            ---|   AAU
  UUAAUACU    GCU CU  CUGUUGUCAUUGU   UUGA   A
  AGUUGUGA    CGA GG  GACAACAGUAACA   AAUU   A
UU        ACAC   C  UU            UUA^   AUU
      80        70        60        50

-----------------------------------------------------
```

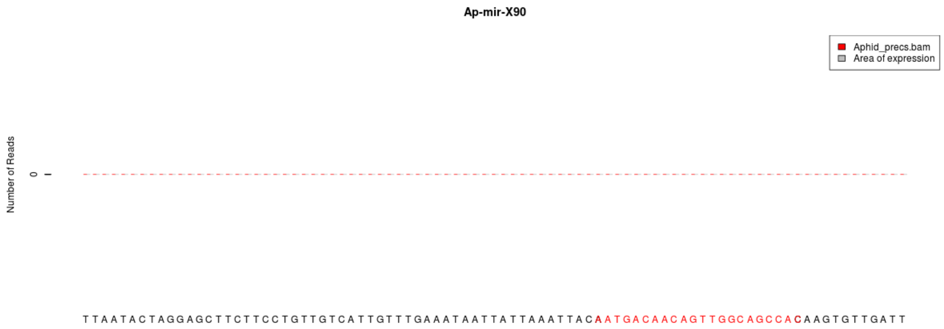

>Ap-mir-X91
CCAAACTTTCGATGCAGGTGTGACAAAAAAGATTTAAGATATAAAATATA**TTTTTGTCATGCCTGCGGCG**AAAGTTTAGTT

>Ap-mir-X91-3p
TTTTTGTCATGCCTGCGGCG

```
CCAAACUUUCGAUGCAGGUGUGACAAAAAAGAUUUAAGAUAUAAAAUAUAUUUUUGUCAUGCCUGCGGCGAAAGUUUAGUU
..((((((((.(((((((((((((((((..((((........))))...)))))))))))))))))).)))))))))....

 Initial dG =    -35.60

          10        20        30
CC--         A                 AG-|   AAG
    AAACUUUCG UGCAGGUGUGACAAAAA   AUUU   A
    UUUGAAAGC GCGUCCGUACUGUUUUU   UAAA   U
UUGA         G                 AUA^   AUA
80        70        60        50        40

-----------------------------------------------------
```

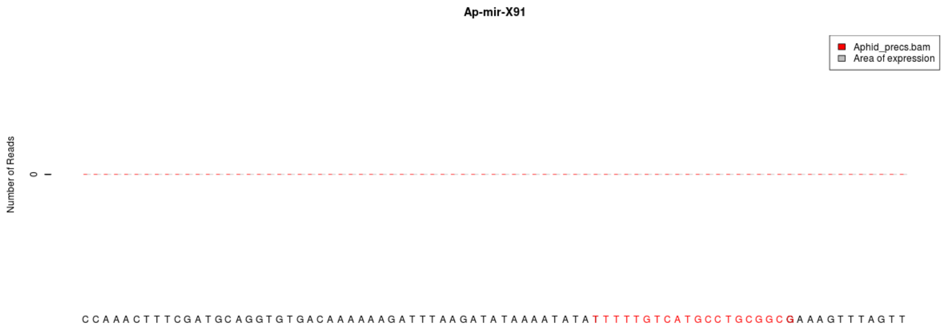

>Ap-mir-X92
TAACTGGGTCGACCTAATATCATGAATTGCTATGTAGTTTAAAATTTA**GCAGTTCGTGATATTCGGCCG**AATCAAGTTTT

>Ap-mir-X92-3p
GCAGTTCGTGATATTCGGCCG

```
UAACUGGGUCGACCUAAUAUCAUGAAUUGCUAUGUAGUUUAAAAUUUAGCAGUUCGUGAUAUUCGGCCGAAUCAAGUUUU
.(((((..(((.((.(((((((((((((((((.((........)).))))))))))))))))).)).)))..)).)))..

 Initial dG =    -30.50

          10        20        30
U-   -| GG   A  U                 U  AGU
  AAC UG  UCG CC AAUAUCAUGAAUUGCUA GU   U
  UUG AC  AGC GG UUAUAGUGCUUGACGAU UA   U
UU   A^ UA   C  C                 U  AAA
.        70        60        50        40

-----------------------------------------------------
```

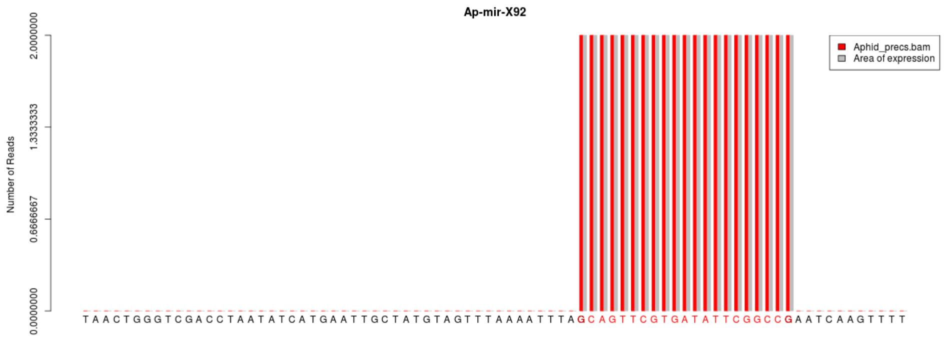

>Ap-mir-X93
TTATAGGGTGG**TAAAATAATCGACCAATGATCGTCTAG**GTCAGGGTTCAACACCTAATGATCGTTGGTAAAATATTTTACCACTTCTTTG
TA

>Ap-mir-X93-5p
TAAAATAATCGACCAATGATCGTCTAG

```
UUAUAGGGUGGUAAAAUAAUCGACCAAUGAUCGUCUAGGUCAGGGUUCAACACCUAAUGAUCGUUGGUAAAAUAUUUUACCACUUCUUUGUA
.....(((((((((((((....((((((((((((.(((((...........)))))))))))))))))....))))))))))))).......

 Initial dG =    -35.60

          10        20        30        40
UUAUA--|            AUCG            C      CAGGG
       GGGUGGUAAAAUA    ACCAAUGAUCGU UAGGU      \
       UUCACCAUUUUAU    UGGUUGCUAGUA AUCCA      U
AUGUUC^             AAAA            -      CAACU
 90        80        70        60         50

-----------------------------------------------------
```

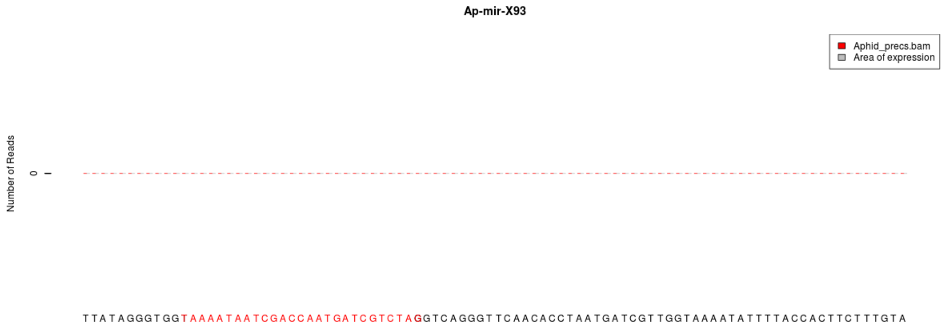

>Ap-mir-X94

TGAATTTGTCGTCAAATCTACTTGAGATTGGCCAGTCCACATATTTTATATTATCCCCCAA**AATCAAGTAGACTTGATG**ACAATTCCAAA

>Ap-mir-X94-3p

AATCAAGTAGACTTGATG

```
UGAAUUUGUCGUCAAAUCUACUUGAGAUUGGCCAGUCCACAUAUUUUAUAUUAUCCCCCAAAAUCAAGUAGACUUGAUGACAAUUCCAAA
.(((((.((((((((.(((((((((..((((..........................))))..))))))))).)))))))))))))....
```

```
 Initial dG =    -29.00

           10         20         30         40
U---|    U         A          GA     CCAGUCCACAUA
    GAAUU GUCGUCAA UCUACUUGA  UUGG            U
    CUUAA CAGUAGUU AGAUGAACU  AACC            U
AAAC^    -         C          AA     CCCUAUUAUAUU
.         80         70         60         50

-----------------------------------------------------
```

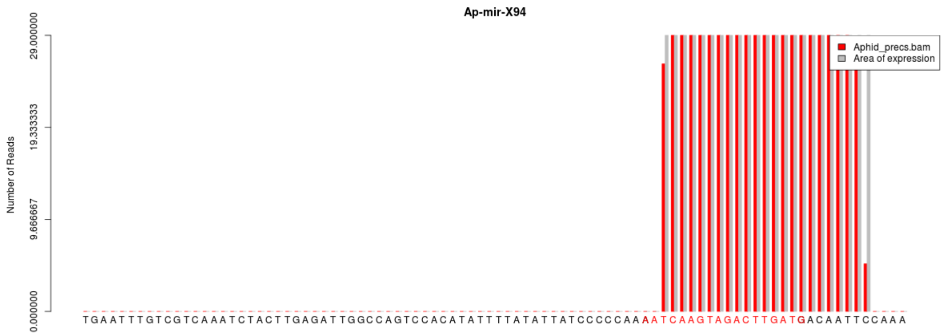

>Ap-mir-X95
ACTGCCCAATCTGGTATGTACACGCATACACAAATCAAAATGCTTGGGCATAT**TTGGCATGCTTGTACATATTGCT**ATTGGGTACTA

>Ap-mir-X95-3p
TTGGCATGCTTGTACATATTGCT

ACUGCCCAAUCUGGUAUGUACACGCAUACACAAAUCAAAAUGCUUGGGCAUAU**UUGGCAUGCUUGUACAUAUUGCU**AUUGGGUACUA
..((((((((..((((((((((.((((...(((((....((((....)))))))))..)))).))))))))))...))))))))...

```
 Initial dG =    -31.60

         10          20         30         40
AC-         CU-|          C     ACA      CAAA    U
   UGCCCAAU    GGUAUGUACA GCAU   CAAAU    AUGC U
   AUGGGUUA    UUAUACAUGU CGUA   GUUUA    UACG G
AUC         UCG^          U     CG-      ----    G
      80         70         60              50
```

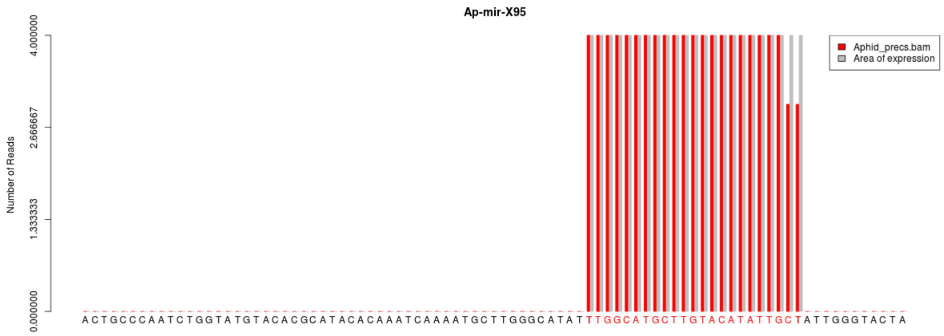

>Ap-mir-X96
GCTTTGTCGAC**AAGCGGCACTTCTCTTTGGCTAT**TTATTTCTTAATAATCTATGGATATAGCCAAATTTTAGTGCTTCTTGATGAAGTAG
CGT

>Ap-mir-X96-5p
AAGCGGCACTTCTCTTTGGCTAT

GCUUUGUCGAC**AAGCGGCACUUCUCUUUGGCUAU**UUAUUUCUUAAUAAUCUAUGGAUAUAGCCAAAUUUUAGUGCUUCUUGAUGAAGUAGCGU
(((...(((.((((.((((((....((((((((((((((....))))).........))))))))).....)))))).)))).)))...)))..

```
 Initial dG =    -29.90

          10        20        30                 40
--   UUG   A    C      UCUC         ---------|    U
  GCU   UCG CAAG GGCACU    UUUGGCUAU         UUAUU C
  CGA   AGU GUUC UCGUGA    AAACCGAUA         AAUAA U
UG   UGA   A    U      UUUU         UAGGUAUCU^    U
  90        80        70        60        50

-----------------------------------------------------
```

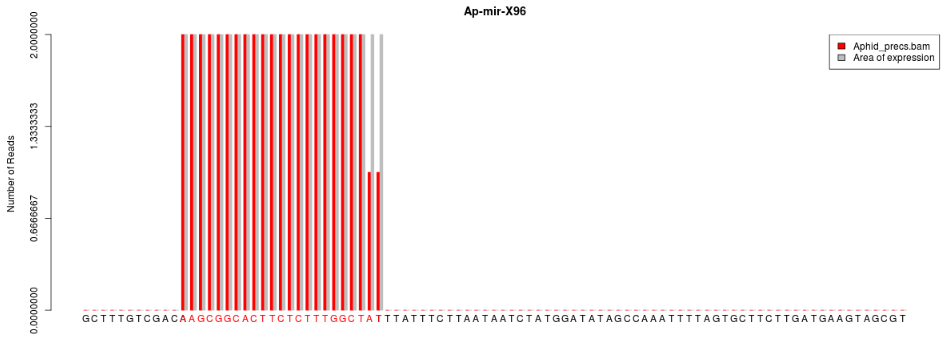

>Ap-mir-X97
GCATAGTCAAT**TTATGTGTCACAAAAGACATGTT**AAACCTTTCTTAAACTATGTCTTATGTGACACACAAATTGACCATGCAT

>Ap-mir-X97-5p
TTATGTGTCACAAAAGACATGTT

GCAUAGUCAAU**UUAUGUGUCACAAAAGACAUGUU**AAACCUUUCUUAAACUAUGUCUUAUGUGACACACAAAUUGACCAUGCAU
((((.((((((((.(((((((((.((((((((((.((......)).))).))))))).))))))))).)))))))).))))..

Initial dG =   -37.90

```
          10         20         30          40
--     A         A          A        -|   A   CC
  GCAU GUCAAUUU UGUGUCACA AAGACAU GUU AA   U
  CGUA CAGUUAAA ACACAGUGU UUCUGUA CAA UU   U
UA     C         C          A        U^   A   CU
  80         70         60         50
```

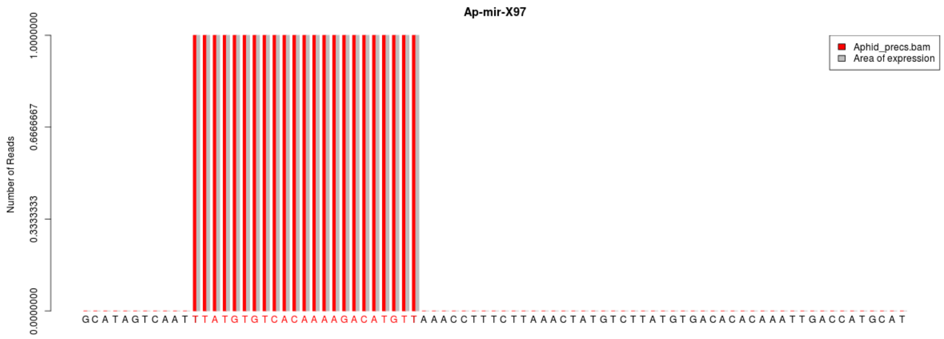

>Ap-mir-X98-1
TATATATGTAT**TTATGTATAGCACTCAACAGGAAGTGGC**CAGGTTCGCCGAGCAAATCCTGTGAGTGCTAGAGCGTGGGTGAGTGTGTAA
A

>Ap-mir-X98-1-5p
TTATGTATAGCACTCAACAGGAAGTGGC

UAUAUAUGUAU**UUAUGUAUAGCACUCAACAGGAAGUGGC**CAGGUUCGCCGAGCAAAUCCUGUGAGUGCUAGAGCGUGGGUGAGUGUGUAAA
```
((((((.(((((((((.((((((((.(((((....((..((....))..))...))))))))))))))..))))))))).))))))..
```

```
 Initial dG =    -40.10

          10         20        30        40
--       G         A-|        A     AGUG   CA   U
  UAUAUAU UAUUUAUGU  UAGCACUCA CAGGA    GC   GG U
  AUGUGUG GUGGGUGCG  AUCGUGAGU GUCCU    CG   CC C
AA       A         AG^        -     AAA-   AG   G
90        80        70         60         50

-----------------------------------------------------
```

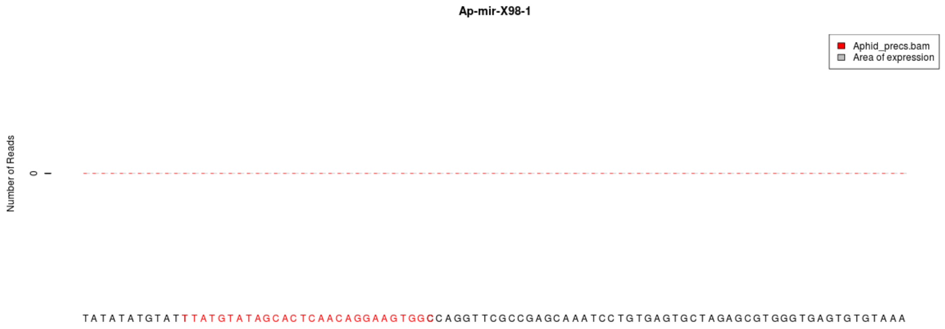

>Ap-mir-X98-2
TATGTATGTAT**TTATGTATAGCACTCAACAGGAAGTGGC**CAGGTTCGCCGAGCAAATCCTGTGAGTGCTAGAGCGTGGGTGAGTGTGTAA
A

>Ap-mir-X98-2-5p
TTATGTATAGCACTCAACAGGAAGTGGC

UAUGUAUGUAU**UUAUGUAUAGCACUCAACAGGAAGUGGC**CAGGUUCGCCGAGCAAAUCCUGUGAGUGCUAGAGCGUGGGUGAGUGUGUAAA
((..(((.((((((((.((((((((.(((((....((..((....))..))...))))))))))))))..))))))))).))))..))..

```
 Initial dG =    -38.40

          10         20        30        40
--  UG   G         A-|        A     AGUG  CA  U
  UA  UAU UAUUUAUGU  UAGCACUCA CAGGA    GC  GG U
  AU  GUG GUGGGUGCG  AUCGUGAGU GUCCU    CG  CC C
AA  GU   A         AG^        -     AAA-  AG  G
90        80        70         60         50

-----------------------------------------------------
```

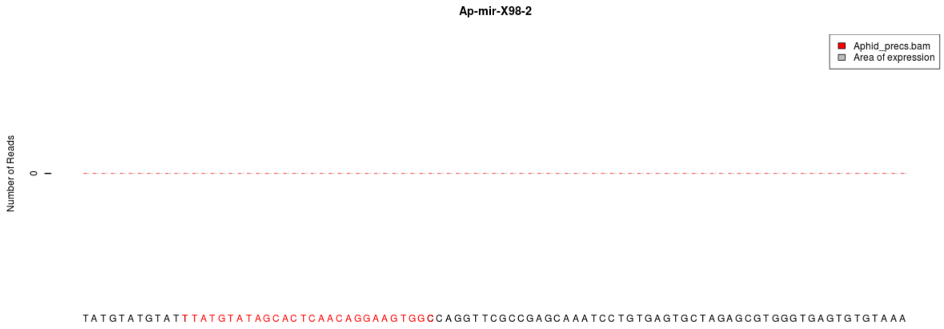

>Ap-mir-X98-3

TATGTATGTAT**TTATGTATAGCACTCAACAGGAAGTGGC**CAGGTTCGCCGAGCAAATCCTGTGAGTGCTAGAGCGTGGGTGAGTGTGTAA
A

>Ap-mir-X98-3-5p

TTATGTATAGCACTCAACAGGAAGTGGC

```
UAUGUAUGUAUUUAUGUAUAGCACUCAACAGGAAGUGGCCAGGUUCGCCGAGCAAAUCCUGUGAGUGCUAGAGCGUGGGUGAGUGUGUAAA
((..(((.(((((((((.((((((((((.(((((....((..((....))..))...))))))))))))))..))))))))).))))..))..

 Initial dG =    -38.40

          10         20        30        40
--  UG   G          A-|        A     AGUG  CA  U
  UA  UAU UAUUUAUGU  UAGCACUCA CAGGA    GC  GG U
  AU  GUG GUGGGUGCG  AUCGUGAGU GUCCU    CG  CC C
AA  GU   A          AG^        -     AAA-  AG  G
90        80        70         60        50

-----------------------------------------------------
```

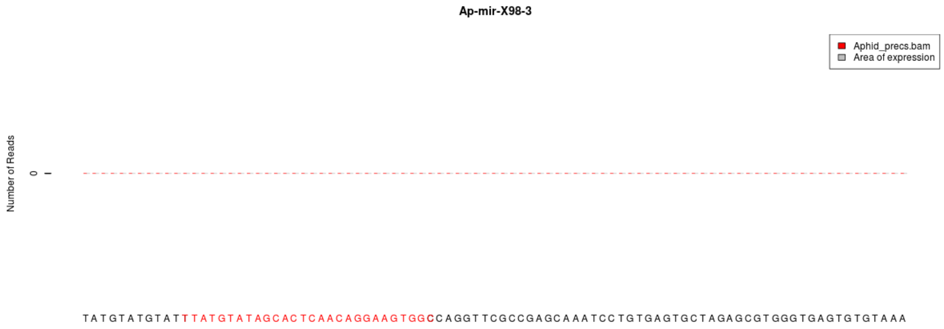

>Ap-mir-X99-1
AGTTAAGTAAGAGGCGTTTTTGTTTAATATGCTCGGCCCAAGT**TAAGCGTTTGTCCAGAAGTAGGCCTAG**GTATTTAACTT

>Ap-mir-X99-1-3p
TAAGCGTTTGTCCAGAAGTAGGCCTAG

```
AGUUAAGUAAGAGGCGUUUUUGUUUAAUAUGCUCGGCCCAAGUUAAGCGUUUGUCCAGAAGUAGGCCUAGGUAUUUAACUU
((((((((..((((.((((((..(((.(((((..((....))..))))))))..))))))...))))...))))))))).
```

```
 Initial dG =    -25.60

         10            20         30
-          AG-    G--|      UU    U      CG   C
 AGUUAAGUA   AGGC   UUUUUG  UAA AUGCU   GC  C
 UCAAUUUAU   UCCG   GAAGAC  GUU UGCGA   UG  A
U          GGA    GAU^      CU    -      AU   A
80         70         60          50         40

-----------------------------------------------------
```

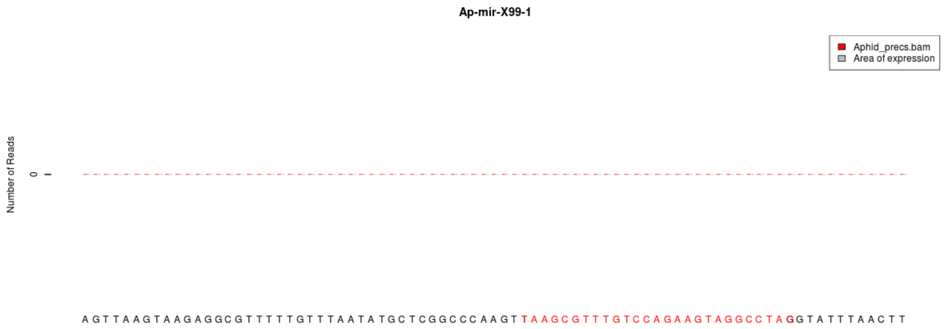

>Ap-mir-X99-2
AGTTAAGTAAGAGGCGTTTTTGTTTAATATGCTCGGCCCAAGT**TAAGCGTTTGTCCAGAAGTAGGCCTAG**GTATTTAACTT

>Ap-mir-X99-2-3p
TAAGCGTTTGTCCAGAAGTAGGCCTAG

AGUUAAGUAAGAGGCGUUUUUGUUUAAUAUGCUCGGCCCAAGU**UAAGCGUUUGUCCAGAAGUAGGCCUAG**GUAUUUAACUU
((((((((..((((.((((((..(((.(((((..((....))..))))))))..))))))...))))...))))))))).

Initial dG = -25.60

```
         10           20         30
-         AG-     G--|     UU   U      CG  C
 AGUUAAGUA   AGGC    UUUUUG  UAA AUGCU  GC C
 UCAAUUUAU   UCCG    GAAGAC  GUU UGCGA  UG A
U         GGA     GAU^     CU   -      AU  A
80        70         60         50         40
```

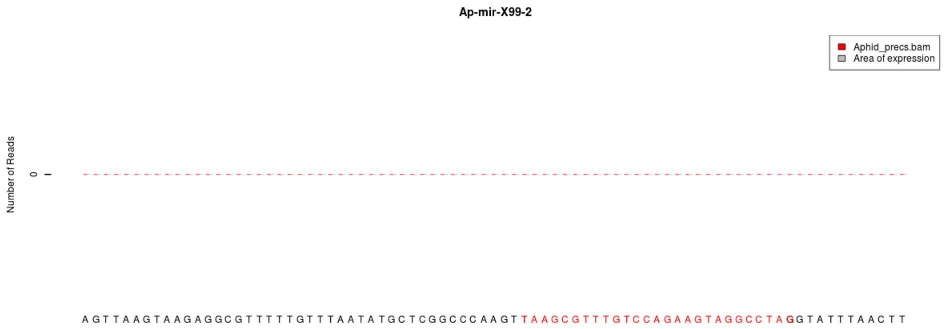

>Ap-mir-X99-3
AGTTAAGTAAGAGGCGTTTTTGTTTAATATGCTCGGCCCAAGT**TAAGCGTTTGTCCAGAAGTAGGCCTAG**GTATTTAACTT

>Ap-mir-X99-3-3p
TAAGCGTTTGTCCAGAAGTAGGCCTAG

AGUUAAGUAAGAGGCGUUUUUGUUUAAUAUGCUCGGCCCAAGU**UAAGCGUUUGUCCAGAAGUAGGCCUAG**GUAUUUAACUU
((((((((..((((.((((((..(((.(((((..((....))..)))))))))..))))))...))))...)))))))).

```
 Initial dG =    -25.60

         10            20         30
-          AG-    G--|     UU   U      CG  C
 AGUUAAGUA   AGGC   UUUUUG  UAA AUGCU  GC C
 UCAAUUUAU   UCCG   GAAGAC  GUU UGCGA  UG A
U          GGA    GAU^     CU   -      AU  A
80         70         60          50         40

-----------------------------------------------------
```

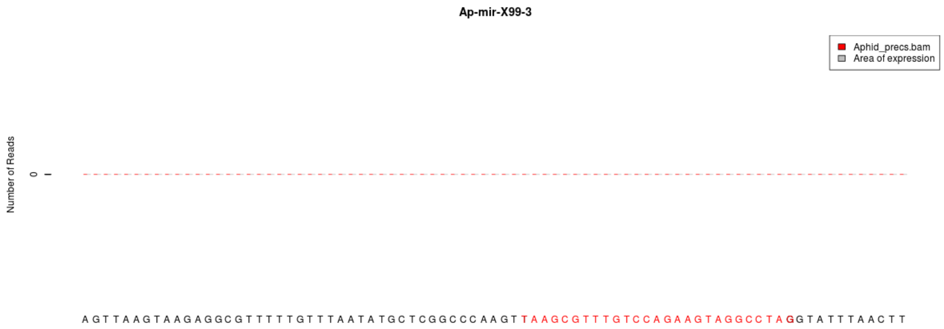

>Ap-mir-iab4
GAATAATGCGTGGCACCCTGT**ACGTATACTAAATGTATCCTGA**GTGCATTATAAAACTGTCCGGTATACCTTCAGTATACGTAACAGGGT
GCCATACCGATCTT

>Ap-mir-iab4-5p
ACGTATACTAAATGTATCCTGA

```
GAAUAAUGCGUGGCACCCUGUACGUAUACUAAAUGUAUCCUGAGUGCAUUAUAAAACUGUCCGGUAUACCUUCAGUAUACGUAACAGGGUGCCAUACCGAUCUU
.........((((((((((((((((((((.((.((((.((((((..........)))...))).)))).)).)))))))))).))))))))))).........
```

```
 Initial dG =    -41.10

        10        20         30          40
GAAUAAUGC           -          .-A|      CC
         GUGGCACCCUG UACGUAUACU   AAUGUAU  \
         UACCGUGGGAC AUGCAUAUGA   UUACGUG  U
UUCUAGCCA          A          \ -^       AG
  100        90        80        50


                                         60
                                 AUAAAACUGUCC  U
                                             GG A
                                             CC U
                                 CUU---------  A
                                            70
```

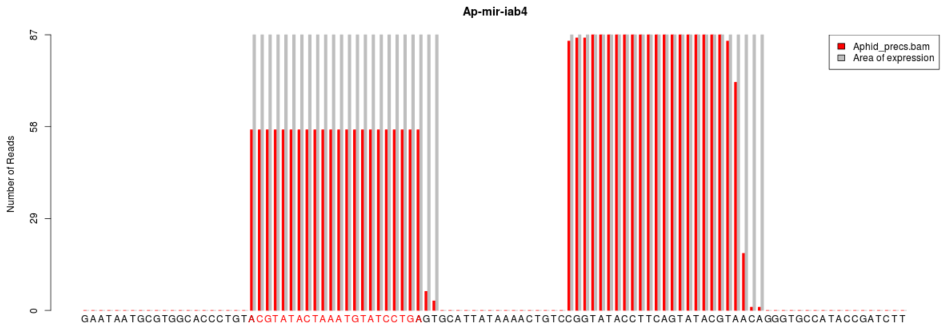

>Ap-mir-133_pre+_11

CGTGTAGCTGT**AGCTGGTTGGATCTGGGCCAAA**TACACATGTATTAATTGAGTTGTGTGCGGTAGCGGCAGCGTGCGTAAAGACAAATAC
AAAGTTTTTGTATGAACCACACTCCGTTCCCAGTTATCGTGTGTATTTTCTCACATAAGTTTCTGGGATTGTGATTACGATGCTGTTGCC
GTTGCTGCCGCAATCACATTTGGTCCCCTTCAACCAGCTGTAGCCGGCACGC

>Ap-mir-133_5p

AGCTGGTTGGATCTGGGCCAAA

```
CGUGUAGCUGUAGCUGGUUGGAUCUGGGCCAAAUACACAUGUAUUAAUUGAGUUGUGUGCGGUAGCGGCAGCGUGCGUAAAGACAAAUACAAAGUUUUUGUAUGAACCACACUCCGUUCC
CAGUUAUCGUGUGUAUUUUCUCACAUAAGUUUCUGGGAUUGUGAUUACGAUGCUGUUGCCGUUGCUGCCGCAAUCACAUUUGGUCCCCUUCAACCAGCUGUAGCCGGCACGC
(((((.((((((((((((((((...((((((((((((....)))))...(((.(((((.((((((((((((((((((((((......(((((((...)))))))......(((.....
((((((..((.(((((........)))))..))..))))))..))).))))).))).))))))))))))))))))))))....))))))..))))))))))))))))..))))).

 Initial dG =    -99.20

          10        20        30
-     A-                UCU       |     A
 CGUGU  GCUGUAGCUGGUUGGA   GGGCCA--AAUAC C
 GCACG  CGAUGUCGACCAACUU   CCUGGU  UUAUG A
C     GC                CC-      \ ^     U
230       220       210                40


                                       50        60        70         80         90
                                    AAU-   G     U             -   -     .-AGACAA      G
                                        UGA UUGUG GCGGUAGCGGCAGC GUG CGUAA        AUACAAA \
                                        ACU AACGC CGUCGUUGCCGUUG CGU GCAUU        UAUGUUU U
                                    UUAC   -     -             U   A     \ ------      U
                                  200        190        180       170              100

-----------------------------------------------------
```

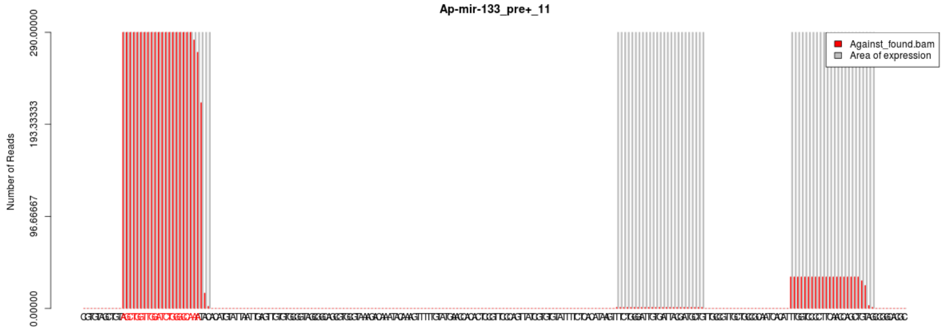

>Ap-mir-193_pre+_11
GTTTTGGTGT**TAGGGATTTAGCGGACCAACGGT**GGTCGACACACATAGATTAAAACAGTCCTACTGGCCTGCTAAGTCCCAAATACCAGC
AAG

>Ap-mir-193_5p
TAGGGATTTAGCGGACCAACGGT

```
GUUUUGGUGUUAGGGAUUUAGCGGACCAACGGUGGUCGACACACAUAGAUUAAAACAGUCCUACUGGCCUGCUAAGUCCCAAAUACCAGCAAG
...((((((((.(((((((((((((.((...(((((..(((.................)))))))))).))))))))))))).)))))))))....
```

Initial dG = -36.50

```
         10        20          30
GUU-        A            A    .-ACG|  GU
    UUGGUGUU GGGAUUUAGCGG CCA      GUG  \
    GACCAUAA CCCUGAAUCGUC GGU      CAC  C
GAAC        A            C    \ ---^  AG
  90        80        70          40


                                      50
                               ACAUA    AA
                                    GAUU  \
                                    CUGA  A
                               CAUC-    CA
                                   60
```

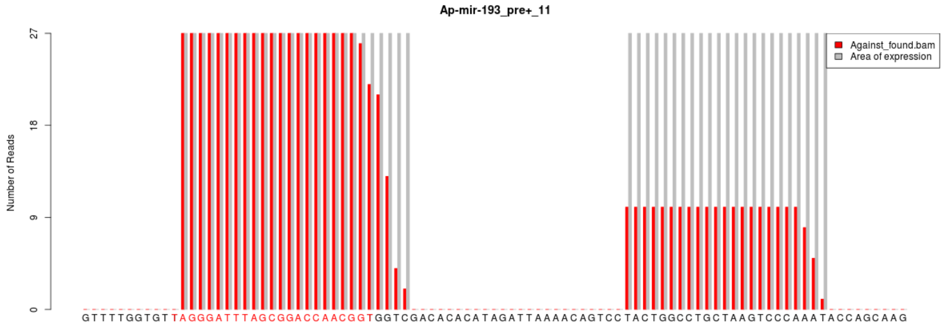

>Ap-mir-210-1_pre+_11
GCTGCTAACGTAGCTGCTGGACACTGCTCAAGATTAATTATAAAAAGTGCGAGCGCGGGCGCACGGCCATAATAAAATATAATGATAAAA
AATATAATATTATAGTAACCGTGCGCGCGGTGGCGACGTACAAATAACTC**TTGTGCGTGTGACAGCGGCTAT**TGTGGCAGCG

>Ap-mir-210-1_3p
TTGTGCGTGTGACAGCGGCTAT

GCUGCUAACGUAGCUGCUGGACACUGCUCAAGAUUAAUUAUAAAAAGUGCGAGCGCGGGCGCACGGCCAUAAUAAAAUAUAAUGAUAAAAAAUAUAAUAUUAUAGUAACCGUGCGCGCGG
UGGCGACGUACAAAUAACUC**UUGUGCGUGUGACAGCGGCUAU**UGUGGCAGCG
(((((((..((((((((((.((((.((.(((((....((((.....(((((.((.((.(((((((((.((((((..
((((...........))))..)))))).)...)))))))).))...))..)))))..)))).))))).))))))..))))))))))..))))))).

```
 Initial dG =    -68.40

         10        20         30        40        50          60          70        80

-       AC          G-|   U  U     UUAA    AAAAA     A-  G--  G         --- C      AA
AAUGA
 GCUGCUA  GUAGCUGCUG  ACAC GC CAAGA    UUAU     GUGCG  GC   CG GCGCACGG   C AUAAUA  AUAU
\
 CGACGGU  UAUCGGCGAC  UGUG CG GUUCU    AAUA     CAUGC  CG   GC CGCGUGCC   G UAUUAU  UAUA
U
G       GU          AG^   -  U     C---    AA---     AG  GUG  G        AAU A      AA
AAAAA
170       160       150        140             130       120       110       100        90
```

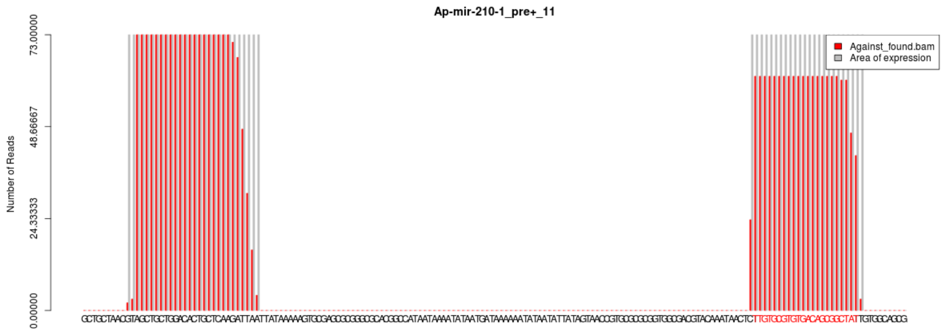

>Ap-mir-210-2_pre+_11
CACATTTGATGTAGCTGCTGGACACTGCACAAGAATTACTTAAAAGTCGCCACTCGGACTTGTACCTATAATATCATATTATTGTACACG
GAGAGTGGTGGCGCCACACGTC**TTGTGCGTGTGACATCGGCT**ATGTCGGGTGTGC

>Ap-mir-210-2_3p
TTGTGCGTGTGACATCGGCT

CACAUUUGAUGUAGCUGCUGGACACUGCACAAGAAUUACUUAAAAGUCGCCACUCGGACUUGUACCUAUAAUAUCAUAUUAUUGUACACGGAGAGUGGUGGCGCCACACGUC**UUGUGCGU**
GUGACAUCGGCUAUGUCGGGUGUGC
((((((((((((((((.((.((((.((((((((...........((((((((((.....(((((..
((((((...))))))).))))).....))))))))))........))))))))))))..)).))))))))))))))))).

```
 Initial dG =    -65.00

        10         20          30         40         50         60         70
-                 C   G-|    U        AUUACUUAAAA          GGACU      CU       U
 CACAUUUGAUGUAGCUG UG   ACAC GCACAAGA           GUCGCCACUC     UGUAC   AUAAUA \
 GUGUGGGCUGUAUCGGC AC   UGUG CGUGUUCU           CGGUGGUGAG     ACAUG   UAUUAU C
C                 U   AG^    -        GCACACCG---          AGGC-      U-       A
   140        130        120        110          100        90          80
```

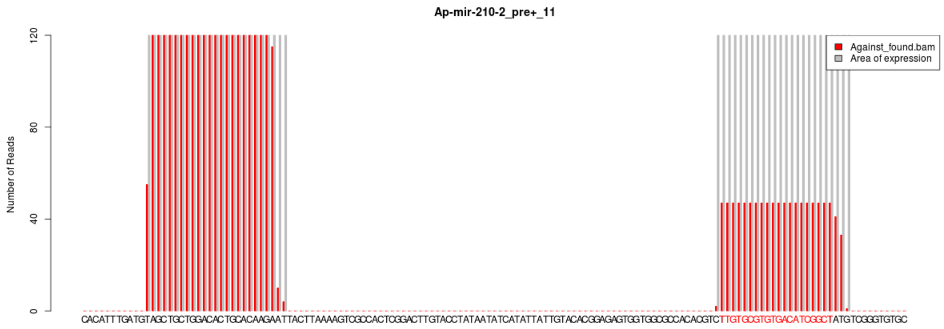

>Ap-mir-31_pre+_11
TGGGTCGGGA**GAGGCAAGATGAGGCAAAGCTGT**ATATAGAATTGCAGTACAATGCAAACTTAACATACATAACAGCTCTGAATCATCGGG
CCACTTCCTACCCGTGA

>Ap-mir-31_5p
GAGGCAAGATGAGGCAAAGCTGT

UGGGUCGGGA**GAGGCAAGAUGAGGCAAAGCUGU**AUAUAGAAUUGCAGUACAAUGCAAACUUAACAUACAUAACAGCUCUGAAUCAUCGGGCCACUUCCUACCCGUGA
.((((.(((((.(((..(((((..((.((((((((.((...(((((......)))))........)).))).))))).))..))))).)).))).))))).)))))....

```
 Initial dG =    -42.40

           10         20         30          40
U---    C     A   AA      GG  A     -   A   GAA-----|    GU
    GGGU GGGAG GGC  GAUGA  CA AGCUG UAU UA          UUGCA  A
    CCCA CCUUC CCG  CUACU  GU UCGAC AUA AU          AACGU  C
AGUG    U     A   GG      AA  C     A   C   ACAAUUCA^    AA
     100        90         80         70         60         50

-----------------------------------------------------
```

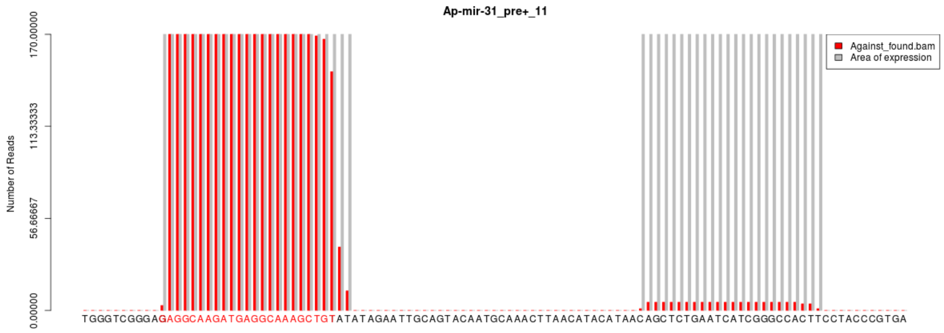

>Ap-mir-375_pre+_11
ACGACCTGATCACTTGAGCCAAGAGGACAAGTTTGTCTAAATTGAAATACAAATCA**ATTTGTTCGTTCGGCTCGAGTCAT**GAGGATCGTC

>Ap-mir-375_3p
ATTTGTTCGTTCGGCTCGAGTCAT

```
ACGACCUGAUCACUUGAGCCAAGAGGACAAGUUUGUCUAAAUUGAAAUACAAAUCAAUUUGUUCGUUCGGCUCGAGUCAUGAGGAUCGUC
((((((.((.(((((((((....((((((((((((...........))))))....))))))....))))))))).)).))).)))).

 Initial dG =    -32.30

          10        20          30
-    -   G  C         .-AAGA|    A
 ACGA CCU AU ACUUGAGCC      GGACA G
 UGCU GGA UA UGAGCUCGG      UCUGU U
C    A   G  C         \ ----^    U
.        80        70

-----------------------------------------------------
```

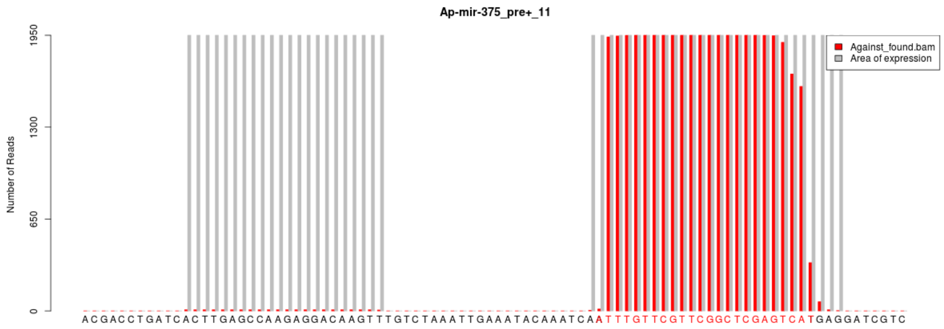

>Ap-mir-750_pre+_11

GTTTGTCCAAT**CAGTTGGAAGTTTGGATCAAGGC**ATGTTTTATGTTTTTACGTTTGGTATAAAATAGAAAATTATCTTTTTAATATCATA
CTGTGCCAGATCTAACTCTTCCAGCTGATTGGACTCGGT

>Ap-mir-750_5p

CAGTTGGAAGTTTGGATCAAGGC

GUUUGUCCAAU**CAGUUGGAAGUUUGGAUCAAGGC**AUGUUUUAUGUUUUUACGUUUGGUAUAAAAUAGAAAAUUAUCUUUUUAAUAUCAUACUGUGCCAGAUCUAACUCUUCCAGCUGAUU
GGACUCGGU

```
....((((((((((((((((.(((((((..(((((.....((((....)))).((((((.(((.
(((......))).))).))))))....))))).)))))))..)))))))))))))))))).....

 Initial dG =    -50.70

         10        20         30          40
GUUU-                 U-        AA      .-GUUUU|   U
     GUCCAAUCAGUUGGAAG  UUGGAUC  GGCAU       AUGU U
     CAGGUUAGUCGACCUUC  AAUCUAG  CCGUG       UGCA U
UGGCU                 UC        A-      \ -----^   U
       120       110       100                50


                                                 60         70
                                        U---      A   U   AA
                                            UGGUAU AAA AGA  A
                                            ACUAUA UUU UCU  U
                                        UCAU      A   U   AU
                                         90        80
```

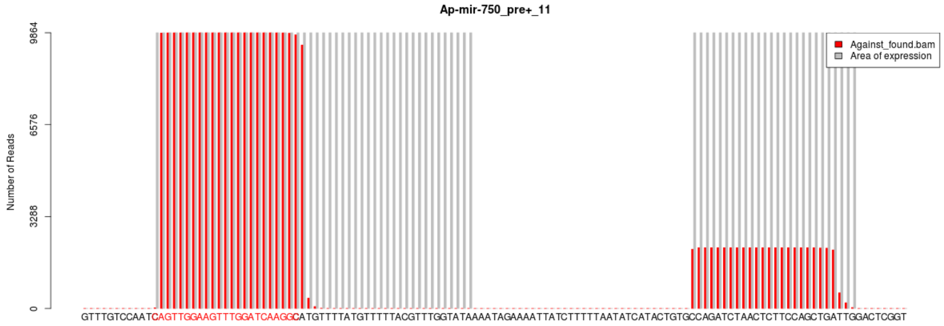

>Novel-5-1_pre+_11
TCGACTACCGG**TGTGATGTGCATGTGGGCTTCTG**TGATATTTACACGAACATTTAAGAACGAGTGGCCCACAGTCACAGCACCTCCAGTG
GTCGGTG

>Novel-5-1_5p
TGTGATGTGCATGTGGGCTTCTG

```
UCGACUACCGGUGUGAUGUGCAUGUGGGCUUCUGUGAUAUUUACACGAACAUUUAAGAACGAGUGGCCCACAGUCACAGCACCUCCAGUGGUCGGUG
((((((((.((.(((.((((..((((((((.((((......................)).)).))))))))..)))).)))..)).))))))))..

 Initial dG =    -38.60

          10         20          30
--        C  U-   A    CA        .-UUC|   AUA
  UCGACUAC GG  GUG UGUG  UGUGGGC     UGUG   \
  GGCUGGUG CC  CAC ACAC  ACACCCG     GCAC   U
GU        A  UC   G    UG        \ ---^   AUU
      90        80        70


                                   50
                                  AA     AA
                                    CAUUU  G
                                    GUGAG  A
                                  --     CA
                                        60

-----------------------------------------------------
```

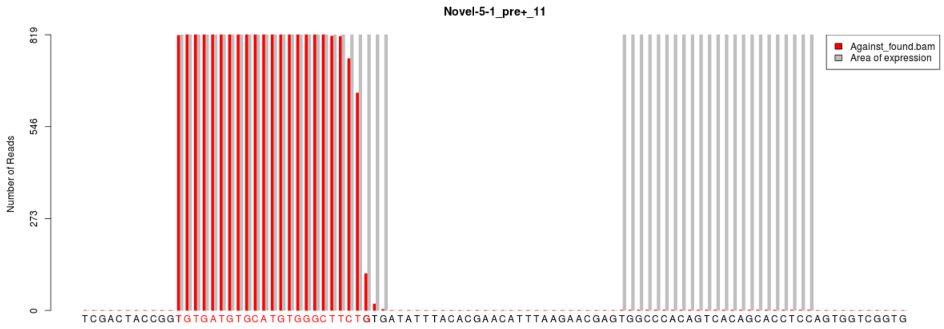

>Novel-5-2_pre+_11
TCGACTACCGG**TGTGATGTGCATGTGGGCTTCTG**TGATATTTACACGAACATTTAAGAACGAGTGGCCCACAGTCACAGCACCTCCAGTG
GTCGGTG

>Novel-5-2_5p
TGTGATGTGCATGTGGGCTTCTG

```
UCGACUACCGGUGUGAUGUGCAUGUGGGCUUCUGUGAUAUUUACACGAACAUUUAAGAACGAGUGGCCCACAGUCACAGCACCUCCAGUGGUCGGUG
((((((((.((.(((.((((..((((((((.((((.......................)).)).))))))))..)))).)))..)).))))))))..

 Initial dG =    -38.60

          10         20          30
--        C  U-    A    CA       .-UUC|   AUA
  UCGACUAC GG  GUG UGUG  UGUGGGC     UGUG   \
  GGCUGGUG CC  CAC ACAC  ACACCCG     GCAC   U
GU        A  UC    G    UG       \ ---^   AUU
      90        80        70


                                  50
                                 AA      AA
                                   CAUUU  G
                                   GUGAG  A
                                 --      CA
                                      60

-----------------------------------------------------
```

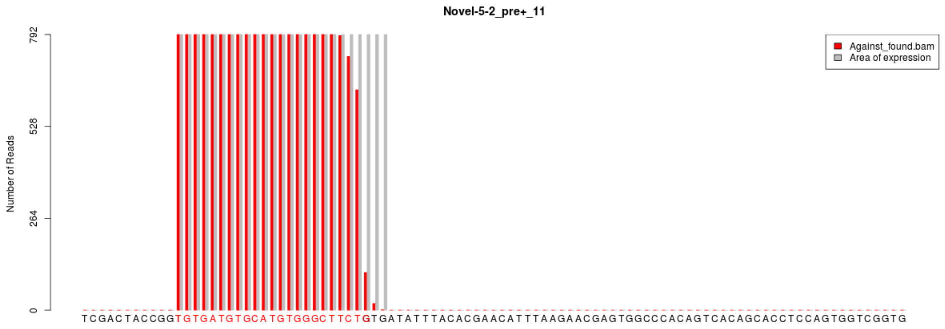

>Ap-mir-1175_pre+_11
AATTCATGAAT**TAAGTGGAGAAGTAGTCTGTTCAC**TATATTTATTAAATATAATAAAATATTTTGCAAATACGCACAAAGTGAGATTCAA
CTCCTCCAACTTAATTCAGTCACT

>Ap-mir-1175_5p
TAAGTGGAGAAGTAGTCTGTTCAC

AAUUCAUGAAU**UAAGUGGAGAAGUAGUCUGUUCAC**UAUAUUUAUUAAAUAUAAUAAAAUAUUUUGCAAAUACGCACAAAGUGAGAUUCAACUCCUCCAACUUAAUUCAGUCACU
......((((((((((((((.(((......((((((...(((((((....)))))))......(((......)))...))))))).....))).))))).)))))))))......

```
 Initial dG =    -29.20

        10         20         30           40
AAUUCA         -      A   AGUCUG      .-AUA|      A
      UGAAUUAAG UGGAG AGU      UUCACU     UUUAUUA A
      ACUUAAUUC ACCUC UCA      GAGUGA     AAAUAAU U
UCACUG         A      C   ACUUA-      \ ---^      A
  110       100        90         80            50


                                        60
                                       AUAUUU   AA
                                             UGC  A
                                             ACG  U
                                       AAC---   CA
                                                 70
```

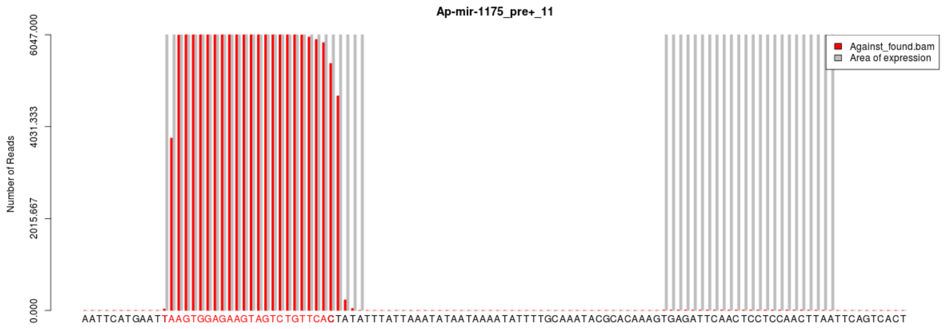

The microRNA toolkit of insects. Guillem Ylla, Bastian Fromm, Maria-Dolors Piulachs, Xavier Belles

Supplementary Data S5. Report generated with MirPlot of the 254 miRNAs of *Apis mellifera* available in miRBase. For each miRBase miRNA the following information is shown;

- The miRNA precursor with mature arm colored red.
- The mature arm colored red and indicating if it is 3' or 5'.
- The precursor folding in Vienna format.
- The free energy of the precursor folding.
- The folding of the miRNA precursor with colored mature arm.
- A plot showing all the expressed nucleotides of the precursor (grey), the reads per million in the available small RNA-seq libraries.

>ame-bantam_prec
TCGAAAACGAAACTGGTTTTCACAATGATTTGACAGATAGATTCGATTC**TGAGATCATTGTGAAAGCTGATT**TTGTTGAAAAGTCGA

>ame-bantam_3p
TGAGATCATTGTGAAAGCTGATT

```
UCGAAAACGAAACUGGUUUUCACAAUGAUUUGACAGAUAGAUUCGAUUCUGAGAUCAUUGUGAAAGCUGAUUUUGUUGAAAAGUCGA
((((.(((((((..((((((((((((((((..((((..........))))))))))))))))))))..))))))).......))))
```

```
 Initial dG =    -30.40

             10        20        30        40
    A-----|      CU                  GA     UAGA
UCGA      AACGAAA  GGUUUUCACAAUGAUUU  CAGA     U
AGCU      UUGUUUU  UCGAAAGUGUUACUAGA  GUCU     U
    GAAAAG^      AG                  --     UAGC
      80        70        60          50

-----------------------------------------------------
```

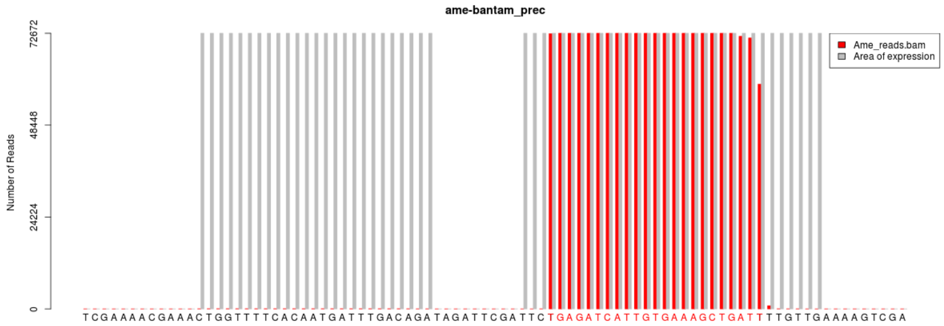

>ame-let-7_prec
ACGATGCCTGGG**TGAGGTAGTAGGTTGTATAGT**AGGGAATGGAAATTCGCGATATACGAAGTCCACTGTACAACTTGCTAACTTTCCCG
GTCGTCGACGC

>ame-let-7_5p
TGAGGTAGTAGGTTGTATAGT

```
ACGAUGCCUGGGUGAGGUAGUAGGUUGUAUAGUAGGGAAUGGAAAUUCGCGAUAUACGAAGUCCACUGUACAACUUGCUAACUUUCCCGGUCGUCGACGC
.(((((.(((((.(((.((((((((((((((((.......(((..((((.......)))).)))))))))))))))))))).))).))))).))))).....
```

```
 Initial dG =    -44.60

           10        20        30          40
A---     C     U   G                A    |   G
    CGAUG CUGGG GAG UAGUAGGUUGUAUAGU GG--GAAU G
    GCUGC GGCCC UUC AUCGUUCAACAUGUCA CC  CUUA A
CGCA     U     U   A                -  \ ^   A
.        90        80        70


                                             50
                                          G---  AU
                                              CG  \
                                              GC  A
                                          UGAA  AU
                                           60
```

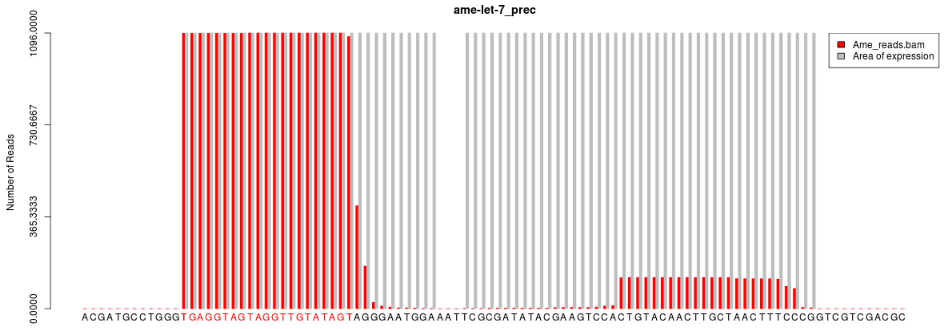

>ame-mir-1-1_prec
CCGGGCGATGCTGTTCCGTGCTTCCTTACTTCCCATAGTGGATGCGACGTA**TGGAATGTAAAGAAGTATGGAG**CTGCGCCCGG

>ame-mir-1-1_3p
TGGAATGTAAAGAAGTATGGAG

```
CCGGGCGAUGCUGUUCCGUGCUUCCUUACUUCCCAUAGUGGAUGCGACGUAUGGAAUGUAAAGAAGUAUGGAGCUGCGCCCGG
((((((.....((((((((((((.((((...(((((.((.......)))))))...)))).)))))))))))))..)))))))

 Initial dG =    -42.00

        10        20        30        40
|      AUGCU           C    UUC     G  GAU
CCGGGCG     GUUCCGUGCUUC UUAC   CCAUA UG   \
GGCCCGC     CGAGGUAUGAAG AAUG   GGUAU GC   G
^      GU---           A    UAA     -  AGC
  80           70        60        50
```

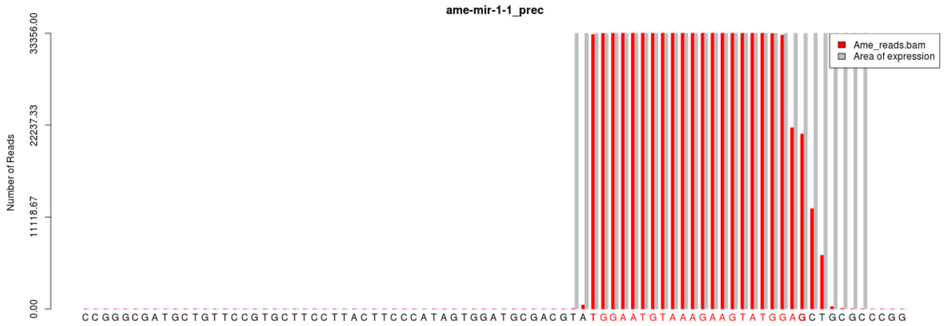

>ame-mir-1-2_prec
CCGGGCGATGCTGTTCCGTGCTTCCTTACTTCCCATAGTGGATGCGACGTA**TGGAATGTAAAGAAGTATGGAG**CTGCGCCCGG

>ame-mir-1-2_3p
TGGAATGTAAAGAAGTATGGAG

```
CCGGGCGAUGCUGUUCCGUGCUUCCUUACUUCCCAUAGUGGAUGCGACGUAUGGAAUGUAAAGAAGUAUGGAGCUGCGCCCGG
((((((.....((((((((((((.((((...(((((.((.......)))))))...)))).)))))))))))))..)))))))

 Initial dG =    -42.00

        10        20        30        40
|      AUGCU            C     UUC      G   GAU
CCGGGCG     GUUCCGUGCUUC UUAC   CCAUA UG   \
GGCCCGC     CGAGGUAUGAAG AAUG   GGUAU GC   G
^      GU---            A     UAA      -   AGC
  80           70        60        50

-----------------------------------------------------
```

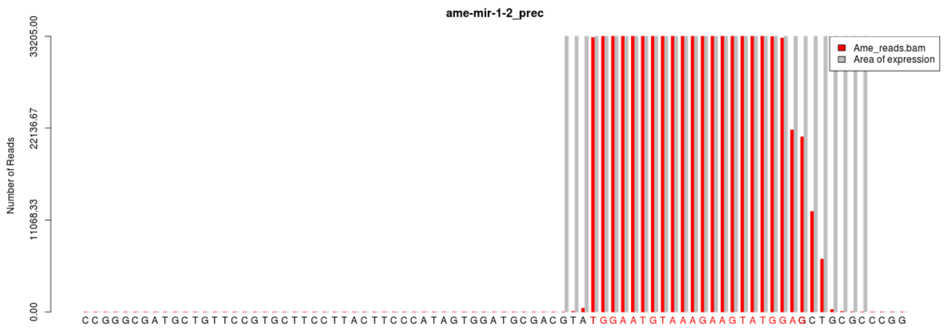

>ame-mir-10_prec
CCCAGTTAATGCTCTACATCT**ACCCTGTAGATCCGAATTTGT**TTGATAAGAGGCGACAAATTCGGTTCTAGAGAGGTTTGTGTGGTGCA
TACAGAGCTAC

>ame-mir-10_5p
ACCCTGTAGATCCGAATTTGT

```
CCCAGUUAAUGCUCUACAUCUACCCUGUAGAUCCGAAUUUGUUUGAUAAGAGGCGACAAAUUCGGUUCUAGAGAGGUUUGUGUGGUGCAUACAGAGCUAC
...((((.((((.((((((..(((((.((((.(((((((((((..........))))))))))).)))).)).))).)).)))))).)))).....)))).. 
```

```
 Initial dG =    -38.40

           10        20         30        40
CCC    A---    U      CU   -| G    U          UGAUA
   AGUU    AUGC CUACAU  ACC CU UAGA CCGAAUUUGUU     \
   UCGA    UACG GGUGUG  UGG GA AUCU GGCUUAAACAG     A
CA-    GACA    U      UU   A^ G    U          CGGAG
.         90        80        70        60        50

-----------------------------------------------------
```

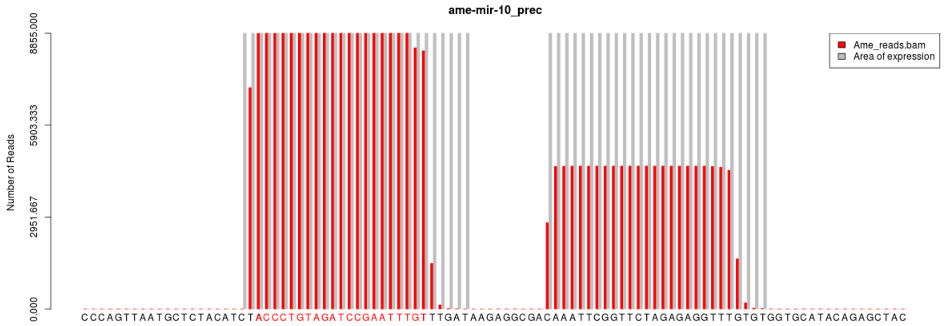

>ame-mir-100_prec
GCTATACTTGATGATACT**AACCCGTAGATCCGAACTTGTG**GGCTTTTTTATATGTATACCGCAAGCTCCTATCTACCGGTACATGTAGT
CAGGCCAGCAT

>ame-mir-100_5p
AACCCGTAGATCCGAACTTGTG

```
GCUAUACUUGAUGAUACUAACCCGUAGAUCCGAACUUGUGGGCUUUUUUAUAUGUAUACCGCAAGCUCCUAUCUACCGGUACAUGUAGUCAGGCCAGCAU
(((...((((((.(((...(((.((((((..((.(((((((.................))))))).))..)))))).)))...))).))))))..)))..

 Initial dG =    -31.30

          10        20        30        40
--|  AUA      G   CUA   C      CC  A       GCUUUUUU
  GCU   CUUGAU AUA   ACC GUAGAU  GA CUUGUGG        \
  CGA   GGACUG UGU   UGG CAUCUA  CU GAACGCC        A
UA^  CC-      A   ACA   C      UC  C       AUAUGUAU
.         90        80        70        60

-----------------------------------------------------
```

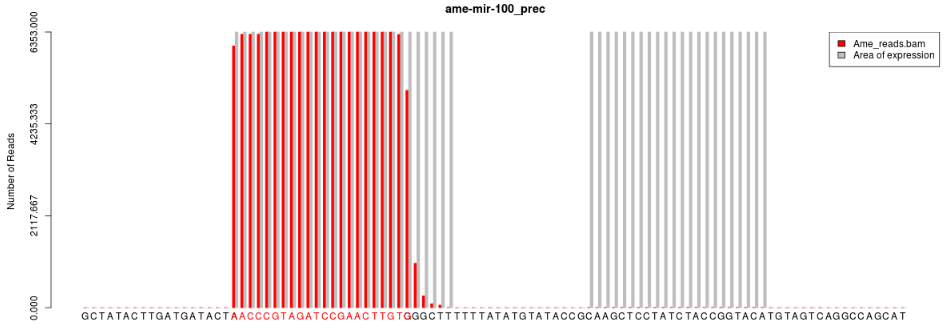

>ame-mir-1000_prec
ACGTCGCCGGGG**ATATTGTCTTGTCACAGCAGT**ACGCCTTTAAAGAGTTACTGGTGTGATGGGACATTGTCCTCGTCCCGTGGCCCTTC
GT

>ame-mir-1000_5p
ATATTGTCTTGTCACAGCAGT

```
ACGUCGCCGGGGAUAUUGUCUUGUCACAGCAGUACGCCUUUAAAGAGUUACUGGUGUGAUGGGACAUUGUCCUCGUCCCGUGGCCCUUCGU
..((((((((((((.(((((..(((((.(((((.((((....)).))))))).)))))..))))).))))))))....)))))).......
```

```
 Initial dG =    -42.70

                 10        20        30         40
AC-----     ----|       U     UG     G      C   -  U
       GUCGC    CGGGGAUA UGUCU  UCACA CAGUA GC CU U
       CGGUG    GCUCCUGU ACAGG  AGUGU GUCAU UG GA A
UGCUUCC     CCCU^       U     GU     G      -  A  A
90        80        70        60        50
```

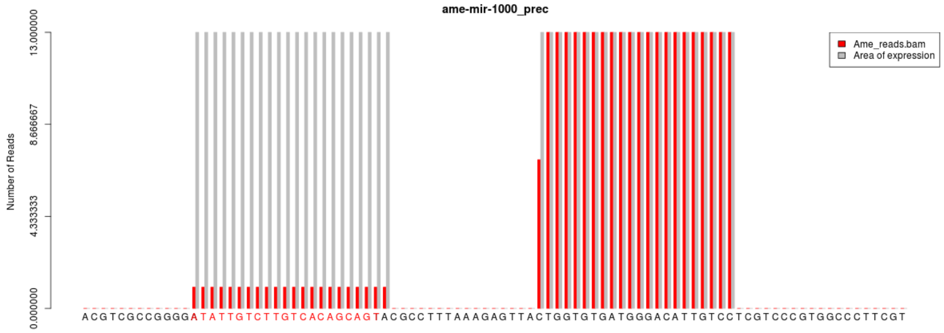

>ame-mir-1006_prec
ATTGGTTCTGAAAAGAATTGAGTAAATTTATATTAAAT**AAAATTCGATTTCTTCTATCATTA**

>ame-mir-1006_3p
AAAATTCGATTTCTTCTATCATTA

```
AUUGGUUCUGAAAAGAAUUGAGUAAAUUUAUAUUAAAUAAAAUUCGAUUUCUUCUAUCAUUA
.(..(((((....)))))..).......................(((.......)))....

 Initial dG =     -8.00

          10
.-A|UG     G
   U  GUUCU A
   A  UAAGA A
\ -^GU     A
   20

-----------------------------------------------------
```

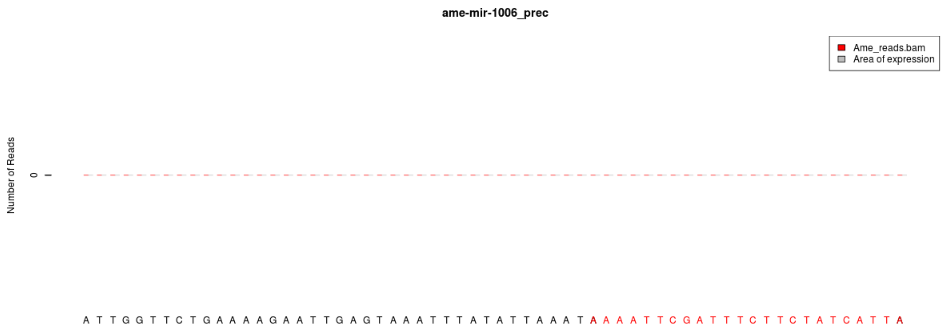

>ame-mir-11_prec
ATGGAGGATAGCCGTACCCTGATGGAGCTCTGGCTGTGACTTGTGTGTTAACTGAATTAAAATCA**CATCACAGGCAGAGTTCTAGTT**AG
GTAACGGTTTCAAACAACA

>ame-mir-11_3p
CATCACAGGCAGAGTTCTAGTT

```
AUGGAGGAUAGCCGUACCCUGAUGGAGCUCUGGCUGUGACUUGUGUGUUAACUGAAUUAAAAUCACAUCACAGGCAGAGUUCUAGUUAGGUAACGGUUUCAAACAACA
.((...((.((((((..(((((((((((((((.((((((..((((..((((.....))))...)))))))))).)))))))))).)))))..))))))))...))...

 Initial dG =    -46.20

          10        20         30        40         50
A--| GAG  U      AC     -          G      CU    UG-    CU
   UG   GA AGCCGU  CCUGA UGGAGCUCUG CUGUGA  UGUG   UUAA  \
   AC   CU UUGGCA  GGAUU AUCUUGAGAC GACACU  ACAC   AAUU  G
ACA^ AAA  -      AU     G          G      --    UAA    AA
      100         90        80        70          60

-----------------------------------------------------
```

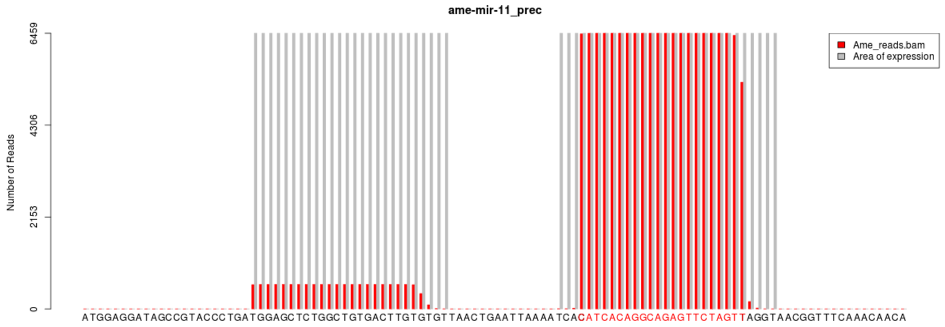

>ame-mir-1175_prec
CAAAAATAAATAATATGCAAGAAAGGGATTATGGTTCAAGTGGAGAAGTGGTCTTTACGCTTTGAATTAAAG**TGAGATTCACTCCTCCA**
ACTTACTCCTGATCCCTAATATTCTTCGTTTTTTTTTTTTTATTTGTTTC

>ame-mir-1175_3p
TGAGATTCACTCCTCCAACTTAC

CAAAAAUAAAUAAUAUGCAAGAAAGGGAUUAUGGUUCAAGUGGAGAAGUGGUCUUUACGCUUUGAAUUAAAG**UGAGAUUCACUCCUCCAACUUAC**UCCUGAUCCCUAAUAUUCUUCGUU
UUUUUUUUUUAUUUGUUUC
...((((((((((...((((((((((((((.((...((((((((.((((((((...
(((((((...))))))))))))).)))).))))).)))...))))))))))....))))).)).........)))))))))).

```
 Initial dG =    -39.20

        10               20            30        40         50         60
CAA          UAU------  -     ----        U  UUC   -     A    -|   UUA       A
   AAAUAAAUAA         GC AAGAA    AGGGAUUA GG   AAG UGGAG AGUG GUCU   CGCUUUG \
   UUUGUUUAUU         UG UUCUU    UCCCUAGU CC   UUC ACCUC UCAC UAGA   GUGAAAU A
C--          UUUUUUUUU  C     AUAA        -  UCA   A     C    U^   ---       U
        130       120       110       100         90        80           70

-----------------------------------------------------
```

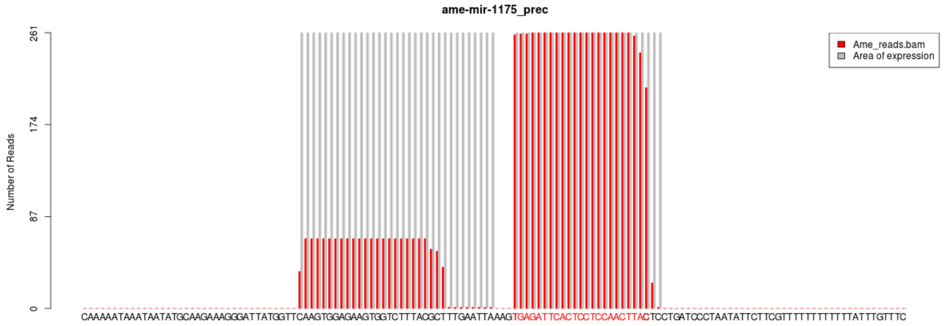

>ame-mir-12_prec
AAGACATGGGTG**TGAGTATTACATCAGGTACTGGT**GTGATATTCAGACAACCAGTACTTGTGTTATACTTACGCTCATGTCTT

>ame-mir-12_5p
TGAGTATTACATCAGGTACTGGT

```
AAGACAUGGGUGUGAGUAUUACAUCAGGUACUGGUGUGAUAUUCAGACAACCAGUACUUGUGUUAUACUUACGCUCAUGUCUU
(((((((((((((((((((.(((.(((((((((((((.........)).)))))))))))))).)))))))))))))))))))

 Initial dG =    -47.00

        10        20        30         40
|                  U   U           -   GAUA
AAGACAUGGGUGUGAGUAU ACA CAGGUACUGGU GU    \
UUCUGUACUCGCAUUCAUA UGU GUUCAUGACCA CA    U
^                  U   -           A   GACU
  80        70         60        50

-----------------------------------------------------
```

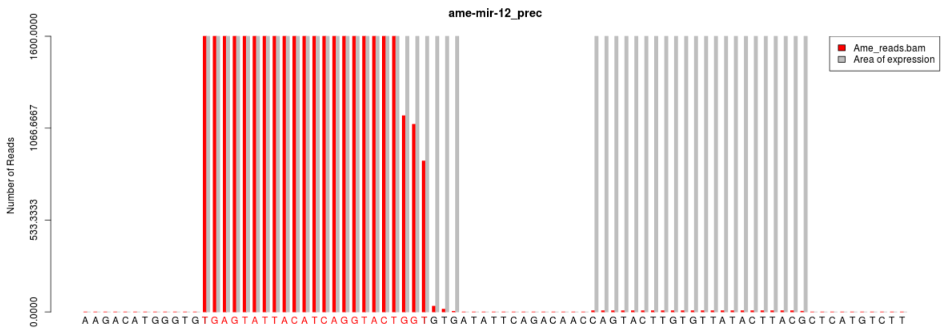

>ame-mir-124_prec
TGCTCCTTGCGTTCACTGCGGGCTTCCATGTGCCAACTTTTCAAAATTCA**TAAGGCACGCGGTGAATGCCAAG**AGCG

>ame-mir-124_3p
TAAGGCACGCGGTGAATGCCAAG

```
UGCUCCUUGCGUUCACUGCGGGCUUCCAUGUGCCAACUUUUCAAAAUUCAUAAGGCACGCGGUGAAUGCCAAGAGCG
.((((...((((((((((((.((((..(((..................))).)))).)))))))))))))...)))).

 Initial dG =    -30.80

        10        20        30
U|   CUU            G    CC   UGCCAACU
 GCUC   GCGUUCACUGCG GCUU  AUG        U
 CGAG   CGUAAGUGGCGC CGGA  UAC        U
G^   AAC            A    A-   UUAAAACU
      70        60         50        40
```

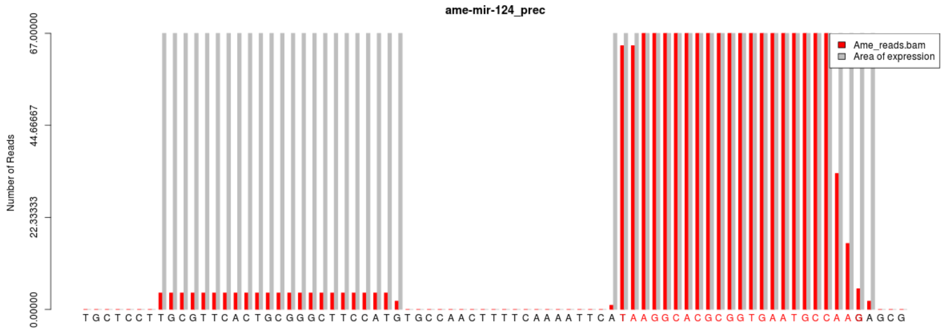

>ame-mir-125_prec
GTAAAGCCTGCCGCGTCGCCGGT**CCCCTGAGACCCTAACTTGTGA**CGTCGCGACCGATATCTCACAGGCTAGATTCTCTGGTATTGGCG
ATGAGTGCTGCCTTTTGC

>ame-mir-125_5p
CCCCTGAGACCCTAACTTGTGA

```
GUAAAGCCUGCCGCGUCGCCGGUCCCCUGAGACCCUAACUUGUGACGUCGCGACCGAUAUCUCACAGGCUAGAUUCUCUGGUAUUGGCGAUGAGUGCUGCCUUUUGC
((((((..((..(((((((((.((..((((..(((.(((((((.((((....))))...))))))).)))..)))).)).))))))))))...)).)).)).)))))

 Initial dG =    -41.20

          10          20         30         40           50
     --  CU  CG-           C  CU    CC    A        C--|   C
GUAAA  GC  GC   CGUCGCCGGU CC  GAGA  CUA CUUGUGA    GUCG G
CGUUU  CG  CG   GUAGCGGUUA GG  CUCU  GAU GGACACU    UAGC A
     UC  U-  UGA          U  U-    UA    C        CUA^   C
     100         90        80         70         60

-----------------------------------------------------
```

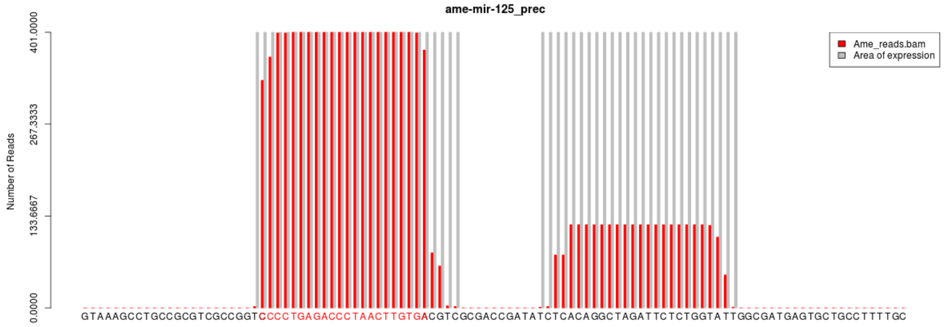

>ame-mir-133_prec
TAATGTTAAGCTTAGCTGGTTGAACACGGGTCAAATATATCGCACGATTGACGCAT**TTGGTCCCCTTCAACCAGCTGT**AGTTGACATTA

>ame-mir-133_3p
TTGGTCCCCTTCAACCAGCTGT

```
UAAUGUUAAGCUUAGCUGGUUGAACACGGGUCAAAUAUAUCGCACGAUUGACGCAUUUGGUCCCCUUCAACCAGCUGUAGUUGACAUUA
((((((((.((((((((((((((...((..(((((...(((......)))...)))))..))..))))))))))))).)))))))))))

 Initial dG =    -35.40

        10         20         30         40
|        G   -            CAC   GU      AUA    CA
UAAUGUUAA CU UAGCUGGUUGAA    GG  CAAAU    UCG   C
AUUACAGUU GA GUCGACCAACUU    CC  GUUUA    AGU   G
^        -  U            CC-  UG      CGC    UA
         80         70         60         50

-----------------------------------------------------
```

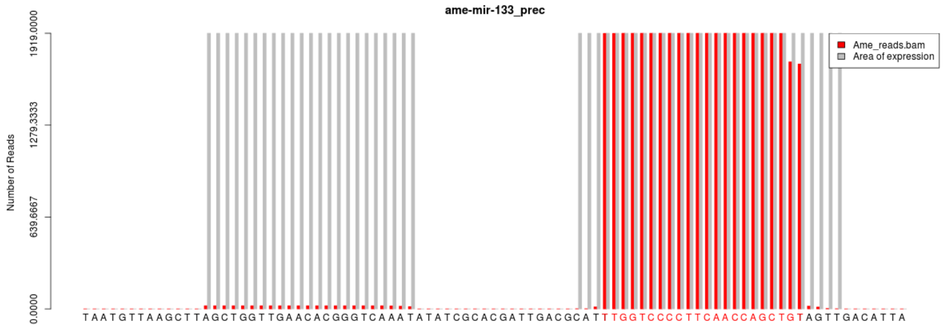

>ame-mir-137_prec
GACTTCATAGGCCAGGTTGGCGACGCGTATTCTTGGGGAATTAACACACATTTGCGCTG**TTATTGCTTGAGAATACACGTA**GTTTGCCT
GGTCGTTCACT

>ame-mir-137_3p
TTATTGCTTGAGAATACACGTA

```
GACUUCAUAGGCCAGGUUGGCGACGCGUAUUCUUGGGGAAUUAACACACAUUUGCGCUGUUAUUGCUUGAGAAUACACGUAGUUUGCCUGGUCGUUCACU
.........((((((((..((.(((.(((((((..((.(((.((((...........))))))).))..))))))).))).)).)).)))))))).......
```

```
 Initial dG =    -39.00

        10        20        30        40        50
GACUUCAUA|       UG  G   C       UG  G   U    CACAU
         GGCCAGGU  GC ACG GUAUUCU  GG AAU AACA     \
         CUGGUCCG  UG UGC CAUAAGA  UC UUA UUGU     U
UCACUUG--^       UU  A   A       GU  G   -    CGCGU
.          90        80        70         60
```

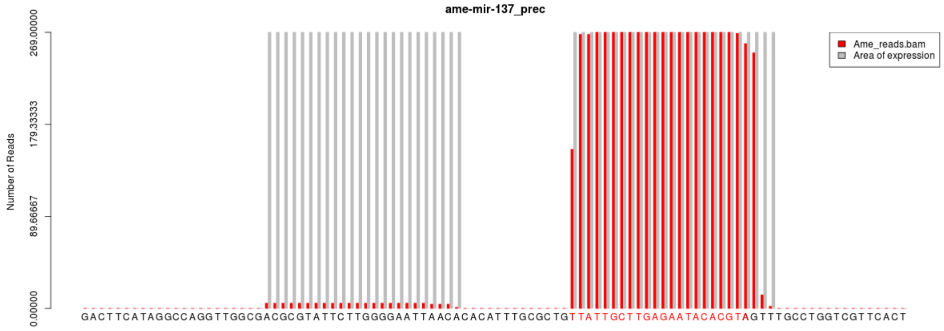

>ame-mir-13a_prec
ACCGAAATGAAAATACCTTTTGCGGTCCGATACATCAAATTGGTTGTGGAATGTTTCGAGTCA**TATCACAGCCATTTTGATGAG**CTTGG
CCCGCAGAATC

>ame-mir-13a_3p
TATCACAGCCATTTTGATGAG

```
ACCGAAAUGAAAAUACCUUUUGCGGUCCGAUACAUCAAAUUGGUUGUGGAAUGUUUCGAGUCAUAUCACAGCCAUUUUGAUGAGCUUGGCCCGCAGAAUC
................(((((((.((((..(((((((.(((((((((.(((........))).))))))))).)))))))...)))).))))))))..

 Initial dG =    -38.90

        10        20        30         40        50
ACCGAAAUGAAAAUACC        U    UA-|      U         A   UUU
                 UUUUGCGG CCGA   CAUCAAA UGGUUGUGG AUG   C
                 AAGACGCC GGUU   GUAGUUU ACCGACACU UAC   G
CU---------------        C    CGA^      U         A   UGA
.                       90        80        70        60

-----------------------------------------------------
```

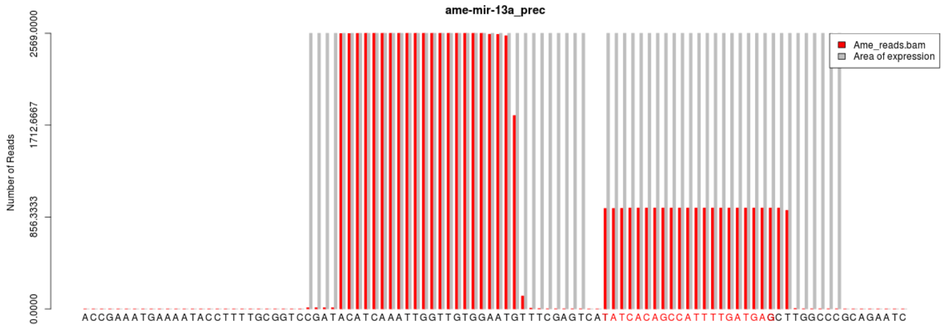

>ame-mir-13b_prec

GCGATCCAATCGTCAAATTGGTTGTGGCGTGTTGCTTTTCTAGATTTCA**TATCACAGCCATTTTTGACGATT**TGGATCG

>ame-mir-13b_3p

TATCACAGCCATTTTTGACGATT

```
GCGAUCCAAUCGUCAAAUUGGUUGUGGCGUGUUGCUUUUCUAGAUUUCAUAUCACAGCCAUUUUUGACGAUUUGGAUCG
.((((((((((((((((.((((((((..(((...((.....))....)))..))))))))..))))))))))).))))))

 Initial dG =    -38.10

         10          20         30
G      -          U-         GC    UUG-| UU
 CGAUCC AAUCGUCAAA  UGGUUGUG  GUG    CU  \
 GCUAGG UUAGCAGUUU  ACCGACAC  UAC    GA  U
-      U          UU         UA    UUUA^ UC
         70        60         50         40
```

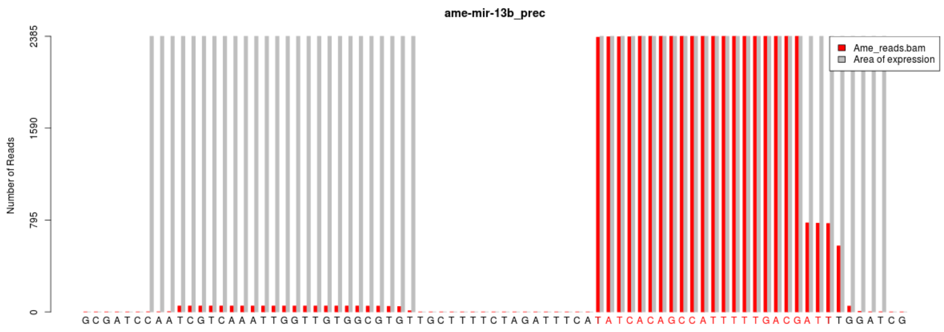

>ame-mir-14_prec
CTTTTTCTCGGTCGCTAGGTCAGTGGGGGTGAGAAACTGGCTTGGCTCTCTGTGCTACGATAG**TCAGTCTTTTTCTCTCTCCTA**TCGGC
TTTGCGACATA

>ame-mir-14_3p
TCAGTCTTTTTCTCTCTCCTA

```
CUUUUUCUCGGUCGCUAGGUCAGUGGGGGUGAGAAACUGGCUUGGCUCUCUGUGCUACGAUAGUCAGUCUUUUUCUCUCUCCUAUCGGCUUUGCGACAUA
..........(((((.(((((.(((((((.((((((..((((.((((.((........)).))))))))..)))))).))))))).))))).)))))...
```

```
 Initial dG =    -43.10

        10        20        30        40        50
CUUUUUCUCG|    U     A       U      CU    U    C  UGU
          GUCGC AGGUC GUGGGGG GAGAAA  GGCU GGCU UC   G
          CAGCG UUCGG UAUCCUC CUCUUU  CUGA CUGA AG   C
AUA-------^    U     C       U      UU    -    U  CAU
.               90        80        70         60

-----------------------------------------------------
```

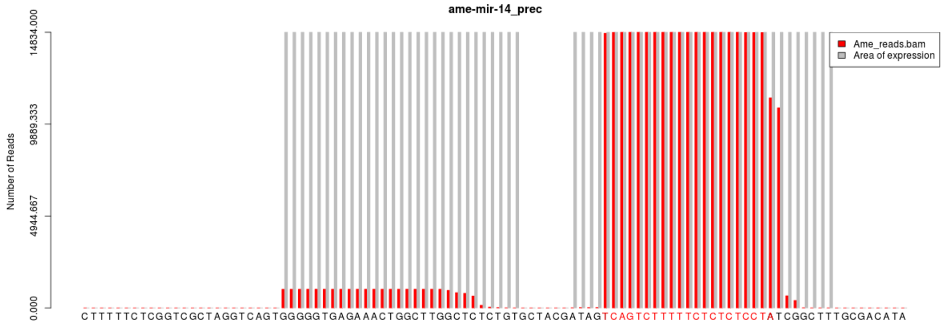

>ame-mir-184_prec
TTCGTGCCCAAAGCCCCTTATCATTCTCCTGTCCGGTGTAGAATTGTTAGACGAC**TGGACGGAGAACTGATAAGGGC**CCGAGGGTCACA
GAA

>ame-mir-184_3p
TGGACGGAGAACTGATAAGGGC

```
UUCGUGCCCAAAGCCCCUUAUCAUUCUCCUGUCCGGUGUAGAAUUGUUAGACGACUGGACGGAGAACUGAUAAGGGCCCGAGGGUCACAGAA
...(((((.....((((((((((((((.(((((((((...........)).))))))))))))).)))))))))).....))).))).....

 Initial dG =    -41.60

          10        20         31         42
UUC-   -   AAAGC         -|     U       -  AGAAU
    GUG CCC     CCCUUAUCA UUCUCC GUCCGGU GU     \
    CAC GGG     GGGAAUAGU AAGAGG CAGGUCA CA     U
AAGA   U   AGCCC         C^     -       G  GAUUG
 90        80        70         60        50

-----------------------------------------------------
```

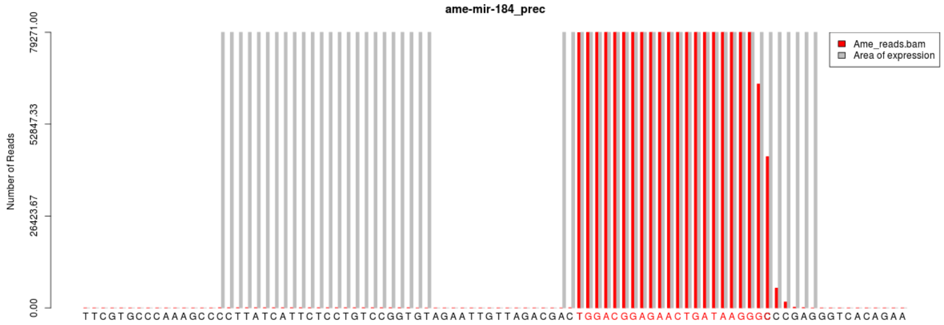

>ame-mir-190_prec
CAAACAAGTCGTCTGGTTTCCGTA**AGATATGTTTGATATTCTTGGTTGTT**TTTTAAAGAATCGACCAGGAATCAAACATATTATTATGG
TGGTCAGAAAA

>ame-mir-190_5p
AGATATGTTTGATATTCTTGGTTGTT

```
CAAACAAGUCGUCUGGUUUCCGUAAGAUAUGUUUGAUAUUCUUGGUUGUUUUUUAAAGAAUCGACCAGGAAUCAAACAUAUUAUUAUGGUGGUCAGAAAA
...........((((..(.((((((((((((((((((..(((((((((.(((.....))).))))))))))))))))))))))).)))))).)..))))...
```

```
 Initial dG =    -37.50

        10       20         30        40        50
CAAACAAGUCG    GU U      -|           AU         U   UU
           UCUG  U CCGUAA GAUAUGUUUGAU  UCUUGGUUG UUU  \
           AGAC  G GGUAUU UUAUACAAACUA  AGGACCAGC AAG  A
AAA--------    UG U      A^           --         U   AA
.                90        80          70        60

-----------------------------------------------------
```

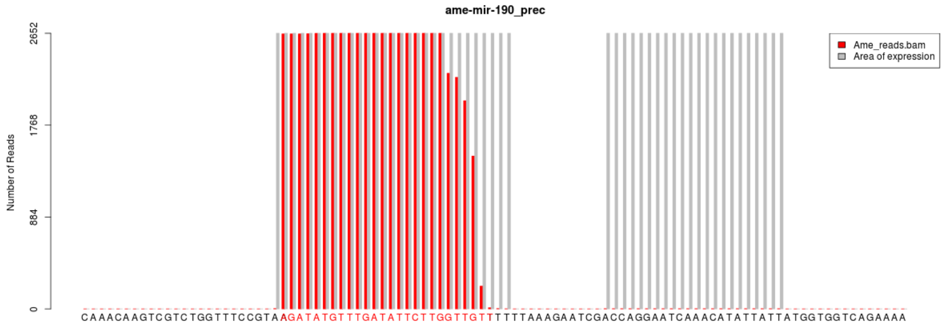

>ame-mir-193_prec
AGGGTCGAAGAGTGAGTAAATGGCCGAGGGTGATTTGGGCCTTAGTGGTCCTGTGGTGGCTGCGTACGAATCC**TACTGGCCTGCTAAGT**
CCCAAGTGATTCTCGGCTCGCGCTGCGATA

>ame-mir-193_3p
TACTGGCCTGCTAAGTCCCAA

```
AGGGUCGAAGAGUGAGUAAAUGGCCGAGGGUGAUUUGGGCCUUAGUGGUCCUGUGGUGGCUGCGUACGAAUCCUACUGGCCUGCUAAGUCCCAAGUGAUUCUCGGCUCGCGCUGCGAUA
...((((.((.((((.......((((((((.(((((((.(((((..(.((.((((.((...........)))))).)).).)..))))).))))))).))))))))))))).)).)))).
```

```
 Initial dG =    -50.80

        10        20        30        40        50        60
AGG|   A  A    GUAAAUG         G       C     UG U  U    U  CUGCG
   GUCG AG GUGA       GCCGAGGGU AUUUGGG CUUAG  G CC GUGG GG     \
   UAGC UC CGCU       CGGCUCUUA UGAACCC GAAUC  C GG CAUC CU     U
A--^   G  G    -------         G       U     GU C  U    -  AAGCA
         110              100        90        80         70

-----------------------------------------------------
```

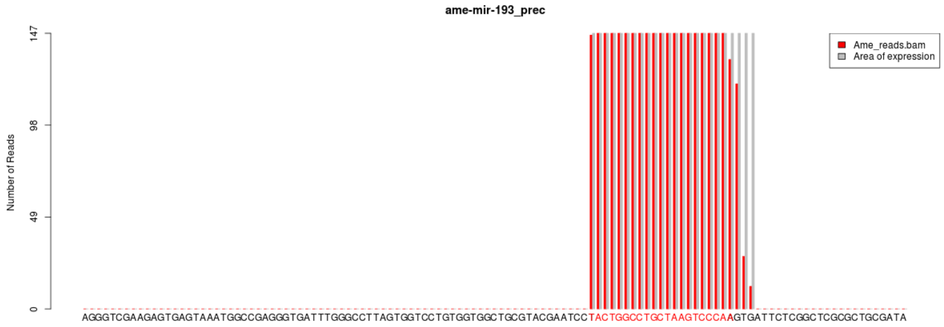

>ame-mir-2-1_prec
GGCGCGTGTGCACCGCTCACAAAGTGGTTGTGATATGCTGATACGAGCGTTCA**TATCACAGCCAGCTTTGATGAGC**GTGGCGTCGCGTC

>ame-mir-2-1_3p
TATCACAGCCAGCTTTGATGAGC

```
GGCGCGUGUGCACCGCUCACAAAGUGGUUGUGAUAUGCUGAUACGAGCGUUCAUAUCACAGCCAGCUUUGAUGAGCGUGGCGUCGCGUC
(((((...((..(((((((((((((((((((((((((......)))....))))))))))))).))))).))))))..))..))))))

 Initial dG =    -46.50

        10         20         30            40
      UGU  AC       -     -            ----|  GA
GGCGCG   GC  CGCUCA CAAAG UGGUUGUGAUAU    GCU  U
CUGCGC   CG  GCGAGU GUUUC ACCGACACUAUA    CGA  A
      UG-  GU       A     G            CUUG^  GC
         80        70        60        50

-----------------------------------------------------
```

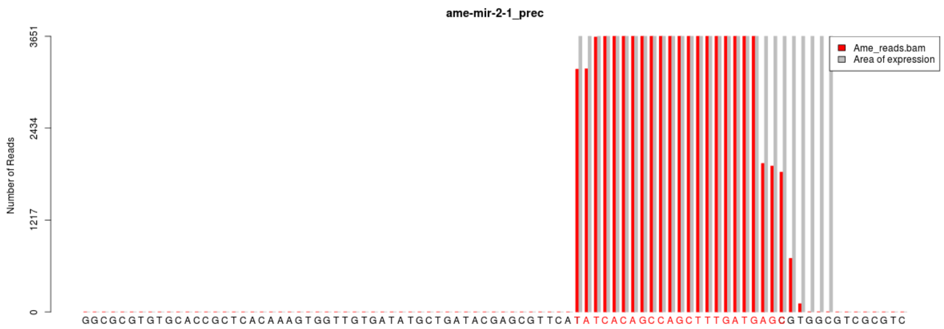

>ame-mir-2-2_prec
TCGACTGTTCCTCCCATCAGAGTGGTTGTGATGTGGTAACTTGGACTCG**TATCACAGCCAGCTTTGATGAGC**GGAACGGTGCGA

>ame-mir-2-2_3p
TATCACAGCCAGCTTTGATGAGC

```
UCGACUGUUCCUCCCAUCAGAGUGGUUGUGAUGUGGUAACUUGGACUCGUAUCACAGCCAGCUUUGAUGAGCGGAACGGUGCGA
((((((((((...((((((((((((((((((((((((.......)).))))))))))))))).))))))))...)))))))).)))

 Initial dG =    -41.40

         10         20          30          40
   -        UCC        -              -| AAC
UCG ACUGUUCC   CAUCAGAG UGGUUGUGAUGUG GU   \
AGC UGGCAAGG   GUAGUUUC ACCGACACUAUGC CA   U
   G        CGA        G              U^ GGU
   80        70         60         50

-----------------------------------------------------
```

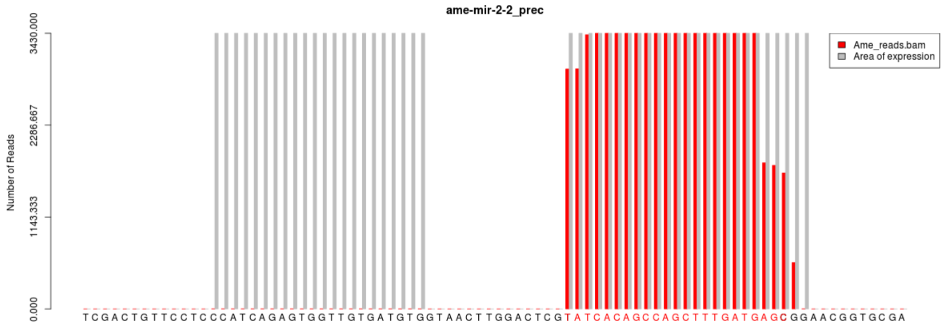

>ame-mir-2-3_prec
AAATATCCCCGGACAAGGACATGCTTTTACCATCAAAGTTGGTTTGTCATAGAGATCGAC**TATCACAGCCAGCTTTGATGAGC**AAAATT
GTGTCCGTCTA

>ame-mir-2-3_3p
TATCACAGCCAGCTTTGATGAGC

```
AAAUAUCCCCGGACAAGGACAUGCUUUUACCAUCAAAGUUGGUUUGUCAUAGAGAUCGACUAUCACAGCCAGCUUUGAUGAGCAAAAUUGUGUCCGUCUA
..........((((..((((((..((((..((((((((((((((.((.((((.......)))).)))))))))))))))))...)))))..)))))))))).

 Initial dG =     -35.30

        10        20        30         40        50
AAAUAUCCCC|   AA      GC     AC-              U  C    AGA
          GGAC  GGACAU  UUUU   CAUCAAAGUUGGUU GU AUAG   \
          UCUG  CCUGUG  AAAA   GUAGUUUCGACCGA CA UAUC   U
A---------^   --      UU     CGA              -  C    AGC
.                   90        80        70         60

-----------------------------------------------------
```

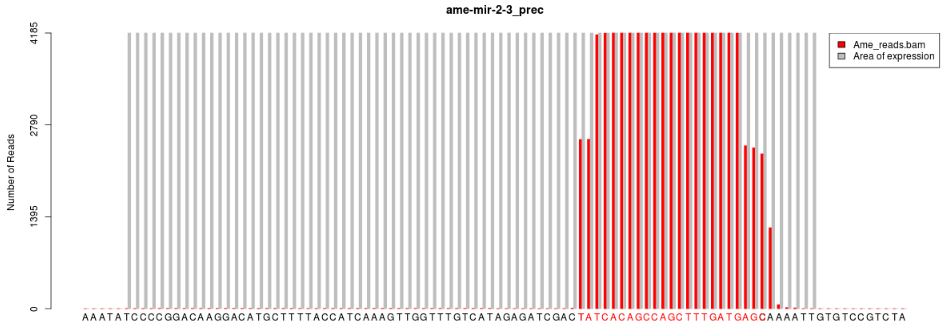

>ame-mir-210_prec
TGGACCCTAATGCAGCTGCTGGCCACTGCACAAGATTAGACATAAGACTC**TTGTGCGTGTGACAGCGGCTA**TGATGGGGTTTCCA

>ame-mir-210_3p
TTGTGCGTGTGACAGCGGCTA

```
UGGACCCUAAUGCAGCUGCUGGCCACUGCACAAGAUUAGACAUAAGACUCUUGUGCGUGUGACAGCGGCUAUGAUGGGGUUUCCA
.((((((((.(..((((((((..(((((((((((((((....)))...))))))))).))).))))))))..).)))))..))).

 Initial dG =    -39.60

          10        20         30           40
U   --     A GC        GC   -         ---|  G
 GGA  CCCUA U  AGCUGCUG  CAC UGCACAAGA   UUA A
 CCU  GGGGU G  UCGGCGAC  GUG GCGUGUUCU   AAU C
A   UU     A UA        A-   U         CAG^  A
    80        70         60        50

-----------------------------------------------------
```

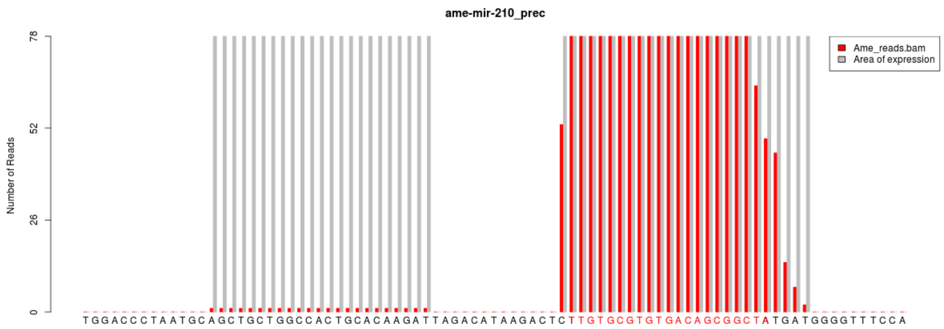

>ame-mir-219_prec
AATTGAATGTCTCAGGCAA**TGATTGTCCAAACGCAATTCTTG**TCTAAACGGTACGAAATCAAGAATTGTGTGGGGACATCAGCGCTCGA
GGTGCGATTCAAC

>ame-mir-219_5p
TGATTGTCCAAACGCAATTCTTG

```
AAUUGAAUGUCUCAGGCAAUGAUUGUCCAAACGCAAUUCUUGUCUAAACGGUACGAAAUCAAGAAUUGUGUGGGGACAUCAGCGCUCGAGGUGCGAUUCAAC
..(((((((((((..((..((((.((((..((((((((((((.................))))))))))))..))))))))..))..)))..)).)))))).
```

```
 Initial dG =     -37.90

         10          20         30         40         50
AA       -  --|  AG  AA    U    AA            UCUAAACG
  UUGAAU GU  CUC  GC  UGAU GUCC  ACGCAAUUCUUG        \
  AACUUA CG  GAG  CG  ACUA CAGG  UGUGUUAAGAAC        G
C-      G  UG^  CU  CG    -    GG            UAAAGCAU
 100        90         80          70         60

-----------------------------------------------------
```

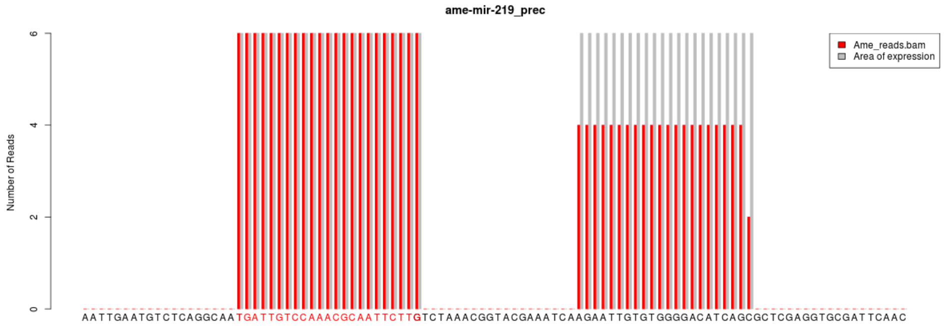

>ame-mir-252a_prec
AATCCTGTCTTGTTC**ATAAGTACTAGTGCCGCAGGAG**TGACTAGGTTGGGTTAGAAATTACCATATCTCCTGCTGCTCAAGTGCTTATC
AATGGGTCCGG

>ame-mir-252a_5p
ATAAGTACTAGTGCCGCAGGAG

```
AAUCCUGUCUUGUUCAUAAGUACUAGUGCCGCAGGAGUGACUAGGUUGGGUUAGAAAUUACCAUAUCUCCUGCUGCUCAAGUGCUUAUCAAUGGGUCCGG
...((...((..((.(((((((((...((.(((((((......(((.............)))....))))))).))...))))))))).))..))...))
```

```
 Initial dG =    -32.20

       10        20        30        40        50
AAU| UGU  UG  C         AGU  C       UGACUA   UGGGUU
   CC   CU  UU AUAAGUACU   GC GCAGGAG      GGU      \
   GG   GG  AA UAUUCGUGA   CG CGUCCUC      CCA      A
---^ CCU  GU  C         ACU  U       UAUA--   UUAAAG
 100       90        80        70          60

-----------------------------------------------------
```

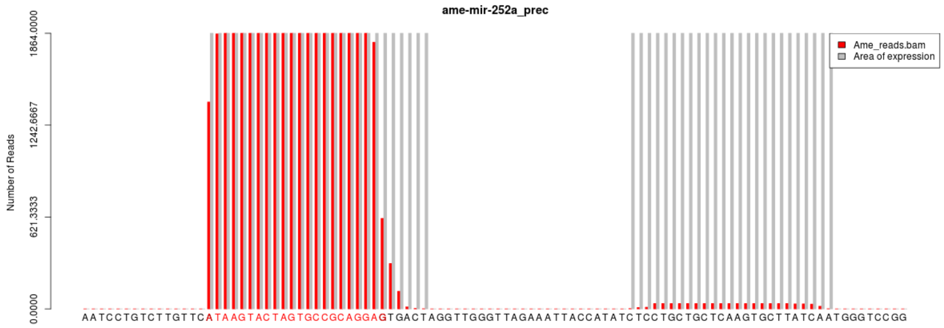

>ame-mir-252b_prec
AATCTGTCTG**TTAAGTAGTAGTGTCGTAGATGA**TGATTTAGAAATTCATCTTGGCACGATTACTTTTCATATAGAAATA

>ame-mir-252b_5p
TTAAGTAGTAGTGTCGTAGATGA

```
AAUCUGUCUGUUAAGUAGUAGUGUCGUAGAUGAUGAUUUAGAAAUUCAUCUUGGCACGAUUACUUUUCAUAUAGAAAUA
..(((((.((..(((((((.((((((.((((((...........))))))))))))).))))))).)).))))).....

 Initial dG =    -26.70

          10        20        30
AA--|    C  UU       A      U      UGAUU
    UCUGU UG  AAGUAGU GUGUCG AGAUGA     U
    AGAUA AC  UUCAUUA CACGGU UCUACU     A
AUAA^    U  UU       G      -      UAAAG
        70        60         50        40

-----------------------------------------------------
```

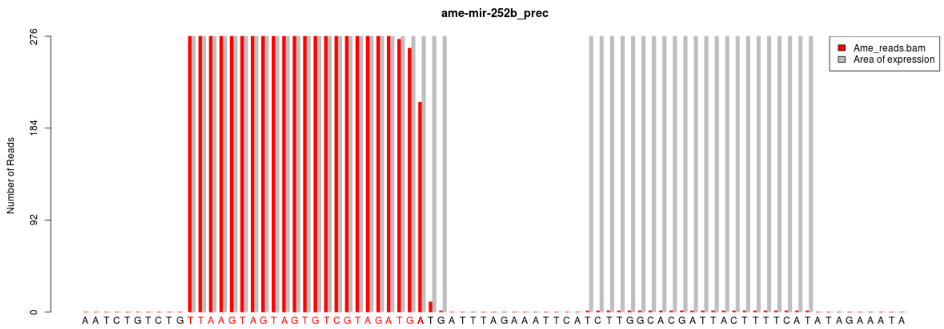

>ame-mir-263a_prec
AGCTTGGACTCT**GTAAATGGCACTGGAAGAATTCAC**GGGGGATTTAAGAAACGGGCCCGTGGAGCTCCCGTGTCATACACAGCGTCCGG
CT

>ame-mir-263a_5p
GTAAATGGCACTGGAAGAATTCAC

```
AGCUUGGACUCUGUAAAUGGCACUGGAAGAAUUCACGGGGGAUUUAAGAAACGGGCCCGUGGAGCUCCCGUGUCAUACACAGCGUCCGGCU
((((.((((.((((..(((((((.(((....((((((((................))))))))..))).)))))))..)))).))))))))

 Initial dG =    -40.20

        10        20        30        40
|   U    U    AA       U   AGAA       GGAUUUA
AGCU GGAC CUGU  AUGGCAC GGA    UUCACGGG       A
UCGG CCUG GACA  UACUGUG CCU    AGGUGCCC       G
^   -    C    CA       C   CG--       GGGCAAA
90         80        70          60        50

-----------------------------------------------------
```

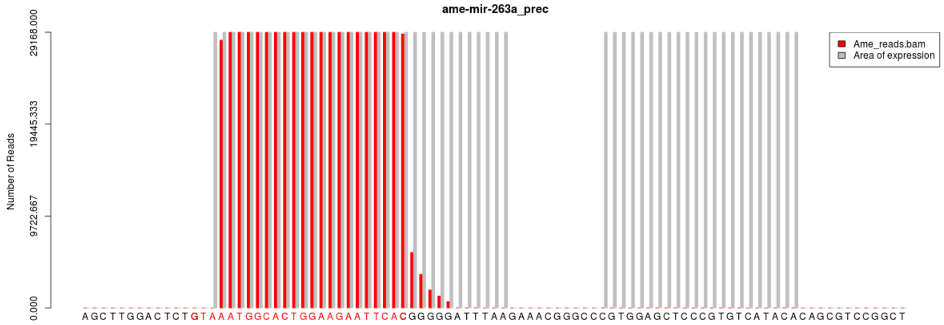

>ame-mir-263b_prec
AAGGTTCTGGCGGGT**CTTGGCACTGGAAGAATTCAC**AGATGTGCAGTGTATAATCGTGGATCTTGCAATGCCATCACTTGCTGGATACC
GA

>ame-mir-263b_5p
CTTGGCACTGGAAGAATTCAC

```
AAGGUUCUGGCGGGUCUUGGCACUGGAAGAAUUCACAGAUGUGCAGUGUAUAAUCGUGGAUCUUGCAAUGCCAUCACUUGCUGGAUACCGA
..((((..((((((..(((((.((.((((.(((((.(((((((...)))).))))))))))))).)).)))))..))))))..)).)))..

 Initial dG =    -39.60

         10        20        30        40
AA   -| UG      CU     C  G    A     A   -    A
  GGU UC  GCGGGU  UGGCA UG AAGA UUCAC GAU GUGC \
  CCA AG  CGUUCA  ACCGU AC UUCU AGGUG CUA UAUG G
AG   U^ GU      CU     A  G    -     -   A    U
90        80        70         60         50

-----------------------------------------------------
```

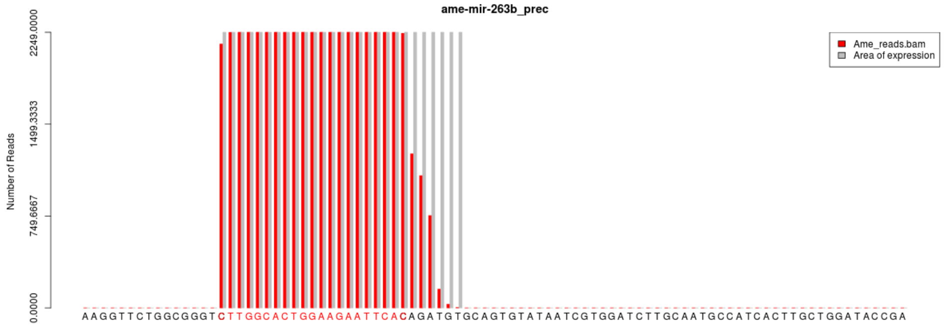

>ame-mir-275_prec
AAACGTTACTTGTCGTGCGCAACGCGCGTTACTCGGGTACTTTAGGCTGTGCCAATTTCGAATCAG**TCAGGTACCTGAAGTAGCGCGCG**
CTGCGGCGAAA

>ame-mir-275_3p
TCAGGTACCTGAAGTAGCGCGCG

```
AAACGUUACUUGUCGUGCGCAACGCGCGUUACUCGGGUACUUUAGGCUGUGCCAAUUUCGAAUCAGUCAGGUACCUGAAGUAGCGCGCGCUGCGGCGAAA
............((((.((((.((((((((((((((((((((..(((((..............)))))))))))))).))))))))))).))))))))..
```

```
 Initial dG =    -48.30

        10        20        30         40        50
AAACGUUACUUG|   G    A           -         UA      UGCCAA
            UCGU CGCA CGCGCGUUACU CGGGUACUU  GGCUG      U
            AGCG GCGU GCGCGCGAUGA GUCCAUGGA  CUGAC      U
AA----------^   -    C           A         --      UAAGCU
.                   90        80        70           60

-----------------------------------------------------
```

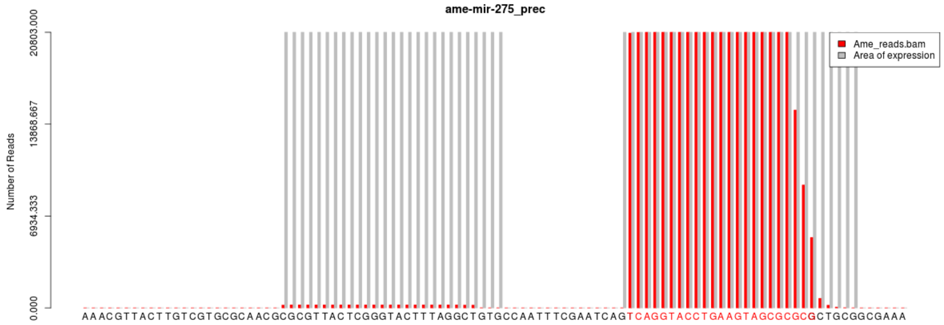

>ame-mir-276_prec
TGGTAGAGATCCAGCAGCGAGGTATAGAGTTCCTACGTAGTGTTCAGAAAG**TAGGAACTTCATACCGTGCTCT**TGGACTTGCCG

>ame-mir-276_3p
TAGGAACTTCATACCGTGCTCT

```
UGGUAGAGAUCCAGCAGCGAGGUAUAGAGUUCCUACGUAGUGUUCAGAAAGUAGGAACUUCAUACCGUGCUCUUGGACUUGCCG
.((((.((.(((((.((((.(((((..(((((((((.............)))))))))..))))).)))).))))))))))).

 Initial dG =    -38.30

        10        20        30        40
U|   G  A     C    A     AG         GUAGUG
 GGUA AG UCCAG AGCG GGUAU  AGUUCCUAC      U
 CCGU UC AGGUU UCGU CCAUA  UCAAGGAUG      U
G^   -  -     C    G     CU         AAAGAC
   80          70        60        50

-----------------------------------------------------
```

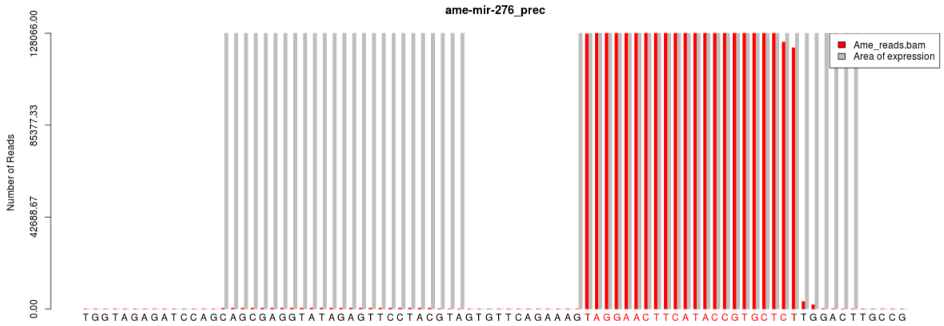

>ame-mir-2765_prec

ATTACTGTGTCGAGGAT**TGGTAACTCCACCACCGTTGGC**GGAATCTAATAGCCAATGGGGTACGAGTTTCCTTCTCTCGGCGCGAAATG

>ame-mir-2765_5p

TGGTAACTCCACCACCGTTGGC

```
AUUACUGUGUCGAGGAUUGGUAACUCCACCACCGUUGGCGGAAUCUAAUAGCCAAUGGGGUACGAGUUUCCUUCUCUCGGCGCGAAAUG
.....((((((((((..((.(((((.(((.((((((((...........)))))))))))..))))).)).)).))))))))).....

 Initial dG =    -39.70

        10         20         30        40
AUUAC         -| UU  U     C-   A        GGAAU
     UGUGUCGAG GA  GG AACUC  ACC CCGUUGGC     \
     GCGCGGCUC CU  CC UUGAG  UGG GGUAACCG     C
GUAAA         U^ U-  U     CA   -        AUAAU
        80         70        60         50

-----------------------------------------------------
```

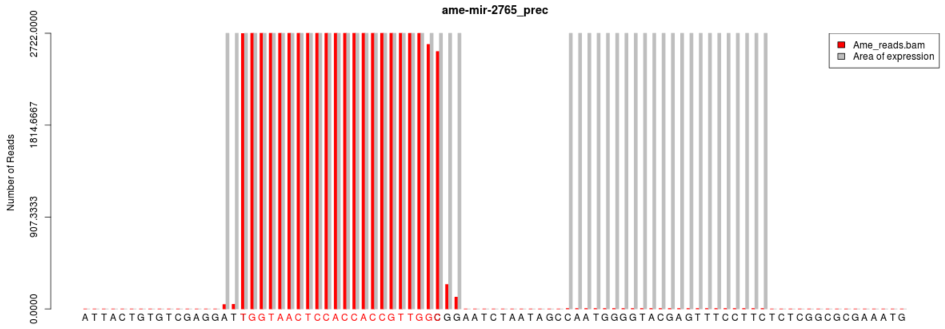

>ame-mir-277_prec
GGCAGTTGGGGCTCGTGCCAGATGCGCGTTTACACGGGCCCTGAATACTG**TAAATGCACTATCTGGTACGACA**TCTCTCCTGTC

>ame-mir-277_3p
TAAATGCACTATCTGGTACGACA

```
GGCAGUUGGGGCUCGUGCCAGAUGCGCGUUUACACGGGCCCUGAAUACUGUAAAUGCACUAUCUGGUACGACAUCUCUCCUGUC
(((((..((((.((((((((((((.((((((((((((...))).....)))))))))..))))))))))))..))))..)))))

 Initial dG =    -35.90

        10         20         30
     UU    C-           C-         -----|  G
GGCAG  GGGG  UCGUGCCAGAUG  GCGUUUACA     CGG \
CUGUC  CUCU  AGCAUGGUCUAU  CGUAAAUGU     GUC  C
     CU    AC           CA         CAUAA^  C
   80        70        60        50        40

-----------------------------------------------------
```

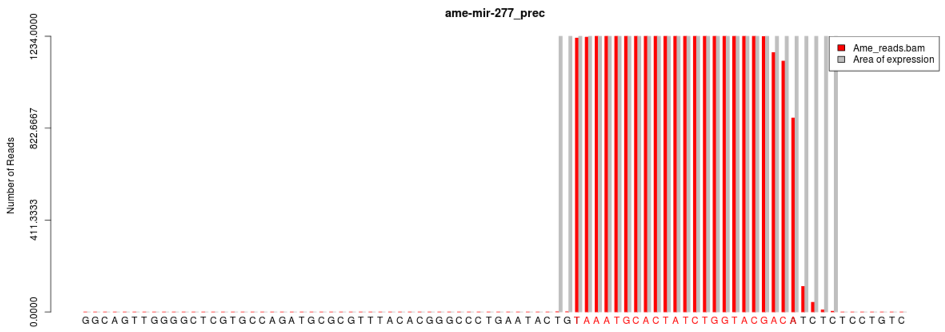

>ame-mir-278_prec
TCACGAGTAGTGTGTCCGGATGAGGTCTTCATCGACCGTGATTTAATTTCATAAGG**TCGGTGGGACTTTCGTCCGTTT**GCAAGACTCGA
GGA

>ame-mir-278_3p
TCGGTGGGACTTTCGTCCGTTT

```
UCACGAGUAGUGUGUCCGGAUGAGGUCUUCAUCGACCGUGAUUUAAUUUCAUAAGGUCGGUGGGACUUUCGUCCGUUUGCAAGACUCGAGGA
...(((((..((((..((((((((((.((((((((((((((.......))))..)))))))))))))).)))))))..))))..))))).....
```

```
 Initial dG =    -44.40

         10         20          30           40
UCA-      AG    UC       -      U          --|   UUU
    CGAGU  UGUG  CGGAUG AGGUC UCAUCGACC   GUGA   \
    GCUCA  ACGU  GCCUGC UUCAG GGUGGCUGG   UACU   A
AGGA      GA    UU       U      -          AA^   UUA
 90        80         70          60         50
```

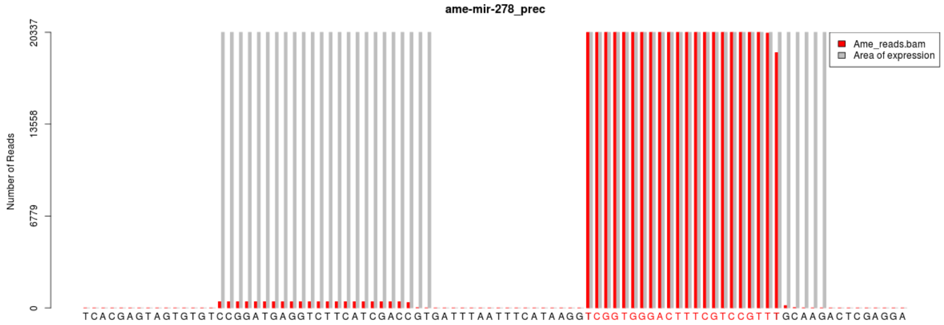

>ame-mir-2788_prec
AGGTTGGAGTTCGTTGCGGCAGGGTTGGGGTTTCTATGCGGCATGTGCCTTGCTTTGAGAAATG**CAATGCCCTTCGAAATCCCAAA**AAT
GTCCAACGCTTATCAACA

>ame-mir-2788_3p
CAATGCCCTTCGAAATCCCAAA

```
AGGUUGGAGUUCGUUGCGGCAGGGUUGGGGUUUCUAUGCGGCAUGUGCCUUGCUUUGAGAAAUGCAAUGCCCUUCGAAAUCCCAAAAAUGUCCAACGCUUAUCAACA
..(((((....(((((.((((...((((((((((.....(((((.((((((.....)))....))))))))....))))))))))...))))))))).....))))).
```

```
 Initial dG =    -38.60

        10        20        30        40            50
AG     AGUU     C    GGG          UAUGC     G    ----|  GC
  GUUGG    CGUUG GGCA   UUGGGGUUUC     GGCAU UGC    CUU  \
  CAACU    GCAAC CUGU   AACCCUAAAG     CCGUA ACG    GAG  U
A-     AUUC     -    AAA          CUUC-     -    UAAA^  UU
      100         90        80         70         60

-----------------------------------------------------
```

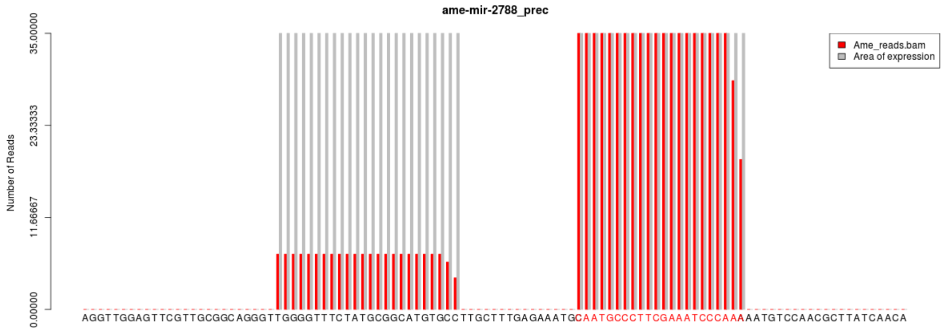

>ame-mir-2796_prec
AAGGAGGGAAGAGAAGGAACGTAGCGATAAGGTTAGGGGTTTCTATCGGCCTCCAGCAGCAACGATGGTT**GTAGGCCGGCGGAAACTAC**
TTGCTCTTGTCGCTAACGATCGTCGGCCCGAAAGATCCA

>ame-mir-2796_3p
GTAGGCCGGCGGAAACTACTTGC

AAGGAGGGAAGAGAAGGAACGUAGCGAUAAGGUUAGGGGUUUCUAUCGGCCUCCAGCAGCAACGAUGGUU**GUAGGCCGGCGGAAACUACUUGC**UCUUGUCGCUAACGAUCGUCGGCCCG
AAAGAUCCA
..((((((....((..((.((((((((((((((.((..((((((..((((((.
((((..........)))).))))))..))))))..)).)).)))))))))).))).)).)).)).)))......))).

```
 Initial dG =    -55.00

              10        20         30         40        50        60
AA   ------|  AAGA  AG  A   -         -  U  GG      AU      C    AGCA
  GGA      GGG    GA  GA CGU AGCGAUAAG GU AG  GUUUCU  CGGCCU CAGC    A
  CCU      CCC    CU  CU GCA UCGCUGUUC CG UC  CAAAGG  GCCGGA GUUG    C
A-   AGAAAG^  GG--  G-  A   A         U  U  AU      CG      U    GUAG
       120          110       100        90        80        70
```

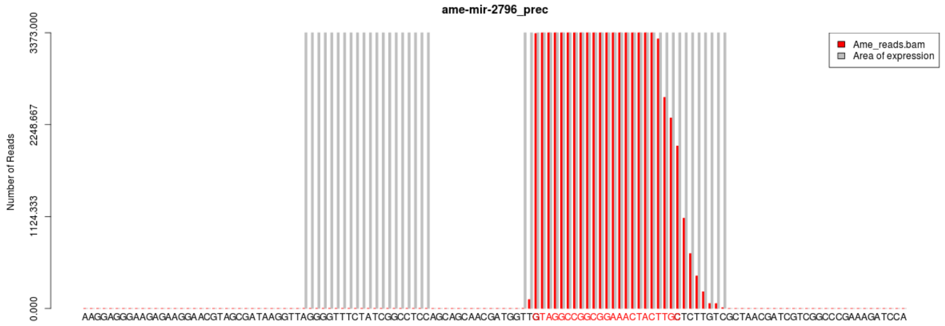

>ame-mir-279a_prec
AGAAAATGAAAAAATTTCCTGAATTTGCCAAATGAGTGAAGGTCTAGTGCACAGAAAATGAAATTG**TGACTAGATCCACACTCATTAA**G
TACGTTCAGGT

>ame-mir-279a_3p
TGACTAGATCCACACTCATTAA

```
AGAAAAUGAAAAAAUUUCCUGAAUUUGCCAAAUGAGUGAAGGUCUAGUGCACAGAAAAUGAAAUUGUGACUAGAUCCACACUCAUUAAGUACGUUCAGGU
...((((......))))(((((((.(((..((((((((..((((((((.(((((.........)))))))))))))..))))))))..))).))))))).
```

```
 Initial dG =    -36.40

        10        20        30        40        50
AGAAAAUGAAAAAAUUU|      U   CA        AA        G     AAAA
                 CCUGAAU UGC  AAUGAGUG  GGUCUAGU CACAG    \
                 GGACUUG AUG  UUACUCAC  CUAGAUCA GUGUU    U
U----------------^      C   AA        AC        -     AAAG
.                        90        80        70         60
```

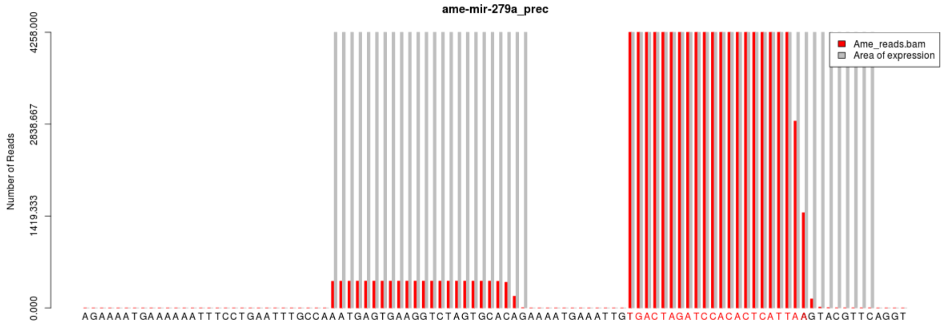

>ame-mir-279b_prec
AAATGTTTGTGAAATTTTATACTCTTGTTATTAGGGACGAAGTATTCATCTAGTGCAACGTTGTAAAGAAATCG**TGACTAGATCGAAAT**
ACTCGTCCCTAATAGAGAGAATCAATAATCATCAACAA

>ame-mir-279b_3p
TGACTAGATCGAAATACTCGTCCC

AAAUGUUUGUGAAAUUUUAUACUCUUGUUAUUAGGGACGAAGUAUUCAUCUAGUGCAACGUUGUAAAGAAAUCG**UGACUAGAUCGAAAUACUCGUCCC**UAAUAGAGAGAAUCAAUAAUC
AUCAACAA
...((((.((((...((.((.(((((..((((((((((((.((((((((((((((.((.
((............))))))))))).).)))))))))))))))))))))))).)).))...)))).)))).

```
 Initial dG =    -41.50

        10         20         30         40           50         60
AAA|   U    AAU  U  A     GU            A     - -       G  A  UUGUA
   UGUU GUGA   UU AU CUCUU  UAUUAGGGACGA GUAUU C AUCUAGU CA CG     A
   ACAA UACU   AA UA GAGAG  AUAAUCCCUGCU CAUAA G UAGAUCA GU GC     A
A--^   C    AAU  C  A     --            -     A C       -  -  UAAAG
       120       110         100         90        80          70

-----------------------------------------------------
```

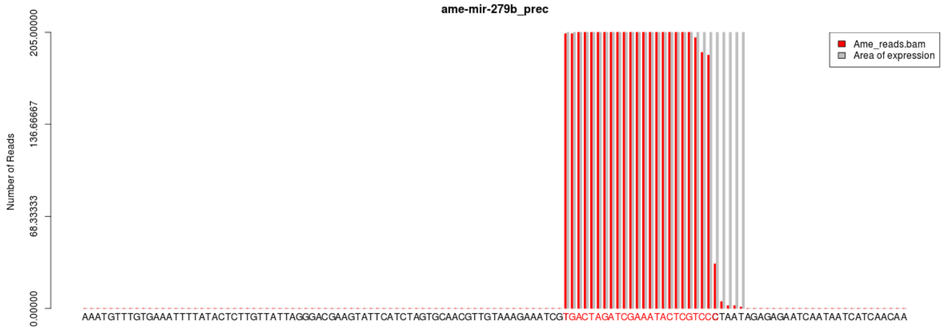

>ame-mir-279c_prec

ATATATTCTAATCGTTGGACGGGTGCACTCTGGTATCATGTGGATTTGCTCA**TGACTAGAGTCACACTCGTCCA**TTGCTTGGAATAC

>ame-mir-279c_3p

TGACTAGAGTCACACTCGTCCA

```
AUAUAUUCUAAUCGUUGGACGGGUGCACUCUGGUAUCAUGUGGAUUUGCUCAUGACUAGAGUCACACUCGUCCAUUGCUUGGAAUAC
...((((((((.((.((((((((((.(((((((..(((((.((.....))))))))))))))..)))))))))).)).)))))))).

 Initial dG =    -37.70

        10        20         30        40
AUA        U  U          C-|      UA      U  AU
   UAUUCUAA CG UGGACGGGUG  ACUCUGG  UCAUG GG  \
   AUAAGGUU GU ACCUGCUCAC  UGAGAUC  AGUAC UC  U
C--        C  U          AC^      --      -  GU
        80        70        60           50

-----------------------------------------------------
```

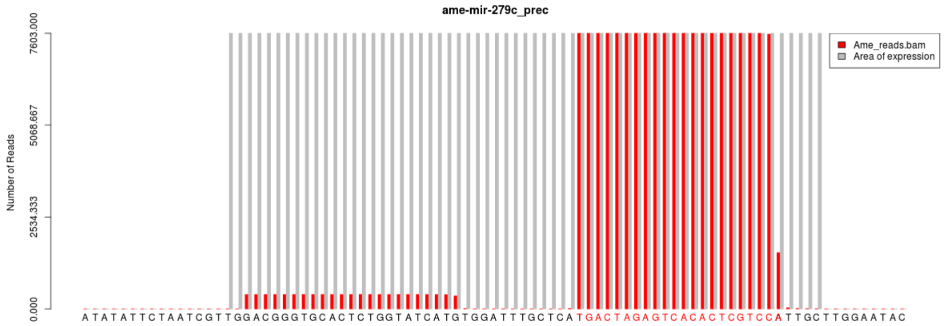

>ame-mir-279d_prec
GATGGCCTTGGATGGGTTTGAATTCAGTCCACGTTTTTTATTTTTATTCG**TGACTAGATCCACACTCATCCA**AGGAAATCGA

>ame-mir-279d_3p
TGACTAGATCCACACTCATCCA

```
GAUGGCCUUGGAUGGGUUUGAAUUCAGUCCACGUUUUUUAUUUUUAUUCGUGACUAGAUCCACACUCAUCCAAGGAAAUCGA
(((..(((((((((((.((.((..((((.(((...............))))))))..)).)).))))))))))))..)))..

 Initial dG =    -33.20

          10        20        30        40
--|  GG           U  A  UC    C   UUUUUUA
  GAU  CCUUGGAUGGGU UG AU  AGUC ACG       \
  CUA  GGAACCUACUCA AC UA  UCAG UGC       U
AG^  AA           C  C  GA    -   UUAUUUU
 80        70        60         50

-----------------------------------------------------
```

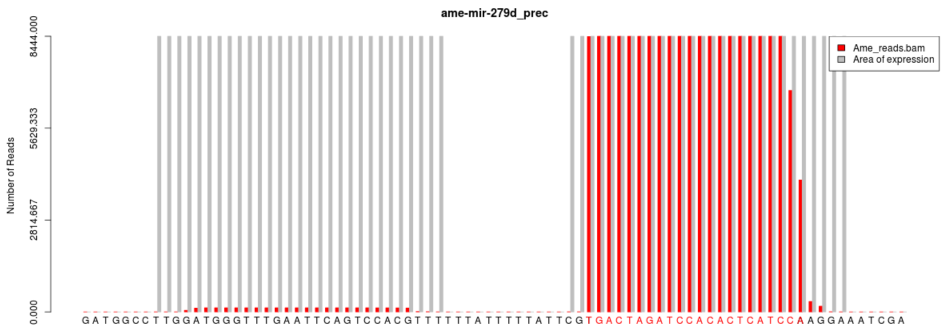

>ame-mir-281_prec
CGACGAAAGGCGCGCGCTATAAAGAGAGCTATCCATCGACAGTATGGTTATAATAGACAC**TGTCATGGAGTTGCTCTCTTTGT**AGACAC
TGCTATGTTCAACG

>ame-mir-281_3p
TGTCATGGAGTTGCTCTCTTTGT

```
CGACGAAAGGCGCGCGCUAUAAAGAGAGCUAUCCAUCGACAGUAUGGUUAUAAUAGACACUGUCAUGGAGUUGCUCUCUUUGUAGACACUGCUAUGUUCAACG
....(((.((((....(((((((((((((..(((((.((((((...(((......))))))))))))))...)))))))))))))....))))...)))....

 Initial dG =    -40.20

          10        20        30         40        50
CGAC   A--    CGCG             UA-|    C      AUG    AU
    GAA   GGCG    CUAUAAAGAGAGC   UCCAU GACAGU   GUU  A
    CUU   UCGU    GAUGUUUCUCUCG   AGGUA CUGUCA   CAG  A
GCAA   GUA    CACA             UUG^    -      ---    AU
 100        90        80        70         60

-----------------------------------------------------
```

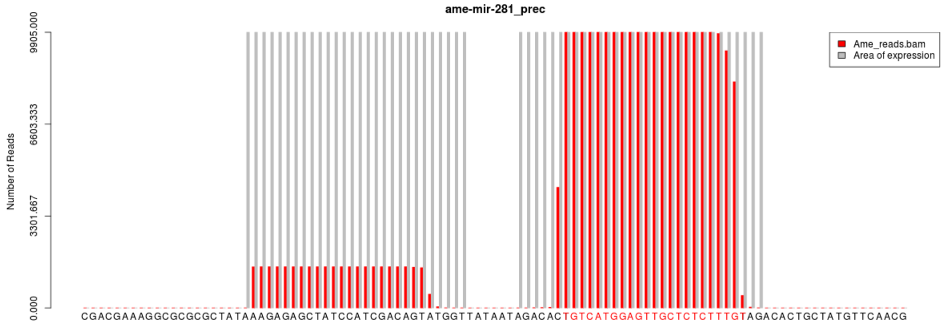

>ame-mir-282_prec
GGACAGAGTAACTT**GATTTAGCCTCTCCTAGGCTTTGTCTGT**ATATAAAGAACGGAGACATAGCCTAGAATAGGTTAGGTCAGGGCTCG
TTC

>ame-mir-282_5p
GATTTAGCCTCTCCTAGGCTTTGTCTGT

```
GGACAGAGUAACUUGAUUUAGCCUCUCCUAGGCUUUGUCUGUAUAUAAAGAACGGAGACAUAGCCUAGAAUAGGUUAGGUCAGGGCUCGUUC
((((.((((..((((((((((((...(((((((.(((((...............))))).)))))))...))))))))))))))))))))))
```

```
 Initial dG =    -39.50

        10        20        30        40
|   A    AA            CUC       U     GUAUAUA
GGAC GAGU  CUUGAUUUAGCCU   CUAGGCU UGUCU       \
CUUG CUCG  GGACUGGAUUGGA   GAUCCGA ACAGA       A
^   -    --            UAA       U     GGCAAGA
 90           80        70        60        50
```

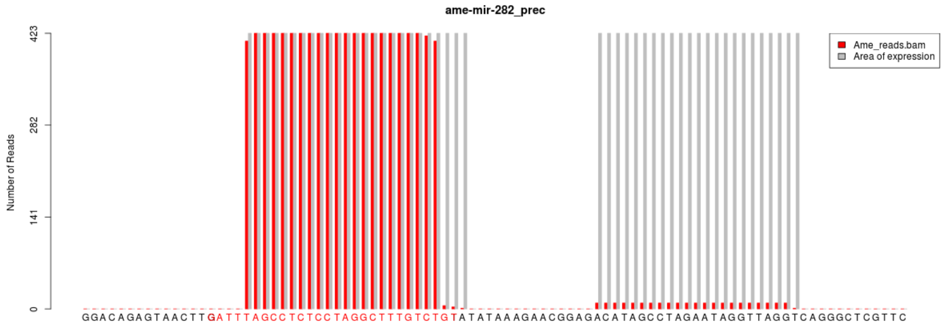

>ame-mir-283_prec
AATAATCTGGTGATGTAGTC**AAATATCAGCTGGTAATTCT**GGGATTTTGACAATAACCCAGGATTCTTGCTGGTATCCGGCTACGAACT
GGACGATCGCC

>ame-mir-283_5p
AAATATCAGCTGGTAATTCT

AAUAAUCUGGUGAUGUAGUC**AAAUAUCAGCUGGUAAUUCU**GGGAUUUUGACAAUAACCCAGGAUUCUUGCUGGUAUCCGGCUACGAACUGGACGAUCGCC
.....((..((..(((((((..((((((((.((.(((((((((.((........)))))))))))).))))))))..))))))).))..))........

```
 Initial dG =    -38.30

           10        20        30        40        50
AAUAA---| UG  GA       AA        U  U         A  UUG
        UC  GU  UGUAGUC  AUAUCAGC GG AAUUCUGGG UU   A
        AG  CA  GCAUCGG  UAUGGUCG UC UUAGGACCC AA   C
CCGCUAGC^ GU  A-       CC        U  -         -  UAA
.        90         80        70         60

-----------------------------------------------------
```

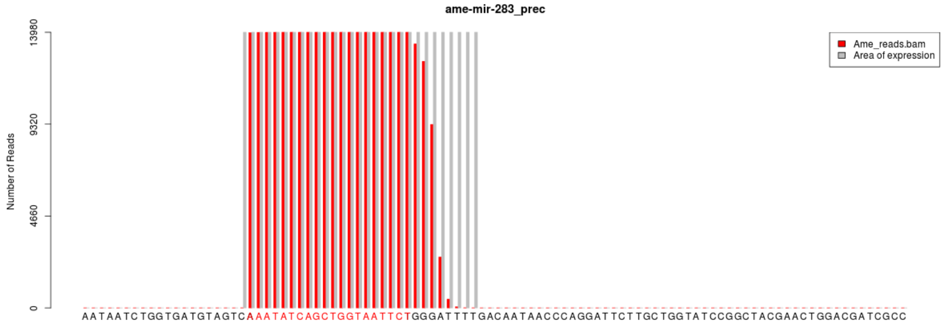

>ame-mir-2944_prec
ACTCCAGAAAATTTAGATGAAAGAATTCGGAGGAACTCTTGCTGTGATATGTTGCCGTAAAAGATTCA**TATCACAGCAGTAGTTACCTG**
GTATTCTTTCGCCTTTCTTCACCTTTACCAAGGAGC

>ame-mir-2944_3p
TATCACAGCAGTAGTTACCTGGT

ACUCCAGAAAAUUUAGAUGAAAGAAUUCGGAGGAACUCUUGCUGUGAUAUGUUGCCGUAAAAGAUUCA**UAUCACAGCAGUAGUUACCUGGU**AUUCUUUCGCCUUUCUUCACCUUUACCA
AGGAGC

```
......(((.....((.((((((((((((...((((.(((((((((((((..............))))))))))))).))))..)))).))))))))).))...))).
((((....))))...
```

Initial dG =   -37.80

```
                     10        20         30        40        50
A    -------------|   AAUUU  A         -    AGG    C             UUGCCGU
 CUCC             AGAA     AG UGAAAGAAU UCGG   AACU UUGCUGUGAUAUG       \
 GAGG             UCUU     UC GCUUUCUUA GGUC   UUGA GACGACACUAUAC       A
C    AACCAUUUCCACU^   -----  C         U    CA-    U             UUAGAAA
   120       110            100        90         80        70        60
```

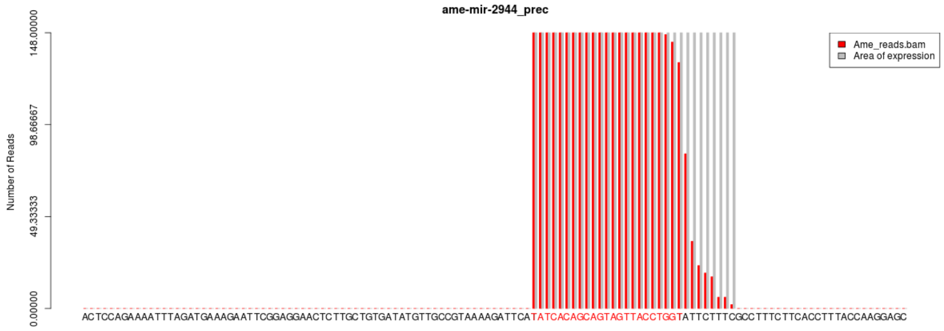

>ame-mir-29b_prec
ATTTAAAGACAATAAGAAGATAGAGGTACTGACTTCTATGCGTGCTGGGGTTTGTGCTAAATCTCC**TAGCACCATTTGAAATCAGT**ACT
ACTCTTCTTAG

>ame-mir-29b_3p
TAGCACCATTTGAAATCAGT

```
AUUUAAAGACAAUAAGAAGAUAGAGGUACUGACUUCUAUGCGUGCUGGGGUUUGUGCUAAAUCUCCUAGCACCAUUUGAAAUCAGUACUACUCUUCUUAG
............((((((((..(.((((((((.(((.(((.(((((((((.............))))))))))))..))).)))))))).))))))))).

 Initial dG =    -38.40

        10        20        30          40        50
AUUUAAAGACAA|       UA A        C   U-   C         UUUGUG
            UAAGAAGA  G GGUACUGA UUC  AUG GUGCUGGGG      \
            AUUCUUCU  C UCAUGACU AAG  UAC CACGAUCCU      C
G-----------^       -- A        A   UU   -         CUAAAU
.                     90        80         70        60

-----------------------------------------------------
```

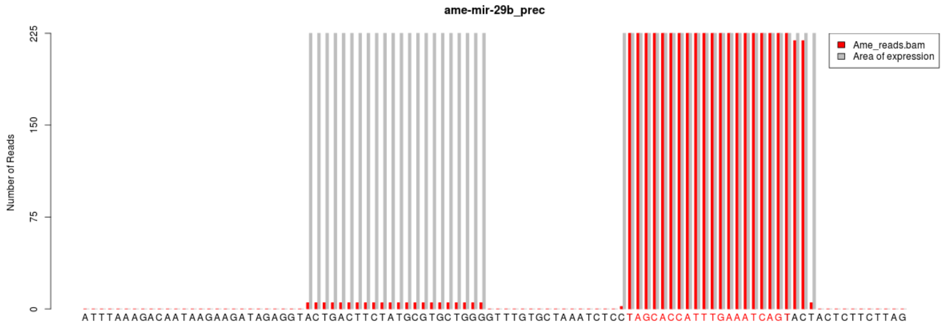

>ame-mir-2b_prec
GACGCCACGC**TCATCAAAGCTGGCTGTGATATGA**ACGCTCGTATCAGCATATCACAACCACTTTGTGAGCGGTGCACA

>ame-mir-2b_5p
TCATCAAAGCTGGCTGTGATATGA

```
GACGCCACGCUCAUCAAAGCUGGCUGUGAUAUGAACGCUCGUAUCAGCAUAUCACAACCACUUUGUGAGCGGUGCACA
...((..((((((.(((((.(((.(((((((((.............))))))))).))))))))))))))..))...

 Initial dG =    -33.30

        10        20        30        40
GAC| CA      U     C   C         AACGCU
   GC  CGCUCA CAAAG UGG UGUGAUAUG      C
   CG  GCGAGU GUUUC ACC ACACUAUAC      G
ACA^ UG      -     -   A         GACUAU
       70          60        50

-----------------------------------------------------
```

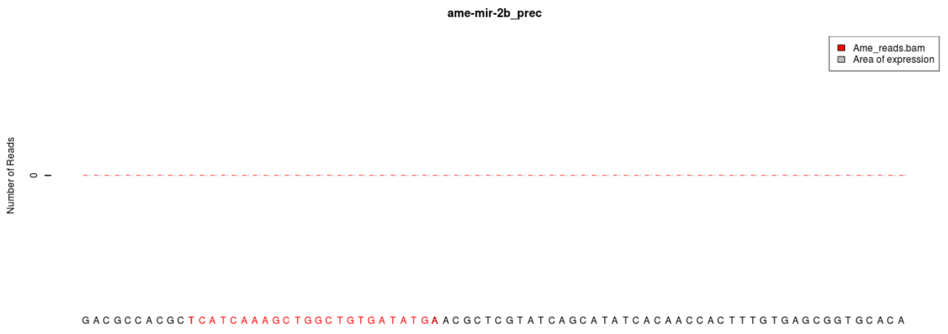

>ame-mir-3049_prec

TGCAGGAGTAAAAGCAATTGAATCGTGTTCCCAGG**TCGGGAAGGTAGTTGCGGCGGAT**TTGGTCAACTGCAAAAGTCCGTCCAACTCCT
TTCCGTCTTGGTGGCGCGAATTCCTCCTCCT

>ame-mir-3049_3p

TCGGGAAGGTAGTTGCGGCGGAT

```
UGCAGGAGUAAAAGCAAUUGAAUCGUGUUCCCAGGUCGGGAAGGUAGUUGCGGCGGAUUUGGUCAACUGCAAAAGUCCGUCCAACUCCUUUCCGUCUUGGUGGCGCGAAUUCCUCCUCC
U
...(((((...........(((((((((..(((((.((((((((.(((((.
(((((((((............))))))))))))))))))))))).))))).. ))))).)))))))))...
ERROR 4: Strange structure
 Initial dG =    -56.20

        10        20          30        40        50        60
UGC|    UAAAAGCAAU     -      UC      U         U      C          GGUCA
   AGGAG          GAAU CGUGU  CCAGG CGGGAAGG AGUUG GGCGGAUUU      A
   UCCUC          CUUA GCGCG  GGUUC GCCUUUCC UCAAC CUGCCUGAA      C
UCC^    -----------    A      GU      U         -      -          AACGU
.                  110       100         90          80         70
```

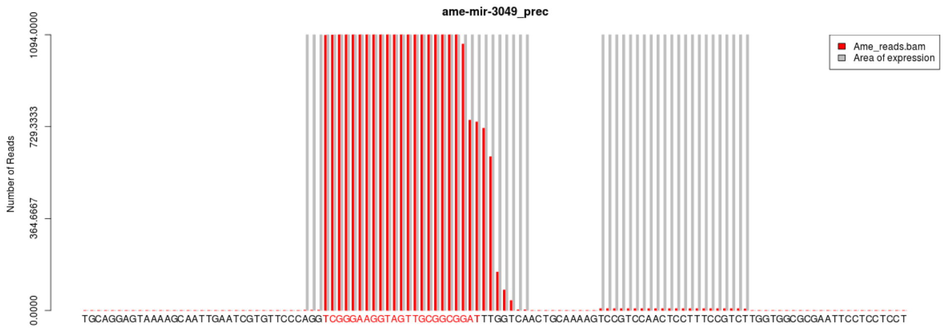

>ame-mir-305_prec
GGAGGCTGCATGTTA**ATTGTACTTCATCAGGTGCTCTG**GTGAACTCGATACCCGGCACCTGTTGGAGCGCAATTCATATGACTGTGCCC
T

>ame-mir-305_5p
ATTGTACTTCATCAGGTGCTCTG

```
GGAGGCUGCAUGUUAAUUGUACUUCAUCAGGUGCUCUGGUGAACUCGAUACCCGGCACCUGUUGGAGCGCAAUUCAUAUGACUGUGCCCU
((.(((..(((((.(((((..(((((.((((((((..((((.......)))).)))))))).))))).)))))..))))).))))).....)))))

 Initial dG =    -37.40

           10        20        30        40
  A   UG---|    U     UA     U        CU     AAC
GG GGC     CAUGU AAUUG  CUUCA CAGGUGCU  GGUG   \
UC CCG     GUAUA UUAAC  GAGGU GUCCACGG  CCAU   U
  -   UGUCA^    C     GC     U        C-     AGC
.         80        70        60         50

-----------------------------------------------------
```

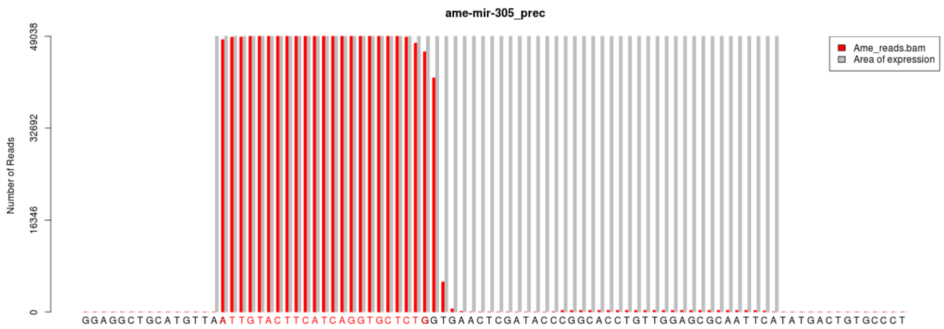

>ame-mir-306_prec
AATTCAGTAAGTAAGGTCAAACAAATATTTACTTTG**TCAGGTACTGAGTGACTCTGAG**TATACATTAAATTACTCAAAGCAGCTTGGTA
CCTAACTGAGTGGATATTGAATACAACAATTTTTGAAA

>ame-mir-306_3p
TCAGGTACTGAGTGACTCTGAG

```
AAUUCAGUAAGUAAGGUCAAACAAAUAUUUACUUUGUCAGGUACUGAGUGACUCUGAGUAUACAUUAAAUUACUCAAAGCAGCUUGGUACCUAACUGAGUGGAUAUUGAAUACAACAAU
UUUUGAAA
..(((((.((.....((......(((((((((((.((.((((((..(((..((.
((((((..........)))))).))..))).))))))).)).)))))))))))....)).....)).))))).
ERROR 4: Strange structure
 Initial dG =    -38.90

        10        20        30        40        50        60
AA|    U  GUAAG  CAAACA           U  C      UG   GA  C      UACA
  UUCAG AA     GU      AAUAUUUACUU GU AGGUAC  AGU  CU UGAGUA    U
  AAGUU UU     CA      UUAUAGGUGAG CA UCCAUG  UCG  GA ACUCAU    U
A-^    U  AACAA  UAAG--           U  A      GU   AC  A      UAAA
      120       110         100        90        80        70
```

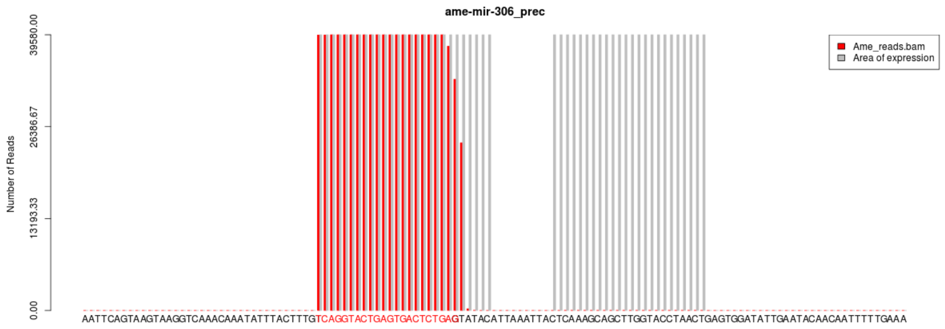

>ame-mir-307_prec
GAGGAGCATCTCGAAAATGGCGGTCACGTGGACTCACTCAACCTGGGTGTGATGCTTGCCTGTGTATCAGGCCCTAGCGGTCA**TCACAA**
CCTTTTTGAGTGAGCGAACGCGACTGTTTTATCGACCATATCCA

>ame-mir-307_3p
TCACAACCTTTTTGAGTGAGCGA

GAGGAGCAUCUCGAAAAUGGCGGUCACGUGGACUCACUCAACCUGGGUGUGAUGCUUGCCUGUGUAUCAGGCCCUAGCGGUCA**UCACAACCUUUUUGAGUGAGCGA**ACGCGACUGUUUU
AUCGACCAUAUCCA
..(((.....((((....(((((((.(((...(((((((((...((((((((((((.
(((((.....)))))......)).))))))).)))..)))))))))...))).)))))))...)))).....))).

Initial dG =   -55.30

```
        10        20        30        40         50              60
GA   GCAUC    AAAU       A   GGA         CCU   -       -  U-----|    UG
  GGA     UCGA    GGCGGUC CGU   CUCACUCAA   GGG UGUGAUG CU      GCCUG  \
  CCU     AGCU    UUGUCAG GCA   GAGUGAGUU   UCC ACACUAC GG      CGGAC  U
A-   AUACC    AUU-       C   AGC         UU-   A       U  CGAUCC^    UA
  130       120        110       100         90        80        70
```

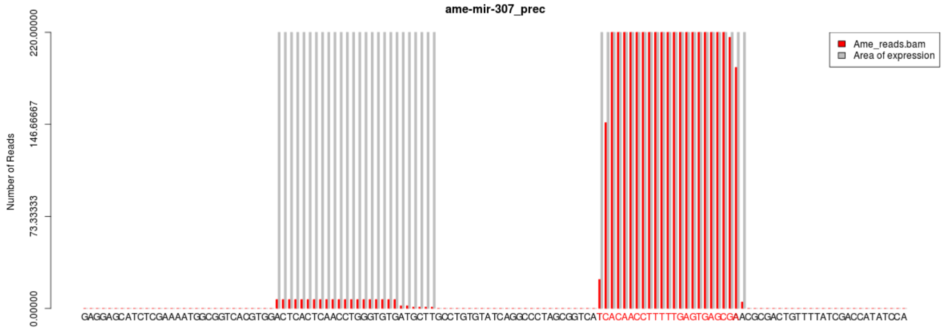

>ame-mir-315_prec

GCTCTTTATGC**TTTTGATTGTTGCTCAGAAAGC**CTTGATTATGATATTGGCTTTCGGGCAATAATCATAATCACGAAAGGGT

>ame-mir-315_5p

TTTTGATTGTTGCTCAGAAAGC

```
GCUCUUUAUGCUUUUGAUUGUUGCUCAGAAAGCCUUGAUUAUGAUAUUGGCUUUCGGGCAAUAAUCAUAAUCACGAAAGGGU
((((((.((.((.(((((((((((.(((((((.............))))))))))))))))))).)).))..)))))))

 Initial dG =    -32.40

        10        20        30        40
       A-| C  U            A       UUGAUU
GCUCUUU  UG UU UGAUUGUUGCUC GAAAGCC      A
UGGGAAA  AC AA ACUAAUAACGGG CUUUCGG      U
       GC^ U  U            -       UUAUAG
 80        70        60         50

-----------------------------------------------------
```

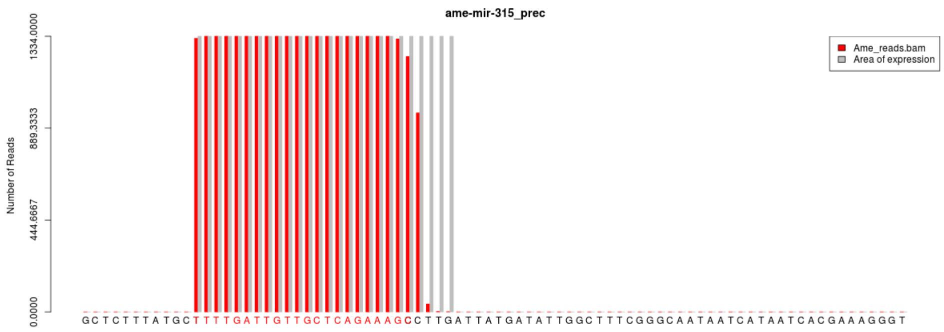

>ame-mir-316_prec
GTGTGGCCGAGGGTG**TGTCTTTTTCCGCTTTGCTGCCG**CTGAAATGGAGTCCGCCAGCAAAGGGGAACAGGCCGACCCTCCCGCACCAA

>ame-mir-316_5p
TGTCTTTTTCCGCTTTGCTGCCG

```
GUGUGGCCGAGGGUGUGUCUUUUUCCGCUUUGCUGCCGCUGAAAUGGAGUCCGCCAGCAAAGGGGAACAGGCCGACCCUCCCGCACCAA
(((((..((((((..((((.(((((.((((((((..((.((.......)).)))))))))))))))).)))))..))))))))))))...

 Initial dG =    -42.30

           10        20        30        40
---|     CC      GU    U     G        CC  U  AAU
   GUGUGG  GAGGGU  GUCU UUUCC CUUUGCUG  GC GA   \
   CACGCC  CUCCCA  CGGA AAGGG GAAACGAC  CG CU   G
AAC^     --      GC    C     -        --  C  GAG
          80        70         60          50

-----------------------------------------------------
```

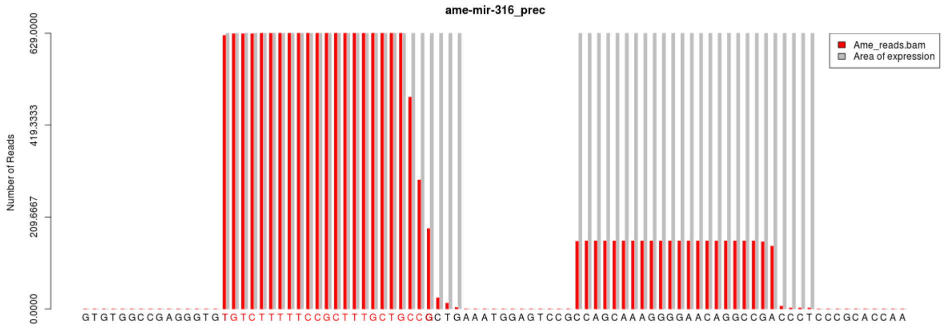

>ame-mir-317_prec
GCTCTCGGAGAACAGGGAGCCACTCTGCGTTCACTCGGTGGGTAATGAAGCGGG**TGAACACAGCTGGTGGTATCTCAGT**TTTCTGAGGG
C

>ame-mir-317_3p
TGAACACAGCTGGTGGTATCTCAGT

```
GCUCUCGGAGAACAGGGAGCCACUCUGCGUUCACUCGGUGGGUAAUGAAGCGGGUGAACACAGCUGGUGGUAUCUCAGUUUUCUGAGGGC
((((((((((((.((((((((((((.(((((((((............))))))))).)))..)))))).)))).)))))))))))))

 Initial dG =    -48.00

        10         20          30        40
             A    -      --|  C         GUGGGU
GCUCUCGGAGAAC GGGA GCCACU  CUG GUUCACUCG      \
CGGGAGUCUUUUG CUCU UGGUGG  GAC CAAGUGGGC      A
             A    A      UC^  A         GAAGUA
.        80         70         60         50

-----------------------------------------------------
```

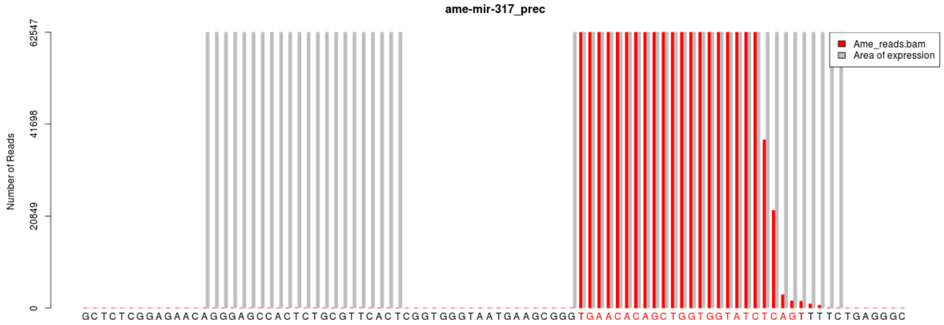

>ame-mir-318_prec
ACGCACTGAATATTATAAGTACATCATGGGATGAACGTGATCCTGTTTGACGGTAATAATTGATCG**TCACTGGGTTATGTTTGTCCC**AT
ACGAGTACTGATTAATCATCGCTATTGCA

>ame-mir-318_3p
TCACTGGGTTATGTTTGTCCC

```
ACGCACUGAAUAUUAUAAGUACAUCAUGGGAUGAACGUGAUCCUGUUUGACGGUAAUAAUUGAUCGUCACUGGGUUAUGUUUGUCCCAUACGAGUACUGAUUAAUCAUCGCUAUUGCA
..(((.(((..((((..(((((.((((((((..((((((((((....(((((((........)))))))..))))))))))..))))))..)))))))...))))..)))....))).
```

```
 Initial dG =    -43.20

           10          20           30         40         50
AC   C---   AU    UA-     A  --|     UG          UGUU       AAU
  GCA    UGA  AUUA   AGUAC UC  AUGGGA  AACGUGAUCC    UGACGGU   A
  CGU    GCU  UAAU   UCAUG AG  UACCCU  UUGUAUUGGG    ACUGCUA   A
A-   UAUC   AC    UAG     -  CA^     GU          UC--       GUU
       110       100         90        80        70          60

-----------------------------------------------------
```

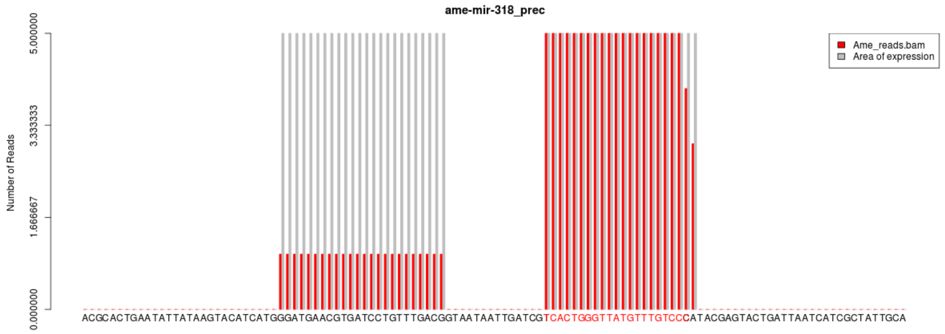

>ame-mir-31a_prec
ATCACGATTCTAACTGGGCGCCTCGAA**GGCAAGATGTCGGCATAGCTGA**TGCGATTTTAAAATTCGGCTGTGTCACATCCAGCCAACCG
AACGCTCAGAC

>ame-mir-31a_5p
GGCAAGATGTCGGCATAGCTGA

```
AUCACGAUUCUAACUGGGCGCCUCGAAGGCAAGAUGUCGGCAUAGCUGAUGCGAUUUUAAAAUUCGGCUGUGUCACAUCCAGCCAACCGAACGCUCAGAC
.............(((((((..(((..(((..(((((.(((((((((((..............))))))))))))))))..)))...))).)))))))..

 Initial dG =    -40.90

        10        20          30        40        50
AUCACGAUUCUAA|      CC    AA-    AA      C           UGCGAU
             CUGGGCG  UCG    GGC  GAUGU GGCAUAGCUGA      U
             GACUCGC  AGC    CCG  CUACA CUGUGUCGGCU      U
CA-----------^      A-    CAA    AC      -           UAAAAU
.                    90        80         70        60

-----------------------------------------------------
```

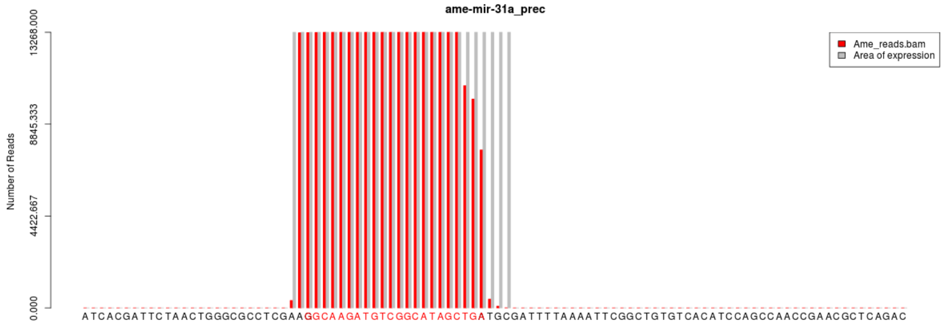

>ame-mir-33_prec
TATTTATTTGATTGCTTACCTGTTACAACT**GTGCATTGTAGTTGCATTG**CATGAAATATAACTATGCAATACTTCTACAGTGCAACTCT
TGTGGCAGATT

>ame-mir-33_5p
GTGCATTGTAGTTGCATTG

```
UAUUUAUUUGAUUGCUUACCUGUUACAACUGUGCAUUGUAGUUGCAUUGCAUGAAAUAUAACUAUGCAAUACUUCUACAGUGCAACUCUUGUGGCAGAUU
...................(((((((((..(((((((((((....((((((((.........))))))))....)))))))))).)..)))))))))...
```

```
 Initial dG =    -33.10

        10        20        30         40        50
UAUUUAUUUGAUUGCUUAC         CU -|         UUGC        AAAU
                   CUGUUACAA  G UGCAUUGUAG    AUUGCAUG    \
                   GACGGUGUU  C ACGUGACAUC    UAACGUAU    A
UUA----------------         CU A^         UUCA        CAAU
.                        90        80        70        60

-----------------------------------------------------
```

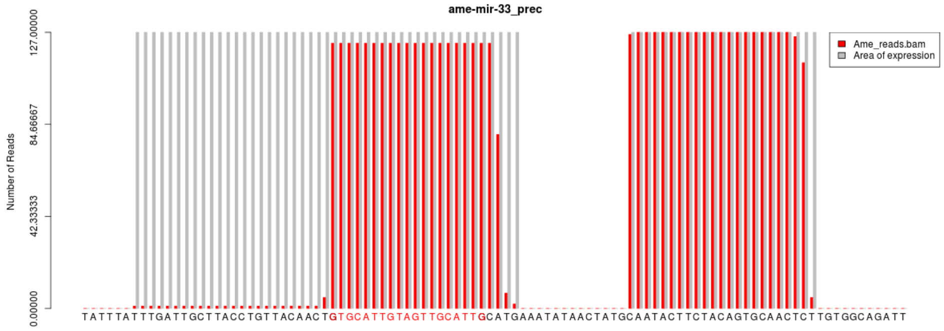

>ame-mir-34_prec
TTTTTTTGCGATTGGCATGTT**TGGCAGTGTTGTTAGCTGGTTG**TGTGGCTACATTGTATATACGACCGCTATCGGCACTGCAATTATGAC
GATCGTAGGGT

>ame-mir-34_5p
TGGCAGTGTTGTTAGCTGGTTG

```
UUUUUUUGCGAUUGGCAUGUUGGCAGUGUUGUUAGCUGGUUGUGUGGCUACAUUGUAUAUACGACCGCUAUCGGCACUGCAAUUAUGACGAUCGUAGGGU
...((((((((((.((((...((((((((.((((.(((((((((((......)).)))))))))))))).))))))))...)))).)))))))))).

 Initial dG =    -47.20

        10        20        30        40         50
UUU|          G    UUG         U    U          -  UA
   UUUUGCGAUUG CAUG   GCAGUGUUG UAGC GGUUGUGUG GC  C
   GGGAUGCUAGC GUAU   CGUCACGGC AUCG CCAGCAUAU UG  A
U--^          A    UAA         U    -          A  UU
.          90        80        70         60

-----------------------------------------------------
```

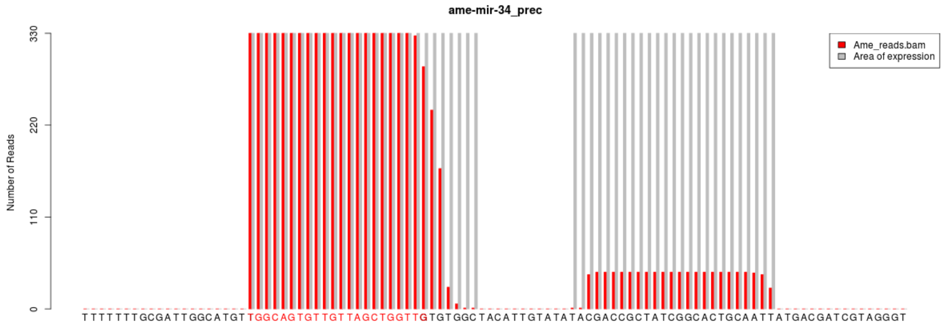

>ame-mir-3477_prec
TTTGGTAGCGTTGGAGTGGG**TAATCTCATGCGGTAACTGTGAG**TGTGTGAATTGTAAAAGCTCATATTACCTCGTGGGGTTTCCCACCC
GTTACCATTA

>ame-mir-3477_5p
TAATCTCATGCGGTAACTGTGAG

```
UUUGGUAGCGUUGGAGUGGGUAAUCUCAUGCGGUAACUGUGAGUGUGUGAAUUGUAAAAGCUCAUAUUACCUCGUGGGGUUUCCCACCCGUUACCAUUA
..(((((((...((.(((((.(((((((((.(((((.(((((((...((.....))...)))))))))))).))))))))).))))))))))))))...

 Initial dG =    -45.00

         10        20        30        40        50
UU-|      GUU   A     U         C     C       GUG   AA
   UGGUAGC   GG GUGGG AAUCUCAUG GGUAA UGUGAGU   UG   \
   ACCAUUG   CC CACCC UUGGGGUGC CCAUU AUACUCG   AU   U
AUU^      ---   -     U         U     -       AAA   GU
        90            80        70         60

-----------------------------------------------------
```

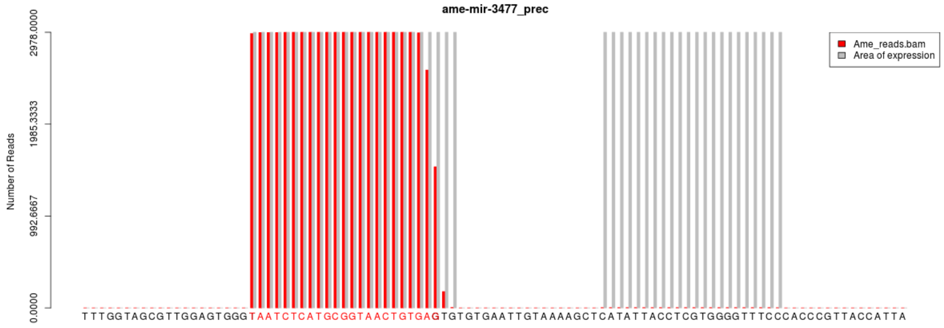

>ame-mir-3478_prec
AGGCGGACATTGGATGAATCCTACCCGGTGAGGTTTTTTTTTTCCAAC**CACACCGGACGAGATTTCAT**CCGACAGCAG

>ame-mir-3478_3p
CACACCGGACGAGATTTCAT

```
AGGCGGACAUUGGAUGAAUCCUACCCGGUGAGGUUUUUUUUUUCCAACCACACCGGACGAGAUUUCAUCCGACAGCAG
..((.....(((((((((..((..((((((.((((..........)))).)))))))...))..)))))))))..))..

 Initial dG =    -29.10

        10        20         30        40
AG| GGACA         UC  AC-       A    UUUU
  GC     UUGGAUGAA  CU   CCGGUG GGUU    U
  CG     AGCCUACUU  GA   GGCCAC CCAA    U
GA^ AC---         UA  GCA      A    CCUU
          70        60        50

-----------------------------------------------------
```

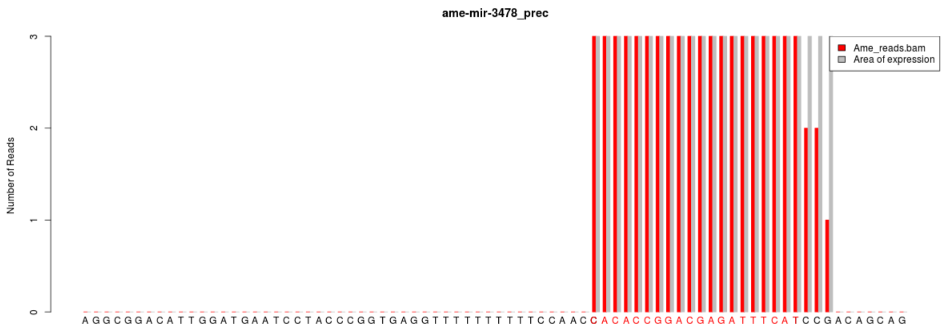

>ame-mir-3715_prec
GTGTGTCGTGCTA**TCGGTAAGCAGAGTATAAGACCT**TTGAGACGCGAAATTTAAAGTATTATGCTCCGTTTATCGTTGGTGCGGCGCGA
AACGAGC

>ame-mir-3715_5p
TCGGTAAGCAGAGTATAAGACCT

```
GUGUGUCGUGCUAUCGGUAAGCAGAGUAUAAGACCUUUGAGACGCGAAAUUUAAAGUAUUAUGCUCCGUUUAUCGUUGGUGCGGCGCGAAACGAGC
.(((((((..(((.((((((((.((((((((...(((((((........)))))))..)))))))).)))))))).)))..))))))).......
```

```
 Initial dG =    -39.80

               10        20        30        40
G-------|      UG   U        A        GAC       ACG
        UGUGUCG  CUA CGGUAAGC GAGUAUAA   CUUUGAG   C
        GCGCGGC  GGU GCUAUUUG CUCGUAUU   GAAAUUU   G
CGAGCAAA^      GU   U        C        AU-       AAA
     90        80        70        60         50

-----------------------------------------------------
```

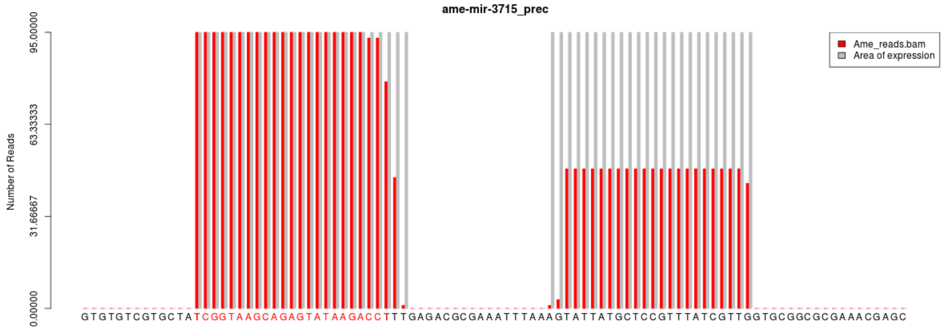

>ame-mir-3716a_prec
TGTAAAGCTAGGCTGTTTTTATTTTATATATTTCGCAATTTTTCATTGGCCTCTGTATGAAATGACCTACATTTACAATACGTATTAAA
TATGTGGATTTGAAATCATATACAATAAGATAAATAAGTAGCTTTAG

>ame-mir-3716a_3p
TATGTGGATTTGAAATCATATA

UGUAAAGCUAGGCUGUUUUUAUUUUAUAUAUUUCGCAAUUUUUCAUUGGCCUCUGUAUGAAAUGACCUACAUUUACAAUACGUAUUAAA**UAUGUGGAUUUGAAAUCAUAUA**CAAUAAGA
UAAAUAAGUAGCUUUAG

```
..(((((((..(((..(((((((.(((((((((((.(((((..(((.......
((((((((((.....))))).)).))))........))).))))))))))).))))).)))))))......)))))))))).
```

```
 Initial dG =    -23.50

        10         20           30         40
UG       AG   GUU          .-AUAUUUCG|   UU
  UAAAGCU  GCU   UUUAUUUUAU          CAAU  \
  AUUUCGA  UGA   AAAUAGAAUA          GUUA  U
G-       --   AU-          \ --------^   CU
     130          120
```

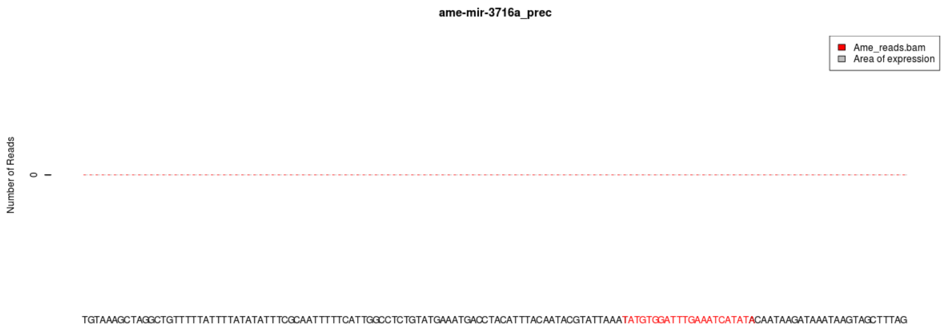

>ame-mir-3716b_prec
CAAACTTGAAATTGAAGAACTTGAATTCGATGGCAATCGTTCTATGTATTTTTGCAATGTATATTTGCAGATACAATTA**CGATGTTATT**
TGAAATCATTGGCAATAATAGTTTA

>ame-mir-3716b_3p
CGATGTTATTTGAAATCATTGGC

CAAACUUGAAAUUGAAGAACUUGAAUUCGAUGGCAAUCGUUCUAUGUAUUUUUGCAAUGUAUAUUUGCAGAUACAAUUA**CGAUGUUAUUUGAAAUCAUUGGC**AAUAAUAGUUUA
.((((....((((..((...(((.(((((((((.((((....((((((..(((((.......))))))))))))...))))))))).))))).))))))..))))...))))).

```
 Initial dG =    -25.70

        10        20        30         40        50        60
C|    UGAA    AA  ACU   A     -     A    UCUA      UU     UGU
 AAACU    AUUG  GA   UGA UUCGA UGGCA UCGU    UGUAUU  UGCAA   \
 UUUGA    UAAC  UU   ACU AAGUU AUUGU AGCA    ACAUAG  ACGUU   A
A^    UAA-    GG  ---   A     U     -    UUA-      --     UAU
  110        100           90         80         70

-----------------------------------------------------
```

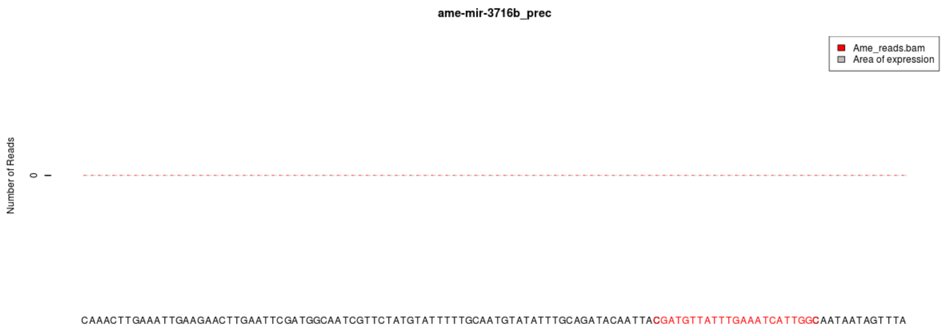

>ame-mir-3717_prec
TTGGAGAGG**TGAGAGAAAAAGAGAGTGTCGG**GGTGGGACGTTTGATAAAATTGCTGTCACACGAATTCTCATTTTTCCTTGTCGCTTG

>ame-mir-3717_5p
TGAGAGAAAAAGAGAGTGTCGG

```
UUGGAGAGGUGAGAGAAAAAGAGAGUGUCGGGGUGGGACGUUUGAUAAAAUUGCUGUCACACGAAUUCUCAUUUUUCCUUGUCGCUUG
...(((.((..((.(((((((((((.(((..(((((..((((....))))..))..))).))))))))).))))))))..)).))).
```

```
 Initial dG =    -24.80

        10        20         30          40
UUG|  A  UG  A      -      G   GG   --  AC    G
   GAG GG  AG GAAAAA GAGAGU UCG  GUG  GG  GUUU A
   UUC CU  UC CUUUUU CUCUUA AGC  CAC  UC  UAAA U
G--^  G  GU  -      A      -   A-   UG  GU    A
         80         70          60        50

-----------------------------------------------------
```

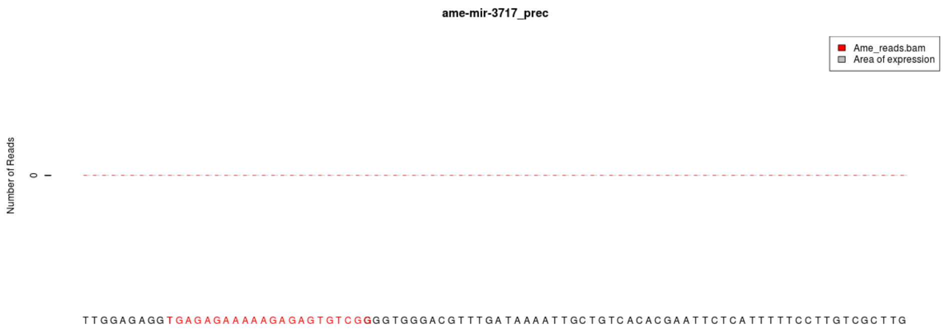

>ame-mir-3718a_prec
TTGAAAGTCGTTCCCGATTTACATTATCGGATCAGGATAGGGTAGGGCAGGGCCATTTCGGTATAACCATTCTG**TCCCCTGTCCTGTCC**
CGATAGTGAGAATCGGTACTGAATCGAAATTATGAACGACTGTTTATTCAT

>ame-mir-3718a_3p
TCCCCTGTCCTGTCCCGATAG

```
UUGAAAGUCGUUCCCGAUUUACAUUAUCGGAUCAGGAUAGGGUAGGGCAGGGCCAUUUCGGUAUAACCAUUCUGUCCCCUGUCCUGUCCCGAUAGUGAGAAUCGGUACUGAAUCGAAAU
UAUGAACGACUGUUUAUUCAU
.((((((((((((((((((.(((((((((..((((((((((...
(((((((((.....)))........))))))))))))))))..))))))))).))))))).................))))))))).....)))).
```

Initial dG =   -58.00

```
             10                         20        30        40        50
U    -----        -----------------       A         AU         GUA       --------|  AU
 UGAA     AGUCGUUC                 CCGAUUU CAUUAUCGG  CAGGAUAGG   GGGCAGG        GCC  \
 ACUU     UCAGCAAG                 GGCUAAG GUGAUAGCC  GUCCUGUCC   CCUGUCU        UGG  U
U    AUUUG        UAUUAAAGCUAAGUCAU       A         CU         ---       UACCAAUA^  CU
.       130       120       110       100        90        80           70        60

-----------------------------------------------------
```

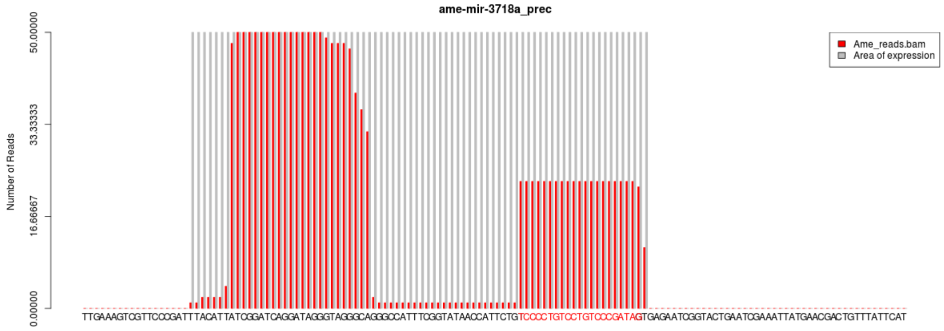

>ame-mir-3718b_prec
TTCGATTCAGTACCGATTCTCACTAT**TCGGGACAGGACAGGGGACAGA**ATGGTTATACCGAAATGGCCCTGCCCTACCCTATCCTGATCC
GATAATGTAAATCGGGAACGAC

>ame-mir-3718b_5p
TCGGGACAGGACAGGGGACAGA

UUCGAUUCAGUACCGAUUCUCACUA**UCGGGACAGGACAGGGGACAGA**AUGGUUAUACCGAAAUGGCCCUGCCCUACCCUAUCCUGAUCCGAUAAUGUAAAUCGGGAACGAC
.(((.(((....(((((..((.(((((((.(((((.((((..(((...((((((......)))))))))).....)))).))))).))))))).))..)))))))))))))).

```
 Initial dG =    -42.60

        10        20        30        40           50
U|  A   AGUA      CU  C       A     C    GA---   AAU      AC
 UCG UUC    CCGAUU  CA UAUCGGG CAGGA AGGG     CAG   GGUUAU  C
 AGC AAG    GGCUAA  GU AUAGCCU GUCCU UCCC     GUC   CCGGUA  G
C^  -   ----      AU  A       A     A    AUCCC   ---      AA
 .            100        90        80        70           60

-----------------------------------------------------
```

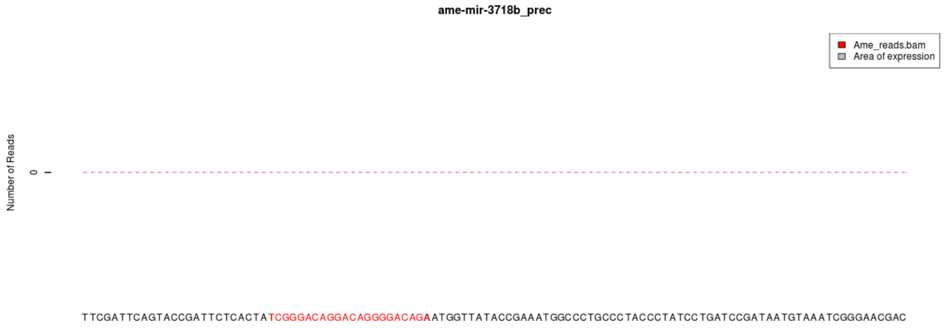

>ame-mir-3718c_prec
CGGTTCGCAT**TATTGGGTCAGGATAGGGCAG**GGCAGGGCCGTTTGGCAGGTACATTTTGTCCCCTGTCCTGTCCCGATAGTGAGAATTG
G

>ame-mir-3718c_5p
TATTGGGTCAGGATAGGGCAG

```
CGGUUCGCAUUAUUGGGUCAGGAUAGGGCAGGGCAGGGCCGUUUGGCAGGUACAUUUUGUCCCCUGUCCUGUCCCGAUAGUGAGAAUUGG
(((((.((((((((((.(((((((((...(((((((((((........)))....)))))))))))))))).))))))))))).)))))).

 Initial dG =    -47.30

         10        20        30            40
-      G          U         GCA       ----|  GUU
 CGGUUC CAUUAUUGGG CAGGAUAGG   GGGCAGG    GCC   U
 GUUAAG GUGAUAGCCC GUCCUGUCC   CCUGUUU    UGG   G
G      A          U         ---       UACA^  ACG
.        80        70            60        50

-----------------------------------------------------
```

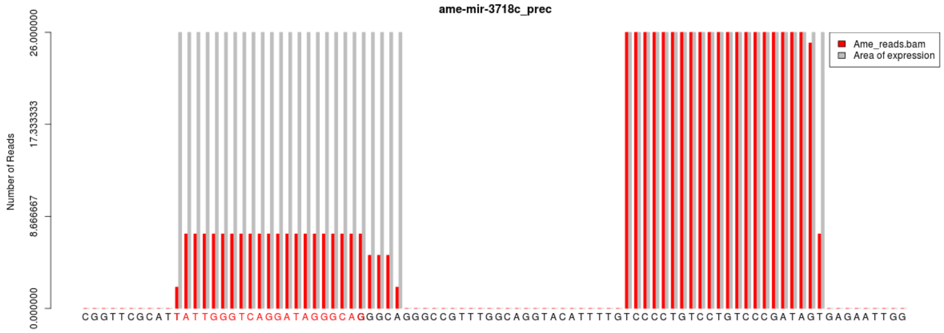

>ame-mir-3719_prec
AATGGAATCGGCGCGGTCCACTATACCGGGTGACCGAAATGTCGCGCAAACTGTATTGAAACGGCGATCGGGA**TACGGATTGCGTGACT
TTTCGA**CCACCCAGTGTGTGAAACGCACCCACCCCCAAAGTCCGTA

>ame-mir-3719_3p
TACGGATTGCGTGACTTTTCGA

AAUGGAAUCGGCGCGGUCCACUAUACCGGGUGACCGAAAUGUCGCGCAAACUGUAUUGAAACGGCGAUCGGGA**UACGGAUUGCGUGACUUUUCGA**CCACCCAGUGUGUGAAACGCACCC
ACCCCCAAAGUCCGUA
.(((((...((.(((...(((.((((.(((((..(((((.(((((((((.(((((((....
((.....)).))))))).)))))))))).)))))..))))).)))))))...))).))...........))))).

```
 Initial dG =    -54.00

                10        20        30        40        50        60
A     AUC--------| C   GUC   U    C     AC     U         A       GAAA  GC
 AUGGA           GG GCG   CAC AUAC GGGUG  CGAAA GUCGCGCAA CUGUAUU    CG  \
 UGCCU           CC CGC   GUG UGUG CCCAC  GCUUU CAGUGCGUU GGCAUAG    GC  G
A     GAAACCCCCAC^ A   AAA   -    A     CA     U         A       G---  UA
   130       120       110        100        90        80           70
```

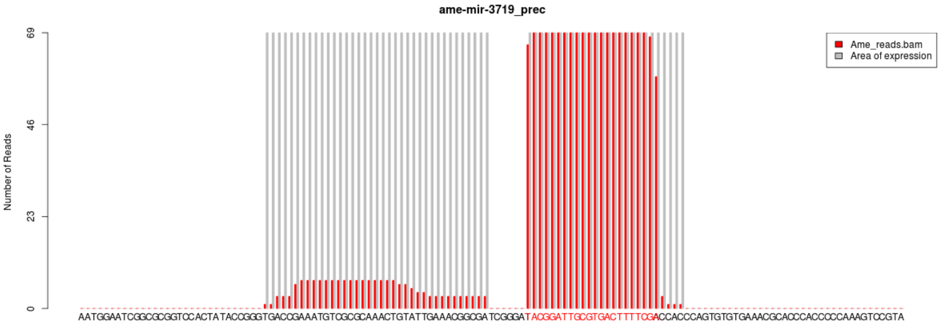

>ame-mir-3720_prec
CTGAGGTTC**ATACGGTGATGAGTTTAAACAG**TTTACACCCGAGTCTGGTTTCGATCGAACCTCAATTTTCTGGGGATCTCGTTCCAGAT
TATGAAAGAGTAAACGTTTGTAGAGTCATAGTTACGCTGAGTATGAACTTC

>ame-mir-3720_5p
ATACGGTGATGAGTTTAAACAG

CUGAGGUUC**AUACGGUGAUGAGUUUAAACAG**UUUACACCCGAGUCUGGUUUCGAUCGAACCUCAAUUUUCUGGGGAUCUCGUUCCAGAUUAUGAAAGAGUAAACGUUUGUAGAGUCAUA
GUUACGCUGAGUAUGAACUUC
..(((((((((((((((((((.((((....((((((.....(((((((...(((..((.
(((((......))))).)))))..)))))))........)))))).....)))).)))).....))))..)))))))))))

Initial dG = -44.80

```
        10               20        30         40           50        60
CU           --    -----    G    AACA-      ACCCG---|      UUU   UC  A     AU
  GAGGUUCAUAC  GGUG     AUGA UUUA     GUUUAC        AGUCUGG   CGA  GA CCUCA  U
  CUUCAAGUAUG  UCGC     UACU AGAU     CAAAUG        UUAGACC   GCU  CU GGGGU  U
--           AG    AUUGA    G    GUUUG      AGAAAGUA^      UU-   --  A     CU
140       130       120       110       100        90         80          70
```

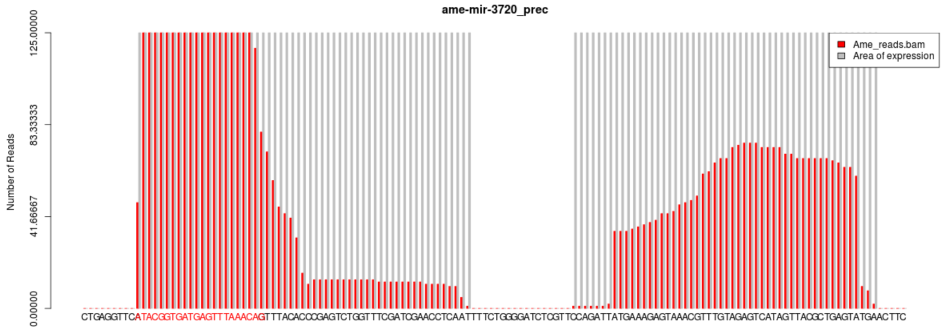

>ame-mir-3721_prec
TGAATCGAGCATTATTTACTCCTTTCACTTCGAGAAGAAAAATAAAAAAGATTTTTATTCACAGA**TCGTTGAAAAAGAGGAGAAGAT**GC
TCCTCCATCATTAATT

>ame-mir-3721_3p
TCGTTGAAAAAGAGGAGAAGAT

```
UGAAUCGAGCAUUAUUUACUCCUUUCACUUCGAGAAGAAAAAUAAAAAAGAUUUUUAUUCACAGAUCGUUGAAAAAGAGGAGAAGAUGCUCCUCCAUCAUUAAUU
......(((((((..((.(((((((...((((((.....((((((((...))))))))......)).))))).))))))))))))))))).............

 Initial dG =    -20.20

                10        20        30         40
UGAAUC--------|      AU  A       CAC      -  AGAAA-        A
              GAGCAUU  UU CUCCUUU   UUCGA GA      AAUAAAAA \
              CUCGUAG  AA GAGGAGA   AAGUU CU      UUAUUUUU G
UUAAUUACUACCUC^      --  -       AA-     G  AGACAC        A
   100        90           80         70        60

-----------------------------------------------------
```

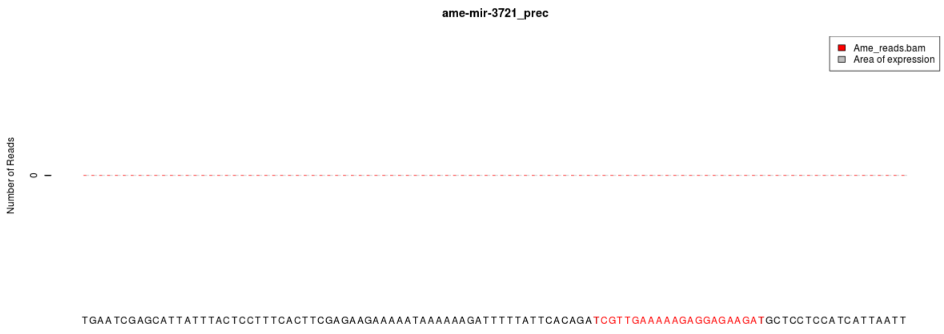

>ame-mir-3722_prec
ATGGAAAGTCGGTGAAACCTTGGGATCGGCCAGAT**CCGATTCGAGGTGTGACGGTT**TCGTTCGAAATTAGCTTGCAACGAATCAGGCCT
CTTTGCGGATTTAAGGGGACCATTAACGATTCT

>ame-mir-3722_3p
CCGATTCGAGGTGTGACGGTT

AUGGAAAGUCGGUGAAACCUUGGGAUCGGCCAGAU**CCGAUUCGAGGUGUGACGGUU**UCGUUCGAAAUUAGCUUGCAACGAAUCAGGCCUCUUUGCGGAUUUAAGGGGACCAUUAACGAU
UCU

```
..((((.((..(((...((((..((((.((.(((.((((((((..(((....
((((((....))))))....)))..)))))).))..)))..)).))))..))))...)))..))..))))
ERROR 4: Strange structure
 Initial dG =    -35.40

         10        20
AU     A-  CG   AAA    .-G| AU
  GGAA  GU  GUG   CCUU   GG  \
  UCUU  CA  UAC   GGGA   CC  C
--     AG  AU   CAG    \ -^ GG
  120       110          30
```

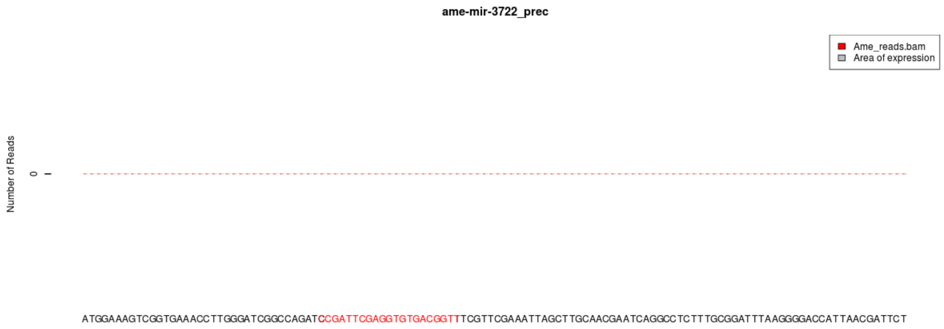

>ame-mir-3723_prec

GACCGGAAGTCACTATTTTCGGAGTT**ACAGCGTTTCAAAGTTTTCGTA**AATGTAAATGTTTAAAAATCGGAAACCCTTTGTTTCACACG
ACGATATCTCCGGAACCGGA

>ame-mir-3723_5p

ACAGCGTTTCAAAGTTTTCGTA

```
GACCGGAAGUCACUAUUUUCGGAGUUACAGCGUUUCAAAGUUUUCGUAAAUGUAAAUGUUUAAAAAUCGGAAACCCUUUGUUUCACACGACGAUAUCUCCGGAACCGGA
..((((..........((((((((......(((..((((((((((((((((......)))))).....)))))))..))))......))).......)))))))))))).
```

```
 Initial dG =    -23.00

        10        20        30               40             50
GA    AAGUCACUAU        UUACAG-   UU----    --       -----|    GU
  CCGG          UUUCGGAG       CGU      CAAA  GUUUUCG     UAAAU  A
  GGCC          AAGGCCUC       GCA      GUUU  CAAAGGC     AUUUG  A
A-    ----------        UAUAGCA   CACUUU    CC       UAAAA^    UA
                  100        90        80        70        60

-----------------------------------------------------
```

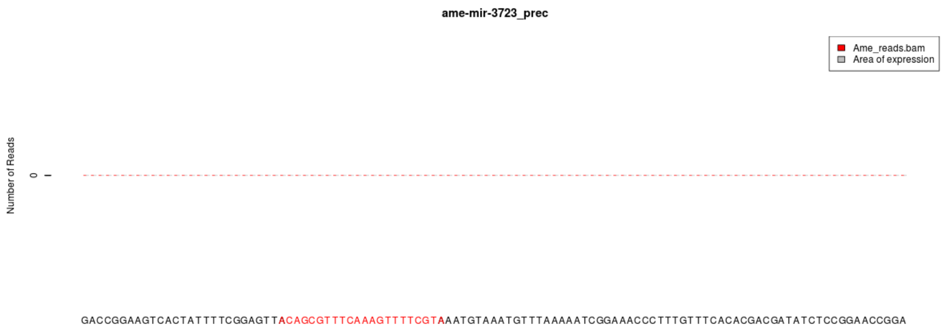

>ame-mir-3724_prec

TTCTGATAATTTAAATCCGTTTTCGATTCATGGATAGAACGAATGAGTAGAATATTTGGTTAATGAATACATAATTAAGCTCAAGTGTT
TTTCAAAATGTTTAATTTGAGAAA**ATCGTGGTAAACTTTGAATATA**TAAGA

>ame-mir-3724_3p

ATCGTGGTAAACTTTGAATATA

UUCUGAUAAUUUAAAUCCGUUUUCGAUUCAUGGAUAGAACGAAUGAGUAGAAUAUUUGGUUAAUGAAUACAUAAUUAAGCUCAAGUGUUUUUCAAAAUGUUUAAUUUGAGAAA**AUCGUG**
GUAAACUUUGAAUAUAUAAGA

```
.(((.((((((((((........((((((.(..((.(((((..((((..
((((((((((((((((.........))).))).))))))))))))))...))))).))..).))..)))).........))))))).))).)))
```

```
 Initial dG =    -19.60

         10        20          30        40          50          60
U   G   -       UCCGUUUU    --  A GG  A     AA-|   UA          -   -   GAAU
 UCU AUA AUUUAAA        CGAU  UC U  AU GAACG   UGAG  GAAUAUUUG GUU AAU    \
 AGA UAU UAAGUUU        GCUA  AG G  UA UUUGU   ACUU  UUUGUGAAC CGA UUA    A
-   A   A       CAAAUGGU    AA  A UU  A     AAA^   --          U   A   AUAC
 .       130       120       110       100          90        80        70

-----------------------------------------------------
```

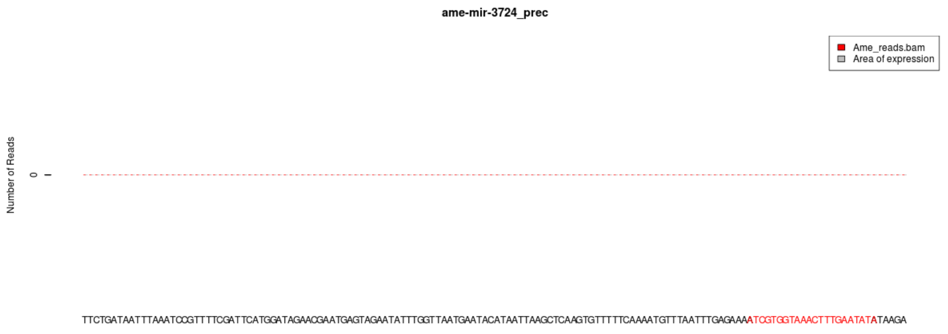

>ame-mir-3725_prec
TATTTGGATAAAGTTATTATATCGGAAGAAGGAAGAGAGAGAGAGAGAGATCTTTTATCTATCTGTGTTTCCT**TTTCTACATTTCTTCGTA**
AAGATGCTGGTCCAATCGTTGG

>ame-mir-3725_3p
TTTCTACATTTCTTCGTAAAG

```
UAUUUGGAUAAAGUUAUUAUAUCGGAAGAAGGAAGAGAGAGAGAGAGAGAUCUUUUAUCUAUCUGUGUUUCCUUUUCUACAUUUCUUCGUAAAGAUGCUGGUCCAAUCGUUGG
...((((((...(((.((((....((((((((((((.((((((.((((.....)))).)))....))))))))))....)))))))))).)))....))))))......
```

```
 Initial dG =    -23.20

            10         20        30            40            50
UAU----      AAA-   A    AUCG      ----       A   ----|  G    CU
       UUGGAU    GUU UUAU    GAAGAA    GGAAGAG GAG    AGA AGAU  \
       AACCUG    UAG AAUG    CUUCUU    UCUUUUC CUU    UCU UCUA  U
GGUUGCU      GUCG   A    ----      UACA       -   UGUG^  A    UU
 .       100        90            80        70          60

-----------------------------------------------------
```

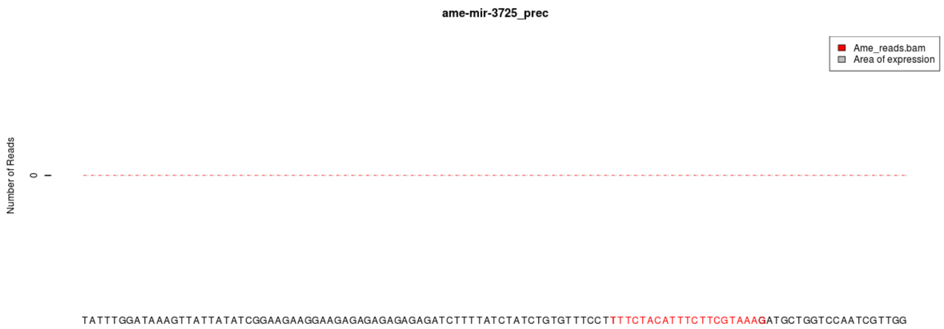

>ame-mir-3726_prec
TGGCGAAAGATAAACAGGCCGAGTGGCACGGCCTTGTCCGCG**AACGAGTGGTGGATGCCAGCGT**TTACATTCCATTAAACGTTGGTCTA
TCGCCACTCGAACAGCAACCGTCTCATTTGCCCTGTGCCG

>ame-mir-3726_3p
AACGAGTGGTGGATGCCAGCGT

```
UGGCGAAAGAUAAACAGGCCGAGUGGCACGGCCUUGUCCGCGAACGAGUGGUGGAUGCCAGCGUUUACAUUCCAUUAAACGUUGGUCUAUCGCCACUCGAACAGCAACCGUCUCAUUUG
CCCUGUGCCG
.(((.........(((((.(((((((.((((..((((.......((((((((((..
((((((((((((........))))))))))))...))))))))))....)))))))).))))))).)))))))).
ERROR 4: Strange structure
 Initial dG =    -53.80

        10        20        30        40        50         60        70
U|  GAAAGAUAA     C       C    CC    CCGCGAA         AU-           CAU
 GGC         ACAGG CGAGUGG ACGG  UUGU       CGAGUGGUGG   GCCAGCGUUUA   U
 CCG         UGUCC GUUUACU UGCC  AACG       GCUCACCGCU   UGGUUGCAAAU   C
G^  ---------     C       C    --    ACAA---         AUC           UAC
                120       110         100          90        80
```

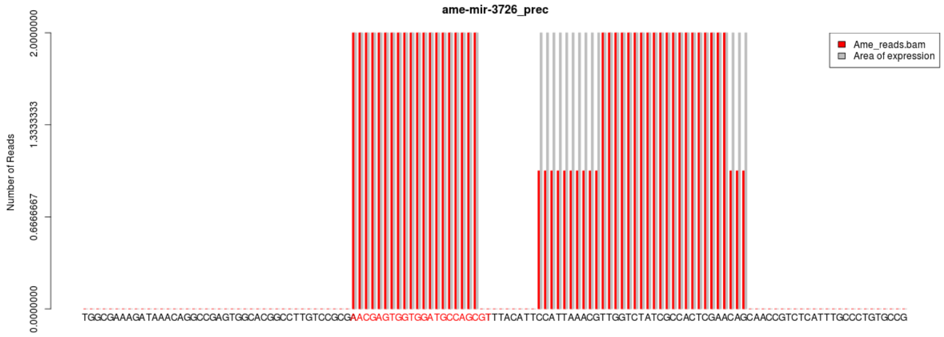

>ame-mir-3727_prec
TCGCCAGTCGGTCGCGCGTTACCGAGCCAAGTGGATGAG**TTCGCGAACGCGCTCCCGCTAA**AAGCT

>ame-mir-3727_3p
TTCGCGAACGCGCTCCCGCTAA

```
UCGCCAGUCGGUCGCGCGUUACCGAGCCAAGUGGAUGAGUUCGCGAACGCGCUCCCGCUAAAAGCU
..((.((.(((..(((((((..((((((((......)).))))))..))))))))..))))).....)).

 Initial dG =    -25.40

           10        20         30
UC  C---| U   UC       AC      -  AG
  GC    AG CGG  GCGCGUU  CGAGC CA  U
  CG    UC GCC  CGCGCAA  GCUUG GU  G
U-  AAAA^ -   CU       GC      A  AG
      60         50        40

-----------------------------------------------------
```

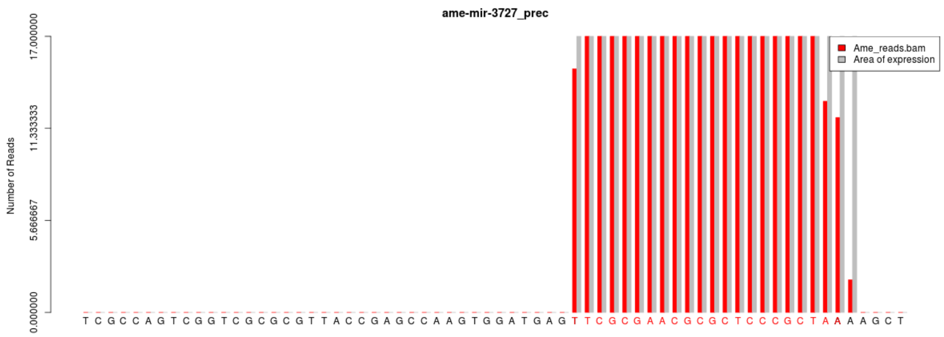

>ame-mir-3728_prec
GAGCGCGCGTGTCTATTAGTAGCGGTAA**CCATCCGTGGGATTCGTAATTTA**GTTTCTAGTTTATCTTGGATCGAAGGTAGGATACGATT
CGCGGCCGCGAAGGCGTAAATACAGGCGTA

>ame-mir-3728_3p
CCATCCGTGGGATTCGTAATTTA

```
GAGCGCGCGUGUCUAUUAGUAGCGGUAACCAUCCGUGGGAUUCGUAAUUUAGUUUCUAGUUUAUCUUGGAUCGAAGGUAGGAUACGAUUCGCGGCCGCGAAGGCGUAAAUACAGGCGUA
....((((.(((......((.((((.......((((((..(((((.((((.((((..((((.....)))).)))).)))).))))).)))))))))))))))))....))......))).)))).
ERROR 4: Strange structure
 Initial dG =    -31.30

        10            20
GAGC    G          .-AGUA|   UAA
    GCGC UGU--CUAUU      GCGG   \
    UGCG ACA  GAUGG      UGCC   C
A---    G   \      \ ----^   UAC
             80               30
```

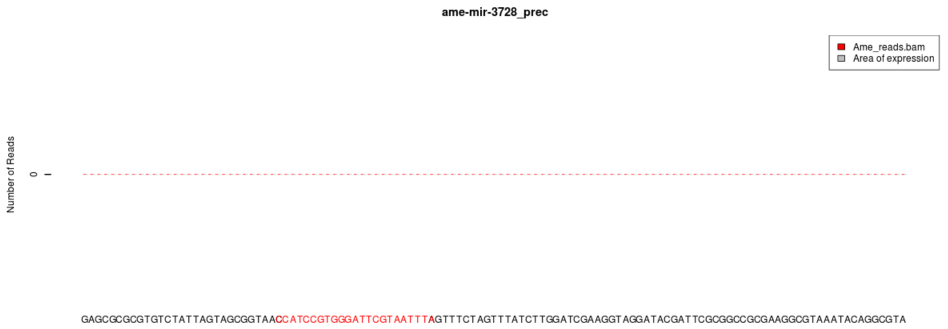

>ame-mir-3729_prec
TCAACGTGTAGTTATCGGAAAAACAGAGTTCGGAAGAGATCAAGAGATCTAATATGACTTTCG**TGTTTTCCCAACTCACTTGCA**AAA

>ame-mir-3729_3p
TGTTTTCCCAACTCACTTGCA

```
UCAACGUGUAGUUAUCGGAAAAACAGAGUUCGGAAGAGAUCAAGAGAUCUAAUAUGACUUUCGUGUUUUCCCAACUCACUUGCAAAA
.(((.(((.((((...((((((((((((.((....(((((....)))))....))))))).)).)))))).))))))))))).....

 Initial dG =    -21.10

            10        20          30        40
U----|  C   U    AUC      -  -     U  GAAG     A
     CAA GUG AGUU   GGAAAA AC AGAGU CG    AGAUC A
     GUU CAC UCAA   CCUUUU UG UUUCA GU    UCUAG G
AAAAC^  -   -    C--      G  C     -  AUAA     A
      80            70        60         50

-----------------------------------------------------
```

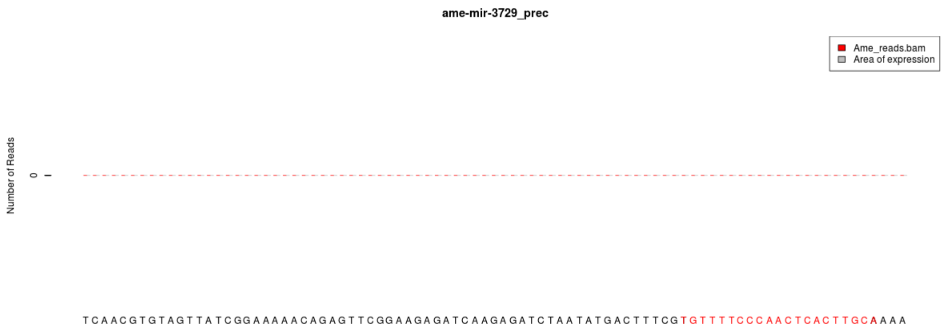

>ame-mir-3730_prec
TAACTTCATTATGTATCGCGATGA**ACAACGAGGGTGAAGATTGCGTG**TATTTGTAAATTCGCATCTCTATCCTCGTTTCTGTATCATCC
CAGCT

>ame-mir-3730_5p
ACAACGAGGGTGAAGATTGCGTG

```
UAACUUCAUUAUGUAUCGCGAUGAACAACGAGGGUGAAGAUUGCGUGUAUUUGUAAAUUCGCAUCUCUAUCCUCGUUUCUGUAUCAUCCCAGCU
................((((((((((((((((((.((((.(((.((........)).))))))).)))))))))...))).)))))...)).

 Initial dG =    -26.40

        10           20             30        40        50
UAACUUCAUUAUGUAUC  ---     -   ---|        A    U   U  AUU
                 GC   GAUGA ACA   ACGAGGGUG AGAU GCG GU   U
                 CG   CUACU UGU   UGCUCCUAU UCUA CGC UA   G
U----------------  ACC     A   CUU^        C    -   U  AAU
                   90        80        70          60

-----------------------------------------------------
```

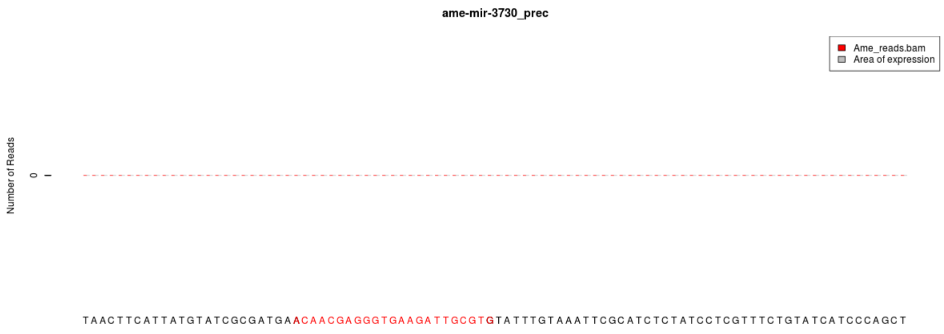

>ame-mir-3731_prec
GGTATAGTAGCTTGAA**GAACATCGACCAAAGCTTGCAAC**TGATGCCTGAAGGAATCGCAAAGCGTTGGTTTGCCTTTCGAAGTTCCACG
AGACATAGTAGTCG

>ame-mir-3731_5p
GAACATCGACCAAAGCTTGCAAC

```
GGUAUAGUAGCUUGAAGAACAUCGACCAAAGCUUGCAACUGAUGCCUGAAGGAAUCGCAAAGCGUUGGUUUGCCUUUCGAAGUUCCACGAGACAUAGUAGUCG
((((.((..((((..((((.((((((((.((((((....(((.((....)).))))))).)).)))).......)))).)))))..)))))..)).))).)).

 Initial dG =    -23.90

          10        20                30         40
-  -   A  AG    AA    A    --------    A  -|    AACU   G  U
 GG UAU GU  CUUG  GAAC UCGA        CCAA GC UUGC    GAU CC G
 CU AUG UA  GAGC  CUUG AGCU        GGUU CG AACG    CUA GG A
G  G   A  CA    AC    A    UUCCGUUU    G  A^    ----   A  A
 100       90        80        70        60            50

-----------------------------------------------------
```

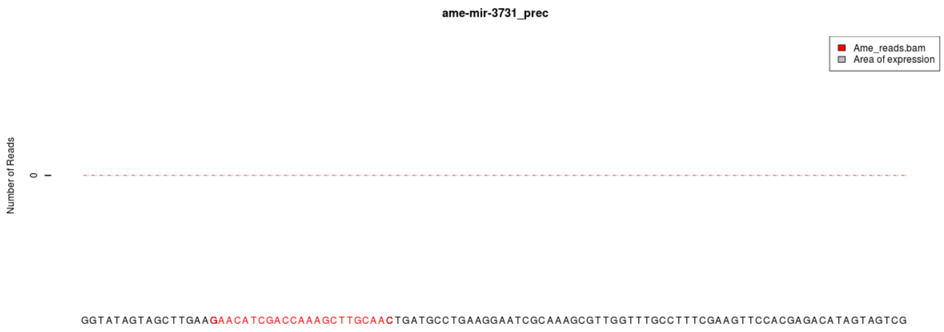

>ame-mir-3732_prec
AATCGTCGGAGCATCATTGTGTGTGTTTGTATGCTTTACTACTTTCTATCAAATTGTCTGGGTTTCTCCTTACCCTTTCAGTGAAAGTT
GGGAAATGCGTTTGG**CGACGCCATGTTTGGTCTCATCT**TTTGATA

>ame-mir-3732_3p
CGACGCCATGTTTGGTCTCATCT

AAUCGUCGGAGCAUCAUUGUGUGUGUUUGUAUGCUUUACUACUUUCUAUCAAAUUGUCUGGGUUUCUCCUUACCCUUUCAGUGAAAGUUGGGAAAUGCGUUUGG**CGACGCCAUGUUUGG**
UCUCAUCUUUUGAUA
.((((...(((.((((.((((.(((((...(((((((.((((((((......((((...
((((........))))...)))))))))).)).))).)))).....)))))))))..)))))))......)))).

Initial dG =   -29.70

```
           10         20        30          40         50        60
A    UCG---   C     U-     U      UGU--     -    A   -|      UAUCAA     UCU     UUC
 AUCG      GAG AUCA  UGUG GUGUU      AUGC UUU CU ACUUUC       AUUG    GGGU   U
 UAGU      CUC UGGU  GUAC CGCAG      UGCG AAA GG UGAAAG       UGAC    CCCA   C
A    UUUCUA   -     UU     -      CGGUU     U    G   U^      ------     UUU     UUC
  130        120        110       100        90              80        70
```

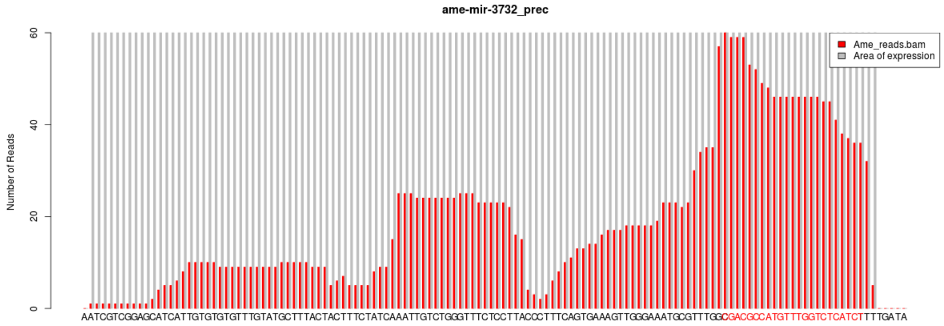

>ame-mir-3733_prec
GCCGTTTCTGCGATTACCAAGT**TTACTAATCTCGAATTTTTCT**CGCGCAACGATCGTTCCTAAGAAGAAATCGAGTAGATGGTAGATAG
AATAATGGAAATGT

>ame-mir-3733_5p
TTACTAATCTCGAATTTTTCT

```
GCCGUUUCUGCGAUUACCAAGUUUACUAAUCUCGAAUUUUUCUCGCGCAACGAUCGUUCCUAAGAAGAAAUCGAGUAGAUGGUAGAUAGAAUAAUGGAAAUGU
..((((((((..(((.....((((((((..(((((.(((((((.....(((....)))....))))))).)))))....))))))))..)))..)))))))).

 Initial dG =    -24.10

        10        20        30          40        50
GC|       CG   ACCAA        AU--     A       CGCGC   G
  CGUUUCUG  AUU     GUUUACUA    CUCGA UUUUUCU     AAC A
  GUAAAGGU  UAA     UAGAUGGU    GAGCU AAGAAGA     UUG U
U-^       AA   GA---        AGAU     A       AUCC-   C
  100        90           80        70        60

-----------------------------------------------------
```

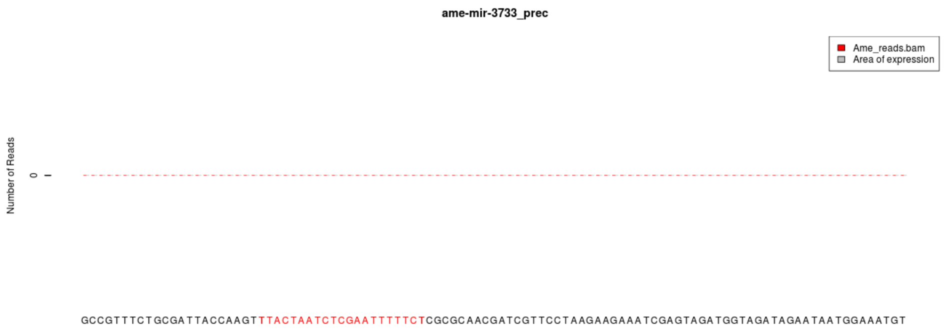

>ame-mir-3734_prec
AAAGGTTAATGCAGTTAGCCGGTCCCCCTTTTGGTGGCCCTTGG**TTTCGCACGGGAGGACGTTAG**CGCTCCTC

>ame-mir-3734_3p
TTTCGCACGGGAGGACGTTAG

```
AAAGGUUAAUGCAGUUAGCCGGUCCCCCUUUUGGUGGCCCUUGGUUUCGCACGGGAGGACGUUAGCGCUCCUC
..(((.....((.((((((..(((((((.....((((((...)))..)))..))).))))))))))))).))).

 Initial dG =    -20.10

        10        20         30          40
AA|  UUAAU  A      CG    -   UUUUG   --   C
  AGG     GC GUUAGC  GUCC CCC     GUG  GCC \
  UCC     CG CGAUUG  CAGG GGG     CGC  UGG U
C-^  U----  -      --    A   CA---   UU   U
   70                60           50

-----------------------------------------------------
```

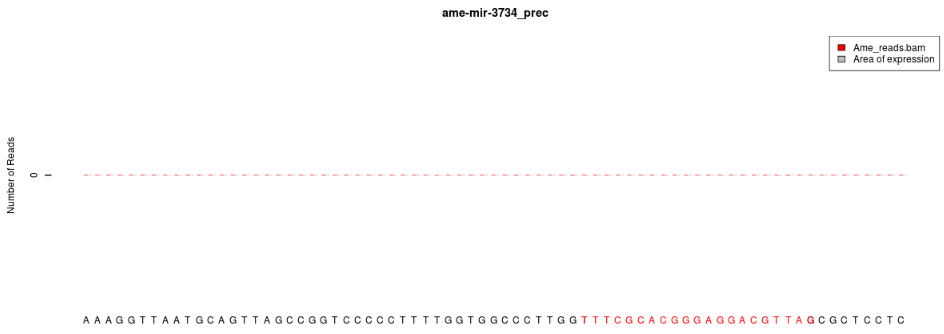

>ame-mir-3735_prec
TAAATA**ACGAGCAAAGTTTCCTCGTGCC**GATAAGATGCAATTTGCTTATCGACGAACAAAACCAATAGAAAAACTGGAAACTGAACCTT
CGTTAA

>ame-mir-3735_5p
ACGAGCAAAGTTTCCTCGTGCC

```
UAAAUAACGAGCAAAGUUUCCUCGUGCCGAUAAGAUGCAAUUUGCUUAUCGACGAACAAAACCAAUAGAAAAACUGGAAACUGAACCUUCGUUAA
....(((((((...((((((((((..((((((..........)))))))))))...................))))))).....))))))).

 Initial dG =    -21.10

        10          20                            30
UAAA       CAA--       ------------------|   GC        AUGC
    UAACGAG     AGUUUCC                    UCGU  CGAUAAG    A
    AUUGCUU     UCAAAGG                    AGCA  GCUAUUC    A
A---       CCAAG       UCAAAAAGAUAACCAAAACA^   --        GUUU
       90        80        70        60          50        40

-----------------------------------------------------
```

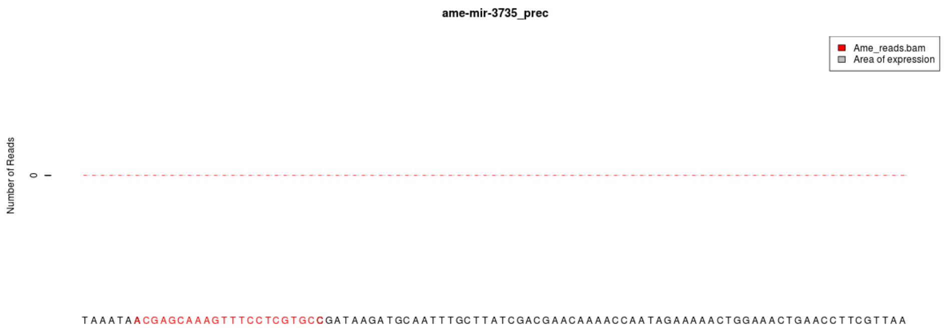

>ame-mir-3736_prec
CGAGATCTGCGTCCTCGG**CGATTACCCTGAGTATGCTCC**GATGCATAGGCGTGTCCTCCGCGACGGTGGCCTCGTACTTGGTCGGGTAG
AAGAGGGGGTTCAGGTCG

>ame-mir-3736_5p
CGATTACCCTGAGTATGCTCC

```
CGAGAUCUGCGUCCUCGGCGAUUACCCUGAGUAUGCUCCGAUGCAUAGGCGUGUCCUCCGCGACGGUGGCCUCGUACUUGGUCGGGUAGAAGAGGGGGUUCAGGUCG
...((((((..((((.....((((((((((((((((((.(((..(((.....)))..))).))).))).)))...)))))))...))))))....)))))..)))))).
```

```
 Initial dG =    -38.90

        10        20            30             40        50
CGA      CG      GGCGA      ---        ---    -|  A   AU   CG
   GAUCUG  UCCUC     UUACCC   UGAGUAU   GCU CCG UGC  AGG  \
   CUGGAC  GGGGG     GAUGGG   GUUCAUG   CGG GGC GCG  UCC  U
G--      UU      AGAA-      CUG        CUC   U^  A   CC   UG
       100        90          80         70         60

-----------------------------------------------------
```

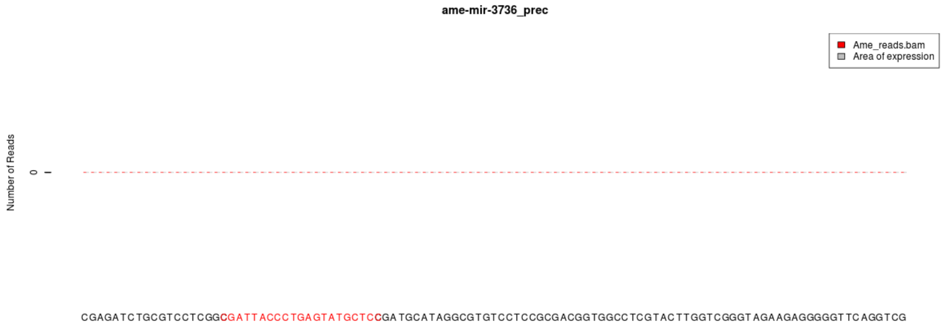

>ame-mir-3737_prec
GATCAAAGTATGGCGAAA**GATCGGAAATTTTGATATGTT**TGTTTGTATATGTATATTTATATAAATTTTAATGATACACCATGTCAAGT
ATTTGAATTTTAAGTTTATACTTTGATG

>ame-mir-3737_5p
GATCGGAAATTTTGATATGTT

GAUCAAAGUAUGGCGAAA**GAUCGGAAAUUUUGAUAUGUU**UGUUUGUAUAUGUAUAUUUAUAUAAAUUUUAAUGAUACACCAUGUCAAGUAUUUGAAUUUUAAGUUUAUACUUUGAUG
.(((((((((((((..(((((((((...(((((((((...(((((((((........)))))))))............)))))))))..))))).))))..))).)))))))))).

Initial dG =   -27.00

```
        10          20          30         40                  50
G          -   GA    -     AAU         UUU----------|        UGU
 AUCAAAGUAU GGC  AAGA UCGGA   UUUGAUAUG             GUUUGUAUA   A
 UAGUUUCAUA UUG  UUUU AGUUU   GAACUGUAC             UAAAUAUAU   U
G          U   AA    A     AU-         CACAUAGUAAUUU^        UUA
     110       100        90          80         70        60
```

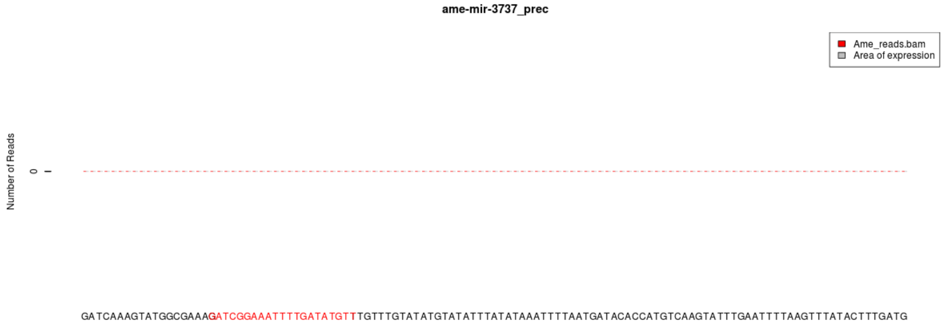

>ame-mir-3738_prec
CGGAATCGC**GTTTACCGGATGTTTTCGCAAT**TTCGCGGGGGAAAAAAATGTGTGAATGCTTGCCGGGGACGAGCTGCGAAGTTGA

>ame-mir-3738_5p
GTTTACCGGATGTTTTCGCAAT

```
CGGAAUCGCGUUUACCGGAUGUUUUCGCAAUUUCGCGGGGGAAAAAAAUGUGUGAAUGCUUGCCGGGGACGAGCUGCGAAGUUGA
(((..((((((((((((....((..(((......)))..))).......)).))))))))..((((....)).))...))..))).

 Initial dG =    -23.30

                 10          20            30
-------  .-GAAUC        -  GAUG---| UU    AA
       CG       GCGUUUAC CG       UU  CGC  U
       GC       CGUAAGUG GU       GG  GCG  U
AGUUGAA  \ -----        U  AAAAAAA^ GG    CU
    80                  50        40



          60
           UU  -  G
             GC CG G
             CG GC G
           GU  A  A
                70

-----------------------------------------------------
```

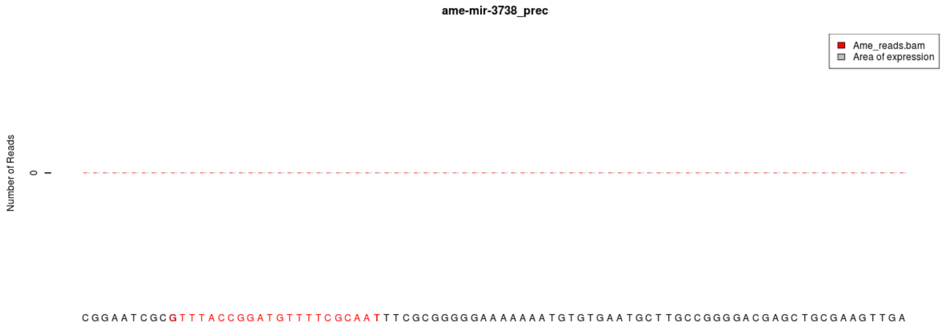

>ame-mir-3739_prec
CTTCTAATCGCCGCCGATCCTCCGCCCGACGAGGATCAATTTCTATCTCTAGCGTGGAGGGAACTTTT**TTGGGAGGGGGAGAGAGCAGC**
TGCCAGAAACTCGAATAAT

>ame-mir-3739_3p
TTGGGAGGGGGAGAGAGCAGCT

```
CUUCUAAUCGCCGCCGAUCCUCCGCCCGACGAGGAUCAAUUUCUAUCUCUAGCGUGGAGGGAACUUUUUUGGGAGGGGGAGAGAGCAGCUGCCAGAAACUCGAAUAAU
.((((....((.((...((((((.(((((.((((.((..(((((((.......))))))))).)))).))))).))))))....)).))....)))).........

 Initial dG =    -31.40

                  10         20        30        40
C----------    AAUC  C  CGA-|     G     C    A  AA       CUC
           UUCU    GC GC    UCCUCC CCCGA GAGG UC  UUUCUAU   \
           AAGA    CG CG    AGGGGG GGGUU UUUC AG  GGAGGUG   U
UAAUAAGCUCA    CCGU  A  AGAG^     A     U    A  --       CGA
      100        90        80        70          60

-----------------------------------------------------
```

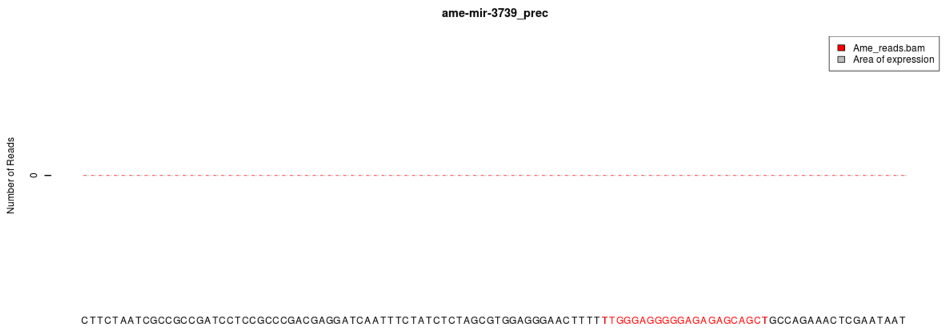

>ame-mir-3740_prec
TTTCAGAGTTGAAAAGA**GGAAAAGGATGACGAGGATTAA**TTTGAGGATGTCCAATTCATAGTCCATCATTTCCTTTTTTTTTTTTGTTAT
CGATGAT

>ame-mir-3740_5p
GGAAAAGGATGACGAGGATTAA

```
UUUCAGAGUUGAAAAGAGGAAAAGGAUGACGAGGAUUAAUUUGAGGAUGUCCAAUUCAUAGUCCAUCAUUUCCUUUUUUUUUUUUGUUAUCGAUGAU
.......(((((((((((((((((((....((((((((...(((((....))...))))))))).))...))))))))))))))....)))))...
```

```
 Initial dG =    -22.40

        10            20        30         40
UUUCAGA     ----|             UGAC  -      AUU   ---  A
       GUUGA    AAAGAGGAAAAGGA    GA GGAUUA   UGA   GG U
       UAGCU    UUUUUUUUUUUUCCU    CU CCUGAU   ACU   CC G
UAG----     AUUG^             UUA-  A      ---   UAA  U
         90        80        70         60           50
```

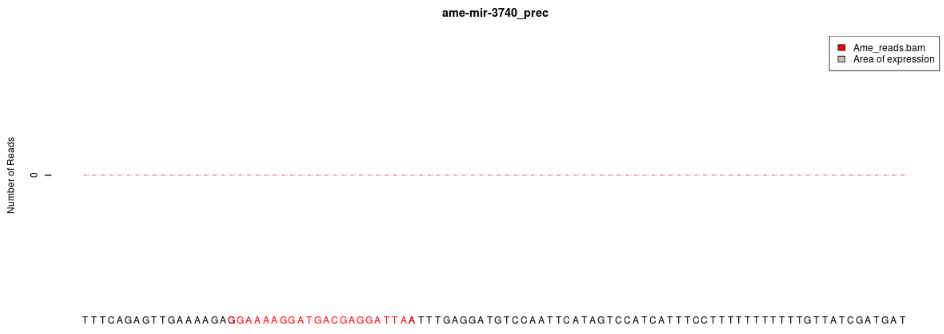

>ame-mir-3741_prec
ACCGATTAATGATACCCACACGGCTCTTTCGATAATATTGATGGCCCCCTCGTGTACCTTTGCAAGAATATCTCGGGGATACGAGAGGG
AAAA**ACGAGAGGGGGAGAGAGAAGTA**TGGGACTTTTATGTAATGGA

>ame-mir-3741_3p
ACGAGAGGGGGAGAGAGAAGTA

ACCGAUUAAUGAUACCCACACGGCUCUUUCGAUAAUAUUGAUGGCCCCCUCGUGUACCUUUGCAAGAAUAUCUCGGGGAUACGAGAGGGAAAA**ACGAGAGGGGGAGAGAGAAGUA**UGGG
ACUUUUAUGUAAUGGA
.(((....((((..((((.((..(((((((.............((((((((...(((...........
(((((......))))))))....))))).)))))))))))..)).)))).....))))....))).

```
 Initial dG =    -41.30

        10          20         30         40          50          60         70
A   AUUA    UA--|   C   GG       GAUAAUAUUGAUGG     -     GUA-   UUGCAAGAAUA     GG
 CCG    AUGA    CCCA AC  CUCUUUC              CCCC CUCGU    CCU           UCUCG  G
 GGU    UAUU    GGGU UG  GAGAGAG              GGGG GAGCA    GGG           AGAGC  A
A   AAUG    UUCA^   A  AA      --------------    A     AAAA   -----------     AU
   130       120       110                     100        90                   80

-----------------------------------------------------
```

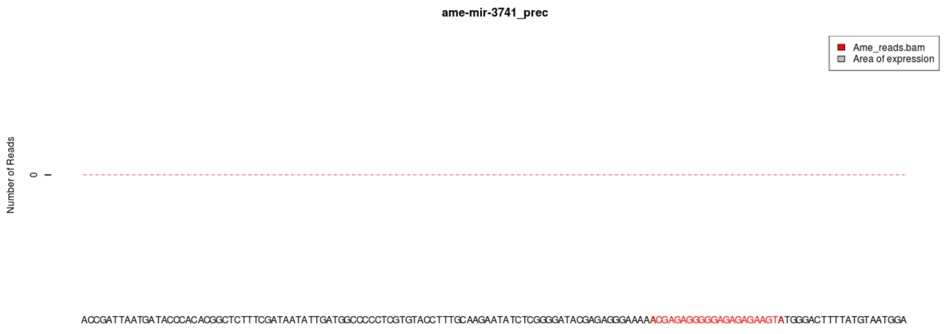

>ame-mir-3742_prec
TCTTTTTATCCGTGTCGTTA**AAATATATTTTAAACGACGGAC**GAATCCCCCTCCGCGTTTTATTTATTATGTTTGCCATAGATTCCACG
GGATTAGGAA

>ame-mir-3742_5p
AAATATATTTTAAACGACGGAC

UCUUUUUAUCCGUGUCGUUA**AAAUAUAUUUUAAACGACGGAC**GAAUCCCCCUCCGCGUUUUAUUUAUUAUGUUUGCCAUAGAUUCCACGGGAUUAGGAA
((((....((((((((....(((((((....(((((.((((..........)))))))))).......))))))).......))...)))))))...)))).

```
 Initial dG =    -20.50

         10           20          30           40
-    UUUA      ---  GUUA--       UUUU---|    A    CGAA
 UCUU    UCCGUG   UC      AAAUAUA       AAACG CGGA    U
 AGGA    GGGCAC   AG      UUUGUAU       UUUGC GCCU    C
A    UUA-      CUU  AUACCG       UAUUUAU^    -    CCCC
         90        80        70        60          50

-----------------------------------------------------
```

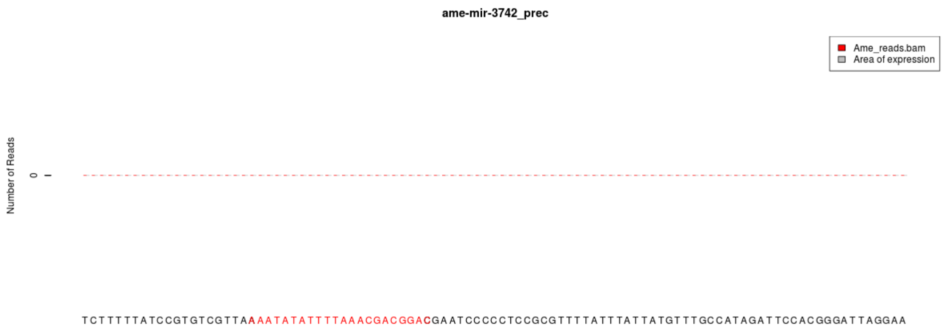

>ame-mir-3743_prec
TACTTGGTCCGAGAGGCGGAGAAGGAAGTTCCGCGGCGCGTTCCGCTCCC**TCGTGACTTTCTTTTCAGCCCCT**TGGCCAGGC

>ame-mir-3743_3p
TCGTGACTTTCTTTTCAGCCCCT

```
UACUUGGUCCGAGAGGCGGAGAAGGAAGUUCCGCGGCGCGUUCCGCUCCCUCGUGACUUUCUUUUCAGCCCCUUGGCCAGGC
..(((((.(((((.(((.(((((((((((..((.((.(((...)))..)).)).))..))))))))))).))).))))))))).
```

```
 Initial dG =    -42.80

        10        20        30         40
UA|    U     A   G           UC  C  C-   U
  CUUGG CCGAG GGC GAGAAGGAAGU  CG GG  GCG \
  GGACC GGUUC CCG CUUUUCUUUCA  GC CC  CGC U
C-^    -     C   A           GU  U  CU   C
  80         70        60        50
```

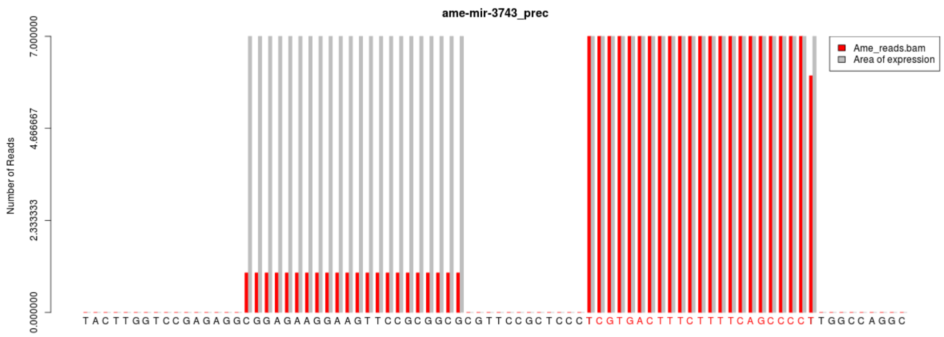

>ame-mir-3744_prec

GAGTATTAAAGTTGA**CGGTATCTTTGTGAAAAATTTCG**AGAAGAGGGAATTTTGAATTTAAATCGTTTCTATCTATGTTCCTCTTGTTA
GAAATTAAGAATAAAATACTGTCTTCATCAACTTTGTTCCATCTCC

>ame-mir-3744_5p

CGGTATCTTTGTGAAAAATTTCG

```
GAGUAUUAAAGUUGACGGUAUCUUUGUGAAAAAUUUCGAGAAGAGGGAAUUUUGAAUUUAAAUCGUUUCUAUCUAUGUUCCUCUUGUUAGAAAUUAAGAAUAAAAUACUGUCUUCAUCA
ACUUUGUUCCAUCUCC
(((...((((((((((((((.(((((....((((((((.((((((((.....
(((..........)))........)))))))).)).))))))....))))))))))))))......)))))))......))).
```

```
 Initial dG =    -30.40

            10              20        30         40        50           60
-   UAU---       ------         C      GAAA      -  G        AUUUU---|  UUUA
 GAG      UAAAGUU      GACGGUAU UUUGU    AAUUUC GA AAGAGGGA        GAA    A
 CUC      GUUUCAA      CUGUCAUA AAAUA    UUAAAG UU UUCUCCUU        CUU    A
C   UACCUU       CUACUU         -      AGAA      A  G        GUAUCUAU^  UGCU
   130       120       110        100        90        80        70

-----------------------------------------------------
```

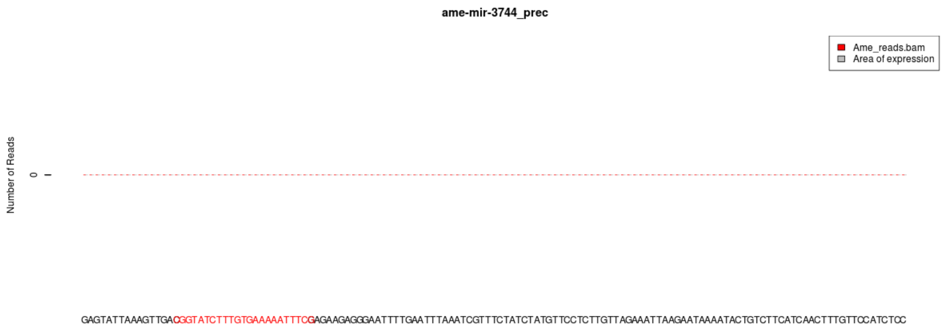

>ame-mir-3745_prec
GCACGCCGGGGGGGTGTGGTCCACAGTTGGTTACGGTGAAACGGGTTTGGCCACCGTCCCCCGTTCTCTGGCCGCTGATTCGTCGAGCC
CGGGTGCCACCCCCCGGCCCGGGTGA

>ame-mir-3745_3p
CGGGTGCCACCCCCCGGCCCGG

```
GCACGCCGGGGGGGUGUGGUCCACAGUUGGUUACGGUGAAACGGGUUUGGCCACCGUCCCCCGUUCUCUGGCCGCUGAUUCGUCGAGCCCGGGUGCCACCCCCCGGCCCGGGUGA
.(((.(((((((((.(((((((......((((.((..(((.(((...((((((...............))))))))).)))..))))))..)).))))).))))..)))))))).

 Initial dG =    -50.50

        10          20         30        40        50        60
G   G     --    U     -| ACAGUU    A  GU   A   GUU      CCGUCCC
 CAC CCGGG  GGGG GUGGU CC      GGUU CG  GAA CGG   UGGCCA       \
 GUG GGCCC  CCCC CACCG GG      CCGA GC  CUU GUC   GCCGGU       C
A   -     GG    C     U^ GC----    -  UG   A   ---      CUCUUGC
    110       100        90             80            70

-----------------------------------------------------
```

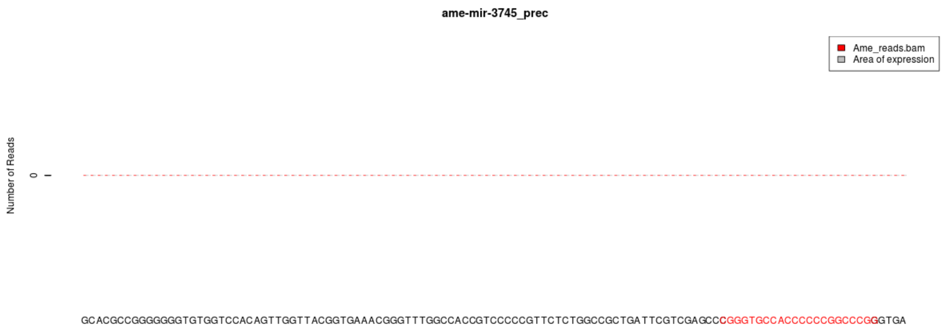

>ame-mir-3746_prec
CTTTCGGATCTTCTTCTCTGACAAGTTTACAGTACAATATTTTCTTTGTTTTTTTTTCTTTTTATGAGGCACAAAGCGAGAAGAAA**CGC**
GGAGAGGAAGGTATGCGTGTCGAGAGAACAGATGCCTCGAAAA

>ame-mir-3746_3p
CGCGGAGAGGAAGGTATGCGT

CUUUCGGAUCUUCUUCUCUGACAAGUUUACAGUACAAUAUUUUCUUUGUUUUUUUUUCUUUUUAUGAGGCACAAAGCGAGAAGAAA**CGCGGAGAGGAAGGUAUGCGU**GUCGAGAGAACA
GAUGCCUCGAAAA
.(((((((((..(((((((((.((.....((((....((((((((((((((((((.((((.
((...)).)))).))))))))).))))))))))..)))))).)))).)))))).))))...)))))).

```
 Initial dG =    -34.40

           10         20        30        40         50        60
C      ---|   UC       -    A  UUACA    AAUA          -         U    A  A
 UUUCGG   AUCU  UUCUCU GACA GU     GUAC    UUUUCUUUGU UUUUUUUUC UUUU UG \
 AAAGCU   UAGA  AAGAGA CUGU CG     UAUG    AGGAGAGGCG AAAGAAGAG GAAA AC G
A      CCG^   C-       G    G  -----    GA--          C         C    C  G
130       120        110            100          90        80        70

-----------------------------------------------------
```

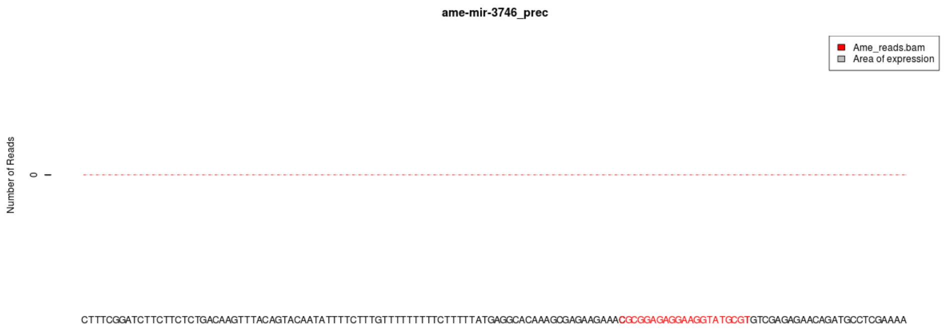

>ame-mir-3747a_prec
GGCCTG**CGAGGCTTTTTTTCAGCGATGA**ATAAAAATATTGTTCATCGTTGGAAAAAAGTCTTGGAGA

>ame-mir-3747a_5p
CGAGGCTTTTTTTCAGCGATGA

```
GGCCUGCGAGGCUUUUUUUCAGCGAUGAAUAAAAAUAUUGUUCAUCGUUGGAAAAAAGUCUUGGAGA
...((.((((((((((((((((((((((((((.....))))))))))))))))))))))))))).)).

 Initial dG =    -37.20

        10        20        30
GGC| G                          AA
   CU CGAGGCUUUUUUUCAGCGAUGAAUAA  \
   GA GUUCUGAAAAAAGGUUGCUACUUGUU  A
A--^ G                          AU
        60        50        40

-----------------------------------------------------
```

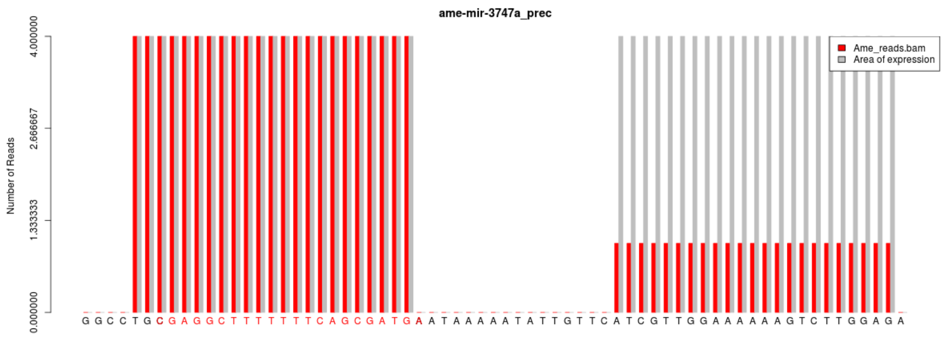

>ame-mir-3747b_prec
TACCCAGTGCCAGTTC**TGCGAGATTTCTTTTCAGTCGTG**AATAAAAATATTATTCACTGCTGGAAAAAAATCTCGCGGAAAAAAATACAC
GATCTGATTGGT

>ame-mir-3747b_5p
TGCGAGATTTCTTTTCAGTCGTG

```
UACCCAGUGCCAGUUCUGCGAGAUUUCUUUUCAGUCGUGAAUAAAAAUAUUAUUCACUGCUGGAAAAAAAUCUCGCGGAAAAAAAUACACGAUCUGAUUGGU
...(((((..(((((((((((((((.((((((((.((((((((.....)))))))).)))))))).))))))))))))))...........)))))))).
```

```
 Initial dG =    -40.10

        10                    20        30        40
UAC     GC   -----------|             C        C        AA
   CCAGU  CAG            UUCUGCGAGAUUU UUUUCAGU GUGAAUAA  \
   GGUUA  GUC            AAGGCGCUCUAAA AAAGGUCG CACUUAUU  A
U--     --   UAGCACAUAAAA^             A        U        AU
 100          90        80        70        60        50

-----------------------------------------------------
```

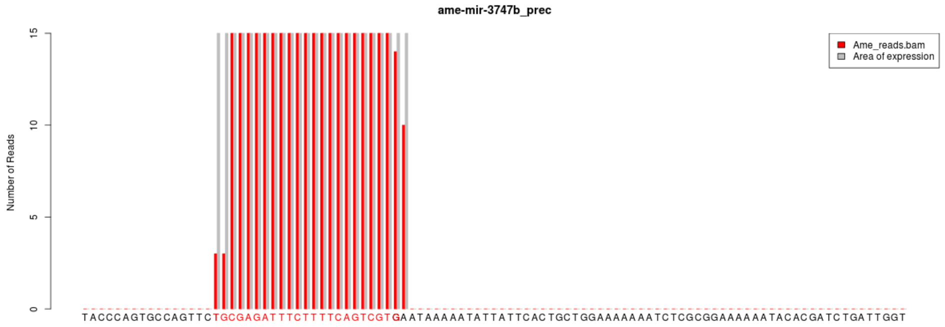

>ame-mir-3748_prec
GTCGGTTACTTTGTGCGGGGAGAATAATTTTACACAGATGGAACGTGTCGATAGCAGTCCTCGTTAGG**CGTCCGTTTCAAAGTAAAACT**
TGCAATTTCCAAATTAAAAGCCTCCC

>ame-mir-3748_3p
CGTCCGTTTCAAAGTAAAACTT

```
GUCGGUUACUUUGUGCGGGGAGAAUAAUUUUACACAGAUGGAACGUGUCGAUAGCAGUCCUCGUUAGGCGUCCGUUUCAAAGUAAAACUUGCAAUUUCCAAAUUAAAAGCCUCCC
...((...((((......(((((.(((((((((..((((((.((((..(((.........)))....))))))))))....)))))).)))...)))))......))))...)).
```

```
 Initial dG =    -20.40

        10        20           30          40          50
GUC  UUA    GUGCGG     A--   -      AC--      A    GU--|  UAGC
   GG   CUUU      GGAGA   UAA UUUUAC    AGAUGG ACGU    CGA    \
   CC   GAAA      CCUUU   GUU AAAAUG    UUUGCC UGCG    GCU    A
C--  UCC    AUUAAA     AAC   C      AAAC      -    GAUU^  CCUG
     110       100        90        80         70        60

-----------------------------------------------------
```

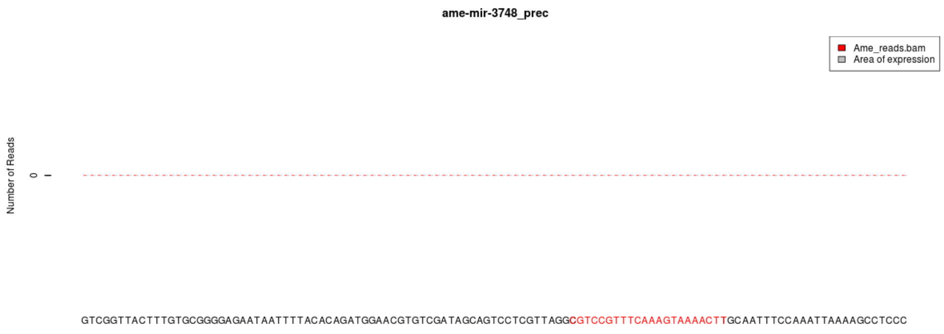

>ame-mir-3749_prec

GACCGATACACGAAGGTGAAAGCCGGCCAGGAGCCGAATCTCGAGGACCTATCTGAAAATC**TGGAATGGCCCTTTCACCTTCCC**TCGAT
A

>ame-mir-3749_3p

TGGAATGGCCCTTTCACCTTCCC

```
GACCGAUACACGAAGGUGAAAGCCGGCCAGGAGCCGAAUCUCGAGGACCUAUCUGAAAAUCUGGAAUGGCCCUUUCACCUUCCCUCGAUA
...(((.....((((((((((..(((((....(((.((.....(((....)))....)).)))..))))))))))))))))..)))...

 Initial dG =    -28.80

        10        20        30        40
GAC|  UACAC           CC      GGAG   A  CUCGA   C
   CGA     GAAGGUGAAAG  GGCCA    CCG AU     GGA C
   GCU     CUUCCACUUUC  CCGGU    GGU UA     UCU U
AUA^  CC---           --      AA--   C  AAAG-   A
.           80          70          60          50

----------------------------------------------------
```

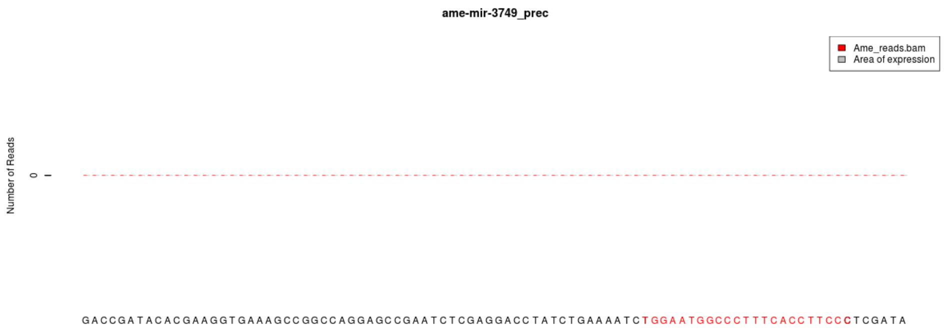

>ame-mir-375_prec
ATCGATTGAATTATCAGTTTGGTGCATCGATCCTAACGATCAACAAACTTTTCACTTGAAAG**TTTGTTCGTTCGGCTCGAGTTA**TCAAA
CTGAATGGATG

>ame-mir-375_3p
TTTGTTCGTTCGGCTCGAGTTA

```
AUCGAUUGAAUUAUCAGUUUGGUGCAUCGAUCCUAACGAUCAACAAACUUUUCACUUGAAAGUUUGUUCGUUCGGCUCGAGUUAUCAAACUGAAUGGAUG
.............(((((((((((..((((.((.(((((...((((((((((.....))))))))))))))).)).))))..))))))))))).......

 Initial dG =    -34.90

        10        20        30        40        50
AUCGAUUGAAUUA|          CA     U  U     UCA          CA
             UCAGUUUGGUG  UCGA CC AACGA   ACAAACUUUU  \
             AGUCAAACUAU  AGCU GG UUGCU   UGUUUGAAAG  C
GUAGGUA------^          UG     C  C     ---          UU
.               90        80        70           60

-----------------------------------------------------
```

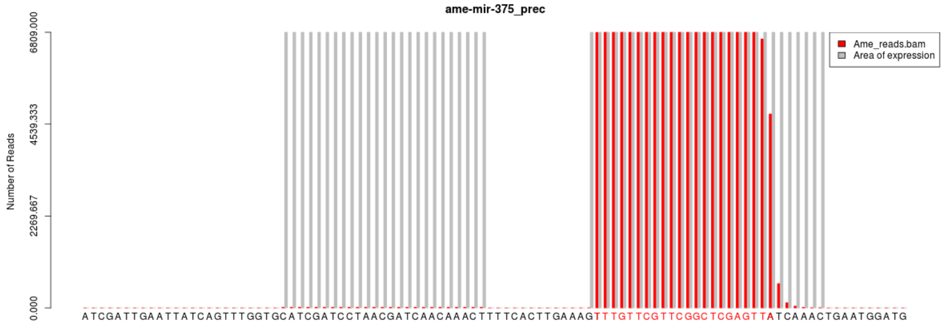

>ame-mir-3750_prec

TGAGCTCATCAG**TAAAGCTTCATCAGCAGGACTG**GTTTTACGATTAAATGGCCTATAGGGACAGTGAAGCTCAAAGGATACATTTGTAT
GAAAGCGGTGGTTAGGTGAGGATTTCCTAGAATTGTA

>ame-mir-3750_5p

TAAAGCTTCATCAGCAGGACTG

```
UGAGCUCAUCAGUAAAGCUUCAUCAGCAGGACUGGUUUUACGAUUAAAUGGCCUAUAGGGACAGUGAAGCUCAAAGGAUACAUUUGUAUGAAAGCGGUGGUUAGGUGAGGAUUUCCUAG
AAUUGUA
((((((((..((((((((...((.....))..)))))))).........((....))....)))).)))))....((((....))))....(((((..
(((((.........))))).))))).
```

```
 Initial dG =    -26.50

             10        20
       -    .-CA|       UCA  AG
--UGAGC UCAU    GUAAAGCU   UC  \
  ACUCG AGUG    CAUUUUGG   AG  C
\      A    \ --^       UC-  GA
    70          40         30



                     50
              GAUUAAAUGG  U
                        CC A
                        GG U
              ACAG------  A
                60
```

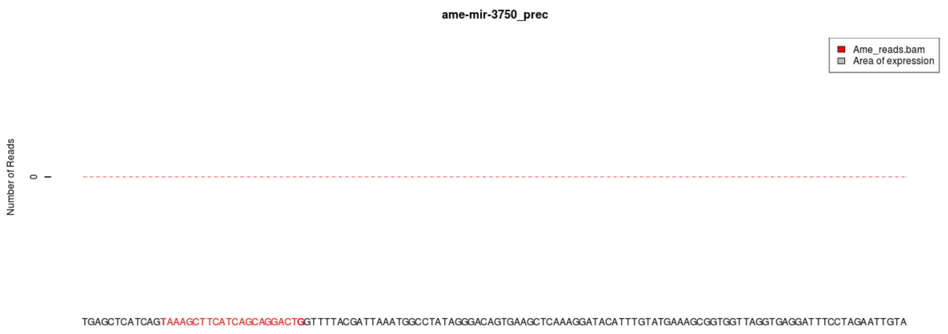

>ame-mir-3751_prec
AATGTTTGTCGCGTATTGACCGGCCATCCAACGCGGTGGCACCCCGAGAATTTCGAAAACTGCAATTGCATAGACAGAGCGATGCATGA
CGGTTTACTTCGG**GGGTGGTGGTGGAAGTCGGGG**GGAGTGAAAAGGAG

>ame-mir-3751_3p
GGGTGGTGGTGGAAGTCGGGG

AAUGUUUGUCGCGUAUUGACCGGCCAUCCAACGCGGUGGCACCCCGAGAAUUUCGAAAACUGCAAUUGCAUAGACAGAGCGAUGCAUGACGGUUUACUUCGG**GGGUGGUGGUGGAAGUC**
GGGGGGAGUGAAAAGGAG
```
........((((.......(((((..((((.(((....((.(((((((....(((.....((((.
((((.........))))))))...))).....))))))))).))).))))).))))).....)))).......
```

Initial dG =   -38.40

```
        10        20        30        40        50         60        70
AAUGUUUG|   GUAUUGA     CA    A   GGUG  A       AAUU-   AAAAC    A    AUAG
        UCGC       CCGGC  UCCA CGC    GC CCCCGAG     UCG     UGCA UUGC    \
        AGUG       GGCUG  AGGU GUG    UG GGGGCUU     GGC     ACGU AGCG    A
GAGGAAA-^   AGGGG--     A-    G   G---  -       CAUUU   AGU--    -    AGAC
      130         120        110           100        90           80
```

```
-----------------------------------------------------
```

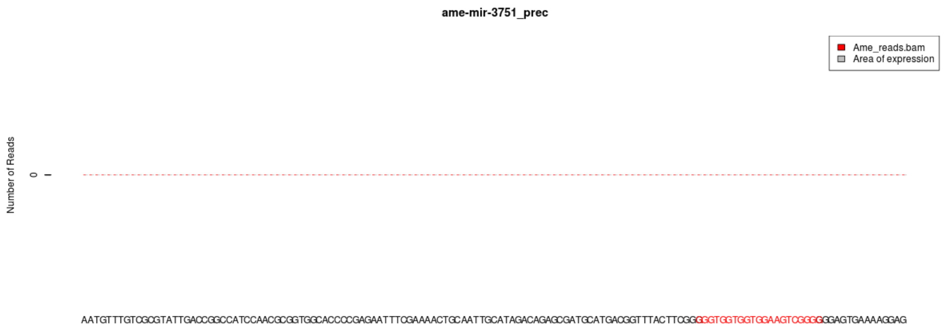

>ame-mir-3752_prec
GTGCGCAACGAATAGCGAAGCTGAGCTATAATTTAGAAAAATTATCGACAACGCGTTTCCCCTTCGATTTTCCATTTCTAAACT**GTGGA TAAAGTTTCCTTGCGAA**CC

>ame-mir-3752_3p
GTGGATAAAGTTTCCTTGCGAA

```
GUGCGCAACGAAUAGCGAAGCUGAGCUAUAAUUUAGAAAAAUUAUCGACAACGCGUUUCCCCUUCGAUUUUCCAUUUCUAAACUGUGGAUAAAGUUUCCUUGCGAACC
((.((((......((.((((((...(((((.((((((((....((((...............)))))......)))))))).))))).....))))))))))))).)).

 Initial dG =    -23.90

         10        20         30        40         50
-| G    ACGAAU  C      GAG-     A        AAUU--     CAACGCG
 GU CGCA      AG GAAGCU    CUAUA UUUAGAAA      AUCGA       \
 CA GCGU      UC CUUUGA    GGUGU AAAUCUUU      UAGCU       U
C^ A    ------  -      AAUA     C        ACCUUU     UCCCCUU
            100         90        80        70        60

-----------------------------------------------------
```

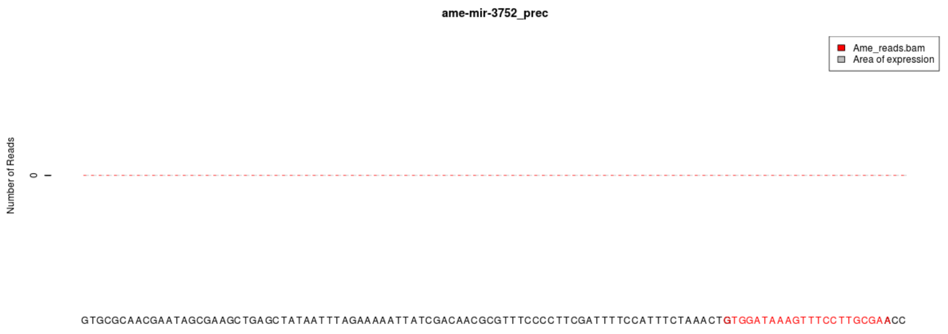

>ame-mir-3753_prec
ACAACAGATCGATCGGCAGCG**TGTCCGTTTCGGGACTCGACCG**TCGGCACAATTGCTCGAGATGCGGGAAGAGTTACAAGAACGCTTAC
ATATTGAAGAGGCATTTGTTGTA

>ame-mir-3753_5p
TGTCCGTTTCGGGACTCGACCG

```
ACAACAGAUCGAUCGGCAGCGUGUCCGUUUCGGGACUCGACCGUCGGCACAAUUGCUCGAGAUGCGGGAAGAGUUACAAGAACGCUUACAUAUUGAAGAGGCAUUUGUUGUA
((((((((...((..(((..(((.(((((...(((((..(((((((((....))).))....))))...)))))....)))))...)))..)))..))...))))))))).
```

```
 Initial dG =    -34.60

         10        20          30         40              50
-         CGA  GG   CG   C--     CGG-     GA-     ----  -|  C
 ACAACAGAU   UC  CAG  UGU   CGUUU    GACUC   CCGU    CG GCA A
 UGUUGUUUA   AG  GUU  ACA   GCAAG    UUGAG   GGCG    GC CGU A
A         CGG  AA   AU   UUC     AACA     AAG     UAGA  U^  U
110       100        90         80        70        60

-----------------------------------------------------
```

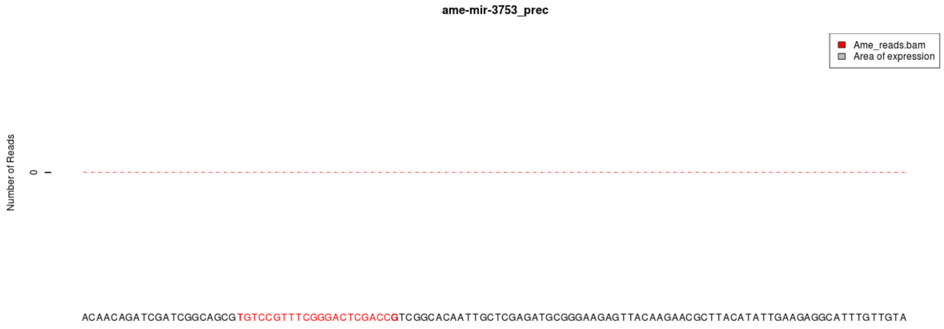

>ame-mir-3754_prec

ACATGTGTGGTGTCTTGACACGATTGCCGGCTT**TCATGGTGAAAAACTCATTCTTT**ACGTGCGCGAGCAACGTGGAATAGGGAATGATT
ATTACCGAAGCCGGTTAGTGTAATAGGACTCGTCGATTGA

>ame-mir-3754_3p

TCATGGTGAAAAACTCATTCTTT

```
ACAUGUGUGGUGUCUUGACACGAUUGCCGGCUUUCAUGGUGAAAAACUCAUUCUUUACGUGCGCGAGCAACGUGGAAUAGGGAAUGAUUAUUACCGAAGCCGGUUAGUGUAAUAGGACU
CGUCGAUUGA
.((..((((.((((((((((....((((((((...(((((.((..
((((((((((((((....)).)))))).....))))))))).)))))))))))))))..)))))..))))))))).))..)).
ERROR 4: Strange structure
 Initial dG =    -45.70

         10          20        30        40        50             60
A  UG  -   U      --|   GAUU         CAU     A  AC        -----     -  G
 CA  UG UGG GUCUUG  ACAC    GCCGGCUUU   GGUGA AA  UCAUUCUU     UACGU GC C
 GU  GC GCU CAGGAU  UGUG    UGGCCGAAG   CCAUU UU  AGUAAGGG     GUGCA CG G
A  UA  U   -      AA^   AU--         ---     A  --        AUAAG     A  A
       120        110         100           90          80        70



-----------------------------------------------------
```

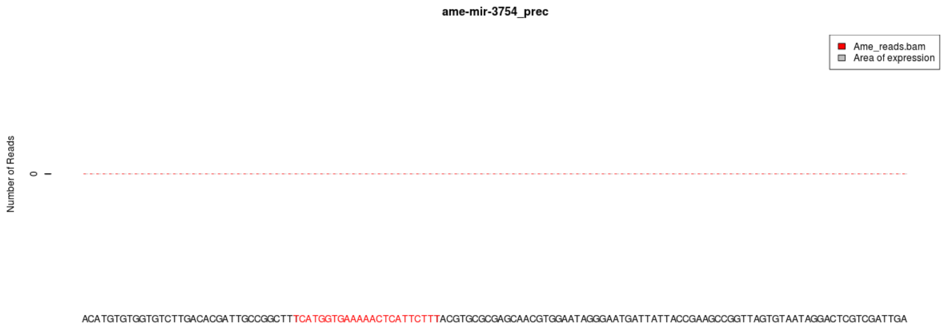

>ame-mir-3755_prec
CATATGTTCAAA**AATGTGTATATATAATTATATCGTG**TTATCGTTCGCAACACAATGCGTTATAATTATATATACAAGATTGCATAG

>ame-mir-3755_5p
AATGTGTATATATAATTATATCGTG

```
CAUAUGUUCAAAAAUGUGUAUAUAUAAUUAUAUCGUGUUAUCGUUCGCAACACAAUGCGUUAUAAUUAUAUAUACAAGAUUGCAUAG
..((((.........(((((((((((((((.((((((...(((...)))..)))))).))))))))))))))))).....))))).

 Initial dG =    -22.30

        10        20        30        40
CA|    UCAAAAAUG                U      AUC   C
  UAUGU         UGUAUAUAUAAUUAUA CGUGUU   GUU \
  AUACG         ACAUAUAUAUUAAUAU GCGUAA   CAA G
G-^    UUAGA----                U      CA-   C
       80             70        60         50

-----------------------------------------------------
```

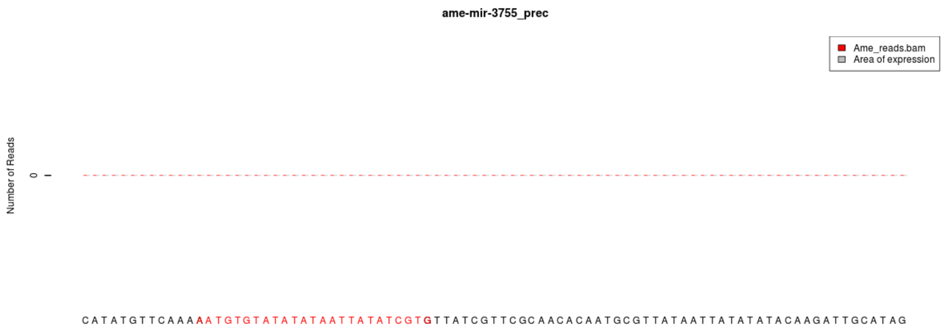

>ame-mir-3756_prec
AAGCATACAAATGTCAT**CTGATTTCTTTCATAAGGAGGA**TTTCAAAAATAATATTGCAATATTGTTTTTTACAATATTGCAAAAAATTT
TTGAAATTCTTCTTATGATAGAAATCAGCTTTATCATTTGTATGCTC

>ame-mir-3756_5p
CTGATTTCTTTCATAAGGAGGA

```
AAGCAUACAAAUGUCAUCUGAUUUCUUUCAUAAGGAGGAUUUCAAAAAUAAUAUUGCAAUAUUGUUUUUUACAAUAUUGCAAAAAAUUUUUGAAAUUCUUCUUAUGAUAGAAAUCAGCU
UUAUCAUUUGUAUGCUC
.((((((((((((....(((((((((.((((((((((((((((((((((((....
((((((((((((.....))))))))))))...))))))))))))))))))))))))).))))))))).......))))))))))))).

 Initial dG =    -67.80

        10          20         30         40         50         60
A            UCAU--|        U                      AAUA            UU
 AGCAUACAAAUG      CUGAUUUCU UCAUAAGGAGGAUUUCAAAAAU    UUGCAAUAUUGU  \
 UCGUAUGUUUAC      GACUAAAGA AGUAUUCUUCUUAAAGUUUUUA    AACGUUAUAACA  U
C            UAUUUC^        U                      AAA-            UU
    130       120        110        100        90         80        70

-----------------------------------------------------
```

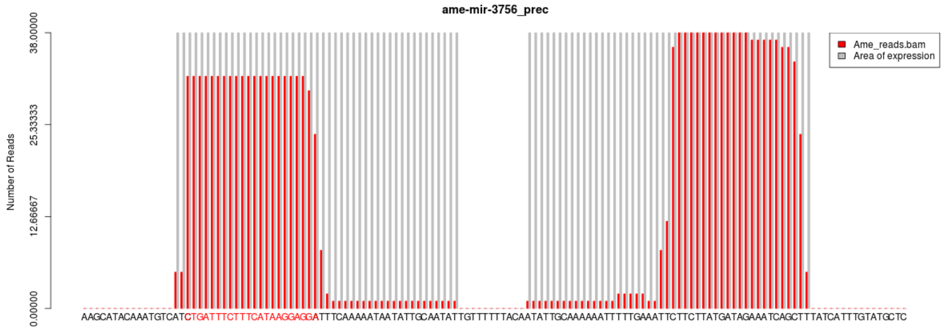

>ame-mir-3757_prec
GGATTGGTAGATGTGAAAGAGAAAGAAGAACTGACTTTAATATGTCTTCTATTATTAAAAATGCATTTTTGTAATCTAATCTGCATATA
T**TGGAATTATTTTTTCATTTTCTT**TAATATTTCAAGTATA

>ame-mir-3757_3p
TGGAATTATTTTTTCATTTTCTT

GGAUUGGUAGAUGUGAAAGAGAAAGAAGAACUGACUUUAAUAUGUCUUCUAUUAUUAAAAAUGCAUUUUUGUAAUCUAAUCUGCAUAUAU**UGGAAUUAUUUUUUCAUUUUCUU**UAAUAU
UUCAAGUAUA
...((((..(((((.((((((((.((((((.(((.(((((((((.................((((..
((........))..))))))))))))).))).)))))).)))))))).))))))))).....

```
 Initial dG =    -21.70

          10        20        30
GGA--    UA     G        A       .-CU|  UUU
     UUGG  GAUGU AAAGAGAA GAAGAA    GAC   A
     AACU  UUAUA UUUCUUUU CUUUUU    CUG   A
AUAUG    --     A        A       \ --^  UAU
         120       110       100         40

-----------------------------------------------------
```

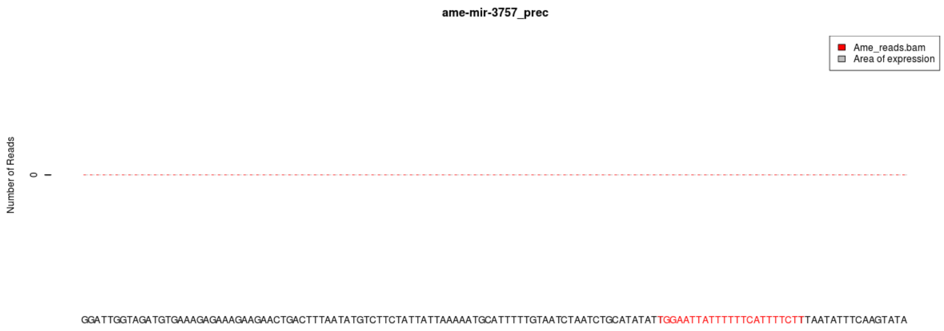

>ame-mir-3758_prec
CCTGGCGGTCCATTAGC**AGTCATATGTCCTGGACCAGGT**GGACCACCTGAAGCCATATGACCTGGTCCTGGTGGCCCAGT

>ame-mir-3758_5p
AGTCATATGTCCTGGACCAGGT

```
CCUGGCGGUCCAUUAGCAGUCAUAUGUCCUGGACCAGGUGGACCACCUGAAGCCAUAUGACCUGGUCCUGGUGGCCCAGU
(((...((((((...(((......)))..)))))))))(((.(((((....((((.......))))...))))).)))..

 Initial dG =    -27.90

          10        20
.-CCUGGC|     UUA   GU
        GGUCCA   GCA  C
        CCAGGU   UGU  A
\ ------^     CC-   AU
            30



     40        50
  AGG   A     UGAA    UAU
     UGG CCACC    GCCA   \
     ACC GGUGG    UGGU   G
  UG-   C     UCC-    CCA
 80         70         60

-----------------------------------------------------
```

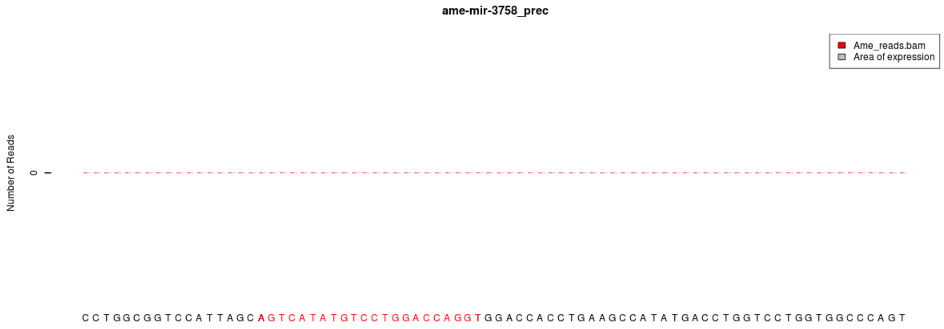

>ame-mir-3759_prec
GGCTACTCGGAGGCTAGTTATGACGGAGGGTCTGAGAGCGGGGGTTCCTGTTGGTGCGG**GACTCACGTCGACTGGGTGTCCGC**GCTAAG
CTC

>ame-mir-3759_3p
GACTCACGTCGACTGGGTGTCCGC

```
GGCUACUCGGAGGCUAGUUAUGACGGAGGGUCUGAGAGCGGGGGUUCCUGUUGGUGCGGGACUCACGUCGACUGGGUGUCCGCGCUAAGCUC
((((...((((.(((((((..(((((((..((((..((((((....))))))....)))).))).))))))))))).).)))).....)))).

 Initial dG =    -33.50

           10          20          30           40
-    ACU--    G -|     AU    -   GG    AG--      G
 GGCU     CGGA G CUAGUU  GACG GAG  UCUG    AGCGGG G
 UCGA     GCCU U GGUCAG  CUGC CUC  GGGC    UUGUCC U
C    AUCGC    G G^     --    A   A-    GUGG      U
 90        80        70            60        50

-----------------------------------------------------
```

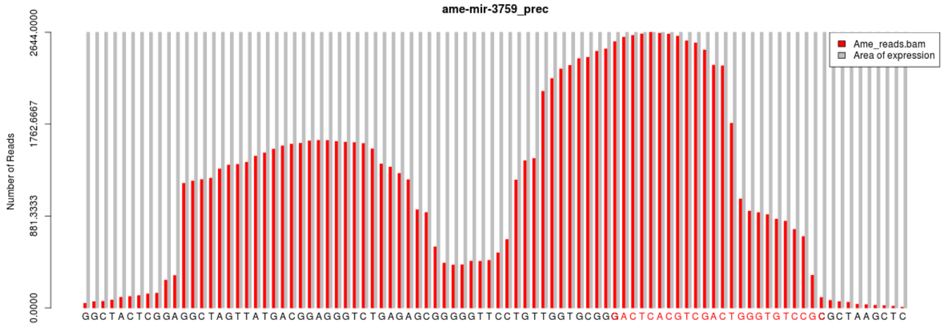

>ame-mir-3760_prec
AAATAAAAATTTGTTTAATATTTTCTTATTCTTA**AATCATATGAATAATCATTTCTAG**AAAAATAGAGAATGTCAAGAAAGATTATTTT
TCATTCAAATTATGATATTATTTATAAATTAGATAAATATTTATA

>ame-mir-3760_3p
AATCATATGAATAATCATTTCTAG

AAAUAAAAAUUUGUUUAAUAUUUUCUUAUUCUUA**AAUCAUAUGAAUAAUCAUUUCUAG**AAAAAUAGAGAAUGUCAAGAAAGAUUAUUUUUCAUUCAAAUUAUGAUAUUAUUUAUAAAUU
AGAUAAAUAUUUAUA
```
..(((((.((((((((((((......((((...((((((((((((......((((((......))))))).......
(((((((...)))))).))))...)))))).)))...)))))))))))))))).))))).
ERROR 4: Strange structure
 Initial dG =    -19.20

        10        20        30         40             50        60
AA     A          AUUUUC    UCU   -      ---    .-UAAUCA|     GA
  AUAAA AUUUGUUUAAU      UUAU   UAA AUCAUA   UGAA        UUUCUA  A
  UAUUU UAAAUAGAUUA      AAUA   AUU UAGUAU   ACUU        AGAGAU  A
A-     A          ------    UUU   A      UAA    \ ------^     AA
   130       120            110       100
```

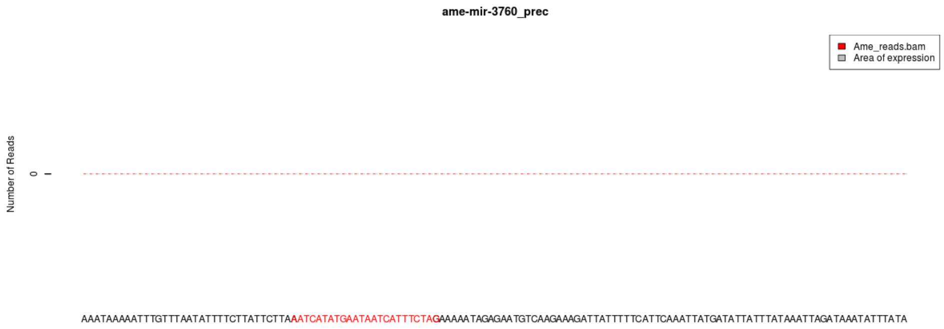

>ame-mir-3761_prec
CTGTCTTTTTCATTATTGGTGGTCGTGTGGG**TCGATTCCCGGGCCTCGTGTGT**TTATATTTTTTTTCATTCTCTTTTCGAGCATCGAGG
CTCCAAGGAAAACGGGCCGAACGACAGCGACGATAGATGGAATAGATAGA

>ame-mir-3761_3p
TCGATTCCCGGGCCTCGTGTGT

CUGUCUUUUUCAUUAUUGGUGGUCGUGUGGG**UCGAUUCCCGGGCCUCGUGUGU**UUAUAUUUUUUUUCAUUCUCUUUUCGAGCAUCGAGGCUCCAAGGAAAACGGGCCGAACGACAGCGA
CGAUAGAUGGAAUAGAUAGA
((((((.(((((((((((...(((((.(((.(((.((((.((((((((.
((((((......................))))))))))))))...)))))..))).))).)))))....)))).))))))).)))))).
ERROR 4: Strange structure
Initial dG = -50.20

```
         10         20          30         40          50        60
-      U       -    GUG--     G   G   A-     C--|       U      AUAUUUUUUUU
 CUGUCU UUUCAUU AUUG     GUCGU UGG UCG  UUCC   GGGCCUCG GUGUUU           \
 GAUAGA AAGGUAG UAGC     CAGCA GCC GGC  AAGG   CUCGGAGC UACGAG           C
A      U       A    AGCGA     A   G   AA    AAC^       -      CUUUUCUCUUA
       130       120       110       100        90         80        70
```

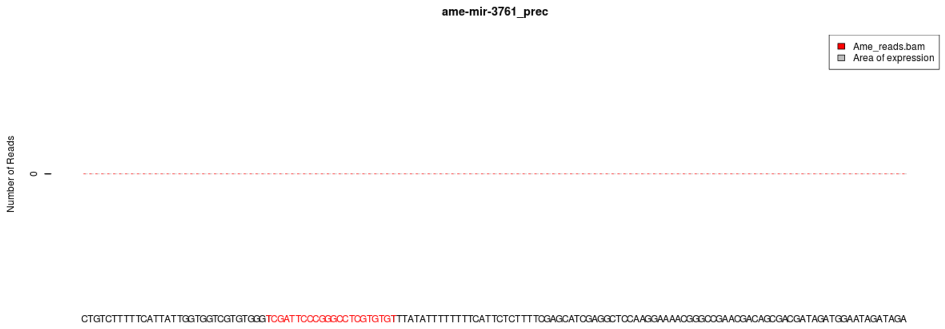

>ame-mir-3762_prec
TGACTTAGTAAAACGGT**AAGATCTGTAAATTAGATGATA**TATGATATACTTATATGATATATTATATGATATATTATATGATATATTAT
ATTATATCTTATATATTTATATCCTTATTATTATATTT

>ame-mir-3762_5p
AAGATCTGTAAATTAGATGATA

UGACUUAGUAAAACGGU**AAGAUCUGUAAAUUAGAUGAUA**UAUGAUAUACUUAUAUGAUAUAUUAUAUGAUAUAUUAUAUGAUAUAUUAUAUUAUAUCUUAUAUAUUUAUAUCCUUAUUA
UUAUAUUU

```
............((((......))))......(((((((...((((((..(((((((.((((.
(((((((((((((....)))))))))))))).)))).))))))))..))))))..)))))))......
```

```
 Initial dG =    -28.40

          10
.-UGACUUAGUAAA|   UA
              ACGG  A
              UGUC  G
\ ------------^   UA
                   20



    30        40        50        60        70
  AAAUUA       UAU      CU       U    U            A
        GAUGAUA   GAUAUA  UAUAUGA AUAU AUAUGAUAUAUU U
        UUAUUAU   CUAUAU  AUAUAUU UAUA UAUAUUAUAUAG A
  UUUAUA       UC-      UU       C    U            U
       120        110       100        90        80

-----------------------------------------------------
```

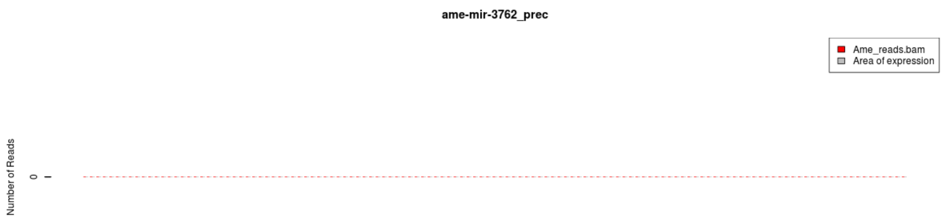

TGACTTAGTAAAACGGTAAGATCTGTAAATTAGATGATATATGATATACTTATATGATATATTATATGATATATTATATGATATATTATATTATATCTTATATATTTATATCCTTATTATTATATTT

>ame-mir-3763_prec
GAAGATTATTCTTGCAATTACCAGCGG**TTCGCATTACTAAAATATCGGTGG**GAATACCTAACAGAATATGCTTGGTATTCCGATGTGCA
TGGTCTAAAGATTTCA

>ame-mir-3763_3p
TTCGCATTACTAAAATATCGGTGG

```
GAAGAUUAUUCUUGCAAUUACCAGCGGUUCGCAUUACUAAAAUAUCGGUGGGAAUACCUAACAGAAUAUGCUUGGUAUUCCGAUGUGCAUGGUCUAAAGAUUUCA
...((....(((......((((((..(((...((((((.(((((..((((....))))...).)))))..))))))...)))..))).))))...)))))..)).
ERROR 4: Strange structure
 Initial dG =    -22.60

        10        20         30        40           50
GAA| UUAU    GCAAUU    -    GU   CAU      A-     - GG-    A
   GA    UCUU      ACCA GCG  UCG   UACUAA  AUAU C   UGGG A
   CU    AGAA      UGGU CGU  AGC   AUGGUU  UAUA G   AUCC U
A--^ UU--    AUC---    A   GU   CUU      CG     A ACA    A
       100           90        80        70        60
```

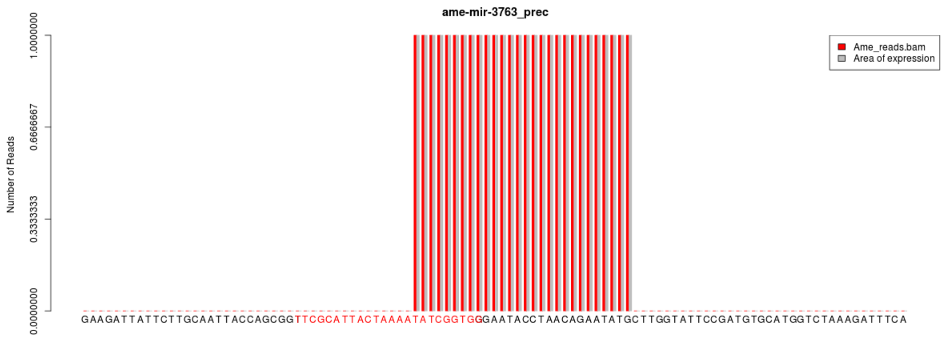

>ame-mir-3764_prec
ACGTGTGATTTTGTGACGTGTTGGGTTTTTGGAAGAATTCTAGGCATTCGTTGCTGGTGGTCTACGCAGGTGAGTAACAATT**TAGATTT**
TTCTTTGATCTTAATTTTAATTGTAATCCATT

>ame-mir-3764_3p
TAGATTTTTCTTTGATCTTAA

ACGUGUGAUUUUGUGACGUGUUGGGUUUUUGGAAGAAUUCUAGGCAUUCGUUGCUGGUGGUCUACGCAGGUGAGUAACAAUU**UAGAUUUUUCUUUGAUCUUAA**UUUUAAUUGUAAUCCA
UU
```
..((((((((..(..((((((..((..((((....)))..))..))).)))..)....)))).)))).)..(((..(.((((((.
(((((.............))))).)))))).)..))).
```

```
 Initial dG =    -22.70

           10             20        30
.-AC    -    UU-- UG   --|  UG  UU   G
    GUGU GAUU    G  ACG  UGU  GG  UUU G
    CGCA CUGG    C  UGC  ACG  UC  AAG A
\ --    U    UGGU GU   UU^  GA  UU   A
          60        50        40
```

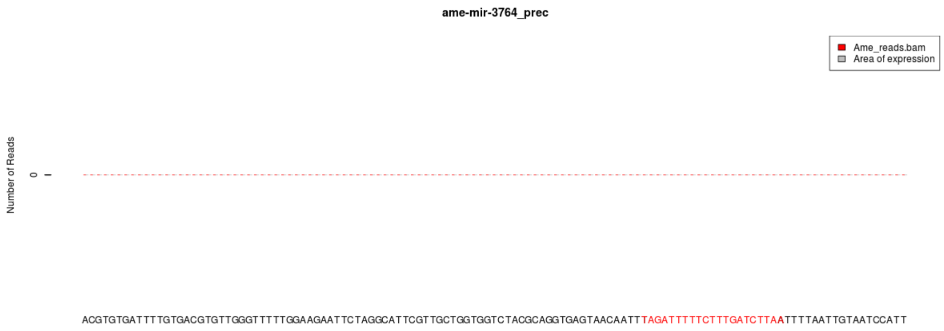

>ame-mir-3765_prec
ACATGACAACAGAAAAT**GAAGAAAAGGCAAGTTGAAAGA**ATCGAAACTGATTTGGATTGAAATATAAATATATCGTAAACATCTTTAAA
TGGAAATTGATTTTAAAAAAACTTGCCTTTCCTTTAC

>ame-mir-3765_5p
GAAGAAAAGGCAAGTTGAAAGA

ACAUGACAACAGAAAAU**GAAGAAAAGGCAAGUUGAAAGA**AUCGAAACUGAUUUGGAUUGAAAUAUAAAUAUAUCGUAAACAUCUUUAAAUGGAAAUUGAUUUUAAAAAAACUUGCCUUU
CCUUUAC
................(((((.(((((((((((...((((((((..((.((((((((((..
((((....))))..))).....)))))))))...))))))))).....)))))))))))).))))).

Initial dG = -25.60

```
        10        20        30          40          50        60
ACAUGACAACAGAAAA     A           GAA--        .-AACU|  UUGG   GAA
                UGAAG AAAGGCAAGUU     AGAAUCGA      GAU    AUU   A
                AUUUC UUUCCGUUCAA     UUUUAGUU      CUA    UAA   U
C---------------     C           AAAAA        \ ----^  UA--   AUA
                   120       110       100             70



                                                    80
                                                  GUAAACA   UU
                                                         UCU  A
                                                         AGG  A
                                                  AA-----   UA
                                                           90

-----------------------------------------------------
```

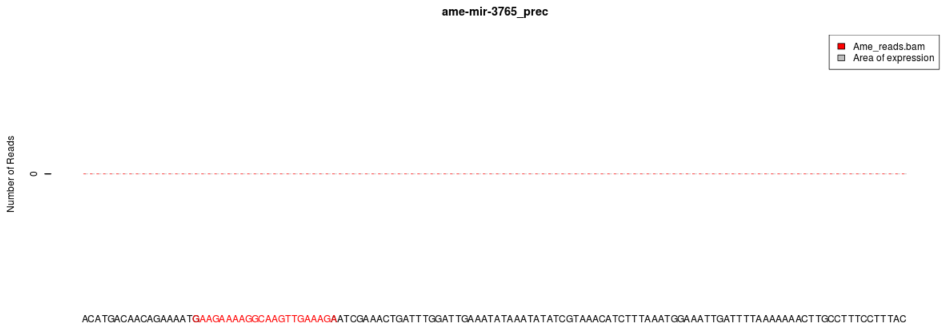

>ame-mir-3766_prec
GAATATTTGATAATGGAATTTTCGTCGATAACAATTTTTCATCGAAATCGATCTTTTTCTAGTCGATATTCGAAAAGTAATGACAG**TAG**
GTGGTTTTGAAAATGACGAAATCGTGAATTAGATTTTATTGGATAA

>ame-mir-3766_3p
TAGGTGGTTTTGAAAATGACG

GAAUAUUUGAUAAUGGAAUUUUCGUCGAUAACAAUUUUUCAUCGAAAUCGAUCUUUUUCUAGUCGAUAUUCGAAAAGUAAUGACAG**UAGGUGGUUUUGAAAAUGACG**AAAUCGUGAAUUAGAUUUUAUUGGAUAA

```
...((((..((((.......((((.((((.....(((.((((((((((.((((....((.((((.
((((.....)))).)))))).))))))))))))...))).)))))))))))).......))))..)))))).
```

Initial dG =   -25.30

```
        10        20        30         40           50
GAA    UG    UGGAAUU    U    AACAAU-             -|    U  UU
   UAUU  AUAA       UUCG CGAU       UUUUCA--UCGAA AUCGA CU  U
   AUAG  UAUU       AAGU GCUA       AAAAGU  AGCUU UAGCU GA  U
A--    GU    UUAGAUU    -    AAGCAGU      \      A^    -  UC
     130       120        110       100       70         60
```

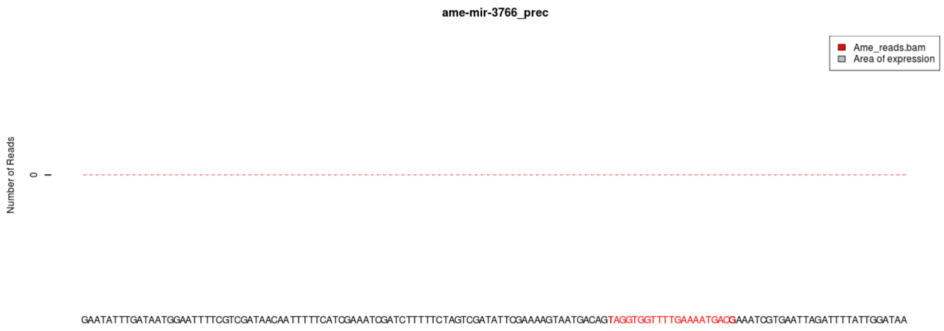

>ame-mir-3767_prec
TGTGGAAATAAACAATTTCAATTAATAATAGTAATGTTTAAAATGTGTAAAATATTTACATGTAATATT**TACACATTATTTACTACTAC**
TAATTGAAATTGTCATATAATATTTACATTCTACAT

>ame-mir-3767_3p
TACACATTATTTACTACTACT

```
UGUGGAAAUAAACAAUUUCAAUUAAUAAUAGUAAUGUUUAAAAUGUGUAAAAUAUUUACAUGUAAUAUUUACACAUUAUUUACUACUACUAAUUGAAAUUGUCAUAUAAUAUUUACAUU
CUACAU
(((((((((.(((((((((((((.((.((((((.......((((((((((.
((((.......))))))))))))))..)))))).)).)))))))))))))..))).........))))))).

 Initial dG =    -29.80

                   10         20        30        40        50
-       ----------   A-|             A  A      UGUUUAA         A    UAC
 UGUGGAA          AUA  ACAAUUUCAAUUA UA UAGUAA       AAUGUGUAAA UAUU   \
 ACAUCUU          UAU  UGUUAAAGUUAAU AU AUCAUU       UUACACAUUU AUAA   A
U       ACAUUUAUAA   AC^             C  C      UA-----         -    UGU
   120       110       100        90        80             70

-----------------------------------------------------
```

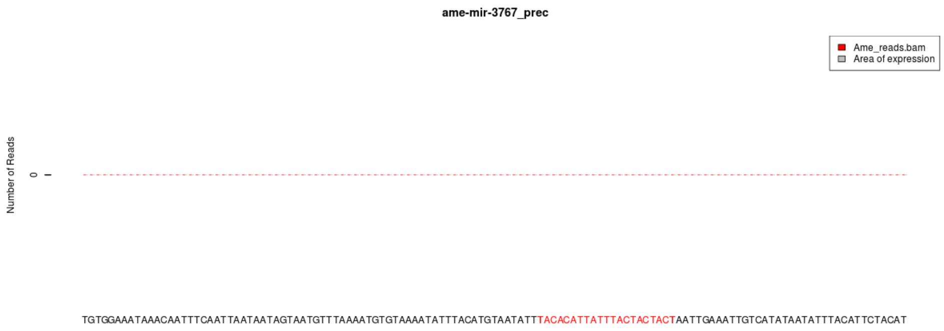

>ame-mir-3768_prec
GCGCTGTGCGGGAAAGTCTTGGGCTTTATGGGGGTGATCAACATGTGCTCGGTCCAGGAGGTCT**CCAACGACGGTCTCTCCAACCGT**T

>ame-mir-3768_3p
CCAACGACGGTCTCTCCAACCGT

```
GCGCUGUGCGGGAAAGUCUUGGGCUUUAUGGGGGUGAUCAACAUGUGCUCGGUCCAGGAGGUCUCCAACGACGGUCUCUCCAACCGUU
......((.((((...(((((((((.((((..((...))..)))).....)))))))))..)))))..((((((.......))))))

 Initial dG =    -25.50

          10        20            30
.-GCGCUG  C    AAG         U----|   GG  U
        UG GGGA   UCUUGGGCU     UAUG  GG \
        AC CUCU   AGGACCUGG     GUAC  CU G
\ ------  -    GG-         CUCGU^   AA  A
               60         50        40

-----------------------------------------------------
```

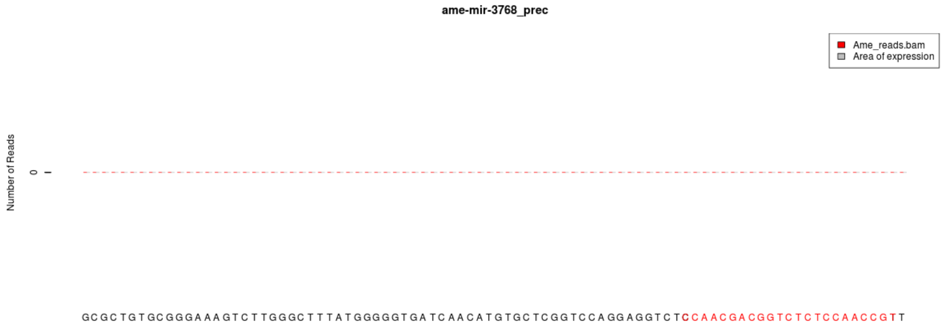

>ame-mir-3769_prec
ATCTTATAGCCTGTTGGTTCGGCTTATGGAAAATTGGGAAAAAAAAAGGAGATCGGAAATCGAGTCGGCCCTCGTAATTTTA**GGTAGCTC
AAGAGAAGGTTGACG**AGAG

>ame-mir-3769_3p
GGTAGCTCAAGAGAAGGTTGACG

AUCUUAUAGCCUGUUGGUUCGGCUUAUGGAAAAUUGGGAAAAAAAAAGGAGAUCGGAAAUCGAGUCGGCCCUCGUAAUUUUA**GGUAGCUCAAGAGAAGGUUGACG**AGAG
.((((.((((((.((..((.((((.((..(((((((.((.......((.(((((.....))).))..)).)).))))))))..)))))).))..))))))))..)))).

```
 Initial dG =    -20.30

         10        20        30        40         50
A    A-|     G  GG  C    U  GG       G  AAAAAAA  A-   -   GA
 UCUU  UAGCCU UU  UU GGCU AU  AAAAUUG GA       GG  GA UCG  \
 AGAG  GUUGGA AG  AA UCGA UG  UUUUAAU CU       CC  CU AGC  A
G    CA^     -  AG  C    -  GA       G  C------  GG  G   UA
      100         90         80        70               60

-----------------------------------------------------
```

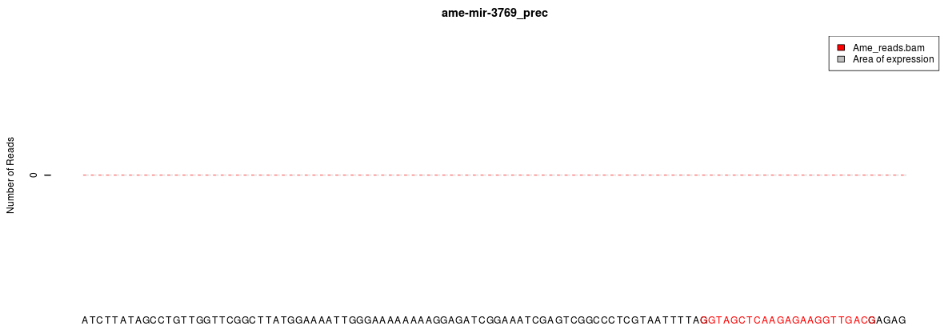

>ame-mir-3770_prec
CAACGCACATGGACGC**AATCCTGCATCAAGTGCGTTGT**TTTGTTTTTCTATAATCAGCAATGCATTGTAAGCAGGATAGTGTTAGTGTG
TTAG

>ame-mir-3770_5p
AATCCTGCATCAAGTGCGTTGT

```
CAACGCACAUGGACGCAAUCCUGCAUCAAGUGCGUUGUUUUGUUUUUCUAUAAUCAGCAAUGCAUUGUAAGCAGGAUAGUGUUAGUGUGUUAG
....((((((.(((((.(((((((....((((((((((((((......))))..)))))))))))....))))))).))))).))))))...
```

```
 Initial dG =    -38.60

        10        20        30           40
CAAC      G      A        AUCA            --|   UU
    GCACAU GACGC AUCCUGC    AGUGCGUUGUU  UUGU  U
    UGUGUG UUGUG UAGGACG    UUACGUAACGA  AAUA  U
GAU-      A      A        AAUG            CU^   UC
   90        80        70        60        50

-----------------------------------------------------
```

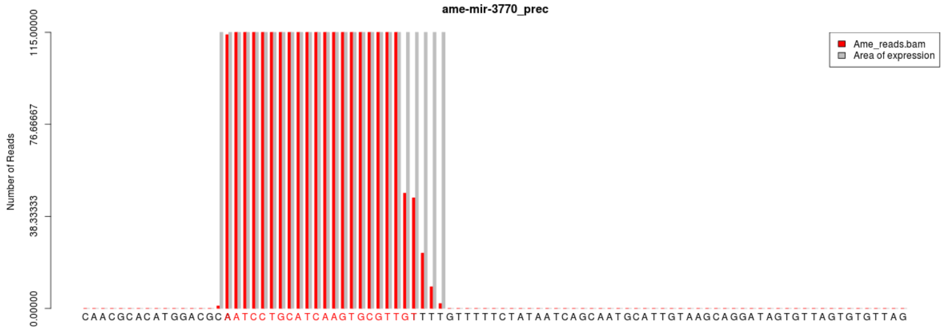

>ame-mir-3771_prec
GATGCTCGTTAATATCGCAGTGTTGAACGTGGCTAAGATTA**TAAAACACAGCGAGTTGCACGAG**CACGT

>ame-mir-3771_3p
TAAAACACAGCGAGTTGCACGAG

```
GAUGCUCGUUAAUAUCGCAGUGUUGAACGUGGCUAAGAUUAUAAAACACAGCGAGUUGCACGAGCACGU
..((((((((((.((((.(((((....((((......))))..))))).)))))))).)))))))...

 Initial dG =    -21.30

          10        20        30
GA-       -|   A    A     GAAC    CU
   UGCUCGU UAAU UCGC GUGUU    GUGG  A
   ACGAGCA GUUG AGCG CACAA    UAUU  A
UGC       C^   -    A     AA--    AG
        60         50          40
```

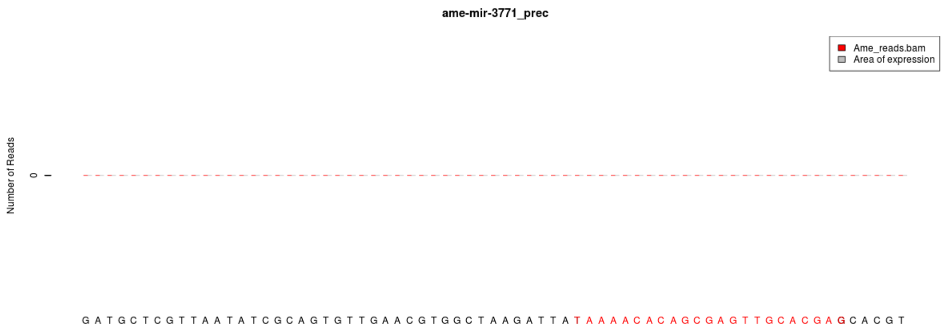

>ame-mir-3772_prec
AAAAGGAGCTGGTGTTCCGGGTTCGATTCCCAGGGCTGACTCGAGAAACGCCGCTTCCCTCGACC**GATCTAGATTGAGAAGGAACGAA**G
GCGCCTA

>ame-mir-3772_3p
GATCTAGATTGAGAAGGAACGAA

```
AAAAGGAGCUGGUGUUCCGGGUUCGAUUCCCAGGGCUGACUCGAGAAACGCCGCUUCCCUCGACCGAUCUAGAUUGAGAAGGAACGAAGGCGCCUA
...(((.(((..((((((...(((((((....((..((..(((((............))))).))..)).)))))))..))))))..))).))).
```

```
 Initial dG =    -29.10

        10        20        30        40        50
AAA|  A   GG      GGG       CCCA  GC  AC     AAACGC
   AGG GCU  UGUUCC   UUCGAUU    GG  UG  UCGAG      \
   UCC CGG  GCAAGG   GAGUUAG    UC  GC  AGCUC      C
A--^  G   AA      AA-       A---  UA  C-     CCUUCG
       90        80             70         60
```

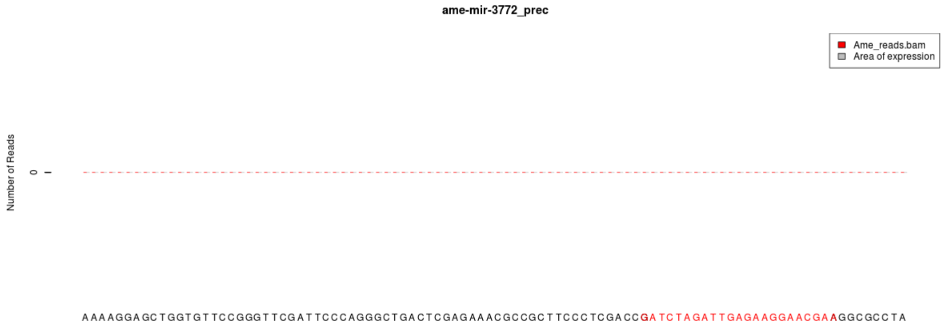

>ame-mir-3773_prec
ATACATCTTCCATTACATGGCAAGTGCTGTTGCAAACTGTCTAAAAGAAGTCAG**TAAGCACAGCTCTTGTCTGTAA**CTGGTAGTGA

>ame-mir-3773_3p
TAAGCACAGCTCTTGTCTGTAA

```
AUACAUCUUCCAUUACAUGGCAAGUGCUGUUGCAAACUGUCUAAAAGAAGUCAGUAAGCACAGCUCUUGUCUGUAACUGGUAGUGA
...((.((.((((((((.((((((.(((((.((..((((.((......)).)))))..))))))).))))))))))))).))).))))).

 Initial dG =    -33.80

        10         20        30        40
AUA| U  U   -     U      U     U  AA    U  AA
   CA CU CCA UUACA GGCAAG GCUGU GC  ACUG CU  A
   GU GA GGU AAUGU CUGUUC CGACA CG  UGAC GA  A
A--^ -  U   C     -      U     -  AA    U  AG
        80         70        60         50

-----------------------------------------------------
```

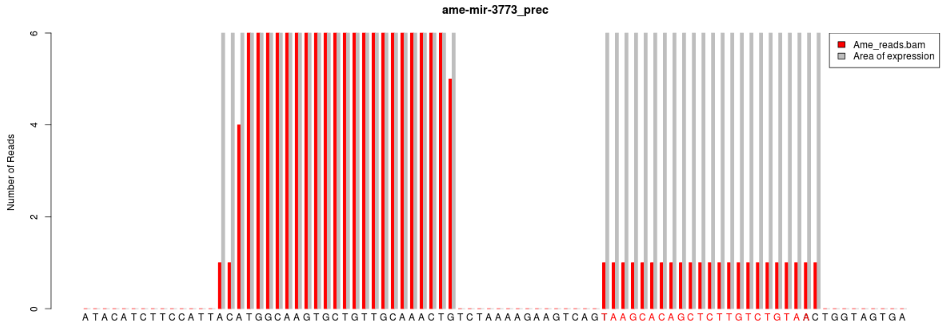

>ame-mir-3774_prec
CTATTTCACAAGTTGGATTAAAATTTTAAGTGAATTTTTCTAAGTTTCTTATCTAACAATAAGTCATTGTACGTTCTTTCTT**GAAAAAT**
TACTTAAGATTGTCAGTTAATTATTTTTTTGAAAAATTGG

>ame-mir-3774_3p
GAAAAATTACTTAAGATTGTC

```
CUAUUUCACAAGUUGGAUUAAAAUUUUAAGUGAAUUUUUCUAAGUUUCUUAUCUAACAAUAAGUCAUUGUACGUUCUUUCUUGAAAAAUUACUUAAGAUUGUCAGUUAAUUAUUUUUUU
GAAAAAUUGG
...(((((.(((...((((((((((((((((((.((((((.(((...........
(((((.....)))))..........)))))))))))))))))))))).....))))))...))).))))).......
```

```
 Initial dG =    -20.50

           10        20             30        40        50        60
CUA---     C   UUG      -----|           A      U   UUUCUUAUCUA     AA
      UUUCA AAG   GAUUAA     AAUUUUAAGUGA UUUUUC AAG           ACAAU  \
      AAAGU UUU   UUAAUU     UUAGAAUUCAUU AAAAAG UUC           UGUUA  G
GGUUAA     U   UUA      GACUG^           -      -   UUUCUUGCA--     CU
       120       110       100        90          80          70

-----------------------------------------------------
```

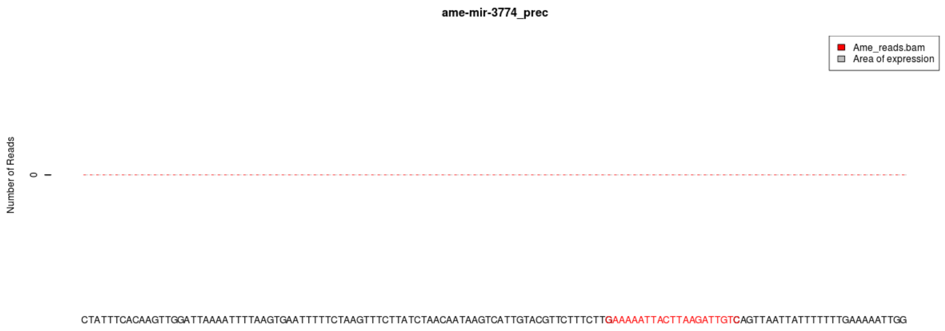

>ame-mir-3775_prec
AATCTTTTTTACTTTT**GAGGCGGATGAGGAGGTTGAATA**GATGATCGTGGTTGCAAAAATTTGTGTACACGTTATGGGTACAAGCATTG
ATTTTATATTTCTCTCTACATATGTGATTACTTGTAT

>ame-mir-3775_5p
GAGGCGGATGAGGAGGTTGAATA

AAUCUUUUUUACUUUU**GAGGCGGAUGAGGAGGUUGAAUA**GAUGAUCGUGGUUGCAAAAAUUUGUGUACACGUUAUGGGUACAAGCAUUGAUUUUAUAUUUCUCUCUACAUAUGUGAUUA
CUUGUAU

```
.((((.(((.((((((..........)))))).))).))))...((((..(((((....)))))..))))......((((((...(((((.
(((((..........))))).))))))))))))
```

```
 Initial dG =    -19.80

          10        20
.-A|   U   U       GAGGC
   AUCU UUU ACUUUU     \
   UAGA AAG UGGAGG     G
\ -^   U   U       AGUAG
    40        30



          50
  .-GAU    GU     A
       CGUG  UGCAA A
       GCAC  GUGUU A
  \ ---    AU     U
       70        60
```

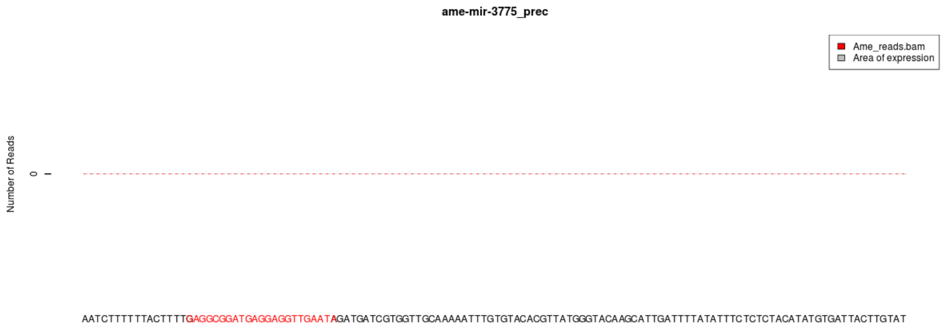

>ame-mir-3776_prec
CGGTGCGTGAAGGGGCAGG**AGGAGGGGGGGAGAGAGAAGCG**TTGTACGAGCGTATTTTGCCCGTTTTTCGGCCACGTTCCTATCGATTC
GCGAACCA

>ame-mir-3776_5p
AGGAGGGGGGGAGAGAGAAGCG

```
CGGUGCGUGAAGGGGCAGGAGGAGGGGGGGAGAGAGAAGCGUUGUACGAGCGUAUUUUGCCCGUUUUUCGGCCACGUUCCUAUCGAUUCGCGAACCA
.(((.((((((..(..(((((...((..((((((....(((..((((....))))..)))...))))))..))...)))))..)..)))))).))).

 Initial dG =    -33.70

        10        20        30        40
C|  G      GG GC     GAG  GG      AGAA   UU    G
 GGU CGUGAA  G  AGGAG   GG  GGAGAG    GCG  GUAC A
 CCA GCGCUU  C  UCCUU   CC  CUUUUU    CGU  UAUG G
A^  A      AG UA     GCA  GG      GCC-   UU    C
      90        80        70         60        50

-----------------------------------------------------
```

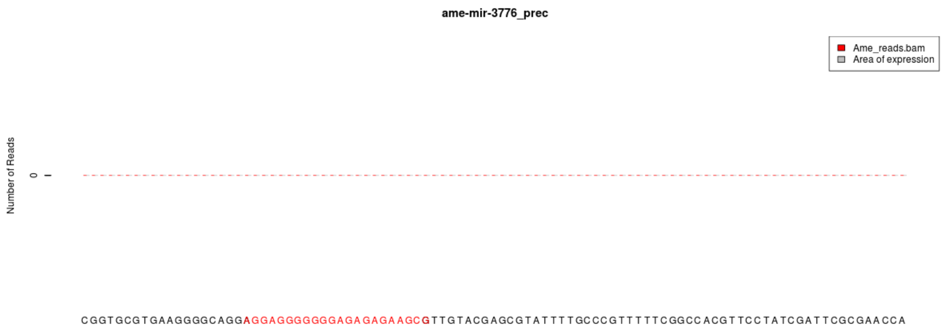

>ame-mir-3777_prec
AATGAAAGGTCTGCGAGGAGGAAAGCTCTCTTTTATTAAACGAAAAATTGGATCGCGCTTGTCGGAATGCAATATTTCGACGGATTCAT TTGG**TGGAAACATTTCTTCCCTGGGA**AAAAATTCAA

>ame-mir-3777_3p
TGGAAACATTTCTTCCCTGGGA

AAUGAAAGGUCUGCGAGGAGGAAAGCUCUCUUUUAUUAAACGAAAAAUUGGAUCGCGCUUGUCGGAAUGCAAUAUUUCGACGGAUUCAUUUGG**UGGAAACAUUUCUUCCCUGGGA**AAAAAUUCAA

```
..((((...(((.((.((((((((......(((((((((........((((((.....
((((((((((...)))))))))))))))))))))))))))...)))))))).))))).....)))).
```

Initial dG = -27.90

```
          10        20        30        40        50        60        70
AA    AGG--|  G  A        GCUCUC          CGAAAAAU      GCGCU          C
  UGAA     UCU CG GGAGGAAA      UUUUAUUAAA        UGGAUC     UGUCGGAAUG \
  ACUU     AGG GU CCUUCUUU      AAGGUGGUUU        ACUUAG     GCAGCUUUAU A
A-    AAAAA^  -  C        ACA---          --------      -----          A
    120        110       100           90                      80
```

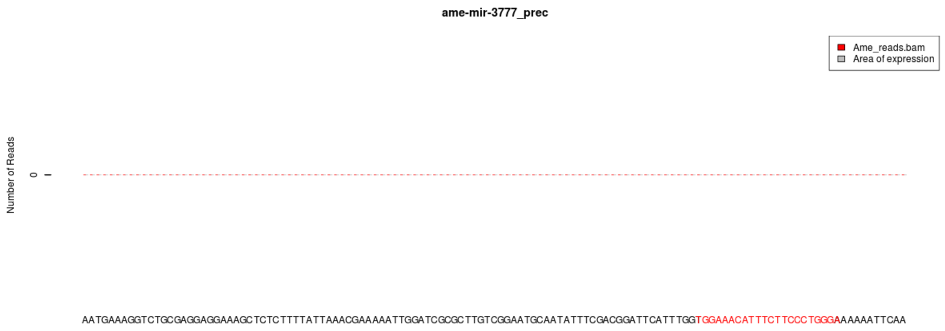

>ame-mir-3778_prec
CTATGAAAGGGTACGATTATAAAGATGTTGCGCTAATTGAATGAATACTTGTTACTTGTGGACCACGAAAGAAAGACGAGTATTT**GTATATAATTGGATGCTTTGTGC**AAATTTTAA

>ame-mir-3778_3p
GTATATAATTGGATGCTTTGTGC

CUAUGAAAGGGUACGAUUAUAAAGAUGUUGCGCUAAUUGAAUGAAUACUUGUUACUUGUGGACCACGAAAGAAAGACGAGUAUUU**GUAUAUAAUUGGAUGCUUUGUGC**AAAUUUUAA
...(((((..((((((.(((....)))..(((((((((.(..((((((((((.(((.((.....)).)))...))))))))))..)...))))))).))).))))))...))))).

```
 Initial dG =    -25.00

        10            20
CUA     GG-      .-U|  A
   UGAAA   GUACGA   UAU A
   AUUUU   CGUGUU   GUA A
A--     AAA      \ -^  G
       110
```

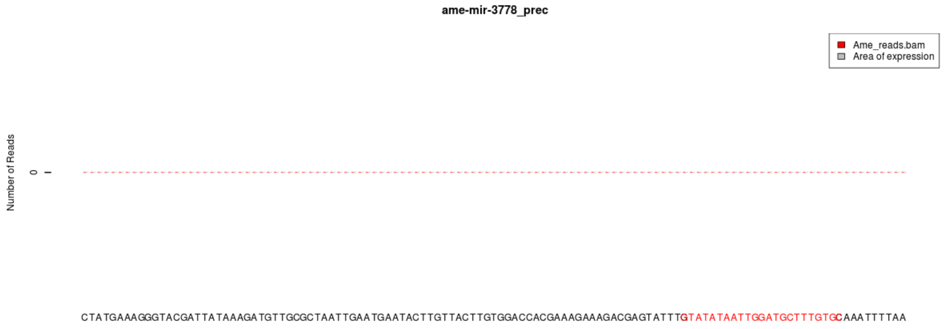

>ame-mir-3779_prec
CACCG**GCCGGGCCTGCCGCTCGACACG**CACCGTCCGCTTCGTTGAACTTGTTGCGCTTCATGGTTCAAAAAGGTTGCTCGATCAACCGG
CATGTCTGGTCGGA

>ame-mir-3779_5p
GCCGGGCCTGCCGCTCGACACG

CACCG**GCCGGGCCUGCCGCUCGACACG**CACCGUCCGCUUCGUUGAACUUGUUGCGCUUCAUGGUUCAAAAAGGUUGCUCGAUCAACCGGCAUGUCUGGUCGGA
..((((((((((.(((((.((((...(((......((((..(((((((((........)).)))))))..))))))))))).....))))).)))))))))).

Initial dG =   -40.60

```
        10            20        30        40         50
CA          C     C----|   CAC   CCGUCC    CG       -  UUG
  CCGGCCGGGC UGCCG     UCGA   GCA      GCUU  UUGAACU UG   C
  GGCUGGUCUG ACGGC     AGCU   CGU      UGGA  AACUUGG AC   G
A-          U     CAACU^   ---   ------    AA       U  UUC
  100        90        80                 70        60
```

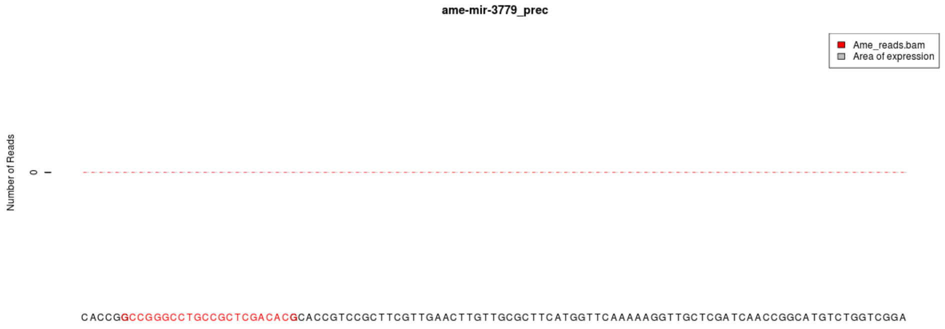

>ame-mir-3780_prec
AACTCCGTCGAGAGTAT**AACTGCGGCATCGTAACTGAC**AAAACTTTCCGTAGCTGCATTTATATGCGTGCCCGGCCGTTGCACGTAAAG
TGATTCCGCTGTTACTCGTTAGAGA

>ame-mir-3780_5p
AACTGCGGCATCGTAACTGAC

```
AACUCCGUCGAGAGUAUAACUGCGGCAUCGUAACUGACAAAACUUUCCGUAGCUGCAUUUAUAUGCGUGCCCGGCCGUUGCACGUAAAGUGAUUCCGCUGUUACUCGUUAGAGA
..(((...((((....((((.((((.((((..((((.(((......(((..(((((((....))))).)).)))...))))).))....)))).)))).))))))))...))).
```

Initial dG =   -29.90

```
        10        20        30           40        50          60
AA|  CGU    AGUA    U    C    UA--  -  A   AACUUU   UA  -     U
  CUC   CGAG    UAAC GCGG AUCG    AC UG CAA      CCG  GC UGCAU U
  GAG   GCUC    AUUG CGCC UAGU    UG AC GUU      GGC  CG GCGUA A
A-^  AUU    ----    U    U    GAAA  C  -   GCC---   C-  U     U
   110           100        90         80            70
```

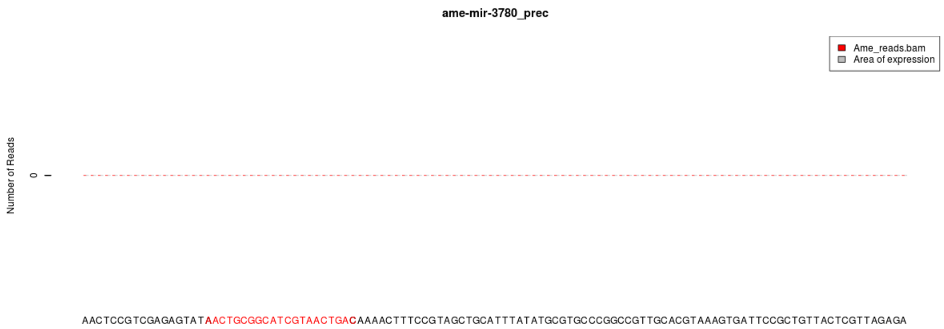

>ame-mir-3781_prec
GAAGAATTGGCTCTATCGTCGGTGATTGATGTCAATTAAACGCGTATACATATGAATATGGCGAGTTGTGTATAAGTG**TAAGTGATTGA**
TCGATCGTGGATTCAAATTCA

>ame-mir-3781_3p
TAAGTGATTGATCGATCGTGGAT

GAAGAAUUGGCUCUAUCGUCGGUGAUUGAUGUCAAUUAAACGCGUAUACAUAUGAAUAUGGCGAGUUGUGUAUAAGUG**UAAGUGAUUGAUCGAUCGUGGAU**UCAAAUUCA
```
...((((((..(((((.((((.........((((((((.((((.((((((((..............)))))))).))))...))))))))))))).))))))..))).))).
```

```
 Initial dG =    -27.20

         10        20          30
GAA   -   GC      C         |    A
   GAA UUG  UCUAU GUCGGU--GAUUG U
   CUU AAC  AGGUG UAGCUA  UUAAC G
A--   A   UU      C      \ ^    U
.         100        90

-----------------------------------------------------
```

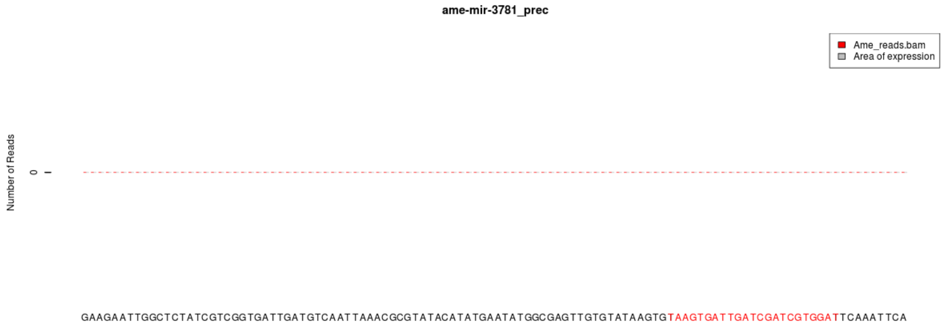

>ame-mir-3782_prec
GCTTTTGCCAGAAGACTTTCTCCCGCGCCTGGTC**CCTGCAGAGACATCTGGCGGACAC**GCATTTTTACGTGCCGCAGTCGCTCTGCTGC
GACCAGTGCGGCAGGAGTTACAAGTCGAGGAACAGTCTGGTCAGCC

>ame-mir-3782_3p
CCTGCAGAGACATCTGGCGGACAC

GCUUUUGCCAGAAGACUUUCUCCCGCGCCUGGUC**CCUGCAGAGACAUCUGGCGGACAC**GCAUUUUUACGUGCCGCAGUCGCUCUGCUGCGACCAGUGCGGCAGGAGUUACAAGUCGAGG
AACAGUCUGGUCAGCC
(((...((((((.(((((.((((.((((((((((...((((((....(((.(((.
((((........))))))))))...)))))))...))))))).)).)).)))).....))))).........)))))).))).

```
ERROR 4: Strange structure
 Initial dG =    -57.70

         10          20        30        40        50        60
-   UUU      .-A|    U  C      C     CCU      ACAU   G   A    CAU
 GCU   GCCAGA   GACUU CU CCGCGC UGGUC   GCAGAG    CUG CGG CACG   U
 CGA   UGGUCU   UUGAG GA GGCGUG ACCAG   CGUCUC    GAC GCC GUGC   U
C   C--      \ -^    -  C      -     CGU      GCU-   -   -    AUU
     130              100         90        80            70
```

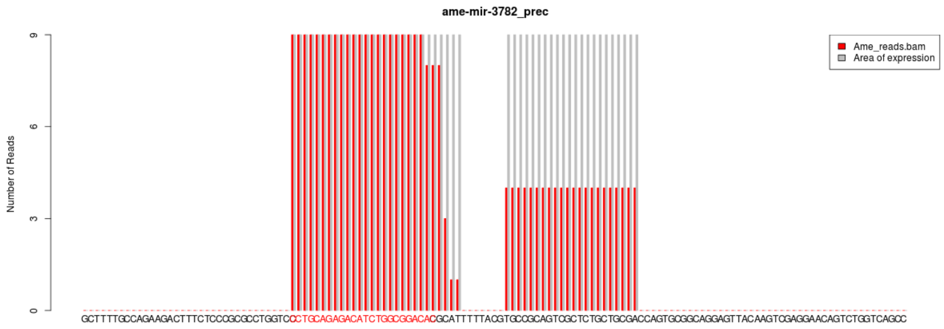

>ame-mir-3783_prec
AAGAGGACGAAGAAGTGAGATGGTGTCATCCCACCAAACAAGTGAGAGTGTGTCAATATAGGTCTA**TACTTTCAATTGTTTGATGAGGT**
GAAATCATTCTAATAATTGACGTTTTCTG

>ame-mir-3783_3p
TACTTTCAATTGTTTGATGAGGT

```
AAGAGGACGAAGAAGUGAGAUGGUGUCAUCCCACCAAACAAGUGAGAGUGUGUCAAUAUAGGUCUAUACUUUCAAUUGUUUGAUGAGGUGAAAUCAUUCUAAUAAUUGACGUUUUCUG
.((((((((..((..((..(((((.(((((.((.(((((((.((((((((((((......))..)))))))))).))))))).)).))))).))))).))..))....))..)))))))).

 Initial dG =    -37.90

        10          20        30        40        50
A        AA  AG--  AG     G     C  C       G          --| AA
 AGAGGACG  GA    UG  AUGGU UCAUC CA CAAACAA UGAGAGUGUG  UC  U
 UCUUUUGC  UU    AU  UACUA AGUGG GU GUUUGUU ACUUUCAUAU  GG  A
G        AG  AAUA  CU     A     A  A       A          CU^ AU
      110       100        90        80        70        60

-----------------------------------------------------
```

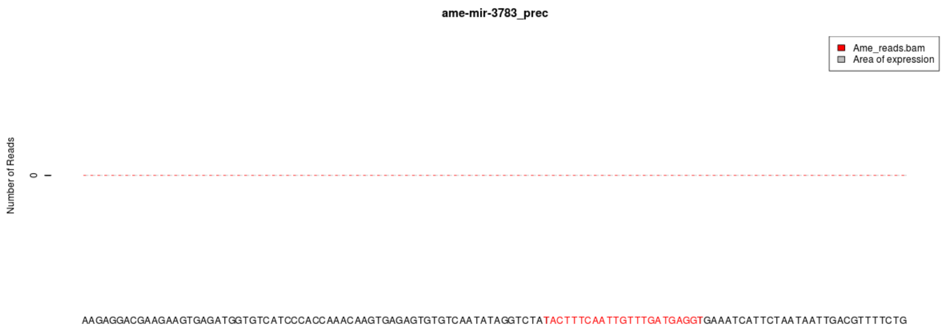

>ame-mir-3784_prec
GCCATTTCCGGCGAATACAGAATAATTCCTCGATCCGTTCGTTCAATCGAGATCTC**TCGCGGAAAATAATTTCTATTT**ATCGATCCTGG
CT

>ame-mir-3784_3p
TCGCGGAAAATAATTTCTATTT

```
GCCAUUUCCGGCGAAUACAGAAUAAUUCCUCGAUCCGUUCGUUCAAUCGAGAUCUCUCGCGGAAAAUAAUUUCUAUUUAUCGAUCCUGGCU
((((....((..(((((.(((.((.((((.(((....((((......)))).....))).))))..)).))).)))))..))....)))).
```

```
 Initial dG =    -20.20

         10        20         30         40
-    UUUC  GC     C   A  A-    U   UCCG-|   UU
 GCCA    CG  GAAUA AGA UA  UUCC CGA     UUCG  C
 CGGU    GC  UUUAU UUU AU  AAGG GCU     GAGC  A
U    CCUA  UA     C   A  AA    C   CUCUA^   UA
90        80        70        60        50
```

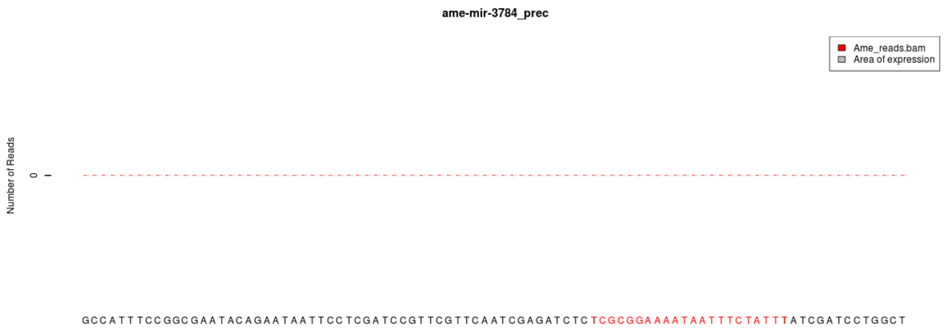

>ame-mir-3785_prec
GCGTAAATGTGTCTCGATTCTCCTTGGGAGGTCTCACGACGTGATAGCGGCGCCCGACTCTCGAAATTGTGG**TACCCTGTAACGTCCTG**
AGACTTCCTGCGGGGATCTGTCGACCCACTTTGTCCT

>ame-mir-3785_3p
TACCCTGTAACGTCCTGAGACT

```
GCGUAAAUGUGUCUCGAUUCUCCUUGGGAGGUCUCACGACGUGAUAGCGGCGCCCGACUCUCGAAAUUGUGGUACCCUGUAACGUCCUGAGACUUCCUGCGGGGAUCUGUCGACCCACU
UUGUCCU
...((((.(((..((((((((((.((((((((((((.(((((.((((.((..
(((((.........))).)).))..)))))).))))).))))))))))))).))))).....))))).)))))))....

 Initial dG =    -53.90

         10            20         30         40         50          60
GCG-    U   UC      ---|    U             C      G     C  CG  -    CUCU
    UAAA GUG  UCGAU   UCUCC UGGGAGGUCUCA GACGU AUAG GG  CC CGA    \
    GUUU CAC  AGCUG   AGGGG GUCCUUCAGAGU CUGCA UGUC CC  GG GUU    C
UCCU    -   CC      UCU^    C             C      A     -  AU  U    AAAG
    120        110       100        90         80          70

-----------------------------------------------------
```

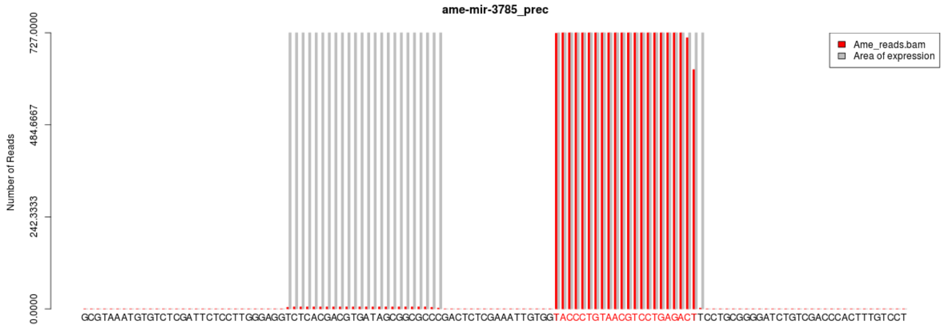

>ame-mir-3786_prec
ACAGGAACAAAACGTGTCCGACGT**CTTGTCCTGGTTCATGTAGGGCG**GAATGTTACGCAAACACTCTGTATGGCTCAGGACGATACGTC
TGGCTCGTCCAACCA

>ame-mir-3786_5p
CTTGTCCTGGTTCATGTAGGGCG

```
ACAGGAACAAAACGUGUCCGACGUCUUGUCCUGGUUCAUGUAGGGCGGAAUGUUACGCAAACACUCUGUAUGGCUCAGGACGAUACGUCUGGCUCGUCCAACCA
...((......(((.(((.(((((.((((((((..((((((((((((........)))......)))))))))..)))))))).))))).))).))).....)).

 Initial dG =    -38.40

        10        20        30        40              50
ACA  AACAAA   U   C     C         GU         ------|  GAA
   GG      ACG GUC GACGU UUGUCCUG  UCAUGUAGG      GCG   U
   CC      UGC CGG CUGCA AGCAGGAC  GGUAUGUCU      CGC   G
A--  AACC--   U   U     U         UC         CACAAA^  AUU
    100          90        80        70        60

-----------------------------------------------------
```

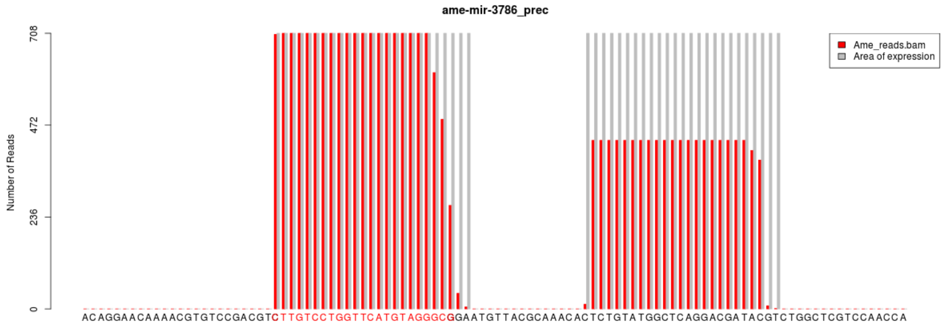

>ame-mir-3787_prec
CATCACGCGTTATATAGGGAATCGATAAAAC**GAGTCGAGCTTGTGTGTGGAA**ACGTTCTAACAACATTGTGGAACAAACCAGCGCGTCA
AAAATTGCTTCAATTTTGTAAATATCGACAGGTTTTGTAACCCGTGATT

>ame-mir-3787_3p
GAGTCGAGCTTGTGTGTGGAA

CAUCACGCGUUAUAUAGGGAAUCGAUAAAAC**GAGUCGAGCUUGUGUGUGGAA**ACGUUCUAACAACAUUGUGGAACAAACCAGCGCGUCAAAAAUUGCUUCAAUUUUGUAAAUAUCGACA
GGUUUUGUAACCCGUGAUU
.((((((.((((((.......((((((..(((((..((((((((((((((....((((((.
((....))))))))...))).)))).))).....))))....)))))...))))))).......)))))).))))))).
ERROR 4: Strange structure
Initial dG =   -34.70

```
        10        20         30           40               50        60
C      C      UAGGGAA      AA-     UC--    -----   -    -|  AAAC      A  A
 AUCACG GUUAUA       UCGAUA   ACGAG    GAGC     UUG UGUG UGG    GUUCUA CA C
 UAGUGC CAAUGU       AGCUAU   UGUUU    UUCG     AAC GCGC ACC    CAAGGU GU A
U      C      UUUGGAC      AAA     UAAC    UUAAA   U    G^  AAA-      -  U
      130       120       110       100        90        80         70
```

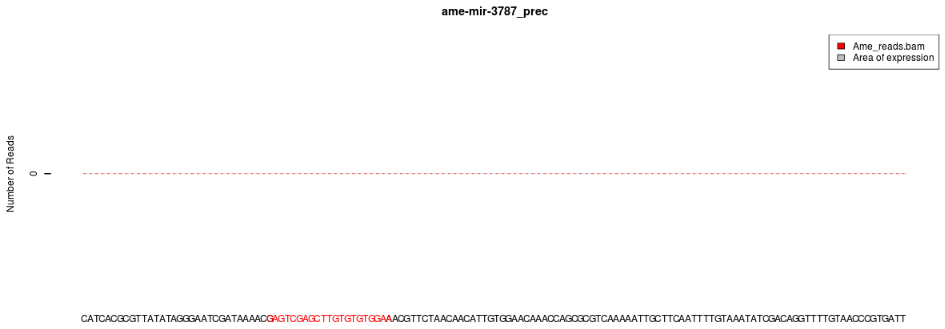

>ame-mir-3788_prec
CTCGACCGCTCCTTAATCAGGGTA**GTTCCGTTACCTCCTGTCCCTC**GCAATCGTCATCGTCCCTGACCAAGAACGACCCAAGAGAGCGG
GAGCCGGAAGCGATCCCAGGGTCA

>ame-mir-3788_5p
GTTCCGTTACCTCCTGTCCCTC

CUCGACCGCUCCUUAAUCAGGGUA**GUUCCGUUACCUCCUGUCCCUC**GCAAUCGUCAUCGUCCCUGACCAAGAACGACCCAAGAGAGCGGGAGCCGGAAGCGAUCCCAGGGUCA
...((((.((.........(((..((((((....(((((((..(((....(((((...(((...)))...).))))).....))).))))))).))).)))...))))))))).

Initial dG = -32.20

```
        10        20          30        40        50          60
CUC|   G  CCUUAAUCA   UA-   -   UUAC       CC   GCAA-    - AUC   C
   GACC CU         GGG   GUU CCG    CUCCUGU  CUC     UCGU C   GUC \
   CUGG GA         CCC   CGA GGC    GAGGGCG  GAG     AGCA G   CAG C
A--^   -  ---------   UAG   A   C---       A-   AACCC    A AAC   U
   110                 100           90         80        70
```

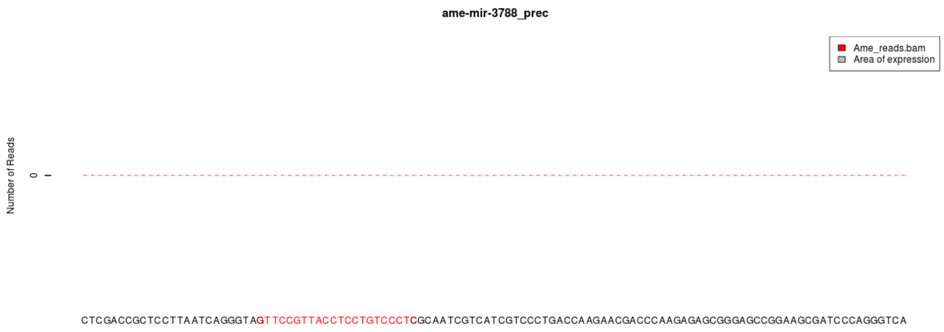

>ame-mir-3789_prec
CCGCTCGAACTCGGCCCTCGTTTGGACCGGCAACAAATTGGCCGCCGCGCGCATGATGCTG**ATCTGGTGCTCGGTGAGGTGAG**GGGGGT
ACGCGGGGT

>ame-mir-3789_3p
ATCTGGTGCTCGGTGAGGTGAG

```
CCGCUCGAACUCGGCCCUCGUUUGGACCGGCAACAAAUUGGCCGCCGCGCGCAUGAUGCUGAUCUGGUGCUCGGUGAGGUGAGGGGGGUACGCGGGGU
((((....((((..((((((..(..(((((((.((.((..(((((...)))......))..)).)).))).)))).)..))))))))))..))))...

 Initial dG =    -35.50

           10        20         30        40
---    UCGA    GG      UU GG    -   A  A  UG  ------|  C
   CCGC    ACUC  CCCUCG  U  ACCG GCA CA AU  GC       CGC \
   GGCG    UGGG  GGGAGU  A  UGGC CGU GU UA  CG       GCG G
UGG    CA--    --      GG G-    U   G  C  GU  UAGUAC^  C
       90            80         70        60        50

-----------------------------------------------------
```

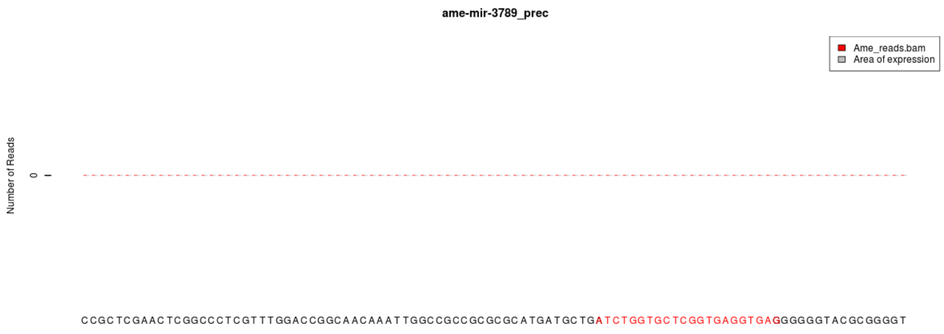

>ame-mir-3790_prec

AGAAAGCGATTAAGCGATCGAGGAGTCTGGGCGGTGAGAAAAATCTGCCAAGACGCTTGGTTACGTTGAAGAGGAAGAGAGAGGGAGGA
TCGCGATCC

>ame-mir-3790_5p

GAAAGCGATTAAGCGATCGAG

```
AGAAAGCGAUUAAGCGAUCGAGGAGUCUGGGCGGUGAGAAAAAUCUGCCAAGACGCUUGGUUACGUUGAAGAGGAAGAGAGAGGGAGGAUCGCGAUCC
.((..(((((..(((((((((..((((.(((((..........))))).)))).)))))))..)))....................))))))..)).

 Initial dG =    -25.60

        10                              20        30
A  AA      AA--------------------   --|     GA    G     UGAG
 GA  GCGAUU                     GCG  AUCGAG  GUCU GGCGG    A
 CU  CGCUAG                     UGC  UGGUUC  CAGA CCGUC    A
C  AG      GAGGGAGAGAGAAGGAGAAGU   AU^     G-    A     UAAA
       90        80        70        60         50        40

-----------------------------------------------------
```

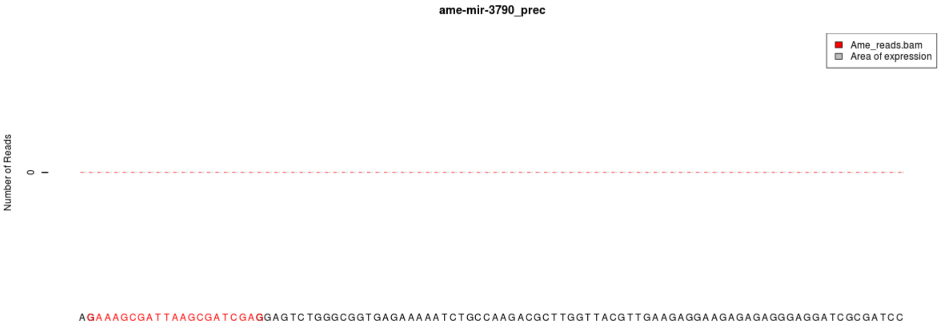

>ame-mir-3791_prec
AATATAGAGACTGCTGTCGGATGAAATCTCGTCCGGTGTGGTTGGAAAAAAAAAACC**TCACCGGGTAGGATTCATCCA**ATGTCCGCCTC
TGTT

>ame-mir-3791_3p
TCACCGGGTAGGATTCATCCA

```
AAUAUAGAGACUGCUGUCGGAUGAAAUCUCGUCCGGUGUGGUUGGAAAAAAAAAACCUCACCGGGUAGGAUUCAUCCAAUGUCCGCCUCUGUU
...(((((...((....((((((.(((..(((((((.((((..........)))).)))))))..))))))))))...))....)))))).

 Initial dG =    -36.40

        10          20         30         40
AAU      ACU-| UGUC       A    CG        U     GGAA
   AUAGAG    GC    GGAUGAA UCU  UCCGGUG GGUU     A
   UGUCUC    UG    CCUACUU AGG  GGGCCAC CCAA     A
U--      CGCC^ UAA-       -    AU        U     AAAA
    90         80          70         60         50

-----------------------------------------------------
```

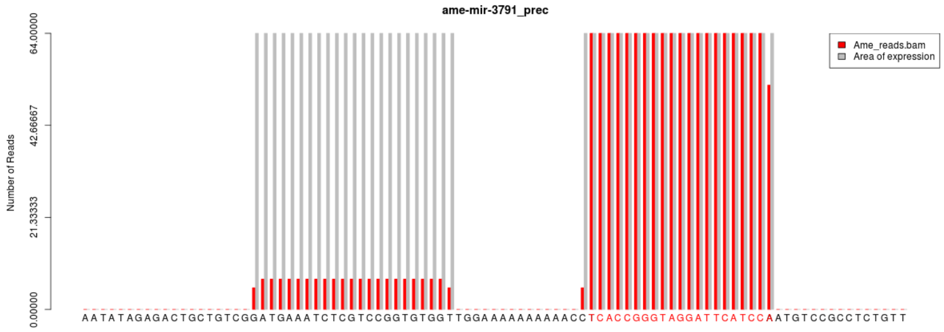

>ame-mir-3792_prec
CGGCCTCTCGTCCGAGTTACGTGTATTCCGTGGAG**GACTGAGGAAAGAGAGAAGGA**TCGGAAACGCAACGCAACCGGATTCCGTTTTCA
TCGATGTTCAACAGTTTTATCTCGCGATCGACATTTCGAACTTGGCCT

>ame-mir-3792_3p
GACTGAGGAAAGAGAGAAGGA

```
CGGCCUCUCGUCCGAGUUACGUGUAUUCCGUGGAGGACUGAGGAAAGAGAGAAGGAUCGGAAACGCAACGCAACCGGAUUCCGUUUUCAUCGAUGUUCAACAGUUUUAUCUCGCGAUCG
ACAUUUCGAACUUGGCCU
.((((....((.((((....((((....(((((((((((..(((...((..((((.(((((.
((.........))..)))))))))..))....)))..))))))..)).))))....)))))))).))..)))).
ERROR 4: Strange structure
 Initial dG =    -35.60

        10        20        30           40         50        60
C|   UCUC  C    UUAC    AUUC    -  --      AG   AGA-  GA    U     A-  CAAC
 GGCC    GU CGAG    GUGU    CGUG GA  GGACUG  GAA    GA  AGGA CGGAA  CG    \
 CCGG    CA GCUU    UACA    GCGC CU  UUUGAC  CUU    CU  UUUU GCCUU  GC    G
U^   UU--  A    ----    GCUA    U  AU      AA   GUAG  AC    -     AG  CAAC
       130           120       110       100        90         80
```

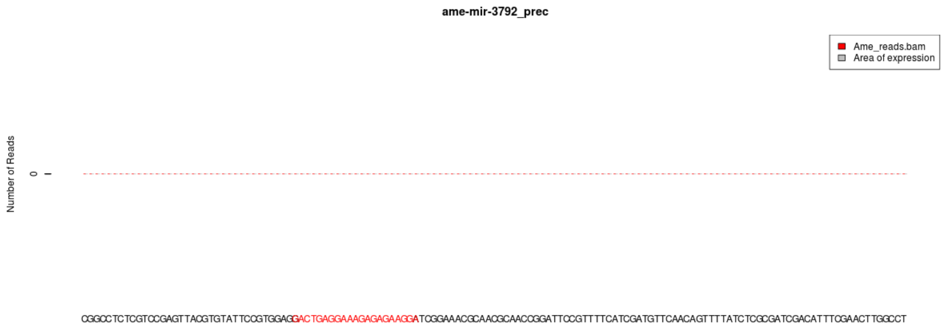

>ame-mir-3793_prec
TT**TGAGCGTGTTTTCCCTGGACTG**GTCTATCTGTCGCGCGATCTTGACGTGGTGGACCTAGCGCCAGAAGAAACTCGTTTC

>ame-mir-3793_5p
TGAGCGTGTTTTCCCTGGACTG

UU**UGAGCGUGUUUUCCCUGGACUG**GUCUAUCUGUCGCGCGAUCUUGACGUGGUGGACCUAGCGCCAGAAGAAACUCGUUUC
...(((((.(((((..((((.(((((((((((((((........))))).))))))))).))..)))).)).))))).))))).

Initial dG =   -31.30

```
       10        20          30         40
UUU     U     CC     A-   -        -|     CGC
   GAGCG GUUUU  CUGG  CU GGUCUAUC UGUCG   G
   UUUGC CAAAG  GACC  GA CCAGGUGG GCAGU   A
C--     U     AA    GC  U        U^    UCU
  80        70        60        50
```

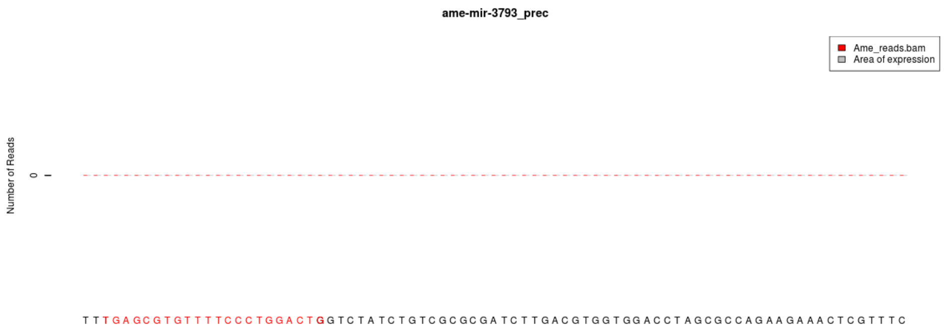

>ame-mir-3794_prec
CGAATTGGAGGATCGATATA**TGGAATGGAAGAGGACGTTTCC**GCGAGTAAGACGAGGAAACATCTTTTCTTTCGCACAATTTGATCGAC
AGTG

>ame-mir-3794_5p
TGGAATGGAAGAGGACGTTTCC

```
CGAAUUGGAGGAUCGAUAUAUGGAAUGGAAGAGGACGUUUCCGCGAGUAAGACGAGGAAACAUCUUUUCUUUCGCACAAUUUGAUCGACAGUG
...((((...((((((....(((((.((((((((..((((((.((.......)).)))))).))))))))))).))....))))))..)))).

 Initial dG =    -26.00

        10        20         30        40
CGA|   GAG      UAUA  -   U        AC      G  AGU
   AUUG   GAUCGA    UG GAA GGAAGAGG  GUUUCC CG   \
   UGAC   CUAGUU    AC CUU UCUUUUCU  CAAAGG GC   A
G--^   AG-      UAAC  G   -        A-      A  AGA
    90         80         70         60        50

-----------------------------------------------------
```

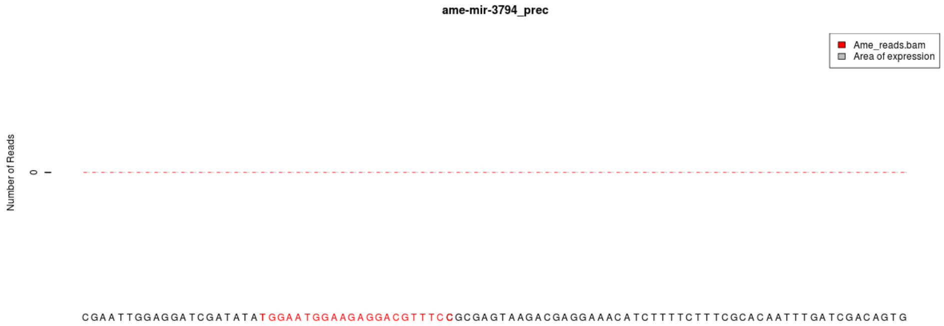

>ame-mir-3795_prec
AATT**GCGTCGACACGTTGGACGTGTT**TAATTAATTCATTCGTGTAGTGAAATTTAATGGACGGTTACACGTGTCTGTCGCAC

>ame-mir-3795_5p
GCGTCGACACGTTGGACGTGTT

AAUU**GCGUCGACACGUUGGACGUGUU**UAAUUAAUUCAUUCGUGUAGUGAAAUUUAAUGGACGGUUACACGUGUCUGUCGCAC
```
...((((.((((((((..(((.((((((.((((((((((.....))))))..))))))))))))))..))))))).).)))).
```

Initial dG = -24.80

```
         10        20        30          40
AAU    U -|      UG   G      A    --      CG
   UGCG C GACACGU  GAC UGUUUA UUAA  UUCAUU  \
   ACGC G CUGUGCA  UUG GCAGGU AAUU  AAGUGA  U
C--    U U^      CA   -      -    UA      UG
   80        70         60         50
```

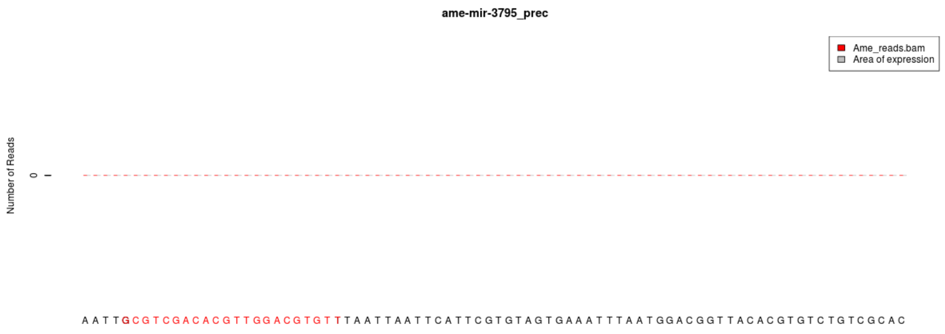

>ame-mir-3796_prec

TCCATCGTGACGAATATTCTTCTCTCGGGGATGCGACTCGAGAGTTATCGGGTAATTAGCTTGGGTTTCTTATGAGTTTGATTC**GATCC TAAGGGGAGGAGAGGATTT**GTAATTGACGTATCGGT

>ame-mir-3796_3p

GATCCTAAGGGGAGGAGAGGATTT

UCCAUCGUGACGAAUAUUCUUCUCUCGGGGAUGCGACUCGAGAGUUAUCGGGUAAUUAGCUUGGGUUUCUUAUGAGUUUGAUUC**GAUCCUAAGGGGAGGAGAGGAUUU**GUAAUUGACGU AUCGGU

```
....(((..((((((.((((((((((.(((((.(((((((.(((...
(((((.....))))))....))).))))......))))))))...)))))))))).))))))...))).........
```

Initial dG =   -36.30

```
              10        20          30              40         50
UCCA-----   UG-      A          G--     G   ------    A   UUA-|     AA
         UCG   ACGAAU UUCUUCUCUC   GGGAU CGA      CUCG GAG    UCGGGU  \
         AGU   UGUUUA GAGAGGAGGG   UCCUA GCU      GAGU UUC    GGUUCG  U
UGGCUAUGC   UAA      G          GAA     -   UAGUUU    A   UUUG^     AU
   120       110       100        90         80        70        60
```

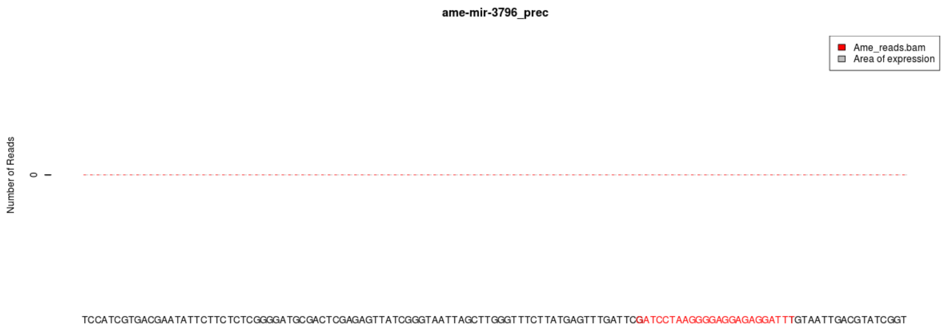

>ame-mir-3797_prec
CGGGA**CAGAACTGGCTCGTATTTTATT**TCAAGTGTCAAACCTCCTCTCGAAACCTCCGCTCCGTGAGAGACGTTTCGCGTCCGAGCGGA
AAGAGGAGAAAGAAAGGAAGAAATAAAGAGAGGAACCGTCGTGTATCGT

>ame-mir-3797_5p
CAGAACTGGCTCGTATTTTATT

CGGGA**CAGAACUGGCUCGUAUUUUAUU**UCAAGUGUCAAACCUCCUCUCGAAACCUCCGCUCCGUGAGAGACGUUUCGCGUCCGAGCGGAAAGAGGAGAAAGAAAGGAAGAAAUAAAGAG
AGGAACCGUCGUGUAUCGU
((..(((..((.((((....((((((((.....((....(((((((.......
(((((((((((((....))))))))...))))))).)))))))...))......))))))))..)))...))))..)))..)).

```
 Initial dG =    -39.50

         10            20         30          40         50         60            70
-  GG    GA  U  ---|  GUAU        AAGUG-  AAAC       CGAAACC       ---        G
 CG  ACA  AC GG   CUC    UUUAUUUC      UC    CUCCUCU       UCCGCUC   CGUGAGA A
 GC  UGU  UG CC   GAG    AAAUAAAG      AG    GAGGAGA       AGGCGAG   GCGCUUU C
U  UA   GC  -  AAG^  AG--        AAGGAA  AAA-       A------       CCU        G
      130        120         110       100         90               80
```

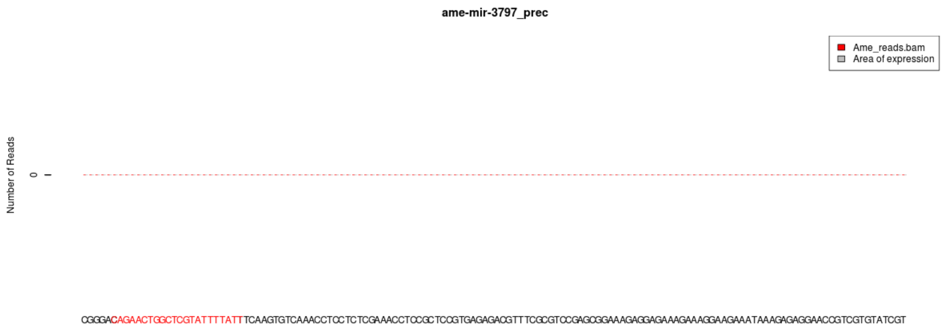

>ame-mir-3798_prec
CTTTTAAATTAAATACACTTATCTCGAATAATTTCTCCAGTATCAAAATGATACAGGGAGAATT**TCATAAATAATCTGTATTTCTTTA**A
GAAT

>ame-mir-3798_3p
TCATAAATAATCTGTATTTCTTTA

```
CUUUUAAAUUAAAUACACUUAUCUCGAAUAAUUUCUCCAGUAUCAAAAUGAUACAGGGAGAAUUUCAUAAAUAAUCUGUAUUUCUUUAAGAAU
.(((((((..((((((...............(((((.(((((.....)))))..))))))..............))))))).))))))..
```

```
 Initial dG =    -19.00

         10        20        30         40
C-|      UU       CUUAUCUCGAAUAAU      A-      AA
  UUUUAAA  AAAUACA               UUCUCC  GUAUC  \
  AGAAUUU  UUUAUGU               AAGAGG  CAUAG  A
UA^      C-       CUAAUAAAUACUUU-      GA      UA
  90         80        70         60        50

-----------------------------------------------------
```

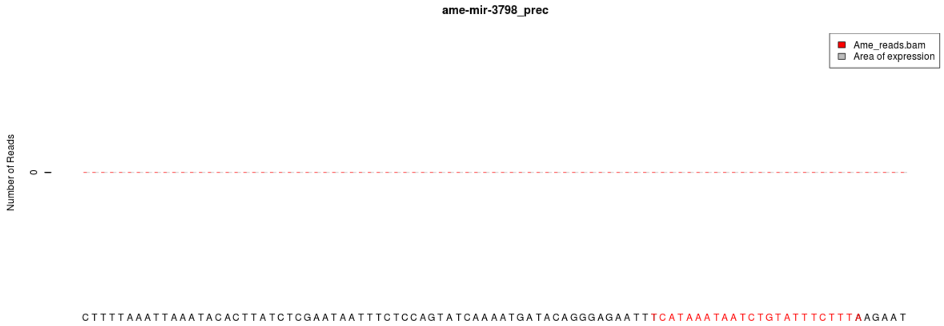

>ame-mir-3799_prec
TCGATCTTAGCGTTGG**AACGATATTGATTGACTCGAT**AGAAAACTTCTGGTCGACTCGATTCCAATCGTTTCCGTGCGAATCA

>ame-mir-3799_5p
AACGATATTGATTGACTCGAT

```
UCGAUCUUAGCGUUGGAACGAUAUUGAUUGACUCGAUAGAAAACUUCUGGUCGACUCGAUUCCAAUCGUUUCCGUGCGAAUCA
..(((....(((..((((((((...((((((.(((((((((...)))).))))).))))))...))))))))..)))..))).

 Initial dG =    -26.20

        10        20        30         40
UC|  CUUA   UU        AUU      C      -    A
  GAU    GCG  GGAACGAU   GAUUGA UCGAU AGAA \
  CUA    CGU  CUUUGCUA   UUAGCU AGCUG UCUU A
A-^  AG--   GC        ACC      C     G    C
   80          70        60        50

-----------------------------------------------------
```

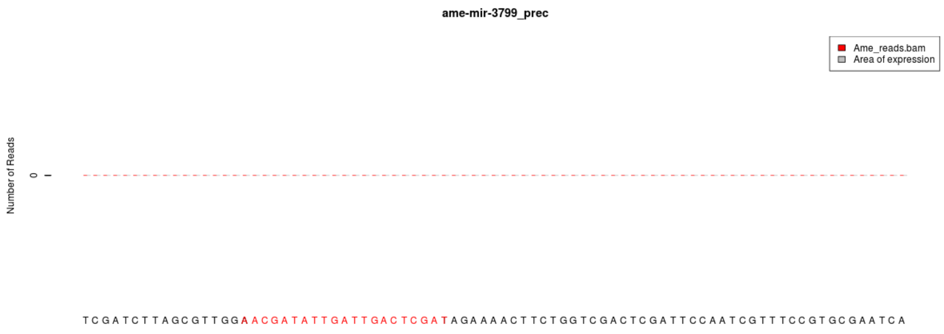

>ame-mir-3800_prec
TACTGTGGTCCACACGTTGACTGTCTCGCGGCCACGTTCCTTAAAAGGCGGTAAATCACGCGTTTCGCCTTCCTTTTTACCGGCCAC**CG**
AGGCCGCTGATTGTGCCATACATAA

>ame-mir-3800_3p
CGAGGCCGCTGATTGTGCCATA

```
UACUGUGGUCCACACGUUGACUGUCUCGCGGCCACGUUCCUUAAAAGGCGGUAAAUCACGCGUUUCGCCUUCCUUUUUACCGGCCACCGAGGCCGCUGAUUGUGCCAUACAUAA
...(((((..((((.((((...((((((.((((..((.......((((((.............)))))).......)).))))..))))))...))))))))))))))).....

 Initial dG =    -31.00

          10        20         30        40        50
UAC--|    UC    C    ACU      C-    AC  UCCUUAA       UAAAUC
     UGUGG  CACA GUUG   GUCUCG  GGCC  GU       AAGGCGG      \
     AUACC  GUGU UAGU   CGGAGC  CCGG  CA       UUCCGCU      A
AAUAC^    --    -    CGC      CA    C-  UUUUUCC       UUGCGC
  110          100        90         80        70        60

-----------------------------------------------------
```

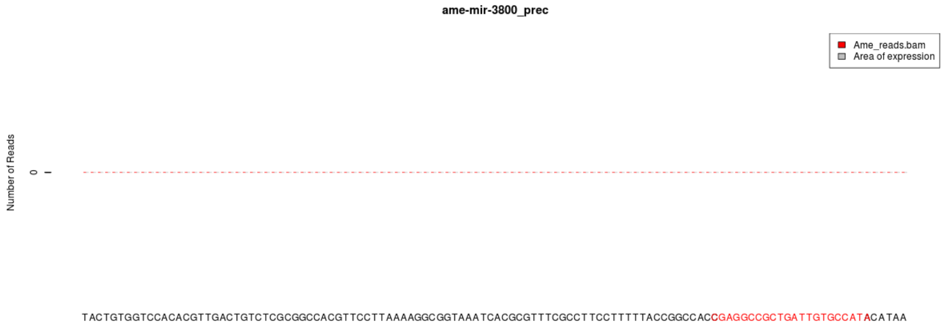

>ame-mir-3801_prec
ATTGGATCCGGTACAGTGTACAGTTTTGCGGTACA**AATCGCGATTGAGAAGGCGAGC**CGTAATGCAGGCGTATGTACGTAACAATCGCA
ACGGTTCGATGTCAGCGGGGTTTATCGAGCTAGAAGGTACGGCCTTGGTCT

>ame-mir-3801_3p
AATCGCGATTGAGAAGGCGAGC

```
AUUGGAUCCGGUACAGUGUACAGUUUUGCGGUACAAAUCGCGAUUGAGAAGGCGAGCCGUAAUGCAGGCGUAUGUACGUAACAAUCGCAACGGUUCGAUGUCAGCGGGGUUUAUCGAGC
UAGAAGGUACGGCCUUGGUCU
...(((((.(((....(((((..(((.((.....(((((.((.((((.....((((((((..(((..
((((....)))).......)))))))))))...)))))).)))))).....)).)))..))))))))..)))))
ERROR 4: Strange structure
 Initial dG =    -40.20

         10        20        30        40        50        60             70
AUU     C-|  ACAG     AG   U  GGUAC     G  A    GAAGG       AA   AG-----    A
   GGAUC  GGU    UGUAC  UUU GC     AAAUC CG UUGA     CGAGCCGU  UGC       GCGU U
   UCUGG  CCG    GCAUG  AGA CG     UUUGG GC GACU     GCUUGGCA  ACG       UGCA G
---     UU^  ----     GA   U  AGCUA     G  -    GUA--       --   CUAACAA    U
 140          130       120       110        100          90          80
```

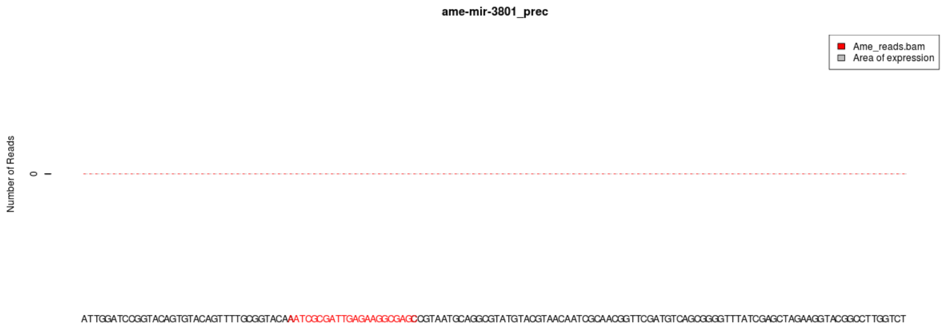

>ame-mir-6000a_prec
TCAGATACTCGTGGTGGTGACGACT**CGTGGGTACTACTCGTCTCTAT**ATCATCAAATTTATAGTACGAGAAGTACCCACAAGACGCATC
ATCAAGTAA

>ame-mir-6000a_5p
CGTGGGTACTACTCGTCTCTAT

```
UCAGAUACUCGUGGUGGUGACGACUCGUGGGUACUACUCGUCUCUAUAUCAUCAAAUUUAUAGUACGAGAAGUACCCACAAGACGCAUCAUCAAGUAA
.....((((..((((((((.((.((.(((((((((.(((((..((((((..........))))).))))).))))))))).)).)))))))))))))).

 Initial dG =    -41.70

        10        20        30        40        50
UCAGA|   CG        A  A  C         A     CU     UCAU
     UACU  UGGUGGUG CG CU GUGGGUACU CUCGU  CUAUA    C
     AUGA  ACUACUAC GC GA CACCCAUGA GAGCA  GAUAU    A
A----^   --        -  A  A         A     U-     UUAA
             90         80        70         60

-----------------------------------------------------
```

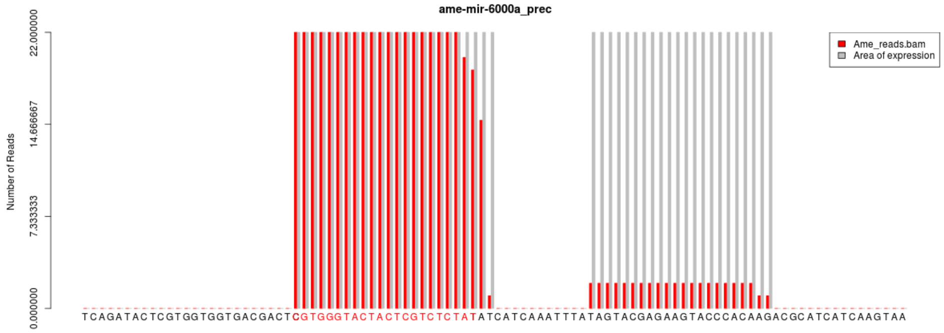

>ame-mir-6000b_prec
TTGATGATGCGTCTTGTGGGTACTTCTCGTACTATAAATTTGATGATA**TAGAGACGAGTAGTACCCACGAG**TCGTCACCAC

>ame-mir-6000b_3p
TAGAGACGAGTAGTACCCACGAG

```
UUGAUGAUGCGUCUUGUGGGUACUUCUCGUACUAUAAAUUUGAUGAUAUAGAGACGAGUAGUACCCACGAGUCGUCACCAC
....((.((((.((((((((((((.(((((.(((((..........)))))..))))).))))))))))))).)).)).)).
```

```
 Initial dG =    -34.80

         10        20        30         40
UUGA  A  -  U            U     A-|    AAUU
    UG UG CG CUUGUGGGUACU CUCGU  CUAUA    U
    AC AC GC GAGCACCCAUGA GAGCA  GAUAU    G
C---  C  U  U            U     GA^    AGUA
   80        70        60        50
```

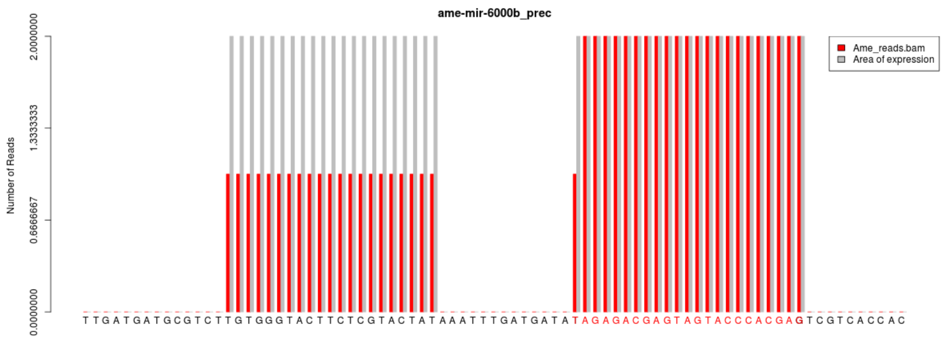

>ame-mir-6001_prec
AG**GTAGGTAACGACTGATGGGAAC**ACTCTGAGATTTTCTTTAATGTTCTCTTTGGTTGTTACCACTAG

>ame-mir-6001_5p
GTAGGTAACGACTGATGGGAAC

```
AGGUAGGUAACGACUGAUGGGAACACUCUGAGAUUUUCUUUAAUGUUCUCUUUGGUUGUUACCACUAG
.(((.(((((((((..(.(((((((....(((.....)))...))))))).)..))))))))))))..
```

```
 Initial dG =    -28.00

         10        20        30
A-|  A         UG U       CUCU   AU
  GGU GGUAACGAC  A GGGAACA    GAG  \
  UCA CCAUUGUUG  U CUCUUGU    UUC  U
GA^  -         GU U       AAU-   UU
        60        50         40
```

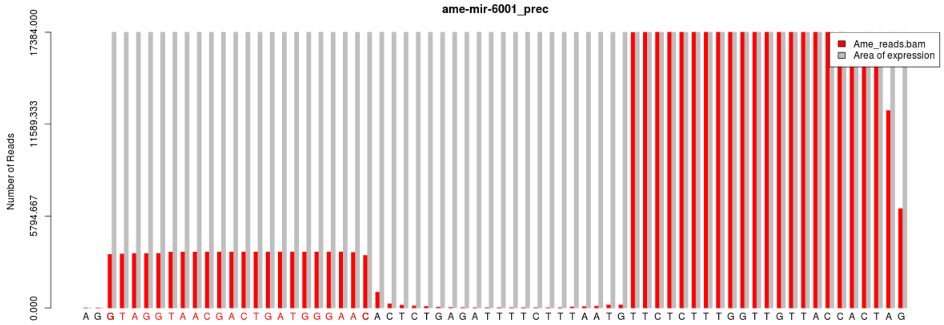

>ame-mir-6002_prec
TCGAAGGATTTTTGCTGA**TCGAAGCGAAGGGTATTCC**GATCGTAATAACAATCGAGAGTAACGTTTCGTGGCACCACTTC

>ame-mir-6002_5p
TCGAAGCGAAGGGTATTCC

```
UCGAAGGAUUUUUGCUGAUCGAAGCGAAGGGUAUUCCGAUCGUAAUAACAAUCGAGAGUAACGUUUCGUGGCACCACUUC
..((((......((((...(((((((.....(((((((((.((....)).)))).))))).))))))).))))...))))

 Initial dG =    -20.00

       10        20        30         40
UC|   GAUUUU    GAU       AAGGG     -    C  A
  GAAG      UGCU   CGAAGCG     UAUUC CGAU GU A
  CUUC      ACGG   GCUUUGC     AUGAG GCUA CA U
--^   ACC---    U--       A----     A    A  A
 80            70              60        50

-----------------------------------------------------
```

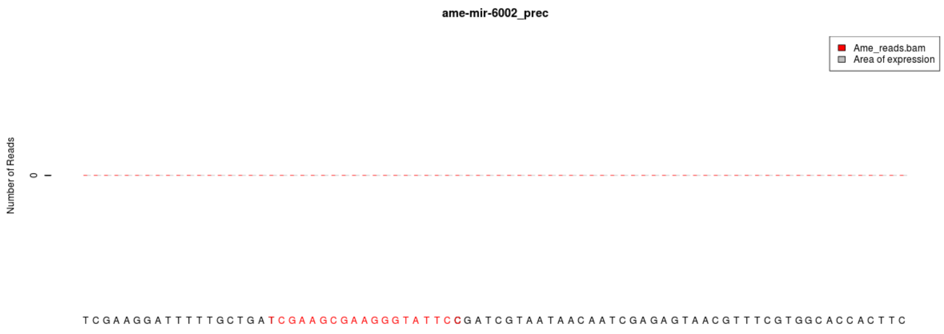

>ame-mir-6003_prec
GTGGCGGCGGGT**CACTCGGGGAAAAGAGGCC**ATTTCACAGGTCTTTGCGTCTCGATTCACCTTCCCTGGA

>ame-mir-6003_5p
CACTCGGGGAAAAGAGGCC

```
GUGGCGGCGGGUCACUCGGGGAAAAGAGGCCAUUUCACAGGUCUUUGCGUCUCGAUUCACCUUCCCUGGA
..((.((.((((...((((((...(((((((........)))))))...)))))))...)))).)))).))...

 Initial dG =    -24.30

         10        20        30
GU-| C  C    CAC      AAA       AUU
   GG GG GGGU   UCGGGG   AGAGGCC   U
   UC CC UCCA   AGCUCU   UUUCUGG   C
AGG^ -  U    CUU      GCG       ACA
.         60        50        40

-----------------------------------------------------
```

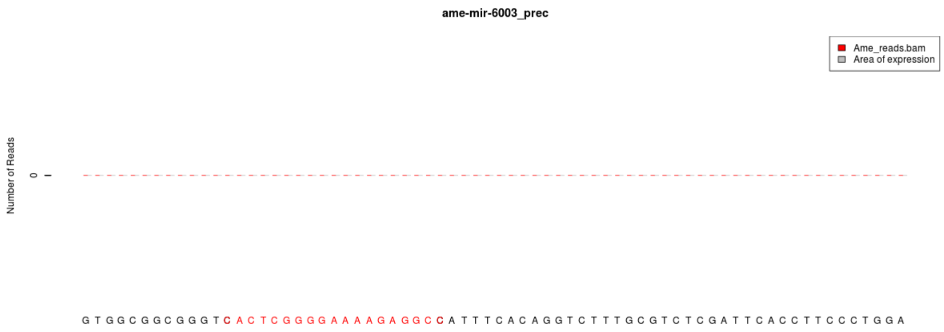

>ame-mir-6004_prec
GGATACGTTAAC**GAATGGAGCGTTAATGATCG**TGTTTCTAAATTGTATCAATGACGAGAGTCACGATTGCGTTGCGTTCTACGGTCTC

>ame-mir-6004_5p
GAATGGAGCGTTAATGATCG

```
GGAUACGUUAACGAAUGGAGCGUUAAUGAUCGUGUUUCUAAAUUGUAUCAAUGACGAGAGUCACGAUUGCGUUGCGUUCUACGGUCUC
((((.(((....(((((.(((((....(((((((.((((..((((...))))....)))).))))))))))))).))))).))))))).

 Initial dG =    -26.00

         10        20        30        40
-|   A   UAAC     G     UAAU       U    AA--    U
 GGAU CGU    GAAUG AGCGU    GAUCGUG UUCU    AUUG \
 UCUG GCA    CUUGC UUGCG    UUAGCAC GAGA    UAAC A
C^   -   U---     G     ----       U    GCAG    U
        80           70            60        50

-----------------------------------------------------
```

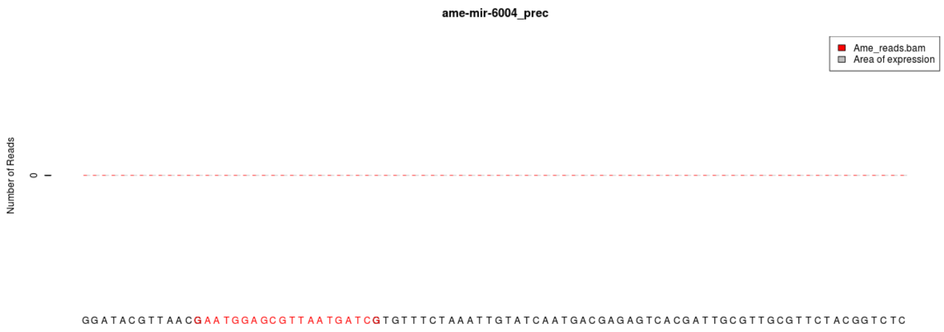

>ame-mir-6005_prec
CGCACTGTCGACTAGTCGTCAAAGATTAGATCGTCAGCAACTTAAGTTTAAGAGAAC**ATATGAACTCTTATGTACGTGA**TTACGATTTC
ACATGCGTAAGAGATTATATGTTTTCTTGAAGCTAATAATCATCGAAGTAGTCATTGATGATAACTAGTCGATA

>ame-mir-6005_3p
ATATGAACTCTTATGTACGTGA

CGCACUGUCGACUAGUCGUCAAAGAUUAGAUCGUCAGCAACUUAAGUUUAAGAGAAC**AUAUGAACUCUUAUGUACGUGA**UUACGAUUUCACAUGCGUAAGAGAUUAUAUGUUUUCUUGA
AGCUAAUAAUCAUCGAAGUAGUCAUUGAUGAUAACUAGUCGAUA
```
.....((((((((((((((((.(((((..(((........(((..((((((((((((((((.((((((((((.
((((........)))).)))))))))).)))))))))))))))))..)))).......)))..))))).)))))))...))))))))))
```
ERROR 4: Strange structure
Initial dG =   -64.50

```
        10           20         30         40         50         60         70         80
CGCAC          ---|      A      GA   UCAGCAAC   AG                 A          C    UUA
     UGUCGACUAG   UCGUCAA GAUUA  UCG        UUA  UUUAAGAGAACAUAUGA CUCUUAUGUA GUGA   C
     AUAGCUGAUC   AGUAGUU CUGAU  AGC        AAU  AAGUUCUUUUGUAUAUU GAGAAUGCGU CACU   G
-----          AAU^      A      GA   UACUAAU-   CG                 A          A    UUA
      160        150        140        130        120        110        100         90
```

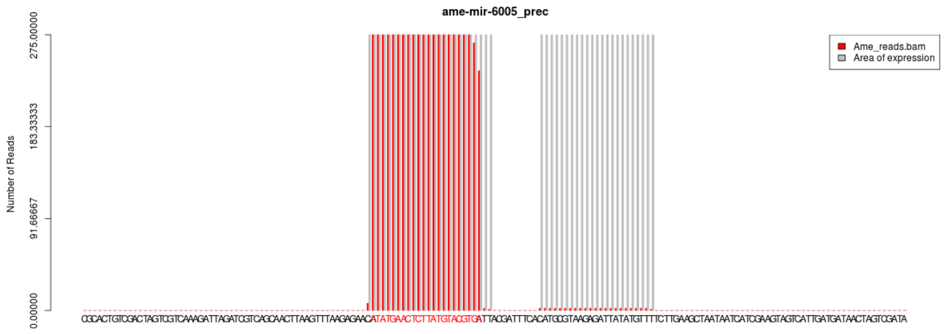

>ame-mir-6006_prec
CAACCCTCGCTAGGGTGAGAAAA**ATAACTGGGAAGAGAGAGG**AATAATTAATTTTCTTTTTCAGTTATAAGTTTGCATTCGAGAAATT

>ame-mir-6006_5p
ATAACTGGGAAGAGAGAGG

```
CAACCCUCGCUAGGGUGAGAAAAAUAACUGGGAAGAGAGAGGAAUAAUUAAUUUUCUUUUUCAGUUAUAAGUUUGCAUUCGAGAAAUU
.....((((.....(..((....((((((((.(((((((((..........)))))))))))))))))...))..)...)))).....
```

Initial dG =   -20.50

```
        10        20        30        40
CAACC|   CUAGG UG  AAAA        G         GAAU
     CUCG     G  AG    AUAACUGG AAGAGAGAG    A
     GAGC     C  UU    UAUUGACU UUUUCUUUU    A
UUAAA^   UUA-- GU  GAA-        -         AAUU
       80          70          60        50
```

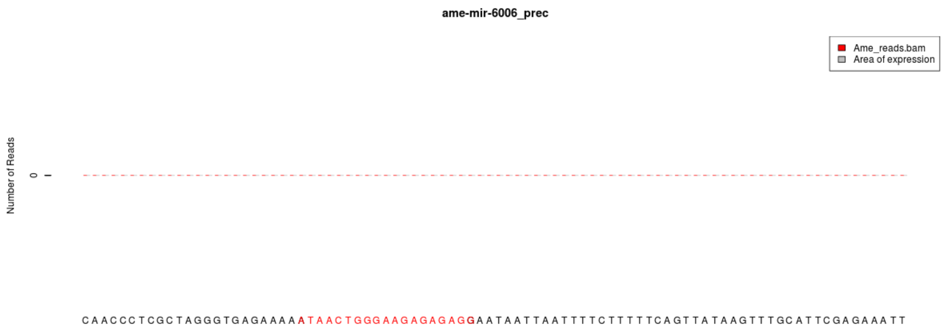

>ame-mir-6012_prec
GGCAGGGTTAGCAGGCTGATATTGTCGGGCGGGGTGTGGAACAAGTAAGTCAT**TTCGGCGATGAGATCAGCCTGT**CGAACCCTGC

>ame-mir-6012_3p
TTCGGCGATGAGATCAGCCTGT

```
GGCAGGGUUAGCAGGCUGAUAUUGUCGGGCGGGGUGUGGAACAAGUAAGUCAUUUCGGCGAUGAGAUCAGCCUGUCGAACCCUGC
.((((((((.((((((((((.(..(((..((..(((..............)))..)).))).).)))))))))))..))))))))

 Initial dG =    -42.70

        10         20        30        40
G        A-|         A UG   GG  GG   UGGAAC
 GCAGGGUU  GCAGGCUGAU U  UCG  CG  GUG      A
 CGUCCCAA  UGUCCGACUA A  AGC  GC  UAC      A
-        GC^         G GU   G-  UU   UGAAUG
     80        70        60         50

-----------------------------------------------------
```

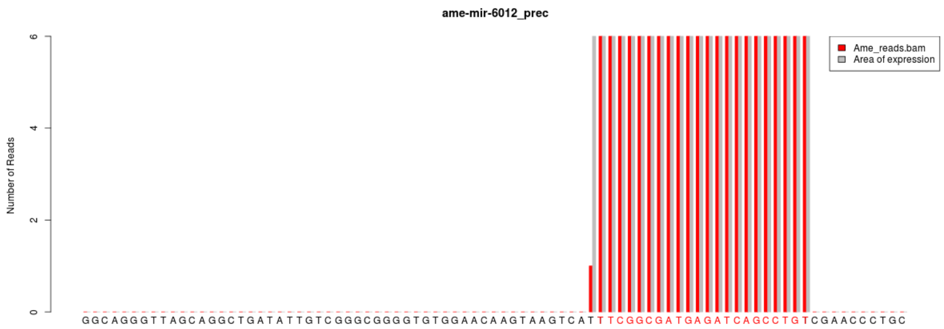

>ame-mir-6037_prec
TATGGTGAAGGTAATAGTCCATAGAGCTTCAAAAAATTGAATCGTGCTTAAACTTT**TAAGCTCTGTGTACTTTTTAC**AGGATCGAAA

>ame-mir-6037_3p
TAAGCTCTGTGTACTTTTTAC

```
UAUGGUGAAGGUAAUAGUCCAUAGAGCUUCAAAAAAUUGAAUCGUGCUUAAACUUUUAAGCUCUGUGUACUUUUUACAGGAUCGAAA
..((((....((((.(((.((((((((((.((((..((((.......))))..))))))))))))))).))).)).))))...)))))...

 Initial dG =    -20.40

         10          20         30         40
UA-|   GAAG    U-   C          C    AA     AUC
   UGGU    GUAA  AGU CAUAGAGCUU AAAA  UUGA   \
   GCUA    CAUU  UCA GUGUCUCGAA UUUU  AAUU   G
AAA^   GGA-    UU   U          -    CA     CGU
      80         70         60         50

-----------------------------------------------------
```

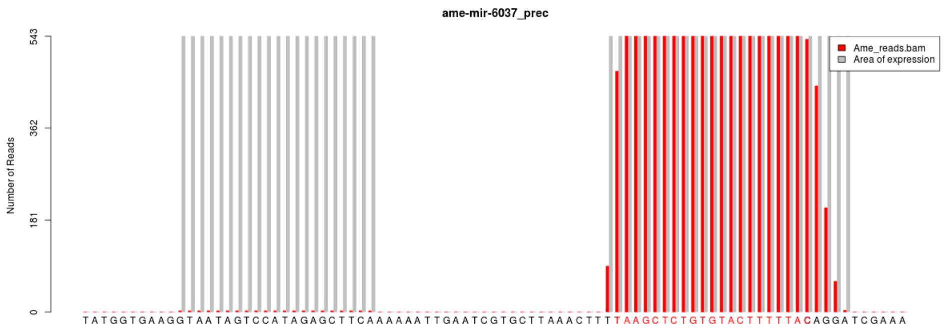

>ame-mir-6038_prec
AAAGCCGTCT**TATGTTTCTGTCTTATTTCATT**GTAAAATTGCGATATGAAATATGACATAAACTTAAGCTGGGTA

>ame-mir-6038_5p
TATGTTTCTGTCTTATTTCATT

```
AAAGCCGUCUUAUGUUUCUGUCUUAUUUCAUUGUAAAAUUGCGAUAUGAAAUAUGACAUAAACUUAAGCUGGGUA
....(((.((((.((((.((((.((((((((((((....))))))..))))))).)))).)))).)))).)))...

 Initial dG =    -22.00

        10        20        30
AAAG   U    U    C    U       --|    A
    CCG CUUA GUUU UGUC UAUUUCA  UUGUA A
    GGU GAAU CAAA ACAG AUAAAGU  AGCGU A
AUG-   C    U    U    U       AU^    U
     70        60        50        40

-----------------------------------------------------
```

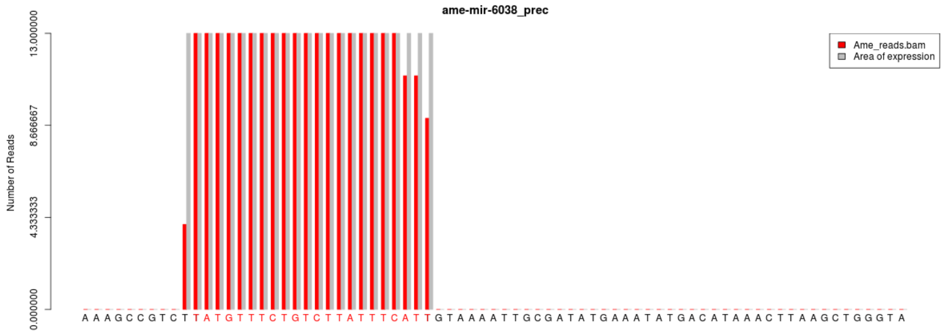

>ame-mir-6039_prec
TTCTACTTTA**AATCGAACGCGTGAGTTTACGT**GTAGCATTGAGCATTTAAGCGACGTAGACACACGTGTTGGACCTTAGAGTAGAATT

>ame-mir-6039_5p
AATCGAACGCGTGAGTTTACGT

```
UUCUACUUUAAAUCGAACGCGUGAGUUUACGUGUAGCAUUGAGCAUUUAAGCGACGUAGACACACGUGUUGGACCUUAGAGUAGAAUU
((((((((((.((.((((((((.((((((((...((.((((....)))))).)))))))).)))))))).))..)))))))))))..

 Initial dG =    -36.60

          10         20        30        40
--           A-| G        A        GUA  A    G
  UUCUACUUUAA  UC AACGCGUG GUUUACGU   GC UUGA C
  AAGAUGAGAUU  AG UUGUGCAC CAGAUGCA   CG AAUU A
UU           CC^ G        A        G--  -    U
       80        70        60           50

-----------------------------------------------------
```

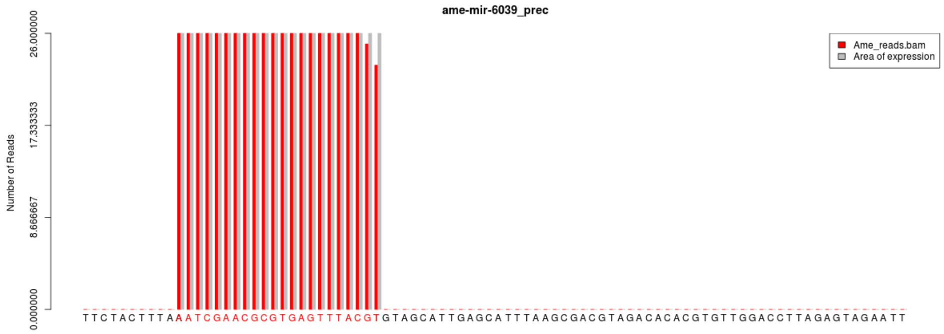

>ame-mir-6040_prec
GGCATATTCGAGACCTCAGTCCAGCCTTGTACTGTGTTGCGTAACTCCA**TAGTACGGGCAGTACTGGGA**AATCGTGTGT

>ame-mir-6040_3p
TAGTACGGGCAGTACTGGGA

```
GGCAUAUUCGAGACCUCAGUCCAGCCUUGUACUGUGUUGCGUAACUCCAUAGUACGGGCAGUACUGGGAAAUCGUGUGU
.(((((..(((...((((((...((((.((((((((...........))))))))))))...))))))...))))))))

 Initial dG =    -27.20

        10        20        30        40
G|    UU   GAC      CCA    U        UUGCG
 GCAUA  CGA   CUCAGU   GCCU GUACUGUG     \
 UGUGU  GCU   GGGUCA   CGGG CAUGAUAC     U
-^    --   AAA      UGA    -        CUCAA
           70        60         50

-----------------------------------------------------
```

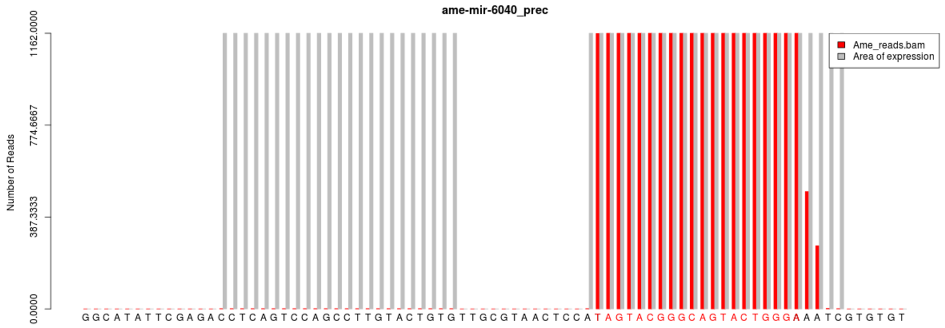

>ame-mir-6041_prec
AAGATATTATTCTTCGATACTGATGATATTTTAATATTA**AAATATCATCGAGATCGAAGGA**TATTTTTCAA

>ame-mir-6041_3p
AAATATCATCGAGATCGAAGGA

```
AAGAUAUUAUUCUUCGAUACUGAUGAUAUUUUAAUAUUAAAAUAUCAUCGAGAUCGAAGGAUAUUUUUCAA
..((...((((((((((..((((((((((((((....))))))))))))))..)))))))))))....))..

 Initial dG =    -28.10

         10        20        30
AA  UAU-|          AC             A
  GA    UAUUCUUCGAU  UGAUGAUAUUUUA U
  CU    AUAGGAAGCUA  GCUACUAUAAAAAU A
AA  UUUU^          GA             U
70        60        50        40

-----------------------------------------------------
```

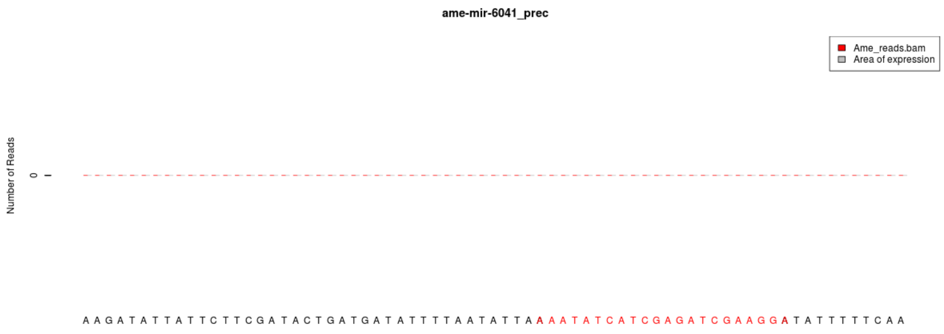

>ame-mir-6042_prec
TCCTTCGACGATACCTACCACTGTACTATCGTCGCCTGCTTGCAACGG**TAGGAACGGTAGTAGTGGAGA**ATTGCTGTGG

>ame-mir-6042_3p
TAGGAACGGTAGTAGTGGAGA

```
UCCUUCGACGAUACCUACCACUGUACUAUCGUCGCCUGCUUGCAACGGUAGGAACGGUAGUAGUGGAGAAUUGCUGUGG
.((..((.((((..((.(((((..((((((((..((((((......)))))).)))))))))))))))).)))).)).))

 Initial dG =    -26.30

        10        20        30        40
U| UU  A    AC  A     GU        CG      UG
 CC  CG CGAU  CU CCACU  ACUAUCGU  CCUGCU  C
 GG  GU GUUA  GA GGUGA  UGAUGGCA  GGAUGG  A
-^ U-  C    A-  -     --        A-      CA
          70            60         50

-----------------------------------------------------
```

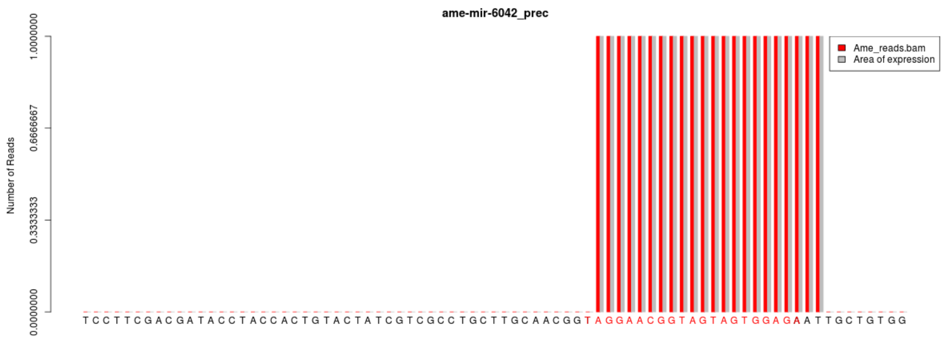

>ame-mir-6043_prec

AATTCCTTTTGATAGACGCGTCCACCATTGTTGCGCGCGACA**ATGGTGACCGTGATCTATTCCA**AAATGCTGAT

>ame-mir-6043_3p

ATGGTGACCGTGATCTATTCCA

```
AAUUCCUUUUGAUAGACGCGUCCACCAUUGUUGCGCGACAAUGGUGACCGUGAUCUAUUCCAAAAUGCUGAU
......((((((((((((((..(((((((((((....)))))))))))..)))).)))))..))))).......

 Initial dG =    -28.80

         10           20         30
AAUUCC-     --     -|   UC           C
       UUUUG  AUAGA CGCG  CACCAUUGUUG G
       AAAAC  UAUCU GUGC  GUGGUAACAGC C
UAGUCGU     CU     A^   CA           G
   70        60        50        40

-----------------------------------------------------
```

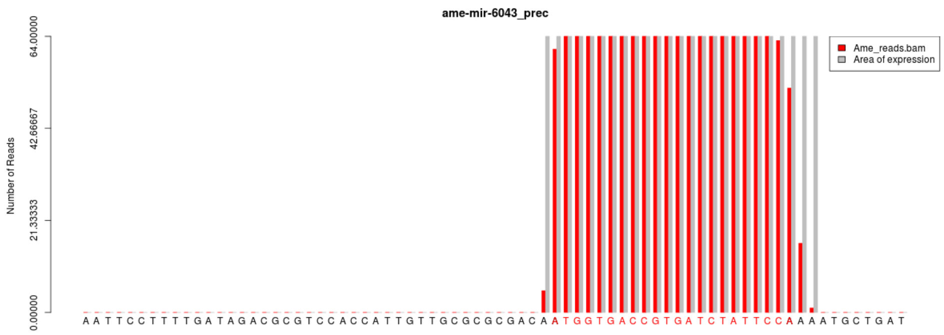

>ame-mir-6044_prec
TATTAATTAT**AAGAGGCTTCTTAGGCAAGTGAT**CAGAGTGTTTGATTGCTAATCTAAGAGTCTCGGGTTCAAATCTCG

>ame-mir-6044_5p
AAGAGGCTTCTTAGGCAAGTGAT

```
UAUUAAUUAUAAGAGGCUUCUUAGGCAAGUGAUCAGAGUGUUUGAUUGCUAAUCUAAGAGUCUCGGGUUCAAAUCUCG
...........((((((.((((((..((..((((((...))))))..))..)))))))))))).((........)).

 Initial dG =    -23.90

          10        20        30
.-UAUUAAUUAUAA|     U      CA  UG      G
              GAGGCU CUUAGG  AG  AUCAGA \
              CUCUGA GAAUCU  UC  UAGUUU U
\ ------------^     -      AA  GU      G
                 60         50        40


      70
  G  UUC
   GG   A
   CU   A
  G  CUA
```

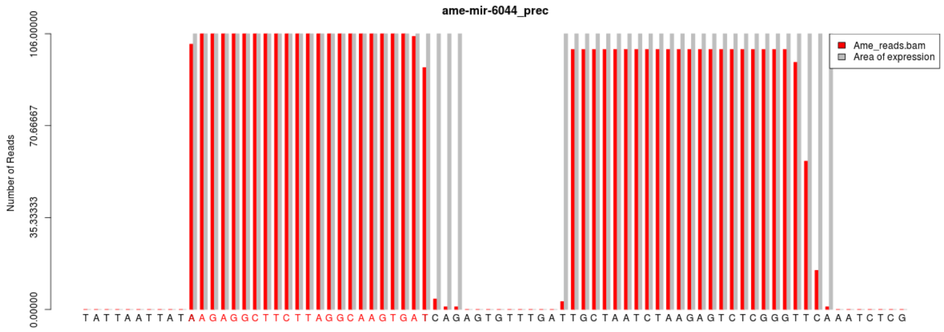

>ame-mir-6045_prec
CTGGTTGGAG**ATTCACTGGACGGCAATGGGCT**TTGTTCTGAACAGCATGTCGCCGTCCAGTCAGAGTCTTCAGCCAGC

>ame-mir-6045_5p
ATTCACTGGACGGCAATGGGCT

```
CUGGUUGGAGAUUCACUGGACGGCAAUGGGCUUUGUUCUGAACAGCAUGUCGCCGUCCAGUCAGAGUCUUCAGCCAGC
((((((((((((((((((((((....(((..((((......)))).)))))))))))))..)))))))))))))).

 Initial dG =    -43.20

         10          20        30        40
-              --|         AAUG   UU    CU
 CUGGUUGGAGAUUC  ACUGGACGGC    GGC  UGUU  G
 GACCGACUUCUGAG  UGACCUGCCG    CUG  ACGA  A
C              AC^         ----   U-    CA
       70        60            50

-----------------------------------------------------
```

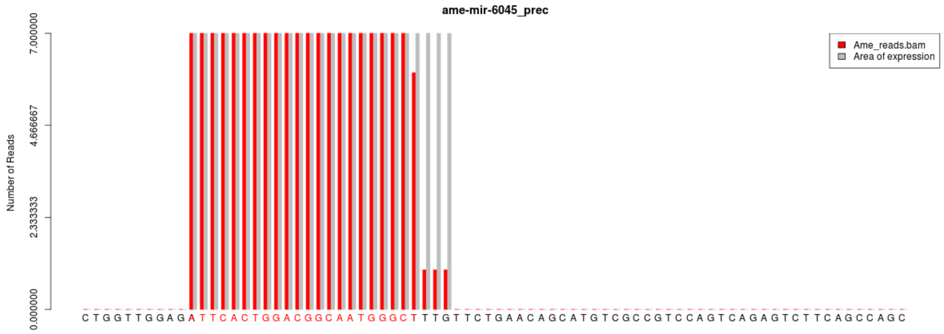

>ame-mir-6046_prec
CACTTAAGGCACGGTTACGGTTCTCTTCGCTGTCCTTCGCGAGTTTAAA**TCGACGAGAATTCCGTGACCGGT**CCTAGGTGTG

>ame-mir-6046_3p
TCGACGAGAATTCCGTGACCGGT

```
CACUUAAGGCACGGUUACGGUUCUCUUCGCUGUCCUUCGCGAGUUUAAAUCGACGAGAAUUCCGUGACCGGUCCUAGGUGUG
(((((.(((.((((((((((((((((((........))))).........)))))..))))))))))))).)))))))..

 Initial dG =    -33.00

          10        20                     30
--       A   A         --      -----------|    UGU
  CACUUA GGC CGGUUACGG  UUCUC             UUCGC   C
  GUGGAU CUG GCCAGUGCC  AAGAG             GAGCG   C
GU       C   -         UU      CAGCUAAAUUU^    CUU
 80         70        60        50        40

-----------------------------------------------------
```

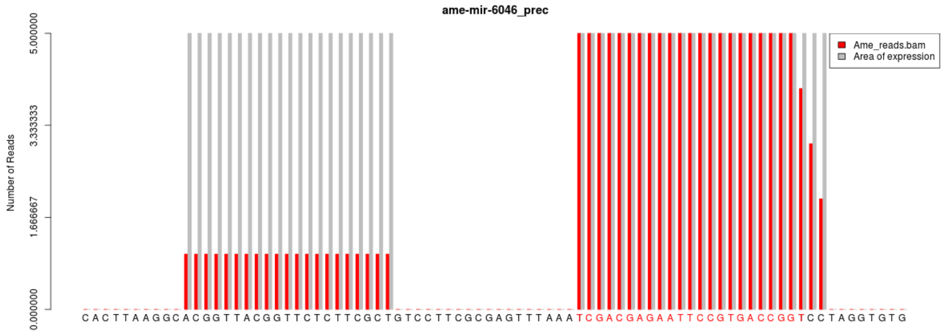

>ame-mir-6047a_prec

TGTTTTGTTTAGTGGACTGACTGGCCTGTGCTGCTTCCCGCAG**TCCACAGCCTACAGTCCACAAT**TTCCTGATCT

>ame-mir-6047a_3p

TCCACAGCCTACAGTCCACAAT

```
UGUUUUGUUUAGUGGACUGACUGGCCUGUGCUGCUUCCCGCAGUCCACAGCCUACAGUCCACAAUUUCCUGAUCU
...........((((((((...(((.((((((((.....))))..))))))))..)))))))).............

 Initial dG =    -28.70

        10          20        30
UGUUUUGUUUA--|       ACU   C    --    UU
             GUGGACUG   GGC UGUG  CUGC  \
             CACCUGAC   CCG ACAC  GACG  C
UCUAGUCCUUUAA^       AU-   -    CU    CC
    70        60         50         40

-----------------------------------------------------
```

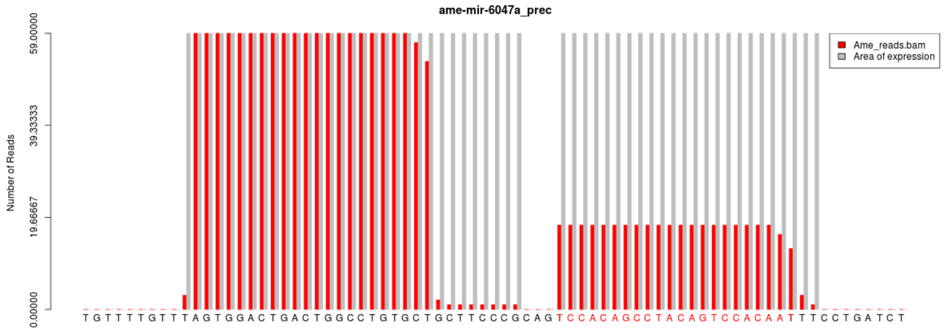

>ame-mir-6047b_prec
ATCAGGAAATTGTGGACTGTAGGCTGTGGACTGCGGGAAGC**AGCACAGGCCAGTCAGTCCACT**AAACAAAACA

>ame-mir-6047b_3p
AGCACAGGCCAGTCAGTCCACT

```
AUCAGGAAAUUGUGGACUGUAGGCUGUGGACUGCGGGAAGCAGCACAGGCCAGUCAGUCCACUAAACAAAACA
...........((((((((..(((((((..((((.....))))))))))).))...))))))))...........

 Initial dG =    -28.80

        10        20          30
AUCAGGAAAUU        UA-   -|    GA    GG
           GUGGACUG   GG CUGUG  CUGC  \
           CACCUGAC   CC GACAC  GACG  G
ACAAAACAAAU        UGA  G^    --    AA
  70        60        50          40

-----------------------------------------------------
```

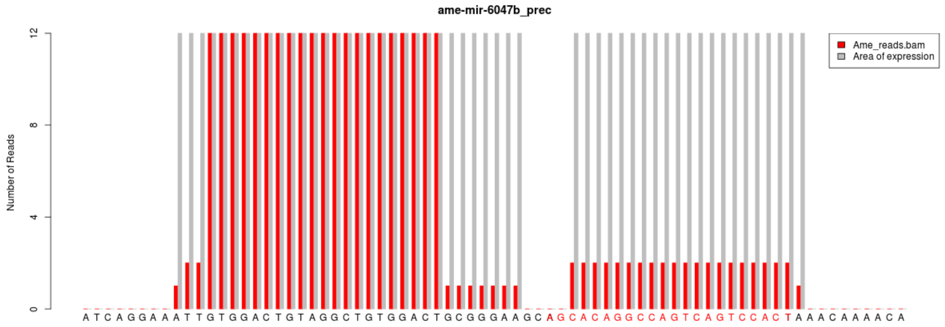

>ame-mir-6048_prec

TGTGAGGGCCAATCAGCGGTCGAACCTCGTCGATGTTAGCTCGGCGTGCCGAGGCGAACTTC**AGAGGCGAGGTGGACCGACTGGT**ATGT
GCAACA

>ame-mir-6048_3p

AGAGGCGAGGTGGACCGACTGGT

```
UGUGAGGGCCAAUCAGCGGUCGAACCUCGUCGAUGUUAGCUCGGCGUGCCGAGGCGAACUUCAGAGGCGAGGUGGACCGACUGGUAUGUGCAACA
.......((((((((((((((..((((((((..((...((((((....))))).)......))..)))))))).))))).))))).)).)).))....
```

```
 Initial dG =    -42.40

         10          20        30            40
UGUGAGG  -  -      -     GA        GA  UUA--- -|    C
       GC CA AUCAG CGGUC  ACCUCGUC  UG      G CUCGG G
       CG GU UGGUC GCCAG  UGGAGCGG  AC      C GAGCC U
ACAA---  U  A     A     G-        AG  UUCAAG G^    G
       90        80         70        60        50

-----------------------------------------------------
```

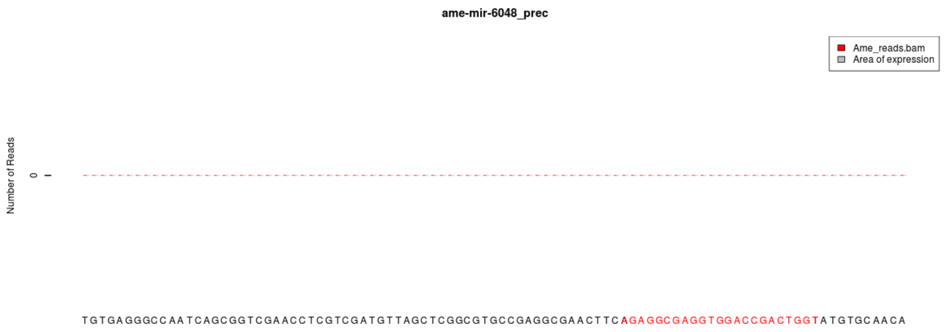

>ame-mir-6049_prec

AGGCGGAGGT**AGGAACGGAGGAATAGACGGT**GGTGCGATACCCGCTACACCACCGACGATCAGCCGTTACCACCACCGCTA

>ame-mir-6049_5p

AGGAACGGAGGAATAGACGGT

```
AGGCGGAGGUAGGAACGGAGGAAUAGACGGUGGUGCGAUACCCGCUACACCACCGACGAUCAGCCGUUACCACCACCGCUA
.(((((.(((.((((((..((.....((((((((((.....)))...))))))))....))..))))).))))).))))).

 Initial dG =    -38.80

        10         20        30
A     A   A  -     AG  AUAGA       ---|  AU
 GGCGG GGU GG AACGG  GA     CGGUGGU   GCG  \
 UCGCC CCA CC UUGCC  CU     GCCACCA   CGC  A
A     A   -  A     GA  AGCA-       CAU^  CC
80         70        60         50

-----------------------------------------------------
```

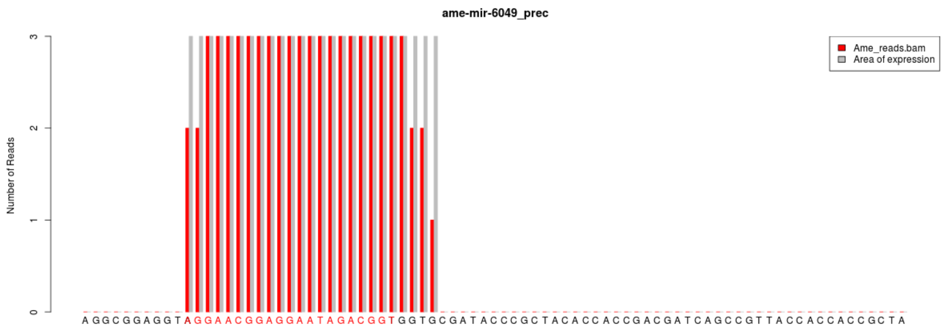

>ame-mir-6050_prec
GCGAGACTCG**GCGGGTTGTGGAGGGTGGAA**CGAAGGTAAAAGTCAAGTCTCTCTTTTATTATCCCCCAATTTTCACGAAGTACGAACAA
ACAAGTCG

>ame-mir-6050_5p
GCGGGTTGTGGAGGGTGGAA

GCGAGACUCG**GCGGGUUGUGGAGGGUGGAA**CGAAGGUAAAAGUCAAGUCUCUCUUUUAUUAUCCCCCAAUUUUCACGAAGUACGAACAAACAAGUCG
....((((...((..(((((((((.(((...((..(((((((..........)))))))..))..))).)))))))))....))........)))).

```
 Initial dG =    -21.00

        10                20         30         40
GCGA    CGG-----  GG--|         G   AAC  AG        UCAA
    GACU        CG    UUGUGGAGG UGG   GA  GUAAAAG    G
    CUGA        GC    AGCACUUUU ACC   CU  UAUUUUC    U
G---    ACAAACAA  AUGA^         A   CC-  AU        UCUC
         90        80         70          60         50

-----------------------------------------------------
```

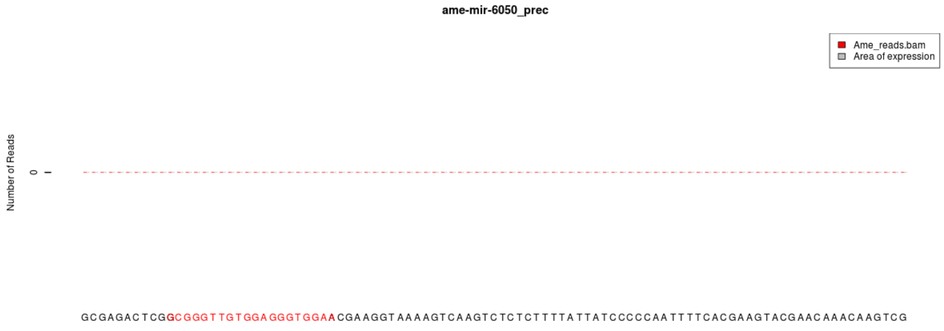

>ame-mir-6051_prec
CTTTATTTGTAATCAGAGCTTGTGTAATTTTCTCTTGATCAAG**AAAATTGCGCACGCTCTGAATAT**AATCTATAAA

>ame-mir-6051_3p
AAAATTGCGCACGCTCTGAATAT

```
CUUUAUUUGUAAUCAGAGCUUGUGUAAUUUUCUCUUGAUCAAGAAAAUUGCGCACGCUCUGAAUAUAAUCUAUAAA
......(((((.(((((((.(((((((((((((........))))))))))))).))))))).))))).........

 Initial dG =    -29.90

          10        20        30
CUUUAU--|    A       U             CUU
        UUGUA UCAGAGC UGUGUAAUUUUCU   G
        AAUAU AGUCUCG ACGCGUUAAAAGA   A
AAAUAUCU^    A       C             ACU
     70        60        50        40
```

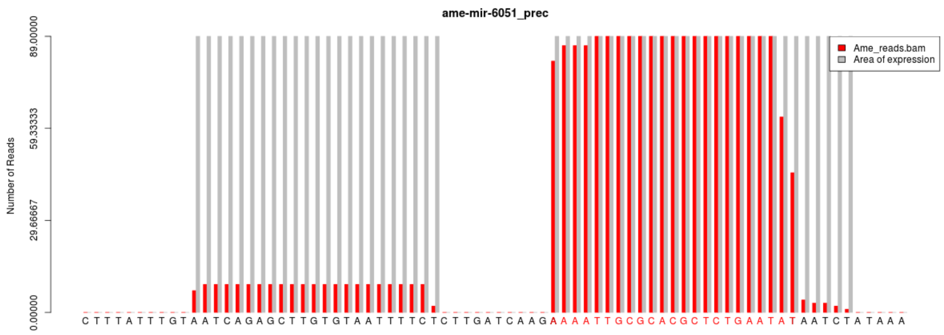

>ame-mir-6052_prec
AAAATAAATT**ATGCAAAAGCTAGAACTCATAGA**ATATAATTTCTTATACATCTATGAGCGTAGATATTTTGCTATAATAATATTG

>ame-mir-6052_5p
ATGCAAAAGCTAGAACTCATAGA

AAAAUAAAUU**AUGCAAAAGCUAGAACUCAUAGA**AUAUAAUUUCUUAUACAUCUAUGAGCGUAGAUAUUUUGCUAUAAUAAUAUUG
```
.......((((((((((.(((...((((((((.(((((....)))))..)))))))))..)))...)))))).))))).......
```

Initial dG =   -17.60

```
        10           20         30          40
AAAAUAA     -      G--|  GAA        A-     U
       AUUAU GCAAAA   CUA   CUCAUAGA  UAUAA U
       UAAUA CGUUUU   GAU   GAGUAUCU  AUAUU U
GUUAUAA     U      AUA^  GC-        AC     C
    80        70        60          50
```

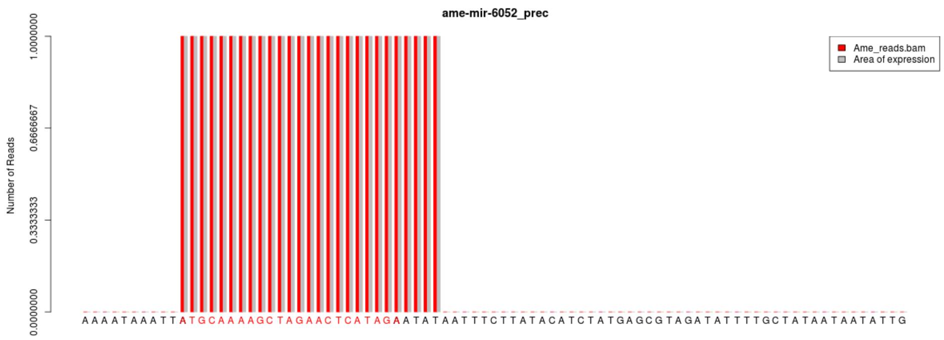

>ame-mir-6053_prec
AATCGTCTCA**ATACGAAAGACCGCGCGGATGT**GTCGTTAGTGTGCACGTGTTCTTTACGCTCGCGCAGTGTTTCGTAAAGAGAAGAGG

>ame-mir-6053_5p
ATACGAAAGACCGCGCGGATGT

```
AAUCGUCUCAAUACGAAAGACCGCGCGGAUGUGUCGUUAGUGUGCACGUGUUCUUUACGCUCGCGCAGUGUUUCGUAAAGAGAAGAGG
..((.((((..((((((.((.(((((..((((......((....))........))))..))))).)).)))))))..)))).))..

 Initial dG =    -31.20

        10        20        30          40
AA  G    AA       G  C     GA    UCGUUA--| G
  UC UCUC  UACGAAA AC GCGCG  UGUG        GU U
  AG AGAG  AUGCUUU UG CGCGC  GCAU        CA G
GG  A    AA       G  A     UC    UUCUUGUG^ C
       80        70        60        50

-----------------------------------------------------
```

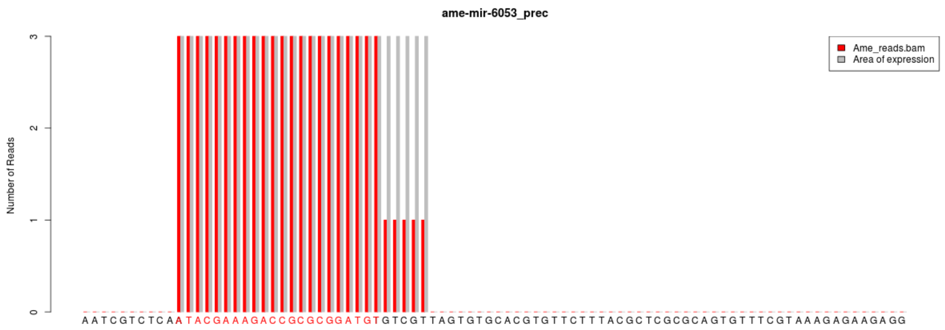

>ame-mir-6054_prec
TGTCGTGGCACGGATTCGAGCGCGTCACAGCGTTGAACCTGTCGCGAAACAATCTGG**CACGGTTGAACGATGTGACGGT**GGGCCGCGCG

>ame-mir-6054_3p
CACGGTTGAACGATGTGACGGT

```
UGUCGUGGCACGGAUUCGAGCGCGUCACAGCGUUGAACCUGUCGCGAAACAAUCUGGCACGGUUGAACGAUGUGACGGUGGGCCGCGCG
.(.((((((.((....)).((.(((((((.((((.(((((((((.((.....))))))).)))).)))).)))))))))..))))))).
```

```
 Initial dG =    -38.50

          10
U U      .-A| G
 G CGUGGC   CG A
 C GCGCCG   GC U
G -      \ -^ U
```

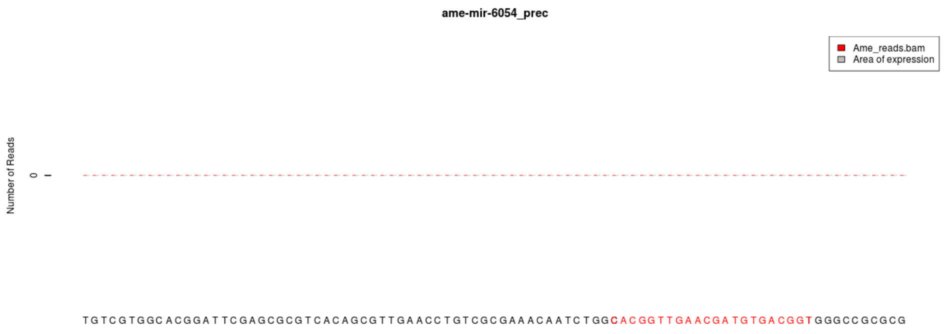

>ame-mir-6055_prec
AACGGAGAGA**AAGACGAATAGCTGTGTGTCGC**GACGGAGTGGAAAGGGACGCGCGGCTATTCCTCTCTTTCTCCCTCC

>ame-mir-6055_5p
AAGACGAATAGCTGTGTGTCGC

```
AACGGAGAGAAAGACGAAUAGCUGUGUGUCGCGACGGAGUGGAAAGGGACGCGCGGCUAUUCCUCUCUUUCUCCCUCC
...(((((((.(((.((((((((((((((((................))))))))))))))))).))).))))))).....

 Initial dG =    -36.50

         10        20        30
AAC-|      A   C               GCGACGGA
    GGAGAGA AGA GAAUAGCUGUGUGUC        \
    CCUCUUU UCU CUUAUCGGCGCGCAG        G
CCUC^      C   C               GGAAAGGU
       70        60        50        40

-----------------------------------------------------
```

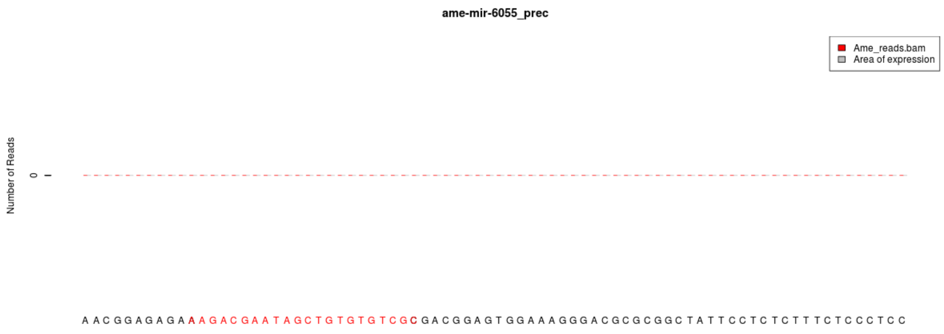

>ame-mir-6056_prec
AAGAAACGTAA**GAGGGACGAGGAGGGAGGTT**TAACGAGGGAAGTTGGCGATCGCTCGTTTCGTTCATTCACGGGGATG

>ame-mir-6056_5p
GAGGGACGAGGAGGGAGGTT

```
AAGAAACGUAAGAGGGACGAGGAGGGAGGUUUAACGAGGGAAGUUGGCGAUCGCUCGUUUCGUUCAUUCACGGGGAUG
......(((..((((((((((((.((...(((((.......)))))...)).)))..))))))).))))))......

 Initial dG =    -18.70

        10          20           30
AAGAAA   AA   -       --|  G  GGU     GAG
      CGU  GAG GGACGAG  GAG GA   UUAAC   \
      GCA  CUU CUUGCUU  CUC CU   GGUUG   G
GUAGGG   --   A       UG^  G  AGC     AAG
       70          60        50        40

-----------------------------------------------------
```

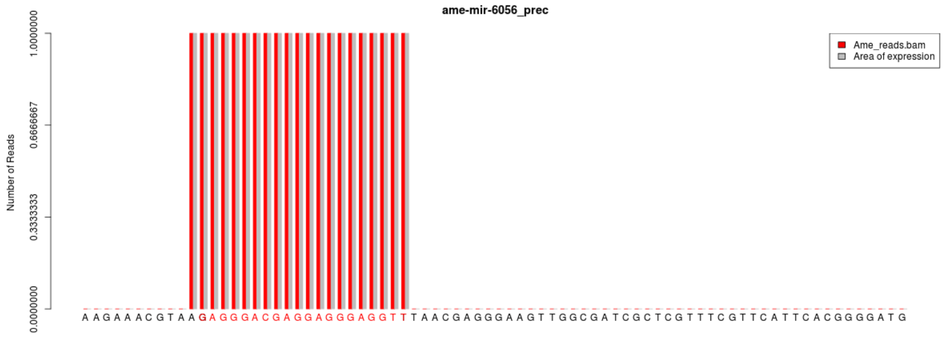

>ame-mir-6057_prec

GATATTTCGA**TTTGTGACTGTAACAACAAATG**GAAAAAAGACATATTTGTTGTTATGTTTGCAAATTAAGCTATC

>ame-mir-6057_5p

TTTGTGACTGTAACAACAAATG

```
GAUAUUUCGAUUUGUGACUGUAACAACAAAUGGAAAAAAGACAUAUUUGUUGUUAUGUUUGCAAAUUAAGCUAUC
((((.((..(((((..(..(((((((((((((...........)))))))))))))..)..))))).)).))))

 Initial dG =     -22.50

        10        20        30
|   U  CG     UG CU              GAAAA
GAUA UU  AUUUG  A  GUAACAACAAAUG     \
CUAU GA  UAAAC  U  UAUUGUUGUUUAU     A
^   C  AU     GU UG              ACAGA
    70        60        50        40

-----------------------------------------------------
```

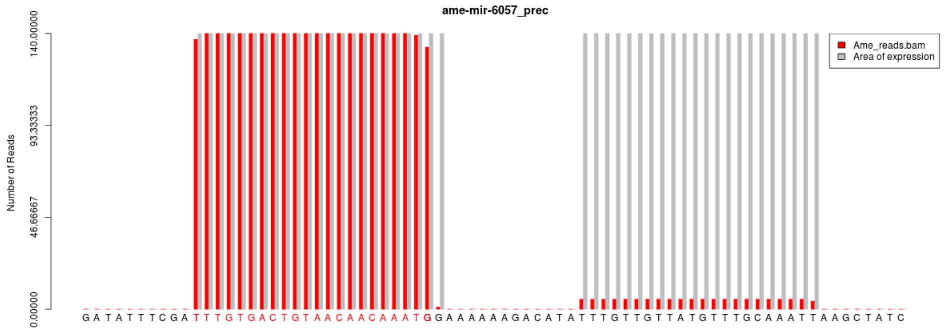

>ame-mir-6058_prec
AGTTGCTGTG**ATGACAATGGTGATGACAACG**TGGATAGAAACGTTAACGTCAACACCAATGCCAACAATAACGACTA

>ame-mir-6058_5p
ATGACAATGGTGATGACAACG

```
AGUUGCUGUGAUGACAAUGGUGAUGACAACGUGGAUAGAAACGUUAACGUCAACACCAAUGCCAACAAUAACGACUA
(((((...((.((.((.(((((.(((((((((........)))))...))))).))))).)).)).))....))))).

 Initial dG =    -23.10

          10         20            30
-     CUG-  A  A  A     A    ---|    GGA
 AGUUG    UG UG CA UGGUG UGAC   AACGU   U
 UCAGC    AC AC GU ACCAC ACUG   UUGCA   A
A     AAUA  A  C  A     A    CAA^    AAG
      70         60         50         40

-----------------------------------------------------
```

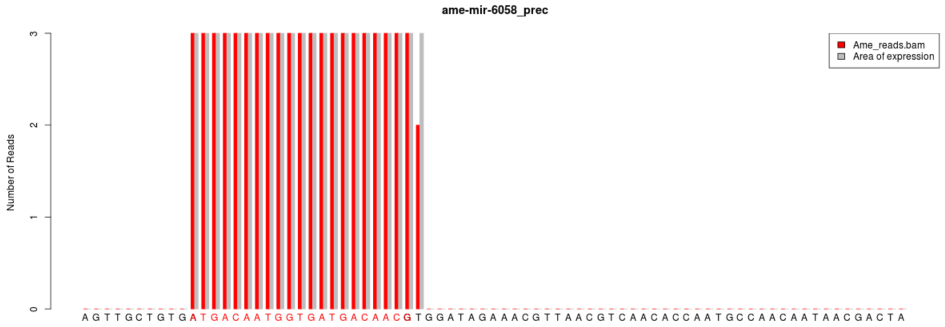

>ame-mir-6059_prec
CGTTTCTGTTCTGTCGTTTCTGCCGAGACTGTCTGG**TAGAGGGAGAGACGGAACCA**CAAAGGAACA

>ame-mir-6059_3p
TAGAGGGAGAGACGGAACCA

```
CGUUUCUGUUCUGUCGUUUCUGCCGAGACUGUCUGGUAGAGGGAGAGACGGAACCACAAAGGAACA
.((((((((((((((..(((((((.((.....)))))))))).....)))))))).....)))))).
```

```
 Initial dG =    -24.60

             10           20
C      -----        GU---|      G  AC
 GUUUCU     GUUCUGUC     UUCUGCC AG  \
 CAAGGA     CAAGGCAG     GAGAUGG UC  U
A      AACAC        AGAGG^      -  UG
     60        50        40
```

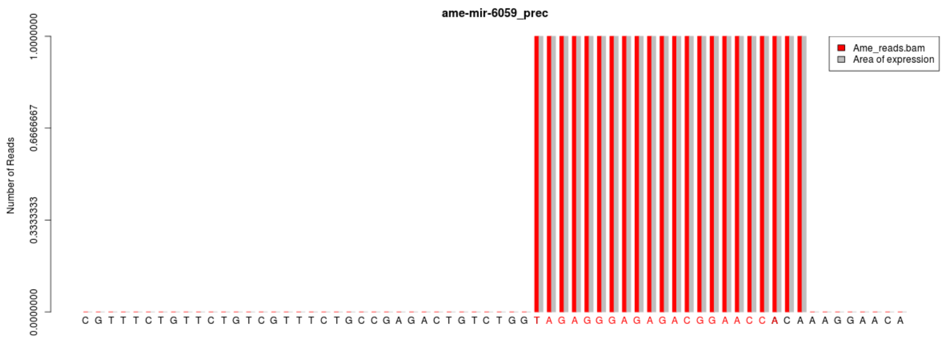

>ame-mir-6060_prec
AGTAAGGAGC**ACGGGTACGACGTTGGCAACGT**GAACAACCGCGTTGCCCCCTTGTCCCCGAGATCCAAACC

>ame-mir-6060_5p
ACGGGTACGACGTTGGCAACGT

```
AGUAAGGAGCACGGGUACGACGUUGGCAACGUGAACAACCGCGUUGCCCCCUUGUCCCCGAGAUCCAAACC
.....(((...((((.((((....(((((((((......)))))))))...)))).))))...))).....

 Initial dG =    -29.70

        10        20        30
AGUAA|  GCA    U    CGUU         AA
     GGA   CGGG ACGA    GGCAACGUG  C
     CCU   GCCC UGUU    CCGUUGCGC  A
CCAAA^  AGA    C    CCC-         CA
70        60        50         40

-----------------------------------------------------
```

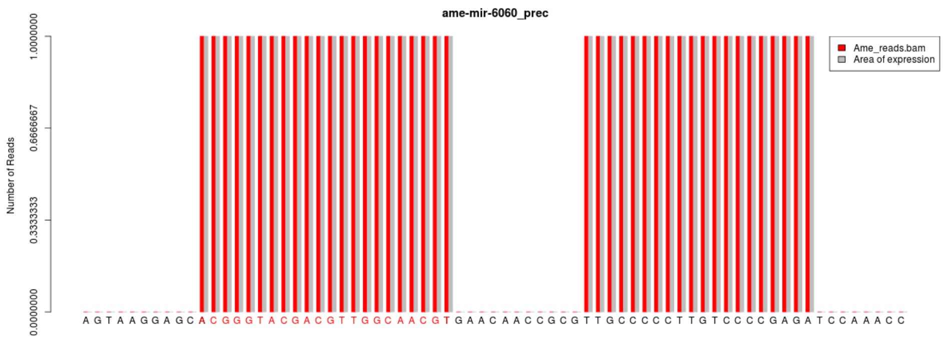

>ame-mir-6061_prec
TTGTCATCGGACGTGGTACATGCCGAACGTTCGTTCTCGTAATAAAT**GACGTTGCACGTTACCACGCCG**GATGTCACTC

>ame-mir-6061_3p
GACGTTGCACGTTACCACGCCG

```
UUGUCAUCGGACGUGGUACAUGCCGAACGUUCGUUCUCGUAAUAAAUGACGUUGCACGUUACCACGCCGGAUGUCACUC
.((.((((((.((((((((.(((..(((((.((((.........)))))))))))).).))))))))))).))).))...
```

```
 Initial dG =    -26.90

           10         20        30
U--  U   -|  A       - A   CG     U    CUCG
   UG CAU CGG CGUGGUA C UGC  AACGU CGUU    \
   AC GUA GCC GCACCAU G ACG  UUGCA GUAA    U
CUC  U   G^  -       U C   --     -    AUAA
        70         60          50
```

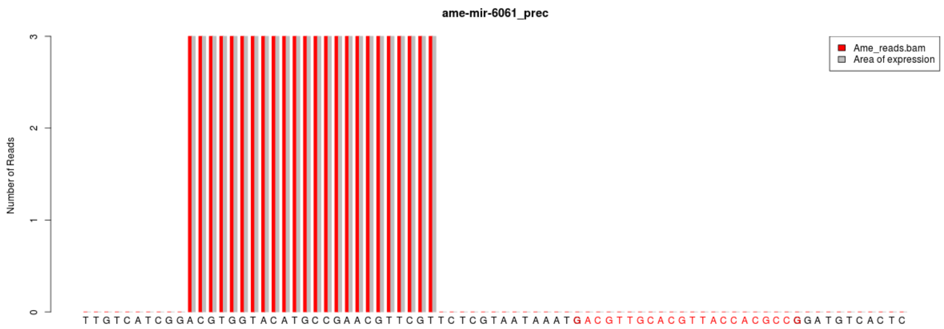

>ame-mir-6062_prec
CGTATACGTTCGTATAAATACCGATATTTGTATATATATTCGTACATA**TACGATATTGGTATTTAGGACA**AACGTAATTT

>ame-mir-6062_3p
TACGATATTGGTATTTAGGACA

CGUAUACGUUCGUAUAAAUACCGAUAUUUGUAUAUAUAUUCGUACAUA**UACGAUAUUGGUAUUUAGGACA**AACGUAAUUU
```
....((((((.((.((((((((((((((.((((((.((....)).))))))))))))))))))))..)).)))))).....
```

```
 Initial dG =    -26.50

        10         20        30        40
CGUA      C  A-|             U      A  U
    UACGUU GU  UAAAUACCGAUAUU GUAUAU UA U
    AUGCAA CA  AUUUAUGGUUAUAG CAUAUA AU C
UUUA      A  GG^             -      C  G
.        70        60         50

-----------------------------------------------------
```

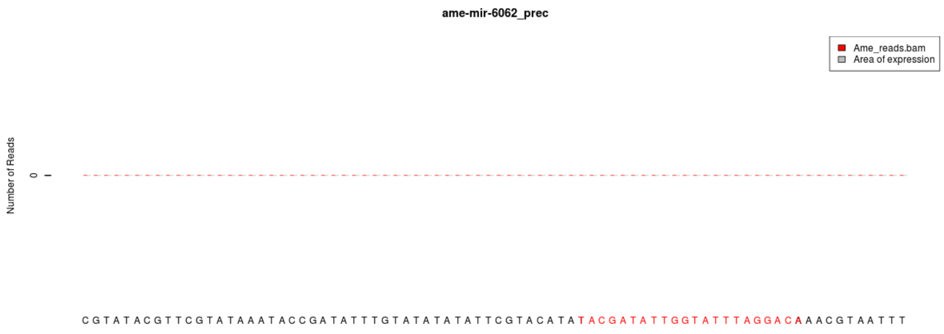

>ame-mir-6063_prec

GCGAAACGCACCGGTGTCTATCGATCGTTGGATTCGCAGCGGCGATGGATCG**ATCGATCGATCGAAGACGCGGT**GAAAAATCTT

>ame-mir-6063_3p

ATCGATCGATCGAAGACGCGGT

```
GCGAAACGCACCGGUGUCUAUCGAUCGUUGGAUUCGCAGCGGCGAUGGAUCGAUCGAUCGAUCGAAGACGCGGUGAAAAAUCUU
..((....((((.((((((((((((((((((..((((....))))....)))).)))))))))...)))))))))).....))..

 Initial dG =    -31.20

         10           20         30
GC  AACG-     -  .-G|      A      GA
  GA     CACCG GU   UCUAUCG UCGUUG  U
  CU     GUGGC CA   AGGUAGC GGCGAC  U
UU  AAAAA     G  \ -^      -      GC
   80        70               40

-----------------------------------------------------
```

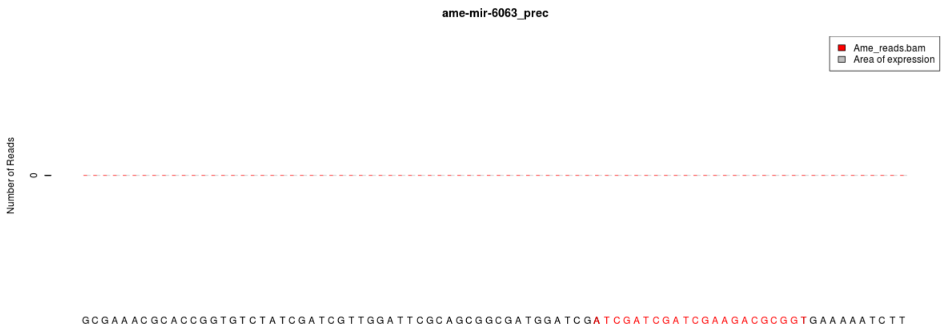

>ame-mir-6064_prec
GAAATGTTCT**TTTGAAATTTTGCAACGAGACG**ATATTAATCCACGTTGCAATGTCTCGTCGCAAAATTCCACGAGGAATCGTTCT

>ame-mir-6064_5p
TTTGAAATTTTGCAACGAGACG

```
GAAAUGUUCUUUUGAAAUUUUGCAACGAGACGAUAUUAAUCCACGUUGCAAUGUCUCGUCGCAAAAUUCCACGAGGAAUCGUUCU
(((..((((((((.(((((((.((((((((....((((....))))...)))))))).)))))))).)).)))))))..))).

 Initial dG =    -26.70

         10         20         30         40
-   AU         -| A         A         AUAU    C
 GAA  GUUCUUU UG AAUUUUGC ACGAGACG    UAAU C
 CUU  UAAGGAG AC UUAAAACG UGCUCUGU    GUUG A
U   GC         C^ C         C         AAC-    C
    80         70         60         50

-----------------------------------------------------
```

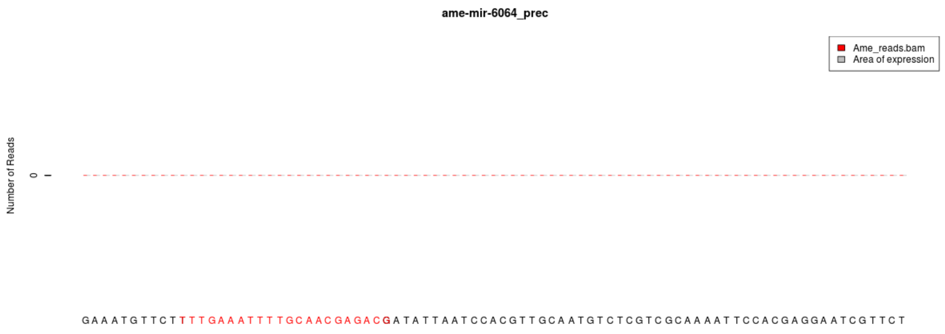

>ame-mir-6065_prec
GTGCGTCAGTGCGGGGGGAACCGCATTCCAGAGTGGGCGGCATC**CTGGAATGCGATCCCCCCGGTA**ACAAGAATTC

>ame-mir-6065_3p
CTGGAATGCGATCCCCCCGGTA

```
GUGCGUCAGUGCGGGGGGAACCGCAUUCCAGAGUGGGCGGCAUCCUGGAAUGCGAUCCCCCCGGUAACAAGAAUUC
...........((((((((..((((((((((..((.....))..)))))))))))).))))))))............

 Initial dG =    -38.10

        10          20        30
GUGCGUCAGUG--|       AC          AG  GG
             CGGGGGGA  CGCAUUCCAG  UG  \
             GCCCCCCU  GCGUAAGGUC  AC  C
CUUAAGAACAAUG^       A-          CU  GG
     70        60         50        40
```

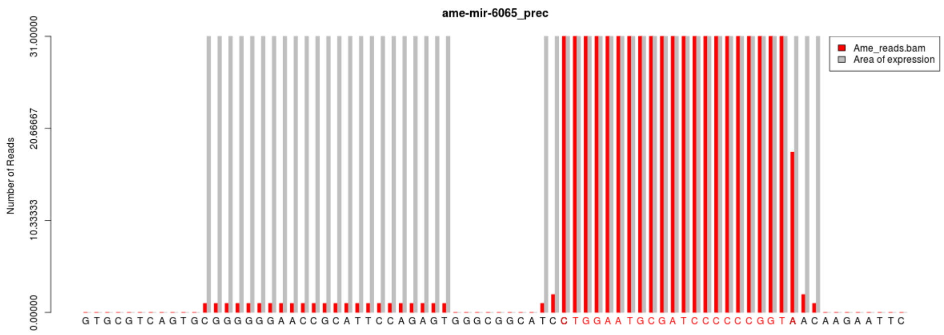

>ame-mir-6066_prec
GCCTCCCGAG**AGGGAGAGGCCTCGTGACCACG**GGGACCTTAATAAAACACTCGTGCACACGCCCTCTTCTCTCTCGTCCTCTCG

>ame-mir-6066_5p
AGGGAGAGGCCTCGTGACCACG

```
GCCUCCCGAGAGGGAGAGGCCUCGUGACCACGGGGACCUUAAUAAAACACUCGUGCACACGCCCUCUUCUCUCUCGUCCUCUCG
......((((((((((((...((((..((((((...............))))))..)))).))))))).))))))........

 Initial dG =    -32.50

          10         20        30        40
GCCUCC--      -|      CCU    AC       GACCUUA
        CGAGAG GGAGAGG   CGUG  CACGGG       \
        GCUCUC CUUCUCC   GCAC  GUGCUC       A
GCUCUCCU      U^      C--    AC       ACAAAAU
   80        70          60        50

-----------------------------------------------------
```

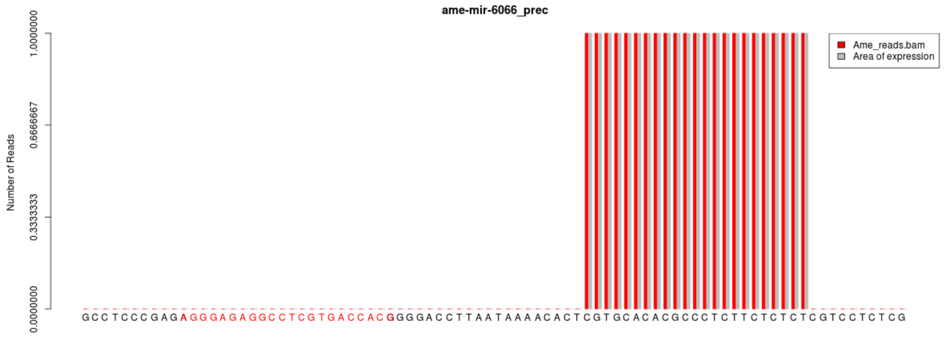

>ame-mir-6067_prec
TTCTGTCGTC**AAACGGATCAAGCTTTTTGTGA**GACTTGGATCGGTCACAAAAAGCGGGATTCGCTTTTCGCATAGGAA

>ame-mir-6067_5p
AAACGGATCAAGCTTTTTGTGA

```
UUCUGUCGUCAAACGGAUCAAGCUUUUUGUGAGACUUGGAUCGGUCACAAAAAGCGGGAUUCGCUUUUCGCAUAGGAA
(((((((..((.((((((..((((((((((((((......))..))))))))))))..)))))).))..)).)))))).

 Initial dG =    -28.40

          10        20        30
-      -  UC  A      AA           --| CU
 UUCUGU CG  AA CGGAUC  GCUUUUUGUGA  GA  U
 AGGAUA GC  UU GCUUAG  CGAAAAACACU  CU  G
A      C  UU  C      GG           GG^ AG
       70        60        50        40

-----------------------------------------------------
```

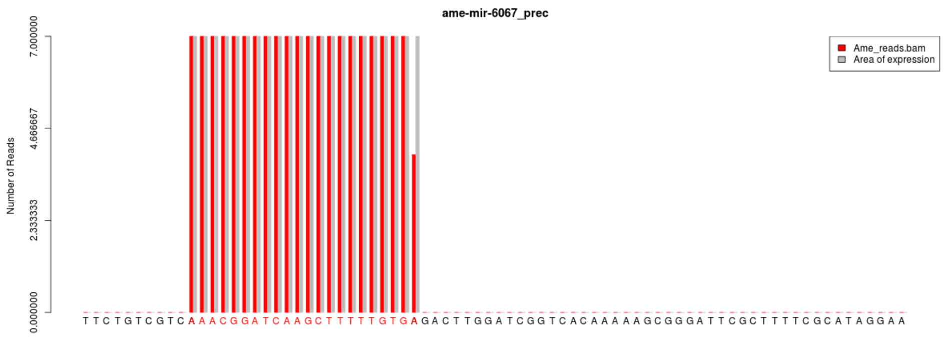

>ame-mir-7_prec
CGAGCGCCGTTGCA**TGGAAGACTAGTGATTTTGTTGT**TCTACTTTCGATATAACAAGGAATCACTAATCATCCTACAAAGGCGCTCG

>ame-mir-7_5p
TGGAAGACTAGTGATTTTGTTGT

```
CGAGCGCCGUUGCAUGGAAGACUAGUGAUUUUGUUGUUCUACUUUCGAUAUAACAAGGAAUCACUAAUCAUCCUACAAAGGCGCUCG
(((((((.(((...(((.((.((((((((((((((((.......)).)))))))..))))))))).)).))).)..))).))))))))

 Initial dG =    -37.20

        10        20        30            40
        G   CAU   A  C         --       -| UAC
CGAGCGCC UUG   GGA GA UAGUGAUUU  UGUUGU UC   \
GCUCGCGG AAC   CCU CU AUCACUAAG  ACAAUA AG   U
        A   AU-   A  A         GA       U^ CUU
      80         70        60        50
```

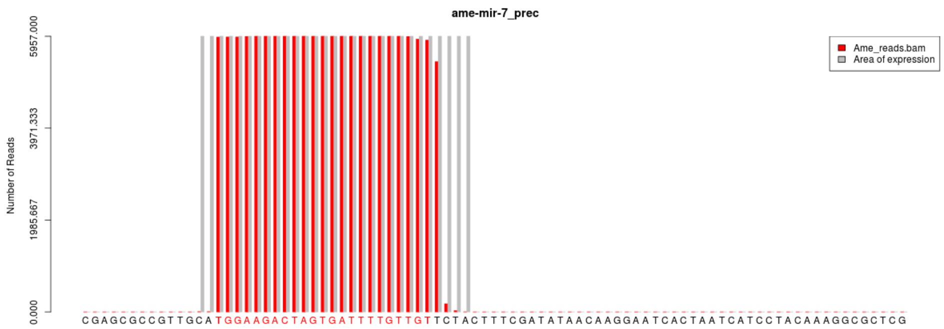

>ame-mir-71_prec
GTCCTCCTTCGGGCGGATTCCGTC**TGAAAGACATGGGTAGTGA**GATGTTCTCACGCTATCGCGTCTCACTATCTTGTCTTTCATCCGGC
GTTCGTTCTGC

>ame-mir-71_5p
TGAAAGACATGGGTAGTGA

```
GUCCUCCUUCGGGCGGAUUCCGUCUGAAAGACAUGGGUAGUGAGAUGUUCUCACGCUAUCGCGUCUCACUAUCUUGUCUUUCAUCCGGCGUUCGUUCUGC
((((.((....)).)))).(((..(((((((((.((((((((((((((............)))))))))))))))))))))))..)))............
```

```
 Initial dG =    -46.80

        10        20        30        40        50
GUCCUCCUUC|       U   UC         U              UCUCA
          GGGCGGAU CCG  UGAAAGACA GGGUAGUGAGAUGU     C
          CUUGCUUG GGC  ACUUUCUGU UCUAUCACUCUGCG     G
CGU-------^       C   CU         -              CUAUC
.               90        80         70        60

-----------------------------------------------------
```

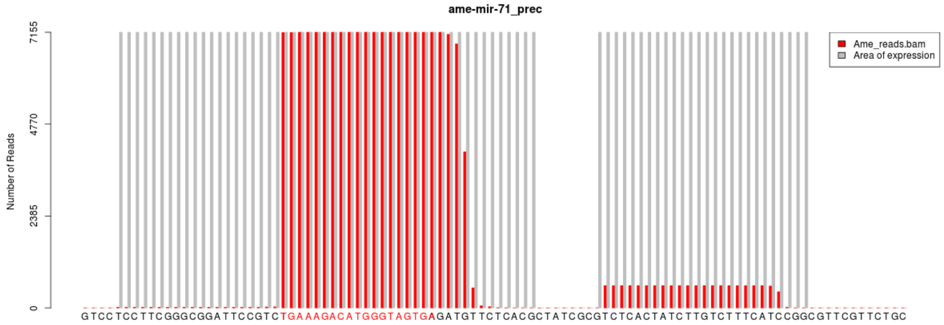

>ame-mir-750_prec
GGGCGTTGCATGGACTGTGATCTGCATGACTTGCAGTTGGAAGTGAGGATCTAGGCAGTGAGCAGCAGCGCTCTGATAACTTG**CCAGAT**
CTAACTCTTCCAGCTCAAGGTCTTGTTGATTATCATCTCCCAAACGA

>ame-mir-750_3p
CCAGATCTAACTCTTCCAGCTC

GGGCGUUGCAUGGACUGUGAUCUGCAUGACUUGCAGUUGGAAGUGAGGAUCUAGGCAGUGAGCAGCAGCGCUCUGAUAACUUG**CCAGAUCUAACUCUUCCAGCUC**AAGGUCUUGUUGAU
UAUCAUCUCCCAAACGA

```
...((((....(((..((((((.(((.(((((..(((((((((...((((((.
(((((((((......)))).....))).)))))))))....)))))))))..))))).))).))))))..)))....)))).
```

Initial dG =   -51.20

```
        10        20        30        40         50              60
GGG    GCAU   CU      U   U     GC         UGA-      A   -   -----|   AG
   CGUU    GGA  GUGAUC GCA GACUU  AGUUGGAAG    GGAUCU GGC AGU     GAGC  C
   GCAA    UCU  UAUUAG UGU CUGGA  UCGACCUUC    UCUAGA CCG UCA     CUCG  A
A--    ACCC   AC      U   U     AC         UCAA      -   U   AUAGU^   CG
      130       120       110       100        90         80        70
```

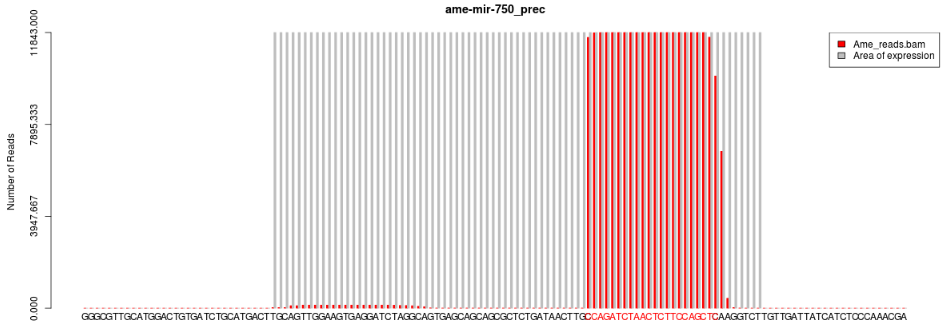

>ame-mir-79_prec
TTCAAGACTGTCAAAGCTTTTGCCTTTACTTTGGTAATATAGCTCTATGATTTTAATTAAGGATCA**TAAAGCTAGATTACCAAAGCA**AA
GAGCACTAGCA

>ame-mir-79_3p
TAAAGCTAGATTACCAAAGCA

```
UUCAAGACUGUCAAAGCUUUUGCCUUUACUUUGGUAAUAUAGCUCUAUGAUUUUAAUUAAGGAUCAUAAAGCUAGAUUACCAAAGCAAAGAGCACUAGCA
..............(((..(((((((.((((((((((.(((((.(((((((((.....))))))))).))))).)))))))))).)))).))).)))..))).
```

```
 Initial dG =    -39.20

        10        20          30        40        50
UUCAAGACUGUCAAA   UU   -|   A          A     C         AA
               GCU  UGC CUUU CUUUGGUAAU UAGCU UAUGAUUUU  \
               CGA  ACG GAAA GAAACCAUUA AUCGA AUACUAGGA  U
A--------------   UC   A^   C          G     A         AU
.                      90        80        70        60

-----------------------------------------------------
```

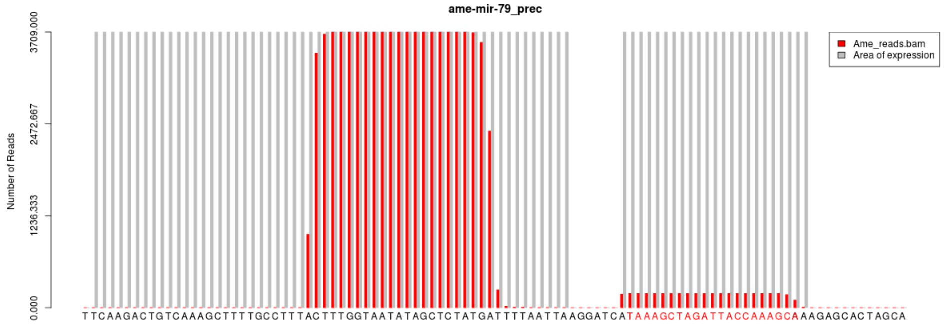

>ame-mir-8_prec

GGAGTATCTGTTCACATCTTACCGGGCAGCATTAGATTGAAGTTGACCTTC**TAATACTGTCAGGTAAAGATGTC**GTCAGGATTCC

>ame-mir-8_3p

TAATACTGTCAGGTAAAGATGTC

GGAGUAUCUGUUCACAUCUUACCGGGCAGCAUUAGAUUGAAGUUGACCUUC**UAAUACUGUCAGGUAAAGAUGUC**GUCAGGAUUCC

```
(((((.((((...(((((((((.(((((.((((((.............)))))).))))).))).)))))))...)))))))))

 Initial dG =    -31.50

        10        20         30        40
|    A    UUC       -   G     C      UUGAAG
GGAGU UCUG   ACAUCUU ACC GGCAG AUUAGA      \
CCUUA GGAC   UGUAGAA UGG CUGUC UAAUCU      U
^    -    UGC       A   A     A      UCCAGU
     80        70        60        50

-----------------------------------------------------
```

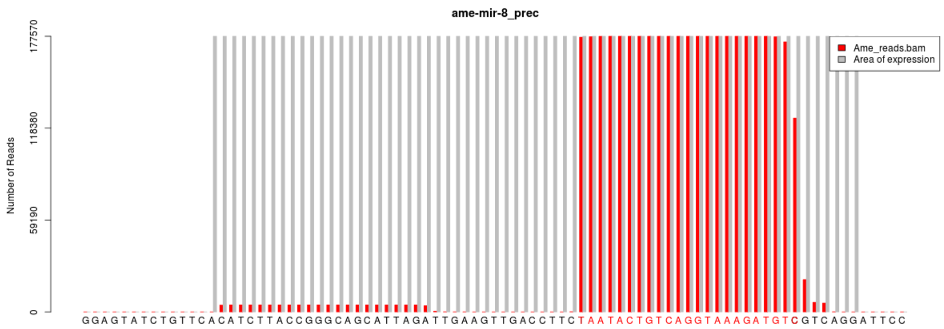

>ame-mir-87-1_prec
ACAGTACTCTTACGCCTGAAATTTGCTTTTTACCTGGCCCTCGTGCTCTCTTACTTTGAGAAAATAAAG**GTGAGCAAAGTTTCAGGTGT**
GTGAGTGCGTT

>ame-mir-87-1_3p
GTGAGCAAAGTTTCAGGTGT

```
ACAGUACUCUUACGCCUGAAAUUUGCUUUUUACCUGGCCCUCGUGCUCUCUUACUUUGAGAAAAUAAAGGUGAGCAAAGUUUCAGGUGUGUGAGUGCGUU
...((((((.((((((((((((((((((...((((.((......))((((.......))))......))))))))))).))))))))))).))))))...

 Initial dG =    -39.20

       10        20         30          40
ACA      U          -       UUU    .-G| CC
   GUACUC UACGCCUGAAA UUUGCUU   ACCU   GC  U
   CGUGAG GUGUGGACUUU AAACGAG   UGGA   CG  C
UUG      U          G       ---    \ -^ UG
.        90        80           70

-----------------------------------------------------
```

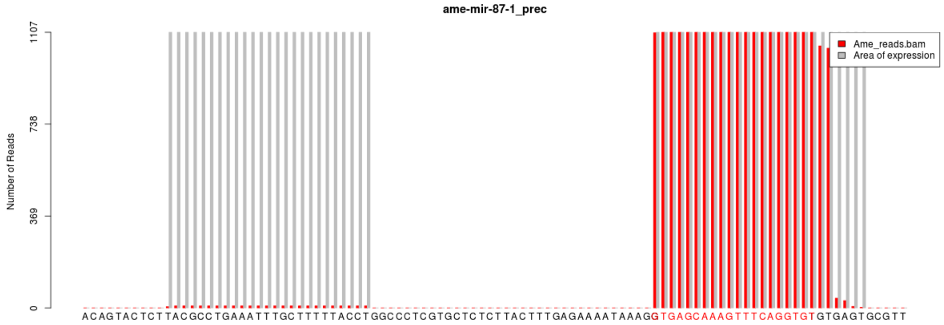

>ame-mir-87-2_prec
TATAAAGCATCCGAACGTTACGGGCGGGCCTGACTCTTTGCTCTGCCCGTATCTTTTTTTCCACTCTGG**GTGAGCAAAGTTTCAGGTGT**
GTCCGTGACAG

>ame-mir-87-2_3p
GTGAGCAAAGTTTCAGGTGT

```
UAUAAAGCAUCCGAACGUUACGGGCGGGCCUGACUCUUUGCUCUGCCCGUAUCUUUUUUUCCACUCUGGGUGAGCAAAGUUUCAGGUGUGUCCGUGACAG
................(((((((((.((((((..((((((((.(((((................)))))))))))))..)))))).))))))))))..
```

```
 Initial dG =    -47.00

        10        20        30        40        50
UAUAAAGCAUCCGAAC|         G      CU         U     UAUCUUUU
                GUUACGGGCG GCCUGA  CUUUGCUC GCCCG        \
                CAGUGCCUGU UGGACU  GAAACGAG UGGGU        U
GA--------------^         G      UU         -     CUCACCUU
.                      90        80          70        60

-----------------------------------------------------
```

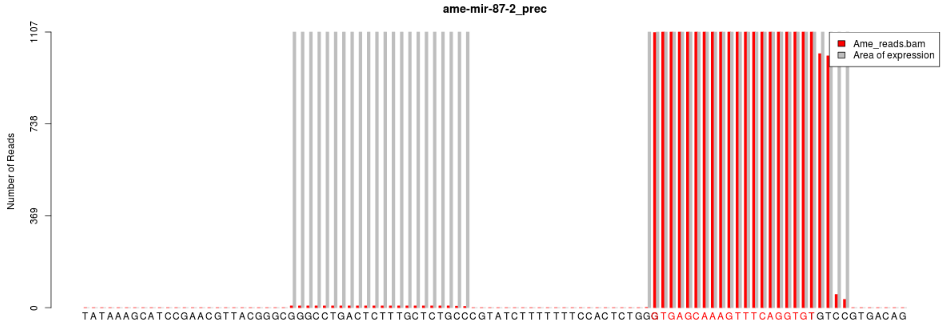

>ame-mir-927a_prec
AGATAAAAGCGTGGTATTTG**TTTTAGAATTCCTACGCTTTACC**GATGTTCGAAGTGGCAAAGCGTTTGAAATCTGAAACGAATGCGCAT
AAACCTTCATC

>ame-mir-927a_5p
TTTTAGAATTCCTACGCTTTACC

```
AGAUAAAAGCGUGGUAUUUGUUUUAGAAUUCCUACGCUUUACCGAUGUUCGAAGUGGCAAAGCGUUUGAAAUCUGAAACGAAUGCGCAUAAACCUUCAUC
..........((((((((((((((((.(((..((((((((.(((..........))).))))))))..))).)))))))))))))))).)))...........
```

Initial dG = -32.20

```
       10          20        30        40
AGAUAAAAGC-   -|             A   CU       A   AUGU
           GUG GUAUUUGUUUUAGA UUC  ACGCUUU CCG    U
           UAC CGUAAGCAAAGUCU AAG  UGCGAAA GGU    C
CUACUUCCAAA   G^             A   UU       C   GAAG
.        90        80        70        60        50
```

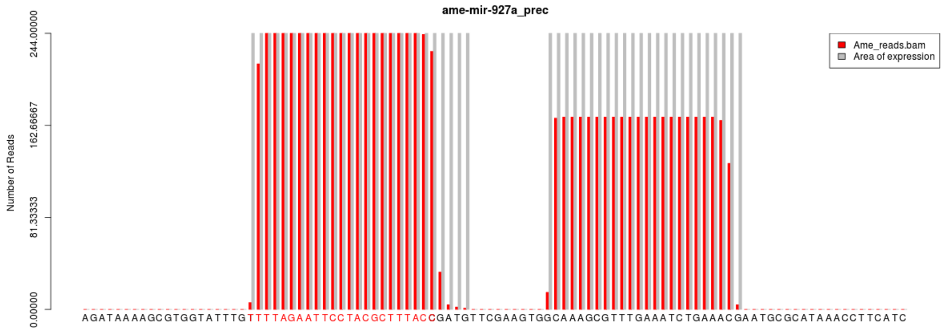

>ame-mir-927b_prec
TTCGACAGTGTGTTACGTTATTTA**TTTTAGAATTTGTACGCTCTGT**TGTCACAAATGATTTCACAGAGCGTACCGATTCTTTAATAAGT
GTCGTCAACAATTATCAACCCTTGAGG

>ame-mir-927b_5p
TTTTAGAATTTGTACGCTCTGT

```
UUCGACAGUGUGUUACGUUAUUUAUUUUAGAAUUUGUACGCUCUGUUGUCACAAAUGAUUUCACAGAGCGUACCGAUUCUUUAAUAAGUGUCGUCAACAAUUAUCAACCCUUGAGG
...((.(((.(((((((.((((((((..((((((.(((((((((((.((((....))))...))))))))))).))))))..)))))))).))).))))))).))..........
```

Initial dG =   -39.50

```
                10         20        30        40           50
UUC--------  C   G    -   U        UU      U           U--|   C
           GA AGU UGUU ACG UAUUUAUU  AGAAUU GUACGCUCUGU   GUCA A
           CU UUA ACAA UGC GUGAAUAA  UCUUAG CAUGCGAGACA   UAGU A
GGAGUUCCCAA  A   -    C   U        UU      C           CUU^   A
    110       100         90        80        70        60
```

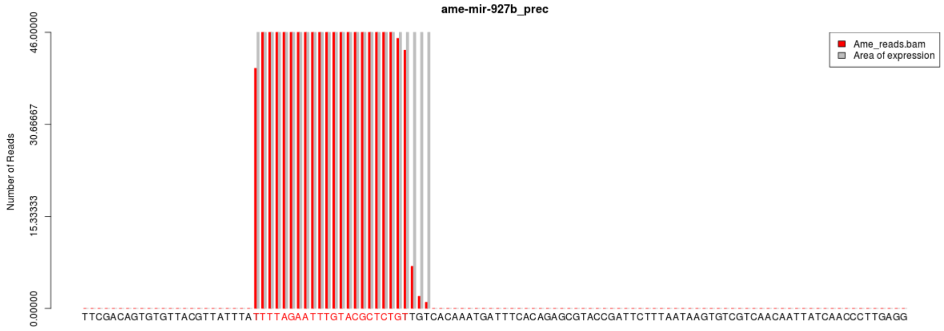

>ame-mir-928_prec
GTCCAGTATTC**CTGGCTGTGGAAGCTGGCGAA**CGTCACCCAGTCCCCGTACAGTTACCTGTGGACCAATACCAGCACCGCGCCTGAATT
AGTTGCGGGAC

>ame-mir-928_5p
CTGGCTGTGGAAGCTGGCGAA

```
GUCCAGUAUUCCUGGCUGUGGAAGCUGGCGAACGUCACCCAGUCCCCGUACAGUUACCUGUGGACCAAUACCAGCACCGCGCCUGAAUUAGUUGCGGGAC
((((.((((((..(((.((((..(((((.(.....).....((((....((((....)))))))).....))))).))))))).)))).....)).))))

 Initial dG =    -30.30

             10        20          30
    A  -----    CU   U    AA      .-C|AA
GUCC GU     AUUC  GGC GUGG  GCUGG    G  \
CAGG CG     UAAG  CCG CGCC  CGACC    C  C
    G  UUGAU    U-   -    A-      \ -^UG
.        90          80


                                   40        50
                                 ACCCA    CCGU    U
                                      GUCC    ACAG U
                                      CAGG    UGUC A
                                 AUAAC    ----    C
                                70            60

-----------------------------------------------------
```

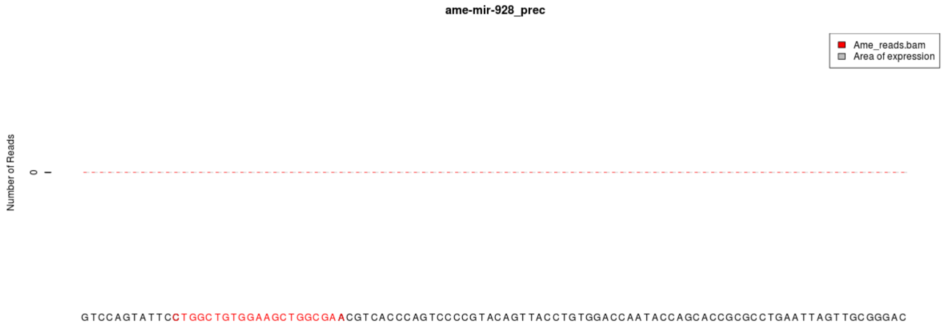

>ame-mir-929_prec
ACTTAACTGGGGTCAA**ATTGACTCTAGTAGGGAGTCC**CTGCATTCAATATGGCGACTTCCTAATAGAGTCAGGCTGACTCCTTTTAAGA
CGTTCAACGGA

>ame-mir-929_5p
ATTGACTCTAGTAGGGAGTCC

ACUUAACUGGGGUCAA**AUUGACUCUAGUAGGGAGUCC**CUGCAUUCAAUAUGGCGACUUCCUAAUAGAGUCAGGCUGACUCCUUUUAAGACGUUCAACGGA
.(((((..(((((((..((((((((.((((((((((((...........)).))))))))).)))))))))..))))))).))))).........

Initial dG = -45.90

```
                   10        20        30         40
A-----------     CU       AA         G         -| UGCAU
            CUUAA  GGGGUCA  UUGACUCUA UAGGGAGUC CC     U
            GAAUU  CCUCAGU  GACUGAGAU AUCCUUCAG GG     C
AGGCAACUUGCA     UU       CG         A         C^ UAUAA
.        90        80        70        60        50
```

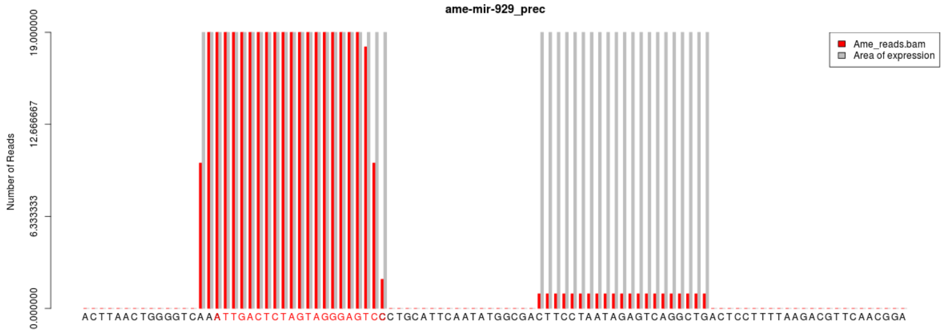

>ame-mir-92a_prec
TTATTTTGCATAGAAGATAGGCCGAGATTTGTGACAATGTTTCGTGATGATGTAATCTTCAAT**ATTGCACTTGTCCCGGCCTAT**CGGAA
TGCATTATATT

>ame-mir-92a_3p
ATTGCACTTGTCCCGGCCTAT

UUAUUUUGCAUAGAAGAUAGGCCGAGAUUUGUGACAAUGUUUCGUGAUGAUGUAAUCUUCAAU**AUUGCACUUGUCCCGGCCUAU**CGGAAUGCAUUAUAUU
.(((..(((((....(((((((((.(((..(((.(((((((....(((......)))...)))))))))).))).)))))))))...)))))..)))..

Initial dG =   -38.90

```
         10        20        30        40        50
U-|  UU     AGAA         A   UU   A       UCGU   GA
  UAU  UGCAU    GAUAGGCCG GAU  GUG CAAUGUU    GAU  U
  AUA  ACGUA    CUAUCCGGC CUG  CAC GUUAUAA    CUA  G
UU^  UU     AGG-         C   UU   -       CUU-   AU
.        90          80        70          60
```

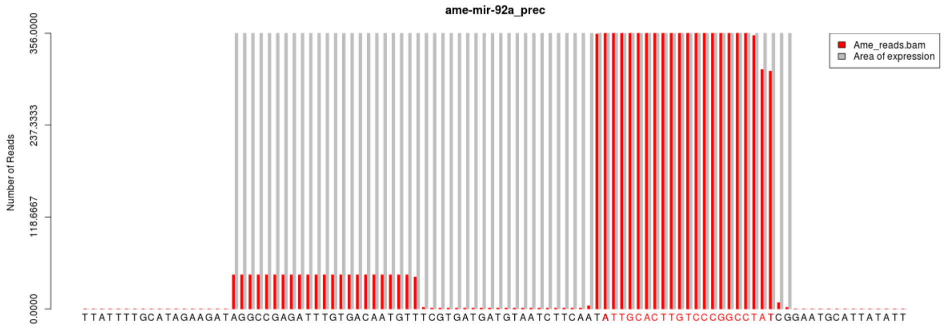

>ame-mir-92b-1_prec
CACGAAGCGTATTCAATTAGGTCAGGACTAATGCAAATATGTAGTGTCGACATCA**AATTGCACCCGTCCCGGCCTGA**TTGAGTGCGCGT
AAATC

>ame-mir-92b-1_3p
AATTGCACCCGTCCCGGCCTGA

```
CACGAAGCGUAUUCAAUUAGGUCAGGACUAAUGCAAAUAUGUAGUGUCGACAUCAAAUUGCACCCGUCCCGGCCUGAUUGAGUGCGCGUAAAUC
......(((((((((((((((((.((((...(((((..((((.......))))....)))))...)))).))))))))))))))))).......
```

```
 Initial dG =    -40.40

         10        20        30          40
CACGAA-                 A    UAA     AU--|   AGU
       GCGUAUUCAAUUAGGUC GGAC   UGCAA    AUGU   \
       CGCGUGAGUUAGUCCGG CCUG   ACGUU    UACA   G
CUAAAUG                 C    CCC     AAAC^   GCU
   90        80        70        60        50
```

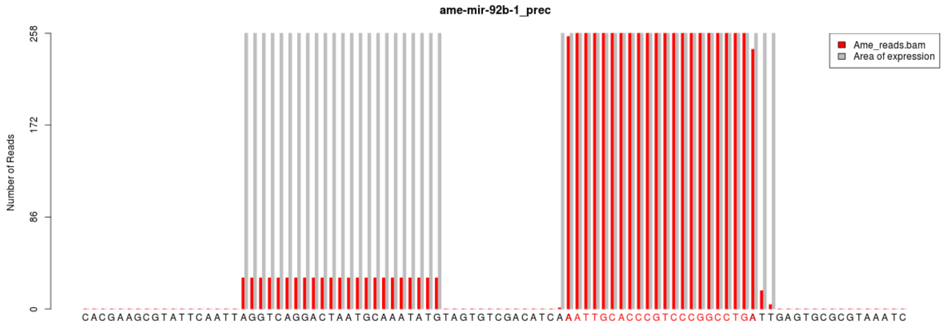

>ame-mir-92b-2_prec
CACGAAGCGTATTCAATTAGGTCAGGACTAATGCAAATATGTAGTGTCGACATCA**AATTGCACCCGTCCCGGCCTGA**TTGAGTGCGCGT
AAATC

>ame-mir-92b-2_3p
AATTGCACCCGTCCCGGCCTGA

```
CACGAAGCGUAUUCAAUUAGGUCAGGACUAAUGCAAAUAUGUAGUGUCGACAUCAAAUUGCACCCGUCCCGGCCUGAUUGAGUGCGCGUAAAUC
......((((((((((((((((.((((...(((((..((((.......))))....)))))...)))).)))))))))))))))))).......
```

```
 Initial dG =    -40.40

         10        20        30          40
CACGAA-                 A    UAA     AU--|   AGU
       GCGUAUUCAAUUAGGUC GGAC   UGCAA    AUGU   \
       CGCGUGAGUUAGUCCGG CCUG   ACGUU    UACA   G
CUAAAUG                 C    CCC     AAAC^   GCU
   90        80        70        60        50
```

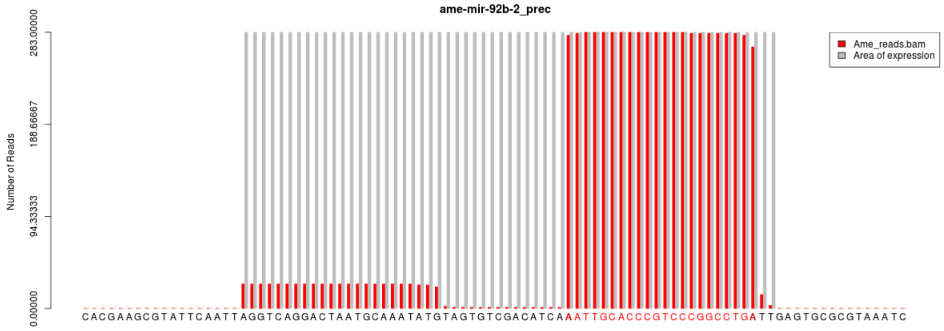

>ame-mir-92c_prec

GGATACTGGC**AGGTTGGGATGTGGGCATTATTTG**TTGCAAGGTTAGATCAAATTGCACTCGTCCCGGCCTGCTGGATCTAGT

>ame-mir-92c_5p

AGGTTGGGATGTGGGCATTATTTG

```
GGAUACUGGCAGGUUGGGAUGUGGGCAUUAUUUGUUGCAAGGUUAGAUCAAAUUGCACUCGUCCCGGCCUGCUGGAUCUAGU
((((.(..(((((((((((((...(((..(((((.............))))))))...)))))))))))))..)))))...

 Initial dG =    -36.80

           10        20        30        40
---|   A UG             UGG   UU     UUGCAA
   GGAU C  GCAGGUUGGGAUG   GCA  AUUUG      G
   UCUA G  CGUCCGGCCCUGC   CGU  UAAAC      G
UGA^   - GU             UCA   --     UAGAUU
 80         70        60          50

-----------------------------------------------------
```

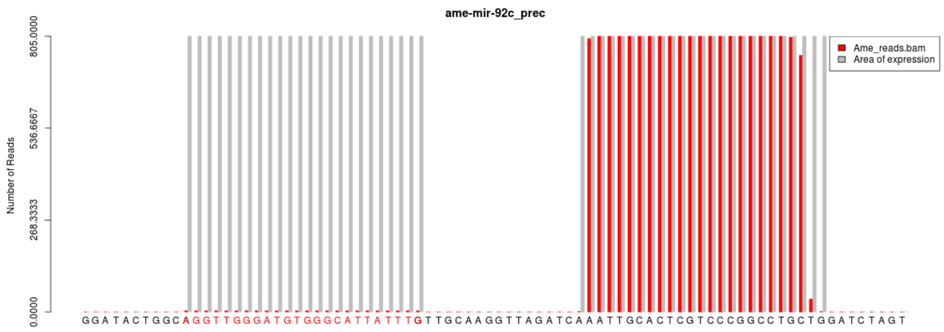

>ame-mir-932_prec
CATCGCGTTGCCTCT**TCAATTCCGTAGTGCATTGCAG**ATGATTGTTCGAATTGACGAGAAAGAACCTGCAAGCACCGCGGGAGTGAGGT
GGCCTCGCGTC

>ame-mir-932_5p
TCAATTCCGTAGTGCATTGCAG

```
CAUCGCGUUGCCUCUUCAAUUCCGUAGUGCAUUGCAGAUGAUUGUUCGAAUUGACGAGAAAGAACCUGCAAGCACCGCGGGAGUGAGGUGGCCUCGCGUC
...((((..(((.(((((.(((((..((((.((((((.......(((......)))).......))))))))))..))))).))))).)))..)))).. 

 Initial dG =    -46.80

        10        20        30        40        50
CAU|   UU   U     A     UA    A      AUGAUUG    AA
   CGCG  GCC CUUCA UUCCG  GUGC UUGCAG       UUCG  U
   GCGC  CGG GGAGU AGGGC  CACG AACGUC       GAGC  U
CU-^   UC   U     G     GC    -      CAAGAAA    AG
.         90        80         70        60

-----------------------------------------------------
```

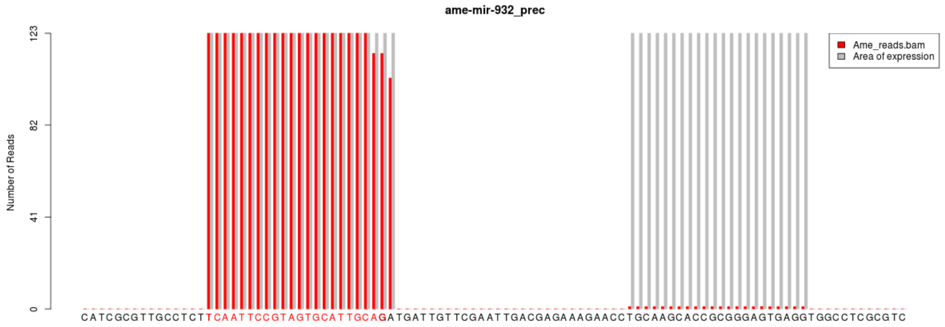

>ame-mir-965_prec
TGGACATATTCCAAG**GGGGAAAGGTTATAGCGATTATG**AGAAATTAAGCCATAAGCGTATAGCTTTTTCCCTTTGGCATTTGTTATT

>ame-mir-965_5p
GGGGAAAGGTTATAGCGATTATG

```
UGGACAUAUUCCAAGGGGGAAAGGUUAUAGCGAUUAUGAGAAAUUAAGCCAUAAGCGUAUAGCUUUUUCCCUUUGGCAUUUGUUAUU
..((((.((.((((((((((((((((((.((.(((((...........))))).)))))))))))))))))))))).)).))))...

 Initial dG =    -34.40

         10        20        30        40
UG-|   U  U                   G  A     AGAAA
   GACA AU CCAAGGGGGAAAGGUUAUA CG UUAUG     \
   UUGU UA GGUUUCCCUUUUUCGAUAU GC AAUAC     U
UUA^   U  C                   -  G     CGAAU
      80        70        60         50

-----------------------------------------------------
```

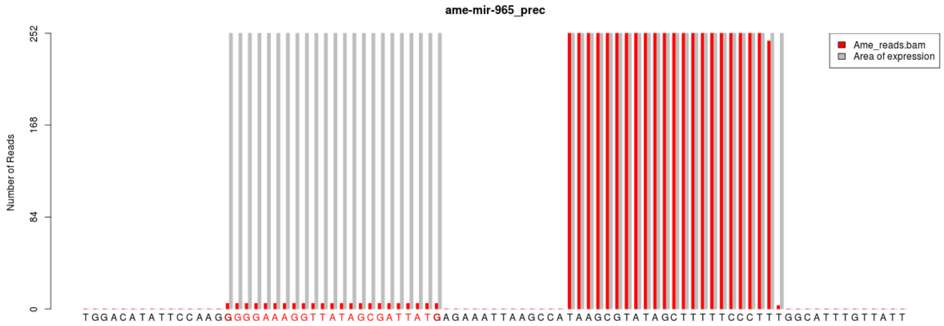

>ame-mir-971_prec
GGAGAGGGAGAGAGACGAGGATGCACCGGGGCCGGTTCACTTTAAGTTCGAATACCAAGCCTGTCTATAAACAGT**TTGGTGTTCTACCT**
TACAGTGAGTCGACCGTGGTATCGTCGACGTTCCAACA

>ame-mir-971_3p
TTGGTGTTCTACCTTACAGTGAG

GGAGAGGGAGAGAGACGAGGAUGCACCGGGGCCGGUUCACUUUAAGUUCGAAUACCAAGCCUGUCUAUAAACAGU**UUGGUGUUCUACCUUACAGUGAG**UCGACCGUGGUAUCGUCGACG
UUCCAACA
```
......((((.....(((.(((..((((.((.((..(((((.((((...(((((((((((.
(((......)))))))))))))))...)))).)))))..)).)).))))))).)))...)))))....
```

```
 Initial dG =    -50.00

        10        20        30        40        50        60
GGAGAG|   AGAGA   G   GC    G  C  GU     U    UUC           C   CU
      GGAG     CGA GAU  ACCG GG CG  UCACU UAAG   GAAUACCAAGC UGU  A
      CCUU     GCU CUA  UGGU CC GC  AGUGA AUUC   CUUGUGGUUUG ACA  U
ACAA--^   GCA--   G   --    G  A  UG     C    CAU           -   AA
       120         110         100        90        80         70

-----------------------------------------------------
```

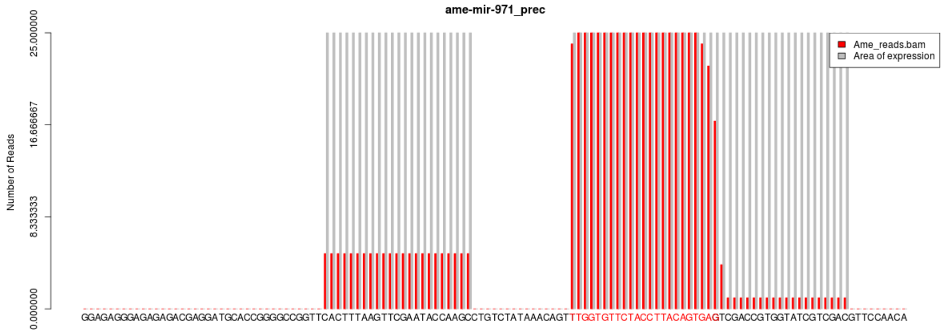

>ame-mir-980_prec
AGCTGATTACGATGCGGCTCGTCATCGGGCACTTTGCTATTTTTTAAACGC**AAGCTGCCTTTTGAAGGGCAACA**CCGTACTCAGTG

>ame-mir-980_3p
AAGCTGCCTTTTGAAGGGCAACA

```
AGCUGAUUACGAUGCGGCUCGUCAUCGGGCACUUUGCUAUUUUUUAAACGCAAGCUGCCUUUUGAAGGGCAACACCGUACUCAGUG
.(((((.((((.((..((((.(((..(((((.(((((...........))))).)))))..))).))))..)).)))).))))).

 Initial dG =     -35.10

        10         20         30         40
A|     U     A   CG     G    UC      C      UAUUU
 GCUGA UACG UG   GCUC UCA   GGGCA UUUGC      U
 UGACU AUGC AC   CGGG AGU   UCCGU GAACG      U
G^     C     C   AA     A    UU      C      CAAAU
     80         70         60         50
```

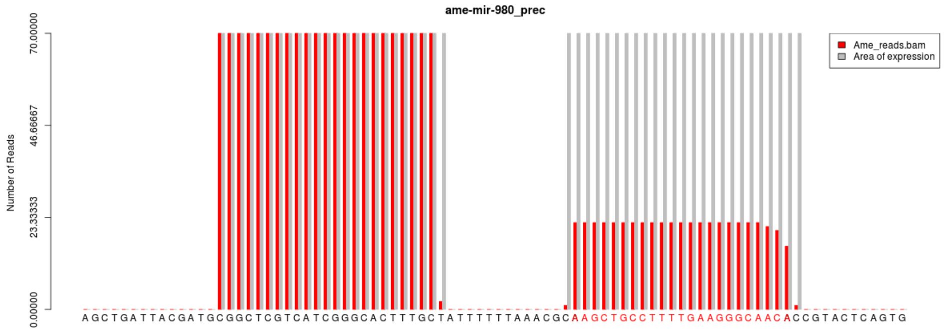

>ame-mir-981_prec
GGGAATGGATGCAAACGTCTGGGTTTCGTGACAGGCGAGCCGTTCTTTACGACTTGG**TTCGTTGTCAACGAAACCTGCA**CGATCTGCAA
CCAAA

>ame-mir-981_3p
TTCGTTGTCAACGAAACCTGCA

```
GGGAAUGGAUGCAAACGUCUGGGUUUCGUGACAGGCGAGCCGUUCUUUACGACUUGGUUCGUUGUCAACGAAACCUGCACGAUCUGCAACCAAA
.....(((.((((..(((.((((((((((((((.(((((((.((.....))..))))))))))))).))))))))).))).....)))).))).
```

```
 Initial dG =    -40.80

        10         20         30        40
GGGAA   A    AA-   C         -|     G       U-  UU
     UGG UGCA   CGU UGGGUUUCG UGACAG CGAGCCG  UC  \
     ACC ACGU   GCA GUCCAAAGC ACUGUU GCUUGGU  AG  U
AA---   A    CUA   C         A^     -       UC  CA
      90        80        70         60        50
```

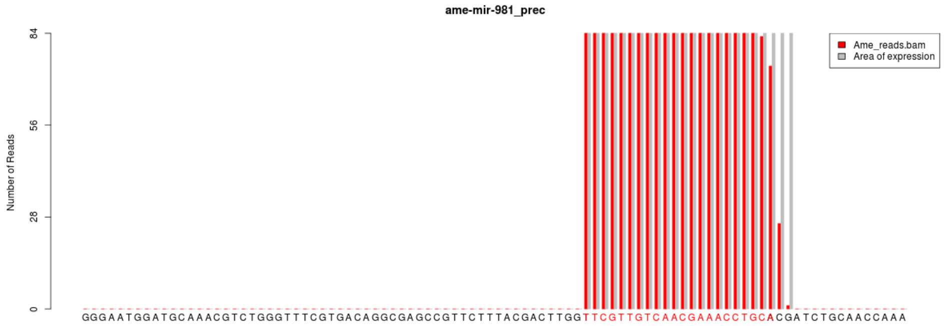

>ame-mir-985_prec
GAAAGGGTAGAAACGATCGATCGATCGACAAATGGCATTTATTACGTTTC**AAAATGTTCCAATATCGGGCA**GGTATTATTTCAGT

>ame-mir-985_3p
AAAATGTTCCAATATCGGGCA

```
GAAAGGGUAGAAACGAUCGAUCGAUCGACAAAUGGCAUUUAUUACGUUUCAAAAUGUUCCAAUAUCGGGCAGGUAUUAUUUCAGU
((((..(((....(((((....)))))).......((...(((((((((....)))))...))))....))...)))..))))...

 Initial dG =    -13.50

             10        20
---    GG   .-GAAA|    G
   GAAA  GUA      CGAUC A
   CUUU  UAU      GCUAG U
UGA    AU   \ ----^    C
    80

-----------------------------------------------------
```

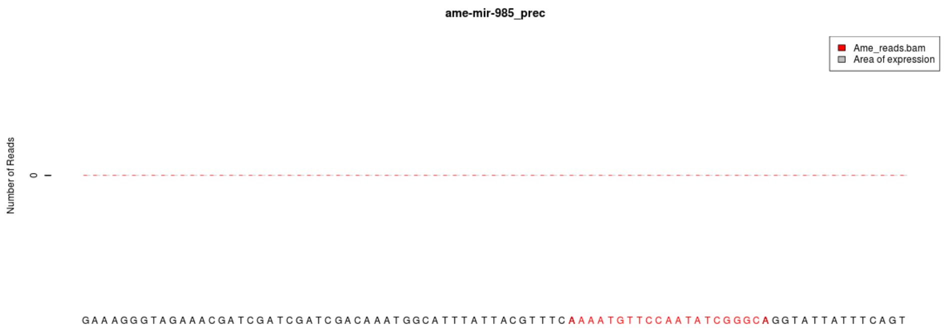

>ame-mir-9864_prec
AGCGTGCCTC**TTTCGAGGCTGTTTAAATGGATT**CACATTGTGCAACTCGACGGCATAAATCCATTTAAGTCGTTCGAAAGAGGCTGGCT
GT

>ame-mir-9864_5p
TTTCGAGGCTGTTTAAATGGATT

AGCGUGCCUC**UUUCGAGGCUGUUUAAAUGGAUU**CACAUUGUGCAACUCGACGGCAUAAAUCCAUUUAAGUCGUUCGAAAGAGGCUGGCUGU
(((..((((((((((((.(..((((((((((((.....(((((.........)))))))))))))))))..)))))))))))))..)))..

```
 Initial dG =    -38.80

          10        20        30        40
--|  GU            G UG            CACAU     AACU
  AGC  GCCUCUUUCGAG C  UUUAAAUGGAUU     UGUGC    \
  UCG  CGGAGAAAGCUU G  GAAUUUACCUAA     AUACG    C
UG^  GU            - CU            -----     GCAG
90        80         70        60             50
```

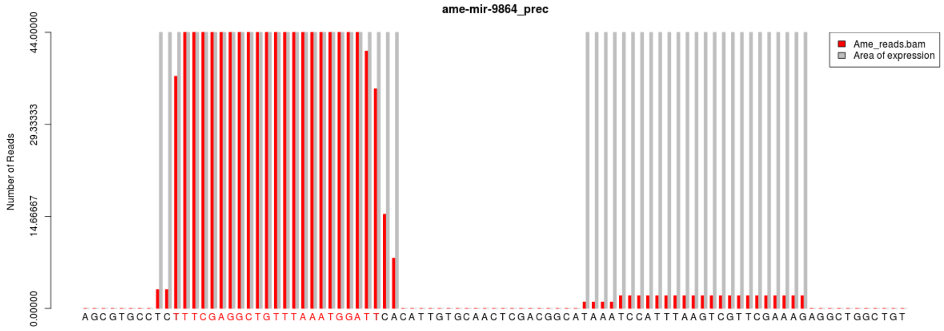

>ame-mir-9865_prec
AAGATGGAAT**TGATTTATGTGGTGATTGTGC**AGTAGCACAATTAGAAGCTGAAAAACCACTGCACGATACATTACATAGATTACTTCCT
ATTAA

>ame-mir-9865_5p
TGATTTATGTGGTGATTGTGC

```
AAGAUGGAAUUGAUUUAUGUGGUGAUUGUGCAGUAGCACAAUUAGAAGCUGAAAAACCACUGCACGAUACAUUACAUAGAUUACUUCCUAUUAA
.....((((.(((((((((((((((((((((((((((..........)))........)))))))))).)))))))))))))).)))).......

 Initial dG =     -34.40

         10        20         30                 40
AAGAU-    U              -          --------|  ACAA
      GGAA UGAUUUAUGUGGUG AUUGUGCAGU        AGC    U
      CCUU AUUAGAUACAUUAC UAGCACGUCA        UCG    U
AAUUAU    C              A          CCAAAAAG^  AAGA
   90        80        70        60        50

-----------------------------------------------------
```

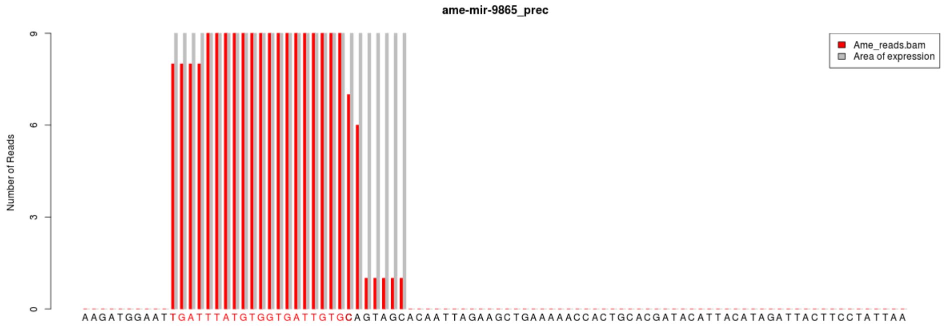

>ame-mir-9866_prec
GATGAGAGGA**TTCGAGTCGGCGGGGTATGAGT**AATACGTTCAGGCATGTACCTCGTATCCCCTTGGCTCGCAATGCCCTCTCCCT

>ame-mir-9866_5p
TTCGAGTCGGCGGGGTATGAGT

```
GAUGAGAGGAUUCGAGUCGGCGGGGUAUGAGUAAUACGUUCAGGCAUGUACCUCGUAUCCCCUUGGCUCGCAAUGCCCUCUCCCU
...(((((((((((((((((.((((((((((...(((((......))))).)))))))))).)))))))).)))..))))))...

 Initial dG =    -37.80

          10         20        30        40
GAU      --   -|       C          UAA     UC
   GAGAGG  AUU CGAGUCGG GGGGUAUGAG   UACGU  A
   CUCUCC  UAA GCUCGGUU CCCUAUGCUC   AUGUA  G
UCC      CG   C^       C          C--     CG
    80        70        60          50

-----------------------------------------------------
```

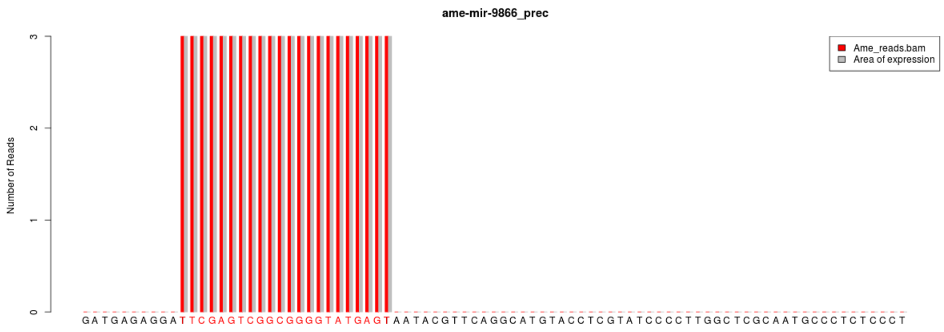

>ame-mir-9867_prec
TCCTGAGGCA**AGACGGTATGGACGGTAGAGACGA**GTCATGATCCTCGTCCTGCCGCGTCACCGTTATCCTCATCC

>ame-mir-9867_5p
AGACGGTATGGACGGTAGAGACGA

UCCUGAGGCA**AGACGGUAUGGACGGUAGAGACGA**GUCAUGAUCCUCGUCCUGCCGCGUCACCGUUAUCCUCAUCC
...(((((...(((((((((..((((((.((((((........))))))))))))))).)))))))..)))))...

Initial dG = -33.70

```
        10         20        30
UCC|    CAA      -   GA      A      UCA
   UGAGG   GACGGU AUG  CGGUAG GACGAG   U
   ACUCC   UUGCCA UGC  GCCGUC CUGCUC   G
CCU^    UA-      C   --      -      CUA
    70         60          50         40
```

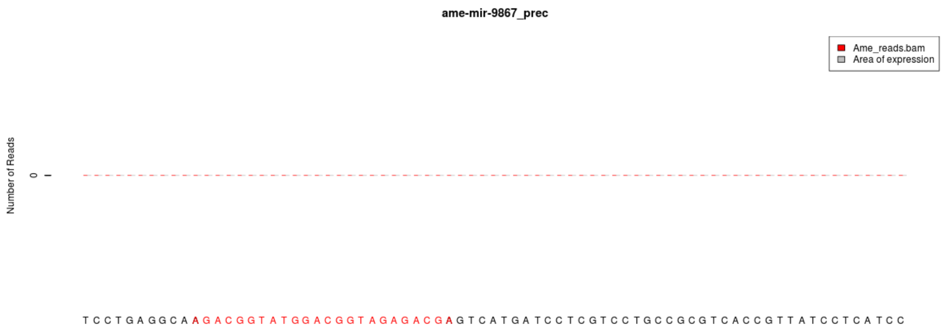

>ame-mir-9868_prec
TCGGCGATAAAAGCTCGGCGTTTCATCGATGCCTGGCCTGCTCTTTCCCCGTGG**TTAAGCTCGATGAAGTCGTCGG**GTTTAGCCGG

>ame-mir-9868_3p
TTAAGCTCGATGAAGTCGTCGG

```
UCGGCGAUAAAAGCUCGGCGUUUCAUCGAUGCCUGGCCUGCUCUUUCCCCGUGGUUAAGCUCGAUGAAGUCGUCGGGUUUAGCCGG
.((((.....((((((((((((((((((.((.(((((..............))))).))))))))))).)))))))))).)))).

 Initial dG =    -38.20

        10        20         30        40
U|   GAUAA           -         U  C     UGCUCU
 CGGC     AAGCUCGGCG UUUCAUCGA GC UGGCC      U
 GCCG     UUUGGGCUGC GAAGUAGCU CG AUUGG      U
G^   A----           U         -  A     UGCCCC
         80        70         60        50
```

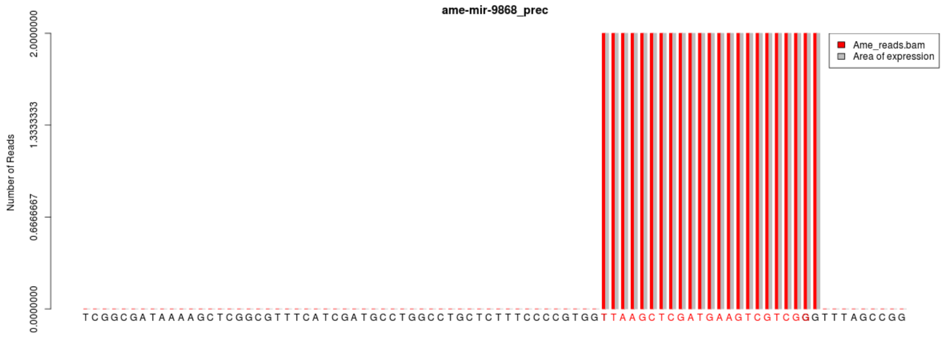

>ame-mir-9869_prec
GATAATTGCA**TCGGGGATCGCGGATGCGGAGAGC**TTGAATGCCATTCAATGCCTACGCTTCTCGTTCCCAGGAGTGATTATCT

>ame-mir-9869_5p
TCGGGGATCGCGGATGCGGAGAGC

```
GAUAAUUGCAUCGGGGAUCGCGGAUGCGGAGAGCUUGAAUGCCAUUCAAUGCCUACGCUUCUCGUUCCCAGGAGUGAUUAUCU
(((((..(..(.((((.((.(((.(((.((.((((((((...)))))).)))).))).))))).)))).)..)..)))))).

 Initial dG =    -32.70

         10        20        30         40
-|     UG AU G    U  C   U   G  A  -      G
 GAUAAU  C  C GGGA CG GGA GCG AG GC UUGAAU \
 CUAUUA  G  G CCCU GC UCU CGC UC CG AACUUA C
U^     GU AG A    U  -   U   A  -  U      C
  80        70         60         50

-----------------------------------------------------
```

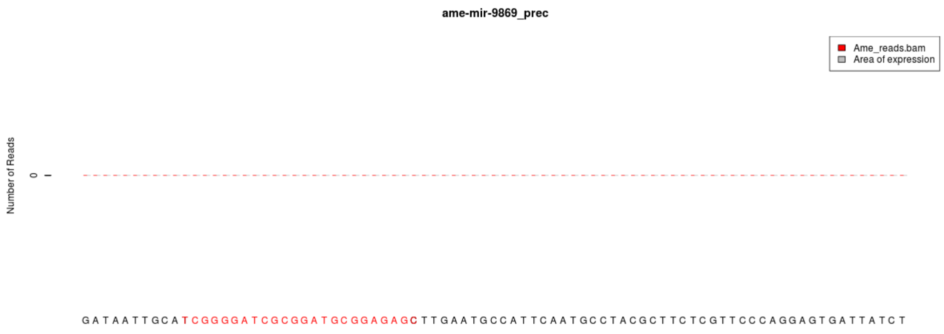

>ame-mir-9870_prec
CGTGCACACG**TGACGACGCCGTCACGTACGTGC**GTTCAACGTGCGCGTGTGTGCGGCGGCGGTGCACGTGTGCGCGT

>ame-mir-9870_5p
TGACGACGCCGTCACGTACGTGC

```
CGUGCACACGUGACGACGCCGUCACGUACGUGCGUUCAACGUGCGCGUGUGUGCGGCGGCGGUGCACGUGUGCGCGU
(((((((((((((..(((((.((((((((((((.......)))))))))))))))))...)).))))))))))))).

 Initial dG =    -49.50

         10           20         30
-             -  GA-|    U            UUC
 CGUGCACACGUG AC   CGCCG CACGUACGUGCG   \
 GCGCGUGUGCAC UG   GCGGC GUGUGUGCGCGU   A
U             G  GCG^    -            GCA
      70         60          50         40

-----------------------------------------------------
```

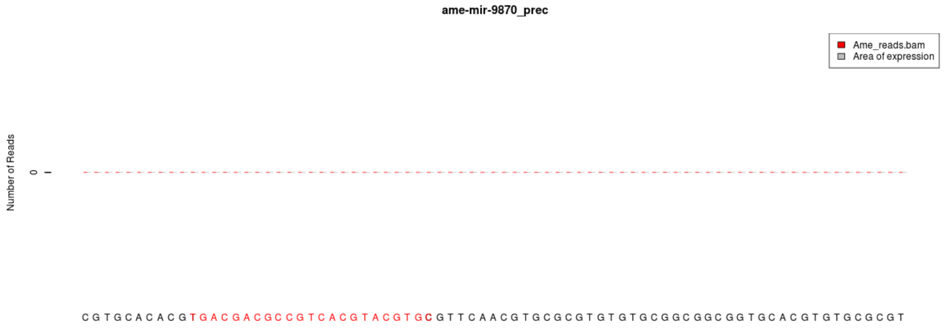

>ame-mir-9871_prec
ACATATTGACGTAATATCATCCGATCTTTAGAGCTTCTATGATTGGA**TGCGGTAAAGGTCGGGTGAGC**GTTCGTAATT

>ame-mir-9871_3p
TGCGGTAAAGGTCGGGTGAGC

```
ACAUAUUGACGUAAUAUCAUCCGAUCUUUAGAGCUUCUAUGAUUGGAUGCGGUAAAGGUCGGGUGAGCGUUCGUAAUU
...(((.(((((....((((((((((((((..((.((((....)))).))..))))))))))))))))))).)))...

 Initial dG =    -32.00

        10        20        30        40
ACA|  U     AAUA              GA  U    U
   UAU GACGU    UCAUCCGAUCUUUA  GC UCUA G
   AUG UUGCG    AGUGGGCUGGAAAU  CG AGGU A
UUA^  C     ----              GG  U    U
       70            60        50

-----------------------------------------------------
```

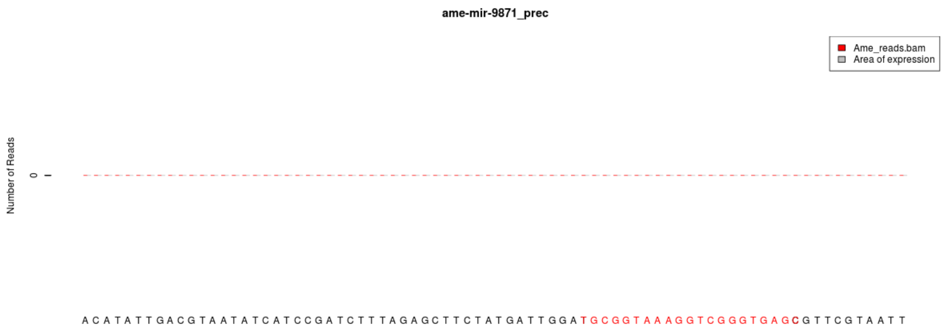

>ame-mir-9872_prec
TATGAGACGA**AACGAGACAGAGATAGAACGA**GAGACAATTTTATCTCTATCATATAACGCAACGTCGTTCTATCTTCGCCTCGCTTCAT
CTAACG

>ame-mir-9872_5p
AACGAGACAGAGATAGAACGA

```
UAUGAGACGAAACGAGACAGAGAUAGAACGAGAGACAAUUUUAUCUCUAUCAUAUAACGCAACGUCGUUCUAUCUUCGCCUCGCUUCAUCUAACG
....(((.(((.((((...((((((((((((((........))))).........(((...)))))))))))))))...)))).))).))).....
```

```
 Initial dG =    -26.40

        10        20        30
UAUG   C   A    ACA            |    CAA
    AGA GAA CGAG   GAGAUAGAACG--AGAGA   U
    UCU CUU GCUC   UUCUAUCUUGC  UCUCU   U
GCAA   A   C    CGC           \ ^    AUU
    90        80        70             40


                                50        60
                                AUCAUAUA   C
                                        ACG \
                                        UGC A
                                --------   A
```

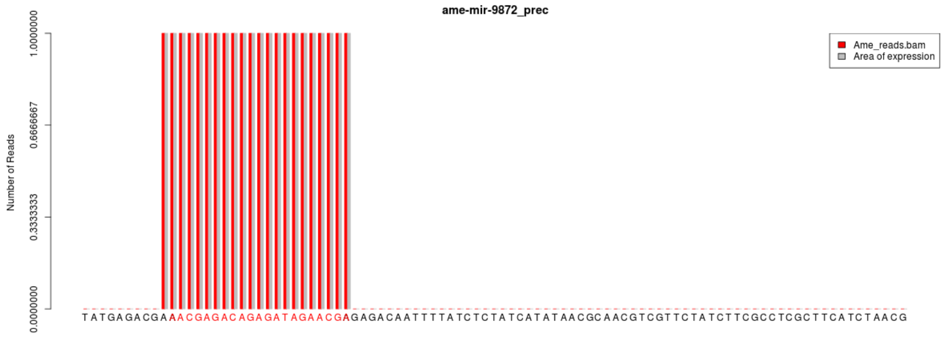

>ame-mir-9873_prec
ACGCCAGCTAATCCGAGGCTGCGGCAAAGATAATTCCCGACGTATTCCAG**GATTATTTGCTGCGGCTTTGGA**TTATTTGGCG

>ame-mir-9873_3p
GATTATTTGCTGCGGCTTTGGA

```
ACGCCAGCUAAUCCGAGGCUGCGGCAAAGAUAAUUCCCGACGUAUUCCAGGAUUAUUUGCUGCGGCUUUGGAUUAUUUGGCG
.((((((.((((((((((((((((((..((((((((..((.....))..)))))))))))))))))))))))))).))))))

 Initial dG =    -44.60

        10        20        30        40
A|     C                  AA        CC  CG
 CGCCAG UAAUCCGAGGCUGCGGCA  GAUAAUUC  GA  \
 GCGGUU AUUAGGUUUCGGCGUCGU  UUAUUAGG  CU  U
-^     U                  --        AC  UA
  80        70        60          50

-----------------------------------------------------
```

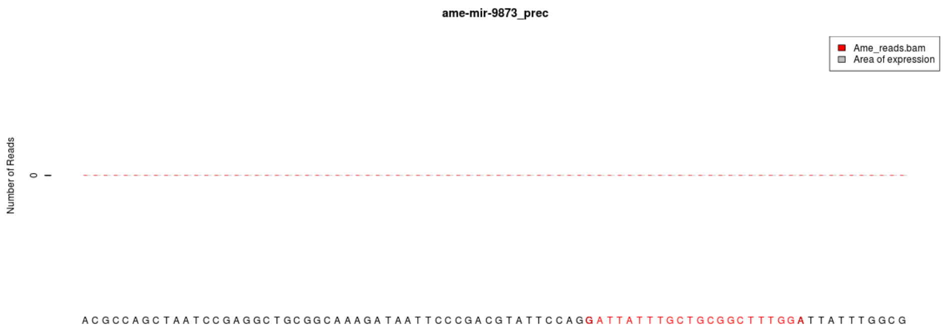

>ame-mir-9874_prec
CGATAAAAGT**AGAATCCTTTGCTCGTCGTGGGA**CGAGCAACGCTCGACTCGTTCCACTCTGTTCCACGACGGGCAAGGAATACGAAACG
TATCT

>ame-mir-9874_5p
AGAATCCTTTGCTCGTCGTGGGA

```
CGAUAAAAGUAGAAUCCUUUGCUCGUCGUGGGACGAGCAACGCUCGACUCGUUCCACUCUGUUCCACGACGGGCAAGGAAUACGAAACGUAUCU
.((((...(((...(((((.((((((((((((((((.((((.......))))...))).))))))))))))))))))).)))......)))).

 Initial dG =    -39.10

           10        20        30           40
C    AAA---|  GAA     U              -   C--    CUC
 GAUA      GUA   UCCUU GCUCGUCGUGGGAC GAG   AACG   \
 CUAU      CAU   AGGAA CGGGCAGCACCUUG CUC   UUGC   G
U    GCAAAG^  A--     -              U   ACC    UCA
   90        80           70        60        50

-----------------------------------------------------
```

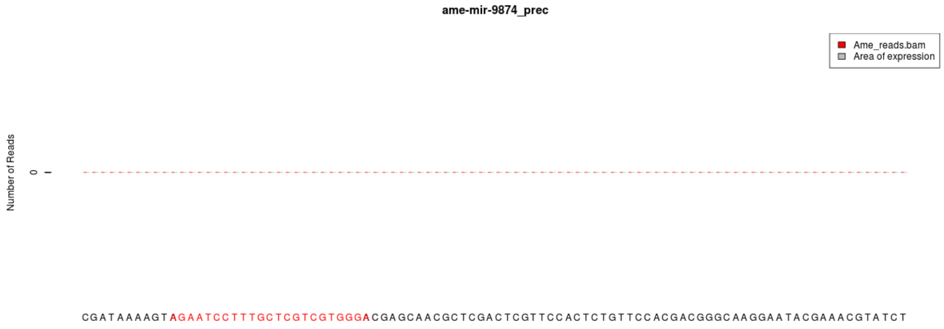

>ame-mir-9875_prec
AAGGGAACACGTGTTCTCTCTGTAATTACGTCGCCGAAGAAAGATATATC**GTGGGATGAGGAAGGGAGGAG**GGAGTCCCTG

>ame-mir-9875_3p
GTGGGATGAGGAAGGGAGGAG

```
AAGGGAACACGUGUUCUCUCUGUAAUUACGUCGCCGAAGAAAGAUAUAUCGUGGGAUGAGGAAGGGAGGAGGGAGUCCCUG
.(((((.......((((((((....((.((((.(((..((........)).)))))))))..)))))))).....))))).

 Initial dG =     -21.60

        10         20         30         40
A|     ACACGUG        GUAA   A     G    AA   AAG
 AGGGA        UUCUCUCU    UU CGUC CCG   GA    A
 UCCCU        AGGAGGGA    GA GUAG GGU   CU    U
G^     GAGGG--        AG--   -     -    G-   AUA
80          70          60           50

-----------------------------------------------------
```

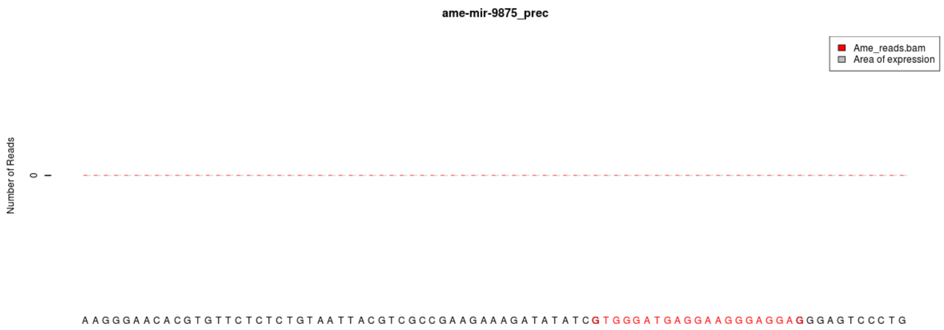

>ame-mir-9876_prec
AGTGGTATGCCCGTTTTATCCATCGATGCACGAAGCTGAAACTTAGCTTTCAACTTCAAGTTTTCCG**TGGGATGGATCGATAGAACGAG**
TGTTCCCTCTT

>ame-mir-9876_3p
TGGGATGGATCGATAGAACGAGT

```
AGUGGUAUGCCCGUUUUAUCCAUCGAUGCACGAAGCUGAAACUUAGCUUUCAACUUCAAGUUUUCCGUGGGAUGGAUCGAUAGAACGAGUGUUCCCUCUU
((.((.((((.((((((((.(((.((.((((((((((((..............))).)))))..))))..)).))).))))))))).)))).))))...
```

Initial dG =   -26.30

```
           10        20         30          40
---  U  U    C         C   G  G-    .-A|     A
   AG GG AUGC CGUUUUAUC AUC AU  CACG   AGCUGA \
   UC CC UGUG GCAAGAUAG UAG UA  GUGC   UCGAUU A
UUC  -  U    A         C   G  GG    \ -^     C
.         90        80        70
```

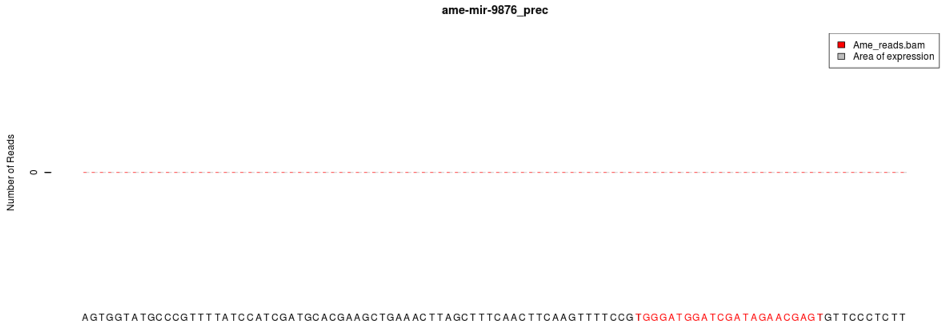

>ame-mir-9877_prec
GCCGTGTGTGAGGTCGTTTGTTGTTTAATAGAAAGGCGGCT**GCGGGACACTGGGTGGGCGGCT**GTATGTATGG

>ame-mir-9877_3p
GCGGGACACTGGGTGGGCGGCT

```
GCCGUGUGUGAGGUCGUUUGUUGUUUAAUAGAAAGGCGGCUGCGGGACACUGGGUGGGCGGCUGUAUGUAUGG
.(((((..((.(((((((..((((((..(((........)))..))))))....)..))))))).))..)))))

 Initial dG =    -21.50

        10        20            30
G     UG  A       UG ----|    AA   AAA
 CCGUG  UG GGUCGUU  U    UGUUU  UAG   G
 GGUAU  AU UCGGCGG  G    ACAGG  GUC   G
-     GU  G       GU GGUC^    GC   GGC
   70        60        50        40

-----------------------------------------------------
```

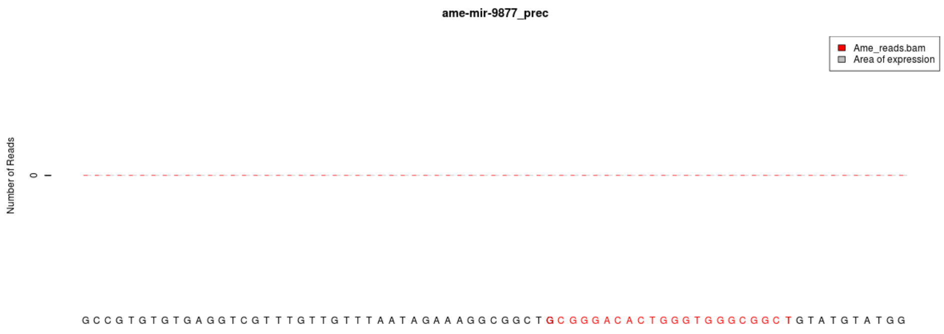

>ame-mir-9878_prec
TAAAAATATCTAAATAGATCCAAACATCCGAATATCTGTAAATAGTTTAT**TGGGATGTTGAATCTGTTGCAG**ACTCGGGTGC

>ame-mir-9878_3p
TGGGATGTTGAATCTGTTGCAG

```
UAAAAAUAUCUAAAUAGAUCCAAACAUCCGAAUAUCUGUAAAUAGUUUAUUGGGAUGUUGAAUCUGUUGCAGACUCGGGUGC
........(((.(((((((...(((((((.((((.(((....)))..)))).)))))))..)))))))..))).........

 Initial dG =     -21.10

         10         20        30         40
UAAAAAUA-   A-|      CCA       G    U-   U
         UCU  AAUAGAU   AACAUCC AAUA  CUG A
         AGA  UUGUCUA   UUGUAGG UUAU  GAU A
CGUGGGCUC   CG^      AG-       G    UU   A
 80        70        60         50

-----------------------------------------------------
```

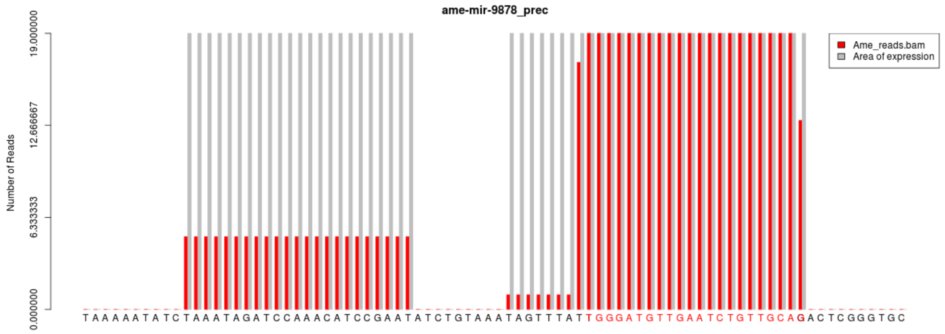

>ame-mir-9879_prec
TGAAATCATTTCACCTTACACTTGTCCACGGTTTCCCGAGGGGATAGG**GTACCGTGGAACAAGGAAGTGGC**AAAGATATTA

>ame-mir-9879_3p
GTACCGTGGAACAAGGAAGTGGC

```
UGAAAUCAUUUCACCUUACACUUGUCCACGGUUUCCCGAGGGGAUAGGGUACCGUGGAACAAGGAAGUGGCAAAGAUAUUA
....(((.((((((......(((((((((((..(((.........))).)))))))).)))))...)))).)).)))....

 Initial dG =    -26.30

              10        20         30        40
UGAAA------|      ACCUUACA     -       UU   GAGG
           UCAUUUC        CUUGU CCACGGU  CCC    \
           GGUGAAG        GAACA GGUGCCA  GGG    G
AUUAUAGAAAC^      --------     A       U-   AUAG
80        70               60        50
```

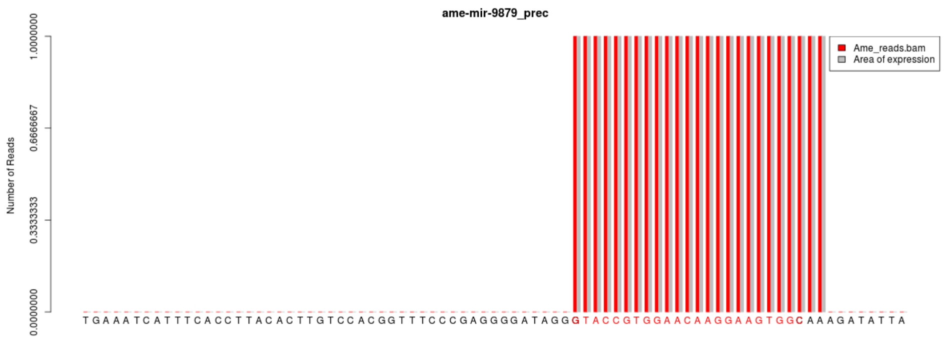

>ame-mir-9880_prec
ATCGAGGGTGAAAACGCTCCACCTCGGCGAAAGCCGATACGACCTCGCCGGCTTGCGTTTTGC**ATCGGCTTTCATCGGATGGGG**TATTG
TCGGC

>ame-mir-9880_3p
ATCGGCTTTCATCGGATGGGG

```
AUCGAGGGUGAAAACGCUCCACCUCGGCGAAAGCCGAUACGACCUCGCCGGCUUGCGUUUUGCAUCGGCUUUCAUCGGAUGGGGUAUUGUCGGC
.((((.(((......(((((.(.((..((((((((((.(((...(((......)))..))).))))))))))..))).)))))))))).)))).
```

Initial dG =   -35.80

```
        10        20        30        40        50
A|   G   GAAAAC      C U  GC          A   CCU   CG
 UCGA GGU      GCUCCA C CG  GAAAGCCGAU CGA   CGC  G
 GGCU UUA      UGGGGU G GC  CUUUCGGCUA GUU   GCG  C
C^   G   ------      A -  UA          C   UU-   UU
   90              80         70         60
```

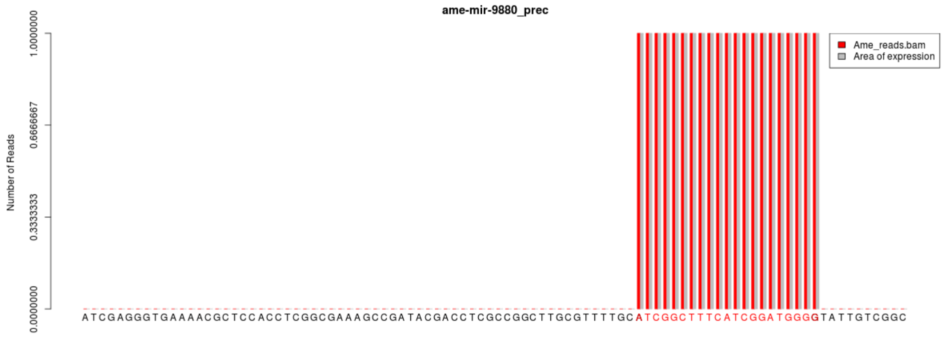

>ame-mir-9881_prec
TAATGCCGCG**TACGTCGCGTTTCGAAAGGGA**AACCGAGAATATCGGGTTATCCCCTCGAAGTGCGCCGTTCTCGGCTTGT

>ame-mir-9881_5p
TACGTCGCGTTTCGAAAGGGA

```
UAAUGCCGCGUACGUCGCGUUUCGAAAGGGAAACCGAGAAUAUCGGGUUAUCCCCUCGAAGUGCGCCGUUCUCGGCUUGU
....((((...(((.((((((((((..((((..((((.....))))....)))).)))))))))).)))...))))....

 Initial dG =    -34.00

        10        20        30
UAAU    CGU   U          AA     AA--|   GA
    GCCG   ACG CGCGUUUCGA  GGGA    CCGA  \
    CGGC   UGC GCGUGAAGCU  CCCU    GGCU  A
UGUU    UCU   C          C-     AUUG^   AU
.        70        60         50

-----------------------------------------------------
```

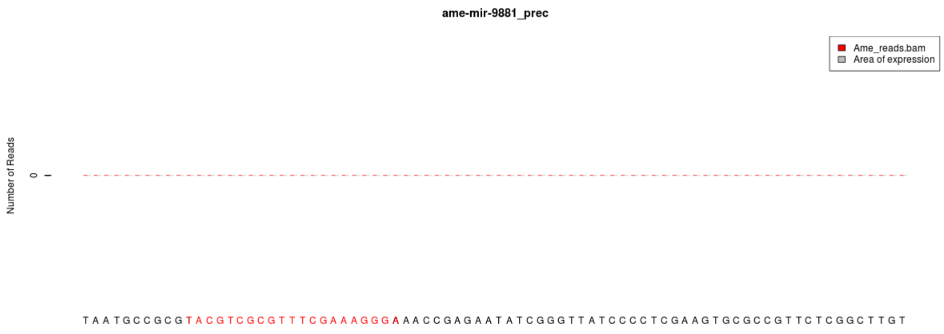

>ame-mir-9882_prec
ATCGAGTTTG**AGCGATGAGACTAGATCTTGGC**TTCGATTCAACGTCAATGACTGGTTTTCATGGTGCTTGAACTG

>ame-mir-9882_5p
AGCGATGAGACTAGATCTTGGC

```
AUCGAGUUUGAGCGAUGAGACUAGAUCUUGGCUUCGAUUCAACGUCAAUGACUGGUUUUCAUGGUGCUUGAACUG
....((((..((((((((((((((.(((((((...........))))).))))))))).)))..))))..)))).

 Initial dG =    -23.80

        10          20         30
AUCG    UG    --   -|      A  -     UUCGA
    AGUU  AGCG  AUG AGACUAG UC UUGGC     \
    UCAA  UCGU  UAC UUUGGUC AG AACUG     U
G---    GU    GG   U^      -  U     CAACU
       70        60         50        40

-----------------------------------------------------
```

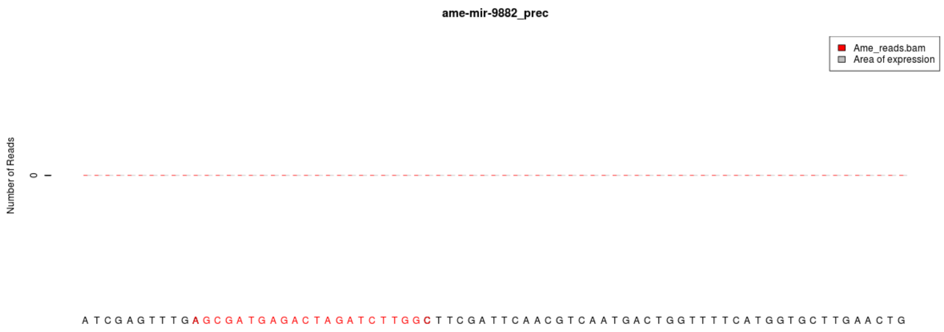

>ame-mir-9883_prec
CTCGGAAAAA**TTCGGGCGGGCTCGGGCGAGA**TCGCGATACCTCGTCCGATCTCGCCCGAATTTACCCGACTCGCTT

>ame-mir-9883_5p
TTCGGGCGGGCTCGGGCGAGA

```
CUCGGAAAAAUUCGGGCGGGCUCGGGCGAGAUCGCGAUACCUCGUCCGAUCUCGCCCGAAUUUACCCGACUCGCUU
.((((..(((((((((((((.(((((((((..........))))))))).)))))))))))))..)))).......

 Initial dG =    -43.90

              10        20        30
C------|   AA              C         AUCG
       UCGG  AAAUUCGGGCGGG UCGGGCGAG    C
       AGCC  UUUAAGCCCGCUC AGCCUGCUC    G
UUCGCUC^   CA              U         CAUA
     70        60        50        40

-----------------------------------------------------
```

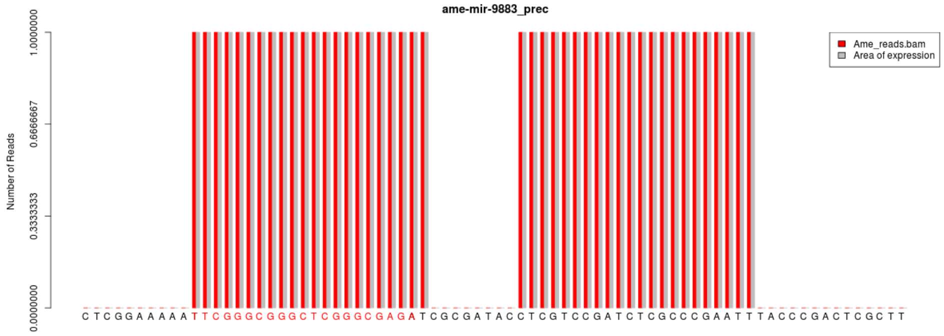

>ame-mir-9884_prec
CCTCCGTTTTCAGTGCTTCAGTCGCGCGACGACGCTCTCGGACGCTTTAGTCAG**TCGGTCGGTGACGAAGCTCCC**AAACGTGATA

>ame-mir-9884_3p
TCGGTCGGTGACGAAGCTCCC

```
CCUCCGUUUUCAGUGCUUCAGUCGCGCGACGACGCUCUCGGACGCUUUAGUCAGUCGGUCGGUGACGAAGCUCCCAAACGUGAUA
..((((((.....(((((.(((((.(((((((...((..(....)..))...))).))))))))))))))....))))).))..

 Initial dG =    -27.30

         10        20        30         40
CC  -|    UCAGU     A     G    -   GCU  CG A
  UC CGUUU     GCUUC GUCGC CGAC GAC   CU  G C
  AG GCAAA     CGAAG CAGUG GCUG CUG   GA  U G
AU  U^    CCCU-     -     -    G   ACU  UU C
    80         70          60        50
```

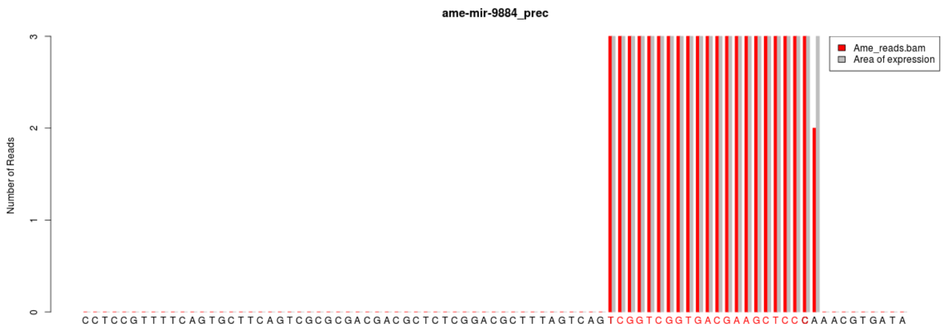

>ame-mir-9885_prec
TCGGCTCGAC**TCGGCAATGATCGGACGTGGTC**GGTTCTGTAGCTCCGAGACCATTTCCGATTATCGCCGAGTCGAGCCGAA

>ame-mir-9885_5p
TCGGCAATGATCGGACGTGGTC

```
UCGGCUCGACUCGGCAAUGAUCGGACGUGGUCGGUUCUGUAGCUCCGAGACCAUUUCCGAUUAUCGCCGAGUCGAGCCGAA
(((((((((((((((.(((((((((.(((((((((......)))....)))))).))))))))).))))))))))))))).
```

```
 Initial dG =    -56.20

         10        20        30
-               A         C       ----|  UC
 UCGGCUCGACUCGGC AUGAUCGGA GUGGUC    GGU  U
 AGCCGAGCUGAGCCG UAUUAGCCU UACCAG    UCG  G
A               C         U       AGCC^  AU
80        70        60        50        40

-----------------------------------------------------
```

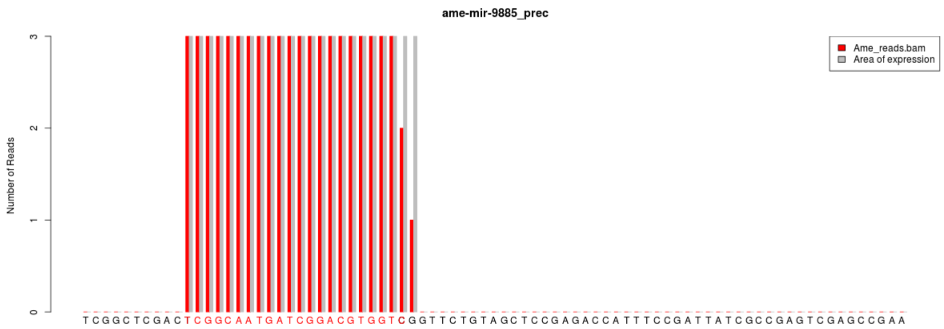

>ame-mir-9886_prec
GTCCTCGTCGTCGCTTCTGCAAGGCGTCGCCTCCGGGGGTGGATGGTGCG**TAGGCGTCACGTTGTGGAACG**GCGACCAGGA

>ame-mir-9886_3p
TAGGCGTCACGTTGTGGAACG

```
GUCCUCGUCGUCGCUUCUGCAAGGCGUCGCCUCCGGGGGUGGAUGGUGCGUAGGCGUCACGUUGUGGAACGGCGACCAGGA
.((((.(((((((.(((..((((((((((((((((....)))).))).....))))))...)))..)))))))))).))))

 Initial dG =    -37.00

        10        20                30
G    C       C   UG   ---      -----   -|   G
 UCCU GUCGUCG UUC  CAA   GGCGUC     GCC UCCG G
 AGGA CAGCGGC AAG  GUU   CUGCGG     UGG AGGU G
-    C       -   GU   GCA      AUGCG   U^   G
 80        70         60        50        40

-----------------------------------------------------
```

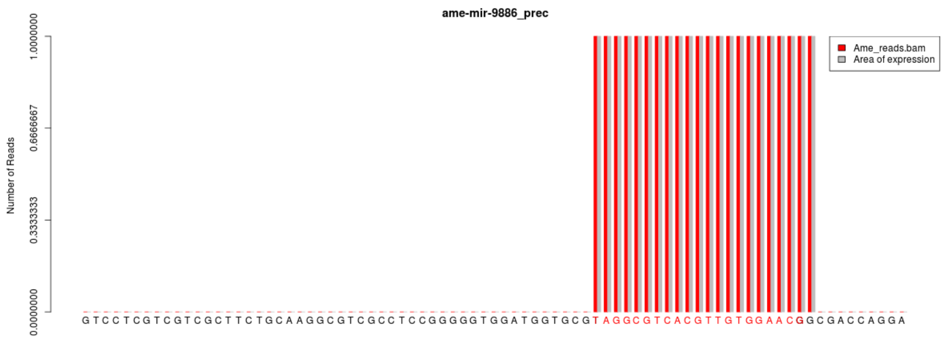

>ame-mir-9887_prec
GGAGAAGAAG**AAGGGCTGGGAAGGGCGGAG**AAGGCGAAGGTGGCTGGTTCGTTAATTACTGATTTTCCTCGGTCCGCGTCACTTTTTA

>ame-mir-9887_5p
AAGGGCTGGGAAGGGCGGAG

```
GGAGAAGAAGAAGGGCUGGGAAGGGCGGAGAAGGCGAAGGUGGCUGGUUCGUUAAUUACUGAUUUUCCUCGGUCCGCGUCACUUUUUA
.((((((.....((((((((((((.(((((..((((((.........))))))..)).))).)))))).))))))......)))))).

 Initial dG =    -24.10

        10          20         30        40
G      AAGAA-      -      G    -| AA      GGUG
 GAGAAG      GGGCUG GGAAGG CGG AG  GGCGAA    \
 UUUUUC      CCUGGC CCUUUU GUC UU  UUGCUU    G
A      ACUGCG      U      A    A^ AA      GGUC
       80        70        60        50

-----------------------------------------------------
```

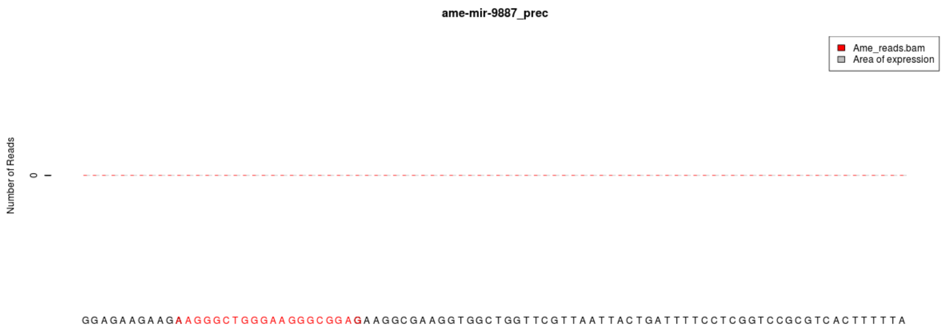

>ame-mir-9888_prec
AGGTGAGCGG**TGGTGGTCAAGCAAGTAGAACGTT**AGAGGAACGTAGACGTTCTATGCTTGACCATCATTGGCCAGTTCC

>ame-mir-9888_5p
TGGTGGTCAAGCAAGTAGAACGTT

```
AGGUGAGCGGUGGUGGUCAAGCAAGUAGAACGUUAGAGGAACGUAGACGUUCUAUGCUUGACCAUCAUUGGCCAGUUCC
.(((...((((((((((((((..(((((((((...........)))))))))))))))))))))))))))))......

 Initial dG =    -37.80

             10        20        30
A-----|  GAG              AG         AGAGG
      GGU   CGGUGGUGGUCAAGCA  UAGAACGUU     \
      CCG   GUUACUACCAGUUCGU  AUCUUGCAG     A
CCUUGA^  ---              --         AUGCA
           70        60          50

-----------------------------------------------------
```

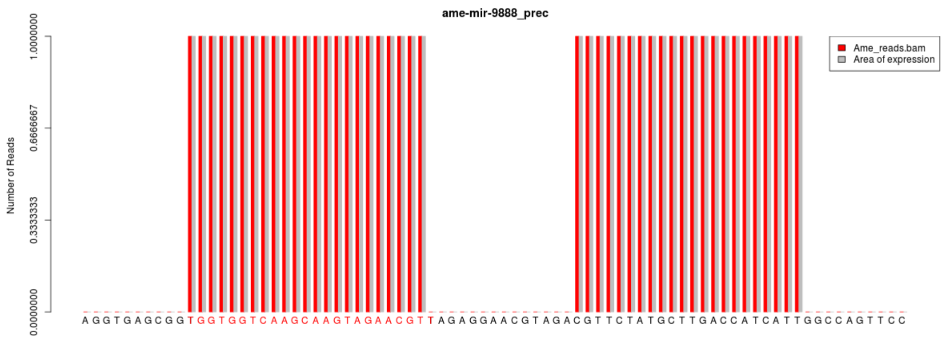

>ame-mir-9889_prec
TCGCACCGTCAGTTTCTTCCGCCGCGCCTGAAAGGCGTTC**AGTGTCGAGCCGAAGAAACGCGC**GCTTCTTCTA

>ame-mir-9889_3p
AGTGTCGAGCCGAAGAAACGCGC

```
UCGCACCGUCAGUUUCUUCCGCCGCGCCUGAAAGGCGUUCAGUGUCGAGCCGAAGAAACGCGCGCUUCUUCUA
..((..(((..((((((((.(((((((.((((.....))))))).)).)).))))))))))).))........

 Initial dG =    -22.90

              10        20          30
UC------| AC   CA        C  -  -   C    AG
        GC  CGU  GUUUCUUC GC CG CGC UGAA  \
        CG  GCG  CAAAGAAG CG GC GUG ACUU  G
AUCUUCUU^ C-   --        C  A  U   -    GC
  70         60          50         40

-----------------------------------------------------
```

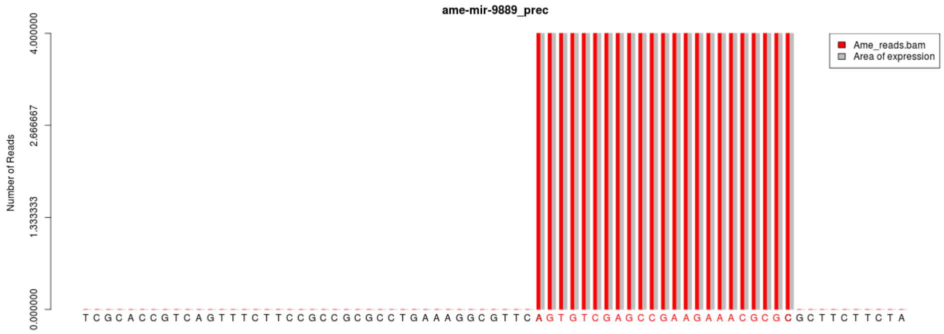

>ame-mir-989_prec
CAGAATCACCGATATTTAAGTATGGGTGGAGAGAGCGGGGAGAGCAAACGATTCGTCGTCCTCCGCCCGG**CGTGATGTGACGTAGTGGT TCT**C

>ame-mir-989_3p
CGTGATGTGACGTAGTGGTTCT

```
CAGAAUCACCGAUAUUUAAGUAUGGGUGGAGAGAGCGGGGAGAGCAAACGAUUCGUCGUCCUCCGCCCGGCGUGAUGUGACGUAGUGGUUCUC
.((((((((((((((...((.(((((((((.((.(((.(((.........))).)))))))))))))))))..))))).)).))..)))))))).

 Initial dG =    -32.90

          10           20         30         40
C        --   --|     UAA   A          A   G    G    AGCA
 AGAAUCAC  CG   AUAUU    GU UGGGUGGAG GA CGG GAG     \
 UCUUGGUG  GC   UGUAG    CG GCCCGCCUC CU GCU CUU     A
C        AU   AG^     UG-   -          -   -    G    AGCA
  90        80          70           60          50

-----------------------------------------------------
```

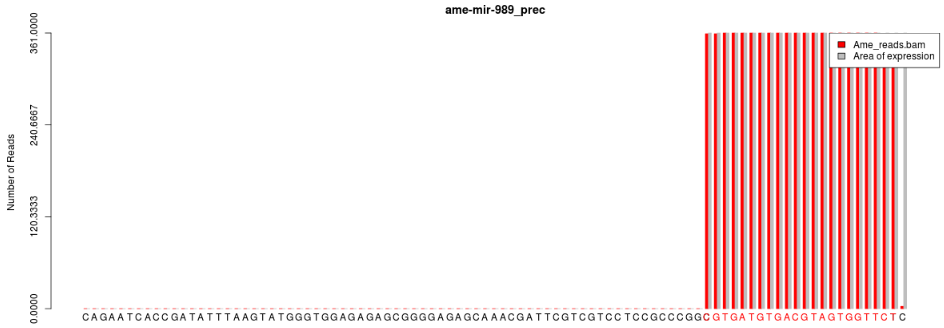

>ame-mir-9890_prec
ATTTTTCTTT**TTCGGAAGAATGTAGAGAAAAAG**ATATACACTTTCTTTTCCTTTCGTTCTTCTTAAACGGAAAGAA

>ame-mir-9890_5p
TTCGGAAGAATGTAGAGAAAAAG

AUUUUUCUUU**UUCGGAAGAAUGUAGAGAAAAAG**AUAUACACUUUCUUUUCCUUUCGUUCUUCUUAAACGGAAAGAA
.(((((((.(((.(((((((((.((((.((((((.........)))))).)))))))))))))).))).))))))).

Initial dG = -24.20

```
       10        20        30
A|      U   C         U    A      UAUA
 UUUUUCU UUU GGAAGAAUG AGAG AAAAGA    \
 AGAAAGG AAA UCUUCUUGC UUUC UUUUCU    C
A^      C   U         -    C      UUCA
    70        60         50        40
```

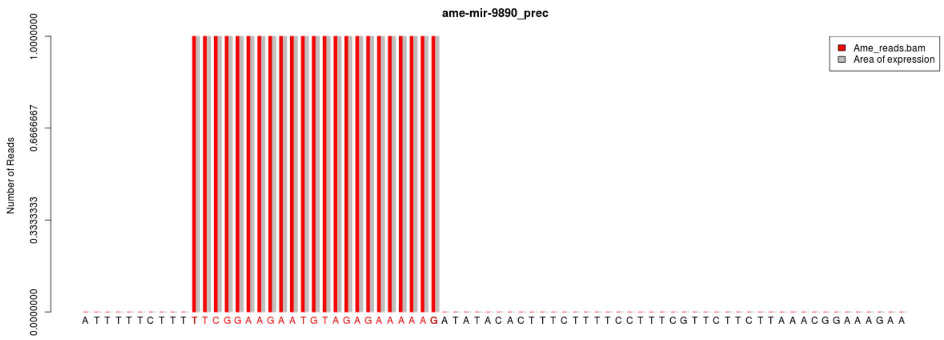

>ame-mir-9891_prec
TGCGTGGCGGCGGCGGCGGCGGCCTGGCCGATGGTGGACCACCC**TCGGCTTCGTCCTCGTCGTCG**TCGACGATGC

>ame-mir-9891_3p
TCGGCTTCGTCCTCGTCGTCG

```
UGCGUGGCGGCGGCGGCGGCGGCCUGGCCGAUGGUGGACCACCCUCGGCUUCGUCCUCGUCGUCGUCGACGAUGC
.((((..(((((((((((.(((..((((((.((((...)))).)))))))..))).))))))))))))...))))

 Initial dG =    -43.30

         10        20        30
U    GG-|           C   CU      U    G
 GCGU   CGGCGGCGGCGG GGC  GGCCGA GGUG \
 CGUA   GCUGCUGCUGCU CUG  UCGGCU CCAC A
-    GCA^           C   CU      C    C
     70        60        50        40

-----------------------------------------------------
```

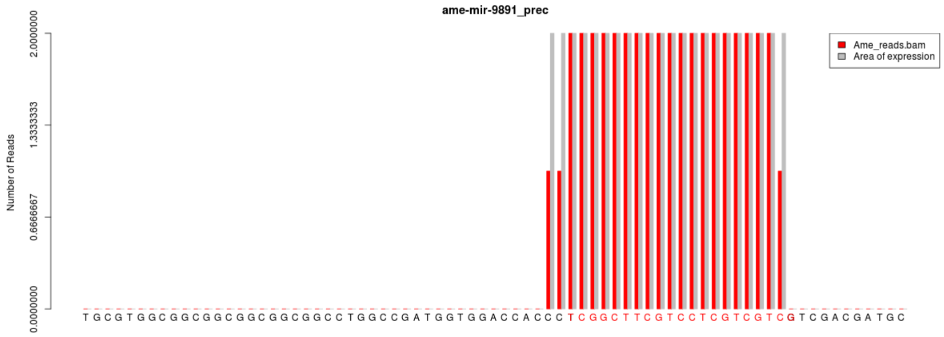

>ame-mir-9892_prec
AATACCGAGGCTGCCTCGATTTACTTTTCGTCGCCAAGTTCAAATCAATCT**TGACGCGATTGTGGAAATCG**AGTTGGTATC

>ame-mir-9892_3p
TGACGCGATTGTGGAAATCG

```
AAUACCGAGGCUGCCUCGAUUUACUUUUCGUCGCCAAGUUCAAAUCAAUCUUGACGCGAUUGUGGAAAUCGAGUUGGUAUC
.((((((......((((((((.((..(((.((.((((...........)))).)))))....)))))))))))))))))).

 Initial dG =    -21.90

        10         20           30         40
A|      GGCUGC        A  UU--   U  C     UUCAA
 AUACCGA      CUCGAUUU CU    UCG CG CAAG     \
 UAUGGUU      GAGCUAAA GG    AGC GC GUUC     A
C^      ------        -  UGUU   -  A     UAACU
80              70         60         50

-----------------------------------------------------
```

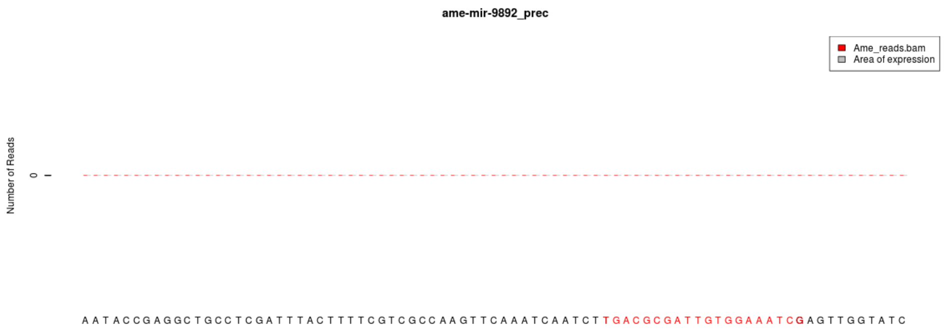

>ame-mir-9893_prec

ATTTCTAGTT**TTATGATCTGGAATACTAGG**TTCATAAGATTAATTATTATAGAATTTAGATCTCCAGGATCGTGAATTAATAATG

>ame-mir-9893_5p

TTATGATCTGGAATACTAGG

```
AUUUCUAGUUUUAUGAUCUGGAAUACUAGGUUCAUAAGAUUAAUUAUUAUAGAAUUUAGAUCUCCAGGAUCGUGAAUUAAUAAUG
.........((((((((((((...(((((((((((.........)))).))))))))...))))).)))))))).........

 Initial dG =    -21.10

        10         20         30         40
AUUUCUAGU        -     AUA         -|    GAUU
         UUUAUGAU CUGGA   CUAGGUUC AUAA    \
         AAGUGCUA GACCU   GAUUUAAG UAUU    A
GUAAUAAUU        G     CUA         A^   AUUA
    80        70        60        50

-----------------------------------------------------
```

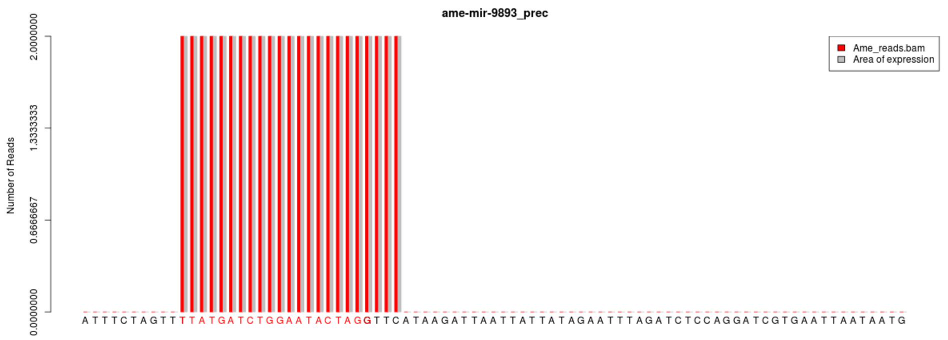

>ame-mir-9894_prec
TATTAACTATTACCTTTTAGCCGTTCACCCTCGCGAGTTGTACGAACGAGTTGTAG**GAGGGCGAGGAGAGGAGGAA**TTAAATCACT

>ame-mir-9894_3p
GAGGGCGAGGAGAGGAGGAA

```
UAUUAACUAUUACCUUUUAGCCGUUCACCCUCGCGAGUUGUACGAACGAGUUGUAGGAGGGCGAGGAGAGGAGGAAUUAAAUCACU
............(((((..((..((.(((((((((.((((....)))).)))))..))))).)))))..)))))...........

 Initial dG =    -25.70

        10        20        30          40
UAUUAACUAUUA|      AG   GU   A      --     G    A
            CCUUUU  CC   UC CCCUC   GCGA UUGU C
            GGAGGA  GG   AG GGGAG   UGUU AGCA G
UCACUAAAUUAA^      GA   --   C      GA     G    A
     80        70          60        50

-----------------------------------------------------
```

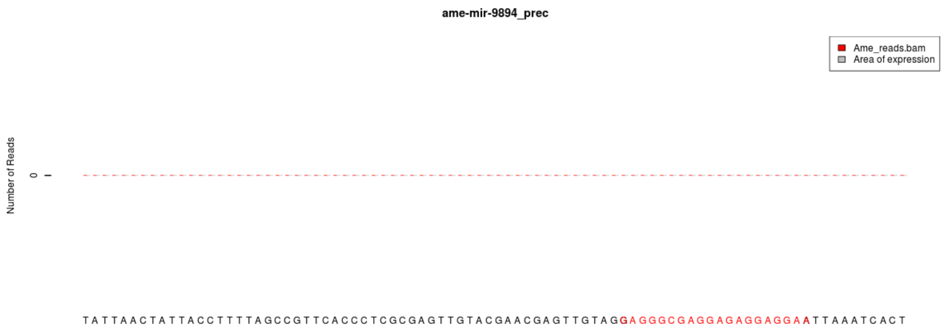

>ame-mir-9895_prec
CGCGAACGATTCGAGAATCGACCGACGATGGATAAACG**TCGTGTCCGTTTCTCGTTTCGA**ATCGAACGTA

>ame-mir-9895_3p
TCGTGTCCGTTTCTCGTTTCGA

CGCGAACGAUUCGAGAAUCGACCGACGAUGGAUAAACG**UCGUGUCCGUUUCUCGUUUCGA**AUCGAACGUA
```
..(((.(((..((((((.((..(.((((((......)))))))..)).)))))))..)))..))).....
```

```
 Initial dG =    -24.40

             10        20        30
CG----   A-|  UU      U  AC G      GA
      CGA  CGA  CGAGAA CG  C ACGAUG  U
      GCU  GCU  GCUCUU GC  G UGCUGC  A
AUGCAA   AA^  UU      U  CU -      AA
.        60        50          40

-----------------------------------------------------
```

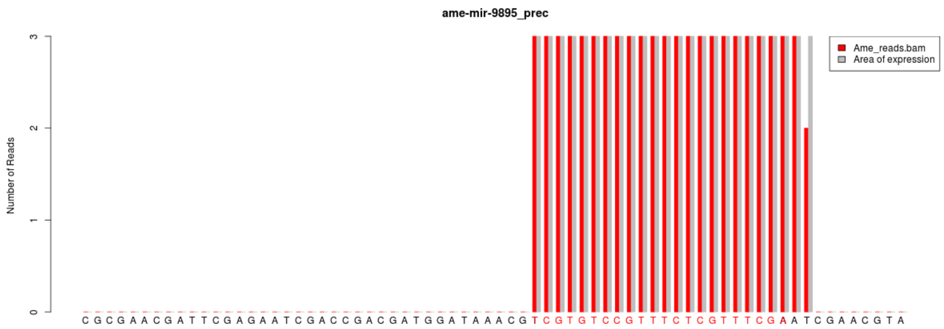

>ame-mir-9896_prec
GATTTATTCG**ACAATAATCGGACACAATCGGC**TAAAGGATCGAATACGAAGGATTGAACCGATGACGTCCGATTATTGCCGAACCGATA

>ame-mir-9896_5p
ACAATAATCGGACACAATCGGC

```
GAUUUAUUCGACAAUAAUCGGACACAAUCGGCUAAAGGAUCGAAUACGAAGGAUUGAACCGAUGACGUCCGAUUAUUGCCGAACCGAUA
.(((..((((.((((((((((((...(((((...((...(((....)))....))...)))))...)))))))))))).)))))..))).

 Initial dG =    -30.10

        10        20        30         40
G   UA    A            ACA     CUA  GGA-|  A
 AUU  UUCG CAAUAAUCGGAC   AUCGG   AA    UCG A
 UAG  AAGC GUUAUUAGCCUG   UAGCC   UU    AGC U
A   CC    C            CAG     AAG  AGGA^  A
        80        70        60        50

-----------------------------------------------------
```

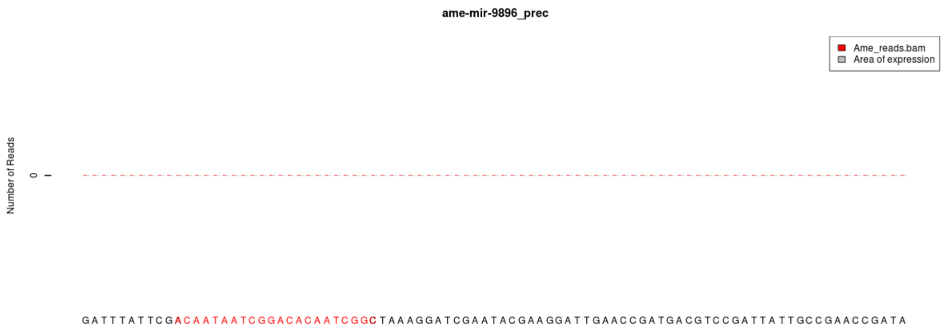

>ame-mir-993_prec
GGCACATTCGTGATCTACCCTGTAGATCCGGGCTTTTGTAGAATTGTAAATATCA**GAAGCTCGTCTCTACAGGTATCT**TACGGATGACA

>ame-mir-993_3p
GAAGCTCGTCTCTACAGGTATCT

```
GGCACAUUCGUGAUCUACCCUGUAGAUCCGGGCUUUUGUAGAAUUGUAAAUAUCAGAAGCUCGUCUCUACAGGUAUCUUACGGAUGACA
....(((((((((..((((.((((((..((((((((((...............))))))))))..))))))))))..)))))))))...
```

```
 Initial dG =    -37.90

        10        20        30        40
GGCA|        UC    C      UC          UAGAAUU
    CAUUCGUGA  UACC UGUAGA  CGGGCUUUUG       \
    GUAGGCAUU  AUGG ACAUCU  GCUCGAAGAC       G
ACA-^        CU    -      CU          UAUAAAU
         80         70        60        50

------------------------------------------------------
```

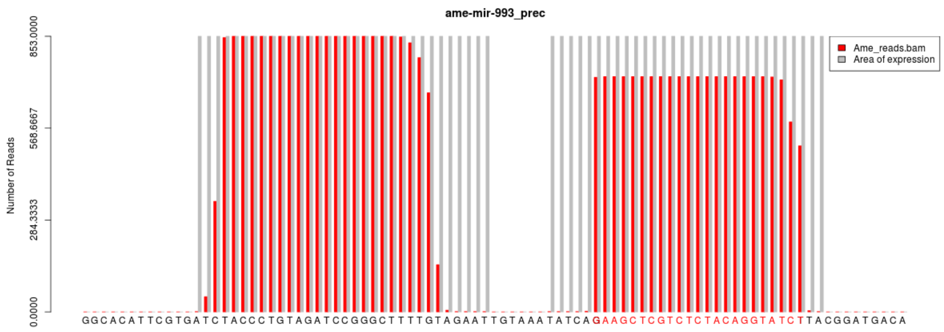

>ame-mir-996_prec
AAACAGATAATTCCATAGGCGAGTATGAATGTGGTGCACGTTTTGATTTATTTCG**TGACTAGATACATACTCGTCT**ACGGTAATCTGTT
TT

>ame-mir-996_3p
TGACTAGATACATACTCGTCT

```
AAACAGAUAAUUCCAUAGGCGAGUAUGAAUGUGGUGCACGUUUUGAUUUAUUUCGUGACUAGAUACAUACUCGUCUACGGUAAUCUGUUUU
(((((((....((.((((((((((((.((.((((.((((.............)))))))).)).))))))))))))).))..)))))))).

 Initial dG =    -37.20

         10        20        30        40
-|       AAUU  A            A  G    G    UUUUGA
 AAACAGAU    CC UAGGCGAGUAUG AU UGGU CACG      \
 UUUGUCUA    GG AUCUGCUCAUAC UA AUCA GUGC      U
U^       AU--  C            A  G    -    UUUAUU
90          80        70        60         50
```

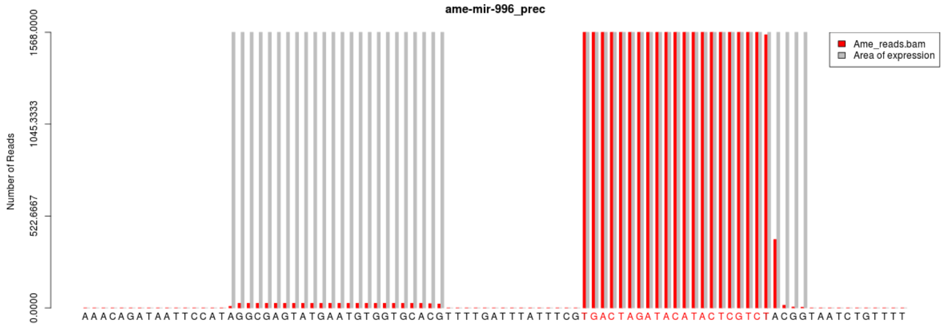

>ame-mir-9a_prec
TGGCGCGGACATTTT**TCTTTGGTTATCTAGCTGTATGA**GTATTATTCGACATCATAAAGCTAGGTTACCGGAGTTAAGCTCCTCGCCA

>ame-mir-9a_5p
TCTTTGGTTATCTAGCTGTATGA

```
UGGCGCGGACAUUUUCUUUGGUUAUCUAGCUGUAUGAGUAUUAUUCGACAUCAUAAAGCUAGGUUACCGGAGUUAAGCUCCUCGCCA
.((((.(((..((..(((((((.((((((((.((((((........)).))))).)))))))).)))))))..))..))).)))).

 Initial dG =    -38.50

        10        20        30         40
U    C   CA  UU       U        G     -| AUU
 GGCG GGA  UU  CUUUGGU AUCUAGCU UAUGA GU   A
 CCGC CCU  AA  GAGGCCA UGGAUCGA AUACU CA   U
A    U   CG  UU       U        A     A^ GCU
      80        70        60        50

-----------------------------------------------------
```

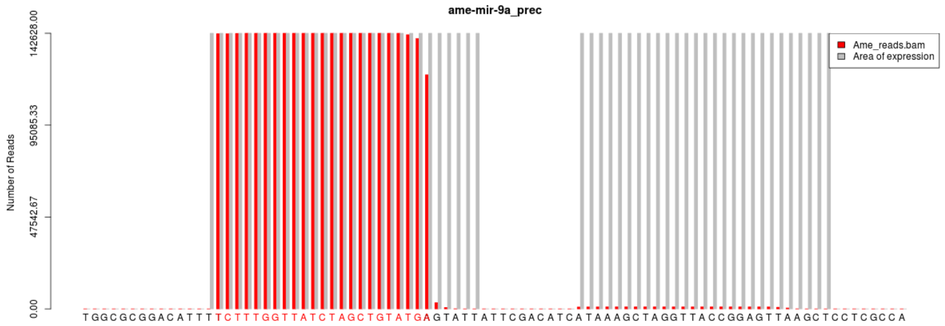

>ame-mir-9b_prec
TAATGCTAGTGCTCTTT**GCTTTGGTAATCTAGCTTTATGA**TCCTTAATTAAAATCATAGAGCTATATTACCAAAGTAAAGGCAAAAGCT
TTG

>ame-mir-9b_5p
GCTTTGGTAATCTAGCTTTATGA

```
UAAUGCUAGUGCUCUUUGCUUUGGUAAUCUAGCUUUAUGAUCCUUAAUUAAAAUCAUAGAGCUAUAUUACCAAAGUAAAGGCAAAAGCUUUG
....(((..(((.((((((((((((((.(((((((((((((..........))))))))))))).)))))))))))))))))..))).....
```

```
 Initial dG =    -43.10

        10        20        30        40
UAAU|  AG   U               C            CCUUA
    GCU  UGC CUUUGCUUUGGUAAU UAGCUUUAUGAU     \
    CGA  ACG GAAAUGAAACCAUUA AUCGAGAUACUA     A
GUUU^  AA   -               U            AAAUU
 90         80        70        60        50
```

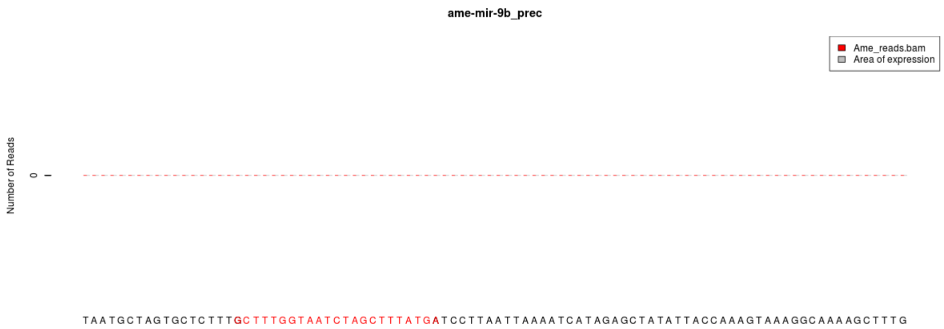

>ame-mir-9c_prec

GCTGTTTCACACATTTCTACCAAATCGTCCCGTTTATTTTTCTTTGGTGAGCTAACTGTATGGTGAATTGAAAGGCCA**TAAAGCTAGTT**
CAGCAAGGCAAGATATTCGAGACATAGCTTCATGAAATGTGACTGAAATT

>ame-mir-9c_3p

TAAAGCTAGTTCAGCAAGGCA

```
GCUGUUUCACACAUUUCUACCAAAUCGUCCCGUUUAUUUUUCUUUGGUGAGCUAACUGUAUGGUGAAUUGAAAGGCCAUAAAGCUAGUUCAGCAAGGCAAGAUAUUCGAGACAUAGCUU
CAUGAAAUGUGACUGAAAUU
...((((((((((((((.........(((.((..((((((.(((((.(((((((.((.
((((((..........)))))).)).))))))).))))).))))))..)).))).........))))))))..)))))).

 Initial dG =    -44.80

          10        20         30        40        50        60
GCU      --        UACCAAAUC-|  C  UU      U     G       A  G      GAAU
   GUUUCA  CACAUUUC          GUC CG  UAUUUU CUUUG UGAGCUA CU UAUGGU    U
   UAAAGU  GUGUAAAG          CAG GC  AUAGAA GGAAC ACUUGAU GA AUACCG    G
U--      CA        UACUUCGAUA^  A  UU      C     G       C  A      GAAA
         130       120       110       100        90        80        70

-----------------------------------------------------
```

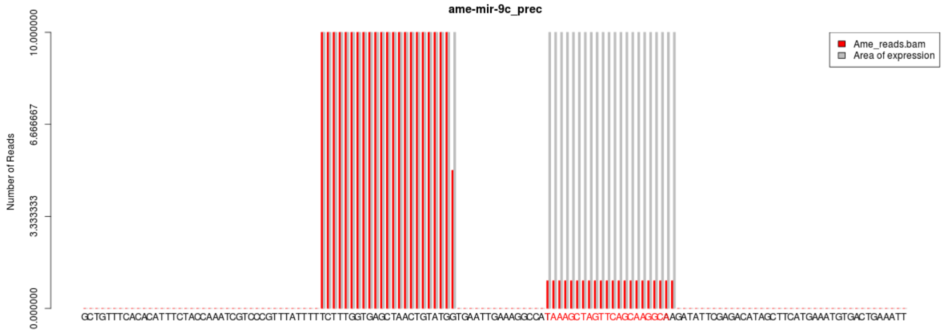

>ame-mir-iab-4_prec
GTGAAACCCCCTGT**ACGTATACTGAATGTATCCTGA**GTGTATTTCTGTCCGGTATACCTTCAGTATACGTAACAGGAGGCTACAC

>ame-mir-iab-4_5p
ACGTATACTGAATGTATCCTGA

```
GUGAAACCCCCUGUACGUAUACUGAAUGUAUCCUGAGUGUAUUUCUGUCCGGUAUACCUUCAGUAUACGUAACAGGAGGCUACAC
(((...((.((((((((((((((((.((((.(((..(........)..))).)))).))))))))))))).)))).))...)))

 Initial dG =    -34.30

        10         20        30        40
   AAA  C    -|            U    C   AG GUA
GUG   CC CCUG UACGUAUACUGAA GUAU CUG  U   U
CAC   GG GGAC AUGCAUAUGACUU CAUA GGC  G   U
   AUC  A    A^            C    U   CU UCU
    80        70        60        50

-----------------------------------------------------
```

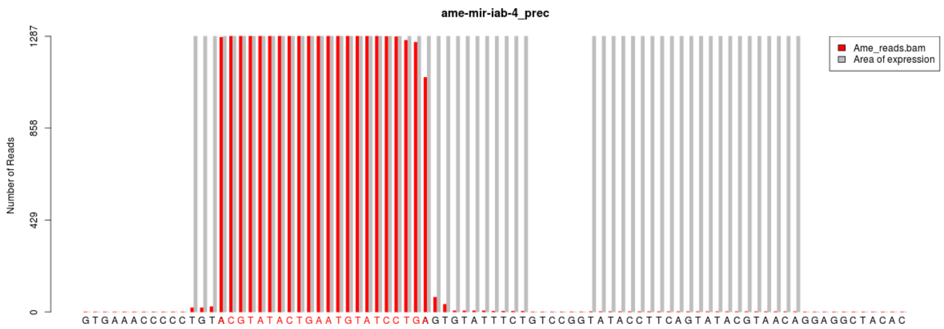

Supplementary Data S6. Report generated with MirPlot for the 342 miRNAs of *Tribolium castaneum* from miRBase and Ninova et al. (2016). For each miRNA, based on the available data, the following information is shown:

- The miRNA precursor.
- The precursor folding in Vienna format.
- The free energy of the precursor folding.
- The folding of the miRNA precursor.
- A plot showing all the expressed nucleotides of the precursor (grey), the reads per million in the available small RNA-seq libraries.

>tca-bantam_prec

```
UCAGCGAGACGAAACUGGUUUUCACAGUGAUUUGACAGAUAUAUUUGAUUCUGAGAUCAUUGUGAAAGCUGAUUUUGUUCA
.....(((.(((((..((((((((((((((((..((((..........)))))))))))))))))))))..)))))))).

 Initial dG =    -30.40

        10        20        30        40
UCAGC|  A     CU                  GA    UAUA
     GAG CGAAA  GGUUUUCACAGUGAUUU  CAGA    U
     CUU GUUUU  UCGAAAGUGUUACUAGA  GUCU    U
A----^  -     AG                  --    UAGU
    80         70        60          50

-----------------------------------------------------
```

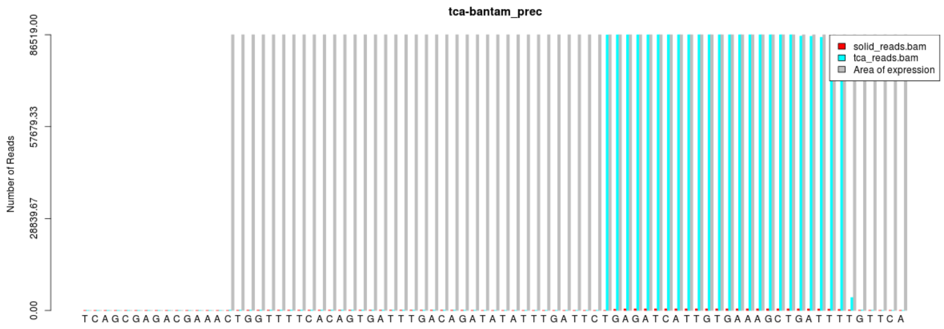

>tca-let-7_prec

```
GUCUGUUUGAGGUAGUAGGUUGUAUAGUAAGUAUUACACCUAUUGGGAGUGCUGUACAGCCUGCUAACUUUCCCAGAC
(((((...(((.(((((((((((((((((.........((....))...))))))))))))))))).)))...)))))

 Initial dG =    -33.60

        10        20        30        40
|    UUU   G                AGUAUUACA  U
GUCUG   GAG UAGUAGGUUGUAUAGUA         CC A
CAGAC   UUC AUCGUCCGACAUGUCGU         GG U
^    CCU   A                GAG------  U
       70        60        50

-----------------------------------------------------
```

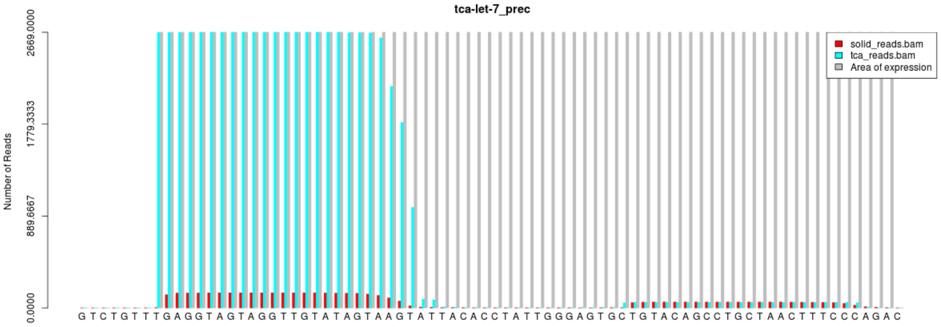

>tca-mir-1_prec

```
UUCGCGAGUUCCGUGCUUCCUUACUUCCCAUAGUGCUUUAAACGUAUGGAAUGUAAAGAAGUAUGGAGCGAA
.......((((((((((((.((((...(((((((.......)).)))))...)))).))))))))))))...

 Initial dG =    -28.20

        10        20        30
UUCGCGA            C    UUC     -| GCU
       GUUCCGUGCUUC UUAC   CCAUA GU   \
       CGAGGUAUGAAG AAUG   GGUAU CA   U
AAG----            A    UAA     G^ AAU
 70             60        50        40

-----------------------------------------------------
```

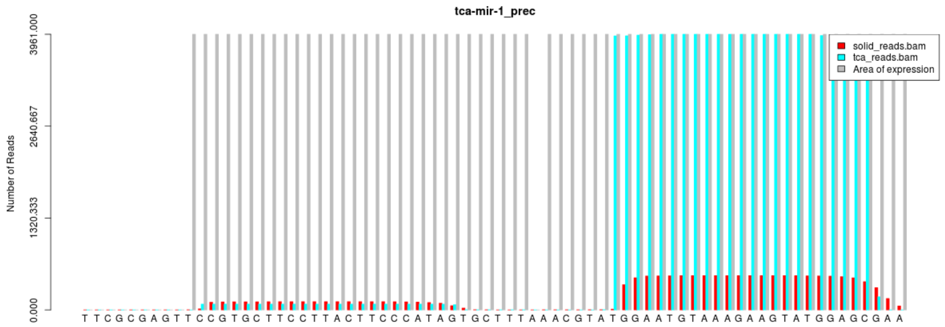

>tca-mir-2a_prec

UUUAUGUGCUGUGUCGCUCAUCAACUGGAUGUGAAAUGGAUUUGUAGCUCAUAUCACAGCCAGCUUUGAUGAGCGCUACAACAUCUAA

```
.(((.(((.((((.((((((((((((((.(((((.(((..(.....)..))).))))).))))..)))))))))).)))).))).)))

 Initial dG =    -35.00

        10        20          30        40
U   U   C    U          --|   A     A   GA UU
 UUA GUG UGUG CGCUCAUCAA  CUGG UGUGA AUG  U  \
 AAU UAC ACAU GCGAGUAGUU  GACC ACACU UAC  G  G
-   C   A    C          UC^   G     A   UC AU
        80        70        60        50

-----------------------------------------------------
```

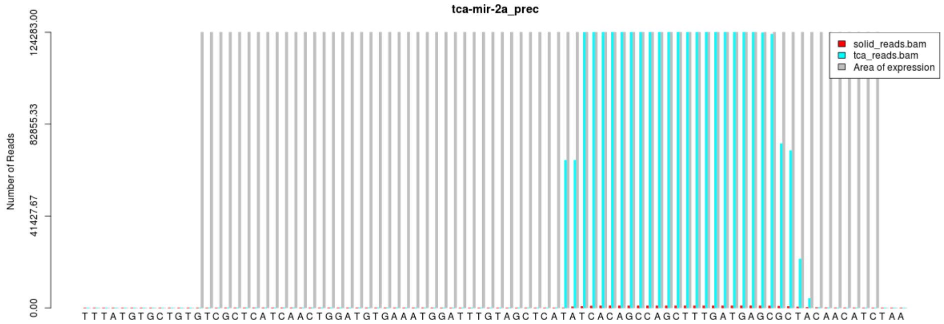

>tca-mir-2b_prec

```
GGUGUAGGCUUUCGCUCAUCAUCUGGUUGUGAAAUGUGAAUUUAUAUCAUAUCACAGCCAGCUUUGAUGAGCGCAAGAGUGCACU
(((((..(((.((((((((.((((((((((..(((((.......)))))))))))))))...))))))))).)))..))))))

 Initial dG =    -42.80

        10        20         30        40
      GG   U         U--|         AA     AUU
GGUGUA  CUU CGCUCAUCA   CUGGUUGUGA  UGUGA   \
UCACGU  GAA GCGAGUAGU   GACCGACACU  AUACU   U
      GA   C         UUC^         --     AUA
    80        70        60          50

-----------------------------------------------------
```

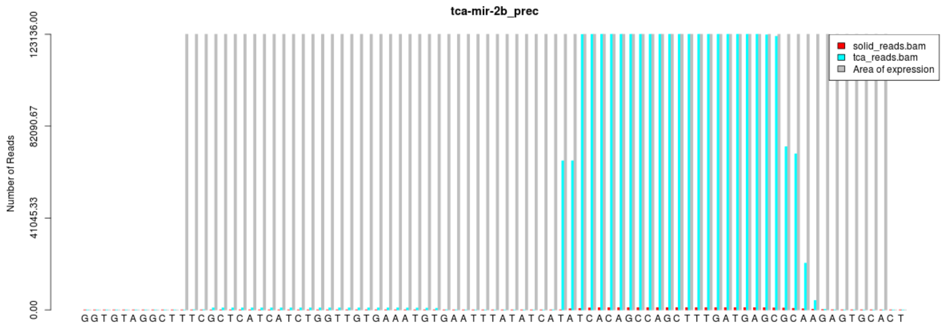

>tca-mir-2c_prec

```
AGUCUUGUCUGUCCUUAUCAGAGUUGGCGGUUUUAGAUUAACUGAUUCUAUCACAGCCAGCUUUGAUGAGCACAGUUGAGAUU
((((((.((((.((((((((((((((.((..((((.........))))..)).)))))))))))))))).)))).)))))))

 Initial dG =    -37.70

        10        20        30        40
|      U    C              G  UU    UUAA
AGUCUUG CUGU CUUAUCAGAGUUGGC GU  UAGA    \
UUAGAGU GACA GAGUAGUUUCGACCG CA  AUCU    C
^      U    C              A  CU    UAGU
  80        70        60        50

-----------------------------------------------------
```

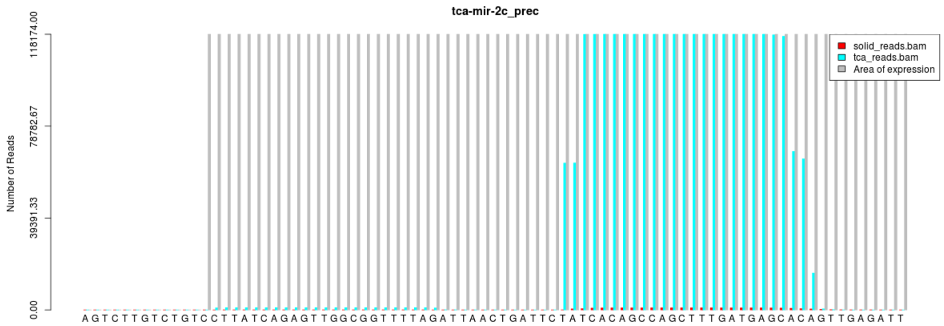

>tca-mir-7_prec

UGGUGUUGUGUGGAAGACUAGUGAUUUUGUUGUUCUAAUUUUUACGUAACAAGGAAUCACUAAUCAUCCCACAAAGCC

.(((.(((((.(((.((.((((((((((.(((((.............)))))))))))))))).)).)).)))))))).)))

```
 Initial dG =    -26.20

        10        20        30        40
U|  G     U   A  C         G     CUAAUU
 GGU UUGUG GGA GA UAGUGAUUUU UUGUU      \
 CCG AACAC CCU CU AUCACUAAGG AACAA      U
-^  A     -   A  A         -     UGCAUU
        70         60         50
```

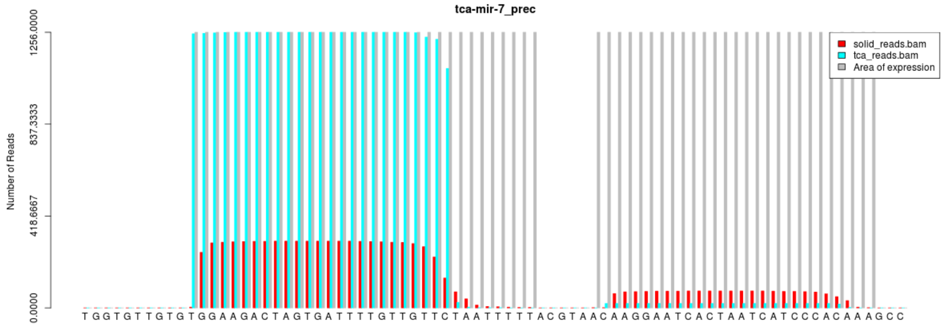

>tca-mir-8_prec

ACUGUUAACAUCUUACCGGGCAGCAUUAGAUUACAUUUUAUACUUCUAAUACUGUCAGGUAAAGAUGUCCUCC

```
.......((((((((((.(((((.((((((..............)))))).))))).))))).))))).....

 Initial dG =    -22.40

        10         20         30
ACUGUUA     -|    G     C      UUACAU
       ACAUC UUACC GGCAG AUUAGA      U
       UGUAG AAUGG CUGUC UAAUCU      U
CCUCC--     A^    A     A      UCAUAU
  70          60        50        40
```

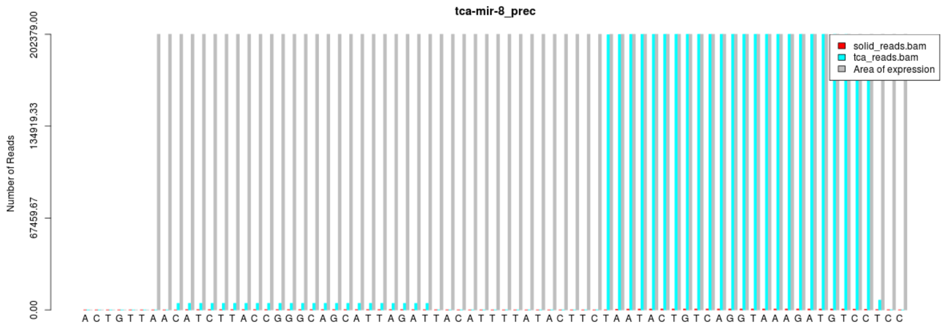

>tca-mir-9a_prec

CUGAUAUUUUCUUUGGUUAUCUAGCUGUAUGAGUAUUGUUUAAUAACAUCAUAAAGCUAGGUUACCGGAGUUAAAACCAGU
(((...((..(((((((.((((((((.(((((....((((....))))))))).)))))))).)))))))..))...))).

```
 Initial dG =    -26.60

         10        20        30        40
-|  AUA  UU       U        G     GUAU    U
 CUG   UU  CUUUGGU AUCUAGCU UAUGA    UGUU A
 GAC   AA  GAGGCCA UGGAUCGA AUACU    ACAA A
U^  CAA  UU       U        A     ----    U
80        70        60        50

-----------------------------------------------------
```

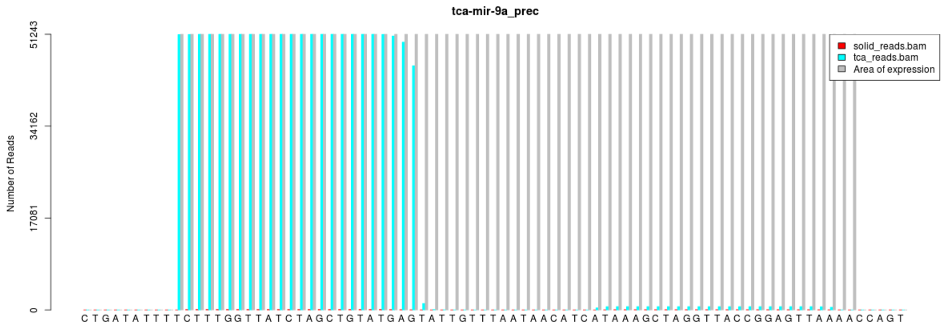

>tca-mir-9b_prec

UUGACGACCGUAAGUUUCUUUGUUUUUGGUUCCAACAGGUGGGGGCCGAUUUUGUUACUUUGGUAAUUUAGCGUUUGUGAUUUUAUUUGAGUCAUAAAGCUAGAUUACCAAAGCAAUGCG
GUUGGCAUUGCGUCGUGGAGAUUGGAGUUGGAAUAAGAAGACGUUCAUU

```
.....((.(((....((((....(((..((((((....(..(..((((((..((((.(((((((((((((((.
((((((((((.....)))))))))))))))))))))))))).)))))..)))))).)..)....)))).))..))).))))).......))).))...

 Initial dG =    -58.90

        10        20        30          40        50        60        70        80
UUGAC  C   AAG      UGUUUUUG        .-AG|UG GG      UU    A               G          UA
     GA CGU   UUUCUU        GUUCCAAC    G  G  GCCGAU  UGUU CUUUGGUAAUUUAGC UUUGUGAUUU  \
     CU GCA   GAAGAA        UAAGGUUG    C  U  CGGUUG  GUAA GAAACCAUUAGAUCG AAAUACUGAG  U
UUA--  U   ---      --------        \ --^GU A-      GC    C               -          UU
            160               150      130        120       110       100         90


                                      G----  GU
                                           UC  \
                                           AG  G
                                      AGGUU  AG
                                          140

-----------------------------------------------------
```

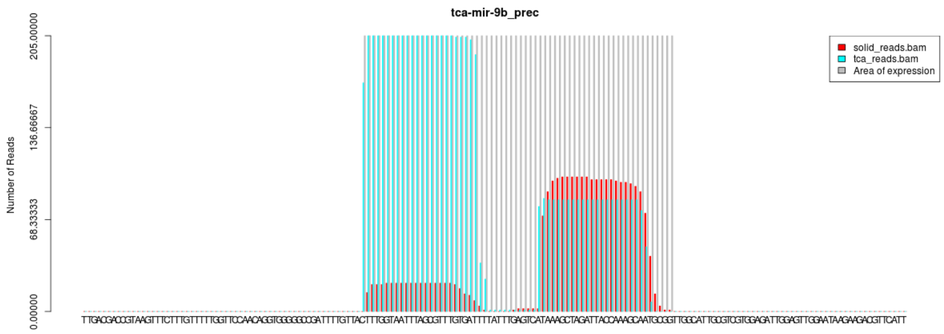

>tca-mir-9c_prec

UGAUUUUGCUAACGCGUUAUCACUUCUUUGGUGAUCUAGCCGUGUGAAUUAUUUUUGAAAUCACGCGUCUAAGUCGCCAAGAUUUGAUGAUGUGUCUAACAACUGGA
.....(((.((((((((((((..((((.(((((..(((.(((((((.((.......)).))))))).)))..)))))))))..))))))))))).)).))).....

```
 Initial dG =    -38.90

        10         20        30        40        50
UGAUU   C  -|          CU    U     UC   C       A  AUU
     UUG UA ACGCGUUAUCA  UCUU GGUGA  UAG CGUGUGA UU   \
     AAC AU UGUGUAGUAGU  AGAA CCGCU  AUC GCGCACU AA   U
AGGUC   A  C^          UU    -     GA   U       A  GUU
     100        90        80         70        60

-----------------------------------------------------
```

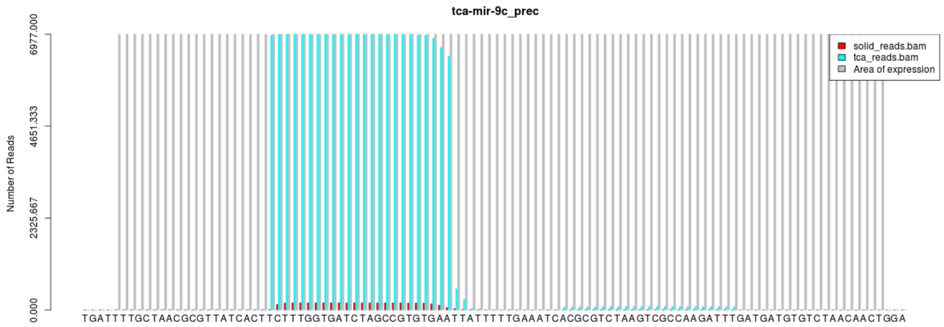

>tca-mir-9d_prec

```
AAUACGUCUACUUUAUAUUUCUUUGGUGAUCUAACUUAUGAUUAACUUUAUAAGUCAUAAAGCUAGAUUCACCAAAGAAAUAUGUGGUUGGCA
.....(((.(((.((((((((((((((((((.(((((((((.........)))))))).).))))).)))))))))))))))).))).))).

 Initial dG =    -36.40

        10        20         30         40
AAUAC   U   U               -     A -|       AACU
     GUC ACU UAUAUUUCUUUGGUG AUCUA C UUAUGAUU    \
     CGG UGG GUAUAAAGAAACCAC UAGAU G AAUACUGA    U
A----   U   U              U     C A^       AUAU
      90        80        70        60        50

-----------------------------------------------------
```

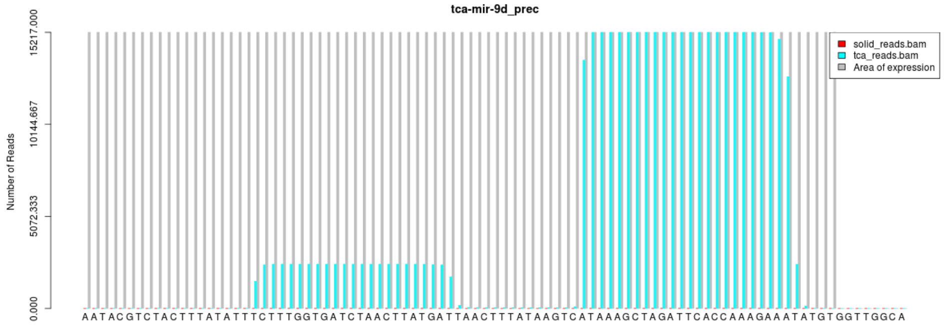

>tca-mir-9e_prec

```
AACAGGUUGCAAGUCGGAUAUAUUCUUCUUUGGUGAUCUAGUUGUAUGAGUGUUUUAUAUAUCAUAAAGCUGGUUCAGCAAAGCAGAUUGCAUUCGACUCUUCGACGAGCC
.....((((..((((((((.((.(((.(((((.(((.((((((.(((((.(((.....)))))))).)))))).))).))))).))).)).))))))))...)))).....

 Initial dG =    -37.00

        10         20        30        40        50
AACAG    CA-|       A  U   U     G   U      G     G   UU
     GUUG   AGUCGGAU UA UCU CUUUG UGA CUAGUU UAUGA UGU  \
     CAGC   UCAGCUUA GU AGA GAAAC ACU GGUCGA AUACU AUA  U
CCGAG    UUC^       C  U   C     G   U      A     -   UA
 .       100        90        80        70         60

-----------------------------------------------------
```

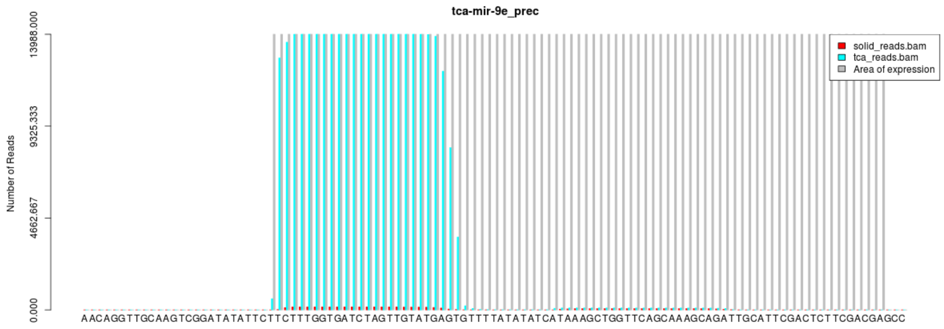

>tca-mir-10_prec

```
CUCUACAUCUACCCUGUAGAUCCGAAUUUGUUUGAAAUCAGGCGACAAAUUCGGUUCUAGAGAGGUUUGUG
.....(((..(((((.((((.((((((((((((((....))...)))))))))))).)))).)).))).)..)))

 Initial dG =    -26.60

        10         20        30
CUCUA   CU   -  G    U           ---| A
     CAU  ACC CU UAGA CCGAAUUUGUU   UG A
     GUG  UGG GA AUCU GGCUUAAACAG   AC A
-----   UU   A  G    U           CGG^ U
     70        60        50        40
```

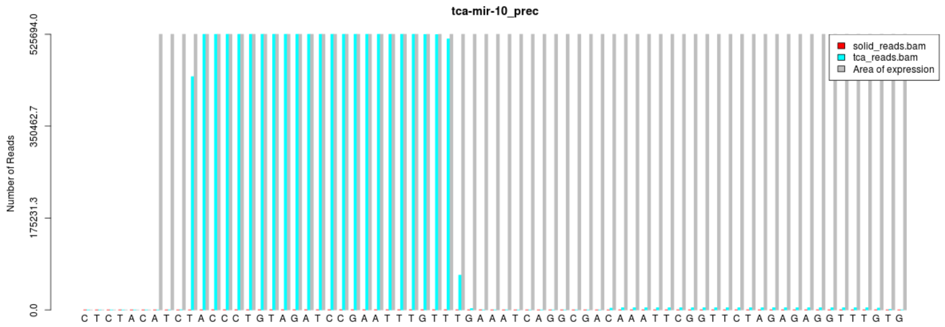

>tca-mir-11_prec

```
CAAACGCUUACAUUGUAAAUGCAUUCUGGGAUUCUGACUGUGACCUGUGUGUGUCUGAUUCACAUCACAGGCAGAGUUCUAGCUAUGCGUUUACAGCUUGCUUCUC
.....((.....(((((((((((.((((((((((.((((((..((((.((.....)).)))))))))).))))))))))..)))))))))))...)).....
```

```
 Initial dG =    -42.00

        10        20         30        40        50
CAAAC| UUACA            U-           A      CC    U  GU
     GC     UUGUAAAUGCAU  CUGGGAUUCUG CUGUGA  UGUG GU  \
     CG     GACAUUUGCGUA  GAUCUUGAGAC GACACU  ACAC UA  C
CUCUU^ UUC--            UC           G      --    U  GU
    100          90        80        70          60

-----------------------------------------------------
```

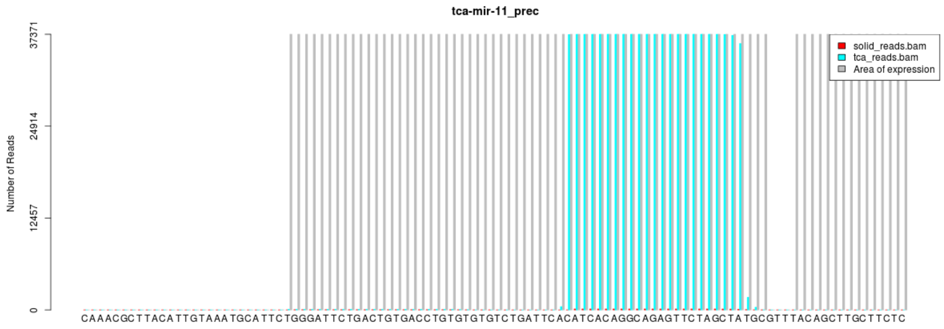

>tca-mir-12_prec

```
UAAAAGUUCAGGGUUUGAGUAUUACAUCAGGUACUGGUUCGGUUUUGAAAAACCGGUCCCUGAGUAACACUUACAUUCUGGCUUUUA
(((((((.((((((.(((((.((((.(((((.(((((((...........))))))).))))))))).))))).)))))))))))))

 Initial dG =    -33.80

        10         20         30         40
|      U      U     A    A     U       CGGUU
UAAAAGU CAGGGU UGAGU UUAC UCAGG ACUGGUU     \
AUUUUCG GUCUUA AUUCA AAUG AGUCC UGGCCAA     U
^      -      C     C    -     C       AAAGU
       80        70         60         50

-----------------------------------------------------
```

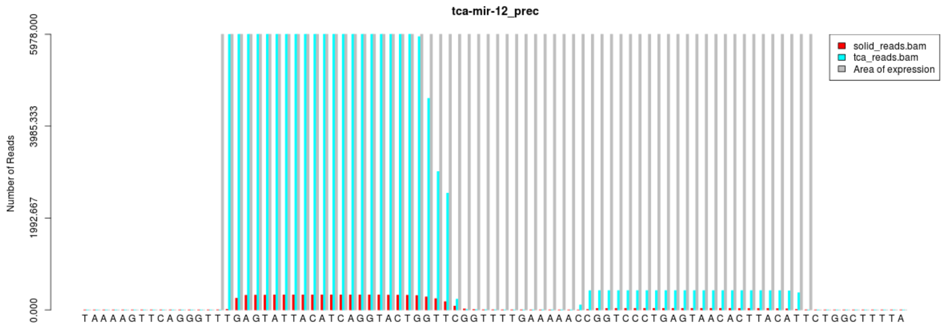

>tca-mir-13a_prec

GAGAUGCCCAAGUCGUCAAUUUGGUUGUGAACUCUUAAUUUAAACGAUAUCACAGCCACUUUGAUGAGCUUGGUUGUCUU

(((((..((((((((((((..(((((((((..((...........))..)))))))))..))))))).))))).)))))

```
 Initial dG =    -33.70

        10         20        30
     GC     -|      UU         AC  UUAAU
GAGAU  CCAAG UCGUCAA  UGGUUGUGA  UC     \
UUCUG  GGUUC AGUAGUU  ACCGACACU  AG     U
     UU     G^      UC         AU  CAAAU
.        70        60        50
```

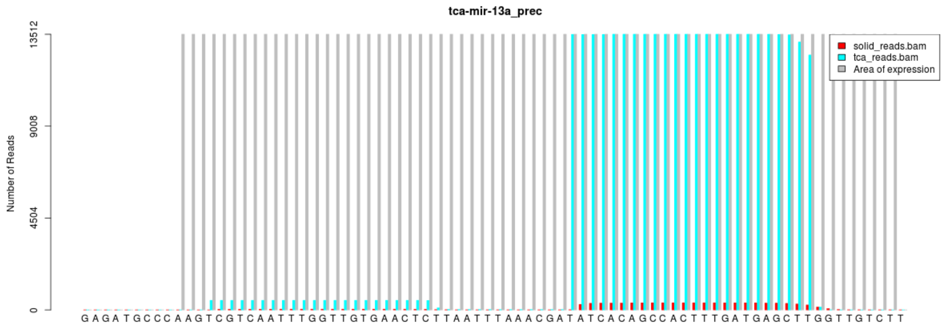

>tca-mir-13b_prec

```
UGCCCUAUCAGUCAAUUUGGUUGUGAAACCUAGUAUUUAUUCGGGUAUCACAGCCAUUUUGACGAGUUUGGUUG
.(((..((..(((((..(((((((((.(((((((....))).)))).)))))))))..)))))..))..)))..

 Initial dG =    -29.50

         10        20        30
U-   CU  CA     UU         A    -|  A
  GCC  AU  GUCAA  UGGUUGUGA ACCU AGU U
  UGG  UG  CAGUU  ACCGACACU UGGG UUA U
GU   UU  AG     UU         A    C^  U
   70        60        50        40

-----------------------------------------------------
```

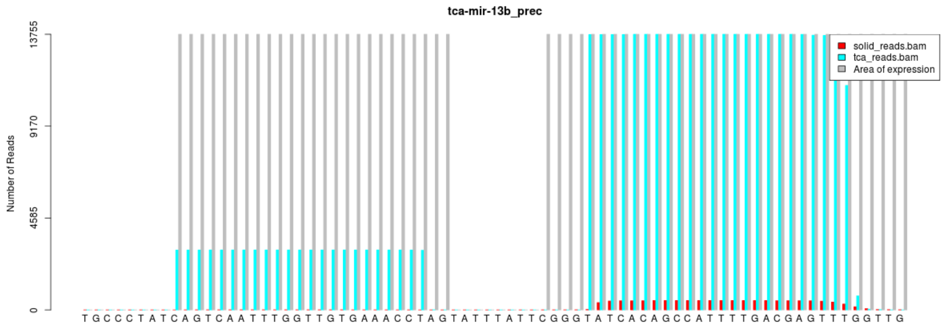

>tca-mir-14_prec

```
GACUCCAAUGGGUGAACAGGGGAGCGGAUUGGACUUACUUUUUUUCAAACAGUCAGUCUUUUUCUCUCUCCUAUUAAUCCAUUCGAU
...((.(((((((.((.(((((((.(((..(((((.(((.(((...))).))).)))))..))).))))))).)).))))))).)).

 Initial dG =    -30.10

        10        20        30        40
GAC| C       G  C       C   UU     U   U   U
   UC AAUGGGU AA AGGGGAG GGA  GGACU ACU UUU \
   AG UUACCUA UU UCCUCUC CUU  UCUGA UGA AAA U
U--^ C       A  A       U   UU     C   C   C
        80        70        60        50

-----------------------------------------------------
```

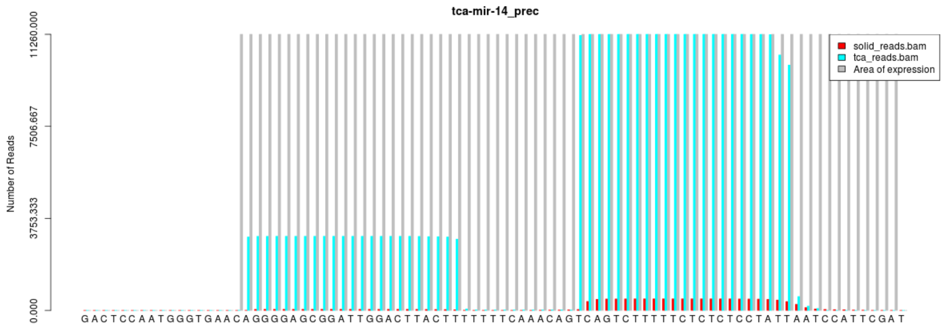

>tca-mir-29_prec

UUUGUGGUGGAACAAUAGUUGUAGGAUUUGGCUUUGGGCACUGUUUUCGUGUGGUGGUGAGAUUUUAUUUUGAUUCUAGCACCAUAUGAAAUCAGUUCUUGUACCAAAUACCAGCGAAAU
ACACCAAGUG

```
....(((((.........((((.((((((((...((((.((((.((((((((((((.((..
(((.......)))..)).)))))))))))).)))).))))..)))))).)).))))....)))))....
```

```
 Initial dG =    -43.60

        10        20         30        40        50        60
UUUG|    GAACAAUAG    A  -      CUU    C    U            G  AG   UUA
    UGGUG         UUGU GG AUUUGG   UGGG ACUG UUUCGUGUGGUG UG  AUU   \
    ACCAC         AGCG CC UAAACC   GUUC UGAC AAAGUAUACCAC AU  UAG   U
GUGA^    AUAA-----    A  A      AU-    U    U            G  CU   UUU
.       120            110        100        90        80        70
```

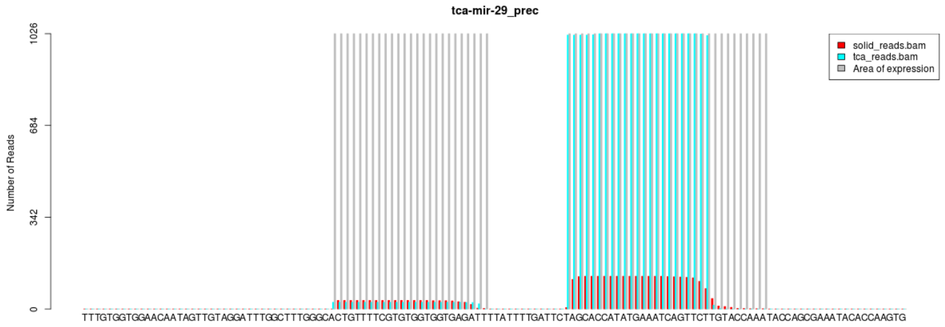

>tca-mir-31_prec

GAGGCAGAGCAGGCAAGAUGUCGGCAUAGCUGAAGGUGUUUGAAACGGCUAUGUCUCAUCCGGCCAACUUAAGCCUU
(((((.(((..(((..((((..((((((((((............)))))))))).))))..)))..)))..)))))

```
 Initial dG =    -30.90

         10        20        30
     A-|  CA   AA    UC          AAGGUG
GAGGC  GAG  GGC  GAUG  GGCAUAGCUG      \
UUCCG  UUC  CCG  CUAC  CUGUAUCGGC      U
     AA^  AA   GC    U-          AAAGUU
      70        60         50        40
```

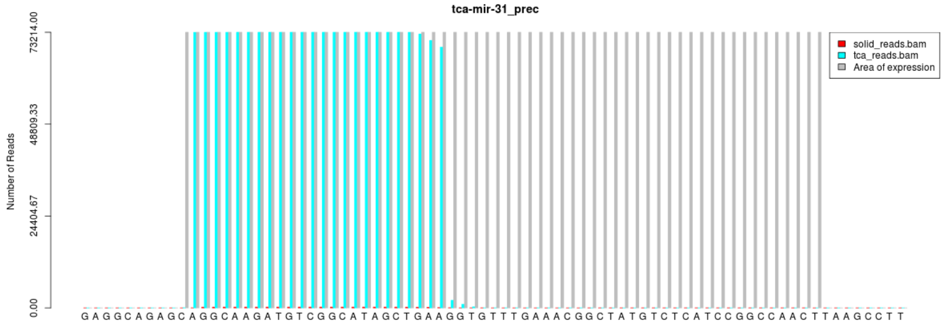

>tca-mir-33_prec

```
AGAAGAUGAAUAUUAUGAUCUUAGGUGCAUUGUAGUUGCAUUGCAUGUUAUUCAAAGUGCAGUGUCUCUCCAGUGCAACUUAGCGCAUAAUUUCACACCUCU
(((...((((.((((((...((((((((((((.((..(((((((((..........)))))))))..)).)))))).))))))..)))))))))))....)))

 Initial dG =    -32.20

         10         20          30         40         50
AGAAGA-|   U       AUC       -       U  UU         GUUA
       UGAA AUUAUG   UUAGGU GCAUUG AG  GCAUUGCAU    U
       ACUU UAAUAC   GAUUCA CGUGAC UC  UGUGACGUG    U
UCUCCAC^   -       GC-       A       C  UC         AAAC
100         90          80         70         60

-----------------------------------------------------
```

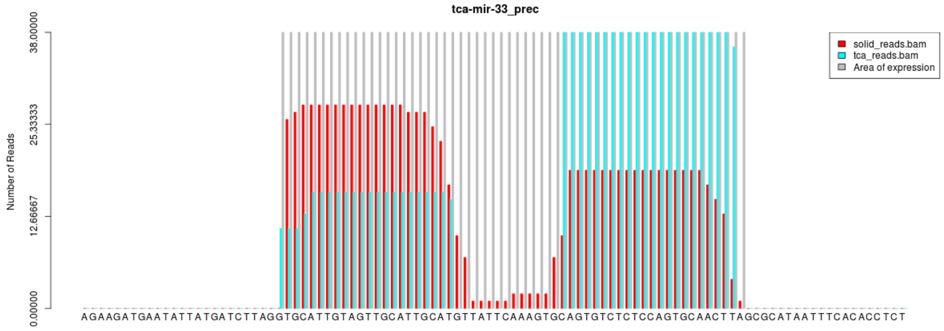

>tca-mir-34_prec

```
AUGUACACGGUGGCAGUGUGGUUAGCUGGUUGUGUUUUUUUGAUCACGACCACUAUCCAUACUCCCUCCAUGAAUAUCG
((((.((.((.((.(((((((.(((.((((((((.((....)).))))))))))).))))))).)).)).)).)))))..

 Initial dG =    -31.80

          10         20         30
--|   A  C  U  C       U   C        U  U
  AUGU CA GG GG AGUGUGG UAG UGGUUGUG UU U
  UAUA GU CC CC UCAUACC AUC ACCAGCAC AG U
GC^   A  A  U  C       U   -        U  U
        70         60          50        40
```

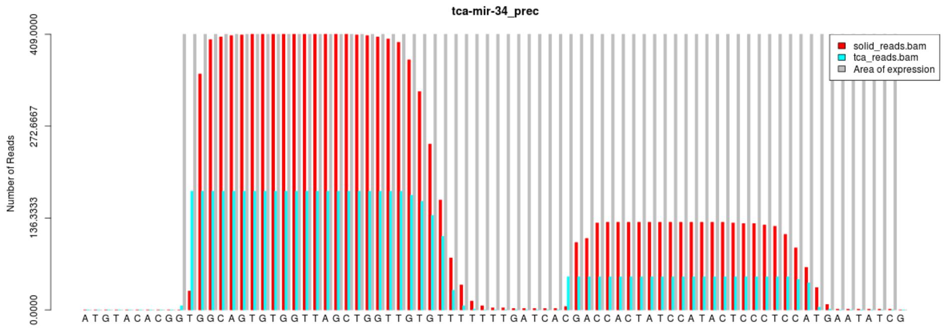

>tca-mir-71_prec

```
GUCUCUUCUUGAAAGACAUGGGUAGUGAGAUGUUUAAUUAAUAAAUUUUGUCUCACUACCUUGUCUUUCAUGUAGCGAU
(((.((...((((((((.((((((((((((((((.....)))))...)))))))))))))))))))))...)).)))

 Initial dG =    -33.90

        10        20        30
   U  UCU         U            ---|    AU
GUC CU   UGAAAGACA GGGUAGUGAGAU   GUUUA  \
UAG GA   ACUUUCUGU UCCAUCACUCUG   UAAAU  U
   C  UGU         -            UUU^    AA
        70         60        50        40

-----------------------------------------------------
```

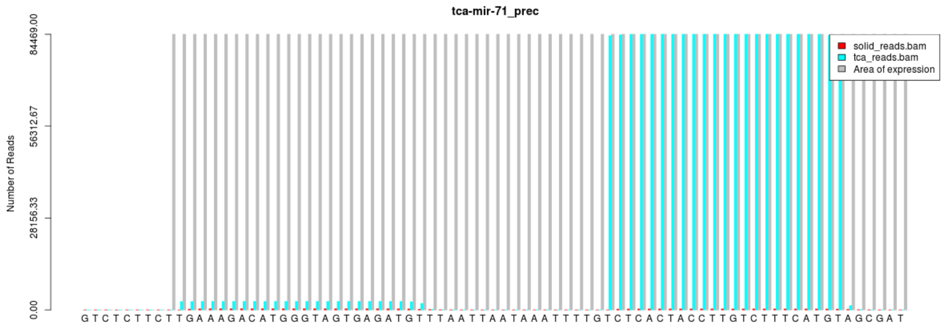

>tca-mir-87a_prec

CAGUCGUUGACGGGCUUGAAAUCUUGCUCUCCCGUGAUUAUUUCGUAAAGGGUGAGCAAAGUUUCAGGUGUGUCAAAGGCUU
.((((.((((((.((((((((..((((((.(((...((......))...))).))))))..)))))))).)))))).)))).

```
 Initial dG =    -39.00

        10        20        30        40
C|   G      G        UC      U   GUG  UA
 AGUC UUGACG GCUUGAAA  UUGCUC CCC   AU  U
 UCGG AACUGU UGGACUUU  AACGAG GGG   UG  U
U^   A      G        GA      U   AAA  CU
 80        70        60        50
```

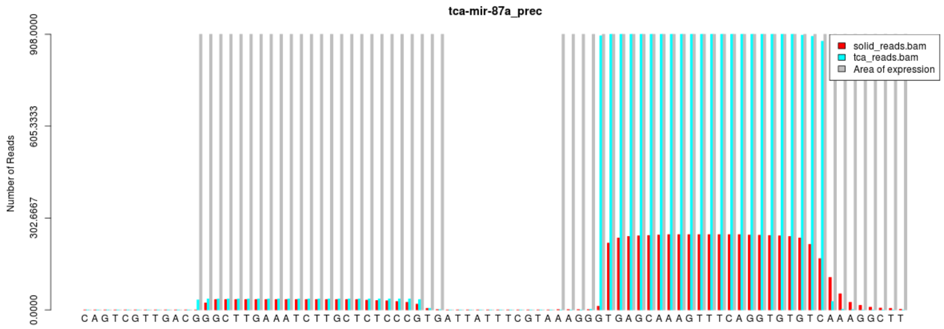

>tca-mir-87b_prec

```
CGGGGGAUGCCUGCCGUCGACACGCUUGAACUUUGUUUUUCCUGUGCAUUAUUCUAGGUGAGCAAAGAUUCAGGUGUGUUGACUAACUCUCUCAUCAG
.((((((........((((((((((((((((((((((..((((..........)))).)))))))).))))))))))))))))....)))))).....

 Initial dG =    -39.00

            10        20        30         40
C----|     UGCCUGCC              -         UU    UGCA
     GGGGGA        GUCGACACGCUUGAA CUUUGUUU  CCUG    U
     CUCUCU        CAGUUGUGUGGACUU GAAACGAG  GGAU    U
GACUA^     CAAU----              A         U-    CUUA
       90            80        70        60          50

-----------------------------------------------------
```

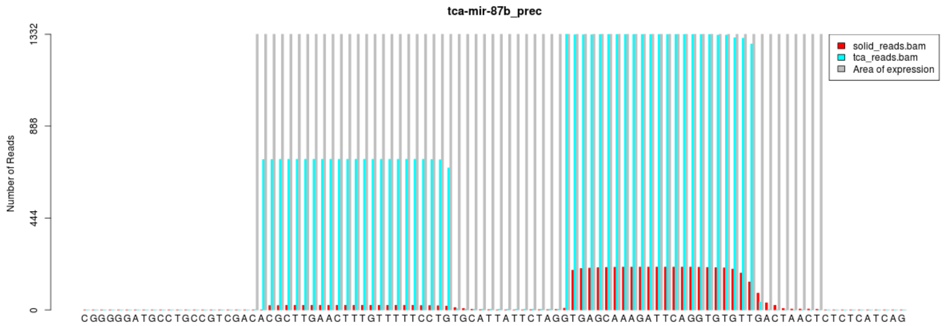

>tca-mir-92a_prec

```
CGCUUUCAGUAGUCCGUGAUGCGUGACAAUAUUUAGUUUUUUUCGAAUAUUGCACUAGUCCCGGCCUAUUGAGGGCGC
((((((((((((.(((.(((..(((.((((((((..........))))))))))..))).))).))))))))))))).

 Initial dG =    -32.30

         10        20        30
-|           U   U   GC   A        AGUU
 CGCUUUCAGUAG CCG GAU  GUG CAAUAUUU    U
 GCGGGAGUUAUC GGC CUG  CAC GUUAUAAG    U
C^           C   C   AU   -        CUUU
       70        60         50        40

-----------------------------------------------------
```

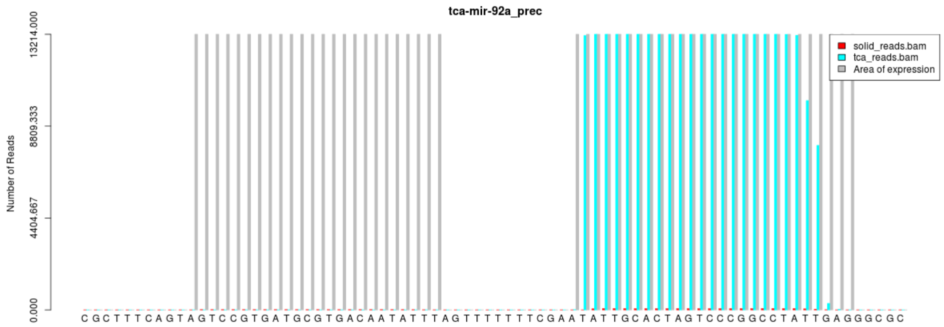

>tca-mir-92b_prec

CUGUGUCGUUUAGGUCGUGUCUUGUGCAACUUGUGGUUUUUCAUUAGUCAAAUUGCACUUGUCCCGGCCUGCAAGGCACAG
((((((...(((((((......((((((((.((((.....)))).))).....))))).......)))))))...)))))))

```
 Initial dG =    -29.60

        10        20             30
       GUU       UGUCUU     -----|  U    UU
CUGUGUC   UAGGUCG      GUGCA     ACU GUGG  \
GACACGG   GUCCGGC      CACGU     UGA UACU  U
       AAC       CCUGUU     UAAAC^  U    UU
 80        70        60        50        40
```

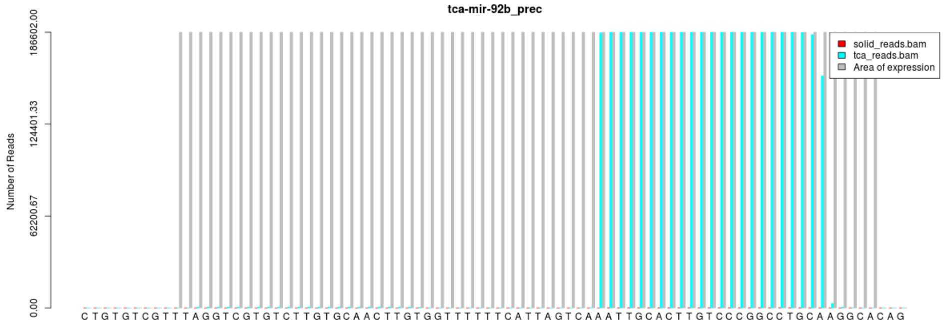

>tca-mir-92c_prec

```
UUUACUUAACAUUUUCGAGGAUGGAACGGUCGGUUGGGUCGUGAUUUGUGCAAAUAUGUUAUUUCGUUCAUAUUGCACCAGUCCCGGCCUGACUGCUCUUCCAGGACCACGCCGGGGUGU
UGGAGGGA
....((((((((((((.((.(((((.((.((((..(((((.((((.((((((.
((((..........))))))))))).)))).)))))..)))))).))))))...)).))..)))))))))).))...

 Initial dG =    -47.00

         10          20          30        40        50        60
UUUA  -         --  A  A--|    C  U    UG      U    U      A    UUAU
    CU UAACAUUUU  CG GG   UGGAA GG CGGU  GGUCG GAUU GUGCAA UAUG    U
    GA GUUGUGGGG  GC CC   ACCUU UC GUCA  CCGGC CUGA CACGUU AUAC    U
AGG-  G         CC  A  AGG^    C  -    GU      C    C      -    UUGC
       120       110       100         90        80         70

-----------------------------------------------------
```

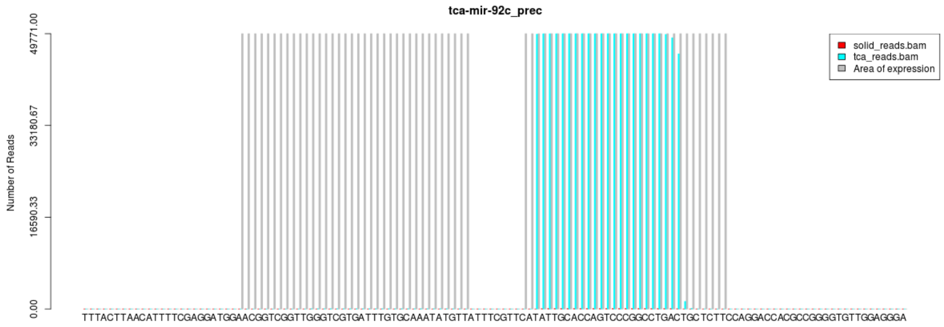

>tca-mir-100_prec

```
AUCCGGUGGAACCCGUAGAUCCGAACUUGUGGGGCUGUGGUCACAAGUUCUUGUUUAUGGGUACCUACUGGUAGAA
..(((((((.((((((((((..((((((((((........))))))))))..)))))))))).)).))))).....

 Initial dG =    -35.70

            10        20        30
AU---     -| A         CC         GGC
     CCGGU GG ACCCGUAGAU  GAACUUGUGG   U
     GGUCA CC UGGGUAUUUG  CUUGAACACU   G
AAGAU     U^ A         UU         GGU
     70        60        50        40

-----------------------------------------------------
```

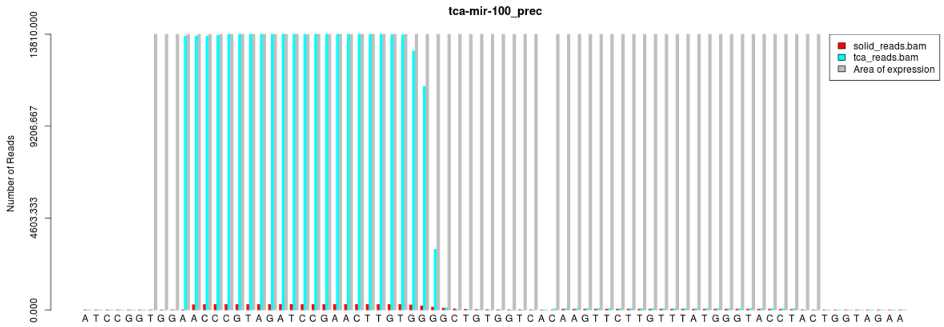

>tca-mir-124_prec

```
GUCAUUCUCCUAGUGUUCACUGUUGGCCUGUAUGUUUUUGUAACGCAUAAGGCACGCGGUGAAUGCCAAGAGCAGACUU
(((...(((...((((((((((..((((.(((((.........)))))))))..)))))))))))...)))..)))..

 Initial dG =    -27.80

          10        20        30
--|  AUU   CUA           UG    G     UUUU
  GUC   CUC   GUGUUCACUGU  GCCU UAUGU    \
  CAG   GAG   CGUAAGUGGCG  CGGA AUACG    G
UU^  AC-   AAC           CA    -     CAAU
         70        60        50

-----------------------------------------------------
```

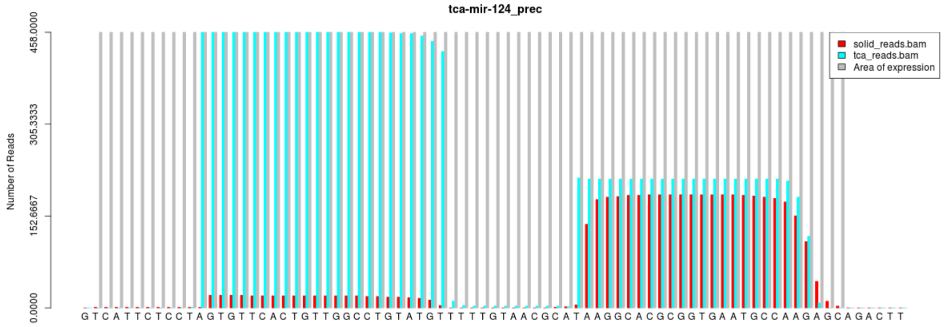

>tca-mir-125_prec

```
AUUUGCCUAUUCCCUGAGACCCUAACUUGUGAUGUUAAAUGGACUCUCACAAGCUAGCGACUCGGGUAUUGGCGGAU
(((((((.....((((((...(((.(((((((.(((.....)))..))))))).)))...))))))....)))))))

 Initial dG =    -27.70

        10        20        30
|      UAUUC     ACC   A       U-   AA
AUUUGCC     CCUGAG   CUA CUUGUGA  GUU  \
UAGGCGG     GGGCUC   GAU GAACACU  CAG  A
^      UUAU-     AGC   C       CU   GU
      70        60        50        40

-----------------------------------------------------
```

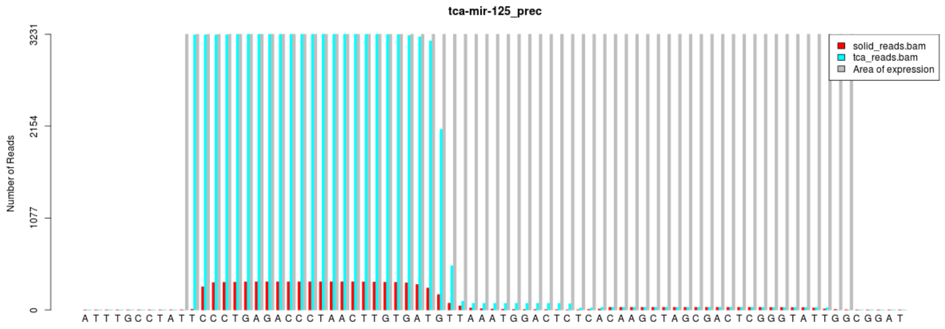

>tca-mir-133_prec

```
GCAAUGUGCAGCUUUAGCUGGUUGAAUCCGGGUCAAAUUGUAAAUGCUAUUUGGUCCCCUUCAACCAGCUGUGGUUGACAUUGC
((((((.(((((.((((((((((((...((..(((((.((....)).)))))..))..)))))))))))).))))))))))))

 Initial dG =    -44.20

        10        20        30        40
|      G     U           UCC  GU     U  A
GCAAUGU CAGCU UAGCUGGUUGAA   GG  CAAAU GU A
CGUUACA GUUGG GUCGACCAACUU   CC  GUUUA CG A
^      -     U           CC-  UG     U  U
   80         70        60         50

-----------------------------------------------------
```

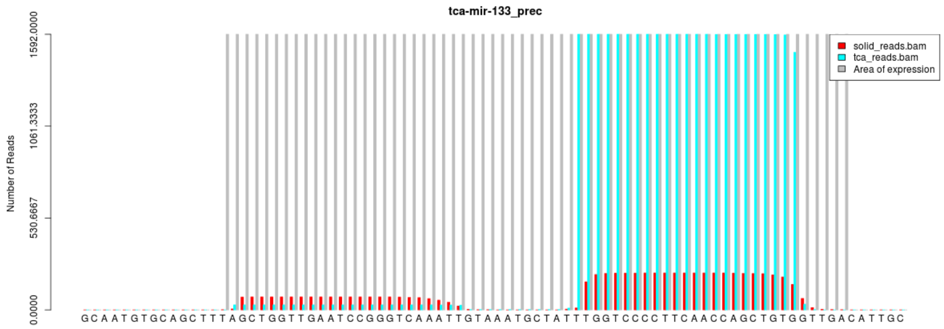

>tca-mir-137_prec

```
UGGUUGGUCACGUGUAUUCUUGGGUAAUUAACACAUAUUCAGGGAUGUUAUUGCUUGAGAAUACACGUAGCUGACAA
..((..(..((((((((((..((((((.((((...........))))))))))..)))))))))))..)..))..

 Initial dG =    -33.90

        10        20        30
UG| UG UC           UG      U    CAUAU
  GU  G  ACGUGUAUUCU  GGUAAU AACA     U
  CA  C  UGCACAUAAGA  UCGUUA UUGU     C
AA^ GU GA           GU      -    AGGGA
      70        60        50         40

-----------------------------------------------------
```

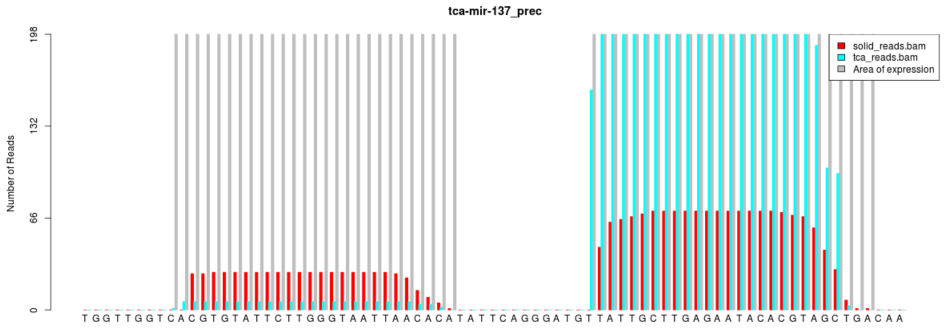

>tca-mir-184_prec

```
UUUUCUUCCAGUCCCUUGUCAUUCUCUCGCCCGGUGUAUUUUAACAACUGGACGGAGAACUGAUAAGGGCUUGUGGGAA
..((((..(((.(((((((((((((.((.(((((((......)).))))).))))))).))))))))).)))..))))

 Initial dG =    -35.30

        10        20         30         40
UU    UC   U         -|    U  C     -  AU
  UUCU  CAG CCCUUGUCA UUCUC CG CCGGU GU  U
  AAGG  GUU GGGAAUAGU AAGAG GC GGUCA CA  U
--    GU   C         C^    -  A     A  AU
          70        60         50

-----------------------------------------------------
```

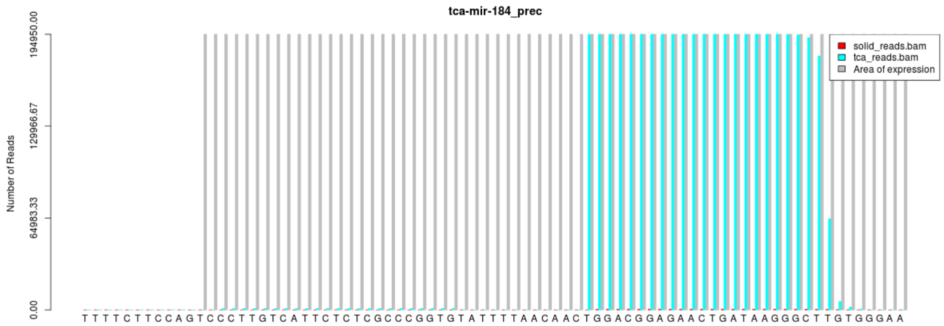

>tca-mir-190_prec

```
GUGAGUUGCUAUAAGAUAUGUUUGAUAUUCUUGGUUGUAUUUUUAAUUCACCCAGGAGUCAAACAUAUUAUUAUAGAAGCGUCACG
((((((.((((((((((((((((((..((((((.((...........)).))))))))))))))))))).))))))).))).)))).

 Initial dG =    -31.60

          10          20         30         40
-    -   G      -|           AU      U  UAUUU
 GUGA GUU CUAUAA GAUAUGUUUGAU  UCUUGG UG      \
 CACU CGA GAUAUU UUAUACAAACUG  AGGACC AC      U
G    G   A      A^           --      C  UUAAU
     80        70        60          50
```

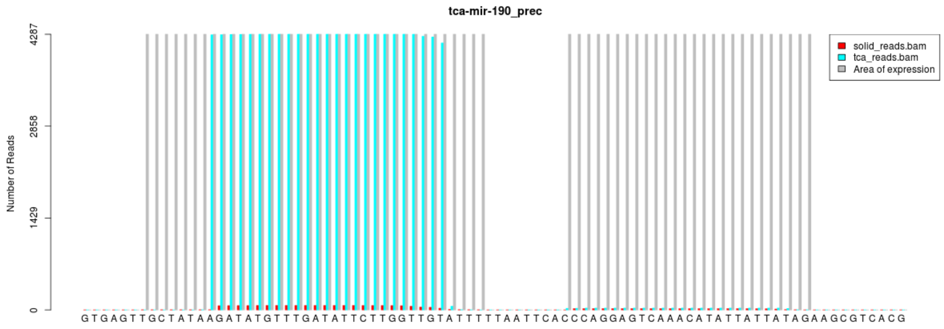

>tca-mir-193_prec

GCAAACUGGUCGUAAUGGUUUGGGCCUUAUAGGUUCAGUUGAUCAUUUUUAAAGCUACUGGCCUGUUAAGUCCCAAGUUAUUACAUUGCCGGAAGCU
((...((((.(((((..((((((.(((((((((.((((.................)))))))))).))).))))))..)))))...))))).)).

```
 Initial dG =    -33.30

         10          20          30         40
-  AAA     C--     GG       C    -|     U    UGAUCAUU
 GC   CUGGU   GUAAU  UUUGGG CUU AUAGGU CAGU        \
 CG   GGCCG   CAUUA  GAACCC GAA UGUCCG GUCA        U
U  AA-     UUA     UU       U   U^     -    UCGAAAUU
       90         80         70          60         50
```

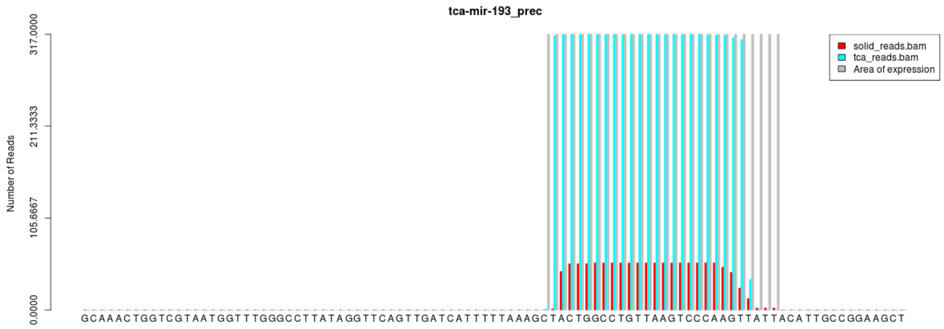

>tca-mir-210_prec

```
CUCACUGCAGCUGCUGGACGCUGCACAAGAUUAGAUAUUUUAGAACUCUUGUGCGUGUGACAGCGGCUAUUGUGGG
(((((...((((((((.((((.((((((((((((...)))))...))))))))))))..))))))))...)))))

 Initial dG =    -34.20

        10         20        30
     UGC        G-    U        ---|    U
CUCAC   AGCUGCUG  ACGC GCACAAGA   UUAGA \
GGGUG   UCGGCGAC  UGUG CGUGUUCU   GAUUU A
     UUA        AG    -        CAA^    U
     70        60         50        40

-----------------------------------------------------
```

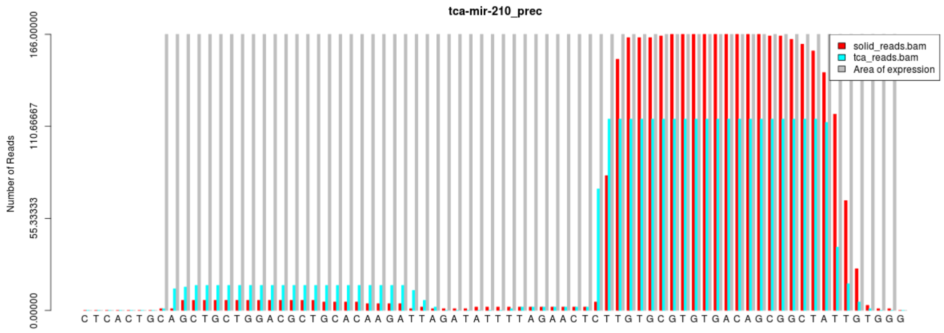

>tca-mir-219_prec

```
UUUCAGGCCCUGAUUGUCCAAACGCAAUUCUUGUUUGCAAUUUUAUCAAGGACUGUGUGUGGACAUCAUCGCUUGAGA
((((((((..((((.(((((.(((((.((((((.............)))))).))))).)))))))))..))))))))

 Initial dG =    -31.40

        10        20        30
|       CC    U     A     A      UUUGCA
UUUCAGGC  UGAU GUCCA ACGCA UUCUUG      \
AGAGUUCG  ACUA CAGGU UGUGU AGGAAC      A
^       CU    -     G     C      UAUUUU
       70        60        50

-----------------------------------------------------
```

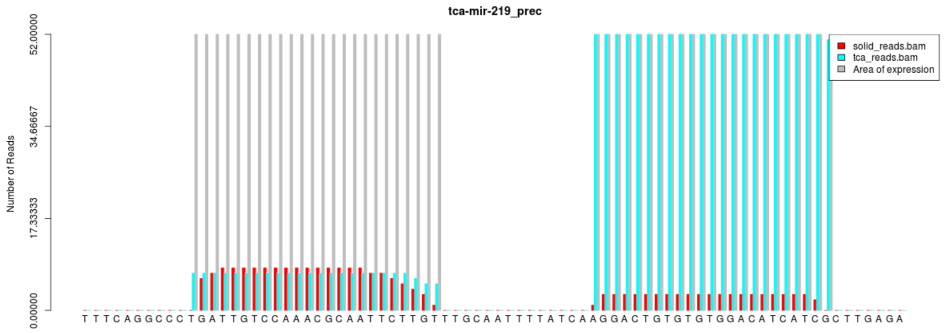

>tca-mir-252a_prec

AAAGACUGUCCUGUUACUAAGUACUAGUGCCGCAGGAGUACAUUCGACCUCCUGCUGCUCAAGUGCUUAUCAAUGGGCACAGAAUGA
.....((((((((((..((((((((...((.(((((((..........))))))).))...))))))))..)))))).)))).....

```
 Initial dG =    -34.50

         10        20        30        40
AAAGA    -|      AC        AGU  C       UACA
     CUGU CCUGUU  UAAGUACU   GC GCAGGAG    U
     GACA GGGUAA  AUUCGUGA   CG CGUCCUC    U
AGUAA    C^      CU        ACU  U       CAGC
      80        70        60        50

-----------------------------------------------------
```

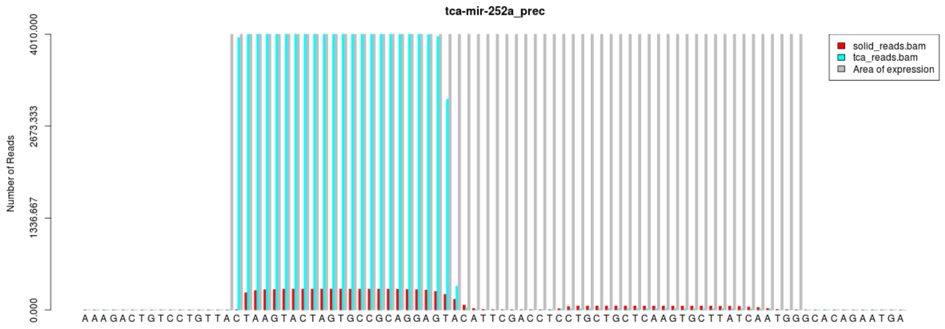

>tca-mir-252b_prec

```
GCCUCUUGUCCACCCGGUAUUAAGUAGUUGCGCCGAAGGUGAUGUUUUUGAAAUCUCCUCGGUCUGGCUGCUUUAUACCGCAUGGAUGAAAAUC
.....(..((((..((((((.(((((((((.(((((.((.(((.........))).))))))).))))))))).))))))..))))..).....
```

```
 Initial dG =    -38.20

        10        20        30        40
GCCUC|UG    CC      U         C     A  U   GUUU
     U  UCCA  CGGUAU AAGUAGUUG GCCGA GG GAU    \
     A  AGGU  GCCAUA UUCGUCGGU UGGCU CC CUA    U
CUAAA^GU    AC      U         C     -  U   AAGU
   90        80        70        60         50
```

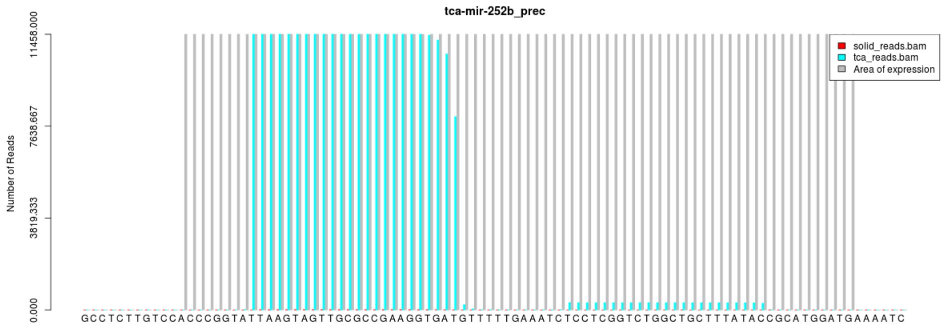

>tca-mir-263a_prec

```
GUGAGCAUAGGCCUGCGAAUGGCACUGGAAGAAUUCACGGGGUUCUUCGCACUCCCGUGGACUUUGAGUGCCAUCCUCAGAGCCCUUGUCC
....(((..((((((...((((((((.((((..((((((((((..........)))))))))))))).))))))))...))).)))..)))..

 Initial dG =    -42.70

        10         20        30        40
GUGA   UA   -|  CGA        G    AA         UUCU
    GCA  GGC CUG   AUGGCACU GAAG  UUCACGGGG    U
    UGU  CCG GAC   UACCGUGA UUUC  AGGUGCCCU    C
CC--   UC   A^  UCC        G    --         CACG
90          80        70          60        50

-----------------------------------------------------
```

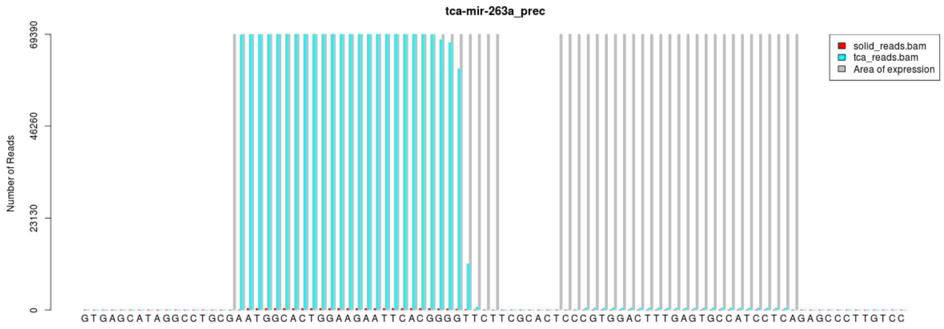

```
>tca-mir-263b_prec
CUUACGAACCUUGGCACUGGAAGAAUUCACAGAUUGUAUUUGAAUCGUGAUUCUUUUAAUGCCAUAUUUCGUGGG
((((((((...(((((.(((((((((.(((.((((.......)))))))))))))))).)))))...))))))))

 Initial dG =    -28.90

        10        20        30
|       CCU     C         U   A    GUA
CUUACGAA   UGGCA UGGAAGAAU CAC GAUU   \
GGGUGCUU   ACCGU AUUUUCUUA GUG CUAA   U
^       UAU     A         -   -    GUU
    70        60        50          40

-----------------------------------------------------
```

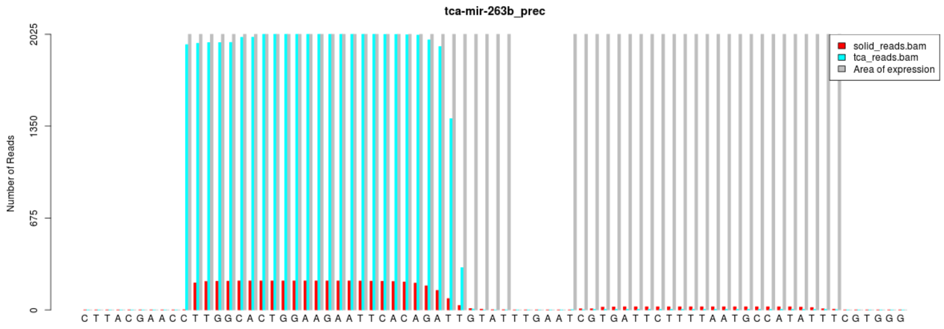

>tca-mir-275_prec

```
GCUACGCGUGUUACCUCAGUACCUAGGCUGGGGUUUUUCGAAAGUCAGGUACCUGAAGUAGCGCGCGCAGC
(((.((((((((((.(((((((((.((((..((....))...)))))))))))).))).)))))))))).)))

 Initial dG =    -37.60

        10         20        30
   A          C   -|     A    GG-  U
GCU CGCGUGUUAC UCA GUACCU GGCU   GG U
CGA GCGCGCGAUG AGU CAUGGA CUGA   CU U
   C          A   C^     -    AAG  U
70        60        50         40
```

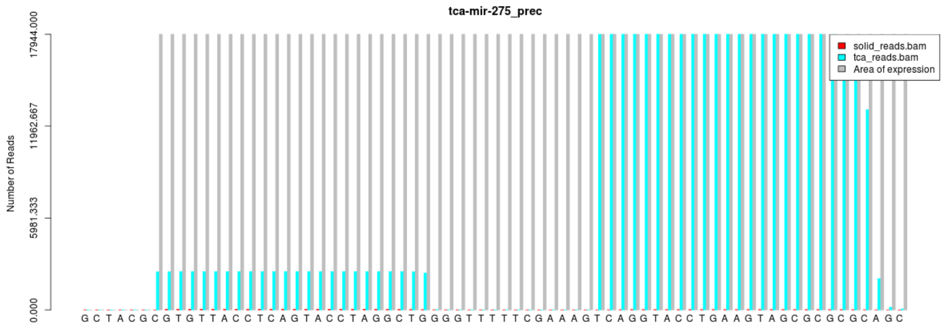

>tca-mir-276_prec

```
UAGACCACUAGCGAGGUAUAGAGUUCCUACGUGAUUUUUCGGCUGUAGGAACUUCAUACCGUGCUCUUGGA
....(((..((((.(((((..((((((((((((....)))..))))))))))..))))).)))).)..))).

 Initial dG =    -31.90

        10        20        30
UAGA   CU    A     AG          --|  U
    CCA  AGCG GGUAU  AGUUCCUACG  UGA U
    GGU  UCGU CCAUA  UCAAGGAUGU  GCU U
A---   UC    G     CU          CG^  U
   70        60        50        40

-----------------------------------------------------
```

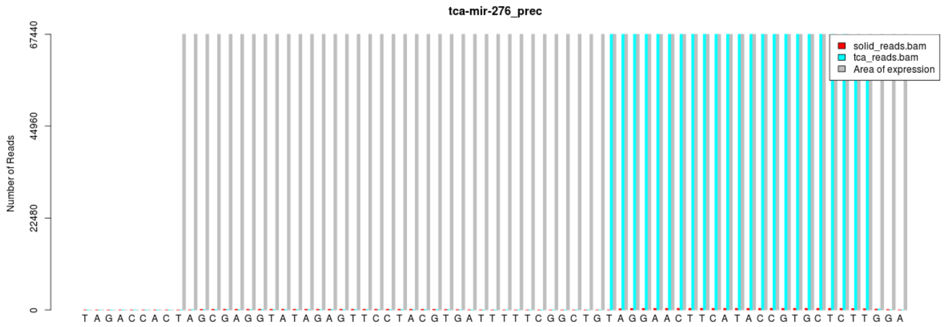

>tca-mir-277_prec

```
GUGGGCUCCGUGCCAGAUGCGCGUUUACACUGCACCGUUCUAAAGAAUUCUGUAAAUGCACUAUCUGGUACGACAGCCCAC
((((((.((((((((((.(((((((((.......((((....))))..)))))))))..))))))))))).)))))))

 Initial dG =    -38.60

        10        20         30        40
       C-           C-|        CUGCACC    U
GUGGGCU  CGUGCCAGAUG  GCGUUUACA       GUUC A
CACCCGA  GCAUGGUCUAU  CGUAAAUGU       UAAG A
       CA           CA^        CU-----    A
80        70        60        50

-----------------------------------------------------
```

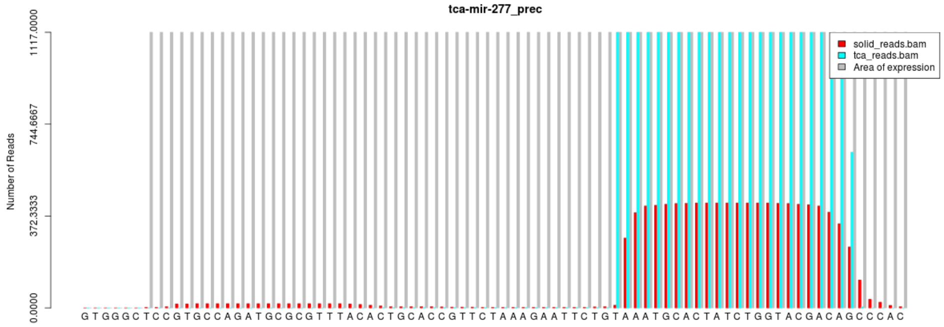

>tca-mir-279a_prec

```
CUUGAUGGGUUCGGGUCUAGUGGCACGGUUUUUUCAACUUCGUGACUAGAUCCACACUCAUUAAG
((((((((((..(((((((((.((..((((.....))))..)).)))))))))..))))))))))

 Initial dG =    -28.40

        10        20        30
|         UC         G  AC    UU
CUUGAUGGGU  GGGUCUAGU GC  GGUU  \
GAAUUACUCA  CCUAGAUCA UG  UCAA  U
^         CA         G  CU    CU
    60        50        40

-----------------------------------------------------
```

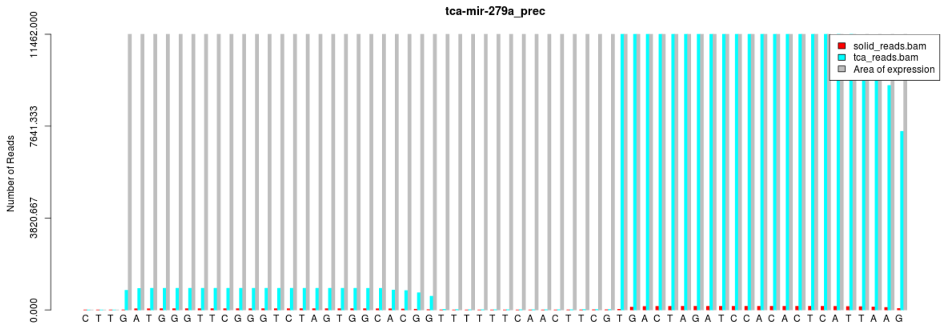

>tca-mir-279b_prec

```
GGUAGUUAUCCUAAAGUUUUUGAUGGGUGAGGGUUUGGUGCAUGUUUUAUUUUAAUCAUGACUAGAUCCACACUCAUCCAAAAAUUUGGUGU
..........(((((.((((.((((((((.(((((((((.((((.((......)).))))))))))))))).)))))))).)))).)))))...

 Initial dG =    -31.40

        10        20        30        40        50
GGUAGUUAUC|    G    U        A         G    U  UA
          CUAAA UUUU GAUGGGUG GGGUUUGGU CAUG UU  U
          GGUUU AAAA CUACUCAC CCUAGAUCA GUAC AA  U
UGU-------^    A    C        A         -    U  UU
 90                80        70         60

-----------------------------------------------------
```

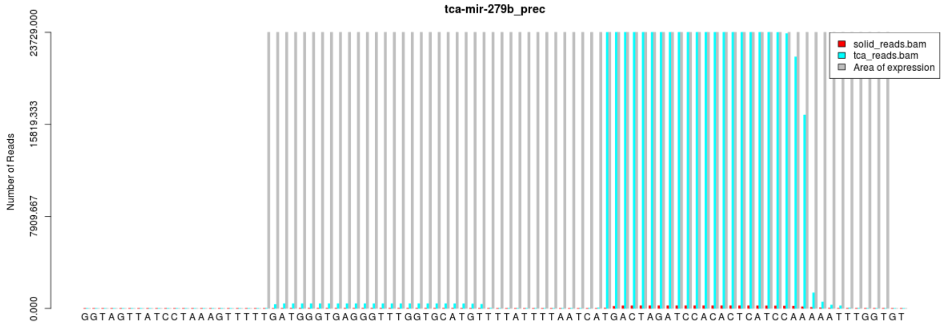

>tca-mir-279c_prec

```
UUUCGCCGCUUAAAUUCAUAUUUUUUUCGGGUGUUCGUUUCGAGUCACAUUGUGAUUGAUUUUGUGACUAGAUCGAACAUUCGCUCGAAGUAUGGAAAAAUCCGCGGGACUU
..((.((((.....((((((((((...((((((((((..((.(((((((..((.....))..))))))).)).))))))))))...))))))))))).......))))))...

 Initial dG =    -39.50

         10          20         30         40         50
UU-  G    UUAAA--|         UUU          UU  G       UU  GA
   UC CCGC       UUCAUAUUUU   CGGGUGUUCG  UC AGUCACA  GU  \
   AG GGCG       AGGUAUGAAG   GCUUACAAGC  AG UCAGUGU  UA  U
UUC  -    CCUAAAA^         CUC          U-  A       UU  GU
110        100        90         80         70         60

-----------------------------------------------------
```

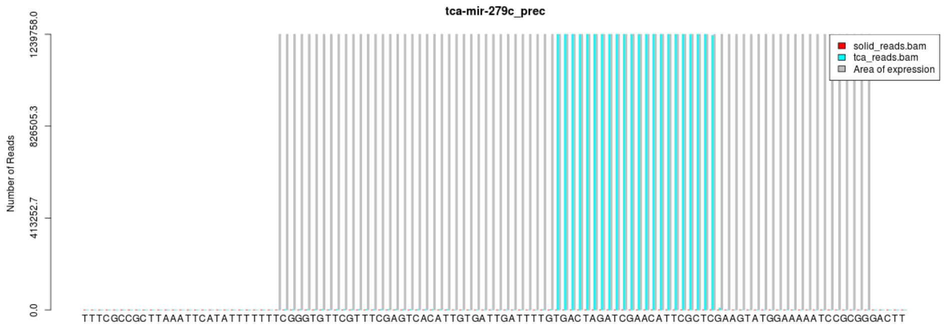

>tca-mir-279d_prec

AUCAAGAGUGGCCCCAAAAACCAAUGAAAACACUGUGUUGGCGAUUGUGGGUUUAGUCCACGUUUUAGUAAACCGUGACUAGAUCCAUACUCGUCUAUAGAGGUUUUUAGCACGACUUGG
GCCCCACAAGCCAACUCCUCCA

```
.....((((((((((((.......(((((((.((.(((.(((((.(((((((((((((.
((((((....))).))))))))))))))))).))))).))).)))))))))).......))))).........)))).))).....

 Initial dG =    -50.90

         10                  20        30        40        50        60
AUCAA   -    --------|    AAACCAA       A   G   U     U             C   -   U
     GAG UGGC        CCCAA       UGAAAAC CU UGU GGCGA UGUGGGUUUAGUC ACG UUU A
     CUC ACCG        GGGUU       AUUUUUG GA AUA CUGCU AUACCUAGAUCAG UGC AAA G
ACCUC   A    AACACCCC^    CAGCACG       -  G   U     C             -   C   U
140       130       120       110        100        90        80        70

-----------------------------------------------------
```

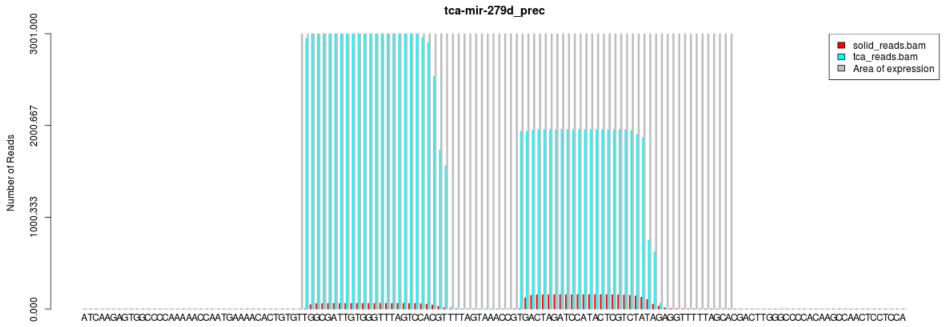

>tca-mir-279e_prec

```
CGGUGGAGCUCUCGGUGUAAGCCAGUGUUCAGUCUAUUGUUUCACAUUGGUUUCGAUUGUGACUAGAUCGAACACUCGCUUGCAACCUGGGUUUG
.....((((((..(((((((((.(((((((.(((((.....((((((((....))).))))).))))).))))))).)))))).))).)))))).

 Initial dG =    -36.50

        10         20        30        40         50
CGGUG|     UC   -      C       A     UUGUU     -   G
     GAGCUC  GGU GUAAGC AGUGUUC GUCUA     UCACA UUG U
     UUUGGG  CCA CGUUCG UCACAAG UAGAU     AGUGU AGC U
G----^     U-   A      C       C     C----     U   U
        90         80        70            60

-----------------------------------------------------
```

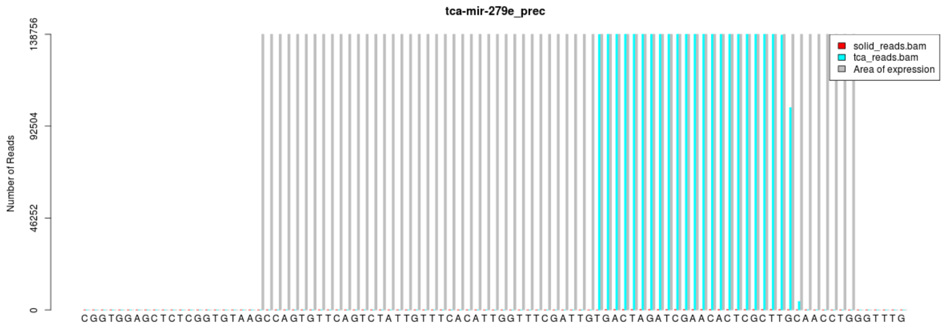

>tca-mir-281_prec

```
UCGAAAAUGAAGAGAGCUAUCCGUCGACAGUAUAUAAUUUACACUGUCAUGGAGUUGCUCUCUUUAU
......((((((((((..(((((.((((((.((.....)).)))))))))))...)))))))))))

 Initial dG =    -30.30

        10         20        30
UCGAAA           UA-|    C      A  UA
      AUGAAGAGAGC   UCCGU GACAGU UA  \
      UAUUUCUCUCG   AGGUA CUGUCA AU  A
------           UUG^    -      C  UU
            60        50         40

-----------------------------------------------------
```

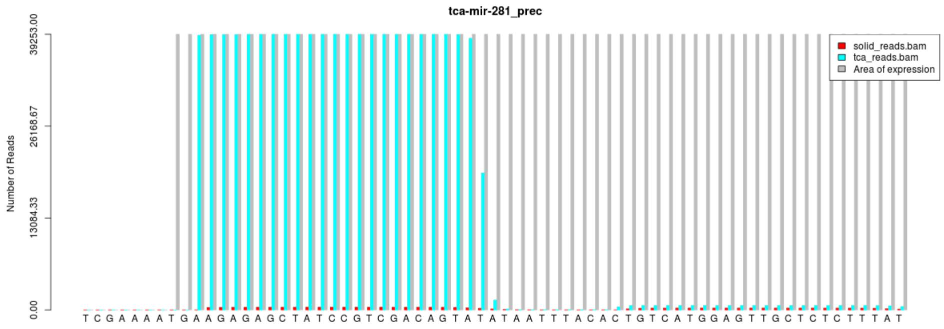

>tca-mir-282_prec

```
UGUAGAGGUAGCCUCUCCUAGGCUUUGUCUGUGAAUUUGGCCGAGACAUAGCCUGAUUGAGGCUAACUUUAGC
..(((((.(((((((...((((((.(((((.((........))))))).))))))...))))))).))))).. 

 Initial dG =    -31.30

        10        20        30
UG|    G       UCC      U     G  AAU
  UAGAG UAGCCUC   UAGGCU UGUCU UG   U
  AUUUC AUCGGAG   GUCCGA ACAGA GC   U
CG^    A       UUA      U     -  CGG
  70        60        50         40

-----------------------------------------------------
```

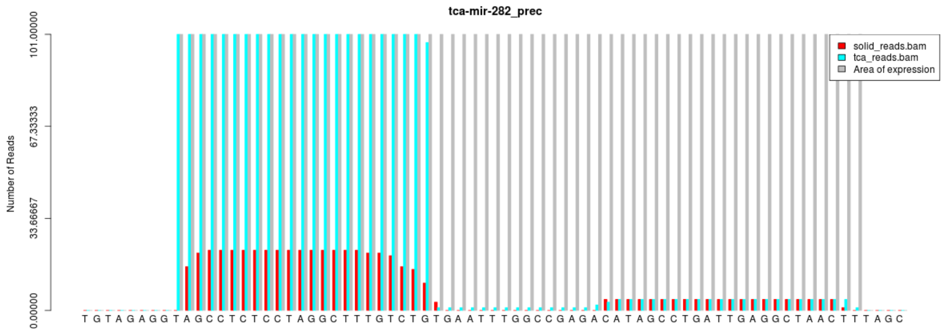

>tca-mir-283_prec

```
GUUGUCUGUGAAAUAUCAGCUGGUAAUUCUGGGUUGAUGAGAUCCCAGAAUAUCGACUGAUAUUAUCAUAAACAAC
(((((.((((((((((((.(((((.(((((((..........)))))))))))).)))))))).))))).)))))

 Initial dG =    -31.60

        10         20        30
     C     -|       C     A       UUGA
GUUGU UGUGA AAUAUCAG UGGUA UUCUGGG    U
CAACA AUACU UUAUAGUC GCUAU AAGACCC    G
     A     A^       A     -       UAGA
     70        60         50        40
```

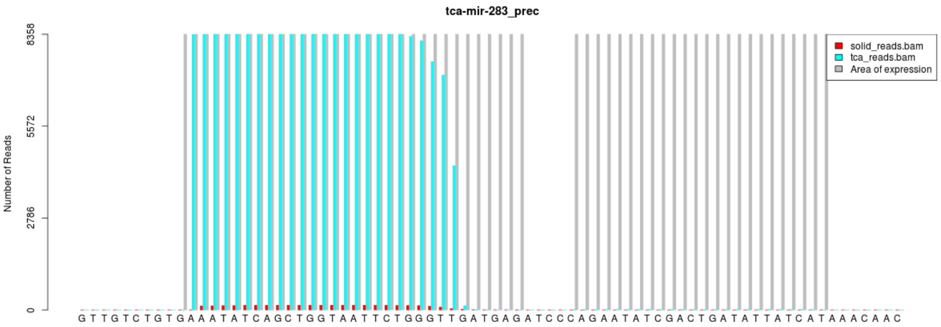

>tca-mir-304_prec

```
AUGCAAUAGACUAAAUCUCAAAUUGUAAAUGUGUACCUAUUGUUUAAAUAAUUUUAAGUACGCCUUUAAUAAAUUGAGAUCGGGACCAUUUCC
..........((..(((((((.(((((((.((((((.((..(((.....)))..)).)))))).)))).))).)))))))..)).........

 Initial dG =    -19.70

        10        20         30        40
AUGCAAUAGA  AA       A   -|   U      C  UU   UA
          CU  AUCUCAA UUG UAAA GUGUAC UA  GUU  \
          GG  UAGAGUU AAU AUUU CGCAUG AU  UAA  A
CCUUUACCA-  GC       A   A^   C      A  UU   UA
  90         80        70        60        50

-----------------------------------------------------
```

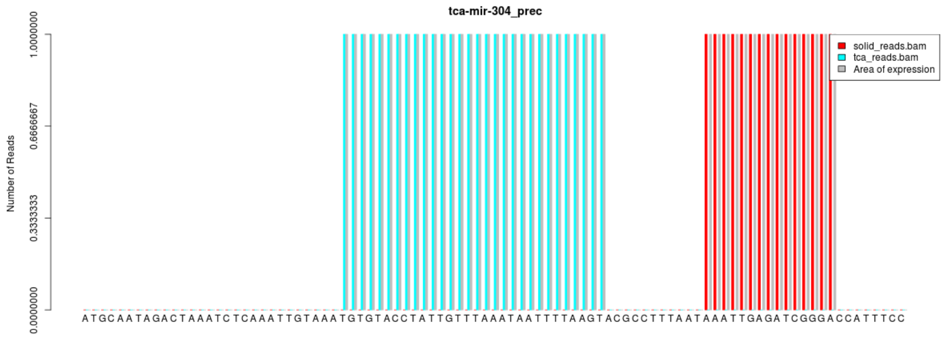

>tca-mir-305_prec

```
CACAUGUCUAUUGUACUUCAUCAGGUGCUCUGGUAUACUUUUUCCCGACACCUGUUGGAGUGCACUAAUUAUGUG
((((((..((.(((((((((.((((((.((.((..........)).)))))))).))))))))).))..))))))

 Initial dG =    -30.90

        10        20        30
|     UC  U         U      C  U  UAUA
CACAUG  UA UGUACUUCA CAGGUG UC GG    C
GUGUAU  AU ACGUGAGGU GUCCAC AG CC    U
^     UA  C         U      -  C  UUUU
    70        60        50         40
```

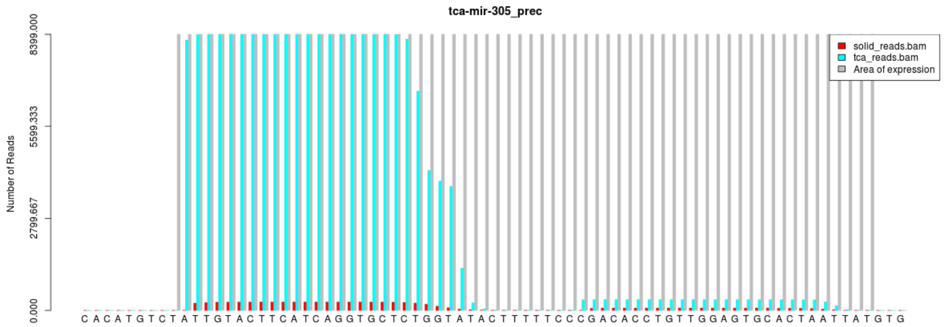

>tca-mir-307_prec

```
GGUGACUCACUCAACCUGGGUGUGAUGUGUUUGCUGUACCAUCACAACCUCCUUGAGUGAGCGACCGG
(((..((((((((...((.(((((((.((.......))))))))).))...)))))))))..))).. 

 Initial dG =    -31.10

          10        20        30
--|  GA         CCU  G       U  UUG
  GGU  CUCACUCAA   GG UGUGAUG GU   \
  CCA  GAGUGAGUU   CC ACACUAC CA   C
GG^  GC         CCU  A       -  UGU
       60        50        40

-----------------------------------------------------
```

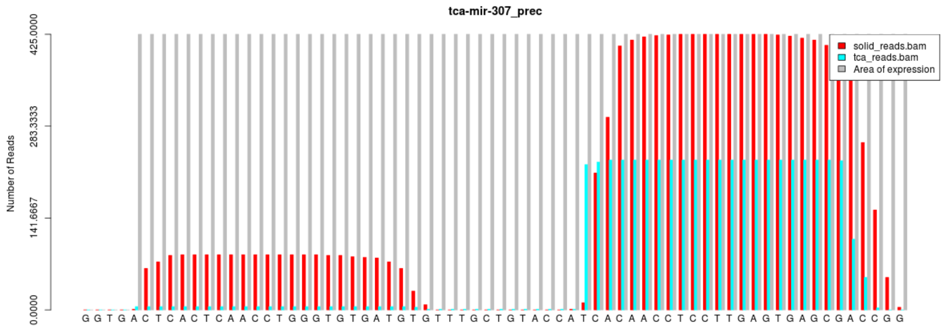

>tca-mir-308_prec

```
UCAAAUGGGGUAAUUGGCGCCCUGCGCAGGAUUUGCUUGUGAAUGGGUGUGCUUGAUUCAAUCACAGGAAAAUUCUGUGCGGGGAGUUGGUUUUGUCACCACA
.....(((...(((..((.((((((((((((((.(((((((.(((((.......))))).))))))).))))))))))))))).))..)))......)))..

 Initial dG =    -47.00

        10            20         30         40         50
UCAAA   GGU---|  UG  G               G        A      GUG
     UGG      AAU  GC CCCUGCGCAGGAUUU CUUGUGA UGGGU   \
     ACC      UUG  UG GGGGCGUGUCUUAAA GGACACU ACUUA   C
AC---   ACUGUU^  GU  A               A        A      GUU
    100        90         80         70         60

-----------------------------------------------------
```

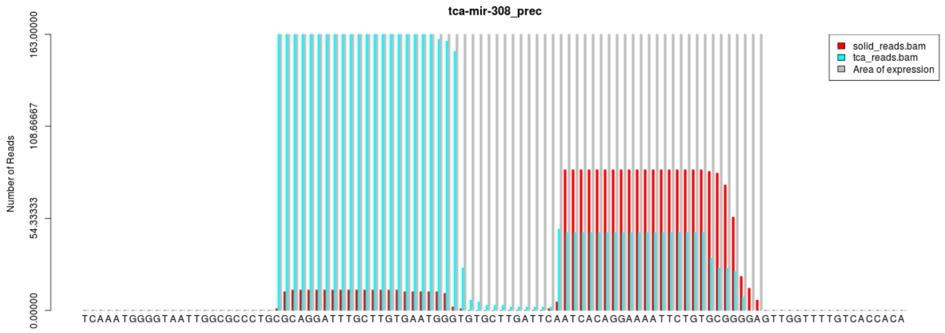

>tca-mir-309a_prec

AAAACGCUAAUCUGUUAGAUGAACUCGACUCGGCUGAUGAUAAAAAAAACAUCACUGGGUUCAGUUUGUCCAAUGGUUUUAGCUAAAA
.....((((.(((((.((..((((.(((((((.(((((..........)))))))))))).)))).)).)))))..))))).....

```
 Initial dG =    -29.90

        10         20        30        40
AAAAC     U-|    A  UG    C       C     AUAA
     GCUAA  CUGUU GA  AACU GACUCGG UGAUG    A
     CGAUU  GGUAA CU  UUGA UUGGGUC ACUAC    A
AAAAU     UU^    C  GU    C       -     AAAA
       80        70        60         50
```

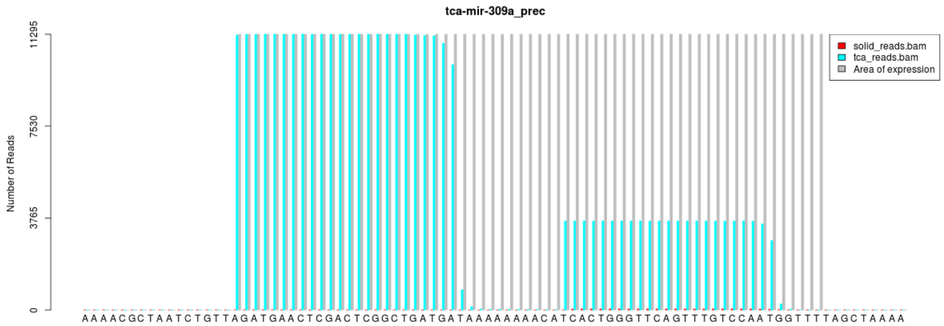

>tca-mir-309b_prec

```
CAUUUGCCCAUCACAUCUCGACUAACUCGAUUCAGUUGAUGUUAAUUAGAUAUCACUGGGUUGAGUUUGUCUCGAUGUUUUGUGCCCAUC
.....((.((..(((((..(((.(((((((((((((.((((((.....)))))))))))))))))))).)))..))))).)).)).....

 Initial dG =    -34.50

        10        20        30        40
CAUUU| C  UC     UC   U             U      AA
     GC CA  ACAUC  GAC AACUCGAUUCAGU GAUGUU  \
     CG GU  UGUAG  CUG UUGAGUUGGGUCA CUAUAG  U
CUACC^ U  UU     CU   U             -      AU
.        80        70        60         50

-----------------------------------------------------
```

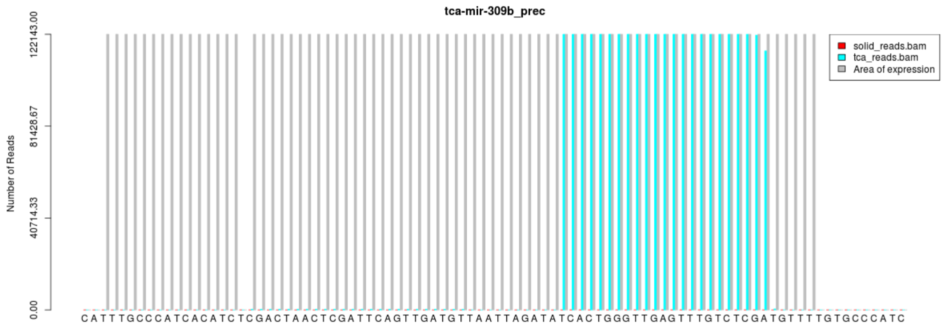

>tca-mir-315_prec

```
GUUCCUUUUGCUUUUGAUUGUUGCUCAGAAAGCCGGGUGUUAGAAAGGCUUUCGGGCAAUAAUCAUUUCCAAAGAGGAC
..(((((((.....((((((((((((.(((((((............))))))))))))))))))))......))))))).

 Initial dG =    -34.10

        10        20        30        40
GU       GCUUU-|           A       GGGUG
  UCCUUUU      UGAUUGUUGCUC GAAAGCC     U
  AGGAGAA      ACUAAUAACGGG CUUUCGG     U
C-       ACCUUU^           -       AAAGA
         70        60         50

-----------------------------------------------------
```

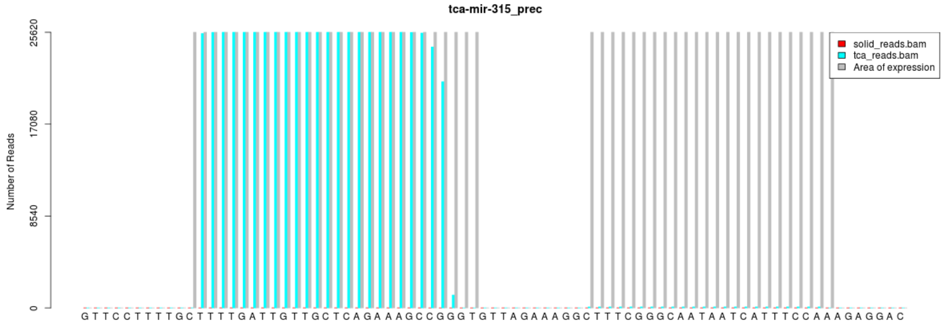

>tca-mir-316_prec

GCCACACUUGUGAACGUGUCUUUUUCCGCUUUGCUGCCGCUGUUCUGUCGUCGCCAGCAGAGAGGAUGAGGCUAGUGCACGAGUUGACC
..((.((((((.((..(((((..(((.((((((((.((.((......)).)).)))))))).))).)))))..)).)))))))))...

```
 Initial dG =    -34.30

         10        20        30        40
GC-| C       A  GU     UU   G        C  C  UU
   CA ACUUGUG AC  GUCUU  UCC CUUUGCUG CG UG  C
   GU UGAGCAC UG  CGGAG  AGG GAGACGAC GC GC  U
CCA^ -       G  AU     U-   A        C  U  UG
         80        70         60        50
```

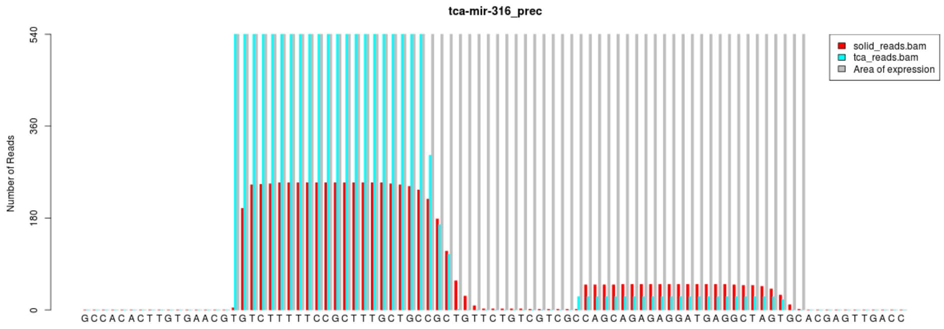

>tca-mir-317_prec

```
GGCGGUGCUGGGGACCACCCUGUGUUUACUCUGUAUUUCGACCAGUGAACACAGCUGGUGGUAUCUCAGUUUCUUCC
((.((.((((((((((((((((((((((..((......)).)))))))))))..)))))).))))))).)).))

 Initial dG =    -34.50

         10           20         30
  C  U-       -      --|          CU  AU
GG GG  GCUGGGG ACCACC  CUGUGUUUACU  GU  U
CC UC  UGACUCU UGGUGG  GACACAAGUGA  CA  U
  U  UU       A      UC^          C-  GC
      70        60        50         40

-----------------------------------------------------
```

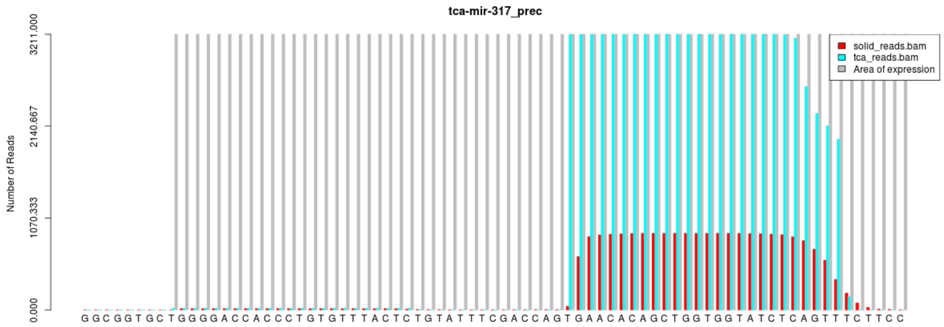

>tca-mir-375_prec

```
UCAAACUGACUUGAGCCUUGCGAAUAAUUCCUAAUUUAAAGAGCGAUUUGUUCGUGUGGCUCGAGUUAGAUAAA
.....((((((((((((.(((((((((.((((.........)).)).))))))))).)))))))))))).....
```

```
 Initial dG =    -31.30

        10         20         30
UCAAA            U          U  -| AAUU
     CUGACUUGAGCC UGCGAAUAA UC CU    \
     GAUUGAGCUCGG GUGCUUGUU AG GA    U
AAAUA            U          U  C^ GAAA
   70         60         50         40

-----------------------------------------------------
```

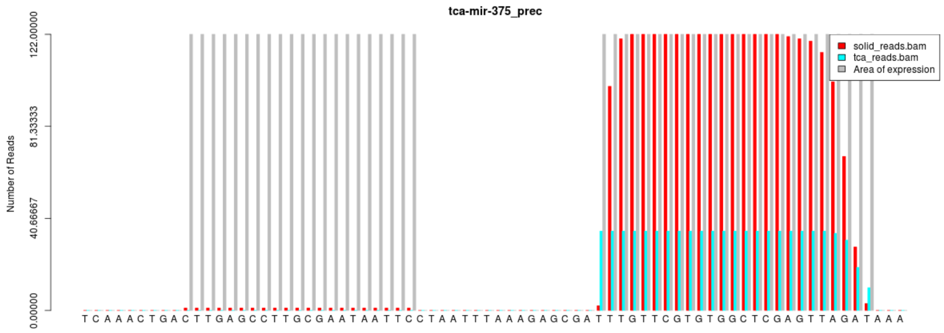

>tca-mir-750_prec

```
AGAAAGCAAAGGGCCCUUGGGUGGAAGGUUGGAUCUUCGCAUGAUUCAAGUUGCCAGAUCUAACUCUUCCAUAUGACGGUCAUUUGCCAACA
.....((((.((((.(((.((((((((((((((..(((..........))).))))))))).))))))).))).)))).))))).....

 Initial dG =    -36.00

        10        20         30        40
AGAAA     G    C   G       -|        UC   UGAU
     GCAAA GGCC UUG GUGGAAG GUUGGAUCU  GCA    U
     CGUUU CUGG AGU UACCUUC CAAUCUAGA  CGU    C
ACAAC     A    C   A       U^        C-   UGAA
 90        80        70        60         50

-----------------------------------------------------
```

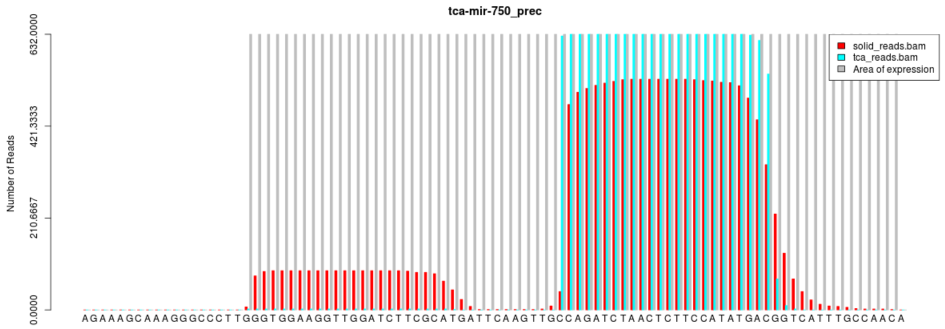

>tca-mir-927a_prec

```
UUUCGGUUUAGAAUUCCUACGCUUUACCGUGUGAAAGGCUGGCAAAGCGUUUGGAUUCUGAAUCGAAC
.((((((((((((((..(((((((.(((.((.....))))).)))))))..)))))))))))))).

 Initial dG =    -31.10

        10        20        30
U|               CU       A   U  GA
 UUCGGUUUAGAAUUC  ACGCUUU CCG GU  \
 AAGCUAAGUCUUAGG  UGCGAAA GGU CG  A
C^               UU       C   -  GA
       60        50        40

-----------------------------------------------------
```

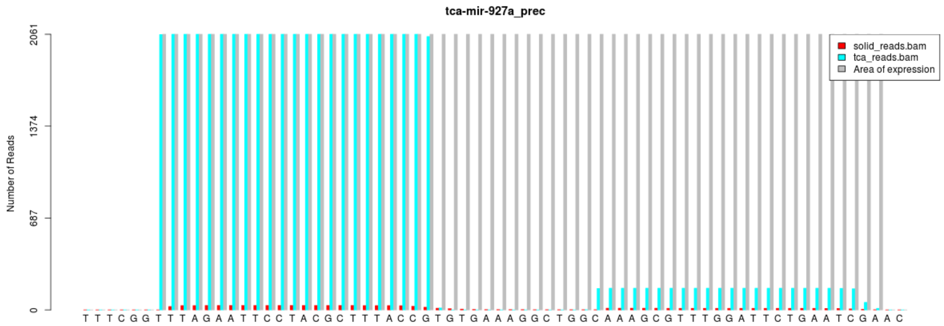

>tca-mir-927b_prec

CGCCCUUUGAGCCCUUCCGGUUUUAGAAUCUGUACGCUUUGUUGUUACUUUAAAAUCACAGAGCUUACGGAUUCUCGAACUGGUGGAGUAUUAAAC
.....(((((((((..(((((((.((((((((((.(((((((.(((.......))).))))))).))))))))))).))))))).)).)).))))).

```
 Initial dG =    -36.30

        10           20         30         40
CGCCC     -  -| UU       U          C       U   ACU
     UUUGA GC CC  CCGGUUU AGAAUCUGUA GCUUUGU GUU   \
     AAAUU UG GG  GGUCAAG UCUUAGGCAU CGAGACA UAA   U
C----     A  A^ U-       C          U       C   AAU
         90          80         70         60

-----------------------------------------------------
```

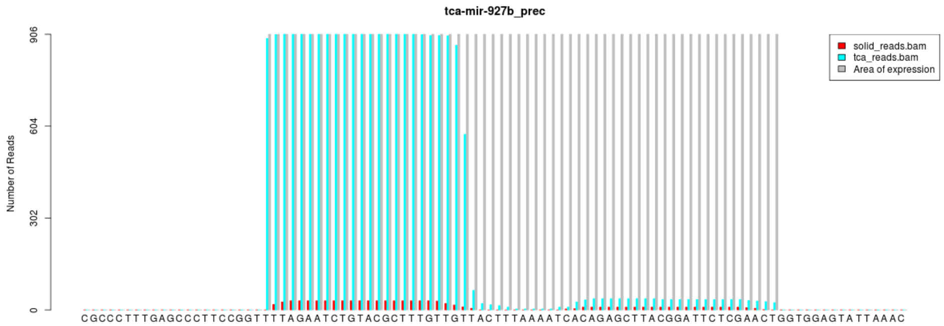

>tca-mir-929_prec

AGACGGCCAUCUUAAUCGGGGUCAAAUUGACUCUAGUAGGGAGUCCGUUACUUUAUAGCGACUCCCUAACGGAGUCAGGUUGACUCCCUUUGAGGGAAAUCUA
(((...((..(((((..((((((((.((((((((..(((((((((.((((.....))))))))))))))..)))))))).))))))))..)))))))...))).

```
 Initial dG =    -49.60

         10        20        30        40        50
-|  CGG  AU     UC        A        AG         C    CU
 AGA   CC  CUUAA  GGGGUCAA UUGACUCU  UAGGGAGUC GUUA  \
 UCU   GG  GAGUU  CCUCAGUU GACUGAGG  AUCCCUCAG CGAU  U
A^  AAA  --     UC        G        CA         -    AU
 100          90        80        70        60

-----------------------------------------------------
```

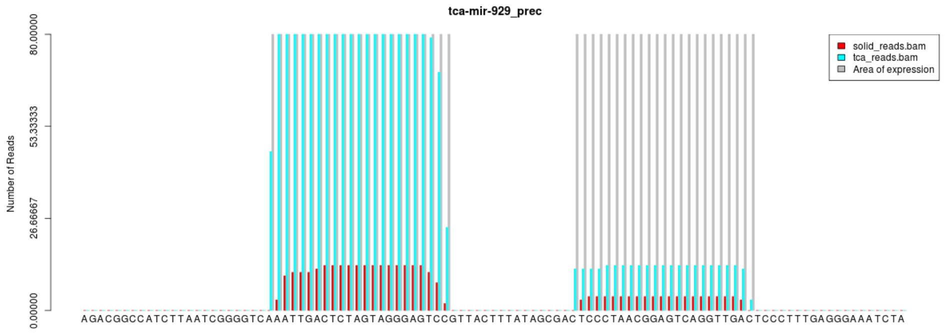

>tca-mir-932_prec

GGAGUGCGUGUCUUCAAUUCCGUAGUGCAUUGCAGUGUUUGUAGCAGCAGUCUGCAAGCAGUGCGGAAGUGAGGAUUCGUCUCU
((((.(((.(((((((.((((((.(((.((((((...((((....)))).))))))))).))))))).))))))).)))))))

```
 Initial dG =    -40.90

        10        20        30        40
|   U   U       A       G   A      UGU    A
GGAG GCG GUCUUCA UUCCGUA UGC UUGCAG   UUGU G
UCUC UGC UAGGAGU AAGGCGU ACG AACGUC   GACG C
^   -   U       G       G   -      U--    A
    80        70        60           50
```

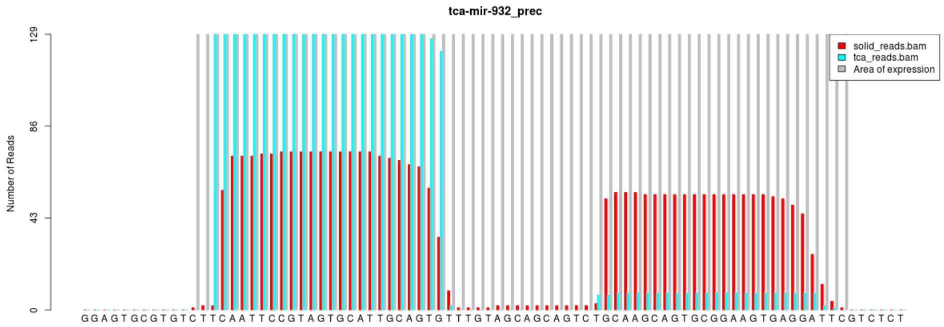

>tca-mir-965_prec

```
GGCGCGCUCCGAUUGGACCCUGGGGUGGAAAGUUGUACCGCUGAUGGUGAUUUUUCUACACAUAAGCGUAUAGCUUUUCCCCUUUGGUUCCAAUCACGACGAUC
..((((....(((((((((..((((.(((((((((((.(((.(((((((....)).)).))).)))))))))))))))))))..)).))))))).)).))...

 Initial dG =    -42.50

          10         20        30        40          50
GG-  -| CUCC       -  CU    U            C   G   -  -  U
   CG CG    GAUUGGA CC  GGGG GGAAAGUUGUAC GCU AUG GU GA U
   GC GC    CUAACCU GG  UCCC CUUUUCGAUAUG CGA UAC CA CU U
CUA  A^ A---       U  UU    -            -   A   A  U  U
  100           90        80         70         60

-----------------------------------------------------
```

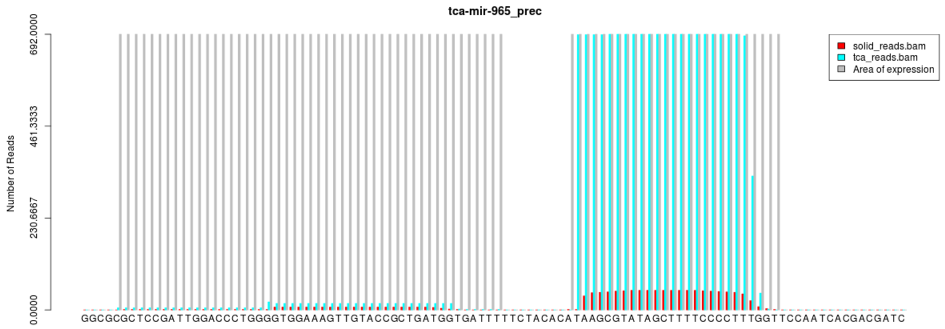

>tca-mir-970_prec

```
AAACGAUGCCGGCGGCAGCUCAGCGUGUGUCGUACUGAUAGUGUUAUCACUAGCUAUCAUAAGACACACGCGGCUAUCGCCGGUCUCUCCU
....((.(((((((..((((..(((((((((....(((((((..........)))))))...))))))))))))))..))))))).))....

 Initial dG =    -46.90

        10        20        30        40
AAAC| U       GC    CA         GUAC       GUUA
    GA GCCGGCG  AGCU  GCGUGUGUC    UGAUAGU    U
    CU UGGCCGC  UCGG  CGCACACAG    ACUAUCG    C
UCCU^ C       UA    --         AAU-       AUCA
90        80          70        60         50

-----------------------------------------------------
```

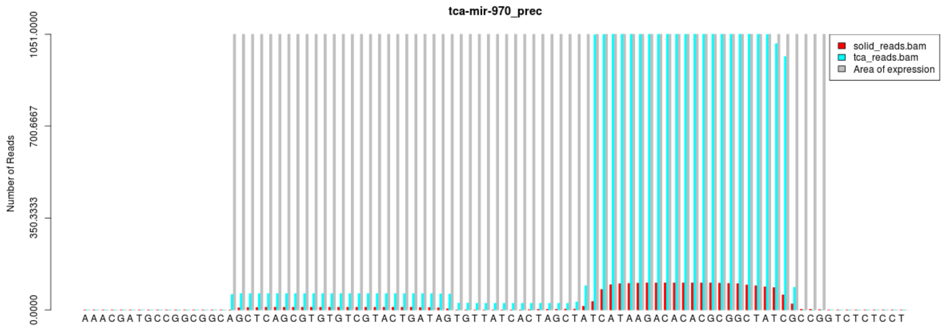

>tca-mir-980_prec

```
UUGACAACUGGGUGCCGCAGGGCUCGUCAUGGGGUAGCUAGCGUGUGAUUAACAGCCAGCUGCCUUUUGAAGGGCUAUAUGGCAUUGGUUUUUUA
.....(((..(.(((((...(((((.(((..((((((((.((.(((.....))))).))))))))..))).)))))...))))))..))).....

 Initial dG =    -41.00

        10        20        30        40
UUGAC|  UG G     CAG     G    UG        A   G    GA
     AAC  G UGCCG   GGCUC UCA  GGGUAGCU GC UGU   \
     UUG  U ACGGU   UCGGG AGU  UCCGUCGA CG ACA   U
AUUUU^  GU -     AUA     A    UU        C   -    AU
    90         80        70        60         50

-----------------------------------------------------
```

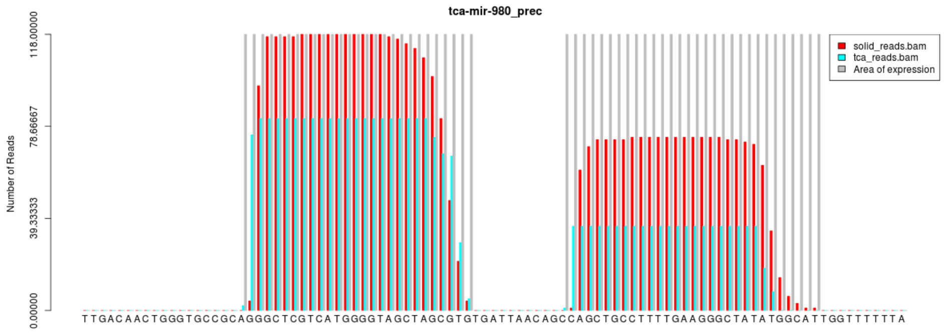

>tca-mir-981_prec

```
UACAAGACUUAUAUCUCAUAAGUUACGGGUUUCGGGGCAUCGGAACCGAUUUUAAGCUGGUUCGUUGUCGACGAAACCUGCAGACUAUGGGACACA
.............((((((.((((.(((((((((.((((...((((((.........))))))..)))).)))))))))..))))))))))....

 Initial dG =    -32.60

        10        20         30         40        50
UACAAGACUUAUA      A    A-         G-|   UCG      AUUU
             UCUCAU AGUU  CGGGUUUCG  GGCA   GAACCG    \
             AGGGUA UCAG  GUCCAAAGC  CUGU   CUUGGU    U
ACAC---------      -    AC         AG^   UG-      CGAA
              90         80        70         60

-----------------------------------------------------
```

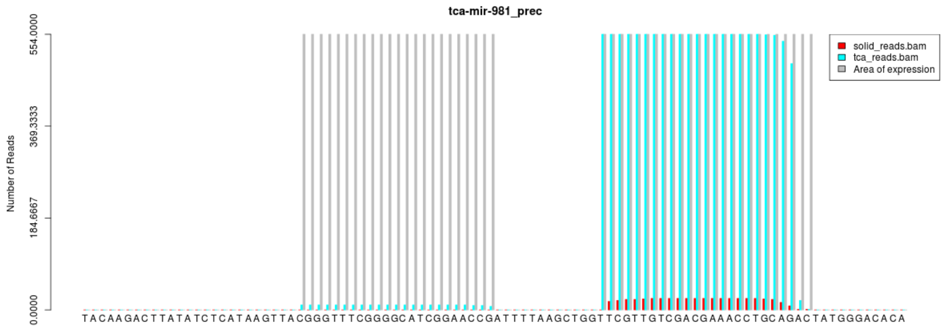

>tca-mir-989_prec

UUUUUGCUCCCAAGGUCCCGCCACUAGGUCGCAUAACACGCAUUGUCAACACCGUGUGAUGUGACGUAGUGGUAUGUCUAGGGGAGUUGCCU
.....((((((..((....(((((((.(((((((.(((((...((....)).))))).))))))).)))))))....))..)))))).....

```
 Initial dG =    -40.60

        10        20        30        40
UUUUU|     AA  UCCC       G        A     CAU  U
     GCUCCC  GG    GCCACUA GUCGCAU ACACG   UG C
     UGAGGG  UC    UGGUGAU CAGUGUA UGUGC   AC A
UCCGU^     GA  UGUA       G        G     C--  A
 90        80        70        60          50
```

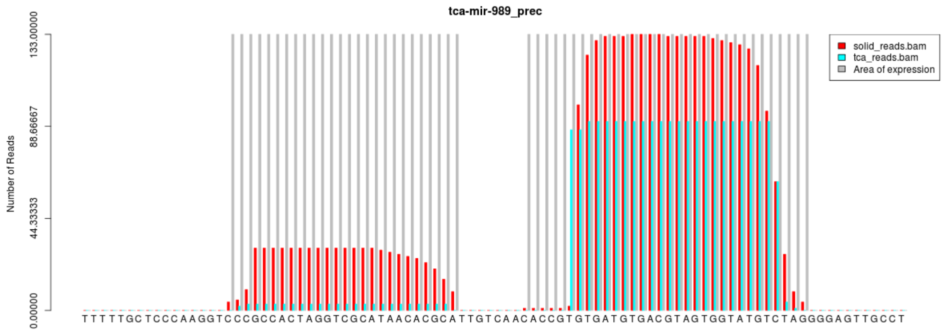

>tca-mir-993_prec

```
GGCACAUCCGUGAUCUACCCUGUAGAUCCGGGCUUUUGUAGAAUUUUUAGAUCAGAAGCUCGUCUCUACAGGUAUCUUACGGAUGGAA
....(((((((((..((((.((((((..((((((((((.............))))))))))..))))))))))..)))))))))...

 Initial dG =    -41.00

        10        20        30        40
GGCA|        UC    C      UC          UAGAAU
    CAUCCGUGA  UACC UGUAGA  CGGGCUUUUG      U
    GUAGGCAUU  AUGG ACAUCU  GCUCGAAGAC      U
AAG-^        CU    -      CU          UAGAUU
        80         70        60        50

-----------------------------------------------------
```

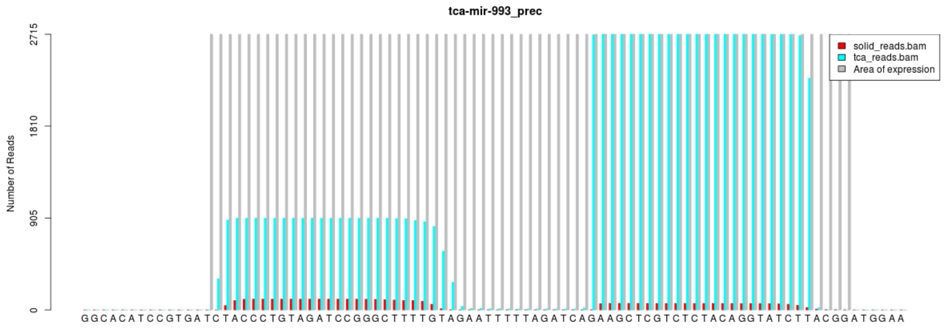

>tca-mir-995_prec

```
CUAAUUUAGCAGAACUCUGAGCUGAAUCUUGAGGUGCUUCCGUGGUUUGGGCUCGUAGCACCACAUGAUUCAGCUUACGUUUCCGCUAACUACC
.....((((.(((...((((((((((.((.((((((..((.(((....))))).)))))).)).)))))))))))...))).))))).....

 Initial dG =    -37.10

        10        20        30        40
CUAAU|    A   CUC          U  A      UC  U   U
     UUAGC GAA   UGAGCUGAAUC UG GGUGCU  CG GGU U
     AAUCG CUU   AUUCGACUUAG AC CCACGA  GC UCG G
CCAUC^    C   UGC          U  A      U-  -   G
   90        80        70        60          50

-----------------------------------------------------
```

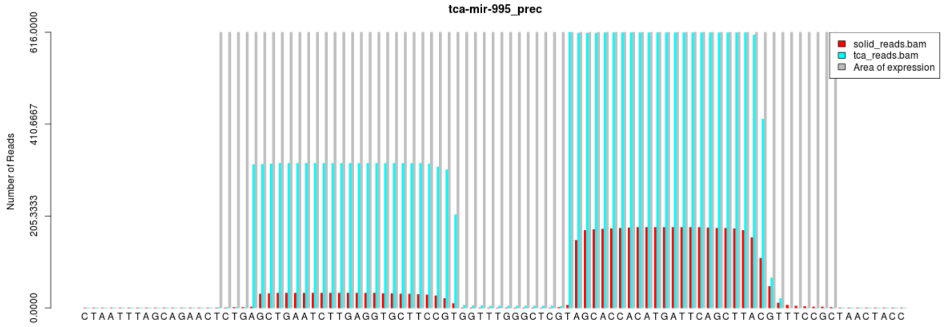

>tca-mir-998_prec

```
GGGACGCUUUGCCACUACGUUGUACUGAAUUUCAUGUGGGCUGCUGUUUUGCGUAGCACCAUGGGAUUCAGCUCAUCGUUUGGUUAUCUGCUAACU
.....((...((((..(((.((..((((((..((((...(((((........)))))..))))..))))))..)).))).))))).....)).....
```

```
 Initial dG =    -33.90

        10          20         30         40
GGGAC  UUU--|   CU   U  UA      UU    UGG     UGU
     GC     GCCA  ACG UG  CUGAAU  CAUG   GCUGC   U
     CG     UGGU  UGC AC  GACUUA  GUAC   CGAUG   U
UCAAU  UCUAU^   U-   U  UC      GG    CA-     CGU
     90        80         70        60          50
```

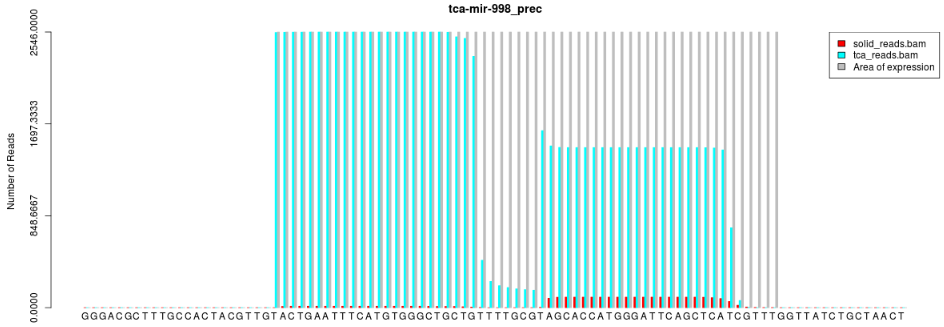

>tca-mir-1000_prec

```
ACGGCUCGGCCAUGAUAUUGUCCUGUCACAGCAGUACUGGUUCCGAGUUACUGCUGCUUCGGGACAUGACCAUGGCCGGGCCUCUUGGCCGACGCCGCCC
..((((((((((((....((((((...(((((((((((....).))).)))))))...)))))))....))))))))))))....(((....)))....

 Initial dG =    -54.50

          10        20        30          40
.-AC          AUAU       UCA       -   -|G
    GGCUCGGCCAUG    UGUCCUG   CAGCAGU ACU G U
    CCGGGCCGGUAC    ACAGGGC   GUCGUCA UGA C U
\ --          CAGU       UUC       U   G^C
     80        70        60        50


        90
  UCUU   C
      GGC G
      CCG A
  CCCG   C
100

-----------------------------------------------------
```

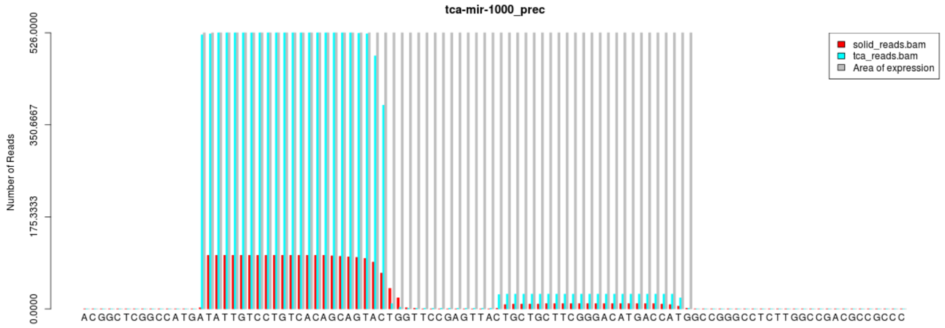

>tca-mir-1175_prec

```
CGCUUUGAUCGAGCUCUGUAAAGUAAGUGGAGCAGUGGUCUCUUCACUUGGUUUUUAUAGUGAGAUUCAACUCCUCCAUCUCAUUUUUUGAGCUCGUUUACAACG
.....(((.(((((((...(((((.(((((((.(((....(((.((((..........)))))))....))).))))).)).))))).)..))))))).))).....

 Initial dG =    -31.60

        10        20         30        40        50
CGCUU|  U       UGU     A   -      C   GGUC   U    UGGU
     UGA CGAGCUC   AAAGU AG UGGAG AGU    UCU CACU    U
     AUU GCUCGAG   UUUUA UC ACCUC UCA    AGA GUGA    U
GCAAC^  U       UU-     C   U      C   ACUU   -    UAUU
   100        90         80        70         60

-----------------------------------------------------
```

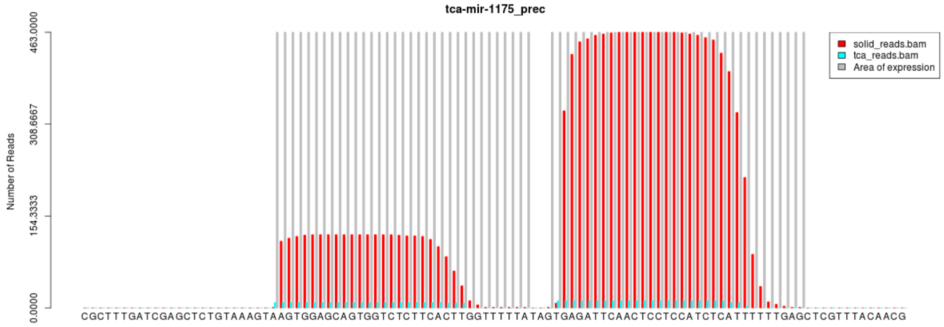

>tca-mir-2765_prec

AAAAGUACCGAAAUUCUUGGAUAUAGUGAGGAGUGGUAACUCCACCACCGUUGGCGUGUGUUGUACUGCCAGCGGUCGAGGAGUUCCUUAACCUCGCUGUAAUUCUCCAAAGGACAAGGU
AAGUUG
.((..((((....((((((((((((((((...((.((((((...((((((((((.
(((...)))))))))))))))...))))))))....))))))))))....))))).)))...))))..)).

```
 Initial dG =    -52.90

        10         20            30         40        50        60
A  AG    GAAA   -     ----         AGU-| U      ACC         U   U
 AA  UACC    UUC UUGGA    UAUAGUGAGG    GG AACUCC   ACCGUUGGCG GUG \
 UU  AUGG    AGG AACCU    AUGUCGCUCC    CC UUGAGG   UGGCGACCGU CAU U
G  GA    AAC-   A     CUUA         AAUU^ -      AGC         -   G
    120        110       100        90         80        70
```

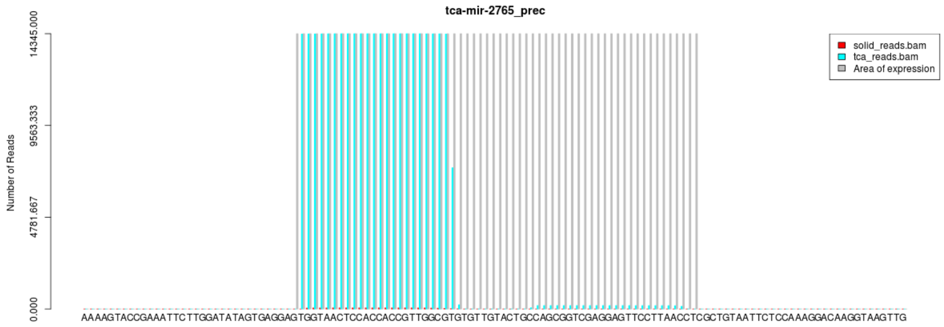

>tca-mir-2788_prec

```
GACAUUUGUUUAUGCAAUUUUGGGGUUUCUUAGCGGCAUUUGCCUCAAAUUACACUGCAAUGCCCUUGGAAAUCCCAAACAUGCAUGACGACUCAA
.....(((((.(((((..((((((((((((.((.((((((.((.............)))))))))).))))))))))))..))))))))))).....

 Initial dG =    -35.30

        10        20        30        40
GACAU|    U     AU            U  C      U  CUCAAA
     UUGUU AUGCA  UUUGGGGUUUCU AG GGCAUU GC      \
     AGCAG UACGU  AAACCCUAAAGG UC CCGUAA CG      U
AACUC^    -     AC            U  -      -  UCACAU
     90         80        70         60

-----------------------------------------------------
```

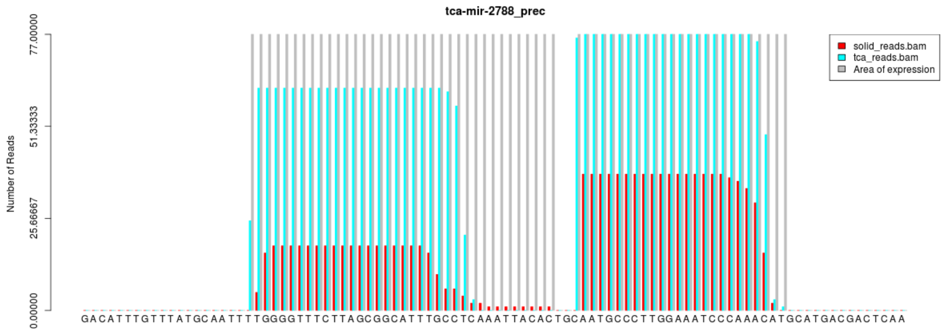

>tca-mir-2796_prec

AUCGAGCUUUCGGUGCAGAAGCCGCUAUUAACGUGGUGACGCAAGAGUAGGGGUUUCUUUCGGCCUCCAGCGGAUAAUAAAGAGUUGUAGGCCGGCGGAAACUACUUGCUUUUACGUUGC
AUUACCUUCGCUGCGGCUUCCCGUCAAUGAUGCGUUUU

```
.....((..(((.(((.((((((((.......(((((((((.(((((((((((((((.(((((((.
((((............)))).))))))).))))))).))))))))).)))..))))))).......))))))))..).)).))).)).....
```

```
 Initial dG =    -61.80

        10          20       30           40        50         60        70
AUCGA  UU   G  - A-        UAUUAAC      --   C         -|      U       C    GGAUA
     GC  UCG UG C  GAAGCCGC       GUGGUG  ACG AAGAGUAGG GGUUUCU UCGGCCU CAGC     A
     CG  AGU AC G  CUUCGGCG       CAUUAC  UGC UUUUCGUUC UCAAAGG GGCCGGA GUUG     U
UUUUG  U-   A  U CC        UCGCUUC      GU   A         A^      C       U    AGAAA
       150       140       130       120       110       100        90        80
```

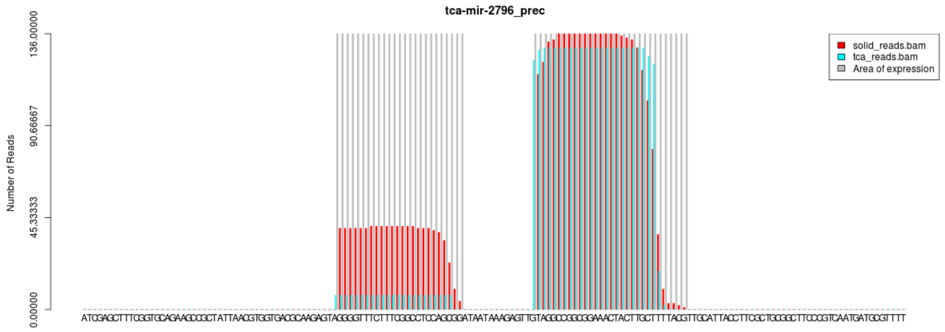

>tca-mir-2944a_prec

```
AACUCGCUGAAACUGUGGAGGAACUAUUGGUGUGAUUUGUUCAUUUAAUCAUAUCACAGCCAUUAGUUACUUCACAGUUUCAGUAUUUU
.....((((((((((((((.(((((.(((((((((.((..........)).)))))).))).))))).))))))))))))))).....

 Initial dG =    -40.50

        10        20        30         40
AACUC               G     U   -|     U  UUCA
     GCUGAAACUGUGGAG AACUA UGG UGUGAU UG    U
     UGACUUUGACACUUC UUGAU ACC ACACUA AC    U
UUUUA               A     U   G^     U  UAAU
        80        70        60        50
```

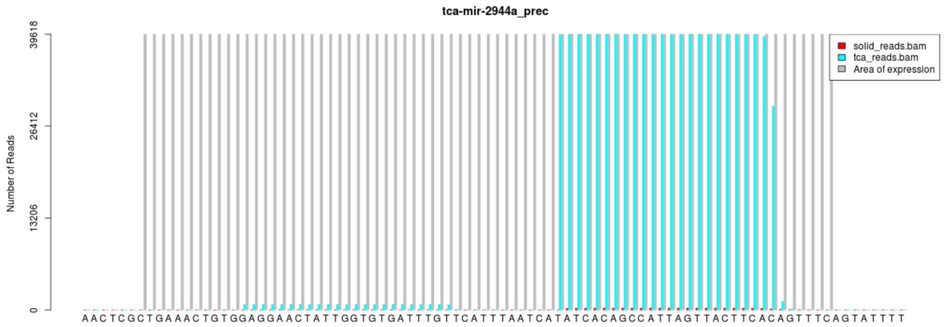

>tca-mir-2944b_prec

```
ACUUCAUUUCGCAUAAUGCAUUUAAGGAACUACAGGUGUGAUGUGUGAUUUCAUUAAACGUAUCACAGCCGUAGUUGCCUUACUGUAUUUGUGAAAUCUCAA
.....(((((((.((((((..(((((((((((.((((((((((((...........)))))))))).)))))))).))))).)))))))))))))).....
```

```
 Initial dG =    -35.40

        10        20         30         40        50
ACUUC|       U      UU     -      A  -          GAUUU
     AUUUCGCA AAUGCA  UAAGG AACUAC GG UGUGAUGUGU     \
     UAAAGUGU UUAUGU  AUUCC UUGAUG CC ACACUAUGCA     C
AACUC^       -      C-     G      -  G          AAUUA
100       90         80         70        60

-----------------------------------------------------
```

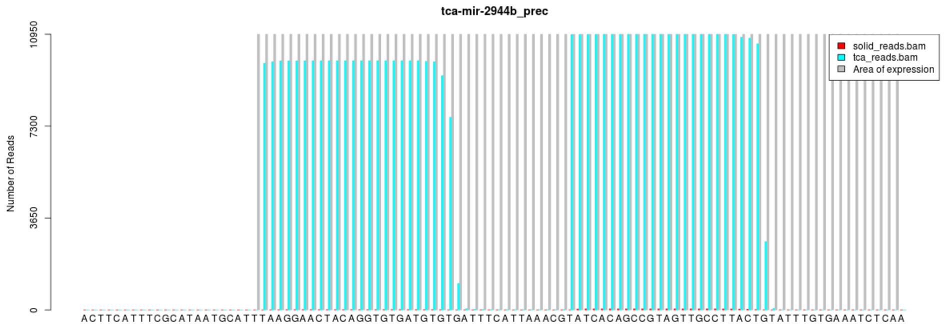

>tca-mir-2944c_prec

```
GCCGAGCGACACUCAGGGAAUCUGGCUUGAAGGAACUUCUGGUGUGAUAUGUAUUUAUCAAUAUAUCACAGCCAGUAGUUACCUACGAAAUGGGUUCCCGAUUGCAACAG
.....((((...((.(((((((((..(((.(((((((.((((((((((((((........)))))))))).)))).)))).))).)))..))))))))))))))).....

 Initial dG =    -43.20

        10        20        30         40         50
GCCGA|   CAC  A         GC   A   -    U    -         AUU
     GCGA   UC GGGAAUCUG  UUG AGG AACU CUGG UGUGAUAUGU   U
     CGUU   AG CCCUUGGGU  AGC UCC UUGA GACC ACACUAUAUA   A
GACAA^   ---  -         AA   A   A    U    G         ACU
.          100         90        80        70        60

-----------------------------------------------------
```

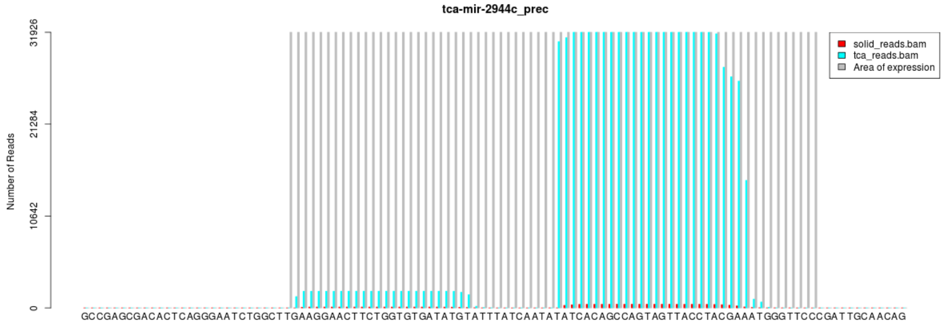

>tca-mir-3049_prec

```
AUGUGGUGGAACGAUACGAUGGUGAGUGGGGGCGCAGGGACAUGGGGGCGCCACAUGGGGUCGGGAAGACAGUUGCGGCGGAUUUCGUUUUUUCAAGUCCGUUCGCUGCAUUCCCAGCCC
GGUGUCGUGCCGAGGCAGCAAGGGGGCGCUCAGGCUAGGAGGUUGUUUACA
.....(((.((((((....((((......((((((.......((..(((((.((((.((((.(((((..((((...
((((((((((.........))))))))).))))..))))).)))).)))).)))))....))........))))))..))))....))))))))).

 Initial dG =    -63.50

        10        20        30        40           50        60        70        80

AUGUG|  G      ACGA    GAGUGG      AGGGACA-  GG--     C    G    C     GA    UGC
CGUU
     GUG AACGAU    UGGU      GGGCGC        UG    GGCGC ACAU GGGU GGGAA  CAGU   GGCGGAUUU
\
     CAU UUGUUG    AUCG      CUCGCG        AC    CCGUG UGUG CCCG CCCUU  GUCG   UUGCCUGAA
U
A----^  -      GAGG    GA----      GGGGAACG  GGAG     C    G    A     AC    C--
CUUU
   170        160           150       140       130       120       110         100


-----------------------------------------------------
```

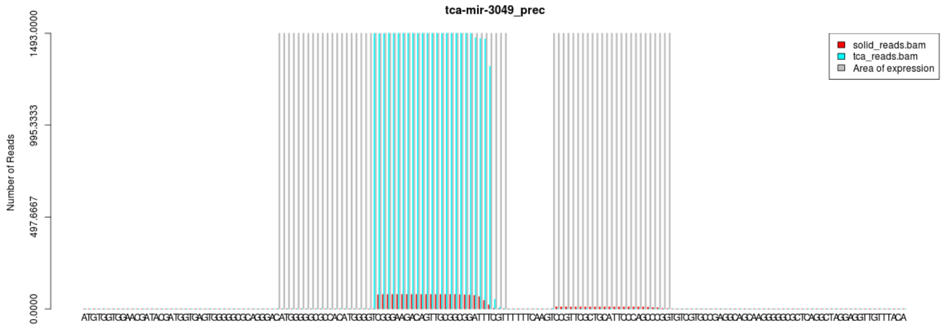

>tca-mir-3477_prec

UGCACGCUUGACUGCGUUUCGUUAAUCUCAUUUGGUAACUGUGAGGUUUGUUUAUUCCUCAGACUACUGAAUGGGAUCGACAAAAUGUGGUUUUGUUUUCA
.....((..(((..(((((.((..((((((((..(((....(((((..........)))))...)))..))))))))..)).)))))..)))..)).....

```
 Initial dG =    -35.20

        10        20        30        40        50
UGCAC| UU   UG     C  UA        UG   ACUG     UUUG
     GC  GAC  CGUUU GU  AUCUCAUU  GUA    UGAGG    U
     UG  UUG  GUAAA CA  UAGGGUAA  CAU    ACUCC    U
ACUUU^ UU   GU     A  GC        GU   CAG-     UUAU
 .        90        80        70         60
```

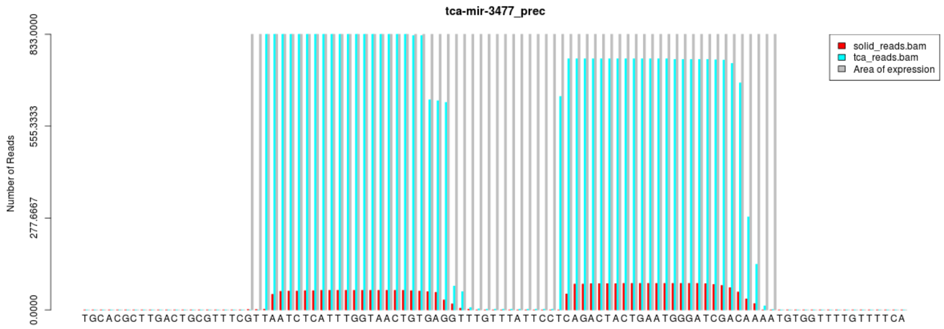

>tca-mir-3791_prec

AUCAAGUUAAACGUUCGGUGGUUUUAUUACGGUGGUUGCGAUUUUUACAAUCACCGGGUAGAAAUCAUCUCAUGUUUAACCAAAU
.....((((((((..(((((((((....(((((((((.........)))))))))....)))))))))..)))))))).....

```
 Initial dG =    -29.80

        10        20        30        40
AUCAA|        UC         AUUA         CGAU
     GUUAAACGU  GGUGGUUUU    CGGUGGUUG    \
     CAAUUUGUA  CUACUAAAG    GCCACUAAC    U
UAAAC^        CU         AUGG         AUUU
    80        70        60        50
```

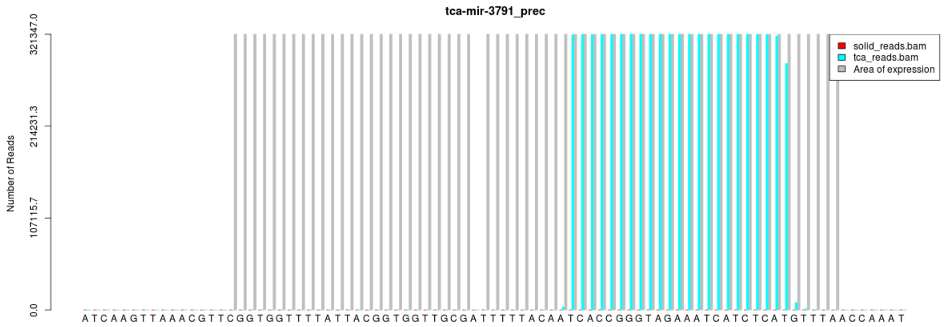

>tca-mir-3802_prec

```
CACUUGAUGUAACUUUAUACAAAAAUCAACAAAAGUAUGGCUUGCUUGAAUGACGAGAUAAAAUCGUUAUAUCGUAGUCGCCAUCUGUCGCUACAAUGUUUUGUUUCAUAUAUUUUUUA
.....((((((.......((((((.........((.(((((..(((((((((((((.......))))))).))).))).)))))))............))))))....)))))).....
```

```
 Initial dG =    -20.30

        10        20        30            40          50
CACUU|     ACUUUAU      AUCAACAAA---  U      UU    -    -       GAU
     GAUGUA      ACAAAA            AG AUGGC  GCU UGA AUGACGA   \
     UUAUAU      UGUUUU            UC UACCG  UGA GCU UAUUGCU   A
AUUUU^     ACUU---      GUAACAUCGCUG  -      C-    U   A       AAA
       110          100        90          80          70
```

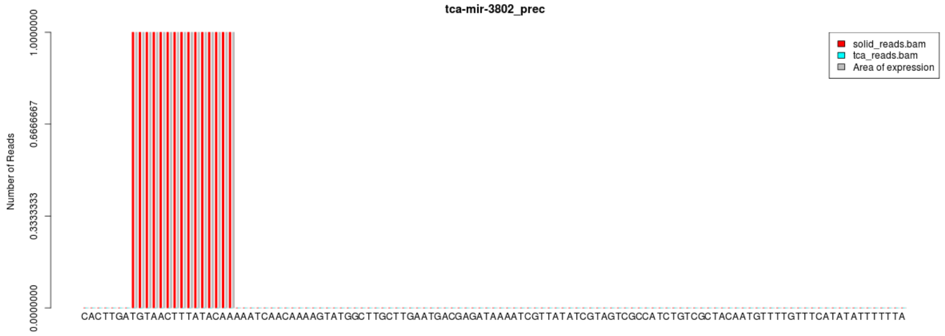

>tca-mir-3803_prec

```
CACACGAGGGAAUAUUAAAAAUGGAGGUACAUAACGUUCUGUUGCGCCAAUAAUGUCCACCAUUGUAACUGGUGUCCCUCUUCCA
.....(((((.((((((..(((((.((.((((..(((......)))......)))))).))))).....)))))))))))).....

 Initial dG =    -22.10

        10           20        30            40
CACAC     A      AA---     A  U    AA----|  UC
     GAGGG AUAUUA     AAUGG GG ACAU      CGU  U
     CUCCC UGUGGU     UUACC CC UGUA      GCG  G
ACCUU     -      CAAUG     A  -    AUAACC^  UU
    80         70        60         50

-----------------------------------------------------
```

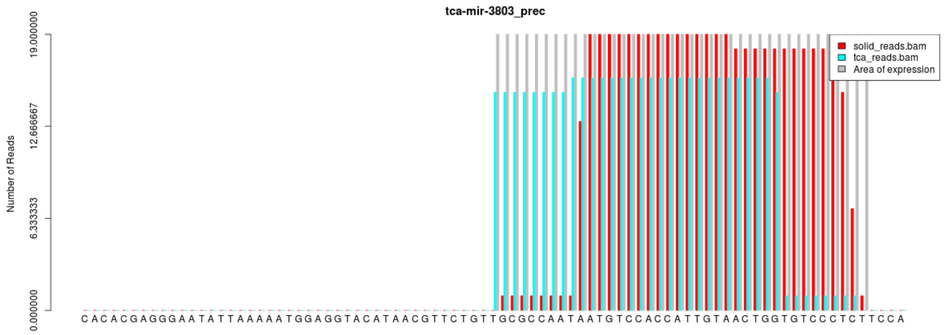

>tca-mir-3804a_prec

AUAUUUUUUGGCACACAUCAAUGUCCACGUUUUUGUGACUUGAUUCGCUGAAAUGUUCCAUAAGUGUGCUAGAACAACC
.....((((((((((....(((...(((((((.((((......)))).)))))))..)))..)))))))))).....

```
 Initial dG =    -22.70

        10        20        30        40
AUAUU|          AUCA   UCC        U    CU
     UUUUGGCACAC    AUG   ACGUUUU GUGA  U
     AAGAUCGUGUG    UAC   UGUAAAG CGCU  G
CCAAC^          AA--   CU-        U    UA
        70          60         50
```

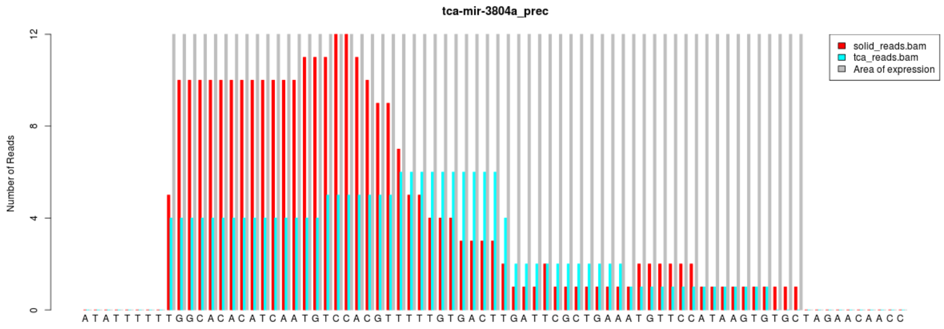

>tca-mir-3804b_prec

```
CUCGGGUUAUCAGCUCAUUGCACACAACAAUGUCCACAUUCUUGCGAUUUCAUACGUCACUGGAAUGUUCCAUAAGUGUGCGAAAUCAGCGGUAGCAAAAG
.....((((((.(((..((((((((....(((...(((((((...(((.......)))...)))))))..)))..))))))))....))))))))).....

 Initial dG =    -26.70

        10          20        30        40        50
CUCGG      A   CA--|       AACA   UCC       UGC   UUC
     GUUAUC GCU    UUGCACAC    AUG   ACAUUCU   GAU   \
     CGAUGG CGA    AGCGUGUG    UAC   UGUAAGG   CUG   A
GAAAA      -   CUAA^       AA--   CU-       UCA   CAU
 .         90        80          70         60

-----------------------------------------------------
```

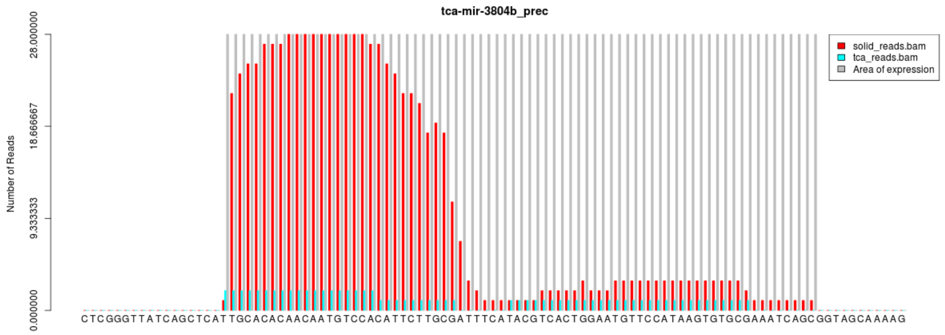

>tca-mir-3805a_prec

```
UUUUGUCCAAGCUCAAGUCCGAUCACAUGUGGGUCGUAUAUUUUCAAUAUUAUCAUAAUAUAUGUCUCGCAUCUGACGGUCUUGGGGUGGGUUGUU
.....((((..((((((.(((.(((.(((((((.((((((((..............)))))))).))))))).)))))).)))))).)))).....

 Initial dG =    -35.70

        10        20        30        40
UUUUG|   AG      U   A   C       U        UUCAAU
     UCCA  CUCAAG CCG UCA AUGUGGG CGUAUAUU      A
     GGGU  GGGUUC GGC AGU UACGCUC GUAUAUAA      U
UUGUU^   G-      U   -   C       U        UACUAU
     90         80         70        60        50

-----------------------------------------------------
```

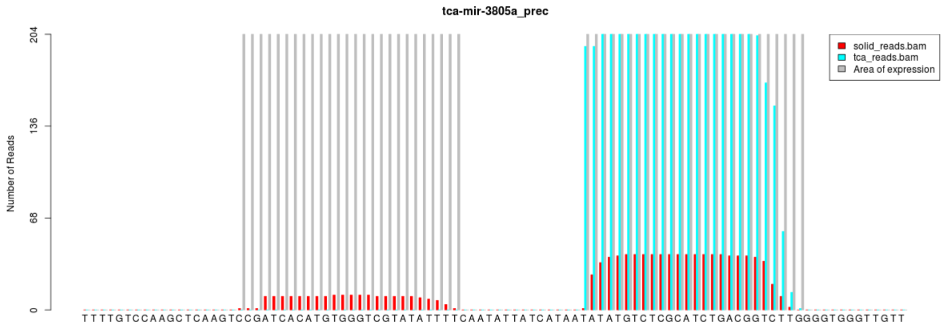

>tca-mir-3805b_prec

AAGAAGAUUUGCGCCUAUUCCCAGGUCCGUUCACAUUCGGAUUUUAUUGUGAAUAUUAAAUAUGAUUCGUCUGUGAUGGUCUUGUGGGUCGGUCCGAUCAACAA
.....((((.(.(((...(((((((.((((.((((..((((((.((((..........)))).))))))..)))))))).)))).))))..))).)))))).....

```
 Initial dG =    -30.90

        10        20         30        40        50
AAGAA|   U C   UAU   -    U    U    UU      U    GUGA
     GAUU G GCC   UCC CAGG CCGU CACA  CGGAUU UAUU    A
     CUAG C UGG   GGG GUUC GGUA GUGU  GCUUAG AUAA    U
AACAA^   - C   CU-   U    U    -    CU      U    AUUA
  100        90         80         70        60

-----------------------------------------------------
```

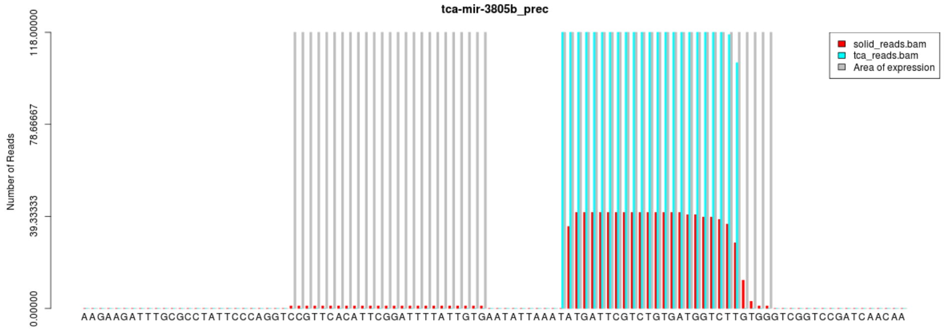

>tca-mir-3806_prec

AACAAAAAGUUUUAUUUUUCAGAGCAUUUCAACCAAUUUCCUGUUUCUGUUCCCGGAUUGUCACAUAUUUUUUAAAUAUUAAAUAUGUGCAUCCGGGAGUAGAUCUAGGGGGUUGGUACA
AUCAACGGCUUCGUAUUUGAUUUUAU
........(((((.......))))).......((((((..((((..(((((((((((((.(.(((((((((..........)))))))))))))))))))))))..)))).))))))..
((((((((....)))...))))))....

```
 Initial dG =    -49.50

          10
.-AACAAAAA|    AUU
          GUUUU   \
          CGAGA   U
\ --------^    CUU
              20



       30        40        50        60        70
  .-AUUUCA      UU    UU            U U         UUUA
          ACCAAU  CCUG  UCUGUUCCCGGAU G CACAUAUUU    A
          UGGUUG  GGAU  AGAUGAGGGCCUA C GUGUAUAAA    A
  \ ------      GG    CU            - -         UUAU
               110       100         90         80

-----------------------------------------------------
```

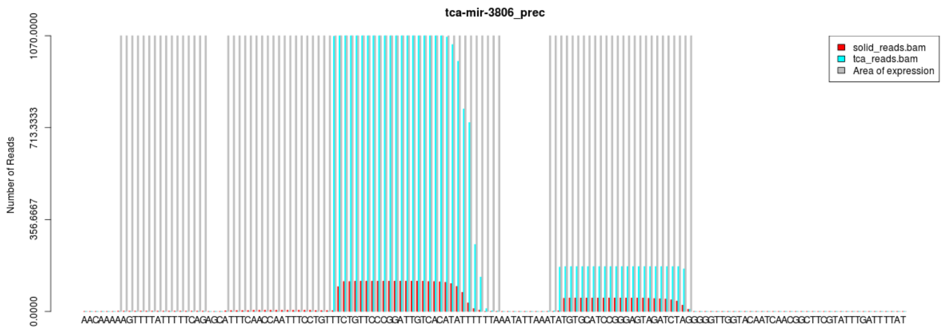

>tca-mir-3807_prec

AUUAAUAAUUAAUUCGGGCGAUGGCGAUGAUGUGCGGCCAAAUCGAAAUUCUGAAAUCAUUUGCCGAACAUCCUCGCCACUAUCCAGUUAAUUACAAGC

```
.....((((((((.(((...((((((.(((((.((((.((((.((..........)))))))))).))))).))))))...))))))))))).....

 Initial dG =    -32.40

        10        20        30        40        50
AUUAA|        C   CGA      U     G    C    C  AAUU
     UAAUUAAUU GGG   UGGCGA GAUGU CGGC AAAU GA    C
     AUUAAUUGA CCU   ACCGCU CUACA GCCG UUUA CU    U
CGAAC^        -   AUC      C     A    -    -  AAAG
        90         80        70         60

-----------------------------------------------------
```

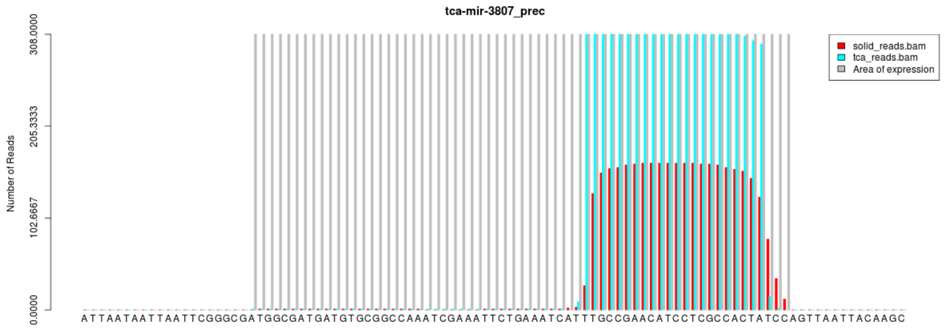

>tca-mir-3808_prec

```
UUUAAGAGGGUUUUAACCAAUUUUUCAGUCUCAUAUUCGCACUUGACAUAUUUUGGAUUAAAUAAAAUAUGUACACGUGUGGAUAUGGGGCCGAAGUUGGUACAGUCAACGG
.....((..((....(((((((((...((((((((((((((.((((((((((((.......))))))))))).)).))))))))))))))).)))))))))))..)).....

 Initial dG =    -41.20

        10        20        30        40         50
UUUAA| GG  UUUA         UCA              U  -          GAU
     GA  GU    ACCAAUUUU   GUCUCAUAUUCGCAC UG ACAUAUUUUG   \
     CU  CA    UGGUUGAAG   CGGGGUAUAGGUGUG AC UGUAUAAAAU   U
GGCAA^ GA  ----         C--              C  A          AAA
110          100          90        80        70        60
```

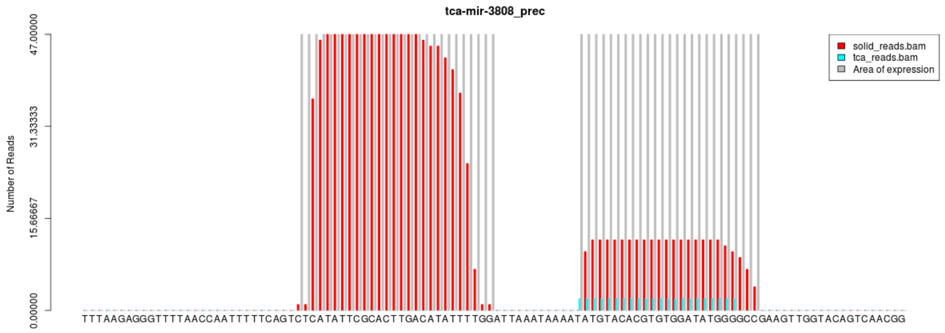

>tca-mir-3809_prec

```
UCAUUAAUGUUUGGCGGAAAUCAUAACUUGCUCGCUGAUGUAAUUUUAACAGACGUCGGUCAGUUGGUUAUGAAUUCUGAUAAAUGUUUGCUC
.....((((((((.(((((.((((((((.(((.((((((((...........)))))))).))).)))))))).))))).)))))))).....

 Initial dG =    -30.70

        10        20        30        40
UCAUU|       G     A        U   C        AAUUU
     AAUGUUUG CGGAA UCAUAACU GCU GCUGAUGU     \
     UUGUAAAU GUCUU AGUAUUGG UGA UGGCUGCA     U
CUCGU^       A     A        U   C        GACAA
  90        80        70        60        50

-----------------------------------------------------
```

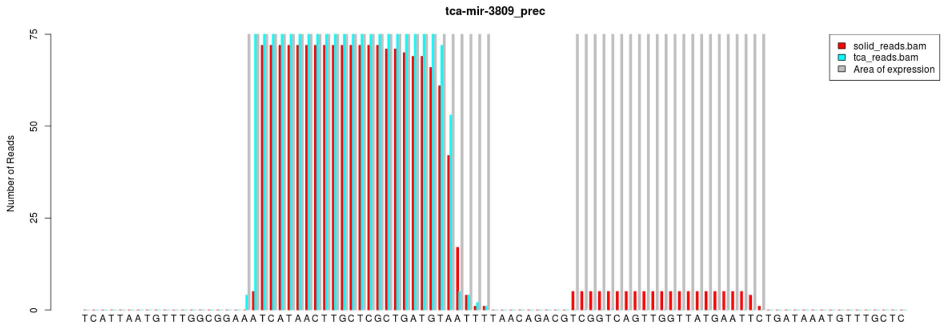

>tca-mir-3810_prec

```
AAAUAGAAGAUAAUGUCCGAUUUGUAGUUUACACGAAUUUGGUCCUUUUUUUUAAUAGGAUCAUUCGUGGGUAAUCUACACAUCGUGCAUUUUUGUCACGUC
.....((.((.((((..((((.(((((....((((((..(((((((.((....)).)))))))))))))......))))).)))).)).))))).)).)).....

 Initial dG =    -32.20

        10        20        30          40        50
AAAUA  A  U    UC    U     UUUA--|     UU       U  U
     GA GA AAUG  CGAU UGUAG      CACGAA  UGGUCCU UU U
     CU UU UUAC  GCUA ACAUC      GUGCUU  ACUAGGA AA U
CUGCA  G  U    GU    C     UAAUGG^     --       U  U
100       90        80        70          60

-----------------------------------------------------
```

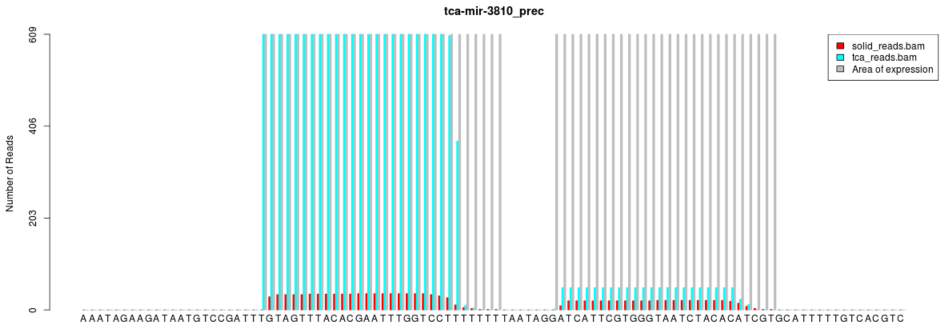

>tca-mir-3811a_prec

```
UUUAUCCAAUUCUCAGGAUUCUUGCUCAGAGUUUACAUAUUUUGGUUUAAAUAUGAAAUAUGUACAACCGUGAGUGGAGACCUGGGGGUUGGUCAGU
.....((((((((((((.(..(..((((..((((((((((((((.........))))))))))).)))..))))..)..)))))))))))))).....

 Initial dG =    -36.00

       10        20        30         40
UUUAU|           A UC UG    GA   -           GUUU
     CCAAUUCUCAGG U  U  CUCA  GUU UACAUAUUUUG    \
     GGUUGGGGGUCC A  G  GAGU  CAA AUGUAUAAAGU    A
UGACU^           - GA GU    GC   C           AUAA
      90         80        70        60        50
```

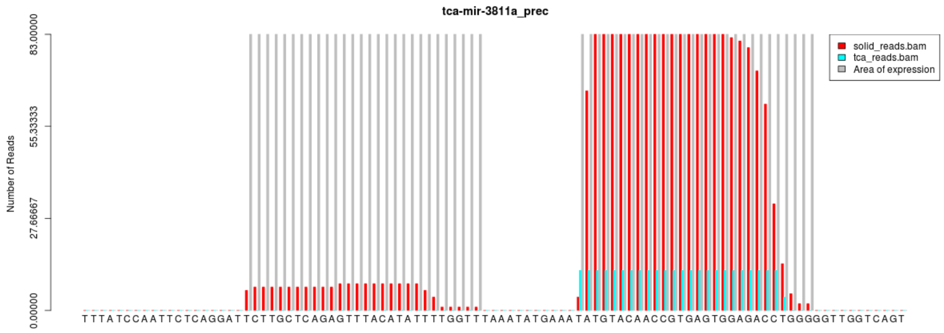

>tca-mir-3811b_prec

UUUAUCCAAUUCUCAGGAUUCUUGCUCAGGGUUUACUAAAUAUGAAAUAUGUACAACCGUGAGUGGAGACCUGAGGGUUGGUCAGU

```
.....((((((((((((.(..(..((((.(((((((..............))).)))).)))))..)..)))))))))))))).....
```

```
 Initial dG =    -34.60

        10        20        30         40
UUUAU|           A UC UG    G    -   UAAAUA
     CCAAUUCUCAGG U  U  CUCA GGUU UAC      U
     GGUUGGGAGUCC A  G  GAGU CCAA AUG      G
UGACU^           - GA GU    G    C   UAUAAA
     80        70         60        50
```

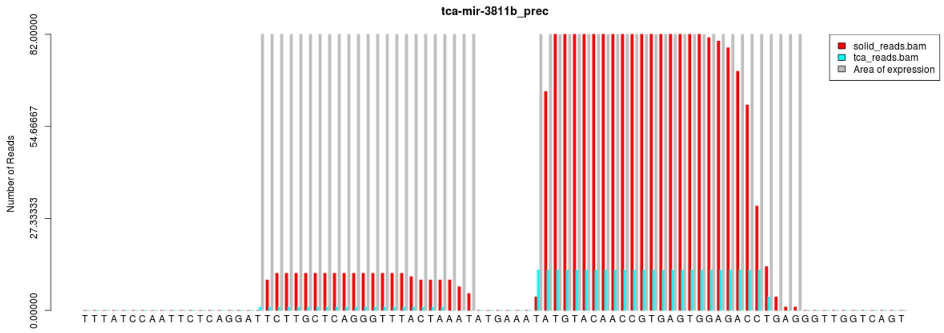

>tca-mir-3811c-1_prec

```
UUUAUCCAAUUCUCAGGAUUCUUGCUCAGGGUUUACAUGUUUUGGUUUAAAUAUGAAAUAUGUACAACCGUGAGUGGAGACCUGGAGGUUGGUCAGU
.....(((((..(((((.(..(..((((.((((((((((((((.........)))))))))))).)))).))))..)..))))))..))))).....

 Initial dG =    -37.60

        10        20        30         40
UUUAU|    UC      A UC UG    G    -           GUUU
     CCAAU  UCAGG U  U  CUCA GGUU UACAUGUUUUG    \
     GGUUG  GGUCC A  G  GAGU CCAA AUGUAUAAAGU    A
UGACU^    GA      - GA GU    G    C           AUAA
      90         80        70        60        50

-----------------------------------------------------
```

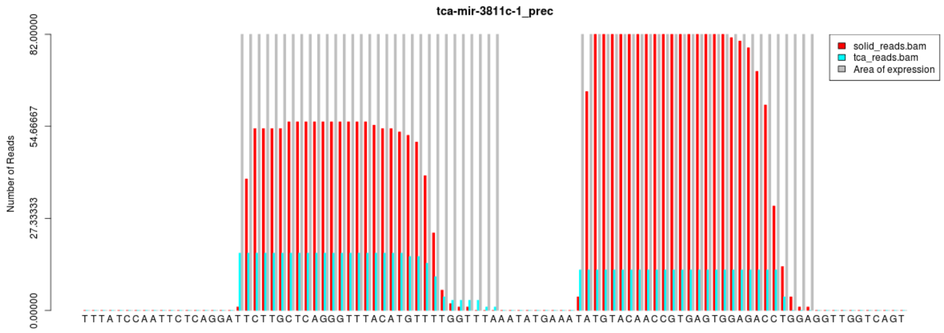

>tca-mir-3811c-2_prec

```
UUUAUCCAAUUCUCAGGAUUCUUGCUCAGGGUUUACAUGUUUUGGUUUAAAUAUGAAAUAUGUACAACCGUGAGUGGAGACCUGGAGGUUGGUCAGU
.....((((..(((((.(..(..((((.((((((((((((((.........)))))))))))).)))).))))..)..))))))..))))).....

 Initial dG =    -37.60

        10        20        30         40
UUUAU|    UC     A UC UG    G    -           GUUU
     CCAAU  UCAGG U  U  CUCA GGUU UACAUGUUUUG    \
     GGUUG  GGUCC A  G  GAGU CCAA AUGUAUAAAGU    A
UGACU^    GA     - GA GU    G    C           AUAA
      90         80        70        60        50

-----------------------------------------------------
```

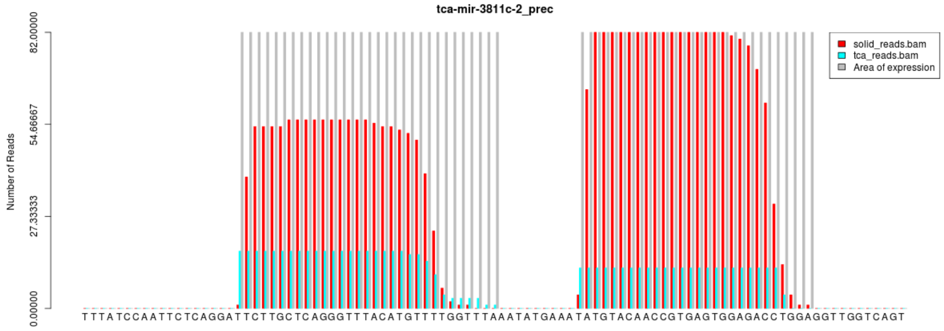

>tca-mir-3811d_prec

```
AAGUUUUAUCCAAUUCUCAGGAUUCUUCUCAGGGUUUACAUGUUUUGGUCUAAAUAUGAAAUAUGUACAACCGUGAGUGGAGACCUGGGGGUUGGUUAGUCAAC
.....(((.((((((((((((.((((.((((.(((((((((((((((.........))))))))))).)))).)))).)))).)))))))))))).))).....

 Initial dG =    -42.40

        10         20         30          40         50
AAGUU    U            A    U    G    -|          GUCU
     UUA CCAAUUCUCAGG UUCU CUCA GGUU UACAUGUUUUG    \
     GAU GGUUGGGGGUCC GAGG GAGU CCAA AUGUAUAAAGU    A
CAACU    U            A    U    G    C^          AUAA
  100        90         80         70         60

-----------------------------------------------------
```

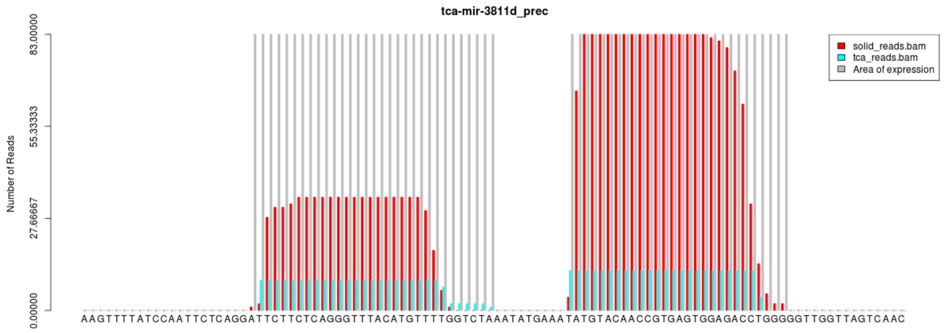

>tca-mir-3811e_prec

AUACGGUUUUAACUAAUUCUCAGGAUUCUUACUUGCGGUGUACAUGUUUUGGUAUGAAUAGUAAAUAUGUACAACCGUGAGUGGAGACUGGGGGUUGGUUAGUCAACAGCUU

.....((((((((((((((((..(((..(((..((((((((((((((..((....))...)))))))))))).))))..))))))..)))))))))))))))..))).....

```
 Initial dG =    -43.70

          10        20        30         40        50
AUACG   --|              GA   UU   UG    -          UG-  A
     GUU  UUAACUAAUUCUCAG  UUC  ACU  CGGU GUACAUGUUU   GU U
     CAA  GAUUGGUUGGGGGUC  AGG  UGA  GCCA CAUGUAUAAA   UA G
UUCGA   CU^              AG   --   GU    A          UGA  A
110       100        90          80        70        60

-----------------------------------------------------
```

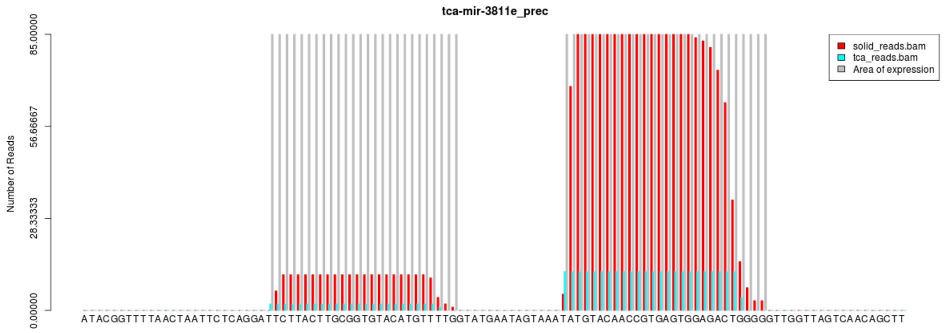

>tca-mir-3812_prec

UUUUUUAGGAUUGUGAGCUGAGGCUCGUUUUAAGUGAUUUUGAUGACACUAUUGGUUAUGAACAAUACUGGCACAUCAUCACUUAGCAUGAGCUUUAGAUUUUCGUCAAGUUCUACCAAA

.....((((((((((((((((((((.(((((((((..((.((.((.(((((........))))).)).))))..))))))))).)))))))))))...)))).))).))))).....

```
 Initial dG =    -38.00

        10             20        30        40        50
UUUUU     -   -    ---|          U         UU  A  A  C     GUU
     UAGGA UUG UGAG   CUGAGGCUCGU UUAAGUGAU  UG UG CA UAUUG   A
     AUCUU AAC GCUU   GAUUUCGAGUA GAUUCACUA  AC AC GU AUAAC   U
AAACC     G   U    UUA^          C         CU  -  G  C     AAG
.       110       100        90        80          70        60

-----------------------------------------------------
```

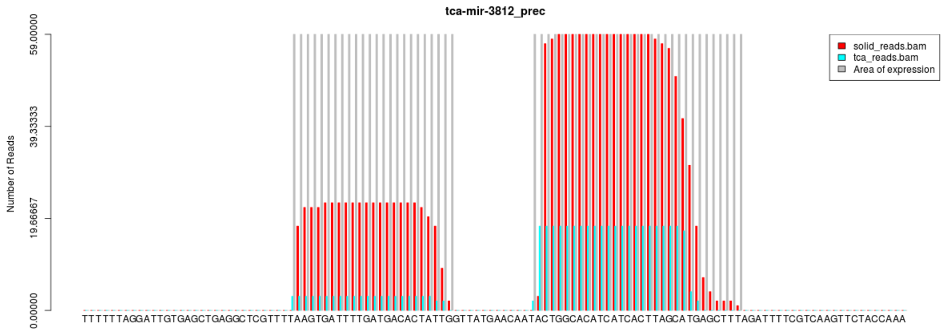

>tca-mir-3813_prec

```
UUUAAGUGUUGCAAAUUUUUAUUCUGCUCUUUUCGACGUUUUAAAAUUUGAAAAACGUCGAAGGAGUCAGGAUAAAAAUUUUUGACACAAAUC
.....(((((.(((((((((((((((((((.(((((((((.........))))))))))))))))).)))))))))))))).)))))).....

 Initial dG =    -38.30

        10        20          30        40
UUUAA     C              -|     U         AAAA
     GUGUUG AAAUUUUUAUUCUG CUCUUU CGACGUUUU    \
     CACAGU UUUAAAAAUAGGAC GAGGAA GCUGCAAAA    U
CUAAA     U              U^     -         AGUU
  90        80        70         60        50

-----------------------------------------------------
```

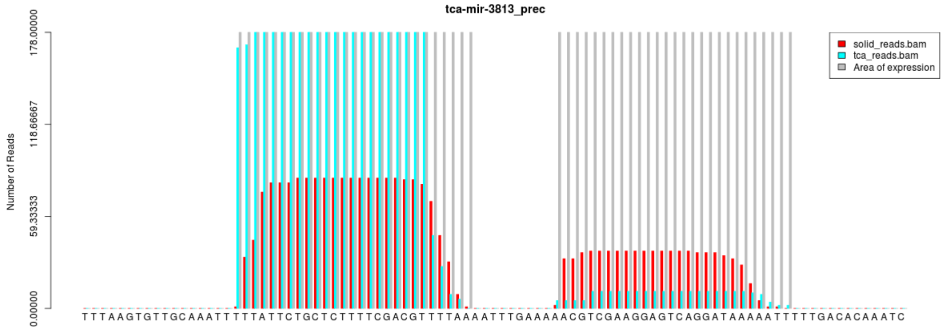

>tca-mir-3814_prec

```
UUCUCUUUGUUGAAAGUUUACAAUUUAGCGCUCUAUUCUAGUUAAAUUUUACACUAGCAUGAGCGUUUGUUGUAGAUUUUUGGCAAAAAUUU
.....((((..(((((((((((..(((((((.((.(((((..........))))).))))))))).))))))))))))..))))).....

 Initial dG =    -33.60

        10        20        30        40
UUCUC|    UG            UU       U  U     UAAA
     UUUGU  AAAGUUUACAAU  AGCGCUC AU CUAGU    U
     AAACG  UUUUAGAUGUUG  UUGCGAG UA GAUCA    U
UUUAA^    GU            U-       -  C     CAUU
 90        80        70          60        50

-----------------------------------------------------
```

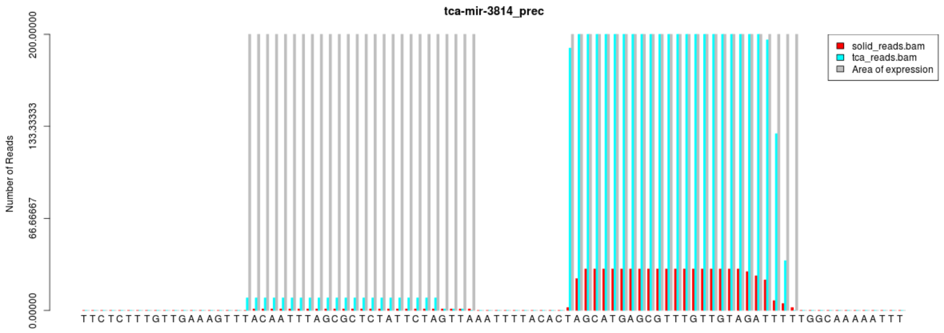

>tca-mir-3815_prec

```
GAAAAAAUUAUUUGCGAUUGGGGGUAGGAUGCUCAAAUGUUUGUGUUUAAUUAAACUUAUGAGUCCUUCUAACCUCAGUUGUAAAUAAUUGCUAU
.....(((((((((((((((((.(((((.(((((...(((((........)))))...)))))..))))).))))))))))))))))))).....

 Initial dG =    -33.80

        10        20        30         40
GAAAA                  G     U-|    AAU     UGU
     AAUUAUUUGCGAUUGGGG UAGGA  GCUCA   GUUUG   U
     UUAAUAAAUGUUGACUCC AUCUU  UGAGU   CAAAU   U
UAUCG                  A     CC^    AUU     UAA
    90        80        70        60        50

-----------------------------------------------------
```

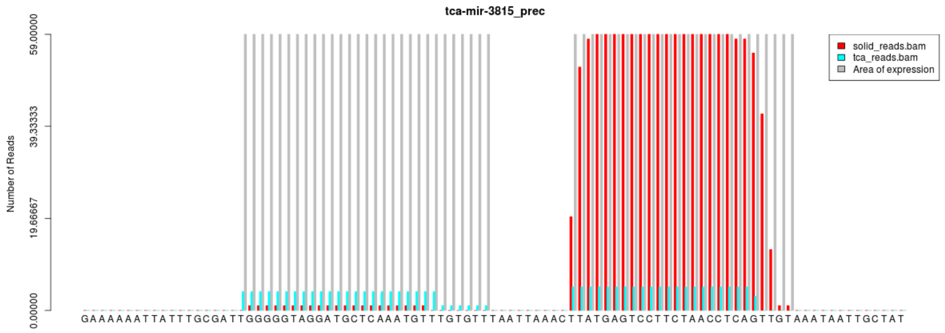

>tca-mir-3816_prec

GAUAGUGCAGAUUUAGAAUUGUAUUUUAGUUGAGCUGGGCAAAUUUAUUGUGGCCGGUUACAAGGCAUGUUUGGCAGCAAUUAGCUUUUGUAAUAUUGCUGCCAUUCAUACCUUACAAUU
GGUCUCAGUGUUUCCAACACCGAUAAACCUUAAUCACUUUUUUAGAAUUGUA

```
.....(((((.(((((((..(((((...(((....(((.......(((((.((((((((..((((.(((..((((((((((..
((....))...))))))))))..))).))))..)))))))).)))))........)))...)))...))).))..))))))).)))))

 Initial dG =    -50.60

        10         20          30         40           50         60         70         80

GAUAG     A        UU   -    UUA    GAGC    GCAAAUU--     U         AC     C    UU           UA-| U

     UGCAG UUUAGAA  GU AUU   GUU     UGG          UAUUG GGCCGGUU  AAGG AUG  UGGCAGCAAU   GC
U
     AUGUU AGAUUUU  CA UAA   CAA     GCC          GUGAC CUGGUUAA  UUCC UAC  ACCGUCGUUA   UG
U
-----     A        UU   C    UUC    AUA-    ACAACCUUU     U         CA     A    UU           UAA^ U

     170        160        150         140        130        120        110        100         90
```

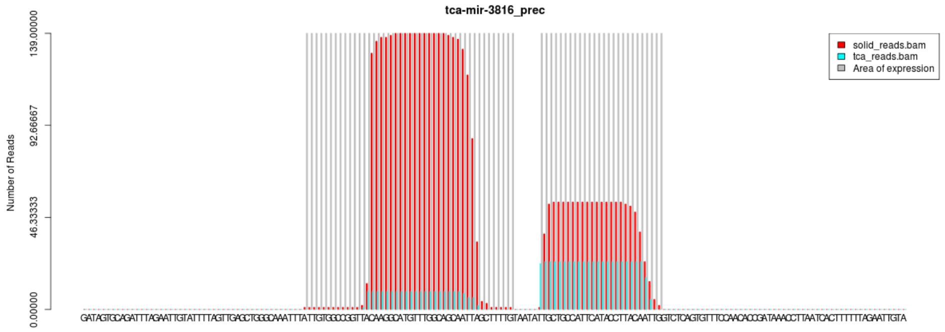

>tca-mir-3817_prec

UCCAAGGUUGCUGCGAGAUAACUGGGGCCAGUUUCGGGGUGUGGGAGUGGUAUUAUUUUUUAAAGUAAUAUUGCUCACUACACCUCCAAAUUGGUCUUGGUACUUCAACCGACUA

.....(((((....(((...((..((((((((((.((((((((((((..((((((((((...))))))))))..))).))))))))).))))))))))..)).)))))))).....

```
 Initial dG =    -54.20

        10        20        30        40         50        60
UCCAA|    CUGC   AUA  UG          C         -   UG         U
     GGUUG    GAG   AC  GGGCCAGUUU GGGGUGUGG GAG  GUAUUAUUUU \
     CCAAC    UUC   UG  UCUGGUUAAA CUCCACAUC CUC  UAUAAUGAAA U
AUCAG^    ----   A--  GU          C         A   GU         U
    110              100        90        80        70

-----------------------------------------------------
```

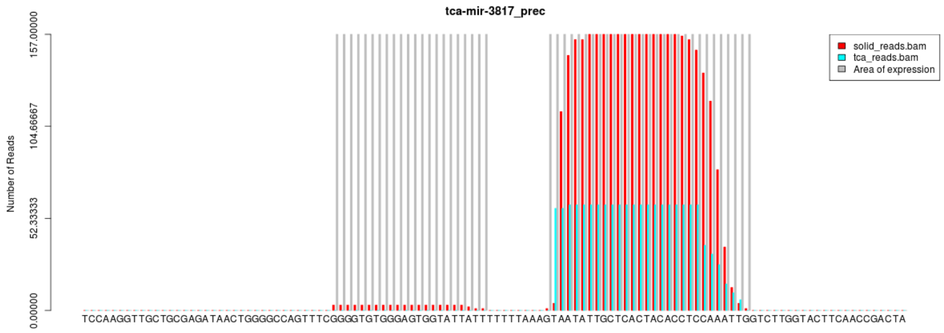

>tca-mir-3818_prec

AGAAAUGUGCUGCAAAUUUUCCUUUCGUUCCAAUGAUGCUUUAAAUUUGAGGAACAUCUGCGGAGCUAAAGCGAAACUUUUUGGCACAAAUAU
.....((((((.(((.((((((((.(((((...(((((((((....)))))..))))...))))).)))).)))).))).))))))).....

```
 Initial dG =    -27.10

        10        20         30          40
AGAAA       C   U    -    C     AAU    --|    A
     UGUGCUG AAA UUUC CUUU GUUCC   GAUG  CUUUA A
     ACACGGU UUU AAAG GAAA CGAGG   CUAC  GGAGU U
UAUAA       U   C    C    U     CGU    AA^    U
  90        80        70         60         50

-----------------------------------------------------
```

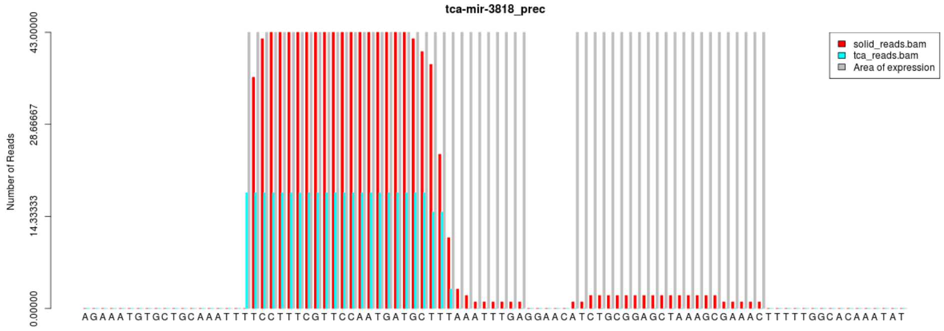

>tca-mir-3819_prec

AGCUAGGAAGCUAACAGGUAAUUCGUAUUAAUAUUCCAUUUAGUUCUGGAGCAAGGCUCUAUUCUUAUGUAAAAUUAUUAUUUGUUUUUGAUGUAAAAAUGCUUCCACUAA
.....(((((..(((...(((..(((.((((.((.(((..((...((((((...))))))..)).))).)).)))).)))..))).....))).......)))))).....

```
 Initial dG =    -20.00

        10              20        30        40        50
AGCUA      UA----   GGU--|  UC   U    A  C   UU  UUC      A
     GGAAGC      ACA     AAU  GUA UAAU UU CAU  AG   UGGAGC \
     CCUUCG      UGU     UUG  UAU AUUA AA GUA  UC   AUCUCG A
AAUCA      UAAAAA   AGUUU^  UU   U    A  U   U-  UU-      G
 .       100        90        80        70          60

-----------------------------------------------------
```

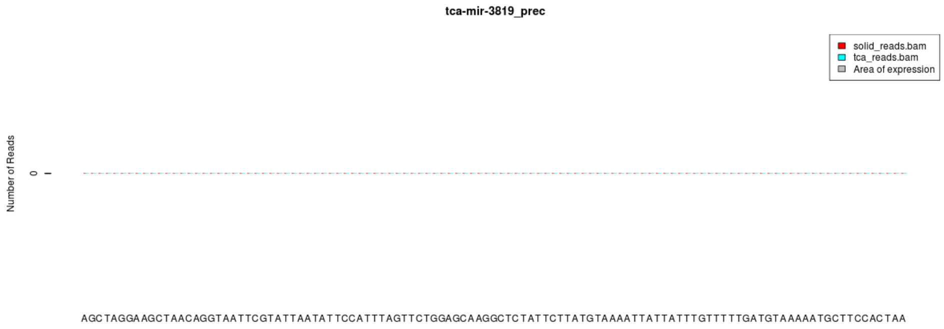

>tca-mir-3820_prec

AAAAAUUGUGUGCAAUUUCUUGCCAAGCCGUUACAGCAUUUUAUAUUUAAGAAAUCCUGUACAGGUUUUGCAAUAAAAUUGUGCACGAACAUU
.....(((((..((((((.((((.(((((..(((((.(((((........))))).)))))..))))).)))))..))))))..))))).....

```
 Initial dG =    -31.00

        10         20         30         40
AAAAA     UG       C-|   C     GU      C     AUA
     UUGUG  CAAUUU  UUGC AAGCC  UACAG AUUUU   U
     AGCAC  GUUAAA  AACG UUUGG  AUGUC UAAAG   U
UUACA     GU       AU^   U     AC      C     AAU
  90        80         70         60         50
```

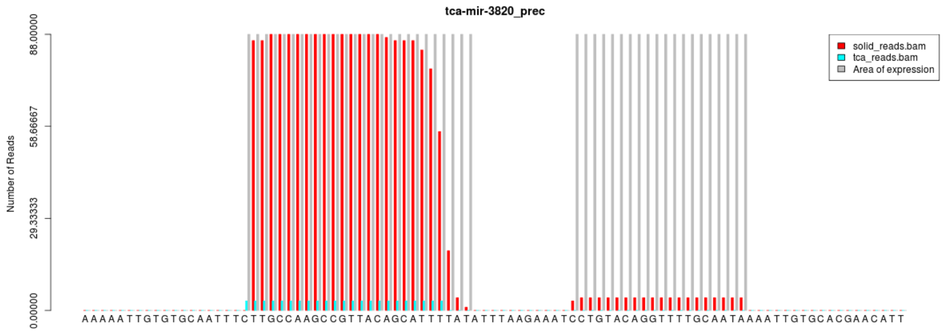

>tca-mir-3821_prec

```
AUGUAUGUGUUGCAAUUUAGCAUCCUCCUCUUAGGAAUUUUAUAUUUGAGAAAUUUCUAGUGAGUGAGGAUGUUAAUUUUUGGCACAAAUCA
.....((((((.((.((((((((((((((((((((((((((........))))))))))).))).)))))))))))).)).))))))).....

 Initial dG =    -35.50

        10        20         30         40
AUGUA       C  U           -   -|          AUA
     UGUGUUG AA UUAGCAUCCUC CUC UUAGGAAUUUU   U
     ACACGGU UU AAUUGUAGGAG GAG GAUCUUUAAAG   U
ACUAA       U  U           U   U^          AGU
 90        80        70        60        50

-----------------------------------------------------
```

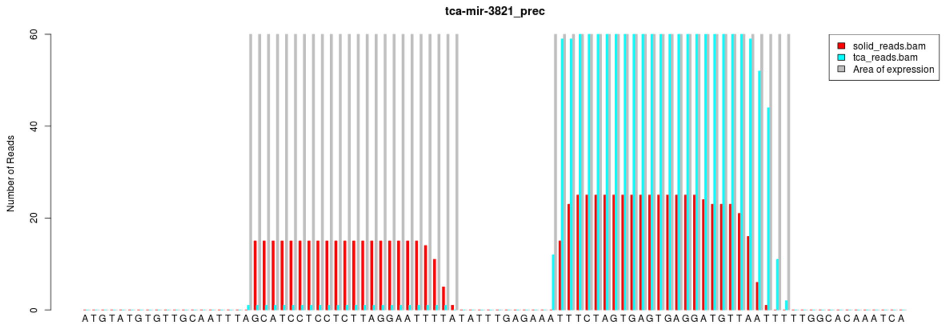

>tca-mir-3822_prec

AAAAUAUAAUAUUUUAUUUUAGAACUGACAAUAGCUGUAGUUCGUUUCGAGGUGUUGCACAGUUAAUAUUGUACCUUAAAGCAAUAUUUUCUGUGUGAACCUCGGAUUGGACUUUGGAUU
UUCAACUUUUGUCCUGGAUUUACGAUUAAAAAUAUUGUUGAUA
.....((((((((((((.((((.(((((((.(((((.((((((.((((((((.(..(((((..
((((((((........))))))))..)))))..))))))))).))))))).)))........)).)))))..)).)))).)))..))))))))))).....

```
 Initial dG =    -46.70

        10          20          30                40        50        60        70
AAAAU          --   U    A  --     U  --------|  U      U        G UG     UU        ACC
     AUAAUAUUUU  AUU UAGA CU  GACAA AG        CUG AGUUCG UUCGAGGU U  CACAG  AAUAUUGU   U
     UGUUAUAAAA  UAG AUUU GG  CUGUU UC        GGU UCAGGU AGGCUCCA A  GUGUC  UUAUAACG   U
AUAGU          AU   C    A  UC     U  AACUUUUA^  U      U        - GU     UU        AAA
 160       150       140       130       120       110       100         90        80
```

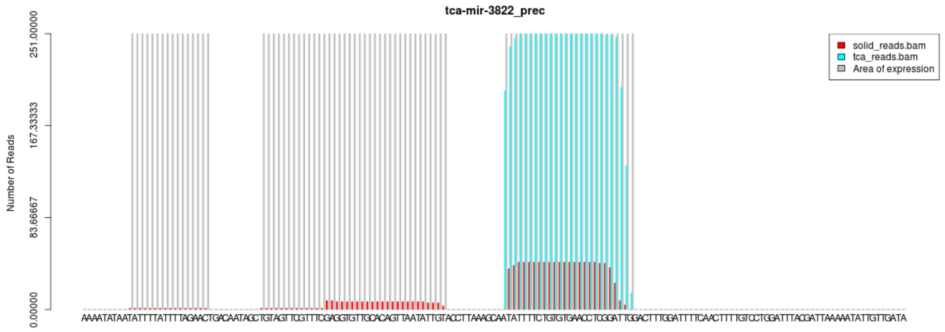

>tca-mir-3823_prec

```
AAUUUUGUGUCUUGUUUAAAGGUCGUGGUUUUGGCAUUUUGCUGUUGAAAUCAGUAUAUCAGACUACGGUCUUUGGAUGAGGCUCAUUUUU
.....((.((((..(((((((..((((((((.((.....(((((.......)))))..))))))))))..))))))).)))).)).....

 Initial dG =    -32.60

        10        20        30        40
AAUUU| U    UG       GU        U  CAUUU     UUG
     UG GUCU  UUUAAAG  CGUGGUUU GG     UGCUG   \
     AC CGGA  AGGUUUC  GCAUCAGA CU     AUGAC   A
UUUUU^ U    GU       UG        -  AU---     UAA
90        80        70         60           50

-----------------------------------------------------
```

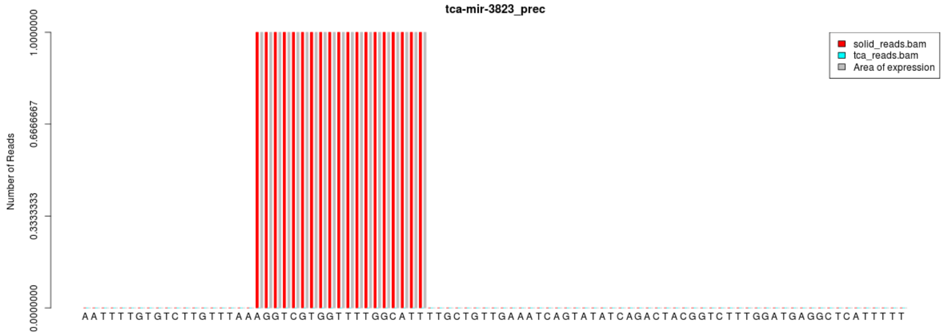

>tca-mir-3824_prec

```
AUGGACAAAGACUGAUACGCGGUGUAAAAUUGGUUCGACUAUUUGUGAACUAAAUAAAUGGUUGAGUCAGUUUUGCACCGUAUAUCAGUUAAUGACA
.....((..((((((((..((((((((((((..((((((((((((.......))))))))))))..))))))))))))))..))))))))..))...

 Initial dG =    -48.30

        10        20        30        40
AUGGA| AA        CG              GU            GAA
     CA  GACUGAUA  CGGUGUAAAAUUG  UCGACUAUUUGU   \
     GU  UUGACUAU  GCCACGUUUUGAC  AGUUGGUAAAUA   C
ACA--^ AA        AU              UG            AAU
        90        80        70        60

-----------------------------------------------------
```

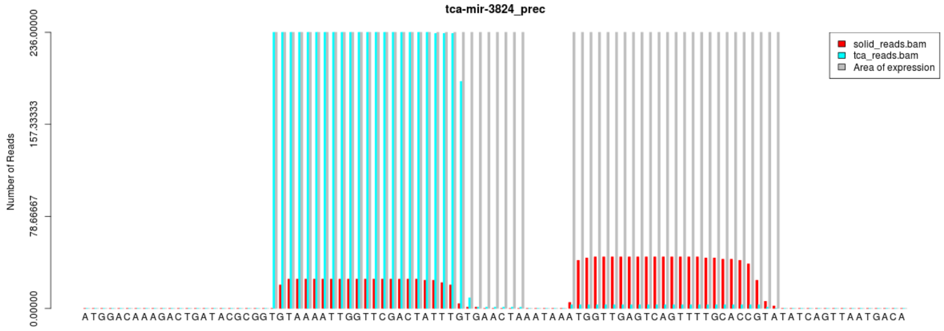

>tca-mir-3825_prec

```
AAAAUGUUUUGGUGAAGAUUUGUUUUCUUCUUCUGCACUAUUUUAUACUUAAAAAACCGUGCGGAGAAGACAACAAGUCUUUAACAAAAUUCUUC
.....((((((.((((((((((((.(((((.(((((((..(((((....)))))....))))))))))))).))))))))))))).)))))).....
```

```
 Initial dG =    -34.50

        10         20         30         40
AAAAU      G            U     U       UA--|    U
     GUUUUG UGAAGAUUUGUU UCUUC UCUGCAC    UUUUA A
     UAAAAC AUUUCUGAACAA AGAAG AGGCGUG    AAAAU C
CUUCU      A            C     -       CCAA^    U
    90         80         70          60         50
```

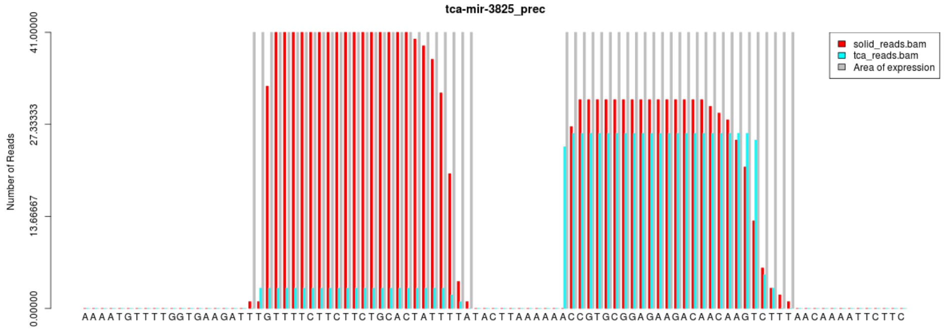

>tca-mir-3826_prec

```
AGGGUUGUGAUGUAACUGGGGCCGGUUUCGAGGCGUGGUGGGCGGUAAUAUUAGUAAAAAUAAUACUAUCCCCGACUACGCCUCGGAAUUGGUUUUGGUGUUUUCACACAAAC
.....(((((....((..((((((((((((((((((((((((.((((.(((((.......))))).))))))).)))))))))))))))))))))..))....))))).....

 Initial dG =    -56.80

        10        20        30         40        50
AGGGU     UGUA  UG                     -|  C    A     GUA
     UGUGA    AC  GGGCCGGUUUCGAGGCGUGGU GGG GGUA UAUUA   \
     ACACU    UG  UUUGGUUAAGGCUCCGCAUCA CCC CUAU AUAAU   A
CAAAC     UUUG  GU                     G^  -    C     AAA
 110       100        90        80         70        60

----------------------------------------------------
```

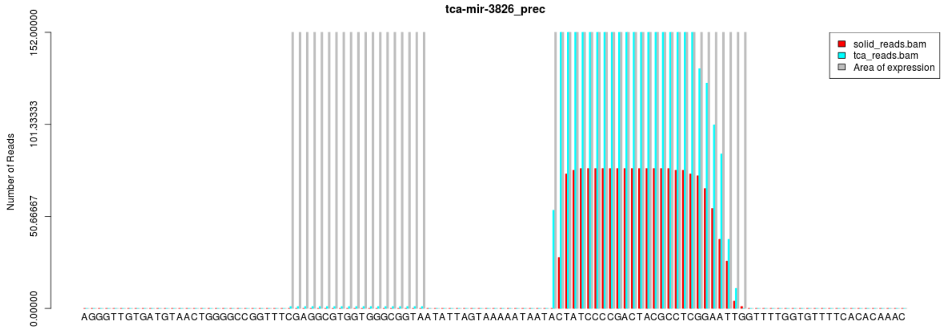

>tca-mir-3827_prec

```
GCUUCUCACUGCGCUGCUGCGGAUGCCGCAAGAUAAAAAAUCUUCCGAUUUUCAUUUGUAGUUGCUUUGAAAUUU
.....(((..(((..((((((((((.((.(((((.....))))).)).....)))))))))))))..))).....

 Initial dG =    -18.20

        10        20            30
GCUUC   CU   CU          C----| C     AA
     UCA  GCG  GCUGCGGAUG     CG AAGAU  \
     AGU  CGU  UGAUGUUUAC     GC UUCUA  A
UUUAA   UU   --          UUUUA^ C     AA
    70          60        50        40

-----------------------------------------------------
```

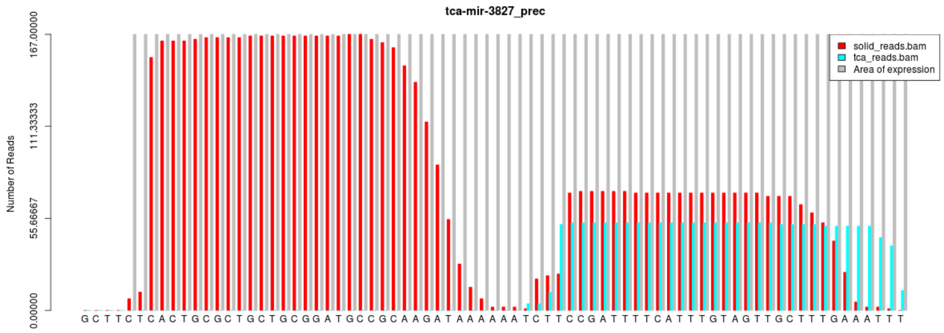

>tca-mir-3828_prec

```
UCUCUGCGAGAACUGAAACUCGGUUCUGAAGUGCAGGAUAGUUAUUUUUAAAUAAAGAAAAUAGCUAGCCUCGUACUCGGAAUCGAAGAUCAGUUAGUGCA
....((((..((((((...((((((((((.(((((((.(((((((((((.......))))))))))).))).))))))))))))))...))))))..))))

 Initial dG =    -43.30

        10        20        30         40        50
UCUC|   AG      AAC          A    -   A           AAA
    UGCG  AACUGA   UCGGUUCUGA GUGC AGG UAGUUAUUUUU   \
    ACGU  UUGACU   AGCUAAGGCU CAUG UCC AUCGAUAAAAG   U
----^   GA      AGA          -    C   G           AAA
   100        90        80         70        60

-----------------------------------------------------
```

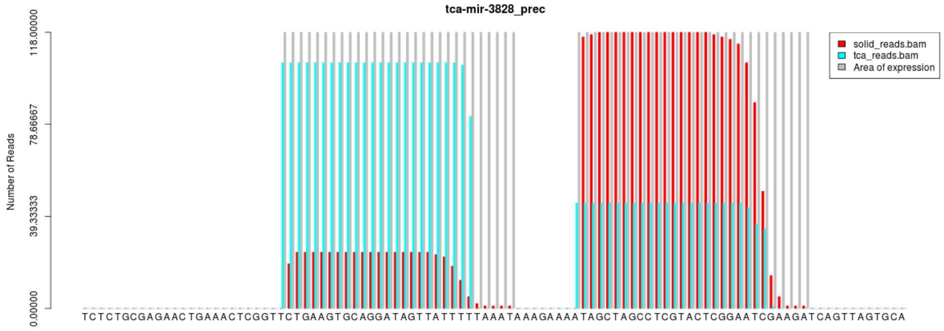

>tca-mir-3829_prec

GUUCAAUCAGCAGCAUACAGAGUUCCUCGAUGCCGUUUCUUCGAGUGGAUUGACUUGGUUUCAAGGAACCCUAAAUGCUGCUGGUGAUUA

..(((.((((((((((..((.((((((.((.((((..((.((.....))..))..)))).)).)))))).))..)))))))))))))...

```
 Initial dG =    -36.40

         10        20        30        40
GU-|  A          AC  A      C  U    UU  U-  GA
   UCA UCAGCAGCAU  AG GUUCCU GA GCCG  UC  UC  \
   AGU GGUCGUCGUA  UC CAAGGA CU UGGU  AG  AG  G
AUU^  -          AA  C      A  U    UC  UU  GU
.         80        70        60        50
```

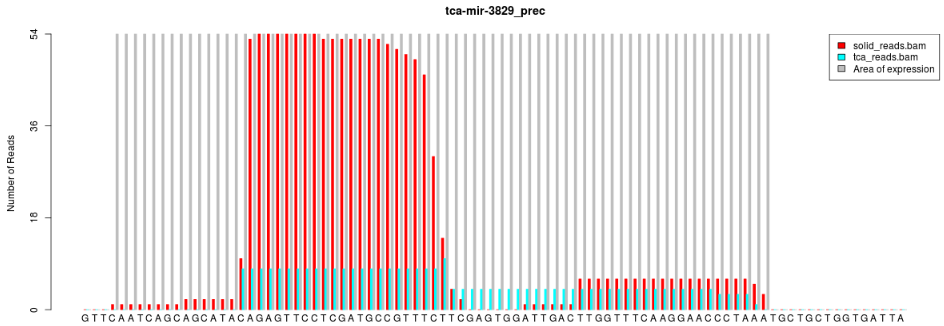

>tca-mir-3830_prec

```
GAAGCGUGGUGCUCGGUGCGGCACCGGCAUUAUGGUGGAAGACAAAUAUUUGGCCUCGGUCGCGUUGGGAGCCGCCAUU
.....((((((.(((..((((((...(((..(((..((.....)).)))....)))...))))))..))).))))))....

 Initial dG =    -29.40

        10        20        30
GAAGC     G   GG      ACC   AU--|  GG  GA
     GUGGU CUC  UGCGGC   GGC    UAU  UG  \
     CGCCG GGG  GCGCUG   CCG    AUA  AC  A
UUAC-     A   UU      GCU   GUUU^  A-  AG
         70        60        50

-----------------------------------------------------
```

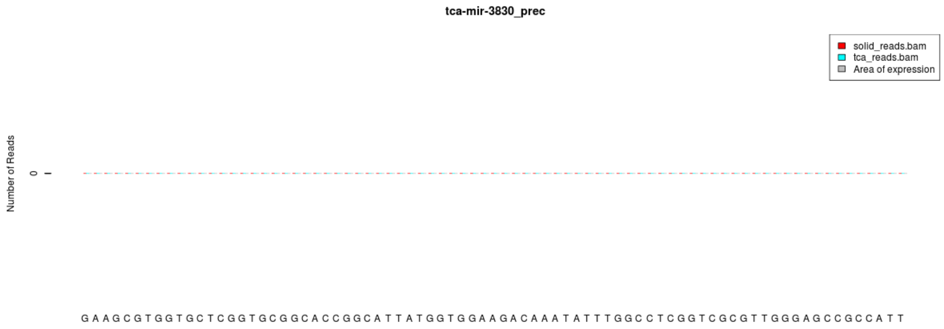

>tca-mir-3831_prec

```
GUGAGCUUUUCCAAACAUUGCUAUUUUGUCGCUCGAUGUCCGCUCCAGUCGUGAUACGACACAACAAAAUUGCAAUGUUUGAAAAGUCAUU
((((.((((((.(((((((((.(((((((...(((.(((((((....).)).)))))))....))))))).))))))))))))))))))).
```

```
 Initial dG =    -31.50

         10        20        30         40
-|   G      C         U       CGC-   A     -  - U
 GUGA CUUUUC AAACAUUGC AUUUUGU    UCG UGUC CG C C
 UACU GAAAAG UUUGUAACG UAAAACA    AGC AUAG GC G C
U^   -      -         U       ACAC   -    U  U A
90          80        70        60          50

-----------------------------------------------------
```

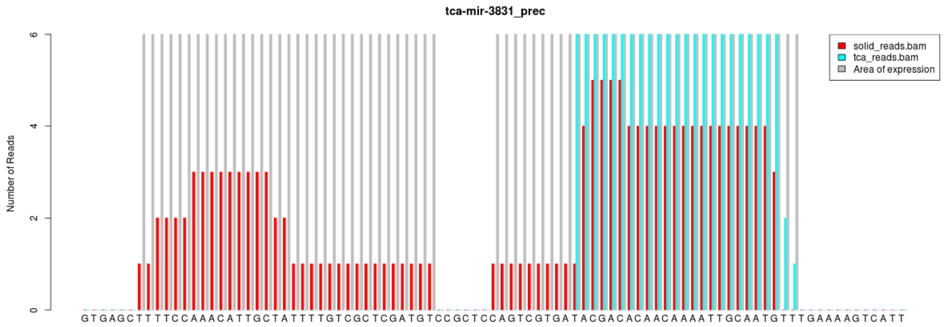

>tca-mir-3832_prec

```
UUAGAUUAACUGGAGUAAAACGGGCGAAAUGAUUCUGGUGUGUAGUUUUGUCCUAUUUCAUACACCAAAAUCGUUUCUCCUGUUUAUUUCAGUUAAGCACG
.....((((((((((.((((((.(((((((((.(((((((((((........)))..)))))))).))))))))).))))))))))))))))))).....

 Initial dG =    -40.60

        10        20        30        40          50
UUAGA          A       C         C        --|  UUU
     UUAACUGGAGU AAACGGG GAAAUGAUU UGGUGUGU  AGU   G
     AAUUGACUUUA UUUGUCC CUUUGCUAA ACCACAUA  UUA   U
GCACG          -       U         A        CU^  UCC
 .        90         80        70        60

-----------------------------------------------------
```

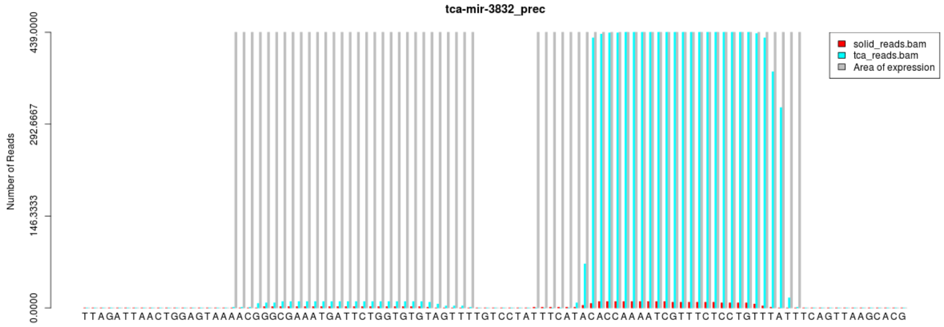

>tca-mir-3833_prec

UUUUGUUAGUUUUAACGGCAAGUCACAGCAGGCUGUUAUGGAAAUCACAGUAACAGCCUUCCUGAAGCUUGCAAGAGAAAAUUGAACUGU

```
.....((((((((....((((((..(((.(((((((((((.......)).)))))))))..)))..)))))).....)))))))).....
```

```
 Initial dG =    -30.20

        10         20         30         40
UUUUG        AACG-      CA   C-         -| GAA
     UUAGUUUU     GCAAGU  CAG  AGGCUGUUA UG   \
     AGUUAAAA     CGUUCG  GUC  UCCGACAAU AC   A
UGUCA        GAGAA      AA   CU         G^ ACU
.        80        70        60        50

-----------------------------------------------------
```

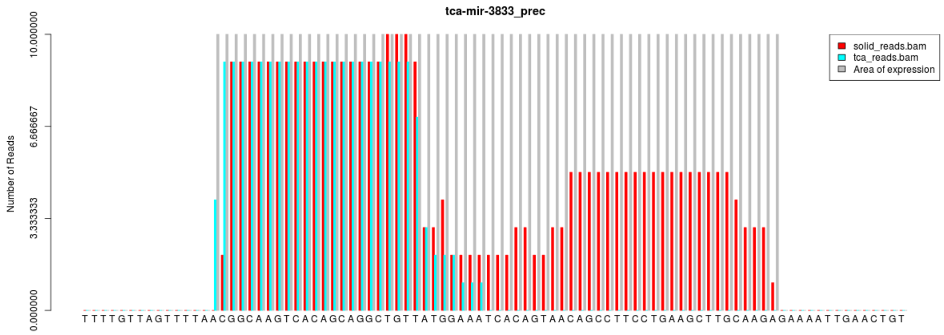

>tca-mir-3834_prec

```
GGAUGGAAUGAUGUUAUUUUCGACCUCCACCACCCUCUCUGGUAGAAAGUACGUCAUUCACCUC
((...((((((((.((((((.(((...............))).)))))))))))))))).))..

 Initial dG =    -18.90

          10         20         30
--| AUG         U      G   UCCACCA
  GG   GAAUGAUGU AUUUUC ACC       \
  CC   CUUACUGCA UGAAAG UGG       C
CU^ A--         -      A   UCUCUCC
   60           50         40

-----------------------------------------------------
```

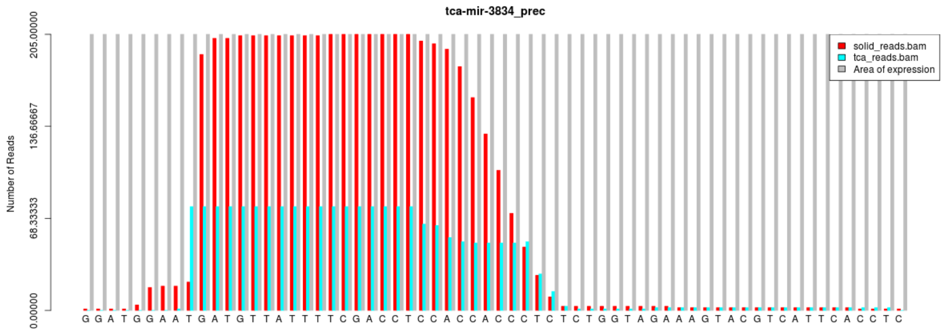

>tca-mir-3835_prec

```
UAAAAUCUUGUUGCCACUGAUGAAUUACUGCUAGAAUCUUCCUUUAAUUUAAGUUGAGGAUUCGCAGUUUCUGAGGCAAAUUAGAAAA
.....(((..(((((......(((..(((((..(((((((((((......)))..))))))))))))))))...)))))...)))...

 Initial dG =    -23.70

        10         20        30        40
UAAAA   UG-|    ACUGAU   UU      UA        --   UA
     UCU   UUGCC      GAA  ACUGC  GAAUCUUC  CUU  A
     AGA   AACGG      CUU  UGACG  CUUAGGAG  GAA  U
AAA--   UUA^    AGU---   --      --        UU   UU
         80           70            60        50

-----------------------------------------------------
```

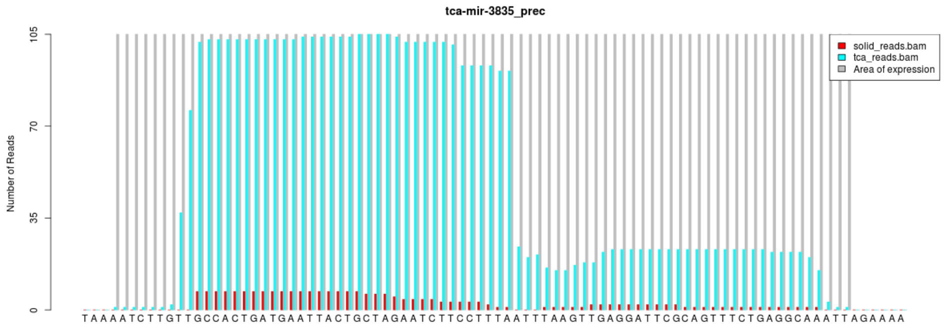

>tca-mir-3836a_prec

```
UUUUUGAAAUGUCUGUCAGGUCUACCUCUGCUUAAGUUAAUAUUUAUUUAAUUAUCACUGGGCAAGGUAGACUUUACAGCCGUUUCUUCAA
.....(((((.((((.(((((((((.(((((((((((.......))))))).....)))))))))))))))).)))).))))))).....

 Initial dG =    -32.30

        10        20        30             40
UUUUU      U    C          C      -----|     UAU
     GAAAUG CUGU AGGUCUACCU UGCUUA     AGUUAA   \
     CUUUGC GACA UUCAGAUGGA ACGGGU     UUAAUU   U
AACUU      C    U          -      CACUA^     UAU
90        80        70         60        50

-----------------------------------------------------
```

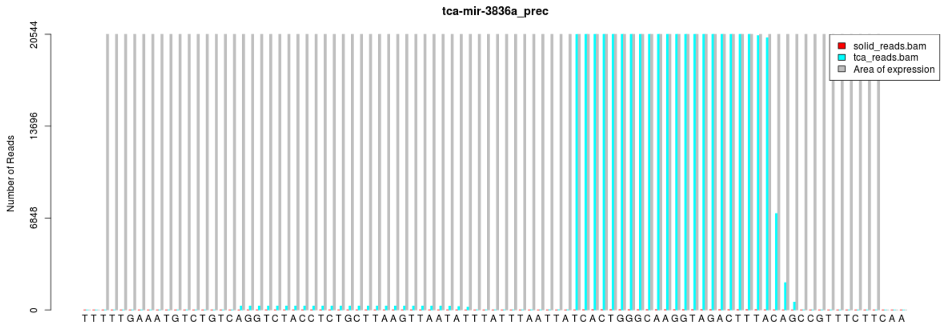

>tca-mir-3836b_prec

UUUUUGAAAUGUCUGCCAGGUCCACCUCUGCUUAAGUUGAUAUUUAUUUAAUUAUCACUGGGCAAGGUAGACUCUACAGCCGUUUCUUCAA
.....((((((.(((...((((.((((.((((((.((.((((..........)))))))))))))))).)))).)))...))).)))))).....

```
 Initial dG =    -26.90

        10        20        30        40
UUUUU|     U   CCA    C    C      A  U    UUUA
     GAAAUG CUG   GGUC ACCU UGCUUA GU GAUA    U
     CUUUGC GAC   UCAG UGGA ACGGGU CA CUAU    U
AACUU^     C   AUC    A    -      -  -    UAAU
90        80        70         60          50
```

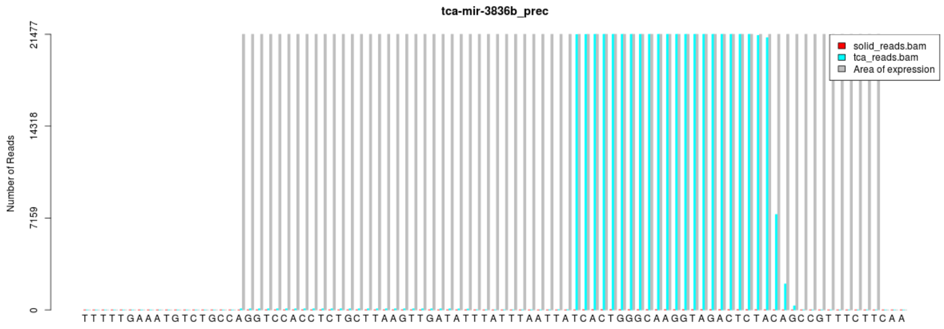

>tca-mir-3837_prec

AAAAACAUUUGUCUAAUAAAUAAAUCUUGAGACGUUUUUUUAAACUUGUUAAGACAUUAUGAGCGAGAGUGAGAUAUUGUCGGAAGAUUGUUUUUAUGAAAGUGAACAG
.....((((.....(((((..((((((..((((...(((((..((((((...........))))))..)))))...))))..))))))...)))))..))))).....

```
 Initial dG =    -22.20

        10        20         30        40        50
AAAAA|    GUCUA     UA-       GA     UUU     AA      AAGAC
     CAUUU     AUAAA   AAUCUU  GACG   UUUUA  CUUGUU     \
     GUGAA     UAUUU   UUAGAA  CUGU   AGAGU  GAGCGA     A
GACAA^    AG---     UUG       GG     UAU     GA      GUAUU
       100           90         80        70        60

-----------------------------------------------------
```

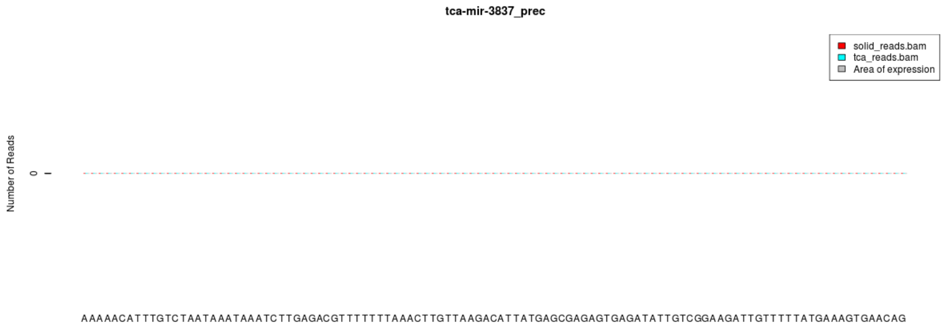

>tca-mir-3838_prec

ACAGAGAUAUUCGCGCAAAGUUAGAGGUUUAGCCUAAGUCGUCUUAAUAAAAUGUUUUUAAUAAUUGAGAAAAUUUUAAAUGUUCUAACAUUUUGAGUCCGAUCCUGUCUACCUGCCGCU
UUGUGAUGAAGGCUUCAAAAU

```
...(((...((((((((((((...((((...((....((((.(((((...((((((...((((.
((((((...)))))).)))).)))))))))))..))))...))..)))))...)))))))).))))...))).....
```

```
 Initial dG =    -29.00

         10         20        30        40         50        60        70
ACA--   AUA    -|       UAG    UUA  CUAA    U-     UAA      UUU    A      A
     GAG   UUCG CGCAAAGU   AGGU   GC    GUCG  CUUAA   AAUGUU   AAUA UUGAGA \
     CUU   AAGU GUGUUUCG   UCCA   UG    UAGC  GAGUU   UUACAA   UUGU AAUUUU A
UAAAA   CGG    A^       CCG    UC-  UCC-    CU     ---      UC-    A      A
 .       130       120       110         100           90          80
```

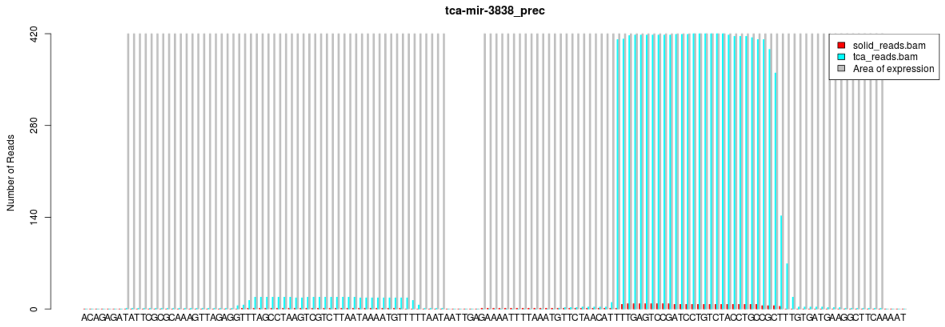

>tca-mir-3839_prec

AUAAUGAGCGAGAUAGUAGUUUUGUCACAGGAAAUUGCGAUAUGUUCGAAUAUUUUAUGAACUUGUUGCCGUUUCCUACGUGCAAAAUUCGAAUCAAUGCUU
.....((((.(((...(((((((.((((((((((.((((((.(((((((....)).))))).)))))).))))))).)))))))..))))))))))...)))..)))))

```
 Initial dG =    -30.30

        10         20         30        40         50
AUAAU     A-   AGU       U   --       U      U     -| U
     GAGCG  GAU   AGUUUUG CAC  AGGAAAU GCGAUA GUUCG AA A
     UUCGU  CUA   UUAAAAC GUG  UCCUUUG CGUUGU CAAGU UU U
-----     AA   AGC       -   CA       C      U     A^ U
     100        90         80        70        60

-----------------------------------------------------
```

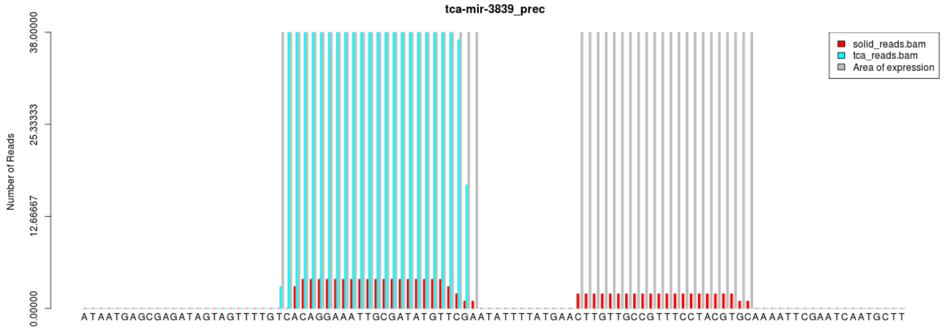

>tca-mir-3840_prec

CAUCAGUGAAUGCUUUGUAGGGGGUACUGUAAGUACGUACGAUUUUUUUAACGUAGUACGAACGGUACUCACUAUGAACGUUACACAGUUU
.....((((((.((..(((.(((((((((..((((.((((..........))))))))..))))))))).)))..)))))).))).....

```
 Initial dG =    -31.80

         10        20        30        40
CAUCA   -|   C  UG   G         AA    G    AUUU
     GUG AAUG UU  UAG GGGUACUGU  GUAC UACG    U
     CAC UUGC AA  AUC CUCAUGGCA  CAUG AUGC    U
UUUGA   A^   -  GU   A         AG    -    AAUU
90        80         70        60         50
```

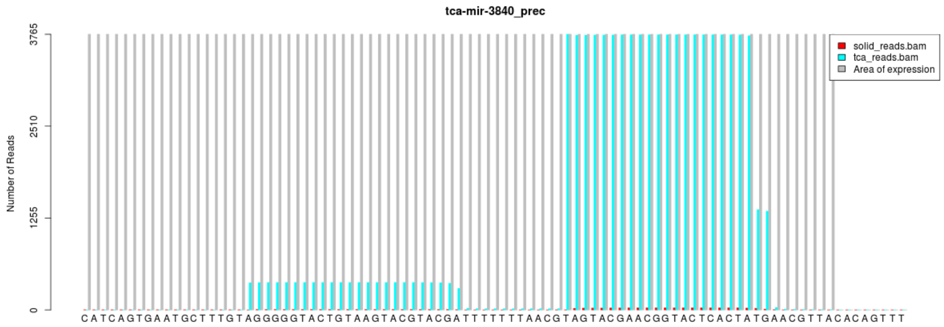

>tca-mir-3841_prec

```
CUCUUUUCGCAUUAUCUUUCGUGCGAUCUUUCCAUUCCUACUUGUUGUUUGAUAAGUAGUACGGAAUUAUCUCACAUUAGAUAUUGGGAGUUUUC
.....(((.((.(((((...(((.(((..((((....(((((((((....)))))))))...))))..))).)))...))))).)).))).....
```

```
 Initial dG =    -28.50

        10        20        30        40
CUCUU|  G  U     UUC   C   CU    AUUC         G
     UUC CA UAUCU   GUG GAU  UUCC    CUACUUGUU U
     GAG GU AUAGA   CAC CUA  AAGG    GAUGAAUAG U
CUUUU^  G  U     UUA   U   UU    CAU-         U
    90        80        70        60         50
```

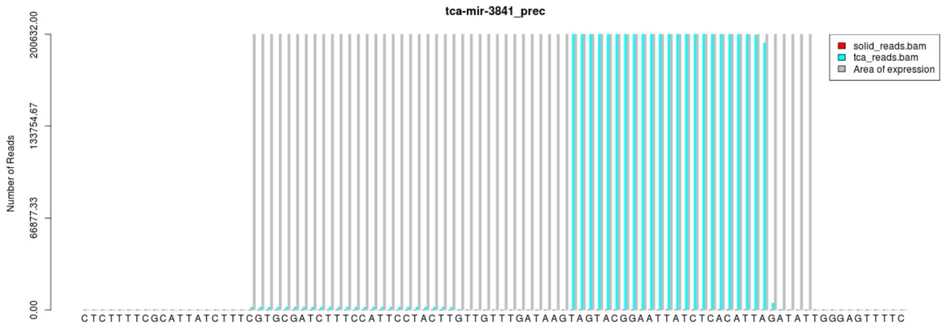

>tca-mir-3842_prec

```
UCCCACAACCUAACAACAAGGAGGAAAAACGAGUCGGUCGGCAUGGCACUGAUGCGAACGCUUCGUUUUUAUCCUUGUUGUUUUAUCGUUGAAAAU
.....((((..(((((((((((..(((((((((.((.(((.(((.......)))))).)).))))))))).)))))))))))).....)))).....

 Initial dG =    -34.10

        10           20         30         40
UCCCA    CU---|          GG          U  G   G   GGC
     CAAC     AACAACAAGGA  AAAAACGAG CG UCG CAU   \
     GUUG     UUGUUGUUCCU  UUUUUGCUU GC AGC GUA   A
UAAAA    CUAUU^          A-          C  A   -   GUC
     90        80         70         60          50

-----------------------------------------------------
```

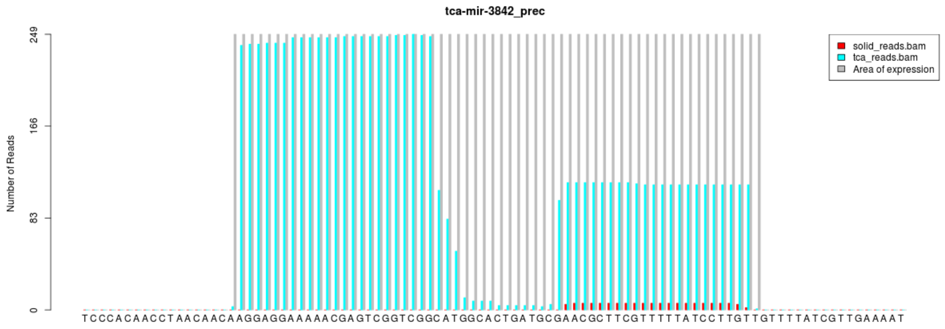

>tca-mir-3843_prec

```
UGUGGCUGACAAGUUUUCAGUUGUUGAUGUGGCUUUAGUGGAGAUUUUACUGAAUUAAAGCUAUGUCAGCAAUUAAAAAAAUGUCGGGUCUU
.....((((((..((((.(((((((((((((((((((((..((......))..))))))))))))))))))))).))))..)))))).....

 Initial dG =    -36.70

        10        20        30        40
UGUGG|     AG    C                    GG  AU
     CUGACA  UUUU AGUUGUUGAUGUGGCUUUAGU  AG  U
     GGCUGU  AAAA UUAACGACUGUAUCGAAAUUA  UC  U
UUCUG^     AA    A                    AG  AU
 90        80        70        60        50

-----------------------------------------------------
```

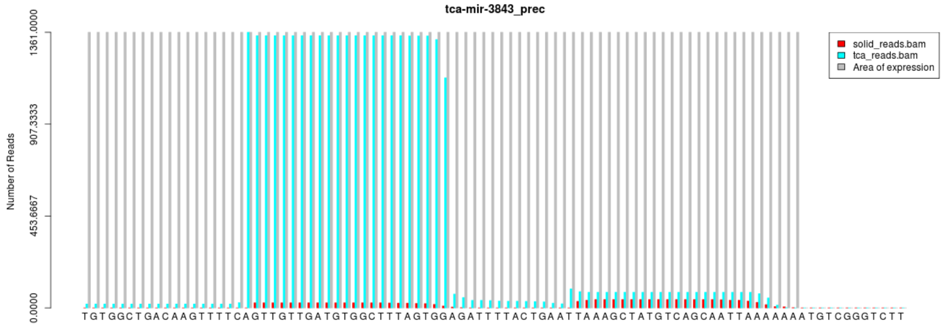

>tca-mir-3844_prec

```
AUUUAUGUUUUGAGAGGCACCUCAGAAAACCCCACGCACAUGUUUGUACAAAUUGUGUGUGAUAAAAUGAGGUGCCAGAGCAAAUUG
.....((((((....(((((((((........((((((((.((((....))))))))))))).......)))))))))))))))).....
```

```
 Initial dG =    -31.20

        10        20        30        40
AUUUA|     GAGA         GAAAACCC        U    G
     UGUUUU    GGCACCUCA        CACGCACA GUUU U
     ACGAGA    CCGUGGAGU        GUGUGUGU UAAA A
GUUAA^     ----         AAAAUA--        -    C
      80            70          60         50

-----------------------------------------------------
```

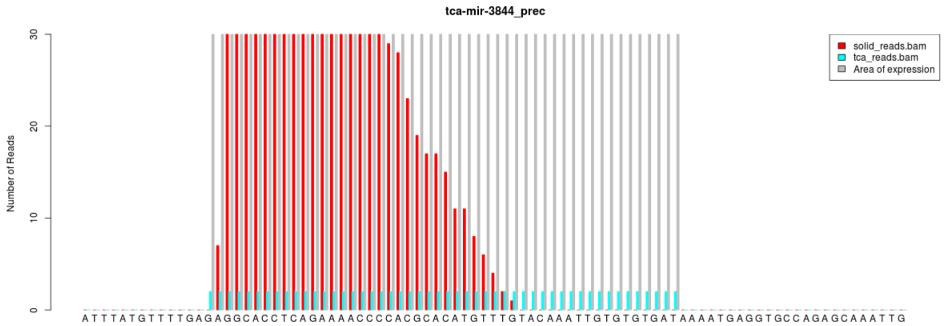

>tca-mir-3845_prec

CGAAAUGGUGAUUUAUGUAAUAAAGUUGGUGGUCAAUGUCCCACGCCUUGGAGUUAUCUUGGCCGGAUUAAGUCGAAUGCUGUGUACGCCAUGGUGGAAAUAAGUGUUGGGACCCACGCA
ACGUCCAUAGAUGGGUCAAUCAGUUAU

```
.....(((((((((((.((.....((((((((.....((((((.((((((...(((((.((((.(.....
(((.....))).....))))).))))))...)))).)))))))))))).)))).....)).)))))))).))).....
```

```
 Initial dG =    -42.30

         10        20         30        40         50        60        70
CGAAA    -        G  AUAAA    -|   UCAAU      C  -    GAG     U    C GAUUA   CG
     UGG UGAUUUAU UA     GUUG GUGG     GUCCCA GC CUUG   UUAUC UGGC G     AGU  \
     ACU ACUGGGUA AU     CAAC CACC     CAGGGU UG GAAU   GGUGG ACCG C     UCG  A
UAUUG   A         G  ACCUG    G^   -----      -  U    AAA     U    - AUGUG   UA
     140       130       120            110        100        90         80
```

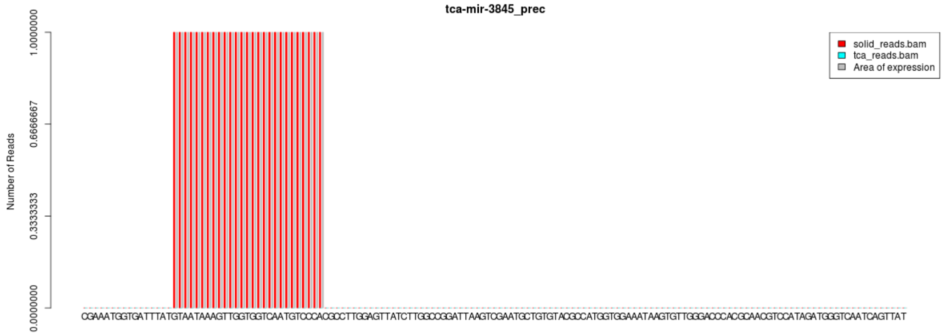

>tca-mir-3846_prec

GGAAUAGAGUUACUUCCAUUGAUGAUAGUAAAUGUUCUUGGCUUAUAAGUCUUUGUGUCAAGAAUAUUGACCACAAUUACGUUGCCUCAAUCAUCGACGUCAUCAACUGACAUGGCAACU
AAUUCUCCAAU

```
(((...((((((.(((((((((((((.((..(((.....(((......((..
((((((((((.....)))).)))))..))...)))......)))..))))))))))......)))).)).)))))))))...

 Initial dG =    -33.30

           10                20        30        40        50         60
---   AUA      C  -    ------|      A  AA   UUCUU   UUAUAA  CU      -    GA
   GGA   GAGUUA UU CCAU      UGAUGAU GU  AUG     GGC      GU  UUGUG UCAA  \
   CCU   CUUAAU AA GGUA      ACUACUG CA  UAC     CCG      CA  AACAC AGUU  A
UAA   ---      C  C    CAGUCA^      -  GC   UAACU   UUG---  UU      C    AU
 .          120       110       100         90           80        70

-----------------------------------------------------
```

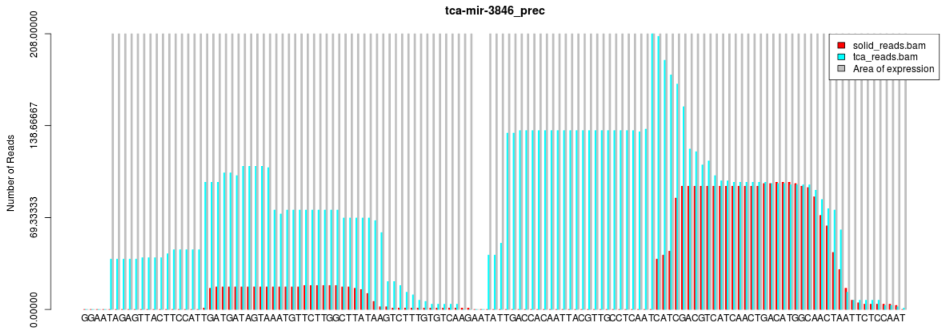

>tca-mir-3847_prec

```
GAAGUGCUGUUUAAAUUCGAACUUUUUAUUUAAUUUGGCAAUGUGUGCGAGUUACACGUCUCGAAUUUCGAUCAGGUUCGAUUUUAACCGGCUUAAA
.....((((.(((((.((((((((.......(((((((..(((((((.....))))))).)))))))......)))))))).))))).)))).....

 Initial dG =    -27.10

        10        20        30        40
GAAGU|   U     U        UUUAUUU       CA       CG
     GCUG UUAAA UCGAACUU       AAUUUGG  AUGUGUG  \
     CGGC AAUUU AGCUUGGA       UUAAGCU  UGCACAU  A
AAAUU^   C     U        CUAGCU-       C-       UG
      90        80        70         60

-----------------------------------------------------
```

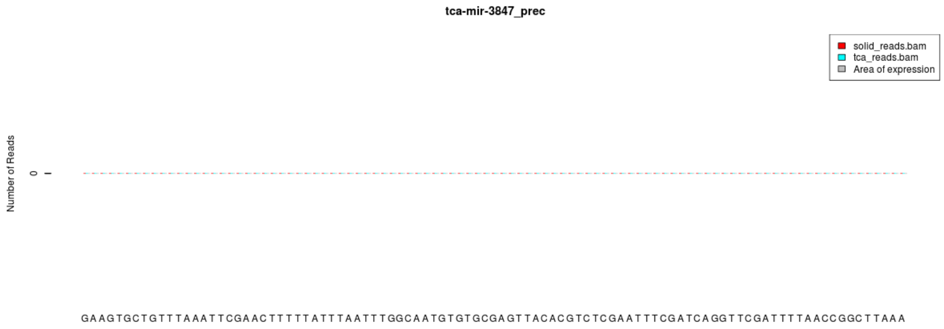

>tca-mir-3848_prec

UAUUAUCCGCUCUAUGUAGCGGUAUAUAAGGUUAGCGCGCUGACCGCGUGUUACCCACUUAAUGUGCUUACAUUGGAAGGAAAUU

.....(((..((((((((..(((((((.((((((((((((.....))))))))...)))).))))))))))))).))).)))....

```
 Initial dG =    -27.50

        10         20         30            40
UAUUA   GC   -     GC        A    ---|       UG
     UCC  UCU AUGUA  GGUAUAU AGGU   UAGCGCGC  \
     AGG  AGG UACAU  UCGUGUA UUCA   AUUGUGCG  A
UUAA-   A-   U     --        A    CCC^       CC
     80         70          60        50
```

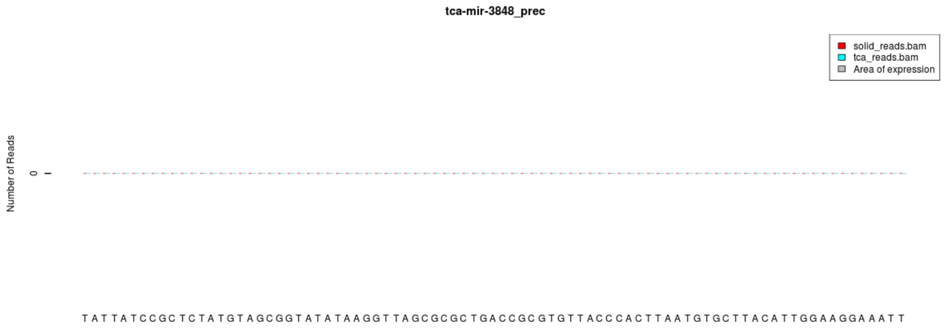

>tca-mir-3849_prec

GUAACUGUUCGAAAUGCUGACAUUUUAACCAUAGUGCUGUUGUUUAUAUUCAAAUUAAUAGCAUUGUGUUGGAAAUGUCGUCUUUUCGAACAAACUA

```
.....((((((((...((((((((...((((((((((((((((......)))).))))))))))))...)))))))))...))))))))).....
```

```
 Initial dG =    -35.30

        10        20        30        40
GUAAC         UGC         AAC           -|   AU
     UGUUCGAAA   UGACAUUUU   CAUAGUGCUGUU GUUU  A
     ACAAGCUUU   GCUGUAAAG   GUGUUACGAUAA UAAA  U
AUCAA         UCU         GUU           U^   CU
      90        80        70        60        50
```

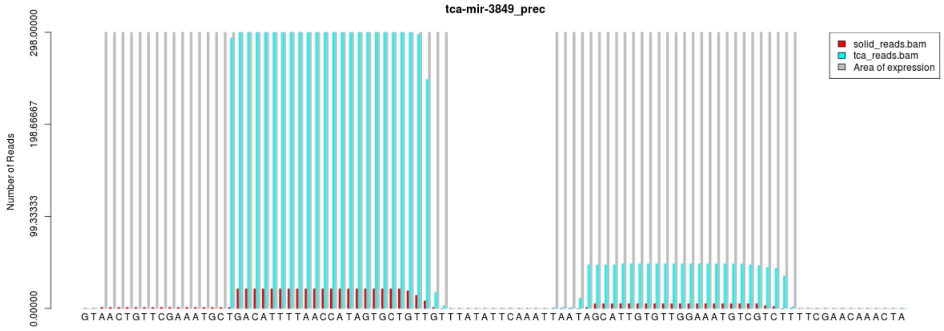

>tca-mir-3850_prec

GUAAUUGCAUUUGAUAGAUUAGGCCGAUAUUAGUCUAAACAAGGUUGAGACUAUUCUAUAAAUUAUUACAAAAUCGCUAAUAAGGUAUAAGGCAGUAAAAUGUUCGAGACUAAUGUUGGU
UUCAUUUUACUCGAAAAUACUUUUUUUUGCAUAAAA

```
.....((((((((((((..((((((((((((((((....((.(((...(((.(..((((..
((((((.........))))))..))))..).)))..))).))..))))))))))))))))...)))).))))).............)))).....
```

```
 Initial dG =    -33.70

                    10         20         30        40        50        60        70
GUAAU    ------------     -    UU-|              AAAC  G   GAG   A UC    AA      CAAA
     UGCA            UUUGA UAGA   AGGCCGAUAUUAGUCU    AA GUU   ACU U  UAUA  UUAUUA    \
     ACGU            AAGCU AUUU   UUUGGUUGUAAUCAGA    UU UAA   UGA G  AUAU  AAUAAU    A
AAAAU    UUUUUUUCAUAA     C    UAC^              GC--  G   AA-   C GA    GG      CGCU
    150       140       130       120       110        100        90        80
```

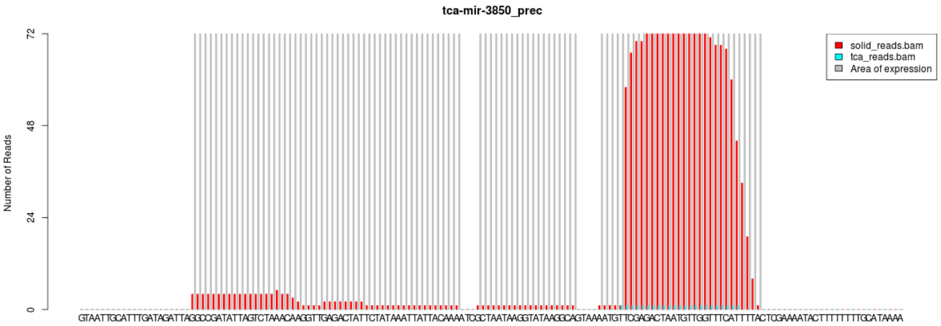

>tca-mir-3851a-1_prec

```
CGGACGAAUUAUGAAUUUGGCGCCGAGUUGAUUUGGAGUAGUACUAUGGUUUUGGGUACUCACAUAGUACAUUCCGAAUCGACUCGUUAACAAAUUUAAAUUCAACAA
.....(((((.((((((((....(((((((((((((((.((((((((..............))))))))))))))))))))))))....))))))))))))).....

 Initial dG =    -44.10

        10        20        30         40        50
CGGAC|    A        GCGC                A        GUUUUG
     GAAUU UGAAUUUG    CGAGUUGAUUUGGAGU GUACUAUG      G
     CUUAA AUUUAAAC    GCUCAGCUAAGCCUUA CAUGAUAC      G
AACAA^    -        AAUU                -        ACUCAU
      100         90        80         70        60

-----------------------------------------------------
```

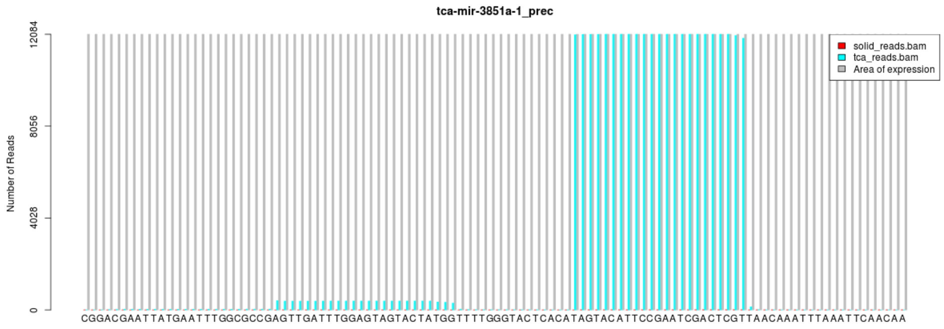

>tca-mir-3851a-2_prec

```
CGGACGAAUUAUGAAUUUGGCGCUGAGUUGAUUUGGAGUAGUACUAUGGUUUUGGGUACUCACAUAGUACAUUCCGAAUCGACUCGUUAACAAAUUUAAAUUCAACAA
.....(((((.((((((((....((((((((((((((((.((((((((..............))))))))))))))))))))))))....))))))))))))).....

 Initial dG =    -42.40

        10        20        30        40        50
CGGAC|    A        GCGC              A        GUUUUG
     GAAUU UGAAUUUG    UGAGUUGAUUUGGAGU GUACUAUG      G
     CUUAA AUUUAAAC    GCUCAGCUAAGCCUUA CAUGAUAC      G
AACAA^    -        AAUU              -        ACUCAU
      100         90        80         70        60

-----------------------------------------------------
```

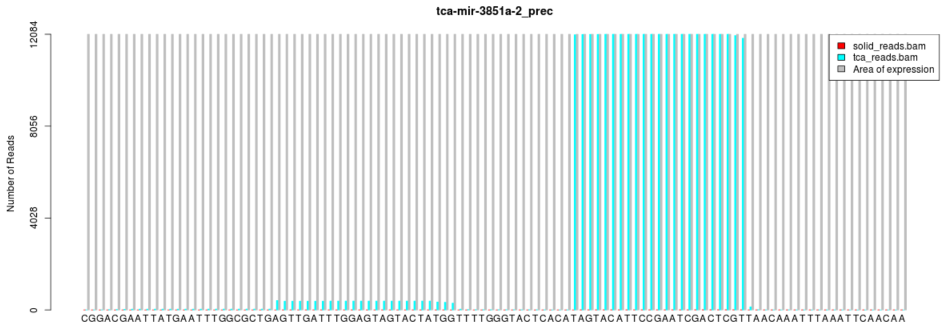

>tca-mir-3851b_prec

CGGACGAAUCAUAAAUUUGAUUACAAGUUGAUUUGGAGUAGUACUAUGGUGUUUGGUACUCACAUAGUACGUUCCGAAUCAACUCGUUAACAAAUUUAAAUUCAACAA
.....((((..((((((((...((.((((((((((((...((((((((..((.....))...))))))))..)))))))))))).))...)))))))).)))).....

```
 Initial dG =    -37.40

        10        20        30        40        50
CGGAC|   CA        AUU  A           GUA        GU-  UU
     GAAU  UAAAUUUG   AC AGUUGAUUUGGA   GUACUAUG   GU  \
     CUUA  AUUUAAAC   UG UCAACUAAGCCU   CAUGAUAC   CA  G
AACAA^   A-        AAU  C           UG-        ACU  UG
      100         90        80         70        60

-----------------------------------------------------
```

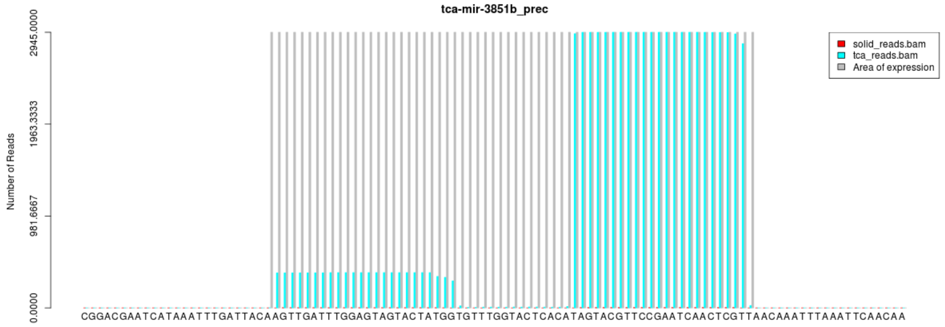

>tca-mir-3851c_prec

```
CCGGACGAAUCAUGAAUUUGGCGCCGAGUUGAUUUGGAGUCGUACUAUGGUUUUUGGUACGCACAUAGUACGUUCCGAAUCGACUCGUUAACAAAUUUUAAAUUCGACAAU
.....(((((...(((((((....(((((((((((((((.(((((((((((.........)).)))))))))))))))))))))))))....)))))))...))))).....

 Initial dG =    -46.00

        10        20        30        40         50
CCGGA|    CAU       GCGC               U         -  UUUU
     CGAAU   GAAUUUG    CGAGUUGAUUUGGAG CGUACUAUG GU    \
     GCUUA   UUUAAAC    GCUCAGCUAAGCCUU GCAUGAUAC CG    G
UAACA^    AAU       AAUU               -         A  CAUG
 .       100        90        80         70        60

-----------------------------------------------------
```

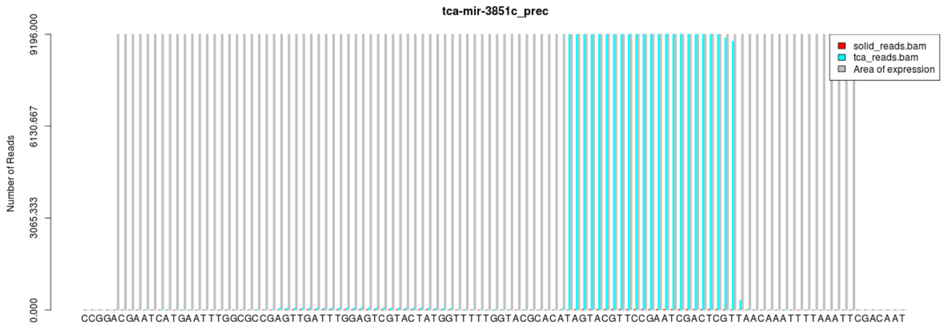

>tca-mir-3851d_prec

```
AUACUGCUAAUGGUUCCGGAGUCGUACUAUGGUUUUUGAUACUCACAUAGUACGUUCCGAAUCGACUCGUUAACAAAUUUUAAAUUCAACAAUUAAACCAAUUAAAUUUAAUAUUGAGUG
GACAUGGGGUGGUACUGUGUAAUUUUUGACCCAUAGUACGGCCCAUGUUGGC
.....((((((((.....((.((((((((((((.((..((.....((((((((((..(((..((.(((((.((..
((((((.......................)))))))..)).))))).))..)))..).))))))))))...))..)).))))))))))))))).)))))))

 Initial dG =    -49.30

          10            20        30
.-AUAC  C     -   C---|    U         GUUUUU
      UG UAAUG GUU    CGGAG CGUACUAUG      G
      AC AUUGC CAG    GCCUU GCAUGAUAC      A
\ ----  A     U   CUAA^    -         ACUCAU
          70        60         50        40


     80        90         100
  AAUUUUAAAU      .-AUUAAA   A    AUU
            UCAACA        CCA UUAA   U
            GGUUGU        GGU AGUU   A
  C---------      \ ------   G    AUA
           170           120       110

-----------------------------------------------------
```

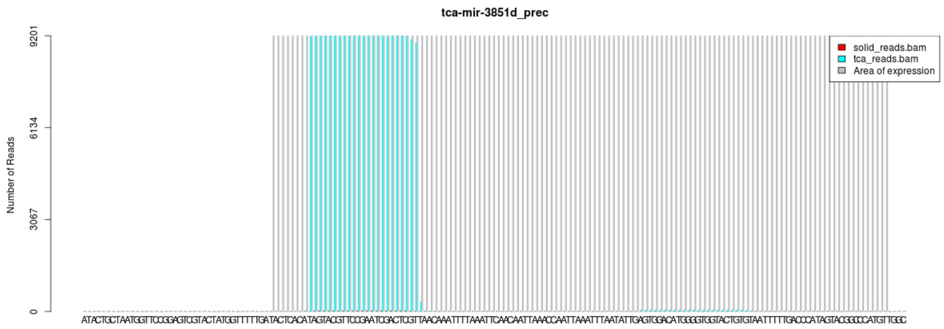

>tca-mir-3851e_prec

```
UGUUUGAAUUUGGCGCCGCGUUGAUUUGGAGUCGUACUAUGGUUUUUGGUACUCACAUAGUACGUUCCGAAUCGACUAGUUAACAAAUUUUAAAU
.((((((((((..((...(((((((((((.(((((((((..............)))))))))))))))))))))..))...)))))))).))))

 Initial dG =    -32.80

         10         20        30        40
U    -       GC-| CGC            U         GUUUUU
 GUUU GAAUUUG   GC   GUUGAUUUGGAG CGUACUAUG      G
 UAAA UUUAAAC   UG   CAGCUAAGCCUU GCAUGAUAC      G
-    U       AAU^ AU-            -         ACUCAU
     90        80         70         60        50

-----------------------------------------------------
```

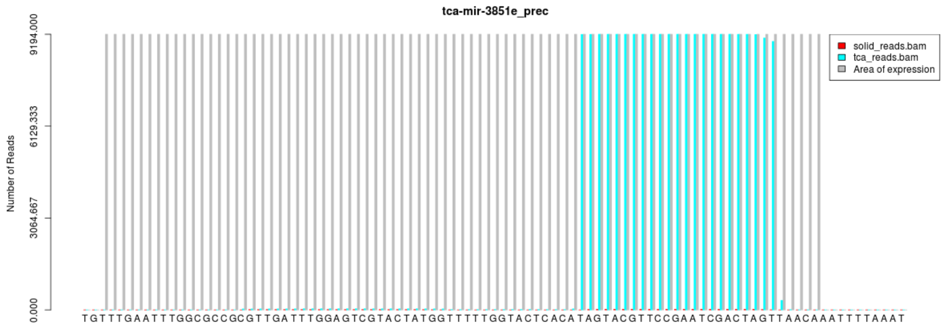

>tca-mir-3851f_prec

```
UGUUUGAAUUUGGCGCCGCGUUGAUUUGGAGUCGUACGAUGGUUUUUGGUACUCACAUAGUACGUUCCGAAUCGACUCGUUAACAAAUUUUAAAU
.(((((((((((....((.((((((((((((.(((((.(((..............))).))))))))))))))))).))....))))))).))))

 Initial dG =    -31.20

         10        20        30        40
U    -|      GCGC  C            U     G   GUUUUU
 GUUU GAAUUUG    CG GUUGAUUUGGAG CGUAC AUG      G
 UAAA UUUAAAC    GC CAGCUAAGCCUU GCAUG UAC      G
-    U^      AAUU  U            -     A   ACUCAU
     90        80        70         60        50
```

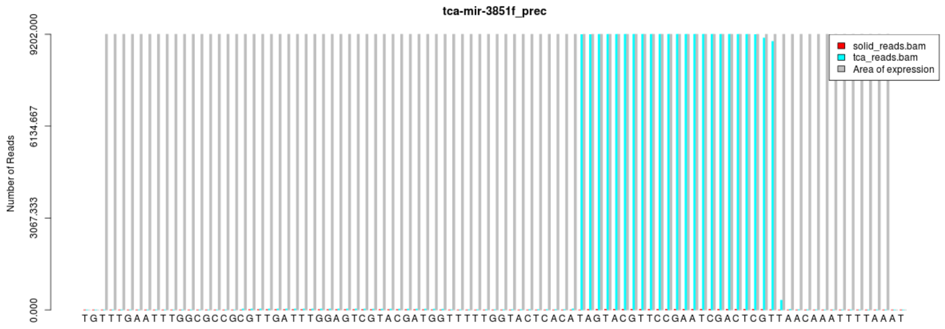

>tca-mir-3852_prec

CUCCAUGACGUCACACUCUUAAUACGGGGCCUUUAAGGAGUUCUGCGGCUUCUUCUGCGCCAUAAUUGUAUUAAUAUCGCGGUGACGCAACGAC

.....((.((((((....((((((((((((....(((((((......)))))))....)))....))))))))).......))))))))).....

```
 Initial dG =    -24.90

        10          20            30        40
CUCCAUGAC   .-ACACUC         ----|  CUUU       UC
         GUC        UUAAUACGG    GGC    AAGGAGU  U
         CAG        AAUUAUGUU    CCG    UUCUUCG  G
---------   \ ------         AAUA^  CGUC       GC
                       70        60        50


              80
              UAUC   G
                  GCG U
                  CGC G
              CAA-   A
              90

-----------------------------------------------------
```

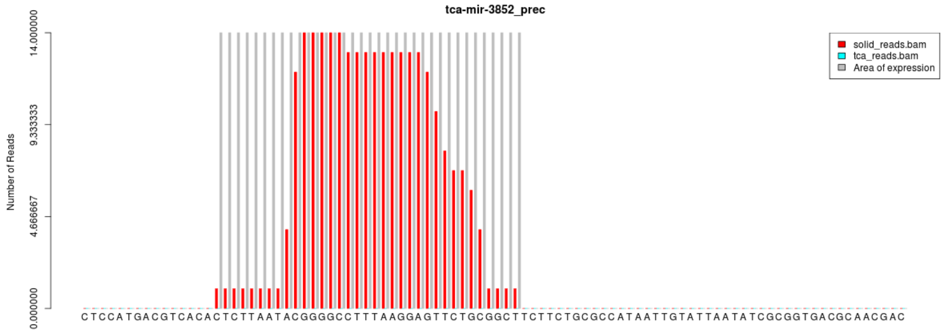

>tca-mir-3853_prec

```
UUUCAUGAAAAAAUAUCCUUGUGCAUCUUGUGUGUAUUUUGUGAAAUGCUAUAGUUGAAUAUUUUAGUUUUUUUCAAAAUAUGCUCAUAAUGUGCAAGGGUGAUUUGUUAUCGGU
..(((((.(((.((((((((..(((..((.((((((((((.((((.((((.(((.....))).)))).)))).)))))))))).))..)))..)))))))).))).)))).)).

 Initial dG =    -36.20

         10        20        30        40        50
UU  -|   A   A        UG   CU  U          U    U    U   UG
  UC AUGA AAA UAUCCUUG  CAU  UG GUGUAUUUUG GAAA GCUA AGU  \
  GG UAUU UUU GUGGGAAC  GUA  AC CGUAUAAAAC UUUU UGAU UUA  A
U-  C^   G   A        GU   AU  U          U    U    U   UA
    110       100        90        80        70        60

-----------------------------------------------------
```

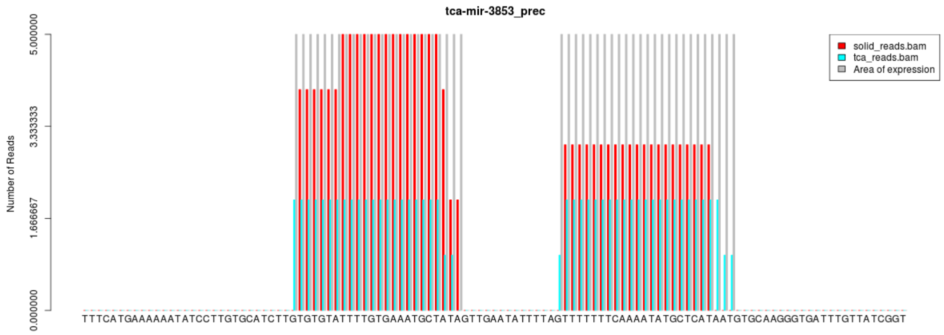

>tca-mir-3854_prec

```
CGACACCACAAUCCCAGCUCCACAUUAUUGCAUUUCAAAAAUAAUAGUGGCGUUUAGUAUUGUGGAAAUU
.....((((((...(((.((((((((((..........)))))).)))).)))....))))))).....

 Initial dG =    -18.40

        10         20         30
CGACA       CCC-   U    -|      GCAU
     CCACAAU    AGC CCAC AUUAUU    U
     GGUGUUA    UUG GGUG UAAUAA    U
UUAAA       UGAU   C    A^      AAAC
.        60         50         40

-----------------------------------------------------
```

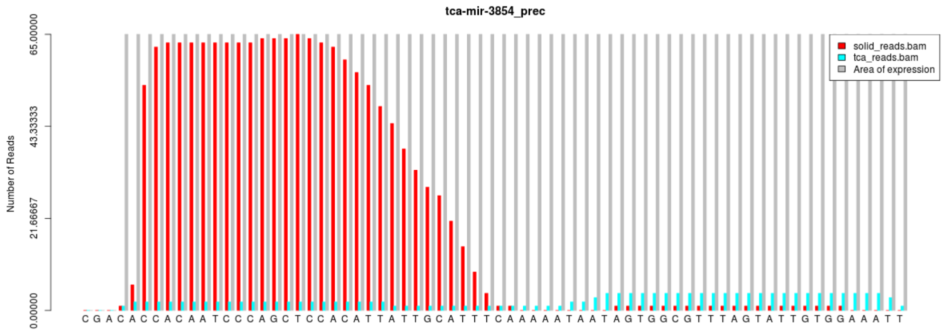

>tca-mir-3855_prec

```
AGUUUUUUUCGUCAGAUUUGUUAAAGGUUUCGCAAUUAACGCGUAAUUUUUGGCAAAGUGACGGAAAUAUCC
.....((((((((..(((((((((((((.(((.......))).)))))))))))))).))))))))).....

 Initial dG =    -25.10

        10        20        30
AGUUU|        GA            U   AAU
     UUUUCGUCA  UUUGUUAAAGGUU CGC   \
     AAAGGCAGU  AAACGGUUUUUAA GCG   U
CCUAU^        G-            U   CAA
 70        60         50        40

----------------------------------------------------
```

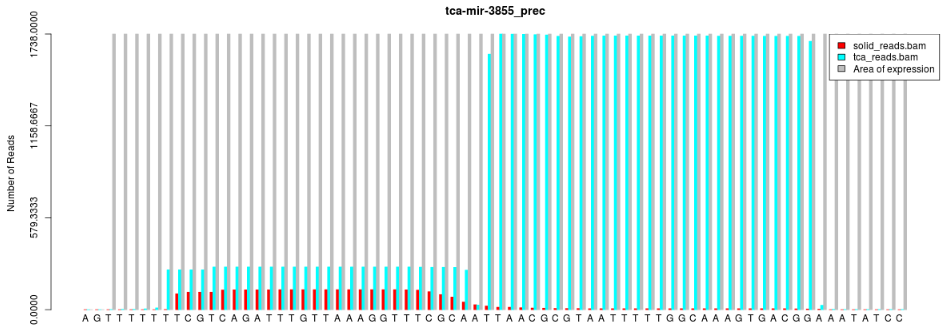

>tca-mir-3856_prec

```
GUGUGGUUUUUUUGCAAGAAAGUCAUUUGAACACUUUCAAUGUUUCGCAAACUGAGUGAUUUUUCGUUGCGGAAAUUCAAGUC
...(((..((((((((((((((((((((((((.......))))).........))))))))))..))))))))).)))....

 Initial dG =    -16.90

         10          20                30
GUG-   UU         --           --------|    CUU
    UGG  UUUUUGCAA  GAAAGUCAUUU        GAACA   \
    ACU  AAAGGCGUU  UUUUUAGUGAG        UUUGU   U
CUGA   U-         GC           UCAAACGC^    AAC
  80         70         60         50         40

-----------------------------------------------------
```

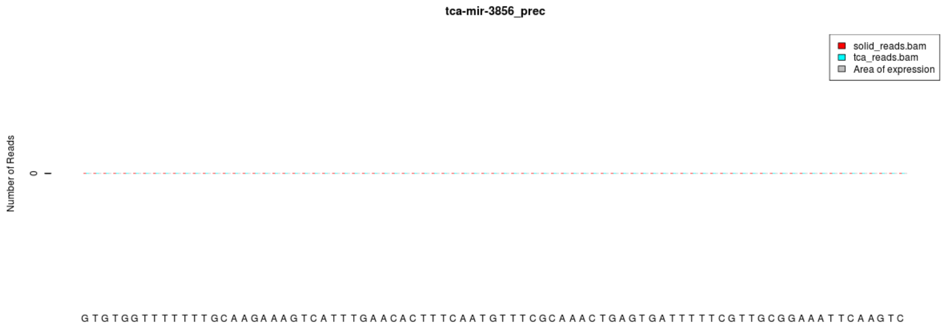

>tca-mir-3857_prec

```
AGAACGAGAAAUUGGGCUGUGGUGUGCCUUGAUACUAUCCCACGUGUUGCAGGAUGGAACAGGUGUGGUGUCUCAAUGCUCAUCAUGGCGCAAUUUUCAUAUG
.....((((.(((.(((((((((.((.((((.((.(((.(((.((((.(.....).)))).))).))))).)))).)).))))))))).))))))))).....

 Initial dG =    -39.80

        10        20        30        40        50
AGAAC|    A   G         U  C    U  U   C   G    G AG
     GAGAA UUG GCUGUGGUG GC UUGA AC AUC CAC UGUU C  \
     CUUUU AAC CGGUACUAC CG AACU UG UGG GUG ACAA G  G
GUAUA^    -   G         U  U    C  -   U   G    G UA
 100         90        80        70         60

-----------------------------------------------------
```

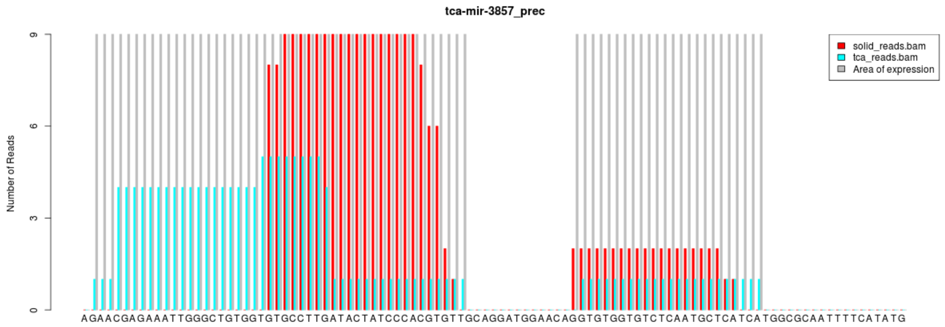

>tca-mir-3858_prec

GUUUUUUAGUGAAGCGAAGUUUUUUCAUUGGCCAAUAAAAAAUGAAACUCUCUUGCUUCUCUAAUAAAU
((((.((((.(((((((((...(((((((...........)))))))..)).))))))).)))).))))

```
 Initial dG =    -17.30

        10         20        30
    U    U       -| UUU       GGCCA
GUUU UUAG GAAGCGA AG   UUUCAUU     \
UAAA AAUC CUUCGUU UC   AAAGUAA     A
    U    U       C^ UC-       AAAAU
        60        50         40
```

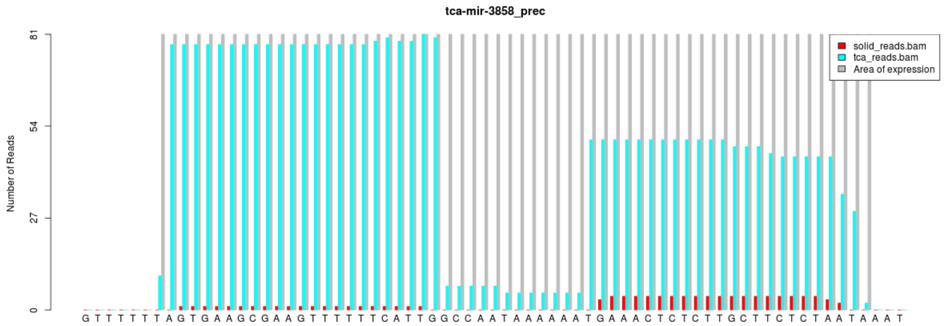

>tca-mir-3859_prec

```
CACACCCCACACAUUUAAGCCGGCCGUUUCAUGUCUUAAAAUGAAGAGGUGUGCUCGUUGUGGGAACAU
.....((((((......(((..(((.((((((........)))))).)))..)))...)))))).....

 Initial dG =    -20.20

        10        20        30
CACAC|     CAUUUA   CG   G      GUC
     CCCACA      AGC  GCC UUUCAU   U
     GGGUGU      UCG  UGG GAAGUA   U
UACAA^     UGC---   UG   A      AAA
        60           50        40

-----------------------------------------------------
```

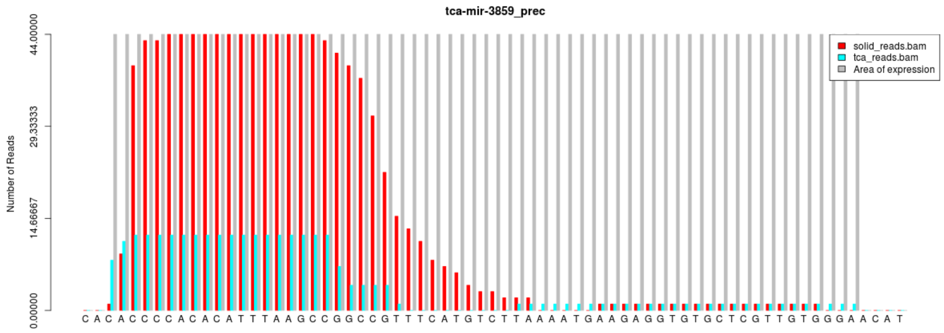

>tca-mir-3860_prec

```
UAGUAGCACUGGACGAUCUAAACAUGUCUUCUUUAAUGAAGUUGUGUAAAGGAAACAGUGCAAAAU
.....((((((.....(((..(((((.((((......)))).)))))..)))...)))))).....

 Initial dG =    -18.00

        10        20        30
UAGUA|     GACGA   AA     U    UU
     GCACUG     UCU  ACAUG CUUC  U
     CGUGAC     GGA  UGUGU GAAG  A
UAAAA^     AAA--   AA     U    UA
     60          50        40

-----------------------------------------------------
```

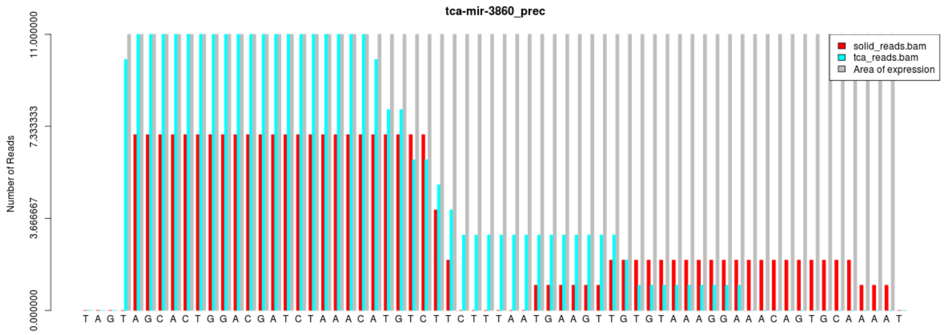

>tca-mir-3861_prec

```
GUUUAGUGUGUUUCGAAGGGUACUUAAUGGAAUUAUUUGGUAUCAAUUGAUUUUCGAUUUAAUUGGUACCAUUUAAUUCCAUUUCGGAAUUUUCGAAAUGUUCAUGUG
.....(((.((((((((((......((((((((((..((((((((((((((........))))))))))))))..))))))))))......))))))))))...)))...

 Initial dG =    -38.70

          10        20        30        40        50
GUUUA   U--|         GUACUU          UU             UUU
     GUG   GUUUCGAAGG      AAUGGAAUUA  UGGUAUCAAUUGA   U
     UAC   UAAAGCUUUU      UUACCUUAAU  ACCAUGGUUAAUU   C
GUG--   UUG^         AAGGCU          UU             UAG
        100        90        80        70        60

-----------------------------------------------------
```

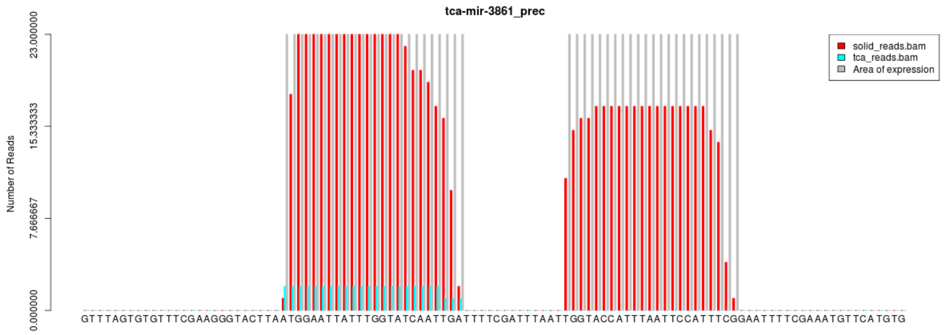

>tca-mir-3862_prec

```
UGCAAGGUUGCUCGAAAUUGGGCAAAAGUCCGCCUGAACAUUUUUCUAAUUGUUCAGGCGCUCUUUUGCAUGACUUUGGCCAACCACAAC
.....(((((..((((.((..(((((((..((((((((((.((....)).))))))))))..))))))))..)).)))).))))).....

 Initial dG =    -38.70

        10        20        30        40
UGCAA|    CU    A  GG       UC           U  U
     GGUUG  CGAA UU  GCAAAAG  CGCCUGAACA UU U
     CCAAC  GUUU AG  CGUUUUC  GCGGACUUGU AA C
CAACA^    CG    C  UA       UC           U  U
.        80        70        60        50

-----------------------------------------------------
```

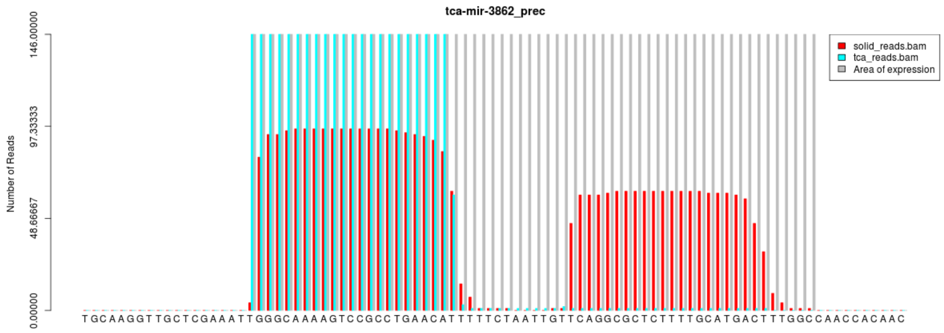

>tca-mir-3863_prec

```
UGCCAGGGUGUGAAGAAUUGAUCAAGUAGCGCCGCUUGGUCUUUGAAUUGAUUGAUCAGCGUCGUUGCUUGAUCCAUUCUCUGCAUCCACAAU
.....((((((..(((((.((((((((((((.((((.((((............)))))))).))))))))))))).))))))..)))))).....

 Initial dG =    -43.70

       10         20         30         40
UGCCA|     GA     U            C    U    UUUGA
     GGGUGU  AGAAU GAUCAAGUAGCG CGCU GGUC     A
     CCUACG  UCUUA CUAGUUCGUUGC GCGA CUAG     U
UAACA^     UC     C            U    -    UUAGU
  90        80         70         60         50

-----------------------------------------------------
```

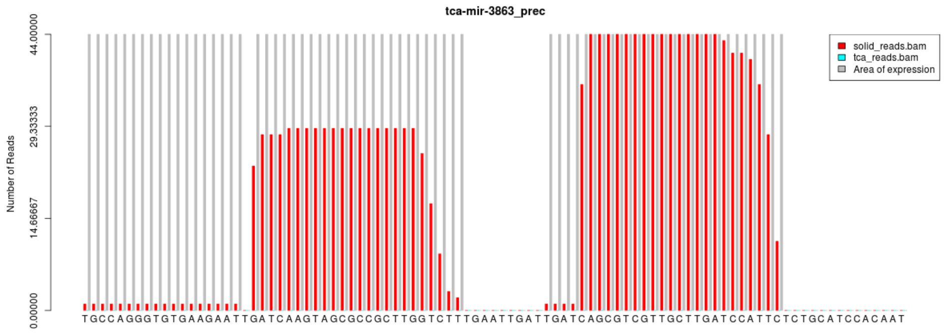

>tca-mir-3864_prec

```
AGUGAUGUGGGGCGACAUAGGCAUGGUUUGAGUUUGUUUAAUAAACUUGAAACCUCCUACUAUGUCGCAGAAUAAUUAG
.....(((...((((((((....((((((((((((...))))))))).)))))).....)))))))))...))).....

 Initial dG =    -26.50

        10        20          30
AGUGA   GGG         GCAU-     -|       U
     UGU   GCGACAUAG     GGUUU GAGUUUGU \
     AUA   CGCUGUAUC     CCAAA UUCAAAUA U
GAUUA   AGA         AUCCU     G^       A
        70        60        50        40

-----------------------------------------------------
```

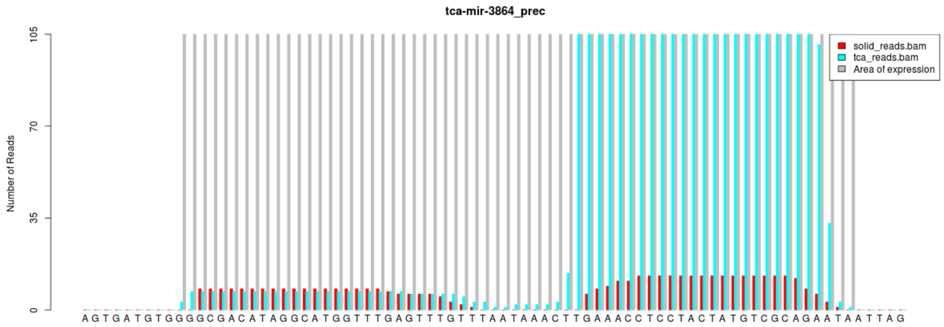

>tca-mir-3865_prec

```
UCCAAAAUUGUUUUUUAAUAAAUUAAGGCAAGAUCUUGAGCUGGUGUGUGCCGUUUUAGCACACCAGUGUGGUUUCCUUGCCAGAAAAAUAGAAAUAAUU
.....(((((((((...........((((((..((...((((((((((.........))))))))))..))....))))))........)))))))))

 Initial dG =    -29.30

        10        20        30          40        50
UCCAA|         UAAUAAAUUAA      AU--  UGA          GCCG
     AAUUGUUUUU           GGCAAG    CU   GCUGGUGUGU    \
     UUAAUAAAGA           CCGUUC    GG   UGACCACACG    U
-----^         UAAAAAGA---      CUUU  UG-          AUUU
   100        90           80        70         60

-----------------------------------------------------
```

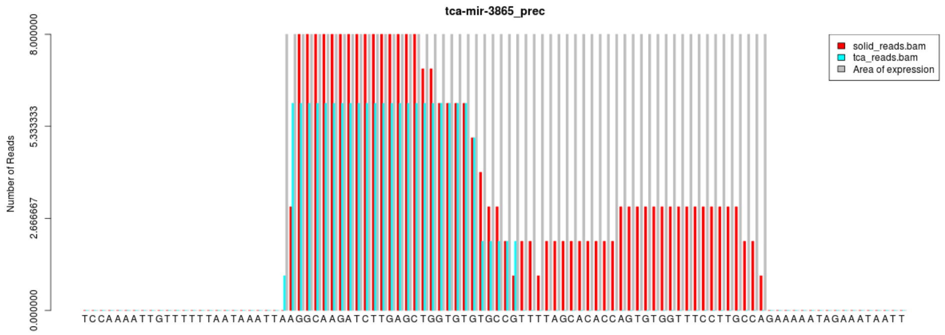

>tca-mir-3866_prec

GUAAUUUUUUUUUCGCACAGUUUGAUCAAUCGGUAACCUUGAUGGCUACUCGGUUAUAAUCCUGUGCUAAAUAAUGGAAGAAAAUGAGC
.....((((((((((((((....((.((((((((.((.....)).))).)))))))....))))))........)))))))))......

```
 Initial dG =    -20.30

        10                 20        30         40
GUAAU         --------|     UUUG  C     -   A  UU
     UUUUUUUUC        GCACAG    AU AAUCG GUA CC  \
     AAAAGAAGG        CGUGUC    UA UUGGC CAU GG  G
CGAGU         UAAUAAAU^     CUAA  -     U   C  UA
        80        70        60         50

-----------------------------------------------------
```

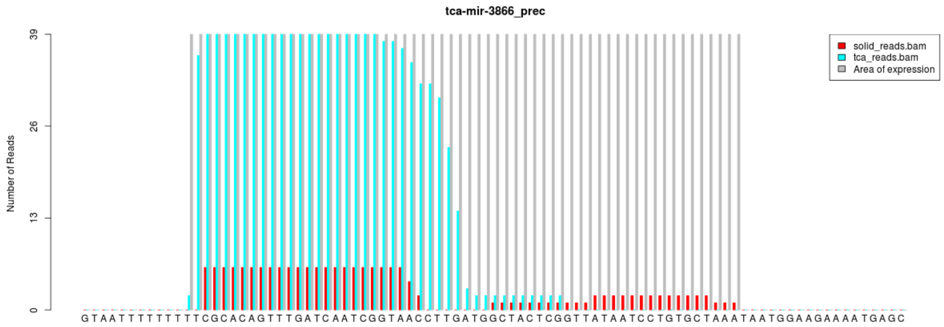

>tca-mir-3867_prec

```
UCCGCCCCUAUAAGGACCACAGUGGAAGCAGGGAACGGUAUCAUUUCGAUUACACCGUUCCCGUUAUUUGCAGCGGGGCUUGUGGGGUACAG
...(((((.(((((..((...(..(((((.((((((((((((.....)))...)))))))))))).))..)...))..))))))))))....

 Initial dG =    -38.00

         10        20         30           40
UCC-     U     GA  ACA UG  -   A          ---|  AU
    GCCCC AUAAG  CC   G  GA AGC GGGAACGGU   AUC  \
    UGGGG UGUUC  GG   C  UU UUG CCCUUGCCA   UAG  U
GACA     -     GG  CGA GU  A   -         CAU^  CU
 90         80        70         60        50

-----------------------------------------------------
```

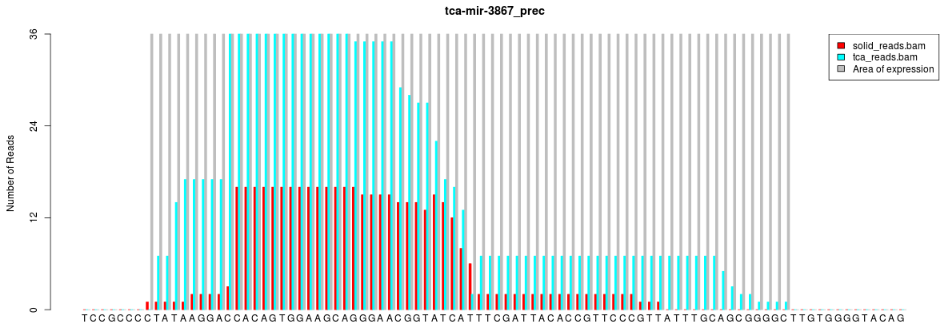

>tca-mir-3868_prec

```
GUAAACGGAAUUAAAGUACCAGCACCAGUAUGUAUUUUGUAUGCAUUCUUUUUACAACAAUUUAUGCAACAAGAAAUCAACUAAAGUUUAAUUUUGAUUCUGAUGGU
.....((((((((((((...(((...(((.((..((((((.(((((.................)))))))))))...)))))...))).)).))))))))))))).....
```

```
 Initial dG =    -20.10

        10        20        30         40        50
GUAAA|           ACC   ACC   A  UA-      A     UCUUUUUA
     CGGAAUUAAAGU   AGC   AGU UG   UUUUGU UGCAU        \
     GUCUUAGUUUUA   UUG   UCA AC   AGAACA ACGUA        C
UGGUA^           AU-   AAA   -  UAA      -     UUUAACAA
     100        90         80         70         60
```

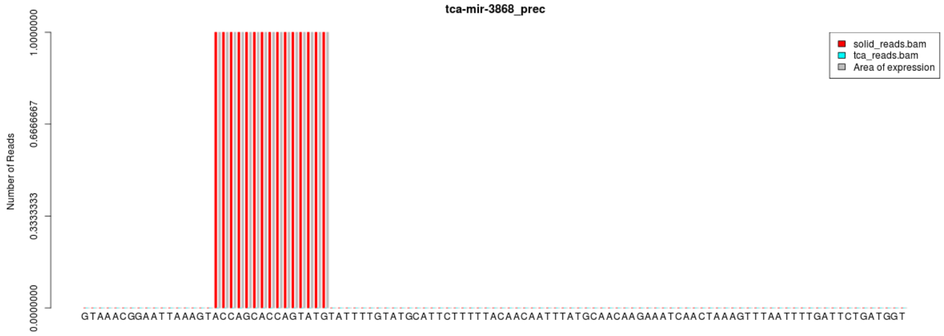

>tca-mir-3869_prec

```
UAAUUACUGAUAGAUGGUCUCGAAAAGCCUUAUAGCGAUGUUGGGGGCAUUCGAGACCCCUGUUAGUUACCU
.....((((((((..(((((((((..(((((.((((...))))))))).))))))))).)))))))).....

 Initial dG =    -28.00

        10         20         30
UAAUU|       AU         AA     A    G
     ACUGAUAG  GGUCUCGAA  GCCUU UAGC \
     UGAUUGUC  CCAGAGCUU  CGGGG GUUG A
UCCAU^       C-         A-     -    U
 70        60         50          40

-----------------------------------------------------
```

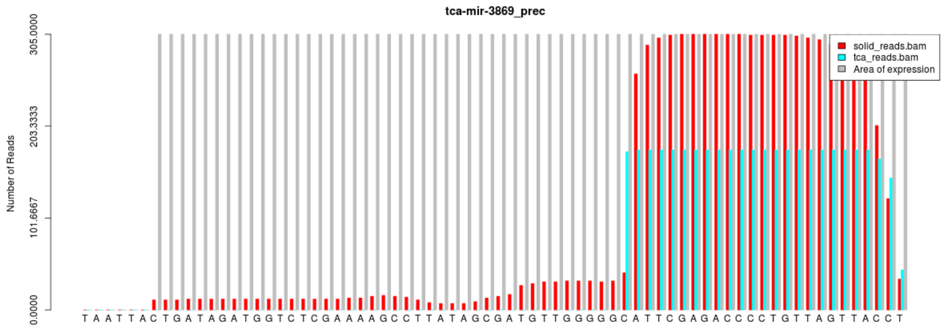

>tca-mir-3870_prec

```
GCCAAUUCGAGGCACAAGAGGAAAAAGAGCGCAAAAUACACAACUUUGGCGGAGCUCGAGCCACAAGUGCCCGGAAUAUU
.....((((.(((((....((.....((((((.(((........))).))...))))...))....))))))))).....

 Initial dG =    -19.70

        10        20        30          40
GCCAA|   A     AAGA  AAAAA    ---  A   UAC
     UUCG GGCAC    GG     GAGC   GC AAA   A
     AGGC CCGUG    CC     CUCG   CG UUU   C
UUAUA^   -     AACA  GAG--    AGG  G   CAA
.         70        60          50

-----------------------------------------------------
```

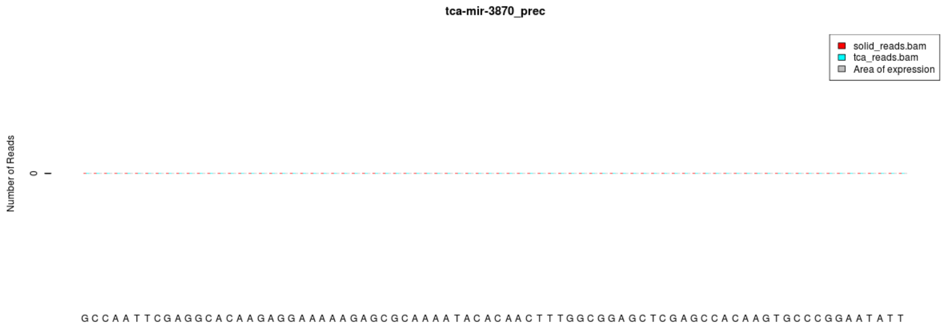

>tca-mir-3871_prec

```
AUCCCACAAUCCCGAUUUCUGCCUUGUGCCGCUCCCUCCUAAUUCCCGCCACAUUUGCCUAAUCGGGCAUGUCCCAC
.....(((..(((((((...((..((((.((...............)).))))...))..)))))))..))).....

 Initial dG =    -16.10

        10        20         30
AUCCC|  AU       UCU  CU-    C  CUCCCUC
     ACA  CCCGAUU   GC   UGUG CG       \
     UGU  GGGCUAA   CG   ACAC GC       C
CACCC^  AC       UC-  UUU    C  CCUUAAU
      70        60         50        40

-----------------------------------------------------
```

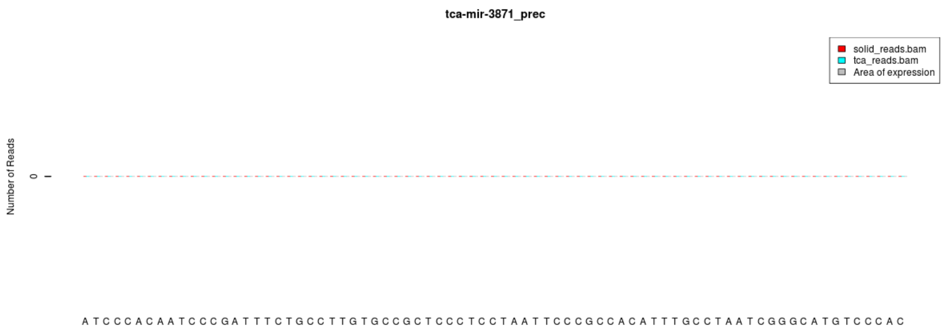

>tca-mir-3872_prec

GUGAAGUGAUCUACAGCCAGAAAUGCAUUAUUAUGCUCUGCGAAGUAAGUGCAUAUCUUAAUUUUUGCAUUUUUUACUAAUUGUUCAUGCUCA
.(((.((((.....((..(((((((((....(((((.((........)).)))))..........)))))))))..))......))))..)))

```
 Initial dG =    -17.00

         10         20        30              40
G   A-    UCUAC-  CC         UUAU------|    U  GCG
 UGA  GUGA      AG  AGAAAUGCA          UAUGC CU   A
 ACU  UACU      UC  UUUUUACGU          AUACG GA   A
-   CG    UGUUAA  AU         UUUUAAUUCU^    U  AUG
   90        80        70        60        50
```

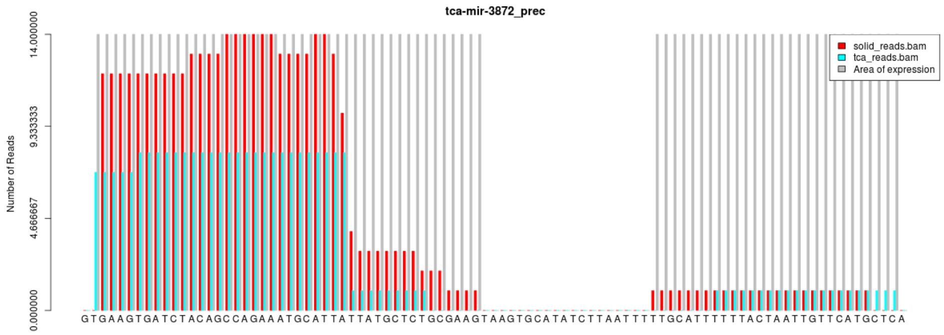

>tca-mir-3873_prec

UUAUUAGUUGUUGCCGAAUGUUAUAAGGUUUGCAUUCGUUAGGAAUAUAUUAAUUGUUAAAACGAAAAUCCAAAUAAAUUGUUCUUGAAUGUAAUUUUAGCUCAUAAUUGUAAUAAUUUA
ACC

```
.....(((((((((...(((...(((((((.(((((((...((((((..(((.
(((..............))).)))..))))))))))))))))))))...)))....))))))))).....
```

```
 Initial dG =    -20.10

        10         20        30        40        50        60
UUAUU         CGA-|  UUA       U       UUA       UA   A   UUAAAA
     AGUUGUUGC    AUG   UAAGGUU GCAUUCG   GGAAUA  UUA UUG      C
     UUAAUAAUG    UAC   AUUUUAA UGUAAGU   UCUUGU  AAU AAC      G
CCAAU         UUAA^  UCG       -       ---       UA   A   CUAAAA
 120       110       100         90           80        70
```

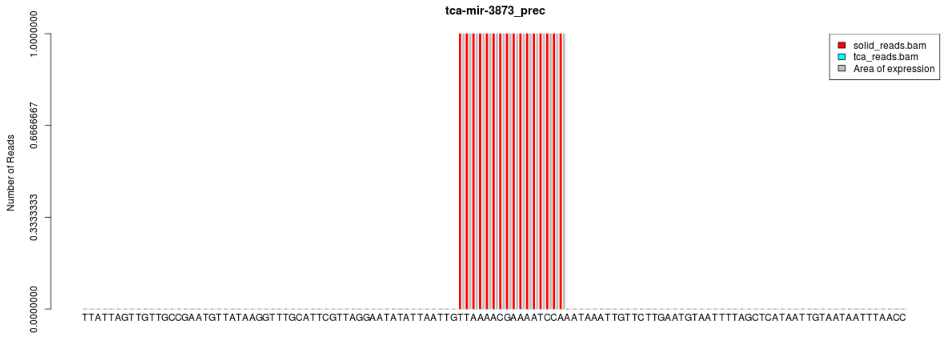

>tca-mir-3874_prec

AGCAAGUGGCAGAUUGGCUGAUGGAUGCUGUCAUAAUAAGGGACUCGACACUGGUUCAGAUAAGAGCAAACACACGCAAAACUUAUCGUGGGAUAAAUCGCCUCAUUGACGACUUAUCCG
AAGCGGAUCGAUUGCACACACA

```
.....((((((((((.(((..((((((..(((.((((.(((....(((......((((((((((.
((........))....)))))).)))).....)))))).)))).)))..))).)))))).))).)))).).)))...))).)))...

 Initial dG =    -35.70

         10           20         30         40         50         60           70
AGCAA   -   ---|   G   GA       CU   A-     A   GACU   CACUGG     -       A---  AAA
     GUG GCA   GAUU GCU  UGGAUG  GUC  UAAU AGG    CGA       UUCA GAUAAG    GC   C
     CAC CGU   CUAG CGA  GCCUAU  CAG  GUUA UCC    GCU       GGGU CUAUUC    CG   A
ACA--   A   UAG^   G   A-       U-    CA     C   ----   AAAUA-     G       AAAA  CAC
140         130        120        110           100          90        80

-----------------------------------------------------
```

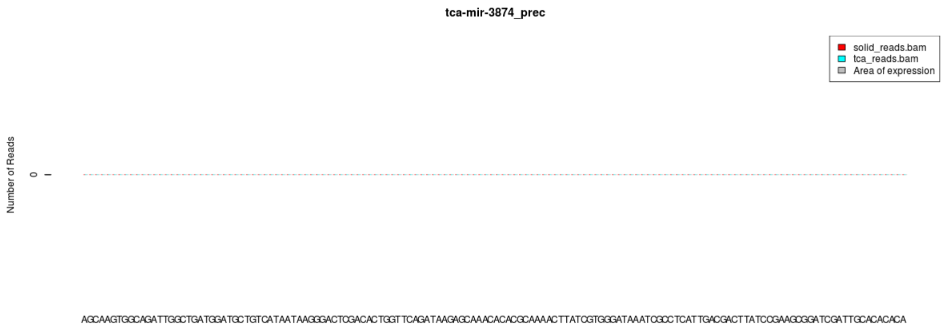

>tca-mir-3875_prec

```
CACUUGCGUCUUUAGCUCAAUAAAUAUUAGAGGUGCUUUAUUUGGGCCAAUAUUUGCGCUAGAUAGCGCGUAUU
....(((((.((((((.(((...(((((...(((.((......)))))))))))))).))))))..))))))....

 Initial dG =    -15.20

        10         20        30        40
CACU     C-|     U   UAA     AGA   G  UU
    UGCGU  UUUAGC CAA   AUAUU   GGU CU  A
    GCGCG  AGAUCG GUU   UAUAA   CCG GG  U
UUAU     AU^     C   ---     ---   -  UU
   70        60            50

-----------------------------------------------------
```

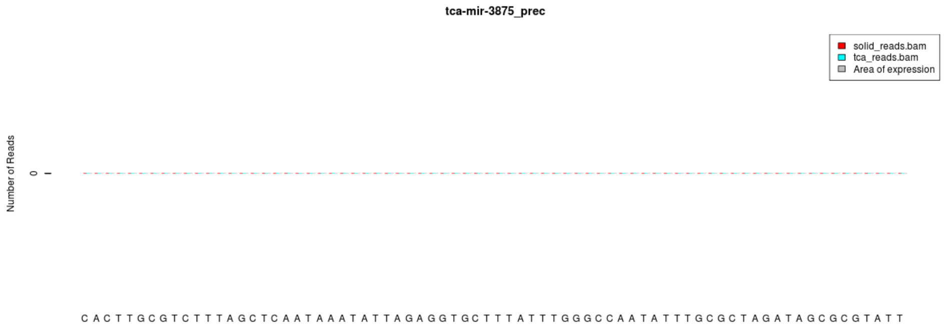

>tca-mir-3876_prec

```
UUUCGUUUUAUUUUACCACUUACUACCAAGUUGACUAGACCAGUGGUAAAAUGAGAAAAAA
.....((((((((((((((..(((..........)))...))))))))))))))).....

 Initial dG =    -15.80

        10        20         30
UUUCG               UA-|  CCAA
     UUUUAUUUUACCACU   CUA    G
     AGAGUAAAAUGGUGA   GAU    U
AAAAA               CCA^  CAGU
60        50        40

-----------------------------------------------------
```

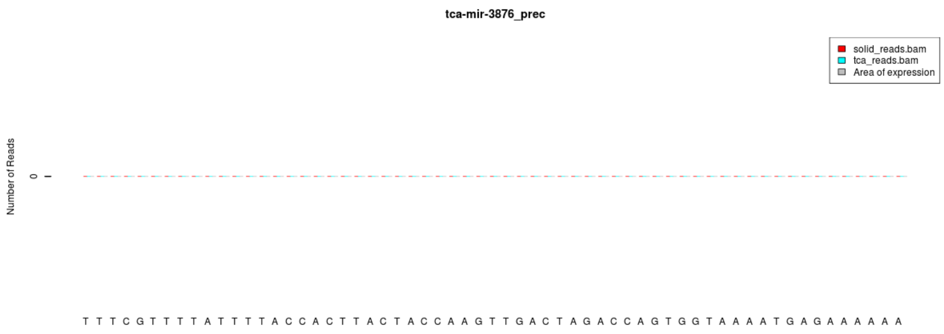

>tca-mir-3877_prec

```
GUCAACCUUCCAAAAAAGCACUCGUUUGGUUACUAAAUAACCAAGUGAGUUGUCACUGGAAGGAGAGA
.....((((((.....((((((((((((((.....)))))))))))).)))...))))))).....

 Initial dG =    -24.40

        10         20          30
GUCAA|      AAAAA   -            CU
     CCUUCCA     GCA CUCGUUUGGUUA  \
     GGAAGGU     UGU GAGUGAACCAAU  A
AGAGA^      CAC--   U            AA
       60          50         40

-----------------------------------------------------
```

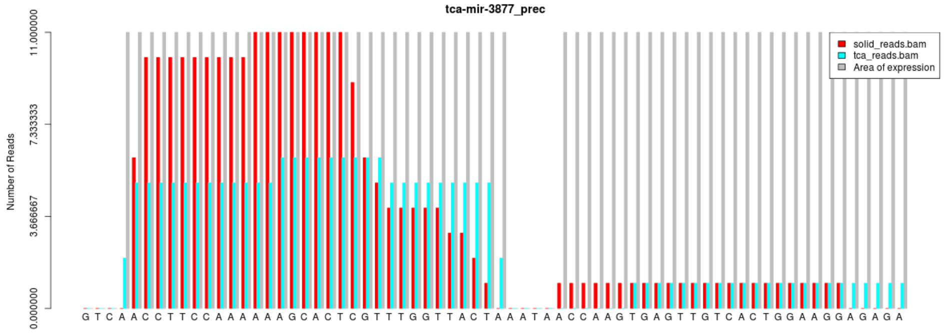

>tca-mir-3878_prec

```
UGAUAGGUGUGGUGGACGGAGAACUAAUUGUAUUAAAAUUGCUACCUACUUGCUUCGAUUUGAAAUUUUUAGUGUCCGUGUUGUUAUCA
........((((..((((((..(((((..((.(((((...((.........)).....))))).))..))))).))))).)..))))..

 Initial dG =    -15.90

        10         20        30        40
UGAUAGGU    UG -     GA     UU  A     AUU--| UACC
        GUGG  G ACGGA  ACUAA  GU UUAAA     GC    \
        UAUU  U UGCCU  UGAUU  UA AGUUU     CG    U
AC------    GU G     G-     UU  A     AGCUU^ UUCA
              80         70        60        50

-----------------------------------------------------
```

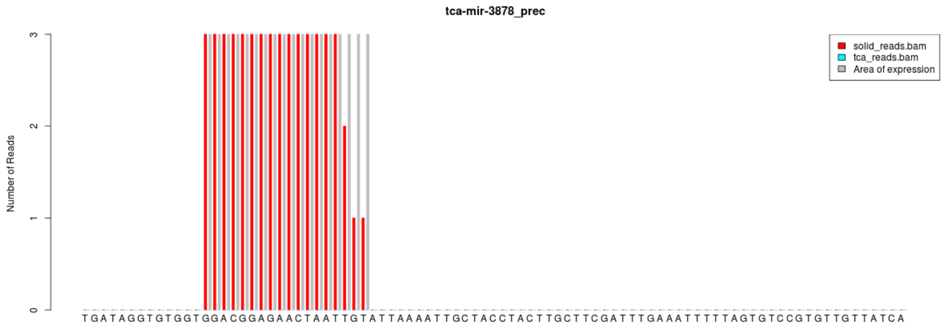

>tca-mir-3879_prec

```
CAAUUGGGUUAUUGUGUGCCGGUCAUUACAUCAUUACAGGUCAUGUAAACCGGCCAUAAAGCCCUCAAC
.....(((((.(((((.((((((..((((((.((.....)).))))))))))))))))))))))).....

 Initial dG =    -26.00

        10        20        30
CAAUU|    A     U      CA      C  UA
     GGGUU UUGUG GCCGGU  UUACAU AU  \
     CCCGA AAUAC CGGCCA  AAUGUA UG  C
CAACU^    -     -      --      C  GA
        60          50          40

-----------------------------------------------------
```

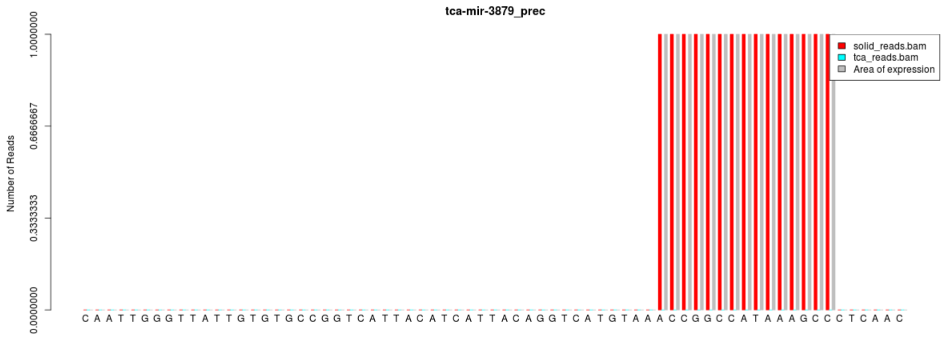

>tca-mir-3880_prec

```
CUUCAUUGGGUCCAAGAAACGAAAGAAUUCGGCGAUUUUUGUCCUCUGAGUCUUAGGUUCCGGGAAUUGAGAUUGCGACUUCCGUCGAUGUUGUCCUGAAAUAC
.....(..((..(((....(((..(((.(((.(((((((..((((..((..(...)..)).))))...)))))))))).)))..)))...))).))..).....

 Initial dG =    -24.10

        10        20        30        40         50
CUUCA|UG  UC   GAAA   AA   U   G       UG-    CU  GU U
     U  GG  CAA    CGA  GAA UCG CGAUUUU   UCCU  GA  C \
     A  CC  GUU    GCU  CUU AGC GUUAGAG   AGGG  CU  G U
CAUAA^GU  U-   GUA-   GC   C   -       UUA    C-  UG A
  100         90         80         70         60

-----------------------------------------------------
```

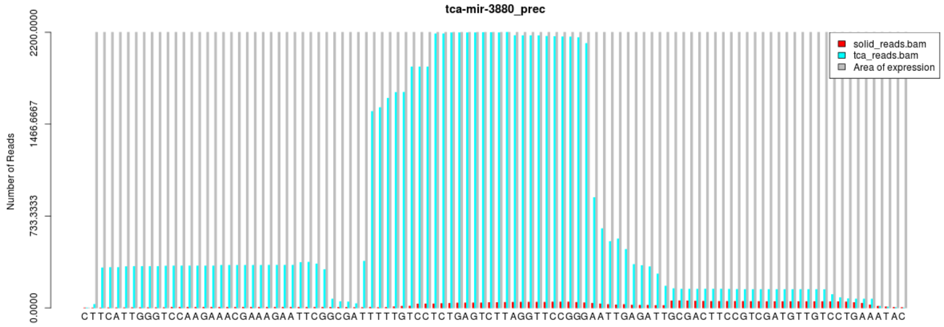

>tca-mir-3881_prec

```
ACGCAGACUCUUCGAGUUUAAAUUAUGUUGAGUCUUUCCGAUAGCGAUCUUUGCUUUAGGGAUCUUUAAUUUCUCGGAUGUCUUUUC
....((((..((((((..........((((((...((((...(((((...)))))...))))..))))))..)))))).))))....

 Initial dG =    -18.20

        10        20        30        40
ACGC|   UC      UUUAAAUUAU      UCU    GAU     U
    AGAC  UUCGAG          GUUGAG   UUCC   AGCGA \
    UCUG  AGGCUC          UAAUUU   AGGG   UCGUU C
CUUU^   U-      UU--------      CU-    AUU     U
      80                  70         60        50

-----------------------------------------------------
```

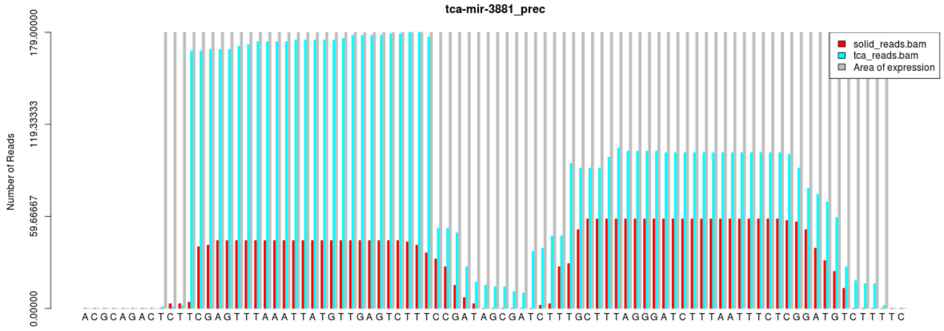

>tca-mir-3882_prec

UCUCCGCACUUGACACAUCGGUGUUCUACCAUGCAGUUCCUCUGUGCGUGGAAGAAUCGCUAACACCGAUGGCACUGAGCUGUUU
.....(((((....(((((((((((.(((((((.........))))))).)))).......))))))))).....))).))).))..

```
 Initial dG =    -26.90

         10         20               30
UCUCC   -   GACA-         -------|  A       GUUC
     GCA CUU     CAUCGGUGU       UCU CCAUGCA    \
     UGU GAG     GUAGCCACA       AGA GGUGCGU    C
UU---   C   UCACG         AUCGCUA^  A       GUCU
       80        70        60        50
```

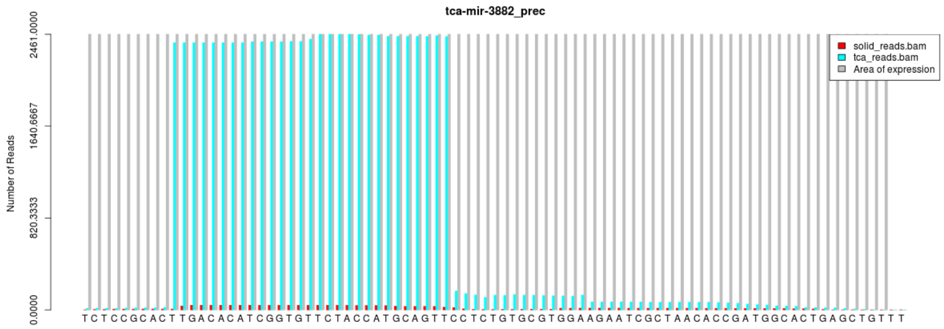

>tca-mir-3883_prec

```
UUCUCCCAUAAGAACCCUCGUUAGCGAGUUUGGCGAAGCUCCAAACCUCUUUAACAGCAUCUUUGGUGGAU
.((..(((.((((...((.(((((.((((((((.......))))).))).)))))))..)))))))..)).

 Initial dG =    -18.20

        10        20         30
U| UC   U    ACC  C     C   -     CGA
 UC  CCA AAGA   CU GUUAG GAG UUUGG   \
 AG  GGU UUCU   GA CAAUU CUC AAACC   A
U^ GU   -    AC-  -     U   C     UCG
70         60          50        40

-----------------------------------------------------
```

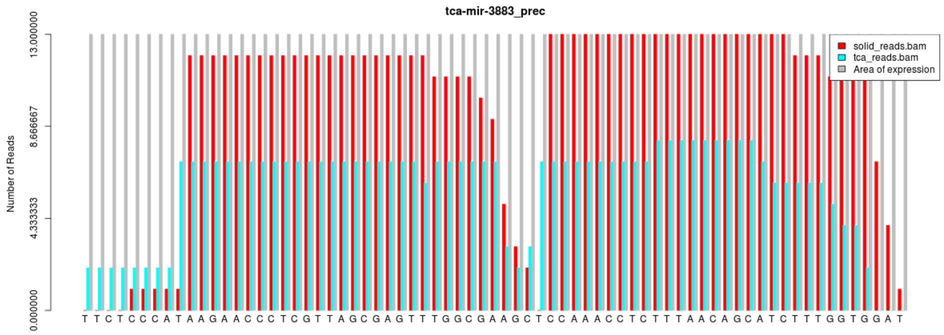

>tca-mir-3884_prec

```
AAAACUGAAGCUGACGAGUUAACUGCCGGGUACUUGUUCCCGGAUUUUUUCCCCAGCCUCAAGAAG
.....(((.((((..(((.......(((((........))))).....)))..)))).))).....

 Initial dG =    -18.10

        10        20        30
AAAAC|  A    AC   UUAACUG     UAC
     UGA GCUG  GAG       CCGGG   U
     ACU CGAC  CUU       GGCCC   U
GAAGA^  C    CC   UUUUA--     UUG
     60        50          40

-----------------------------------------------------
```

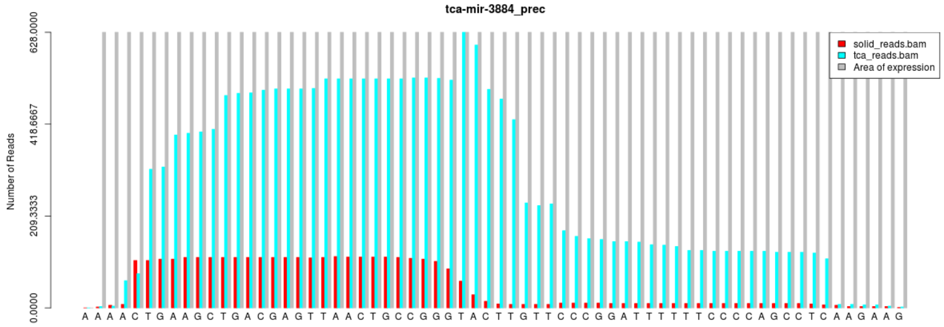

>tca-mir-3885_prec

```
AACAACGAAGGCGGCGGCGGCGGCCGAUGCGCUUUUUUUUCACUGUUAAUGAAUGGCAAGUGUGCGGGUUUUGUUUUUACGUUUUCGGGUAA
.....((((((((..(((((...(((.(((((((.(..((((.......))))..).)))))))))))...))))).....))))))))).....
```

```
 Initial dG =    -23.90

        10          20
AACAA        .-G|   G
     CGAAGGCG   CGGC G
     GCUUUUGC   GCCG C
AAUGG        \ -^   G
 90        80


                               30        40
               AU--------------      U UU    CUG
                               GCGCUU U  UUCA   \
                               UGUGAA G  AAGU   U
               AUUUUUGUUUUGGGCG      C GU    AAU
                       70        60        50
```

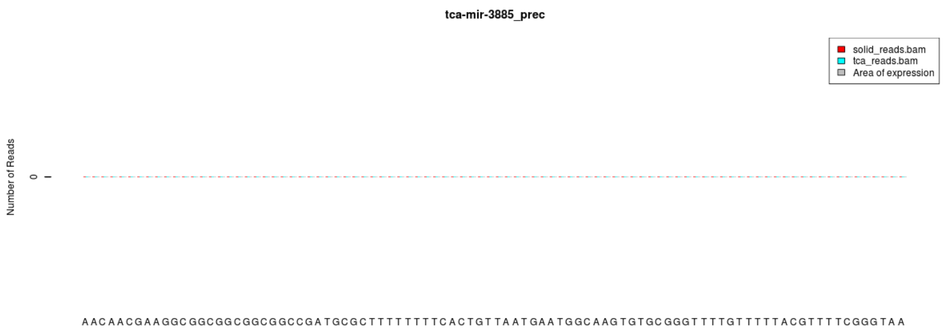

>tca-mir-3886_prec

UACAACUCACAGCGAGCUUUGACAGGUGAUGAGGGUGGUGAGGAUGGCGAUUAUUAUCGGUUAUAUAUCAGUGGUAGGGAGCGG
.....((((.((((....((((.((((((((..((.((....)).))..)))))))).))))....)).)).)).))).....

```
 Initial dG =    -17.80

         10         20        30        40
UACAA   -  A  -| GCUU    A        GG  G  G
     CUC AC GC GA    UGAC GGUGAUGA  GU GU A
     GGG UG UG CU    AUUG CUAUUAUU  CG UA G
GGCGA   A  G  A^ AUAU    G        AG  G  G
   80        70        60        50
```

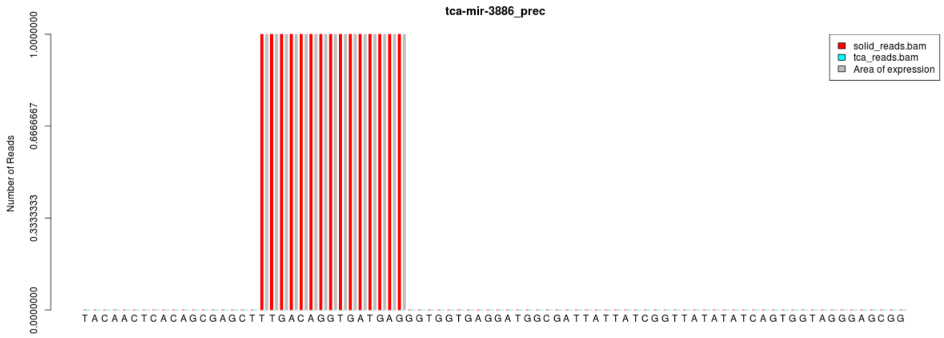

>tca-mir-3887_prec

```
GAGAUGGGGUUGUCAUAAAAACGCACCCCUUAUUUAACAACGAAUAUAACCUUGUGGCGCCUUUUUAUUCCAACUCCUAAUG
.....(((((((..(((((((.((.((............((((........)))))).)).)))))))..))))))).....

 Initial dG =    -18.60

        10        20        30        40
GAGAU|      UC       C  A  CCUUAUUUAACA    AUA
     GGGGUUG  AUAAAAA GC CC            ACGA   U
     CCUCAAC  UAUUUUU CG GG            UGUU   A
GUAAU^      CU       C  C  ------------    CCA
 80        70        60                    50

-----------------------------------------------------
```

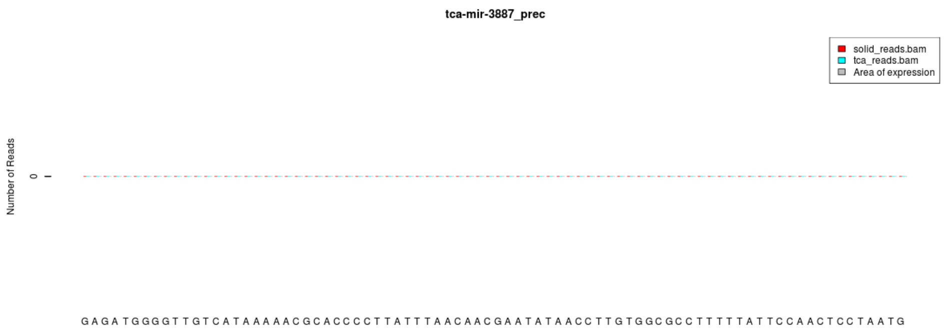

>tca-mir-3888_prec

```
GGAAAAUUUCGCUGUGCUUUGAAUGUCACGCCGAUAAAAAUAAUUUUUGUAUAUUGAAUAAAUGAGAUACGAAAAAAUUCAAUUUCCGCGUGACGUAGUUAAGGUUAGCGUGAAGUAUCA
C
.((...((((((((.((((((((((((((((.((.........((((((((.
((........)).)))))))))).........)).)))))))))..))))))))))))).)))...))..

 Initial dG =    -32.30

          10        20          30        40        50
G-  AAA   -     U       --|        C  UAAAAAUAA         A  GAA
  GA   UUU CGCUG GCUUUGA  AUGUCACGC GA         UUUUUGUAU UU   U
  CU   AAG GCGAU UGGAAUU  UGCAGUGCG CU         AAAAGCAUA AG   A
CA  AUG   U     -       GA^        C  UUAACUUAA         G  UAA
 .       110        100        90        80        70        60

-----------------------------------------------------
```

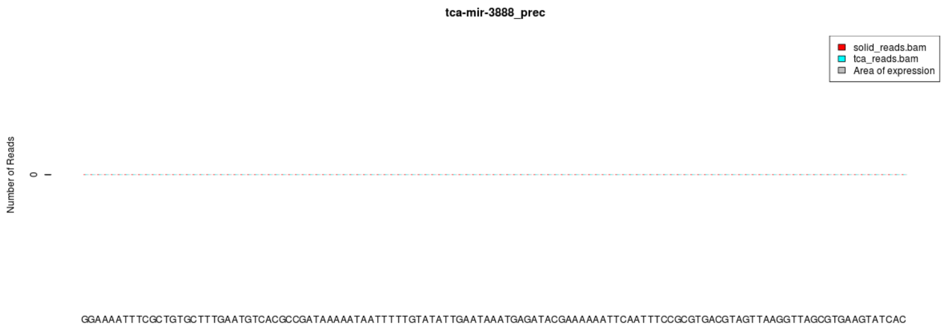

>tca-mir-3889_prec

```
UGUAAUAAACUCACAAAAUGCGACUUGUUCUCUCUGUAUUGUGUGGAGUUGCUCAUAGUACGGAUUGAACACGUCGCUGUUUUUGAGUUUACGCUU
.....((((((((.(((((((((.(((((..((((((((((((.((.....))))))))))))))..))))).))))).))))))))))))).....

 Initial dG =    -38.40

        10         20        30        40
UGUAA|       C     -     U     UC          U  AG
     UAAACUCA AAAAU GCGAC UGUUC  UCUGUAUUGUG GG  \
     AUUUGAGU UUUUG CGCUG ACAAG  AGGCAUGAUAC UC  U
UUCGC^       -     U     C     UU          -  GU
     90         80        70        60         50

-----------------------------------------------------
```

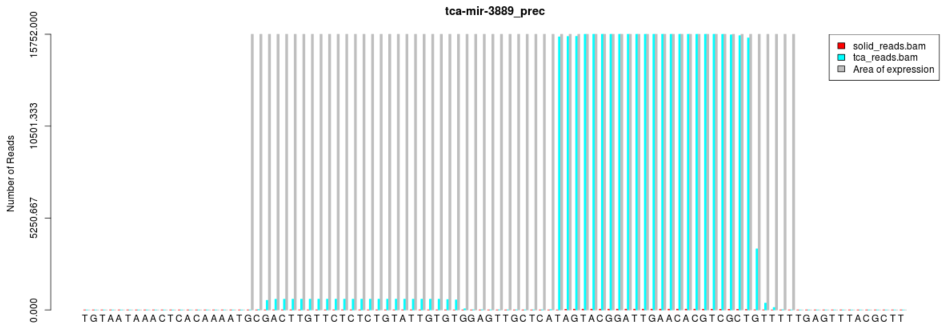

>tca-mir-3890_prec

CACCCUGUAGUUAUAACAUGGGAUCGAAAAUAUACUGAAUUAGAGCAGCUGUAUCUGCGGAACUUUAGUUACAUUUUAGCCGGUUAUGUACGGCUGCACUUUUAAAGUUAUGUUGUCUUAUGUUGGUUUUACACACAU

```
.....((((...((((((((((....((((((((.....((.((((((((((......((((...((((.....)))).))))..)))))))))).))....)).))))))))))))))))...))))).....
```

```
 Initial dG =    -36.20

        10        20        30         40        50        60        70
CACCC|    UUA           CGAA      -  GAAUU  A          CUGCGG    UUA    CA
     UGUAG   UAACAUGGGAU    AAUAUA CU     AG GCAGCUGUAU      AACU   GUUA  \
     ACAUU   GUUGUAUUCUG    UUGUAU GA     UC CGUCGGCAUG      UUGG   CGAU  U
UACAC^    UUG           ----      U  AAUUU  A          UA----    C--    UU
      130       120           110       100        90              80

-----------------------------------------------------
```

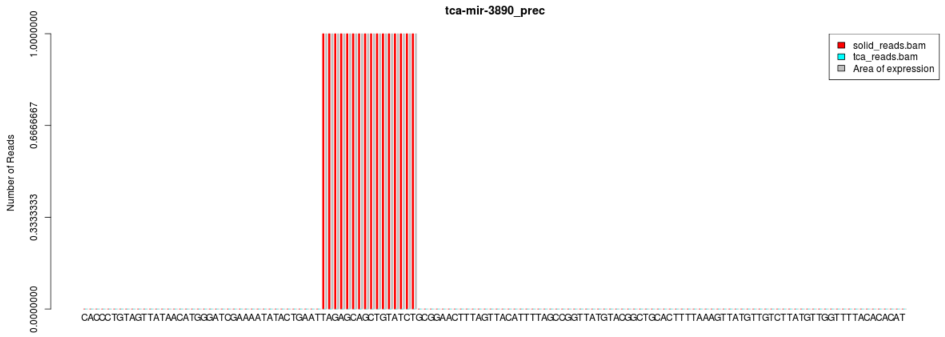

>tca-mir-3891_prec

UCGGAGAUGCAGAGGUUAAUGUGUCGUAAAACACUUACUGUAAUAAAAGUUAAUAAAAUGUGUGUAGUGUAAAUACCUCACCGCAUCAACCA

```
..((.(((((.(((((...((..(..(...((((......((((....)))).......)))))..)..))...)))))...)))))..)).

 Initial dG =    -18.20

         10          20         30         40
UC  A-     A--|    UAA  UG CG AAA    UUACUG-    A
  GG  GAUGC   GAGGU   UG  U  U   ACAC       UAAU A
  CC  CUACG   CUCCA   AU  G  G   UGUG       AUUG A
A-  AA     CCA^    UAA  GU AU ---    UAAAAUA    A
  90        80        70            60        50

-----------------------------------------------------
```

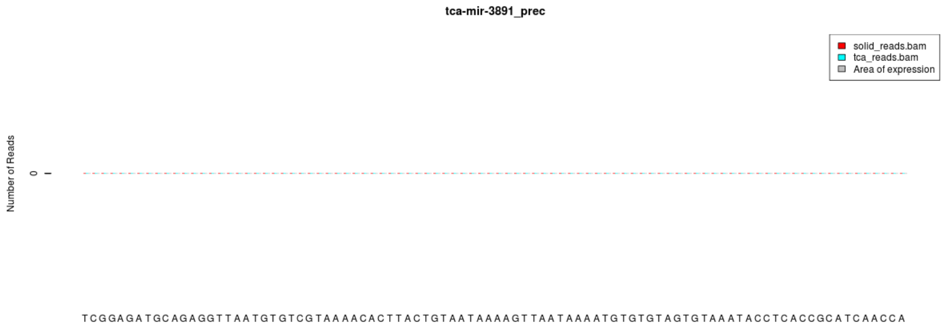

>tca-mir-3892_prec

```
AUUUCGUUAACCCCUGGUGGAAAACCCAUACAUUUGUUUAAGUGUGGUUAAUUCACAUUGGGGGCAUAAUAAUUC
.....((((.((((..((((((...((((((..........))))))....))).))).)))..)))).)))).....

 Initial dG =    -22.50

        10          20         30
AUUUC    A-    UG   -   AAC-|     AUUU
     GUUA  CCCC  GUG GAA    CCAUAC    G
     UAAU  GGGG  UAC CUU    GGUGUG    U
CUUAA    AC    GU   A   AAUU^     AAUU
    70         60        50        40

-----------------------------------------------------
```

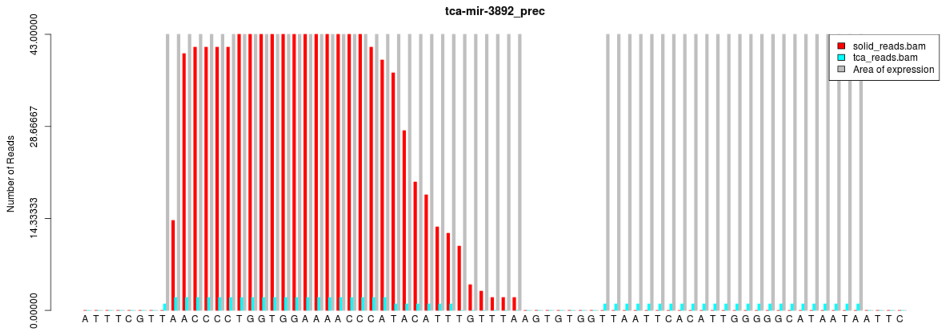

>tca-mir-3893_prec

```
GGUUUUAUUUUUCCGAUGUUACGACGGCUUUACAUGAAAUUUGAAUCAUAAAGCUGUCAGUAACGUGGACAAAUAUUACU
(((..(((((.(((.((((((((((((((((..(((........)))))))))))))).)))))))))).))))).)))

 Initial dG =    -29.60

        10        20         30        40
|  UU     U   G       -          CA   AAU
GGU  UAUUU UCC AUGUUAC GACGGCUUUA  UGA   U
UCA  AUAAA AGG UGCAAUG CUGUCGAAAU  ACU   U
^  UU     C   -       A          --   AAG
.        70         60        50

-----------------------------------------------------
```

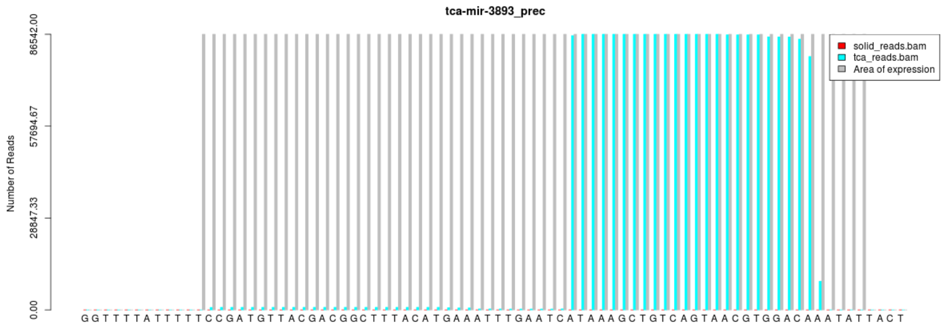

>tca-mir-3894_prec

```
UGAAAUUUGCGGUUUUAAUUCCAUAAUGCAGACGAUUUUUACGACGGCGGAACGUGAGCGGUUUAUUGGAAUUAAUUGUAAACAAAA
.....((((((((..((((((((...(((..(((.(((...((....))))))))..)))......))))))))))))))))).....

 Initial dG =    -16.60

        10        20            30        40
UGAAA        UU        UAA---|  AG   A   UUA  A
     UUUGCGGU  UAAUUCCA      UGC  ACG UUU   CG C
     AAAUGUUA  AUUAAGGU      GCG  UGC AAG   GC G
AAAAC        --        UAUUUG^  AG   -   ---  G
      80          70        60         50

-----------------------------------------------------
```

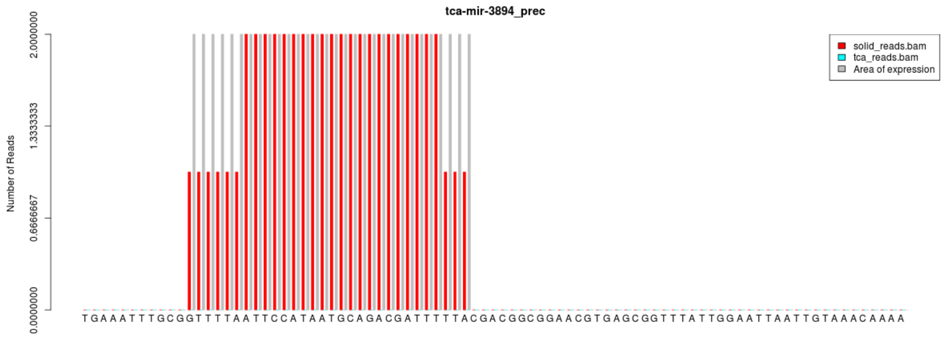

>tca-mir-3895_prec

```
CGAUUUAUUGAGUUGAUUACGAUGGUAUCGGUCUAGCUCUCGAAGUUCCGCUGUAAUCGGAACCGGAACCGGAAUCGCCUUGAUAAUAGU
....((((..((.((((((((.(((..((((........))))....))).))))))))...(((....)))......))..))))....

 Initial dG =    -21.50

        10          20          30
CGAU    UG  .-U        A   UA--|   UCU
    UUAU  AG   UGAUUACG UGG    UCGG   A
    AAUA  UC   GCUAAUGU GCC    AGCU   G
UGAU    GU  \ -        C   UUGA^   CUC
.        80            50        40


             60
              GAA---   G
                    CCG A
                    GGC A
              CGCUAA   C
                     70

-----------------------------------------------------
```

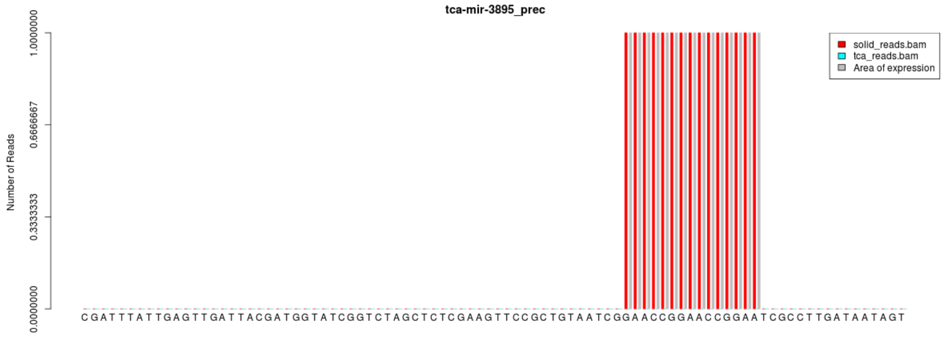

>tca-mir-3896_prec

```
CCGAUUUUGACAUAUCUCGAGUGAAAUUCCAACGACGGGCUGGGUCUCCAACCGGAGCUAACAGACACUAGAUUAGAACUGCAAACGGAUAAGGUCAAAUUUUU
.....((((((.((((.((..((...(((..........(((((.((((....))))))..)))...........)))...))..))))))..)))))).....

 Initial dG =    -19.60

        10         20        30         40          50
CCGAU      A-    U  AG  AAA   CAACGACGGG-   --| U    A
     UUUGAC  UAUC CG  UG   UUC           CUG  GG CUCC A
     AAACUG  AUAG GC  AC   AAG           GAC  UC GAGG C
UUUUU      GA    -  AA  GUC   AUUAGAUCACA   AA^ -    C
  100        90         80        70        60

-----------------------------------------------------
```

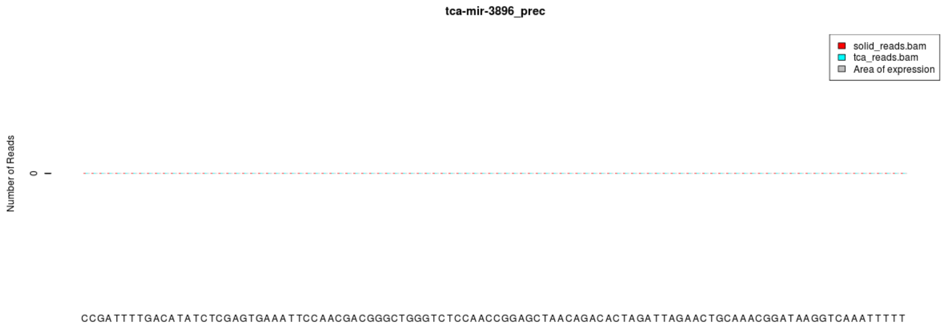

>tca-mir-3897_prec

```
CAAAAGUGUUUACUUCACUCUCGCCUUCGCCGGCAUGGCGCUGAUGGCCGCCGUGGUGGUGGGGGUGGGGGUGGCACGGCGC
.....((((....(((((((((((((((((.((((((((.....).))))))).))))))))))))))))))...)))))

 Initial dG =    -46.70

        10         20         30         40
CAAAA|    UACU                  G       - UG
     GUGUU    UCACUCUCGCCUUCGCCG CAUGGCG C  \
     CGCGG    GGUGGGGGUGGGGGUGGU GUGCCGC G  A
-----^    CAC-                  G       C GU
      80         70         60         50

-----------------------------------------------------
```

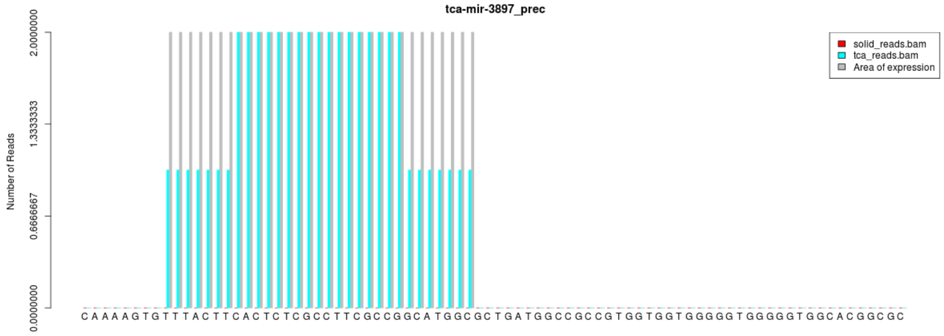

>tca-mir-3898_prec

```
GACCUUGCGAUUGCUGUACCGGCUCAUAGCAUGUUUAAUUAUCUGCGCGCUAUUGAUGGAGAAGUUGUAAUUAUGAACCGGGAUGUUACUUGCAUGCAG
.....(((((..((....((((.((((((......(((((.(((.((((....)).)).))))))))...)))))).))))...))...))))).....

 Initial dG =    -20.50

        10         20        30        40         50
GACCU     UU-| UGUA    C      CAUGUU     A   G  -  C
     UGCGA   GC    CCGG UCAUAG      UAAUU UCU CG CG U
     ACGUU   UG    GGCC AGUAUU      GUUGA AGA GU GU A
GACGU     CAU^ UAG-    A      AAU---     -   G  A  U
        90         80        70            60

----------------------------------------------------
```

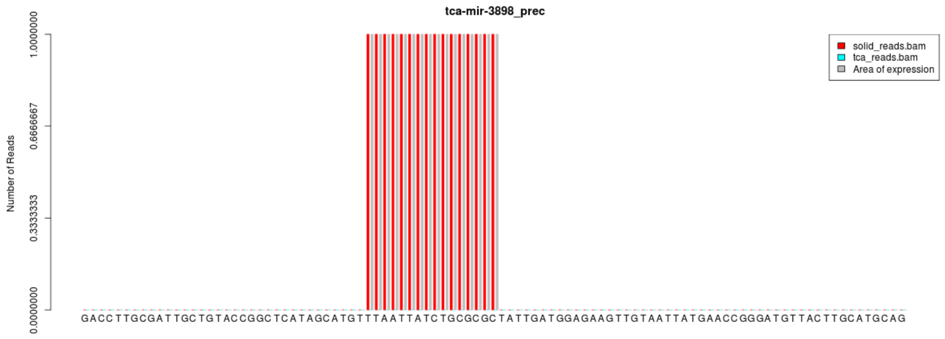

>tca-mir-3899_prec

```
UAUAACCCACUUCGAGAGCCCGCCUUGGGACAGACUUCGGCGACACUCGAUAGGGAUAAA
.....(((...(((((....((((..((......))..))))...)))))..))).....

 Initial dG =    -19.50

        10        20        30
UAUAA|  ACU     AGCC    UU  GA
     CCC   UCGAG    CGCC  GG  C
     GGG   AGCUC    GCGG  UC  A
AAAUA^  AU-     ACA-    CU  AG
.         50         40

-----------------------------------------------------
```

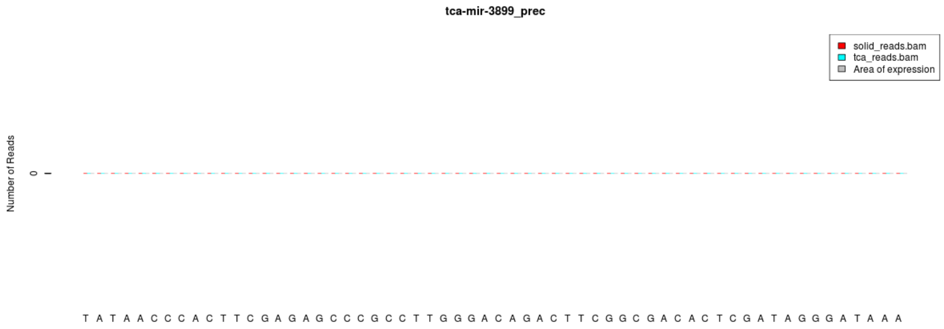

>tca-mir-3900_prec

```
AUUUUGUCCCAUUUUUCUGGGGGUGGCACCUCCUUGCGAAGGAGGUGUGAUCGCUUUGAAUGGCACUACUG
.....((.((((((....((.(((.((((((((((...)))))))))).))).))..)))))).)).....

 Initial dG =    -29.80

        10        20        30
AUUUU| C      UUCU  G   G          G
     GU CCAUUU    GG GGU GCACCUCCUU \
     CA GGUAAG    UC CUA UGUGGAGGAA C
GUCAU^ C      UU--  G   G          G
70        60          50        40

-----------------------------------------------------
```

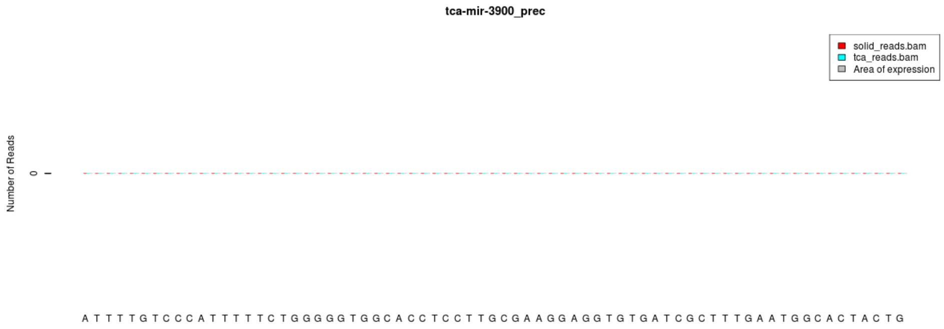

>tca-mir-3901_prec

```
CUUAUGUGCUAAGCAUUUUGAUUUUAUGAGUCAAAAAUCUAAUAAAACGAUAUUUGUAAUAAAUUGACUUCGUUAUCUUUGUAUUUUUUAUUAUUUUUUCAUUUAUAUUUCAAUUACGUU
UUCACUAAAU
.....(((..((((...((((...(((((((.(((((..((((((((.(((((..((((..
((.....))..))))....))))))))))))).))))).))))))))..))))....)))).))).....
```

```
 Initial dG =    -18.00

        10         20        30        40        50          60
CUUAU|  CU    AUU-    UUU       C     UC        C     UU--    UA  UU
     GUG  AAGC    UUGA   UAUGAGU AAAAA  UAAUAAAA GAUAU    GUAA  AA  \
     CAC  UUUG    AACU   AUAUUUA UUUUU  AUUAUUUU UUAUG    UAUU  UU  G
UAAAU^  U-    CAUU    UU-       C     U-        -     UUUC    GC  CA
.        120       110        100         90         80        70
```

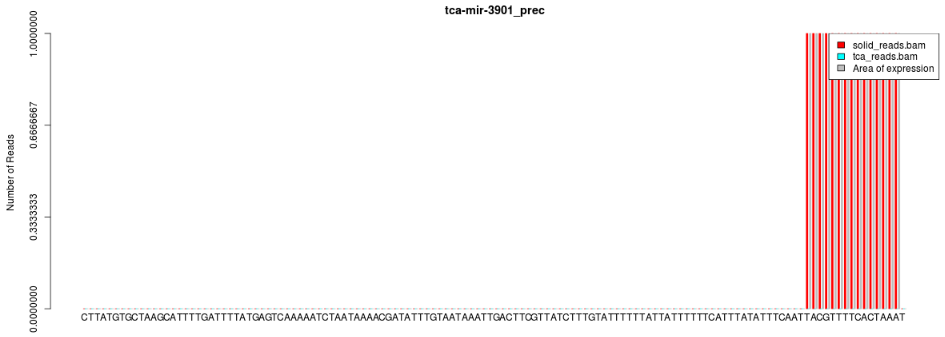

>tca-mir-3902_prec

```
CCGUCAUAUAACCGUGGUUAUAUAACCGGCAUAUUACCAUGGUUAUAUAACCAGCAUAUAACCAUGGUUAUAUAAUCGGCAUAUAACCAUGGUUAUAUAACCG
..((.((((((((((((((((((..((((.((((.(((((((((((((.......))))))))))))).))))..)))).)))))))))))))))))).))..
```

```
 Initial dG =    -47.30

        10        20        30         40        50
CC| C                  AA    C-    U             AAC
  GU AUAUAACCGUGGUUAUAU  CCGG  AUAU ACCAUGGUUAUAU   \
  CA UAUAUUGGUACCAAUAUA  GGCU  UAUA UGGUACCAAUAUA   C
GC^ A                  C-    AA    U             CGA
 100        90        80         70        60

----------------------------------------------------
```

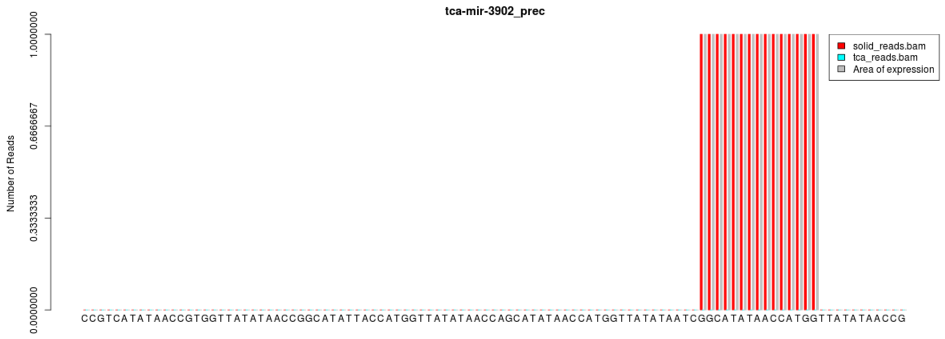

>tca-mir-3903_prec

```
CUUCUAUUUAUUUAUGUUGGGGAGUGAAGUGCCAUUUUACUUUUAGUAAAACAUAUUACAUACUAUUCCAACCGUAAUUAAGUCUAGA
..((((((((.(((((((((((((((..(((...((((((.....))))))).....))).)))).))))))).)))).))))))..)))

 Initial dG =    -15.50

          10         20         30          40
CU   --     U    -       -    AA   CCA--|     UU
  UCU  AUUUA UUAU GUUGGGG AGUG  GUG     UUUUAC  \
  AGA  UGAAU AAUG CAACCUU UCAU  CAU     AAAAUG  U
--   UC     U    C       A    A-   UAUAC^     AU
         80        70        60          50

-----------------------------------------------------
```

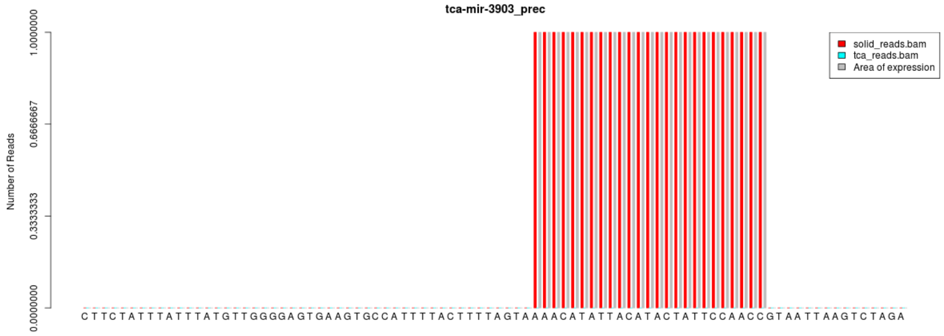

>tca-mir-3904_prec

AUAAAAUCAUUAGUUAUCUUAAAAGAUAUUAUUAAUAAGUGCUGUAAUCUAUUUAAGCUCAUUAAUAGAAAUGACUUAUUUAAAAAUAAUGUUUUAAUUUUUGGUGUGACUGUGAUAAAC
A

```
.....((((.(((((((.((((((((((((((..((((((..((..
((((((..........))))))....)).))))))..))))))))).....)))))).))))))))))).....
```

```
 Initial dG =    -20.50

        10        20             30        40          50
AUAAA    U       C      -----         AA      CU  AA--|     UAAG
     AUCA UAGUUAU UUAAAA     GAUAUUAUU  UAAGUG  GU    UCUAUU    C
     UAGU GUCAGUG GGUUUU     UUGUAAUAA  AUUUAU  CA    AGAUAA    U
ACAAA    -       U      UAAUU         AA      U-  GUAA^     UUAC
 .        110       100        90        80         70        60
```

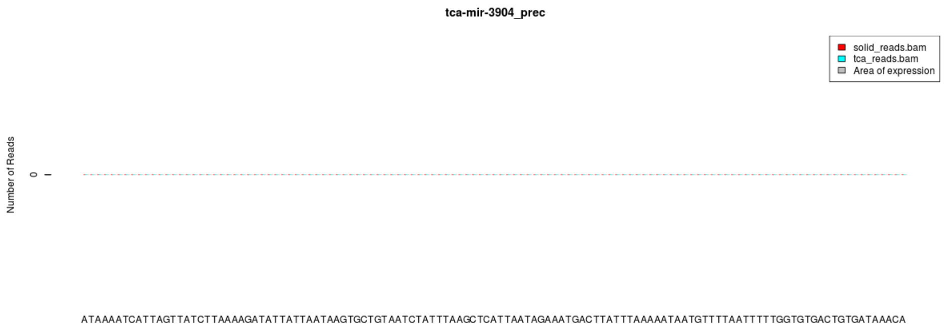

>tca-mir-3905_prec

UUAUCGGGUUCUCAAACGGCGGAACUGGCGGGGAAGGGGGAGCCGCCUUGCCUUCGGCCGGGCCGCACCAAGGCAAGCAGCACUUCCAAACCCCGACCAGCGUGGCGAGUUACUCACCCC
UCAA
.....((((.....(((.((....(((((((((..(((((.((.(.
(((((((((((((...))))....))))))))).)).)))))...))))).))))....))..)))....)))).....

```
 Initial dG =    -49.80

        10         20         30         40        50
UUAUC    UCUCA   G-  GGAA    -     AA-     A  C C        ----|   C
     GGGU     AAC  GC    CUGG CGGGG   GGGGG GC G CUUGCCUU    CGGC \
     CCCA     UUG  CG    GACC GCCCC   CCUUC CG C GAACGGAA    GCCG G
AACUC    CUCA-   AG  GUGC    A     AAA     A  A -        CCAC^   G
  120        110       100        90        80         70

-----------------------------------------------------
```

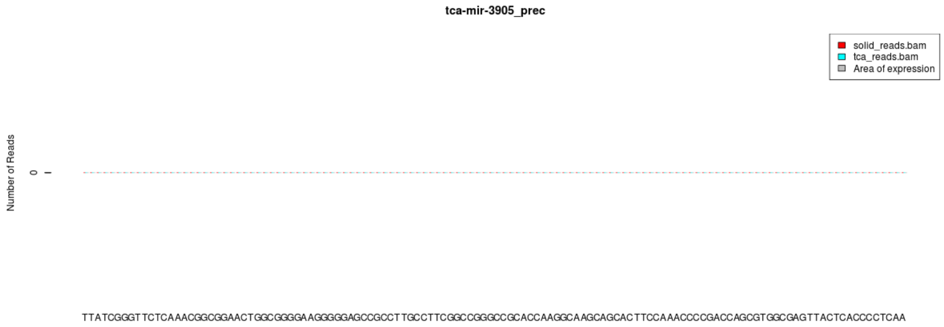

>tca-mir-6007_prec

AUUGGUCUUAUGGUACAAAGACGUUAGAAACAGGACUACGAAAUUAAUUAUGGUUCUGUCUCUUUCGUUUUGCAUCAUGGGAACAUA
..((.((((((((...((((((..(((.(((((((((..(......)..))))))))).)))..))))))..))))))))).))..

```
 Initial dG =    -30.40

        10        20        30        40
AU| G         ACA      UU   A         CG AA
  UG UCUUAUGGU   AAGACG  AGA ACAGGACUA  A  U
  AC AGGGUACUA   UUUUGC  UCU UGUCUUGGU  U  U
AU^ A         CG-      UU   C         AU AA
      80        70        60        50
```

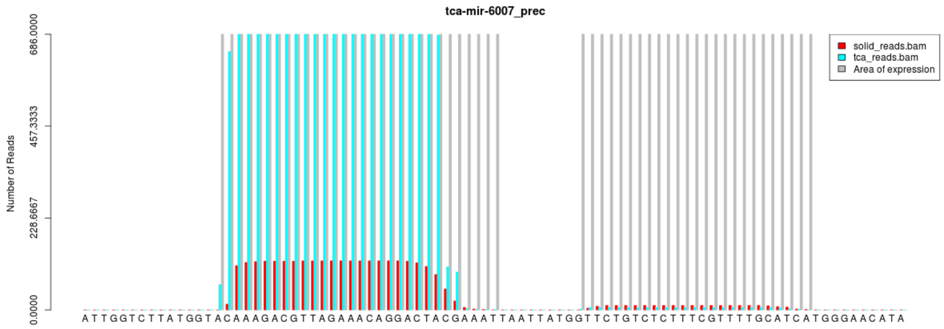

>tca-mir-6008_prec

GUAUUUAGCUGGACUGCUGGUGUCAUGUAUUCGACUCUUUUCACCAUGCCGUACCUCCUAGUCGCCCAUUACCACGCUAGCGGAACGGUUGUUACU
(((..((((((..((((((((((...(((..(((((......((......)).......))))).....))).))))))))))..)))))).))).

```
 Initial dG =    -26.80

         10        20        30           40
-   UU      GA          CAU   UU---     CUUUUC-| CA
 GUA  UAGCUG  CUGCUGGUGU   GUA     CGACU       AC  U
 CAU  GUUGGC  GGCGAUCGCA   CAU     GCUGA       UG  G
U   U-      AA          C--   UACCC     UCCUCCA^ CC
      90        80          70        60        50

-----------------------------------------------------
```

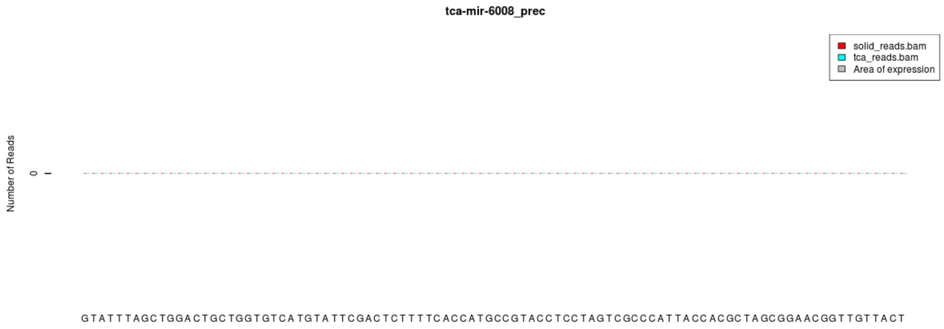

>tca-mir-6009_prec

CGACAGUUGUUUGGCUUCGUUGGCUCAAAUGUUGAGCGAGACCCUGACGCUCUUUGCGUGGGCGCUCGACGAGUCCAAGUGCGACAGGCG

```
........((((...(((((((((....((((((((...(((..((((.....)))))))))))))))))))).)))...))))))))).

 Initial dG =    -32.40

        10        20            30        40        50
CGACAGUU     GCU    ---    -|  AAAU        AGA   UG    UC
        GUUUG   UCGU   UGG CUC    GUUGAGCG   CCC  ACGC  \
        CGGAC   AGCG   ACC GAG    CAGCUCGC   GGG  UGCG  U
G-------     ---    UGA   U^  ----        ---   --    UU
.                  80            70           60

-----------------------------------------------------
```

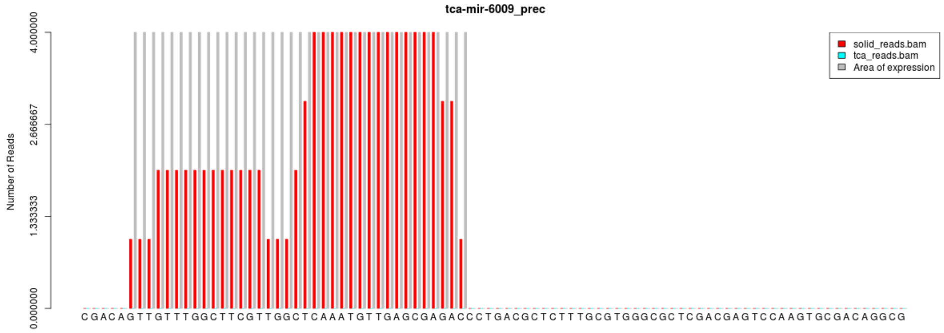

>tca-mir-6010_prec

```
AUUUUUUGCAGUUUGUUUUUGUCGCCUUUUAGGCACUUGGCGUAUUUUAACAAGUAGCUGUAAGGGUGCUCGACUUGUAAAUCAA
....(((((((...(((...(.(((((((..((((((((...........))))).)))..)))))))).))))))))))....

 Initial dG =    -20.40

        10        20        30         40
AUUU|      UUU   UUU U       UA   -     GCGUA
    UUUGCAG   GUU   G CGCCUUU  GGC ACUUG     \
    AAAUGUU   CAG   C GUGGGAA  UCG UGAAC     U
AACU^      ---   CU- -       UG   A     AAUUU
    80           70          60         50

-----------------------------------------------------
```

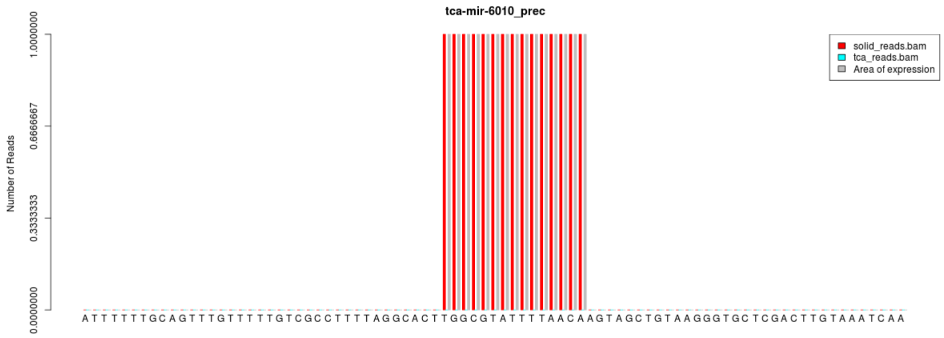

>tca-mir-6011_prec

```
CGAGUCCUCGAUGAUGUAAGCGGAACCGUCCGGGUUGUAGACGAUCUUGCCGGAGAACUGCUCCACGUCCGAGGUGU
.....(((((..(((((.(((((..(((...((((((....))))))...)))....)))))..)))))))))))...

 Initial dG =    -25.30

        10         20          30
CGAGU     AU     A-     AA--|  UCC      U
     CCUCG  GAUGU  AGCGG    CCG   GGGUUG A
     GGAGC  CUGCA  UCGUC    GGC   UCUAGC G
UGU--     --     CC     AAGA^  CGU      A
        70          60        50        40

-----------------------------------------------------
```

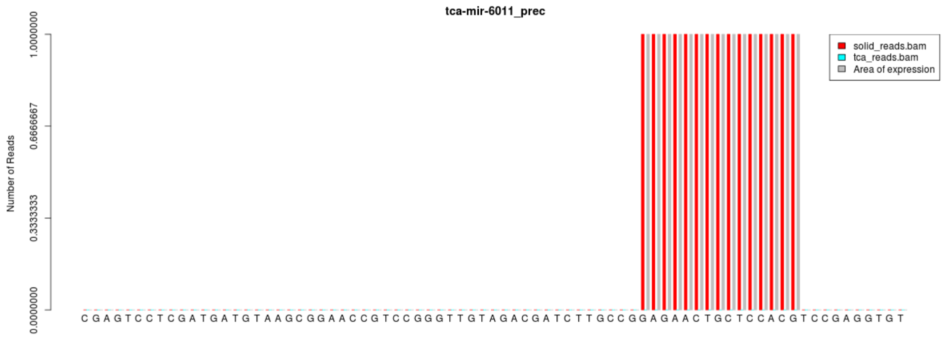

>tca-mir-6012_prec

CGAUUGUGACUGGGUCACGUAGAGGAAGGAAAGACAAUCUGGUGGUGUGCAGGGUGAACAGACUGAUCUUGUUUCUGCGGAAAUGUUAGUUACAGUCAUUUCGGCGAUGAGAUCAGCCUG
UCGAACCCAGCUCGAGUUCGUUCAACACUUUCUUCUCUAGUAAUAUGCAGCGC
.....(((.(((.((.((.((((((..((((((......(((...((.((.((((..((((.(((((((..((...((.
((((((...........)))))).)))))..))))))).))))...)))).)).))...)))).......))))))))))))))...)).))))))

```
 Initial dG =    -53.40

        10          20         30          40         50          60         70         80
90
CGAUU   A   G  C--| G      AA       ACAAUC-   UGG  U  A    GA-    A       UG  UCU  G
UUAGU
     GUG CUG GU   AC UAGAGG  GGAAAG       UGG   UG GC GGGU   ACAG CUGAUCU  UU   GC GAAAUG
\
     CGC GAC UA   UG AUCUCU  UCUUUC       GCU   GC CG CCCA   UGUC GACUAGA  AG   CG CUUUAC
U
-----   -   G  UAA^ -      --       ACAACUU   UGA  U  A    AGC    C       GU  ---  G
UGACA
       170       160          150       140       130       120       110          100
```

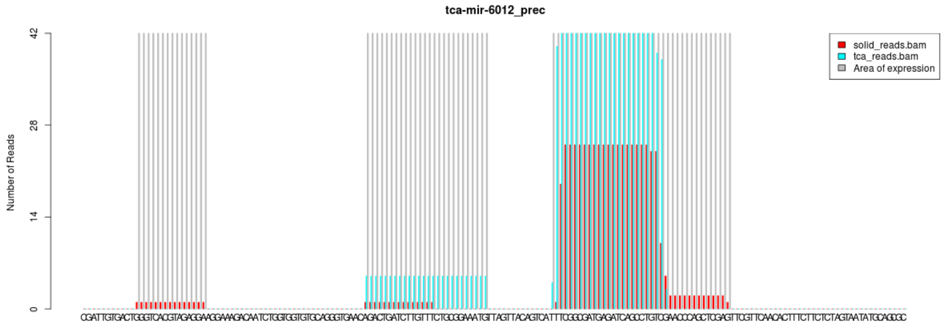

>tca-mir-6013_prec

```
GCUCAGGCGUCGUCCAAAGCGCUCCAGGAAGCGCAAGCCGAGGUGAAGCUGCUGACGACCGCCUUGCA
((..((((((((((...((((((.((....((....)).....)).))).)))))))).))))).)).

 Initial dG =    -27.90

          10        20         30
-| UC     -     CAA   -   C  GGAA-  G
 GC  AGGCG UCGUC   AGC GCU CA     GC C
 CG  UCCGC AGCAG   UCG CGA GU     CG A
A^ U-     C     ---   U   A  GGAGC  A
        60           50        40

-----------------------------------------------------
```

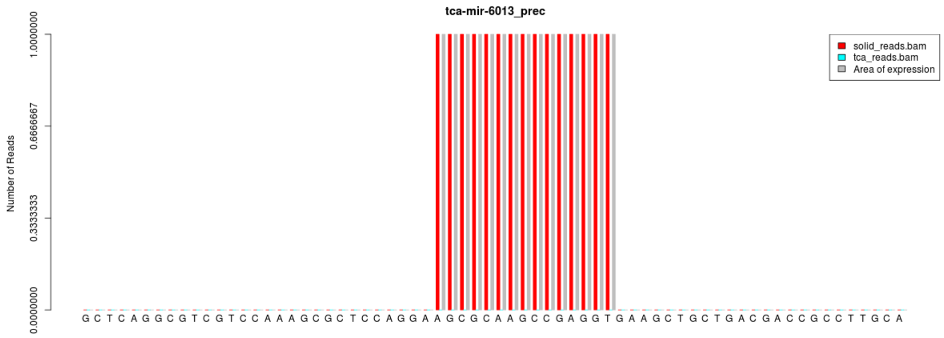

>tca-mir-6014_prec

CAUUAAAUUGGGUCACUCUCUUGACGUAUUCCAAAUAAAGCAUGAUUUGGAAUUUCCCGUGUUGAGUGGCAUAAAUUAUUUG
.....((((..(((((((.....(((.(((((((((........)))))))))....)))...)))))))...)))).....

```
 Initial dG =    -20.40

        10         20           30        40
CAUUA    GG-       UCUUG   U---|        AAA
     AAUU   GUCACUC     ACG    AUUCCAAAU   G
     UUAA   CGGUGAG     UGC    UAAGGUUUA   C
GUUUA    AUA       UUG--   CCUU^        GUA
 80        70          60         50

-----------------------------------------------------
```

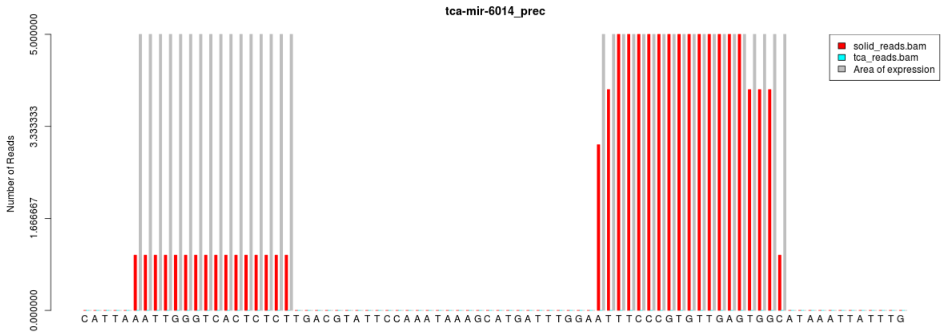

>tca-mir-6015_prec

```
CUUUUCGUCAUUUAAUAAUCGCGAUUGGCACACCUGCCGCUCCAAUAUUCAGAAAUAUAGGCAGAUGCAGCGCCGGUUGUGGUGAUGUGUGU
........(((...((.((((((((((((....((((..((((.(((((....))))).)).)).)).)))).))))))))))))).)).))).

 Initial dG =    -26.40

        10        20        30        40         50
CUUUUCGU|  UUA  A            ACAC    CG  -  A     C
        CAU   AU AUCGCGAUUGGC    CUGC  CU CC AUAUU A
        GUG   UA UGGUGUUGGCCG    GACG  GA GG UAUAA G
U-------^  UG-  G            C---    UA  C  A     A
        90         80          70        60

-----------------------------------------------------
```

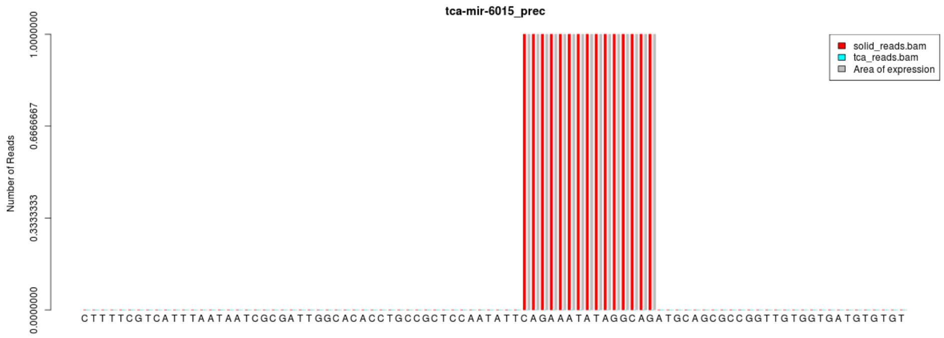

>tca-mir-6016_prec

```
GGCGCAAGAGUAGAAGAACAUGUGGUCGUGGUUAUUCGGCAAUGCCACGCCCACAAGUCCUCCUACUUUUACGAC
..((.((((((((.((.((.(((((.((((((...........)))))).))))).)).)).)))))))).))..

 Initial dG =    -30.10

        10        20        30
GG| C        A  A  A     U      UAUUC
  CG AAGAGUAG AG AC UGUGG CGUGGU     \
  GC UUUCAUC UC UG ACACC GCACCG     G
CA^ A        C  C  A     C      UAACG
    70        60        50        40

-----------------------------------------------------
```

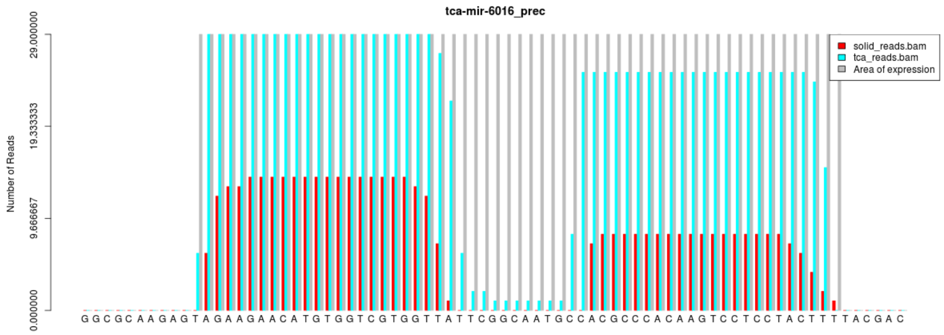

>tca-mir-6017_prec

AUAAGAGUUUGUUGUUUCCACCCUGUCGUGUUCCUAAUUGGUAUGUUGUAUUUUUUAUUGGGCUGAUUCGUGUUUACGAUGAGUCUUCCUGUAUCCAAGCCGAGUGCCUCUAAUCAAUUC
CAAUUAGUCGGAUGACACGGACAAUUUACUGGUCU

```
.....(((..(((((.....((.((((((....((((((((...((((.(((...((((.
((((((((((((.......))))))).............)))).)))).....))))))).))))))))....)))))).))))))..))).....

 Initial dG =    -35.50

        10        20        30        40        50          60                    70
AUAAG   UU     UUCCA  C      GUUC        UAU    U   UUU--    G    ------------|      GUU
     AGU  GUUGU     CC UGUCGU    CUAAUUGG   GUUG AUU     UAUU GGCU            GAUUCGU   \
     UCA  UAACA     GG ACAGUA    GAUUAACC   UAAC UAA     GUGA CCGA            CUGAGUA   U
UCUGG   UU     -----  C      GGCU        U--    -   UCUCC    G    ACCUAUGUCCUU^      GCA
   150            140       130       120          110       100        90         80
```

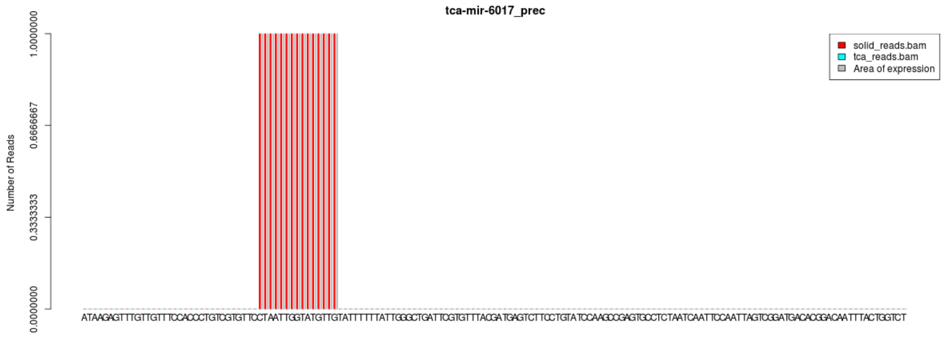

>tca-mir-6018_prec

```
AAACAAUGGUAGGCAGCUAGUUUCAAUGGAAUUAAUAAUUUUUUUAAAAAACAAUAGAAUUCAACUUGUGACUAGUCGACCCUACCAUCUCCA
.....((((((((..(((((((.(((..(((((......((((....))))......)))))...))).)))))))....)))))))).....

 Initial dG =    -22.80

        10          20         30        40
AAACA        CA--       U   UG-|    AAUAAU    U
     AUGGUAGG    GCUAGUU CAA   GAAUU      UUUU U
     UACCAUCC    UGAUCAG GUU   CUUAA      AAAA A
ACCUC        CAGC       U   CAA^    GAUAAC    A
  90        80        70        60        50

-----------------------------------------------------
```

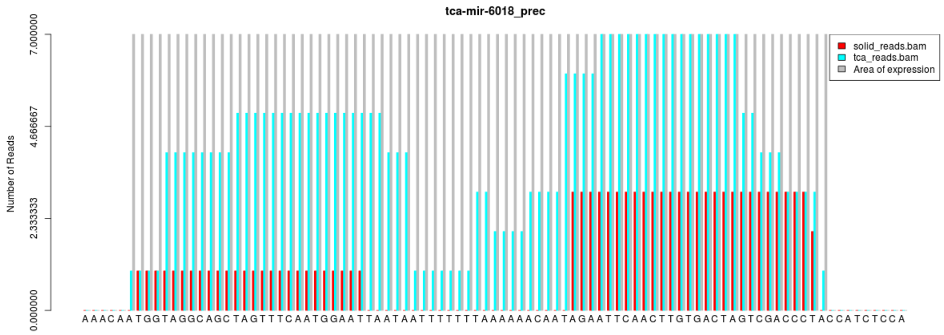

>tca-mir-iab-4_prec

```
UUAAACCCCCUGUACGUAUACUGAAUGUAUCCUGAGUGUUUUCUGUCCGGUAUACCUUCAGUAUACGUAACAGGAGGCCAC
.....((.((((((((((((((((.((((.(((..(.......)..))).)))).))))))))))))).)))).))....

 Initial dG =    -32.60

        10         20        30        40
UUAAA  C    -|            U    C   AG GUU
     CC CCUG UACGUAUACUGAA GUAU CUG  U   \
     GG GGAC AUGCAUAUGACUU CAUA GGC  G   U
CACC-  A    A^            C    U   CU UCU
80         70        60        50

-----------------------------------------------------
```

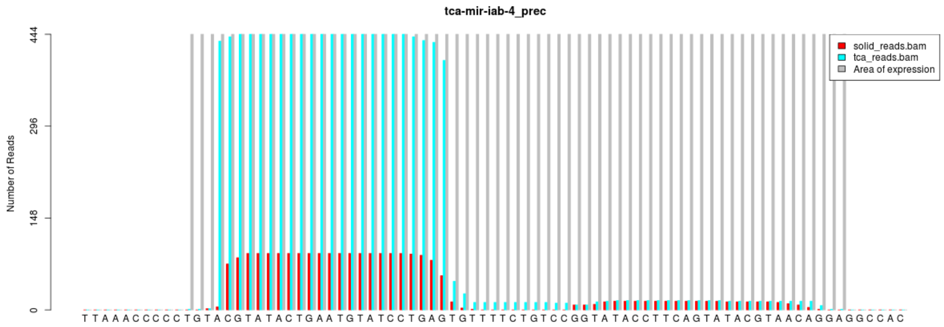

>tca-mir-iab-8_prec

UGAAAUGUGGCCUCCUGUUACGUAUACUGAAGGUAUACCGGACAGAAAACACUCAGGAUACAUUCAGUAUACGUACAGGGGGUUUAACAUUCUG

...(((((((((((((((.((((((((((((.((((.((.((..........)).)))))).))))))))))))))))))))))..)))))...

```
 Initial dG =    -40.80

          10        20        30        40
UGA     --|         U            G    A  G  CAGA
   AAUGU  GGCCUCCUGU ACGUAUACUGAA GUAU CC GA    A
   UUACA  UUGGGGGACA UGCAUAUGACUU CAUA GG CU    A
GUC     AU^         -            A    -  A  CACA
   90        80         70        60         50
```

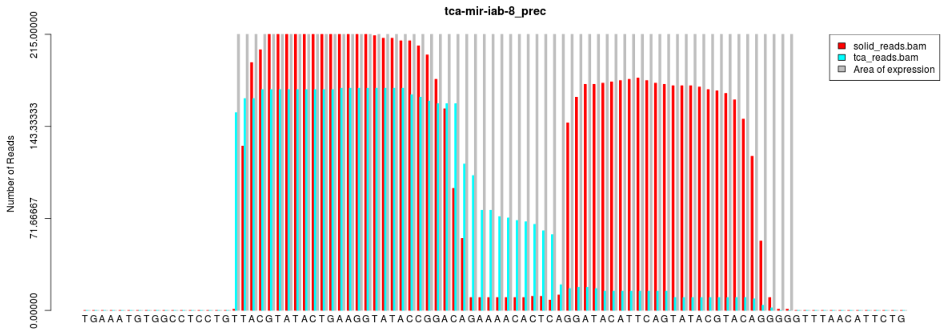

>Tca-Mir-306_candA_prec

UCUCUAAUGGAAUAGGAUUCACGUACUUUACAGUUUUGGGGCCCCAAUGCUUCAGGCAAGAUUAUCCUAUAAUCGGUUGCCUAAAAAAAGAUAUCAGGUACUUAGUGCCAAAUAAUUGAA
UUUAGUUCAUCAUUUUUUGUAAUUCUAGAGGCAGGUGCUUGGAAUACAAAACAAACGAAACUCAGGAGAAAAUUUAUGAA
(((((....((((..((((((....((((..(((.((((....)))).)))..((((((((((((...))))))..))))))....))))......
(((((...))))).......))))))..))))......((((((.(((((..(((....))))))))))))))).............))))).........

```
 Initial dG =    -46.70

                     10         20             30         40
-----------     .-AAUG    AG      .-CGUA    .-AC|  U    G
           UCUCU      GAAU  GAUUCA      CUUU    AGU UUGG G
           AGAGG      CUUG  UUAAGU      GAAA    UCG AACC C
AAGUAUUUAAA     \ ----    AU      \ ----    \ --^  U    C
.       190                120        90        50


                                                          60
                                              UC--      --      C
                                                  AGGCAA  GAUUAU \
                                                  UCCGUU  CUAAUA C
                                              AAAA      GG      U
                                                   80         70

-----------------------------------------------------
```

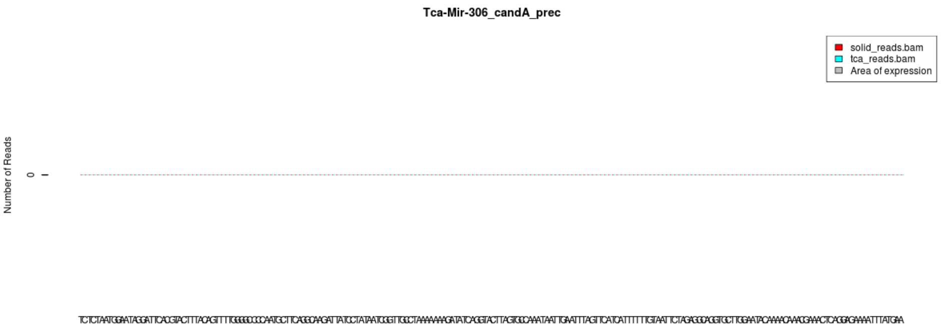

>Tca-Mir-306_cand_B_prec

AAACAAAAAUGAUCGCCCACUUGAAGGACAAGAAGAAGAUUAGGGCGAAAAAGCGCUGGUCUCAGUGCAUGUACAUGUACUUUUUGCUCGGAUUCAGGUACUUAGUGCGGCUGAAAUAUG
GUUGGUAAAACUUUUCUAUUUUUUCUUGCAGAUUGCAAGCCAACGACGAACUCUCCGCCCACAGCAAGGAAAUCCGCGGC
............((((...((((.....))))..((.((((((.((......)).)))))))).((((((....))))))((((((((((((((((((((...)))).)))))).....
(((((...................(((((.....))))))))))).........)))).....))))))))).....)))).

```
 Initial dG =    -48.30

        10          20            30           40
AAACAAAAAUGAUC   .-C       --- GA     .-AGA|      G  GA
              GCC   ACUUGAA   G  CAAGA     AGAUUAG GC  A
              CGG   UGGACUU   C  GUUUU     UCUGGUC CG  A
--------------   \ -       AGG UC     \ ---^      G  AA
            200             90               60         50


                                                 70
                                          C       G
                                           AGUGCAU U
                                           UCAUGUA A
                                          -       C
                                           80

-----------------------------------------------------
```

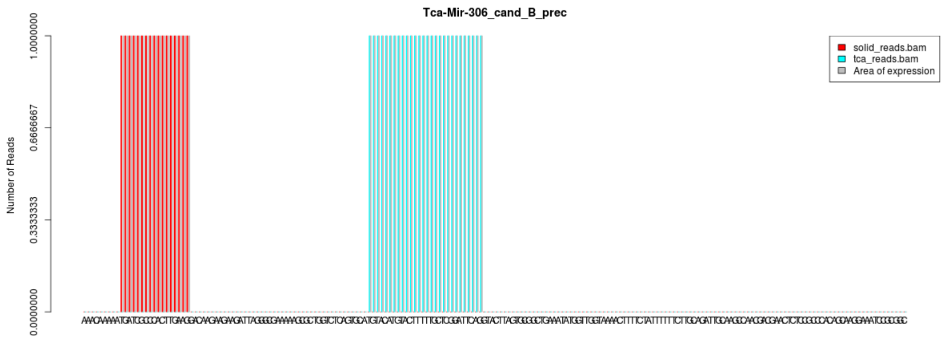

>Tca-Mir-274_prec

AUUCUUGUCGGAAUGUAAACAGCUGCGGAGAUUUUGUGACCGACACUUGAAGUAUUUUUUCACGAGAUUUUGUUAUAACACACUGAAAAGUCACUGACUUGAACCGGCAAUAAUACGUUAUUGAAUUUAUUAUUUUCUUUUUUUUUUGGUCUAGGAUGUUUUUUUUAAUCACAAAAGCU

.(((..(((((...............((((((((((((..((.((.....)).))...))))))))))))..........(((....)))..)))))..))).((.(((((((.(((....))).))))))).))...(((((.((((.(((((......))))))))).)))))..

```
 Initial dG =    -27.80

          10          20        30        40
.-A   UU     .-AAUGUAAACAGCUGCG            CC-| C  UU
   UUC  GUCGG                GAGAUUUUGUGA   GA AC  \
   AAG  CAGUC                UUUUAGAGCACU   UU UG  G
\ -   UU     \ ----------------            UUU^ A  AA
    100                      70        60        50



                      80
               GUUAUAACAC   G
                         ACU A
                         UGA A
               AC--------   A
                         90
```

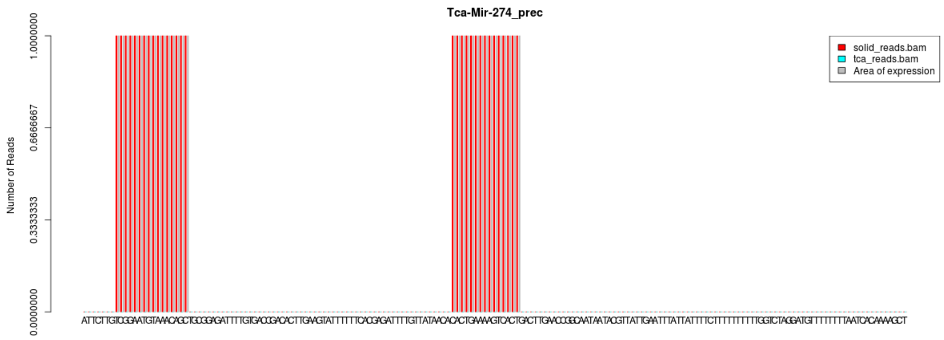

>Tca-Mir-274candb_prec

AUUUCUGUUGGAAUGUAAACAGCUCCGGAAAUUUUGUGACCGACACUUGACACAAAUGUCUAGAGAGAAAUCUCAGAUGUAAACGUAGCAUAAAGGAUGAUGGUUUUCUGUUAUUUUUUU
UGUGUUUAGGUGUCAUAUUAAAAUUUACAUGCAUCUUUACAAAAAAUGUACAAAUAGAA
..(((((((....((((((..((.....((((((((.....(((((((((((((.((((((.((((....)))))))))))...........
(((((((((((((....))))))))))))).)))).)))))))))....))))))))....))...))))))...........)))))))

```
 Initial dG =    -43.00

          10
AU            A-| U
  UUCUGUU--GGA  UG A
  AAGAUAA  CCU  AC A
--       \    CG^ A
                20



               30        40        50           60
           .-GG        UGACC         -    .-A      A    G
               AAAUUUUG     GACACUUGA CACA   AUGUCU GAGA A
               UUUAAAAU     CUGUGGAUU GUGU   UGUAGA CUCU A
           \ --        UAUA-         U    \ -      -    A
                   140        130           80         70

-----------------------------------------------------
```

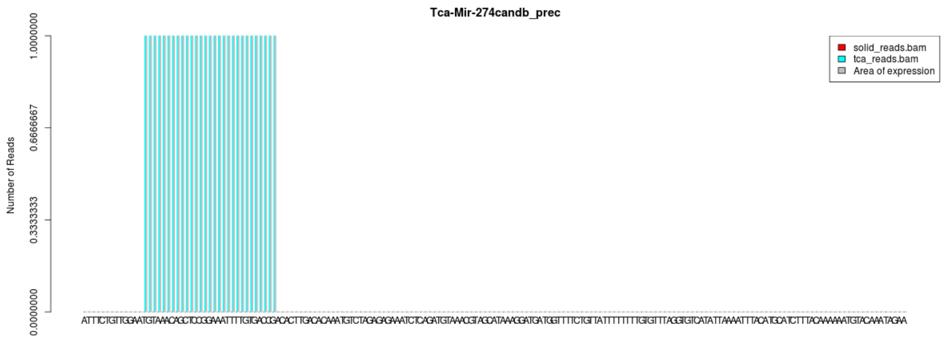

```
>Tca-mir_1006cand_prec
UGCAGAAUUUUAUUAGACAAAUUUAAACCGAGAAGAAAUCGAAUUUAAUUCUGCGGAUUAUGUGUUUAAUUUAGGUAUGAACACUGGAAAAAAGGGACGUUUUAGUCCAAUUUCAUACUU
CACUGAGCUAGAAGGAAGUAG
(((((((((..(((.((....(((.......)))....)).)))..)))))))))......(((((((.........)))))))..((((....((((......))))..)))).
((((((.((........)))))))).

 Initial dG =    -30.40

          10        20
  |        UU   A  CAAA   AAA
--UGCAGAAUU  AUU GA    UUU   \
  GCGUCUUAA  UAA CU    AAG   C
\ ^        UU   G  AAAG   AGC
      50        40        30



       60        70
  .-GAUUAU       AUUU
          GUGUUUA    \
          CACAAGU    A
  \ ------       AUGG
             80

-----------------------------------------------------
```

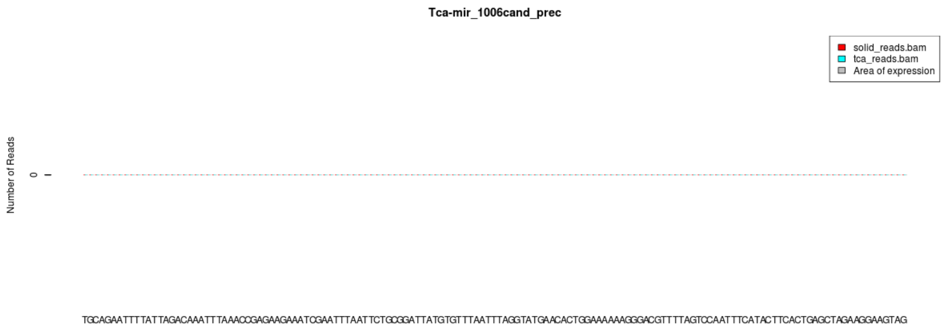

>Tca-Mir-278candB_prec

ACCAGUUCACUGGUGUUUAUAUUCGGGGGUAAACAUUGCAUCGACGGAUGAAAGCGGCGUAAUUUUCCAUGAUGGGACUUUCGUCCGGUGAAACUACGAUGGUAUUUACCUCGAUUUGAG
CCCAUAGUUCUCAAAAGCACACAAAAACGUUUAUUUCGGAAGUAAUGCG
(((((....)))))((.....(((.(..((((((..........((((((((((..........((((.....)))))))))))))))(((......(((.(((....)))))).
((((((.........))))))....))).......))))))..).))).....)).

```
 Initial dG =    -45.40



  |    U
--ACCAG U
  UGGUC C
\ ^    A
     10



       20        30           40        50        60        70
  -  UUAUA   G GG      .-AUUGCAUCGA          CGGCGUAAUU    AU
   GU     UUC G  GUAAAC            CGGAUGAAAG          UUCC  \
   CG     AAG C  UAUUUG            GCCUGCUUUC          AGGG  G
  G  UAAUG   G UU      \ ----------          ----------    UA
         160       150                  80

-----------------------------------------------------
```

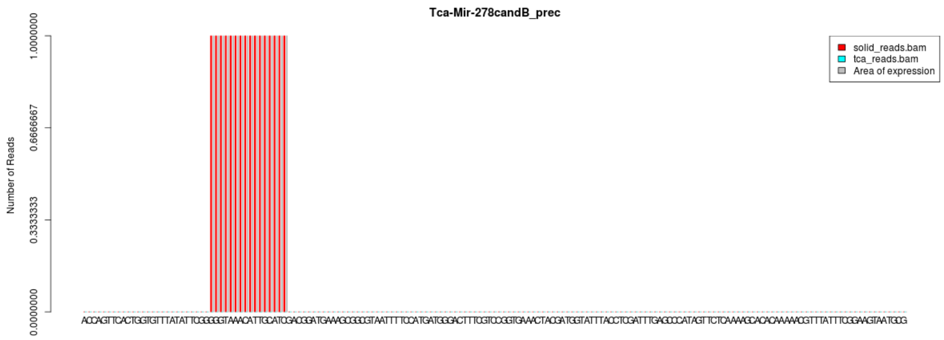

>Tca-Mir-278candA_prec

GAAAGCCGCUAAGUUUUUGUAAGUUUUUCCACAAUGCUAGUCCCAAGUCACGACCAAACCGUUUGGCUUCUGUCGUGUGUGCGGUCGGUGGGACUGUCGUCUUCUACACGGCCUUUAAAU
GCCGACAAACCCAAAGCAUACAUGCCUCCCAAUUGAAAGUAAGUUUGG
((((((......))))))...............(((.((((((((.....(((((..((....((((....))))...))..))))).)))))))).)))........
((((........)))).(((((.....(((....))).((......))......))))).

```
 Initial dG =    -36.10



  |     CG
--GAAAGC  C
  UUUUUG  U
\ ^     AA
         10



    20          30        40        50        60
       A     .-UCCACA   C        AGUCA     AA  CGUU    U
  --GUA GUUUU        AUG UAGUCCCA     CGACC  AC    UGGC U
    CAU CGAAA        UGC GUCAGGGU     GCUGG  UG    GCUG C
  \    A     \ ------   U        G----     CG  UGU-    U
   140             100        90            80         70

-----------------------------------------------------
```

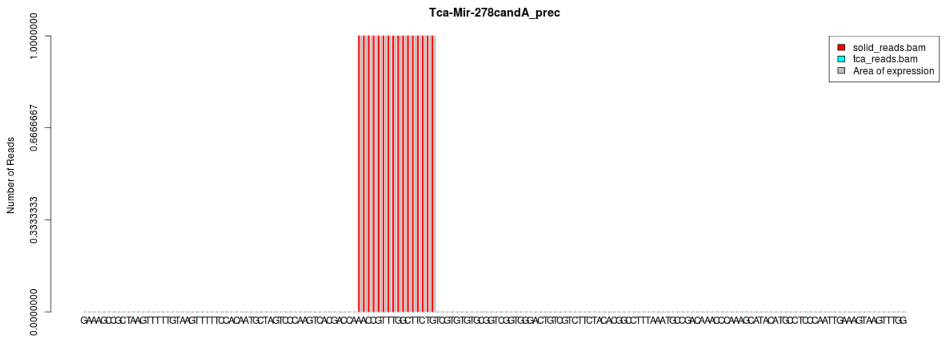

>tca-mir-11614_prec

```
UUAGCGUUGACUGUGUGGAUGAUCGUGUGGUAGCCGAUGGAAGUAACGGUAGUGCGAUCAUCCGCUGUCAAAGCUUUACUACC
..(((.(((((...(((((((((((..(....((((..........)))).)..))))))))))).))))).)))........

 Initial dG =    -31.80

              10        20        30        40
UU------|  G     UGU           UG GGUA    AUGG
        AGC UUGAC   GUGGAUGAUCG  U    GCCG    A
        UCG AACUG   CGCCUACUAGC  G    UGGC    A
CCAUCAUU^  A     U--           GU A---    AAUG
  80        70          60          50

-----------------------------------------------------
```

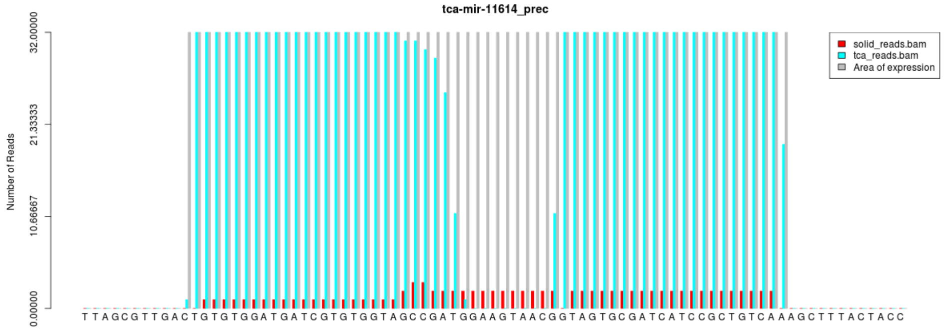

>tca-mir-11615_prec

```
ACACUUACGACGAUUCGUCCUUUUCGGACUCGGACUACUCCGGCGAUGAGUCCGAGUCCGAAUGGGGCGGACUGCGUAUCGAA
.....((((.((.((((((((.((((((((((((((..((....))..)))))))))))))).)))))))).))))))).....

 Initial dG =    -44.20

        10         20         30         40
ACACU|   A  A        U              AC  C
     UACG CG UUCGUCCU UUCGGACUCGGACU  UC G
     AUGC GU AGGCGGGG AAGCCUGAGCCUGA  AG G
AAGCU^   -  C        U              GU  C
  80         70         60         50

-----------------------------------------------------
```

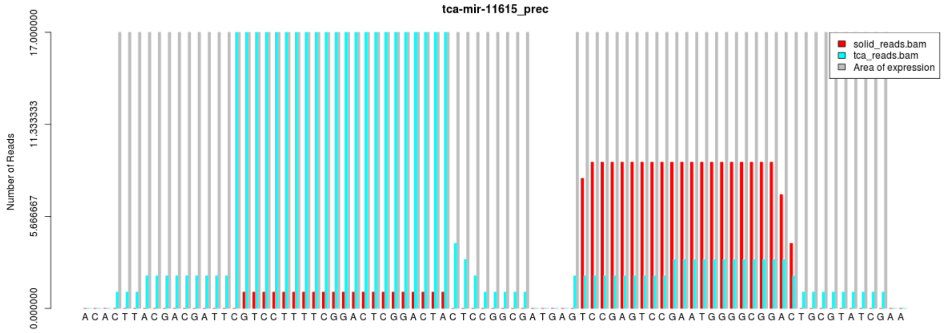

>tca-mir-11619e_prec

AACAAAACUAUCGGAUGAGAGAUGAGAGUUUGCGAUAAUCUUCAUUUUAGAAUUUAACAAAUGAGGAGUCACAGACUUGAUUUUUCGCUCGAUUGUUCGACA

.....(((.((((..((((((((..(((((((.(((..((((((((((...........))))))))))).))))))).))))))))..)))).))).....

```
 Initial dG =    -31.10

        10        20        30        40        50
AACAA|  U    GA        GA        C   AA         UAGAA
     AAC AUCG  UGAGAGAU  GAGUUUG GAU  UCUUCAUUU     \
     UUG UAGC  GCUUUUUA  UUCAGAC CUG  AGGAGUAAA     U
ACAGC^  U    UC        G-        A   --         CAAUU
100        90        80         70          60

-----------------------------------------------------
```

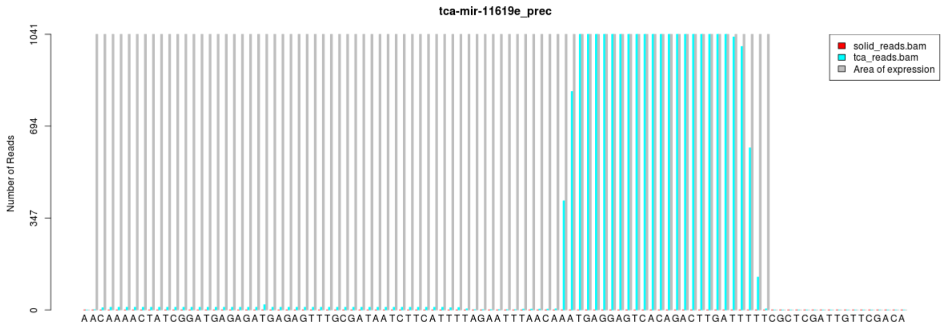

>tca-mir-11619f_prec

```
AAAUUAUCGAAUGAGAGAUGAGAGUUUGUGAUAAUCUUCAUUUUAGAAUUUAACAAAUGAGGAGUCACAGACUUGAUCUUUCGCUCGAUUGCUC
.....((((.((((((((..(((((((((((..(((((((((...........)))))))))))))))))))).)))))))).))))).....

 Initial dG =    -35.30

        10        20        30        40
AAAUU|    A        GA            AA         UAGAA
     AUCGA UGAGAGAU  GAGUUUGUGAU  UCUUCAUUU     \
     UAGCU GCUUUCUA  UUCAGACACUG  AGGAGUAAA     U
CUCGU^    C        G-            --         CAAUU
   90        80         70          60        50

-----------------------------------------------------
```

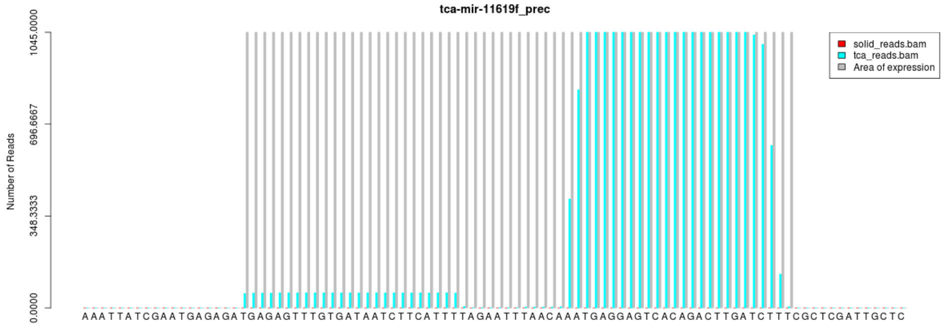

>tca-mir-11620_prec

```
CGGCGGUGCCCCAGGUCAACGGAGCCGUGCAUCCUGACGAUAUCAACGUGAUGCCCGGACCGGUGCCCCAUGUCAACGCCGCG
..((((((...((((.((.(((..(((.(((((...(((.......)))))))).))).))).)).)).))..))....)))))).
```

```
 Initial dG =    -29.10

        10           20         30         40
CG      CCC-  --| U  A   AG   U     CUG   AUA
  GCGGUG    CA  GG CA CGG  CCG GCAUC   ACG   \
  CGCCGC    GU  CC GU GCC  GGC CGUAG   UGC   U
G-      AACU  AC^ C  G   A-   C     ---   AAC
   80        70        60         50
```

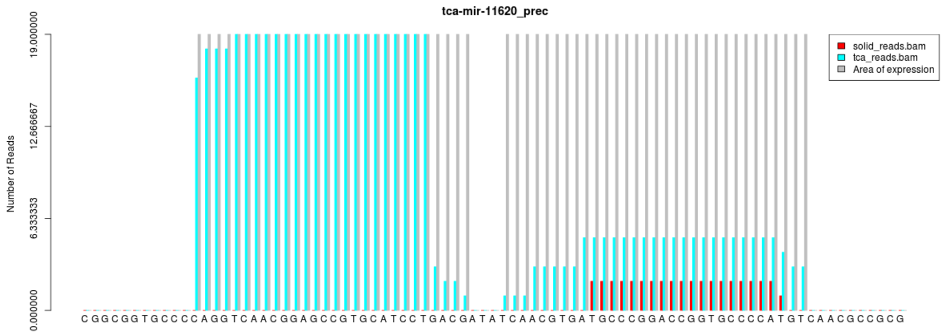

>tca-mir-11621_prec

```
CAACCAACUUUCCUUGAGAAUAAAGCGUAUUUGUCGUUGCAUCCUUAACAUAAACGCAACGAUUCAGGUGCUUUUCUUUCAAUCAAAGUUAAUAC
.....((((((..((((((..(((((((....((((((((...............))))))))....)))))))..))))))..)))))).....

 Initial dG =    -25.40

        10        20        30        40
CAACC|     CC      AU       AUUU       AUCCUUA
     AACUUU  UUGAGA  AAAGCGU    GUCGUUGC       \
     UUGAAA  AACUUU  UUUCGUG    UAGCAACG       A
CAUAA^     CU      CU       GACU       CAAAUAC
    90        80        70        60        50

-----------------------------------------------------
```

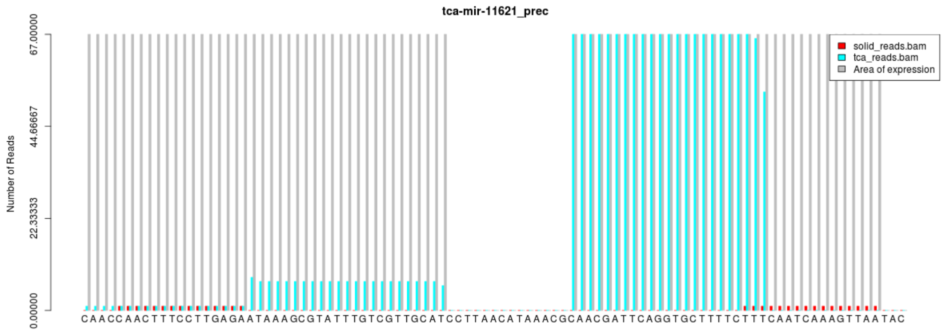

>tca-mir-11622_prec

```
ACUAAAUCAGUUGAUGUAAAGGAAUUGCAGGAUUGAAAUGUCUGCAGCGUUUUAGUCUUGUAAAUUCCUUUGGGUUUACUGAUAAAAA
.....((((((.(((.(((((((((((((((((((((((((.....))))))))))))))))).)))))))).))).)))))).....

 Initial dG =    -38.20

        10        20         30        40
ACUAA      U   G        -|                CU
     AUCAGU GAU UAAAGGAA UUGCAGGAUUGAAAUGU  \
     UAGUCA UUG GUUUCCUU AAUGUUCUGAUUUUGCG  G
AAAAA      U   G        A^                AC
       80        70        60        50

-----------------------------------------------------
```

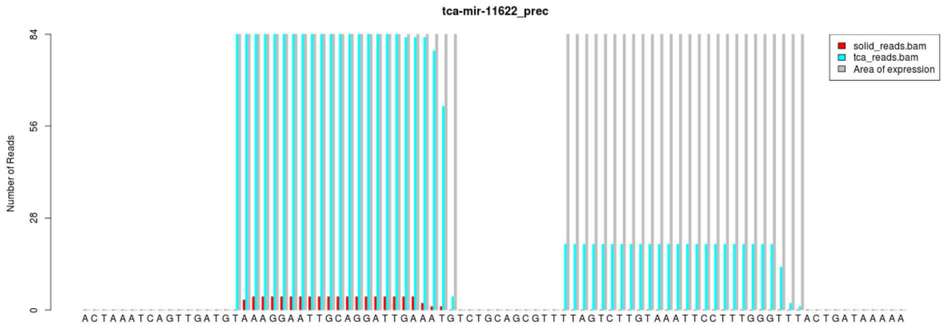

>tca-mir-11623_prec

```
ACACUCGCGUCCGAACGAUCACGGCGGAGGGUUGAUUUCGCCGGAUCGGCUCAUGGACGUGGACGA
....(((((((((.(((((.((((((((......))))))))))))))..))..)))))))))....

 Initial dG =    -32.50

        10            20         30
ACAC        --  A-|    A         GG
    UCGCGUCC  GA  CGAUC CGGCGGAG  U
    GGUGCAGG  CU  GCUAG GCCGCUUU  U
AGCA        UA  CG^    -         AG
     60        50         40

-----------------------------------------------------
```

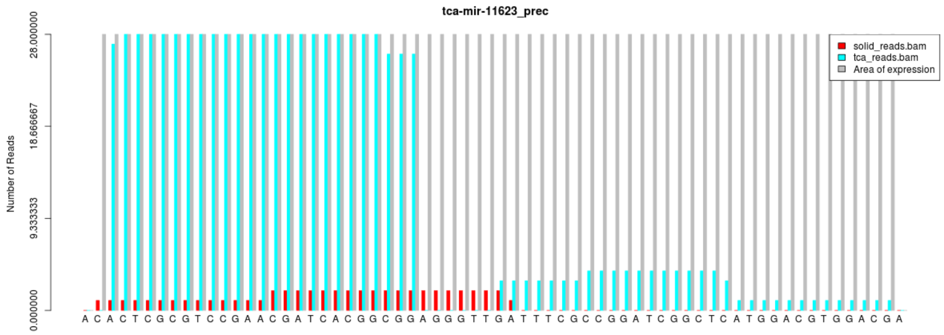

>tca-mir-11627_prec

```
AGUGUAGGCGAUUUUGACCCAUCUUCAGACAACUCUGGAUUGUUUUAAAUGUCCACGAGUUUGCUUUUGAUGGGAGAUCAAUAGUCUUUUAG
.....((((....((((((((((...((..((((((((((..........))))).)))))..))...))))))...)))))..)))).....

 Initial dG =    -25.70

        10           20         30          40
AGUGU    GAUU    ---      UUC  AC     -|    UGUU
     AGGC    UUGA   CCCAUC   AG  AACUC UGGAU    U
     UCUG    AACU   GGGUAG   UC  UUGAG ACCUG    U
GAUUU    AU--    AGA      UUU  GU     C^    UAAA
 90          80         70         60         50

-----------------------------------------------------
```

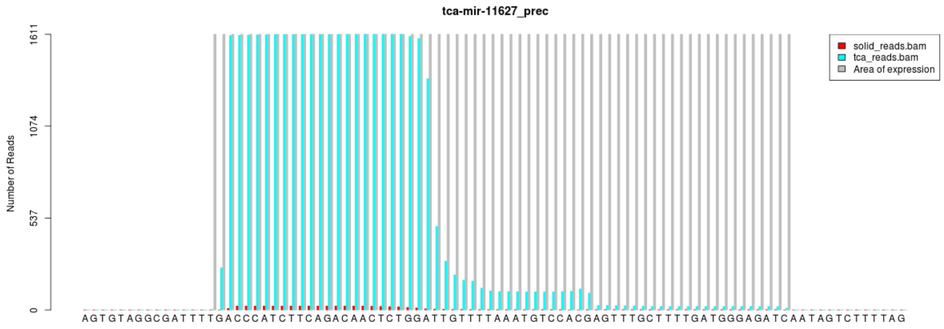

>tca-mir-11628_prec

```
GCAUCAAAAUAAGGCGUGAAAUUGCAGGAACUCUACUUACGUAAUGUUUAAUUUUAUCGUGAGUAGAGUUCUUGCAGUCAGUUUUAUUUUUUUAA
.....((((((((((.(((..(((((((((((((((((((((((((.........))).)))))))))))))))))))))))))))))))))))).....

 Initial dG =    -39.30

        10        20        30        40
GCAUC|         G   AA                    -   UGUU
     AAAAUAAGGC UGA  UUGCAGGAACUCUACUUACG UAA    \
     UUUUAUUUUG ACU  GACGUUCUUGAGAUGAGUGC AUU    U
AAUUU^         -   --                    U   UUAA
    90         80          70        60        50

-----------------------------------------------------
```

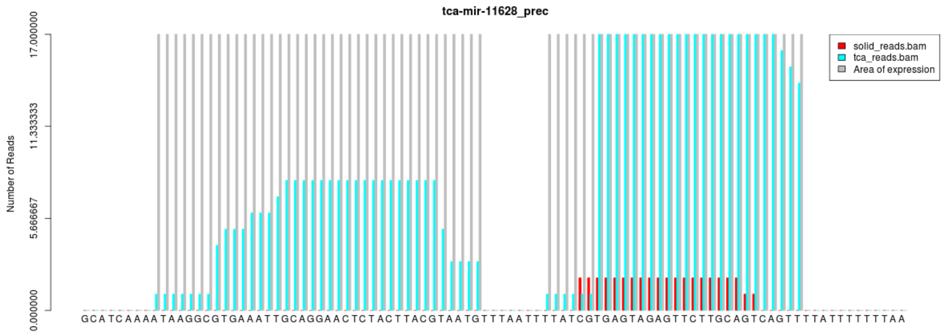

>tca-mir-11629_prec

AUUAAAUGUCAACAAUCGGAUCAUUUUGGACUUUUAUAAACAUUAAUAAAACAGAUGUUUAGAAAAGGACAAAUUGACCCGAUUAUUGAUAUAAUAU
.....(((((((.((((((.(((.((((..(((((.((((((((.........)))))))).))))).)))).))).)))))).))))))).....

```
 Initial dG =    -33.50

        10        20        30        40
AUUAA|      C      A   U    GA     A        AAUA
     AUGUCAA AAUCGG UCA UUUG  CUUUU UAAACAUU    \
     UAUAGUU UUAGCC AGU AAAC  GAAAA AUUUGUAG    A
UAUAA^      A      C   U    AG     G        ACAA
      90        80        70        60        50

-----------------------------------------------------
```

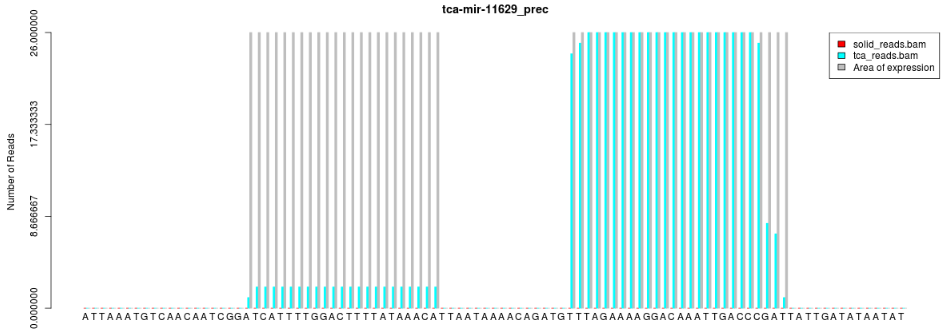

>tca-mir-11630_prec

```
CAGCUACUGGUCUAUUAGUCAGAUAUGGGUGGAAUGGUGAUGGUAAUCAUGAACACCCAUCAAAUUCAGUUAUGUCUGGCUGGAUACCAGUC
.....((((((...((((((((((((((.((((....((((((.............))))))..)))).))))))))))))))..)))))).
```

```
 Initial dG =    -34.40

        10        20        30        40
CAGCU|     CUA             G    AUGG      UAAUCA
     ACUGGU   UUAGUCAGAUAUGG UGGA    UGAUGG      \
     UGACCA   GGUCGGUCUGUAUU ACUU    ACUACC      U
C----^     UA-             G    AA--      CACAAG
     90         80        70          60

-----------------------------------------------------
```

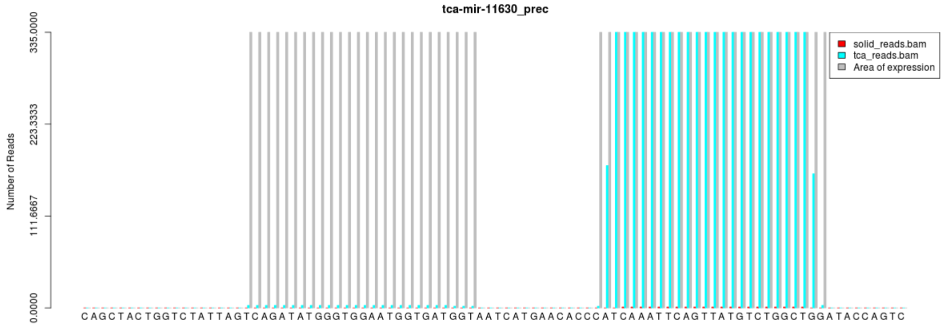

>tca-mir-11635_prec

```
GGCGUGCUUCGCUCCAUAGAUUUUGUAUUCGAGAAAUCUUUGGAGUGUUGAACGUCACA
((((....((((((.(((((((........))))))).)))))))....)))))...

 Initial dG =    -25.30

           10        20
---|    GCUU       U       GUA
   GGCGU    CGCUCCA AGAUUUU   U
   CUGCA    GUGAGGU UCUAAAG   U
ACA^    AGUU       U       AGC
        50        40        30

-----------------------------------------------------
```

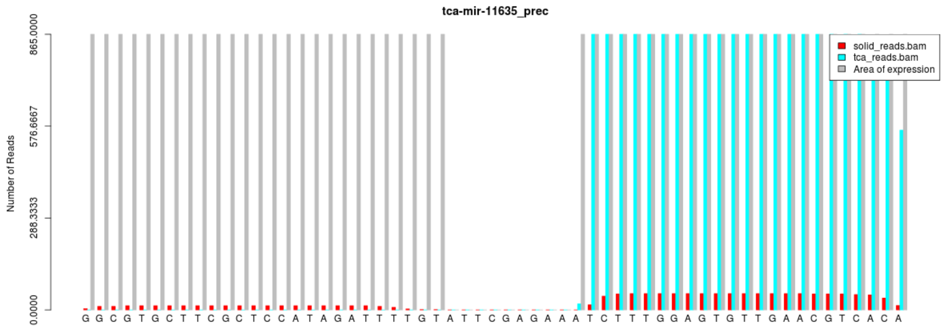

>tca-mir-11636_prec

```
UGAGGUCUCGUUUGCUAAAUUUUAAUUUAACAAAAUUUAGGAACAUGAUUCUCAGAC
((((((..((((.(((((((((........))))))))))))..))).))))...

 Initial dG =    -13.40

            10        20
---    -|  UC    G         AAU
   UGAG GUC  GUUU CUAAAUUUU   U
   ACUC UAG  CAAG GAUUUAAAA   U
CAG    U^  UA    -         CAA
      50         40        30

-----------------------------------------------------
```

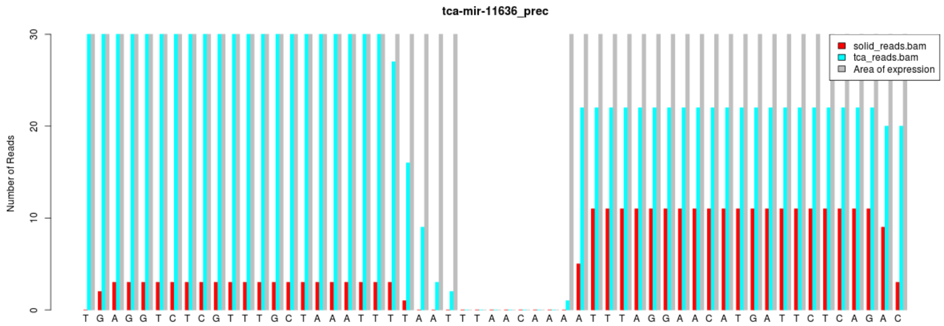

>tca-mir-11637_prec

UUUUAUAAAGGUUGAUUUGGCGCUCGAAUCCCAAGUUACUAUUCUAUGGGUUGAGUGCGCCAAAACCCUUAUAAAACGA
((((((((.((((...((((((((......((((((........)).))))......))))))))))).))))))))...

```
 Initial dG =    -26.40

           10        20        30
---|       A    GAU       UCGAAU    -  UUA
   UUUUAUAA GGUU   UUGGCGC      CCCA AG   C
   AAAAUAUU CCAA   AACCGCG      GGGU UC   U
AGC^       C    ---       UGAGUU    A  UUA
        70           60        50        40

-----------------------------------------------------
```

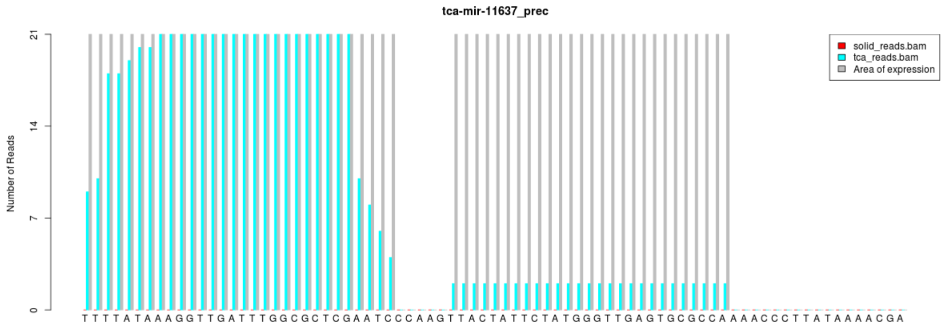

>tca-mir-11638_prec

```
UGAAAUCGUGCAUGUUUCUGUAAUGUUGGUUGCAUAGCAAAACCAUUUCAGGGCAUGACACGGGUUCGAGU
((((..(((((((((.(((.((((....((((...))))...)))).))))))))).))))..))))...

 Initial dG =    -23.00

           10         20        30
---    AU    -|     U   U    UUGG    A
   UGAA  CGUG CAUGUU CUG AAUG    UUGC \
   GCUU  GCAC GUACGG GAC UUAC    AACG U
UGA    GG    A^     -   U    CAA-    A
70        60         50         40

-----------------------------------------------------
```

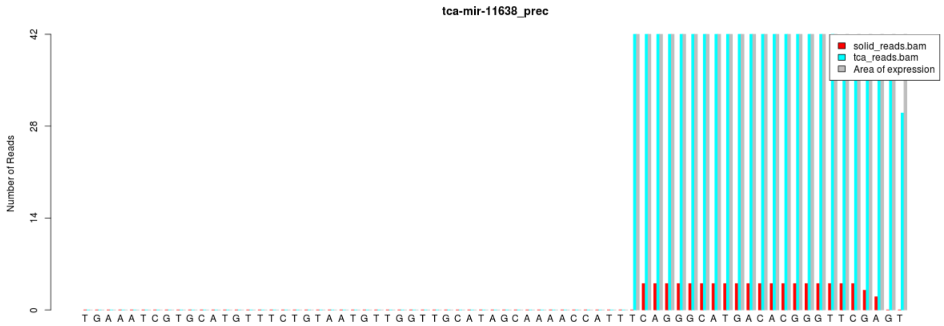

>tca-mir-11639_prec

```
GGGUAAGCAGAGGUUUUACAUUUUGUUUUUGAAGGAUGAGAUCCUCUUGCUUUCCCAAA
(((.(((((((((.(((.((((((........))))))))).))))).)))).)))...

 Initial dG =    -22.70

            10        20
---   U    -|    U   A      GUU
   GGG AAGC AGAGG UUU CAUUUU   U
   CCC UUCG UCUCC AGA GUAGGA   U
AAA   U    U^    U   -      AGU
        50        40         30

-----------------------------------------------------
```

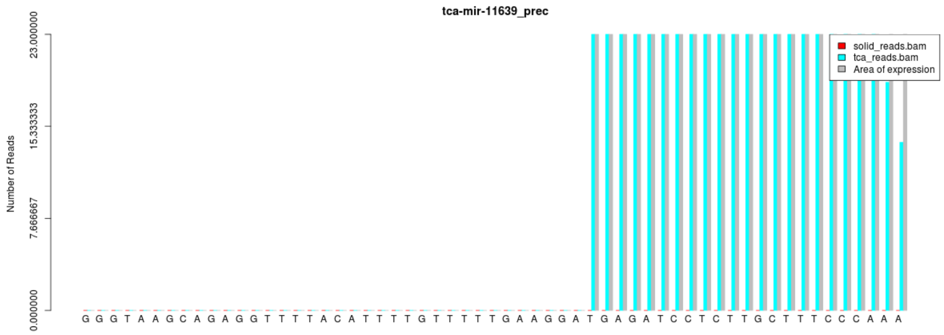

>tca-mir-11641_prec

UAAAUCUUUGAUCAAAGUGGCCUUCCAAUCACAGUCUACUUUGAUCGGAAAUUUAAUU
(((((.((((((((((((((.((.........)).))))))))))))))).))))).

```
 Initial dG =    -18.60

           10        20
---|    C              C  UCCA
   UAAAU UUUGAUCAAAGUGG CU    \
   AUUUA AGGCUAGUUUCAUC GA    A
UUA^    A              U  CACU
       50        40        30
```

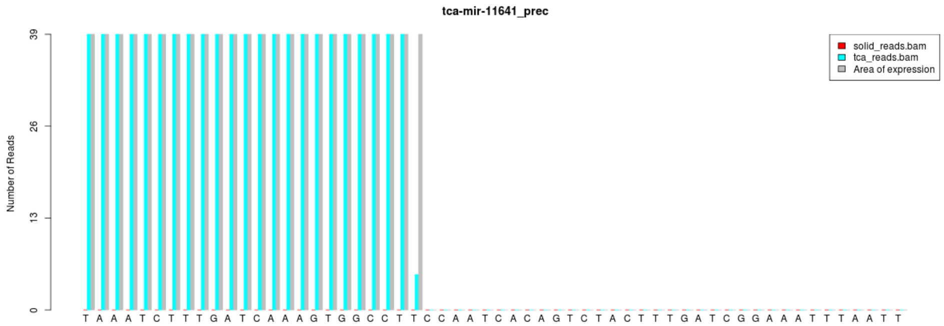

>tca-mir-11642_prec

```
UGGAAACAUUUGAUCGUACCAUGUUUCAGUUUCAAUCAUGGUACGAUUGAUGUUGCCUUA
.((.((((.(..(((((((((((.............)))))))))))..))))).))...

 Initial dG =    -23.70

          10        20
U--| A    U UG           UUUCAG
   GG AACA U  AUCGUACCAUG      \
   CC UUGU A  UAGCAUGGUAC      U
AUU^ G    - GU           UAACUU
.         50        40

-----------------------------------------------------
```

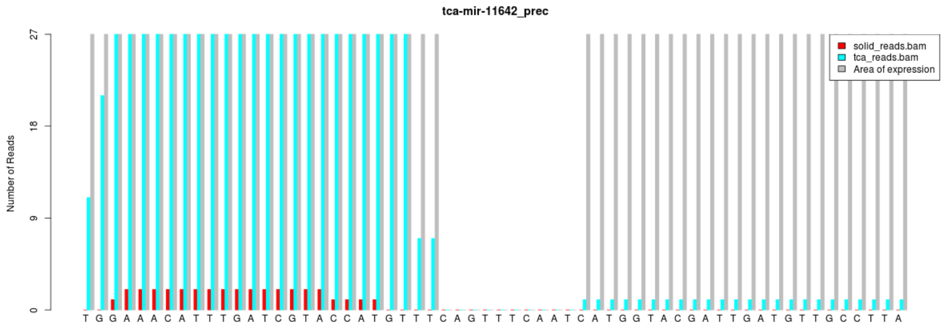

>tca-mir-11643_prec

```
UGGGGCUGUCGGUGCACGGCUCGCAAAUAAAAGUAGUGUCGUUGCGCCGGAAGCCCCGGGGGC
((((((..((((((((((((..((........))...))))).)))))))..)))))))).....

 Initial dG =    -33.00

             10         20
-----       GU      -     UC-| AAA
     UGGGGCU  CGGUGC ACGGC   GC   U
     GCCCCGA  GCCGCG UGCUG   UG   A
CGGGG       AG      U     UGA^ AAA
  60         50        40        30

-----------------------------------------------------
```

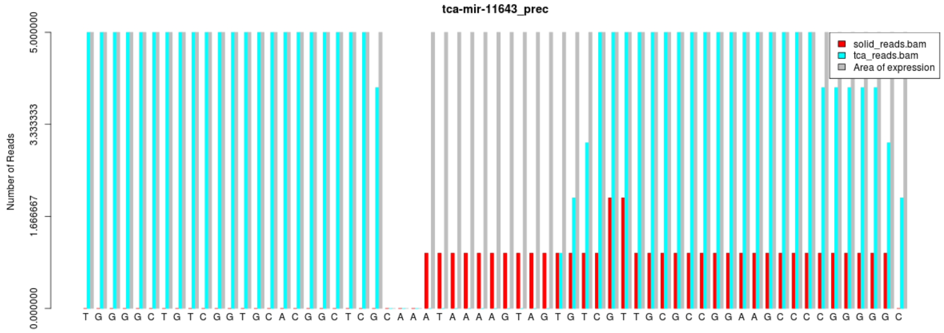

>tca-mir-11644_prec

```
UGUUACGUCCAAGGCGUCGGUAAGUAAAUUAGGCGUCGACGCCUUGGACGUAGGAACC
..((((((((((((((((((...((.......)).))))))))))))))))))).....

 Initial dG =    -31.40

           10        20
UG---|                 UAA  AAA
     UUACGUCCAAGGCGUCGG   GU   \
     GAUGCAGGUUCCGCAGCU   CG   U
CCAAG^                 G--  GAU
       50        40          30

-----------------------------------------------------
```

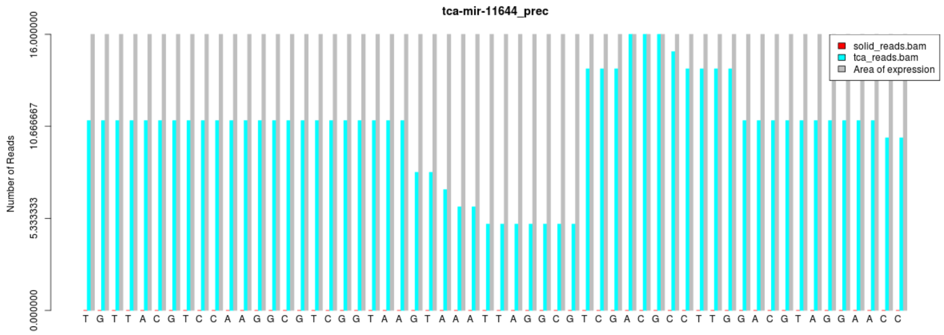

>tca-mir-11645_prec

```
UAUAGCCCGGACACUAACGAAAUCGCCAAACCGUCCGAGCUUUUGGUUAGCGUCCGCGCCUACAU
....((.(((((.(((((.(((..((............)).))).))))).))))).))......

 Initial dG =    -19.10

          10        20        30
UAUA--| C     A     G   UC  CAAAC
      GC CGGAC CUAAC AAA  GC     C
      CG GCCUG GAUUG UUU  CG     G
UACAUC^ C     C     G   U-  AGCCU
    60        50         40

-----------------------------------------------------
```

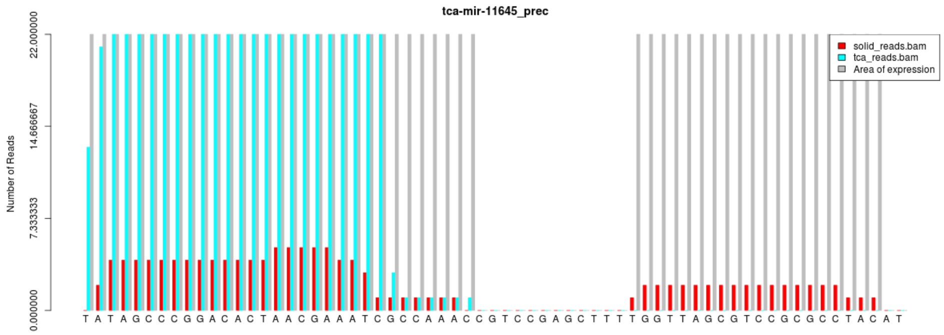

>tca-mir-11646_prec

```
CCAUGUUUGACUCCACCUCCAACCGCGACCACAUCCAAAACCACCAGGACCAUGGGGGUGUCGCUUGUAUAGAGAAAGAGGAAACAUGAUUU
.(((((((..(((...(((..((.(((((.((..(((...((....))....)))..)))))))..))...)))...))).)))))))....

 Initial dG =    -25.80

           10        20         30        40
C---       GA   CAC   CA-  C-|    C  AU   AAA-  A
    CAUGUUU  CUC   CUC   AC  GCGAC AC  CCA    CC C
    GUACAAA  GAG   GAG   UG  CGCUG UG  GGU    GG C
UUUA       G-   AAA   AUA  UU^    -  GG   ACCA  A
 90         80        70        60         50

-----------------------------------------------------
```

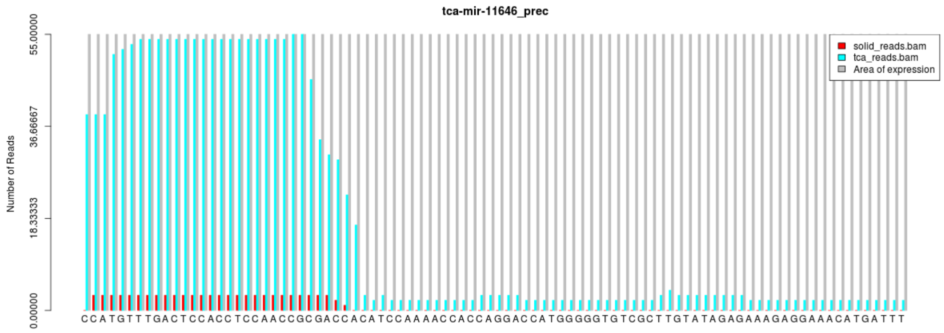

>tca-mir-11647_prec

```
CACCCGACACACCAAACUUUUAUUUCUAACACAGAUAAUAAAAAUAAAAGUUUGGAGUGUCGGGUGUUA
((((((((((.((((((((((((((.((..........)).))))))))))))))).))))))))))...

 Initial dG =    -34.20

           10         20         30
---|         A              C  ACAC
   CACCCGACAC CCAAACUUUUAUUU UA    A
   GUGGGCUGUG GGUUUGAAAAUAAA AU    G
AUU^         A              A  AAUA
        60         50         40

-----------------------------------------------------
```

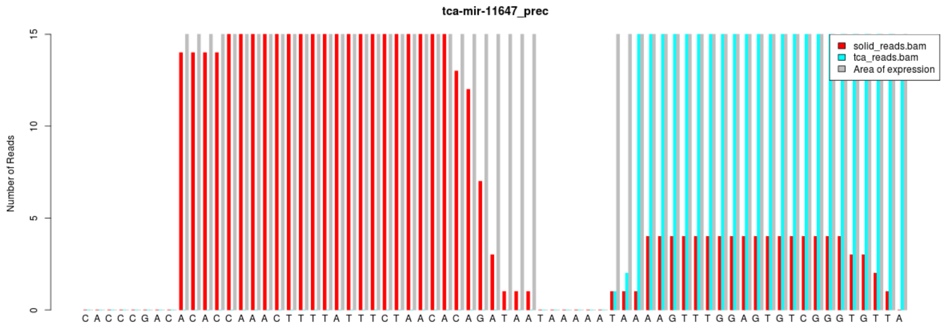

>tca-mir-11648_prec

```
UCACAAGAUUGAAUAGGACCGGGCUAAGAGGGUCACCCUGUCUCACUCCACGGAGCAUCCUUAUCGGUCUUGU
..((((((((((..(((((((((...(((.((.....)).)))....)).)))....)))).))))))))))

 Initial dG =    -23.50

        10            20          30
UC          AU    ----   -  CUA-|  G  UC
  ACAAGAUUGA  AGGA    CCG GG    AGA GG  \
  UGUUCUGGCU  UCCU    GGC CC    UCU UC  A
--          AU    ACGA   A  UCAC^  G  CC
    70        60        50        40

-----------------------------------------------------
```

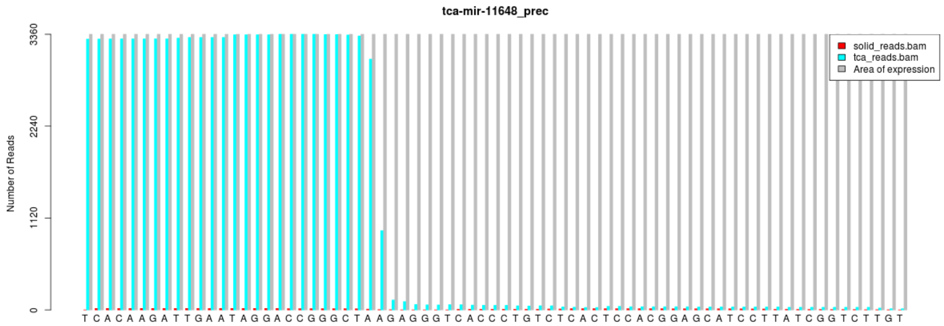

>tca-mir-11649_prec

```
CGGCCGGUCGCAGGCCAGAAAUUAUACCGUUGAGAGAUUUCUGCCUUUGACGGGUUCGCUU
(((((.((((.((((.(((((((...........))))))))))).)))).))).))...

 Initial dG =    -21.60

            10        20
---  -|  G    C    C       AUACC
   CG GCC GUCG AGGC AGAAAUU     G
   GC UGG CAGU UCCG UCUUUAG     U
UUC  U^  G    U    -       AGAGU
60        50         40        30

-----------------------------------------------------
```

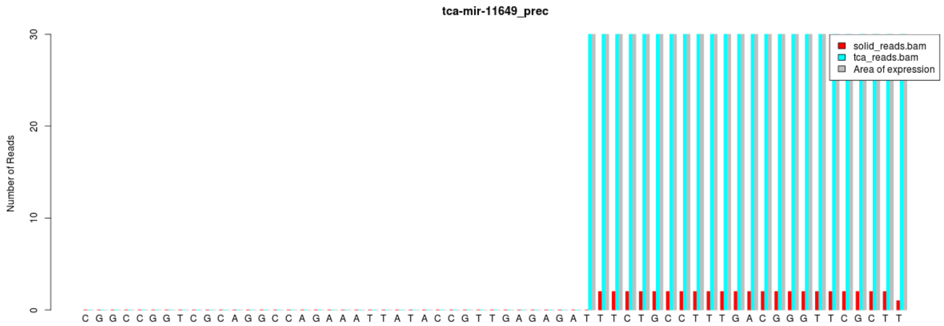

>tca-mir-11650_prec

```
CCCGAAAACGUCCUCGAAGUAACGGCACGCGCCAUCACUUACUACCUGGACGUCUCGGCGGA
.((((..((((((....(((((.(((....))).....)))))....)))))).)))))....

 Initial dG =    -19.60

           10        20
C---    AA      UCGA     C----|  A
    CCGA  ACGUCC    AGUAA     GGC C
    GGCU  UGCAGG    UCAUU     CCG G
AGGC    C-      UCCA     CACUA^  C
 60         50        40        30

-----------------------------------------------------
```

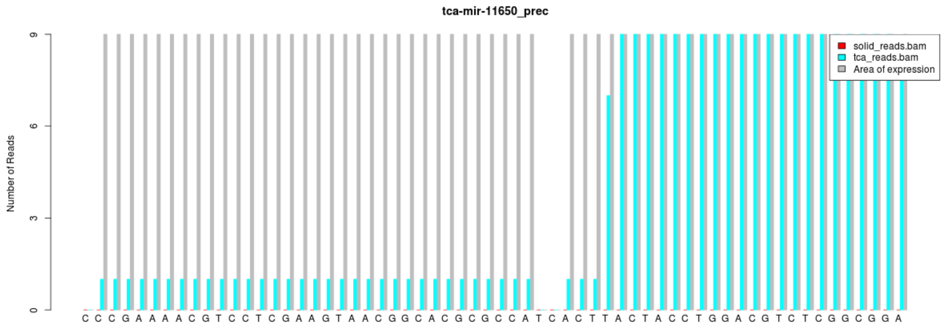

>tca-mir-276b_prec

```
AGCACGGUAUGAAGUUCCUACAGCCGAAAAAUCACGUAGGAACUCUAUACCUCGCUAGUG
(((..(((((.(((((((((.............))))))))).))))))..)))....

 Initial dG =    -22.30

            10        20
----|  AC      A         AGCCGA
    AGC  GGUAUG AGUUCCUAC      A
    UCG  CCAUAU UCAAGGAUG      A
GUGA^  CU      C         CACUAA
.        50        40        30

-----------------------------------------------------
```

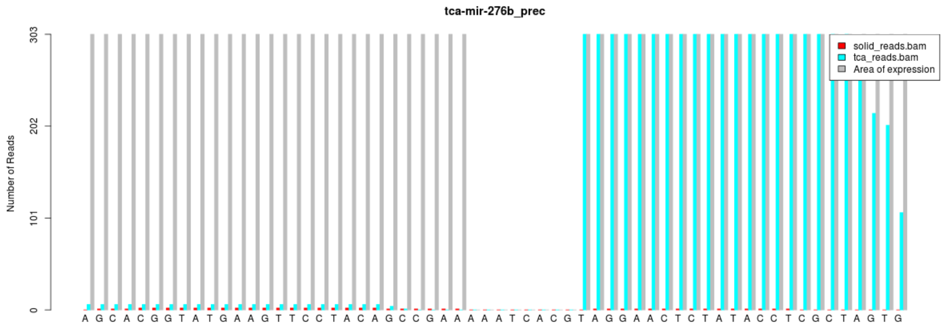

>tca-mir-307b_prec

```
UCACUCAAGGAGGUUGUGAUGGUACAGCAAACACAUCACACCCAGGUUGAGUGAGUCA
((((((((...((.(((((((((.......)).))))))).))...))))))))....
```

```
 Initial dG =    -22.30

            10        20
----        GGA  U       -| ACA
    UCACUCAA   GG UGUGAUG GU   \
    AGUGAGUU   CC ACACUAC CA   G
ACUG        GGA  C       A^ AAC
       50        40        30
```

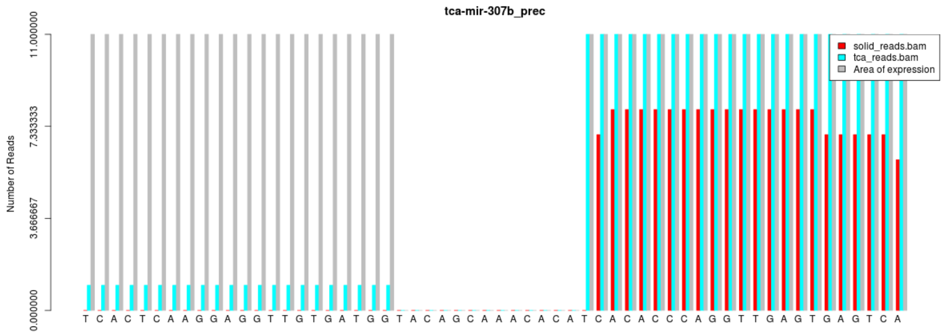

>tca-mir-309c_prec

```
AUUCCGGUACCACUUUUGGAUAAACCAUACUCAGUUGAUGUAUUAUAAAGCAUCACUGGGUAGAGUUUGUCCAAUAGUUUUACCACCAA
.....((((..(((.((((((((((..((((((((.(((((........)))))))))))))..)))))))))).)))..)))).....

 Initial dG =    -38.30

        10        20        30        40
AUUCC|   CC   U          CA        U     AUU
     GGUA  ACU UUGGAUAAAC  UACUCAGU GAUGU   A
     CCAU  UGA AACCUGUUUG  AUGGGUCA CUACG   U
AACCA^   UU   U          AG        -     AAA
        80        70        60          50

-----------------------------------------------------
```

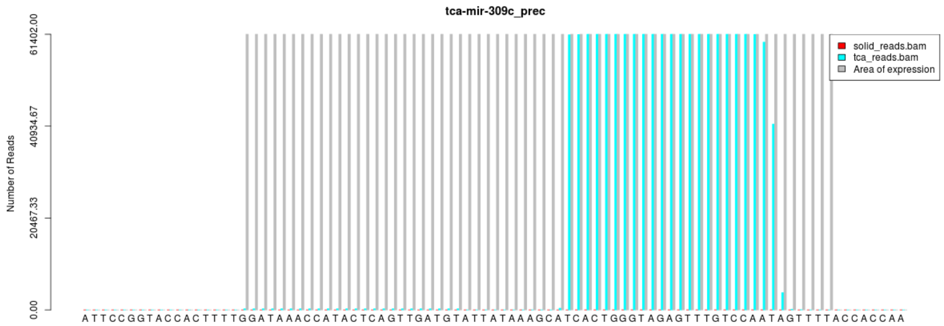

>tca-mir-3806b-1_prec

```
CUCAGUAAAUUUUCCUGUUUCUACGUACGGAUUCGUACAUAUUUUUUGAACAAUCAAAAUAUGUGUAUUCGUGCGUAGCAAUAGGGGGGUUUGGUGCA
..((.((((((..((((((.((((((((((....((((((((.((((((....)))))).))))))))))))))))))).)))))))..)))))).))..

 Initial dG =    -42.70

        10        20        30        40        50
CU| G      UU      U          AUUC        U      A
  CA UAAAUU  CCUGUU CUACGUACGG    GUACAUAU UUUUGA C
  GU GUUUGG  GGAUAA GAUGCGUGCU    UAUGUGUA AAAACU A
AC^ G      GG      C          ----        U      A
       90        80        70            60

-----------------------------------------------------
```

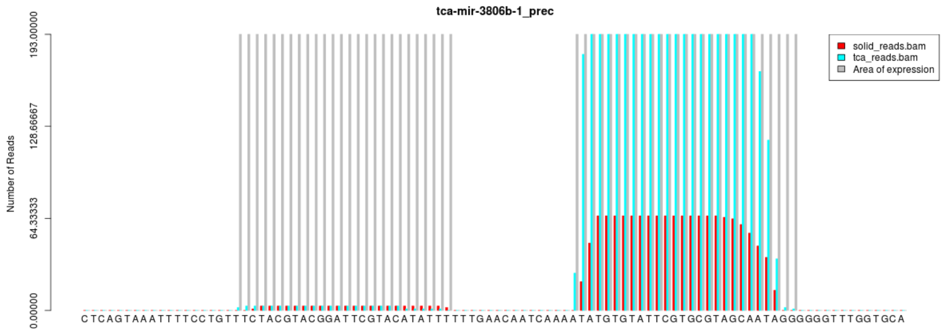

>tca-mir-3806b-2_prec

```
CAGUCAAUUUUCCUGUUUCUACGUACGGAUUCGUACAUAUUUUUUGAAUAAUCAAAAUAUGUGUAUUCGUGCGUAGCAAUAGGGGGGUUUGGUG
...((((((..((((((.((((((((((....((((((((.((((((....)))))).))))))))))))))))))).)))))))..)))).))..

 Initial dG =    -37.90

         10        20        30        40
CAG  -|   UU      U          AUUC        U      A
   UC AAUU  CCUGUU CUACGUACGG    GUACAUAU UUUUGA U
   GG UUGG  GGAUAA GAUGCGUGCU    UAUGUGUA AAAACU A
GU-  U^   GG      C          ----        U      A
    90        80        70            60        50

-----------------------------------------------------
```

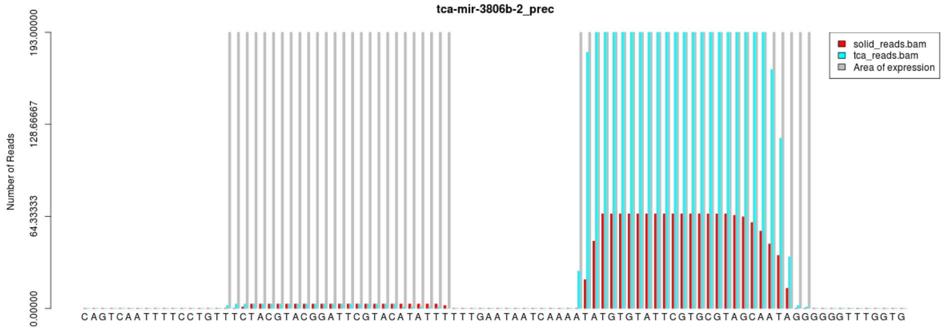

>tca-mir-3806b-3_prec

UUGGUCAGUCAACAGCUUUGCUGUGUAGGUUGAGGAGCAUCUCAGUCAAUUUUCCUGUUUCUACGUACGGAUUCGUACAUAUUUUUUGAACAAUUAAAAUAUGUGUAUUCGUGCGUAGCA
AUAGGGGGGUUUGGUGCAAUCAACGGCAUCGUAUAUUUUAUGUUGGUUGUAAUC

```
.....((..(((((....(((.((((..(((((...(((((......((((..((((((.((((((((((((...
((((((((((..........)))))))))).))))))))))))).))))))..)))).)))))..))))).)))).))).......)))))..)).....

 Initial dG =    -59.60

        10           20         30         40         50         60         70         80
90
UUGGU  GU     GCUU---|  U    AG     GGA     UCAGUC    UU      U            UCG
UUGA
     CA  CAACA       UGC GUGU  GUUGA   GCAUC      AAUU  CCUGUU CUACGUACGGAU   UACAUAUUUU
A
     GU  GUUGU       AUG UACG  CAACU   CGUGG      UUGG  GGAUAA GAUGCGUGCUUA   GUGUAUAAAA
C
CUAAU  UG     AUUUUAU^  C    G-     AA-     U-----    GG      C            U--
UUAA
  170       160       150        140              130       120       110         100
```

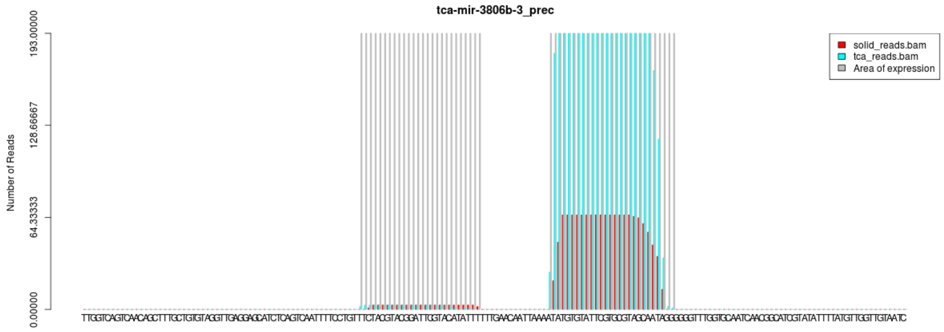

>tca-mir-3806b-4_prec
UGUAGGUUGAGGAGCAUCUCAGUCAAUUUUCCUGUUUCUACGUACGGAUUCGUACAUAUUUUUUAAACAAUCAGAAUAUGUGUAUUCGUGCGUAGCAAUAGGGGGAUUUGGUGCAAUCAA
CGGCGU
.((..(((((...(((((......((((..((((((.((((((((((((...
((((((((((..........)))))))))).))))))))))))).))))))..)))).)))))..))))).))..

```
 Initial dG =    -46.80

         10        20        30        40        50        60
U-| AG     GGA     UCAGUC    UU      U            UCG           UUAA
  GU  GUUGA   GCAUC      AAUU  CCUGUU CUACGUACGGAU   UACAUAUUUU    A
  CG  CAACU   CGUGG      UUAG  GGAUAA GAUGCGUGCUUA   GUGUAUAAGA    C
UG^ G-     AA-     U-----    GG      C            U--           CUAA
     120        110            100        90          80        70

-----------------------------------------------------
```

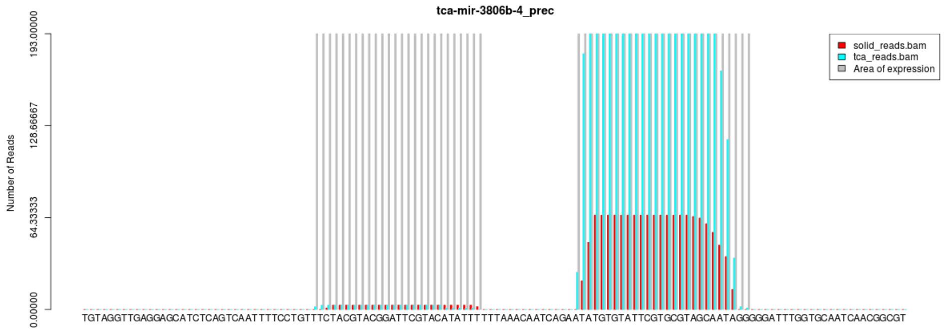

>tca-mir-3806c_prec

```
CUACGUACGGAUACGUACAUAUUUUUUGAACAAUCAAAAUAUGUGUAUUCGUGCGUAGCAU
((((((((((((((..((((((((..(((....)))))))))))))))))))))))))...

 Initial dG =    -25.60

           10         20         30
---|             GU         UU   A
   CUACGUACGGAUAC  ACAUAUUU  UGA C
   GAUGCGUGCUUAUG  UGUAUAAA  ACU A
UAC^             --         --   A
60         50           40

-----------------------------------------------------
```

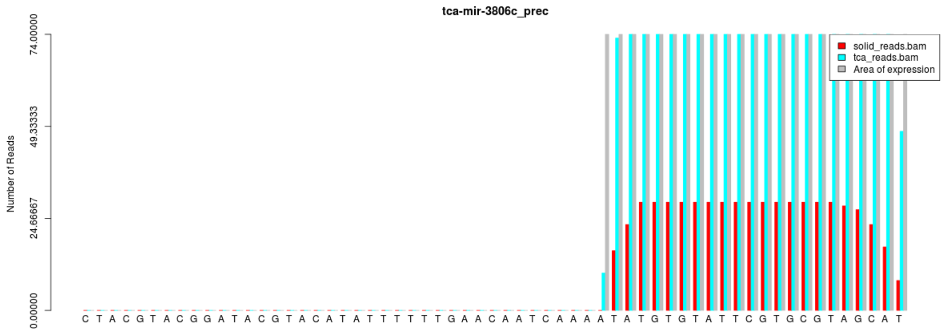

>tca-mir-3836c-1_prec

```
UUUCCGAAAUUGCUGCCAGGUCCACCUUUUGCUUUUGUUGAUAUUUAUUUAACUAUCACUGGGCAAGGUAGACCUUGGAGUUGUUUCUUAAA
.....((((.(((.(((((((.((((..(((((..((.((((.((....)).)))))).))))))))).)))).)))))).))))).....

 Initial dG =    -28.40

        10          20         30         40
UUUCC|    U   G   -    C    UU     UU  U    U  A
     GAAAU GCU CCA GGUC ACCU  UGCUU  GU GAUA UU U
     CUUUG UGA GGU CCAG UGGA  ACGGG  CA CUAU AA U
AAAUU^    U   -   U    A    --     U-  -    C  U
 90        80         70          60          50

-----------------------------------------------------
```

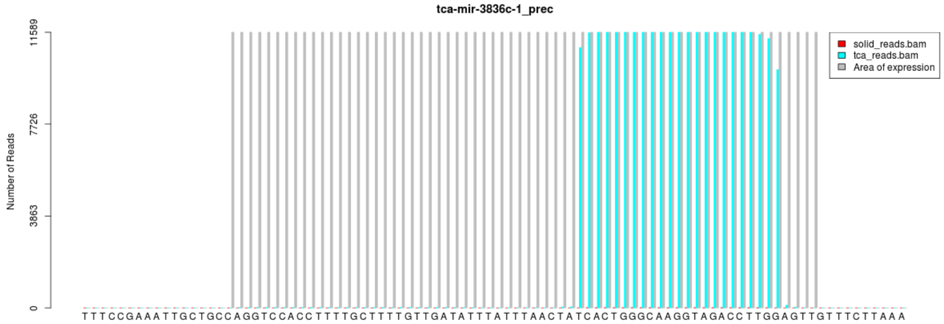

>tca-mir-3836d_prec

UUUUCGUAAUUUCUGCUCGGUCUAUCCCUGCUUUGGUUGAUAUUUAUUUAAUUAUCACUGGGCAAAGUAGGCCCUGCAGUAGUUUCUUCAA
.......((((.((((..(((((((...(((.(..((.((((..........))))))..)))).)))))))..)))).)))).......

```
 Initial dG =    -24.20

        10        20        30        40
UUUUCGU|   U    UC       CCC   U UG  U    UUUA
       AAUU CUGC  GGUCUAU   UGC U  GU GAUA    U
       UUGA GACG  CCGGAUG   ACG G  CA CUAU    U
AACUUCU^   U    UC       AA-   - GU  -    UAAU
90        80        70          60         50

-----------------------------------------------------
```

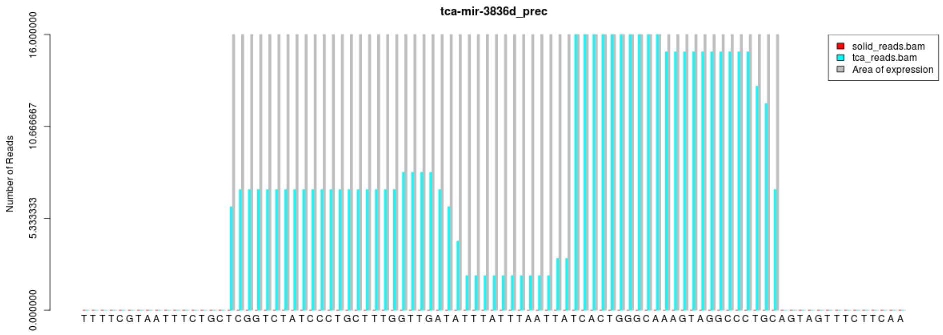

>tca-mir-3836c-2_prec

```
UUUUCGAAAUUUCUGCCAGGUCCACCUUUUGCUUUUGUUGAUAUUUAUUUAUCUAUCAUUGGGCAAGGUAGACCCUGGAGUGGUUUCUUAAA
.....(((((..((.(((((((.((((..(((((.((..((((......))))...))..))))))))).)).)))))))..))))).....

 Initial dG =    -29.10

        10        20         30          40
UUUUC|    UU  G     -  C    UU     U-  UU-    UU
     GAAAU  CU CCAGG UC ACCU  UGCUU  UG   GAUA  U
     CUUUG  GA GGUCC AG UGGA  ACGGG  AC   CUAU  A
AAAUU^    GU  -     C  A    --     UU  UAU    UU
 90        80         70          60        50

-----------------------------------------------------
```

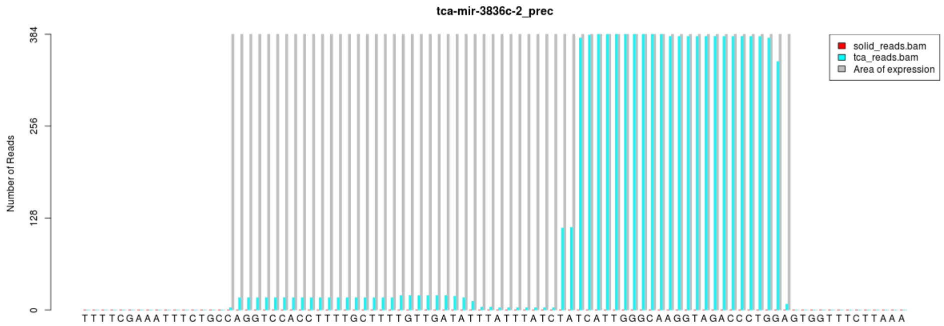

>tca-mir-3836e_prec

AUUUUCUGCUUGGUCUAUCCUUGCUUUGGUUGAUAUUUAUUUAAUUAUCACUGGGCAAAGUAGACCUUGCAGUAGUU
.....((((..(((((((..((((.(..((.((((..........))))))..))))).))))))).)))).....

```
 Initial dG =    -26.30

        10        20        30        40
AUUUU|   UU        CC    U UG  U    UUUA
     CUGC  GGUCUAU  UUGC U  GU GAUA    U
     GACG  CCAGAUG  AACG G  CA CUAU    U
UUGAU^   UU        A-    - GU  -    UAAU
      70        60          50
```

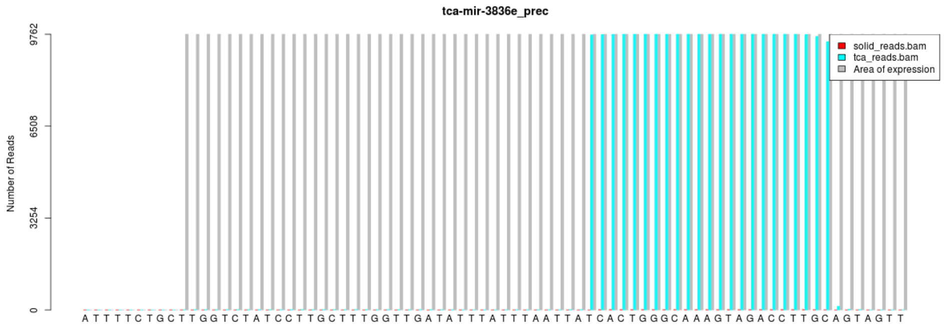

>tca-mir-3836c-3_prec

```
UUUUCGAAAUUUCUGCCAGGUCCACCUUUUGCUUUCGUUGAUAAUUAUUUAACUAUCACUGGGCAAGGUAGACCCUGGAGUGGUUUCUUAAA
.....(((((..((.(((((((.((((..(((((....(((((.((....)).)))))..))))))))).)).))))))).)))))).....

 Initial dG =    -27.40

        10        20         30        40
UUUUC|    UU  G     -  C    UU     UCGU     A  A
     GAAAU  CU CCAGG UC ACCU  UGCUU    UGAUA UU U
     CUUUG  GA GGUCC AG UGGA  ACGGG    ACUAU AA U
AAAUU^    GU  -     C  A    --     UC--     C  U
 90        80         70          60          50

-----------------------------------------------------
```

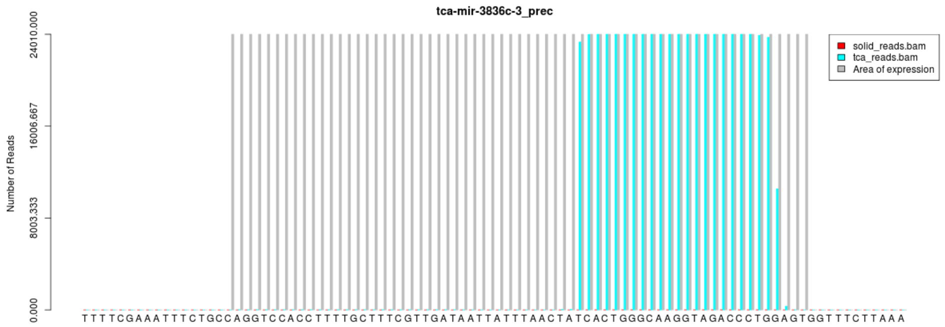

>tca-mir-3836f_prec

```
AUUUUCUGCUCGGUCUAUCCUUGCUUCGGUUGAUAUUUAUUUAAUUAUCACUGGGGAAAGUAGACCUUGCAGUAGUU
.....((((..((((((..(((.(((((((.((((..........))))))))))).)))))))))..)))).....

 Initial dG =    -25.20

        10        20        30        40
AUUUU|   UC      UC   G       U    UUUA
     CUGC  GGUCUA  CUU CUUCGGU GAUA    U
     GACG  CCAGAU  GAA GGGGUCA CUAU    U
UUGAU^   UU      --   A       -    UAAU
      70          60        50

-----------------------------------------------------
```

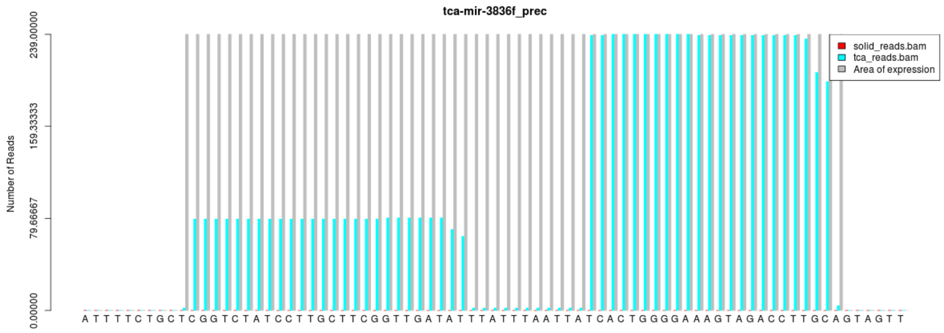

>tca-mir-3836h_prec

GGUCUAUCCUUGCUUAGAUUGAUAUUUAUUUUAUUGUCACUGGGCAAGGUAGACCCUA

```
((((((.((((((((((..(((((..........)))))))))))))))))))))...

 Initial dG =    -26.20

           10        20
---|     U          AU     UUUA
   GGUCUA CCUUGCUUAG  UGAUA    U
   CCAGAU GGAACGGGUC  ACUGU    U
AUC^     -          --     UAUU
       50         40          30
```

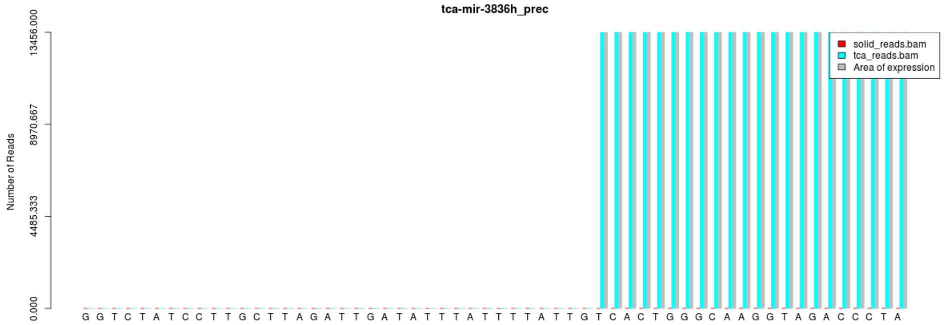

>tca-mir-3836i-1_prec

CGGUCUAUCUUUGUUUAGACUGAUAUUUAUUUAAUUAUCACUGGGCAAAGUGGAUCUUGC
.((((((.((((((((((..(((((..........)))))))))))))))))))))....

```
 Initial dG =    -19.10

           10        20        30
C---|     U          AC     UUUA
    GGUCUA CUUUGUUUAG  UGAUA    U
    CUAGGU GAAACGGGUC  ACUAU    U
CGUU^     -          --     UAAU
.         50          40
```

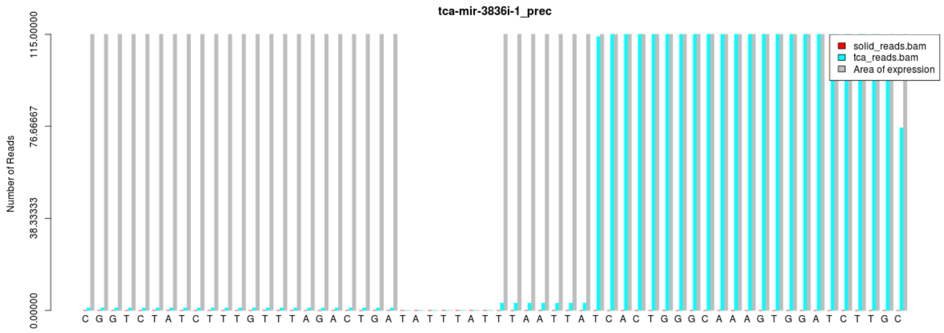

>tca-mir-3836i-2_prec

```
CGGUCUAUCUUUGUUUAGACUGAUAUUUAUUUAAUUAUCACUGGGCAAAGUGGAUCUUGC
.((((((.((((((((((..(((((..........))))))))))))))))))))))....

 Initial dG =    -19.10

           10        20        30
C---|     U          AC     UUUA
    GGUCUA CUUUGUUUAG  UGAUA    U
    CUAGGU GAAACGGGUC  ACUAU    U
CGUU^     -          --     UAAU
.         50          40

-----------------------------------------------------
```

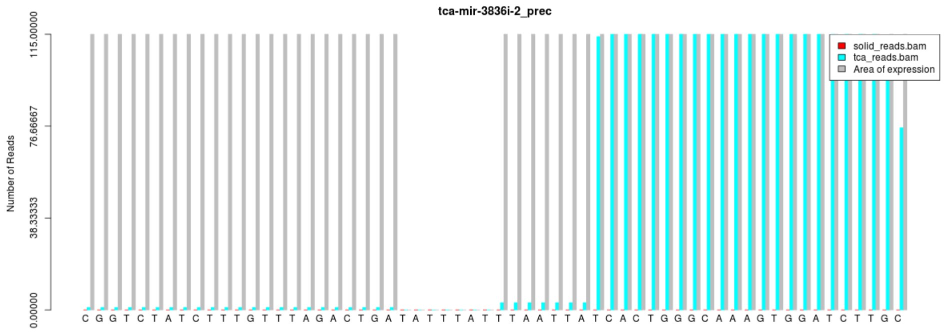

>tca-mir-3836j_prec

```
GGCCUCUCCUUGCUUUGGUUGAUAUUUAUUUAAUUAUCACUGGGCAAAGUAGACCCUGC
((..(((.(((((.(..((.((((..........))))))..))).))).))).))...

 Initial dG =    -11.00

           10         20
---| CC   C   -  U UG  U    UUUA
   GG  UCU CUU GC U  GU GAUA    U
   CC  AGA GAA CG G  CA CUAU    U
CGU^ C-   U   A  - GU  -    UAAU
         50         40         30

-----------------------------------------------------
```

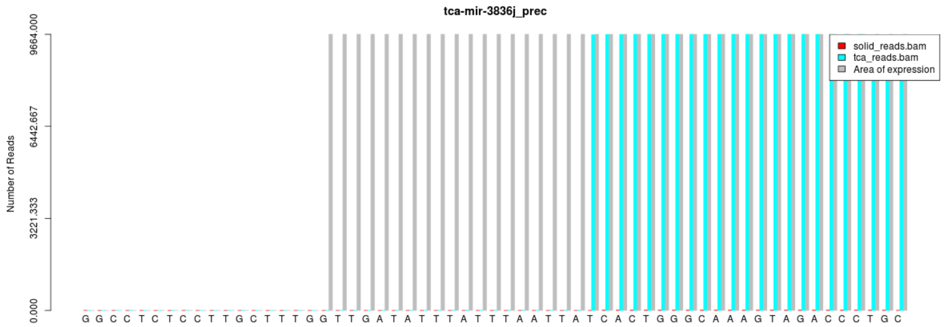

>tca-mir-3851m-1_prec

```
UUUUUUGAGUAAAUUUAGUACUGGGUUGGCACAGAGUAGUACUAUGUAAAAUUCUAUUCUAUAGUACGACCCAUGUCGGCCCAGUAAGAGAUUUCAAUUCACCAGC
.....(((((((((((..(((((((((((((....((.((((((((.((.......)).)))))))).))...)))))))))))))..))))))..))))).....
```

```
 Initial dG =    -34.50

        10          20         30         40         50
UUUUU     --|     AG             CAGA  A         U  AAU
     UGAGU  AAAUUU  UACUGGGUUGGCA    GU GUACUAUG AA   \
     ACUUA  UUUAGA  AUGACCCGGCUGU    CA CAUGAUAU UU   U
CGACC     AC^     GA             ACC-  G         C  AUC
    100        90         80         70         60
```

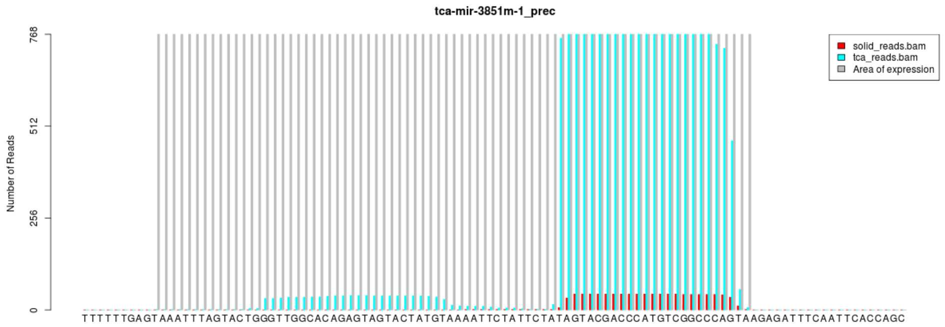

>tca-mir-3851l-1_prec

```
GAAUAAAUUUAGUGCUGGGUUGGCAUAAGGUGGUACCAUGUAACAUUUUAACCCAUAGUACGACCCAUGCCGACUCAGUAAUAAAUUACAAU
.....((((((.((((((((((((((..(((.((((.(((.............))).)))).))).)))))))))))))).)))))).....

 Initial dG =    -35.40

        10        20        30        40
GAAUA|     G              AA   G    C   UAACAU
     AAUUUA UGCUGGGUUGGCAU  GGU GUAC AUG      \
     UUAAAU AUGACUCAGCCGUA  CCA CAUG UAC      U
UAACA^     A              C-   G    A   CCAAUU
 90        80        70         60        50

-----------------------------------------------------
```

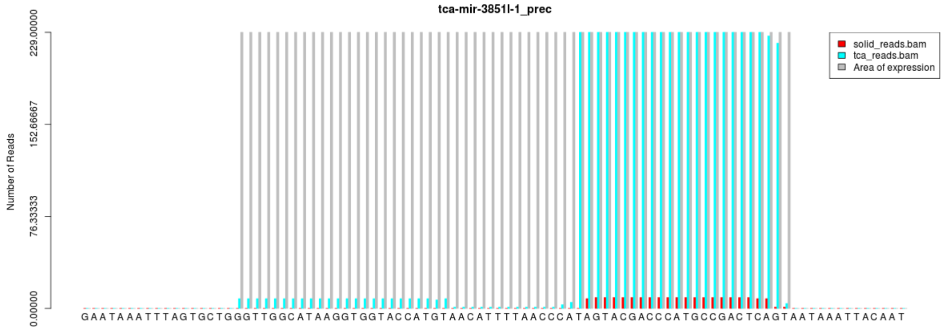

>tca-mir-3851n-1_prec

AUUUUUGAGUAAAUUUAGUACUGGGUUGGCACAGAGUAGUACUGUGUAAAAUUCUAUUUCAUAGUACGACCCAUGCCGGCCUAGUAAGAAAUUUCAAUUCACCAGC
.....((((((((((..(((((((((((((....((.((((((((.(((......)))))))))))).))...)))))))))))))..))))))..))))).....

```
 Initial dG =    -37.60

        10         20        30        40        50
AUUUU     --|     AG             CAGA  A        U   AU
     UGAGU  AAAUUU  UACUGGGUUGGCA    GU GUACUGUG AAA  U
     ACUUA  UUUAAA  AUGAUCCGGCCGU    CA CAUGAUAC UUU  C
CGACC     AC^     GA             ACC-  G        -   AU
    100        90        80         70        60
```

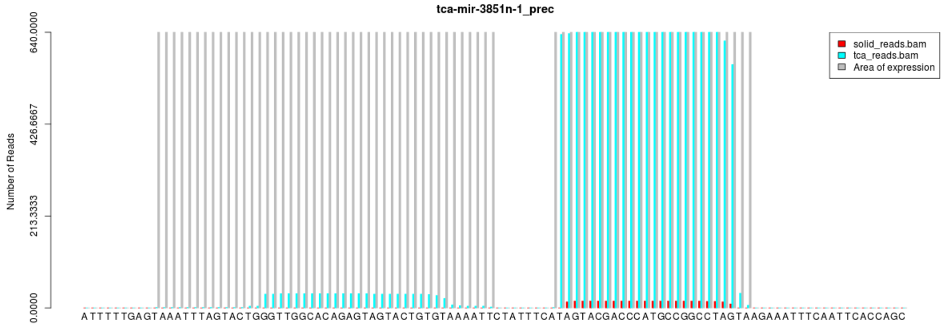

>tca-mir-3851o-2_prec

UAUUAAAUUUAGUGCUUGGUUGGCGUGGAGUGGUACCAUGUAAAAUUUUAAUCCAUAGUACGACCCAUUCCGGCCCAGUAAUAAAUUAAAAU
.....((((((.((((.((((((.((((.((.((((.(((.............))).)))).)))))).)))))).)))).))))))).....

```
 Initial dG =    -29.80

        10        20        30        40
UAUUA|     G    U      C    A  G    C   UAAAAU
     AAUUUA UGCU GGUUGG GUGG GU GUAC AUG      \
     UUAAAU AUGA CCGGCC UACC CA CAUG UAC      U
UAAAA^     A    C      U    -  G    A   CUAAUU
 90        80        70         60        50
```

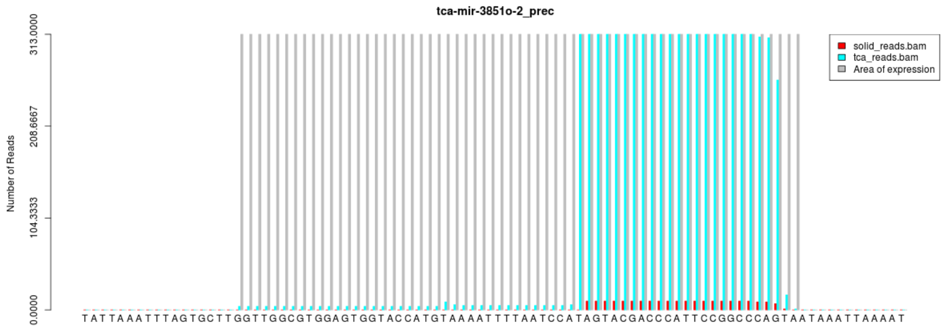

>tca-mir-3851k-1_prec

```
GCAUAAAUUUAGUGCUGGGUUGGCAAAAGGUGGUACCAUGUAACAUUUUAACCCAUAGUACGACCCAUGCCGGCUCAGUAAUAAAUUACAAU
.....((((((.(((((((((((((...(((.((((.(((.............))).)))).)))..))))))))))))).))))))).....

 Initial dG =    -33.90

        10        20        30        40
GCAUA|     G             AAA   G    C   UACAU
     AAUUUA UGCUGGGUUGGCA   GGU GUAC AUG      \
     UUAAAU AUGACUCGGCCGU   CCA CAUG UAC      U
UAACA^     A             AC-   G    A   CCAAUU
 90        80        70         60        50

-----------------------------------------------------
```

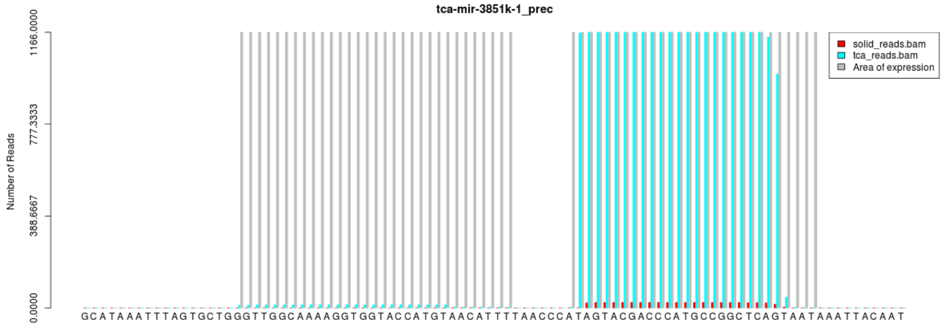

>tca-mir-11618b-1_prec

```
CAAAAUGUUUUCGAUACUUAGUUAGCAUUGGCUGUUUCUAUGCUAUUUUAUGGUCAUAGUACAGCCAGUGCUCACUUCAUUUCGGAUUCAAUAAC
.....((..(((((.....(((.((((((((((((..(((((((((...)))).))))).))))))))))))).))).....)))))..)).....

 Initial dG =    -37.40

        10        20        30        40
CAAAA| UU     UACUU   U            UU     -    U
     UG  UUCGA     AGU AGCAUUGGCUGU  CUAUG CUAU \
     AC  AGGCU     UCA UCGUGACCGACA  GAUAC GGUA U
CAAUA^ UU     UUACU   C            U-     U    U
    90        80        70        60         50

-----------------------------------------------------
```

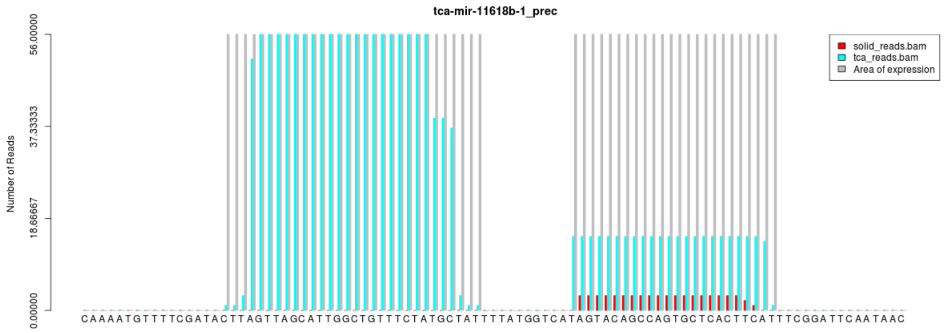

>tca-mir-3851o-1_prec

UAUUAAAUUUAGUGCUUGGUUGGCGUAGAGUGGUACCAUGUAAAAUUUUAAUCCAUAGUACGACCCAUUCCGGCCCAGUAAUAAAUUAAAAU
.....((((((.((((.((((((.((...((.((((.(((.............))).)))).))..)).)))))).)))).)))))).....

```
 Initial dG =    -21.90

        10        20        30        40
UAUUA|     G    U      C  AGA  G    C   UAAAAU
     AAUUUA UGCU GGUUGG GU   GU GUAC AUG      \
     UUAAAU AUGA CCGGCC UA   CA CAUG UAC      U
UAAAA^     A    C      U  CC-  G    A   CUAAUU
 90        80        70         60        50
```

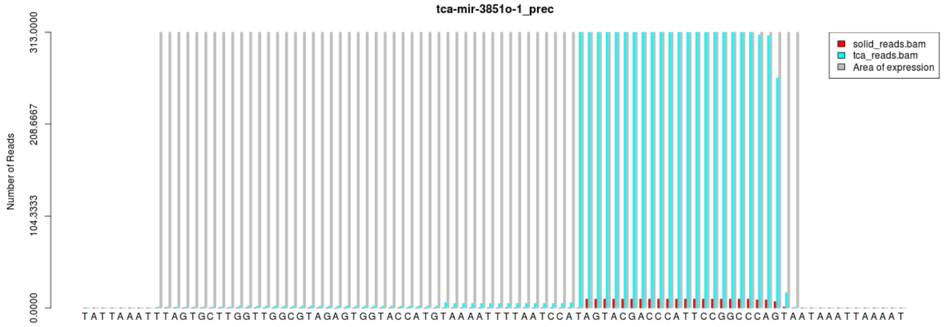

>tca-mir-3851l-2_prec

```
GAAUAAAUUUAAUGCUGGGUUGGCAUAAGGUGGUACCAUGUAACAUUUUAACCCAUAGUACGAUCCAUGCCGACUCAGUAAUAAAUUACAAU
.....((((((.((((((((((((((..(((.((((.(((.............))).)))).))).)))))))))))))).))))))).....

 Initial dG =    -32.80

        10        20        30        40
GAAUA|     A              AA   G    C   UAACAU
     AAUUUA UGCUGGGUUGGCAU  GGU GUAC AUG      \
     UUAAAU AUGACUCAGCCGUA  CUA CAUG UAC      U
UAACA^     A              C-   G    A   CCAAUU
 90        80        70         60        50

-----------------------------------------------------
```

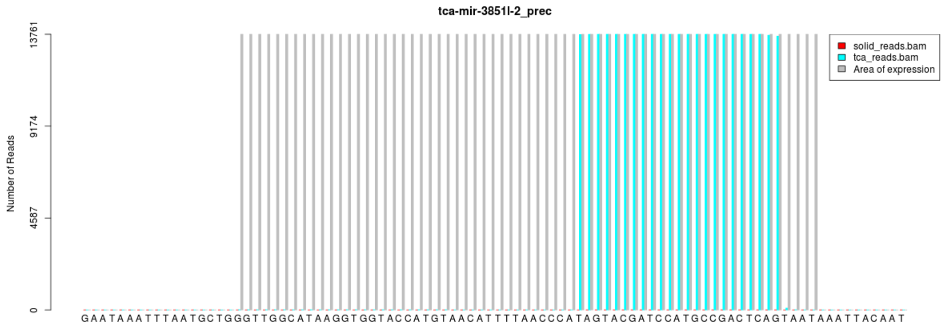

>tca-mir-11618b-2_prec

CAAAAUGUUUUCGAUACUUAGUUAGCAUUGGCUGUUUCUAUGCUAAUUUAUGGUCAUAGUACAGCCAGUGCUCACUUCAUUUCGGAUUCAAUAAC
.....((..((((.....(((.((((((((((((..((((((((.....))).)))))).))))))))))))).))).....)))))..)).....

```
 Initial dG =    -37.20

        10        20        30        40
CAAAA| UU     UACUU   U            UU     -   AU
     UG  UUCGA     AGU AGCAUUGGCUGU  CUAUG CUA  \
     AC  AGGCU     UCA UCGUGACCGACA  GAUAC GGU  U
CAAUA^ UU     UUACU   C            U-     U   AU
    90        80        70        60         50
```

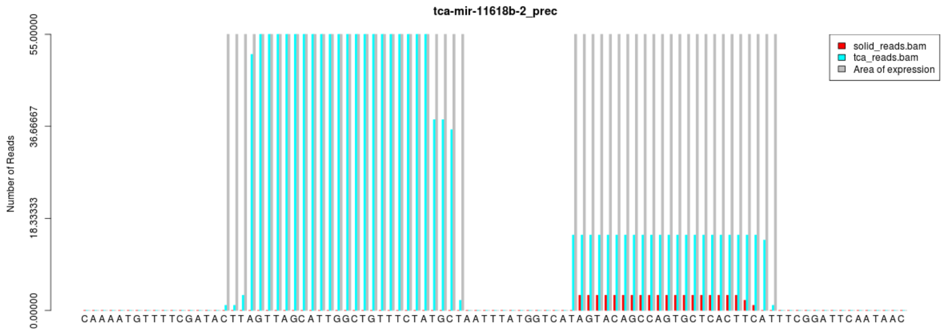

>tca-mir-3851k-2_prec

GCAUAAAUUUAGUGCUGGGUUGGCAAAAGGUGGUACCAUGUAACAUUUUAACCCAUAGUACGACCCAUGCCGGCUCAGUAAUAAAUUAUAAU
.....((((((.(((((((((((((...(((.((((.(((.............))).)))).)))..))))))))))))).))))))).....

```
 Initial dG =    -33.90

        10        20        30        40
GCAUA|     G             AAA   G    C   UAACAU
     AAUUUA UGCUGGGUUGGCA   GGU GUAC AUG      \
     UUAAAU AUGACUCGGCCGU   CCA CAUG UAC      U
UAAUA^     A             AC-   G    A   CCAAUU
 90        80        70         60        50
```

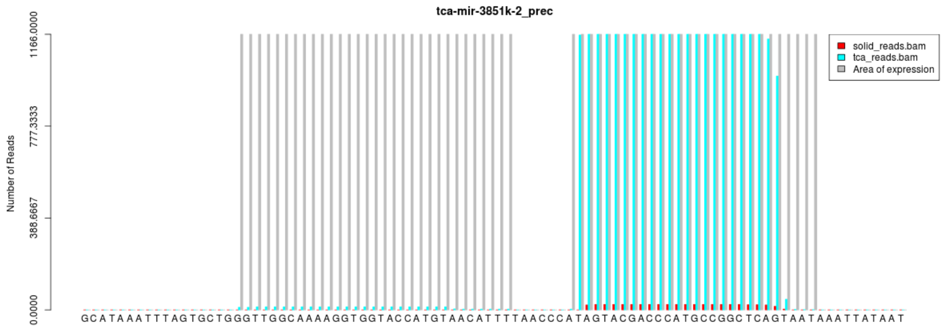

>tca-mir-3851g-1_prec

AAUUAAAUUUAAUACUGAGUGGACAUGGGGUGAUACUGUGUAAUUUUUGACUCAUAGUACGACCCAUGUUGGCUCAGUAAUAAAUUACAUU
.....((((((.((((((((.((((((((.((.(((((((............))))))))).)))))))).)))))))).))))))).....

```
 Initial dG =    -38.00

        10        20        30        40
AAUUA|     A        G        G  A       UAAUU
     AAUUUA UACUGAGU GACAUGGG UG UACUGUG     U
     UUAAAU AUGACUCG UUGUACCC GC AUGAUAC     U
UUACA^     A        G        A  -       UCAGU
90        80        70        60         50
```

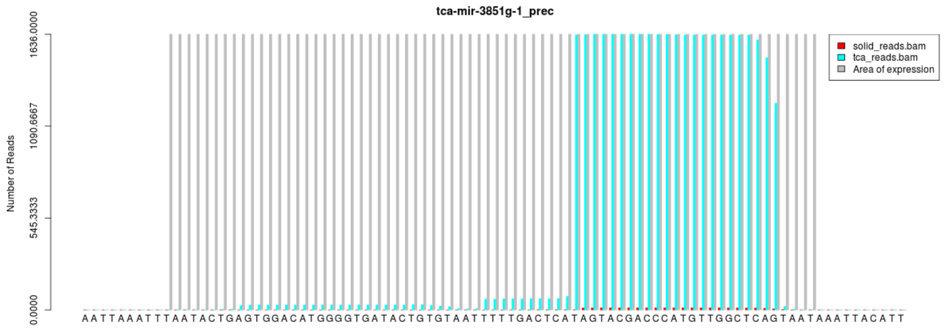

>tca-mir-3851h_prec

```
AAUUAAAUUUAAUAUUGAGUGGACAUGGGUGGUACUGUGUAAUUUUUGACUCAUAGUACGGUCCAUGUUGGCUCAGUAAUAAAUUACAUU
.....((((((.((((((((.(((((((((.((((((((...........)))))))).))))))))).)))))))).))))))).....

 Initial dG =    -37.10

        10        20        30        40
AAUUA|     A        G         G        UAAUU
     AAUUUA UAUUGAGU GACAUGGGU GUACUGUG     U
     UUAAAU AUGACUCG UUGUACCUG CAUGAUAC     U
UUACA^     A        G         G        UCAGU
.        80        70        60        50

-----------------------------------------------------
```

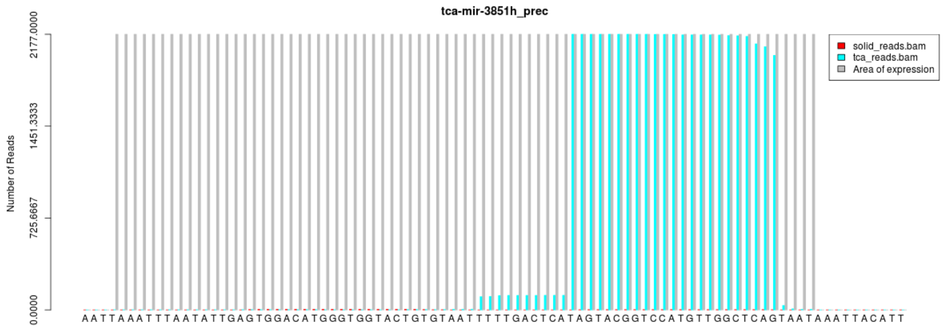

>tca-mir-3851i_prec

```
UCAUUGAAUUUAACAUUGAGUGGAUAUGGGGUGGUACUGUGAAUUUUUUGAACAUAGUACGGCCCAUGUCGGCUCACUGAUAAAUUCCAUUU
.....(((((((.((.(((((.((((((((.(.((((((((...........)))))))).))))))))).))))).)).))))))).....

 Initial dG =    -38.80

        10        20        30        40
UCAUU|      A  U     G        G G        AAUUU
     GAAUUUA CA UGAGU GAUAUGGG U GUACUGUG     \
     CUUAAAU GU ACUCG CUGUACCC G CAUGAUAC     U
UUUAC^      A  C     G        - G        AAGUU
 90        80        70         60        50

-----------------------------------------------------
```

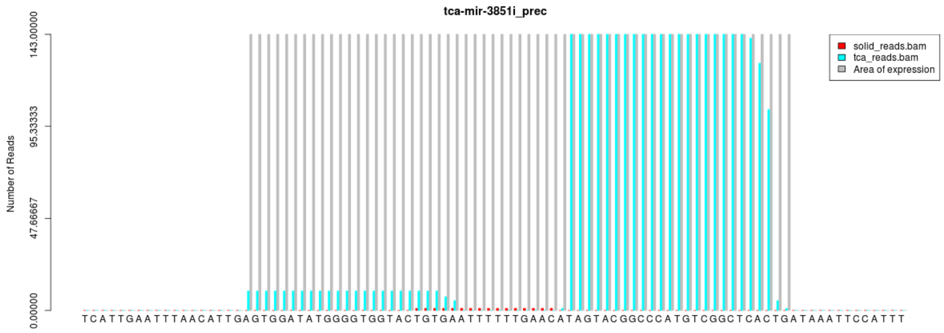

>tca-mir-3851j_prec

```
AAUAAUAAAUAUGGUGAUGAGUUGAUUUGGGGUGGUACUAUGUUUGAAGAUACGCAUAGUACGUUCCGAAUCAACUUGUUAACAAAUUUAAAUUC
.....(((((.((.(((..(((((((((((..((.((((((((..........))))))))))..)))))))))))..))).)).))))).....
```

```
 Initial dG =    -35.70

        10         20         30         40
AAUAA|    A  G   UG          GG  G        UUGA
     UAAAU UG UGA  AGUUGAUUUGG  UG UACUAUGU    A
     AUUUA AC AUU  UCAACUAAGCC  GC AUGAUACG    G
CUUAA^    A  A   GU          UU  -        CAUA
    90         80         70          60         50
```

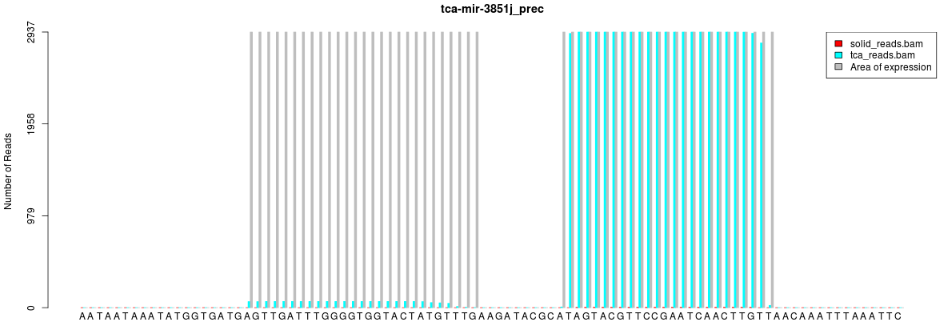

>tca-mir-3851n-2_prec

AUUUUUGAGUAAAUUUAGUACUGGGUUGGCACAGAGUAGUACUGUGUAAAAUUCUAUUUCAUAGUACGACCCAUGCCGGCCUAGUAAGAGAUUUCAUUUCACCAGC
.....((((.((((((..((((((((((((((....((.((((((((.(((......))))))))))).))...)))))))))))))..))))))...)))).....

```
 Initial dG =    -36.00

        10          20        30        40        50
AUUUU    U--|     AG             CAGA  A        U   AU
     UGAG   AAAUUU  UACUGGGUUGGCA    GU GUACUGUG AAA  U
     ACUU   UUUAGA  AUGAUCCGGCCGU    CA CAUGAUAC UUU  C
CGACC    UAC^     GA             ACC-  G        -   AU
    100        90        80         70        60
```

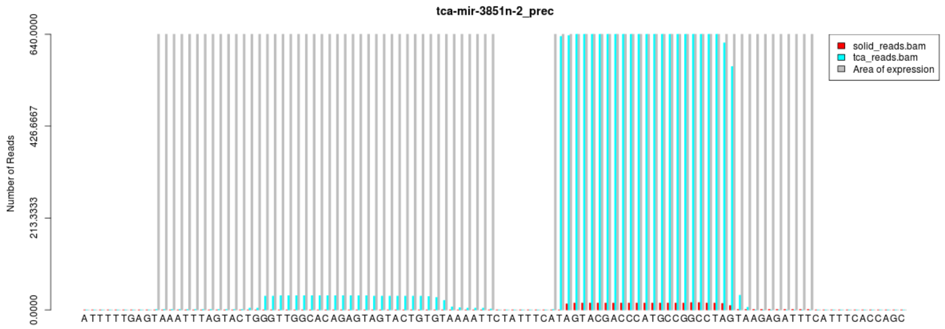

>tca-mir-3851l-3_prec

UUUAAGCUAAAUUUUAGUGAAUUUAUUGCUGUGUUGGCAUAAGUAGUACCAUGAAAAAUUUGAAUCUAUAGUACGACCCAUGCCGACUCAGUAAUAAAUUACAAUUCCAGCAAGUG

```
.....(((.((((...((((.((((((((((.((((((((..((.((((.(((.............))).)))).))..)))))))).))))))))))))))))))..))).....
```

```
 Initial dG =    -32.70

         10        20        30        40        50
UUUAA   A-|   UUA    A          U        AA  A    C   AAAAAU
     GCU  AAUU   GUGA UUUAUUGCUG GUUGGCAU  GU GUAC AUG      \
     CGA  UUAA   CAUU AAAUAAUGAC CAGCCGUA  CA CAUG UAU      U
GUGAA   CC^   ---    -          U        CC  G    A   CUAAGU
    110          100         90        80        70
```

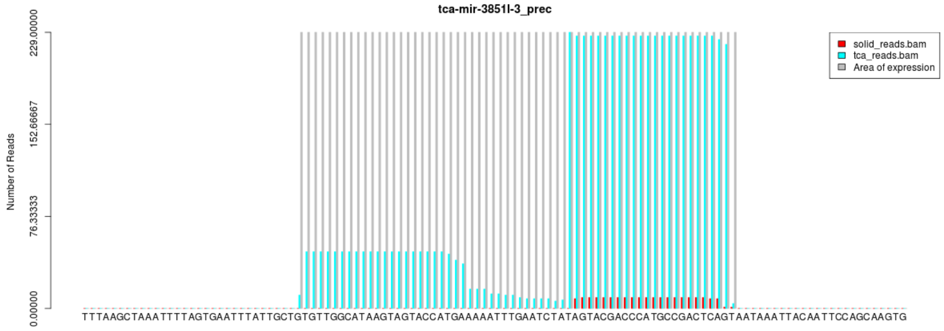

>tca-mir-3851k-3_prec

```
GAAUAAAUUUAGUGCUGGGUUGGCAAAAGGUGGUACCAUGUAACAUUUUAACCCAUAGUACGACCCAUGCCGGCUCAGUAAUAAAUUACAAU
.....((((((.((((((((((((((...(((.((((.(((.............))).)))).)))..))))))))))))).)))))).....
```

```
 Initial dG =    -33.90

        10        20        30        40
GAAUA|     G             AAA   G    C   UAACAU
     AAUUUA UGCUGGGUUGGCA   GGU GUAC AUG      \
     UUAAAU AUGACUCGGCCGU   CCA CAUG UAC      U
UAACA^     A             AC-   G    A   CCAAUU
 90        80        70         60        50

-----------------------------------------------------
```

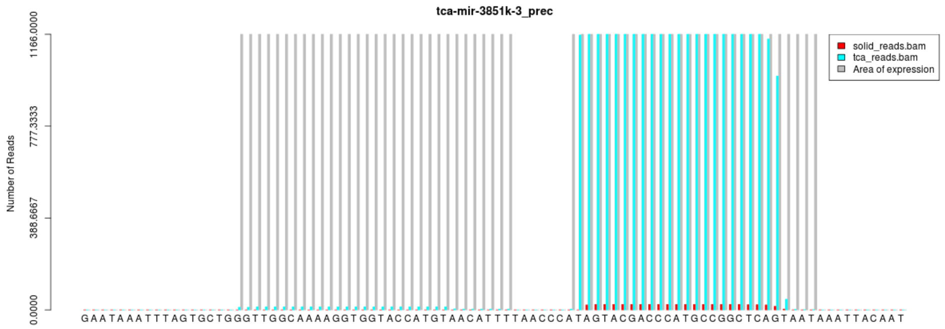

>tca-mir-11618b-3_prec

```
CAAAAUGUUUUCGAUACUUAGUUAGCAUUGGCUGUUUCUAUGCUAAUUUAUGGUCAUAGUACAGCCAAUGUUUGCUUCAGUUCGGAUUCAAUAAC
.....((..(((((.(((.(((.((((((((((((..((((((((.....))).))))).))))))))))))).))).)).))))))))..)).....

 Initial dG =    -35.80

        10         20        30        40
CAAAA| UU     U   U-   U            UU     -   AU
     UG  UUCGA ACU  AGU AGCAUUGGCUGU  CUAUG CUA  \
     AC  AGGCU UGA  UCG UUGUAACCGACA  GAUAC GGU  U
CAAUA^ UU     -   CU   U            U-     U   AU
    90         80        70        60         50
```

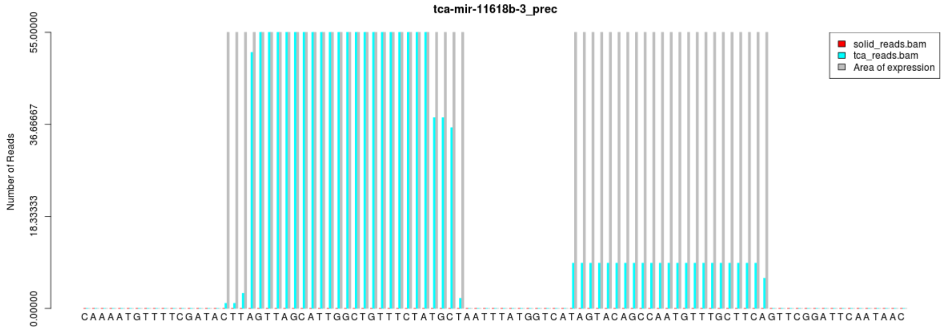

>tca-mir-3851m-3_prec

```
UAAGUAAAUUUAGUACUGGGUUGGCACAGAGUAGUACUAUGUAAAAUUCUAUUCCAUAGUACGACCCAUGUCGGCCCAGUAAGAGAUUUCAAUU
.....((((((..(((((((((((((....((.((((((((.............)))))))).))...)))))))))))))..)))))).....

 Initial dG =    -33.10

        10        20        30        40
UAAGU|     AG             CAGA  A        UAAAAU
     AAAUUU  UACUGGGUUGGCA    GU GUACUAUG      \
     UUUAGA  AUGACCCGGCUGU    CA CAUGAUAC      U
UUAAC^     GA             ACC-  G        CUUAUC
   90        80        70         60        50

-----------------------------------------------------
```

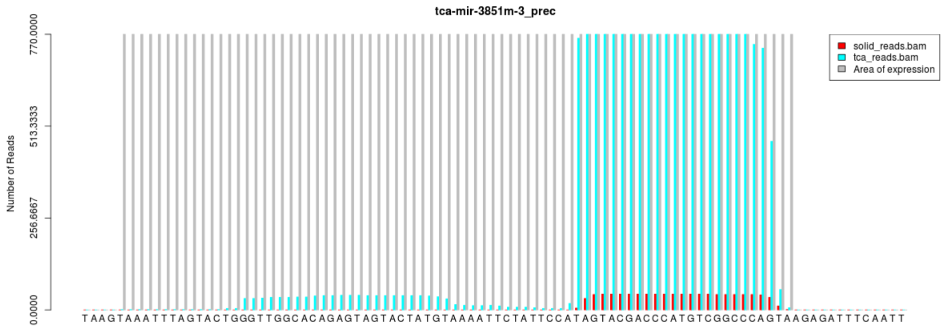

>tca-mir-3851m-2_prec

AAGUAAAUUUAGUACUGGGUUGGCACAGAGUAGUACUAUGUAAAAUUCUAUUCCAUAGUACGACCCAUGUCGGCCCAGUAAGAGAUUCCAAU
.....(((((..(((((((((((((....((.((((((((.............)))))))).))...)))))))))))))..))))).....

```
 Initial dG =    -32.30

        10        20        30        40
AAGUA|    AG             CAGA  A        UAAAAU
     AAUUU  UACUGGGUUGGCA    GU GUACUAUG      \
     UUAGA  AUGACCCGGCUGU    CA CAUGAUAC      U
UAACC^    GA             ACC-  G        CUUAUC
 90        80        70         60        50
```

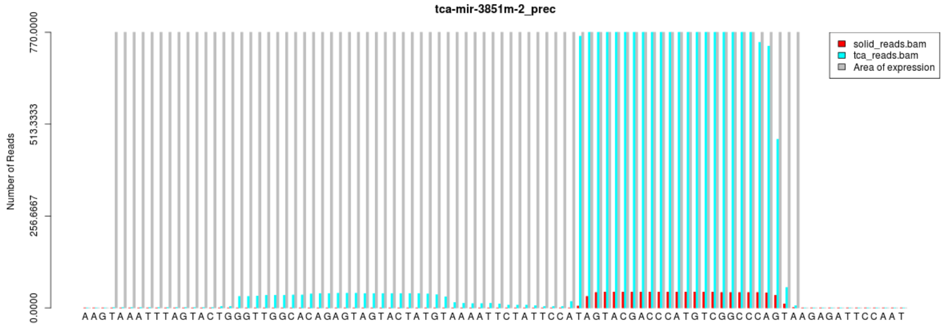

>tca-mir-11618a-5_prec

AUGUUUUCGAUAAAUAGUUAGCAUUGGUCGUUUCUAUGCUUCUUCGUGGUCAUUGAUCGGCCAAUGUUCGCUUUAUUUCGAAUCCUC
.....((((.....(((.(((((((((((..((.(((((.......)).))).)).))))))))))).))).....))))).....

```
 Initial dG =    -26.90

        10        20        30         40
AUGUU|    UAAAU   U           UU  U   -  UCU
     UUCGA     AGU AGCAUUGGUCG  UC AUG CU   \
     AAGCU     UCG UUGUAACCGGC  AG UAC GG   U
CUCCU^    UUAUU   C           U-  U   U  UGC
      80        70        60         50
```

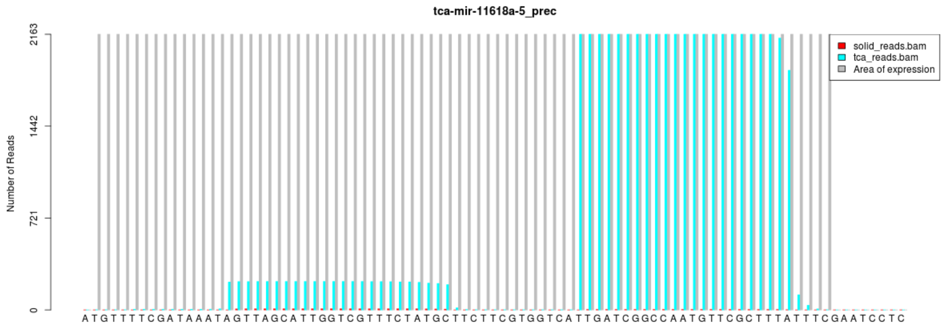

>tca-mir-3851k-4_prec

```
GAAUAAAUUUAGUGCUGGGUUGGCAAAAGGUGGUACCAUGUAACAUUUUAACCCAUAGUACGACCCAUGCCGGCUCAGUAAUAAAUUACAAU
.....((((((.(((((((((((((...(((.((((.(((.............))).)))).)))..))))))))))))).))))))......

 Initial dG =    -33.90

        10        20        30        40
GAAUA|     G             AAA   G    C   UACAU
     AAUUUA UGCUGGGUUGGCA   GGU GUAC AUG      \
     UUAAAU AUGACUCGGCCGU   CCA CAUG UAC      U
UAACA^     A             AC-   G    A   CCAAUU
 90        80        70         60        50

-----------------------------------------------------
```

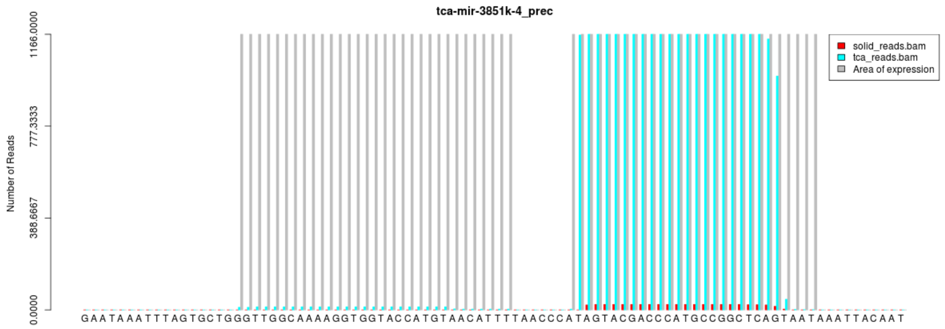

>tca-mir-3851g-3_prec

```
UUGACAUGGGGUGGUACUGUGUAAUUUUUGACUCAUAGUACGACCCAUGUUGGCUCAG
(..(((((((.(.((((((((............)))))))).))))))))..).....

 Initial dG =    -23.50

             10        20
-----|UG       G G        UAAUU
     U  ACAUGGG U GUACUGUG     U
     G  UGUACCC A CAUGAUAC     U
GACUC^GU       - G        UCAGU
       50         40        30

-----------------------------------------------------
```

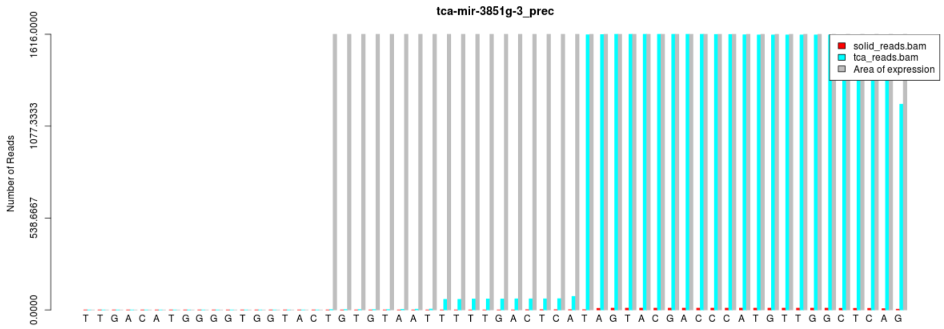

>tca-mir-3851p_prec

```
GGGUUAGCAUAAGUGGUACCAUAAAAAUUUUUAAUCCAUAGUACGACAAUGCCAACUCAGU
(((((.((((..((.((((.....................)))).)).)))).)))))...

 Initial dG =    -13.30

           10        20
---|    A    AA  G    CAUAAAAAUU
   GGGUU GCAU  GU GUAC          \
   CUCAA CGUA  CA CAUG          U
UGA^    C    A-  G    AUACCUAAUU
60        50         40
```

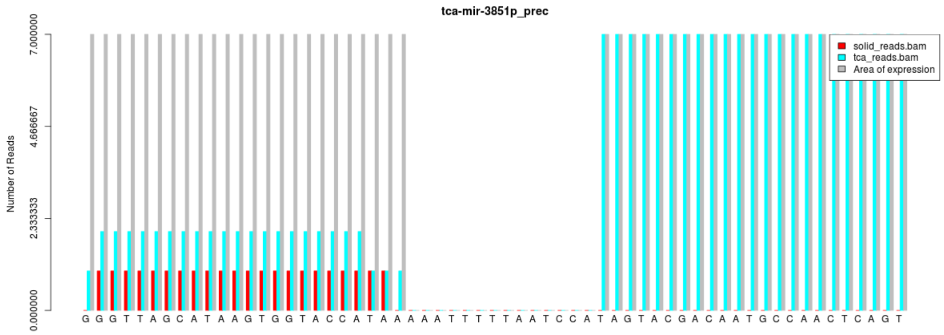

>tca-mir-3851l-4_prec

```
GGUUGGCAUAAGGUGGUACCAUGUAACAUUUUAACCCAUAGUACGAUCCAUGCCGACUCAG
((((((((..(((.((((.(((.............))).)))).))).)))))))))...

 Initial dG =    -19.30

           10        20
---|        AA   G    C   UAACAU
   GGUUGGCAU  GGU GUAC AUG      \
   UCAGCCGUA  CUA CAUG UAC      U
GAC^        C-   G    A   CCAAUU
60        50         40

-----------------------------------------------------
```

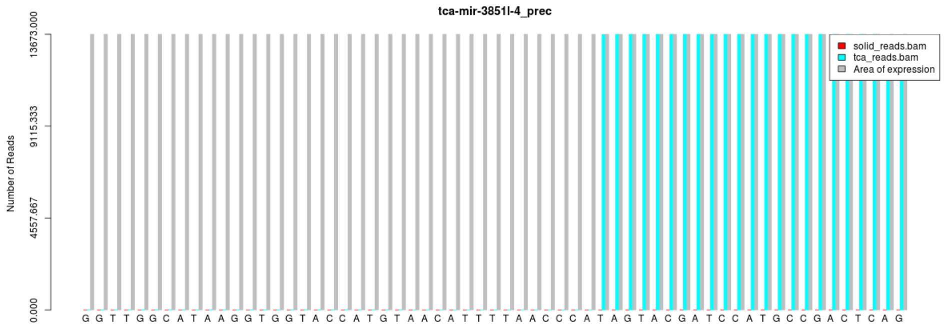

>tca-mir-6016b_prec

```
AGGAGGACUUGUGGGCGUGGCAUUGCCGAAUAACCACGACCACAUGUUCUUCUACUCU
((((((((.(((((.(((((.............))))).))))).)))))))).....

 Initial dG =    -24.70

             10        20
-----|       U     G     CAUUGC
     AGGAGGAC UGUGG CGUGG      \
     UCUUCUUG ACACC GCACC      C
UCUCA^       U     A     AAUAAG
       50        40        30

-----------------------------------------------------
```

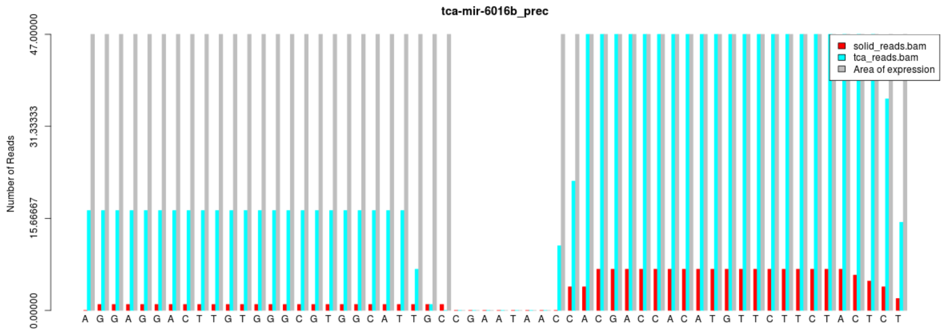

>tca-mir-927c_prec

```
UUCAGAAUCCAAACGCUUUGCCAGCCUUUCACACGGUAAAGCGUAGGAAUUCUAAACCG
...(((((((..((((((((((............)))))))))).))).))))......

 Initial dG =    -19.20

            10        20
UUC---    -|  AA          AGCCU
      AGAA UCC  ACGCUUUGCC     U
      UCUU AGG  UGCGAAAUGG     U
GCCAAA    A^  A-          CACAC
        50         40        30
```

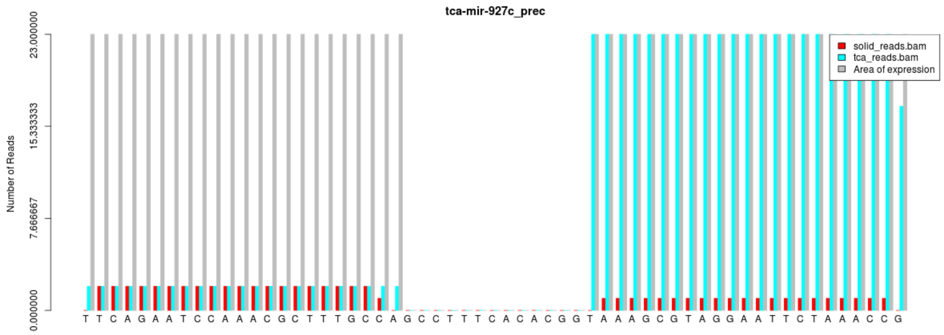

>tca-mir-971b_prec

```
AGCUUUGAGUGUGUAUAGUCGGGUCACUCUAAGUUUGAACACCAAGCUACAUUUAGCUCGUUUGGUGUUCUACCUUACAGUGAGCGGACUGUACACCAAAUCAUCACU
.....(((..((((((((((...(((((.((((...((((((((((((....)))))....))))))))...)))).)))))...)))))))))))....))).....

 Initial dG =    -40.60

        10          20        30        40          50
AGCUU   GU--          GGG     C    UUU        ----|    C
     UGA    GUGUAUAGUC   UCACU UAAG   GAACACCA    AGCUA A
     ACU    CACAUGUCAG   AGUGA AUUC   CUUGUGGU    UCGAU U
UCACU   AAAC          GCG     C    CAU        UUGC^    U
      100        90        80        70        60

-----------------------------------------------------
```

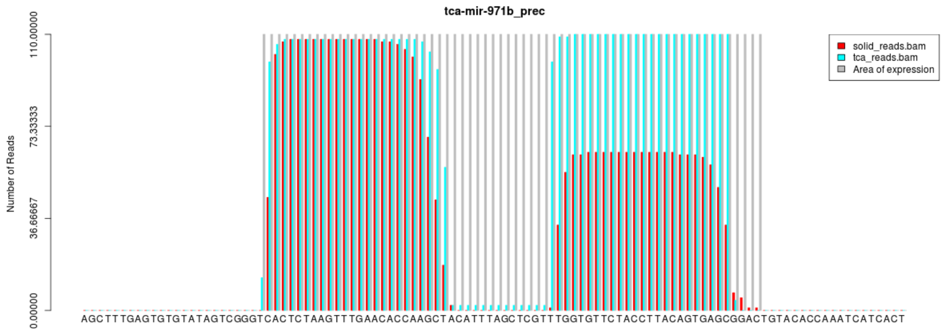

>tca-mir-11619b_prec

```
UUGUCGGGUGUGACACAAAGUCUAUGAUAUUCUUCAUUCUAUAAUUCACCAAAUGAAGGGUCACAGACUUUGUCUUCGACCACACUUCAUUG
.....(((((((..(((((((((.((((..((((((((............)))))))))))).))))))))).......))))))).....

 Initial dG =    -25.20

        10             20        30        40
UUGUC       AC-----|        A    AU        CUAUAA
     GGGUGUG       ACAAAGUCU UGAU  UCUUCAUU      \
     UUCACAC       UGUUUCAGA ACUG  GGAAGUAA      U
GUUAC       CAGCUUC^        C    --        ACCACU
 90        80        70        60          50

-----------------------------------------------------
```

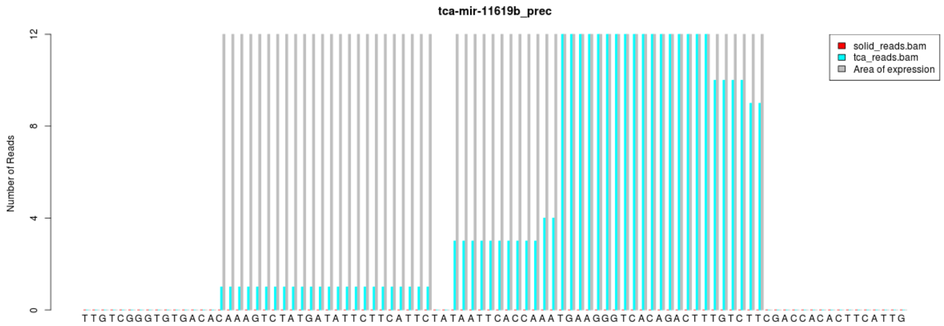

>tca-mir-11619a-3_prec

```
CUUUCGAACGGAACACGAAGUCUGUGAUAUUCUUCAUUUUAUAAUUUAACAAAUGAAGCGUCAUUGGCUUGGUUUUUCGUUCAACAC
.....((((((((.((.(((((.(((((...((((((((...........)))))))).))))).))))).)).))))))))).....

 Initial dG =    -28.80

        10        20        30        40
CUUUC|       C  G     U     AUU        UAUAA
     GAACGGAA AC AAGUC GUGAU   CUUCAUUU     \
     CUUGCUUU UG UUCGG UACUG   GAAGUAAA     U
CACAA^       U  G     U     C--        CAAUU
      80        70        60          50

-----------------------------------------------------
```

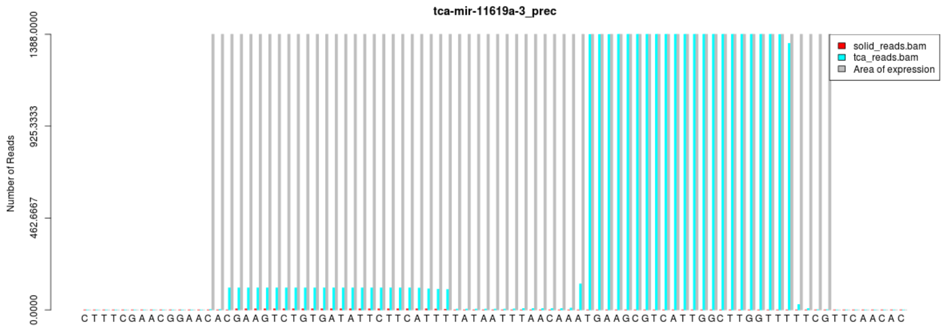

>tca-mir-11619a-1_prec

CUUUCGAACGGAACACGAAGUCUGUGAUAUUCUUCAUUUUAUAAUUUAACAAAUGAAGAGUCAUUGGCUUGGUUUUUCGUUCAACAC

.....((((((((.((.(((((.(((((..((((((((((..........))))))))))))))).))))).)).)))))))).....

```
 Initial dG =    -30.80

        10        20        30        40
CUUUC|       C  G     U     AU         UAUAA
     GAACGGAA AC AAGUC GUGAU  UCUUCAUUU     \
     CUUGCUUU UG UUCGG UACUG  AGAAGUAAA     U
CACAA^       U  G     U     --         CAAUU
      80        70        60          50
```

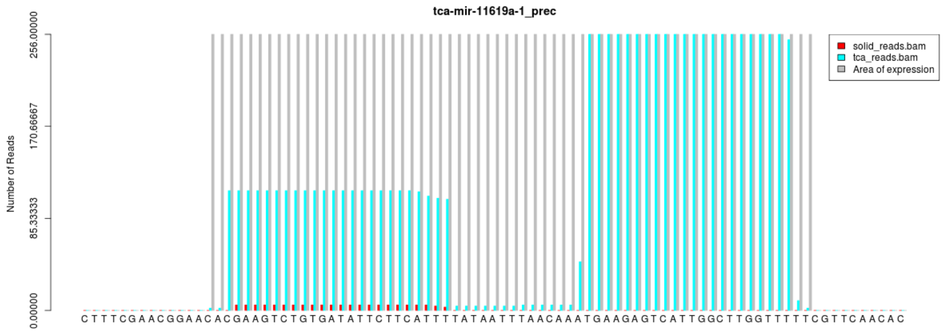

>tca-mir-11619a-2_prec

```
CUUUCGAACGGAACACGAAGUCUGUGAUAUUCUUCAUUUUAUAAGUUAACUAAUGAAGAGUCAUUGGCUUGGUUUUUCGUUCAACAC
.....((((((((.((.(((((.(((((..((((((((.............))))))))))))).))))).)).)))))))).....

 Initial dG =    -30.40

        10        20        30        40
CUUUC|       C  G     U     AU        UUAUAA
     GAACGGAA AC AAGUC GUGAU  UCUUCAUU      \
     CUUGCUUU UG UUCGG UACUG  AGAAGUAA      G
CACAA^       U  G     U     --        UCAAUU
      80        70        60          50

-----------------------------------------------------
```

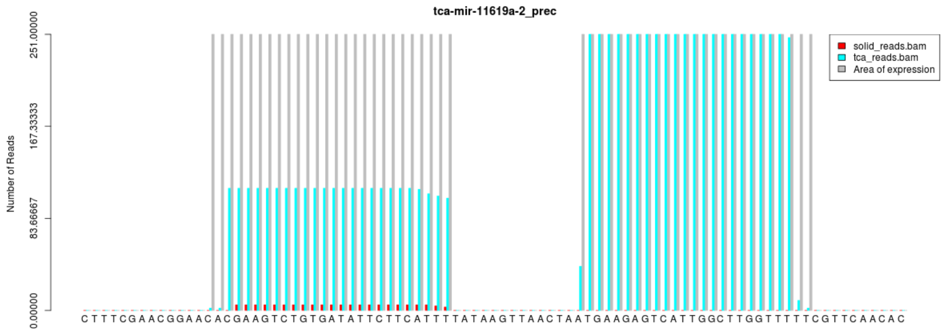

>tca-mir-11624a-1_prec
UAAUAGAUAAGUGUAAAAUUGAGACUUUAACAUUUUCAUGCAAUUUAUAUCAACAUGAUAUGCUAGAGUGUCGGUUCUACAACUAUCACAAA
.....((((..((((.((((((.((((((.(((..(((((.............))))).))).)))))).)))))).)))).)).)))).....

```
 Initial dG =    -25.10

        10        20        30        40
UAAUA|   AG    A      G      A   UU     CAAUUU
     GAUA  UGUA AAUUGA ACUUUA CAU  UCAUG      \
     CUAU  ACAU UUGGCU UGAGAU GUA  AGUAC      A
AAACA^   CA    C      G      C   U-     AACUAU
 90        80        70        60         50
```

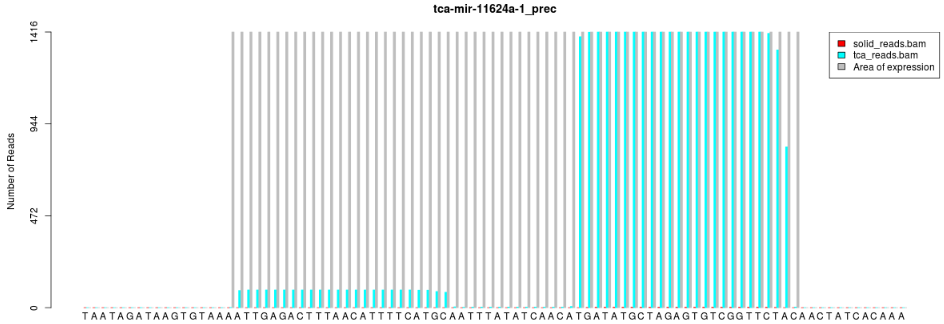

>tca-mir-11624a-2_prec

UUUUAUGUAGUUUUAAAUUAUAAUAGAUUGGUGUAAAAUUGAGACUUUAACAUUUUCAUGCAAUUUAUAUCAACAUGAUAUGCUAGAGUGUCGGUUCUACAACUAUCACAAAUCAAUCUU
CGAACUAAGGCUCAAACUU

```
.....((.(((((((.........(((((((((((.((((((.((((((.(((..
(((((.............))))).))).)))))).)))))).))))...........)))))))......))))))))).....
```

```
 Initial dG =    -27.90

        10        20        30                   40        50        60
UUUUA  U       AAUUAUAAU       -----------|   A      G      A   UU     CAAUUU
     UG AGUUUUA         AGAUUGG           UGUA AAUUGA ACUUUA CAU  UCAUG      \
     AC UCGGAAU         UCUAACU           ACAU UUGGCU UGAGAU GUA  AGUAC      A
UUCAA  -       CAAGCU---       AAACACUAUCA^   C      G      C   U-     AACUAU
        130       120          110       100        90        80         70

-----------------------------------------------------
```

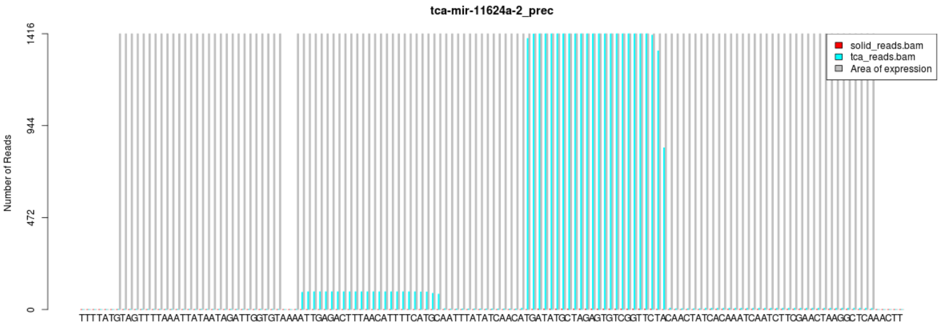

>tca-mir-11624a-3_prec

```
AUAAUAGAUUGAUGUAAAAUUGAGACUUUAACAUUUUCAUGCAAUUUAUAUCAACAUGAUAUGCUAGAGUGUCGGUUUUACAACUAUCACAAAUCAAUCUUCGAA
.....((((((((((((((((.((((((.(((..(((((.............))))).))).)))))).)))))))))))...........))))))).....

 Initial dG =    -28.10

        10                    20        30        40
AUAAU       -----------|          G      A   UU     CAAUUU
     AGAUUGA           UGUAAAAUUGA ACUUUA CAU  UCAUG      \
     UCUAACU           ACAUUUUGGCU UGAGAU GUA  AGUAC      A
AAGCU       AAACACUAUCA^          G      C   U-     AACUAU
   100        90        80        70        60         50

-----------------------------------------------------
```

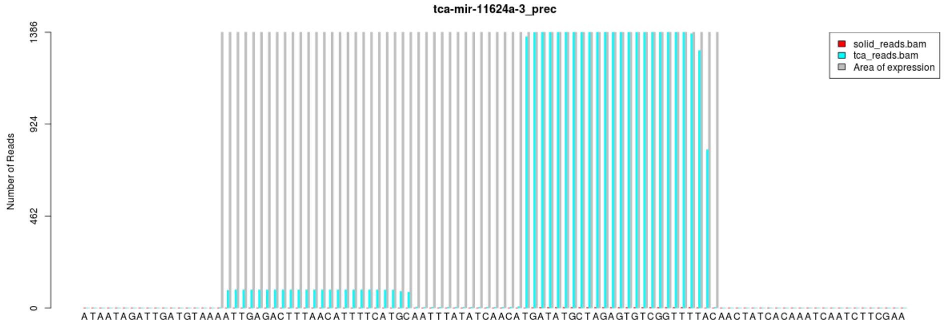

>tca-mir-11624b-1_prec

```
UAAUAGAUUGGAGUAAAAUUGAGACUUUAGCAUUUUCAUGCAAUUUAUAUCAACAUGAUGUGCUAAAGUGUCGGUUUUACAACAAUCACAAA
.....(((((..((((((((((.((((((((((..(((((.............))))).)))))))))).))))))))))..))))).....
```

```
 Initial dG =    -36.70

        10        20        30        40
UAAUA|    GA          G          UU     CAAUUU
     GAUUG  GUAAAAUUGA ACUUUAGCAU  UCAUG      \
     CUAAC  CAUUUUGGCU UGAAAUCGUG  AGUAC      A
AAACA^    AA          G          U-     AACUAU
 90        80        70        60         50
```

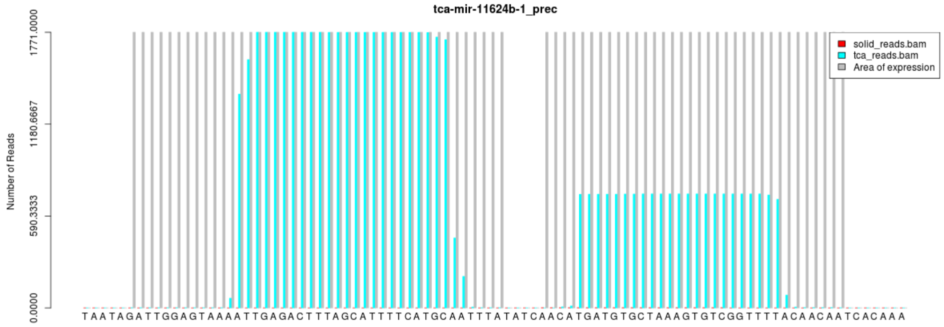

>tca-mir-11624d_prec

UAAUAGAUUGGUGUAAAAUUGGGACUUUAGCAUUUUCAUGCAAUUUAUAUCAUCAUGAUGUGCUAAUGUGUCGGUUUUACAACAAUCACAAA

```
.....((((.(((((((((((.((.(((((((..(((((.............))))).))))))).)).))))))))))).))))).....

 Initial dG =    -31.80

        10        20        30        40
UAAUA|    G           G  U       UU     CAAUUU
     GAUUG UGUAAAAUUGG AC UUAGCAU  UCAUG      \
     CUAAC ACAUUUUGGCU UG AAUCGUG  AGUAC      A
AAACA^    A           G  U       U-     UACUAU
 90        80        70        60         50

-----------------------------------------------------
```

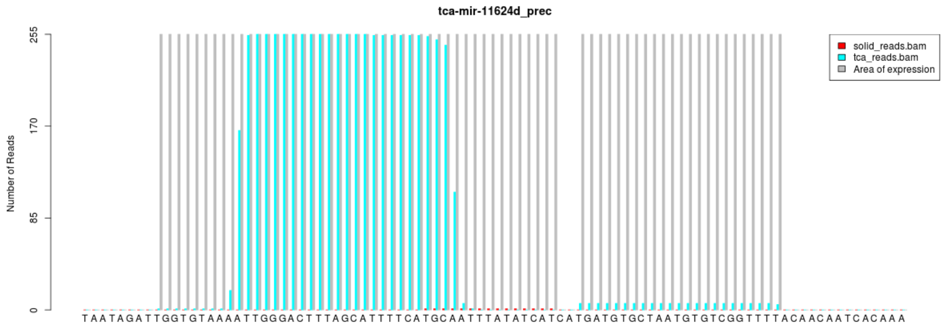

>tca-mir-11624c_prec

UAAUAGAUUGGUGUAAAAUUGAGACUUUAGUAUUUUCAUGCAAUUUAUGUCAACAUGAUAUGCUAAAGUGUCGGUUCUACAACAAUCACAAA

.....((((.((((.((((((.((((((((((..(((((.............))))).)))))))))).)))))).)))).))))).....

```
 Initial dG =    -31.60

        10        20        30        40
UAAUA|    G    A      G         UU     CAAUUU
     GAUUG UGUA AAUUGA ACUUUAGUAU  UCAUG      \
     CUAAC ACAU UUGGCU UGAAAUCGUA  AGUAC      A
AAACA^    A    C      G         U-     AACUGU
 90        80        70        60         50
```

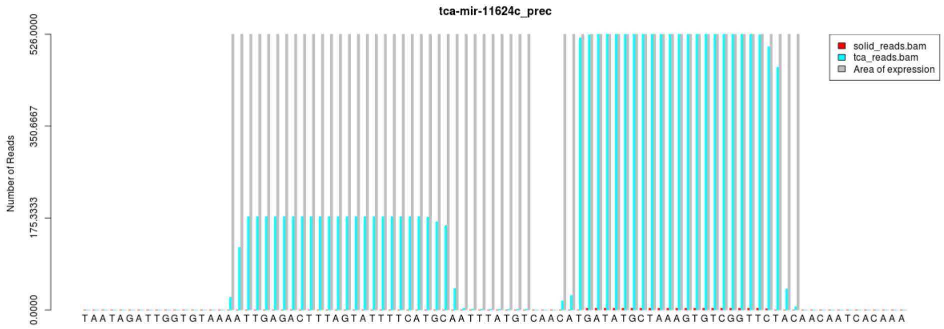

>tca-mir-11624b-2_prec

```
AUUGGGACUUUACCAUUUUCAUGCAAUUUAUAUCAUCAUGAUGUGCUAAAGUGUCGGUUUU
(((((.((((((.(((..(((((.............))))).))).)))))).)))))...

 Initial dG =    -13.30

           10        20
---|    G      C   UU     CAAUUU
   AUUGG ACUUUA CAU  UCAUG      \
   UGGCU UGAAAU GUG  AGUAC      A
UUU^    G      C   U-     UACUAU
60        50        40
```

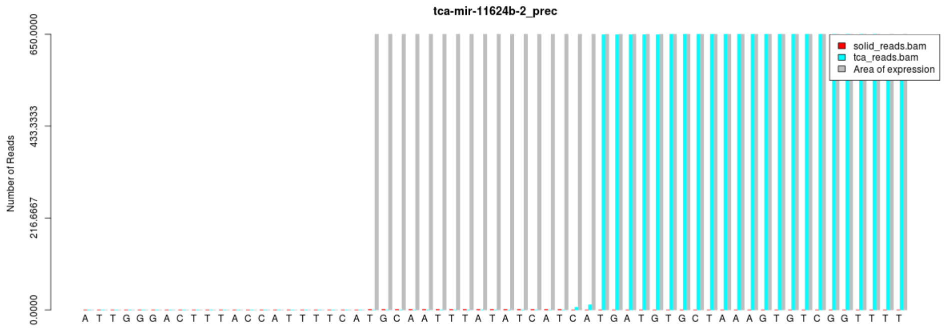

>tca-mir-11617b_prec

UUUCCGAAUUCACUGUUAAGUUGUCAUGGAGUAGUACUGUAUGGAAUAUAAUAAUUAUUGUACUUCCUAUGGCAGCUUACUAAUGAAUUCAAAUU

.....((((((.((.(((((((((((((...(((((.(((..............))).)))))..))))))))))))).)).))))))).....

```
 Initial dG =    -30.50

        10        20        30        40
UUUCC|      C  U             AGU     U   UGGAAU
     GAAUUCA UG UAAGUUGUCAUGG   AGUAC GUA      A
     CUUAAGU AU AUUCGACGGUAUC   UCAUG UAU      U
UUAAA^      A  C             CU-     U   UAAUAA
    90        80        70         60        50
```

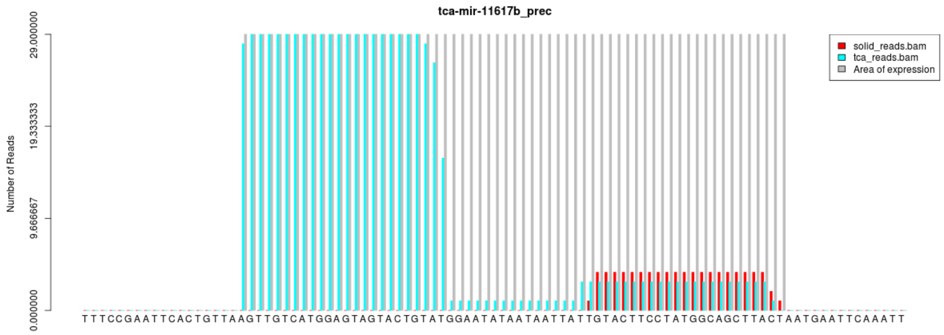

>tca-mir-11617c_prec

```
UUUCCGAAUUCACUGUUGAGUUGGCAUAGAGUAGUACUGUAUGGAAUAUGAUAAUUAUUGUACUUCCUAUGACAGCUUACUAAUGAAUUCAAAUU
.....(((((((.((.(((((((.(((((...(((((.(((..............))).)))))..))))).))))))).)).))))))).....

 Initial dG =    -26.50

        10        20        30        40
UUUCC|      C  U       G     AGU     U   UGGAAU
     GAAUUCA UG UGAGUUG CAUAG   AGUAC GUA      A
     CUUAAGU AU AUUCGAC GUAUC   UCAUG UAU      U
UUAAA^      A  C       A     CU-     U   UAAUAG
    90        80        70         60        50

-----------------------------------------------------
```

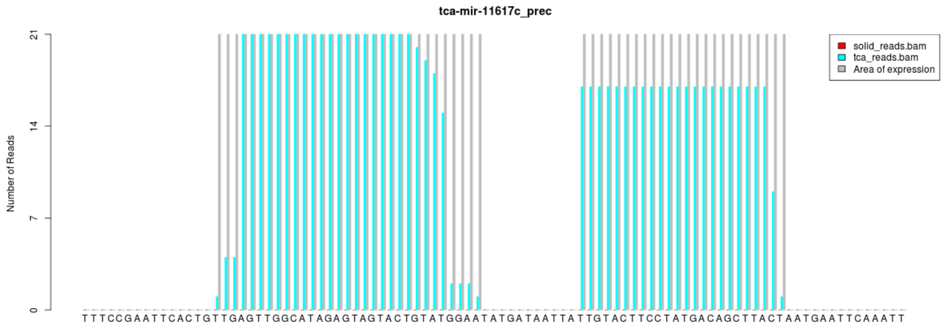

>tca-mir-11617a-1_prec

UUUUGGAAUUCACCAUUGAGUUGGCAUAGGGUAGUACUGCAGACCAAUGUAAUUGUAGUACUUCCUAGGGCAGCUUAUUAAGGAAUUCAAAUU

```
.....((((((.....((((((.((.(((((.((((((((((((....))..))))))))))))))))..)))))))).....)))))).....
```

```
 Initial dG =    -31.40

        10        20         30        40
UUUUG      ACCAU      G  A-      U          --| C
     GAAUUC     UGAGUU GC  UAGGG AGUACUGCAG  AC A
     CUUAAG     AUUCGA CG  AUCCU UCAUGAUGUU  UG A
UUAAA      GAAUU      -  GG     -          AA^ U
  90        80         70         60        50

----------------------------------------------------
```

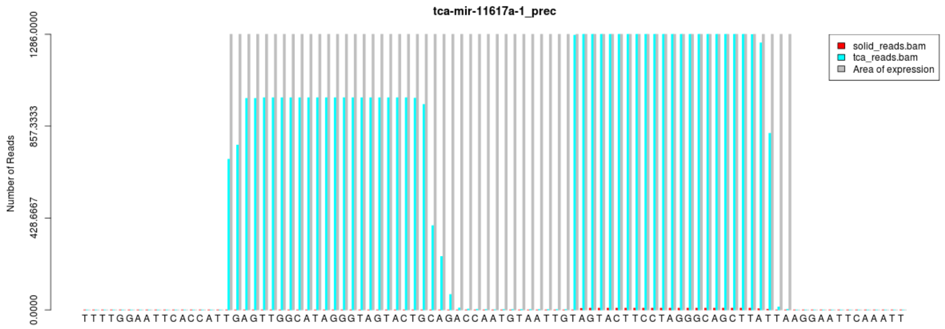

>tca-mir-11617a-2_prec

UUUUGGAAUUCACCACUGAGUUGGCAUAGGGUAGUACUGCAGACCAUUGUAAUUGUAGUACUUCCUAGGGCAGCUUAUUAAUGAAUUCAUAUU
.....((((((....((((((.((.(((((.((((((((((((....))..)))))))))))))))..)))))))))....))))))).....

```
 Initial dG =    -31.20

        10         20          30         40
UUUUG       CCAC      G  A-      U          --| C
     GAAUUCA    UGAGUU GC  UAGGG AGUACUGCAG  AC A
     CUUAAGU    AUUCGA CG  AUCCU UCAUGAUGUU  UG U
UUAUA       AAUU      -  GG      -          AA^ U
  90        80          70          60         50
```

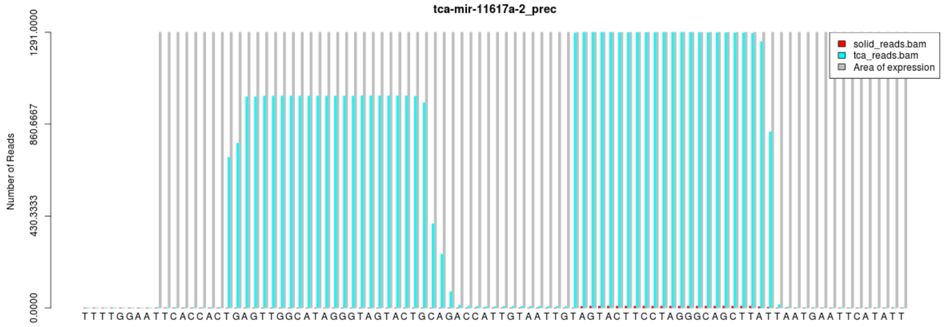

>tca-mir-11617a-3_prec

UUUUGGAAUUCACCAUUGAGUUGGCAUAGGGUAGUACUGCAGACCAUUGUAAUUGUAGUACUCCCUAGGGCAGCUUAUUAAUGAAUUCAAAUU

.....((((((....((((((.((.(((((.((((((((((((....))..))))))))))))))))..))))))))....))))))).....

```
 Initial dG =    -34.80

        10        20         30        40
UUUUG       CCAU      G  A-     U          --| C
     GAAUUCA    UGAGUU GC  UAGGG AGUACUGCAG  AC A
     CUUAAGU    AUUCGA CG  AUCCC UCAUGAUGUU  UG U
UUAAA       AAUU      -  GG     -          AA^ U
  90        80         70         60        50

-----------------------------------------------------
```

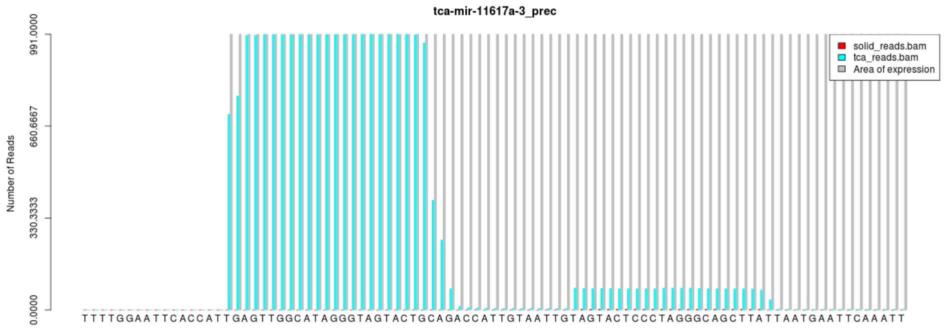

>tca-mir-11617a-4_prec

```
UUUUGGAAUUCACCAUUGAGUUGGCAUAGGGUAGUACUGCAGACCAUUGUAAUUGUAGUACUCCCUAGGGCAGCUUAUUAAUGAAUUCAAAUU
.....(((((((....((((((.((.(((((.((((((((((((....))..)))))))))))))))..)))))))))....))))))).....

 Initial dG =    -34.80

        10        20         30        40
UUUUG       CCAU      G  A-     U          --| C
     GAAUUCA    UGAGUU GC  UAGGG AGUACUGCAG  AC A
     CUUAAGU    AUUCGA CG  AUCCC UCAUGAUGUU  UG U
UUAAA       AAUU      -  GG     -          AA^ U
  90        80         70         60        50

-----------------------------------------------------
```

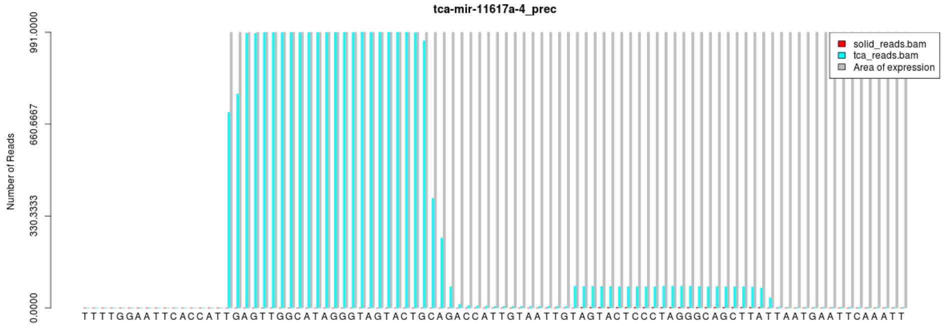

>tca-mir-11617d-1_prec

```
AGUUGGCAUGGGUGGUACUAUAUAUAUCAUAGUAAUUAUAGUACUUUCUAUGGCAACUUA
(((((.((((((.(((((((((..(((....)))..))))))))).)))))).)))))..

 Initial dG =    -23.30

          10        20
--|    G      U         UA   C
  AGUUG CAUGGG GGUACUAUA  UAU A
  UCAAC GUAUCU UCAUGAUAU  AUG U
AU^    G      U         UA   A
.        50        40        30

-----------------------------------------------------
```

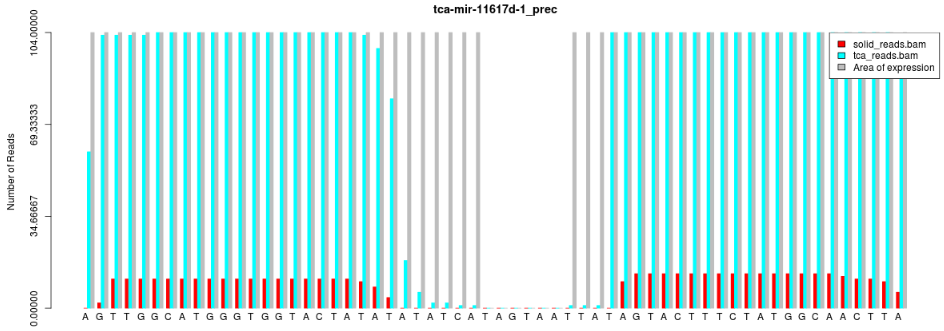

>tca-mir-11617e_prec

```
AGUUGGCAUGGAUGGUACUGUAUAGAUCAUAGUAAUUAUAGUACUUUCUAUGGCAACUUA
(((((.((((((.(((((((((...((....))...))))))))).)))))).))))).. 

 Initial dG =    -24.00

          10        20
--|    G      U         UAG  C
  AGUUG CAUGGA GGUACUGUA   AU A
  UCAAC GUAUCU UCAUGAUAU   UG U
AU^    G      U         UAA  A
.        50        40        30
```

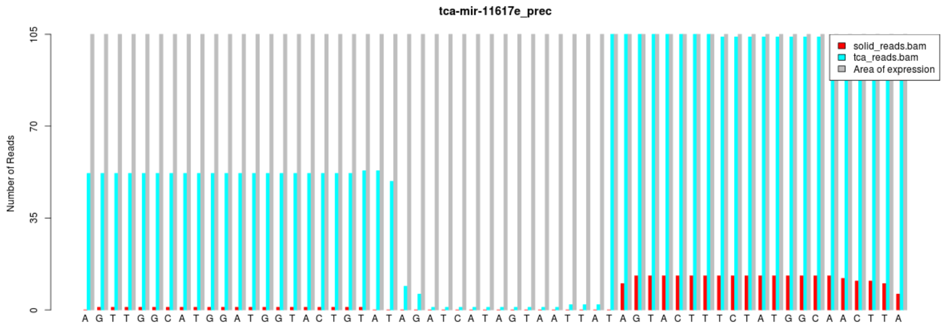

>tca-mir-11617f-1_prec

CUUGGCAUGGGUGGUACUAUAUGGAUUAUAGUAAUUAUAGUACUUUCUAUGGCAACUUA

.(((.((((((.(((((((((...((....))...))))))))).)))))).)))....

```
 Initial dG =    -19.70

          10        20
C---|  G      U         UGG  U
    UUG CAUGGG GGUACUAUA   AU A
    AAC GUAUCU UCAUGAUAU   UG U
AUUC^  G      U         UAA  A
        50        40        30
```

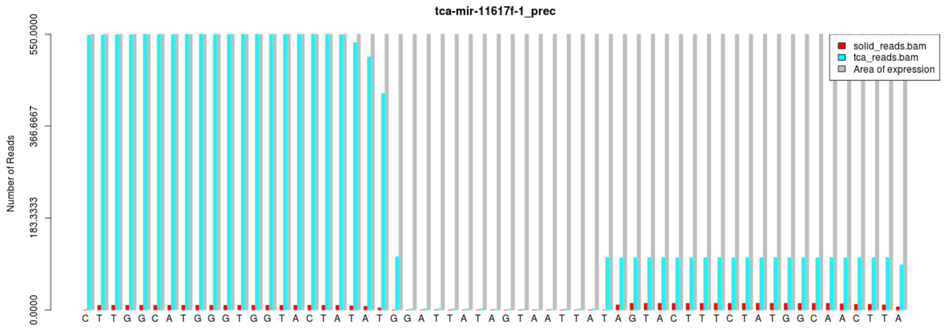

>tca-mir-11617f-2_prec

```
CUUGGCAUGGGUGGUACUAUAUGGAUCAUAGUAAUUAUAGUACUUUCUAUGGCAACUUA
.(((.((((((.(((((((((...((....))...))))))))).)))))).)))....

 Initial dG =    -19.30

           10        20
C---|  G      U         UGG  C
    UUG CAUGGG GGUACUAUA   AU A
    AAC GUAUCU UCAUGAUAU   UG U
AUUC^  G      U         UAA  A
        50        40        30

-----------------------------------------------------
```

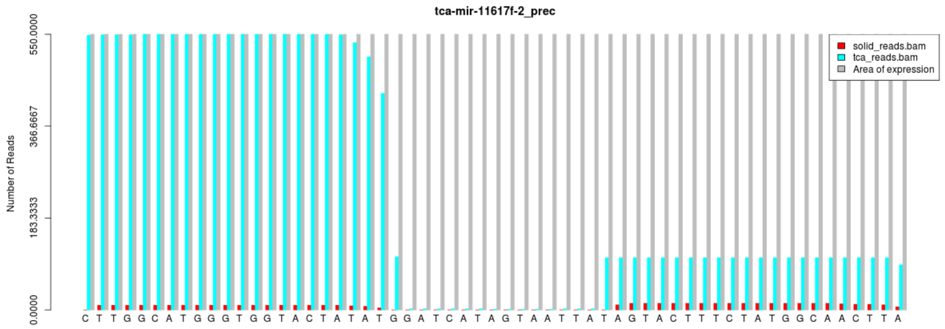

>tca-mir-11617d-2_prec

AGUUGGCAUGGGUGGUACUAUAUAGAUCAUAGUAAUUAUAGUACUUUCUAUGGCAACUUA
(((((.((((((.(((((((((...((....))...))))))))).)))))).)))))..

```
 Initial dG =    -23.10

          10        20
--|    G      U         UAG  C
  AGUUG CAUGGG GGUACUAUA   AU A
  UCAAC GUAUCU UCAUGAUAU   UG U
AU^    G      U         UAA  A
.        50        40        30
```

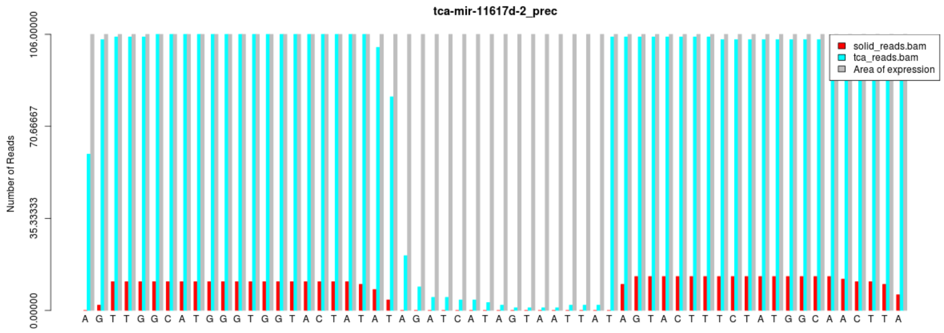

>tca-mir-11617d-3_prec
AGUUGGCAUGGGUGGUACUAUAUAGAUCAUAGUAAUUAUAGUACUUUCUAUGGCAACUUA
(((((.((((((.(((((((((...((....))...))))))))).)))))).)))))..

```
 Initial dG =    -23.10

          10        20
--|    G      U         UAG  C
  AGUUG CAUGGG GGUACUAUA   AU A
  UCAAC GUAUCU UCAUGAUAU   UG U
AU^    G      U         UAA  A
.        50        40        30
```

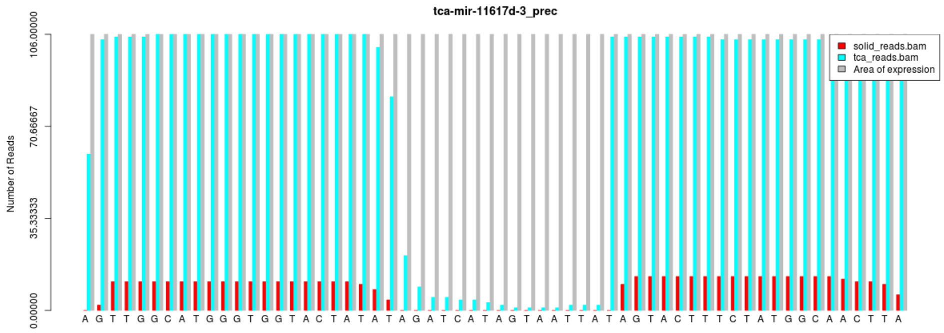

>tca-mir-11618a-1_prec

```
ACAAAAUGUUUUCGAUGUAUAGUUAGCAUUGGUCGUUUCUAUGCUUUUUUGUGAUCAUAUACCGAUCGAUGCUCGCUUUAUUUCGAGUCACAU
.....((((.(((((.(((.(((.(((((((((((....((((.((......)).))))...))))))))))).))).))).)))))..))))

 Initial dG =    -28.00

        10          20         30         40
ACAAA    U-|    U   U   U           UUUC    C  UU 
     AUGU  UUCGA GUA AGU AGCAUUGGUCG    UAUG UU  U
     UACA  GAGCU UAU UCG UCGUAGCUAGC    AUAC AG  U
-----    CU^    U   U   C           CAU-    U  UG 
       90        80         70        60          50

-----------------------------------------------------
```

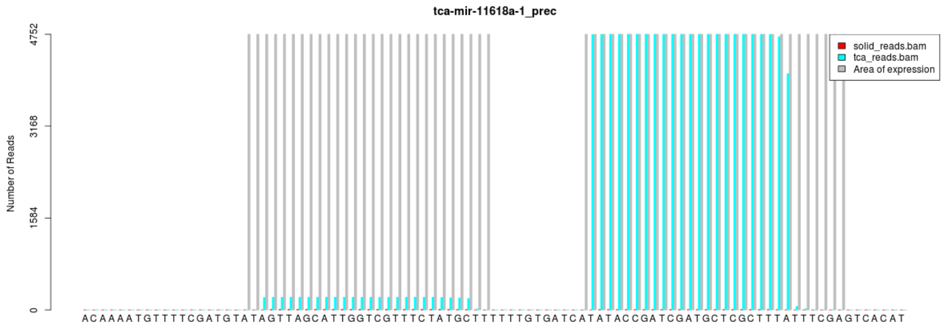

>tca-mir-11618a-4_prec

UGUUUUCGAUGUAUAGUUAGCAUUGGUCGUCUCUAUGCUUUUUUAUGAUCAUAUACCGAUCGAUGCUCGCUUUAUUUCGAAUCAC
....(((((.(((.(((.(((((((((((....((((.((......)).))))...))))))))))).))).))).))))).....

```
 Initial dG =    -26.60

        10        20        30        40
UGUU|    U   U   U           UCUC    C  UU
    UUCGA GUA AGU AGCAUUGGUCG    UAUG UU  U
    AAGCU UAU UCG UCGUAGCUAGC    AUAC AG  U
CACU^    U   U   C           CAU-    U  UA
    80        70        60         50

-----------------------------------------------------
```

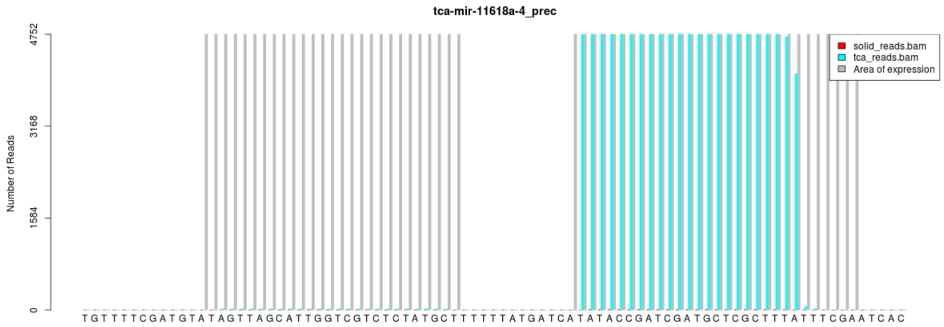

>tca-mir-11618a-3_prec
AAAAUGUUUUCGAUAAAUAGUUAGCAUUGGUCGUUUCUAUGCUUCUUCGUGGUCAUUGAUCGGCCAAUGUUCGCUUUAUUUCGAAUCCUCUAG
```
........((((.....(((.((((((((((..((.(((((.......)).))).)).))))))))))).))).....)))))........
```

```
 Initial dG =    -26.90

        10        20        30        40
AAAAUGUU|    UAAAU   U           UU  U   -  UCU
        UUCGA     AGU AGCAUUGGUCG  UC AUG CU   \
        AAGCU     UCG UUGUAACCGGC  AG UAC GG   U
GAUCUCCU^    UUAUU   C           U-  U   U  UGC
  90        80        70        60         50

-----------------------------------------------------
```

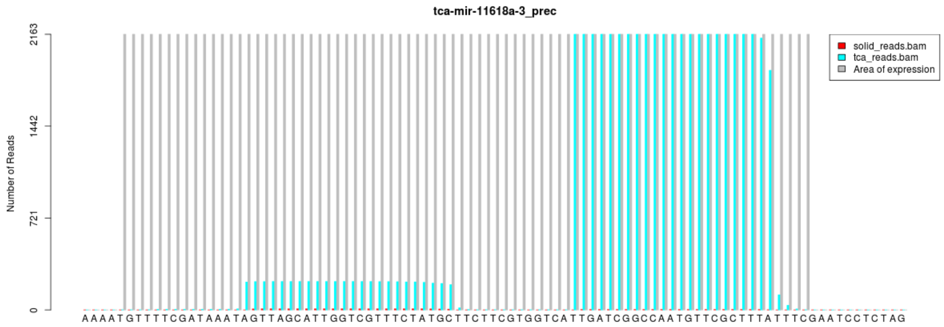

>tca-mir-11618b-4_prec

```
CAAAAUGUUUUCGAUACUUAGUUAGCAUUGGCUGUUUCUAUGCUAAUUUAUGGUCAUAGUACAGCCAAUGUUCGCUUCAGUUCGGAUUCAAUAAC
.....((..(((((.(((.(((.((((((((((((..((((((((.....))).))))).))))))))))))).)))..))))))))..)).....

 Initial dG =    -35.80

        10         20        30        40
CAAAA| UU     U   U-   U            UU     -   AU
     UG  UUCGA ACU  AGU AGCAUUGGCUGU  CUAUG CUA  \
     AC  AGGCU UGA  UCG UUGUAACCGACA  GAUAC GGU  U
CAAUA^ UU     -   CU   C            U-     U   AU
    90         80        70        60         50

-----------------------------------------------------
```

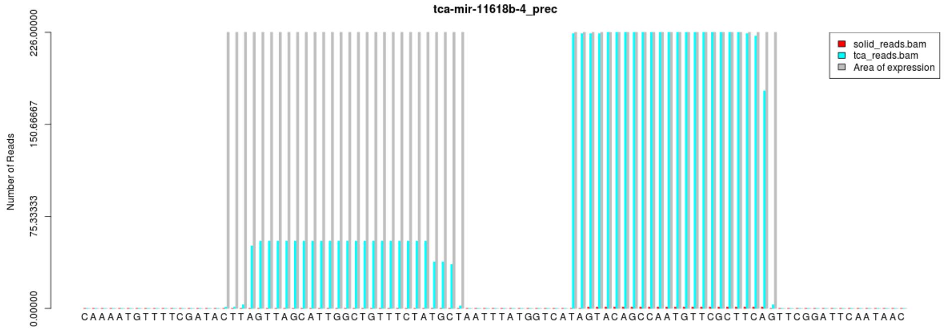

>tca-mir-11618a-2_prec

```
ACAAAAUGUUUUCGAUGUAUAGUUAGCAUUGGUCGUUUCUAUGCUUUUUAUGAUCAUAUACCGAUCGAUGCUCGCUUUAUUUCGAAUCACAUAACUG
.....((((.(((((.(((.(((.(((((((((((....((((.((.....)).))))...))))))))))).))).))).)))))..)))).....

 Initial dG =    -28.80

        10         20        30        40
ACAAA    U-|    U   U   U           UUUC    C  UU
     AUGU  UUCGA GUA AGU AGCAUUGGUCG    UAUG UU  \
     UACA  AAGCU UAU UCG UCGUAGCUAGC    AUAC AG  U
GUCAA    CU^    U   U   C           CAU-    U  UA
      90        80        70        60          50

-----------------------------------------------------
```

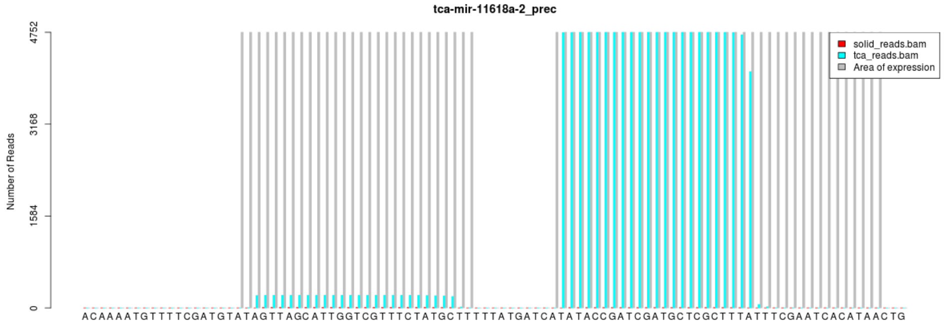

>tca-mir-11625a_prec

```
AUUAACCAACUUGAUUCAAAAUUGAUUUUAAGUAGACAUGAGUGGUGUUUCUGUGGCAUUAUUCAUAAUACUGCUAAUGUCUCCUGAGACUCGCUUUUGAAUACUCCUGGAAUAC
.....(((.....((((((((.(((.(((.((.((((((.((..((((...(((((......)))))))))..)).)))))).)).))).))).)))))))).....))).....
```

```
 Initial dG =    -30.90

        10        20        30        40        50
AUUAA|  ACUUG        U   U   A  U      G  UG    UUC     CA
     CCA     AUUCAAAA UGA UUU AG AGACAU AG  GUGU   UGUGG  U
     GGU     UAAGUUUU GCU AGA UC UCUGUA UC  CAUA   AUACU  U
CAUAA^  CCUCA        C   C   G  C      A  GU    ---     UA
   110       100       90        80        70            60

-----------------------------------------------------
```

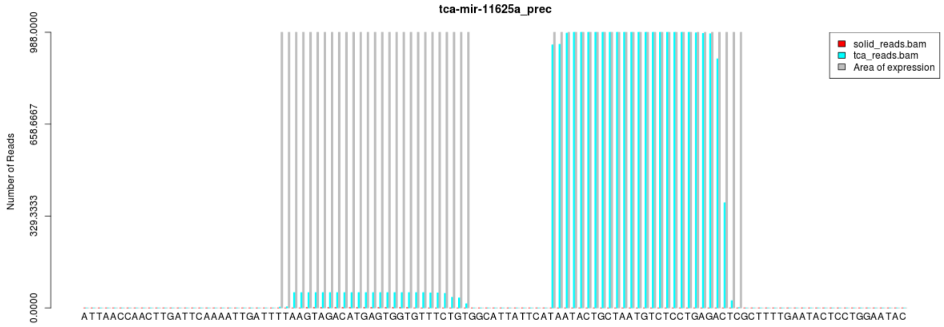

>tca-mir-11626a_prec

```
UUUUGUUUGUUGGCAUGAUUGAUUUAGCUCAUGUUGAAUUGGUUCAUGAGUUGAUCAAGUAGGCCGAGAAACGGAGCCA
((((((((.(((((....((((((.((((((((...........))))))))))))))....))))).))))))))...

 Initial dG =    -29.60

           10        20        30
---|       G     AUGA      U        UUGAA
   UUUUGUUU UUGGC    UUGAUU AGCUCAUG     \
   GAGGCAAA AGCCG    AACUAG UUGAGUAC     U
ACC^       G     GAUG      -        UUGGU
        70        60         50        40

-----------------------------------------------------
```

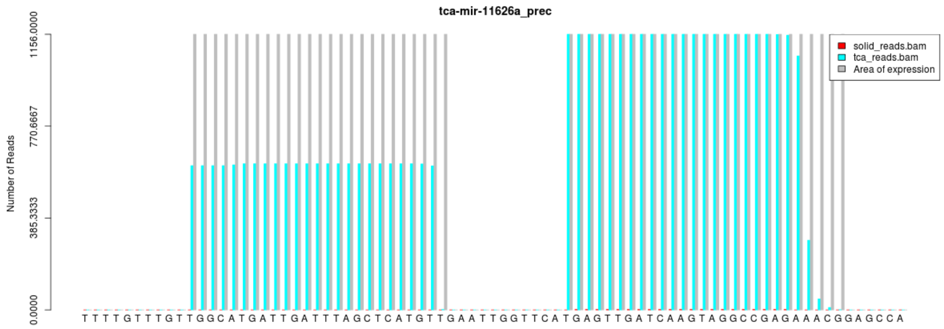

>tca-mir-11626b_prec

```
AAUUUUGGUUUUGUUUGUGGGCAUGAUUGUUUUAGCUCAUGUCUCAUGAUUCAUGAUGAUUGGAAAGCAUGAGUUGAUCAAUCAGGCCAGGAAACAGAACCACCUUC
.....((((((((((...(((.((((((..(((((((((((....(((((......)))))....))))))))))).)))))).)))...)))))))))))).....

 Initial dG =    -40.40

        10        20        30        40        50
AAUUU|          GUG   A      UU          CUCA     CA
     UGGUUUUGUUU   GGC UGAUUG  UUAGCUCAUGU    UGAUU  U
     ACCAAGACAAA   CCG ACUAAC  AGUUGAGUACG    GUUAG  G
CUUCC^          GGA   G      U-          AAAG     UA
     100        90        80        70        60

-----------------------------------------------------
```

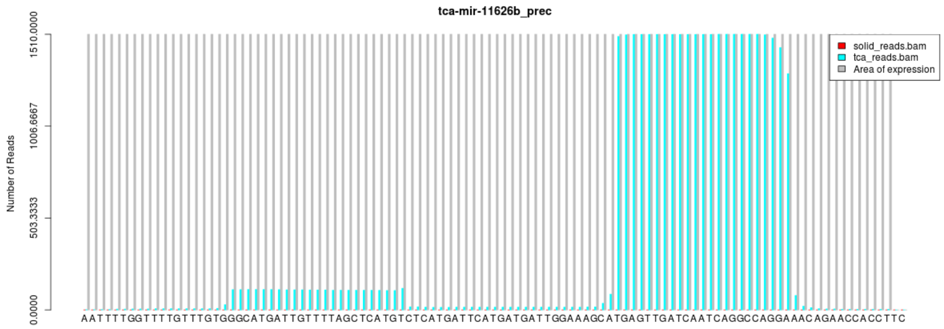

>tca-mir-11631_prec

```
GGCAAUGGGAGGGGGAUUGGCAGCUGGAGUUGACUCUUGAUGGACAGCCCCUAGACCACCAAGGGUGGACUUAUGCUGUUGAUUUCCCGUCACAAUAUU
.....(((..(((..((..(((((..(((((.(((((((.(((.............))).))))))).)))))..)))))..))..)))..).)).....
```

```
 Initial dG =    -42.70

        10        20        30        40
GGCAA  -|GA   GG  UG     UG     G       A   ACAGC
     UG G  GGG  AU  GCAGC  GAGUU ACUCUUG UGG     C
     AC C  CCC  UA  UGUCG  UUCAG UGGGAAC ACC     C
UUAUA  A^UG   UU  GU     UA     G       C   AGAUC
       90        80        70        60        50
```

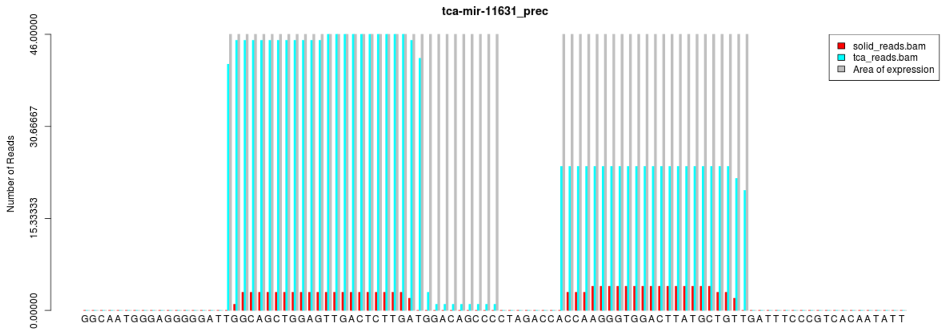

>tca-mir-11632_prec

AAUUCUCUAAACAUGCUAUACUUUGAUUUUAGUAAGGAUUUUAAUAAGGUUCCCCGCAGGUUUUUUAAUAAAUAACUCAAAAUUAAGUGGUUUAUCAGACCCCCUGUUGUCAUGGGUAAA
AAGUUUUUUAACCAAUCAAAGUUUUGCGGUGAAAGAUA
.....(((..((.(((...(((((((((.....(((((((((.......((.((((((((...(((.((((((.
(((........))).)))))).)))...)))))......))).)))))))))))).....)))))))))...)))))...)))..

```
 Initial dG =    -28.80

        10         20        30        40        50              60        70        80
AAUUC   AA-| A   UAU         UUAGU         AAUAAGG  C   ------     UUU   A      A   CAA
     UCU   AC UGC   ACUUUGAUU     AAGGAUUUU       UU CCC      GCAGG   UUU AUAAAU ACU   A
     AGA   UG GCG   UGAAACUAA     UUUUUGAAA       AA GGG      UGUCC   AGA UAUUUG UGA   A
AU---   AAG^ -   UUU         CCAAU         -------  U   UACUGU     CCC   C      G   AUU
         150        140       130       120              110       100        90
```

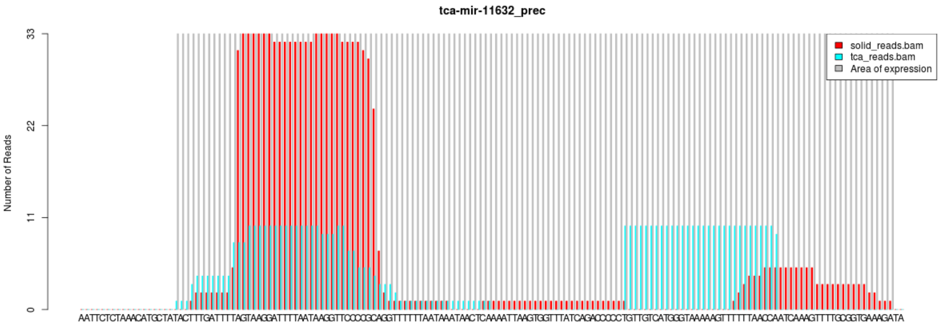

>tca-mir-11633_prec

```
CCAUUUCGGCCGGAGACUUCAACGCUCGCGCAAGCGACGAGCGCCUCCGGGCGAUGAAA
.....(((.((((((.......((((((((....)).)))))).)))))).))).....

 Initial dG =    -26.90

        10        20         30
CCAUU|  G      ACUUCAA      -  C
     UCG CCGGAG       CGCUCG CG A
     AGC GGCCUC       GCGAGC GC A
AAAGU^  G      C------      A  G
        50              40

-----------------------------------------------------
```

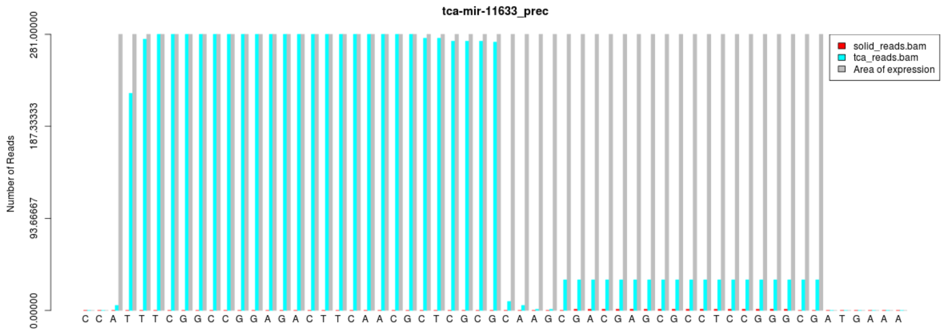

>tca-mir-3836g-1_prec

UUUUUGAAAUGUCUGUCAGGUCUACCUCUGCUUAAGUUGAUAUUUAUUUAAUUAUCACUGGGCAAGGUAGACUCUACAGCCGUUUCUUCAA
.....((((((.((((..(((((((((.((((((.((.((((..........)))))))))))))))))))))))..)))).))))))).....

```
 Initial dG =    -34.30

        10        20        30        40
UUUUU|     U    CA         C      A  U    UUUA
     GAAAUG CUGU  GGUCUACCU UGCUUA GU GAUA    U
     CUUUGC GACA  UCAGAUGGA ACGGGU CA CUAU    U
AACUU^     C    UC         -      -  -    UAAU
90        80        70         60          50
```

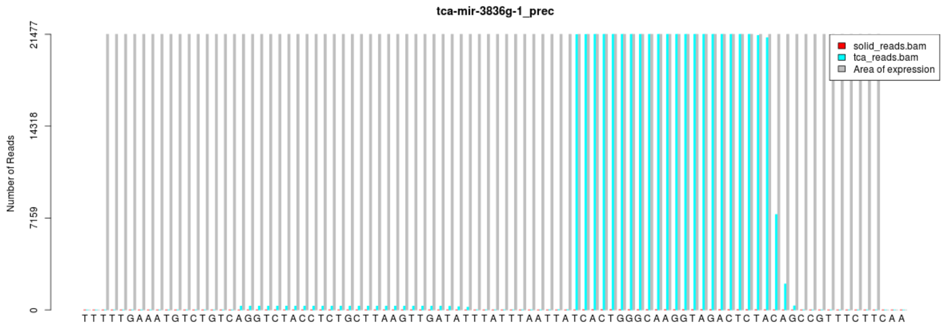

>tca-mir-3836g-2_prec

```
AAUGUCUGUCAGGUCUACCUCUGCUUAAGUUGAUAUUUAUUUAAUUAUCACUGGGCAAGGUAGACUCUACAGCCGUU
((((.((((..(((((((((.((((((.((.((((..........))))))))))))))))))))).)))).))))

 Initial dG =    -29.30

        10        20        30        40
|   U    CA         C      A  U    UUUA 
AAUG CUGU  GGUCUACCU UGCUUA GU GAUA    U
UUGC GACA  UCAGAUGGA ACGGGU CA CUAU    U
^   C    UC         -      -  -    UAAU 
      70        60          50

-----------------------------------------------------
```

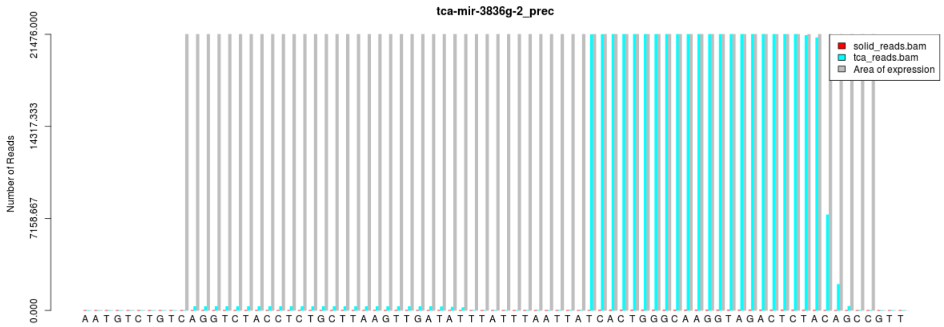

>tca-mir-3851q_prec

AUUUUGUGAGUACAUUUAGUUCUGAGUUGGCAUAGAGUAGUACUAUGUAAAAUUCUUCUCCAUUGUACGACCCAUGCUGGCCCAGUGAAAGAUUUCAAUUCACUAACA

.....((((((..((((....(((.((..((((...((.((((.(((.............))).)))).))..))))..)).)))....))))....)))))).....

```
 Initial dG =    -22.80

        10          20         30         40         50
AUUUU      AC--|   AGUU   A  UG    AGA  A    U   UAAAAU
     GUGAGU    AUUU    CUG GU  GCAU   GU GUAC AUG      \
     CACUUA    UAGA    GAC CG  CGUA   CA CAUG UAC      U
ACAAU      ACUU^   AAGU   C  GU    CC-  G    U   CUCUUC
      100        90         80         70         60
```

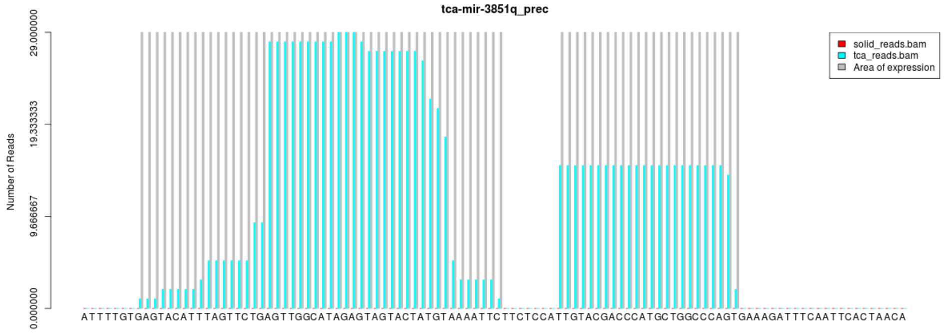

>tca-mir-3851g-2_prec

```
UUUAAUACUAAGUGGACAUGGGGUGAUACUGUGUAAUUUUGACUCAUAGCACGACCCGUGUUGGCUCAGUAAUAUA
.....((((.(((.((((((((((...(((((...........))))))))..)))))))).))).)))).....

 Initial dG =    -24.20

        10        20          30
UUUAA    A   G        --|  AUA     UAAUU
     UACU AGU GACAUGGG  GUG   CUGUG     U
     AUGA UCG UUGUGCCC  CAC   GAUAC     U
AUAUA    C   G        AG^  ---     UCAGU
      70        60          50        40

-----------------------------------------------------
```

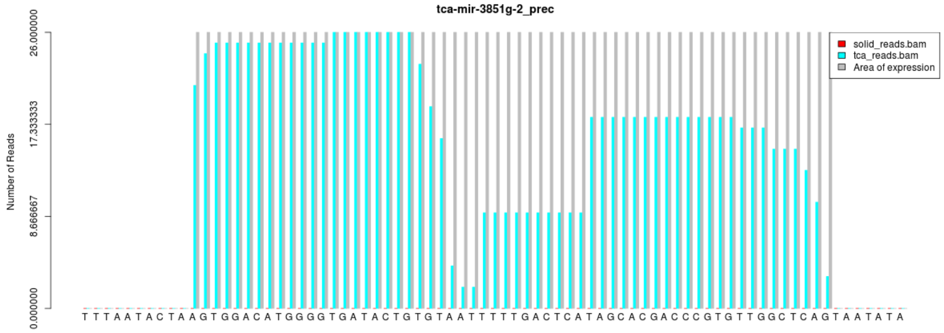

>tca-mir-3851d-1_prec

AAUUAAAUUUAAUAUUGAGUGGACAUGGGGUGGUACUGUGUAAUUUUUGACCCAUAGUACGGCCCAUGUUGGCUCAGUAAUAAAUUACAUU
.....((((((.((((((((.((((((((.(.((((((((............)))))))).))))))))).)))))))).))))))).....

```
 Initial dG =    -38.40

        10        20        30        40
AAUUA|     A        G        G G        UAAUU
     AAUUUA UAUUGAGU GACAUGGG U GUACUGUG     U
     UUAAAU AUGACUCG UUGUACCC G CAUGAUAC     U
UUACA^     A        G        - G        CCAGU
90        80        70         60        50
```

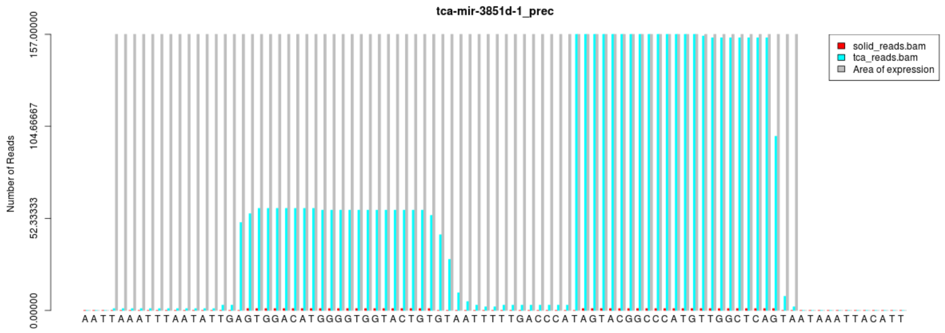

>tca-mir-3851d-2_prec

AAUUAAAUUUAAUAUUGAGUGCACAUGGGGUGGUACUGUGUAAUUUUUGACUCAUAGUACGGCCCAUGUUGGCUCAGUAAUAAAUUACAUU
.....((((((.((((((((..(((((((.(.((((((((............)))))))).))))))))..)))))))).)))))).....

```
 Initial dG =    -34.50

        10        20        30        40
AAUUA|     A        GC       G G        UAAUU
     AAUUUA UAUUGAGU  ACAUGGG U GUACUGUG     U
     UUAAAU AUGACUCG  UGUACCC G CAUGAUAC     U
UUACA^     A        GU       - G        UCAGU
90        80        70          60        50
```

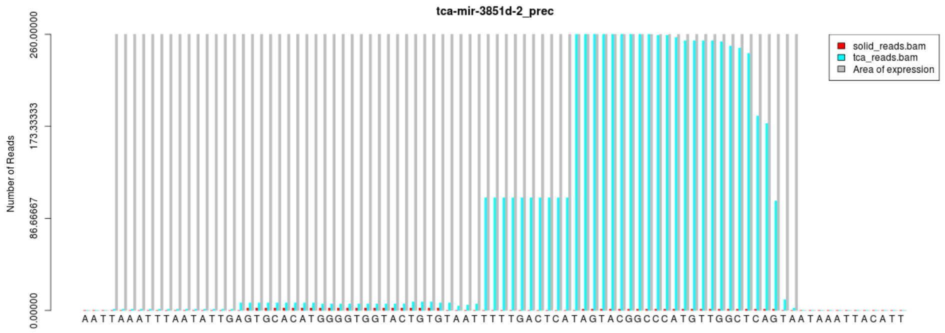

>tca-mir-3851m-4_prec

```
UUAGUACUGGGUUGGCACAGAGUAGUACUAUGUAAAAUUCUAUUUCAUAGUACGACCCGUGUCGGCCCAGUAAGAG
....((((((((((((((...((.((((((((.(((......)))))))))))).))..))))))))))))))....

 Initial dG =    -33.90

        10         20         30
UUAG|              AGA  A         U   AU
    UACUGGGUUGGCAC   GU GUACUAUG AAA  U
    AUGACCCGGCUGUG   CA CAUGAUAC UUU  C
GAGA^              CC-  G         -   AU
     70         60          50          40

-----------------------------------------------------
```

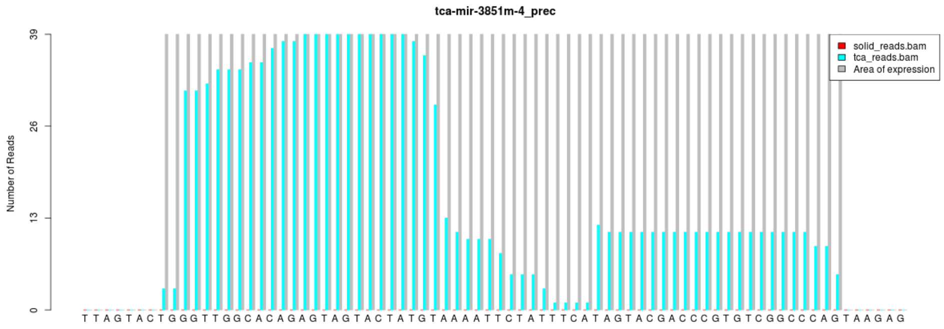

>tca-mir-3851r_prec

```
UAGUUCUGAGUUGGCAUAGAGUAGUACUAUGUAAAAUUUUUCUCCAUAGUACGACCCAUGCCGGCCCAGUGAAA
.....(((.((((((((...((.((((((((.............)))))))).))..)))))))).))).....

 Initial dG =    -23.50

        10        20        30
UAGUU|  A        AGA  A        UAAAAU
     CUG GUUGGCAU   GU GUACUAUG      \
     GAC CGGCCGUA   CA CAUGAUAC      U
AAAGU^  C        CC-  G        CUCUUU
   70        60         50        40

-----------------------------------------------------
```

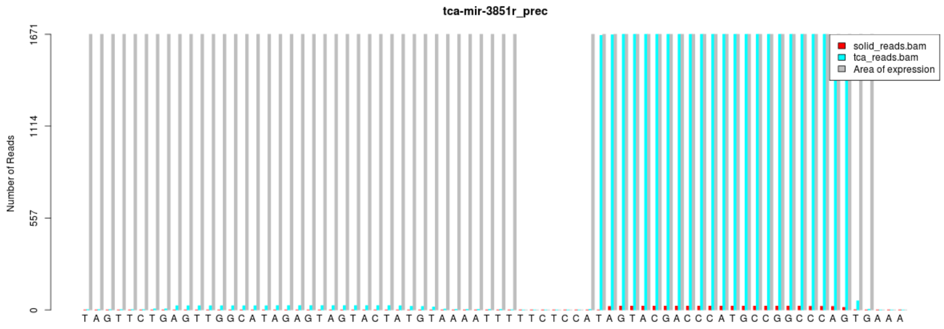

>tca-mir-3858a-2_prec

```
CUGCAACUGUAACAGUUUGUUGUUUUUUAGUGAAGCGAAGUUUUUUCAUUUUAAUAAAAAUGAAACUCUUUUGCUUCUCUAAUAAAUACAAAUUCAUUCUUCCUAACAGUAUAUA
.....(((((...(((((((.((((.((((.(((((((((...(((((((((....)))))))))...))))))))).)))).))))))))))).........))))).....

 Initial dG =    -28.00

        10                 20        30        40        50
CUGCA     AAC--------|      U    U    U         UUU         A
     ACUGU           AGUUUGU GUUU UUAG GAAGCGAAG   UUUCAUUUU A
     UGACA           UUAAACA UAAA AAUC CUUCGUUUU   AAAGUAAAA U
AUAUA     AUCCUUCUUAC^      -    U    U         CUC         A
   110       100        90         80        70        60

-----------------------------------------------------
```

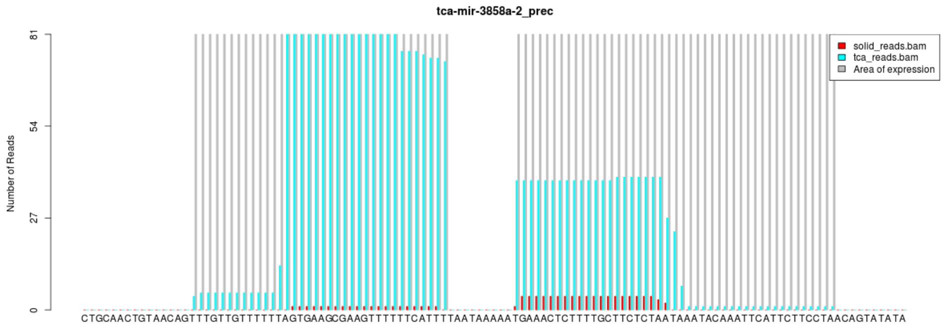

>tca-mir-3858b_prec

ACACAGUUUGUUGUUUUUAGAGAAGCGAAGAUUUUUCGUUGUGUAAAAAAGAAUGAAACUCUUUAGCUUCUCUAAUAAAUGCAAAUUUAUU
....(((((((.....(((((((((((((((..(((((((...........))))))).))))).)))))))))).....)))))))....

```
 Initial dG =    -26.00

        10        20         30        40
ACAC       UGUUU          -|    UU       GUGUA
    AGUUUGU     UUAGAGAAGC GAAGA  UUUCGUU     \
    UUAAACG     AAUCUCUUCG UUUCU  AAAGUAA     A
UUAU       UAAAU          A^    C-       GAAAA
90        80        70        60         50
```

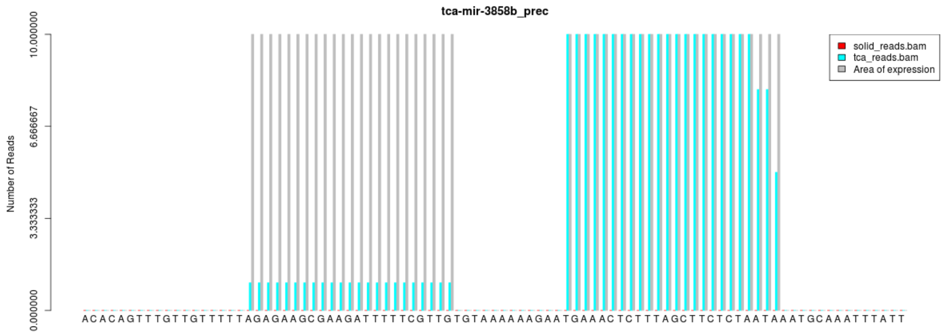

>tca-mir-11619c_prec

```
AACUAUCGGAUGAGUGAUGAUAGUCUGCGAUAUUCUUCAUUUUAUAAUUUAACAAAUGAAGAGUCUCAGACUUGGUCUUACGCUCGAUUGCUCGACACAA
.....((((.((((((..((((((((.(((..(((((((((...........))))))))))))).))))))..)))...))))))).....)))).....

 Initial dG =    -26.60

        10             20          30        40
AACUA    A----      AU-   --|     C   AU         UAUAA
     UCGG     UGAGUG   GAU  AGUCUG GAU  UCUUCAUUU     \
     AGCU     GCUCGC   CUG  UCAGAC CUG  AGAAGUAAA     U
AACAC    CGUUA      AUU   GU^     U   --         CAAUU
.        90         80         70          60        50

-----------------------------------------------------
```

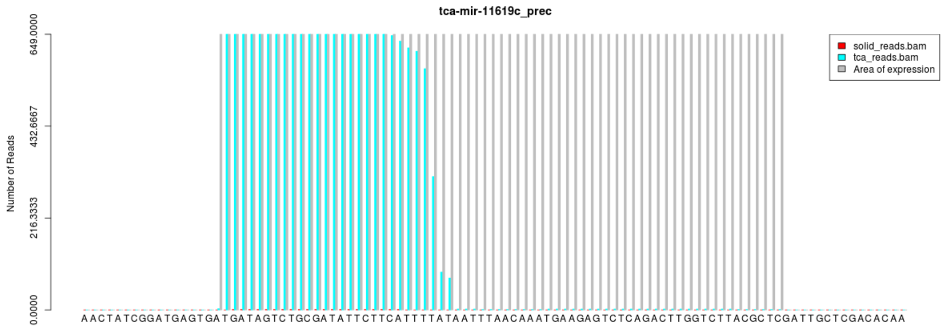

>tca-mir-11619d_prec

```
AAACUAUCGGAUGAGAGAUGAUAGUCUGCGAUAUUCUUCAUUUUAUAAUUUAACAAAUGAAGAACCUCAGACUUGGUCUUAGGCUCGAUUGCU
.....((((....(((((...((((((.....((((((((((...........))))))))))...))))))..)))))....))))).....
```

```
 Initial dG =    -21.80

        10        20        30        40
AAACU|    AUGA     GAU      CGAUA          UAUAA
     AUCGG    GAGAU   AGUCUG     UUCUUCAUUU     \
     UAGCU    UUCUG   UCAGAC     AAGAAGUAAA     U
UCGU-^    CGGA     GU-      UCC--          CAAUU
  90         80         70          60        50

-----------------------------------------------------
```

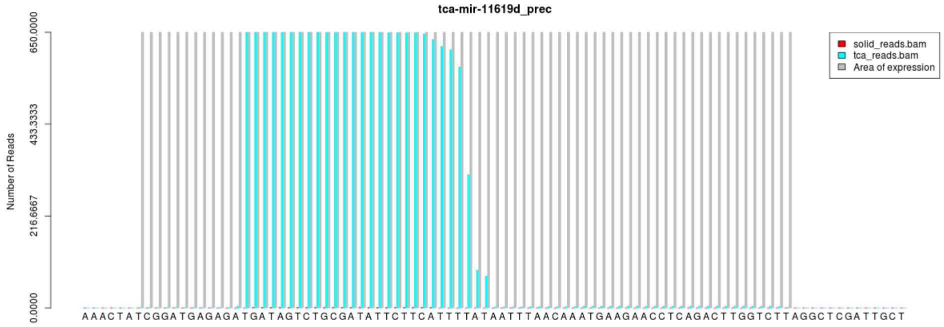

>tca-mir-11625b_prec

AAGGCUCAAACUUGAUUUUAAGUAGACAUGAGUGGUAUUUAUGUUAUACAUCAUUCAUAAUACUGCUAAUGUUUCCUGUGACUCGCUGUUUGAAAACU

.....((((((.(((.....((.((((((.((..(((((.(((............))))))))..)).)))))).)).....)))..)))))).....

```
 Initial dG =    -21.90

        10         20        30        40
AAGGC      U-|  UUUUA  U      G  UG     U   UUAUA
     UCAAAC  UGA     AG AGACAU AG  GUAUU AUG     C
     AGUUUG  GCU     UC UUUGUA UC  CAUAA UAC     A
UCAAA      UC^  CAGUG  C      A  GU     -   UUACU
       90        80        70        60         50
```

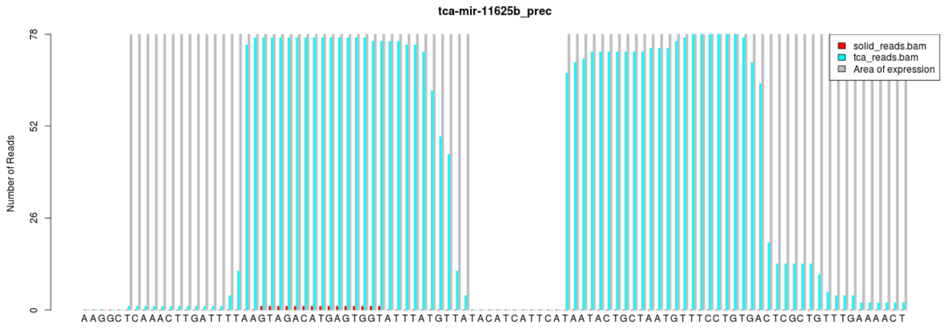

>tca-mir-11634_prec

```
AUUGGGAGUUCAGGGCGCGGCUGUGACCUGCAGCCACUCGGAGAGGCCGCGCCCCGCACUCGCGAGGUC
.(((.((((...((((((((((....((...........))...))))))))))...)))).)))....

 Initial dG =    -31.80

           10        20        30
A---|  G    UCA          GUGA  UGCAG
    UUG GAGU   GGGCGCGGCU    CC     \
    AGC CUCA   CCCGCGCCGG    GG     C
CUGG^  G    CGC          AGA-  CUCAC
        60        50         40

-----------------------------------------------------
```

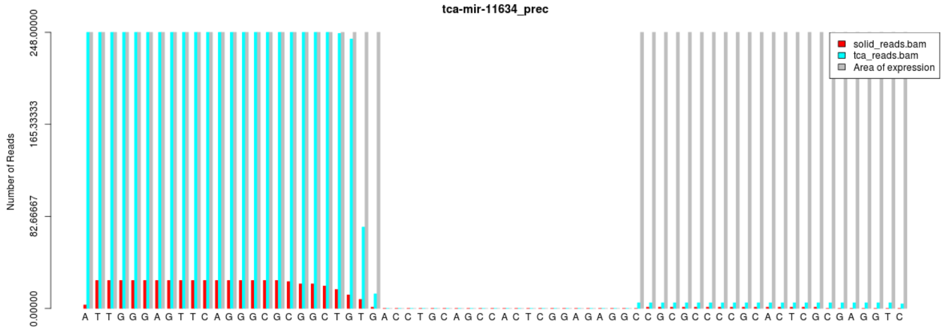

>tca-mir-3836c-4_prec

```
AGGUCCACCUUUUGCUUUUGUUGAUAUUUAUUUAACUAUCACUGGGCAAGGUAGACCCUGA
.((((.((((..(((((..((.((((.((....)).)))))).))))))))).)))).... 

 Initial dG =    -17.10

           10        20        30
A---|   C    UU     UU  U    U  A
    GGUC ACCU  UGCUU  GU GAUA UU U
    CCAG UGGA  ACGGG  CA CUAU AA U
AGUC^   A    --     U-  -    C  U
60        50            40

-----------------------------------------------------
```

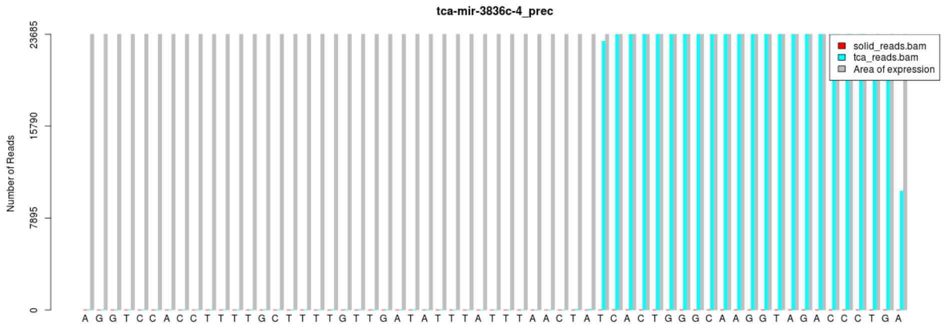

>tca-mir-3836c-5_prec

```
AGGUCCACCUUUUGCUUUUGUUGAUAUUUAUUUAACUAUCACUGGGCAAGGUAGACCCUGG
.((((.((((..(((((..((.((((.((....)).)))))).))))))))).)))).....

 Initial dG =    -17.10

           10        20        30
A---|   C    UU     UU  U    U  A
    GGUC ACCU  UGCUU  GU GAUA UU U
    CCAG UGGA  ACGGG  CA CUAU AA U
GGUC^   A    --     U-  -    C  U
60        50            40

-----------------------------------------------------
```

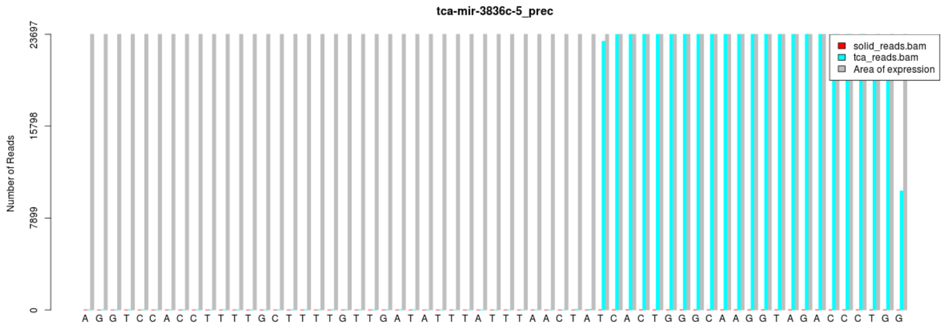

The microRNA toolkit of insects. Guillem Ylla, Bastian Fromm, Maria-Dolors Piulachs, Xavier Belles

Supplementary Data S7. Report generated with MirPlot for the 554 miRNAs of *Bombyx mori* available in miRBase. For each miRNA, based on the available data, the following information is shown;

- The miRNA precursor.
- The precursor folding in Vienna format.
- The free energy of the precursor folding.
- The folding of the miRNA precursor.
- A plot showing all the expressed nucleotides of the precursor (grey), the reads per million in the available small RNA-seq libraries.

bmo-bantam_prec

```
UAAAAGGAACUACGAAACUGGUUUUCAUAAUGAUUUGACAGAUUGUUUUGUAUUCUGAGAUCAUUGUGAAAGCUAAUUUUGUUCCUGGUA
....((((((...((((.((((((((((((((((((..((((.((.....)).)))))))))))))))))))))))).))))))))))....
```

```
 Initial dG =    -31.40

        10        20        30        40
UAAA|     UAC    C                  GA    U  UU
    AGGAAC   GAAA UGGUUUUCAUAAUGAUUU  CAGA UG  \
    UCCUUG   UUUU AUCGAAAGUGUUACUAGA  GUCU AU  U
AUGG^     ---    A                  --    U  GU
.           80        70        60          50
```

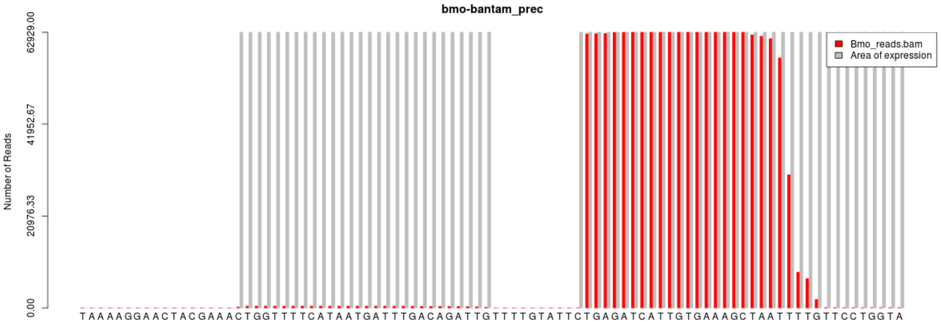

>bmo-let-7_prec

```
GGUACUGCCGUCGGCUUGUUGAGGUAGUAGGUUGUAUAGUACGGAAAUACAACACAUAGGUGCGACUGUAUAGCCUGCUAACUUUCCGAGCUGACGGAAUGACA
.((....((((((((((..(((.(((((((((((((((.((.........(((....))))))))))))))))))))))).))).)).)))))))))))....)).

 Initial dG =    -49.50

        10        20        30        40        50
G| ACUG           UU   G                A  GAAAUACAA   A
 GU    CCGUCGGCUUG  GAG UAGUAGGUUGUAUAGU CG         CAC U
 CA    GGCAGUCGAGC  UUC AUCGUCCGAUAUGUCA GC         GUG A
A^ GUAA           CU   A                -  ---------   G
  100        90        80        70                  60

-----------------------------------------------------
```

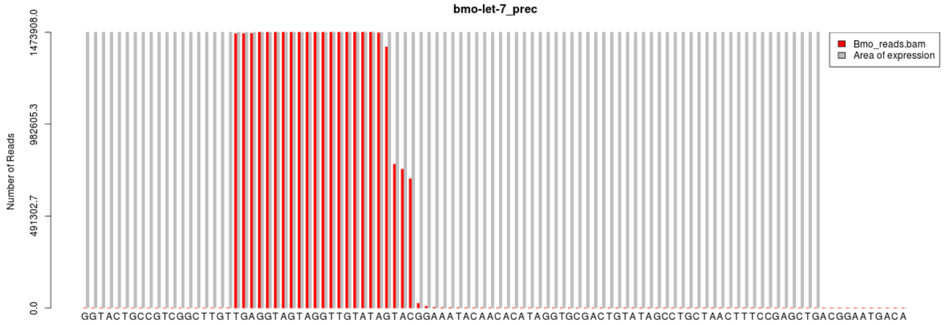

>bmo-mir-1a_prec

```
AGCCUUGCGCAAGUUCCGUGCUUCCUUACUUCCCAUAGUCAUUGUAAUCAUAUGGAAUGUAAAGAAGUAUGGAGCUGCGCGGGCG
.((((.(((((.(((((((((((.((((...(((((.............)))))...)))).)))))))))))))))))))))).

 Initial dG =    -42.50

        10        20        30        40
A|   U     A            C    UUC     GUCAUU
 GCCU GCGCA GUUCCGUGCUUC UUAC   CCAUA      \
 CGGG CGCGU CGAGGUAUGAAG AAUG   GGUAU      G
G^   -     -            A    UAA     ACUAAU
     80         70        60        50
```

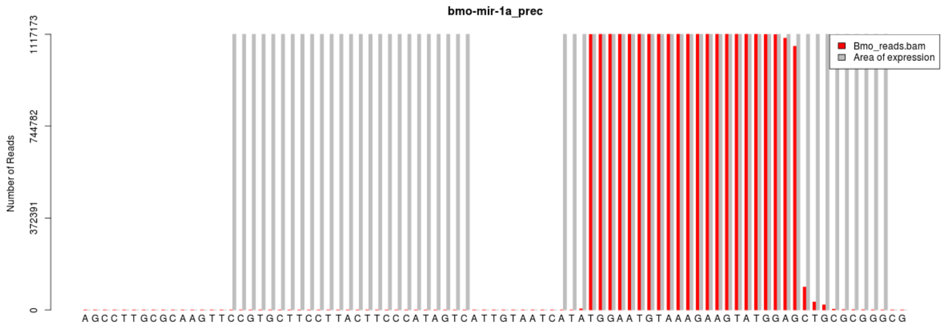

>bmo-mir-1b_prec

```
CCGCGCAGCUCCAUACUUCUUUACAUUCCAUAUGAUUACAAUGACUAUGGGAAGUAAGGAAGCACGGAACUUGCGCAAG
..((((((.(((...((((((((((.(((((((.............))))))).))))))))))...))).)).))))...

 Initial dG =    -29.70

          10         20         30
CC-     -| C   AUA         A       UGAUUA
   GCGC AG UCC   CUUCUUUAC UUCCAUA      \
   CGCG UC AGG   GAAGGAAUG AGGGUAU      C
GAA     U^ A   CAC         A       CAGUAA
        70         60         50         40

-----------------------------------------------------
```

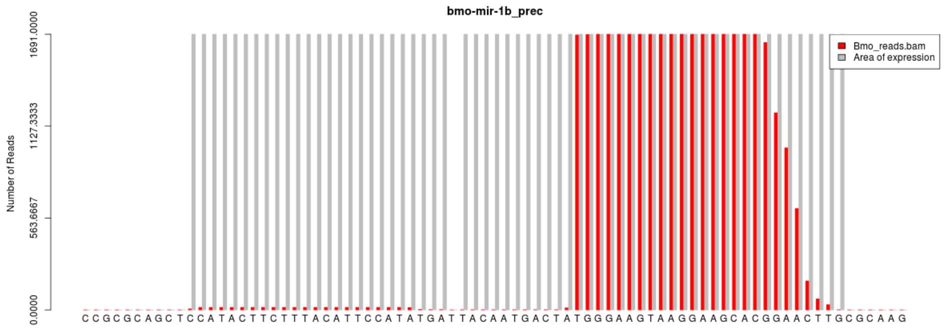

>bmo-mir-2a-1_prec

```
GGCGGAAUGUAGUCGUGGCAUCAAAGUCGGUUUGUCAUAGGUCAUAACGUAACUAUCACAGCCAGCUUUGAUGAGCACGACUUCAUCUGU
.(((((.((.(((((((.(((((((((.((((.((.((((............)))).)))))).)))))))))..))))))).)))))))

 Initial dG =    -37.60

        10          20         30         40
G|    A  U       G-         C    U  C    GUCAU
 GCGGA UG AGUCGUG  CAUCAAAGU GGUU GU AUAG     A
 UGUCU AC UCAGCAC  GUAGUUUCG CCGA CA UAUC     A
-^    -  U       GA         A    -  C    AAUGC
90         80         70         60          50
```

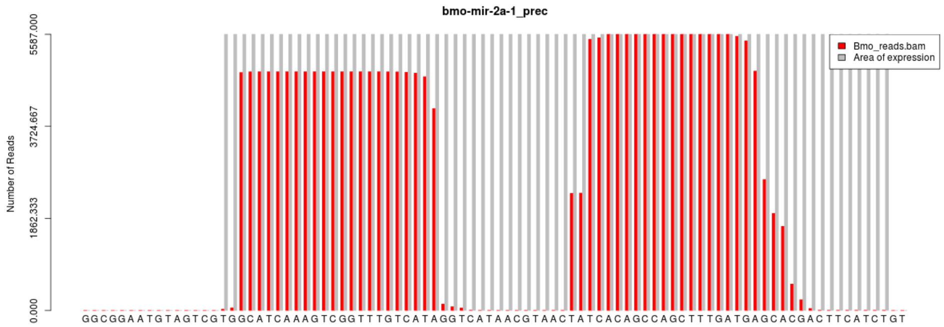

>bmo-mir-2a-2_prec

```
AAUGUUUCGUCGCUCACAAAGUGGUUGUCGUAUGCGUUUUUACUUUGGCCAUAUCACAGCCAGCUUUGAUGAGCGAUGAA
.....(((((((((((((((((((((.(((((.(((........)))))))).))))))).))))).)))))))))))

 Initial dG =    -38.10

        10         20         30        40
AAUGU           -     -|      C     C   UUU
     UUCGUCGCUCA CAAAG UGGUUGU GUAUG GUU   A
     AAGUAGCGAGU GUUUC ACCGACA UAUAC CGG   C
-----           A     G^      C     -   UUU
    80        70        60        50

-----------------------------------------------------
```

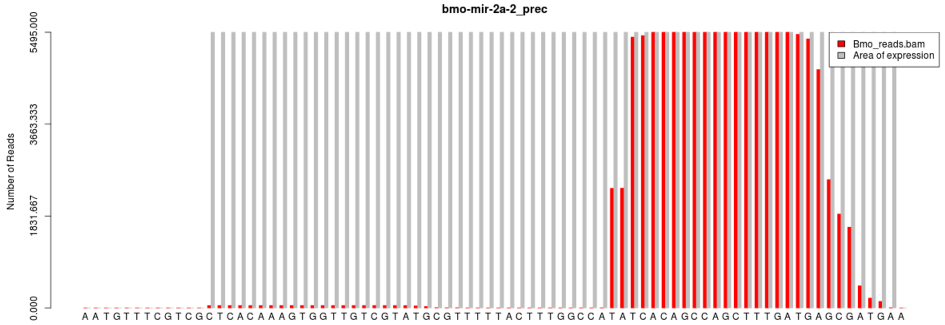

>bmo-mir-2b_prec

```
CGCUAGAACUCGACAAGGUGGUUGUGACAUGGGGGCUCAGGAUCAUAUCACAGCCAGCUUUGUUGAGUUCUCGGCU
.((((((((((((((((((((((((((.((((..........)))).))))))))).))))))))))))))).))).

 Initial dG =    -40.00

         10         20         30
C   -              -|         C    GGGC
 GCU AGAACUCGACAAGG UGGUUGUGA AUGG    U
 CGG UCUUGAGUUGUUUC ACCGACACU UACU    C
U   C              G^         A    AGGA
     70         60         50         40

-----------------------------------------------------
```

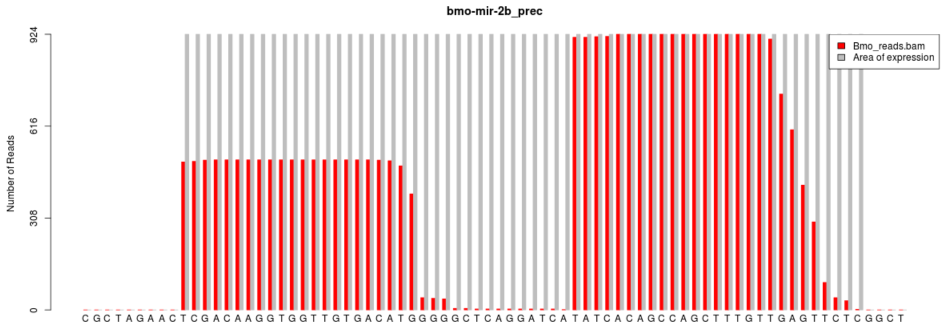

>bmo-mir-7_prec

```
CGCUUCGUGUUGUAUGGAAGACUAGUGAUUUUGUUGUUUUUGUUGACUAACAAGAAAUCACUAAUCUGCCUACAAAGCGACAGCA
.(((((((.(((((.((.(((.((((((((((((((((......))).)))))..)))))))).))).))))))).)))).))).

 Initial dG =    -31.60

         10        20        30           40
C   -    G     U  A   C        --     -|  UU
 GCU UCGU UUGUA GG AGA UAGUGAUU  UUGUU GUU  U
 CGA AGCG AACAU CC UCU AUCACUAA  AACAA CAG  G
A   C    A     -  G   A        AG     U^  UU
    80         70        60        50

-----------------------------------------------------
```

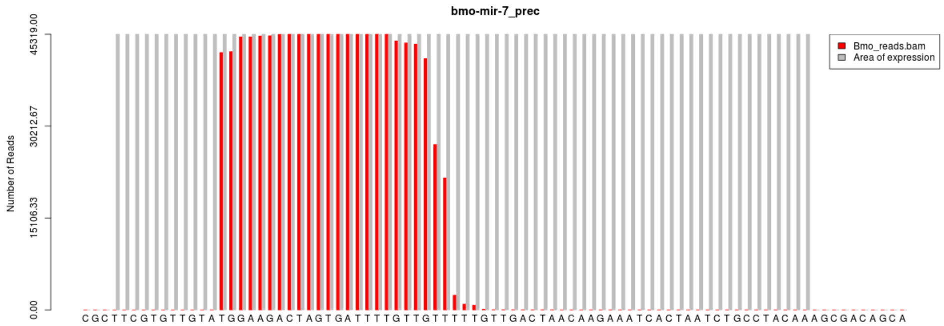

>bmo-mir-8_prec

CACGACGGAGUAACGGUUCGCAUCUUACCGGGCAGCAUUAGAGUCCUGUCUAUAUUUUCUAAUACUGUCAGGUAAAGAUGUCGUCCGCGCUCCACGUUCGUC
.((((.(((((..(((..(((((((((((.(((((.(((((((.............))))))).))))).))).)))))).)).))).))))).....)))).

```
 Initial dG =    -37.60

           10        20          30        40
C    C---|    AA   UU  -      -   G     C       UCCUGU
 ACGA    GGAGU  CGG  CG CAUCUU ACC GGCAG AUUAGAG      \
 UGCU    CCUCG  GCC  GC GUAGAA UGG CUGUC UAAUCUU      C
C    UGCA^    C-   U-  U      A   A     A       UUAUAU
100        90          80        70        60
```

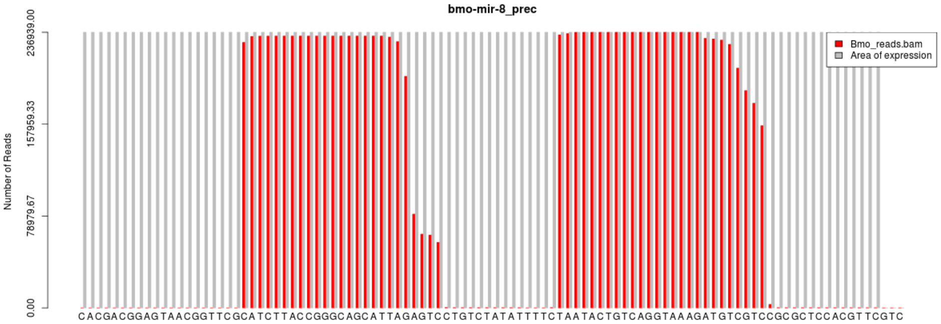

>bmo-mir-9a_prec

```
AGUAGAUUGGUUAAUUAUCUUUGGUUAUCUAGCUGUAUGAGUAUUACUGACGUCAUAAAGCUAGGUUACCGGAGUUAAGUGCCGUCUACA
.((((((.(((...(((.(((((((.((((((((.(((((((.......)).))))).)))))))).))))))).)))..))))))))).

 Initial dG =    -37.30

        10         20         30         40
A|      U   UAA   U       U         G      -  AUU
 GUAGAU GGU   UUA CUUUGGU AUCUAGCU UAUGA GU   \
 CAUCUG CCG   AAU GAGGCCA UGGAUCGA AUACU CA   A
A^      -   UG-   U       U         A      G  GUC
.         80         70         60         50

-----------------------------------------------------
```

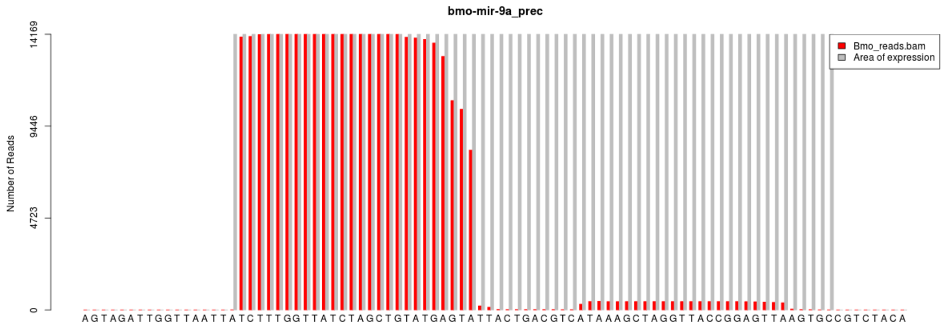

>bmo-mir-9b_prec

```
GACGGCGCGACGCGAUGCUUUGGUAAUCUAGCUUUAUGAACGGAAUGAUGUCACGGAGCUAAAUCGCCAAAGCGUCGCAGCGCGCCCGGC
...((((((..((((((((((((..((.((((((..(((.((......)))))..)))))).))..))))))))))))..))))))....

 Initial dG =    -50.40

         10        20        30        40
GAC-|     AC            UA  C      UA   A  GA
    GGCGCG  GCGAUGCUUUGG  AU UAGCUU  UGA CG  A
    CCGCGC  CGCUGCGAAACC  UA AUCGAG  ACU GU  U
CGGC^     GA            GC  A      GC   -  AG
.        80        70        60         50

-----------------------------------------------------
```

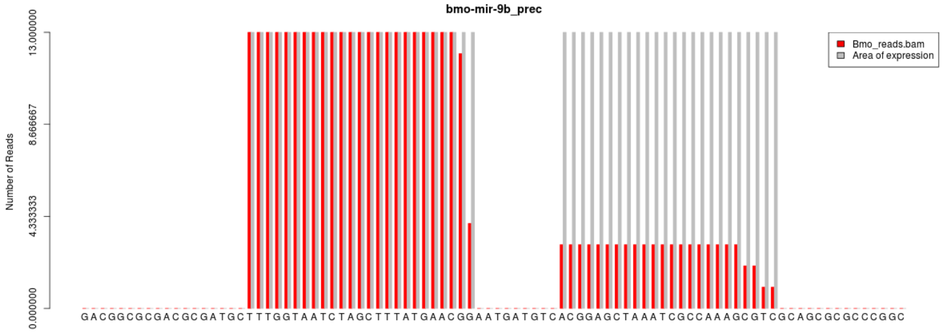

>bmo-mir-9c_prec

```
CAGCGCGCCGAGGGUCGCGGGCCGUCUCUUUGGUAUCCUAGCUGUAGGCGUGCGCGGCCCUAAAGUUAUGGUACCGAAGUUACGGCUCGUGACGUAUCAGUCGACGUC
..(((((.((..((((((((((((..((((((((((.(((((.((((.((.....)))))).))))).)))))))))))..))))))))))))...)).).)).))).

 Initial dG =    -54.00

          10         20         30         40         50
CA   -  - C  GG-|           CU          C     G    C  GC
  GCG CG C GA   GUCGCGGGCCGU  CUUUGGUAUC UAGCU UAGG GU  \
  UGC GC G CU   CAGUGCUCGGCA  GAAGCCAUGG AUUGA AUCC CG  G
C-   A  U A  AUG^           UU          U     A    -  GC
       100        90         80         70         60

-----------------------------------------------------
```

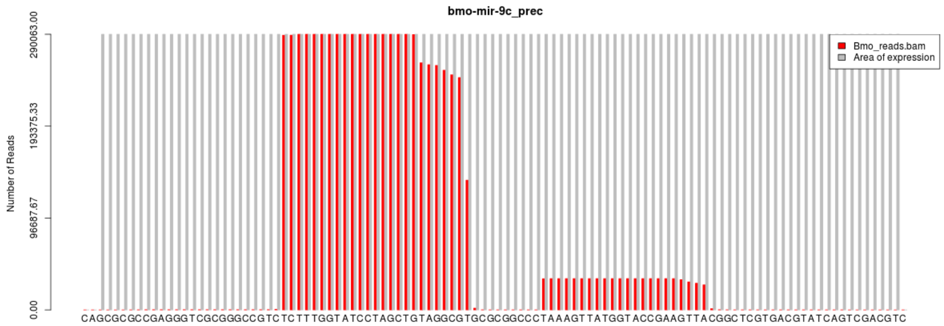

>bmo-mir-9d_prec

GCUCAAUUGGAGUAUUUGGUGAUUCAGCUGAUGUGAUGAAAACUUUUUAAUUCAUAAAGCUAAUUCACUGAGUGUUCGAGUUGAG
.(((((((.(((((((..((((...((((..(((((..(((....)))...))))).))))...))))..))))))).)))))))

```
 Initial dG =    -29.50

        10        20        30         40
G|      G       UG     UUC    GA      UG-   A
 CUCAAUU GAGUAUU  GUGA   AGCU  UGUGA   AAA C
 GAGUUGA CUUGUGA  CACU   UCGA  AUACU   UUU U
-^      G       GU     UAA    A-      UAA   U
     80        70        60         50
```

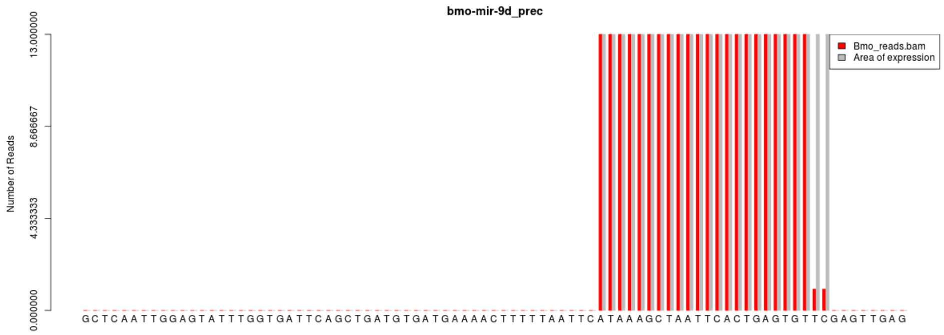

>bmo-mir-10_prec

```
AGUGCCCUACAUCUACCCUGUAGAUCCGAAUUUGUUUGAAGUGAGGCGACAAAUUCGGUUCUAGAGAGGUUUGUGUGGUGCACG
.((((.((((((..(((((.((((.(((((((((((...........))))))))))).)))).)).))).)..)))))).)))).

 Initial dG =    -39.60

        10         20        30        40
A    C      CU   -| G    U           UGAAG
 GUGC CUACAU  ACC CU UAGA CCGAAUUUGUU     \
 CACG GGUGUG  UGG GA AUCU GGCUUAAACAG     U
G    U      UU   A^ G    U           CGGAG
   80        70        60        50

-----------------------------------------------------
```

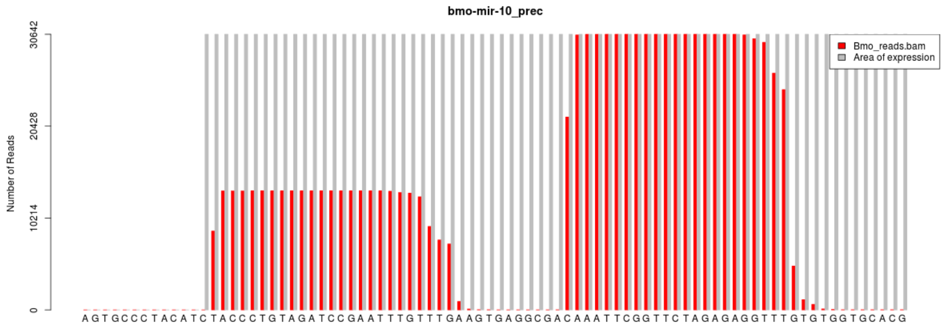

>bmo-mir-11_prec

```
GUUUUGUGGCUAGCACUCGACAUGUGACCUGUGUUCGGUAAUCACAUCACAGUCAGAGUUCUAGCUACACGGCGCG
((..(((((((((.(((((((.(((((..((((.........)))))))))))).)))).)))))))))..))...

 Initial dG =    -33.40

           10          20         30
---  UU          C     -|  A      CC    UUCG
   GU  UGUGGCUAG ACUC GAC UGUGA  UGUG    \
   CG  ACAUCGAUC UGAG CUG ACACU  ACAC    G
GCG  GC          U    A^  -      --    UAAU
     70         60          50          40

-----------------------------------------------------
```

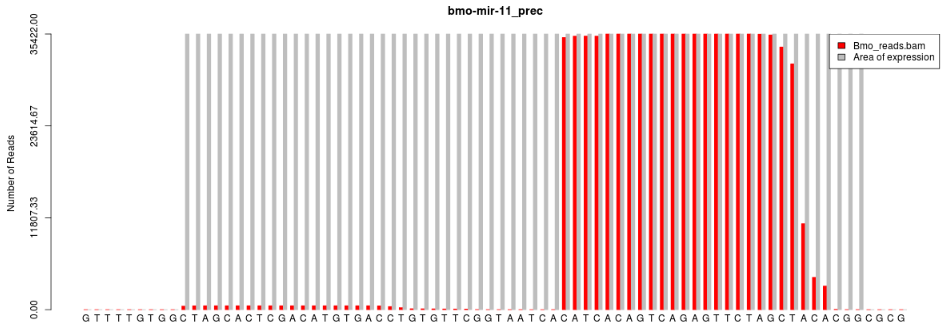

>bmo-mir-12_prec

```
UUCUGAGUAUUACUUCAGGUACUGGUUGUAUUAAAUAAAAAACGGAAUAAGUUAUUAACACAGCCUAAUAGAGAUAUUACGGAUUUACGA
.((((((((((.((..(((..(((((((...........................)))).))))))...)).)))))).)))).......

 Initial dG =    -12.50

               10         20         30        40
U------    -      A  UC-|  UA   -    UAUUAAAUAAAAA
       UCUG AGUAUU CU   AGG  CUG GUUG             \
       AGGC UUAUAG GA   UCC  GAC CAAU             A
AGCAUUU    A      A  UAA^  --   A    UAUUGAAUAAGGC
.        80        70         60        50

-----------------------------------------------------
```

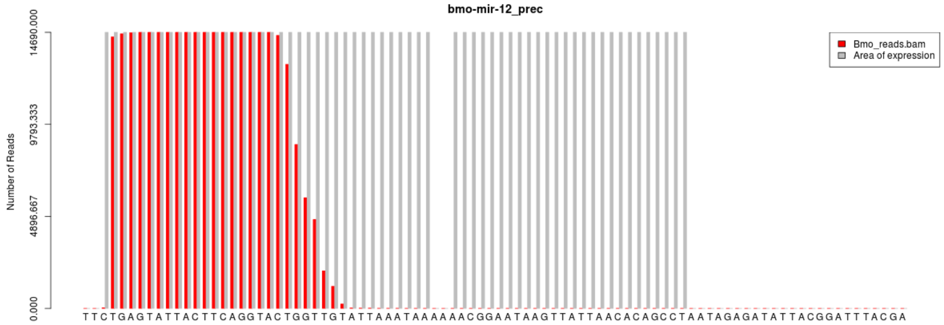

>bmo-mir-13a_prec

```
GAUAAUCUCGAUCCUGUCAAAGCGGCGGUGAAAUGUCUCCUCCCUUUCAUAUCACAGCCACUUUGAUGUGGUCGGUUUA
.......((((((.((((((((.(((.((((.(((............))).)))).))).)))))))).))))))....

 Initial dG =    -27.50

        10        20        30        40
GAUAAUC|     C        C   G    A   UCUCC
       UCGAUC UGUCAAAG GGC GUGA AUG     U
       GGCUGG GUAGUUUC CCG CACU UAC     C
AUUU---^     U        A   A    A   UUUCC
           70        60        50

-----------------------------------------------------
```

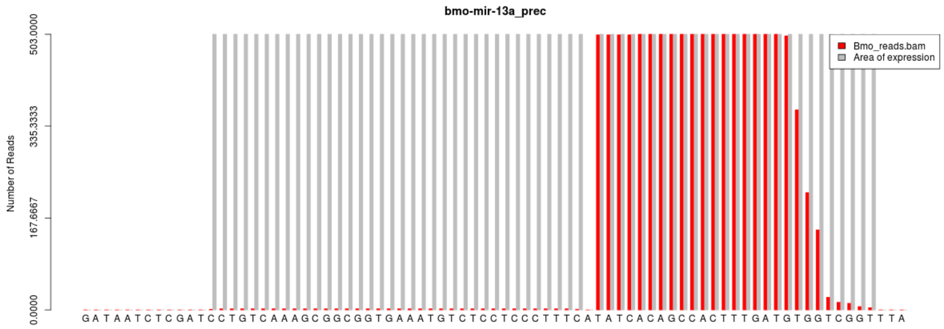

>bmo-mir-13b_prec

```
GUCCAUGGCCCACUCGUAAAAAUGGCUGUGUCGUGUAGCAACCCUCAUAUCACAGCCAUUUUUGACGAGUUGGGCUGUGU
...((((((((((((((((((((((((((..(((.((.....)))))..))))))))))))))).))))).)))))))).

 Initial dG =    -43.50

        10          20         30         40
GUC         -     -|             UC    U   CA
   CAUGGCCC ACUCG UAAAAAUGGCUGUG  GUG AG  \
   GUGUCGGG UGAGC GUUUUUACCGACAC  UAC UC  A
U--         U     A^             UA    -   CC
.          70         60         50

-----------------------------------------------------
```

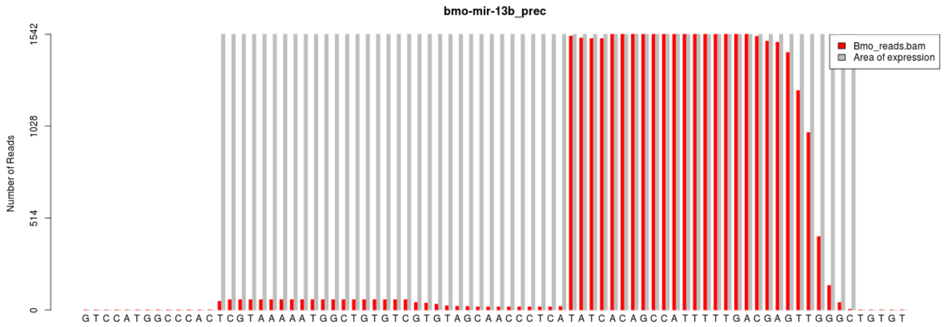

>bmo-mir-14_prec

```
UUGUCAUUUGUGUCGGGGAGAGAAAUCGACGAGGCUGUUUUAUUUAGUCAGUCUUUUUCUCUCUCCUAUAUGAGUGACAU
.(((((((..(((.((((((((((((..(((..(((((.......))))).)))..)))))))))))).)))..))))))).

 Initial dG =    -39.90

        10         20         30         40
U|       UG   C           UC   GA      UUU
 UGUCAUU  UGU GGGGAGAGAAA  GAC  GGCUG   \
 ACAGUGA  AUA CCUCUCUCUUU  CUG  CUGAU   U
U^       GU   U           UU   A-      UUA
.         70         60         50

-----------------------------------------------------
```

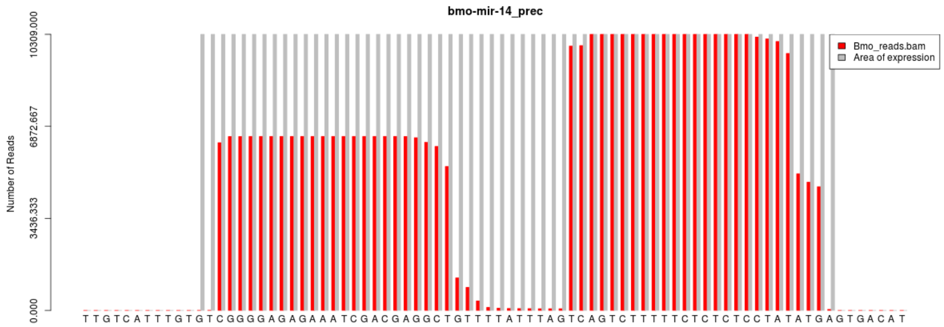

>bmo-mir-31_prec

GUCGAGCCGGUGGCUGGGAAGGCAAGAAGUCGGCAUAGCUGUUUGAAUAAGAUACACGGCUGUGUCACUUCGAGCCAGCUCAAUCCGCCGGCUUUCUUCAAUUUCAAGAUUUGCGGAUGC
U

```
...((((((((((.((((..(((..(((((.(((((((((((............)))))))))))))))))..)))..)))))..))))))))))(((.
(((.........))).)))....
```

```
 Initial dG =    -51.60

          10          20         30         40
.-GUC          C-|   AA   AA      C           UUGAAU
     GAGCCGGUGG  UGGG  GGC  GAAGU GGCAUAGCUGU      \
     UUCGGCCGCC  ACUC  CCG  CUUCA CUGUGUCGGCA      A
\ ---          UA^   GA   AG      -           CAUAGA
        90        80         70         60         50



         100
  ----   U   UUUC
      UCU CAA    \
      AGG GUU    A
  UCGU   C   UAGA
 120       110
```

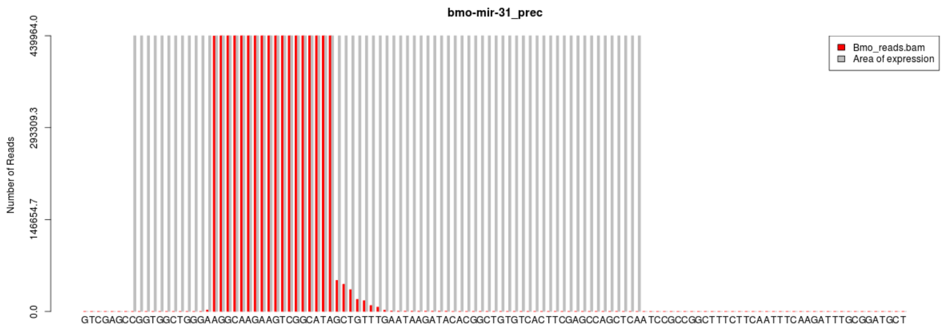

>bmo-mir-33_prec

UAAAGCGAGAGUGCAUUGUAGUUGCAUUGCACCGGUUCCAUUAACGUGCAAUAUGACUACAAGGCAAAUCCCGAAUUAGU
(((..((.((.(((.((((((((..(((((((..(((.....))))))))))..)))))))).))))..)).))..)))..

```
 Initial dG =    -24.90

          10          20         30
--   AG  A  G-|  A         GC        CG   CC
  UAA  CG GA  UGC UUGUAGUU  AUUGCAC  GUU  \
  AUU  GC CU  ACG AACAUCAG  UAACGUG  CAA  A
UG   AA  C  AA^  G         UA        --   UU
.        70         60         50
```

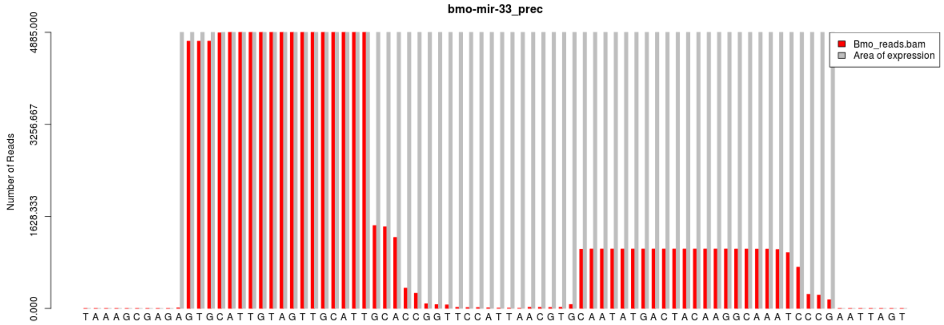

```
>bmo-mir-34_prec
AGAAUCAGGGUAGACCGCGUUGGCAGUGUGGUUAGCUGGUUGUGUAUGGAAAUGACAACAGCCACUAACGACACUGCUCCUGCGUGCACCCUAAAUCA
.((...(((((....((((..((((((((.(((((.(((((((((((....)).)).))))))))))))).))))))))..))))...)))))...)).

 Initial dG =    -44.70

        10        20        30        40
A| AUC     AGAC    UU        G     C       -  -  G
 GA   AGGGU    CGCG  GGCAGUGU GUUAG UGGUUGU GU AU G
 CU   UCCCA    GCGU  UCGUCACA CAAUC ACCGACA CA UA A
A^ AAA     CGU-    CC        G     -       A  G  A
       90         80        70         60        50

-----------------------------------------------------
```

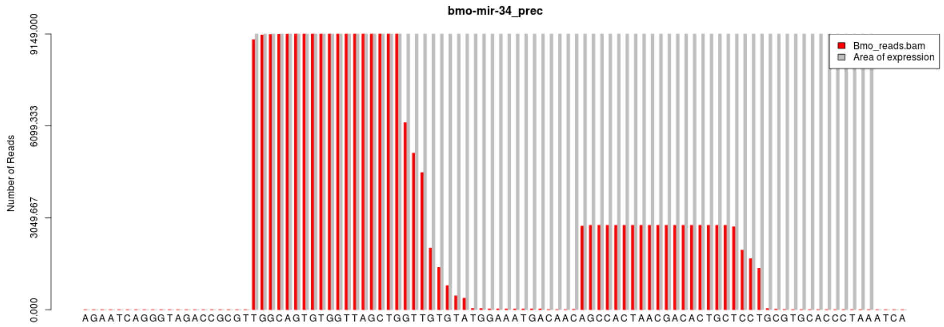

>bmo-mir-71_prec

```
GCAAGAUGCUCUCUACCUCUUGAAAGACAUGGGUAGUGAGAUGUCCCGUACAUCUAAAAUUCUCACUACCUUGUCUUUCAUGCGGAUUGGGCAAAUAUCUUCACUCGGCUUCGGAAGAU
......(((((....((...(((((((((.(((((((((((..................))))))))))))))))))))...))...)))))...((((((...........))))))

 Initial dG =    -40.50



  | AA
--GC  \
  CG  G
\ ^ UA




 10           20         30         40         50
  UC    A   .-UCU          U           UGUCCCGUA
    UCU CC      UGAAAGACA GGGUAGUGAGA          \
    AGA GG      ACUUUCUGU UCCAUCACUCU          C
  U-    A   \ ---          -           UAAAAUCUA
              80          70         60

-----------------------------------------------------
```

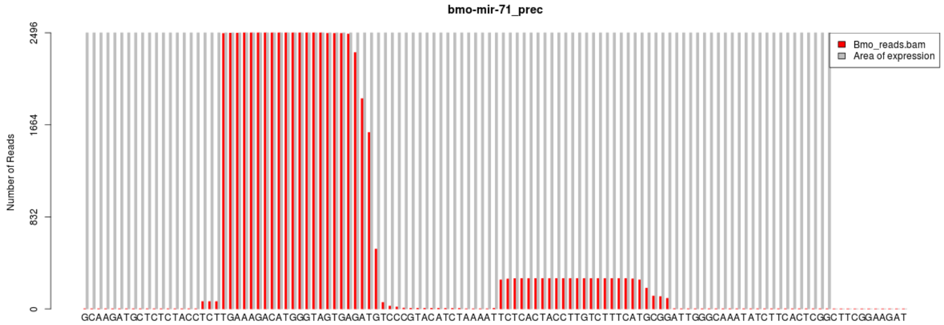

>bmo-mir-79_prec

```
CGCCGCCGGGCGCGCUGCGACGCUUUGGCGAUUUAGCUCCGUGACAUCAUUCCGUUCAUAAAGCUAGAUUACCAAAGCAUCGCGUCGCGCCGUCUCAACG
........((((((.(((((.(((((((..((((((((..(((((........)).)))..))))))))..))))))).))))).))))))).........
```

```
 Initial dG =    -47.40

         10        20        30        40
CGCCGCCG-      C     C       CG        CC   -| AUC
         GGCGCG UGCGA GCUUUGG  AUUUAGCU  GUG AC   A
         CCGCGC GCGCU CGAAACC  UAGAUCGA  UAC UG   U
GCAACUCUG      U     A       AU        AA   U^ CCU
.        90        80        70        60        50
```

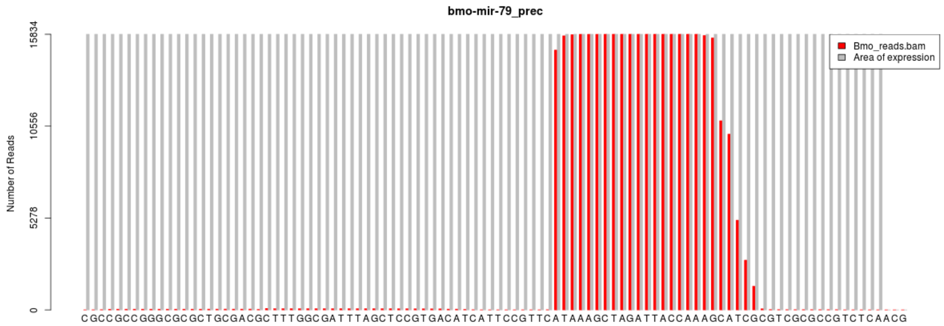

>bmo-mir-87_prec

```
UGAAGAAGUUCGGUACACUAAUGGGCCUGAAUCGUUGCUCAGACCUGUAUUUUCGACCAGGUGAGCAAACUUUCAGGUGUGUUAGUGCCGGCUGGUCUACA
.((...(((.(((..((((((((.(((((((...((((((..(((((..........)))))))))))...))))))).))))))))))))))..))....

 Initial dG =    -40.90

           10        20        30        40        50
U---| AGA   U   UA        G        UCG      AG     UAUU
    GA   AGU CGG  CACUAAUG GCCUGAA   UUGCUC  ACCUG    U
    CU   UCG GCC  GUGAUUGU UGGACUU   AACGAG  UGGAC    U
ACAU^ GG-    -   --        G        UCA      --     CAGC
 .          90          80        70          60

-----------------------------------------------------
```

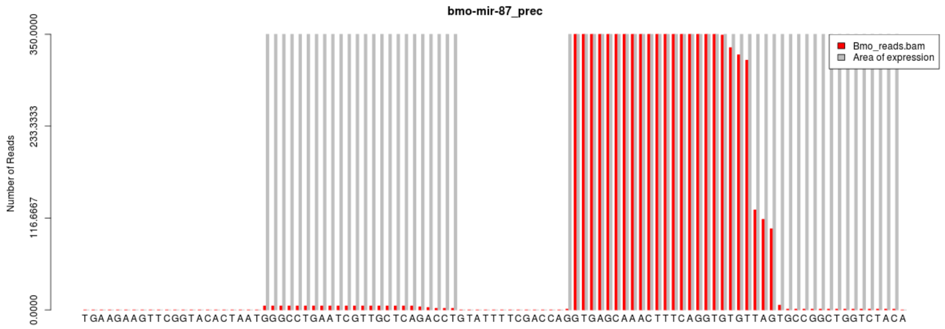

>bmo-mir-92a_prec

```
GGCCCGUUUGGCUGGGCAGUGACUGGCGCCAUAUUCGGUACGUGUGCGAUAUUGCACCAGUCCCGGCCUAUCCGAGCGGGC
.((((((((((.(((((...((((((.((.(((((..(((....)))))))).)).))))))...))))).))))))))))

 Initial dG =    -45.00

       10        20        30        40
G|         C     AGU       C  C      CG   C
 GCCCGUUUGG UGGGC   GACUGG GC AUAUU  GUA G
 CGGGCGAGCC AUCCG   CUGACC CG UAUAG  CGU U
-^         U     GCC       A  U      --   G
 80        70        60        50

-----------------------------------------------------
```

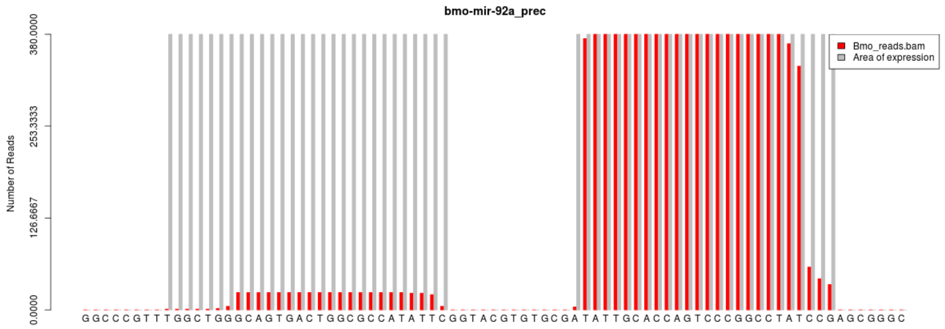

>bmo-mir-92b_prec

```
GCGGUGGCCACGCUUAGGACGCGAUUAGGUGUAAACGUUUGUGUUUCAUAUCAAAUUGCACCAAUCCCGGCCUGCCUGUGGCCACAGC
((.(((((((((..((((.((.((((.(((((((.....((((...)))).....))))))))))).)).)))))..))))))))).))

 Initial dG =    -39.00

        10         20         30         40
| G         CU    A  C    A       ACGUU    U
GC GUGGCCACG  UAGG CG GAUU GGUGUAA      UGUG \
CG CACCGGUGU  GUCC GC CUAA CCACGUU      AUAC U
^ A         CC    G  C    -       AAACU    U
       80        70         60         50

-----------------------------------------------------
```

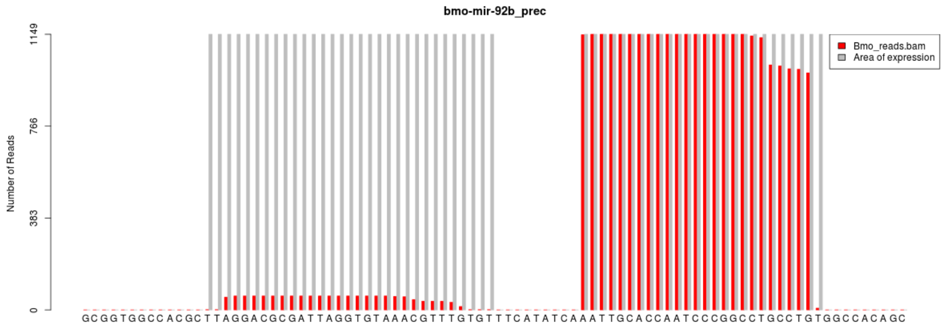

>bmo-mir-100_prec

CAUUAAGCCUCUUUAACUGGAACCCGUAGAUCCGAACUUGUGCGCUAUUGUCGUUGGGGACUCCGUCCAAUCGCGCGUAACGAUAGCGACUAUUCAAUGGCAACAAUAUCGAUUCUAAAA
GUUGCCGGCAAUCAAAACCAAA

```
................(((((((...((((..(((..((((..(((((((..((((.((((...)))).
((((.......))))..))))....))))))).)))))..)))..))))...))).)))).............
```

```
 Initial dG =    -33.70

        10        20         30        40        50          60
CAUUAAGCCUCUUUAA    -   CCG    UC   AC    GC       .-UC|     GAC
                CUGG AAC   UAGA  CGA  UUGU  GCUAUUG    GUUGGG   \
                GGCC UUG   AUCU  GCU  AACA  CGGUAAC    UAACCU   U
AAACCAAAACUAAC--    G   AAA    UA   AU    A-       \ --^     GCC
140       130         120       110        100         70


                                                               80
                                                     CG----   GUAA
                                                           CGC    \
                                                           GCG    C
                                                     UUAUCA   AUAG
                                                        90

-----------------------------------------------------
```

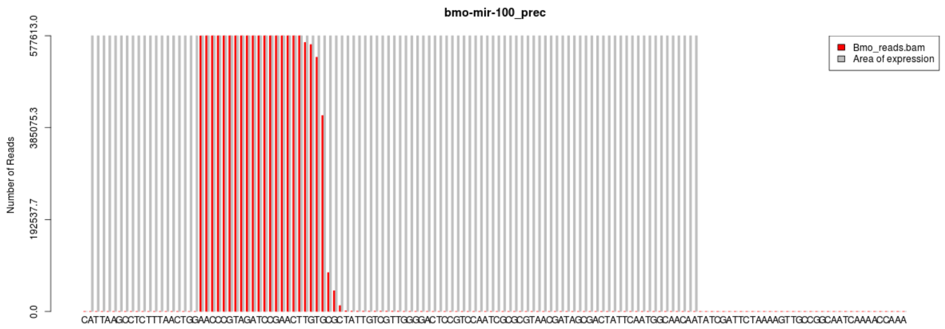

>bmo-mir-124_prec

```
CAGUCCACCUCCUCGCGUUCACUGCCGGAGCCGUUAUGUAUAUUUAAAAUUCAUAAGGCACGCGGUGAAUGCCAAGAGCGGACUC
.(((((..(((...((((((((((((.(..(((.(((((.............)))))))).)))))))))))))...))).))))).

 Initial dG =    -33.80

        10        20        30        40
C|    AC   CUC           C GA   G     UAUAUU
 AGUCC  CUC   GCGUUCACUGC G  GCC UUAUG      \
 UCAGG  GAG   CGUAAGUGGCG C  CGG AAUAC      U
C^    C-   AAC           - A-   -     UUAAAA
    80         70         60          50

-----------------------------------------------------
```

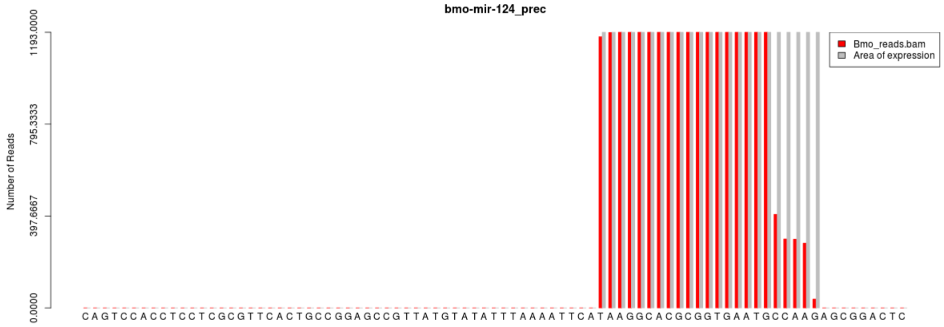

>bmo-mir-133_prec

AAAGCGAGAGCGUUGUUCGCUUUAGCUGGUUGACUUCGGGUCAAAUUGUCAUUUUCAUAUCAUUUGGUCCCCUUCAACCAGCUGUAGUUAACAUCUGCUUU
......((((((.((((.(((.(((((((((((....((..(((((.((.........)).)))))..))...))))))))))).))).)))).))..))))))

```
 Initial dG =    -35.80

        10          20         30         40         50
AAAGCG      U-|   C   U          CUUC  GU      U  CAUU
      AGAGCG  UGUU GCU UAGCUGGUUGA    GG  CAAAU GU    \
      UUUCGU  ACAA UGA GUCGACCAACU    CC  GUUUA UA    U
------      CU^   U   U          UCC-  UG      C  UACU
     100        90         80         70         60

-----------------------------------------------------
```

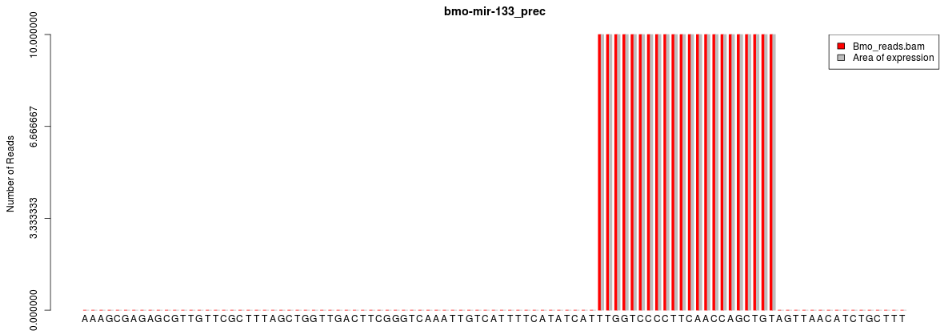

>bmo-mir-137_prec

```
UAACACGCUUGGAGGUCACGCGUAUUCUUGGGGAAUUAACACAUAUGUGAGAUGUUAUUGCUUGAGAAUACACGUAGCUGACAAGGAGUA
....((.((((..((..(((.(((((((..((.(((.((((...........))))))).))..))))))).))).)).))..)))).)).

 Initial dG =    -30.20

         10        20        30        40
UAAC  G-|   GA  UC   C       UG  G   U    CAUAU
    AC  CUUG  GG  ACG GUAUUCU  GG AAU AACA     \
    UG  GAAC  UC  UGC CAUAAGA  UC UUA UUGU     G
A---  AG^   AG  GA   A       GU  G   -    AGAGU
.           80        70        60         50

-----------------------------------------------------
```

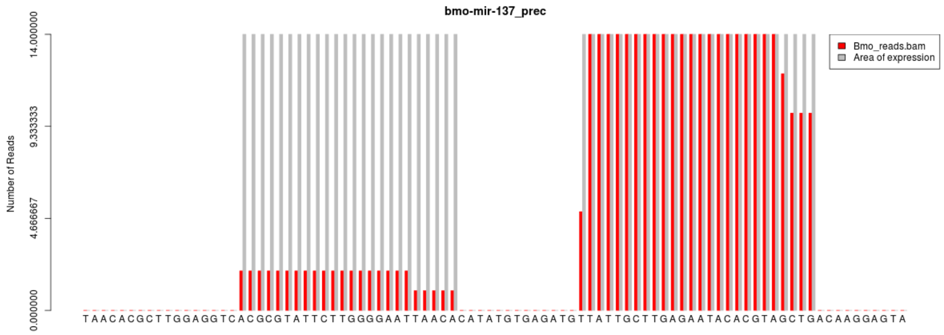

>bmo-mir-184_prec

```
GUGCAGUGACGUGCCCUUGUCAUUCUUCAGGCCCUGUGUAUUUACAACUACUGGACGGAGAACUGAUAAGGGCACGCCGUGUAC
(((((.((.((((((((((((((((((...((..((((.......)).)).))..)))))).))))))))))))).)))))))

 Initial dG =    -36.60

        10        20         30         40
|    G  A             -      AGG  CU  -  AUU
GUGCA UG CGUGCCCUUGUCA UUCUUC   CC  GU GU   \
CAUGU GC GCACGGGAAUAGU AAGAGG   GG  CA CA   U
^    -  C             C      CA-  U-  U  ACA
   80         70        60          50

-----------------------------------------------------
```

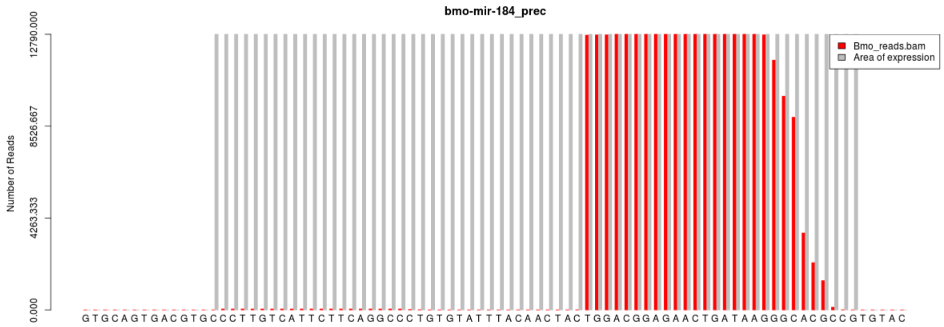

>bmo-mir-190_prec

```
GAGCCGCGCGUCGUCGAGAUAUGUUUGAUAUUCUUGGUUGUUUGUUUUGGAUCACCCGGGAAUCAAACAUAUUACUCUACGACCGCGGCC
..((((((.(((((.((((((((((((((.((((.(((.((((.....)))).))).)))))))))))))))..))).))))))))))).

 Initial dG =    -42.90

        10          20         30         40
GA      C     C   --|          A    U   U    GU
  GCCGCG GUCGU GAG  AUAUGUUUGAU UUCU GGU GUUU  \
  CGGCGC CAGCA CUC  UAUACAAACUA AGGG CCA UAGG  U
C-      -     U   AU^          -    C   C    UU
.          80         70         60         50

-----------------------------------------------------
```

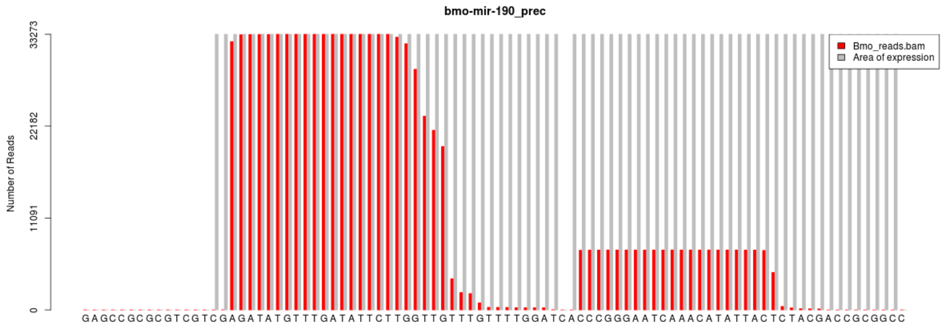

>bmo-mir-210_prec

```
AAUGAAGUCCAAAGCAGCUGCUGGCCACUGCACAAGAUUAGUCAAGUUUAUACUCUUGUGCGUGUGACAGCGGCUAUUGCGGGCAUUUCAUU
((((((((((..((.((((((((..((((((((((((.((..........)).))))))))).))).)))))))).))...)).))))))))

 Initial dG =    -38.30

         10          20          30         40
        -  AA-| C        GC    -          U  GUCA
AAUGAAGU CC   AG AGCUGCUG  CAC UGCACAAGA UA     A
UUACUUUA GG   UU UCGGCGAC  GUG GCGUGUUCU AU     G
        C  GCG^ A        A-    U          C  AUUU
 90        80        70          60        50

-----------------------------------------------------
```

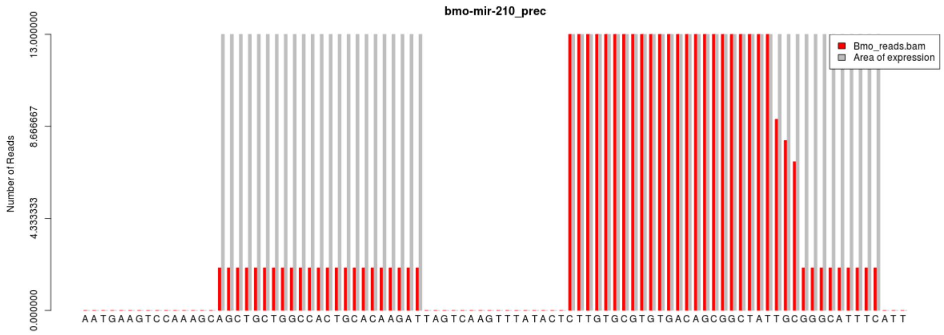

>bmo-mir-252_prec

AACUAAACCUUGUUCCUAAGUACUAGUGCCGCAGGAGCGUUUUGUAGUAUCUCCUGCUGCCUAAGUGCUUAUCAAUGAGGGUUUA
.......(((..((..((((((((((.((.(((((((.((........))))))))).))))).))))))))..))..))).....

```
 Initial dG =    -32.00

        10        20         30        40
AACUAAA   UG  CC       -|  U  C       C  UUU
       CCU  UU  UAAGUAC UAG GC GCAGGAG GU   G
       GGA  AA  AUUCGUG AUC CG CGUCCUC UA   U
AUUUG--   GU  CU       A^  -  U       -  UGA
      80        70         60         50

-----------------------------------------------------
```

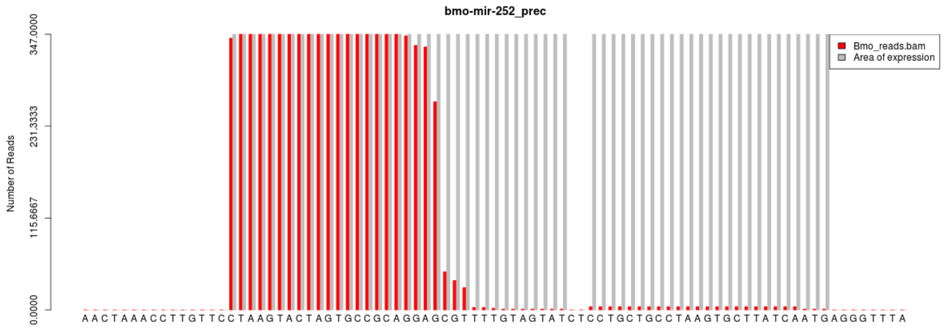

>bmo-mir-263a_prec

```
AUCGAUCCAAGCACAGGCAAUGGCACUGGAAGAAUUCACGGGUUCAGUUUAUAUAUUCCCGUGAUCUCUUAGUGGCAUCACUGGUGCAGGACGAC
.(((.(((..((((.((..(((.(((((..(((..(((((((...............)))))))))).))))).))).)).)))).)))))).

 Initial dG =    -36.90

        10        20        30        40
A|  A   AA    A  CA   G     GA   AU       UUCAGUU
 UCG UCC  GCAC GG  AUG CACUG  AGA  UCACGGG       \
 AGC AGG  CGUG UC  UAC GUGAU  UCU  AGUGCCC       U
C^  -   A-    G  AC   G     UC   --       UUAUAUA
     90         80        70          60

-----------------------------------------------------
```

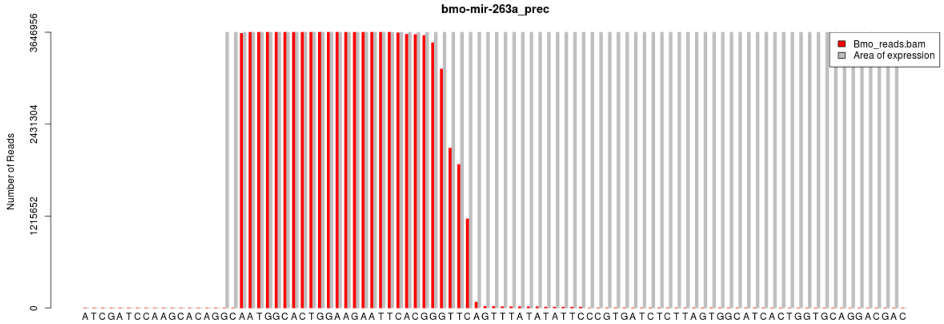

>bmo-mir-263b_prec

AGCCGACGCUGUUCCUUGGCACUGGGAGAAUUCACAGGAGUUGUAAUUCAUACCCGUGAAUUUCCCGAUGCCUUAGCUCAGUGUGGUCA

```
.(((.((((((......((((.(((((((.(((((.((.((((.....)).)))))))))))))))).)))).......))))))))).. 

 Initial dG =    -36.00

         10        20        30        40
A-|  G      UUCCUU    C       A     A  A  -  UA
  GCC ACGCUG      GGCA UGGGAGA UUCAC GG GU UG  \
  UGG UGUGAC      CCGU GCCCUUU AAGUG CC CA AC  A
AC^  -      UCGAUU    A       -     -  -  U  UU
         80        70         60          50

-----------------------------------------------------
```

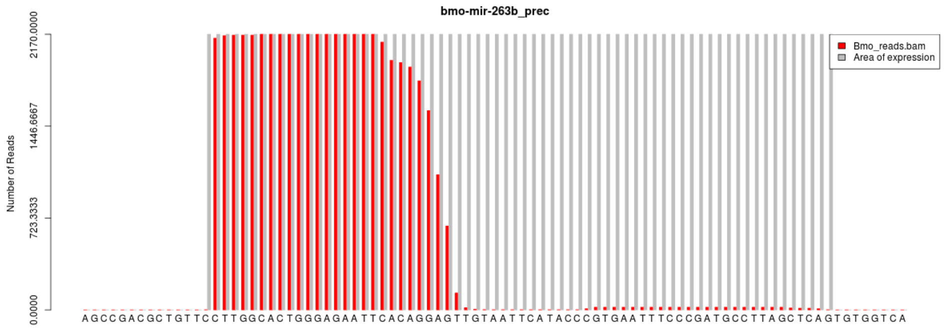

>bmo-mir-274_prec

```
GGCUGUUGUUAGGCGAAGUUGGUUUGUGACCGUCACUAACGGGCAGUGUUAAUACGGUUGCUCGUUUUGGCGAUCGCAAAAUGAACUCGCUUGAC
.......(((((((((.(((.((((((((.(((((..(((((((((...........))))))))).))))).)))).)))).))))))))))))

 Initial dG =    -38.60

        10        20         30        40        50
GGCUGUU|        A   G    -    C     CU         UGUUA
       GUUAGGCGA GUU GUUU GUGA CGUCA  AACGGGCAG     \
       CAGUUCGCU CAA UAAA CGCU GCGGU  UUGCUCGUU     A
-------^        -   G    A    A     U-         GGCAU
           90         80        70         60

----------------------------------------------------
```

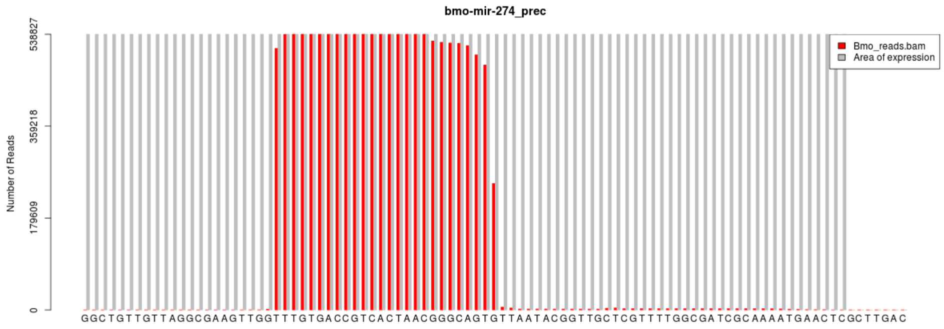

>bmo-mir-275_prec

```
CAGGCGGCAGCGCCGCGCGCUACUCCGGCGCCAGGACUGUCCUCACCGAGUCAGGUACCUGAAGUAGCGCGCGGUGUCUCCUCCUA
.(((.((..((((((((((((((((.((..((..((((..........)))).))..)).)).)))))))))))))))..)).))).

 Initial dG =    -48.50

        10         20          30         40
C   C  CA              -| C  CG  AG    GUCC
 AGG GG  GCGCCGCGCGCUAC UC GG  CC  GACU    U
 UCC CC  UGUGGCGCGCGAUG AG CC  GG  CUGA    C
A   U  UC              A^ U  AU  A-    GCCA
     80         70         60          50

-----------------------------------------------------
```

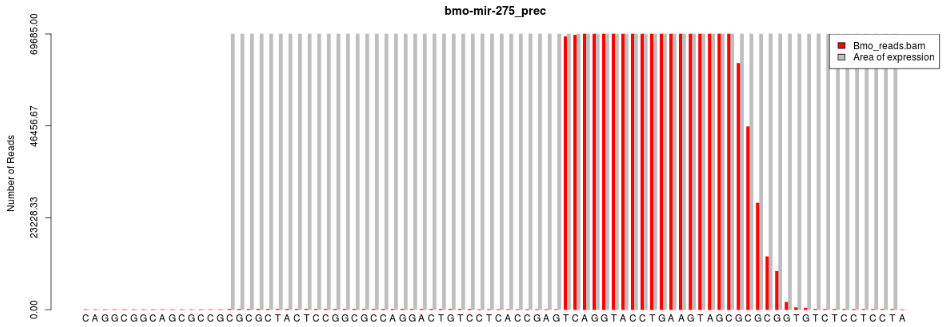

>bmo-mir-276_prec

```
CUGGUAAUUACCACUAGCGAGGUAUAGAGUUCCUACGUAUGCUAACACUGUAGGAACUUCAUACCGUGCUCUUGGGUUUGCCAA
.((((((...(((..((((.(((((..(((((((((((........)).)))))))))..))))).)))))..)))..)))))).

 Initial dG =    -37.90

        10         20         30          40
C|      UUA   CU     A      AG          -  AUG
 UGGUAA   CCA  AGCG GGUAU  AGUUCCUAC GU   C
 ACCGUU   GGU  UCGU CCAUA  UCAAGGAUG CA   U
A^      UG-   UC     G      CU          U  CAA
   80          70         60         50

-----------------------------------------------------
```

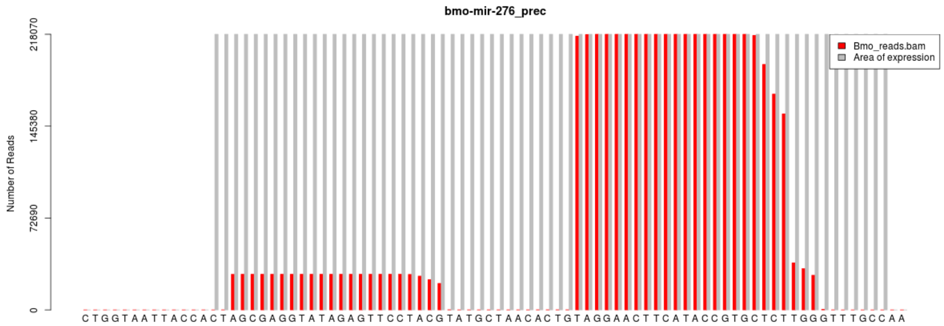

>bmo-mir-277_prec

```
UUGAUUACGCCUCGGGGUUCGUGCCAGGAGUGCGUUUGCAAAGAGGCCGACAUGUUUGCGUUAUUGCAAUGUUAACACUGUAAAUGCACUAUCUGGUACGACAUCCCGGGGCGUGUAGCA
AC
(((..((((((((((((((((((((((((((((((((((((........(((((...
((((....)))))))))).....))))))))))))).))))))))))).))))))))))))))...))).

 Initial dG =    -62.90

          10         20         30        40        50        60
-   AU-            -          -|          AAGAGGCC     GUU    U
 UUG   UACGCCUCGGGGU UCGUGCCAGG AGUGCGUUUGCA        GACAU   UGCG U
 AAC   GUGCGGGGCCCUA AGCAUGGUCU UCACGUAAAUGU        UUGUA   ACGU A
C   GAU            C          A^          CACAA---     ---    U
120       110       100        90        80          70

-----------------------------------------------------
```

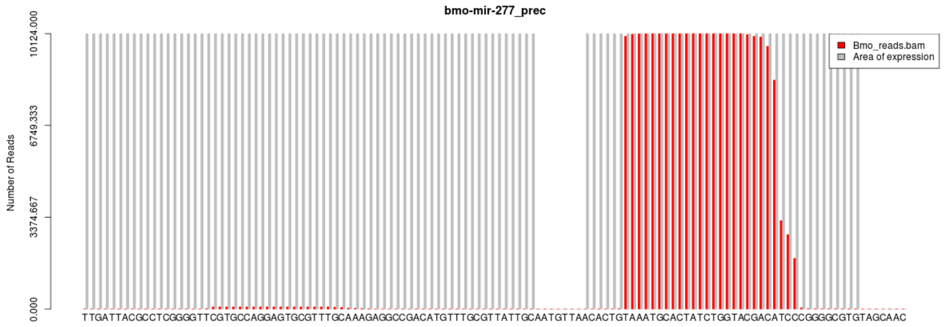

>bmo-mir-278_prec

```
GUAUCAAGUGCUACGCCCGGACGAACUUCCCAGCUCGGCCGACAAUCAGGGGUCGGUGGGAUCUUCGUCCGUUUGUAUCGCUUAUAC
((((.(((((.((((..((((((((..(((((.(.(((((..........)))))))))))..))))))))..)))).)))))))))

 Initial dG =    -40.60

        10        20        30        40
|   C     C    CC        CU      G U     GACA
GUAU AAGUG UACG  CGGACGAA  UCCCA C CGGCC    A
CAUA UUCGC AUGU  GCCUGCUU  AGGGU G GCUGG    U
^   -     U    UU        CU      - -     GGAC
       80        70        60          50
```

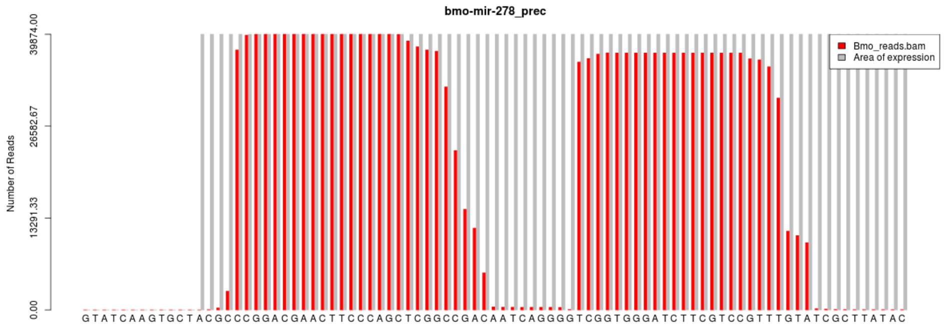

>bmo-mir-279a_prec

ACGUCAAUUUCUUUCGAUGAGUGGAGGUUUAGUGCAUGUUUAUUUACACCAUGACUAGAUCCACACUCAUCCAUGGAAGUUGCGA
.((.((((((((...((((((((..((((((((.((((...........))))))))))))..))))))))...)))))))))).

```
 Initial dG =    -35.80

        10        20        30        40
A| U       UUC       GA        G    UUUAU
 CG CAAUUUCU   GAUGAGUG  GGUUUAGU CAUG     \
 GC GUUGAAGG   CUACUCAC  CUAGAUCA GUAC     U
A^ -       UAC       AC        -    CACAU
     80        70        60         50
```

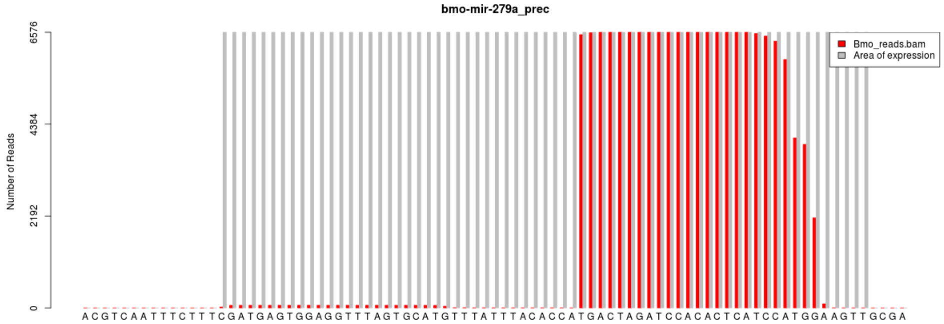

>bmo-mir-279b_prec

UUUGUAGUUAAUGGGUAUAGGUCUAGUAUCACCGGGCUGAUUCUGUGACUAGAUCUACACUCAUUGAGUGCUUAUGG
...(((.(((((((((.(((((((((..((((.(((....))).)))))))))))))).))))))))).)))......

```
 Initial dG =    -31.50

           10        20        30
UUU---|  G         A         UA    C   C
      GUA UUAAUGGGU UAGGUCUAG  UCAC GGG U
      CGU AGUUACUCA AUCUAGAUC  AGUG CUU G
GGUAUU^  G         C         --    U   A
      70        60        50          40
```

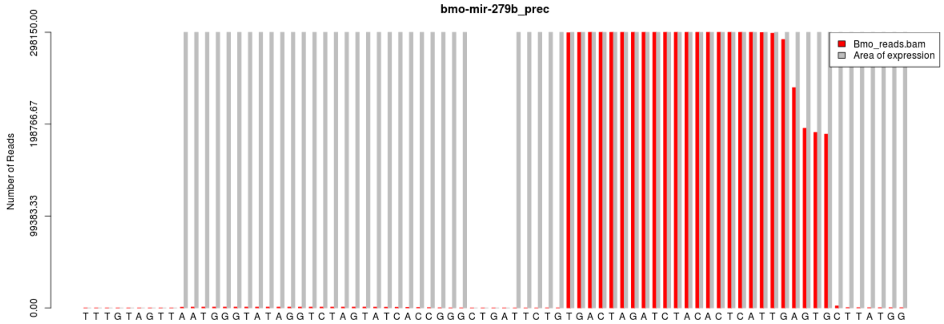

>bmo-mir-279c_prec

```
UGAUAUUUGUGGGCGAGUUUGCUUCUGGUUCAUGUUUUAUAUUGAUCAUGACUAGAUCCAUACUCGUCUGCAGCCAUCAA
((((..(((..(((((((.((..((((((.((((.((......)).))))))))))..)).)))))))..)))..)))).

 Initial dG =    -32.80

         10        20        30        40
-|   AU   UG       U  CU      U    U  UA
 UGAU  UUG  GGCGAGU UG  UCUGGU CAUG UU  U
 ACUA  GAC  CUGCUCA AC  AGAUCA GUAC AG  A
A^   CC   GU       U  CU      -    U  UU
.        70        60         50

-----------------------------------------------------
```

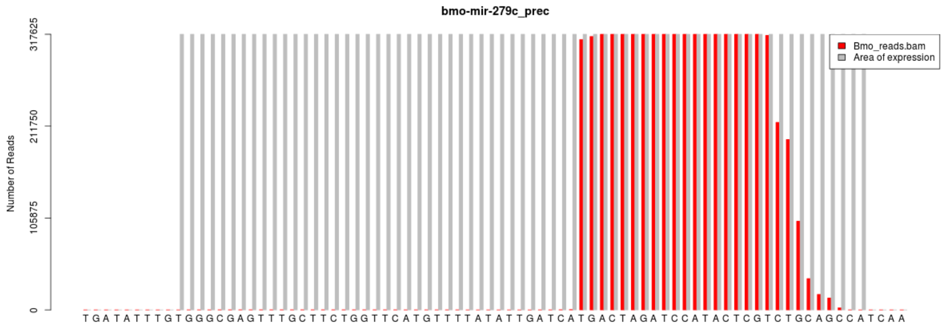

```
>bmo-mir-279d_prec
UUCCUCAGACGAUGAGUGACCGUUUAGUUCAAGUUGUCAAUUCUUGACUAGAUUUUCACUUAUCCUCUGCGGGCAC
.(((.((((.(((((((((..(((((((.((((.........)))))))))))..))))))))).)))).)))...

 Initial dG =    -30.30

          10        20        30
U--|  U    C         CC        U    UUGU
   UCC CAGA GAUGAGUGA  GUUUAGU CAAG    \
   GGG GUCU CUAUUCACU  UAGAUCA GUUC    C
CAC^  C    C         UU        -    UUAA
     70        60        50          40

-----------------------------------------------------
```

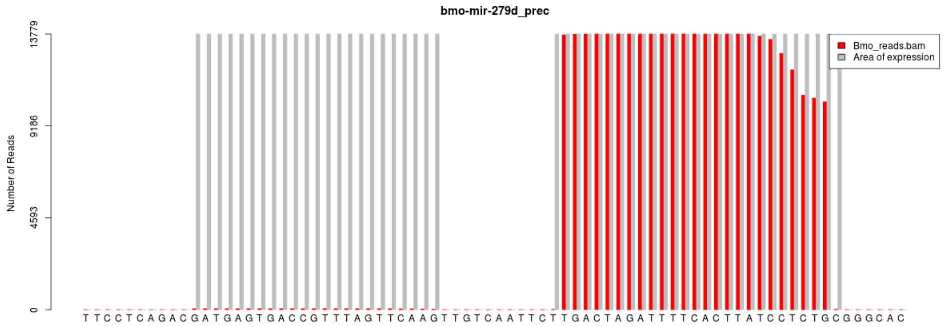

>bmo-mir-279e_prec

UAGUUUCGAGAUUAUUCUAAUCAGCUGUUAGGAGAAUAGUGUUGCGAUGUGUACUCAAUUUAUUAGUGACUAGCGAUACACUCCGGCUCCGCUCGGGUCUUUAACA
(((((...(((....)))....)))))...((((....(((((((...((.((((.........))))))..))))))).))))(((.((....))))).......

```
 Initial dG =    -22.00

        10        20        30        40        50
UA|  UC      AUUCUAAUC    GUUA    AAUA       GAU  G    CAAU
  GUU  GAGAUU         AGCU    GGAG    GUGUUGC   GU UACU    \
  CAA  UUCUGG         UCGG    CCUC    CAUAGCG   CA GUGA    U
A-^  U-      GCUCGCC--    ----    A---       AU-  -    UUAU
      100        90               80            70

-----------------------------------------------------
```

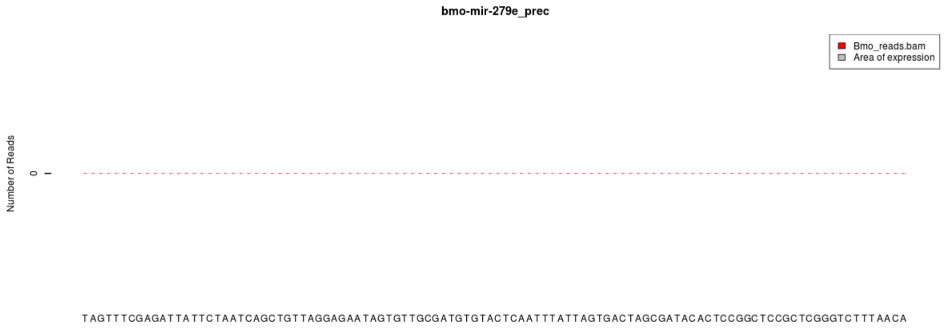

>bmo-mir-281_prec

UCUGUUAAUGAAGAGAGCUAUCCGUCGACAGUAUUGCUGAUAAACACUGUCAUGGAGUUGCUCUCUUUAUGAACGGA
((((((.(((((((((((..(((((.((((((.((.......)).)))))))))))...))))))))))).))))))

```
 Initial dG =    -36.90

        10        20         30
      A           UA-|    C      A  GCU
UCUGUU AUGAAGAGAGC   UCCGU GACAGU UU   \
AGGCAA UAUUUCUCUCG   AGGUA CUGUCA AA   G
      G           UUG^    -      C  AUA
      70        60         50        40

-----------------------------------------------------
```

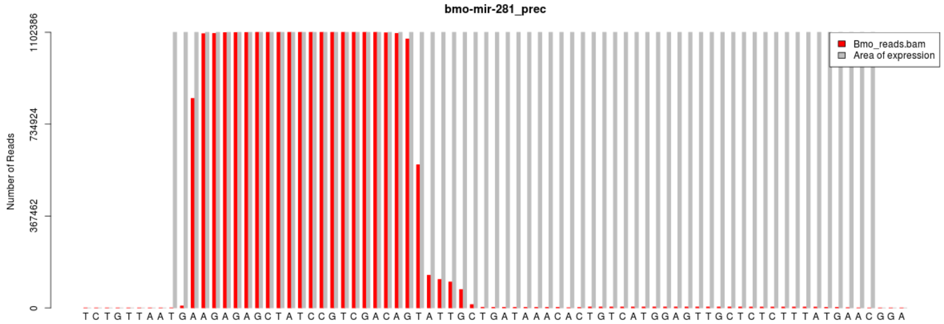

>bmo-mir-282_prec

```
CGGGACUUUGACCUAGCCUCUCCUUGGCUUUGUCUGUUUCGUUAGUAGCUCAGACAUAGCCUGAUAGAGGUUACGUUUUGUCCCU
.(((((...(((.((((((((....((((.((((((..............)))))).))))....)))))))).)))..))))).

 Initial dG =    -38.30

        10        20        30        40
C|    UUU   C        CCUU    U      UUUCGU
 GGGAC   GAC UAGCCUCU    GGCU UGUCUG      U
 CCCUG   UUG AUUGGAGA    CCGA ACAGAC      A
U^    UU-   C        UAGU    U      UCGAUG
    80         70        60        50

-----------------------------------------------------
```

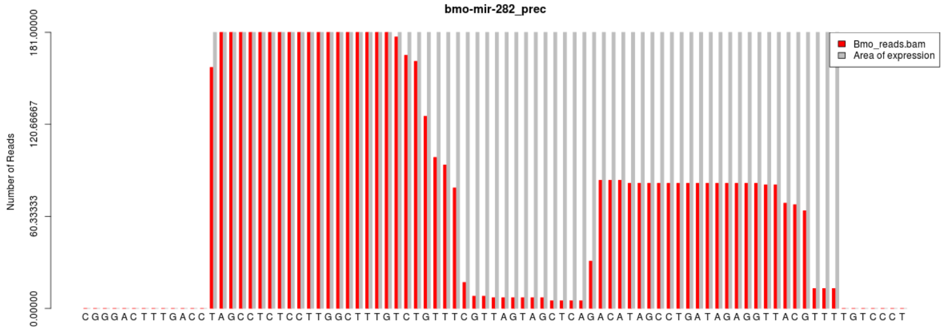

>bmo-mir-283_prec

AACGUUUCCCGACUAAAUAUCAGCUGGUAAUUCUGGGCUUUAUUAAUCCCAGGCUAUCAGCUGGUAUACAGUUAUGAUACGUA

```
.((((.((..((((..(((((((((((((..((((((.((....)).)))))).)))))))))))))..))))..)).)))).
```

```
 Initial dG =    -33.80

        10        20        30        40
A|   U  CC    AA            AU      C  U
 ACGU UC  GACU  AUAUCAGCUGGUA  UCUGGG UU A
 UGCA AG  UUGA  UAUGGUCGACUAU  GGACCC AA U
A^   U  UA    CA            C-      U  U
  80        70        60         50
```

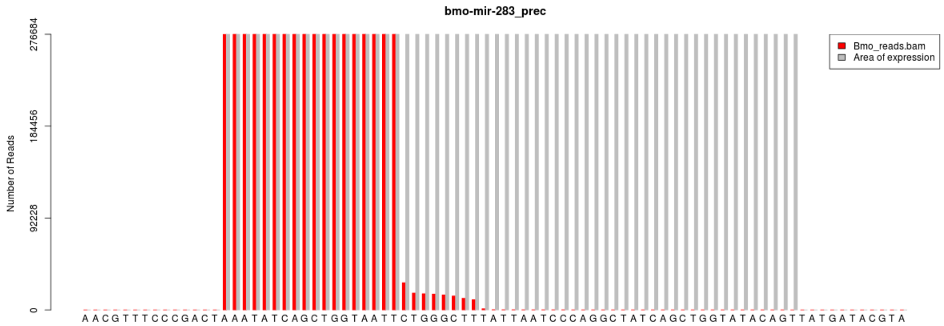

>bmo-mir-285_prec

```
UGUCCAUGUUUUGACAACUGUAUUCGAGUGUGUGGAAGAGUUUUAAAAAUCCUCUAGCACCAUUCGAAUUCAGUGCUCGAACGUGGUUA
...((((((((.((..((((.(((((((((.(((..((((..((...))..))))..))))))))))))).))))..))))))))))...

 Initial dG =    -33.00

        10        20        30        40
UGU|       U  CA    U         U   GA    UU  A
   CCAUGUUU GA  ACUG AUUCGAGUG GUG  AGAG  UU \
   GGUGCAAG CU  UGAC UAAGCUUAC CAC  UCUC  AA A
AUU^       -  CG    U         -   GA    CU  A
        80         70         60        50
```

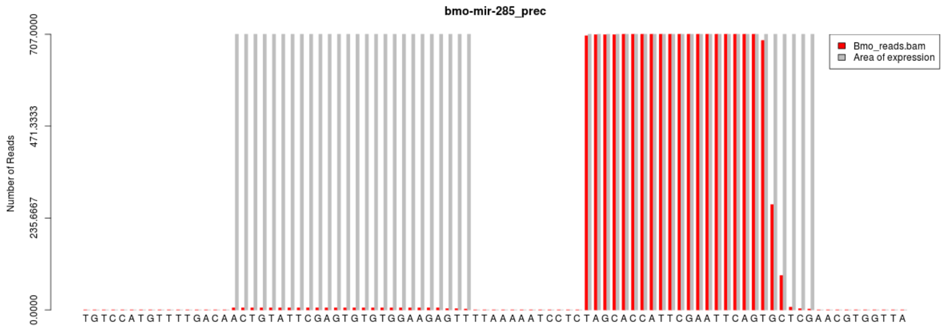

>bmo-mir-305_prec

```
CGACCGCCGCACGCCCAUUGUACUUCAUCAGGUGCUCUGGUGAUCAUAGUUCCAGGCGCUUGUUGGAGUACACUUACCGUGUCGGCGGUUA
.(((((((((((.....(((((((((.(((((((.((((.(((....))))))))))))))).)))))))))).....)))).)))))))).

 Initial dG =    -49.80

         10        20        30        40
C        -|   CCCAU         U       U    U   C
 GACCGCCG CACG     UGUACUUCA CAGGUGC CUGG GAU A
 UUGGCGGC GUGC     ACAUGAGGU GUUCGCG GACC UUG U
A        U^   CAUUC         U       -    -   A
90        80        70        60          50

-----------------------------------------------------
```

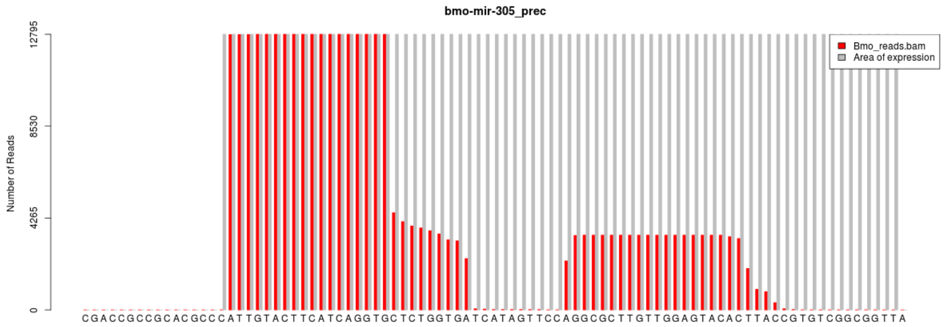

>bmo-mir-306a_prec

```
CCGCGGGAGACGGAGGCGGGCGGCCGCCUCAGGUACUAGGUGACUCUGAGUGGUGCCGGUCUCAGAGCCGCCUCGUGCCUCAGCGCGGGCUCCGCCGACACGUCCGCC
..((((...(((..(((((((..((((((.((((((.(((((.(((((((((....))..))))))).))))).)))))).)).)))))).))))).....)))))))).
```

```
 Initial dG =    -59.30

        10           20          30         40         50
CC     GAG    GA--       -   GG     -   C       U      A       --| G
  GCGG    ACG     GGCGG GC  CCGC CU AGGUAC AGGUG CUCUGAG  UG U
  CGCC    UGC     CCGCC CG  GGCG GA UCCGUG UCCGC GAGACUC  GC G
C-     ---    ACAG       U  --     C  C       C      C       UG^ C
          100        90          80         70         60

-----------------------------------------------------
```

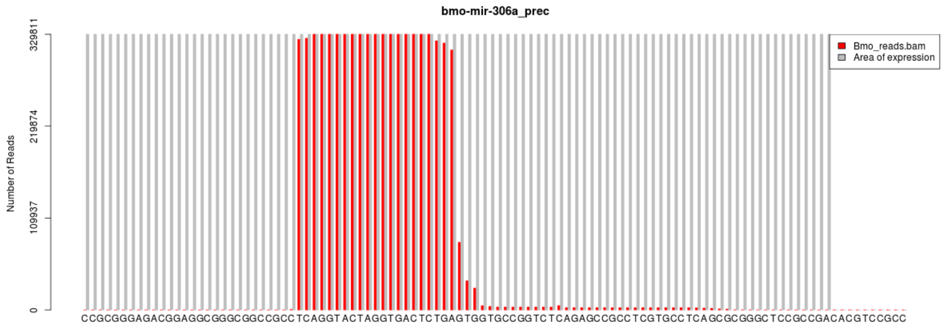

>bmo-mir-306b_prec

```
GAGCCCGCGCUGAGGCACGAGGCGGCUCUGAGACCGGCACCACUCAGAGUCACCUAGUACCUGAGGCGGCCGCCCG
..((((((.((.(((.((.(((.(((((((((..........))))))))).))).)).))).))))))..))...

 Initial dG =    -35.70

           10        20        30
GA-  --|   G  G   C  G   C         ACCG
   GC  CCGC CU AGG AC AGG GGCUCUGAG    G
   CG  GGCG GA UCC UG UCC CUGAGACUC    C
GCC  CC^   -  G   A  A   A         ACCA
     70         60        50        40
```

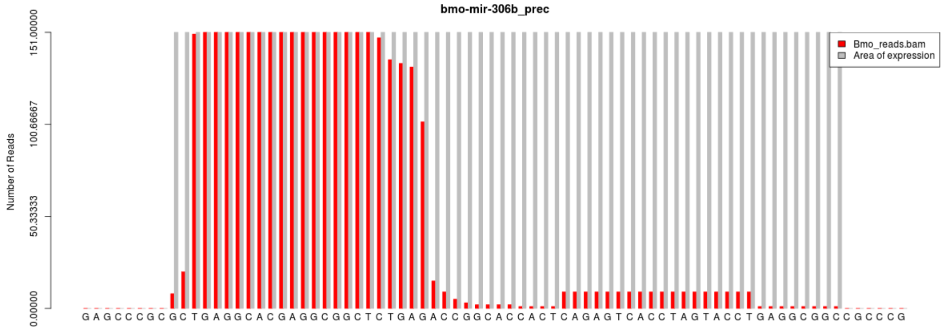

>bmo-mir-307_prec

```
AGCUCGUUCCCGGUUACUCACUCAACCUGGGUGUGAUGUGUGCACUCGUUGCUCGGCCCAUCACAACCUCCUUGAGUGAGCGAUCGUCUAUGAGCC
.((((((...((((..(((((((((...((.(((((((.(((((.....)))...)).))))))).))...)))))))))..))))...)))))).
```

```
 Initial dG =    -42.80

        10        20        30        40
A      UCC    UA         CCU  G       U  ---|  CU
 GCUCGU   CGGU  CUCACUCAA   GG UGUGAUG GU    GCA  \
 CGAGUA   GCUA  GAGUGAGUU   CC ACACUAC CG    CGU  C
C      UCU    GC         CCU  A       C  GCU^  UG
     90        80        70        60        50
```

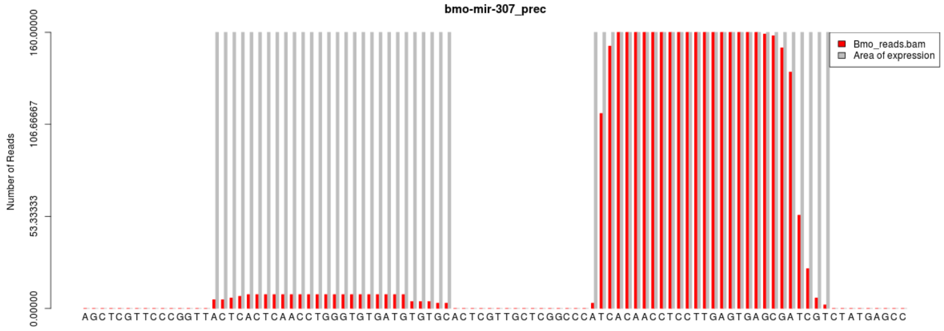

>bmo-mir-308_prec
CGUUUCCACGCGUGGUAUUAUUCUUGUGAAUGUGUUUUAGCUACAAUCACAGGAUAAUACUGCGAGUGGCGGUGUC
(((..((((.((..(((((((.(((((((.(((((....)).))).))))))))))))))..)).)))).)))..

```
 Initial dG =    -31.50

          10        20        30
--|  UU    G  UG       U       A   -  U
  CGU  CCAC CG  GUAUUAU CUUGUGA UGU GU U
  GUG  GGUG GC  CAUAAUA GGACACU ACA CG U
CU^  GC    A  GU       -       A   U  A
     70        60         50        40
```

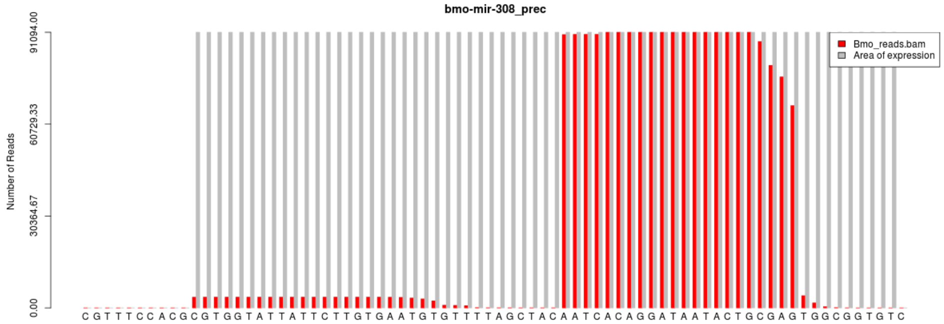

>bmo-mir-316_prec

```
UGCAUAAAUUUGUCUUUUUCCGCUUUGCUGCUGAUUUUUUUCGUACGUCAACGGCAAAGUGAAAAGGUCUCCUUUAUGAGAA
..((((((.....(((((((.(((((((((.((((...........)))).))))))))))))))))).....)))))).... 

 Initial dG =    -24.20

          10        20        30        40
UG--|     UUUGU       C         C    UUUUU
    CAUAAA     CUUUUUC GCUUUGCUG UGAU     \
    GUAUUU     GGAAAAG UGAAACGGC ACUG     U
AAGA^     CCUCU       -         A    CAUGC
 80        70         60        50

-----------------------------------------------------
```

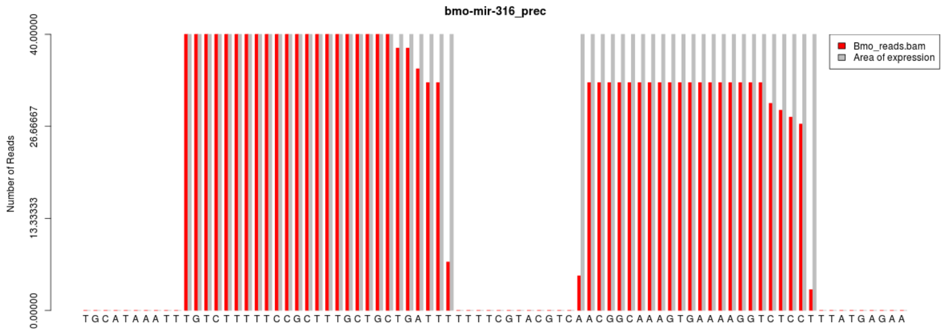

>bmo-mir-317_prec

```
GGGCUAUCGAACGACCGGGUGCCACGCUGUGCUCUCUCGAAAAUUAUGGCGGAGUGAACACAGCUGGUGGUAUCUCAGUUGUUCCCUAGUUC
((((((..(((((((.((((((((((((((.((.(((.............))).)).)))))))..)))))))))..)))))))..))))))

 Initial dG =    -39.50

        10         20          30        40
      UC        C-          --|     C  U   GAAAAU
GGGCUA  GAACGAC  GGGUGCCAC  GCUGUG UC CUC      \
CUUGAU  CUUGUUG  UCUAUGGUG  CGACAC AG GAG      U
      CC        AC          GU^     A  U   GCGGUA
 90        80         70        60        50

-----------------------------------------------------
```

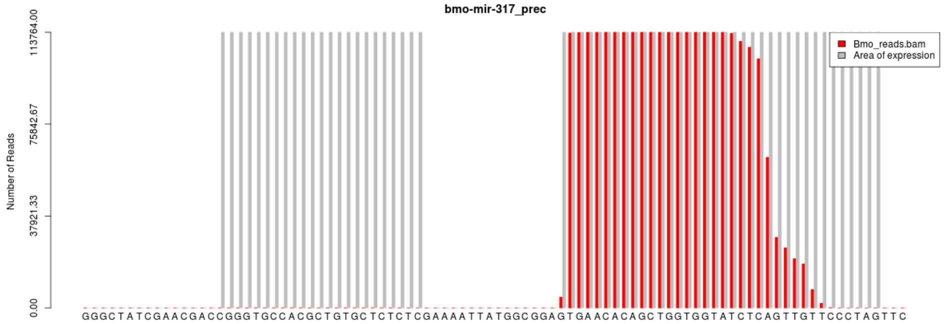

>bmo-mir-375_prec

```
CAGCAAGGCGACCCGAGCGGUCUGAGCAAACUGUGACAAUAUCUAGUUUGUUCGCCCCGGCUCGUGUCGCCAAGCGUCA
..((..((((((.(((((((..(((((((((((.((.....))))))))))))))..))).)))).))))))..))....

 Initial dG =    -40.10

          10         20        30
CA--  AA      C    -|  UC           U  CA
    GC  GGCGAC CGAG CGG  UGAGCAAACUG GA  \
    CG  CCGCUG GCUC GCC  GCUUGUUUGAU CU  A
ACUG  AA      U    G^  CC           -  AU
        70        60        50         40
```

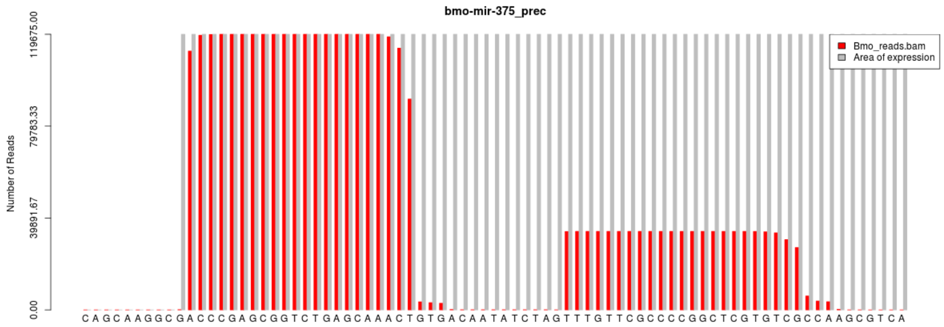

>bmo-mir-745_prec

```
UAGUUGGUUGCGGCUCAUCGUGUGGCAGUUUGCUAGGUUAGAAUGCCAGCUGCCUAGCGAAGGGCAACGAACAACUGAG
((((((.(((..((((.((((..(((((((.((...........)).)))))))..)))).)))).)))..))).))))))..

 Initial dG =    -33.90

          10        20        30
--|     G   CG    A    GU       U  UAGGU
  UAGUUG UUG  GCUC UCGU  GGCAGUU GC     \
  GUCAAC AGC  CGGG AGCG  CCGUCGA CG     U
GA^     A   AA    A    AU       C  UAAGA
        70        60        50        40

-----------------------------------------------------
```

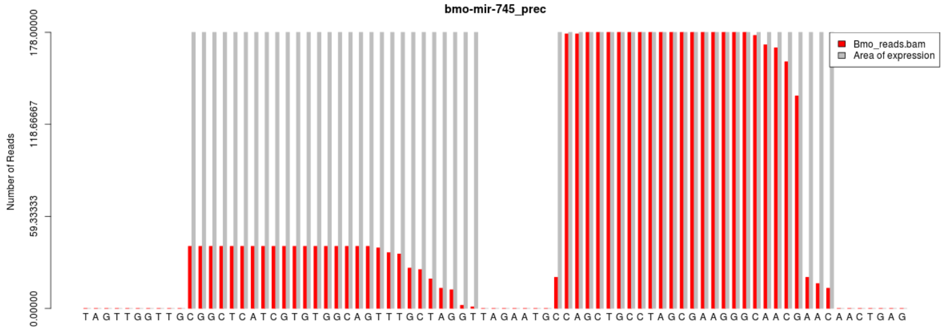

>bmo-mir-750_prec

```
CUUCAUGUCUGAGUUGGACAGGGGAUCUUGACAGUUUCAUGGUUCGCUGCCAGAUCUAUCUUUCCAGCUCACGCGUGGA
.(((((((.(((((((((.(((((((((.(.((((.........)))).))))))).))).))))))))).)))))))

 Initial dG =    -32.50

        10        20         30        40
C       C         C   -|     U A    UUCA
 UUCAUGU UGAGUUGGA AGG GGAUCU G CAGU    U
 AGGUGCG ACUCGACCU UCU UCUAGA C GUCG    G
-       C         U   A^     - C    CUUG
         70        60         50

-----------------------------------------------------
```

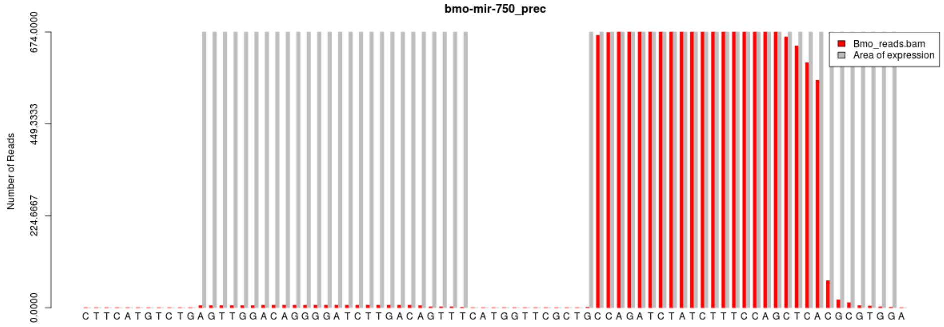

>bmo-mir-927_prec

```
UGCAGCCUUUUUGCUUUAGAAUUCCUACGCUUUACCGUUGCCAAGUAGUUGGGCAAAGCGUUUGGAUUCUAAAACGAAAUAACUGCCA
.((((...(((((.((((((((((..(((((((.((...((......))..)).)))))))..)))))))))).)))))...))))..

 Initial dG =    -30.90

         10        20        30        40
U-|   CCU     C          CU        A  GUU  CA
  GCAG   UUUUG UUUAGAAUUC  ACGCUUU CC   GC  A
  CGUC   AAAGC AAAUCUUAGG  UGCGAAA GG   UG  G
AC^   AAU     A          UU        C  GU-  AU
       80        70        60        50

-----------------------------------------------------
```

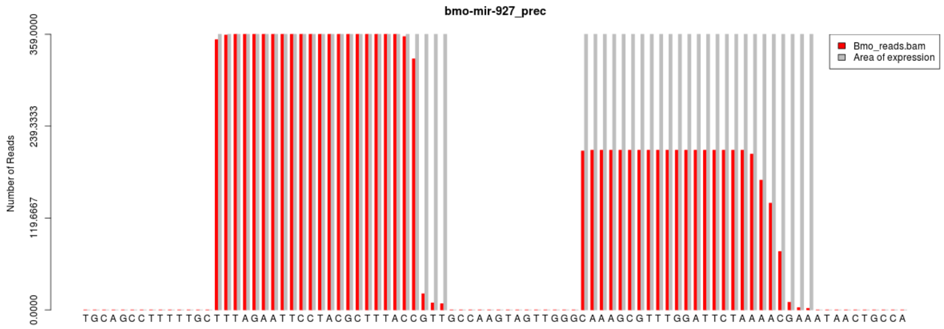

>bmo-mir-929_prec

```
UGUUAAAUUGACUCUAGUAGGGAGUCCGUUCCGCGCGACUCCCUAAUCGAGUCAGGUUGACUUCUUUGAUGCUAAAUCGACG
.(((((.(((((((...((((((((((.....)).)))))))))...))))))).))))).....(((((.....)))))..

 Initial dG =    -34.90

          10        20         30
.-U     A       UAG         -| UU
   GUUAA UUGACUC   UAGGGAGUC CG  \
   CAGUU GACUGAG   AUCCCUCAG GC  C
\ -     G       CUA         C^ GC
   60        50        40


         70
  UUCU     GC
      UUGAU  \
      AGCUA  U
  GC--     AA
    80

-----------------------------------------------------
```

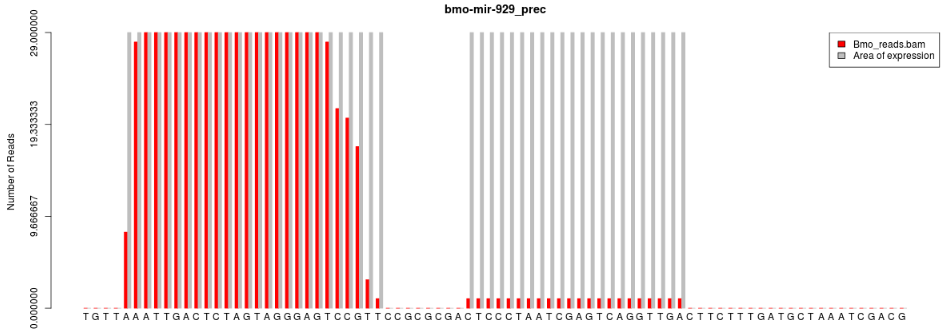

>bmo-mir-932_prec

```
GUGUUGAGGCUUCAAUUCCGUAGUGCAUUGCAGUGCAUGCUGCCACCUGCAAGCAGUGCGGGAGUGAGGCUGAGGCAUGCCUUAACUAC
(((((..(((((((.(((((((.(((.((((((.((.....))...))))))))).))))))).)))))))..))))).........

 Initial dG =    -40.70

                   10        20        30
-----------     GA       A       G   A      U--| AU
           GUGUU  GGCUUCA UUCCGUA UGC UUGCAG   GC  \
           UACGG  UCGGAGU AGGGCGU ACG AACGUC   CG  G
CAUCAAUUCCG     AG       G       G   -      CAC^ UC
        80        70        60         50        40

-----------------------------------------------------
```

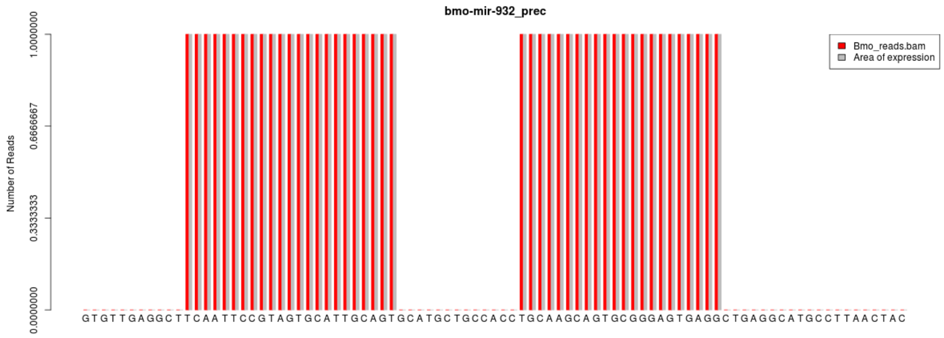

>bmo-mir-965_prec

```
AUAUAAAUGGCUGCAUAGCUAAACGGGAGAAGCUAUAGCGCUAUAUGUCAAUAUUAAAAUCAUAAGCGUAUAGCUUUUCCCCUUGGUUAUGCAUGCAUAUUCUC
.........(((((((((((((..(((((((((((((.((((.((((.............))))))))))))))))))))).))))))))))).)).........

 Initial dG =    -43.20

        10         20         30         40         50
AUAUAAAUG  -|          AC              G    A    UCAAUA
         GC UGCAUAGCUAA  GGGAGAAGCUAUA CGCU UAUG      \
         CG ACGUAUUGGUU  CCCUUUUCGAUAU GCGA AUAC      U
CUCUUAUA-  U^          C-              -    -    UAAAAU
  100         90         80        70          60

-----------------------------------------------------
```

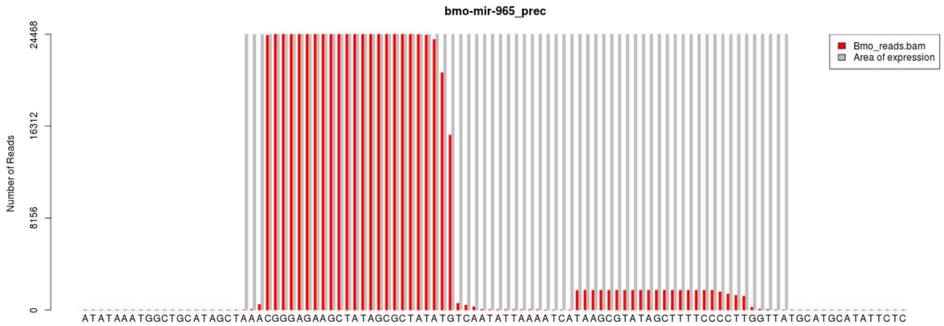

>bmo-mir-970_prec

```
UGGAGGAAUAGACAGAGCCUUGCGUGUGCUCUUAUUGGUAGUGUGACUGAUAAUCUAUCAUAAGACACACGCGGCUCUGUCUGACUCCAGCG
(((((...(((((((((((..(((((((.((((((.(((((.((........))))))))))))))))))))))))))))))).)))))...

 Initial dG =    -47.20

           10        20        30        40
---|    GAA           UU       C      U     U  GAC
   UGGAG   UAGACAGAGCC  GCGUGUG UCUUAU GGUAG GU   U
   ACCUC   GUCUGUCUCGG  CGCACAC AGAAUA CUAUC UA   G
GCG^    A--           --       -      -     -  AUA
 90          80          70         60          50

-----------------------------------------------------
```

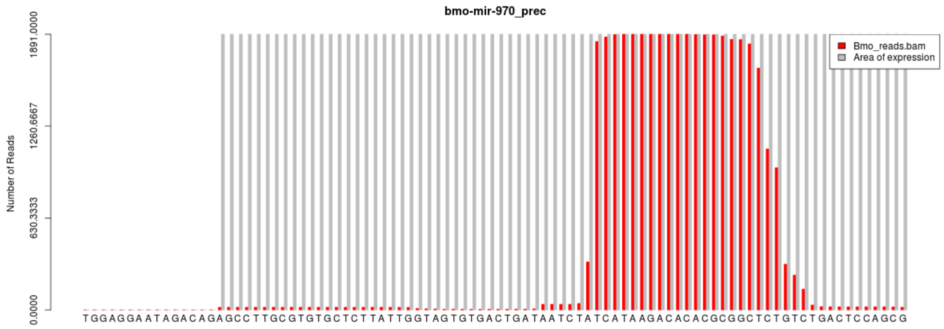

>bmo-mir-989a_prec

```
GGUUCAGGGUUGAAAUGGCCAUUACCUCACAGUCACGUGGUCAUGAUGGUACAACGUGUGAUGUGACGUAGUGGAAGUCUCGACCUGGAUA
.(((((((.((((.((..(((((((.(((((..(((((.((.........)).)))))...))))).))))))).)).))))))))))))).

 Initial dG =    -36.80

        10        20        30         40
G|      G    A  GG       C     GU-     G  CAUG
 GUUCAGG UUGA AU  CCAUUAC UCACA   CACGU GU    \
 UAGGUCC AGCU UG  GGUGAUG AGUGU   GUGCA CA    A
A^      -    C  AA       C     AGU     A  UGGU
90         80        70        60        50

-----------------------------------------------------
```

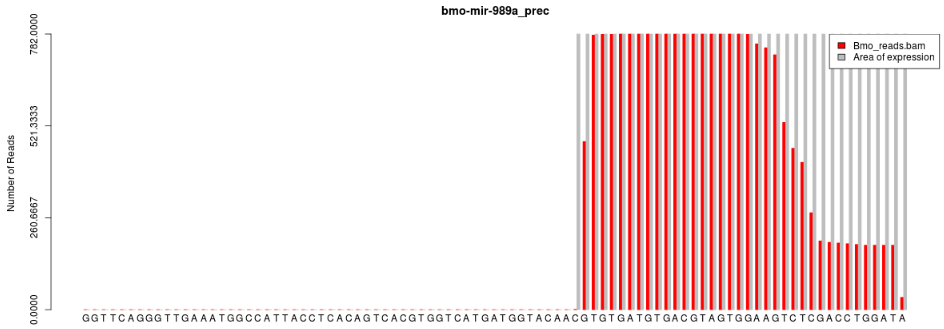

>bmo-mir-989b_prec

```
CACUACAUGUAACUUGUGACGUACAGUGUGAUGUGACGUGAACAAGUAGCGACCAAGUCAUAUUUUACCACGAGCAUAUUAUUCUUAACUCUUCGACUUAAUGUCACGUAUACGUACAGU
A
.(((..(((((...((((((((..(((((((((((.((((...(((((..
(((...))).)))))...))))..))))))))).............)))..))))))))..)))))..))).

 Initial dG =    -26.50

        10        20                     30         40        50
C   AC     ACU        AC   -------------        A-|   AAC     GC   C
 ACU  AUGUA   UGUGACGU  AGU             GUGAUGUG  CGUG   AAGUA  GAC \
 UGA  UGCAU   GCACUGUA  UCA             UAUUAUAC  GCAC   UUUAU  CUG A
A   CA     AU-        AU   GCUUCUCAAUUCU        GA^   CAU     A-   A
 .       110        100        90        80        70        60

-----------------------------------------------------
```

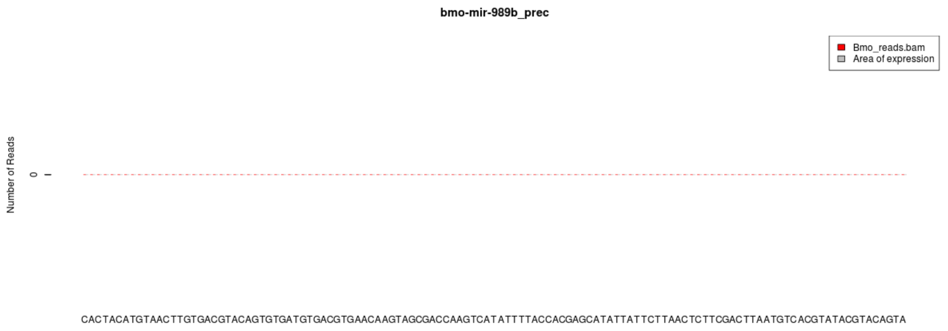

>bmo-mir-993a_prec

```
GGCACAUUCGUGAUCUACCCUGUAGAUCCGGGCUUUUGUAGAUUAAUUACAUCAGAAGCUCGUCUCUACAGGUAUCUCACGGCUGGAA
....((..(((((..((((.((((((..((((((((((..............))))))))))..))))))))))..)))))..))...

 Initial dG =    -36.30

        10        20        30        40
GGCA| UU     UC    C      UC         UAGAUU
    CA  CGUGA  UACC UGUAGA  CGGGCUUUUG      A
    GU  GCACU  AUGG ACAUCU  GCUCGAAGAC      A
AAG-^ CG     CU    -      CU         UACAUU
        80         70        60        50

-----------------------------------------------------
```

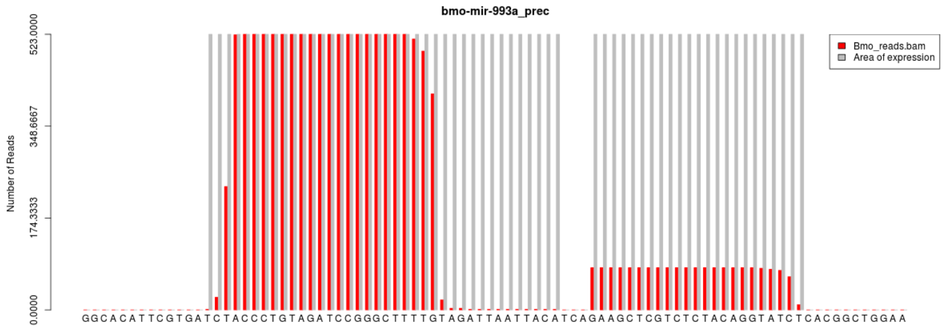

>bmo-mir-993b_prec

```
GGCACAUUCGUGAUCUACCCUGUAGAUCCGGGCUUUCGUAAAAAACAAAUCGAAAGCUCGUCUCUACAGGUAUAUCACGGCUGGCCCACGCCACCUUUUCUACAAUUAGUCAAGAACUG
(((.((..((((((.((((.((((((..((((((((((............))))))))))..)))))))))).))))))..)))))........(((..(((....)))..)))).....

 Initial dG =    -46.30

          10        20        30        40
  |  A  UU      C    C      UC           UAAAA
--GGC CA  CGUGAU UACC UGUAGA  CGGGCUUUCG      A
  CCG GU  GCACUA AUGG ACAUCU  GCUCGAAAGC      A
\ ^  -  CG      U    -      CU           UAAAC
        80        70         60        50


    90       100
  CACGCCAC   UU   C
          CUU  CUA A
          GAA  GAU A
  GUCAA---   CU   U
            110

-----------------------------------------------------
```

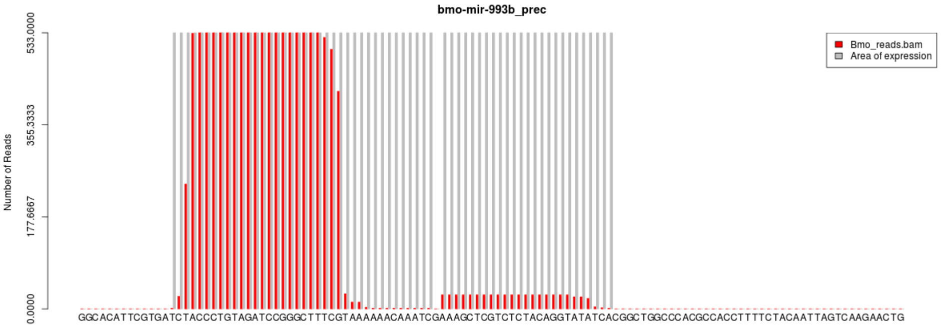

>bmo-mir-998_prec

```
CUCGGUCGCGAGCUGCCUCCCGUGCGGCUGCAGUGCUCGCGUGUAGCACCAUGGGAUUCAGCUCACGGCCGCU
..((((((.((((((..(((((((..((((((((....)).))))))..)))))))..)))))).)))))).. 

 Initial dG =    -45.50

        10        20        30
CU      C      CC       CG      -| G
  CGGUCG GAGCUG  UCCCGUG  GCUGCA GU C
  GCCGGC CUCGAC  AGGGUAC  CGAUGU CG U
UC      A      UU       CA      G^ C
  70        60        50        40

-----------------------------------------------------
```

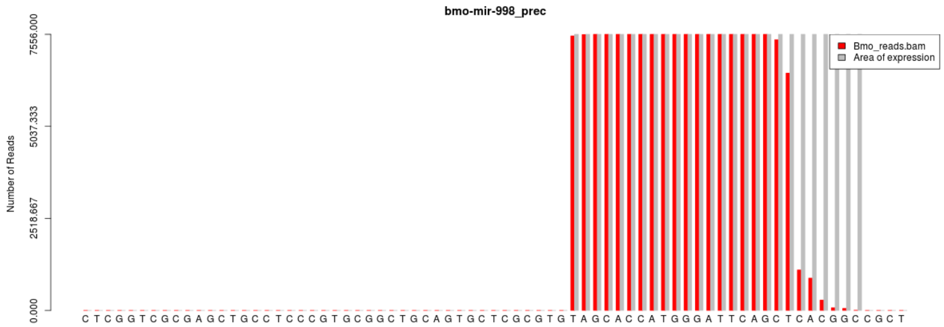

>bmo-mir-1000_prec

```
GGCGCGUCGCGGCCAUAUUGUCCUGUCACAGCAGUACUUGGAAGCUACUGCUGCGUCUGGACAAGCCGGCCGCGGCGCCGCC
((((((((((((((...((((((.....((((((((((....)).)))))))).....))))))...))))))))))).)))

 Initial dG =    -51.30

         10        20        30         40
   -          AUA      UGUCA        -| U
GGC GCGUCGCGGCC   UUGUCC     CAGCAGUA CU G
CCG CGCGGCGCCGG   AACAGG     GUCGUCAU GA G
   C          CCG      UCUGC        C^ A
 80        70        60        50

-----------------------------------------------------
```

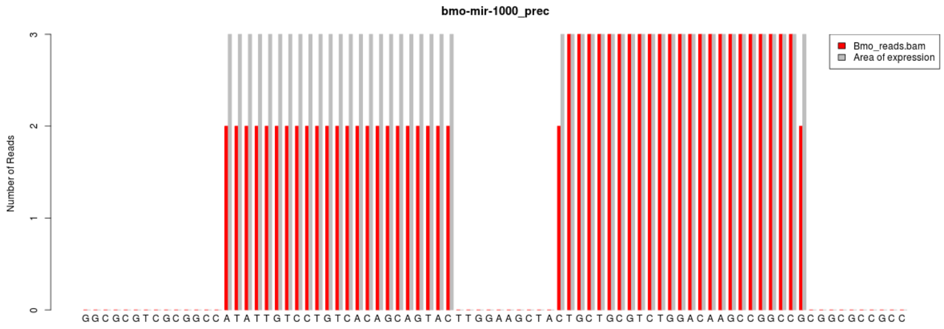

>bmo-mir-1175_prec

```
GUGACAGAUUAAGUGGAGGUGUGAUCUCUUCACUUUUUAUGUUUAAAGUGAGAUUCAACUCCUCCAACUUAACCCUGGAUCG
....(((.(((((((((((..(((..(((.((((((........))))))))).)))...)))))).)))))..))).....

 Initial dG =    -26.90

          10         20         30        40
GUGA-   A-      -      UG-|  UC   U      UUA
     CAG  UUAAG UGGAGG   UGA  UCU CACUUU   U
     GUC  AAUUC ACCUCC   ACU  AGA GUGAAA   G
GCUAG   CC      A      UCA^  U-   -      UUU
 80        70        60          50

-----------------------------------------------------
```

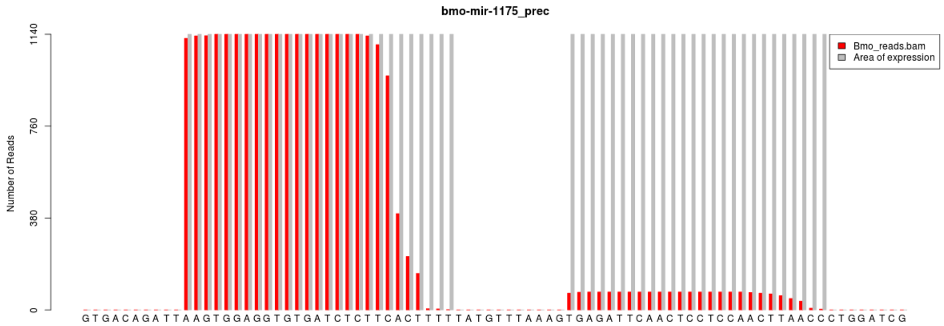

>bmo-mir-1923_prec

UCGAAACUUGCCAUUUAAAAUGGUGCCUUCGAUAACUUUUUUCAUUAAUUUUUAAUGACUAAUCGCGUACCGUUGCAUAGCCGUGGCGG
(((((...(((((((...)))))))..)))))........(((((((...)))))))...........((((..(......)..))))

```
 Initial dG =    -19.30

          10
  |    ACU       U
--UCGAA   UGCCAUU \
  AGCUU   GUGGUAA A
\ ^    CC-       A
   30         20


          40
  .-UAACUUUUU       U
             UCAUUAA \
             AGUAAUU U
  \ ---------       U


-----------------------------------------------------
```

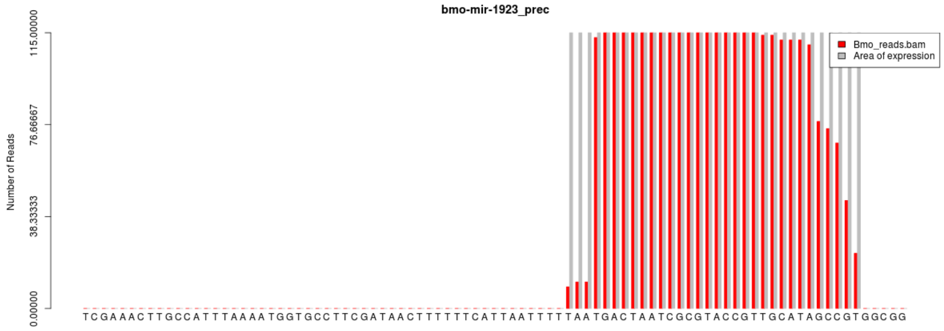

>bmo-mir-1926_prec

```
GUGGCAGUUAUUUCGUUUUAGAAUCCAAACGCUUUGCCCAACUACUUGGCAACGGUAAAGCGUAGGAAUUCUAAAGCAAAAAGGCUGCAU
...(((((..(((.((((((((((((..((((((((((.........(....))))))))))).))).))))))))).)))..)))))..

 Initial dG =    -36.10

        10        20         30        40        50
GUG     UA   C         -|  AA         CAACUACUU G
   GCAGU  UUU GUUUUAGAA UCC  ACGCUUUGCC         G C
   CGUCG  AAA CGAAAUCUU AGG  UGCGAAAUGG         C A
UA-     GA   A         A^  A-         --------- A
.         80        70         60

-----------------------------------------------------
```

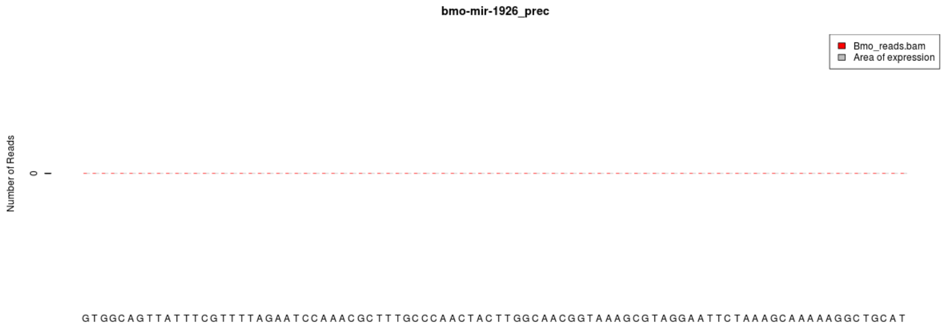

>bmo-mir-2723_prec

CUGGAUACAAGCAGCAGACCAAUCGUUCUGGCUAGGCUUGGGGUAGGUAGUCCAGCAGUGGACGUCUGUGGGCUGAUAGAUAGA

```
(((..((..(((.((((((.....((((..(((.((((..........)))).)))...))))))))))..)))..))..))).
```

```
 Initial dG =    -24.60

         10         20        30         40
-   GA  CA   A-|     CAAUC    UG-   A    UGGG
 CUG  UA  AGC  GCAGAC     GUUC   GCU GGCU    G
 GAU  AU  UCG  UGUCUG     CAGG   CGA CUGA    U
A   AG  AG   GG^     -----    UGA   C    UGGA
   80        70             60        50

-----------------------------------------------------
```

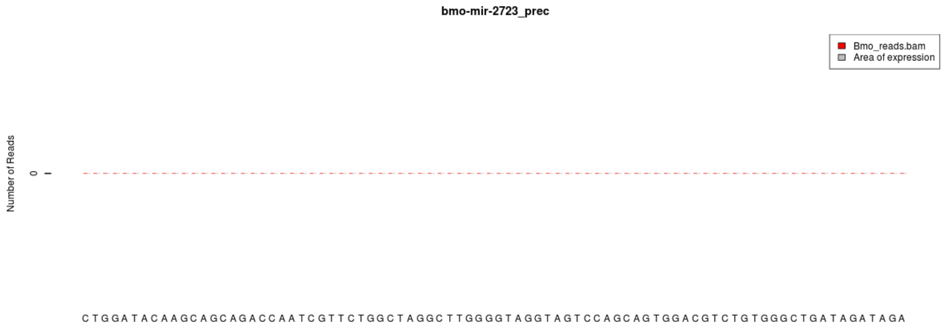

>bmo-mir-2724_prec

```
CCGAGCAUCCCCCGGUGUGAUAGCUCCCUGCCGGAUAUCUGCUUCACACAGUGUGCAGUCAGGCGGGAGCUCCGACUCCUGGACGUUCA
..((((.(((...((.((...(((((((.(((.((...((((..(((...))).))))))).)))))))))))...)).)).))).)))).

 Initial dG =    -37.00

        10        20        30        40
CC|   A   CCC  U  GAU       U   G  UAU    UU   A
  GAGC UCC   GG GU   AGCUCCC GCC GA   CUGC  CAC \
  CUUG AGG   CC CA   UCGAGGG CGG CU   GACG  GUG C
A-^   C   U--  U  GCC       -   A  ---    U-   A
         80          70         60             50

-----------------------------------------------------
```

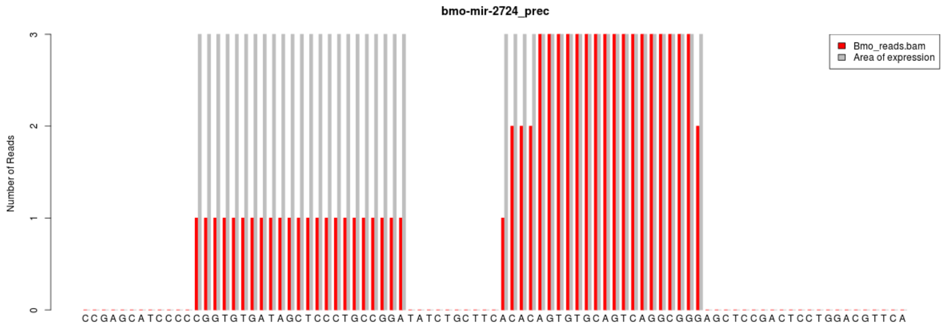

>bmo-mir-2729_prec

```
AGCAAGCAUUUAUGCUGGAGUGAGUAACAGAUUUUAUGAGGUCGGUCCAUCGUGUUGGAGAUCGUCCGCGUGAUCUUUUGCCCUCGACUUUCCCUUGCA
.(((.(((....))).((((((((...((((..(((((.((.(((((((......))))..))).)).)))))...)))))..))))...))))..))).
```

```
 Initial dG =    -25.70

          10
A   .-A|  U
 GCA   GCA U
 CGU   CGU U
A   \ -^  A



          20           30          40           50
      U-    ---    UAA    UU-     A  U   --    UC
        GGAG   UGAG   CAGA   UUAUG GG CGG  UCCA  G
        CCUU   GCUC   GUUU   AGUGC CC GCU  AGGU  U
      UC    UCA    CC-    UCU     G  U   AG    UG
          90          80         70         60

-----------------------------------------------------
```

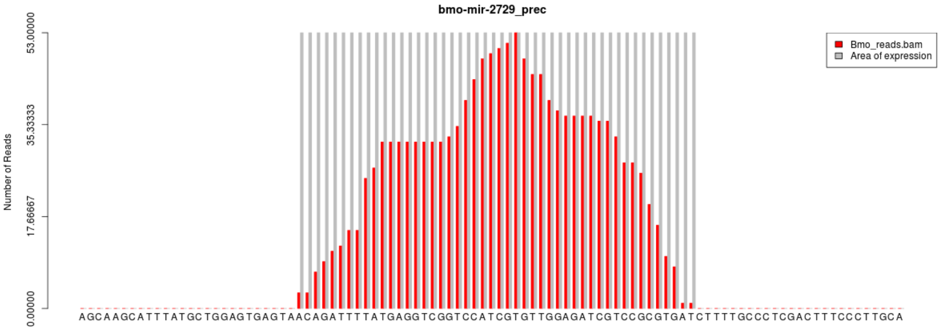

>bmo-mir-2731-1_prec

```
AAACCUAACAGAUGCGAGACCAUGGUAUGUGGAAAUAAAAGCCACCCGGUUUUUAUCUUUCCACACCAAAAGAUCCACAUCUCCCGAGGUG
..((((...(((((.((.....((((..(((((((((((((((....)))))))))..))))))))))).....))..)))))....)))).

 Initial dG =    -29.00

         10          20         30           40
AA    AAC-     C-| GACCA    AU      --          A
  ACCU    AGAUG  GA     UGGU  GUGGAA  AUAAAAGCC C
  UGGA    UCUAC  CU     ACCA  CACCUU  UAUUUUUGG C
G-    GCCC     AC^ AGAAA    --      UC          C
 90         80         70          60         50
```

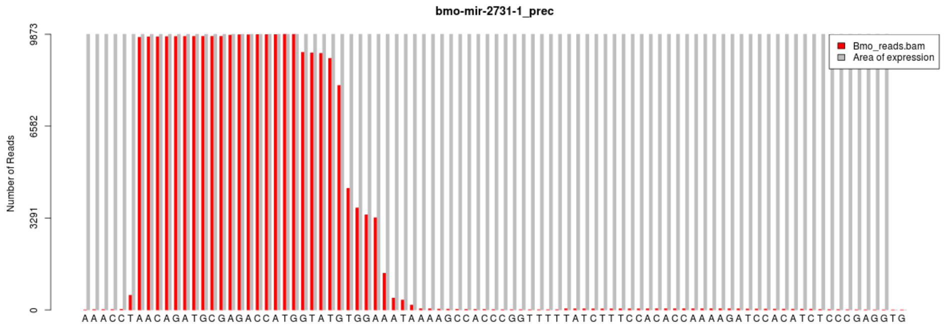

>bmo-mir-2731-2_prec

```
AAACCUAACAGAUGCGAGACCAUGGUAUGUGGAAAUAAAAGCCACCCGGUUUUUAUCUUUCCACACCAAAAGAUCCACAUUUCCCGAGGUG
..((((...(((((.((.....((((..((((((((((((((....)))))))))..))))))))))).....))..)))))....)))).

 Initial dG =    -26.40

         10          20         30           40
AA    AAC-     C-| GACCA    AU      --          A
  ACCU    AGAUG  GA     UGGU  GUGGAA  AUAAAAGCC C
  UGGA    UUUAC  CU     ACCA  CACCUU  UAUUUUUGG C
G-    GCCC     AC^ AGAAA    --      UC          C
 90        80        70           60        50

-----------------------------------------------------
```

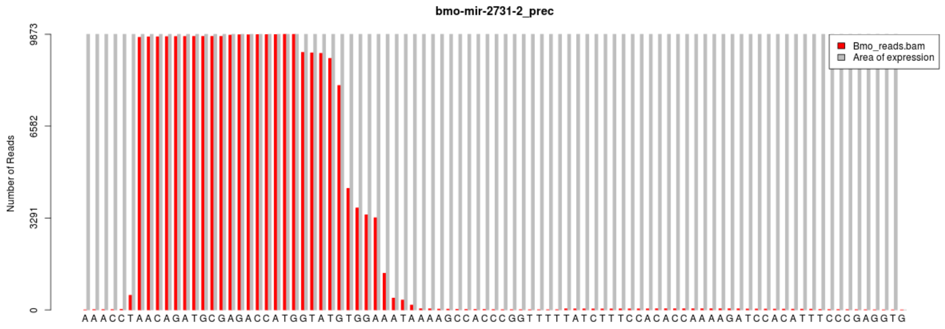

>bmo-mir-2733a-1_prec

```
UUUCUCUCUAAUACAGUCAUCACACUCUCAGUUGAUAUGUGUAACAUUCUAUCACUGGGUGCAUGAUGAUUGUGGUACAGAGAU
..(((((.((.((((((((((((....((((((.((((.(((...)))..))))))))))....))))))))))).)).))))).

 Initial dG =    -30.00

        10        20        30         40
UU|    C  A           CACU      U    U-   U
  UCUCU UA UACAGUCAUCA    CUCAGU GAUA  GUG \
  AGAGA AU GUGUUAGUAGU    GGGUCA CUAU  UAC A
U-^    C  G           ACGU      -    CU   A
    80        70        60         50

-----------------------------------------------------
```

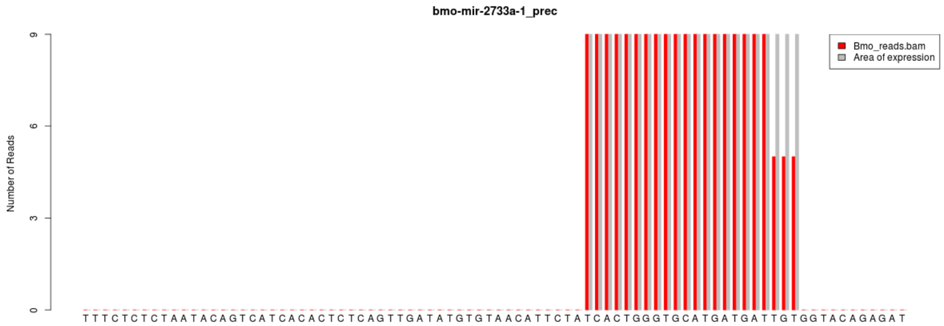

>bmo-mir-2733a-2_prec

```
UAUAUCACUGGGUGCAUGAUGAUUGUGAUAGAGAGAUCACCACAUCACGCUGCCAUUACAUGUGACAUCUCUUCUAUAUCUCUCUCUAAUGCAGUCAUCAUACUCUCAGUUGAUAUG
((((((((((((...((((((((((((.((((((((........(((((.((......))))))).............)))))))).))))))))))))...)))))).))))))

 Initial dG =    -39.00

         10         20         30         40                50
      -      UGC            A        UCACCACA-------|    C   CC
UAUAUC ACUGGG   AUGAUGAUUGUG UAGAGAGA               UCACG UG   A
GUAUAG UGACUC   UACUACUGACGU AUCUCUCU               AGUGU AC   U
      U      UCA            A        CUAUAUCUUCUCUAC^    -   AU
     110       100         90         80         70          60

-----------------------------------------------------
```

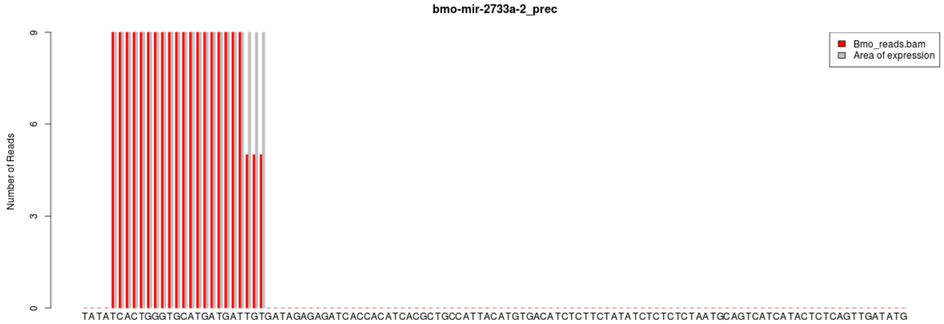

>bmo-mir-2733a-3_prec

GUUGAUAUGUGGAAAAUUCUAUCACUGGGUGCAUGAUGAUUGUGGUAGAGAGAUCAUCUCAUCACGCCGCCAUCACAUGUAACAUCUCUUCUAUUUCUCUCUAAUACAGUCAUCACACUC
UCAGUUGAUAUGUGUAACAUUAUAUCAC

```
..((((((((....((..((((((((((....(((((((((((.((((((((...............
((........))...............)))))))).))))))))))....))))).))))).))....))).)))))).

 Initial dG =    -40.90

         10        20         30        40        50        60        70
GU       -|  GAAA  UC     -     UGCA           G        UCAUCUCAUCACGCC  CAU
  UGAUAU GUG    AU  UAUCA CUGGG    UGAUGAUUGUG UAGAGAGA               GC   C
  ACUAUA UAC    UG  AUAGU GACUC    ACUACUGACAU AUCUCUCU               UG   A
C-       U^  AAUG  U-     U     UCAC           A        UUAUCUUCUCUACAA  UAC
       140        130       120       110       100        90        80

-----------------------------------------------------
```

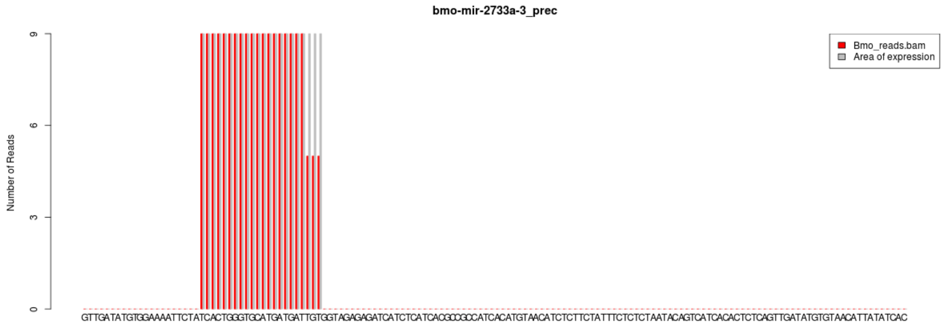

>bmo-mir-2733b-1_prec

UCUCUCUAAUAUAGUCAUCACACUCUCAGUUGAUAUGUGUAACAUUCUAUCACUGGGUGCGUGAUGAUUGUGGUAGAGAU

```
..((((((.((((((((((((...((((((.((((.(((...)))..)))))))))))...)))))))))))).)))))).
```

```
 Initial dG =    -32.90

        10        20        30         40
UC|     A            ACU      U    U-   U
  UCUCUA UAUAGUCAUCAC   CUCAGU GAUA  GUG \
  AGAGAU GUGUUAGUAGUG   GGGUCA CUAU  UAC A
U-^     G            CGU      -    CU   A
.         70        60         50

-----------------------------------------------------
```

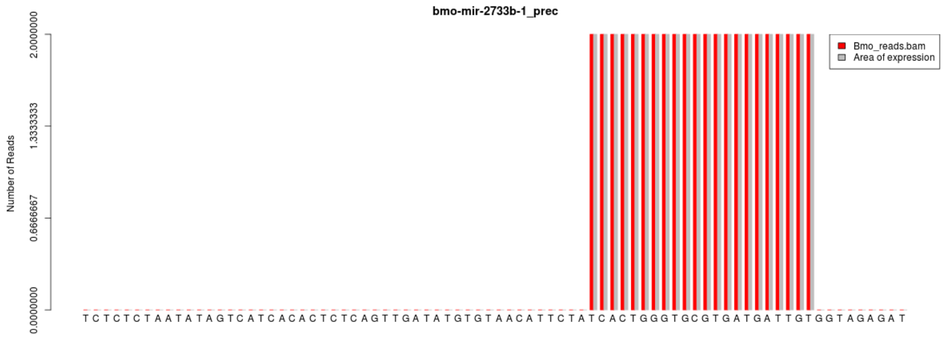

>bmo-mir-2733b-2_prec

UAACAUUCUAUCACUGGGUGCGUGAUGAUUGUGGUAGAGAUUUUCACAUAACGCCACCAUCACAUGUGACAUCUCUCCUGUCUCUCUGUAAUGCAGUCAUCAUACUCACAGUUGAUAUGU
UUGACAUUC

```
.((((...((((((((.....(((((((((((((.((((((..
((((((..............)))))).)))))))).............))))))))))).....)))).))))))))........

 Initial dG =    -32.10

               10         20        30                       40        50
U-------    UUC    -    GGUGC          -------------| U      UU      AACGCC
        AACA   UAUC ACUG     GUGAUGAUUGU              GG AGAGAU  UCACAU      A
        UUGU   AUAG UGAC     UACUACUGACG              CC UCUCUA  AGUGUA      C
CUUACAGU    ---    U    ACUCA          UAAUGUCUCUCUGU^ -      C-      CACUAC
       120          110       100        90        80         70         60

-----------------------------------------------------
```

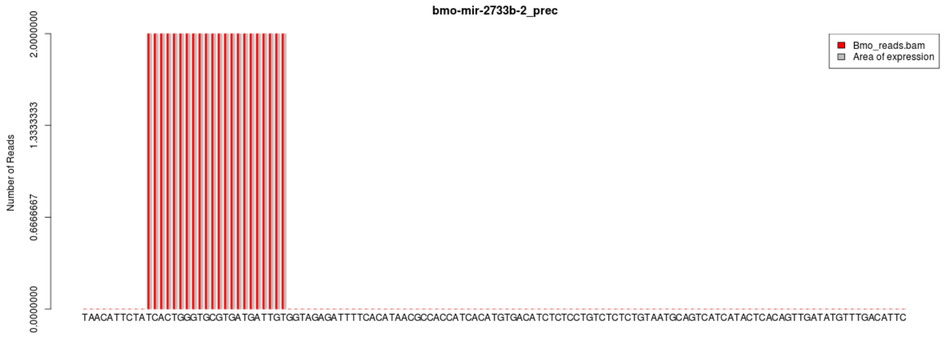

>bmo-mir-2733c_prec

```
GAUUGUCACUGUCAGUUGUCACUAUCGCCAGUGUCAGUGUCGUAAUUCUGUCACUGGGAGAGUGAUGAUUGCCGGUGACGAU
.((((((((((.((((..(((((.((.((((((.(((..........))).)))))))).)))))..)))).))))))))))

 Initial dG =    -43.00

        10        20        30        40
G|         U    UG     A  G      U   UGUC
 AUUGUCACUG CAGU  UCACU UC CCAGUG CAG    G
 UAGCAGUGGC GUUA  AGUGA AG GGUCAC GUC    U
-^         C    GU     G  -      U   UUAA
  80        70        60         50

-----------------------------------------------------
```

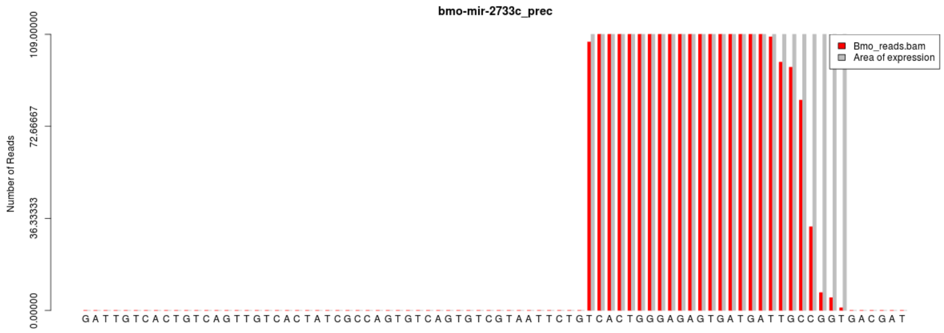

>bmo-mir-2733d_prec

```
CUCUCUGUAAUGCAGUCAUCAUACUCACAGUUGAUAUGUUUGACAUUCUAUCACUGGGUGUAUGAAGAUUGUGGUACAGAGA
.((((((((..((((((.((((((...(((.(((((((.....))...)))))))))...)))))).))))))..))))))))

 Initial dG =    -32.90

        10        20        30           40
C        AU       A      UCA   U     ---| UU
 UCUCUGUA  GCAGUC UCAUAC   CAG UGAUA   UG  \
 AGAGACAU  UGUUAG AGUAUG   GUC ACUAU   AC  U
-        GG       A      UGG   -     CUU^ AG
  80        70        60         50

-----------------------------------------------------
```

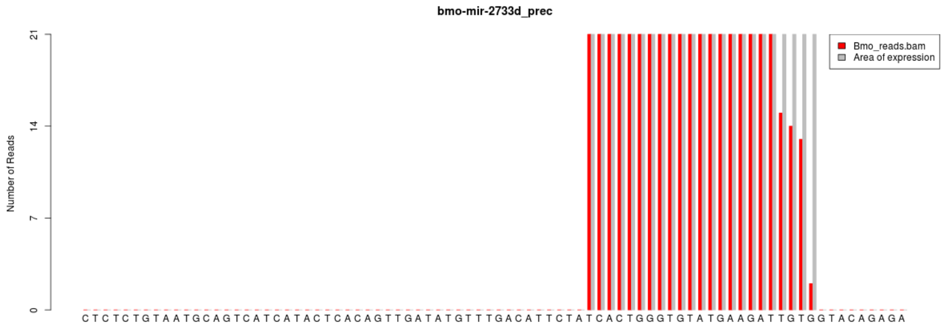

>bmo-mir-2733e-1_prec

ACUGUCUACAACAAUGUCUCUCAACAAUAGUUAUCACUUCCUCAGUCGACAUGUUUUCACAUUCUGUCACUGGGAAUGUAAUAGCUAUUCUGGAUAGA

.(((((((......((.....))..(((((((((.((((((.((((.(((((((....)))...)))))))))))).)).))))))))).))))))).

```
 Initial dG =    -27.30

          10
A       .-CAACAA| UC
 CUGUCUA        UG  \
 GAUAGGU        AC  U
A       \ ------^ UC
                  20



               30         40        50
          AC         C  -    U    C    ---   U
            AAUAGUUAU AC UUCC CAGU GACA   UGU U
            UUAUCGAUA UG AAGG GUCA CUGU   ACA U
          C-         A  U    -    -    CUU   C
         90         80         70         60
```

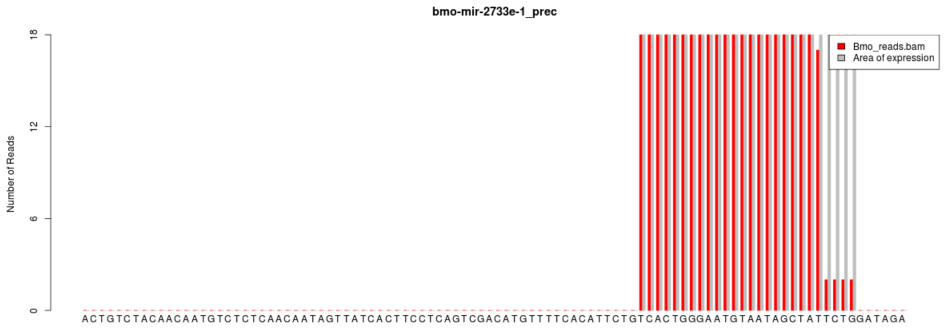

>bmo-mir-2733e-2_prec

```
UUUGCUGGACUGUCUAUAAUGUCUUUCAACAAUAGCUAUUACUUCUCCAGAAGACAUUCACAUUCUGUCACUGGGAAUGUAAUAGCUAUUUCCAAAAUU
.(((..((((..........))))..))).((((((((((((((.((((..((((.........)))).))))))).)))))))))))))).........
```

```
 Initial dG =    -28.50

          10
.-U|  CU    UGUC
   UUG  GGAC    U
   AAC  UCUG    A
\ -^  UU    UAAU
           20



 30                 40          50         60
  C--------            -   U    AA    UUCA
         AAUAGCUAUUAC UUC CCAG  GACA    \
         UUAUCGAUAAUG AAG GGUC  CUGU    C
  UUAAAACCU            U   -    A-    CUUA
          90        80         70

-----------------------------------------------------
```

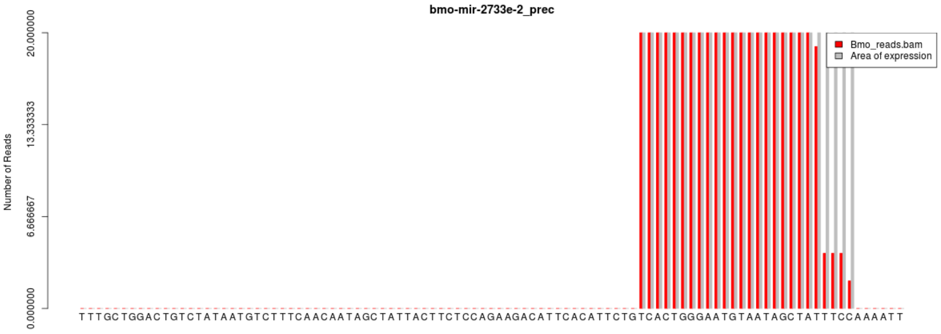

>bmo-mir-2733e-3_prec

UUUGCUGGACUGUCUAUAAUGUCUUUCAACAAUAGCUAUUACUUCUCCAGAAGACAUUCACAUUCUGUCACUGGGAAUGUAAUAGCUAUUUCCAAAAUU

```
.(((..((((..........))))..))).((((((((((((((.((((..((((.........)))).))))))).))))))))))))).........

 Initial dG =    -28.50

          10
.-U|  CU    UGUC
   UUG  GGAC    U
   AAC  UCUG    A
\ -^  UU    UAAU
           20


 30                 40         50        60
  C--------            -   U    AA    UUCA
           AAUAGCUAUUAC UUC CCAG  GACA    \
           UUAUCGAUAAUG AAG GGUC  CUGU    C
  UUAAAACCU            U   -    A-    CUUA
          90        80         70

-----------------------------------------------------
```

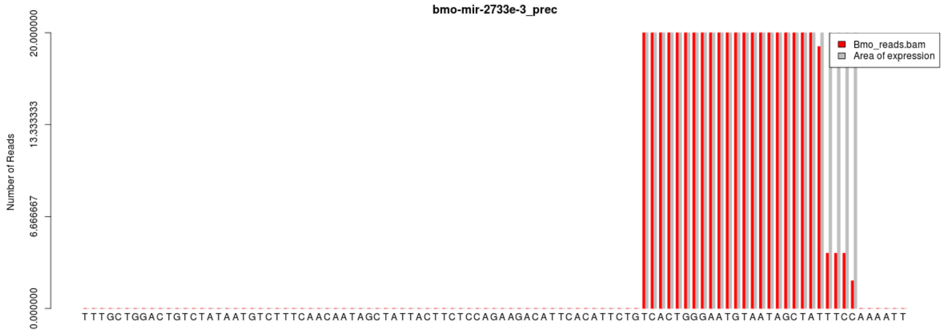

>bmo-mir-2733f_prec

```
ACGUCUACAUUGCUAAUCUGUCUAUAAAAGACAGUUAUUACUAACUCGGUAGACACGUCACUGUGUCACUGGGUAUGUAAUGACAGUCGUUGAUAGAUAAUCGCGUCGUACCAAACGU
((((.(((..(((.....(((((((....(((.((((((((..(((((((.((((((....))))))))))))))..)))))))).)))....)))))))...)))..)))....))))

 Initial dG =    -39.20

           10         20         30         40         50
    C---|  AU   UAAUC       AAAA   A         UA        A      U
ACGU    UAC  UGC     UGUCUAU    GAC GUUAUUAC  ACUCGGU GACACG C
UGCA    AUG  GCG     AUAGAUA    CUG CAGUAAUG  UGGGUCA CUGUGU A
    AACC^  CU   CUA--       GUUG   A         UA        -      C
      110       100          90         80         70         60

-----------------------------------------------------
```

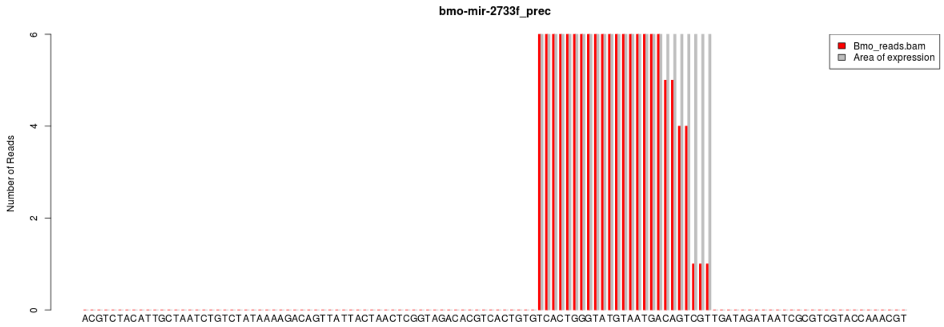

>bmo-mir-2733g_prec

UAUCACUGGGUGCAUGAAGAUUGUGGUACAGAGAUCAUCACAUCACGCCGCCGUGACAUCUCUUCUAGCUGUCUUUCUCUCUCUAAUGCAGUCAUCAUACUCUGAGUUGAUA
((((((.((...((((.((((((..((.(((((.....((((((((....)))))).............)))).....))))).))..)))))).))))...)).))).))))

```
 Initial dG =    -26.10

         10        20        30        40
    -   G  UGC    A      GG  C     UCAUC   -------------|    C
UAUC ACU GG   AUGA GAUUGU  UA AGAGA     ACA             UCACG C
AUAG UGA UC   UACU CUGACG  AU UCUCU     UGU             AGUGC G
    U   G  UCA    A      UA  C     CUUUC   CGAUCUUCUCUAC^    C
110       100        90        80        70        60        50
```

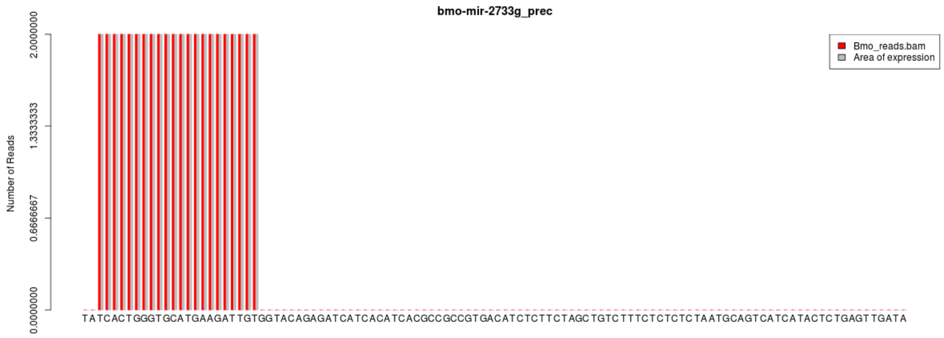

>bmo-mir-2733h_prec

```
UCUCUCUAAUGCAGUCAUCAUACUCUGAGUUGAUAUGUGGAAAAUUCUAUCACUGGGUGUAUGAUGAUUGUGGUAGAGAGA
((((((((..((((((((((((((.((.(((.((((.((.....))..))))))).)).))))))))))))))..))))))))

 Initial dG =    -38.00

        10        20        30         40
|       AU              U  G   U    U-  GG
UCUCUCUA  GCAGUCAUCAUAC CU AGU GAUA  GU  \
AGAGAGAU  UGUUAGUAGUAUG GG UCA CUAU  UA  A
^       GG              U  G   -    CU  AA
80        70        60         50

-----------------------------------------------------
```

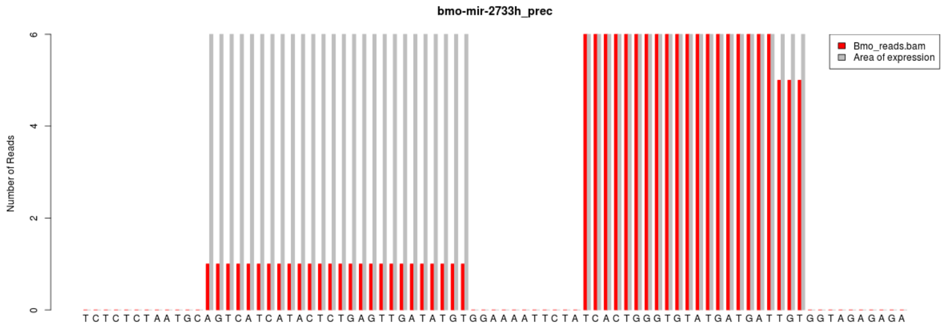

>bmo-mir-2733i_prec

ACGUUGUCUACAGUGUCUGUUAAUAAUAGUUGUUACUUCCUCAGAUGACAUUAUAUAUUGUCACUGGGAAUGUAAUGACUAUUUUCAACAGACCGUAACUGCAAACCG
..((.((.(((...(((((((...((((((..((((((((.(((.(((((........)))))))))))).)))))..))))))...))))))).))))).))......

```
 Initial dG =    -38.50

            10         20         30          40         50
AC----| U  C   AGU       AAU      UG    -    U   A     UUA
      GU GU UAC   GUCUGUU   AAUAGU  UUAC UUCC CAG UGACA   U
      CG CA AUG   CAGACAA   UUAUCA  AAUG AAGG GUC ACUGU   A
GCCAAA^ U  -   C--       CUU      GU    U    -   -     UAU
      100           90         80         70           60
```

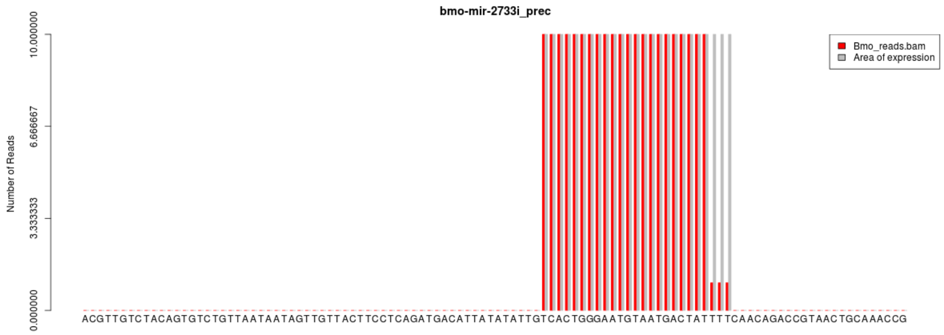

>bmo-mir-2733j_prec

```
CUUCUAUAUCUCUCUCUAAUGCAGUCAUCAUACUCUCAGUUGAUAUGUGUAACAUUCUAUCACUGGGUGCAUGAUGAUCGUGGUAGAGAGAUCAUCACAUCACGC
..........((((((((..((.((((((((...((((((.((((.(((...)))..))))))))))...)))))))).))..)))))))).............

 Initial dG =    -32.90

        10             20         30         40          50
CUUCUAUAUC----|       AU  A         ACU       U    U-   U
              UCUCUCUA  GC GUCAUCAU   CUCAGU GAUA  GUG \
              AGAGAGAU  UG UAGUAGUA   GGGUCA CUAU  UAC A
CGCACUACACUACU^       GG  C         CGU       -    CU   A
   100        90         80         70          60

-----------------------------------------------------
```

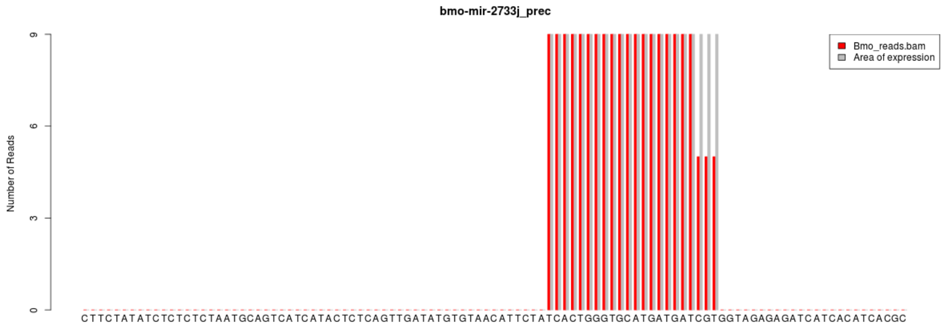

>bmo-mir-2734_prec

```
CAGUUUUAGAGCUUUGUCGUGUCUGGUAGUCGCGUUUUCCUUUUAGGAGGAUAGCUAGUUUUAACCGGCGACAAGUGCUUCUUUUGCUC
.(((...(((((((((((((...(((((....(((((.(((((....))))).)))..))....)))))))))))).))).))...))).

 Initial dG =    -25.10

         10          20         30           40
C   UUU  -    -|      UGU      AGUC  --   U     U
 AGU   AG AGC UUUGUCG   CUGGU    GC  GUU UCCUU U
 UCG   UC UCG GAACAGC   GGCCA    UG  CGA AGGAG A
C   UUU  U    U^      ---      AUUU  AU   U     G
        80         70            60         50

-----------------------------------------------------
```

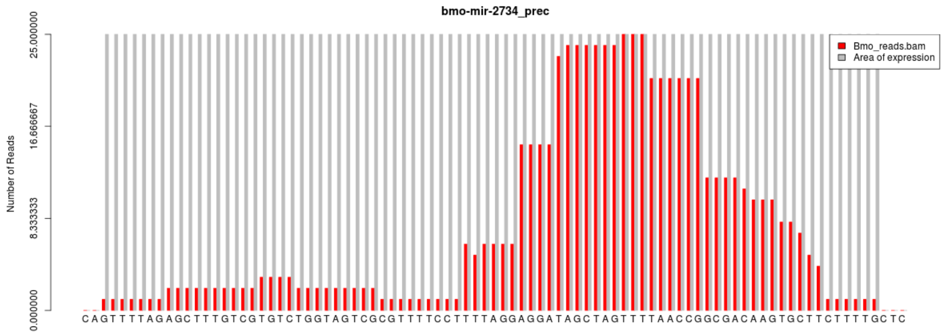

>bmo-mir-2736_prec

UGUUACCAAAACGUCGAUGGUACCAGCAGAGCCUUGUAUCUACGCUGGAGCGGCUUCGGGAUCGUUGACGCCAGCGCGGGGACU
.((..((....((((((((((.((....(((((..((.((......)).)))))))..)))))))))))).......))..)).

```
 Initial dG =    -24.40

        10            20         30         40
U   UA  AAAA---|         A  AGCA     UU  A  UA
 GU  CC       CGUCGAUGGU CC    GAGCC  GU UC  C
 CA  GG       GCAGUUGCUA GG    UUCGG  CG GG  G
U   GG  CGCGACC^         -  GC--     --  A  UC
   80        70         60            50

-----------------------------------------------------
```

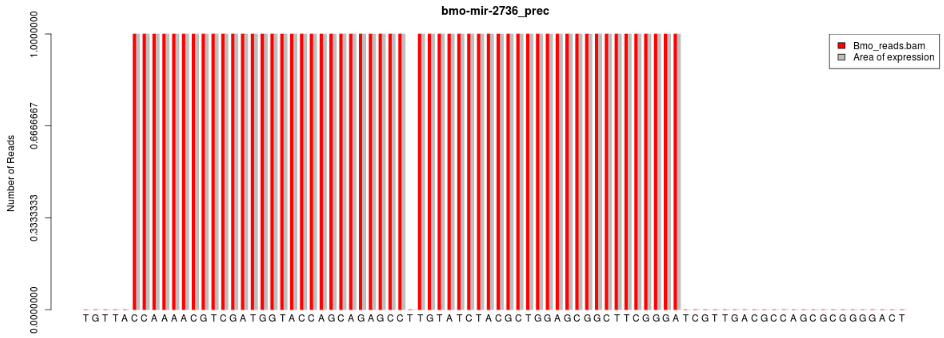

>bmo-mir-2737_prec

```
CUGUGGCGUCUAUGUUUUUAUACUGUCCAGAUUUGUGCUGCCUUUACCAUAUUUUUCGAUUGUGUAAAAUUGACGCAUUCACC
..((((((((.....((((((((.(((.(((..((((.((....)).))))..))).))).))))))))..)))))...))).

 Initial dG =    -19.80

           10        20        30        40
CU   ---|    UAUGU        U   C   UU    C  C
  GUG   GCGUC     UUUUAUAC GUC AGA  UGUG UG C
  CAC   CGCAG     AAAAUGUG UAG UUU  AUAC AU U
C-   UUA^    UU---        U   C   UU    C  U
   80        70           60        50

-----------------------------------------------------
```

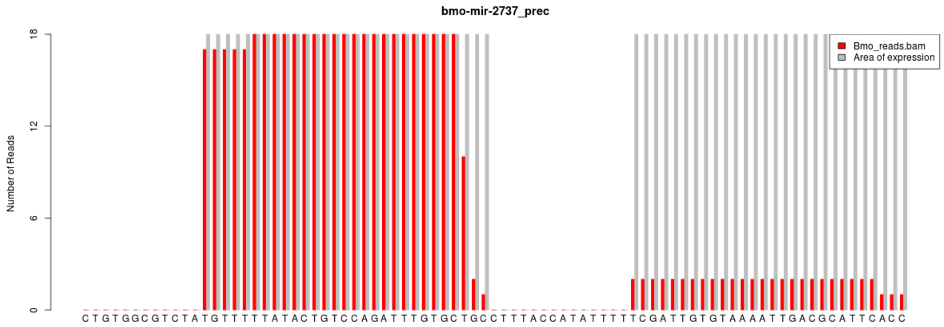

>bmo-mir-2738_prec

AUUCUCUGAUCUAAAUGAUGAAUGUUCUUUACCAUUAAAAGACUGAUAUGAAUAUUGAAGAUCUUUUAGAACGGCCAUCUGAUGAAUCAUAAAAACUAAAUUUGGGAAUA
((((((((((.((...((((..((((((........((((((((((((....))))..)).))))))))))))..))))...)).)))).............)))))).

```
 Initial dG =    -15.90

                       10        20        30        40          50
-      --------------|   C  AAU    AA      UUACCAUU      -  --    U
 AUUCUC              UGAU UA   GAUG  UGUUCU        AAAAGA CU  GAUA G
 UAAGGG              ACUA GU   CUAC  GCAAGA        UUUUCU GA  UUAU A
A      UUUAAAUCAAAAAU^   A  AGU    CG      --------      A  AG    A
.       100        90        80        70                60
```

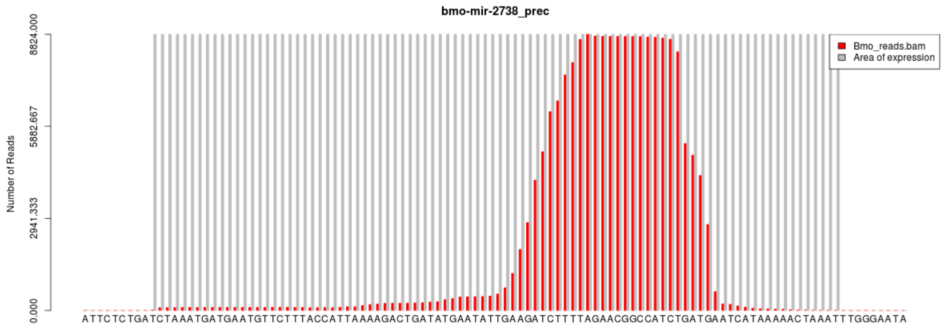

>bmo-mir-2739_prec

```
GCUGCACCUCGGGAGAUGUGGAUAUUAUGGUGUGGAGGGAUAGAAACCGCGUGGCUUUAAUUUCAACAUACCAUGGUCUCGUAACUACCAGGUGUGGC
((..(((((.((...(((.((((..(((((((((..(..((.(((.((....)).))).))..)..))))))))))))))))).....)))))))..))

 Initial dG =    -37.20

        10           20         30         40         50
  UG     C  GAG--|  U    AU          GA GG  A   A  G
GC  CACCU GG     AUG GGAU  UAUGGUGUG  G  AU GAA CC C
CG  GUGGA CC     UGC UCUG  GUACCAUAC  C  UA UUU GG G
  GU     -  AUCAA^  -    --          AA UU  A   C  U
       90         80            70         60

-----------------------------------------------------
```

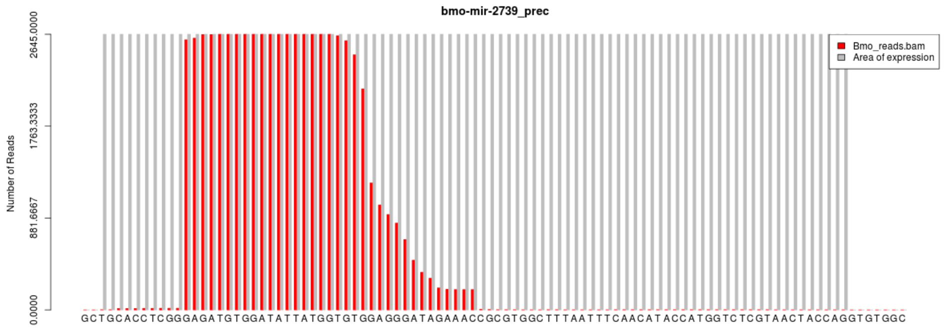

>bmo-mir-2742_prec

```
UGAGAAUGUCAUUAGACUUCUGAUUGAAAAACUUUGUAAAGAUUUUCAUUGGAUUAGUGUUGUCUUU
.((((....((((((...((..((.(((((.(((....))).)))))))..)))))))))...)))).

 Initial dG =    -12.80

        10        20        30
U|   AUGU      ACU  UG  U     A   U
 GAGA    CAUUAG   UC  AU GAAAA CUU G
 UUCU    GUGAUU   AG  UA CUUUU GAA U
U^   GUU-      ---  GU  -     A   A
      60            50         40

-----------------------------------------------------
```

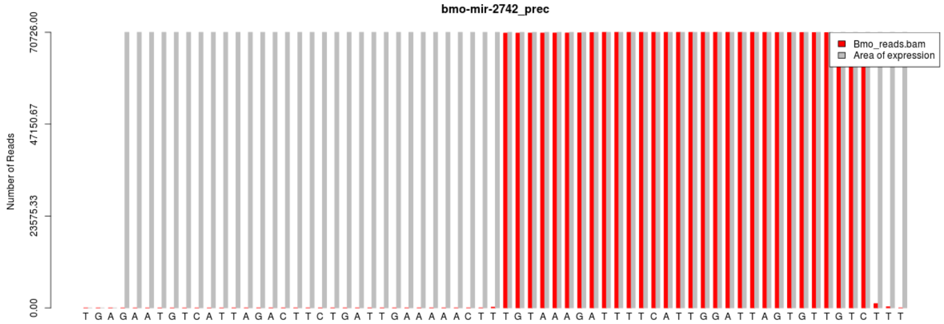

>bmo-mir-2744_prec

```
UCGGCAGCAGCUCAGCCUUGUCAUCGUCAUCGAAUGCCGGUGGUUGGCCUGAAGGCGUACAGGGAGGCAUAGUACGGUAUUCGGAUUUGAGAGUCCUAGCUAGUCGCCA
..(((.((((((.((..((.(((..(((..(((((((((...(((..((((........))))..)))......)))))))))))).))).))..)))))).)).))).

 Initial dG =    -36.90

         10        20        30        40           50
UC   A  -|   C  CC  G   UC   AU         GUG---   GG    AAG
  GGC GC AGCU AG  UU UCA  GUC  CGAAUGCCG      GUU  CCUG   G
  CCG UG UCGA UC  GA AGU  UAG  GCUUAUGGC      CGG  GGAC   C
A-   C  A^   -  CU  G   U-   --         AUGAUA   AG    AUG
        100         90           80         70         60

-----------------------------------------------------
```

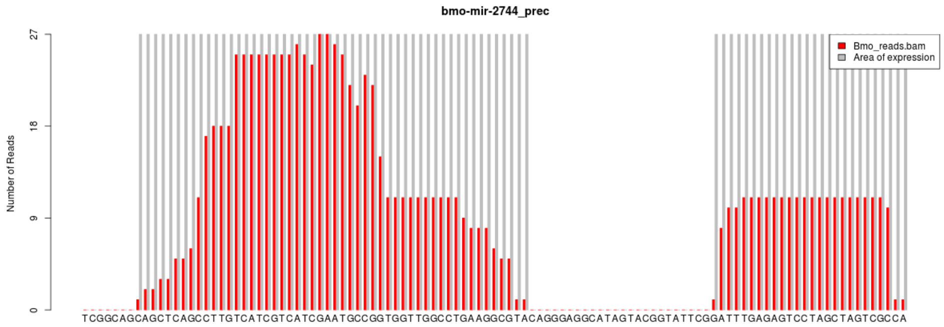

>bmo-mir-2745_prec

```
AGUGCCGUUGAUGGCCCGAAAUUCCGAAUUUUAGCUUUAUUAAUGCUAAAUUCGGUCUUUCGGGCAUAUCAAAGGCACC
.(((((.((((((((((((((..((((((((.(((.........)))))))))))..)))))))).)))))).))))).

 Initial dG =    -43.00

        10          20         30
A     G      -|       UU        U   UUUA
 GUGCC UUGAUG GCCCGAAA  CCGAAUUU AGC    \
 CACGG AACUAU CGGGCUUU  GGCUUAAA UCG    U
C     A      A^       CU        -   UAAU
        70         60         50

-----------------------------------------------------
```

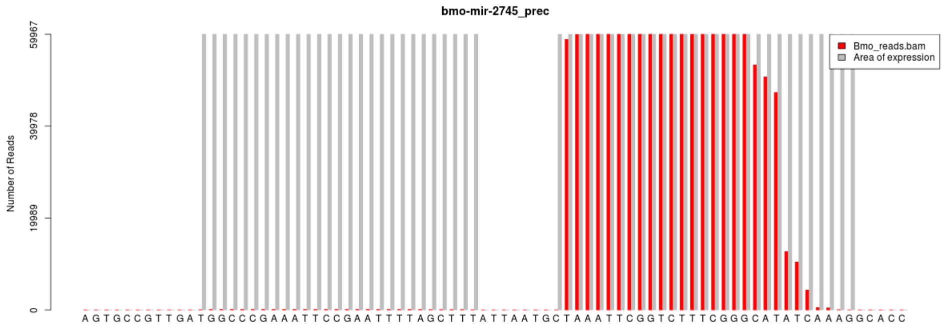

>bmo-mir-2748_prec

```
UCGGAAACGCGACCCACUGAGAAGAUCCGGCGAGAAAACUCAGUGGGCUGUGUCUAUGGGUUUAACAUCCUCGCCGAGCCCUUCGUCGCAAGCG
........((((.......((((...(((((((.(((((((.((((.....)))))))))))......))))))).....)))))))))).....

 Initial dG =    -30.80

        10        20         30             40
UCGGAAAC|    CCACUGA    AUC-       A-----       G    CU
        GCGAC       GAAG    CGGCGAG      AAACUCA UGGG  \
        CGCUG       CUUC    GCCGCUC      UUUGGGU AUCU  G
GCGAA---^    -------    CCGA       CUACAA       -    GU
   90                  80         70         60

-----------------------------------------------------
```

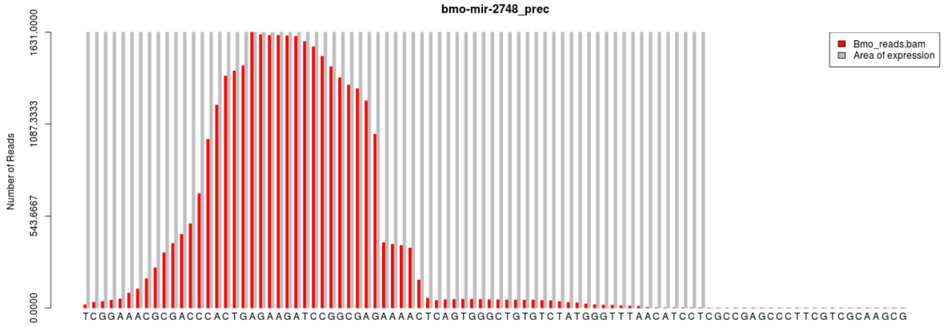

>bmo-mir-2751_prec

```
ACCUAUCGAGUAUGCUAGCCGCCUACUCACCGCAGCGGAGCGCAACUACUCUACUACAGAACGGGAAGCGCUUGCUGUAGUCUGGGCCGUGGAGCGUUUCAGGC
.(((...(((.(((((.((.(((((...((..(((((.(((((..((..(((.....)))...))..))))))))))..)).))))).))..))))))))))).

 Initial dG =    -37.40

        10         20        30        40         50
A|  AUC   U      A-  C     CUC  CG     G     AA  AC-   AC
 CCU   GAG AUGCU  GC GCCUA   AC  CAGCG AGCGC  CU   UCU  \
 GGA   CUU UGCGA  UG CGGGU   UG  GUCGU UCGCG  GG   AGA  U
C^  ---   -      GG  C     C--  AU     -     AA  GCA   CA
     100         90          80         70        60

-----------------------------------------------------
```

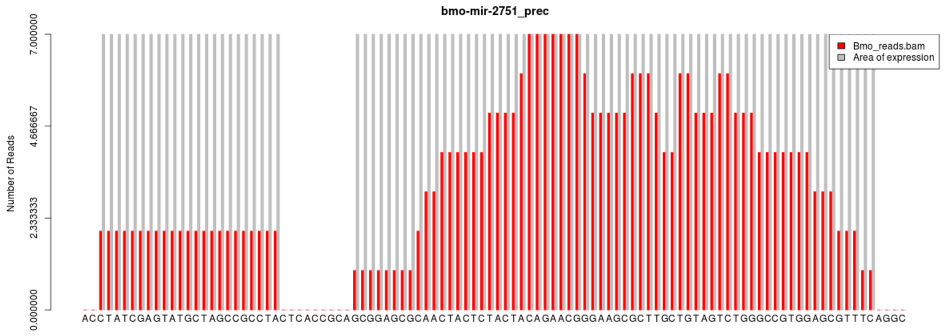

>bmo-mir-2752_prec

GGGUAACGCCGCGGACUGUCUCAACACGAUGGCCACGGAGCCGUCGACGUCCCCGAUCCAGCAGUCCUGGUCGGUGGGGACGAAGUACGGCUCCGCGGCGACAGCGACGUCGAUUGACCA
CUCCGCCAUGGUGUGGACG

```
..((.((((((((((.((..((((..((((((((.(((((((((...((((((((..
((((.....))))....))))))))....))))))))).)))........))))).)))).)).)))))...)))))..)).
```

```
 Initial dG =    -67.00

         10            20         30                  40          50           60
GG   A-      ---     C  UC     CA      --------   A         CGA-         AU--|   CA
  GU  ACGCC   GCGGA UG  UCAA  CGAUG        GCC CGGAGCCGU    CGUCCCCG    CCAG  \
  CA  UGUGG   CGCCU AC  AGUU  GCUGC        CGG GCCUCGGCA    GCAGGGGU    GGUC  G
G-  GG     UAC     C  C-     A-     AGCGACAG   C         UGAA         GGCU^   CU
        130        120          110        100        90         80         70

-----------------------------------------------------
```

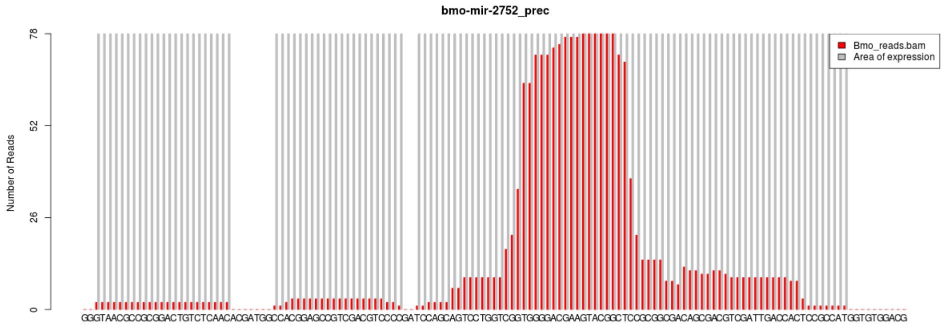

>bmo-mir-2753_prec

```
AACUGAAGUCCCUCCGCAGACCCAGAGGUUCCGGCCCUCUUCUGUAUGGAGUCAAAUGGAAGAAGCUGGUAUUUGAAUGGUAUUUGGUAAUGGAAGAAGCCGGUA
.((((...(((..(((...(((((((....(((((..((((((((.((....)).)))))))).)))))..))))...)))...)))....)))......)))).

 Initial dG =    -27.60

           10          20           30        40        50
A    AAG---   CU--   CAG   ---|   GGUU     CC        A  G
 ACUG      UCC    CCG   ACC   CAGA    CCGGC  UCUUCUGU UG A
 UGGC      AGG    GGU   UGG   GUUU    GGUCG  AGAAGGUA AC G
A    CGAAGA   UAAU   UUA   UAA^   AU--     A-        A  U
   100        90        80        70           60

-----------------------------------------------------
```

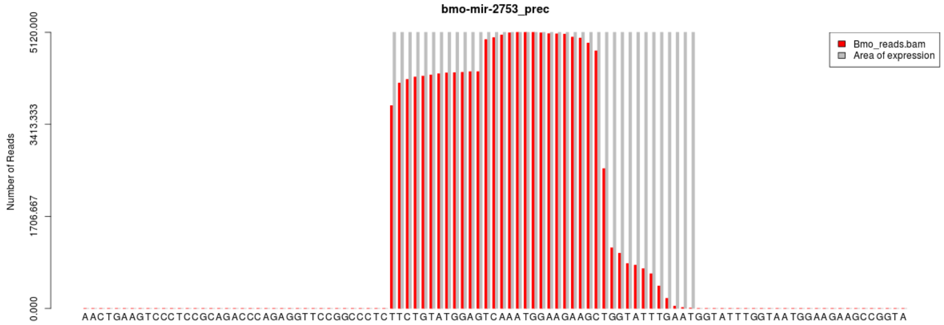

>bmo-mir-2754_prec

```
UGGGUUUGGACAUGUUGCUACGCAAUGCCGGUCAAAAGCGCGUUGUUAAAUCUGUGCGACAUCUCACUCUGGUAUUGACUGUAAUAGUUUAUCUCCA
.(((..(((((.((((((....(((((((((......(((((..........))))).........)))))))))...)))))))))))..))).

 Initial dG =    -27.40

        10        20        30             40
U   UU     A     UACG         UCAAAA-----|    UUGU
 GGG  UGGAC UGUUGC    CAAUGCCGG           GCGCG    U
 CCU  AUUUG AUAAUG    GUUAUGGUC           CGUGU    A
A   CU     -     UCA-         UCACUCUACAG^    CUAA
      90         80         70        60        50

-----------------------------------------------------
```

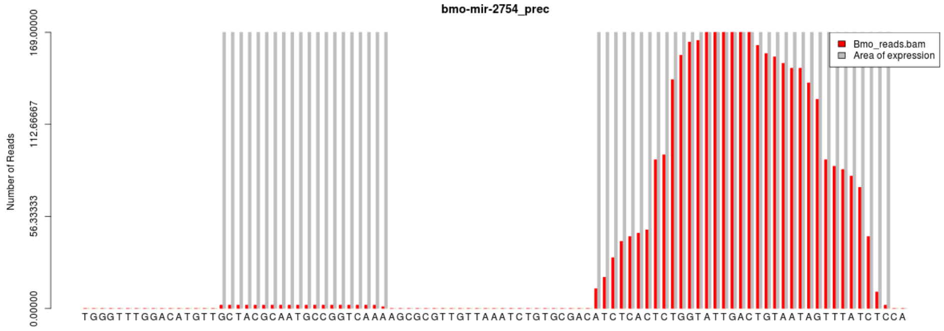

>bmo-mir-2755_prec

```
UUUGGUGAAGCAAGGUGGCCUAGCAGAGUGUUUUCAACAAAGCUUGCACCCUGUCAGACCAUACUUGUUUUAACUAACC
.(((((((((((((((((.((..(((.((((..............)))).)))..)).)))).))))))))).)))).. 

 Initial dG =    -25.80

          10         20        30
U-    -         -|   C  AG   A    UUUCAA
  UUGG UGAAGCAAG GUGG CU  CAG GUGU      C
  AAUC AUUUUGUUC UACC GA  GUC CACG      A
CC    A         A^   A  CU   C    UUCGAA
        70        60        50        40

-----------------------------------------------------
```

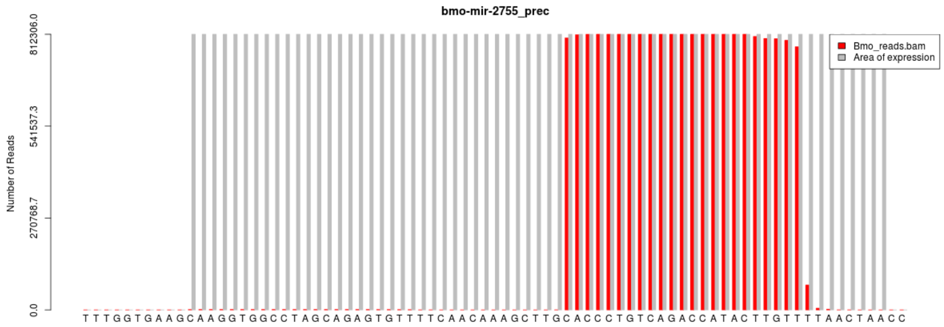

>bmo-mir-2756_prec

```
UCAGUUGCACACCCUGUAGCUGCCAAGGGGCGCAGCUUAAUUCCGCCCCUCGGCUGCUACAUUGUAUGCUACUGAAU
(((((.(((.((..((((((.(((.(((((((...........))))))).))).))))))..)).))).))))).. 

 Initial dG =    -40.40

          10        20        30
--|    U   C  CC      U   A       CAGCU 
  UCAGU GCA AC  UGUAGC GCC AGGGGCG     \
  AGUCA CGU UG  ACAUCG CGG UCCCCGC     U
UA^    U   A  UU      U   C       CUUAA 
      70        60        50        40

-----------------------------------------------------
```

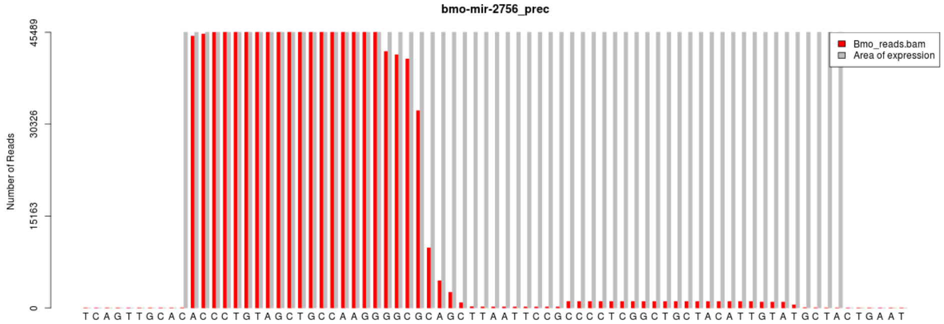

>bmo-mir-2757_prec

```
AAUUAUUGAAACGGGAAUGGUCACUUACAACUGUUUUUUCUUUCUAGCUGAAAGUGUCCAUUUCCGUUUUGAUACUAU
...(((..((((((((((((.(((((.((.(((...........))).)).))))).))))))))))))..)))....

 Initial dG =    -30.10

         10        20        30
AAU-|  UG           U     A  A   UUUUU
    UAU  AAACGGGAAUGG CACUU CA CUG     \
    AUA  UUUGCCUUUACC GUGAA GU GAU     U
UAUC^  GU           U     A  C   CUUUC
       70        60        50        40

-----------------------------------------------------
```

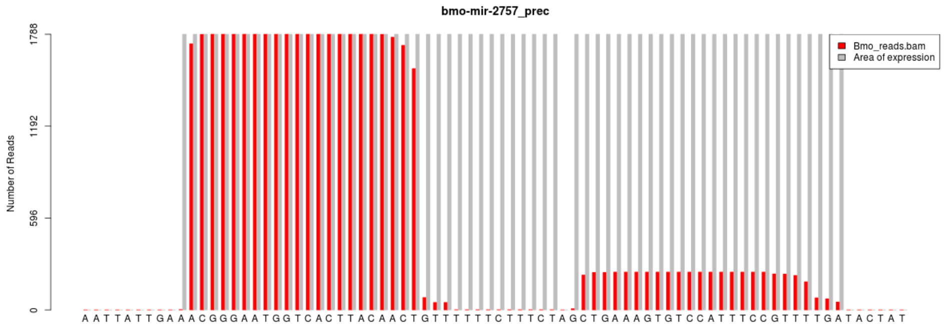

>bmo-mir-2758_prec

```
AGUUUAGAAGACUUGGUAGAACACGUAGUAAGCAGGUGGAAUCUCCCUGCGUGUUCUACCAAGUUAUCUAGGUUGG
((..((((.(((((((((((((((((((..((..........))..))))))))))))))))))).)))))..))..

 Initial dG =    -37.00

          10        20        30
--| UU    A                   UA  CAGG
  AG  UAGA GACUUGGUAGAACACGUAG  AG    U
  UU  AUCU UUGAACCAUCUUGUGCGUC  UC    G
GG^ GG    A                   CC  UAAG
     70        60        50        40

-----------------------------------------------------
```

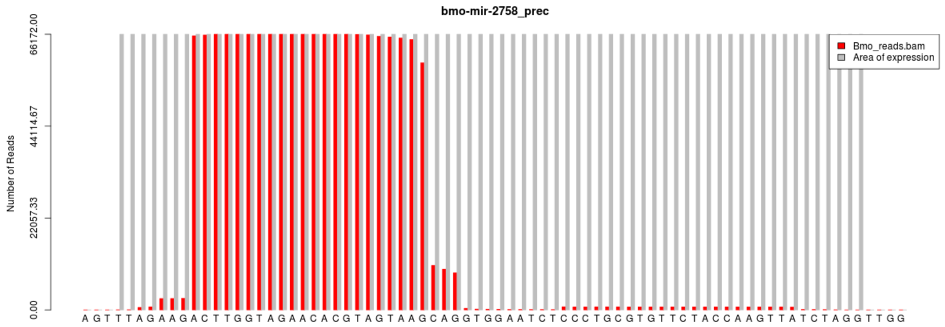

>bmo-mir-2759_prec

UUCAAUGACUCUCCGUGUUCUUUAAUCACUCAAGACUUAUGUUCUUGAAAGAUCAUAGAAUGCGAAAUGUCCUUGUA
..(((.(((....((((((((...(((..(((((((....)).)))))..)))...))))))))....))).)))..

```
 Initial dG =    -20.60

        10        20        30
UU   U   UCUC        UUA   AC     -| U
  CAA GAC    CGUGUUCU   AUC  UCAAG AC U
  GUU CUG    GCGUAAGA   UAG  AGUUC UG A
AU   C   UAAA        UAC   AA     U^ U
      70        60        50        40

-----------------------------------------------------
```

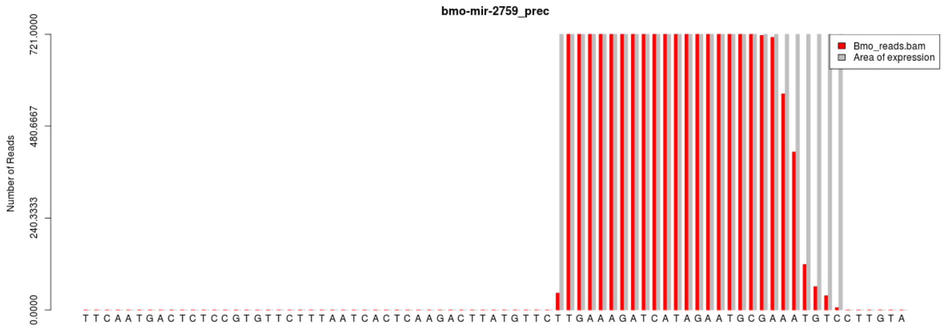

>bmo-mir-2760_prec

```
CCGAAUGAAACGAGGCUUAAUUGAACCAAAAAGCGCAUCCACUUGAUGUUUAAAACAGUUUUUUGGUUGUUAAGUUCCAUUUUAUUUGUAG
.(((((((((...((((((((..((((((((((((((((.....)))))........)))))))))))))))))))...)))))))))...

 Initial dG =    -25.40

          10        20        30                 40
C--         CGA        UG           --------|    CA
   CGAAUGAAA   GGCUUAAU  AACCAAAAAGC        GCAUC  \
   GUUUAUUUU   UUGAAUUG  UUGGUUUUUUG        UGUAG  C
GAU         ACC        --           ACAAAAUU^    UU
90        80        70          60        50

-----------------------------------------------------
```

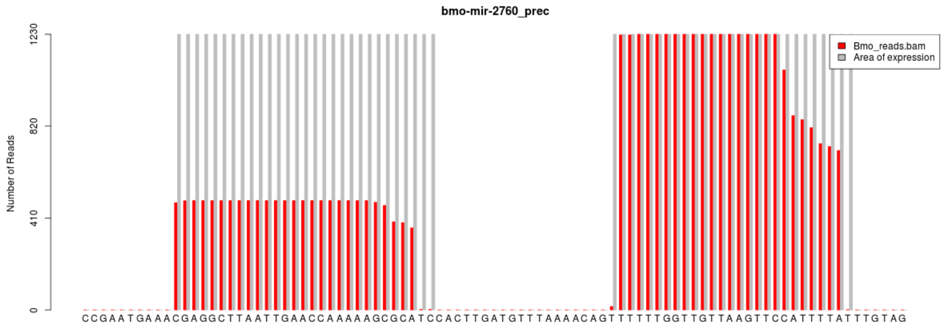

>bmo-mir-2761_prec

AUAGACGCUGCAUCGAGACGGUUCCAUACAAGCUGCACCAGACUUGUGUGGAACCGUCGUCGAUGGAAAAAAUAAUU
..........((((((((((((((((((((((((...))).))))))))))))))))).))))))...........

```
 Initial dG =    -39.20

        10           20        30
AUAGACGCUG--      -                -|  C
            CAUCGA GACGGUUCCAUACAAG CUG \
            GUAGCU CUGCCAAGGUGUGUUC GAC A
UUAAUAAAAAAG      G                A^  C
      70        60        50        40

-----------------------------------------------------
```

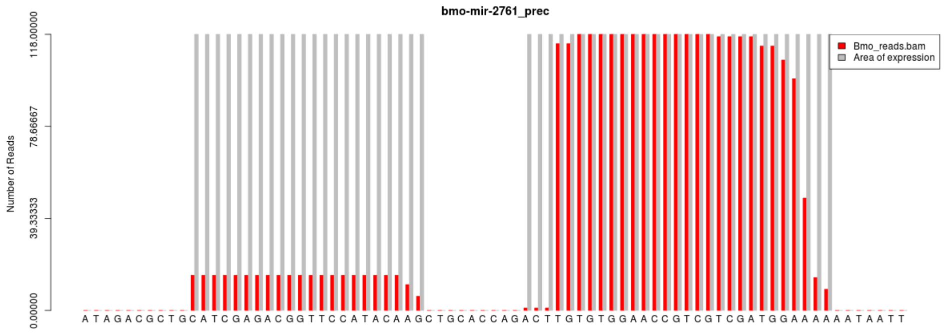

>bmo-mir-2762_prec

```
UUUACUAUUUUACCGUCACAUUUCUUUAUGUAUCACGGAUGGAGACACGUGGUACGUCGGGAAAUGUACGGUAAAAUAUGAUU
.....(((((((((((.((((((((..((((((((((..((....))))))))))))..))))))))))))))))))).....

 Initial dG =    -39.70

        10        20        30        40
UUUAC|          C        UU          GA  G
     UAUUUUACCGU ACAUUUCU  AUGUAUCACG  UG A
     AUAAAAUGGCA UGUAAAGG  UGCAUGGUGC  AC G
UUAGU^          -        GC          --  A
  80        70         60        50

-----------------------------------------------------
```

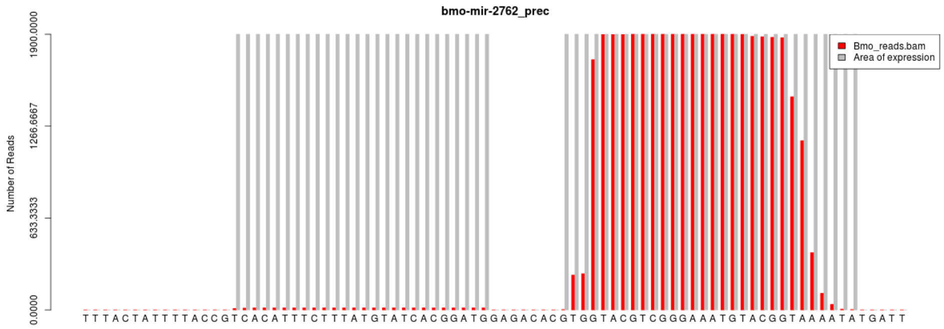

>bmo-mir-2763_prec

```
GGCAAUGUACUGCAAAGUAGUGAACAUAAUUUAUCGUGAGAUUUAUUAUUAGAUAUUAUGCUCAUUUCUUUGGAUAUGUUGUACG
.((((((((...(((((.(((((.((((((..(((....)))............)))))).))))).))))).))))))))...

 Initial dG =    -20.90

          10         20         30
G--        CUG     U     A      UU----------|  G
   GCAAUGUA   CAAAG AGUGA CAUAAU            AUC U
   UGUUGUAU   GUUUC UUACU GUAUUA            UAG G
GCA        AG-     U     C      UAGAUUAUUAUU^  A
    80         70         60         50         40

-----------------------------------------------------
```

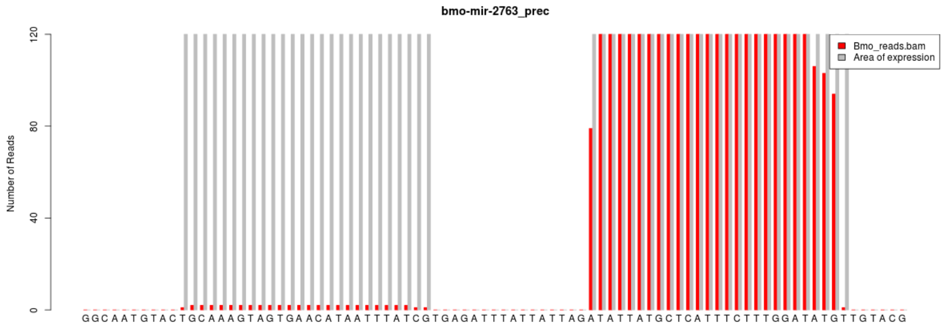

>bmo-mir-2764_prec

```
CAGUUCUUCUUUAGUAGCUACAAUAUCUAUGACGUUGAUUCGAAUUAUUUCGUAGAUAUUGUAGUUACUGGAGAAUUAUCU
......((((((((((((((((((((((((((...((((....))))..))))))))))))))))))))))))))......

 Initial dG =    -35.30

        10        20        30        40
CAGUUC|                         CGU    U
      UUCUUUAGUAGCUACAAUAUCUAUGA   UGAU C
      AAGAGGUCAUUGAUGUUAUAGAUGCU   AUUA G
UCUAUU^                         UU-    A
80        70        60        50

-----------------------------------------------------
```

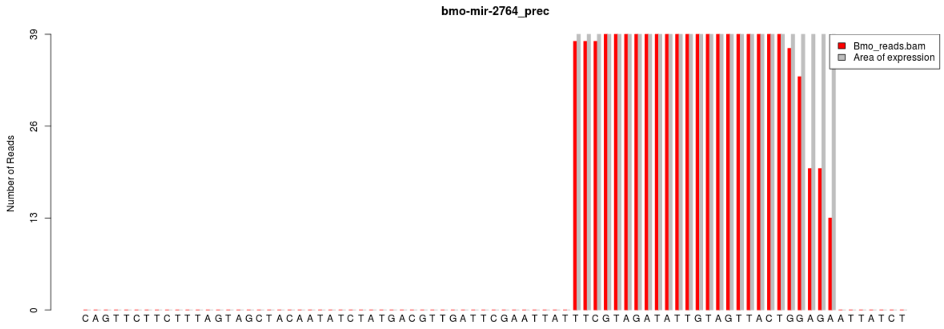

>bmo-mir-2765_prec

```
UAUCUGGGCCUGGUAACUCCACCACCGUUGGCGUUAUCAAUCCGCCAACGGGGUAAGAGUUCCCUUUCCUAGACG
..((((((...((.(((((.(((.(((((((((.........))))))))))))..))))).))...)))))).. 

 Initial dG =    -36.20

        10        20         30
UA      CCU  U     C-|  A         UUAU
  UCUGGG   GG AACUC  ACC CCGUUGGCG    \
  AGAUCC   CC UUGAG  UGG GGCAACCGC    C
GC      UUU  C     AA^  -         CUAA
    70        60         50        40
```

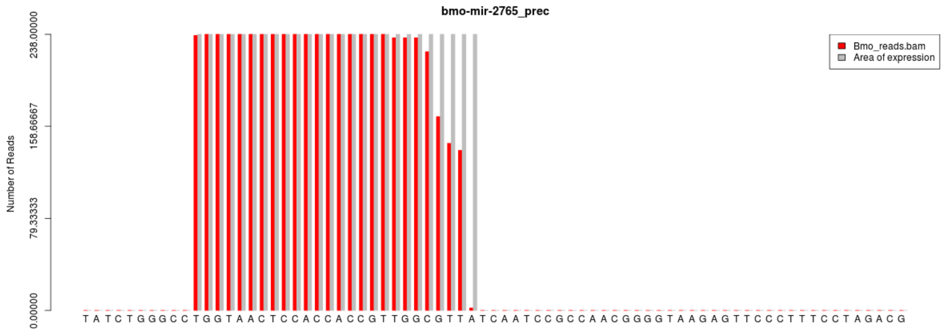

>bmo-mir-2766_prec

```
CAUUACUCGUCCGCCCUUCGUCUUGACUGGCGCUGUCGCUUGCAUCAGUCUUGUCGAAUGGUGGGUGAGUUUC
....((((..(((((.((((....(((((..((........))..)))))....)))).)))))..))))...

 Initial dG =    -32.30

        10        20        30
CAUU|   GU      C    UCUU     GC  UGU
    ACUC  CCGCC UUCG    GACUG  GC   C
    UGAG  GGUGG AAGC    CUGAC  CG   G
CUU-^   UG      U    UGUU     UA  UUC
   70        60        50        40

-----------------------------------------------------
```

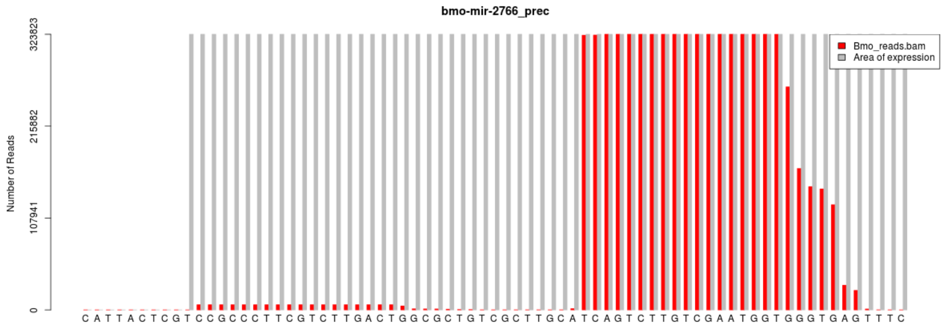

>bmo-mir-2767_prec

```
ACCGUUGUUUCAAGUAAAUCUCGUGCGGUUUGUCUCAUUCCGAACCGUAUGAGAUUUACUUAAAAUGAUUGUG
((.((..(((.(((((((((((((((((((((........))))))))))))))))))))).)))..)).)).

 Initial dG =    -34.80

         10        20        30
-| C  UG   C                     UCU
 AC GU  UUU AAGUAAAUCUCGUGCGGUUUG   C
 UG UA  AAA UUCAUUUAGAGUAUGCCAAGC   A
G^ U  GU   A                     CUU
  70        60        50        40

-----------------------------------------------------
```

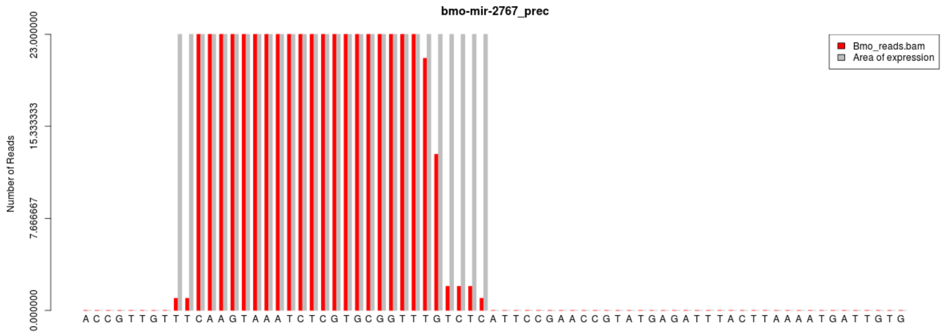

>bmo-mir-2768_prec

```
GUCGGGCGACCGGUGAAAUAUUUUGACCAAUUUUAUAUAUCGGUCUGAUCGUUGACUCAAGGUGAAUUGGUUAAGAUAUUGCAUCGUUUGCUCGAUC
((((((((.(((((.(((((((((((((((((.((.....((((........))))....))))))))))))))))))).))))).))))))))).

 Initial dG =    -40.50

         10        20        30        40
-|        C     A                 U  AUAUC    UGA
 GUCGGGCGA CGGUG AAUAUUUUGACCAAUUU AU     GGUC   U
 UAGCUCGUU GCUAC UUAUAGAAUUGGUUAAG UG     UCAG   C
C^        U     G                 -  GAAC-    UUG
      90        80        70         60         50

-----------------------------------------------------
```

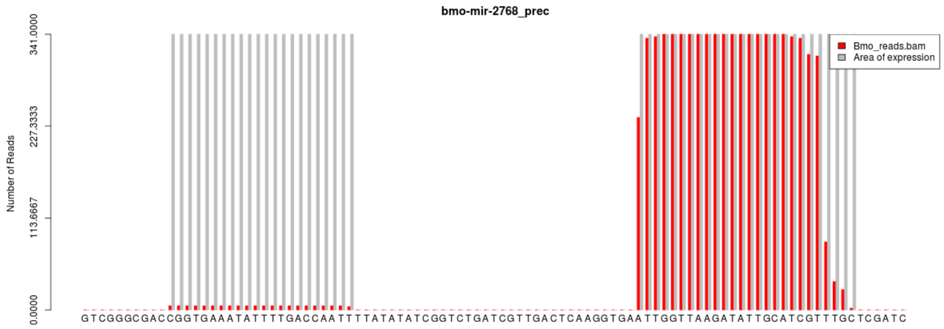

>bmo-mir-2769_prec

```
UAAAUUACACGACCGAACAUCUGAUAAUAUACUAUAUGAUAUGAUAAUAUAUUAUCAGAUUUUCGGUCGCGUUAUUUU
.((((.((.((((((((.(((((((((((((.(((........))).))))))))))))).)))))))).)).)))).

 Initial dG =    -31.70

        10        20        30
U|   U  A        C             C   AUG
 AAAU AC CGACCGAA AUCUGAUAAUAUA UAU   A
 UUUA UG GCUGGCUU UAGACUAUUAUAU AUA   U
U^   U  C        U             A   GUA
       70        60        50        40

-----------------------------------------------------
```

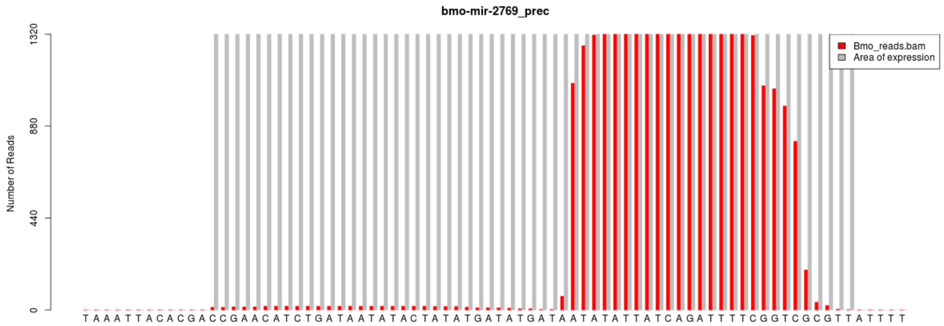

>bmo-mir-2770_prec

```
UCAGUCUUCCUAGAUAGAAUUCGUCGGUGGAUAACCUGGUUAUCCCCGUGUACUGUUAGGGUGGGCACG
...((((.(((.(((((....((.(((.(((((((...)))))))))))))..))))).))).))))...

 Initial dG =    -24.90

        10        20        30
UCA|   U   A     AAUU  U   U       C
   GUCU CCU GAUAG    CG CGG GGAUAAC \
   CGGG GGG UUGUC    GU GCC CCUAUUG U
GCA^   U   A     AU--  -   -       G
        60          50          40
```

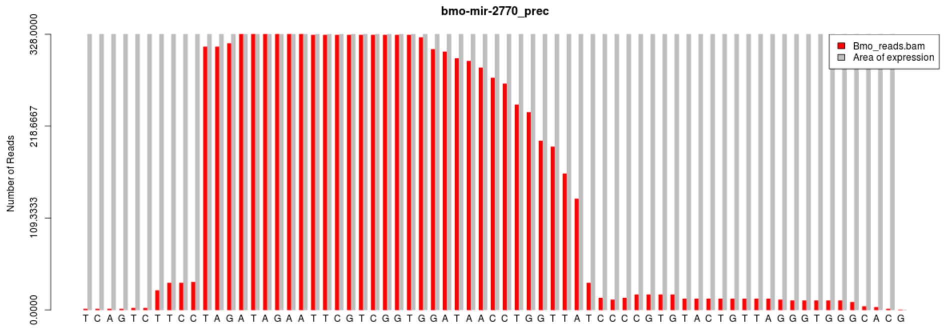

>bmo-mir-2771_prec

UCCCAACUUUUAACAUUACGAGGAUGGGUUUAGUUAGGGGAGUCCCAACUGAGUGGUCCCCUGAAGAUAUUAUGCAGAACACUAAUGUACCCAUUGGG
.((((.((((.........)))).)))).....(((((((((...(((......))))))))))))................((((((....)))))).

```
 Initial dG =    -24.30

          10
U    .-A|  U  A
 CCCA   CUU UA C
 GGGU   GAG AU A
-    \ -^  C  U
         20



               30        40        50
       G      .-UUAG        GUC   AC
        AUGGGU      UUAGGGGA   CCA  U
        UACCCA      AGUCCCCU   GGU  G
       -      \ ----        ---   GA
           90          60

-----------------------------------------------------
```

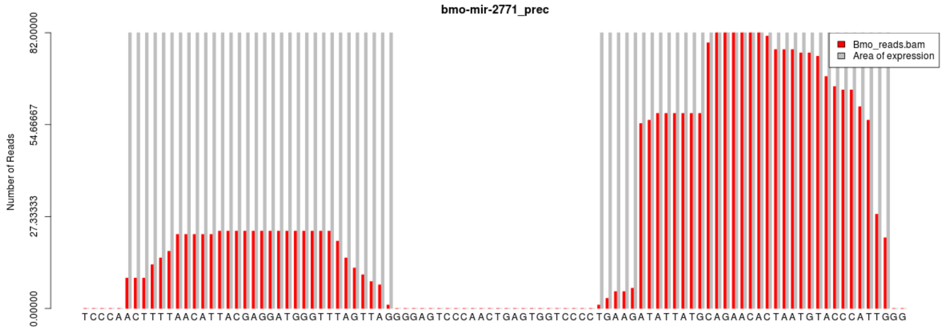

>bmo-mir-2772a-1_prec

```
UUCUUUGCCUUAUCUCCAAACUCAAAGUGGUACCAGCAGUGUGGAUGGUCAUCUGGUCGUUGCCGUGUUUGAGUUCUGGACCUGGAGCGUAUGUGG
.....(((((...(((((((((((..((((((((..(((........))))))))....)))...)))))))).)))))....))).))).....

 Initial dG =    -24.10

         10          20         30            40
UUCUU   -   AUC-    -        GU-   ----|    CA   UGG
     UGC CUU    UCCA AACUCAAA   GGU    ACCAG  GUG   A
     AUG GAG    AGGU UUGAGUUU   CCG    UGGUC  UAC   U
GGUGU   C   GUCC    C        GUG   UUGC^    --   UGG
     90        80        70        60          50

-----------------------------------------------------
```

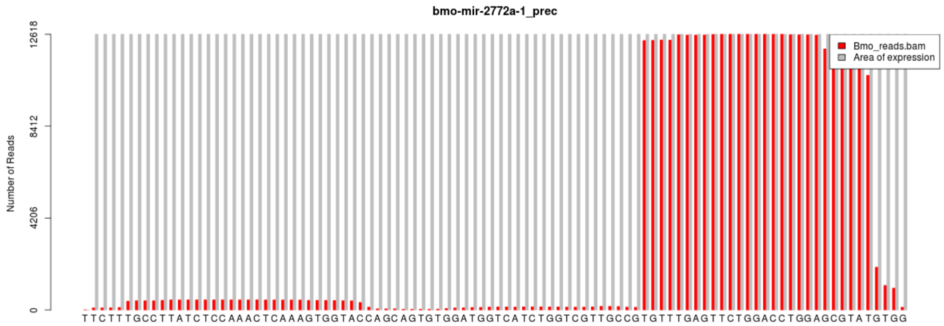

>bmo-mir-2772a-2_prec

```
UUCUUUGCCUUAUCUCCAAACUCAAAGUGGUACCAGCAGUGUGGAUGGUCGUCUGGUCGUUGCCGUGUUUGAGUUCUGGACCUGGAGCGUAUGUGG
.....(((((...((((((((((((..(((((((((.............).)))))....)))...)))))))).)))))....))).))).....

 Initial dG =    -22.30

         10          20         30             40
UUCUU   -   AUC-    -         GU-   ----     -|AGUGUG
     UGC CUU    UCCA AACUCAAA   GGU    ACCAG C      \
     AUG GAG    AGGU UUGAGUUU   CCG    UGGUC G      G
GGUGU   C   GUCC    C         GUG   UUGC     U^CUGGUA
     90        80        70        60        50

-----------------------------------------------------
```

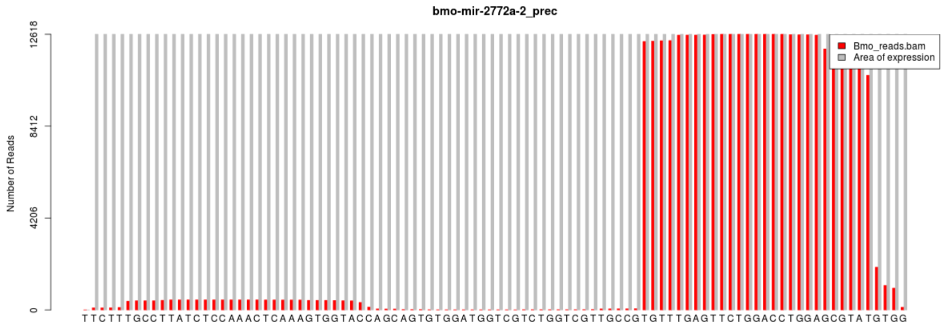

>bmo-mir-2772b_prec

```
UUCUUUGCCUUAUCUCUAAACUCAAAGUGGUACCAGCAGUGUGGAUGGUCAUCUGGUCGUUGCCGUGUUUGAGUUCUGGAUCUGGAGCGUAUGUGG
.....(((((...(((((((((((..((((((((..(((........))))))))....)))...)))))))).))))....))).))).....

 Initial dG =    -21.80

         10          20         30            40
UUCUU   -   AUC-    -        GU-   ----|    CA   UGG
     UGC CUU    UCUA AACUCAAA   GGU    ACCAG  GUG   A
     AUG GAG    AGGU UUGAGUUU   CCG    UGGUC  UAC   U
GGUGU   C   GUCU    C        GUG   UUGC^    --   UGG
     90        80        70        60           50

-----------------------------------------------------
```

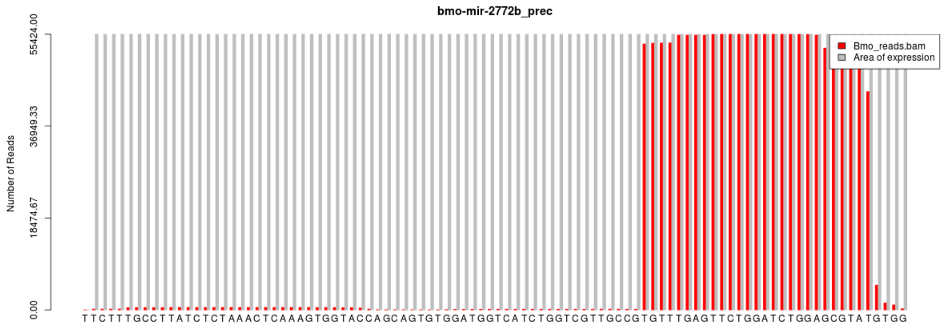

>bmo-mir-2774a-1_prec

```
AAGACGAUAAGGGAUCGCCGGUCUACCGAGCAAUAUCACCCGCUCGGUGGAAGAUCGUCCCUUAAUCUUCAC
..((.((((((((.((....((((((((((..........))))))))))....))))))))).)).))..

 Initial dG =    -31.40

         10        20        30
AA  C  -|      U  CCGG          AAUA
  GA GA UAAGGGA CG    UCUACCGAGC    U
  CU CU AUUCCCU GC    AGGUGGCUCG    C
CA  U  A^      -  UAGA          CCCA
 70        60         50        40

-----------------------------------------------------
```

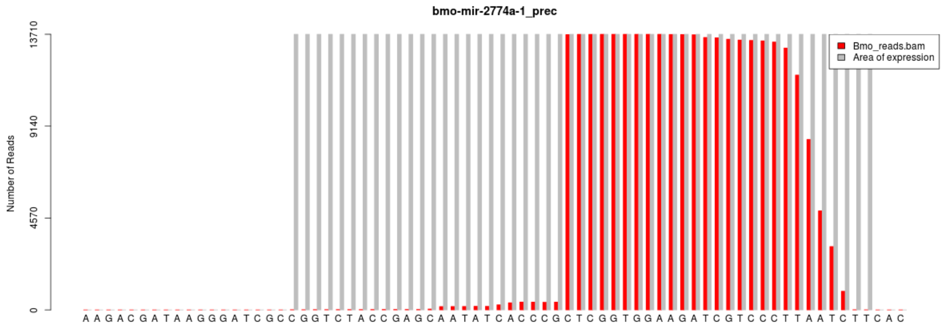

>bmo-mir-2774a-2_prec

```
ACAACAACAUUCGGACCGUCGGUCUACCGAGCAAUAUCACCCGCUCGGUGGAAGAUCGUCCCUUUGUCAUUAU
......(((...((((.(((..((((((((((..........)))))))))).))).))))...)))......

 Initial dG =    -27.90

        10        20        30
ACAACA|  UUC    C   GG          AAUA
      ACA   GGAC GUC  UCUACCGAGC    U
      UGU   CCUG UAG  AGGUGGCUCG    C
UAUUAC^  UUC    C   A-          CCCA
  70        60         50        40

-----------------------------------------------------
```

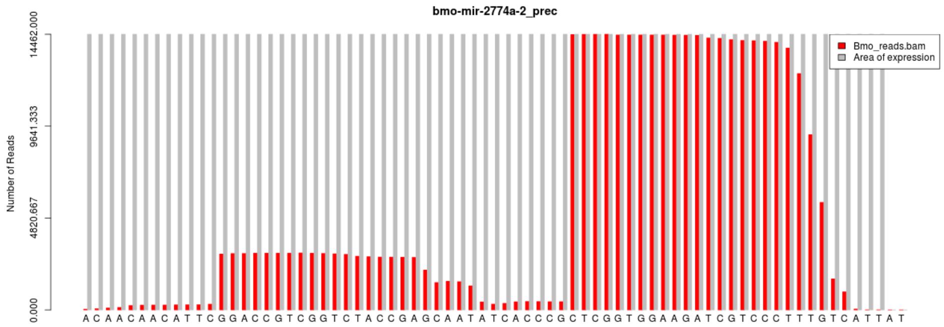

>bmo-mir-2774a-3_prec

```
AAAAAAACAUUCGGACCAUCGGUCUACCGAGCAAUAUCACCCGCUCGGUGGAAGAUCGUCCCUCCGUCGUUAA
............((((.(((..((((((((((..........)))))))))).))).))))............

 Initial dG =    -26.50

        10        20        30
AAAAAAACAUUC|   C   GG          AAUA
            GGAC AUC  UCUACCGAGC    U
            CCUG UAG  AGGUGGCUCG    C
AAUUGCUGCCUC^   C   A-          CCCA
  70        60         50        40

-----------------------------------------------------
```

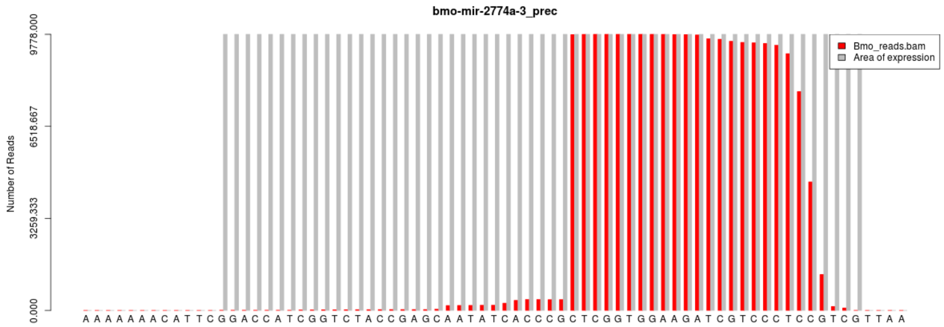

>bmo-mir-2774a-4_prec

```
ACAACAACAUUCGGACCGUCGGUCUACCGAGCAAUAUUACCUGCUCGGUGGAAGAUCGUCCCUUUGUCGUAUA
...((.(((...((((.(((..(((((((((((........))))))))))).))).))))...))).))...

 Initial dG =    -31.60

        10        20        30
ACA| A   UUC    C   GG          AUA
   AC ACA   GGAC GUC  UCUACCGAGCA   U
   UG UGU   CCUG UAG  AGGUGGCUCGU   U
AUA^ C   UUC    C   A-          CCA
  70        60         50        40

-----------------------------------------------------
```

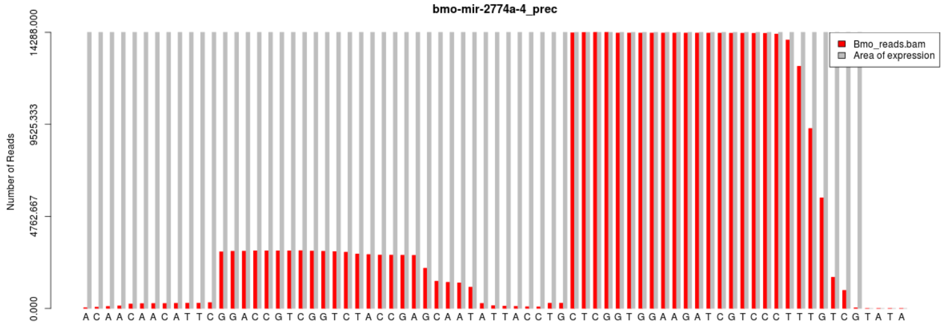

>bmo-mir-2774b-1_prec

```
GAACAACAACACCCGGACCGUCGGUCUACCGAGCAAUAUCACCCGCUCGGUAGAAGAUCUUCCCUUUGUCGUAUAUA
.....((.(((...(((..(((..((((((((((..........)))))))))).)))..)))...))).)).....

 Initial dG =    -25.60

        10        20        30
GAACA| A   CCC   CC   GG          AAUA
     AC ACA   GGA  GUC  UCUACCGAGC    U
     UG UGU   CCU  UAG  AGAUGGCUCG    C
AUAUA^ C   UUC   UC   A-          CCCA
      70        60         50        40

-----------------------------------------------------
```

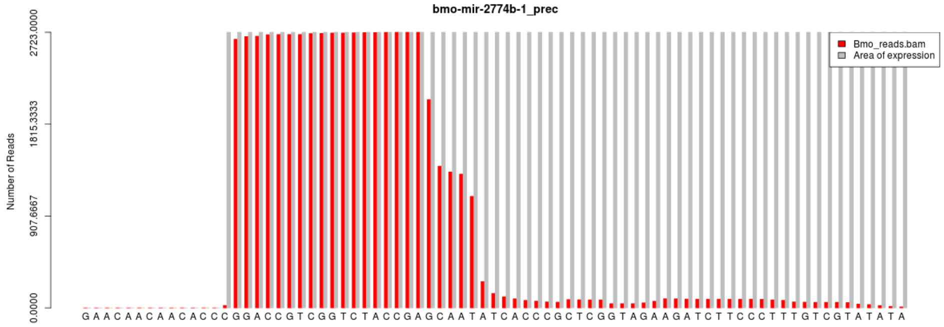

>bmo-mir-2774b-2_prec

```
GAGCAACAACAUUCGGACCGUCGGUCUAGCGAGCAAUUACACCCGCUCGGUAGAAGAUCUUCCCUUUGUCGUAAACC
.....((.(((...(((..(((..((((.(((((..........))))).)))).)))..)))...))).)).....

 Initial dG =    -21.80

       10        20        30
GAGCA| A   UUC   CC   GG    G     AAUU
     AC ACA   GGA  GUC  UCUA CGAGC    A
     UG UGU   CCU  UAG  AGAU GCUCG    C
CCAAA^ C   UUC   UC   A-    G     CCCA
      70        60         50        40

-----------------------------------------------------
```

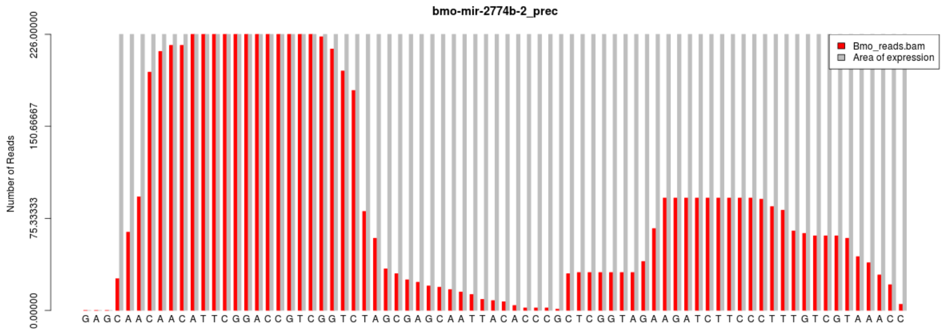

>bmo-mir-2774c_prec

```
AACAACAACAUUCGGACCGUCGGUCUAUAGAGCAAUAUCACCCGCUCGGUGGAAGACCUUCCCUUCGUCGUUAUC
...(((.((....(((..(((..(((((.((((..........)))).))))).)))..)))....)).)))...

 Initial dG =    -18.30

        10        20        30
AAC|  A  AUUC   CC   GG     A    AAUA
   AAC AC    GGA  GUC  UCUAU GAGC    U
   UUG UG    CCU  CAG  AGGUG CUCG    C
CUA^  C  CUUC   UC   A-     G    CCCA
    70        60         50        40

-----------------------------------------------------
```

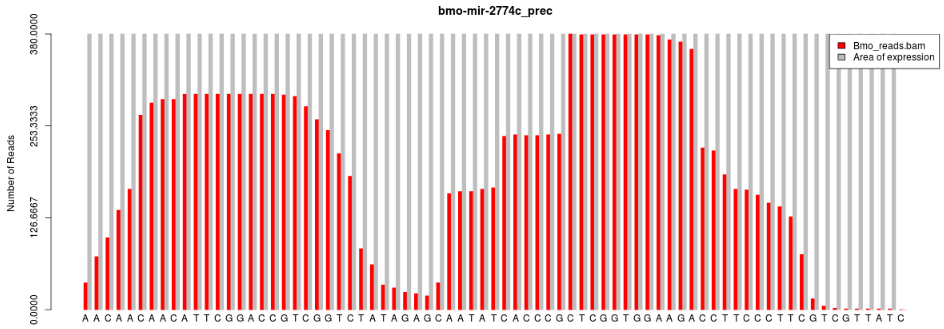

>bmo-mir-2775a_prec

```
CAUUGUGCACCGCGGGAGAAAAGUAGGACAUUUCCUCCCACGCGGGAUGAGAUGUCCUACUUUUUCCCGCGGUGCACAUAC
...((((((((((((((((.(((((((((((((.((((....)))).)))))))))))))))))))))))))))))))...

 Initial dG =    -61.70

        10        20        30        40
CAU|                 A            C    A
   UGUGCACCGCGGGAGAAA GUAGGACAUUUC UCCC C
   ACACGUGGCGCCCUUUUU CAUCCUGUAGAG AGGG G
CAU^                 -            U    C
80        70         60        50

-----------------------------------------------------
```

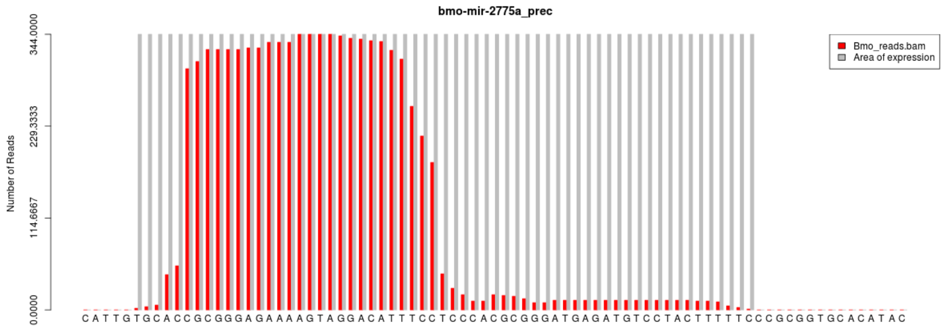

>bmo-mir-2775b_prec

```
AAGGGAGAAAAGUAGGACGUUUCCUCCCGCGUGUAAAAUUUCAAACACGCGGGAUGAGAUGUCCUACUUUUUUCCCGC
..((((((((((((((((((((.((((((((((..........)))))))))).))))))))))))))))))))))..

 Initial dG =    -54.80

        10        20        30
AA|                    C          AAAA
  GGGAGAAAAGUAGGACGUUUC UCCCGCGUGU    U
  CCCUUUUUUCAUCCUGUAGAG AGGGCGCACA    U
CG^                    U          AACU
       70        60        50        40

-----------------------------------------------------
```

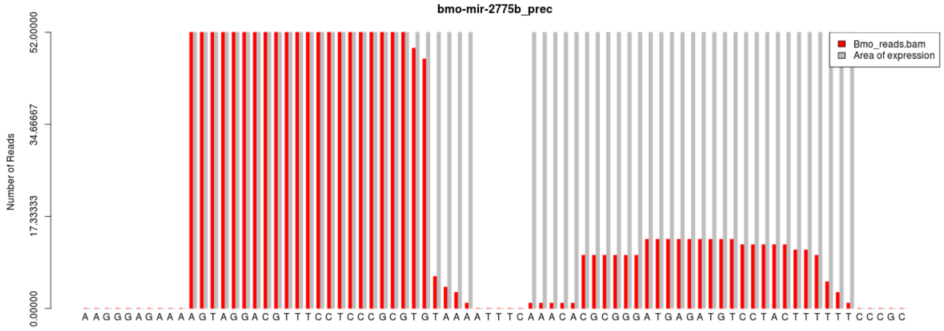

>bmo-mir-2776_prec

```
CUCCUAUUAGAGAGUACGCAAAAAAACAAUUUGGUUUUACCCGAAAUUGCUUUUUUGCAAGCUCAUUAUCAUCUA
...........((((..((((((((.((((((((......)).)))))).))))))))..))))...........

 Initial dG =    -18.50

        10        20        30
CUCCUAUUAGA    AC         A       -| UU
           GAGU  GCAAAAAA CAAUUU GG  U
           CUCG  CGUUUUUU GUUAAA CC  U
AUCUACUAUUA    AA         C      G^ CA
    70        60        50        40
```

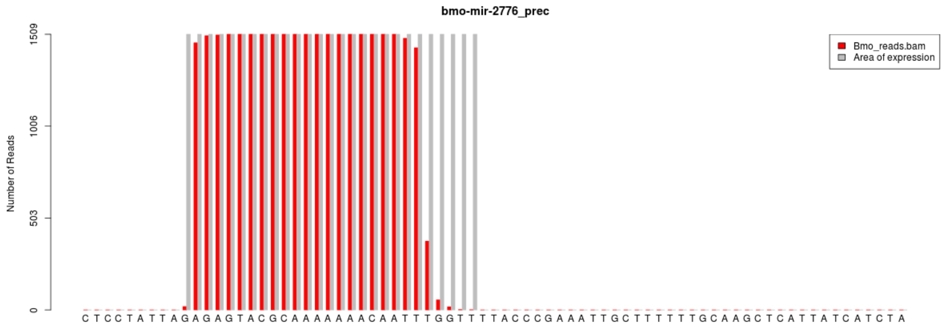

>bmo-mir-2777_prec

```
CGAAACCCCUAGGGACCUUGCCUGGGUUAUCUCGGUGUCGGACGAGAGCGCAGGGGAGGUGCUUAGUGGGGACGC
.....(((((((.(((((.((((.((..(((((........))))))).)))).))))).))))).))).....

 Initial dG =    -38.90

         10         20         30
CGAAA   -|    G      G     G  UA     GUG
     CCC CUAGG ACCUU CCUG GU  UCUCG   U
     GGG GAUUC UGGAG GGAC CG  AGAGC   C
CGCAG   U^    G      G     G  --     AGG
    70         60         50          40

-----------------------------------------------------
```

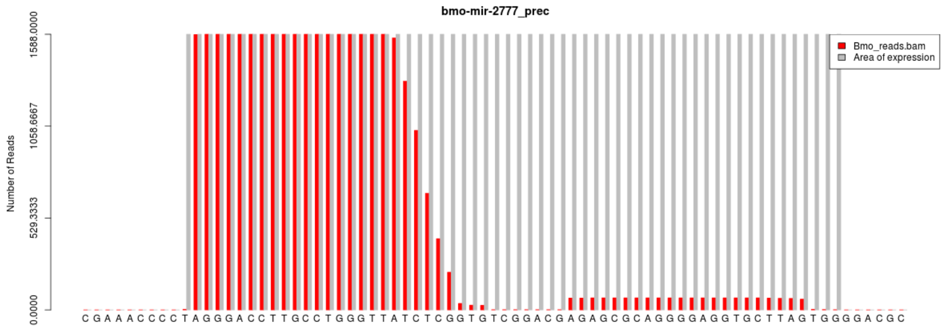

>bmo-mir-2778a-1_prec

```
CACGGCAAAAGUUUUUUUGCAUAUCCUGCACAGUUUCAGUUCUCGCAGAGUACGCAAAAAAACAAUUUUGACGUGAG
((((.((((((((((((((.((..((((..((........)).))))..)).)))))))))))..))))).)))).. 

 Initial dG =    -25.40

          10           20         30
--    G     --|         A  UC    AC  UUU
  CACG CAAAA  GUUUUUUUGC UA  CUGC  AG   C
  GUGC GUUUU  CAAAAAAACG AU  GACG  UC   A
GA    A     AA^         C  GA    C-  UUG
      70        60        50          40

-----------------------------------------------------
```

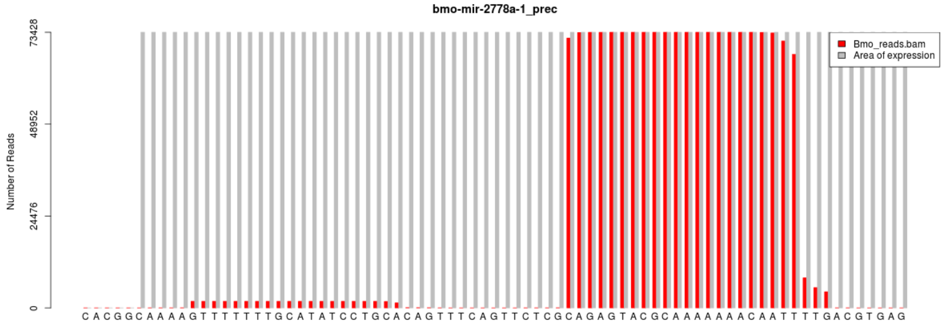

>bmo-mir-2778a-2_prec

```
ACUCACGGCAAAAGUUUUUUUGUAUAUCCUGCACAGUUUCAGUUCUCGCAGAGUACGCAAAAAAACAAUUUUGACGUGAG
.((((((.((((((((((((((..((..((((..((........)).))))..))..)))))))))..))))).))))))

 Initial dG =    -28.30

        10          20        30        40
A      G     --|        UA  UC    AC  UUU
 CUCACG CAAAA  GUUUUUUUG  UA  CUGC  AG   C
 GAGUGC GUUUU  CAAAAAAAC  AU  GACG  UC   A
-      A     AA^        GC  GA    C-  UUG
80        70        60        50

-----------------------------------------------------
```

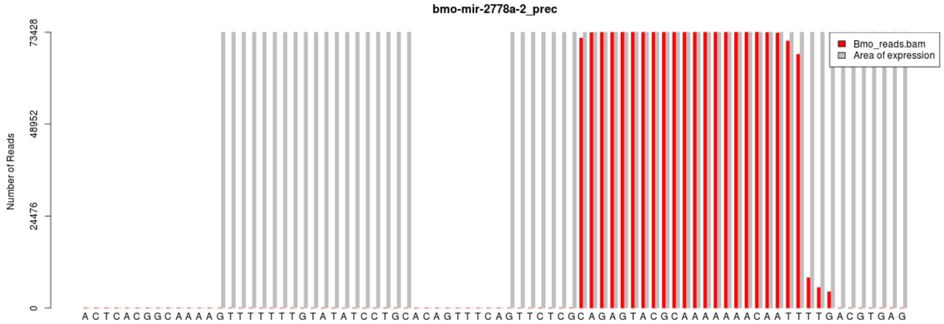

>bmo-mir-2778a-3_prec

```
CACGGCAAAAGUUUUUUUUGCAUAUCCUGCACAGUUUCAGUUCUCGCAGAGUACGCAAAAAAACAAUUGAGUAUUUAC
.((..(((..((((((((((.((..((((..((........)).))))..)).))))))))))..))).))......

 Initial dG =    -19.80

            10        20        30
C-----| GG   AA         A  UC    AC  UUU
      AC  CAA  GUUUUUUUGC UA  CUGC  AG   C
      UG  GUU  CAAAAAAACG AU  GACG  UC   A
CAUUUA^ A-   AA         C  GA    C-  UUG
      70         60        50         40

-----------------------------------------------------
```

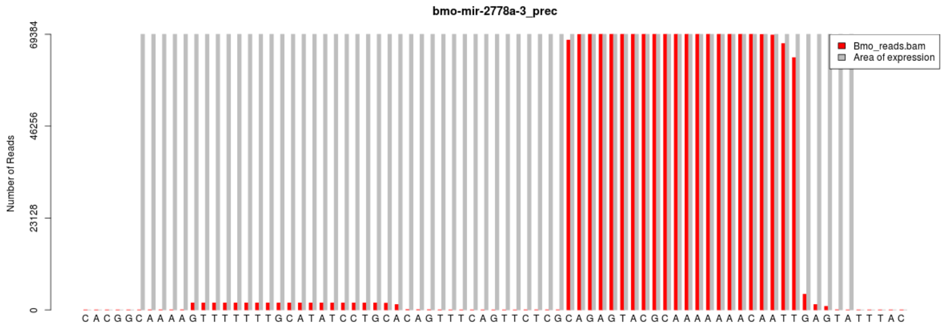

>bmo-mir-2778a-4_prec

```
CACGGCAAAAGUUAUUUUGCAUAUCCUGCACAGUUUCAGUUCUCGCAGAGUACGCAAAAAAACAAUUUUGACGUGAG
((((.((((((((.((((((.((..((((..((........)).))))..)).)))))).)))..))))).)))))..

 Initial dG =    -21.90

          10          20         30
--    G     --|  A      A  UC    AC  UUU
  CACG CAAAA  GUU UUUUGC UA  CUGC  AG   C
  GUGC GUUUU  CAA AAAACG AU  GACG  UC   A
GA    A     AA^  A      C  GA    C-  UUG
      70        60        50         40

-----------------------------------------------------
```

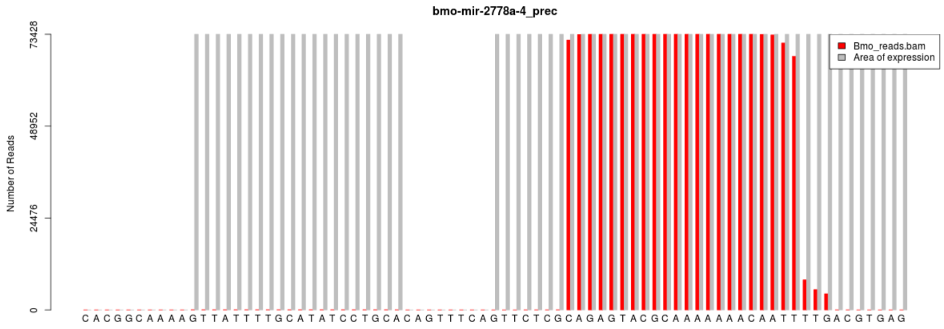

>bmo-mir-2778b_prec

```
CACGGCAAAAGUUUUUUUGCAUAUCCUGCACAGUUUCAGUUCUCGCAGAGUAGGCAAAAAAACAAUUUUGACGUGAG
((((.((((((((((((((.((..((((..((........)).))))..)).))))))))))..))))).)))))..

 Initial dG =    -25.40

          10          20        30
--    G     --|         A  UC    AC  UUU
  CACG CAAAA  GUUUUUUUGC UA  CUGC  AG   C
  GUGC GUUUU  CAAAAAAACG AU  GACG  UC   A
GA    A     AA^         G  GA    C-  UUG
      70        60        50         40

-----------------------------------------------------
```

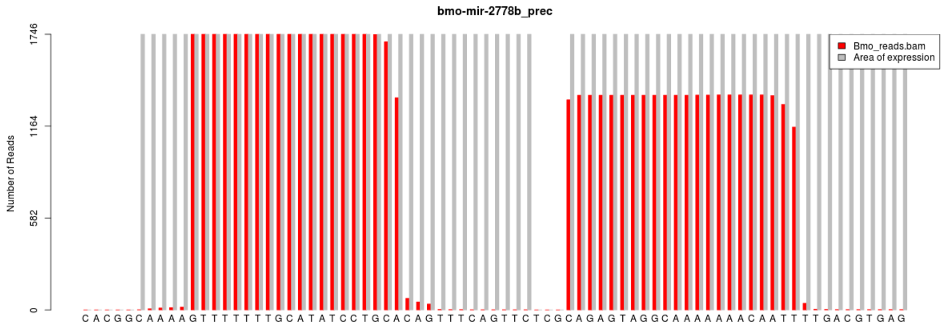

>bmo-mir-2778c_prec

```
CACGGCAAAAGUUUUUUUGCAUAUCCUGCACAGUUUCAGUUCUCGCAGAGUACGCAAAAAAUCAAUUUUGACGUGAG
((((.(((((..((((((((.((..((((..((........)).))))..)).))))))))....))))).)))).. 

 Initial dG =    -23.00

          10          20         30
--    G     GU--|       A  UC    AC  UUU
  CACG CAAAA    UUUUUUGC UA  CUGC  AG   C
  GUGC GUUUU    AAAAAACG AU  GACG  UC   A
GA    A     AACU^       C  GA    C-  UUG
      70        60        50         40

-----------------------------------------------------
```

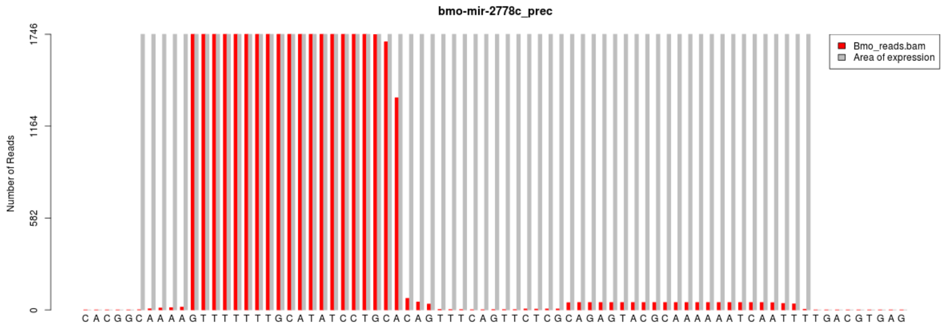

>bmo-mir-2778d_prec

```
CACGGCAAAAGUUUUUUUGCAUAUCCUGCACAGUUUCAGUUCUCGCAGAGUACGCAAAAAACAAUUUUGACGUGAG
((((.(((((..((((((((.((..((((..((........)).))))..)).))))))))...))))).))))..

 Initial dG =    -23.00

          10         20        30
--    G     GU-|       A  UC    AC  UUU
  CACG CAAAA   UUUUUUGC UA  CUGC  AG   C
  GUGC GUUUU   AAAAAACG AU  GACG  UC   A
GA    A     AAC^       C  GA    C-  UUG
     70        60        50         40

-----------------------------------------------------
```

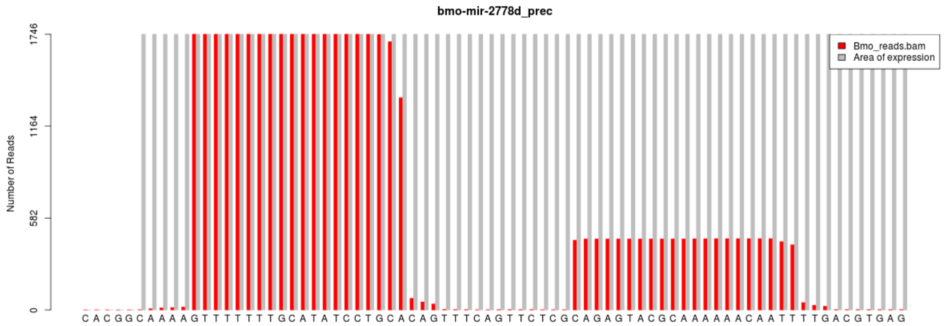

>bmo-mir-2779_prec

UUGCUGGUUUAUAUCCGGCUCGAAGGACCAUAUGUUGCGUUGUGGUAAUUGAGCGUGGACUGUAUUUGUUAUU
.........(((((((((((((...((((((........))))))..)))))).)))).)))).........

```
 Initial dG =    -20.20

        10          20         30
UUGCUGGUU    -    -|     AGG       UGU
         UAUA UCCG GCUCGA   ACCAUA   U
         AUGU AGGU CGAGUU   UGGUGU   G
UUAUUGUUU    C    G^     AA-       UGC
  70        60         50          40
```

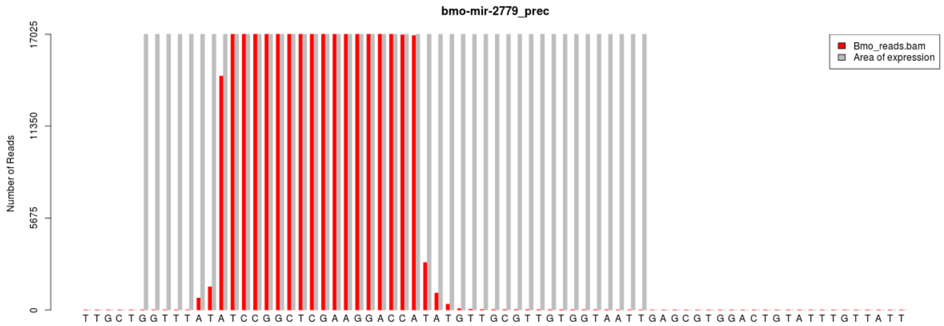

>bmo-mir-2780a-1_prec

```
CCACGGUGAAAAGGGUGUUAAAUGAAGACUUAUUAGGGCUUCGGGCAUAUCGAAGAAAAACAGGAAAUCGUUUGAAUGCUCGUCUAAUGGA
(((....((..((.((.((((((((...(((.......((((((.....))))))......)))...)))))))))).))..))...))).

 Initial dG =    -17.70

         10        20        30        40
-|  CGGU  AA  G  G        AGA   AUUAGGG      GC
 CCA    GA  AG GU UUAAAUGA   CUU       CUUCGG  \
 GGU    CU  UC UA AGUUUGCU   GGA       GAAGCU  A
A^  AAU-  GC  G  -        AAA   CAAAAA-      AU
90         80         70        60         50

-----------------------------------------------------
```

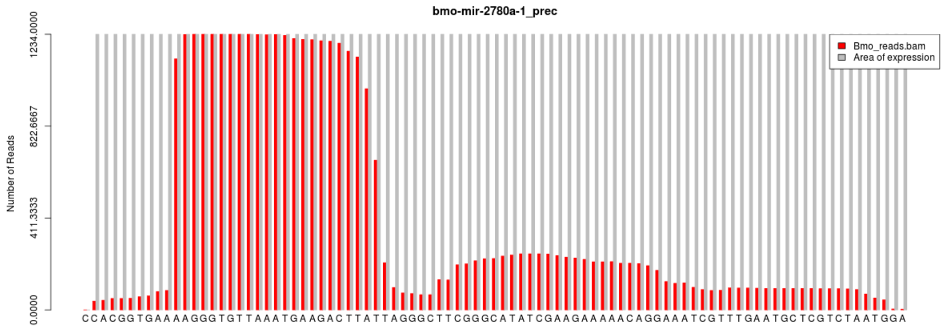

>bmo-mir-2780a-2_prec

```
CCACGGUGAAAAGGGUGUUAAAUGAAGACUUAUGGCUUCGGGCAUGUCGAAGAAAAACAGGACAUCGUUUGAAUGCUCGUCUAAUGGA
(((....((..((.((.((((((((...((.....((((((.....))))))......))....)))))))))).))..))...))).

 Initial dG =    -17.10

         10        20         30         40
-|  CGGU  AA  G  G        AGA-  UAUGG-      GC
 CCA    GA  AG GU UUAAAUGA    CU      CUUCGG  \
 GGU    CU  UC UA AGUUUGCU    GA      GAAGCU  A
A^  AAU-  GC  G  -        ACAG  CAAAAA      GU
        80         70        60        50

-----------------------------------------------------
```

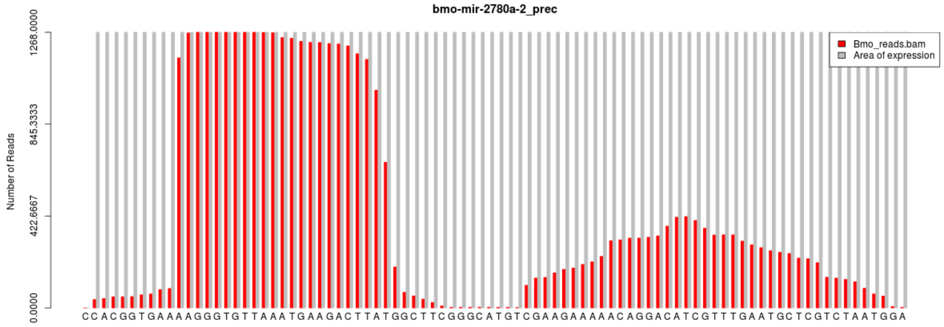

>bmo-mir-2780a-3_prec

```
CCACGGUGAAAAGGGUGUUAAAUGAAGACUUAUGGCUUCGGUCAUAUCGAAGUUAAACAGGACAUCGUUUGAAUGCUCGUCAAAUGGA
(((...(((..((.((.((((((((...((..((((((((((...)))))))))))...))....))))))))))).))..)))..))).

 Initial dG =    -22.80

         10        20         30         40
-|  CGG   AA  G  G        AGA-  UA-          C
 CCA   UGA  AG GU UUAAAUGA    CU   UGGCUUCGGU \
 GGU   ACU  UC UA AGUUUGCU    GA   AUUGAAGCUA A
A^  AA-   GC  G  -        ACAG  CAA          U
        80         70        60        50

-----------------------------------------------------
```

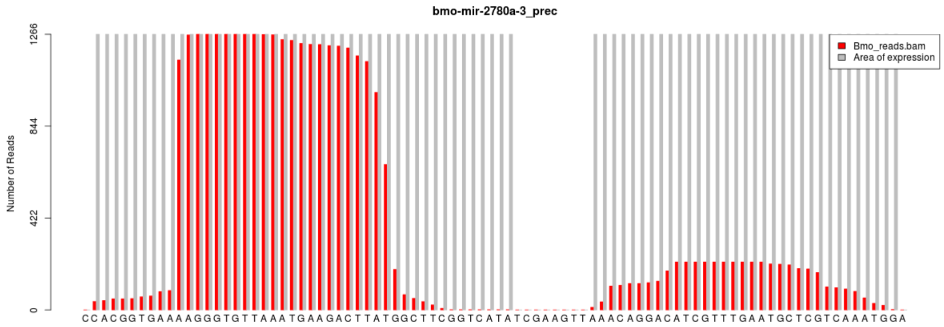

>bmo-mir-2780b-1_prec

```
CCACGGUGAAAAGGGUGUUAAAUGAAGACUUAAGGCUUCGGGCAUAUCGAAGAAAAACAGGACAUCGUUUGAAUGCUCGUCUAAUGGA
(((....((..((.((.((((((((...((.....((((((.....))))))......))....)))))))))).))..))...))).

 Initial dG =    -17.70

         10         20          30          40
-|  CGGU  AA  G  G        AGA-  UAAGG-      GC
 CCA    GA  AG GU UUAAAUGA    CU      CUUCGG  \
 GGU    CU  UC UA AGUUUGCU    GA      GAAGCU  A
A^  AAU-  GC  G  -        ACAG  CAAAAA      AU
        80          70         60         50

-----------------------------------------------------
```

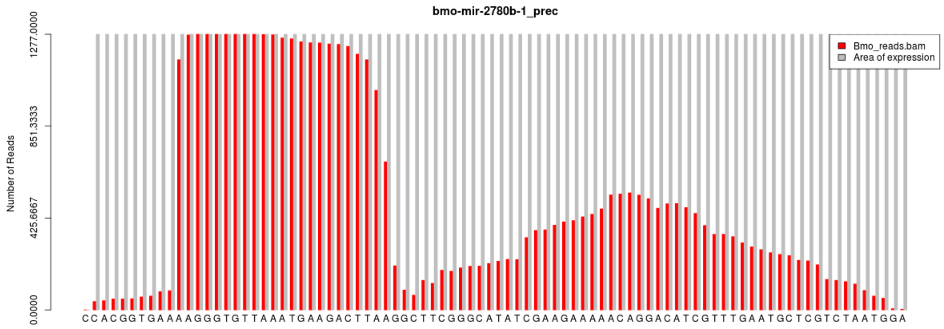

>bmo-mir-2780b-2_prec

```
CCACGGUGAAAAGGGUGUUAAAUGAAGACUUAAGAGCUUCGGGCAUAUCGGAGAAAAACAGGACAUCGUUUGAAUGCUCGUCUAAUGGA
(((....((..((.((.((((((((...((......((((((.....))))))......))....)))))))))).))..))...))).

 Initial dG =    -17.60

         10        20         30        40
-|  CGGU  AA  G  G        AGA-  UAAGAG      GC
 CCA    GA  AG GU UUAAAUGA    CU      CUUCGG  \
 GGU    CU  UC UA AGUUUGCU    GA      GAGGCU  A
A^  AAU-  GC  G  -        ACAG  CAAAAA      AU
         80         70        60        50

-----------------------------------------------------
```

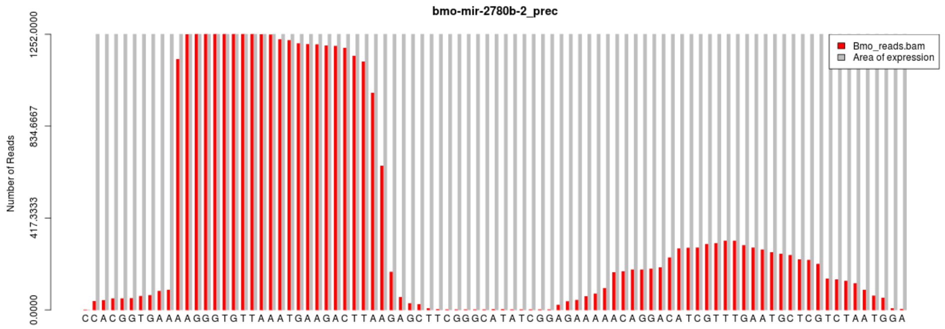

>bmo-mir-2780c_prec

```
CCACGGUGAAAAGGGCGUUAAAUGAAGACUUAGGGCUUCGGGCAUAUCCAAGAAAAACAGGACAUCGUUUGAAUGCUCGUCUAAUGGA
(((....((...((((((((((((...((.....(((.(((....))))))......))....))))))).)))))).))...))).

 Initial dG =    -18.00

         10         20         30         40
-|  CGGU  AAA       -       AGA-  UAGGG-   C   C
 CCA    GA   GGGCGU UAAAUGA    CU      CUU GGG A
 GGU    CU   CUCGUA GUUUGCU    GA      GAA CCU U
A^  AAU-  G--      A       ACAG  CAAAAA   -   A
        80          70         60         50

-----------------------------------------------------
```

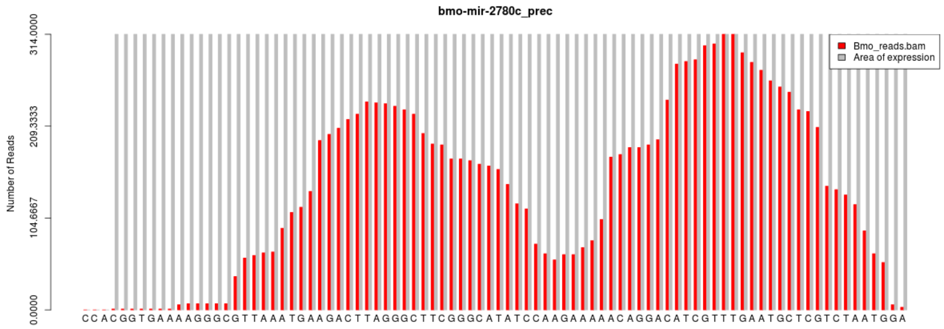

>bmo-mir-2780d_prec

```
ACCACGGUGAAAAGGUGUUAAAUGAAGACUUAGGGCUUCGAGCAUAUCGAAGAAAAACAGGACAUCGUUUGAAUGCUCGUCUAAUGGA
.(((....((..((...((((((((...((.....((((((.....))))))......))....))))))))...))..))...))).

 Initial dG =    -18.20

        10        20         30         40
A   CGGU  AA  GUG        AGA-  UAGGG-|     GC
 CCA    GA  AG   UUAAAUGA    CU      CUUCGA  \
 GGU    CU  UC   AGUUUGCU    GA      GAAGCU  A
A   AAU-  GC  GUA        ACAG  CAAAAA^     AU
        80        70        60        50

-----------------------------------------------------
```

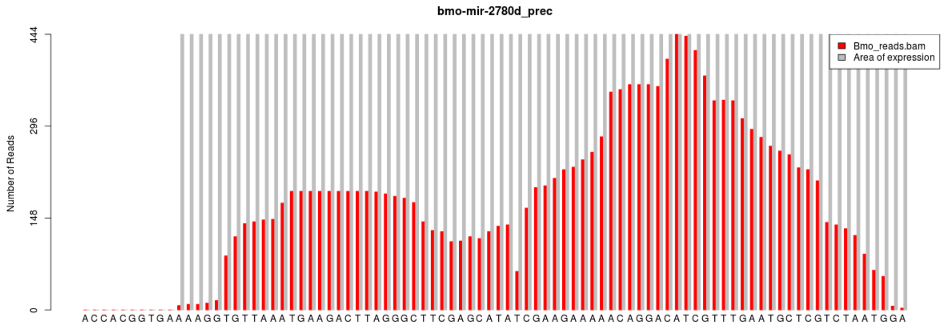

>bmo-mir-2781_prec

```
AUGGCUGAUUUACCAGAUGUUAGAUUGAAGGAGGUCAGUCGACCUUUCACUUACACCGGGUUAGAUUAUUUCGGUCCAA
.((((((((((((...(((.((..(((..((((((....))))))))))).)))...))).)))).....)))).))).

 Initial dG =    -19.00

              10         20        30
A   -    -----   -|  AGA   U  AU   AG       A
 UGG CUGA     UUU ACC   UGU AG  UGA  GAGGUC G
 ACC GGCU     AGA UGG   ACA UC  ACU  UUCCAG U
A   U    UUAUU   U^  GCC   U  --   --       C
        70         60        50            40

-----------------------------------------------------
```

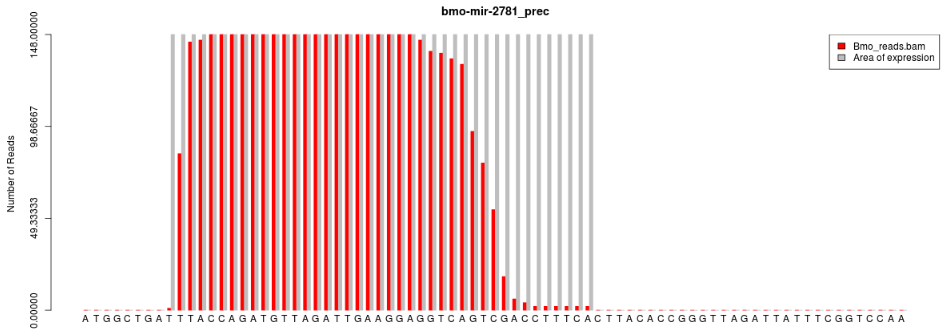

>bmo-mir-2782_prec

```
AUCUACGGAUUGCGUCAUGUUUGUAUACGUAAGUUCUAAAUUAAAAUUCACUUGCUGUAUGAAAUUGGCGACGAUCGUGCGC
...((((.(((((((((..((..((((.((((((...............))))))))))..))..))))).))))))))...

 Initial dG =    -21.30

        10         20        30        40
AUC|   G    -     UG  UG    C      UCUAAAU
   UACG AUUG CGUCA  UU  UAUA GUAAGU       \
   GUGC UAGC GCGGU  AA  AUGU CGUUCA       U
CGC^   -    A     UA  GU    -      CUUAAAA
 80         70        60          50

-----------------------------------------------------
```

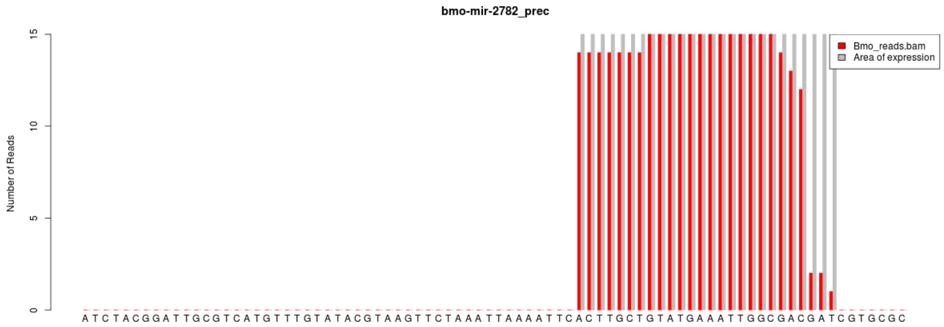

>bmo-mir-2783_prec

```
GAUGACGUAACGGGCGAGGGUGUGGGUGUGGCGCAGCAAGCCGGAGCGGCUGGCGGACGGGCGGCGCGUGCGCGUGUGCGUGGAGGUGUUCG
((..((....((.(((...(((((.((((.((...((.(((((...))))).))......)).)))).)))))...))).))...))..)).

 Initial dG =    -31.20

         10        20        30            40
-  UG  GUAA  G   AGG     G    G  GCA---| A     G
 GA  AC    CG GCG   GUGUG GUGU GC      GC AGCCG \
 CU  UG    GU CGU   CGCGU CGCG CG      CG UCGGC A
G  UG  GAG-  G   GUG     G    G  GGCAGG^ G     G
 90         80        70        60        50

-----------------------------------------------------
```

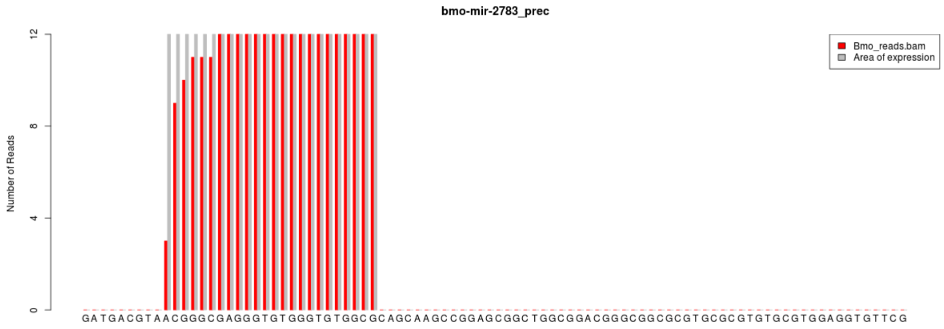

>bmo-mir-2784_prec

AGCGCUUCGCACUCGGCGGCGAGUGUGUGUGUGGGGAGGCAGCGCGCGCACACCGCCUCCGGUCGAUACUCUA
......(((.((.(((.((((.((((((((((((.........))))))))))))))).))))))))).......

```
 Initial dG =    -35.00

         10        20        30
AGCGCU-|  C  U   C    A           GGGA
       UCG AC CGG GGCG GUGUGUGUGUG    \
       AGC UG GCC CCGC CACACGCGCGC    G
AUCUCAU^  -  -   U    -           GACG
  70          60         50        40
```

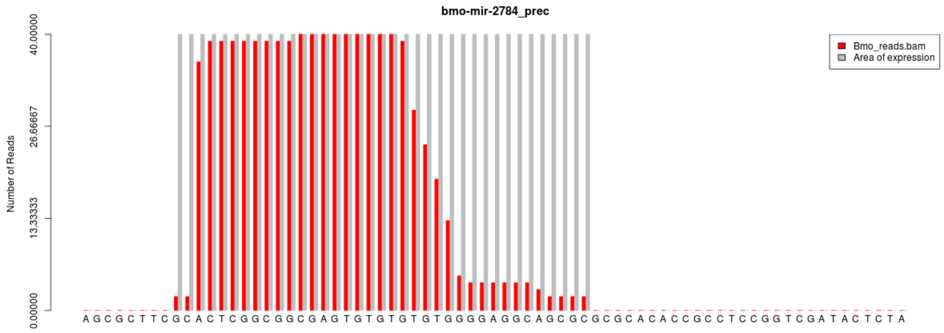

>bmo-mir-2785_prec

ACCGGAUGUCUUAGGCCUCGUACCGCACUGAAUUAAUAUAAUUUUAAUGCAGUGCGGAACGAGGCUAAAACGUCCCAU
...((((((.((((.((((((.((((((((.(((((.......))))).)))))))).)))))))))).))))))...

```
 Initial dG =    -41.20

        10         20         30
ACC|     C    G      A         A     UAU
   GGAUGU UUAG CCUCGU CCGCACUG AUUAA   \
   CCUGCA AAUC GGAGCA GGCGUGAC UAAUU   A
UAC^     A    -      A         G     UUA
       70         60         50
```

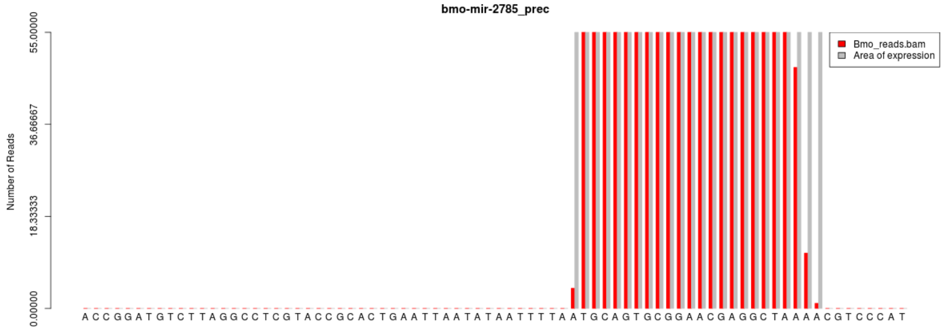

>bmo-mir-2786_prec

```
UCAUUUCUUCAGGUUAUGCAGGCGAAGGAAUAUCAGCUAGCGUACUCUCUCGCCUUCAUGACCUGGUGAAUUGU
.((.(((.((((((((((.((((((..((.(((........))).))..)))))).)))))))))).))).)).

 Initial dG =    -31.70

        10        20        30
U| U   U          C      AG  A   CAG
 CA UUC UCAGGUUAUG AGGCGA  GA UAU   C
 GU AAG GGUCCAGUAC UCCGCU  CU AUG   U
U^ U   U          U      CU  C   CGA
   70        60        50        40

-----------------------------------------------------
```

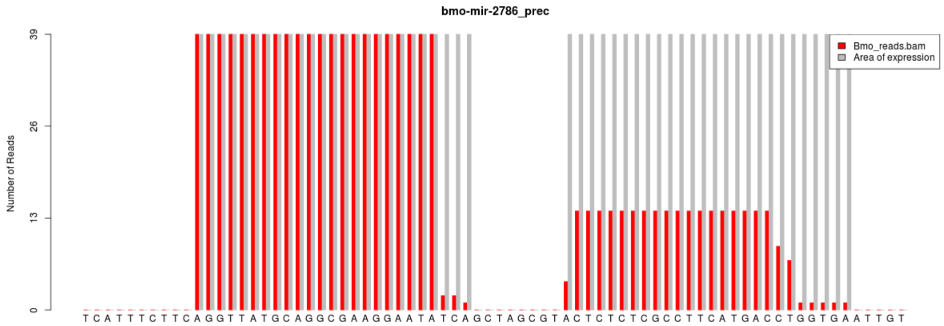

>bmo-mir-2787_prec

AAAGAAAACAUAUCUUGAAUUUGAUUAGCACUAAAUGUGUUGAUAAUAUUAUCACUAAUGUCGCUGAGAAAGUUCCACGAUGUGCAUGUCAAAUGAAU
...((...((((((..((((((..(((((((.....(((.((((...)))).)))....)).)))))..))))))...))))))....))........

```
 Initial dG =    -20.70

              10         20         30        40
AAA-----  AAA-      UU-       GA      -| UAAAU   U    A
        GA    CAUAUC   GAAUUU  UUAGC AC     GUG UGAU \
        CU    GUGUAG   CUUGAA  AGUCG UG     CAC AUUA A
UAAGUAAA  GUAC      CAC       AG      C^ UAAU-   U    U
       90        80        70        60         50

-----------------------------------------------------
```

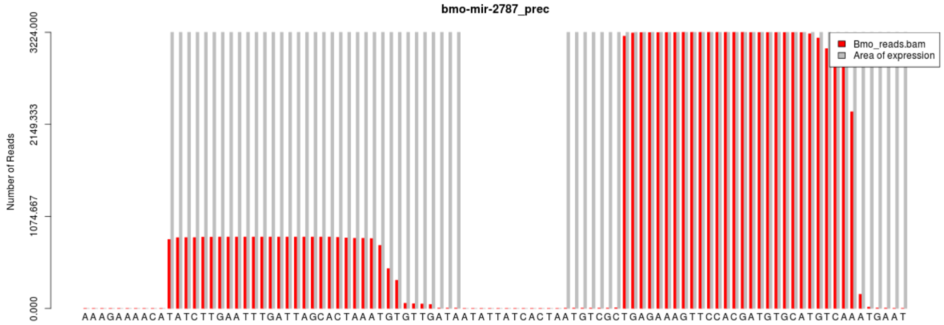

>bmo-mir-2788_prec

GCCGUAGAACUGGGGUUUCAAAGCGGUAUGUGCCUUCGAUUAUGCAAUGCCCUUGGAAAUCCCAAACGUGCUGGCG
((((((....(((((((((.(((.(((((.(((..........))))))))))).)))))))))....))).))).

```
 Initial dG =    -28.80

          10        20        30
-   -|  GAAC         A   C     G   CUUC
 GCC GUA    UGGGGUUUC AAG GGUAU UGC    G
 CGG CGU    ACCCUAAAG UUC CCGUA ACG    A
G   U^  GCAA         G   -     -   UAUU
     70        60         50         40

-----------------------------------------------------
```

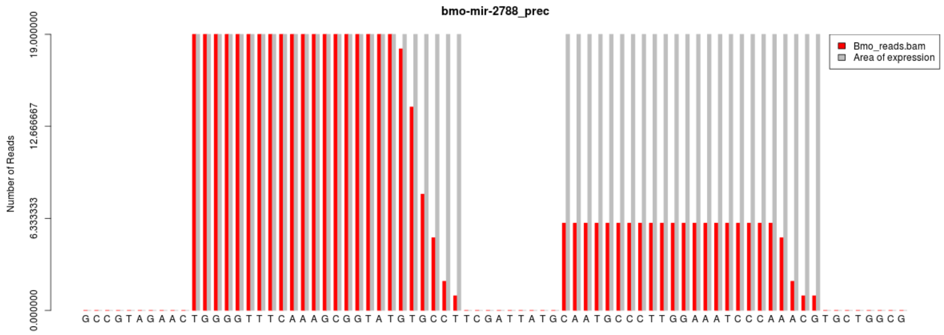

>bmo-mir-2789_prec

CCGUGUUUAUUUCUGUACUCUUUAUUUUCUGUCACAGUUGUUCUGUCGUGGAAAUGUCUGUCGUAGAAAGUUUGGAGGUGGAUUCGUACAGCG
.((((.....((..(.((((..((((((((..((((...((((.....))))....))))..))))))))..)))))..))......))).))

```
 Initial dG =    -24.90

         10        20        30        40
C  -|  UUAUU  UG A    UU        UC    UUG-    GU
 CG UGU     UC  U CUCU  AUUUUCUG  ACAG    UUCU  \
 GC ACA     AG  G GAGG  UGAAAGAU  UGUC    AAGG  C
-  G^  UGCUU  GU -    UU        GC    UGUA    UG
   90        80         70        60        50

-----------------------------------------------------
```

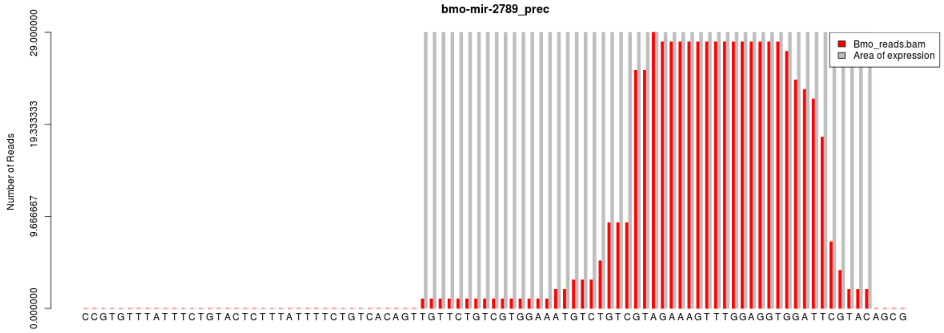

>bmo-mir-2790_prec

```
CAAUUCUAUUUGGAUAAAGAACAAUUCUUAAAAAUAUAUAAUAAUAGUUGACUUCAGUUGUGUUAAGAACUGUAUUUUGUCCAAAUAGAAAAUUG
...((((((((((((((((.(((.((((((.....(((((((...((....))...))))))))))))).))).)))))))))))))))).....

 Initial dG =    -29.10

          10        20        30        40
CAA--|               A   A      AAAAU       AAU  U
     UUCUAUUUGGAUAAAG ACA UUCUUA     AUAUAAU   AG U
     AAGAUAAACCUGUUUU UGU AAGAAU     UGUGUUG   UC G
GUUAA^               A   C      -----       ACU  A
    90        80        70              60        50

-----------------------------------------------------
```

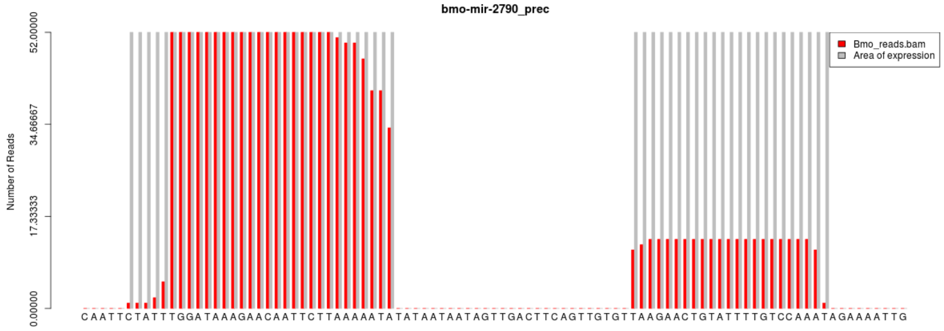

>bmo-mir-2791_prec
CUCGAUGUAUUUUUCAUAACAGCUCUGUCAGCCGACGAAAGCACACAGGUCCGUUAUGAAAAUAUCACAGAGU
(((..((...((((((((((.((.((((..((........))..))))))..)))))))))))...))..))).

```
 Initial dG =    -19.90

         10        20         30
-   GA  UAU          A-| U    CA  CGA
 CUC  UG   UUUUCAUAAC  GC CUGU  GC   C
 GAG  AC   AAAAGUAUUG  UG GACA  CG   G
U   AC  UAU          CC^ -    CA  AAA
  70        60        50         40
```

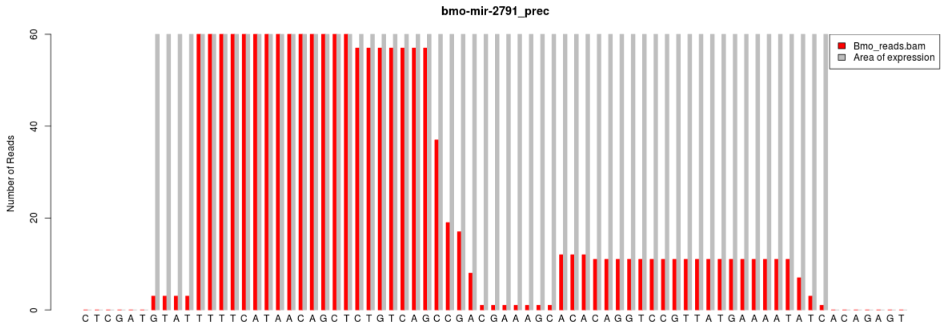

>bmo-mir-2792_prec

UGAACUGCUGUGGUAUCGUUCUUUAAUCAACACUUUAAUUUGAAUAAUGUAGAUUAGAUAGCGAUUCCAUUUACAGUGUGC
...((((..((((.((((((.(((((((.(((...............))).))))))).)))))).))))...))))....

```
 Initial dG =    -21.60

          10        20        30
UGA-    CU-|   U      C       A   CUUUAAU
    ACUG   GUGG AUCGUU UUUAAUC ACA       \
    UGAC   UACC UAGCGA AGAUUAG UGU       U
CGUG    AUU^   U      U       A   AAUAAGU
80        70        60        50
```

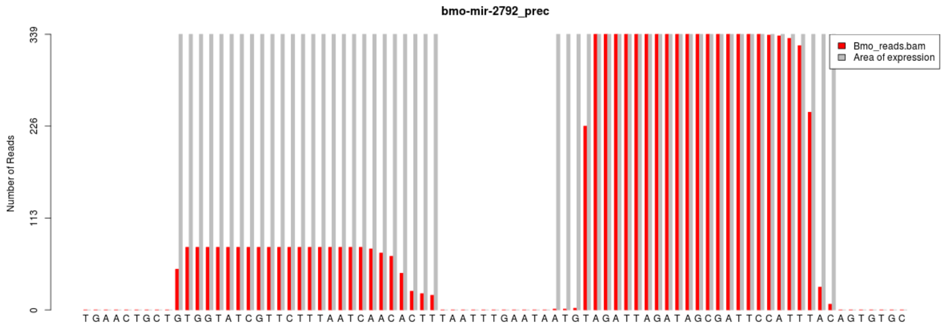

>bmo-mir-2794_prec

AGGGGCACGCGACUCCGCGCCGAAGUCAAUUGUGAUGCGGUCGGCUUUUUAGGGACCCCGUUCACGGCGACUUUUCGUAGCGAUGCUGCCGCG
...((((.(((....(((..(((((((..((((((.((((..(((((....))).))))))))))))).)))))..)))..))).)))))))...

```
 Initial dG =    -31.10

        10         20           30         40
AGG|   C   ACUC   GC   --    AA      U    UC  -   U
   GGCA GCG    CGC  CGA  AGUC  UUGUGA GCGG  GG CUU U
   CCGU CGU    GCG  GCU  UCAG  GGCACU UGCC  CC GGG U
GCG^   -   A---   AU   UU    C-      -    --  A   A
  90            80        70          60          50

----------------------------------------------------
```

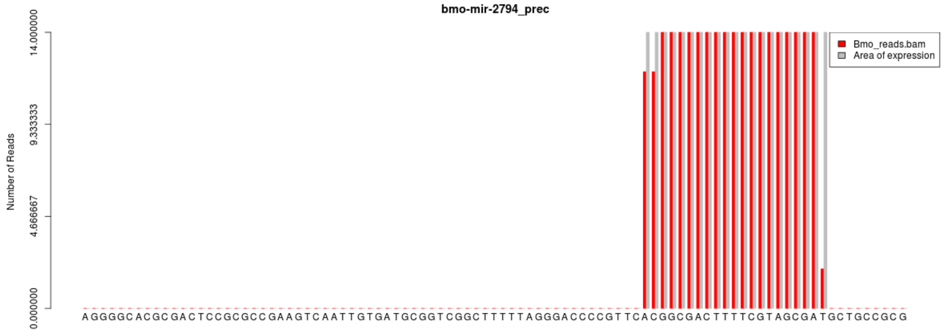

>bmo-mir-2795_prec

```
CGUCCCCACACAAGUUUGGUGAUACGCGGGCGCAGUUUUAGUGGCCCGACAUCGCUCGCUCUUUCUUCGUUACGU
(((........(((..(((((((...(((((.((.......)))))))..))))).))..)))........))).

 Initial dG =    -17.70

         10         20        30
-   CCCCACAC   UU  -|    ACG     G  GUU
 CGU        AAG  UG GUGAU   CGGGC CA   \
 GCA        UUC  GC CGCUA   GCCCG GU   U
U   UUGCUUCU   UC  U^    CA-     -  GAU
    70        60        50          40

-----------------------------------------------------
```

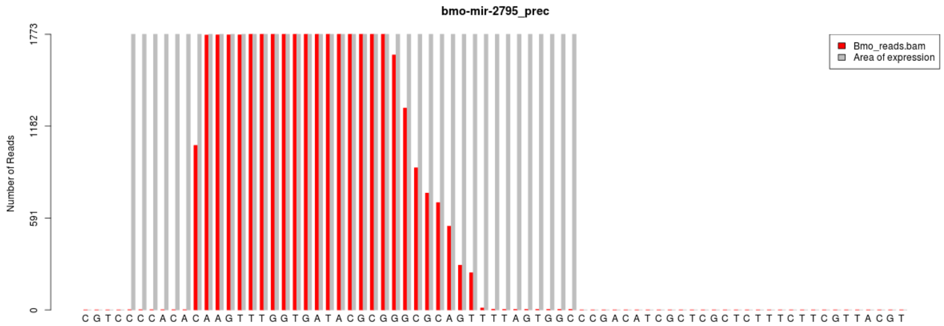

>bmo-mir-2796_prec

```
UACGGUCAGCAGGGGUUUCUUUCGGCCUUCAGAUGCGUCAUAUCCUGUAGGCCGGCGGAAACUACUUGCCGACUCUACU
...((((.(((((((((((.(((((((.((((((.....)))).)).))))))).))))))).))))).)))).....

 Initial dG =    -35.30

          10         20         30
UAC--    A     -       U        U  -|   CG
     GGUC GCAGG GGUUUCU UCGGCCU CA GAUG   \
     UCAG CGUUC UCAAAGG GGCCGGA GU CUAU  U
UCAUC    C     A       C        U  C^   AC
        70         60         50         40

-----------------------------------------------------
```

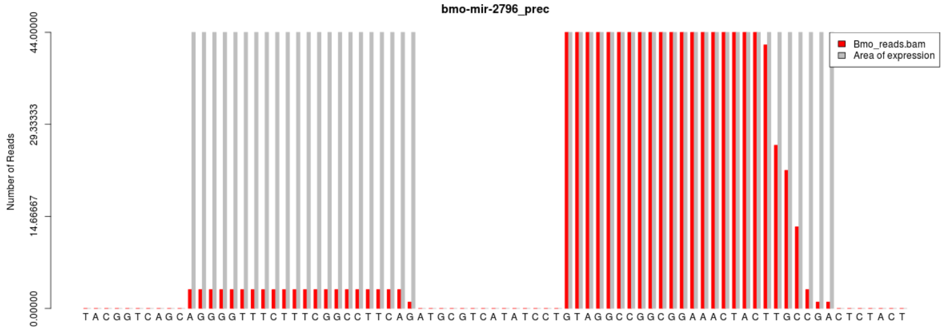

>bmo-mir-2797a_prec

UGUUGUGAUCAUAAGUAGGUUUUAUUCGACUUUGUUCUAUAUUCAAGUCGCGUGAAUCCUACUUUUGAUGACUGCGA

(((.((.((((.(((((((.(((((.((((((.((.....))..)))))).))))).))))))).)))).)).))).

```
 Initial dG =    -27.60

         10        20        30
-   U  G    U       U     U      U-| UC
 UGU GU AUCA AAGUAGG UUUAU CGACUU  GU  \
 GCG CA UAGU UUCAUCC AAGUG GCUGAA  UA  U
A   U  G    U       U     C      CU^ UA
      70        60        50        40
```

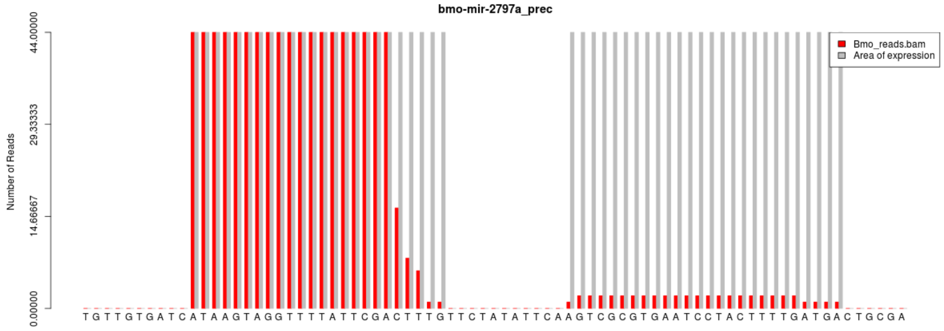

>bmo-mir-2797b_prec

```
UAUCGUAAGCAUAAGUAGGUUCUUAUUCGGCUAUGUUUUCAAUUCAAGCCGCAUUAAGCCUACUUAGACUGGCGACA
..((((.((..((((((((((...((.(((((.((.........))))))).))..))))))))))..)).))))..

 Initial dG =    -26.20

        10        20        30
UA|   A  CA         CUU  U     A  UUUU
  UCGU AG  UAAGUAGGUU   AU CGGCU UG    \
  AGCG UC  AUUCAUCCGA   UA GCCGA AC    C
AC^   G  AG         AU-  C     -  UUAA
      70        60         50
```

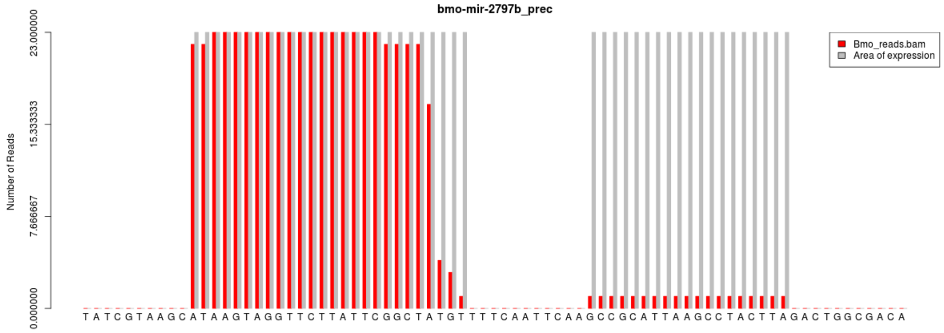

>bmo-mir-2797c_prec

```
UAUGAUUAACAUAAGUAGGUUUUAUUCAGUUUGUGUUUCAUUCAAACCGUAUAAACCCUACUUUUGCUGAUGAUAA
(((.((((.((.(((((((.(((((...(((((.........)))))...))))).))))))).)).)))).))).

 Initial dG =    -20.20

         10        20        30
-|  G    A  U       U     UCA     UGUU
 UAU AUUA CA AAGUAGG UUUAU   GUUUG    \
 AUA UAGU GU UUCAUCC AAAUA   CAAAC    U
A^  G    C  U       C     UGC     UUAC
     70        60        50        40

-----------------------------------------------------
```

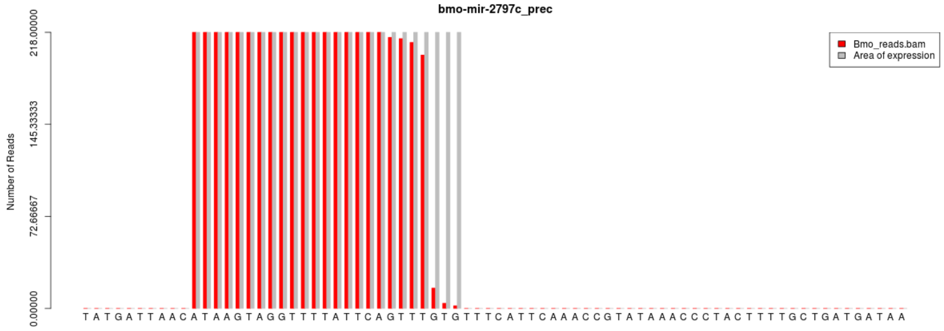

>bmo-mir-2797d_prec

```
UGUCGUGUUUAUAAGUAGACAUUGUCCGGCUUGUUGUUCAUCAAGUCGAACAUAAUCUGCUUACAACCGUGACGAGAUGCAGUGCGCGGCG
...........((((((((...(((.(((((((........))))))).)))...))))))))...(((((((........)).))))))..

 Initial dG =    -29.80

          10        20        30
U       .-UUA|       CAU   C       UUG
 GUCGUGU     UAAGUAGA   UGU CGGCUUG   U
 CGGCGCG     AUUCGUCU   ACA GCUGAAC   U
G       \ ---^       AAU   A       UAC
90             60        50        40


               70
          CAACC  GAC
               GU   G
               CG   A
          UGA--  UAG
              80

-----------------------------------------------------
```

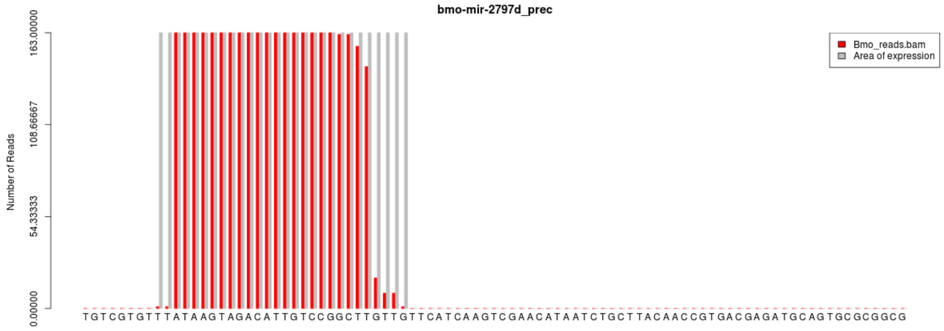

>bmo-mir-2798_prec

```
UUCAGCCACGUAAAGCGCUAUCUCUGUCGCACACUAUGUGAUGUGUGCGACAGAGAUAGCGCUCGAUGUGCAAUA
....((.((((..(((((((((((((((((((((........)))))))))))))))))))))..)))))).....

 Initial dG =    -49.60

        10        20        30
UUCA| C    AA                    UAU
    GC ACGU  AGCGCUAUCUCUGUCGCACAC   G
    CG UGUA  UCGCGAUAGAGACAGCGUGUG   U
AUAA^ -    GC                    UAG
    70         60        50        40

-----------------------------------------------------
```

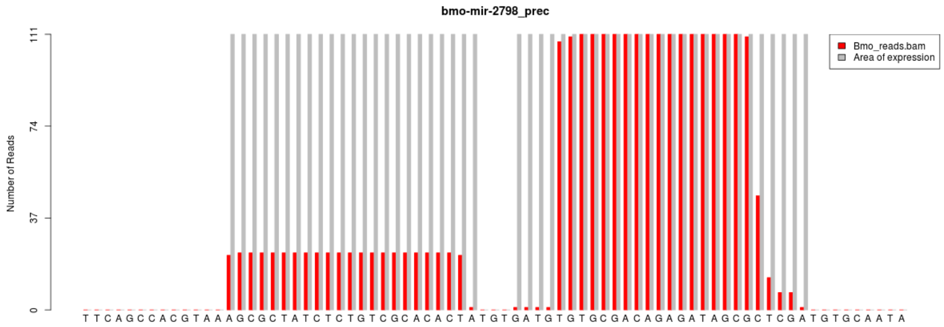

>bmo-mir-2799_prec

```
AUAUGGAAUUAGAGGUUUAUGAACAUGAUGAGUUUACGAUACUCAUCAUGUUCAUAAACCUCUAAUUCCAAAC
...((((((((((((((((((((((((((((((.......)))))))))))))))))))))))))))))))...

 Initial dG =    -49.00

        10        20        30
AUA|                             UUA
   UGGAAUUAGAGGUUUAUGAACAUGAUGAGU   \
   ACCUUAAUCUCCAAAUACUUGUACUACUCA   C
CAA^                             UAG
  70        60        50        40

-----------------------------------------------------
```

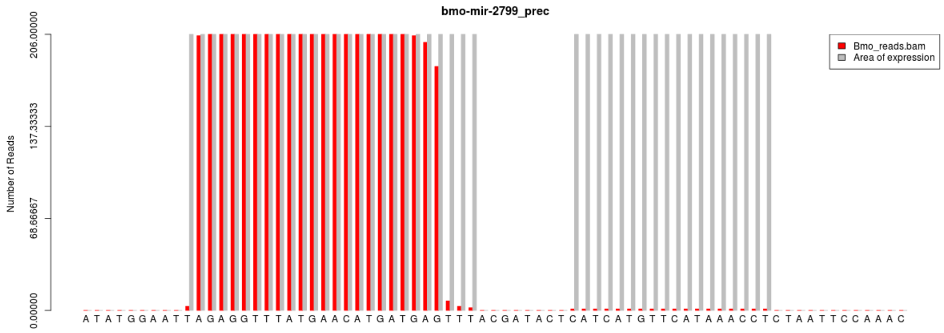

>bmo-mir-2800_prec

```
AAUUUGUAGUAGAAUAUUGUGUCUUGUAAACUUAAUUCAGUUGAGCACAUGAAAAGGGUUUCAGGAACCAUAUUUGUGCACAAUUU
...((((.(((((((((.((.(((((.((((((..((((((.....)).))))..))))))))))))).)))))).)))))))...

 Initial dG =    -18.00

        10         20        30         40
AAU|   A   -      U  G      U      AA    -  UG
   UUGU GUA GAAUAU GU UCUUG AAACUU  UUCA GU  \
   AACA CGU UUUAUA CA AGGAC UUUGGG  AAGU CA  A
UUU^   -   G      C  -      -      AA    A  CG
     80         70          60        50

-----------------------------------------------------
```

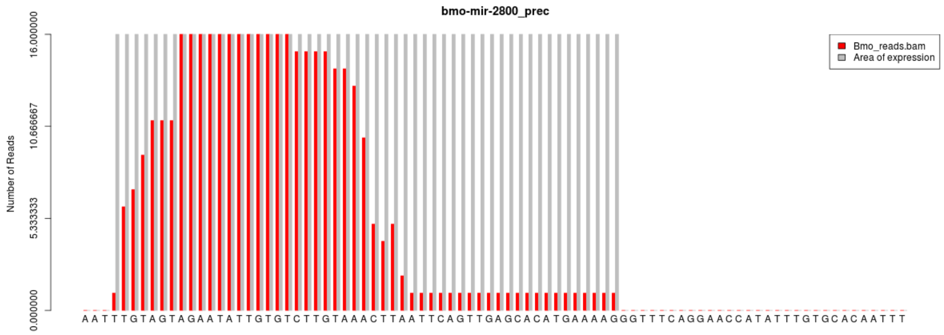

>bmo-mir-2801_prec

```
CACAUGUUUAGUGGAAUCUUGUGACUUUGUAAAGUGCUUGCAAUCAGGCAUGCGUGUGAUGGCUGACAGAGGUUUUAAGCAUGUGG
((((((((((...(((((((((..(((..((..(((((((....)))))))...))..).))...))).)))))))))))))))).

 Initial dG =    -25.10

         10        20          30
-          GUG       .-GUGACU|     AG
 CACAUGUUUA   GAAUCUU        UUGUAA  \
 GUGUACGAAU   UUUGGAG        AACGUU  U
G          ---       \ ------^     CG
     80           70           40


                                 50
                       --     G   GCG
                         UCAG CAU   \
                         AGUC GUA   U
                       AC     G   GUG
                                 60
```

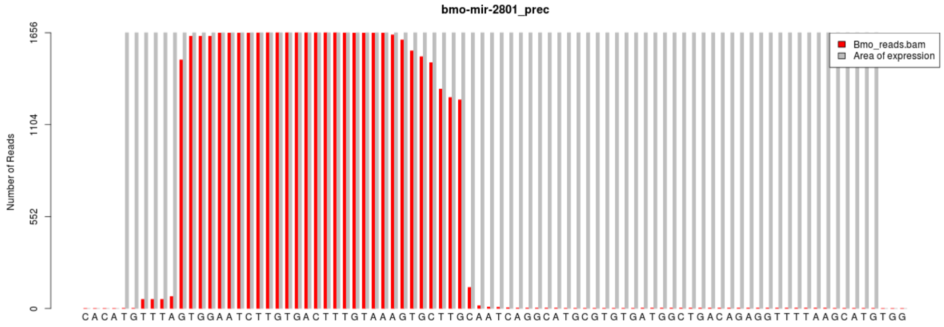

>bmo-mir-2803_prec

```
CCAUGGCUUAUGGACGUUACCAAUGCCAGGGACAGAGCCAAGCCGCUUCCUAGCAUUGAGUACUCUCCGCAGGCCUCGU
....(((((.((((...((((((((.(((....((((......))))))).)))))).)))...)))).))))).....

 Initial dG =    -27.70

        10        20         30        40
CCAU     A    CGU   -|      C   GACA    CA
    GGCUU UGGA   UAC CAAUGC AGG    GAGC  A
    CCGGA GCCU   AUG GUUACG UCC    UUCG  G
UGCU     C    CUC   A^     A   ----    CC
        70        60        50

-----------------------------------------------------
```

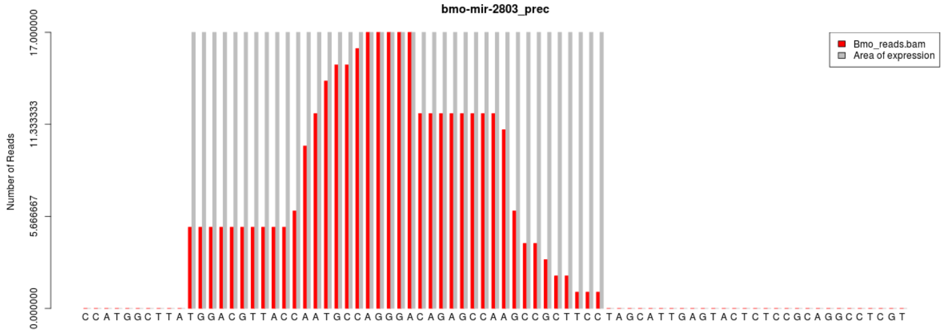

>bmo-mir-2804-1_prec

```
GAAGACAUUACAGUAGUGUAUUACAAUACAAAGUGUGAACUUUGCAUUGUAAUACACUGUUAUAUUACUUGA
.............((((((((((((((.((((((....))))))).))))))))))))))............

 Initial dG =    -23.70

        10         20         30
GAAGACAUUACAG|              A      G
             UAGUGUAUUACAAU CAAAGU U
             GUCACAUAAUGUUA GUUUCA G
AGUUCAUUAUAUU^              C      A
 70         60         50         40

-----------------------------------------------------
```

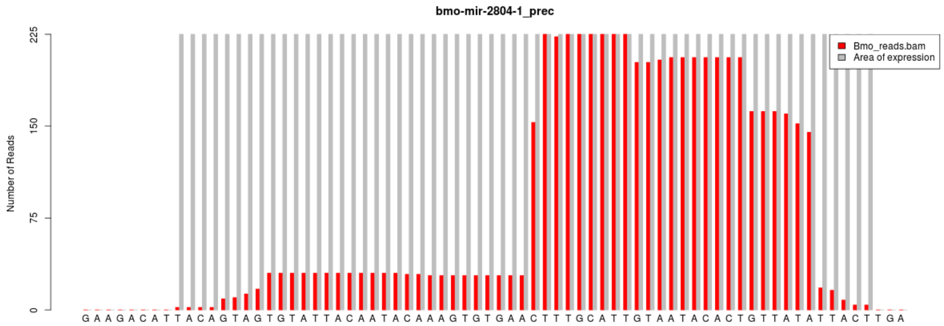

>bmo-mir-2804-2_prec

```
UUCGGGUGUGUCAUUACAGUAGUGUAUUACAAUACAAAGUAUGAACUUUGCAUUGUAAUACACUGUUAUAUUACUUGA
.((((((((....)))..((((((((((((((.((((((....)))))).))))))))))))))).......))))))

 Initial dG =    -26.60

          10
U         |  U
 UCGGGU--GUG C
 AGUUCA  CAU A
-      \ ^  U



               20        30        40
         AG-----              A      A
                UAGUGUAUUACAAU CAAAGU U
                GUCACAUAAUGUUA GUUUCA G
         UUAUAUU              C      A
          70        60        50

-----------------------------------------------------
```

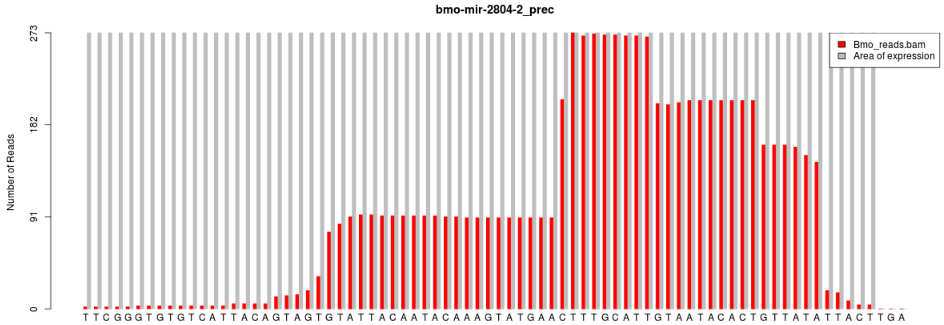

>bmo-mir-2804-3_prec

```
UUCGGGUGUUUCAUUACAGUAGUGUAUUACAAUACAAAGUAUGAACUUUGCAUUGUAAUACACUGUUAUAUUACUUGA
.(((((((.......(((((...((((((((((.((((((....)))))).))))))))))))))).....)))))))

 Initial dG =    -25.60

        10        20        30        40
U|      UUUCAUU     AGU          A      A
 UCGGGUG       ACAGU   GUAUUACAAU CAAAGU U
 AGUUCAU       UGUCA   CAUAAUGUUA GUUUCA G
-^      UAUAU--     ---          C      A
        70             60        50

-----------------------------------------------------
```

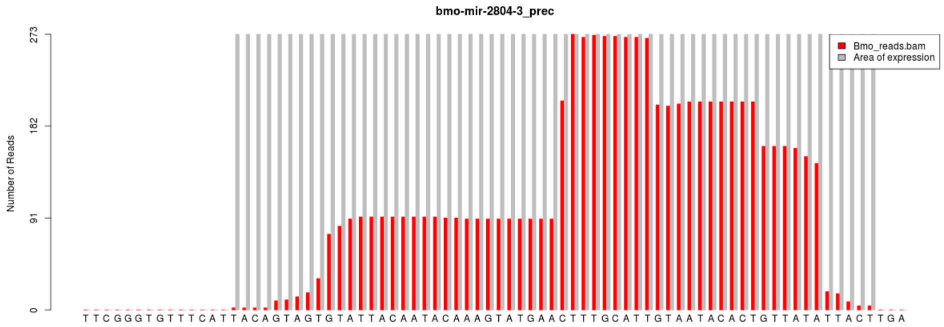

>bmo-mir-2804-4_prec

```
UUCGGGUUUUUCAUUACAGUAGUGUAUUACAAUACAAAGUAUGAACUUUGCAUUGUAAUACACUGUUAUAUUACUUGA
.((((((.......((.((((((((((((((((.((((((....)))))).))))))))))))))))).))..))))))

 Initial dG =    -24.80

        10         20         30         40
U|      UUUUCAU   C                A      A
 UCGGGU        UA AGUAGUGUAUUACAAU CAAAGU U
 AGUUCA        AU UUGUCACAUAAUGUUA GUUUCA G
-^      UU-----   A                C      A
             70         60         50

-----------------------------------------------------
```

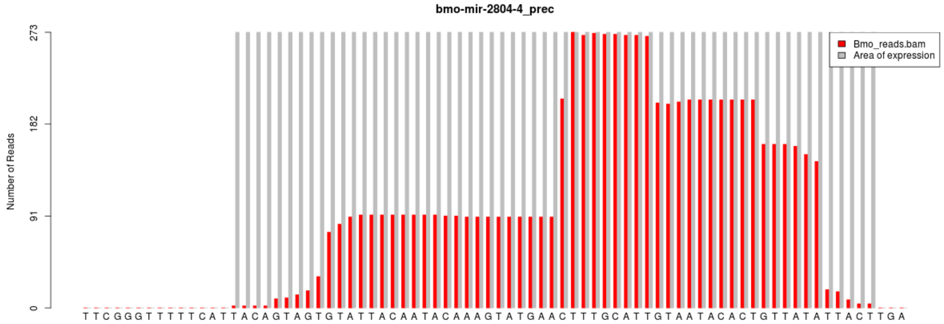

>bmo-mir-2805_prec

```
CUGUUGGACUCUAAGUCCAGGUCGUCAUGGAGGUCGACGUUCCUGACGAACCACGGAAAUAUCCAACAA
.(((((((...((..(((.((((((((.((((.......))))))))).)))..)))..))))))))).

 Initial dG =    -24.70

        10         20         30
C|      CUC  AG   A-   -     U    GUC
 UGUUGGA   UA  UCC  GGU CGUCA GGAG   \
 ACAACCU   AU  AGG  CCA GCAGU CCUU   G
A^      ---  AA   CA   A     -    GCA
           60        50         40

-----------------------------------------------------
```

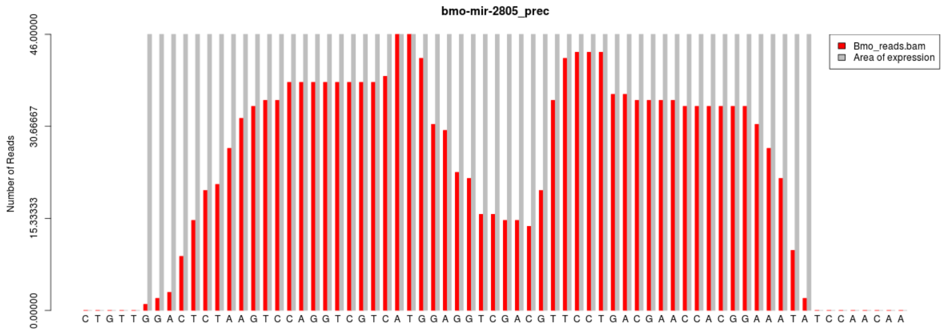

>bmo-mir-2806-2_prec

```
AACUUUAUUCUAUUGCAUGUCGGGUUAGAGAAGCUGUGCAAGCUUCUCUAACCGAUACUGCAUGGAAUAAAUAG
...(((((((((.((((((((.((((((((((.....)))))))))))))))))).)))))))))))...

 Initial dG =    -37.40

        10         20        30
AAC|         U   -      G           GU
   UUUAUUCUAU GCA UGUCGG UUAGAGAAGCU  \
   AAAUAAGGUA CGU AUAGCC AAUCUCUUCGA  G
GAU^         -   C      -           AC
   70         60         50        40

-----------------------------------------------------
```

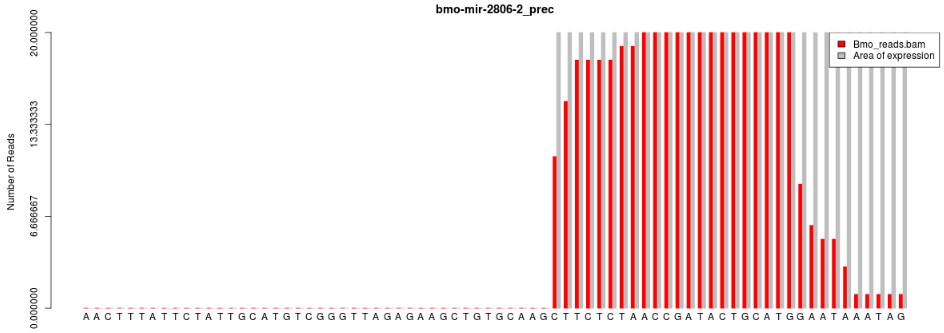

>bmo-mir-2807a-1_prec

UAAAAGUUAAGUCAAAUUUGUAUGAAGUUGGAACGCUUGCCUAUGUUUGCCGCUAGGGGCACUGUUCCAGCUGCAUACAAAUUUCAGGUAACUUUU
.((((((((....(((((((((((.((((((((((..(((((..((.....))...))))).)))))))))).))))))))))))....))))))))

```
 Initial dG =    -39.20

        10        20        30        40
U|       AGUC          A          CU     AU-  UU
 AAAAGUUA    AAAUUUGUAUG AGUUGGAACG  UGCCU   GU  \
 UUUUCAAU    UUUAAACAUAC UCGACCUUGU  ACGGG   CG  G
-^       GGAC          G          C-     GAU  CC
      90        80        70         60        50
```

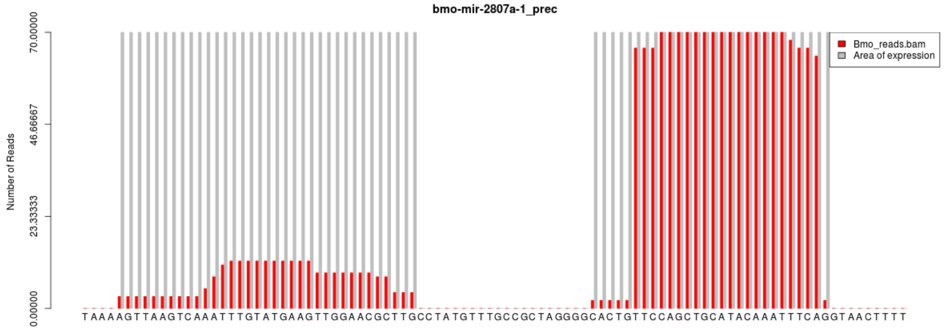

>bmo-mir-2807a-2_prec

```
AAAAGUUAAGUCAAAUUUGUAUGGAGUUGGAACGUUUGCCUAUGUUUGCCGCUAGGGGCACUGUUCCAGCUGCAUACAAAUUUCAGGUAACUCUU
...(((((....(((((((((((.((((((((((..(((((..((.....))...))))).)))))))))).)))))))))))....)))))...

 Initial dG =    -38.80

        10        20        30        40
AAA|    AGUC          G          UU     AU-  UU
   AGUUA    AAAUUUGUAUG AGUUGGAACG  UGCCU   GU  \
   UCAAU    UUUAAACAUAC UCGACCUUGU  ACGGG   CG  G
UUC^    GGAC          G          C-     GAU  CC
    90        80        70         60        50

-----------------------------------------------------
```

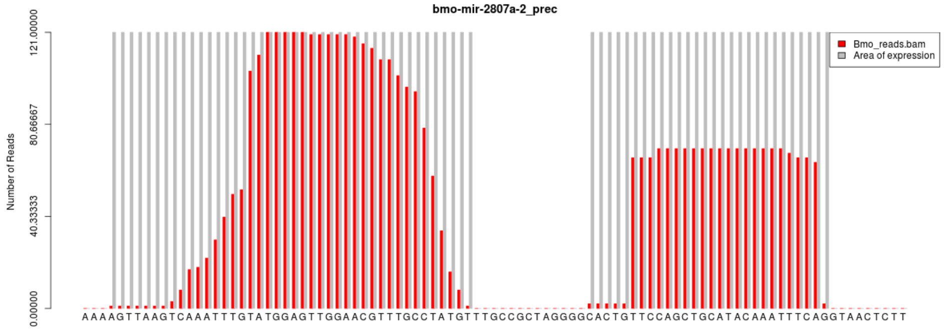

>bmo-mir-2807a-3_prec

```
UAAAAGUUAAGUCAAAUUUGUAGGGAGUUGGAACGUUUGCCUAUGUUAGCCGCUAGGGGCGCUGUUCCAGCUGCAUACAAAUUUCAGGUAAUUUUU
.((((((((....((((((((((...((((((((((..(((((..((.....))...))))).))))))))))...))))))))))....))))))))

 Initial dG =    -31.80

        10        20        30        40
U|       AGUC         GGG          UU      AU-  UA
 AAAAGUUA    AAAUUUGUA   AGUUGGAACG  UGCCU   GU  \
 UUUUUAAU    UUUAAACAU   UCGACCUUGU  GCGGG   CG  G
-^       GGAC         ACG          C-      GAU  CC
      90        80        70         60        50

-----------------------------------------------------
```

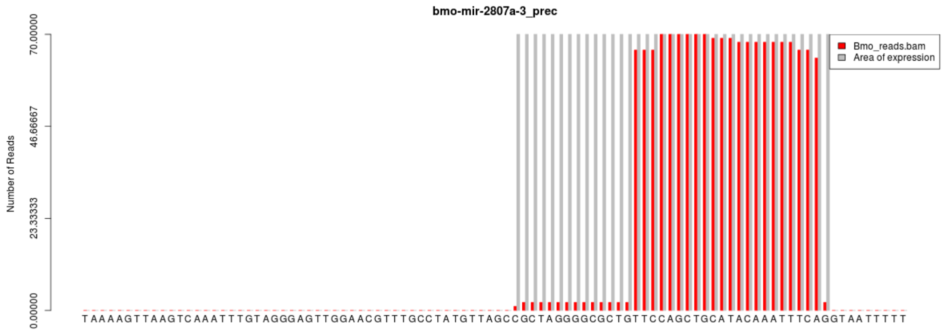

>bmo-mir-2807a-4_prec

```
UAAAAGCUAACUCAAAUUUGUAGGGAGUUGGAACGUUUGCCUAUGUUUGUCGCUAGGGGCACUGUUCCAGCUGCAUACAAAUUUCAGGUAACUUUU
.(((((.((.((.(((((((((...((((((((((..(((((..((.....))...))))).))))))))))...))))))))).)).)).)))))

 Initial dG =    -32.50

        10        20        30        40
U|    C  A  C         GGG          UU      AU-  UU
 AAAAG UA CU AAAUUUGUA   AGUUGGAACG  UGCCU   GU  \
 UUUUC AU GA UUUAAACAU   UCGACCUUGU  ACGGG   CG  G
-^    A  G  C         ACG          C-      GAU  CU
      90        80        70         60        50

-----------------------------------------------------
```

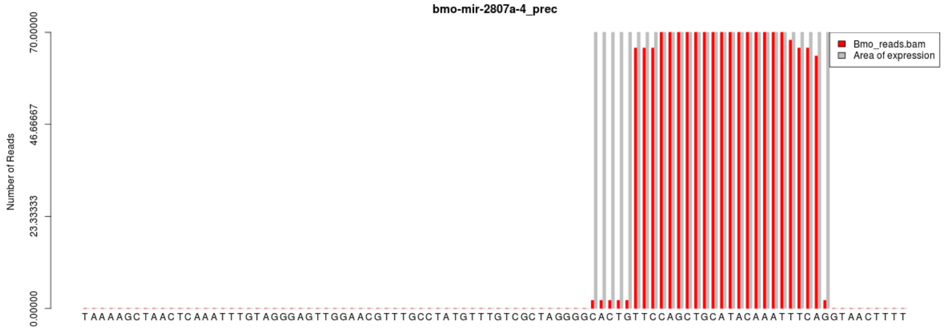

>bmo-mir-2807a-5_prec

```
AAAAGUUAAGUCAAAUUUGUAUGGAGUUGGAAUGUUUGCCUAUGUUUGCCCCGGGGGUCAUUGUUCCAGCUGCAUACAAAUUUCAGGUAACCUUU
....((((....((((((((((.((((((((((...((((.((.......)).))))...)))))))))).))))))))))))....))))....

 Initial dG =    -32.30

        10        20        30        40
AAAA|   AGUC          G          UUU    A  UUU
    GUUA    AAAUUUGUAUG AGUUGGAAUG   GCCU UG   \
    CAAU    UUUAAACAUAC UCGACCUUGU   UGGG GC   G
UUUC^   GGAC          G          UAC    G  CCC
    90        80        70        60        50

-----------------------------------------------------
```

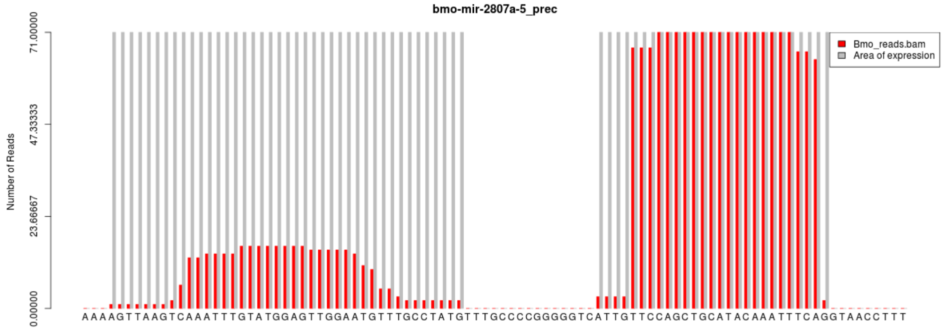

>bmo-mir-2807a-6_prec

```
AGCGUAAAACUAAAAUUUGUAUGGAGUUGGAACGUUUGCUUAUGUUUGCCACUAGGGGCGCUGUUCCAGCUGCAUACAAAUUUCAGUUAACUUUU
.......((((.(((((((((((.((((((((((..(((((..((.....))...))))).)))))))))).))))))))))).)))).......

 Initial dG =    -35.20

        10        20        30        40
AGCGUAA|   A           G          UU     AU-  UU
       AACU AAAUUUGUAUG AGUUGGAACG  UGCUU   GU  \
       UUGA UUUAAACAUAC UCGACCUUGU  GCGGG   CA  G
UUUUCAA^   C           G          C-     GAU  CC
    90        80        70         60        50

-----------------------------------------------------
```

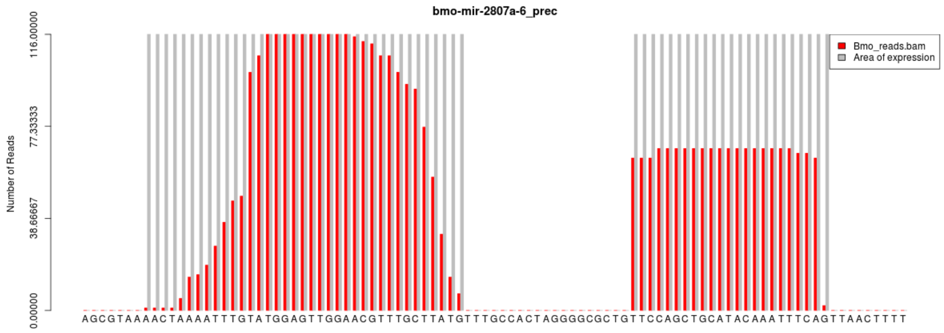

>bmo-mir-2807b_prec

UUAACUCAAAUUUGUAUGCAGUUGGAACAGCGUUCCUAGCGGCAAACGUAGGCAACUGUUUCAACUCCAUACAAAUUUGAGUUA
.(((((((((((((((((.((((((((((((....((((.((.....))))))...))))))))))))).))))))))))))))))))

```
 Initial dG =    -42.10

        10        20        30        40
U|                C           CGUU    G  GC
 UAACUCAAAUUUGUAUG AGUUGGAACAG    CCUA CG  \
 AUUGAGUUUAAACAUAC UCAACUUUGUC    GGAU GC  A
-^                C           AAC-    -  AA
    80        70        60         50
```

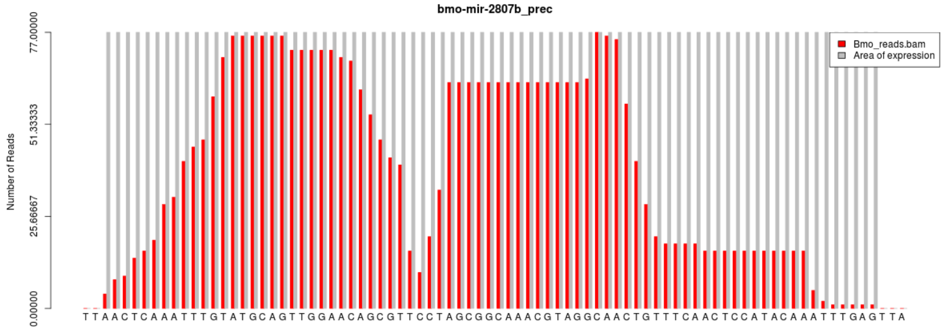

>bmo-mir-2807c_prec

```
UAACCAGACGUACGACGAUGCGACAAAUAUGACAUGUUUACACGUGUCAUAUUUCGCAUUGUUGACGUUUUGUUGU
((((.((((((.(((((((((((..((((((((((((....)))))))))))))))))))))))))))))).)))).

 Initial dG =    -38.30

        10        20        30
-|   C      A          CA           U
 UAAC AGACGU CGACGAUGCGA  AAUAUGACAUGU U
 GUUG UUUGCA GUUGUUACGCU  UUAUACUGUGCA A
U^   U      -          --           C
     70         60          50        40

-----------------------------------------------------
```

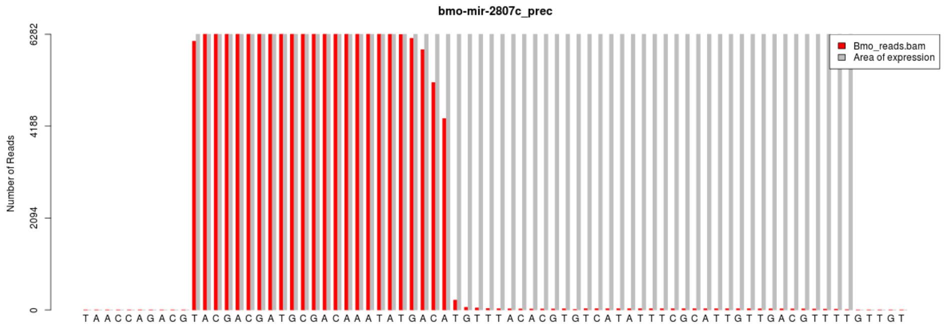

>bmo-mir-2807d_prec

AGUGCGAGUUAUUUAACAUUUUCGAUAGCGUAAAAGUUAGUUCAUAUUUGUAUGGAAUGGGACCGUUUGCCGCUAGAGGCGCUGUUCCAGCUGCAUACAAAAUUGGGCUAAUUUUUACGC
UAUCGAGAACGUUAAAAAACUCGCACUAA

```
((((((((((.((((((.((((((((((((((((((((((((((..((((((((...((((((.((..
(((......))))).))))))....))))))))..)))))))))))))))))))))))))).)))))).))))))))))..
```

```
 Initial dG =    -81.10

          10         20         30         40         50          60         70
--           A      A                          UA         GAA-|      C  UU   GC
  AGUGCGAGUU UUUAAC UUUUCGAUAGCGUAAAAGUUAGUUCA  UUUGUAUG    UGGGAC GU  GCC  U
  UCACGCUCAA AAAUUG AAGAGCUAUCGCAUUUUUAAUCGGGU  AAACAUAC    ACCUUG CG  CGG  A
AA           A      C                          UA         GUCG^      U  --   AG
       140        130        120        110        100         90          80

-----------------------------------------------------
```

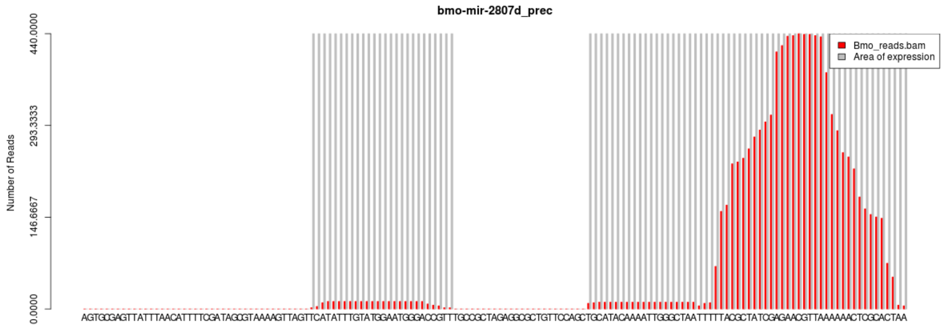

>bmo-mir-2808a-1_prec

```
AGCCGUGCUUCGUAGAAUCUACCAUCGGAUCGGAAACGCGAUCCACUGAGAAGAUCCGGCGAGAAACUCAGUGGGCUG
((((.((((((((...((((..((..((((((......))))))..))...))))...)))).......)))))))).

 Initial dG =    -22.00

           10
.-A    UG-       |   A
   GCCG   CUUC--GUAG \
   CGGC   GAAG  CAUC A
\ -    CUA    \ ^   U
  60        50   20



                        30
                CAUC-      GA
                     GGAUCG  A
                     CCUAGC  A
                AGUCA      GC
                         40

-----------------------------------------------------
```

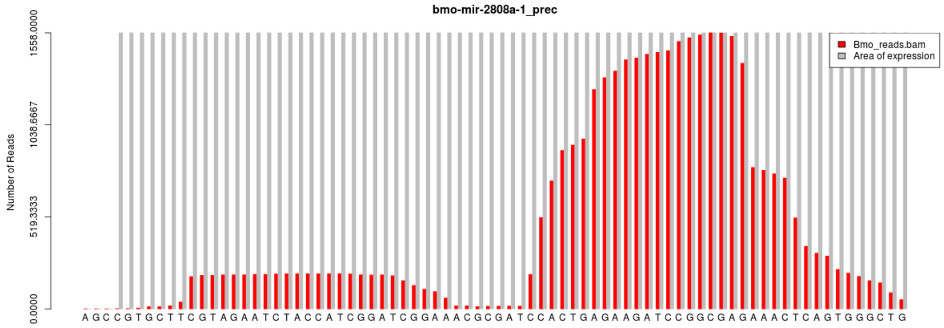

>bmo-mir-2808a-2_prec

```
GGACGUGCUUCGUAGAAUCUACCAUCGGAUCGGAAACGCGAUCCGGUGGUAGAUUCUGCGAAGUACGGCUCUUGCUA
...(((((((((((((((((((((((((((((......)))))))))))))))))))))))))))))..........

 Initial dG =    -54.00

               10        20        30
GGA-------|                            GA
          CGUGCUUCGUAGAAUCUACCAUCGGAUCG  A
          GCAUGAAGCGUCUUAGAUGGUGGCCUAGC  A
AUCGUUCUCG^                            GC
      70        60        50        40

-----------------------------------------------------
```

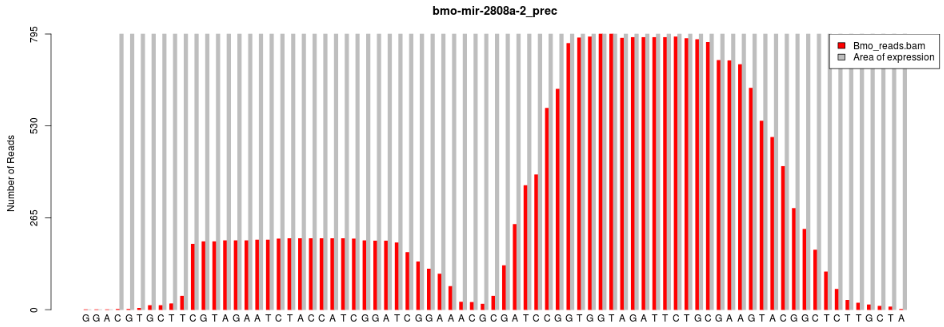

>bmo-mir-2808b_prec

```
CUUUUUUUUUGGCUACCGAUGCUGGUAAUCUCGGCUAGUUAGUGCUUCGUAGAAUCUACCAUCGGAUCAGAAACGCGC
.....(((((((...((((((.(((...((((((..(((....)))))).)))..))).)))))).))))))).....

 Initial dG =    -18.10

        10        20        30         40
CUUUU|      CUA      C    UAA   -   CU   U
     UUUUUGG   CCGAUG UGG   UCU CGG  AGU A
     AAAGACU   GGCUAC AUC   AGA GCU  UCG G
CGCGC^      A--      C    UA-   U   --   U
       70         60         50

-----------------------------------------------------
```

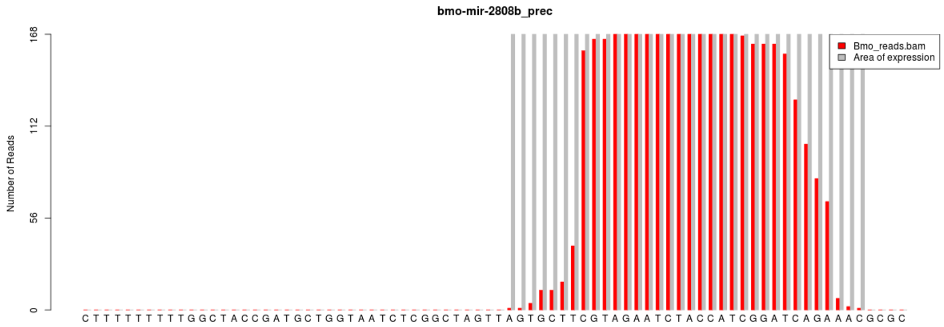

>bmo-mir-2808c_prec

```
CACAGAGUUUCUCGACGGAUCUUCGCGGGUUUGGGUCGCUAUUCGAUCUGGUGGUAGAUUCAGCGAAACAAUGCUCUUUU
...(((((...(((..((((((((((.((.(((((......))))).)).)))).))))))..)))......)))))...

 Initial dG =    -23.90

        10           20          30
CAC     UUC---   AC      -|   G  U     UC
   AGAGU      UCG  GGAUCU UCGC GG UUGGG  G
   UCUCG      AGC  CUUAGA GGUG UC AGCUU  C
UUU     UAACAA   GA      U^   G  U     AU
.        70        60        50        40

-----------------------------------------------------
```

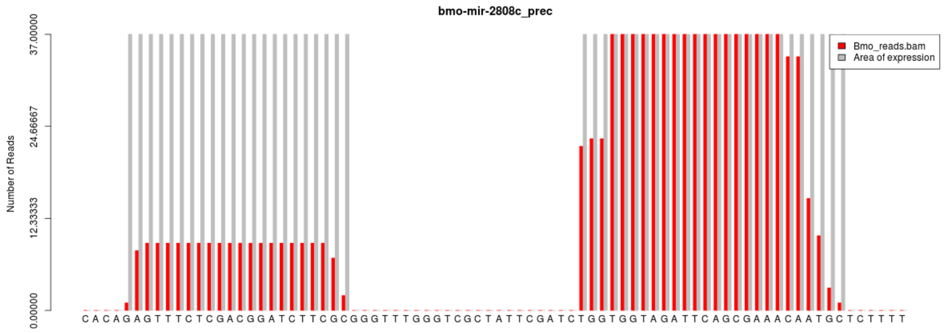

>bmo-mir-2808d_prec

GGAGUAUUGCUAACACUGGUCCUAGCAAAAGCAGUGCUUCGCAGAAUCUACGACCGGAUUGGAAAUGCGACCCACUGAGAAGAUCCGGCGAGAAGCUCGGUGUGCUGCUUGGAAUAGUUC
UACGUAUACUUA
.((((((.((((..((((.(((......((((((..(.(((.((..(((.((.(((((((.......
((......))....))))))))))))..))))).)..))))))))).))))..)).)))))))).

```
 Initial dG =    -40.80

        10         20        30        40        50        60        70
G|      U  -  AC    G   UAGCAA      UG U    C  AA    A  A       GGAAAUG  AC
 GAGUAU GC UA  ACUG UCC      AAGCAG  C UCG AG  UCU CG CCGGAUU       CG  C
 UUCAUA UG AU  UGAU AGG      UUCGUC  G GGC UC  AGA GC GGCCUAG       GU  C
A^      -  C  CU    A   ------      GU U    -  GA    -  -       AAGA---  CA
130        120             110       100          90         80

-----------------------------------------------------
```

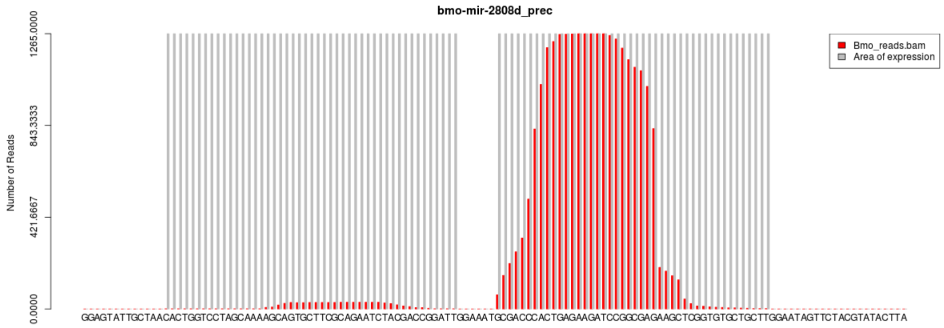

>bmo-mir-2808e_prec

```
AGCUCACGGGGCUCAAACCGAAGUGCUUCGCAGAAUCUACCACCGGGUCGGUAGCGCGACCCACUGAGAAGAUCCGGUGAGUAACUCAGUGGGCUGUGUCUAUGGGUUA
.((((((.(((.((.......((((..((((.....(((((........))))).))))..)))).....))))).))))))((((((.((((.....)))))))))).
```

```
 Initial dG =    -38.20

          10              20
.-A      G   ----    .-AAC|   G
   GCUCAC GGG    CUCA     CGAA U
   UGAGUG CCU    GAGU     GCUU G
\ -      G   AGAA    \ ---^   C
    80        70         30


                                40        50
                       CAGAAUCUACCACC      GU
                                     GGGUCG  A
                                     CCCAGC  G
                       CA------------      GC
                                      60

-----------------------------------------------------
```

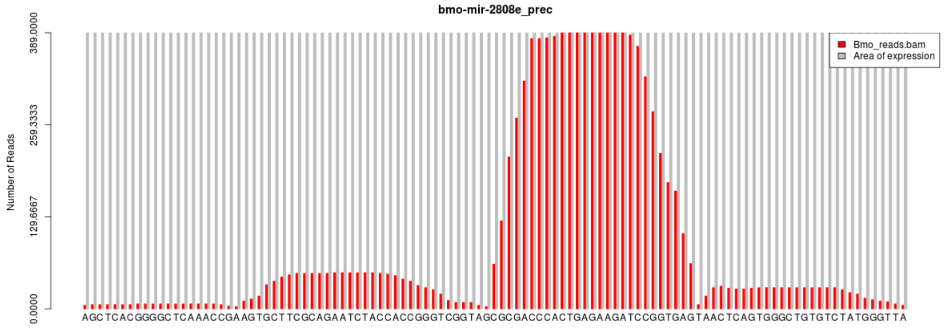

>bmo-mir-2809_prec

```
UACUCGAAGCGAAACAGCGAACGGAUCACCUGAUGCGAACCGGAAGAAGCGAAUUGAGAGUUUUAAAGUGUUUGUUGAGUGUUUUAAGCA
..((..(((((...(((((((((......(((........)))..(((((.........)))))....)))))))))..))))).))..

 Initial dG =    -17.70

        10        20          30
UA  CG     AAA         .-GAUCAC|  AUG
  CU  AAGCG   CAGCGAACG        CUG   C
  GA  UUUGU   GUUGUUUGU        GGC   G
AC  AU     GA-         \ ------^  CAA
.        80         70           40


                                 50
                         AA--     GAAU
                             GAAGC    \
                             UUUUG    U
                         GAAA     AGAG
                                 60

-----------------------------------------------------
```

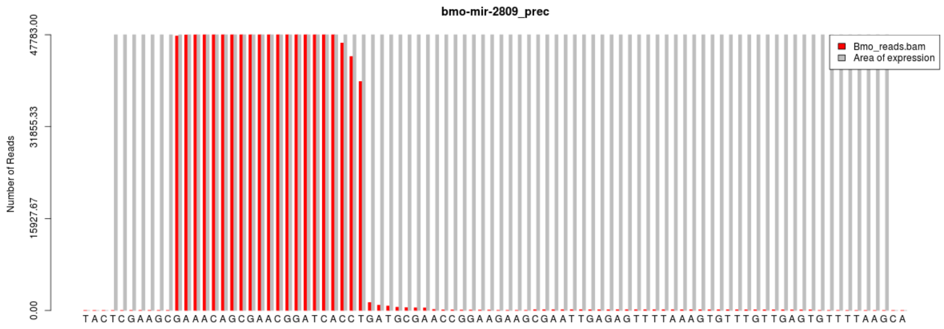

>bmo-mir-2810_prec

```
AAGUAAAGAGAGAUUUUCGAGAACAGCUAAUUCGGCUGCGUGGCUGUUGCGCAGAAGAUGCUCAGCGUUGCCU
..((((...((((((((((...((((((((...((....))))))))))).....)))))).))).....)))))..

 Initial dG =    -17.90

         10          20           30
AA    AGA-   -      GAG--|       AUU  G
  GUAA    GAG AUUUUC     AACAGCUA   CG C
  CGUU    CUC UAGAAG     UUGUCGGU   GC U
UC    GCGA   G      ACGCG^       ---  G
  70        60        50           40

-----------------------------------------------------
```

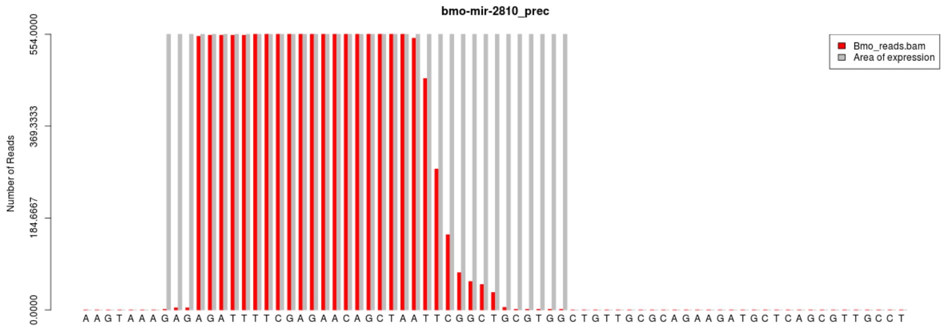

>bmo-mir-2812_prec

```
CCCUGGCCCUGUCAGAAUCGAGUUACUACAGCCUGGACGAGAUUAUGGGCCGCACCGUCCAGGCUGUAAUAGAAAGGCUGAGUGCCGGG
.((((((....((((.......(((.(((((((((((((................))))))))))))).))).....)))).))))))

 Initial dG =    -38.10

        10        20        30        40
C|     CCUG    AAUCGAG   C             AGAUUAU
 CCUGGC    UCAG       UUA UACAGCCUGGACG       G
 GGGCCG    AGUC       GAU AUGUCGGACCUGC       G
-^     UG--    GGAAA--   A             CACGCCG
           80          70        60        50

-----------------------------------------------------
```

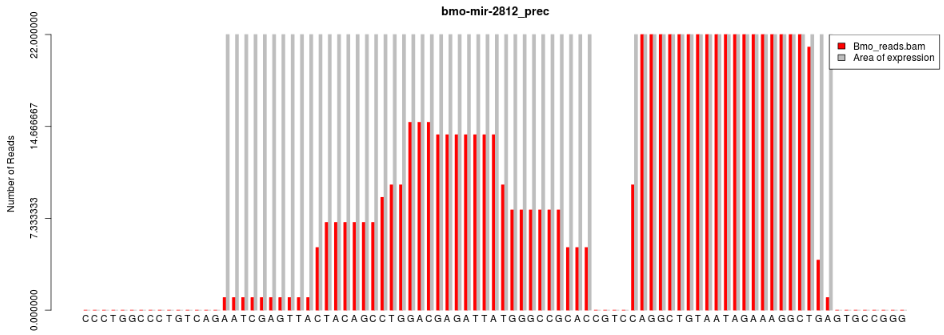

>bmo-mir-2813-1_prec

```
CCAAGCCAAAUUCUAGAUUAAGCCGUUGGAAAGACGUCACCAGUGACGAAAUCCUACGGUUUUUAGCAGUUUUGAUGUU
.......((((((((((...((((((.(((....(((((....)))))...))).))))))))))).)))))).......

 Initial dG =    -20.60

        10          20        30        40
CCAAGCC      -|    UUA      U   AAGA     C
       AAAUU CUAGA   AGCCGU GGA    CGUCA C
       UUUGA GAUUU   UUGGCA CCU    GCAGU A
UUGUAGU      C^    ---      U   AAA-     G
        70            60        50

-----------------------------------------------------
```

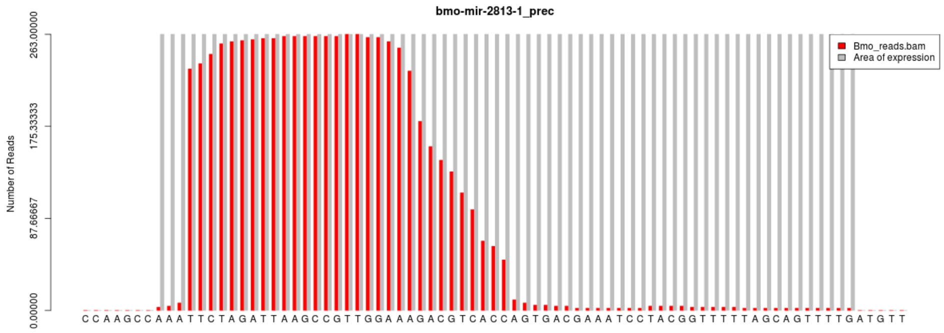

>bmo-mir-2813-2_prec

```
CCAAGCCAAAUUCUAGAUUAAGCCGUUGGAAAGACGUCACCAGUGACGAAAUCCUACGGUUUUUAGCAGUUUUAAUGUU
.......((((((((((...((((((.(((....(((((....)))))...))).))))))))))).))))).......

 Initial dG =    -20.60

        10          20        30        40
CCAAGCC     -|    UUA      U   AAGA     C
       AAAUU CUAGA   AGCCGU GGA    CGUCA C
       UUUGA GAUUU   UUGGCA CCU    GCAGU A
UUGUAAU     C^    ---      U   AAA-     G
        70            60        50

-----------------------------------------------------
```

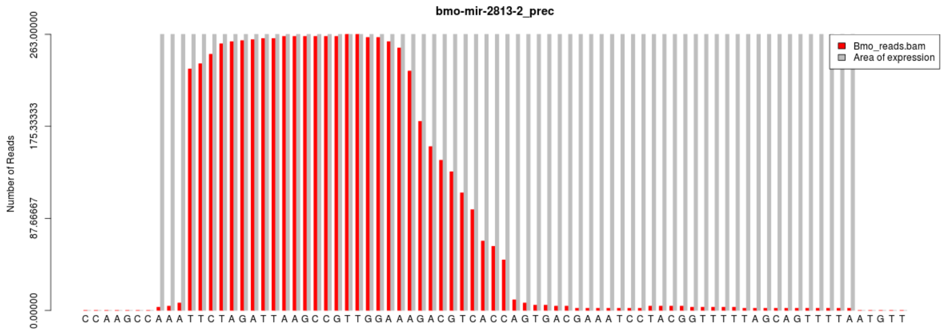

>bmo-mir-2814-2_prec

```
ACUUGUCAGCUCUGCAACAAGAAAGGGCACUUUGCCACUGUUUGUAGAAGCUCUUCUAAAUCUACAAAUUCUGUGGUAGUAGGUACUGAAGAUC
.......((((((((((......(((((.....))).))..))))))).)))((((...((((((.............))))))...))))...
```

```
 Initial dG =    -22.70

          10
ACUU   .-A|    GCAA
    GUC   GCUCU    C
    UAG   CGGGA    A
C---   \ -^    AAGA
                 20



                30           40         50
         A-------  -       U--         AGCUC
                CU UUGCCAC   GUUUGUAGA     U
                GA GAUGGUG   UAAACAUCU     U
         AAGUCAUG  U       UCU         AAAUC
        90        80        70        60

-----------------------------------------------------
```

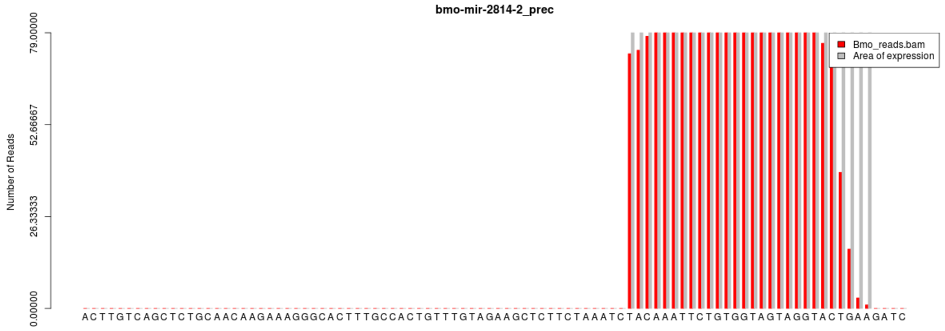

>bmo-mir-2814-3_prec

```
AGAUCUUCAGUACCUACUACCACUGUUUGUAGAAGCUCUUCUAAAUCUACAAAUUCUGUGGUAGUAGGUACUGAAGAUC
.((((((((((((((((((((((.(((((((((............)))))))))...))))))))))))))))))))))

 Initial dG =    -47.00

        10        20           30
A                      U--|        AGCUC
 GAUCUUCAGUACCUACUACCAC   GUUUGUAGA     U
 CUAGAAGUCAUGGAUGAUGGUG   UAAACAUCU     U
-                      UCU^        AAAUC
         70        60        50        40

-----------------------------------------------------
```

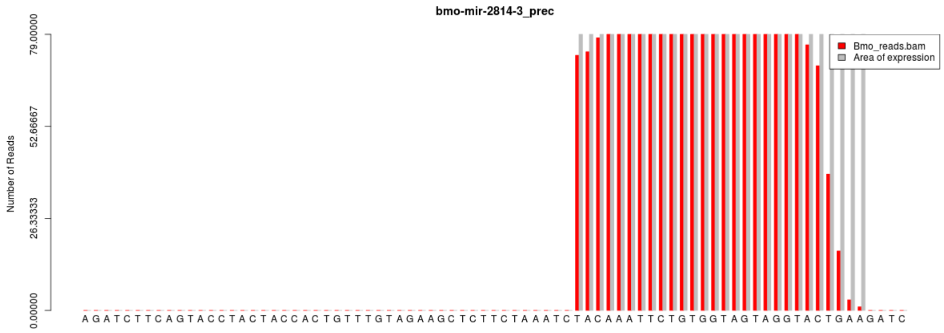

>bmo-mir-2816-1_prec

```
CUCAACUGUCCUCGAGUGAGGUAGAGUCGUGUUAUUGCGUCACCACGACAGCAUCAGUUUGAGGAAAAUUUGAU
.((((...((((((((((.((...((((((............)))))).)).))).))))))))....)))).

 Initial dG =    -21.80

         10         20        30
C    CUG-        -|  G  AGA      UUAUU
 UCAA    UCCUCGAG UGA GU   GUCGUG     G
 AGUU    AGGAGUUU ACU CG   CAGCAC     C
U    UAAA        G^  A  A--      CACUG
   70        60        50          40

-----------------------------------------------------
```

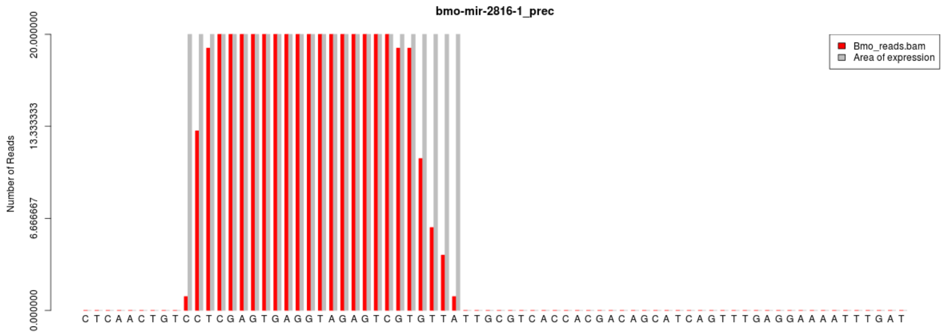

>bmo-mir-2816-2_prec

CUCAACUGUCCUCGAGUGAGGUAGAGUCGUGUUAUUGCGUCACCACGACAACAUCAGUUUGAGGAAAAUUUGAU
.((((...(((((((((((.((...((((((............)))))).)).))).)))))))))....)))).

```
 Initial dG =    -21.50

         10          20         30
C    CUG-        -|  G  AGA      UUAUU
 UCAA    UCCUCGAG UGA GU   GUCGUG     G
 AGUU    AGGAGUUU ACU CA   CAGCAC     C
U    UAAA        G^  A  A--      CACUG
   70        60         50          40
```

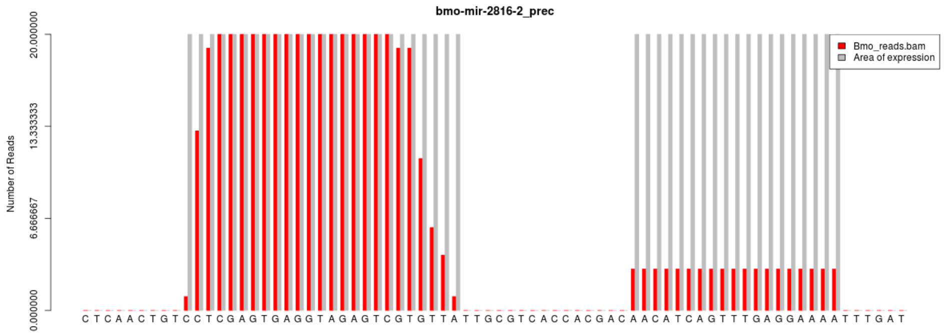

>bmo-mir-2816-3_prec

```
CUCAACUGUCCUCGAGUGAGGUAGAGUCGUGUUAUUGCGUCACCACGACAACAUCAGUUUGAGGAAAAUUUUA
........((((((((((.((...((((((............)))))).)).))).))))))))........

 Initial dG =    -19.40

        10         20        30
CUCAACUG        -|  G  AGA      UUAUU
        UCCUCGAG UGA GU   GUCGUG     G
        AGGAGUUU ACU CA   CAGCAC     C
AUUUUAAA        G^  A  A--      CACUG
  70        60        50          40

-----------------------------------------------------
```

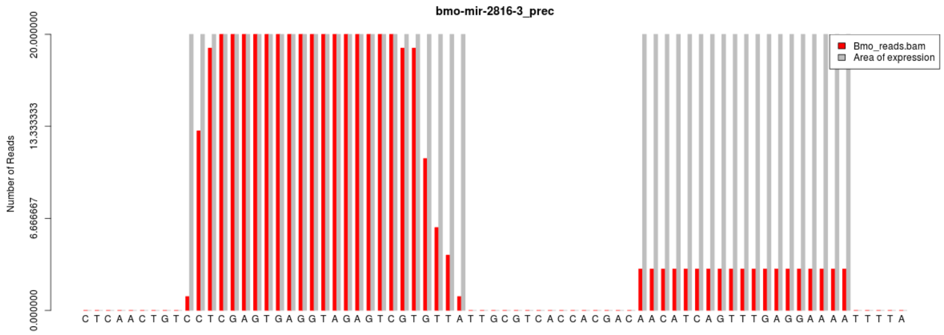

>bmo-mir-2817_prec

```
UUCGUUUCCAAACAAGACUGCGUAGACCGAGCUUUAUGACCUGGCGCCGAGUCUUGCUGGAAGGAC
.((.((((((..(((((((((((((...............))).))).)))))))).)))))))).

 Initial dG =    -17.80

        10          20         30
U  G      AA       --   -|  ACCGAGC
 UC UUUCCA  CAAGACU  GCG UAG       \
 AG GAAGGU  GUUCUGA  CGC GUC       U
C  -      C-       GC   G^  CAGUAUU
      60          50         40

-----------------------------------------------------
```

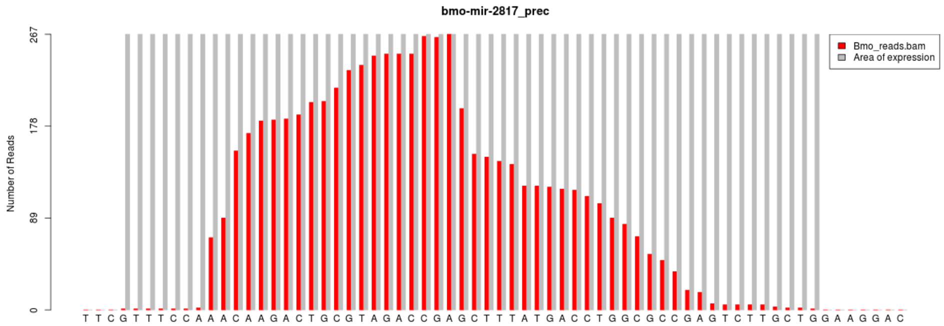

>bmo-mir-2818-1_prec

```
CGAAUCCCGCCAAGGGAAGAUAUCUGUAUGAUAAAUAUAAAUGUCUUUUCCAGGGUUAUGGAUGUA
...((((.(((..(..(((((((.(((((.....))))).))))))))..)...)))...))))...

 Initial dG =    -19.80

          10         20        30
CGA    C--   AA-|GG       C     GA
   AUCC   GCC   G  AAGAUAU UGUAU  \
   UAGG   UGG   C  UUCUGUA AUAUA  U
AUG    UAU   GAC^UU       A     AA
     60        50        40

-----------------------------------------------------
```

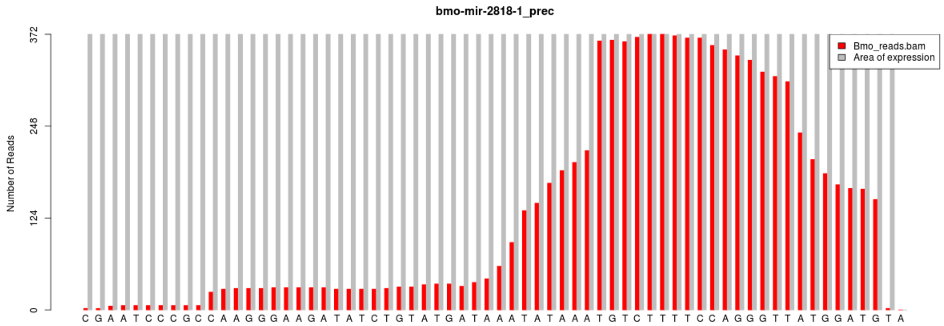

>bmo-mir-2818-2_prec

```
CGAAUCCCGCCAAGGGAAGAUAUCUGUAUGAUAAAUAUAAAUGUCUUUUCCAGGGUUAUGGAUGUCU
.((((((.(((..(..(((((((.(((((.....))))).))))))))..)...)))...)))).)).

 Initial dG =    -20.30

           10         20        30
C  -    C--   AA-|GG       C     GA
 GA AUCC   GCC   G  AAGAUAU UGUAU  \
 CU UAGG   UGG   C  UUCUGUA AUAUA  U
U  G    UAU   GAC^UU       A     AA
      60        50        40

-----------------------------------------------------
```

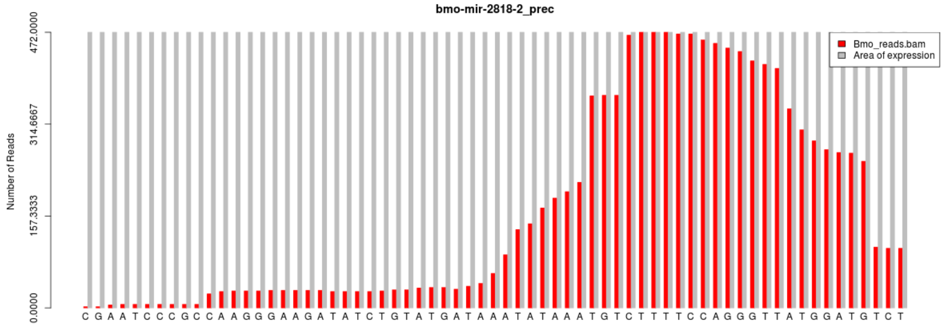

>bmo-mir-2819_prec

```
AUAAAUUGGGUGAGGAUUGAUACUUACGGGUUCCAUGAACGCUUACUUCGCUCCGUCAAUGCCUGCUCUAUCGGUUCCCCAGCGGGC
.....((((...(((((((((....(((((....(((..((.......))....)))..)))))...)))))))))))))).....

 Initial dG =    -20.30

        10        20        30        40
AUAAA|    UGA         CUUA      UCCA   AC--  UUA
     UUGGG   GGAUUGAUA    CGGGU    UGA    GC   \
     GACCC   CUUGGCUAU    GUCCG    ACU    CG   C
CGGGC^    ---         CUC-      UA--   GCCU  CUU
      80            70         60           50

----------------------------------------------------
```

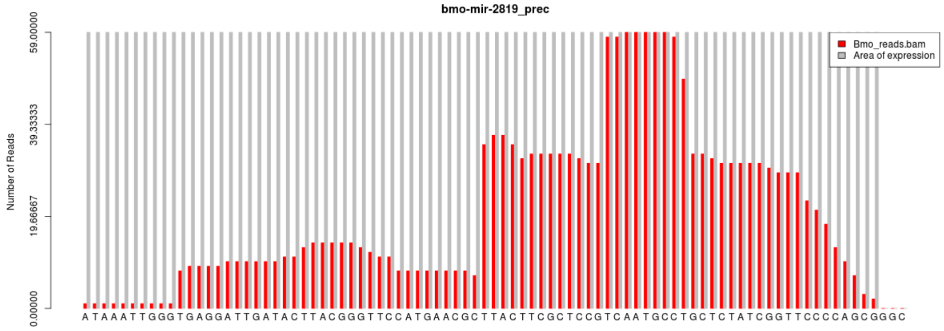

>bmo-mir-2820-1_prec

```
CGUCAAGCCUAUUCAUGAUCCUUUUGCAUCGUGCAUCGUACAUGGCCUUGGCGGCACGCACAGCAAGAUGGAACGUGAAUAAGGCGCUUAA
......((((((((((.(((((((((..(((((..(((.((......)))))))))))....)))))).))).)))))))).)))......

 Initial dG =    -33.40

         10        20           30        40
CGUCAA   -        A   -       AU--|    AU   A  UG
      GCC UAUUCAUG UCC UUUUGC    CGUGC  CGU CA  G
      CGG AUAAGUGC AGG AGAACG    GCACG  GCG GU  C
AAUUCG   A        A   U       ACAC^    --   -  UC
90        80        70        60           50

-----------------------------------------------------
```

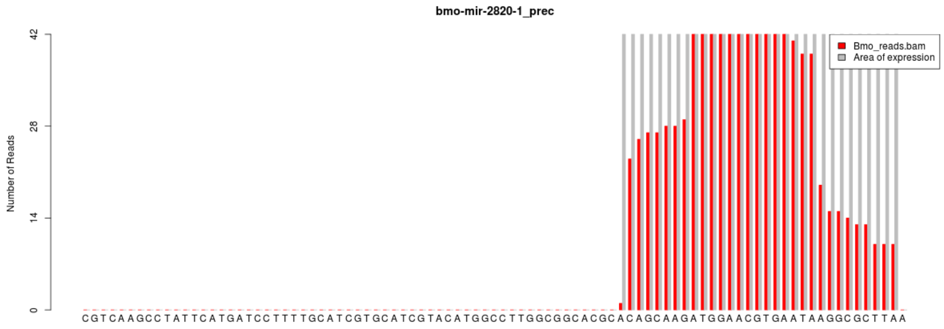

>bmo-mir-2820-2_prec

```
CGUCAAGCCUAUUCAUGAUCCUUUUGCAUCGUGCAUCGUGCAUGGCCUUAGCGGGACGUACAGCAAGAUGGAACGUGAAUAAGGCGCUUUA
......((((((((((.(((((((((...((((.((.(((.........))).)).)))).)))))).))).))))))))).)))......

 Initial dG =    -33.90

         10        20         30        40
CGUCAA   -         A   -|     AUC    A  G   AUGG
      GCC UAUUCAUG UCC UUUUGC   GUGC UC UGC    \
      CGG AUAAGUGC AGG AGAACG   CAUG AG GCG    C
AUUUCG   A         A   U^     A--    C  G   AUUC
90        80        70          60        50

-----------------------------------------------------
```

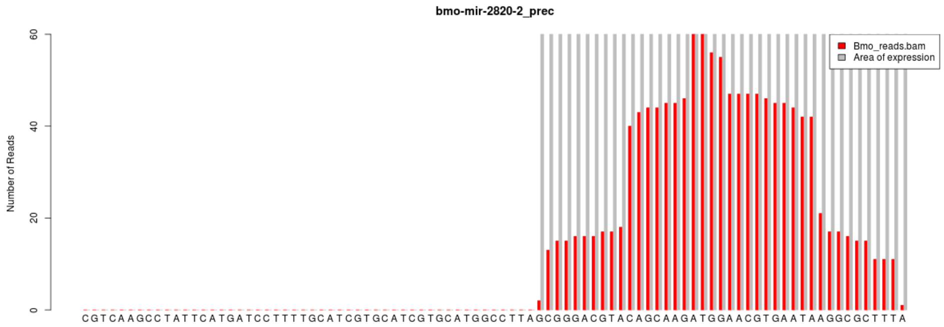

>bmo-mir-2821_prec

```
AGUCUAGUAGAUAUUCCGGCGCUUUAGUAACAGAAAUCGAUAUAAUUACUUUAAUGCGUCACGUAGGGCACCGGUGACCUACAUGAC
.(((..((((.....((((.((((((((.((..........................)).)).)))))).))))....))))..)))

 Initial dG =    -19.10

        10         20          30         40
A|   UA    AUAUU    C       -  A  AGAAAUCGAUAU
 GUC  GUAG     CCGG GCUUUA GU AC             A
 CAG  CAUC     GGCC CGGGAU CA UG             A
-^  UA    CAGU-    A      G  C  CGUAAUUUCAUU
       80          70         60         50

-----------------------------------------------------
```

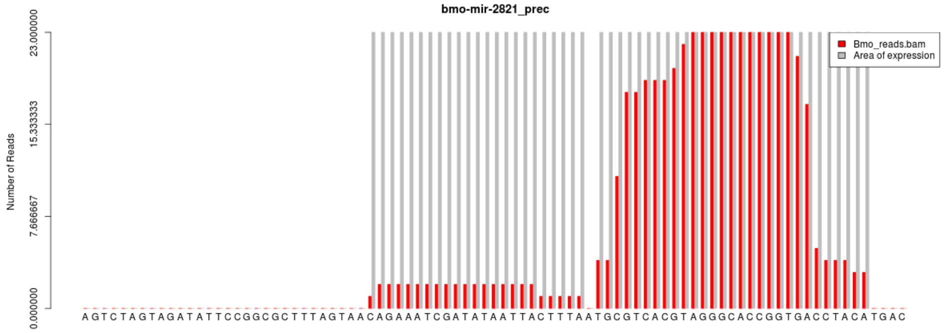

>bmo-mir-2822-1_prec

```
GGUCUUAUGCAAGUUUUGUAAAGUGUCACCUUAUUCUGAAGGGAUAUUUUACUCCGCUUGCAAAUUAUG
.......(((((((...((((((((((.((((......))))))))))))))...))))))).......

 Initial dG =    -24.80

        10        20        30
GGUCUUA|      UUU          A    AU
       UGCAAGU   GUAAAGUGUC CCUU  U
       ACGUUCG   CAUUUUAUAG GGAA  C
GUAUUAA^      CCU          -    GU
        60        50         40

----------------------------------------------------
```

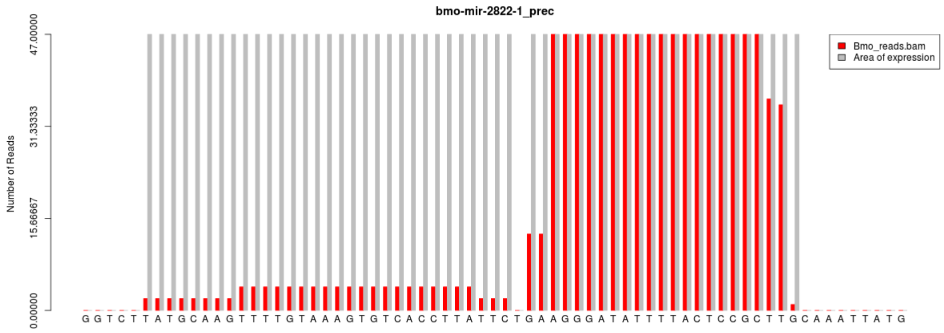

>bmo-mir-2822-2_prec

```
ACGGGUCUUAUGCAAGUUUUGUAAAGUGUCACCUUAUUCUGAAGGGAUAUUUUACUCCGCUUACAGAUCAUA
...(((((...(.((((...((((((((((.((((......))))))))))))))...)))).))))))...

 Initial dG =    -20.90

        10        20        30
ACG|    UAU C    UUU          A    AU
   GGUCU   G AAGU   GUAAAGUGUC CCUU  U
   CUAGA   C UUCG   CAUUUUAUAG GGAA  C
AUA^    --- A    CCU          -    GU
 70          60        50         40

-----------------------------------------------------
```

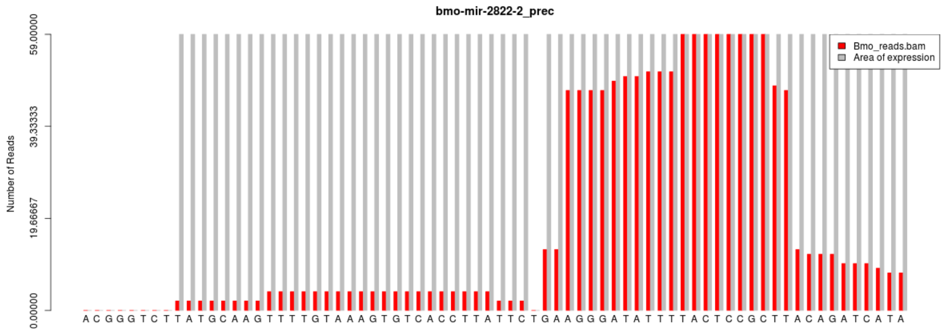

>bmo-mir-2824_prec

CACGAGAGCUGGUACUGUGCCAGAAAUUGGAUCGUCCUGUGAAGCCGAGAUGUCAUGGCCAGCGUGAACUUCAAACCGCCACAGGUAACCACUCUGCGAU
..((((((.((((.(((.((((((..((((.(((.....)))..))))....)).))))))).............(((......))).)))))))).))..

```
 Initial dG =    -27.20

         10          20           30
CA  -    C    .-A   U    -  AA--    A-|  UC
  CG AGAG UGGU   CUG GCCA GA    UUGG  UCG  \
  GC UCUC ACCA   GAC CGGU CU    AGCC  AGU  C
UA  G    -    \ -   -    A  GUAG    GA^  GU
.         90      60         50        40


                      70        80
                CGUGAACUUCAA   GC
                            ACC  C
                            UGG  A
                A-----------   AC


-----------------------------------------------------
```

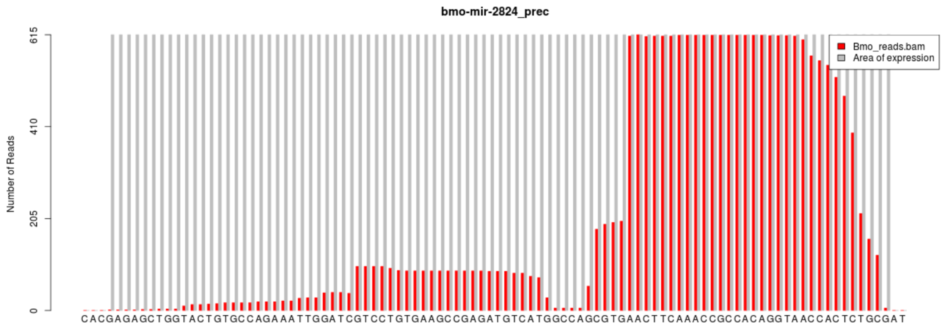

>bmo-mir-2825_prec

```
AAGUGCUGGAGCAGCUUUCAGAUUGGGAUAAUGAGGAUACUGAUGGAGACGAAGGUCCAUCAGGAUCACGAAGAAGCUAUUGCCGUAUUG
.(((((.((...((((((....(((.(((..(((((((.((..((....)).)))))).)))..))).))).))))))....))))))).

 Initial dG =    -24.40

        10         20        30         40
A|    U  AGC-      CAGA   G   AA   -    A  GA  G
 AGUGC GG    AGCUUU    UUG GAU  UGA GGAU CU  UG A
 UUAUG CC    UCGAAG    AGC CUA  ACU CCUG GA  GC G
G^    -  GUUA      A---   A   GG   A    -  A-  A
.         80           70        60           50

-----------------------------------------------------
```

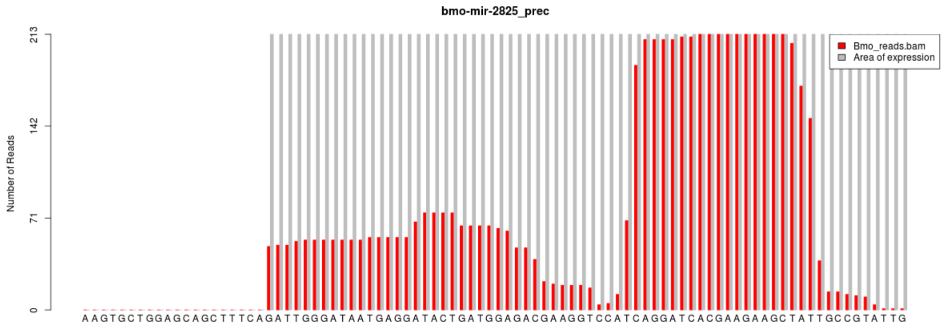

>bmo-mir-2826_prec

```
GAUCGCUGAAAAAAGAUCGAGGAUCCGAUAUUGGGACCGGCCGUUAGUGGGAUCUCUCGUCUGCUCGCCCGAUUGGGGGUCG
((((...((....(((.(((((((((.((..(((......)))...)).))))).))))))).)).)).(((....))))))).

 Initial dG =    -25.60

           10        20         30
-    .-GCU| AAAA   U    -     G  AU-   GA
 GAUC     GA    AGA CGAG GAUCC AU   UGG  C
 CUGG     CU    UCU GCUC CUAGG UG   GCC  C
G    \ ---^ CG--   -    U     G  AUU   GG
 80              60         50        40


         70
       G   G
        CCC A
        GGG U
       -   U

-----------------------------------------------------
```

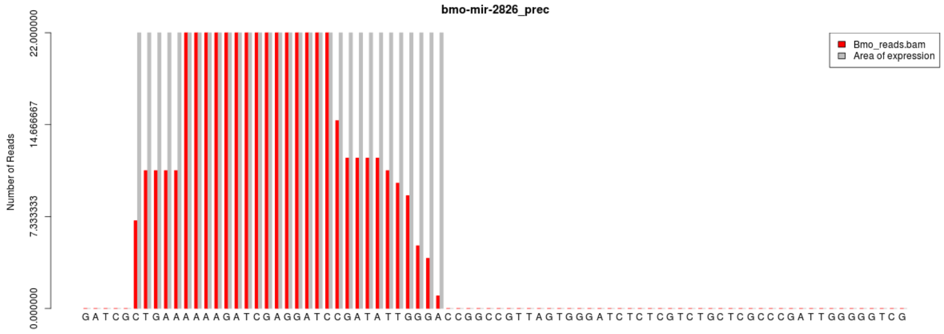

>bmo-mir-2827_prec

```
UUUUAUUACUCAGACUAUCAGUACGUACGCUGCGUGCGAUCCAGCCGAGCCCGUCUGGUGGCCCAUGAAAA
.........(((..(((((((.(((...(((.((.((......))))))).))))))))))....)))...

 Initial dG =    -19.20

        10          20        30        40
UUUUAUUAC   GA--|      U   UAC   G  U  GA
         UCA    CUAUCAG ACG   GCU CG GC  U
         AGU    GGUGGUC UGC   CGA GC CG  C
AAA------   ACCC^      -   C--   -  -  AC
70              60          50

-----------------------------------------------------
```

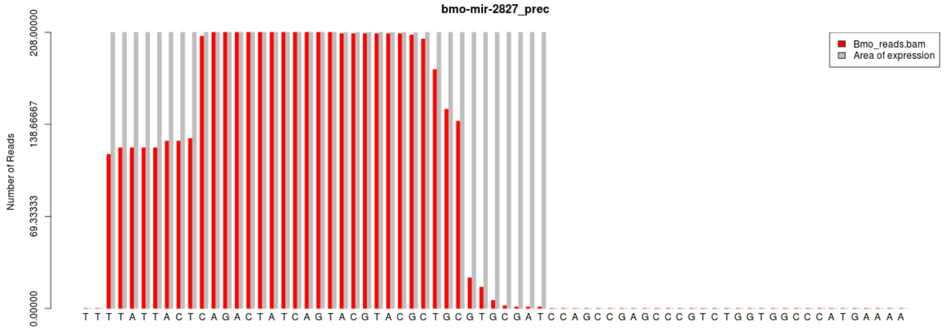

>bmo-mir-2828_prec

```
UUCACAACUGAUAUUCGAUAUGUGAACGGUUCCGAGAACGUAGUUGCGGACUUCUUAUCGCGCAUAGAGACUGUAACGUGUUCCACGAACAUCGACUA
..........(((.((..((((((..((((...(((..(((....)))..)))...))))))))))..)).)))..(((((((...))))).))....

 Initial dG =    -20.10

          10        20        30        40
.-UUCACAACUG|  U  GA      AA    UCC   AA   A
            AUA UC  UAUGUG  CGGU   GAG  CGU G
            UGU AG  AUACGC  GCUA   UUC  GCG U
\ ----------^  C  AG      --    UUC   AG   U
               70          60         50


         80
  AA--  -     C
      CG UGUUC \
      GC ACAAG A
  AUCA  U     C
         90

-----------------------------------------------------
```

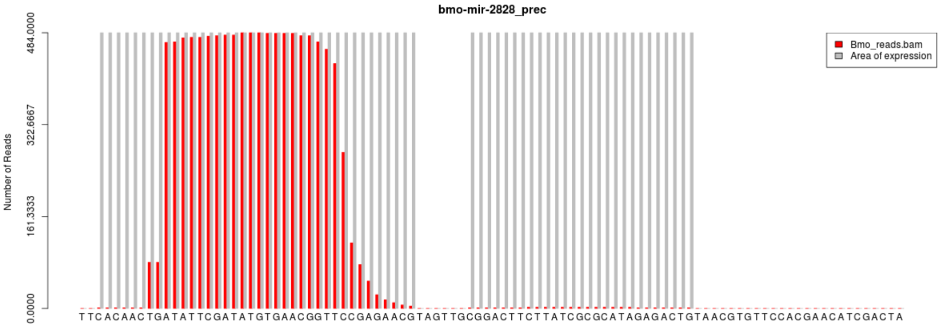

>bmo-mir-2830-1_prec

```
GAAUGGAGGAGUGCGGCCUGGGGCAGCCGCUGAACCCUUGGUUCAGCGACUAUCUCAGGGCGCGCUCUUAUCGUGU
..((((((((((((.((((((..((.(((((((((...))))))))).))..)))))).))))))))).))))..

 Initial dG =    -47.80

         10        20        30
GA    -|        G      GC  C         C
  AUGG AGGAGUGCG CCUGGG  AG CGCUGAACC \
  UGCU UUCUCGCGC GGACUC  UC GCGACUUGG U
UG    A^        G      UA  A         U
     70        60        50        40

-----------------------------------------------------
```

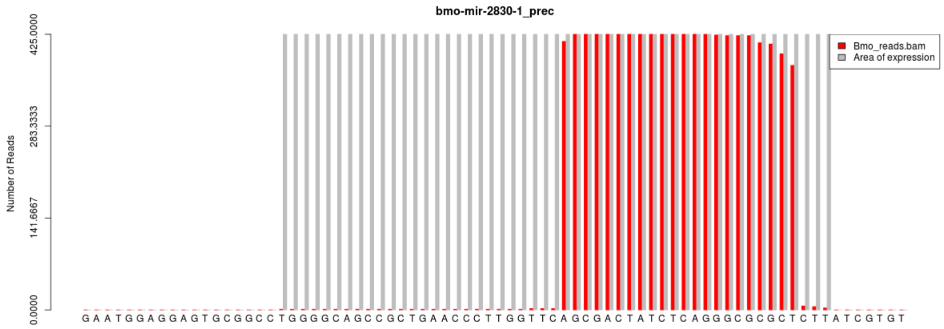

>bmo-mir-2830-2_prec

```
GAAUGGAGGAGUGCGGCCUGGGGCAGCCGCUGAACCCUUGGUUCAGCGACUAUCUCAGGGCGCGCUCUUACCGUGU
..((((((((((((.((((((..((.(((((((((...))))))))).))..)))))).))))))))).))))..

 Initial dG =    -50.70

         10        20        30
GA    -|        G      GC  C         C
  AUGG AGGAGUGCG CCUGGG  AG CGCUGAACC \
  UGCC UUCUCGCGC GGACUC  UC GCGACUUGG U
UG    A^        G      UA  A         U
      70        60        50        40

-----------------------------------------------------
```

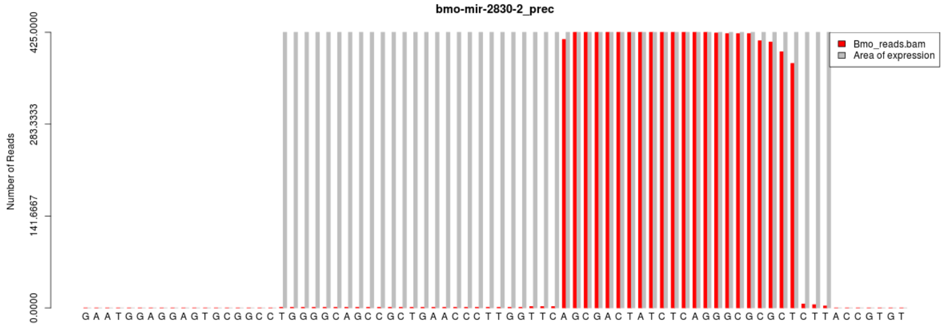

>bmo-mir-2831-1_prec

```
GCACAGAUAGUCAAGCAAAUGCUUUAUUUGUAUGUUUCCUACAUACAAACAAAGCAUUUGUUUGACCAUCUGUGA
.(((((((.((((((((((((((((.(((((((((.....))))))))).)))))))))))))))).))))))).

 Initial dG =    -43.60

        10        20        30
G|      A                 A         UU
 CACAGAU GUCAAGCAAAUGCUUU UUUGUAUGU  \
 GUGUCUA CAGUUUGUUUACGAAA AAACAUACA  C
A^      C                 C         UC
    70        60        50        40

-----------------------------------------------------
```

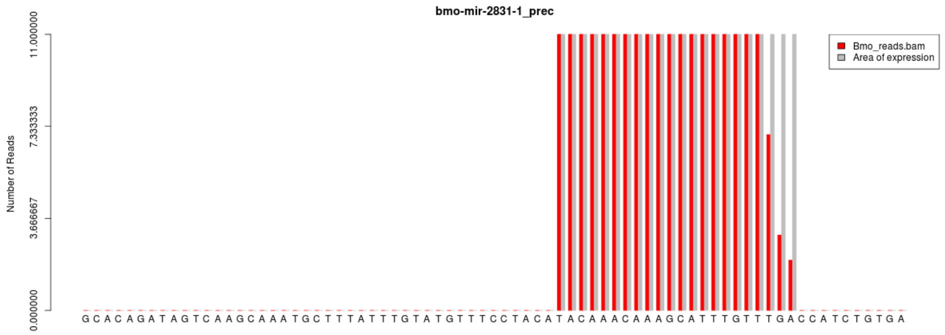

>bmo-mir-2831-2_prec

```
GCACAGAUGGUCAAGCGAAUGUUUUGUUUGUAUGUUCUUACAUACAAACAAAGCAUUUGUUUGACCAUCGGUGA
.(((.((((((((((((((((((((((((((((((....)))))))))))))))))))))))))))))).))).

 Initial dG =    -48.80

        10         20         30
G|   A                            U
 CAC GAUGGUCAAGCGAAUGUUUUGUUUGUAUGU C
 GUG CUACCAGUUUGUUUACGAAACAAACAUACA U
A^   G                            U
   70         60         50         40

-----------------------------------------------------
```

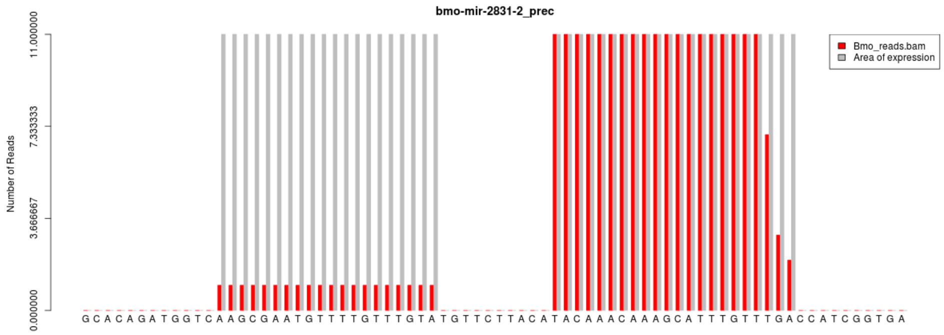

>bmo-mir-2832_prec

```
UUUAUGAAGGUUGGAUAGUGCGUUUUGGAUGUCCCGUUAGGUUAACUAGUGACCAAGGUACCCAGUUCGAGAGUCAUCUCUUCAACUCACUUCUAAAG
((((.(((((((((.((((..((((((((((.((....(((((.....)))))..)).))...)))))))))..)).)).))))))...)))))))).

 Initial dG =    -21.10

            10          20         30           40
-    U    ---      U  -  CG        ---| C  GUUA      AC
 UUUA GAAG   GUUGGA AG UG  UUUUGGAU   GU CC    GGUUA  \
 AAAU CUUC   CAACUU UC AC  AGAGCUUG   CA GG    CCAGU  U
G    -    ACU      C  U  UG        ACC^ U  AA--      GA
        90         80         70         60           50

-----------------------------------------------------
```

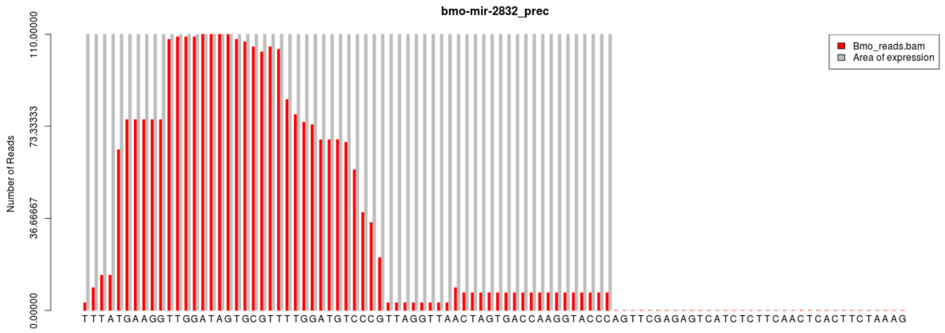

>bmo-mir-2833-1_prec

AAGAAUUCGCGGUAAACAGUGGAAACUGGUCCUCAUAUGUUGAGAGAUUGGAAAUGUACUUUUUGGUGAAUAAAG

```
....((((((..((((.((((....(..(((((((.....)))).)))..).....)))).))))))))))....

 Initial dG =    -17.20

        10         20          30
AAGA|     GG     C     GAAA- UG    -     UA
    AUUCGC  UAAA AGUG      C  GUC CUCA  \
    UAAGUG  GUUU UCAU      G  UAG GAGU  U
GAAA^     --     U     GUAAA GU    A     UG
    70           60         50         40
```

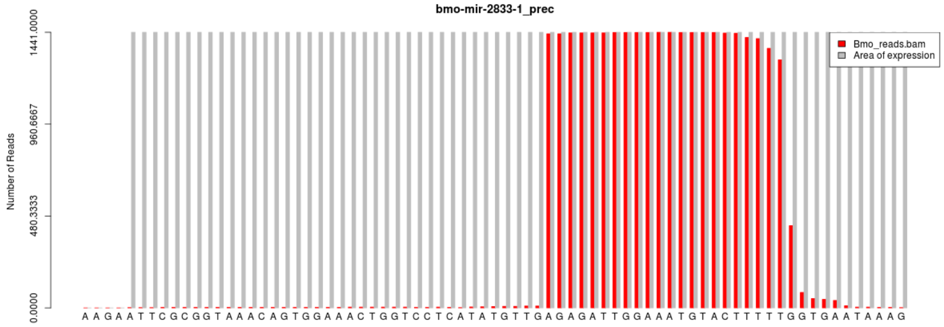

>bmo-mir-2833-2_prec

```
AAGAAUUCGCGGUAAACAGUGGAAACUGGUCCUCAUAUGUUGAGAGAUUGGAAAUGUACUUUUUGGUGAAUAAAU
....((((((..((((.((((....(..(((((((.....)))).)))..).....)))).))))))))))....

 Initial dG =    -17.20

        10        20         30
AAGA|     GG    C    GAAA- UG   -    UA
    AUUCGC  UAAA AGUG     C  GUC CUCA  \
    UAAGUG  GUUU UCAU     G  UAG GAGU  U
UAAA^     --    U    GUAAA GU   A    UG
    70          60        50        40
```

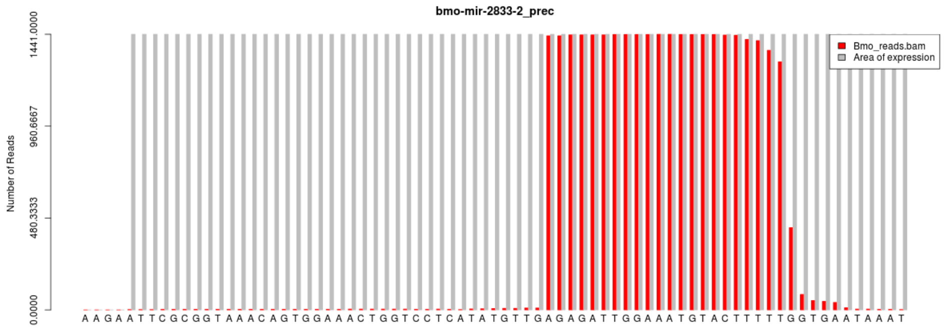

>bmo-mir-2833b_prec

```
AGAAUUCGCGGUAAACAGUGGAAACUGGUCCUCAUAUGUUGAGAGAUUGGAAAUGUACUUUUUAGUGAAUAAA
...((((((..((((.((((....(..(((((((.....)))).)))..).....)))).))))))))))...

 Initial dG =    -17.50

        10        20         30
AGA|     GG    C    GAAA- UG   -    UA
   AUUCGC  UAAA AGUG     C  GUC CUCA  \
   UAAGUG  AUUU UCAU     G  UAG GAGU  U
AAA^     --    U    GUAAA GU   A    UG
  70         60        50        40

----------------------------------------------------
```

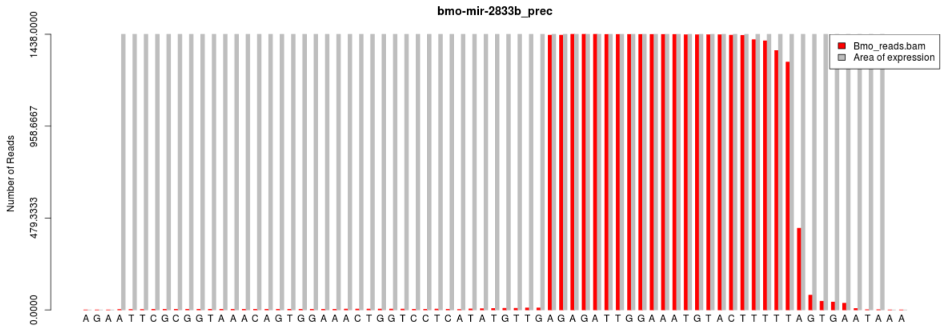

>bmo-mir-2834-1_prec

```
UCCAGUGGAUUAAUAUCGGGACUAGACGACGGCCAGUCUUCAGCUCUGAUGAAGUCCGAAACG
.....((((((..((((((..((.((.(((.....))).))))..))))))).)))))).....

 Initial dG =    -18.10

        10        20        30
UCCAG|     AA      GA  A  C   GG
     UGGAUU  UAUCGG  CU GA GAC  \
     GCCUGA  GUAGUC  GA CU CUG  C
GCAAA^     A-      UC  -  U   AC
  60        50        40

-----------------------------------------------------
```

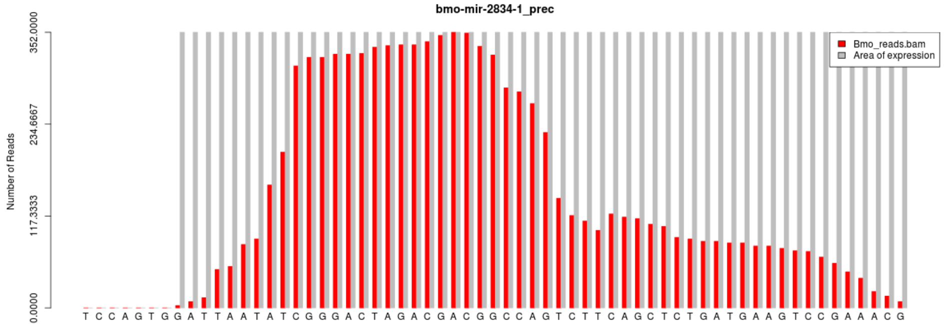

>bmo-mir-2834-2_prec

```
UCCAGCGGAUUAAUAUCGGGACUAGACGACGGCCAGUCUUCAGCUCUGAUGAAGUCCGAAACG
.....((((((..((((((..((.((.(((.....))).)))).)))))).)))))).....

 Initial dG =    -19.90

        10        20        30
UCCAG|     AA      GA  A  C   GG
     CGGAUU  UAUCGG  CU GA GAC  \
     GCCUGA  GUAGUC  GA CU CUG  C
GCAAA^     A-      UC  -  U   AC
  60        50        40

-----------------------------------------------------
```

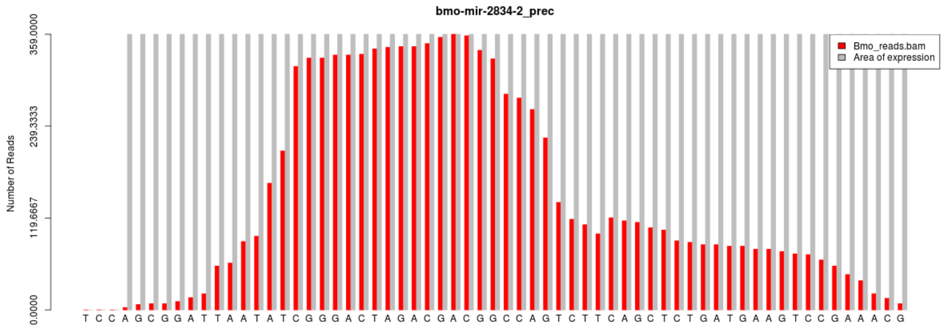

>bmo-mir-2835_prec

```
AGUAUUGUCUCCCAUAACAUUCAUGAAAGUGGGCAUCCCGGACCCUGGUUUUUCACACAUUAUGAGCUUUAGGAGACAGGUAUA
.((((((((((..(((..(((((((..(((((....((((...))))...)))))...))))))).)))))))))))).))).

 Initial dG =    -22.10

         10        20         30        40
A   -|       CA   CA       AA-     CAUC    A
 GUA UUGUCUCC  UAA  UUCAUGA   GUGGG    CCGG \
 UAU GACAGAGG  AUU  GAGUAUU   CACUU    GGUC C
A   G^       --   UC       ACA     UUU-    C
   80          70        60        50

-----------------------------------------------------
```

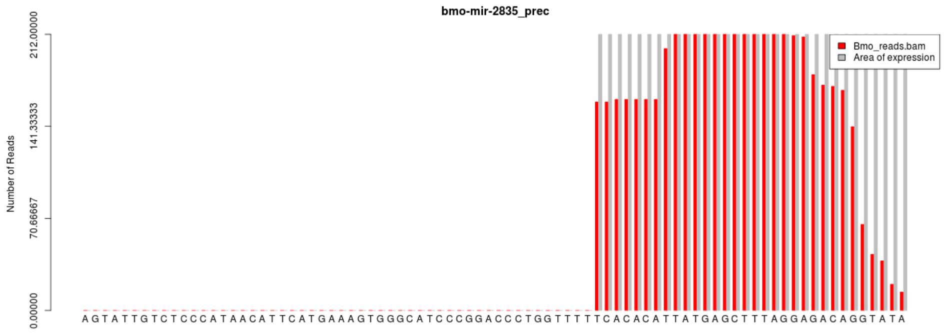

>bmo-mir-2836_prec

```
UGAGAUUUGACAUCCCGCUGUAGACGACGCGCCCAUGUAAGAUUGUGACUUGGCACUGUAUGUACCUAGGGUUGAUGUCGUCUCG
.(((((..((((((..(((.(((((((((.(((((((......))))....)))..))).))))..))).))).))))))))))).

 Initial dG =    -22.70

        10         20            30              40
U     UU       CC    G    --    -    C-    ----|    UA
 GAGAU  GACAUC  GCU UAG  ACG ACG  GCC     CAUG  A
 CUCUG  CUGUAG  UGG AUC  UGU UGU  CGG     GUGU  G
G     --       U-    G    CA    A    CA    UUCA^    UA
    80            70         60         50

-----------------------------------------------------
```

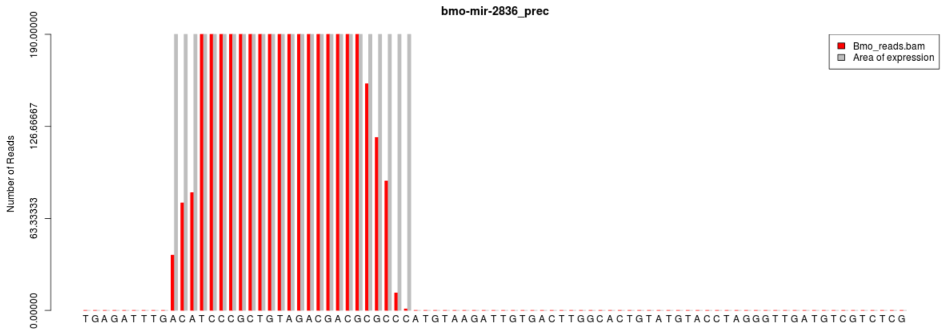

>bmo-mir-2837_prec

```
CAAAAUGAAUAGAUGGUACUACGUGGGAAUGUGGCGUUCGCUCCUGGCCUUUUCAUUUCCAUUAUUAUUAUUAUUAUUUUU
.(((((((...((((((.((.(((((((((.(((...........)))....))))))))))).))))))).))))))).

 Initial dG =    -17.00

        10         20         30            40
C|      AUA        C  C          U---   GUUCG
 AAAAUGA   GAUGGUA UA GUGGGAAUG    GGC      \
 UUUUAUU   UUAUUAU AU UACCUUUAC    CCG      C
U^      A--        U  -          UUUU   GUCCU
80          70         60         50

-----------------------------------------------------
```

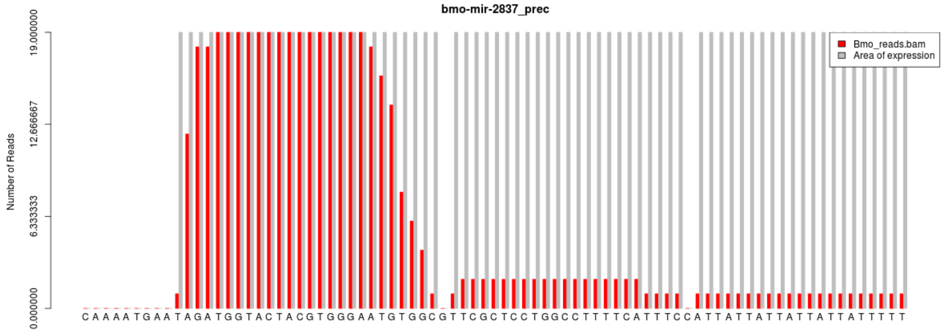

>bmo-mir-2838_prec

```
AAAUGAUUUAAAUUCAGCAAACUCACGGGAUAAACUCGCUAUGGUUUUAUAUGGGAGAUCUGUGGUAGCUGCAUUCUUCAAAUCUUCG
....(((((.(((.((((..((.((((((.....(((.(((((......)))))))).)))))))).)))).)))....)))))....

 Initial dG =    -18.10

        10           20         30         40
AAAU     A---|  U    AA  U       AUAAA   G     GU
    GAUUU    AAU CAGC  AC CACGGG     CUC CUAUG  U
    CUAAA    UUA GUCG  UG GUGUCU     GAG GGUAU  U
GCUU     CUUC^  C    A-  -       A----   -     AU
       80        70          60              50

-----------------------------------------------------
```

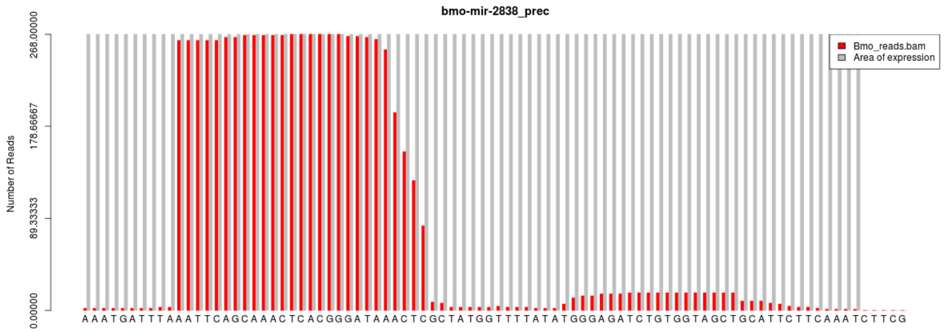

>bmo-mir-2839_prec

```
AAUCAUACCGUCAAACAGAGCGCCAGCGCUAUGAGCGCUAUAGCGCUGGCGCACUGUUUGACGGUAUGUUACCGU
...((((((((((((((.((((((((((((((.....))))))))))))))).)))))))))))))))).......

 Initial dG =    -53.90

            10         20         30
AAU----|              A              AG
       CAUACCGUCAAACAG GCGCCAGCGCUAUG  \
       GUAUGGCAGUUUGUC CGCGGUCGCGAUAU  C
UGCCAUU^              A              CG
    70         60         50         40

-----------------------------------------------------
```

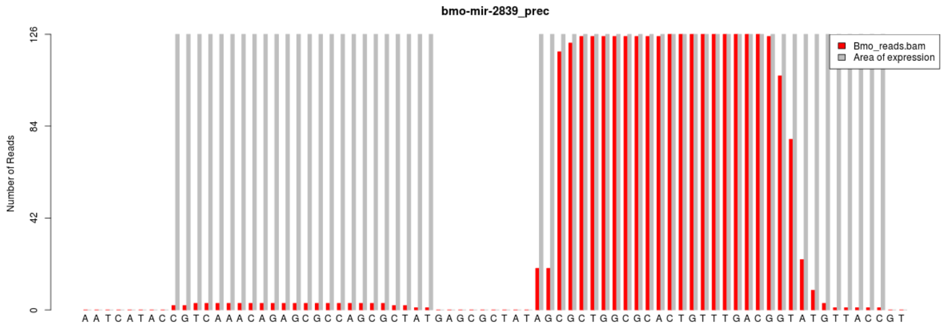

>bmo-mir-2840_prec

```
AGUCUCCAGGUAGGAACUGGAAGAAGAGGAGGAUUUUGGUCUUCCUCUUCUCCCUCUUCUUCUCUGGAAGCG
.(..(((((..(((((..((.((((((((((((((....))))))))))))))).))..)))))..)))))..).

 Initial dG =    -39.40

        10        20        30
A|UC     GU     CU  A             U
 G  UCCAG  AGGAA  GG AGAAGAGGAGGAU U
 C  AGGUC  UUCUU  CC UCUUCUCCUUCUG U
G^GA     UC     CU  C             G
 70        60        50        40

-----------------------------------------------------
```

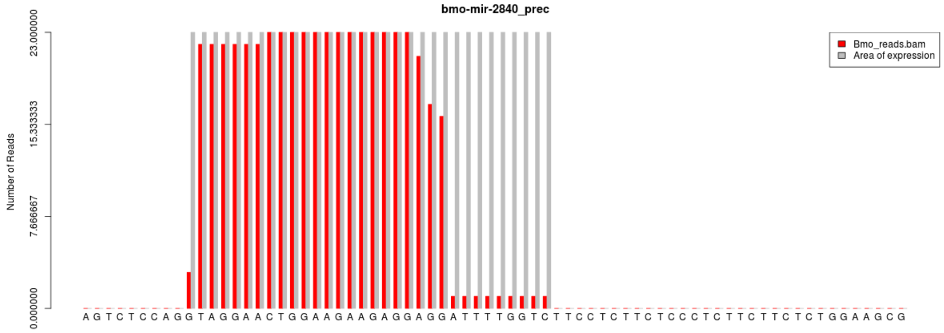

>bmo-mir-2841_prec

```
UGCGGCCUGUUAUGUAUUUUAGCCGUGUUACUAGGAAGGAAGAGAGUGUGUAUCAUAGUUAAGAGCUCAAAACCUGGCGGUC
.((.(((.(((.((..(((((((..((.(((..................))).))..)))))))...)).))).)))..))).)).

 Initial dG =    -18.60

         10         20         30         40
U  G   U-   A  UA-|      CG  U   UAGGAAGG
 GC GCC  GUU UG   UUUUAGC  UG UAC        A
 UG CGG  CAA AC   AGAAUUG  AC AUG        A
C  G   UC   A  UCG^      AU  U   UGUGAGAG
 80        70        60        50

-----------------------------------------------------
```

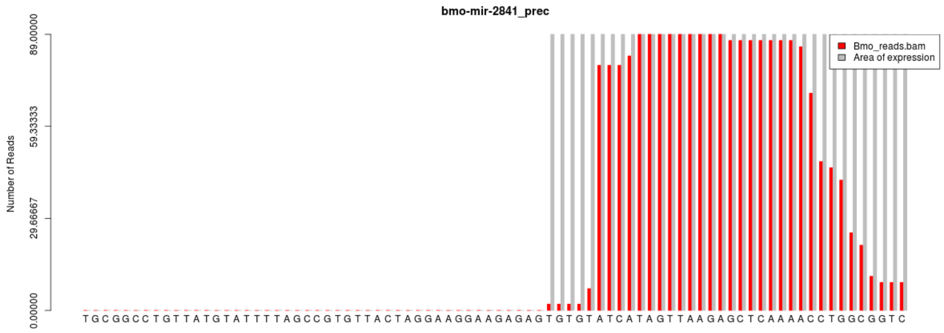

>bmo-mir-2842_prec

```
UCGACUUUCUUGAAGAUCCUCGUACUGGUGGCGCCAUCCCGUCGCGGAGUGUGGAGCCUUCUUCUGUCGUCGAG
(((((......((((.(((.((..(((.(((((......))))))))..)).)))..))))).......))))).

 Initial dG =    -23.10

         10           20         30
-     UUUCUU-    A-|  U  UA    G      CC
 UCGAC       GAAG  UCC CG  CUG UGGCG  A
 AGCUG       CUUC  AGG GU  GGC GCUGC  U
G     CUGUCUU    CG^  U  GA    -      CC
   70        60         50          40

-----------------------------------------------------
```

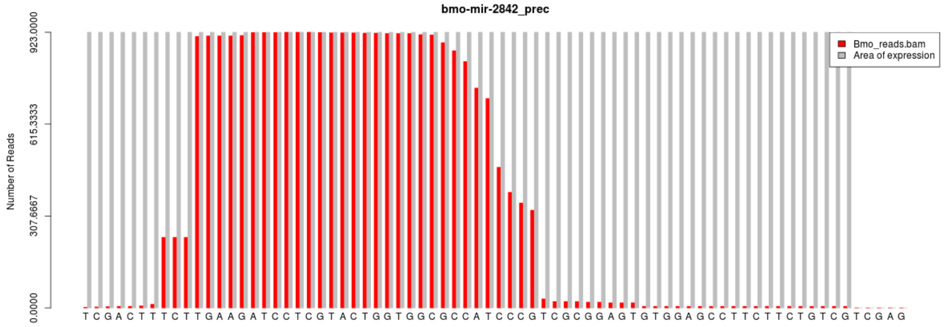

>bmo-mir-2843-1_prec

```
GGCUUUGGAGUCUAAGGAAAUUAGGUCGGAUACAUACGAAUUAGUGUGUUUGCGACGCUGUCUUCGUGAUCAAGCCU
(((((....(((....(((..(((((((...((((((......))))))...)))).))).)))..)))..))).))))).

 Initial dG =    -22.80

         10        20         30        40
-|    UGGA   UAAG   AU   -    GAU       GA
 GGCUU    GUC    GAA  UAG GUCG   ACAUAC  A
 CCGAA    UAG    CUU  GUC CAGC   UGUGUG  U
U^    C---   UG--   CU   G    GUU       AU
         70           60        50

-----------------------------------------------------
```

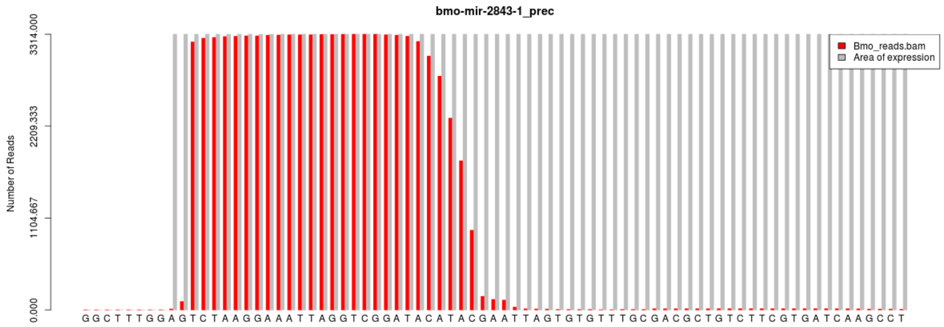

>bmo-mir-2843-2_prec

GGCUUUGGAGUCUAAGGAAAUUAGGUCGGAUACAUACGAAUUAGUGCGUAUGCGUGUCUGUCUUCGUGAUCAAGCCUGACCCCUUAAUCUAUCGGAAA

...(((((((..(((((...((((((..(((...((((((.(((((((....)))).)))..)))))))))..))))))..)))))..)).)))))..

```
 Initial dG =    -25.90

         10        20        30        40          50
GGC     -| UC     AAA      CG   ACA      U-   -    U
   UUUGG AG  UAAGG   UUAGGU  GAU   UACGAA  UAG UGCG A
   AGGCU UC  AUUCC   AGUCCG  CUA   GUGCUU  GUC GUGC U
AA-     A^ UA     CC-      AA   ---      CU   U    G
        90        80         70           60

-----------------------------------------------------
```

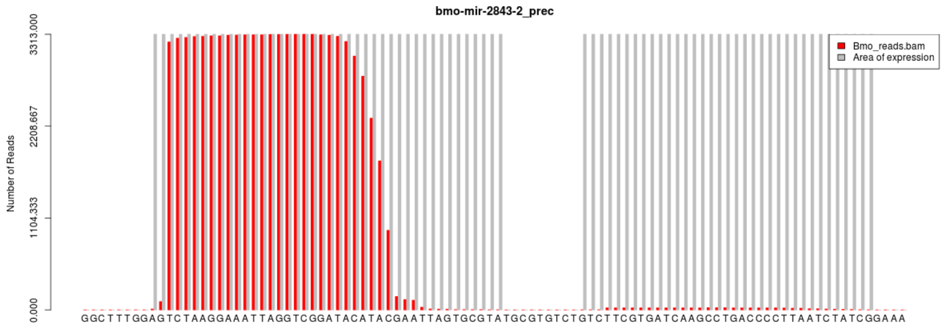

>bmo-mir-2845_prec

UGCCAAGAAGUAAUCCGGCUUCUGGGAUGGAUCGUCCCACUCUACAACAUGACCGUUGCCAGCUGCUGUGCGUAAGUGUUGGUU

.....((((((......))))))((((((...)))))))......((((((......((((((...))).)))...))))))...

```
 Initial dG =    -22.60

          10
.-UGCCA|     AA
       AGAAGU  U
       UCUUCG  C
\ -----^     GC
         20


         30
          G
  --GGGAUG \
    CCCUGC A
  \       U
```

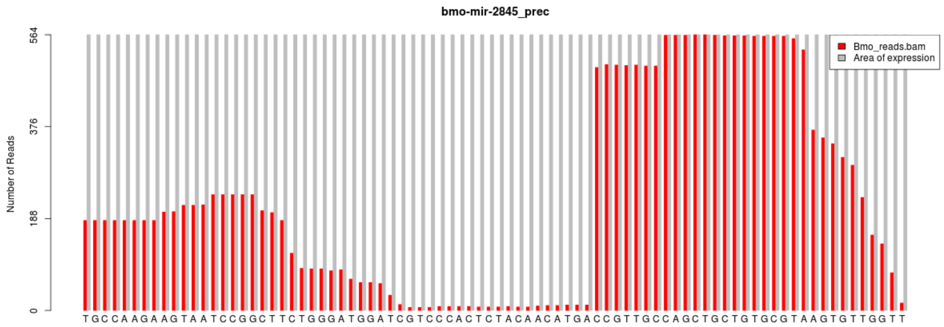

>bmo-mir-2846_prec

```
AAUAUAUCGUGGGAUUACUAAUGGCGUAGUGCGAACGCGUGAACCGAGACGUGGAAACGAGUCGUAUGGUGCGAAGAAGUCCCGGCGGAGACAG
......((((((((((.((....(((..((((((...(((...(((.....)))..)))..))))))..)))..)).))))))).)))......

 Initial dG =    -27.60

         10         20         30         40
AAUAUA   -|      A  AAUG   UA      ACG   GAA   AG
      UCG UGGGAUU CU    GCG  GUGCGA   CGU   CCG  \
      GGC GCCCUGA GA    CGU  UAUGCU   GCA   GGU  A
GACAGA   G^      A  AG--   GG      GA-   AA-   GC
   90        80          70         60          50

-----------------------------------------------------
```

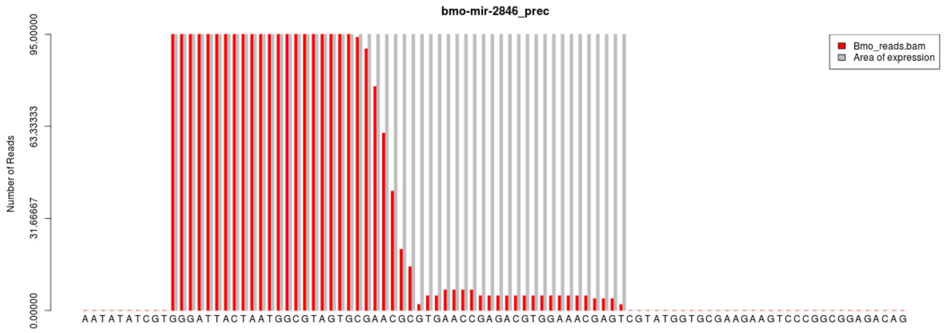

>bmo-mir-2847_prec

ACCCAGCUUCCAUCGGCUUGGAUAAUAUCCUGUGCGUAGAGUGUAGUUUAUUGUGUUUGUCAGUGCGGUAUAAUAUGCUGGG
.((((((.((((......))))...(((.(((..(...(((..(((....)))..)))....)..))).))).....))))))

```
 Initial dG =    -24.30

          10
A      .-U|   UC
 CCCAGC   UCCA  G
 GGGUCG   AGGU  G
-      \ -^   UC
  80       20



                30         40
         UAA-   C   UG GUA-   UG   U
             UAU CUG  C    GAG  UAG U
             AUA GGC  G    UUU  GUU U
         UAUA   U   GU ACUG   GU   A
              70        60        50
```

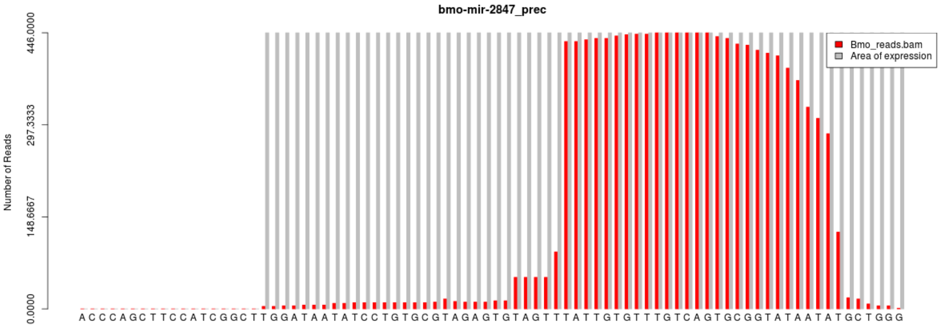

>bmo-mir-2848_prec

```
UGCCGCUACCAACCUUGAGAUAUGAGUAAAUGGCUGCCCUACCCUUCAAACCGGAACGCAUUACUGCUUCGCGGCAGAAAUAGGCAAAGUGGUGGUAC
((((((((....(((.......)))........((((.((...(((...((((((.(((....)))))).)))..))).))))))..)))))))))).
```

```
 Initial dG =    -24.20

           10
-         .-CAAC|  GAG
 UGCCGCUAC      CUU   \
 AUGGUGGUG      GAG   A
C         \ ----^  UAU
       90



              30        40        50         60
            UAAAUGGC    C  CCC   AAA   -   C   U
                    UGCC UA   UUC   CCG GAA GCA U
                    ACGG AU   AAG   GGC CUU CGU A
            AA------    -  A--   AC-   G   -   C
                          80           70

-----------------------------------------------------
```

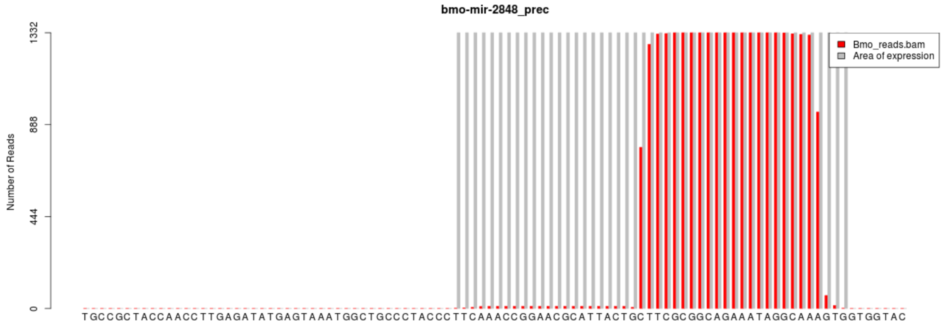

>bmo-mir-2849-1_prec

```
GUGGACGUUUAACUUUACGAUUAUGGACUCAGUUCCAAAAGAUAACUGGUGUUUUAUGUGGGACGAAACCGUGGCGGGUUGAGGUGGAAAUGAAAUG
.....(((((.((((((((.((((((..((.((((((......(((....))).....))))))))..))))))))...))))))..))))).....

 Initial dG =    -22.70

        10            20        30        40
GUGGA     A-       ---| A      AC  A      AAAGAU   U
     CGUUU  ACUUUA   CG UUAUGG  UC GUUCCA      AAC G
     GUAAA  UGGAGU   GC GGUGCC  AG CAGGGU      UUG G
GUAAA     GG       UGG^ -      AA  -      GUAUU-   U
      90        80         70         60         50

----------------------------------------------------
```

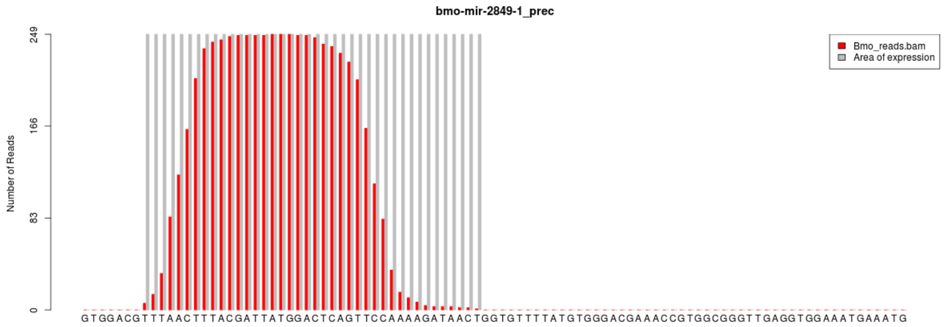

>bmo-mir-2849-2_prec

```
GUGGACGUUUAACUUUACGAUUAUGGACUCAGUUCCAAAAGAUAGCUGGUGUCUUAUGUGGGACGAAACCGUGGCGGGUUGAGGUGGAAAUGAAAUG
.....(((((.((((((((.((((((..((.((((((.((((((.....))))))...))))))))..))))))))...))))))..))))).....

 Initial dG =    -24.20

        10             20         30           40
GUGGA     A-       ---| A      AC  A      A--      GC
     CGUUU  ACUUUA   CG UUAUGG  UC GUUCCA   AAGAUA  \
     GUAAA  UGGAGU   GC GGUGCC  AG CAGGGU   UUCUGU  U
GUAAA     GG       UGG^ -      AA  -      GUA      GG
      90         80          70          60         50

-----------------------------------------------------
```

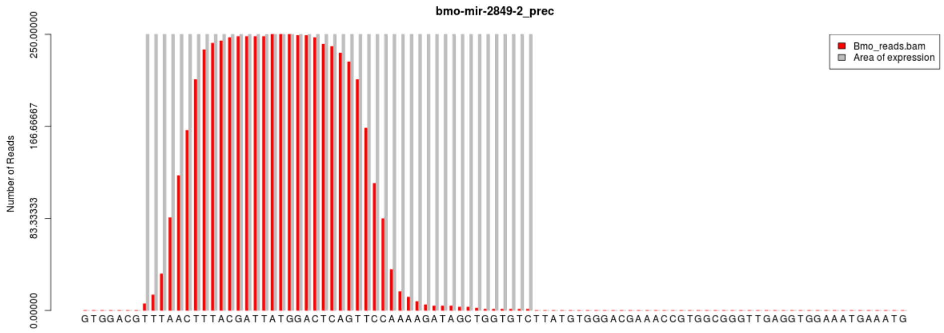

>bmo-mir-2849-3_prec

```
GUGGACGUUUAACUUUACGAUUAUGGACUCAGUUCCAAAAGAUAACUGAUGUUUUAUGUGGGACGAAACCAUGGCGGGUUGAGGUGGAAAUGAAAUG
.....((((((.((((((((.((((((..((.((((((......(((....))).....))))))))..))))))))...))))))..))))).....

 Initial dG =    -23.10

        10            20        30        40
GUGGA     A-       ---| A      AC  A      AAAGAU   U
     CGUUU  ACUUUA   CG UUAUGG  UC GUUCCA      AAC G
     GUAAA  UGGAGU   GC GGUACC  AG CAGGGU      UUG A
GUAAA     GG       UGG^ -      AA  -      GUAUU-   U
      90        80         70         60         50

-----------------------------------------------------
```

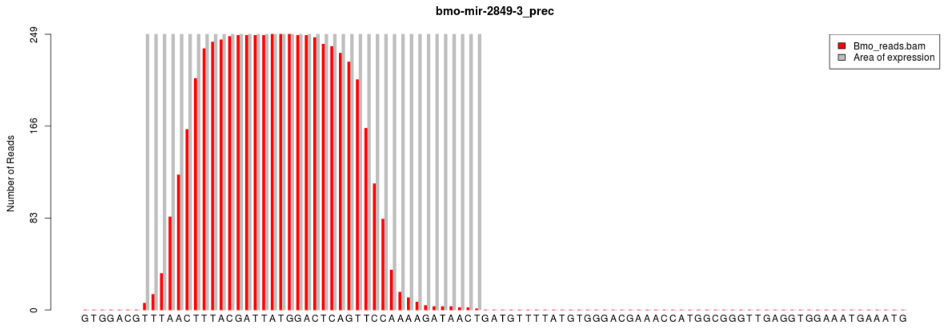

>bmo-mir-2851-1_prec

UGUGAUAAUCUCUGUACCACAAUCAUCAUGCACCCAGUGAUAGAAUGUUACACAUAUCAACUGAGAGUGUGAUGACUGUAUUAGAGAGAAAUAGAAGAG

........(((((.((..(((.(((((((((...((((((((............))))).)))...))))))))).)))..)).)))))..........

```
 Initial dG =    -27.00

          10        20        30         40
UGUGAUAA--     G  CC   A         ACC   -|    GAAUG
          UCUCU UA  ACA UCAUCAUGC   CAG UGAUA     U
          AGAGA AU  UGU AGUAGUGUG   GUC ACUAU     U
GAGAAGAUAA     G  UA   C         AGA   A^    ACACA
        90        80        70        60        50
```

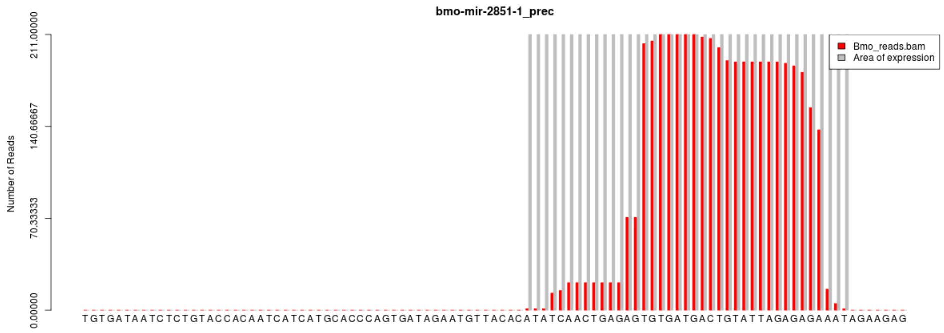

>bmo-mir-2851-2_prec

```
UGUGGUGAUCUCUCUAUCACAAUCAUCAUGCACCCAGUGAUAUAAUGUUACACAUAUCAACUGAGAGUGUGAUGACUGUAUUAGAGAGAAAUAGAAGAG
........((((((((..(((.(((((((((...(((((((((..........)))))).)))...))))))))).)))..))))))))..........

 Initial dG =    -32.40

          10        20        30         40
UGUGGUGA--        UC   A         ACC   -|     AAUG
          UCUCUCUA  ACA UCAUCAUGC   CAG UGAUAU    U
          AGAGAGAU  UGU AGUAGUGUG   GUC ACUAUA    U
GAGAAGAUAA        UA   C         AGA   A^     CACA
        90        80        70        60        50

-----------------------------------------------------
```

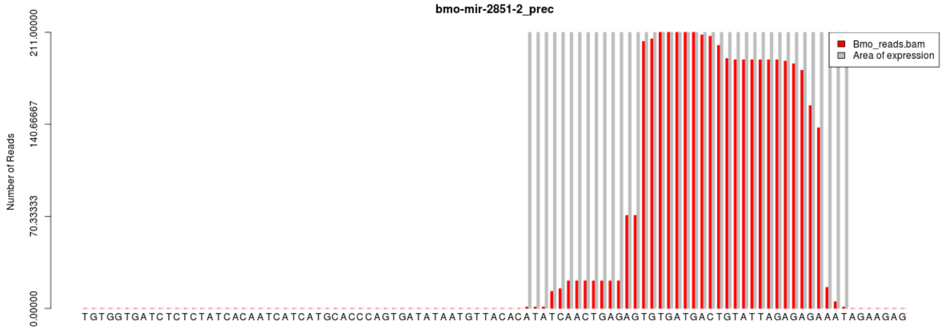

>bmo-mir-2852_prec

```
AGCAUAGAACCAGAUUUCGGAUUUUUUGUAUAAAUUGCUGUAUGAGCGUGAACUACAACAGAUGAAUUUUUUGAGAACCGUCUAUGCUA
((((((((......((((((((((.(((((.....((((.....)))).....))))).)))).......)))))))....)))))))).

 Initial dG =    -18.30

         10        20                30        40
-        ACCAGA       -------|  U     UAAAU    GU
 AGCAUAGA      UUUCGGA       UUU UUGUA     UGCU  \
 UCGUAUCU      AGAGUUU       AGA AACAU     GCGA  A
A        GCCA--       UUUAAGU^  C     CAAGU    GU
        80          70        60        50

-----------------------------------------------------
```

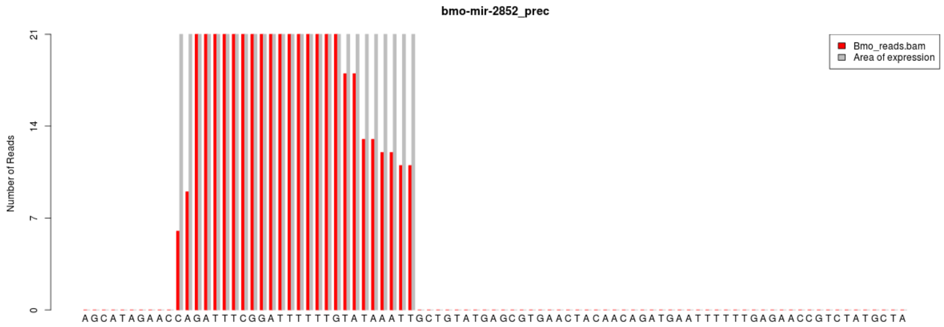

>bmo-mir-2854_prec

AAGCCCUUUUUCUUUCAAAACUGCAGGACGGCCUUUAUACGUGGCCUUUUCCAGUUUACGCAGCUAAAUGAAGUGGUUUC

((((((((..((....((((((.((((.((((.........)))))))).))))))....))......)))).))))).

```
 Initial dG =    -18.20

          10            20        30
-     -    UU----| UUCA      C    C    UUUA
 AAGCC CUUU      CU    AAACUG AGGA GGCC    \
 UUUGG GAAG      GA    UUUGAC UUUU CCGG    U
C     U    UAAAUC^ CGCA      C    -    UGCA
.        70        60        50         40
```

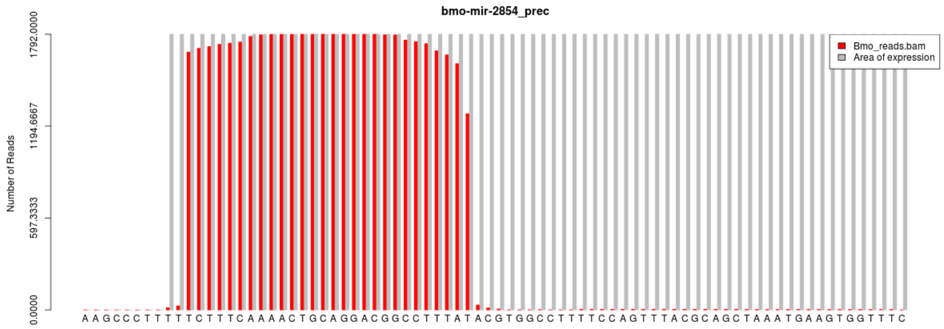

>bmo-mir-2855_prec

```
UGUUGUGUGACGGGUAACUUUGCAUCGCUGGCAGUGCAAGCAAUGCCUCGACCAAUCUCUGCAGUCACGACG
....(((((((..(((...(((..(((..((((.((....)).)))).))).)))....))).))))).)).

 Initial dG =    -20.10

         10         20        30
UGUU  -|    GG   ACU-   CA   CU    G  C
    GU GUGAC  GUA    UUG  UCG  GGCA UG A
    CA CACUG  CGU    AAC  AGC  CCGU AC A
G---  G^    A-   CUCU   C-   U-    A  G
    70         60         50         40
```

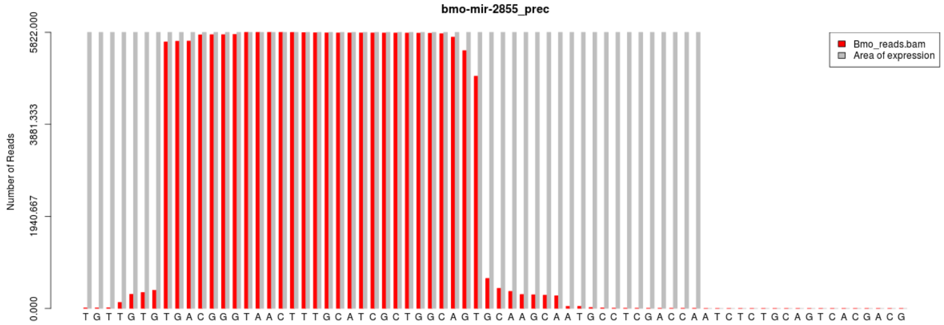

>bmo-mir-2856-1_prec

ACUGUACUAUCUUGUCUGUGGUUCGAGGGUGAGAUUGUCACAGCAGUAUAGGGGGUACAUUCGAGAACCGUAAGACAAUUAAUAUAAA

```
..((((.((..((((((..(((((..(((((..(((.((...........)).))).)))))..)))))...)))))).)).)))).. 

 Initial dG =    -19.60

       10         20        30        40
AC|   C  UC      GU-      GA      AG   G  ACAGC
  UGUA UA  UUGUCU   GGUUC  GGGUG  AUU UC      \
  AUAU AU  AACAGA   CCAAG  CUUAC  UGG GG      A
AA^   A  U-      AUG      AG      A-   G  AUAUG
      80         70        60         50

-----------------------------------------------------
```

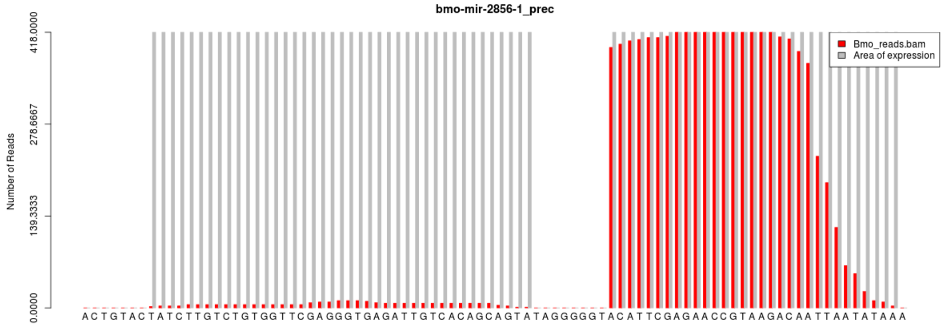

>bmo-mir-2856-2_prec

```
CUGUGCUAUCUUGUCUGUGGUUCGAGGGUGAGAUUGUCACAGCACUAUAGGUGGUACAUUCGAGAACCGUAAGACAAUUAAUAUAAA
.((((.((..((((((..(((((..(((((..((..((...........))..)).)))))..)))))...)))))).)).))))..

 Initial dG =    -20.90

         10         20        30        40
C-|   C  UC      GU-     GA      AG  UG  ACAGC
  UGUG UA  UUGUCU   GGUUC  GGGUG  AU  UC     \
  AUAU AU  AACAGA   CCAAG  CUUAC  UG  GG     A
AA^   A  U-      AUG     AG      A-  GU  AUAUC
      80         70        60         50

-----------------------------------------------------
```

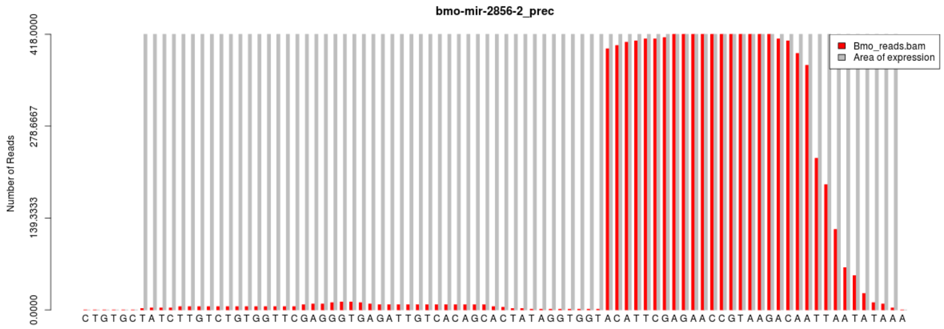

>bmo-mir-2857_prec

```
CUGUAUGACCUGAUGGGACACAUACGGAACAUGCUGGAGCUAAAGAAGCUGAUCGUGGUCGUCUCACUGGACAUACAGG
((((((.((...((((((((.(((((..((.(((...........))))).))))))).))))))..)).))))))).

 Initial dG =    -26.10

         10          20         30         40
-|      A  UGA       -  A      AA  U   GGAGC
 CUGUAUG CC   UGGGAC AC UACGG  CA GCU     \
 GACAUAC GG   ACUCUG UG GUGCU  GU CGA     U
G^      A  UC-       C  -      A-  -   AGAAA
        70          60          50

-----------------------------------------------------
```

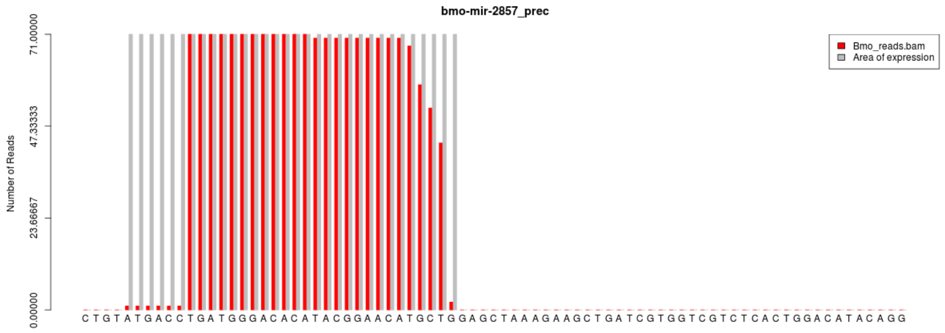

>bmo-mir-2859_prec

```
CGCACCUGAGGAGCCUGGCGGACCAGUUAUGUUACGAUGAACAAUCCCCCCAACUAUUGAUCGGGCAGGACAACUGGGGACUC
....(((.((...((((..(((....((((......))))....))).(((...........)))))))....)).)))....

 Initial dG =    -17.60

        10            20         30
CGCA   G  GAG-    .-GC|  CCAG    GU
    CCU AG    CCUG    GGA    UUAU  U
    GGG UC    GGAC    CCU    AGUA  A
CUCA   G  AACA    \ --^  AACA    GC
  80        70              40


                     50
                    C   AACUA
                     CCC     \
                     GGG     U
                    -   CUAGU
                         60

-----------------------------------------------------
```

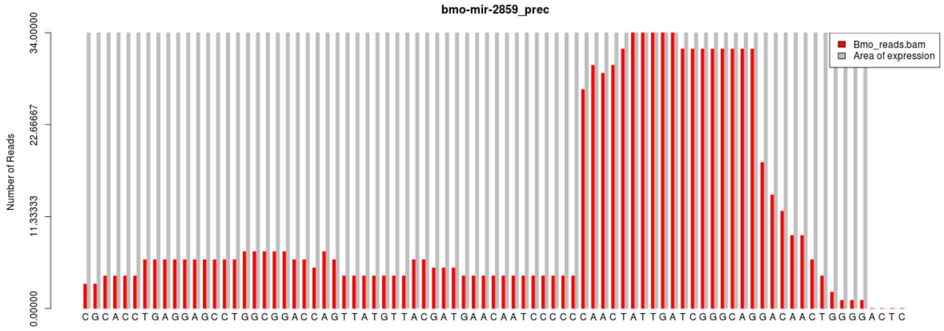

>bmo-mir-2860_prec

```
GAAGGACACCCGCUGUAUGAUUGUCCGAGGUACGAGAGAGCUACGGUGCAGGAACGAUGGAGGAUGGCAAAGGAGUCACGC
.........((.((((....(((((((.(((........))).))).)))).....)))).)).((((......))))...

 Initial dG =    -15.40

        10            20         30
GAAG   A  C--     AUG--    -|  GAG    - -  A
    GAC CC   GCUGU     AUUG UCC   GUAC G AG G
    CUG GG   CGGUA     UAGC AGG   CGUG C UC A
CGCA   A  AAA     GGAGG    A^  A--    G A  G
80        70        60        50          40

-----------------------------------------------------
```

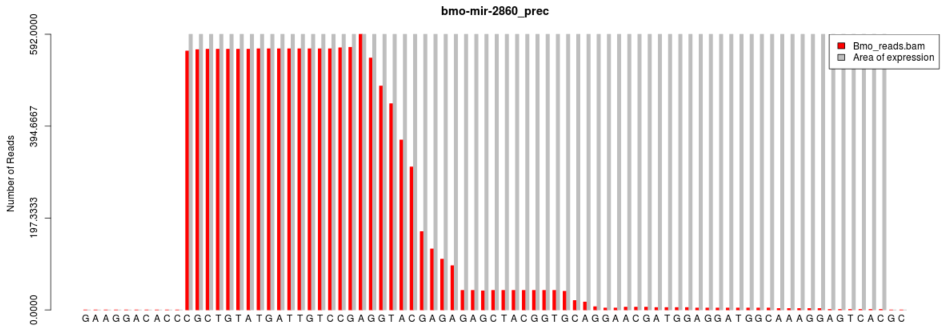

>bmo-mir-2998_prec

AAGAACAGGAUGAGGUAGAUAAACAGGUUAAGGAGUGGCUGGAACAAGGAGUUAUUCGGGUAAGCUUUUCUGAAUAUACUAGCCCAAUUGUUCUU
((((((((..((.((((((((..((((..((((..((.(((((((.....))..))))).))..)))))))))..))).))).)))).))))))))

```
 Initial dG =    -23.50

        10          20        30
        GA  A   .-AGAUAAACAG|  AA  AG
AAGAACAG  UG GGU            GUU  GG  \
UUCUUGUU  AC CCG            CAA  UC  U
        A-  -   \ ----------^  GG  GG
    90                          40


                     50         60
                  AAGG   -        UAA
                      AGU UAUUCGGG   G
                      UCA AUAAGUCU   C
                  A---   U        UUU
                     80        70
```

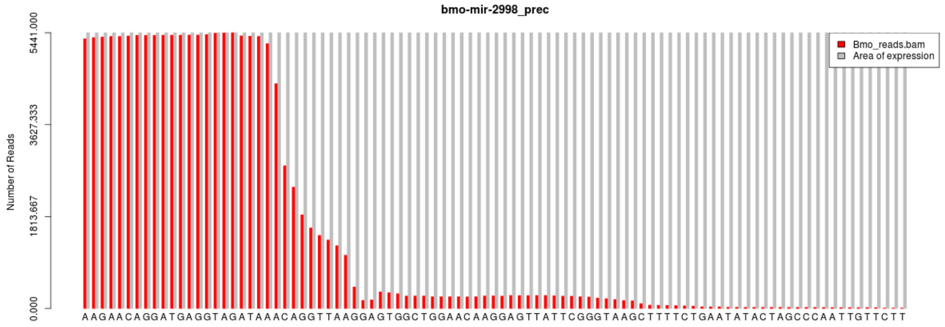

>bmo-mir-2999_prec

```
CGCCCGCGCGGCUGCGACGGACUAGACGCGCAUUUUCCUCGGGCUGCUGUUGAACCUUGCGCAGUUUCAGUUAUUUGUUUAGUUAUUGUUUUAGUUAUUACGUUGUGGGGG
..(((((((((((.((((((((((((((((..(((..(((....)))..)))...))))).............))))))))..)))).)))).....))).))))).. 

 Initial dG =    -31.40

         10                20                        30         40
CG     -   -----    C    --        --------------     UU-|  CU   G
  CCCGC GCG     GCUG GACG  GACUAGAC              GCGCA   UUC  CGG C
  GGGUG UGC     UGAU UUGU  UUGAUUUG              CGCGU   AAG  GUC U
GG     U   AUUAU    U    UA        UUUAUUGACUUUGA     UCC^  UU   G
 .       100        90        80        70        60        50

-----------------------------------------------------
```

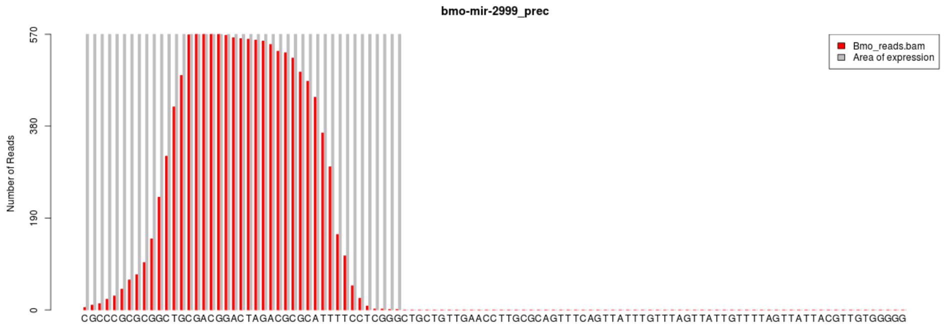

>bmo-mir-3000_prec

AUAAGGUACUCGUAGUAAUAGUCUGUUGGGUCUACUGCGCUUAGAUGAAGACACUACAUUAUGUAUUAGUGUUGUGUUCACCAUGUAAGCGCUACAGAAAUGACUGCUCAAAGUGCAGUG
U
(((..(((((.(((((.....(((((.((.....))(((((((.(((..(((((.
((((((.....)))))).))))).)...))).))))))).)))))....)))))....)))))..)))

```
 Initial dG =    -39.00

        10           20          30
   AG      C---     AAUAG     .-U| GU
AUA  GUACU    GUAGU     UCUGU   GG  \
UGU  CGUGA    CGUCA     AGACA   UC  C
   GA      AACU     GUAA-     \ -^ AU
 .       110       100



                                            40         50        60
                                         -       G   AA-     U      UG
                                          GCGCUUA AUG   GACAC ACAUUA  \
                                          CGCGAAU UAC   UUGUG UGUGAU  U
                                         U       G   CAC     U      UA
                                           90        80        70
```

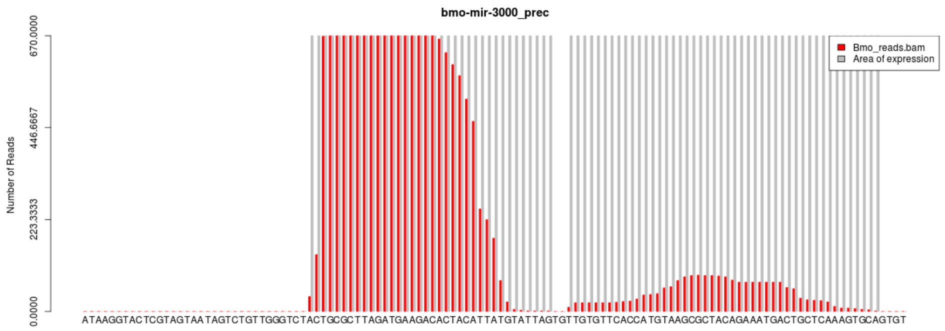

>bmo-mir-3001_prec

```
GUAUGUGAUUCAGAUGCGUUUAAGUUGAAAGAAUUGUAGAUUUUGAUUCCGUUUUCGCCAAUAAAAUUCAGCAGAAUUGAUAUUUUUCUUUUGUCUUUGGCGUCAUCAAGGCCAUUAGC
............((((((..(((.((((((((..((((((((((......(((........)))......))))))))..)))..))))))))).)))..))).)))).............

 Initial dG =    -22.40

        10         20        30          40        50
GUAUGUGAUUCA    -   UU   U        UU   --|      AUUCCG   UCG
            GAUG CGU  AAG UGAAAGAA  GUA  GAUUUUG      UUU   C
            CUAC GCG  UUC GUUUUCUU  UAU  UUAAGAC      AAA   C
CGAUUACCGGAA    U   GU   U        UU   AG^      GACUUA   UAA
       110       100        90        80        70        60

-----------------------------------------------------
```

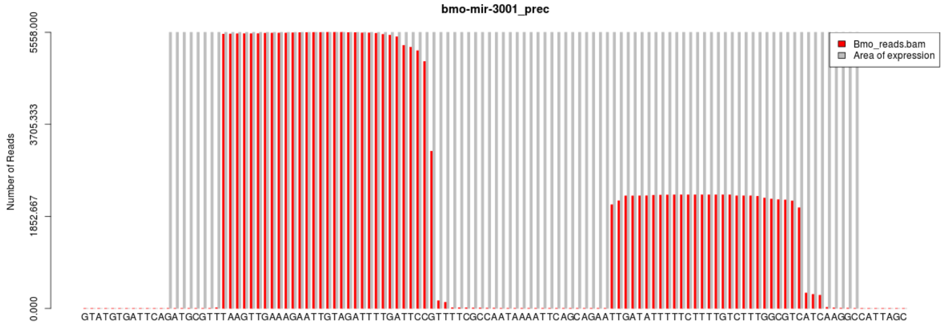

>bmo-mir-3203_prec

```
ACUUGUGAAUUCUCAGCAUGUAUUGAAAUAGCUUGACGUGCAAUUUCACGGAGUAAACCACGUAUGUCAGCUCAGUCAGUACACGCUGAGAAGCUCGGAC
.....(((.((((((((.((((((((...((((.(((((((....................)))))))))))...)))))))).))))))))..)))...

 Initial dG =    -37.40

         10        20        30        40        50
ACUUG   A-|       A         AAU    U       AAUUUCACG
     UGA  UUCUCAGC UGUAUUGA   AGCU GACGUGC         G
     GCU  AAGAGUCG ACAUGACU   UCGA CUGUAUG         A
CAG--   CG^       C         GAC    -       CACCAAAUG
.          90        80        70         60

-----------------------------------------------------
```

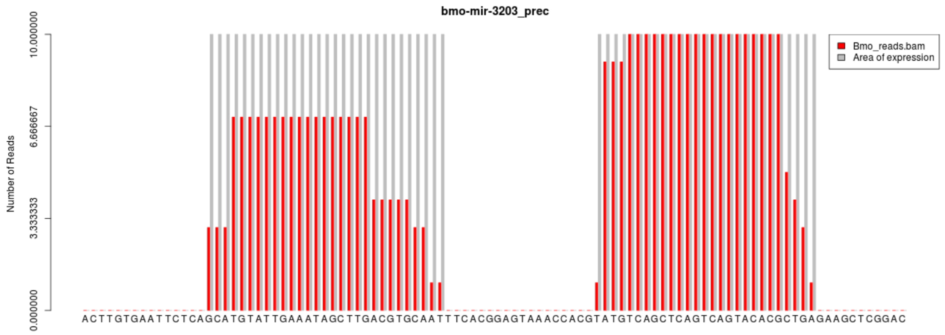

>bmo-mir-3204a_prec

```
GCAAGAAUCAAUGUAGACAUCAAGGCCCGCGUCAUAAACUUCCCAUAUGAUUUCCCAUAUGGGAGUUUAUGACUUAGGCUUCGUUGCCUCUGUCGUCACCGUCUUGA
.(((((....(((((((((...(((((...((((((((((.((((((((......))))))))))))))))))...)))))...))..)))).))).....))))).
```

```
 Initial dG =    -40.30

        10            20        30        40        50
G     AUCA-   -    --| UCA     CGC          U        AU
 CAAGA     AUG UAGA  CA   AGGCC   GUCAUAAACU CCCAUAUG  U
 GUUCU     UGC GUCU  GU   UUCGG   CAGUAUUUGA GGGUAUAC  U
A     GCCAC   U    CC^ UGC     AUU          -        CC
     100        90        80        70         60

-----------------------------------------------------
```

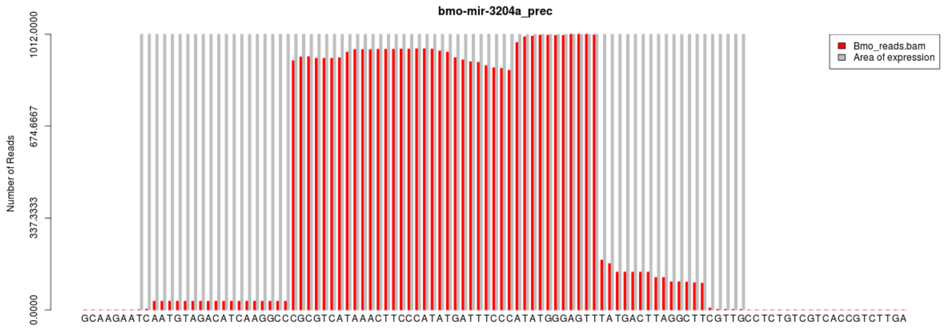

>bmo-mir-3204b_prec

```
UGAAUGUAGACAUCAAGGCCUGCGUCAUAAACUUCCCAUAUGAUUUCCCAUAUGGGAGUUUAUGACUUGGGCUUCGUUGUCUUUGUCC
.((....(((((...(((((((.((((((((((.((((((((......)))))))))))))))))).)))))))...)))))...)).

 Initial dG =    -41.30

        10         20         30         40
U| AUGU     UCA        C          U        AU
 GA    AGACA   AGGCCUG GUCAUAAACU CCCAUAUG  U
 CU    UCUGU   UUCGGGU CAGUAUUUGA GGGUAUAC  U
C^ GUU-     UGC        U          -        CC
        80         70         60          50

-----------------------------------------------------
```

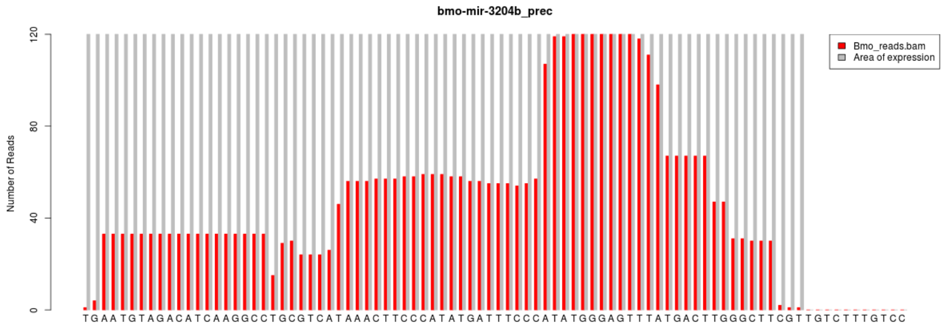

>bmo-mir-3205_prec

```
ACGCCCGUAAACAUUUUUGUUAACUGCUUGAGUUGCUUAUCUAUAACUAAUUUAUGUACAUUAUCUAUGUAUUUAUUUCAGCUCGAGCAGUCUAACAGAGCGGAGCUCUCUGUU
..((((((.......(((((((((((((((((((....................((((((.....))))))......)))))))))))))).))))))))))).)).........

 Initial dG =    -33.60

               10        20         30        40        50        60
AC------  -|   AAACAUU       -             CUUAUCUAUAACUAAUUUAU      UA
        GC CCGU       UUUGUUA ACUGCUUGAGUUG                    GUACAU  \
        CG GGCG       AGACAAU UGACGAGCUCGAC                    UAUGUA  U
UUGUCUCU  A^   -------       C             UUUAUU--------------      UC
  110       100                90        80                      70

-----------------------------------------------------
```

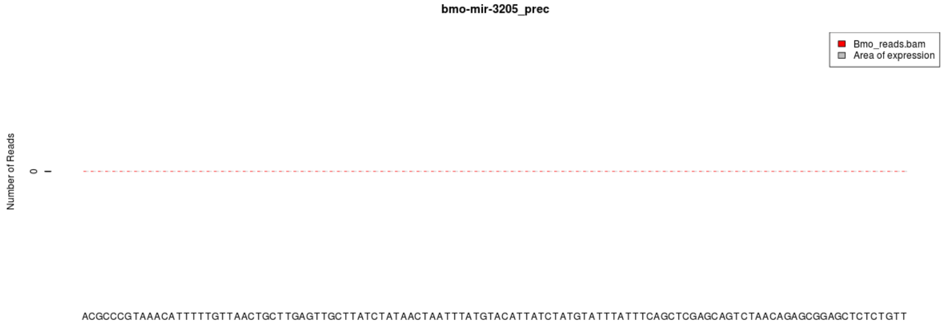

>bmo-mir-3206_prec

AUAGUUAACGAUAUCCAUCAGUUUGUAGGUAAAAUAUUCUCUUUUACUAUUAGUUUUUCUGAGCAUGAAUAUCAGCAACAUCUAGAUGGGUUAACUAA
.((((((((.....(((((((.((((.((((...(((.(((....((.....))......))).)))..)))).))))...)).))))))))))))).

```
 Initial dG =    -19.80

        10         20          30
A        GAUAU     -  UU   .-A|      UAU
 UAGUUAAC     CCAUC AG  UGU   GGUAAAA   \
 AUCAAUUG     GGUAG UC  ACA   UCAUUUU   U
A        -----     A  U-   \ -^      CUC
       90              80            40


                             50        60
                             AUUAGUUUUUCUGA  A  AA
                                           GC UG  \
                                           CG AC  U
                             A-------------  -  UA
```

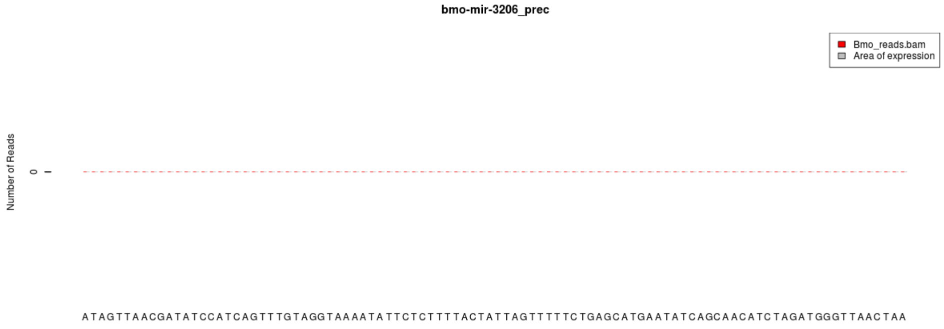

>bmo-mir-3207_prec

AGUGUGAAGUGACGUAGGGAAGGCUUAGUUUUAGUCAACGUACAACUGAAUAAAAGGGCGGGUUACUGAACGCUUGUUAGCUUGUUCCCCCUGCAAUCCCCAUGGUGCACG
.(((((..(((.....((((...(((...((((((.........))))))...))).(((((.....(((((((....)))..))))..)))))..))))))))..))))).

```
 Initial dG =    -27.80

        10              20
A     AA   AC----      .-AA|   UAG
 GUGUG  GUG      GUAGGG    GGCU   \
 CACGU  UAC      CGUCCC    CUGA   U
G     GG   CCCUAA      \ --^   UUU
 .       100        90          30


                           40        50        60
                         AACGUACAACU      AAG       UAC
                                    GAAUAA   GGCGGGU   U
                                    CUUGUU   UUGUUCG   G
                         C----------      CGA       CAA
                                          80        70

-----------------------------------------------------
```

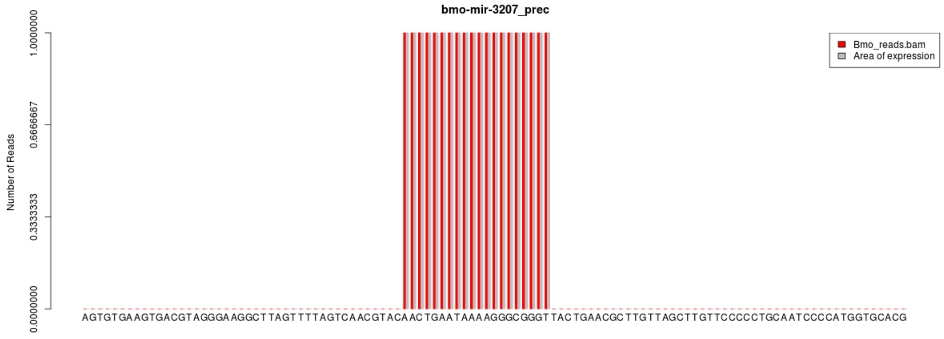

>bmo-mir-3208_prec

```
CGGGUGUCACGUCGAGGAAAGAGAGAGAGAUAGAGAGGUACUACGGGUGUCAUUUUUAUUUGUUCUUUUUUAUUGCUUAGAUGGGGGACGAGCUCA
.(((((((.((((((((.((.((((((((((((((((((..((.....))..).)))))))))...)))))))).)).))).)))))...))).)))).

 Initial dG =    -24.90

            10         20            30         40
C    --   A--    -   G  A        ---        -|GU  UA
 GGGU  GUC   CGUC GAG AA GAGAGAGA   GAUAGAGA G  AC  \
 CUCG  CAG   GUAG UUC UU UUUUUUCU   UUAUUUUU C  UG  C
A    AG   GGG    A   G  A        UGU        A^UG  GG
     90        80         70         60         50

-----------------------------------------------------
```

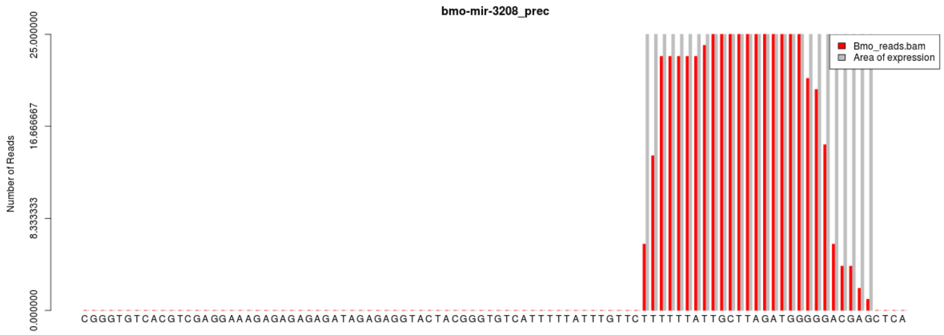

>bmo-mir-3209_prec

AGCAACAUGUAGCUCAGUGUUCAGCGAAUUCUUUUGGACUAUUUAAUCGGGCCUCUUAGUCUUCUUCCAUGAGGCUUUUCAAAAACCAUCAGCUAUUCGAGGCGACGUGAGUCUAUUCCG
CC
.((.....((((((((.((((...(((((.((..(((...........
((((((...............)))))))........)))..))..)))))....)))))))).))))...)).

```
 Initial dG =    -26.30

        10         20         30         40        50        60
A| AACAU    -    G    CAG-     U-  UU   ACUAUUUAAUC       UUAGUCU
 GC     GUAG CUCA UGUU    CGAAU  CU  UGG           GGGCCUC       \
 CG     UAUC GAGU GCAG    GCUUA  GA  ACC           UUCGGAG       U
C^ CCU--    U    -    CGGA     UC  CU   AAAAACUU---       UACCUUC
120         110        100        90        80           70
```

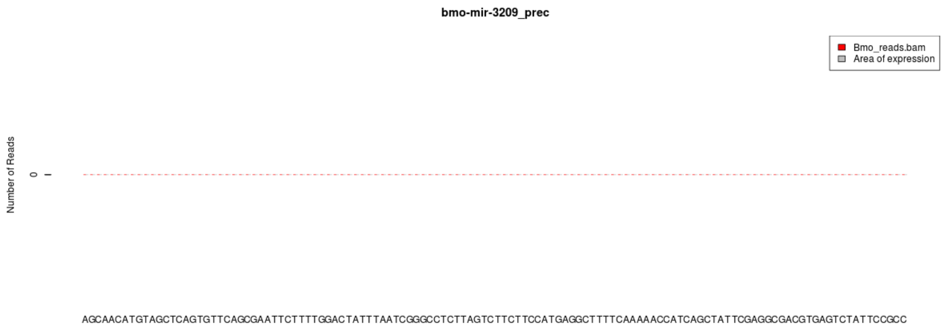

>bmo-mir-3210_prec

```
GUGGCUGCUCUUGAACUGUUAGGGUUGCAUCAGAGGCGCUUGGGCAACUAUCAGGAAAUUCACUCACUCCUCCUGACUAAGCCCCUGCUCACCCACCUGUCUUGGAGAUGCCGA
.((((..((((.((...((..((((.(...(((.((.((((((.......((((((..............)))))))))))))))))..)))))))...))..))))..)))).

 Initial dG =    -33.10

        10         20        30        40        50        60
G    UG    U-| ACU  UA    U CAU   A  C      GCAACUA      AAUUCA
 UGGC  CUCU  GA   GU  GGGU G   CAG GG GCUUGG       UCAGGA      C
 GCCG  GAGG  CU   CA  CCCA C   GUC CC CGAAUC       AGUCCU      U
A    UA    UU^ GUC  --    - UC-   -  -      -------      CCUCAC
  110       100            90           80               70

-----------------------------------------------------
```

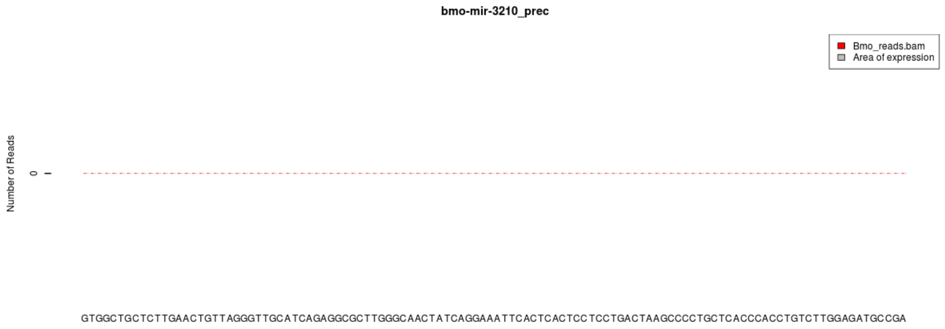

>bmo-mir-3211_prec

AACAUACGGCUGAGUCCGCGUGCGGCAGUCGACUGACUCGUUUUUUAGGCUACGUGUUCGAUAUUUUUUCUUCGUUUCGUCGCCAGCCAGUUGUCCGACUCCUUGGUCACCGGUCAGUCC
GGUAUGUC
.((((((((((((..(((.((((((.(((((((.((((.(((.....(((.(((....
(((..........)))...))).))))))).)))))).)))))))...).)))))))))).))).))))).

```
 Initial dG =    -40.30

          10        20            30         40        50        60
A     -   -    GU   C   - ---| C     -  U    C   UUUUA   U   UGUU   UAUU
 ACAUA CGG CUGA  CCG GUG C   GG AGUCG AC GACU GUU     GGC ACG    CGA    U
 UGUAU GCC GACU  GGC CAC G   CC UCAGC UG UUGA CGA     CCG UGC    GCU    U
C     G   U    --   -   U GUU^ -     C  -    C   -----   C   UUU-   UCUU
      120          110        100         90             80         70

-----------------------------------------------------
```

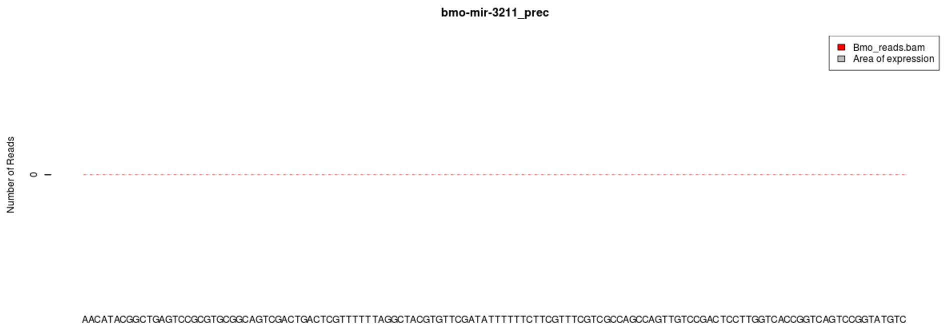

>bmo-mir-3212_prec

UUCAGGUCACUCUUCGUUCAUGAAAAUGUCUUUUGUCUUUGAAAGAGAUCGCAUCGAAUAAAAAAAUGAAAGUUAUUUUAAUUUAUUUCACUGUCUUUCUUAAGGUACUUUUUAAGUAUU
UUUUUAUGUUCUGAU
.((((..((......(((((((...((.((((((......)))))).))..))).))))(((((((((((((.
((((((((..................))))))))))))).....)))))))).))..)))).

```
 Initial dG =    -19.30

          10
U     .-UC| UC
 UCAGG    AC  \
 AGUCU    UG  U
U     \ --^ CU
   130


         20         30            40        50        60          70
        U        U  -   UU---  -      A      CGCAUC       --       A
         CAUGAAAA GU CUU     GU CUUUGA AGAGAU      GAAUAAA  AAAAUGA \
         GUAUUUUU UA GAA     CA GGAAUU UUUCUG      UUUAUUU  UUUUAUU A
        U        U  U   UUUUU  U      C      UCAC--       AA       G
              120       110       100        90          80

-----------------------------------------------------
```

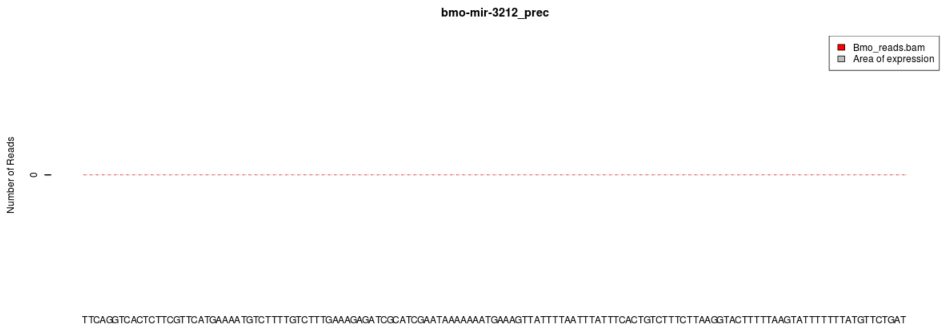

>bmo-mir-3213_prec

AAUUGUACUACUUUACAGGGAAUGCUCAGCUUGUUUCUAAGUACCGAAAUCAGUAAGUUGUCGUAGGCAGAUUUUAUAUCUAGGAAAGCUGAUAUGACCGAGUGUUUGUUAGAAAAUGCA
AUC
.((((((((((..(((.((..(((.((((((..((((((..((..((((((.......
(((.....))))))))).))..))))))))))))))))..))..)))...)).))....)))))).

```
 Initial dG =    -25.30

        10        20          30        40
A      CUAC   AC  G        .-A|  U     UAA
 AUUGUA    UUU  AG GAAUGCUC   GCU GUUUC   G
 UAACGU    AAA  UU UUUGUGAG   UGA UAAAG   U
C      A---   GA  G        \ -^  C     CCA
 120          110       100      50


                                 60        70
                             AAGUU      GG   A    AUA
                                  GUCGUA  CAG UUUU   U
                                  CAGUAU  GUC AAAG   C
                             C----      A-   G    GAU
                                          90        80
```

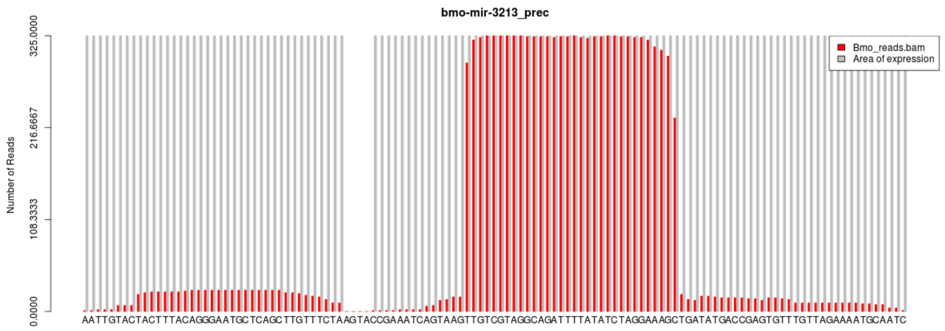

>bmo-mir-3214_prec

AGGUCUCCAUGGCCUACAUCUCGAGUAGUUUGCUUUCUGGGUCAUCCAUCGGCAAUGCUUUUAUAACCGAUAUGGCUAUUCAUGGUUCUGUUGUAUGAAGAUAGAUAGAAAGGUCGAAUG
UAGGAGGGUAACUA

```
.(((..((....(((((((.((((........((((((.((((((..(((((..((((....))))..)))))))))))).........
((((((.......)))))).)))))).)))))))))))..))..))).
```

```
 Initial dG =    -35.00

        10        20            30        40
A   CU  AUGG       C    .-GU   -|    UUC    U
 GGU  CC    CCUACAU UCGA    AGU UUGCU   UGGG \
 UCA  GG    GGAUGUA AGCU    UCG AACGG   ACCU C
A   AU  GA--       -    \ --   U^    CU-    A
  130         120                   50


                                       60        70        80
                          --------------      AC      UG     U
                                        UUUAUA  CGAUA  GCUA U
                                        AAGUAU  GUUGU  UGGU C
                          GGAAAGAUAGAUAG      --      CU     A
                           110       100          90

-----------------------------------------------------
```

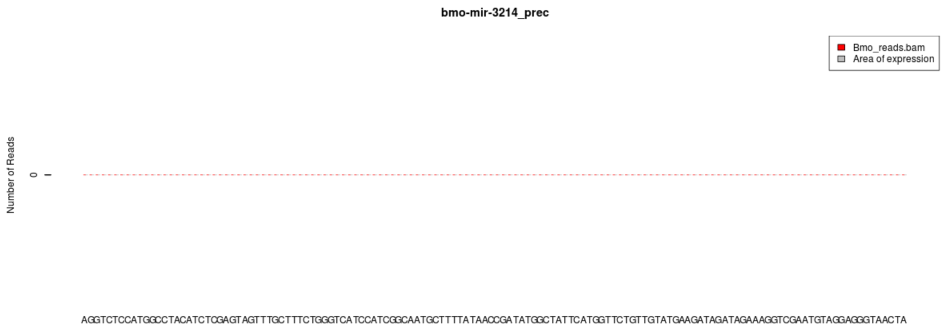

>bmo-mir-3215_prec

AUUGCAGUACAUUGCGGGUCGCACGCGGUUCGUUGUAAAUAAAUAUUUUUGGAAGCGAUCGAUUUCCGAAAUCCAUUCUGUUCCCUCGCGCUGUCGCCGCGGCCGCGCGUCGGCUCACUG
U
...((((((((..(((..(((....)))..))))))).........(((((((((.......)))))))))......)))).((..(((((.
((((...)))).)))))..))........

```
 Initial dG =    -35.10

          10        20
AUU       |   UU   GG   C
   GCAG--UACA  GCG  UCG A
   UGUC  AUGU  UGC  GGC C
---    \ ^   --   UU   G
  120            30


            40        50
         .-AAUAAAUAU       CGA
                    UUUUGGAAG   \
                    AAAGCCUUU   U
         \ ---------         AGC
                    70        60

-----------------------------------------------------
```

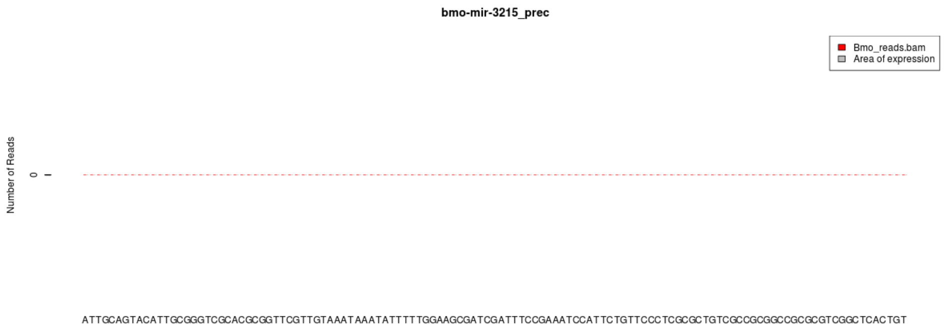

>bmo-mir-3216_prec

```
CCUUUAAGCCACAUCGACUCAAAUUCGUUUUGCUUUAUCUUUUUGGGAAAGUUGACGGACGCAUUUCUGUAUCGCUCAAAGAUAUCAGGCAAAACAUUAGUCAAAUGUGAUCUUGGAA
..((((((.(((((.((((..(((..(((((((((.((.((((((((.......(((((......)))))....)))))))).)).)))))))))))))))))..)))))..)))))).

 Initial dG =    -30.50

         10         20        30        40        50        60
CC      C-     C-|   CA   UC         U  C        AAAGUUG     CG
  UUUAAG  CACAU  GACU  AAU  GUUUUGCUU AU UUUUUGGG       ACGGA  C
  AGGUUC  GUGUA  CUGA  UUA  CAAAACGGA UA AGAAACUC       UGUCU  A
A-      UA     AA^   --   --         C  U        GCUA---     UU
       110       100            90        80          70

-----------------------------------------------------
```

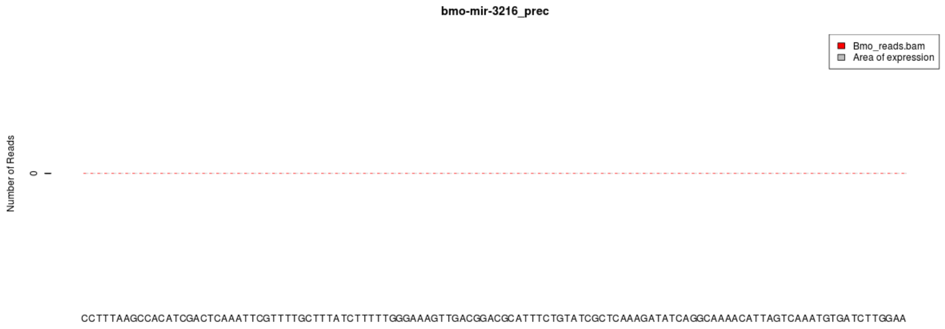

>bmo-mir-3217_prec

UGCUCAGAGUAUAUGUUUUUUGUUAUUCUGUUUUAGGUUGUGUUGCGUUUUUAUCUAUUAACCGAACUGCGAAAUACGAAUAUCCUUUGCUUUUUAGAGAUCACGUAUACCGCAACUUGA
GUGAAAAUGGCAGAGAAAUAUUCUACGCU
.((..(((((((...((((((((((((....(((((((((((.(((((.....((((..((.((((...
((.....)).......)))).))..)))).....)))))...)))))))))))....)))))))))))))))))))..)).

```
 Initial dG =    -35.40

        10        20        30        40          50        60            70
U  UC       AUG            CUGU           U--     UUUUA    UU  C    CUG----| AA
 GC  AGAGUAU   UUUUUUGUUAUU    UUUAGGUUGUG   UGCGU     UCUA  AA CGAA       CG  \
 CG  UCUUAUA   AAGAGACGGUAA    GAGUUCAACGC   AUGCA     AGAU  UU GUUU       GC  A
U  CA       ---            AAGU           CAU     CUAG-    UU  C    CCUAUAA^ AU
       140          130       120       110       100          90        80

-----------------------------------------------------
```

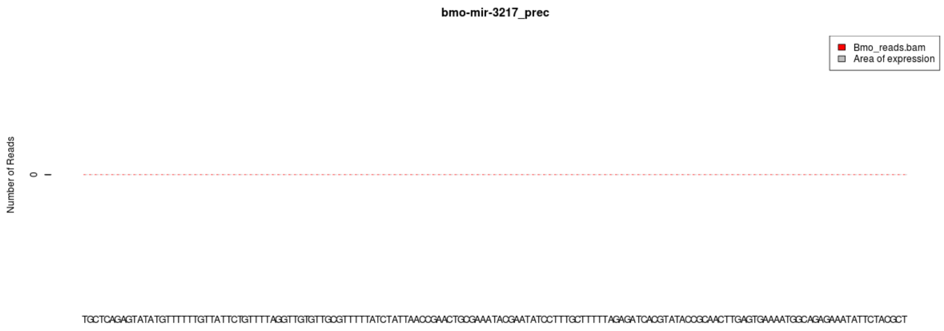

>bmo-mir-3218_prec

GAUCCAUAAUAAUUUGAUAUGAGACAAGUCAUACACAGAGUGAGCAGCGUUGAGGUAUGGUCGAGGCAUCACUCCCCUCCUGAGCAUGACUCUGCGCUAUGAACUGACCCUUAUUAUUUA
GGUC
((((.(((((((..((.(((((......)))))))...(((....(((((.(((.((((.
((((((.........))))..)).)))).))).)))))....))).....)))))))...))))

```
 Initial dG =    -28.40

          10          20
    C--       .-UU| A     GA
GAUC   AUAAUAA    UG UAUGA  C
CUGG   UAUUAUU    AC AUACU  A
    AUU       \ --^ -     GA
  120                  30


                     40        50        60          70
                CAG--   GAGC     U   G     G  --     CAUC
                     AGU    AGCGU GAG UAUG UC  GAGG      \
                     UCA    UCGCG CUC GUAC AG  CUCC      A
                CCCAG   AGUA     U   A     G  UC     CCUC
              110       100        90        80

-----------------------------------------------------
```

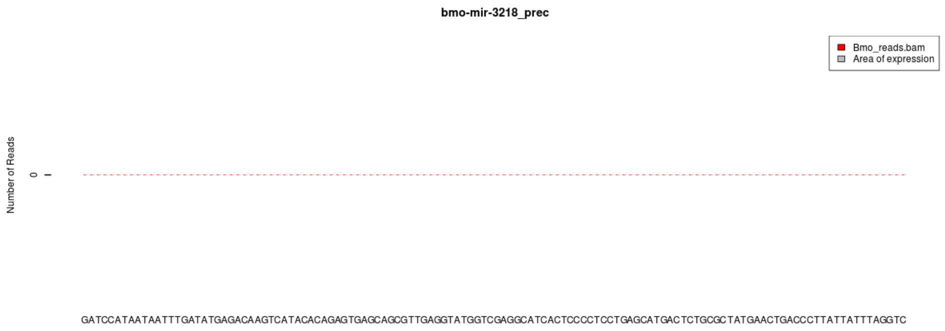

>bmo-mir-3219_prec

CGACGAUCCUGAUCAGGUCACCGGUGAGACUAAUAUAUCCAUGGUGUUAUUUUUUUUAAUGAUCAAUUAUUUCUUUCAUUGGUCUACACCAUGCAUUUUUAAUCUCAGGGUUCUCUGAAGAAUAUCAUCU

```
.((.(((.....(((((..(((..(((((.(((...((.((((((((.........
((((((((.............)))))))...))))))))).))..))).))))).))).)))..))))).....))).)).

 Initial dG =    -29.40

        10        20        30        40        50        60
C| C   CCUGA     UC   GG     C   UAU  C        UAUUUUUUU       UCAAUU
 GA GAU     UCAGG  ACC  UGAGA UAA   AU CAUGGUGU         UUAAUGA      \
 CU CUA     AGUCU  UGG  ACUCU AUU   UA GUACCACA         GGUUACU      A
U^ A   UAAGA     CU   G-     A   UU-  C        UCU------       UUCUUU
 .       120       110        100         90              80

-----------------------------------------------------
```

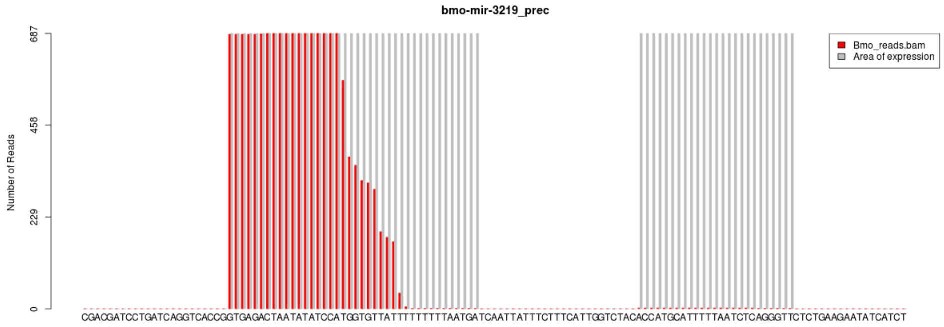

>bmo-mir-3220_prec

```
GCGUUUCUUUCCGCGCGUGACGGUUUGGAACCCAUCGGAGGUUCCGGGUUCGGGGCUGGCGGCCGACUCGAACUCCAUC
..((((...((.((.(((..((((((.((((((...((.....)))))))).))))))))))).))...)))).......

 Initial dG =    -25.70

            10        20        30        40
GC----|   CUU  C  G   GA      G      AUC  AG
      GUUU   UC GC CGU  CGGUUU GAACCC   GG  \
      CAAG   AG CG GCG  GUCGGG CUUGGG   CC  G
CUACCU^   CUC  C  -   --      G      ---  UU
        70         60          50

-----------------------------------------------------
```

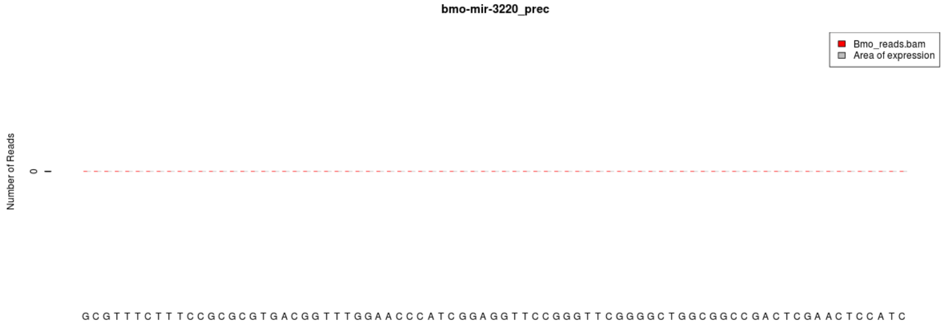

>bmo-mir-3221_prec

```
CAUCUUAACAGGAAAUUUAGCUUCGGGCAAAUAGAGACAGGAAAAUGAAGCUUUCUUUAGACAGGCUCUCAUCUAAGCCCGACGUUUUAGAUUGAUUACGAUACU
.(((.((((((.......(((.((((((...((((..........((((((((((....)).))))).))))))).))))))).)))).....))).))).)))...

 Initial dG =    -21.60

           10        20        30        40         50
C--   U   -|  GAAAUUU   U      AAA    GACAGGAAAA   -     -  U
   AUC UAA CAG       AGC UCGGGC   UAGA          UGA AGCUU UC U
   UAG AUU GUU       UUG AGCCCG   AUCU          ACU UCGGA AG U
UCA   C   A^  AGAUU--   C      A--    ----------   C     C  A
   100        90          80                    70        60

-----------------------------------------------------
```

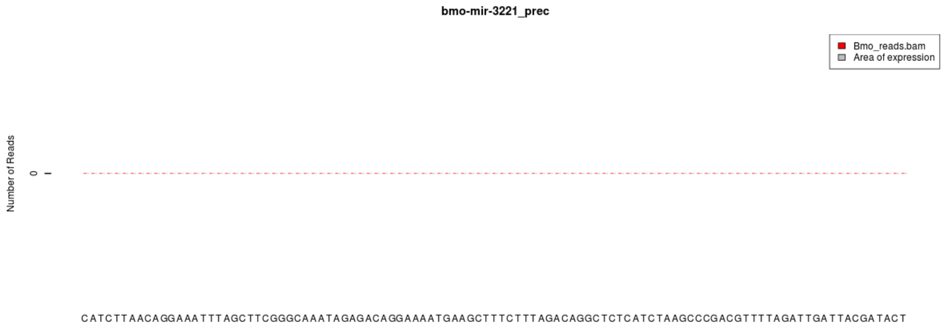

>bmo-mir-3222_prec

GCGUGGCGUGGCGUGGCUAGCUAUGGUCUGUGAAUUCGUUUACUUUGUUAAUUUAGUGGUCCUUCAGGUUAAAACGUGUAAUAGUGGAGAUUUACUUAUCAUAGUAUCUAUGAAAGCGCA
UAAAUGU

```
.....((((..(((((...((((((((..(((((((..((((((..((((.....((...
((....)).....))...)))))))))))))))))..))))))))..)))))...))))........
```

```
 Initial dG =    -31.70

           10         20        30        40        50        60
GCGUG---    GG-|    CUA        CU       CG      UU    AUUUA  GGU--  U
        GCGU   CGUGG   GCUAUGGU  GUGAAUU  UUUACU  GUUA     GU     CC U
        CGCG   GUAUC   UGAUACUA  CAUUUAG  AGGUGA  UAAU     CA     GG C
UGUAAAUA    AAA^    UA-        UU       --      --    GUG--  AAAUU  A
     120       110        100        90            80          70
```

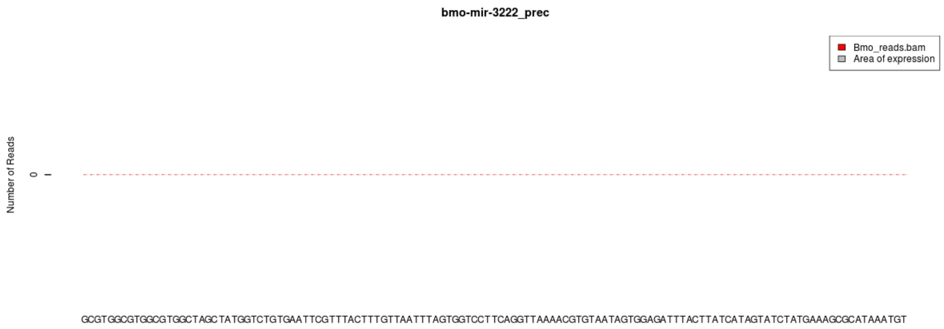

>bmo-mir-3223_prec

```
ACUUUUACCUUUUACCUUUACCACAGACACUUGUACCUUUUCUAAGCCCUAAUUGGCUGGGAGAGAGGUUGACCUGGCUCUCUGGGGAGGAUAAUUUGGUGC
.....((((..((((((((.(((.(((((.....(((((((((.((((......)))).))))).)))).....))..))).)))))))).)))...)))).

 Initial dG =    -28.00

        10          20          30         40        50
ACUUU    UU-   -     A   C   --  CUUGU    -|    A    CU
     UACC   UUA CCUUU CCA AGA  CA     ACCU UUUCU AGCC  A
     GUGG   AAU GGAGG GGU UCU  GU     UGGA AGAGG UCGG  A
C----    UUU   A     -   C   CG  CCAGU    G^    G    UU
    100        90         80        70        60

-----------------------------------------------------
```

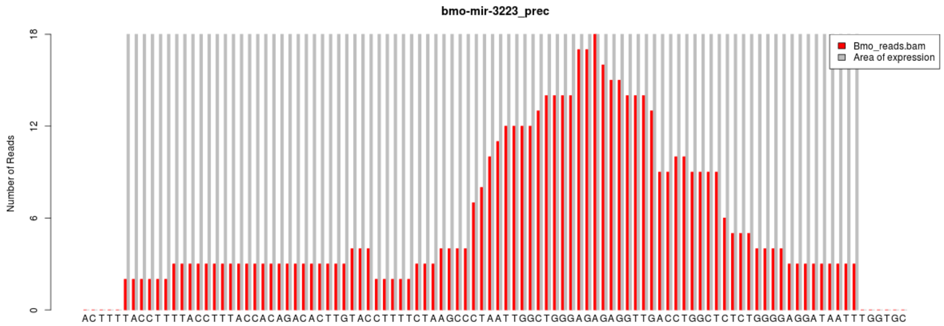

>bmo-mir-3224_prec

UGGCGCAGGAGGAGGGACGGUUACUCCCAUCAUCCUGGCCUUCUUGCGGUAGACCGGACACCGCCUAUCAACGGCGGCGUGGUCCUUCCCGCAAUUGGCGCAGGAGAAUGGGCCGUCCCU
CGGCCUCGGGCA
...((.(((..(((((((((.....(((((..((((((((...((((((..((((..((.
(((((.......))))).))))))....))))))..))).))))).)..)))))))))))))..))))).....

```
 Initial dG =    -72.20

         10        20        30         40        50          60
UGG-| C   AG         UUACU     CA     -   UUC      UA--    GG  A     UAU
    CG AGG  GAGGGACGG     CCCAU  UCCUG GCC   UUGCGG    GACC  AC CCGCC   \
    GC UCC  CUCCCUGCC     GGGUA  AGGAC CGG   AACGCC    CUGG  UG GGCGG   C
ACGG^ -   GG         -----     AG     G   UU-      CUUC    --  C     CAA
130        120            110       100         90          80        70

-----------------------------------------------------
```

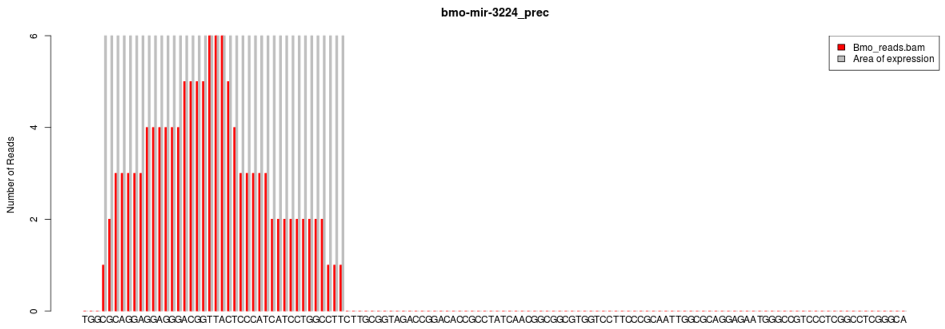

>bmo-mir-3225_prec

GAAAGUUCAGUUUAGUUUGUUGACACUCCAUUAAAAUACAUAUGACUGUUUAUAACACUGAUUAGGCGUAGUACGAACUUGUUUCGAGGUUAAUUAUAUUCAAUGUGAUGUGAAGUUGUU
AAUAAAGAUUUGCUGCAUU

```
...(((.((((....((((((((((((.(((.....(((((.(((.(((...((((..(((.
((((((.....))..)))))..))).)))))...))).))))))))..))).))).))))))))).....)))).)))
```

```
 Initial dG =    -24.20

        10         20         30        40        50        60          70
GAA   U    UUAG-         -|  C   UAAAA     A   C   UUA    AC   U-    --  UA
   AGU CAGU     UUUGUUGAC ACU CAU     UACAU UGA UGU   UAAC  UGA  UAGG  CG  \
   UUA GUCG     AAAUAAUUG UGA GUG     GUGUA ACU AUA   AUUG  GCU  GUUC  GC  G
---   C    UUUAG         U^  A   UA---     -   U   UUA    GA   UU    AA  AU
          130       120       110           100        90        80

-----------------------------------------------------
```

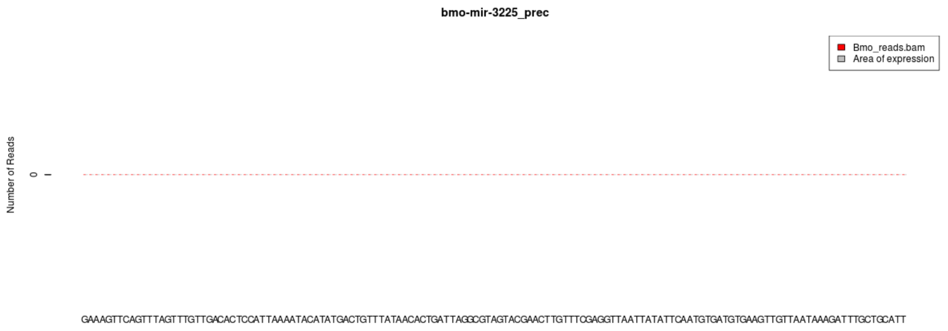

>bmo-mir-3226_prec

AGAUGACGUAGGUGUUCUAUUUUUCUACGGGAACUUCUCUACUAUGACCGGGUUCUGACGGAGAUUGAACGUCGGGUGAGAGGGUCGGGGAGUCGUUUGGUGCGGUAGGGCCCUAAAAAU
AUC
.((((...((((.((((((((....(((.((((((((((......((((....
((((((((.........)))))))......))))))))))..))).))).))))))))))))....))))

```
 Initial dG =    -33.90

         10        20        30          40        50          60
A    ACG-|   U        UUUC   G   --       UACUAU    GGGU--       GAGA
 GAUG    UAGG GUUCUAUU    UAC GGA  ACUUCUC      GACC      UCUGACG    \
 CUAU    AUCC CGGGAUGG    GUG UUU  UGAGGGG      CUGG      GGGCUGC    U
-    AAAA^   -        C---   G   GC       ------    GAGAGU       AAGU
  120        110          100        90              80        70

-----------------------------------------------------
```

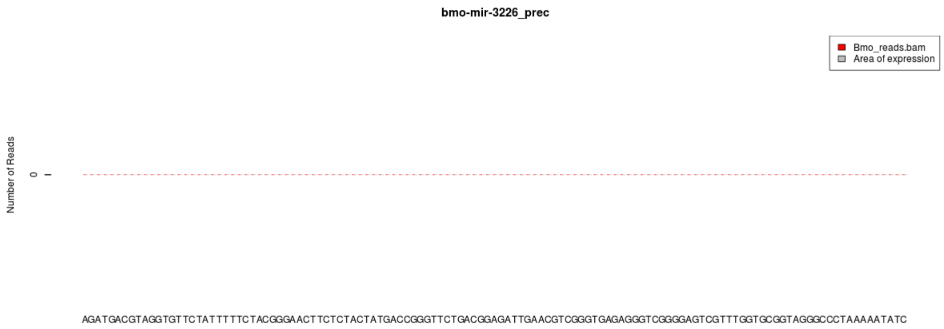

>bmo-mir-3227_prec

UAAAAUCUGGCAAAAUAAUAUAGUCAUUAGCAAUUUUUCAGGACGCUCGGACCCCAAAGACGUCAUCAGUGUGUUUAGUGUGAGUUAACGUAAAAGUUAAUGCUUUGUAUGGAAUUGUAA
CGUUUGCCUAAGUUUGC

```
((((....((((((....((((((((((((((.........((((.((..........))))))).......)))).)))).........
((((((((((....))))).))))..))))))...))))))....)))).
```

```
 Initial dG =    -23.10

         10        20           30        40        50
-    AUCU      AUAA            -|   AUUUUUCAG    C  GGAC
 UAAA    GGCAAA    UAUAGU--CAUU AGCA         GACG UC    C
 GUUU    CCGUUU    AUGUUA  GUGA UUGU         CUGC AG    C
C    GAAU      GCA-      \     U^   GUGACUA--    -  AAAC
     130       120        80        70           60


                                  90
                          UGAGUUAA    -     U
                                  CGUA AAAGU A
                                  GUAU UUUCG A
                          AG------    G     U
                                 110       100

-----------------------------------------------------
```

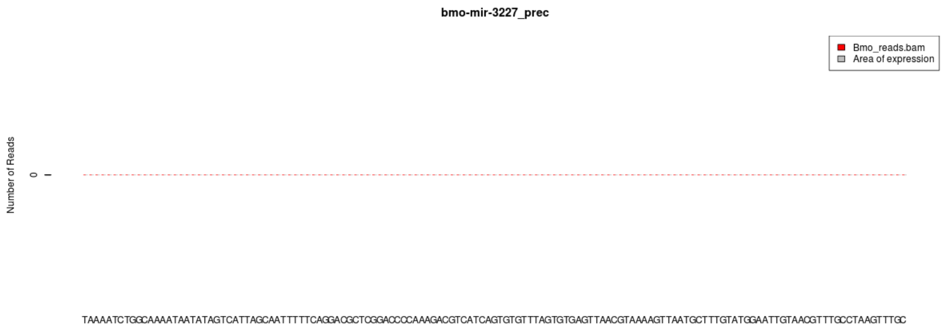

>bmo-mir-3228_prec

```
UAUUUGAGCACCUUGAGAUCAAAUGUUGCUGUCCUUGGAUCUUGAAGUUCUGAAUUUUCAAGAUCUUCAGAUUCGUGGCUUCGAGCUGUCAUUACUUGAGAUGGAGGAAUCU
....(((.((.((((((.......((..(.(((...(((((((((((........)))))))))))...)))..)..)))))))).))))).......((((......))))

 Initial dG =    -26.60

         10        20        30         40        50
.-UAUU|  G  C      AUCAAAU  UG U-   CUU          UUC
      UGA CA CUUGAG       GU  C  GUC   GGAUCUUGAAG   U
      ACU GU GAGCUU       CG  G  UAG   UCUAGAACUUU   G
\ ----^  -  C      -------  GU CU   ACU          UAA
      90         80               70        60



        100
  UUACUUG    GG
         AGAU  A
         UCUA  G
  -------    AG
         110

-----------------------------------------------------
```

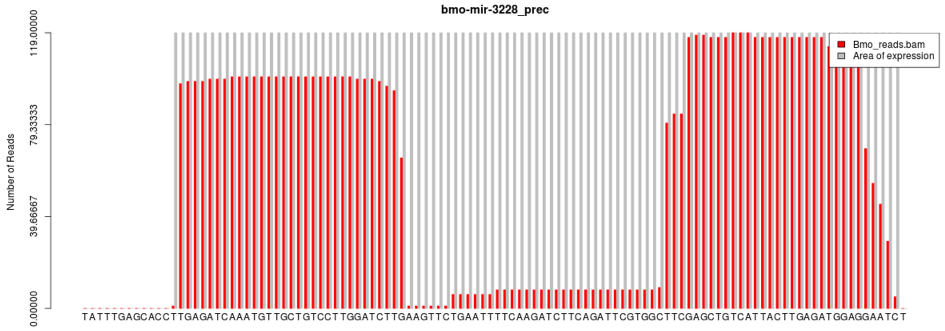

>bmo-mir-3229_prec

UUCAAUUUUUCGUAUUUGCCGGUAGCGGGUGGAUUUUUUAUGAUAUCUUUAAUUUCGCGGAUGUACCUCUGUUCCAGCUGCGAGAUAAUGUGGUAAAACUUUGUAGUAUCUUCCUUGAUA
CCGGAUAUUUGGAAUUGU
..(((((((..((((...((((((..(((.((((.((((((.((((.....(((((((((.((.
((....))..)).))))))))).)))).))))))........)))).)))...))))))))))..))))))).

```
 Initial dG =    -33.10

        10        20         30                40        50        60
UU       UC    UUG      GC-   U    U--------|     G    CUUUA         A  U-  C
  CAAUUUU  GUAU   CCGGUA   GGG GGAU         UUUUAU AUAU     AUUUCGCGG UG  AC U
  GUUAAGG  UAUA   GGCCAU   UCC UCUA         AAAAUG UGUA     UAGAGCGUC AC  UG C
U-       UU    ---      AGU   U    UGAUGUUUC^     G    A----         G  CU  U
       130          120       110       100        90            80        70

-----------------------------------------------------
```

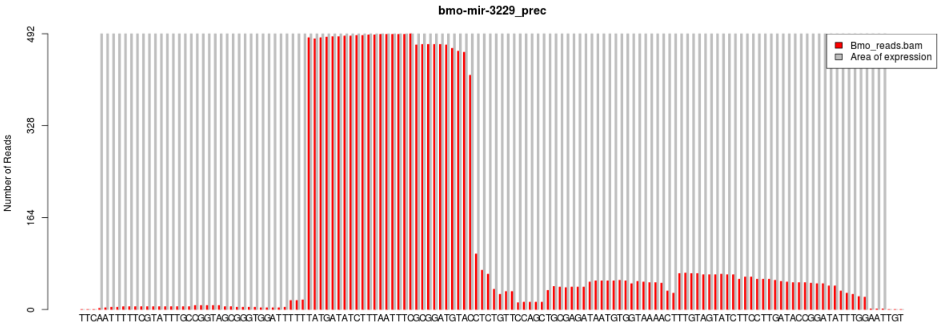

>bmo-mir-3230_prec

AUGCUUGGAGAGAAAUCAGUAGUGAAAUGGGAGUCCAUGUGCCGAAGUUAAAGAAAAAAUUGUGUCUUCUACCACUUCAAACUGCAUAUCUCUGAUAAGUGUCACUCCAUACUGCAUUCU
UUUUCGGUAC
.((((..(((((((..(((((((((.((.((((...((((((.(((((...
((((...........))))...)))))).....)))))))))).)).....)))))....))))..)))))))..)))).

```
 Initial dG =    -29.10

        10        20                  30        40            50        60
A    UG        AU    ----     A----  G    UCC      C----|    UAA    AAAAU
 UGCU  GAGAGAA  CAGU    AGUGA     AU GGAG   AUGUGC     GAAGU   AGAA     \
 AUGG  UUUUCUU  GUCA    UCACU     UA UCUC   UAUACG     CUUCA   UCUU     U
C    CU        AC    UACC     GUGAA  G    ---      UCAAA^    CCA    CUGUG
.       120       110       100        90           80        70

-----------------------------------------------------
```

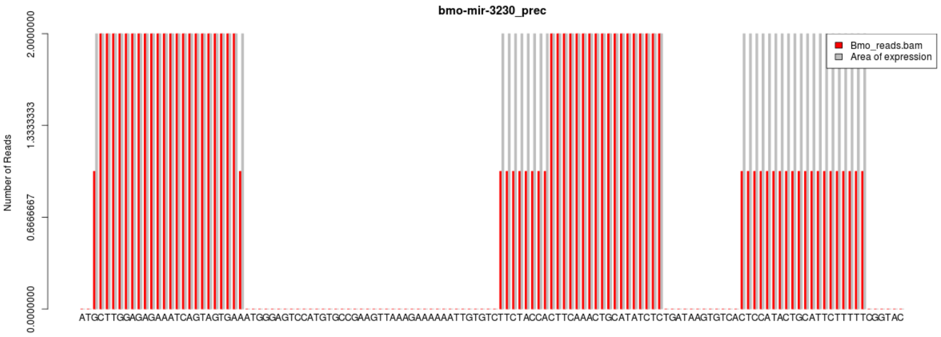

>bmo-mir-3231_prec

GUAUAGGUUAGUUUGAUGUAUAGCACACAUAAGUUUCUUAGUAAGCUAUAGCACGUUACCGCAUAUUAGCUUGAUAUUGAUUGGUCACCUUACUGAAAUAUAUCUGUACA

((((((((..((((....((((((..((.((((...))))))..))))))....((.((((.(((((.....)))))....)))).)).......))))..)))))))).

```
 Initial dG =    -19.20

         10          20        30
-        UA    .-GAUG|     AC  A    U
 GUAUAGGU  GUUU      UAUAGC  AC UAAG \
 CAUGUCUA  UAAA      AUAUCG  UG AUUC U
A        UA    \ ----^     AA  -    U
.       100         50         40


                            60
                 GCAC---  U    C---     AG
                        GU ACCG    AUAUU  \
                        CA UGGU    UAUAG  C
                 GUCAUUC  C    UAGU     UU
                     90        80
```

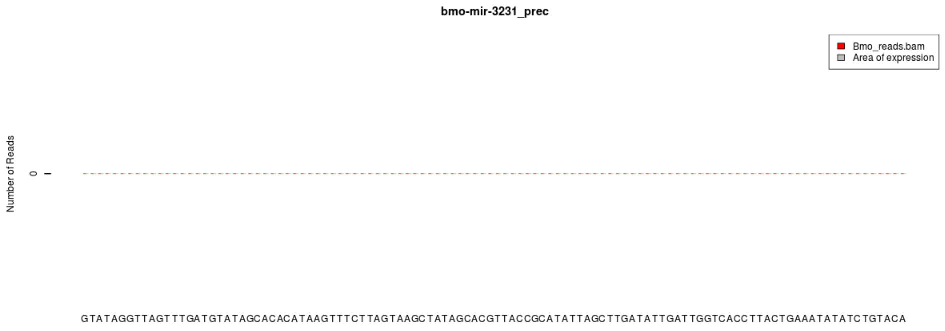

>bmo-mir-3232_prec

UGUGAUCUGUGACAAUCUGCUUUCAUAUUGUUUCUUAAUUAAACUGCUCAUUGCUUUCUGUAUUGCUCAUUUGUUGAGUAUGUGAUAGGGUAUUUUGAUUGACUUUAGAUAGUUUGUCUU
GGUGUUUCUUCACU

.((((.....(((.(((......((.(((((((.(((((((((.(((((..(((.....))).
((((((.....))))))).......))))).))))))))))....))))))).))....))))))...)))).

```
 Initial dG =    -26.40

        10         20         30            40           50
U    UCUGU   A   UGCUUU  U       C---         C     .-AU|  UU
 GUGA     GAC AUC      CA AUUGUUU    UUAAUUAAA UGCUC    UGC  \
 CACU     UUG UGG      GU UGAUAGA    AGUUAGUUU AUGGG    AUG  U
U    UCU--   -   UUCU--  U       UUUC         U     \ --^  UC
  130          120         110       100        90       60


                                                                    70
                                                       U------      UU
                                                              UGCUCA  \
                                                              AUGAGU  U
                                                       AUAGUGU      UG
                                                             80

-----------------------------------------------------
```

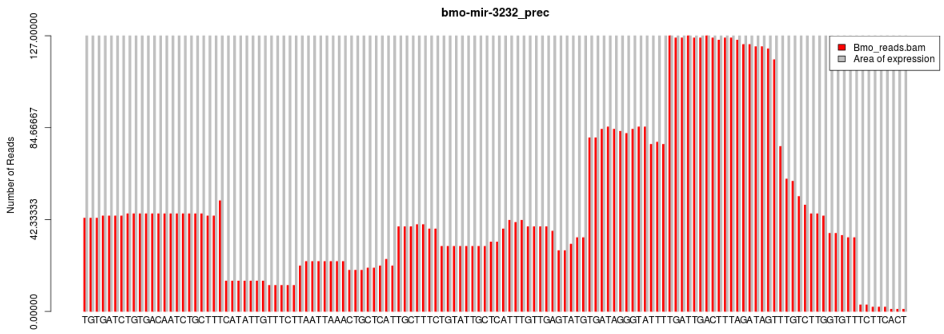

>bmo-mir-3233_prec

AAUGUGCCUUUUGGUUCUUCGUGUUCCAUUACUCUUACUAUUCUUAUAAUAUAAUCUUUUAUAGUUUAAAUGUUAAGGGUUUCUGAAACUGAAUUAUAUGAUCAGGGCACGG
..(((((((..(((((.((((.(((.((..(((((((.((((.......(((((....))))).....)))).)))))))...)).)))))))......))))))))))))).

```
 Initial dG =    -23.10

        10             20        30         40        50
AA       UU     C-----|   U   C  UU-       C    CUUAUAA     U
  UGUGCCU  UGGUU      UUCG GUU CA   ACUCUUA UAUU       UAUAA C
  GCACGGG  ACUAG      AAGU CAA GU   UGGGAAU GUAA       AUAUU U
G-       --     UAUAUU^   -   A  CUU       U    AUUUG--     U
 110         100        90         80        70          60

-----------------------------------------------------
```

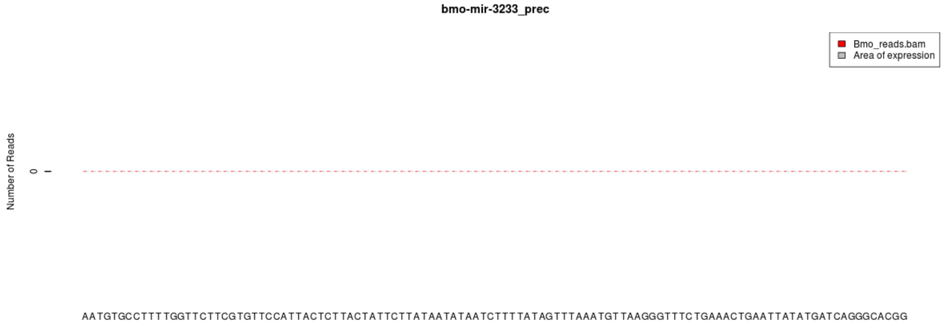

>bmo-mir-3234_prec

AAAACCUAAAUCGCAGAAUCCUCUAGCCUCAUCGUGCAUGGGAACGGUCAUCUCGUGAACGGAACCUGACAUGUCACGUUCCGGCUCGCGCAAAUUACAAGCCGGCGUUCAUACGUGCAU
UGACAGCUA

```
...........(((((....))).)).(((..(((((((.(((((.((..(((((((.((((((.
(((....))).))))))..)))))).........))..)))))))...))))))))))......

 Initial dG =    -34.40

        10        20         30        40         50                  60        70
AAAACCUAAAUCGCAGAAUCCUC    C-    UC       G--     G    AU  ---------     A-|     C   C
                       UAGC  UCA  GUGCAUG   GAAC GUC  CU         CGUGA  CGGAAC UGA A
                       AUCG  AGU  UACGUGC   CUUG CGG  GA         GCGCU  GCCUUG ACU U
-----------------------    AC    --       AUA     -   CC  ACAUUAAAC     CG^     C   G
                                120       110        100        90        80

-----------------------------------------------------
```

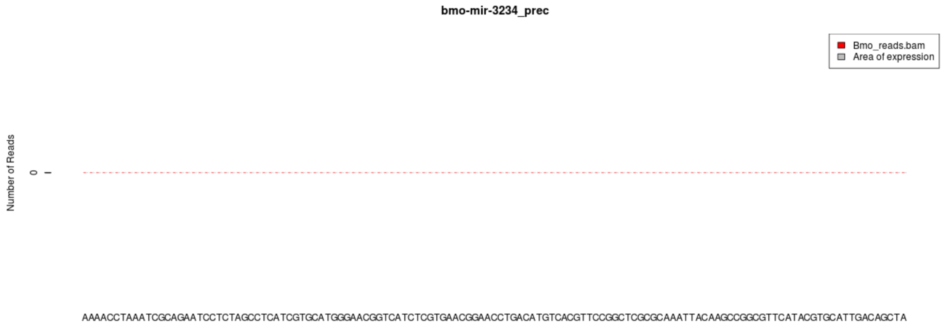

>bmo-mir-3235_prec

```
GGCAGGGCGGUAGUACCUACCCGUGCGGACUCAGAAGAGGUCUUACCACCAUAUCUGGUCGGUCCAACGUCUCGGGCCUCUACCUCGUGG
..((.((.(((((..(((...(((..(((((((((...(((......)))...)))))...))))).))).....)))...))))))).)).

 Initial dG =    -27.30

        10         20         30           40
GG  G  C     UA-   ACC-   GC     ---|   AGA   CU
  CA GG GGUAG   CCU    CGU  GGACU   CAGA   GGU  U
  GU CU CCAUC   GGG    GCA  CCUGG   GUCU   CCA  A
G-  G  -     UCC   CUCU   A-     CUG^   AUA   CC
.          80        70         60         50

-----------------------------------------------------
```

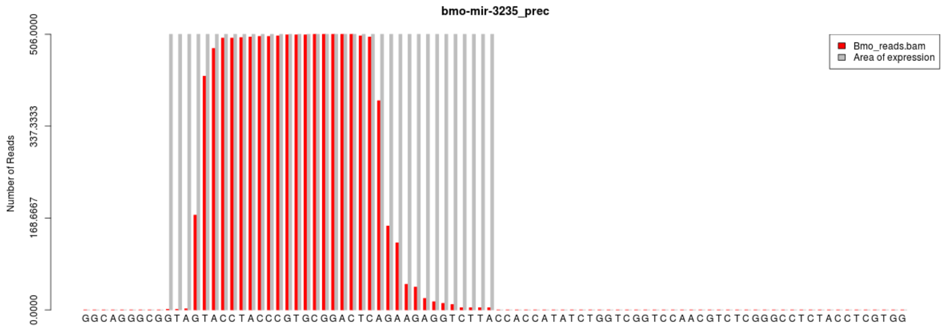

>bmo-mir-3236_prec

CAUUCUAAAACAAGUCAUUUUGUUUGUAAAAAACGUGCUUUGAAAUAUUCAAACCUGUAAACUUUUGCAGUUCAGUGCUAUGUUAUGUUAUUUAAAAUAUUAGAACAGUGGUUUCGUUGG
GAUA
.(((((((...((..(((..(((((((((..(((((((.(((((..........
((((((....))))))))))))).)).)))))...)))).........))))))))..))..))))))).

```
 Initial dG =    -20.40

        10        20                    30          40        50        60
C       AAC  GU   UU     -----------    AA-      -| U     AUAUUCAAAC      A
 AUUCUAA   AA  CAU  UGUUU           GUAA   AACGU GC UUGAA          CUGUAA C
 UAGGGUU   UU  GUG  ACAAG           UAUU   UUGUA CG GACUU          GACGUU U
A       GC-  UG   --     AUUAUAAAAUU    GUA      U^ U     ----------      U
  120        110        100        90        80                   70

-----------------------------------------------------
```

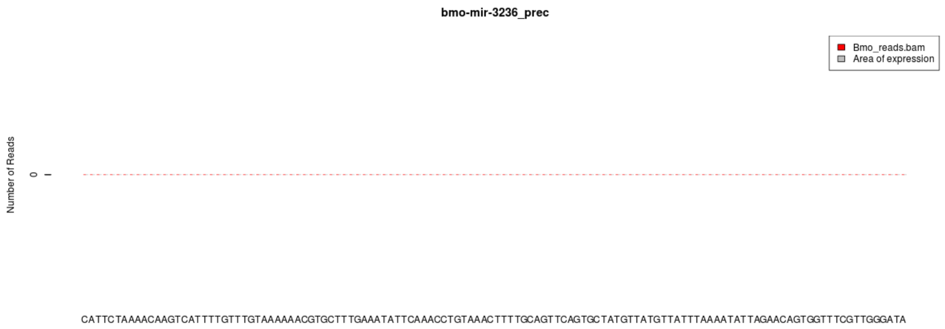

>bmo-mir-3237_prec

```
GAUCCAUAUGUUGUUUAAGUGGUCACCGGAUUCGAUACUCAUCCAAAUGUGUAUUUCGCCAUACGCAUUGAGGCAUGGAGUCAAAAUGAUUGUAGACUGUCAUAGGU
...((.((((..((((..(..((((...(((((.((.((((.....((((((((......))))))))))))..)).)))))....)))).)))))...)))))).

 Initial dG =    -27.80

        10         20         30         40        50
GAU| A    UU-    AA UG    CCG-     G  A-    UCCAA        UU
   CC UAUG   GUUU  G  GUCA    GAUUC AU  CUCA     AUGUGUAU  C
   GG AUAC   CAGA  U  UAGU    CUGAG UA  GAGU     UACGCAUA  G
U--^ -    UGU    -- GU    AAAA     G  CG    -----        CC
        100          90        80        70             60

-----------------------------------------------------
```

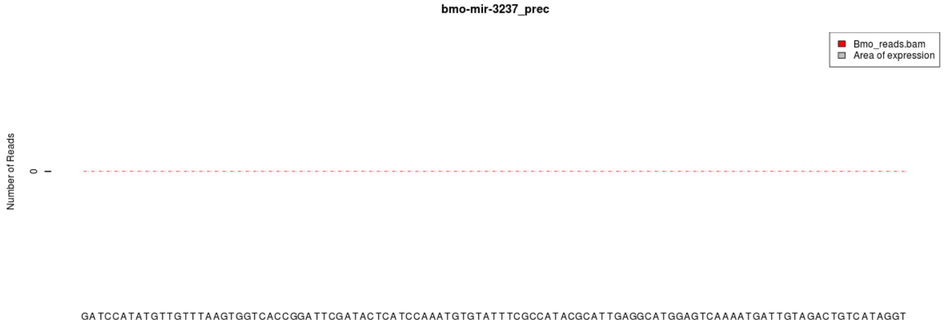

>bmo-mir-3238_prec

```
AAAGUCAAAUGAAUCAUGCAUUGACUACAUGUUUGCCAUGAAAGAGCUUGGACGCCGAGGCAAAAUGGCGGACUACGUGGCUAUAAAAUAUGUUGUAGAUGGUAUACAAGAUCAA
..((((((.((.......))))))))(((((((((((((.....(((.....((....)).....)))......))))))....))))))))((((.......)))).......

 Initial dG =    -24.50

          10
.-AA|     A  AAU
    AGUCAA UG   \
    UCAGUU AC   C
\ --^     -  GUA
          20


        30          40         50         60
  ACA----   U      .-GAAAGA  U   A-     AGG
         UGU UGCCAU        GC UGG  CGCCG   C
         ACA AUGGUA        UG AUC  GCGGU   A
  AACUAGA   U      \ ------  C   AG     AAA
     110       100                70

-----------------------------------------------------
```

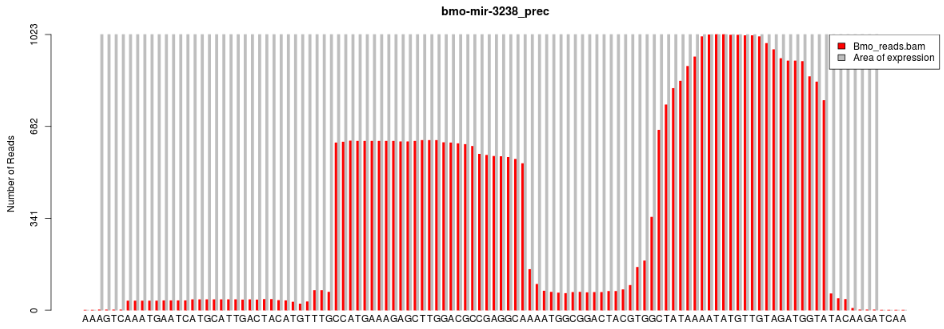

>bmo-mir-3239_prec

```
AUCCGUUGUGCUAUAUCGAUAACUUUGGUUUUCUGUUGGAUCACCUCGUUCUGGAUUCGAUCCUUCAGAGAAAAAGAGAUUUUUAUUACACGAUGU
...(((((((..(((..(((..((((..(((((((..(((((.((.......))....)))))..))))))).)))))))..))).)))))))).

 Initial dG =    -27.10

        10        20        30        40
AUC       CU   UC   AA    GG       UU     A---| UCG
   CGUUGUG  AUA  GAU  CUUU  UUUUCUG  GGAUC    CC   \
   GUAGCAC  UAU  UUA  GAGA  AAGAGAC  CCUAG    GG   U
U--       AU   UU   --    AA       UU     CUUA^ UCU
       90        80           70        60        50

----------------------------------------------------
```

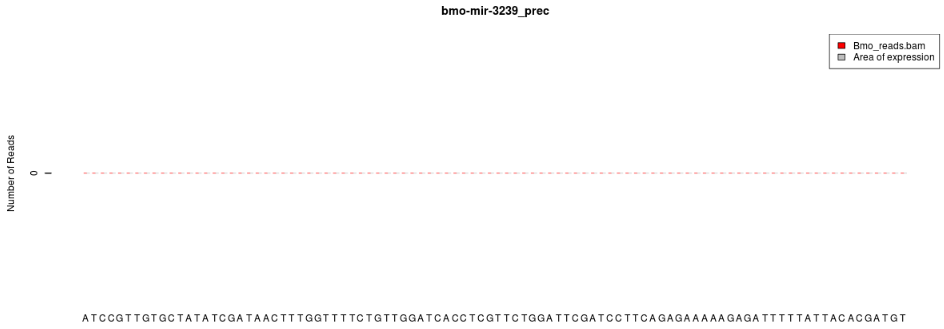

>bmo-mir-3241_prec

```
CACUACCGCAUAGAAUAUAAAGGAUCUUUACCAAAGCAGUCUUGCCUCAUAAUAAAAUUGCCAAUAAUAUCUUGAGCUCCAGACUACAUUGAAAGAUAAUUUGUUCUGCAAUGAAAGGUA
C
...((((...(((((((......((((((..(((...(((((...((((..
(((.............)))..)))))....)))))...))))))))))....))))))).........)))).

 Initial dG =    -18.30

        10              20        30        40         50        60
CAC    GCA-----|      UAAAGG      AC   AGC     UGC-    UA   AAAUUG
   UACC        UAGAAUA      AUCUUU  CAA   AGUCU    CUCA  AUA      \
   AUGG        GUCUUGU      UAGAAA  GUU   UCAGA    GAGU  UAU      C
C--    AAAGUAAC^      UUAA--      --   ACA     CCUC    UC   AAUAAC
 120       110       100             90         80        70

-----------------------------------------------------
```

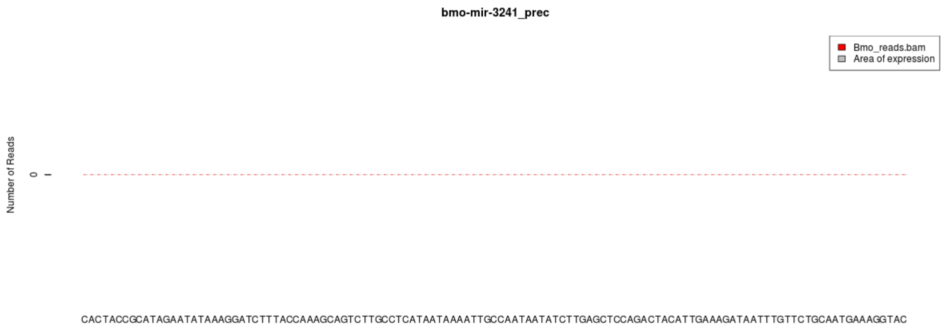

>bmo-mir-3242_prec

CCAGAAGUCUUCCAUAAUCAUUCUAGUUGCUUCAGGGAAACCUUUGCCUUCAUCUUCUGCAACUUGUUGUAGAGUUCUUACAGCAAGGAACGGAGCCGAAGCAGUUCCAAAUGUUACCCA
AAGCAAUCUGAAAUCUUUGACUUUA

```
...((((((...........(((..(((((((..(((.(((.((((.((((...(((((...
((((((((((.....))))))))))...)))))..)))).....)))).))).))).))))))).)))......)))))).

 Initial dG =    -38.30

        10        20        30        40             50        60        70
CCA|     UUCCAUAAUCA   UA       CA   A   C    C-----    AUC     CAA          AG
   GAAGUC           UUC  GUUGCUU  GGG AAC UUUG      CUUC   UUCUG   CUUGUUGUAG  \
   UUUCAG           AAG  UAACGAA  CCC UUG AAAC      GAAG   GAGGC   GAACGACAUU  U
A--^     UUUCUA-----   UC       A-   A   U    CUUGAC    CC-     AAG          CU
     140            130       120        110       100         90        80

-----------------------------------------------------
```

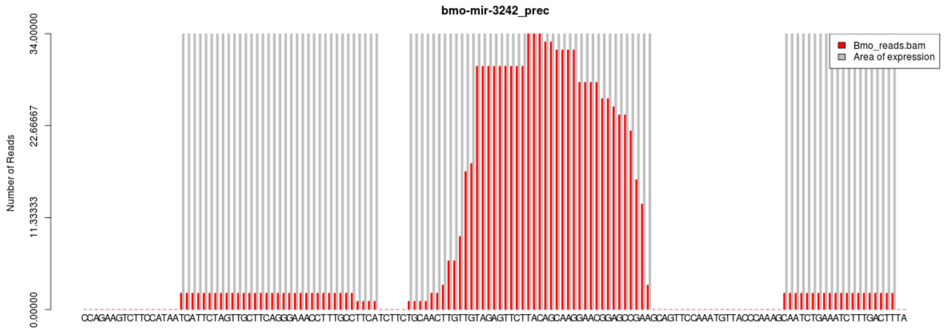

>bmo-mir-3243_prec

GUAUCUAAAUGUUUUCACGAACGACUUUUACGGUGAAGGAAUAACAUCAGAUUAUUCUACGUCUUAUAUGUGUAAUGCUGUAUUGACGGAAAUUUAGUUUGGAACAUUCCAGUCGUUCGU
AGCGCCAUUUUGUUUUUAGAUGU

```
.(((((((((((...((((((((((..........(((((....((((((((.....((((.((((.
((.....)))))))..))))......))))))))...))))))))))))))))))))..........)))))))).

 Initial dG =    -33.10

                  10        20        30        40        50         60         70
G        ----------|   UUC          UUUACGGUGAA     AACA        UUCUA-    U-    U  GU
 UAUCUAAA          UGUU   ACGAACGACU           GGAAU    UCAGAUUA      CGUC  UAUA GU  \
 GUAGAUUU          GCGA   UGCUUGCUGA           CCUUA    GGUUUGAU      GCAG  AUGU CG  A
U        UUGUUUUACC^   ---          -----------     CAA-        UUAAAG    UU    -  UA
 140       130          120                    110        100        90        80

-----------------------------------------------------
```

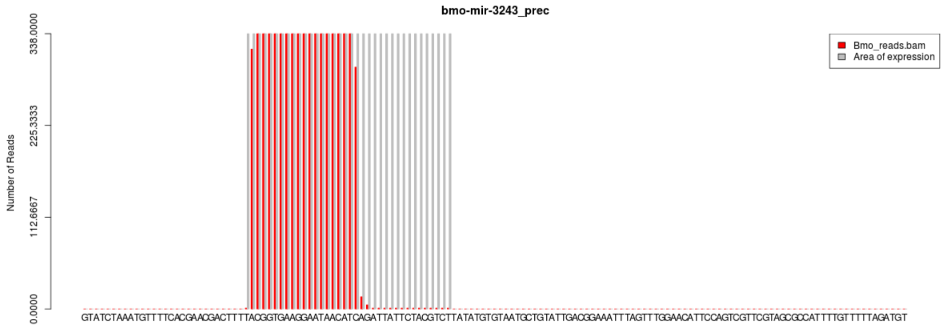

>bmo-mir-3244_prec

CUUUUUAUCCCGAGUAGGACGAAUGGCAGUACUUCCAUUGAGUUUUCGUUACUGUGACAAAUUGAUAGAGAUUUUUAGCUGACGAUGGAAUCUUUCUAUGGCGCCAUUACAUUGCGGGUG
GU

```
....((((((((((((((.((.((((.((...((((((((((((...(((.
((((.........)))).)))....))))..))))))))...))))))..))))..))).))).)))))).
```

```
 Initial dG =    -24.90

        10            20         30        40           50
CUUU      -   -   --  A  A-    C  UAC        --    UUC-|  A    GACA
    UUAUCC CGA GUA  GG CG  AUGG AG   UUCCAUUG  AGUU    GUU CUGU    \
    GGUGGG GUU CAU  CC GC  UAUC UU   AAGGUAGC  UCGA    UAG GAUA    A
U---      C   A   UA  -  GG    -  UCU        AG    UUUU^  A    GUUA
   120       110        100         90        80         70

-----------------------------------------------------
```

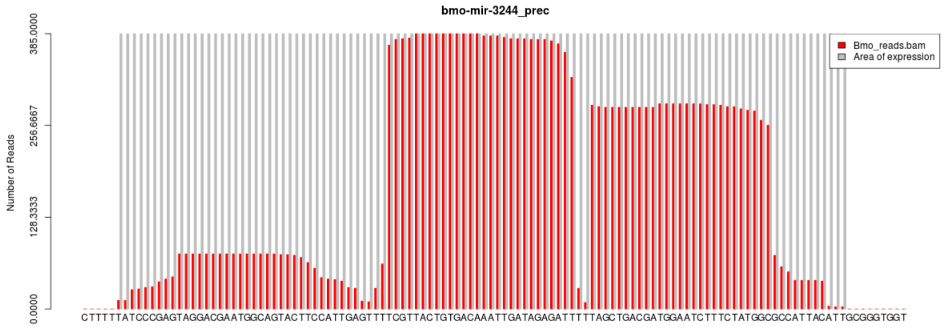

>bmo-mir-3245_prec

```
GACGAAGGUGUUGUUCCGUUGGUGAUGGUGAGUCCAGUCCAGUCGCGGGAAGAAACACUUUUCCACUUUUGUGUCACUUUGGUUCACUAGUCACUUGGGAGACGUCCUGGGA
.....(((((((.(((((..((((((((((((.((((......((((((((((.....)))))).....)))).....))))))))))).)))))))))))))).)))....
```

```
 Initial dG =    -33.00

         10        20         30        40             50
GACGA   -    G     UU     -       U    UCCAGU    -----|     AA
     AGG UGUU UUCCG  GGUGA UGGUGAG CCAG      CGCG     GGAAGA  \
     UCC GCAG AGGGU  UCACU AUCACUU GGUU      GUGU     CCUUUU  C
AGGG-   U    -     --     G       -    UCACU-    UUUCA^     CA
110         100          90         80         70        60

-----------------------------------------------------
```

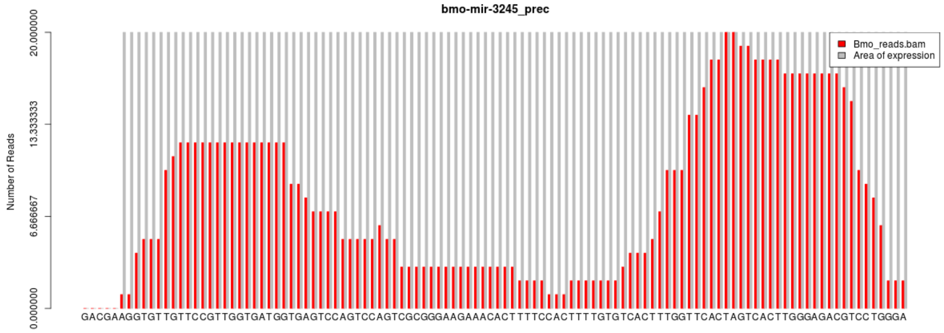

>bmo-mir-3246_prec

CUUGUUUUAACUUGAUAUUUUGCUAUCAGAACAUUGUGUCAUUCUCGACAAGUACUCGUCUUUUACUUGCGCGUCAGCAAGUGCAUCGAAGUCGAGUACCGUCGGGCACAUAAUUGUUUC
UAUGCGGGAUAAUGUGUCGGCUACA
..(((.(..((....((((((((.((..(((((((((((....((((((..(((((((.
((((((((((((......)))))))....)))).))))))).)))))).)))))).))))))..))))))))))....))..)..)))

```
 Initial dG =    -45.70

         10        20        30         40        50        60             70
CU   U-|UA  UUGA         U   CA      -      CAUU      AA       U    ----       GC
  UGU  U  AC     UAUUUUGC AU  GAACA UUGUGU    CUCGAC  GUACUCG CUUU    UACUUGC  G
  ACA  G  UG     AUAGGGCG UA  UUUGU AAUACA    GGGCUG  CAUGAGC GAAG    GUGAACG  U
--   UC^GC  UGUA         -  UC      U      C---      C-       U    CUAC       AC
     140       130        120       110          100         90        80
```

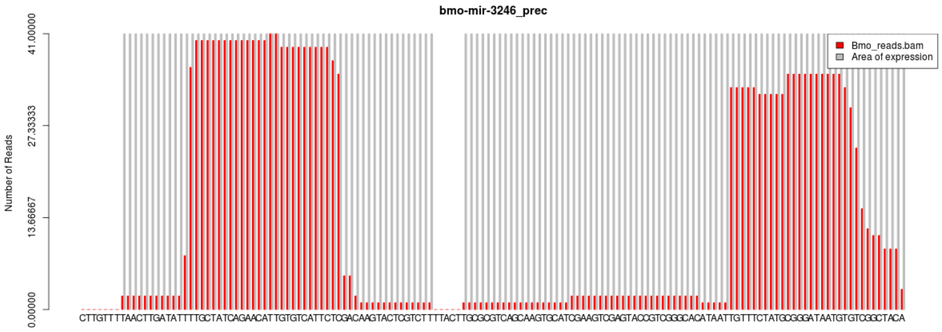

>bmo-mir-3247_prec

UAUUAAAUUGUAAAAAAGCGUGGGUUGCCGUUCAGGAUAUUAUCAAAAUAACCCUUCUACUCAUAUCUGUUCAUAAAAUAUUUAUAACUACUGAUACCAUGCACCCCAUGUUUUUUUUUA
GUUG

```
.....(((((.(((((((((((((.(((.((((((....((((..(((((........
((........))........)))))))))...)))).))...))).))))))))))))).))))).

 Initial dG =    -25.70

        10        20           30         40        50        60
UAUUA     U             U   C--  -|   GAUA    CA     ACCCUUCU  UCA
     AAUUG AAAAAAGCGUGGG UGC   GU UCAG    UUAU  AAAUA        AC   U
     UUGAU UUUUUUUGUACCC ACG   CA AGUC    AAUA  UUUAU        UG   A
G----     U             C   UAC  U^   AUC-    --     AAAAUACU  UCU
      120       110       100        90           80        70

-----------------------------------------------------
```

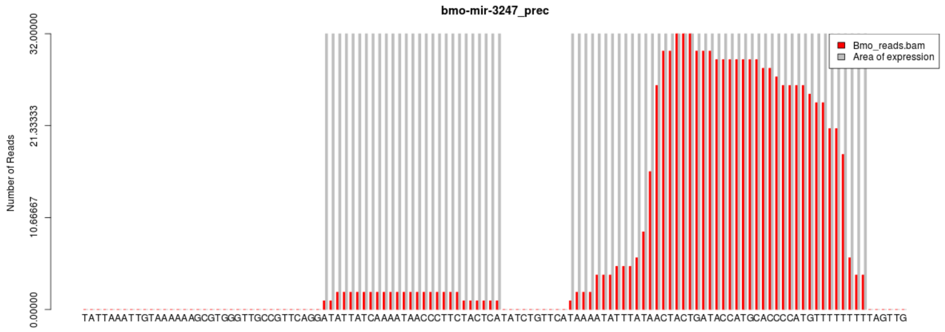

>bmo-mir-3248_prec

```
GAGCUGGUGUUGGCAAACACUGCUGUAAAUCAAAUGUGUACACCUGUAACGUGUCAUCAUUCUUGCACCUUAGCGUGUCAGCUCGUUUCAUUUCAUAGGCUUGAUCAGCUAGCG
..((((((.....(((...(((.((.((((.(((((.(((((((((....(((.((.......)))))..))).))))..)).))))).)))))))))..)))....)))))).

 Initial dG =    -26.20

        10        20        30          40         50
GA|     GUUGG   ACA   C  U    C     U  --    -   UAAC   U  UCA
  GCUGGU     CAA   CUG UG AAAU AAAUG GU  ACAC CUG    GUG CA   \
  CGAUCG     GUU   GAU AC UUUA UUUGC CG  UGUG GAU    CAC GU   U
G-^     ACUA-   CG-   -  -    C     U  AC    C   UC--   -  UCU
   110        100           90        80        70

-----------------------------------------------------
```

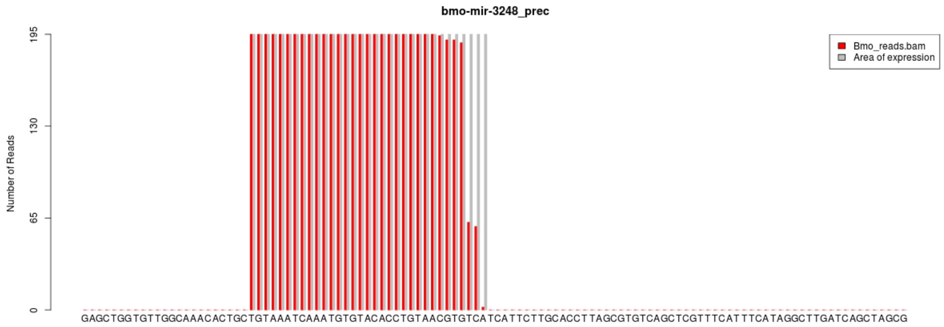

>bmo-mir-3249_prec

GAGGUUUGCACCUGAUGUCCUAACUCUGCAACGAUAUUUACUAUGCAUCUGUUAUCUUAACUAGCAACUGAACAACAACUUUAUUAGCUGGUUUAUCGUUUUACGUUCGAAGAAAAUAUC
GUGUCCGAUAUCGUGUCGUAGCUUCU
((((((.((((.(((((((........(((.((((((((.((.((.((.((..((...(((((((...
((((.......))))...))))))).....))..)).)).)).)).))).))))))))))))..))))))).)).)))))))).

```
 Initial dG =    -29.10

          10        20        30        40        50         60        70
-|     U  -  C       CUAACUCU   A        A  A  C  C  UU  CUU-       AAC    CAA
 GAGGUU GC AC UGAUGUC        GCA CGAUAUUU CU UG AU UG  AU    AACUAGC   UGAA   \
 CUUCGA UG UG GCUAUAG        UGU GCUAUAAA GA GC UG AU  UG    UUGGUCG   AUUU   C
U^     -  C  U       CC------   -        A  A  U  C  UU  CUAU       AUU    CAA
    140        130              120       110       100        90        80

-----------------------------------------------------
```

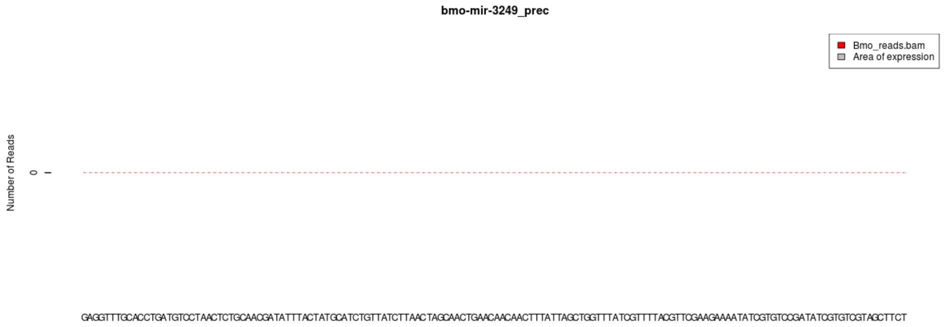

>bmo-mir-3250_prec

```
CAGUACAGUACCGUACAGUACAGUGCAGUGUAGUGCAGCACAAUACAGUACCGUACAGUACAGUGCUGUGUAGUGCAGCACAGUACAGUACAGUACAGUGCAGUGCAGUGCA
..((((.((((.((((.((((.((((.(((..((((.((((.(((((((((.(((...))).))))))))).)))).)))).)))..))).)))).)))).)))).)))).)))).

 Initial dG =    -52.80

        10        20        30        40        50
CA|   A    C    A    A    A   UA    A    A         C   C
  GUAC GUAC GUAC GUAC GUGC GUG  GUGC GCAC AUACAGUAC GUA \
  CGUG CGUG CGUG CAUG CAUG CAU  CACG CGUG UGUGUCGUG CAU A
A-^   A    A    A    A    A   GA    A    A         A   G
 110       100        90        80        70        60

-----------------------------------------------------
```

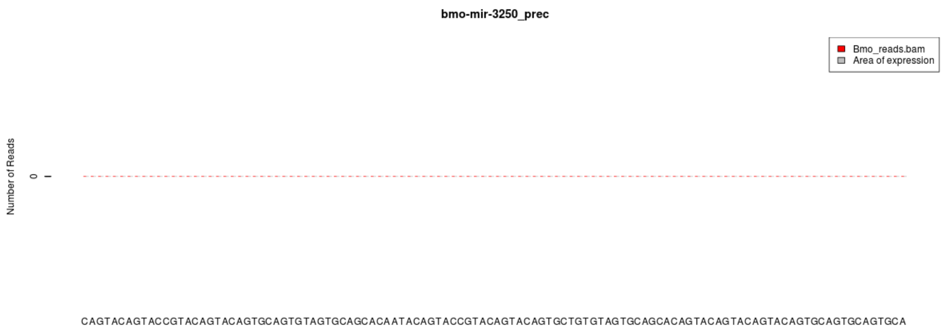

>bmo-mir-3251_prec

CCUCUUCGCACGUUUCCAGACUGGUUUCUUCUAAGGAGCCAUUUAUAUAUUCACUUAUAUGCUCUUCCAUAAGCUAGAAGCAGUUAGUCAGAUAGUUGCCAGAU

..(((..((((...((..((((..((.((((((((((((.....(((((......)))))))))))).........)))))).))..)))).))..)).)).))).

```
 Initial dG =    -22.10

         10        20        30                40        50
CC   UC  -  GUU  CA    GG  U      --------|     CAUUU     UU
  UCU  GC AC   UC  GACU  UU CUUCUA        AGGAGC     AUAUA  C
  AGA  CG UG   AG  CUGA  GA GAAGAU        UUCUCG     UAUAU  A
U-   C-  U  AU-  A-    UU  C      CGAAUACC^     -----     UC
   100          90         80        70              60
```

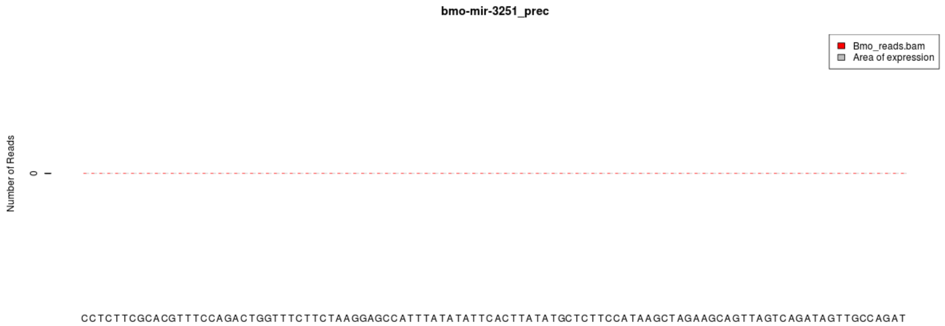

>bmo-mir-3252_prec

```
GCAGCUUAGUCGUCAGUGACCACGAAAACUAUGAAGAAUUCCGAAUAUCUGAAACAAUAUACCUAAUAGCAAAAACAAAGCGAGAUUAAACCGUAAAAGUAGUUUUAAUUACAUUGGACU
CUGUUAUUGACUAUGAUAAAAGCG
...((((.((((((((((((........((....))...(((((.................................(((.........)))....
(((((....))))).)))))....)))))))))...)))..)))).

 Initial dG =    -19.90

         10           20          30
GCA    A-   ---         .-CACGAAAA| A
   GCUU  GUC   GUCAGUGAC          CU U
   CGAA  UAG   CAGUUAUUG          GA G
G--    AA   UAU         \ --------^ A
    140       130


                             40           50         60         70         80
                          AAU-     .-AUAUCUGAAACAAUAUACCUAAUAGCAAAAACAAA   AGAU
                              UCCGA                                     GCG    \
                              AGGUU                                     UGC    U
                          UCUC     \ -----------------------------------   CAAA
                          120                                              90

-----------------------------------------------------
```

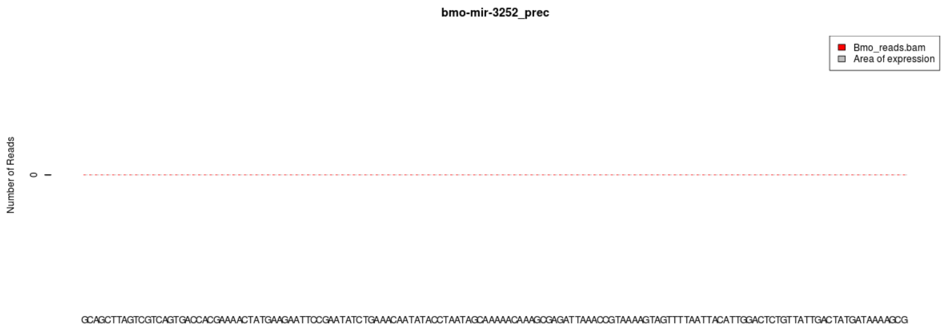

>bmo-mir-3253_prec

```
GCUUUAAUAGUUGUGCUCGUUGAUAGUAAGUUGAUAAAGGAUUAUUUUGUUCUAAGAUACCUUUAACUCACUGUGUACGGUAUACCUUAAAAAGAAGAAAAAAAA
.((((...((.(((((.(((..(((((.((((((...(((..(((((((...)))))))))))))))).))))).)))))))).))...))))...........
```

```
 Initial dG =    -21.60

                  10        20        30        40        50
G----------|   AAU  U     U   UG     A      UAA   AU       U
           CUUU   AG UGUGC CGU  AUAGU AGUUGA   AGG  UAUUUUG \
           GAAA   UC AUAUG GCA  UGUCA UCAAUU   UCC  AUAGAAU U
AAAAAAAAGAA^   AAU  C     -   UG     C      ---   --       C
   100        90        80         70          60

-----------------------------------------------------
```

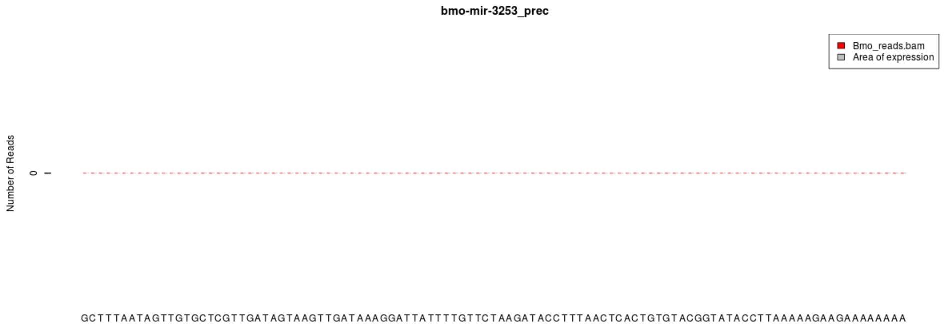

>bmo-mir-3254_prec

```
AGGCCGGCCUUGUAAGCUUCGAACUUCGCGAUUAUGUCCGGACAGACGUCGCGGACGAACUUAAAGAAGAGCGCGCCA
.(((..(((((.((((.((((...((((((((..(((....)))...))))))))))))))))...))).))..))).

 Initial dG =    -25.40

         10          20         30
A   CG  -   G--|   C    AAC        UA-   C
 GGC  GC CUU   UAAG UUCG   UUCGCGAU   UGU C
 CCG  CG GAA   AUUC AAGC   AGGCGCUG   ACA G
A   CG  A   GAA^   -    ---        CAG   G
       70        60             50        40

-----------------------------------------------------
```

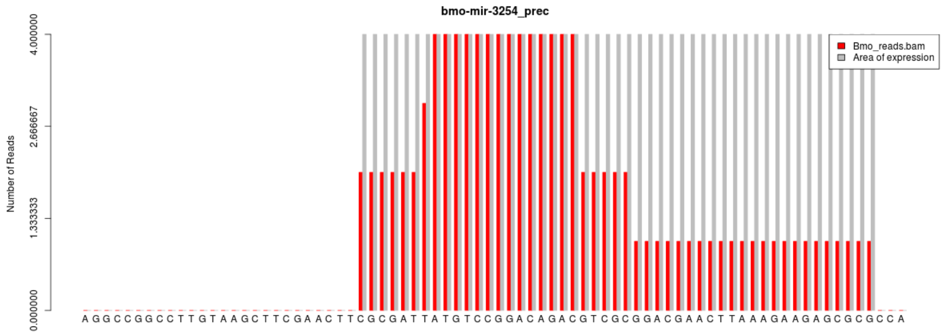

>bmo-mir-3255_prec

```
ACUUGAAGGUAGUAGCUUUGUUUUGCCUGUUGUAUUAUACCAAUCUUGAUGGAUACUGGCUAUCAUUGCCAUAAUAUGAUGUGAAUUAAAGUUACUUUCAAA
..((((((((...((((((.((..(..((..((((((..(((...((((((.......)))))))))..))))))..)))..)).))))))))))))))).
```

```
 Initial dG =    -26.50

        10        20        30        40        50
AC|        GUA      U  UG CU  UG      AC   UCU      AUA
  UUGAAGGUA   GCUUUG UU  C  GU  UAUUAU  CAA   UGAUGG   \
  AACUUUCAU   UGAAAU AA  G  UA  AUAAUA  GUU   ACUAUC   C
A-^        ---      U  GU --  GU      CC   ---      GGU
 100          90          80        70          60
```

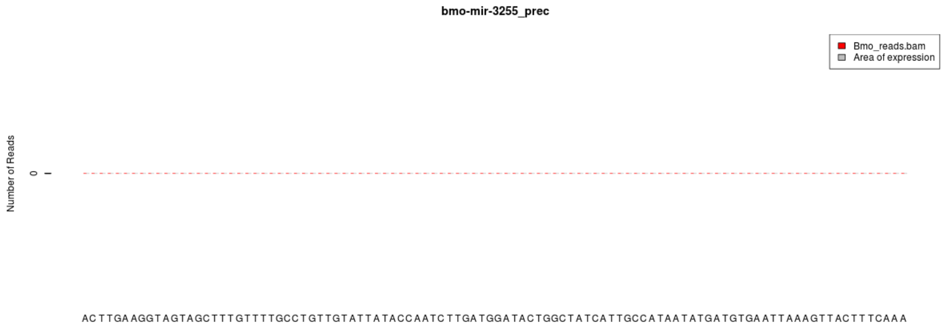

>bmo-mir-3256_prec
UGAAGGAUGGCUCUGAUUUCCGCUUGUAAUGGUAUCUACAGAAAAUCCUUACGCUAUGGAGCUCAGCUCAGCAUCAAGACAAUUUUUUUUUCCGGAGCGCCCCUUAACAAGUGGUAGACA
ACCAAUCUAGUGCGAACAUUCUG
```
...((((((((.((((((.((((((((...(((.(((..((((((........((.((..
(((......)))..)))).......))))))..))).)))).....))))))))).........)))).)).))...)))))).
```

```
 Initial dG =    -27.80

           10                  20        30           40        50        60
UGA      ---  U  -    U--------        AAU--   A-|  AC      UCCUUACG  A  GA   CA
   AGGAUG   GC CU GAUU         CCGCUUGU     GGU  UCU  AGAAAA        CU UG  GCU  G
   UCUUAC   CG GA CUAA         GGUGAACA     CCG  AGG  UUUUUU        GA AC  CGA  C
G--      AAG  U  U    CCAACAGAU        AUUCC   CG^  CC      UUUAACA-  -  UA   CU
   140       130       120       110       100        90        80          70
```

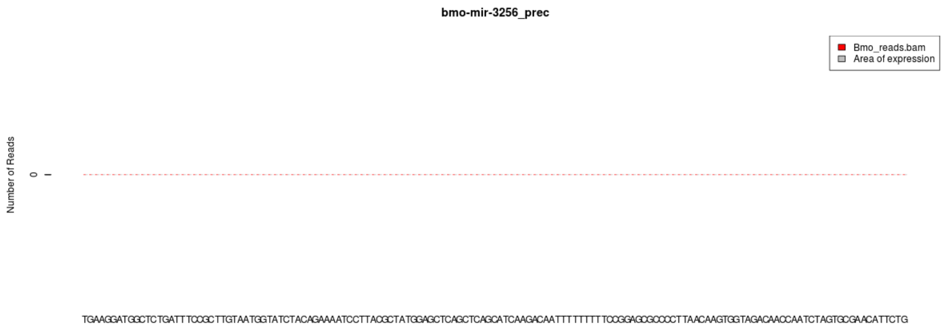

>bmo-mir-3257_prec

GUUGGCUGGCGAUUACGCGAUUUGCCGUGACCAGAGGACAAAUGUACGCUUCAUAUACGUUAUGAGUCGUCGCGUUAAUCGGACCGAA
.((((....((((((((((((..((((((((..((((((....))...))))......)))))).)).))))))).)))))..)))).

```
 Initial dG =    -23.70

        10           20          30
G    CUGG     -            -| GUGA
 UUGG    CGAUU ACGCG--AUUUG CC    \
 AGCC    GCUAA UGCGC  UAAAC GG    C
A    AG--     U     \      A^ AGAC
         80             40


                           50
                      GU   C      UA
                        ACG UUCAUA  \
                        UGC GAGUAU  C
                      --   U      UG
                       70        60
```

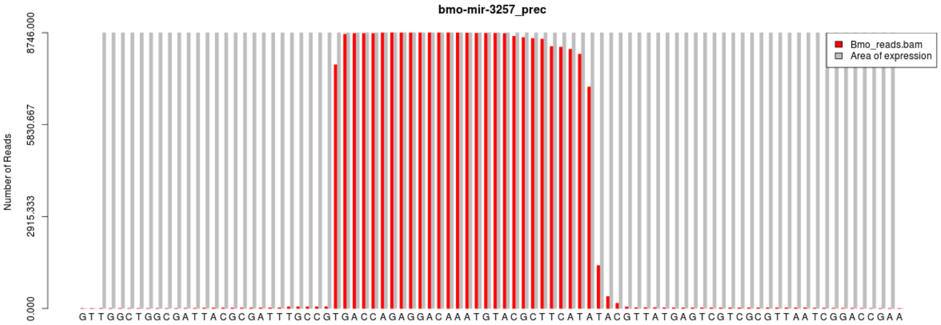

>bmo-mir-3258_prec

```
AUUGGAUUAGAAAACCACCGCGUUGGGUACGUCUAAAAUGCCACGCGGCUCACUGGAUCUAAAAUGCCACGUGGUUUGUUUACUAGCUGCAAUC
...((.((((.((((.(((((((..((((.(((((....(((....)))....)))))......)))))))))))..)))).))))))......

 Initial dG =    -23.70

           10          20         30             40
AUU---  A    A    C-       UG    C-----|    AAAU   A
      GG UUAG AAAC  ACCGCGU  GGUA      GUCUA    GCC C
      UC GAUC UUUG  UGGUGCA  CCGU      UAGGU    CGG G
CUAACG  -    A    UU       --    AAAAUC^    CACU   C
   90          80         70          60         50

-----------------------------------------------------
```

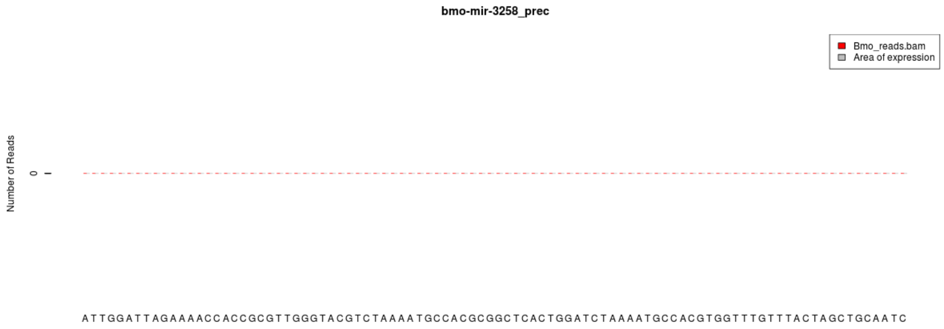

>bmo-mir-3259_prec

```
CAAGAGAAGCUCUUGUAUUGAGUACAAUUGACAAGAAAAGCUCUUUGAUUGAGUACAAUUGACAAGAAAAGCCCUUUUUUAGUGCAAUUGACAAGAAAAUCUUUUU
.((((((...((((((........((((((((.(((((((..((((..(((...........)))..))))..))))))).)).)))))))))))))...)))))).

 Initial dG =    -21.70

        10        20        30         40        50
C|     AGC      AUUGAGUA      -  A       CU    GA   AGUAC
 AAGAGA   UCUUGU        CAAUUG AC AGAAAAG  CUUU  UUG     \
 UUUUCU   AGAACA        GUUAAC UG UUUUUUC  GAAA  AAC     A
U^     AAA      --------      G  A       CC    AG   AGUUA
    100                90        80        70        60

-----------------------------------------------------
```

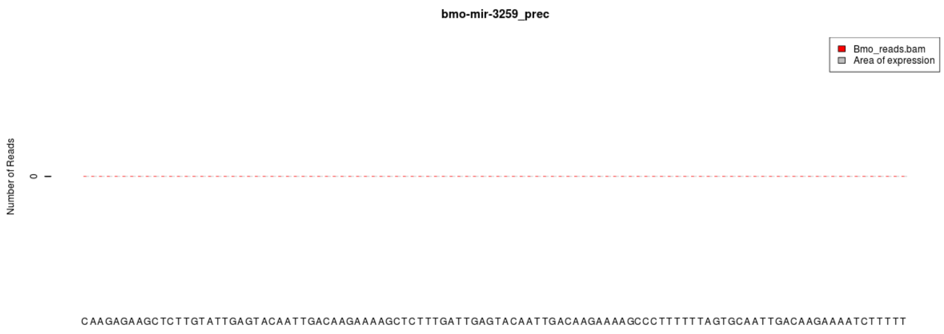

>bmo-mir-3260_prec

AUGCAAGAUAGAAGAAGUAGAAUGAAAGAUAGUAAGAAUGAAAGAAAAUAAAUCCGCAAUCUGGGGGUUUACUUUUCGGUUUAAUGUUCACUGUCUCUUGAUUACUUUCGUGAGUUGA
............((((((...((.(((((((.((..((((.(((((((((((.(.....).))))))).)))))..))))...)).))))))).))..)))))))..........

```
 Initial dG =    -23.30

        10        20        30         40          50
AUGCAAGAUAGAA       AAU  A       A  AA-    A-     -|      G AA
             GAAGUAG   GA AGAUAGU AG   UGAA  GAAAA UAAAUCC C  \
             UUUCAUU   UU UCUGUCA UU   AUUU  CUUUU AUUUGGG G  U
AGUUGAGUGC---       AG-  C       C  GUA    GG     C^      G UC
      110           100         90        80        70

-----------------------------------------------------
```

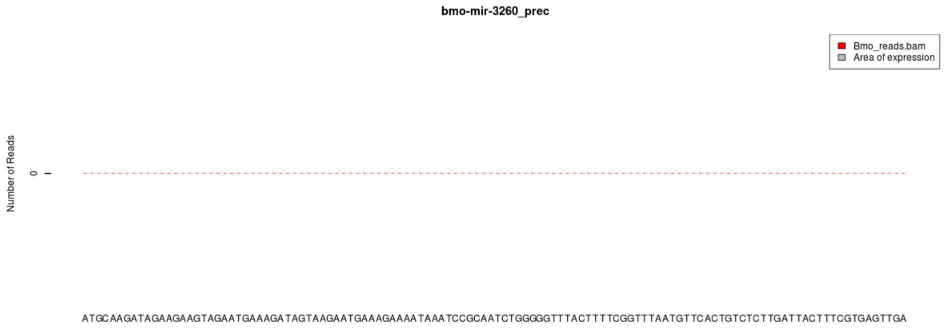

>bmo-mir-3261_prec

UAAUAGUCCUGCAUACUUAUGCCAUGAAAACGUAGAAUUUAAGGAUAAGGUAGGGUAAAUUUUACUCCAUUACAAUAUAGAUUUAAAUUUUAUAGCUCAUGGUAGAGUAGCUUAUUUAUC
..((((....((.(((((.((((((((....((((((((((((..((..(((((((((...))))))...)))....))..))))))))))))...))))))))))))))))....)))).

```
 Initial dG =    -28.30

        10        20        30        40          50
UA    UCCU  A     A        AAAC            GA  AG--   ---|     A
  AUAG    GC UACUU UGCCAUGA    GUAGAAUUUAAG  UA    GUA   GGGUAA \
  UAUU    CG AUGAG AUGGUACU    UAUUUUAAAUUU  AU    CAU   CUCAUU U
C-    UAUU  -     -        CGA-            AG  AUAA   UAC^     U
.        110         100         90        80        70
```

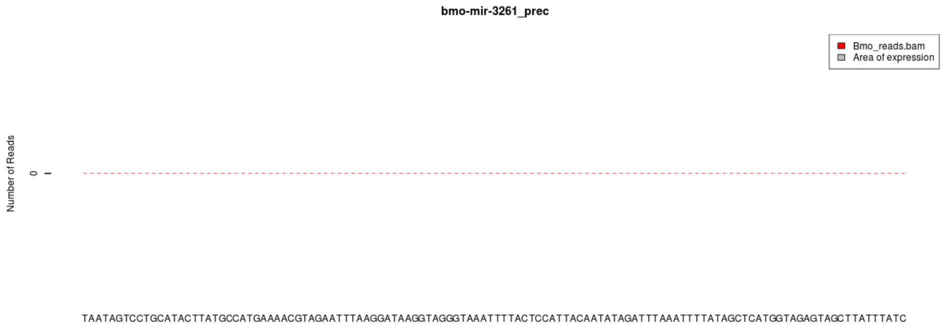

>bmo-mir-3262_prec

UUAUCUGUGGGUUUGCUUGCAAGUCAAUACUAUCAAGCGGGUUUCUAUAAUGUAAACGCAGCACGCACGAAGUUGCACUAAGCGUGGGCUCGGAAUAGUGUCAAGAAGUAAAGAGGCGCA
GUCGGAUAC

```
.((((((((.((((..((((......(((((((....(((((((.....((((....
(((((.........)))))).....))))))))))))..)))))))......)))))..)))).))..)))))).
```

```
 Initial dG =    -26.90

          10        20        30        40        50         60
U      --| G    GC    AAGUCA       CAAG       CUAUA    AAAC-     ACGC
 UAUCUG  UG GUUU  UUGC      AUACUAU    CGGGUUU     AUGU     GCAGC    \
 AUAGGC  AC CGGA  AAUG      UGUGAUA    GCUCGGG     UGCG     CGUUG    A
C      UG^ G    GA    AAGAAC       AG--       -----    AAUCA     AAGC
       120       110       100          90             80        70

-----------------------------------------------------
```

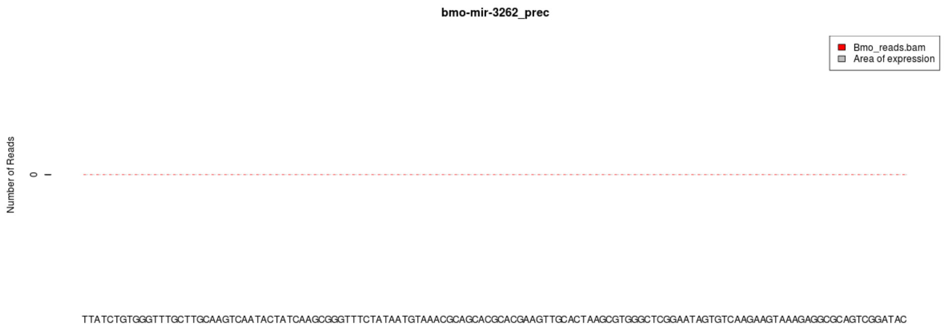

>bmo-mir-3263_prec

AUAAUAUGUAUACAUAUUGCGCGUCUUGUAGCUGUUGAGUCCCGUUGAGAAGUGAAGGUGGUUCUGUAUUUUUUAUGCGUCGCGGAGUUAGCACCGUUCCGCCAGUUUGC
.((((((....))))))).(((.((.((((((((((.((((((((((((((.(((....))).))))))))))).)))....))).))))))..))).)).))..)))

```
 Initial dG =    -23.60

          10
.-A|      U
   UAAUAUG A
   GUUAUAC U
\ -^      A



 20           30          40             50        60
  C   U-  U  --   --      G   ----   -         A   U
   GCG  CU GU  AGC  UGUUGA UCC    CGU UGAGAAGUG AGG G
   CGU  GA CG  UUG  ACGAUU AGG    GCG AUUUUUUAU UCU G
  -   UU  C  CC   CC      G   CGCU   U         G   U
 110       100        90        80        70

-----------------------------------------------------
```

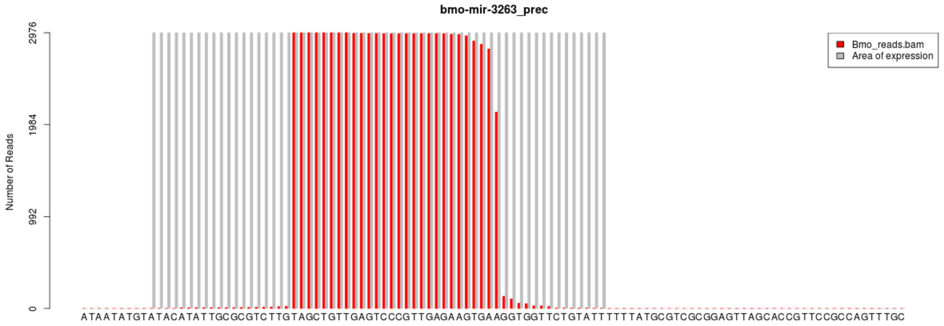

>bmo-mir-3264_prec

AAACAACUGAAACAAAUUAUUUUUUUGUCGUUGUGGAUGAUUGUGGAAGACAUAUAUCGAGGGGUGAAAGGGUAUUUGGUUUAAGCAACAUUUUCGCGACAUCACAGGAGAGCCAUUUAG
AGUUUAAAAGUUGG
...(((((.((((((((..(((((((((.((((((((((.((((..(((.((.
(((((............))))).)).)))..))))))))..))))))...)))))))))..))))...))))...))))).

```
 Initial dG =    -25.90

          10           20          30          40        50        60
AAA     G--    ---    UA         C--      --|   A    GG   A  U     GAGGG
   CAACU   AAAC   AAAU  UUUUUUUGU   GUUGUG  GAUG UUGU  AAG CA AUAUC     G
   GUUGA   UUUG   UUUA  GAGAGGACA   CAGCGC  UUAC AACG  UUU GU UAUGG     U
G--     AAA    AGA    CC         CUA      UU^   -    AA   G  U     GAAAG
    130       120       110       100        90         80        70

-----------------------------------------------------
```

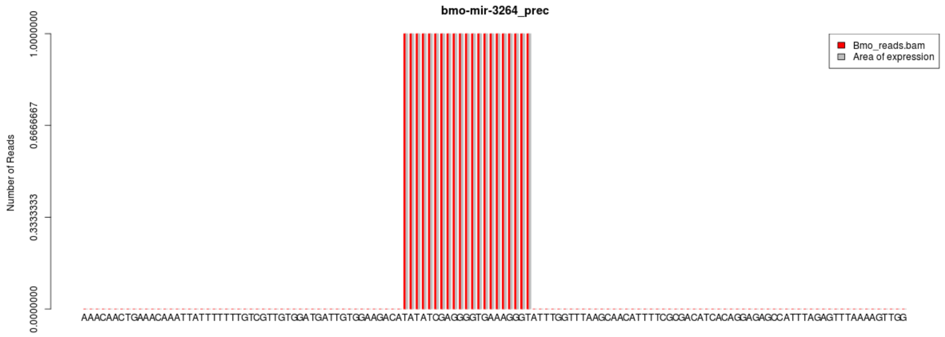

>bmo-mir-3265_prec

```
GUAGAGCUAAGACUGAGAGCUCGUGUCUUAAGGUGGGUGGCGGCAGUUACAUUGUUGAUGUCCAUGGGCUCCGGUAACUAUUUAACUCA
...(((.((((((((.(((((((((......((..((((((....))))).)..))......)))))))))))))).....)))).))).

 Initial dG =    -26.50

        10             20        30         40
GUA   C    -----|   A         UCUUAA  UG -     G
   GAG UAAG     ACUG GAGCUCGUG      GG  G GUGGC G
   CUC AUUU     UGGC CUCGGGUAC      UU  U CAUUG C
A--   A    AUCAA^   -         CUGUAG  GU A     A
          80         70        60        50

-----------------------------------------------------
```

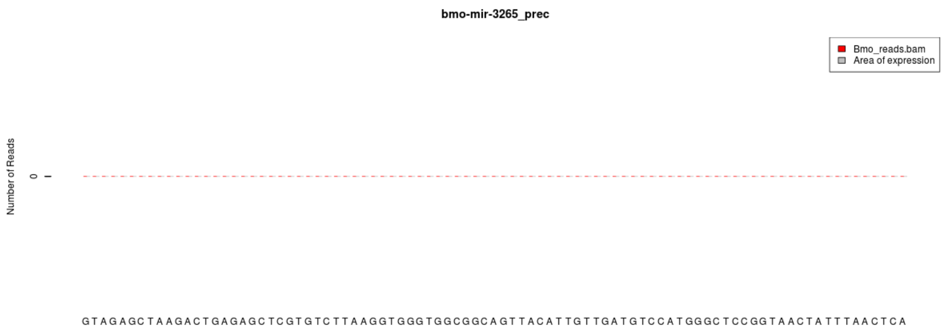

>bmo-mir-3266_prec

UACAUUGCUGAAGUUACAUUGGGAAGUGAGACCUCGUAGCCUCGCAGCCCACUGUGAUGGUGACUCCUGGGGUAGUUGUGUGGCGGCAUAAAAAUGAUGACUCUCGCCAAAGCCCACUGA UUUUGGAGUGA

```
...........((((((.((((..(((((..........)))))..)))).))))))....(((((..((((..(((.((((.
(((((.......))).)).))))))).)))).........)))))..
```

```
 Initial dG =    -38.40

        10        20           30              40
UA    G UG      CAU    AA---            ----  A   -|     C
  CAUU C  AAGUUA   UGGG     GUGAGA--CCUC    GU GCC UCGCAG C
  GUGA G  UUUAGU   ACCC     CGCUCU  GGGG    CA UGG AGUGUC C
A-    - GU      C--    GAAAC      \     UCCU  G   U^     A
130        120         110           70        60        50


                                                 80
                                    UAG-------      G
                                              UUGUGU G
                                              AAUACG C
                                    CAGUAGUAAA      G
                                   100        90

-----------------------------------------------------
```

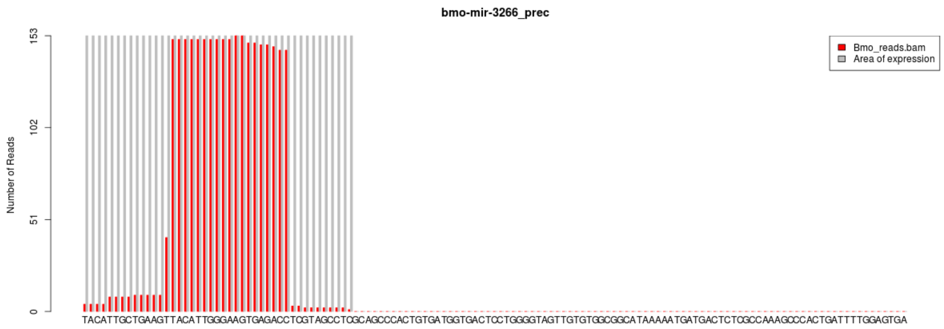

>bmo-mir-3267a_prec

```
CCGCCGAAGAUGGGUGGCGGCAUUAAGUAUUGAUGUUUUUGAGAUCCGGCAACCACUUAAAAUCAGAUUGGCUUUGAGCUCGUCCACCCAUUGAAGCA
..((....(((((((((((((...((((.....((.(((((((............))))))).)).....))))...)).)).)))))))))...)).
```

```
 Initial dG =    -31.10

        10          20        30        40
CC  CGAA         -  -| AUU    AUUGA  U       AUCCG
  GC    GAUGGGUGG CG GC   AAGU     UG UUUUGAG     G
  CG    UUACCCACC GC CG   UUCG     AC AAAAUUC     C
A-  AAG-         U  U^ AGU    GUUAG  U       ACCAA
         90        80        70        60        50
```

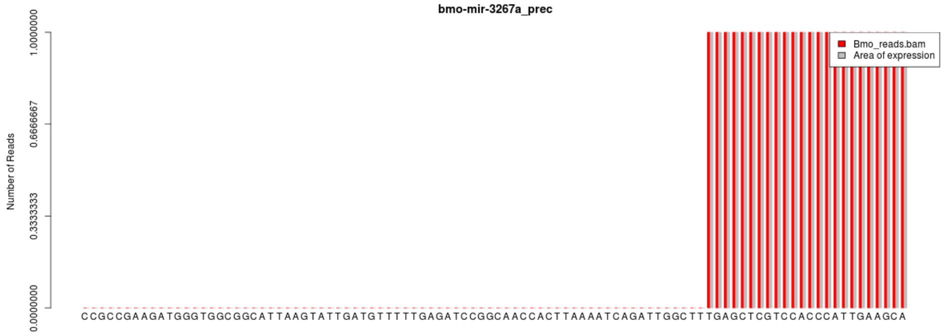

>bmo-mir-3267b_prec

```
UCAACACAUGUCUCAAGGUGGGUGGUGGAUUAUCGUUUUUAAUGCUUAUGGUCUACAGUGACCAUUGAAAACCAGUUAAGCCAUGAGCUCGUCUAUUCAUUUUGACAGUAAAAAUAAAAU
............((((((((((((((((.((((.((((....((...((((((......)))))).......))...)))).))))..))).)))))))))))))...............
```

```
 Initial dG =    -25.40

        10           20           30        40            50
UCAACACAUGUC---             -   A-    C    UUAA  CUU----|     UA
               UCAAGGUGGGUGG UGG  UUAU GUUU    UG       AUGGUC  C
               AGUUUUACUUAUC GCU  AGUA CGAA    AC       UACCAG  A
UAAAAUAAAAAUGAC             U   CG    C    UUG-  CAAAAGU^     UG
.       110       100        90        80         70        60

-----------------------------------------------------
```

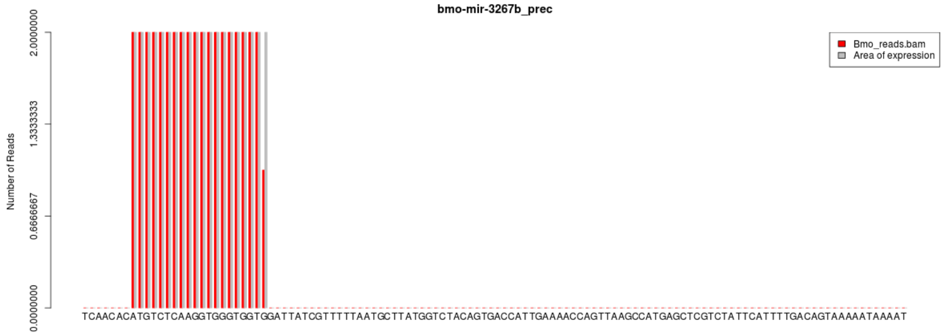

>bmo-mir-3267c_prec

```
CAUUUACGUUGUAGAUGUCUAUGGGCUUCAGUAACCACUUAACACCAGGUGGACUGAGCUCGUCCAUUCAUCUAAACAAUAAAUAAAAUAAUC
.......(((.((((((...(((((((.((((..((((((......)))))))))))))))))).....)))))))))................

 Initial dG =    -26.70

                 10        20          30        40
CAUUUAC---------   G      UCU--|      U    AA      AA
                GUU UAGAUG     AUGGGCU CAGU  CCACUU  C
                CAA AUCUAC     UGCUCGA GUCA  GGUGGA  A
CUAAUAAAAUAAAUAA   -      UUACC^      -    --      CC
  90        80         70        60           50

-----------------------------------------------------
```

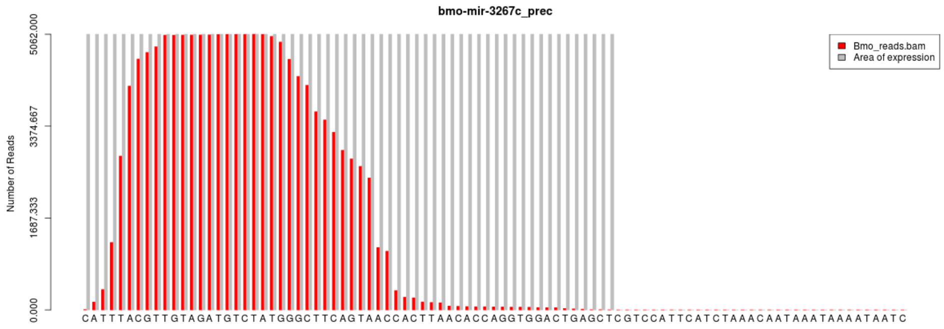

>bmo-mir-3268_prec

AAUGAACAGGUGAAUUGAAGUAUGAUUCAUUAGUAGACUAUACUCGUAAUUAUUCUAAUAGUGAUGGGAAUUGUGUUUGUCGAUGAAUUAUUGGUACUCAAAAAACCUUCGA
..(((..((((...(((.((((((((((((..(((((....(((((.(((((....))))).)))))......)))))..))))))))....))))))))...))))))).

```
 Initial dG =    -25.90

        10        20            30        40          50
AA   AC    GAA   A     ----        AG     UAUA--|    A     U
  UGA  AGGU   UUG AGUAU    GAUUCAUU  UAGAC      CUCGU AUUAU C
  GCU  UCCA   AAC UCAUG    UUAAGUAG  GUUUG      GGGUA UGAUA U
A-   --    AAA   -     GUUA        CU     UGUUAA^    G     A
 110         100         90        80        70        60

-----------------------------------------------------
```

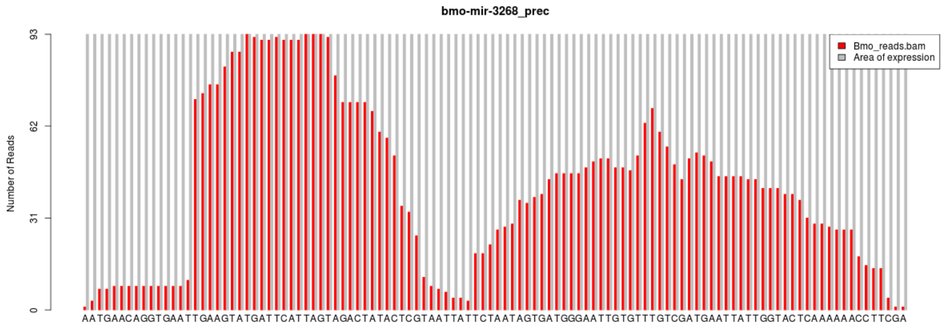

>bmo-mir-3269_prec

ACGCUUCCUACUAGUUGCUUUGUUAUCAGUUACUGCUAUAUGGCAACAGAUGGCGUUAUUCAUACAGGCAUAACAAUAUCUAGUUGCUACCUACAGUCUAGCUUUGUCUGCAUACAAAAA
ACAGAAAGU
..((((.((....(((..(((((...(((.((..((((.((((((((((((..
((((((.....)).)))))..))))).))))))).......)).))))..)).)))...))))).))))).))))

```
 Initial dG =    -26.30

        10        20        30        40                  50         60
AC    C  ACUA   GC     UAU   U  CU    U  -------      -     GC     -| AU
  GCUU CU    GUU  UUUGU   CAG UA  GCUA AU       GGCAAC AGAUG  GUUAU UC  \
  UGAA GA    CAA  AAACA   GUC GU  CGAU UG       UCGUUG UCUAU  CAAUA GG  A
--    A  ----   A-     UAC   U  UU    C  ACAUCCA      A     AA     C^ AC
             120        110       100        90        80        70

-----------------------------------------------------
```

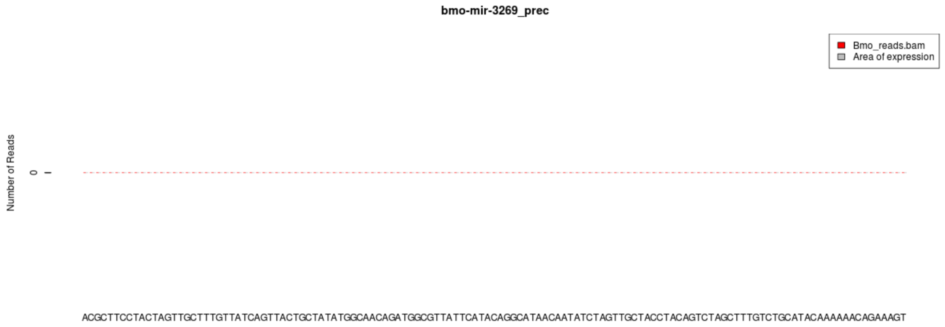

>bmo-mir-3270_prec

```
CGGCAUUCUUAUGUCUUUUUCCUAAAACGAUUCAUUUAAUAGGAAUGAUUUUCAUACCUAUUAAAUGAAUCGUUUUAGGAAACAAAACUCUGAAUGG
...(((((....((.((((((((((((((((((((((((((((.(((.....))).))))))))))))))))))))))))))).)).))...))))).

 Initial dG =    -43.80

        10         20        30        40
CGG|    UUAU  C  -                         A   AU
   CAUUC    GU UU UUUCCUAAAACGAUUCAUUUAAUAGG AUG  \
   GUAAG    CA AA AAAGGAUUUUGCUAAGUAAAUUAUCC UAC  U
G--^    UCU-  A  C                         A   UU
        90         80        70        60

-----------------------------------------------------
```

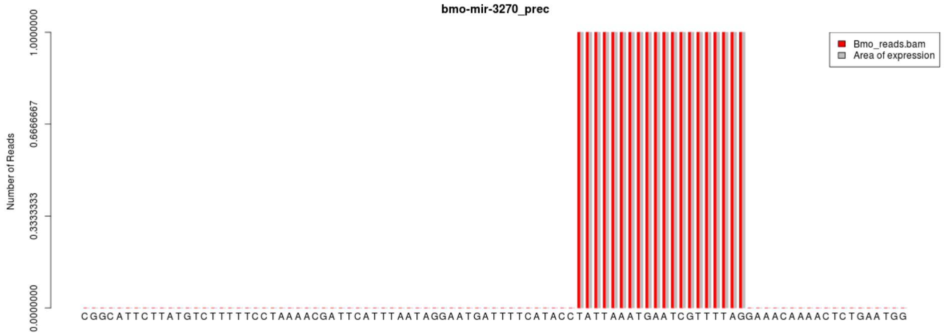

>bmo-mir-3271_prec

GCGGUGGAUUCGUGGAUUUUCGUGUGGCAAACAGAACAGUGCGUGACAUCAUGUGCGCGUGCUGCUGAGUAACAAUAGCAGCAGCACCCCACGUGUUUUCACGCUCGUAGUCUGUGAAGC
AACAAGGCAGCAGUGCAGUGCG

```
(((.((.(((.((.........(((.((..(((((.....((((((...((((((...
((((((((((...........))))))))))..))))))...))))))......)))))...)).))).....))))).)).))).

 Initial dG =    -51.80

         10        20        30         40         50        60        70
-|  G  G   C  GGAUUUUCG   G  AA-     ACAGU-      CAU      CGC        AGUAA
 GCG UG AUU GU         UGU GC   ACAGA      GCGUGA   CAUGUG   GUGCUGCUG     \
 CGU AC UGA CG         ACA CG   UGUCU      CGCACU   GUGCAC   CACGACGAC     C
G^  G  G   -  ACGGA----   A  AAG     GAUGCU      UUU      CC-        GAUAA
140        130             120       110       100        90         80

-----------------------------------------------------
```

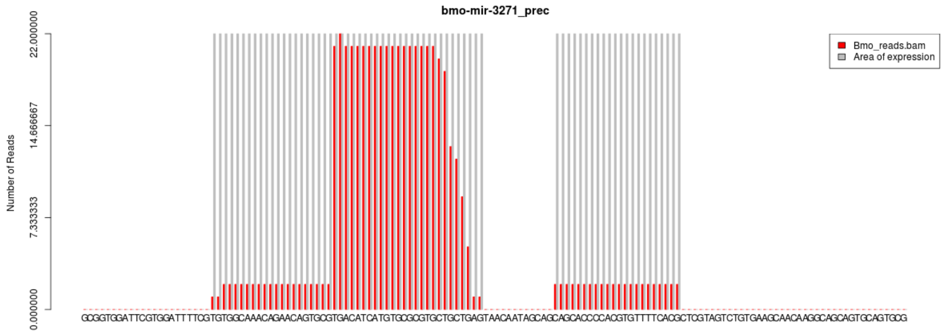

>bmo-mir-3272_prec

AUAAUUCAGUGUGAAGUUUGGGUGUCCAUAUUUUCAACUAAUUUCUUAUAAUUGGGGAUAUAUUUAGAGUUCCCGGUCCUUAAACAGUUAUCUAAAUGUGAGUCAUUAAGCUUUGUCUGG
UAUUGA

```
.((((((((.(..((((((((..(..(((((((.....(((((..(((..
((((((((((.........)))))))))...)))..)))))...))))))))..)..))))))))..))))).)))).

 Initial dG =    -26.30

         10        20        30        40        50         60
A    -|   U UG        GU UC       UCAAC     UC   UA-         AUAU
 UAAU UCAG G  AAGUUUGG  G  CAUAUUU     UAAUU  UUA   AUUGGGGAU    \
 GUUA GGUC U  UUCGAAUU  U  GUGUAAA     AUUGA  AAU   UGGCCCUUG    U
A    U^   - GU        AC GA       UCU--     CA   UCC         AGAU
    120        110       100          90        80        70

-----------------------------------------------------
```

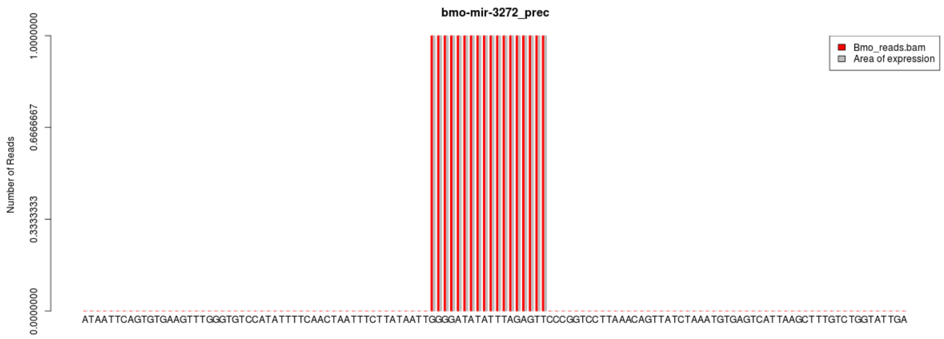

>bmo-mir-3273_prec

UUAAUUGAGGGUAUUUUUUUAUUGCUUAGAUGGGUGGACGAGCUCGCAGCCCAUCUGAUGUUAAGUGGUUACCGGAGCCCGUAGACAUCUACAACGUAAAUGCCUCCACCCAUCUUAGAUAUGAGUUCUAAGGUCUCAGUA

```
...(((((((.......(((((..((.(((((((((((...((.....((......((((((....
((((.....))))....))))))......))....)).))))))))))).))..))))).......))))))).
```

```
 Initial dG =    -40.10

        10         20        30          40        50
UUA       GUAUUUU-     UG  U           .-CGA    GCAGC-|  U  GAU
   AUUGAGG        UUUAU  CU AGAUGGGUGGA     GCUC      CCA CU   \
   UGACUCU        GAGUA  GA UCUACCCACCU     CGAG      GGU GA   G
A--       GGAAUCUU     UA  U           \ ---    GCCAUU^  -  AUU
 140       130       120       110                  70


                                         80
                                         CC----------    A
                                                     GUAG C
                                                     CAUC A
                                         CCGUAAAUGCAA    U
                                           100        90
```

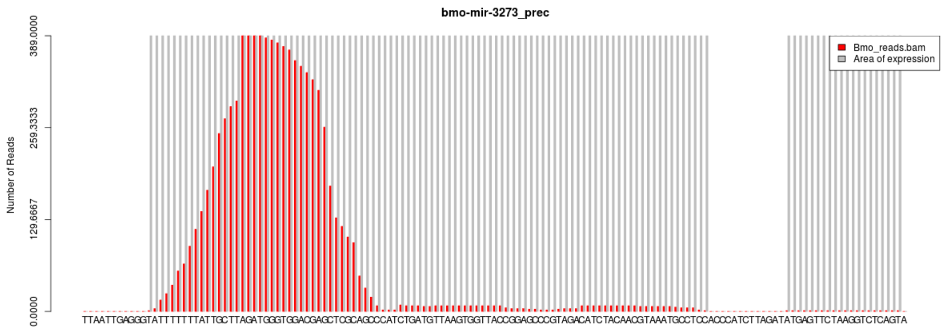

>bmo-mir-3274_prec

```
CACUGCAUAUGAUGCGUGAAUGUAUAUUUCAUCUAAGAAAACUAUUGGCCAACCUUCUACACGAUUUUGCUUCAUAGUAUCGAUAUACUUGCGUCUCCAUGCAA
...(((((..((.(((..(..(((((((....(((.(((....((((..............)))).....))).))).....))))))))..))).)).))))).
```

```
 Initial dG =    -19.60

        10        20        30        40         50
CAC|    AU  U   UG AU       UCAU   A   AACU-    GCCAAC
   UGCAU  GA GCG  A  GUAUAUU    CUA GAA     AUUG      C
   ACGUA  CU UGC  U  CAUAUAG    GAU CUU     UAGC      U
A--^    C-  C   GU --       CUAU   A   CGUUU    ACAUCU
    100         90          80        70         60

-----------------------------------------------------
```

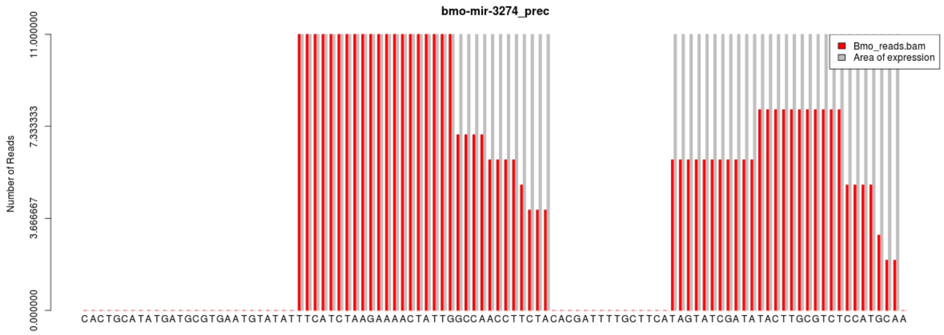

>bmo-mir-3275_prec

CGAAUUAGUGAUUAUACAAUUAAGUCGGUGAAUUAUCAAGUUCCUUAUCUUAGGACUUACAGUACGAGGAGCAUCUCGAAGUUGGAGCACGAAGAACUGGGAGACGGGAUGAUGAUGAUU
UGAUUCUUGAGUAGGACUUCCUUCA

```
.(((..(((..((((.(((((((((((....((((((..((((((..((((....(((......
((((......))))......)))....))))...)))).))..))))))..)))))))))...)).)))).)))...))).
```

```
 Initial dG =    -32.30

         10        20        30         40        50          60        70
C   UU-   GA    ACAA     UCGG          A-     UUA      GA   .-ACAGUA|   GA
 GAA   AGU  UUAU    UUAAG    UGAAUUAUCA  GUUCC   UCUUAG  CUU        CGAG  G
 CUU   UCA  GAUG    AGUUC    GUUUAGUAGU  UAGGG   AGGGUC  GAA        GCUC  C
A   CCU   G-    ----     UUA-          AG     CAG      AA   \ ------^   UA
   140            130        120       110       100



                                                              80
                                                              AA-  U
                                                                 GU   G
                                                                 CG   G
                                                              GCA  A
                                                              90
```

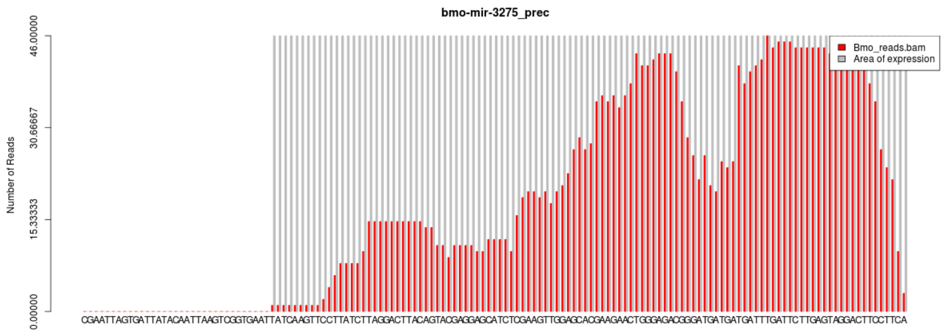

>bmo-mir-3276_prec

UGUUAUACAACUGAAGUCAAUUUAUGAUUGGUGUUUGUGAAAAGUGCAGGAAAACAUUUGCAGCUAAAAAUUUGUUUCCAUAACAUCUUUAGAAACAUACAUUUAUAGCAUUGCUAUUAU
UUGAUUGACC
.((((..(((.(((((.((((((((((.((((((((.((((..((...(((((..
((((........))))...)))))...))...)))).)))))).)).)))))).)))))).))).)))..)))).

```
 Initial dG =    -23.50

        10         20         30         40         50         60
U    UA   C   -  U    -      U  -      G    AA-  GCA     AC-|   GCA
 GUUA  CAA UGA AG CAAU UUAUGA UG GUGUUU UGAA   GU   GGAAA   AUUU   G
 CAGU  GUU AUU UC GUUA GAUAUU AC UACAAA AUUU   CA   CCUUU   UAAA   C
C    UA   U   A  -    C      U  A      G    CUA  AUA     GUU^   AAU
.       120        110       100        90        80        70

-----------------------------------------------------
```

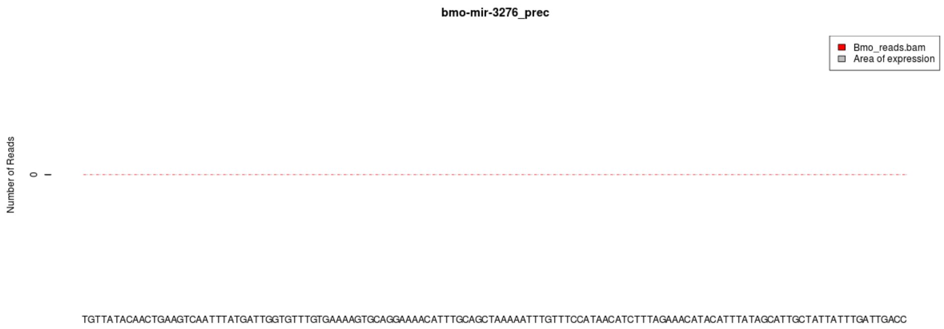

>bmo-mir-3277_prec

CAAAUGUUUGUUGUGGGCAUGUUUCUGAAAUUCGAUCUACGUUAAUAAACCAAGUAUGUAGUAGUGGUAACUCUCGCUAAGAAAUAGUUUAUUAUGGAAAUGUAAAAUUUGAGUUUCGUC
UGACUUGUAUUUGUGAACAUA

```
...(((((..(.....(((.(((..((((((((((..((((((((((((((....(((....
((((((......))))))....))))))))))))).....)))))....))))))))))...))).)))....)..))))).
```

```
 Initial dG =    -30.20

        10        20         30          40             50        60        70
CAA     UG UGUGG   U   UC-          UC--     -----|       CAAG   GUAG      UA
   AUGUU  U     GCA GUU   UGAAAUUCGA    UACGU     UAAUAAAC    UAU    UAGUGG  A
   UACAA  G     UGU CAG   GCUUUGAGUU    AUGUA     AUUAUUUG    AUA    AUCGCU  C
A--     GU UUUA-   U   UCU          UAAA     AAGGU^       ----   AAGA      CU
 140       130        120       110       100        90            80

-----------------------------------------------------
```

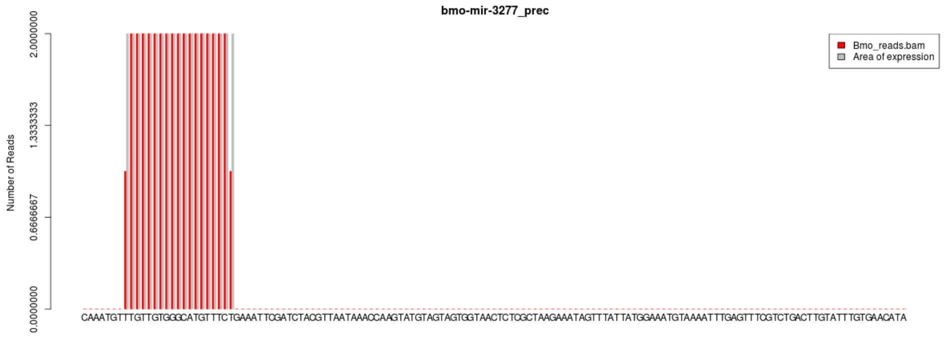

>bmo-mir-3278_prec

```
CACUGUUGGUGGUGUUUGGAUCUUACAACAUUACACAUGAUGUAAGAUCAAAAGCAGAAAUAUACCACAAUAGG
..(((((.(((((((...(((((((((.(((.....))).)))))))))...........)))))))))))).

 Initial dG =    -23.90

        10                 20        30
CA      G       UUG---------|        A   UA
  CUGUU GUGGUGU             GAUCUUACA CAU  \
  GAUAA CACCAUA             CUAGAAUGU GUA  C
G-      -       UAAAGACGAAAA^        A   CA
    70         60        50        40

-----------------------------------------------------
```

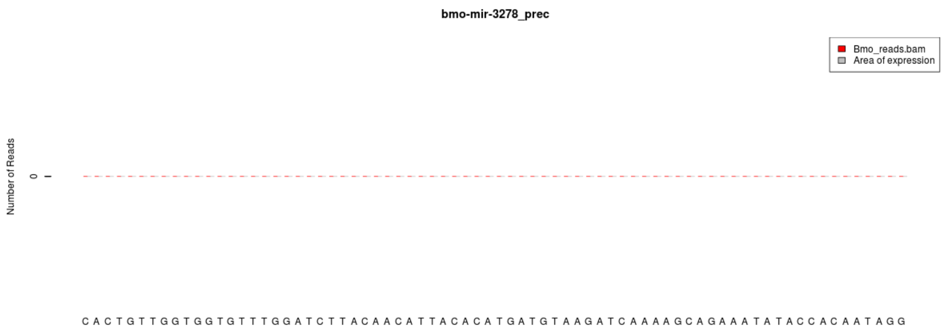

>bmo-mir-3279_prec

AAUUAACACAAAUGCUUUCUUUUCAAAAUAGUCAGGUGGAUAAGAAGCAAGAAUUAUUUGAACUAAUAUCAAUAAUGUUUAUUCAAGAAAUAAAGCACAUGGUAUCGACUAUUCCGGACU
AACUUUGGGAAGUCAAGCCGU

```
.............((((.(((..((((.(((((.(((.((((....((....(((.((((((..
(((((.....)))))..)))))).)))...))......)))).)))......))))).)))..)))..))))...
```

```
 Initial dG =    -26.00

        10         20         30          40         50         60         70
AAUUAACACAAAU    U-   UU    A-     A  .-UGGAUAAGAA| AAGA   A      CU     CA
             GCUU  CUU  CAAA  UAGUC GG            GC    AUU UUUGAA  AAUAU  \
             CGAA  GAA  GUUU  AUCAG CC            CG    UAA GAACUU  UUGUA  A
UGC----------    CU   GG    CA     G  \ ----------^ AAA-   A      AU     AU
 .                 130       120                        90         80


                                         100
                                        AC     A
                                          AUGGU U
                                          UAUCA C
                                        U-     G
                                          110

-----------------------------------------------------
```

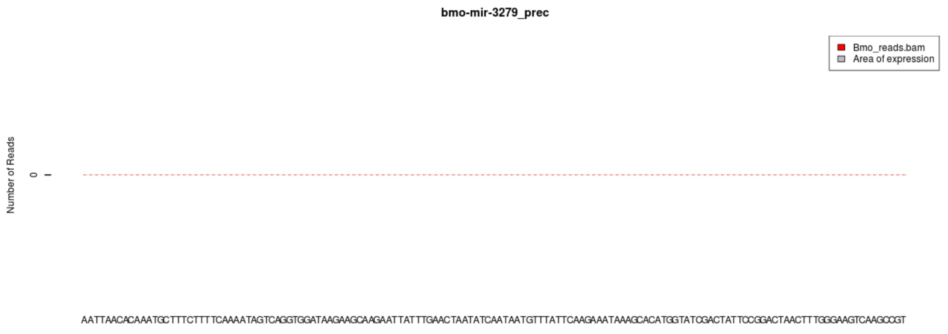

>bmo-mir-3280_prec

UACUGCUGACAUCUAGGUGGUCAUGGUUUUUGAUUGAACUGUAUUCACGUUGGCUGCGAUAGAACCAGUCAAUCGAUAUAAUUACUUUAAAUUUAGACUCACCUGGUGUCAAGUGGUU
.((..(((((.((((((((((.(((..(((((...((.((((((...((((((((.........)))))))).)))))).))...))))).)))))).))))))))))).)..)).

```
 Initial dG =    -36.50

         10         20        30        40        50        60
U  UG -|    U       -   A   UU     UUG  C      CAC       CGAU
 AC  C UGACA CUAGGUG GUC UGG  UUUGA   AA UGUAUU   GUUGGCUG    \
 UG  G ACUGU GGUCCAC CAG AUU  AAAUU   UU AUAUAG   UAACUGAC    A
U  GU A^    -       U   -   U-     UCA  A      C--       CAAG
      110        100          90        80          70
```

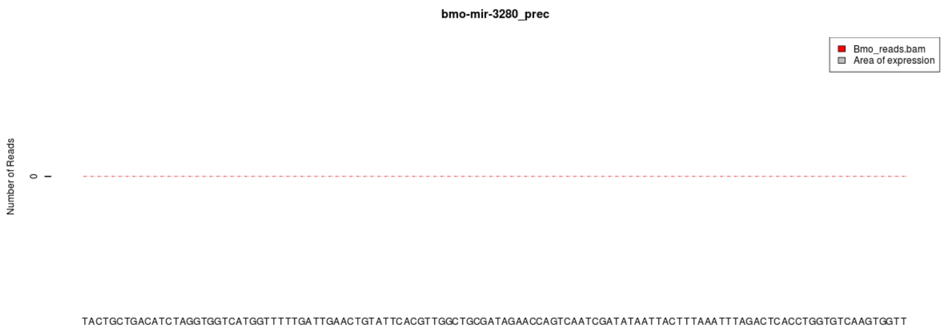

>bmo-mir-3282_prec

```
ACGAAGCAGUCAUGCGUUUUUUUUGGGGAAGAAAGGAAGGAUUACUGUUACGAAGGCCUUUCCAGCUUCACCAGGAUCUGAAAGCGUCUAGUGUUGAGA
......(((.((((((((((((((((.((((...((((((....((.......))..))))))..)))).))))))....)))))))...))))))...

 Initial dG =    -23.80

        10           20            30        40
ACGAAG   U   ---       ----|      G    AAA      AUUA  GUU
      CAG CAU   GCGUUUU    UUUUGG GAAG   GGAAGG    CU   \
      GUU GUG   UGCGAAA    AGGACC CUUC   CCUUUC    GA   A
AGA---   -   AUC       GUCU^      A    GA-      CG--  AGC
            90        80        70          60

-----------------------------------------------------
```

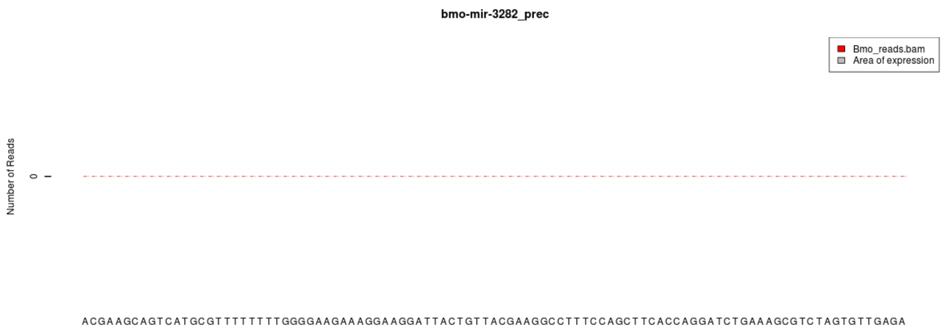

>bmo-mir-3283_prec

CAAGUCACCCCAUCCCACAACAGGGAGUUCCUUAACUUUCUUUAAACGAAAUGAUAAUAAAUGAUCAUUUCUGUUUGUUGUCAGGUUCCGAAGUCCAUUGAGGGUUGUGGAGGACG

...(((..((((.(((.(((...(((.(((...(((((....((((((((((((........)))))))).)))))....)))))..))).))).))).))).)).)).)))..

```
 Initial dG =    -35.30

        10         20        30        40          50
CAA   AC  -| U   A   CAG   G   CUU     UCUU-     -        AAU
   GUC  CC CA CCC CAA   GGA UUC   AACUU     UAAAC GAAAUGAU   A
   CAG  GG GU GGG GUU   CCU AAG   UUGGA     GUUUG CUUUACUA   A
G--   GA  U^ U   A   A--   G   CC-     CUGUU     U        GUA
      110       100          90         80        70        60

-----------------------------------------------------
```

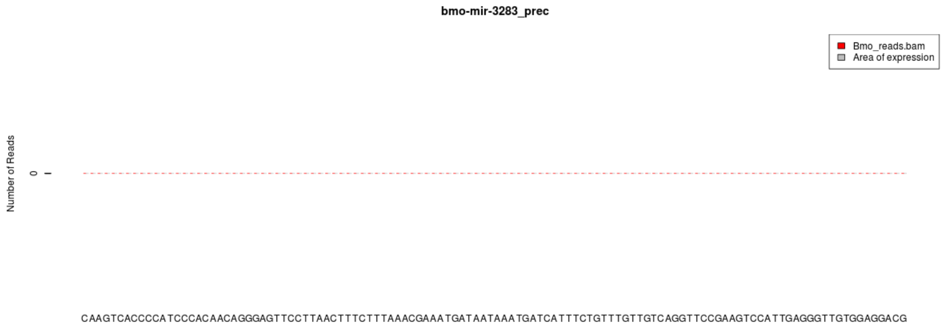

>bmo-mir-3284_prec

```
ACAUUCGCGAGCUCCUUAAUAUUUGUAAGGUUAAGGACGUAUUAAUGAUUUUAUUGAAAGAAAUAAAGAUGUUGACGUCGCCUUUGGGGCUUGAAGAAC
...(((.((((((((...........(((((....(((((..(((((.(((((((......))))))).))))))))))))))).))))))))..))).

 Initial dG =    -23.70

         10        20        30        40        50
ACA   G-|       UUAAUAUUUGU     UAAG     AU     A       GA
   UUC  CGAGCUCC           AAGGU    GACGU  UAAUG UUUUAUU  A
   AAG  GUUCGGGG           UUCCG    CUGCA  GUUGU GAAAUAA  A
C--   AA^       U----------     ----     --     A       AG
          90                  80               70        60

-----------------------------------------------------
```

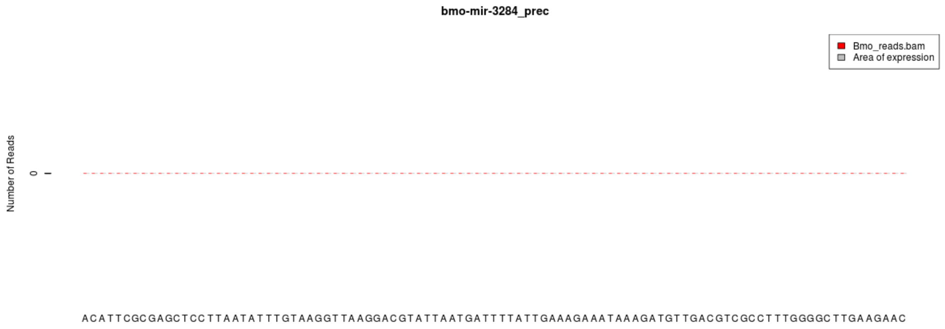

>bmo-mir-3285_prec

AACUUAUCGCUAUAUUCCAAUUAAUGUUGGAAAUAUUCAGCGGCUCGCGACGCCAUGUUUUAUUAACCUUUCAUAAUGUACGAGCUUUAUCUUGUUGUAUUAAAUCAGCUUUAAUGUGGA
ACGGUUUUGAGA
..((((..(((...((((((((((.(((((.......((((((((((..(((..
(((..............)))..))).))))))).......)))).......))))).))))).))))).)))..)))).

```
 Initial dG =    -26.50

        10         20        30        40               50        60
AA    UC   AUA     -     U     AAAUAUU    -------|     CG   CC   UUUUAU
  CUUA  GCU   UUCCA AUUAA GUUGG       CAGC       GGCUCG  ACG  AUG      U
  GAGU  UGG   AAGGU UAAUU CGACU       GUUG       UCGAGC  UGU  UAC      A
A-    UU   C--     G     U     AAAUUAU    UUCUAUU^     A-   AA   UUUCCA
 130         120       110       100        90        80         70

-----------------------------------------------------
```

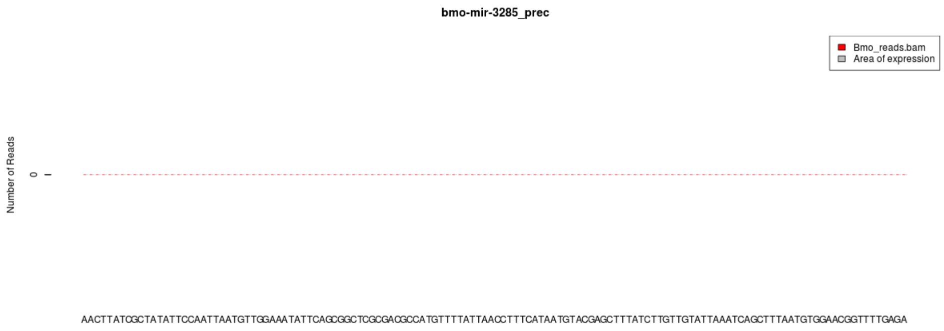

>bmo-mir-3286_prec

AUGAUUGGCGAAUAAUCGAAGCCCAAAACAACUAUUGGUCACUGCACAGGAAUAGUGAAACCUGCAUACUCUUGUGCGUGUUCCAAUAGUUAUUGUGGGUGCUUCAAAAUCGACAUGUCA
.((((((.(((......((((((..((.(((((((((.(((.((((((((.((((.......)).)).)))))))))))..)))))))))).))...)).)))))....))).)).))))

```
 Initial dG =    -40.50

         10        20         30         40        50
A    -  G   AUAAUC     -  AA-  C         U-|  U        A  -  GAA
 UGAU UG CGA      GAAGC CC   AA AACUAUUGG  CAC GCACAGGA UA GU   \
 ACUG AC GCU      CUUCG GG   UU UUGAUAACC  GUG CGUGUUCU AU CG   A
-    U  A   AAAA--     U  GUG  A         UU^  -        C  A  UCC
 .       110         100        90         80         70

-----------------------------------------------------
```

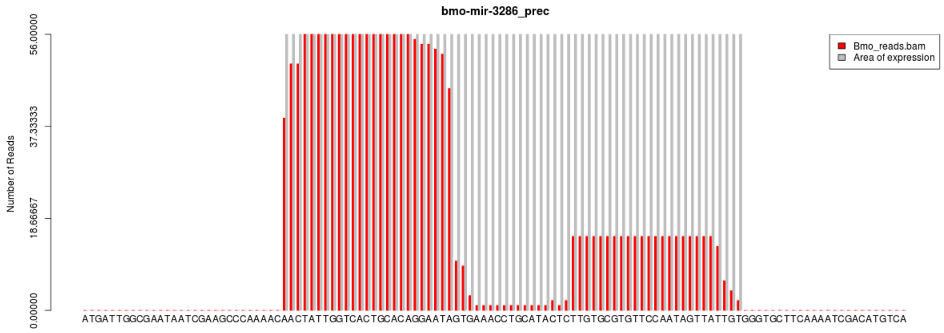

>bmo-mir-3287_prec

```
GAGUCCGCACGGGUAGGUACCACCGCCUGUCUAUUUCUGCCGUGAAGCAUAAUGCGUUUCGGUUUUGAAGGGUGGCGUAACCGUUGUAACUAU
.(((..((((((.((.(.....((((((.((.......((((....((.....))....))))...)).))))))).)).))).))).)))..

 Initial dG =    -24.40

          10        20        30        40        50
G-|  CC   -   G  G UACCA      G  UAUUUCU    UGAA  AU
  AGU  GCA CGG UA G     CCGCCU UC       GCCG    GC  \
  UCA  UGU GCC AU C     GGUGGG AG       UGGC    CG  A
UA^  A-   U   A  G -----      A  UUU----    UUUG  UA
  90          80             70            60

-----------------------------------------------------
```

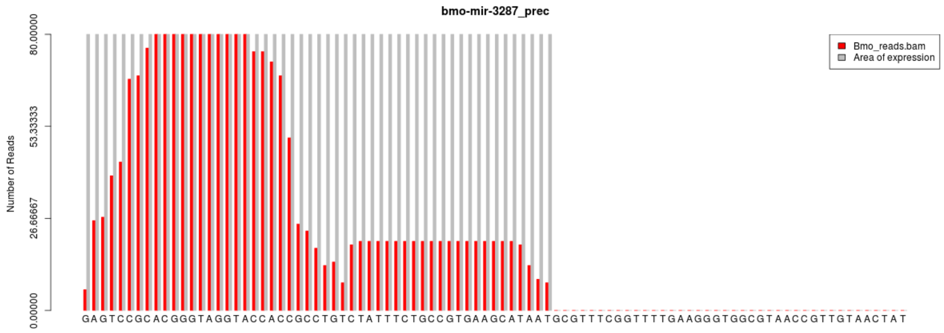

>bmo-mir-3288_prec

UAGCUUGCUUUGAGCUUGGAGUAGCUUGCUUGGAGUAGCUUGCUUGGAGCUUGGAGUAGCUUGCUUGGAGGUUGGAGUAGCUUGUUUGGAGCUUGGAGUAGCUCUUGUGAGUUCGCGCGG
GUA

```
..((((((..((((((((((((.((((((((.((..((((.((((.((.(((.
((((.....)))).))).)).))))))))...)).))))...)))).)))))...))))))).)))))).

 Initial dG =    -46.60

        10            20            30          40         50
UA      UU       ---     A     ---     G   GU-|    U     G   G    G     AG
  GCUUGC  UGAGCUU   GGAGU GCUU   GCUU GA   AGCU GCUU GA CUU GAGU  \
  UGGGCG  GCUUGAG   UCUCG UGAG   CGAG UU   UCGA UGAG UU GAG UUCG  C
A-      C-       UGU     A     GUU     G   UGU^    -     G   G    G     UU
  120        110       100         90         80         70

-----------------------------------------------------
```

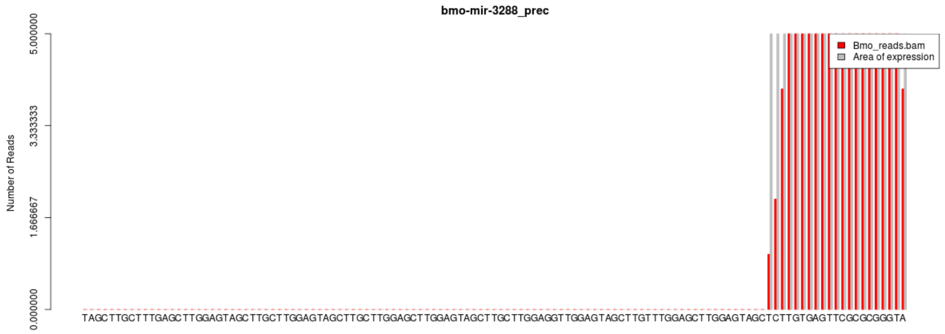

>bmo-mir-3289_prec

```
CUAAUAUAUCCGUUUUGUAUGUGGCCUUGCGGGGAUUACUCAGCAAGAACGGUCUGGUUUAGUUGAAGUAAUUGUUACGCGGUCACUAUUUAACA
...........(((..(((.((((((.((..(.((((((((((((..((((......)))).)))).))))))).)..)).)))))))))..))).

 Initial dG =    -28.30

        10         20         30         40          50
CUAAUAUAUCC|  UU   U       U  CG G       -    AA    GG
           GUU  GUA GUGGCC UG  G GAUUACU CAGC  GAAC  U
           CAA  UAU CACUGG GC  U UUAAUGA GUUG  UUUG  C
A----------^  UU   -       C  AU G       A    A-    GU
              90          80         70          60

-----------------------------------------------------
```

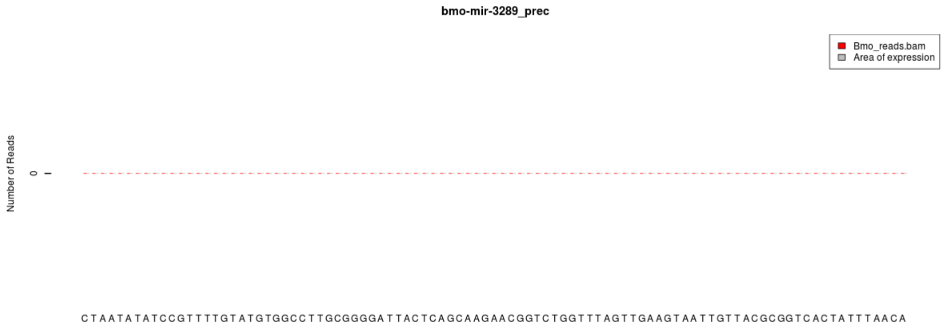

>bmo-mir-3290_prec

```
AUCGUCCGAAUCAUAGUUUGUAAUUAAAUUUAUGAUUUUGACCAGUAGUCGCAAAUAGUCGCAAAUGUGGGCAACUAAUUGAAAUCGUCCGUUUUAGUCACGAUCUGGUGAGGCGGGCA
...(((((..((((((.((((.((((((...(((((((..(....((((.((..(((........)))..)).)))).)..)))))))....)))))).)))).))).))).)))..))))).

 Initial dG =    -33.40

        10         20        30         40        50        60
AUC     AA   -   U    A      UUU-|      UG CCAG    C  AA   GUC
   GUCCG  UCA UAG UUGU AUUAAA    AUGAUUU  A    UAGU GC  AUA   G
   CGGGC  AGU GUC AGCA UGAUUU    UGCUAAA  U    AUCA CG  UGU   C
A--     GG   G   U    C      UGCC^      GU A---    A  GG   AAA
         110       100        90        80           70

-----------------------------------------------------
```

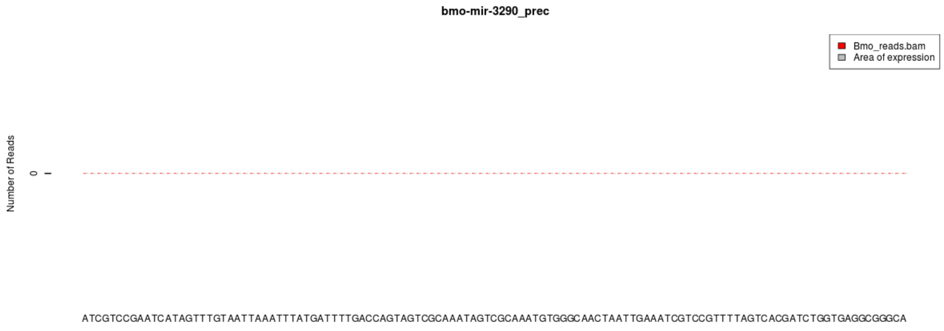

>bmo-mir-3291_prec

GUUUGUGGGGAAAUUUACUGAAGAUAAGCUGGGGCAAAAACAUUCAAUACGCCCCCUGUUAGUGUUACGAUUUCUGAGUAGUAAUACGUGAUAGUGAGAGUGUCCUGGCUAUUUCUUUUU
UUUUUUCAUUACU

```
((..(((..((((......(((((..(((..(((((......((((.........((((((((((((.
(((....))).))))))).))))))))))..)))))..)))...))))).)))))..)))).))).)).

 Initial dG =    -34.40

         10        20         30        40        50        60         70
-| UU   GG    UUUACU     UA-   UG     AAAACA    AUACGCCCC      -       G   U
 GU  GUG  GAAA      GAAGA   AGC  GGGCA      UUCA         CUGUUA GUGUUAC AUU C
 CA  UAC  UUUU      UUUCU   UCG  CCUGU      GAGU         GAUAGU CAUAAUG UGA U
U^ U-   UU    UU----     UUA   GU     GA----    ---------      G       A   G
 130        120           110       100                     90        80

-----------------------------------------------------
```

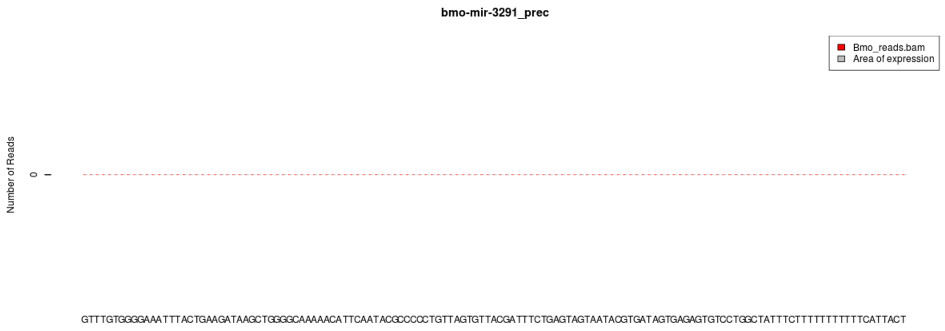

>bmo-mir-3292_prec

AUCUUUUUCUACAGUCAUAUUUCUUUGGACGCAGAAGUCACGAAUGAUGGAAUUACCGGUUGAAAAUGAGAUUGUCUAGUGUCUUAAGAAAAGUAGUUGCAUGUGUGAGAGAA
.((((((..((((((..((((((((.((((((((((((.((.....(((.....)))........)).)))).))).))))))).))))).)))...)).)))).)))))).

```
 Initial dG =    -23.60

        10          20          30          40           50
A      UC    -  CA-   --     U      -   -    A  AAUGA---|  AA
 UCUUUU  UACA GU   UAU  UUCUU GGACGC AGA AGUC CG        UGG  \
 AGAGAG  GUGU CG   AUG  AAGAA UCUGUG UCU UUAG GU        GCC  U
A      U-    A  UUG   AA     U      A   G    A  AAAAGUUG^  AU
 110        100        90        80        70        60
```

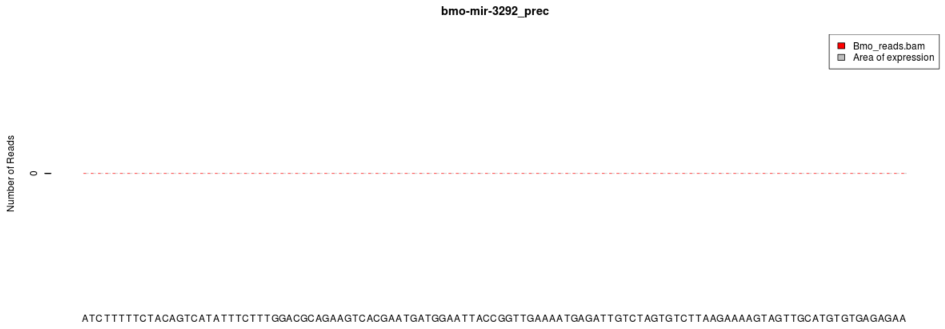

>bmo-mir-3293_prec

```
AUUAUUUUGACGCAUAGCGGAUGGUACGUGAAAAAUAGAUUUCUAGAAACUUCACUAUUCAAUUCAAGUGAACAUUCAUCUUGAAUAAGUCUAGAAUCCGAAAAUUAAGUUAUUUUUACG
CACAAUCUUUAGGCUGUUUCUUUAAAAUC
...(((((((...(((((((((.((.((((.(((((((..(((((((..(((.........
((((((((((....))).)))))))))))))))))............))))))))))).)).))))....)))))....))))))).

 Initial dG =    -26.60

        10              20        30        40                    50        60         70
AUU       CGC-     ----    G  A    A       AU-----------|      AA   CACUAUUCA       -   A
   AUUUUGA    AUAGC    GGAU GU CGUG AAAAUAG             UUCUAGA  CUU         AUUCAAG UGA C
   UAAAAUU    UGUCG    UCUA CA GCAU UUUUAUU             AAGAUCU  GAA         UAAGUUC ACU A
C--       UCUU     GAUU    A  C    -       GAAUUAAAAGCCU^      --   ---------       U   U
         140       130       120        110       100        90                    80

-----------------------------------------------------
```

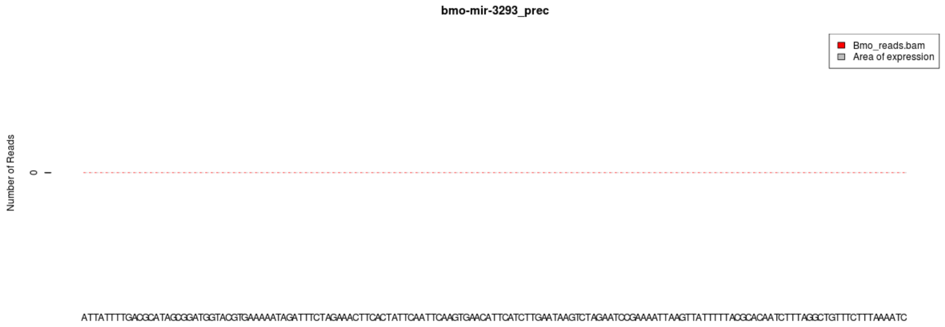

>bmo-mir-3294_prec

```
AAUCUUUUGACAUAUAUCGCGUGUUCACGGUAGGUAUUGGCUCGUGGCACUAUGUAGUACCACAGACAGUAUCUAACCUGAACACAGAUAUUUCAUCGUGAA
..((...(((...(((((..(((((((.(((((((((((.((.((((.(((....))).)))))).)))))))).)))))))))).))))).))).....)).
```

```
 Initial dG =    -36.00

         10        20        30         40        50
AA  UUU-|  CAU     GC        C   -        G  C    C   A
  UC    UGA   AUAUC  GUGUUCA GGU AGGUAUUG CU GUGG ACU U
  AG    ACU   UAUAG  CACAAGU CCA UCUAUGAC GA CACC UGA G
A-  UGCU^  U--     A-        -   A        A  -    A   U
 100          90         80         70         60

-----------------------------------------------------
```

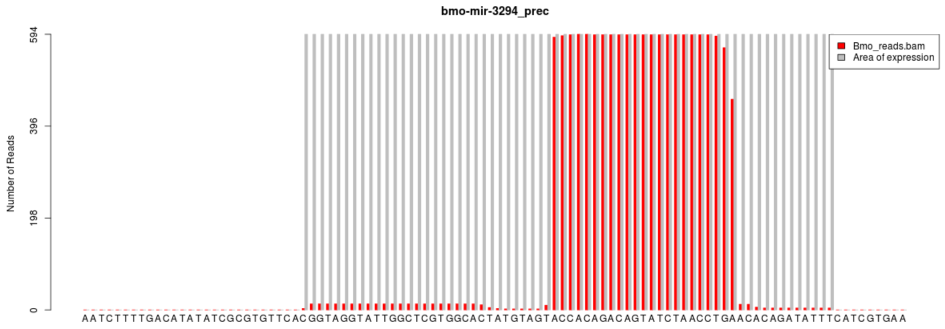

>bmo-mir-3295_prec

```
AAUGUCGAGCGGUAGUUGUUAUACAGUUUUCCUUCGUUUCCGCUAUGGGCGCUGUUCCAAUUCCAUACGUUACUUUUACGCGAUCGAGAAGUUAAAUAACUCGCGCUAAGCAC
..(((..((((..(((((((..((..(((((..((((...((.((((((............))))))))..........)))).)))))))..)))))))..))))..))).
```

```
 Initial dG =    -24.60

        10        20        30        40                50
AA   CG    GU       AU  AG     CU    UUC-------| C      CGCUG
  UGU  AGCG  AGUUGUU  AC  UUUUC  UCGU          CG UAUGGG     U
  ACG  UCGC  UCAAUAA  UG  AAGAG  AGCG          GC AUACCU     U
C-   AA    GC       AU  --     CU    CAUUUUCAUU^ -      UAACC
  110       100           90        80        70         60

-----------------------------------------------------
```

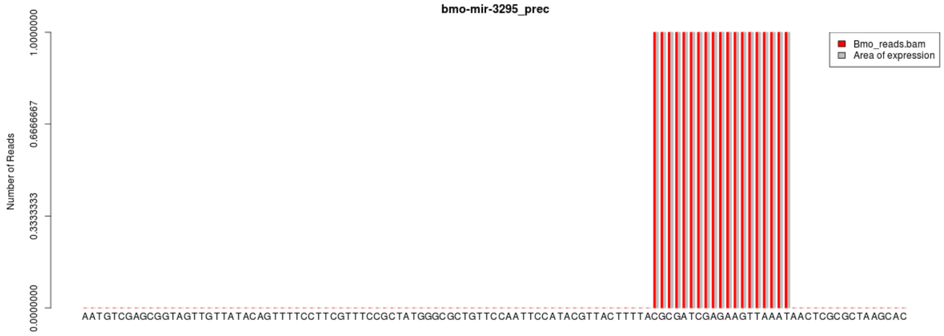

>bmo-mir-3296_prec

UGGGCAGAUUUUUGAGUAUGCCCGCUGUACAAUUCCAGUCGCCGUUUUGAGGUUAGCUACUCGGGAUAUGCUGUCUUUGCCA

```
..((((((......((((((((((..(((.(((..(((........)))..)))...))).)))).))))))...)))))).

 Initial dG =    -21.90

               10        20          30
U-----      AU--         U  .-CC|   UAC
      GGGCAG    UUUUGAGUA GC    GCUG   A
      UCUGUC    AGGGCUCAU CG    UGAC   A
ACCGUU      GUAU         -  \ --^   CUU
 80        70        60


                             40
                              C-   GUU
                                GCC   \
                                UGG   U
                              AU   AGU
                                  50

-----------------------------------------------------
```

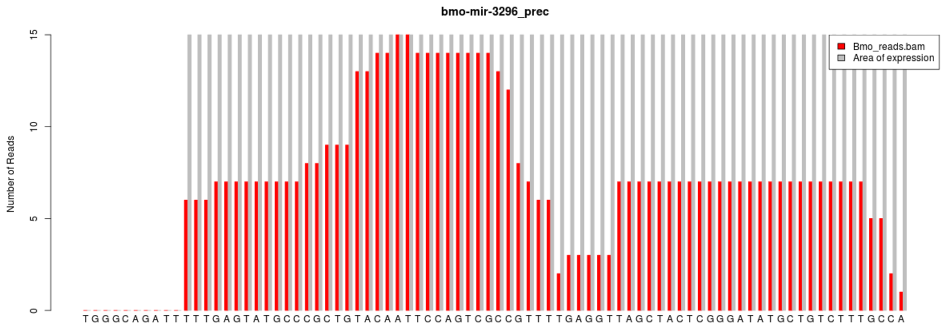

>bmo-mir-3297_prec

UUAGUUAUAACGACAAUUGCCUAGCACAUCUAGUCAAAAAUAUUGGAGGCAAACCAUCUGGACCUGGGCCUUUAUUUAAGUCUAGUUUUUUUAACUUAUGCAAUAUUUCUGACUCAGGUA
UGAUCAGAGUGUUUAACG

...((((....((((.((......((.(((((((((.(((((((((((..(((....((((((.
((((......)))).))))))....)))..)))...)))))))).))))).)))).))....)).)))))))).

```
 Initial dG =    -29.90

        10        20        30         40           50        60        70
UUA|   UAAC    A  GCCUAG  C    -     A        ---   GC   CCAU      C    CC
   GUUA    GACA UU      CA AUCU AGUCA AAAUAUUG   GAG  AAA    CUGGAC UGGG  U
   CAAU    UUGU AG      GU UGGA UCAGU UUUAUAAC   UUC  UUU    GAUCUG AUUU  U
G--^   ----    G  ACUA--  A    C     C        GUA   AA   UUUU      A    AU
            130         120       110       100        90        80
```

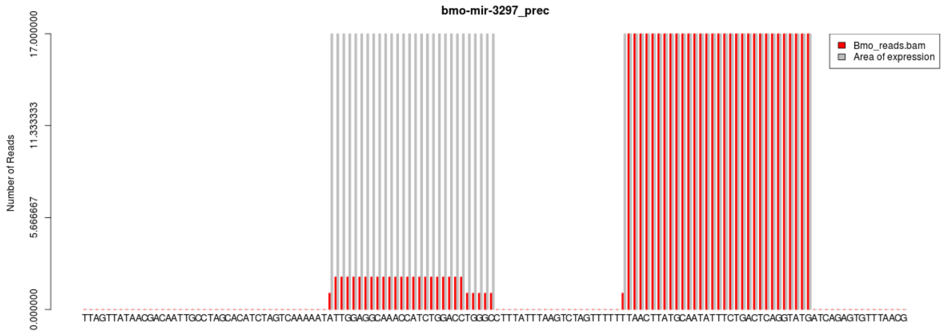

>bmo-mir-3298_prec

```
UAUCGUUCCCUCACUGCUGAGGAUUGCGAACACAUGUGACGUUCUUCCAUUCUGUUCGAUCAUGGCGGUAUGGACCGC
...((.(((...((((((...(((..((((((.(((.((......)))))..))))))))))..))))))..))).)).

 Initial dG =    -20.50

        10        20        30         40
UAU| U   CUC      GAG   UG      C-   U  CG
   CG UCC   ACUGCU   GAU  CGAACA  AUG GA  U
   GC AGG   UGGCGG   CUA  GCUUGU  UAC CU  U
C--^ C   UA-      UA-   --      CU   -  UC
         70          60          50

-----------------------------------------------------
```

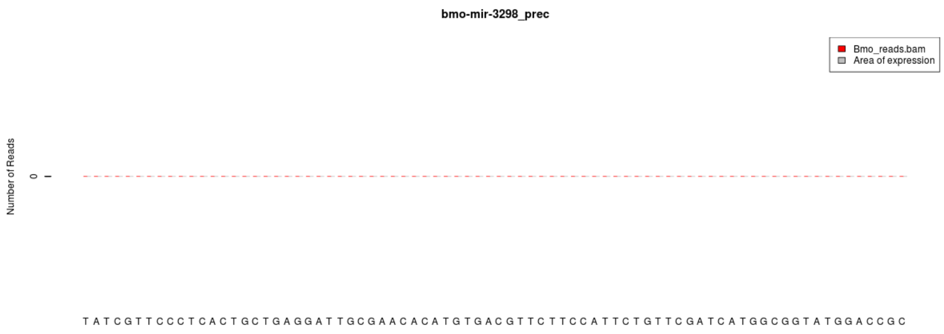

>bmo-mir-3299_prec

```
CUUAAUACUCGAACAUGCGACUUUAACUCGAACAGAAUUUAUGCGUGUGUGUGUCACAUAAUUAUACUCUUCUGUUCAUUGUUAAAGUCUGUAGUCUAAUUGAGAAUUGAU
.(((((.(((((((.((((((((((((..((((((((..((((..((((((...))))))..))))...))))))))...))))))))).))))))....))))).))))).

 Initial dG =    -33.50

        10             20         30         40        50
C     A     ----  A   -         UC-        UU-|   CG      U
 UUAAU CUCGA    AC UGC GACUUUAAC   GAACAGAA   UAUG  UGUGUG \
 AGUUA GAGUU    UG AUG CUGAAAUUG   CUUGUCUU   AUAU  AUACAC G
U     A     AAUC  -   U         UUA        CUC^   UA      U
 .       100        90        80        70        60

-----------------------------------------------------
```

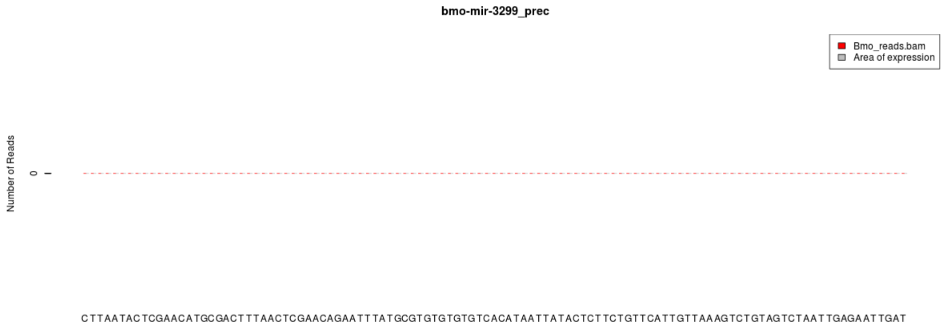

>bmo-mir-3301_prec

```
UAAGUAUGUGUUUCAUUAUAAAUUUCGGUGAUAGUAACCCGUUGAGUUUCUCGCCGGAUAUUCUCAGUGGGUCGCGAUUCCAAUCCGAUAGUAAAUUCUGCG
...(((...((((.(((((....((.((.(((.(..((((((((((..(((....)))....))))))))))..).)))))))....)))))))))..))).

 Initial dG =    -21.40

        10        20        30        40          50
UAA|  UGU    C     AAAU  C  U   A UA          UU--   C
   GUA   GUUU AUUAU    UU GG GAU G  ACCCGUUGAG    UCU G
   CGU   UAAA UGAUA    AA CC UUA C  UGGGUGACUC    AGG C
G--^  CU-    -     GCCU  -  -   G GC          UUAU   C
  100         90         80         70         60

-----------------------------------------------------
```

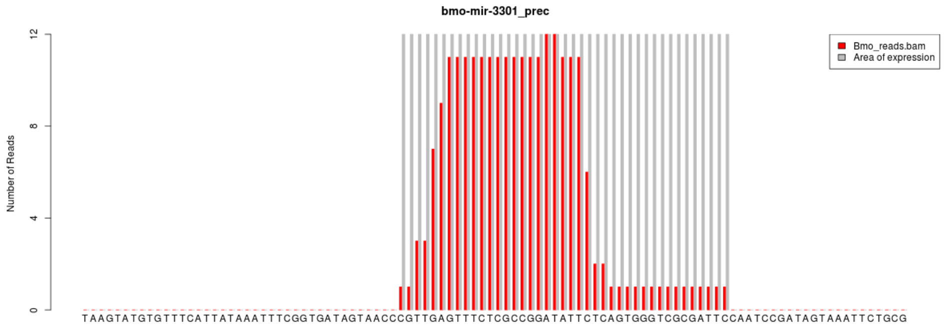

>bmo-mir-3302_prec

CUAAUGGUGGUGUUACGAAUUUCAGUCUGAUUGCAAAGCCUGUUACAUGCUUCCAGAUGUACUUGCGAUAAAAGUCUGGACAAUCACAGCAACCAGUAAUCCAGUCGGUCUGAUUGUUUG
GUAGUGUUA

```
.......(((..((((.((..((((.(((((((......(((.....
((((((((((((...............)))))))).......))))..))).....))))))).)))))....)).))))..)))

 Initial dG =    -29.90

        10        20          30        40        50               60
CUAAUGG   UG    G  UU--    U       CAAAGC   UUACA    -------|      GUACUUG
       UGG  UUAC AA    UCAG CUGAUUG      CUG     UGCU       UCCAGAU       \
       AUU  GAUG UU    AGUC GGCUGAC      GAC     ACGA       AGGUCUG       C
-------   GU    G  UGUU    U       CUAAU-   CA---    CACUAAC^      AAAAUAG
              120       110       100             90        80        70

-----------------------------------------------------
```

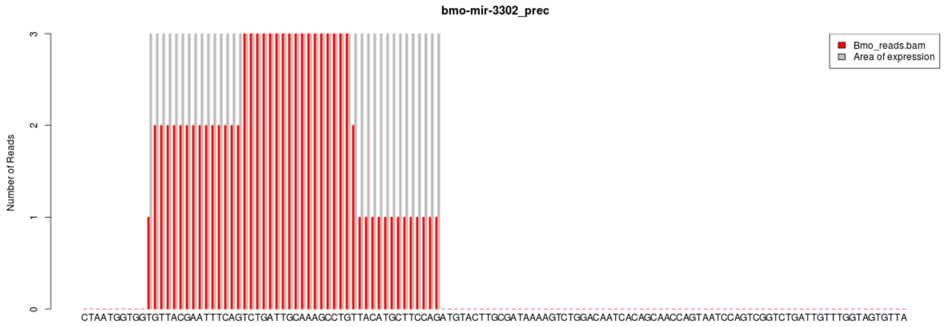

>bmo-mir-3303_prec

```
GGUUUGAGGAGUUGGAUUACGCCCUCUGACACCUCCCGAUGGAAUGCAACCAUCGAGGGUGCAGAGACAUUCGGAGUGAACUG
............((.((((.(((((((.(((((..((((((.......)))))).)))))))))))......)).)))).)).

 Initial dG =    -26.90

        10        20              30        40
GGUUUGAGGAGUU  A    G  ------|    A     CC      AAU
             GG UUAC CC       CUCUG CACCU  CGAUGG   \
             UC AGUG GG       GAGAC GUGGG  GCUACC   G
G------------  A    A  CUUACA^    -     A-      AAC
              80        70         60         50

-----------------------------------------------------
```

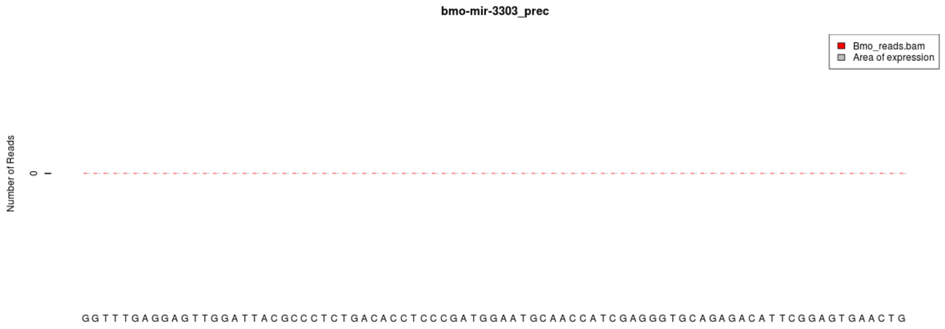

>bmo-mir-3304a_prec

CAGUCUCCCUAUUAAUGAUAUUCCAAGUCAUUUUAAUUUUGUCAGGUGCAUUUUUUAUAAGAUUGCCUAAGUGCAAUGACUUGGCCAAUGUACAGACUUUUUUAAACAGCUUGGAAUAUU
UACAGACA
..((((..........((((((((((((...(((((....(((..(((((((.....
(((((((((......)))))..))))...))))))).)))....)))))..))))))))))))...)))).

```
 Initial dG =     -32.60

        10         20         30         40         50         60
CA|   CCCUAUUAAU            CAU      UUUU   AG        UUUUA    --     CU
  GUCU          GAUAUUCCAAGU   UUUAA     GUC  GUGCAUU     UAAG  AUUGC  A
  CAGA          UUAUAAGGUUCG   AAAUU     CAG  CAUGUAA     GUUC  UAACG  A
A-^   CAU-------            AC-      UUUU   A-        CCG--    AG     UG
              120        110         100          90           80         70
```

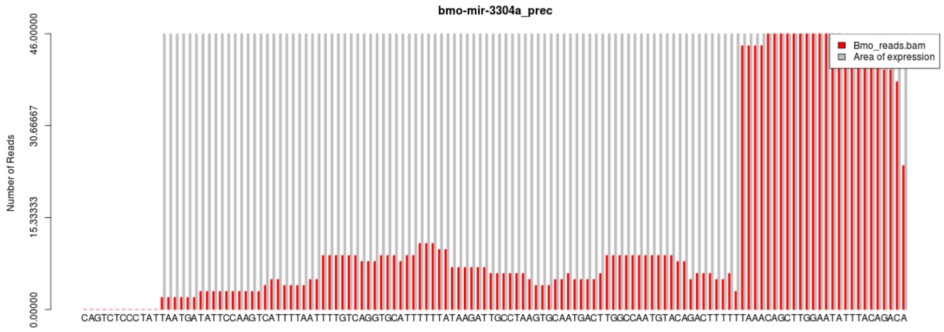

>bmo-mir-3305_prec

UGAGCUCUUGUCUAAUAUUUUUUGAUAAAAUUUUCGUGGUAUUAGAUGAAAGAAUAUAUGUUUUUAUUAUUACUUCCAUCCGUUUACAUAUGGUUAUUACGUCACUAGAUGGAAAUAUAA
GGCCG
.(.((.(((((....(((((..((((..(((..((((((((...((((.(((((((.
(((....))))))).))).))))....))).)))))..)))..))))..)))))....))))))))).

```
 Initial dG =    -21.50

        10         20        30         40         50         60
U A  U     CUAA-     UU    AA   UU     -   UUA-    A   -|   U   U
 G GC CUUGU     UAUUU  UGAU  AAU  UCGUG GUA    GAUG AAG AAUA AUG U
 C CG GAAUA     GUAGA  ACUG  UUA  GGUAU CAU    CUAC UUC UUAU UAU U
G -  -     UAAAG     UC    CA   UU     A   UUGC    C   A^   -   U
     120       110       100        90        80        70

-----------------------------------------------------
```

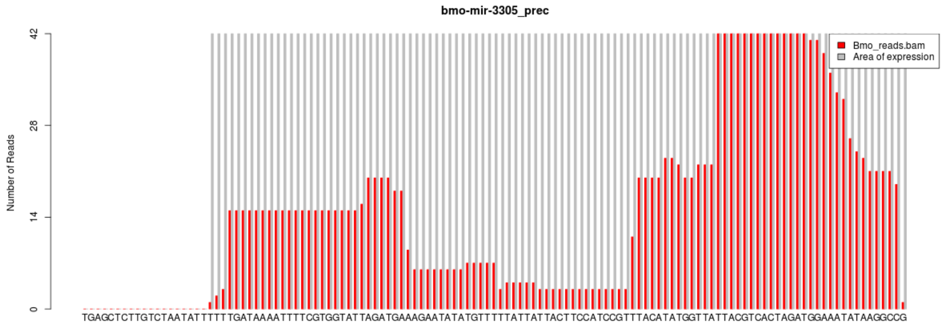

>bmo-mir-3306_prec

GGCCGUCGGGAUAGGCCGACAUUAUAUAUUUUUUGAUAUUUUAUUUUUAUUUUAACACCUUAUUAUAUUCACAGUUACAAUUUUACACUAAUGUUAUAUAUAUAUAAUCGAUAGAUUUUG
ACGAGAGCUA
((((((((((((....(((..((((((((....(((((((........(((.
((((.................)))).)))........)))))))...)))))))))))....)))))))))...))).

```
 Initial dG =     -21.60

            10        20        30        40        50        60
-   ---|        AGGC   CA        UUUU       UUAUUUUU   U    ACCUUAUU
 GGC   CGUCGGGAU    CGA  UUAUAUAU    UGAUAUU        AUU UAAC        \
 UCG   GCAGUUUUA    GCU  AAUAUAUA    AUUGUAA        UAA AUUG        A
A   AGA^        GAUA   --        UAU-       UCACAUUU   C    ACACUUAU
.       120       110         100         90        80        70

-----------------------------------------------------
```

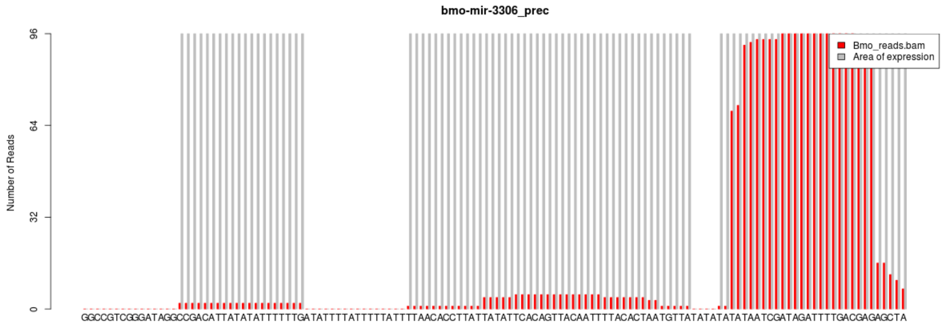

>bmo-mir-3307_prec

CAUGAUGGCGAUCUGUUUAAUUGGUAAACUGCACAUUGUAUCGCUUCGCACCGCAGAUGUUUUGGCAAUUUGGUUUCCGCUAAUAGCGUCCUCGUUUUGUCGCAUAAUGUGCGGUUCAUC
CGUUCUGAUAUGCCAUUAA

```
..((((((((((.....(((.(((.(((((((((((((...((..((...((..
(((((((((((............))))).)))))))..))...))..))))))))))))))).))).)))..))).)))))))).
```

```
 Initial dG =    -41.60

        10         20        30        40        50        60         70
CA        -|  UGUUU   U   A             AUC  UU  CAC  CA      -     AAUUU
  UGAUGGCG AUC     AAU GGU AACUGCACAUUGU   GC  CG   CG  GAUGUU UUGGC     G
  AUUACCGU UAG     UUG CUA UUGGCGUGUAAUA   CG  GU   GC  CUGCGA AAUCG     G
A-        A^  UC---   C   C             ---  CU  UUU  UC      U     CCUUU
        130          120       110          100        90        80
```

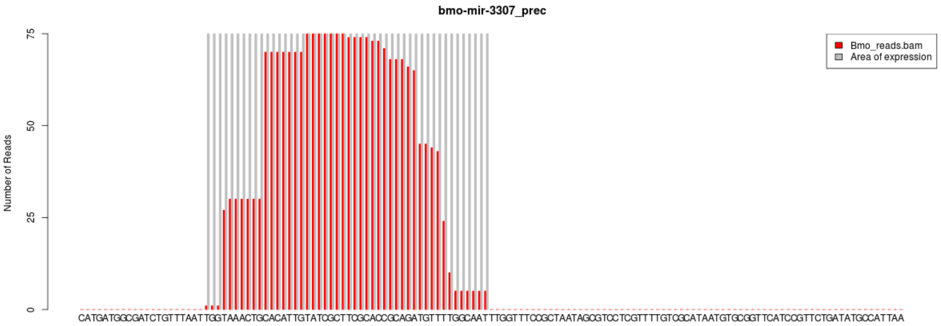

>bmo-mir-3308_prec

```
AUCAUACUUUAUCUUGGUAAUUGGUUGUCUGGAUUUAUGAAGCGAAGGCGGACUGUUAAUCAAAGACGUGAUCUUCCUUGGUCUUCUAGGCAAUGGGACAAAAUCAUCUGAAAAGUGGAG
.((.((((((....((((..((.((((((((((...((.(((.(((((((..((.........)).)))...))))))).))..)))))))))).)).....)))).....)))))))).

 Initial dG =    -25.60

        10         20           30        40           50
A  A      AUCU-    AA---  G          UUU  G   C    ---|  GA  GUUA
 UC UACUUU     UGGU     UU GUUGUCUGGA   AU AAG GAAG   GCG  CU    \
 AG GUGAAA     ACUA     GG UAACGGAUCU   UG UUC CUUC   UGC  GA    A
G  -      AGUCU    AAACA  G          UC-  G   -    UAG^  A-  AACU
.        110       100        90         80         70         60

-----------------------------------------------------
```

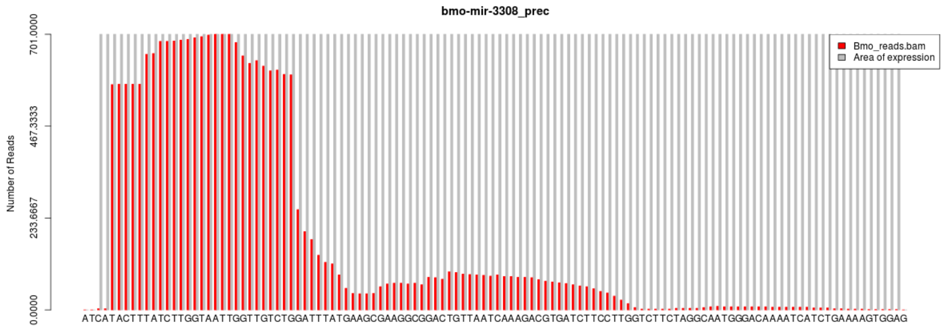

>bmo-mir-3309_prec

```
ACAAUUUGGAGAACACUUUAUAAAUAUUCGACAUAAGGCUUAUAGGCCUGUAGUUUUCUAUAAUGUUCCUAUCCCCUUUUUUGUGUAAUAGUAUAAUUGUGCUCUUGGUC
........((((.(((.(((((..(((.(((...((((...(((((..(((((....)))))......)))))..))))..))).)))....)))))..))).))))....

 Initial dG =    -18.60

        10         20          30        40           50
ACAAUUUG    A   U-     AA--   U   CAU    CUU     CC---|    U
        GAGA CAC  UUAUA    UAU CGA   AAGG   AUAGG     UGUAG U
        UUCU GUG  AAUAU    AUG GUU   UUCC   UAUCC     AUAUC U
CUGG----    C   UU     GAUA   U   UU-    CC-     UUGUA^    U
.           100        90        80          70        60

-----------------------------------------------------
```

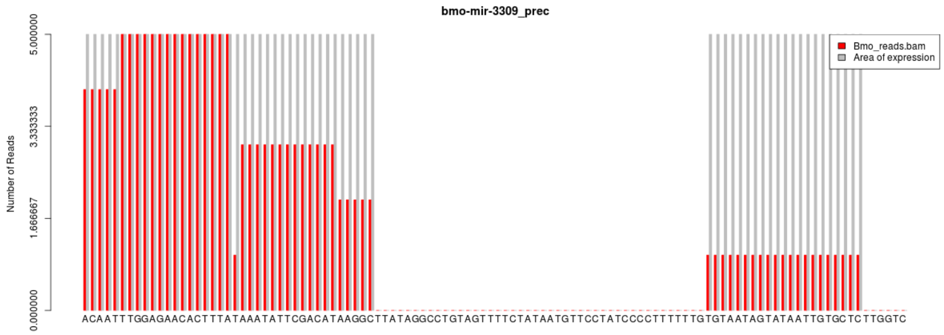

>bmo-mir-3310_prec

AGCGGUAGACCCGCAUCCCGCAGCUUGUAAACCAGCCCCUCGUGCCACACACGGUCGAAGGCUUUCUCGAUGUCGAGAAAGACCGCGACCGUGUAUUGGCCCGAGUUGUACCGGUCCGCC
AAGUGAUGCAGGACGCGAGUCAGUUGCA
.((((..(((.(((.(((.((((((((...(((.((..((((.((((.(((((((((..
((((((((((....)))))))).)).))))))))))..)))).)))))..))...)))....)))))..))).))).))).))).)))..)))).

```
 Initial dG =    -71.30

        10         20          30           40          50         60          70
A    UA   C   A   C   --      UAA-   A--  CC    U    C-|         AA  -         A
 GCGG  GAC CGC UCC GCA  GCUUG     ACC   GC  CUCG GCCA  ACACGGUCG  GG CUUUCUCG U
 CGUU  CUG GCG AGG CGU  UGAAC     UGG   UG  GAGC CGGU  UGUGCCAGC  CC GAAAGAGC G
A    GA   A   C   A   AG      CGCC   CCA  UU    C    UA^         G-  A         U
      140        130        120        110        100         90         80
```

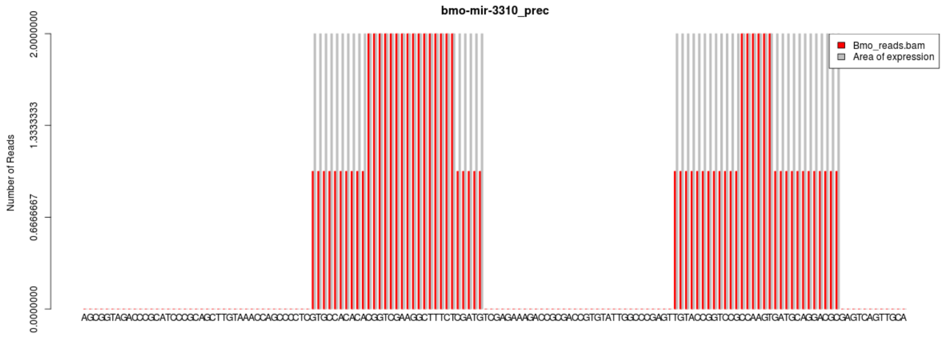

>bmo-mir-3311_prec

```
AAAAGGAACGCCCACAGAACUGGCAGGUCCAACCUGGCGCAUCCUGACCCACAGGAUCUGCGCCGGCGGAACCCCGCACAGUCGGGUUAUCGCCUG
...(((...((((...((.(((((.(((((...(((((((((((((.....)))))..)))))))).)).))).)).))).))))))))).....))).

 Initial dG =    -39.50

          10        20            30        40
AAA   AAC--|   ACA  A    -  A-    -  AAC        --     AC
   AGG     GCCC   GA CUG GC  GGU CC   CUGGCGCA  UCCUG  \
   UCC     UGGG   CU GAC CG  CCA GG   GGCCGCGU  AGGAC  C
G--   GCUAU^   ---  -   A  CC   A  C--        CU     AC
       90            80         70          60        50

-----------------------------------------------------
```

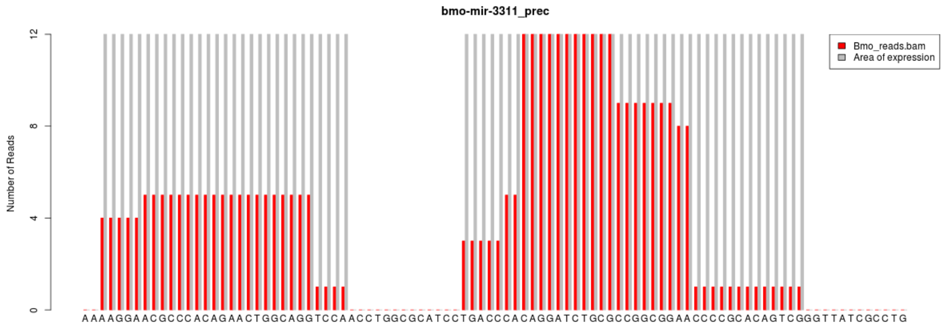

>bmo-mir-3312_prec

GGCCGCUCUCUUGAAUUCGGGACAAUCUUGUGGGAUGUGCCCCGGCUUCUAACAUGCUGCGCAUUUCCUUUCUUGCUUCGCUUGCGUGGUG

```
............(((..((((.((((((....)))).)).)))))..)))...(((((.(((((..........)))...)).)))))...

 Initial dG =    -23.10

          10        20         30
G     .-UCUCUU   UU    A  -|   U
 GCCGC        GAA  CGGG CA AUCU G
 UGGUG        CUU  GCCC GU UAGG U
G     \ ------   CG    C  G^   G
90                    40


       50        60
        UAA  U  UGC   UUUC
           CA GC   GCA    C
           GU CG   CGU    U
        C--  U  CUU   UCUU
              80        70

-----------------------------------------------------
```

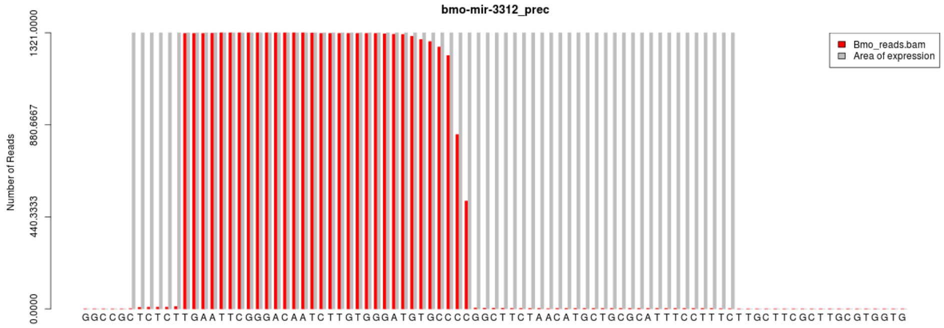

>bmo-mir-3314_prec

```
GGGCAUGGCGGCAUCGCAAAUGUUUGCCAUGGUGCCCCGGAACCAGCCGACCUCCGCAUCCACGUCCGCGAGCCGCGCGGGUUUUGGCGCCCACGCAGCAACAGCGGCCGCCUCCA
(((...((((((..(((...((((.((..(((.((.((((((((.((((..(((.((..........))))).)).)).)))))))).))))).))))))...))))))))))).
```

```
 Initial dG =    -47.70

         10        20        30        40         50        60
-|  CAU      AU   AAA    U  CA   U  C        A  -  AC   C  AUCC
 GGG   GGCGGC  CGC   UGUU GC  UGG GC CCGGAACC GC CG  CUC GC    A
 CCU   CCGCCG  GCG   ACGA CG  ACC CG GGUUUUGG CG GC  GAG CG    C
A^  ---      --   ACA    -  C-   -  C        G  C  C-   -  CCUG
       110        100           90        80         70
```

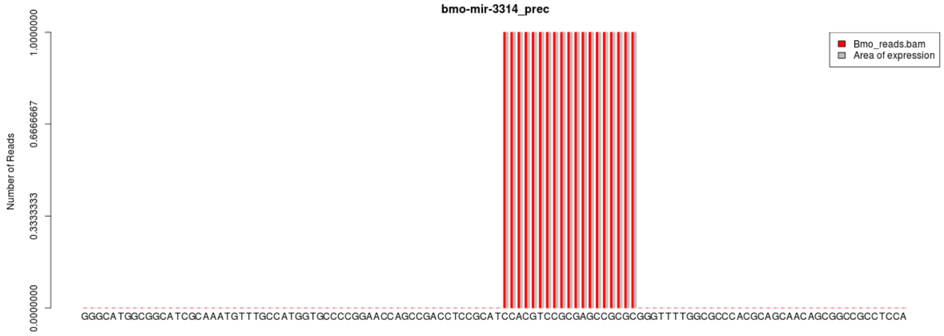

>bmo-mir-3315_prec

GCACCUCGGGUUGAGUCUACCGGGAUUGGAGAUAGCGUUGGGGAAGGAGAGCGAGGAGGGGAUCUUACUUGCCUUGGGGCUGUUCCUGUCCUGGCGGAGCCCUAGGC
...(((.((((....((..((((((.(((.(((((.(..((((((((((...........)))).))).)))..).)))))))).)))))))..)))))).))).

```
 Initial dG =    -46.00

        10        20        30        40          50
GCA|  C    UGAG  UA       U   G     G UG   -   -    GCGAG
   CCU GGGU    UC  CCGGGAU GGA AUAGC U  GGG AAG GAGA     G
   GGA CCCG    AG  GGUCCUG CCU UGUCG G  UCC UUC UUCU     A
C--^  U    ----  GC       U   -     G GU   G   A    AGGGG
       100            90         80        70        60

-----------------------------------------------------
```

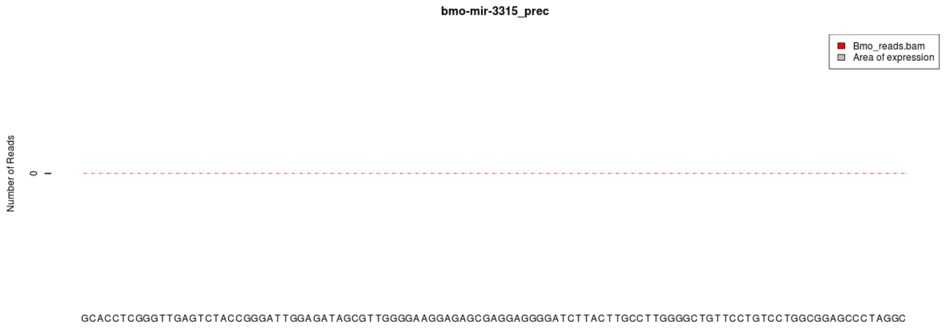

>bmo-mir-3316_prec

CGGUGCGACAGGUACGGCAAUCAGUGCCGCGGCAGGGGCCGGUAGCUUCAGCUGUUCUAUUGGUGCGGGAAGUUCCAGUUGCGGUUGCUCGAGGGUGGCUGGUGAUCCAUGUGUUAGCUG
CAUUACUGUUUCUUCGACUCCGU

```
(((..(((.(((.((((.(((((((...((.((((((((((((.(((((((((((...(((((.
((.....)).))))).)))))))).....)))).))))))..))).)))))..)))).))).)))).))))))...))).
```

```
 Initial dG =    -46.10

          10           20         30         40
-   UG-   C   U    .-CA|      CC     A     CG
 CGG   CGA AGG ACGG    AUCAGUG  GCGGC GGGGC  \
 GCC   GCU UCU UGUC    UGGUUAU  UGUCG CUUCG  G
U   UCA   -   U    \ --^      CU     A     AU
 140        130           60         50


                           70
                     .-GC   A
                         GGG A
                         CCU G
                     \ --   U
```

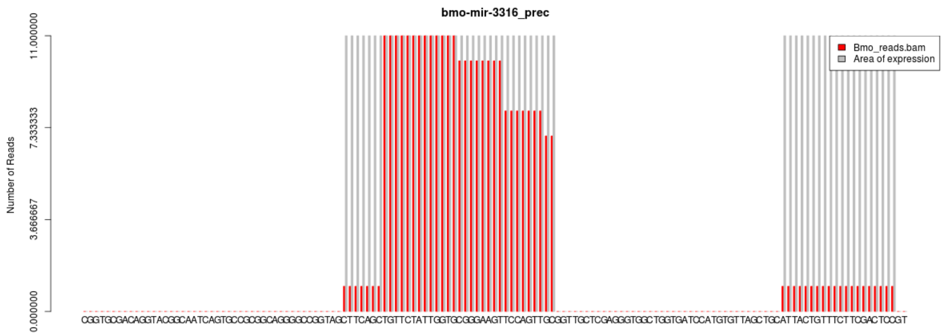

>bmo-mir-3317_prec

AAUCGUUGAAAGGUUUGGGCUGCAUUUCUAAGACCAAAACUCAUUCGCGGAAAUUCAUAUAGACCGAACGGCGUGAUAAUUGCCGUCUUCUCUAUGUCGUCGGGGUUUACAGGAAUGUUG
UGAUAAGCUCUGCAGACGU

```
...((((...(((((((.((.((((((((.(((((.........(((((.......(((((((....
((((((.......))))))....))))))))).))))))))..)))))))).))..))))).))...)))).
```

```
 Initial dG =    -35.30

        10           20         30          40
AAU    GAA   -      G-   U         A-      AAA  .-UCA|  G
   CGUU   AG GUUUG  GC GCAUUUCU  AGACC   AC      UUC \
   GCAG   UC CGAAU  UG UGUAAGGA  UUUGG   UG      AAG C
U--    ACG  U      AG   U         CA      GGC  \ ---^  G
         130       120        110       100       50


                                                      60        70
                                              AUUC        CCGA       UGA
                                                  AUAUAGA     ACGGCG    \
                                                  UGUAUCU     UGCCGU    U
                                              C---        CUUC       UAA
                                                         90        80
```

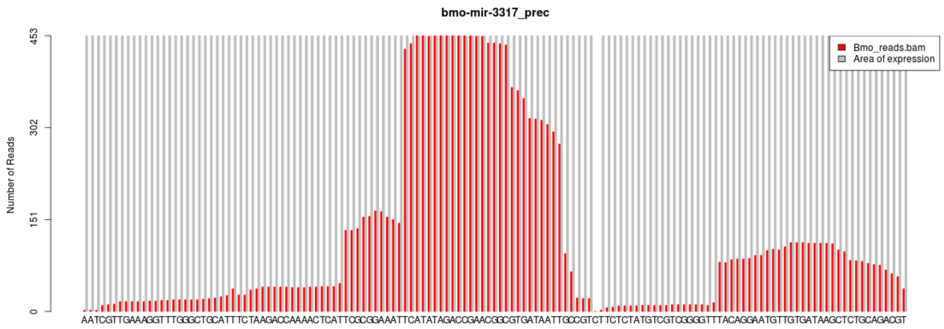

>bmo-mir-3318_prec

```
AUGACCUAUCGGAUUUUACAUUUUUGCAUGUUGCGCUUAAAAAAUUAGACACAGACACUGCCCGUGCGUUUGAAUCCUAUCAGCGCUUAAAUAAUAAGGGAUGCGAAAUACCAUUGUUUG
UAGAUCUAGUGUCU
..((.(((.(((((.......((((((((.(((((((.......((((((.
((...........)).))))))........)))))...........)).))))))))......)))))))).))........

 Initial dG =    -22.10

          10        20
.-AUGACCUA| G      ACAUUUU   AU
          UC GAUUUU       UGC  \
          AG UUAAAA       GCG  G
\ --------^ A      AAUUC--   UU
                  40



 50        60          70
  CAC        .-CCCGU     U  AU
     AGACACUG       GCGUU GA  C
     UCUGUGAU       CGCGA CU  C
  ---        \ -----     -  AU
       130               80

-----------------------------------------------------
```

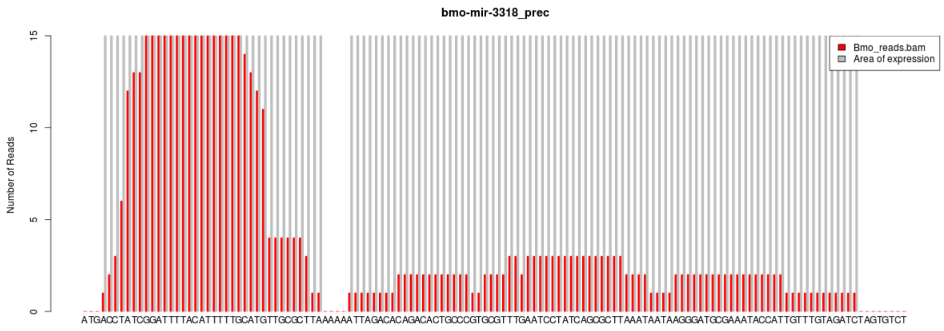

>bmo-mir-3319_prec

UGGCCUCCGAGUCCGGCACACCGCCAGCGGGGCCCGGUUGCUGGAGUGUCCUGGUGCCAACACCAGUGCGGCCGCGGACAAGCUAGCAACCCGGCUCCGUGAAUCCUGCCGGACCCAGAA
GUGGGCGUA

```
..((((....((((((((........((((((((.(((((((((..(((((((((((..
((....))))).))).)))))..))))))))).))))))))......))))))))........))))...
```

```
 Initial dG =    -67.20

         10            20        30        40        50
UG-    CCGA----        CACCGCCA        C         AG      |     C
   GCCU        GUCCGGCA        GCGGGGCC GGUUGCUGG  UGUC--CUGGUG C
   CGGG        CAGGCCGU        UGCCUCGG CCAACGAUC  ACAG  GACCAC A
AUG    UGAAGACC        CCUAAG--        C         GA    \ ^     A
       120       110         100        90        80          60


                                                           70
                                                         U  G
                                                          GC G
                                                          CG C
                                                         G  C
```

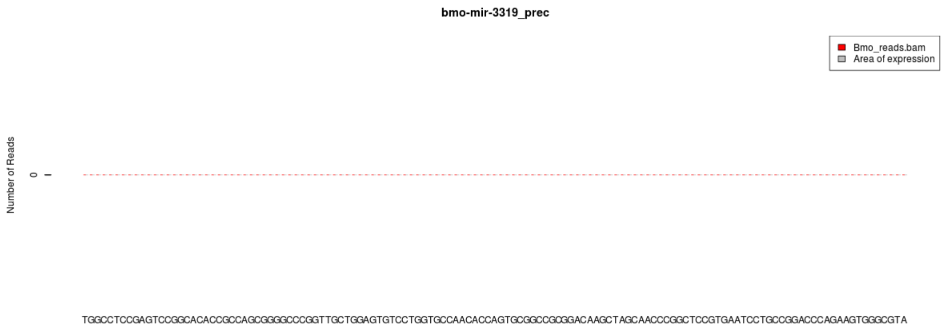

>bmo-mir-3321_prec

```
CGACGGGGGCGGGCCUGUGCAUCUCUCACGCGCCCCGCCGUCUCCUUUUGCGAGAUGGUGCACUCGCAGAAGUCAAGCAUCGCCUUCCACGA
.(((((.((..(((.((((.......)))).))))).))))).((((((((((........))))))))))...................

 Initial dG =    -33.60

          10        20
.-C|    G  CG   C    CAU
   GACGG GG  GGC UGUG   \
   CUGCC CC  CCG GCAC   C
\ -^    G  --   C    UCU
    40          30



                          50
  UC------------------          AUG
                      CUUUUGCGAG   G
                      GAAGACGCUC   U
  AGCACCUUCCGCUACGAACU          ACG
   90        80        70        60

-----------------------------------------------------
```

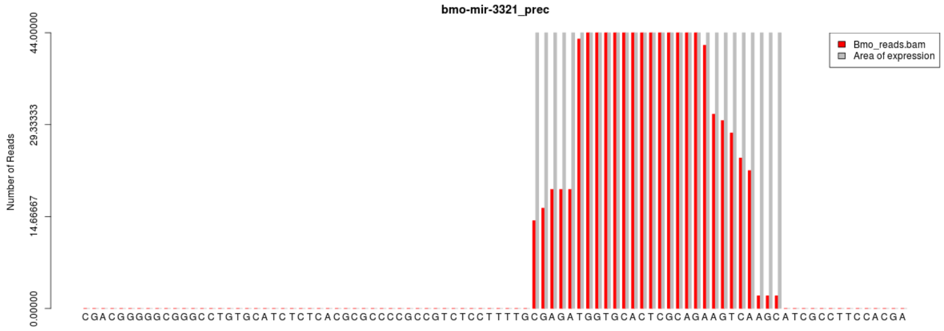

>bmo-mir-3322_prec

CAGGCCUCGUUUGUGUUCCAAUUGGAGUGUGGUUGGUGUGUCUGUAAUGCCGGUGGGUUUCGUGACUUCAAUCUCGUCGGUUUGAAGUUGGCUAGGUCC
..(((((((.(((..((((....))))..))).)).....(((......((((..(((.....((....))..)))..))))........))).))))).

```
 Initial dG =    -23.90

        10          20
CA      C  .-UU|UG    A
  GGCCU GU     G  UUCC A
  CUGGA CG     U  GAGG U
C-      U  \ --^GU    U
             30



                    40
            .-GG       GUC
                UUGGUGU   \
                GGCCGUA   U
            \ --       AUG
                  50

-----------------------------------------------------
```

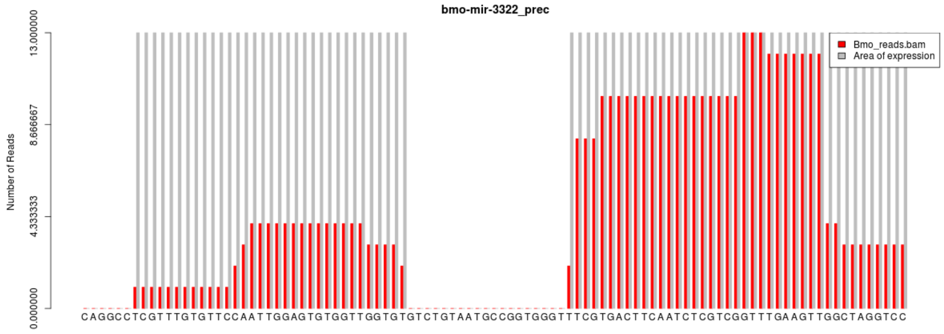

>bmo-mir-3323_prec

```
UGAGAUCCCUUUGAAGGGGCGUACUUUGUUAGCGUCGACAAUUUUUUGUAUAUUAGAUACAUCAUUAUCAAUACGAAUAAUGUUAAUGCCUUUACAGGUGACUUUUAAACAAUCAAC
((((((((((.(((((.((((.....)))).((((..(((.((.(((((((....((((......)))).))))))).)))))..))))))))).))).)).))))).........

 Initial dG =    -21.60

          10
.-UGAGA|    U
       UCCCU U
       GGGGA G
\ -----^    A



 20        30          40        50        60
  CGUACU     .-A    CG   A  U       AUUA    CA
        UUGUU   GCGU  ACA UU UUUGUAU    GAUA  U
        AACAA   CGUA  UGU AA AAGCAUA    CUAU  C
  CAACU-     \ -    AU   -  U       A---    UA
        110              80        70

-----------------------------------------------------
```

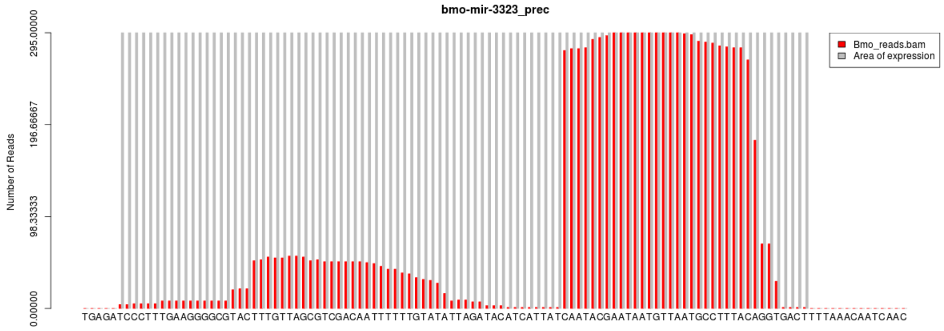

>bmo-mir-3324_prec

ACUCCGCAAAUACAGCGUCCAUGAUCCCUGAGUGCCUAGGUGGGGCGGCUUAAAAACUCUCGCCUUGCGGCGAGGCCCCAACUUCUCGGACCUGAGCGGUUCUAUUGAACGCAGGUGGCG
AUGCGGC
...(((((..(((.((((.((.((.((((.((..((.(((((((((............
((((((....))))))))))))))...))).))..)).)).)).))...)).)))).))).....))))).

```
 Initial dG =    -45.80

        10           20           30        40           50        60
ACU     AA--   A-    C  U--  U  -  G  UG  U   ---|     GGCUUAAAAACU      U
   CCGCA    UAC  GCGU CA   GA CC CU AG  CC AGG   UGGGGC            CUCGCC U
   GGCGU    GUG  CGCA GU   CU GG GA UC  GG UCU   ACCCCG            GAGCGG G
C--     AGCG   GA    A  UAU  U  C  G  CA  C   UCA^     ------------      C
       120       110       100        90        80                    70
```

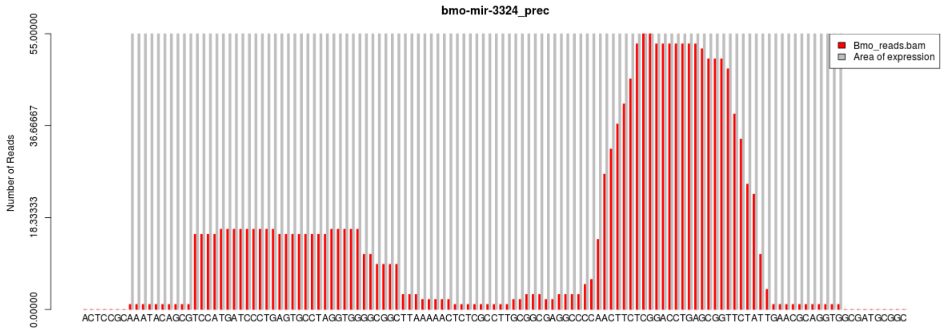

>bmo-mir-3325_prec

GGCAAGUGUGUAUUGGCCAGAUCACCCCUCAUUAUAUCUGAAGCAAGUACAAGUAAACAAUCAGUACCAGCAAAAAGAAAACUGUUGUUACUUGAGGAUAAAUUUGACAGUGAUGAGUCA
AAUACACCAGCA

```
.((..((((((.(((((...(((((...(((...(((((..........(((((((.(((.
((((...............))))))))))))))).)))))....)))..)))))..))))))))))..)).
```

```
 Initial dG =    -29.70

        10        20        30         40        50        60        70
G| AA      A     CAG      CCC   UUA-     GAAGCAAGUA      A   U    ACCAGCA
 GC  GUGUGU UUGGC   AUCAC   UCA    UAUCU          CAAGUAA CAA CAGU       \
 CG  CACAUA AACUG   UAGUG   AGU    AUAGG          GUUCAUU GUU GUCA       A
A^ AC      -     AG-      AC-   UUAA     A---------      -   -    AAAGAAA
130        120        110        100                   90          80

-----------------------------------------------------
```

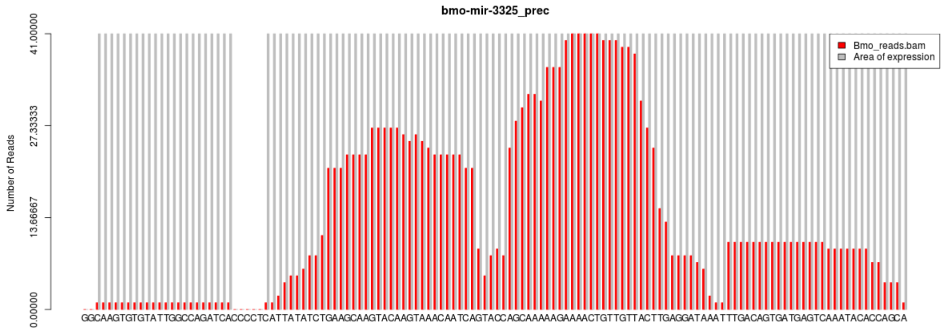

>bmo-mir-3326_prec

```
UGAGUGGACUGUAGGUUUUGUGGUUCUUGUGAAAUAUAAACACUUUAUCACUAUUACUGGGUGUUUAUUGGUAUUUAUAGAAAGUACAAAGGUUUUUACAGGCACA
...(((..((((((((((((((.((..((((((..((((((((((.............))))))))))).....))))))..)).)))))))...)))))))).))).

 Initial dG =    -26.60

        10           20        30           40        50
UGA   GA       ---       G  CU      AU---|         UAUCAC
   GUG  CUGUAGG   UUUUGUG UU  UGUGAA     AUAAACACUU      \
   CAC  GACAUUU   GAAACAU AA  AUAUUU     UAUUUGUGGG      U
A--   G-       UUG       G  AG      AUGGU^         UCAUUA
       100        90        80        70        60

-----------------------------------------------------
```

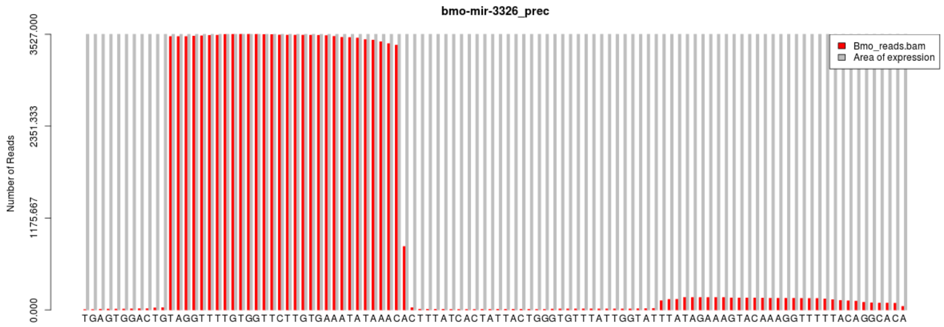

>bmo-mir-3327_prec

```
AGAACAUUUUGGGUAAUGUAUAAACAACAGGAAUGUUAUGUACAGUAUACAGAUAAUUCUAUAUGUAACGUUUUUGUUGUCCUAAAUUACUCUACUACCUCAACU
.((.......(((((((......(((((((((((((((((((.((.............)).))))))))))))))))))).....))))))).......))....

 Initial dG =    -26.00

           10         20         30         40         50
A---| ACAUUUU       GUAUAA                   C  UAUACA
    GA       GGGUAAU      ACAACAGGAAUGUUAUGUA AG      \
    CU       CUCAUUA      UGUUGUUUUUGCAAUGUAU UC      G
UCAA^ CCAUCAU       AAUCC-                   A  UUAAUA
   100        90         80         70        60

-----------------------------------------------------
```

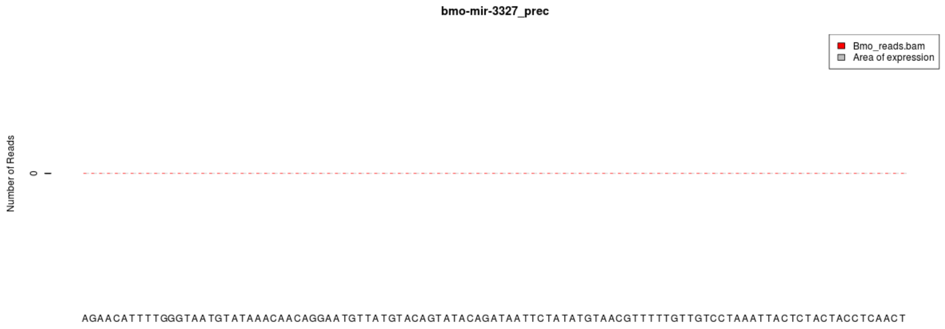

>bmo-mir-3328_prec

UCCAGAUUGGAGAGGACCAUGGAGAUGUAGAAGAAUUGAUGAUCCCUUCAUCCAACAUUUUAGAAAUUUGGGAUUGGACUUCUUGUUUGUGCACUUCAGGGAAUCUAUAUGAUUUU
(((.....)))((...((.(((((.((((.(((....((...(((..((..((((.((((...))))))))))..)))..))...))).))))))))))))...))...........

```
 Initial dG =    -22.00

           10        20        30        40        50        60
UC---| GAU    GAGGA  A     A    G   AAUU  UGA   CU  AU    C    U
     CA   UGGA     CC UGGAG UGUA AAG    GA   UCC  UC  CCAA AUUU \
     GU   AUCU     GG ACUUC ACGU UUU    CU   AGG  AG  GGUU UAAA A
UUUUA^ AU-    AAG--  -     -    G   GUU-  UC-   UU  --    -    G
    110          100          90         80           70

-----------------------------------------------------
```

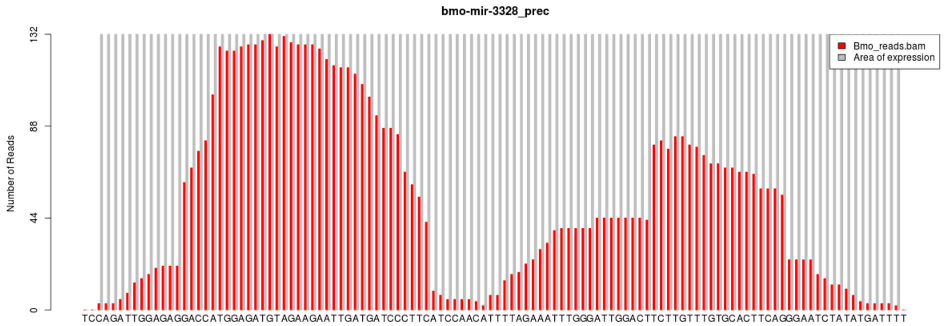

>bmo-mir-3329_prec

```
CCCAAUCAUUAUGACUAUUGUUGUGUUAGGGCAAUGAGUCACAUUUAAAAUUUUAUUGAGUAUGUGGGUACAUGAAGCCCCAGGCAUACAAUAAUUUAUGACAGAUUGU
..(((((.((((((.(((((.((((((.((((.(((..((((((....(((...)))....))))))...)))...)))).)))))))))))..)))))).)))))).

 Initial dG =    -24.60

         10         20         30           40        50
CC     A-      C-     U      A-     A--   AG-|     UUAA   U
  CAAUC  UUAUGA  UAUUG UGUGUU  GGGC   AUG   UCACAU    AAU \
  GUUAG  AGUAUU  AUAAC AUACGG  CCCG   UAC   GGUGUA    UUA U
U-     AC      UA     -      AC     AAG   AUG^     UGAG   U
        100        90         80         70         60

-----------------------------------------------------
```

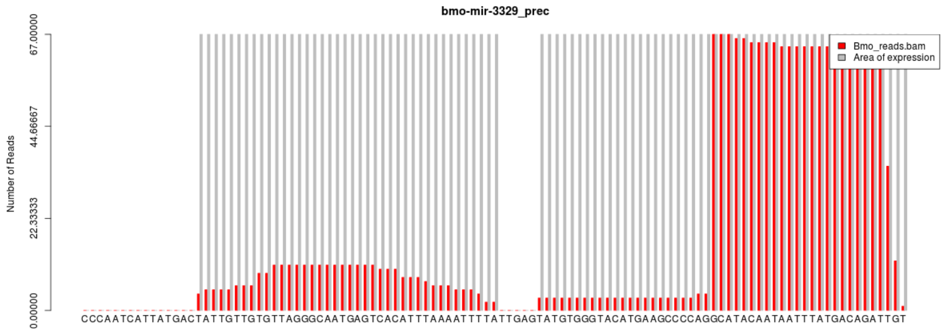

>bmo-mir-3330_prec

UUUCAGUACCCCAUCUCGCGACGCGCUAUCGACGCGUCACUUUCGGGGUGUCCCUUGAUCUGUAUGUUACCGUAGUGAAAGCGCUCCACGAAUUGUUCCUAUUGGU

..((((((...((..(((.((.(((((.((((((.((.((..((((((....))))))).......)).)))))..))).)))))))..)))..))....)))))).

```
 Initial dG =    -25.00

        10          20        30          40             50
UU      CCC-  UC   C-  C     A   --   C  C  UU-----|     U
  UCAGUA    CA  UCG  GA GCGCU UCG  ACG GU AC       UCGGGG G
  GGUUAU    GU  AGC  CU CGCGA AGU  UGC CA UG       AGUUCC U
U-      CCUU  UA   AC  -     A   GA   -  U  UAUGUCU^     C
     100        90         80         70        60

-----------------------------------------------------
```

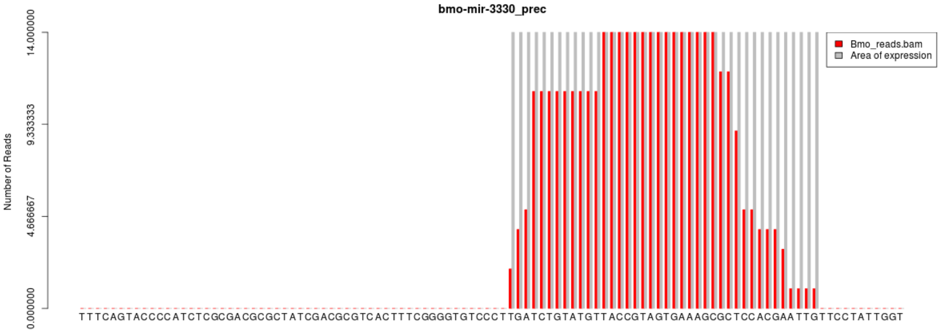

>bmo-mir-3332_prec

```
CACUGACCCCGCUCAGCGAGCAACCUGGCCUCGGUGCGAGCGAGGAGGUCGAUUUCCUCGCCAUGCCACCGCCGCUUCACCAUCAUCGGAGCGGCAUCCGUA
.((.((..((((((..(((.......(((..(((((((.(((((((((....)))))))))..))).))))..))).........)))))))))..)).)).

 Initial dG =    -39.10

        10        20          30          40        50
C  U  CC      AG   GCAACCU--   CU    -   A-|         U
 AC GA  CCGCUC  CGA         GGC  CGGU GCG  GCGAGGAGG C
 UG CU  GGCGAG  GCU         UCG  GCCA CGU  CGCUCCUUU G
A  C  AC      --   ACUACCACU   CC    C   AC^         A
100       90          80        70        60

-----------------------------------------------------
```

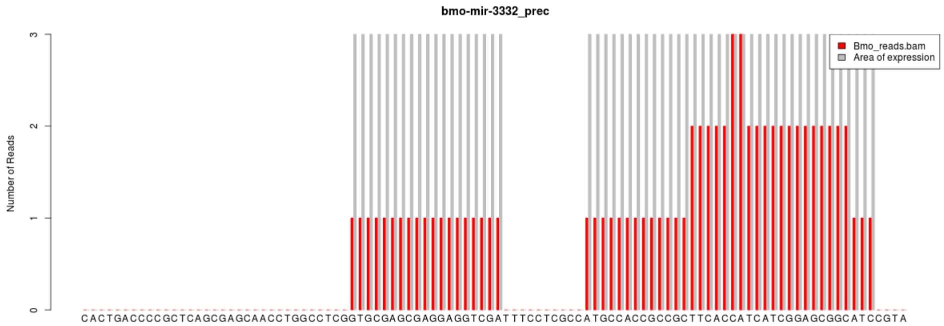

>bmo-mir-3333_prec

AUACAGGUACCGUCCCGUUCGAGAAGCAAGACAAAGUGCCAGAUGUCAAAUCGCGACGGCACCGUCAAAUCGCGACCCGUGUUGCCGUGGUAAGAAUUCAAAAACUAACGGAUUGGUUUC
GAACAGUCACCCGCCCGG
.....(((((.(((..(((.....)))..)))...))))).((..((..((((((.((((((.(((.......)))..)))))).))))))..))..))...........
(((((........)))))..........

```
 Initial dG =    -31.50

          10          20
.-AUACA     C--|  CC   CG
       GGUAC   GUC  GUU  \
       CCGUG   CAG  CGA  A
\ -----     AAA^  AA   AG
      40        30



            50        60
  .-A  UG  AA     A      C-   AAA
     GA  UC  AUCGCG CGGCAC  GUC   \
     CU  AG  UGGUGC GUUGUG  CAG   U
  \ -  UA  AA     C      CC   CGC
             90        80

-----------------------------------------------------
```

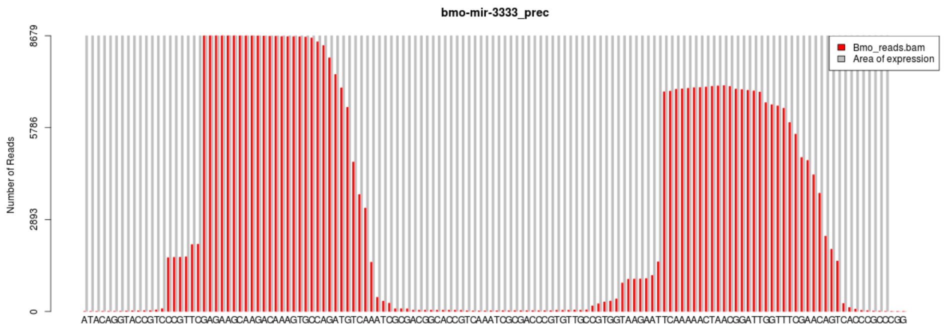

>bmo-mir-3334_prec

CGUUGCUGCUCUUCUAGUUUCCCUCCACGUGAACCAGAAUGAUGGAAGGACAGGGCGGCAGGUGCUCUUCGUCCGACGACGUCGAUGUUCCUUUGGUUUGAUUGUUCCAGGUUCUUCAUCGUGUAGCUCAAGUAUUGCUUU

```
....((....(((..((((......(((((((...(((((..(((((.((((((.(((.(((.((((..
((((....))))..)).)).))))))).)))).)).))))).)))))))).)))).)))).)))....))...

 Initial dG =    -34.50

        10        20         30        40        50         60         70
CGUU| UGCU   CU    UCCCUC    -   ACC     GA     G  -    G   C   U  -  UU    C
    GC    CUU  AGUU      CACG UGA   AGAAU  UGGAA GA CAGG CGG AGG GC UC  CGUC G
    CG    GAA  UCGA      GUGC ACU   UCUUG  ACCUU UU GUUU GUU UCC UG AG  GCAG A
UUU-^ UUAU   C-    U-----    U   ---     G-     G  A    G   -   U  U  CU    C
 .        130             120          110        100         90        80
```

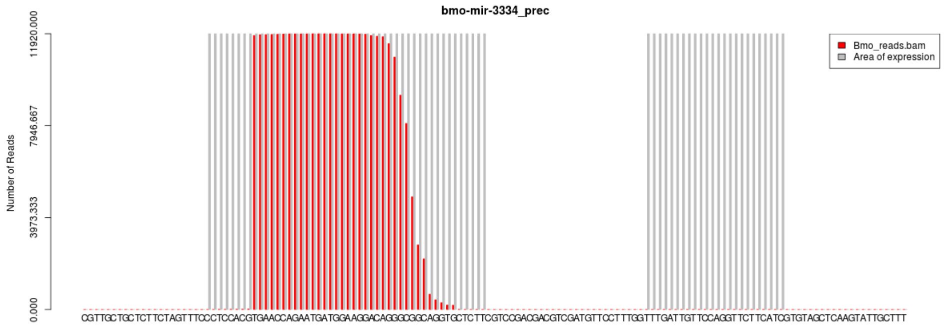

>bmo-mir-3335_prec

AAGUCGUCGAAUUAUAUGAGCCGUGUCCGUACCGCUUUCAGGUUGGUUACAAUAAGCAGUUAAUGUUUUAUAAUGUUCCAUUUAAAAGUGGUUCUUGAAUAUCCGUCUAAUAUCAACGUC
GAU

```
..((((.((....((((.((.((..((...((((((((.(((.(((..(((..
(((((.....)))))).....))).)))))).))))))))....)).....)).)).))))...)).))))
```

```
 Initial dG =    -23.20

        10        20            30         40        50            60
AA    U  AAUU    G  C  UG---  CGU-        C   U   UU   AU---|    GU
  GUCG CG    AUAU AG CG     UC    ACCGCUUU AGG UGG  ACA     AAGCA  \
  UAGC GC    UAUA UC GC     AG    UGGUGAAA UUU ACC  UGU     UUUGU  U
--    U  AAC-    A  U  CUAUA  UUCU        A   -   U-   AAUAU^    AA
   120        110       100        90         80         70
```

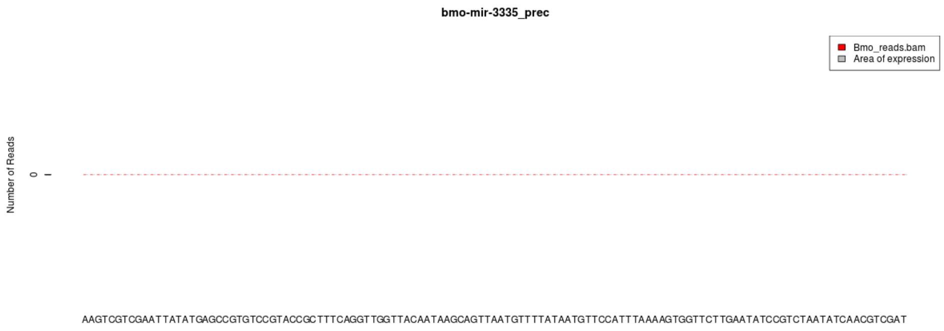

>bmo-mir-3336_prec

```
CUUGCUUAAGGCAUAGACACAGCUUGGGGGUUCCGUCGCGUCCUUAACACGCGAACAUUCUGUGGUUGCGUGAUCGCCGAGGCAU
..(((((..(((((..((.(((((..(((((....((((((.......))))))..)))))..))))).)).)).))).))))).

 Initial dG =    -26.60

        10         20        30        40
CU|    AA   -  AG  A     UG     UCCG      CCU
  UGCUU  GGC AU  AC CAGCU  GGGGU    UCGCGU   \
  ACGGA  CCG UA  UG GUUGG  UCUUA    AGCGCA   U
U-^    G-   C  G-  C     UG     CA--      CAA
     80          70        60          50

-----------------------------------------------------
```

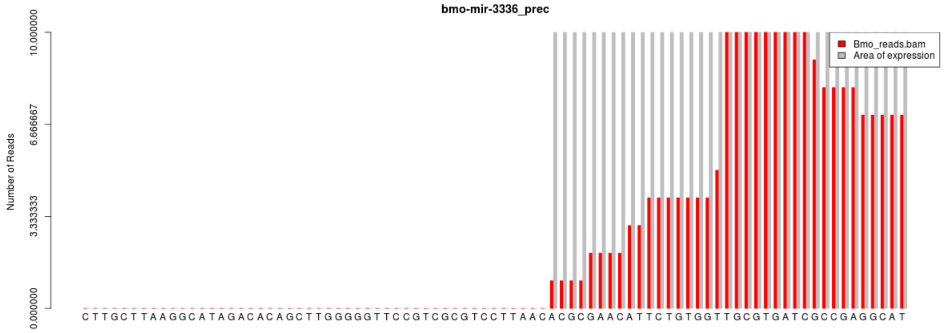

>bmo-mir-3337_prec

```
GAGCACCUUCGCCUCCAUAUCCGUGCUCGGACGCUUGGGUGCGACUCUAUUGCGAUGUGCUCCUUCACCAGUCUGUCCAAGGCCUCUUCGCGCAUCGAUACAUGGGCGCAGGUGA
...(((((.(((((...((((.((((.((((.((((((..(.((((.....((.....)).........)))))..))))).)...)))).)))).)))).)))...))))).))))).

 Initial dG =    -39.30

          10        20        30
  |      CU    CUCCAUAUC  UG      A
--GAGCAC  UCGC         CG  CUCGG C
  CUCGUG  AGCG         GC  GGGUU G
\ ^     U-    UUAUCUCA-  GU      C
   60         50         40


          70
  CU     .-A    GU
    UCACC   GUCU  \
    AGUGG   CGGA  C
  --     \ -    AC
              80

-----------------------------------------------------
```

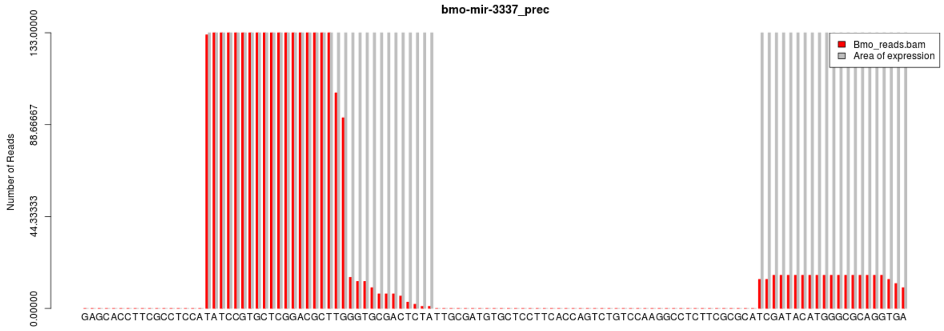

>bmo-mir-3338_prec

```
ACCAAGUGACAAUAGAGCGUCUCAAAGCAAACACAGUAGUGUGCAAUGCUAAAGUCCUAACGUGCAUGUACUUACUUUGUUUGUUCUGAUACACUCUUAUGAGCUUC
...((((..((..((((.((.(((.((((((((.(((((.(((((.(((....((....))..))))))))))))).)))))))).))).)).)))).)).)))).

 Initial dG =    -33.40

        10        20        30        40        50
ACC|   GA  AU    C  C   A        C     U     A   UAAA  C
   AAGU  CA  AGAG GU UCA AGCAAACA AGUAG GUGCA UGC    GU C
   UUCG  GU  UCUC CA AGU UUGUUUGU UCAUU CAUGU ACG    CA U
C--^   A-  AU    A  U   C        U     -     -   UG--  A
        100        90        80         70           60

-----------------------------------------------------
```

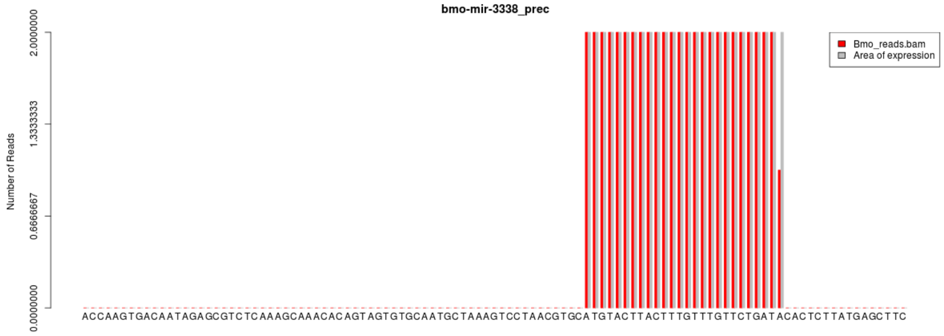

>bmo-mir-3339_prec

```
UGGUUUUUAGACUUUUUAGAUGUUGGUCAAGCUCGUGAUCUGUGUCCGAUCAUUCCGACAUGUUGACCGAUUAAUUAGAAAUUGUCC
.........((((((((((..(((((((((..((((((((.......)))))...)))....)))))))))...)))))))..))).

 Initial dG =    -20.10

        10          20         30             40
UGGUUUUUA   --        AU-         GC--   ---|    UGU
         GAC  UUUUUAG   GUUGGUCAA    UCG   UGAUC   \
         CUG  AAAGAUU   UAGCCAGUU    AGC   ACUAG   G
C--------   UU        AAU         GUAC   CUU^    CCU
              80         70         60         50

-----------------------------------------------------
```

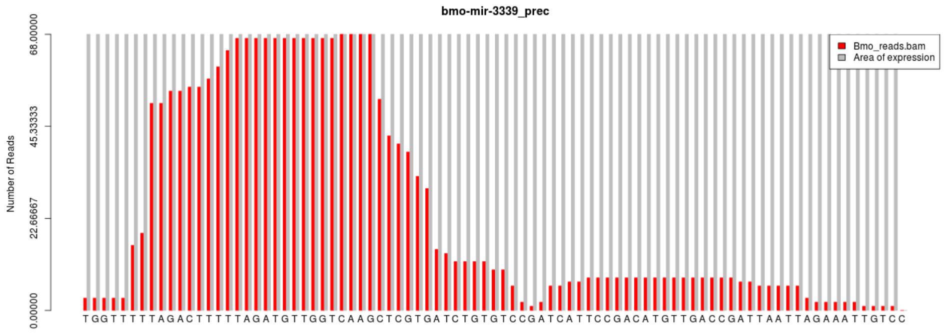

>bmo-mir-3340_prec

```
GCAGUGUCUCGAGGUCACUGCUCCGGAGGGGUUUGUCAGGAGGGAGAUAUACGGAUGCGAUGACGAUCGGCUGG
.((((....(((.((((.(((((((.....((((.((....)).))))...)))).))).)))).)))))))).

 Initial dG =    -22.80

        10         20         30
G|   GUCU   G-    C   -    GAGGG    G  A
 CAGU    CGA  GUCA UGC UCCG     GUUU UC G
 GUCG    GCU  CAGU GCG AGGC     UAGA GG G
G^   ----   AG    A   U    AUA--    G  A
   70            60        50          40

-----------------------------------------------------
```

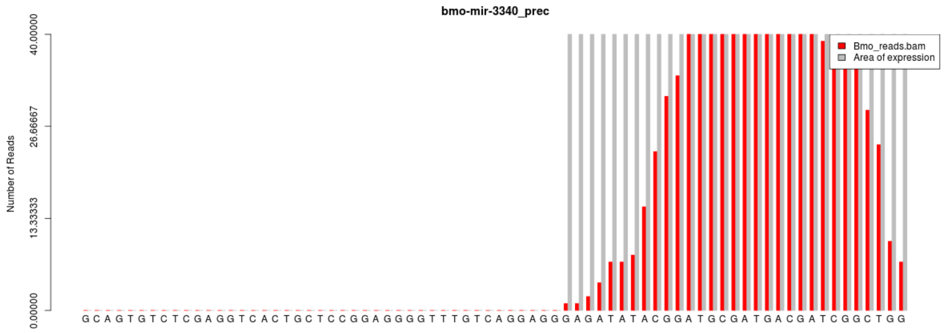

>bmo-mir-3342_prec

AUCAGAUUUGGCGGUGGCUCUUGCCACUGACCAUGGUGACGAGUCGAGCCCCCGAAACGCCUCACAAUGAGGUCCGGGAUGGUCGCUCGCACCGAUGGGAUCGCUAAGCCGCCGUCCUG
AA
.((((....((((((((((...((......((((((((.((((.(((.((.((((....
(((((.....))))).)))).)))))))))))))).)))).....))..)))))))))))))).

```
 Initial dG =    -59.00

        10        20        30        40        50         60
A|   AUUU          CUU  CACUGA    -    A    U   G  C-    AAAC     AC
 UCAG    GGCGGUGGCU   GC      CCAU GGUG CGAG CGA CC  CCCG    GCCUC  \
 AGUC    CUGCCGCCGA   CG      GGUA CCAC GCUC GCU GG  GGGC    UGGAG  A
A^   ----          AU-  CUAG--    G    -    -   -  UA    C---     UA
120           110        100           90          80           70

-----------------------------------------------------
```

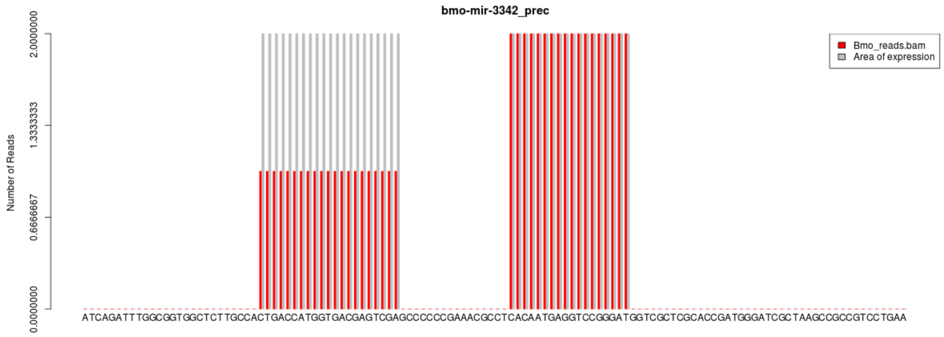

>bmo-mir-3343_prec

```
GACUCCUUGGCAAUUCUCCAUCGUUCCUGCACCGUAGCUCCCUUGUAUAUCGGACAGUCGUACAACGGGUGCCCUUCCUUGCAUUGCGGGCACGCCGGCUUGUCU
........(((((....((..(((.((((((..(((((.(((((((((...........)))))).))).)).......)))..)))))).)))..)).))))).
```

```
 Initial dG =    -28.30

        10        20        30               40         50
GACUCCUU     UUCU  AU   U      CC   -------  U   -|     AUCGG
        GGCAA    CC  CGU CCUGCA  GUA       GC CCC UUGUAU     \
        CUGUU    GG  GCA GGGCGU  CGU       CG GGG AACAUG     A
U-------     C---  CC   C      UA   UCCUUCC  U   C^     CUGAC
          100            90        80        70        60
```

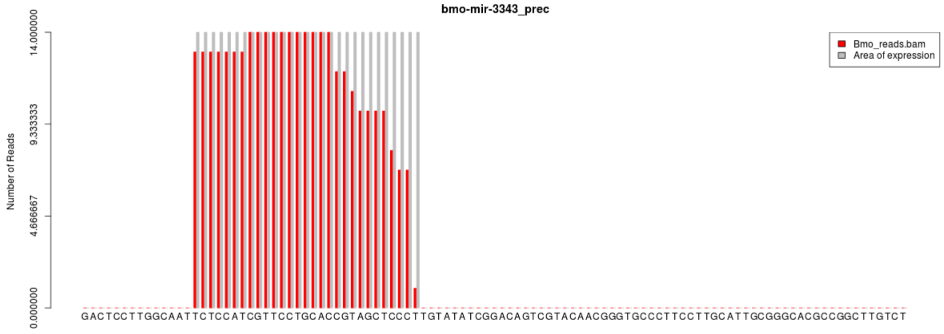

>bmo-mir-3344_prec

AAAUCGUUGACCUAUUGCAAGAAGGACUCAGCCAGCGAGUUGUCGCUCGUCGGCUCCACAUAAGCCAGUCCUGUGUUUCGAAAGCU
...(((..(((...........((((((.((((.((((((....)))))).)))).......... .))))))..))).))).....

```
 Initial dG =    -20.70

        10        20                  30        40
AAAUC   GACCUAUU   AGA      C----------|   A      U
     GUU        GCA   AGGACU           AGCC GCGAGU G
     CGA        UGU   UCCUGA           UCGG UGCUCG U
U----   AAGCUU--   G--      CCGAAUACACC^   C      C
         80             70         60         50

-----------------------------------------------------
```

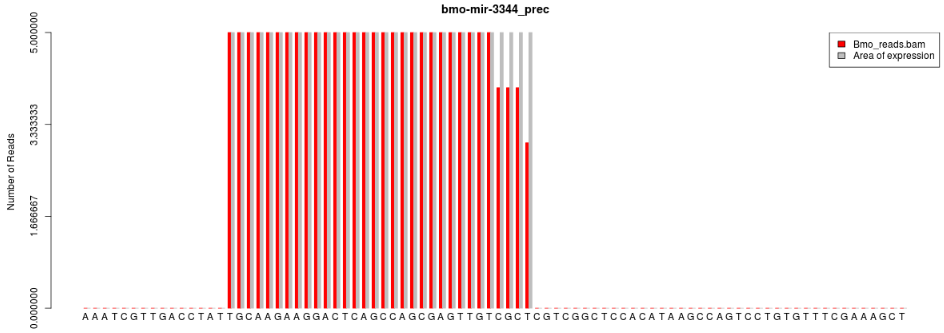

>bmo-mir-3345_prec

```
CUAUGCCAAUUAUCGUUACGCGCGUCGCUUAGGCGGGAGUGGACUUCGCGUUGUAGACGACGCAUCCGAAUUCUGUUUGAAUGCGUGGGAAGACGAUCGUAGGCCCGGA
((..(((.((.((((((.(((((((...((((((((((.((((....((((((....)))))).))))..)))))))))))))))))....)))))).)).)))..)).

 Initial dG =    -39.20

         10           20        30         40        50
-  AU   A  U      A---|      CGC          G-    CUUC      U
 CU  GCC AU AUCGUU    CGCGCGU   UUAGGCGGGA  UGGA    GCGUUG A
 GG  CGG UG UAGCAG    GUGCGUA   AGUUUGUCUU  GCCU    CGCAGC G
A  CC   A  C      AAGG^      ---          AA    A---      A
       100        90           80        70           60

-----------------------------------------------------
```

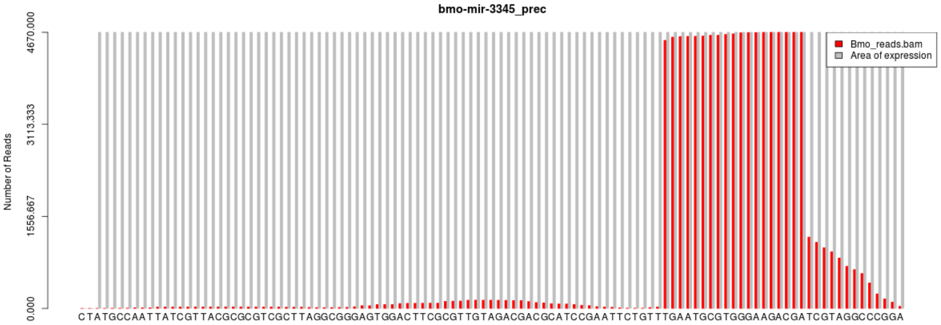

>bmo-mir-3347a_prec

GCGGAGAUUUAUCUAAACUUGUGGGACCUGUUCUGUUUAGUAUGUAUGCGGCGGUAUUAAUGAGCUCUGCCCAUAGCACUUGCGGUAAUGGCUCAUGAGCGUACAUCAUUGAUCUUGCUG
UCUCAACUAGGGUUCUGUUCUCCC

```
..(((((...((((.......((((((..((.....((((((((((((..........(((((((.
((((((.......)).))))..))))))))..)))))))).)))))....)).))))))....)))).....))))).
```

```
 Initial dG =    -40.80

        10          20        30        40         50        60         70
GC     UUU--|   AAACUUG      CU  UCUGU     -        GGCGGUAUUA       C-    -  UAG
  GGAGA     AUCU       UGGGAC  GU     UUAGU AUGUAUGC          AUGAGCU  UGCC CA   \
  CCUCU     UGGG       ACUCUG  CG     AGUUA UACAUGCG          UACUCGG  AUGG GU   C
C-     UGUCU^   AUCA---      U-  UUCU-     C        AG--------       UA    C  UCA
   140       130          120        110       100                90        80
```

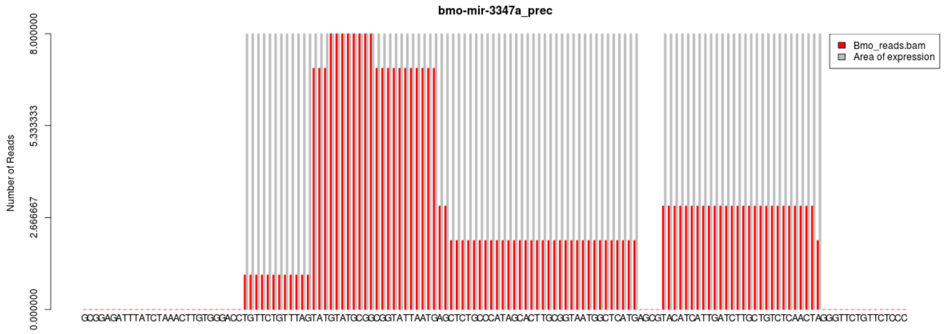

>bmo-mir-3347b_prec

ACUUGUGGGACCUGUUCUGUUCAGUAUGUAUGCGGCGGUAUUAAUGAGCUCUGCCCAUAGCACUUGCGGUAAUGGCUCAUGAGCGUACAUCAUUGAUCUUGCUAUCUCCG
....(.((((...((.....((((((((((((..........(((((((.((((((.......)).)))).)))))))..)))))))).)))))....))..))))).

```
 Initial dG =    -30.30

        10        20         30        40        50          60
ACUU|U    CCU  UCUGU     -        GGCGGUAUUA       C-    -  UAG
    G GGGA   GU     UCAGU AUGUAUGC          AUGAGCU  UGCC CA   \
    C CUCU   CG     AGUUA UACAUGCG          UACUCGG  AUGG GU   C
G---^-    AU-  UUCU-     C        AG--------       UA    C  UCA
.            100         90                80        70

-----------------------------------------------------
```

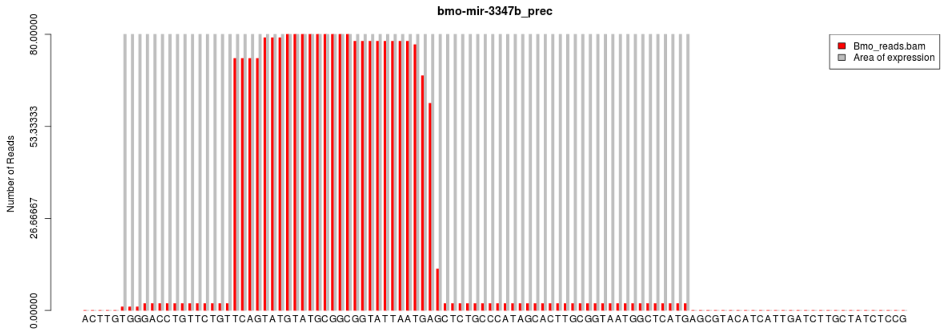

>bmo-mir-3348_prec

```
CACGCGUAAUCCCCGGCGAGCACCUCCGCCCCCGGGUCUCAGGCCCAGCGAGCCUCCCGGCGAGCAUGCA
...((((..((.((((.(((...(((.((....((((.....)))).))))).))))))).))..)))).

 Initial dG =    -27.30

        10        20        30
CAC|   AA  C    C   CAC   C  CCCC    CU
   GCGU  UC CCGG GAG   CUC GC    GGGU  \
   CGUA  AG GGCC CUC   GAG CG    CCCG  C
A--^   CG  C    -   C--   -  A---    GA
.          60           50

-----------------------------------------------------
```

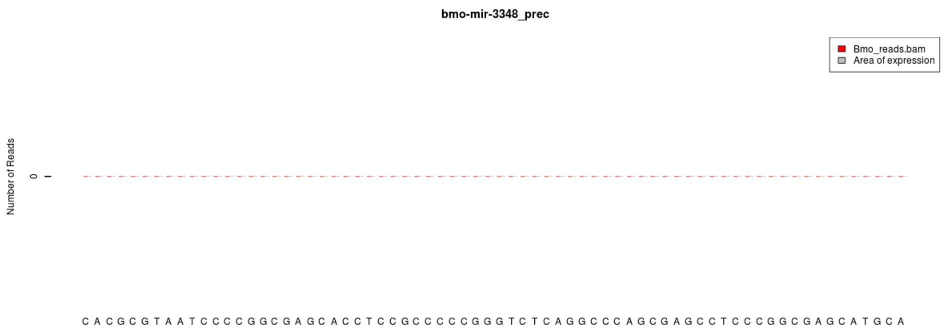

>bmo-mir-3350_prec

UACGUACAAAGAUUUAUUAAAAAUAAAAAUAAGAAUUGUCAUACAUGCAUGUGUUUUAAAUUGUCGGUAGUCGGUGAUAAUAAUAUGUUUCAUGUAUUCUAAUUUUUCUUUAUAUGCUUA
UAUUUAUAUUGUAAUUGUAU

```
((((((((........(((..(((.(((.((((((((..(((((((.(((((((....((..
(((.....)))..))...)))))))..)))))))..)))))))).))).)))..)))........)))))..)))).

 Initial dG =    -22.00

           10        20        30        40         50        60
-    --     AGAUUUAU   AA   A   U        UC       C-|      UUAA  UG   GU
 UACG  UACAA        UAA  AUA AAA AAGAAUUG  AUACAUG  AUGUGUU    AU  UCG  \
 AUGU  AUGUU        AUU  UAU UUU UUUUUAAU  UAUGUAC  UGUAUAA    UA  GGC  A
U    UA     AUAUUUAU   CG   A   C        CU       UU^      UAA-  GU   UG
.       130       120       110       100        90        80         70

-----------------------------------------------------
```

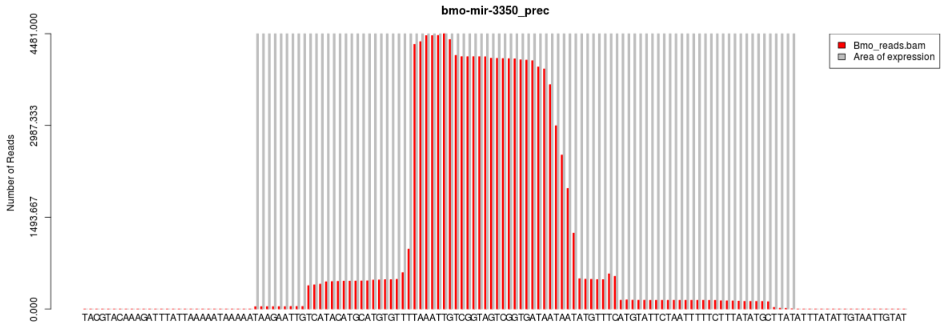

>bmo-mir-3351_prec

```
GUAGCGCAUUUACGUUGUGGAUGUCUAUGGGCGCCAGUAACUACUUAACAGCUGGUGGGCUGUUUAAAACAAUCGUGAUUAUUUUCUACU
((((.....((((((((((((((((((.(((...(((....))).....))).)))))).))))...)))).))))).......)))).

 Initial dG =    -21.40

           10           20
-    CGCAU--     -      | A    U
 GUAG       UUACG UUGU--GG UGUC A
 CAUC       AGUGC AACA  CC GCGG U
U    UUUUAUU     U    \ ^ -    G
.        80        70         30


                           40        50
                        AGUAACUACUU       G
                                   AACAGCU G
                                   UUGUCGG U
                        AAAU-------       G
                                      60

-----------------------------------------------------
```

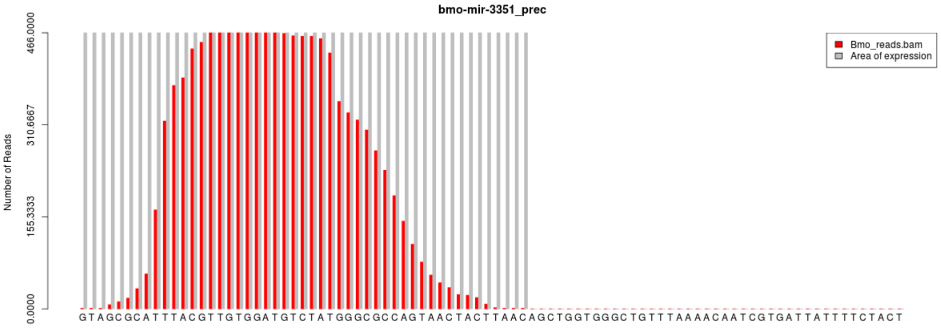

>bmo-mir-3352_prec

UACUACGGCGCCACCGAGUCGAUCAAGAAUUAUAAAGAAGAUUAAUUGUUUUCAAAUAUUUUUGGUUAUAUUGGUCUUCUGUUCUAUUUGUGCUGUGUCUAACUGUAGAGC

..((((((...(((..((((((...(((((.....((((((((((.((..(((((....)))))..))))))))))))))))))))..))).)))))).....))))))...

```
 Initial dG =    -26.00

          10
.-UAC        .-CACC| GU
     UACGGCGC      GA  \
     GUGUCGUG      CU  C
\ ---        \ ----^ AG
           90


                    30        40        50
               A---     UAUAA          U  UU     U
                   AGAAU     AGAAGAUUAAU GU  UCAAA A
                   UCUUG     UCUUCUGGUUA UA  GGUUU U
               UUUA     -----          -  UU     U
                          80        70         60

-----------------------------------------------------
```

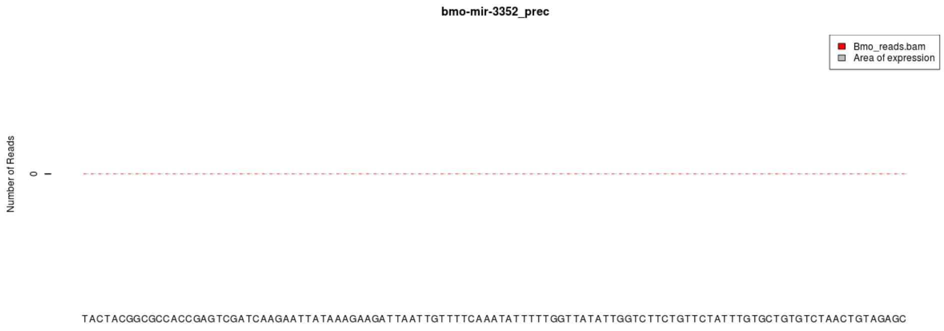

>bmo-mir-3353_prec

AGGAAGUCAGGCUCUAUUUUUUCCCAAGAUUCAGAUAAUUUUUAAACCAAAGUUCGACAAGUUGGGUUCGCUGGCUUAGGUGUAAAAAUGUAACUGAUAAACAGAGUUUGAUUCACA
..(.((((((((((......((....)).((((...(((((((.(((.((((((((((.......).)))..))))).))).))))))))....)))).....)))))))))))))...

```
 Initial dG =    -25.50

         10          20
AG- A           .-AUUUUU| C
   G AGUCAGGCUCU        UC C
   C UUAGUUUGAGA        AG A
ACA -           \ ------^ A
      110


                 30             40        50           60
                  U----    AUA-       A   A     --   - AAG
                       UCAG    AUUUUUA ACC AAGUU  CGA C   \
                       AGUC    UAAAAAU UGG UUCGG  GCU G   U
                  CAAAU    AAUG       G   A     UC   U GGU
                  100        90        80        70

-----------------------------------------------------
```

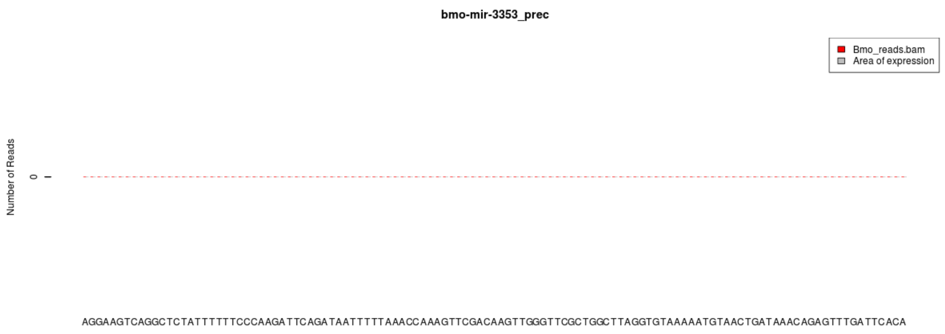

>bmo-mir-3354_prec

GAAGUCGUUCAGCAGUUUCUGUGUGUAUUGCCAACGUGUCAGGUUGCUUAUUUUAGCGUCUUGCAGGUUCACAUCAGGCACAGUUACAGGGGGUUCA
(((.((.((.....((..((((((....((..(((.((.((((.((((......)))).)))))).)))..)).....))))))..)))).)).))).

```
 Initial dG =    -25.90

         10         20         30         40         50
-|  G   G  CAGCA  UU       GUAU  CC   G  U     U     UA
 GAA UC UU     GU  CUGUGU    UG  AAC UG CAGG UGCU  U
 CUU GG GA     CA  GACACG    AC  UUG AC GUUC GCGA  U
A^  G   G  -----  UU       GACU  AC   G  -     U     UU
      90              80         70          60
```

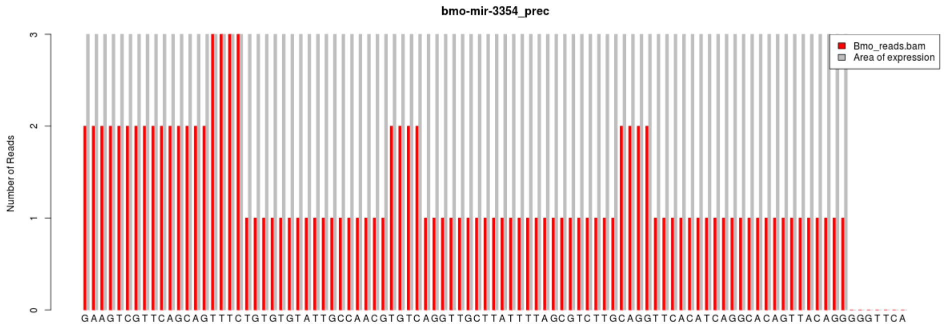

>bmo-mir-3355_prec

GAGAAUCUCUAUUAUGAGGAGAGUUCAGUUUUAAAAUUUGAAACUUUCAGUAGAAUUUUACAGCAAACUUCGAUGGCGACGCAUUGUUAAAUUUAAUACUAUAAAGGUUCAAGUCAGCAG
GAAAGCCUGGUUUUUGAGAAUGUCAU

```
((...((((....((.(((....(((.(((.....(((((((.((((.((((((((((.((((......
(((....)))....)))).))))))..))))..)))).))))))).))).)))...))).))....))))...))..
```

```
 Initial dG =    -25.20

           10
.-GA   -|    AUU
    GAA UCUCU   \
    CUU AGAGG   A
\ --   G^    AGU
         20



         30          40         50          60        70
  AG-----        .-U     A    C-    --      U    CAAACU   A
         UUUUAAAA   UUGAA CUUU  AGUA  GAAUUU ACAG      UCG U
         AGAGUUUU   AACUU GAAA  UCAU  UUUAAA UGUU      AGC G
  UACUGUA        \ -     G    UA    AA      U    ACGC--   G
      140           110       100        90        80

-----------------------------------------------------
```

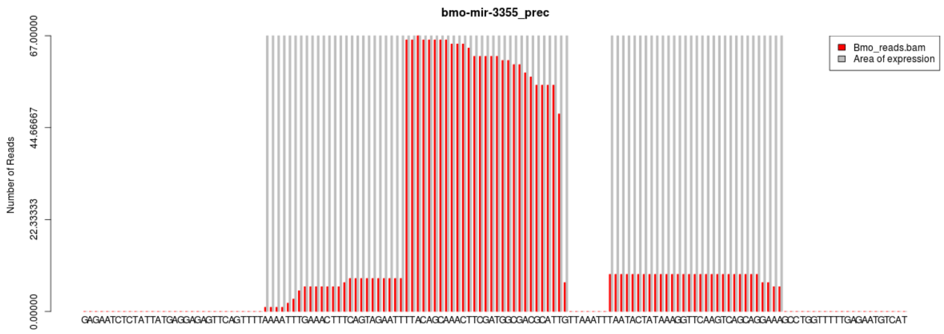

>bmo-mir-3356_prec

```
UAUUGUUUUUUUUUUCUUCUAUAAUCGAACCCGCAAAAAUUGAAAUUAGCGUAAAAUCAAACCCGCAAAAUAUUAAUGUUUUAAGCUUUCACCGGUGGGUUCCGUGUUGAAGAAAGAAAG
GACGGC
..(((((((((((((((((.(((...((((((((......((((...(((.
((((((....................)))))).)))))))...))))))))..))).))))))))))))))))).

 Initial dG =    -35.80

        10        20        30        40        50        60
UA|                U   AUC        AAAAAU    AUU   G      CAAACCCGC
  UUGUUUUUUUUUUCUUC AUA   GAACCCGC      UGAA   AGC UAAAAU         A
  GGCAGGAAAGAAAGAAG UGU   CUUGGGUG      ACUU   UCG AUUUUG         A
C-^                U   GC-        GCC---    ---   A      UAAUUAUAA
     120       110        100           90            80        70

-----------------------------------------------------
```

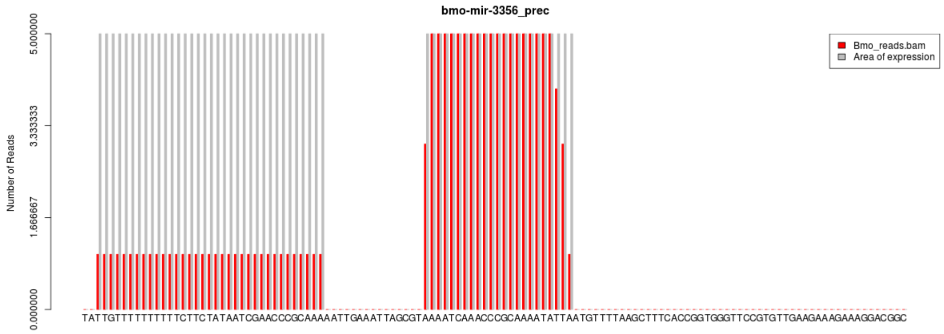

>bmo-mir-3357_prec

ACGACAAUGAGCGACCGGUCGGAGUGGUAUGGCGUUCUAACCCGACAACCUGGUUCUGGUCUUUUACCUCUCCCUGGUUCGUCAUCUCUCCCUGCUCGA
.(((((..(((.((..(((..(((((......)))))..))).(((((((.((....(((.....)))....)).)))).))).)).)))..)).))).

```
 Initial dG =    -26.30

         10          20
A   -  AU   C  .-CC|  CG     GU
 CGA CA  GAG GA    GGU  GAGUG  A
 GCU GU  CUC CU    CCA  CUUGC  U
A   C  CC   U  \ --^  AU     GG
        90          40        30


                        50        60
                 C   -    U  UUCU   CU
                  GAC AACC GG    GGU  \
                  CUG UUGG CC    CCA  U
                 A   C    U  CUCU   UU
                    80        70

-----------------------------------------------------
```

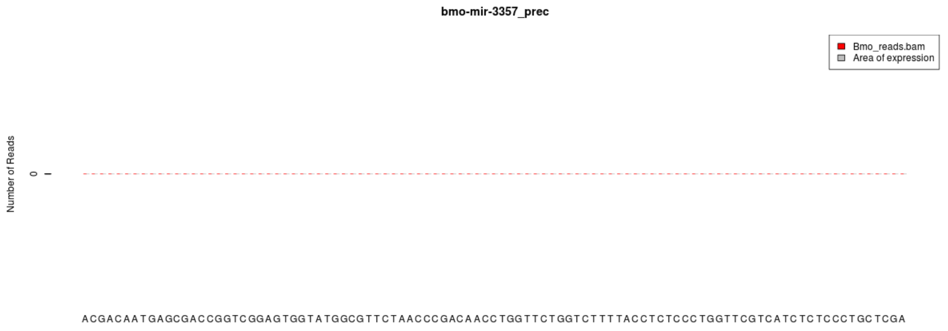

>bmo-mir-3358_prec

```
CGAUGGGUAAAUAUUUGUUAAUCCUCCUCCGUGCGUCACGAUCCUCAGUGCUGAGGGUCGUGACCUUUACGGAGCAGUUUUGUUGGGUCUAUGUUCCUCA
.((.(..((...((..(.(((..((.(((((((.(((((((((((((....)))))))))))))...))))))).))..))).)..))...))..).)).

 Initial dG =    -40.50

        10        20        30          40
C  U GG  AAU  UU U   UC  C       C--|            G
 GA G  UA   AU  G UAA  CU CUCCGUG   GUCACGAUCCUCA U
 CU C  GU   UG  U GUU  GA GAGGCAU   CAGUGCUGGGAGU G
A  C UU  AUC  GG U   UU  C       UUC^            C
.        90        80        70        60        50

-----------------------------------------------------
```

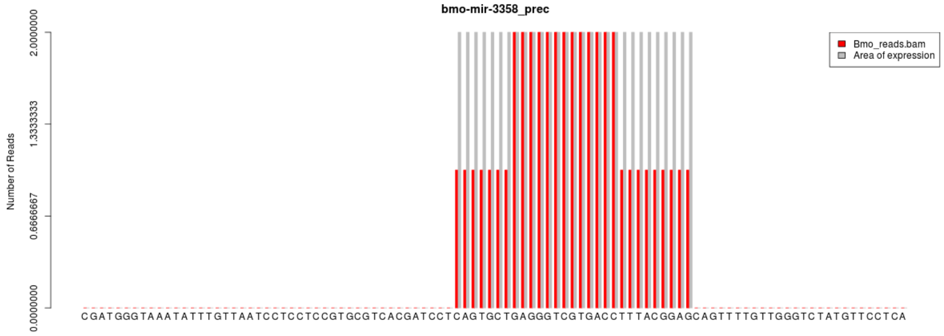

>bmo-mir-3359_prec

```
ACAGUGCGGUAUCCGUGAACCGCCCUGACCGCAAUGGCGCGCUGCGGAUGUCGCAACGAUGAGACGUUGUCGCGGGUCAGGGCGUGGCACCAAAUGGGACCCCCGUACA
...((((((..((((((...((((((((((((.(..(((((.((((.....)))).))......)))..).)))).))))))))...))).......)))...)))))).

 Initial dG =    -48.20

        10               20          30              40
ACA      UA-   ------   AAC         -    A UG   ------| C    GA
   GUGCGG   UCC      GUG   CGCCCUGA CCGC A  GCG      CG UGCG  \
   CAUGCC   AGG      CAC   GCGGGACU GGCG U  UGC      GC ACGC  U
A--      CCC   GUAAAC   GGU        G    C GU   AGAGUA^ A    UG
         100        90        80        70        60        50

-----------------------------------------------------
```

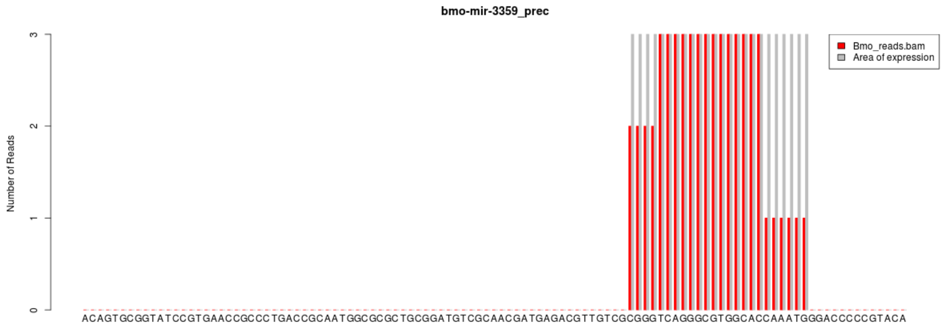

>bmo-mir-3360_prec

CUAUUACUAGGAUUUGCAUUUUGAUAAUUAUAUGAAAAUAGCUGCUCCCUAUUACAUUUUCUUAGCAGUUCAUUUCGGUAAUUAUGGAAAUGUUCAAAUGUACAUAAUAA

```
....(((...((...(((((((.((((((((.(((((..(((((((.................)))))))..)))))))))))))).)))))))))....))).......
```

```
 Initial dG =    -23.10

            10         20        30        40        50
CUAU----   UAG-| UUU       G        A     AU       CCCUAUUA
        UAC    GA   GCAUUUU AUAAUUAU UGAAA  AGCUGCU        \
        AUG    CU   UGUAAAG UAUUAAUG GCUUU  UUGACGA        C
AAUAAUAC   UAAA^ ---       G        -     AC       UUCUUUUA
.       100           90        80         70        60

-----------------------------------------------------
```

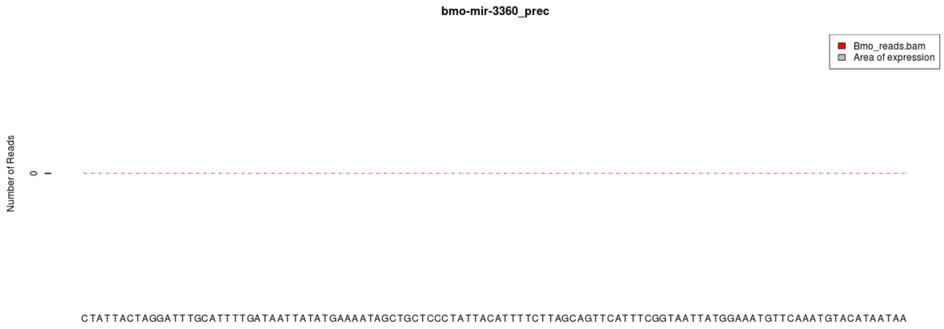

>bmo-mir-3361_prec

AAGUCGCCACUCCAAAGCAUUGACUAGUUUGCGUGUCAAACUCGGAUAUCCACGUCUCGUCACCAGUAACAACGCGUUCCGUGAAUGAUGGGUCGGAAUUUAUACGUUCUAGUAUGUUCU
CAGCGAG
...((((........(((((..(((((...((((((.(((.((.((...((.((((...((((....
(((.....)))..)))))..)))))))).)).)))))))))).))))))))))....)))).

```
 Initial dG =    -28.50

        10        20        30        40        50        60        70
AAG|   CACUCCAA     UG     UUU      C   C  G  UAU  A    UCG    CAGU   AA
   UCGC        AGCAU  ACUAG   GCGUGU AAA UC GA   CC CGUC   UCAC    AAC  \
   AGCG        UUGUA  UGAUC   UGCAUA UUU AG CU   GG GUAG   AGUG    UUG  C
G--^   ACUC----     --     U--      -   A  G  ---  -    UA-    CC--   CG
       120              110          100             90         80

-----------------------------------------------------
```

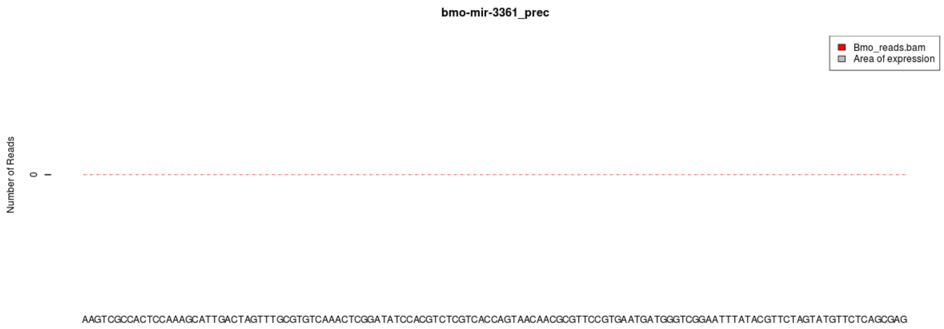

>bmo-mir-3362_prec

```
AAGCCGUUUGUGAUGCUGAUUUGAAGUUUAUGAAUGUUGUAGCCCGGUGGCCUGGUUCUGCACAUGAUGCUACUAUAUUUAAUAAUUCUAUUUUAAGAGGUUUUAUAGACA
.....((((((((.(((..((((((((((((((((((.(((((...(((((........)).)))...))))).))))))).)))....)))))))).))).)))))))).

 Initial dG =    -26.30

        10        20            30         40         50
AAGCC        U   GA        ----   -       U      CCG   -| CUG
     GUUUGUGA GCU  UUUGAAGU    UUA UGAAUGU GUAGC   GUG GC   G
     CAGAUAUU UGG  GAAUUUUA    AAU AUUUAUA CAUCG   UAC CG   U
A----        U   A-        UCUU   A       U      UAG   A^ UCU
   110       100         90        80        70        60

-----------------------------------------------------
```

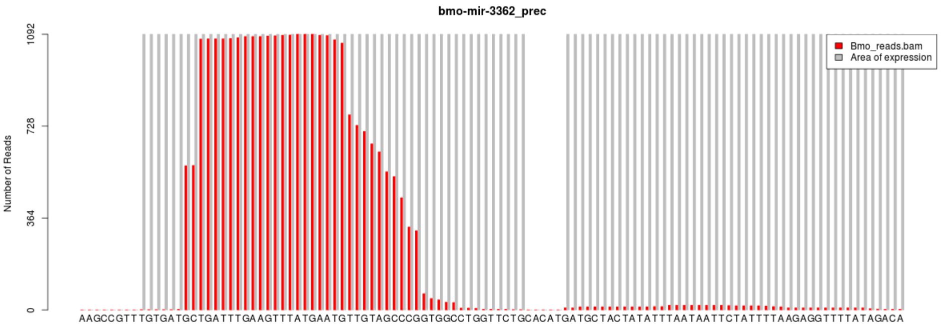

>bmo-mir-3363_prec

UCAAAUGUUAAGUUCACUGUGUAUCCCUUGUAGUUAAAAUAAAUAAAUAUAAUAACCUUUAUGUUAUUCAUACGUAUUUGUGUACUGCGUUUACGGGAAUUCUUUUUAAGAUCUUCCCAU
UUU

```
...........................((((((....(((((((..(((((((((......)))))).)))..))))))).))))))......(((((.
((((...))))..)))))).....

 Initial dG =    -21.80

          10        20        30        40        50
.-UCAAAUGUUAAGUUCACUGUGUAUCCCU|     UAAA       AA    -      CU
                              UGUAGU    AUAAAUA  UAU AAUAAC  U
                              GCGUCA    UGUUUAU  AUA UUAUUG  U
\ ----------------------------^     UG--       GC    C      UA
                                        80        70        60



 90       100
  UUUAC     U-    U
       GGGAA  UCUU \
       CCCUU  AGAA U
  UUUUA     CU    U
   120       110

-----------------------------------------------------
```

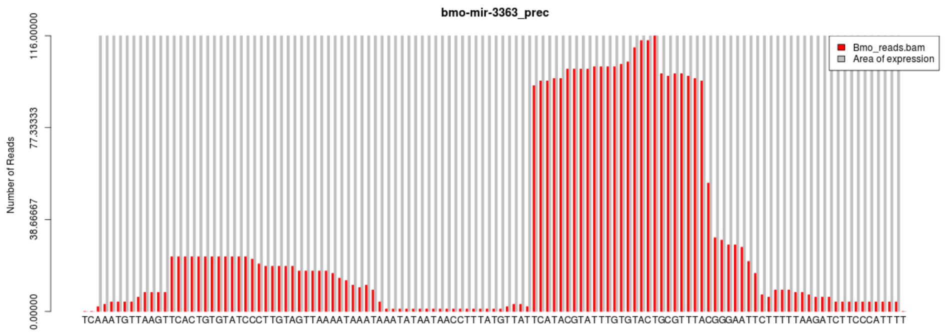

>bmo-mir-3364_prec

```
GCUUUCCGUGACUAUCACUUCCAUAAUUUCACGACCCCAUAUGGCAAUAAAUGAUUCGUAUGGAGUCGCGAAAGAAACCGUUGGAGCACUGAUUUCGGAGAAAGG
.(((((..(((..((((.(((((...((((.((((.((((((((...........)))))))).)))).)))).........)))))...)))).)))..))))).

 Initial dG =    -29.80

        10          20             30        40
G     CG   CU    C--     UAA-----|   A    C        CAAUA
 CUUUC  UGA  AUCA   UUCCA        UUUC CGAC CCAUAUGG     \
 GAAAG  GCU  UAGU   GAGGU        AAAG GCUG GGUAUGCU     A
G     AG   U-    CAC     UGCCAAAG^   C    A        UAGUA
   100         90        80        70        60

-----------------------------------------------------
```

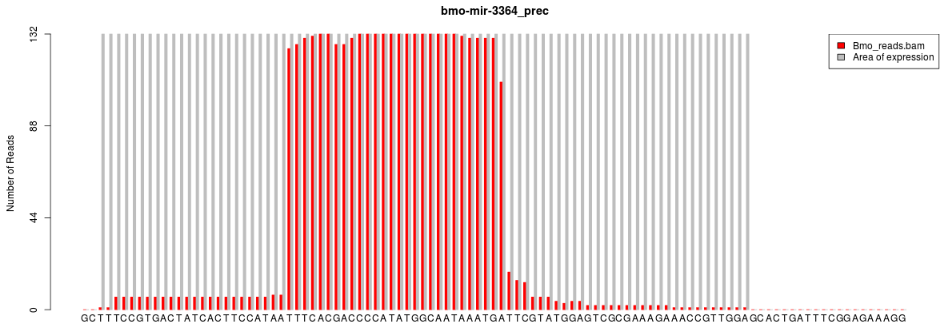

>bmo-mir-3365_prec

```
CAAUGGCACCAUUAGAUACUGAUGCGUGUGUUAGUCAAUUAGUCAAGAUAACGGUGCGUCUGGCAUUAUAUGACUAGAUCUACCACAGCAACAGGGCUGGGUCGA
.....(((((((((...)))))).)))).((((((((..((((.(((........)))))))...)).))))))).((.(((.((((......))))))).))

 Initial dG =    -28.40

        10        20        30        40        50
CAA|   A   UUA AUA   A   G    U   UCAA       AG   ACG    GU
   UGGC CCA   G   CUG UGC UGUG UAG    UUAGUCA  AUA   GUGC  \
   GCUG GGU   C   GAC ACG ACAC AUC    GAUCAGU  UAU   UACG  C
A--^   -   --- GG-   A   -    C   UA--       A-   ---    GU
      100            90          80          70

-----------------------------------------------------
```

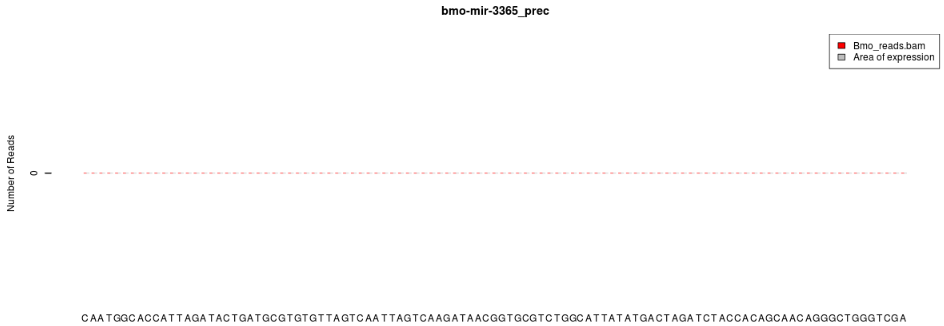

>bmo-mir-3366_prec

AAGAAAAGCGUCUUAACCAAACUGAUGUACUAGAAAACAAGUUUUGGUAAAUACAGUUCCCUUGCUCCCUACCGAAACAAUUGUUCAUUUUAGUAUGAAGGGUGGUUGGUACUAGCUCAU
GUCUC
.(((..(((((.(((((((..((...((((((((((((((((((((((.....
(((......)))...)))))))))..))))).))))))))))..))..))))))).))..)))....))).

```
 Initial dG =    -33.80

            10        20        30          40          50        60
A   AA--   --  C       AA  GAU         --     --|        AAUAC   UC
 AGA    AGC  GU UUAACCA  CU   GUACUAGAA  AACAA  GUUUUGGUA     AGU  C
 UCU    UCG  CA GGUUGGU  GA   UAUGAUUUU  UUGUU  CAAAGCCAU     UCG  C
C   GUAC   AU  U       GG  AG-         AC     AA^        CCC--   UU
   120       110       100         90        80        70
```

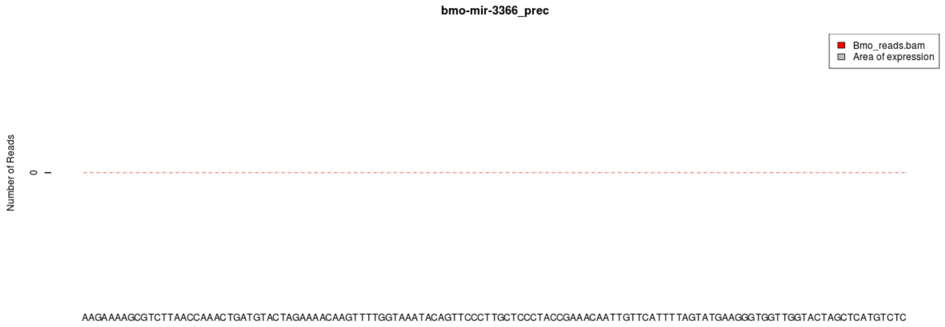

>bmo-mir-3367_prec

```
AAAGUUAAUGUUAUCUUGUGCAUAUUUUCUUAUCUACGCAUUUGUCAUUUAAAAUGAUGGUAAACAGUUUGUUUGGGUGACGCUGCGCUGAUAUUAGCC
...((((((((((....(((((.......(((((((.((((((..((((......))))..))).....))).)))))))...))))))))))))))).

 Initial dG =    -21.60

        10        20        30        40             50
AAA|         UCUU     UAUUUUC       C   -----   GU    UA
   GUUAAUGUUA    GUGCA       UUAUCUA GCA     UUU  CAUU  A
   CGAUUAUAGU    CGCGU       AGUGGGU UGU     AAA  GUAG  A
C--^         ----     CGC----       U   UUGAC   UG    UA
          90                80        70        60

-----------------------------------------------------
```

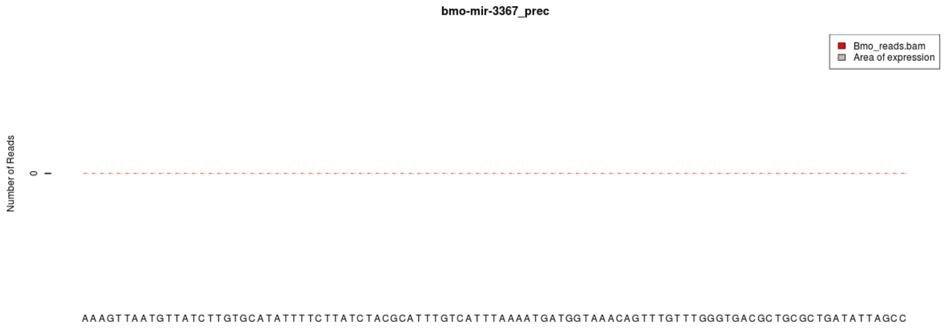

>bmo-mir-3368_prec

AUACCGUCGCCGCUUCAGUCUUUUAGAGAAGAAGUGGAAGGCGCAGUGAUAUGCUUUCAUAUCUCAAUUGUUUACCAGCUAUUCAAAAGAUUUGGAACUAAAGCGGCCGAAAUGUCAGGA

...((...(((((((((((((((.(((.((...(((.(((((....((((((....))))))....))))).))).)).))))))))))).))......)))))).((....)).)).

```
 Initial dG =    -27.70

          10                20        30        40          50
AUA  .-GUC      ------  -|       A   A  AAG   A     CAGU-      C
   CC     GCCGCU      UC AGUCUUUU GAG AG   UGG AGGCG     GAUAUG U
   GG     CGGCGA      GG UUAGAAAA CUU UC   ACC UUUGU     CUAUAC U
A--  \ ---      AAUCAA  U^       -   A  G--   A     UAACU      U
.              100        90         80          70        60


       110
       C  A
        GA A
        CU U
       A  G
```

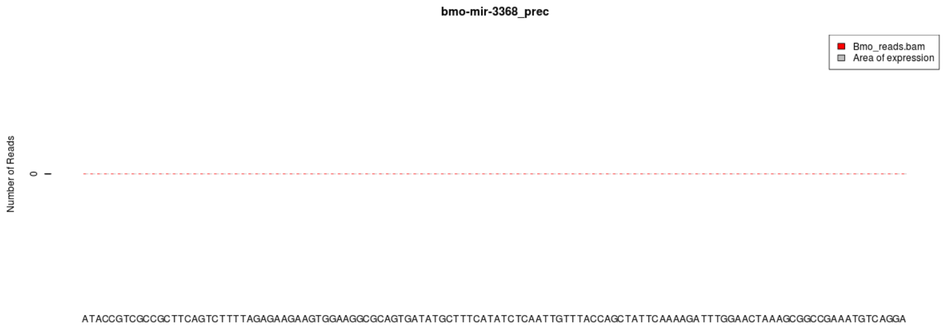

>bmo-mir-3369_prec

```
UGCUGGUGGCGGGUGAAAAAUGAGAUCUAAUUAUAAAGAGUAUUGUUUUGCGGCCAAGAAAUAGGUACAUCGGUGCUACUUACAGAUAGAUCGUUUUCAAAUUUGGGGUCAAAAGCG
.(((..((((...((((((....((((((.((.(((..(((((((.......(((........)))....)))))))..))).)).)))))).))))))........))))..))).

 Initial dG =    -26.00

        10             20        30        40        50
U   GG    GGG-----|     AUGA      A  A   AG       UUUUGCG   AAG
 GCU  UGGC        UGAAAA    GAUCUA UU UAA  AGUAUUG       GCC   A
 CGA  ACUG        ACUUUU    CUAGAU GA AUU  UCGUGGC       UGG   A
G   AA    GGGUUUAA^     G---      A  C   CA       UACA---   AUA
     110       100           90        80        70            60

-----------------------------------------------------
```

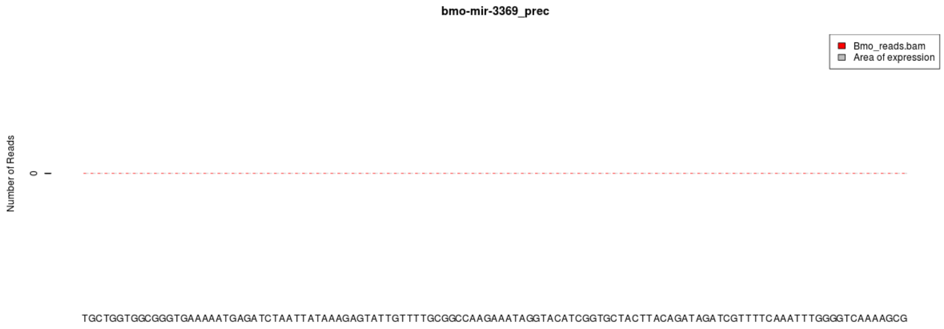

>bmo-mir-3370_prec

```
UCGUCAGAUUAUAGAAACAGAUUUAUCAGCAACAUGCUUUGUCAACUGUACAAUACUUACUUUCAGCUGCAAGCGUUGUCAGCAUUUUGUAAUUGUAUACUUGUACAAUGUCUUGAAACG
UCUUGCUAAAAUCUGAA
...(((((((.(((....((((...((((..((((....((((((.((((((((...(((.....
(((((((...))).)))).....))))))))))).))).))))))).))))...))))..))).))))))).

 Initial dG =    -31.00

        10        20        30        40         50        60         70
UCG|      A   AAAC    UUA    CA    GCUU   -   C        ACU   UUUCA    -   G
   UCAGAUU UAG    AGAU   UCAG  ACAU    UGU CAA UGUACAAU   UAC     GCUG CAA \
   AGUCUAA AUC    UCUG   AGUU  UGUA    ACA GUU AUAUGUUA   AUG     CGAC GUU C
A--^      A   GU--    CAA    C-    ----   U   C        ---   UUUUA    U   G
       130         120        110           100           90        80

-----------------------------------------------------
```

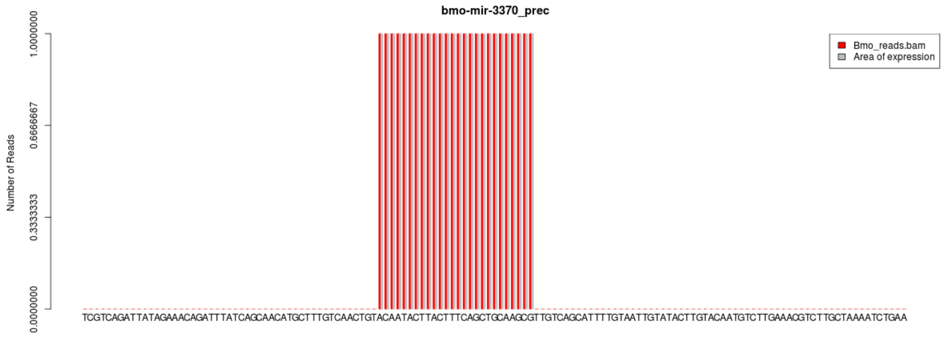

>bmo-mir-3371_prec

```
GAAUUGAAUUUCUCAUCUACCAUGCACAUGCAUAAUAACAACAUAUACGCAUAGCCAAAGCUGGCAUCGUAACGUUGUUAUAGUACAAUUAAUGUAUGGACCAGUGAGUGAGUUAAG
...((((....(((((...(((((((..((.((.((((((((...((((.((.((((....))))))))))..)))))))).)).)).....)))))))....)))))....)))).
```

```
 Initial dG =    -30.20

        10         20            30         40         50
GAA    AUUU     CUA-        CA---| C  A         AUA    C  A    A
   UUGA    CUCAU    CCAUGCA     UG AU AUAACAAC   UACG AU GCCA A
   AAUU    GAGUG    GGUAUGU     AC UG UAUUGUUG   AUGC UA CGGU G
G--    GAGU     ACCA        AAUUA^ A  A         CA-    -  -    C
       110       100         90         80          70           60

-----------------------------------------------------
```

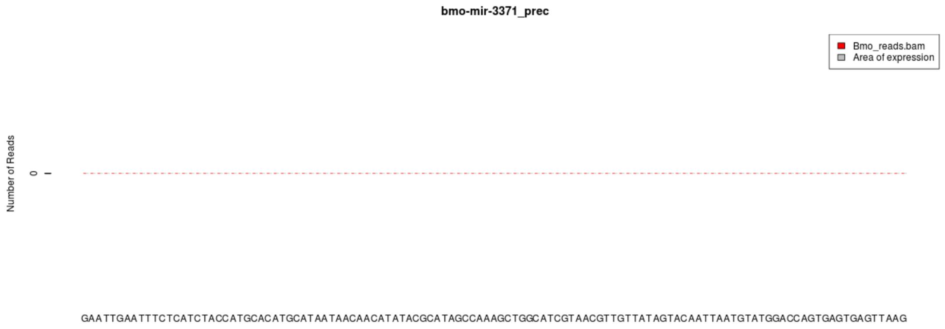

>bmo-mir-3372_prec

AACUCAUUUGUUUUUAUUAUGUUCUUGGCGUCAGAGCCAAAUGAUUUUUUGUUUACAAUAAUUUGAGCCUAUCGCCAAGCUAUUUUAAGCAAUGAC

```
...(((((.((((.....(((..(((((((..((.(((((((......(((....)))..))))).)))))..))))))).)))...))))))))).

 Initial dG =    -22.70

        10        20        30         40        50
AAC|    U    UUAUU   UU        UC  A  -     GAUUUU   U
   UCAUU GUUU     AUG  CUUGGCG  AG GC CAAAU      UUG U
   AGUAA CGAA     UAU  GAACCGC  UC CG GUUUA      AAC U
C--^    -    UUU--   C-       UA  -  A     AU----   A
        90         80         70         60

-----------------------------------------------------
```

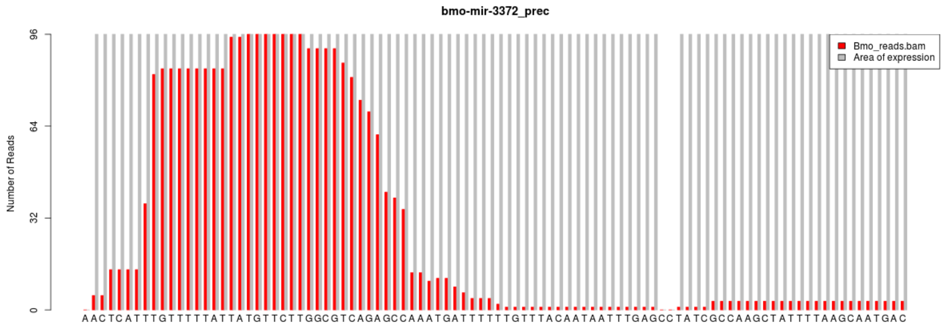

>bmo-mir-3373_prec

AUAGGUGAGCAAUAUAGCCGCAGAACUGUUCGGGCAUGCCCCUCAACCUCAGCAAUUCGACAAGUGCCUCGUGAGACACAGUGUCGAAUGCCUUGGCGAAGUCUAGCACCGCCACGUGAC
AUUCCCGCAGCUUCUUCCUGCUAUCCCCA

```
...((..((((....(((.((.(((.(((((((((.(((..((...((...(((.(((((((.(((..
((....)))))..))))))))))...))...))....)))..))).)).)))))))..)).)))......))))....)).
```

```
 Initial dG =    -42.50

          10          20         30           40          50         60         70
AUA  UG--    AUAU--   C  A-    C     -  -   A-    CC--| CAA  UCA   A       A-   CC  G
   GG    AGCA      AGC GC  GAA UGUU CG GGC  UGC     CU    CC   GCA UUCGACA  GUG  UC U
   CC    UCGU      UCG CG  CUU ACAG GC CCG  ACG     GA    GG   CGU AAGCUGU  CAC  AG G
A--  CCUA    CCUUCU   A  CC    -     U  A    CC    AUCU^ AGC  UUC    -       GA   --  A
         140       130        120        110        100         90         80
```

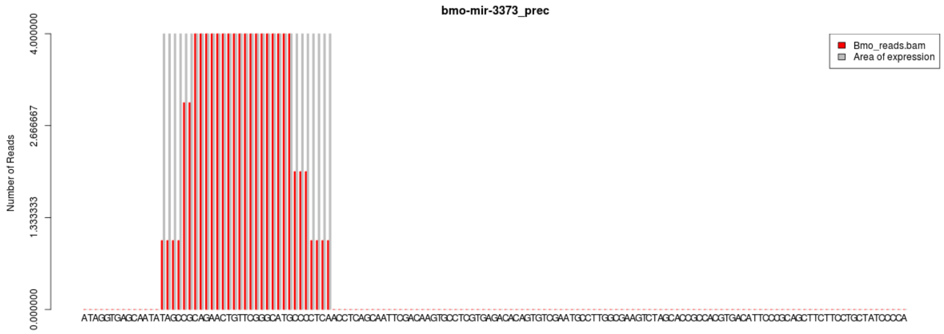

>bmo-mir-3374_prec

```
UUGACAGCCGUUGAAGAUGCACCAGAUGUUGAGAGGCCUGAAUGGAAACGCCAGGAUGCUUUAUUUGCUCGCACUCAACA
.........(((((.(.(((..((((((..(((...((((..((....)).))))...)))))))))...))))))))).

 Initial dG =    -21.00

        10        20         30        40
UUGACAGCC|    A A   AC-       UU   AGG    AA  G
         GUUGA G UGC   CAGAUG  GAG   CCUG  UG A
         CAACU C ACG   GUUUAU  UUC   GGAC  GC A
A--------^    - -   CUC       --   GUA    C-  A
.                  70          60         50

-----------------------------------------------------
```

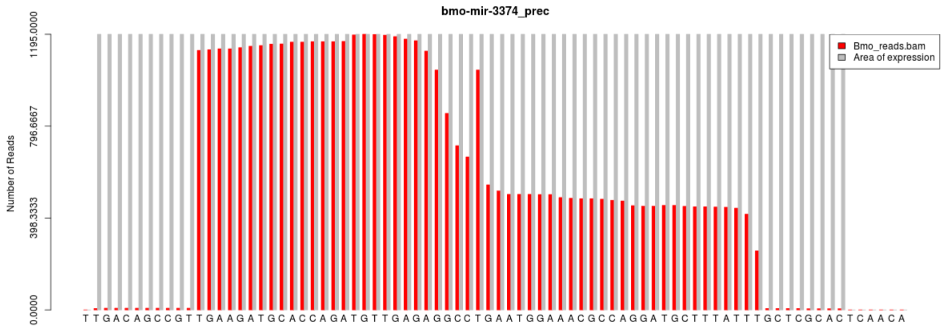

>bmo-mir-3375_prec

```
UGUGUUUGUUCAGGUCCCCAGCUCCAUGUGUAAUUCUUCUUGGUUAGUCGAGUAAGCGGUUCUGCUAUCUUUGAAAAGUUUGGAAUAAACUUCCUGAACCAAGAGCAUG
.((((((((((((........((((.....((((.(((..((.((((.(((.......))).)))).))..))).)))))))).......)))))))...)))))).

 Initial dG =    -25.00

           10        20        30        40        50
U      ---|      UCCCCAGC    UGUGU    C   UU  U    C   UAA
 GUGUUU   GUUCAGG        UCCA     AAUU UUC  GG UAGU GAG   \
 UACGAG   CAAGUCC        AGGU     UUGA AAG  UC AUCG CUU   G
G      AAC^      UUCAAAUA    -----    A   UU  U    U   GGC
       100        90             80        70        60

-----------------------------------------------------
```

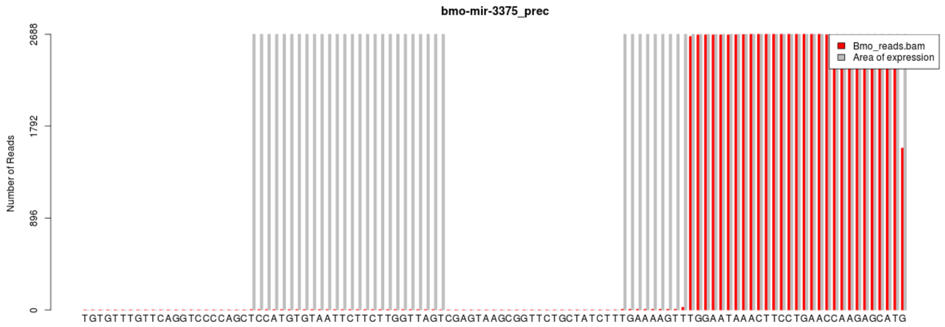

>bmo-mir-3377_prec

```
CUGGGGAUAUUUAAAUGAAGUUAUGAAGUCAGGAUACGUAAAUUUACGUCUUAUCCUUUCAUAGGUUCUUGAUUUGUAACCG
..((..(((.......(((.(((((((...((((((((((....)))))...)))))))))))).)))......)))..)).

 Initial dG =    -21.10

        10        20        30          40
CU| GG   UUUAAAU   G       GUC     ---     A
  GG  AUA       GAA UUAUGAA   AGGAU   ACGUA A
  CC  UGU       CUU GAUACUU   UCCUA   UGCAU U
G-^ AA   UUAGUU-   G       ---     UUC     U
  80        70         60           50

-----------------------------------------------------
```

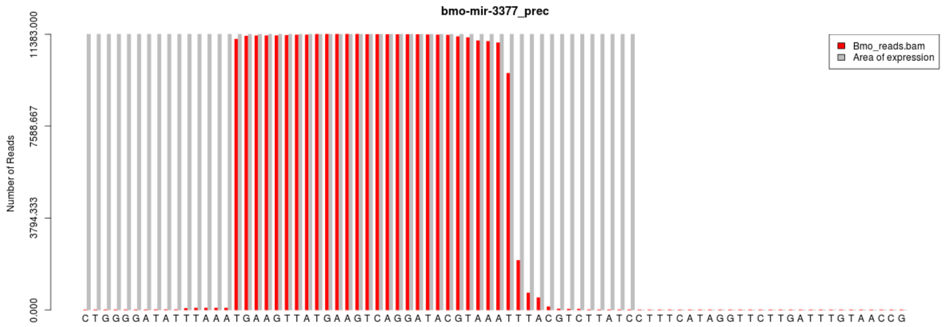

>bmo-mir-3378_prec

```
GUCCUUAUCUUCCAUGUUGAUCGGGGCUGCUUCGCUCGCGUCGUCCUCCACGUGGGAUCUUGCUGCUCCAAUUGUCUGGACACGCAGG
..(((.....((((...((((.(((((.((....((((((((........))))))).....)).))))).))))..)))).....)))

 Initial dG =    -26.20

        10        20        30         40
GU|  UAUCU    UGU    C     U  UUCG-       CGU
  CCU     UCCA   UGAU GGGGC GC     CUCGCGU   C
  GGA     AGGU   GUUA CCUCG CG     GGGUGCA   C
--^  CGCAC    CU-    A     U  UUCUA       CCU
         80         70        60         50

-----------------------------------------------------
```

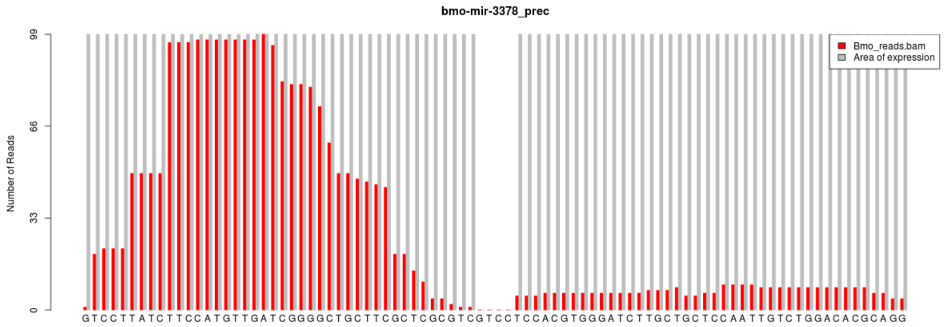

>bmo-mir-3381_prec

GAGAUCAAGAUUCCUCGCAAAGGUUCACUCCCCCAGGAUCAUUCGAUGCCGAAGAUGAAGGAGUAUUCAUACCUGGCCGAUGGCGUCAAGAUA
...(((..(((.(((((...((((..(((((..((...((.((((....))))))))..)))))......))))...))).)).)))..))).

```
 Initial dG =    -22.10

        10         20            30        40
GAG   AA   U  -   CAA    UC----|    CC  GGA  A    A
   AUC  GAU CC UCG   AGGU      ACUCC  CA   UC UUCG U
   UAG  CUG GG AGC   UCCA      UGAGG  GU   AG AAGC G
A--   AA   C  U   CGG    UACUUA^    AA  ---  -    C
    90        80        70        60            50
```

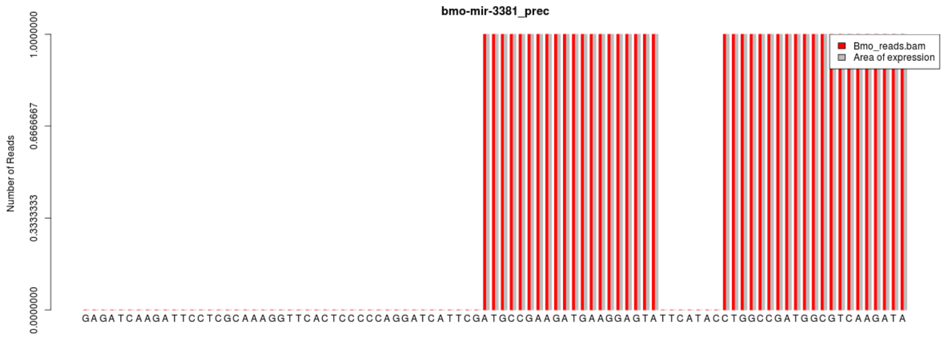

>bmo-mir-3382_prec

```
UCCGCGUCGUUGCCUCUUCAAGUUUUGUGUUCUGGGUCGUGCCGGUCGCGGAGCUCUCGUAUGUUUCGUACGACGUCGCUUCUGUUGCCUCCGGACUGGAUUCUUGAACCGCAGGUUCGA
GUGGC
.((((.(((..((((.((((((..((..((.(((((..((..(((..((((.....
(((((((...))))))))..))))..)))..)).)))))))..))..))))))....)))).))))))).

 Initial dG =    -40.50

        10           20        30        40        50        60
U    G   UU    C---|     UU  GU  U     UC  GC   UC    AGCUC       U
 CCGC UCG  GCCU    UUCAAG  UU  GU CUGGG  GU  CGG  GCGG     UCGUAUG \
 GGUG AGC  UGGA    AAGUUC  AG  CA GGCCU  CG  GUC  CGCU     AGCAUGC U
C    -   U-    CGCC^     UU  GU  -     C-  UU   UU    GC---       U
    120        110       100         90         80           70

-----------------------------------------------------
```

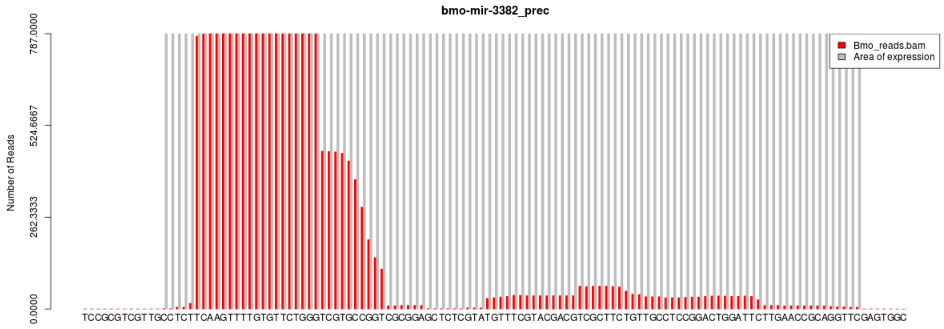

>bmo-mir-3383_prec

```
GAAAUAGUGCCUGAGGGCUUCGUCCAUGUUCGCGUCGGCCGUCGUCGCGUGUGCCACGAAGUUGACGCGCUGUCUCUU
((.(((((((.....((((((((....((.((((.((((....)))))))).)).))))))))...))))))).)).. 

 Initial dG =    -27.70

          10         20         30         40
--| A       CUGAG        CCAU  U    U    C
  GA AUAGUGC     GGCUUCGU    GU CGCG CGGC G
  CU UGUCGCG     UUGAAGCA    CG GUGC GCUG U
UU^ C       CAG--        C---  U    -    C
       70          60           50

-----------------------------------------------------
```

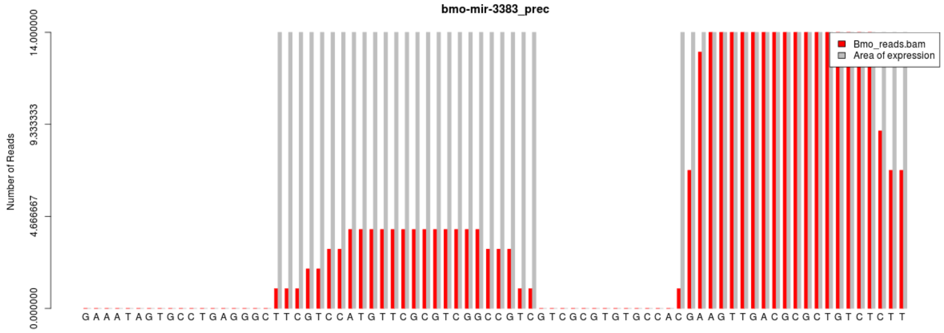

>bmo-mir-3384_prec

AUGAAAUGUGUCCUCUAUCCGGCUCGAAUGGACCAUGAAAUGUACACGACCCGGUCUGUAUGGCUGUUUCCGAGUCGUGUAAUAAUAUUUUCUGCUGUCUGUUUGUGCGCUGGUAGAAGA
CGGCUGUUUCU

```
..((((((((((.((((.(((((.(((((((((((.((((((((((((((.(((...
(.....).....))).)))))))....))))))).))..)))))))))...))))))))).)))).).))))).
```

```
 Initial dG =    -44.30

          10        20          30          40             50          60
AU     - -    C    U     U--         --  U       ----       C   UCU--|UA
  GAAAU G UGUC UCUA CCGGC   CGAAUGGAC  CA GAAAUGU    ACACGAC CGG     G  \
  CUUUG C GCAG AGAU GGUCG   GUUUGUCUG  GU UUUUAUA    UGUGCUG GCC     C  U
U-     U G    A    -     CGU         UC  C       AUAA       A   UUUGU^GG
130       120        110       100        90        80        70

-----------------------------------------------------
```

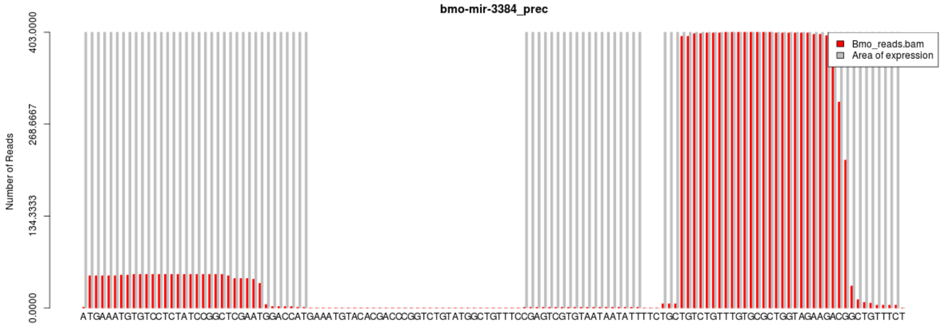

>bmo-mir-3385_prec

UUAGUUUUGCAUGCAAACCUUACCUUCAAAGGAUAUCCACUGUCCCCUAAGAGAUAUGCAACAUUAUAAGCUCCACUUUCAAACACACGGUGUCUUUGACUGUUUUGGUGUAUGGUAGAG
UCA

```
....((((((((((..(((..((..(((((((.....((((((......((((....
((..........))....))))).......)))))))))))))..))...)))))))).)))))...

 Initial dG =    -21.70

         10        20         30        40         50        60
UUAG     -|    AA   UU-  CU       AUAUC      CCCCUA-    AUAU  AACA
    UUUUG CAUGC  ACC   AC  UCAAAGG     CACUGU       AGAG    GC    U
    GAGAU GUAUG  UGG   UG  AGUUUCU     GUGGCA       UUUC    CG    U
ACU-     G^    --   UUU  UC       -----      CACAAAC    ACCU  AAUA
  120       110        100             90         80        70

-----------------------------------------------------
```

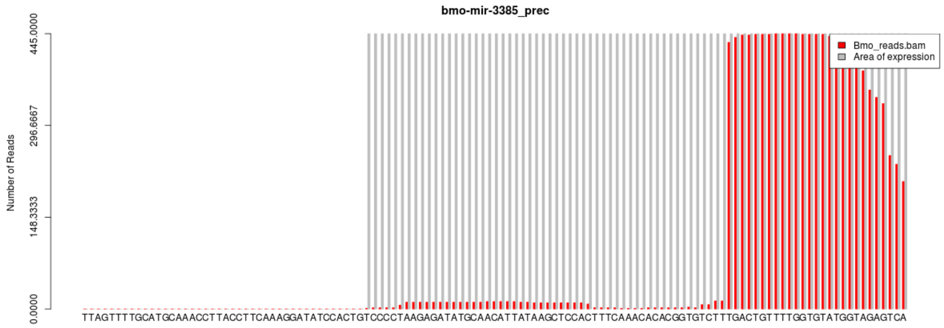

>bmo-mir-3386_prec

GAUCUGAAAAAUUAUUAAUGAGGGCAGUACGGAUACCUAACCAGUUCUCUGGUAUGCCAUUUGAAUUUAUGGUCCUGGCGGCAGGACCAGUUAUUUUAUUUAAUAUACCAUUUAUUAAGC
UGAGGUUAUUUAAUUCGUUGCCAGGAUCU

```
(((((......(((....))).((((..((((((((((...(((((...(((((((......((((...
((((((((....))))))))...)))).......)))))))).......)))))))))......))))))))))).))))).
```

```
 Initial dG =    -39.50

           10
-     .-GAAAAA|  U
 GAUCU        UUA U
 CUAGG        AGU A
U     \ ------^  A
              20



              30              40            50         60         70
        G    GU      ------    AAC     CUC----       CCAUUU-    UUA        G
         GGCA  ACGGAU      ACCU   CAGUU       UGGUAUG       GAAU   UGGUCCUG C
         CCGU  UGCUUA      UGGA   GUCGA       ACCAUAU       UUUA   ACCAGGAC G
        A    --      AUUUAU    ---     AUUAUUU       AAUUUAU    UUG        G
         140         130          120       110        100        90         80

-----------------------------------------------------
```

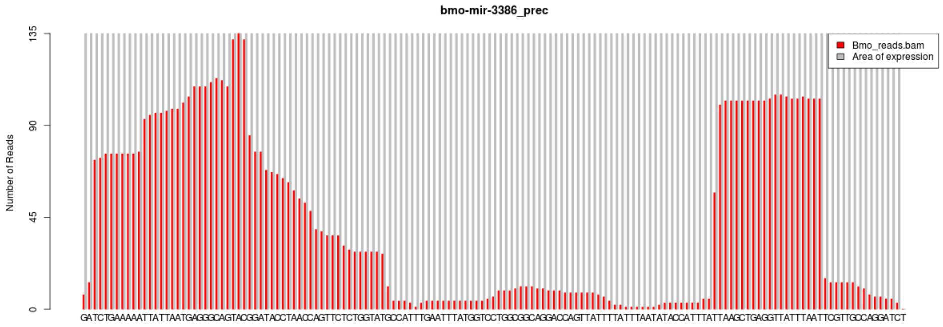

>bmo-mir-3387_prec

UUGCUGCCUUUGUUGACUAACAACAUUAAUGAAAACGAUCGUGGACUACCUUUAACGCCGAUAUUACAAGUGCUAAUUGCUUUGAGAUUUUACGCAACAGGCAAUUUUCAGGUUCGUGCA
UAAUGUGCUUUUGUGUCUACAUAAUAGAA

```
.(((.(((..(((((.....)))))....((((((..((((((............)).))))........((((..
((((.............))))..)))).)))))))))....))).......((.(((((....))))).))..
```

```
 Initial dG =    -22.80

          10
UUG   .-CCUU|    AC
   CUG      UGUUG  \
   GAU      ACAAC  U
AA-   \ ----^    AA
                20


           30        40          50         60        70         80
        UUAAUGAAAACGAUC      .-UACCUUUAAC  -   UAUUACAAG-    AA    UUUGAG
                       GUGGAC            GC CGA          UGCU  UUGC      \
                       CAUCUG            CG GCU          ACGG  AACG      A
        AAUA-----------      \ ----------  U   UGGACUUUUA    AC    CAUUUU
                     140                        110       100        90

-----------------------------------------------------
```

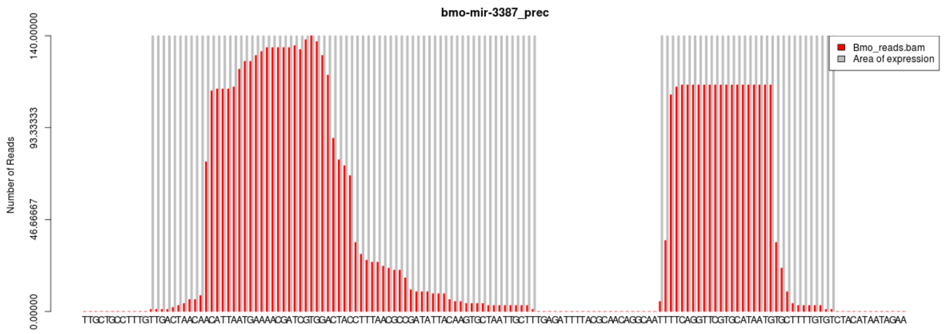

>bmo-mir-3388_prec

UUAUCUUCGCACGCUGACAAGUCAAACAUGCCUUGGCCCAUUCACGGCAGUUCUUACGCAUCAGUGGCCAAACGAAGCGUUGCGCAACGAGUUUAGCUGUCGCAUUGGGACCUGGAUGAC
UGAGCGAGUGGAGGC

```
...(((((((.((((....(((((..((......((((((....(((((((((((((((.((.
((......)))).)))).........)))...))))))))...)))).)))).)))))..))))).)))).))))))).

 Initial dG =    -42.30

        10        20        30         40        50          60
UUA       A    GACA     AA  UGCCUU  -    UUCA        .-CUUA|  A
   UCUUCGC CGCU    AGUCA  CA      GG CCCA    CGGCAGUU      CGC U
   GGAGGUG GCGA    UCAGU  GU      CC GGGU    GCUGUCGA      GUG C
C--       A    G---     AG  ------  A    UAC-        \ ----^  A
     130          120            110        100


                                                          70        80
                                                        GCCAAA  AA   U
                                                              CG  GCG \
                                                              GC  CGC U
                                                        UUUGA-  AA   G
                                                           90

-----------------------------------------------------
```

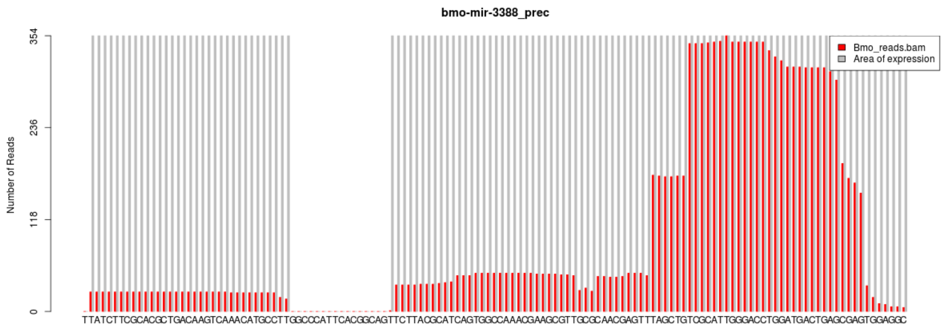

>bmo-mir-3389_prec

```
AGGGGACCCCGAAUGACGCCCCGCAGGACCCAGUCGUAGCCGAUGUUCCACAGCAGGGGGCCCAGGACAGACCCCUGCGGAACACCGCGCUCGACGGGGAAGCGGAACAUCGCCCCA
.((((....(((.((.....((((....(((.((((.(((((.((((((...(((((((............))))))))))))).)).))))))))))...)))).)))))))))).

 Initial dG =    -54.20

        10        20        30        40         50        60
A|   ACCC   A  ACGCC    AGGA   A    U   -  A      ACA       GCCCA
 GGGG    CGA UG     CCGC    CCC GUCG AGC CG UGUUCC   GCAGGGG     G
 CCCC    GCU AC     GGCG    GGG CAGC UCG GC ACAAGG   CGUCCCC     G
A^   ----   -  AA---    AAG-   -    -   C  C      ---       AGACA
         110        100         90        80           70

-----------------------------------------------------
```

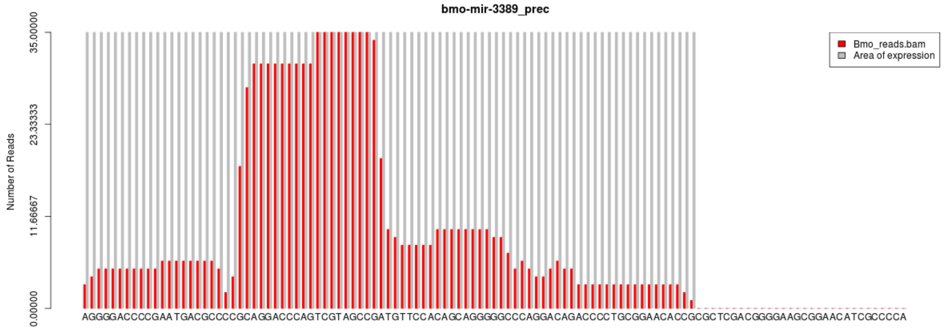

>bmo-mir-3391_prec

AGGUCGUGGUAUUUCGUACCUUCAUGGCUCAGUACUGUGCGAACGGAUUGUUGUUCGUCGAUCCGUCCCCGGUGUGGUGCUGUGACAAAAAGUGAUGUGAGUCCGUCGAG
...((((((.(((((((..(((..((...((((((..(((..((((((((........)))))))).....)))..))))))...))..)))..))).))))))).))).

```
 Initial dG =    -33.60

         10         20        30        40          50
AGG   -   U    -   AC   CA  GCU       UG   GA---|       UUG
   UCG UGG AUUU CGU  CUU  UG   CAGUAC  UGC      ACGGAUUG   U
   AGC GCC UGAG GUA  GAA  AC   GUCGUG  GUG      UGCCUAGC   U
G--   U   -    U   GU   AA  AGU       GU   GCCCC^       UGC
.          100        90        80        70        60
```

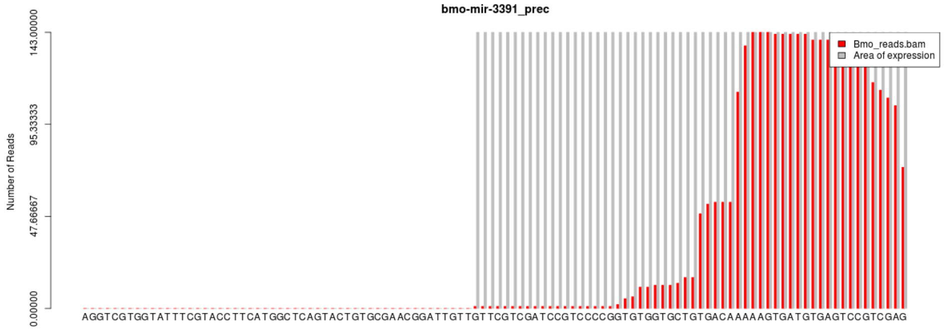

>bmo-mir-3392_prec

```
CACGAAUAUUUACUCGAUGCGGGAAUCGAACCCACGACCCUCGGCGCAACAGUUAGGGGCACUAACUACUACACCACCGAGUCAGUCGAUUUCUUCGAAUUACUAUUUGAU
..((((((.....((((...(((((((((......(((..((((......((((((.....)))))).........)))))))..))))))))))))).....))))))..

 Initial dG =    -26.50

        10        20        30        40        50
CA|     UUUAC    UGC         ACCCAC   CC    CGCAAC---      GG
  CGAAUA     UCGA   GGGAAUCGA      GAC  UCGG         AGUUAG  \
  GUUUAU     AGCU   UCUUUAGCU      CUG  AGCC         UCAAUC  G
UA^     CAUUA    ---         GA----   --    ACCACAUCA      AC
 .       100           90              80         70        60

-----------------------------------------------------
```

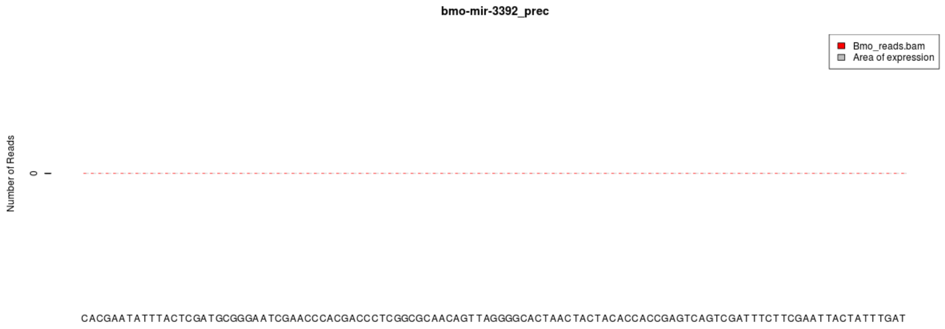

>bmo-mir-3393_prec

CGAACGUUUGUAGGAGGAGAUUUAUCUGGAUAAUUCGUCGAUUCAGUCAAUAGGUCGAUUUUCUAACGAGAAGACAAAUACUAAUUAAUCGAUAUACGAAUUAGCAUAAAAUCGAUUUUU GCAAAUA

.....((((((((((..(((((...((..((((((((((((..........(((.. (((((....)))))))).........))))))....))))))).))..)))))..))))))))))).

```
 Initial dG =    -29.60

        10        20        30             40        50         60
CGAACGA           GA     AUC  GA       ----      CAGUCAAUAG-|  GA     U
     GUUUGUAGGAG  GAUUU   UG  UAAUUCG    UCGAUU           GUC  UUUUC A
     UAAACGUUUUU  CUAAA   AC  AUUAAGC    AGCUAA           CAG  AAGAG A
A----           AG     AU-  G-       AUAU      UUAAUCAUAAA^  --     C
         120       110         100        90        80          70
```

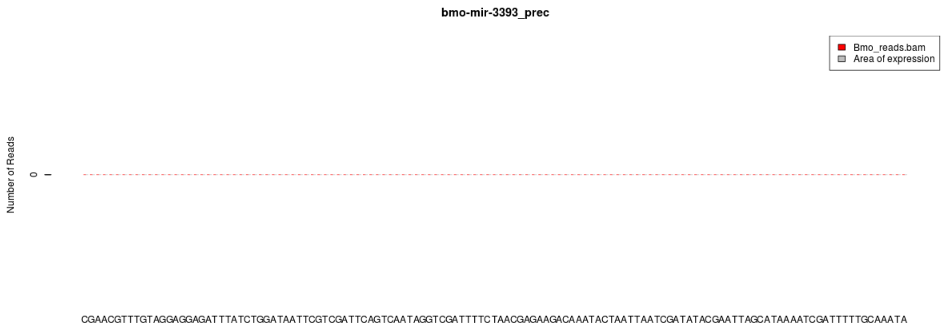

>bmo-mir-3394_prec

UCGGCGUCCUCGUACAAUCGCCUUCCGGGUGCCUUGAUGAUUCGGGAGCCCCAAGCGACAUGUUUCGCAUUGAGGUCCCCAACCACCAAGGCCGGAUGGCGAUCAUGGUUCAAGGCCCGU
CU
..((((.(((....((((((((.((((...((((((.((.((.(((..((.
(((((((......)))).))).))..))))).)).)))))))))).))))))..)).....)))..)))).

```
 Initial dG =    -50.10

         10           20        30        40        50         60
UC    U-   CGUA-  --|     U    GGU      A  A  C   AG  C   -    CA
  GGCG  CCU     CA  AUCGCC UCCG   GCCUUG UG UU GGG  CC CAA GCGA  U
  CUGC  GGA     GU  UAGCGG AGGC   CGGAAC AC AA CCC  GG GUU CGCU  G
U-    CC   ACUUG  AC^     U    ---      C  C  -   CU  A   A    UU
 120       110       100           90         80        70
```

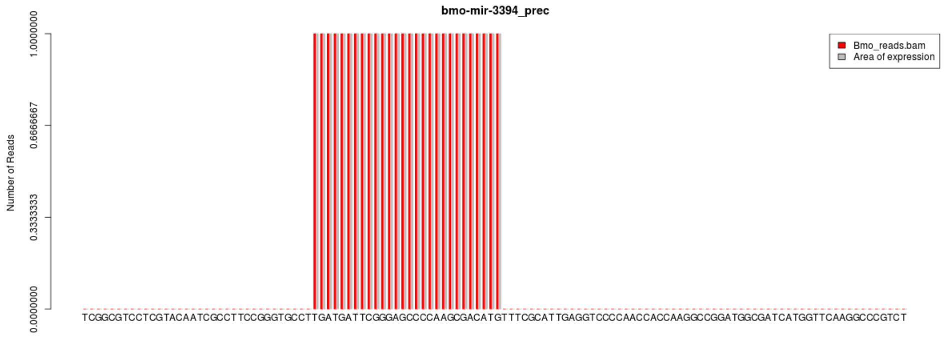

>bmo-mir-3395_prec

GACUGGUAGCUCCCCGUAGUACCGGUGCGAUGGUCCGGGCCUGGCUUGGCGGGCGGCCGUCGCGAUGGCAGCAGCUAGUUCGGCGAUGUCGCUCUUCGGUGCGACAGGUACGGCAAUCGG
UG

```
.((((((.(((.((.((.(((((((.((((((..((((((.(((((......((.
(((((....))))).)))))))))))))...))))))...))))))).)).))...))).)))))).
```

```
 Initial dG =    -53.00

        10          20          30        40
G      A   C--  C  A       .-U|        CGG     GC
 ACUGGU GCU   CC GU GUACCGG   GCGAUGGUC   GCCUG  U
 UGGCUA CGG   GG CA CGUGGCU   CGCUGCCGG   CGGGC  U
G      A   CAU  A  G       \ -^        ---     GG
120       110       100         60           50


                                    70
                             GAU--    GCA   AG
                                  GGCA   GCU  \
                                  CUGU   CGG  U
                             UCUCG    AG-   CU
                                 90         80
```

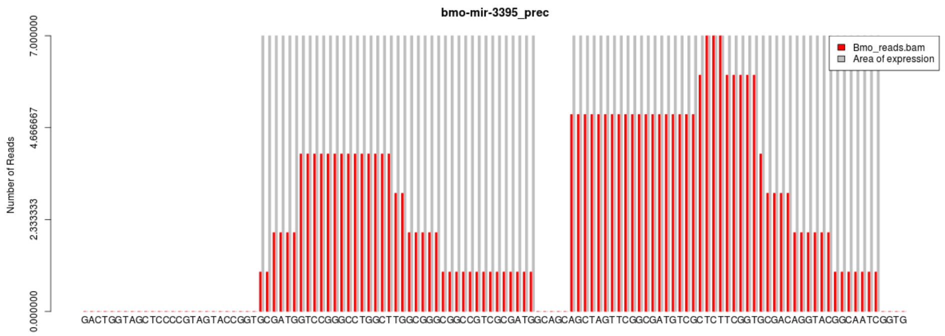

>bmo-mir-3396_prec

UUCGGAUCCUUCUCCAGUUUCUAUUUCACGGACGUCAGGCGAUUUAAUGCGGCUUCAAAGUUGUUGGGCAUCUUCGGGUUCUCCUCUCCUCUCCCCGCAGUGCGGUCUCGUAGCGGCCGU
CGGGUCGUUGCUGGGUGGAAUCCC

```
...(((......)))..............(((.(((..(((((((...........)))))))..))).)))..((((((..(((........((((...))))....
(((((((((....))))))))).))).)).)))))).
```

```
 Initial dG =    -42.30


.-UUC|  UC
     GGA  C
     CCU  U
\ ---^  CU
         10



       20        30        40        50
  .-AGUUUCUAUUUCAC   C   AG       AAUGC
                  GGA GUC  GCGAUUU     G
                  UCU CGG  UGUUGAA     G
  \ --------------   A   GU       ACUUC
                    70        60

-----------------------------------------------------
```

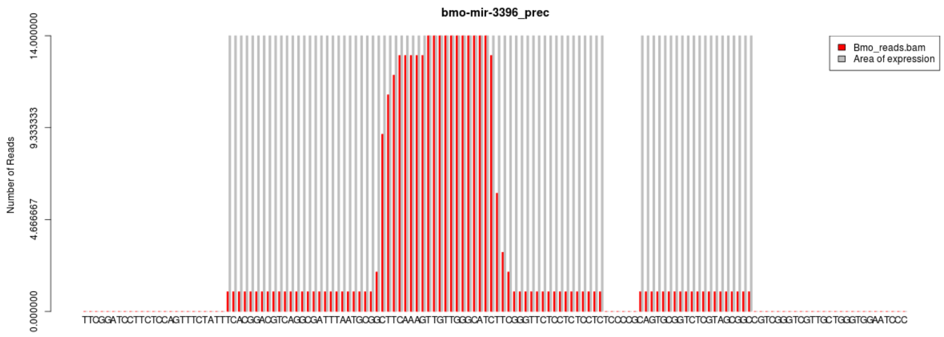

>bmo-mir-3397_prec

```
GGAUCUUCUACCCCGCAGUUAGGCUGAAUAUCUCGGUAGAAUCAGUCAGAAUGCAAUUGUUUGAAAGUUGGAUGAUGUGAUAUAUUGUGAUAUUUGUACAAGCAAACUCA
((..........))..((((..(((((((((.(((((..((((..((..((.(((((.......))))).))..)))))).))))).)))))))).....))).))))..

 Initial dG =    -20.20



  | AUCU
--GG    U
  CC    C
\ ^ CCAU
     10



      20             30        40        50        60
  GC    AG   -----       U     GA    GU  GA  G     GUU
    AGUU  GCU     GAAUAUC CGGUA  AUCA  CA  AU CAAUU   \
    UCAA  CGA     UUUAUAG GUUAU  UAGU  GU  UA GUUGA   U
  AC    A-   ACAUG       U     A-    --  AG  G     AAG
110        100        90         80          70

-----------------------------------------------------
```

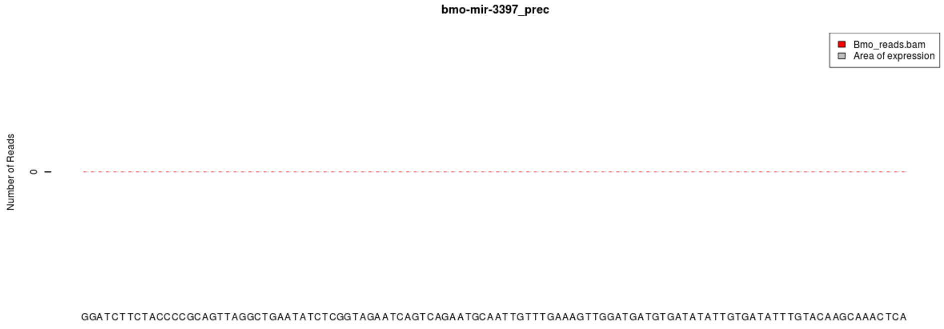

>bmo-mir-3398_prec

```
AGUUUAUGUUGACAGUGGCUUGGUUGUCGUCAUUCGUGACGCUUUAAAACACCAAUGAACUGCAGAUCAGACUAUAUGUAAACAUGAUUGAUUCAUUUU
...(((((((.(((((((..(((((((..(((((.(((...........))).)))))...))).))))..)))).))).)))))))...........

 Initial dG =    -20.70

                 10         20          30         40
AGU---------       G   -    CU    -   CG-|    C   ACGCU
            UUAUGUU ACA GUGG  UGGU UGU   UCAUU GUG     \
            AGUACAA UGU UAUC  ACUA ACG   AGUAA CAC     U
UUUUACUUAGUU       A   A    AG    G   UCA^    C   AAAAU
        90         80         70         60         50

-----------------------------------------------------
```

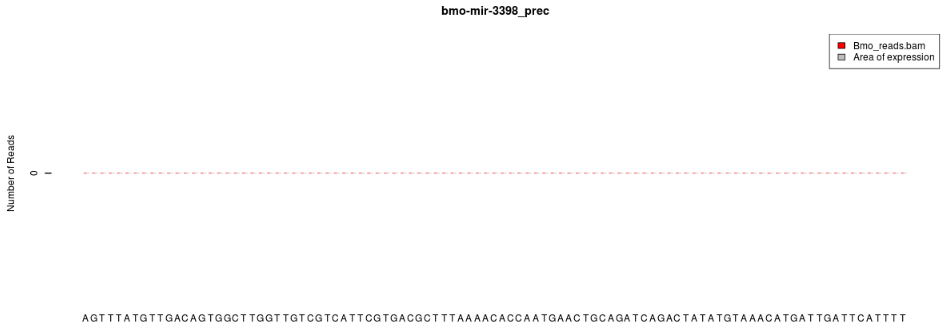

>bmo-mir-3399_prec

```
UUUCAAUAUCUACAUUGAAUACCUGGCUAUGAGGCGUUGUCUCGACCUGGGUCUCGCUGGAUGUGCUUGGUUCUGUAUAUAUUCGAC
.((((((......)))))).(((..(...((((((...))))))..)..))).(((....((((((........))))))...))).

 Initial dG =    -19.40

         10        20         30        40
UU  -|    C    UU  AU   U   -   GA    UUGU   GA
  UC AAUAU UACA  GA  ACC GGC UAU  GGCG    CUC  \
  AG UUAUA AUGU  CU  UGG UCG GUA  UCGC    GGG  C
C-  C^    U    --  --   U   U   GG    UCU-   UC
       80            70        60          50
```

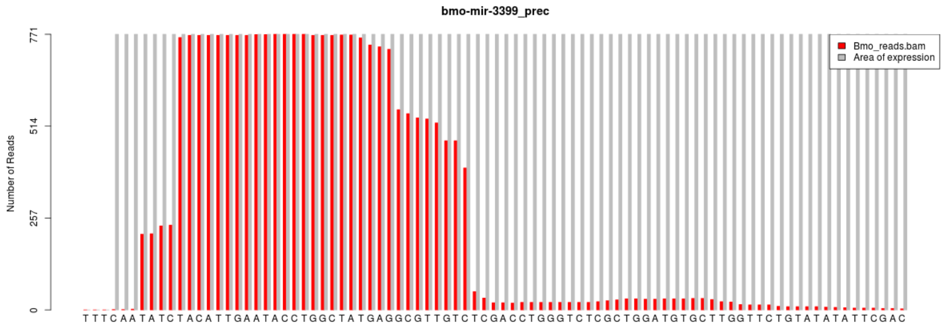

>bmo-mir-3400_prec

```
CGUGCAGAGCAGCUUGCAAGUCAUUUUCUUAGGCCCCCCUCGCCGGUCCCCAGACCGACAUGUGAGGGGGACCCGAAACACUUAGAGGGUUGUUGUACCUGGGCGG
(((.((((((((((..((((...((((...((.((((((((((((((....)))))....))))))))).)).)))).)))).)))...))))))))....))).))).

 Initial dG =    -44.80

             10         20         30         40            50
-   G   ----        GC-|   CAU    UUA  C         ----     C
 CGU CAG    AGCAGCUU   AAGU   UUUC   GG CCCCCUCGC    CGGUC C
 GCG GUC    UUGUUGGG   UUCA   AAAG   CC GGGGGAGUG    GCCAG C
G   G   CAUG        AGA^   C--    C--  A         UACA     A
    100        90         80            70         60

-----------------------------------------------------
```

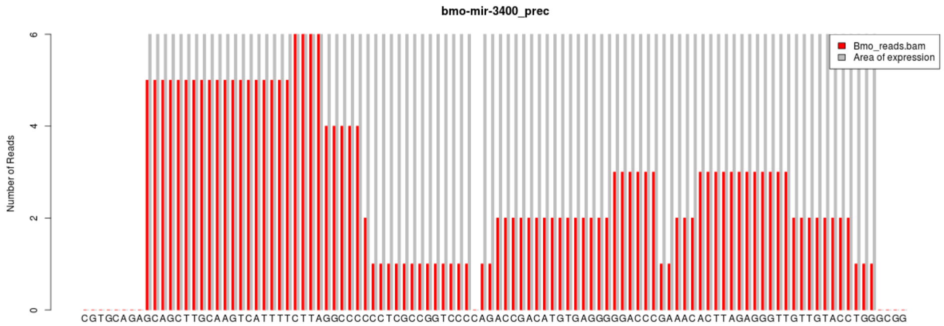

>bmo-mir-3401_prec

```
CCUUUACAGUGUAAAUCGUUCGUGAGCACAUACCAAGUACGCAUCUACUCAAAAAAUUUGGUACCAGCUCGCGGGAUUUGAACAUCGUGGU
...((((.((((((((..(((((((...((((((((.................))))))))...))))))))))))))..)))).)))).

 Initial dG =    -25.00

        10          20        30        40
CCU    A    --|    GU        ACA        ACGCAUCU
   UUAC GUGU  AAAUC  UCGUGAGC   UACCAAGU        \
   GGUG UACA  UUUAG  GGCGCUCG   AUGGUUUA        A
U--    C    AG^    --        ACC        AAAAACUC
  90        80          70        60        50

-----------------------------------------------------
```

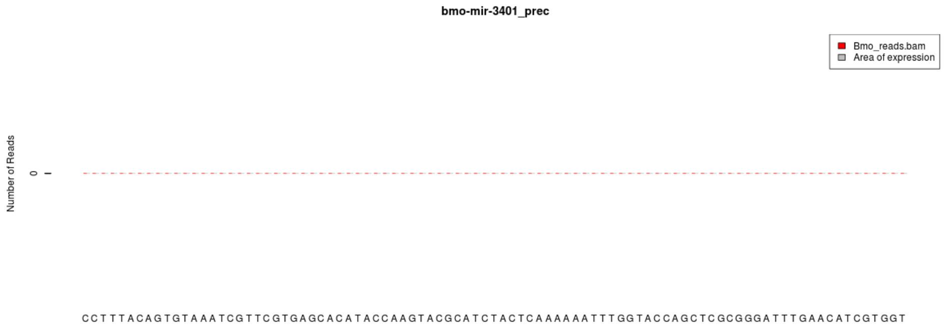

>bmo-mir-3402_prec

```
GGGUAAAGGCCCGAAAAGAUCCACUGCAAUAACUUUAAACCGCUGAGCUGGAACUGGAGUCUGGAGUAGUCCCGCUCGCUUAAAGUUAGACGGCUGGUACCGCUGGCACUCUAUACA
..(((.(((((.........((((((...(((((((((...((.((((.(((.(((..........)))))).))))))))))))))))..))).)))).......))).)).)))...

 Initial dG =    -30.20


  |   A
--GGGU A
  CCCG A
\ ^   G
   10



           20        30        40        50        60
  GAAA-   .-UCCACUGCAA         ACC  U    U   A   GAGU
       AGA            UAACUUUAA   GC GAGC GGA CUG    C
       UCU            AUUGAAAUU   CG CUCG CCU GAU    U
  ACAUA   \ ----------         ---  -    C   -   GAGG
       110                   80            70

-----------------------------------------------------
```

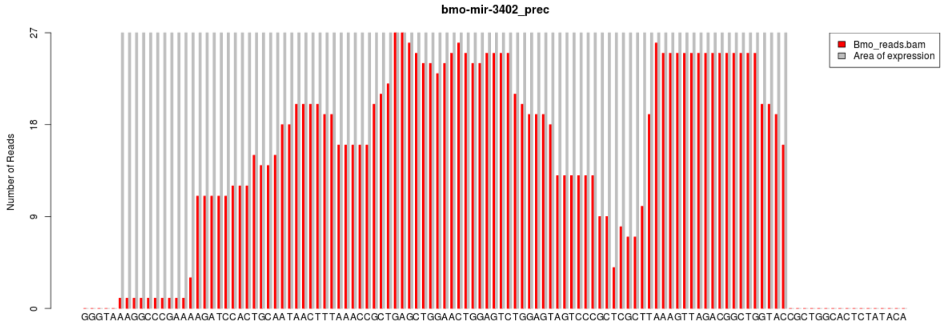

>bmo-mir-3403_prec

GCAAGCGGACUUCUCUGGCUUCGUCUGAUUCUGAGUCCCGGAUCUCAGCCCUGGAAUGACGCACUUGCUGCAAACAUGAUGAUGCUGAAAUAGUGUUCUGAAUCUAGUCUGAAGUCUGAU
UUGG

```
.((((((((((((...((((..(((((...........)))))...))))(((((..((.(((((....
(((..........)))......)))))))....)))))...))))))))).)))).

 Initial dG =    -31.30

         10          20          30
G    -        U--    C   .-G|   AUU     UCC
 CAAG CGGACUUC   CUGG UUC   UCUG   CUGAG   C
 GUUU GUCUGAAG   GAUC AAG   AGGU   GACUC   G
G    A        UCU    U   \ -^   CCC     UAG
  120       110       100       50        40


                              60          70
                           AU  C     UGCU--   AACA
                             GA GCACU      GCA    U
                             CU UGUGA      CGU    G
                           U-  -     UAAAGU   AGUA
                                     90        80

-----------------------------------------------------
```

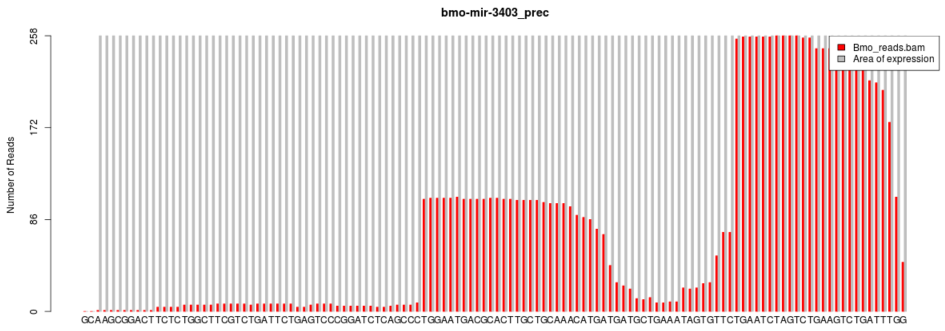

>bmo-mir-3404_prec

UGAAAGACAUUGUUUUUACAUUCCCAUCGUCACAAUAUUUAGUGUAAAUGCGGUAAAGAUUUGAAAUGUUUUUAAACUCACUUGGUAAAUAAAGUCGAGUUGAAUCUUUUCUGCAAUAGU
GUGACGGUACUAAAGGCAGUAACUUA

```
...(((..((((((((((.......(((((((((.(((..........(((((.((((((((............
((((((((........)))).))))))))))))).)))))))).)))))))))..))))))))))..))).
```

```
 Initial dG =    -35.30

        10        20        30        40        50        60        70         80
UGA|  AC          CAUUCCC         A   UUAGUGUAAA     U        GAAAUGUUUUUA     -    GGU
   AAG  AUUGUUUUUA       AUCGUCACA UAU          UGCGG AAAGAUUU            AACUC ACUU   A
   UUC  UGACGGAAAU       UGGCAGUGU AUA          ACGUC UUUCUAAG            UUGAG UGAA   A
A--^  AA          CA-----         G   ----------     U        ------------     C    AUA
      140       130            120                 110                   100        90

-----------------------------------------------------
```

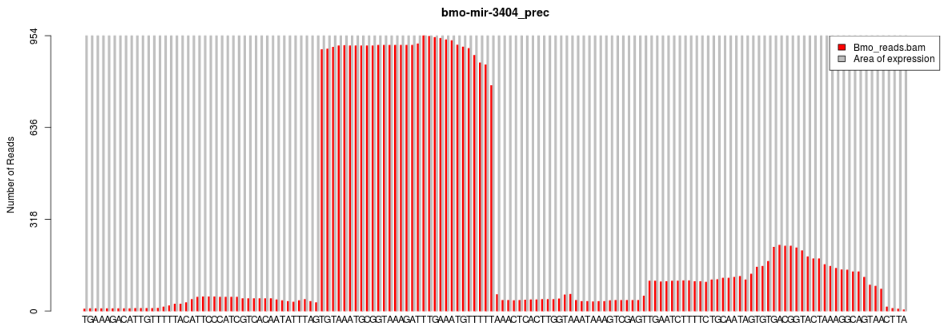

>bmo-mir-3405_prec

UCUUUUGUGACAAAUCACCAUAAUUUUCCAUUGUGUUCUUGAAACUCUCUAGAUUUCUUCGUAACGGUGUUGACCGUUUCUUUUUCACAAUAAAGCGAUUGAGGGUACGUUCUGAAUAAC
UGGUAGAAACUGGAGGUGUGUCUUGGAU

```
(((.....((((.(((.(((...(((((((...(((((..(((((((((..((((.(((..
((((((((....))))))...........)..))).))))))))))...))).)))))..))).)))).))).))).)))..))).

 Initial dG =    -31.40

         10         20          30         40            50         60                     70
-   UUUGU    A    A    UAA     -    UUG      UU    ---       UA     U    CG -----------|     G
 UCU      GACA AUC CCA    UUUU CCA    UGUUC   GAA    ACUCUC  GAUU CUU   U            AACGGU U
 AGG      CUGU UGG GGU    AAGA GGU    AUAAG   CUU    UGGGAG  UUAG GAA   A            UUGCCA U
U   UU---     G    A    CA-     U    CA-      U-    GCA       --     C    AU ACACUUUUUCU^     G
         140        130         120          110          100          90         80

-----------------------------------------------------
```

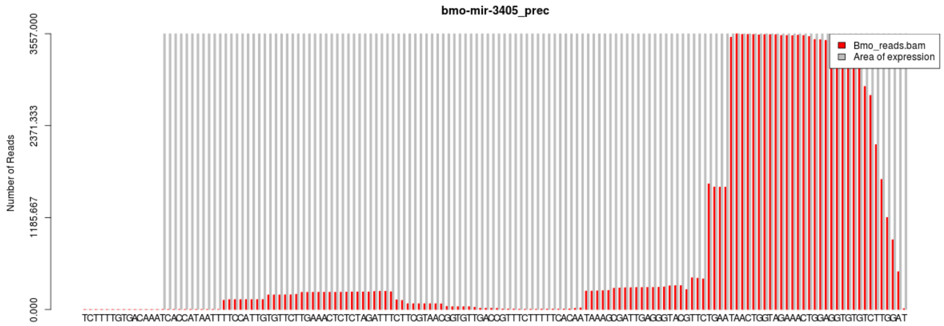

>bmo-mir-3406_prec

CGUUCGCAUAUUUAGAUGAUGUGAUAAUAGUAAGUAGCACGUUUGAUGAGCAUGUUAGUCUUCUUCUUCGCGUGUUAGACAAGCUGCGCCAUGCUAAUCUAACAAUUAAUUUAGAAAAGU
GUGAAUU

```
.((((((((.(((((((..(((.....(((((....(((.(((((...(((((((.
((.......))..)))))))...))))))))....)))))....)))....)))))))...)))))))).

 Initial dG =    -30.60

        10            20         30         40         50          60
C        A--        GA--|  GAUAA     AGUA   C     AUG        U-  UCU
 GUUCGCAU   UUUAGAU    UGU     UAGUA    GCA GUUUG   AGCAUGU  AG   \
 UAAGUGUG   AGAUUUA    ACA     AUCGU    CGU CGAAC   UUGUGCG  UC   U
U        AAA        AUUA^  AUCUA     ACCG   -     AGA        CU  UUC
     120       110       100        90          80        70

-----------------------------------------------------
```

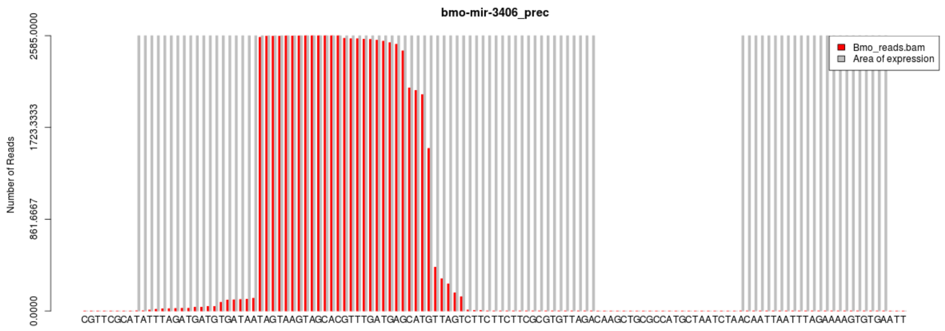

>bmo-mir-3407_prec

UGAACCGAUUUCACUUCUAAUUUUAUUACGUUUAUUCGGUUUUAGGUAUUAUGAUCACUGGGAGAGACGGGAAGUUGUCGGUUUCACCUGCUCGCGAAU
.((((((((...(((((..........(((((.((((((.(((.......)))..))))))..)))))))))))).))))))))..............

```
 Initial dG =    -19.30

                      10        20        30         40
U--------------|       UUC      AAUUUUAUUA     A-       U-   GGU
               GAACCGAU   ACUUCU          CGUUU  UUCGGU  UUA   \
               UUUGGCUG   UGAAGG          GCAGA  GGGUCA  AGU   A
UAAGCGCUCGUCCAC^       U--      ----------     GA       CU   AUU
        90        80          70                  60        50
```

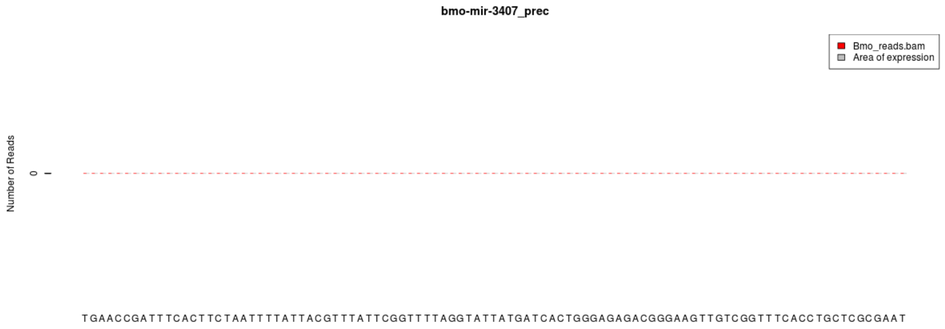

>bmo-mir-3408_prec

UAAUUGUGACUCGAUCGCCUUCUCUGUUCACGGGUAAGUGACGCUAAAAACCUGUUCAUUUGAGGUUCGCGACUAUCCGGUGUGAGUAGCGGACGUAUAUGAA

...(..((...((.((((...(((......((((((.((..(((....(((((.........))))).))))))))))))....)))..)))).))..))..).

```
 Initial dG =    -23.30

        10        20        30        40        50
UAA|UG  ACU  A    CUU   UGUUCA      A  GA   UAAA     GUUC
   U  UG   CG UCGC   CUC      CGGGUA GU  CGC    AACCU    \
   A  AU   GC GGCG   GAG      GCCUAU CA  GCG    UUGGA    A
A--^GU  AU-  A    AU-   UGUG--      -  --   C---     GUUU
   100         90         80             70            60

-----------------------------------------------------
```

bmo-mir-3408_prec

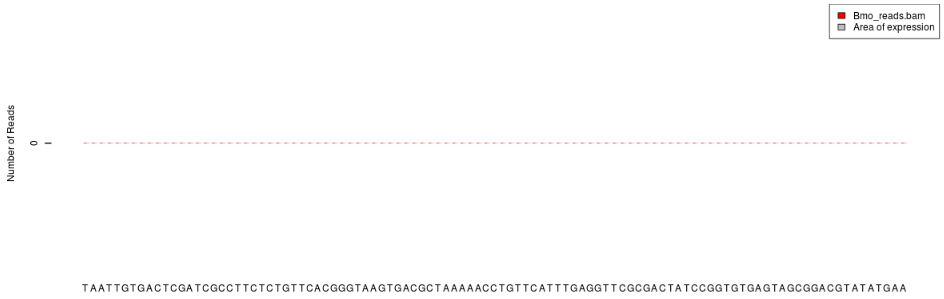

>bmo-mir-3409_prec

```
AGUAAGCUCCGUGAACAAGCUUCCGCAAGUGCCAUUCCUUUCAUACGAAGCUCUUAAACACCGCCACGGAAGCCUAUGUUCACC
..........(((((((.(((((((...(((.......((((....))))........))).....)))))))...))))))).

 Initial dG =    -20.70

        10          20          30          40
AGUAAGCUCC       A--       CAA--   CCAUUCC-|   A
          GUGAACA   GCUUCCG     GUG        UUUC U
          CACUUGU   CGAAGGC     CAC        GAAG A
C---------       AUC       ACCGC   AAAUUCUC^   C
            80        70        60        50

-----------------------------------------------------
```

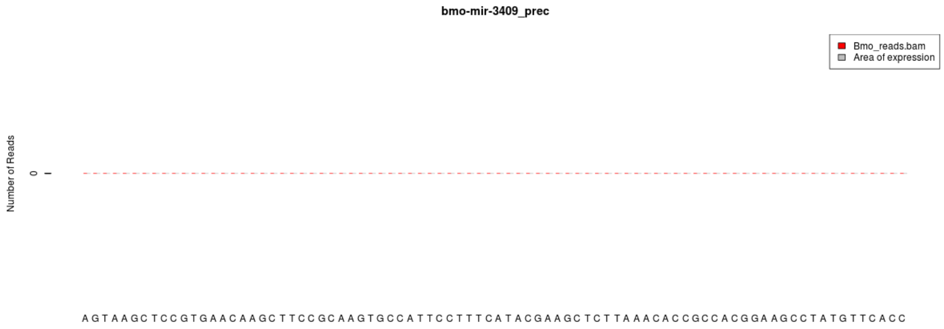

>bmo-mir-3410_prec

AAGUAUAAAGAUCCUACAUUGAGUGUAAAUGUUCAUGAAGCAAAAAAUCCCGAUUUUUUUUUGAUAAUAAUGAUUUAUCUCGAUAGUGGCGUUGUAAGUACUA
.(((((...(((.((((((((((.(((((...((((....(((((((.........)))))))......))))))))))))))))).)))).)))....))))).

```
 Initial dG =    -20.90

         10         20        30        40          50
A     AAA-   C    -|     U     UGU    GAAG--       UCCC
 AGUAU    GAU CUAC AUUGAG GUAAA   UCAU      CAAAAAA    \
 UCAUG    UUG GGUG UAGCUC UAUUU   AGUA      GUUUUUU    G
A     AAUG   C    A^     -     ---    AUAAUA       UUUA
 100        90        80            70        60

-----------------------------------------------------
```

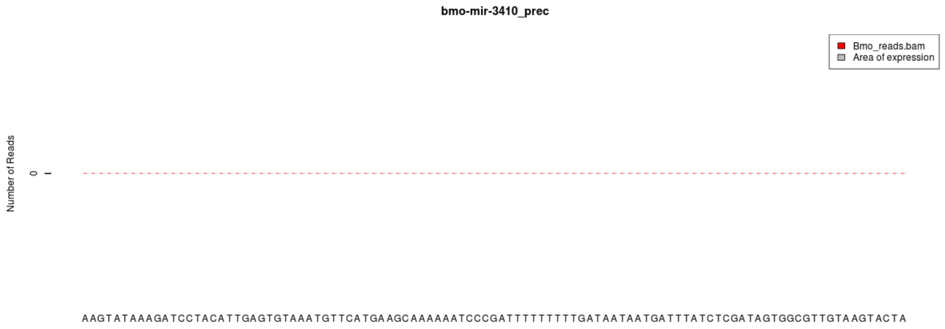

>bmo-mir-3411_prec

```
GGCUAUCAGCGAAUAGGUAGGGGAAAAAAAAGCUGCCUAAGUUACUUUGUGUAGUAUACAAUAGUAGCGUCGGUAUCCUCAUCUGUACUCGUGAGGUU
((((.(((.(((((((((.(((((.........((((...((((((((((((...)))))).))))))...)))))))))))))))..))))))))))

 Initial dG =    -27.60

        10          20        30        40         50
|   A   G   --      A     AAAAAAAGC    UAA      -      A
GGCU UCA CGA  AUAGGU GGGGA         UGCC   GUUACU UUGUGU \
UUGG AGU GCU  UGUCUA CUCCU         AUGG   CGAUGA AACAUA G
^   -   -   CA      -     ---------    CUG      U      U
         90         80                 70        60

-----------------------------------------------------
```

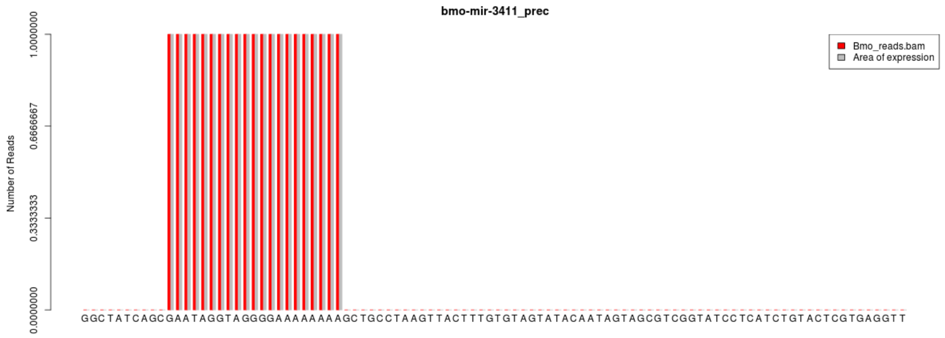

>bmo-mir-3412_prec

```
UAAACAAACGCUUAUGUCAAAUAAAUUACAGAACGACGUCACAUUCUUUAGAUUCAAGUCUCGUGGAAUGUAUUCUUCAUUAGUUUAAUUAAUAUAGUCGCUCGUGUUUC
.(((((..((.((((((.((.(((((((..(((.((.((.(((((((..((((....))))...)))))))))))))))..))))))).)).))))))).))....))))).

 Initial dG =    -20.00

          10        20        30        40         50
U     AA--| C      C  A       CA   C  C  C       UU-    U
 AAACA    CG UUAUGU AA UAAAUUA  GAA GA GU ACAUUCU   AGAU C
 UUUGU    GC GAUAUA UU AUUUGAU  CUU CU UA UGUAAGG   UCUG A
C     GCUC^ U      A  A       UA   -  -  -       UGC    A
.       100        90        80            70        60

-----------------------------------------------------
```

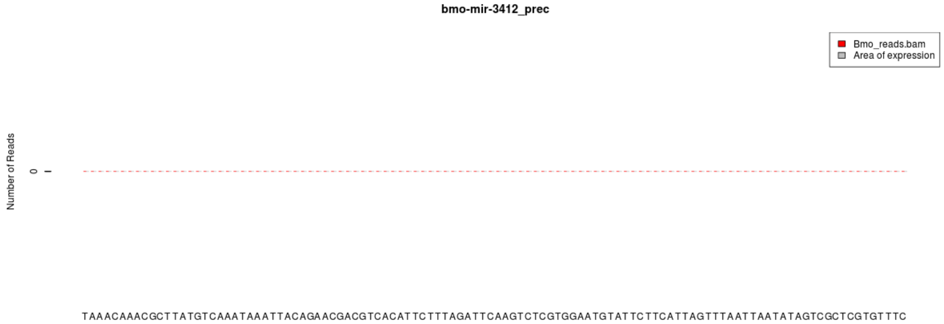

>bmo-mir-3413_prec

UUACAUGUGAAAAUGAGUACCCGCUGCGCCCGAGAGAGAGCAGGUUGAUUUUACGAUCCUUAUAUAGUUUCUGCGCGUUUUGUUCAUUUUAUAUGUAC
.((((((((((.((((((...((.((((..((((......((((((......))).))).......)))).))))))..))))))))))))))))).

```
 Initial dG =    -21.70

        10        20        30        40          50
U|         A      CCC  U    CC    GAGAGC-   -   AU
 UACAUGUGAAA UGAGUA   GC GCGC  GAGA       AGG UUG  U
 AUGUAUAUUUU ACUUGU   UG CGCG  CUUU       UCC AGC  U
C^          -      UU-  -    U-    GAUAUAU   U   AU
       90        80           70        60

-----------------------------------------------------
```

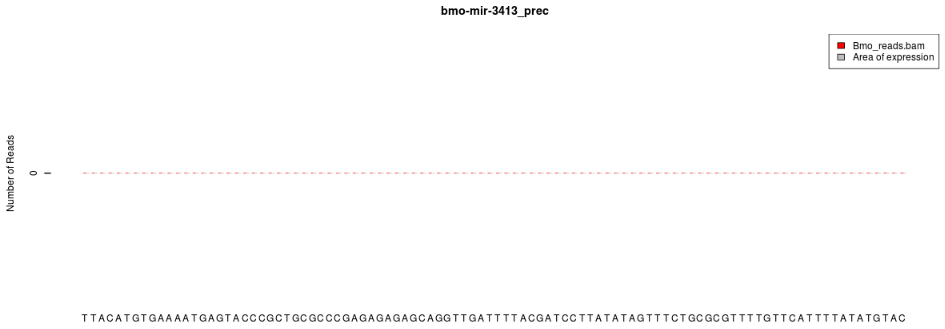

>bmo-mir-3414_prec

AAAGAUUCCUUAAGUUUUUUGAACGCAGUGUCGGCCUCAUCUGUCCACUGGAAGGGAGGUGUUGAUUUUUUGUUCGAUGUCAACCUAUUAAGCGGCCCUG
..((..((((((((((..(((((((.((.(((((((((.(((.(((...)))))))))))..)))).)).)))))))....)))...))))).))..)).

```
 Initial dG =    -20.50

         10             20        30          40
AA  AU  -     ---   UU--       C  U    --|    A   G   A
  AG  UC CUUAA   GUU    UUGAACG AG GUCG  GCCUC UCU UCC \
  UC  GG GAAUU   CAA    AGCUUGU UU UAGU  UGGAG GGA AGG C
G-  CC  C     AUC   CUGU       U  U    UG^    -   -   U
.          90        80        70        60          50
```

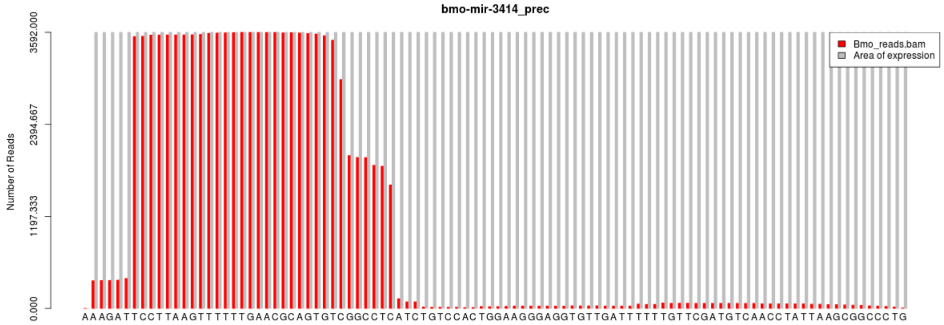

>bmo-mir-3415_prec

```
UUGGUAACGAUGGAAAUAAGCAAUCUUGUAAUCUUCUCGCUAUCUCCUCUCAGAGUCGUUCCAAGGAUGCCACUGUUUCUCAGUCGUACCUCAGUAAUG
..(((.(((((((((((.(((.(((((.(((...(((..............)))..))).))))).)))...)))))))..))))))))).........

 Initial dG =    -23.20

               10            20        30        40
UU-------   A     --       A--|  A     U   CUU   GCUAUC
         GGU ACGAU  GGAAAUA   GCA UCUUG AAU   CUC      U
         CCA UGCUG  UCUUUGU   CGU GGAAC UUG   GAG      C
GUAAUGACU   -     AC       CAC^  A     C   CU-   ACUCUC
        90          80        70        60          50

-----------------------------------------------------
```

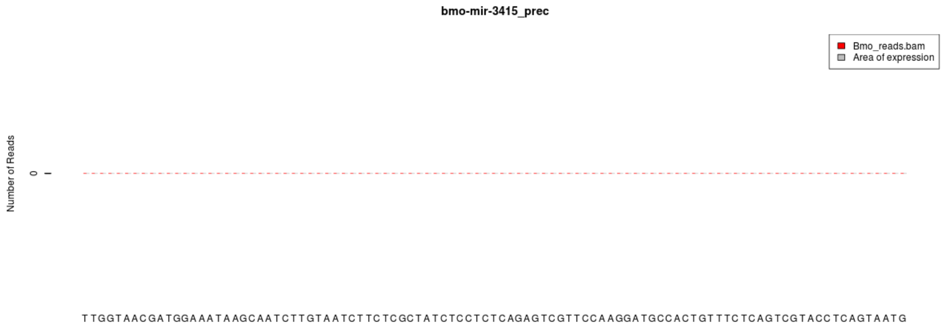

>bmo-mir-3416_prec

GGCGAGAAACUCAGUGGGCUGUUUGUUUUUUCUGCGGAUCGAUUUUAUGUGAGGCACCUGUUUUUUAUUGACGCGUUUCCUUGGUGCUAUUGUGGUUCUUCUACAUGAAUUCGAAUGUCG
CAGGACGUGCUGGCUGGGUUAUCUUGUG
.((((((((((((((.(((((((........((((((.((((.(((((((((((.(((.......(((..
(.........)..))).......))).)))).))))))).)))))...))))))))))).))).)))))))).)))))).

```
 Initial dG =    -50.70

         10         20         30           40         50          60         70
G      -        G    -|   UGUUUUUU      A--    U        -    C   UGUUUUU    UG CGCG
 GCGAGA AACUCAGU GGC UGUU        CUGCGG   UCGA UUUAUGU GAGG ACC       UAU   A    \
 UGUUCU UUGGGUCG UCG GCAG        GACGCU   AGCU AAGUACA CUUC UGG       GUG   U    U
G      A        G    U^   --------      GUA    U        U    U   UGUUAUC    GU CCUU
      140       130                 120       110        100         90        80
```

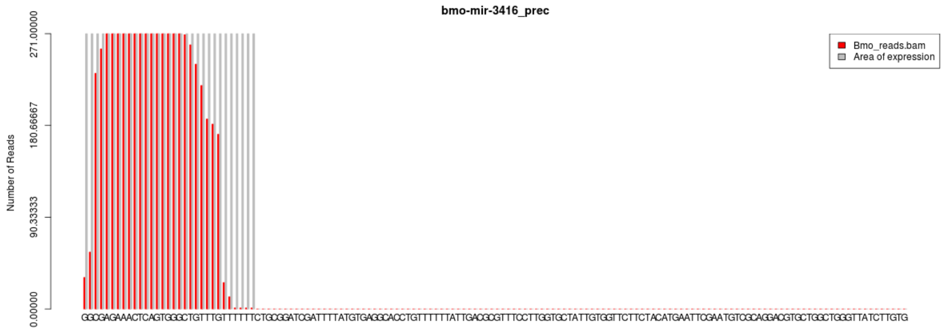

>bmo-mir-3417_prec

```
GUAAAGGAAUCGUUACGUUUUGUUUGUCGCUAAAUGAGUACGUUUUAUGAUCUAUAUCUUACUGGAUGGAUGUUGGCCGUGAAUUAUAUUCAGCUGCAGUAGAGAUAUUCGAUAAUGUUU
UGUUUUUACG
(((((((...((..((((((((..((((.(((..((((((...((((((..(.
((((((........)))))).)..)))))))...)))))))......))).)))))..))).)))))..))))))))).

 Initial dG =    -29.70

         10         20        30              40        50        60
-       AAU  UU     -   UU    G   AA-----|      CGU      AU U      UAC
 GUAAAGG   CG  ACGUU UUG  UGUC CUA       UGAGUA   UUUAUG  C AUAUCU   U
 CAUUUUU   GU  UGUAA AGC  AUAG GAU       ACUUAU   AAGUGC  G UGUAGG   G
G       ---  UU     U   UU    A   GACGUCG^     AUU      CG U      UAG
.          120       110       100        90        80        70

-----------------------------------------------------
```

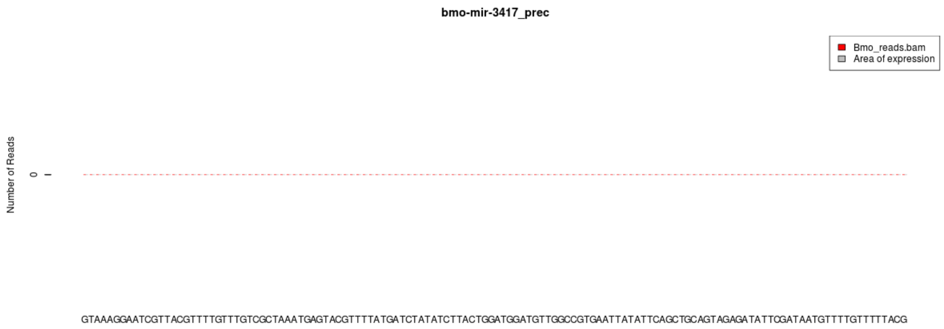

>bmo-mir-3418_prec

AUCCGUCUCCUCUGAAAGCUAACUCCAAAAGAAGCUUACGAGGCUUUGUACAAUAUCUAUAGACGUAAAGUACACUCCGGCGCCUCUUAGAAUCUUUCUGGUCAGACAUGAGACGAAAAU
GUG

```
...((((((.(((((.........((((((((..((...(((((..(((((..
(((........)))..))))).......)))))..))..))))).))))))))...))))))........

 Initial dG =    -29.70

             10          20          30        40              50        60
AUC-----      C--|    AAGCUAACU   -     AG  UAC     UU-----     AA   CUA
        CGUCUC   UCUGA         CCA AAAGA  CU   GAGGC       UGUAC  UAU   U
        GCAGAG   AGACU         GGU UUUCU  GA   CUCCG       ACAUG  AUG   A
GUGUAAAA      UAC^    ---------   C     AA  UU-     CGGCCUC     AA   CAG
 120       110               100        90         80        70

-----------------------------------------------------
```

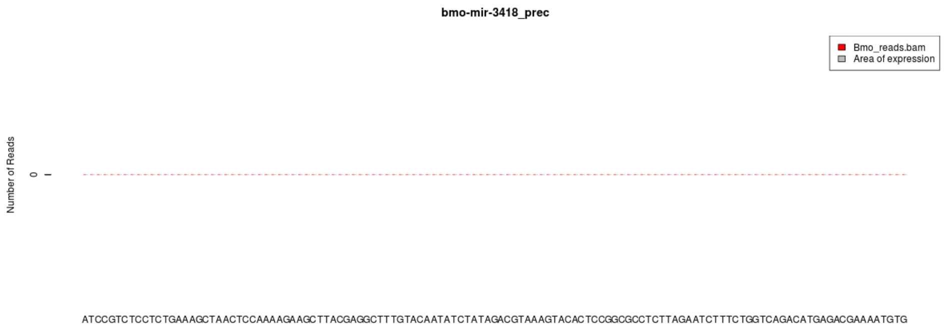

>bmo-mir-3419_prec

UAUUUUAUUUGAACAACAGAAUGAGCGCACGUUUUCUAAAAAGUAAGAACUGUAACUAGCAGGCUAACAUUAAAUGUUAGCCUUUAAUACUGUAAAACUAUUAAAACGUAAGUCUAUAAU
AAAAA
..(((((((...........(((..(..(((((((...((.(((....((.(((..(((.
((((((((((...)))))))))))))).))).))...))).)))))))))..)..))))))))))).

```
 Initial dG =    -20.90

        10        20        30        40        50        60        70
UA|      UGAACAACAGA   AG GC       CUA  A   AAGA  U   AC   C          U
  UUUUAUU           AUG  C  ACGUUUU   AA AGU    AC GUA  UAG AGGCUAACAU \
  AAAAUAA           UAU  G  UGCAAAA   UU UCA    UG CAU  AUU UCCGAUUGUA A
A-^      -----------   CU AA       ---  A   AAA-  U   A-   -          A
    120                  110          100         90          80

-----------------------------------------------------
```

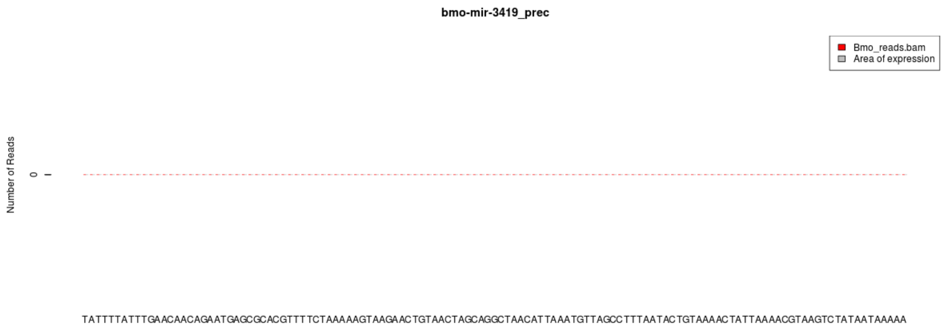

>bmo-mir-3420_prec

```
AGCGAUCAGAAUUUACUGUUAUUGCCUGGCCUCGCUCAUUUUCGAAGAAAUGGGGUCCGAUGAUUCUAUGAGCCGACAUGGCCGCCCAAACCGUAACUUUAAGCGAAUGCA
.(((.((.(((.((((.((..(((..((((((((((((............((((((....)))))))))))).))...)))))..))))).)))).))).....)).))).

 Initial dG =    -24.50

           10        20        30             40        50
A   A  A---   U    U  UA   CC     ---  -|     UUUCGAAGAAAU      C
 GCG UC    GAA UUAC GU  UUG  UGGCC   UC GCUCAU            GGGGUC G
 CGU AG    UUU AAUG CA  AAC  GCCGG   AG CGAGUA            UCUUAG A
A   A  CGAA   C    C  --   CC     UAC  C^     ------------      U
 .       100        90         80        70                     60

-----------------------------------------------------
```

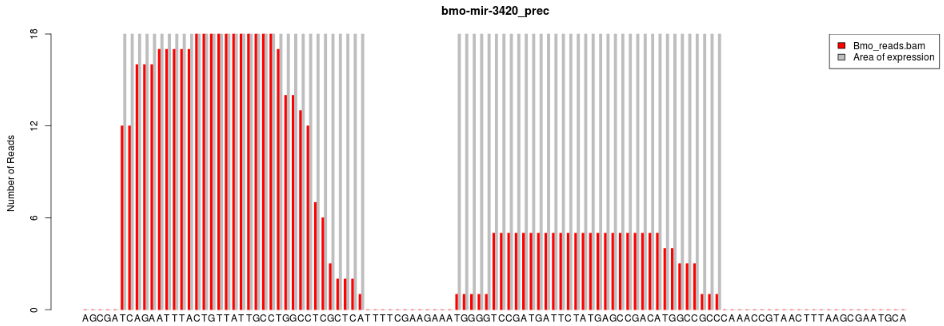

>bmo-mir-3421_prec

AAACAGCCGAAUUCGCAAUAGUUGUUGGCGUGCUUUCAAACGAAUUAAAUCAGAGGUCUUUGGUAGUGAAGCGACACUCAUUACUGCACCAAUGUUAUUAGGUAUGUUUGAUGUUGAAAC
AAAAGUUACUAUUGUAAGUGUCGCUAGUG

```
..(((((...(((..(((((((..(((....((..(((((((..((((..((..(((...
((((((((((........))))))))).)))..))...))))...))))))).)).....))).....)))))))..)))...))).)).
```

```
 Initial dG =    -33.70

         10        20           30         40         50         60        70
AA  -   CGA   CG       UG---   GCGU-  UU       AA-    AU-| GA   CUU         AGC
  AC AGC   AUU  CAAUAGU     UUG     GC  UCAAACG   UUAA   CA  GGU   UGGUAGUGA   G
  UG UCG   UGA  GUUAUCA     AAC     UG  AGUUUGU   GAUU   GU  CCA   GUCAUUACU   A
G-  A   CUG   AU       UUGAA   AAAGU  U-       AUG    AUU^ AA   C--         CAC
        140       130       120        110       100        90          80
```

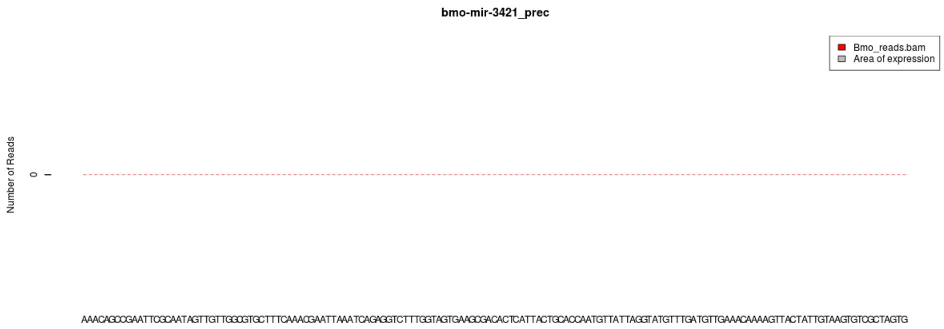

>bmo-mir-3422_prec

```
UAGGUGGCAGACGAGAAUGGAUCUACUGUGAAGGACUGAGAAUAUAUAUUCCCGAUCUGAUUAACAGAUAGAUUCUGAUUAAUGCUUUUGUUGCUGUCG
..((..(((.((((((..(((((((((((...(((.((.((((....)))).)).)))).....)))).))))))).........)))))))))..)).

 Initial dG =    -25.40

        10                  20          30           40
UA  UG   G      AU--------       -    GAA--|  C  A    A
  GG  GCA ACGAGA          GGAUCUA CUGU     GGA UG GAAU U
  CU  CGU UGUUUU          CUUAGAU GACA     UCU GC CUUA A
G-  GU   -      CGUAAUUAGU       A    AUUAG^  A  C    U
          90         80         70         60         50

-----------------------------------------------------
```

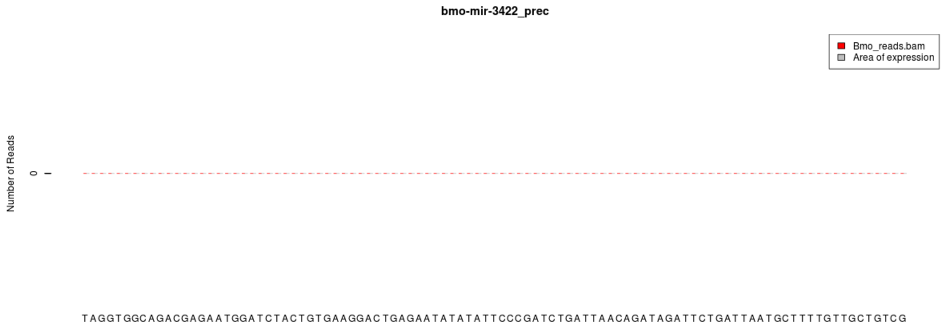

>bmo-mir-3423_prec

UCCACCACCCACAUUAAUACUUUAGGUCUAUUGCAUGAUAAAUGCGAAAAUUAUUAUUAUUUUUUGCGGGUUUGAUUUUUAUUACAUGAUGCUAUUUAUUGAUCGUGGAAGGGUC
...(((..(((((((((((...(((...(((((..(((((((((((((((.((....)).)))))))).........)))))))..))))))))..))))))).))))...))).

```
 Initial dG =    -20.80

         10         20        30        40                 50
UCC   AC-    -       CUU   GUC     CA       ---------|       U  U
   ACC   CCAC AUUAAUA   UAG   UAUUG  UGAUAAA         UGCGAAAA UA U
   UGG   GGUG UAGUUAU   AUC   GUAGU  AUUAUUU         GCGUUUUU AU A
C--   GAA    C       UU-   ---     AC       UUAGUUUGG^       U  U
     110       100            90        80        70        60

-----------------------------------------------------
```

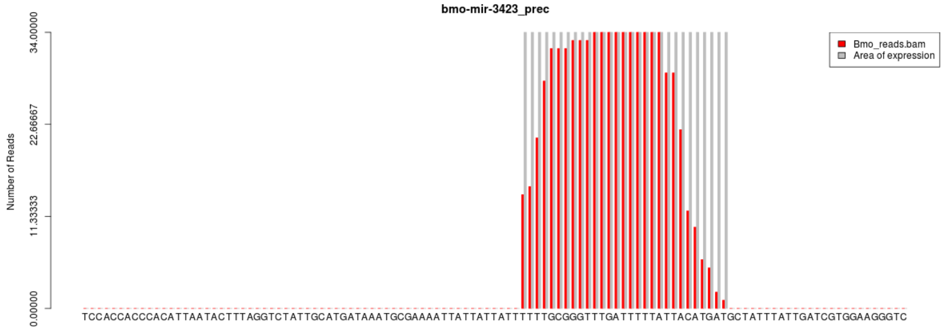

>bmo-mir-3424_prec

GUCUGGCUUGGUAGUUCCAUUAUAACCUCAGUCAUAUGUAGUAUUUUACCUAUGUUUUGUUGCCCUUGUAGACAGACGAGCUGGUGAGUAGUUACUGUCGCUGUGGAUAUUAACAAUGCC
ACGGGCAUGGCC
(((((.(((((((........(((.((.((((.(((.((((...((((((..(((((..
((((....))))..)))))....))))))...))))))).)))).)).)))......))))).)).)).))).

```
 Initial dG =    -31.30

           10        20        30        40        50         60
-   -  G  -|    GUUCCAUU   A  U    C   U    UAU      UA--     UG    C
 GUC UG CU UGGUA        AUA CC CAGU AUA GUAG   UUUACC    UGUUU  UUGC C
 CGG AC GG ACCGU        UAU GG GUCG UGU CAUU   GAGUGG    GCAGA  GAUG U
C   U  G  C^    AACAAU--   A  U    C   -    GAU      UCGA     CA    U
130       120         110       100         90        80        70

-----------------------------------------------------
```

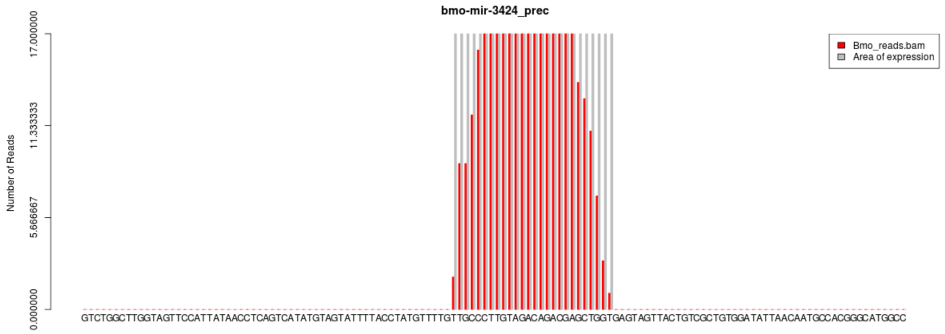

>bmo-mir-3425_prec

```
AUCGGUCUAAUUAUCUCAUUAAAACAUUUUUUUCGAAUUUUUACCCUUUUUUGGUCUGAGGUAUAUCGGAUGUAAAGCAAGGAAAAAUUGAGGACGGUUUUCUGAAAGAGCGACCC
...((((...((...(((..(((((....(((((.(((((((..(((((((((((((((......)))))).))))).))))))))))))))))..))))).)))..))..)))).

 Initial dG =    -22.50

        10         20         30         40          50         60
AUC|   UAA  AUC   UU     AUUU     G        AC    -     -      GG
   GGUC   UU   UCA  AAAAC    UUUUC AAUUUUU  CCUU UUUUG GUCUGA  U
   CCAG   AG   AGU  UUUUG    AGGAG UUAAAAA  GGAA GAAAU UAGGCU  A
C--^   CG-  AA-   C-     GC--     -       --    C     G      AU
      110          100          90           80         70

-----------------------------------------------------
```

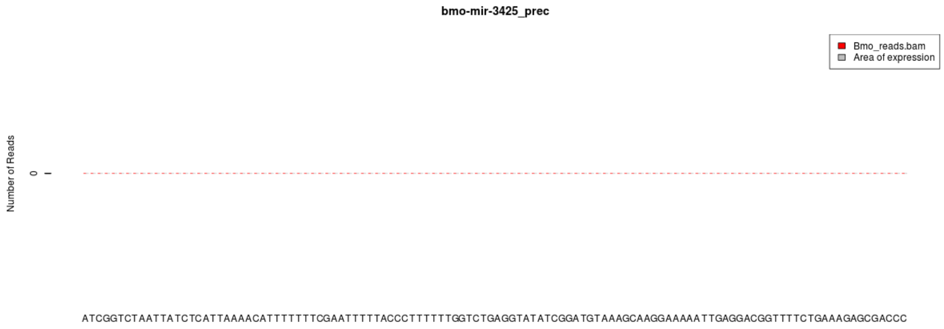

>bmo-mir-3426_prec

UUAAGUCUUAUGGUCUUCAUUUAAUUUUGAUGUACGUAUAUGUACGAAUAUCCCUGCUAUUUGAUCAAGAUGUGCAUAUGUUGAGUGAAUUUAAUUUCUUAGUAUUAGUAAAAUAAC
(((((....((....((((((((((....((((((((...((..((((((.......))))))..))..))))))))..))))))))))....))..))))).............

```
 Initial dG =    -20.70

                       10        20        30        40        50
---------------|    UCUU  GGUC          UUUG        AUA  UA       UCC
               UUAAG    AU    UUCAUUUAAU    AUGUACGU   UG  CGAAUA   \
               GAUUC    UA    AAGUGAGUUG    UACGUGUA   AC  GUUUAU   C
CAAUAAAAUGAUUAU^    UU--  AUUU          UA--        GA-  UA       CGU
     110       100          90        80          70         60

-----------------------------------------------------
```

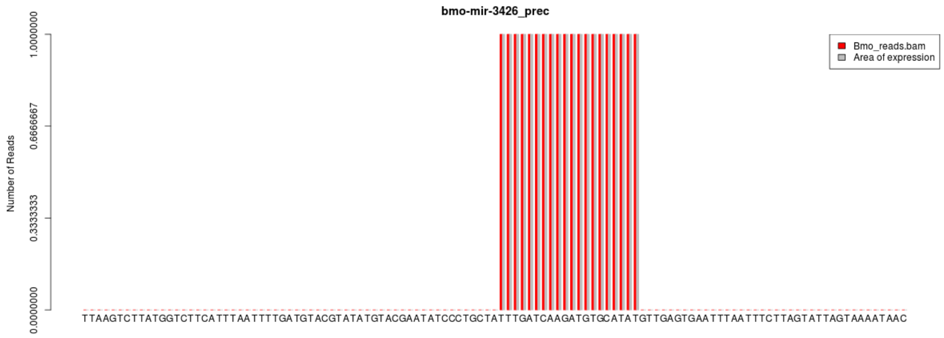

>bmo-mir-3427_prec

AGUAUCGGUUUACCAGUUUAGGGUGUAGCUGGUGAUAAGUGAUUUUUAAAUCAUCAAUUGUAUCACGAAAUAAAAGUCUUCAUUAUAAACGCUUUUGCGGUAGAAAUUGCC
.(((....((((((.((..(((((((...((((((.....((((((((..((..............))..)))))))).))))))...))))))).))))))))...))).

```
 Initial dG =    -20.90

          10        20
.-AGUAUCGGU|        UAG
           UUACCAGUU   G
           AGUGGUCGA   G
\ ---------^        UGU
               30


     40          50        60
  UAA UG       .-AAUCAUCAA   UA
     G  AUUUUUA           UUG  \
     C  UAAAGAU           AGC  U
  C-- GU       \ ---------   AC
   110

-----------------------------------------------------
```

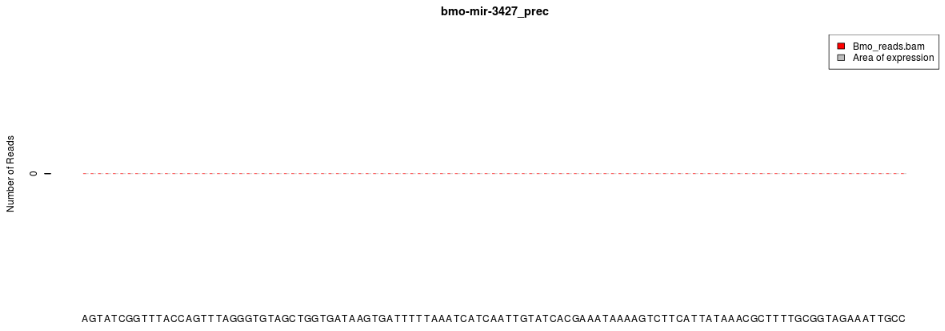

>bmo-mir-3428_prec

```
CUAGUUUUUAAUCUUACUGCUCAGACGUGUCAUUUUUCCUAUUAAGGUGAAACUUGAACAAUAACUCGGGAAGAGCUGUUGCGCUUUGAGGAAGGUUUAUGACUU
..((((...((((((....(((((((((.((((((((((.(((((......)))))..........)))))))).)).)))).)))))).))))))...)))).

 Initial dG =    -22.80

        10        20         30         40
CU    UUU      ACUG      -    U  -        A---------|    GU
  AGUU   AAUCUU    CUCAGA CGUG CA UUUUUCCU          UUAAG  G
  UCAG   UUGGAA    GAGUUU GCGU GU GAGAAGGG          AGUUC  A
U-    UAU      G---      C    U  C        CUCAAUAACA^    AA
    100           90        80        70        60        50

-----------------------------------------------------
```

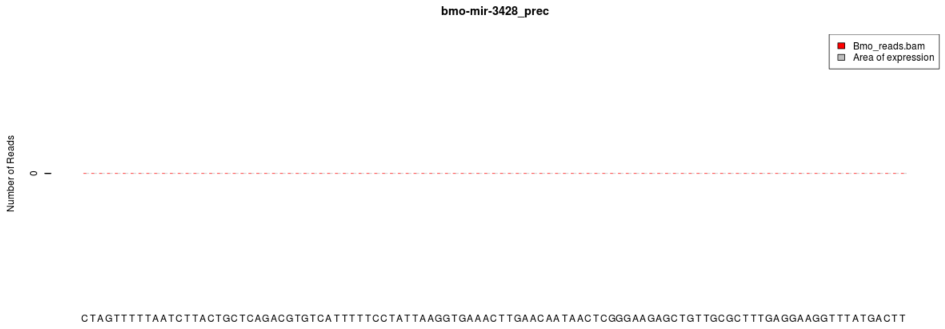

>bmo-mir-3429_prec

```
AAUAUCUUAAUAAUUUCUUAACGAUUUGUAGUUGCUAAAAAUAUAUCGUUUUAUACAACGUAGCACAUACGGGCGACGGACAAACGUAUGAUUUUGUUGGAA
....(((.(((((..((...(((.(((((.((((((..........((((......))))...........))))))..))))))))..))..)))))))).

 Initial dG =    -21.80

        10        20        30         40         50
AAUA|  U     UU  UUA   A     A-      AAAAAUAUAU-    UU
    UCU AAUAA  UC   ACG UUUGU  GUUGCU           CGUU  A
    AGG UUGUU  AG   UGC AAACA  CAGCGG           GCAA  U
A---^  -     UU  UA-   -     GG      GCAUACACGAU    CA
   100         90          80        70        60

-----------------------------------------------------
```

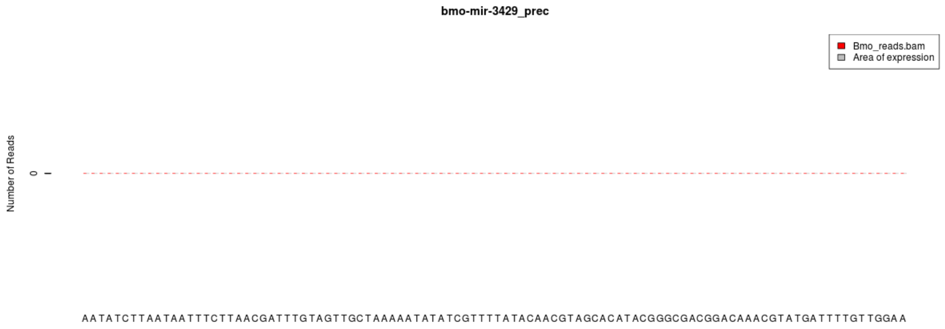

>bmo-mir-3430_prec

GUUAUUCAAGGCUACGUCUUUUGAAGGUUAUUAUAUUGCUUAUUAAGAAUUUGUGCUCAUCCAAAGAGACUGAUAAGCGGAAGUUGCGUGUCGGGUUAGCCUUAUUAUUGAAGGUUUUGU
CAUAUUUCAAACCAAACUUUGAAUAU

```
..(((((((((........(((((((..(((.(((..((((.((((.(((....(((.((((.....(((.....
(((....)))....))))))).)))...))).))))))))..))).))))))))))).....))))))))).
```

```
 Initial dG =    -28.70

        10           20
GU         .-CUACG| U
  UAUUCAAGG       UC U
  AUAAGUUUC       AG U
U-         \ -----^ U
     140



                     30        40         50
             A--    .-AUUAUAUU       AGAA-    UGC
                GGUU          GCUUAUUA     UUUG   U
                CCAA          CGAAUAGU     AAAC   C
             AAA    \ --------       CAGAG    CUA
                 130                70        60

-----------------------------------------------------
```

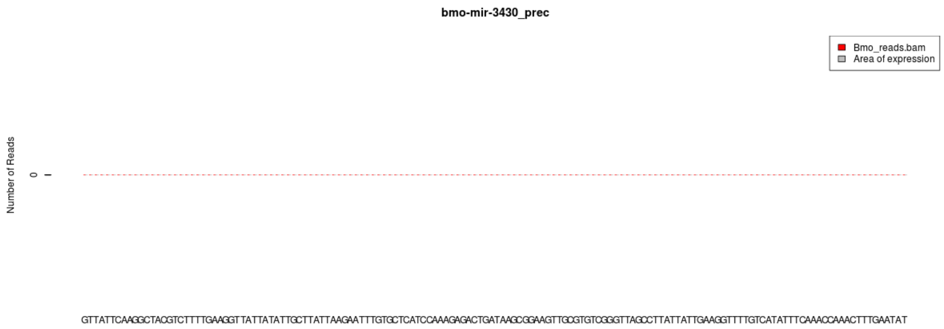

>bmo-mir-6497_prec

```
GGAUCCGUAACUUCGGAAUAAGGAUUGGCUCUGAGGACCGGGGCGUGUCGGGUUUGGACGGGAAGCGGAUGCGGCCGGUGCCGGGUCUGGUCGAUGUUCGUGCGUUAUGUUCG
...((((......)))).....(((..((((((.....))))))..)))..((.((((((.((..(((((.((((....)))).))))).))..)))))).))..........

 Initial dG =    -36.90

          10
.-GGA|   UA
     UCCG  A
     AGGC  C
\ ---^   UU



     20        30
  .-AUAAG   UG       AG
         GAU  GCUCUG  \
         CUG  CGGGGC  G
  \ -----   UG       CA
                 40

-----------------------------------------------------
```

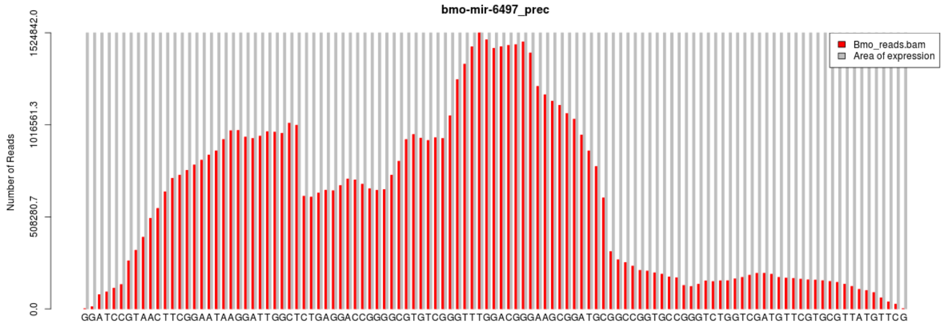

>bmo-mir-6498_prec

```
AGUGCGGACGCGAGUGCGACGCGUCUGUUGUCGCAGCCGUGCAGUCUCGGACUAGUGCGUGUUUGUUUGAACGUCUGCGAUGAUACAGUUUCGGGCACUCGCAGGACCCGUC
...((((..((((((((..((...((((((((((((.(((.(((...(((((........))))).))).))).))))))))..))))...)).))))))))).....)))).

 Initial dG =    -43.00

           10        20          30          40
AGU    AC---        GA  CGU    --        .-C  UG -|   U
   GCGG     GCGAGUGC  CG   CUGU  UGUCGCAG   CG  C AGUC \
   UGCC     CGCUCACG  GC   GACA  GUAGCGUC   GC  G UCAG C
C--    CAGGA        G-  UUU    UA        \ -  GU A^   G
  110       100         90        80                 50


                                          60
                                           U-    G
                                             GUUU U
                                             CAAG U
                                           UG    U
                                              70

-----------------------------------------------------
```

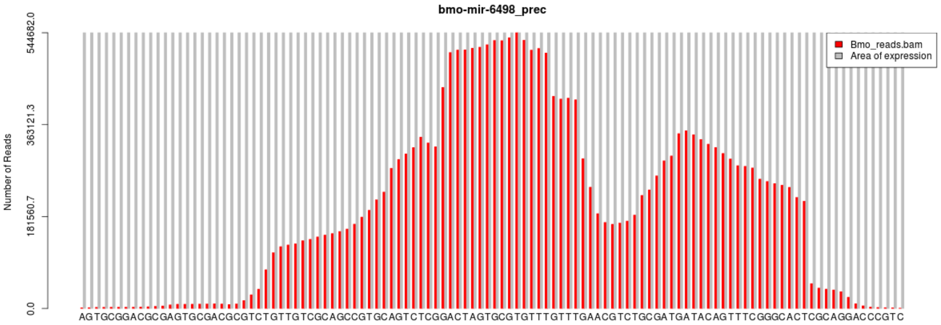

>bmo-mir-iab-4_prec

```
GCGUCGCCUUCUGUACGUAUACUGAAUGUAUCCUGAGUGCGCGAUUCUUGUCCGGUAUACCUUCAGUAUACGUAACAGAAGAUGACAC
..((((.(((((((((((((((((((.((((((.((..............)).)).)))).))))))))))))))).)))))).))))..

 Initial dG =    -37.10

        10         20        30         40
GC    C      -             U    -| U  GUGCGC
  GUCG CUUCUG UACGUAUACUGAA GUAU CC GA      G
  CAGU GAAGAC AUGCAUAUGACUU CAUA GG CU      A
CA    A      A             C    U^ C  GUUCUU
       80        70        60        50

-----------------------------------------------------
```

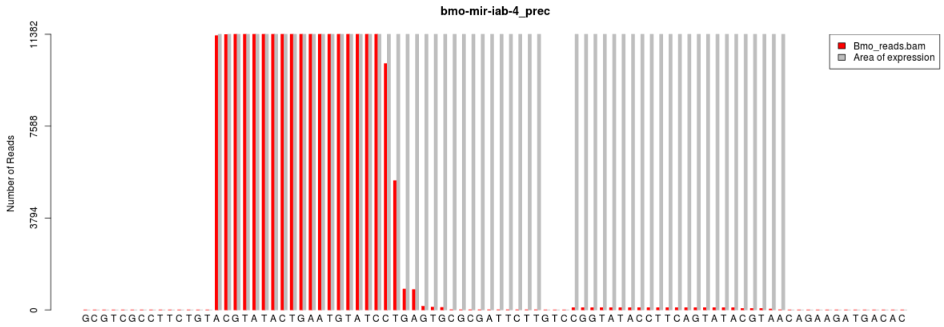

>bmo-mir-iab-8_prec

```
GUGUCAUCUUCUGUUACGUAUACUGAAGGUAUACCGGACAAGAAUCGCGCACUCAGGAUACAUUCAGUAUACGUACAGAAGGCGACGC
(((((..(((((((.((((((((((((.((((.((.((..............)).)))))).)))))))))))))))))))..)))))

 Initial dG =    -39.30

        10        20        30        40
|    AU       U            G    A  G   CAAGAA
GUGUC  CUUCUGU ACGUAUACUGAA GUAU CC GA      U
CGCAG  GAAGACA UGCAUAUGACUU CAUA GG CU      C
^    CG       -            A    -  A   CACGCG
       80         70        60          50

-----------------------------------------------------
```

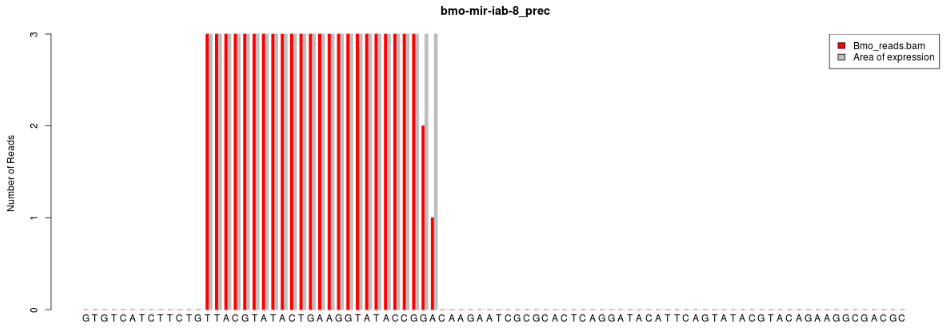

>bmo-mir-76_prec

```
AAGAAUCCGCACAUUCUUCGGGUUUCGGGACGAUGUAACCGUGAAUAAAUUGGUUCGUUGUCGUCGAAACCUGCAAAAUGUUCGUUGAAUUUCGUCGCAG
..(((((((.(((((...((((((((((((((((.(((((.........))))))))))).))))))))))...))))).))..))..))).......

 Initial dG =    -32.50

                 10        20         30        40
AA-----   --  --  C     CUU          -|      U     UGAA
       GAA  UC  CG ACAUU   CGGGUUUCGG GACGAUG AACCG    \
       CUU  AG  GC UGUAA   GUCCAAAGCU CUGUUGC UUGGU    U
GACGCUG   UA  UU  U     AAC          G^      -     UAAA
.        90        80        70        60         50

-----------------------------------------------------
```

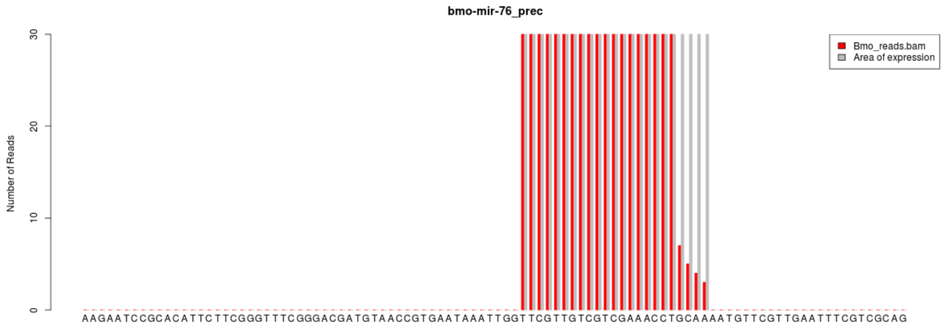

>bmo-mir-971_prec

```
UAAUGAGAUGCGCUUCACUCUAAGUAUGAACACCAAGCGUAUUUUCAUAGAGUUUGGUGUUCUACCUUACAGUGAAAUGAACUCAUU
.((((((...((.((((((.((((...(((((((((((.............)))))))))))...)))).)))))).))..))))))

 Initial dG =    -32.40

        10         20         30         40
U|     AUG  C       C    UAU            GUAUUU
 AAUGAG   CG UUCACU UAAG   GAACACCAAGC      \
 UUACUC   GU AAGUGA AUUC   CUUGUGGUUUG      U
-^     AA-  A       C    CAU            AGAUAC
       80         70         60         50

-----------------------------------------------------
```

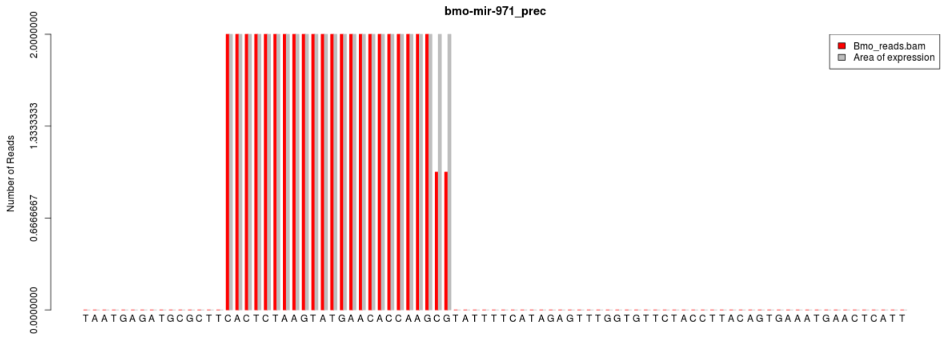

The microRNA toolkit of insects. Guillem Ylla, Bastian Fromm, Maria-Dolors Piulachs, Xavier Belles

Supplementary Table S4. Currently available data on insect miRNAs in miRBase 21.

Insect	Precursors	Mature
Bombyx mori	487	563
Drosophila melanogaster	256	466
Apis mellifera	254	259
Tribolium castaneum	220	430
Drosophila pseudoobscura	210	273
Drosophila simulans	135	178
Drosophila virilis	134	237
Plutella xylostella	133	128
Acyrthosiphon pisum	123	103
Aedes aegypti	101	124
Manduca sexta	98	93
Heliconius melpomene	92	97
Drosophila grimshawi	82	72
Drosophila erecta	81	78
Drosophila sechellia	78	76
Drosophila willistoni	77	72
Drosophila ananassae	76	75
Drosophila yakuba	76	75
Drosophila persimilis	75	69
Culex quinquefasciatus	74	93
Drosophila mojavensis	71	71
Anopheles gambiae	66	65
Nasonia vitripennis	53	53
Nasonia giraulti	32	32
Nasonia longicornis	28	28
Locusta migratoria	7	14

The microRNA toolkit of insects. Guillem Ylla, Bastian Fromm, Maria-Dolors Piulachs, Xavier Belles

Supplementary Table S9. Description of the *Blattella germanica* small RNA-seq libraries used for the miRNA predictions. The accession number of each library, the number of raw reads and the number of mapped reds is shown.

Accession	Tissue	Reads	Mapped reads
SRR3668533	Ovary nymph 6	48,394,142	39,974,355
SRR3668535	Whole body nymph 5 days 3-5	36,987,112	34,214,212
SRX1846178	Whole body nymph 6 days 5-7	23,689,204	21,965,103
SRR3657068	Ovary control 1	24,890,711	13,041,921
SRR3657068	Ovary control 2	31,896,704	14,254,683
SRR060166	Ovary Adult	8,381,086	4,589,056
SRR060167	Whole body nymph 6 days 0-8	11,070,372	7,605,497
		185,309,331	135,644,827

The microRNA toolkit of insects. Guillem Ylla, Bastian Fromm, Maria-Dolors Piulachs, Xavier Belles

Initial ΔG = -35.75

```
           10        20        30        40        50        60        70        80        90       100       110
     A-                UCU       AUACACAUGUAUUAAUUGAGUUGUGUGCGGUAGCGGCAGCGUGCGUAAAGACAAAUACAAAGUUUUUGUAUGAACCACACUCC
CGUGU  GCUGUAGCUGGUUGGA   GGGCCAA                                                                                   \
GCACG  CGAUGUCGACCAACUU   CCUGGUU                                                                                   G
     GC                CC-       UACACUAACGCCGUCGUUGCCGUUGUCGUAGCAUUAGUGUUAGGGUCUUUGAAUACACUCUUUUAUGUGUGCUAUUGACCCUU
   230       220       210        200       190       180       170       160       150       140       130       120
```

Supplementary Figure S1. Structure of the Mir-133 precursor in *Acyrtosyphon pisum*. In red the mature miRNA, in blue the star miRNA. Note the hairpin loop comprising about 200 nucleotides.

The microRNA toolkit of insects. Guillem Ylla, Bastian Fromm, Maria-Dolors Piulachs, Xavier Belles

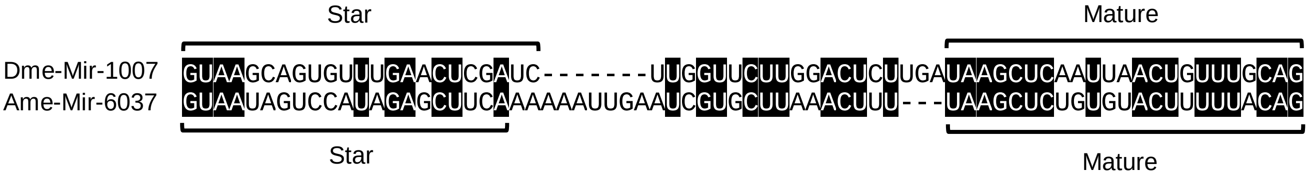

Supplementary Figure S2. Alignment of Mir-1007 precursor of *Drosophila melanogaster* (Dme-Mir-1007) and Mir-6037 of *Apis mellifera* (Ame-Mir-6037).
